# Supplementary material for: Dysfunctional mitochondria trap proteins in the intermembrane space
Source: EMBO J. 2025 Jun 16;44(15):4352–77. doi: 10.1038/s44318-025-00486-1 (PMC12317151; doi:10.1038/s44318-025-00486-1)
Supplement: Supplementary file 1 — Appendix [file 44318_2025_486_MOESM1_ESM.pdf]

Appendix for

Dysfunctional mitochondria trap proteins in the intermembrane space

Tamara Flohr, Markus Räschle, Johannes M. Herrmann*

*Corresponding author Email: hannes.herrmann@biologie.uni-kl.de

Table of Content:

**Appendix Figure S1**	**page 2**
**Appendix Figure S2**	**page 4**
**Appendix Figure S3**	**page 5**
**Appendix Figure S4**	**page 6**
**Appendix Figure S5**	**page 7**
**Appendix Figure S6**	**page 8**
**Appendix Figure S7**	**page 9**
**Appendix Table S1**	**page 10**
**Appendix Table S2**	**page 12**

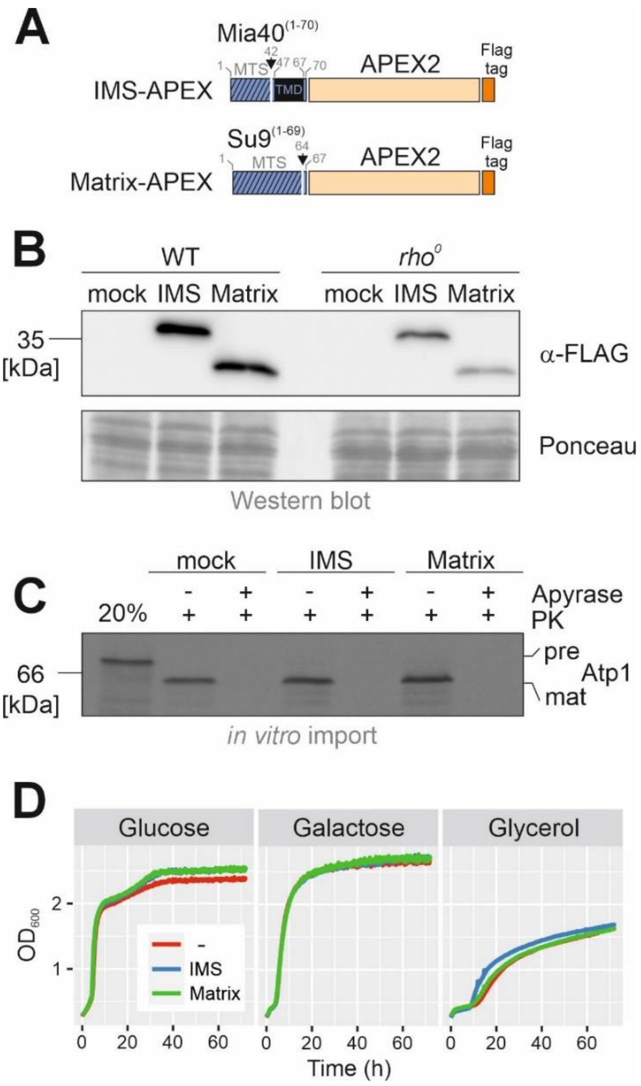

Appendix Figure S1. APEX2 fusion proteins can be targeted to the IMS and the matrix of mitochondria.

(**A**) Schematic representation of the structure of the fusion proteins used in this study. MTS, mitochondrial targeting signal of Mia40 and of subunit 9 of the ATPase of *N. crassa*; TMD, transmembrane domain. (**B**) IMS-APEX or matrix-APEX was integrated into the genome of wild type (WT) and *rho^0* cells. Cell extracts were analyzed by Western blotting. (**C**) Mitochondria were isolated from wild type cells expressing none, IMS-APEX or matrix-APEX fusion constructs. Radiolabeled Atp1 precursor was incubated for 5 min in the presence or absence of apyrase. Non-imported protein was removed by protease treatment. (**D**) The three indicated strains were cultured on full media with the indicated carbon sources at 30°C. Cell growth was recorded continuously. Shown are mean values of three technical replicates.

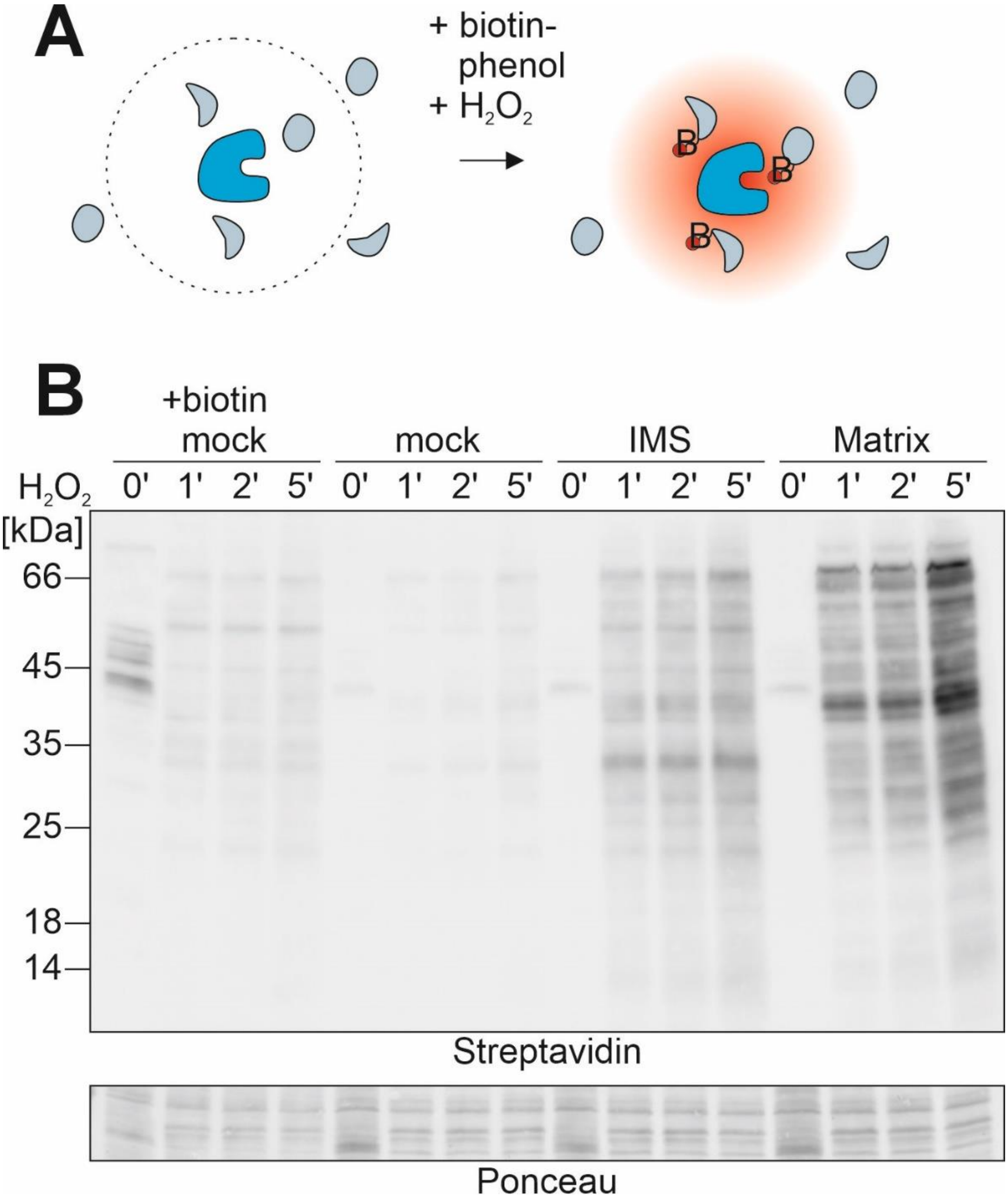

Appendix Figure S2. Mitochondria-targeted APEX2 leads to subcompartment-specific biotinylation patterns.

(**A**) Scheme of the APEX2-induced biotinylation of proximal proteins upon addition of biotin-phenol and hydrogen peroxide (H_2O_2). (**B**) Control cells of the W303 wild type strain not expressing any APEX fusion protein (mock), or cells with genomically integrated genes for the expression of IMS-APEX or matrix-APEX were grown in media with (+biotin) or without biotin. Mitochondria were isolated from these strains and incubated with 100 µM biotin-phenol for 30 min before hydrogen peroxide (1 mM) was added for the times indicated. Proteins from cell extracts were resolved by SDS-PAGE, transferred to nitrocellulose and probed for biotinylation using Streptavidin-coupled peroxidase. Ponceau staining was used as loading control. This figure relates to the experiment shown in Fig. 1D, but this panel shows a larger section of the blot, including the samples derived from cells grown in the presence of 2 µg/l biotin.

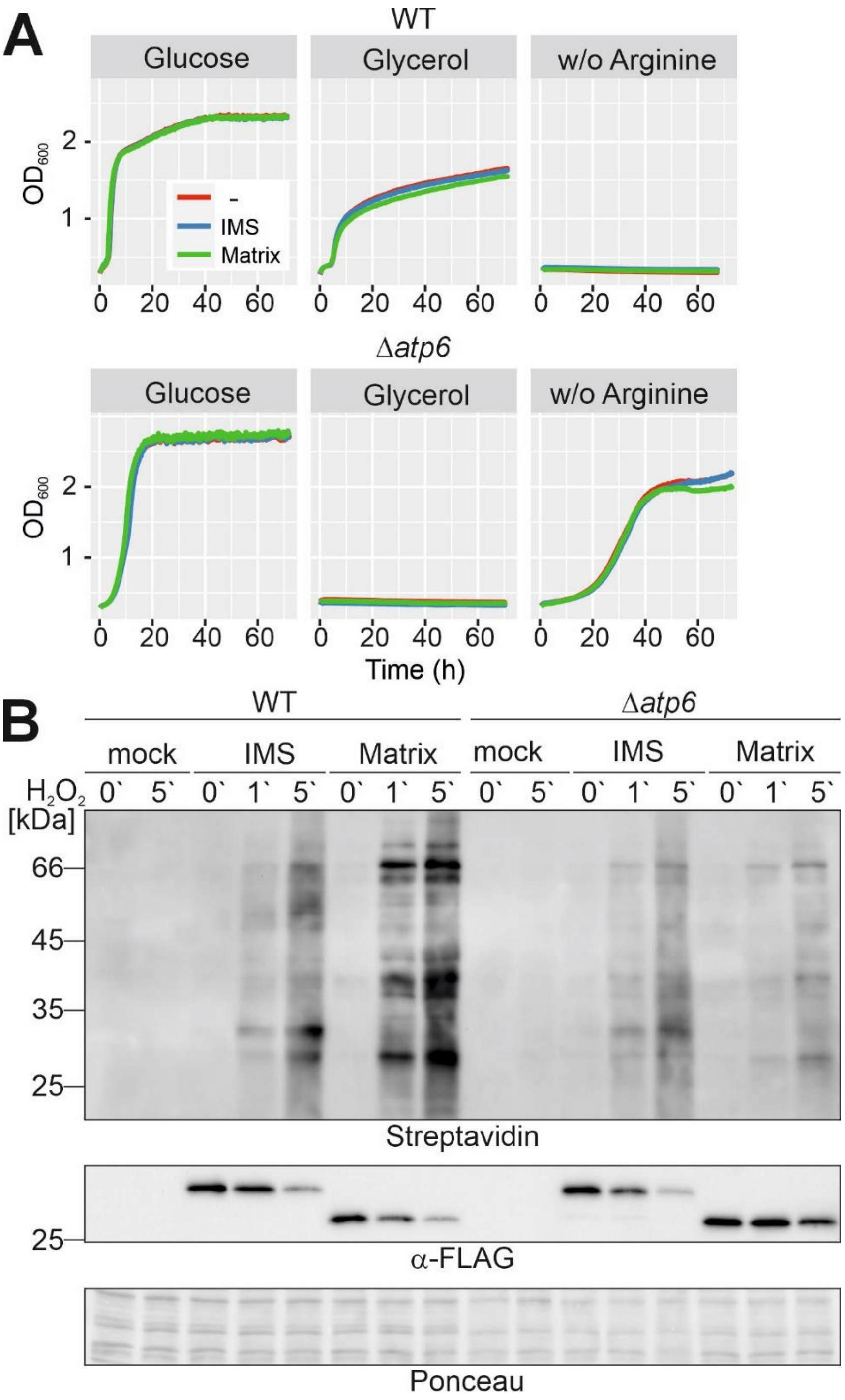

Appendix Figure S3. Expression of the APEX fusion reporters in the *Δatp6* model does not compromise cellular fitness.

(**A**) The *Δatp6* mutant and the corresponding wild type contain genomically integrated expression cassettes for IMS-APEX or matrix-APEX as indicated. Growth in different synthetic media was constantly recorded. Medium without Arginine contained galactose as carbon source. Shown are mean values from three technical replicates. (**B**) Isolated mitochondria of the indicated strains were incubated as described for Appendix Fig. S1B. The presence of the FLAG-tagged APEX reporters was validated by probing the nitrocellulose membrane with FLAG-specific antibodies. Ponceau serves as loading control.

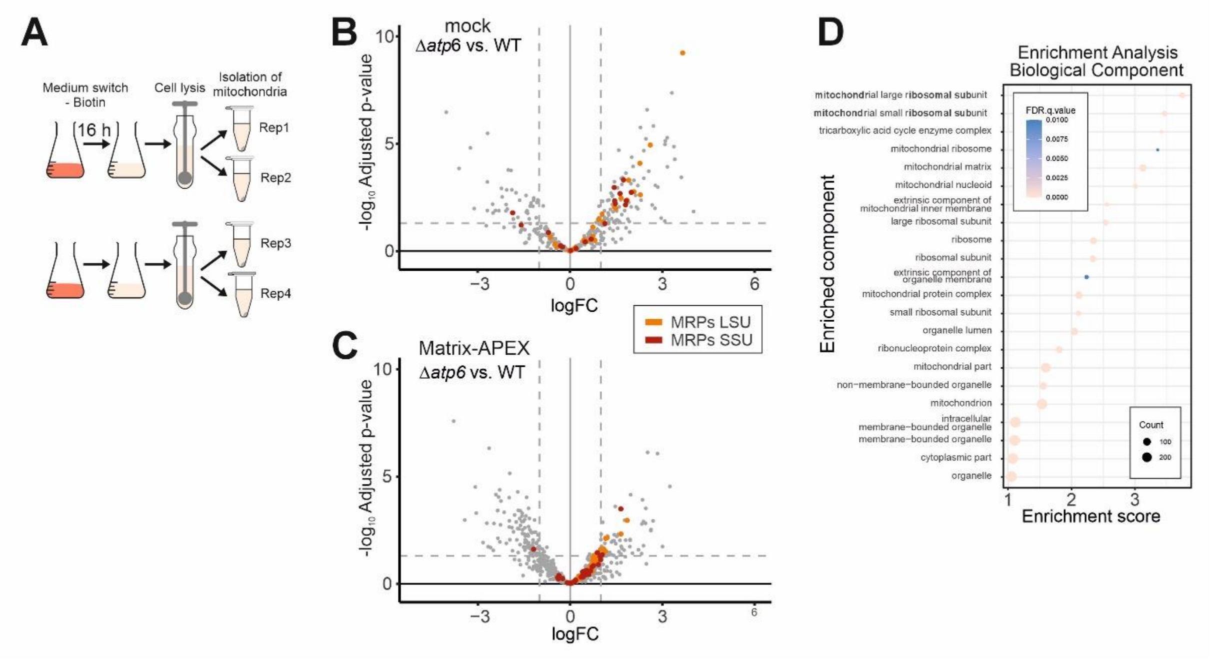

Appendix Figure S4. Many mitoribosomal proteins accumulate in the IMS of the *Δatp6* mutant.

(**A**) Schematic representation of the proximity labeling experiment. The four replicates (Rep1 to Rep4) were generated from two preparations of isolated mitochondria each. (**B**, **C**) Volcano plots showing the proteins purified on streptavidin beads from mitochondrial extracts of the indicated samples. See legend to Fig. 2f for details. Proteins of the small and large subunit of the mitochondrial ribosome are shown in red or orange. (**D**) The proteins that were specifically enriched (logFC>1, p-value<0.05) in the IMS of *Δatp6* mitochondria (corresponding to Fig. 2f) were further analyzed by gene ontology (GO) enrichment, using the GOrilla tool (http://cbl-gorilla.cs.technion.ac.il/).

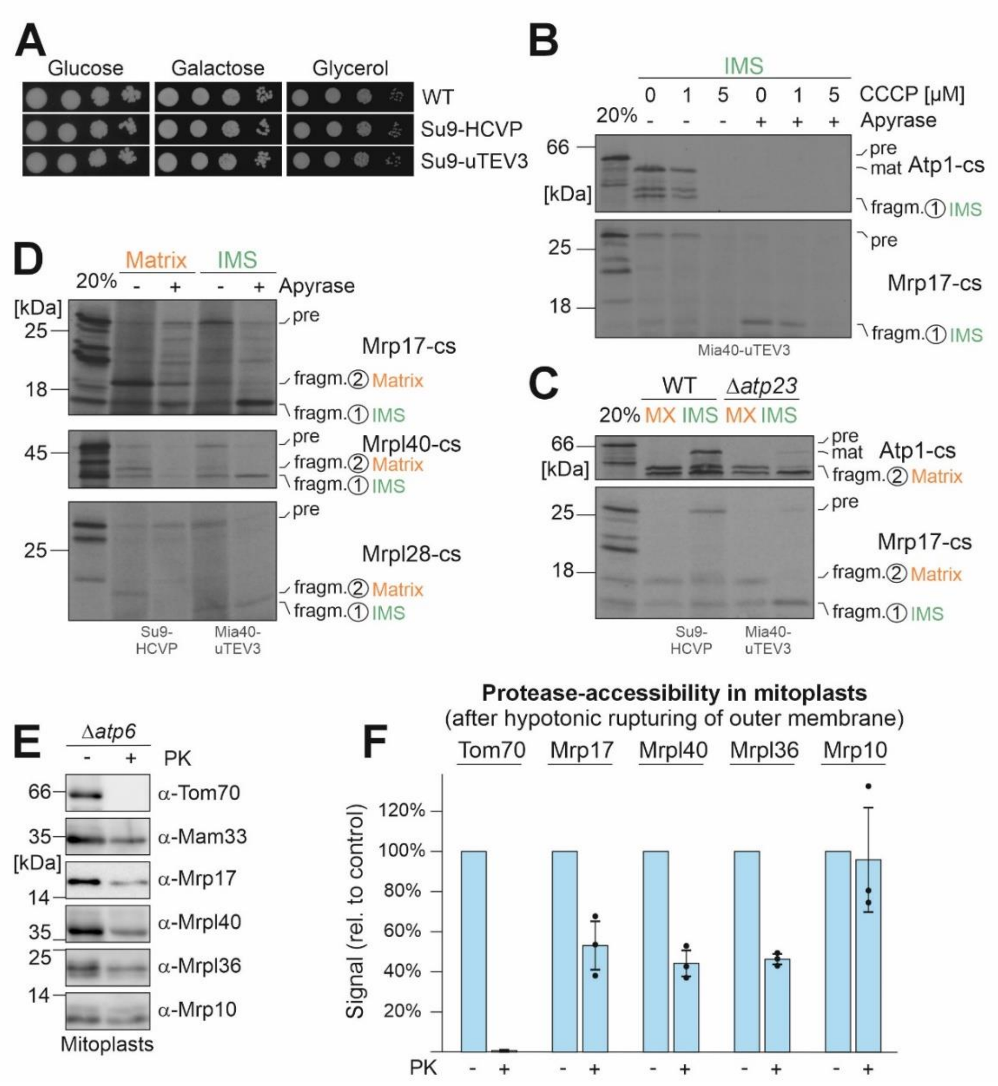

Appendix Figure S5. Intramitochondrial proteases are powerful reporters in protein localization experiments.

(**A**) Wild type cells expressing no protease, Su9-HCVP or Su9-uTEV3 were grown to mid-log phase on galactose containing medium before tenfold serial dilutions were dropped on media containing the indicated carbon sources. (**B-D**) Mitochondria were isolated from wild type or *Δatp23* cells expressing the proteases Su9-HCVP (Matrix, MX) or Mia40-uTEV3 (IMS). Mitochondria were incubated with 40 U/ml apyrase and carbonyl cyanide m-chlorophenyl hydrazone (CCCP) as indicated. The indicated radiolabeled proteins were imported for 5 min before non-imported proteins were removed by protease treatment. (**E**, **F**) Mitochondria were isolated from *Δatp6* cells and incubated for 30 min in hypotonic swelling buffer containing 60 mM sorbitol in the presence or absence of 100 µg/ml proteinase K (PK). Proteins were lysed in sample buffer and subjected to SDS-PAGE and Western blotting with the indicated antibodies. The outer membrane protein Tom70 served as control for a surface-exposed protein. Mrp10 is a MRP that is imported by a two-step import pathway via the IMS into mitochondria and was found to be well protected from protease. For Mrp17, Mrpl36 and Mrpl40 about half of the endogenous protein was protease-accessible, compatible with their partial trapping in the IMS. (**F**) The amounts of protease-accessible proteins were quantified from three biological replicates. Mean values and standard deviations are shown.

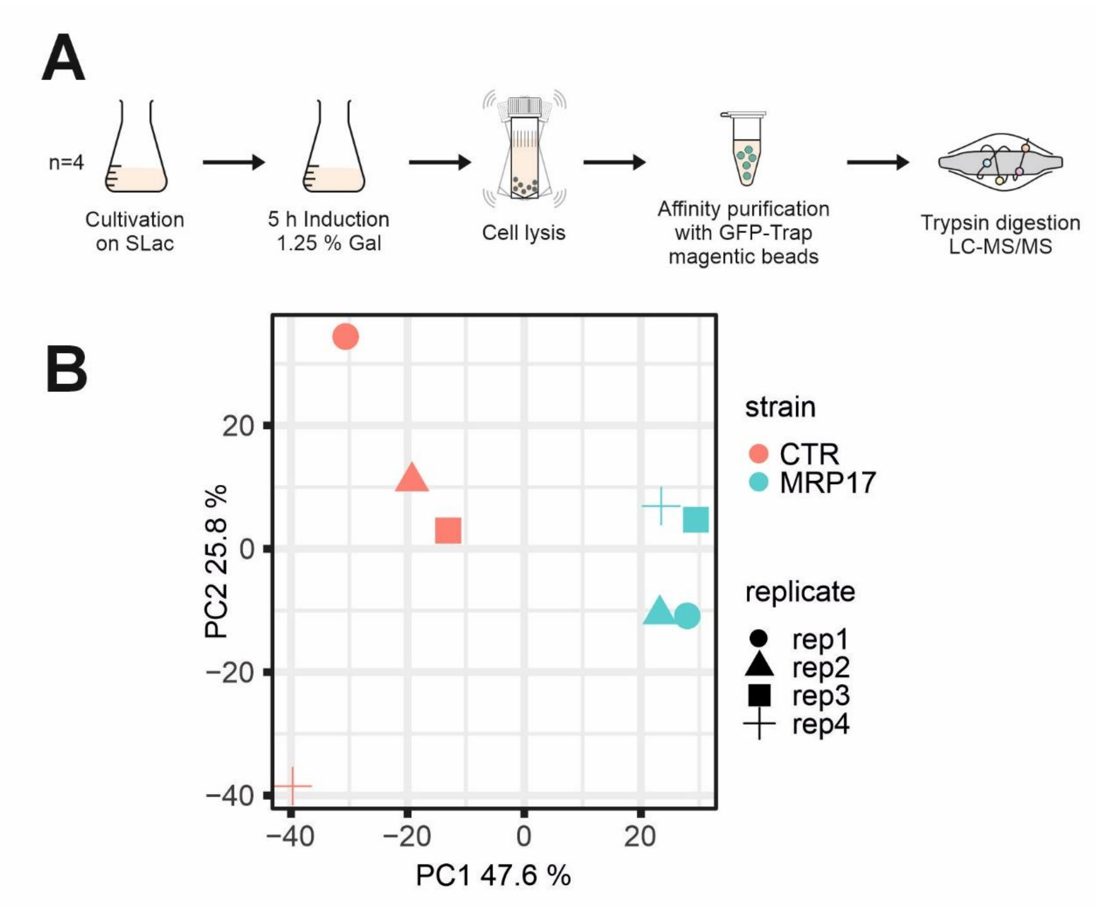

Appendix Figure S6. Identification of the interactome of GFP-Mrp17.

(**A**) Schematic representation of the GFP-Mrp17 purification. Wild type cells containing plasmids for GFP-Mrp17 expression or an empty vector control were grown and processed as shown. (**B**) Principal Component Analysis of the proteomes of cells expressing GFP-Mrp17 and the corresponding empty vector control.

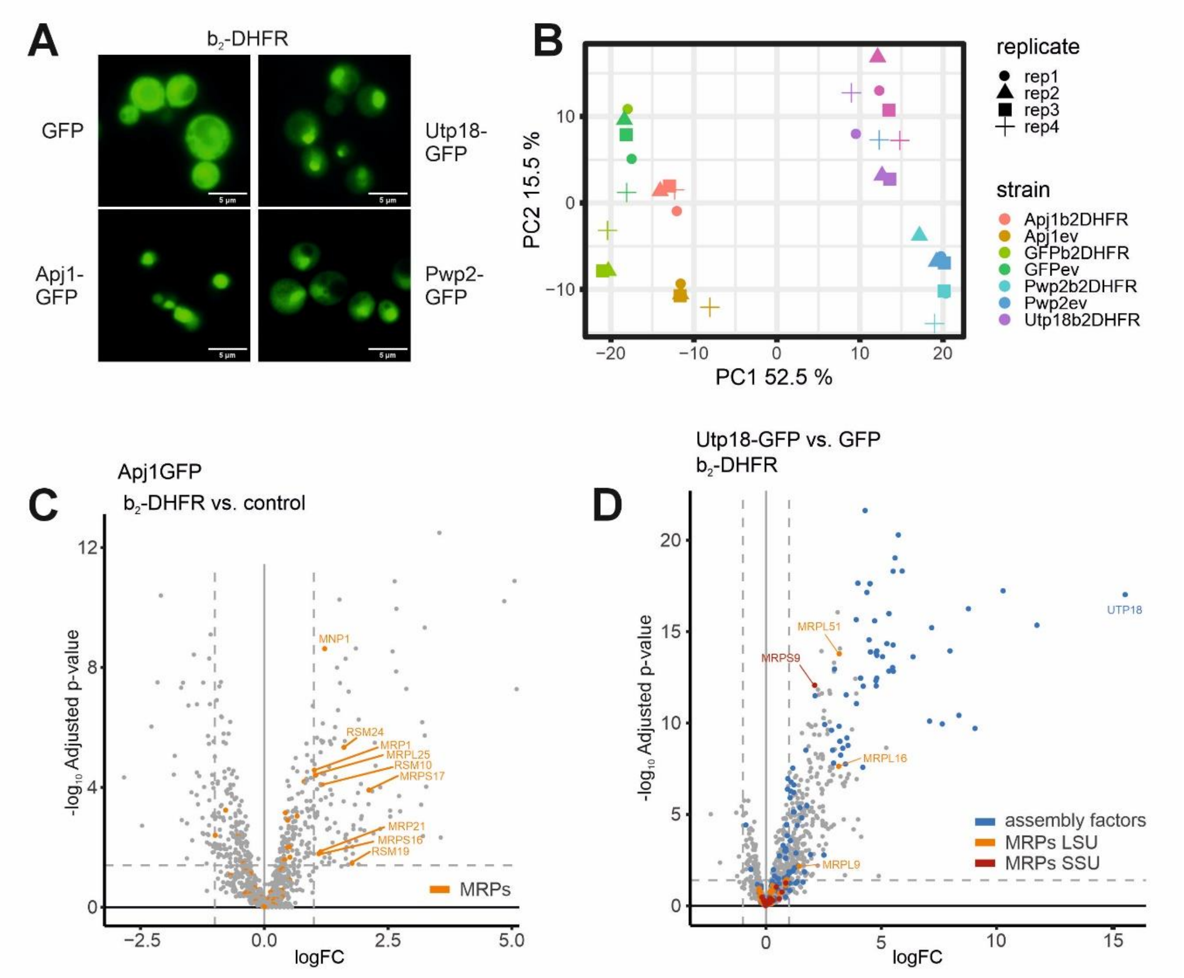

Appendix Figure S7. MRPs are found as interactors of nuclear proteins.

(**A**) Fluorescence microscopy of wild type cells expressing the indicated GFP proteins and the b_2-DHFR clogger for 4.5 h. Whereas GFP shows a cytosolic distribution, Apj1-GFP, Pwp2-GFP and Utp18-GFP localized to the nucleus. (**B**) Principal Component Analysis of the proteomes of cells expressing the indicated GFP fusion proteins. Cells contained either plasmids for the expression of the b_2-DHFR clogger or an empty vector control. Four biological replicates were used per sample. (**C**) Volcano plots showing the Apj1 interactomes with and without clogger expression. The $\log_2$ fold change (logFC) values were calculated from n=4 samples. The distribution of MRPs is indicated. Significantly enriched MRPs (logFC>1, p-value<0.05) are labeled. (**D**) Proteins associated with a GFP-tagged version of the ribosome biogenesis factor Utp18 were identified by mass spectrometry in extracts from clogger-inducing wild type cells. Extracts from GFP-containing cells served as controls. The $\log_2$ fold change (logFC) values were calculated from n=4 samples. MRPs and proteins of the ribosome assembly complex in the nucleus are highlighted. Significantly enriched MRPs (logFC>1, p-value<0.05) are labeled.

Appendix Table S1. A list of yeast strains used in this study.

Database	Strain	Genotype	Reference
HHY0084	W303 (WT)	MATα {leu2-3,112 trp1-1 can1-100 ura3-1 ade2-1 his3-11,15} [phi^+]	PMID: 6310324
HHY3713	W303 Su9-APEX2-FLAG	W303 YPRCD15C::Su9-APEX2-FLAG-Hygromycin	This study
HHY3714	W303 IMS-APEX2-FLAG	W303 YPRCD15C::IMS-APEX2-FLAG-Hygromycin	This study
HHY0621	MR6 (WT)	D273-10B MATα ade2-1 his3-11,15 trp1-1 leu2-3,112 ura3-1 CAN1 arg8::HIS3 *rho+*	PMID: 17261589
HHY3886	MR6 Su9-APEX2-FLAG	MR6 YPRCD15C::Su9-APEX2-FLAG-Hygromycin	This study
HHY3887	MR6 IMS-APEX2-FLAG	MR6 YPRCD15C::IMS-APEX2-FLAG-Hygromycin	This study
HHY0622	MR10 (*Δatp6)*	D273-10B MATα ade2-1 his3-11,15 trp1-1 leu2-3,112 ura3-1 CAN1 arg8::HIS3; atp6::ARG8m *rho+*	PMID: 17261589
HHY3888	MR10 *Δatp6* Su9-APEX2-FLAG	MR10 YPRCD15C::Su9-APEX2-FLAG-Hygromycin	This study
HHY3889	MR10 *Δatp6* IMS-APEX2-FLAG	MR10 YPRCD15C::IMS-APEX2-FLAG-Hygromycin	This study
HHY0730	*Δatp23*	W303-1B MATα ATP23::HIS3MX	PMID: 17135288
HHY3890	*Δatp23* Su9-APEX2-FLAG	*Δatp23* YPRCD15C::Su9-APEX2-FLAG-Hygromycin	This study
HHY3891	*Δatp23* IMS-APEX2-FLAG	*Δatp23* YPRCD15C::IMS-APEX2-FLAG-Hygromycin	This study
HHY4405	*Δatp23* Su9-NeonGreen,Cox4MTS-mScarlet	*Δatp23* cHHYTK279-Su9-NeonGreen, Cox4MTS-mScarlet	This study
HHY4406	*Δatp23* Su9-Neongreen, Mrp17-mScarlet	*Δatp23* cHHYTK281-Su9-NeonGreen, Mrp17-mScarlet	This study
HHY3989	W303 IMS-APEX-GFP11, IMS-GFP1-10	W303, cHHYTK257-IMS-APEX-GFP11,IMS-GFP1-10	This study
HHY3990	W303 IMS-APEX-GFP11, Su9-GFP1-10	W303, cHHYTK258-IMS-APEX-GFP11,Su9-GFP1-10	This study
HHY3992	W303 Su9-APEX-GFP11, IMS-GFP1-10	W303, cHHYTK260-Su9-APEX-GFP11,IMS-GFP1-10	This study
HHY3993	W303 Su9-APEX-GFP11, Su9-GFP1-10	W303, cHHYTK261-Su9-APEX-GFP11,Su9-GFP1-10	This study
HHY4407	MR10 IMS-APEX-GFP11, IMS-GFP1-10	MR10, cHHYTK257-IMS-APEX-GFP11,IMS-GFP1-10	This study
HHY4408	MR10 IMS-APEX-GFP11, Su9-GFP1-10	MR10, cHHYTK258-IMS-APEX-GFP11,Su9-GFP1-10	This study
HHY4409	MR10 Su9-APEX-GFP11, IMS-GFP1-10	MR10, cHHYTK260-Su9-APEX-GFP11,IMS-GFP1-10	This study
HHY4410	MR10 Su9-APEX-GFP11, Su9-GFP1-10	MR10, cHHYTK261-Su9-APEX-GFP11,Su9-GFP1-10	This study
HHY3715	W303 rho^0	W303 rho^0	PMID: 38551959
HHY3716	rho0 Su9-APEX2-FLAG	W303 rho^0 YPRCD15C::Su9-APEX2-FLAG-Hygromycin	This study
HHY3717	rho0 IMS-APEX2-FLAG	W303 rho^0 YPRCD15C::IMS-APEX2-FLAG-Hygromycin	This study
HHY1819	*Δtom20*	W303 TOM20::HIS3	PMID: 21460184
HHY1653	*Ssc1-3*	MATa ade2-101 lys2 ura3-52 leu2-3,112 Atrpl sscl-3(LEU2)	PMID: 8408191
HHY1767	YPH500	MATα ura3-52 lys2-801_amber ade2-101_ochre trp1-Δ63 his3-Δ200 leu2-Δ1	PMID: 2659436
HHY1768	*Δtom5*	YPH500 TOM5::HIS3	PMID: 9217162
HHY1764	YPH499 Tom40-WT	YPH499 TOM40::ADE2 pFL39-Tom40-WT	PMID: 21825073
HHY1765	*tom40-18*	YPH499 TOM40::ADE2, pFL39-Tom40-18	PMID: 21825073
HHY3430	*Δhsp78*	W303 HSP78::NAT	PMID: 38551959
HHY3351	W303 Su9-HCVP	W303 YPRCD15C::Su9-HCVP-Hygromycin	This study
HHY3353	W303 Su9-uTEV3	W303 YPRCD15C::Su9-uTEV3-Hygromycin	This study
HHY3473	W303 IMS-uTEV3	W303 YPRCD15C::IMS-uTEV3-Hygromycin	This study
HHY 3979	*Δatp23* Su9-HCVP	*Δatp23* YPRCD15C::Su9-HCVP-Hygromycin	This study
HHY3980	*Δatp23* IMS-uTEV3	*Δatp23* YPRCD15C::IMS-uTEV3-Hygromycin	This study
HHY0495	YPH499	MATa ura3-52 lys2-801_amber ade2-101_ochre trp1-Δ63 his3-Δ200 leu2-Δ1	PMID: 2659436
HHY3373	CRISPRi-ev	YPH499, pKR366-dCas9-Mxi	This study
HHY3376	CRISPRi-TIM44	YPH499, pKR366-dCas9-Mxi-TIM44	This study
HHY4411	W303 Mrp17-GFP, Su9-RFP	W303 pYX233-Mrp17-GFP, pYX112-Su9-RFP	This study
HHY4412	W303 GFP-Mrp17, Su9-RFP	W303 pYX233-GFP-Mrp17, pYX112-Su9-RFP	This study

HHY4413	W303 GFP-Mrp17, NLS-Tomato	W303 pYX233-GFP-Mrp17, pYX112-NLS-Tomato	This study
HHY4414	W303 GFP-Mrp17	W303 pYX233-GFP-Mrp17	This study
HHY4415	W303 GFP	W303 pYX233-GFP	This study
HHY4416	W303 Mrp17-GFP, Su9-RFP	W303 pYX142-Mrp17-GFP, pYX112-Su9-RFP	This study
HHY4417	W303 GFP-Mrp17, Su9-RFP	W303 pYX142-GFP-Mrp17, pYX112-Su9-RFP	This study
HHY4792	Δ*cox19* Su9-Neongreen, Mrp17-mScarlet	BY4742 Δ*cox19* cHHYTK281-Su9-NeonGreen, Mrp17-mScarlet	This study
HHY4553	W303 Mrpl40-GFP, Su9-RFP	W303 pYX233-Mrpl40-GFP, pYX112-Su9-RFP	This study
HHY4554	W303 GFP-Mrpl40, Su9-RFP	W303 pYX233-GFP-Mrpl40, pYX112-Su9-RFP	This study
HHY4555	W303 GFP-Mrpl40, NLS-Tomato	W303 pYX233-GFP-Mrpl40, NLS-Tomato	This study
HHY4556	W303 Mrpl28-GFP, Su9-RFP	W303 pYX233-Mrpl28-GFP, pYX112-Su9-RFP	This study
HHY4557	W303 GFP-Mrpl28, Su9-RFP	W303 pYX233-GFP-Mrpl28, pYX112-Su9-RFP	This study
HHY4558	W303 GFP-Mrpl28, NLS-Tomato	W303 pYX233-GFP-Mrpl28, NLS-Tomato	This study
HHY4559	W303 Apj1-GFP, ev	W303 pYX142-Apj1-GFP, pYX233-ev	This study
HHY4560	W303 Pwp2-GFP, ev	W303 pYX142-Pwp2-GFP, pYX233-ev	This study
HHY4561	W303 Utp18-GFP, ev	W303 pYX142-Utp18-GFP, pYX233-ev	This study
HHY4562	W303 GFP, ev	W303 pYX142-GFP, pYX233-ev	This study
HHY4563	W303 Apj1-GFP, b2-DHFR	W303 pYX142-Apj1-GFP, pYX233-b2-DHFR	This study
HHY4564	W303 Pwp2-GFP, b2-DHFR	W303 pYX142-Pwp2-GFP, pYX233-b2-DHFR	This study
HHY4565	W303 Utp18-GFP, b2-DHFR	W303 pYX142-Utp18-GFP, pYX233-b2-DHFR	This study
HHY4566	W303 GFP, b2-DHFR	W303 pYX142-GFP, pYX233-b2-DHFR	This study
HHY4570	*Δatp23* pYX232-ev	*Δatp23* pYX232-ev	This study
HHY4571	*Δatp23* pYX232-Mrp17	*Δatp23* pYX232-Mrp17	This study
HHY4572	*Δatp23* pYX232-Mrp17K-A	*Δatp23* pYX232-Mrp17K-A	This study
HHY4585	W303 Mrp17K-A-GFP, Su9-RFP	W303 pYX233-Mrp17K-A-GFP, pYX112-Su9-RFP	This study
HHY4586	W303 GFP-Mrp17K-A, Su9-RFP	W303 pYX233-GFP-Mrp17K-A, pYX112-Su9-RFP	This study
HHY4587	W303 GFP-Mrp17K-A, NLS-Tomato	W303 pYX233-GFP-Mrp17K-A, NLS-Tomato	This study

Appendix Table S2. Plasmids used in this study.

Database	Plasmid	Description	Reference
cHHYTK159	IMS-APEX2-FLAG	YPRCD15-pRNR1-IMS-APEX2-FLAG-tTDH1-HygromycinR	This study
cHHYTK160	Su9-APEX2-FLAG	YPRCD15-pRNR1-Su9-APEX2-FLAG-tTDH1-HygromycinR	This study
cHHYTK257	IMS-APEX2-GFP11, IMS-GFP1-10	Split-GFP reporter, pRNR2-IMS-APEX-GFP11-tENO2, pRNR1-IMS-GFP1-10-tADH1, ARS/CEN, URA3	This study
cHHYTK258	IMS-APEX2-GFP11, Su9-GFP1-10	Split-GFP reporter, pRNR2-IMS-APEX-GFP11-tENO2, pRNR1-Su9-GFP1-10-tADH1, ARS/CEN, URA3	This study
cHHYTK260	Su9-APEX2-GFP11, IMS-GFP1-10	Split-GFP reporter, pRNR2-Su9-APEX-GFP11-tENO2, pRNR1-IMS-GFP1-10-tADH1, ARS/CEN, URA3	This study
cHHYTK261	Su9-APEX2-GFP11, Su9-GFP1-10	Split-GFP reporter, pRNR2-Su9-APEX-GFP11-tENO2, pRNR1-Su9-GFP1-10-tADH1, ARS/CEN, URA3	This study
HHB2046	pGem4-Oxa1	in vitro expression, pSP6-Oxa1	PMID: 35231030
HHB1398	pGem4-Atp1	in vitro expression, pSP6-Atp1	PMID: 29382700
HHB1648	pGem4-Mrp17-DHFRmut	in vitro expression, pSP6-Mrp17-DHFRmut	PMID: 34786732
HHB1362	pGem4-Mrp17-DHFR	in vitro expression, pSP6-Mrp17-DHFR	This study
HHB2180	pKR366-dCas9-Mxi-TIM44	CRISPRi plasmid for TIM44 knockdown, ARS/CEN, pTEF-dCas9-Mxi-TIM44, URA3	This study
HHB1598	pKR366-dCas9-Mxi-ev	CRISPRi empty vector, ARS/CEN, pTEF-dCas9-Mxi, URA3	PMID: 33826901
cHHYTK279	Su9-NeonGreen, Cox4MTS-mScarlet	pTEF2-Su9-NeonGreen-tTDH1, pTEF1-Cox4MTS-mScarletI-tENO2, ARS/CEN, URA3	This study
cHHYTK281	Su9-NeonGreen, Mrp17-mScarlet	pTEF2-Su9-NeonGreen-tTDH1, pTEF1-Mrp17-mScarletI-tENO2, ARS/CEN, URA3	This study
HHB2035	pGem4-Mrp17-cs	in vitro expression, pSP6-Mrp17- cleavage site	This study
HHB2036	pGem4-Atp1-cs	in vitro expression, pSP6-Atp1- cleavage site	This study
HHB2192	Su9-HCVP-Hygro	YPRCD15-TDH3p-Su9-HCVP-TDH2t-HygromycinR	This study
HHB2194	Su9-uTEV3-Hygro	YPRCD15-TDH3p-Su9-HCVP-TDH2t-HygromycinR	This study
HHB2195	IMS-uTEV-Hygro	YPRCD15-TDH3p-Su9-HCVP-TDH2t-HygromycinR	This study
HHB2369	pYX233-Mrp17-GFP	2μ, pGAL-Mrp17-GFP, TRP1	This study
HHB2370	pYX233-GFP-Mrp17	2μ, pGAL-GFP-Mrp17, TRP1	This study
HHB2371	pYX233-GFP	2μ, pGAL-GFP, TRP1, Control for GFP-Mrp17 Pulldown	This study
HHB2165	pYX112-Su9-RFP	ARS/CEN, pTPI-Su9-RFP, URA3	This study
HHB2381	NLS-Tomato	CEN/ARS, pGPD-NLS-Tomato, URA3	PMID: 34786732
HHB1645	pGem4-Hsp60	in vitro expression, pSP6-Hsp60	PMID: 30213914
HHB679	pGem4-b2-DHFR	in vitro expression, pSP6-b2-DHFR	PMID: 30886345
HHB1249	pGem4-Mrp17	in vitro expression, pSP6-Mrp17	PMID: 34786732
HHB2374	pGem4-Su9-DHFRmut	in vitro expression, pSP6-Su9-DHFRmut	This study
HHB2375	pGem4-Su9-DHFR	in vitro expression, pSP6-Su9-DHFR	This study
HHB2376	pGem4-Mrpl40-cs	in vitro expression, pSP6-Mrpl40-cleavage site	This study
HHB2377	pGem4-Mrpl28-cs	in vitro expression, pSP6-Mrpl28-cleavage site	This study
HHB2372	pYX142-Mrp17-GFP	ARS/CEN, pTPI-Mrp17-GFP, LEU2	This study
HHB2373	pYX142-GFP-Mrp17	ARS/CEN, pTPI-GFP-Mrp17, LEU2	This study
HHB2501	pYX233-Mrpl40-GFP	2μ, TRP1, pGAL-Mrpl40-GFP	This study
HHB2502	pYX233-GFP-Mrpl40	2μ, TRP1, pGAL-GFP-Mrpl40	This study
HHB2503	pYX233-Mrpl28-GFP	2μ, TRP1, pGAL-Mrpl28-GFP	This study
HHB2504	pYX233-GFP-Mrpl28	2μ, TRP1, pGAL-GFP-Mrpl28	This study
HHB2505	pYX142-Apj1-GFP	ARS/CEN, LEU2, pTPI-Apj1-GFP	This study
HHB2506	pYX142-Pwp2-GFP	ARS/CEN, LEU2, pTPI-Pwp2-GFP	This study
HHB2507	pYX142-Utp18-GFP	ARS/CEN, LEU2, pTPI-Utp18-GFP	This study
HHB2508	pYX142-GFP	ARS/CEN, LEU2, pTPI-GFP	This study
HHB1717	pYX233-b2-DHFR	2μ, TRP1, pGAL-b2-DHFR	This study
HHB1491	pYX232-Mrp17	2μ, TRP1, pTPI-Mrp17	This study
HHB1492	pYX232-Mrp17K-A	2μ, TRP1, pTPI-Mrp17K-A	This study
HHB2511	pYX233-Mrp17K-A-GFP	2μ, TRP1, pGAL-Mrp17K-A-GFP	This study
HHB2512	pYX233-GFP-Mrp17K-A	2μ, TRP1, pGAL-Mrp17K-A-GFP	This study
