# Supplementary figures and images for: Dysfunctional mitochondria trap proteins in the intermembrane space
Source: EMBO J. 2025 Jun 16;44(15):4352–77. doi: 10.1038/s44318-025-00486-1 (PMC12317151; doi:10.1038/s44318-025-00486-1)

Fig-1-B

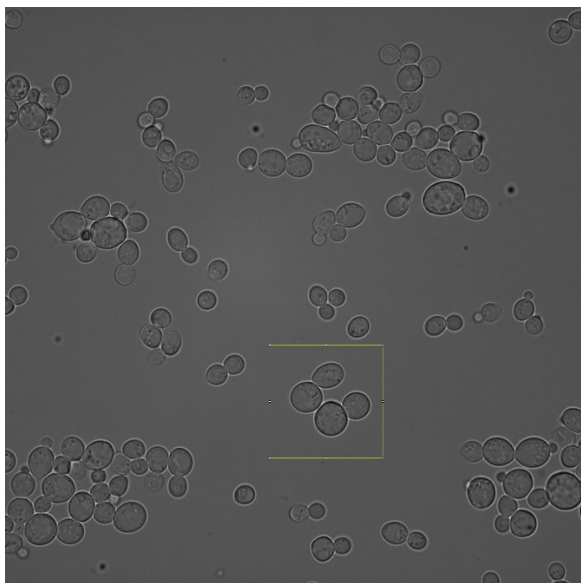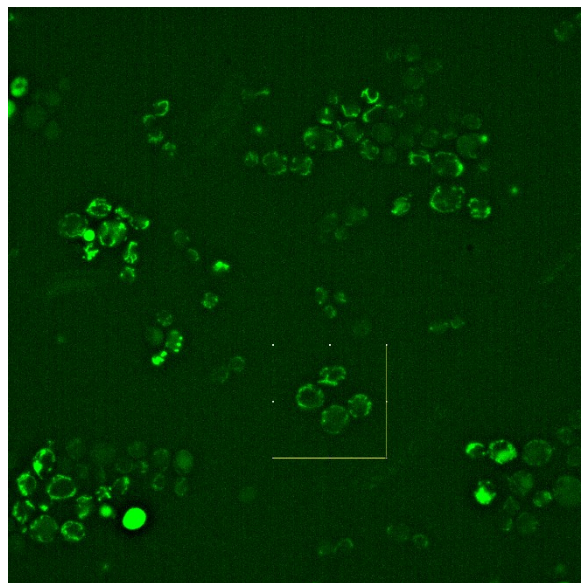

IMS-APEX-GFP11, Mia40-GFP1-10

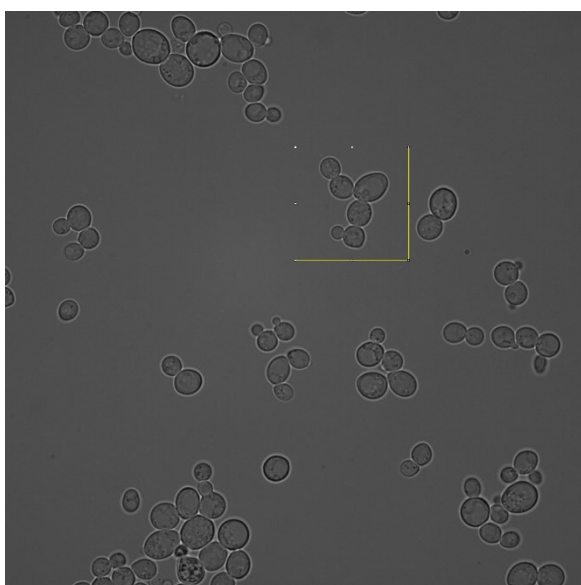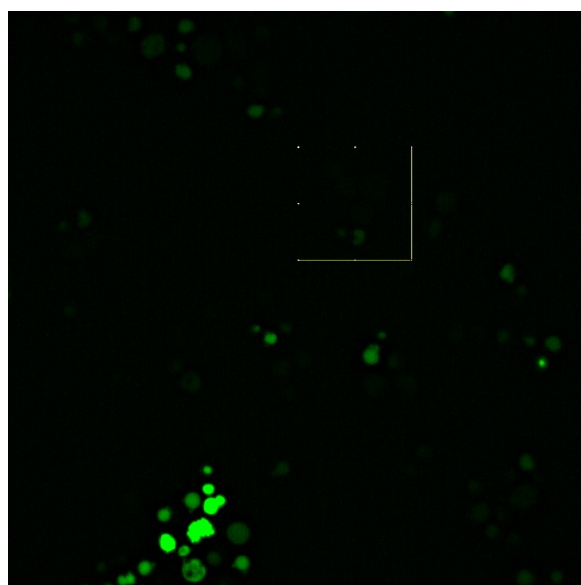

IMS-APEX-GFP11, Su9-GFP1-10

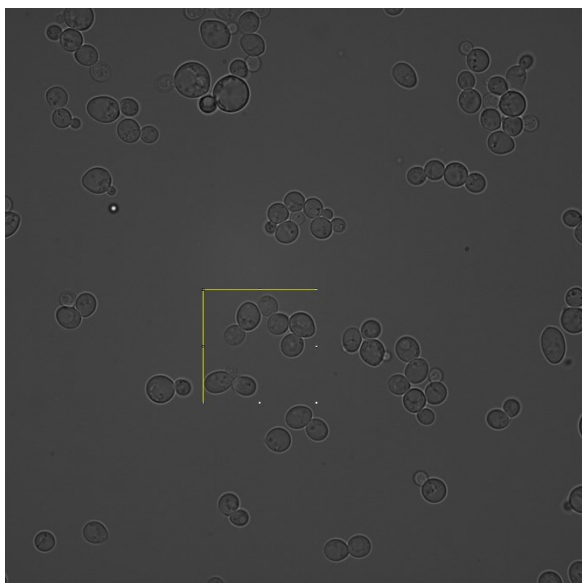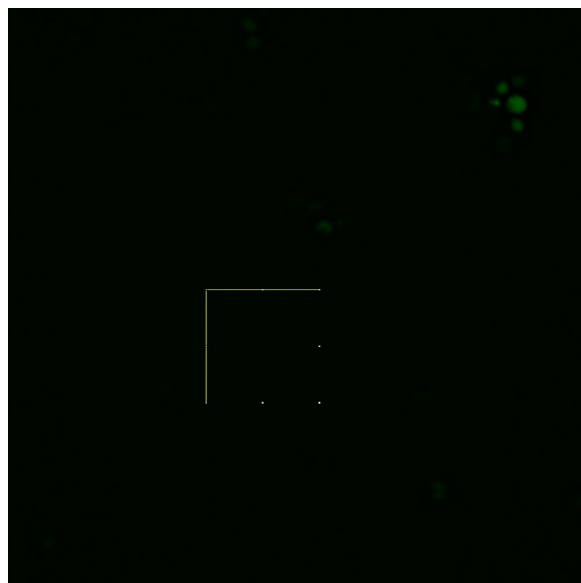

Matrix-APEX-GFP11, Mia40-GFP1-10

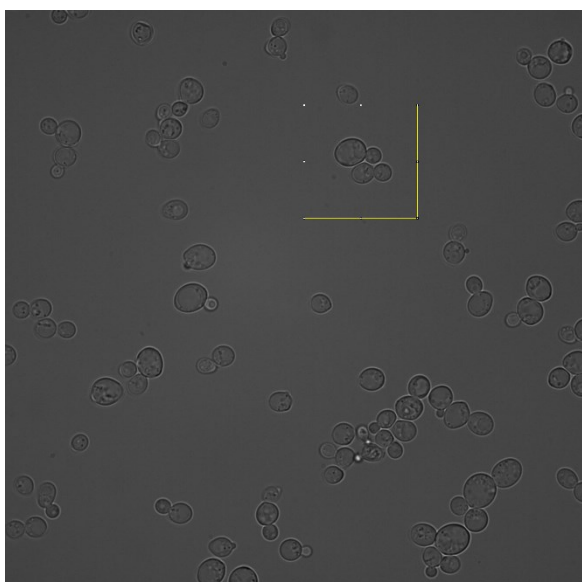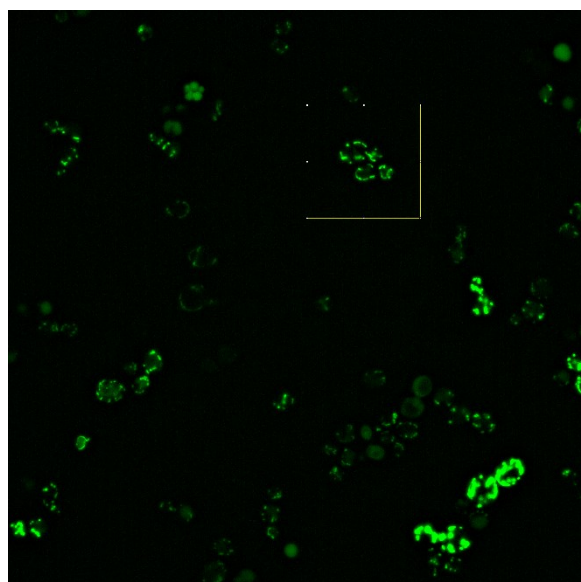

Matrix-APEX-GFP11, Su9-GFP1-10

Supplement: Supplementary file 8 — Source data Fig. 1 [file 44318_2025_486_MOESM8_ESM.zip › SD figure 1/SD figure 1B.pdf]

Fig-1-C

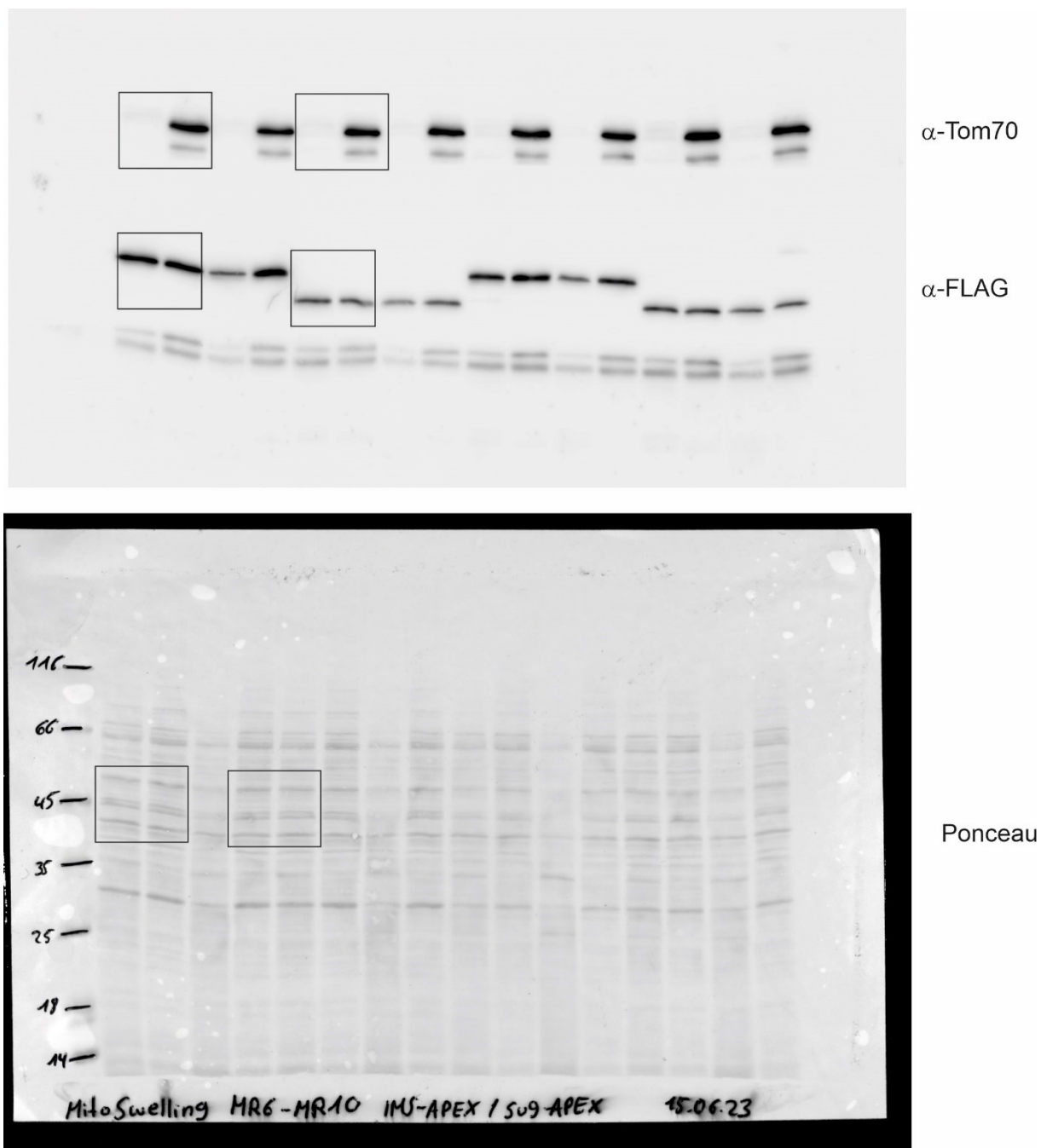

Supplement: Supplementary file 8 — Source data Fig. 1 [file 44318_2025_486_MOESM8_ESM.zip › SD figure 1/SD figure 1C.pdf]

Fig-1-D

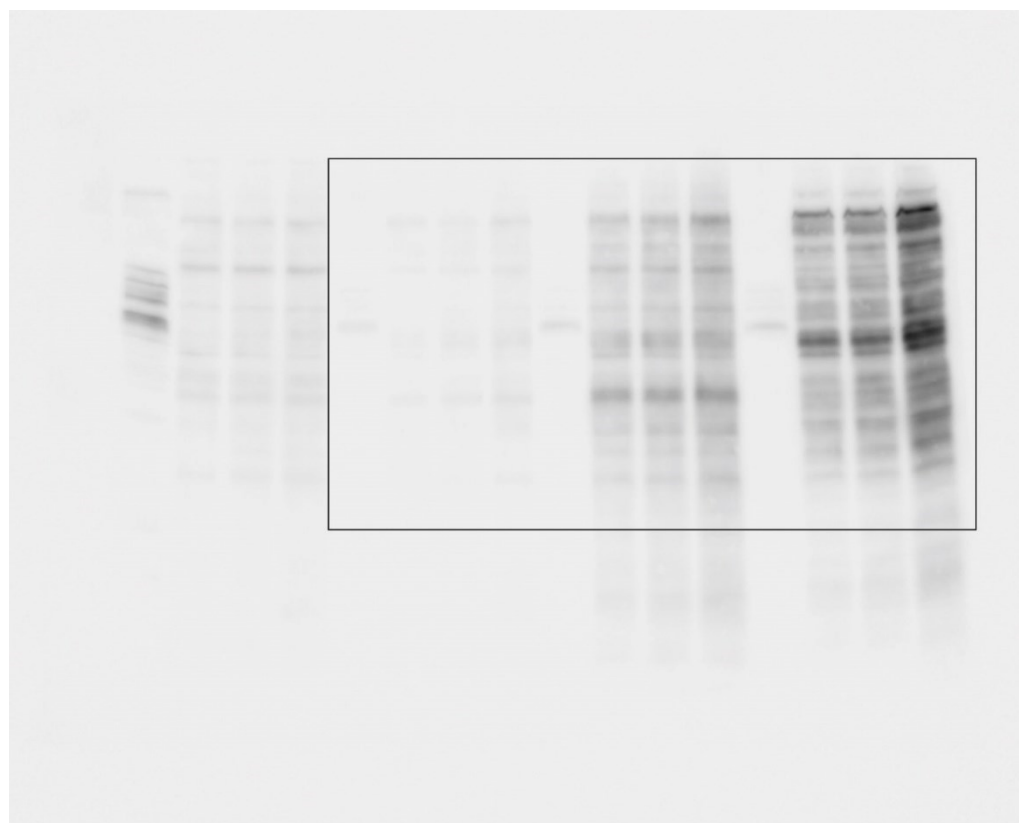

Streptavidin

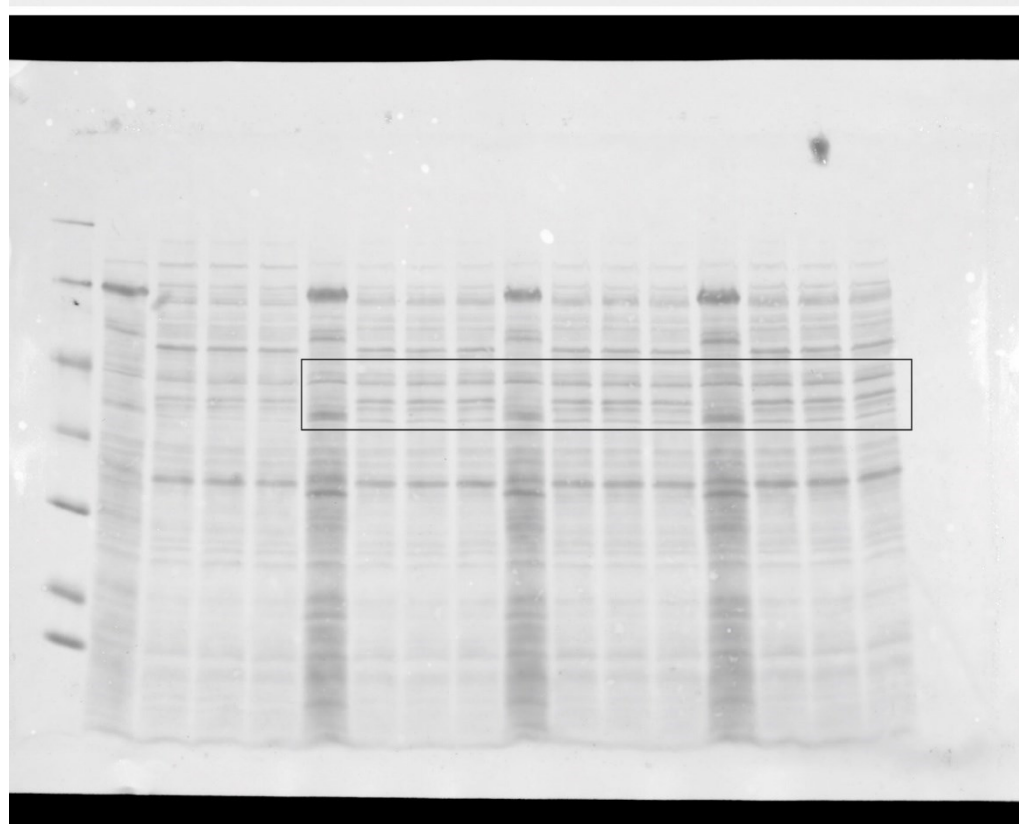

Ponceau

Supplement: Supplementary file 8 — Source data Fig. 1 [file 44318_2025_486_MOESM8_ESM.zip › SD figure 1/SD figure 1D.pdf]

Fig-1-E

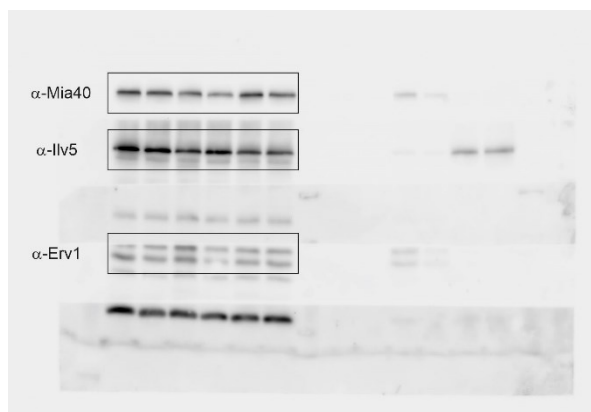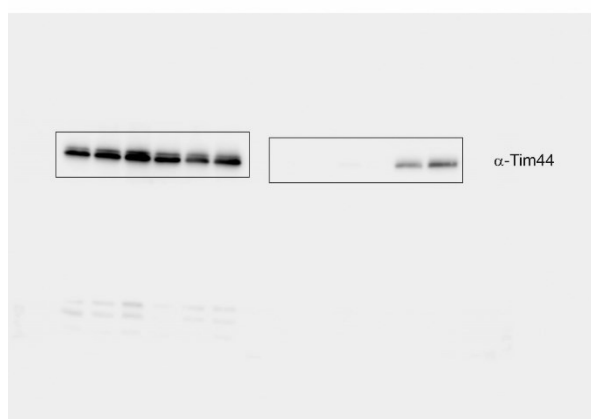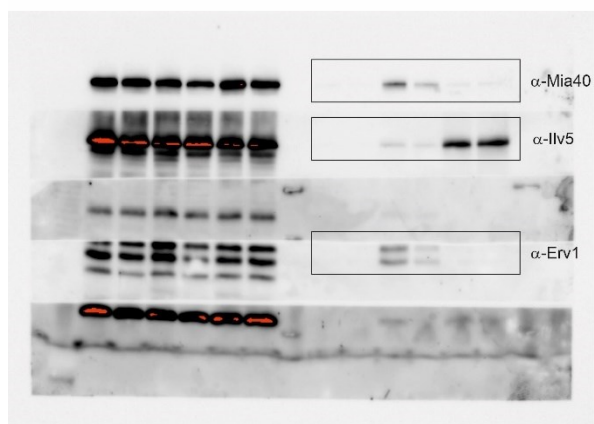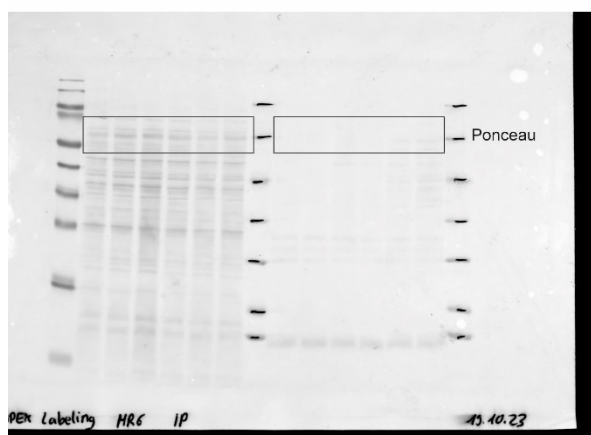

Supplement: Supplementary file 8 — Source data Fig. 1 [file 44318_2025_486_MOESM8_ESM.zip › SD figure 1/SD figure 1E.pdf]

Fig-2-C

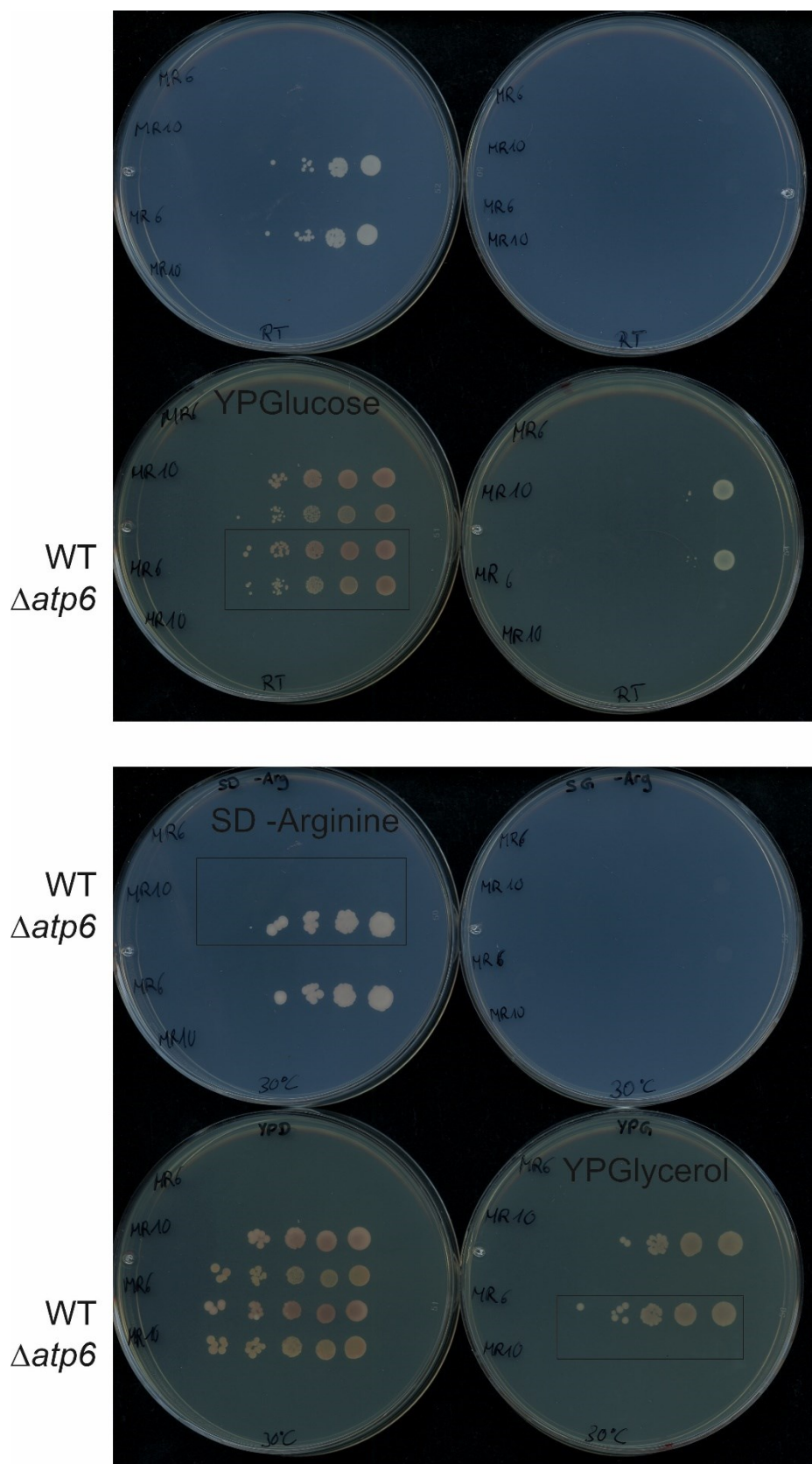

Supplement: Supplementary file 9 — Source data Fig. 2 [file 44318_2025_486_MOESM9_ESM.zip › SD figure 2/SD figure 2C.pdf]

Fig-2-D

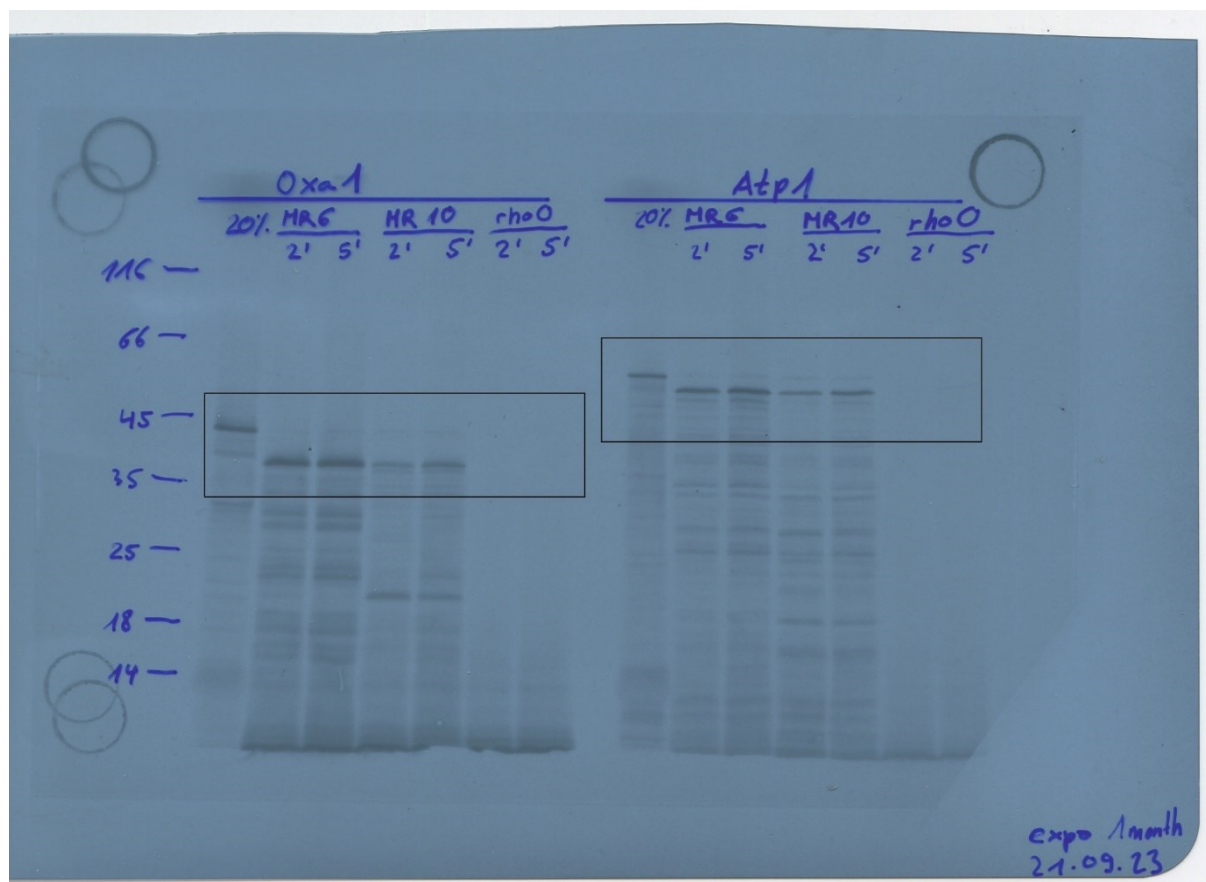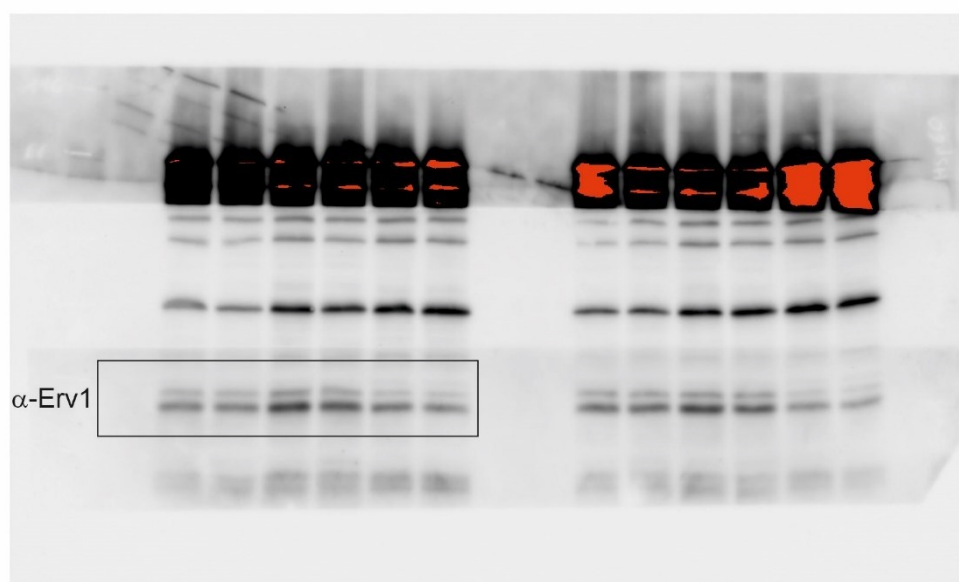

Supplement: Supplementary file 9 — Source data Fig. 2 [file 44318_2025_486_MOESM9_ESM.zip › SD figure 2/SD figure 2D.pdf]

Fig-3-A

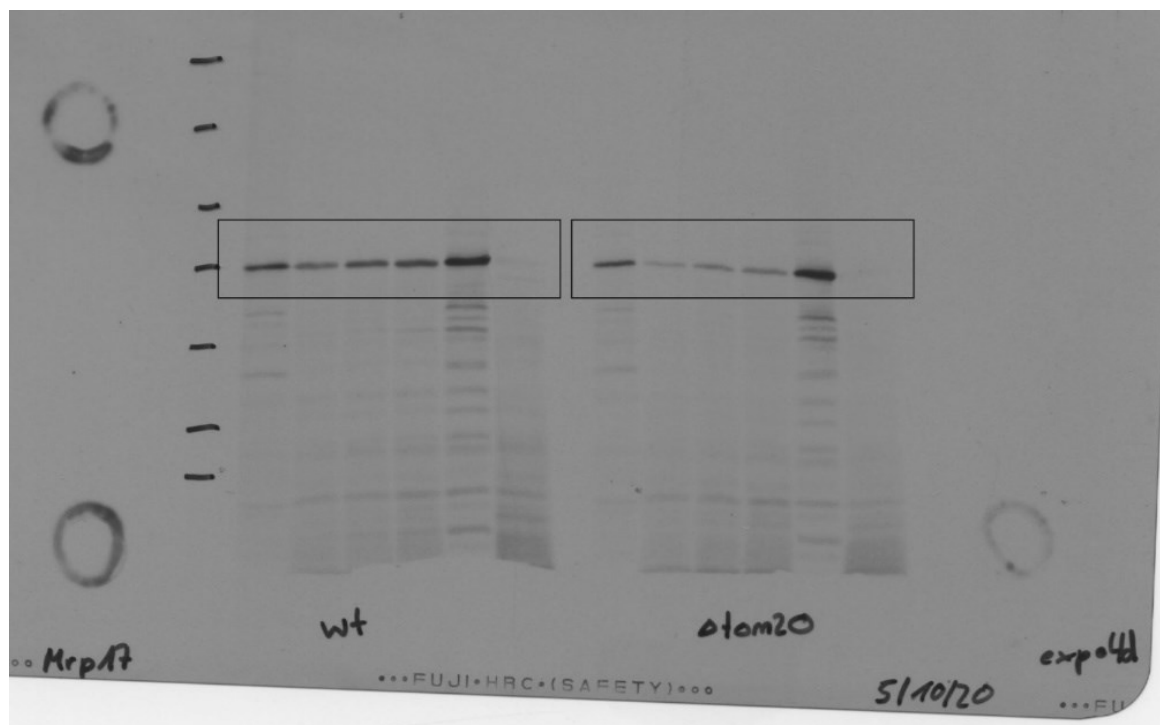

Supplement: Supplementary file 10 — Source data Fig. 3 [file 44318_2025_486_MOESM10_ESM.zip › SD figure 3/SD figure 3A.pdf]

Fig-3-B

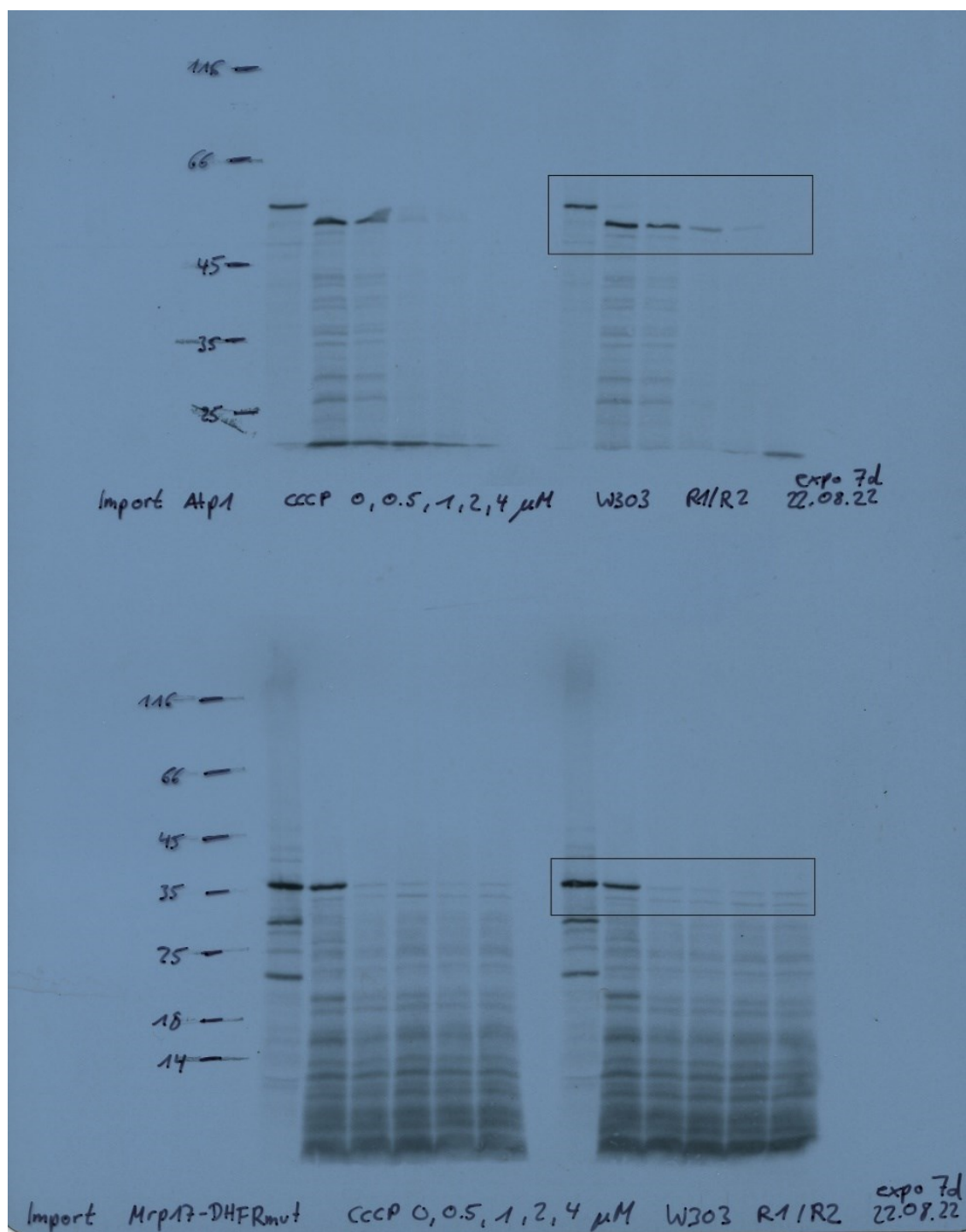

Supplement: Supplementary file 10 — Source data Fig. 3 [file 44318_2025_486_MOESM10_ESM.zip › SD figure 3/SD figure 3B.pdf]

Fig-3-C

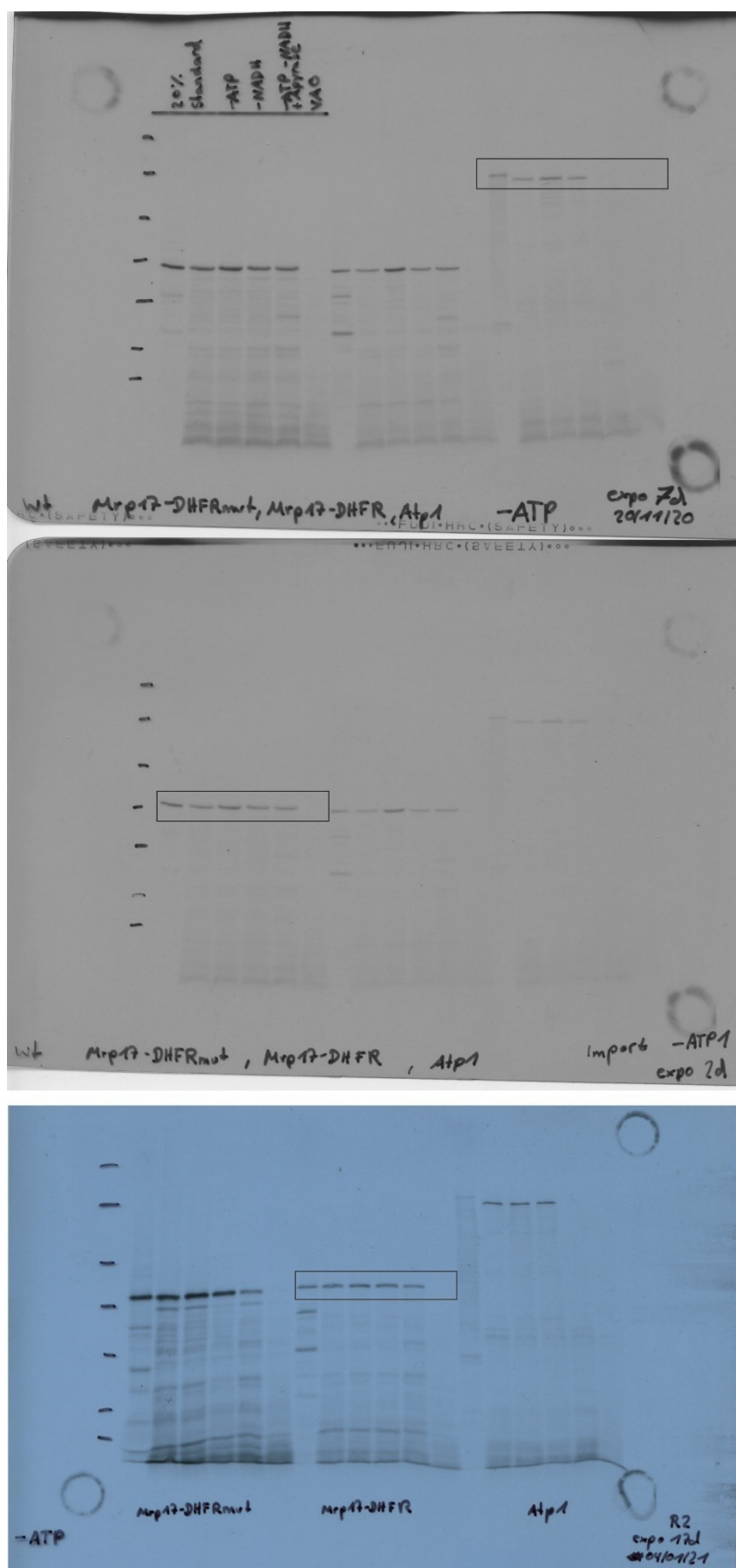

Supplement: Supplementary file 10 — Source data Fig. 3 [file 44318_2025_486_MOESM10_ESM.zip › SD figure 3/SD figure 3C.pdf]

Fig-3-D

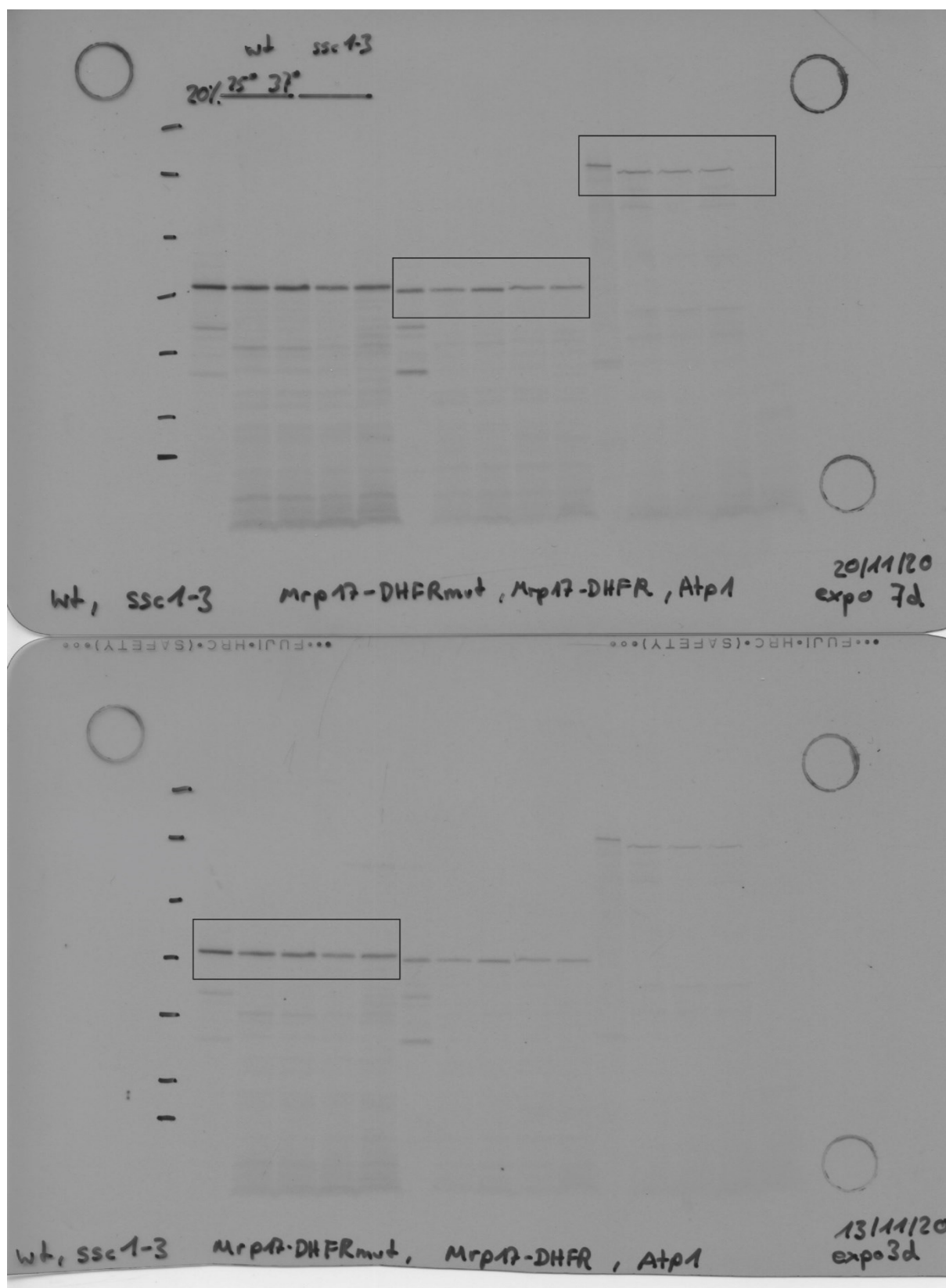

Supplement: Supplementary file 10 — Source data Fig. 3 [file 44318_2025_486_MOESM10_ESM.zip › SD figure 3/SD figure 3D.pdf]

Fig-3-F

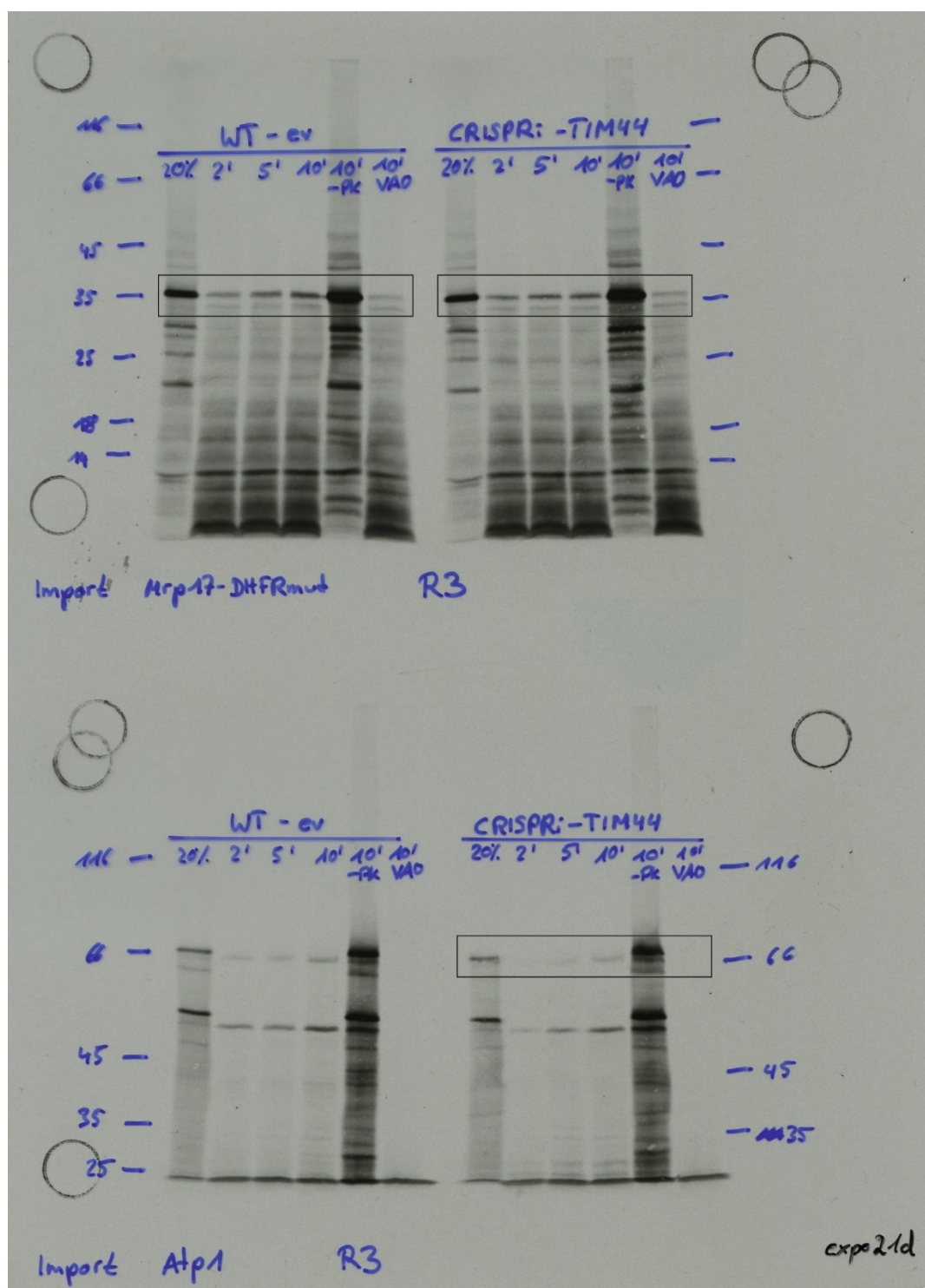

Supplement: Supplementary file 10 — Source data Fig. 3 [file 44318_2025_486_MOESM10_ESM.zip › SD figure 3/SD figure 3F.pdf]

Fig-3-H

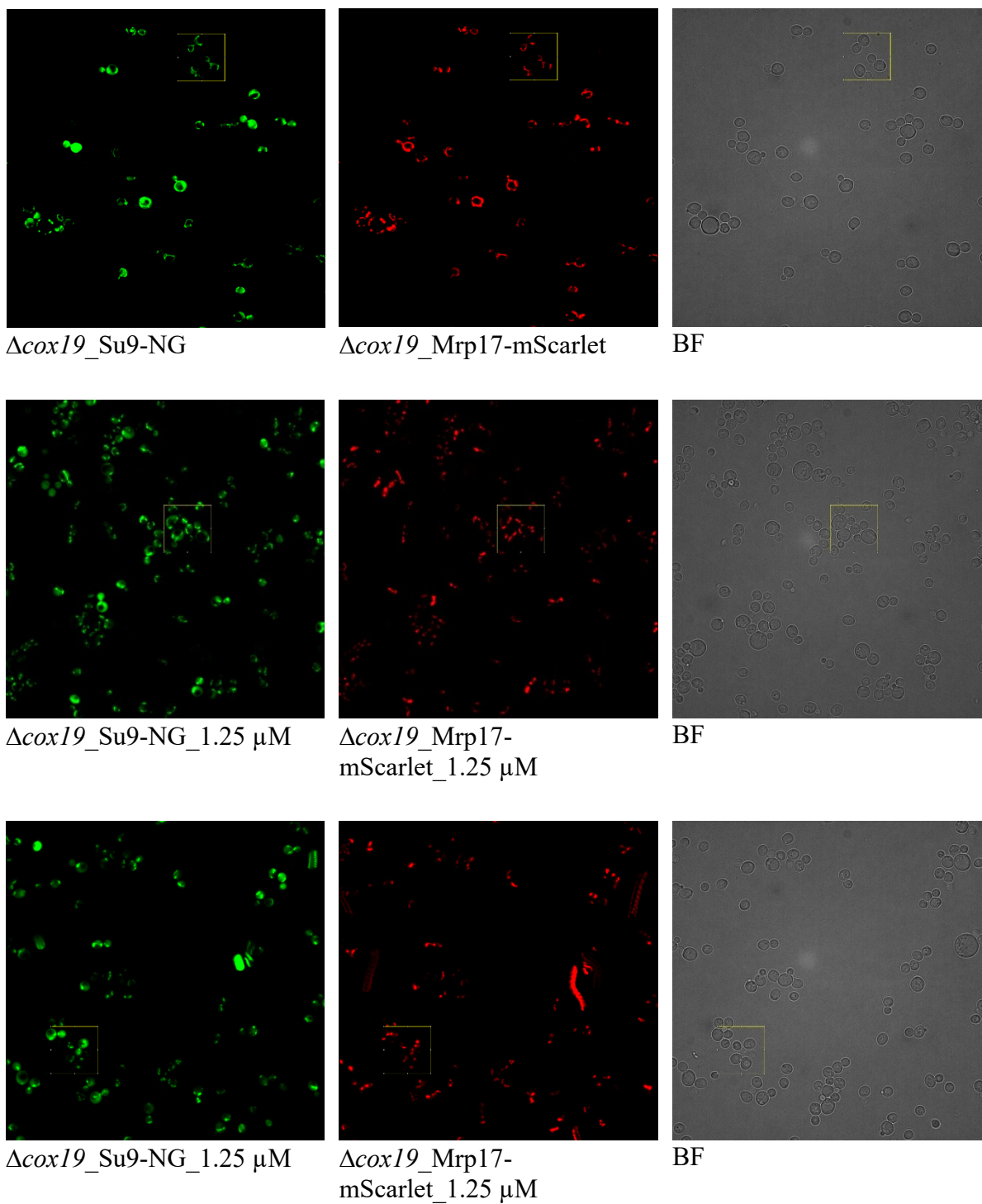

Supplement: Supplementary file 10 — Source data Fig. 3 [file 44318_2025_486_MOESM10_ESM.zip › SD figure 3/SD figure 3H.pdf]

Fig-4-A

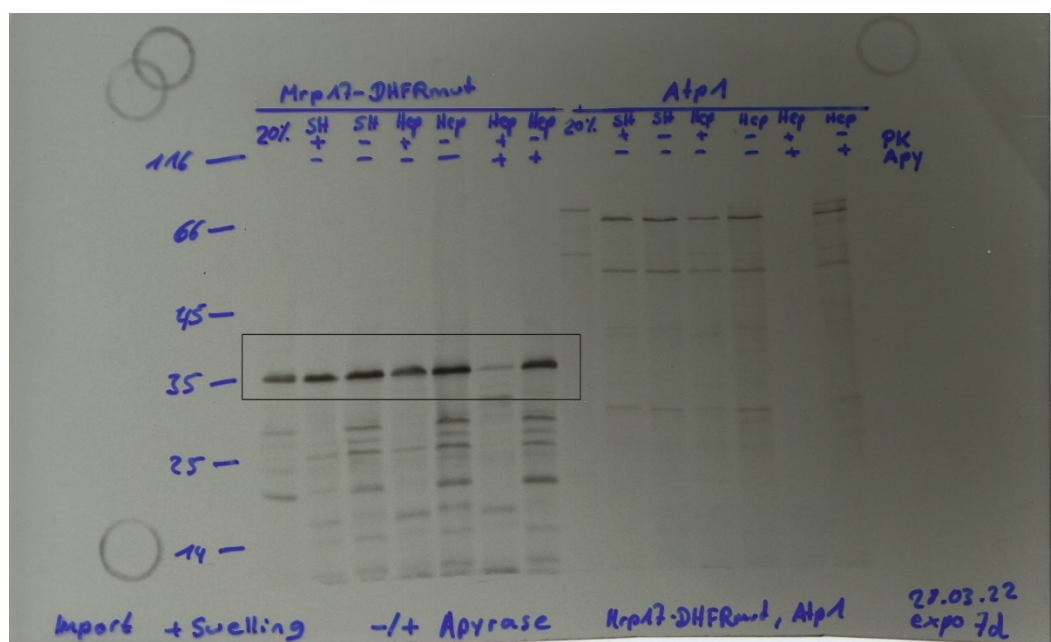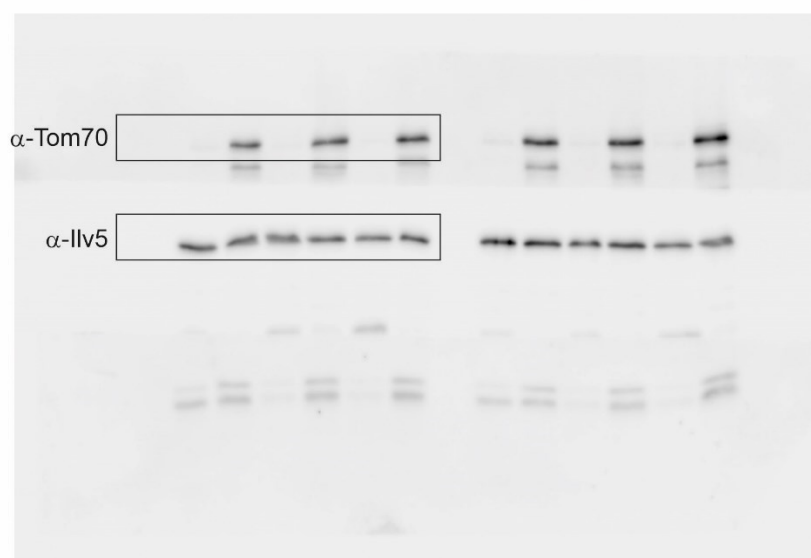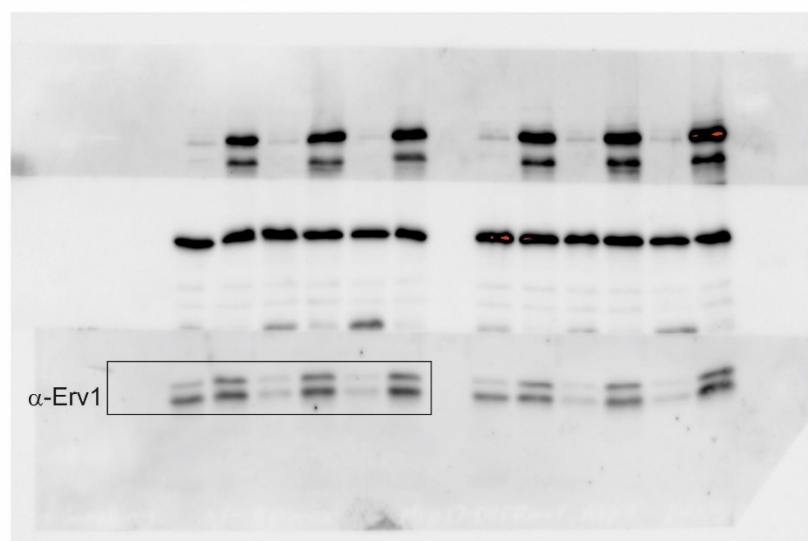

Supplement: Supplementary file 11 — Source data Fig. 4 [file 44318_2025_486_MOESM11_ESM.zip › SD figure 4/SD figure 4A.pdf]

Fig-4-C

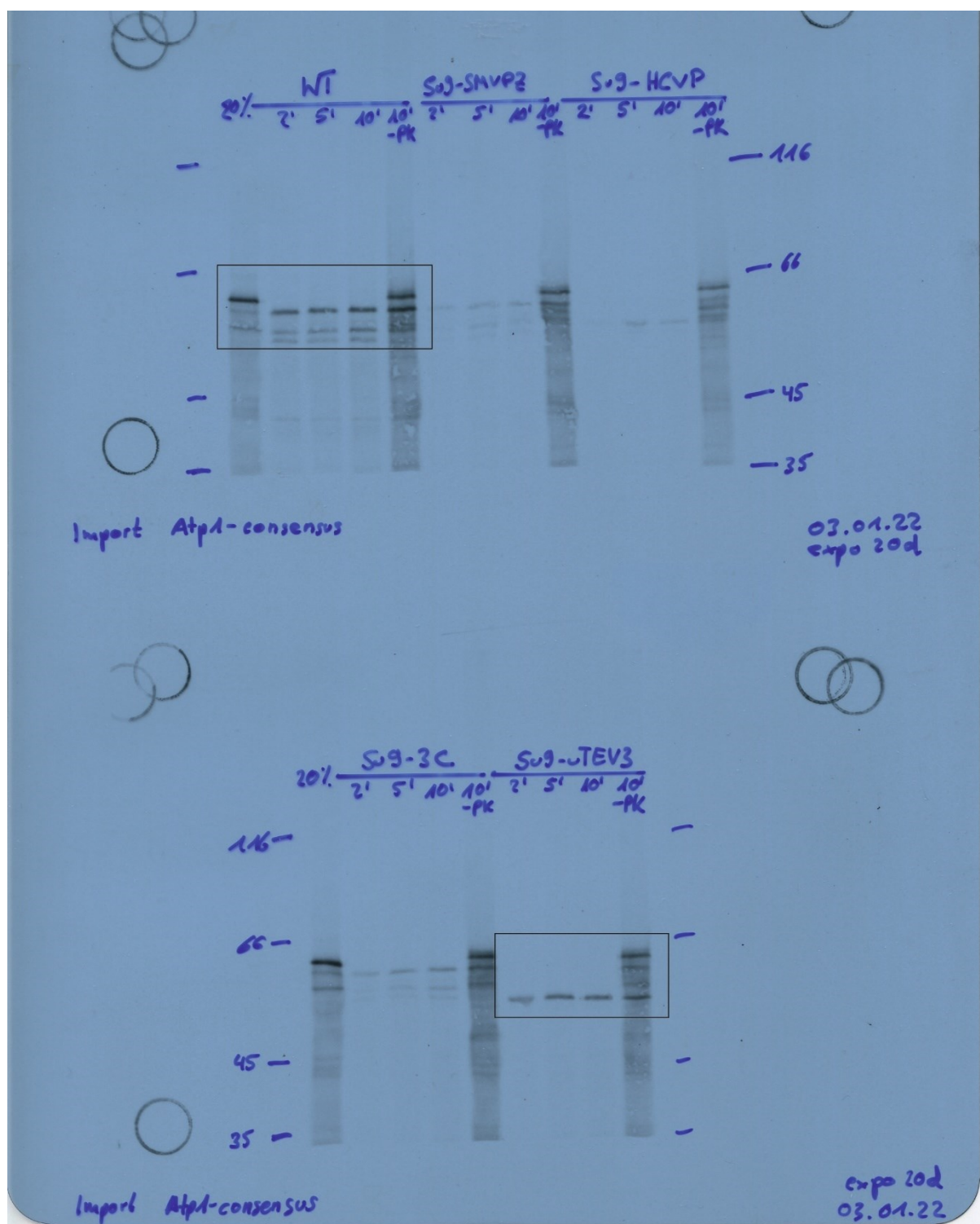

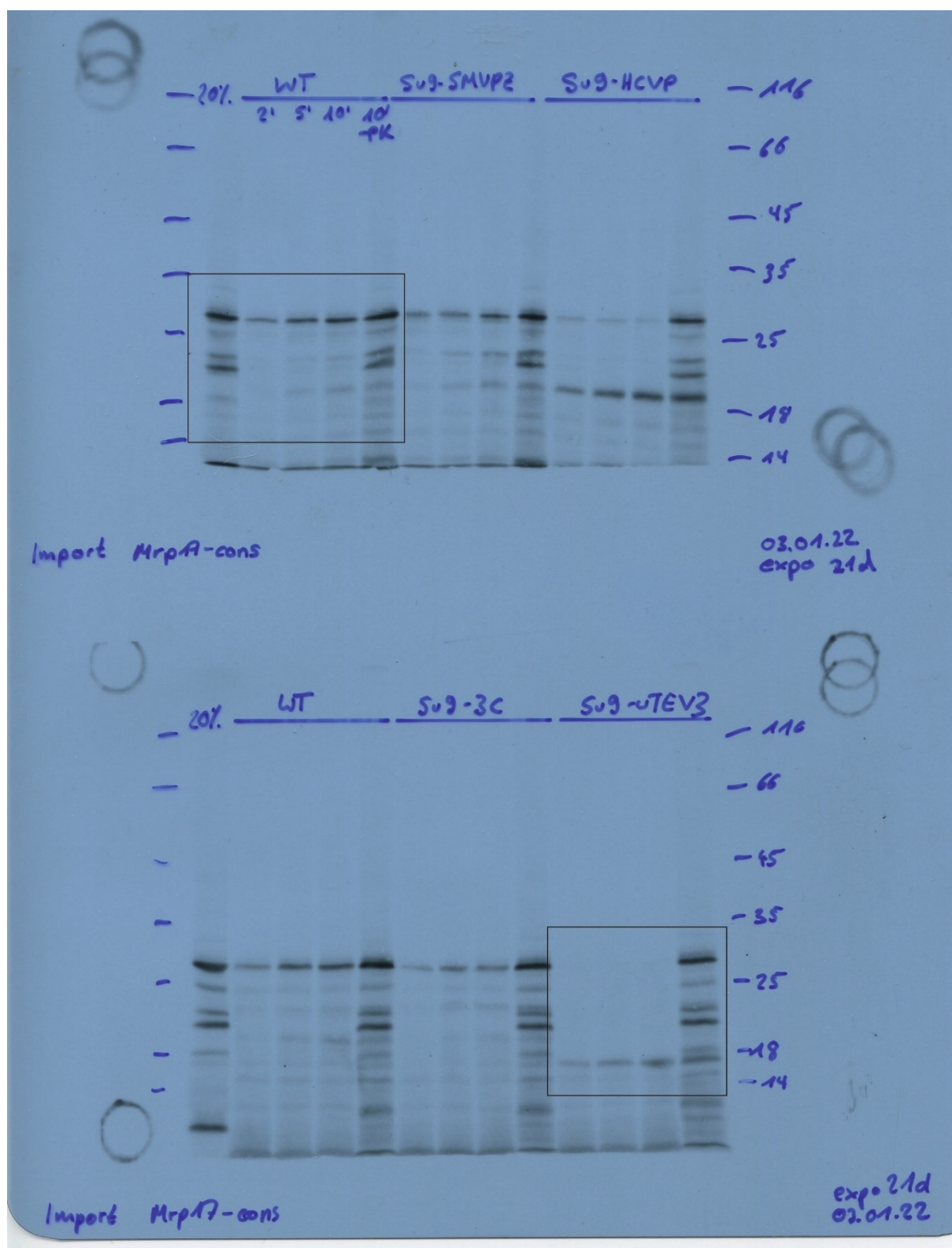

Supplement: Supplementary file 11 — Source data Fig. 4 [file 44318_2025_486_MOESM11_ESM.zip › SD figure 4/SD figure 4C.pdf]

Fig-4-D

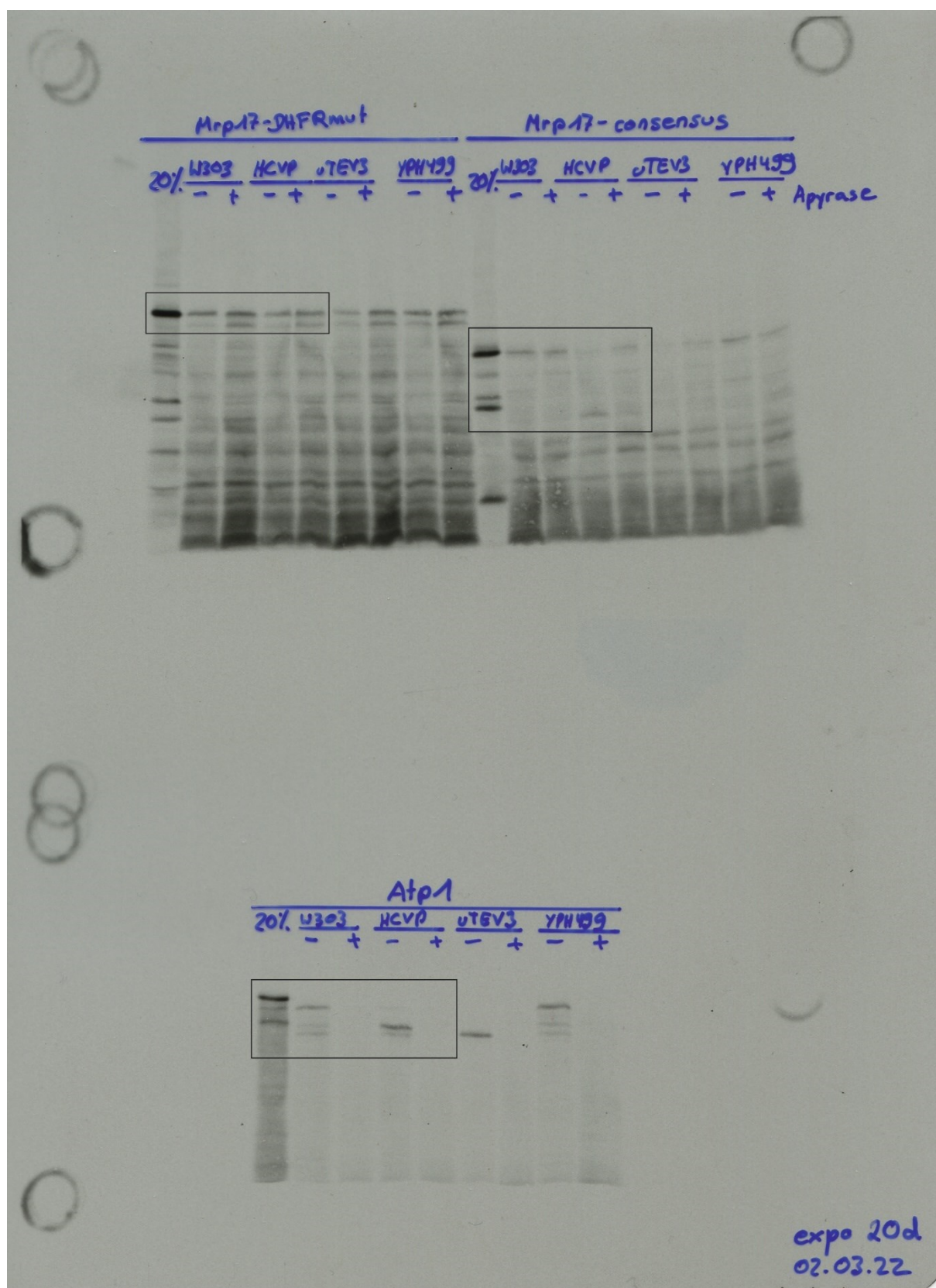

Supplement: Supplementary file 11 — Source data Fig. 4 [file 44318_2025_486_MOESM11_ESM.zip › SD figure 4/SD figure 4D.pdf]

Fig-4-E

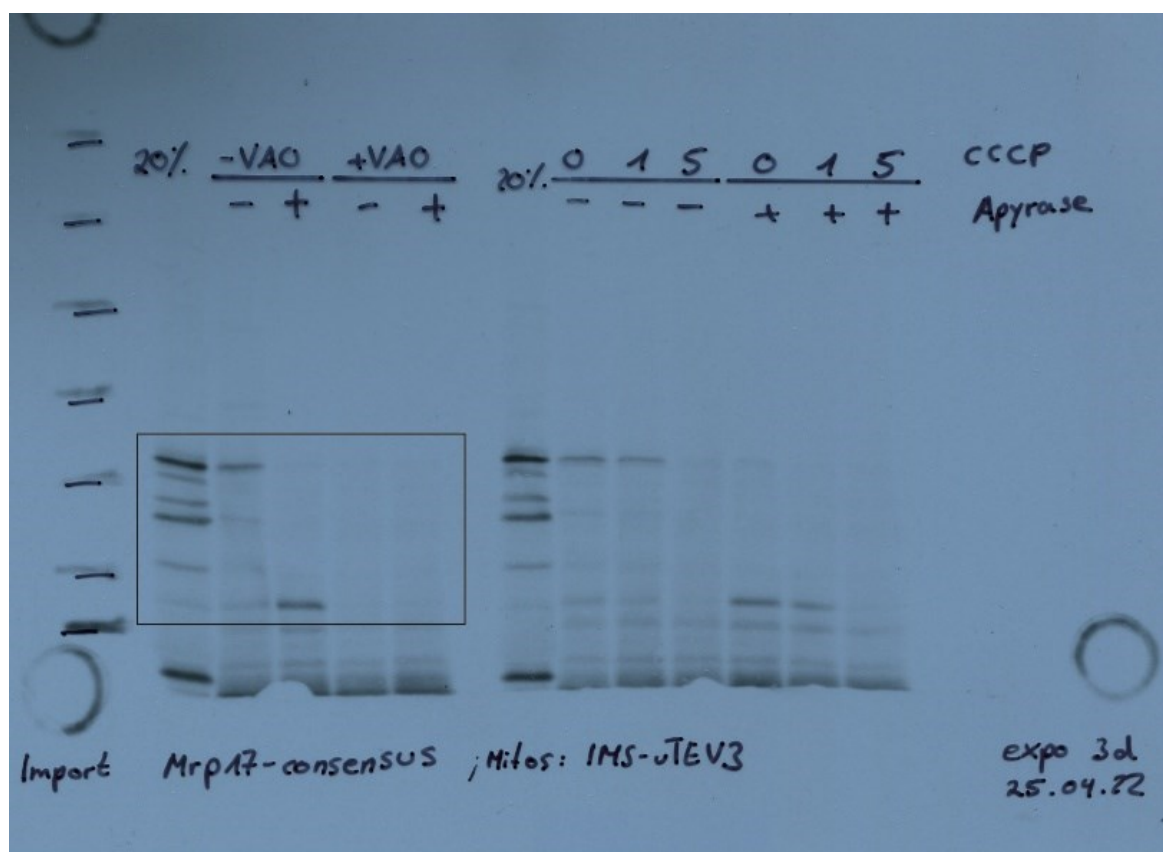

Supplement: Supplementary file 11 — Source data Fig. 4 [file 44318_2025_486_MOESM11_ESM.zip › SD figure 4/SD figure 4E.pdf]

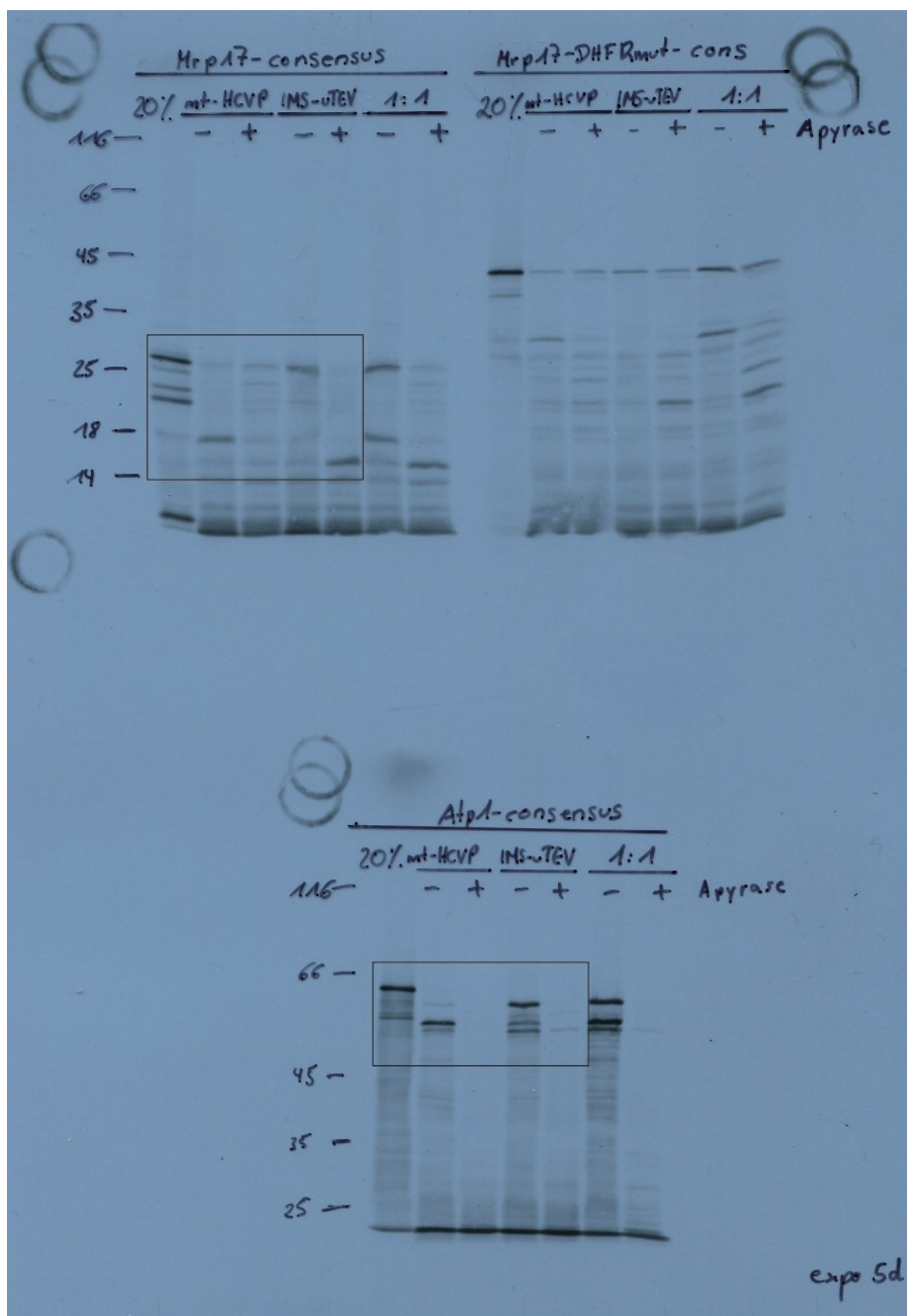

Supplement: Supplementary file 11 — Source data Fig. 4 [file 44318_2025_486_MOESM11_ESM.zip › SD figure 4/SD figure 4F.pdf]

Fig-5-B

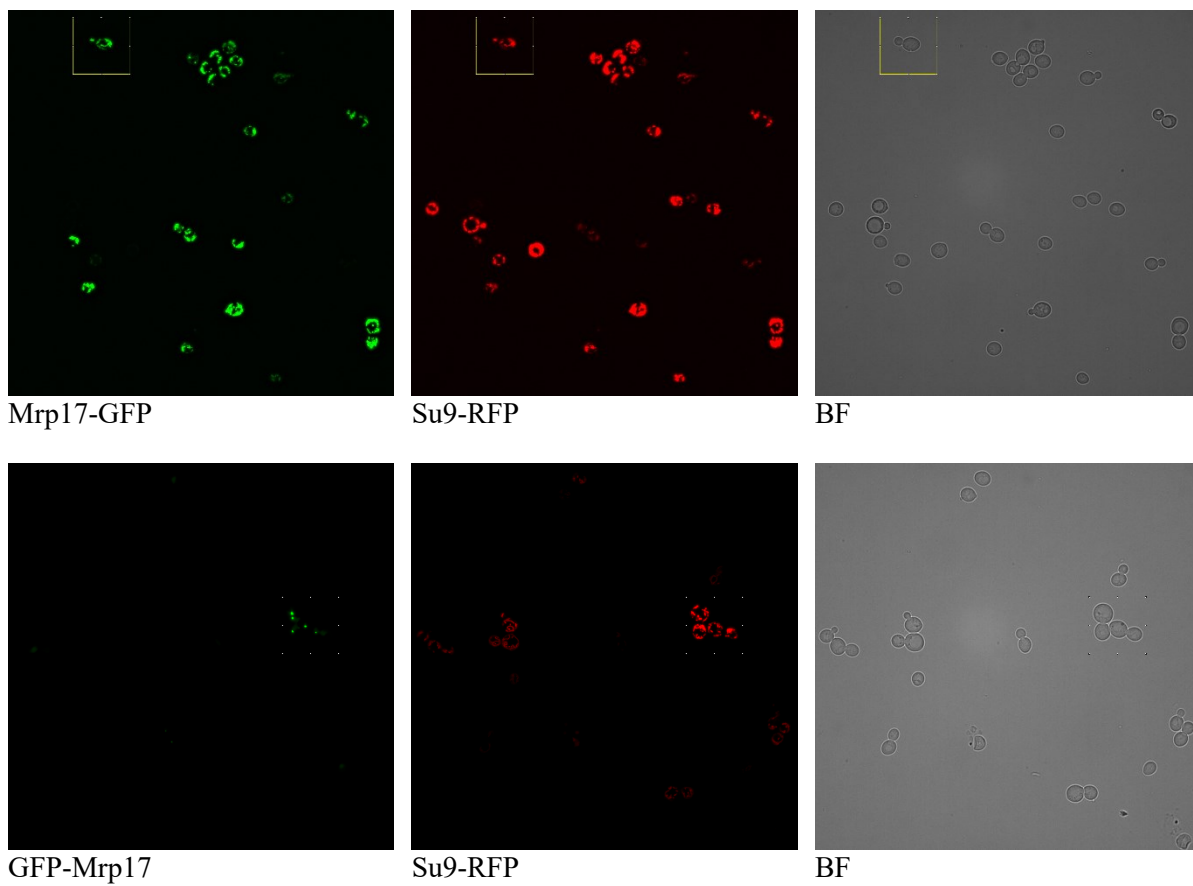

Supplement: Supplementary file 12 — Source data Fig. 5 [file 44318_2025_486_MOESM12_ESM.zip › SD figure 5/SD figure 5B.pdf]

Fig-5-C

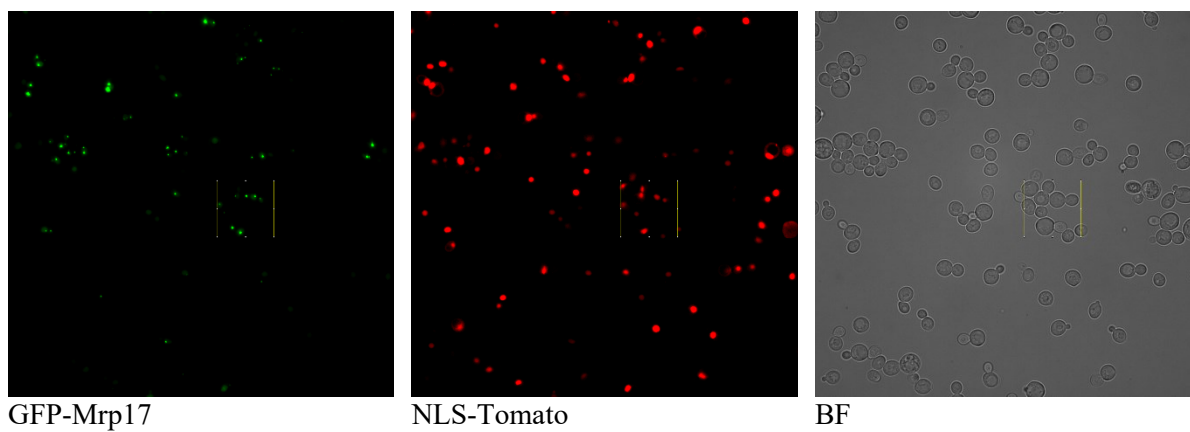

Supplement: Supplementary file 12 — Source data Fig. 5 [file 44318_2025_486_MOESM12_ESM.zip › SD figure 5/SD figure 5C.pdf]

Fig-5-E

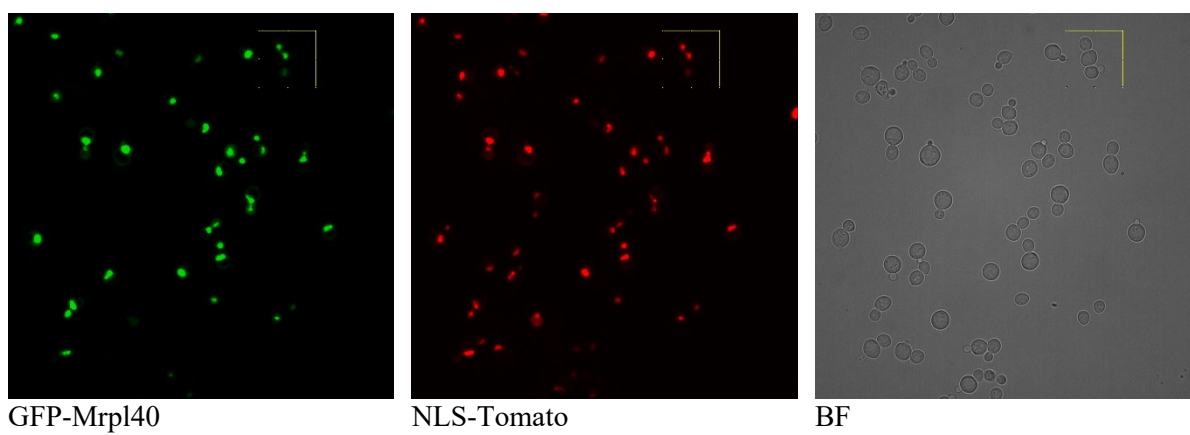

Supplement: Supplementary file 12 — Source data Fig. 5 [file 44318_2025_486_MOESM12_ESM.zip › SD figure 5/SD figure 5E.pdf]

Fig-5-F

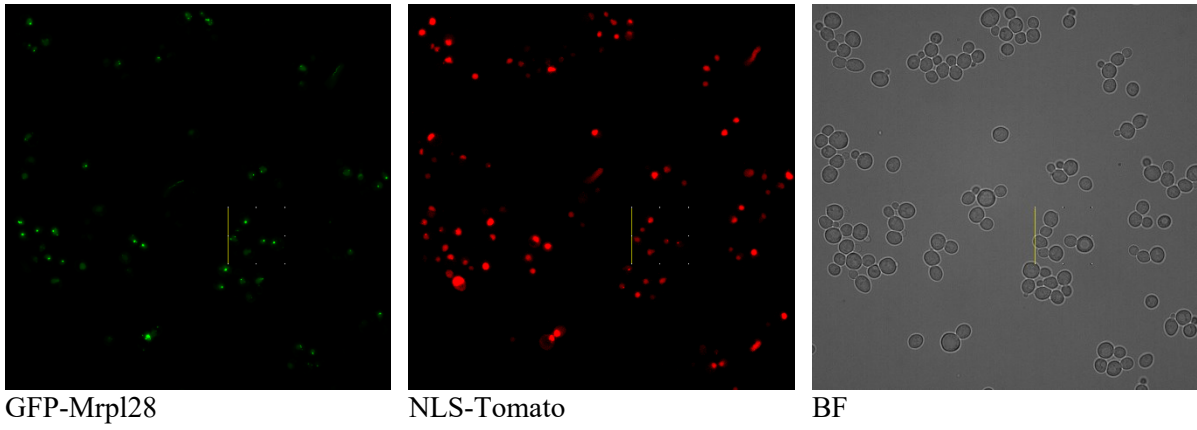

Supplement: Supplementary file 12 — Source data Fig. 5 [file 44318_2025_486_MOESM12_ESM.zip › SD figure 5/SD figure 5F.pdf]

Fig-5-G

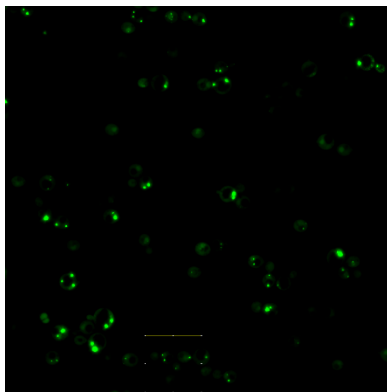

GFP-Mrp17K-A

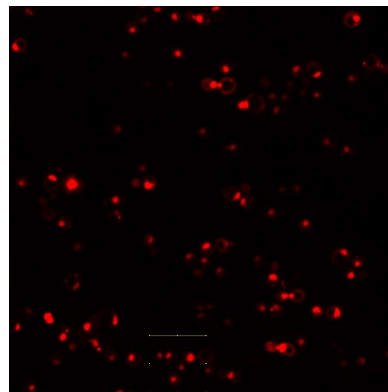

NLS-Tomato

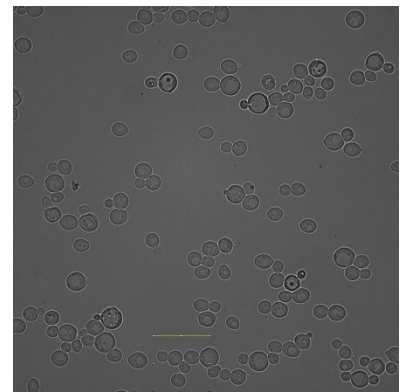

BF

Supplement: Supplementary file 12 — Source data Fig. 5 [file 44318_2025_486_MOESM12_ESM.zip › SD figure 5/SD figure 5G.pdf]

Fig-5-H

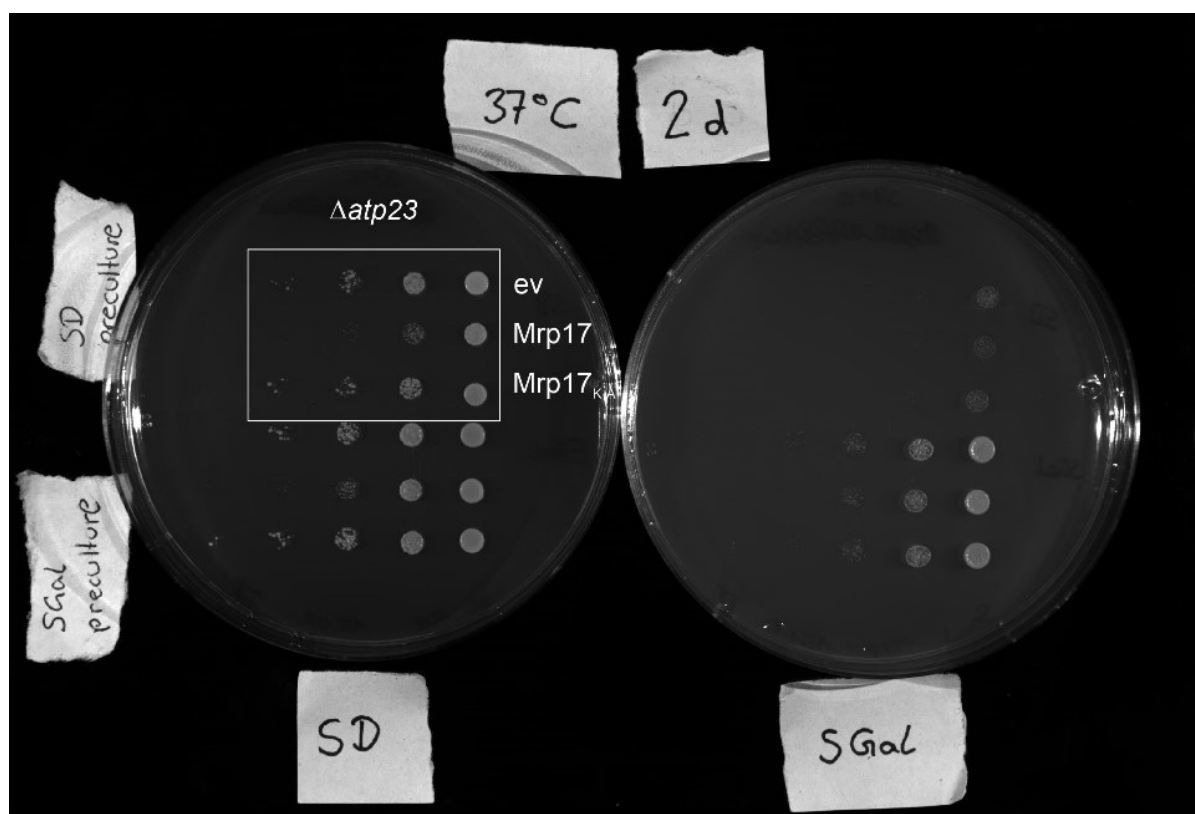

Supplement: Supplementary file 12 — Source data Fig. 5 [file 44318_2025_486_MOESM12_ESM.zip › SD figure 5/SD figure 5H.pdf]

Fig-EV2-B

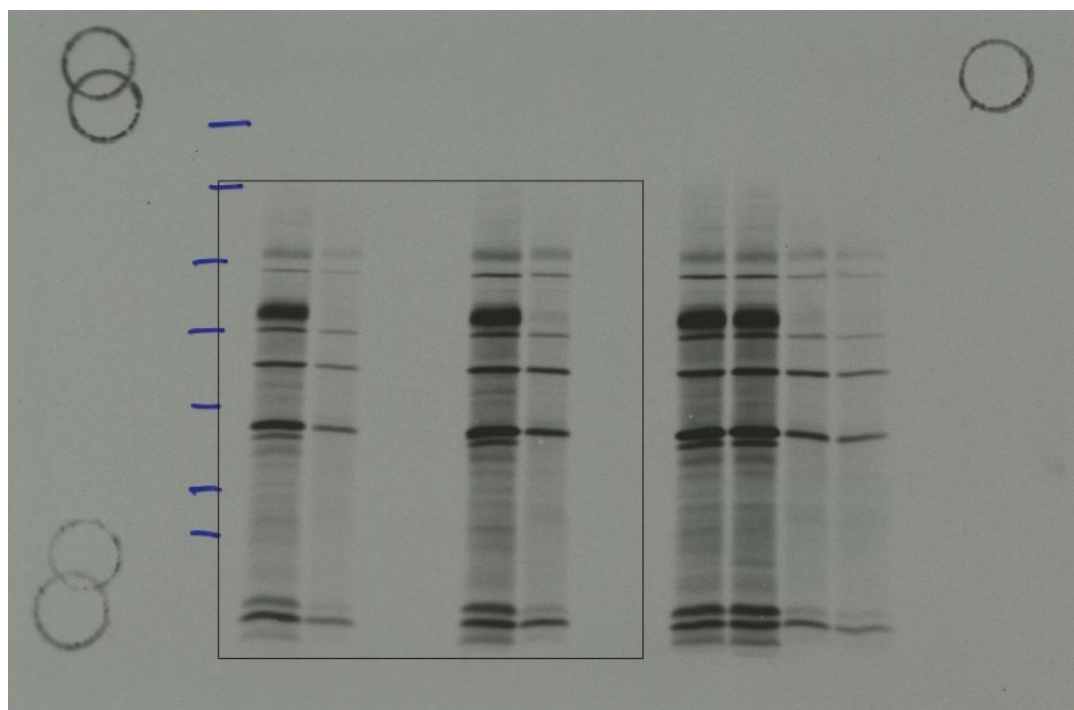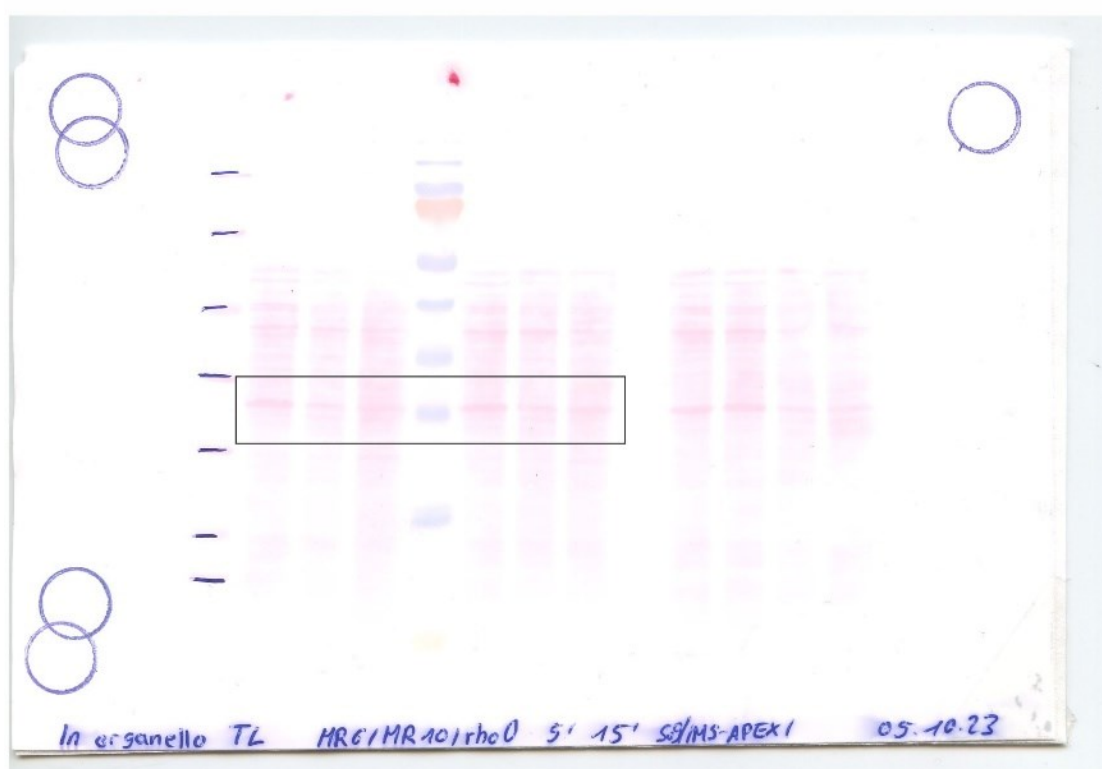

Supplement: Supplementary file 14 — Figure EV and Appendix Source Data [file 44318_2025_486_MOESM14_ESM.zip › SD figure EV2/SD figure EV2B.pdf]

Fig-EV3-A

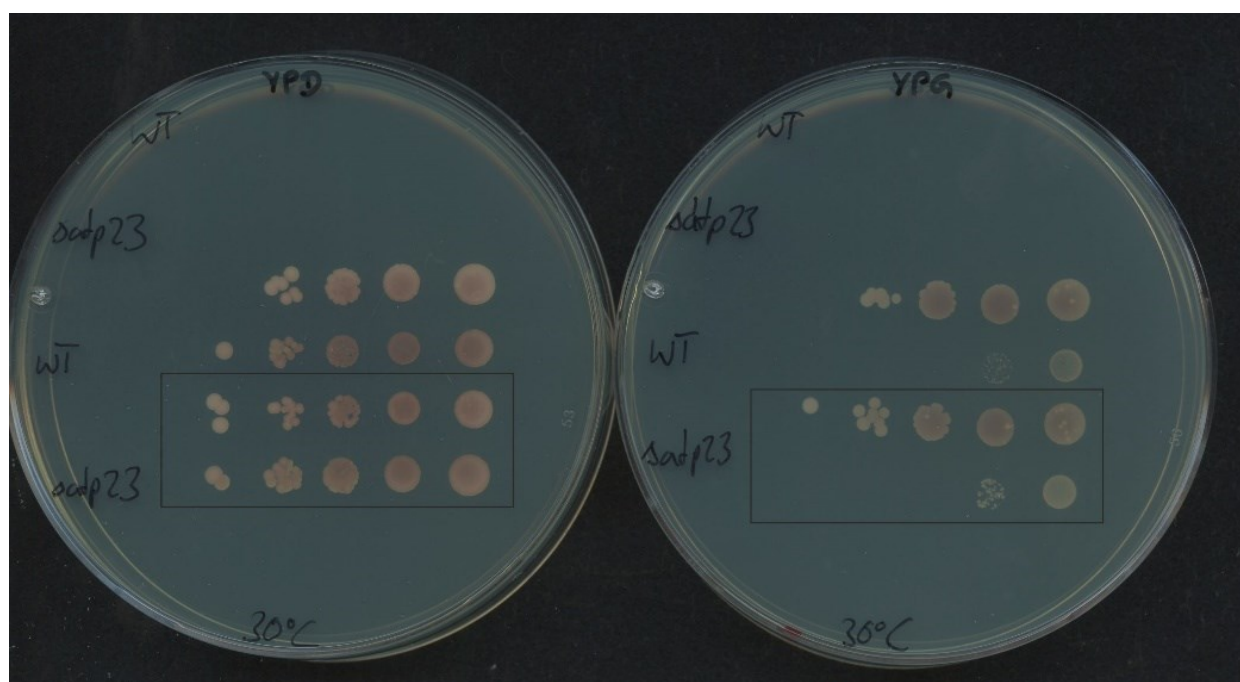

Supplement: Supplementary file 14 — Figure EV and Appendix Source Data [file 44318_2025_486_MOESM14_ESM.zip › SD figure EV3/SD figure EV3A.pdf]

Fig-EV3-C

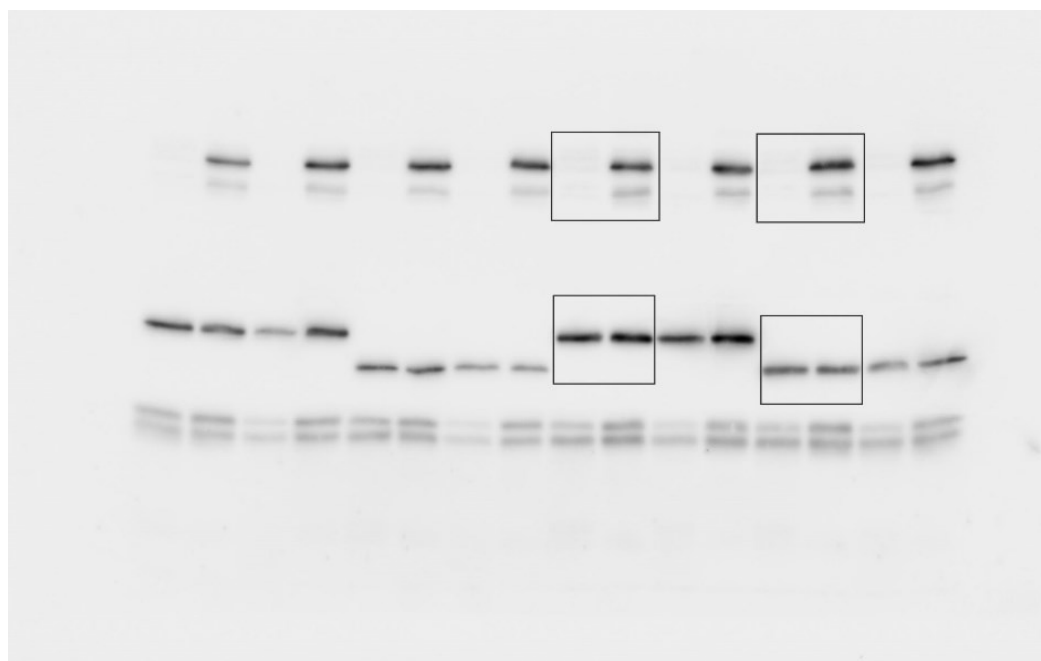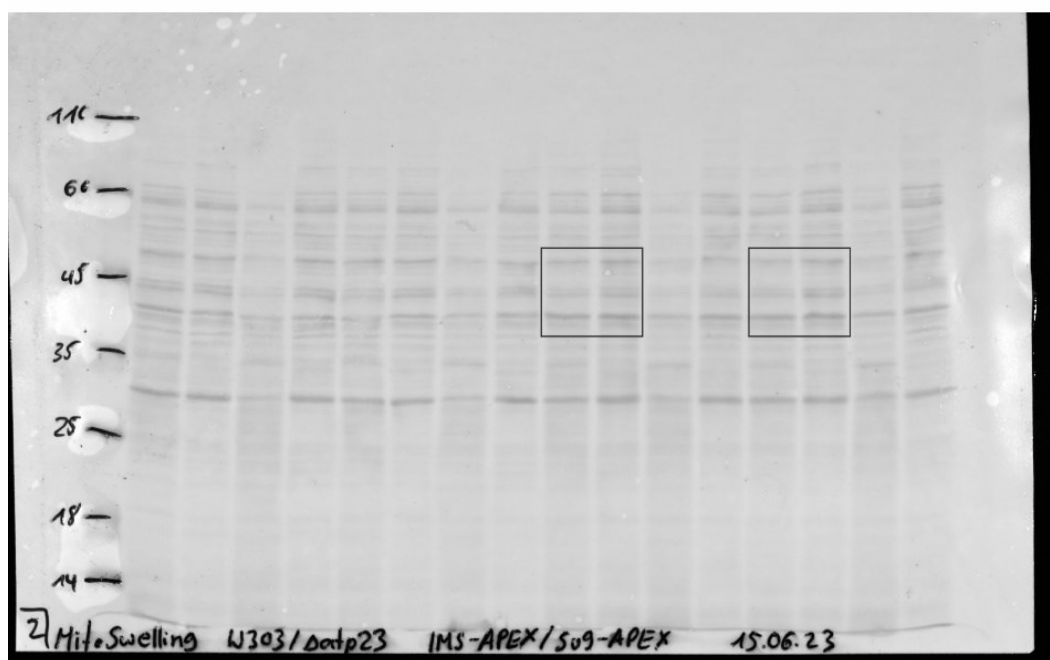

Supplement: Supplementary file 14 — Figure EV and Appendix Source Data [file 44318_2025_486_MOESM14_ESM.zip › SD figure EV3/SD figure EV3C.pdf]

Fig-EV4-A

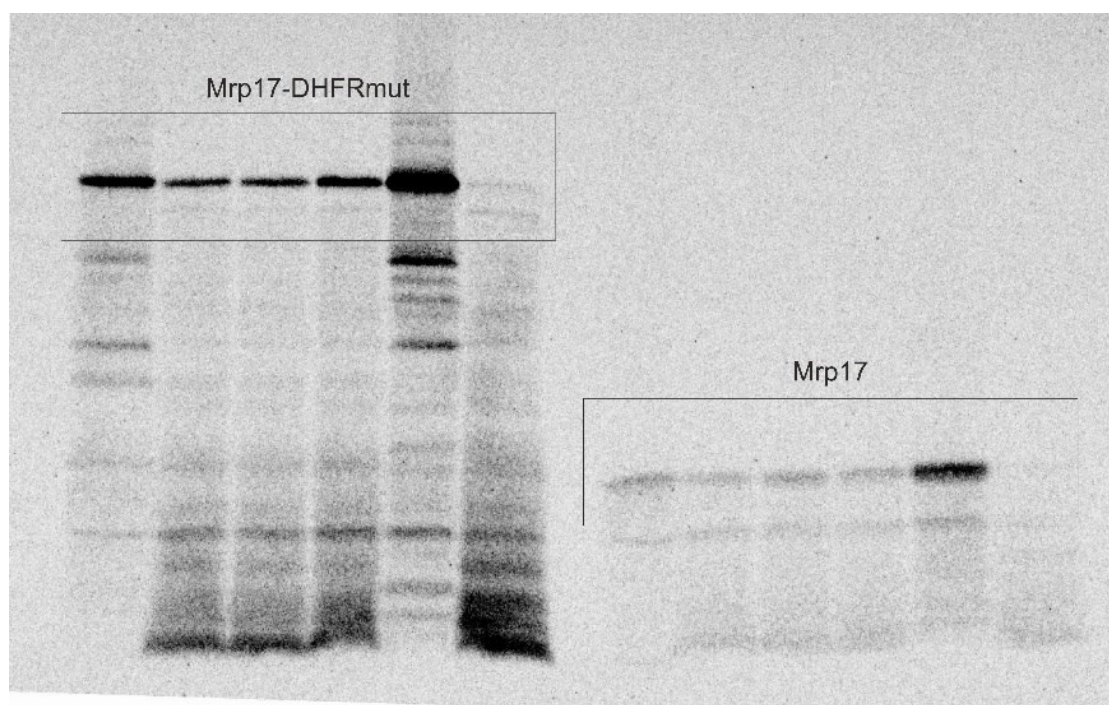

Supplement: Supplementary file 14 — Figure EV and Appendix Source Data [file 44318_2025_486_MOESM14_ESM.zip › SD figure EV4/SD figure EV4A.pdf]

Fig-EV4-B

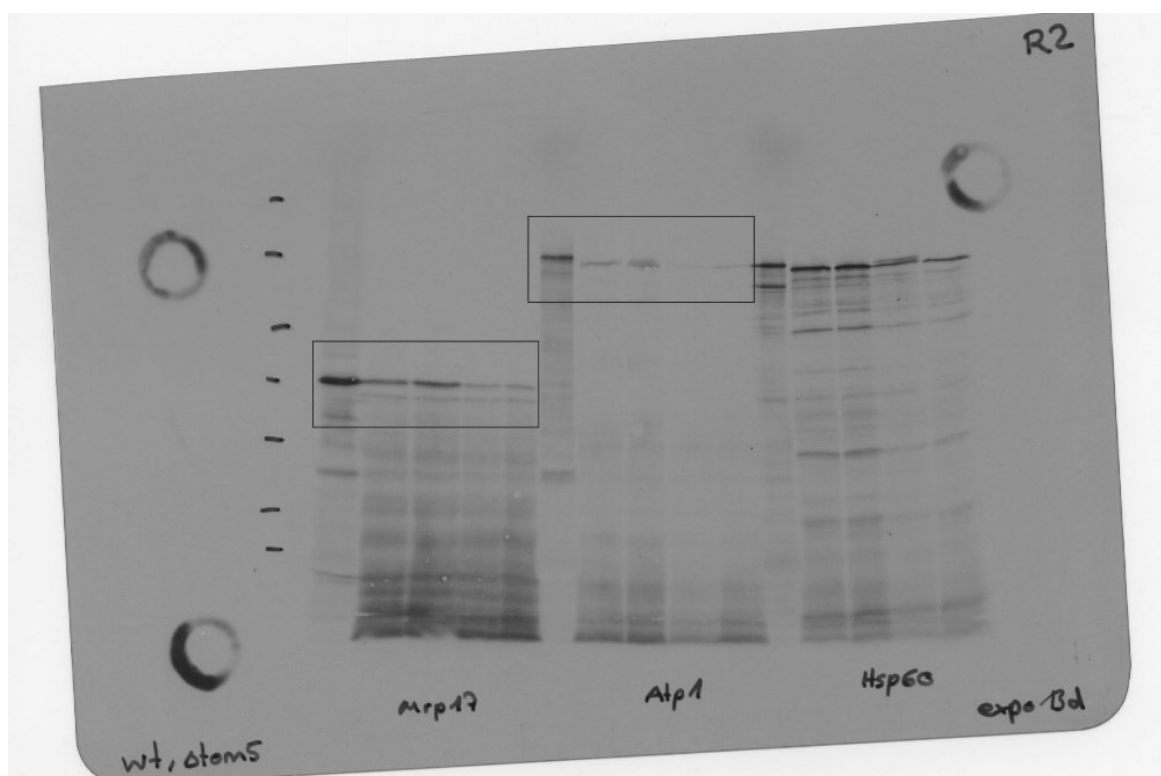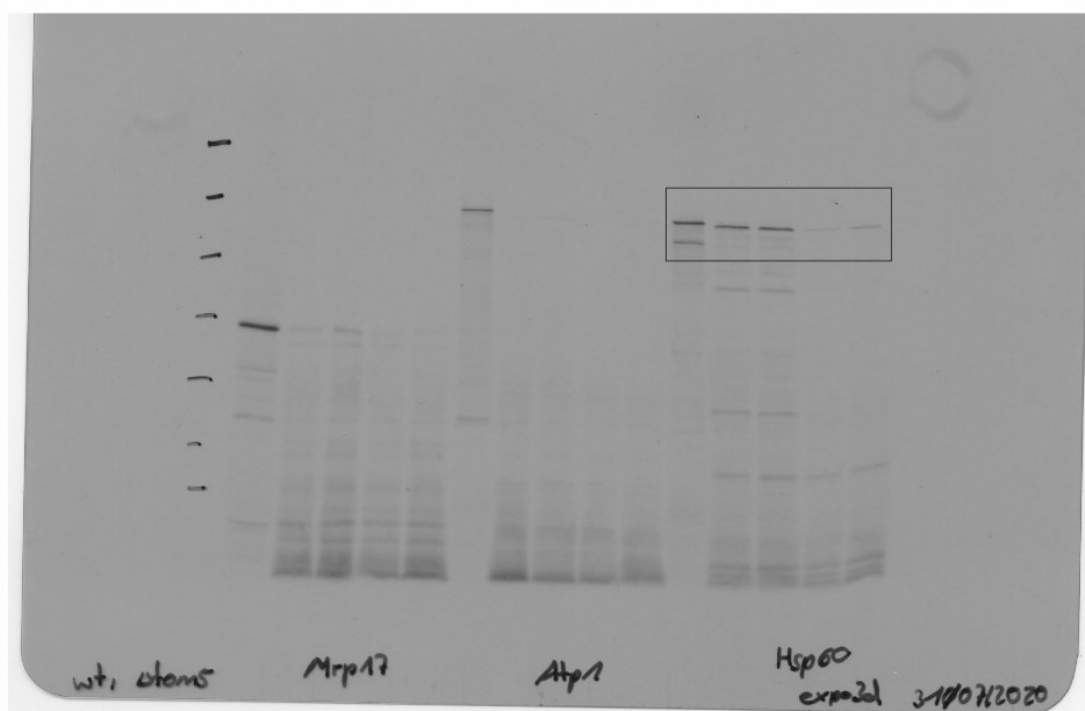

Supplement: Supplementary file 14 — Figure EV and Appendix Source Data [file 44318_2025_486_MOESM14_ESM.zip › SD figure EV4/SD figure EV4B.pdf]

Fig-EV4-C

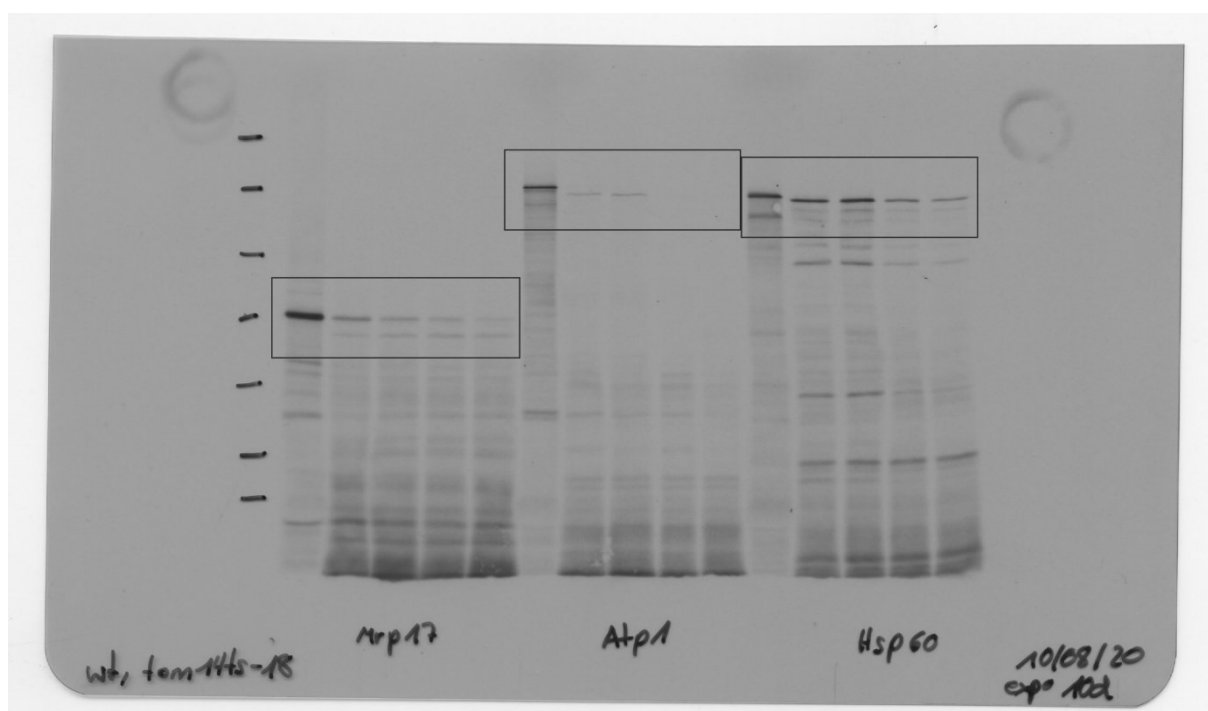

Supplement: Supplementary file 14 — Figure EV and Appendix Source Data [file 44318_2025_486_MOESM14_ESM.zip › SD figure EV4/SD figure EV4C.pdf]

Fig-EV4-D

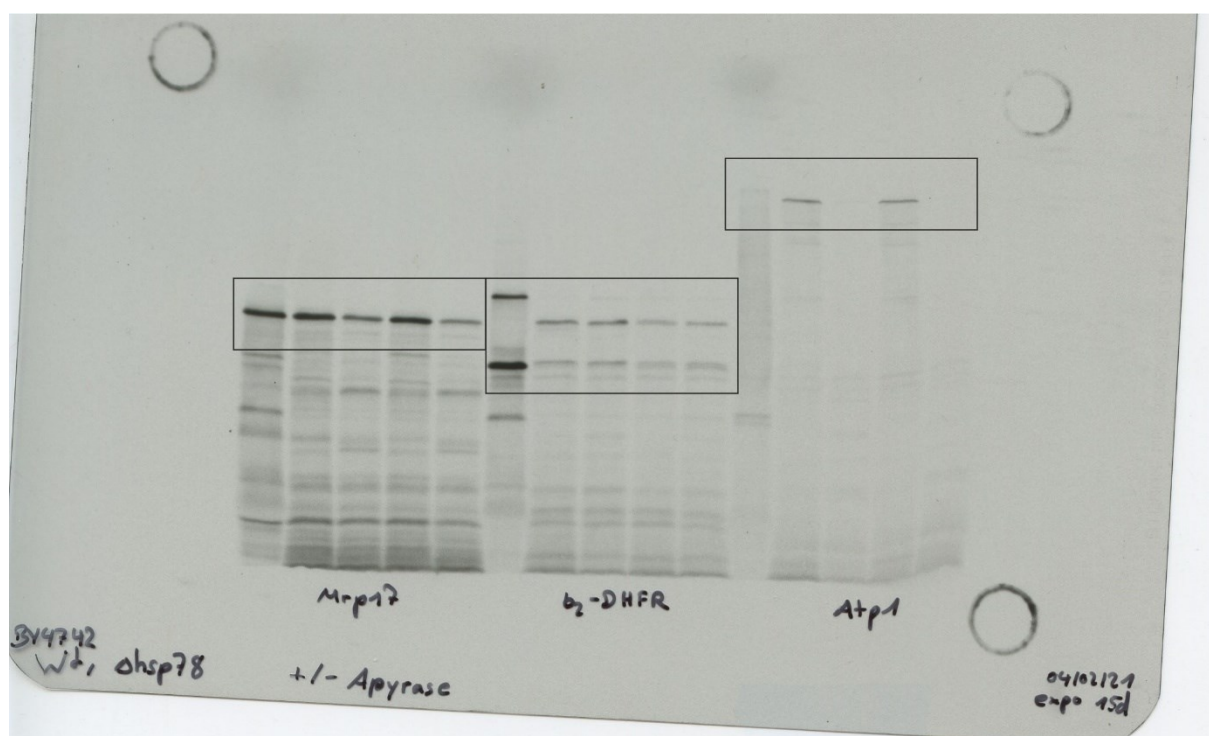

Supplement: Supplementary file 14 — Figure EV and Appendix Source Data [file 44318_2025_486_MOESM14_ESM.zip › SD figure EV4/SD figure EV4D.pdf]

Fig-EV4-E

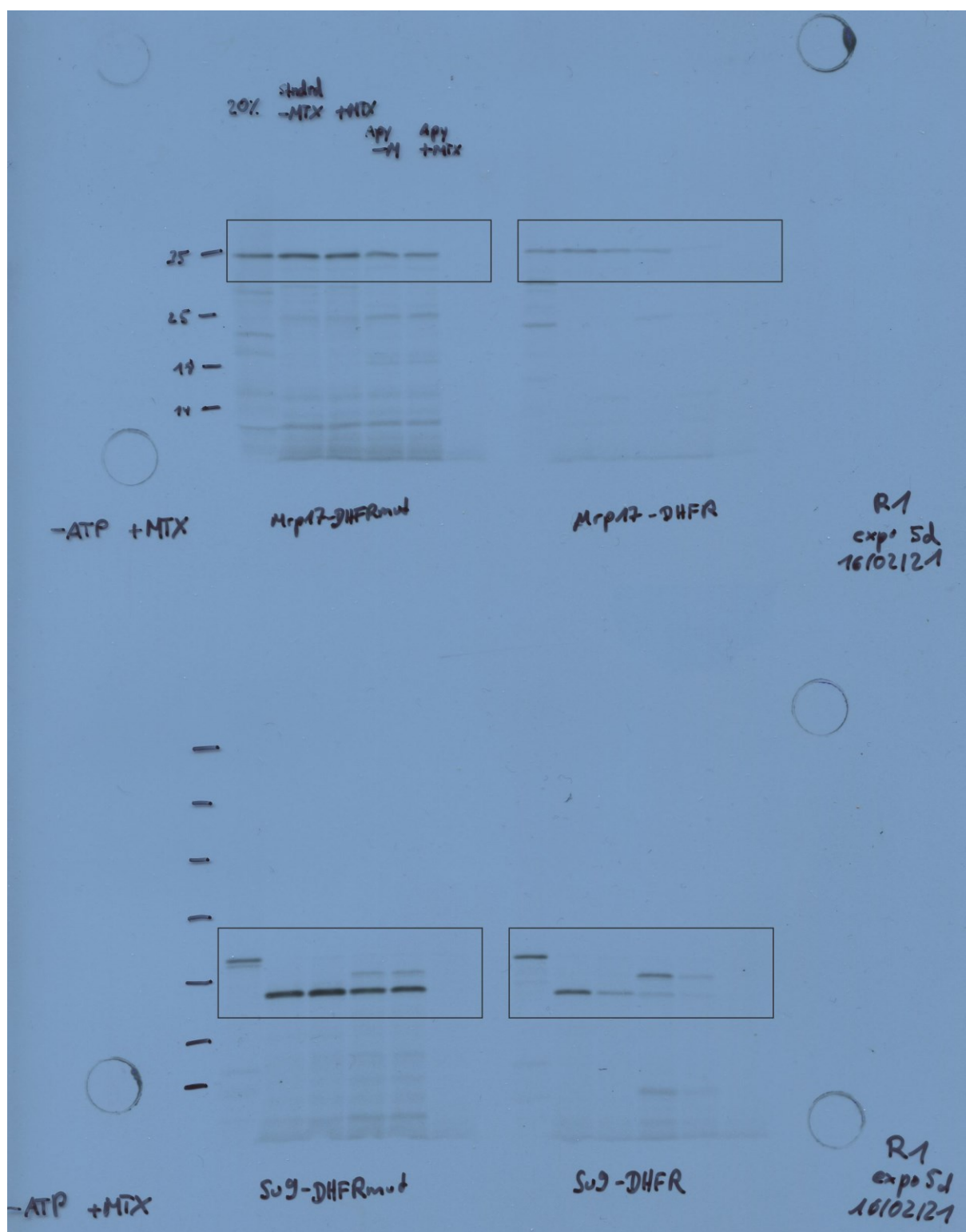

Supplement: Supplementary file 14 — Figure EV and Appendix Source Data [file 44318_2025_486_MOESM14_ESM.zip › SD figure EV4/SD figure EV4E.pdf]

Fig-EV4-G

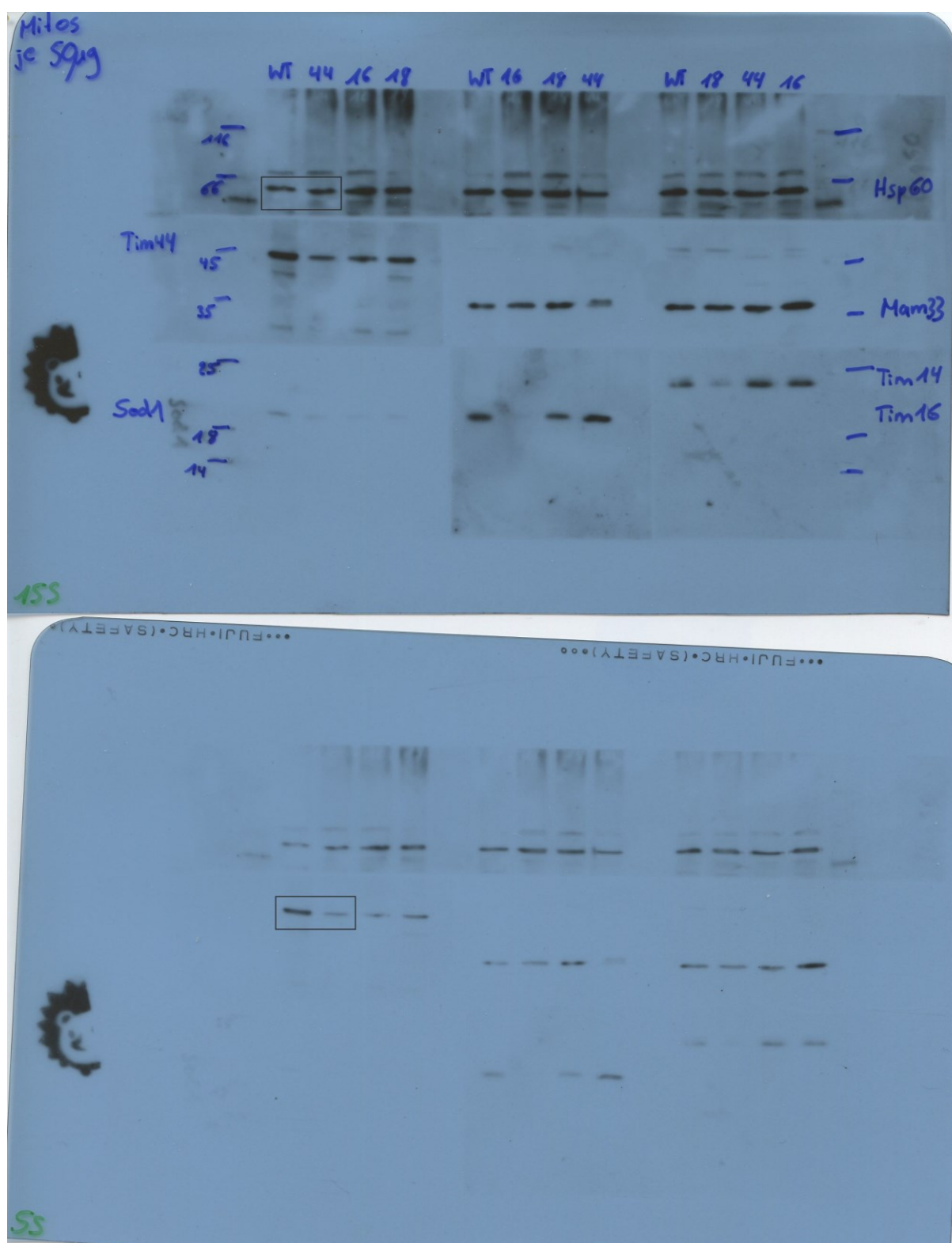

Supplement: Supplementary file 14 — Figure EV and Appendix Source Data [file 44318_2025_486_MOESM14_ESM.zip › SD figure EV4/SD figure EV4G.pdf]

Fig-EV4-H

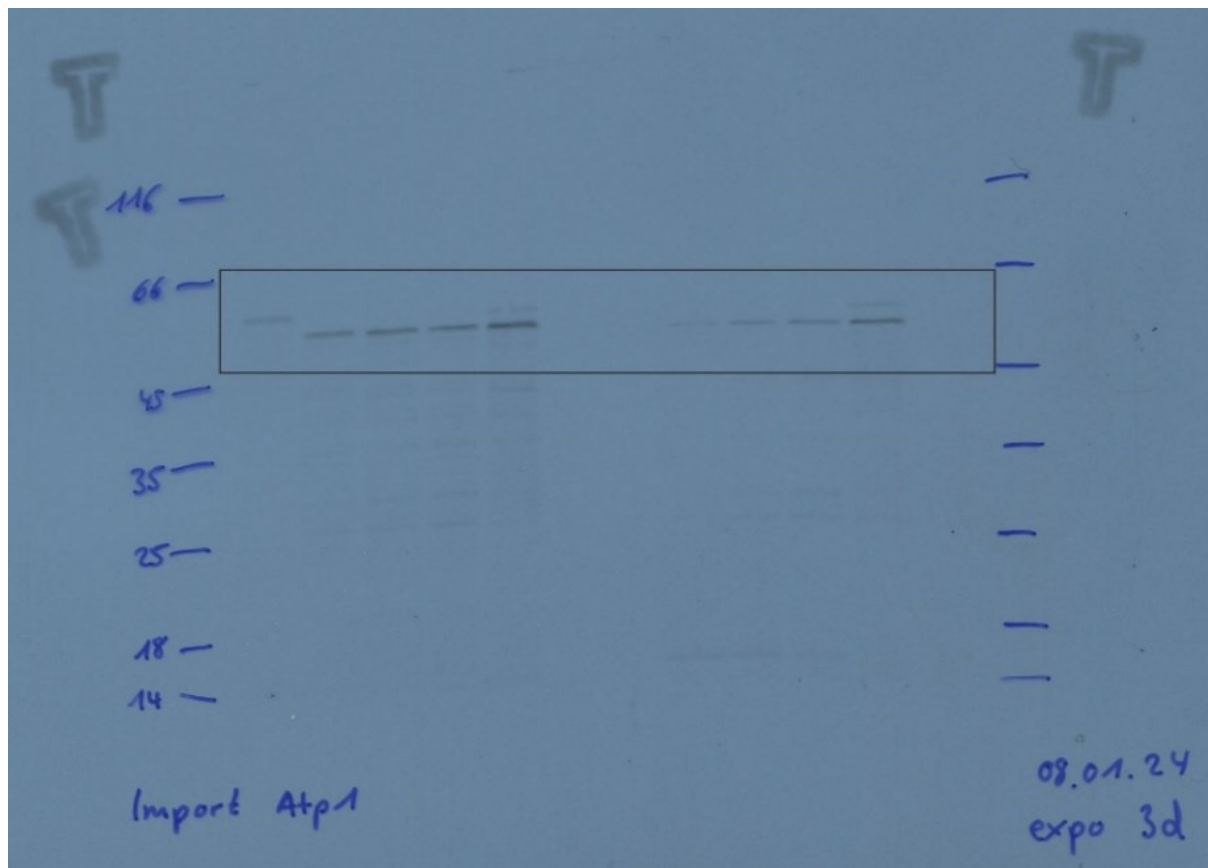

Supplement: Supplementary file 14 — Figure EV and Appendix Source Data [file 44318_2025_486_MOESM14_ESM.zip › SD figure EV4/SD figure EV4H.pdf]

Fig-S1-B

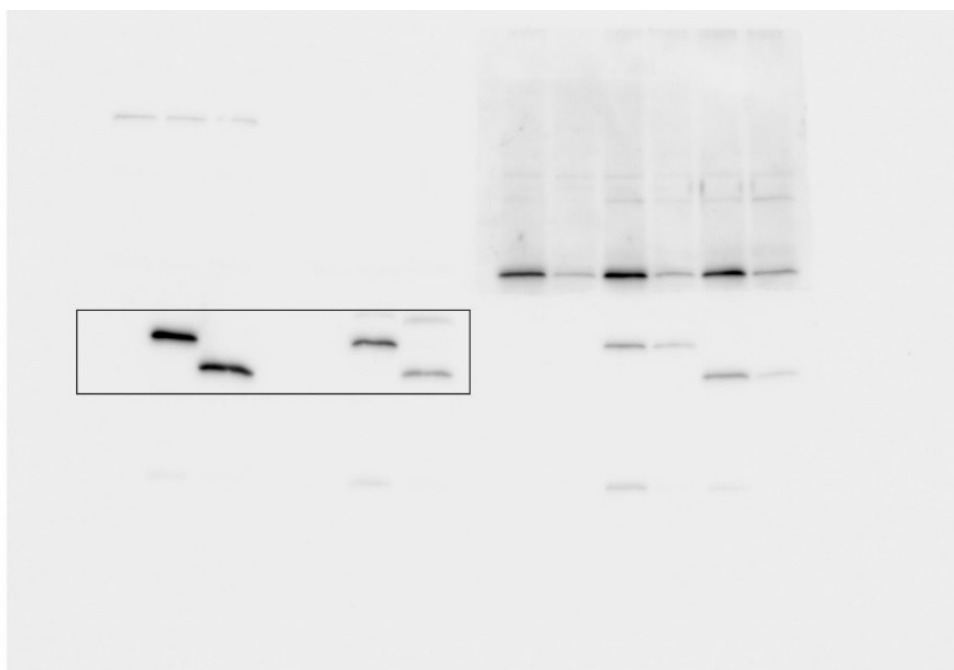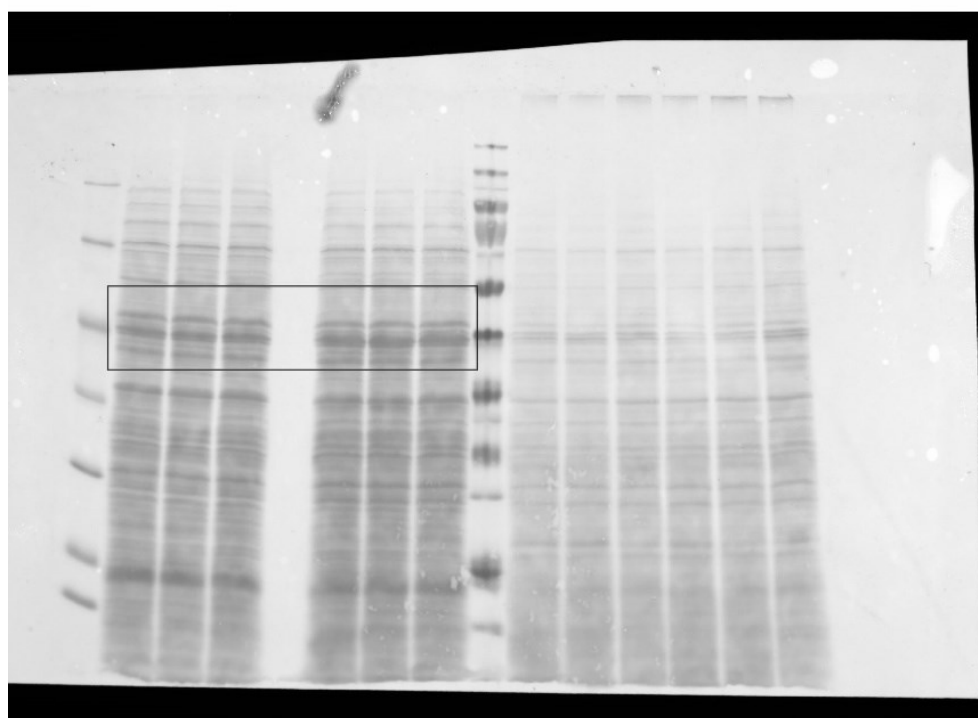

Supplement: Supplementary file 14 — Figure EV and Appendix Source Data [file 44318_2025_486_MOESM14_ESM.zip › SD figure S1/SD figure S1B.pdf]

Fig-S1-C

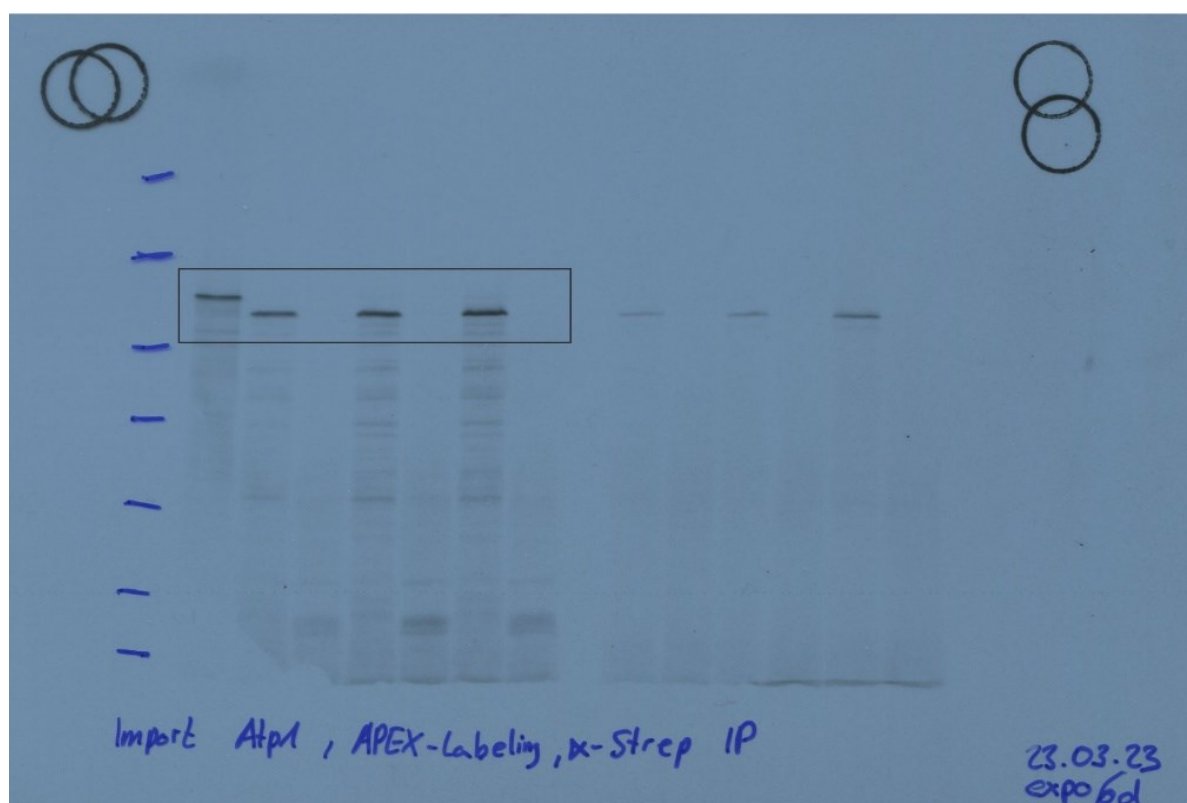

Supplement: Supplementary file 14 — Figure EV and Appendix Source Data [file 44318_2025_486_MOESM14_ESM.zip › SD figure S1/SD figure S1C.pdf]

Fig-S2-B

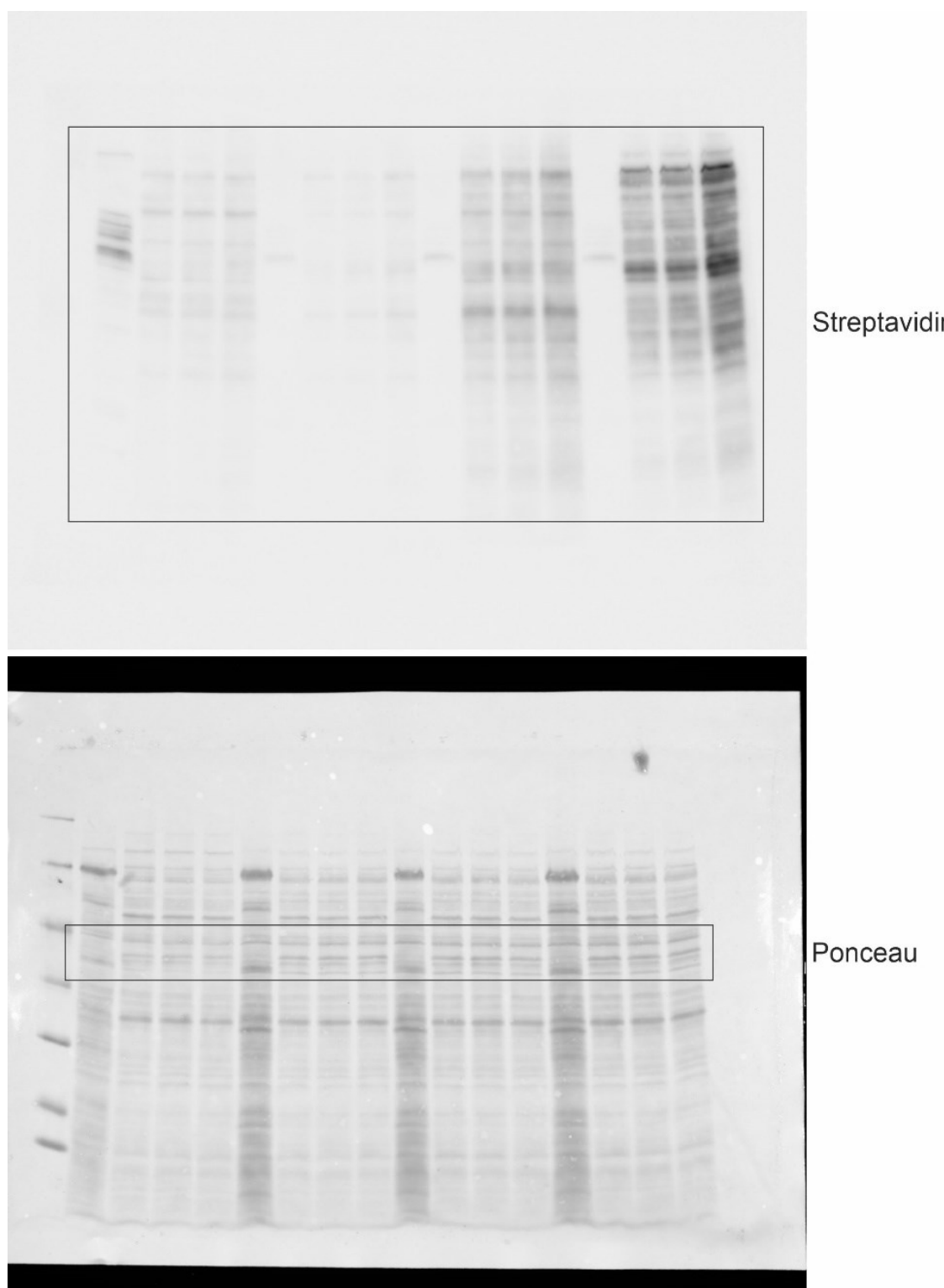

Supplement: Supplementary file 14 — Figure EV and Appendix Source Data [file 44318_2025_486_MOESM14_ESM.zip › SD figure S2/SD figure S2B.pdf]

Fig-S3-B

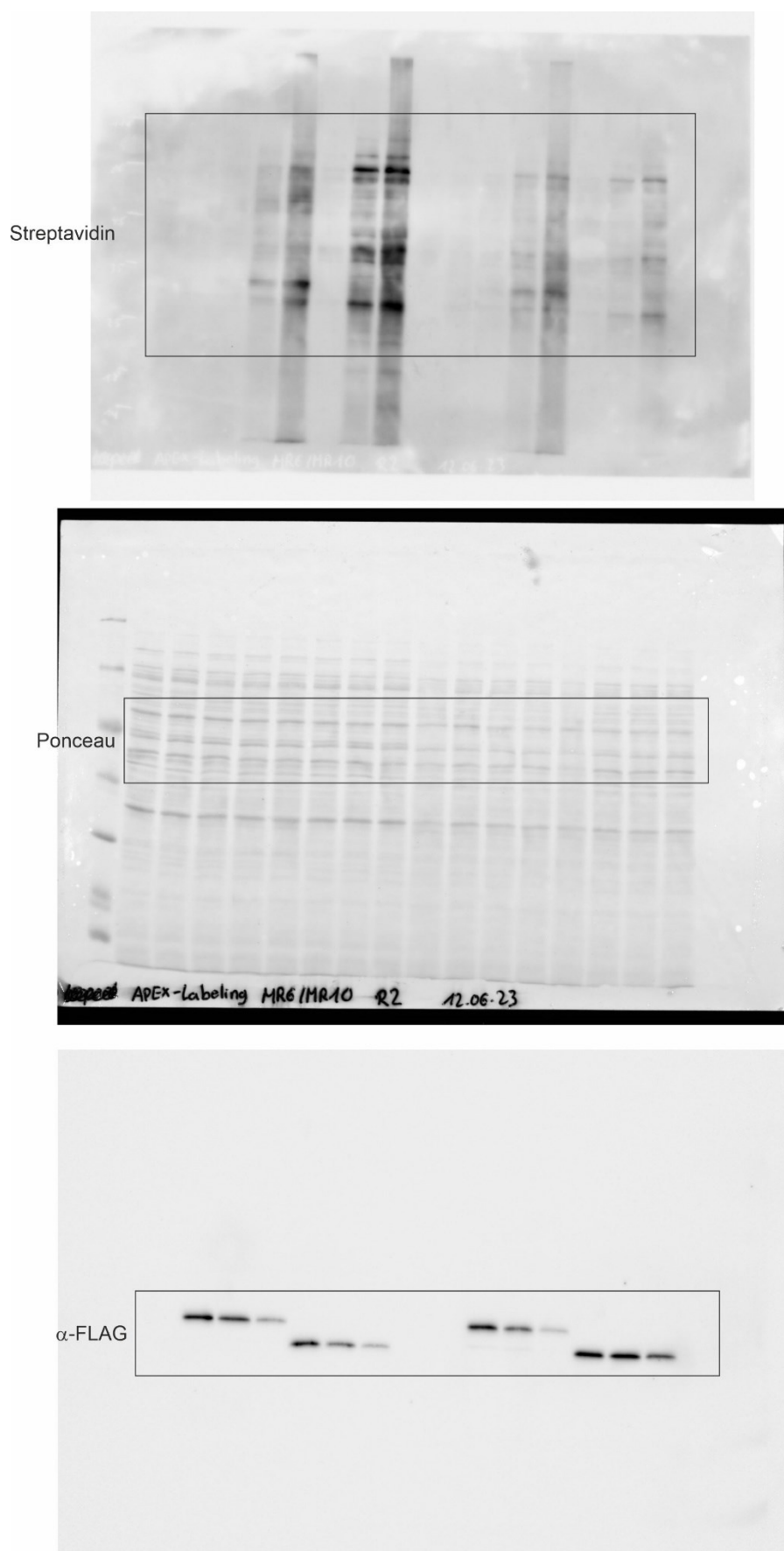

Supplement: Supplementary file 14 — Figure EV and Appendix Source Data [file 44318_2025_486_MOESM14_ESM.zip › SD figure S3/SD figure S3B.pdf]

Fig-S5-A

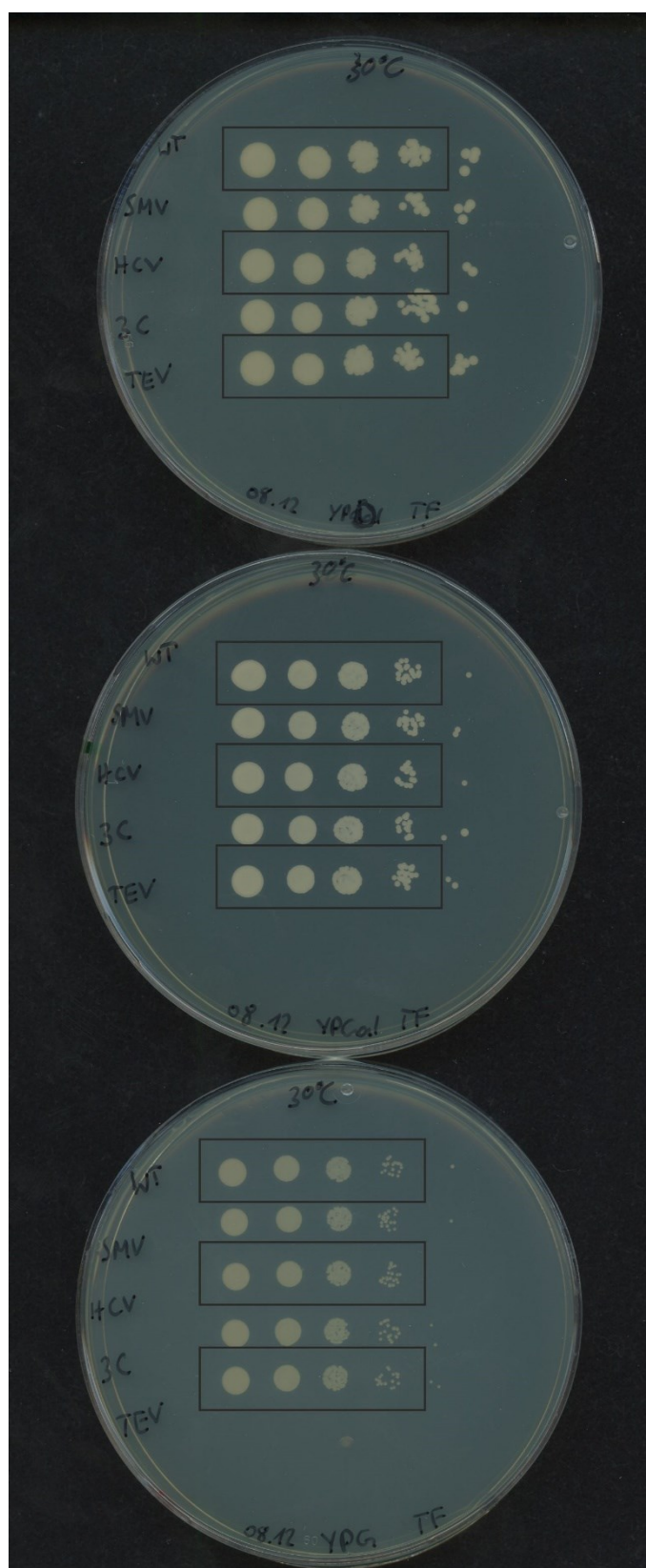

Supplement: Supplementary file 14 — Figure EV and Appendix Source Data [file 44318_2025_486_MOESM14_ESM.zip › SD figure S5/SD figure S5A.pdf]

Fig-S5-B

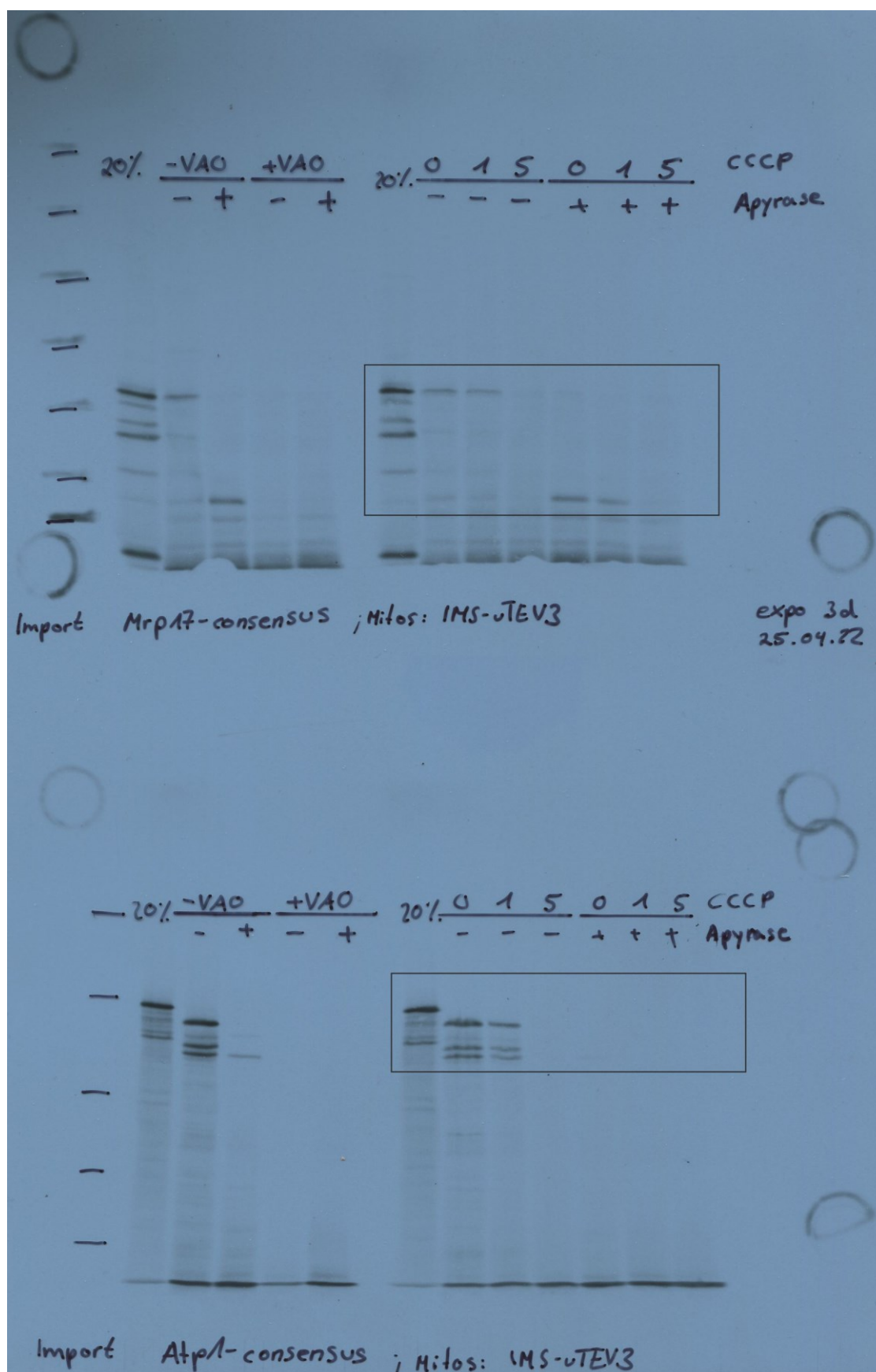

Supplement: Supplementary file 14 — Figure EV and Appendix Source Data [file 44318_2025_486_MOESM14_ESM.zip › SD figure S5/SD figure S5B.pdf]

Fig-S5-C

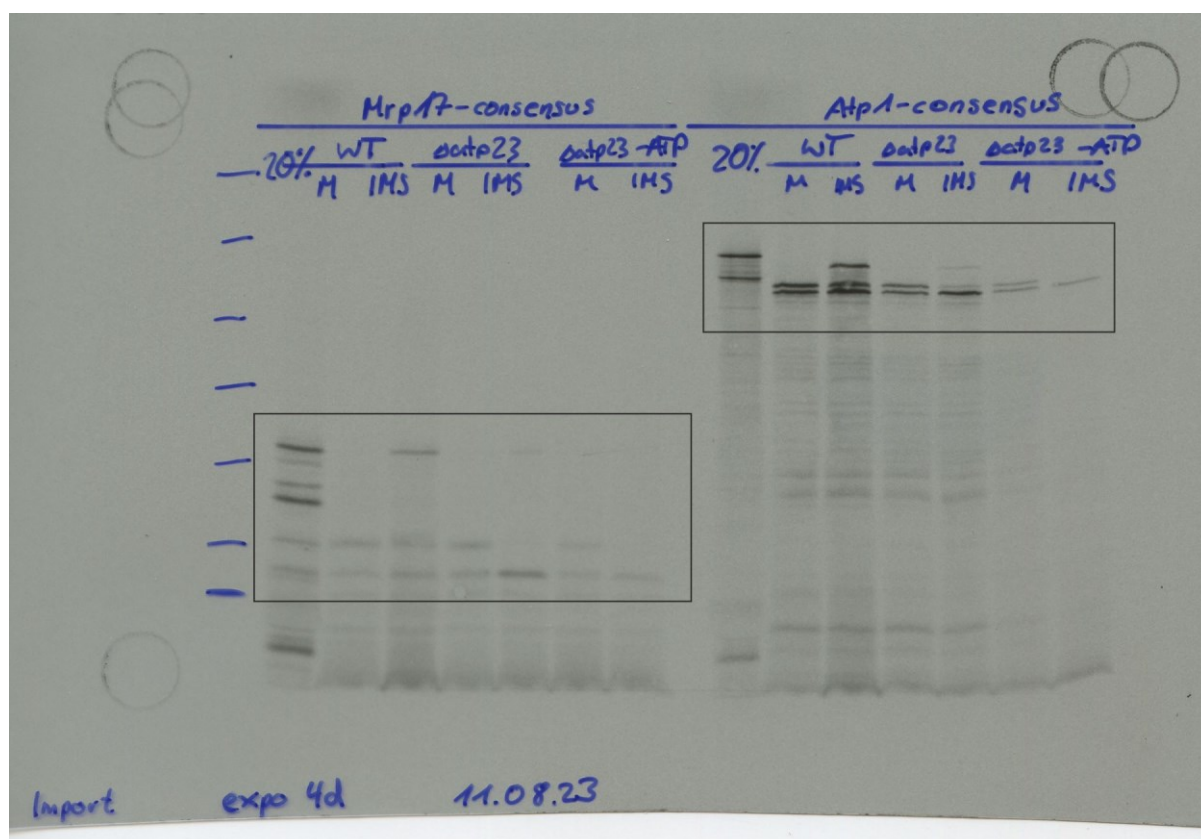

Supplement: Supplementary file 14 — Figure EV and Appendix Source Data [file 44318_2025_486_MOESM14_ESM.zip › SD figure S5/SD figure S5C.pdf]

Fig-S5-D

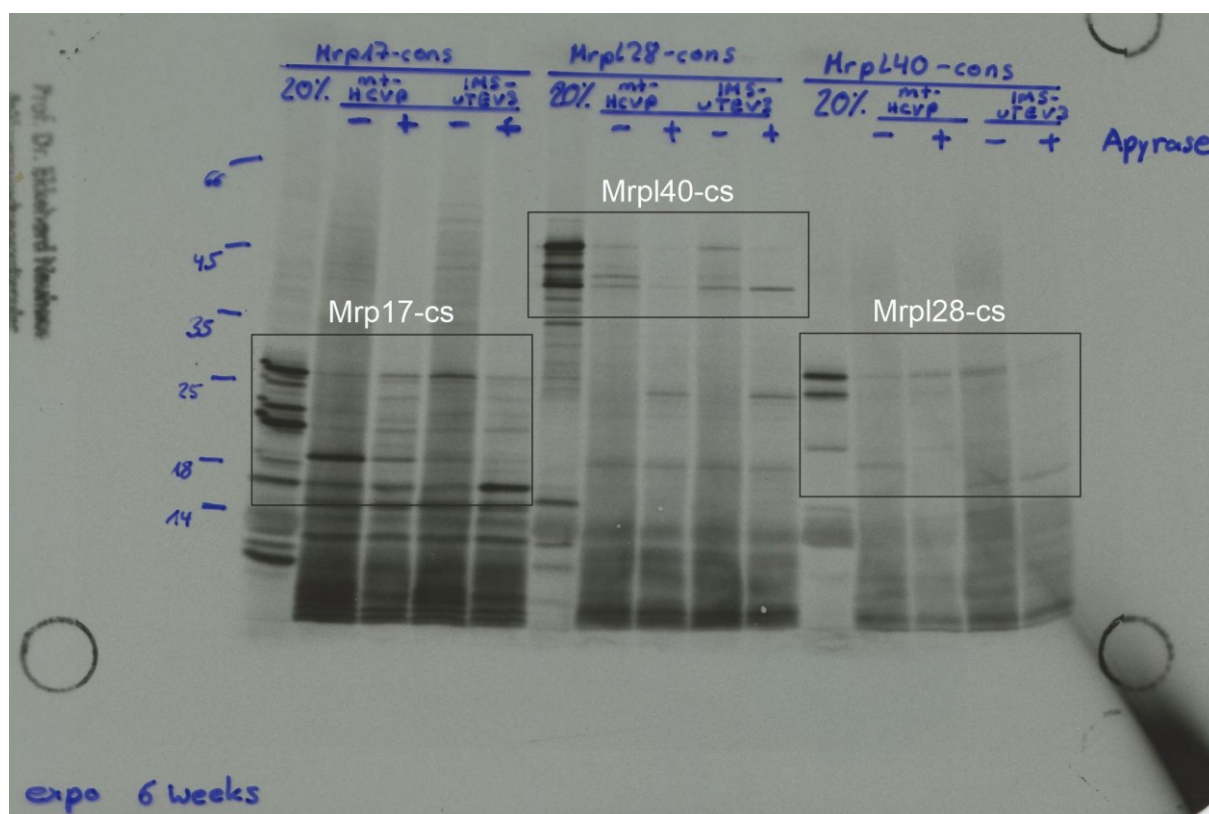

Supplement: Supplementary file 14 — Figure EV and Appendix Source Data [file 44318_2025_486_MOESM14_ESM.zip › SD figure S5/SD figure S5D.pdf]

Fig-S5-E

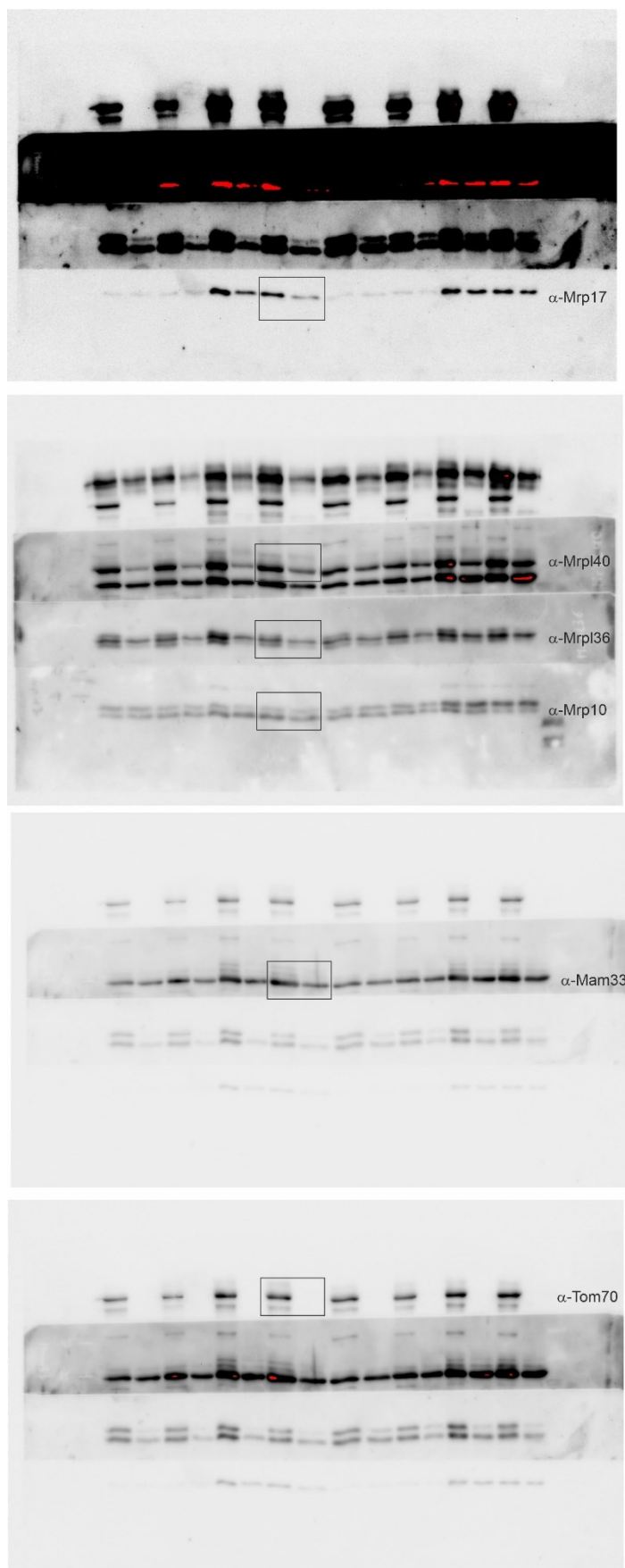

Supplement: Supplementary file 14 — Figure EV and Appendix Source Data [file 44318_2025_486_MOESM14_ESM.zip › SD figure S5/SD figure S5E.pdf]
