# Supplementary material for: Dysfunctional mitochondria trap proteins in the intermembrane space
Source: EMBO J. 2025 Jun 16;44(15):4352–77. doi: 10.1038/s44318-025-00486-1 (PMC12317151; doi:10.1038/s44318-025-00486-1)
Supplement: Supplementary file 10 — Source data Fig. 3 [file 44318_2025_486_MOESM10_ESM.zip › SD figure 3/SD figure 3E.pdf]

Fig-3-E

Tim44	Rep 1	Rep 2	Rep 3	Mean	Std. Dev	GOI-HG	GOI-Ref	Ratio
WT	18.37	18.73	19.2	18.76667	0.339837	4.173333	0	1
guide1	21.2	21.46	21.97	21.54333	0.319826	7.133333	2.96	0.128514
