# Supplementary material for: Dysfunctional mitochondria trap proteins in the intermembrane space
Source: EMBO J. 2025 Jun 16;44(15):4352–77. doi: 10.1038/s44318-025-00486-1 (PMC12317151; doi:10.1038/s44318-025-00486-1)
Supplement: Supplementary file 12 — Source data Fig. 5 [file 44318_2025_486_MOESM12_ESM.zip › SD figure 5/SD figure 5D.pdf]

Source Data for Fig. 5D (15S) and 6D (35S)
Shown are the measured Cq values

Replicate 1

		tech. rep 1	tech. rep 2	tech. rep 3
**GFP**	35S	12.18	12.45	12.74
	15S	11.53	11.55	13.09
**GFP-Mrp17**	35S	10.08	10.4	11.2
	15S	10.16	10.04	10.28
**Mrp17-GFP**	35S	10.94	11.46	10.94
	15S	3.61	4.4	3.46
**GFP-Mrp17K-**	35S	11.52	11.7	11.69
	15S	10.63	10.64	10.66
**Pwp2**	35S	8.76	8.91	9.01
	15S	10.4	10.46	10.53
**Utp18**	35S	9.05	9.16	
	15S	10.73	10.81	

Replicate 2

		tech. rep 1	tech. rep 2	tech. rep 3
**GFP**	35S	13.09	12.9	12.88
	15S	11.98	11.96	12.07
**Mrp17-GFP**	35S	12.97	10.8	11.61
	15S	5.81	6.25	5.43
**GFP-Mrp17**	35S	10.83	11.12	11.15
	15S	9.65	9.56	9.75
**GFP-Mrp17K-**	35S	11.41	11.62	11.59
	15S	9.7	9.58	9.79
**Pwp2**	35S	8.91	9.23	9.39
	15S	9.63	38.03	35.88
**Utp18**	35S	9.74	9.93	9.83
	15S	10.35	10.68	10.73

Replicate 3

		tech. rep 1	tech. rep 2	tech. rep 3
**GFP**	35S	12.54	12.71	12.78
	15S	11.48	11.77	11.85
**Mrp17-GFP**	35S	11.24	11.24	10.85
	15S	4.63	4.34	4.3
**GFP-Mrp17**	35S	10.34	10.57	10.31
	15S	9.32	9.69	9.26
**GFP-Mrp17K-**	35S	11.74	12.12	11.58
	15S	9.25	9.65	9.7
**Pwp2**	35S	8.49	8.8	8.98
	15S	10.38	10.82	10.75
**Utp18**	35S	9.14	9.27	9.27
	15S	9.41	9.78	10.22
