# Supplementary material for: Dysfunctional mitochondria trap proteins in the intermembrane space
Source: EMBO J. 2025 Jun 16;44(15):4352–77. doi: 10.1038/s44318-025-00486-1 (PMC12317151; doi:10.1038/s44318-025-00486-1)
Supplement: Supplementary file 15 — Expanded View Figures [file 44318_2025_486_MOESM15_ESM.pdf]

Expanded View Figures

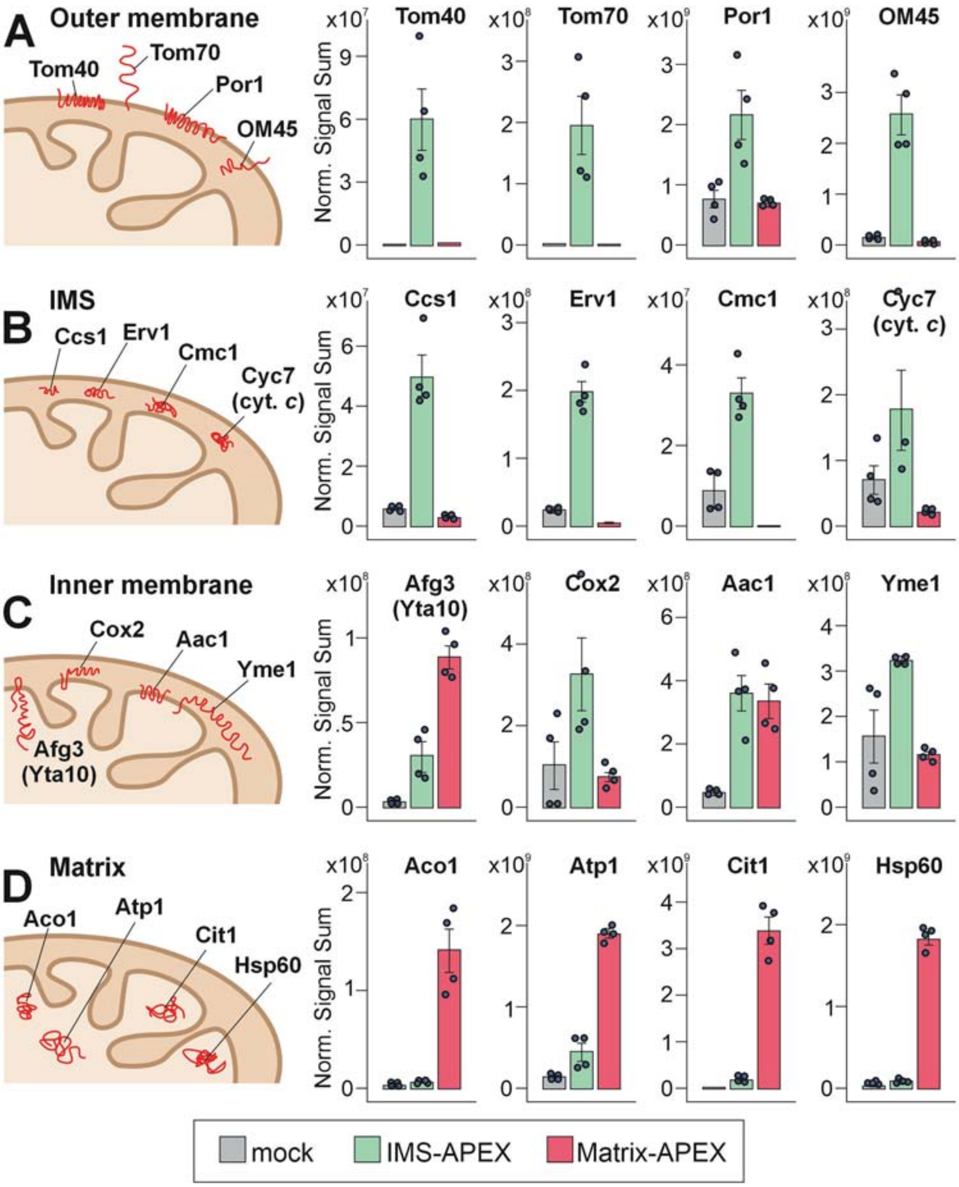

Figure EV1. APEX-mediated biotinylation patterns reveal intramitochondrial distribution of proteins.

(**A–D**) Protein levels were detected by mass spectrometry in the streptavidin pulldowns from lysed isolated mitochondria, derived from cells expressing no APEX (gray), or IMS-APEX (green) or matrix-APEX (red). Shown are the normalized signal sums of mitochondrial proteins of the four different mitochondrial subcompartments. Plotted were mean values and standard deviations from four replicates. The intramitochondrial localizations of the proteins are sketched on the left. See Dataset EV1 for details.

© The Author(s)

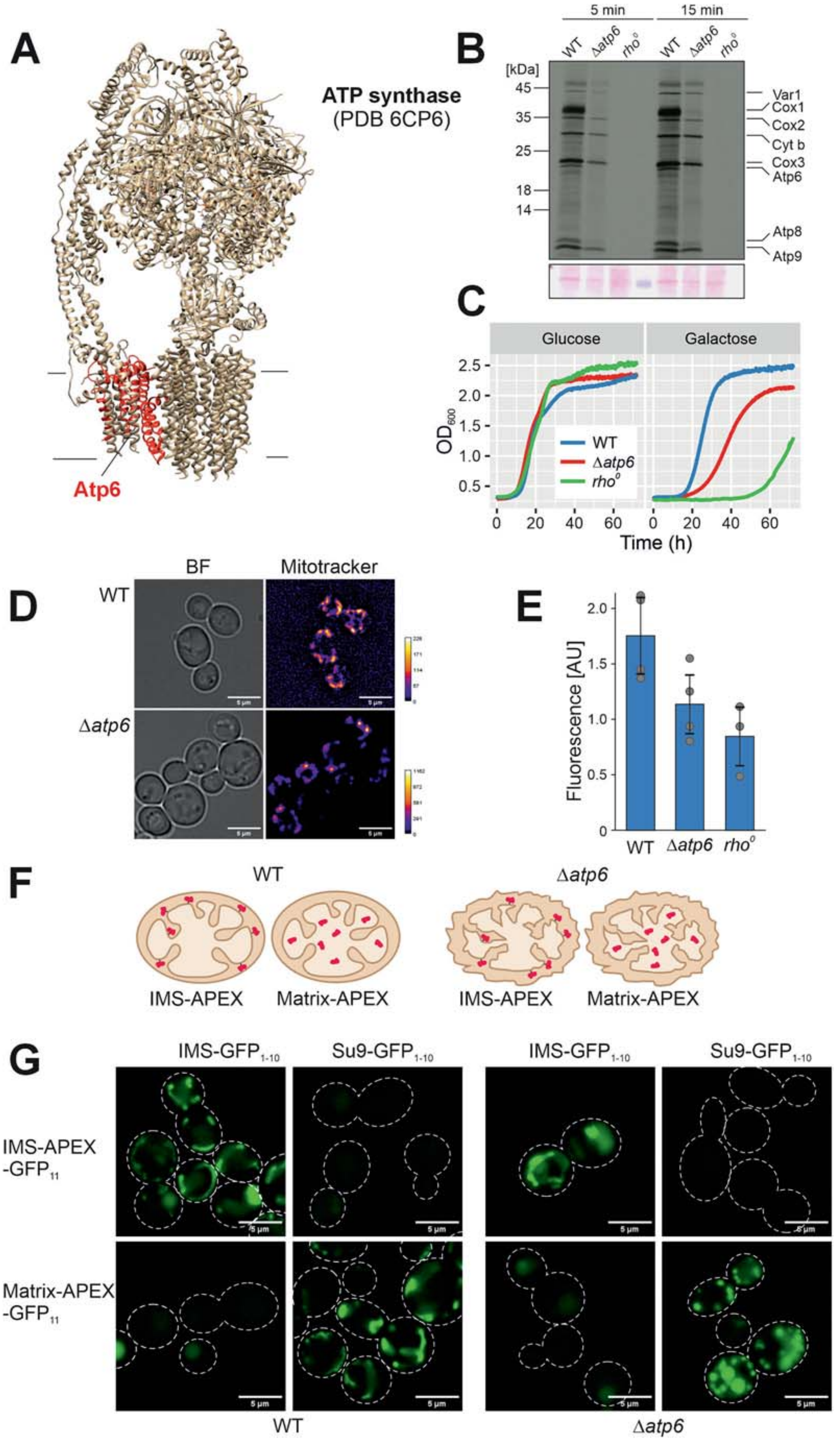
A
ATP synthase
(PDB 6CP6)
Atp6
B
5 min
15 min
WT
Δatp6
rho°
[kDa]
45
35
25
18
14
Var1
Cox1
Cox2
Cyt b
Cox3
Atp6
Atp8
Atp9
C
Glucose
Galactose
OD600
Time (h)
WT
Δatp6
rho°
D
BF
Mitotracker
WT
Δatp6
E
Fluorescence [AU]
F
WT
Δatp6
IMS-APEX
Matrix-APEX
G
IMS-GFP1-10
Su9-GFP1-10
IMS-APEX -GFP11
Matrix-APEX -GFP11
5 μm

 © The Author(s)

Figure EV2. A mutant lacking the mitochondrially encoded *ATP6* gene serves as example for mitochondrial dysfunction.

(**A**) Structure of the ATPase complex of baker's yeast (Srivastava et al, 2018). Atp6 (shown in red) is a subunit of the F_o part that plays a crucial role in proton pumping. (**B**) Mitochondria were isolated from the indicated strains and incubated in *in organello* translation buffer in the presence of ^{35}S-methionine for 5 or 15 min. Radiolabeled translation products were separated by SDS-PAGE and visualized by autoradiography. (**C**) Growth of the indicated strains in synthetic medium containing the indicated carbon sources was recorded continuously and plotted. Shown are mean values of three technical replicates. (**D**) Wild-type and *Δatp6* cells were grown in galactose medium. Mitochondria were stained with the membrane potential-dependent dye mitotracker CMXRos. Intensity values were calculated to indicate the degree of mitochondrial energization in the two strains. (**E**) The fluorescence intensities of the mitotracker-stained mitochondria were quantified as a measure of the mitochondrial membrane potential in the strains. Shown are mean values and standard deviations from four biological replicates. (**F**) Schematic representation of intramitochondrial distribution of the APEX fusion proteins in the different strains used in this study. (**G**) The IMS-APEX and matrix-APEX reporters were fused to the eleventh beta sheet of GFP (GFP_{11}) and coexpressed with IMS- and matrix-targeted fragments of the first 10 beta sheets of GFP ($GFP_{1\text{-}10}$). Signals indicate colocalization with these split-GFP reporters (Cabantous and Waldo, 2006). Source data are available online for this figure.

© The Author(s)

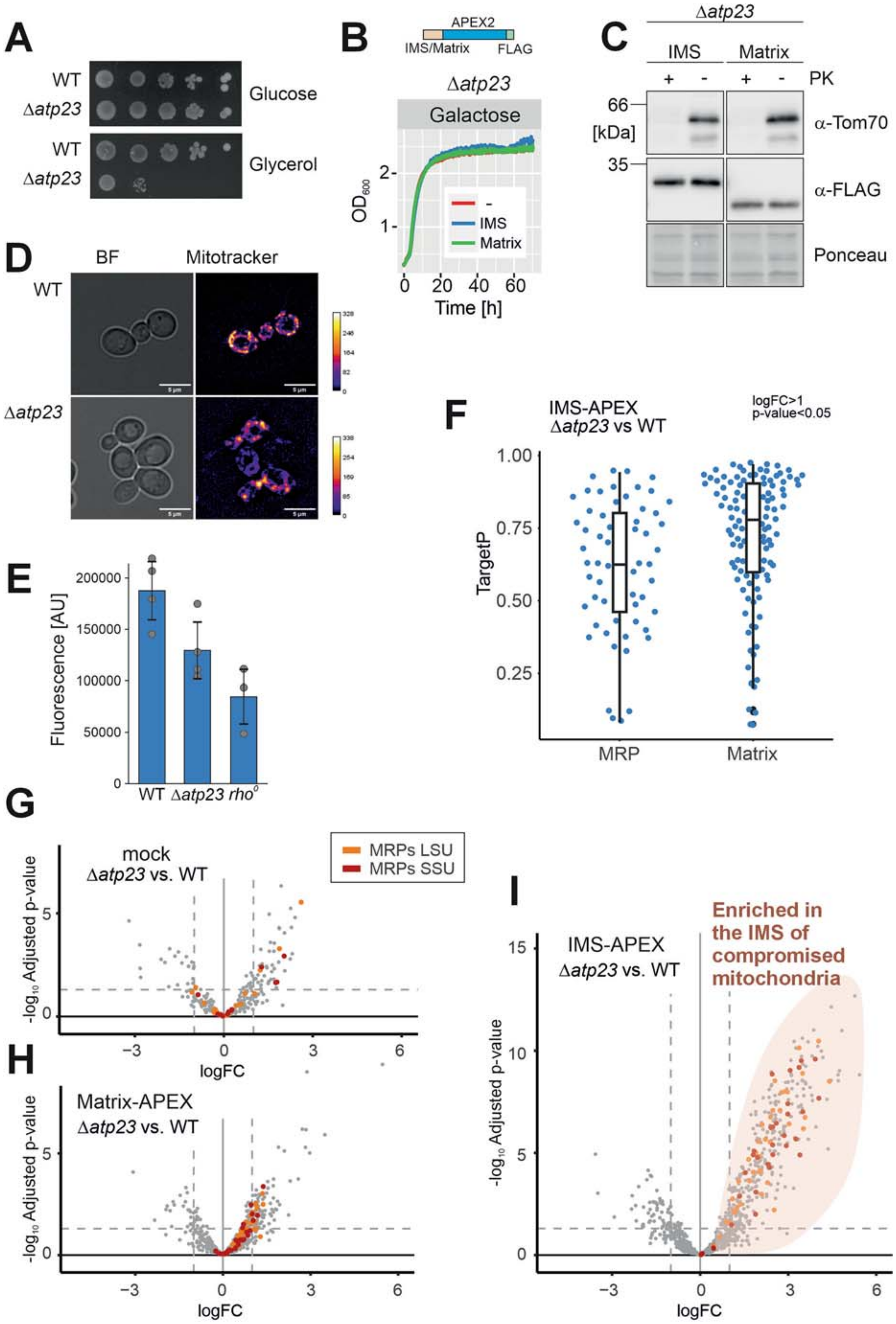
A
WT
Δatp23
Glucose
WT
Δatp23
Glycerol
B
APEX2
IMS/Matrix
FLAG
Δatp23
Galactose
OD600
Time [h]
-
IMS
Matrix
C
Δatp23
IMS
Matrix
PK
66
[kDa]
35
α-Tom70
α-FLAG
Ponceau
D
BF
Mitotracker
WT
Δatp23
E
Fluorescence [AU]
WT
Δatp23
rho0
F
IMS-APEX
Δatp23 vs WT
logFC>1
p-value<0.05
TargetP
MRP
Matrix
G
mock
Δatp23 vs. WT
MRPs LSU
MRPs SSU
-log10 Adjusted p-value
logFC
H
Matrix-APEX
Δatp23 vs. WT
I
IMS-APEX
Δatp23 vs. WT
Enriched in the IMS of compromised mitochondria

Figure EV3. A nuclear model for mitochondrial dysfunction phenocopies the situation in *Δatp6* cells.

(**A**) Wild-type (WT) and *Δatp23* cells were grown to log phase in galactose-containing medium before tenfold serial dilutions were dropped onto medium containing glucose or glycerol as carbon sources. (**B**) The growth of *Δatp23* cells expressing the indicated APEX reporters was measured continuously. (**C**) Mitochondria were isolated from wild-type and *Δatp23* cells and incubated with or without proteinase K (PK) to remove non-imported proteins. The presence of the surface protein Tom70 and of the FLAG-tagged APEX fusion proteins was verified by Western blotting. (**D**) Wild-type and *Δatp23* cells were grown in galactose medium. Mitochondria were stained with the membrane potential-dependent dye mitotracker CMXRos. Intensity values were calculated to indicate the degree of mitochondrial energization in the two strains. (**E**) The fluorescence intensities of the mitotracker-stained mitochondria were quantified as a measure of the mitochondrial membrane potential in the strains. Shown are mean values and standard deviations from four biological replicates. (**F**) Comparison of the TargetP values of IMS-located MRPs in comparison to other matrix proteins that are enriched in the IMS of *Δatp23* mitochondria. Shown are proteins that are significantly enriched logFC > 1, P value < 0.05. (**G–I**) Volcano plots showing the relative distribution of proteins purified with streptavidin beads in mitochondria from wild-type and *Δatp23* cells. The $\log_2$ fold change (logFC) values were calculated from $n = 4$ samples. Proteins of the small subunit of the mitoribosome are colored in red and of the large subunit in orange. Significantly enriched proteins are indicated by the dashed lines (logFC > 1, P value < 0.05). Please note the strong accumulation of MRPs (shown in color) in the IMS of *Δatp23* cells, that is reminiscent to the situation in *Δatp6* cells (Fig. 2F). Source data are available online for this figure.

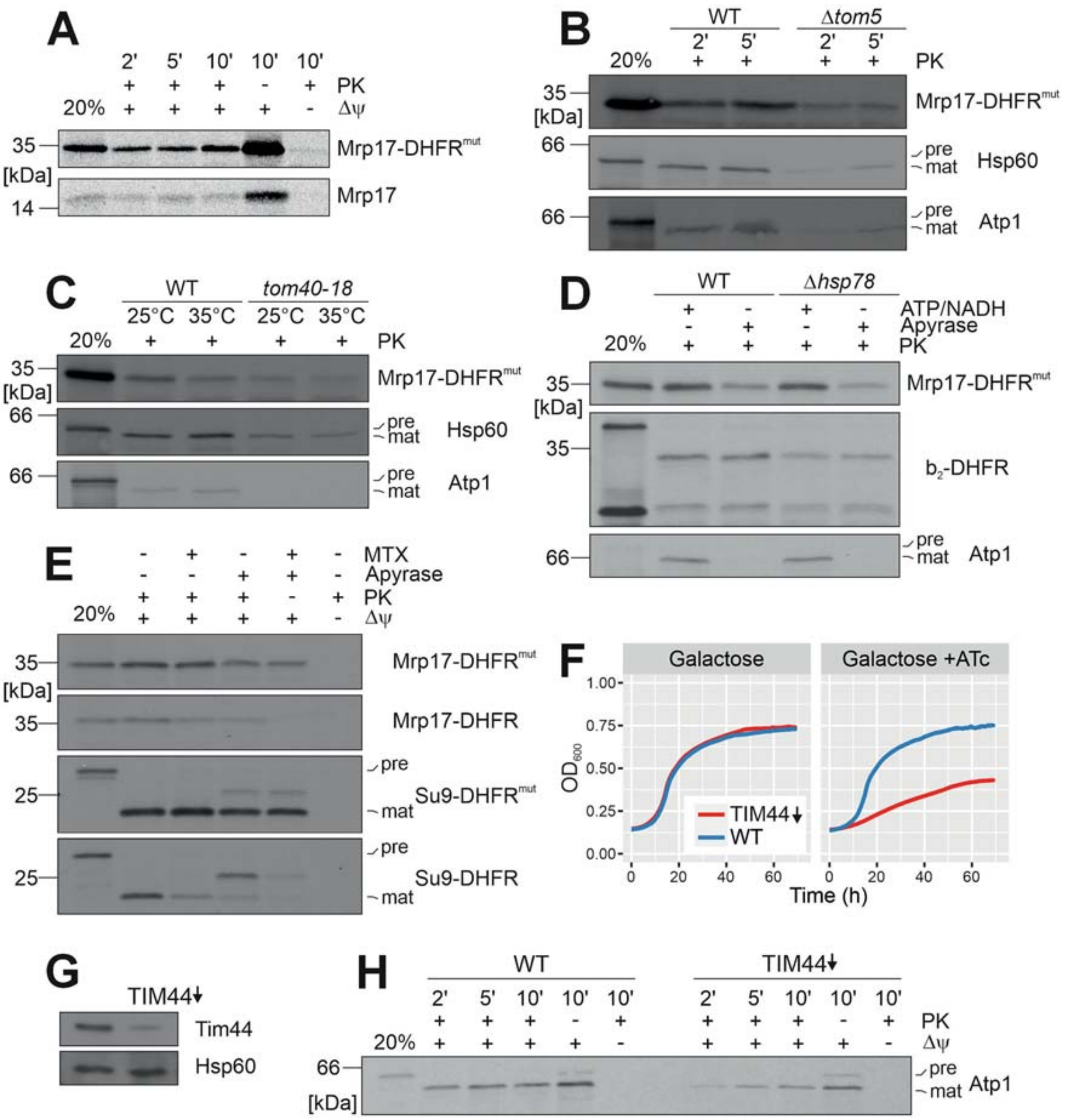

Figure EV4. Mrp17 is imported into mitochondria independent on the function of the import motor.

(**A**) Radiolabeled Mrp17-DHFR^{mut} and Mrp17 were incubated with isolated wild-type mitochondria for the times indicated. Mrp17 has only four methionine residues (including the N-terminal one); the fusion to the permanently unfolded DHFR domain (Vestweber and Schatz, 1988) increased the number of methionine residues leading to considerably enhanced radiolabeled signals of the protein (Bykov et al, 2022). The membrane potential (Δψ) was dissipated where indicated. Mitochondria were reisolated and incubated with proteinase K (PK). 20% of the radiolabeled protein used per import lane was loaded for control. (**B–D**) Radiolabeled proteins were incubated with mitochondria isolated from the indicated strains. In case of the temperature-sensitive *tom40-18* cells and the corresponding wild type (**C**), mitochondria were pretreated for 10 min at 35 °C before the experiment. (**E**) The indicated proteins were synthesized in reticulocyte extract in the presence or absence of 5 μM methotrexate (MTX) to stabilize the folding of DHFR, before import experiments into wild-type mitochondria were carried out for 5 min. (**F**) Wild-type cells containing the TIM44-CRISPRi plasmid were grown in the absence or presence of 960 ng/μl anhydrotetracycline (ATc) at 30 °C. Cells were preincubated for 6 h with ATc before the growth curve measurement. Shown are mean values of three technical replicates. (**G**) The expression of Tim44 was repressed by induction of the Tim44-CRISPRi plasmid for 16 h before mitochondria were isolated and analyzed by Western blotting. (**H**) Mitochondria were isolated from cells in which the expression of Tim44 had been repressed for 16 h. Radiolabeled Atp1 was imported into these mitochondria for the times indicated. Source data are available online for this figure.

 © The Author(s)

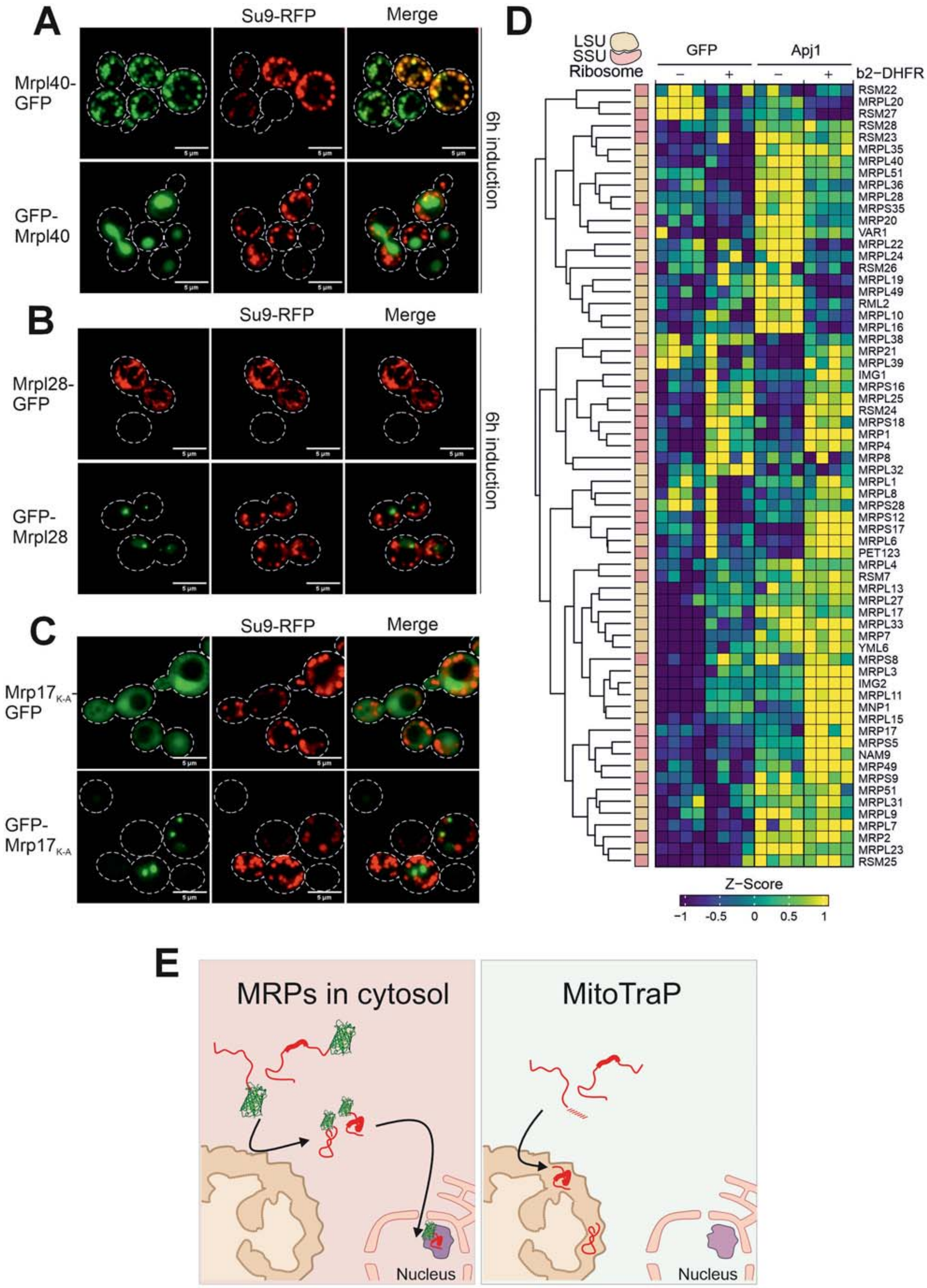
A
Su9-RFP
Merge
Mrpl40-GFP
GFP-Mrpl40
6h induction
B
Su9-RFP
Merge
Mrpl28-GFP
GFP-Mrpl28
6h induction
C
Su9-RFP
Merge
Mrp17K-A-GFP
GFP-Mrp17K-A
D
LSU
SSU
Ribosome
GFP
Apj1
b2-DHFR
RSM22
MRPL20
RSM27
RSM28
RSM23
MRPL35
MRPL40
MRPL51
MRPL36
MRPL28
MRPS35
MRP20
VAR1
MRPL22
MRPL24
RSM26
MRPL19
MRPL49
RML2
MRPL10
MRPL16
MRPL38
MRP21
MRPL39
IMG1
MRPS16
MRPL25
RSM24
MRPS18
MRP1
MRP4
MRP8
MRPL32
MRPL1
MRPL8
MRPS28
MRPS12
MRPS17
MRPL6
PET123
MRPL4
RSM7
MRPL13
MRPL27
MRPL17
MRPL33
MRP7
YML6
MRPS8
MRPL3
IMG2
MRPL11
MNP1
MRPL15
MRP17
MRPS5
NAM9
MRP49
MRPS9
MRP51
MRPL31
MRPL9
MRPL7
MRP2
MRPL23
RSM25
Z-Score
−1 -0.5 0 0.5 1
E
MRPs in cytosol
MitoTraP
Nucleus
Nucleus

Figure EV5. The non-importable GFP-Mrp17 variant associates with components of the 90S pre-ribosome assembly complexes.

(**A–C**) GFP-Mrpl40, GFP-Mrpl28 and GFP-Mrp17$_{K\text{-}A}$ were expressed and visualized by fluorescence microscopy 6 h after induction in galactose-containing medium. Mitochondria were stained by expression of Su9-RFP. (**D**) Heat map showing the intensities of MRPs there were co-isolated with GFP or Apj1-GFP in the presence or absence of the clogger b_2-DHFR. Proteins were filtered for small and large subunits of the mitochondrial ribosomes. Imputed LFQ intensities were z-score normalized across rows and subject to non-supervised hierarchical clustering. $n = 4$. (**E**) Mitochondrial dysfunction leads to the trapping of MRPs in the IMS. The accumulation of MRPs in the IMS might prevent their targeting to the nucleus and, thus, their interference with the assembly of ribosomes in the nucleolus.

 © The Author(s)
