# Supplementary material for: Dysfunctional mitochondria trap proteins in the intermembrane space
Source: EMBO J. 2025 Jun 16;44(15):4352–77. doi: 10.1038/s44318-025-00486-1 (PMC12317151; doi:10.1038/s44318-025-00486-1)
Supplement: Supplementary file 5 — Dataset EV3 [file Dataset EV3.pdf › Dataset EV3.pdf]

Sheets

**Name**	**Description**
Normalized signal sums	Processed LFQ protein intensities for all samples

Samples

Name	Description
S1_WT	Isolated mitochondria of WT cells. APEX labeling was done for 5 min, biotinylated proteins were affinity purified with Streptavidin beads
S1_WT_MATRIX	Isolated mitochondria of WT cells containing Matrix-APEX. APEX labeling was done for 5 min, biotinylated proteins were affinity purified with Streptavidin beads
S1_WT_IMS	Isolated mitochondria of WT cells containing IMS-APEX. APEX labeling was done for 5 min, biotinylated proteins were affinity purified with Streptavidin beads
S1_atp23	Isolated mitochondria of *Δatp23* cells. APEX labeling was done for 5 min, biotinylated proteins were affinity purified with Streptavidin beads
S1_atp23_MATRIX	Isolated mitochondria of *Δatp23* cells containing Matrix-APEX. APEX labeling was done for 5 min, biotinylated proteins were affinity purified with Streptavidin beads
S1_atp23_IMS	Isolated mitochondria of Δ*atp23* cells containing IMS-APEX. APEX labeling was done for 5 min, biotinylated proteins were affinity purified with Streptavidin beads
S2_WT	Isolated mitochondria of WT (MR6) cells. APEX labeling was done for 5 min, biotinylated proteins were affinity purified with Streptavidin beads
S2_WT_MATRIX	Isolated mitochondria of WT (MR6) cells containing Matrix-APEX. APEX labeling was done for 5 min, biotinylated proteins were affinity purified with Streptavidin beads
S2_WT_IMS	Isolated mitochondria of WT (MR6) cells containing IMS-APEX. APEX labeling was done for 5 min, biotinylated proteins were affinity purified with Streptavidin beads
S2_atp6	Isolated mitochondria of *Δatp6* (MR10) cells. APEX labeling was done for 5 min, biotinylated proteins were affinity purified with Streptavidin beads
S2_atp6_MATRIX	Isolated mitochondria of Δ*atp6* (MR10) cells containing Matrix-APEX. APEX labeling was done for 5 min, biotinylated proteins were affinity purified with Streptavidin beads
S2_atp6_IMS	Isolated mitochondria of Δ*atp6* (MR10) cells containing IMS-APEX. APEX labeling was done for 5 min, biotinylated proteins were affinity purified with Streptavidin beads

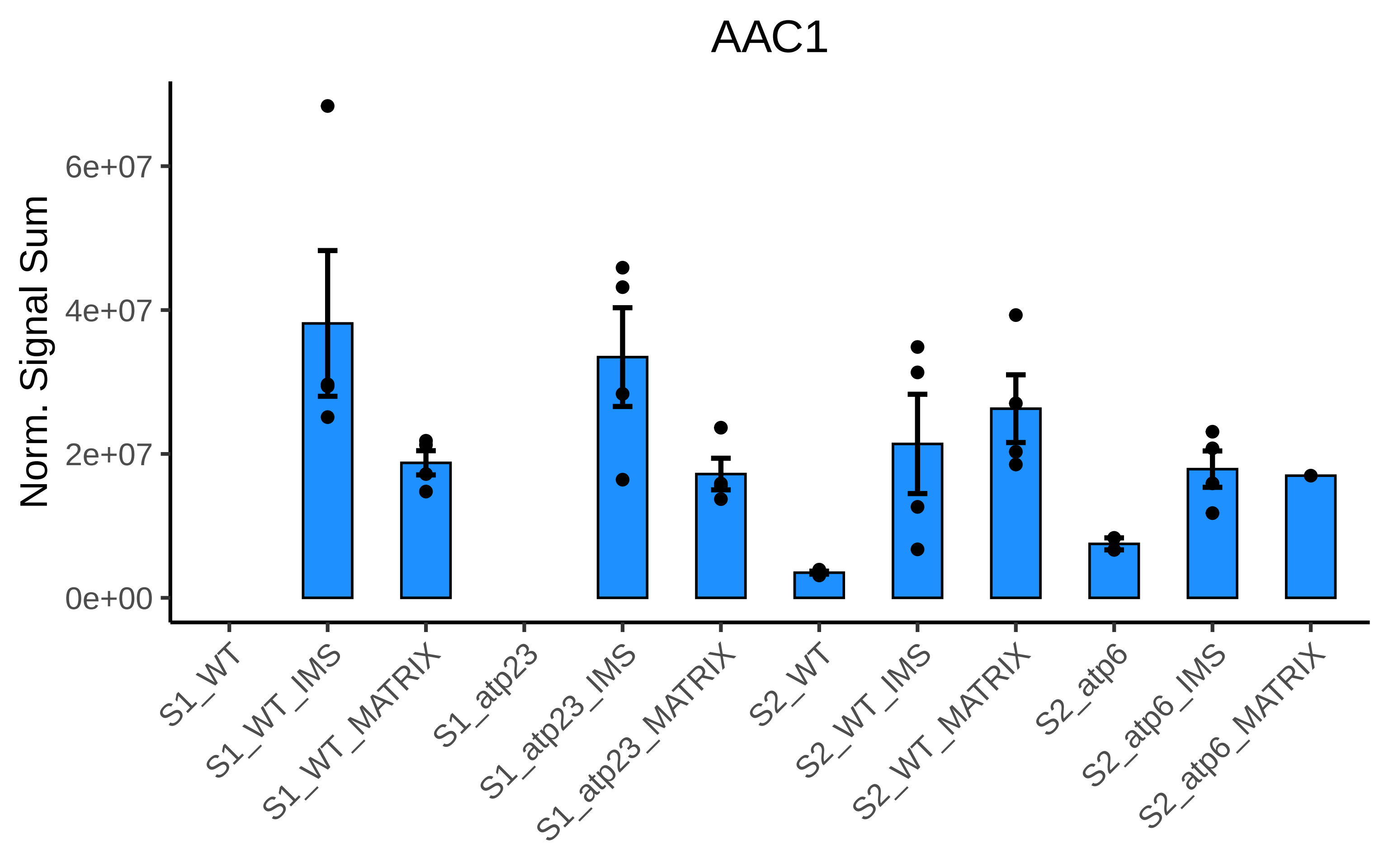

AAC1
Norm. Signal Sum
6e+07
4e+07
2e+07
0e+00
S1_WT
S1_WT_IMS
S1_WT_MATRIX
S1_atp23
S1_atp23_IMS
S1_atp23_MATRIX
S2_WT
S2_WT_IMS
S2_WT_MATRIX
S2_atp6
S2_atp6_IMS
S2_atp6_MATRIX

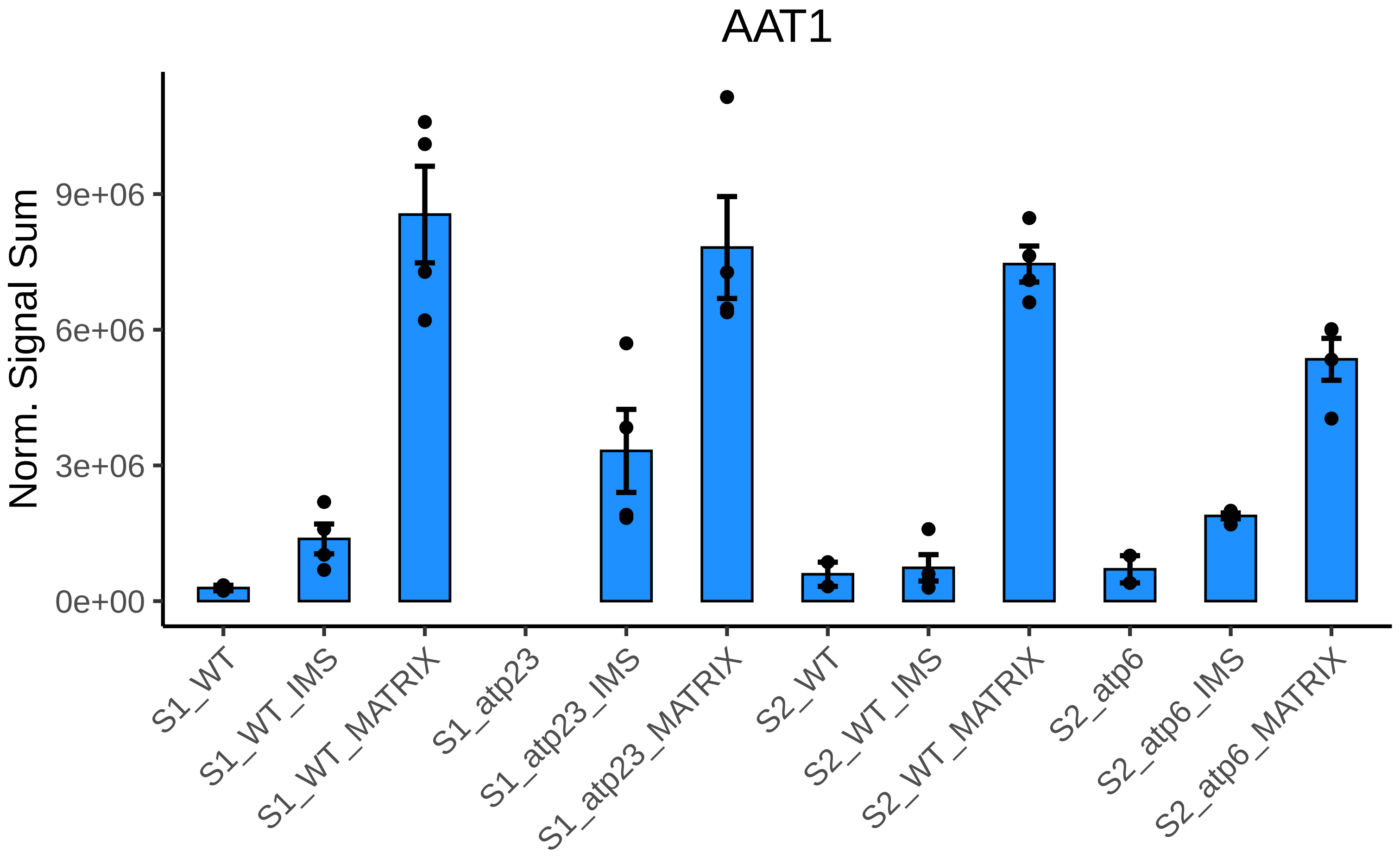
AAT1
Norm. Signal Sum
9e+06
6e+06
3e+06
0e+00
S1_WT
S1_WT_IMS
S1_WT_MATRIX
S1_atp23
S1_atp23_IMS
S1_atp23_MATRIX
S2_WT
S2_WT_IMS
S2_WT_MATRIX
S2_atp6
S2_atp6_IMS
S2_atp6_MATRIX

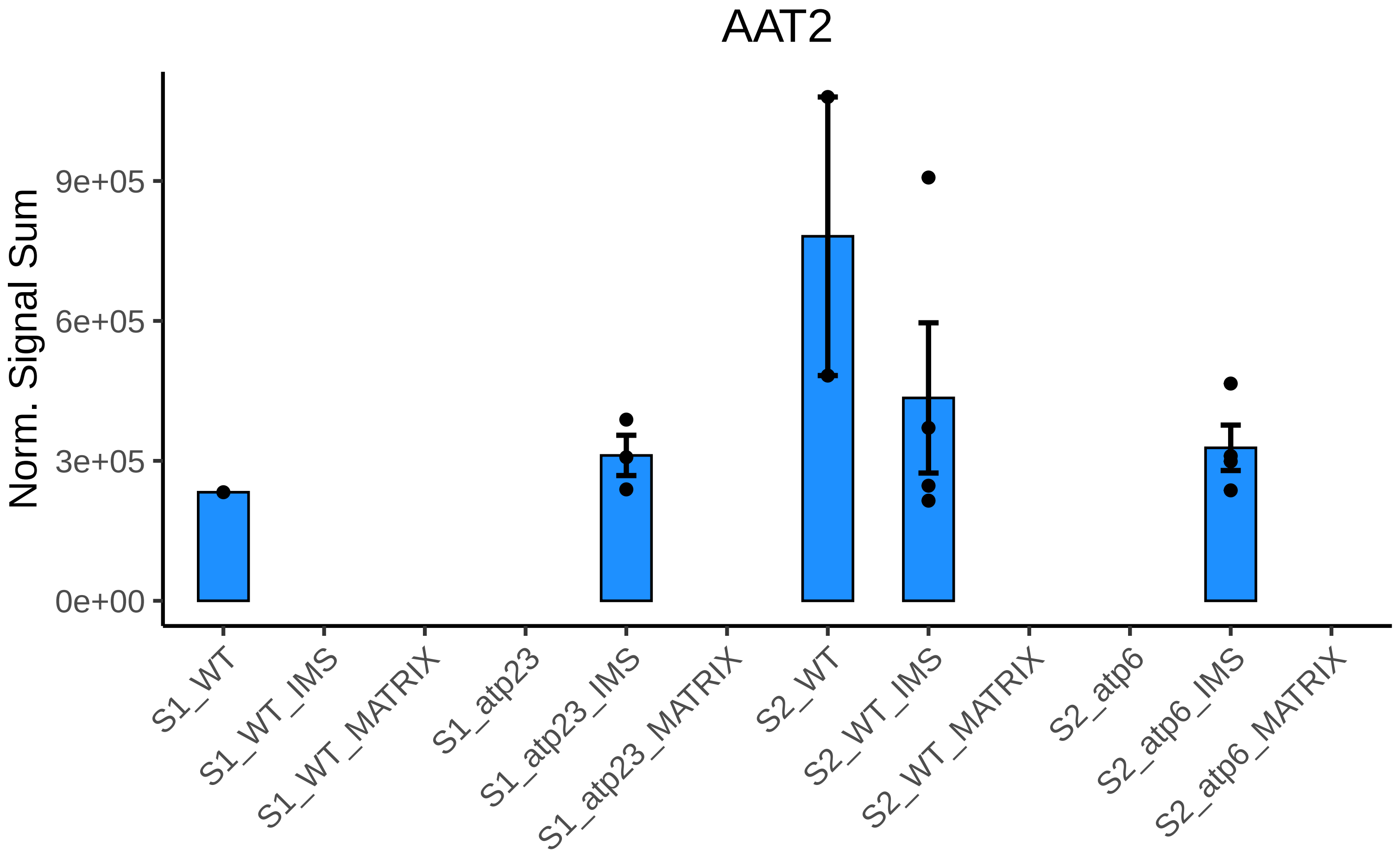

AAT2
Norm. Signal Sum
9e+05
6e+05
3e+05
0e+00
S1_WT
S1_WT_IMS
S1_WT_MATRIX
S1_atp23
S1_atp23_IMS
S1_atp23_MATRIX
S2_WT
S2_WT_IMS
S2_WT_MATRIX
S2_atp6
S2_atp6_IMS
S2_atp6_MATRIX

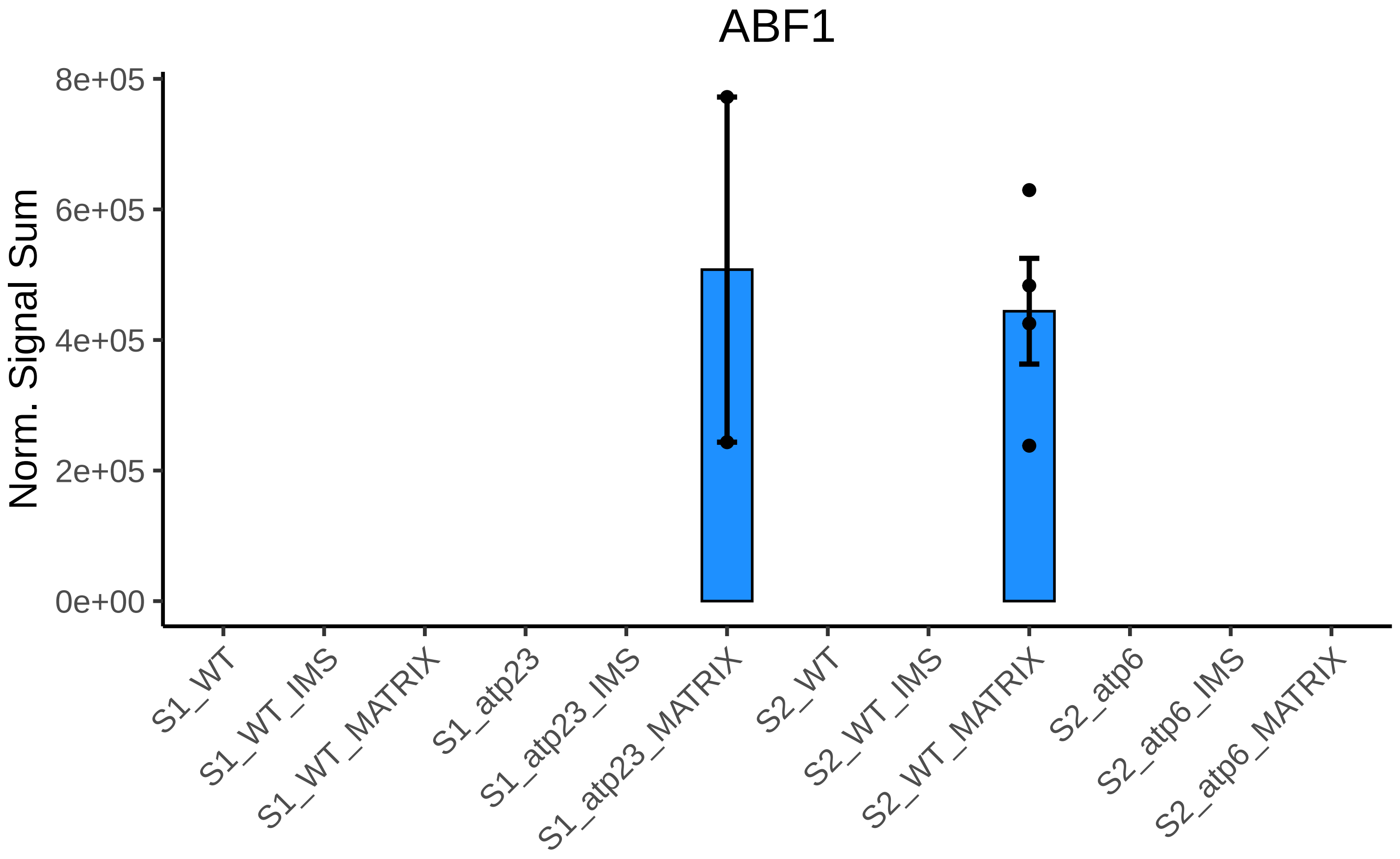

ABF1
Norm. Signal Sum
8e+05
6e+05
4e+05
2e+05
0e+00
S1_WT
S1_WT_IMS
S1_WT_MATRIX
S1_atp23
S1_atp23_IMS
S1_atp23_MATRIX
S2_WT
S2_WT_IMS
S2_WT_MATRIX
S2_atp6
S2_atp6_IMS
S2_atp6_MATRIX

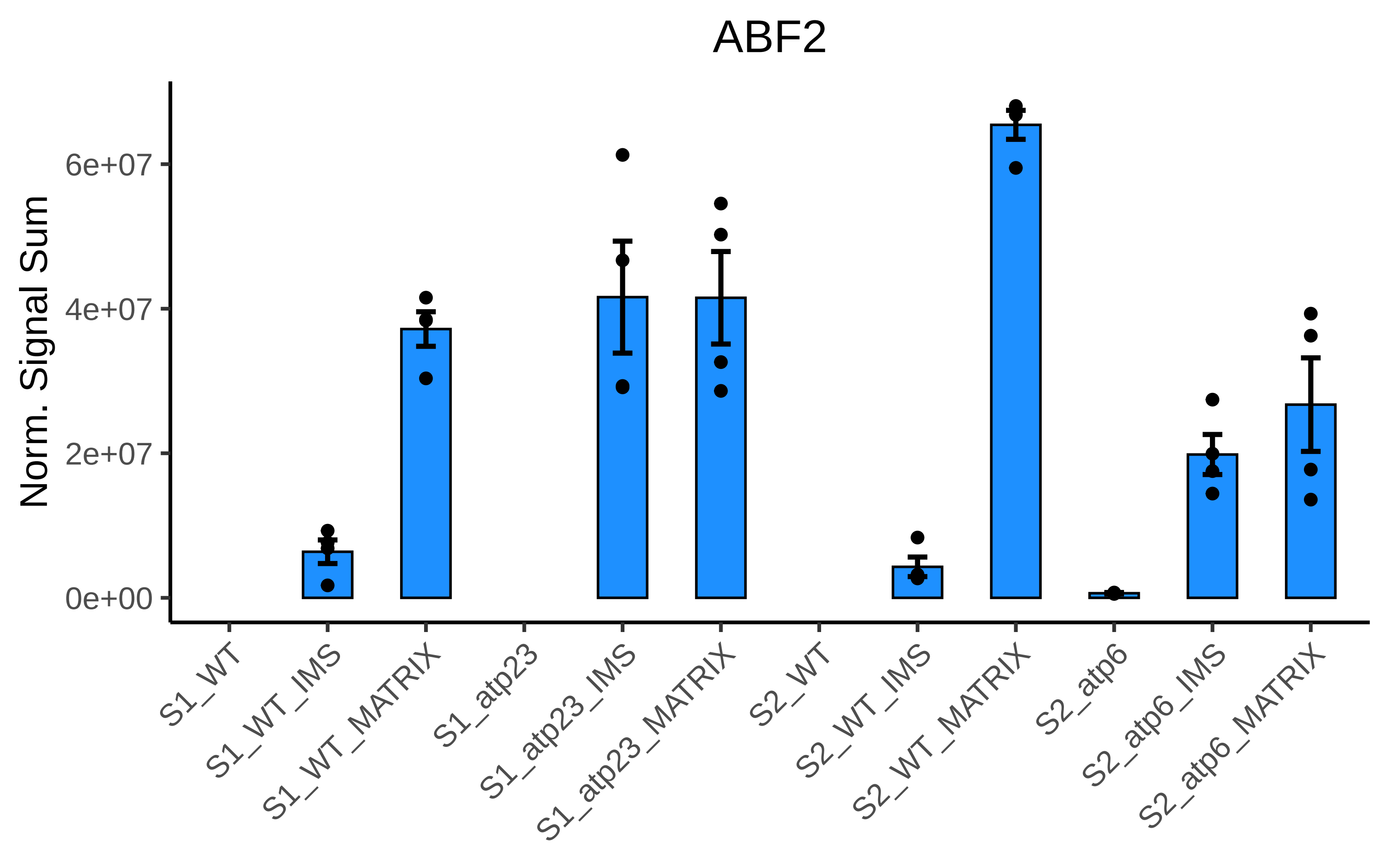
ABF2
Norm. Signal Sum
6e+07
4e+07
2e+07
0e+00
S1_WT
S1_WT_IMS
S1_WT_MATRIX
S1_atp23
S1_atp23_IMS
S1_atp23_MATRIX
S2_WT
S2_WT_IMS
S2_WT_MATRIX
S2_atp6
S2_atp6_IMS
S2_atp6_MATRIX

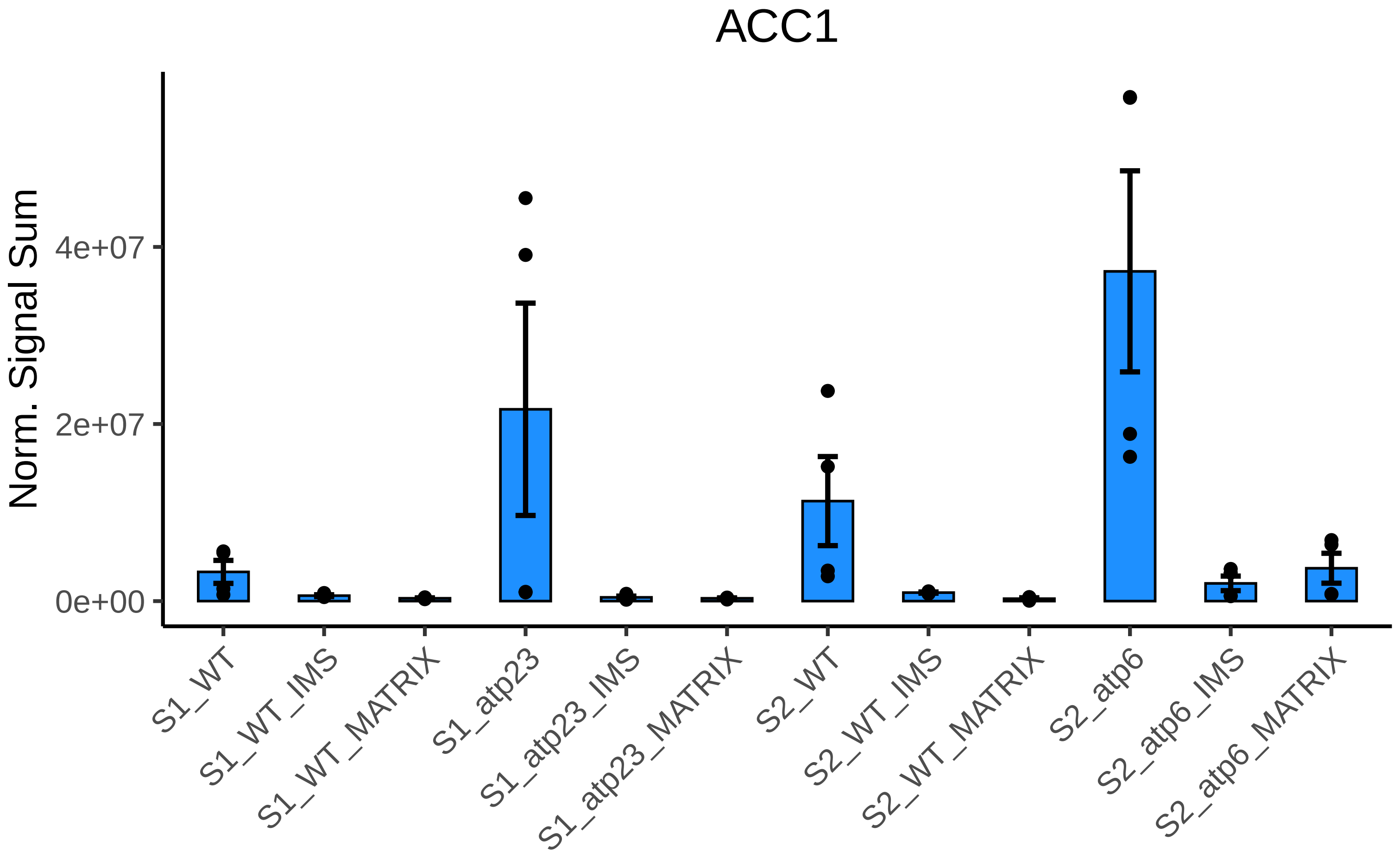

ACC1
Norm. Signal Sum
0e+00
2e+07
4e+07
S1_WT
S1_WT_IMS
S1_WT_MATRIX
S1_atp23
S1_atp23_IMS
S1_atp23_MATRIX
S2_WT
S2_WT_IMS
S2_WT_MATRIX
S2_atp6
S2_atp6_IMS
S2_atp6_MATRIX

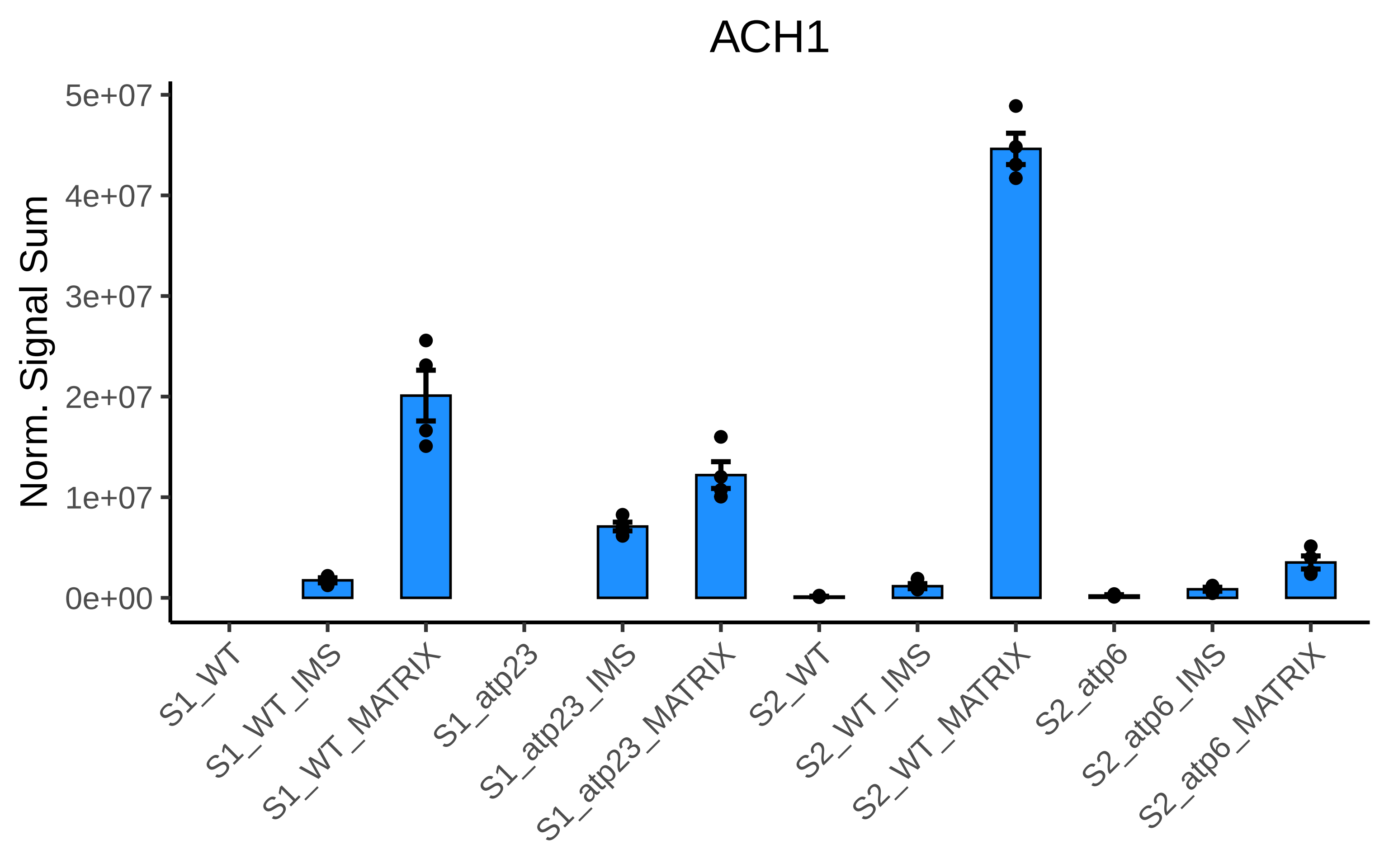

ACH1
Norm. Signal Sum
5e+07
4e+07
3e+07
2e+07
1e+07
0e+00
S1_WT
S1_WT_IMS
S1_WT_MATRIX
S1_atp23
S1_atp23_IMS
S1_atp23_MATRIX
S2_WT
S2_WT_IMS
S2_WT_MATRIX
S2_atp6
S2_atp6_IMS
S2_atp6_MATRIX

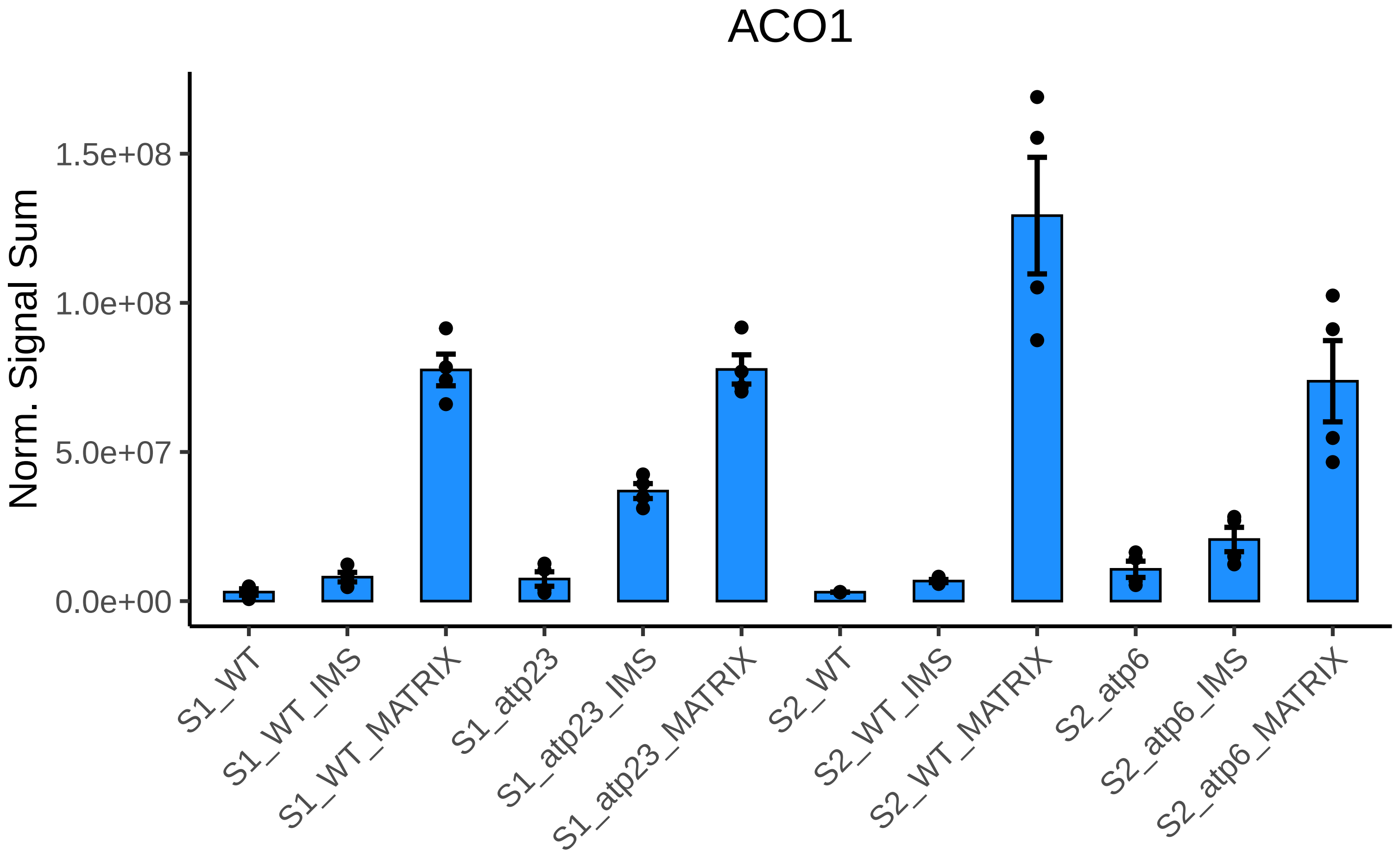
ACO1
Norm. Signal Sum
1.5e+08
1.0e+08
5.0e+07
0.0e+00
S1_WT
S1_WT_IMS
S1_WT_MATRIX
S1_atp23
S1_atp23_IMS
S1_atp23_MATRIX
S2_WT
S2_WT_IMS
S2_WT_MATRIX
S2_atp6
S2_atp6_IMS
S2_atp6_MATRIX

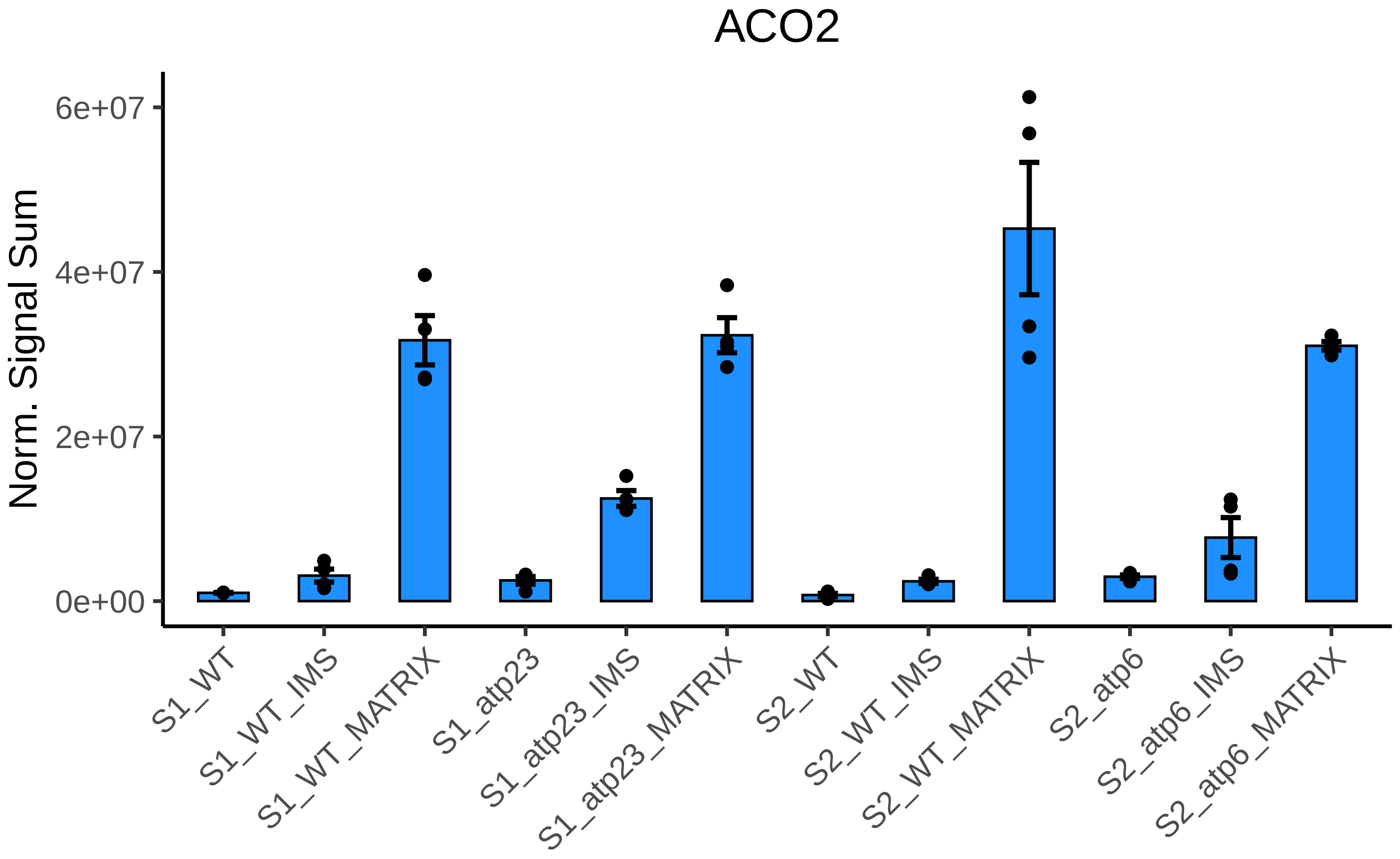

ACO2
Norm. Signal Sum
6e+07
4e+07
2e+07
0e+00
S1_WT
S1_WT_IMS
S1_WT_MATRIX
S1_atp23
S1_atp23_IMS
S1_atp23_MATRIX
S2_WT
S2_WT_IMS
S2_WT_MATRIX
S2_atp6
S2_atp6_IMS
S2_atp6_MATRIX

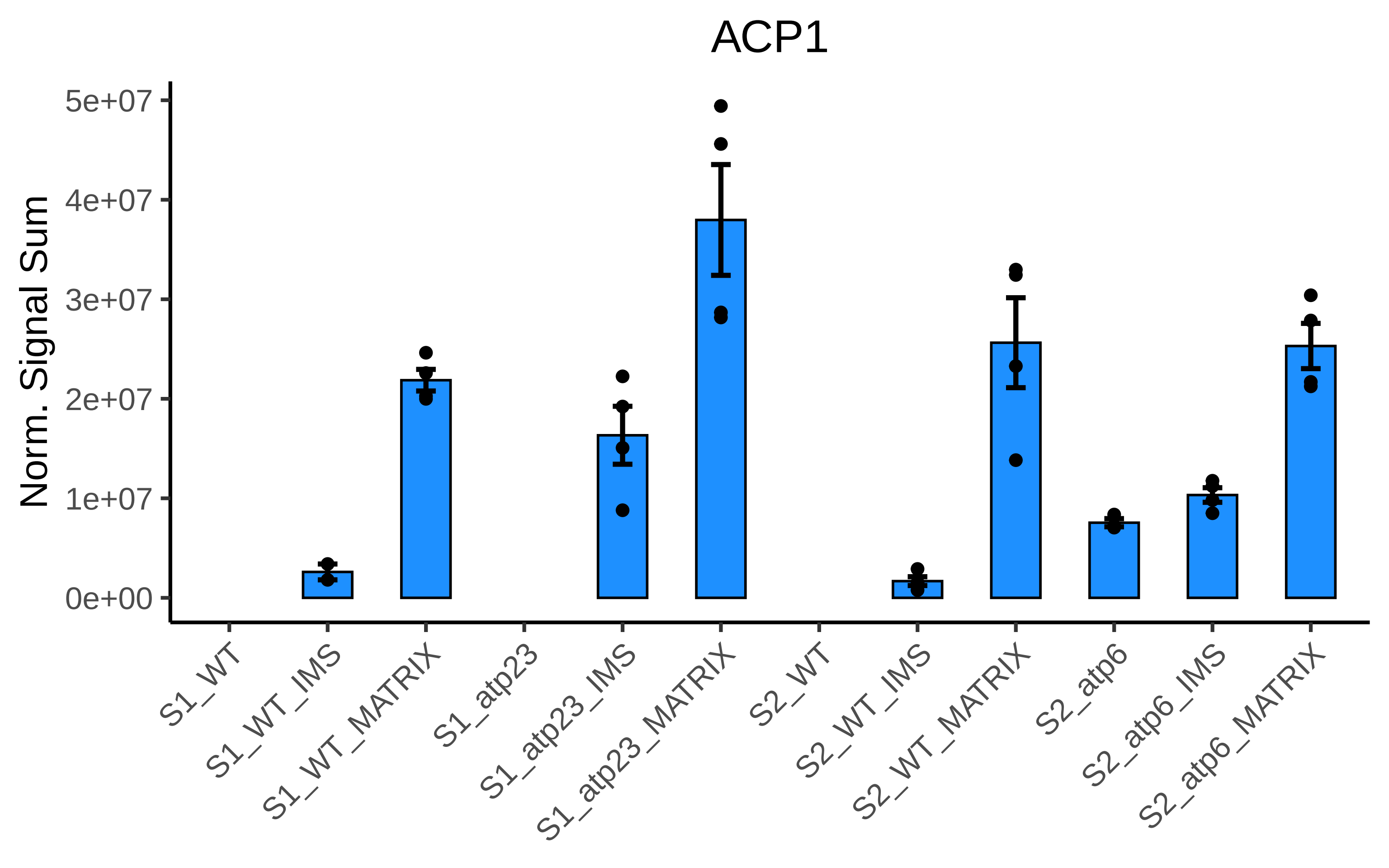

ACP1
Norm. Signal Sum
5e+07
4e+07
3e+07
2e+07
1e+07
0e+00
S1_WT
S1_WT_IMS
S1_WT_MATRIX
S1_atp23
S1_atp23_IMS
S1_atp23_MATRIX
S2_WT
S2_WT_IMS
S2_WT_MATRIX
S2_atp6
S2_atp6_IMS
S2_atp6_MATRIX

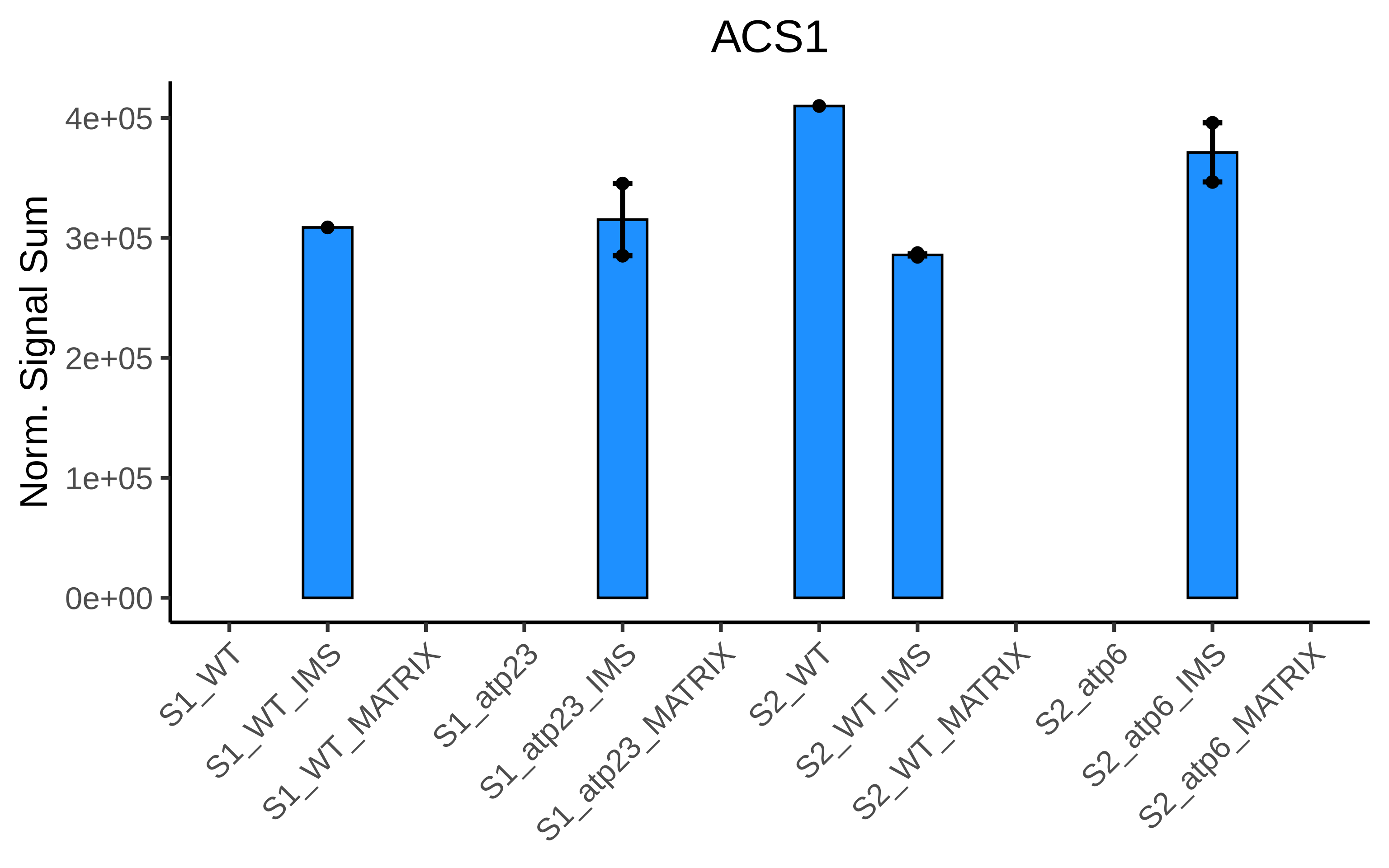

ACS1
Norm. Signal Sum
4e+05
3e+05
2e+05
1e+05
0e+00
S1_WT
S1_WT_IMS
S1_WT_MATRIX
S1_atp23
S1_atp23_IMS
S1_atp23_MATRIX
S2_WT
S2_WT_IMS
S2_WT_MATRIX
S2_atp6
S2_atp6_IMS
S2_atp6_MATRIX

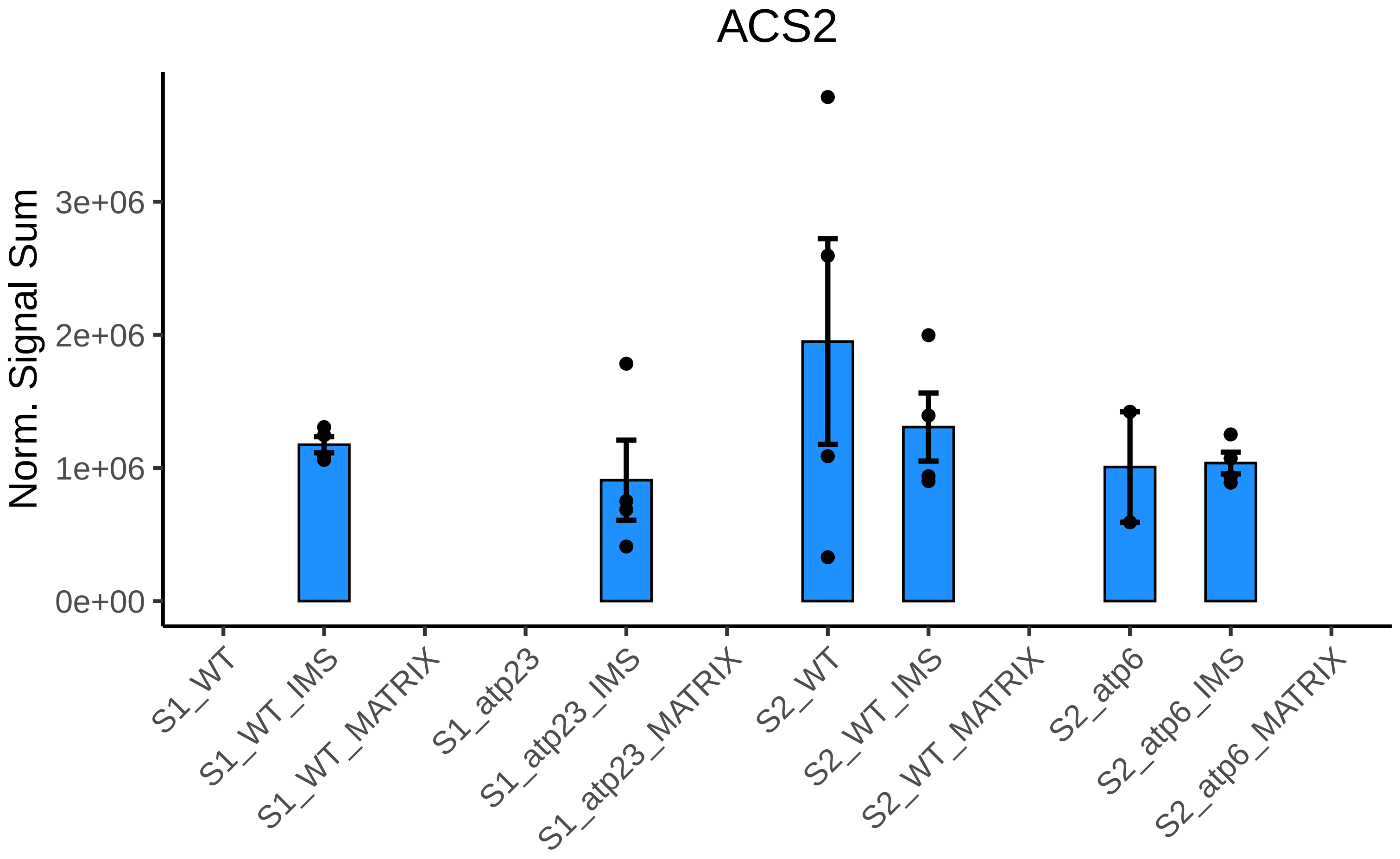

ACS2
Norm. Signal Sum
3e+06
2e+06
1e+06
0e+00
S1_WT
S1_WT_IMS
S1_WT_MATRIX
S1_atp23
S1_atp23_IMS
S1_atp23_MATRIX
S2_WT
S2_WT_IMS
S2_WT_MATRIX
S2_atp6
S2_atp6_IMS
S2_atp6_MATRIX

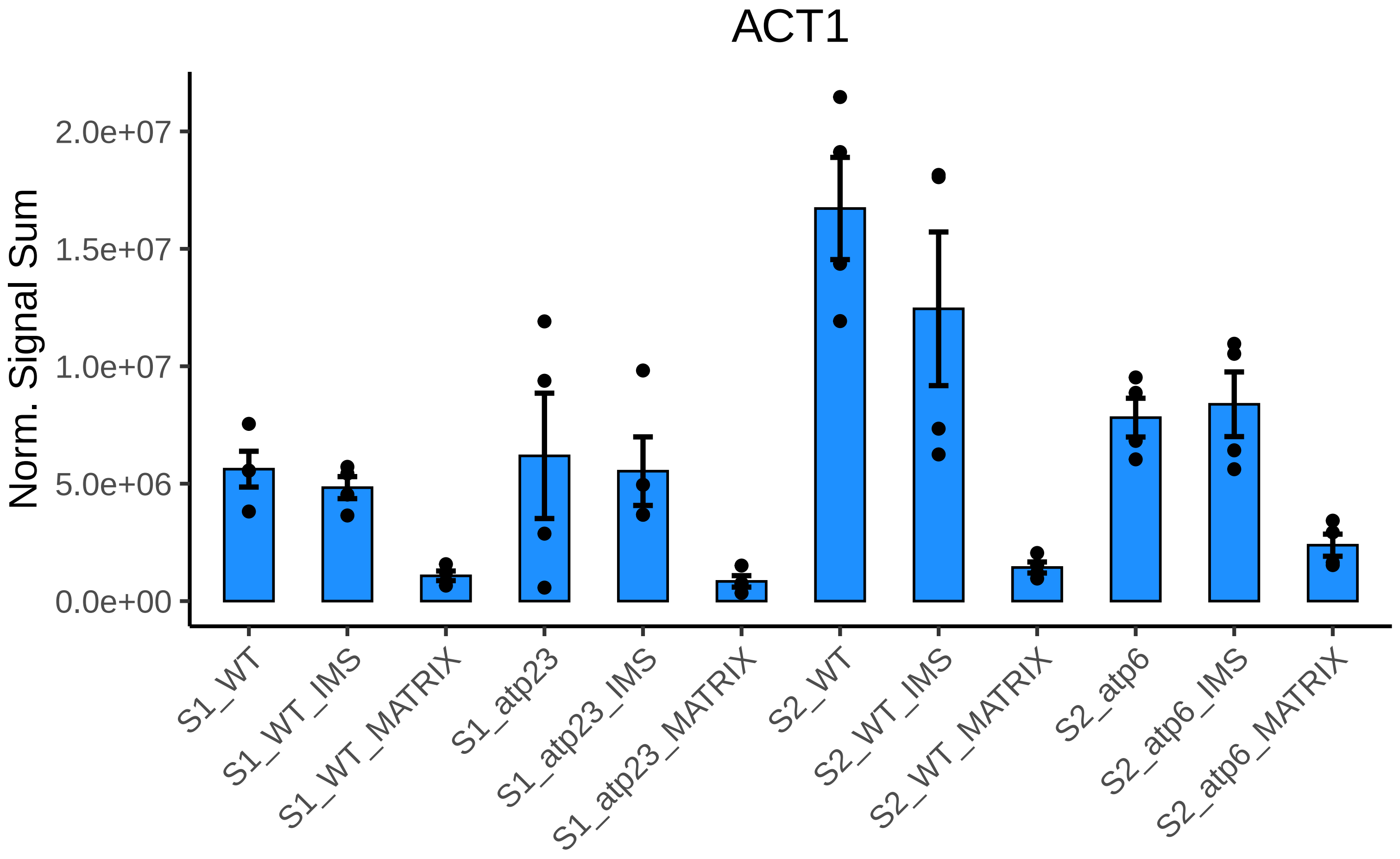
ACT1
Norm. Signal Sum
2.0e+07
1.5e+07
1.0e+07
5.0e+06
0.0e+00
S1_WT
S1_WT_IMS
S1_WT_MATRIX
S1_atp23
S1_atp23_IMS
S1_atp23_MATRIX
S2_WT
S2_WT_IMS
S2_WT_MATRIX
S2_atp6
S2_atp6_IMS
S2_atp6_MATRIX

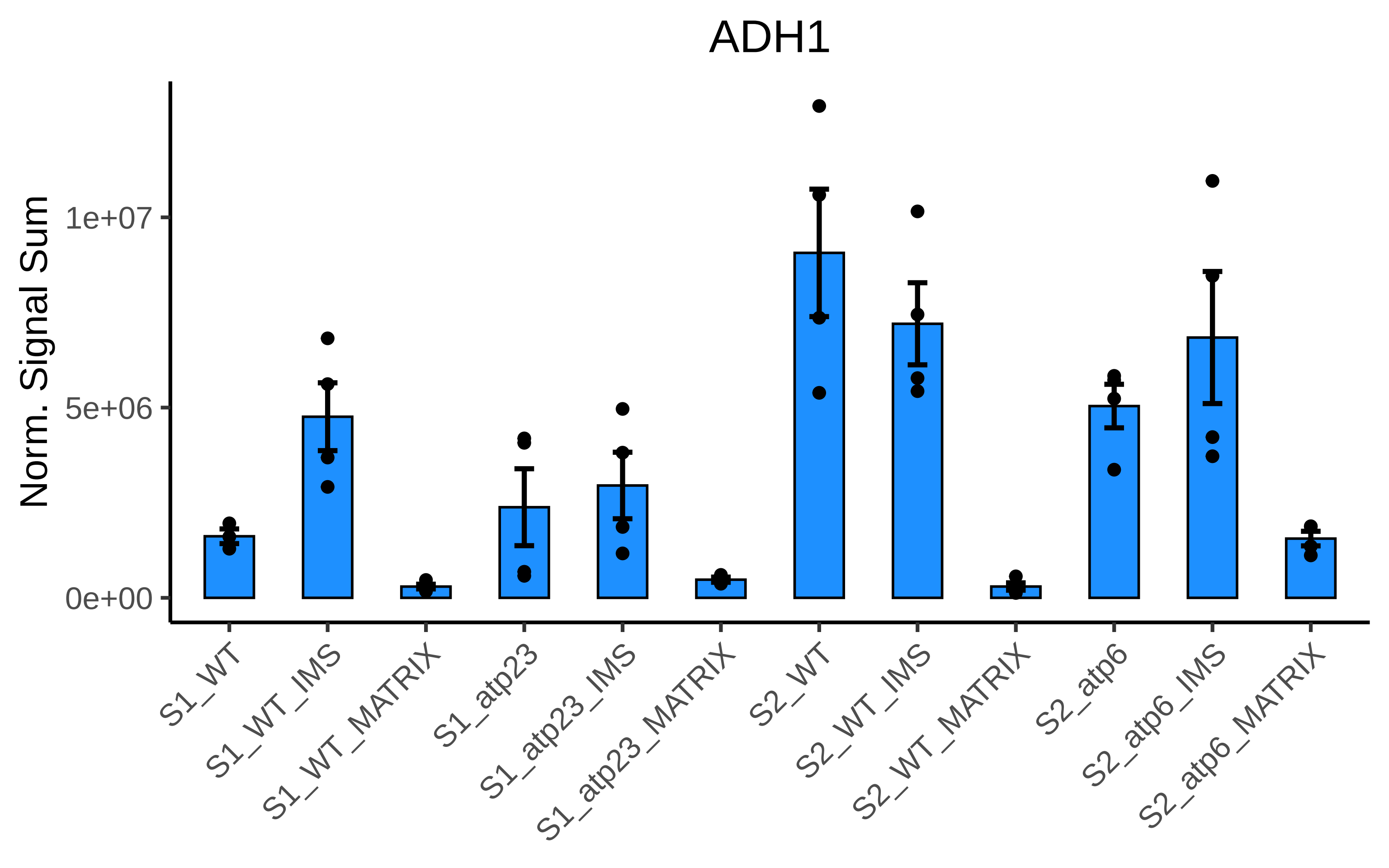
ADH1
Norm. Signal Sum
1e+07
5e+06
0e+00
S1_WT
S1_WT_IMS
S1_WT_MATRIX
S1_atp23
S1_atp23_IMS
S1_atp23_MATRIX
S2_WT
S2_WT_IMS
S2_WT_MATRIX
S2_atp6
S2_atp6_IMS
S2_atp6_MATRIX

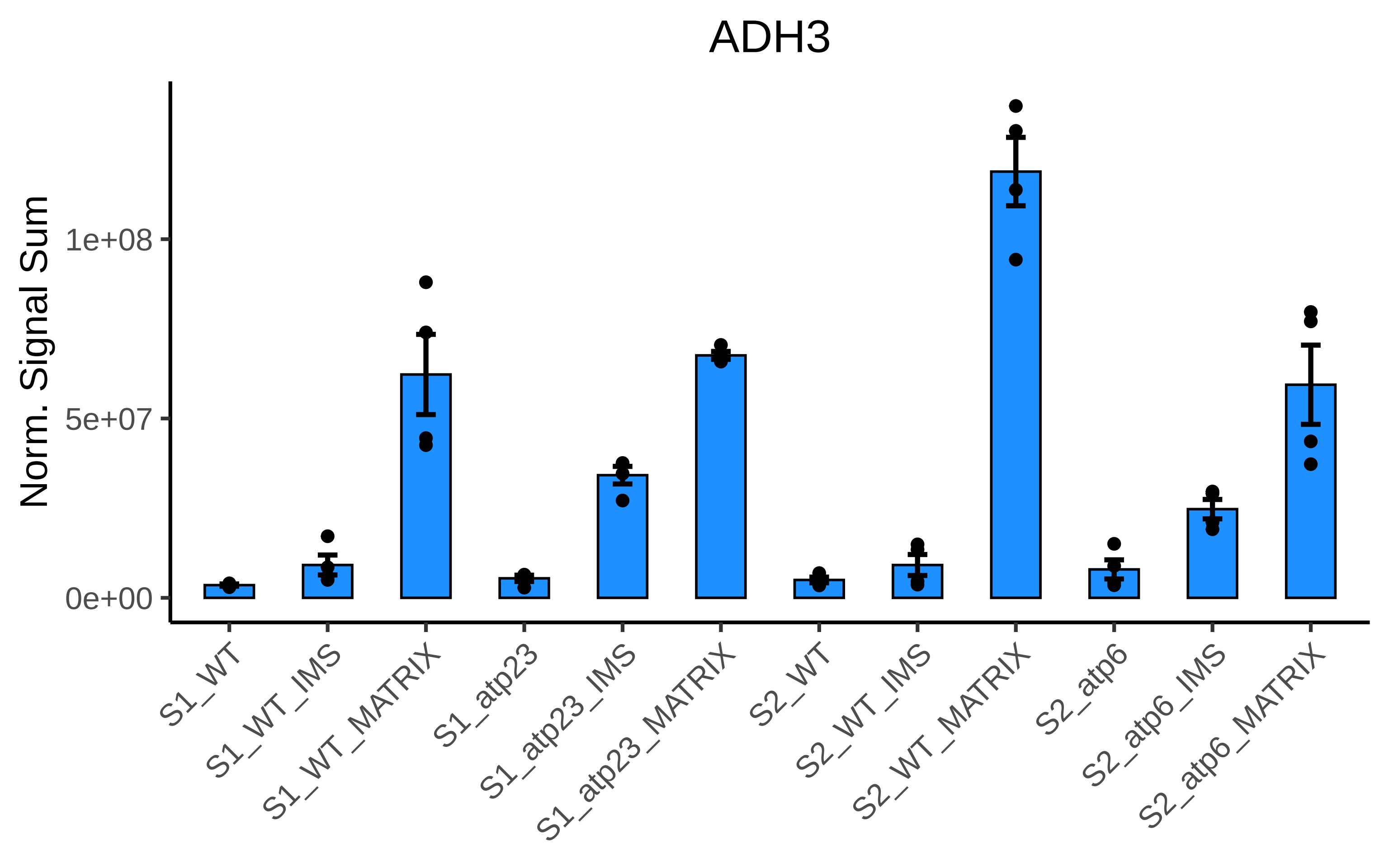

ADH3
Norm. Signal Sum
1e+08
5e+07
0e+00
S1_WT
S1_WT_IMS
S1_WT_MATRIX
S1_atp23
S1_atp23_IMS
S1_atp23_MATRIX
S2_WT
S2_WT_IMS
S2_WT_MATRIX
S2_atp6
S2_atp6_IMS
S2_atp6_MATRIX

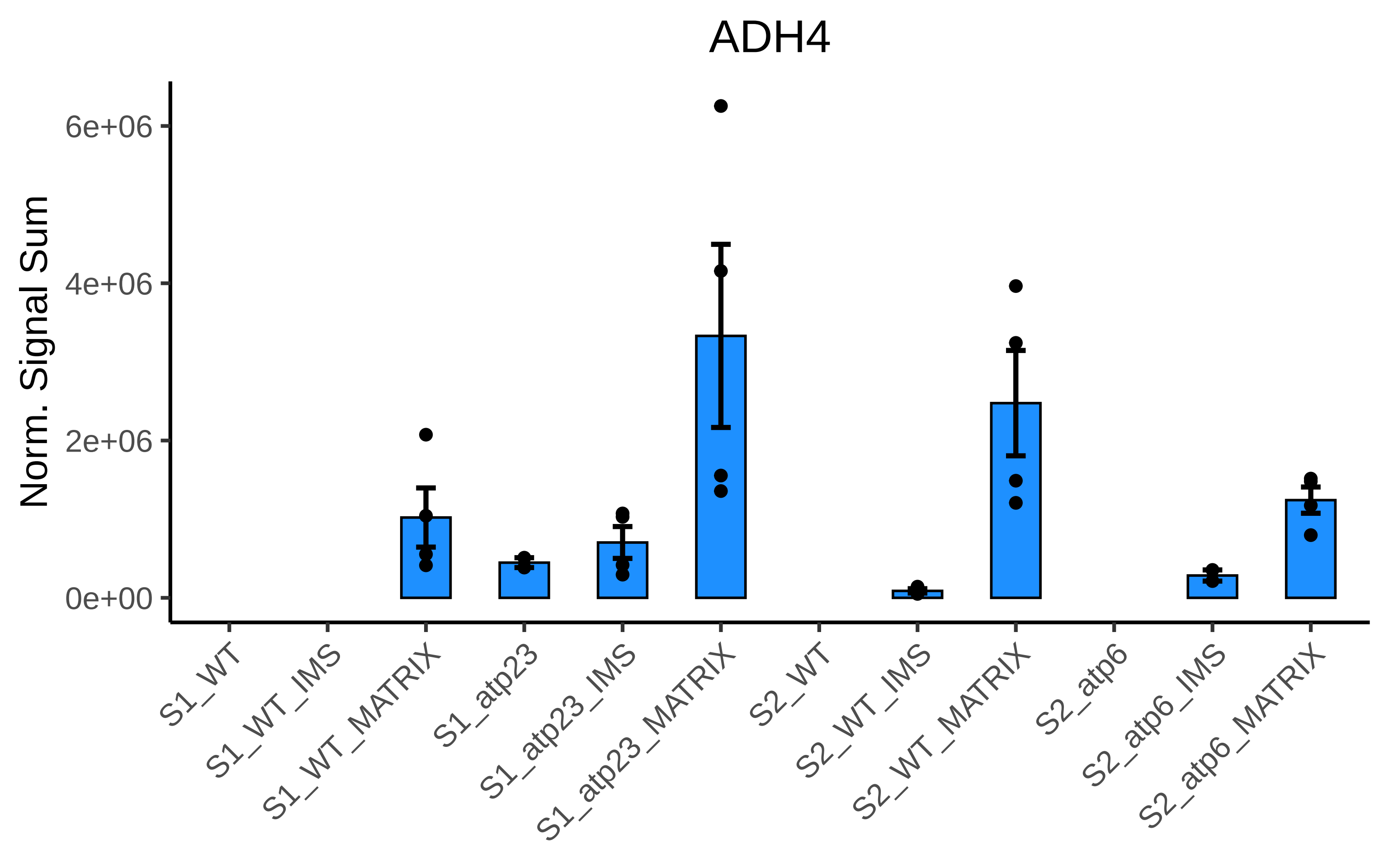
ADH4
Norm. Signal Sum
6e+06
4e+06
2e+06
0e+00
S1_WT
S1_WT_IMS
S1_WT_MATRIX
S1_atp23
S1_atp23_IMS
S1_atp23_MATRIX
S2_WT
S2_WT_IMS
S2_WT_MATRIX
S2_atp6
S2_atp6_IMS
S2_atp6_MATRIX

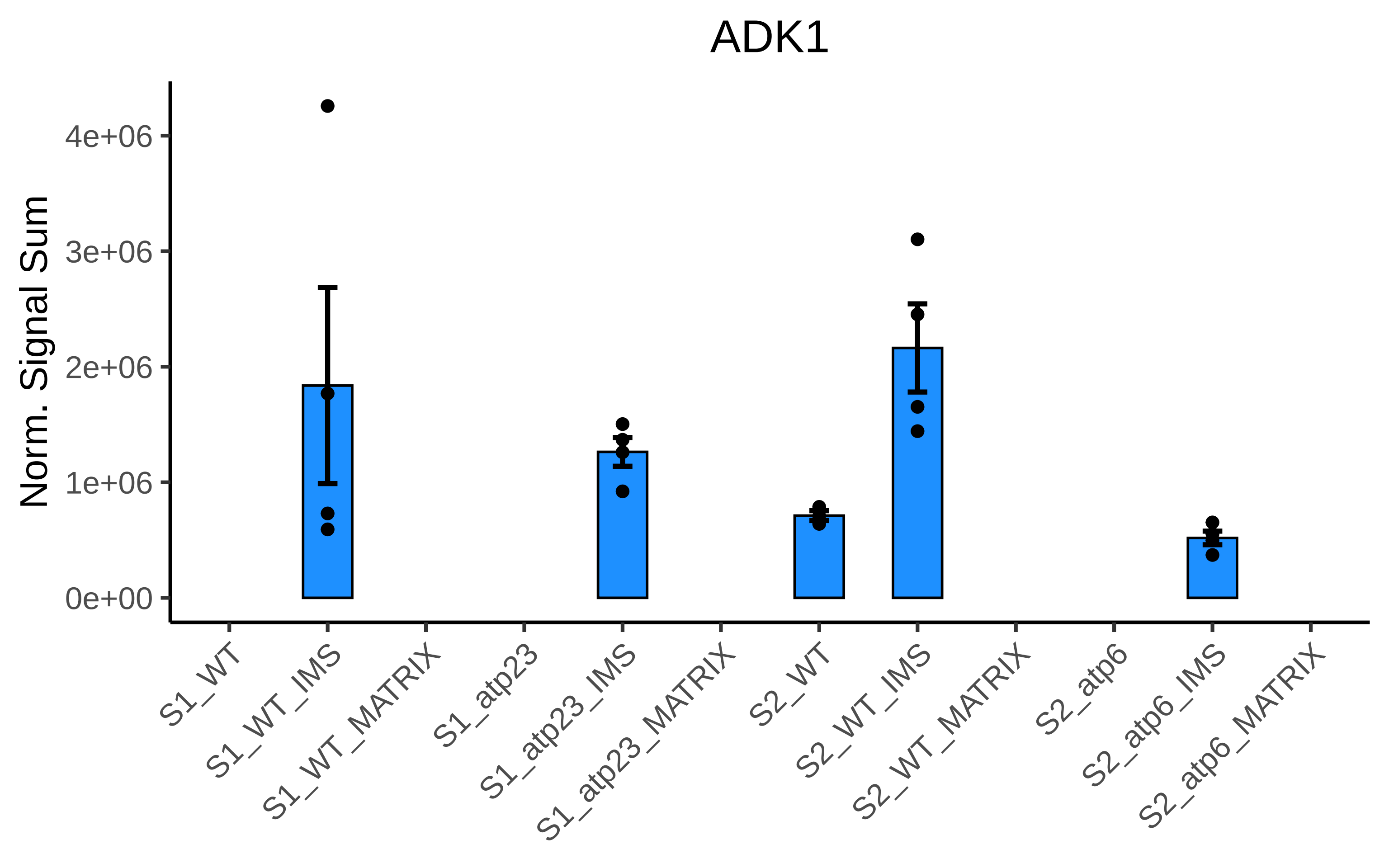

ADK1
Norm. Signal Sum
4e+06
3e+06
2e+06
1e+06
0e+00
S1_WT
S1_WT_IMS
S1_WT_MATRIX
S1_atp23
S1_atp23_IMS
S1_atp23_MATRIX
S2_WT
S2_WT_IMS
S2_WT_MATRIX
S2_atp6
S2_atp6_IMS
S2_atp6_MATRIX

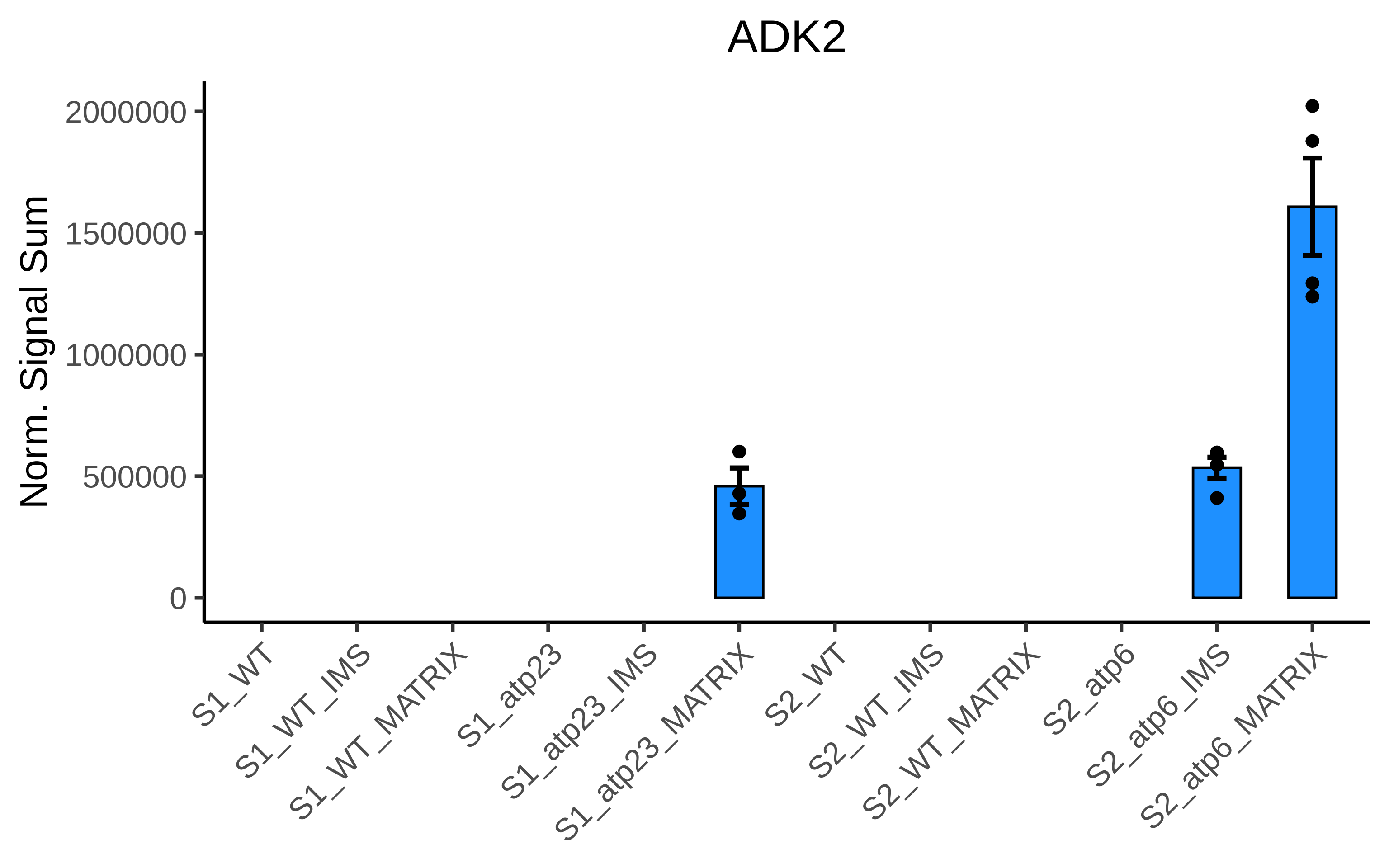

ADK2
Norm. Signal Sum
2000000
1500000
1000000
500000
0
S1_WT
S1_WT_IMS
S1_WT_MATRIX
S1_atp23
S1_atp23_IMS
S1_atp23_MATRIX
S2_WT
S2_WT_IMS
S2_WT_MATRIX
S2_atp6
S2_atp6_IMS
S2_atp6_MATRIX

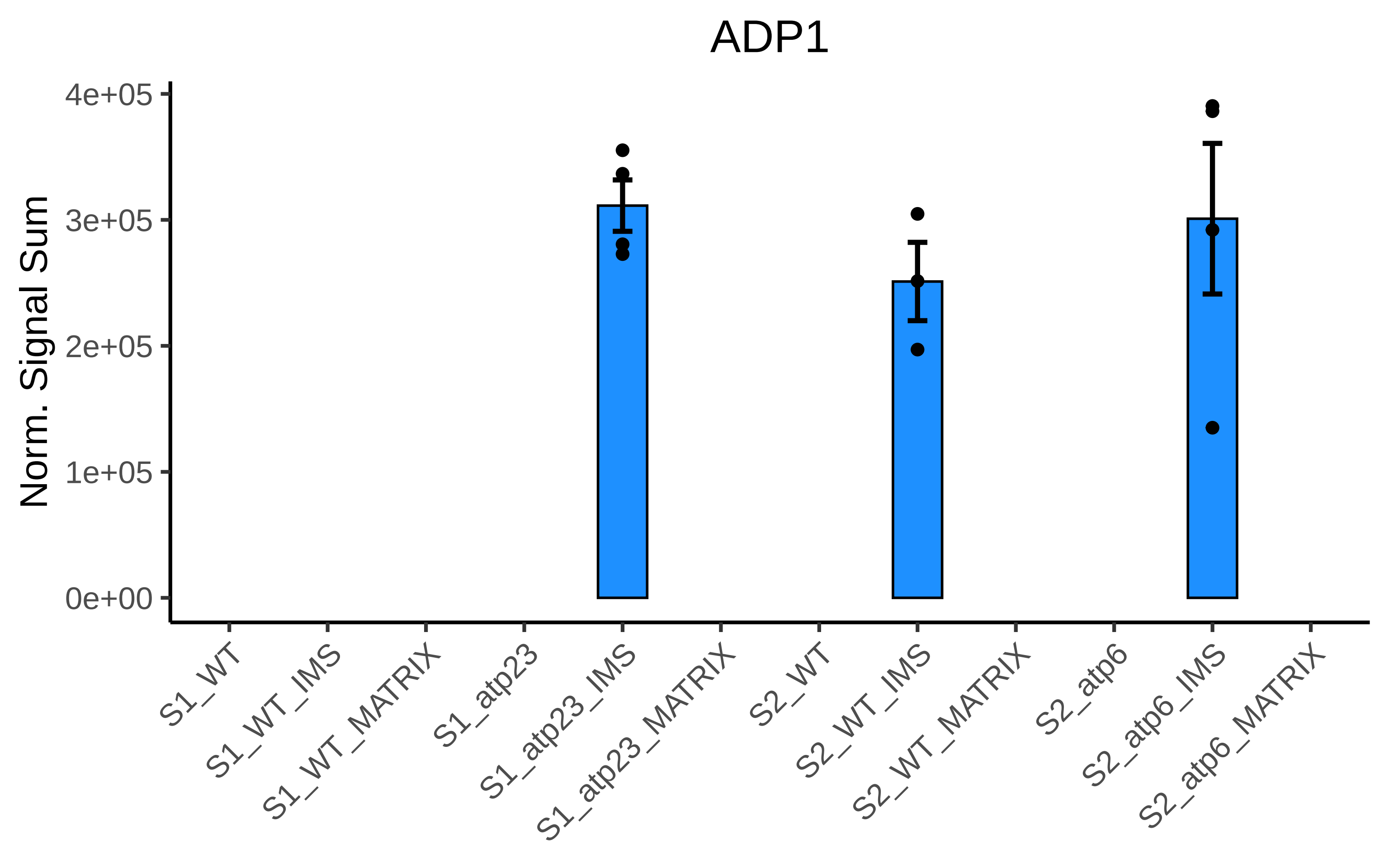

ADP1
Norm. Signal Sum
4e+05
3e+05
2e+05
1e+05
0e+00
S1_WT
S1_WT_IMS
S1_WT_MATRIX
S1_atp23
S1_atp23_IMS
S1_atp23_MATRIX
S2_WT
S2_WT_IMS
S2_WT_MATRIX
S2_atp6
S2_atp6_IMS
S2_atp6_MATRIX

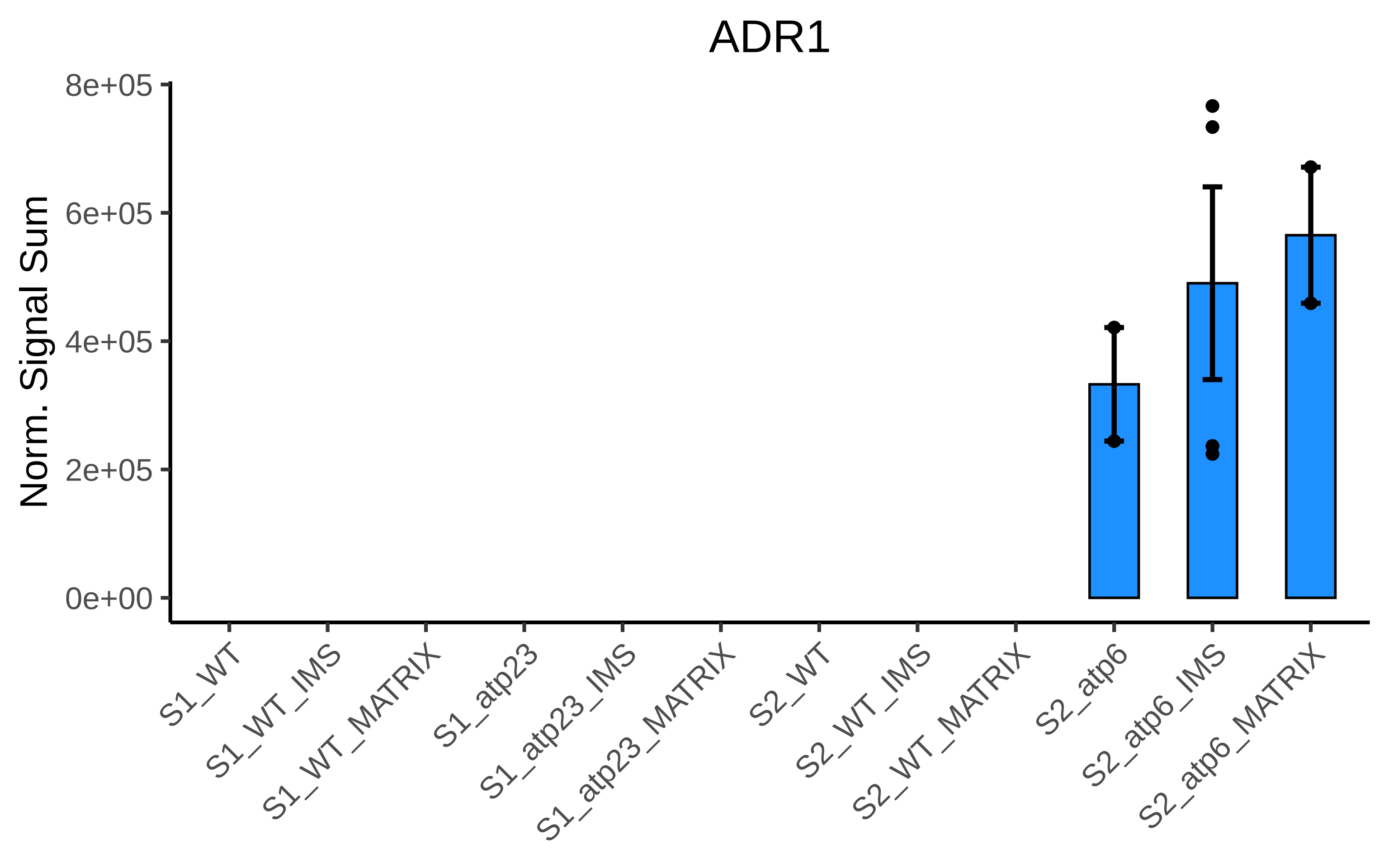
ADR1
Norm. Signal Sum
8e+05
6e+05
4e+05
2e+05
0e+00
S1_WT
S1_WT_IMS
S1_WT_MATRIX
S1_atp23
S1_atp23_IMS
S1_atp23_MATRIX
S2_WT
S2_WT_IMS
S2_WT_MATRIX
S2_atp6
S2_atp6_IMS
S2_atp6_MATRIX

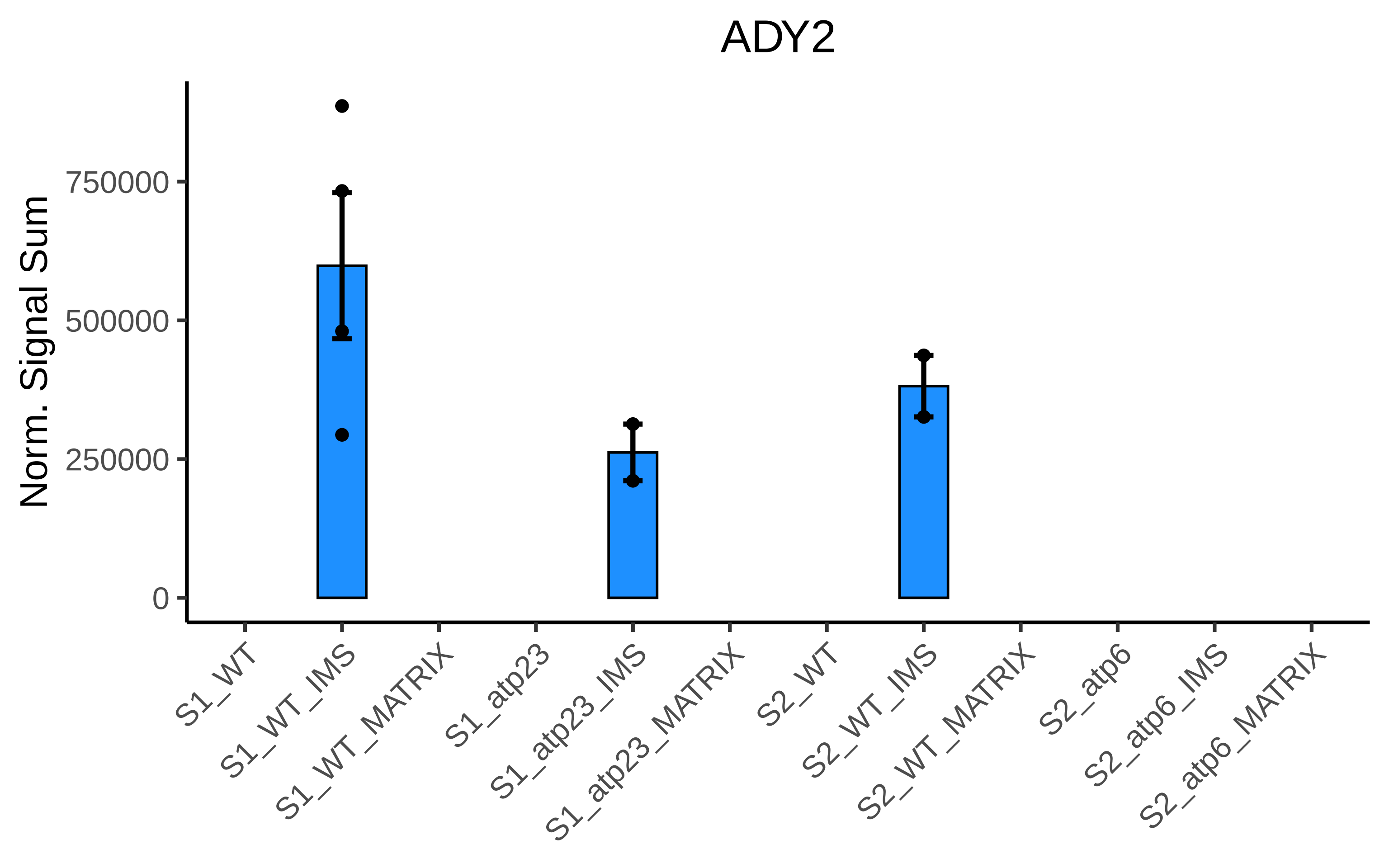

ADY2
Norm. Signal Sum
750000
500000
250000
0
S1_WT
S1_WT_IMS
S1_WT_MATRIX
S1_atp23
S1_atp23_IMS
S1_atp23_MATRIX
S2_WT
S2_WT_IMS
S2_WT_MATRIX
S2_atp6
S2_atp6_IMS
S2_atp6_MATRIX

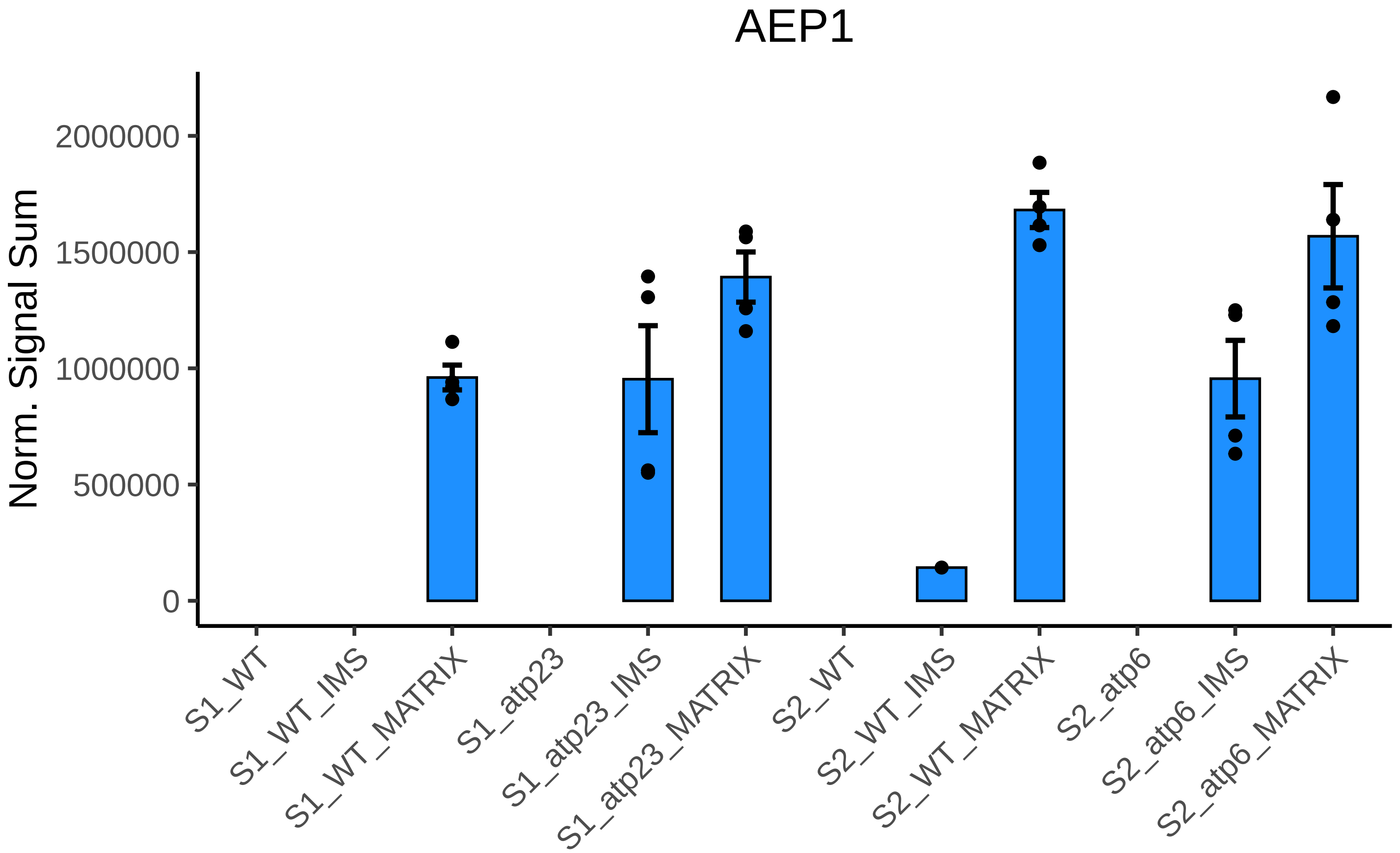
AEP1
Norm. Signal Sum
2000000
1500000
1000000
500000
0
S1_WT
S1_WT_IMS
S1_WT_MATRIX
S1_atp23
S1_atp23_IMS
S1_atp23_MATRIX
S2_WT
S2_WT_IMS
S2_WT_MATRIX
S2_atp6
S2_atp6_IMS
S2_atp6_MATRIX

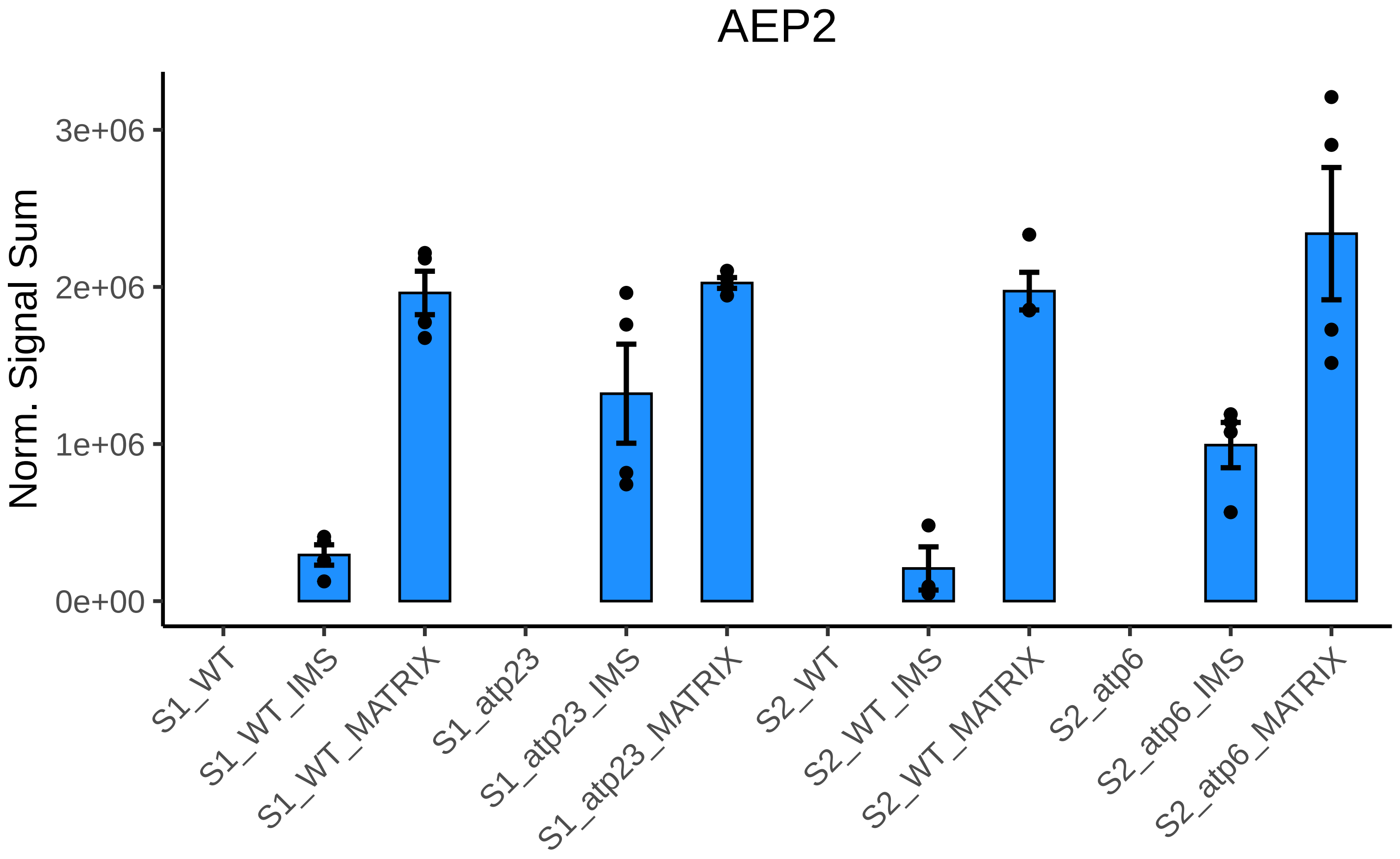

AEP2
Norm. Signal Sum
3e+06
2e+06
1e+06
0e+00
S1_WT
S1_WT_IMS
S1_WT_MATRIX
S1_atp23
S1_atp23_IMS
S1_atp23_MATRIX
S2_WT
S2_WT_IMS
S2_WT_MATRIX
S2_atp6
S2_atp6_IMS
S2_atp6_MATRIX

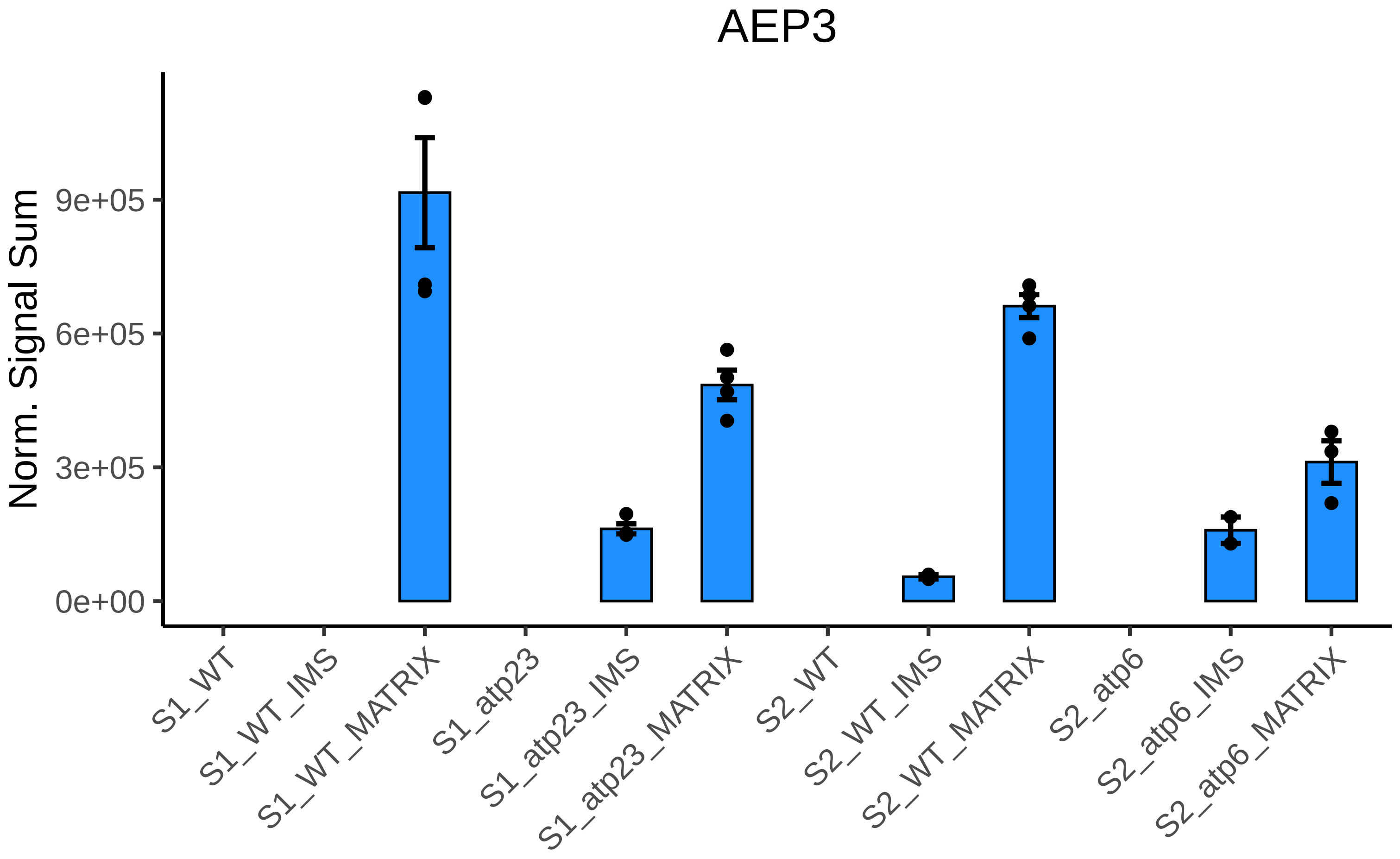
AEP3
Norm. Signal Sum
9e+05
6e+05
3e+05
0e+00
S1_WT
S1_WT_IMS
S1_WT_MATRIX
S1_atp23
S1_atp23_IMS
S1_atp23_MATRIX
S2_WT
S2_WT_IMS
S2_WT_MATRIX
S2_atp6
S2_atp6_IMS
S2_atp6_MATRIX

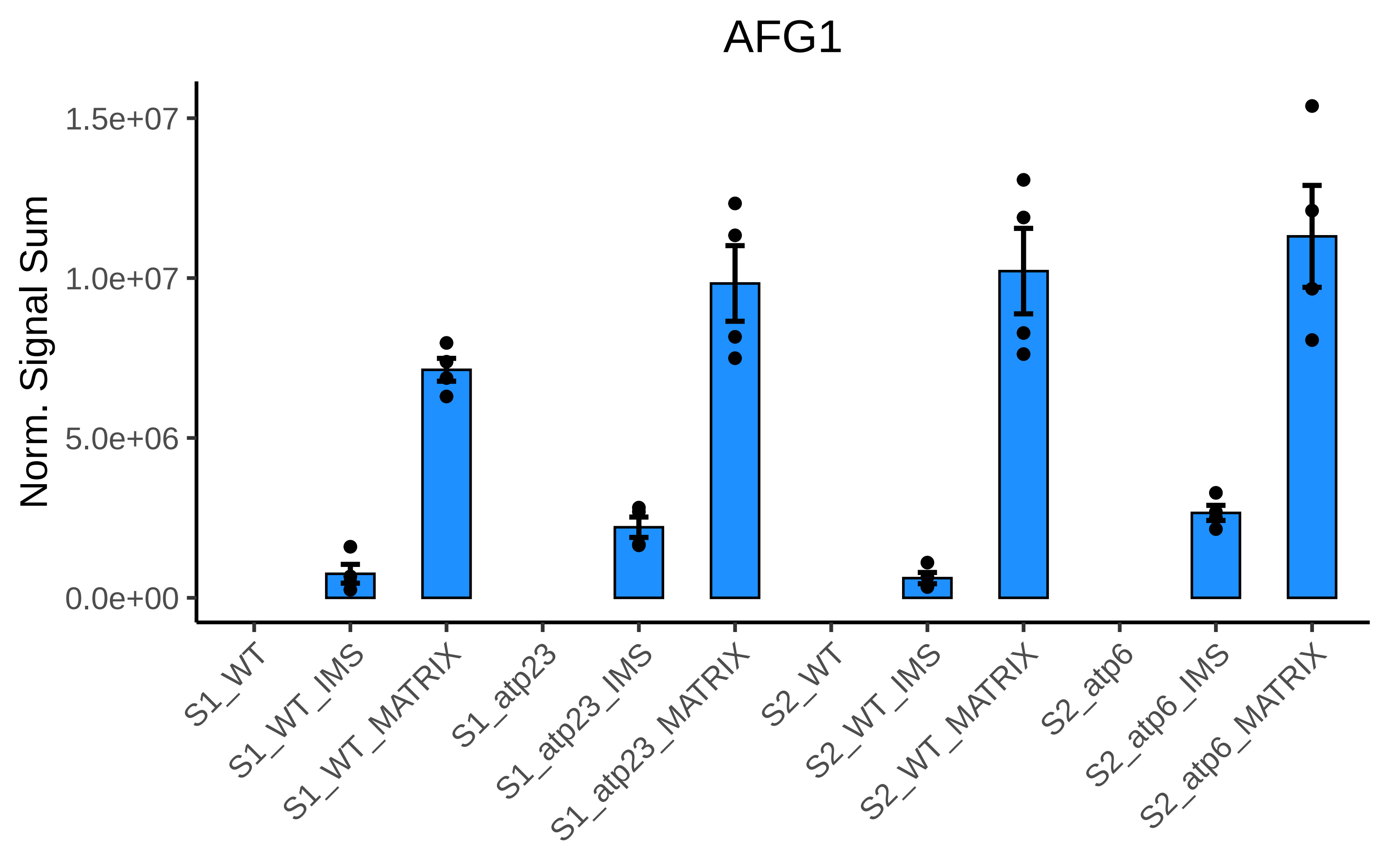
AFG1
Norm. Signal Sum
1.5e+07
1.0e+07
5.0e+06
0.0e+00
S1_WT
S1_WT_IMS
S1_WT_MATRIX
S1_atp23
S1_atp23_IMS
S1_atp23_MATRIX
S2_WT
S2_WT_IMS
S2_WT_MATRIX
S2_atp6
S2_atp6_IMS
S2_atp6_MATRIX

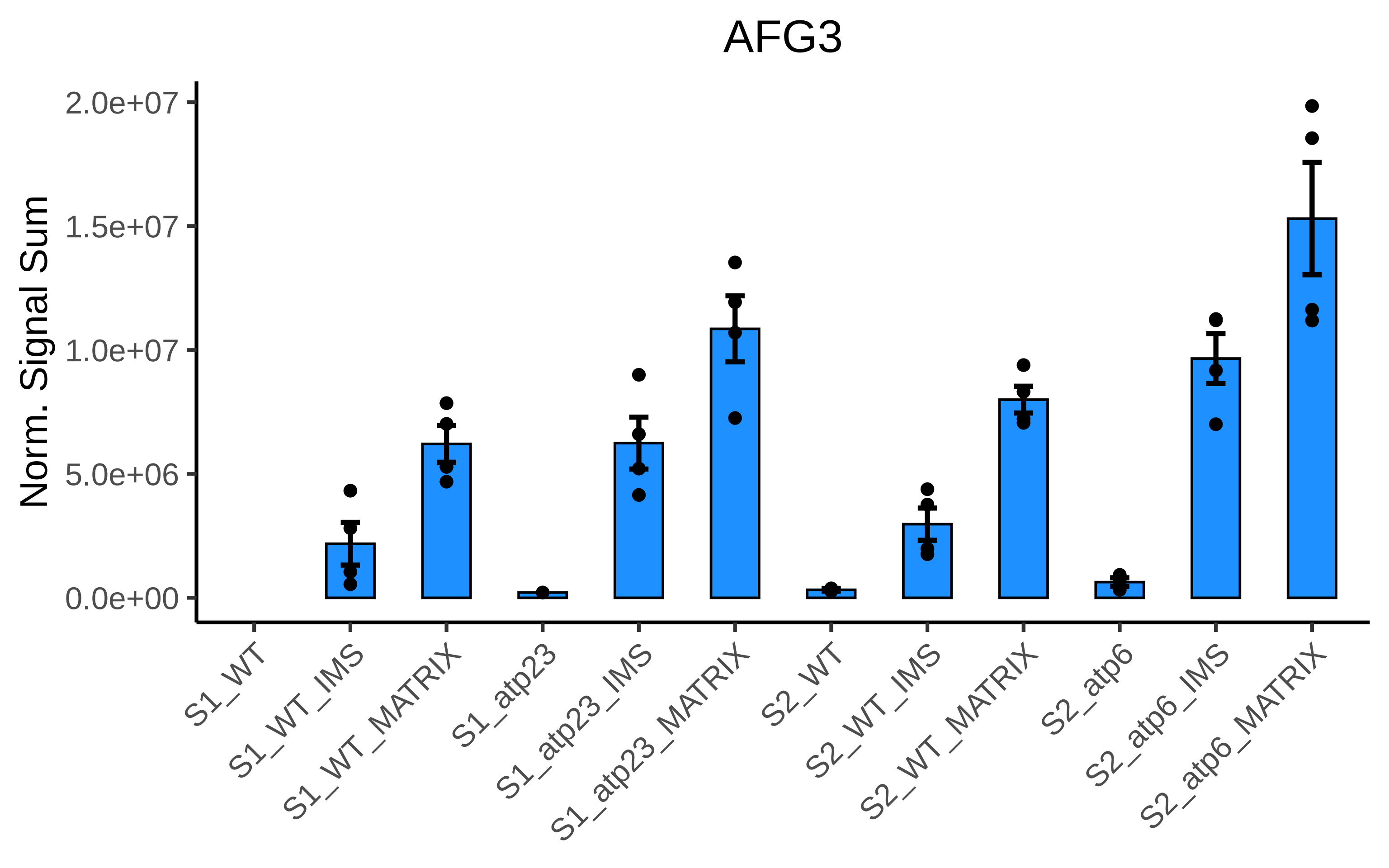
AFG3
Norm. Signal Sum
2.0e+07
1.5e+07
1.0e+07
5.0e+06
0.0e+00
S1_WT
S1_WT_IMS
S1_WT_MATRIX
S1_atp23
S1_atp23_IMS
S1_atp23_MATRIX
S2_WT
S2_WT_IMS
S2_WT_MATRIX
S2_atp6
S2_atp6_IMS
S2_atp6_MATRIX

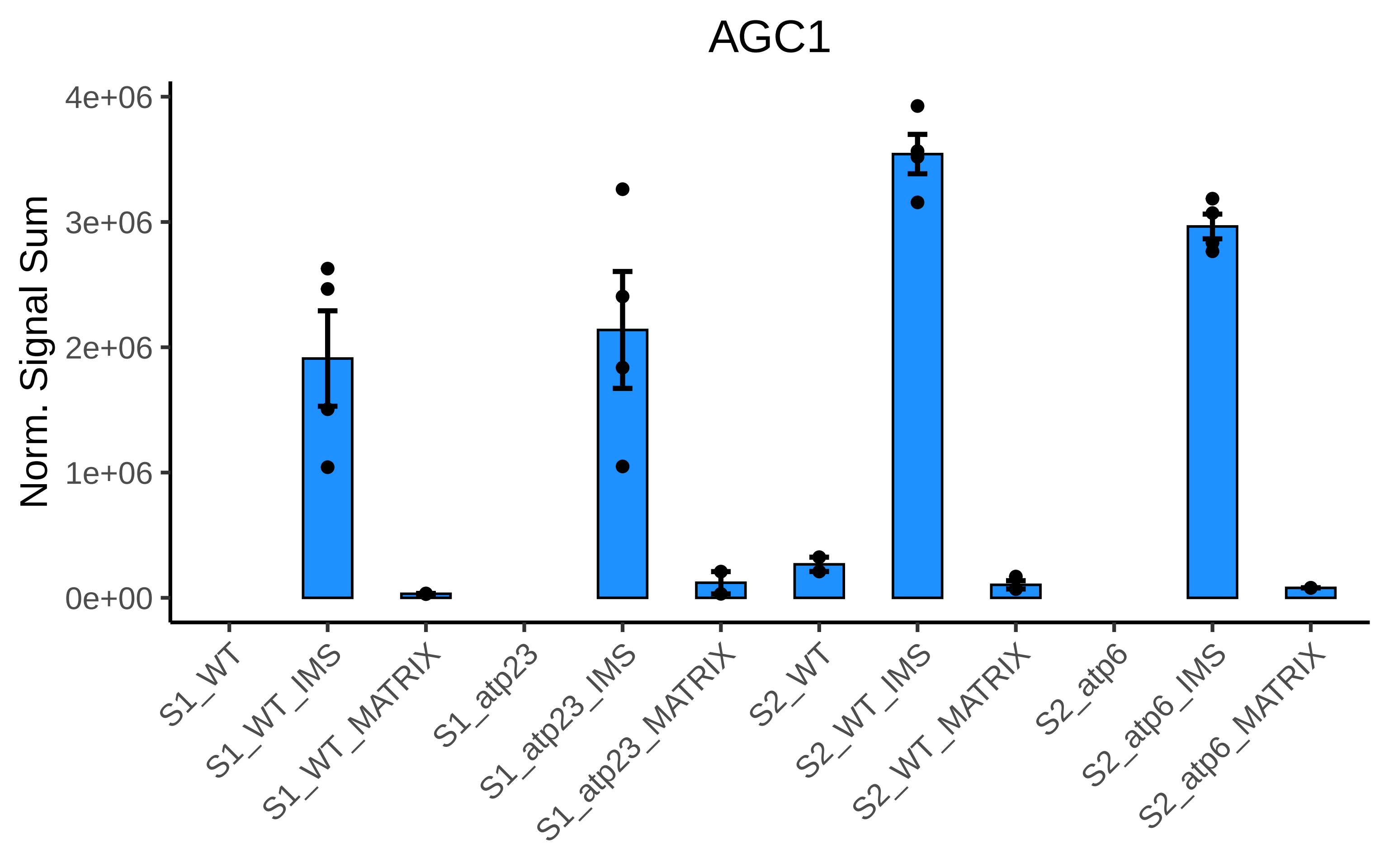

AGC1
Norm. Signal Sum
4e+06
3e+06
2e+06
1e+06
0e+00
S1_WT
S1_WT_IMS
S1_WT_MATRIX
S1_atp23
S1_atp23_IMS
S1_atp23_MATRIX
S2_WT
S2_WT_IMS
S2_WT_MATRIX
S2_atp6
S2_atp6_IMS
S2_atp6_MATRIX

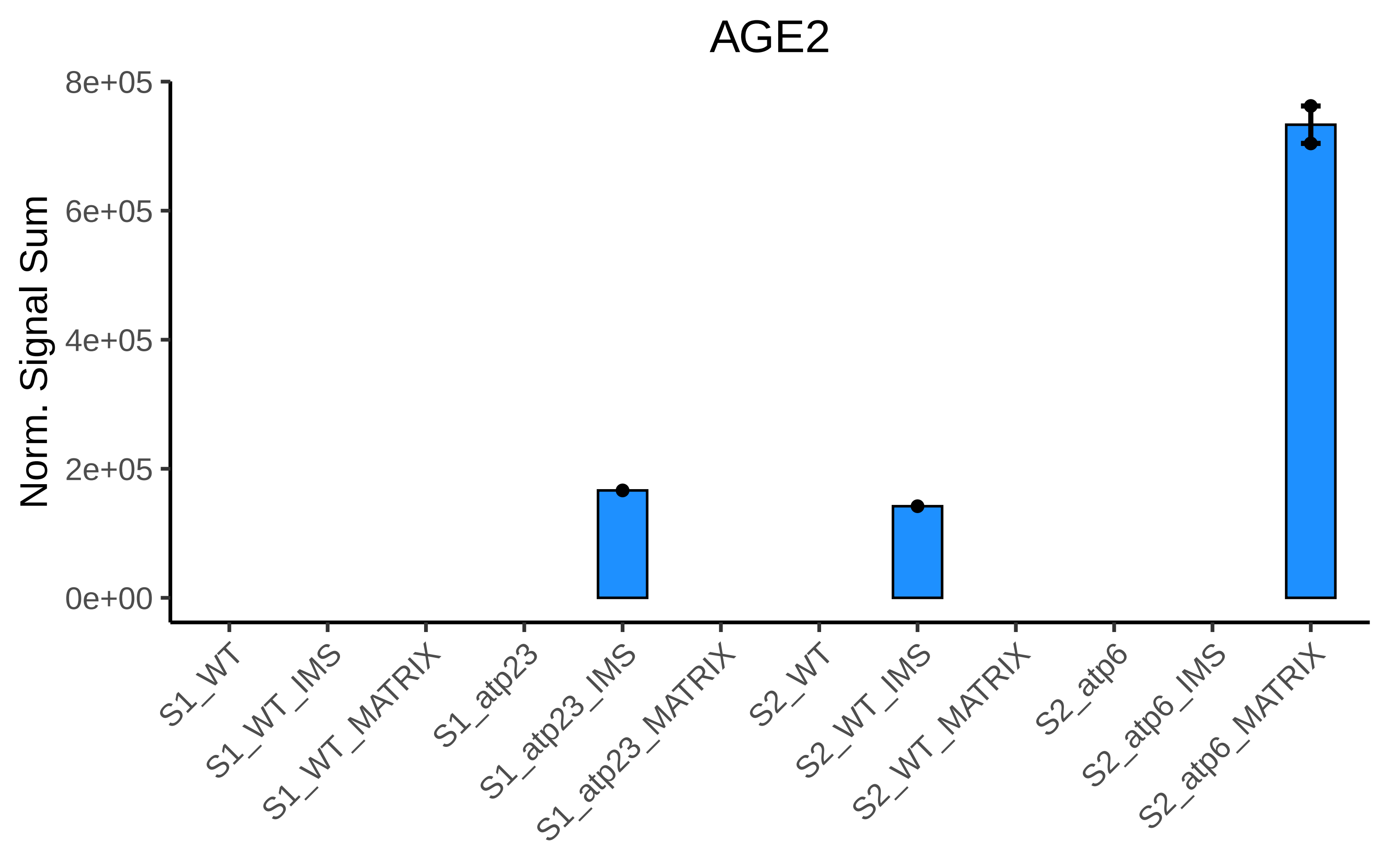

AGE2
Norm. Signal Sum
8e+05
6e+05
4e+05
2e+05
0e+00
S1_WT
S1_WT_IMS
S1_WT_MATRIX
S1_atp23
S1_atp23_IMS
S1_atp23_MATRIX
S2_WT
S2_WT_IMS
S2_WT_MATRIX
S2_atp6
S2_atp6_IMS
S2_atp6_MATRIX

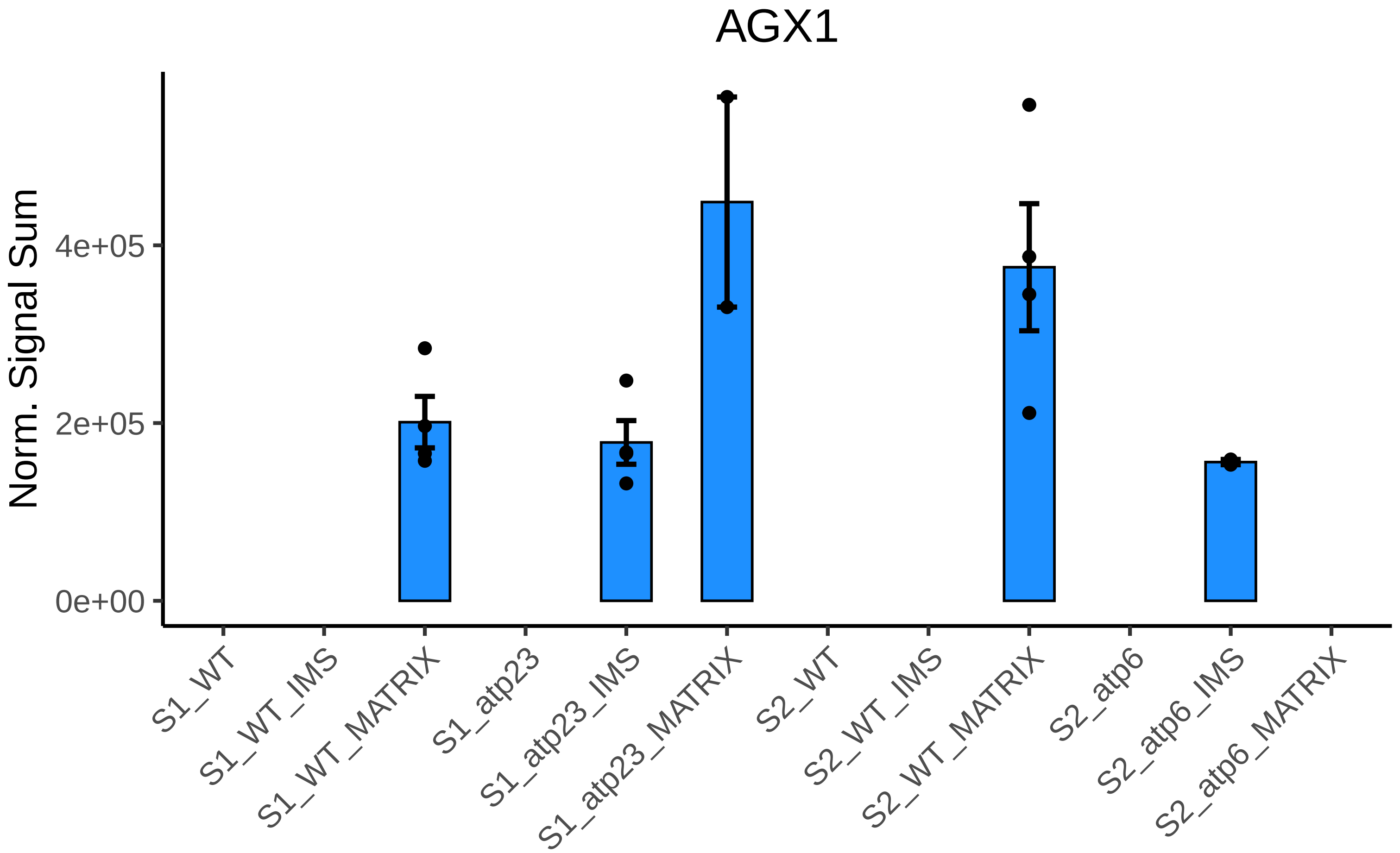

AGX1
Norm. Signal Sum
4e+05
2e+05
0e+00
S1_WT
S1_WT_IMS
S1_WT_MATRIX
S1_atp23
S1_atp23_IMS
S1_atp23_MATRIX
S2_WT
S2_WT_IMS
S2_WT_MATRIX
S2_atp6
S2_atp6_IMS
S2_atp6_MATRIX

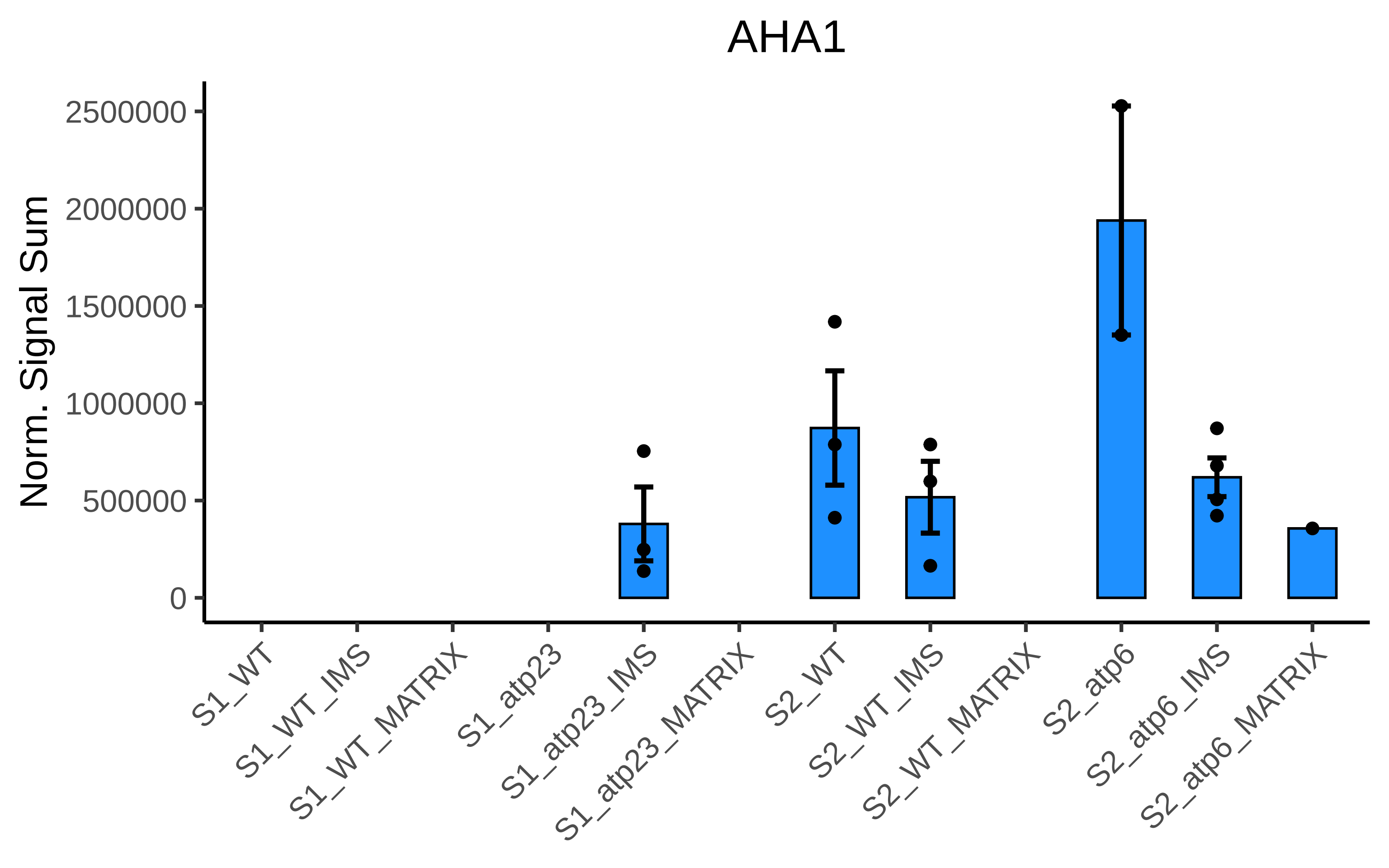
AHA1
Norm. Signal Sum
2500000
2000000
1500000
1000000
500000
0
S1_WT
S1_WT_IMS
S1_WT_MATRIX
S1_atp23
S1_atp23_IMS
S1_atp23_MATRIX
S2_WT
S2_WT_IMS
S2_WT_MATRIX
S2_atp6
S2_atp6_IMS
S2_atp6_MATRIX

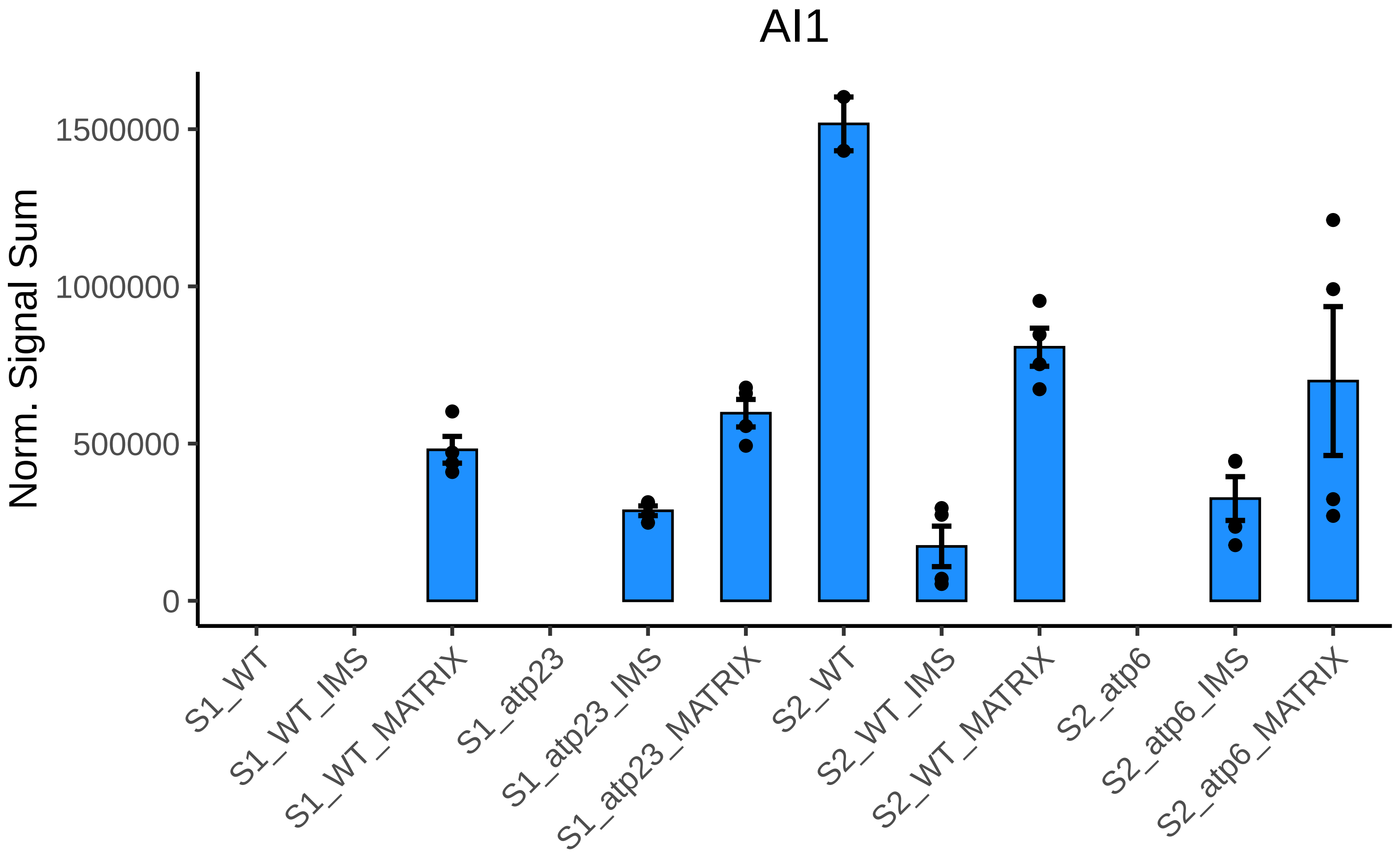
AI1
Norm. Signal Sum
1500000
1000000
500000
0
S1_WT
S1_WT_IMS
S1_WT_MATRIX
S1_atp23
S1_atp23_IMS
S1_atp23_MATRIX
S2_WT
S2_WT_IMS
S2_WT_MATRIX
S2_atp6
S2_atp6_IMS
S2_atp6_MATRIX

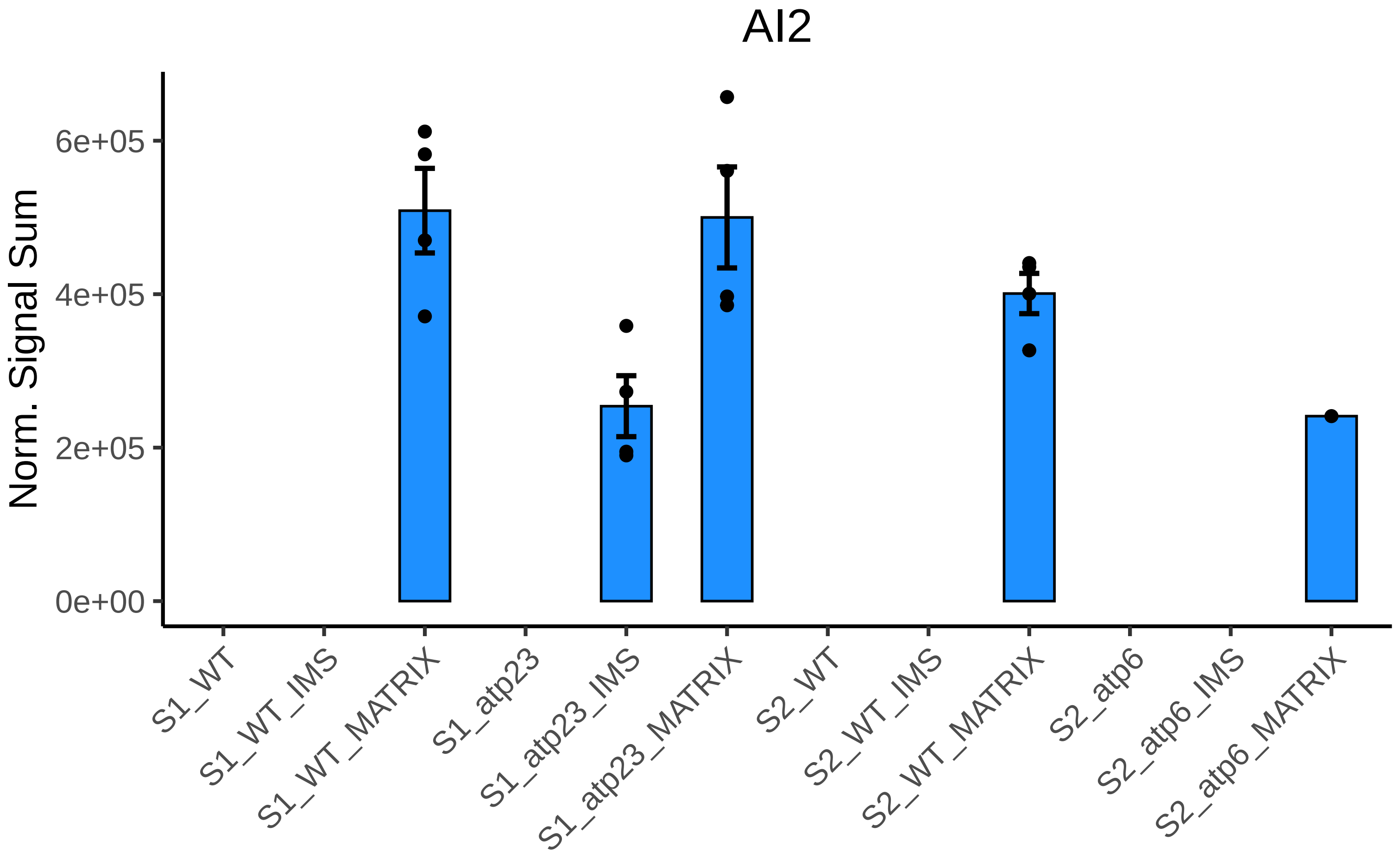

AI2
Norm. Signal Sum
6e+05
4e+05
2e+05
0e+00
S1_WT
S1_WT_IMS
S1_WT_MATRIX
S1_atp23
S1_atp23_IMS
S1_atp23_MATRIX
S2_WT
S2_WT_IMS
S2_WT_MATRIX
S2_atp6
S2_atp6_IMS
S2_atp6_MATRIX

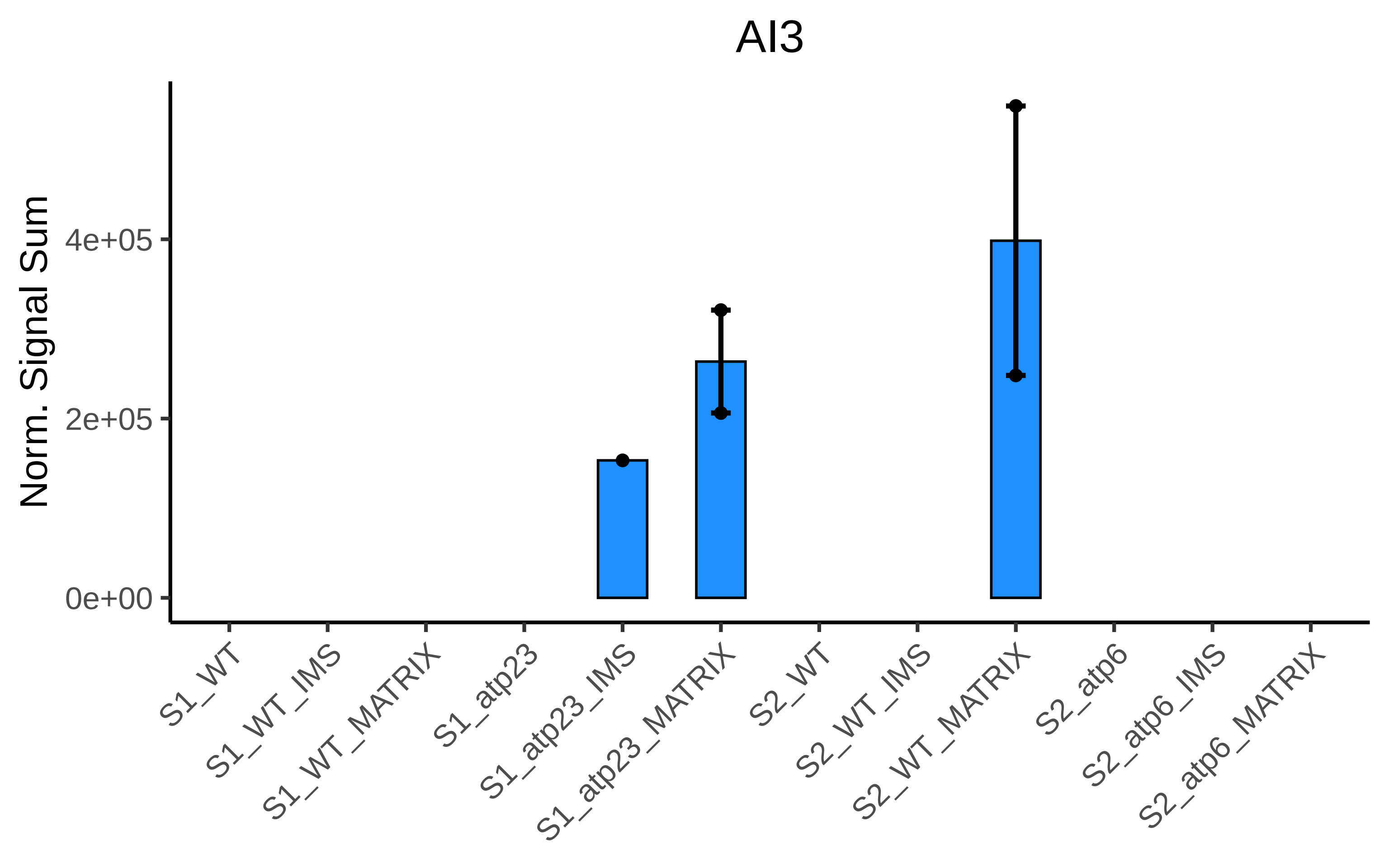
AI3
Norm. Signal Sum
0e+00
2e+05
4e+05
S1_WT
S1_WT_IMS
S1_WT_MATRIX
S1_atp23
S1_atp23_IMS
S1_atp23_MATRIX
S2_WT
S2_WT_IMS
S2_WT_MATRIX
S2_atp6
S2_atp6_IMS
S2_atp6_MATRIX

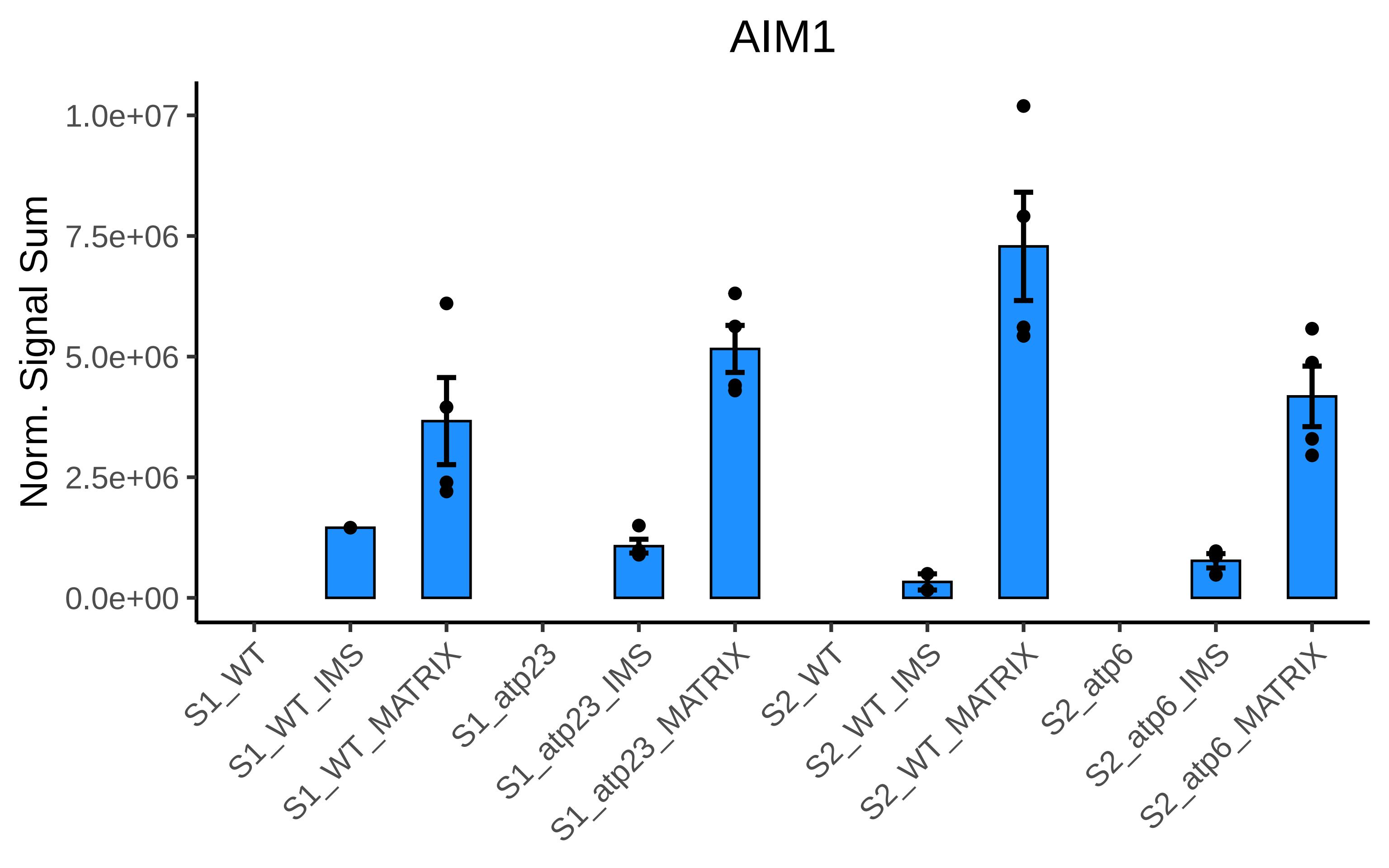
AIM1
Norm. Signal Sum
1.0e+07
7.5e+06
5.0e+06
2.5e+06
0.0e+00
S1_WT
S1_WT_IMS
S1_WT_MATRIX
S1_atp23
S1_atp23_IMS
S1_atp23_MATRIX
S2_WT
S2_WT_IMS
S2_WT_MATRIX
S2_atp6
S2_atp6_IMS
S2_atp6_MATRIX

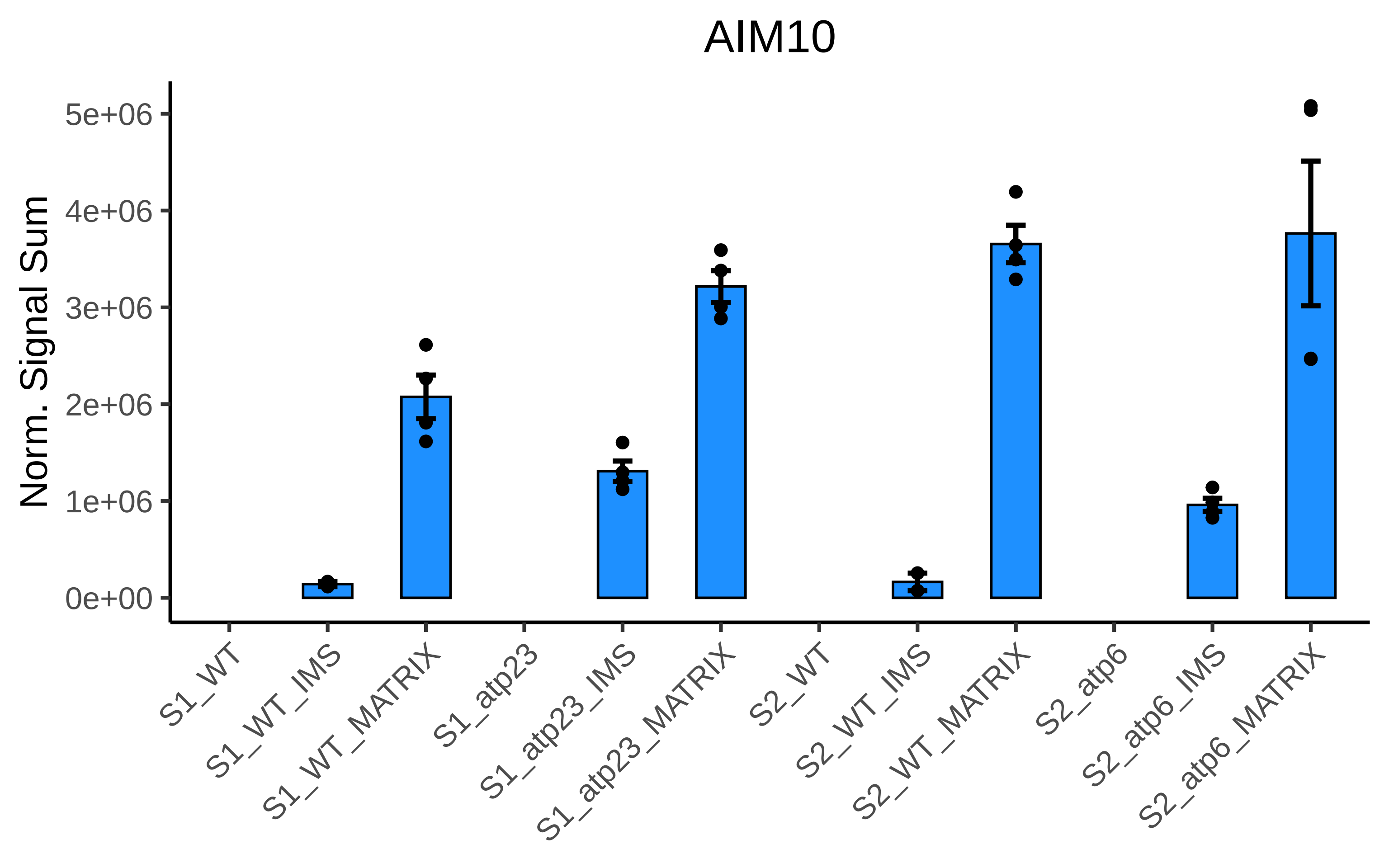
AIM10
Norm. Signal Sum
5e+06
4e+06
3e+06
2e+06
1e+06
0e+00
S1_WT
S1_WT_IMS
S1_WT_MATRIX
S1_atp23
S1_atp23_IMS
S1_atp23_MATRIX
S2_WT
S2_WT_IMS
S2_WT_MATRIX
S2_atp6
S2_atp6_IMS
S2_atp6_MATRIX

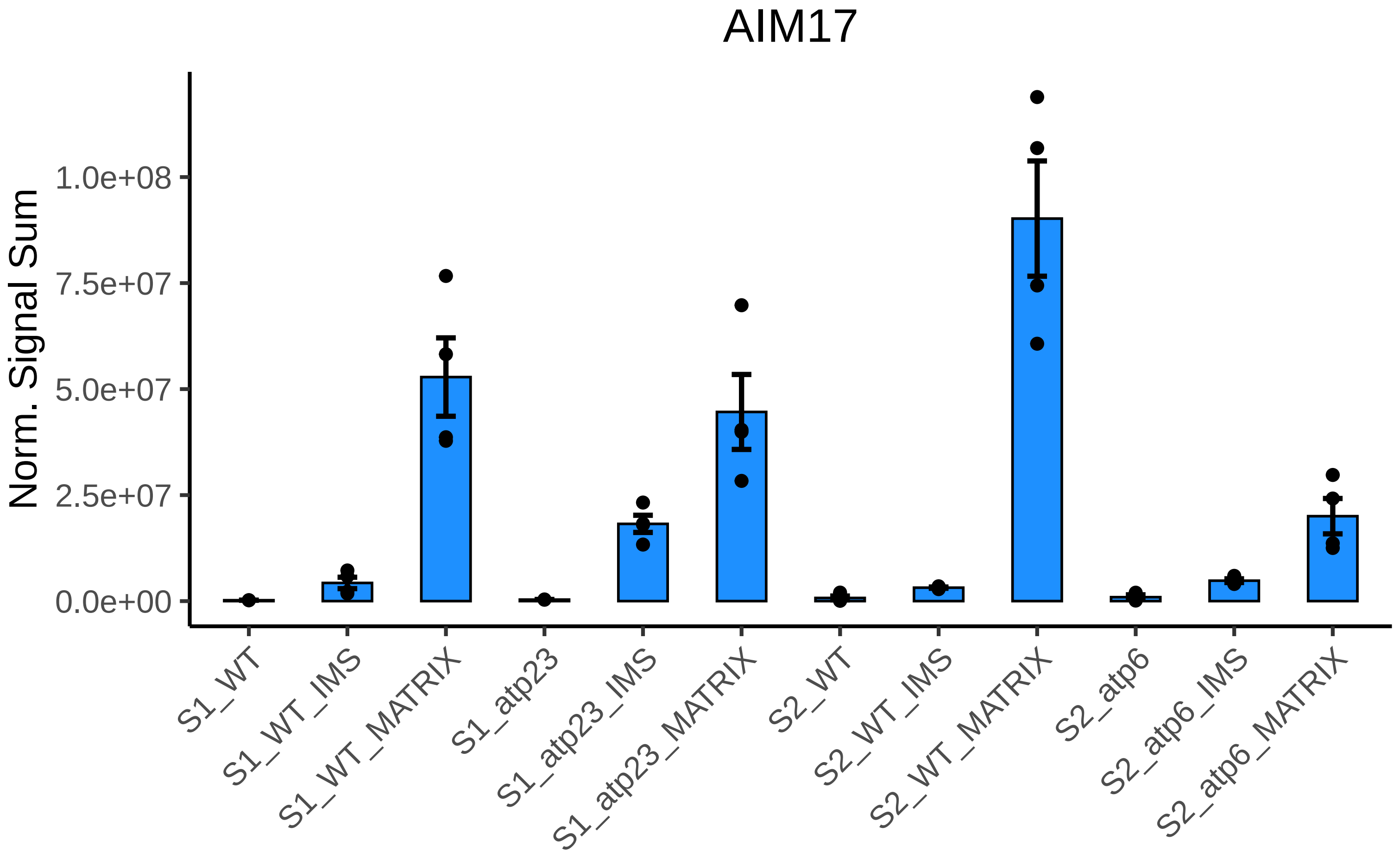

AIM17
Norm. Signal Sum
1.0e+08
7.5e+07
5.0e+07
2.5e+07
0.0e+00
S1_WT
S1_WT_IMS
S1_WT_MATRIX
S1_atp23
S1_atp23_IMS
S1_atp23_MATRIX
S2_WT
S2_WT_IMS
S2_WT_MATRIX
S2_atp6
S2_atp6_IMS
S2_atp6_MATRIX

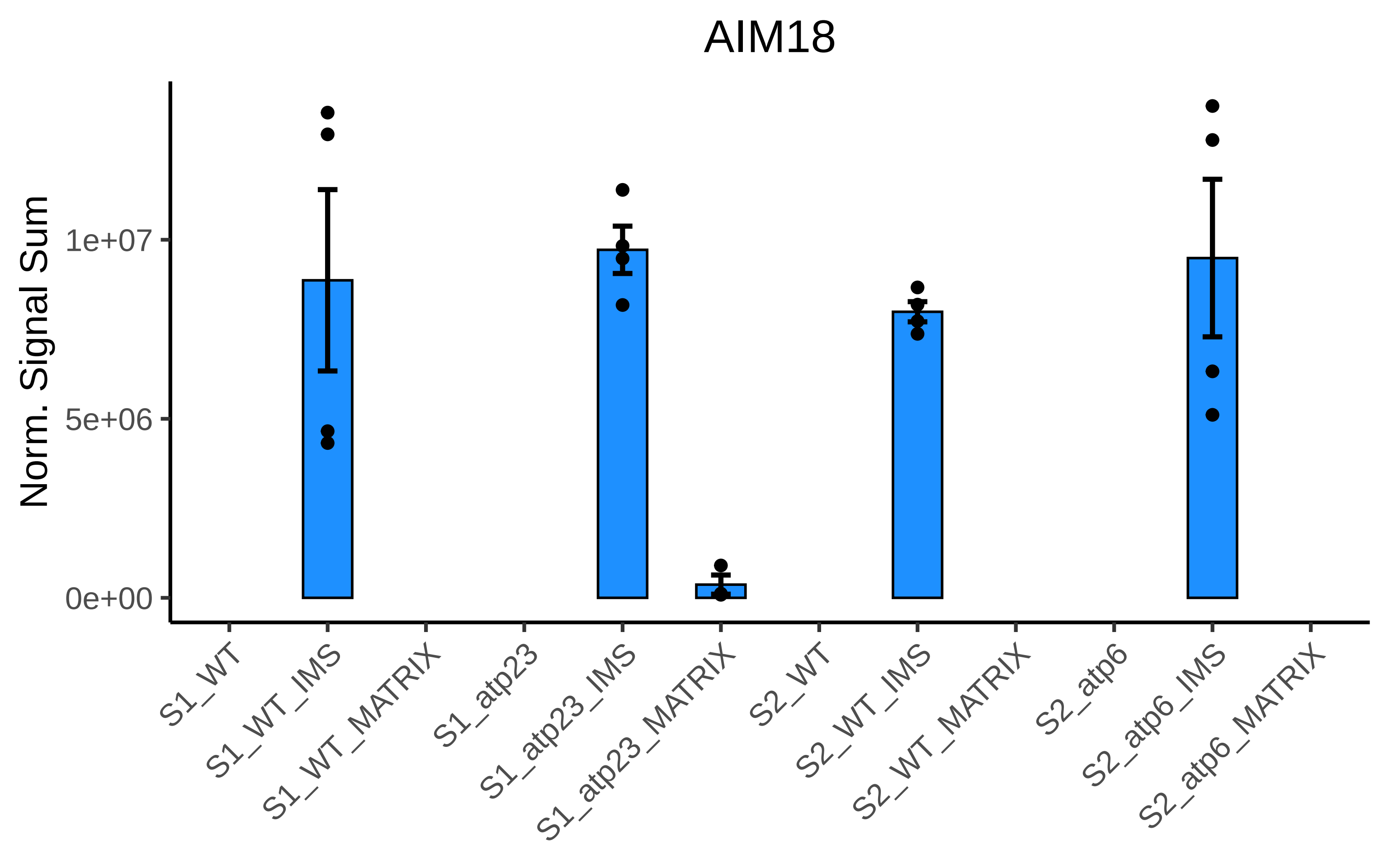
AIM18
Norm. Signal Sum
1e+07
5e+06
0e+00
S1_WT
S1_WT_IMS
S1_WT_MATRIX
S1_atp23
S1_atp23_IMS
S1_atp23_MATRIX
S2_WT
S2_WT_IMS
S2_WT_MATRIX
S2_atp6
S2_atp6_IMS
S2_atp6_MATRIX

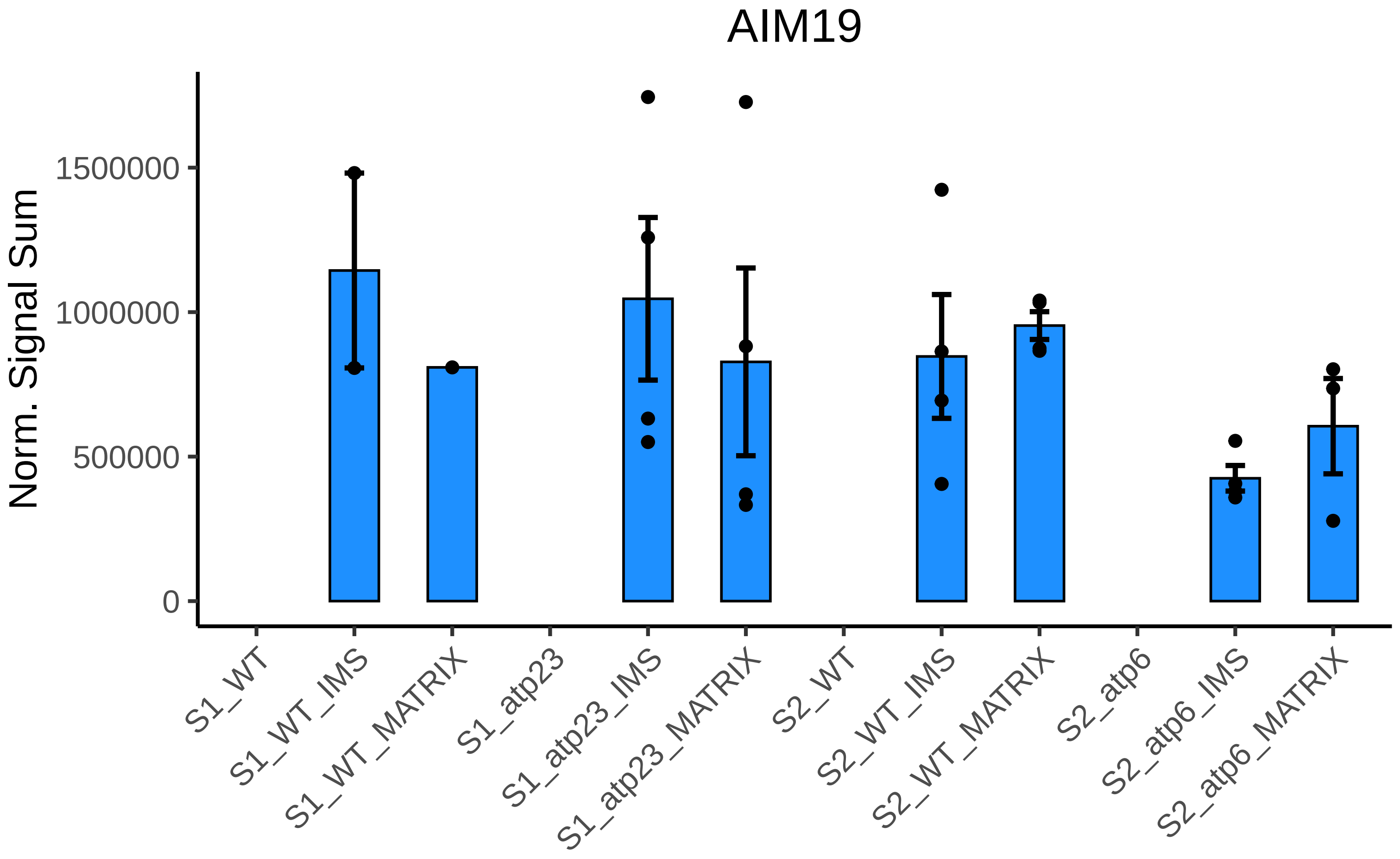
AIM19
Norm. Signal Sum
1500000
1000000
500000
0
S1_WT
S1_WT_IMS
S1_WT_MATRIX
S1_atp23
S1_atp23_IMS
S1_atp23_MATRIX
S2_WT
S2_WT_IMS
S2_WT_MATRIX
S2_atp6
S2_atp6_IMS
S2_atp6_MATRIX

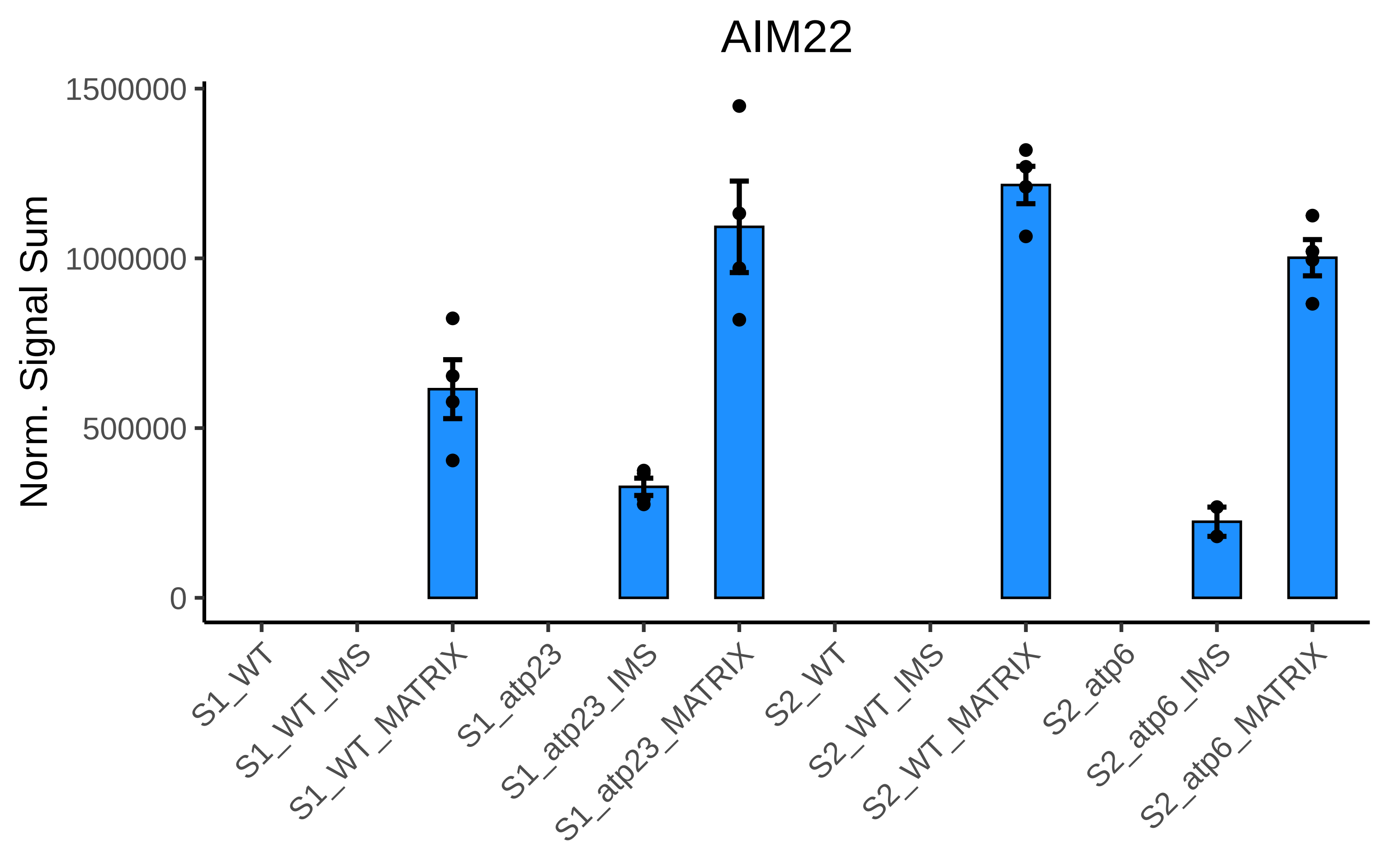

AIM22
Norm. Signal Sum
1500000
1000000
500000
0
S1_WT
S1_WT_IMS
S1_WT_MATRIX
S1_atp23
S1_atp23_IMS
S1_atp23_MATRIX
S2_WT
S2_WT_IMS
S2_WT_MATRIX
S2_atp6
S2_atp6_IMS
S2_atp6_MATRIX

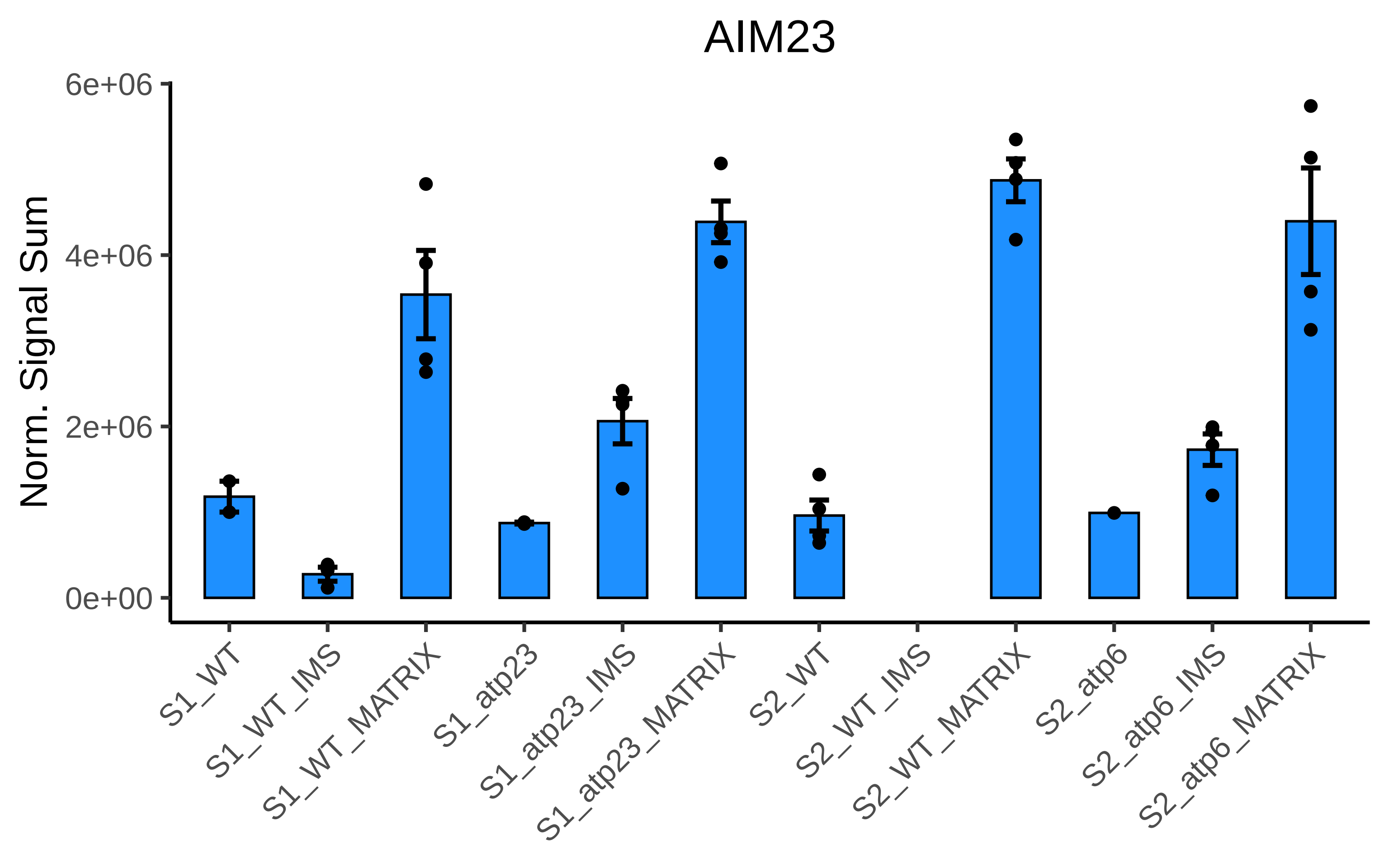
AIM23
Norm. Signal Sum
6e+06
4e+06
2e+06
0e+00
S1_WT
S1_WT_IMS
S1_WT_MATRIX
S1_atp23
S1_atp23_IMS
S1_atp23_MATRIX
S2_WT
S2_WT_IMS
S2_WT_MATRIX
S2_atp6
S2_atp6_IMS
S2_atp6_MATRIX

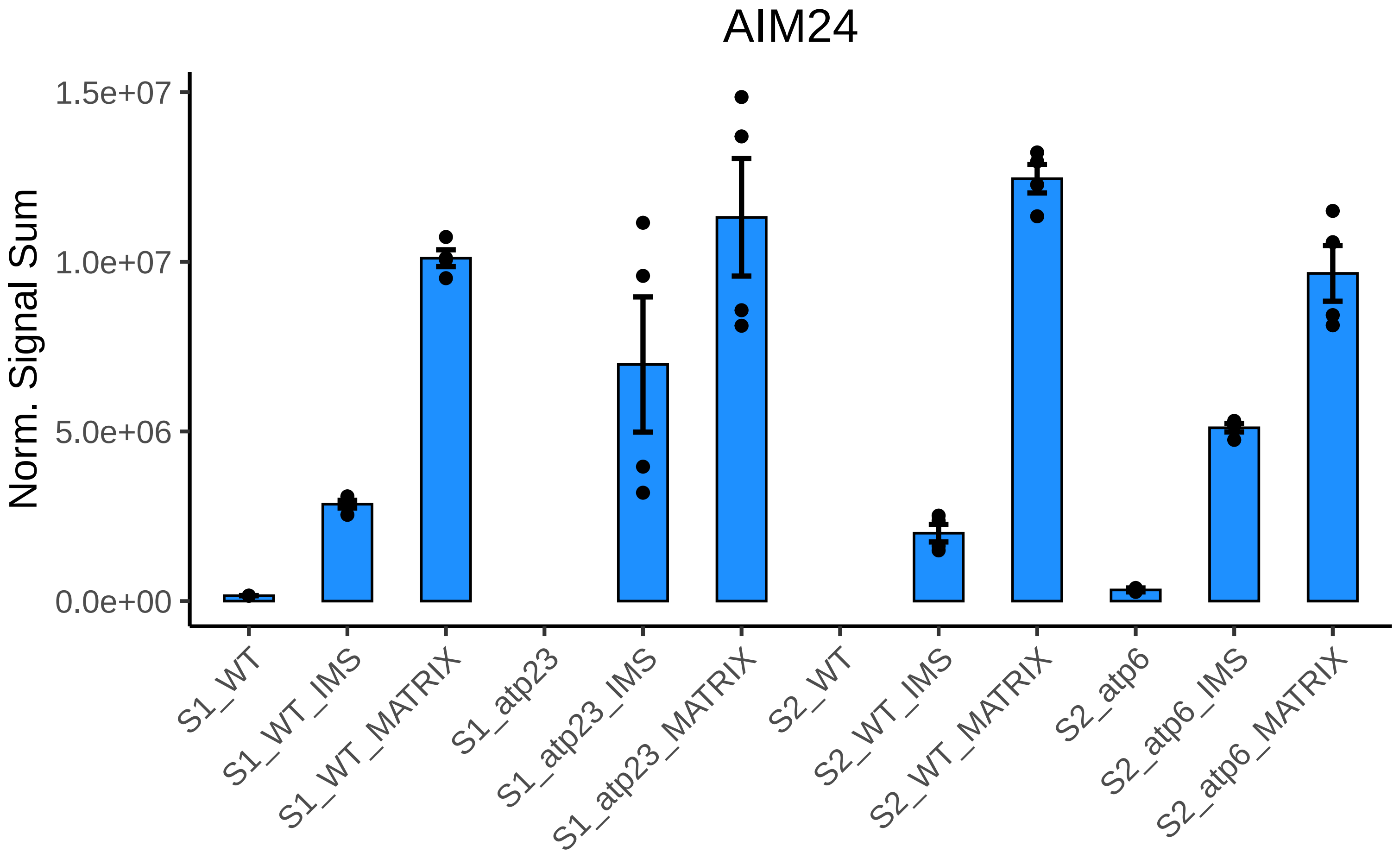

AIM24
Norm. Signal Sum
1.5e+07
1.0e+07
5.0e+06
0.0e+00
S1_WT
S1_WT_IMS
S1_WT_MATRIX
S1_atp23
S1_atp23_IMS
S1_atp23_MATRIX
S2_WT
S2_WT_IMS
S2_WT_MATRIX
S2_atp6
S2_atp6_IMS
S2_atp6_MATRIX

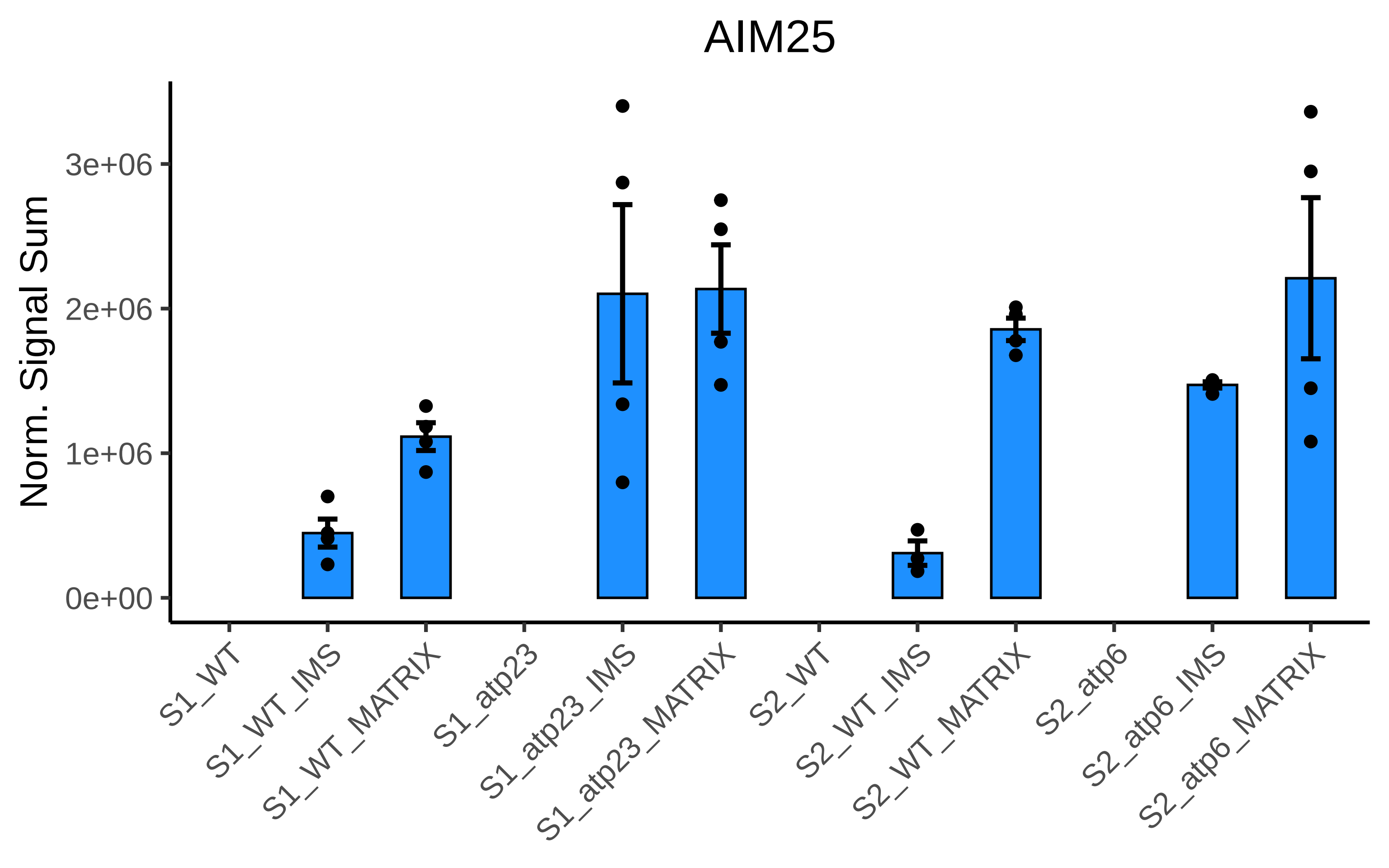
AIM25
Norm. Signal Sum
3e+06
2e+06
1e+06
0e+00
S1_WT
S1_WT_IMS
S1_WT_MATRIX
S1_atp23
S1_atp23_IMS
S1_atp23_MATRIX
S2_WT
S2_WT_IMS
S2_WT_MATRIX
S2_atp6
S2_atp6_IMS
S2_atp6_MATRIX

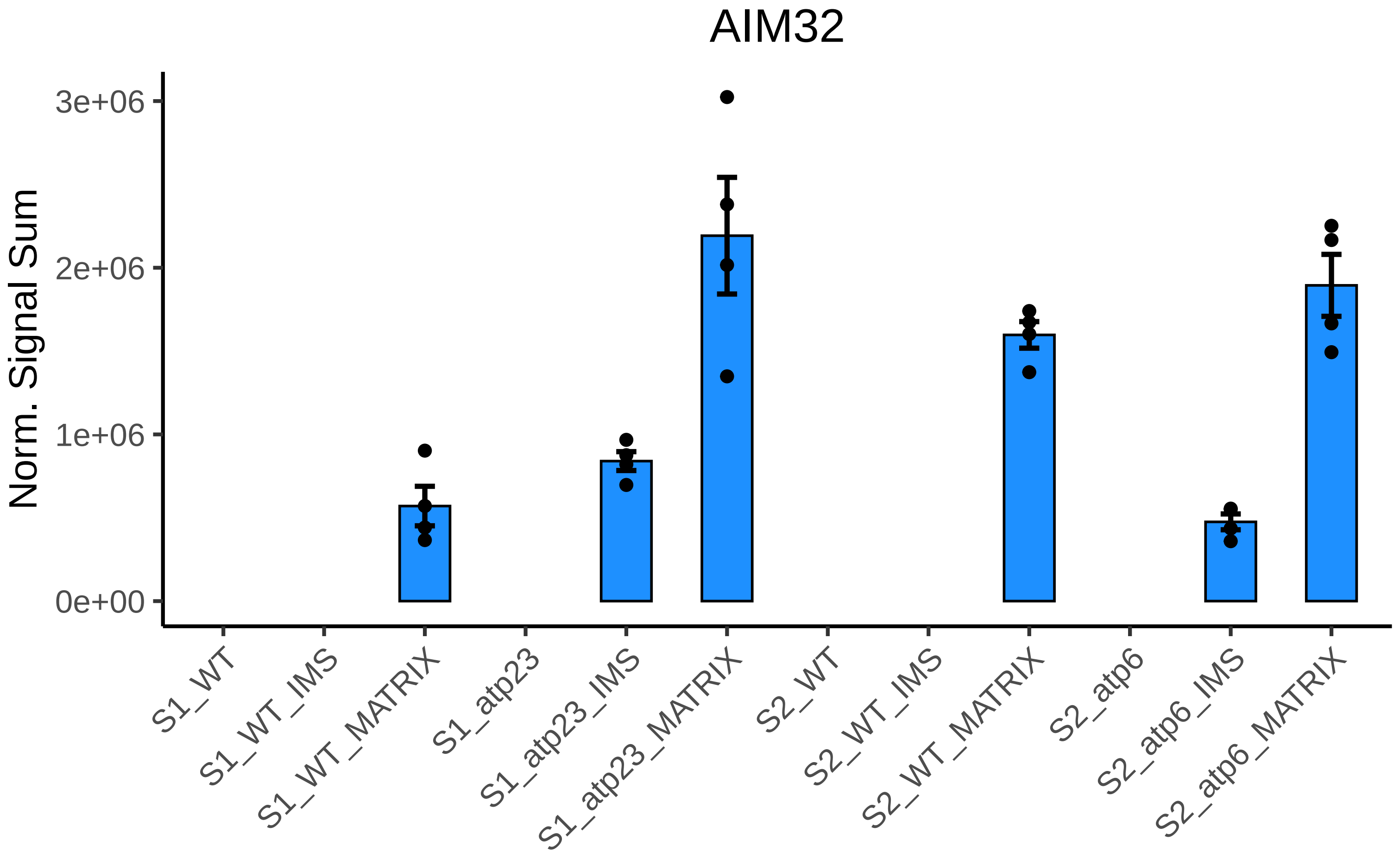

AIM32
Norm. Signal Sum
3e+06
2e+06
1e+06
0e+00
S1_WT
S1_WT_IMS
S1_WT_MATRIX
S1_atp23
S1_atp23_IMS
S1_atp23_MATRIX
S2_WT
S2_WT_IMS
S2_WT_MATRIX
S2_atp6
S2_atp6_IMS
S2_atp6_MATRIX

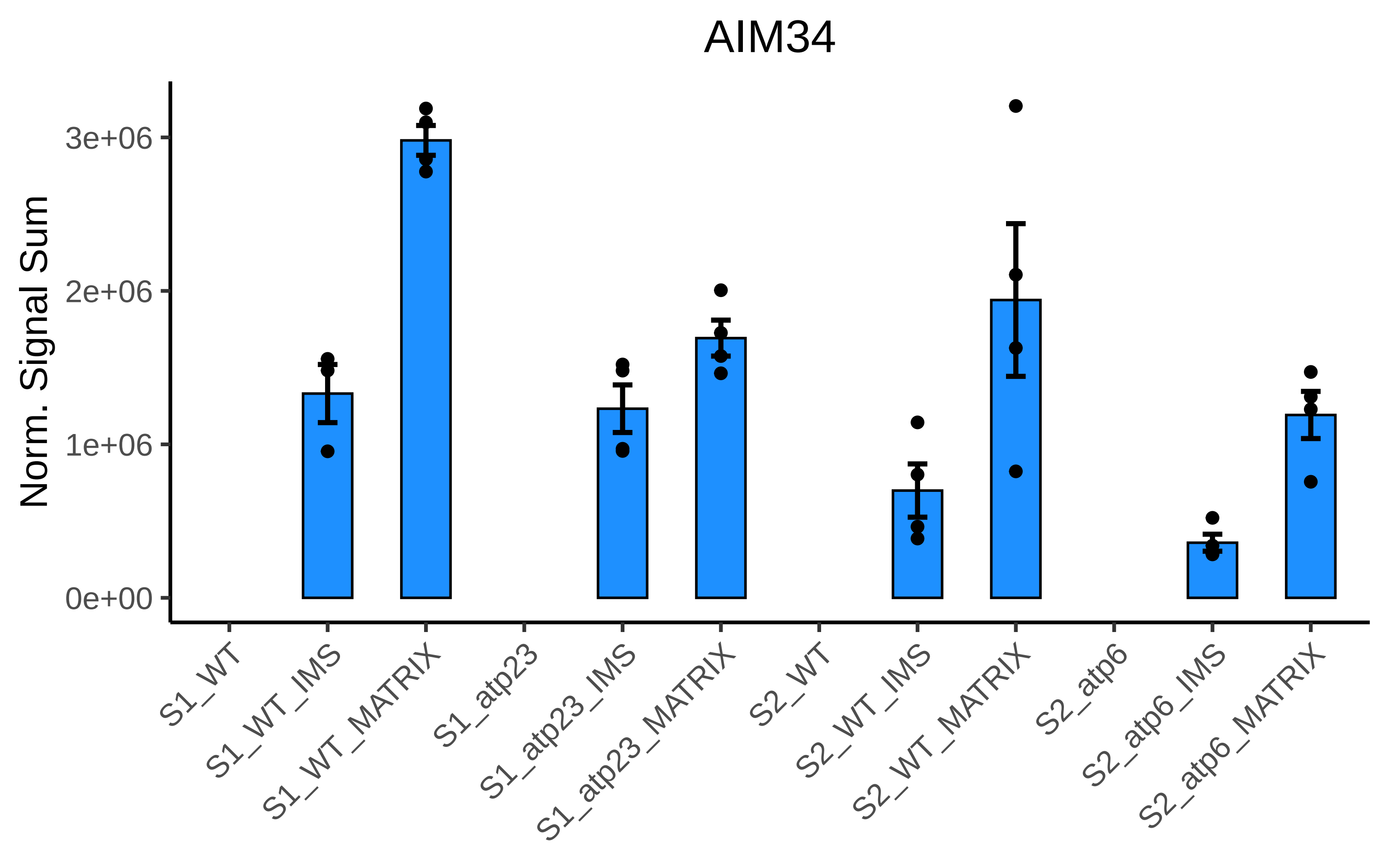

AIM34
Norm. Signal Sum
3e+06
2e+06
1e+06
0e+00
S1_WT
S1_WT_IMS
S1_WT_MATRIX
S1_atp23
S1_atp23_IMS
S1_atp23_MATRIX
S2_WT
S2_WT_IMS
S2_WT_MATRIX
S2_atp6
S2_atp6_IMS
S2_atp6_MATRIX

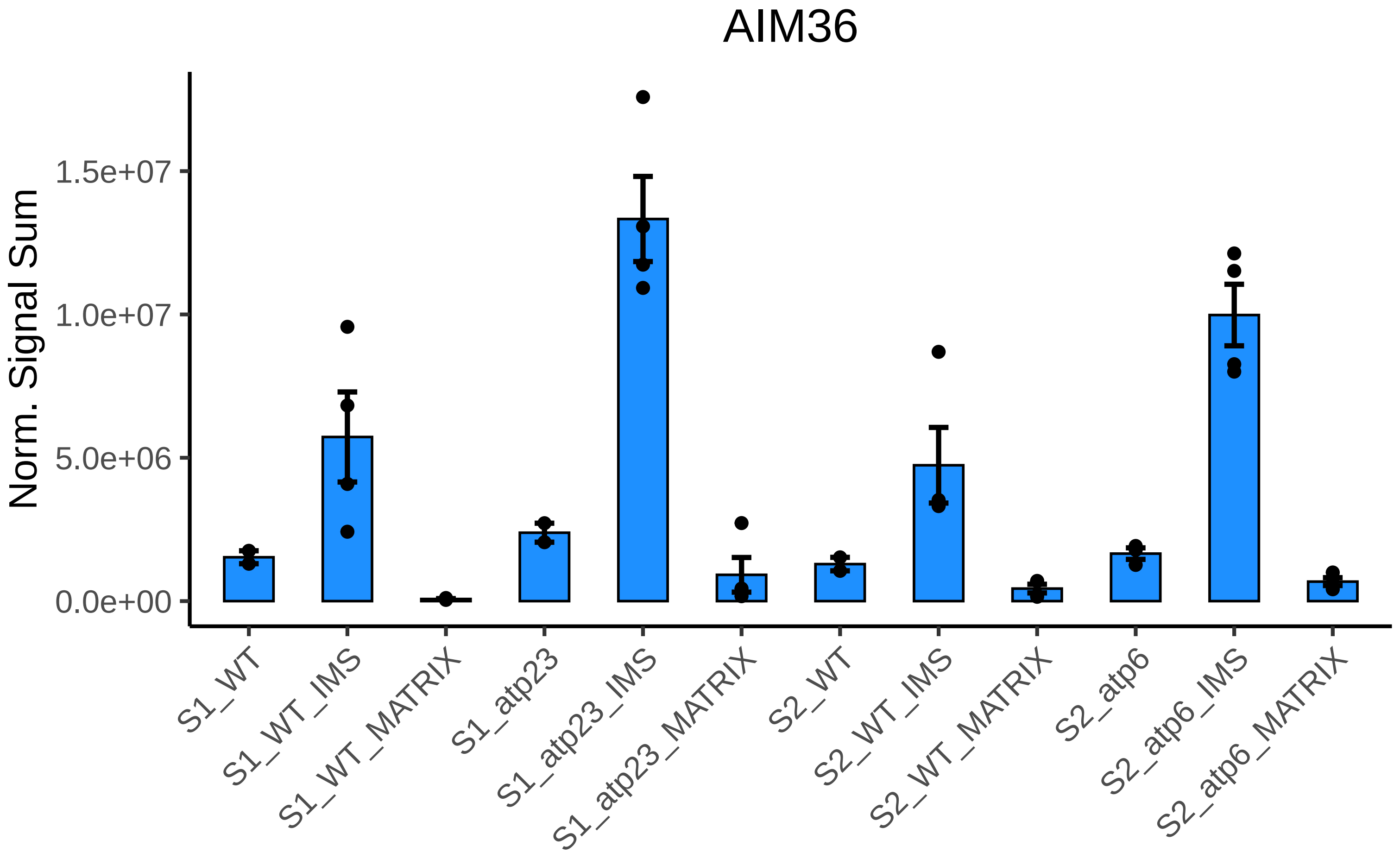

AIM36
Norm. Signal Sum
1.5e+07
1.0e+07
5.0e+06
0.0e+00
S1_WT
S1_WT_IMS
S1_WT_MATRIX
S1_atp23
S1_atp23_IMS
S1_atp23_MATRIX
S2_WT
S2_WT_IMS
S2_WT_MATRIX
S2_atp6
S2_atp6_IMS
S2_atp6_MATRIX

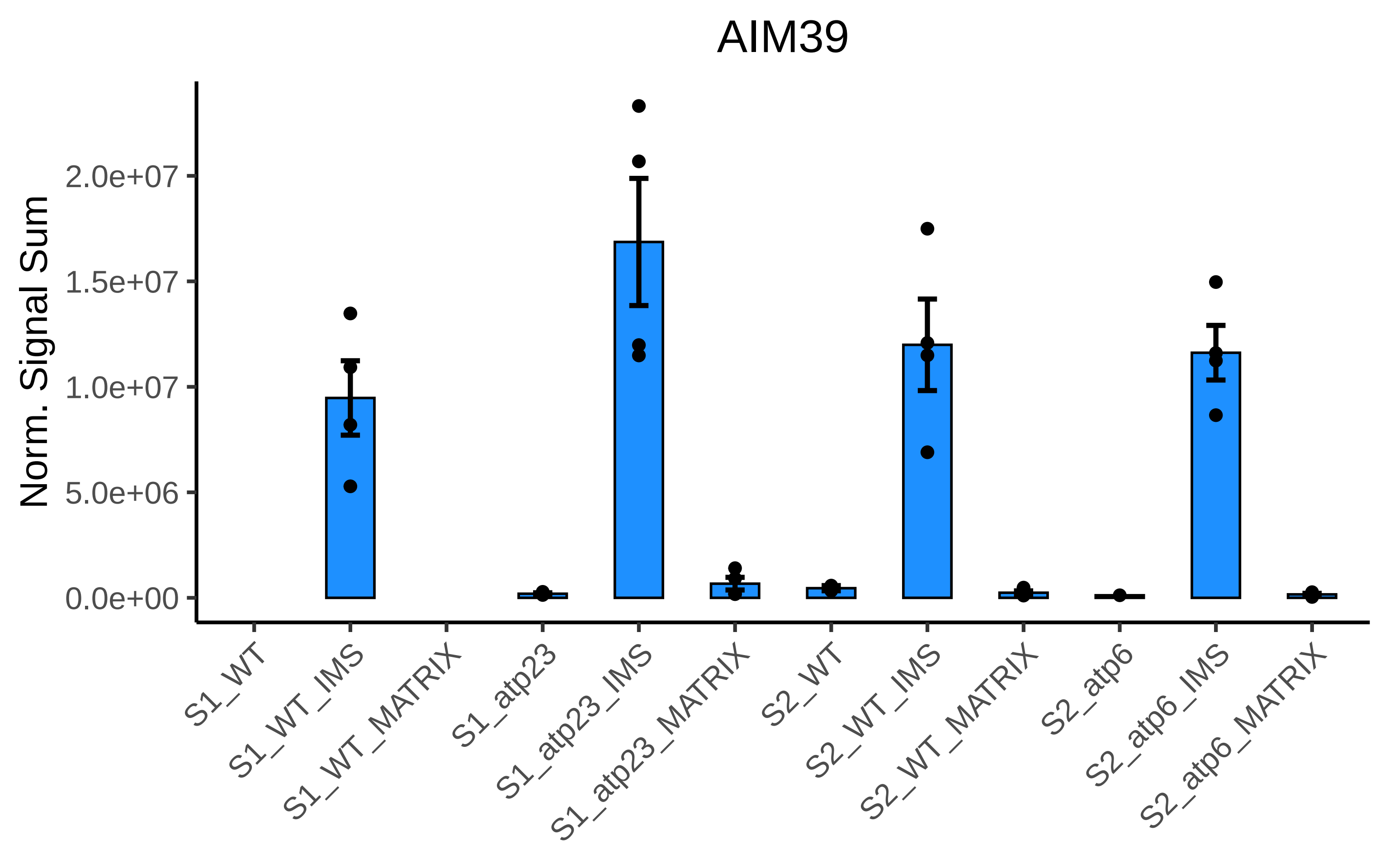
AIM39
Norm. Signal Sum
2.0e+07
1.5e+07
1.0e+07
5.0e+06
0.0e+00
S1_WT
S1_WT_IMS
S1_WT_MATRIX
S1_atp23
S1_atp23_IMS
S1_atp23_MATRIX
S2_WT
S2_WT_IMS
S2_WT_MATRIX
S2_atp6
S2_atp6_IMS
S2_atp6_MATRIX

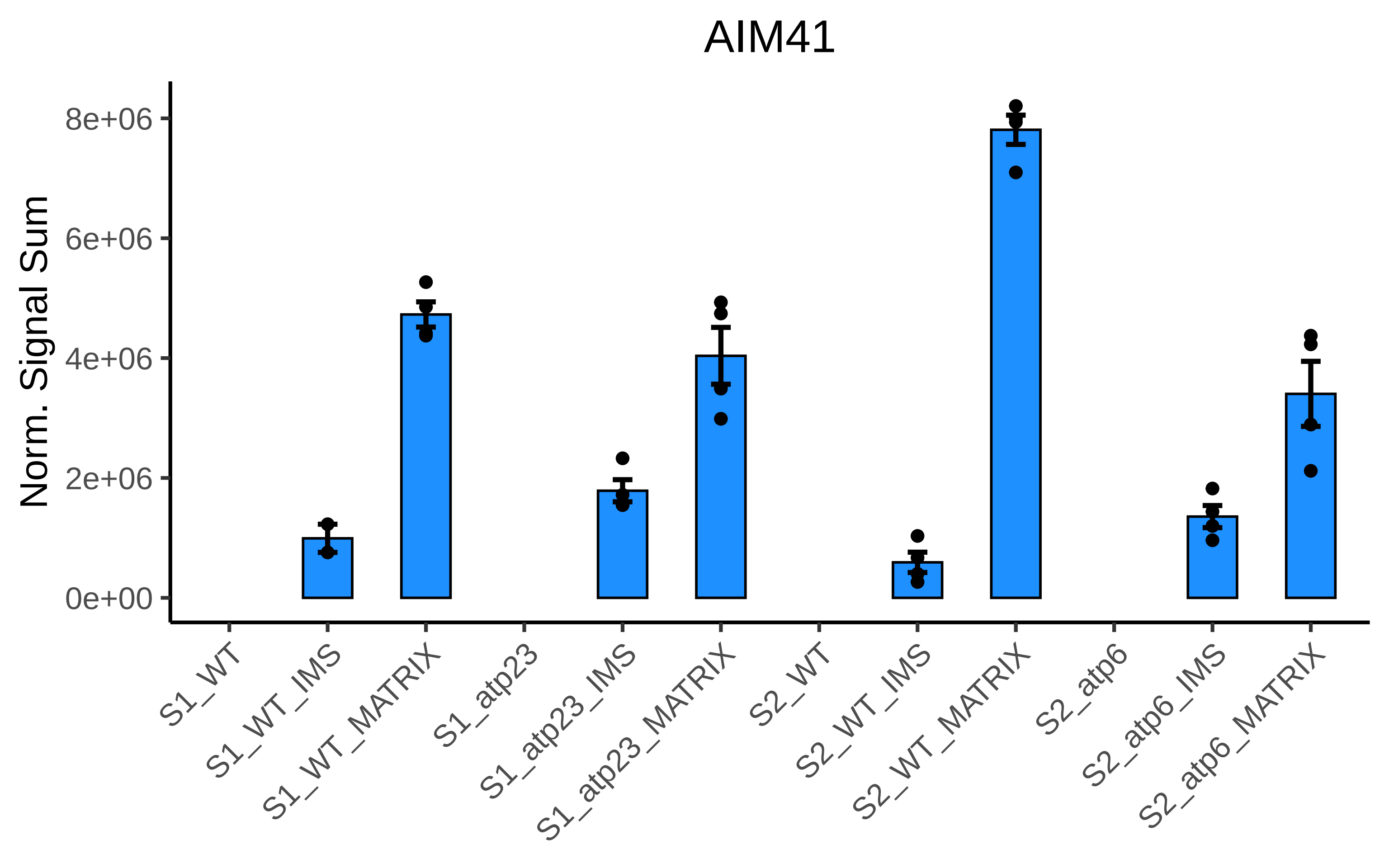

AIM41
Norm. Signal Sum
8e+06
6e+06
4e+06
2e+06
0e+00
S1_WT
S1_WT_IMS
S1_WT_MATRIX
S1_atp23
S1_atp23_IMS
S1_atp23_MATRIX
S2_WT
S2_WT_IMS
S2_WT_MATRIX
S2_atp6
S2_atp6_IMS
S2_atp6_MATRIX

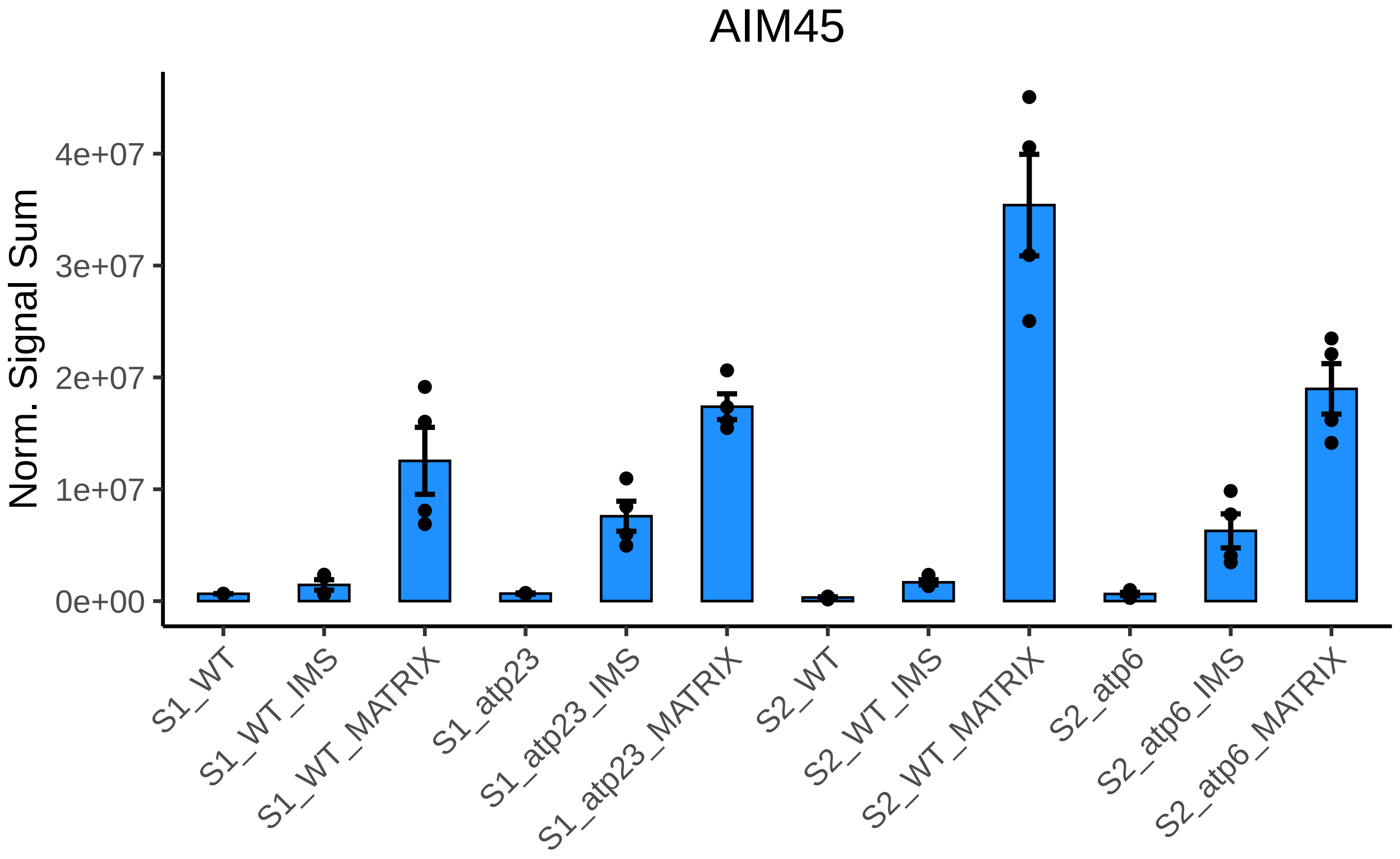
AIM45
Norm. Signal Sum
4e+07
3e+07
2e+07
1e+07
0e+00
S1_WT
S1_WT_IMS
S1_WT_MATRIX
S1_atp23
S1_atp23_IMS
S1_atp23_MATRIX
S2_WT
S2_WT_IMS
S2_WT_MATRIX
S2_atp6
S2_atp6_IMS
S2_atp6_MATRIX

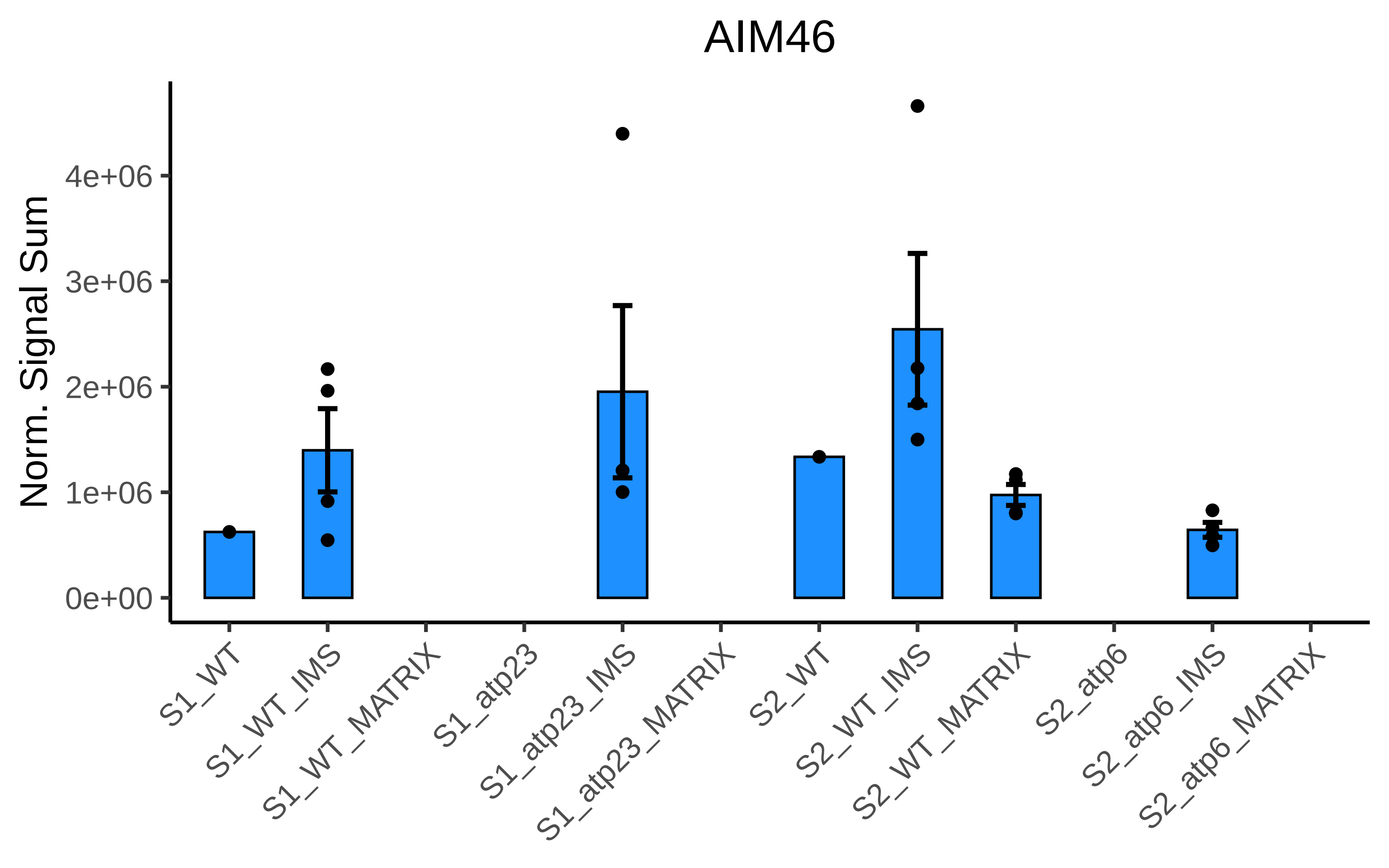
AIM46
Norm. Signal Sum
4e+06
3e+06
2e+06
1e+06
0e+00
S1_WT
S1_WT_IMS
S1_WT_MATRIX
S1_atp23
S1_atp23_IMS
S1_atp23_MATRIX
S2_WT
S2_WT_IMS
S2_WT_MATRIX
S2_atp6
S2_atp6_IMS
S2_atp6_MATRIX

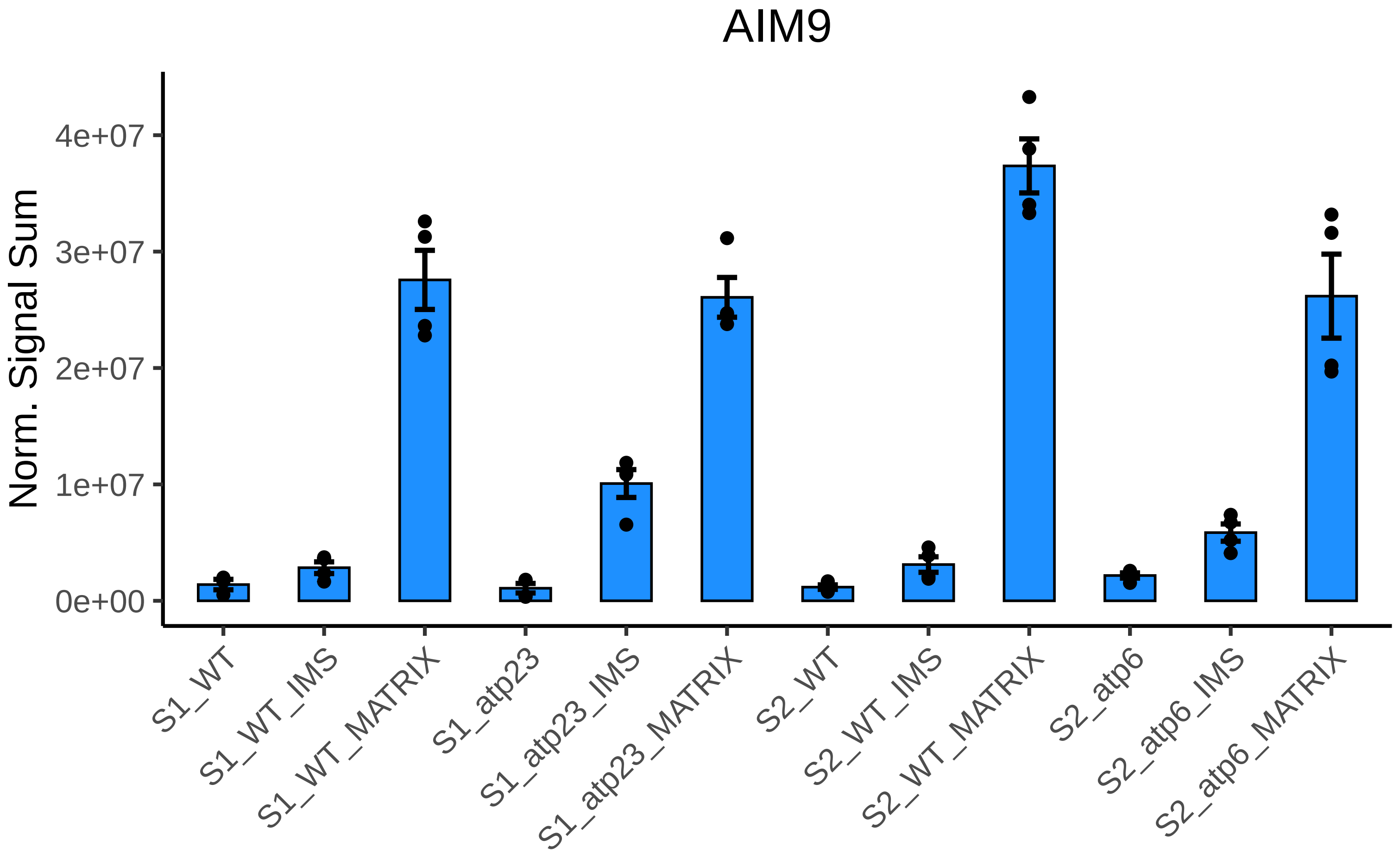
AIM9
Norm. Signal Sum
4e+07
3e+07
2e+07
1e+07
0e+00
S1_WT
S1_WT_IMS
S1_WT_MATRIX
S1_atp23
S1_atp23_IMS
S1_atp23_MATRIX
S2_WT
S2_WT_IMS
S2_WT_MATRIX
S2_atp6
S2_atp6_IMS
S2_atp6_MATRIX

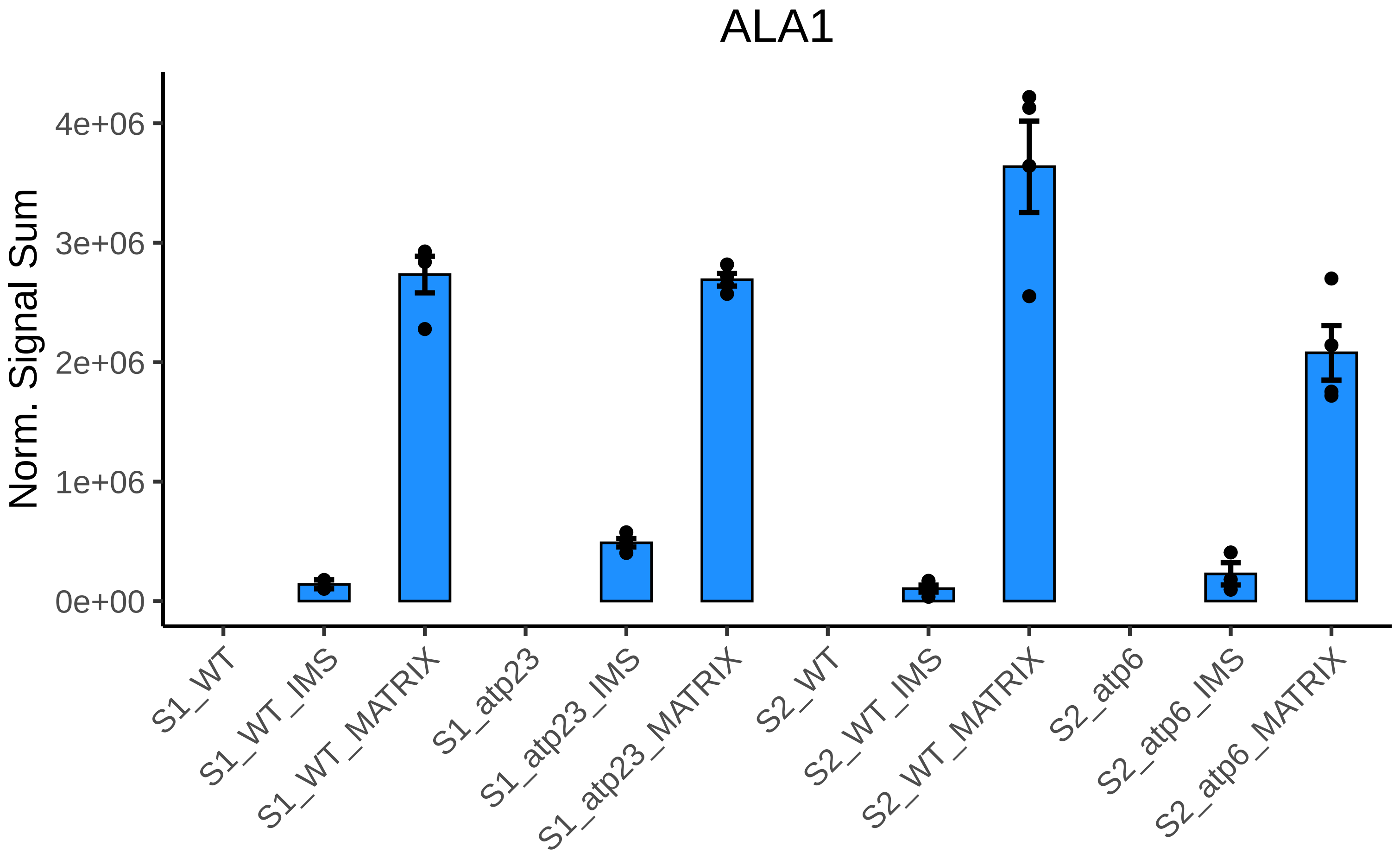

ALA1
Norm. Signal Sum
4e+06
3e+06
2e+06
1e+06
0e+00
S1_WT
S1_WT_IMS
S1_WT_MATRIX
S1_atp23
S1_atp23_IMS
S1_atp23_MATRIX
S2_WT
S2_WT_IMS
S2_WT_MATRIX
S2_atp6
S2_atp6_IMS
S2_atp6_MATRIX

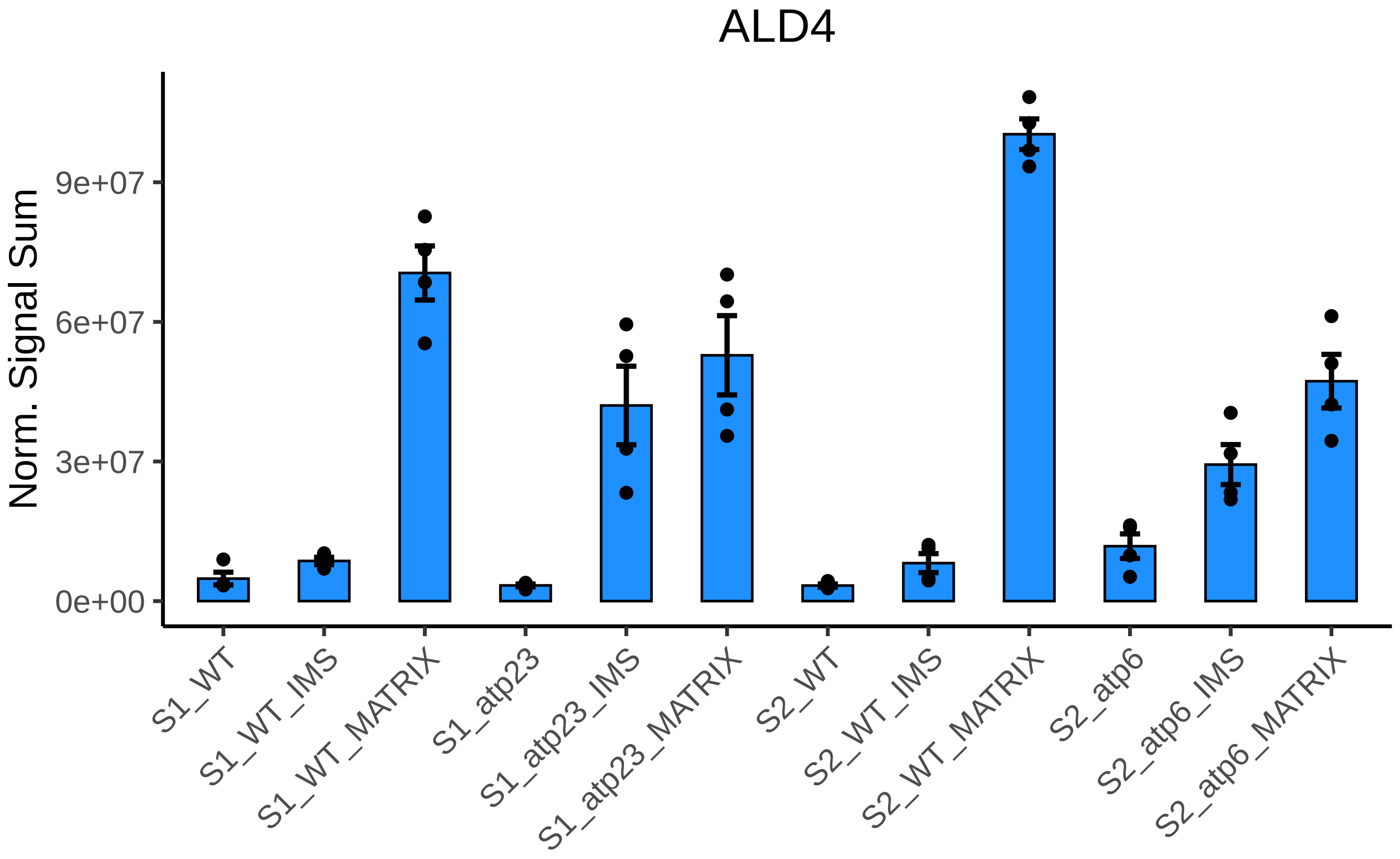
ALD4
Norm. Signal Sum
9e+07
6e+07
3e+07
0e+00
S1_WT
S1_WT_IMS
S1_WT_MATRIX
S1_atp23
S1_atp23_IMS
S1_atp23_MATRIX
S2_WT
S2_WT_IMS
S2_WT_MATRIX
S2_atp6
S2_atp6_IMS
S2_atp6_MATRIX

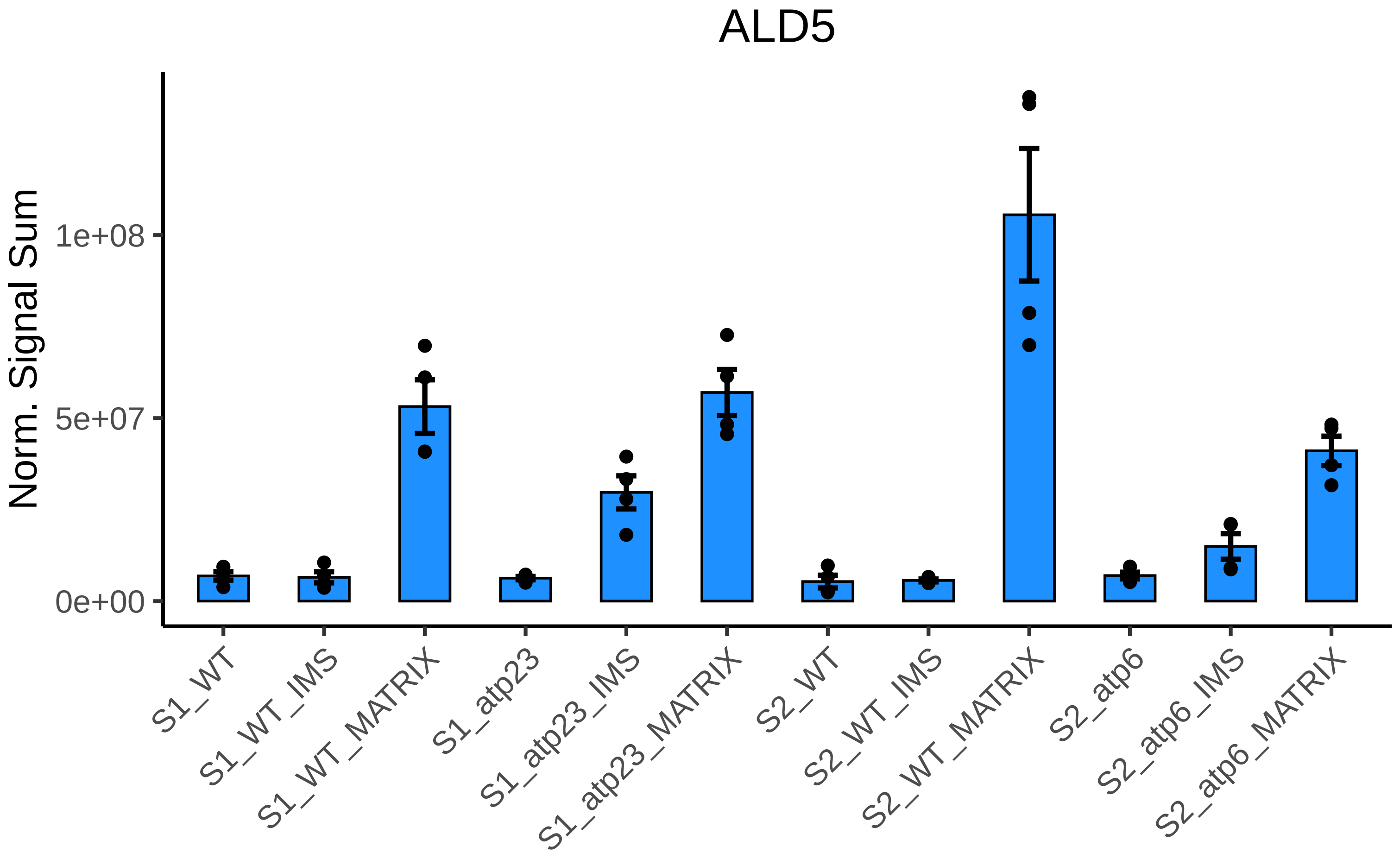

ALD5
Norm. Signal Sum
1e+08
5e+07
0e+00
S1_WT
S1_WT_IMS
S1_WT_MATRIX
S1_atp23
S1_atp23_IMS
S1_atp23_MATRIX
S2_WT
S2_WT_IMS
S2_WT_MATRIX
S2_atp6
S2_atp6_IMS
S2_atp6_MATRIX

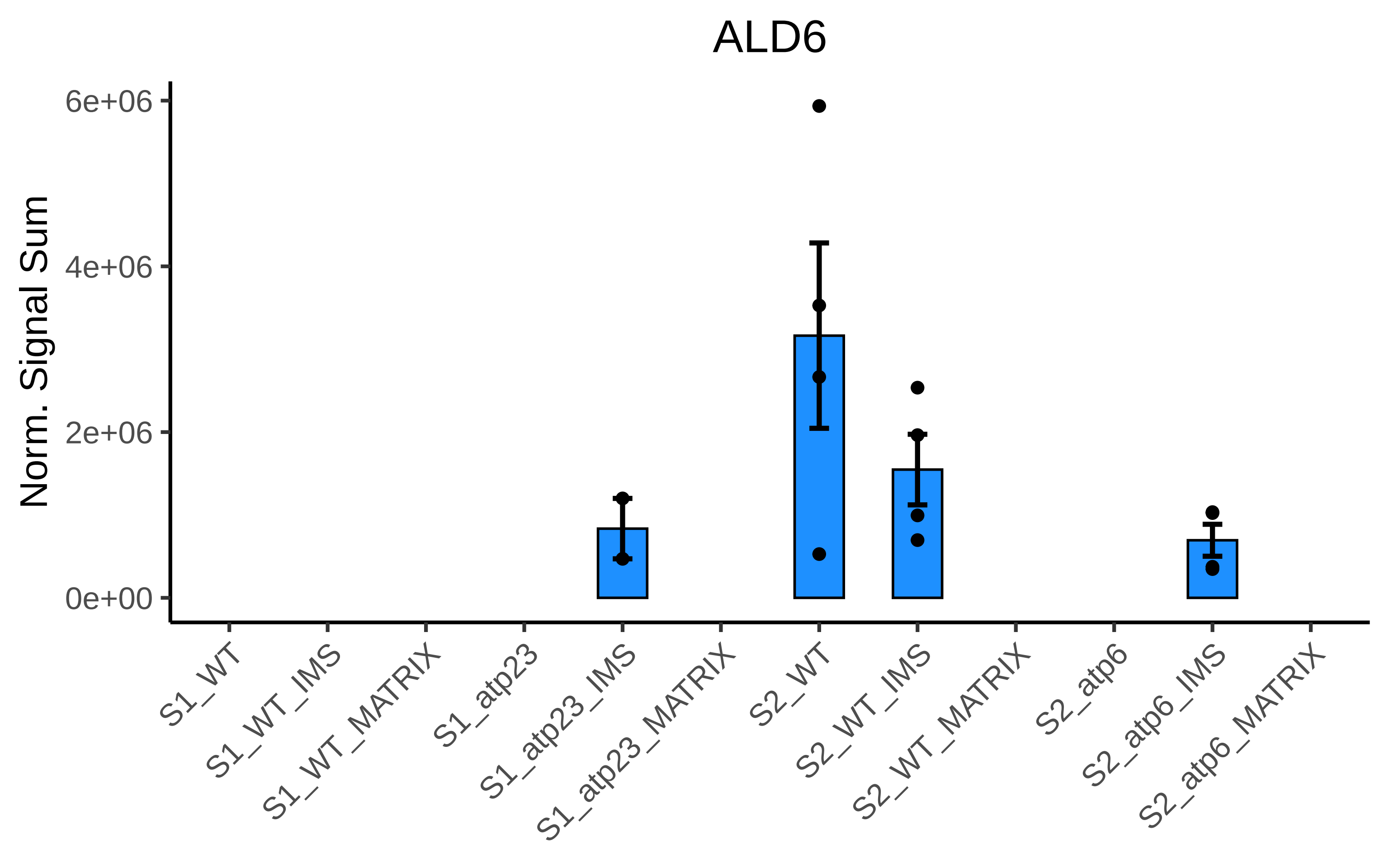

ALD6
Norm. Signal Sum
6e+06
4e+06
2e+06
0e+00
S1_WT
S1_WT_IMS
S1_WT_MATRIX
S1_atp23
S1_atp23_IMS
S1_atp23_MATRIX
S2_WT
S2_WT_IMS
S2_WT_MATRIX
S2_atp6
S2_atp6_IMS
S2_atp6_MATRIX

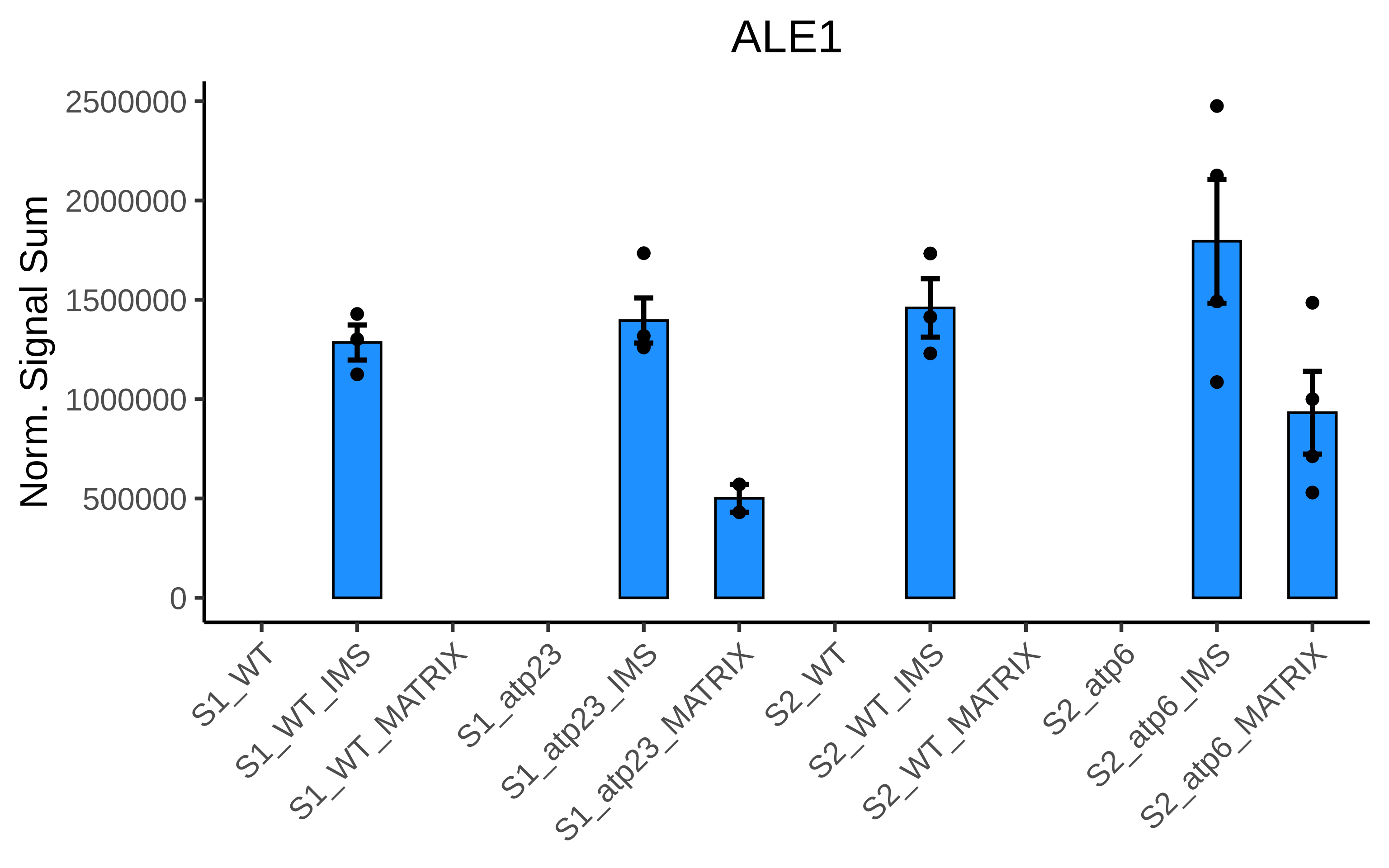
ALE1
Norm. Signal Sum
2500000
2000000
1500000
1000000
500000
0
S1_WT
S1_WT_IMS
S1_WT_MATRIX
S1_atp23
S1_atp23_IMS
S1_atp23_MATRIX
S2_WT
S2_WT_IMS
S2_WT_MATRIX
S2_atp6
S2_atp6_IMS
S2_atp6_MATRIX

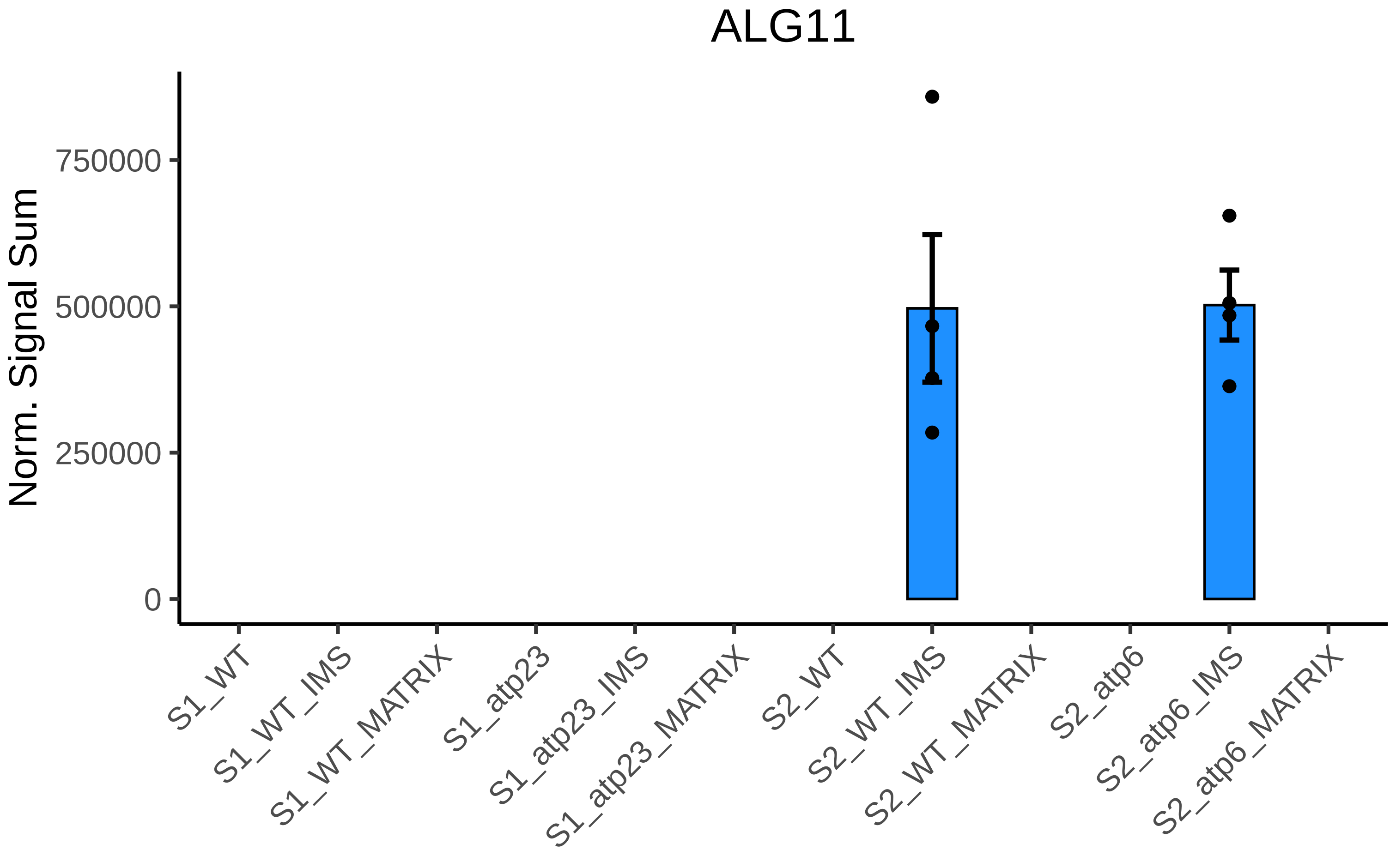
ALG11
Norm. Signal Sum
750000
500000
250000
0
S1_WT
S1_WT_IMS
S1_WT_MATRIX
S1_atp23
S1_atp23_IMS
S1_atp23_MATRIX
S2_WT
S2_WT_IMS
S2_WT_MATRIX
S2_atp6
S2_atp6_IMS
S2_atp6_MATRIX

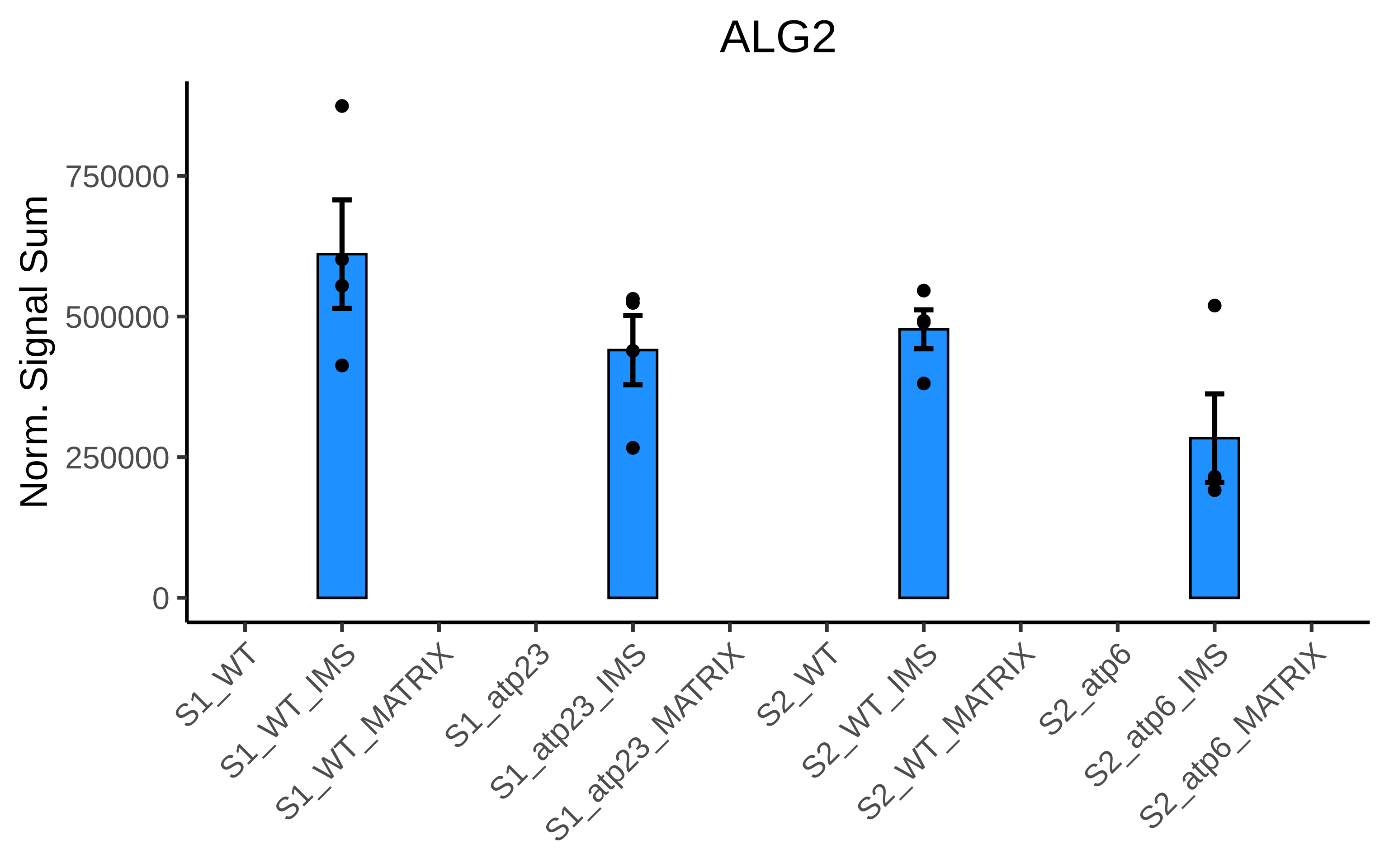

ALG2
Norm. Signal Sum
750000
500000
250000
0
S1_WT
S1_WT_IMS
S1_WT_MATRIX
S1_atp23
S1_atp23_IMS
S1_atp23_MATRIX
S2_WT
S2_WT_IMS
S2_WT_MATRIX
S2_atp6
S2_atp6_IMS
S2_atp6_MATRIX

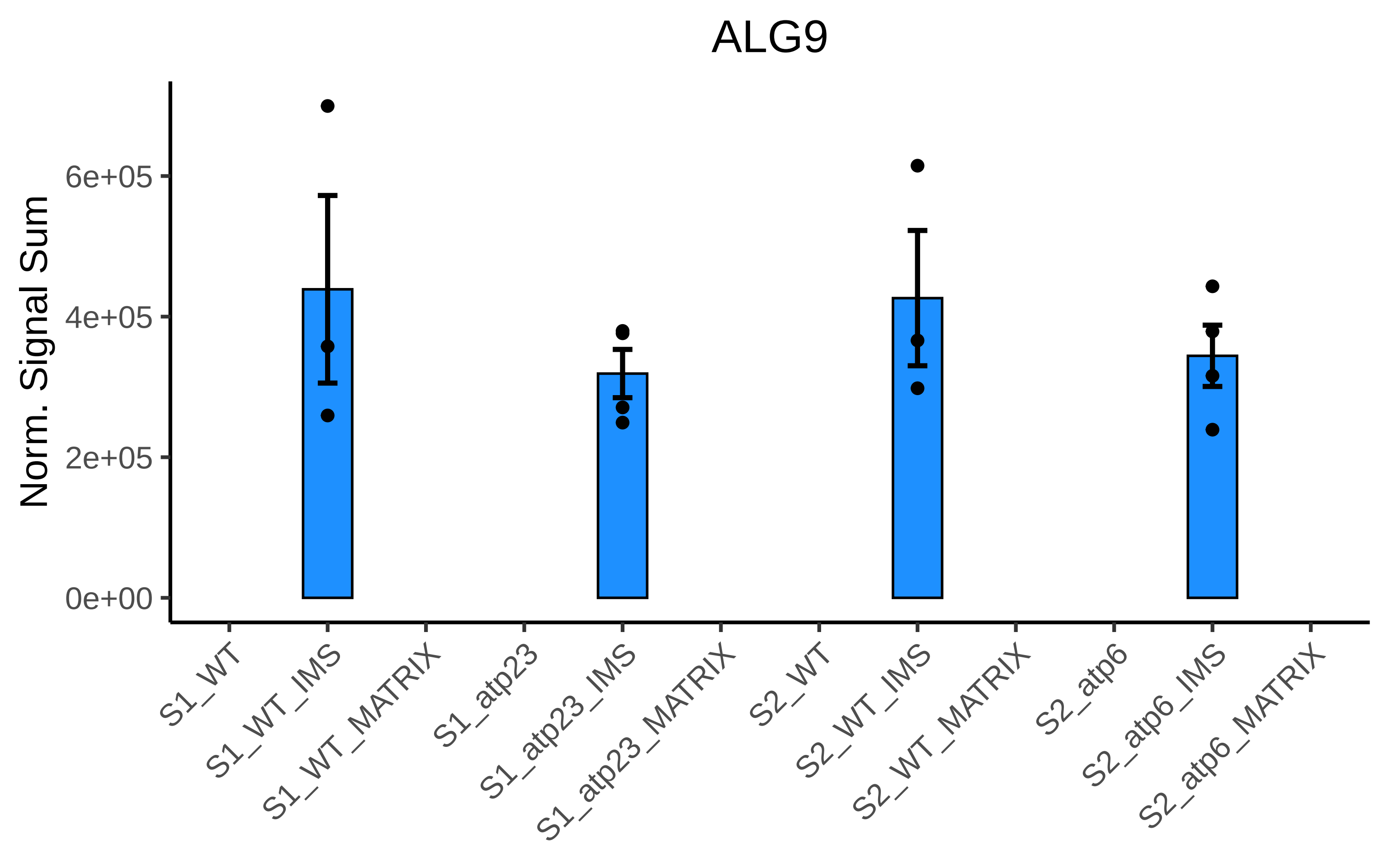

ALG9
Norm. Signal Sum
0e+00
2e+05
4e+05
6e+05
S1_WT
S1_WT_IMS
S1_WT_MATRIX
S1_atp23
S1_atp23_IMS
S1_atp23_MATRIX
S2_WT
S2_WT_IMS
S2_WT_MATRIX
S2_atp6
S2_atp6_IMS
S2_atp6_MATRIX

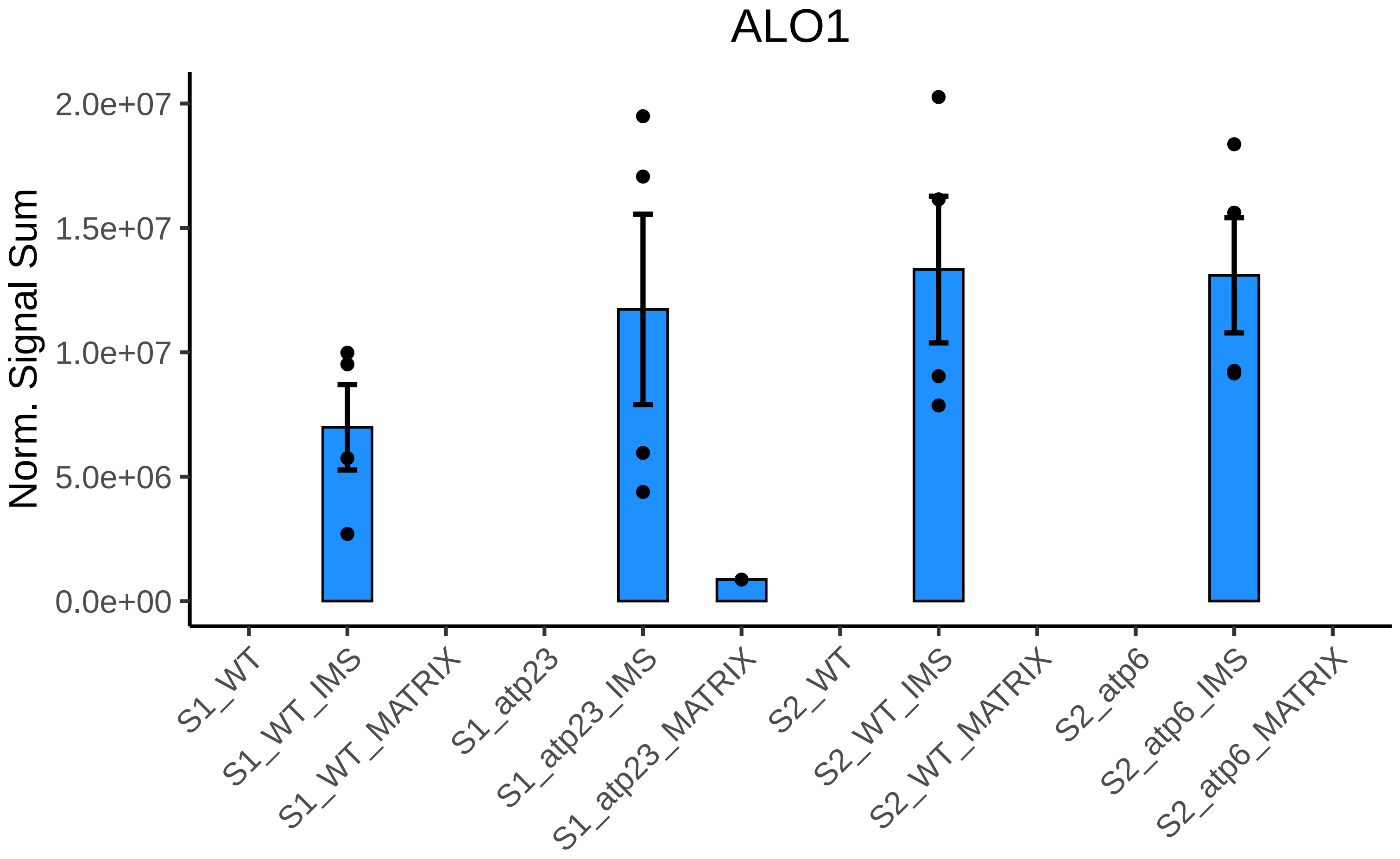

ALO1
Norm. Signal Sum
2.0e+07
1.5e+07
1.0e+07
5.0e+06
0.0e+00
S1_WT
S1_WT_IMS
S1_WT_MATRIX
S1_atp23
S1_atp23_IMS
S1_atp23_MATRIX
S2_WT
S2_WT_IMS
S2_WT_MATRIX
S2_atp6
S2_atp6_IMS
S2_atp6_MATRIX

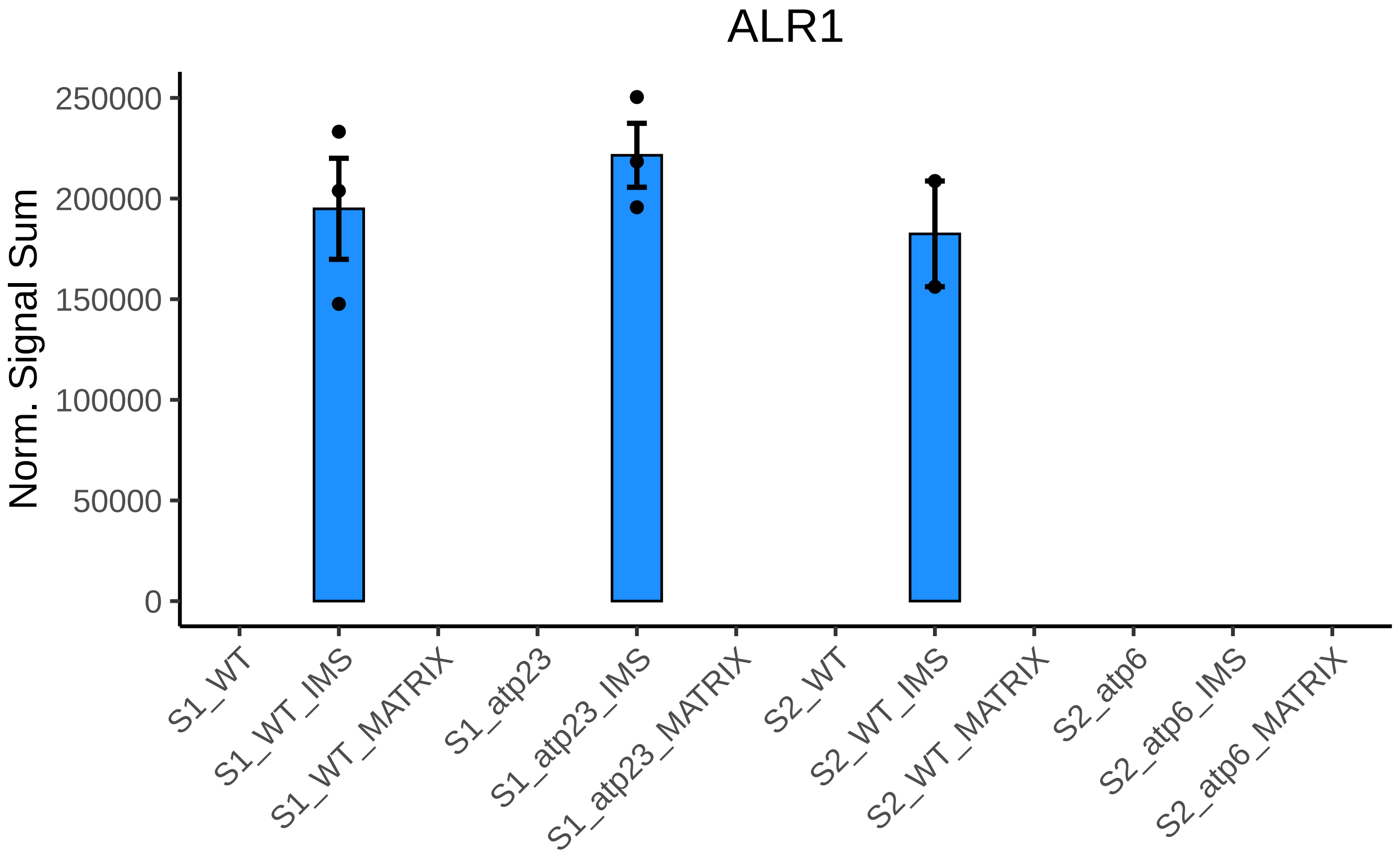

ALR1
Norm. Signal Sum
250000
200000
150000
100000
50000
0
S1_WT
S1_WT_IMS
S1_WT_MATRIX
S1_atp23
S1_atp23_IMS
S1_atp23_MATRIX
S2_WT
S2_WT_IMS
S2_WT_MATRIX
S2_atp6
S2_atp6_IMS
S2_atp6_MATRIX

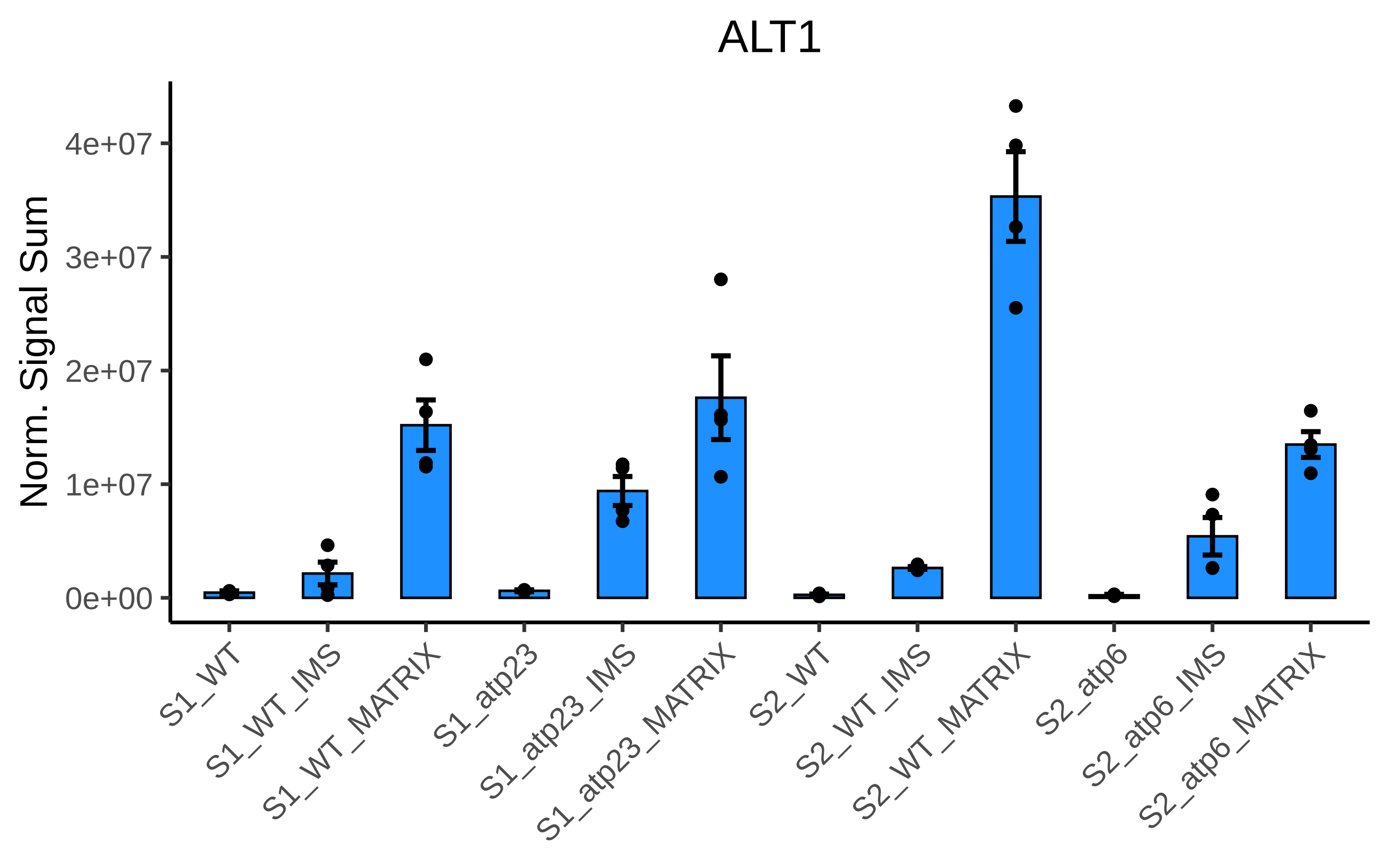
ALT1
Norm. Signal Sum
4e+07
3e+07
2e+07
1e+07
0e+00
S1_WT
S1_WT_IMS
S1_WT_MATRIX
S1_atp23
S1_atp23_IMS
S1_atp23_MATRIX
S2_WT
S2_WT_IMS
S2_WT_MATRIX
S2_atp6
S2_atp6_IMS
S2_atp6_MATRIX

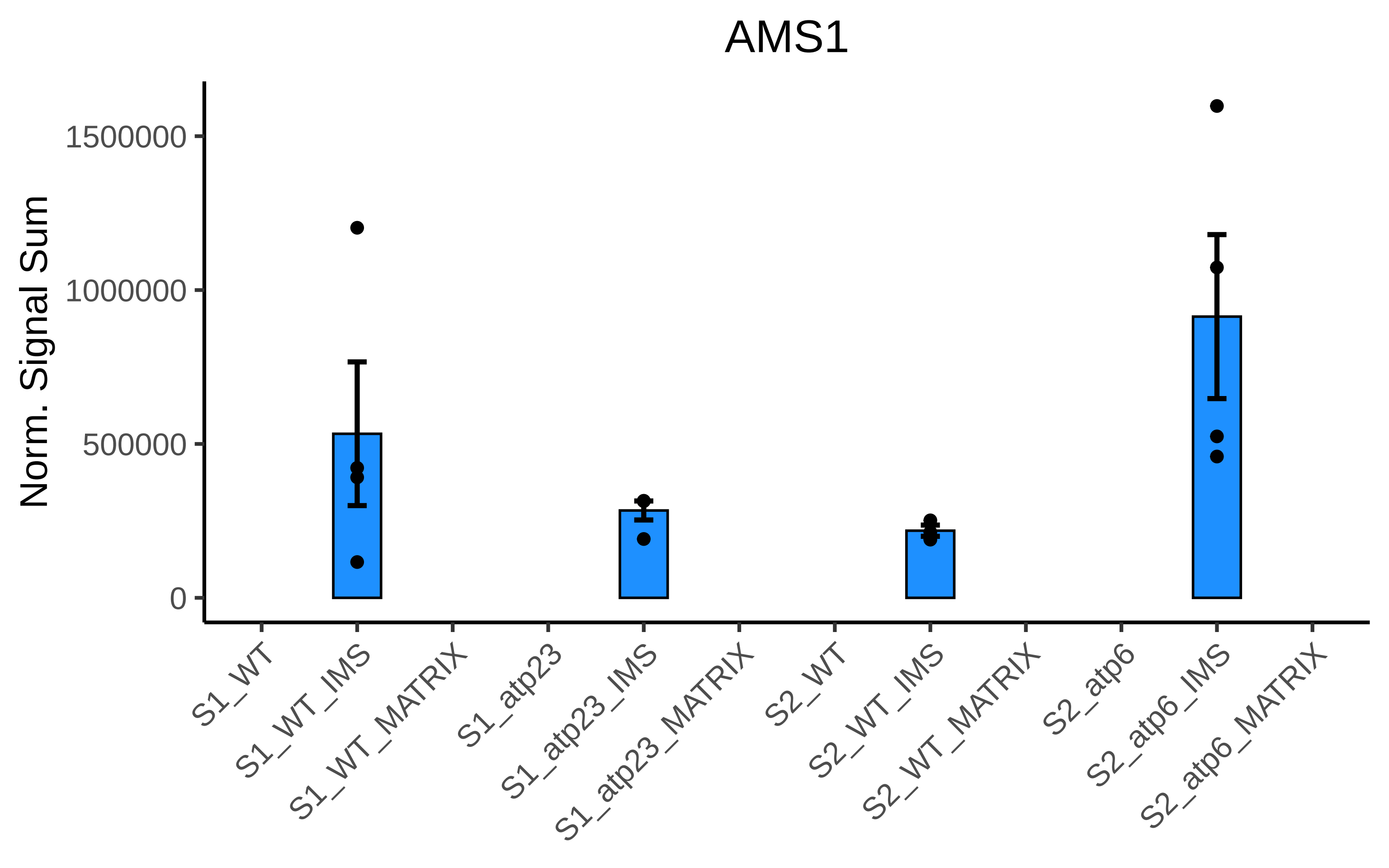

AMS1
Norm. Signal Sum
1500000
1000000
500000
0
S1_WT
S1_WT_IMS
S1_WT_MATRIX
S1_atp23
S1_atp23_IMS
S1_atp23_MATRIX
S2_WT
S2_WT_IMS
S2_WT_MATRIX
S2_atp6
S2_atp6_IMS
S2_atp6_MATRIX

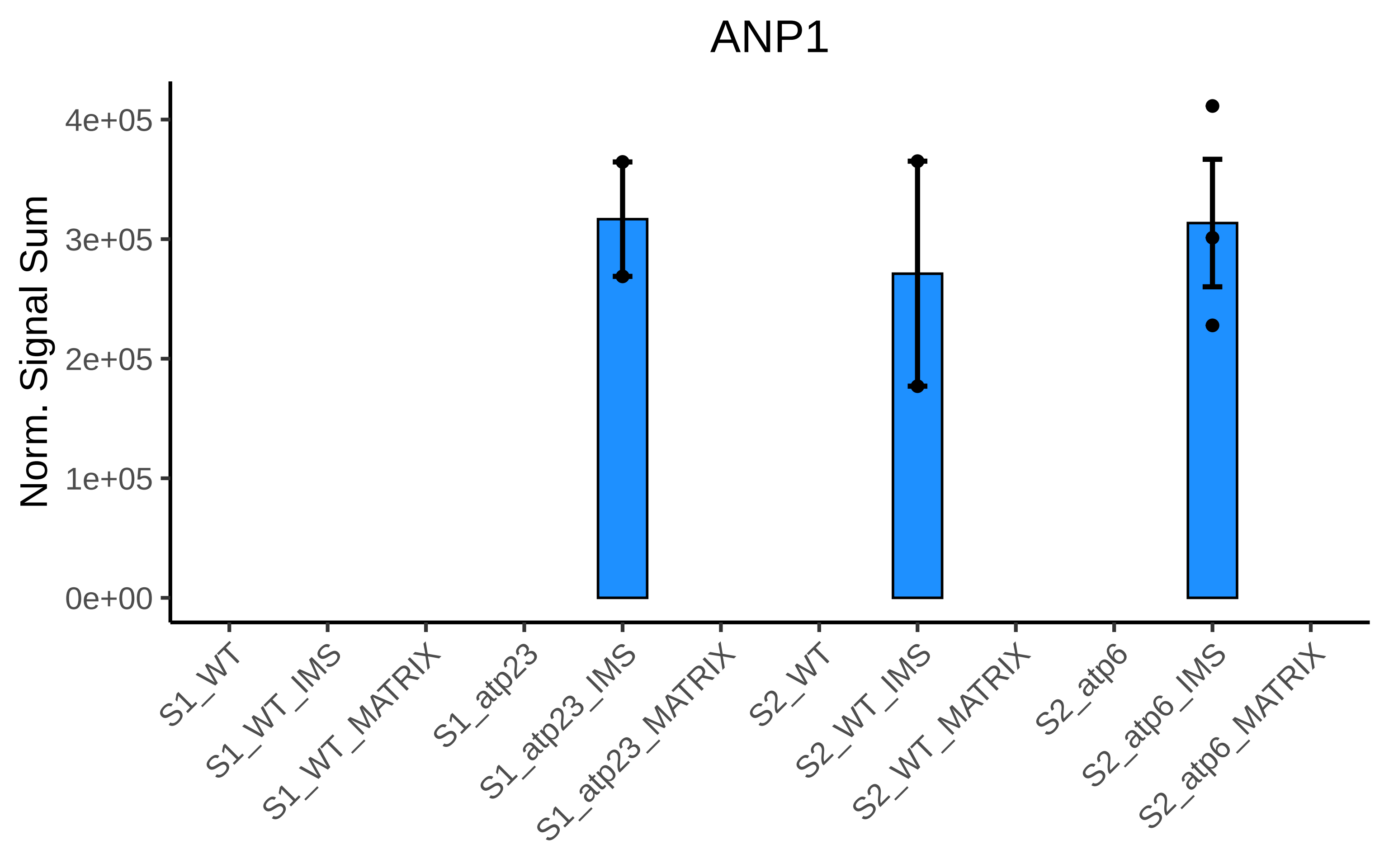

ANP1
Norm. Signal Sum
4e+05
3e+05
2e+05
1e+05
0e+00
S1_WT
S1_WT_IMS
S1_WT_MATRIX
S1_atp23
S1_atp23_IMS
S1_atp23_MATRIX
S2_WT
S2_WT_IMS
S2_WT_MATRIX
S2_atp6
S2_atp6_IMS
S2_atp6_MATRIX

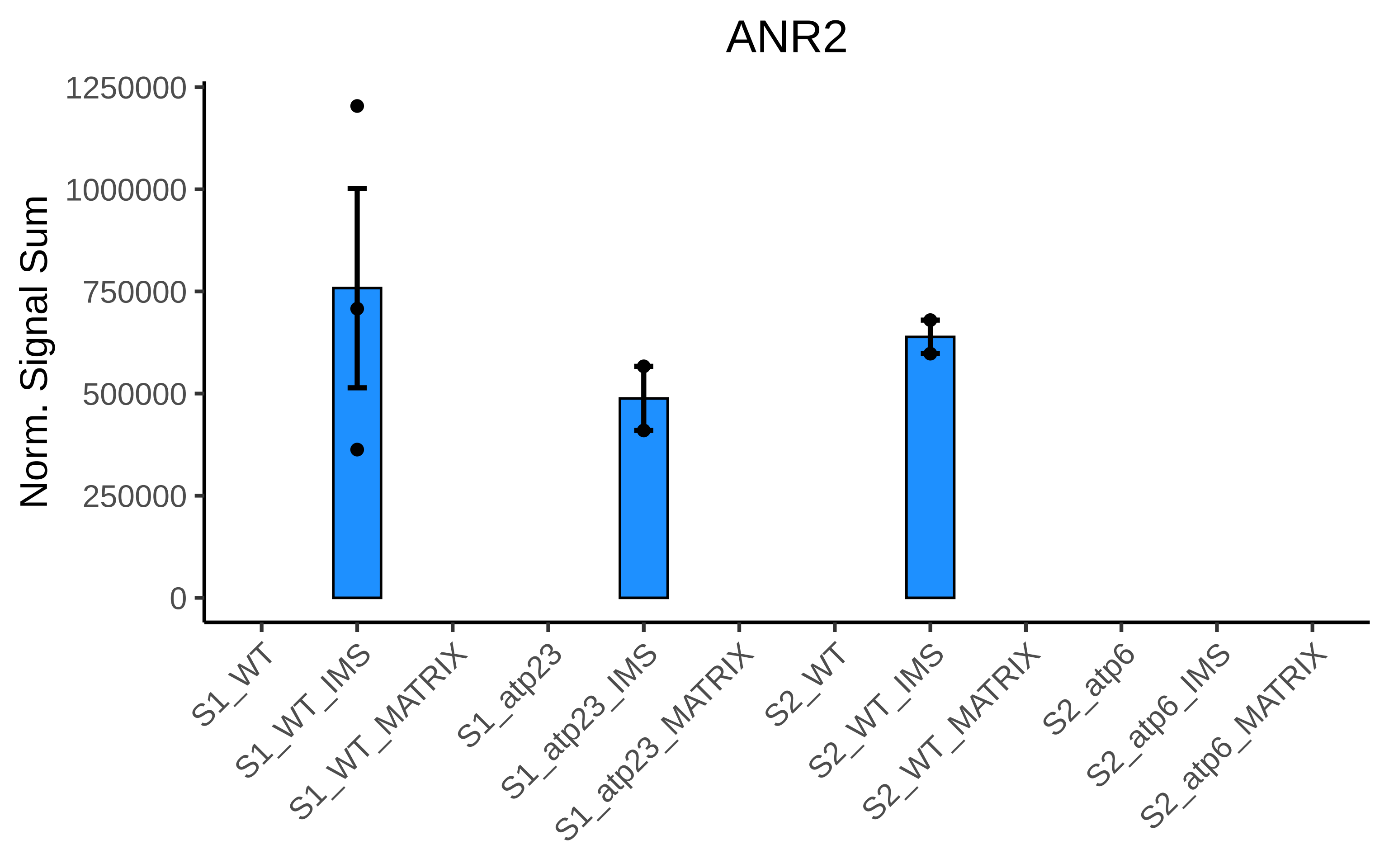

ANR2
Norm. Signal Sum
1250000
1000000
750000
500000
250000
0
S1_WT
S1_WT_IMS
S1_WT_MATRIX
S1_atp23
S1_atp23_IMS
S1_atp23_MATRIX
S2_WT
S2_WT_IMS
S2_WT_MATRIX
S2_atp6
S2_atp6_IMS
S2_atp6_MATRIX

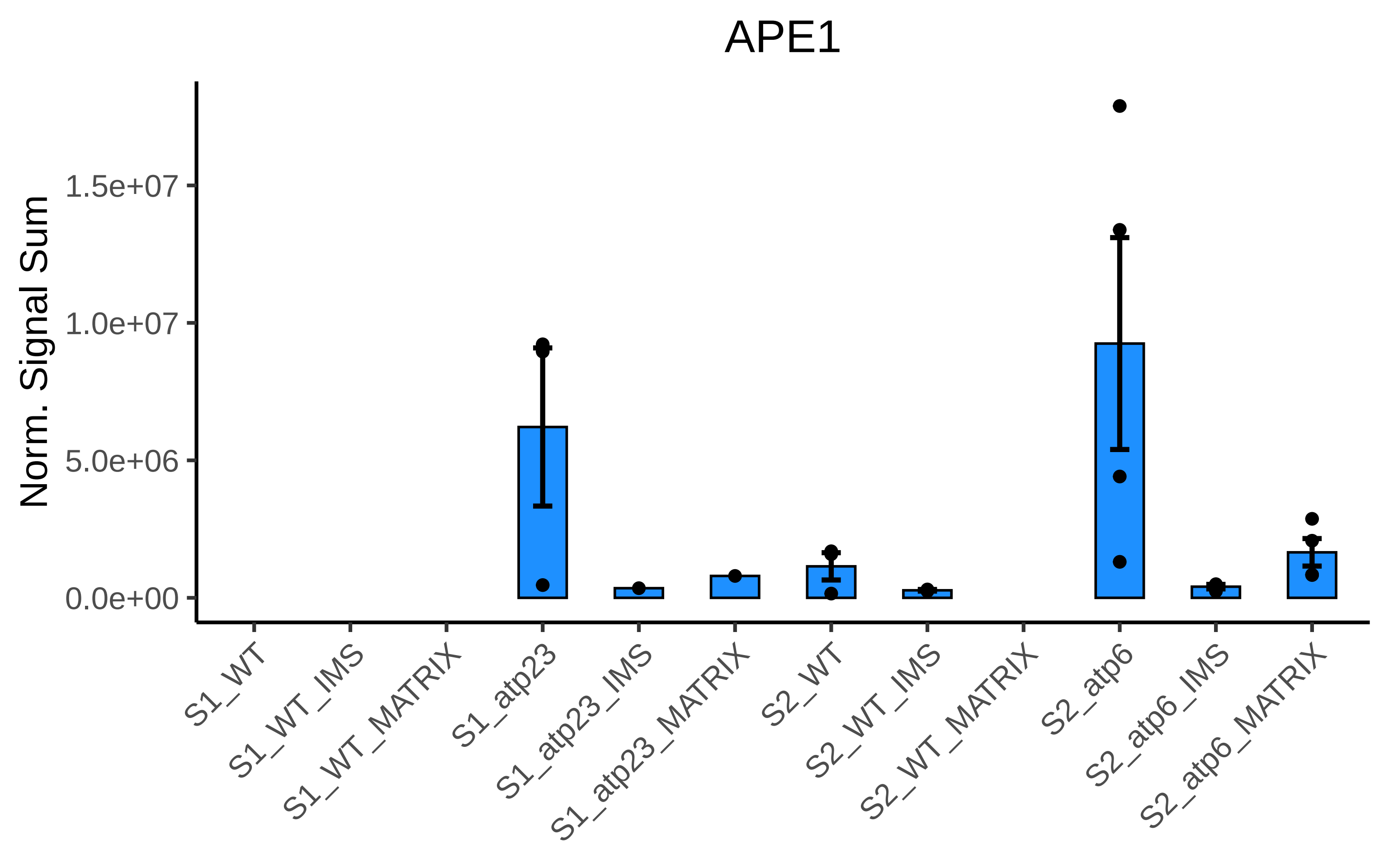
APE1
Norm. Signal Sum
1.5e+07
1.0e+07
5.0e+06
0.0e+00
S1_WT
S1_WT_IMS
S1_WT_MATRIX
S1_atp23
S1_atp23_IMS
S1_atp23_MATRIX
S2_WT
S2_WT_IMS
S2_WT_MATRIX
S2_atp6
S2_atp6_IMS
S2_atp6_MATRIX

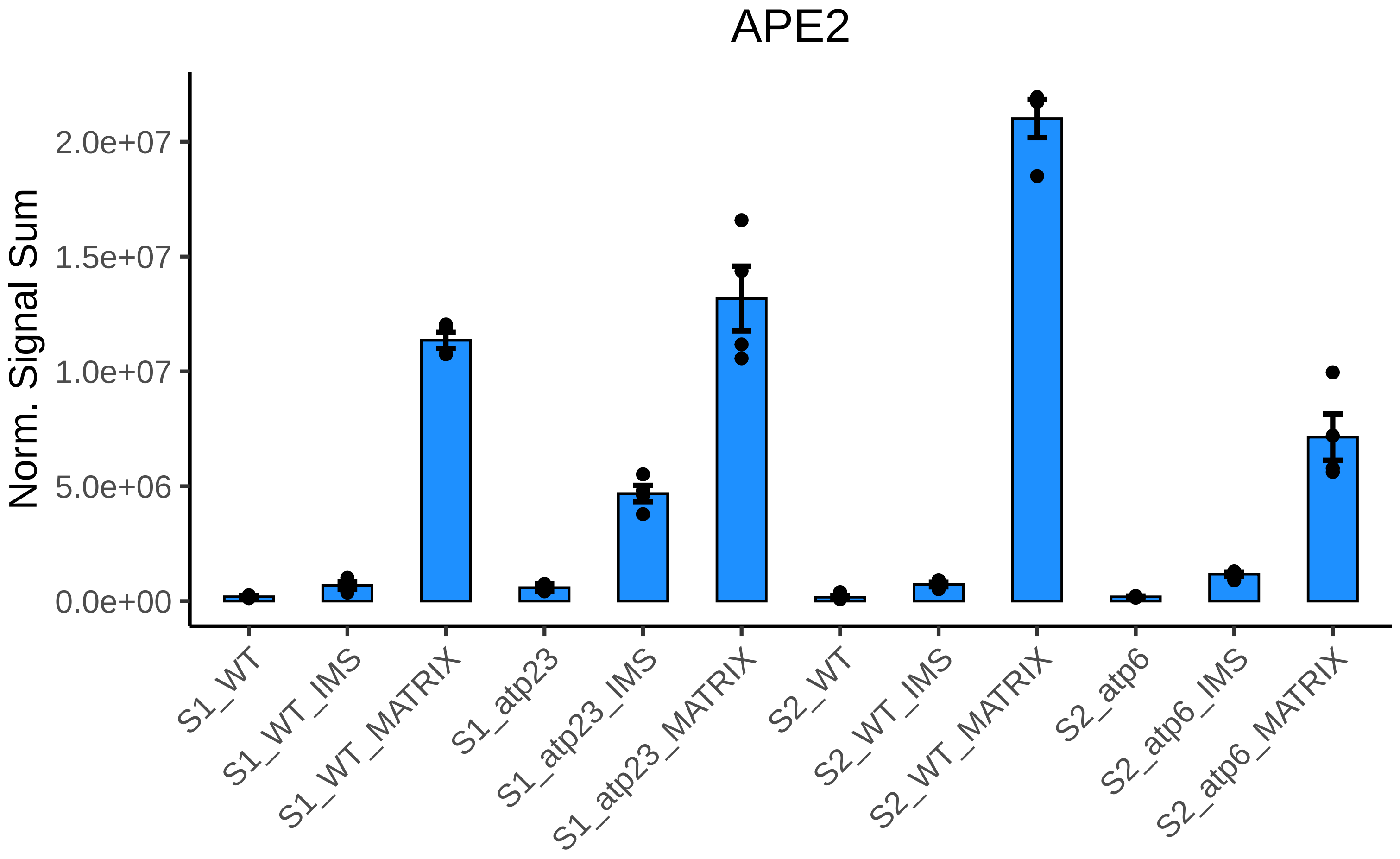

APE2
Norm. Signal Sum
2.0e+07
1.5e+07
1.0e+07
5.0e+06
0.0e+00
S1_WT
S1_WT_IMS
S1_WT_MATRIX
S1_atp23
S1_atp23_IMS
S1_atp23_MATRIX
S2_WT
S2_WT_IMS
S2_WT_MATRIX
S2_atp6
S2_atp6_IMS
S2_atp6_MATRIX

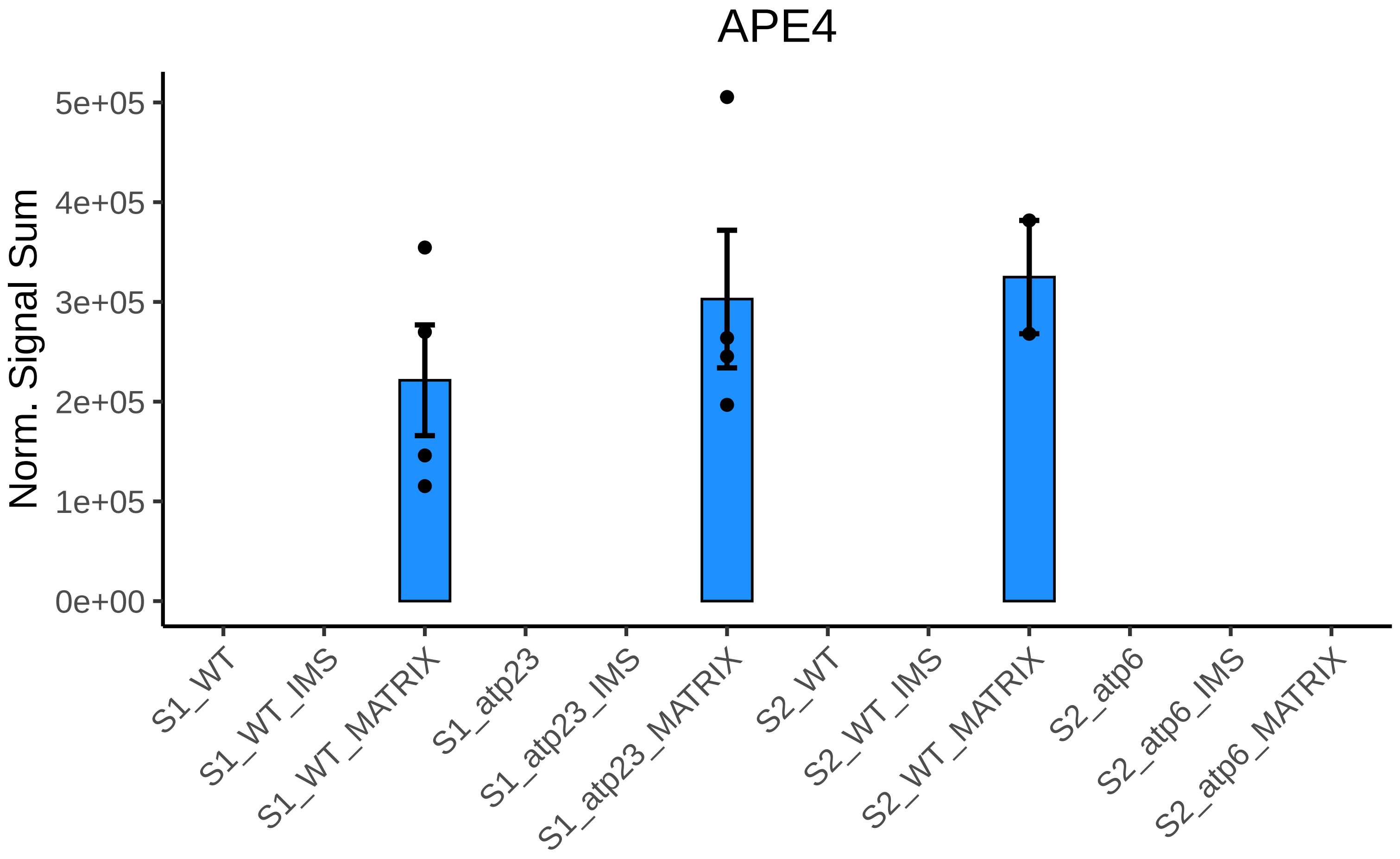
APE4
Norm. Signal Sum
5e+05
4e+05
3e+05
2e+05
1e+05
0e+00
S1_WT
S1_WT_IMS
S1_WT_MATRIX
S1_atp23
S1_atp23_IMS
S1_atp23_MATRIX
S2_WT
S2_WT_IMS
S2_WT_MATRIX
S2_atp6
S2_atp6_IMS
S2_atp6_MATRIX

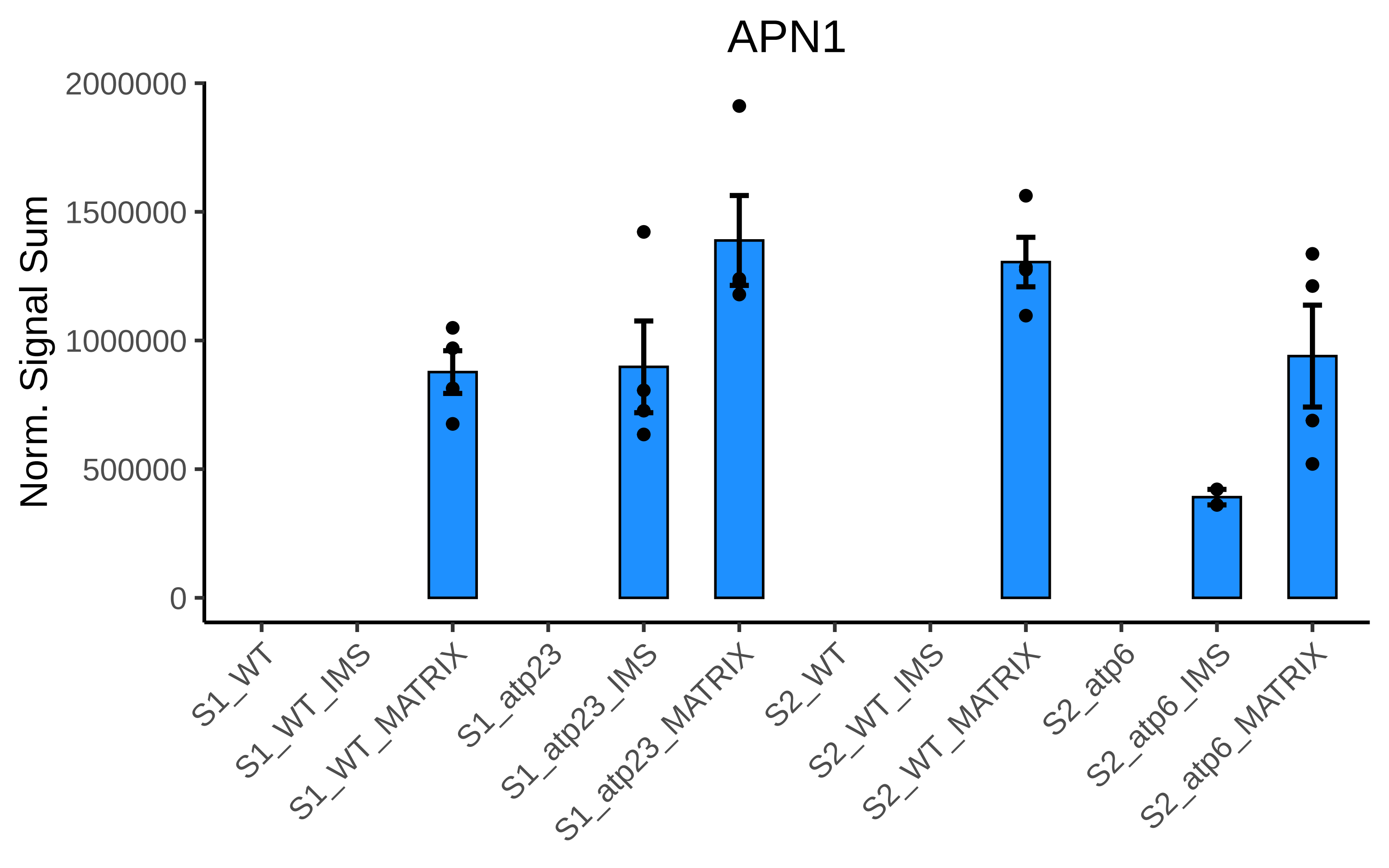
APN1
Norm. Signal Sum
2000000
1500000
1000000
500000
0
S1_WT
S1_WT_IMS
S1_WT_MATRIX
S1_atp23
S1_atp23_IMS
S1_atp23_MATRIX
S2_WT
S2_WT_IMS
S2_WT_MATRIX
S2_atp6
S2_atp6_IMS
S2_atp6_MATRIX

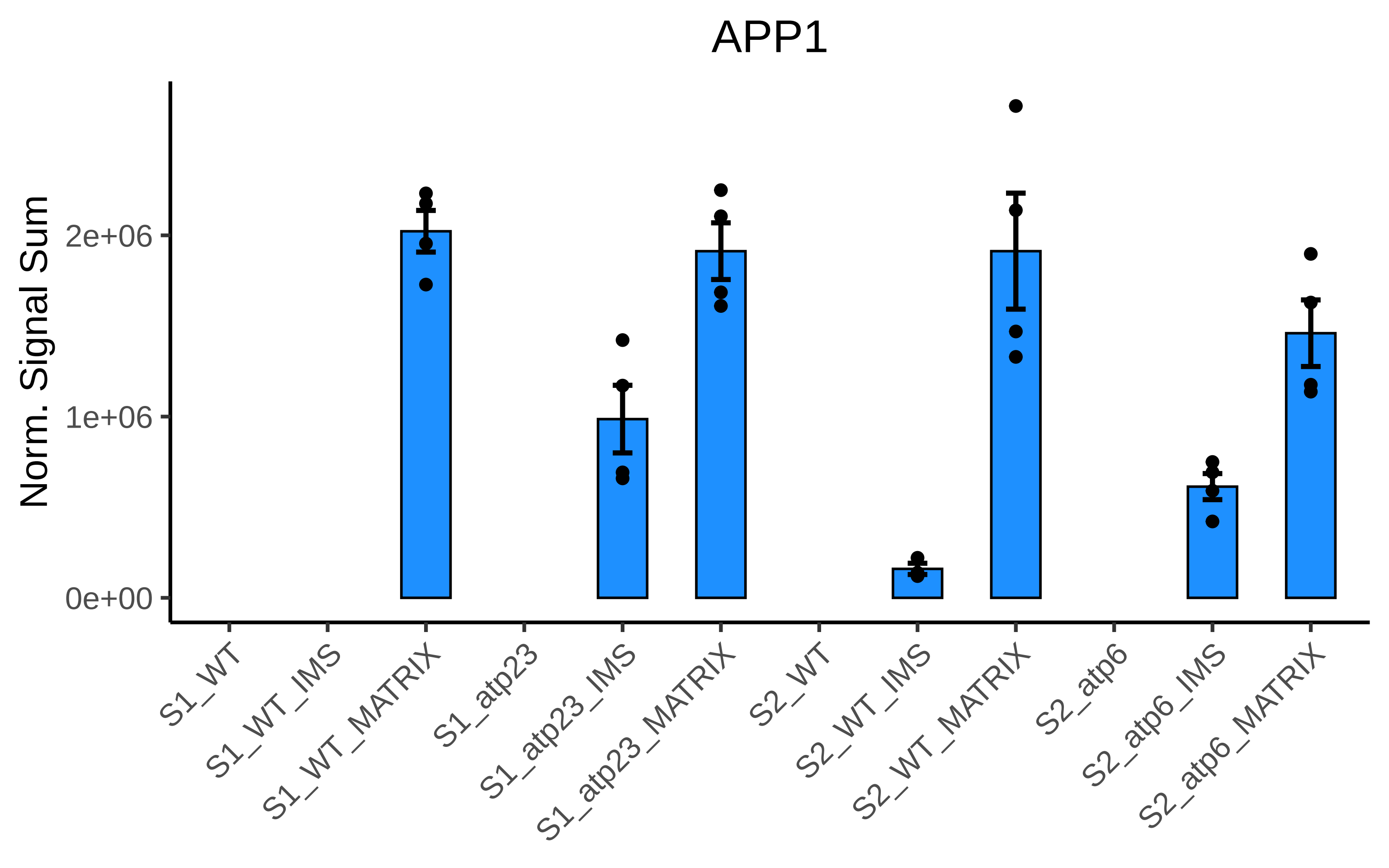
APP1
Norm. Signal Sum
2e+06
1e+06
0e+00
S1_WT
S1_WT_IMS
S1_WT_MATRIX
S1_atp23
S1_atp23_IMS
S1_atp23_MATRIX
S2_WT
S2_WT_IMS
S2_WT_MATRIX
S2_atp6
S2_atp6_IMS
S2_atp6_MATRIX

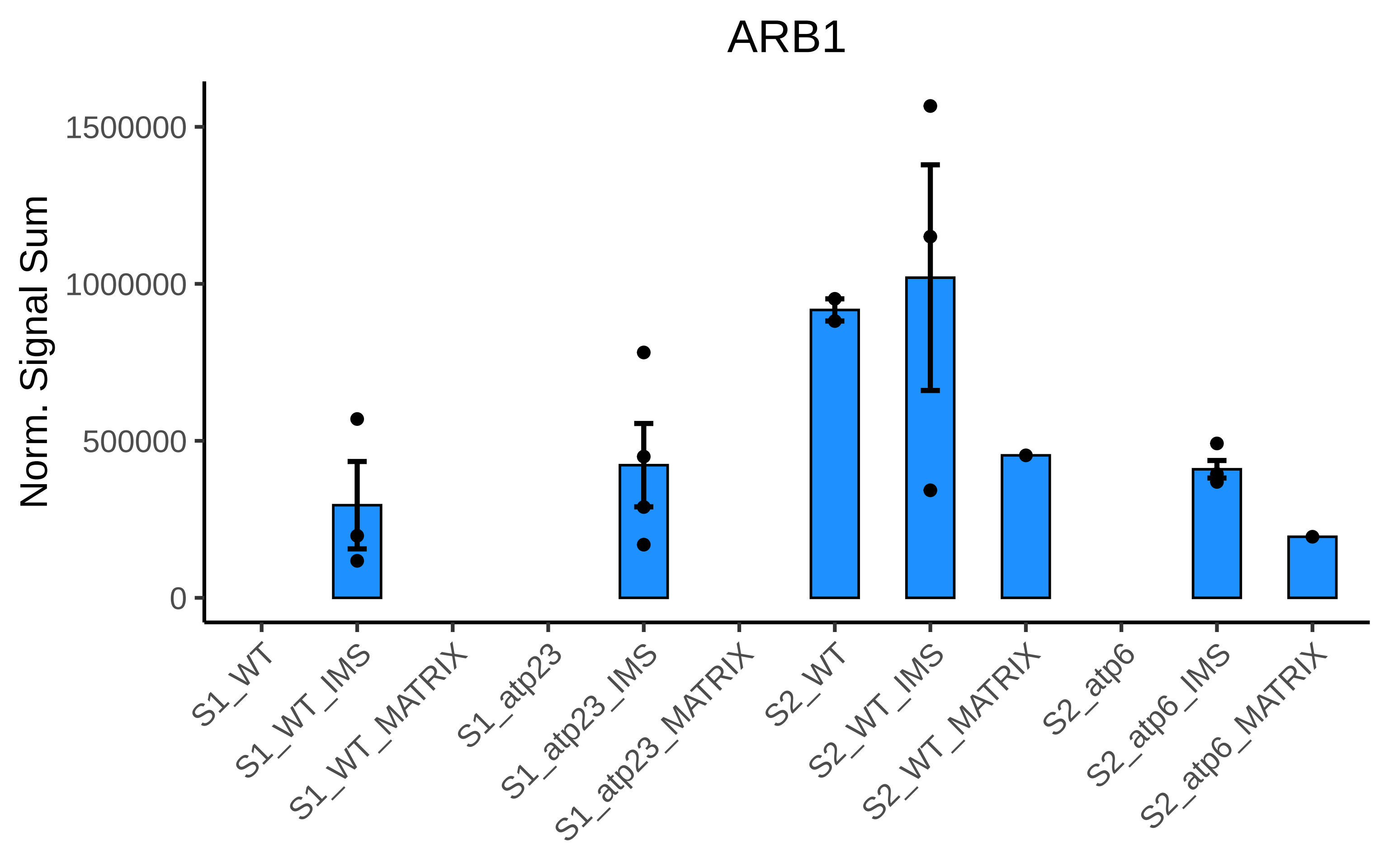
ARB1
Norm. Signal Sum
1500000
1000000
500000
0
S1_WT
S1_WT_IMS
S1_WT_MATRIX
S1_atp23
S1_atp23_IMS
S1_atp23_MATRIX
S2_WT
S2_WT_IMS
S2_WT_MATRIX
S2_atp6
S2_atp6_IMS
S2_atp6_MATRIX

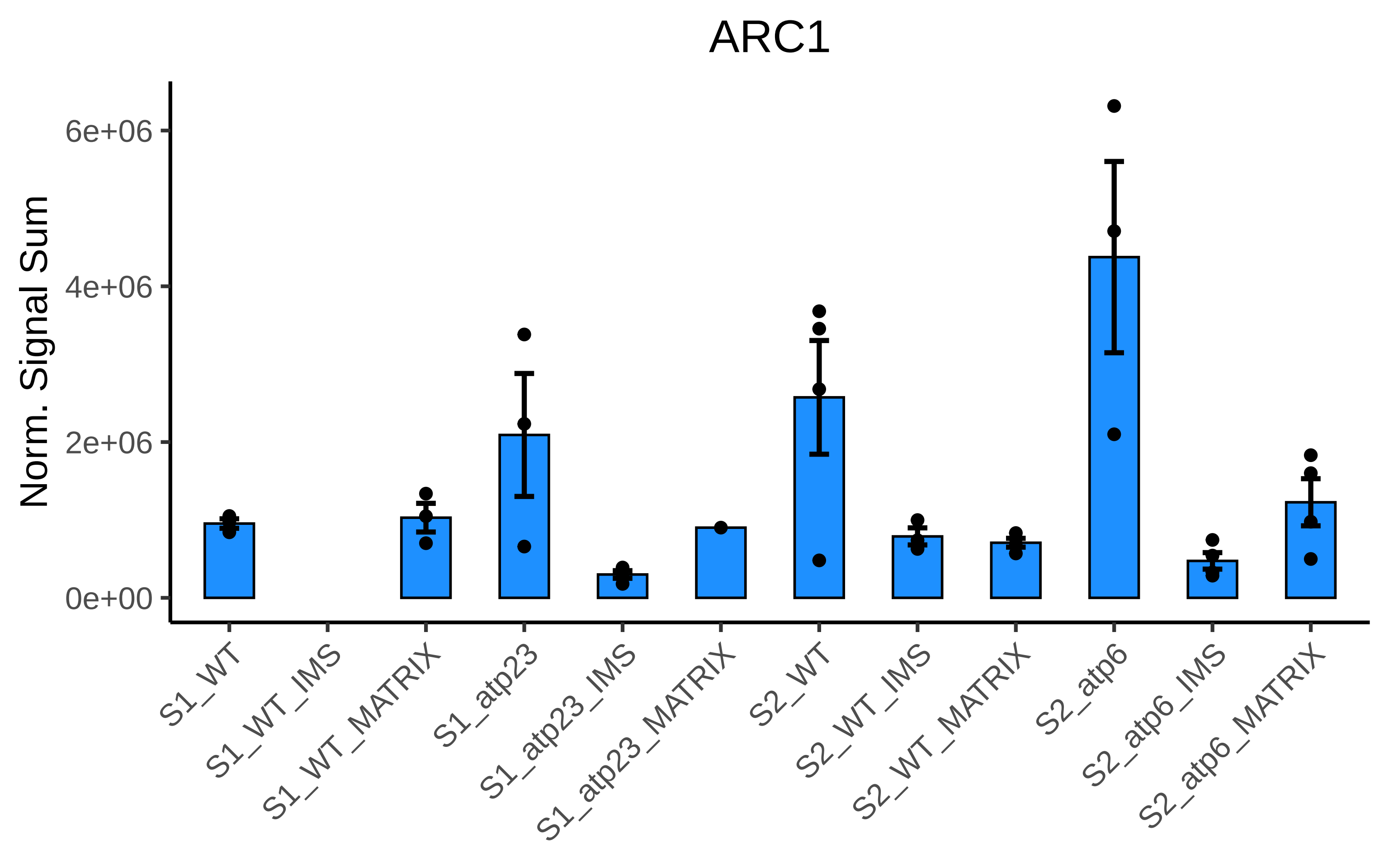

ARC1
Norm. Signal Sum
6e+06
4e+06
2e+06
0e+00
S1_WT
S1_WT_IMS
S1_WT_MATRIX
S1_atp23
S1_atp23_IMS
S1_atp23_MATRIX
S2_WT
S2_WT_IMS
S2_WT_MATRIX
S2_atp6
S2_atp6_IMS
S2_atp6_MATRIX

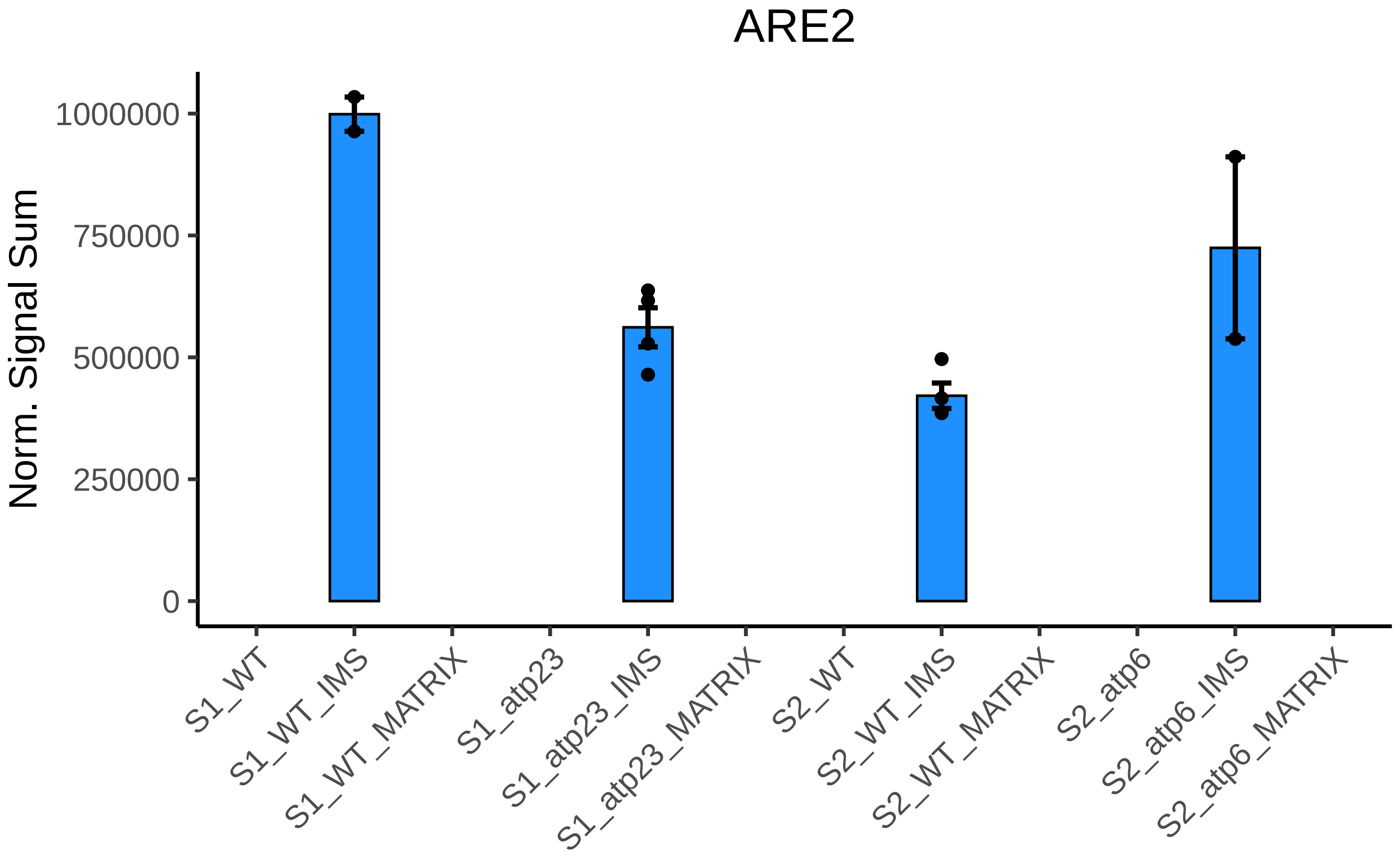

ARE2
Norm. Signal Sum
1000000
750000
500000
250000
0
S1_WT
S1_WT_IMS
S1_WT_MATRIX
S1_atp23
S1_atp23_IMS
S1_atp23_MATRIX
S2_WT
S2_WT_IMS
S2_WT_MATRIX
S2_atp6
S2_atp6_IMS
S2_atp6_MATRIX

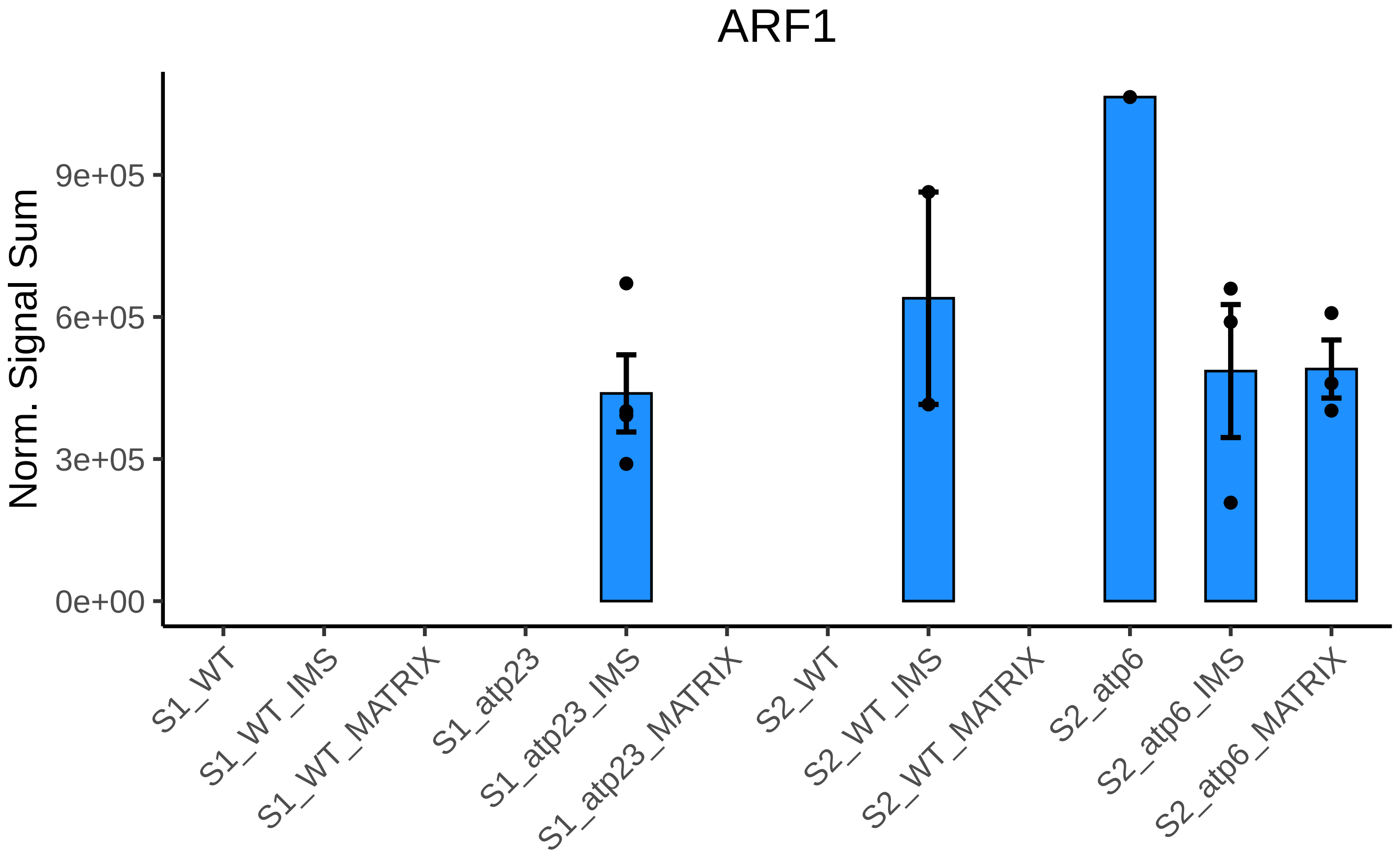

ARF1
Norm. Signal Sum
9e+05
6e+05
3e+05
0e+00
S1_WT
S1_WT_IMS
S1_WT_MATRIX
S1_atp23
S1_atp23_IMS
S1_atp23_MATRIX
S2_WT
S2_WT_IMS
S2_WT_MATRIX
S2_atp6
S2_atp6_IMS
S2_atp6_MATRIX

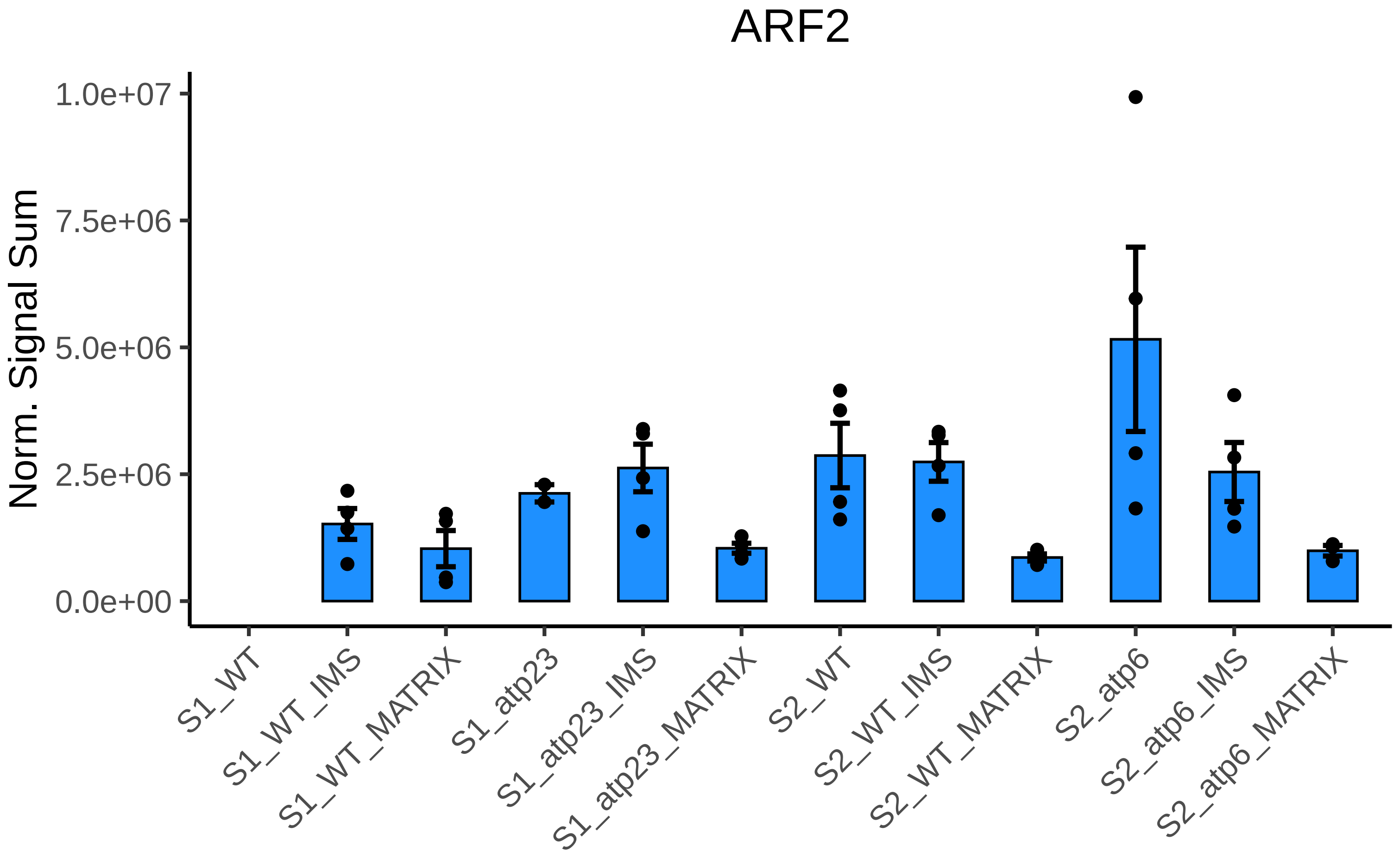
ARF2
Norm. Signal Sum
1.0e+07
7.5e+06
5.0e+06
2.5e+06
0.0e+00
S1_WT
S1_WT_IMS
S1_WT_MATRIX
S1_atp23
S1_atp23_IMS
S1_atp23_MATRIX
S2_WT
S2_WT_IMS
S2_WT_MATRIX
S2_atp6
S2_atp6_IMS
S2_atp6_MATRIX

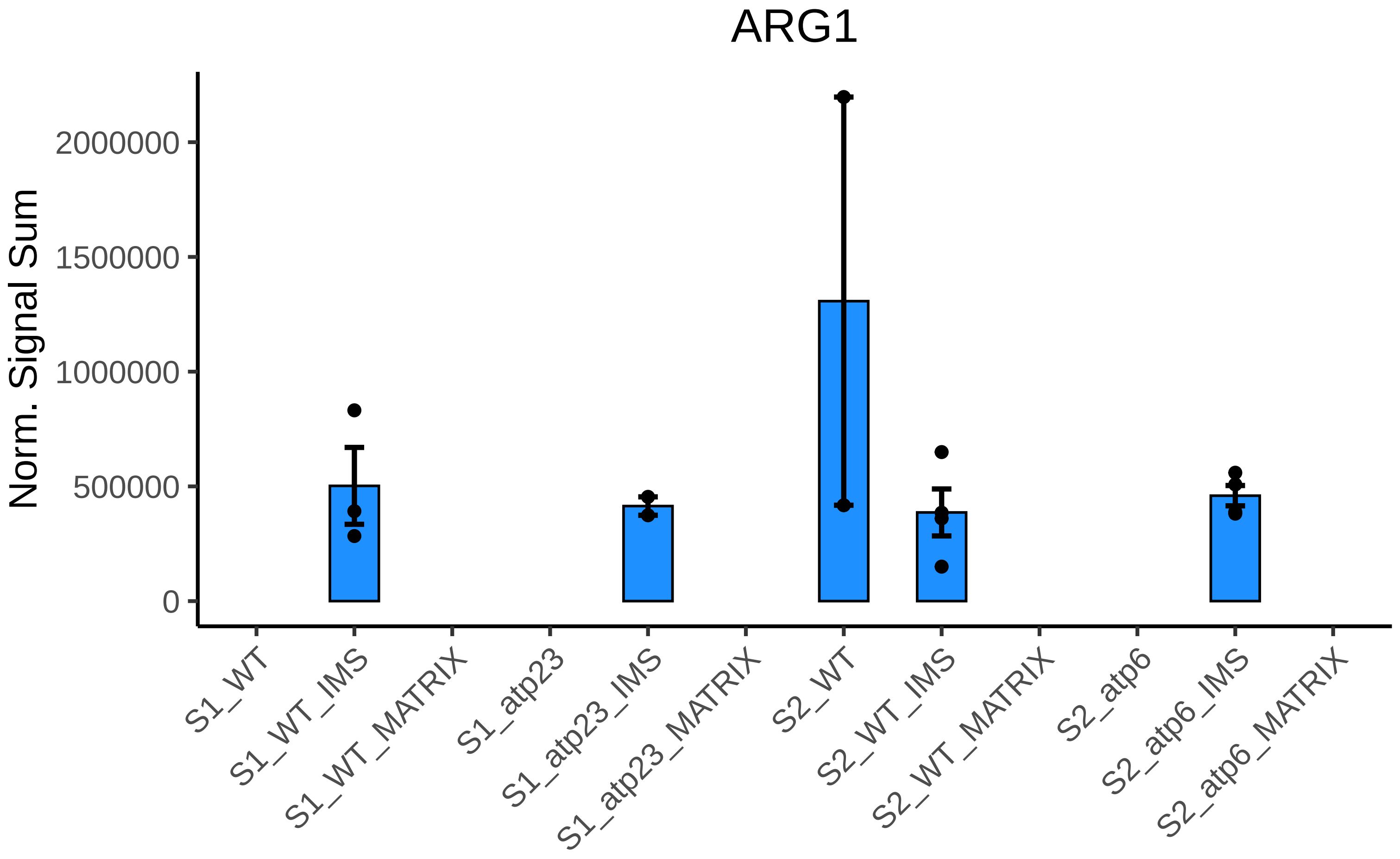
ARG1
Norm. Signal Sum
2000000
1500000
1000000
500000
0
S1_WT
S1_WT_IMS
S1_WT_MATRIX
S1_atp23
S1_atp23_IMS
S1_atp23_MATRIX
S2_WT
S2_WT_IMS
S2_WT_MATRIX
S2_atp6
S2_atp6_IMS
S2_atp6_MATRIX

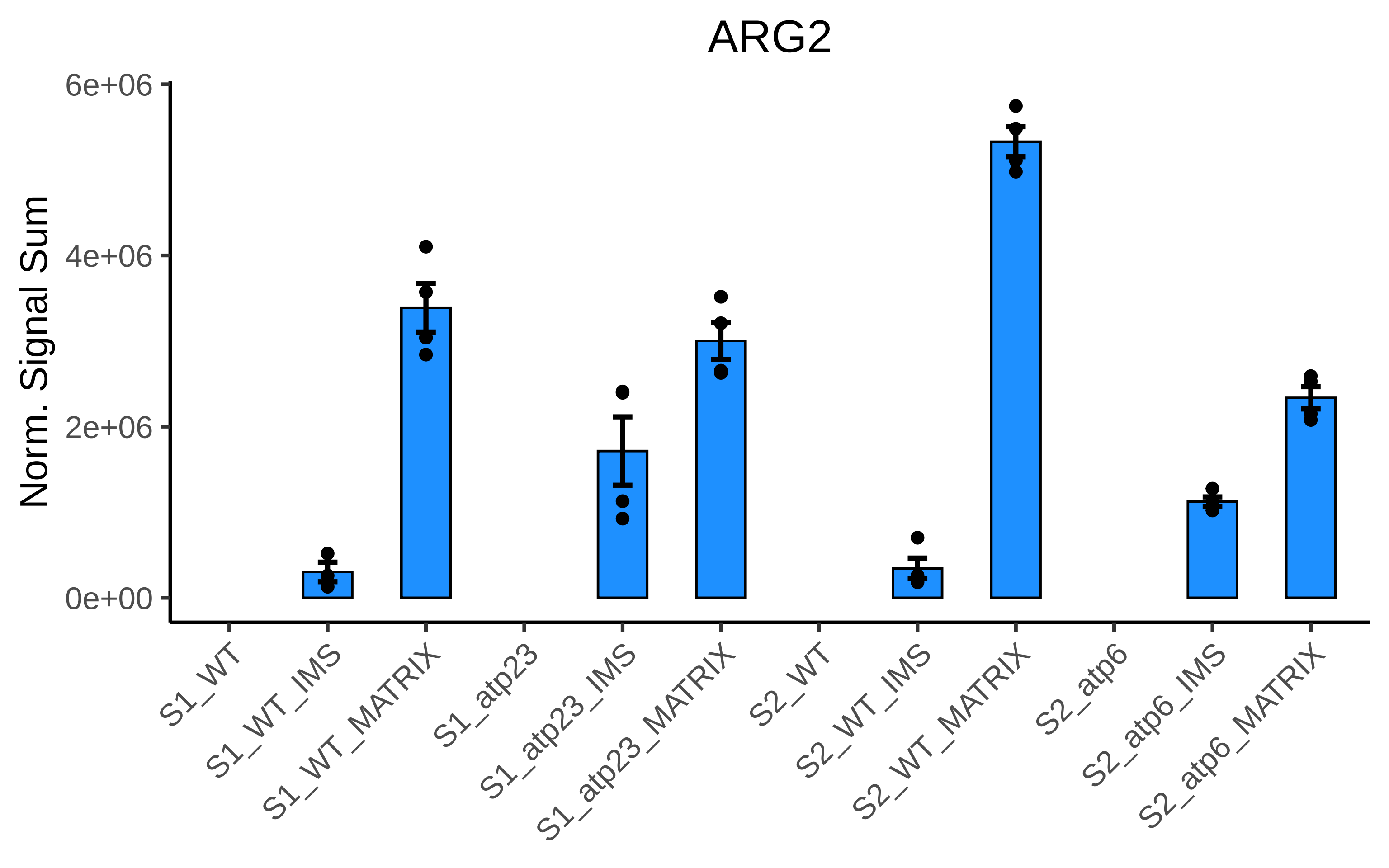
ARG2
Norm. Signal Sum
6e+06
4e+06
2e+06
0e+00
S1_WT
S1_WT_IMS
S1_WT_MATRIX
S1_atp23
S1_atp23_IMS
S1_atp23_MATRIX
S2_WT
S2_WT_IMS
S2_WT_MATRIX
S2_atp6
S2_atp6_IMS
S2_atp6_MATRIX

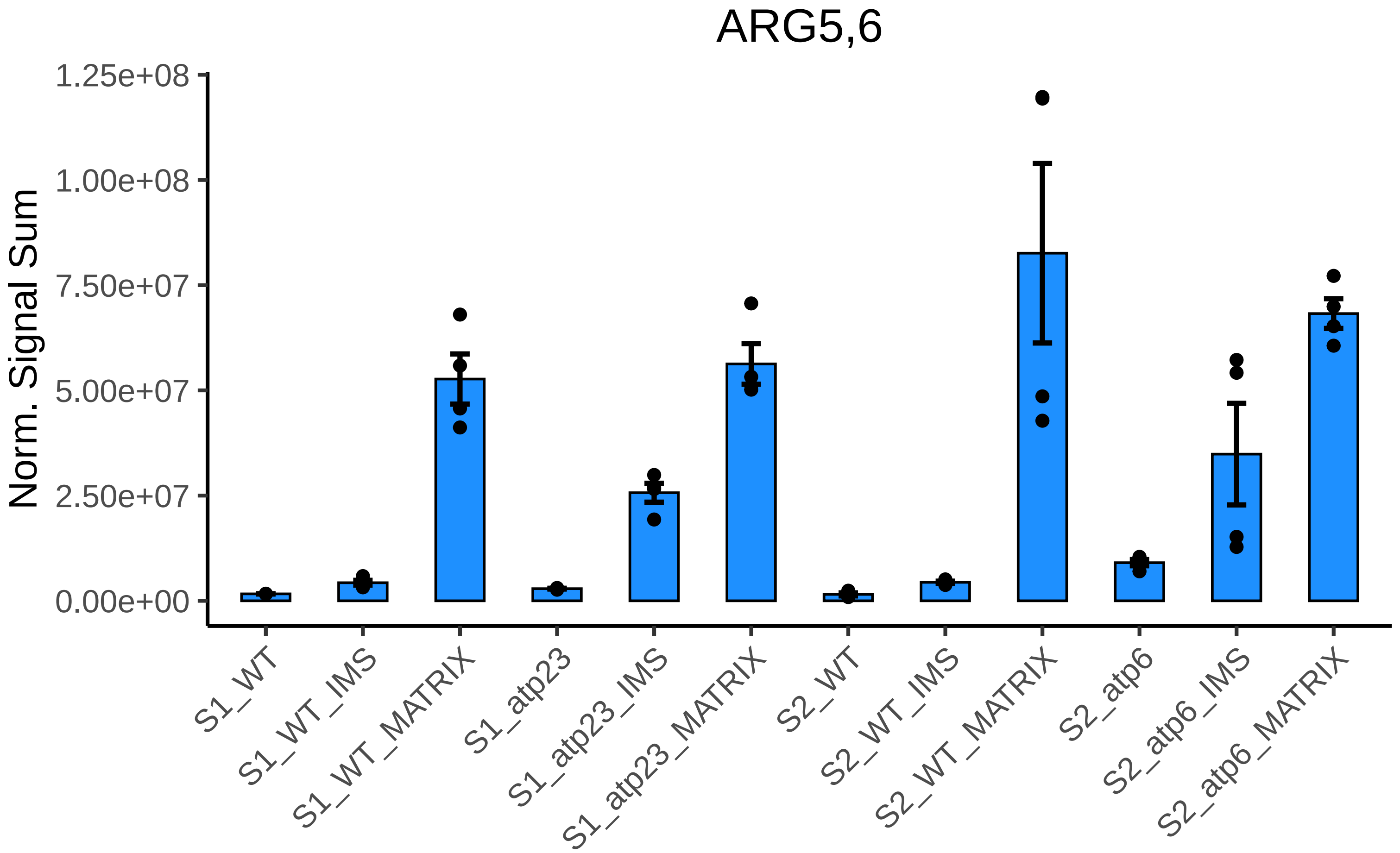

ARG5,6
Norm. Signal Sum
1.25e+08
1.00e+08
7.50e+07
5.00e+07
2.50e+07
0.00e+00
S1_WT
S1_WT_IMS
S1_WT_MATRIX
S1_atp23
S1_atp23_IMS
S1_atp23_MATRIX
S2_WT
S2_WT_IMS
S2_WT_MATRIX
S2_atp6
S2_atp6_IMS
S2_atp6_MATRIX

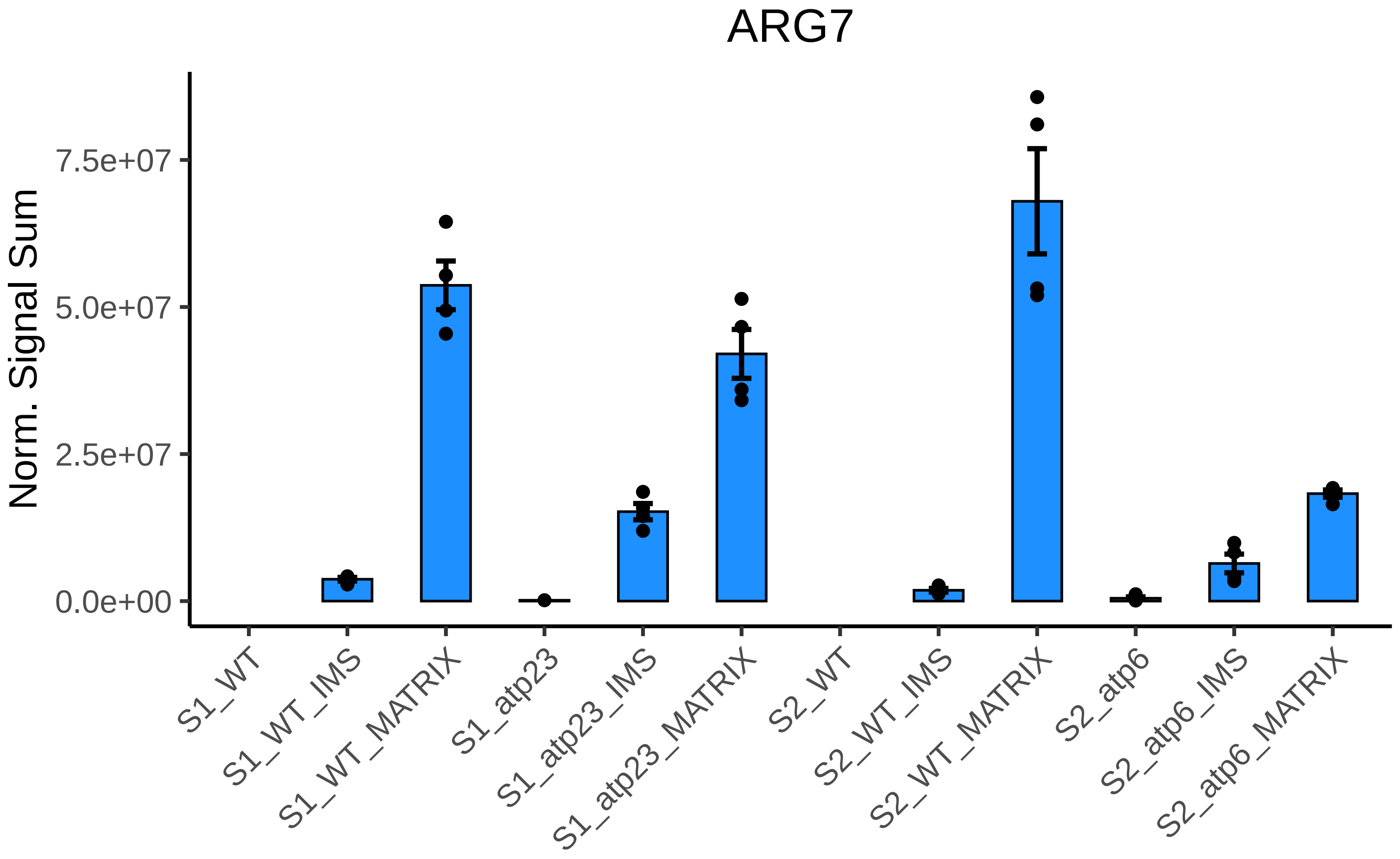

ARG7
Norm. Signal Sum
7.5e+07
5.0e+07
2.5e+07
0.0e+00
S1_WT
S1_WT_IMS
S1_WT_MATRIX
S1_atp23
S1_atp23_IMS
S1_atp23_MATRIX
S2_WT
S2_WT_IMS
S2_WT_MATRIX
S2_atp6
S2_atp6_IMS
S2_atp6_MATRIX

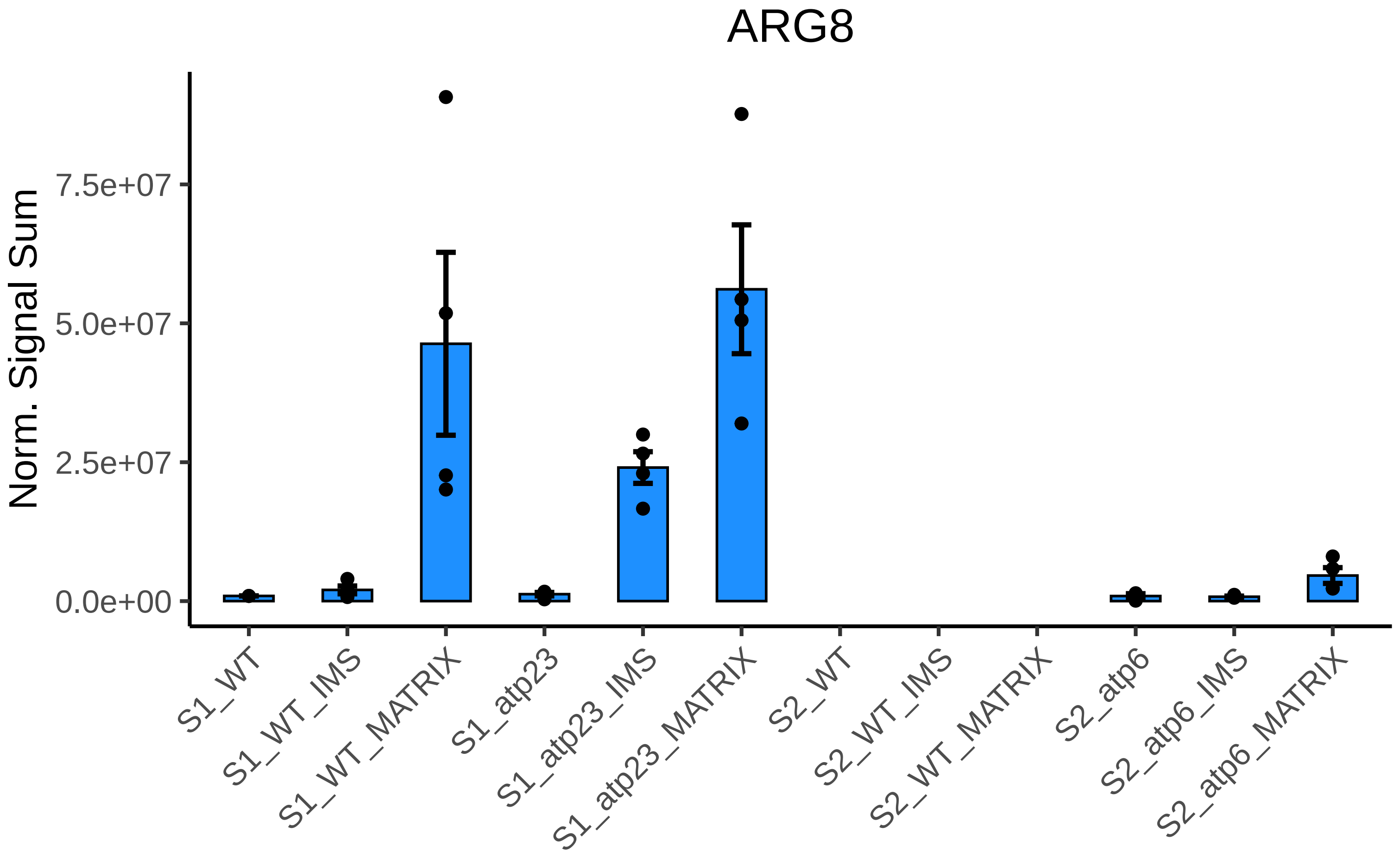

ARG8
Norm. Signal Sum
7.5e+07
5.0e+07
2.5e+07
0.0e+00
S1_WT
S1_WT_IMS
S1_WT_MATRIX
S1_atp23
S1_atp23_IMS
S1_atp23_MATRIX
S2_WT
S2_WT_IMS
S2_WT_MATRIX
S2_atp6
S2_atp6_IMS
S2_atp6_MATRIX

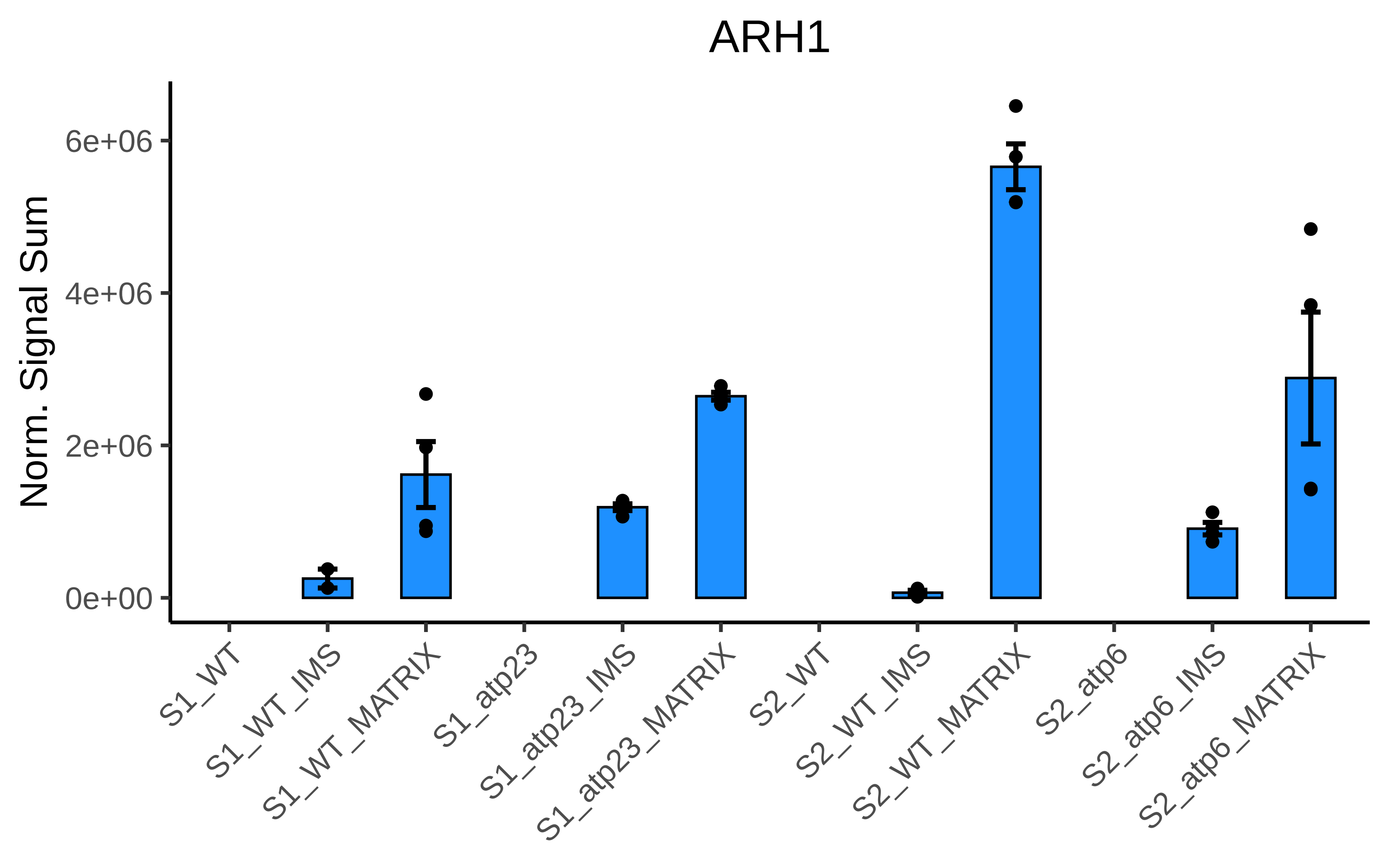
ARH1
Norm. Signal Sum
6e+06
4e+06
2e+06
0e+00
S1_WT
S1_WT_IMS
S1_WT_MATRIX
S1_atp23
S1_atp23_IMS
S1_atp23_MATRIX
S2_WT
S2_WT_IMS
S2_WT_MATRIX
S2_atp6
S2_atp6_IMS
S2_atp6_MATRIX

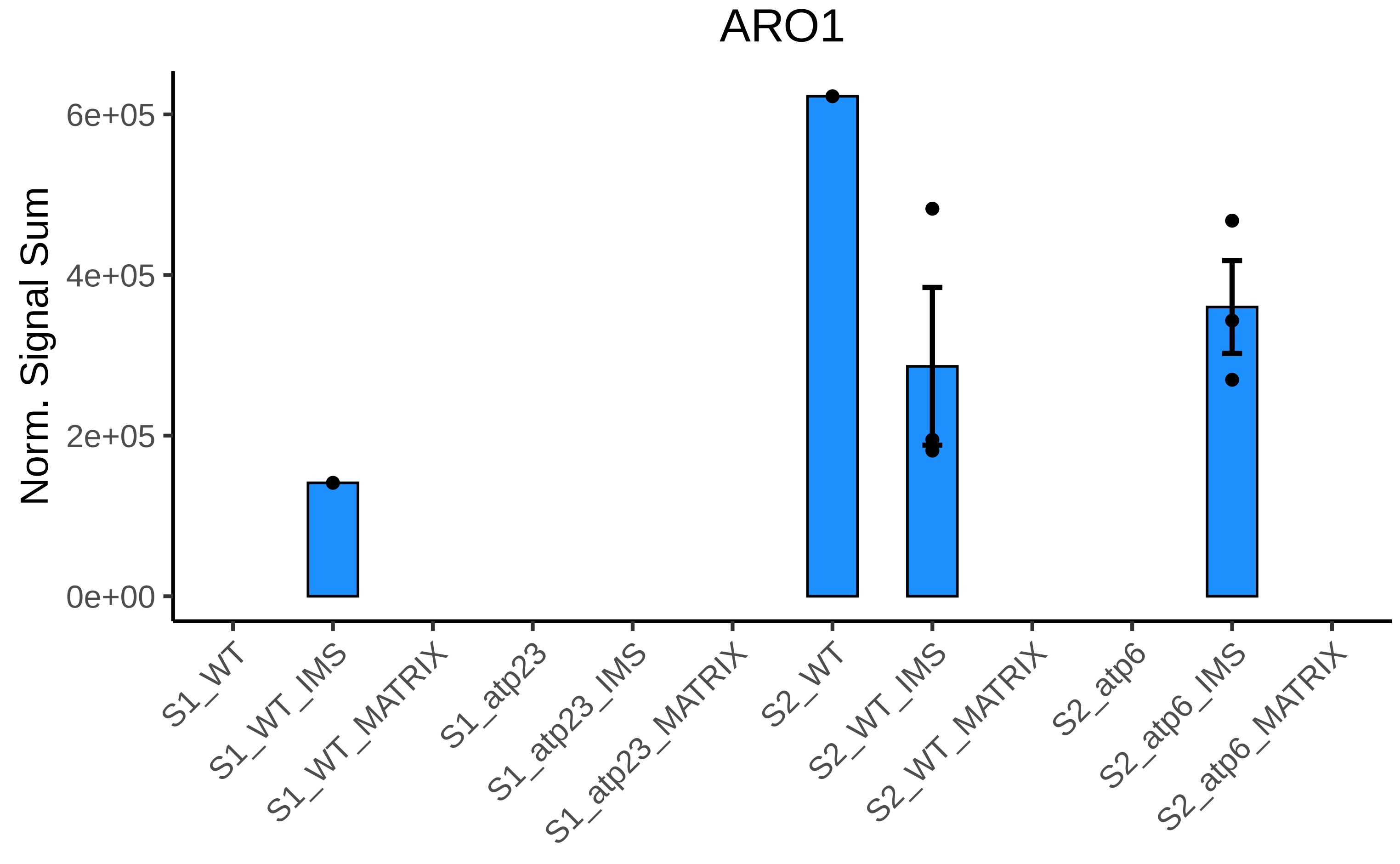

ARO1
Norm. Signal Sum
6e+05
4e+05
2e+05
0e+00
S1_WT
S1_WT_IMS
S1_WT_MATRIX
S1_atp23
S1_atp23_IMS
S1_atp23_MATRIX
S2_WT
S2_WT_IMS
S2_WT_MATRIX
S2_atp6
S2_atp6_IMS
S2_atp6_MATRIX

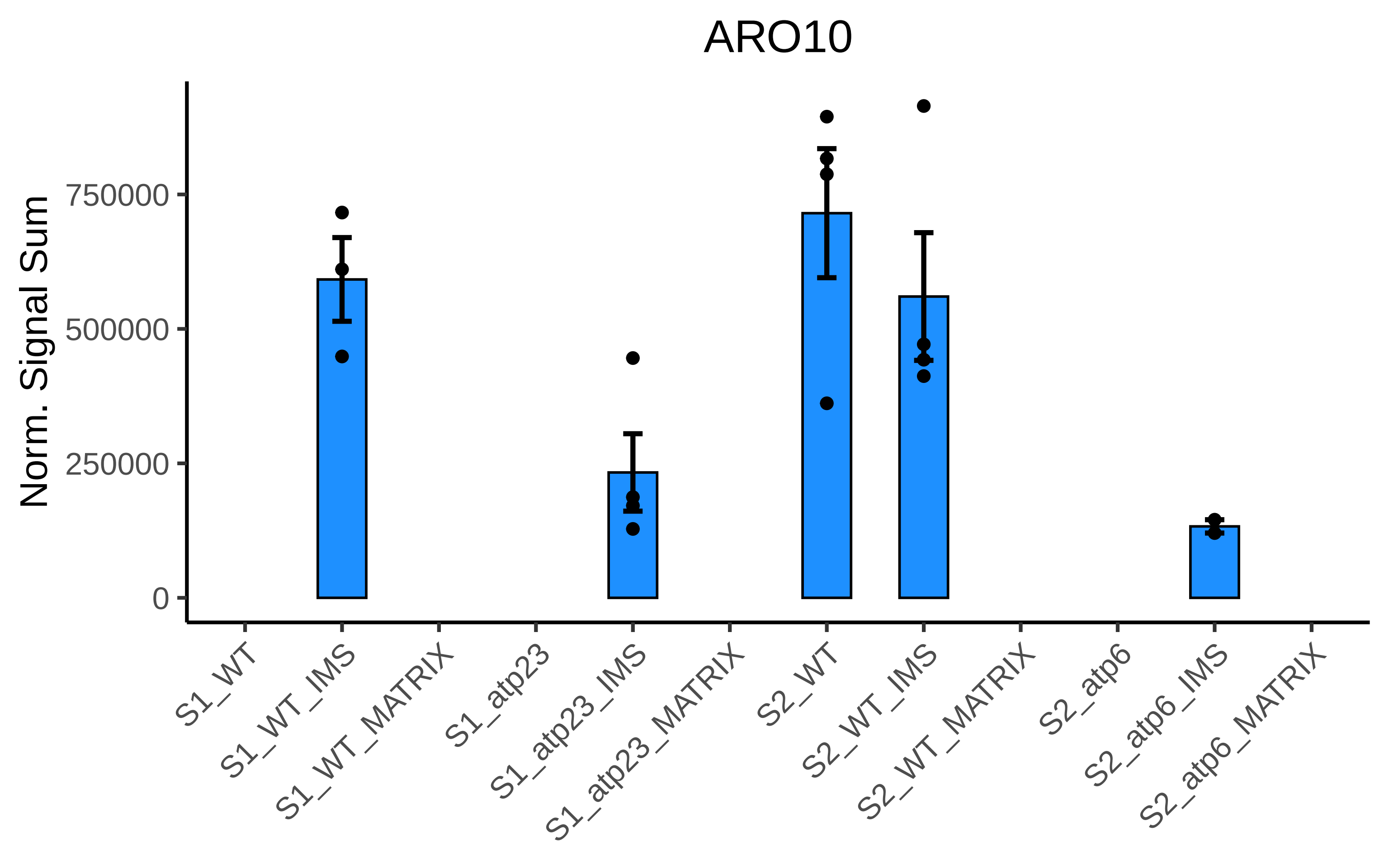

ARO10
Norm. Signal Sum
750000
500000
250000
0
S1_WT
S1_WT_IMS
S1_WT_MATRIX
S1_atp23
S1_atp23_IMS
S1_atp23_MATRIX
S2_WT
S2_WT_IMS
S2_WT_MATRIX
S2_atp6
S2_atp6_IMS
S2_atp6_MATRIX

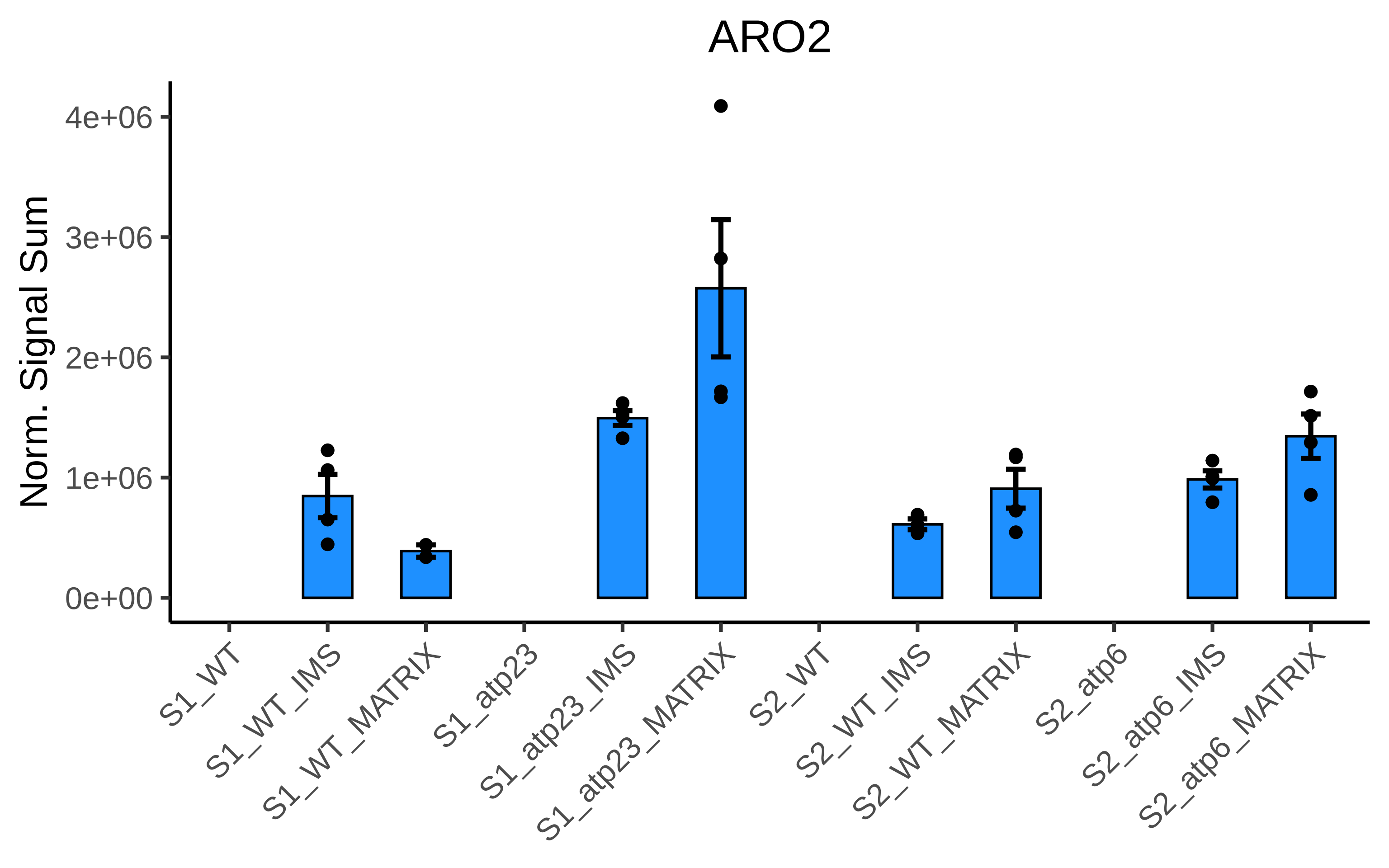
ARO2
Norm. Signal Sum
4e+06
3e+06
2e+06
1e+06
0e+00
S1_WT
S1_WT_IMS
S1_WT_MATRIX
S1_atp23
S1_atp23_IMS
S1_atp23_MATRIX
S2_WT
S2_WT_IMS
S2_WT_MATRIX
S2_atp6
S2_atp6_IMS
S2_atp6_MATRIX

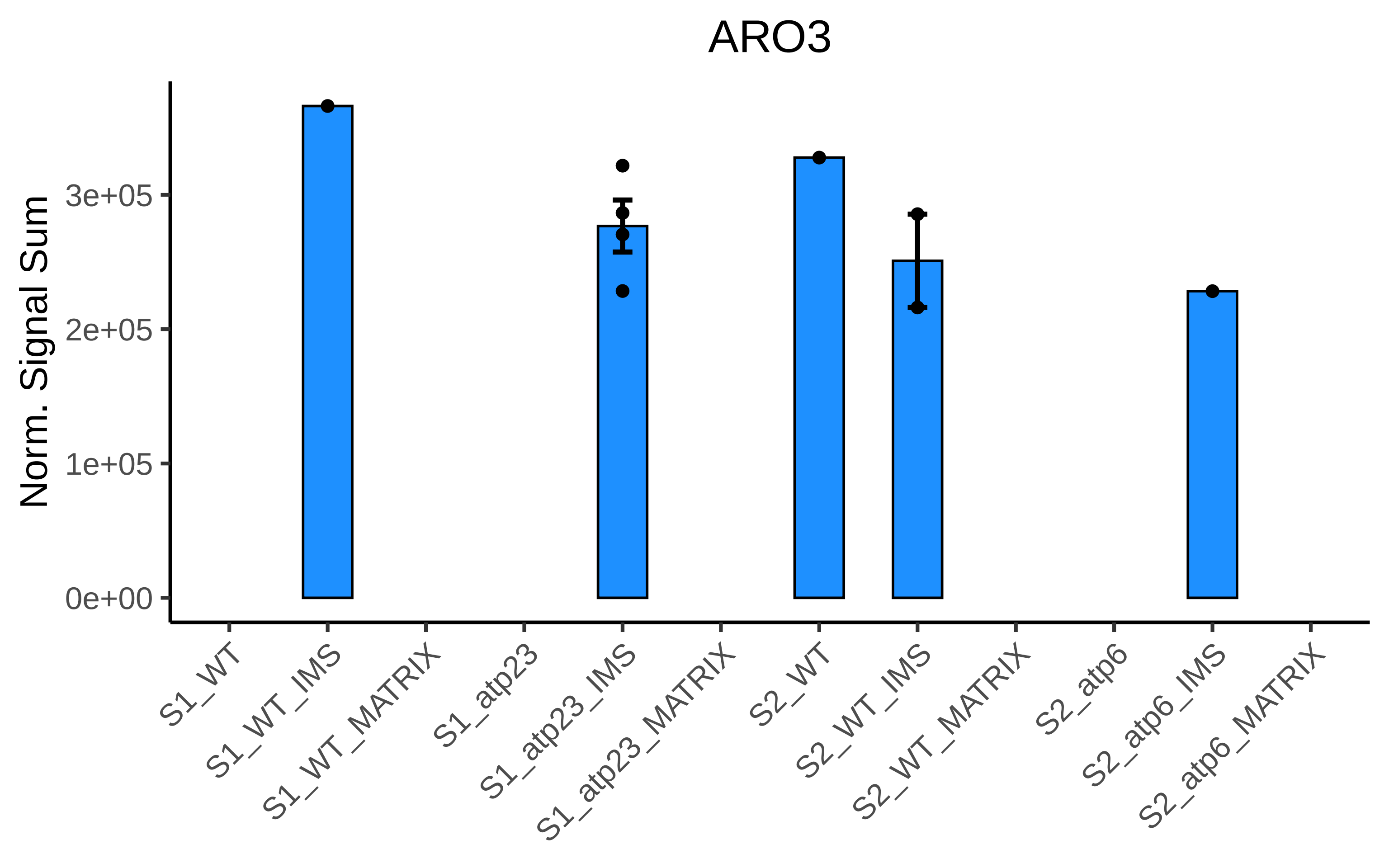
ARO3
Norm. Signal Sum
0e+00
1e+05
2e+05
3e+05
S1_WT
S1_WT_IMS
S1_WT_MATRIX
S1_atp23
S1_atp23_IMS
S1_atp23_MATRIX
S2_WT
S2_WT_IMS
S2_WT_MATRIX
S2_atp6
S2_atp6_IMS
S2_atp6_MATRIX

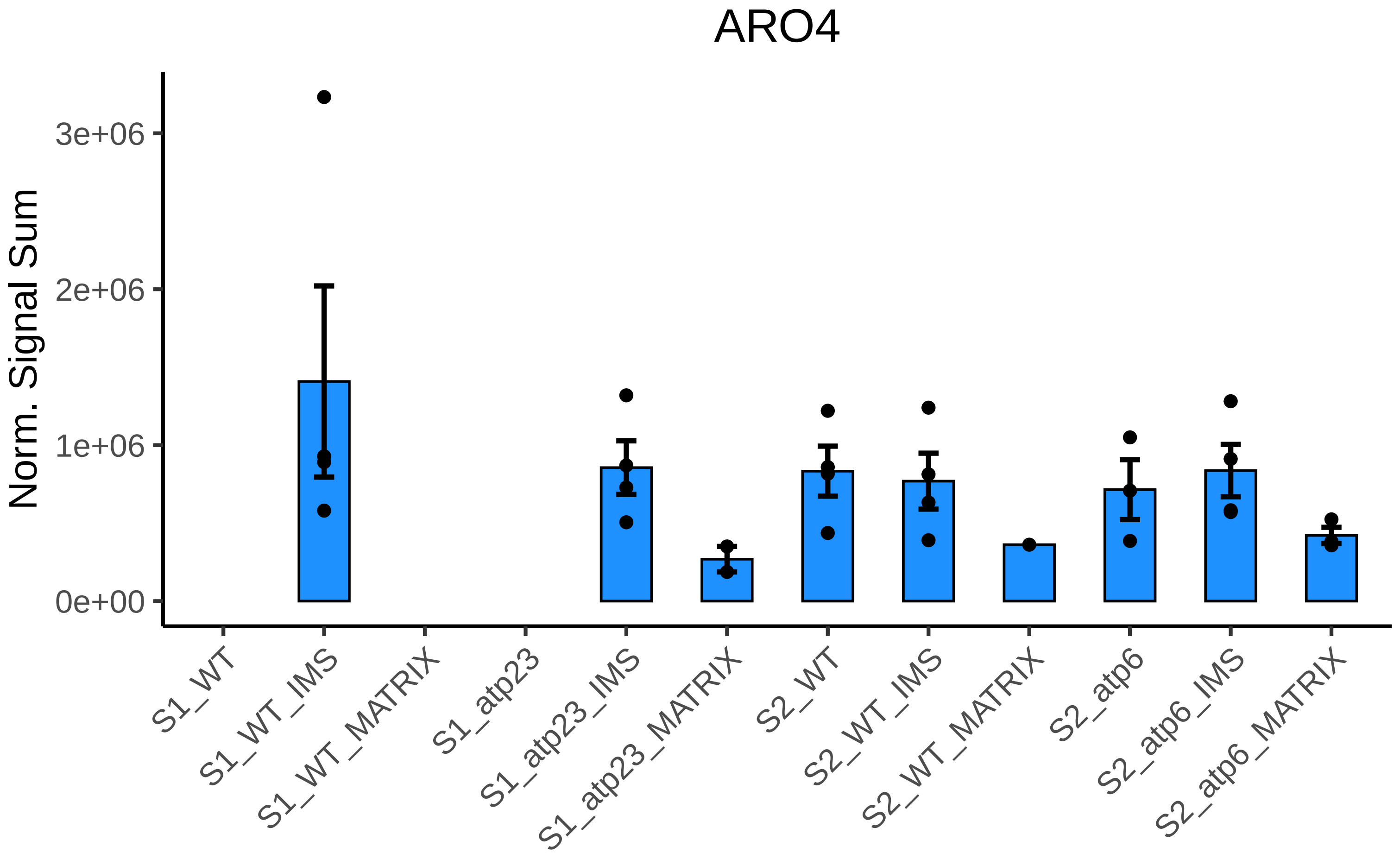

ARO4
Norm. Signal Sum
3e+06
2e+06
1e+06
0e+00
S1_WT
S1_WT_IMS
S1_WT_MATRIX
S1_atp23
S1_atp23_IMS
S1_atp23_MATRIX
S2_WT
S2_WT_IMS
S2_WT_MATRIX
S2_atp6
S2_atp6_IMS
S2_atp6_MATRIX

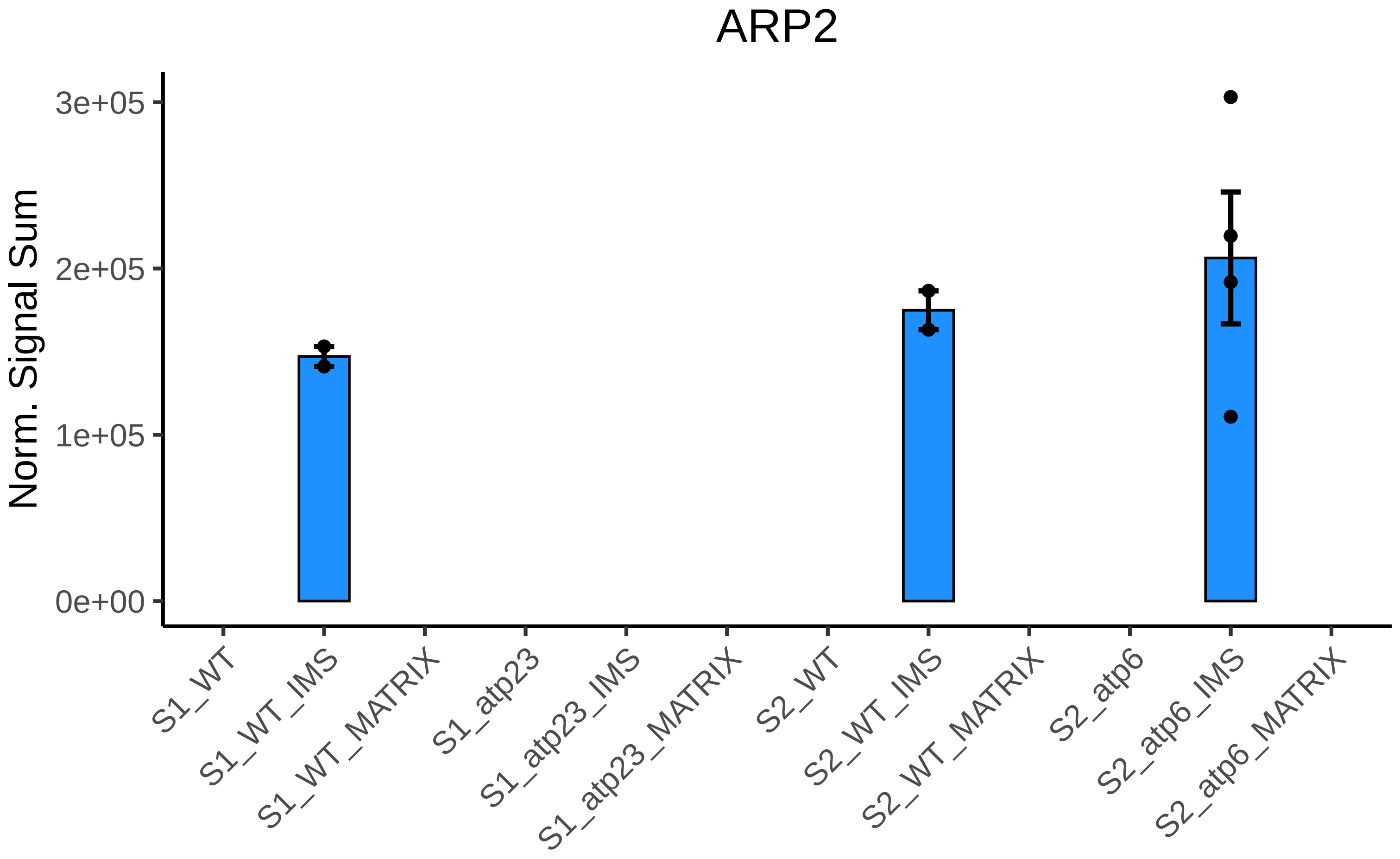
ARP2
Norm. Signal Sum
3e+05
2e+05
1e+05
0e+00
S1_WT
S1_WT_IMS
S1_WT_MATRIX
S1_atp23
S1_atp23_IMS
S1_atp23_MATRIX
S2_WT
S2_WT_IMS
S2_WT_MATRIX
S2_atp6
S2_atp6_IMS
S2_atp6_MATRIX

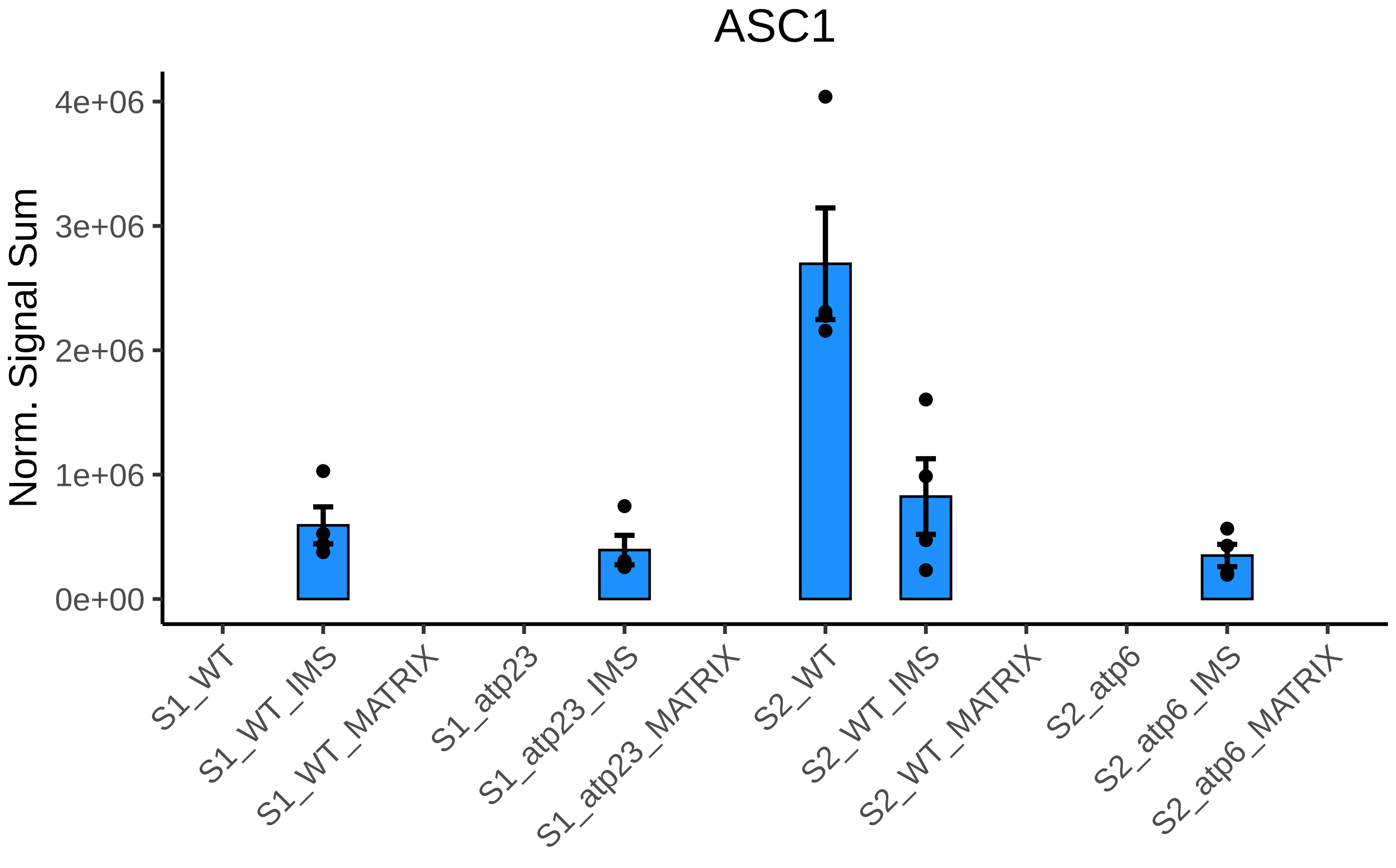
ASC1
Norm. Signal Sum
0e+00
1e+06
2e+06
3e+06
4e+06
S1_WT
S1_WT_IMS
S1_WT_MATRIX
S1_atp23
S1_atp23_IMS
S1_atp23_MATRIX
S2_WT
S2_WT_IMS
S2_WT_MATRIX
S2_atp6
S2_atp6_IMS
S2_atp6_MATRIX

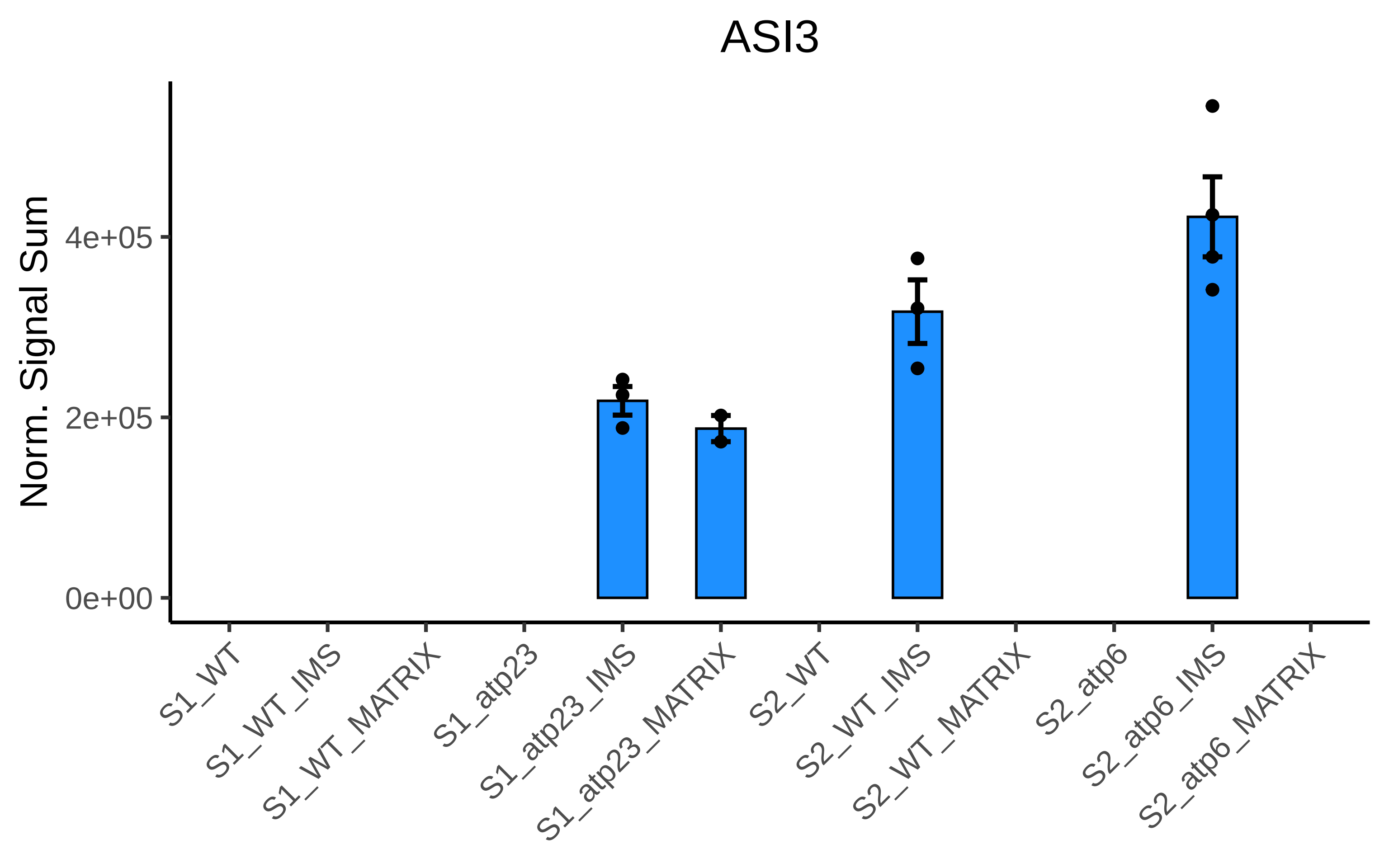

ASI3
Norm. Signal Sum
4e+05
2e+05
0e+00
S1_WT
S1_WT_IMS
S1_WT_MATRIX
S1_atp23
S1_atp23_IMS
S1_atp23_MATRIX
S2_WT
S2_WT_IMS
S2_WT_MATRIX
S2_atp6
S2_atp6_IMS
S2_atp6_MATRIX

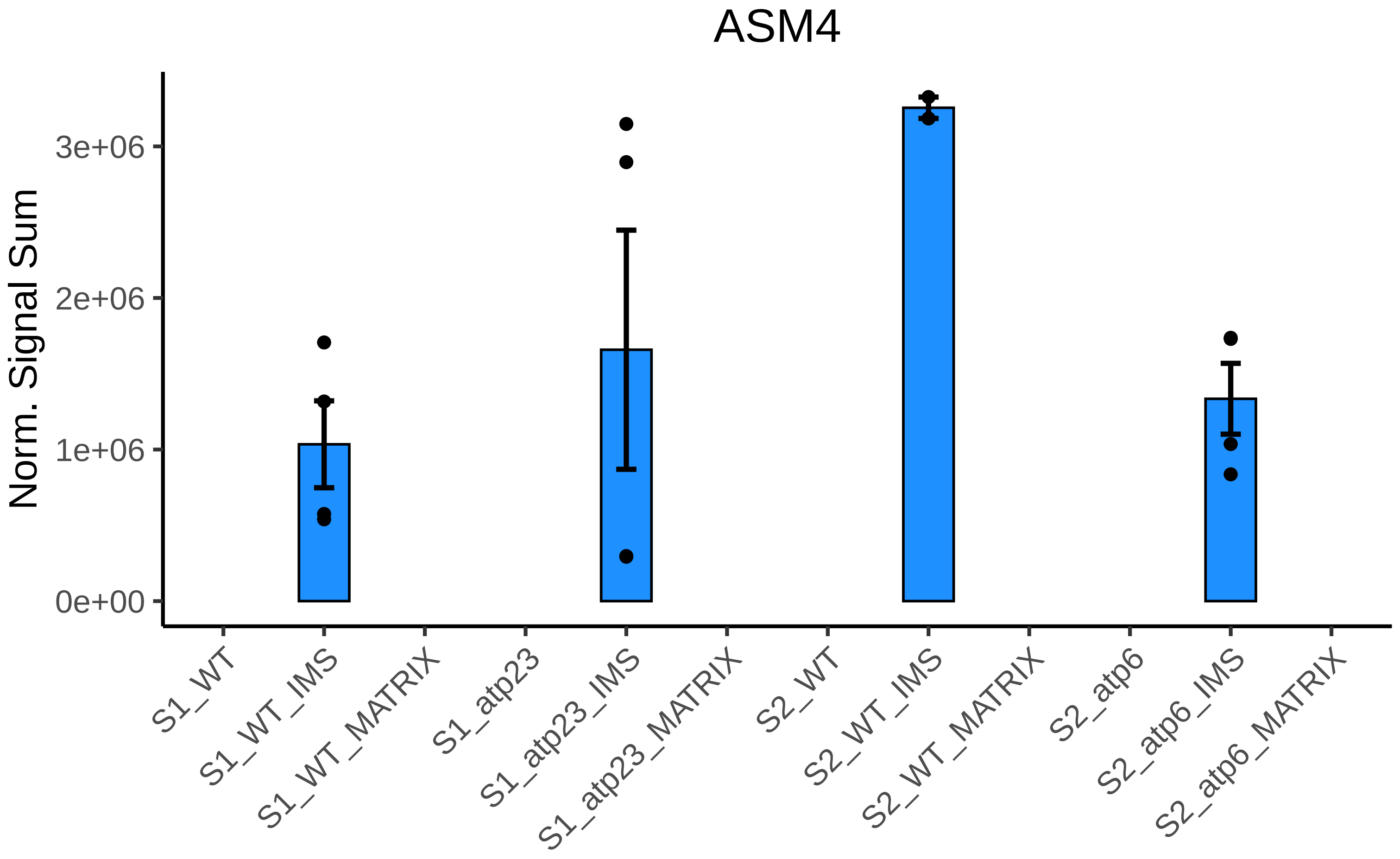
ASM4
Norm. Signal Sum
0e+00
1e+06
2e+06
3e+06
S1_WT
S1_WT_IMS
S1_WT_MATRIX
S1_atp23
S1_atp23_IMS
S1_atp23_MATRIX
S2_WT
S2_WT_IMS
S2_WT_MATRIX
S2_atp6
S2_atp6_IMS
S2_atp6_MATRIX

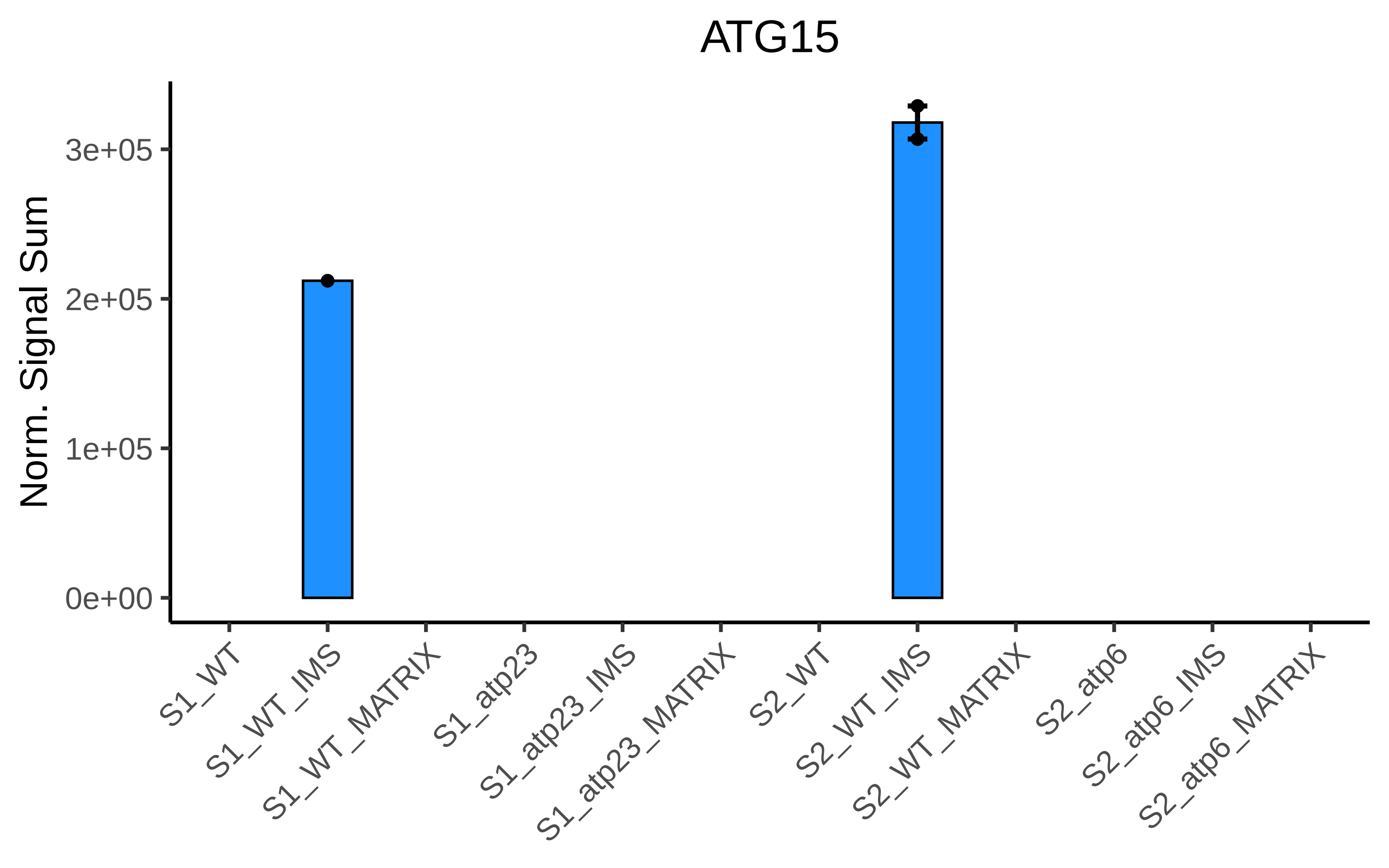
ATG15
Norm. Signal Sum
3e+05
2e+05
1e+05
0e+00
S1_WT
S1_WT_IMS
S1_WT_MATRIX
S1_atp23
S1_atp23_IMS
S1_atp23_MATRIX
S2_WT
S2_WT_IMS
S2_WT_MATRIX
S2_atp6
S2_atp6_IMS
S2_atp6_MATRIX

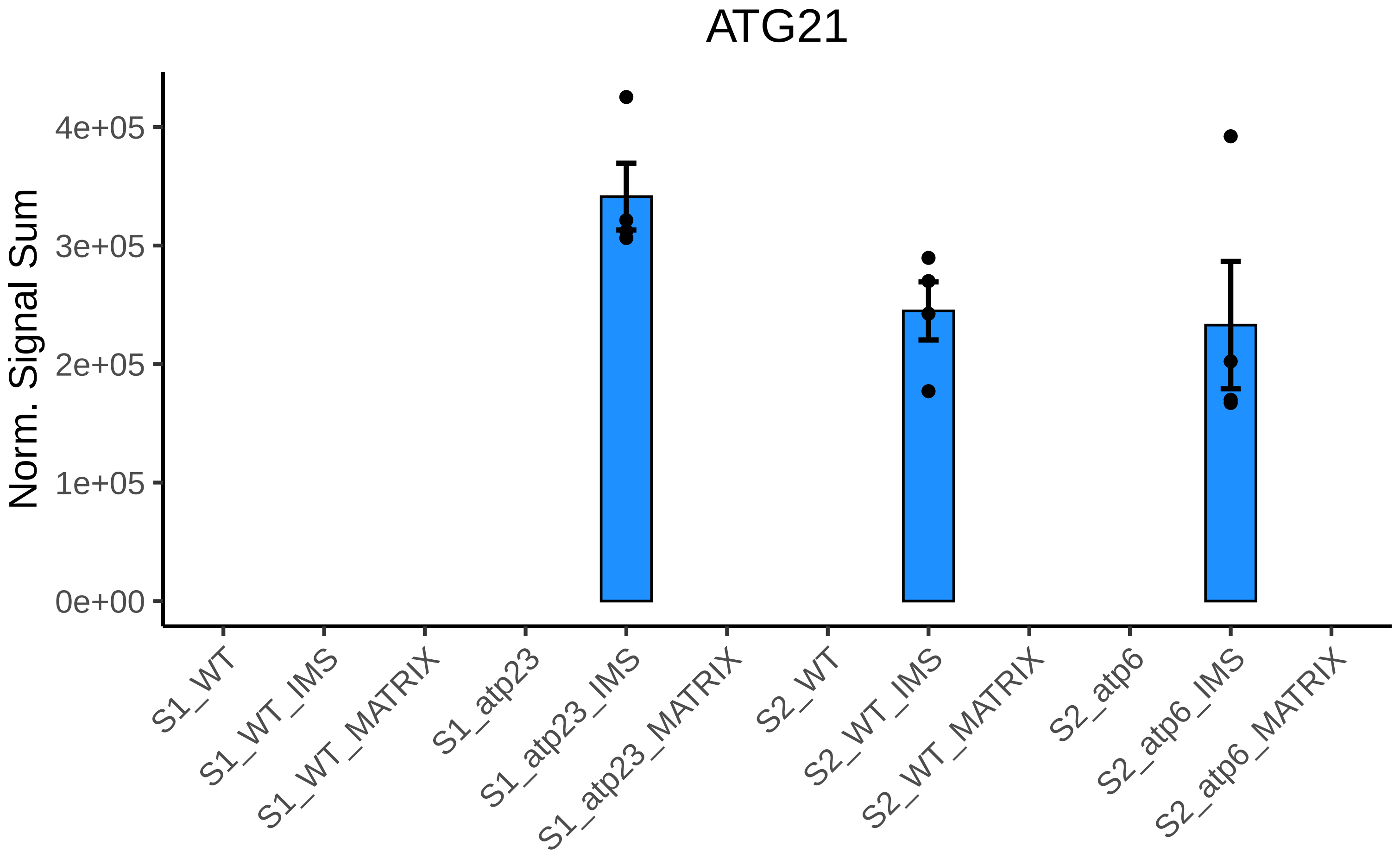

ATG21
Norm. Signal Sum
4e+05
3e+05
2e+05
1e+05
0e+00
S1_WT
S1_WT_IMS
S1_WT_MATRIX
S1_atp23
S1_atp23_IMS
S1_atp23_MATRIX
S2_WT
S2_WT_IMS
S2_WT_MATRIX
S2_atp6
S2_atp6_IMS
S2_atp6_MATRIX

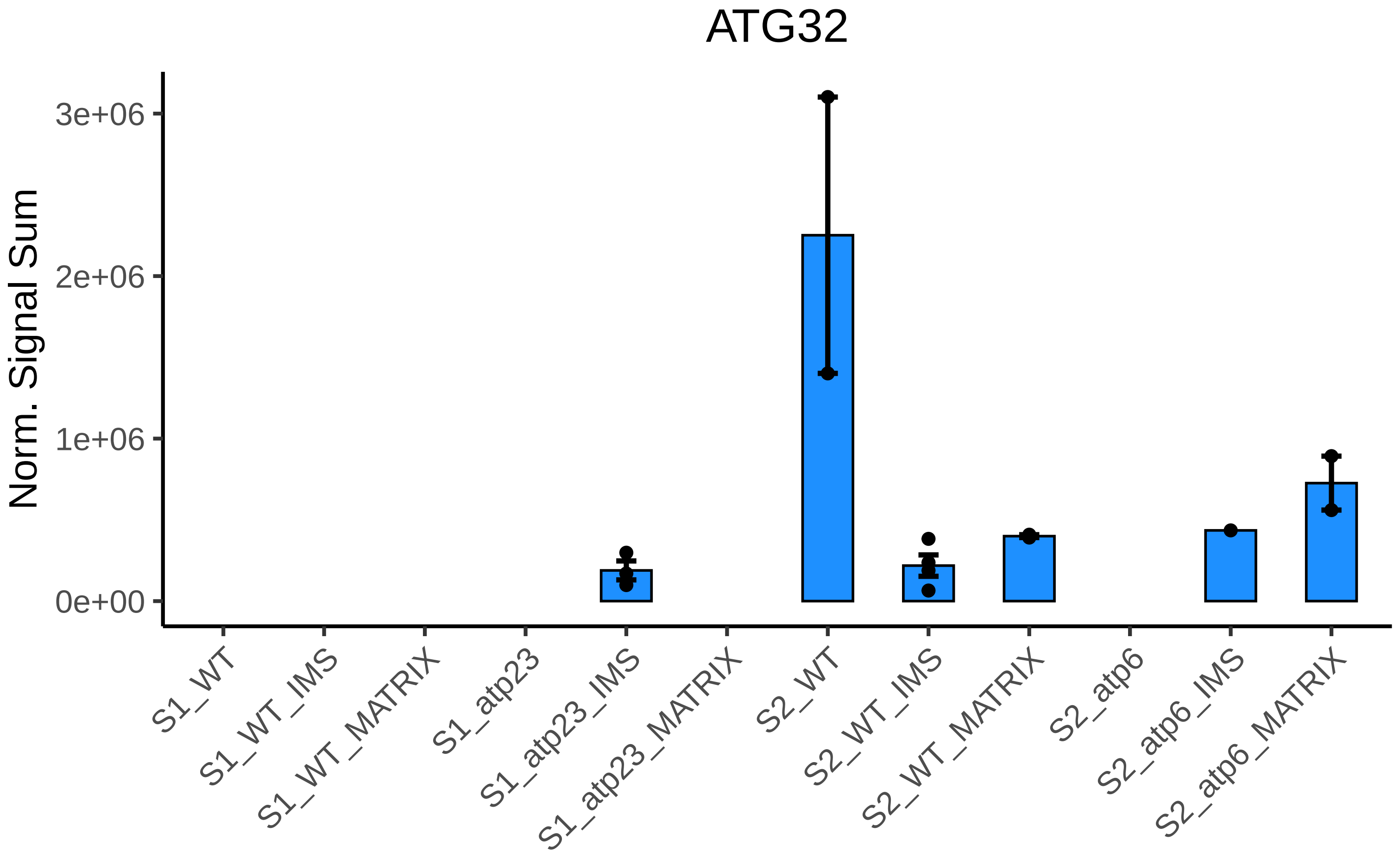
ATG32
Norm. Signal Sum
3e+06
2e+06
1e+06
0e+00
S1_WT
S1_WT_IMS
S1_WT_MATRIX
S1_atp23
S1_atp23_IMS
S1_atp23_MATRIX
S2_WT
S2_WT_IMS
S2_WT_MATRIX
S2_atp6
S2_atp6_IMS
S2_atp6_MATRIX

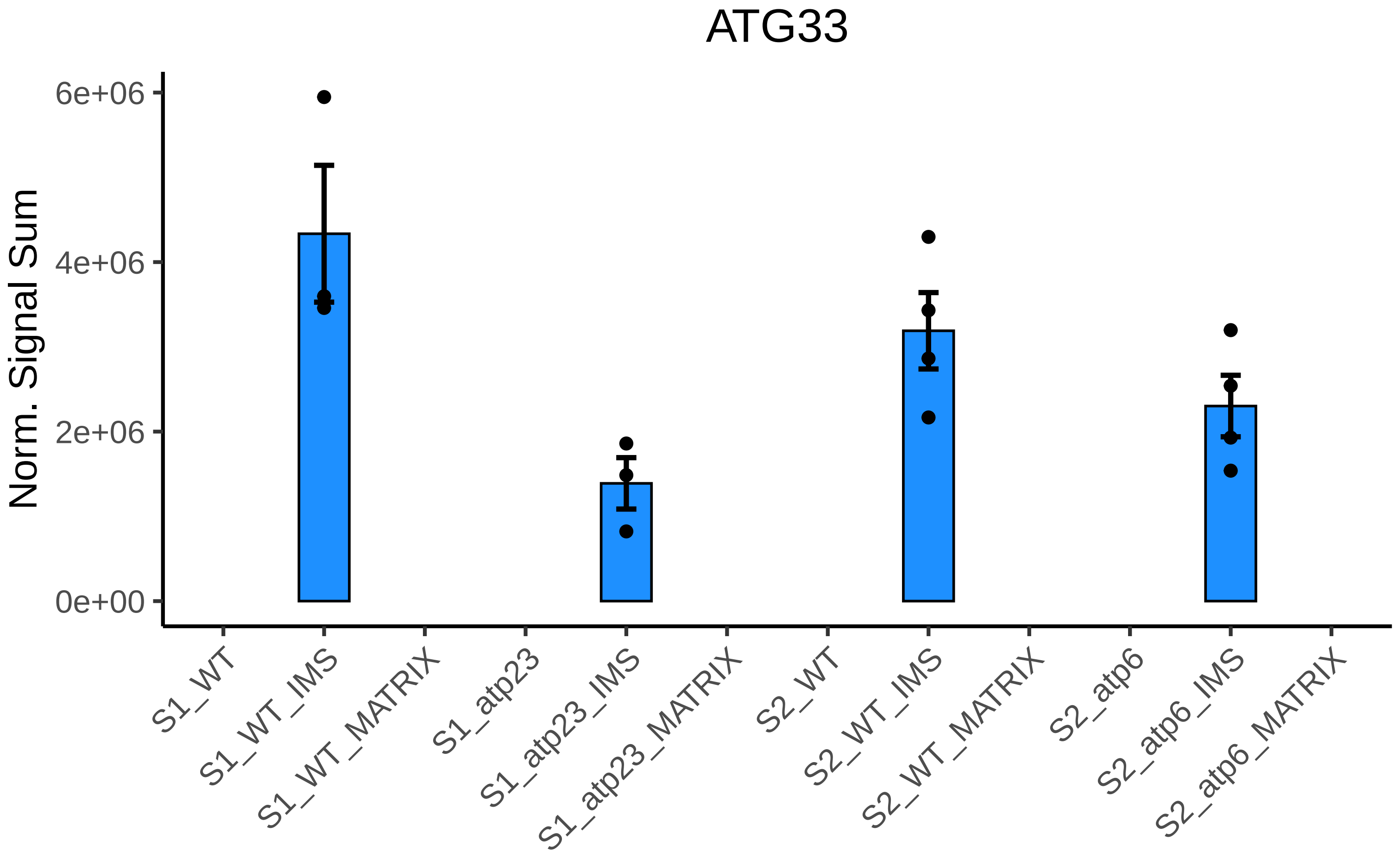

ATG33
Norm. Signal Sum
6e+06
4e+06
2e+06
0e+00
S1_WT
S1_WT_IMS
S1_WT_MATRIX
S1_atp23
S1_atp23_IMS
S1_atp23_MATRIX
S2_WT
S2_WT_IMS
S2_WT_MATRIX
S2_atp6
S2_atp6_IMS
S2_atp6_MATRIX

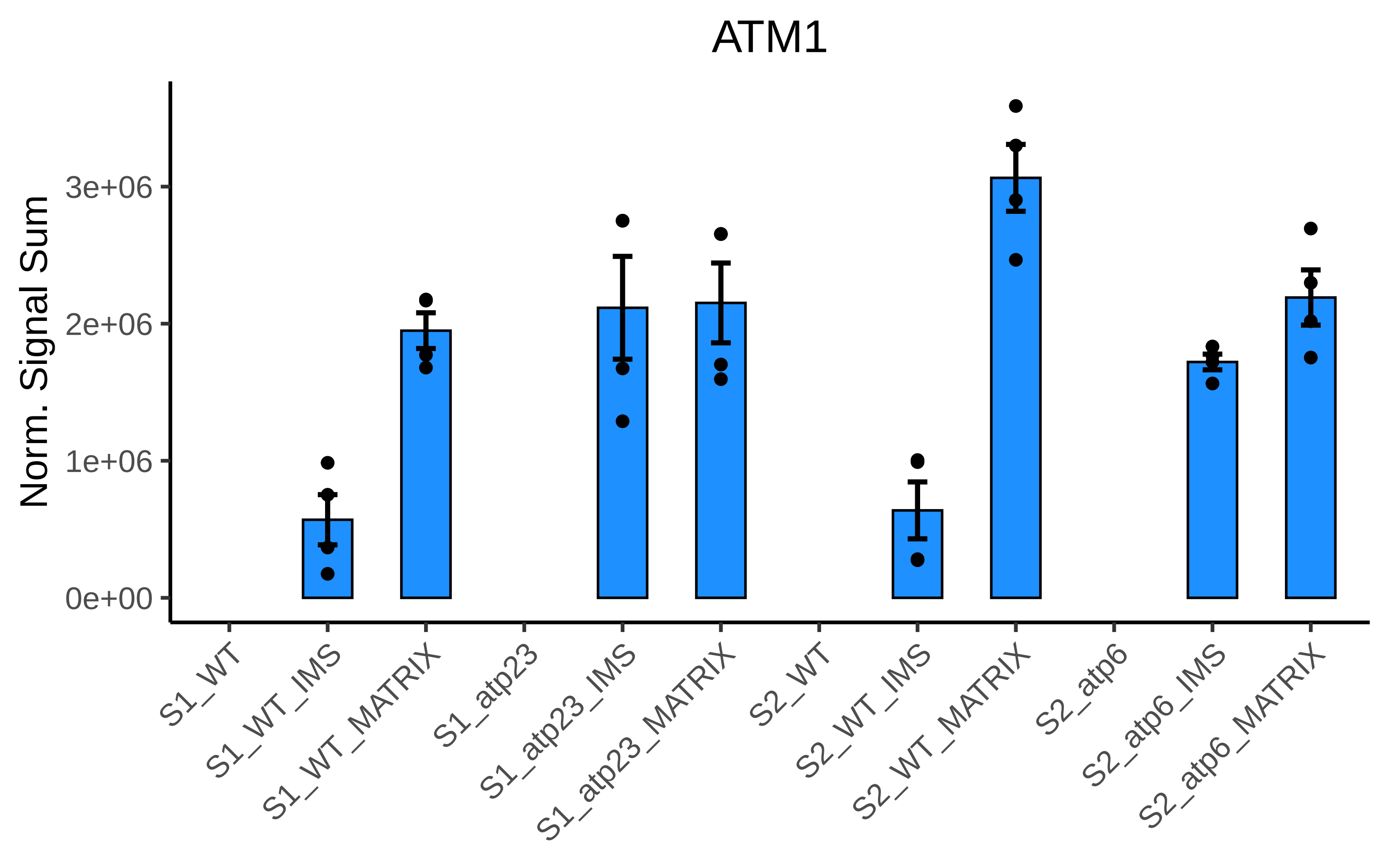
ATM1
Norm. Signal Sum
3e+06
2e+06
1e+06
0e+00
S1_WT
S1_WT_IMS
S1_WT_MATRIX
S1_atp23
S1_atp23_IMS
S1_atp23_MATRIX
S2_WT
S2_WT_IMS
S2_WT_MATRIX
S2_atp6
S2_atp6_IMS
S2_atp6_MATRIX

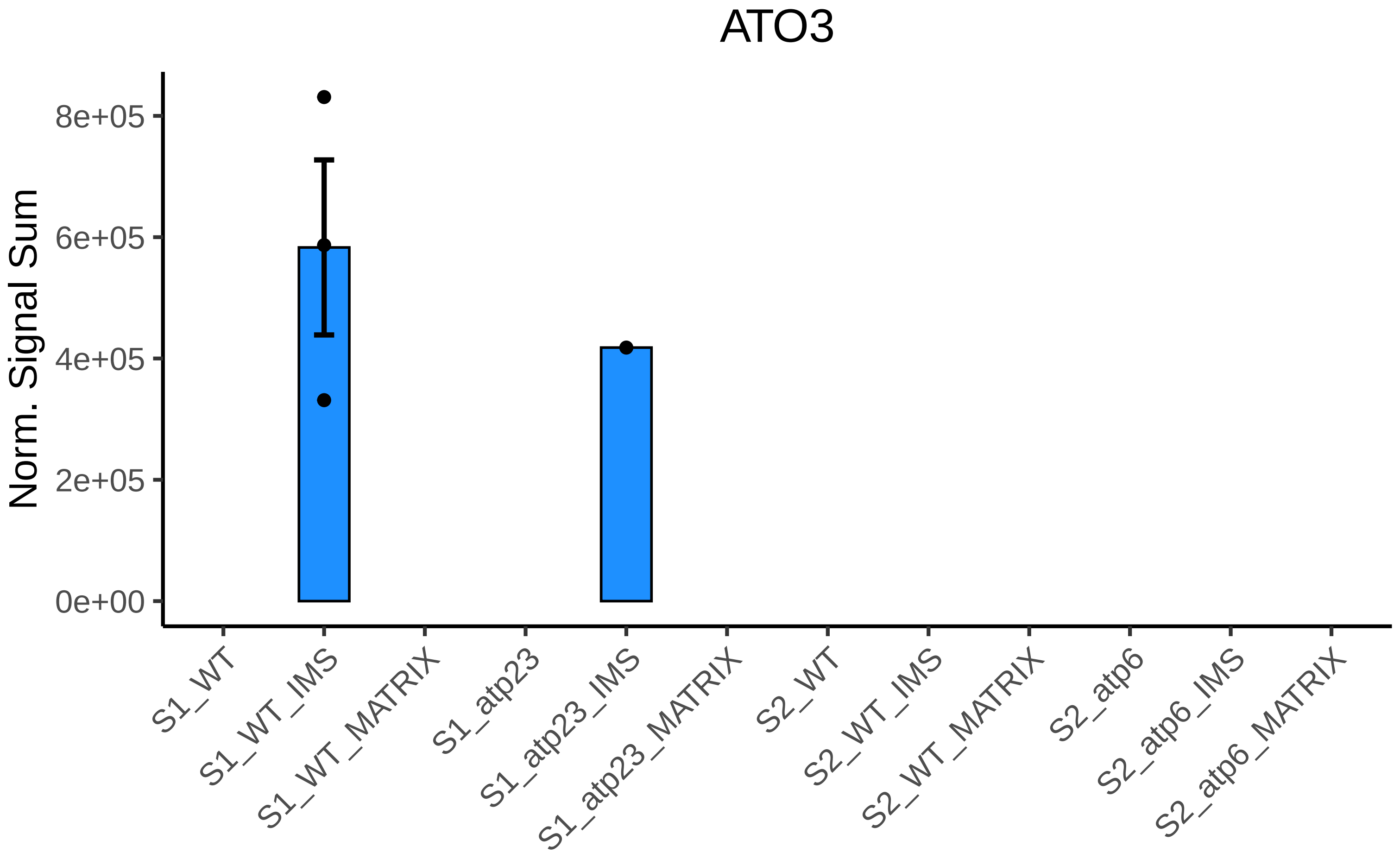
ATO3
Norm. Signal Sum
8e+05
6e+05
4e+05
2e+05
0e+00
S1_WT
S1_WT_IMS
S1_WT_MATRIX
S1_atp23
S1_atp23_IMS
S1_atp23_MATRIX
S2_WT
S2_WT_IMS
S2_WT_MATRIX
S2_atp6
S2_atp6_IMS
S2_atp6_MATRIX

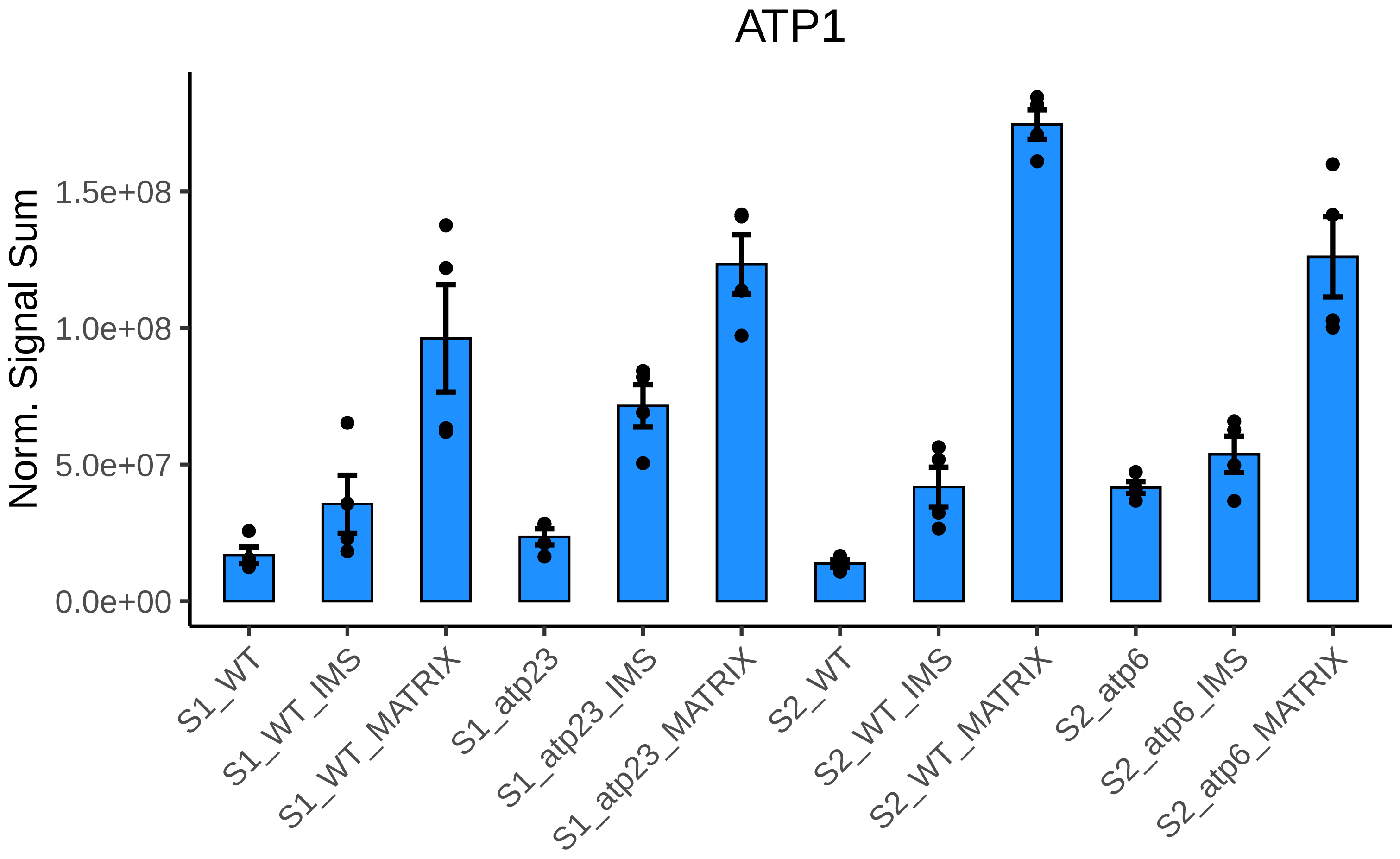

ATP1
Norm. Signal Sum
1.5e+08
1.0e+08
5.0e+07
0.0e+00
S1_WT
S1_WT_IMS
S1_WT_MATRIX
S1_atp23
S1_atp23_IMS
S1_atp23_MATRIX
S2_WT
S2_WT_IMS
S2_WT_MATRIX
S2_atp6
S2_atp6_IMS
S2_atp6_MATRIX

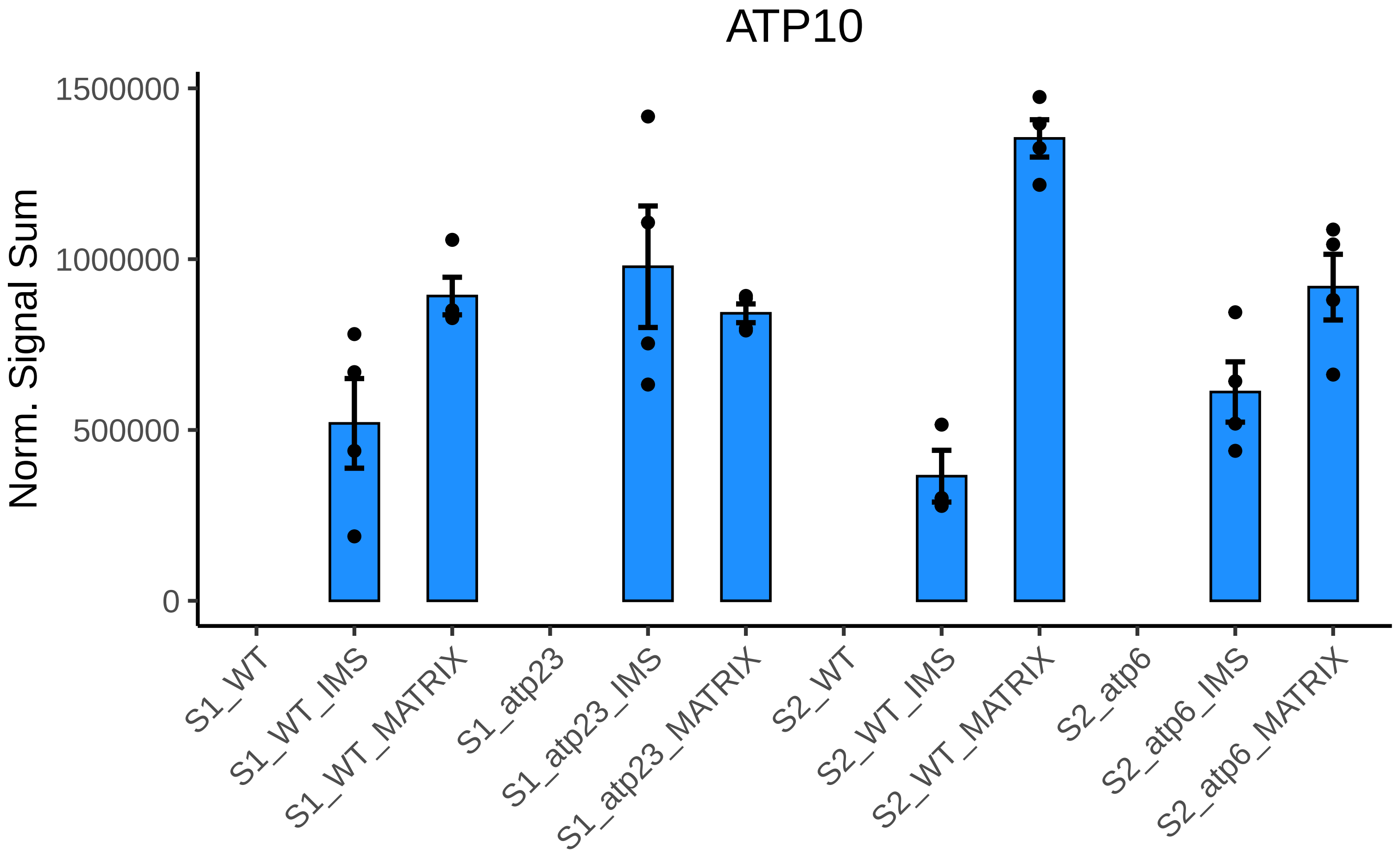

ATP10
Norm. Signal Sum
1500000
1000000
500000
0
S1_WT
S1_WT_IMS
S1_WT_MATRIX
S1_atp23
S1_atp23_IMS
S1_atp23_MATRIX
S2_WT
S2_WT_IMS
S2_WT_MATRIX
S2_atp6
S2_atp6_IMS
S2_atp6_MATRIX

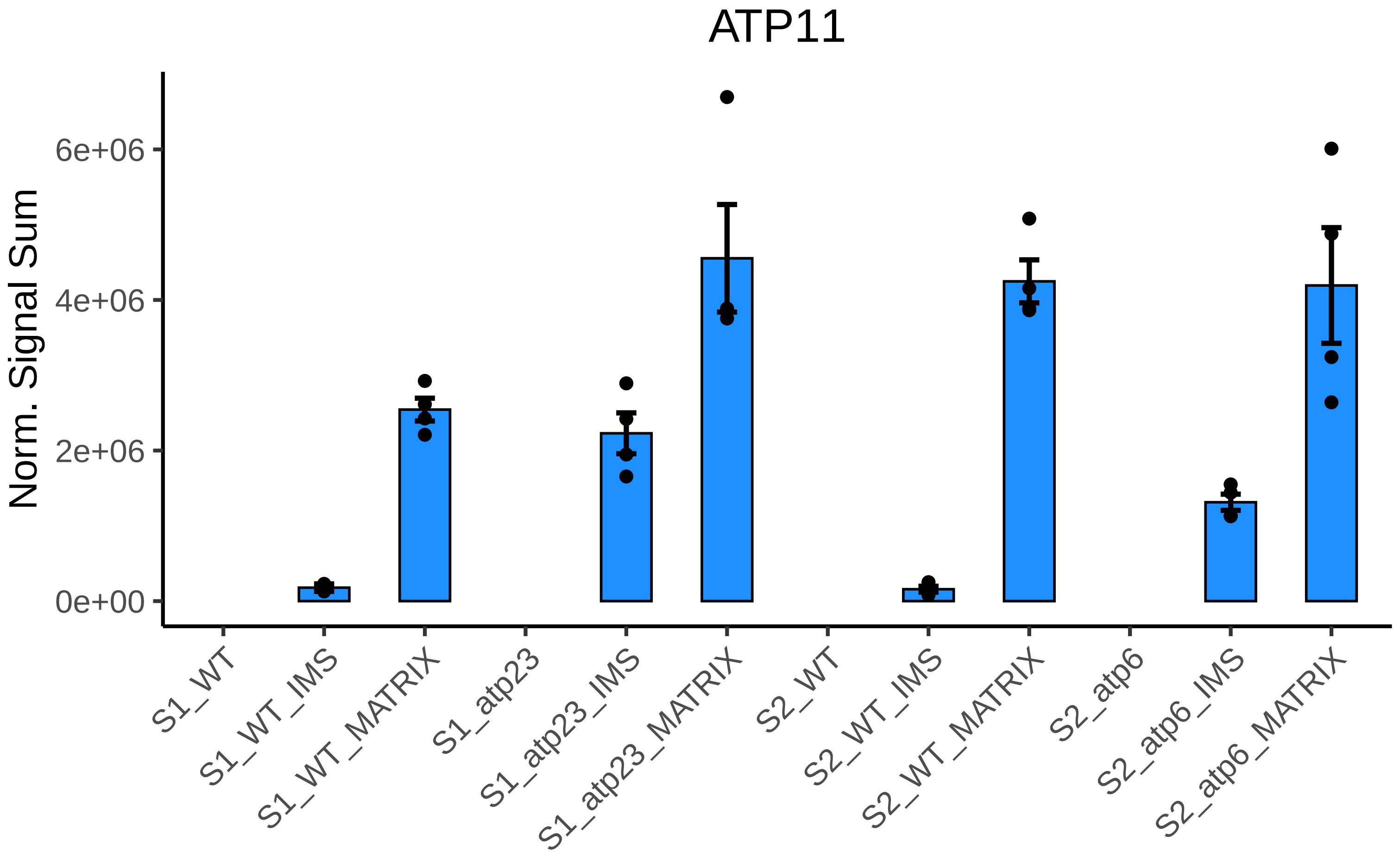
ATP11
Norm. Signal Sum
6e+06
4e+06
2e+06
0e+00
S1_WT
S1_WT_IMS
S1_WT_MATRIX
S1_atp23
S1_atp23_IMS
S1_atp23_MATRIX
S2_WT
S2_WT_IMS
S2_WT_MATRIX
S2_atp6
S2_atp6_IMS
S2_atp6_MATRIX

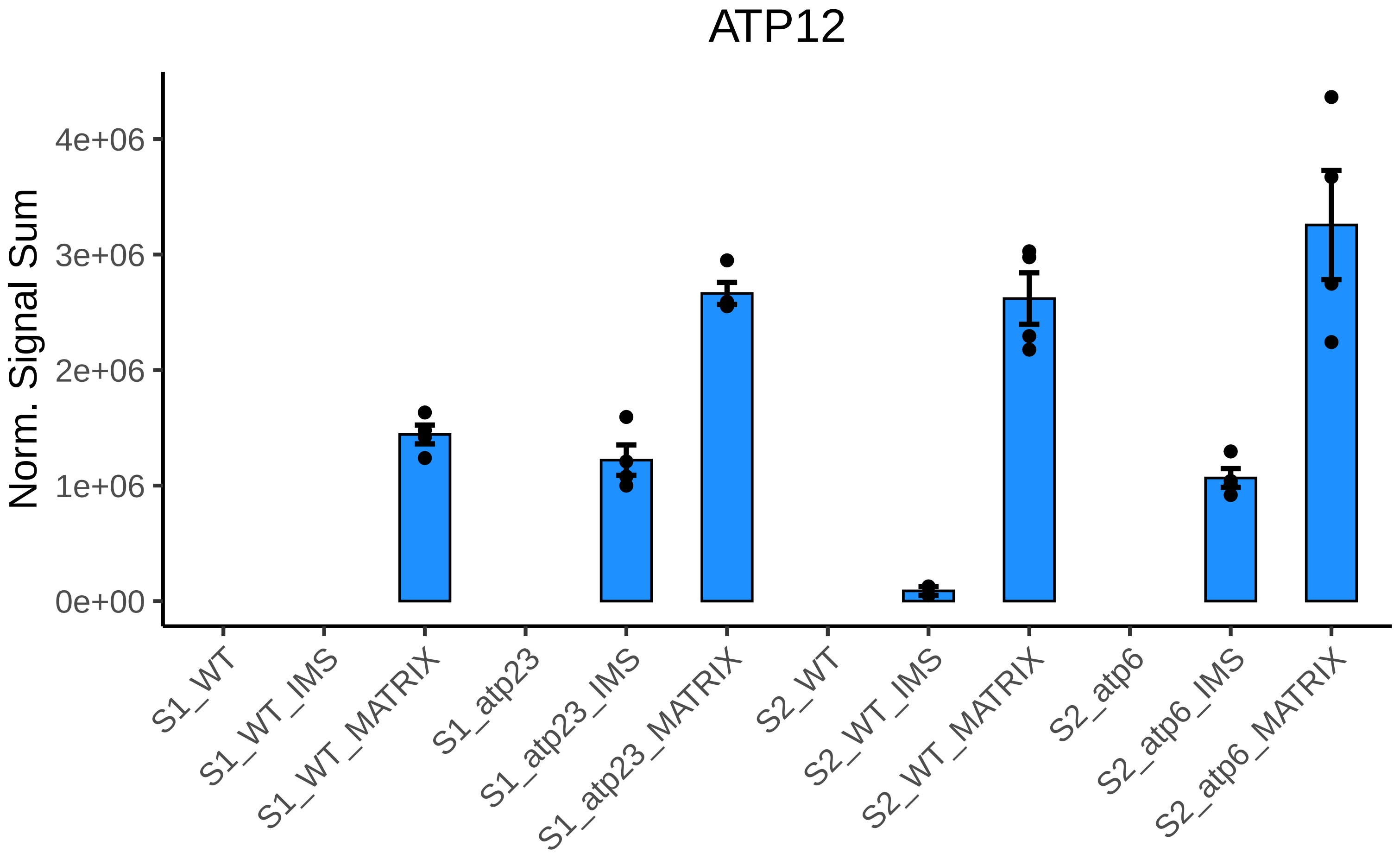
ATP12
Norm. Signal Sum
4e+06
3e+06
2e+06
1e+06
0e+00
S1_WT
S1_WT_IMS
S1_WT_MATRIX
S1_atp23
S1_atp23_IMS
S1_atp23_MATRIX
S2_WT
S2_WT_IMS
S2_WT_MATRIX
S2_atp6
S2_atp6_IMS
S2_atp6_MATRIX

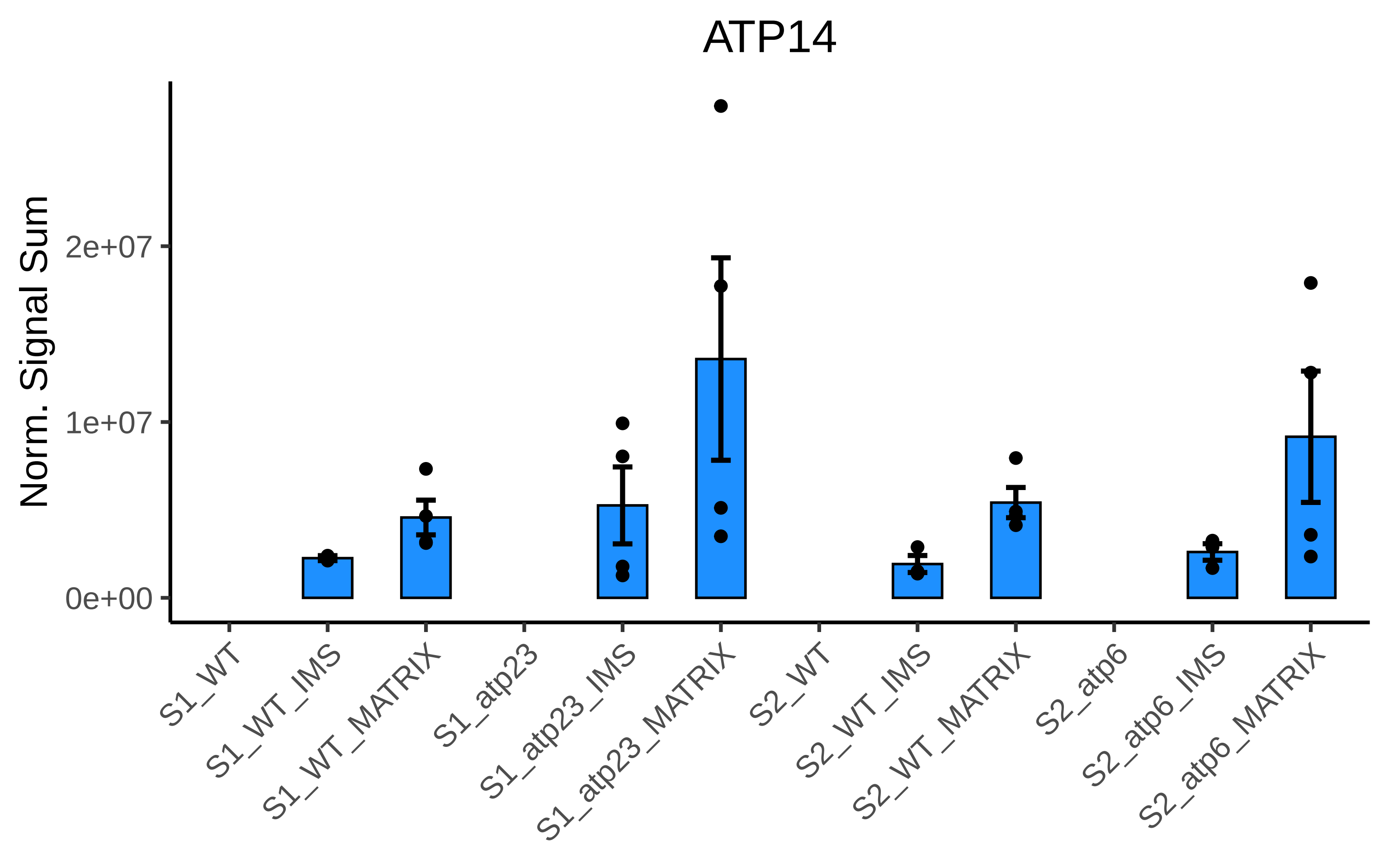
ATP14
Norm. Signal Sum
2e+07
1e+07
0e+00
S1_WT
S1_WT_IMS
S1_WT_MATRIX
S1_atp23
S1_atp23_IMS
S1_atp23_MATRIX
S2_WT
S2_WT_IMS
S2_WT_MATRIX
S2_atp6
S2_atp6_IMS
S2_atp6_MATRIX

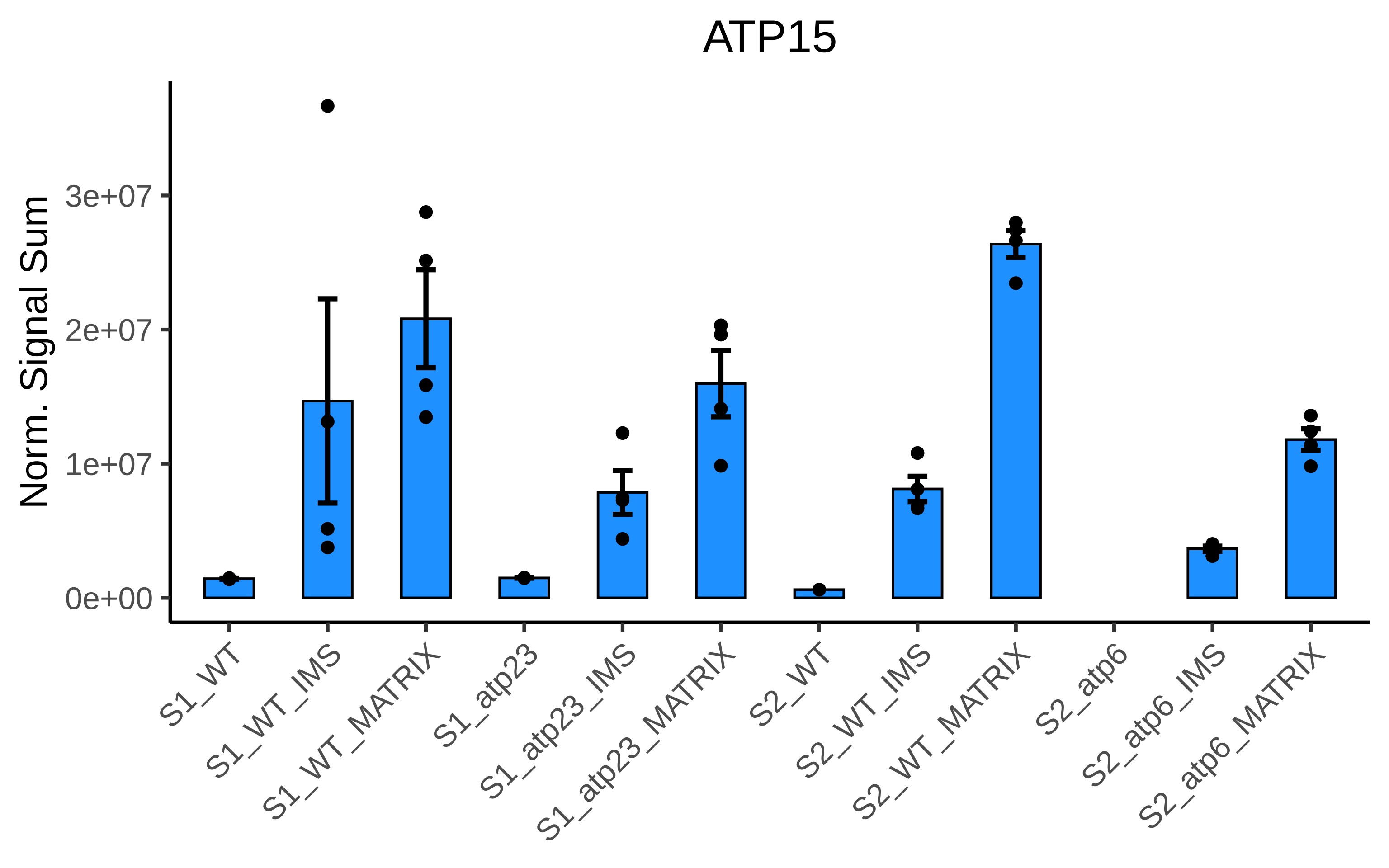

ATP15
Norm. Signal Sum
0e+00
1e+07
2e+07
3e+07
S1_WT
S1_WT_IMS
S1_WT_MATRIX
S1_atp23
S1_atp23_IMS
S1_atp23_MATRIX
S2_WT
S2_WT_IMS
S2_WT_MATRIX
S2_atp6
S2_atp6_IMS
S2_atp6_MATRIX

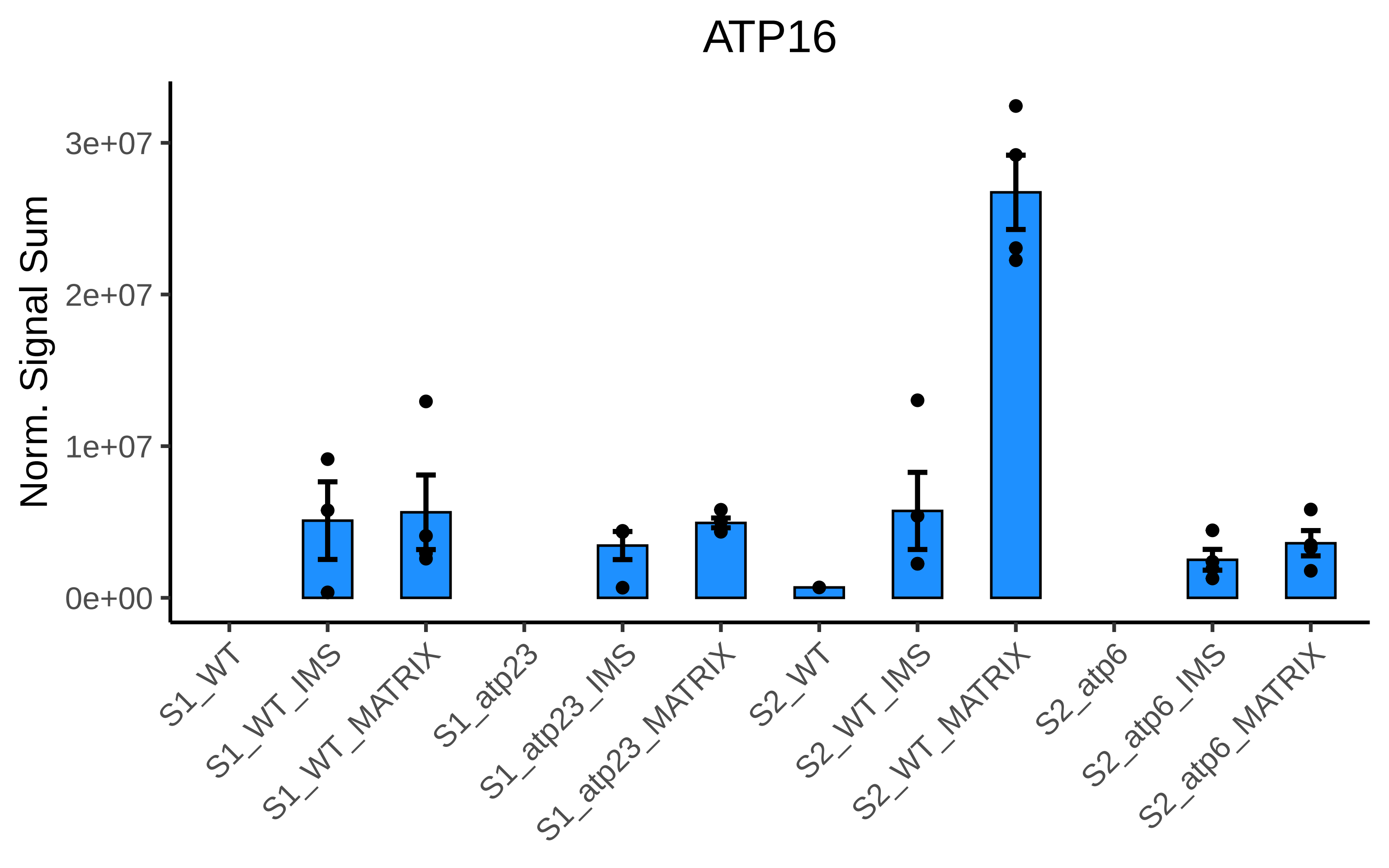

ATP16
Norm. Signal Sum
3e+07
2e+07
1e+07
0e+00
S1_WT
S1_WT_IMS
S1_WT_MATRIX
S1_atp23
S1_atp23_IMS
S1_atp23_MATRIX
S2_WT
S2_WT_IMS
S2_WT_MATRIX
S2_atp6
S2_atp6_IMS
S2_atp6_MATRIX

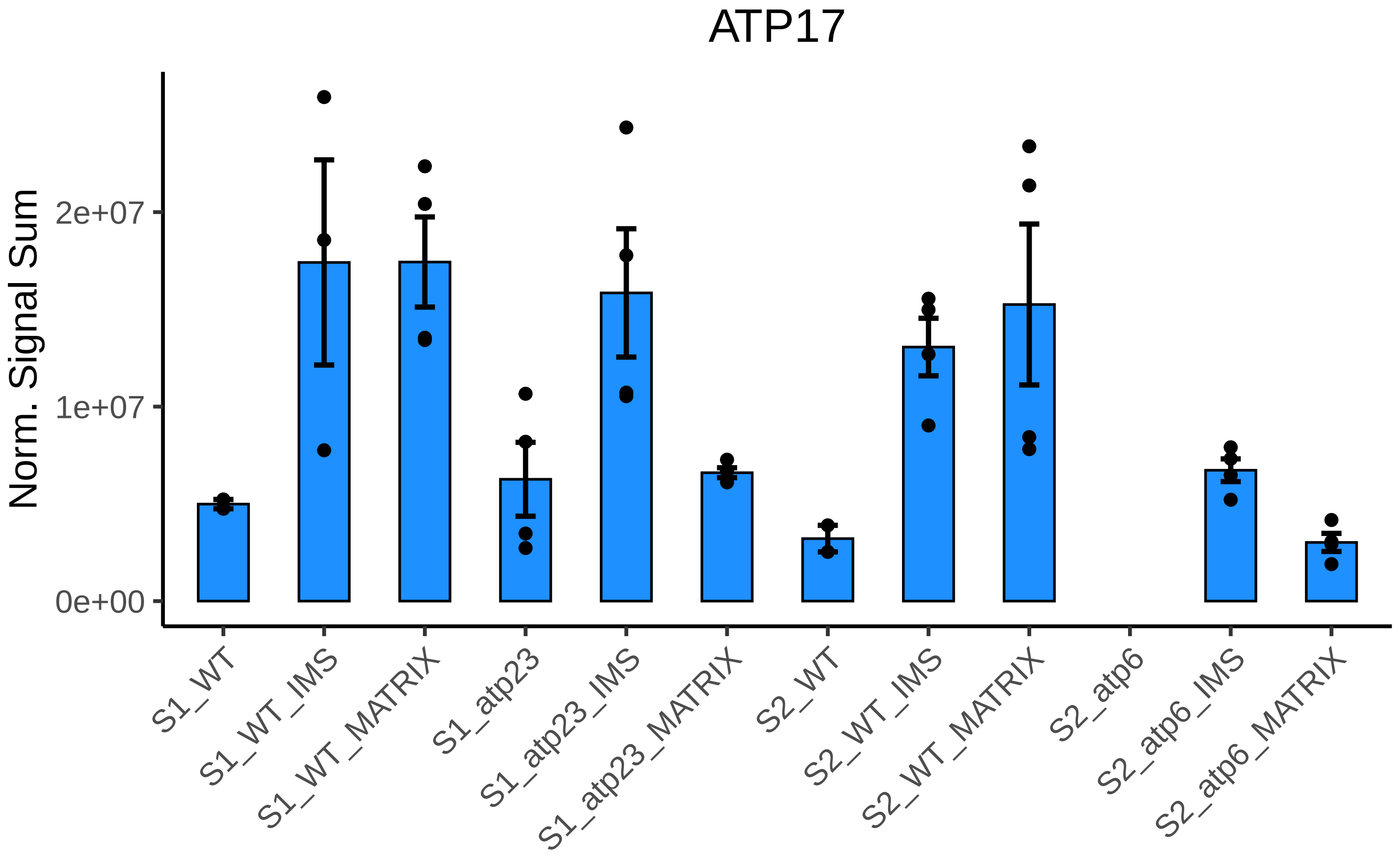

ATP17
Norm. Signal Sum
2e+07
1e+07
0e+00
S1_WT
S1_WT_IMS
S1_WT_MATRIX
S1_atp23
S1_atp23_IMS
S1_atp23_MATRIX
S2_WT
S2_WT_IMS
S2_WT_MATRIX
S2_atp6
S2_atp6_IMS
S2_atp6_MATRIX

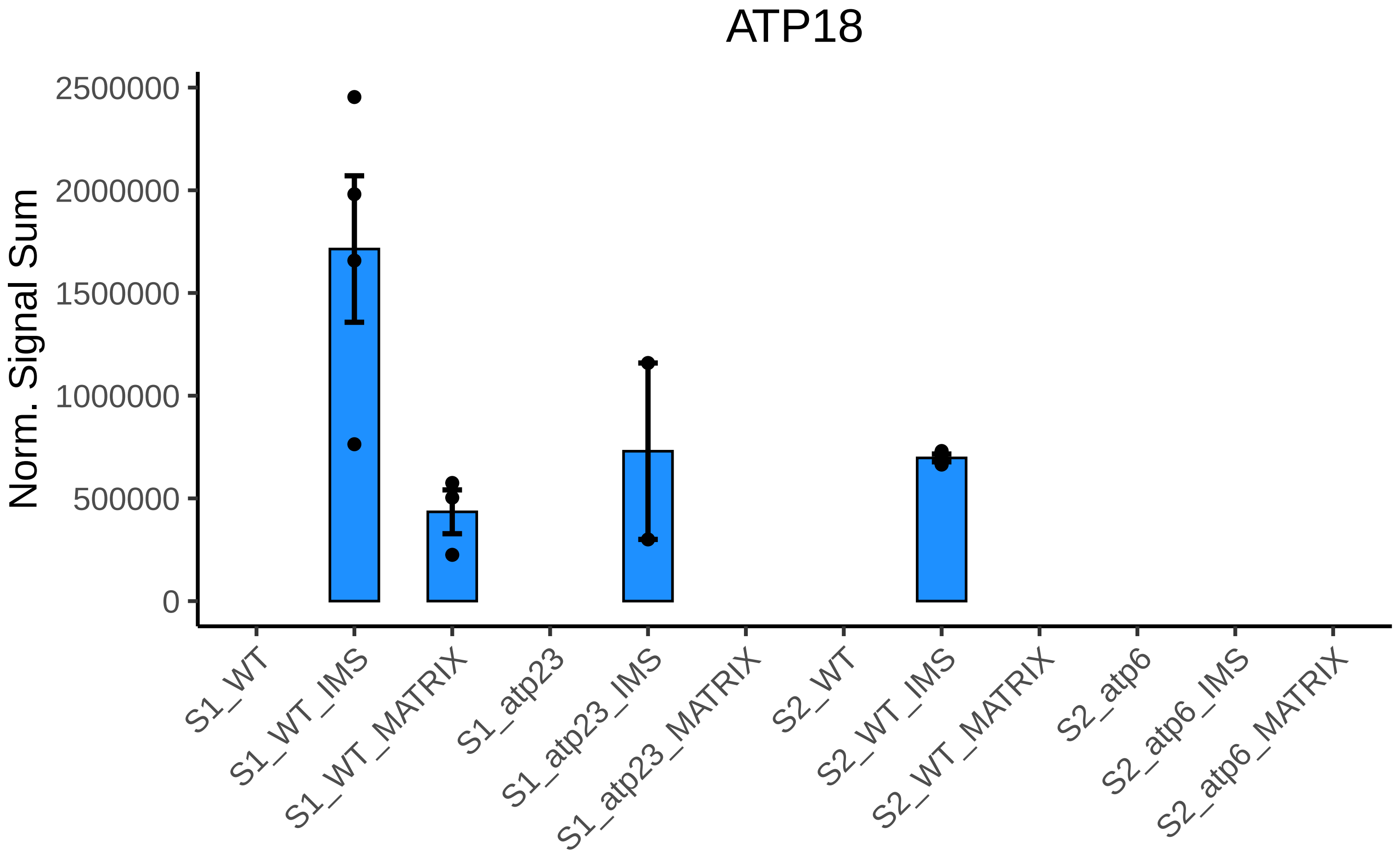
ATP18
Norm. Signal Sum
2500000
2000000
1500000
1000000
500000
0
S1_WT
S1_WT_IMS
S1_WT_MATRIX
S1_atp23
S1_atp23_IMS
S1_atp23_MATRIX
S2_WT
S2_WT_IMS
S2_WT_MATRIX
S2_atp6
S2_atp6_IMS
S2_atp6_MATRIX

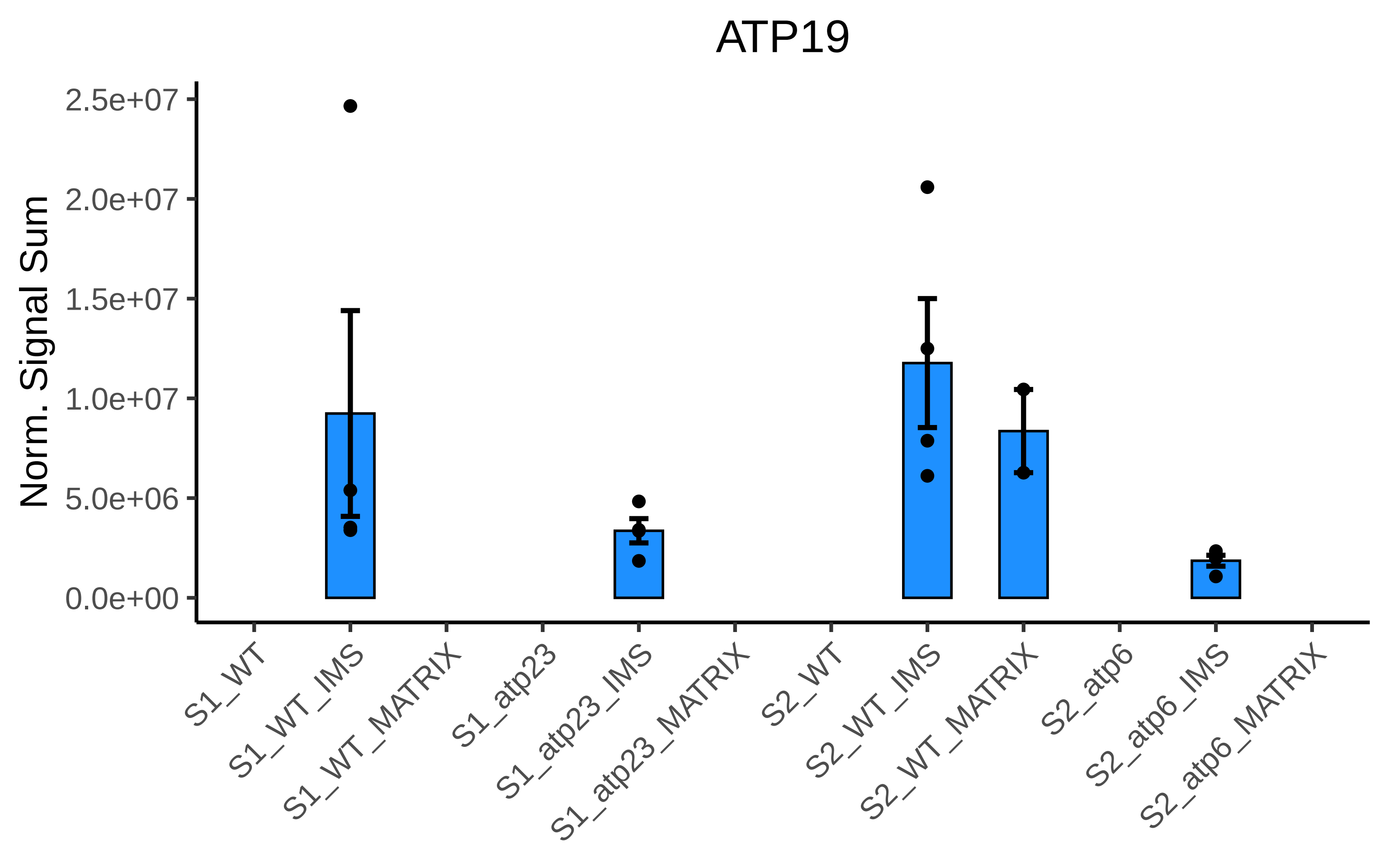

ATP19
Norm. Signal Sum
2.5e+07
2.0e+07
1.5e+07
1.0e+07
5.0e+06
0.0e+00
S1_WT
S1_WT_IMS
S1_WT_MATRIX
S1_atp23
S1_atp23_IMS
S1_atp23_MATRIX
S2_WT
S2_WT_IMS
S2_WT_MATRIX
S2_atp6
S2_atp6_IMS
S2_atp6_MATRIX

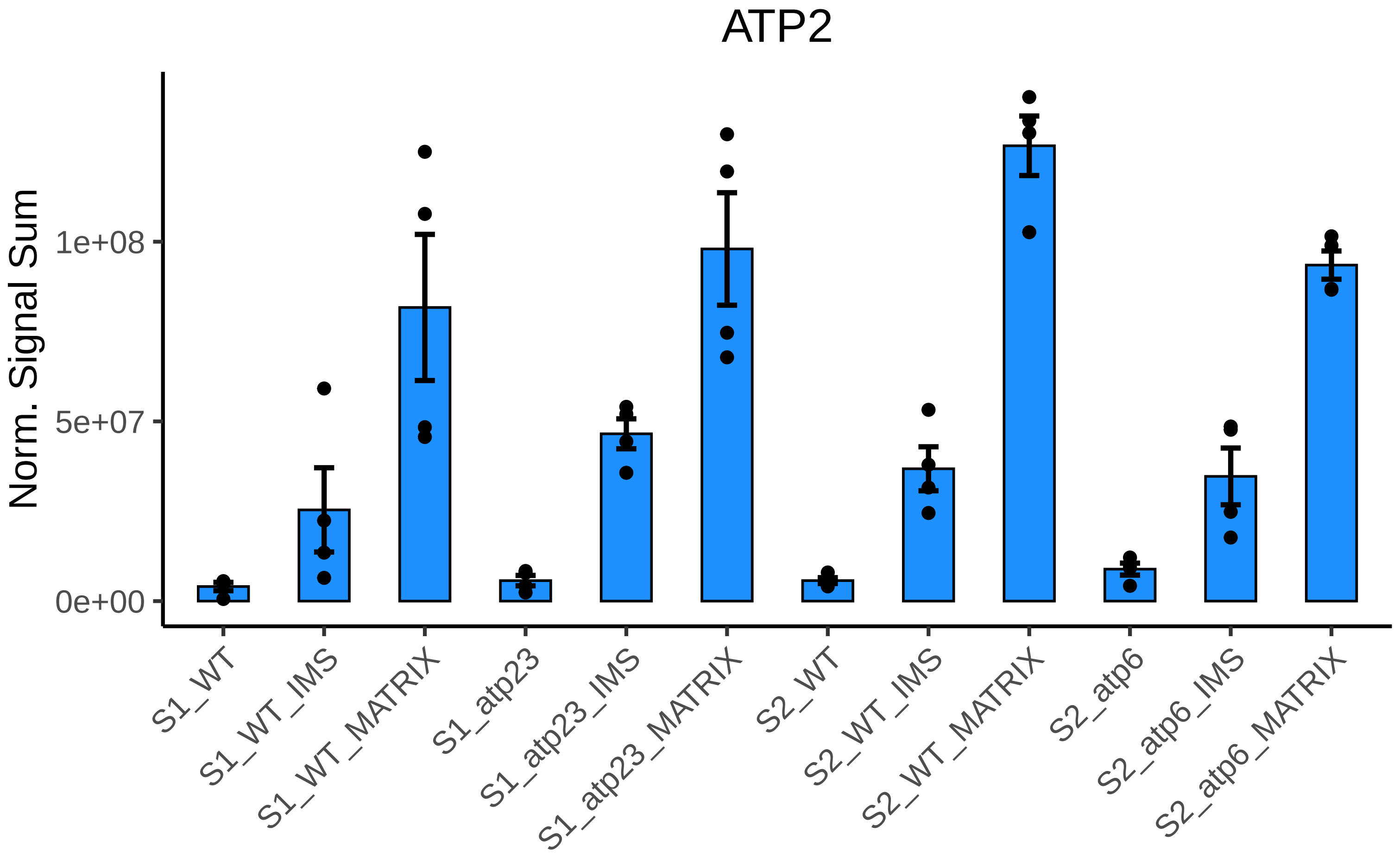
ATP2
Norm. Signal Sum
1e+08
5e+07
0e+00
S1_WT
S1_WT_IMS
S1_WT_MATRIX
S1_atp23
S1_atp23_IMS
S1_atp23_MATRIX
S2_WT
S2_WT_IMS
S2_WT_MATRIX
S2_atp6
S2_atp6_IMS
S2_atp6_MATRIX

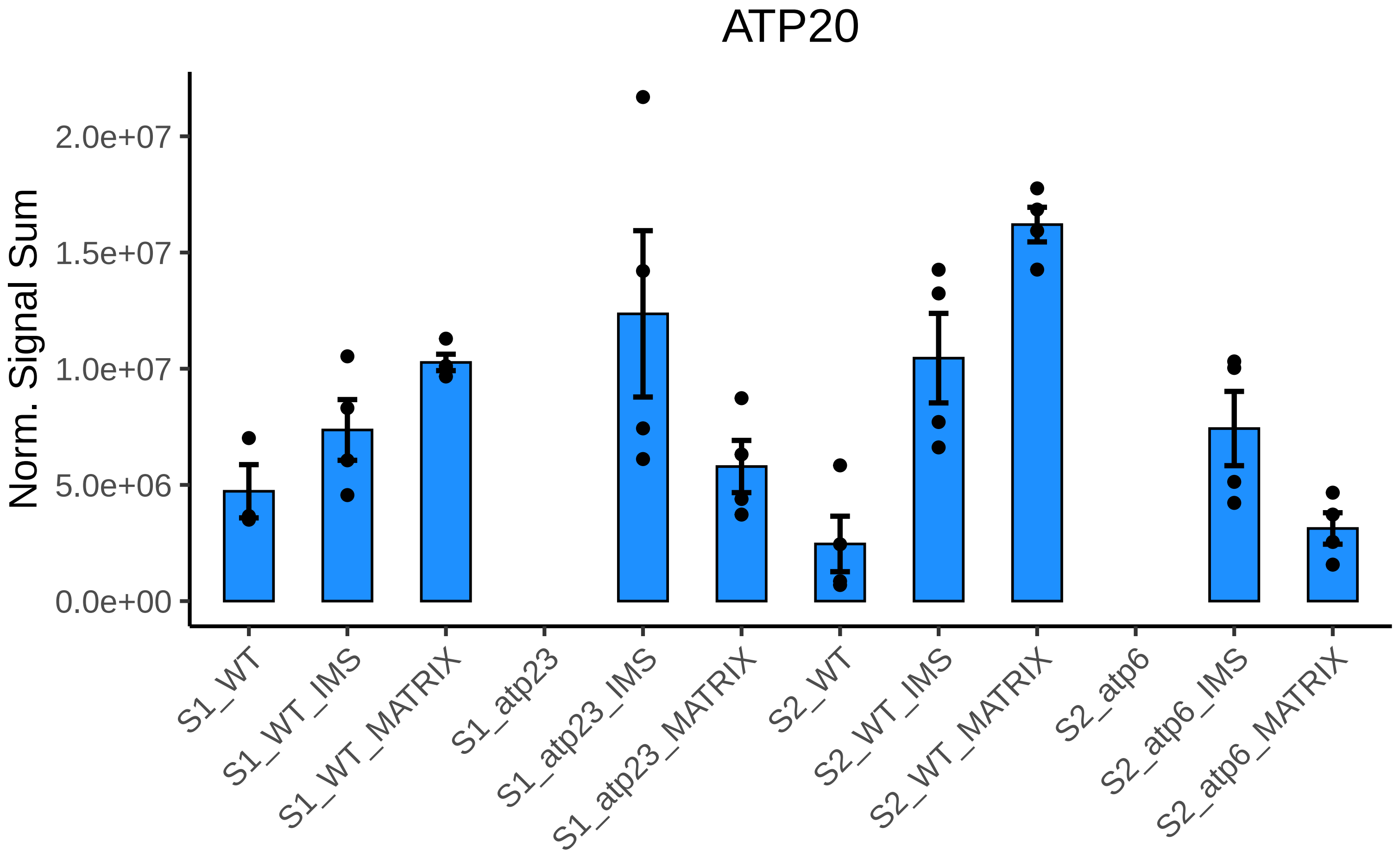

ATP20
Norm. Signal Sum
2.0e+07
1.5e+07
1.0e+07
5.0e+06
0.0e+00
S1_WT
S1_WT_IMS
S1_WT_MATRIX
S1_atp23
S1_atp23_IMS
S1_atp23_MATRIX
S2_WT
S2_WT_IMS
S2_WT_MATRIX
S2_atp6
S2_atp6_IMS
S2_atp6_MATRIX

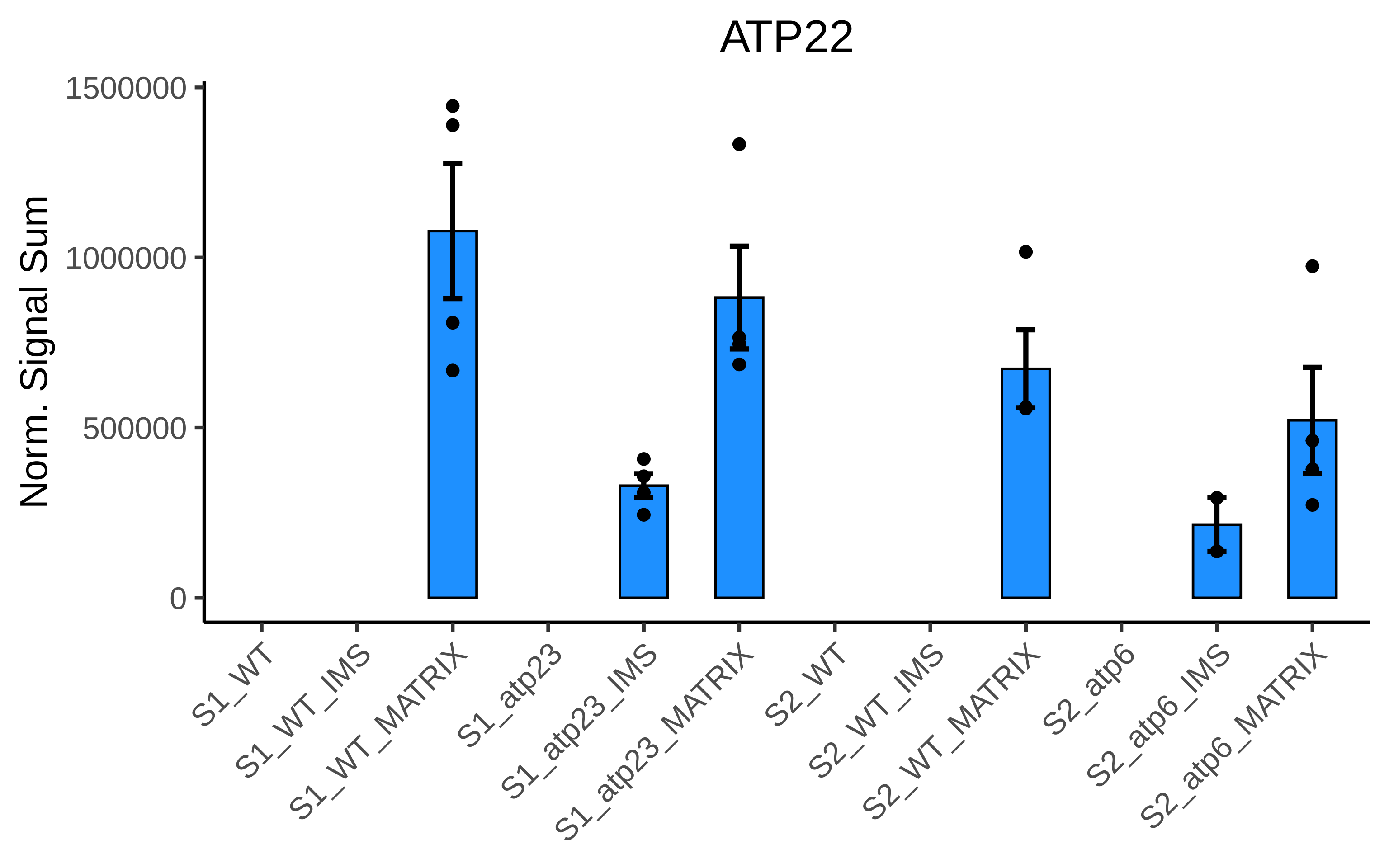

ATP22
Norm. Signal Sum
1500000
1000000
500000
0
S1_WT
S1_WT_IMS
S1_WT_MATRIX
S1_atp23
S1_atp23_IMS
S1_atp23_MATRIX
S2_WT
S2_WT_IMS
S2_WT_MATRIX
S2_atp6
S2_atp6_IMS
S2_atp6_MATRIX

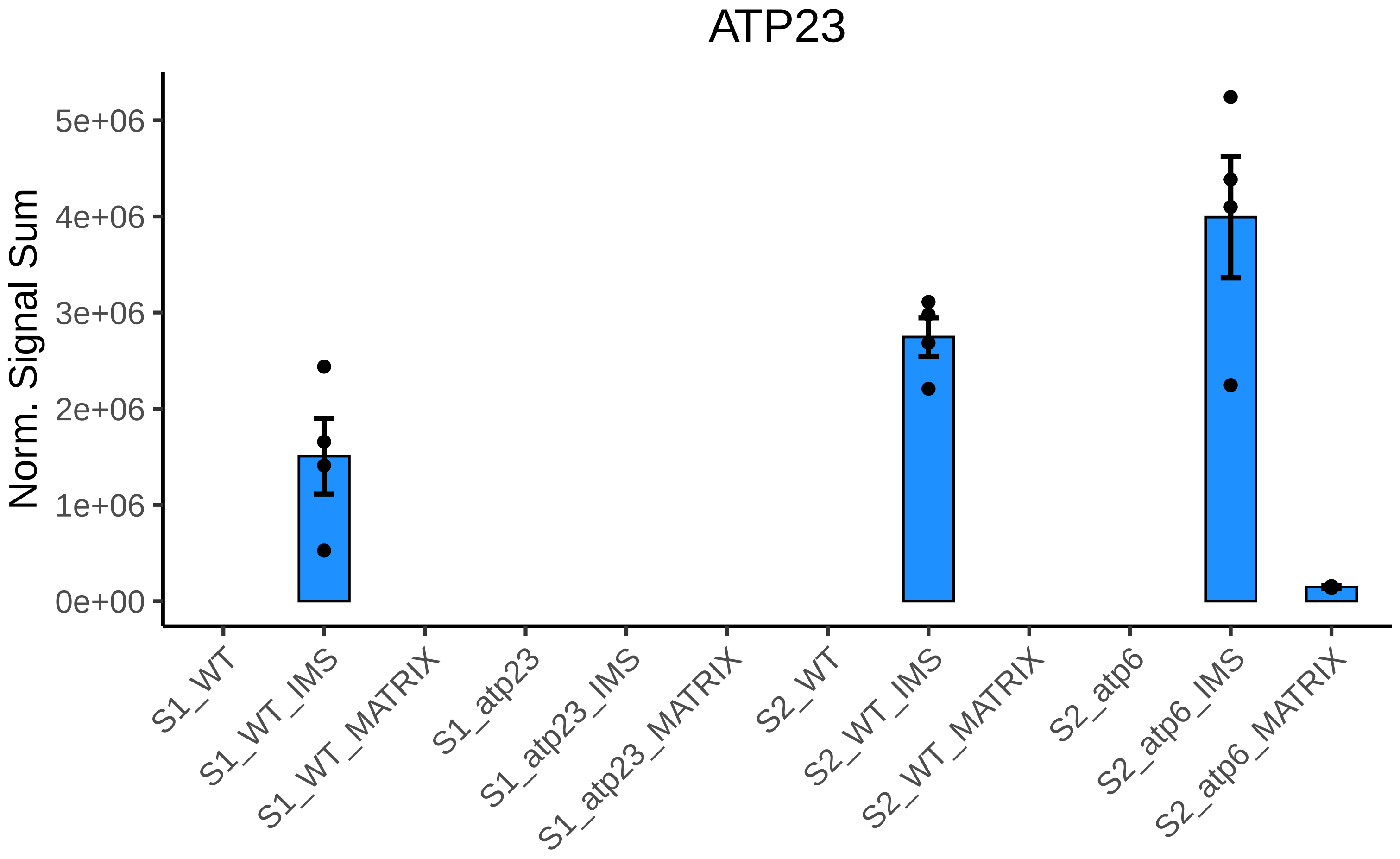

ATP23
Norm. Signal Sum
5e+06
4e+06
3e+06
2e+06
1e+06
0e+00
S1_WT
S1_WT_IMS
S1_WT_MATRIX
S1_atp23
S1_atp23_IMS
S1_atp23_MATRIX
S2_WT
S2_WT_IMS
S2_WT_MATRIX
S2_atp6
S2_atp6_IMS
S2_atp6_MATRIX

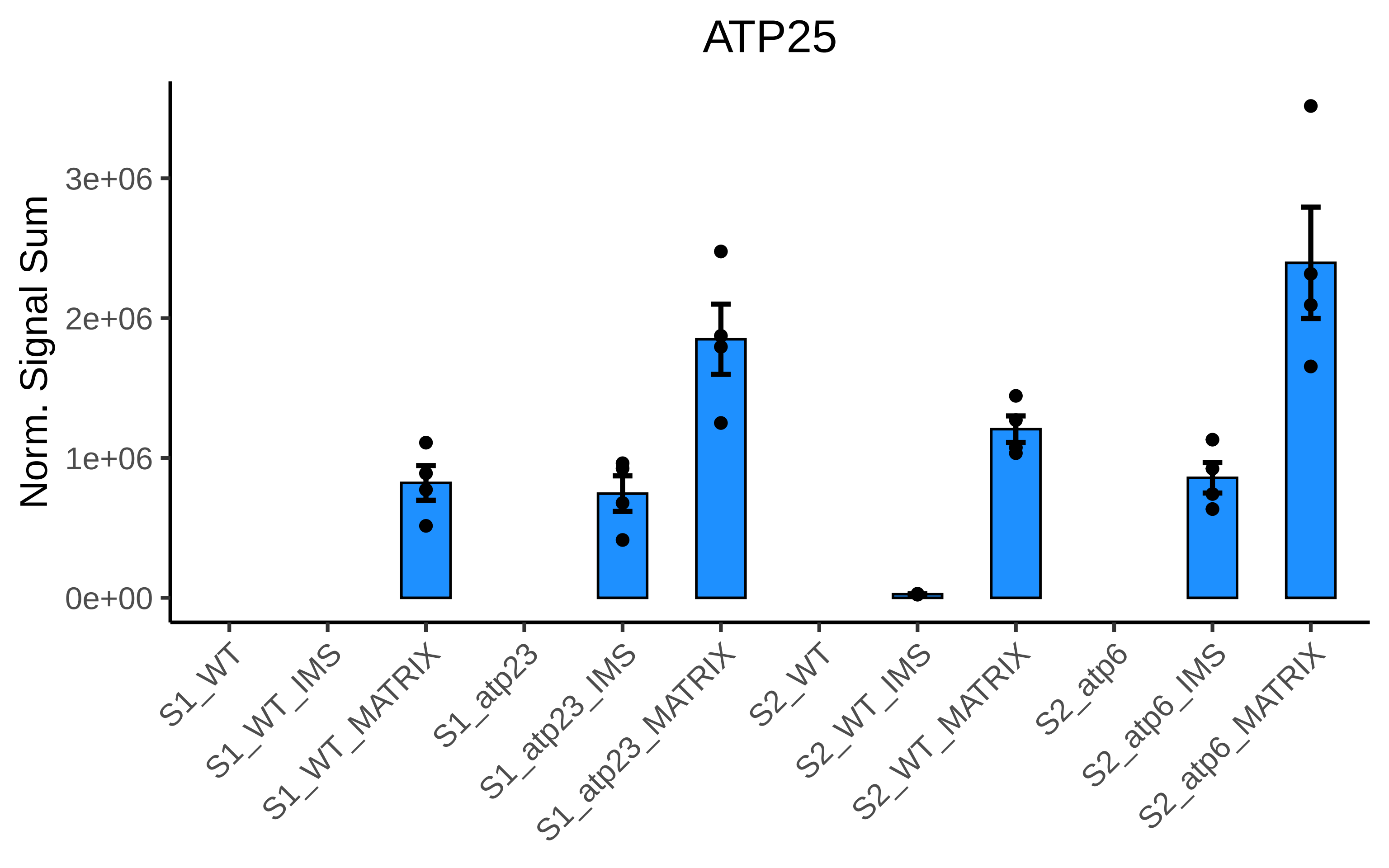
ATP25
Norm. Signal Sum
3e+06
2e+06
1e+06
0e+00
S1_WT
S1_WT_IMS
S1_WT_MATRIX
S1_atp23
S1_atp23_IMS
S1_atp23_MATRIX
S2_WT
S2_WT_IMS
S2_WT_MATRIX
S2_atp6
S2_atp6_IMS
S2_atp6_MATRIX

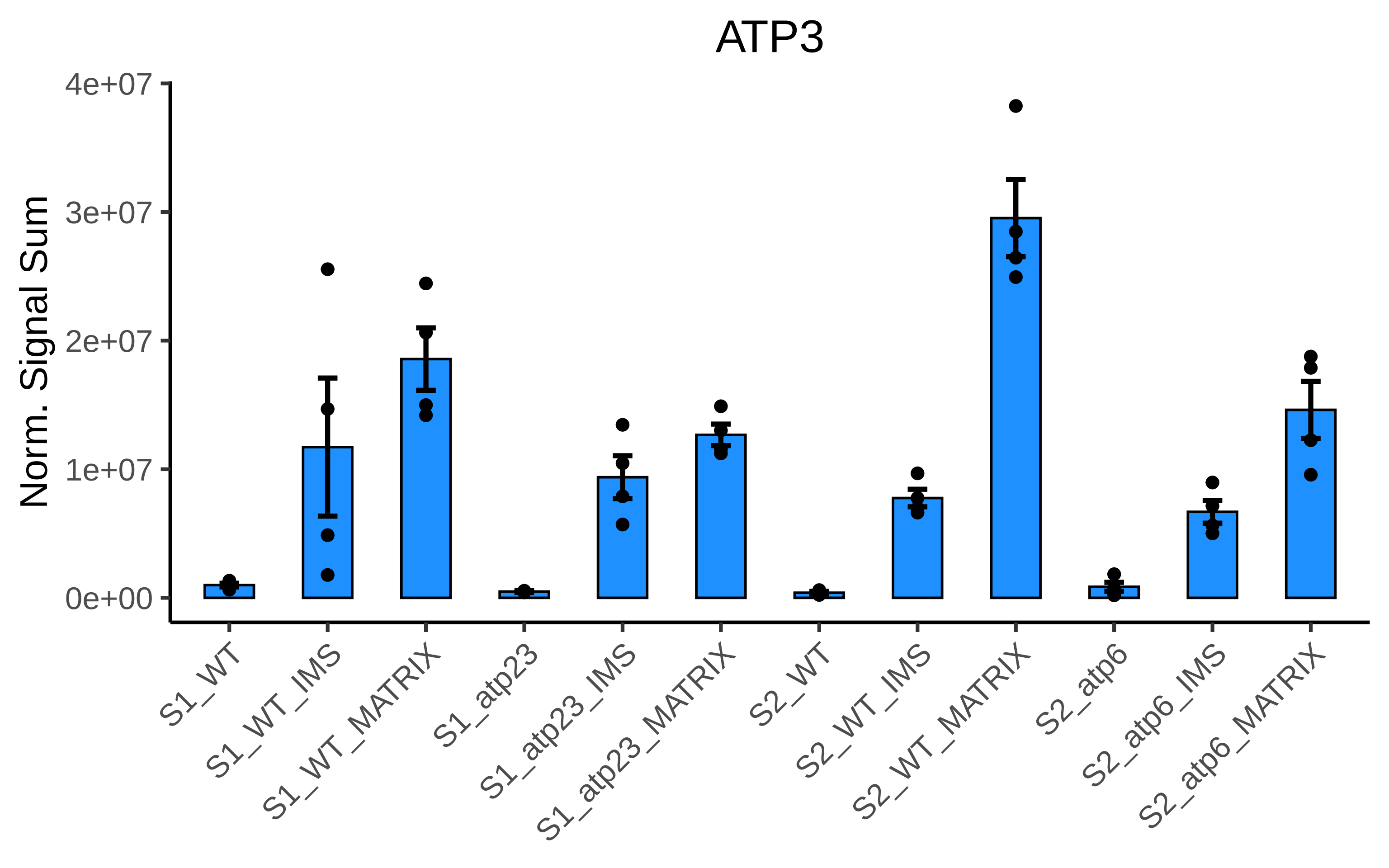

ATP3
Norm. Signal Sum
4e+07
3e+07
2e+07
1e+07
0e+00
S1_WT
S1_WT_IMS
S1_WT_MATRIX
S1_atp23
S1_atp23_IMS
S1_atp23_MATRIX
S2_WT
S2_WT_IMS
S2_WT_MATRIX
S2_atp6
S2_atp6_IMS
S2_atp6_MATRIX

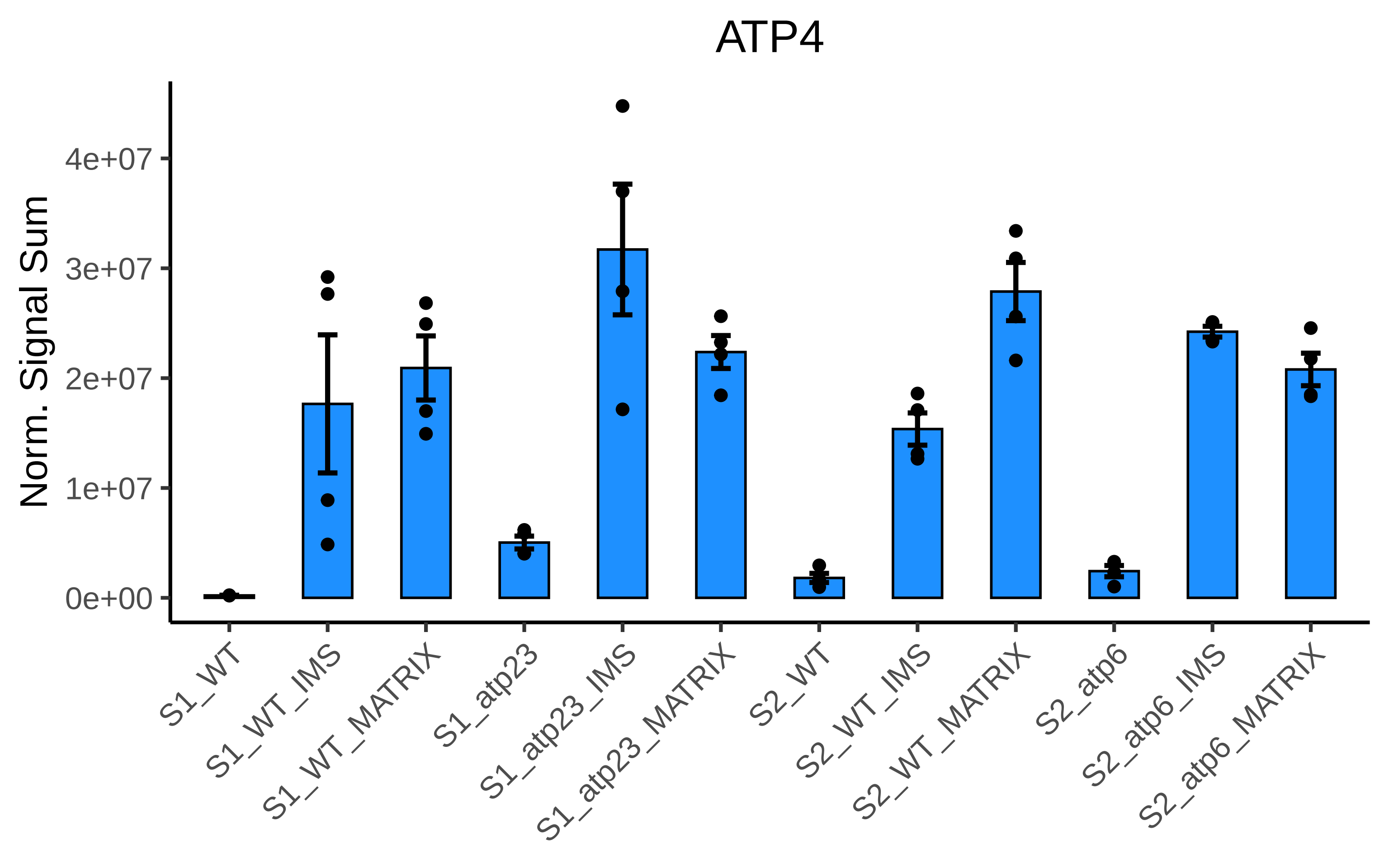

ATP4
Norm. Signal Sum
4e+07
3e+07
2e+07
1e+07
0e+00
S1_WT
S1_WT_IMS
S1_WT_MATRIX
S1_atp23
S1_atp23_IMS
S1_atp23_MATRIX
S2_WT
S2_WT_IMS
S2_WT_MATRIX
S2_atp6
S2_atp6_IMS
S2_atp6_MATRIX

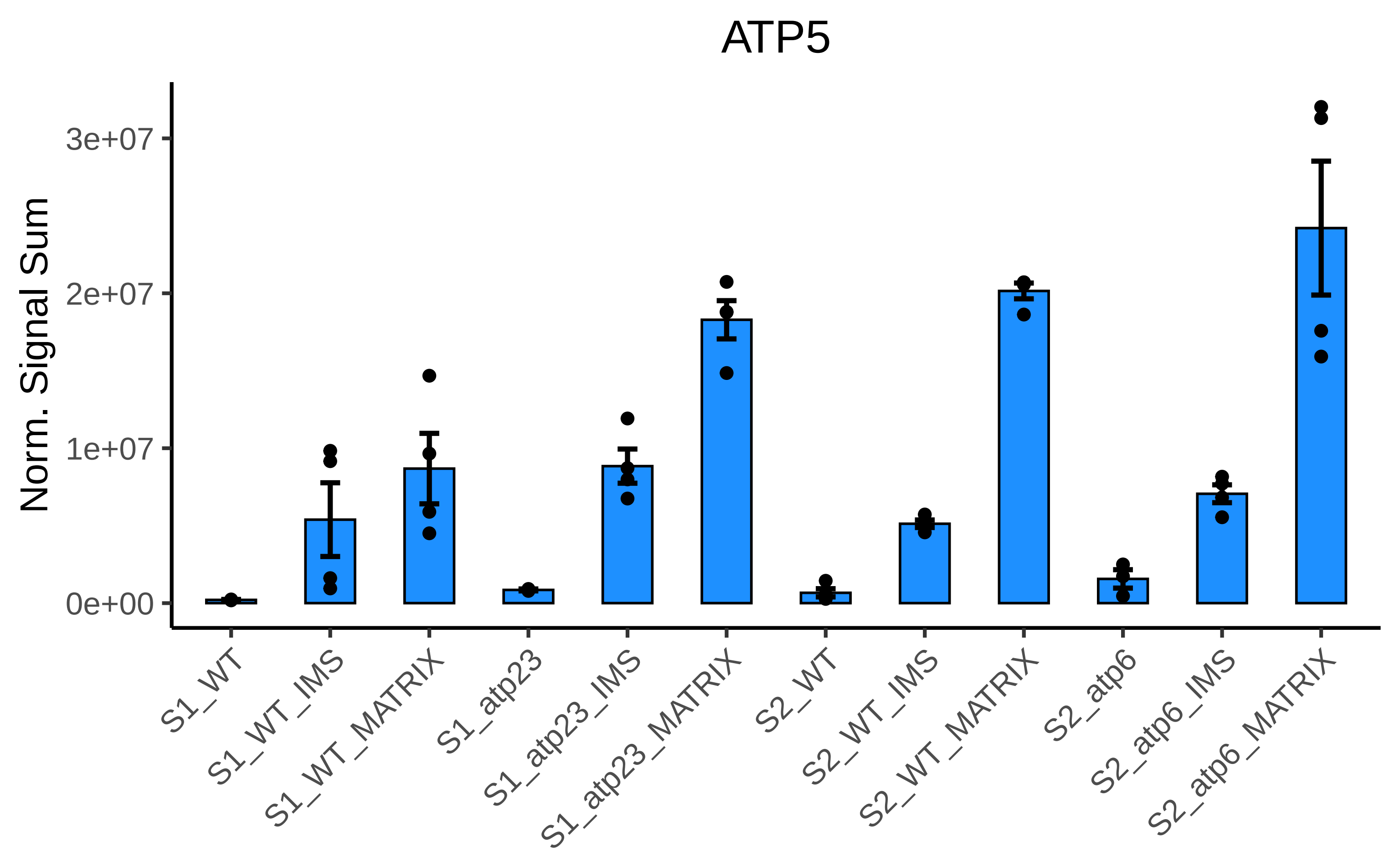
ATP5
Norm. Signal Sum
3e+07
2e+07
1e+07
0e+00
S1_WT
S1_WT_IMS
S1_WT_MATRIX
S1_atp23
S1_atp23_IMS
S1_atp23_MATRIX
S2_WT
S2_WT_IMS
S2_WT_MATRIX
S2_atp6
S2_atp6_IMS
S2_atp6_MATRIX

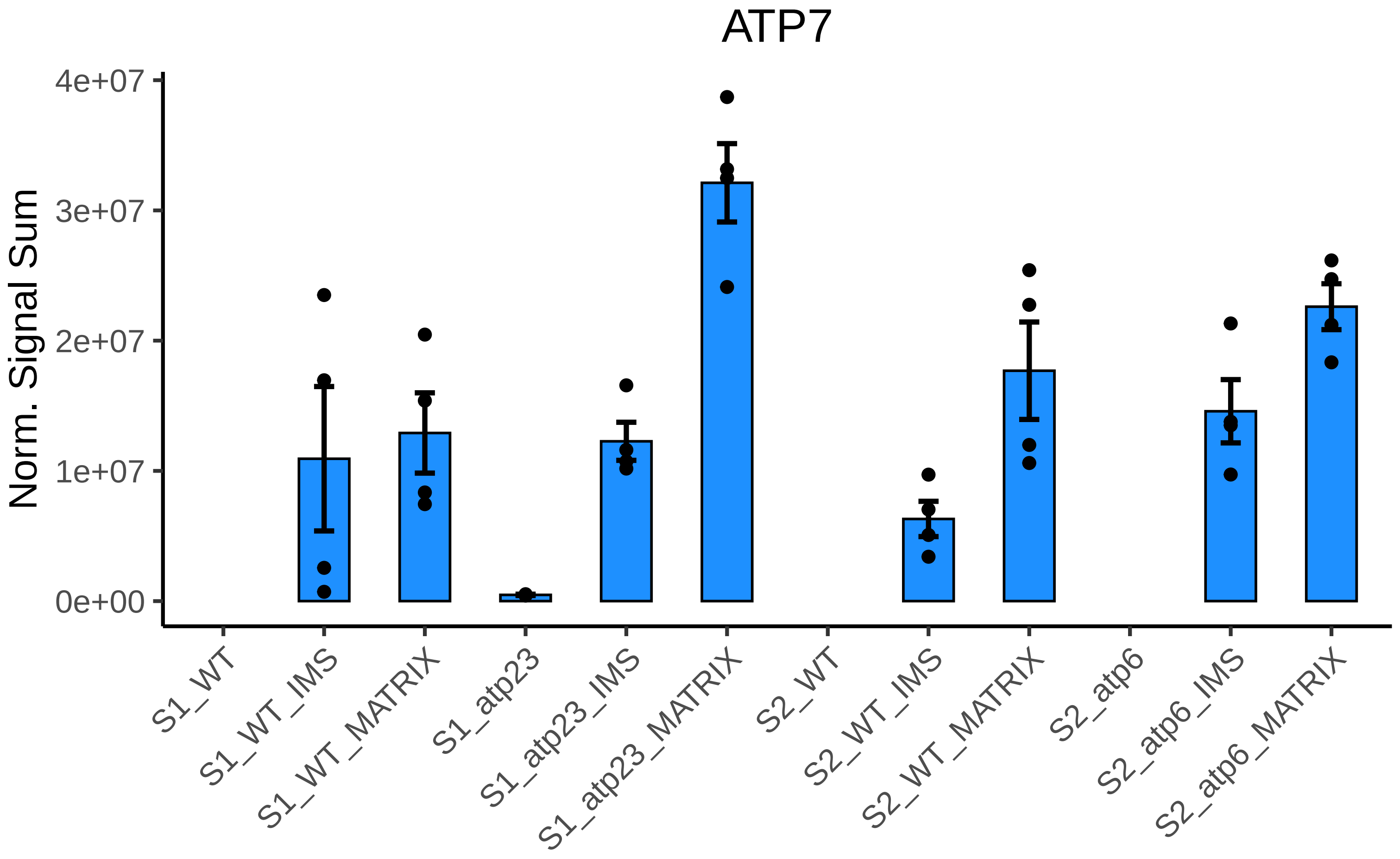
ATP7
Norm. Signal Sum
4e+07
3e+07
2e+07
1e+07
0e+00
S1_WT
S1_WT_IMS
S1_WT_MATRIX
S1_atp23
S1_atp23_IMS
S1_atp23_MATRIX
S2_WT
S2_WT_IMS
S2_WT_MATRIX
S2_atp6
S2_atp6_IMS
S2_atp6_MATRIX

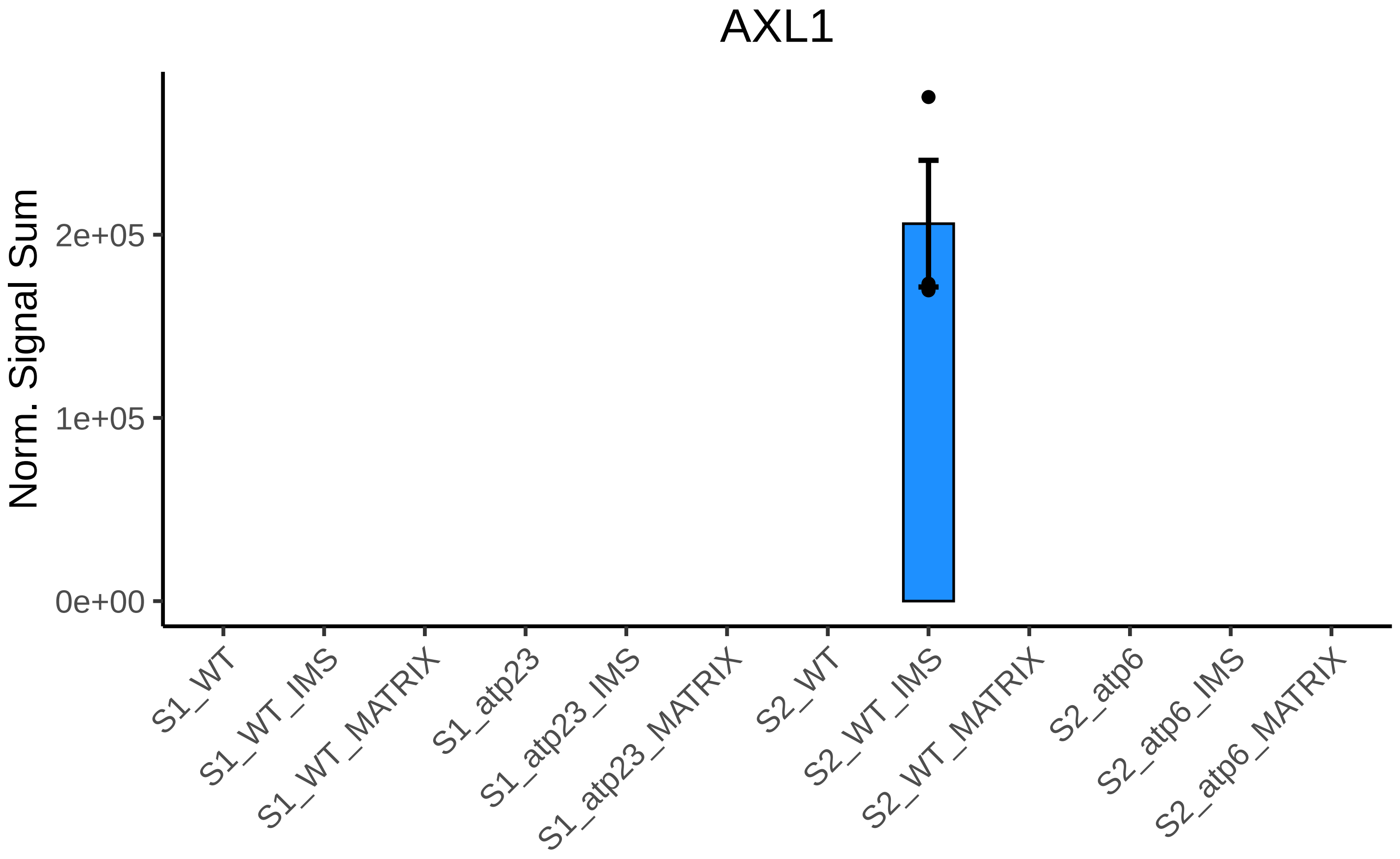
AXL1
Norm. Signal Sum
2e+05
1e+05
0e+00
S1_WT
S1_WT_IMS
S1_WT_MATRIX
S1_atp23
S1_atp23_IMS
S1_atp23_MATRIX
S2_WT
S2_WT_IMS
S2_WT_MATRIX
S2_atp6
S2_atp6_IMS
S2_atp6_MATRIX

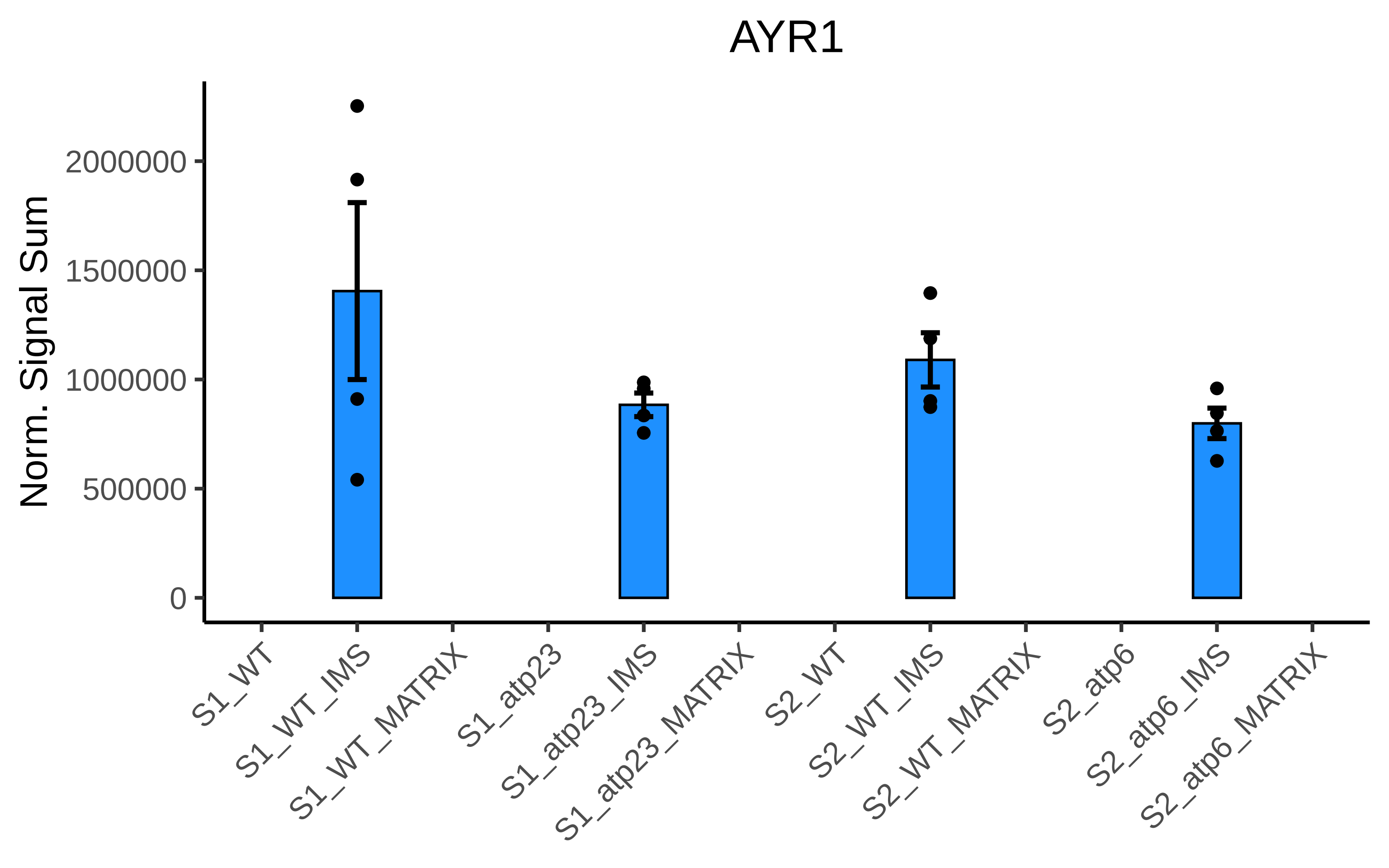

AYR1
Norm. Signal Sum
2000000
1500000
1000000
500000
0
S1_WT
S1_WT_IMS
S1_WT_MATRIX
S1_atp23
S1_atp23_IMS
S1_atp23_MATRIX
S2_WT
S2_WT_IMS
S2_WT_MATRIX
S2_atp6
S2_atp6_IMS
S2_atp6_MATRIX

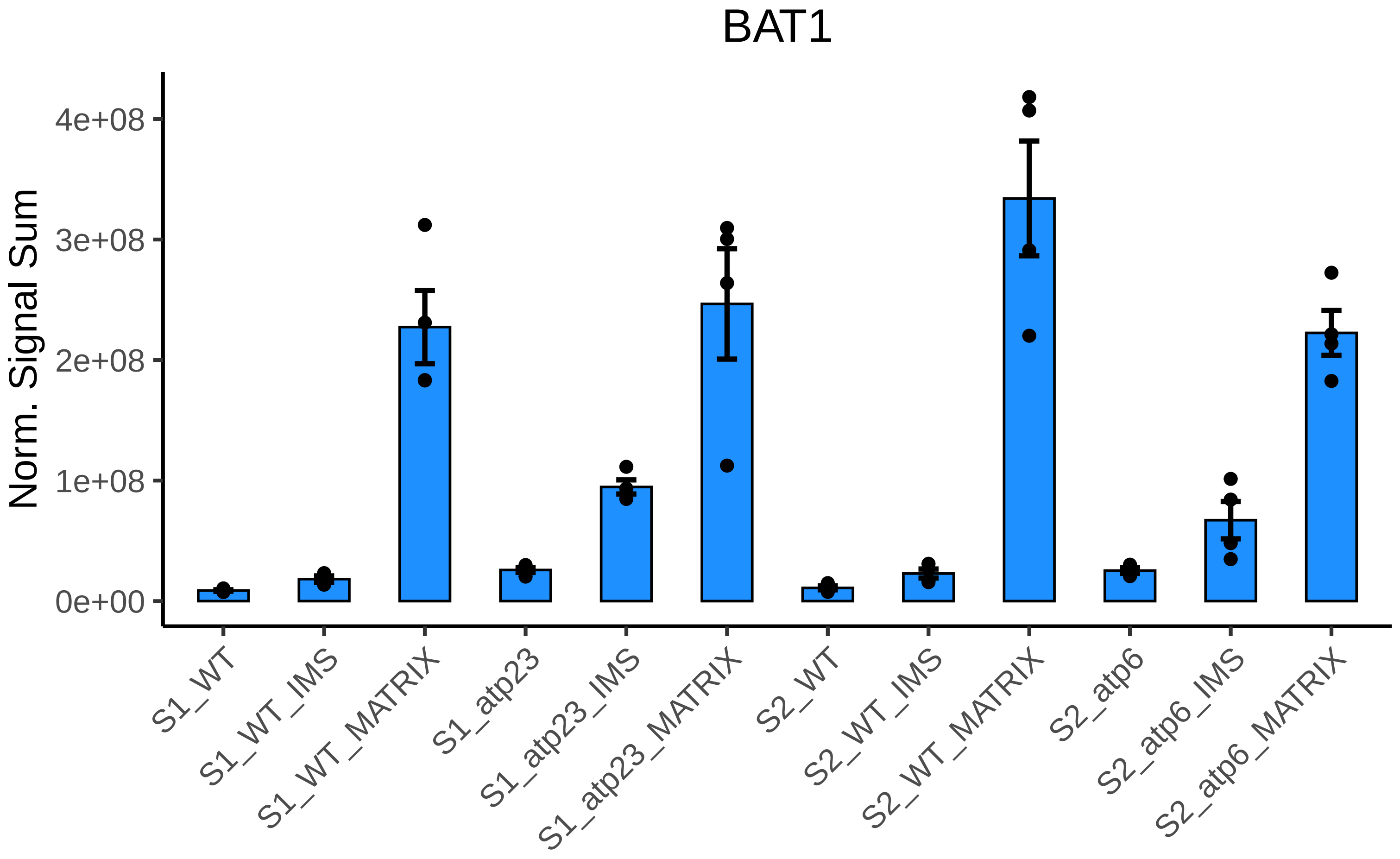

BAT1
Norm. Signal Sum
4e+08
3e+08
2e+08
1e+08
0e+00
S1_WT
S1_WT_IMS
S1_WT_MATRIX
S1_atp23
S1_atp23_IMS
S1_atp23_MATRIX
S2_WT
S2_WT_IMS
S2_WT_MATRIX
S2_atp6
S2_atp6_IMS
S2_atp6_MATRIX

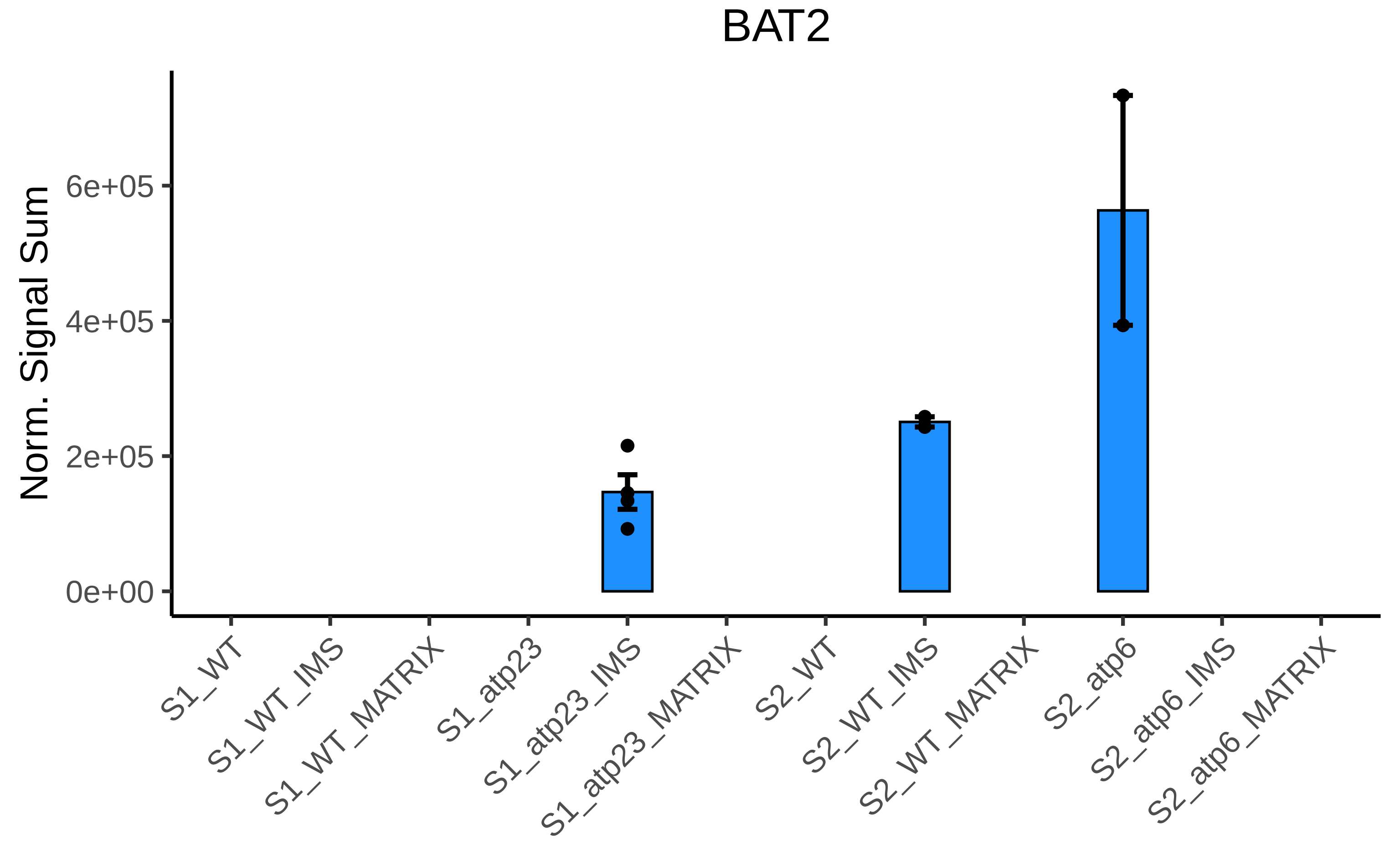

BAT2
Norm. Signal Sum
6e+05
4e+05
2e+05
0e+00
S1_WT
S1_WT_IMS
S1_WT_MATRIX
S1_atp23
S1_atp23_IMS
S1_atp23_MATRIX
S2_WT
S2_WT_IMS
S2_WT_MATRIX
S2_atp6
S2_atp6_IMS
S2_atp6_MATRIX

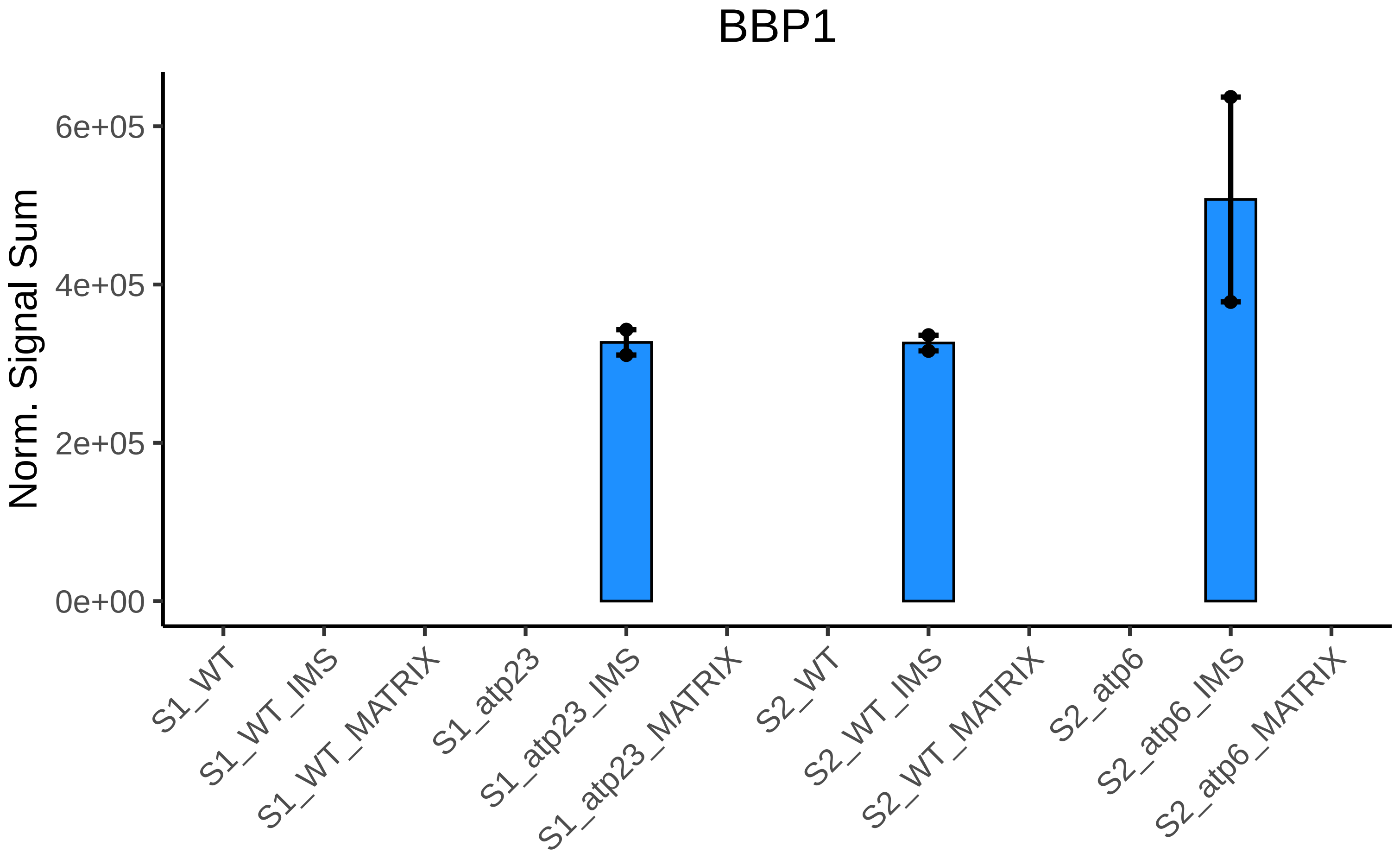
BBP1
Norm. Signal Sum
6e+05
4e+05
2e+05
0e+00
S1_WT
S1_WT_IMS
S1_WT_MATRIX
S1_atp23
S1_atp23_IMS
S1_atp23_MATRIX
S2_WT
S2_WT_IMS
S2_WT_MATRIX
S2_atp6
S2_atp6_IMS
S2_atp6_MATRIX

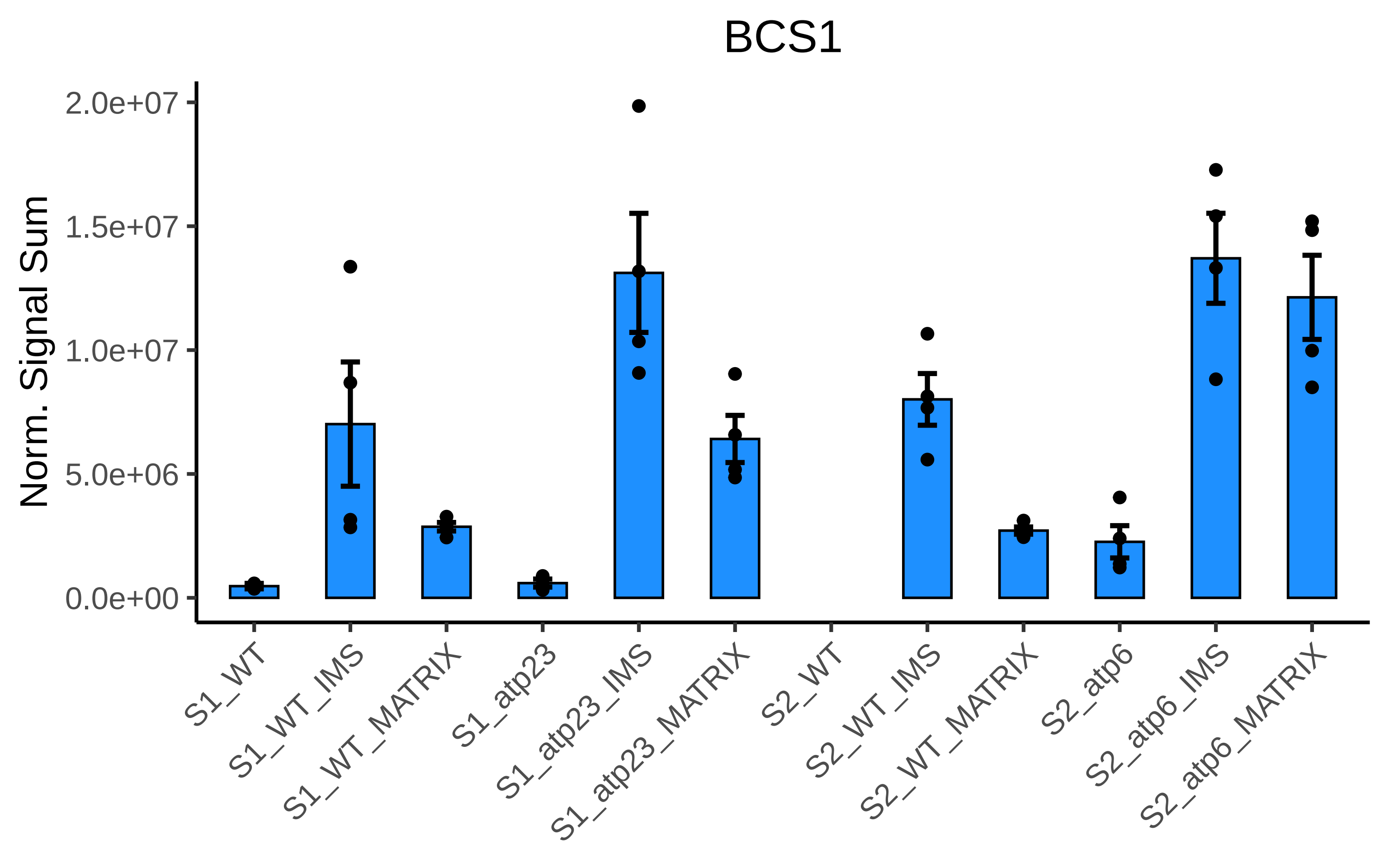

BCS1
Norm. Signal Sum
2.0e+07
1.5e+07
1.0e+07
5.0e+06
0.0e+00
S1_WT
S1_WT_IMS
S1_WT_MATRIX
S1_atp23
S1_atp23_IMS
S1_atp23_MATRIX
S2_WT
S2_WT_IMS
S2_WT_MATRIX
S2_atp6
S2_atp6_IMS
S2_atp6_MATRIX

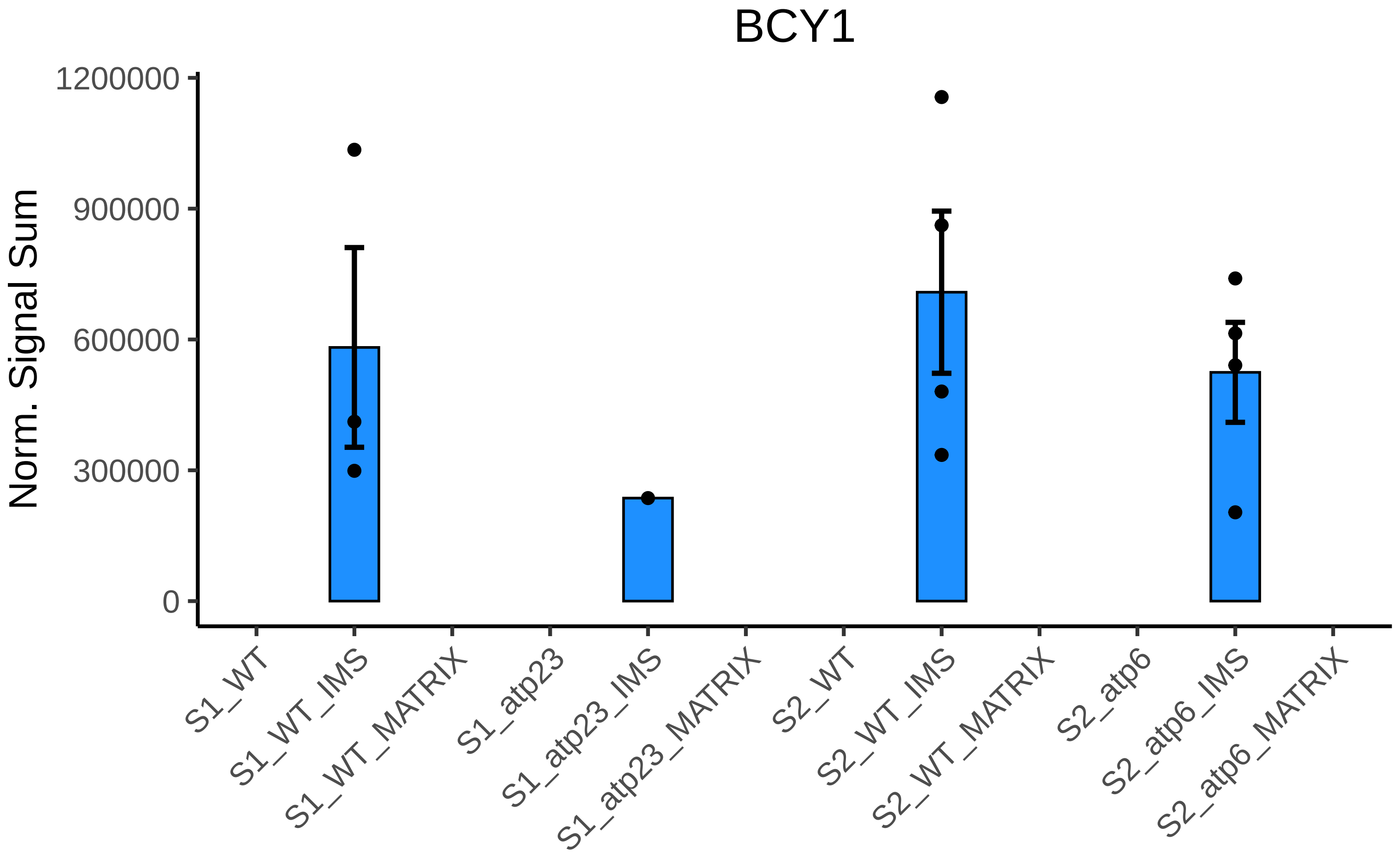
BCY1
Norm. Signal Sum
1200000
900000
600000
300000
0
S1_WT
S1_WT_IMS
S1_WT_MATRIX
S1_atp23
S1_atp23_IMS
S1_atp23_MATRIX
S2_WT
S2_WT_IMS
S2_WT_MATRIX
S2_atp6
S2_atp6_IMS
S2_atp6_MATRIX

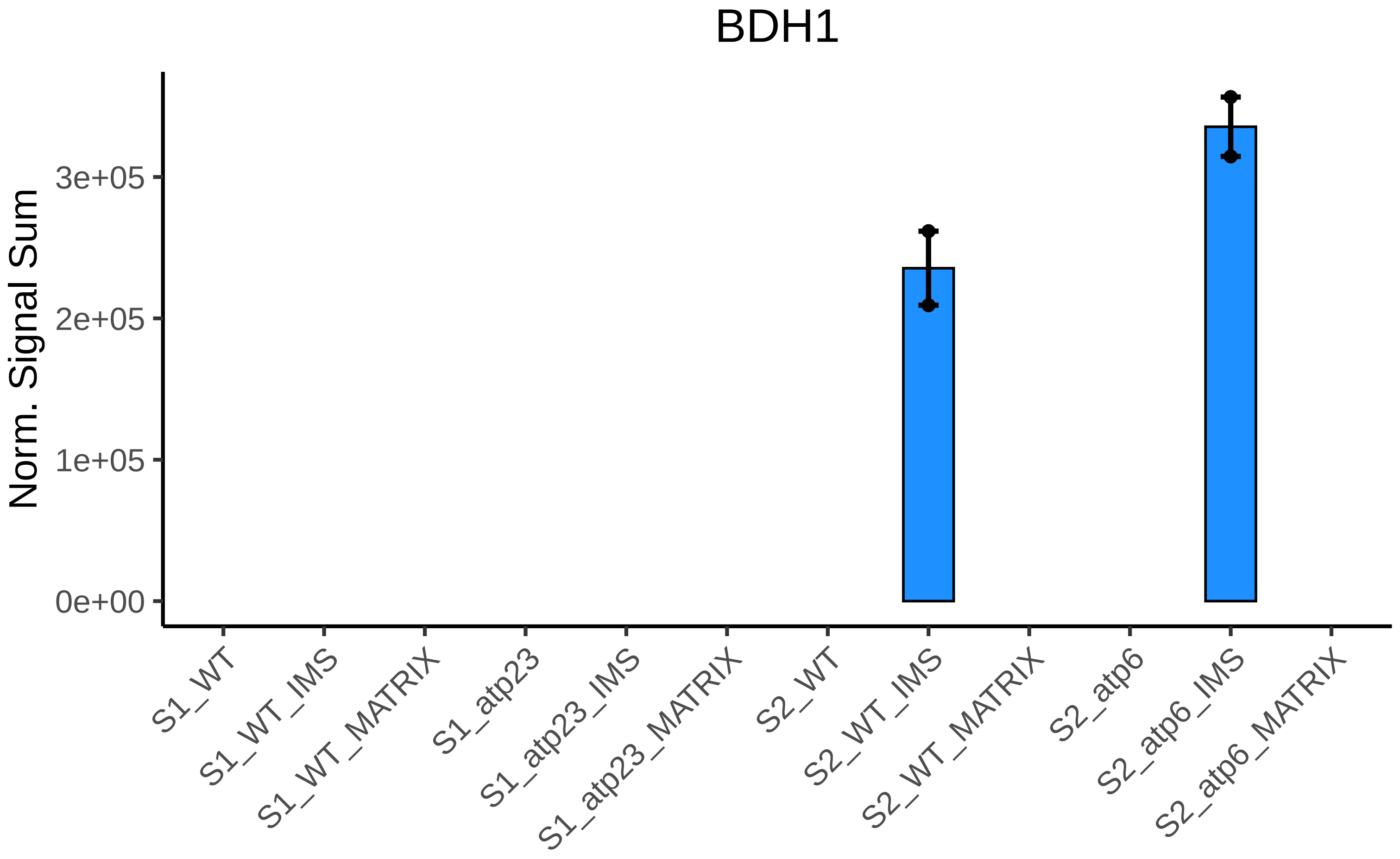

BDH1
Norm. Signal Sum
3e+05
2e+05
1e+05
0e+00
S1_WT
S1_WT_IMS
S1_WT_MATRIX
S1_atp23
S1_atp23_IMS
S1_atp23_MATRIX
S2_WT
S2_WT_IMS
S2_WT_MATRIX
S2_atp6
S2_atp6_IMS
S2_atp6_MATRIX

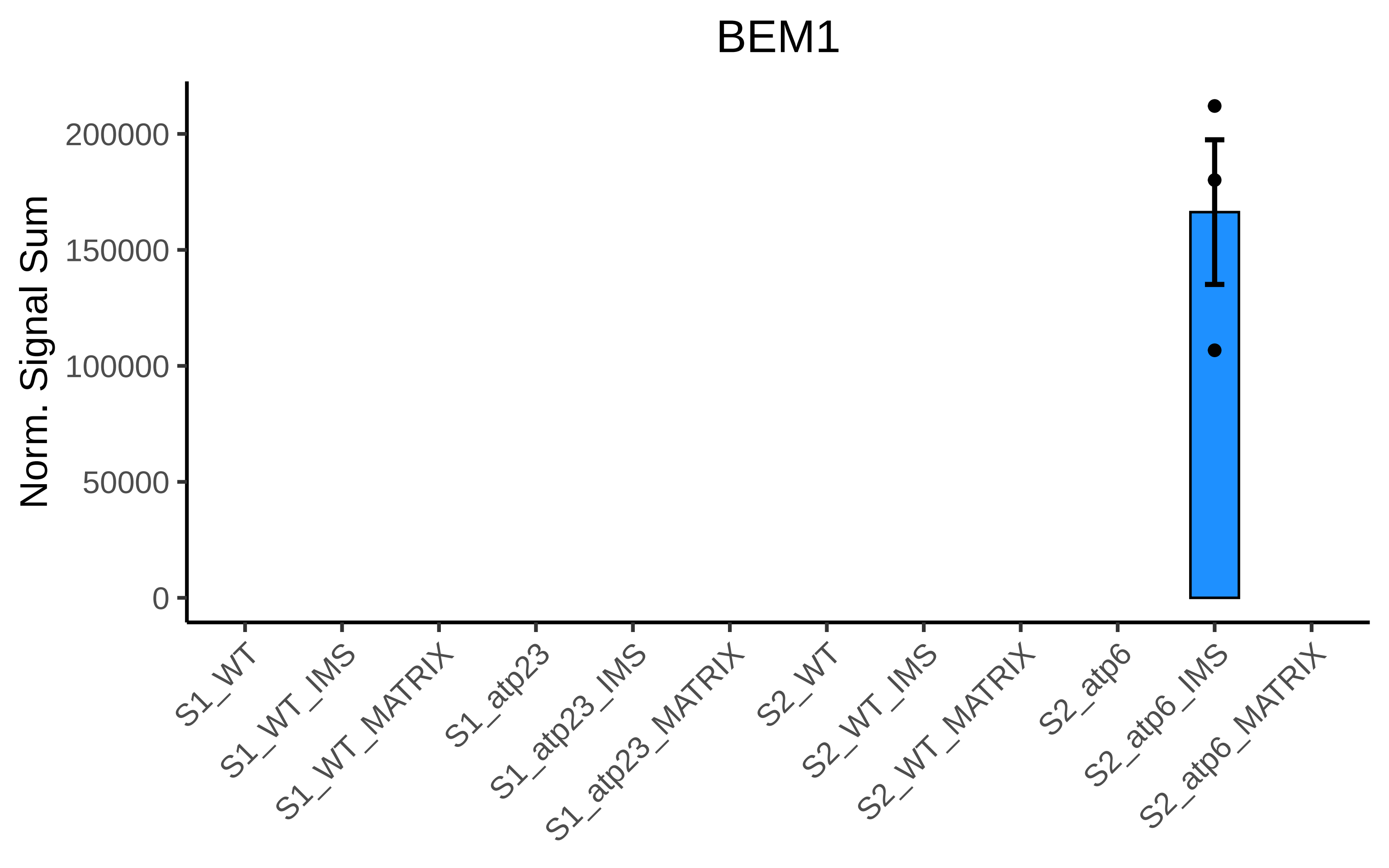
BEM1
Norm. Signal Sum
200000
150000
100000
50000
0
S1_WT
S1_WT_IMS
S1_WT_MATRIX
S1_atp23
S1_atp23_IMS
S1_atp23_MATRIX
S2_WT
S2_WT_IMS
S2_WT_MATRIX
S2_atp6
S2_atp6_IMS
S2_atp6_MATRIX

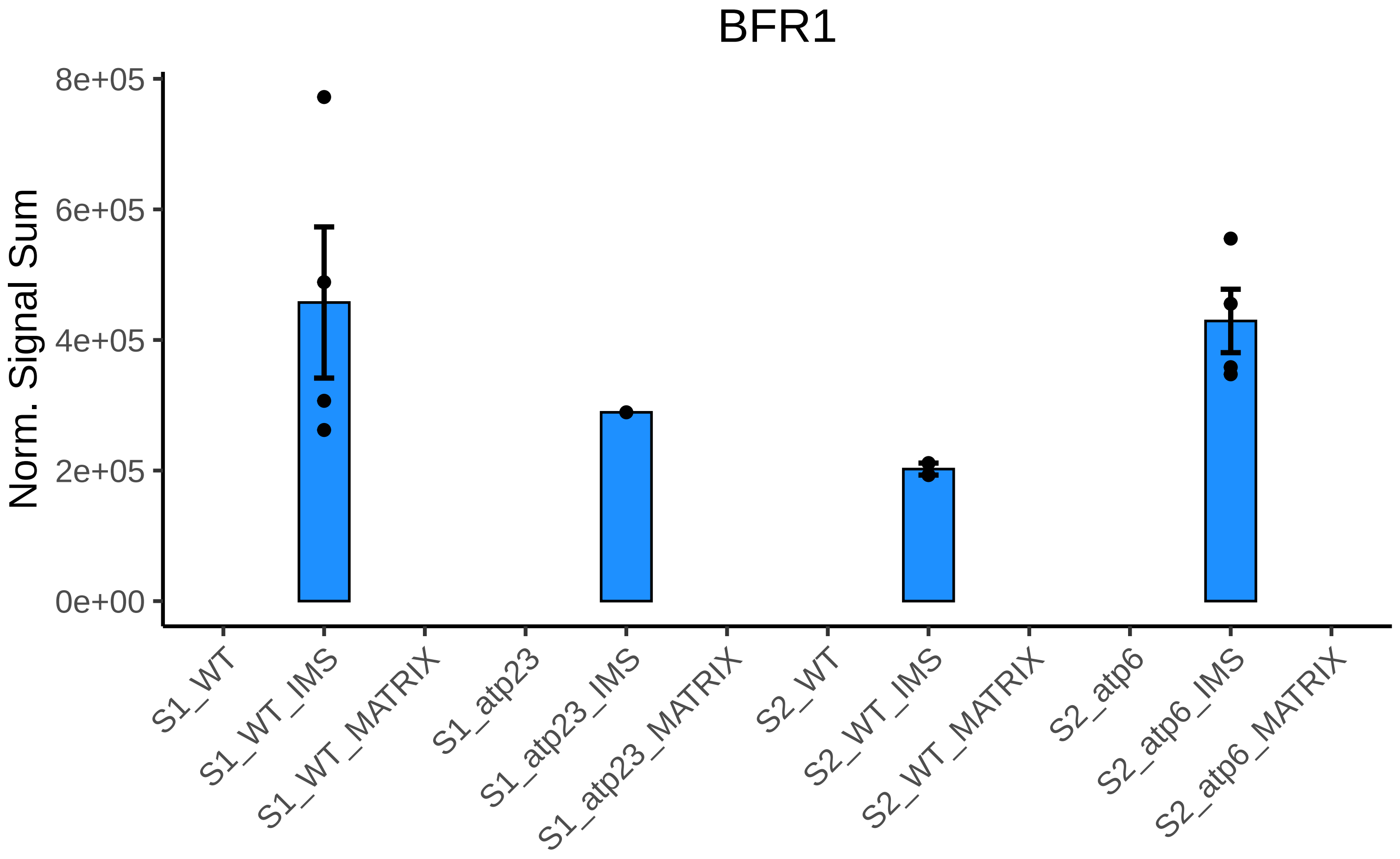

BFR1
Norm. Signal Sum
8e+05
6e+05
4e+05
2e+05
0e+00
S1_WT
S1_WT_IMS
S1_WT_MATRIX
S1_atp23
S1_atp23_IMS
S1_atp23_MATRIX
S2_WT
S2_WT_IMS
S2_WT_MATRIX
S2_atp6
S2_atp6_IMS
S2_atp6_MATRIX

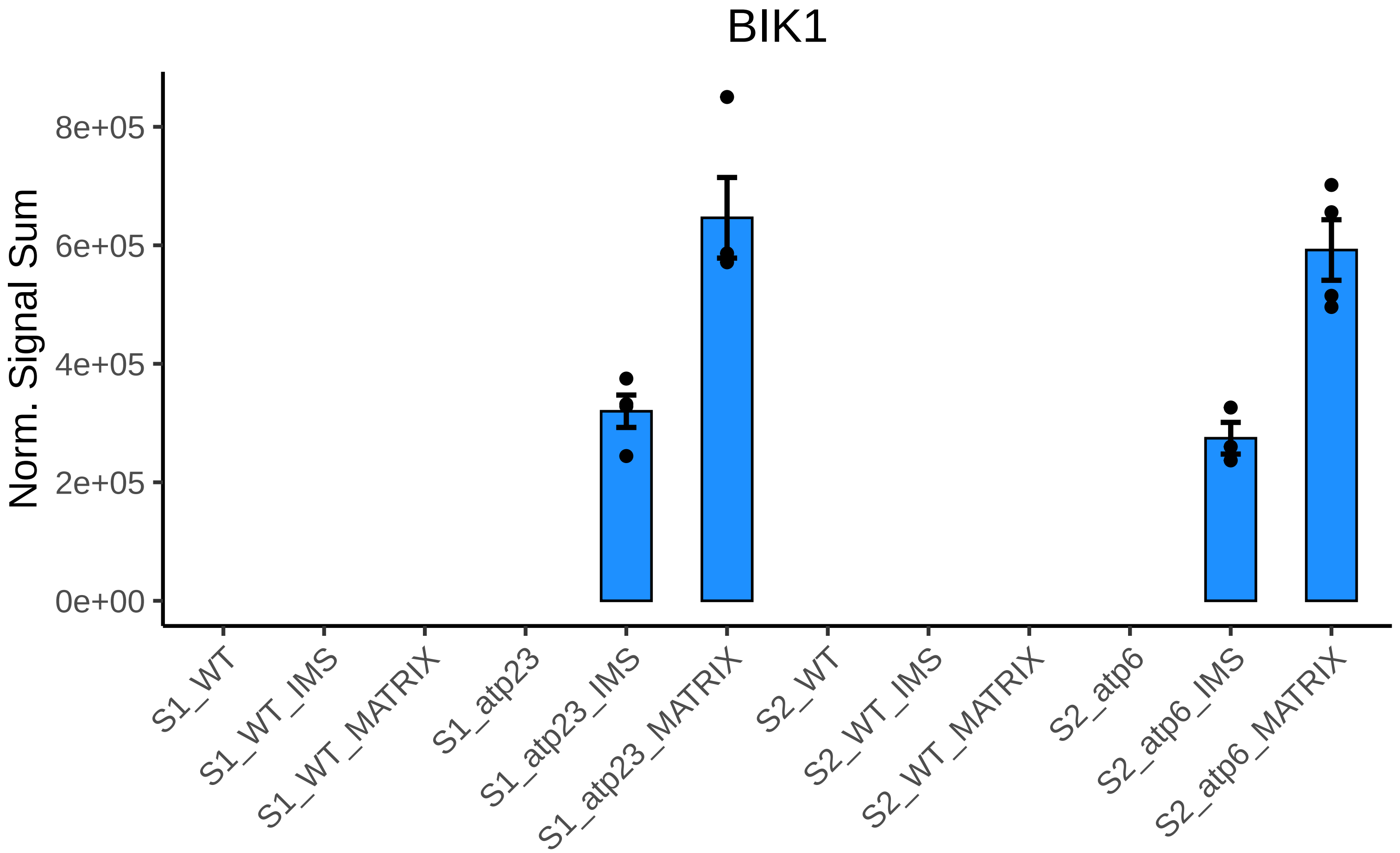

BIK1
Norm. Signal Sum
8e+05
6e+05
4e+05
2e+05
0e+00
S1_WT
S1_WT_IMS
S1_WT_MATRIX
S1_atp23
S1_atp23_IMS
S1_atp23_MATRIX
S2_WT
S2_WT_IMS
S2_WT_MATRIX
S2_atp6
S2_atp6_IMS
S2_atp6_MATRIX

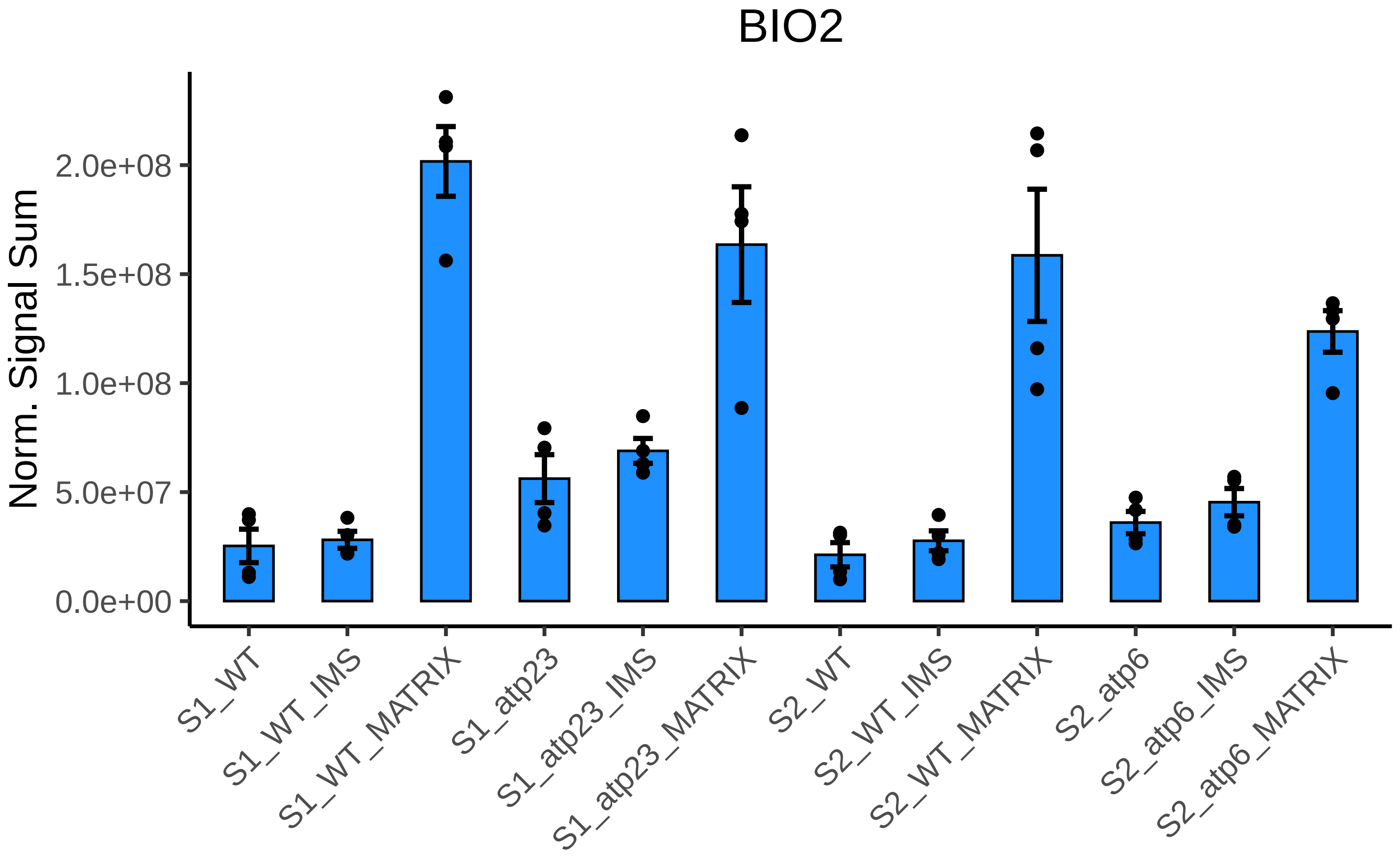

BIO2
Norm. Signal Sum
2.0e+08
1.5e+08
1.0e+08
5.0e+07
0.0e+00
S1_WT
S1_WT_IMS
S1_WT_MATRIX
S1_atp23
S1_atp23_IMS
S1_atp23_MATRIX
S2_WT
S2_WT_IMS
S2_WT_MATRIX
S2_atp6
S2_atp6_IMS
S2_atp6_MATRIX

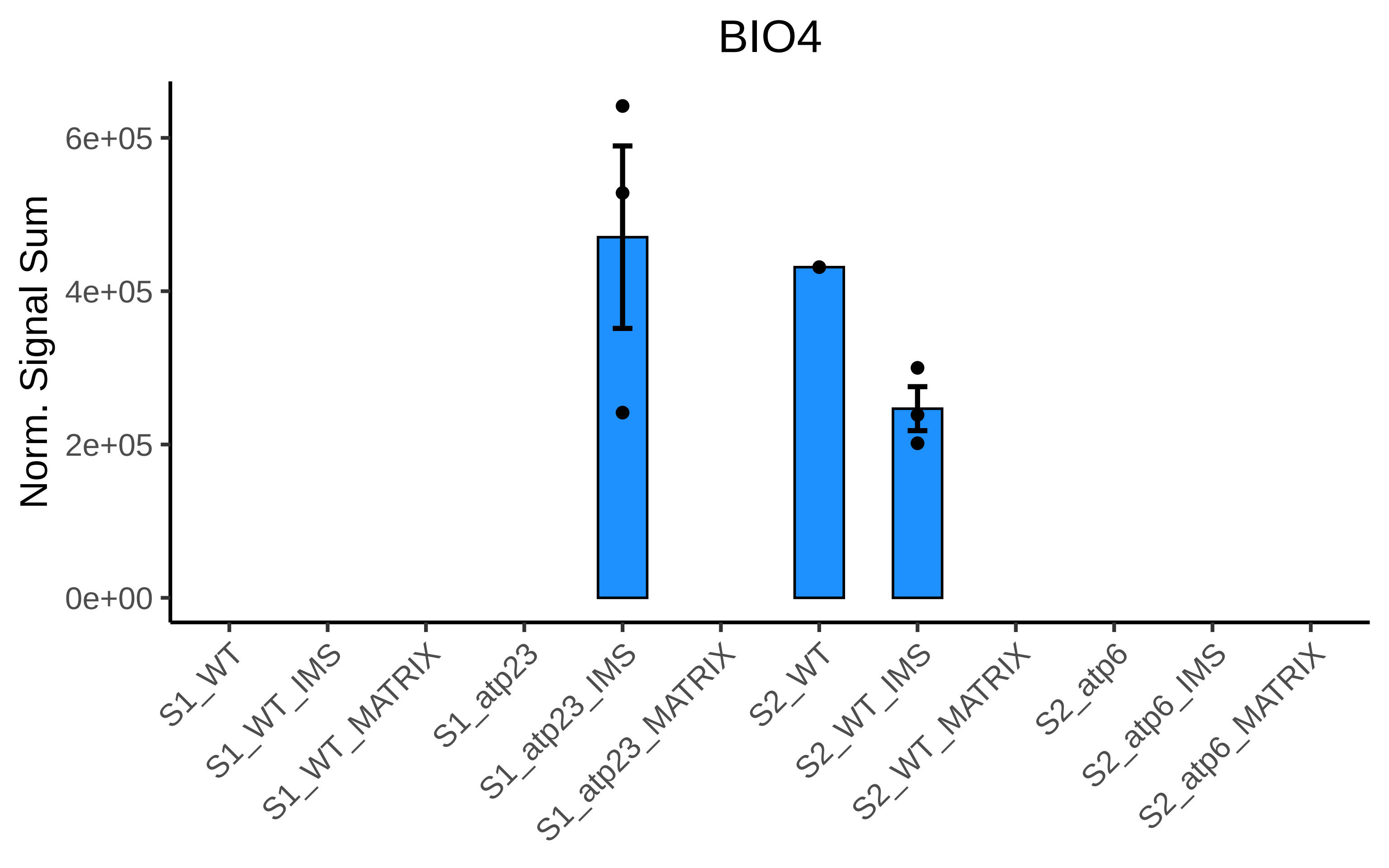
BIO4
Norm. Signal Sum
6e+05
4e+05
2e+05
0e+00
S1_WT
S1_WT_IMS
S1_WT_MATRIX
S1_atp23
S1_atp23_IMS
S1_atp23_MATRIX
S2_WT
S2_WT_IMS
S2_WT_MATRIX
S2_atp6
S2_atp6_IMS
S2_atp6_MATRIX

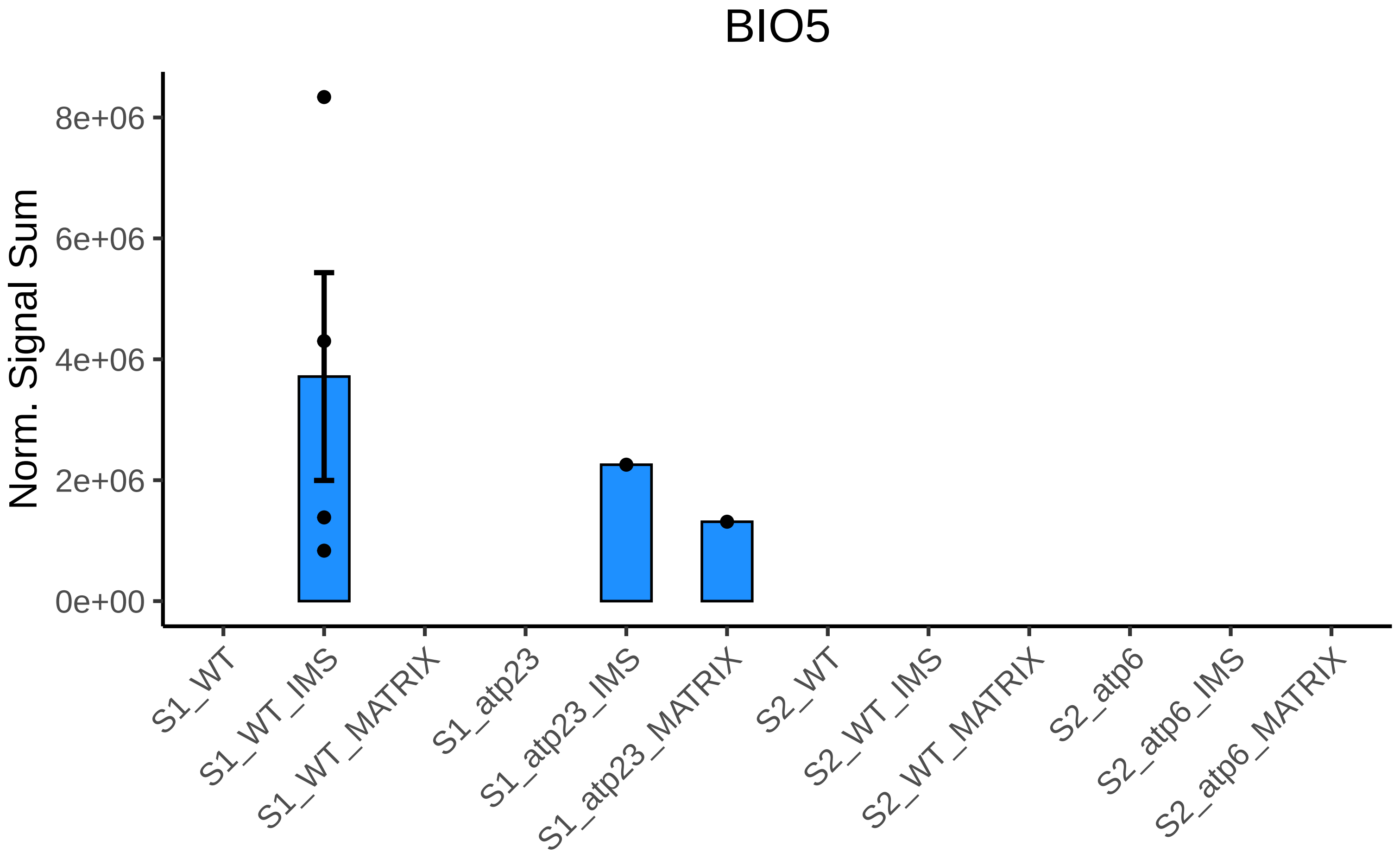

BIO5
Norm. Signal Sum
0e+00
2e+06
4e+06
6e+06
8e+06
S1_WT
S1_WT_IMS
S1_WT_MATRIX
S1_atp23
S1_atp23_IMS
S1_atp23_MATRIX
S2_WT
S2_WT_IMS
S2_WT_MATRIX
S2_atp6
S2_atp6_IMS
S2_atp6_MATRIX

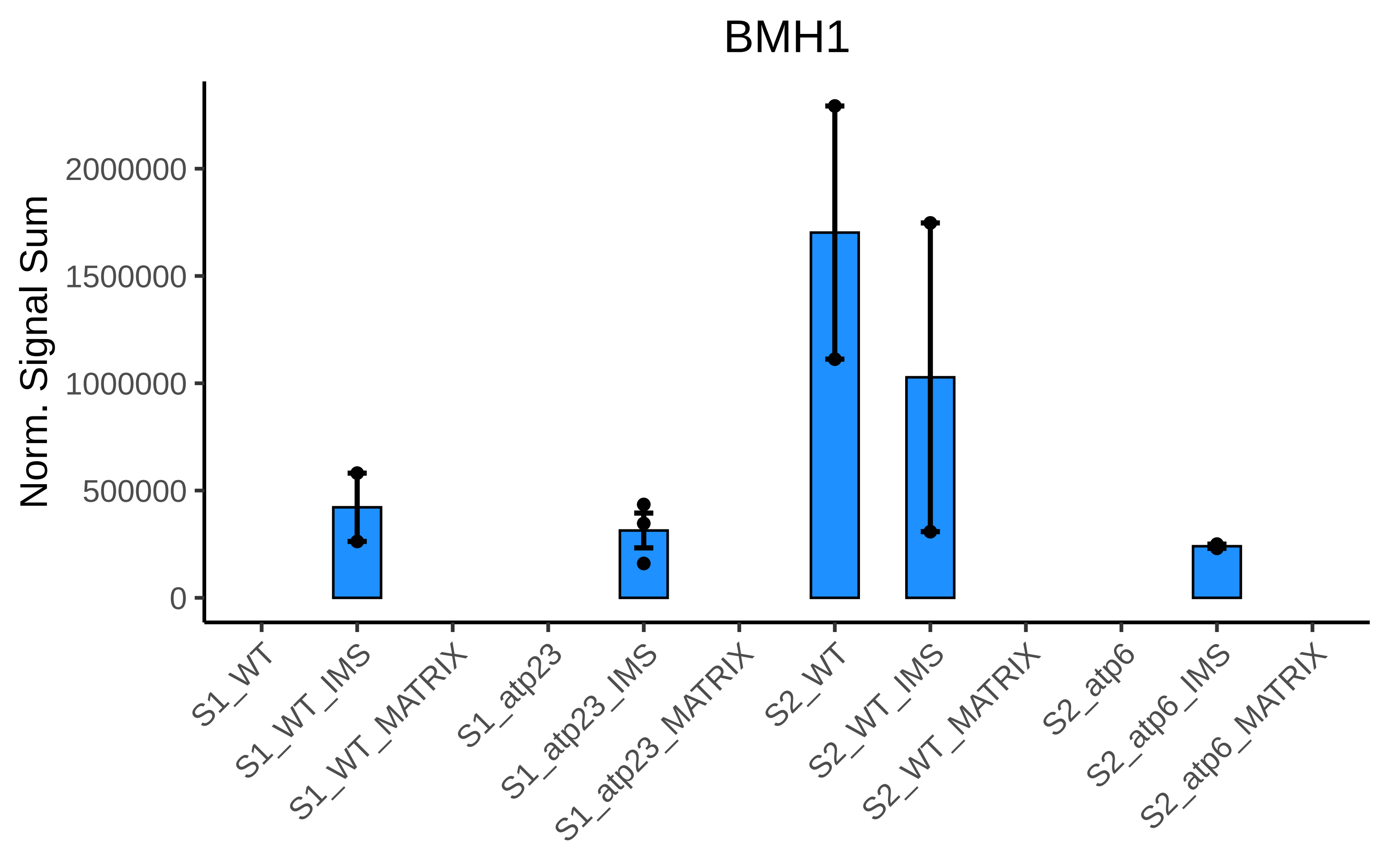

BMH1
Norm. Signal Sum
2000000
1500000
1000000
500000
0
S1_WT
S1_WT_IMS
S1_WT_MATRIX
S1_atp23
S1_atp23_IMS
S1_atp23_MATRIX
S2_WT
S2_WT_IMS
S2_WT_MATRIX
S2_atp6
S2_atp6_IMS
S2_atp6_MATRIX

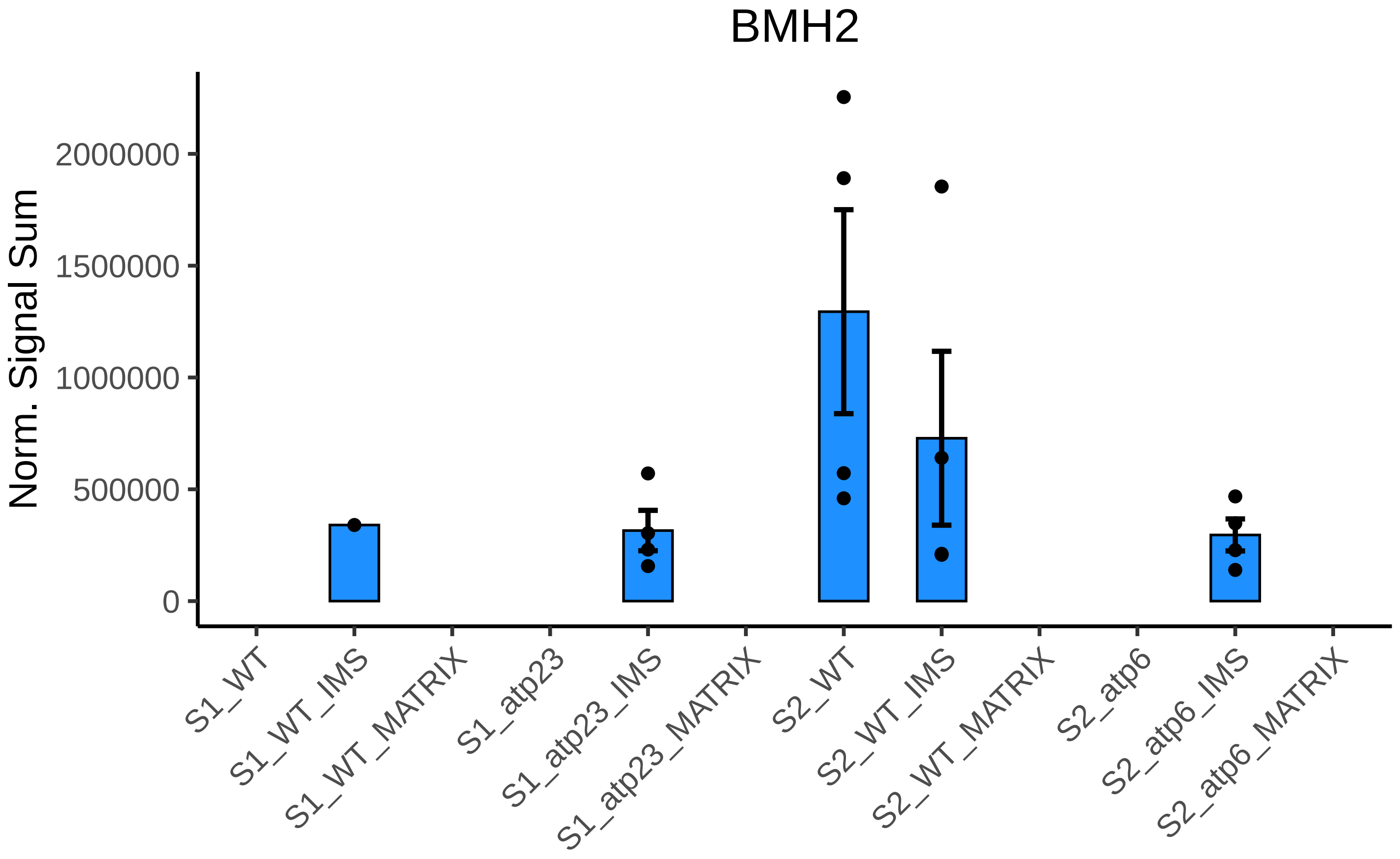

BMH2
Norm. Signal Sum
2000000
1500000
1000000
500000
0
S1_WT
S1_WT_IMS
S1_WT_MATRIX
S1_atp23
S1_atp23_IMS
S1_atp23_MATRIX
S2_WT
S2_WT_IMS
S2_WT_MATRIX
S2_atp6
S2_atp6_IMS
S2_atp6_MATRIX

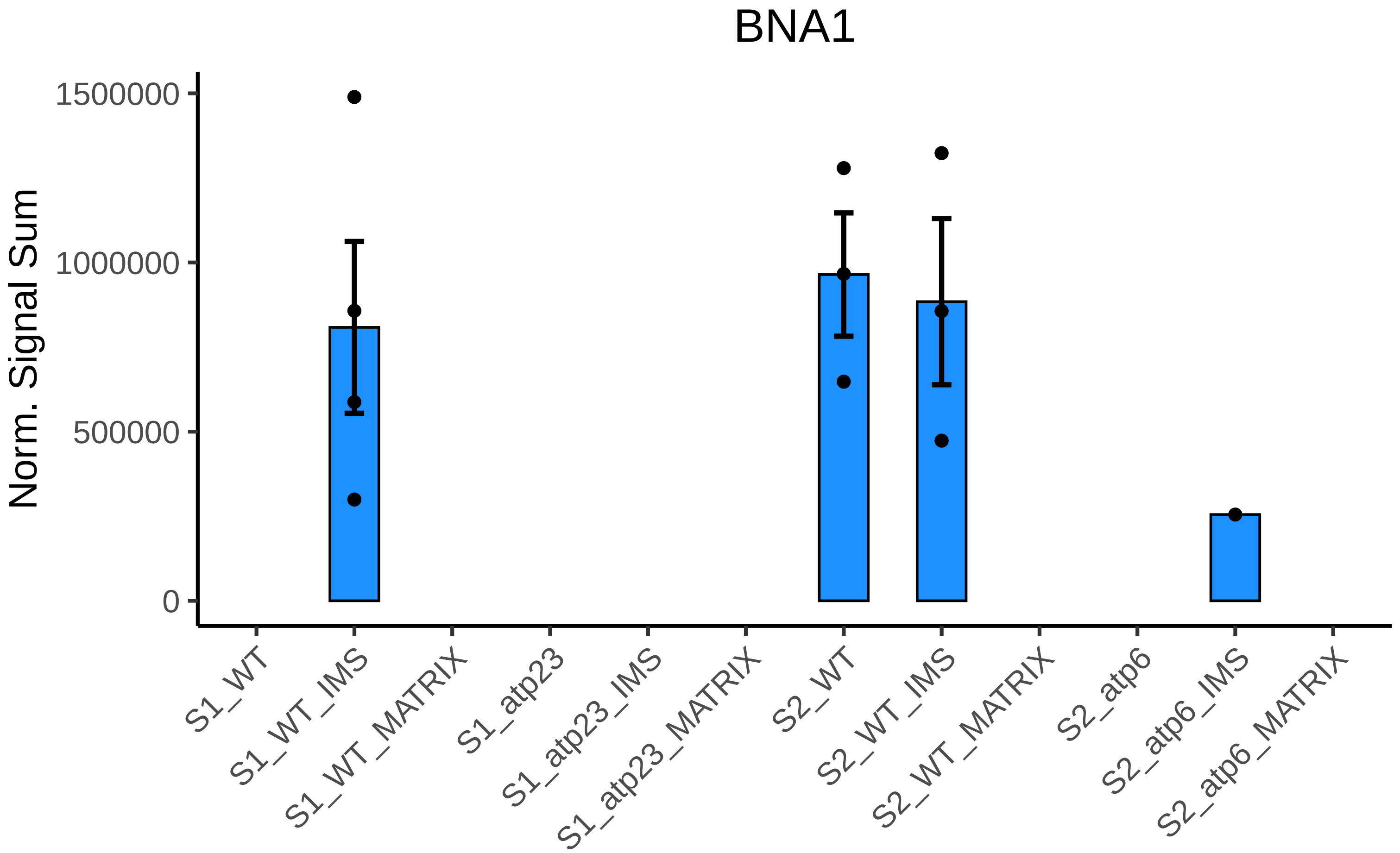
BNA1
Norm. Signal Sum
1500000
1000000
500000
0
S1_WT
S1_WT_IMS
S1_WT_MATRIX
S1_atp23
S1_atp23_IMS
S1_atp23_MATRIX
S2_WT
S2_WT_IMS
S2_WT_MATRIX
S2_atp6
S2_atp6_IMS
S2_atp6_MATRIX

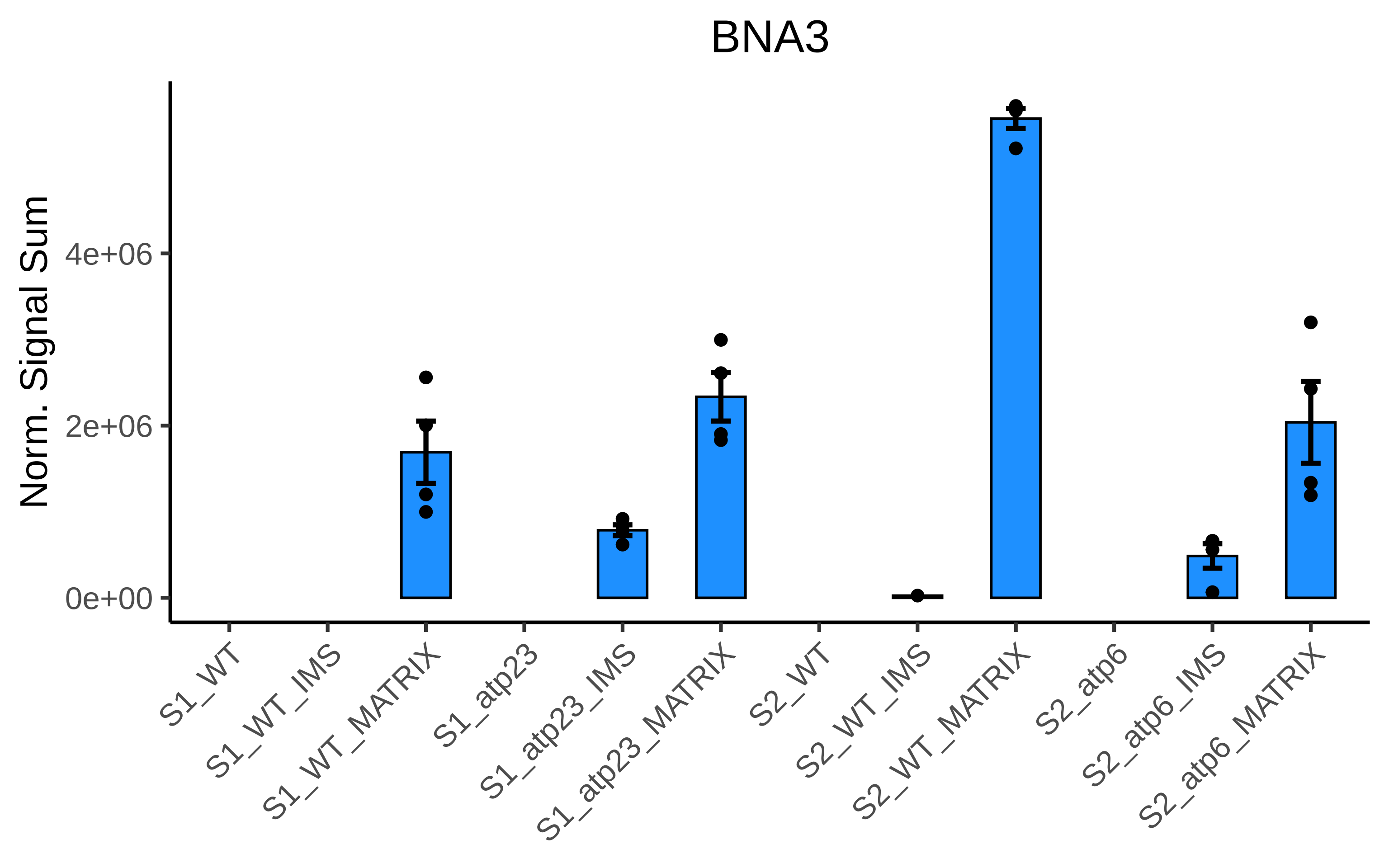

BNA3
Norm. Signal Sum
4e+06
2e+06
0e+00
S1_WT
S1_WT_IMS
S1_WT_MATRIX
S1_atp23
S1_atp23_IMS
S1_atp23_MATRIX
S2_WT
S2_WT_IMS
S2_WT_MATRIX
S2_atp6
S2_atp6_IMS
S2_atp6_MATRIX

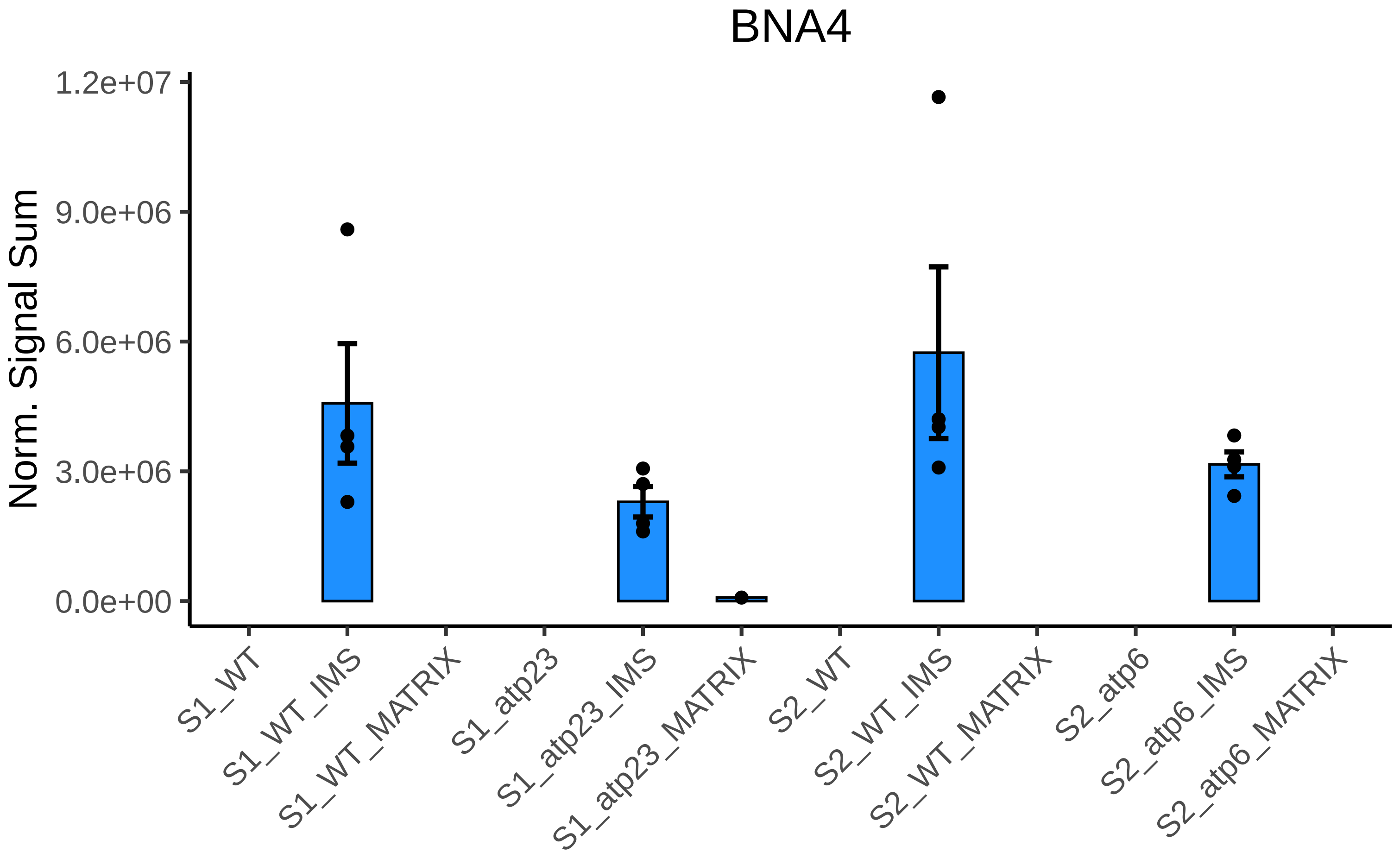
BNA4
Norm. Signal Sum
1.2e+07
9.0e+06
6.0e+06
3.0e+06
0.0e+00
S1_WT
S1_WT_IMS
S1_WT_MATRIX
S1_atp23
S1_atp23_IMS
S1_atp23_MATRIX
S2_WT
S2_WT_IMS
S2_WT_MATRIX
S2_atp6
S2_atp6_IMS
S2_atp6_MATRIX

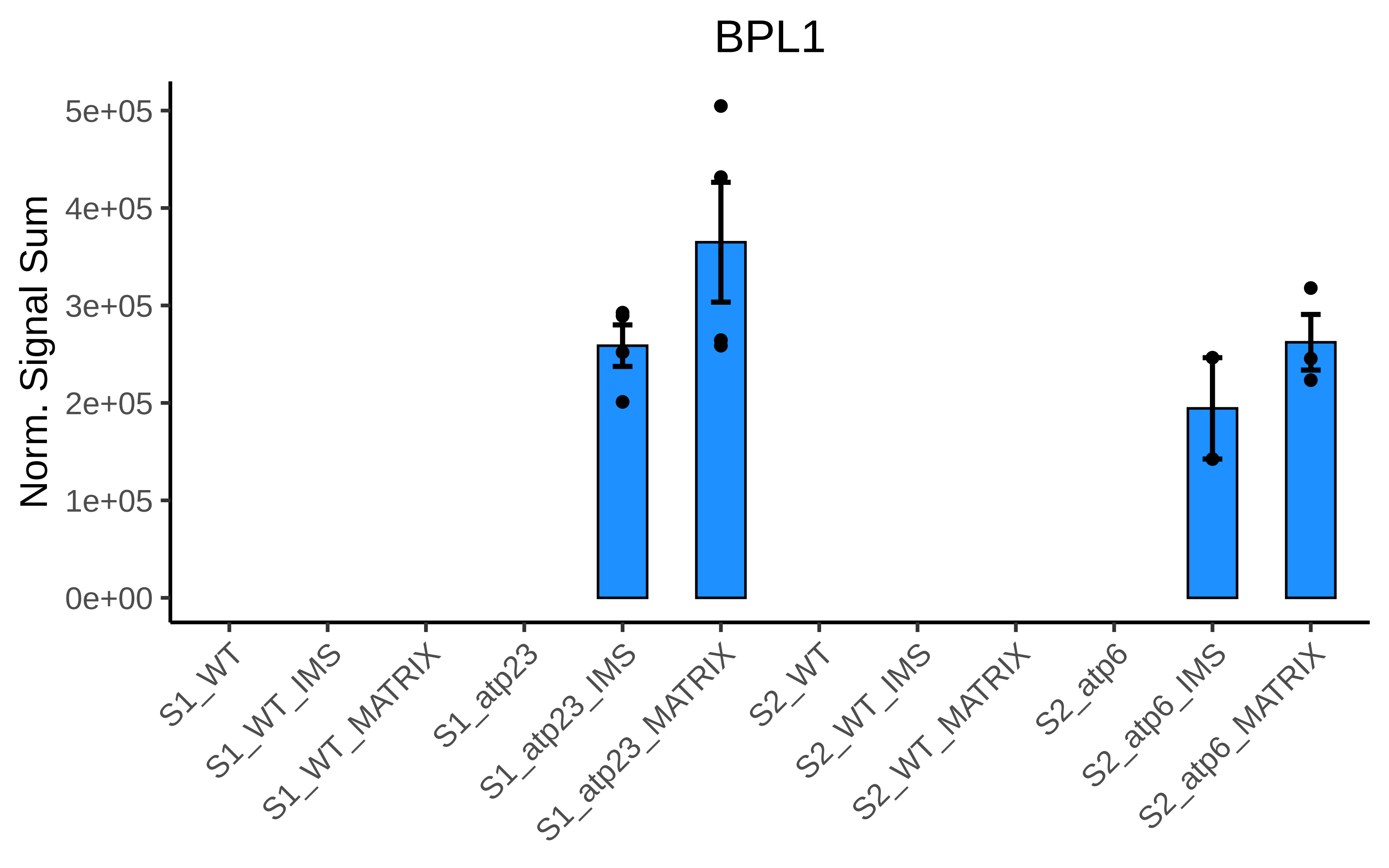
BPL1
Norm. Signal Sum
5e+05
4e+05
3e+05
2e+05
1e+05
0e+00
S1_WT
S1_WT_IMS
S1_WT_MATRIX
S1_atp23
S1_atp23_IMS
S1_atp23_MATRIX
S2_WT
S2_WT_IMS
S2_WT_MATRIX
S2_atp6
S2_atp6_IMS
S2_atp6_MATRIX

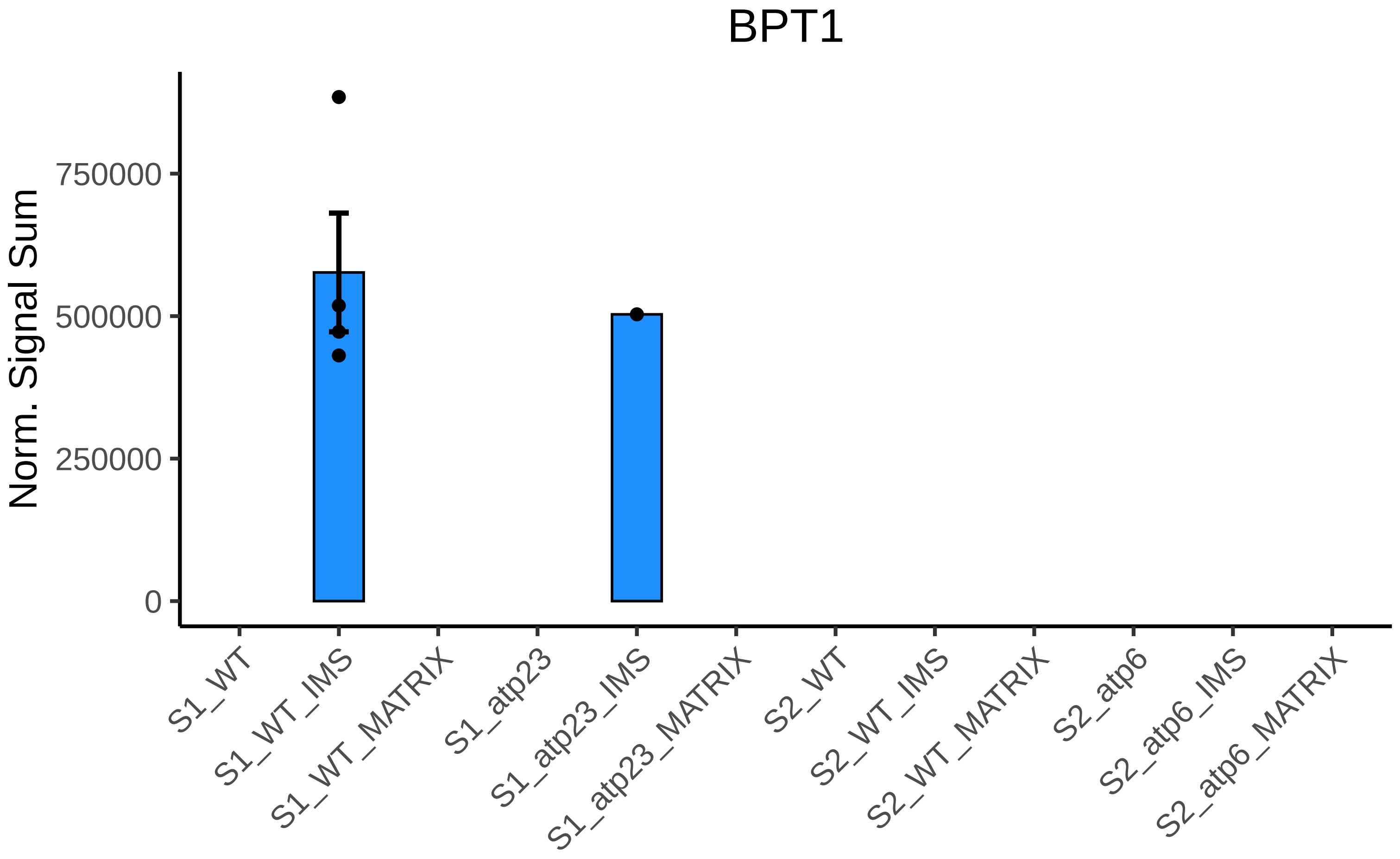
BPT1
Norm. Signal Sum
750000
500000
250000
0
S1_WT
S1_WT_IMS
S1_WT_MATRIX
S1_atp23
S1_atp23_IMS
S1_atp23_MATRIX
S2_WT
S2_WT_IMS
S2_WT_MATRIX
S2_atp6
S2_atp6_IMS
S2_atp6_MATRIX

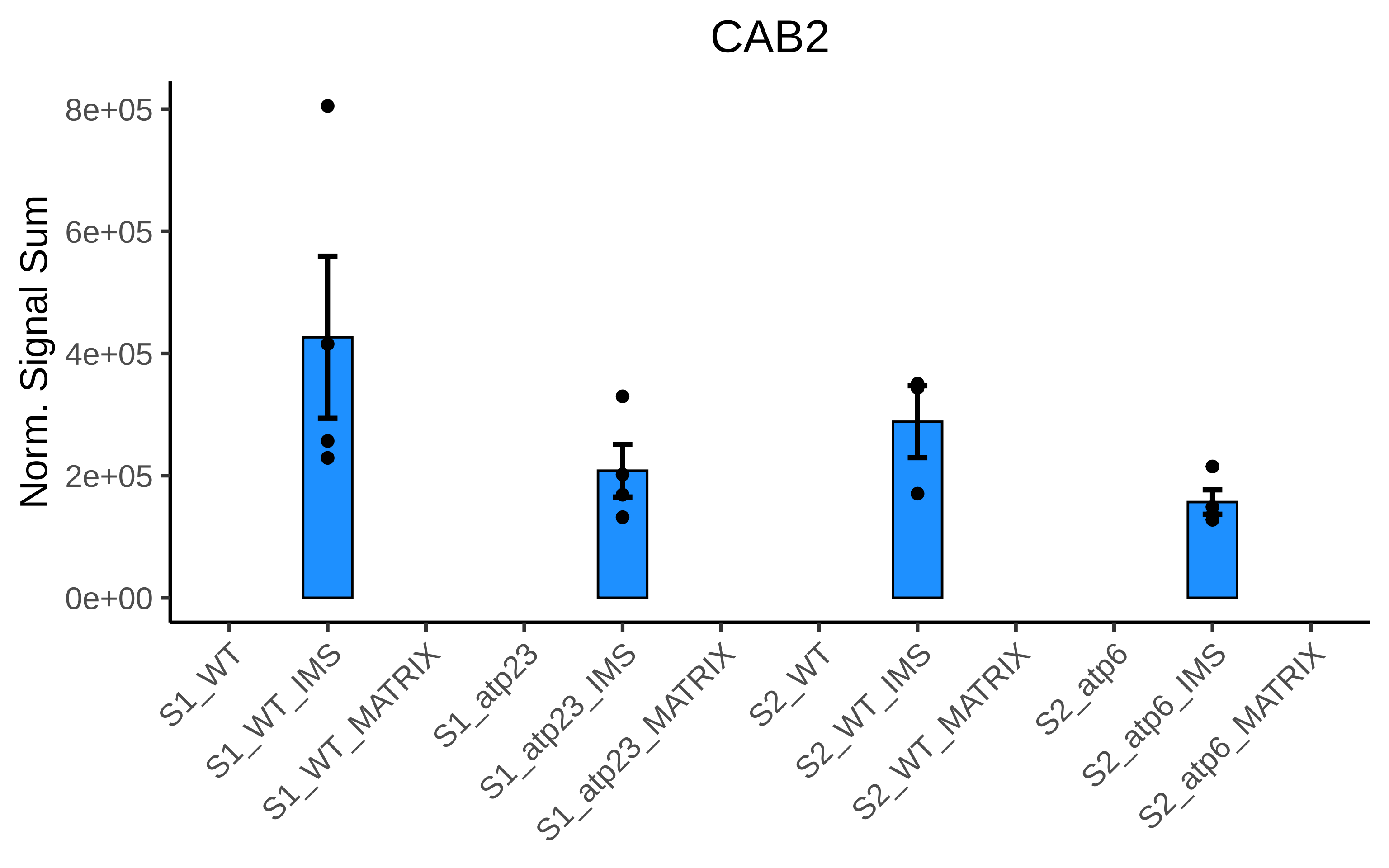

CAB2
Norm. Signal Sum
8e+05
6e+05
4e+05
2e+05
0e+00
S1_WT
S1_WT_IMS
S1_WT_MATRIX
S1_atp23
S1_atp23_IMS
S1_atp23_MATRIX
S2_WT
S2_WT_IMS
S2_WT_MATRIX
S2_atp6
S2_atp6_IMS
S2_atp6_MATRIX

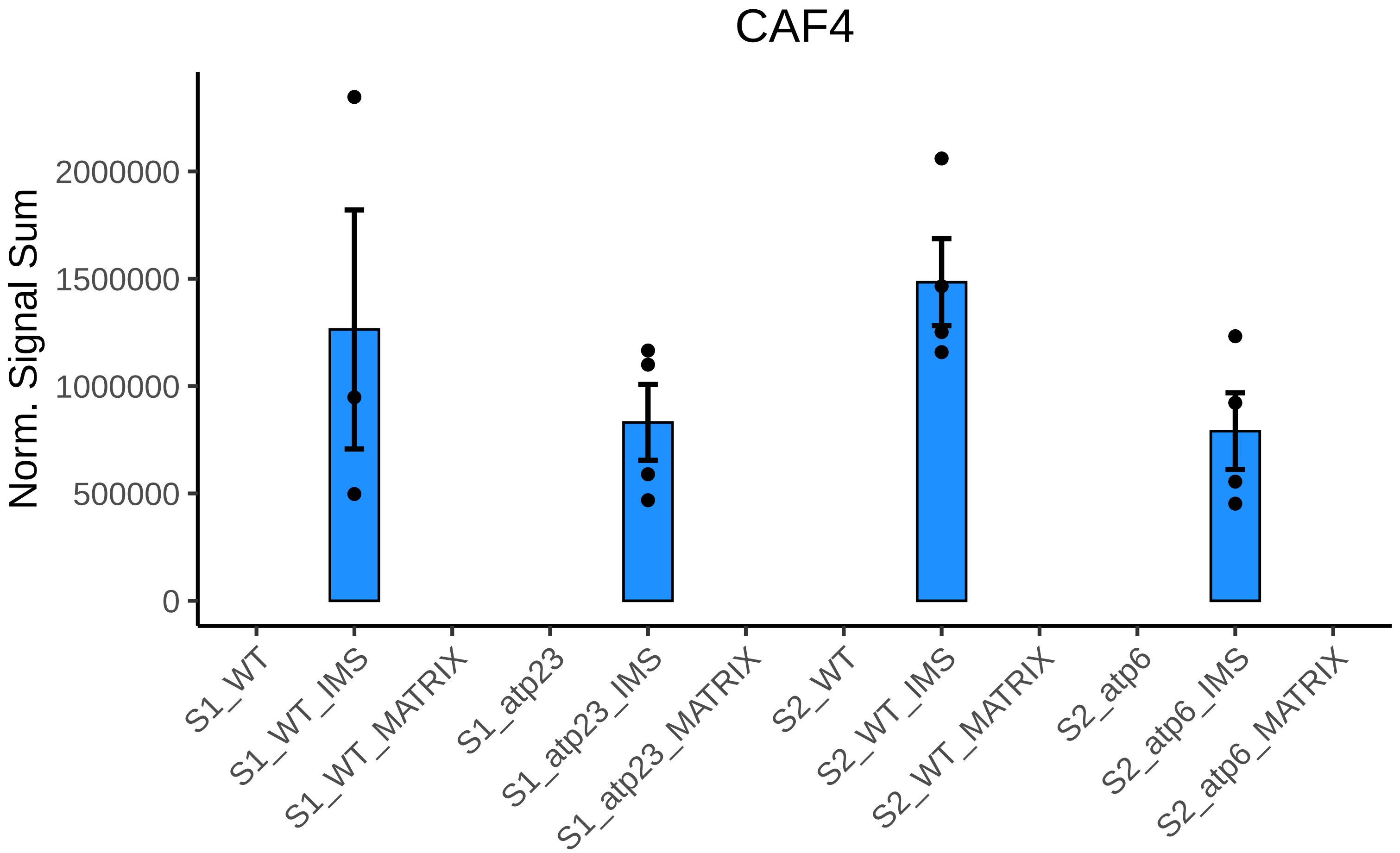
CAF4
Norm. Signal Sum
2000000
1500000
1000000
500000
0
S1_WT
S1_WT_IMS
S1_WT_MATRIX
S1_atp23
S1_atp23_IMS
S1_atp23_MATRIX
S2_WT
S2_WT_IMS
S2_WT_MATRIX
S2_atp6
S2_atp6_IMS
S2_atp6_MATRIX

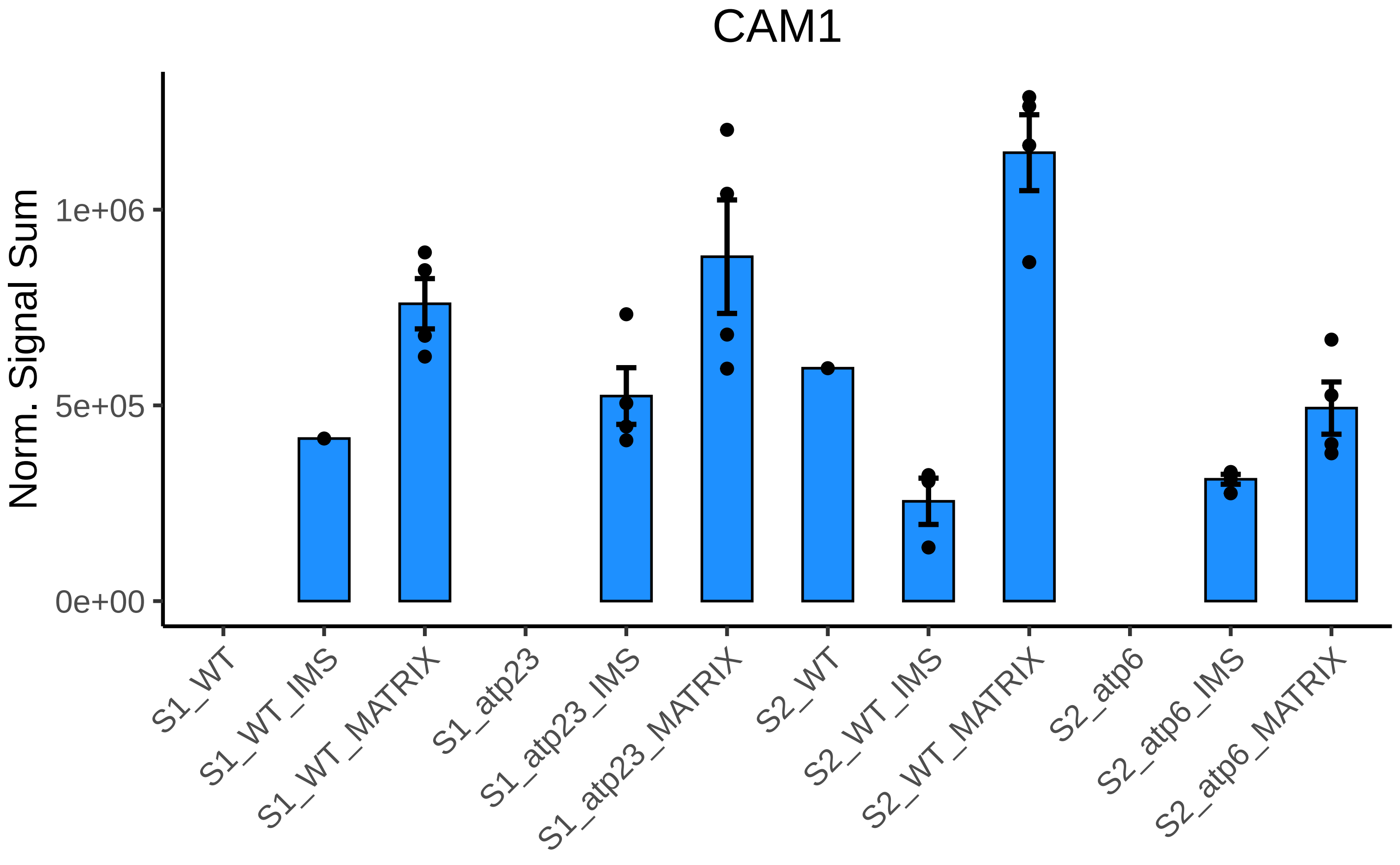

CAM1
Norm. Signal Sum
1e+06
5e+05
0e+00
S1_WT
S1_WT_IMS
S1_WT_MATRIX
S1_atp23
S1_atp23_IMS
S1_atp23_MATRIX
S2_WT
S2_WT_IMS
S2_WT_MATRIX
S2_atp6
S2_atp6_IMS
S2_atp6_MATRIX

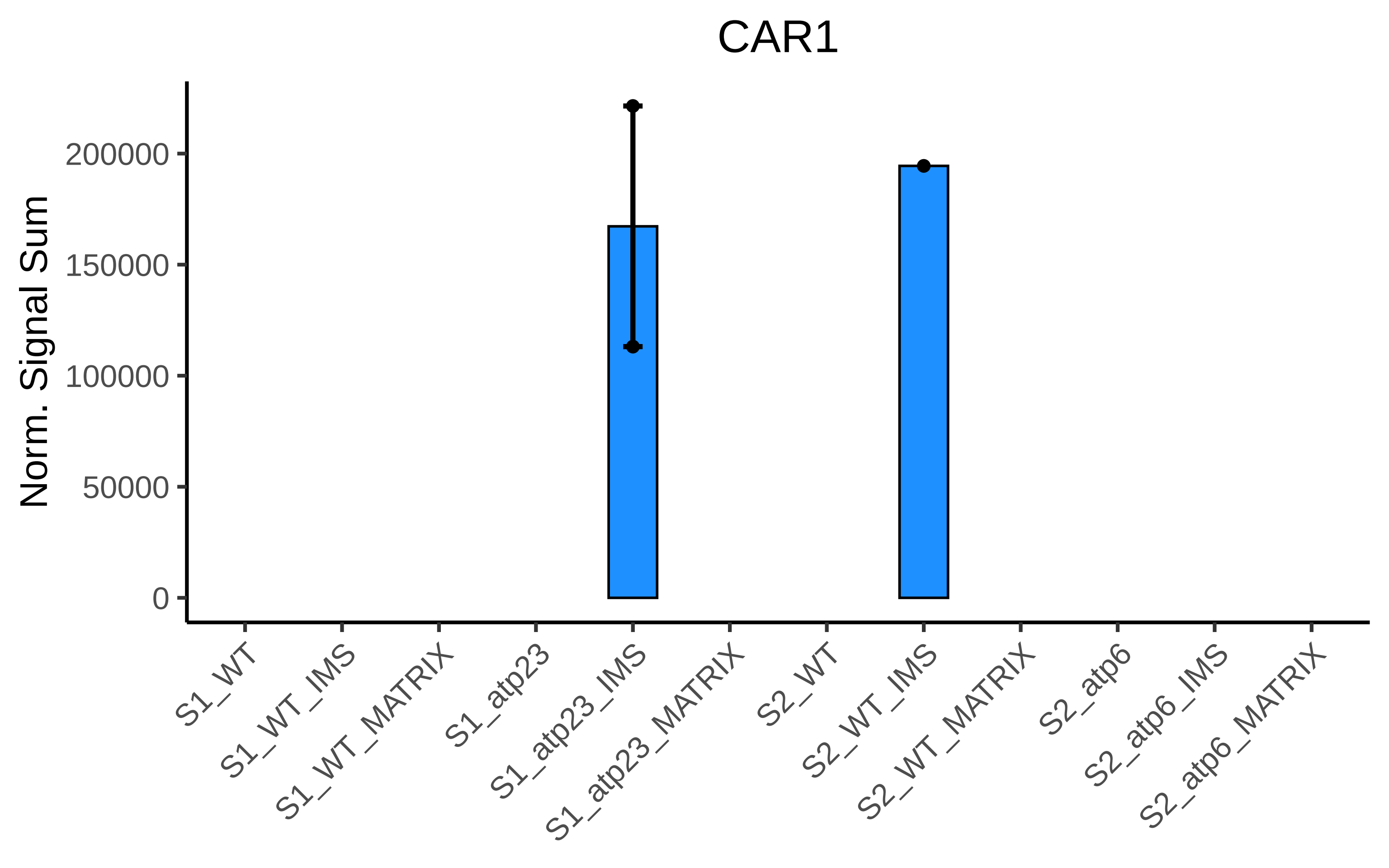

CAR1
Norm. Signal Sum
200000
150000
100000
50000
0
S1_WT
S1_WT_IMS
S1_WT_MATRIX
S1_atp23
S1_atp23_IMS
S1_atp23_MATRIX
S2_WT
S2_WT_IMS
S2_WT_MATRIX
S2_atp6
S2_atp6_IMS
S2_atp6_MATRIX

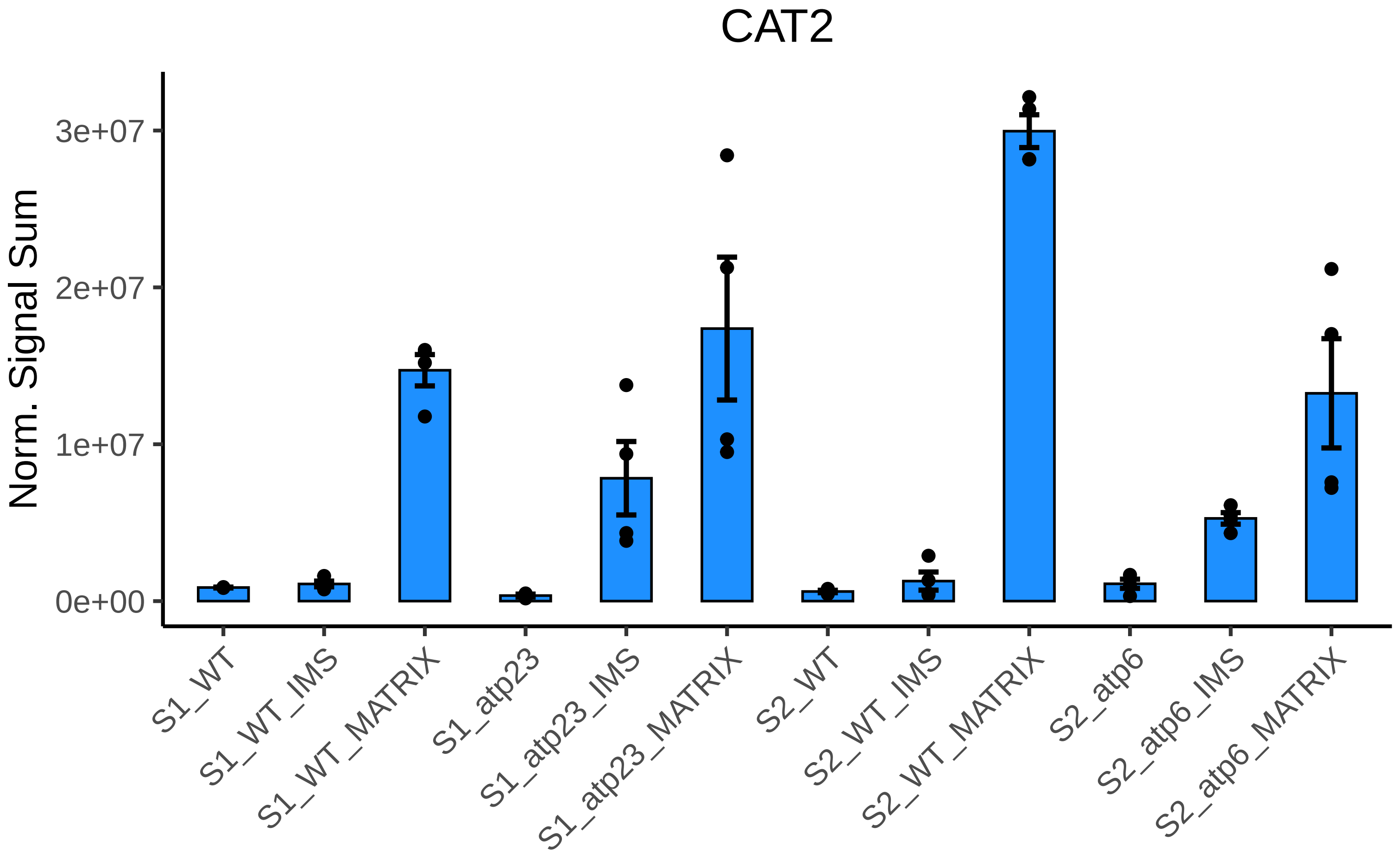

CAT2
Norm. Signal Sum
3e+07
2e+07
1e+07
0e+00
S1_WT
S1_WT_IMS
S1_WT_MATRIX
S1_atp23
S1_atp23_IMS
S1_atp23_MATRIX
S2_WT
S2_WT_IMS
S2_WT_MATRIX
S2_atp6
S2_atp6_IMS
S2_atp6_MATRIX

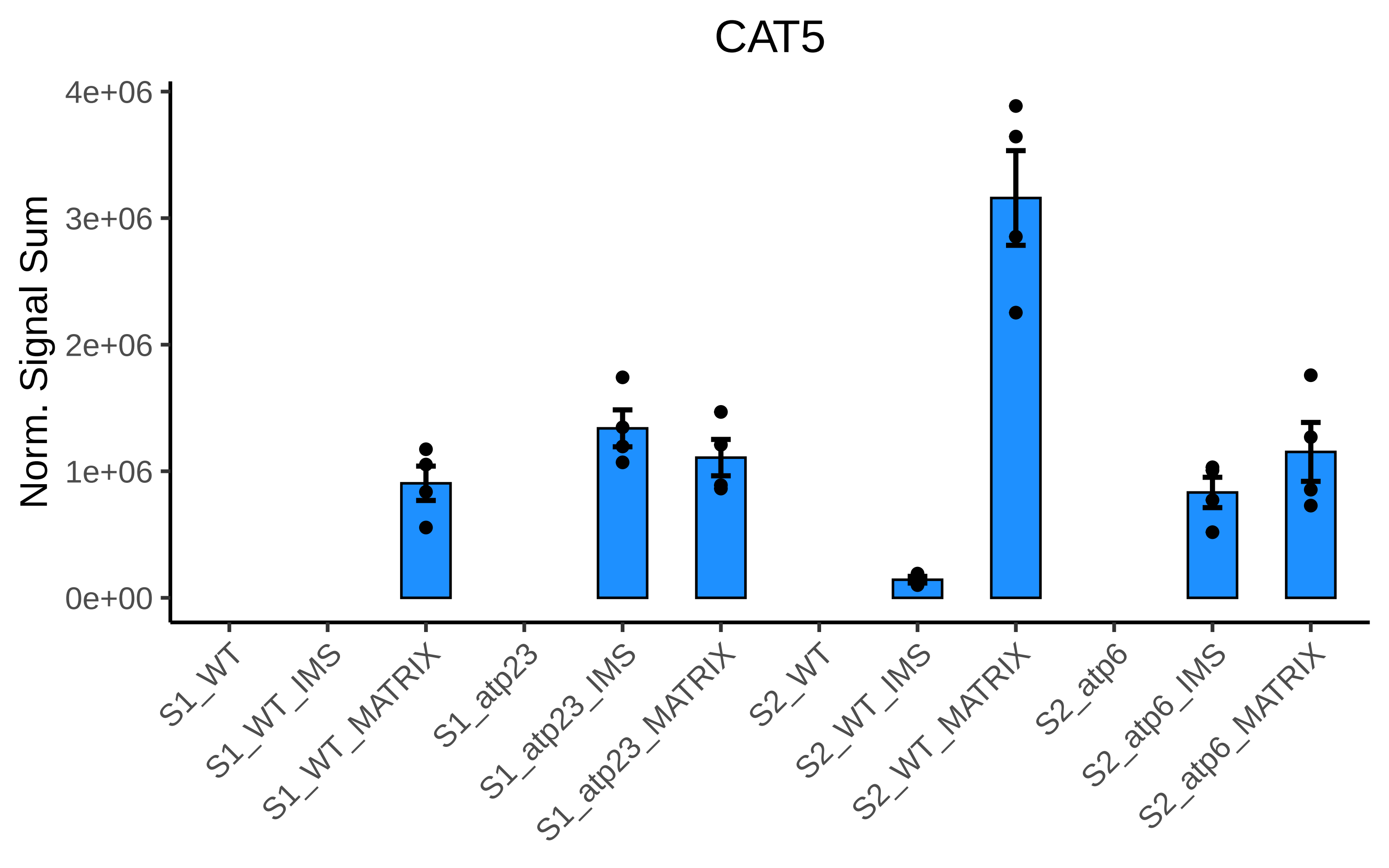
CAT5
Norm. Signal Sum
4e+06
3e+06
2e+06
1e+06
0e+00
S1_WT
S1_WT_IMS
S1_WT_MATRIX
S1_atp23
S1_atp23_IMS
S1_atp23_MATRIX
S2_WT
S2_WT_IMS
S2_WT_MATRIX
S2_atp6
S2_atp6_IMS
S2_atp6_MATRIX

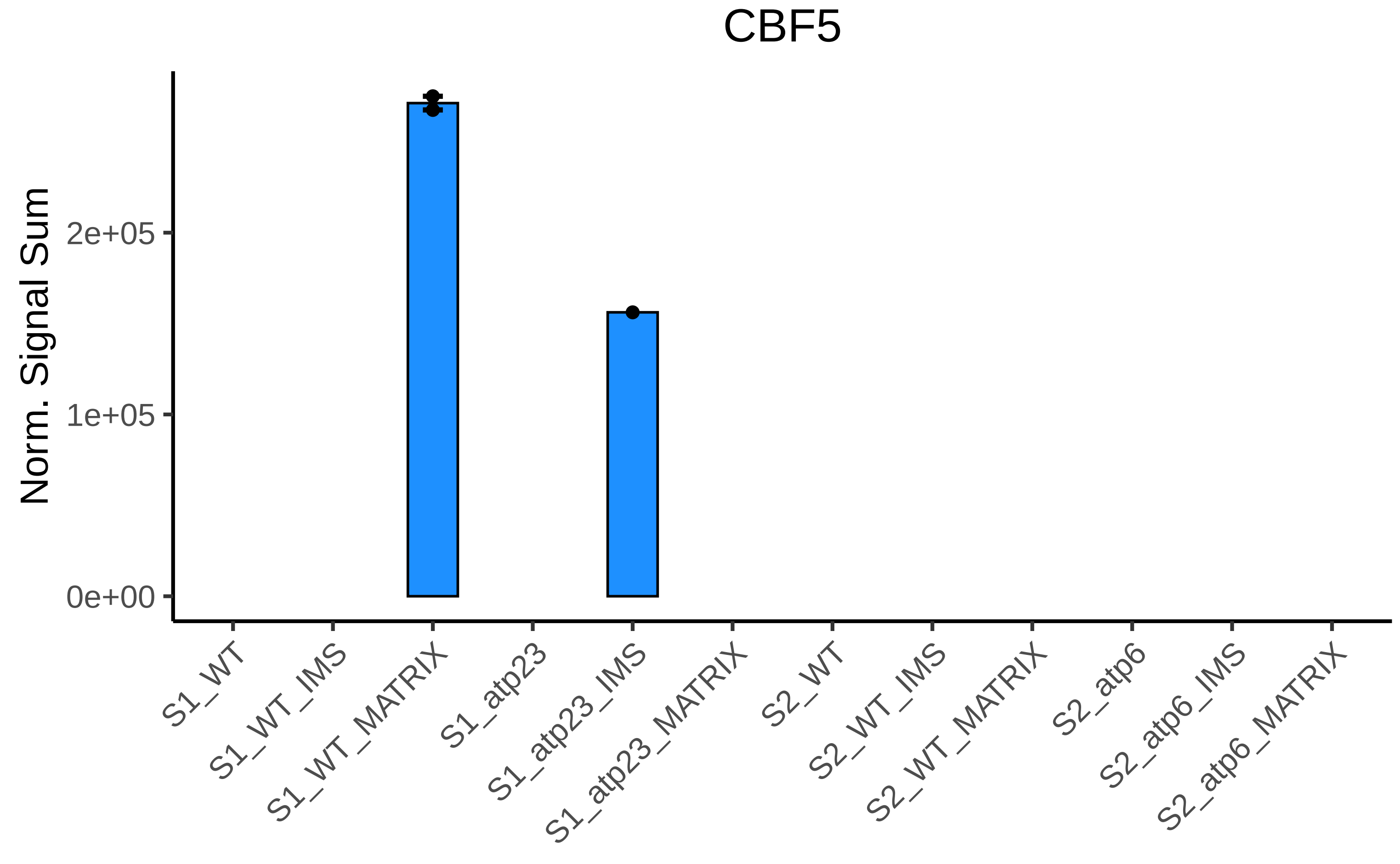
CBF5
Norm. Signal Sum
2e+05
1e+05
0e+00
S1_WT
S1_WT_IMS
S1_WT_MATRIX
S1_atp23
S1_atp23_IMS
S1_atp23_MATRIX
S2_WT
S2_WT_IMS
S2_WT_MATRIX
S2_atp6
S2_atp6_IMS
S2_atp6_MATRIX

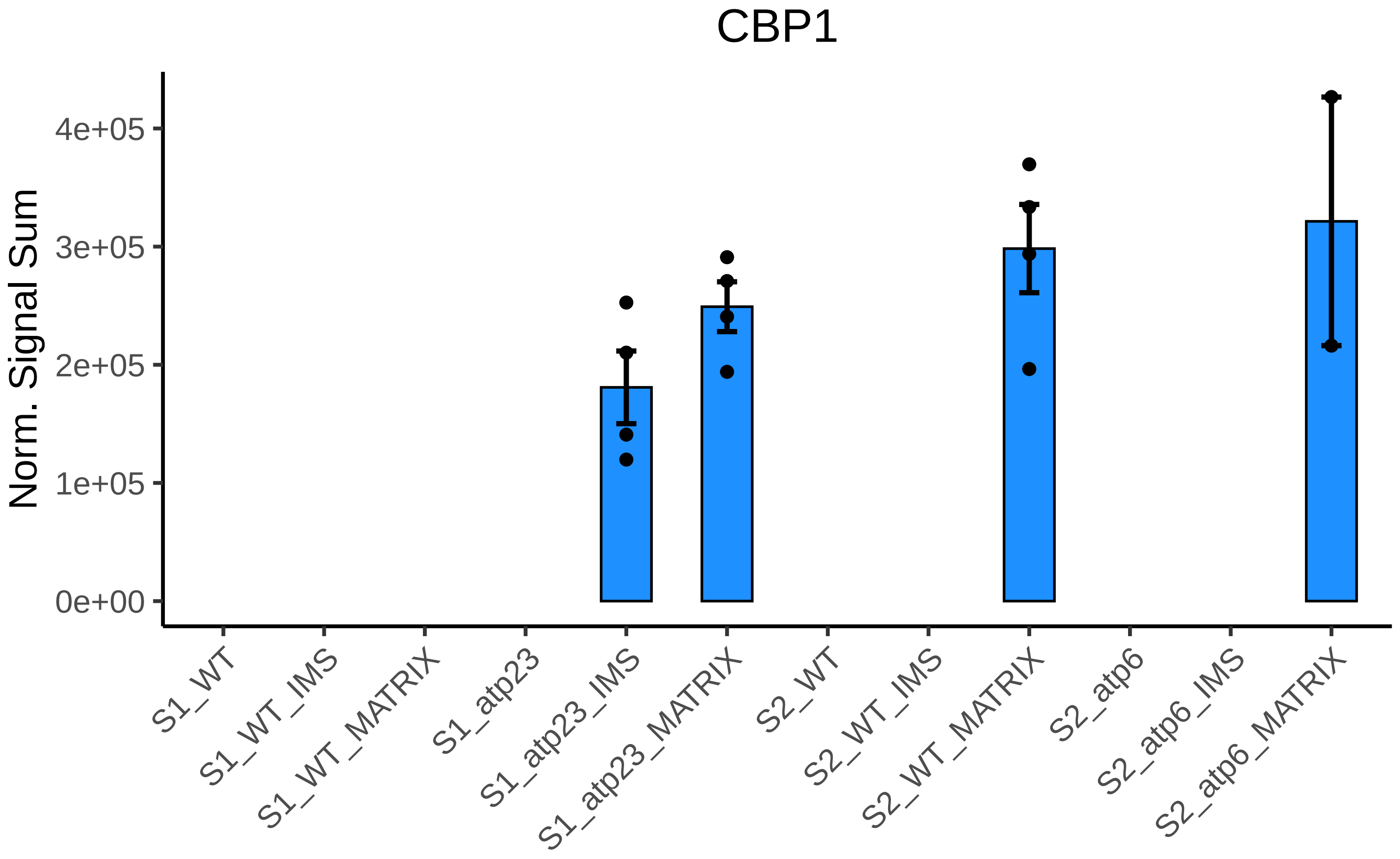

CBP1
Norm. Signal Sum
4e+05
3e+05
2e+05
1e+05
0e+00
S1_WT
S1_WT_IMS
S1_WT_MATRIX
S1_atp23
S1_atp23_IMS
S1_atp23_MATRIX
S2_WT
S2_WT_IMS
S2_WT_MATRIX
S2_atp6
S2_atp6_IMS
S2_atp6_MATRIX

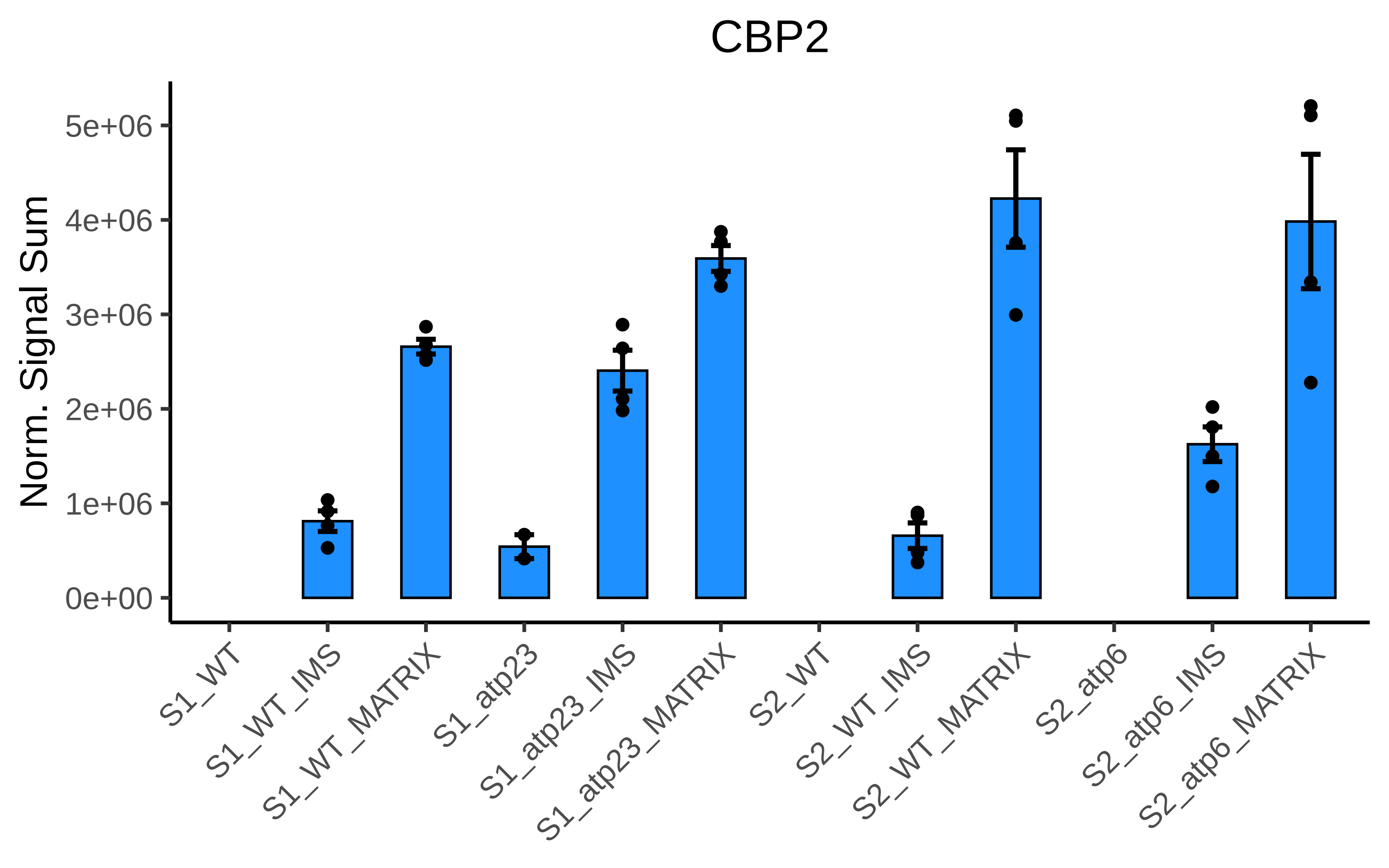

CBP2
Norm. Signal Sum
0e+00
1e+06
2e+06
3e+06
4e+06
5e+06
S1_WT
S1_WT_IMS
S1_WT_MATRIX
S1_atp23
S1_atp23_IMS
S1_atp23_MATRIX
S2_WT
S2_WT_IMS
S2_WT_MATRIX
S2_atp6
S2_atp6_IMS
S2_atp6_MATRIX

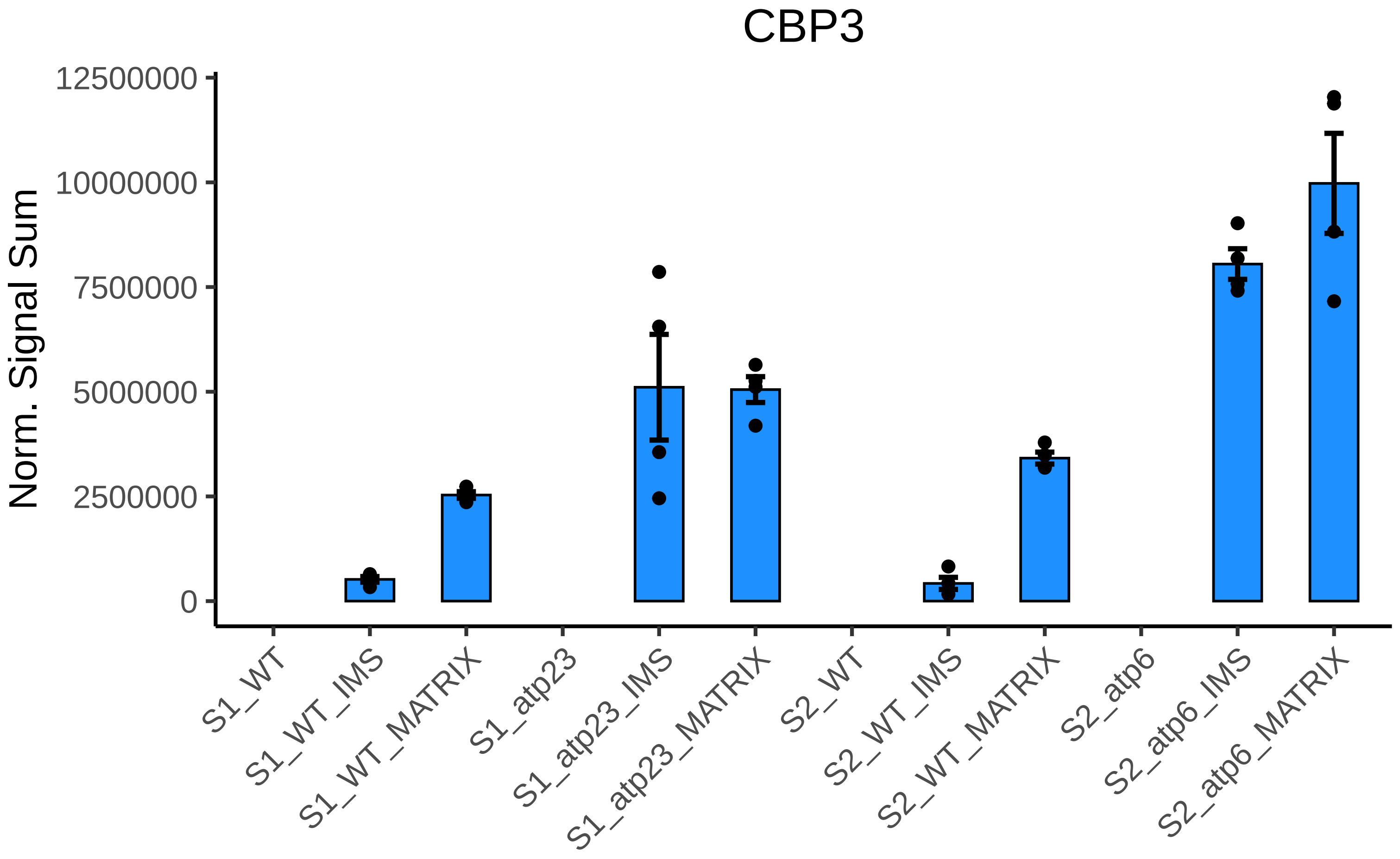

CBP3
Norm. Signal Sum
12500000
10000000
7500000
5000000
2500000
0
S1_WT
S1_WT_IMS
S1_WT_MATRIX
S1_atp23
S1_atp23_IMS
S1_atp23_MATRIX
S2_WT
S2_WT_IMS
S2_WT_MATRIX
S2_atp6
S2_atp6_IMS
S2_atp6_MATRIX

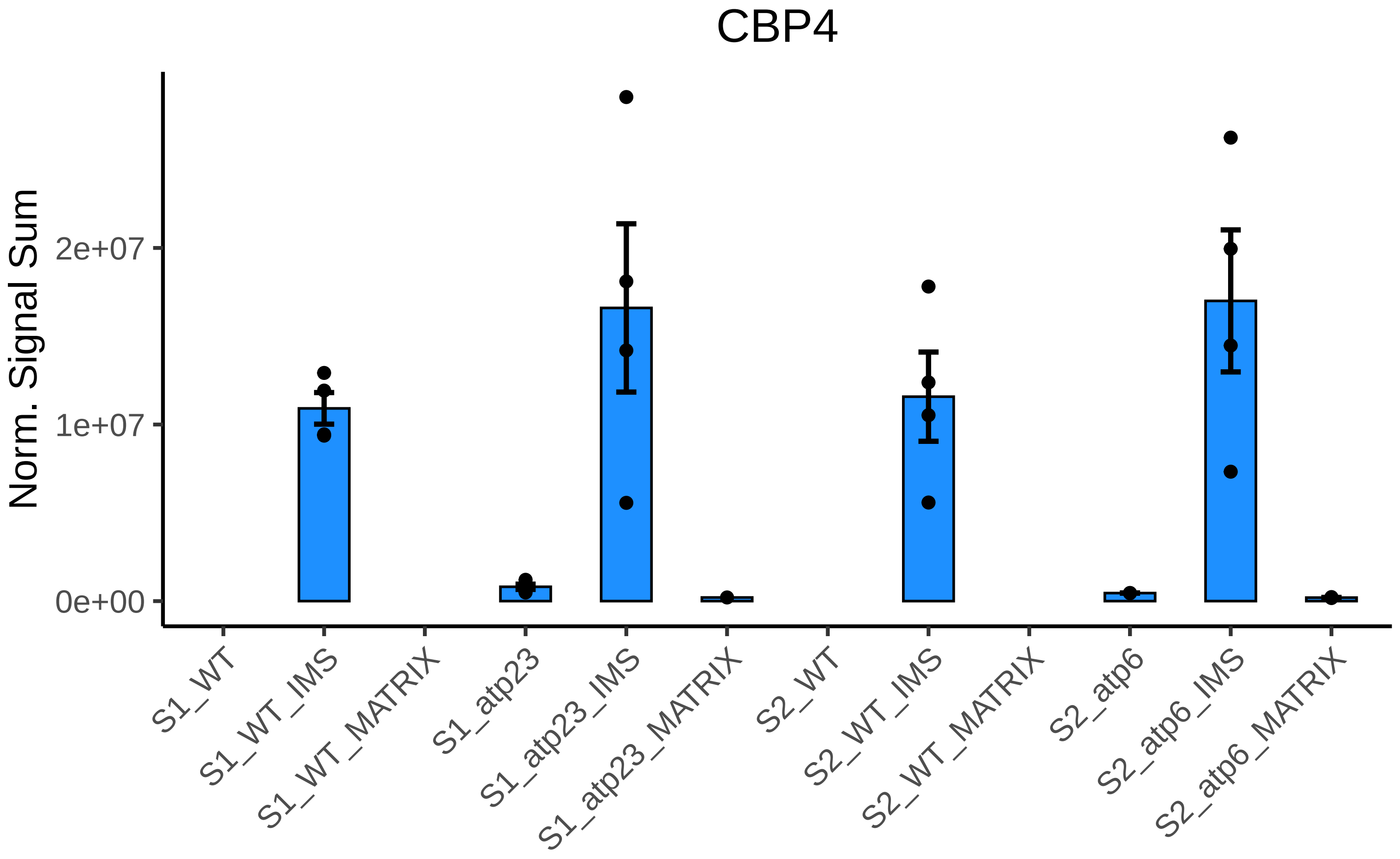

CBP4
Norm. Signal Sum
2e+07
1e+07
0e+00
S1_WT
S1_WT_IMS
S1_WT_MATRIX
S1_atp23
S1_atp23_IMS
S1_atp23_MATRIX
S2_WT
S2_WT_IMS
S2_WT_MATRIX
S2_atp6
S2_atp6_IMS
S2_atp6_MATRIX

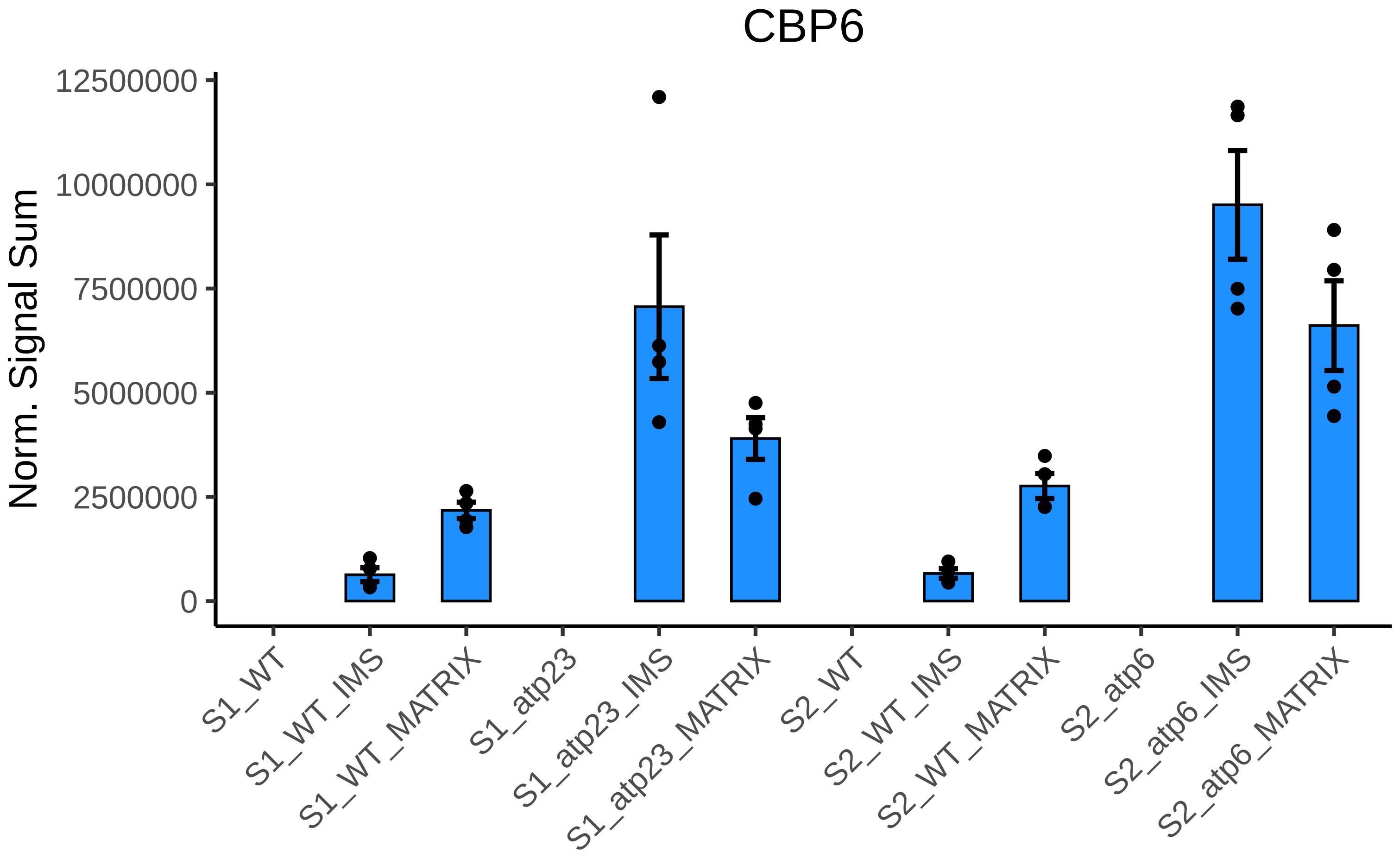

CBP6
Norm. Signal Sum
12500000
10000000
7500000
5000000
2500000
0
S1_WT
S1_WT_IMS
S1_WT_MATRIX
S1_atp23
S1_atp23_IMS
S1_atp23_MATRIX
S2_WT
S2_WT_IMS
S2_WT_MATRIX
S2_atp6
S2_atp6_IMS
S2_atp6_MATRIX

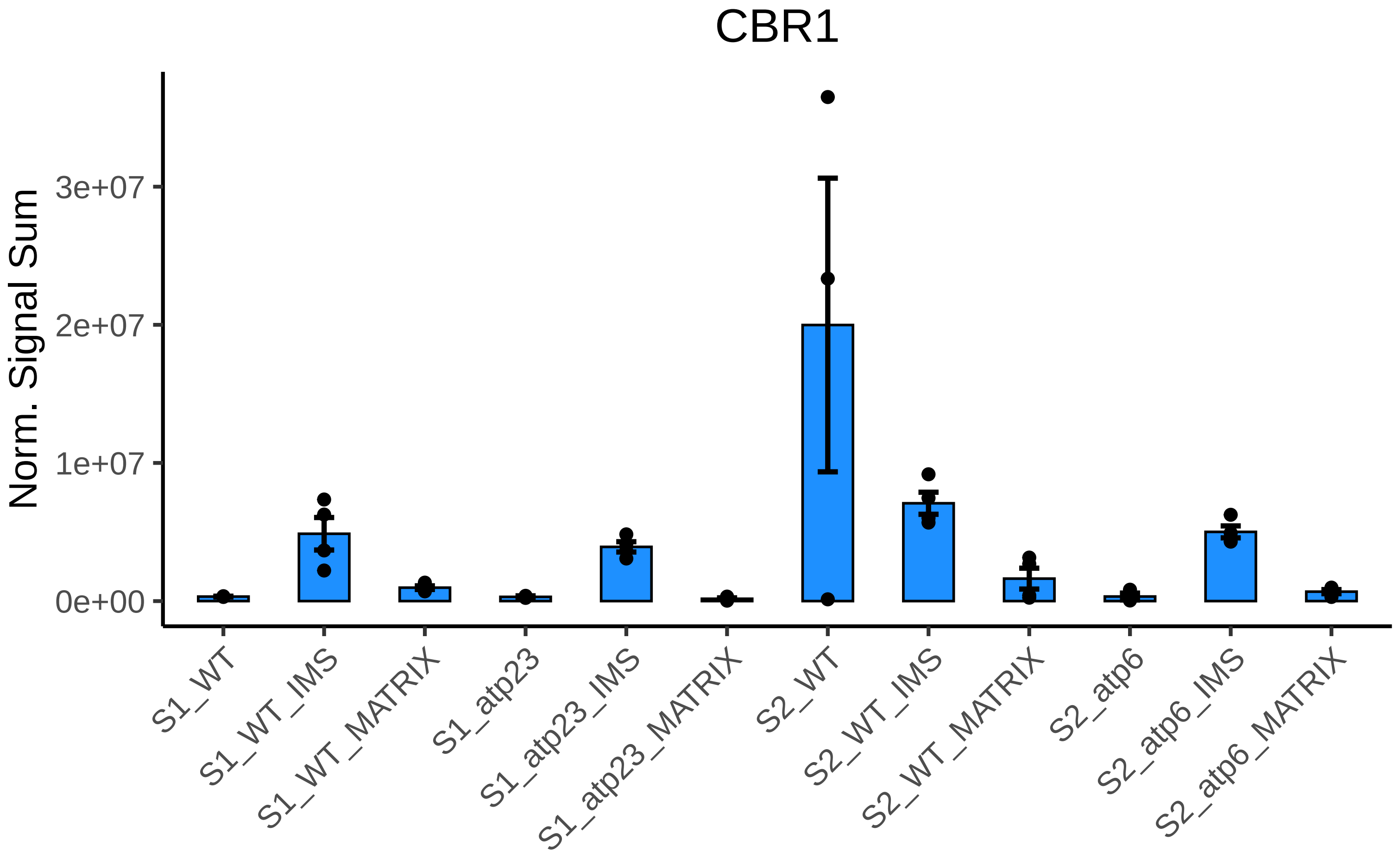
CBR1
Norm. Signal Sum
3e+07
2e+07
1e+07
0e+00
S1_WT
S1_WT_IMS
S1_WT_MATRIX
S1_atp23
S1_atp23_IMS
S1_atp23_MATRIX
S2_WT
S2_WT_IMS
S2_WT_MATRIX
S2_atp6
S2_atp6_IMS
S2_atp6_MATRIX

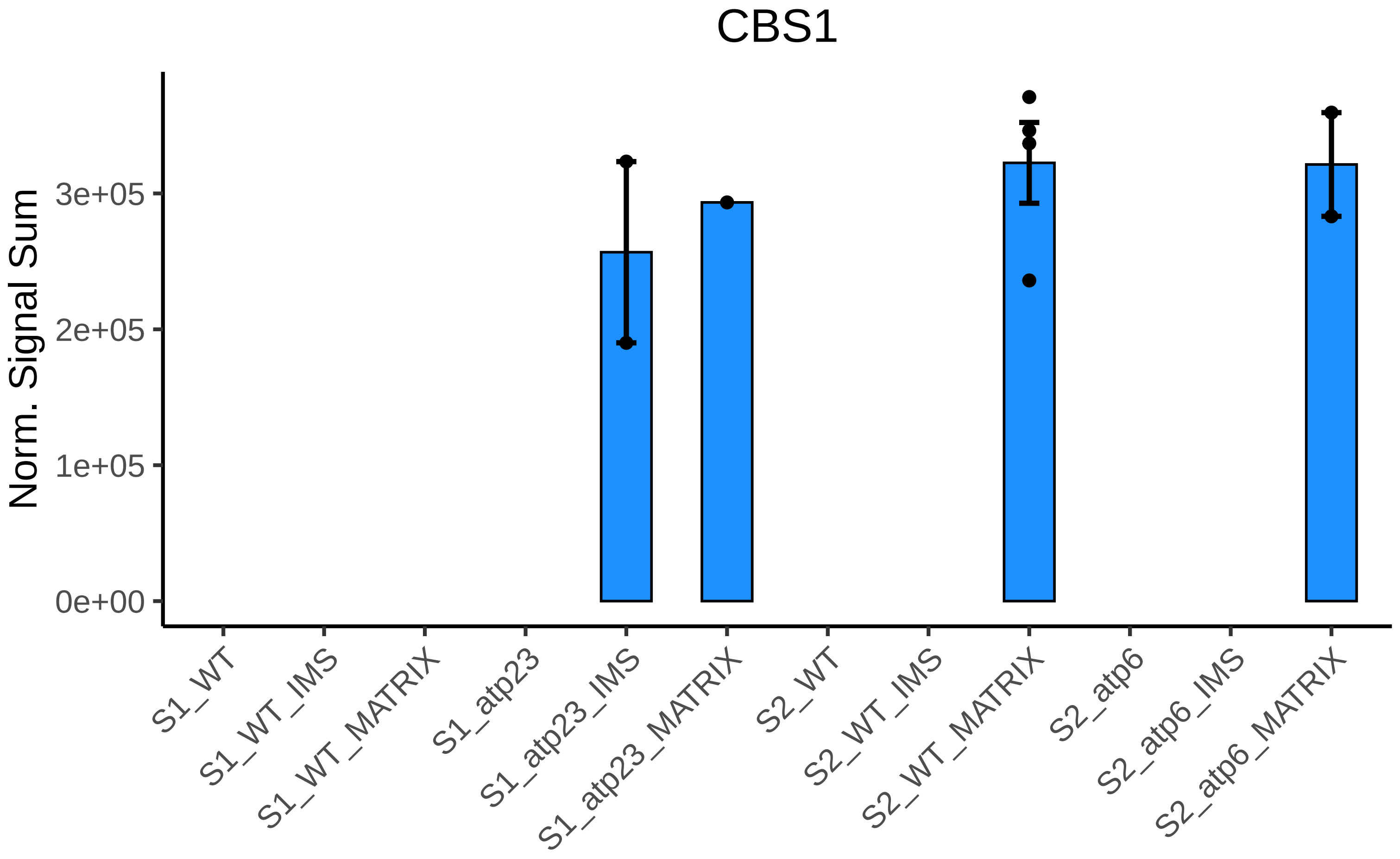
CBS1
Norm. Signal Sum
3e+05
2e+05
1e+05
0e+00
S1_WT
S1_WT_IMS
S1_WT_MATRIX
S1_atp23
S1_atp23_IMS
S1_atp23_MATRIX
S2_WT
S2_WT_IMS
S2_WT_MATRIX
S2_atp6
S2_atp6_IMS
S2_atp6_MATRIX

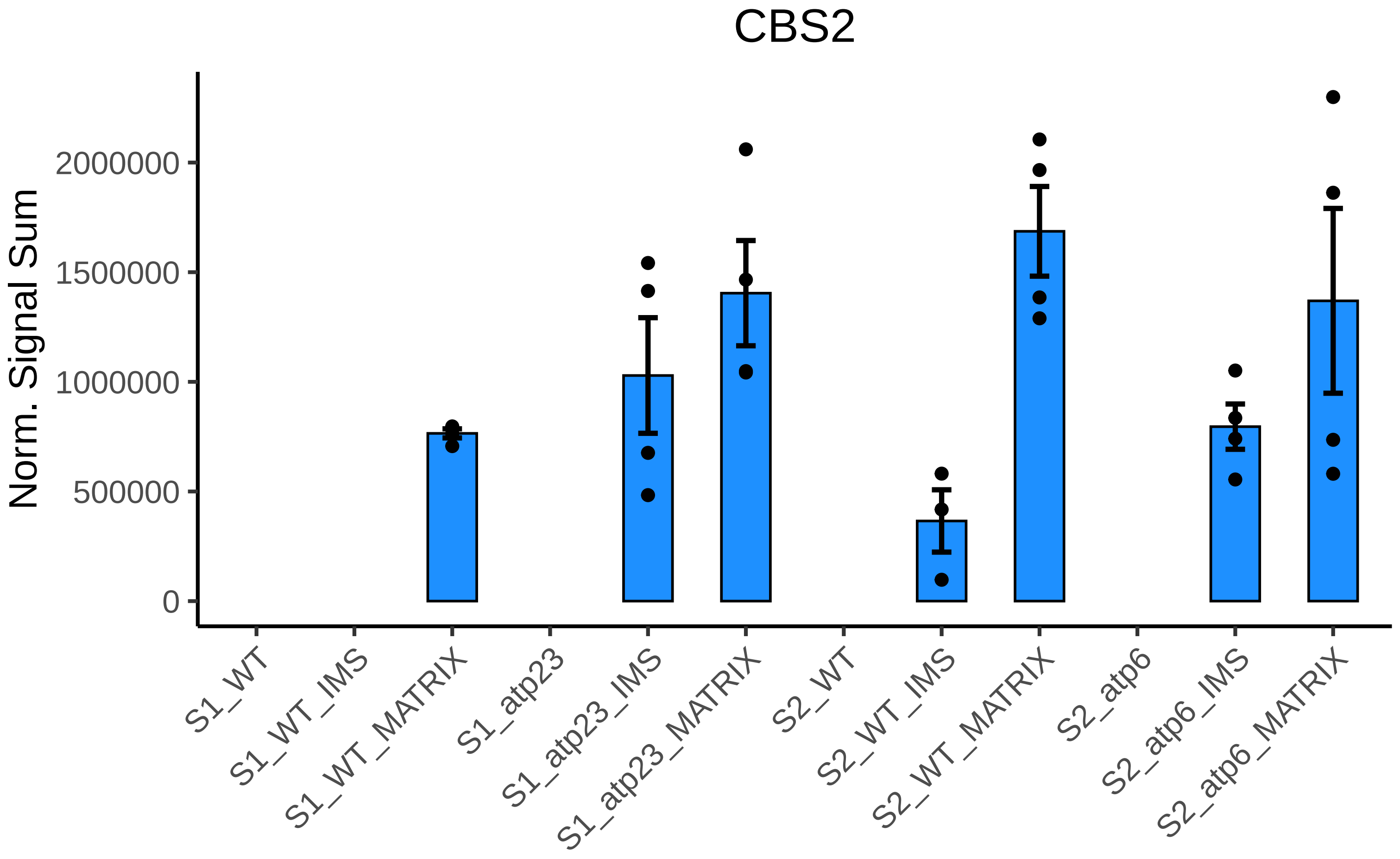

CBS2
Norm. Signal Sum
2000000
1500000
1000000
500000
0
S1_WT
S1_WT_IMS
S1_WT_MATRIX
S1_atp23
S1_atp23_IMS
S1_atp23_MATRIX
S2_WT
S2_WT_IMS
S2_WT_MATRIX
S2_atp6
S2_atp6_IMS
S2_atp6_MATRIX

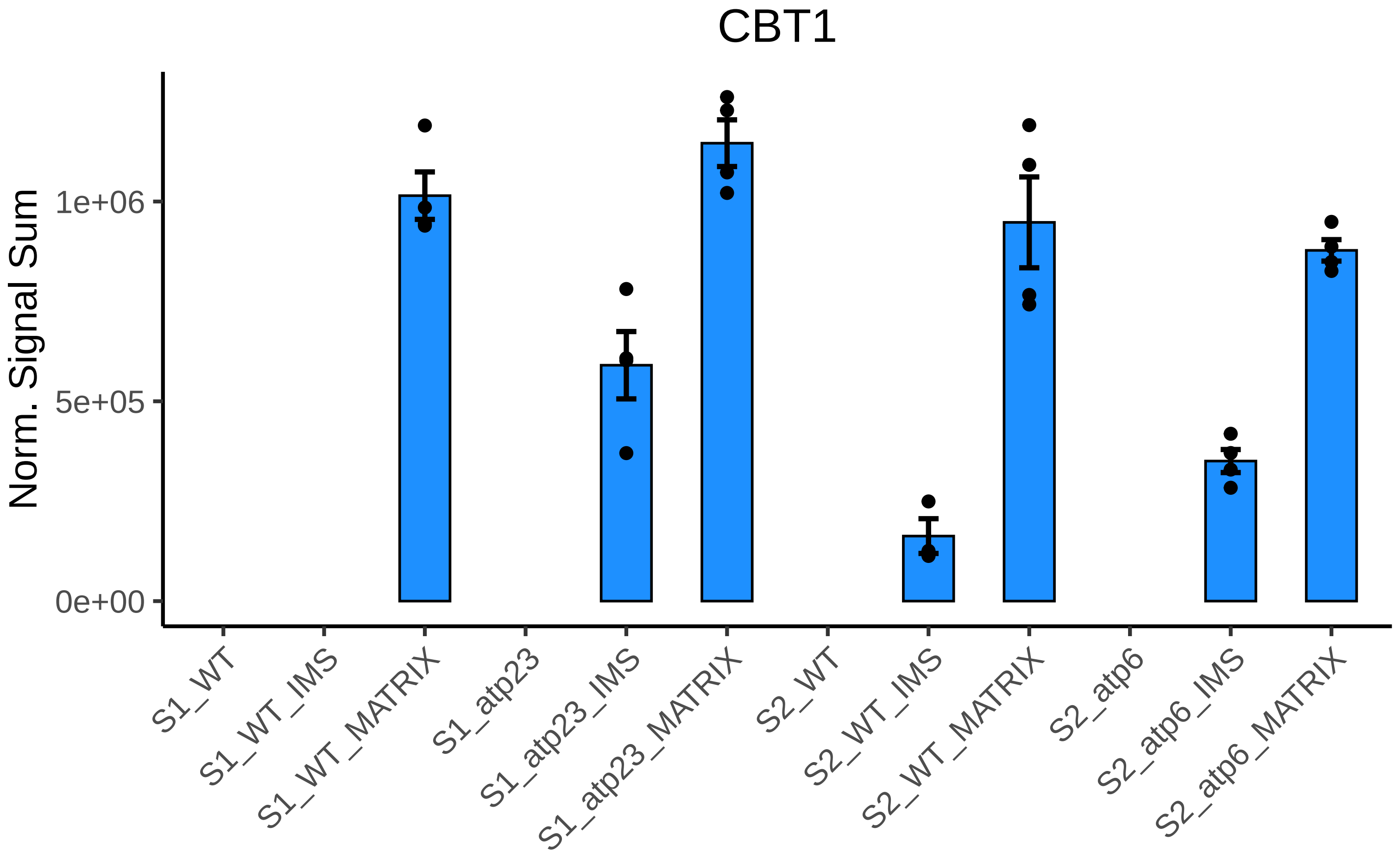
CBT1
Norm. Signal Sum
1e+06
5e+05
0e+00
S1_WT
S1_WT_IMS
S1_WT_MATRIX
S1_atp23
S1_atp23_IMS
S1_atp23_MATRIX
S2_WT
S2_WT_IMS
S2_WT_MATRIX
S2_atp6
S2_atp6_IMS
S2_atp6_MATRIX

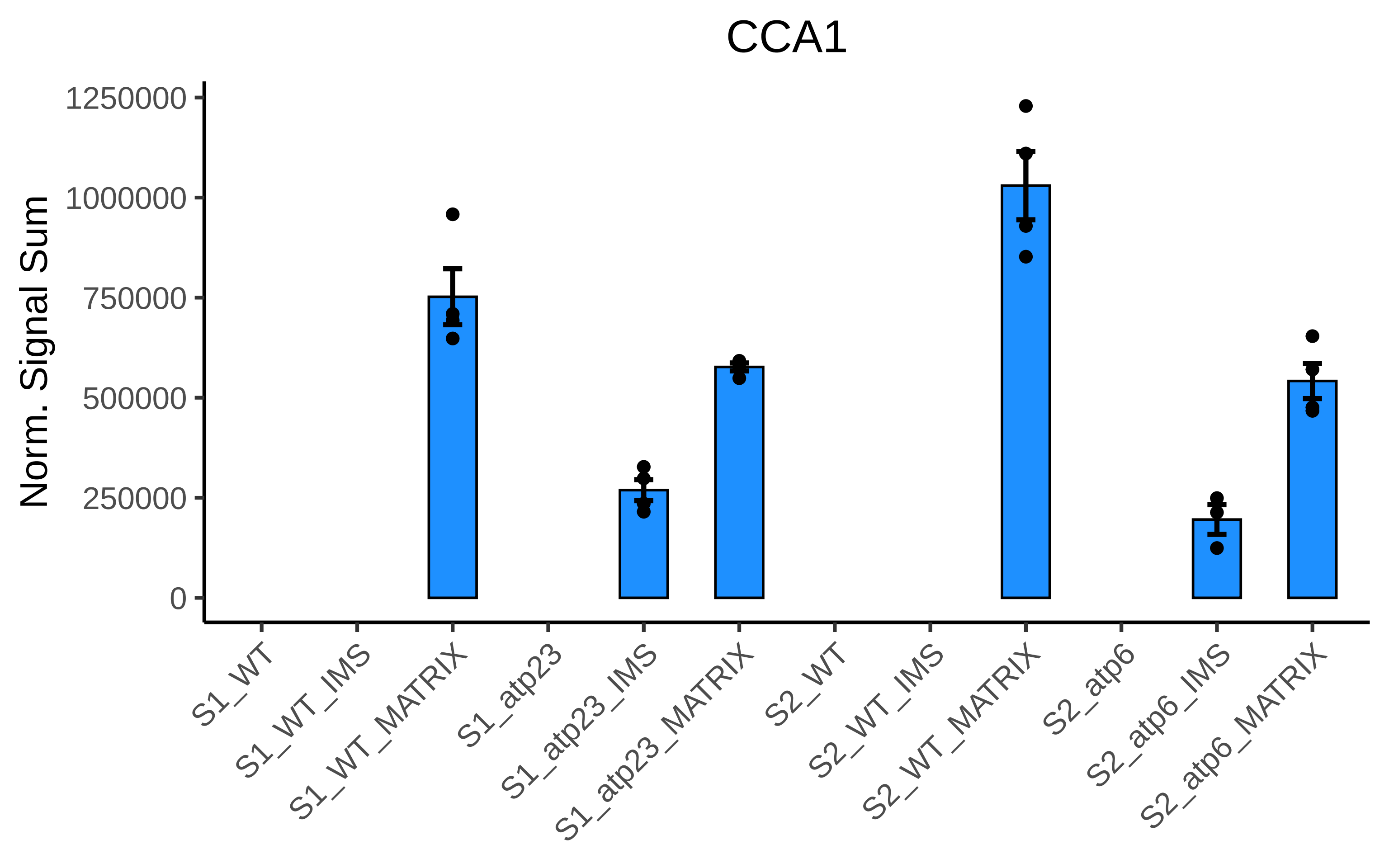

CCA1
Norm. Signal Sum
1250000
1000000
750000
500000
250000
0
S1_WT
S1_WT_IMS
S1_WT_MATRIX
S1_atp23
S1_atp23_IMS
S1_atp23_MATRIX
S2_WT
S2_WT_IMS
S2_WT_MATRIX
S2_atp6
S2_atp6_IMS
S2_atp6_MATRIX

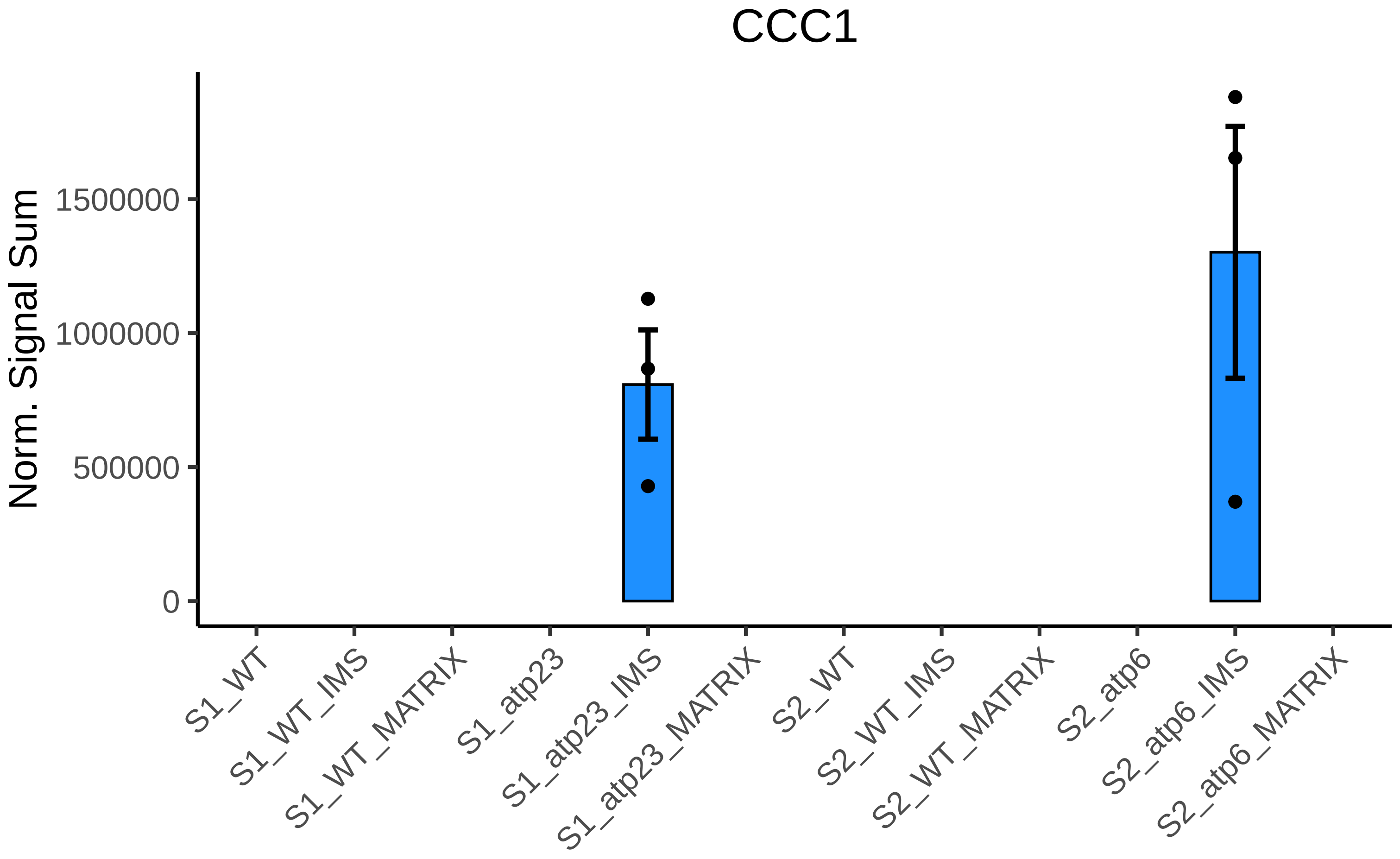
CCC1
Norm. Signal Sum
1500000
1000000
500000
0
S1_WT
S1_WT_IMS
S1_WT_MATRIX
S1_atp23
S1_atp23_IMS
S1_atp23_MATRIX
S2_WT
S2_WT_IMS
S2_WT_MATRIX
S2_atp6
S2_atp6_IMS
S2_atp6_MATRIX

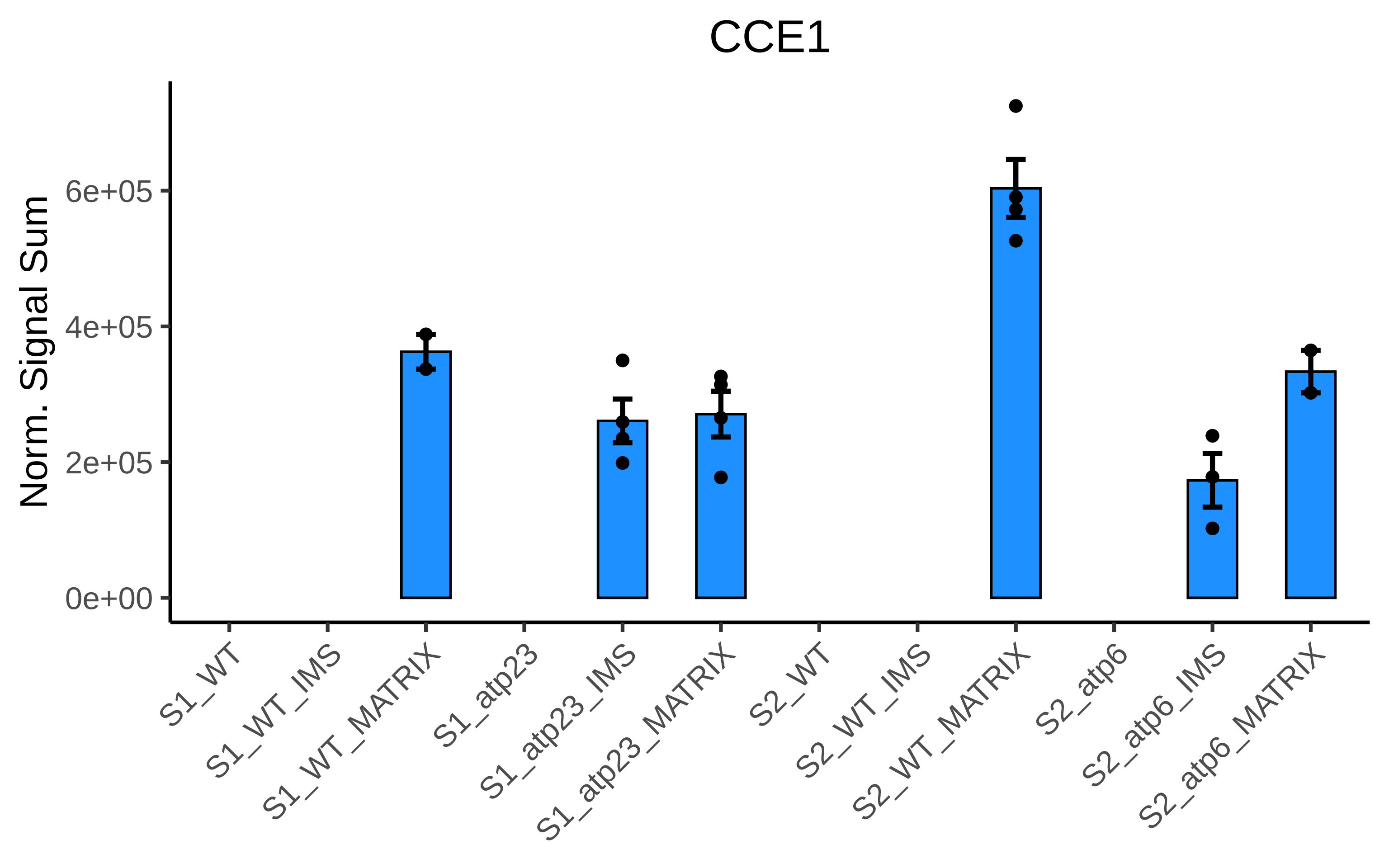
CCE1
Norm. Signal Sum
6e+05
4e+05
2e+05
0e+00
S1_WT
S1_WT_IMS
S1_WT_MATRIX
S1_atp23
S1_atp23_IMS
S1_atp23_MATRIX
S2_WT
S2_WT_IMS
S2_WT_MATRIX
S2_atp6
S2_atp6_IMS
S2_atp6_MATRIX

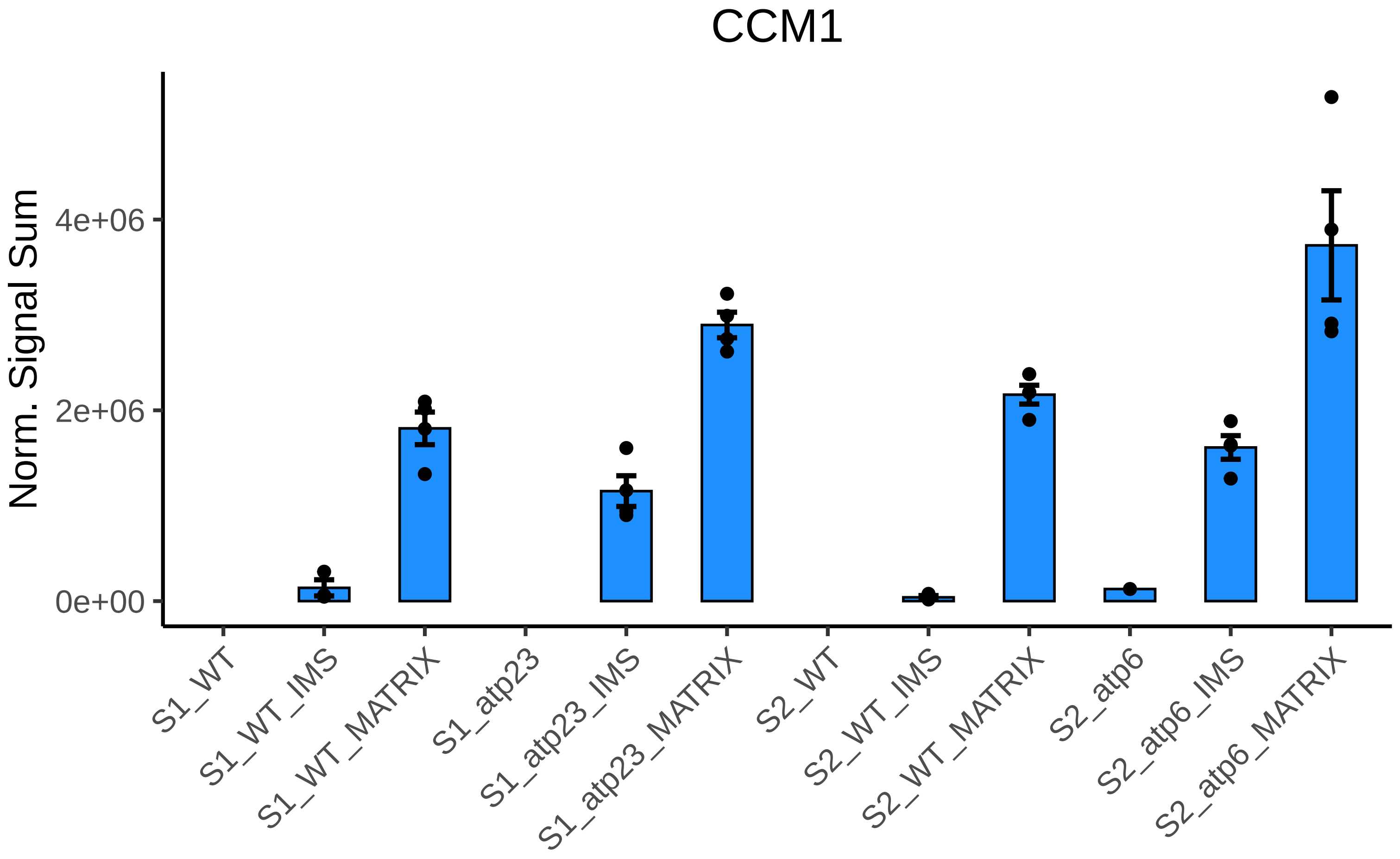

CCM1
Norm. Signal Sum
4e+06
2e+06
0e+00
S1_WT
S1_WT_IMS
S1_WT_MATRIX
S1_atp23
S1_atp23_IMS
S1_atp23_MATRIX
S2_WT
S2_WT_IMS
S2_WT_MATRIX
S2_atp6
S2_atp6_IMS
S2_atp6_MATRIX

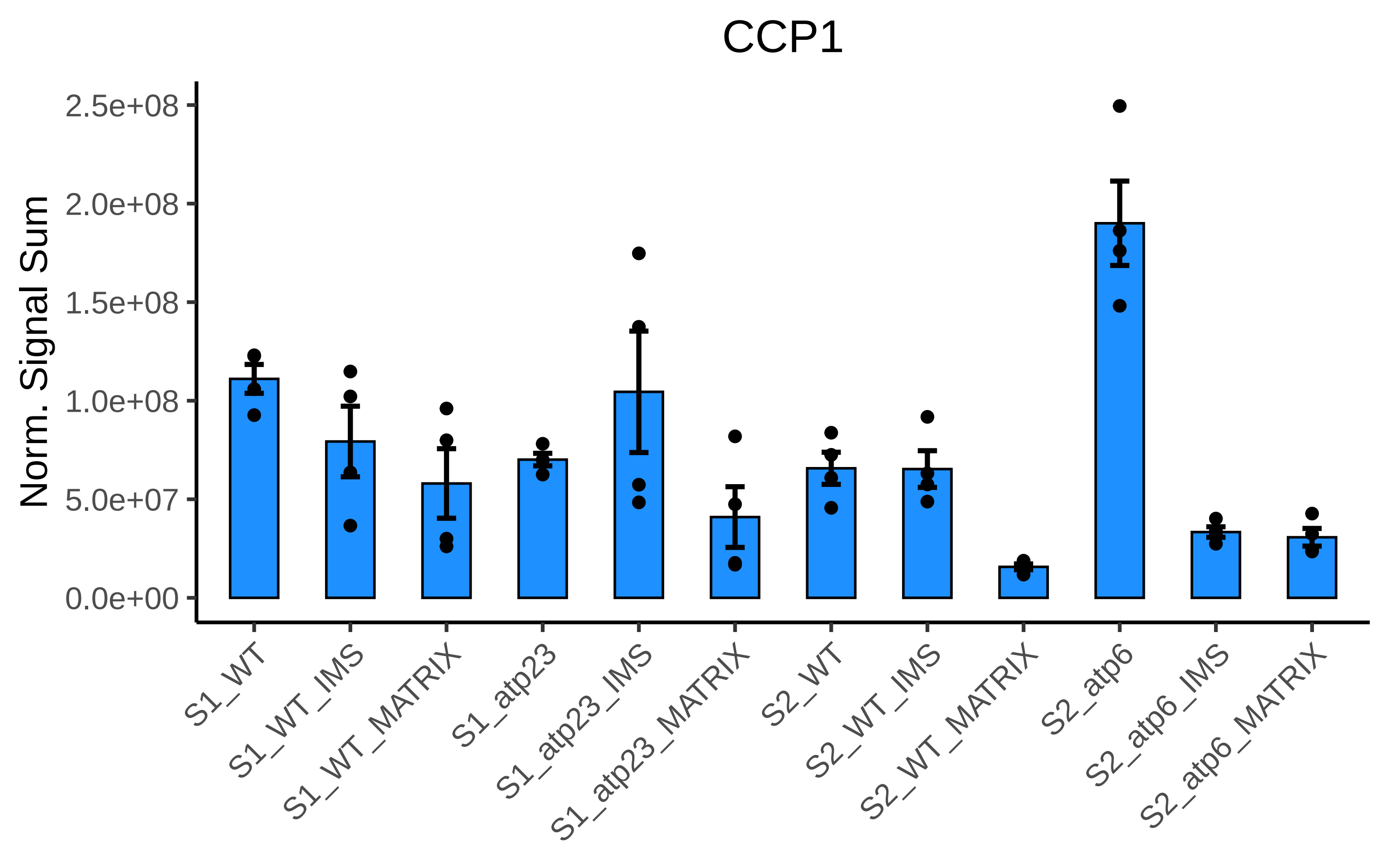
CCP1
Norm. Signal Sum
2.5e+08
2.0e+08
1.5e+08
1.0e+08
5.0e+07
0.0e+00
S1_WT
S1_WT_IMS
S1_WT_MATRIX
S1_atp23
S1_atp23_IMS
S1_atp23_MATRIX
S2_WT
S2_WT_IMS
S2_WT_MATRIX
S2_atp6
S2_atp6_IMS
S2_atp6_MATRIX

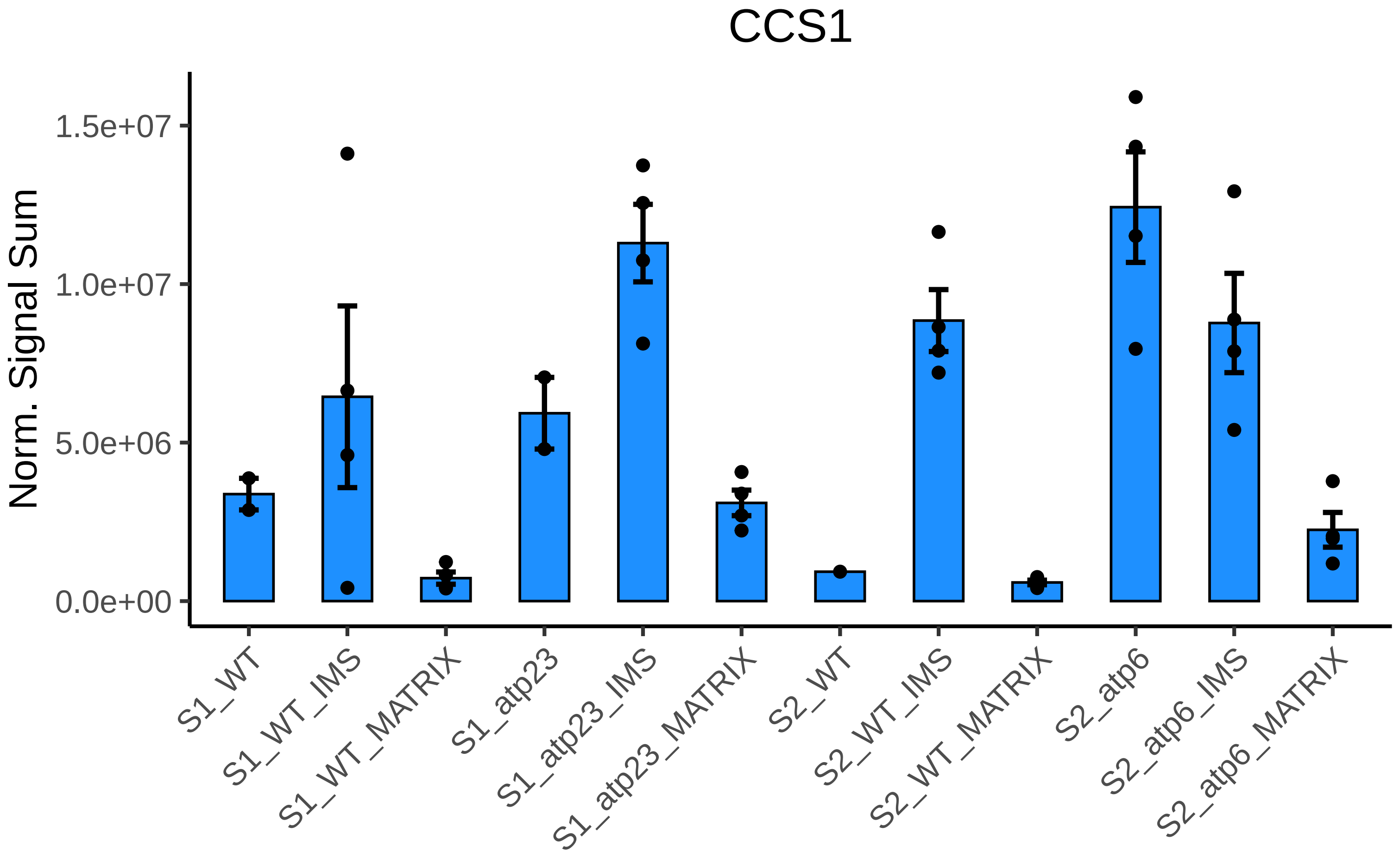

CCS1
Norm. Signal Sum
1.5e+07
1.0e+07
5.0e+06
0.0e+00
S1_WT
S1_WT_IMS
S1_WT_MATRIX
S1_atp23
S1_atp23_IMS
S1_atp23_MATRIX
S2_WT
S2_WT_IMS
S2_WT_MATRIX
S2_atp6
S2_atp6_IMS
S2_atp6_MATRIX

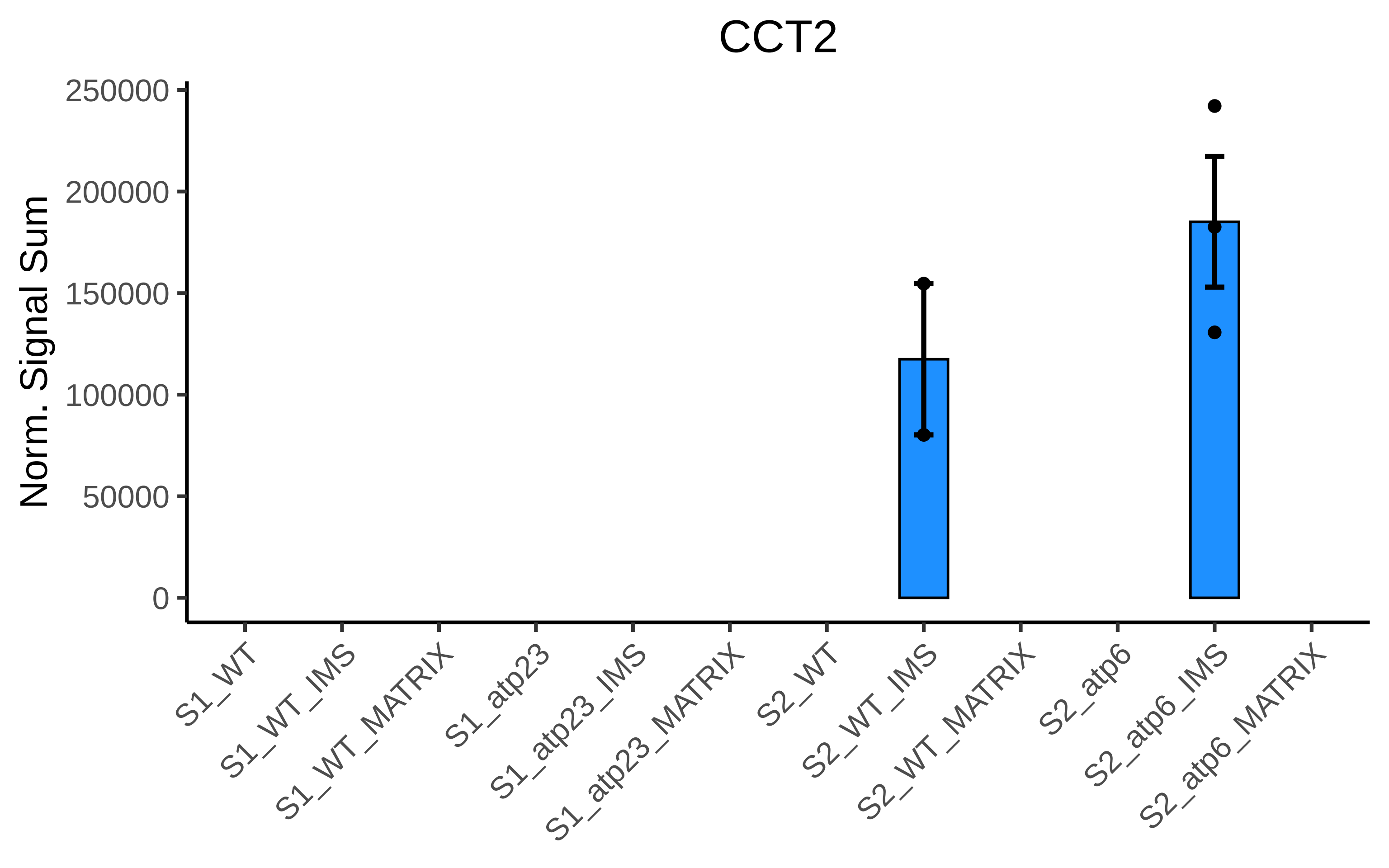

CCT2
Norm. Signal Sum
250000
200000
150000
100000
50000
0
S1_WT
S1_WT_IMS
S1_WT_MATRIX
S1_atp23
S1_atp23_IMS
S1_atp23_MATRIX
S2_WT
S2_WT_IMS
S2_WT_MATRIX
S2_atp6
S2_atp6_IMS
S2_atp6_MATRIX

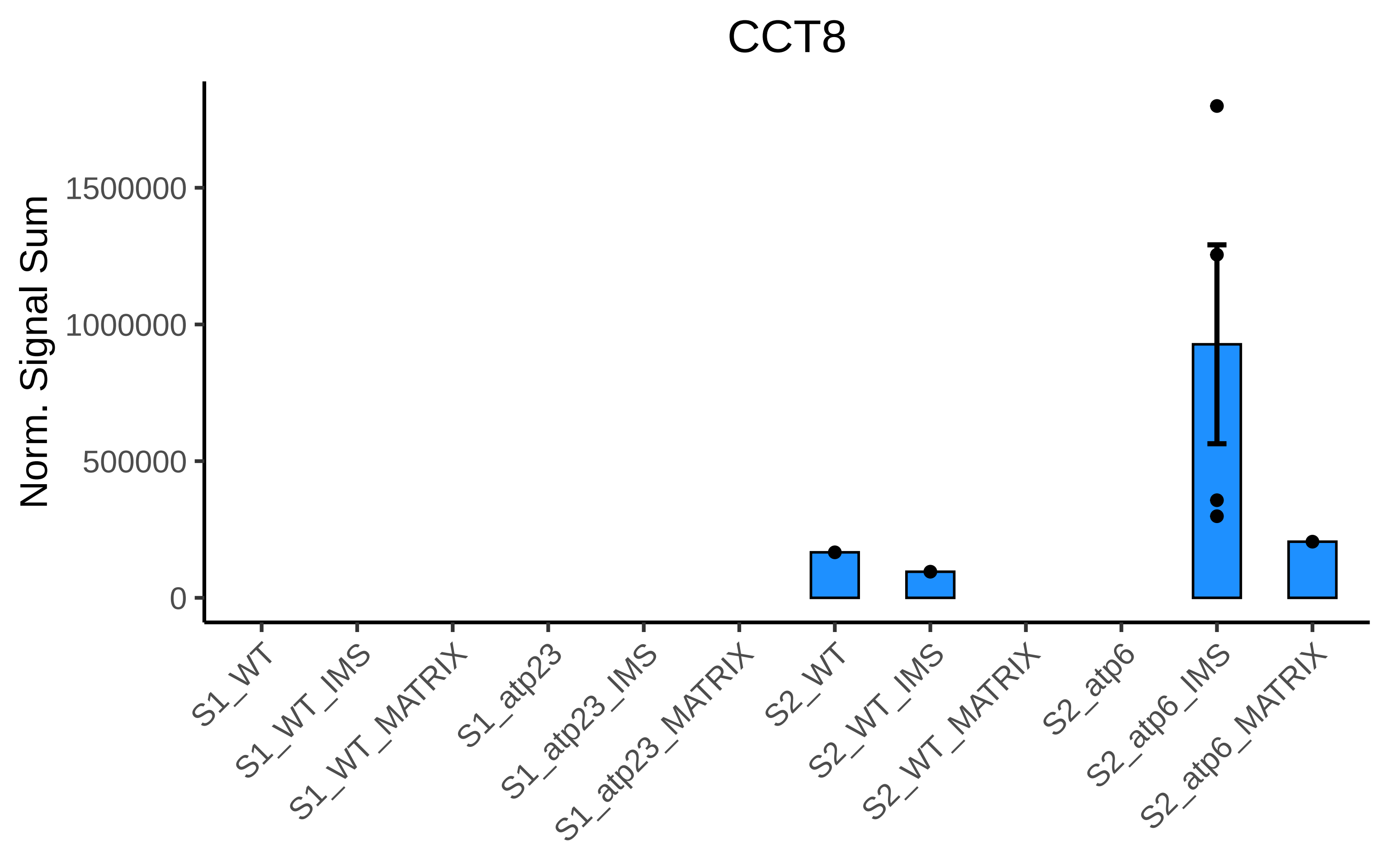
CCT8
Norm. Signal Sum
1500000
1000000
500000
0
S1_WT
S1_WT_IMS
S1_WT_MATRIX
S1_atp23
S1_atp23_IMS
S1_atp23_MATRIX
S2_WT
S2_WT_IMS
S2_WT_MATRIX
S2_atp6
S2_atp6_IMS
S2_atp6_MATRIX

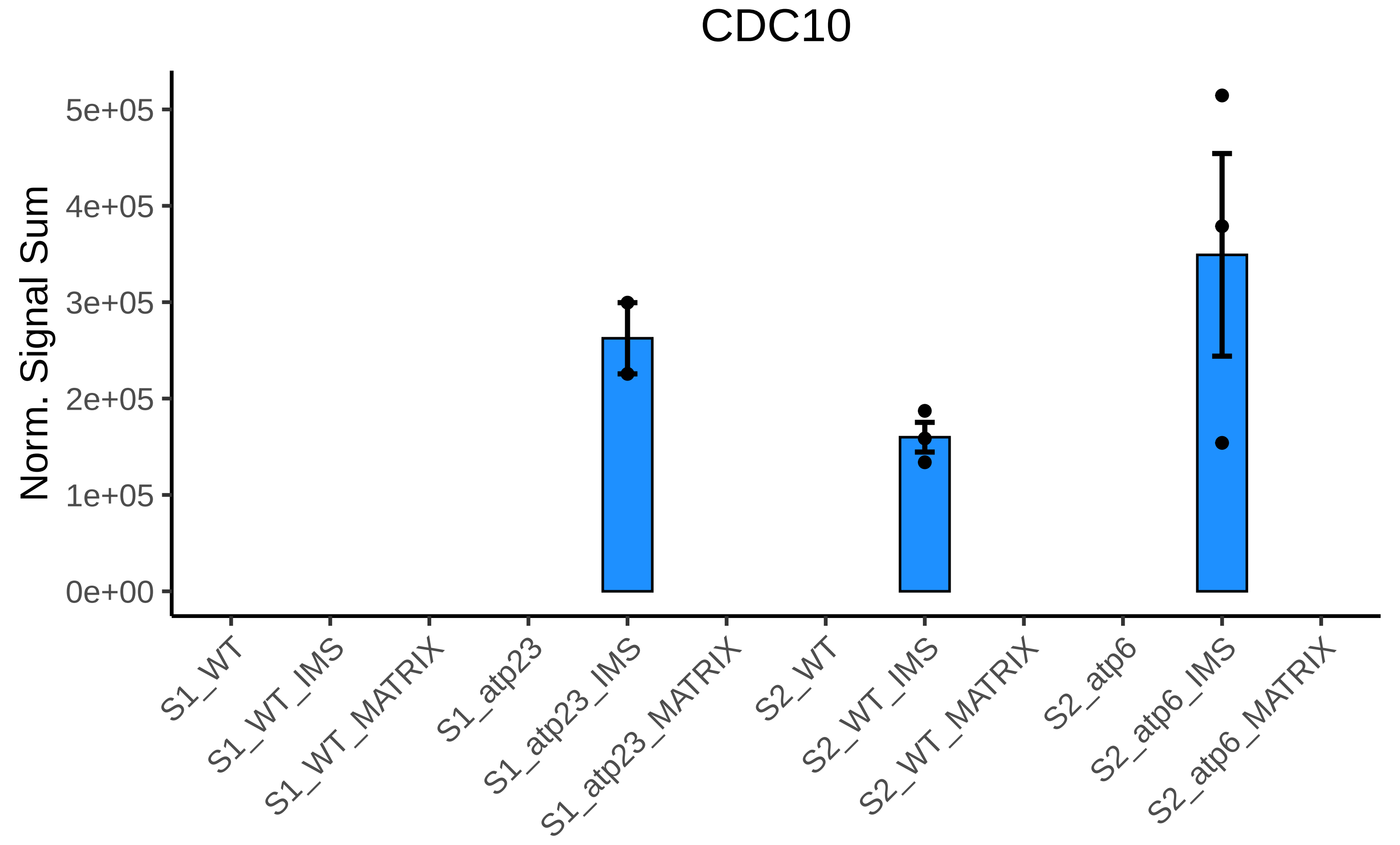

CDC10
Norm. Signal Sum
5e+05
4e+05
3e+05
2e+05
1e+05
0e+00
S1_WT
S1_WT_IMS
S1_WT_MATRIX
S1_atp23
S1_atp23_IMS
S1_atp23_MATRIX
S2_WT
S2_WT_IMS
S2_WT_MATRIX
S2_atp6
S2_atp6_IMS
S2_atp6_MATRIX

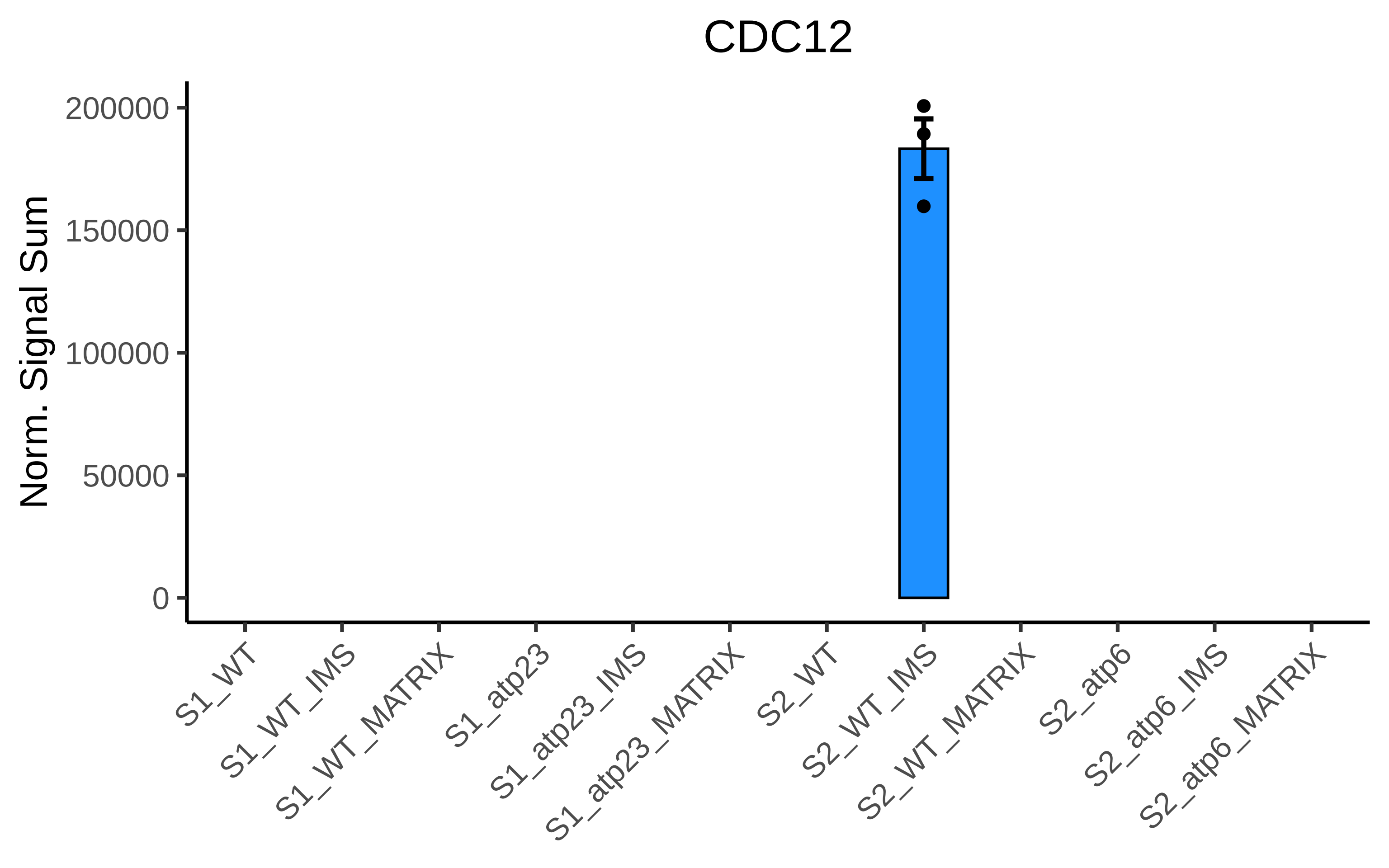
CDC12
Norm. Signal Sum
200000
150000
100000
50000
0
S1_WT
S1_WT_IMS
S1_WT_MATRIX
S1_atp23
S1_atp23_IMS
S1_atp23_MATRIX
S2_WT
S2_WT_IMS
S2_WT_MATRIX
S2_atp6
S2_atp6_IMS
S2_atp6_MATRIX

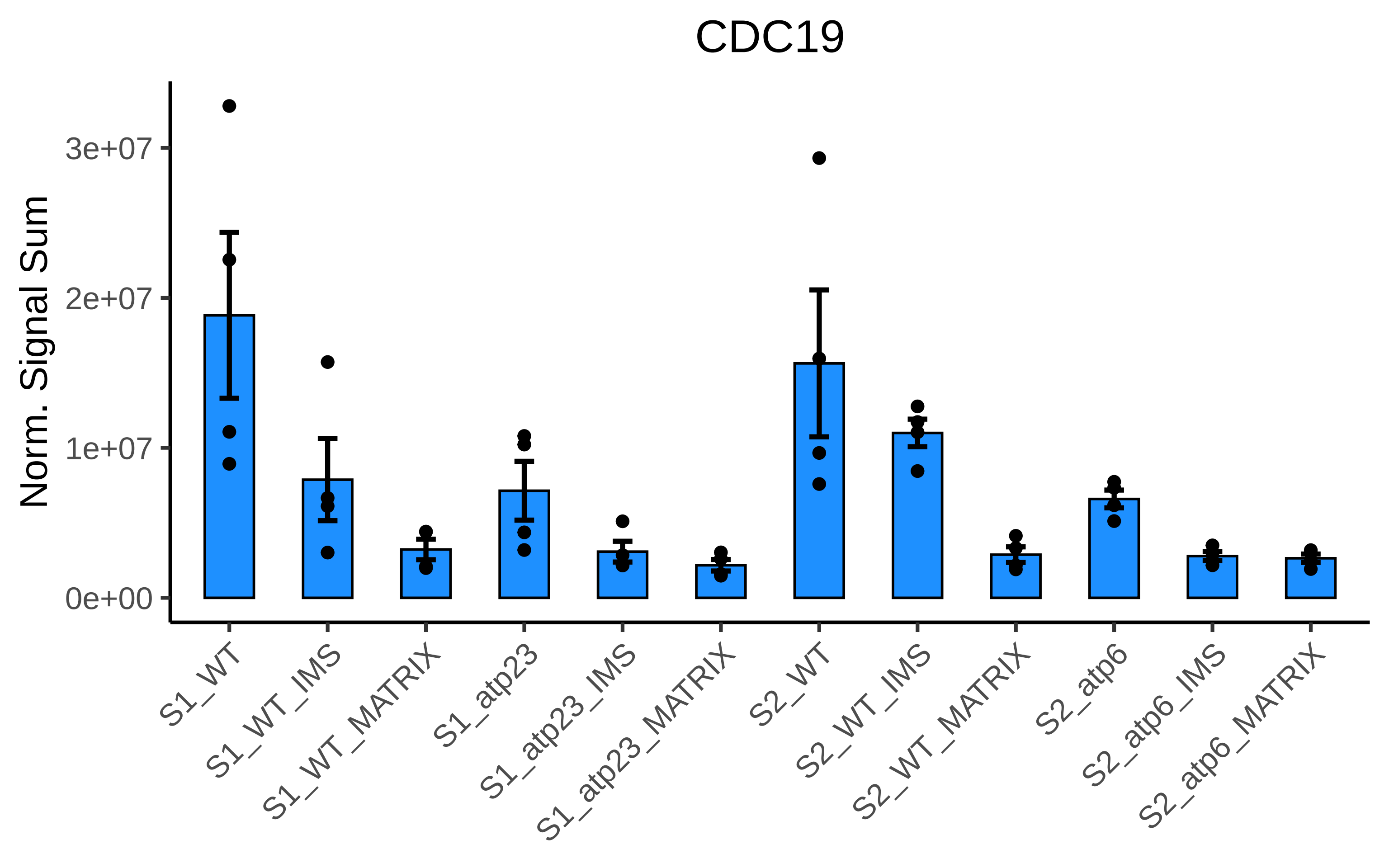

CDC19
Norm. Signal Sum
3e+07
2e+07
1e+07
0e+00
S1_WT
S1_WT_IMS
S1_WT_MATRIX
S1_atp23
S1_atp23_IMS
S1_atp23_MATRIX
S2_WT
S2_WT_IMS
S2_WT_MATRIX
S2_atp6
S2_atp6_IMS
S2_atp6_MATRIX

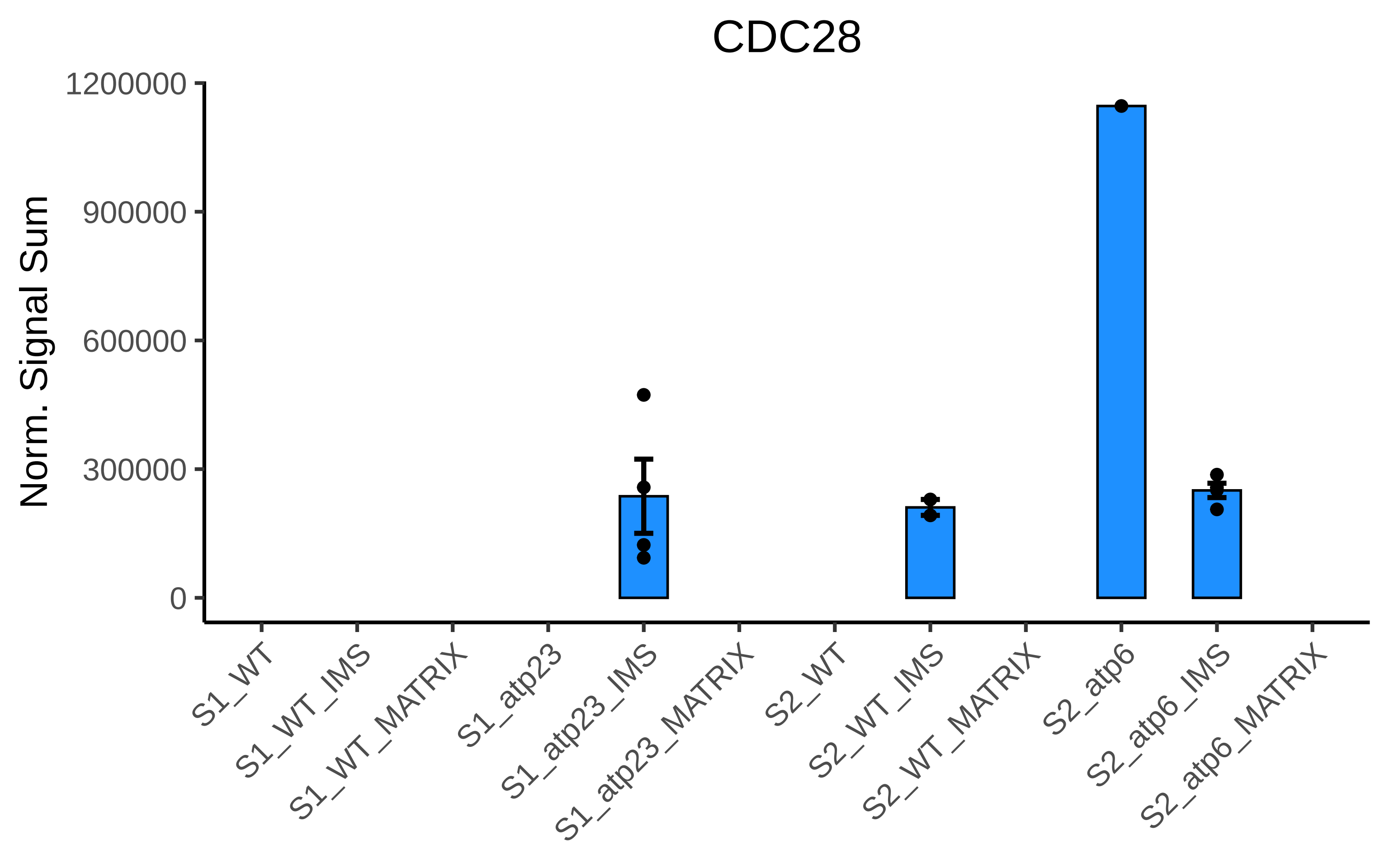

CDC28
Norm. Signal Sum
1200000
900000
600000
300000
0
S1_WT
S1_WT_IMS
S1_WT_MATRIX
S1_atp23
S1_atp23_IMS
S1_atp23_MATRIX
S2_WT
S2_WT_IMS
S2_WT_MATRIX
S2_atp6
S2_atp6_IMS
S2_atp6_MATRIX

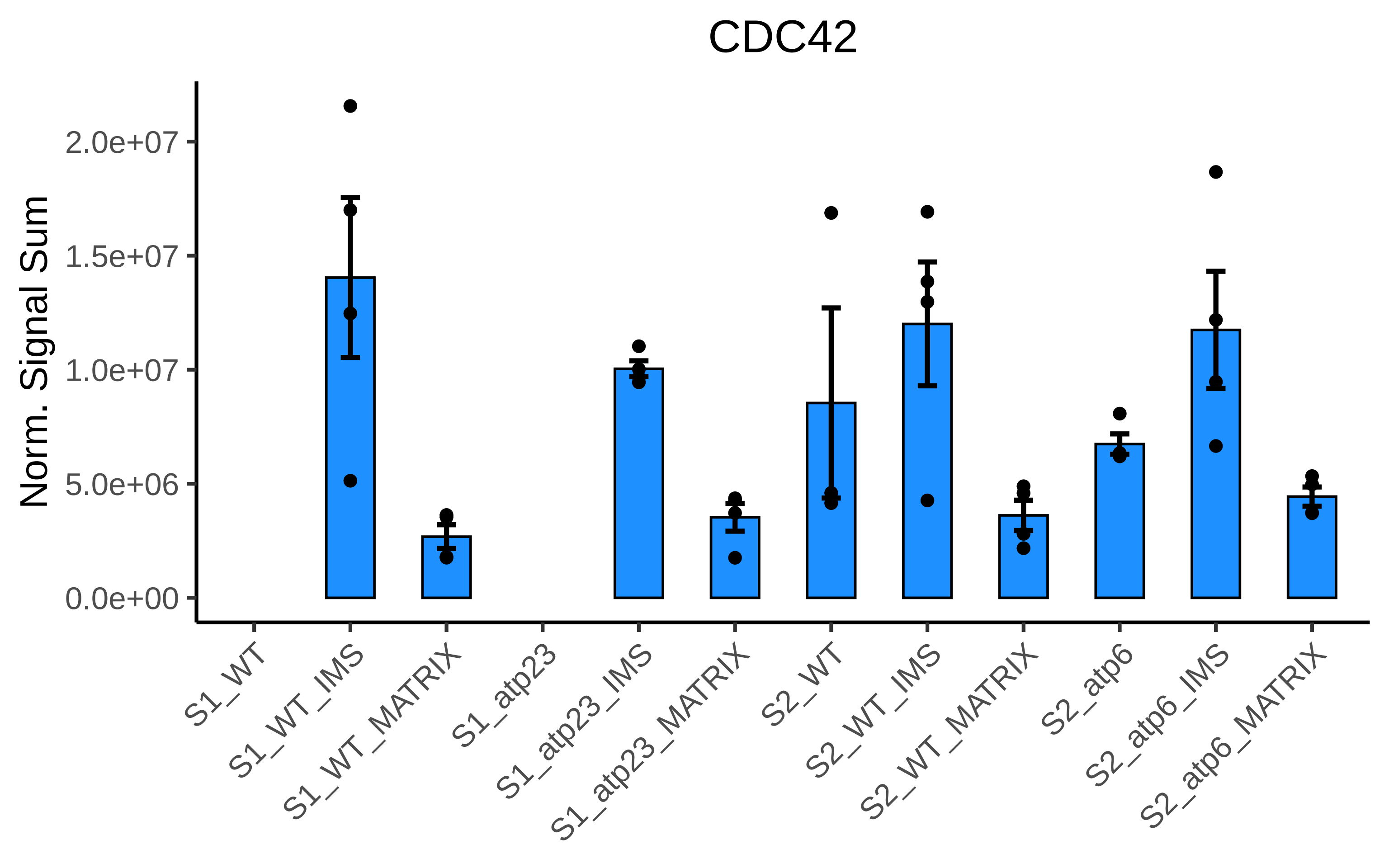
CDC42
Norm. Signal Sum
2.0e+07
1.5e+07
1.0e+07
5.0e+06
0.0e+00
S1_WT
S1_WT_IMS
S1_WT_MATRIX
S1_atp23
S1_atp23_IMS
S1_atp23_MATRIX
S2_WT
S2_WT_IMS
S2_WT_MATRIX
S2_atp6
S2_atp6_IMS
S2_atp6_MATRIX

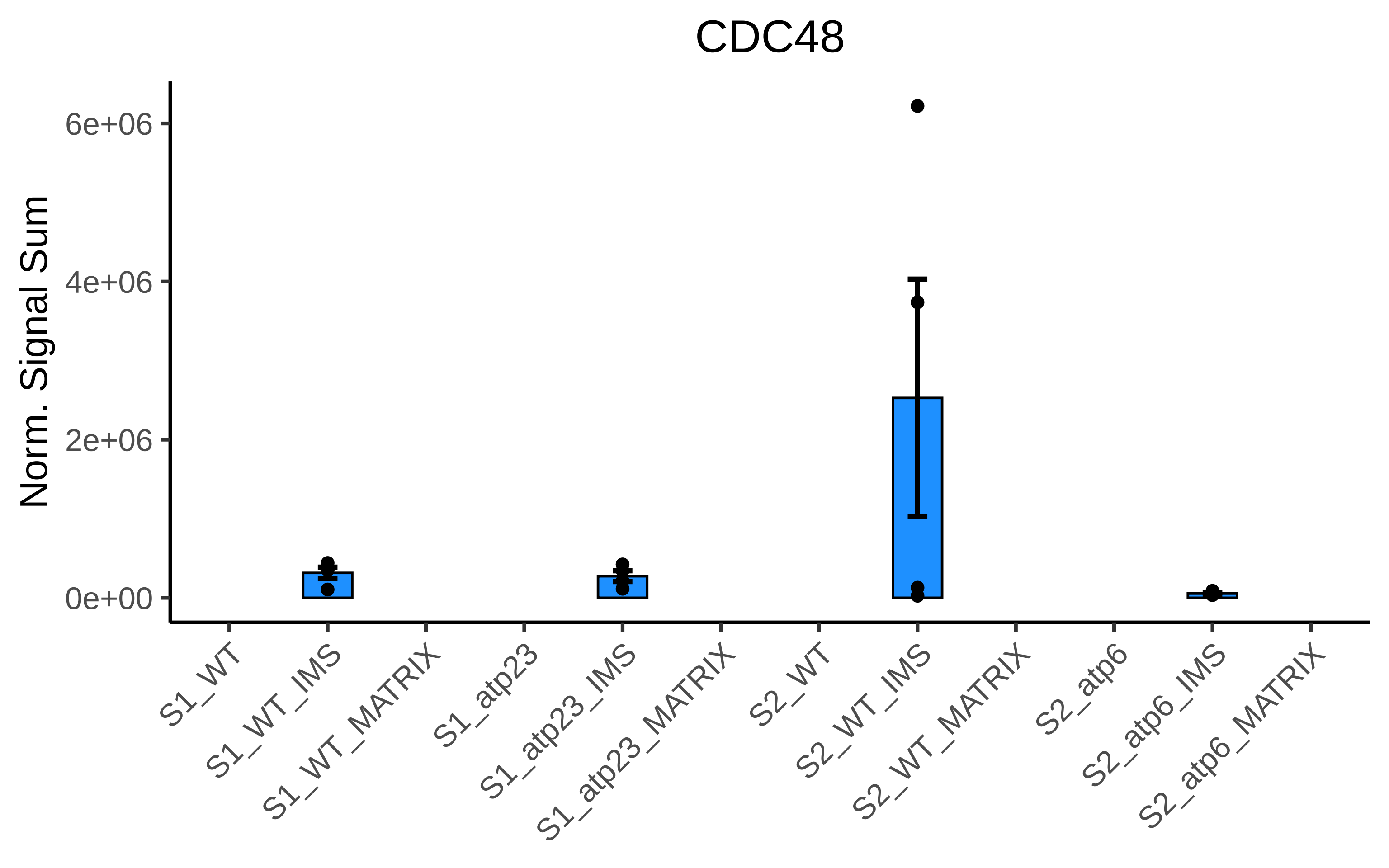

CDC48
Norm. Signal Sum
6e+06
4e+06
2e+06
0e+00
S1_WT
S1_WT_IMS
S1_WT_MATRIX
S1_atp23
S1_atp23_IMS
S1_atp23_MATRIX
S2_WT
S2_WT_IMS
S2_WT_MATRIX
S2_atp6
S2_atp6_IMS
S2_atp6_MATRIX

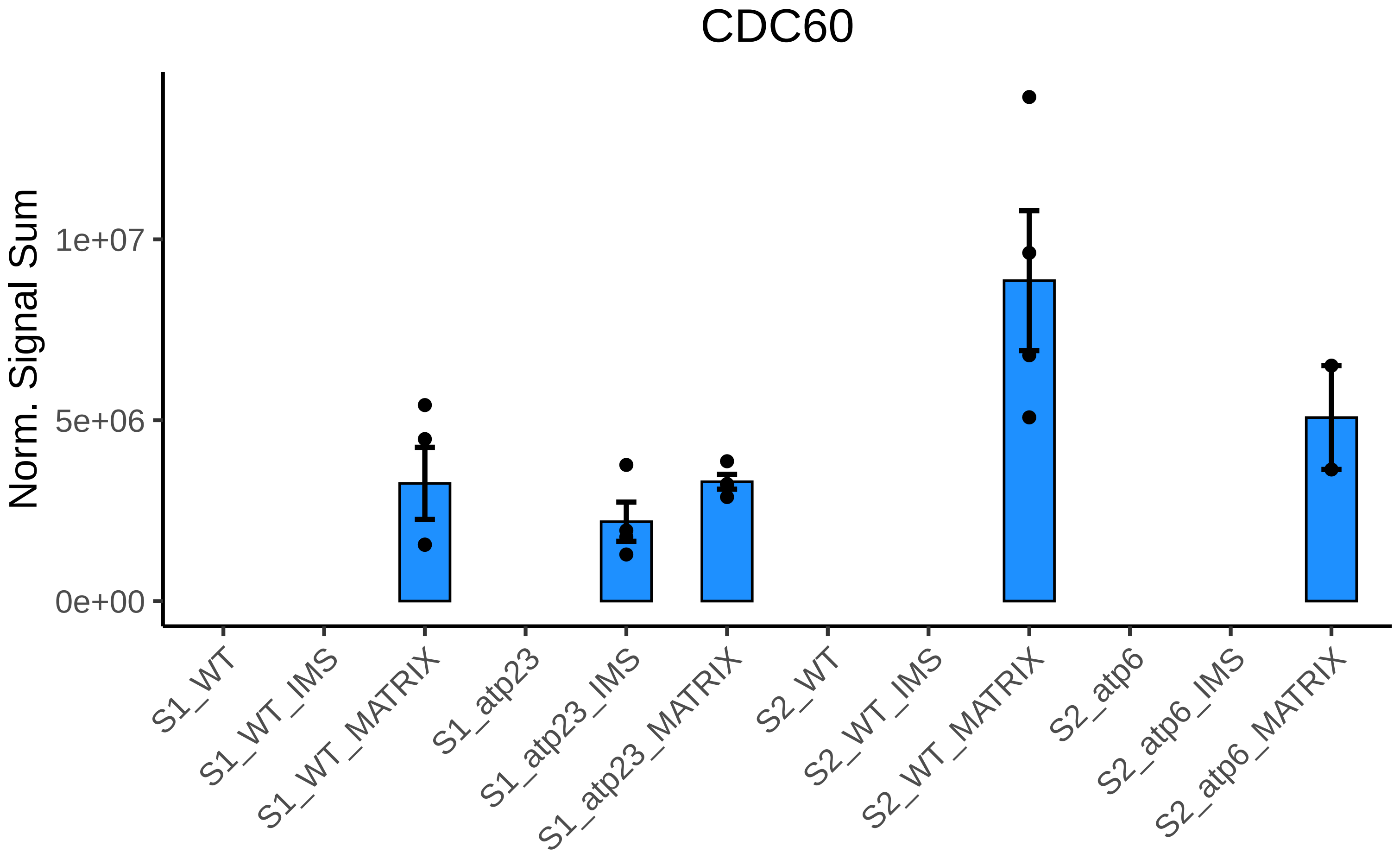
CDC60
Norm. Signal Sum
1e+07
5e+06
0e+00
S1_WT
S1_WT_IMS
S1_WT_MATRIX
S1_atp23
S1_atp23_IMS
S1_atp23_MATRIX
S2_WT
S2_WT_IMS
S2_WT_MATRIX
S2_atp6
S2_atp6_IMS
S2_atp6_MATRIX

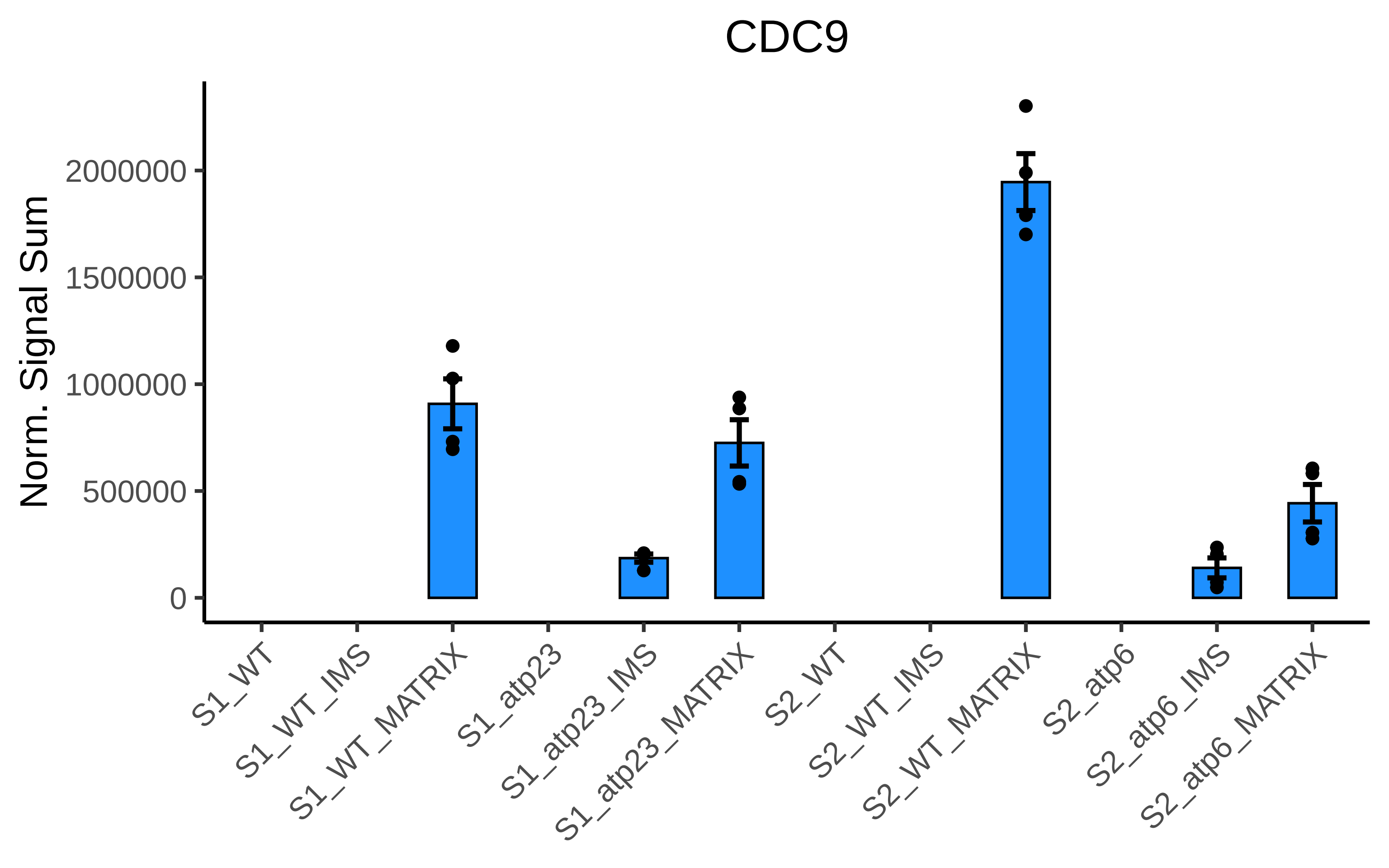
CDC9
Norm. Signal Sum
2000000
1500000
1000000
500000
0
S1_WT
S1_WT_IMS
S1_WT_MATRIX
S1_atp23
S1_atp23_IMS
S1_atp23_MATRIX
S2_WT
S2_WT_IMS
S2_WT_MATRIX
S2_atp6
S2_atp6_IMS
S2_atp6_MATRIX

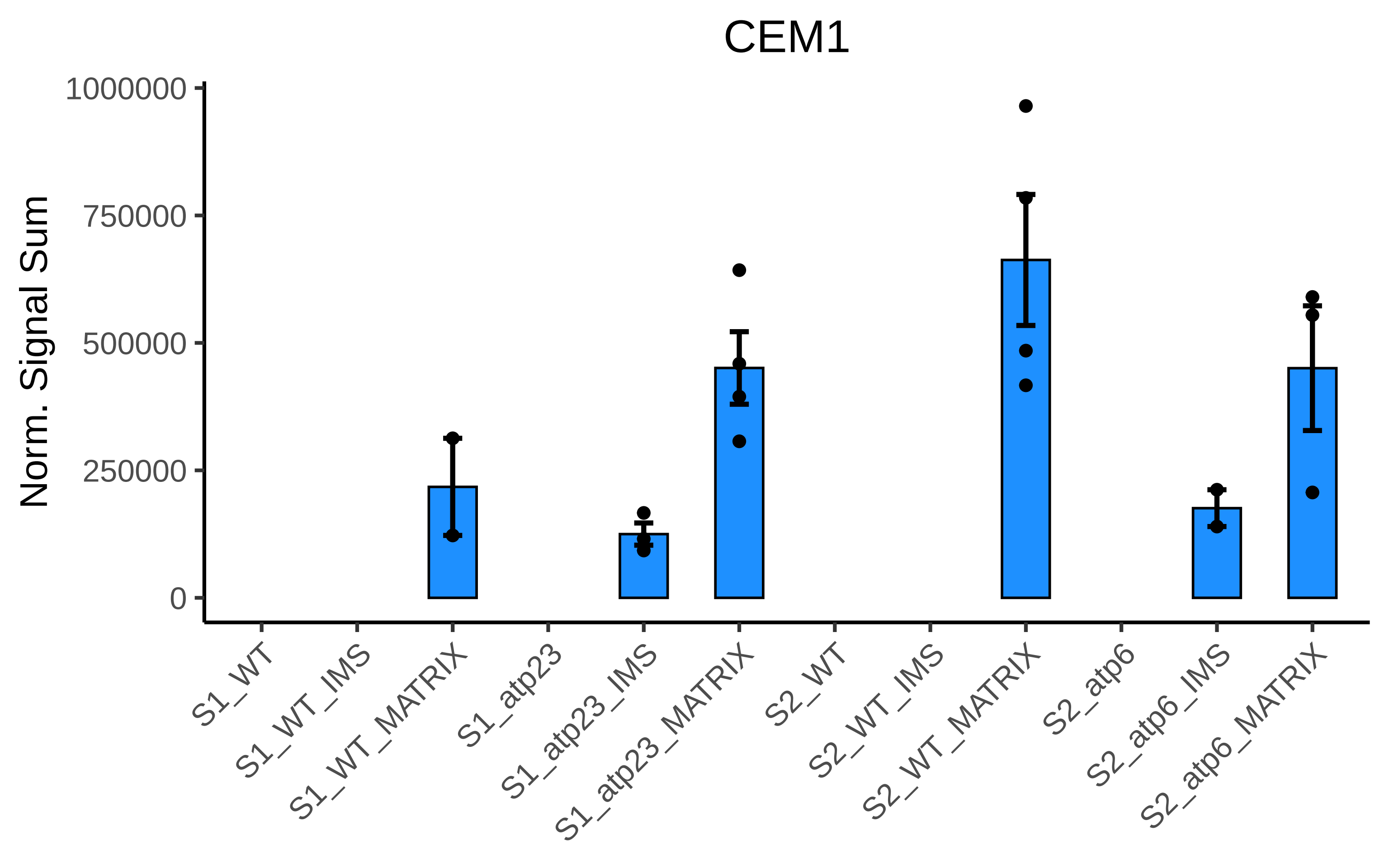

CEM1
Norm. Signal Sum
1000000
750000
500000
250000
0
S1_WT
S1_WT_IMS
S1_WT_MATRIX
S1_atp23
S1_atp23_IMS
S1_atp23_MATRIX
S2_WT
S2_WT_IMS
S2_WT_MATRIX
S2_atp6
S2_atp6_IMS
S2_atp6_MATRIX

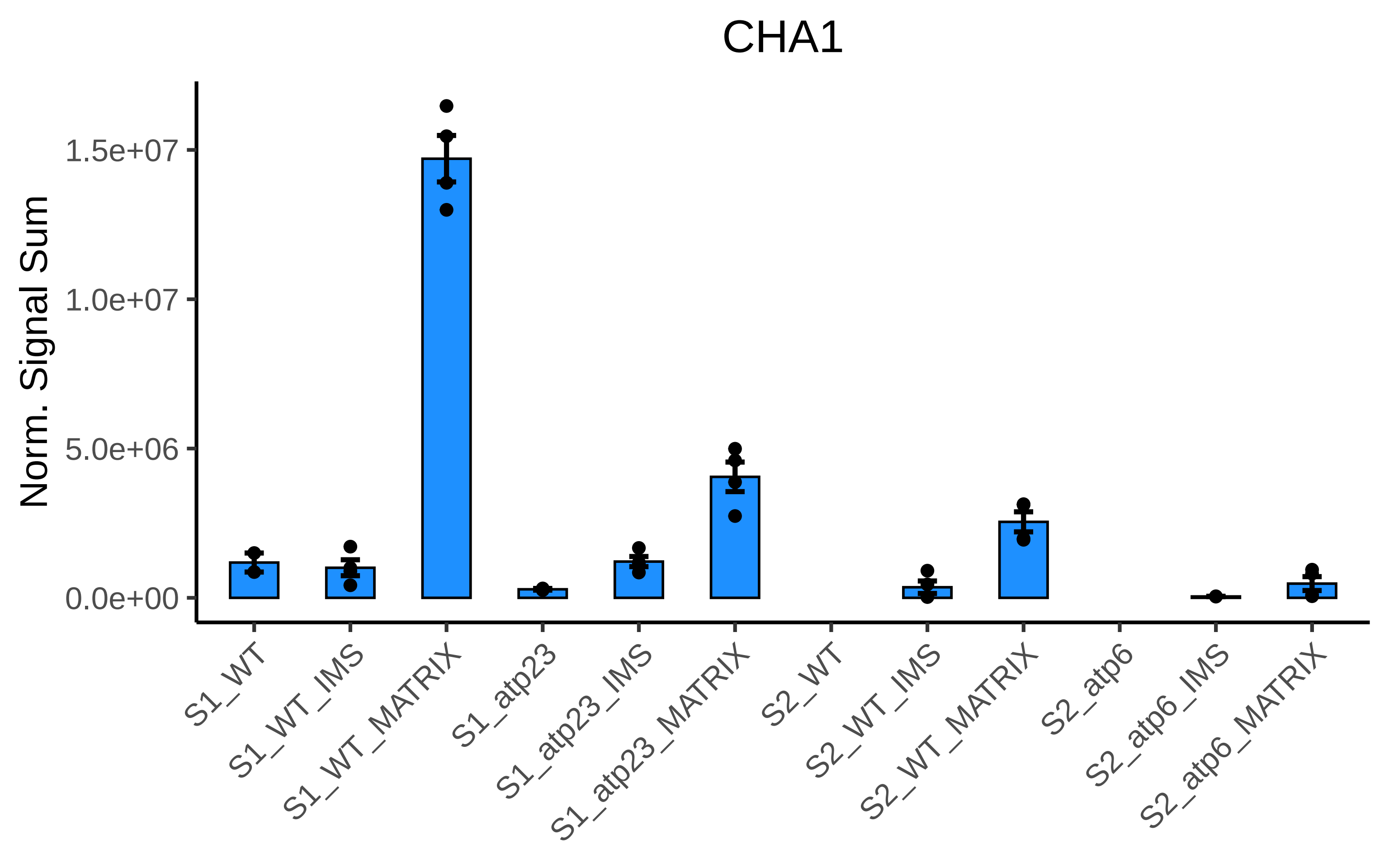

CHA1
Norm. Signal Sum
1.5e+07
1.0e+07
5.0e+06
0.0e+00
S1_WT
S1_WT_IMS
S1_WT_MATRIX
S1_atp23
S1_atp23_IMS
S1_atp23_MATRIX
S2_WT
S2_WT_IMS
S2_WT_MATRIX
S2_atp6
S2_atp6_IMS
S2_atp6_MATRIX

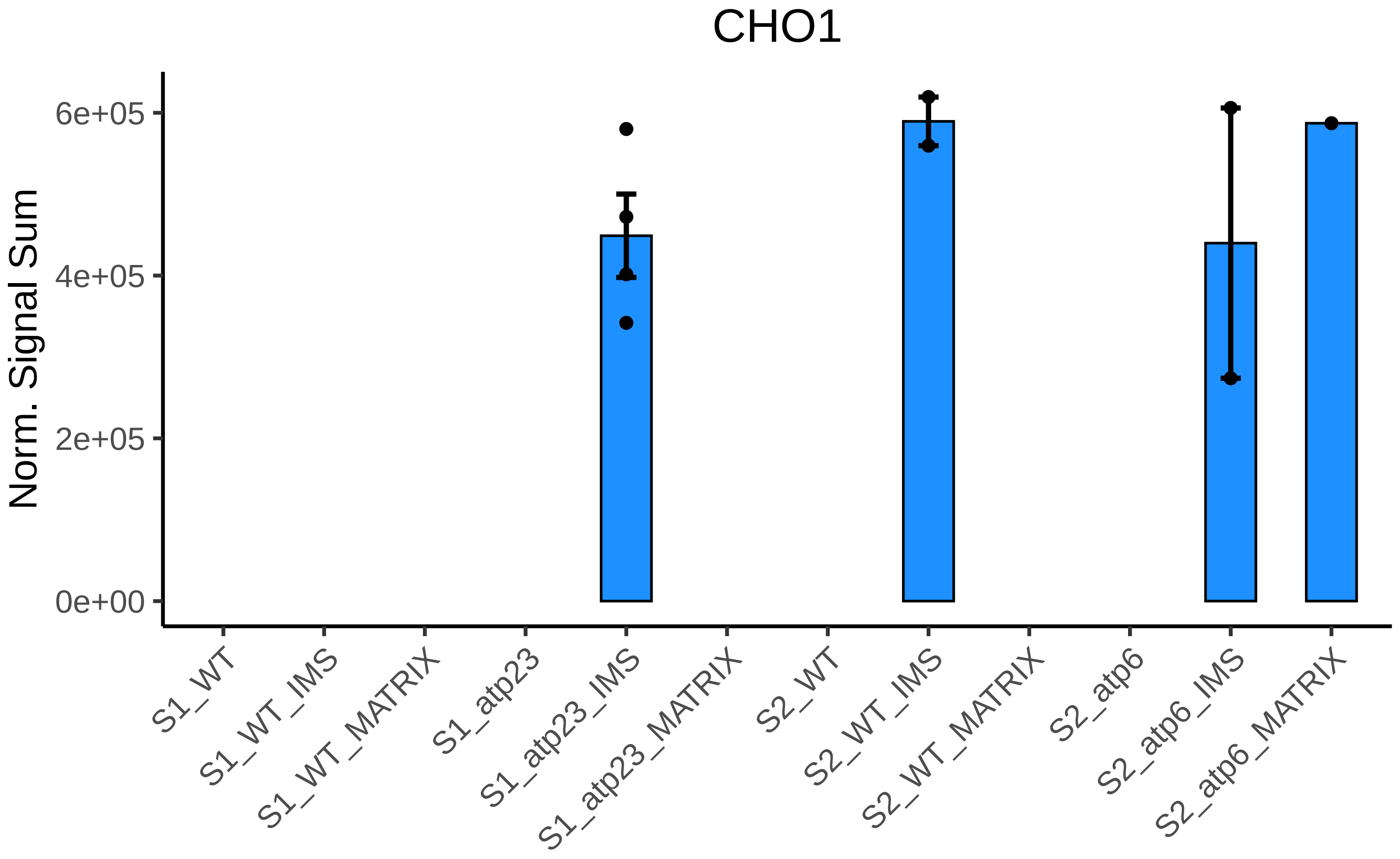

CHO1
Norm. Signal Sum
6e+05
4e+05
2e+05
0e+00
S1_WT
S1_WT_IMS
S1_WT_MATRIX
S1_atp23
S1_atp23_IMS
S1_atp23_MATRIX
S2_WT
S2_WT_IMS
S2_WT_MATRIX
S2_atp6
S2_atp6_IMS
S2_atp6_MATRIX

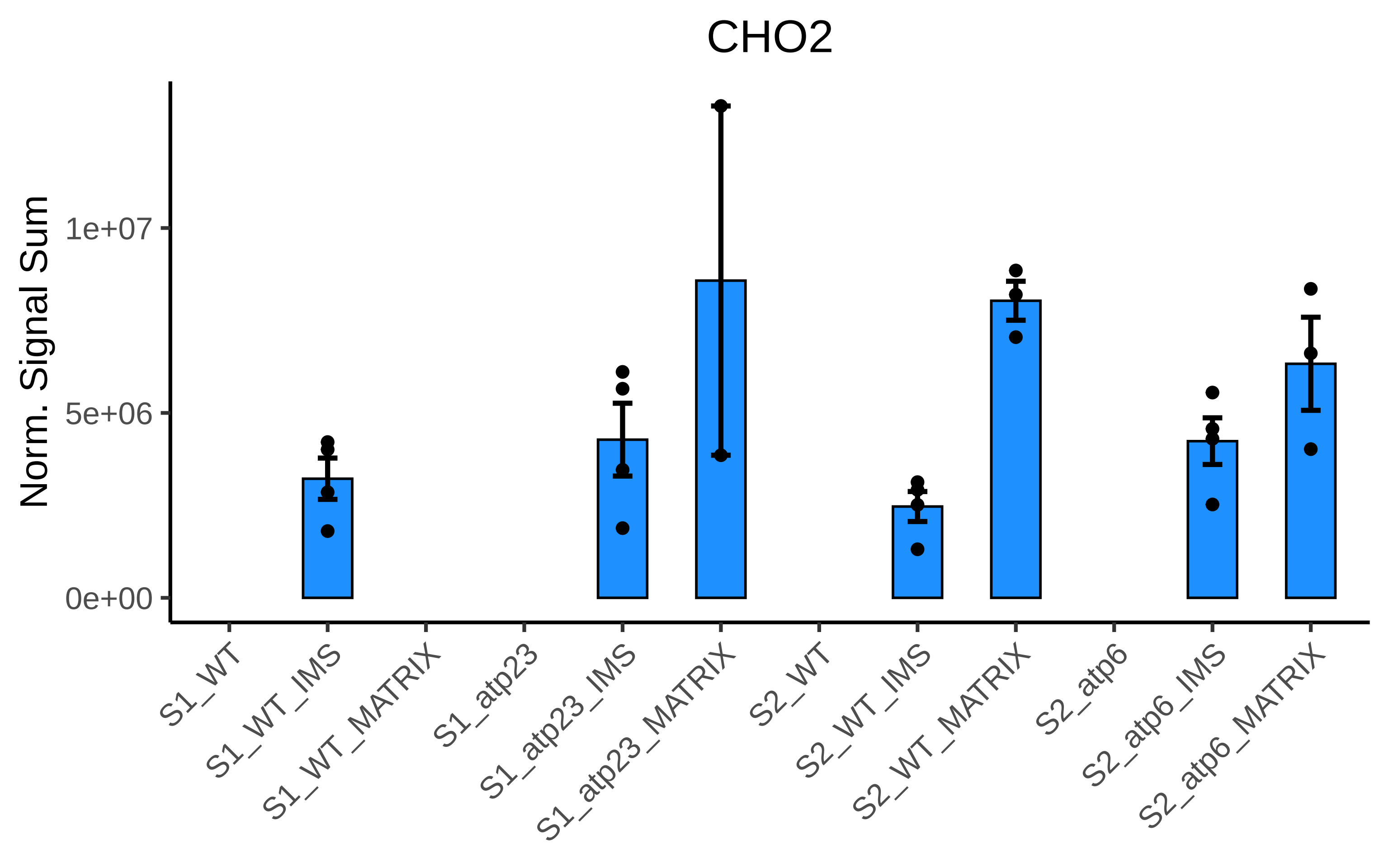
CHO2
Norm. Signal Sum
1e+07
5e+06
0e+00
S1_WT
S1_WT_IMS
S1_WT_MATRIX
S1_atp23
S1_atp23_IMS
S1_atp23_MATRIX
S2_WT
S2_WT_IMS
S2_WT_MATRIX
S2_atp6
S2_atp6_IMS
S2_atp6_MATRIX

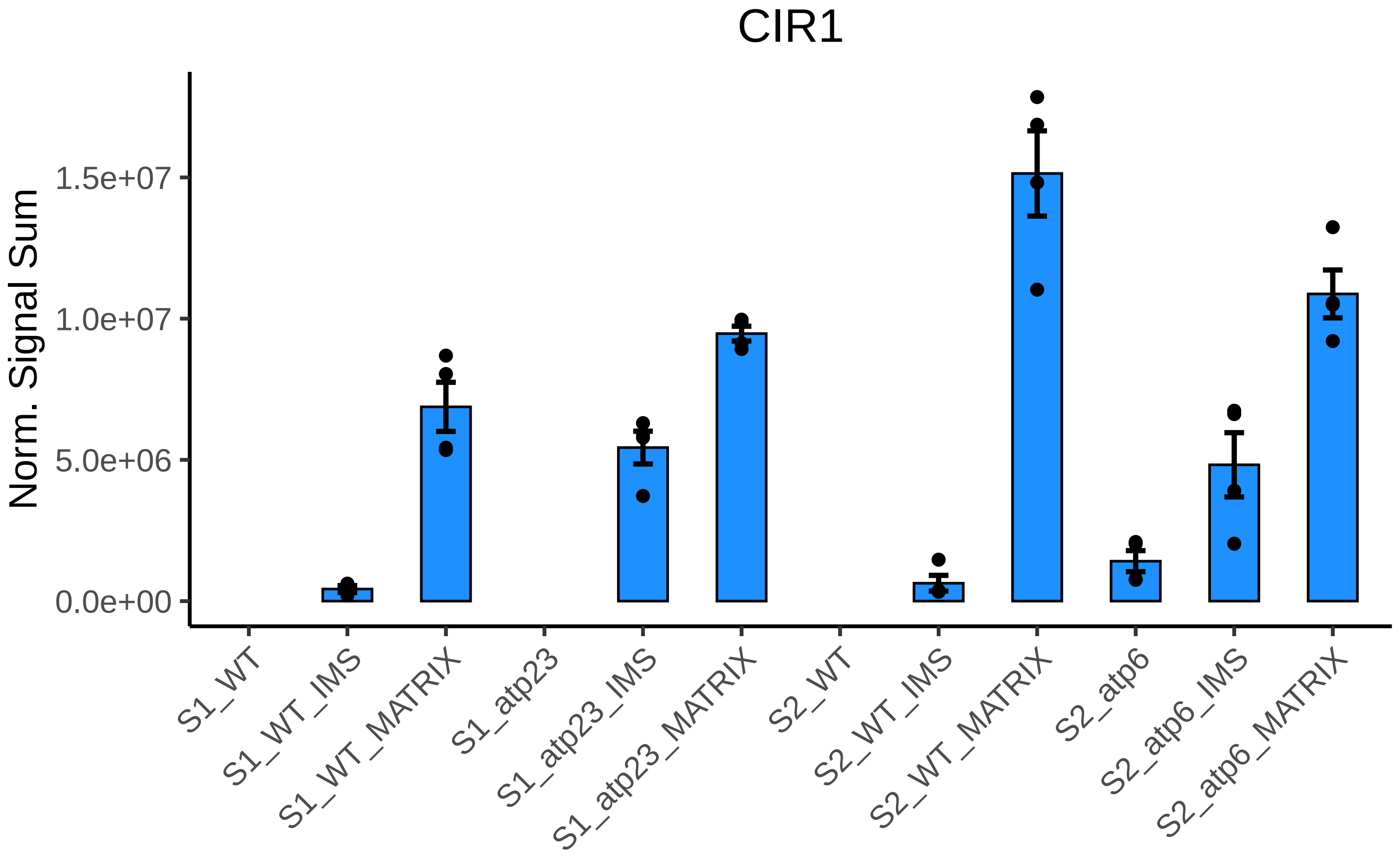

CIR1
Norm. Signal Sum
1.5e+07
1.0e+07
5.0e+06
0.0e+00
S1_WT
S1_WT_IMS
S1_WT_MATRIX
S1_atp23
S1_atp23_IMS
S1_atp23_MATRIX
S2_WT
S2_WT_IMS
S2_WT_MATRIX
S2_atp6
S2_atp6_IMS
S2_atp6_MATRIX

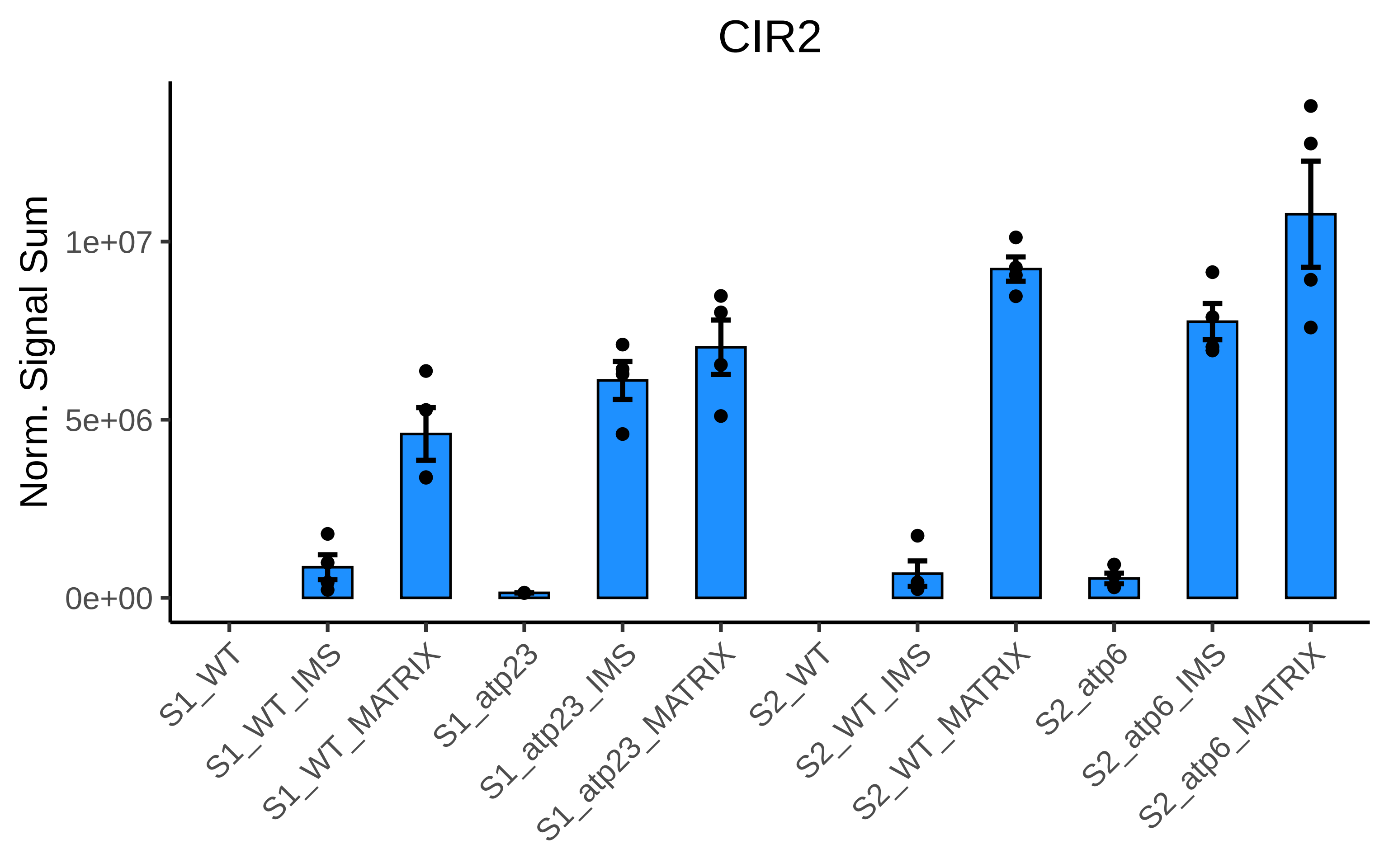

CIR2
Norm. Signal Sum
1e+07
5e+06
0e+00
S1_WT
S1_WT_IMS
S1_WT_MATRIX
S1_atp23
S1_atp23_IMS
S1_atp23_MATRIX
S2_WT
S2_WT_IMS
S2_WT_MATRIX
S2_atp6
S2_atp6_IMS
S2_atp6_MATRIX

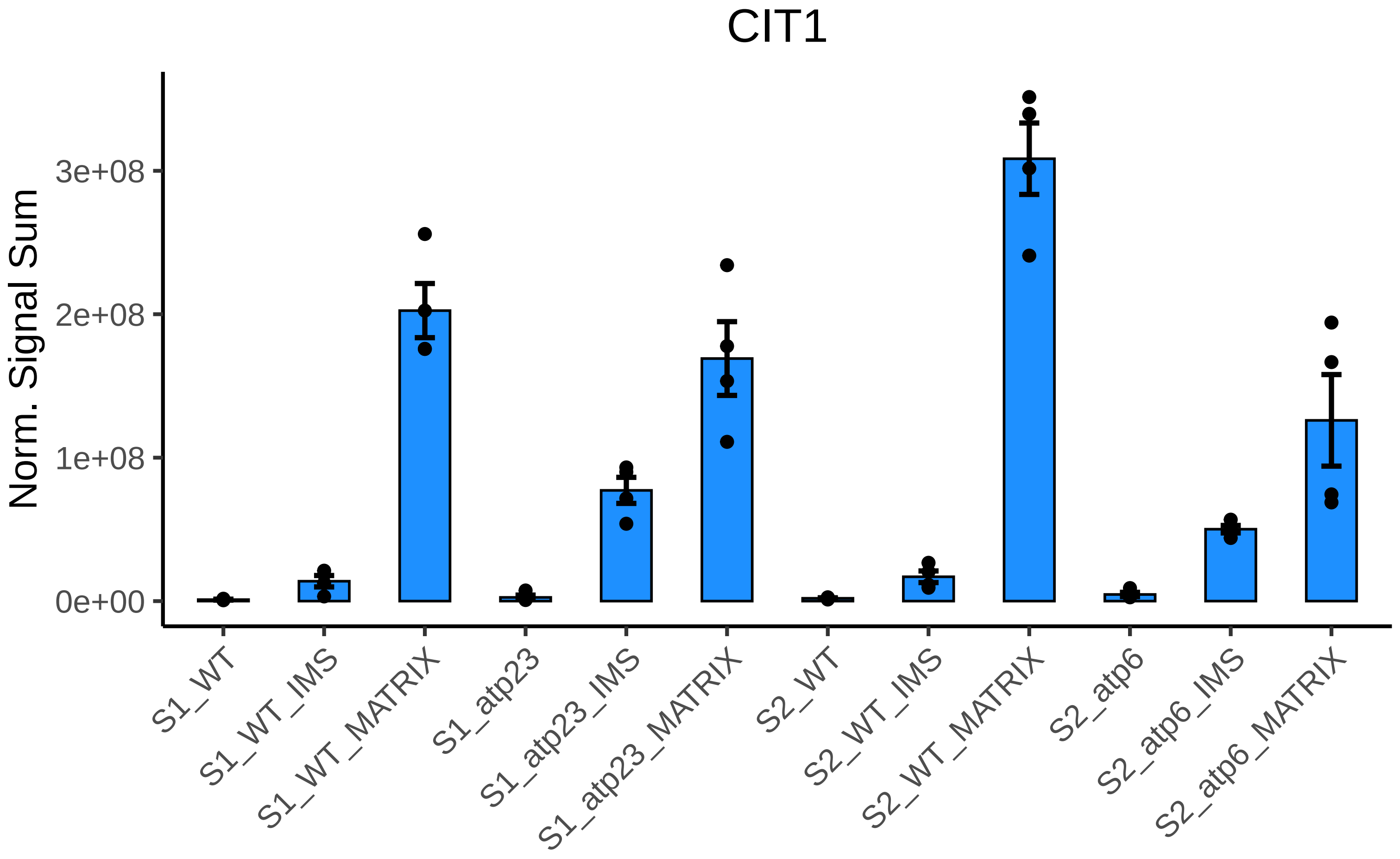
CIT1
Norm. Signal Sum
3e+08
2e+08
1e+08
0e+00
S1_WT
S1_WT_IMS
S1_WT_MATRIX
S1_atp23
S1_atp23_IMS
S1_atp23_MATRIX
S2_WT
S2_WT_IMS
S2_WT_MATRIX
S2_atp6
S2_atp6_IMS
S2_atp6_MATRIX

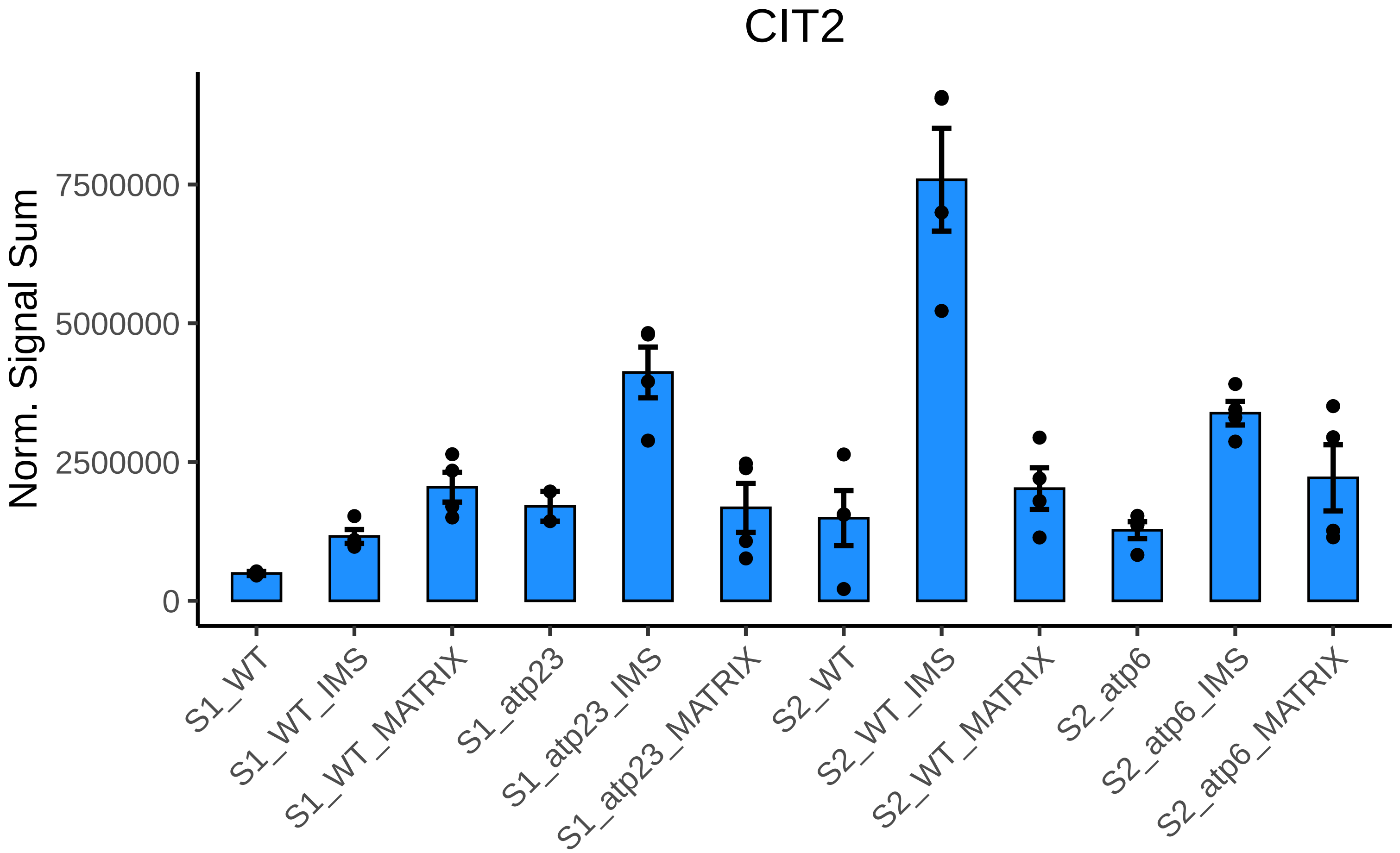
CIT2
Norm. Signal Sum
7500000
5000000
2500000
0
S1_WT
S1_WT_IMS
S1_WT_MATRIX
S1_atp23
S1_atp23_IMS
S1_atp23_MATRIX
S2_WT
S2_WT_IMS
S2_WT_MATRIX
S2_atp6
S2_atp6_IMS
S2_atp6_MATRIX

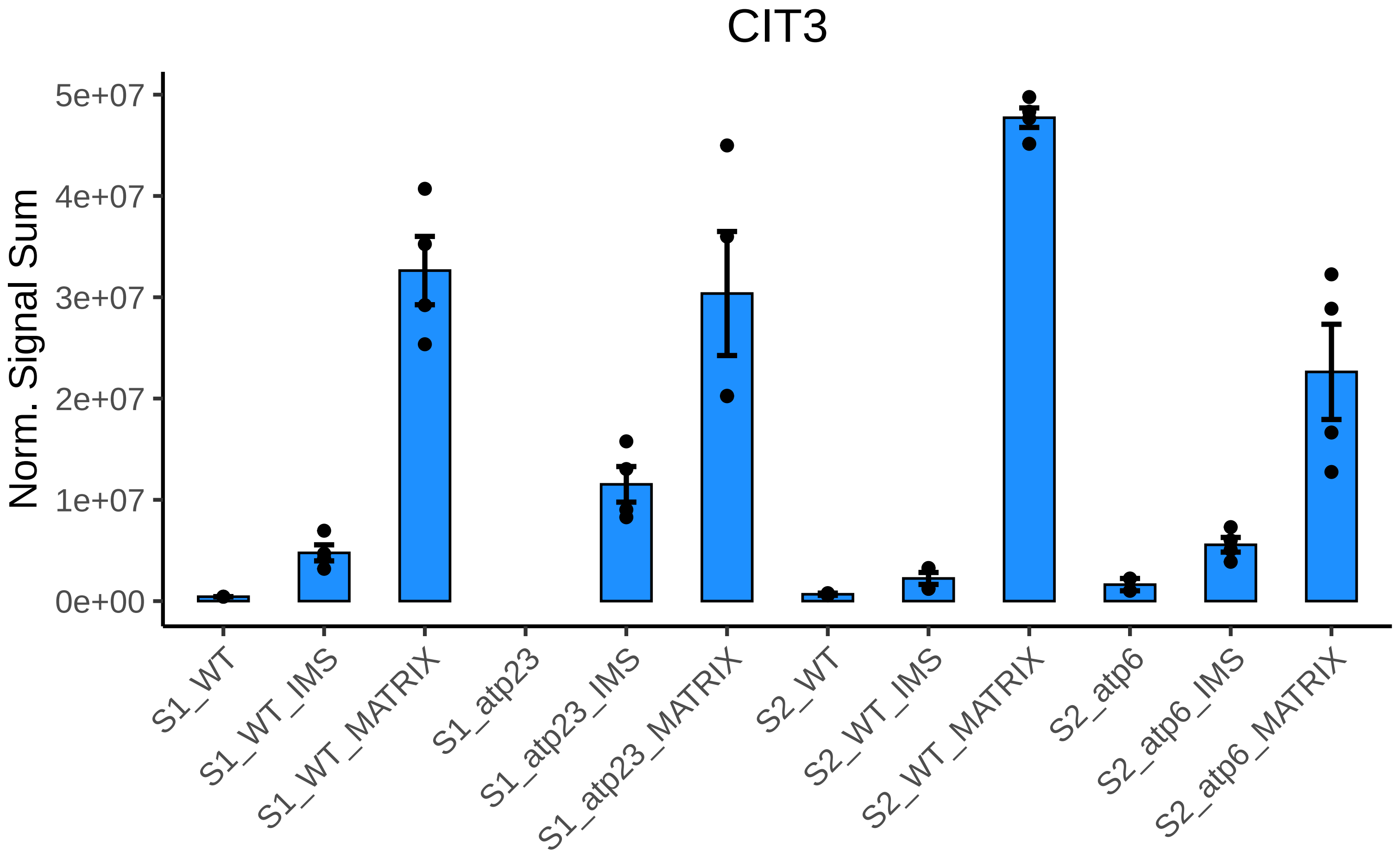
CIT3
Norm. Signal Sum
5e+07
4e+07
3e+07
2e+07
1e+07
0e+00
S1_WT
S1_WT_IMS
S1_WT_MATRIX
S1_atp23
S1_atp23_IMS
S1_atp23_MATRIX
S2_WT
S2_WT_IMS
S2_WT_MATRIX
S2_atp6
S2_atp6_IMS
S2_atp6_MATRIX

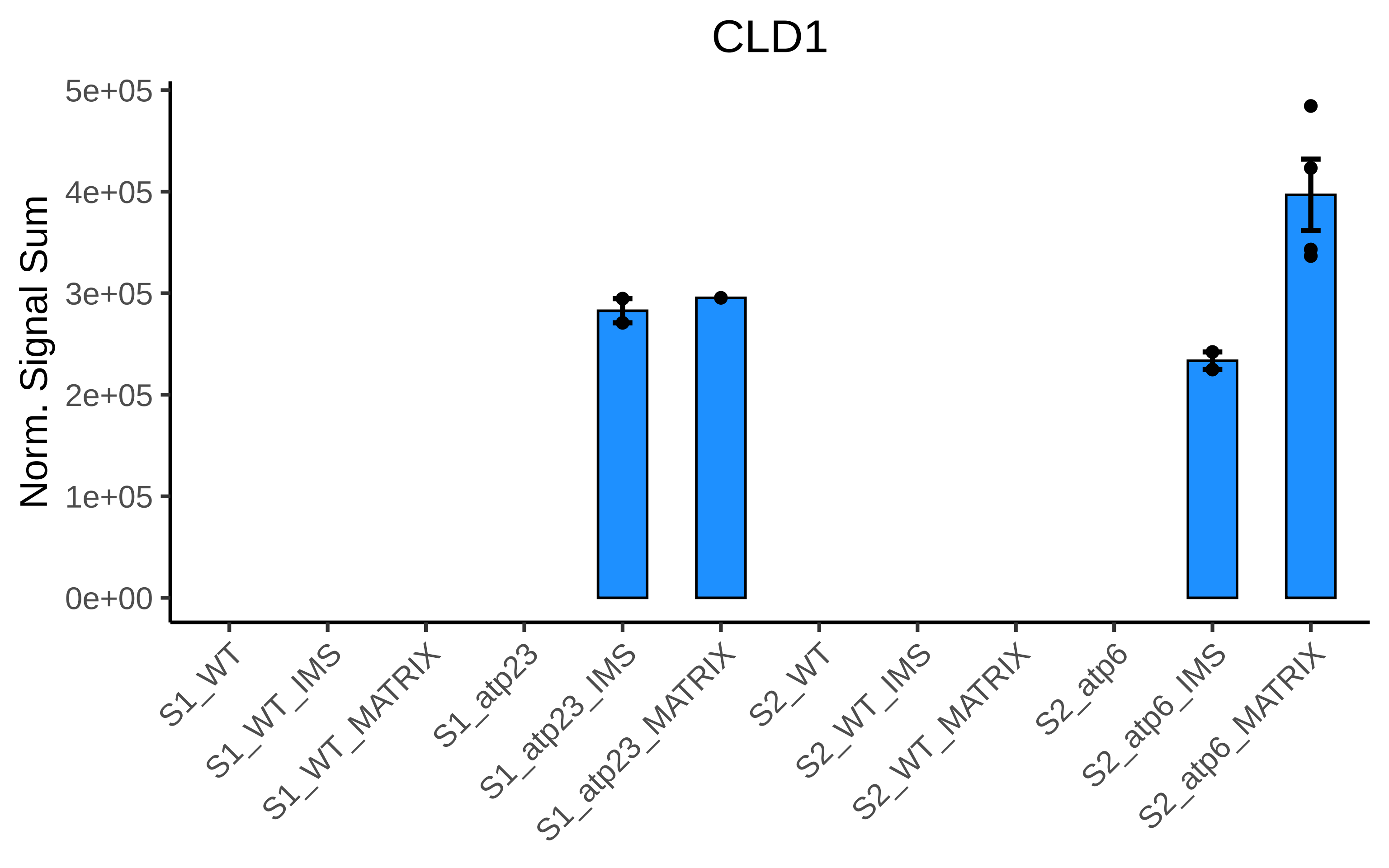

CLD1
Norm. Signal Sum
5e+05
4e+05
3e+05
2e+05
1e+05
0e+00
S1_WT
S1_WT_IMS
S1_WT_MATRIX
S1_atp23
S1_atp23_IMS
S1_atp23_MATRIX
S2_WT
S2_WT_IMS
S2_WT_MATRIX
S2_atp6
S2_atp6_IMS
S2_atp6_MATRIX

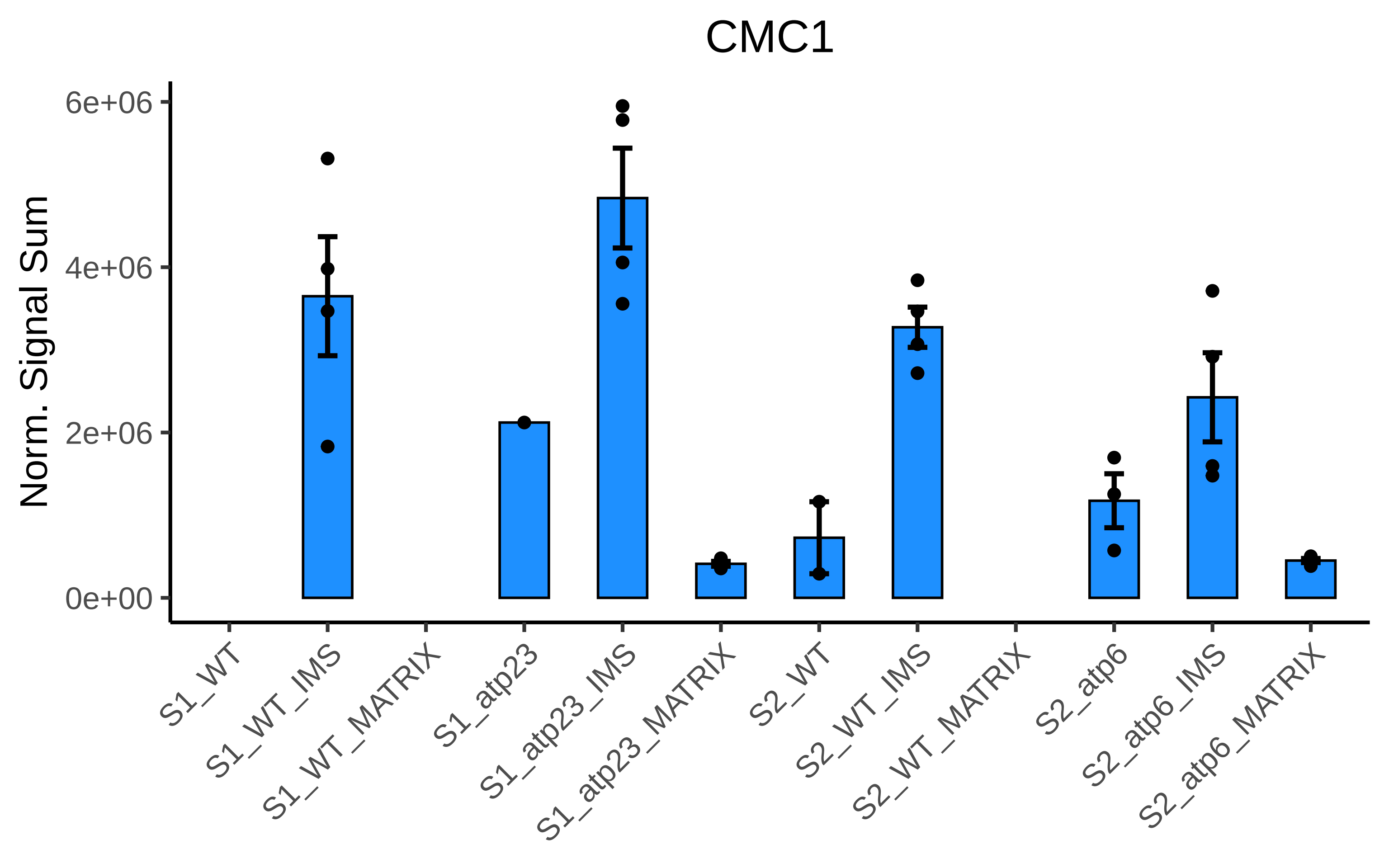
CMC1
Norm. Signal Sum
6e+06
4e+06
2e+06
0e+00
S1_WT
S1_WT_IMS
S1_WT_MATRIX
S1_atp23
S1_atp23_IMS
S1_atp23_MATRIX
S2_WT
S2_WT_IMS
S2_WT_MATRIX
S2_atp6
S2_atp6_IMS
S2_atp6_MATRIX

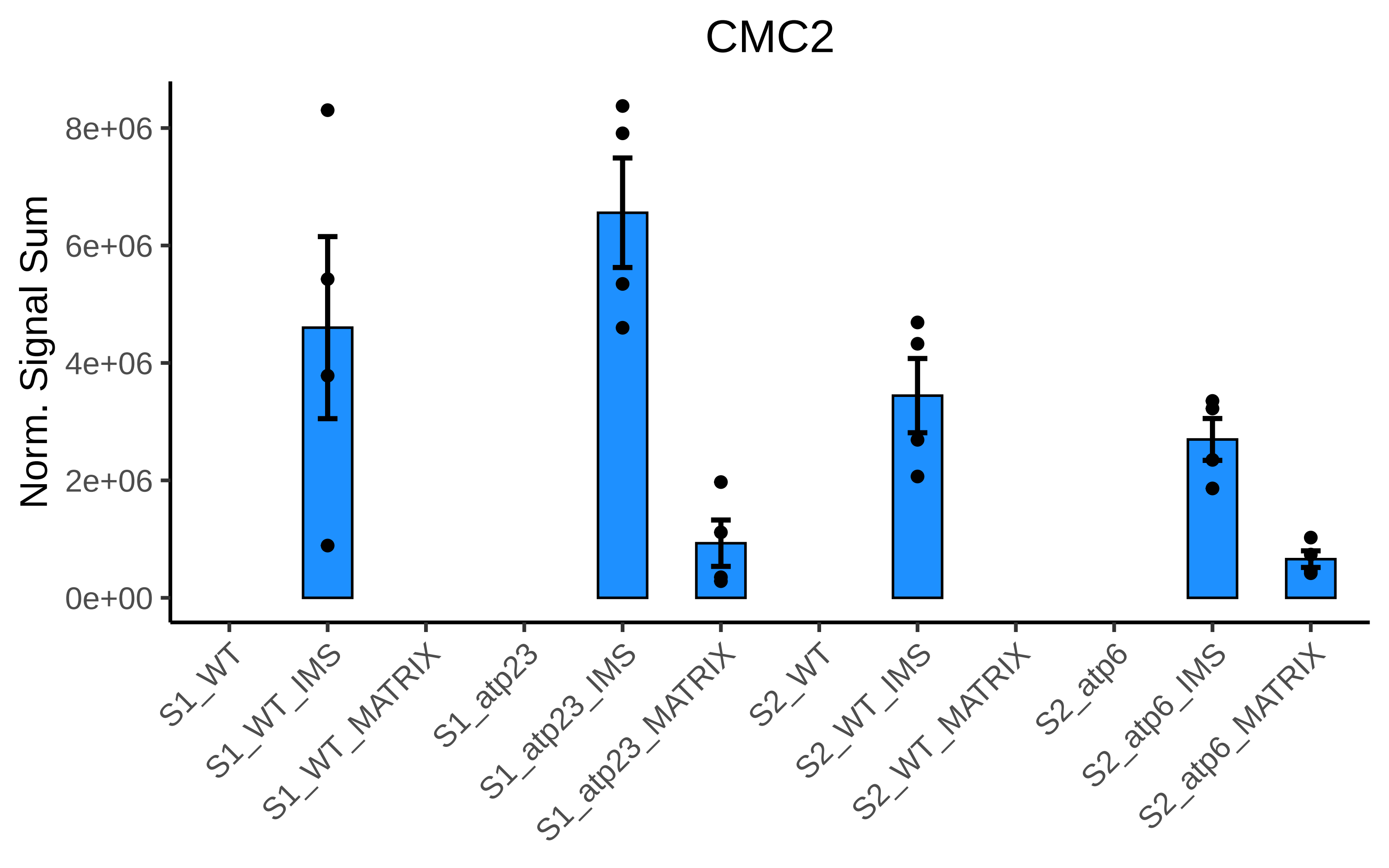

CMC2
Norm. Signal Sum
8e+06
6e+06
4e+06
2e+06
0e+00
S1_WT
S1_WT_IMS
S1_WT_MATRIX
S1_atp23
S1_atp23_IMS
S1_atp23_MATRIX
S2_WT
S2_WT_IMS
S2_WT_MATRIX
S2_atp6
S2_atp6_IMS
S2_atp6_MATRIX

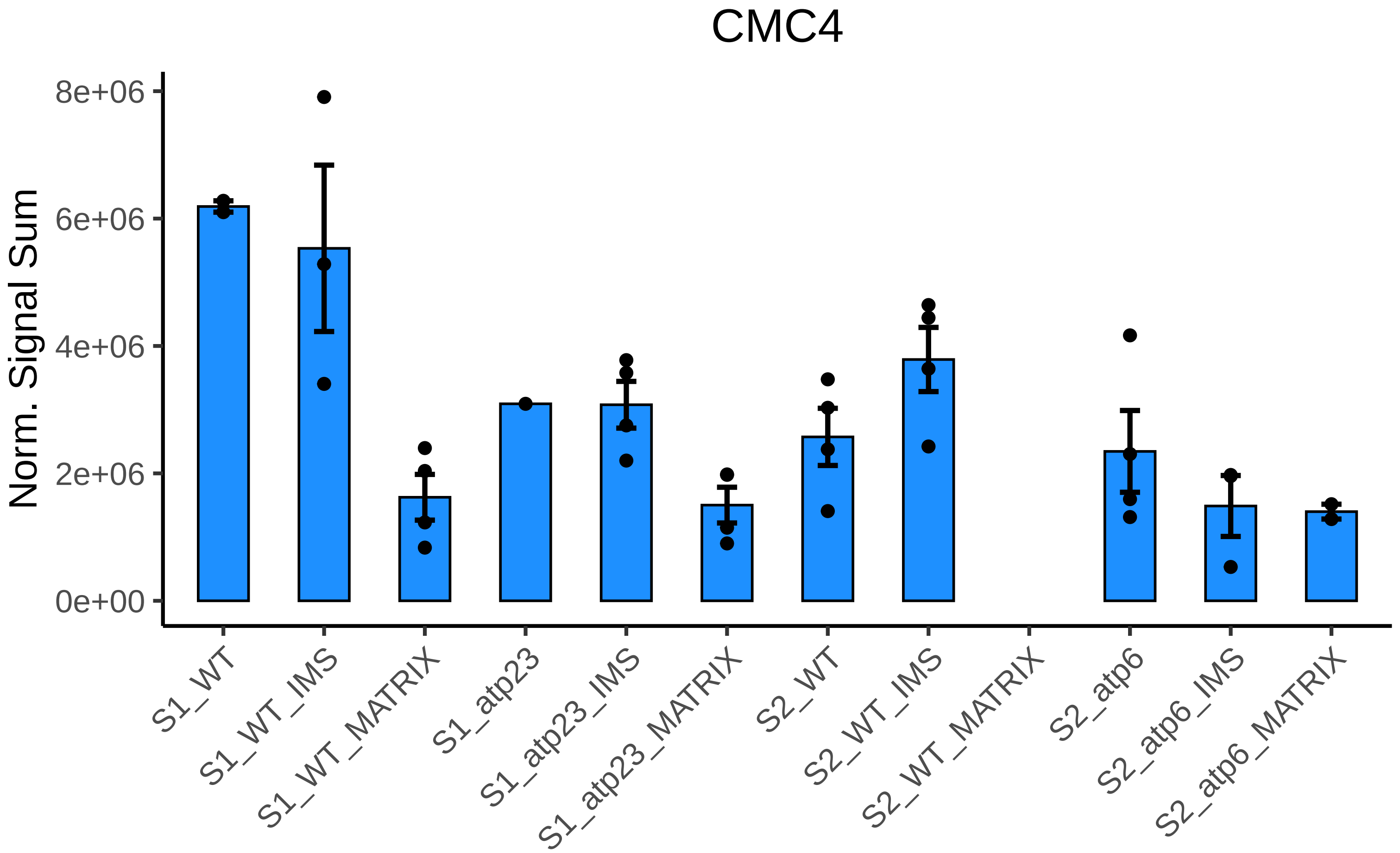

CMC4
Norm. Signal Sum
8e+06
6e+06
4e+06
2e+06
0e+00
S1_WT
S1_WT_IMS
S1_WT_MATRIX
S1_atp23
S1_atp23_IMS
S1_atp23_MATRIX
S2_WT
S2_WT_IMS
S2_WT_MATRIX
S2_atp6
S2_atp6_IMS
S2_atp6_MATRIX

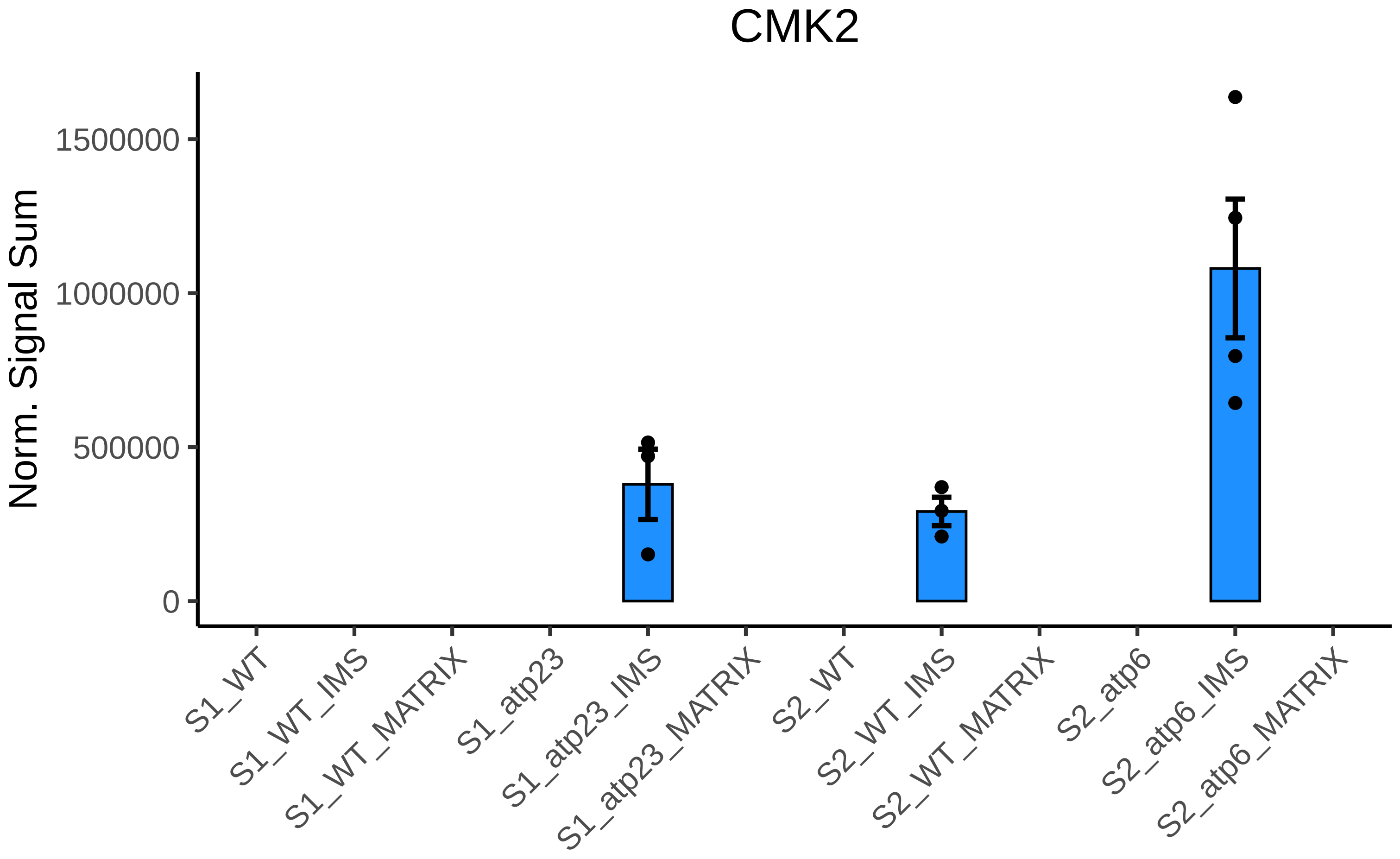

CMK2
Norm. Signal Sum
1500000
1000000
500000
0
S1_WT
S1_WT_IMS
S1_WT_MATRIX
S1_atp23
S1_atp23_IMS
S1_atp23_MATRIX
S2_WT
S2_WT_IMS
S2_WT_MATRIX
S2_atp6
S2_atp6_IMS
S2_atp6_MATRIX

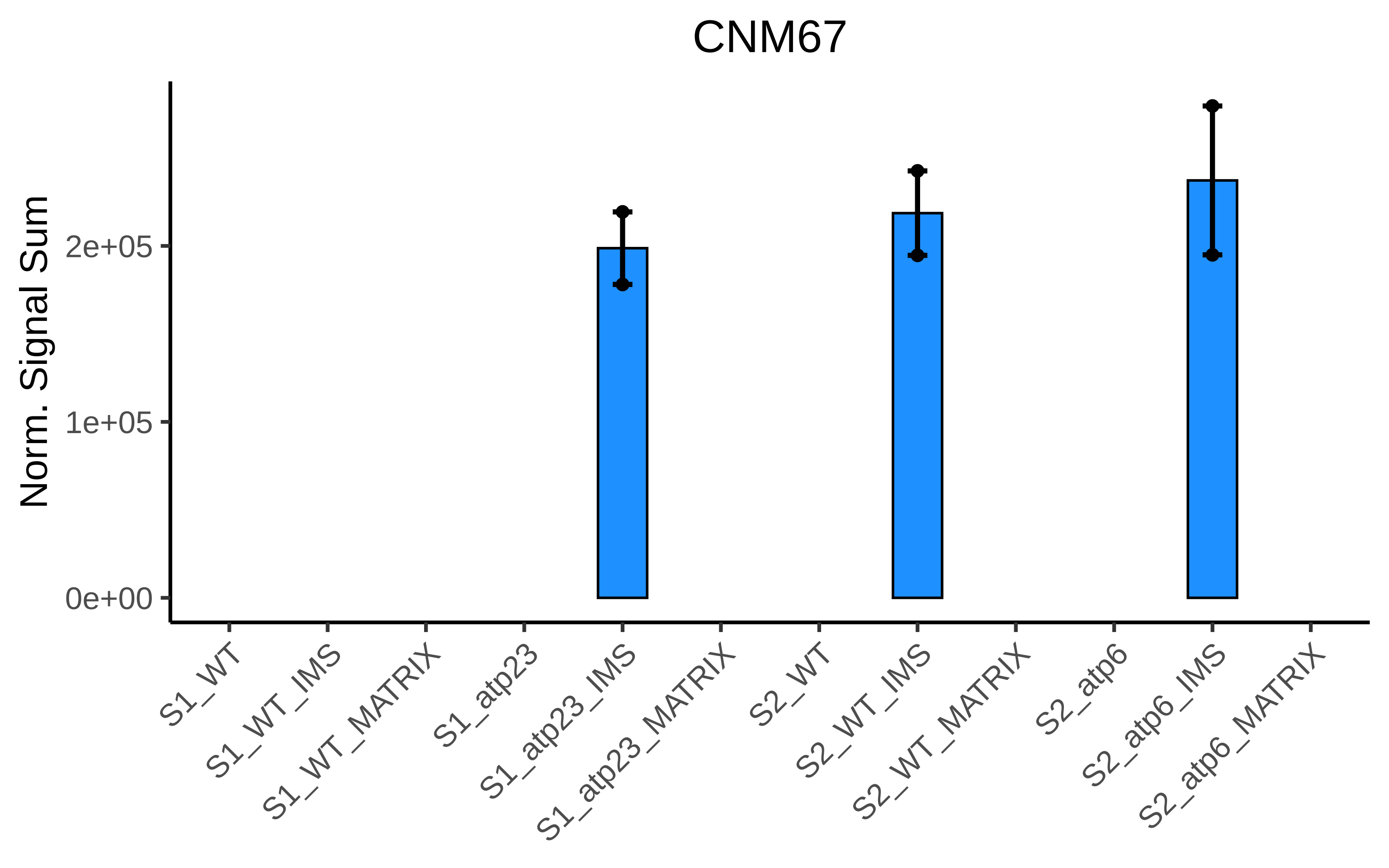
CNM67
Norm. Signal Sum
2e+05
1e+05
0e+00
S1_WT
S1_WT_IMS
S1_WT_MATRIX
S1_atp23
S1_atp23_IMS
S1_atp23_MATRIX
S2_WT
S2_WT_IMS
S2_WT_MATRIX
S2_atp6
S2_atp6_IMS
S2_atp6_MATRIX

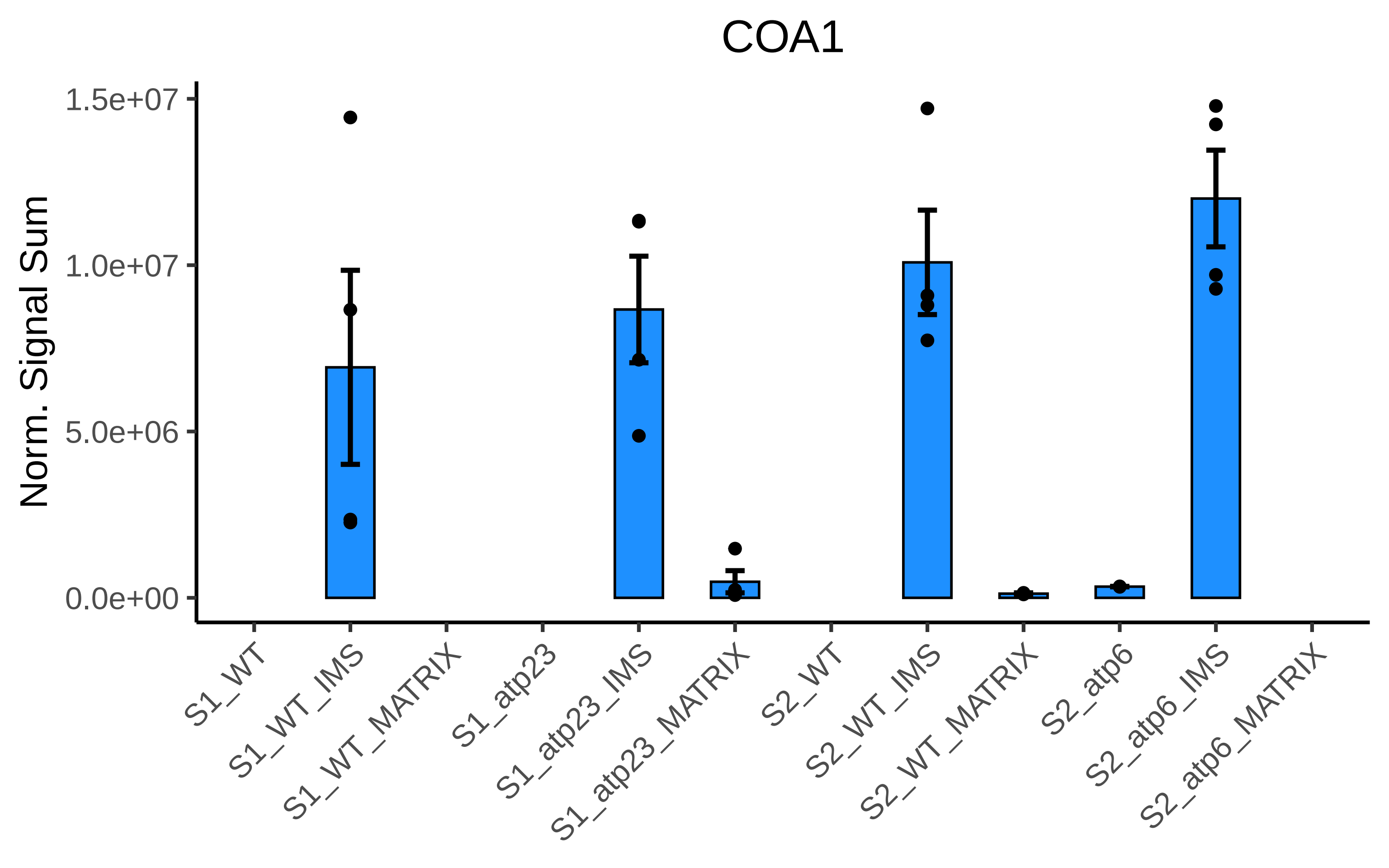
COA1
Norm. Signal Sum
1.5e+07
1.0e+07
5.0e+06
0.0e+00
S1_WT
S1_WT_IMS
S1_WT_MATRIX
S1_atp23
S1_atp23_IMS
S1_atp23_MATRIX
S2_WT
S2_WT_IMS
S2_WT_MATRIX
S2_atp6
S2_atp6_IMS
S2_atp6_MATRIX

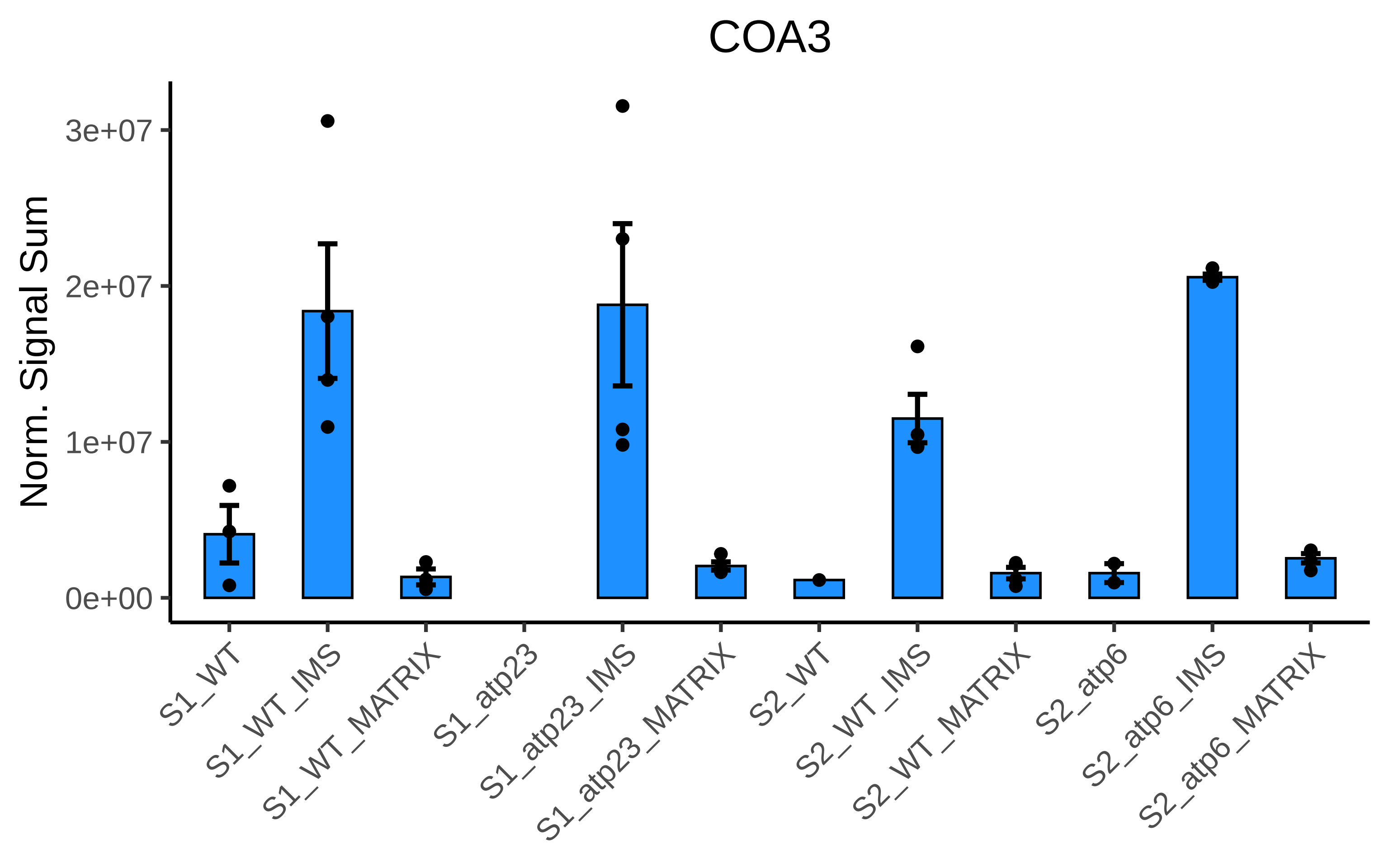

COA3
Norm. Signal Sum
3e+07
2e+07
1e+07
0e+00
S1_WT
S1_WT_IMS
S1_WT_MATRIX
S1_atp23
S1_atp23_IMS
S1_atp23_MATRIX
S2_WT
S2_WT_IMS
S2_WT_MATRIX
S2_atp6
S2_atp6_IMS
S2_atp6_MATRIX

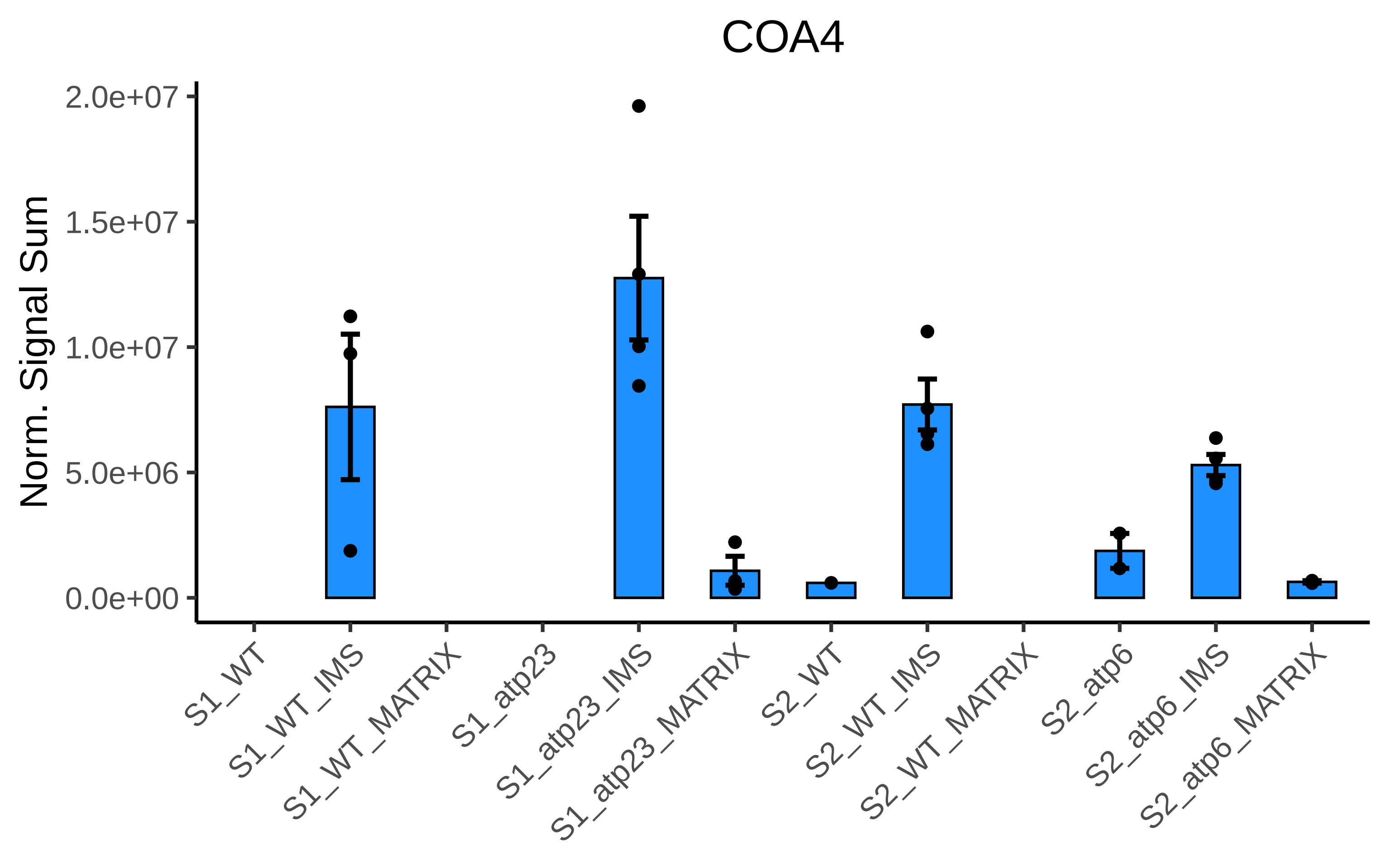

COA4
Norm. Signal Sum
2.0e+07
1.5e+07
1.0e+07
5.0e+06
0.0e+00
S1_WT
S1_WT_IMS
S1_WT_MATRIX
S1_atp23
S1_atp23_IMS
S1_atp23_MATRIX
S2_WT
S2_WT_IMS
S2_WT_MATRIX
S2_atp6
S2_atp6_IMS
S2_atp6_MATRIX

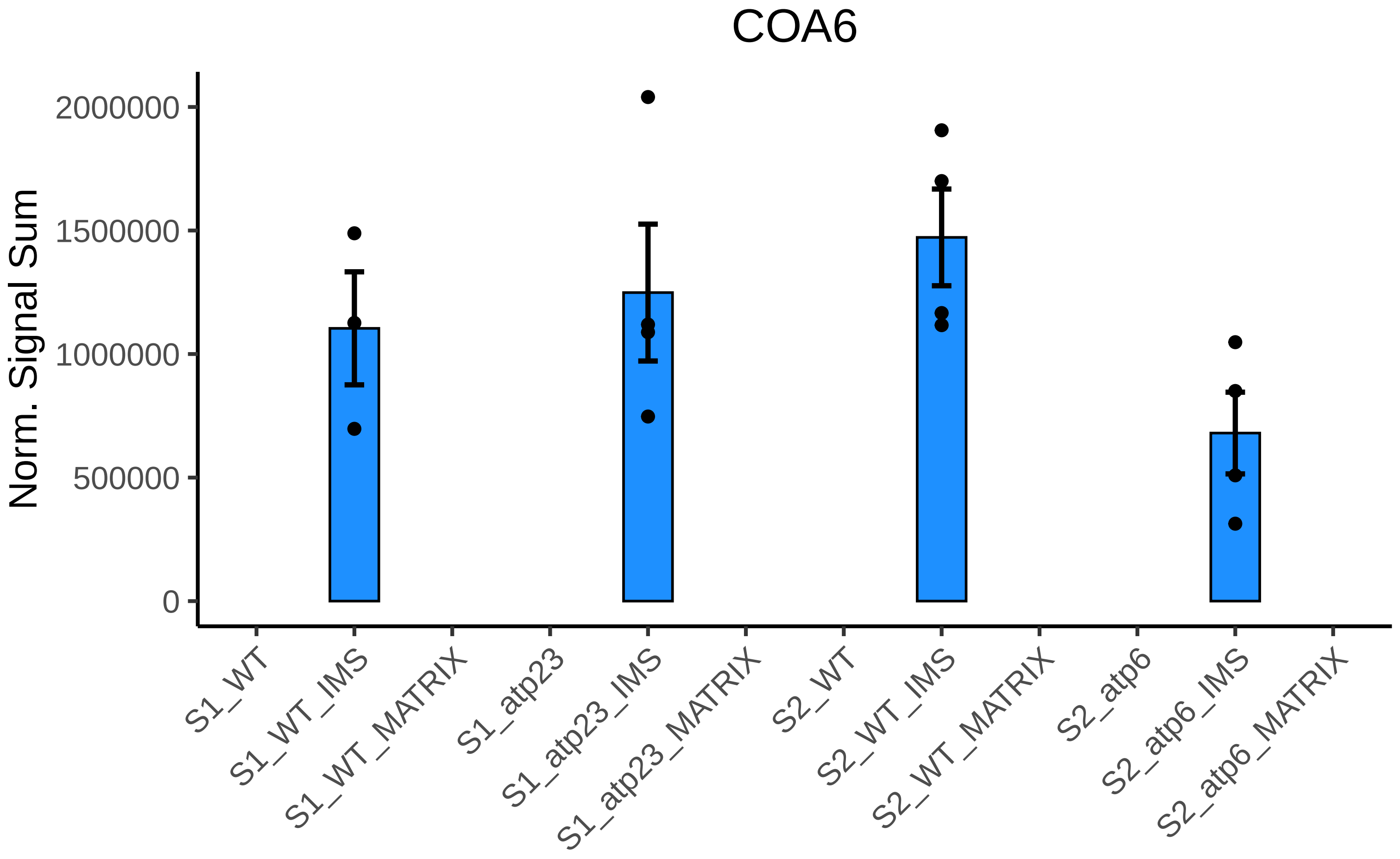
COA6
Norm. Signal Sum
2000000
1500000
1000000
500000
0
S1_WT
S1_WT_IMS
S1_WT_MATRIX
S1_atp23
S1_atp23_IMS
S1_atp23_MATRIX
S2_WT
S2_WT_IMS
S2_WT_MATRIX
S2_atp6
S2_atp6_IMS
S2_atp6_MATRIX

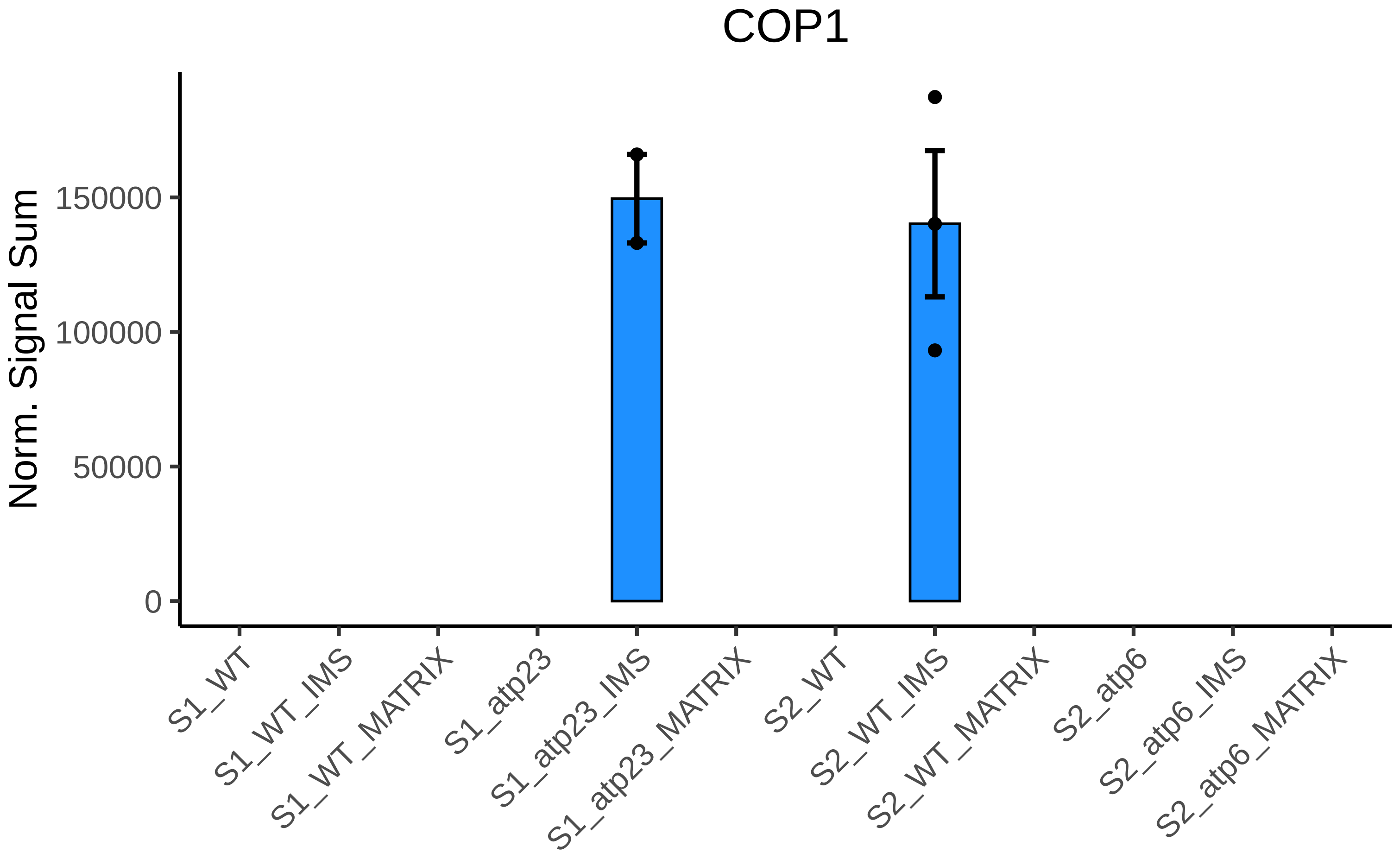

COP1
Norm. Signal Sum
150000
100000
50000
0
S1_WT
S1_WT_IMS
S1_WT_MATRIX
S1_atp23
S1_atp23_IMS
S1_atp23_MATRIX
S2_WT
S2_WT_IMS
S2_WT_MATRIX
S2_atp6
S2_atp6_IMS
S2_atp6_MATRIX

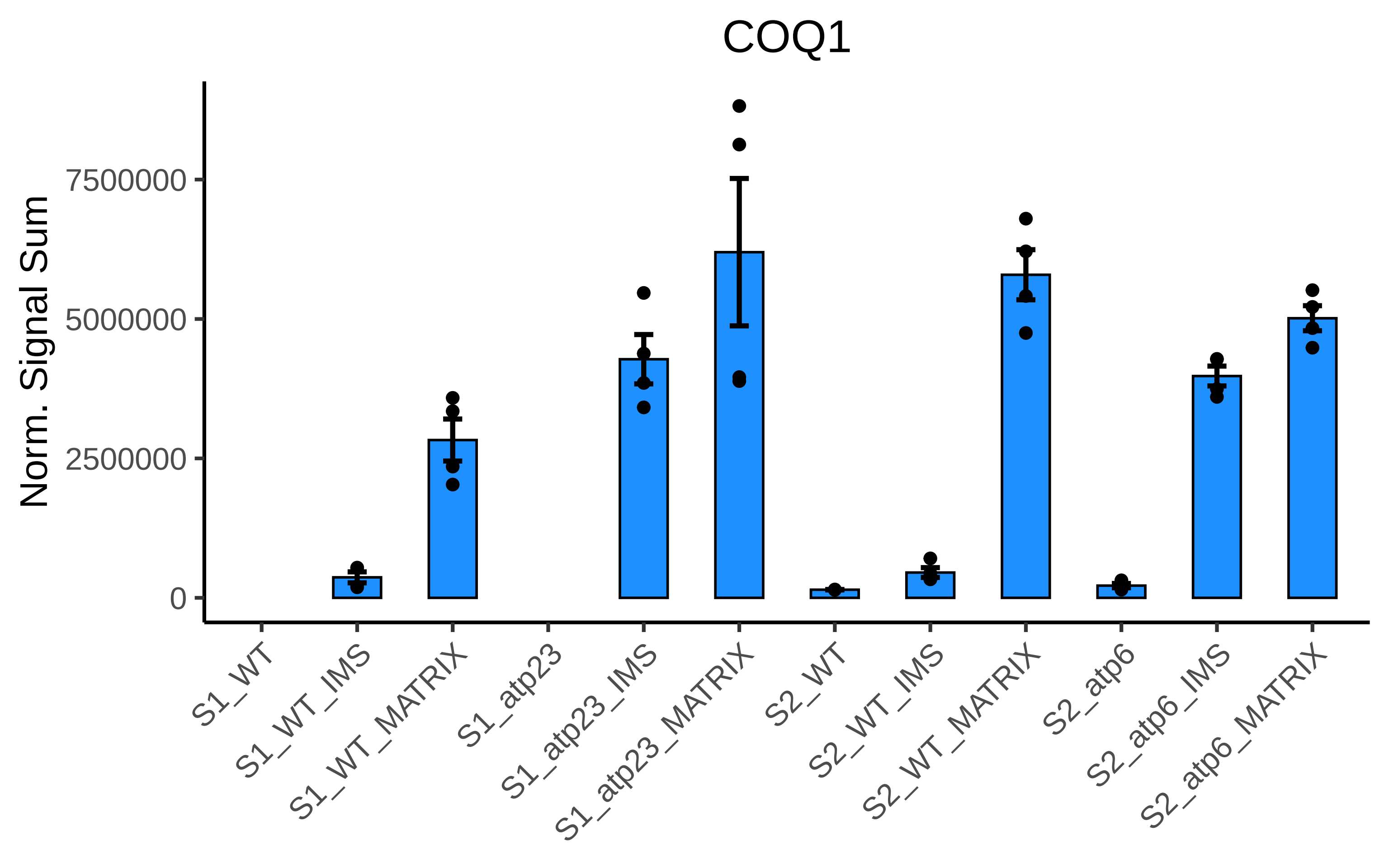

COQ1
Norm. Signal Sum
7500000
5000000
2500000
0
S1_WT
S1_WT_IMS
S1_WT_MATRIX
S1_atp23
S1_atp23_IMS
S1_atp23_MATRIX
S2_WT
S2_WT_IMS
S2_WT_MATRIX
S2_atp6
S2_atp6_IMS
S2_atp6_MATRIX

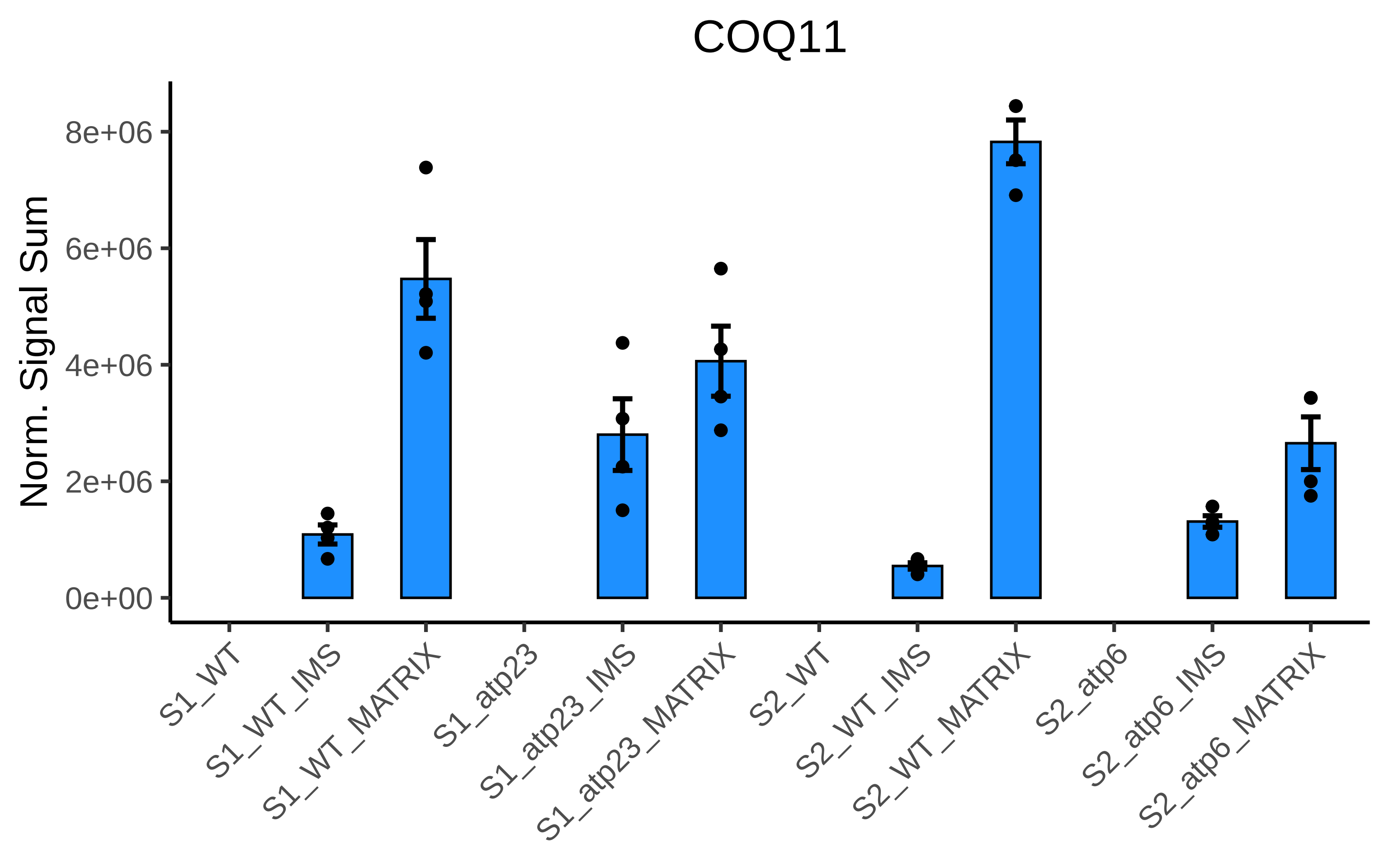

COQ11
Norm. Signal Sum
8e+06
6e+06
4e+06
2e+06
0e+00
S1_WT
S1_WT_IMS
S1_WT_MATRIX
S1_atp23
S1_atp23_IMS
S1_atp23_MATRIX
S2_WT
S2_WT_IMS
S2_WT_MATRIX
S2_atp6
S2_atp6_IMS
S2_atp6_MATRIX

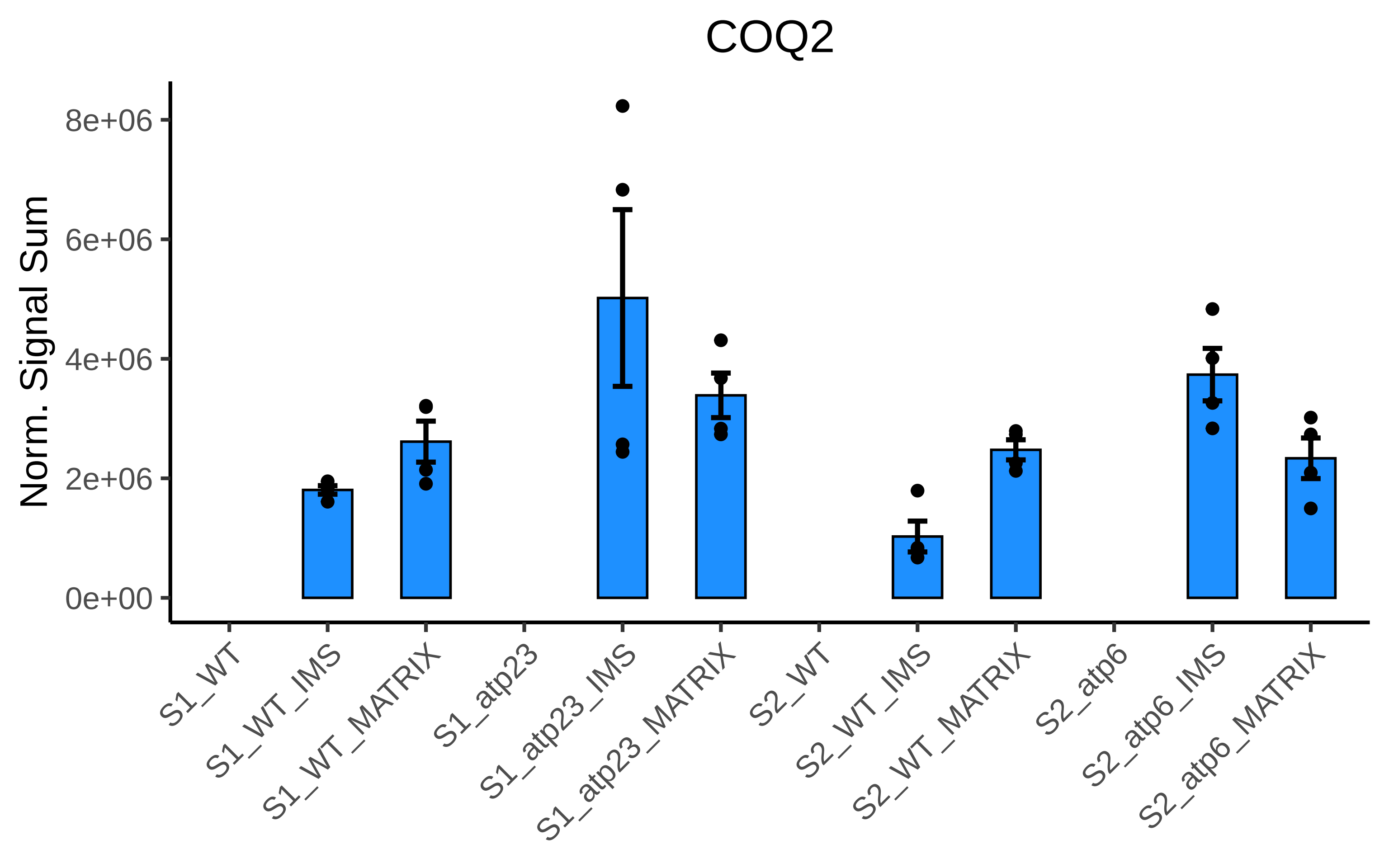
COQ2
Norm. Signal Sum
8e+06
6e+06
4e+06
2e+06
0e+00
S1_WT
S1_WT_IMS
S1_WT_MATRIX
S1_atp23
S1_atp23_IMS
S1_atp23_MATRIX
S2_WT
S2_WT_IMS
S2_WT_MATRIX
S2_atp6
S2_atp6_IMS
S2_atp6_MATRIX

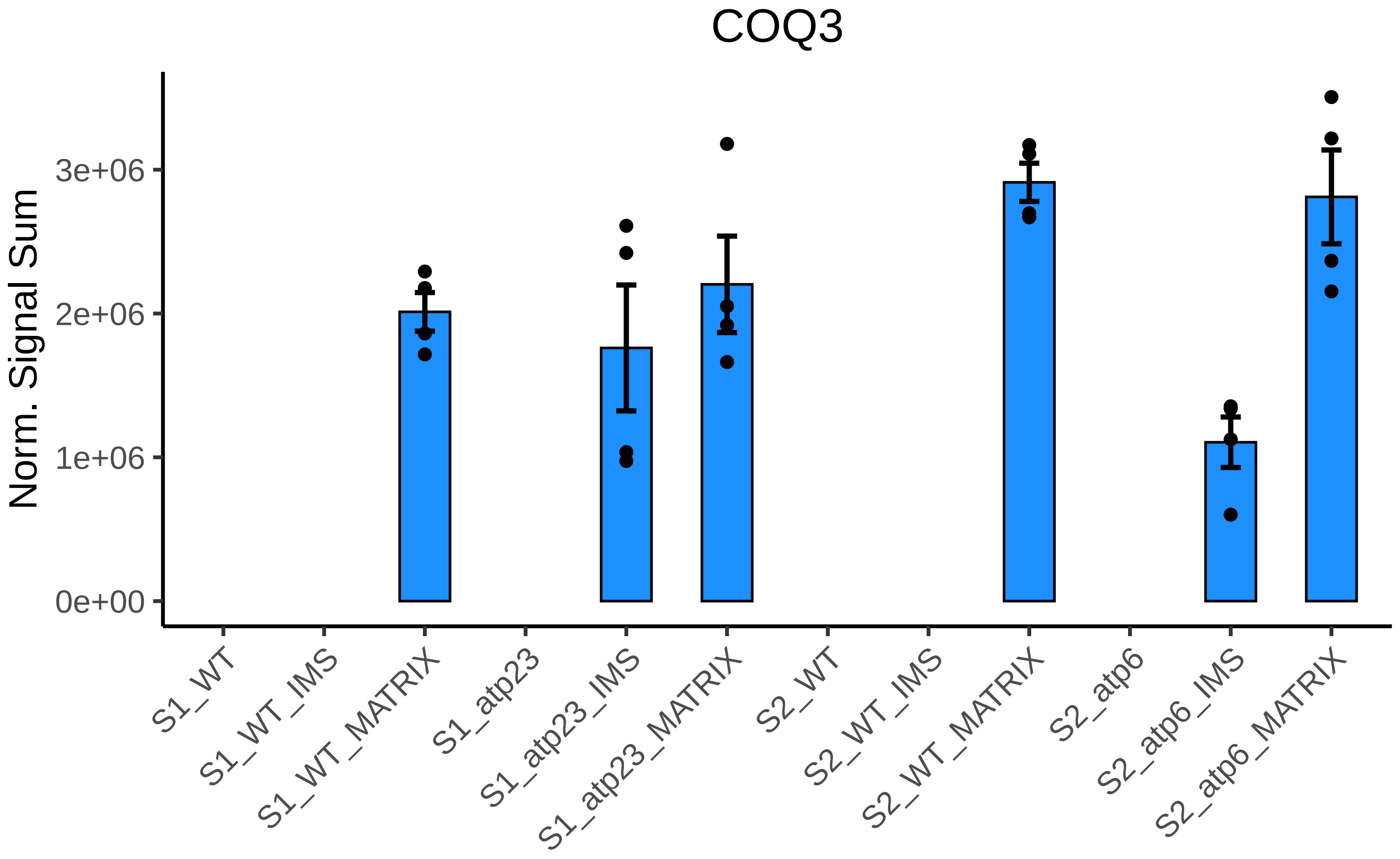

COQ3
Norm. Signal Sum
3e+06
2e+06
1e+06
0e+00
S1_WT
S1_WT_IMS
S1_WT_MATRIX
S1_atp23
S1_atp23_IMS
S1_atp23_MATRIX
S2_WT
S2_WT_IMS
S2_WT_MATRIX
S2_atp6
S2_atp6_IMS
S2_atp6_MATRIX

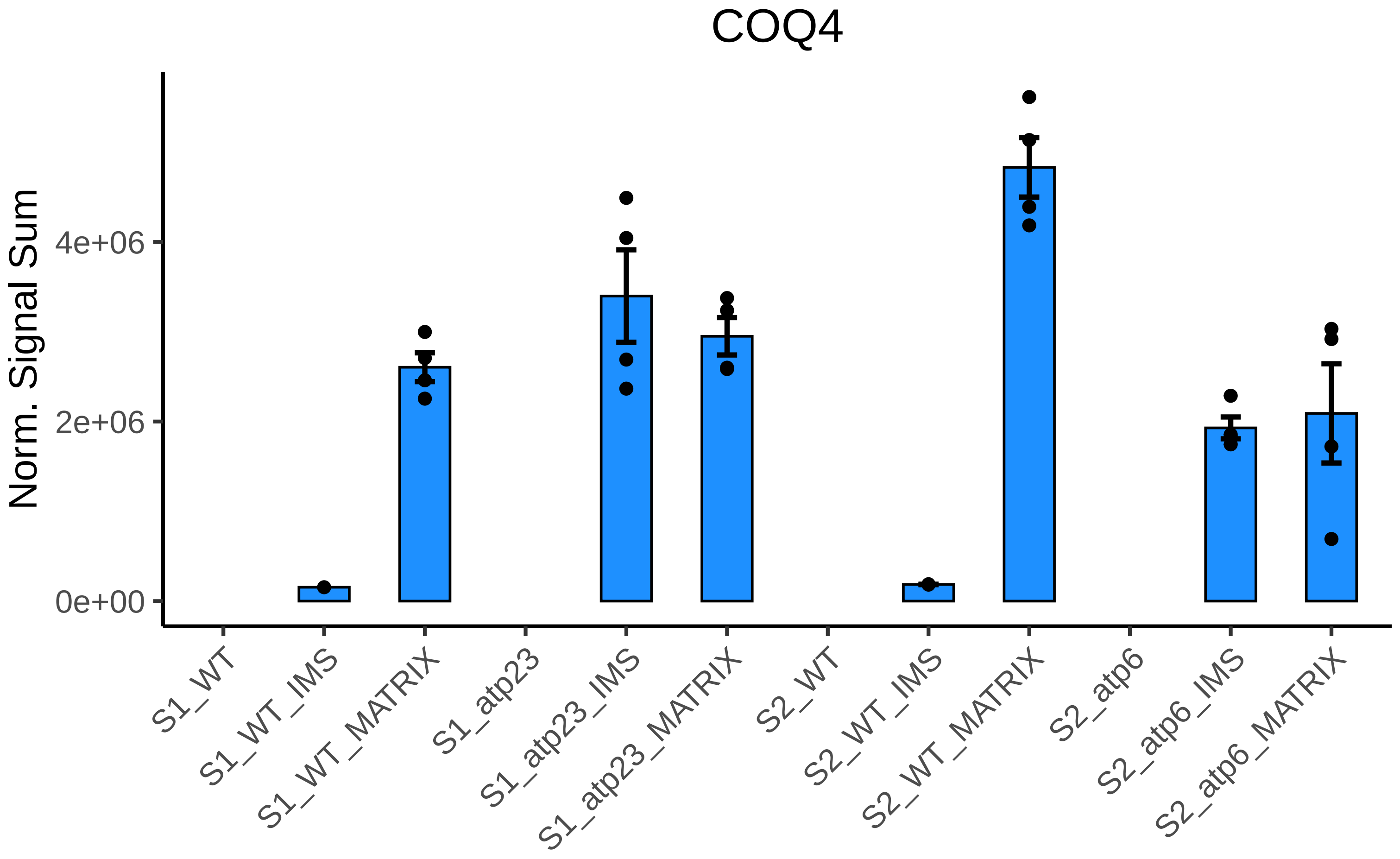

COQ4
Norm. Signal Sum
4e+06
2e+06
0e+00
S1_WT
S1_WT_IMS
S1_WT_MATRIX
S1_atp23
S1_atp23_IMS
S1_atp23_MATRIX
S2_WT
S2_WT_IMS
S2_WT_MATRIX
S2_atp6
S2_atp6_IMS
S2_atp6_MATRIX

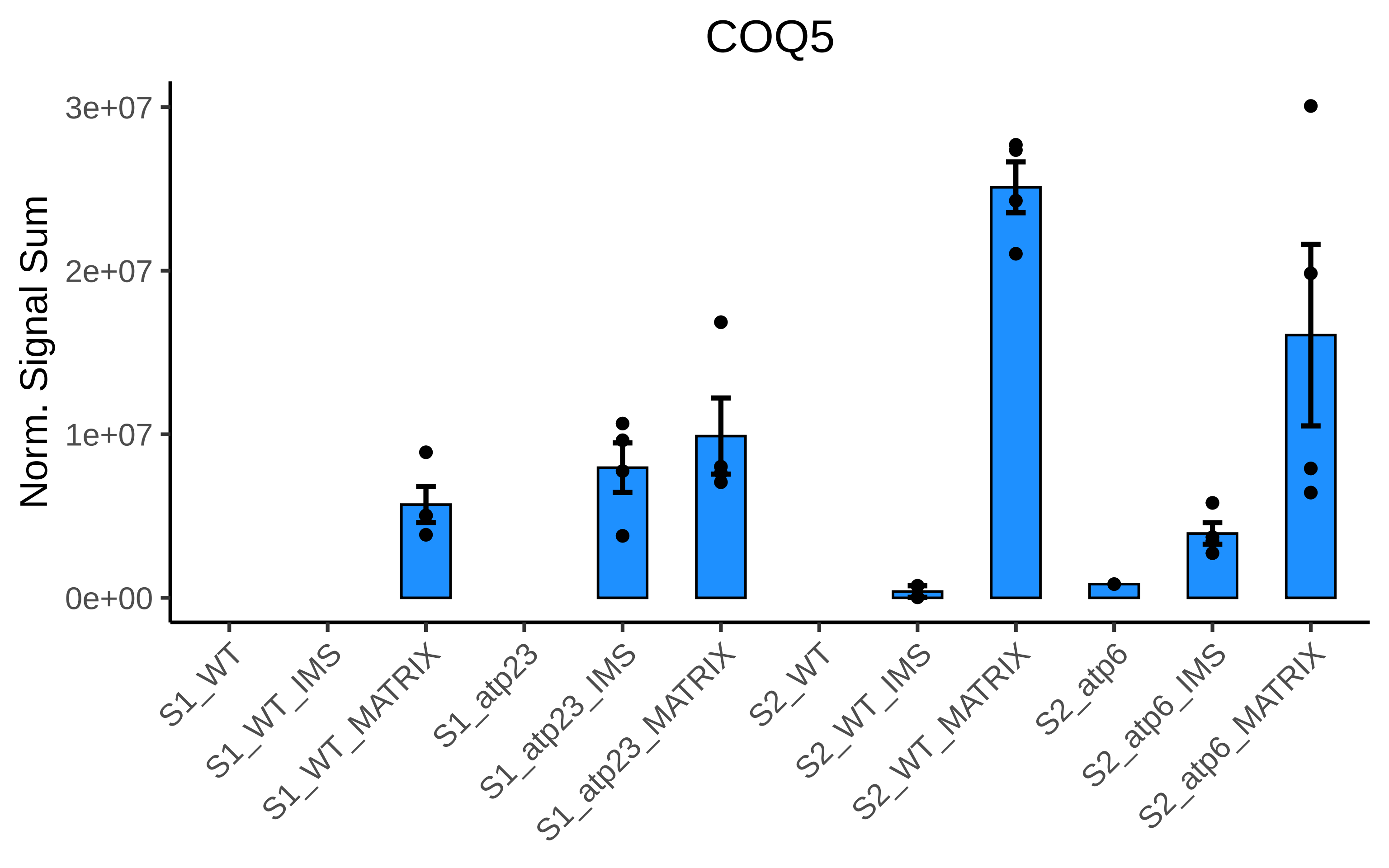

COQ5
Norm. Signal Sum
3e+07
2e+07
1e+07
0e+00
S1_WT
S1_WT_IMS
S1_WT_MATRIX
S1_atp23
S1_atp23_IMS
S1_atp23_MATRIX
S2_WT
S2_WT_IMS
S2_WT_MATRIX
S2_atp6
S2_atp6_IMS
S2_atp6_MATRIX

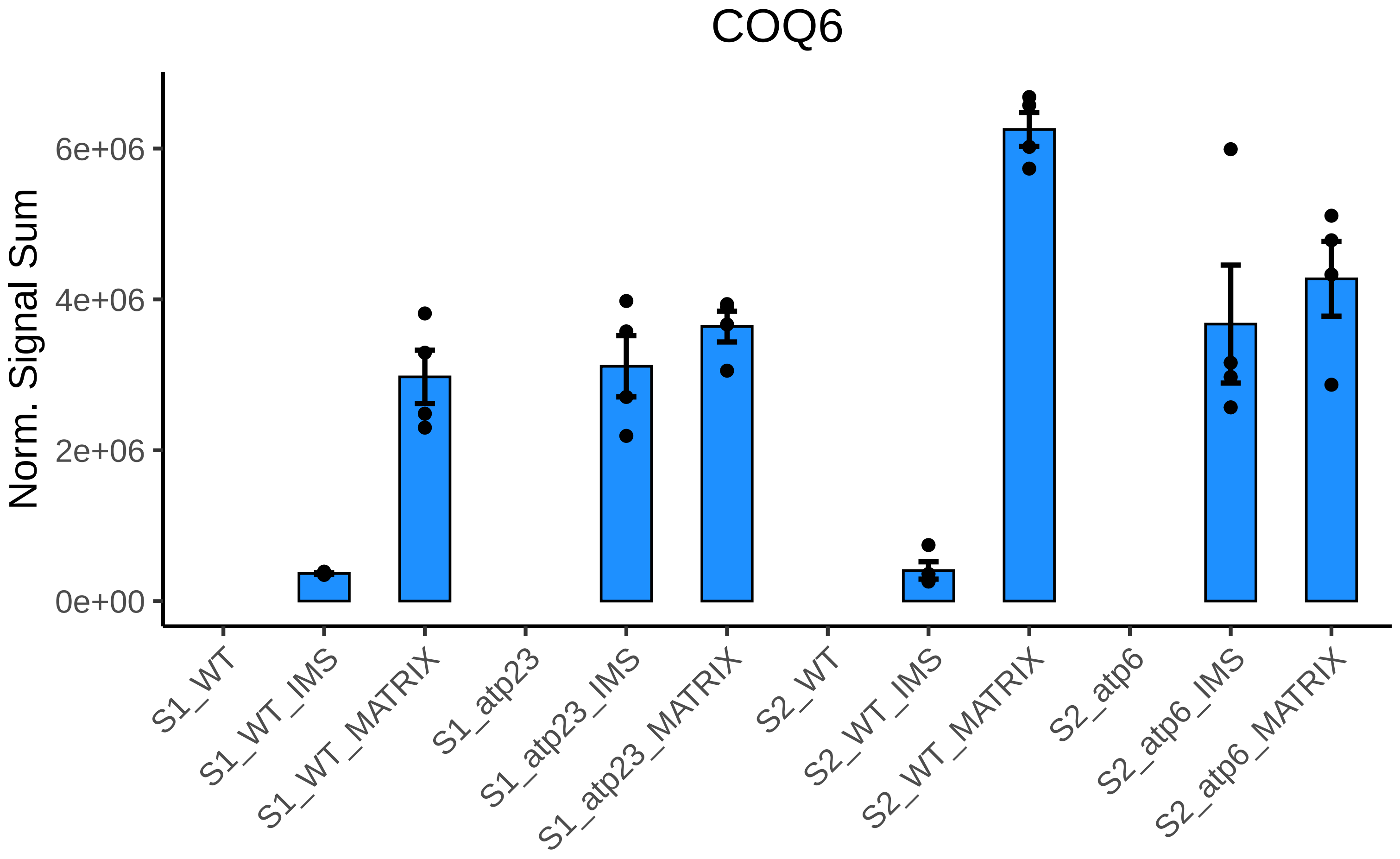
COQ6
Norm. Signal Sum
6e+06
4e+06
2e+06
0e+00
S1_WT
S1_WT_IMS
S1_WT_MATRIX
S1_atp23
S1_atp23_IMS
S1_atp23_MATRIX
S2_WT
S2_WT_IMS
S2_WT_MATRIX
S2_atp6
S2_atp6_IMS
S2_atp6_MATRIX

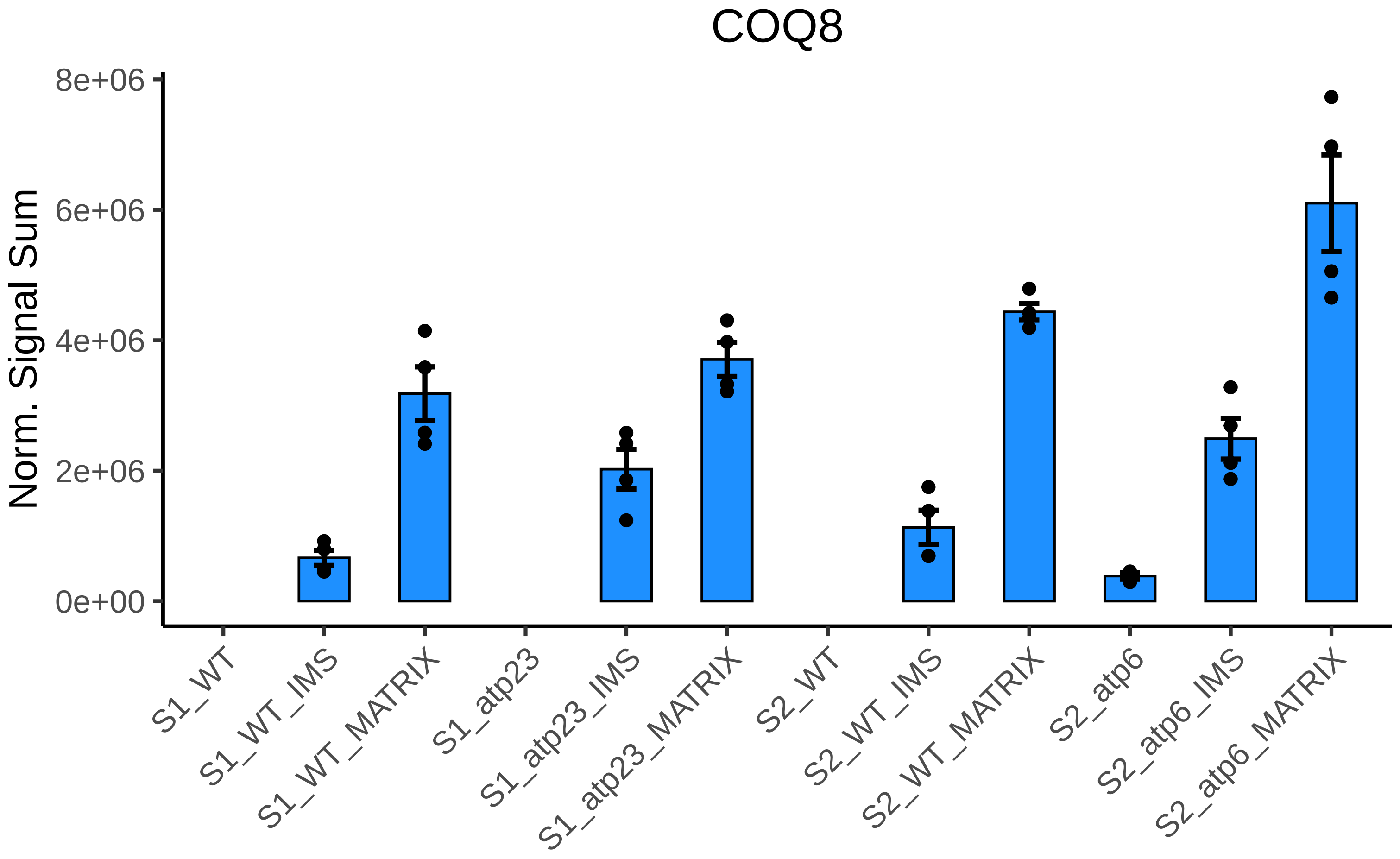
COQ8
Norm. Signal Sum
8e+06
6e+06
4e+06
2e+06
0e+00
S1_WT
S1_WT_IMS
S1_WT_MATRIX
S1_atp23
S1_atp23_IMS
S1_atp23_MATRIX
S2_WT
S2_WT_IMS
S2_WT_MATRIX
S2_atp6
S2_atp6_IMS
S2_atp6_MATRIX

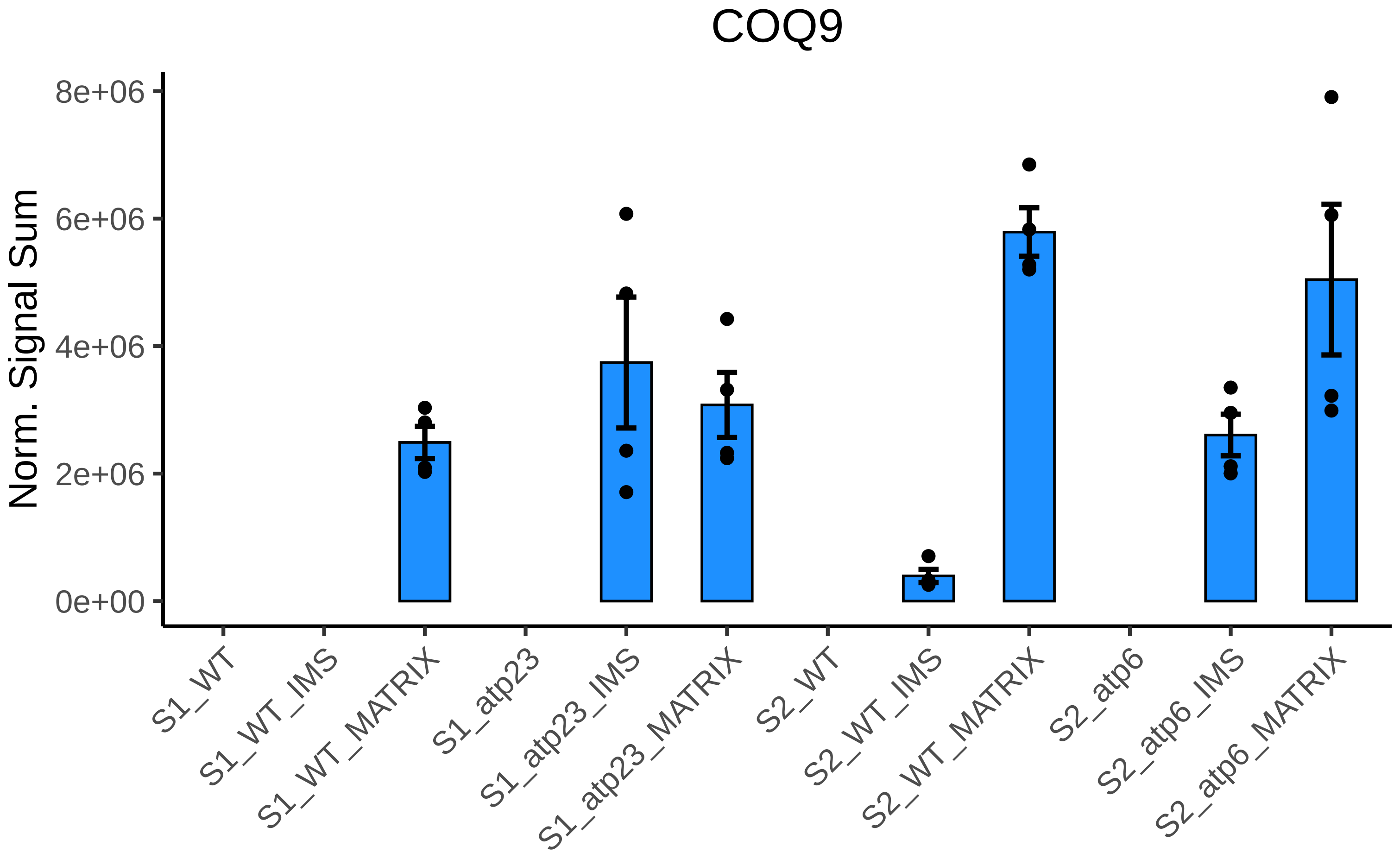

COQ9
Norm. Signal Sum
8e+06
6e+06
4e+06
2e+06
0e+00
S1_WT
S1_WT_IMS
S1_WT_MATRIX
S1_atp23
S1_atp23_IMS
S1_atp23_MATRIX
S2_WT
S2_WT_IMS
S2_WT_MATRIX
S2_atp6
S2_atp6_IMS
S2_atp6_MATRIX

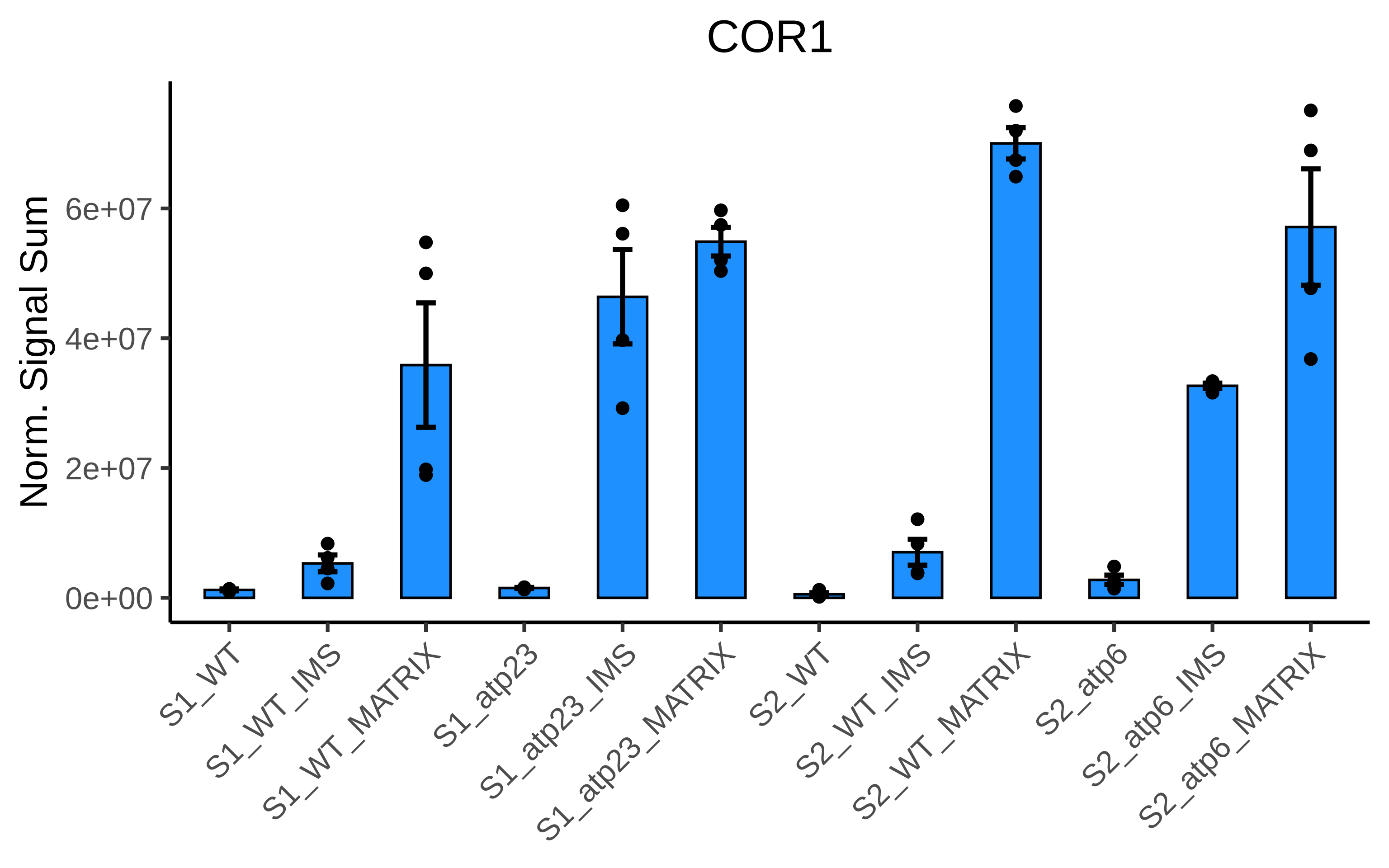
COR1
Norm. Signal Sum
6e+07
4e+07
2e+07
0e+00
S1_WT
S1_WT_IMS
S1_WT_MATRIX
S1_atp23
S1_atp23_IMS
S1_atp23_MATRIX
S2_WT
S2_WT_IMS
S2_WT_MATRIX
S2_atp6
S2_atp6_IMS
S2_atp6_MATRIX

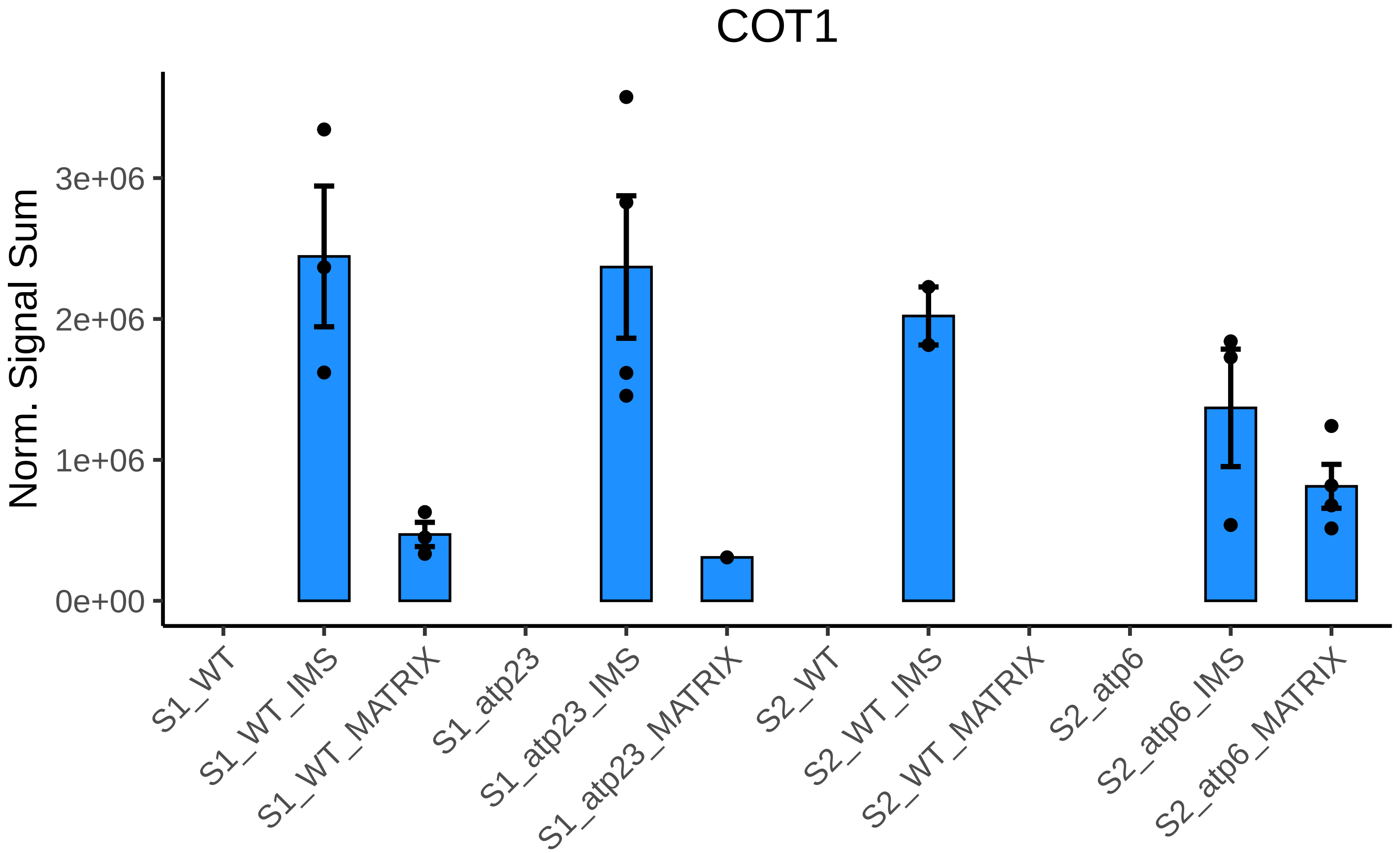
COT1
Norm. Signal Sum
3e+06
2e+06
1e+06
0e+00
S1_WT
S1_WT_IMS
S1_WT_MATRIX
S1_atp23
S1_atp23_IMS
S1_atp23_MATRIX
S2_WT
S2_WT_IMS
S2_WT_MATRIX
S2_atp6
S2_atp6_IMS
S2_atp6_MATRIX

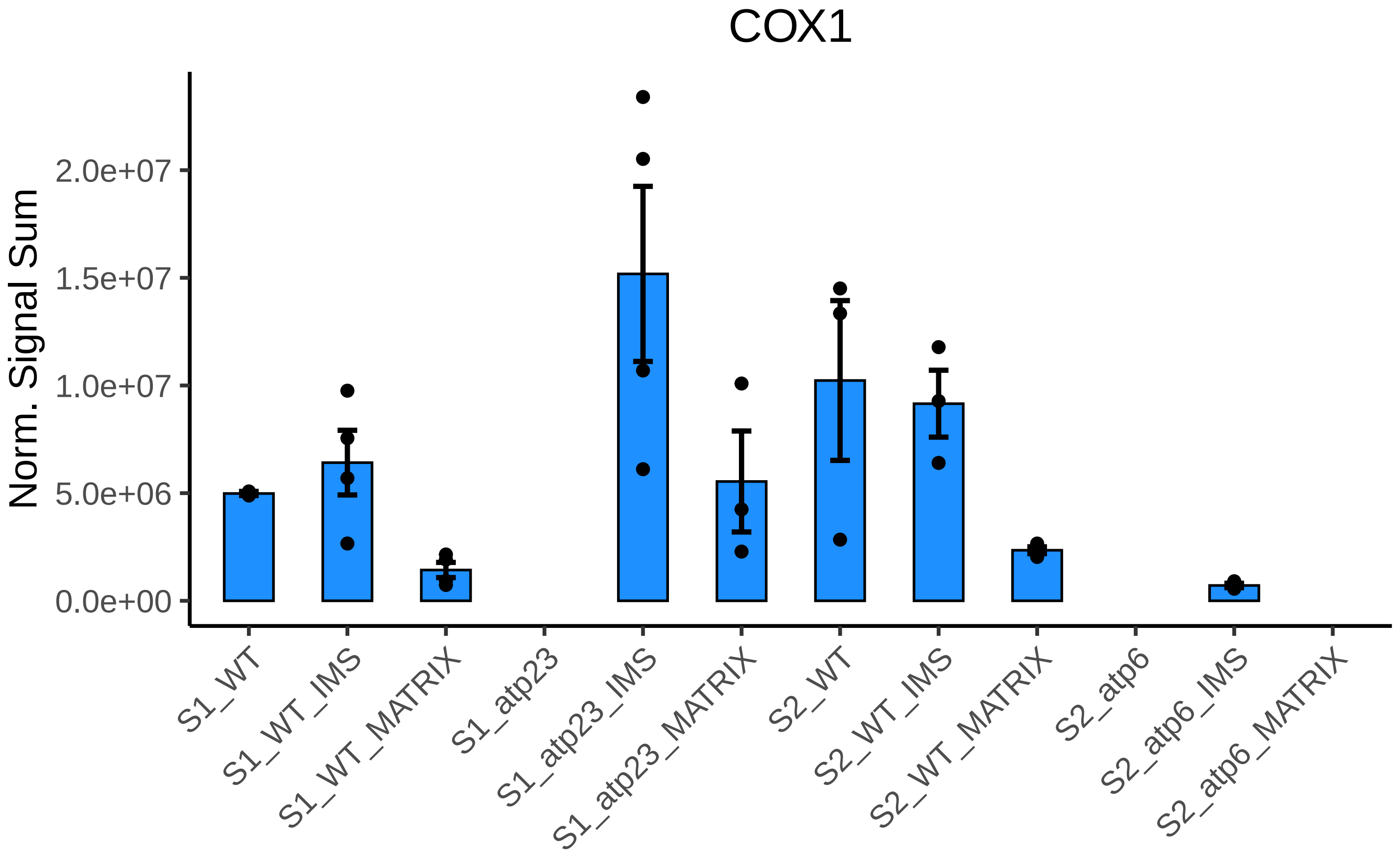

COX1
Norm. Signal Sum
2.0e+07
1.5e+07
1.0e+07
5.0e+06
0.0e+00
S1_WT
S1_WT_IMS
S1_WT_MATRIX
S1_atp23
S1_atp23_IMS
S1_atp23_MATRIX
S2_WT
S2_WT_IMS
S2_WT_MATRIX
S2_atp6
S2_atp6_IMS
S2_atp6_MATRIX

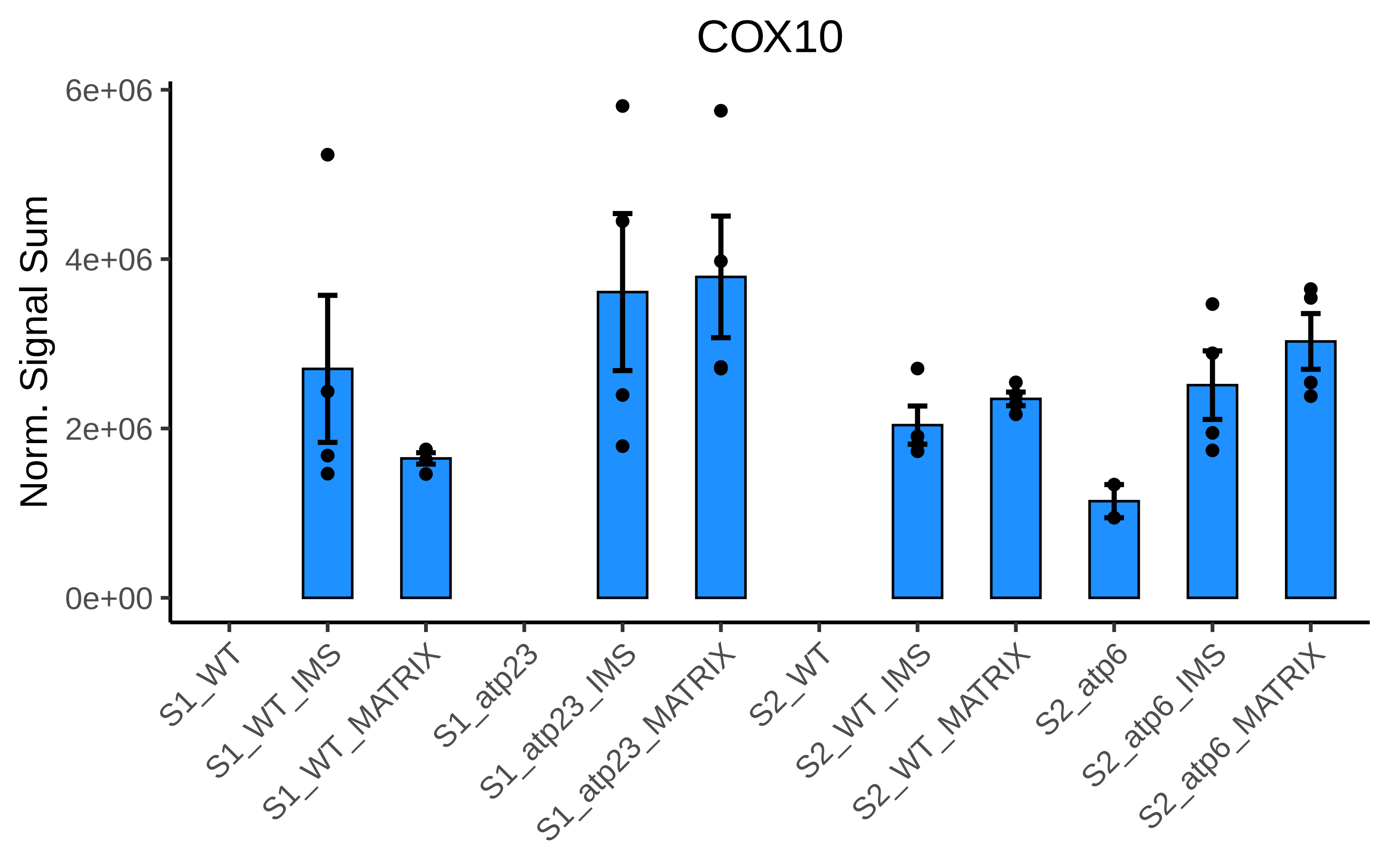

COX10
Norm. Signal Sum
6e+06
4e+06
2e+06
0e+00
S1_WT
S1_WT_IMS
S1_WT_MATRIX
S1_atp23
S1_atp23_IMS
S1_atp23_MATRIX
S2_WT
S2_WT_IMS
S2_WT_MATRIX
S2_atp6
S2_atp6_IMS
S2_atp6_MATRIX

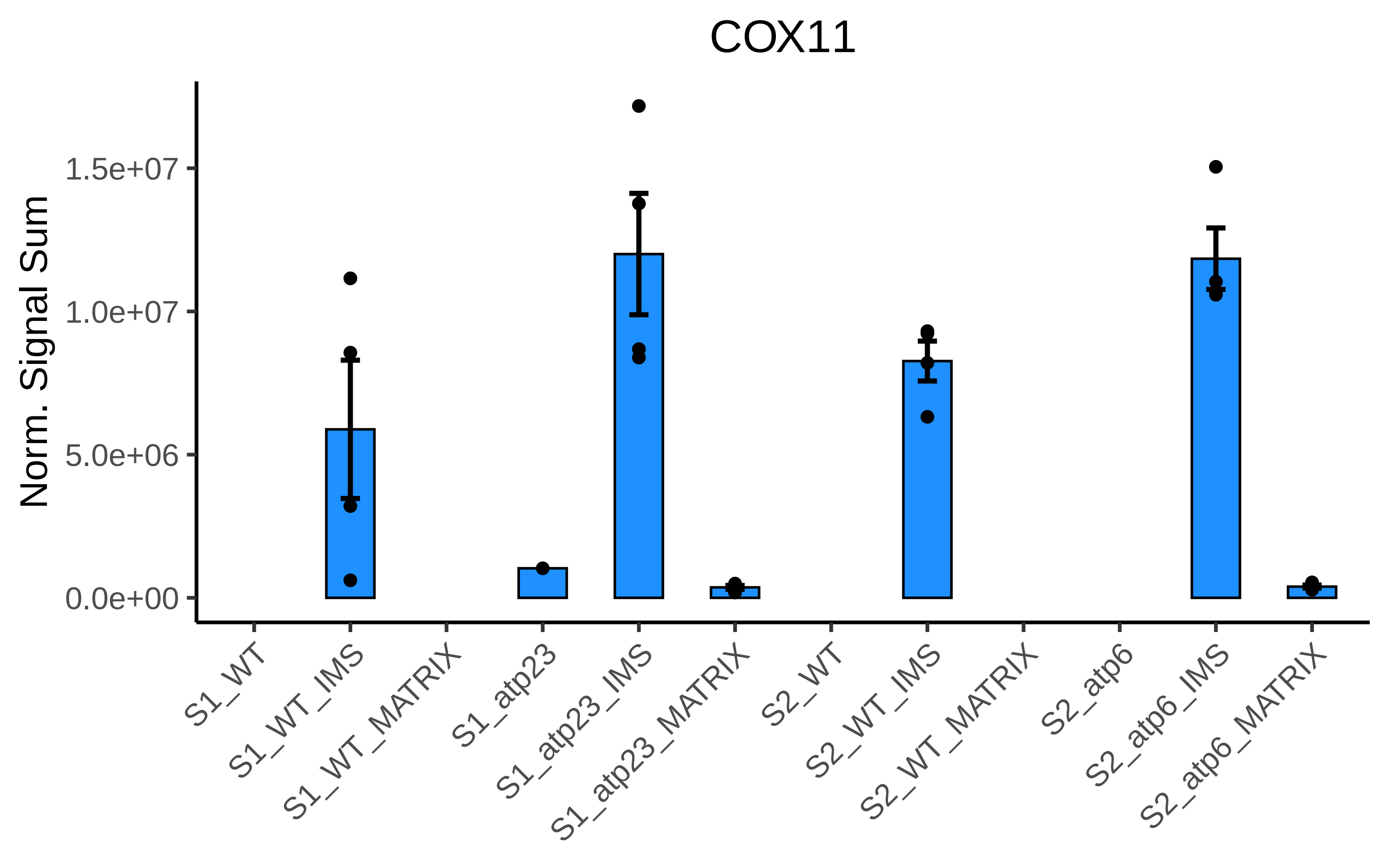
COX11
Norm. Signal Sum
1.5e+07
1.0e+07
5.0e+06
0.0e+00
S1_WT
S1_WT_IMS
S1_WT_MATRIX
S1_atp23
S1_atp23_IMS
S1_atp23_MATRIX
S2_WT
S2_WT_IMS
S2_WT_MATRIX
S2_atp6
S2_atp6_IMS
S2_atp6_MATRIX

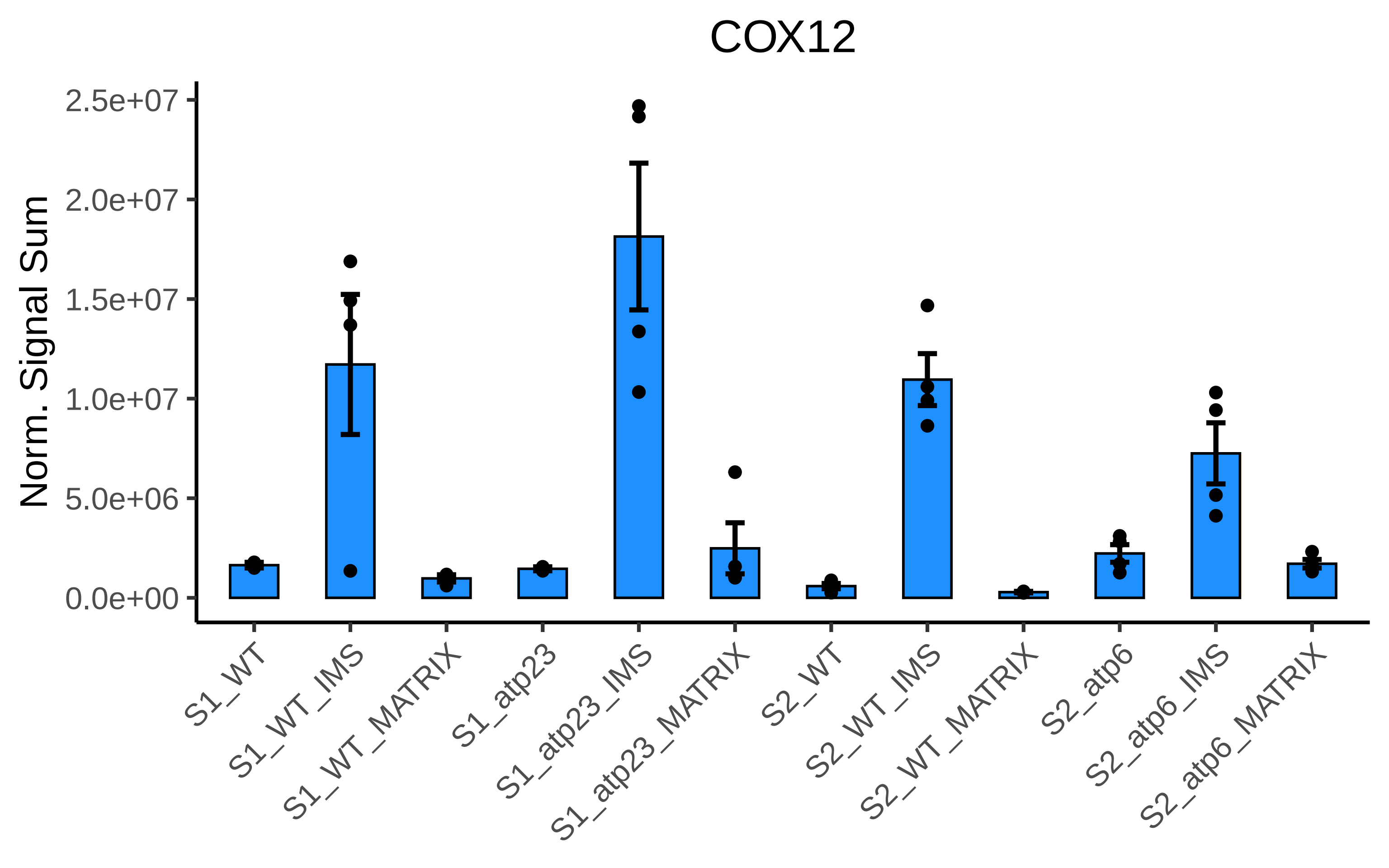

COX12
Norm. Signal Sum
2.5e+07
2.0e+07
1.5e+07
1.0e+07
5.0e+06
0.0e+00
S1_WT
S1_WT_IMS
S1_WT_MATRIX
S1_atp23
S1_atp23_IMS
S1_atp23_MATRIX
S2_WT
S2_WT_IMS
S2_WT_MATRIX
S2_atp6
S2_atp6_IMS
S2_atp6_MATRIX

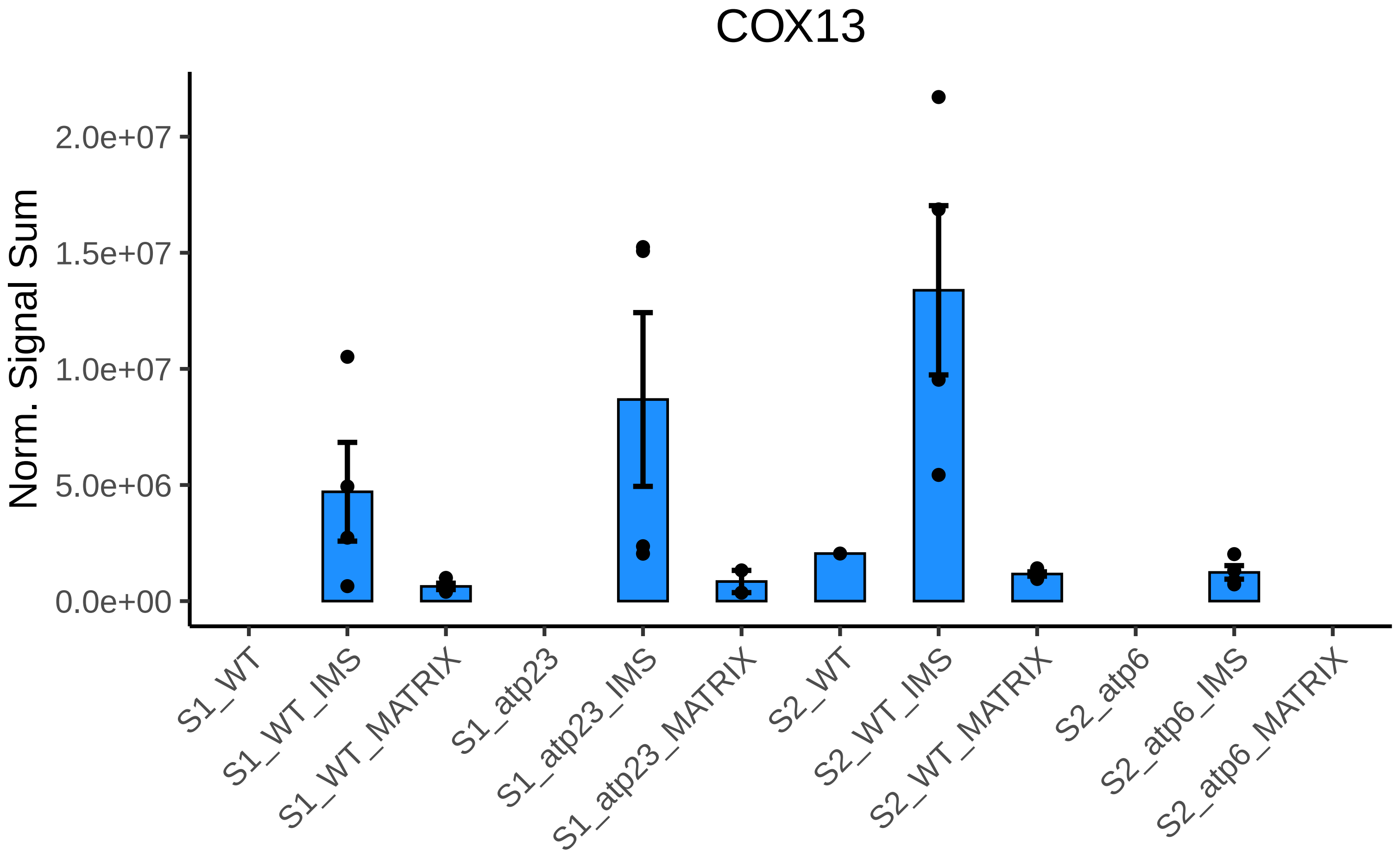
COX13
Norm. Signal Sum
2.0e+07
1.5e+07
1.0e+07
5.0e+06
0.0e+00
S1_WT
S1_WT_IMS
S1_WT_MATRIX
S1_atp23
S1_atp23_IMS
S1_atp23_MATRIX
S2_WT
S2_WT_IMS
S2_WT_MATRIX
S2_atp6
S2_atp6_IMS
S2_atp6_MATRIX

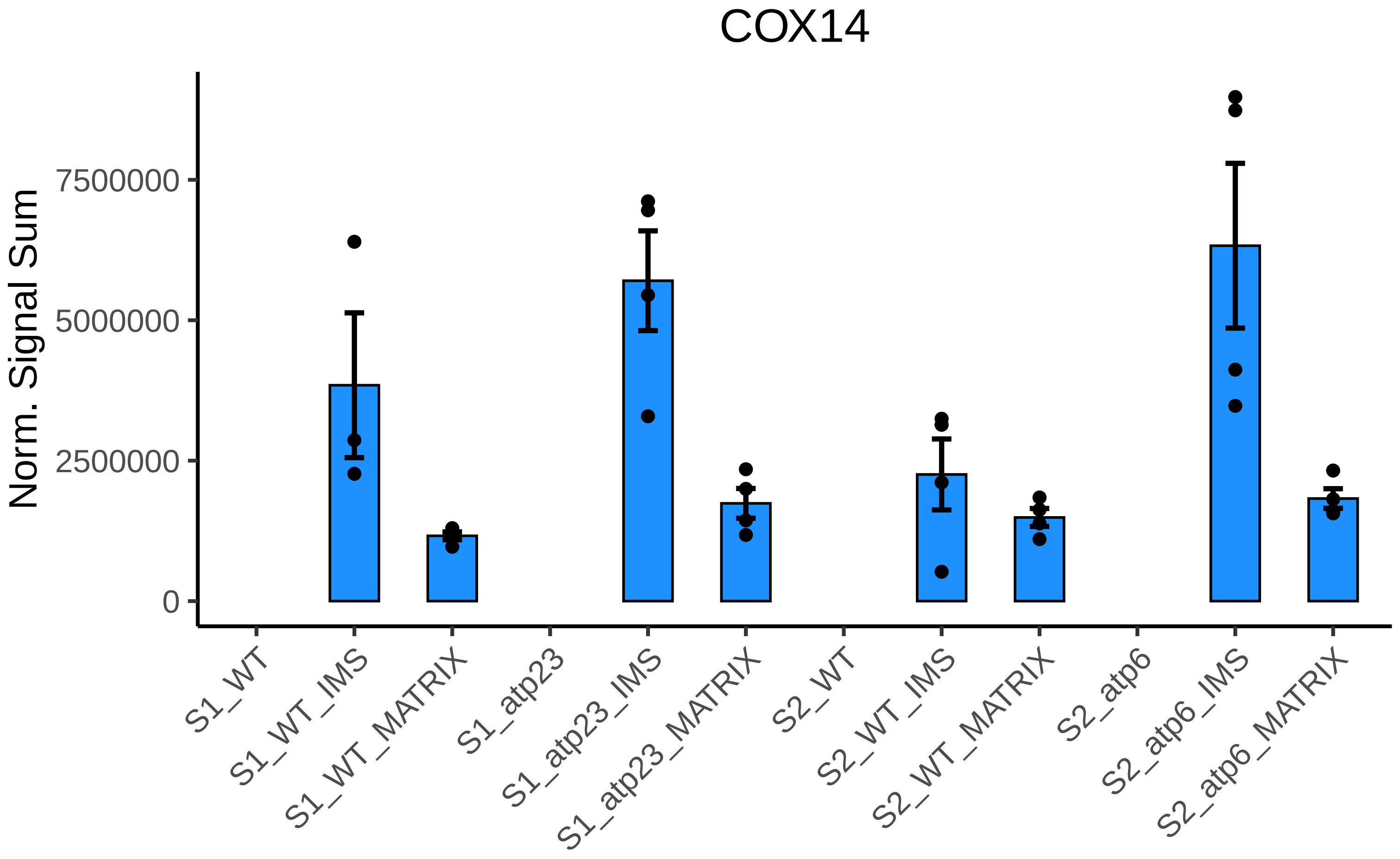

COX14
Norm. Signal Sum
7500000
5000000
2500000
0
S1_WT
S1_WT_IMS
S1_WT_MATRIX
S1_atp23
S1_atp23_IMS
S1_atp23_MATRIX
S2_WT
S2_WT_IMS
S2_WT_MATRIX
S2_atp6
S2_atp6_IMS
S2_atp6_MATRIX

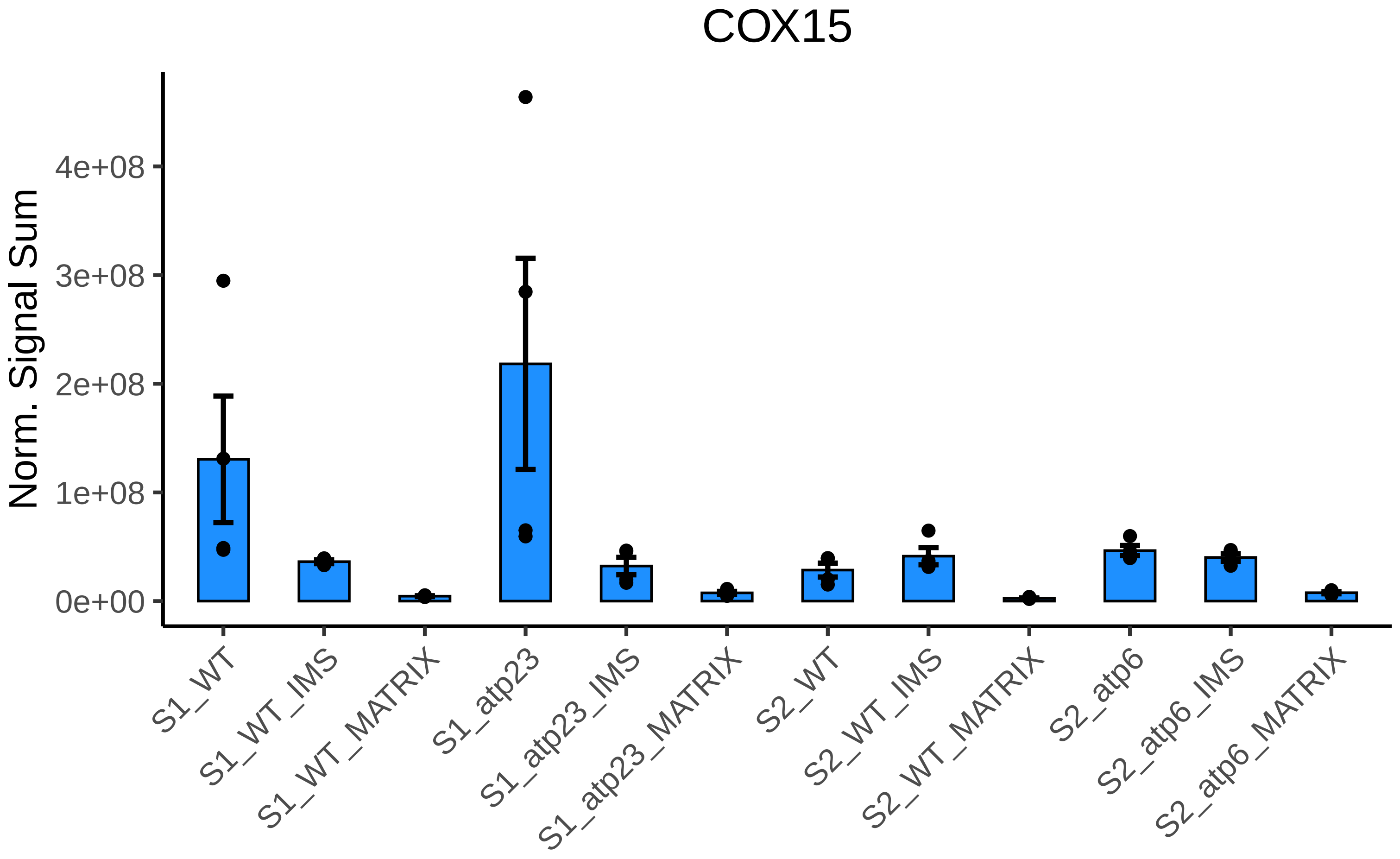
COX15
Norm. Signal Sum
4e+08
3e+08
2e+08
1e+08
0e+00
S1_WT
S1_WT_IMS
S1_WT_MATRIX
S1_atp23
S1_atp23_IMS
S1_atp23_MATRIX
S2_WT
S2_WT_IMS
S2_WT_MATRIX
S2_atp6
S2_atp6_IMS
S2_atp6_MATRIX

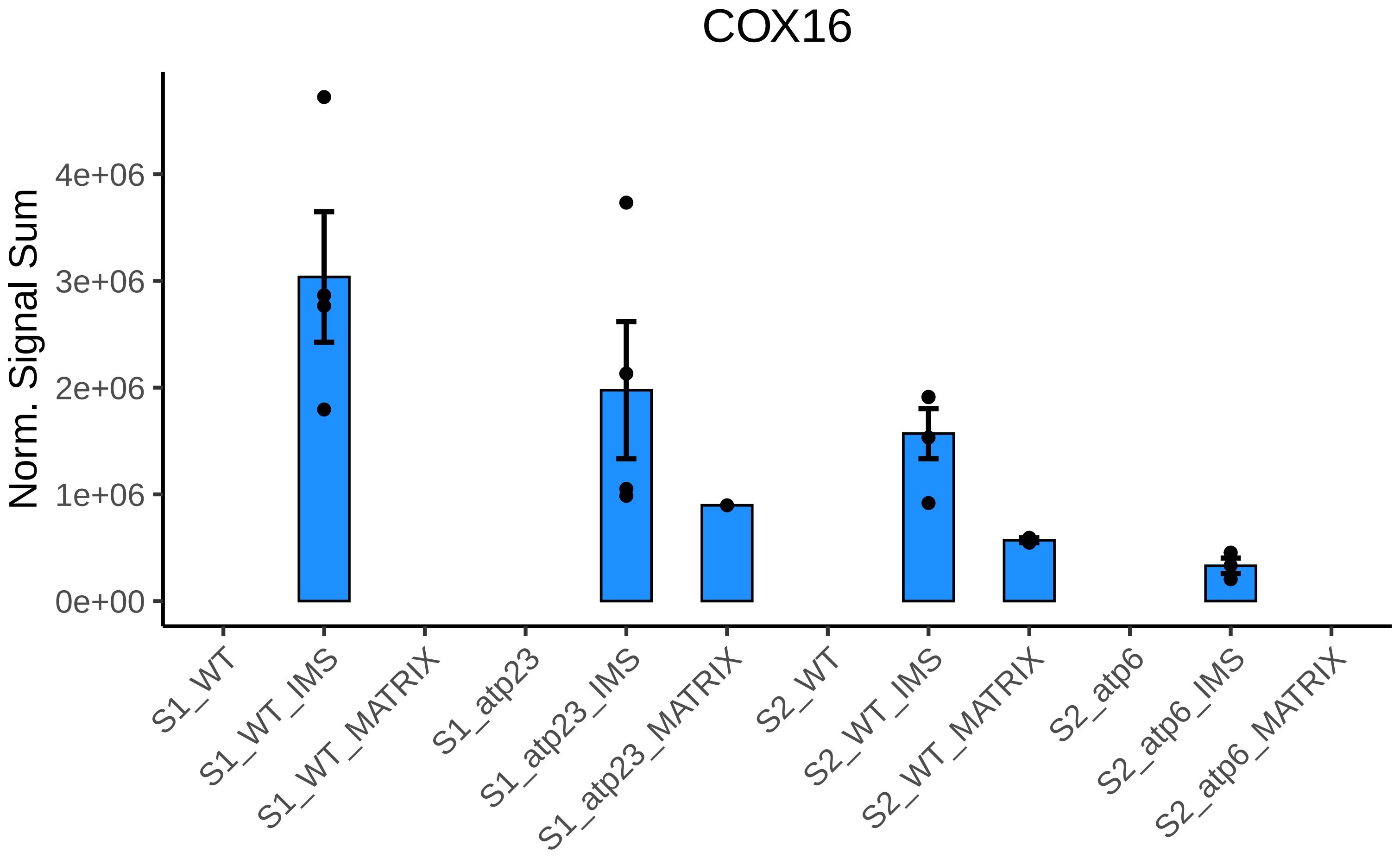
COX16
Norm. Signal Sum
4e+06
3e+06
2e+06
1e+06
0e+00
S1_WT
S1_WT_IMS
S1_WT_MATRIX
S1_atp23
S1_atp23_IMS
S1_atp23_MATRIX
S2_WT
S2_WT_IMS
S2_WT_MATRIX
S2_atp6
S2_atp6_IMS
S2_atp6_MATRIX

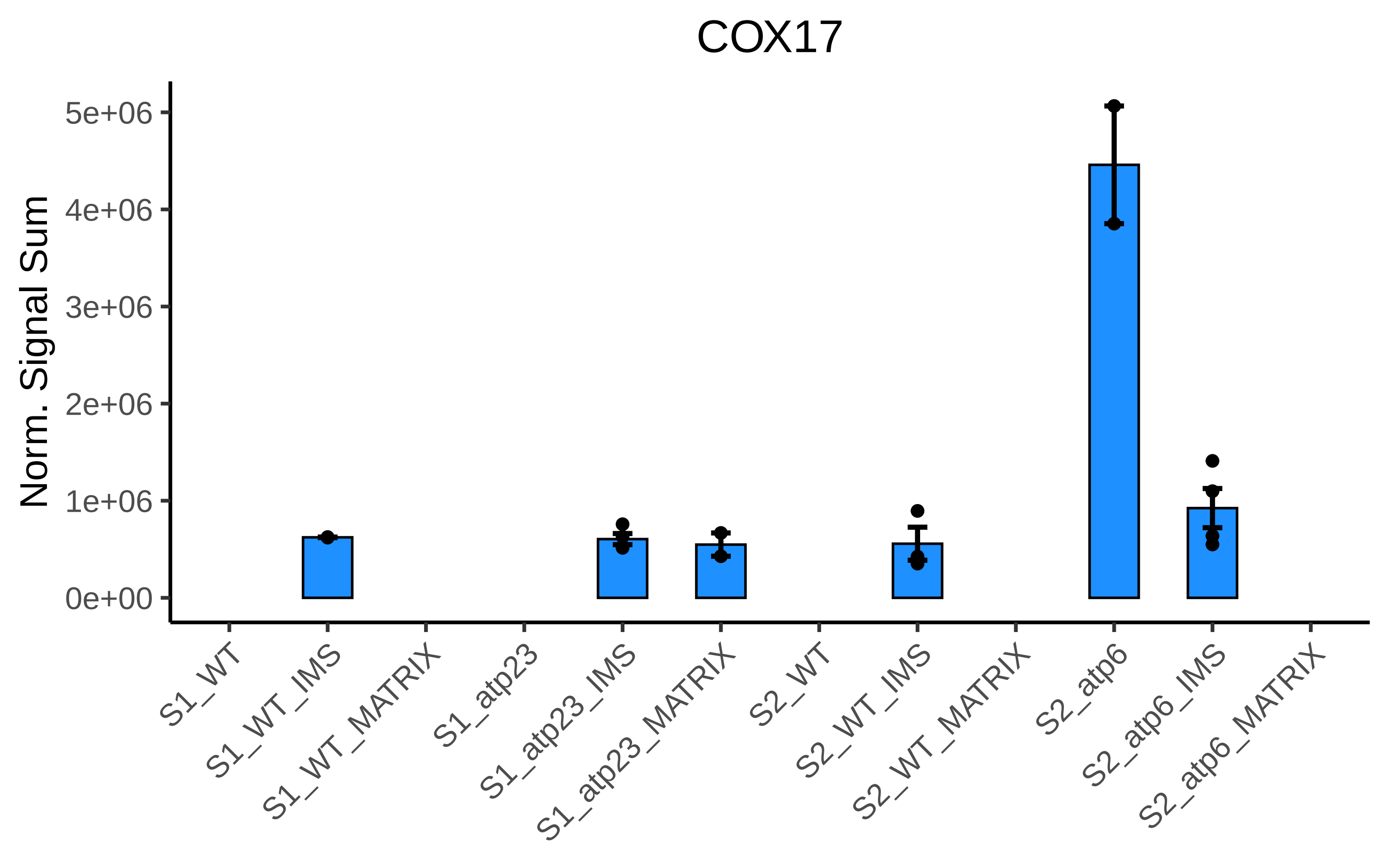
COX17
Norm. Signal Sum
5e+06
4e+06
3e+06
2e+06
1e+06
0e+00
S1_WT
S1_WT_IMS
S1_WT_MATRIX
S1_atp23
S1_atp23_IMS
S1_atp23_MATRIX
S2_WT
S2_WT_IMS
S2_WT_MATRIX
S2_atp6
S2_atp6_IMS
S2_atp6_MATRIX

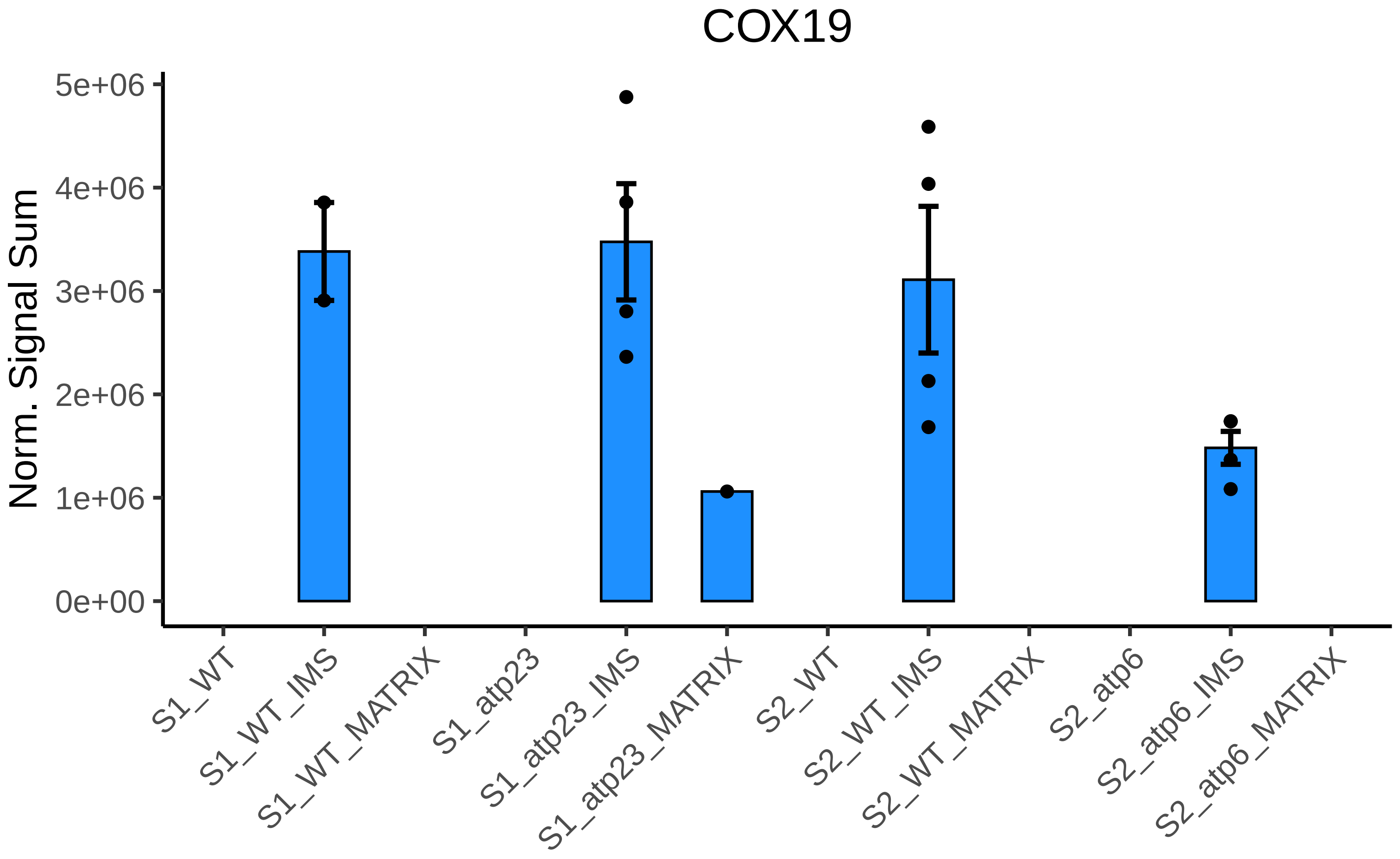
COX19
Norm. Signal Sum
5e+06
4e+06
3e+06
2e+06
1e+06
0e+00
S1_WT
S1_WT_IMS
S1_WT_MATRIX
S1_atp23
S1_atp23_IMS
S1_atp23_MATRIX
S2_WT
S2_WT_IMS
S2_WT_MATRIX
S2_atp6
S2_atp6_IMS
S2_atp6_MATRIX

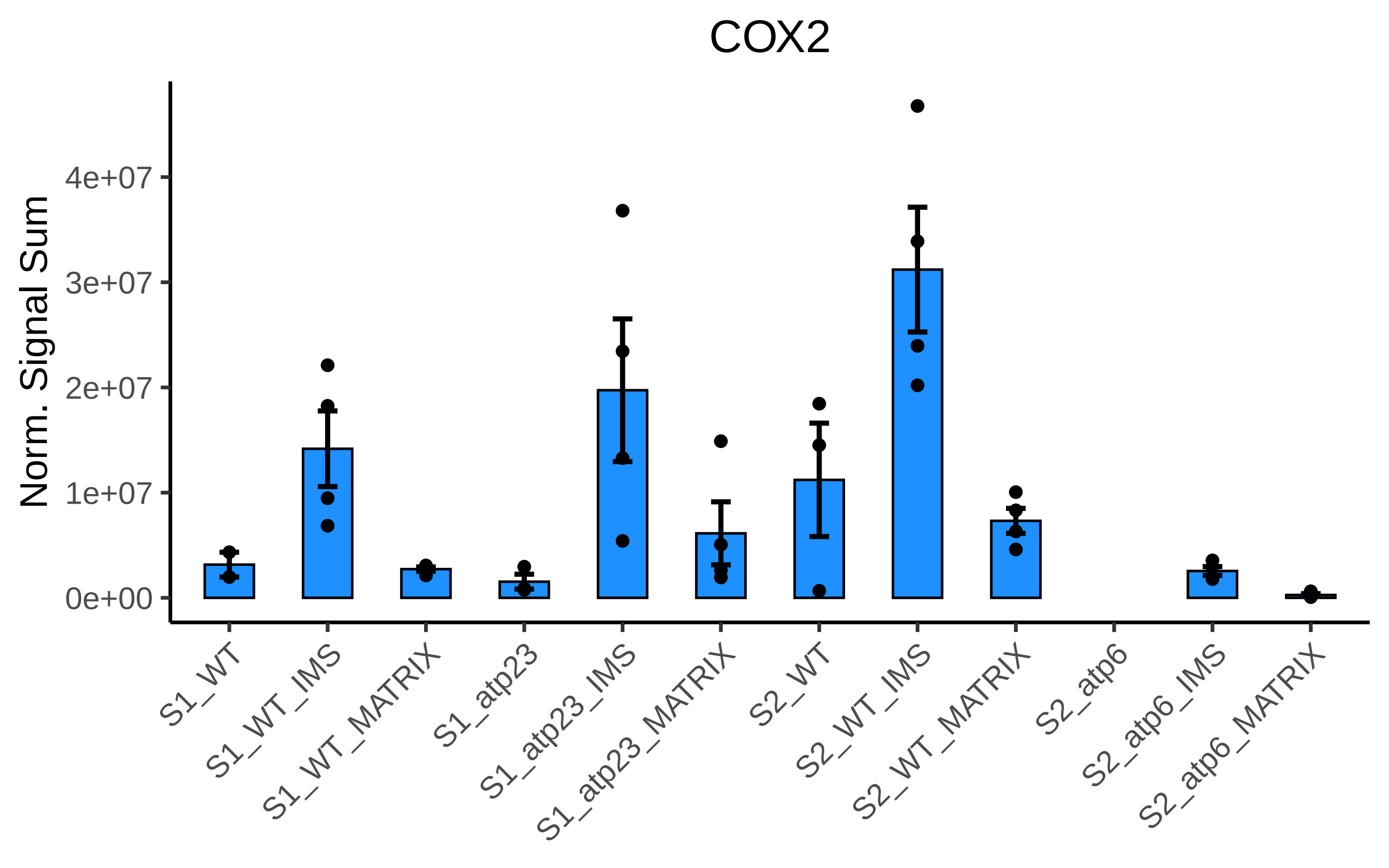
COX2
Norm. Signal Sum
4e+07
3e+07
2e+07
1e+07
0e+00
S1_WT
S1_WT_IMS
S1_WT_MATRIX
S1_atp23
S1_atp23_IMS
S1_atp23_MATRIX
S2_WT
S2_WT_IMS
S2_WT_MATRIX
S2_atp6
S2_atp6_IMS
S2_atp6_MATRIX

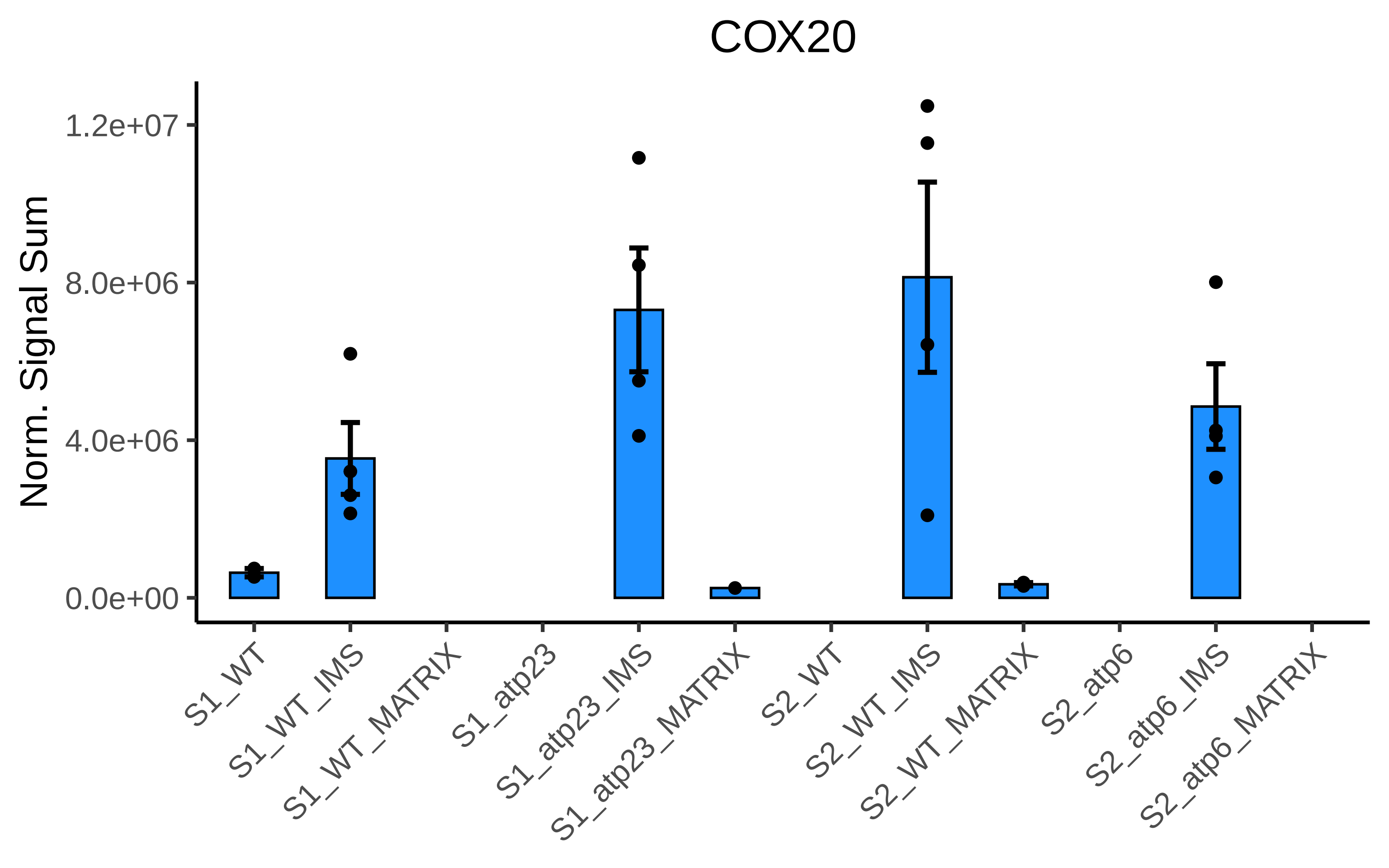
COX20
Norm. Signal Sum
1.2e+07
8.0e+06
4.0e+06
0.0e+00
S1_WT
S1_WT_IMS
S1_WT_MATRIX
S1_atp23
S1_atp23_IMS
S1_atp23_MATRIX
S2_WT
S2_WT_IMS
S2_WT_MATRIX
S2_atp6
S2_atp6_IMS
S2_atp6_MATRIX

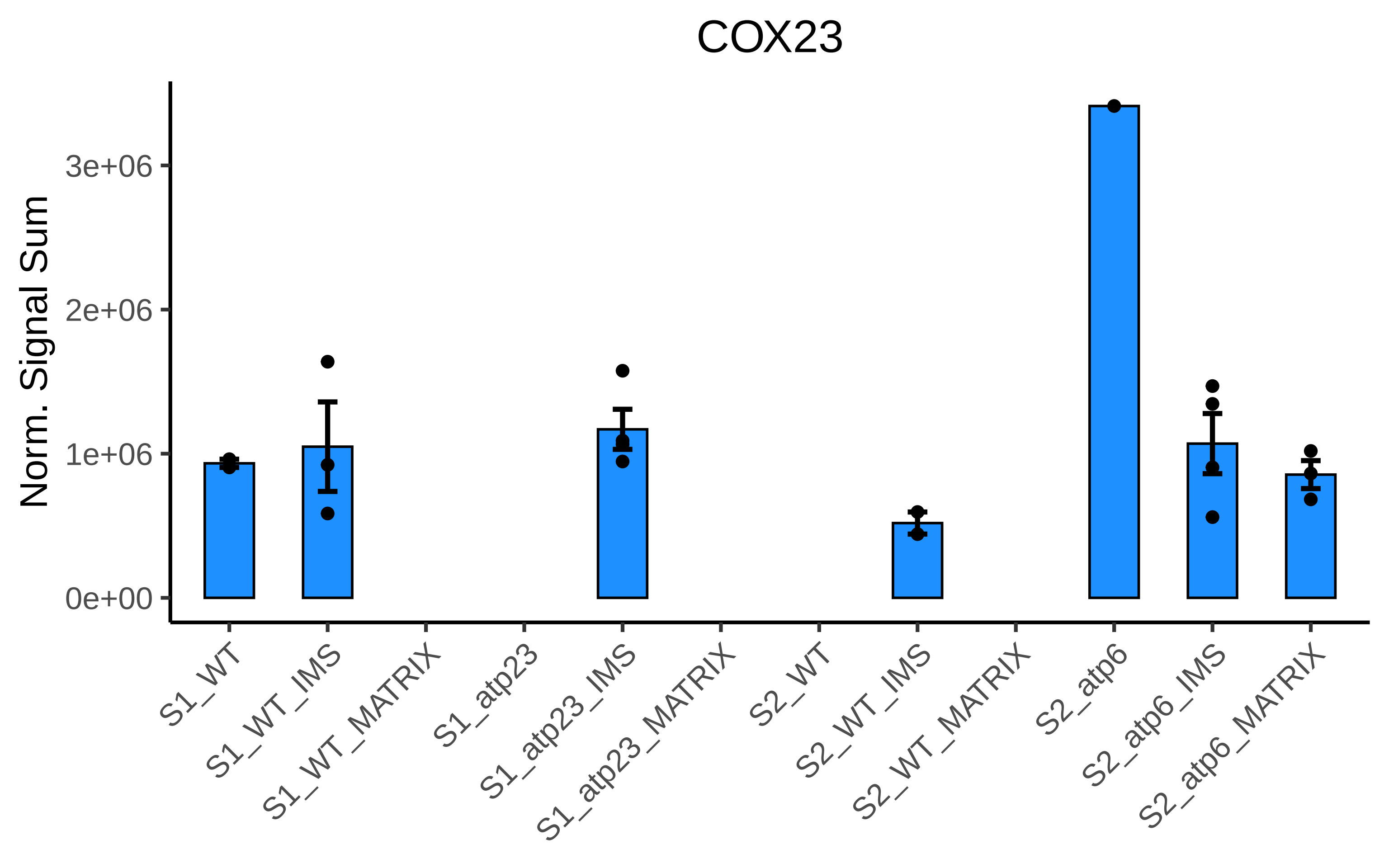

COX23
Norm. Signal Sum
3e+06
2e+06
1e+06
0e+00
S1_WT
S1_WT_IMS
S1_WT_MATRIX
S1_atp23
S1_atp23_IMS
S1_atp23_MATRIX
S2_WT
S2_WT_IMS
S2_WT_MATRIX
S2_atp6
S2_atp6_IMS
S2_atp6_MATRIX

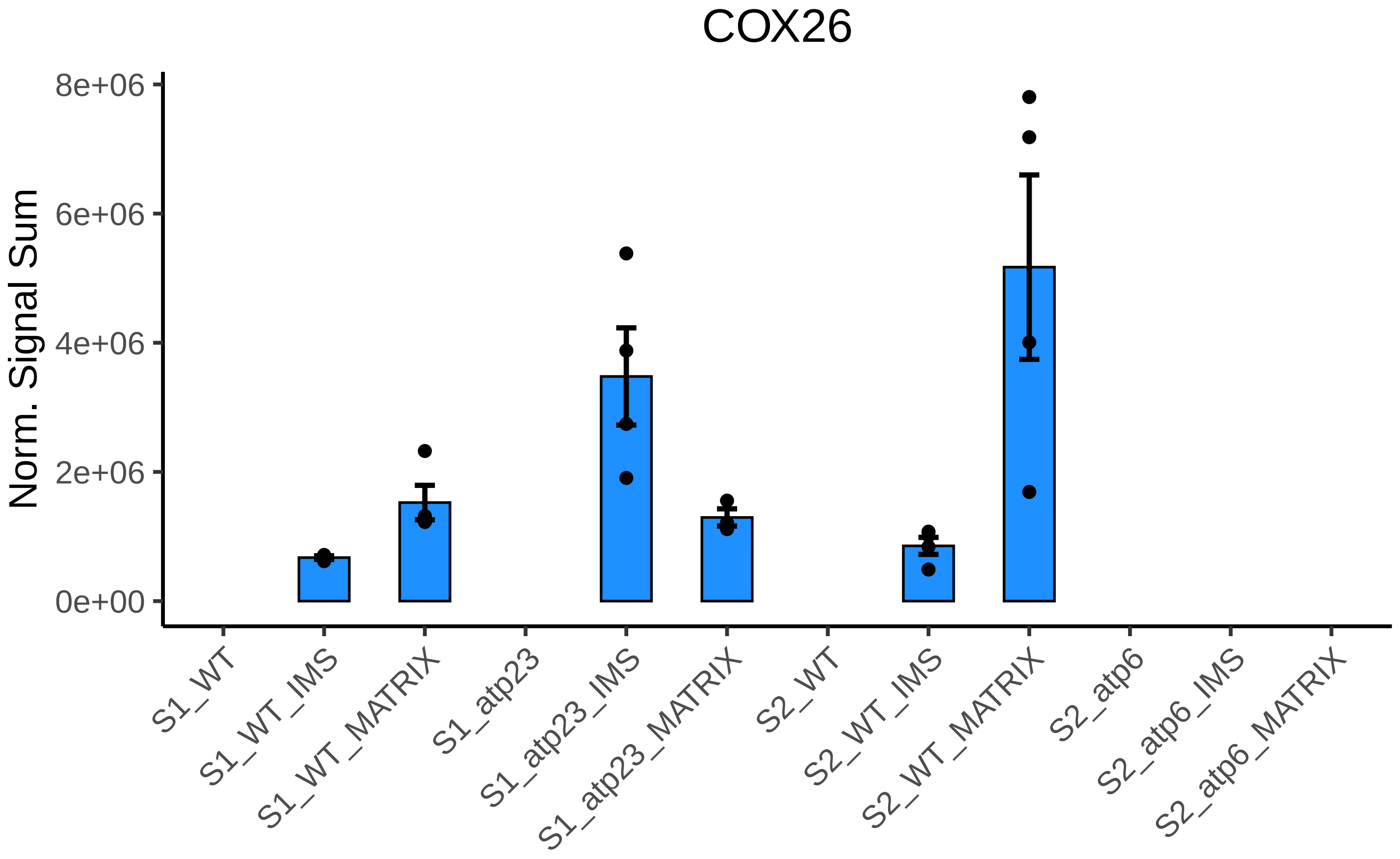
COX26
Norm. Signal Sum
8e+06
6e+06
4e+06
2e+06
0e+00
S1_WT
S1_WT_IMS
S1_WT_MATRIX
S1_atp23
S1_atp23_IMS
S1_atp23_MATRIX
S2_WT
S2_WT_IMS
S2_WT_MATRIX
S2_atp6
S2_atp6_IMS
S2_atp6_MATRIX

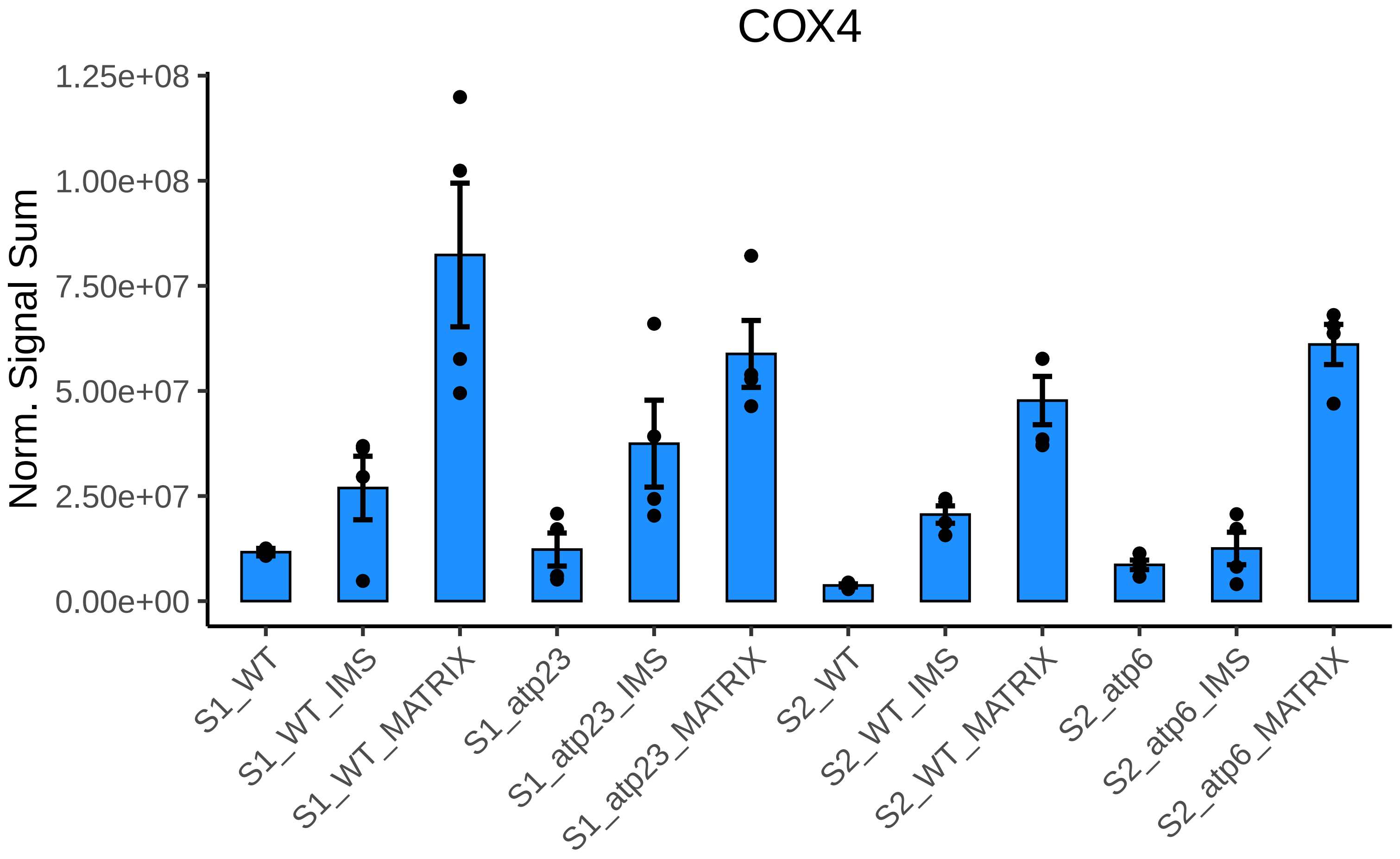

COX4
Norm. Signal Sum
1.25e+08
1.00e+08
7.50e+07
5.00e+07
2.50e+07
0.00e+00
S1_WT
S1_WT_IMS
S1_WT_MATRIX
S1_atp23
S1_atp23_IMS
S1_atp23_MATRIX
S2_WT
S2_WT_IMS
S2_WT_MATRIX
S2_atp6
S2_atp6_IMS
S2_atp6_MATRIX

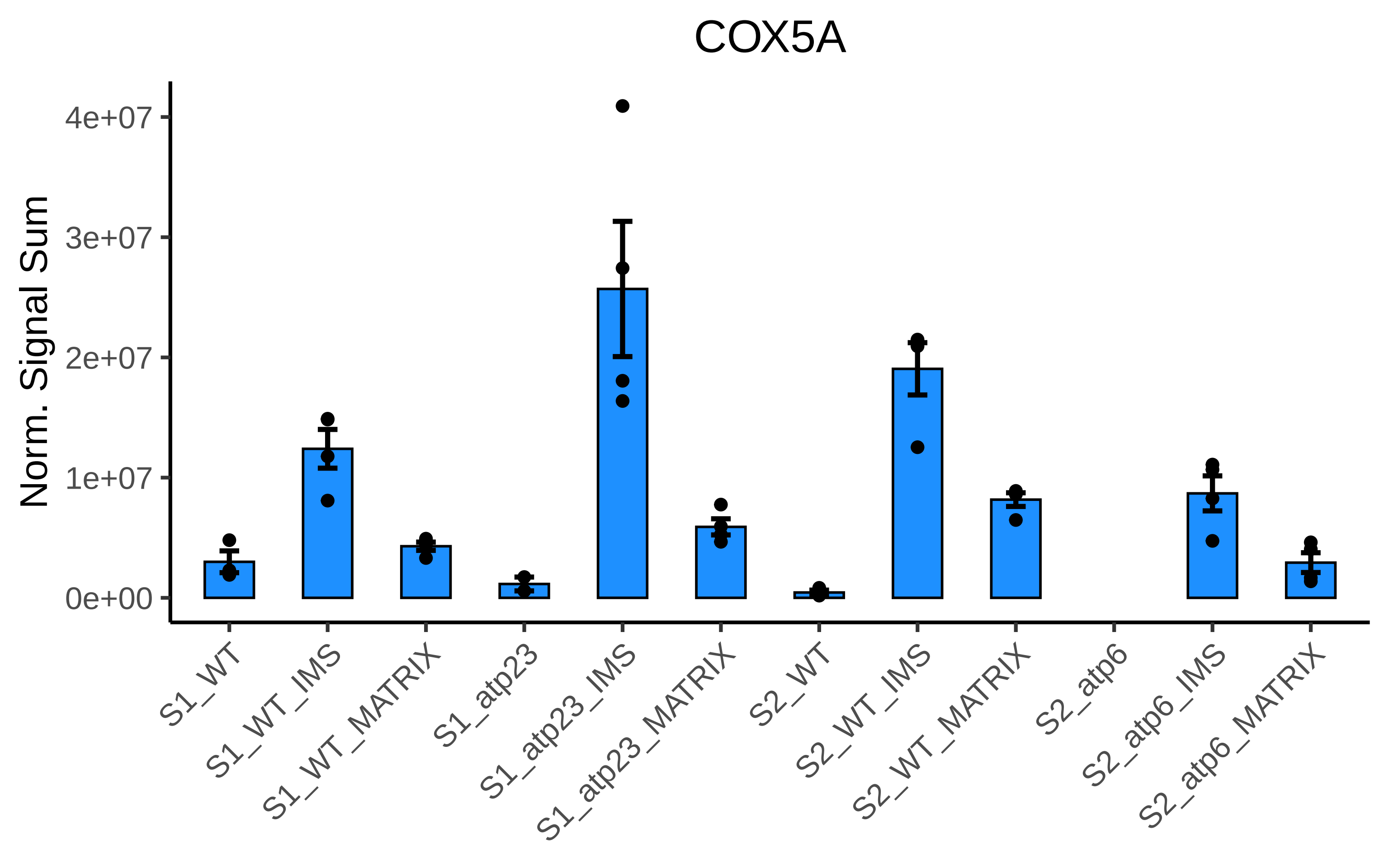
COX5A
Norm. Signal Sum
4e+07
3e+07
2e+07
1e+07
0e+00
S1_WT
S1_WT_IMS
S1_WT_MATRIX
S1_atp23
S1_atp23_IMS
S1_atp23_MATRIX
S2_WT
S2_WT_IMS
S2_WT_MATRIX
S2_atp6
S2_atp6_IMS
S2_atp6_MATRIX

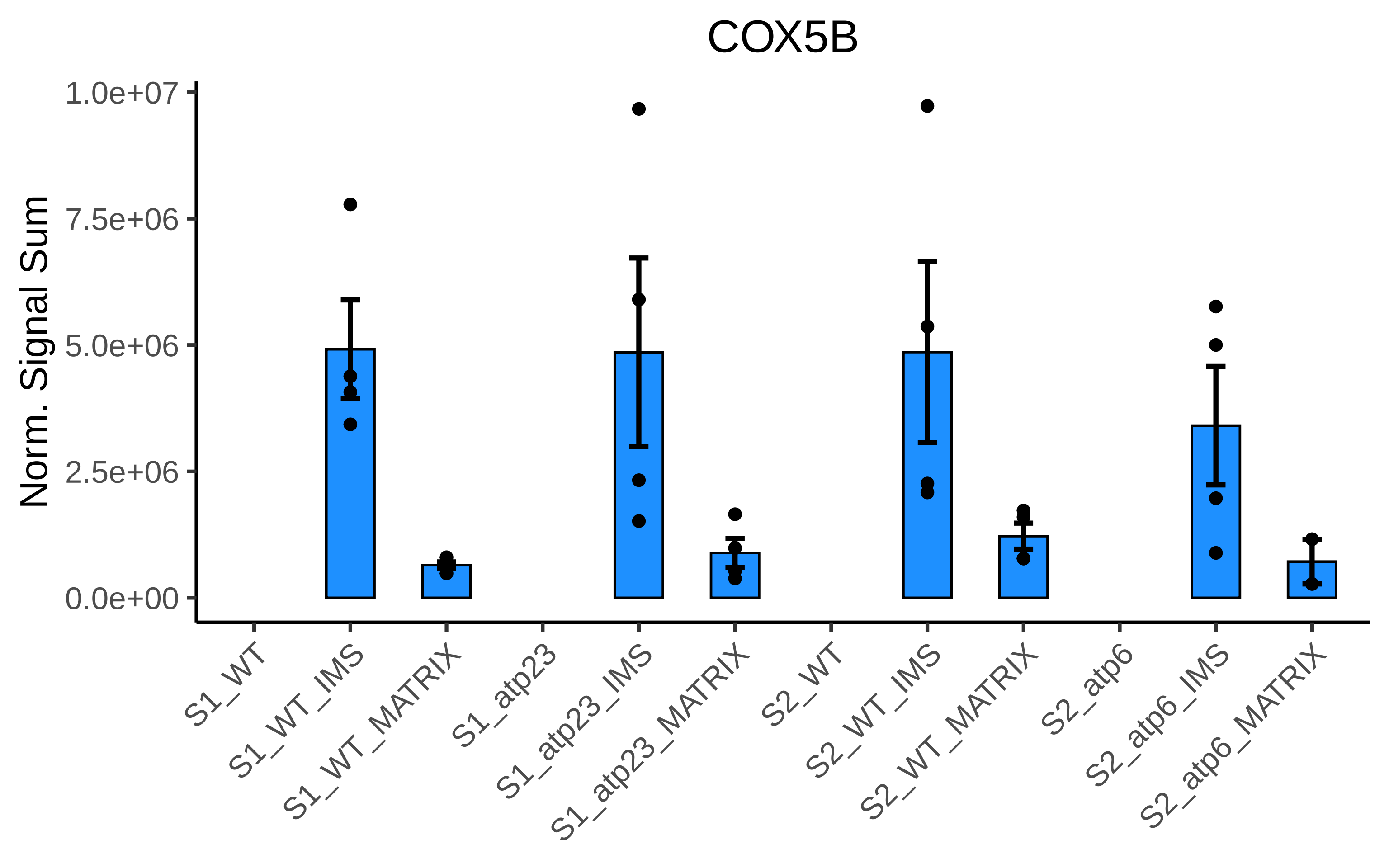

COX5B
Norm. Signal Sum
1.0e+07
7.5e+06
5.0e+06
2.5e+06
0.0e+00
S1_WT
S1_WT_IMS
S1_WT_MATRIX
S1_atp23
S1_atp23_IMS
S1_atp23_MATRIX
S2_WT
S2_WT_IMS
S2_WT_MATRIX
S2_atp6
S2_atp6_IMS
S2_atp6_MATRIX

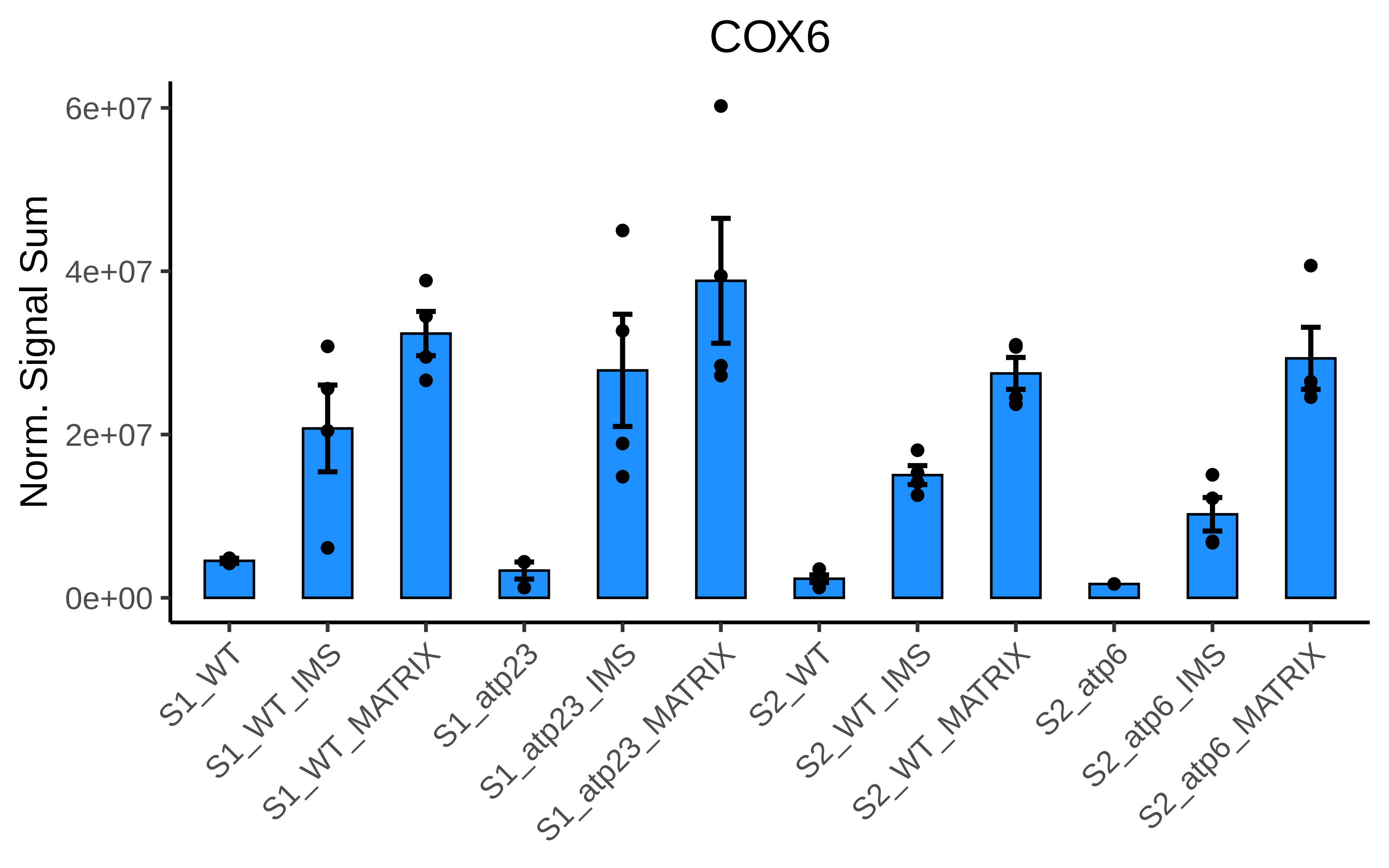
COX6
Norm. Signal Sum
6e+07
4e+07
2e+07
0e+00
S1_WT
S1_WT_IMS
S1_WT_MATRIX
S1_atp23
S1_atp23_IMS
S1_atp23_MATRIX
S2_WT
S2_WT_IMS
S2_WT_MATRIX
S2_atp6
S2_atp6_IMS
S2_atp6_MATRIX

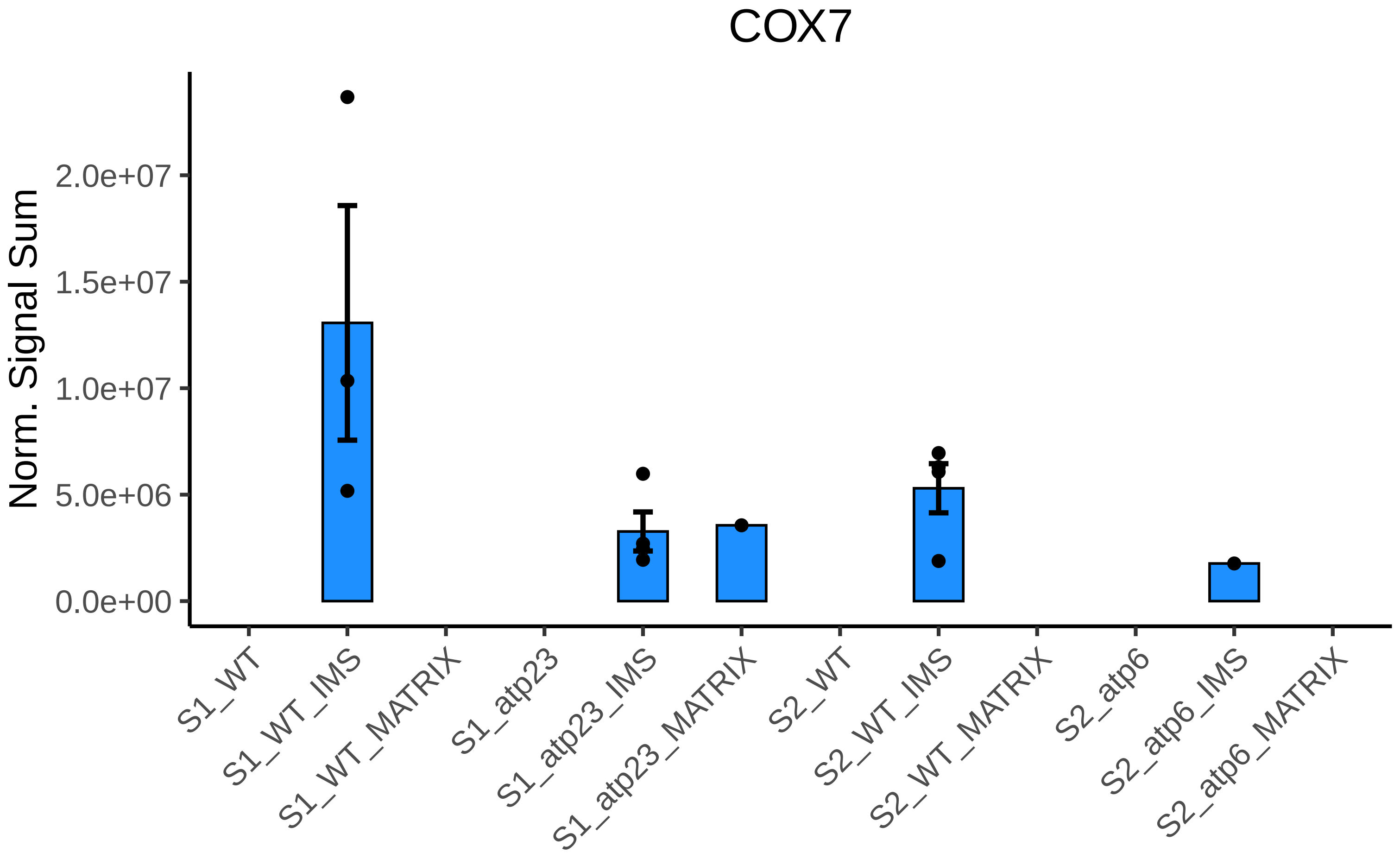

COX7
Norm. Signal Sum
2.0e+07
1.5e+07
1.0e+07
5.0e+06
0.0e+00
S1_WT
S1_WT_IMS
S1_WT_MATRIX
S1_atp23
S1_atp23_IMS
S1_atp23_MATRIX
S2_WT
S2_WT_IMS
S2_WT_MATRIX
S2_atp6
S2_atp6_IMS
S2_atp6_MATRIX

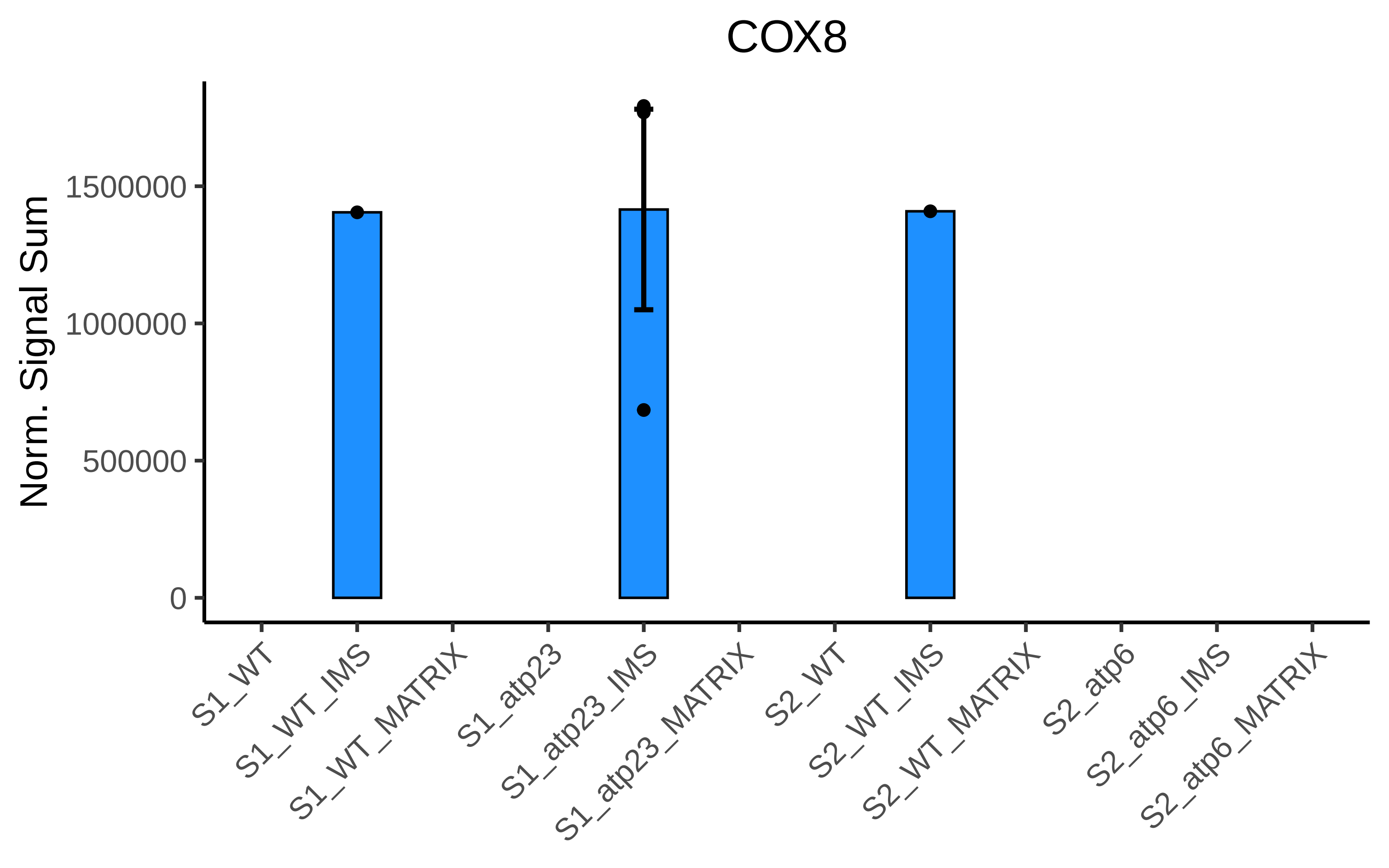
COX8
Norm. Signal Sum
1500000
1000000
500000
0
S1_WT
S1_WT_IMS
S1_WT_MATRIX
S1_atp23
S1_atp23_IMS
S1_atp23_MATRIX
S2_WT
S2_WT_IMS
S2_WT_MATRIX
S2_atp6
S2_atp6_IMS
S2_atp6_MATRIX

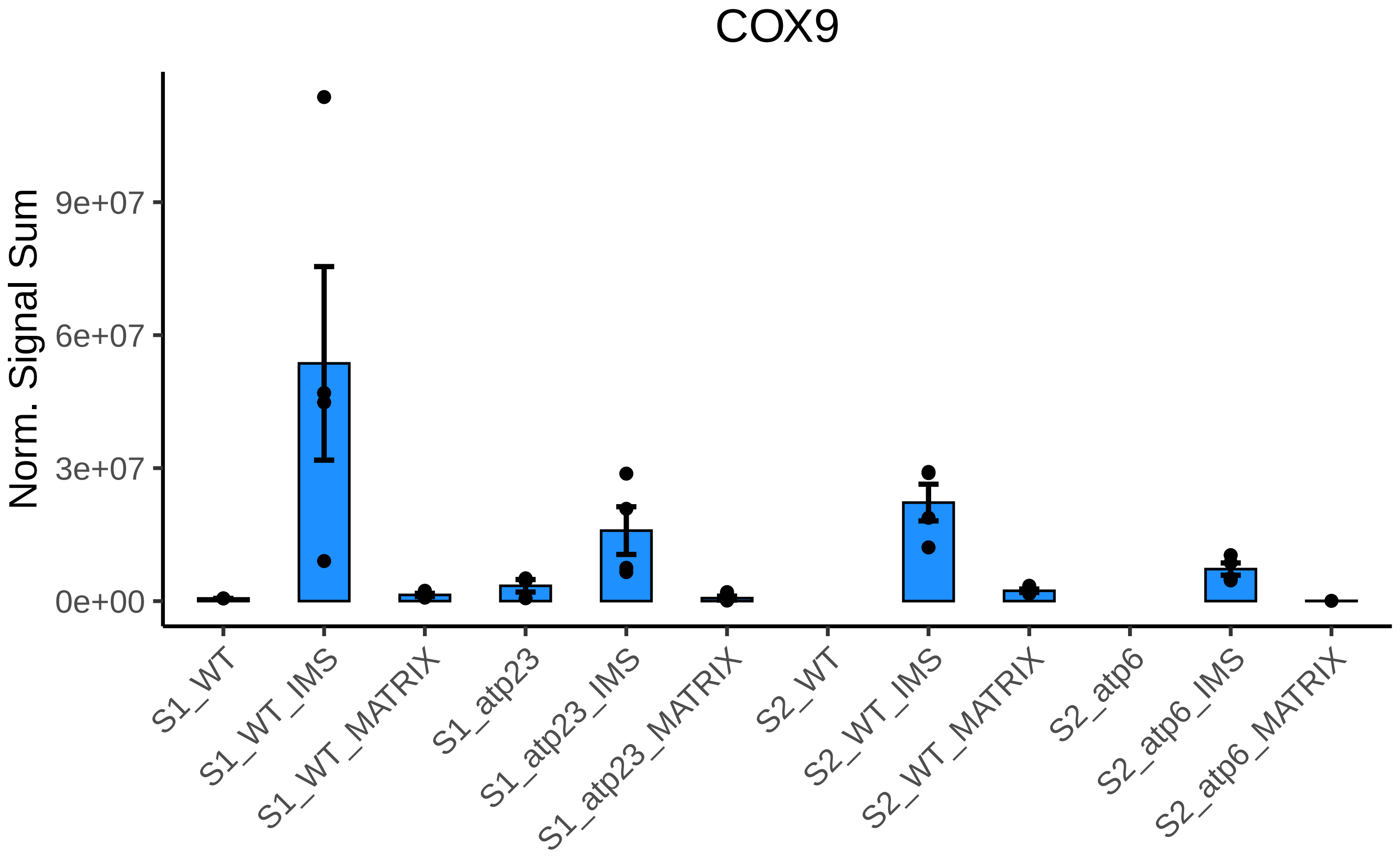
COX9
Norm. Signal Sum
9e+07
6e+07
3e+07
0e+00
S1_WT
S1_WT_IMS
S1_WT_MATRIX
S1_atp23
S1_atp23_IMS
S1_atp23_MATRIX
S2_WT
S2_WT_IMS
S2_WT_MATRIX
S2_atp6
S2_atp6_IMS
S2_atp6_MATRIX

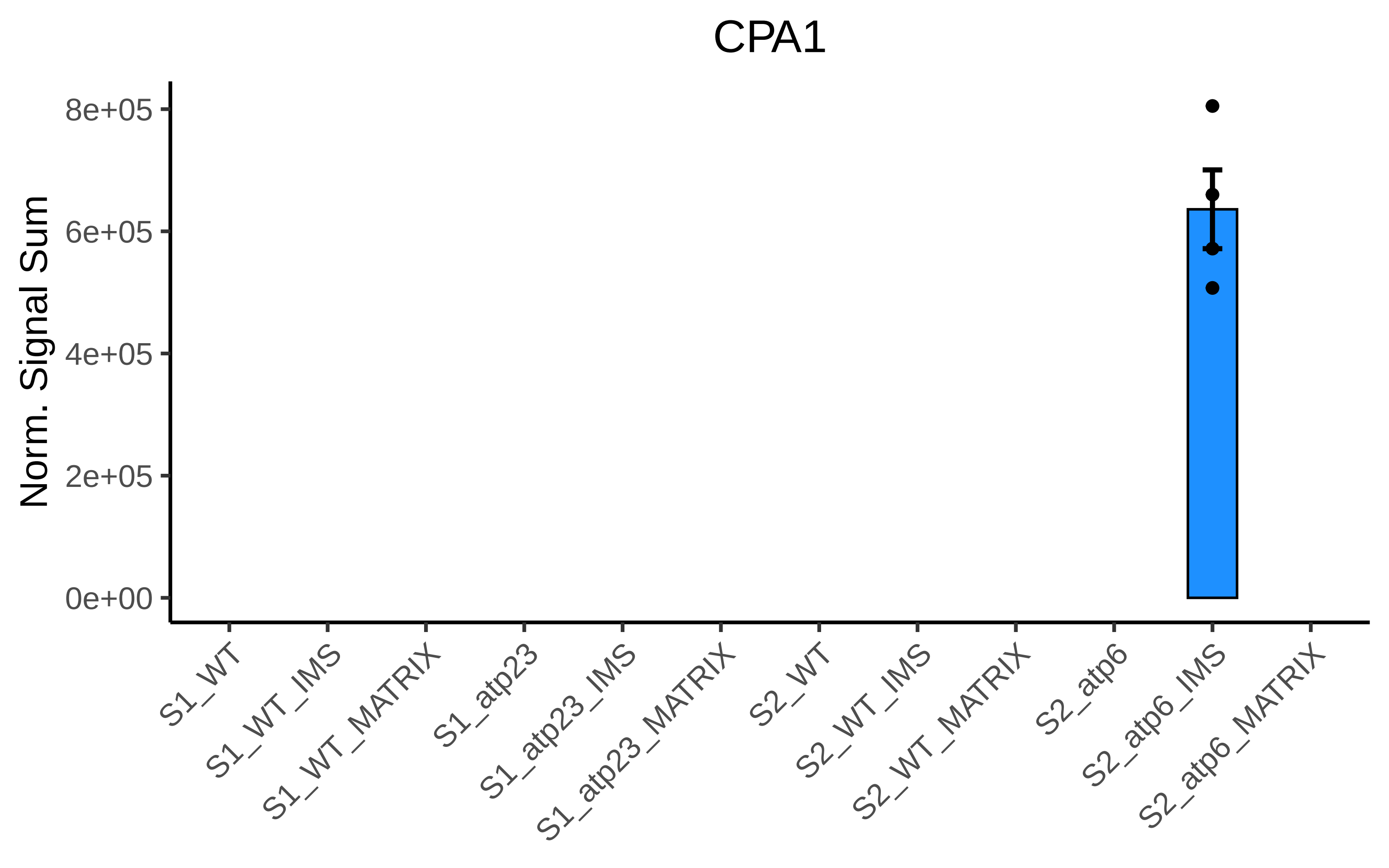
CPA1
Norm. Signal Sum
8e+05
6e+05
4e+05
2e+05
0e+00
S1_WT
S1_WT_IMS
S1_WT_MATRIX
S1_atp23
S1_atp23_IMS
S1_atp23_MATRIX
S2_WT
S2_WT_IMS
S2_WT_MATRIX
S2_atp6
S2_atp6_IMS
S2_atp6_MATRIX

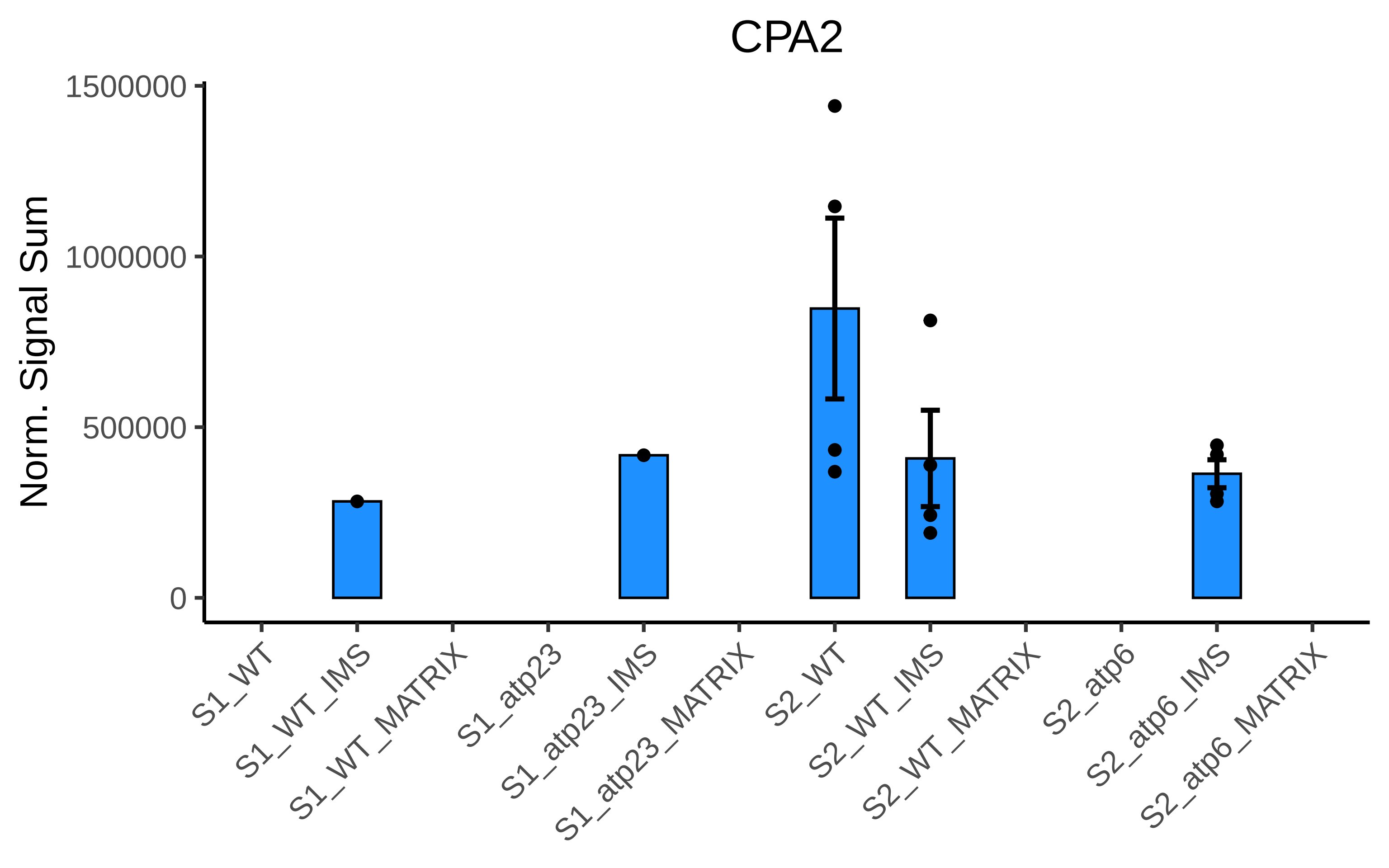

CPA2
Norm. Signal Sum
1500000
1000000
500000
0
S1_WT
S1_WT_IMS
S1_WT_MATRIX
S1_atp23
S1_atp23_IMS
S1_atp23_MATRIX
S2_WT
S2_WT_IMS
S2_WT_MATRIX
S2_atp6
S2_atp6_IMS
S2_atp6_MATRIX

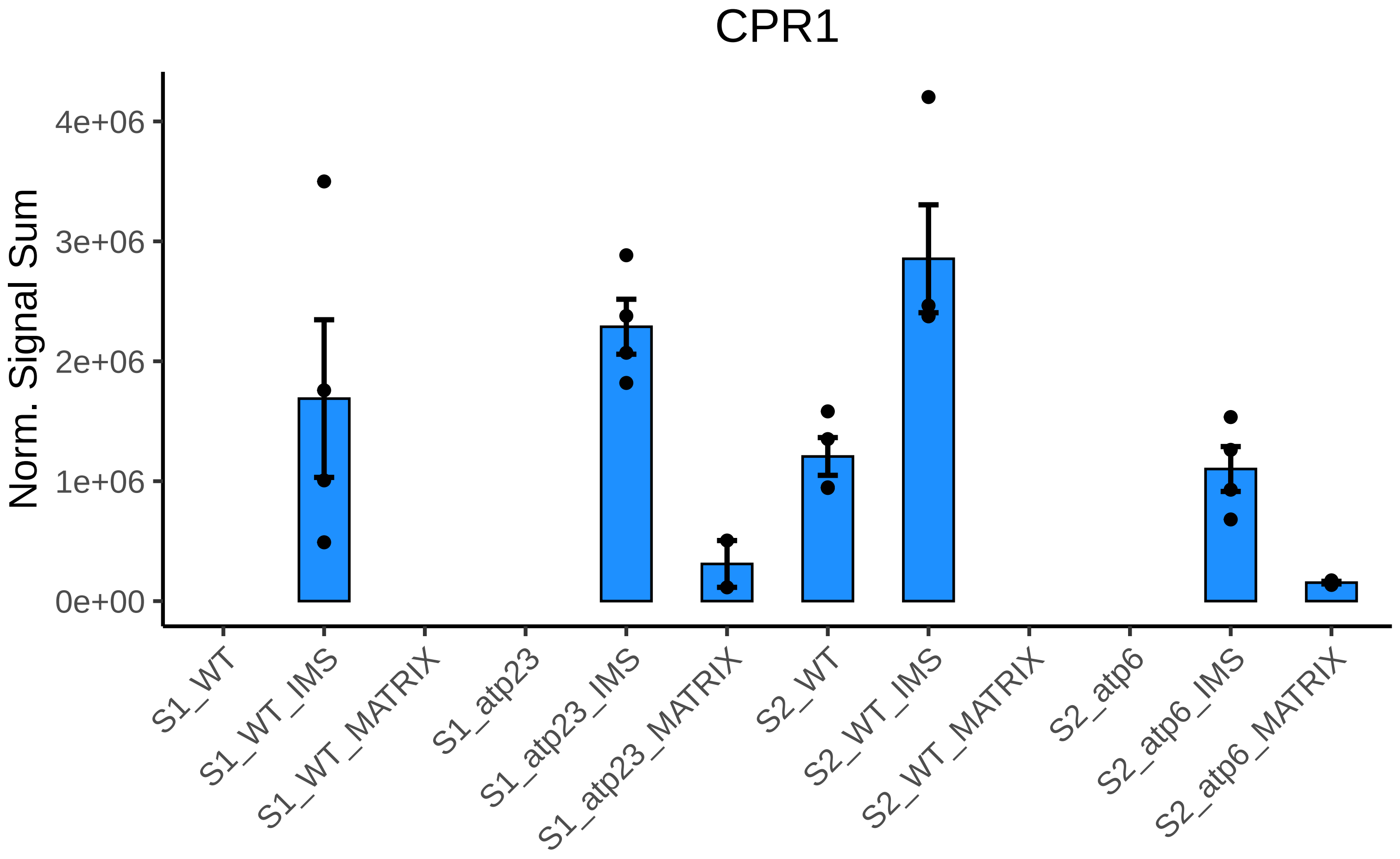
CPR1
Norm. Signal Sum
4e+06
3e+06
2e+06
1e+06
0e+00
S1_WT
S1_WT_IMS
S1_WT_MATRIX
S1_atp23
S1_atp23_IMS
S1_atp23_MATRIX
S2_WT
S2_WT_IMS
S2_WT_MATRIX
S2_atp6
S2_atp6_IMS
S2_atp6_MATRIX

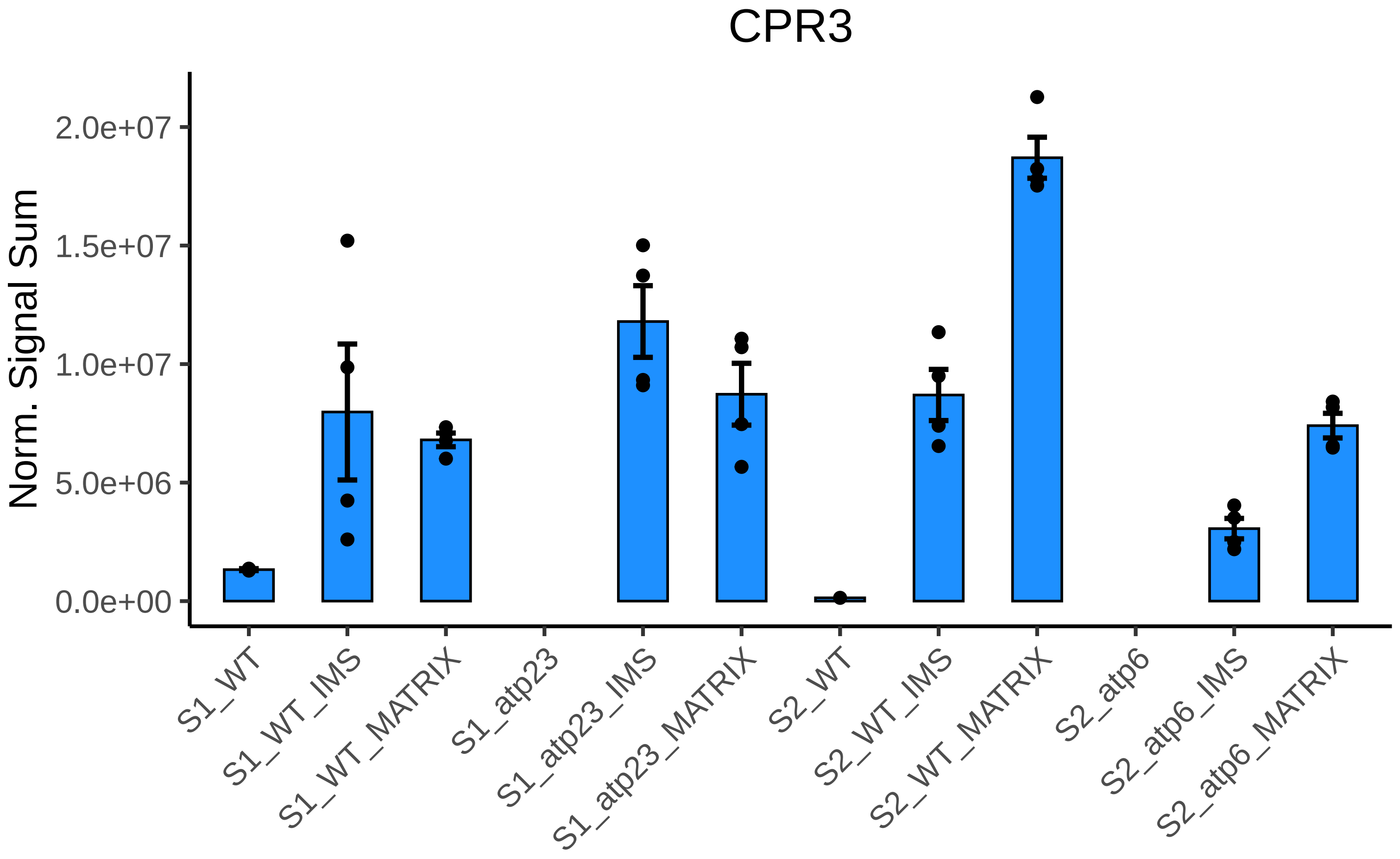

CPR3
Norm. Signal Sum
2.0e+07
1.5e+07
1.0e+07
5.0e+06
0.0e+00
S1_WT
S1_WT_IMS
S1_WT_MATRIX
S1_atp23
S1_atp23_IMS
S1_atp23_MATRIX
S2_WT
S2_WT_IMS
S2_WT_MATRIX
S2_atp6
S2_atp6_IMS
S2_atp6_MATRIX

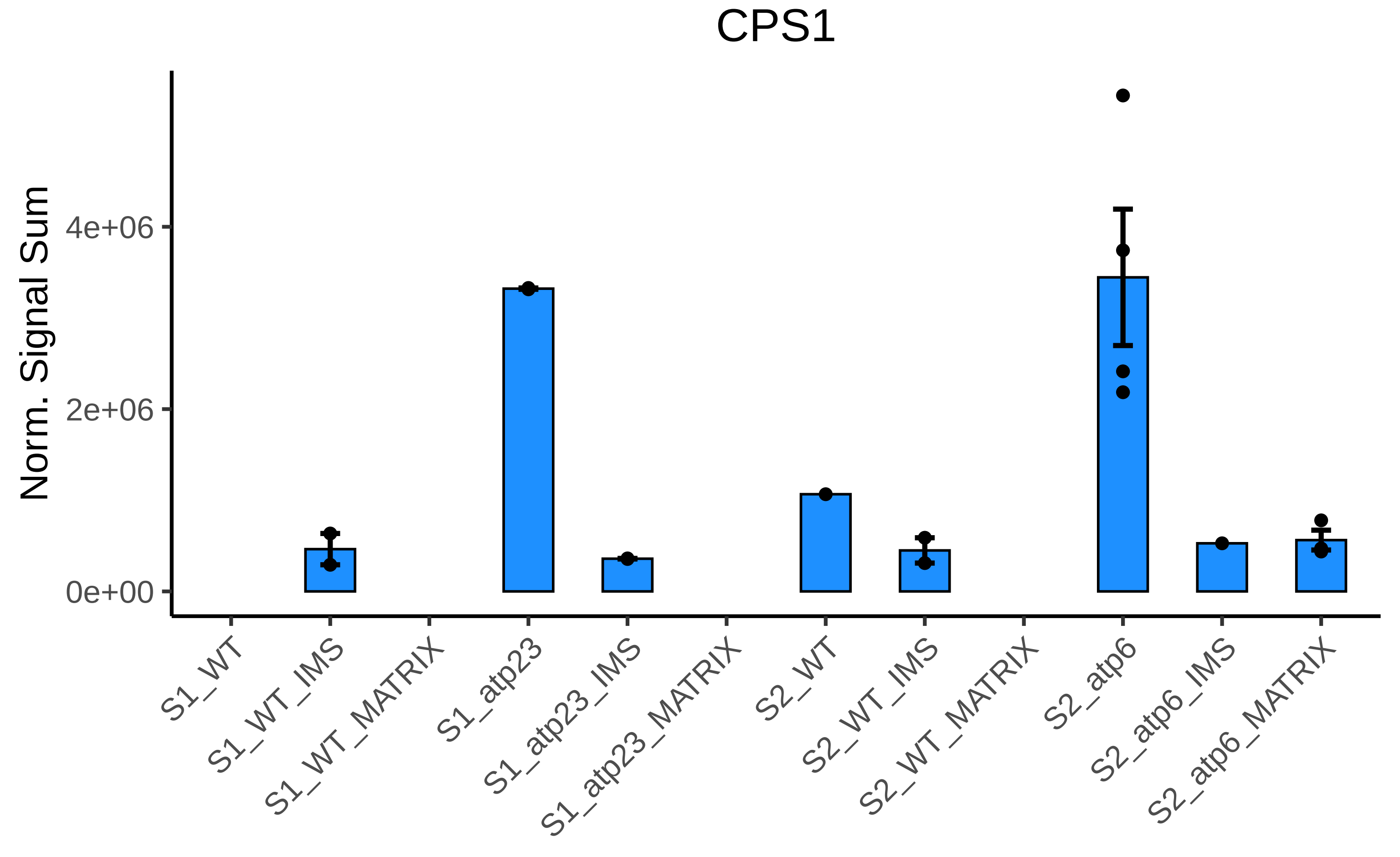
CPS1
Norm. Signal Sum
4e+06
2e+06
0e+00
S1_WT
S1_WT_IMS
S1_WT_MATRIX
S1_atp23
S1_atp23_IMS
S1_atp23_MATRIX
S2_WT
S2_WT_IMS
S2_WT_MATRIX
S2_atp6
S2_atp6_IMS
S2_atp6_MATRIX

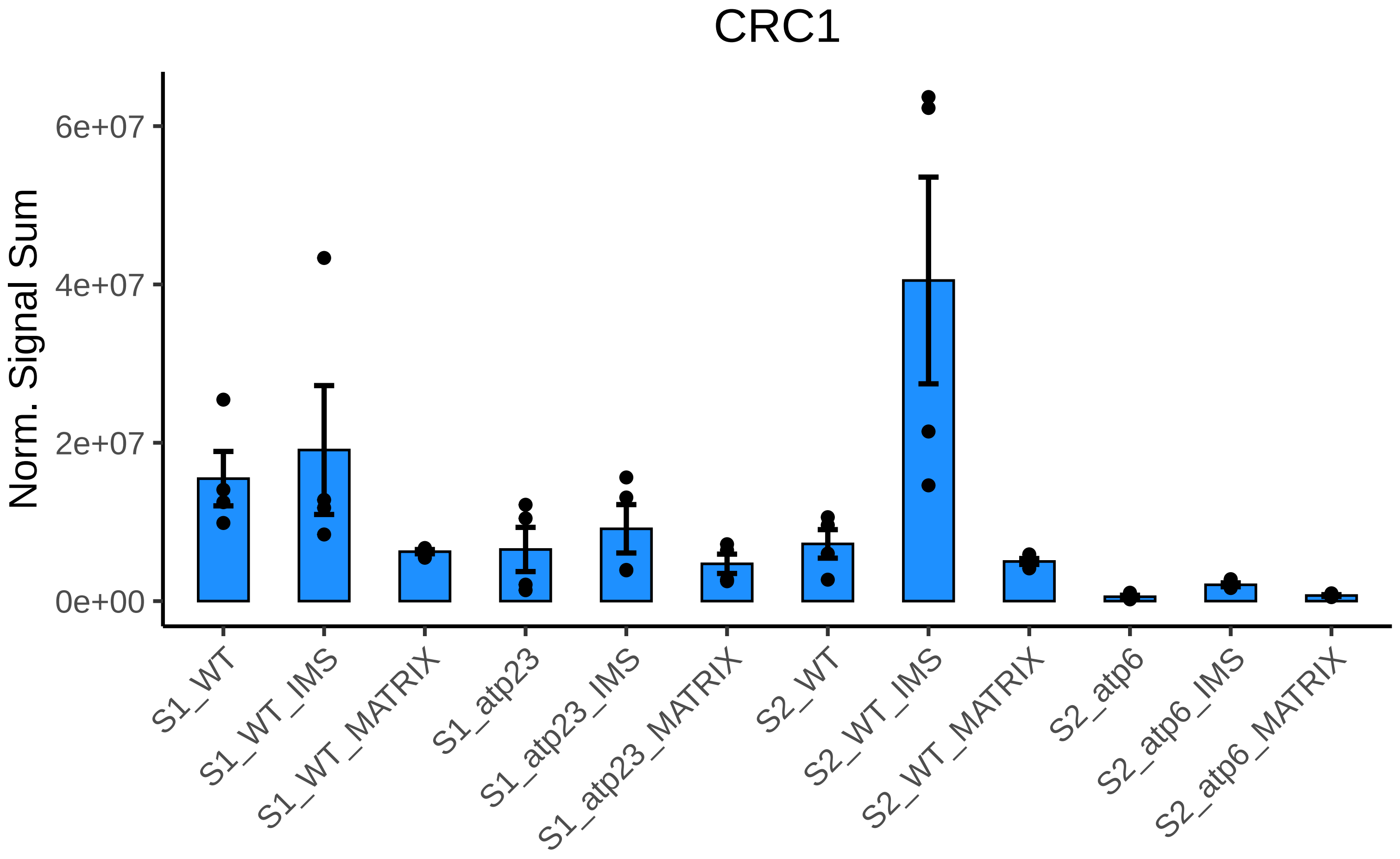
CRC1
Norm. Signal Sum
6e+07
4e+07
2e+07
0e+00
S1_WT
S1_WT_IMS
S1_WT_MATRIX
S1_atp23
S1_atp23_IMS
S1_atp23_MATRIX
S2_WT
S2_WT_IMS
S2_WT_MATRIX
S2_atp6
S2_atp6_IMS
S2_atp6_MATRIX

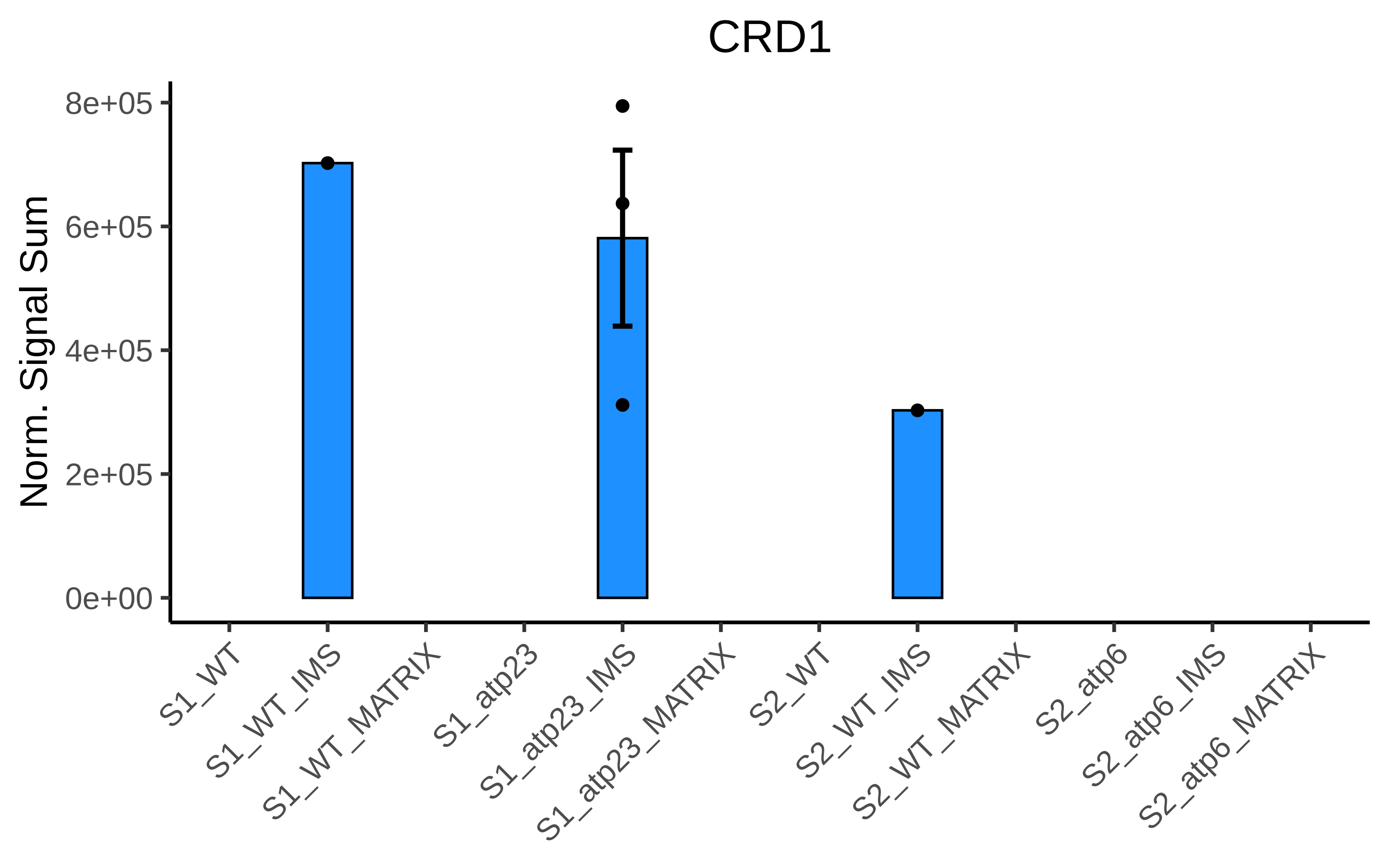

CRD1
Norm. Signal Sum
8e+05
6e+05
4e+05
2e+05
0e+00
S1_WT
S1_WT_IMS
S1_WT_MATRIX
S1_atp23
S1_atp23_IMS
S1_atp23_MATRIX
S2_WT
S2_WT_IMS
S2_WT_MATRIX
S2_atp6
S2_atp6_IMS
S2_atp6_MATRIX

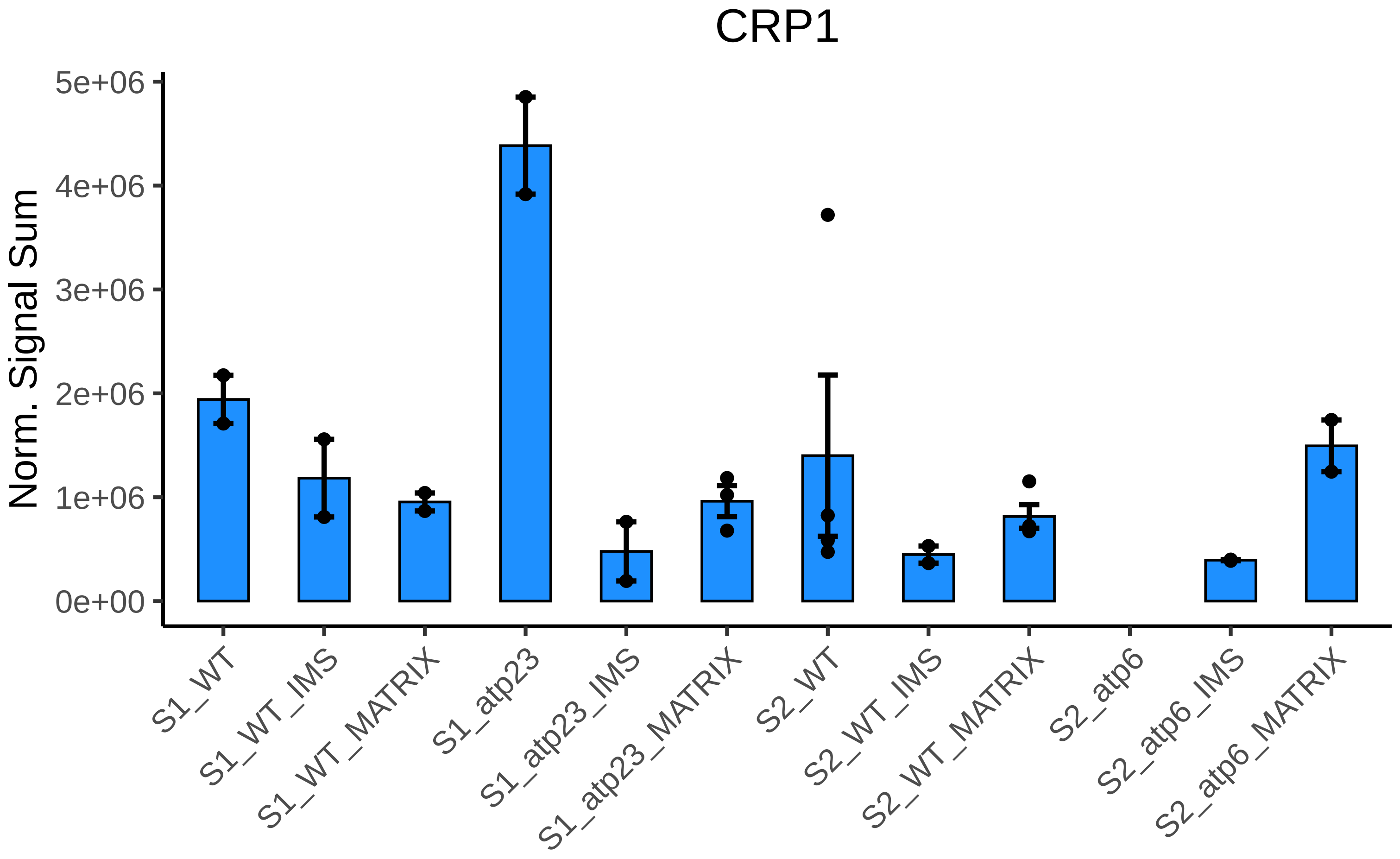
CRP1
Norm. Signal Sum
5e+06
4e+06
3e+06
2e+06
1e+06
0e+00
S1_WT
S1_WT_IMS
S1_WT_MATRIX
S1_atp23
S1_atp23_IMS
S1_atp23_MATRIX
S2_WT
S2_WT_IMS
S2_WT_MATRIX
S2_atp6
S2_atp6_IMS
S2_atp6_MATRIX

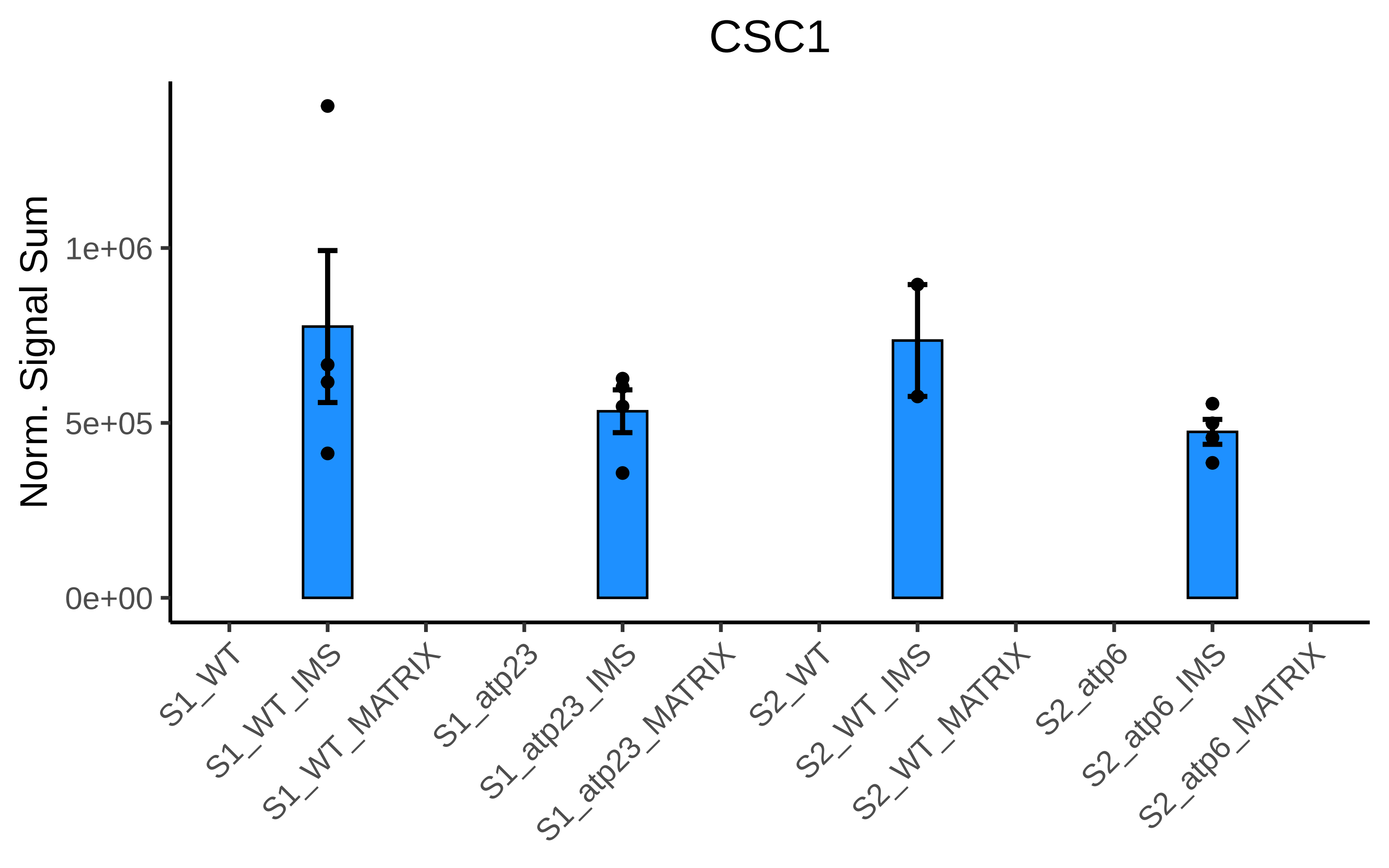

CSC1
Norm. Signal Sum
1e+06
5e+05
0e+00
S1_WT
S1_WT_IMS
S1_WT_MATRIX
S1_atp23
S1_atp23_IMS
S1_atp23_MATRIX
S2_WT
S2_WT_IMS
S2_WT_MATRIX
S2_atp6
S2_atp6_IMS
S2_atp6_MATRIX

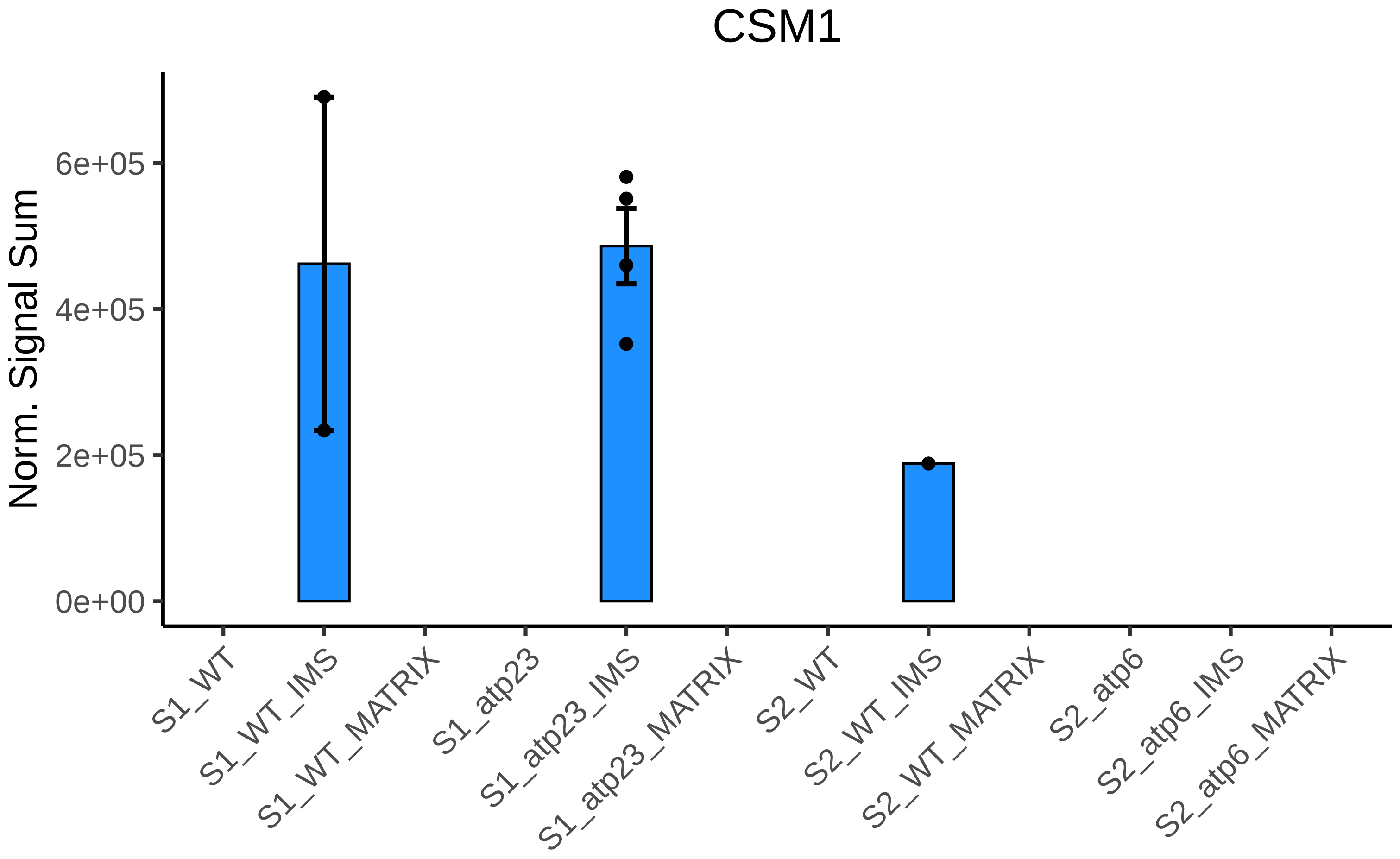
CSM1
Norm. Signal Sum
6e+05
4e+05
2e+05
0e+00
S1_WT
S1_WT_IMS
S1_WT_MATRIX
S1_atp23
S1_atp23_IMS
S1_atp23_MATRIX
S2_WT
S2_WT_IMS
S2_WT_MATRIX
S2_atp6
S2_atp6_IMS
S2_atp6_MATRIX

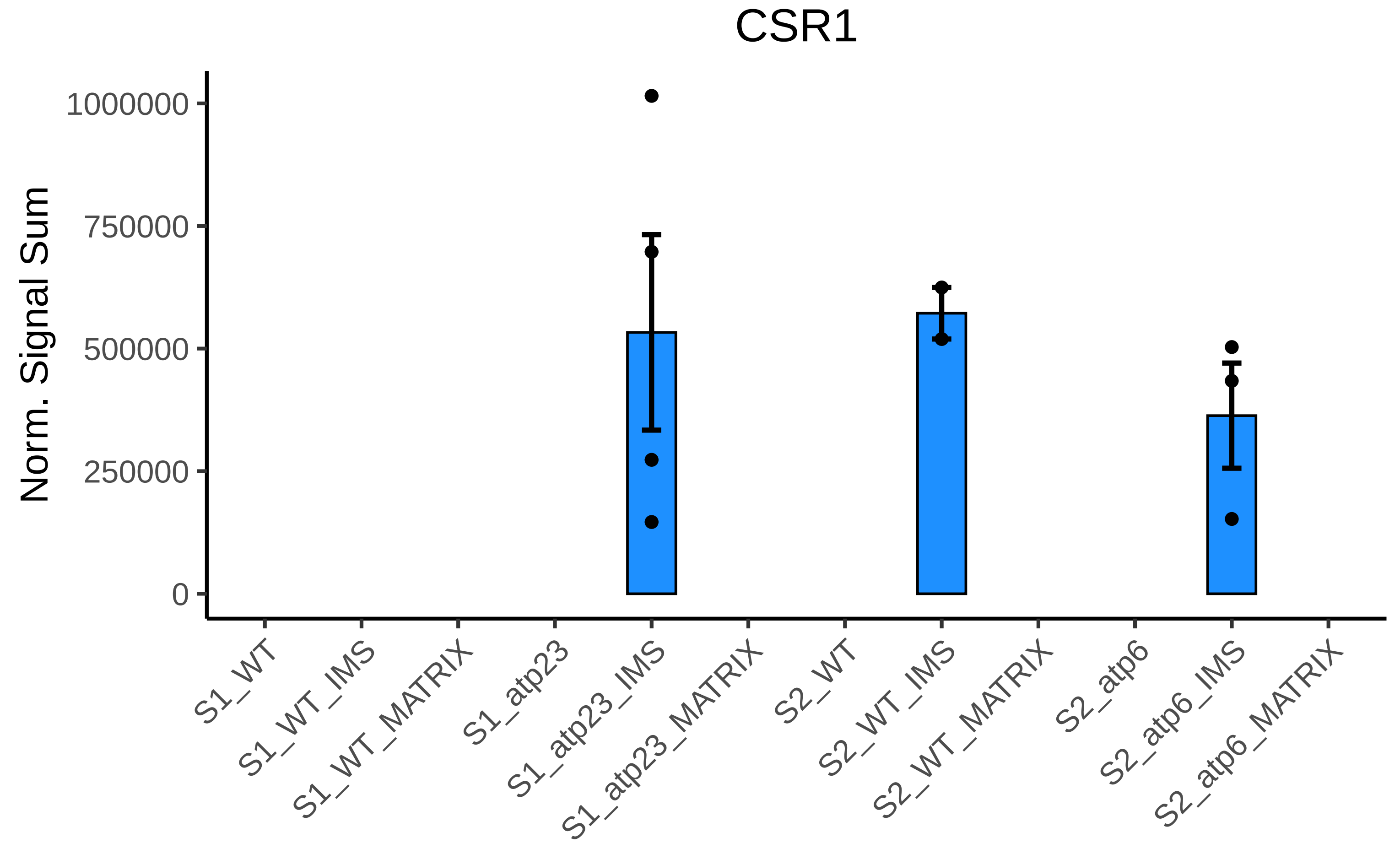
CSR1
Norm. Signal Sum
1000000
750000
500000
250000
0
S1_WT
S1_WT_IMS
S1_WT_MATRIX
S1_atp23
S1_atp23_IMS
S1_atp23_MATRIX
S2_WT
S2_WT_IMS
S2_WT_MATRIX
S2_atp6
S2_atp6_IMS
S2_atp6_MATRIX

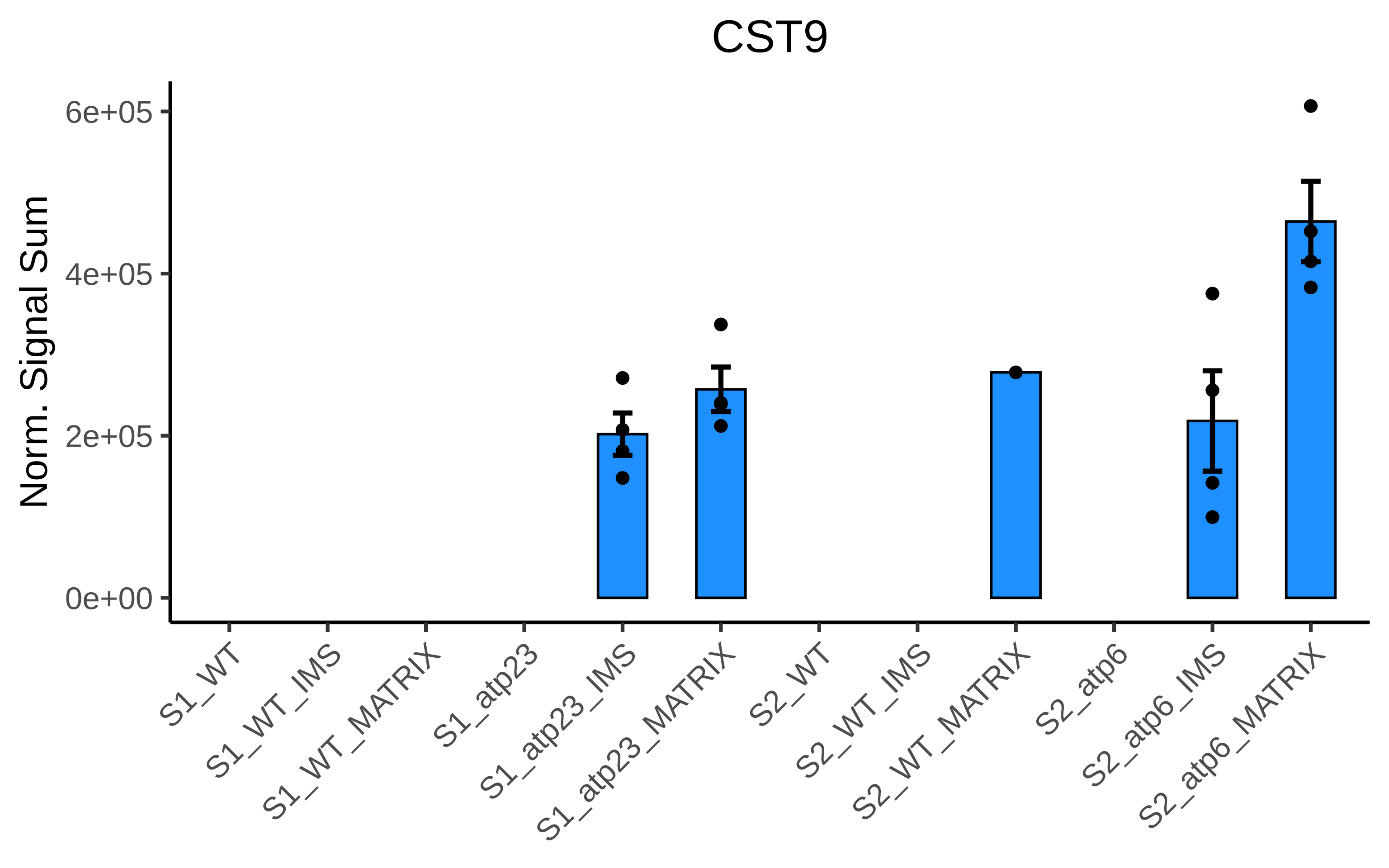
CST9
Norm. Signal Sum
6e+05
4e+05
2e+05
0e+00
S1_WT
S1_WT_IMS
S1_WT_MATRIX
S1_atp23
S1_atp23_IMS
S1_atp23_MATRIX
S2_WT
S2_WT_IMS
S2_WT_MATRIX
S2_atp6
S2_atp6_IMS
S2_atp6_MATRIX

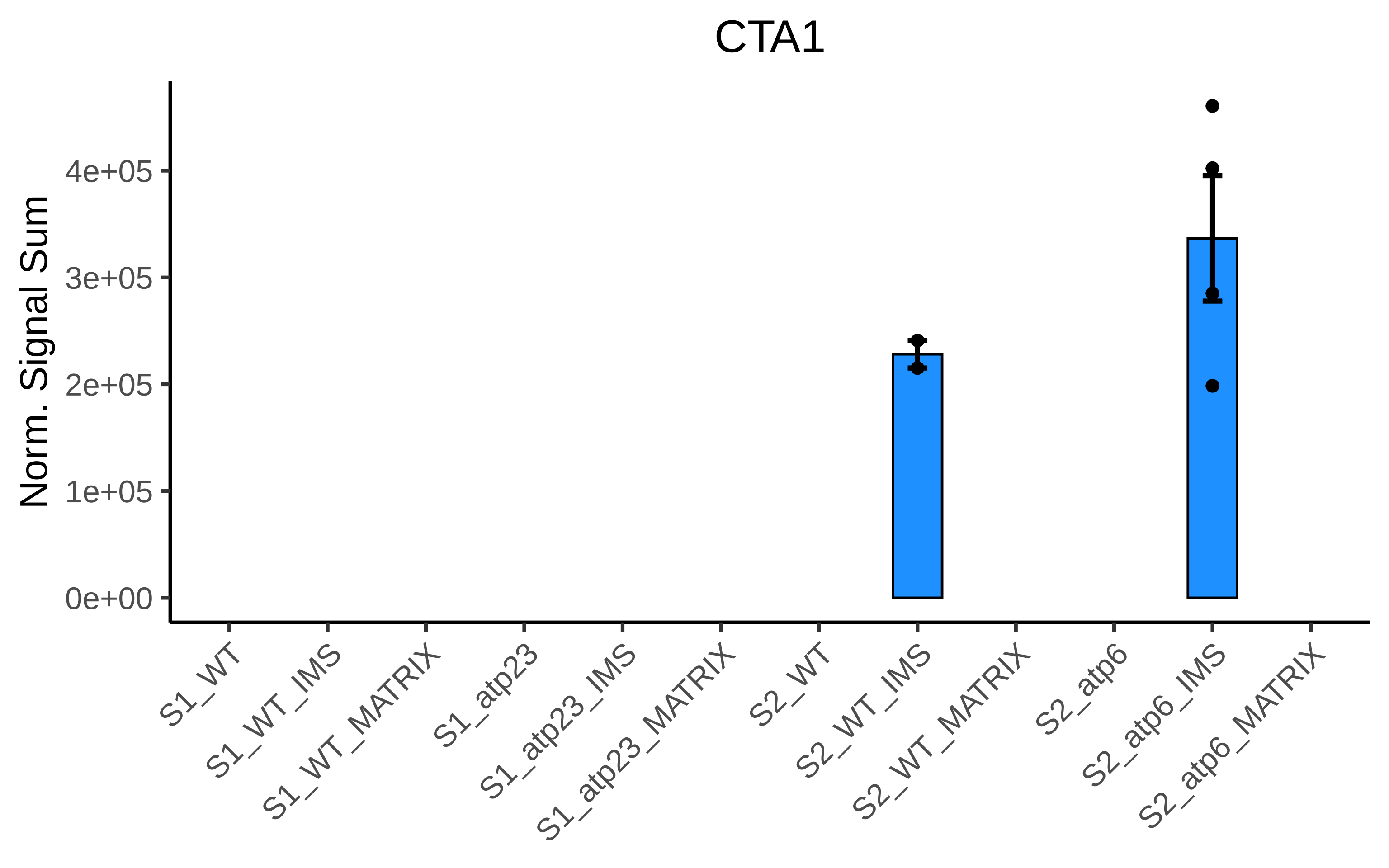
CTA1
Norm. Signal Sum
4e+05
3e+05
2e+05
1e+05
0e+00
S1_WT
S1_WT_IMS
S1_WT_MATRIX
S1_atp23
S1_atp23_IMS
S1_atp23_MATRIX
S2_WT
S2_WT_IMS
S2_WT_MATRIX
S2_atp6
S2_atp6_IMS
S2_atp6_MATRIX

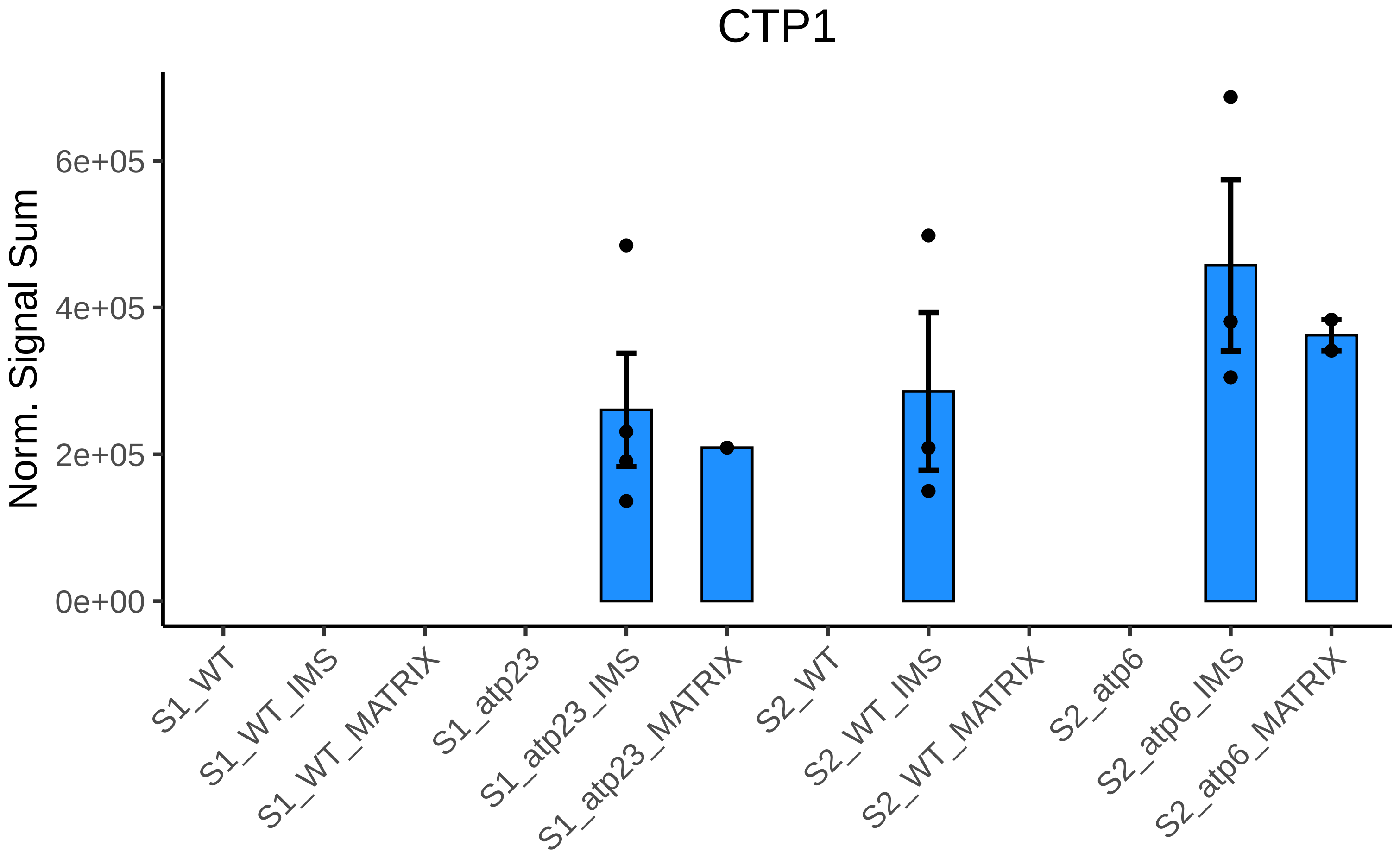

CTP1
Norm. Signal Sum
6e+05
4e+05
2e+05
0e+00
S1_WT
S1_WT_IMS
S1_WT_MATRIX
S1_atp23
S1_atp23_IMS
S1_atp23_MATRIX
S2_WT
S2_WT_IMS
S2_WT_MATRIX
S2_atp6
S2_atp6_IMS
S2_atp6_MATRIX

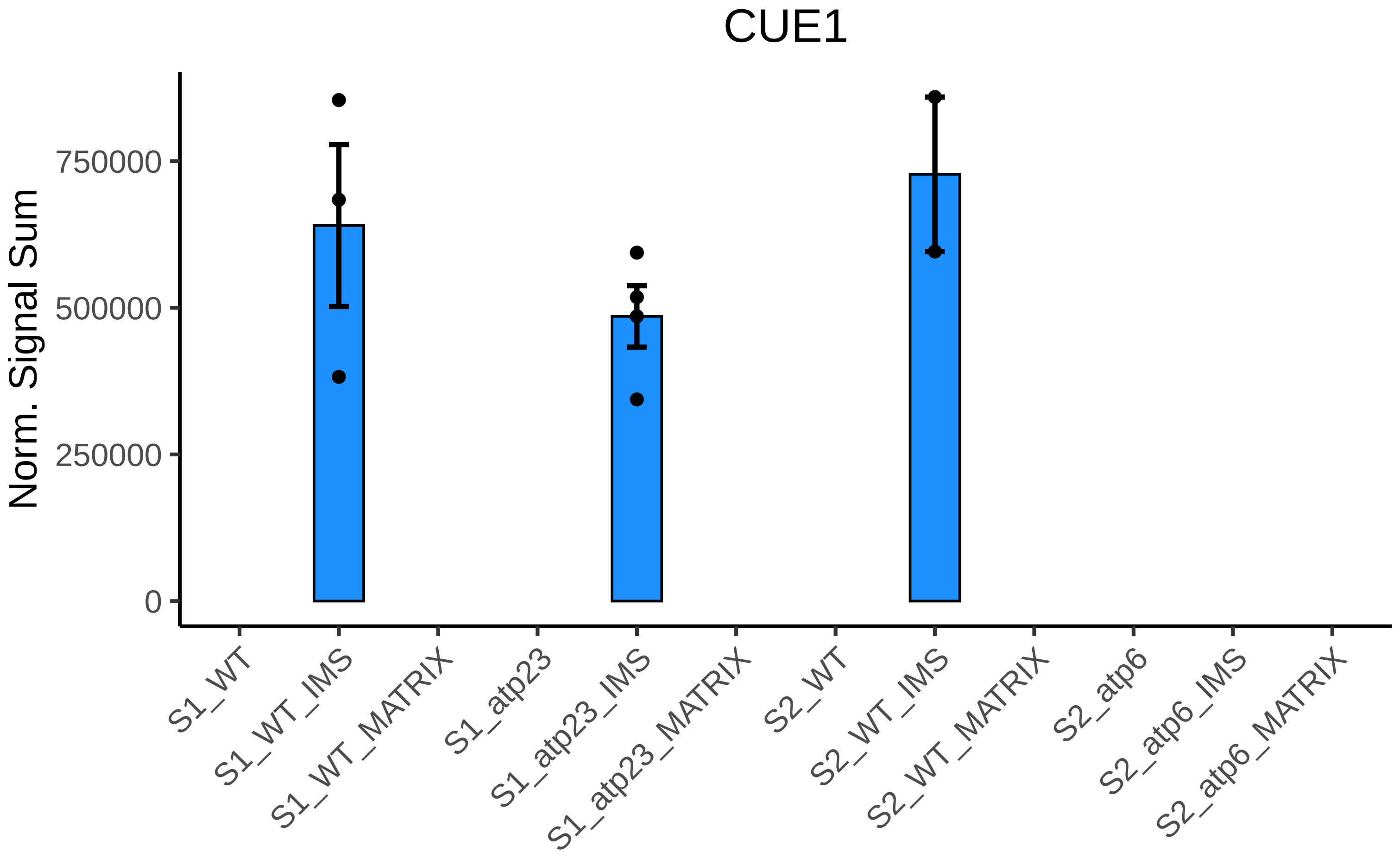
CUE1
Norm. Signal Sum
750000
500000
250000
0
S1_WT
S1_WT_IMS
S1_WT_MATRIX
S1_atp23
S1_atp23_IMS
S1_atp23_MATRIX
S2_WT
S2_WT_IMS
S2_WT_MATRIX
S2_atp6
S2_atp6_IMS
S2_atp6_MATRIX

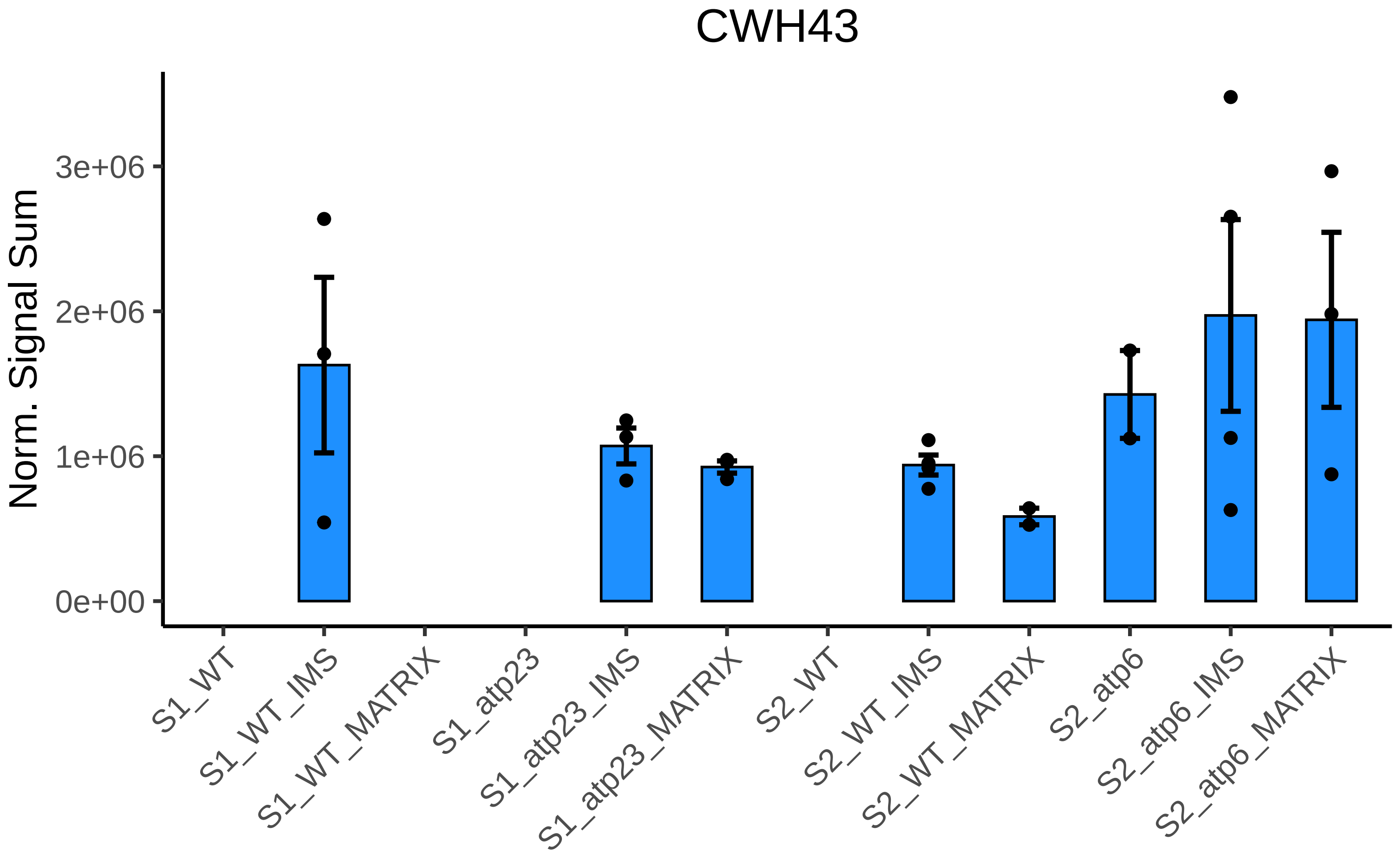

CWH43
Norm. Signal Sum
3e+06
2e+06
1e+06
0e+00
S1_WT
S1_WT_IMS
S1_WT_MATRIX
S1_atp23
S1_atp23_IMS
S1_atp23_MATRIX
S2_WT
S2_WT_IMS
S2_WT_MATRIX
S2_atp6
S2_atp6_IMS
S2_atp6_MATRIX

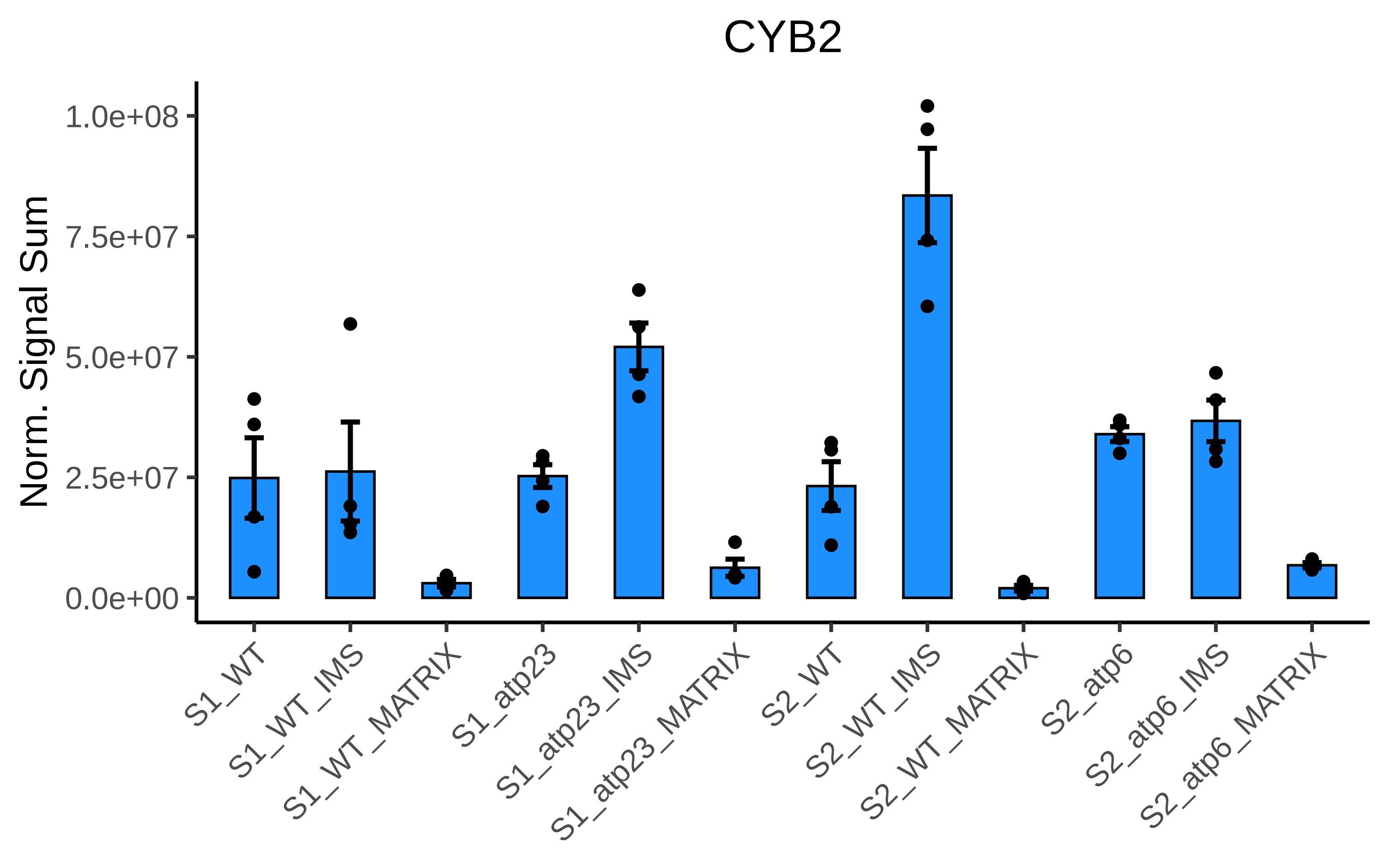
CYB2
Norm. Signal Sum
1.0e+08
7.5e+07
5.0e+07
2.5e+07
0.0e+00
S1_WT
S1_WT_IMS
S1_WT_MATRIX
S1_atp23
S1_atp23_IMS
S1_atp23_MATRIX
S2_WT
S2_WT_IMS
S2_WT_MATRIX
S2_atp6
S2_atp6_IMS
S2_atp6_MATRIX

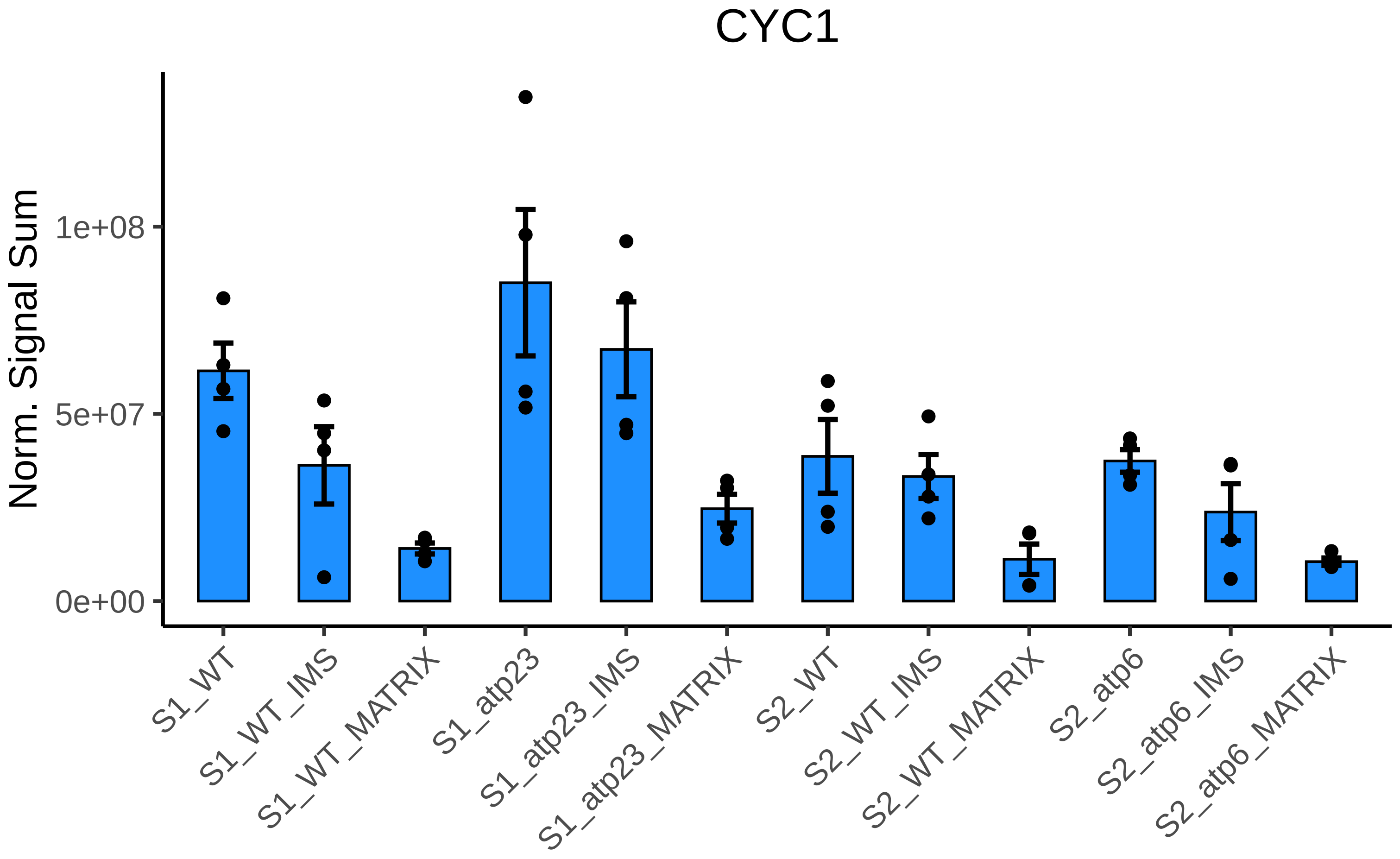

CYC1
Norm. Signal Sum
1e+08
5e+07
0e+00
S1_WT
S1_WT_IMS
S1_WT_MATRIX
S1_atp23
S1_atp23_IMS
S1_atp23_MATRIX
S2_WT
S2_WT_IMS
S2_WT_MATRIX
S2_atp6
S2_atp6_IMS
S2_atp6_MATRIX

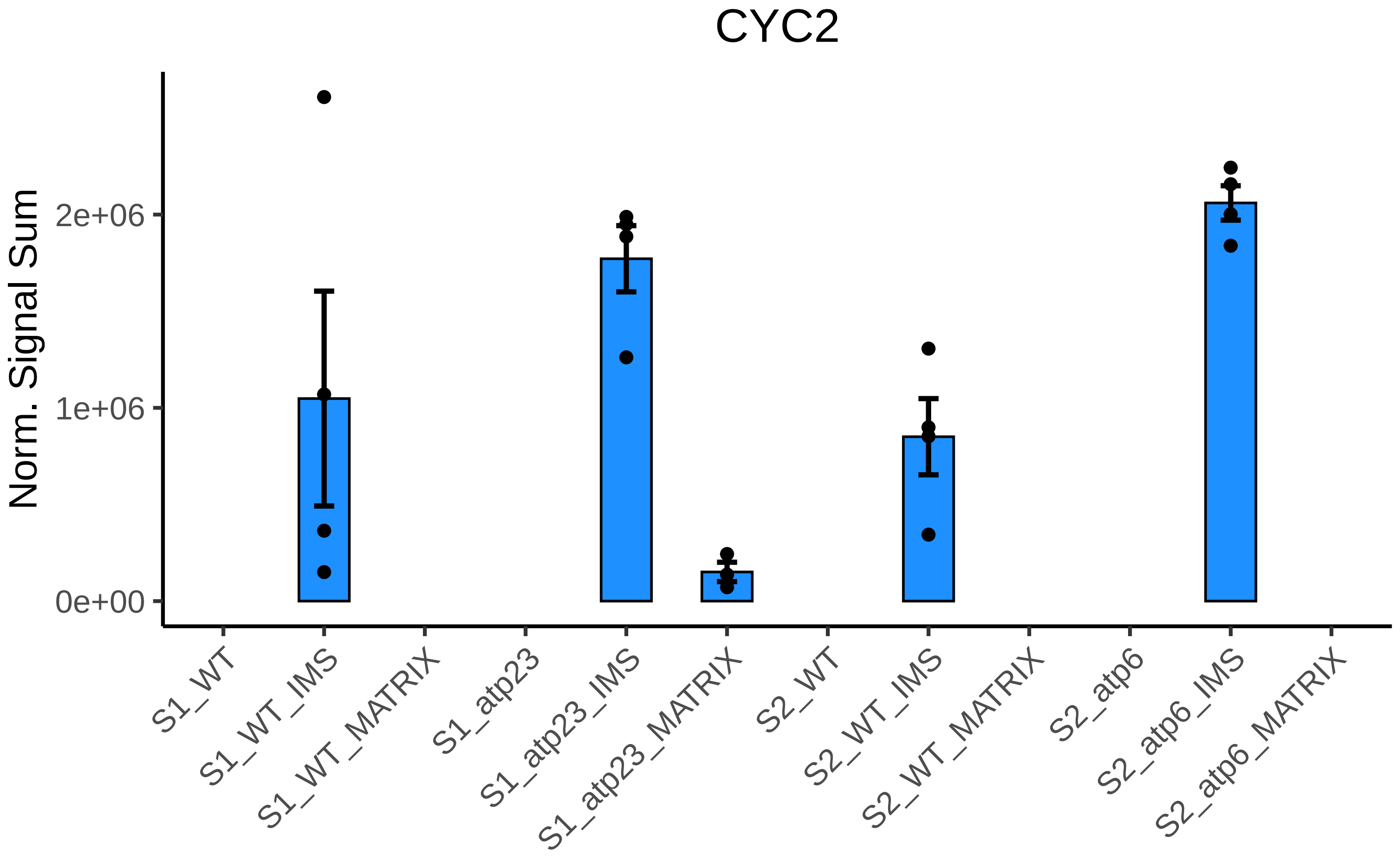
CYC2
Norm. Signal Sum
2e+06
1e+06
0e+00
S1_WT
S1_WT_IMS
S1_WT_MATRIX
S1_atp23
S1_atp23_IMS
S1_atp23_MATRIX
S2_WT
S2_WT_IMS
S2_WT_MATRIX
S2_atp6
S2_atp6_IMS
S2_atp6_MATRIX

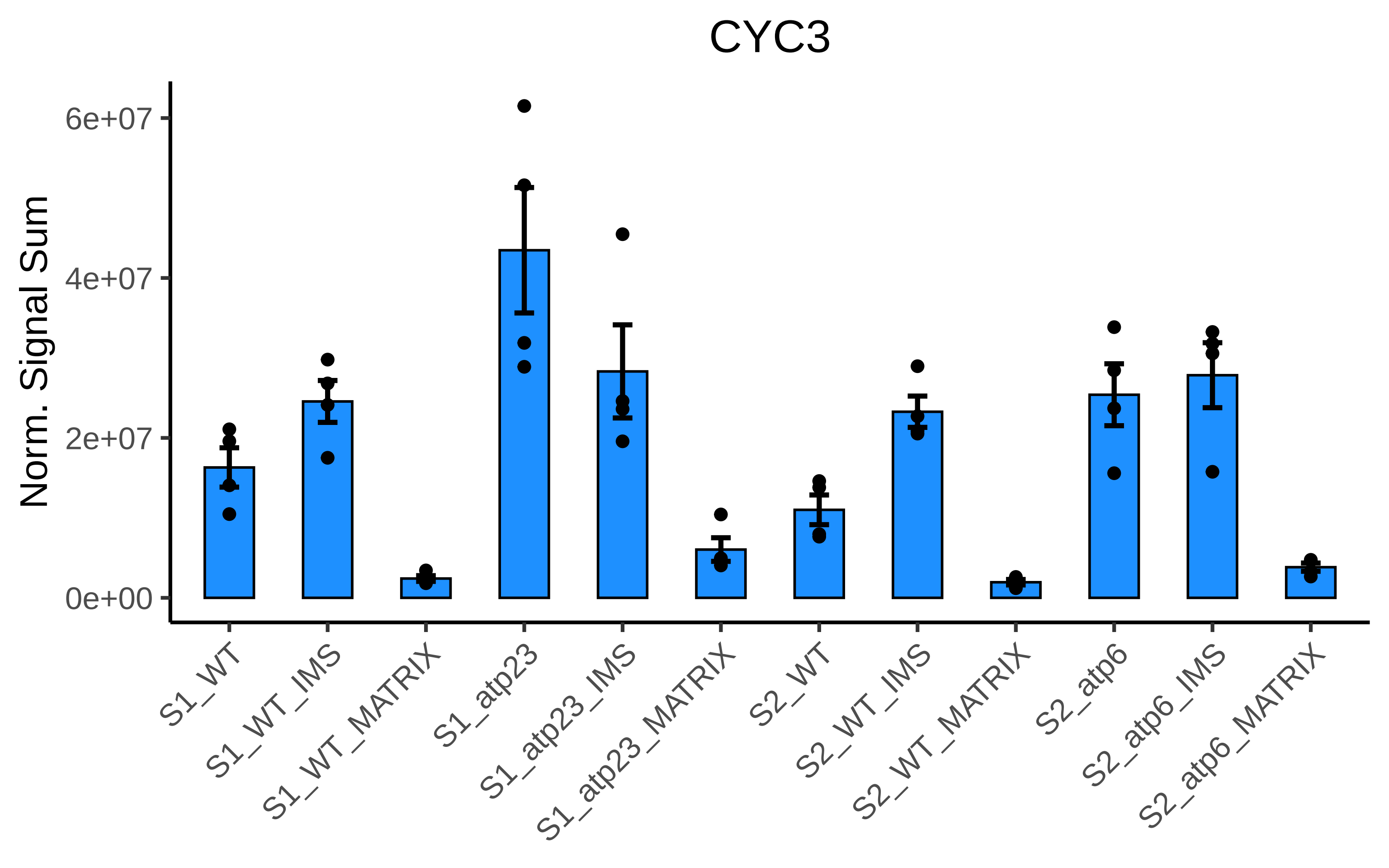

CYC3
Norm. Signal Sum
6e+07
4e+07
2e+07
0e+00
S1_WT
S1_WT_IMS
S1_WT_MATRIX
S1_atp23
S1_atp23_IMS
S1_atp23_MATRIX
S2_WT
S2_WT_IMS
S2_WT_MATRIX
S2_atp6
S2_atp6_IMS
S2_atp6_MATRIX

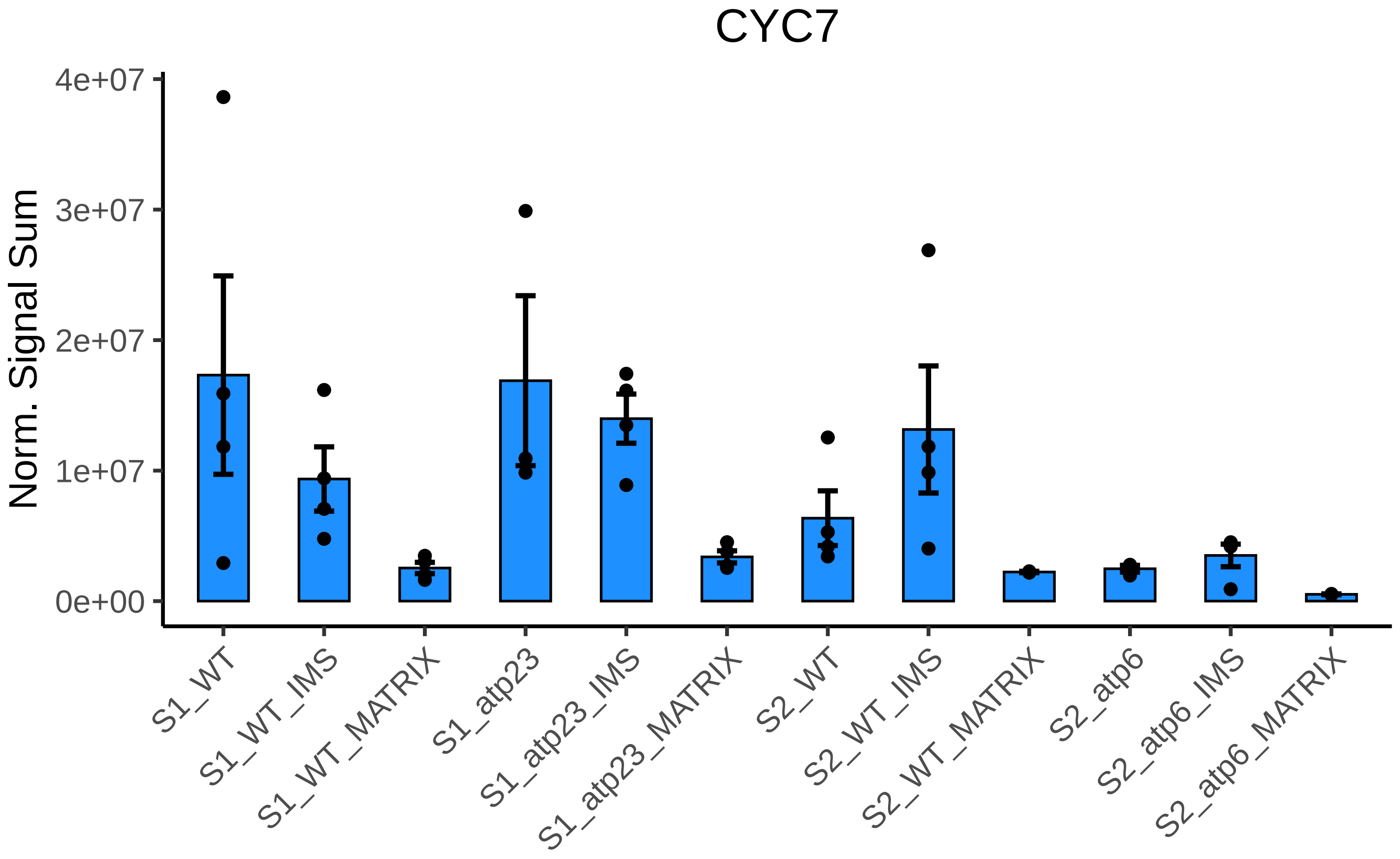

CYC7
Norm. Signal Sum
4e+07
3e+07
2e+07
1e+07
0e+00
S1_WT
S1_WT_IMS
S1_WT_MATRIX
S1_atp23
S1_atp23_IMS
S1_atp23_MATRIX
S2_WT
S2_WT_IMS
S2_WT_MATRIX
S2_atp6
S2_atp6_IMS
S2_atp6_MATRIX

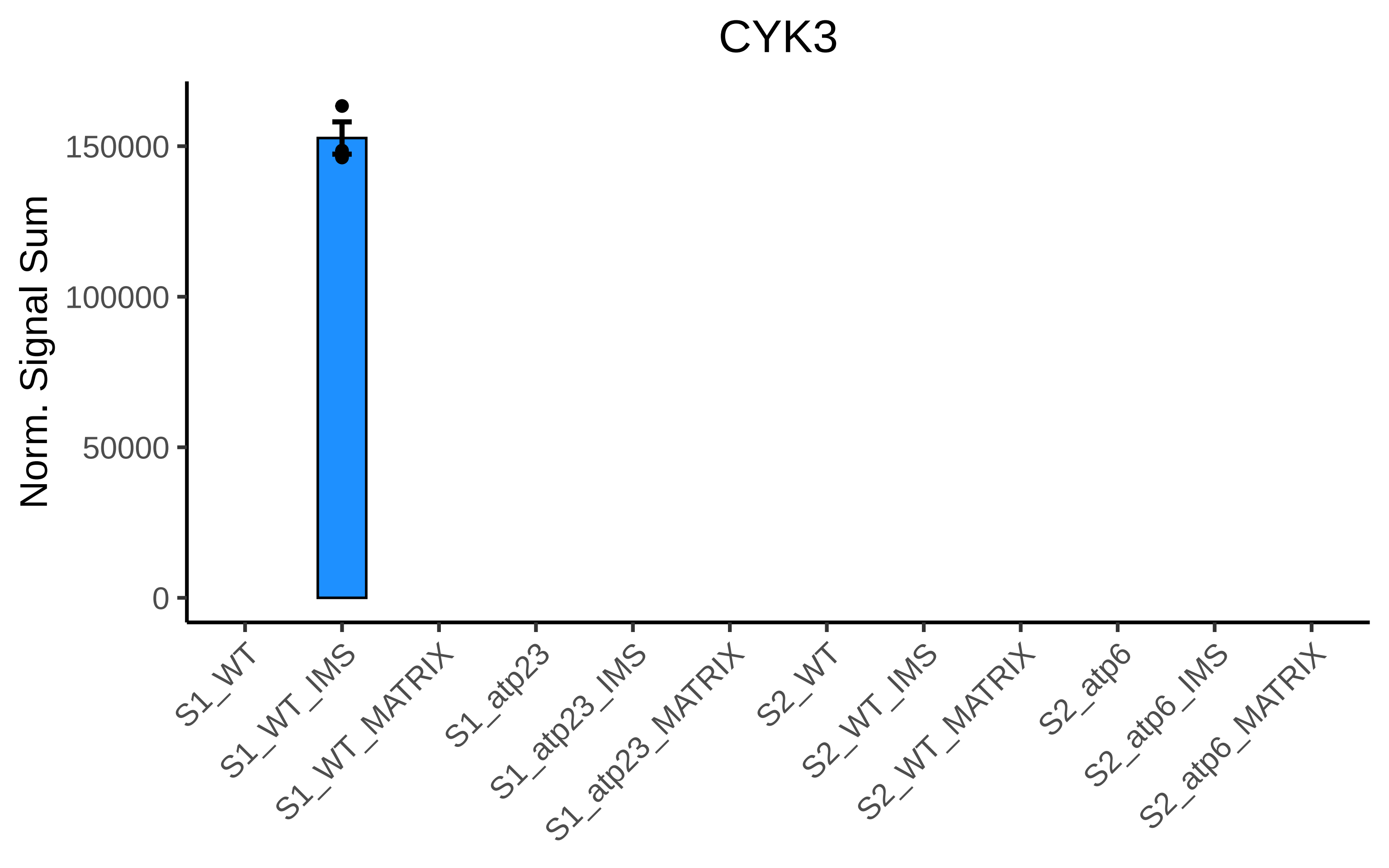

CYK3
Norm. Signal Sum
150000
100000
50000
0
S1_WT
S1_WT_IMS
S1_WT_MATRIX
S1_atp23
S1_atp23_IMS
S1_atp23_MATRIX
S2_WT
S2_WT_IMS
S2_WT_MATRIX
S2_atp6
S2_atp6_IMS
S2_atp6_MATRIX

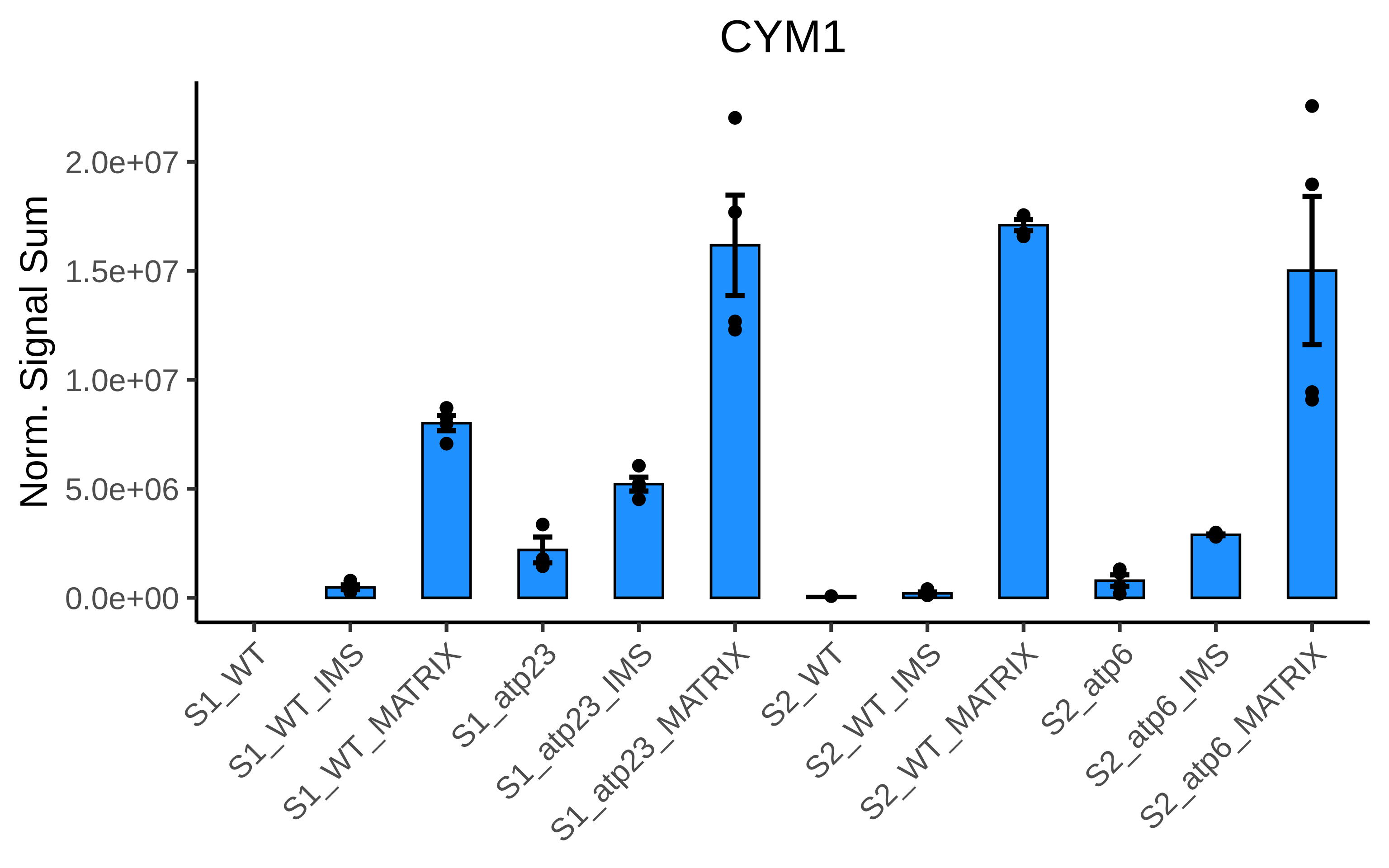
CYM1
Norm. Signal Sum
2.0e+07
1.5e+07
1.0e+07
5.0e+06
0.0e+00
S1_WT
S1_WT_IMS
S1_WT_MATRIX
S1_atp23
S1_atp23_IMS
S1_atp23_MATRIX
S2_WT
S2_WT_IMS
S2_WT_MATRIX
S2_atp6
S2_atp6_IMS
S2_atp6_MATRIX

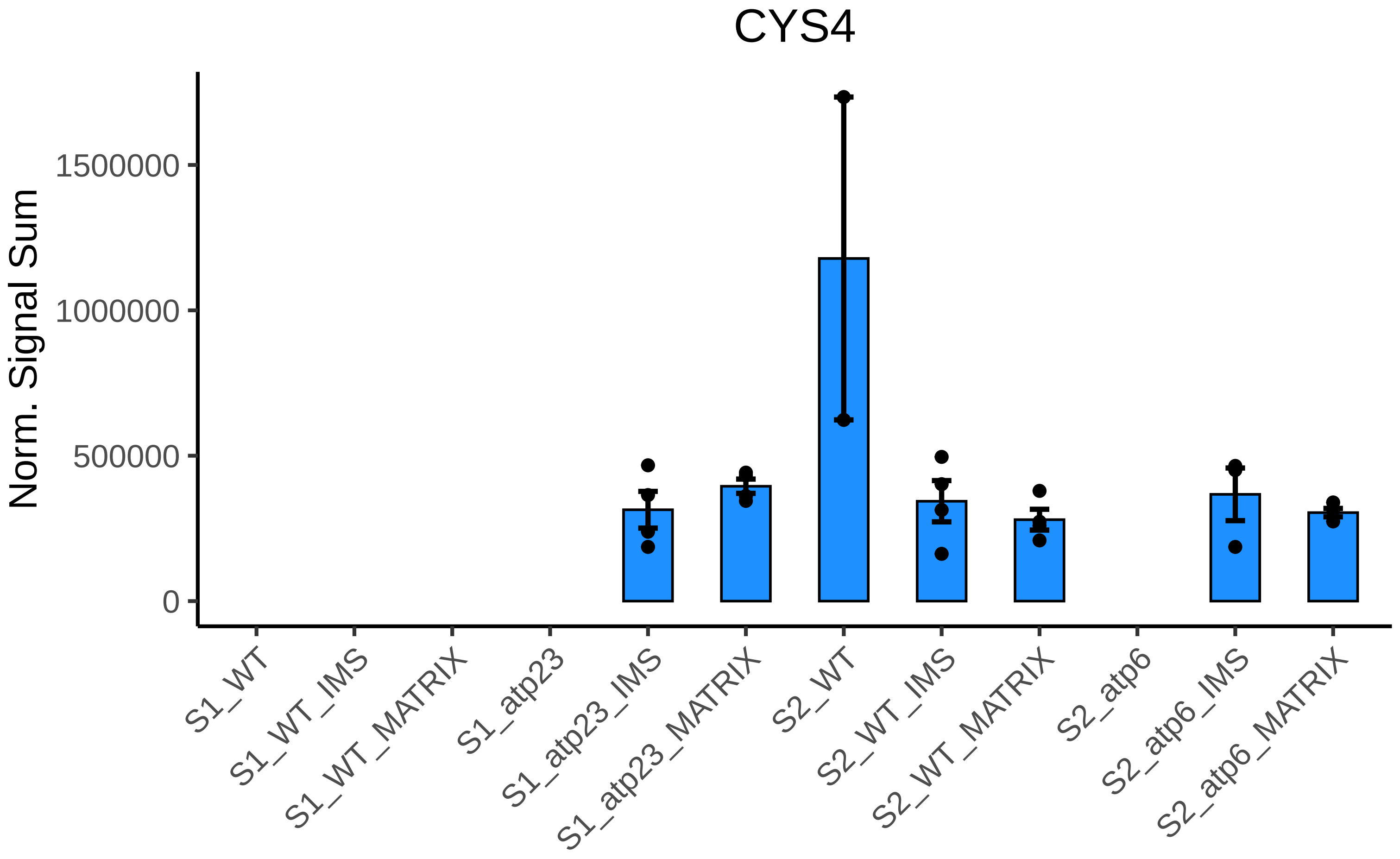

CYS4
Norm. Signal Sum
1500000
1000000
500000
0
S1_WT
S1_WT_IMS
S1_WT_MATRIX
S1_atp23
S1_atp23_IMS
S1_atp23_MATRIX
S2_WT
S2_WT_IMS
S2_WT_MATRIX
S2_atp6
S2_atp6_IMS
S2_atp6_MATRIX

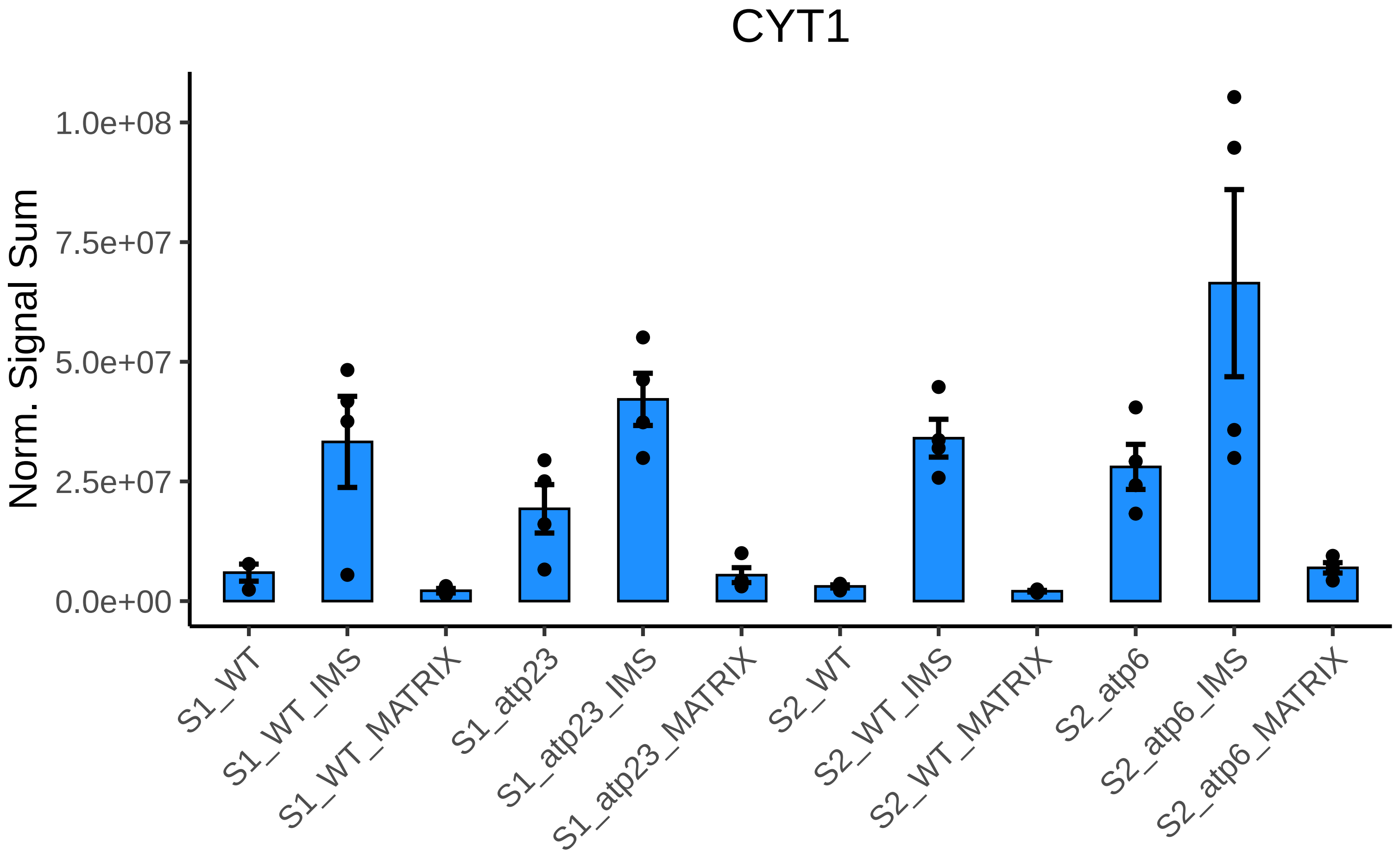

CYT1
Norm. Signal Sum
1.0e+08
7.5e+07
5.0e+07
2.5e+07
0.0e+00
S1_WT
S1_WT_IMS
S1_WT_MATRIX
S1_atp23
S1_atp23_IMS
S1_atp23_MATRIX
S2_WT
S2_WT_IMS
S2_WT_MATRIX
S2_atp6
S2_atp6_IMS
S2_atp6_MATRIX

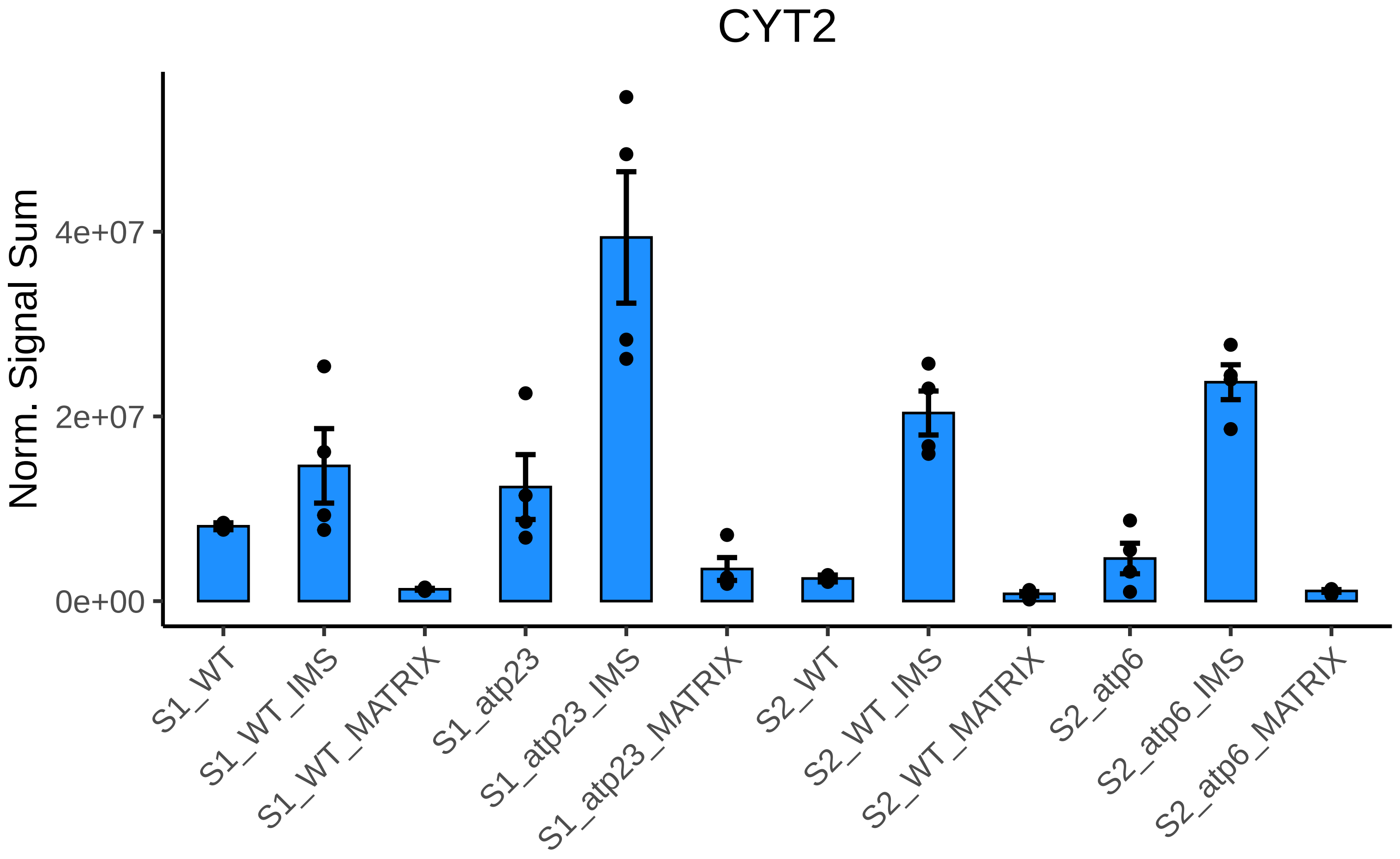

CYT2
Norm. Signal Sum
4e+07
2e+07
0e+00
S1_WT
S1_WT_IMS
S1_WT_MATRIX
S1_atp23
S1_atp23_IMS
S1_atp23_MATRIX
S2_WT
S2_WT_IMS
S2_WT_MATRIX
S2_atp6
S2_atp6_IMS
S2_atp6_MATRIX

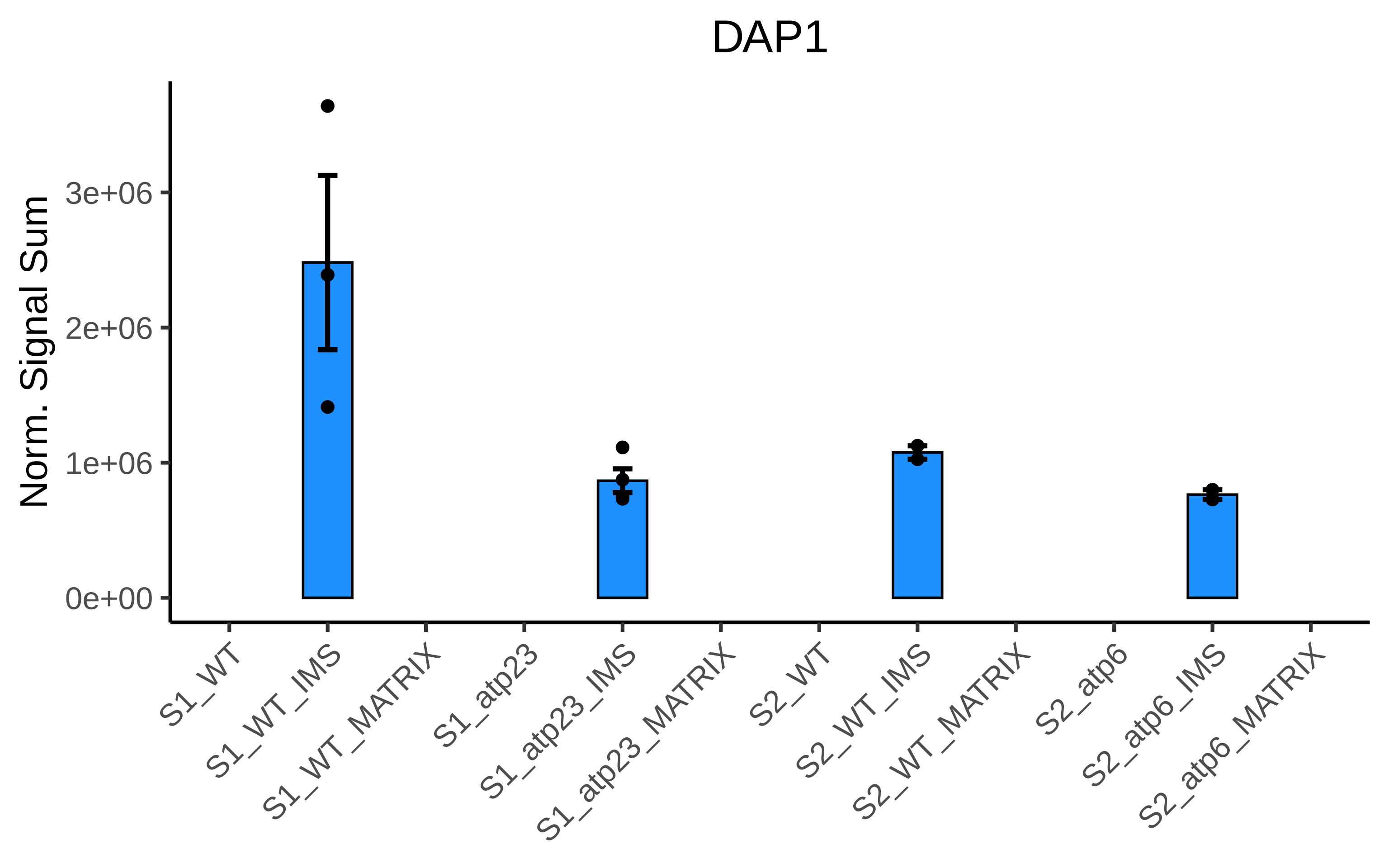

DAP1
Norm. Signal Sum
3e+06
2e+06
1e+06
0e+00
S1_WT
S1_WT_IMS
S1_WT_MATRIX
S1_atp23
S1_atp23_IMS
S1_atp23_MATRIX
S2_WT
S2_WT_IMS
S2_WT_MATRIX
S2_atp6
S2_atp6_IMS
S2_atp6_MATRIX

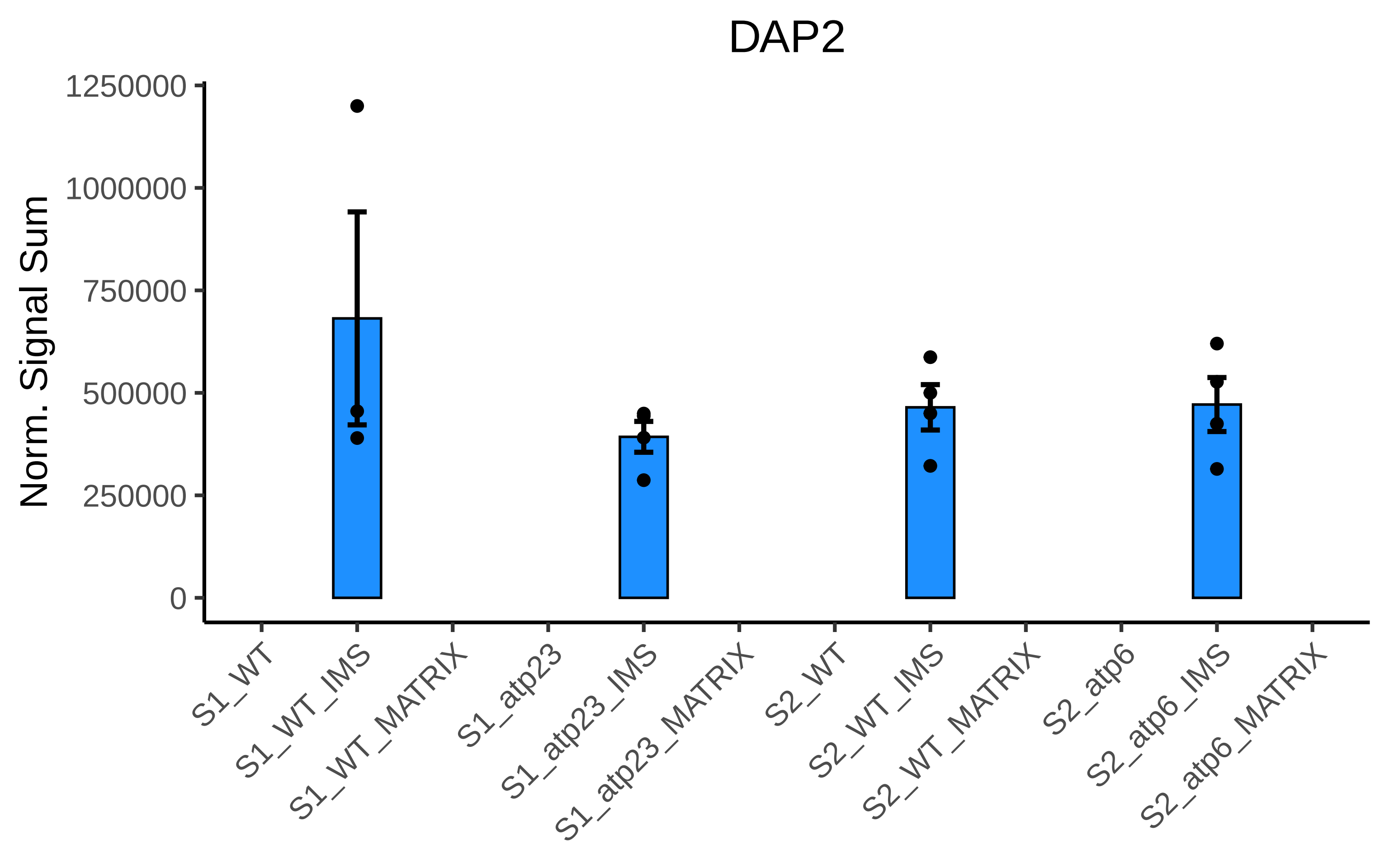

DAP2
Norm. Signal Sum
1250000
1000000
750000
500000
250000
0
S1_WT
S1_WT_IMS
S1_WT_MATRIX
S1_atp23
S1_atp23_IMS
S1_atp23_MATRIX
S2_WT
S2_WT_IMS
S2_WT_MATRIX
S2_atp6
S2_atp6_IMS
S2_atp6_MATRIX

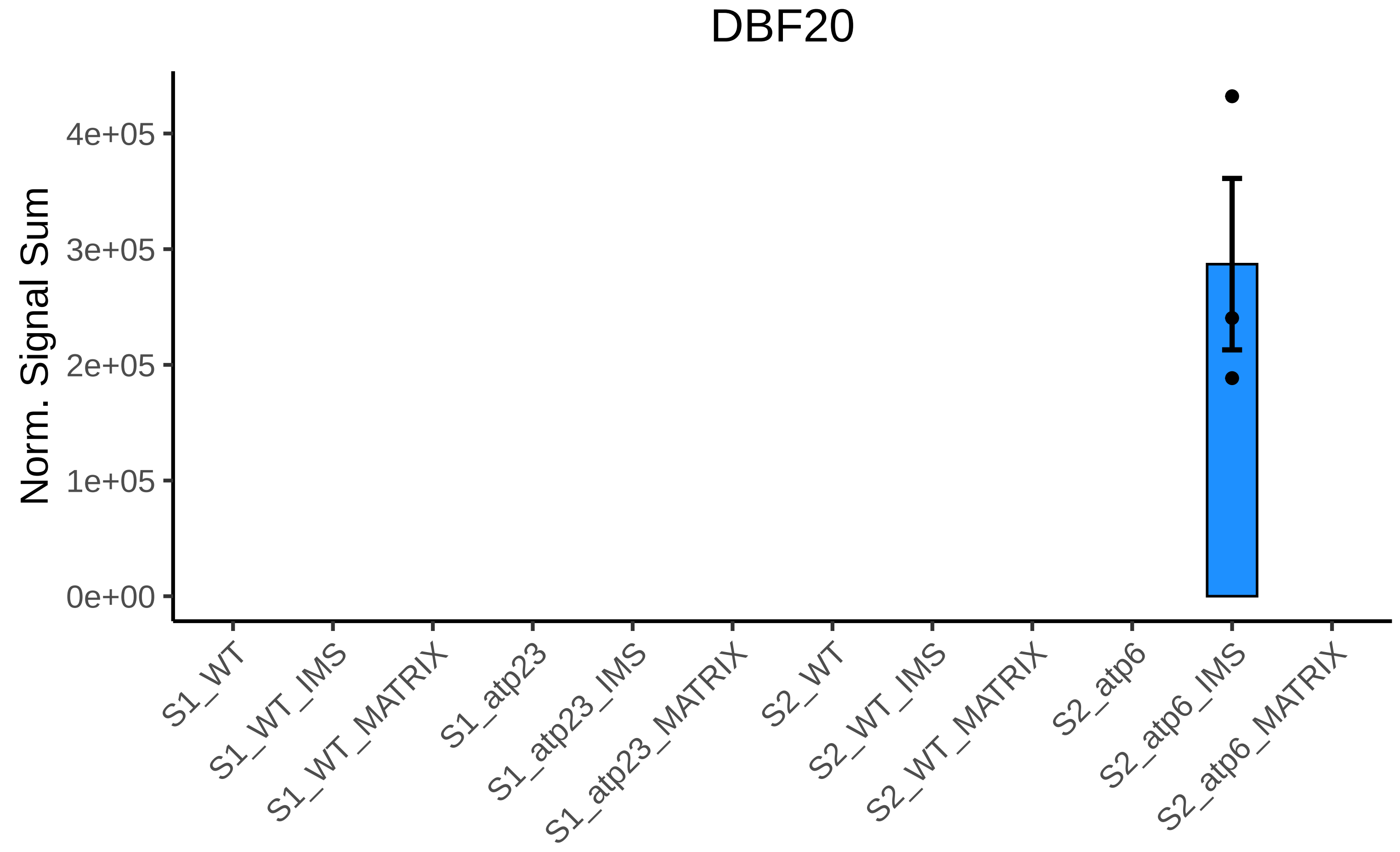

DBF20
Norm. Signal Sum
4e+05
3e+05
2e+05
1e+05
0e+00
S1_WT
S1_WT_IMS
S1_WT_MATRIX
S1_atp23
S1_atp23_IMS
S1_atp23_MATRIX
S2_WT
S2_WT_IMS
S2_WT_MATRIX
S2_atp6
S2_atp6_IMS
S2_atp6_MATRIX

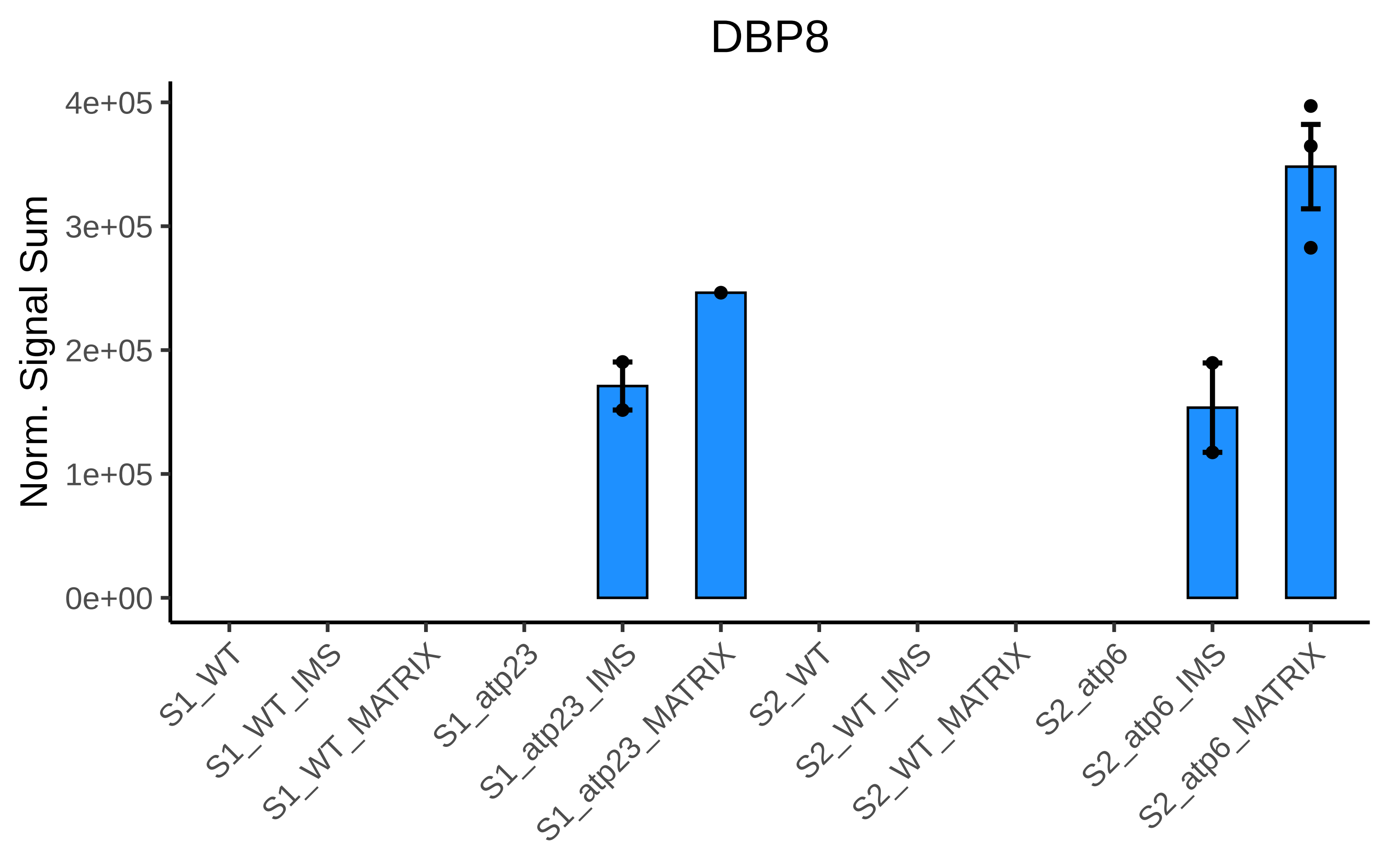
DBP8
Norm. Signal Sum
4e+05
3e+05
2e+05
1e+05
0e+00
S1_WT
S1_WT_IMS
S1_WT_MATRIX
S1_atp23
S1_atp23_IMS
S1_atp23_MATRIX
S2_WT
S2_WT_IMS
S2_WT_MATRIX
S2_atp6
S2_atp6_IMS
S2_atp6_MATRIX

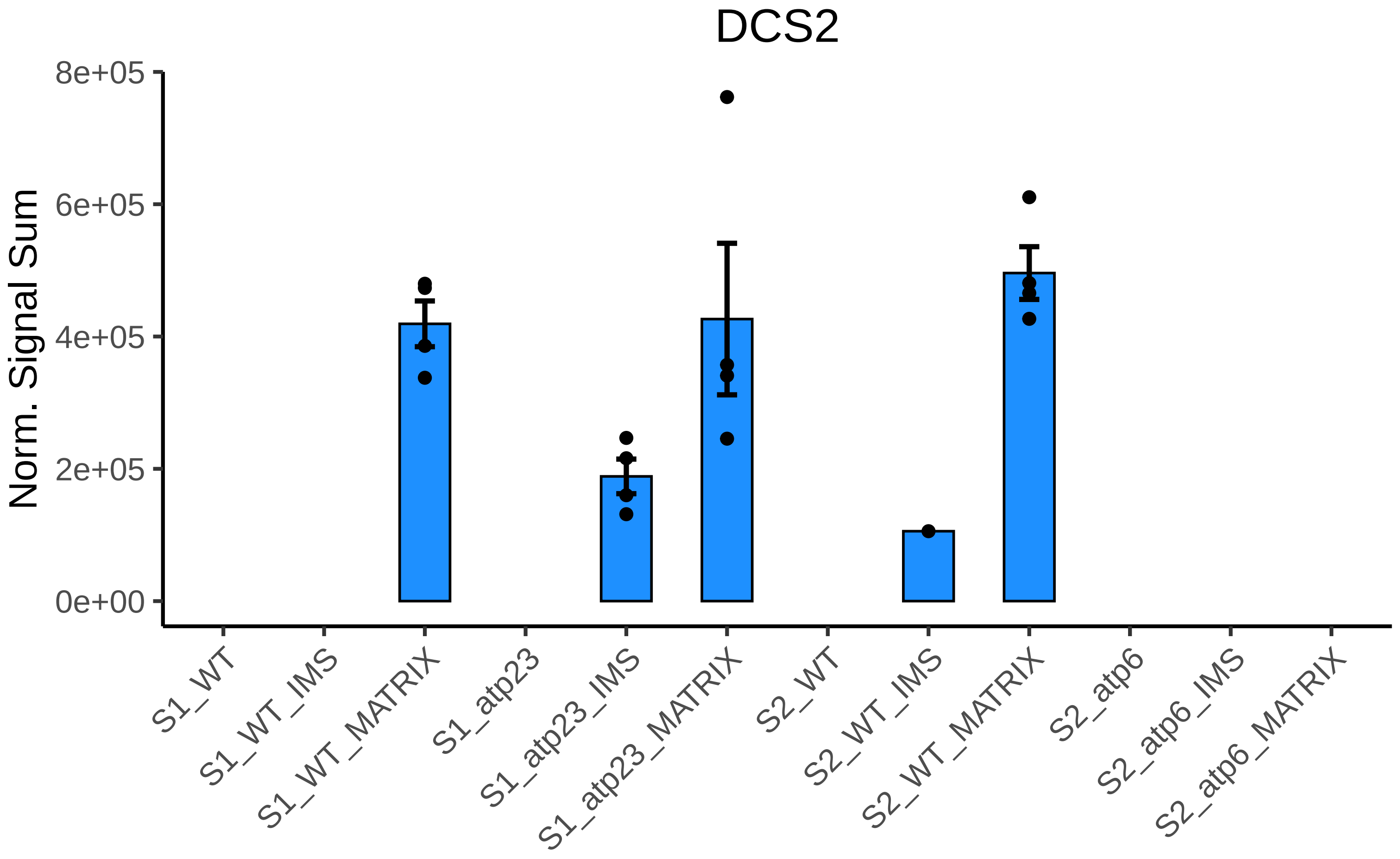
DCS2
Norm. Signal Sum
8e+05
6e+05
4e+05
2e+05
0e+00
S1_WT
S1_WT_IMS
S1_WT_MATRIX
S1_atp23
S1_atp23_IMS
S1_atp23_MATRIX
S2_WT
S2_WT_IMS
S2_WT_MATRIX
S2_atp6
S2_atp6_IMS
S2_atp6_MATRIX

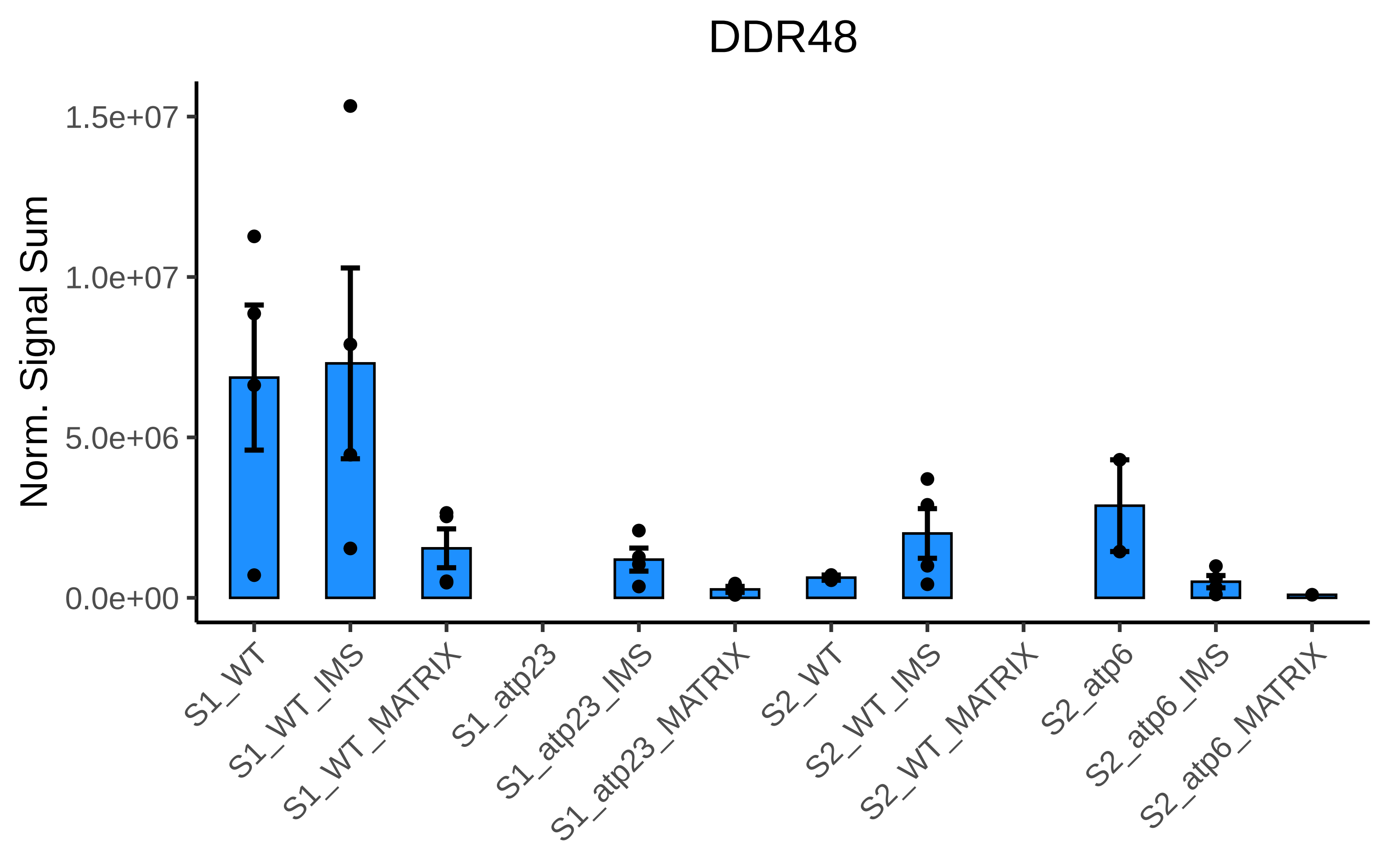

DDR48
Norm. Signal Sum
1.5e+07
1.0e+07
5.0e+06
0.0e+00
S1_WT
S1_WT_IMS
S1_WT_MATRIX
S1_atp23
S1_atp23_IMS
S1_atp23_MATRIX
S2_WT
S2_WT_IMS
S2_WT_MATRIX
S2_atp6
S2_atp6_IMS
S2_atp6_MATRIX

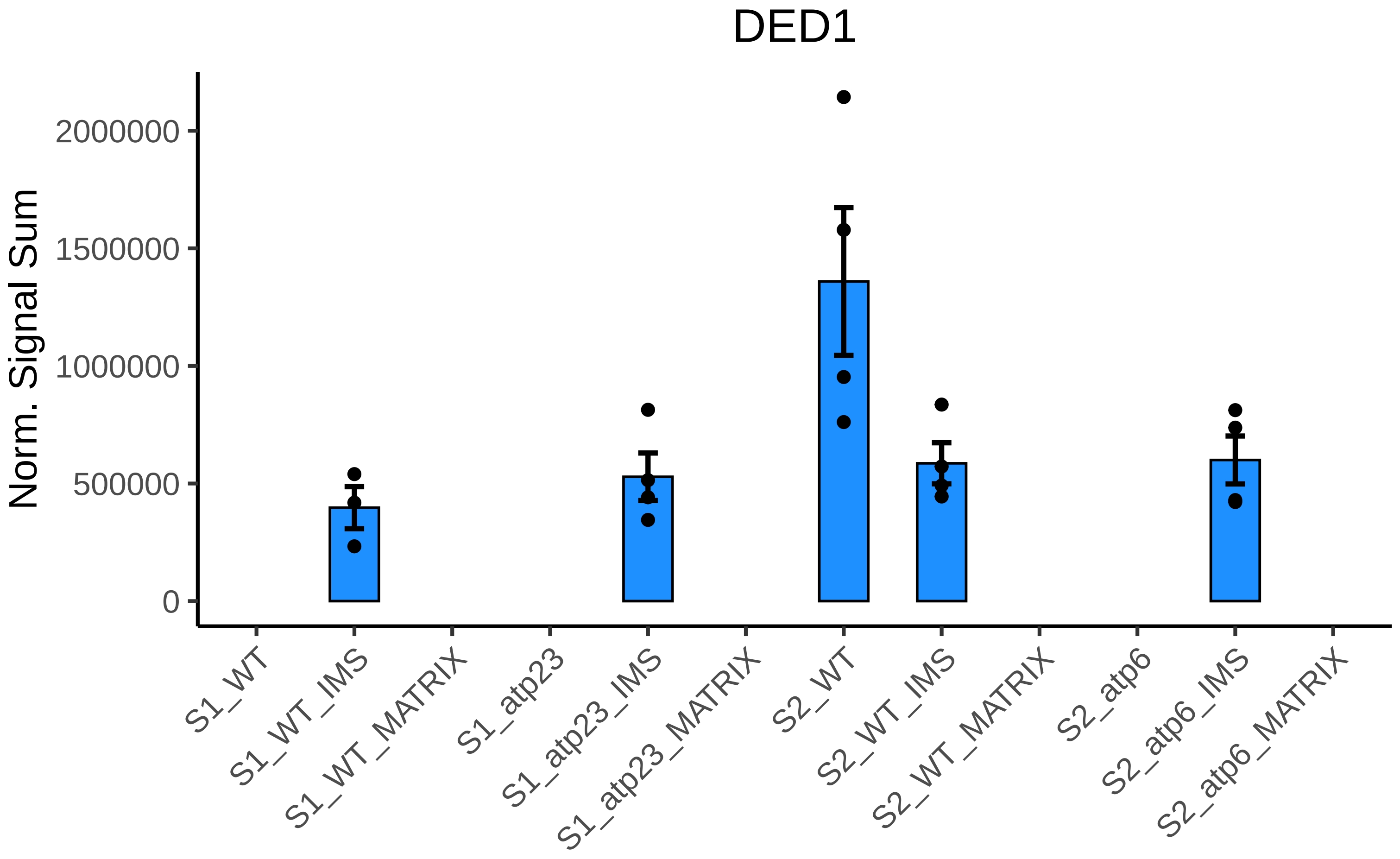

DED1
Norm. Signal Sum
2000000
1500000
1000000
500000
0
S1_WT
S1_WT_IMS
S1_WT_MATRIX
S1_atp23
S1_atp23_IMS
S1_atp23_MATRIX
S2_WT
S2_WT_IMS
S2_WT_MATRIX
S2_atp6
S2_atp6_IMS
S2_atp6_MATRIX

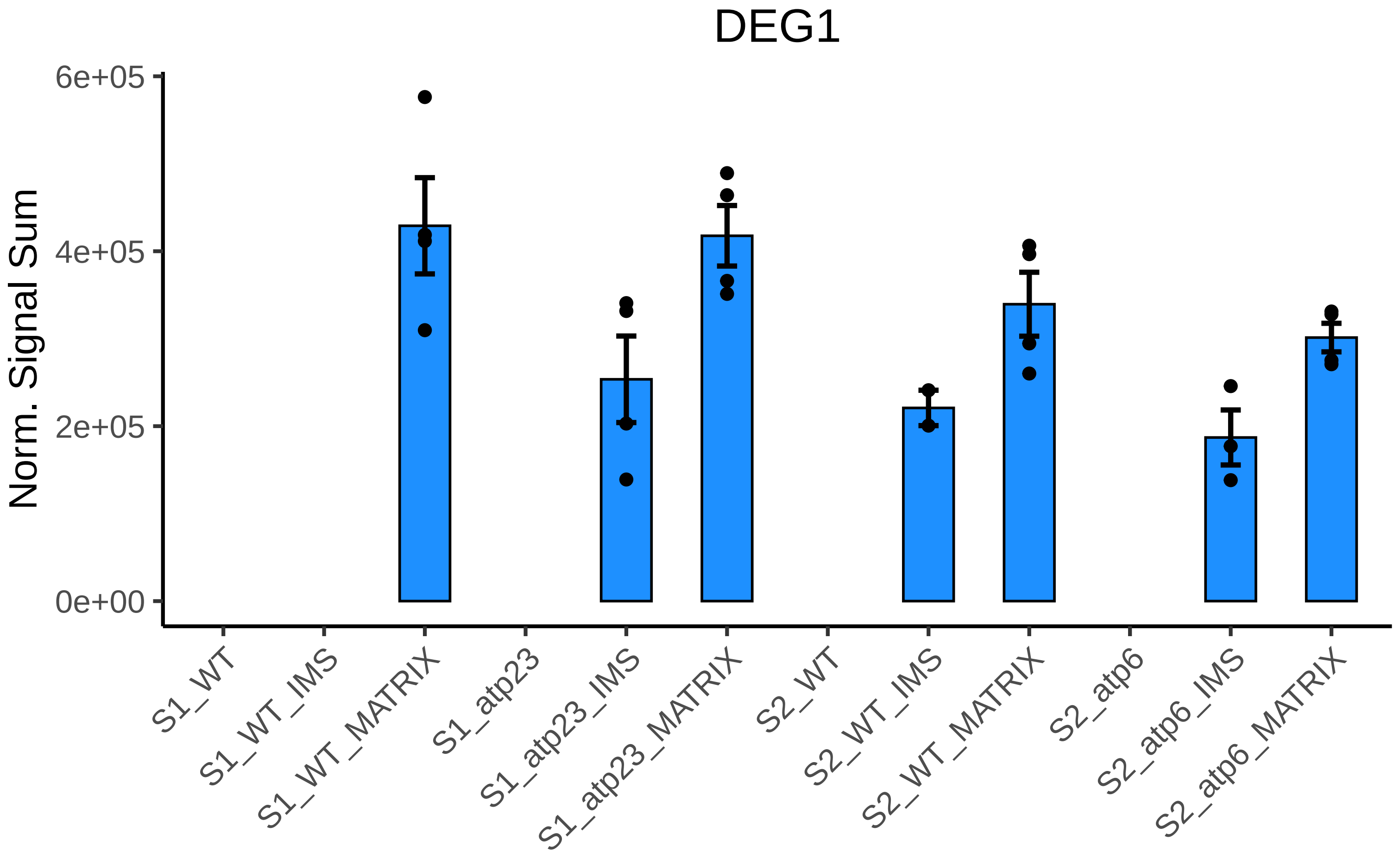

DEG1
Norm. Signal Sum
6e+05
4e+05
2e+05
0e+00
S1_WT
S1_WT_IMS
S1_WT_MATRIX
S1_atp23
S1_atp23_IMS
S1_atp23_MATRIX
S2_WT
S2_WT_IMS
S2_WT_MATRIX
S2_atp6
S2_atp6_IMS
S2_atp6_MATRIX

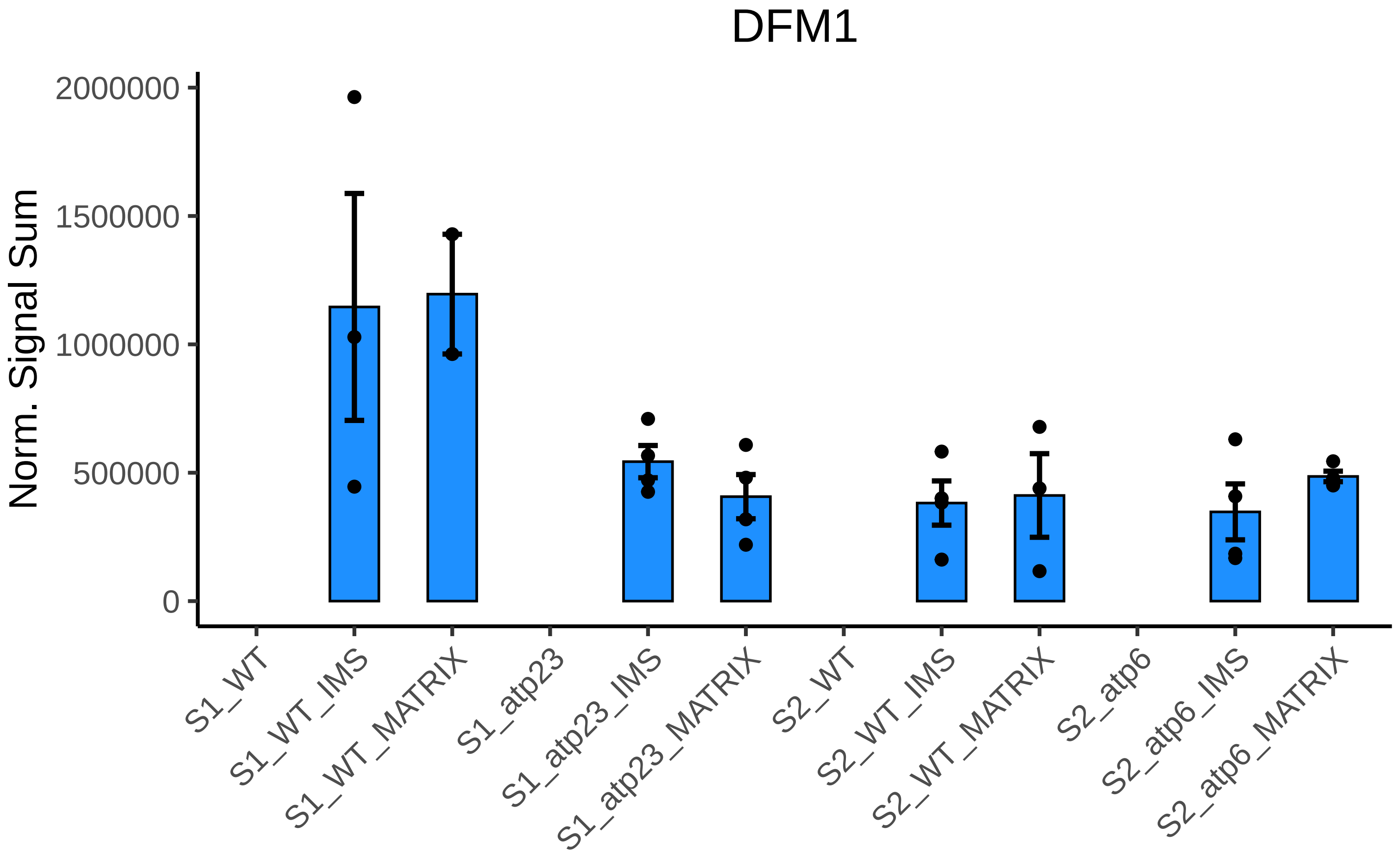

DFM1
Norm. Signal Sum
2000000
1500000
1000000
500000
0
S1_WT
S1_WT_IMS
S1_WT_MATRIX
S1_atp23
S1_atp23_IMS
S1_atp23_MATRIX
S2_WT
S2_WT_IMS
S2_WT_MATRIX
S2_atp6
S2_atp6_IMS
S2_atp6_MATRIX

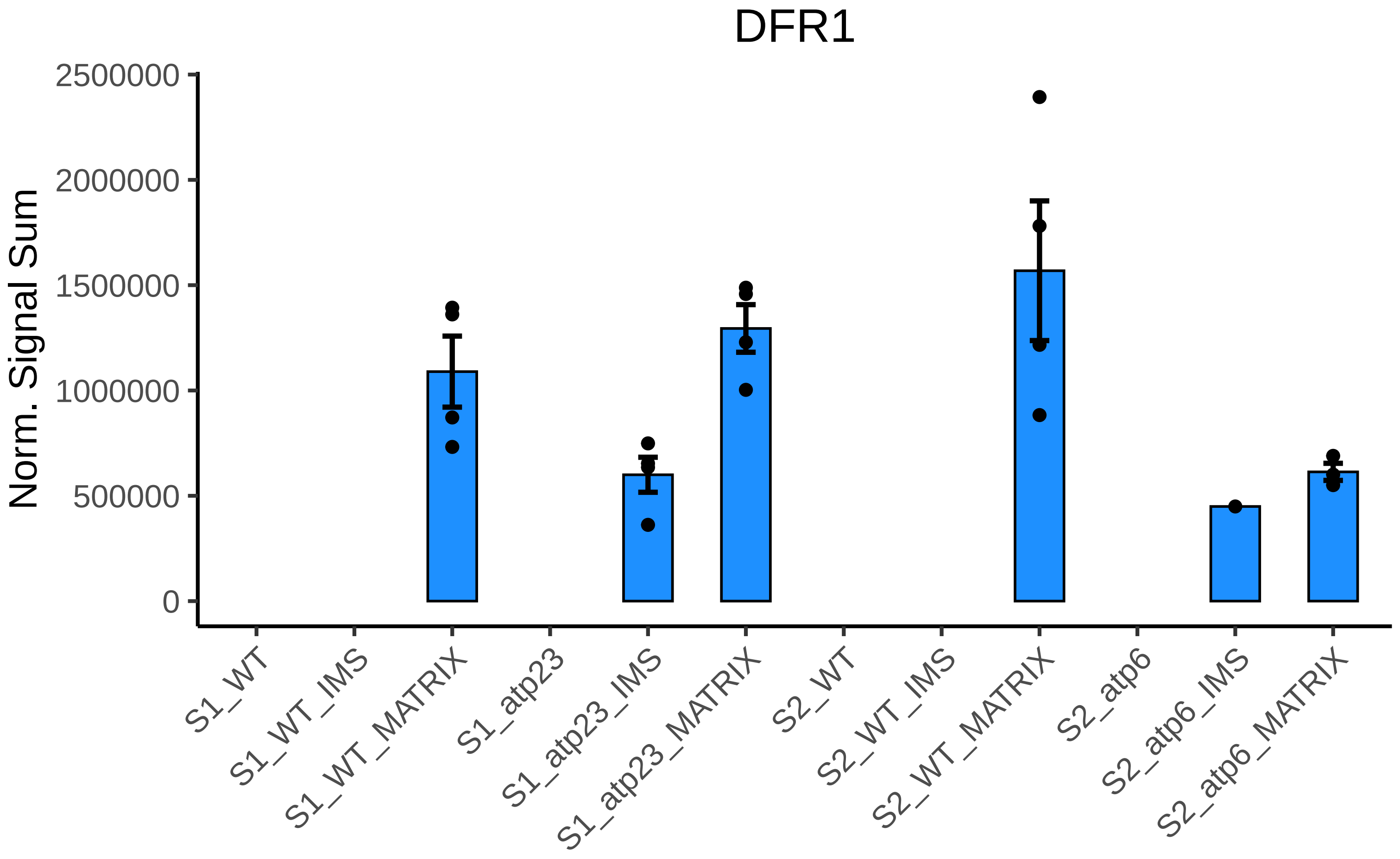

DFR1
Norm. Signal Sum
2500000
2000000
1500000
1000000
500000
0
S1_WT
S1_WT_IMS
S1_WT_MATRIX
S1_atp23
S1_atp23_IMS
S1_atp23_MATRIX
S2_WT
S2_WT_IMS
S2_WT_MATRIX
S2_atp6
S2_atp6_IMS
S2_atp6_MATRIX

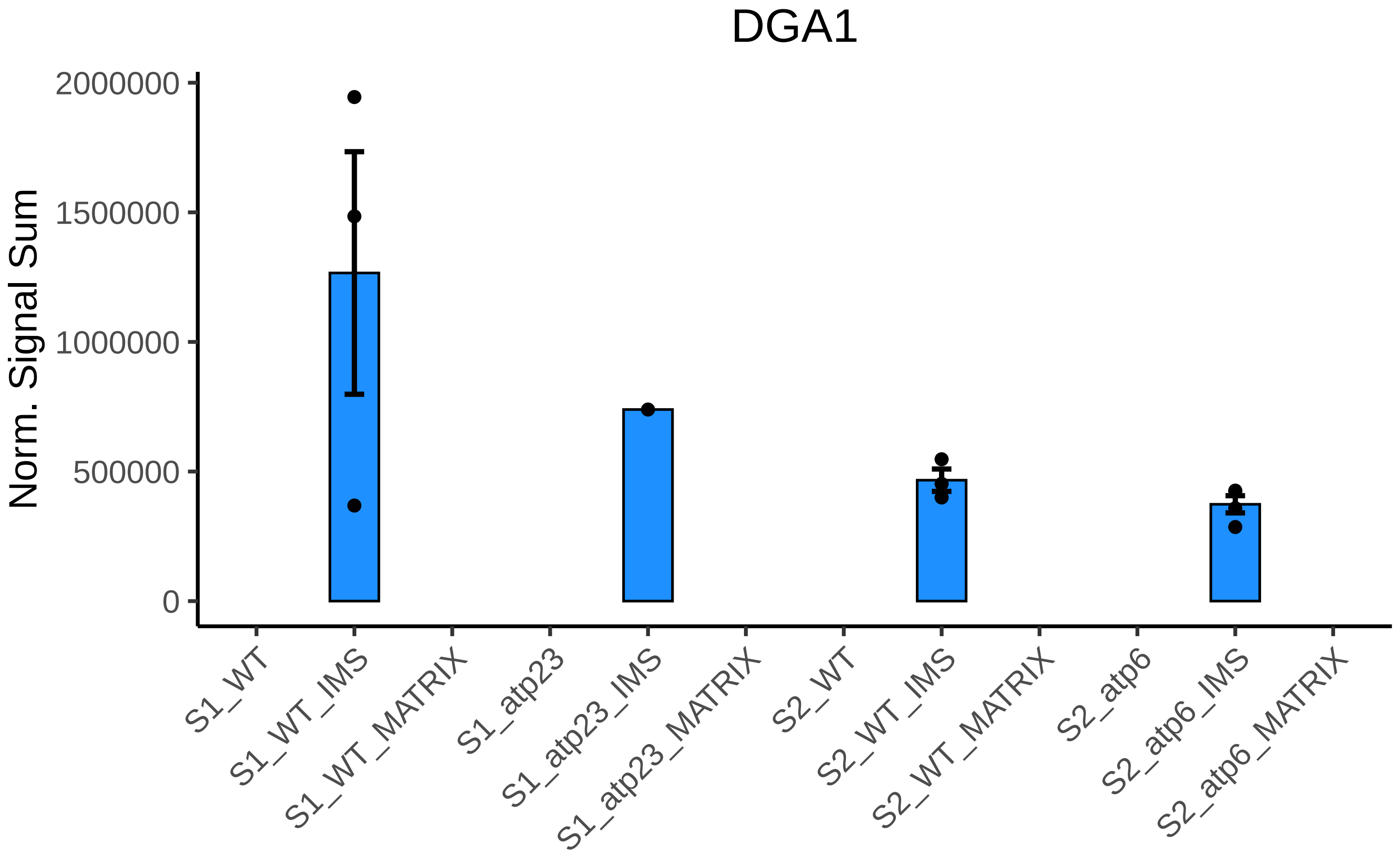

DGA1
Norm. Signal Sum
2000000
1500000
1000000
500000
0
S1_WT
S1_WT_IMS
S1_WT_MATRIX
S1_atp23
S1_atp23_IMS
S1_atp23_MATRIX
S2_WT
S2_WT_IMS
S2_WT_MATRIX
S2_atp6
S2_atp6_IMS
S2_atp6_MATRIX

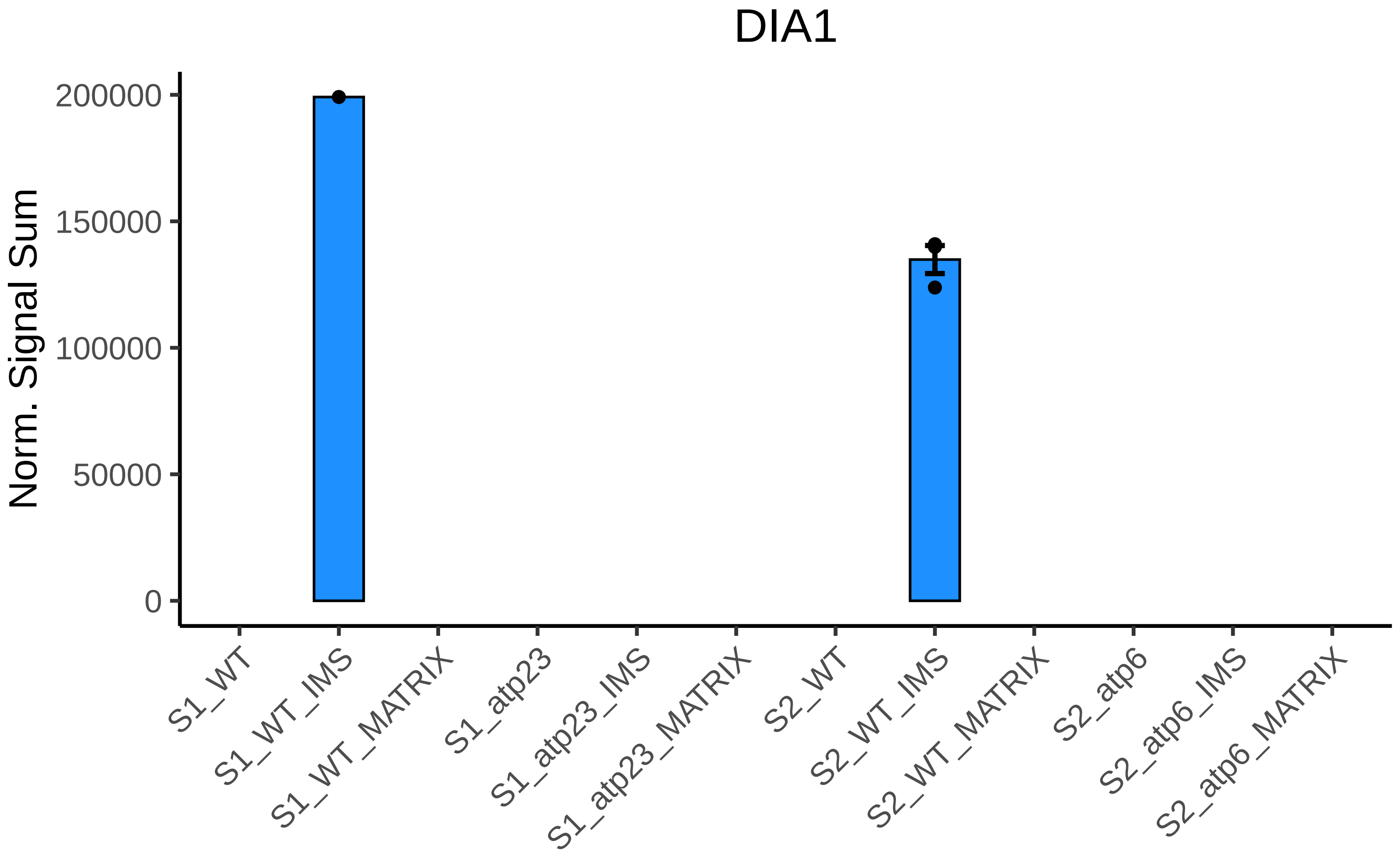
DIA1
Norm. Signal Sum
200000
150000
100000
50000
0
S1_WT
S1_WT_IMS
S1_WT_MATRIX
S1_atp23
S1_atp23_IMS
S1_atp23_MATRIX
S2_WT
S2_WT_IMS
S2_WT_MATRIX
S2_atp6
S2_atp6_IMS
S2_atp6_MATRIX

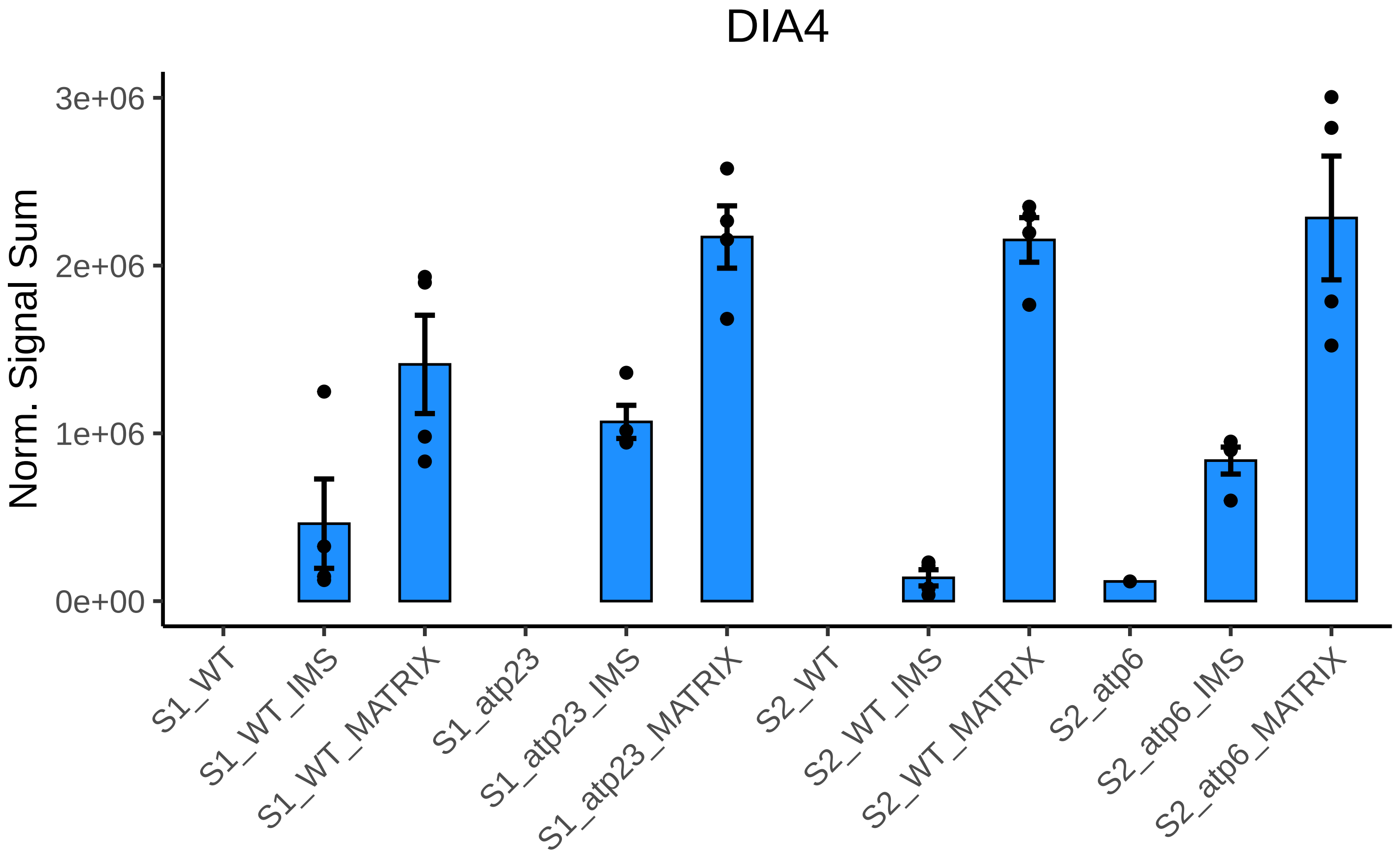
DIA4
Norm. Signal Sum
3e+06
2e+06
1e+06
0e+00
S1_WT
S1_WT_IMS
S1_WT_MATRIX
S1_atp23
S1_atp23_IMS
S1_atp23_MATRIX
S2_WT
S2_WT_IMS
S2_WT_MATRIX
S2_atp6
S2_atp6_IMS
S2_atp6_MATRIX

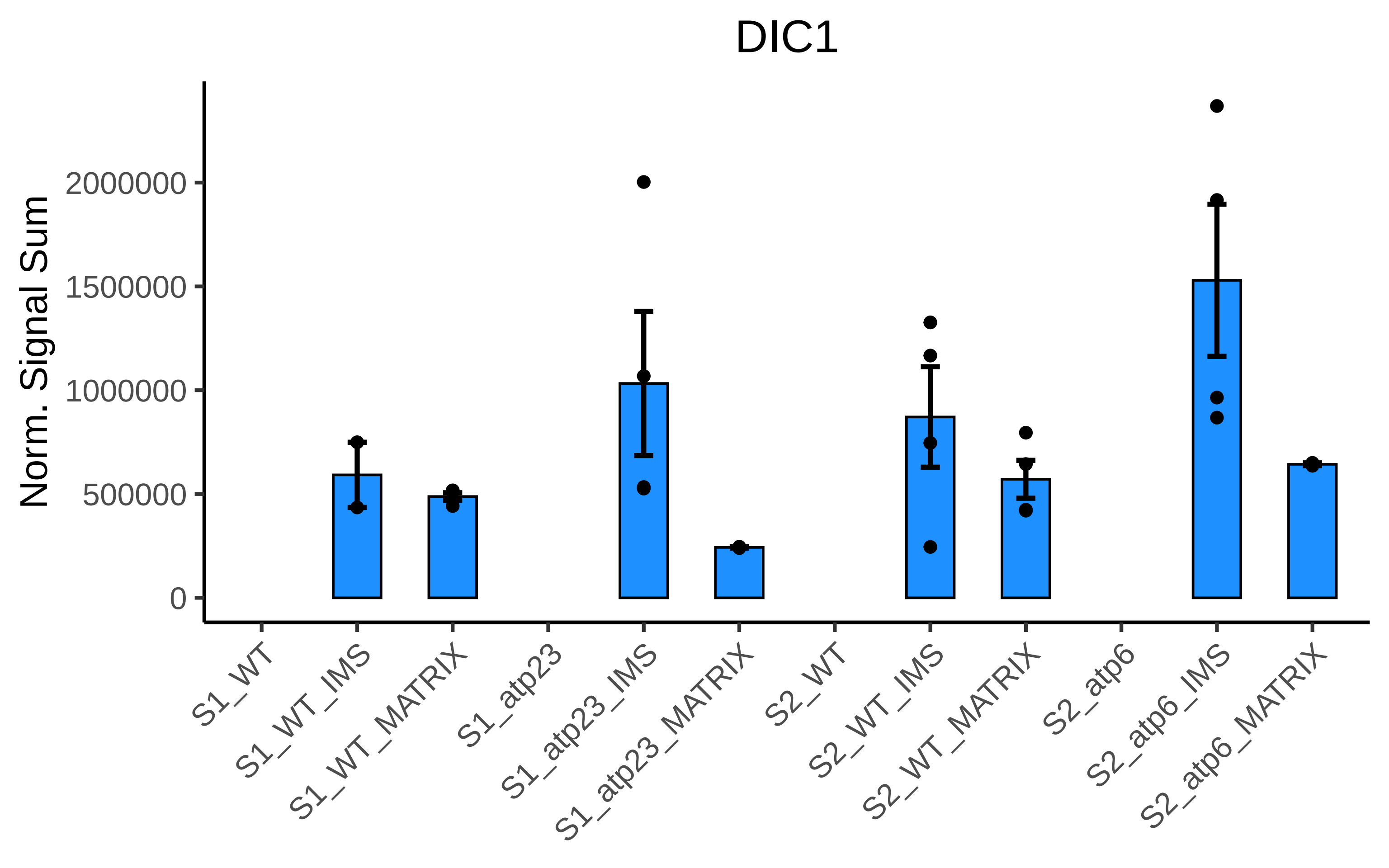

DIC1
Norm. Signal Sum
2000000
1500000
1000000
500000
0
S1_WT
S1_WT_IMS
S1_WT_MATRIX
S1_atp23
S1_atp23_IMS
S1_atp23_MATRIX
S2_WT
S2_WT_IMS
S2_WT_MATRIX
S2_atp6
S2_atp6_IMS
S2_atp6_MATRIX

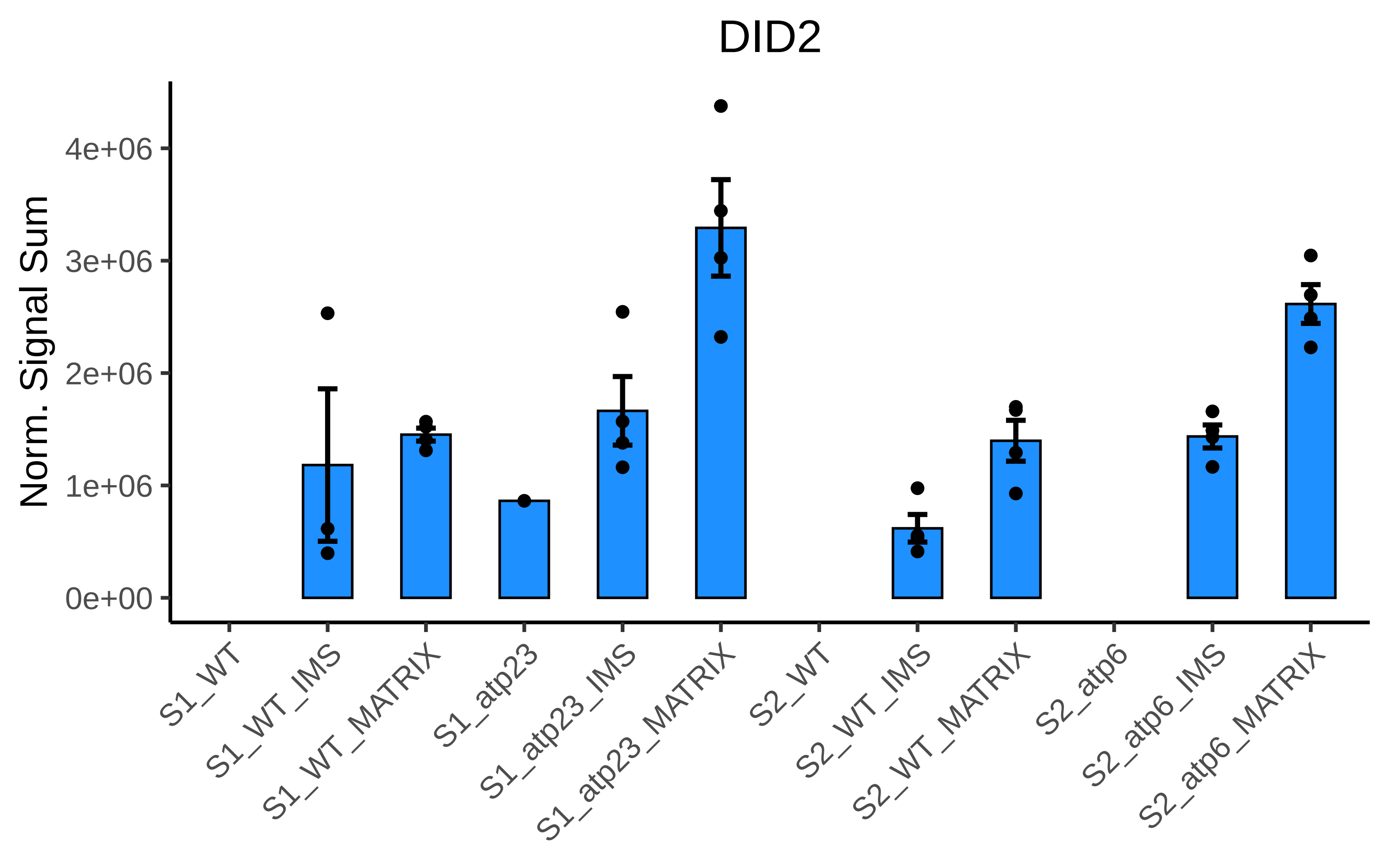
DID2
Norm. Signal Sum
4e+06
3e+06
2e+06
1e+06
0e+00
S1_WT
S1_WT_IMS
S1_WT_MATRIX
S1_atp23
S1_atp23_IMS
S1_atp23_MATRIX
S2_WT
S2_WT_IMS
S2_WT_MATRIX
S2_atp6
S2_atp6_IMS
S2_atp6_MATRIX

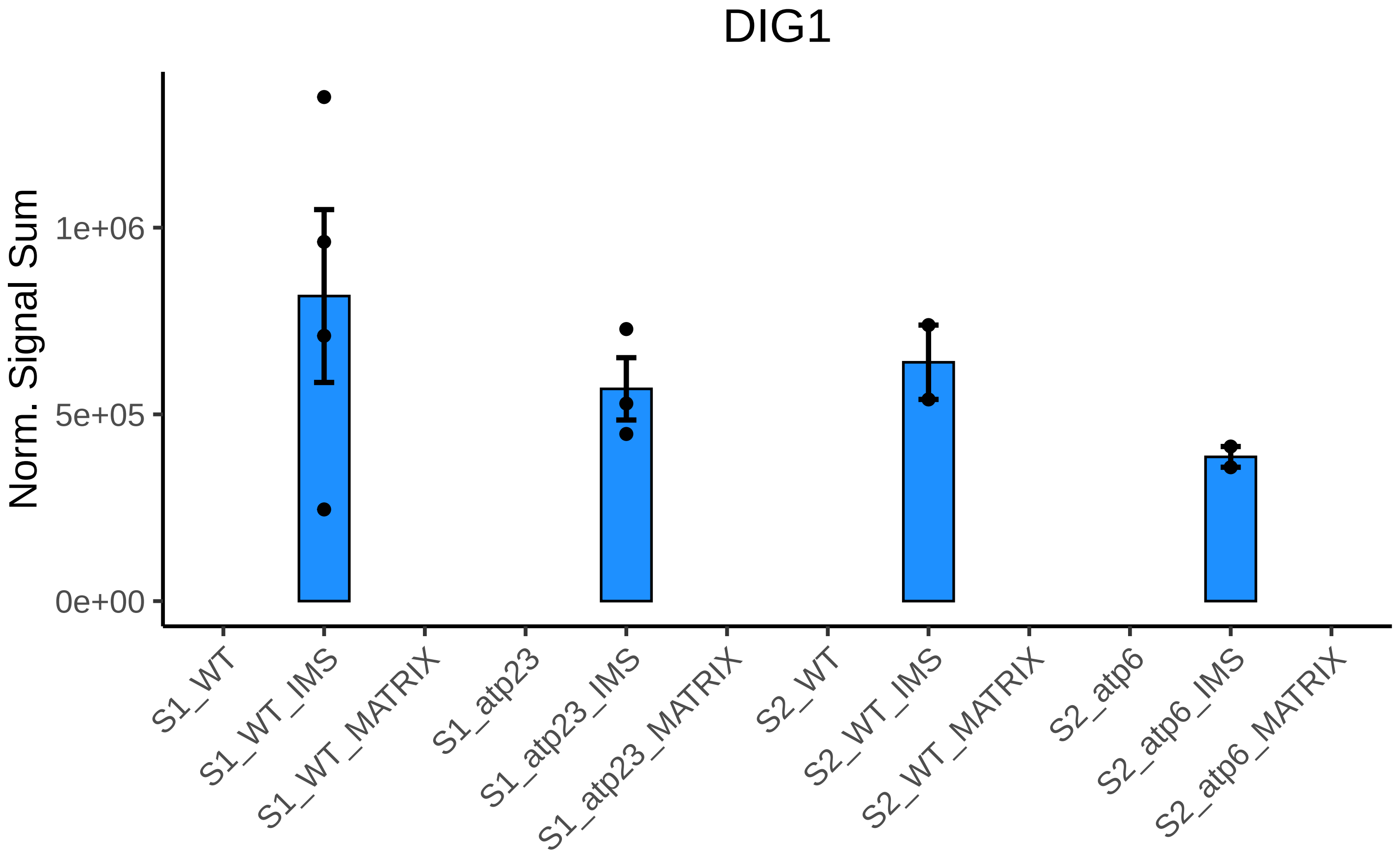

DIG1
Norm. Signal Sum
1e+06
5e+05
0e+00
S1_WT
S1_WT_IMS
S1_WT_MATRIX
S1_atp23
S1_atp23_IMS
S1_atp23_MATRIX
S2_WT
S2_WT_IMS
S2_WT_MATRIX
S2_atp6
S2_atp6_IMS
S2_atp6_MATRIX

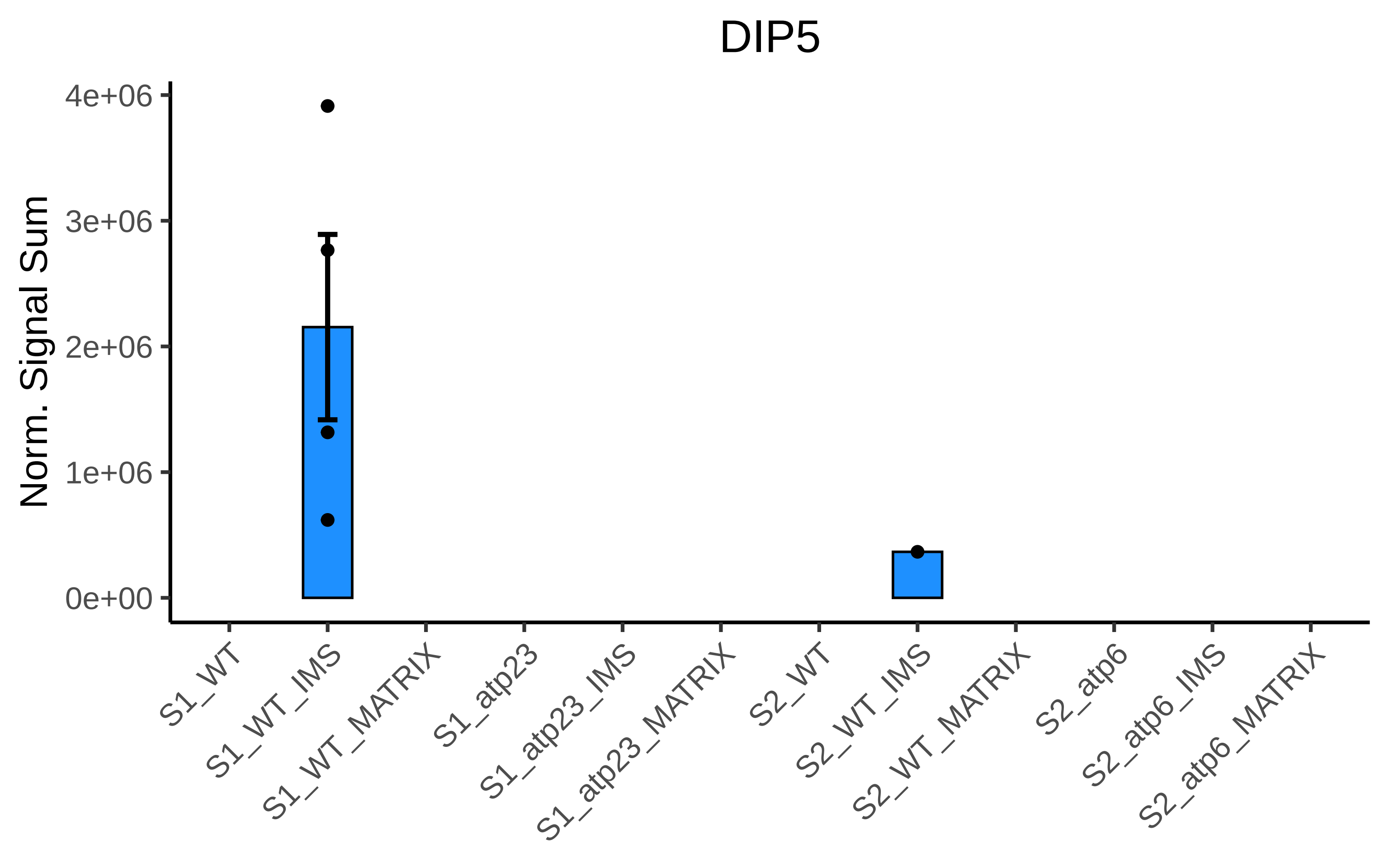

DIP5
Norm. Signal Sum
4e+06
3e+06
2e+06
1e+06
0e+00
S1_WT
S1_WT_IMS
S1_WT_MATRIX
S1_atp23
S1_atp23_IMS
S1_atp23_MATRIX
S2_WT
S2_WT_IMS
S2_WT_MATRIX
S2_atp6
S2_atp6_IMS
S2_atp6_MATRIX

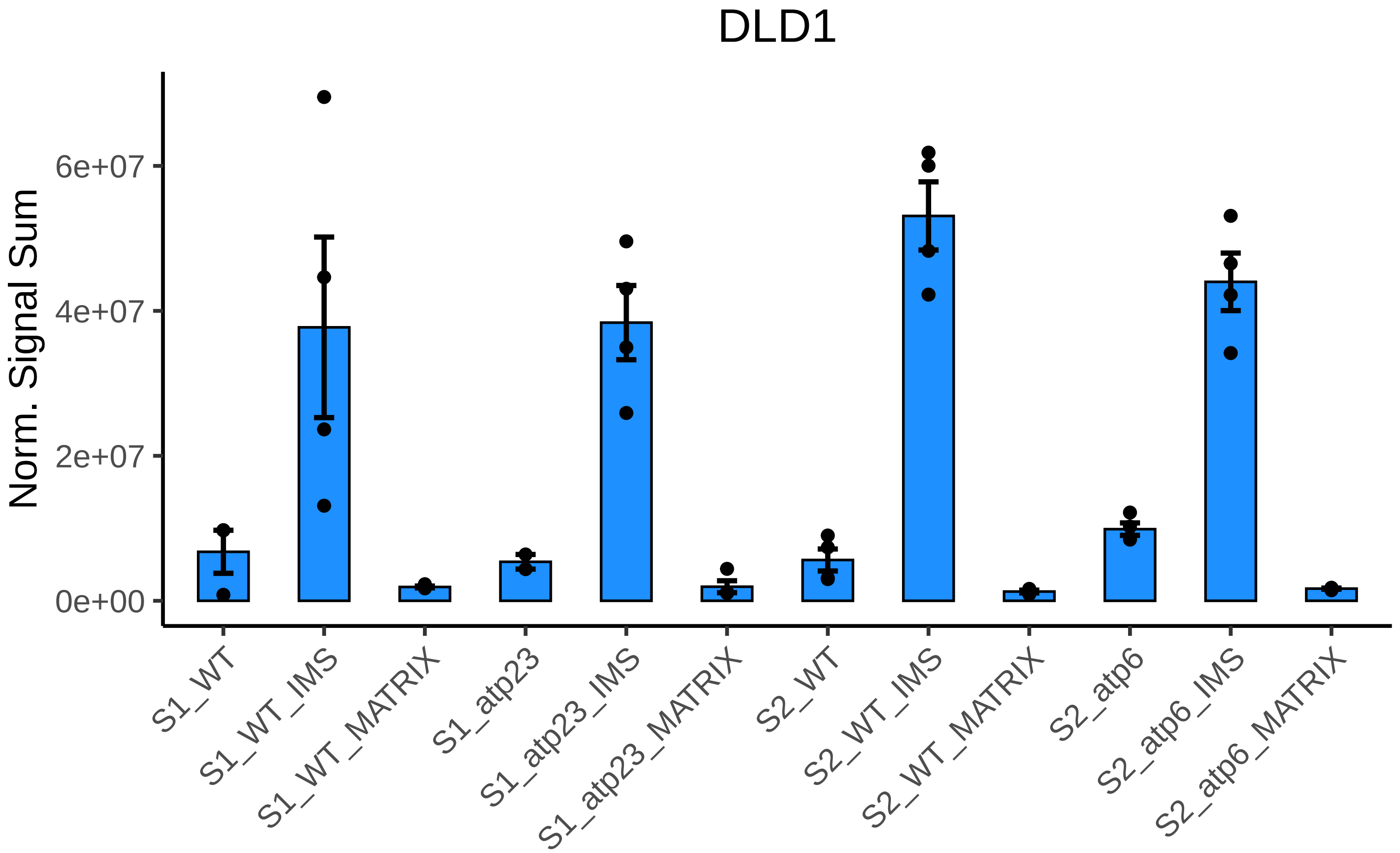
DLD1
Norm. Signal Sum
6e+07
4e+07
2e+07
0e+00
S1_WT
S1_WT_IMS
S1_WT_MATRIX
S1_atp23
S1_atp23_IMS
S1_atp23_MATRIX
S2_WT
S2_WT_IMS
S2_WT_MATRIX
S2_atp6
S2_atp6_IMS
S2_atp6_MATRIX

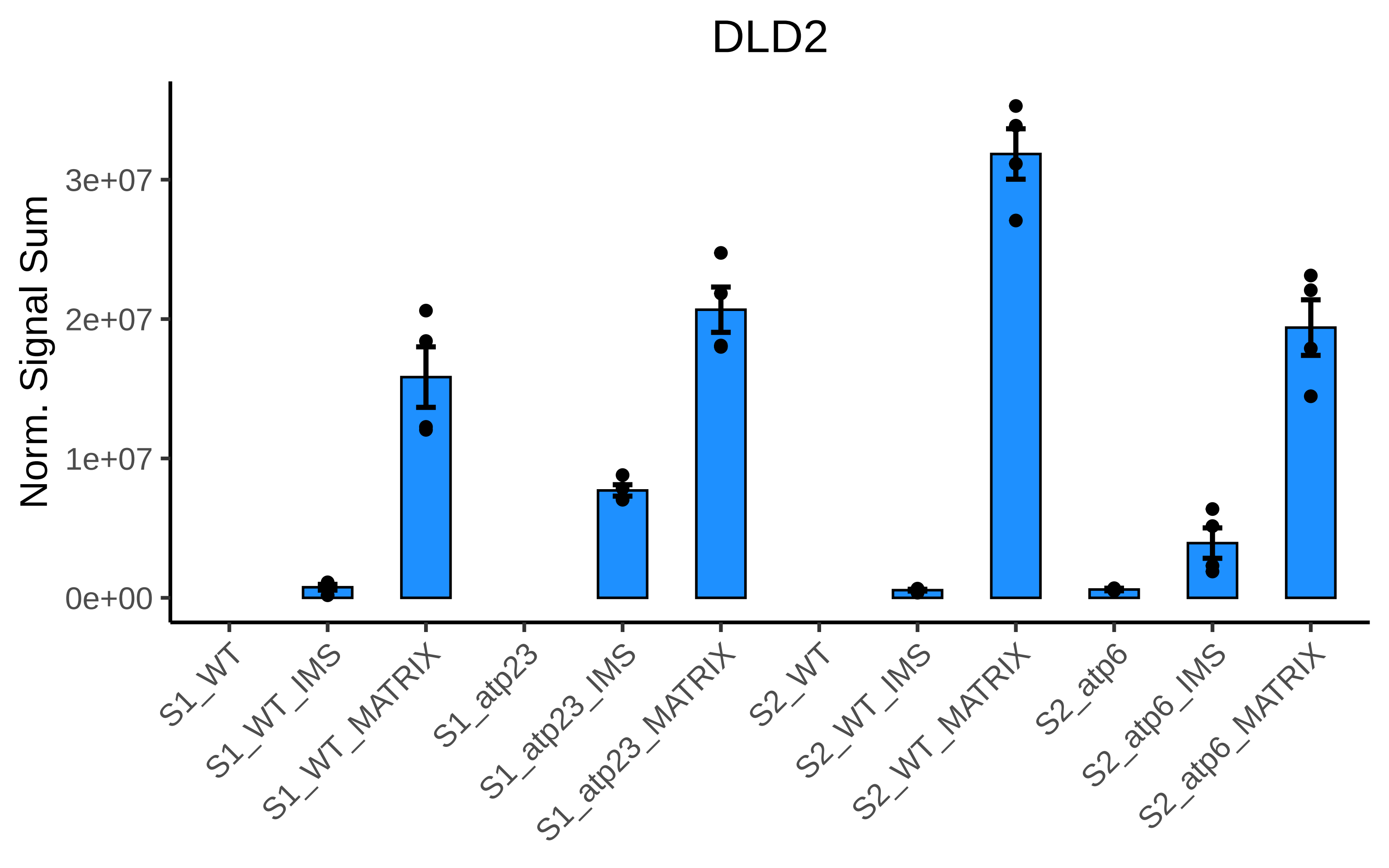

DLD2
Norm. Signal Sum
3e+07
2e+07
1e+07
0e+00
S1_WT
S1_WT_IMS
S1_WT_MATRIX
S1_atp23
S1_atp23_IMS
S1_atp23_MATRIX
S2_WT
S2_WT_IMS
S2_WT_MATRIX
S2_atp6
S2_atp6_IMS
S2_atp6_MATRIX

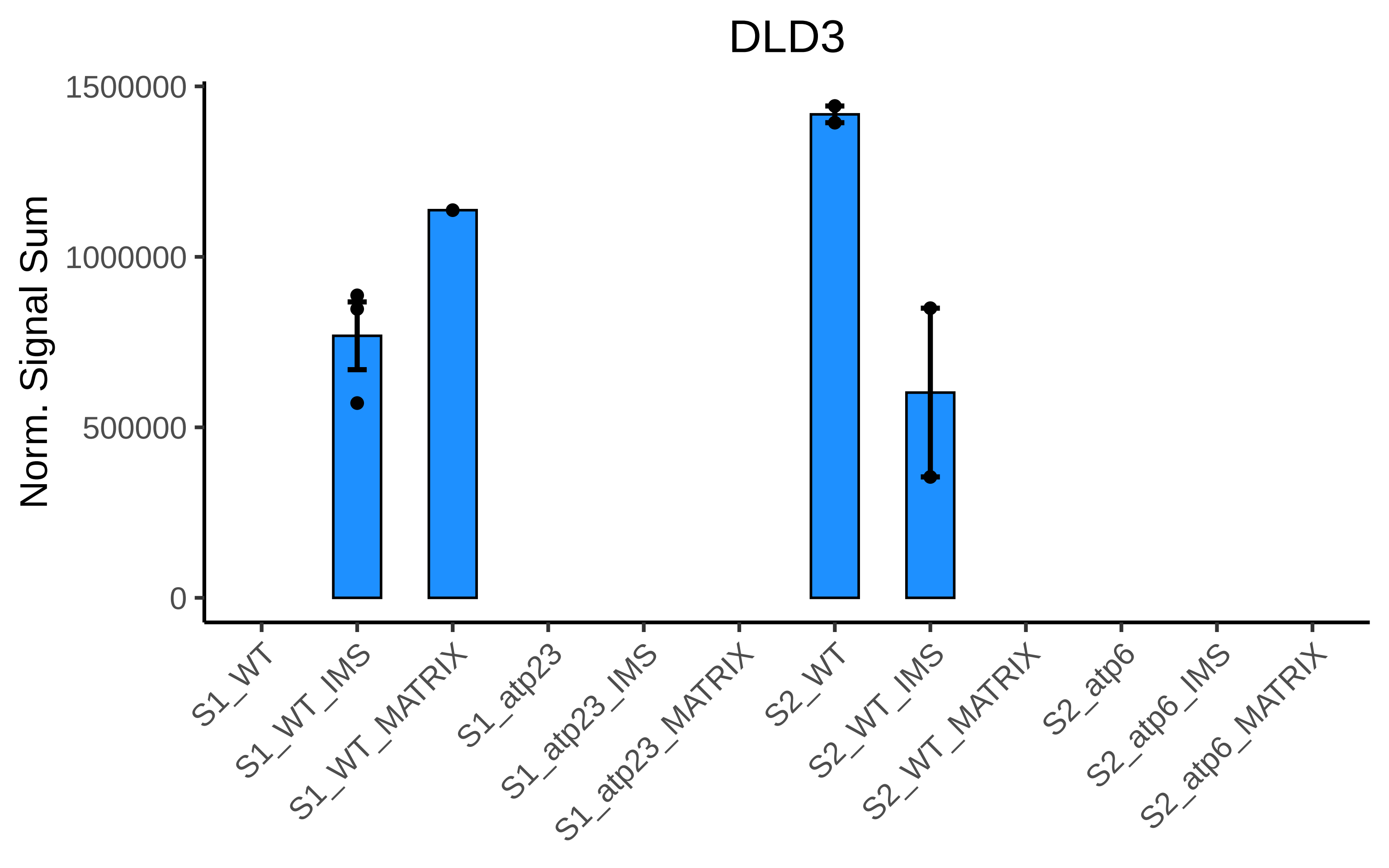

DLD3
Norm. Signal Sum
1500000
1000000
500000
0
S1_WT
S1_WT_IMS
S1_WT_MATRIX
S1_atp23
S1_atp23_IMS
S1_atp23_MATRIX
S2_WT
S2_WT_IMS
S2_WT_MATRIX
S2_atp6
S2_atp6_IMS
S2_atp6_MATRIX

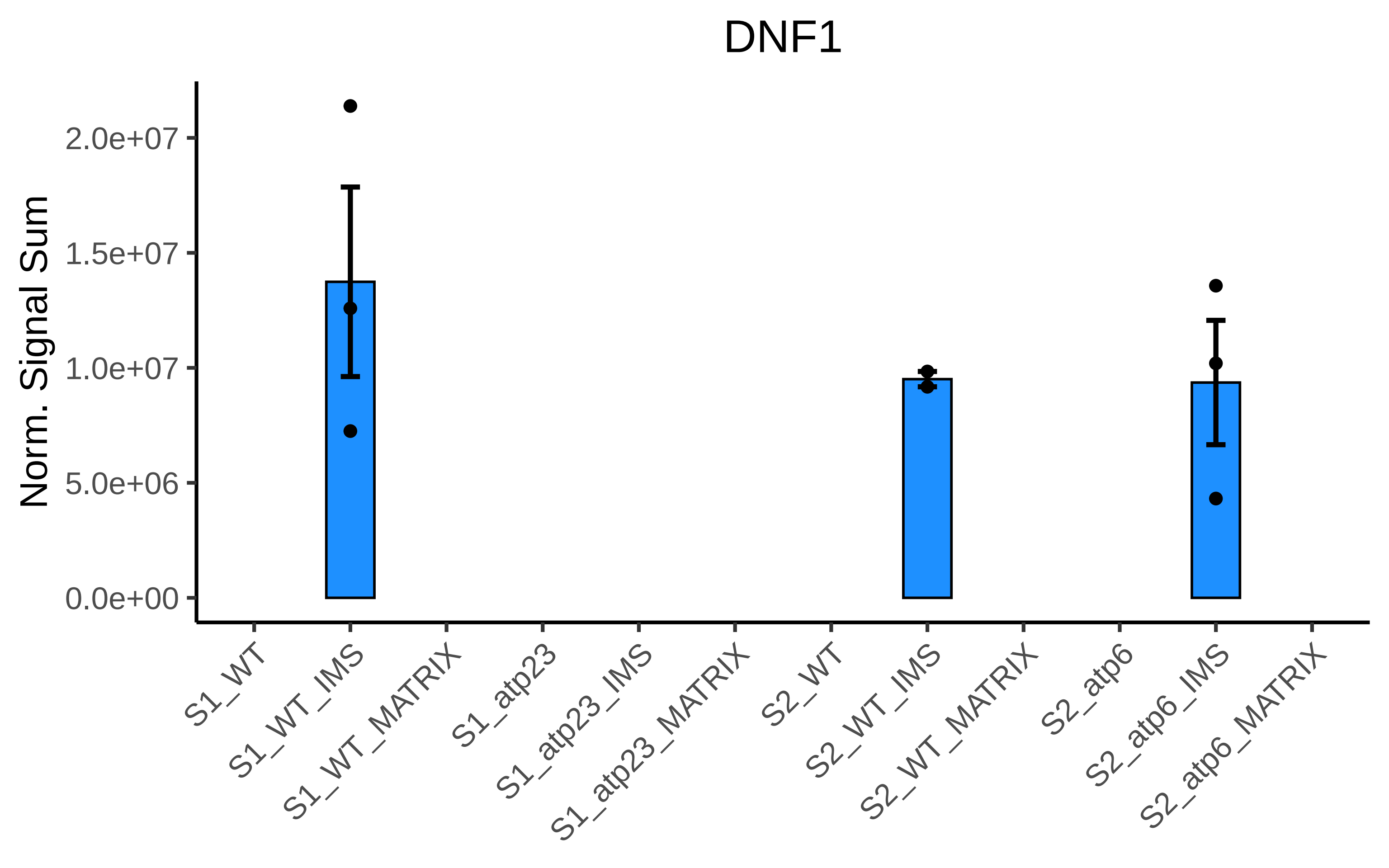

DNF1
Norm. Signal Sum
2.0e+07
1.5e+07
1.0e+07
5.0e+06
0.0e+00
S1_WT
S1_WT_IMS
S1_WT_MATRIX
S1_atp23
S1_atp23_IMS
S1_atp23_MATRIX
S2_WT
S2_WT_IMS
S2_WT_MATRIX
S2_atp6
S2_atp6_IMS
S2_atp6_MATRIX

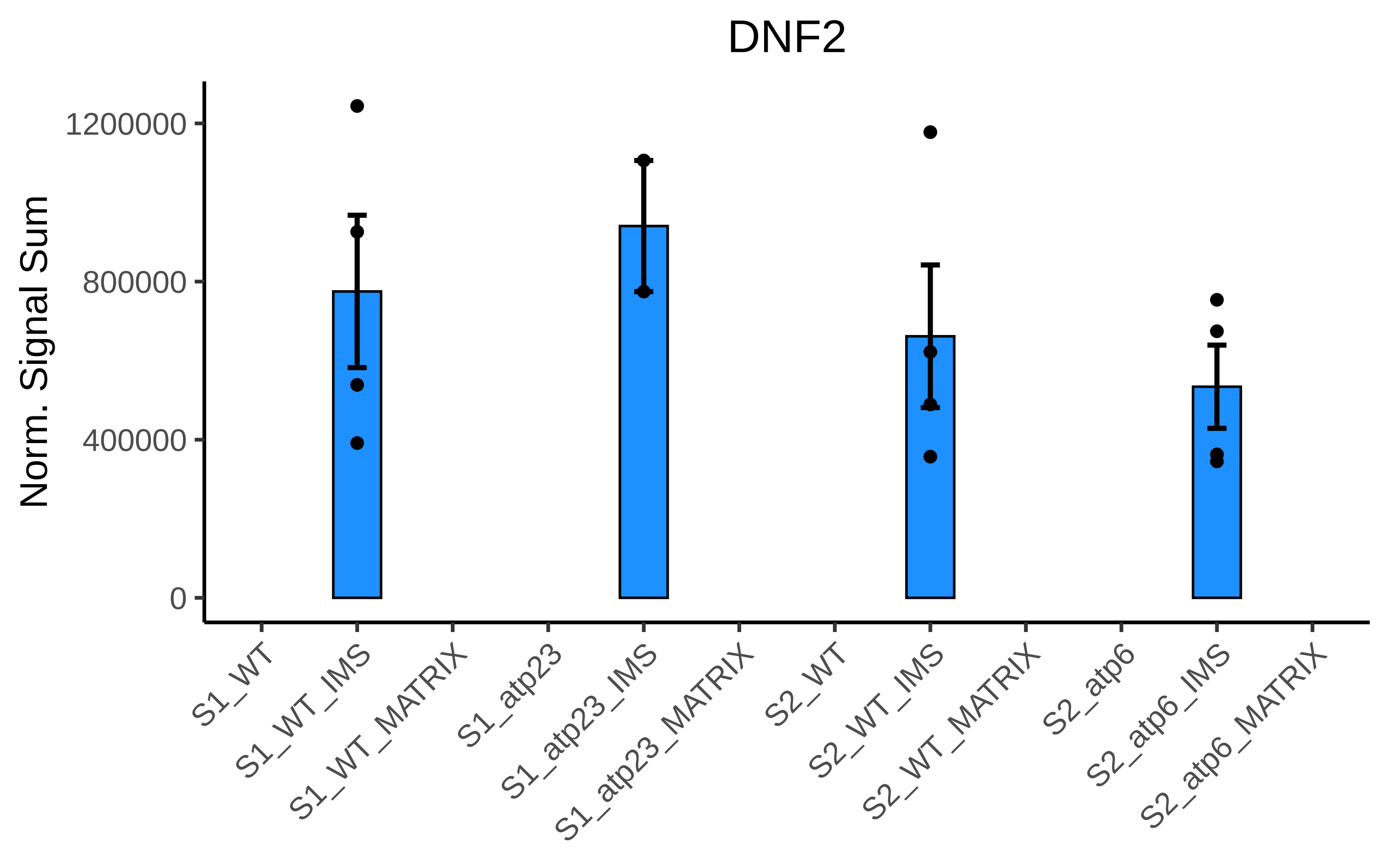
DNF2
Norm. Signal Sum
1200000
800000
400000
0
S1_WT
S1_WT_IMS
S1_WT_MATRIX
S1_atp23
S1_atp23_IMS
S1_atp23_MATRIX
S2_WT
S2_WT_IMS
S2_WT_MATRIX
S2_atp6
S2_atp6_IMS
S2_atp6_MATRIX

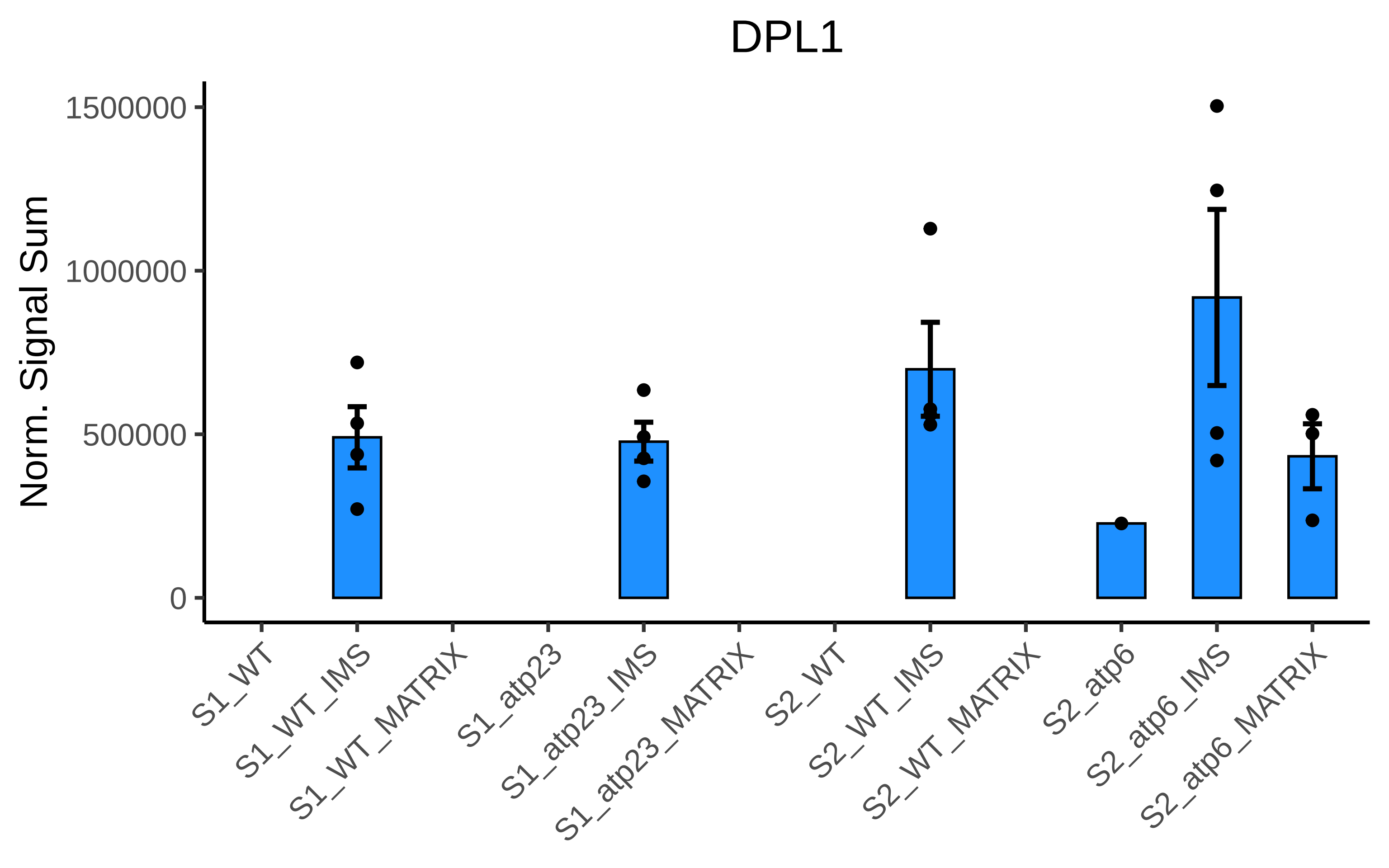
DPL1
Norm. Signal Sum
1500000
1000000
500000
0
S1_WT
S1_WT_IMS
S1_WT_MATRIX
S1_atp23
S1_atp23_IMS
S1_atp23_MATRIX
S2_WT
S2_WT_IMS
S2_WT_MATRIX
S2_atp6
S2_atp6_IMS
S2_atp6_MATRIX

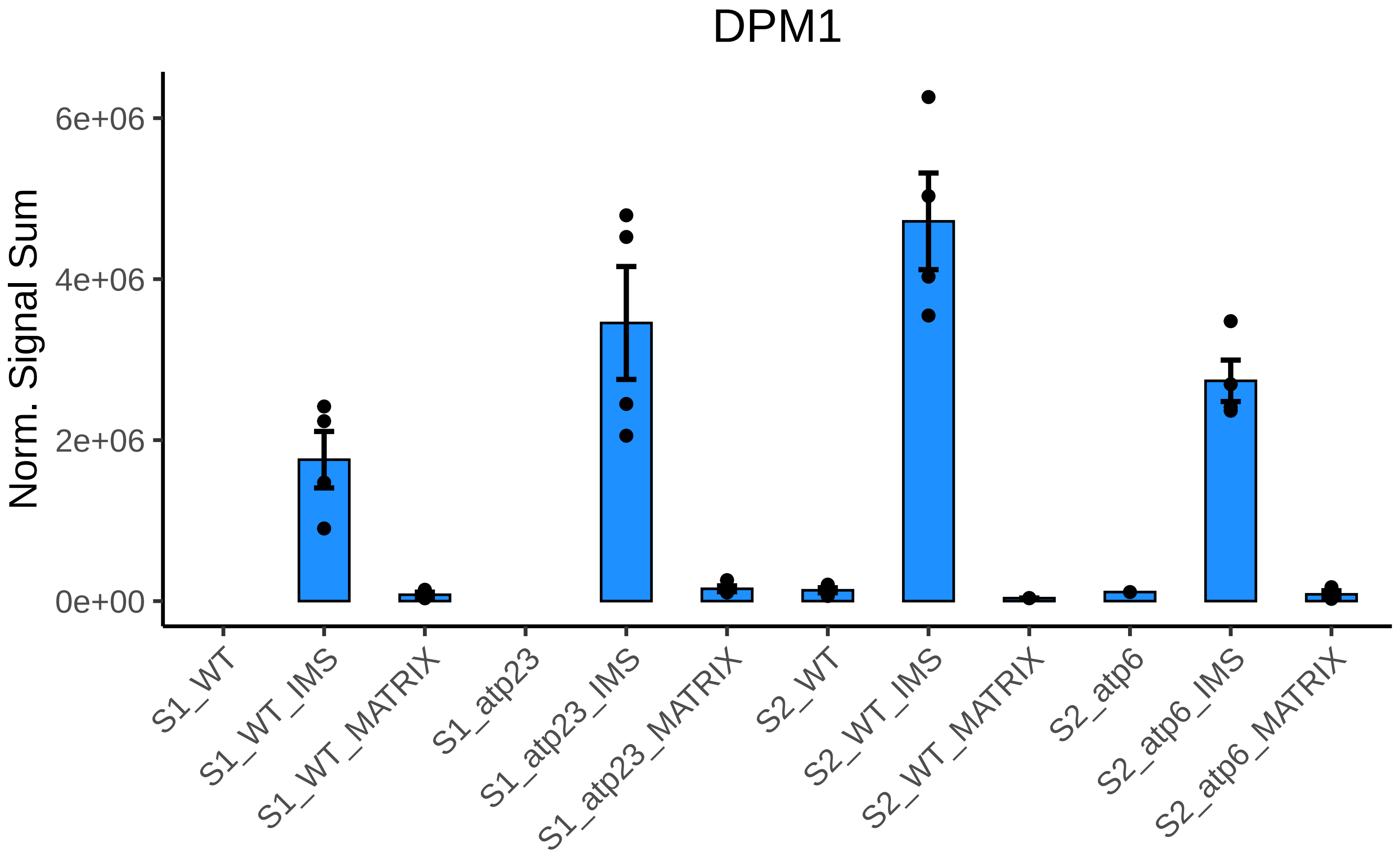
DPM1
Norm. Signal Sum
6e+06
4e+06
2e+06
0e+00
S1_WT
S1_WT_IMS
S1_WT_MATRIX
S1_atp23
S1_atp23_IMS
S1_atp23_MATRIX
S2_WT
S2_WT_IMS
S2_WT_MATRIX
S2_atp6
S2_atp6_IMS
S2_atp6_MATRIX

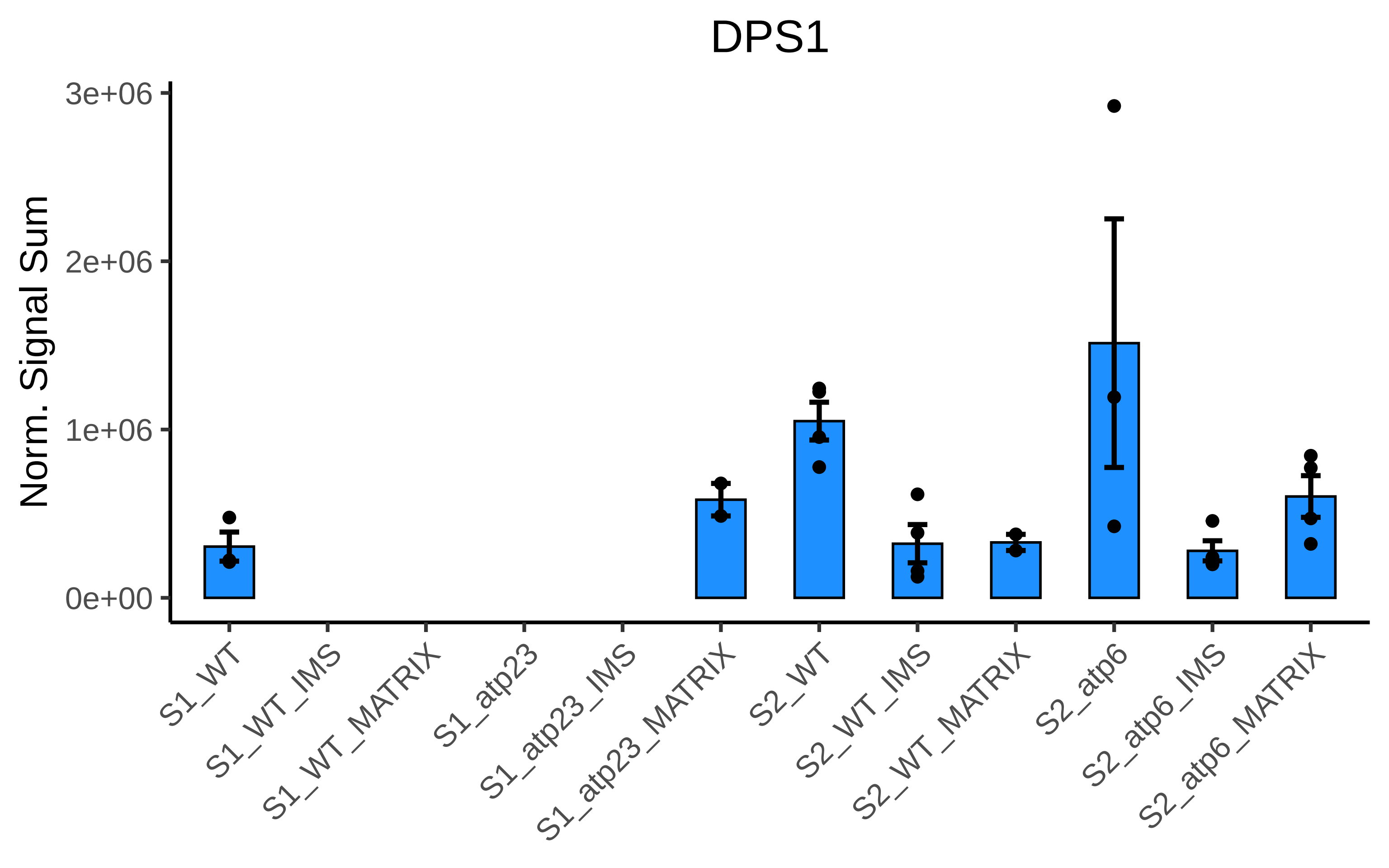
DPS1
Norm. Signal Sum
3e+06
2e+06
1e+06
0e+00
S1_WT
S1_WT_IMS
S1_WT_MATRIX
S1_atp23
S1_atp23_IMS
S1_atp23_MATRIX
S2_WT
S2_WT_IMS
S2_WT_MATRIX
S2_atp6
S2_atp6_IMS
S2_atp6_MATRIX

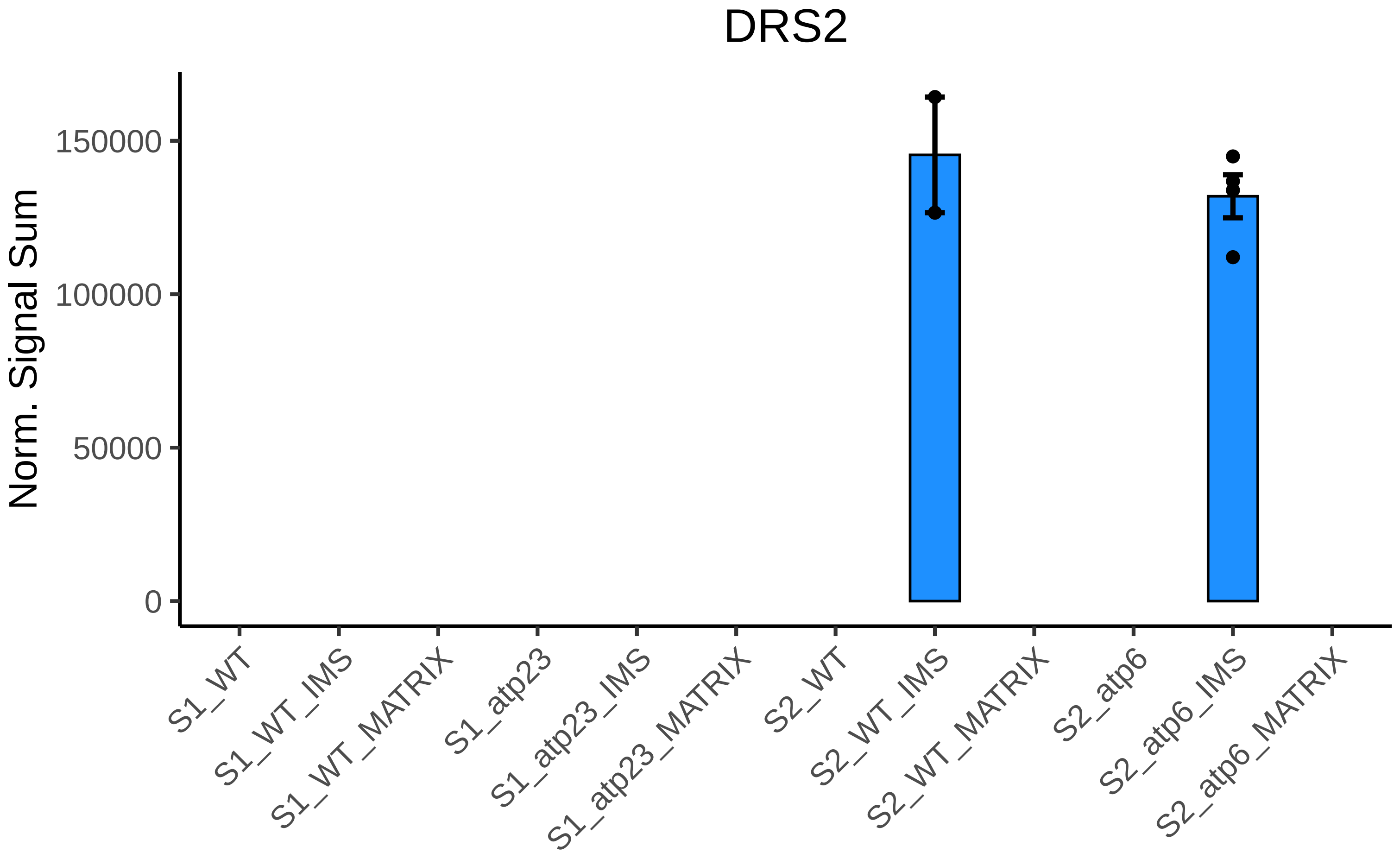
DRS2
Norm. Signal Sum
150000
100000
50000
0
S1_WT
S1_WT_IMS
S1_WT_MATRIX
S1_atp23
S1_atp23_IMS
S1_atp23_MATRIX
S2_WT
S2_WT_IMS
S2_WT_MATRIX
S2_atp6
S2_atp6_IMS
S2_atp6_MATRIX

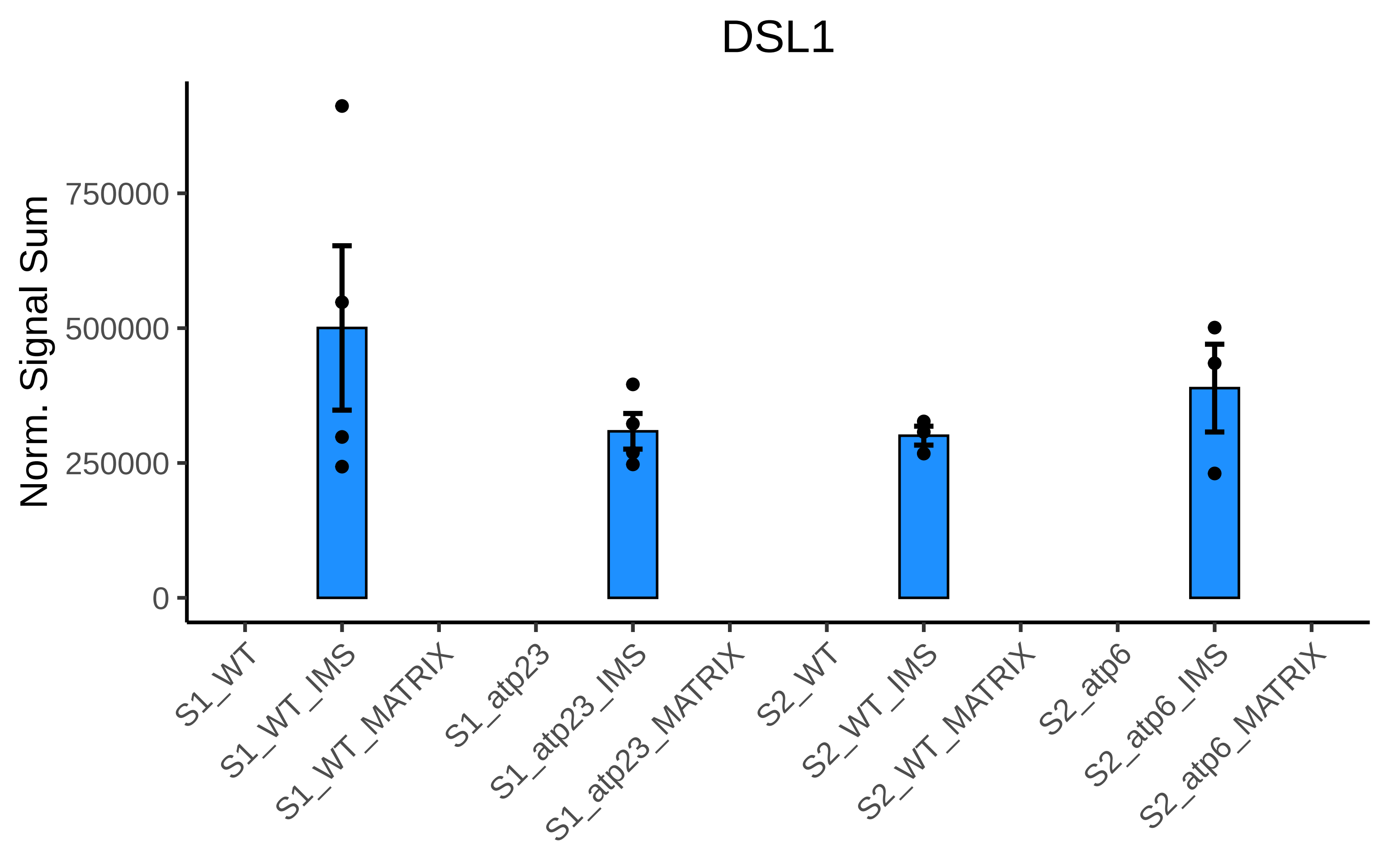

DSL1
Norm. Signal Sum
750000
500000
250000
0
S1_WT
S1_WT_IMS
S1_WT_MATRIX
S1_atp23
S1_atp23_IMS
S1_atp23_MATRIX
S2_WT
S2_WT_IMS
S2_WT_MATRIX
S2_atp6
S2_atp6_IMS
S2_atp6_MATRIX

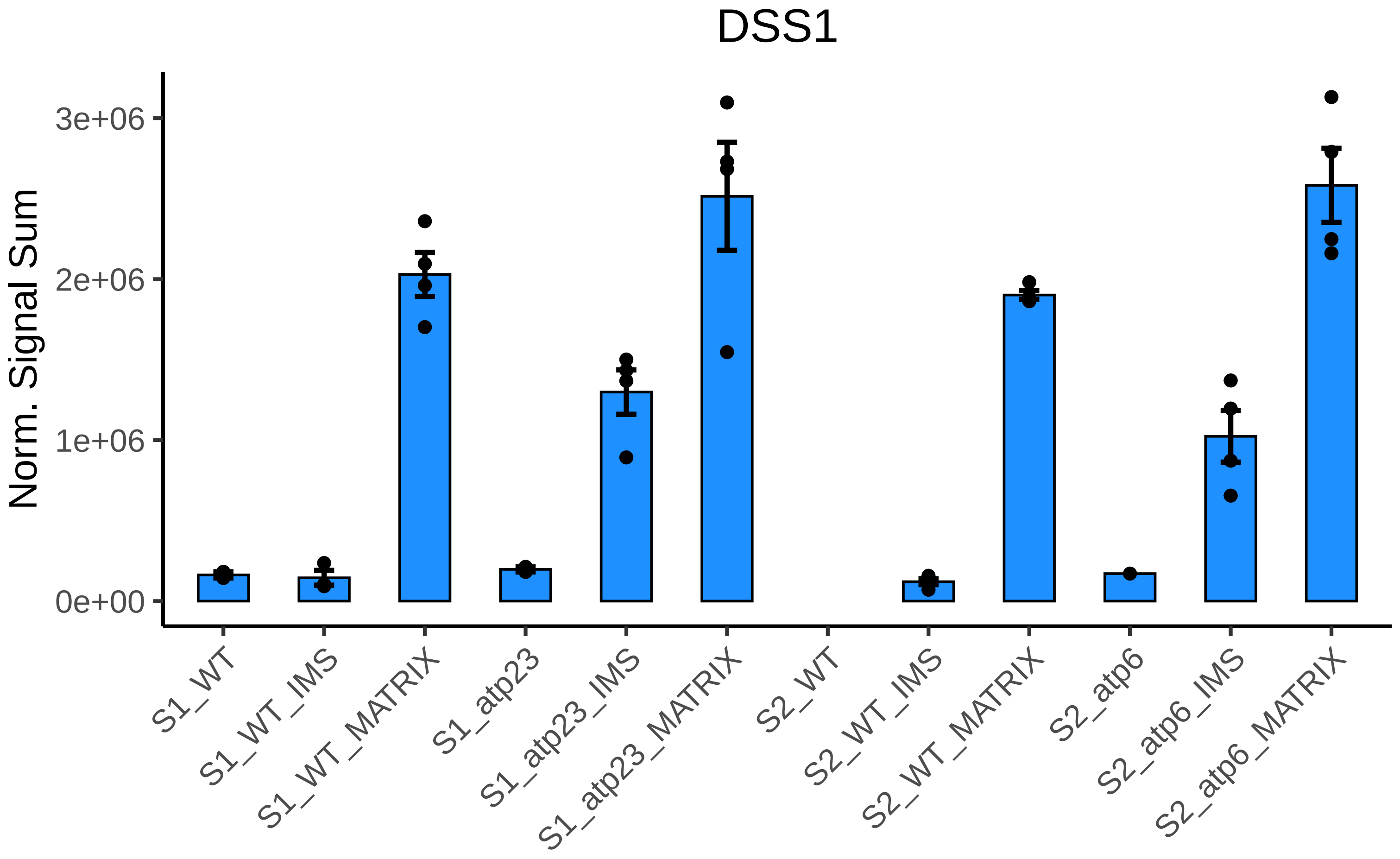
DSS1
Norm. Signal Sum
3e+06
2e+06
1e+06
0e+00
S1_WT
S1_WT_IMS
S1_WT_MATRIX
S1_atp23
S1_atp23_IMS
S1_atp23_MATRIX
S2_WT
S2_WT_IMS
S2_WT_MATRIX
S2_atp6
S2_atp6_IMS
S2_atp6_MATRIX

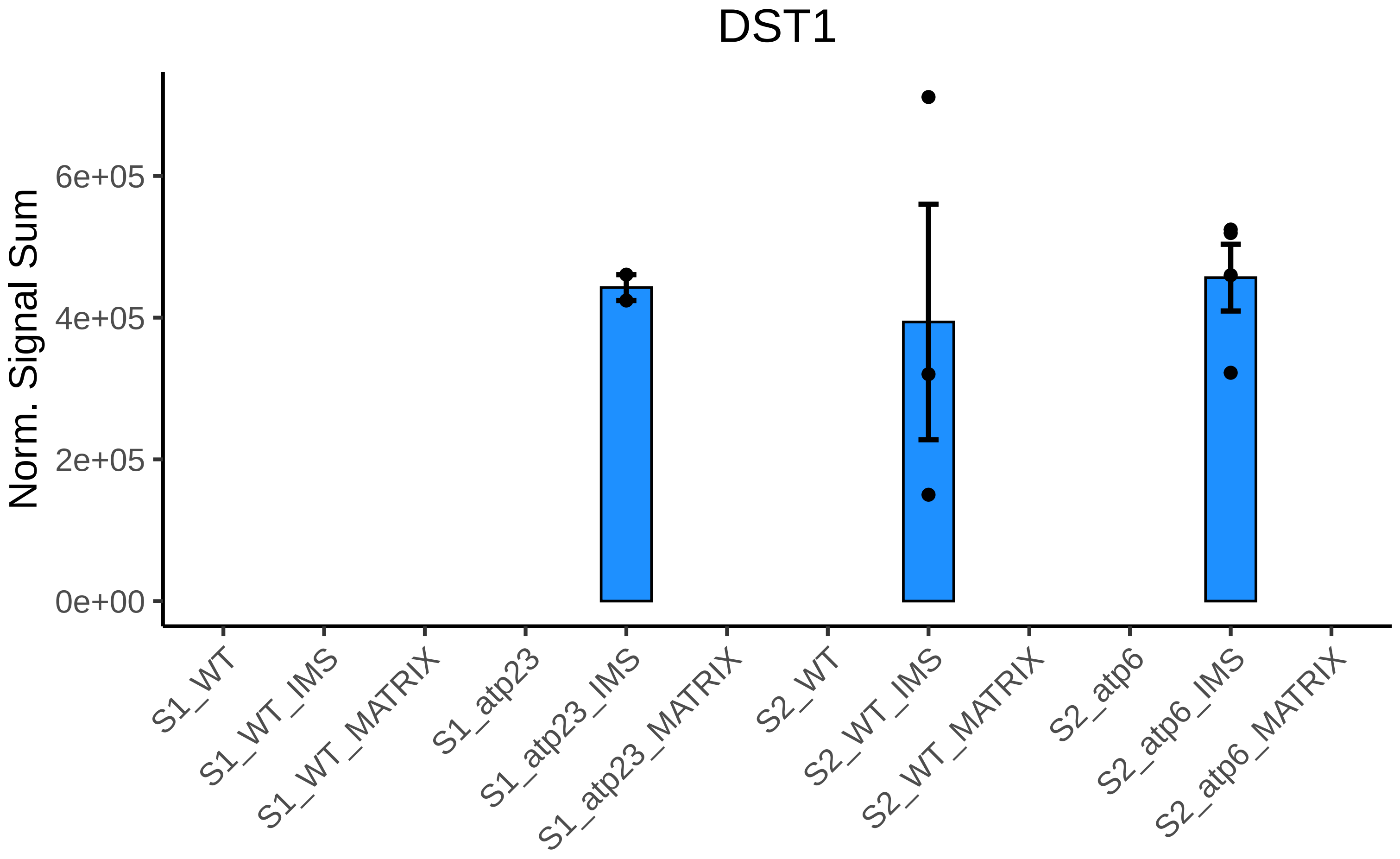

DST1
Norm. Signal Sum
6e+05
4e+05
2e+05
0e+00
S1_WT
S1_WT_IMS
S1_WT_MATRIX
S1_atp23
S1_atp23_IMS
S1_atp23_MATRIX
S2_WT
S2_WT_IMS
S2_WT_MATRIX
S2_atp6
S2_atp6_IMS
S2_atp6_MATRIX

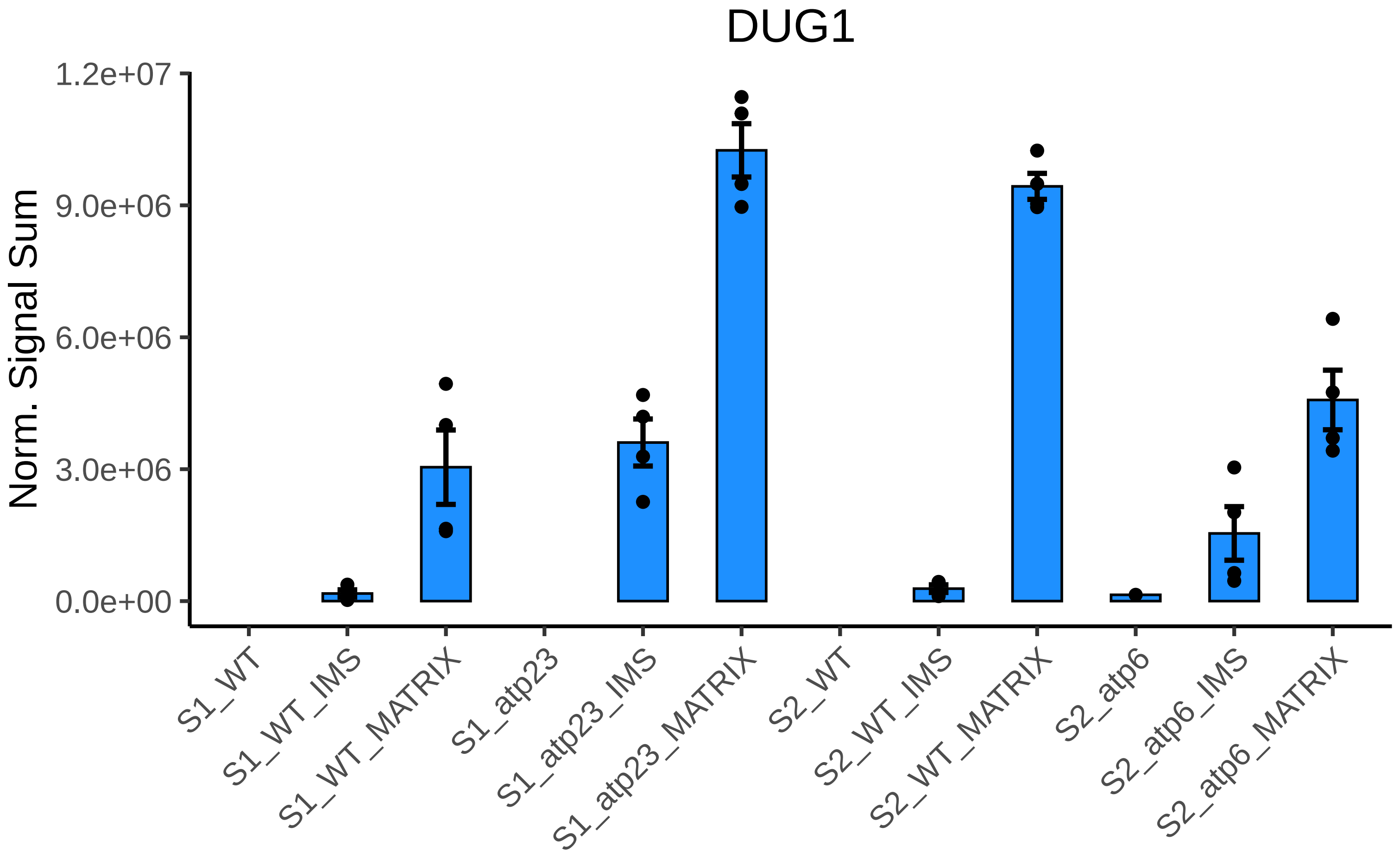
DUG1
Norm. Signal Sum
1.2e+07
9.0e+06
6.0e+06
3.0e+06
0.0e+00
S1_WT
S1_WT_IMS
S1_WT_MATRIX
S1_atp23
S1_atp23_IMS
S1_atp23_MATRIX
S2_WT
S2_WT_IMS
S2_WT_MATRIX
S2_atp6
S2_atp6_IMS
S2_atp6_MATRIX

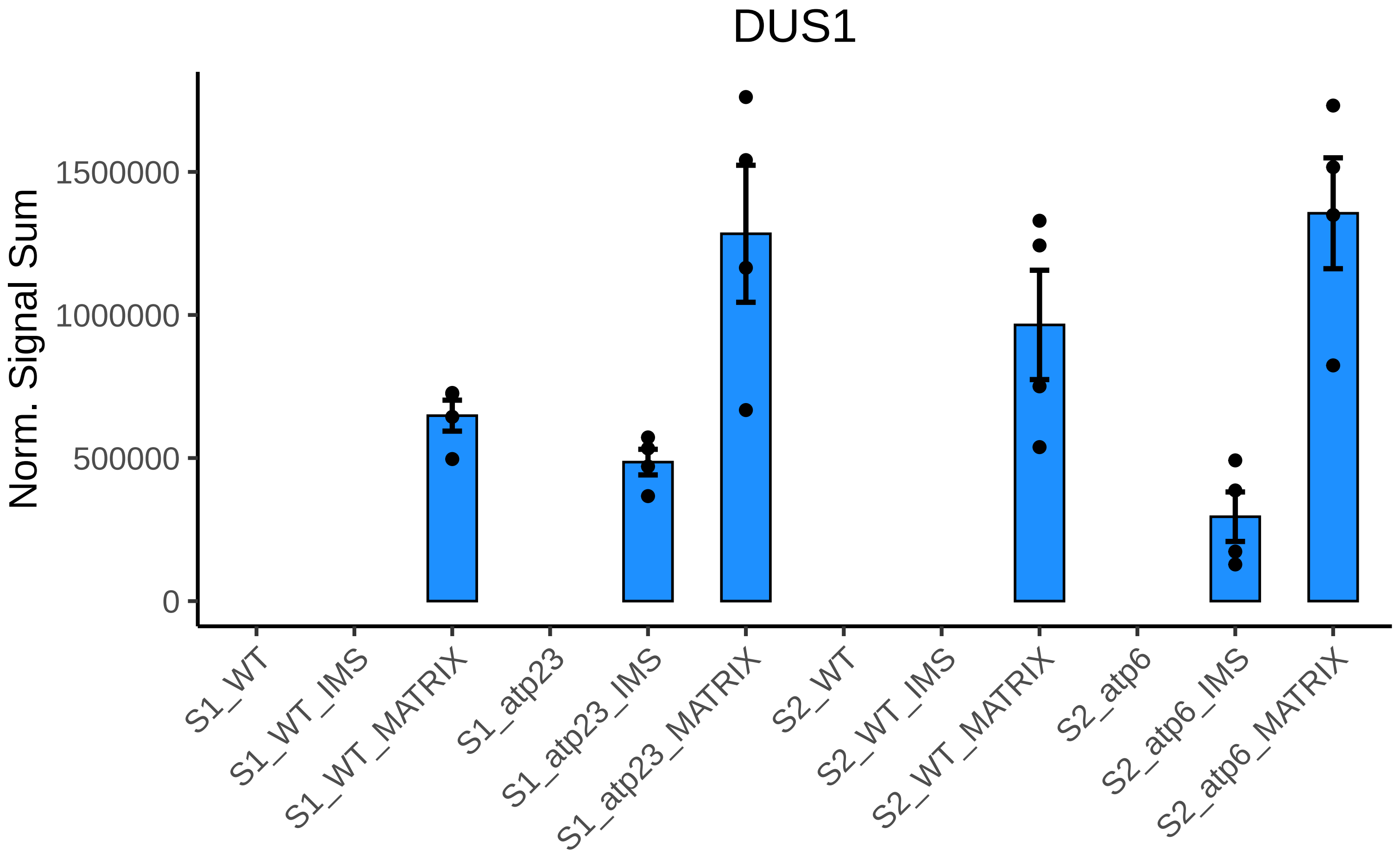
DUS1
Norm. Signal Sum
1500000
1000000
500000
0
S1_WT
S1_WT_IMS
S1_WT_MATRIX
S1_atp23
S1_atp23_IMS
S1_atp23_MATRIX
S2_WT
S2_WT_IMS
S2_WT_MATRIX
S2_atp6
S2_atp6_IMS
S2_atp6_MATRIX

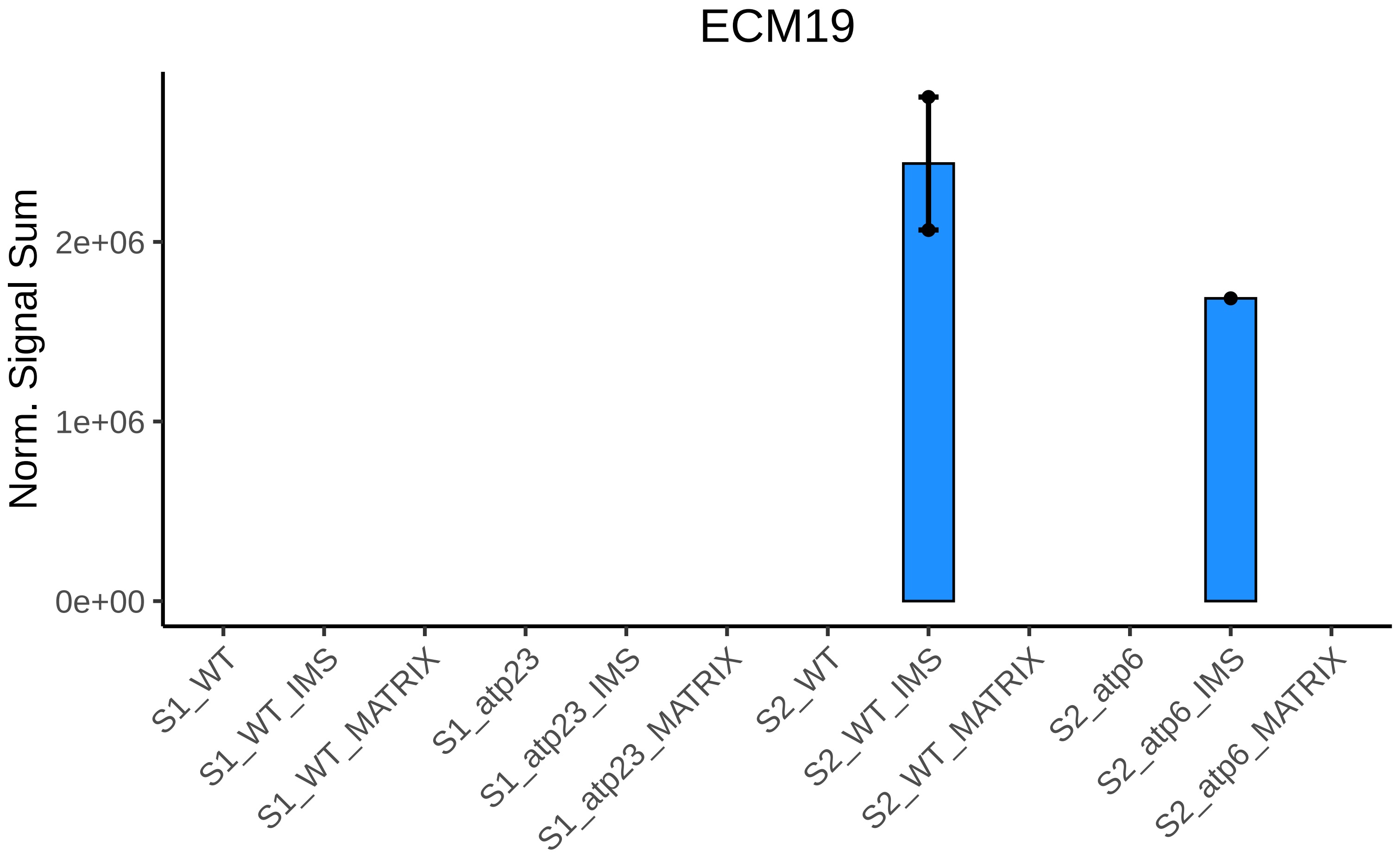

ECM19
Norm. Signal Sum
2e+06
1e+06
0e+00
S1_WT
S1_WT_IMS
S1_WT_MATRIX
S1_atp23
S1_atp23_IMS
S1_atp23_MATRIX
S2_WT
S2_WT_IMS
S2_WT_MATRIX
S2_atp6
S2_atp6_IMS
S2_atp6_MATRIX

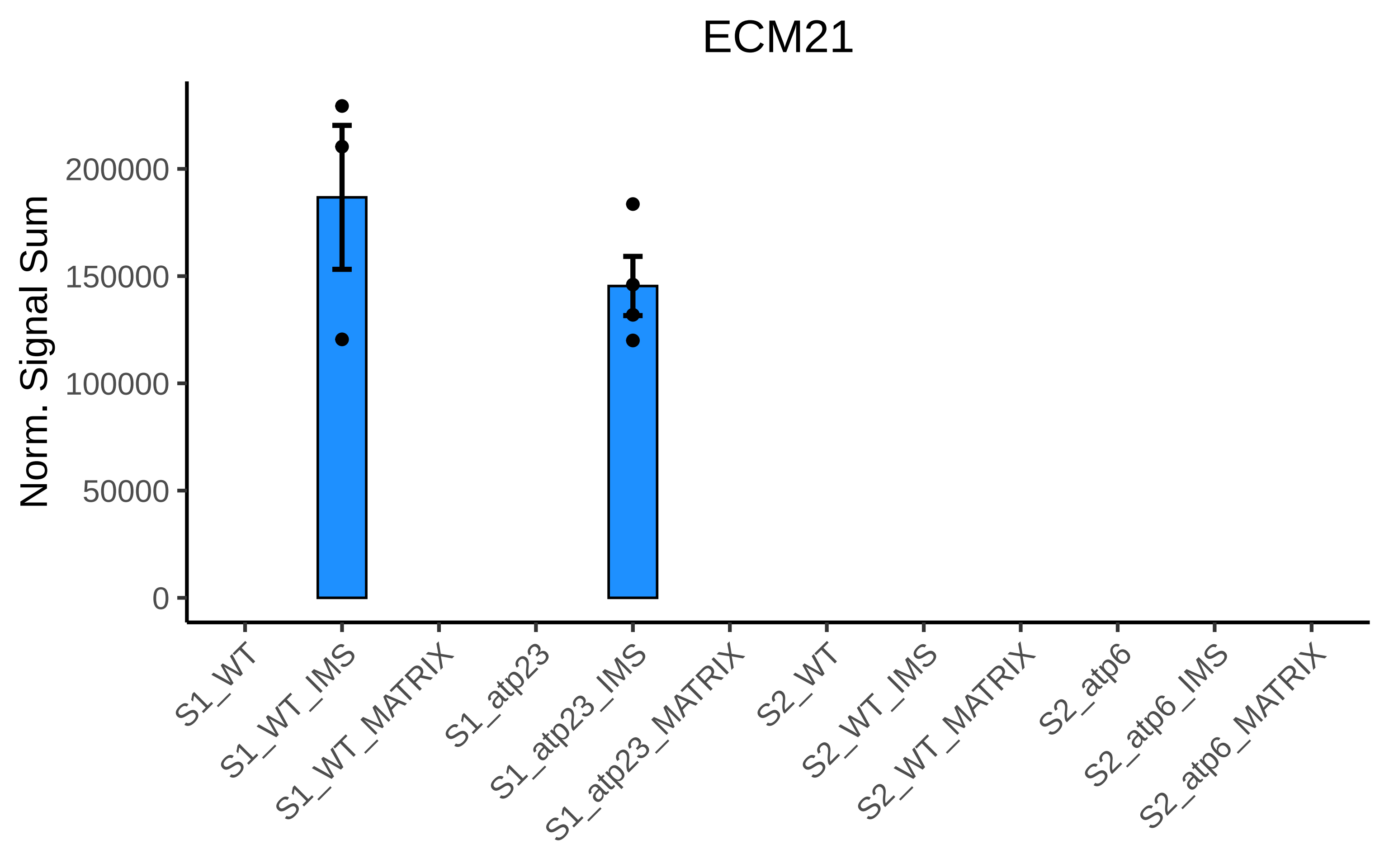

ECM21
Norm. Signal Sum
200000
150000
100000
50000
0
S1_WT
S1_WT_IMS
S1_WT_MATRIX
S1_atp23
S1_atp23_IMS
S1_atp23_MATRIX
S2_WT
S2_WT_IMS
S2_WT_MATRIX
S2_atp6
S2_atp6_IMS
S2_atp6_MATRIX

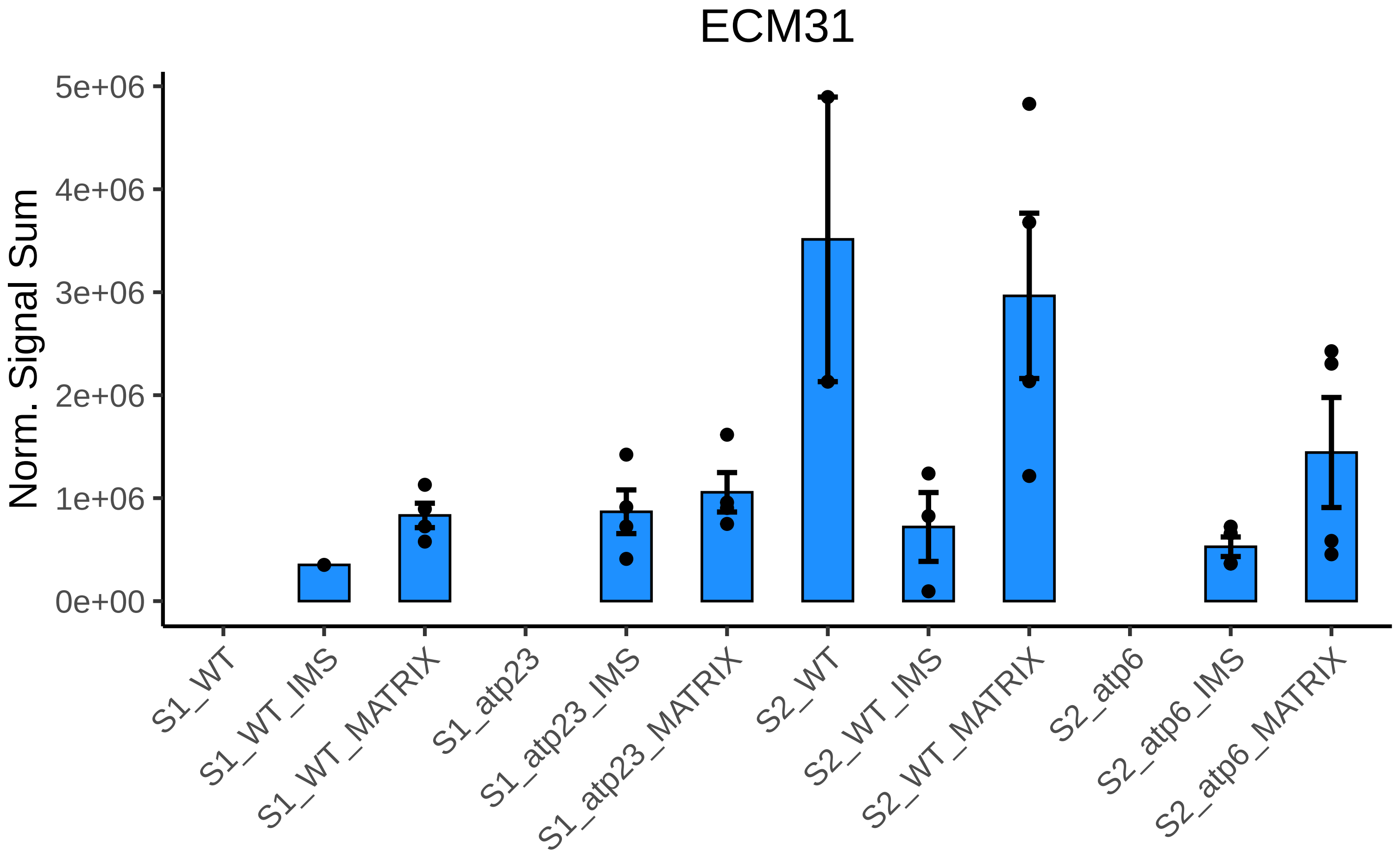
ECM31
Norm. Signal Sum
5e+06
4e+06
3e+06
2e+06
1e+06
0e+00
S1_WT
S1_WT_IMS
S1_WT_MATRIX
S1_atp23
S1_atp23_IMS
S1_atp23_MATRIX
S2_WT
S2_WT_IMS
S2_WT_MATRIX
S2_atp6
S2_atp6_IMS
S2_atp6_MATRIX

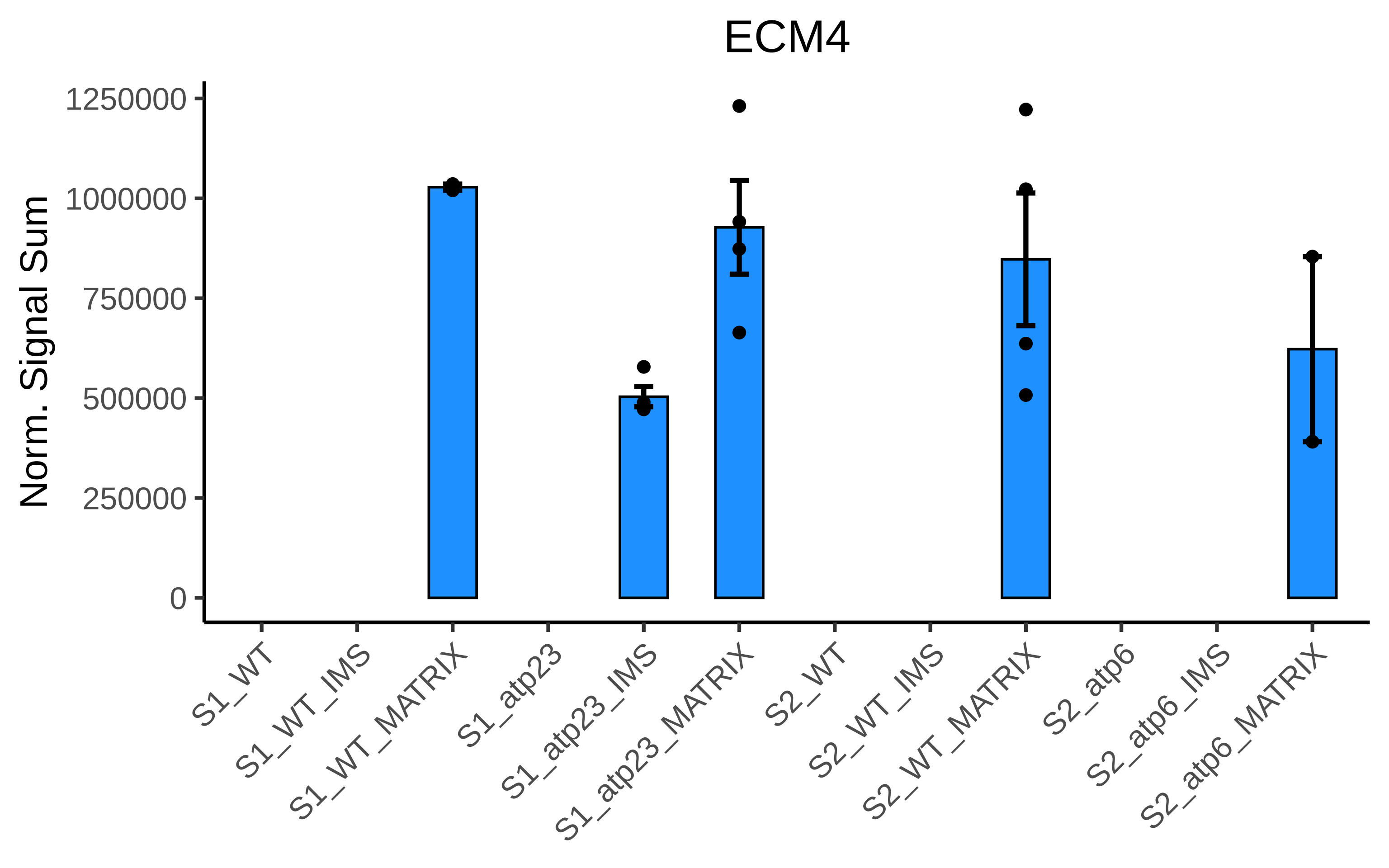
ECM4
Norm. Signal Sum
1250000
1000000
750000
500000
250000
0
S1_WT
S1_WT_IMS
S1_WT_MATRIX
S1_atp23
S1_atp23_IMS
S1_atp23_MATRIX
S2_WT
S2_WT_IMS
S2_WT_MATRIX
S2_atp6
S2_atp6_IMS
S2_atp6_MATRIX

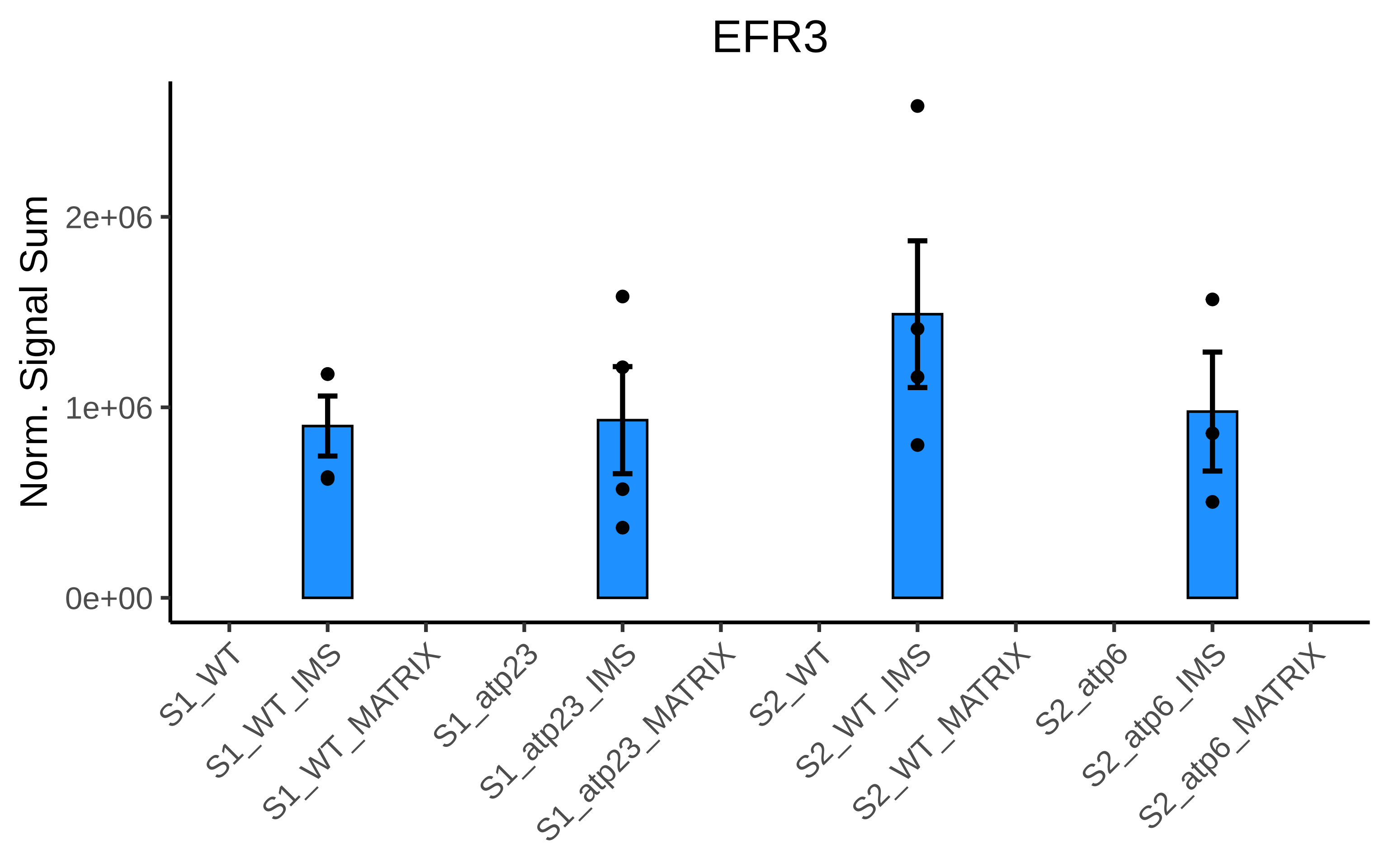
EFR3
Norm. Signal Sum
2e+06
1e+06
0e+00
S1_WT
S1_WT_IMS
S1_WT_MATRIX
S1_atp23
S1_atp23_IMS
S1_atp23_MATRIX
S2_WT
S2_WT_IMS
S2_WT_MATRIX
S2_atp6
S2_atp6_IMS
S2_atp6_MATRIX

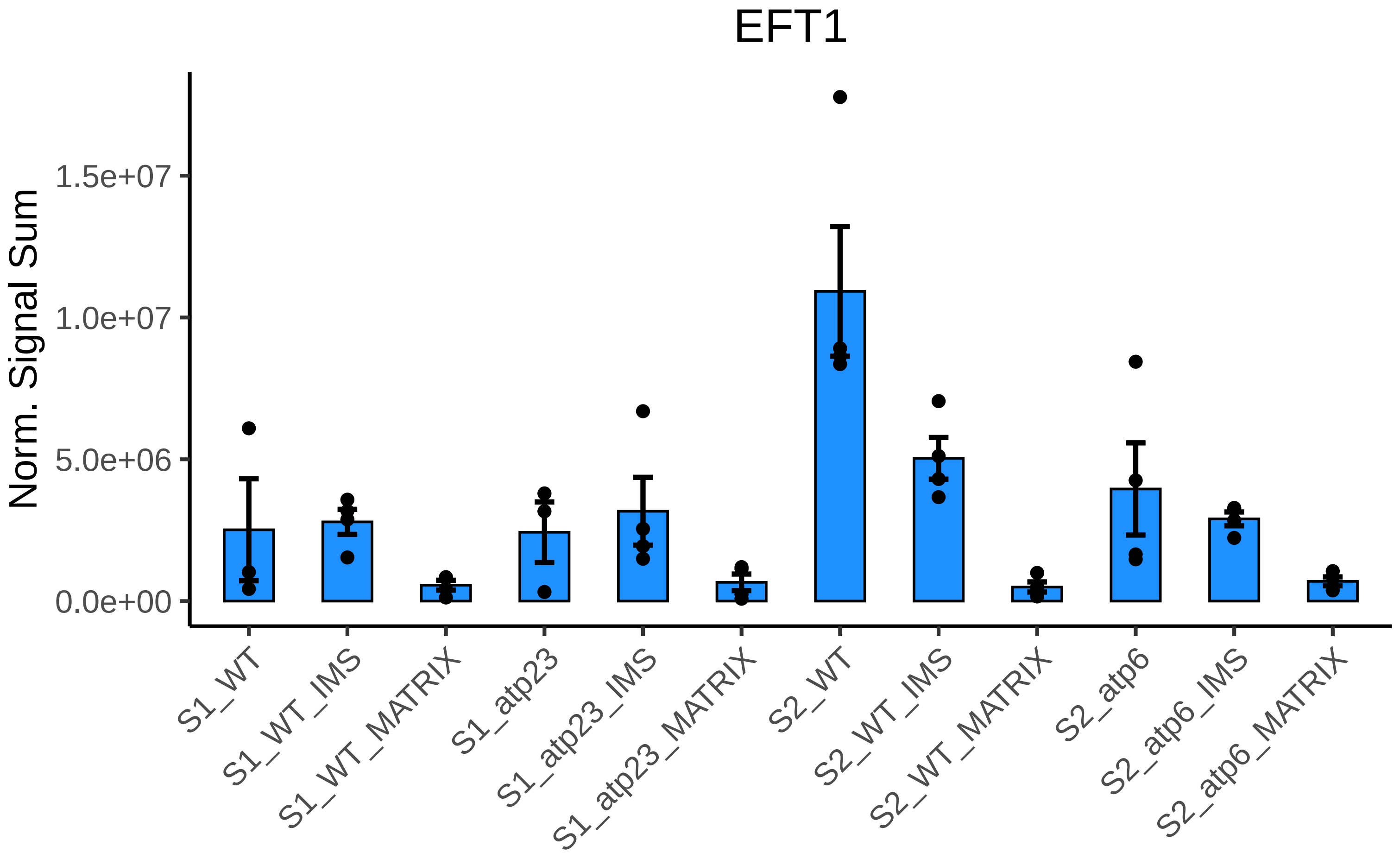

EFT1
Norm. Signal Sum
1.5e+07
1.0e+07
5.0e+06
0.0e+00
S1_WT
S1_WT_IMS
S1_WT_MATRIX
S1_atp23
S1_atp23_IMS
S1_atp23_MATRIX
S2_WT
S2_WT_IMS
S2_WT_MATRIX
S2_atp6
S2_atp6_IMS
S2_atp6_MATRIX

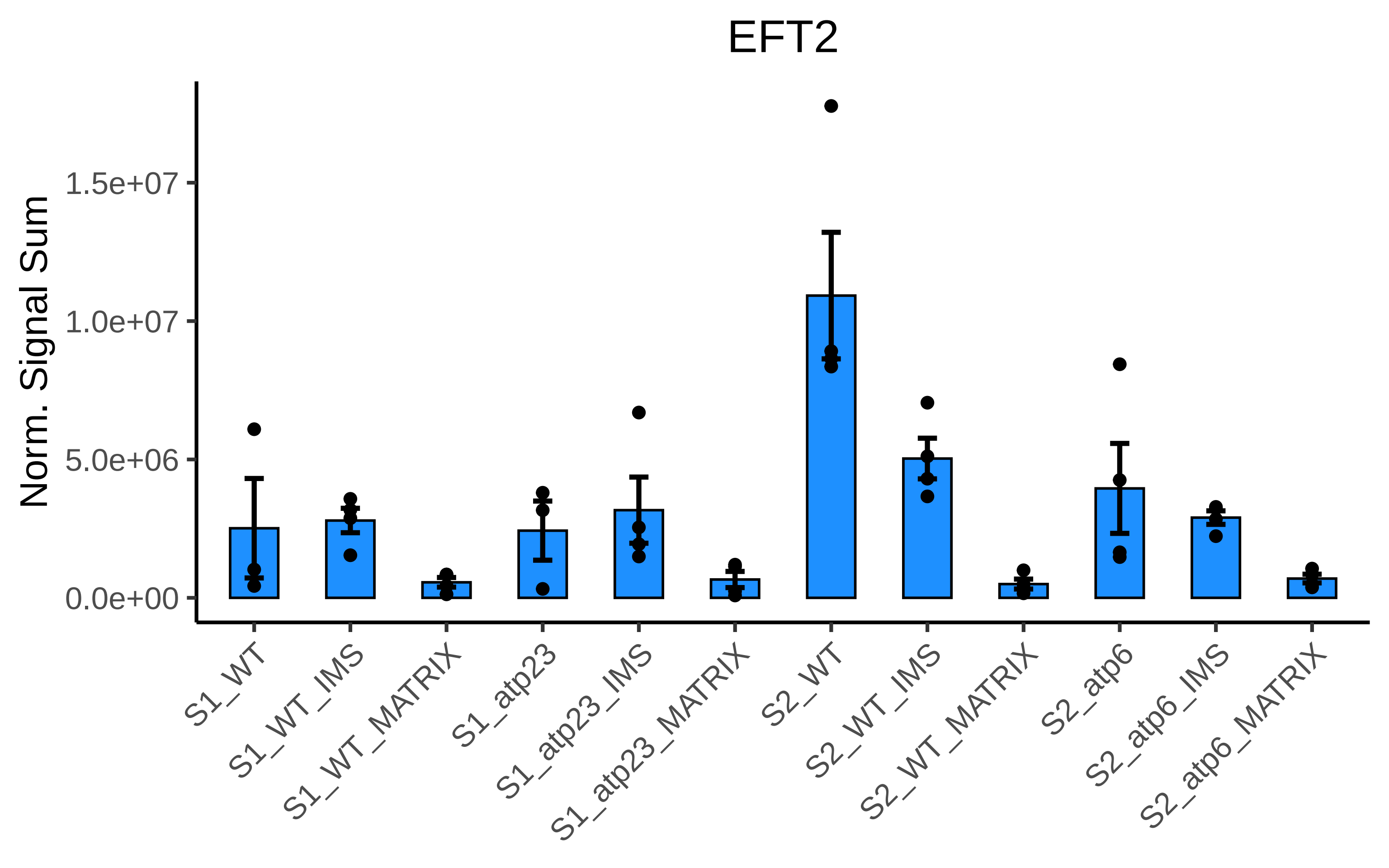
EFT2
Norm. Signal Sum
1.5e+07
1.0e+07
5.0e+06
0.0e+00
S1_WT
S1_WT_IMS
S1_WT_MATRIX
S1_atp23
S1_atp23_IMS
S1_atp23_MATRIX
S2_WT
S2_WT_IMS
S2_WT_MATRIX
S2_atp6
S2_atp6_IMS
S2_atp6_MATRIX

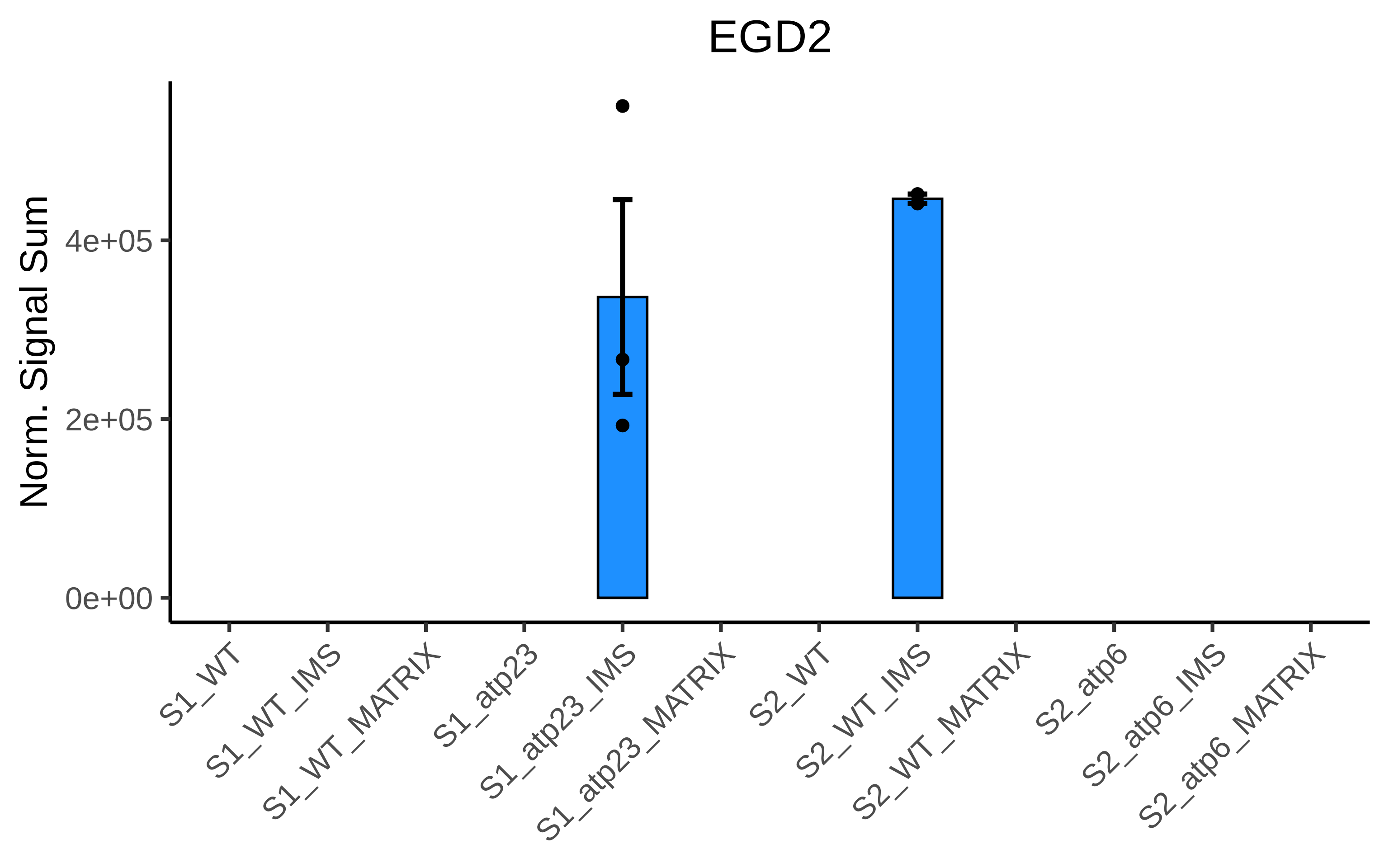

EGD2
Norm. Signal Sum
4e+05
2e+05
0e+00
S1_WT
S1_WT_IMS
S1_WT_MATRIX
S1_atp23
S1_atp23_IMS
S1_atp23_MATRIX
S2_WT
S2_WT_IMS
S2_WT_MATRIX
S2_atp6
S2_atp6_IMS
S2_atp6_MATRIX

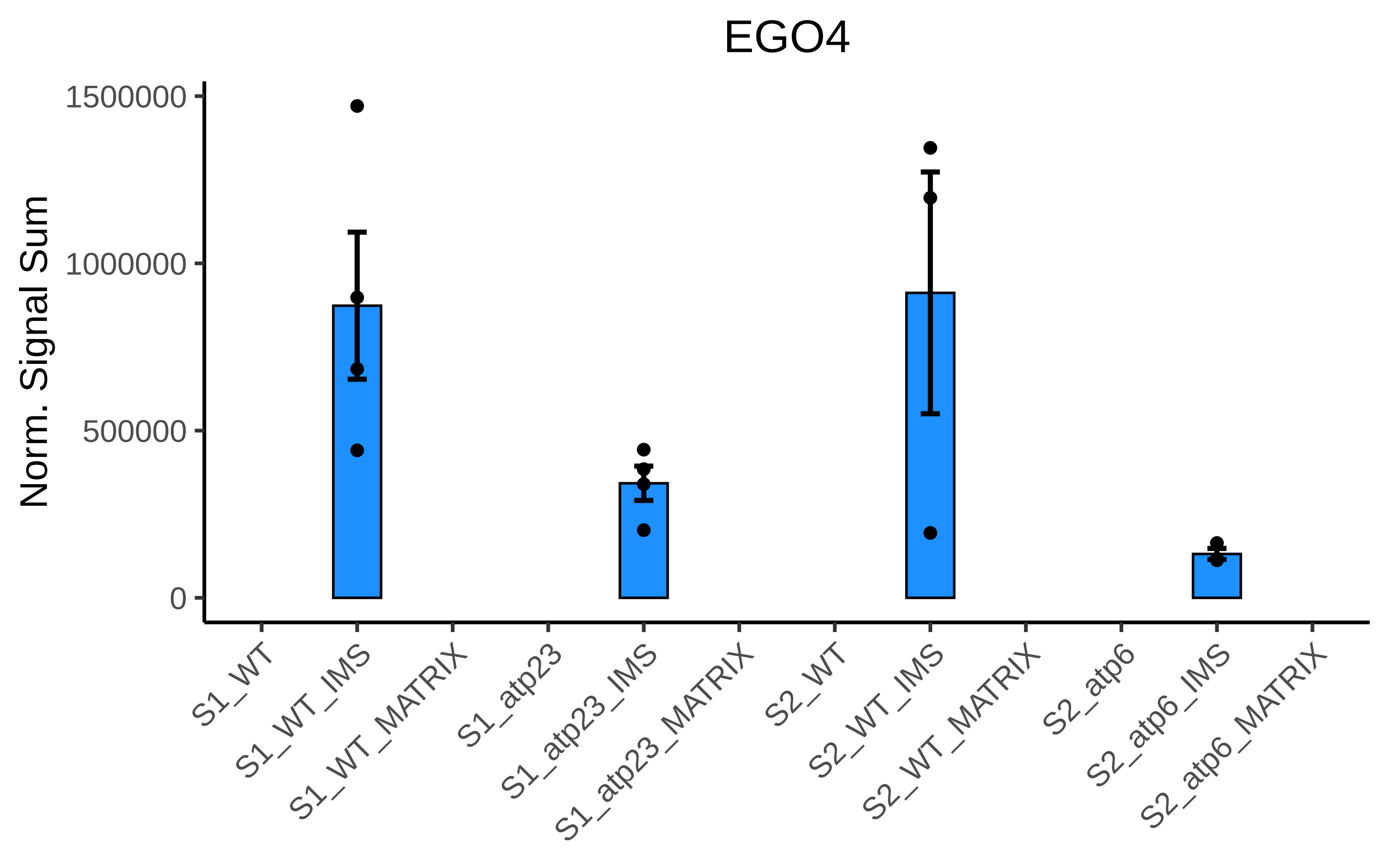

EGO4
Norm. Signal Sum
1500000
1000000
500000
0
S1_WT
S1_WT_IMS
S1_WT_MATRIX
S1_atp23
S1_atp23_IMS
S1_atp23_MATRIX
S2_WT
S2_WT_IMS
S2_WT_MATRIX
S2_atp6
S2_atp6_IMS
S2_atp6_MATRIX

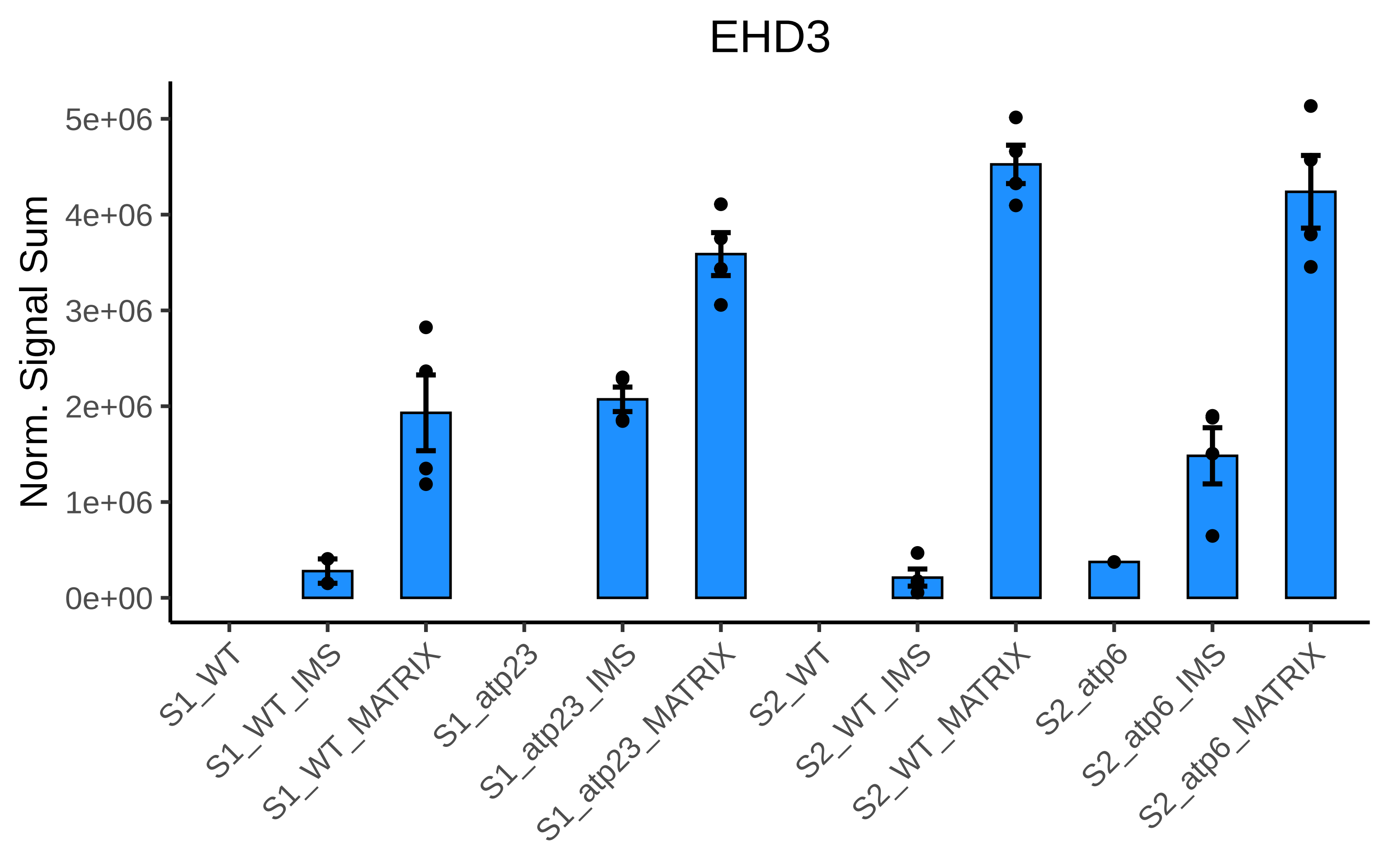

EHD3
Norm. Signal Sum
5e+06
4e+06
3e+06
2e+06
1e+06
0e+00
S1_WT
S1_WT_IMS
S1_WT_MATRIX
S1_atp23
S1_atp23_IMS
S1_atp23_MATRIX
S2_WT
S2_WT_IMS
S2_WT_MATRIX
S2_atp6
S2_atp6_IMS
S2_atp6_MATRIX

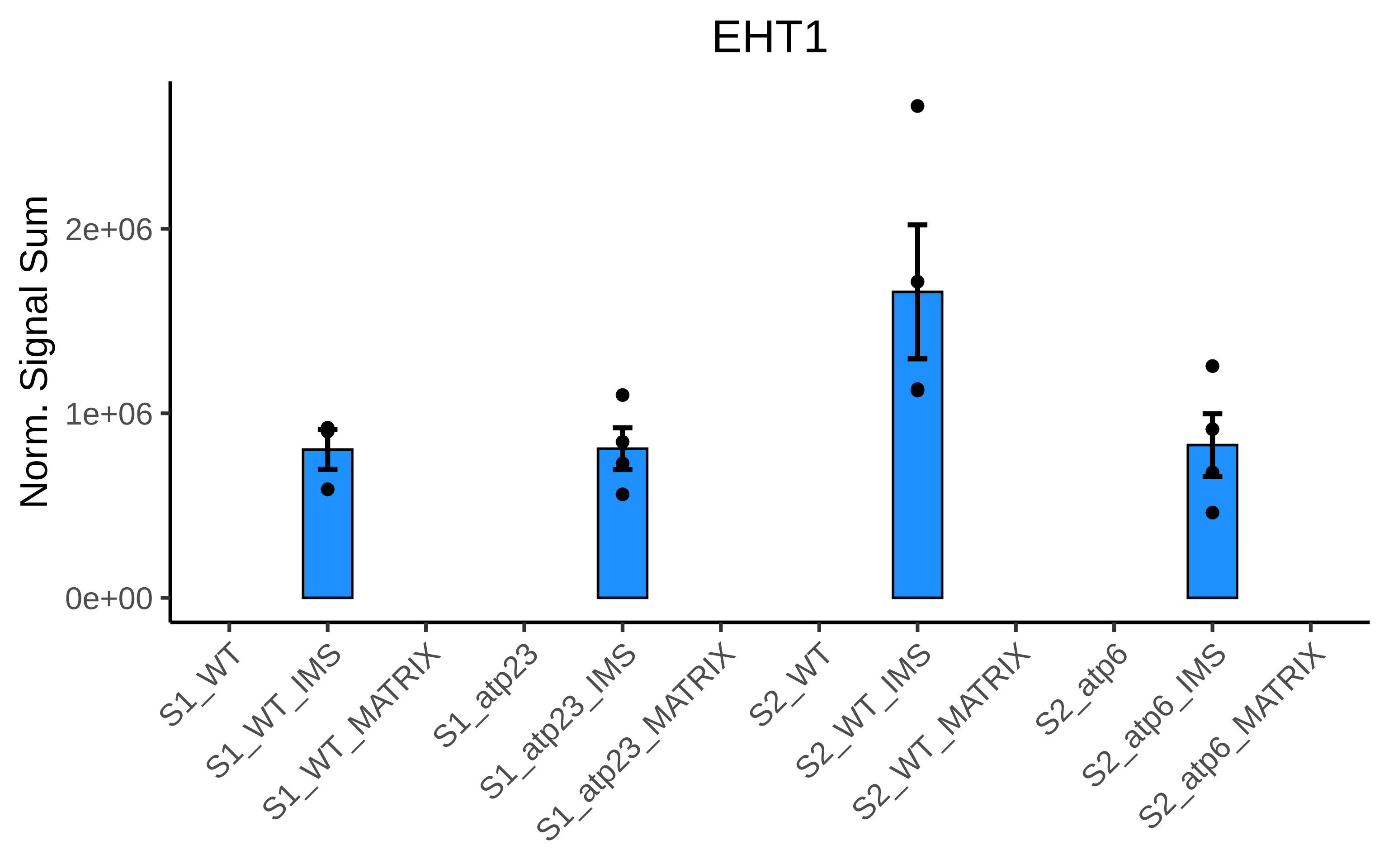

EHT1
Norm. Signal Sum
2e+06
1e+06
0e+00
S1_WT
S1_WT_IMS
S1_WT_MATRIX
S1_atp23
S1_atp23_IMS
S1_atp23_MATRIX
S2_WT
S2_WT_IMS
S2_WT_MATRIX
S2_atp6
S2_atp6_IMS
S2_atp6_MATRIX

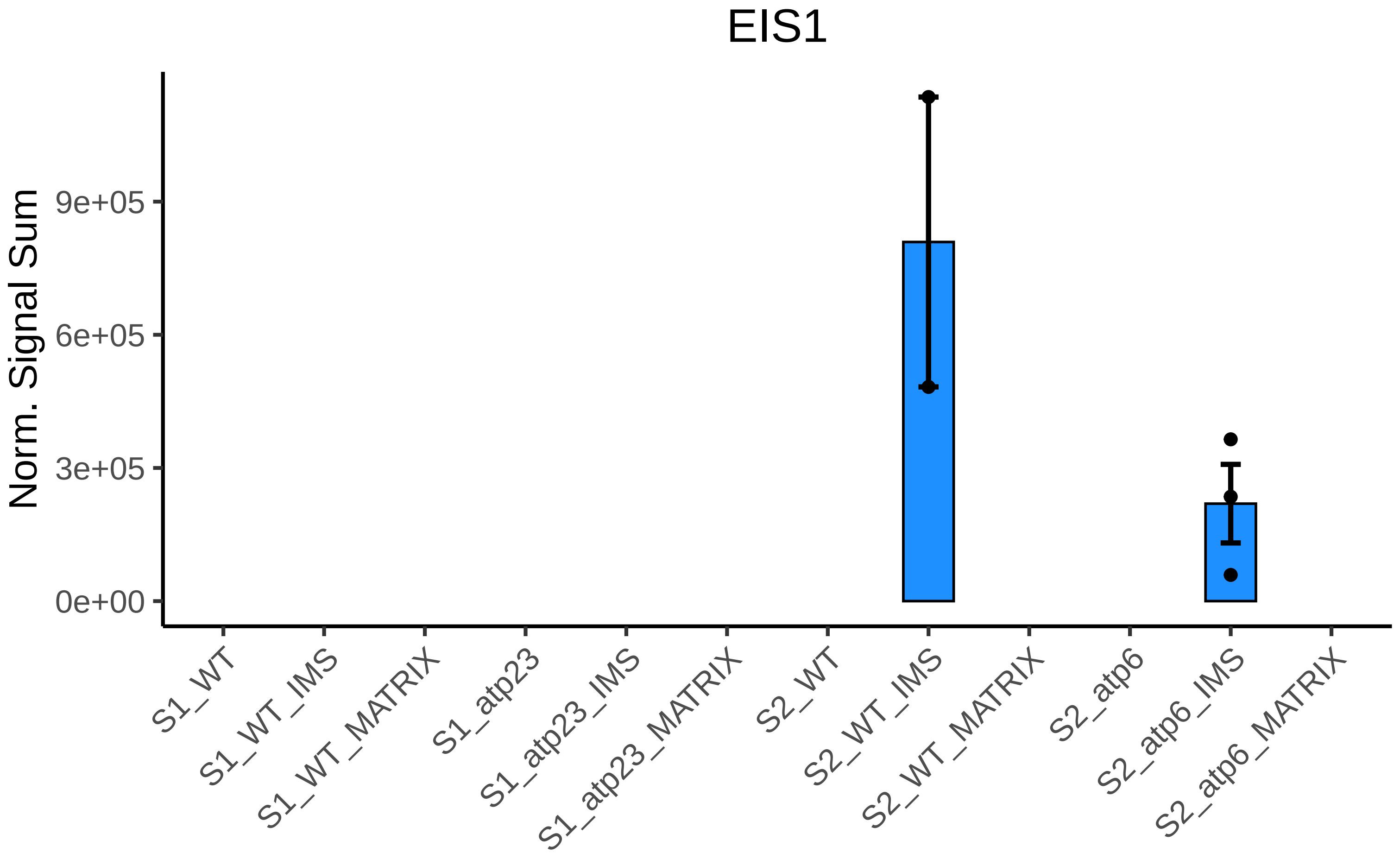
EIS1
Norm. Signal Sum
9e+05
6e+05
3e+05
0e+00
S1_WT
S1_WT_IMS
S1_WT_MATRIX
S1_atp23
S1_atp23_IMS
S1_atp23_MATRIX
S2_WT
S2_WT_IMS
S2_WT_MATRIX
S2_atp6
S2_atp6_IMS
S2_atp6_MATRIX

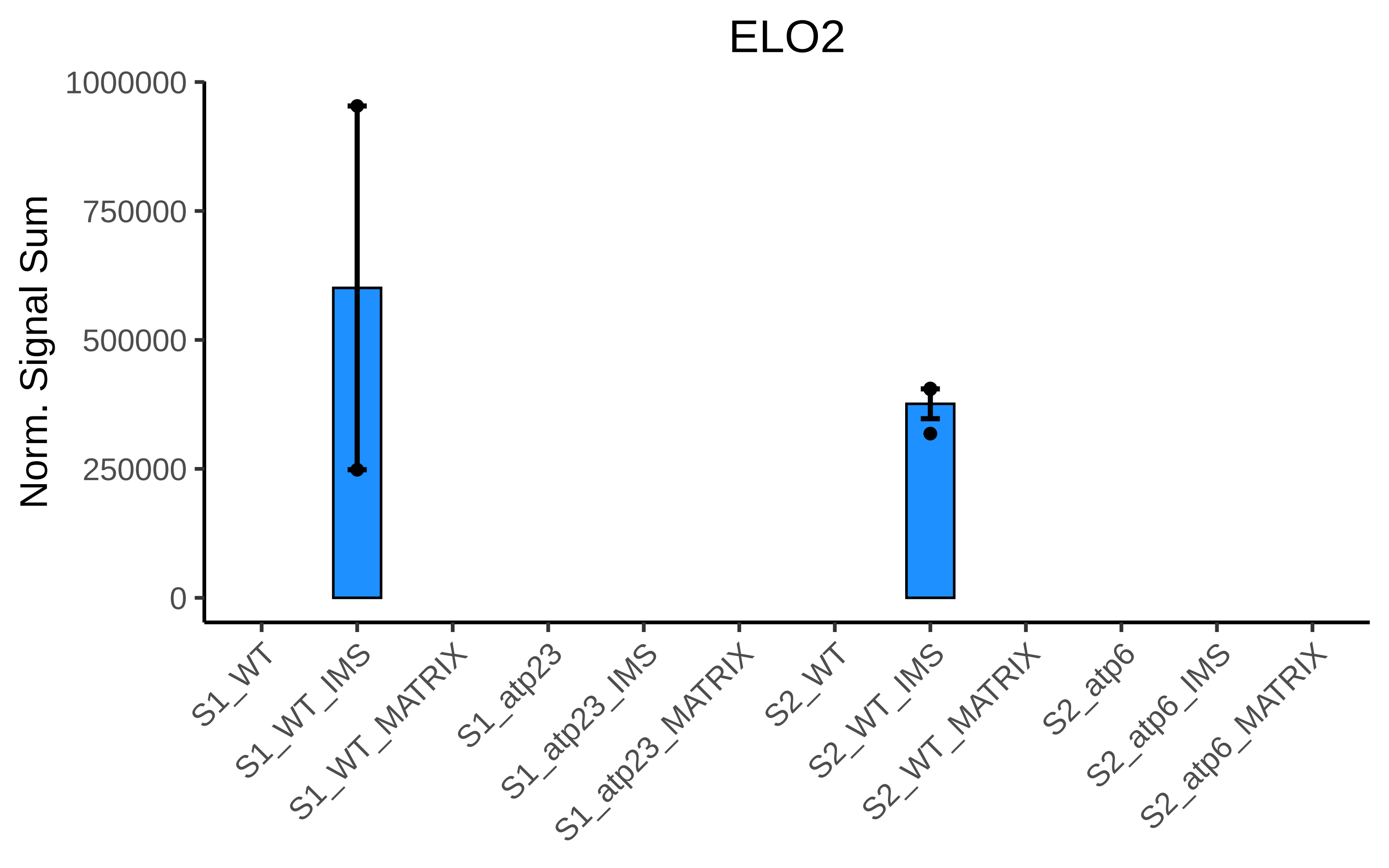
ELO2
Norm. Signal Sum
1000000
750000
500000
250000
0
S1_WT
S1_WT_IMS
S1_WT_MATRIX
S1_atp23
S1_atp23_IMS
S1_atp23_MATRIX
S2_WT
S2_WT_IMS
S2_WT_MATRIX
S2_atp6
S2_atp6_IMS
S2_atp6_MATRIX

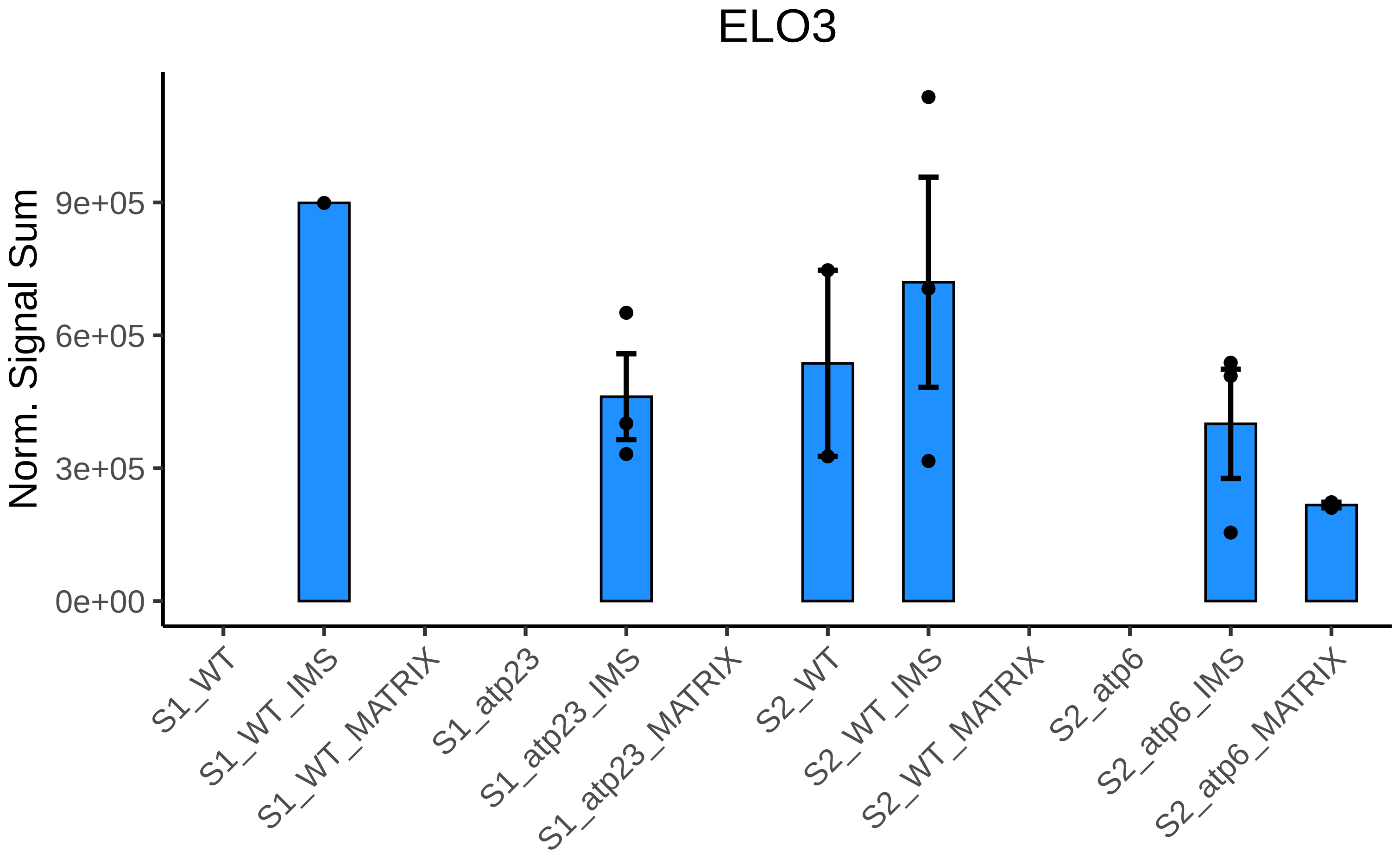

ELO3
Norm. Signal Sum
9e+05
6e+05
3e+05
0e+00
S1_WT
S1_WT_IMS
S1_WT_MATRIX
S1_atp23
S1_atp23_IMS
S1_atp23_MATRIX
S2_WT
S2_WT_IMS
S2_WT_MATRIX
S2_atp6
S2_atp6_IMS
S2_atp6_MATRIX

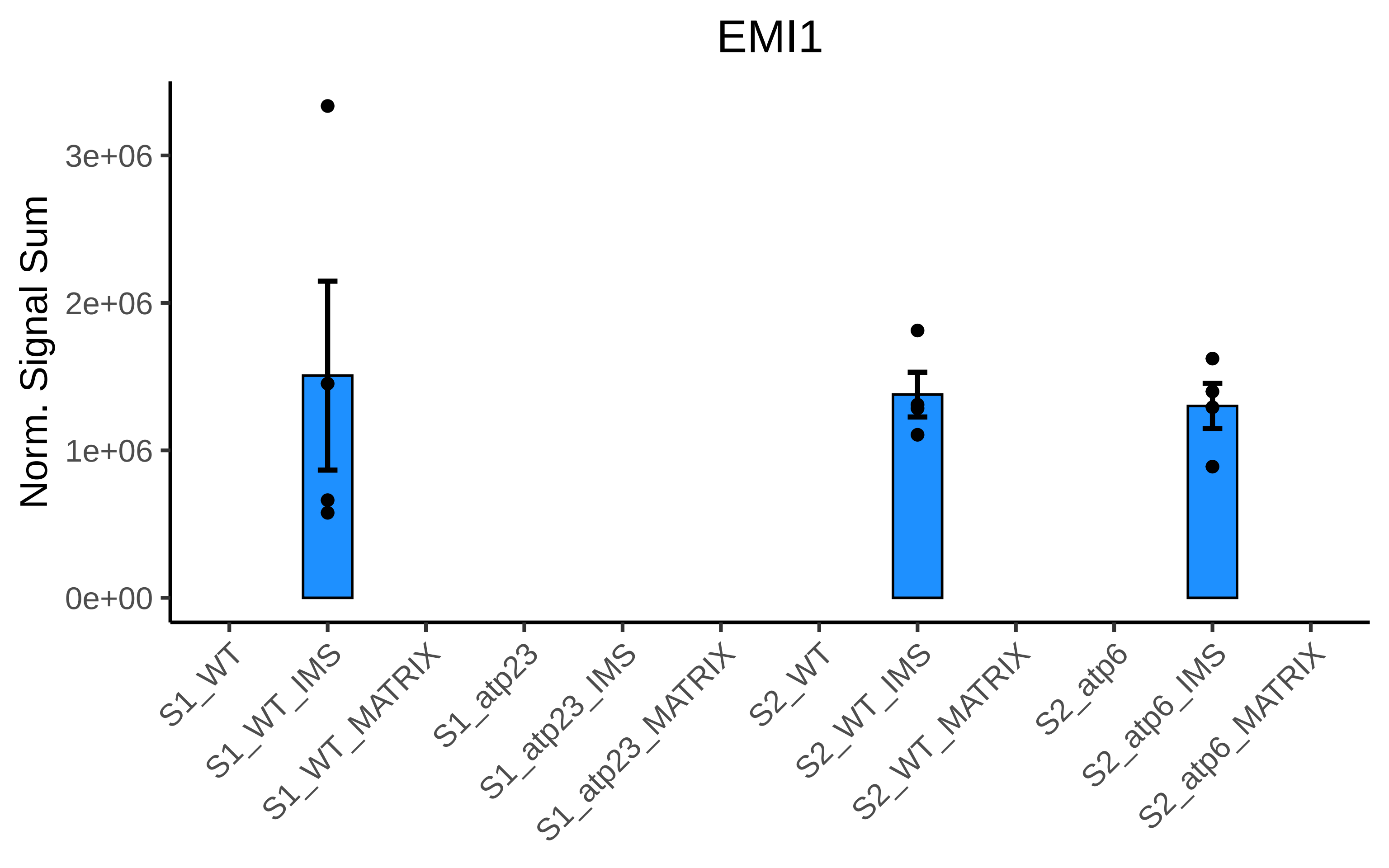
EMI1
Norm. Signal Sum
3e+06
2e+06
1e+06
0e+00
S1_WT
S1_WT_IMS
S1_WT_MATRIX
S1_atp23
S1_atp23_IMS
S1_atp23_MATRIX
S2_WT
S2_WT_IMS
S2_WT_MATRIX
S2_atp6
S2_atp6_IMS
S2_atp6_MATRIX

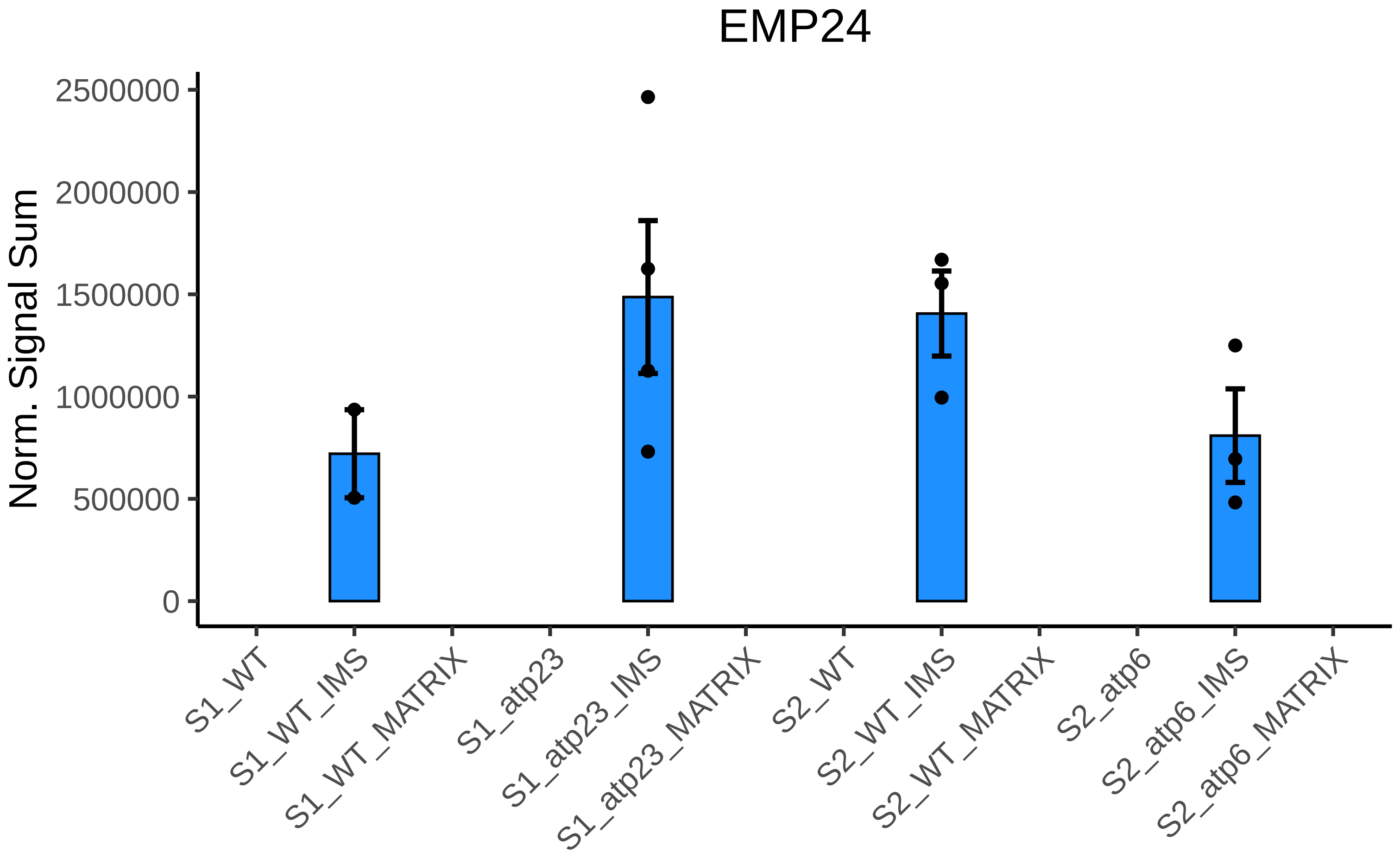

EMP24
Norm. Signal Sum
2500000
2000000
1500000
1000000
500000
0
S1_WT
S1_WT_IMS
S1_WT_MATRIX
S1_atp23
S1_atp23_IMS
S1_atp23_MATRIX
S2_WT
S2_WT_IMS
S2_WT_MATRIX
S2_atp6
S2_atp6_IMS
S2_atp6_MATRIX

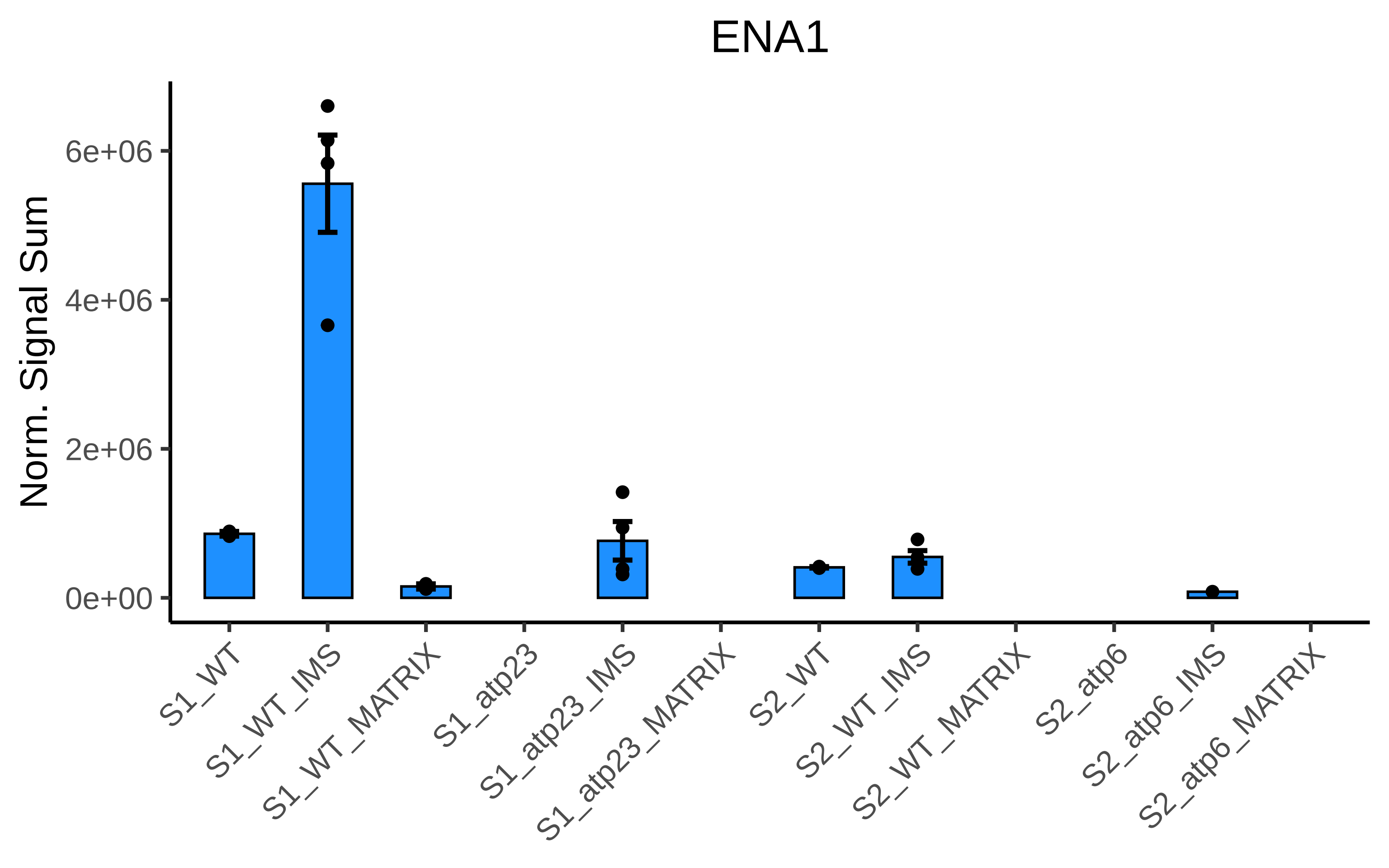

ENA1
Norm. Signal Sum
6e+06
4e+06
2e+06
0e+00
S1_WT
S1_WT_IMS
S1_WT_MATRIX
S1_atp23
S1_atp23_IMS
S1_atp23_MATRIX
S2_WT
S2_WT_IMS
S2_WT_MATRIX
S2_atp6
S2_atp6_IMS
S2_atp6_MATRIX

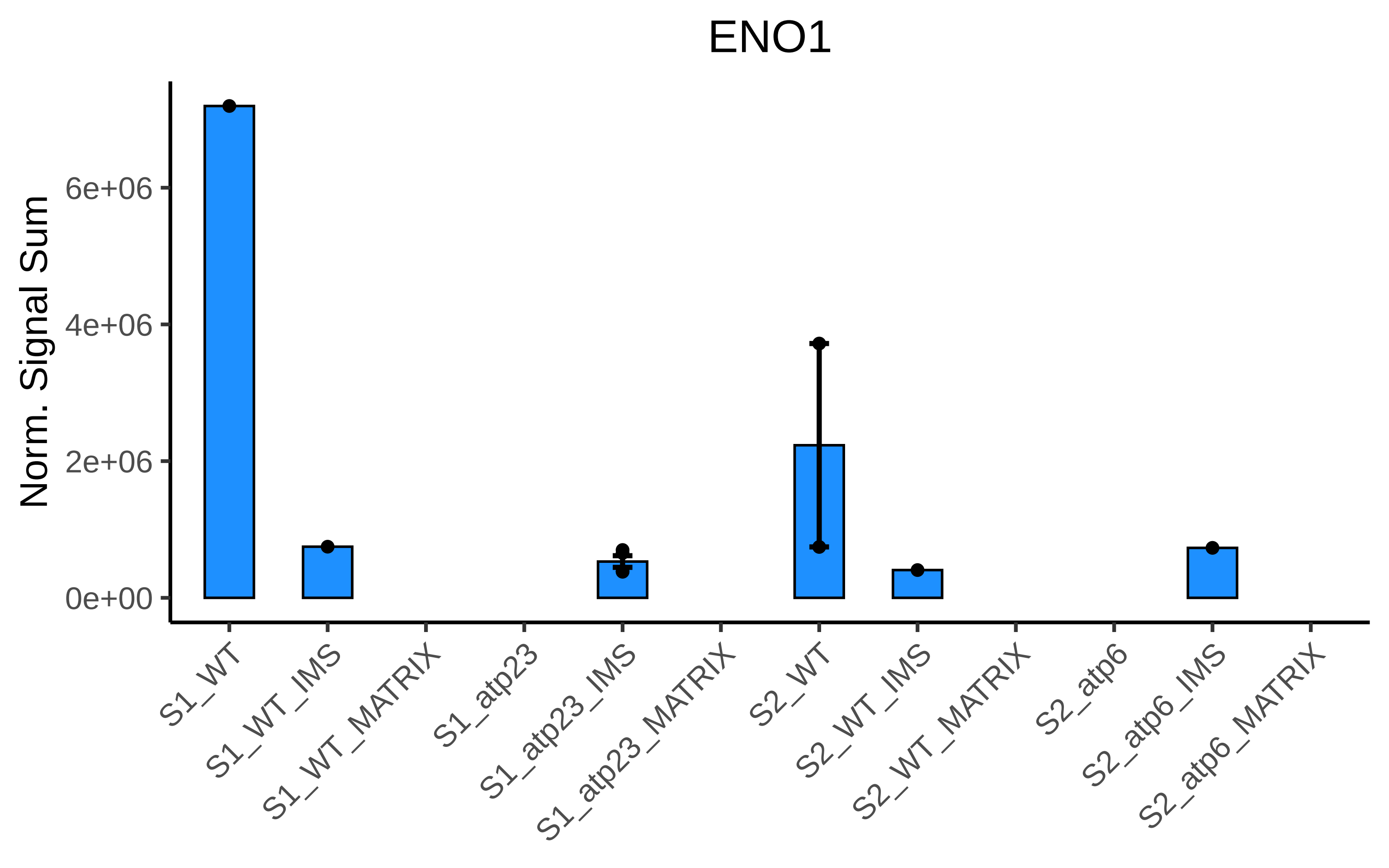

ENO1
Norm. Signal Sum
6e+06
4e+06
2e+06
0e+00
S1_WT
S1_WT_IMS
S1_WT_MATRIX
S1_atp23
S1_atp23_IMS
S1_atp23_MATRIX
S2_WT
S2_WT_IMS
S2_WT_MATRIX
S2_atp6
S2_atp6_IMS
S2_atp6_MATRIX

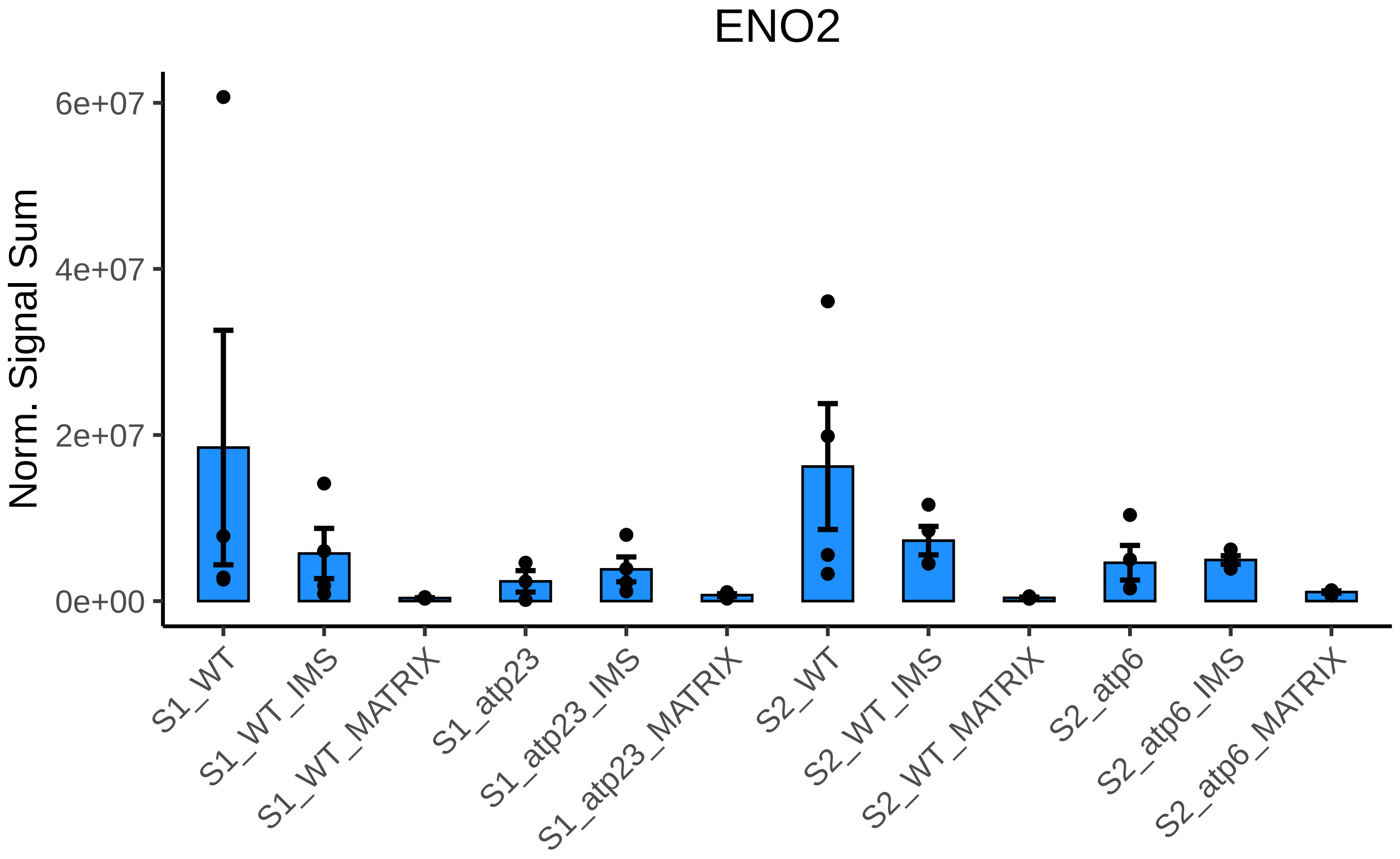
ENO2
Norm. Signal Sum
6e+07
4e+07
2e+07
0e+00
S1_WT
S1_WT_IMS
S1_WT_MATRIX
S1_atp23
S1_atp23_IMS
S1_atp23_MATRIX
S2_WT
S2_WT_IMS
S2_WT_MATRIX
S2_atp6
S2_atp6_IMS
S2_atp6_MATRIX

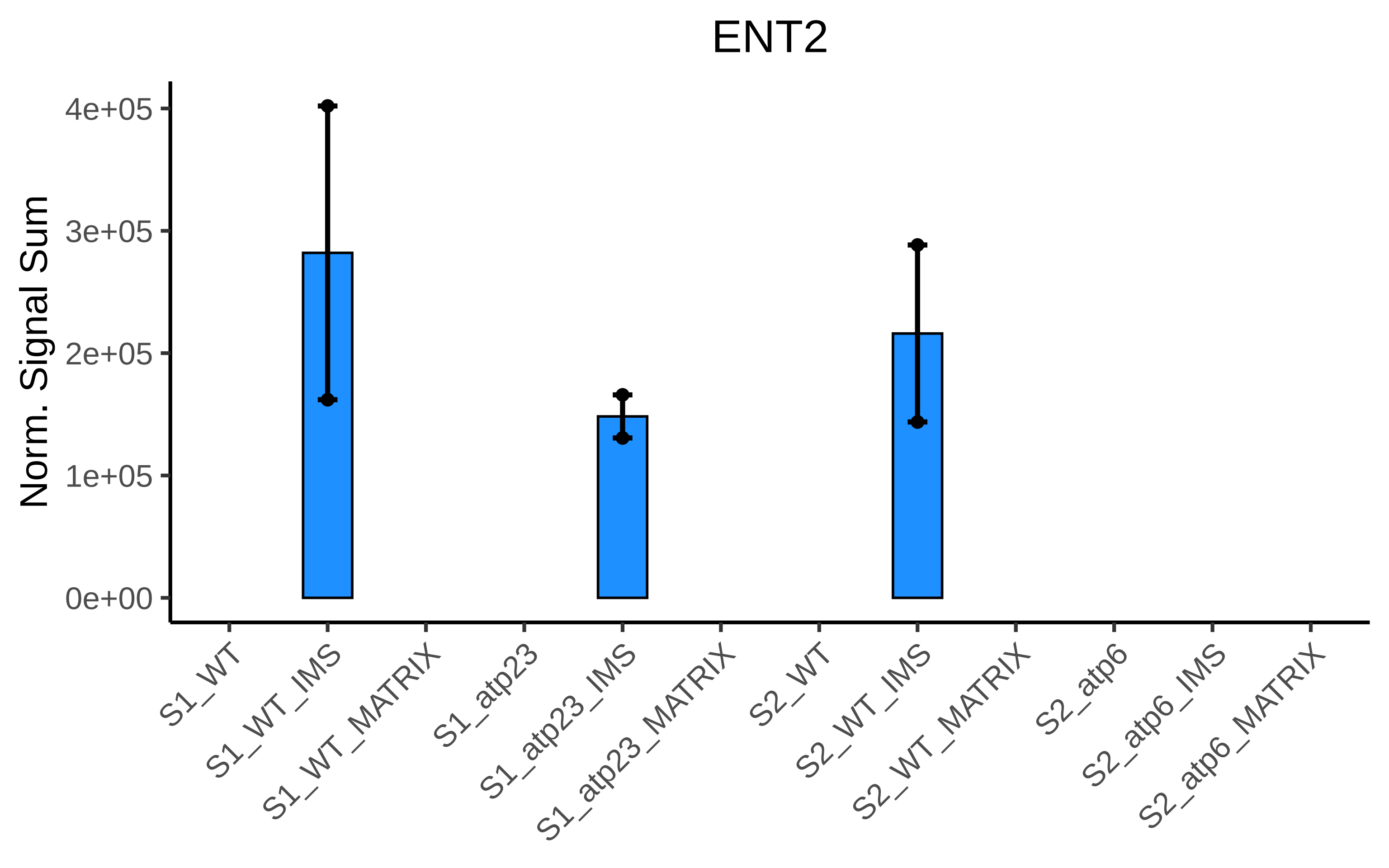

ENT2
Norm. Signal Sum
4e+05
3e+05
2e+05
1e+05
0e+00
S1_WT
S1_WT_IMS
S1_WT_MATRIX
S1_atp23
S1_atp23_IMS
S1_atp23_MATRIX
S2_WT
S2_WT_IMS
S2_WT_MATRIX
S2_atp6
S2_atp6_IMS
S2_atp6_MATRIX

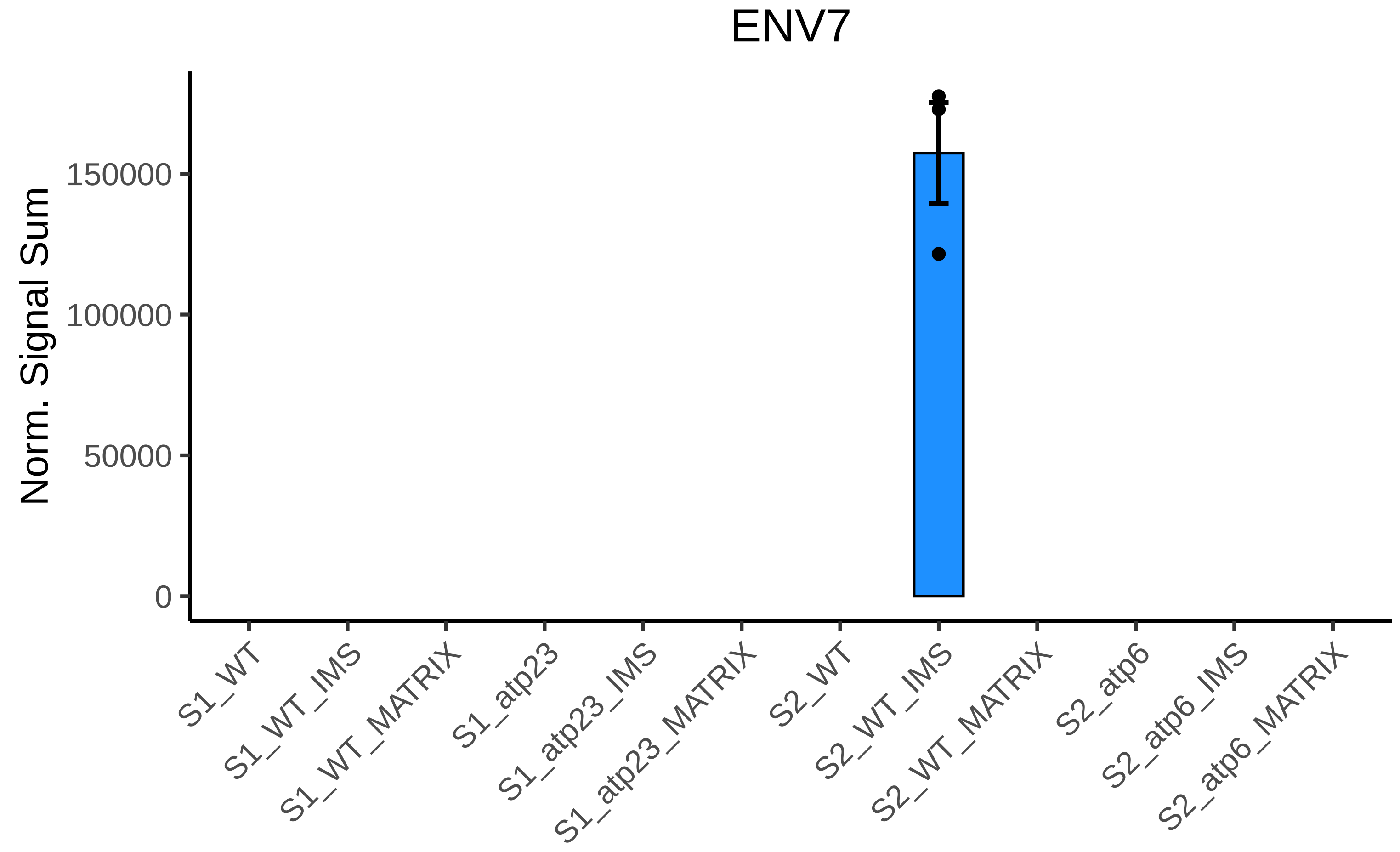

ENV7
Norm. Signal Sum
150000
100000
50000
0
S1_WT
S1_WT_IMS
S1_WT_MATRIX
S1_atp23
S1_atp23_IMS
S1_atp23_MATRIX
S2_WT
S2_WT_IMS
S2_WT_MATRIX
S2_atp6
S2_atp6_IMS
S2_atp6_MATRIX

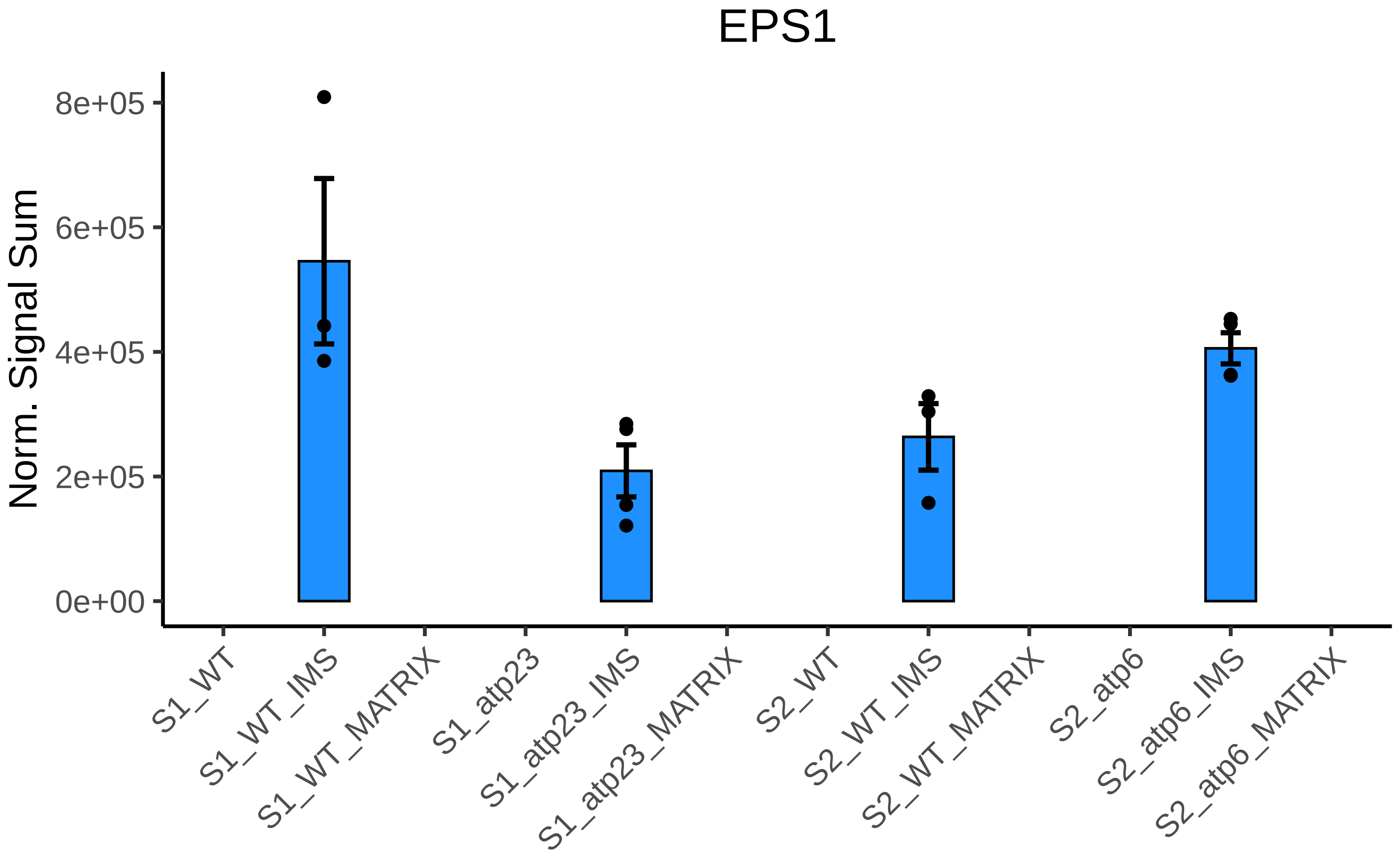

EPS1
Norm. Signal Sum
8e+05
6e+05
4e+05
2e+05
0e+00
S1_WT
S1_WT_IMS
S1_WT_MATRIX
S1_atp23
S1_atp23_IMS
S1_atp23_MATRIX
S2_WT
S2_WT_IMS
S2_WT_MATRIX
S2_atp6
S2_atp6_IMS
S2_atp6_MATRIX

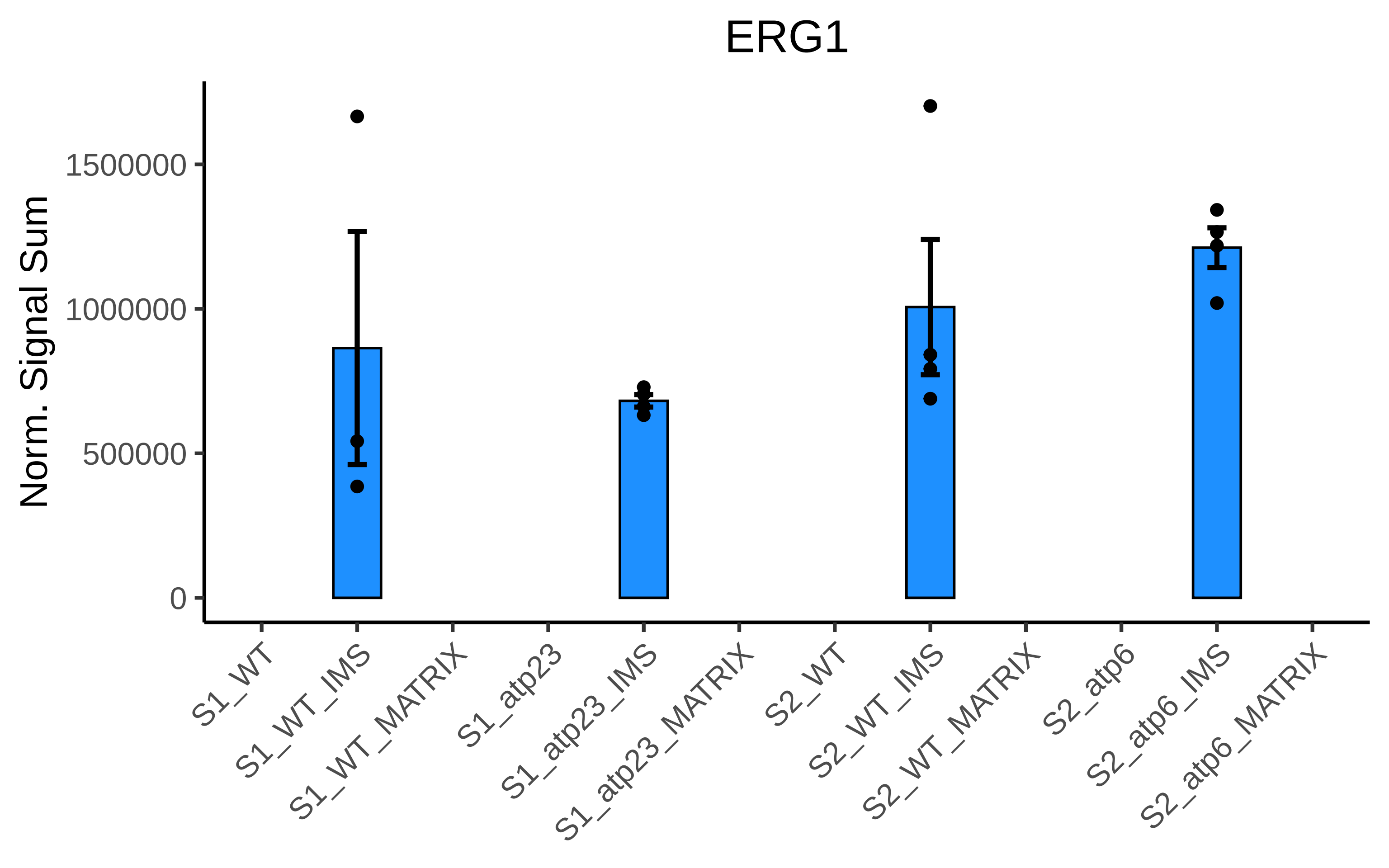

ERG1
Norm. Signal Sum
1500000
1000000
500000
0
S1_WT
S1_WT_IMS
S1_WT_MATRIX
S1_atp23
S1_atp23_IMS
S1_atp23_MATRIX
S2_WT
S2_WT_IMS
S2_WT_MATRIX
S2_atp6
S2_atp6_IMS
S2_atp6_MATRIX

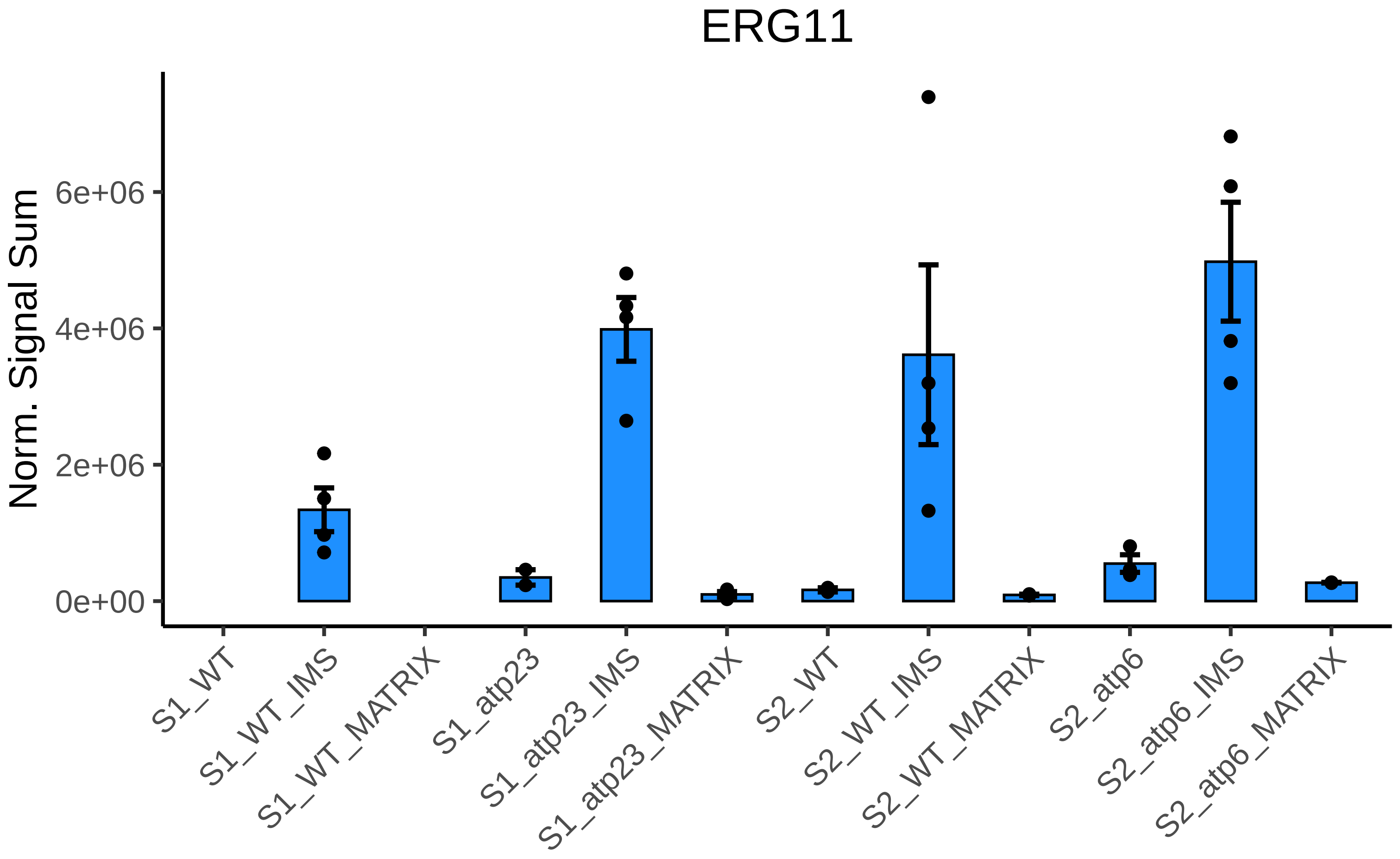

ERG11
Norm. Signal Sum
6e+06
4e+06
2e+06
0e+00
S1_WT
S1_WT_IMS
S1_WT_MATRIX
S1_atp23
S1_atp23_IMS
S1_atp23_MATRIX
S2_WT
S2_WT_IMS
S2_WT_MATRIX
S2_atp6
S2_atp6_IMS
S2_atp6_MATRIX

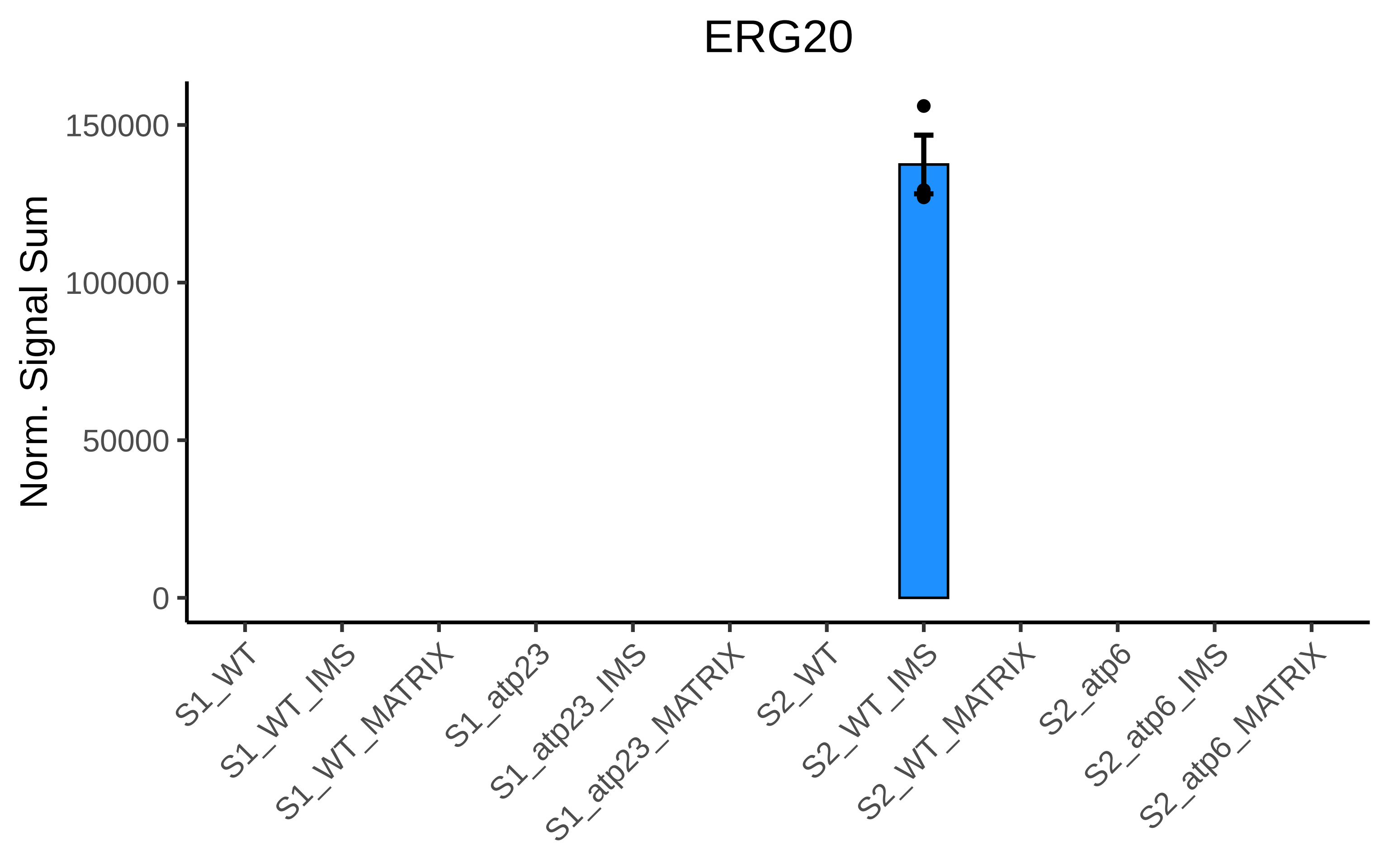

ERG20
Norm. Signal Sum
150000
100000
50000
0
S1_WT
S1_WT_IMS
S1_WT_MATRIX
S1_atp23
S1_atp23_IMS
S1_atp23_MATRIX
S2_WT
S2_WT_IMS
S2_WT_MATRIX
S2_atp6
S2_atp6_IMS
S2_atp6_MATRIX

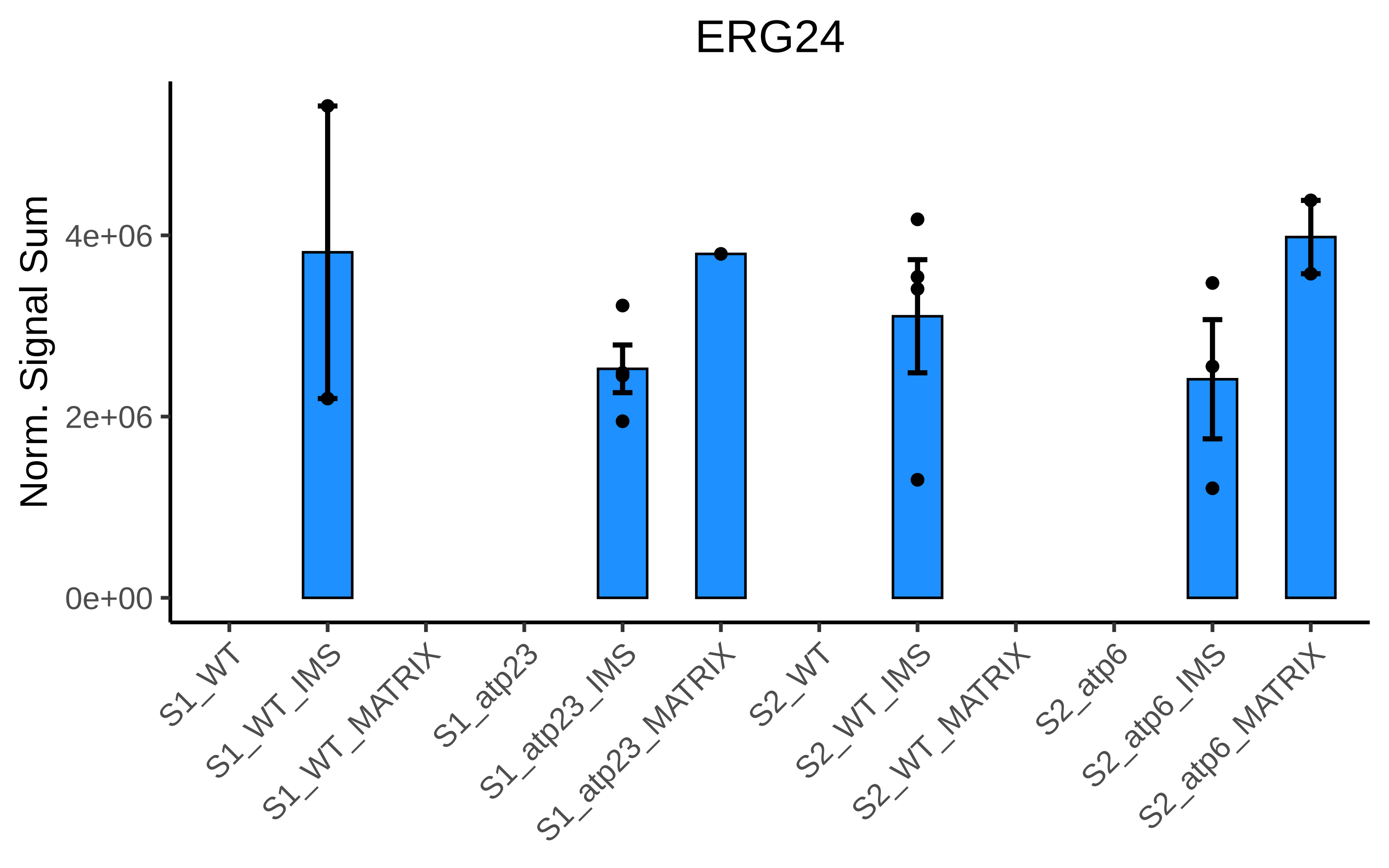

ERG24
Norm. Signal Sum
4e+06
2e+06
0e+00
S1_WT
S1_WT_IMS
S1_WT_MATRIX
S1_atp23
S1_atp23_IMS
S1_atp23_MATRIX
S2_WT
S2_WT_IMS
S2_WT_MATRIX
S2_atp6
S2_atp6_IMS
S2_atp6_MATRIX

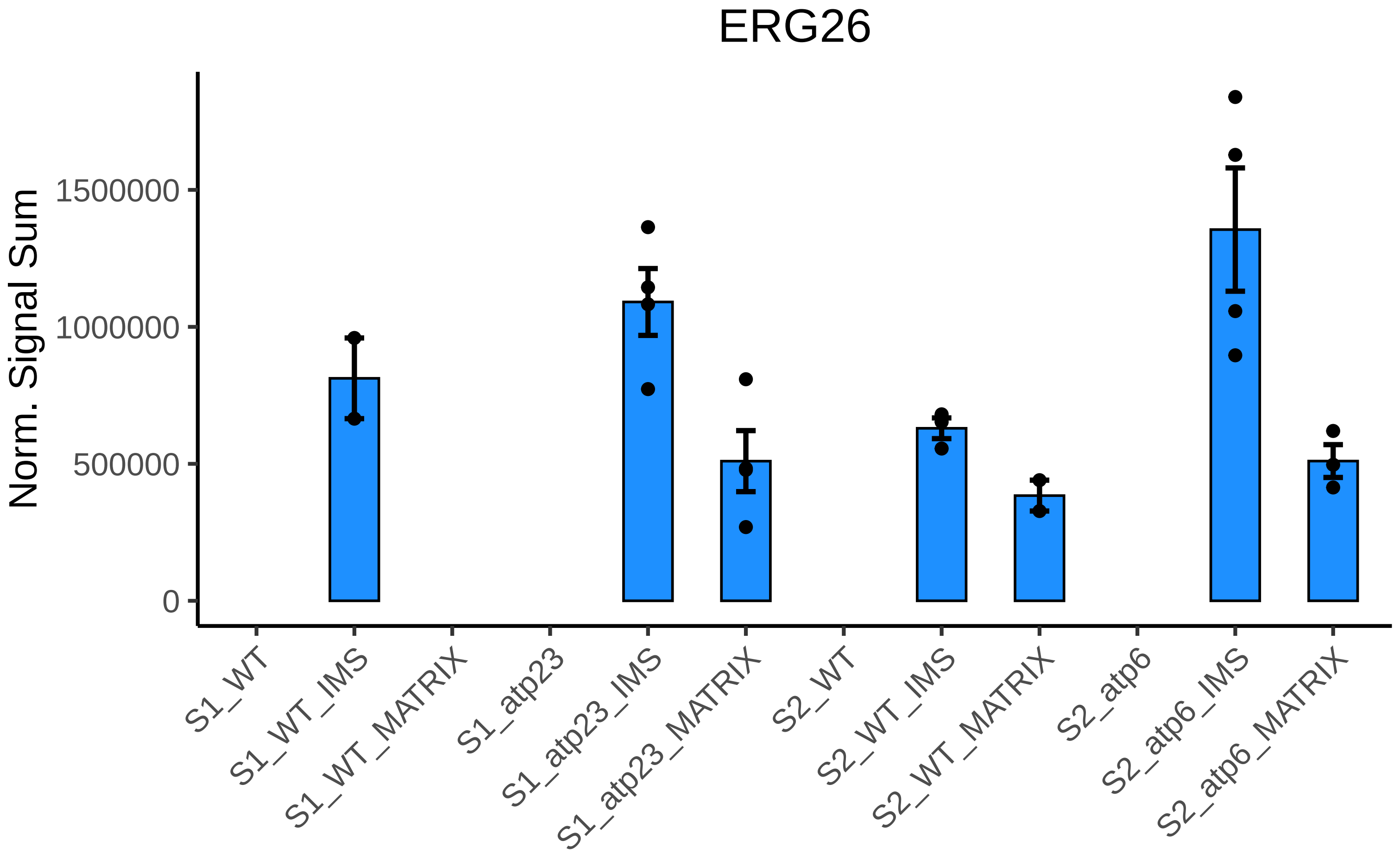

ERG26
Norm. Signal Sum
1500000
1000000
500000
0
S1_WT
S1_WT_IMS
S1_WT_MATRIX
S1_atp23
S1_atp23_IMS
S1_atp23_MATRIX
S2_WT
S2_WT_IMS
S2_WT_MATRIX
S2_atp6
S2_atp6_IMS
S2_atp6_MATRIX

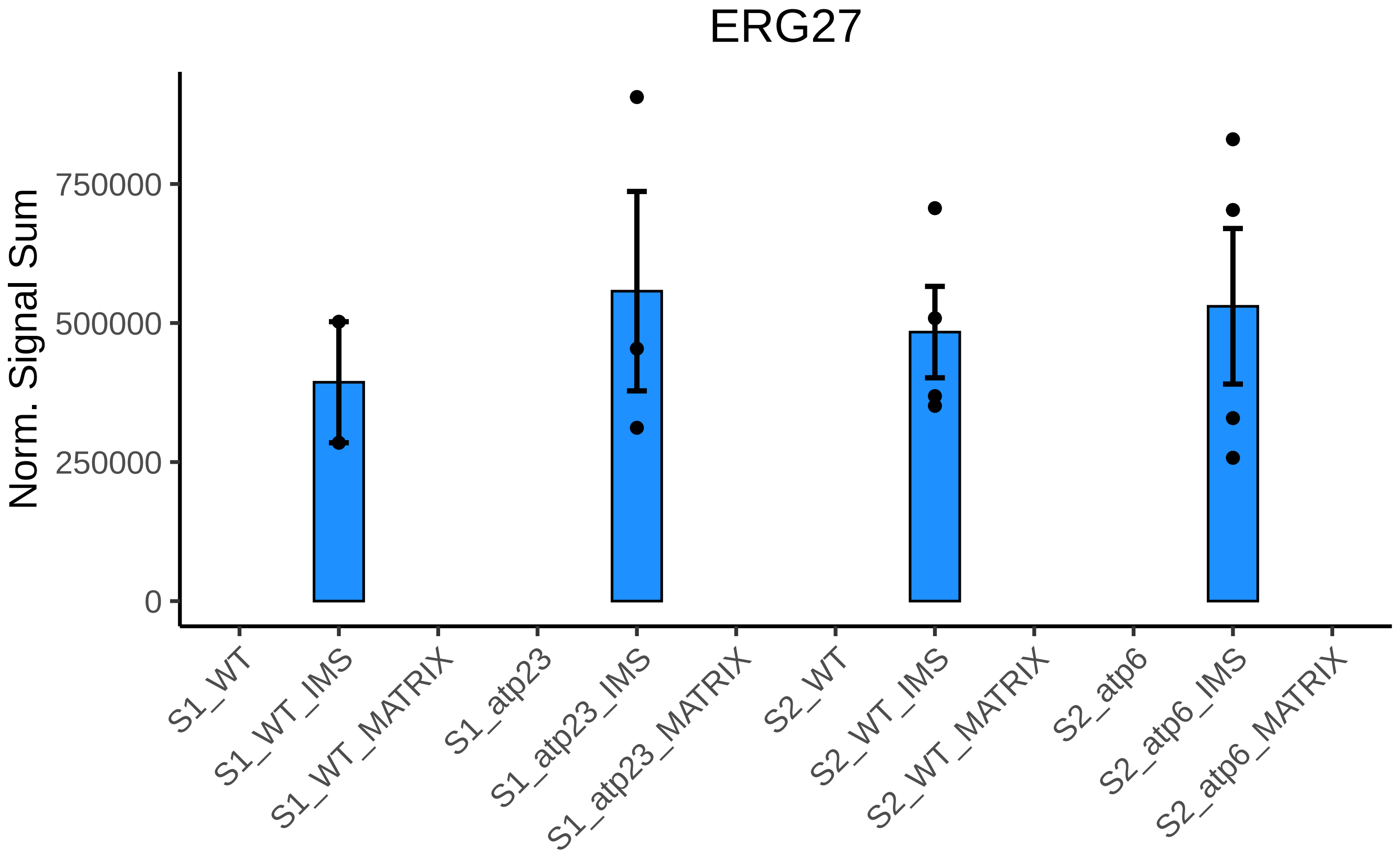

ERG27
Norm. Signal Sum
750000
500000
250000
0
S1_WT
S1_WT_IMS
S1_WT_MATRIX
S1_atp23
S1_atp23_IMS
S1_atp23_MATRIX
S2_WT
S2_WT_IMS
S2_WT_MATRIX
S2_atp6
S2_atp6_IMS
S2_atp6_MATRIX

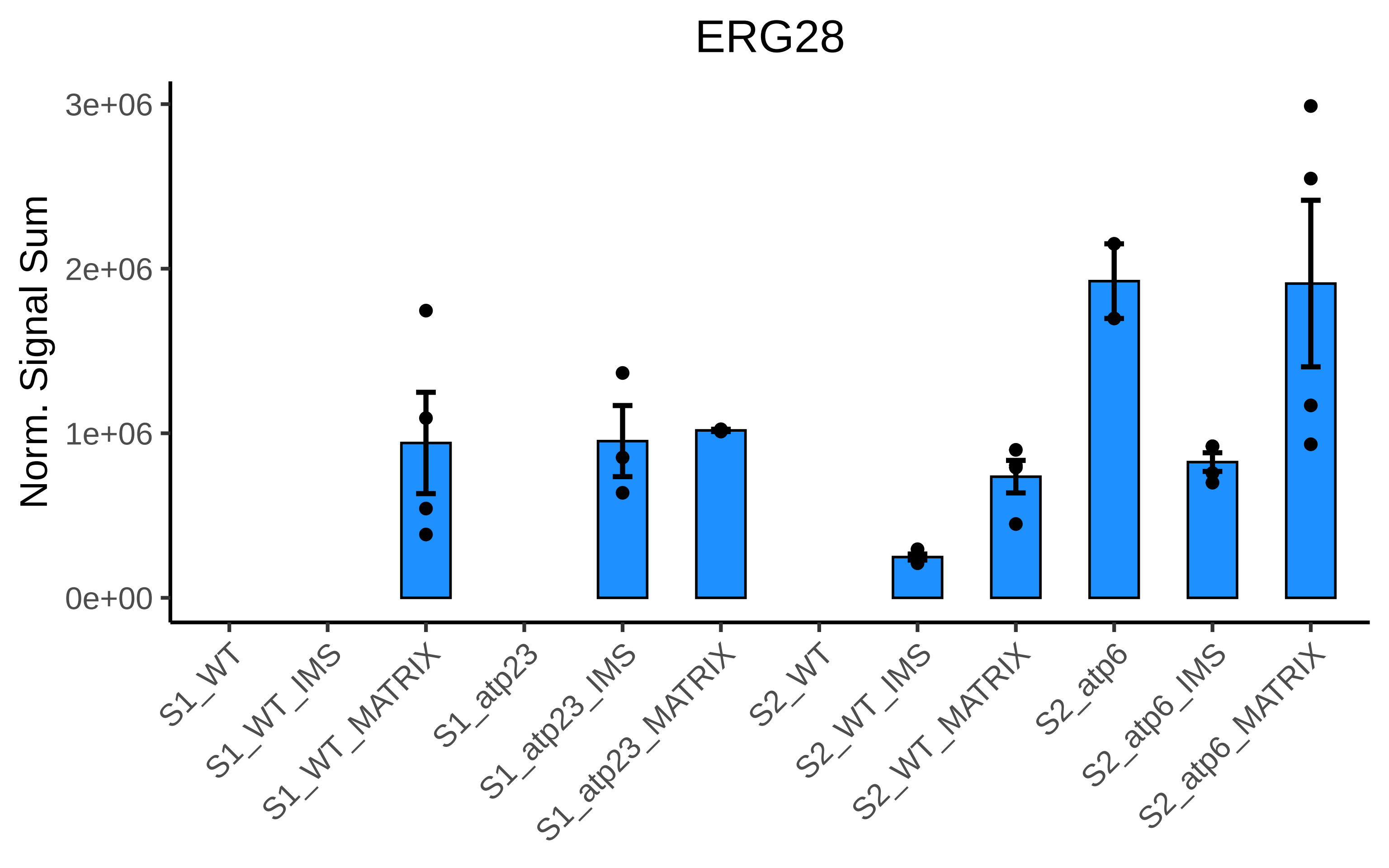

ERG28
Norm. Signal Sum
3e+06
2e+06
1e+06
0e+00
S1_WT
S1_WT_IMS
S1_WT_MATRIX
S1_atp23
S1_atp23_IMS
S1_atp23_MATRIX
S2_WT
S2_WT_IMS
S2_WT_MATRIX
S2_atp6
S2_atp6_IMS
S2_atp6_MATRIX

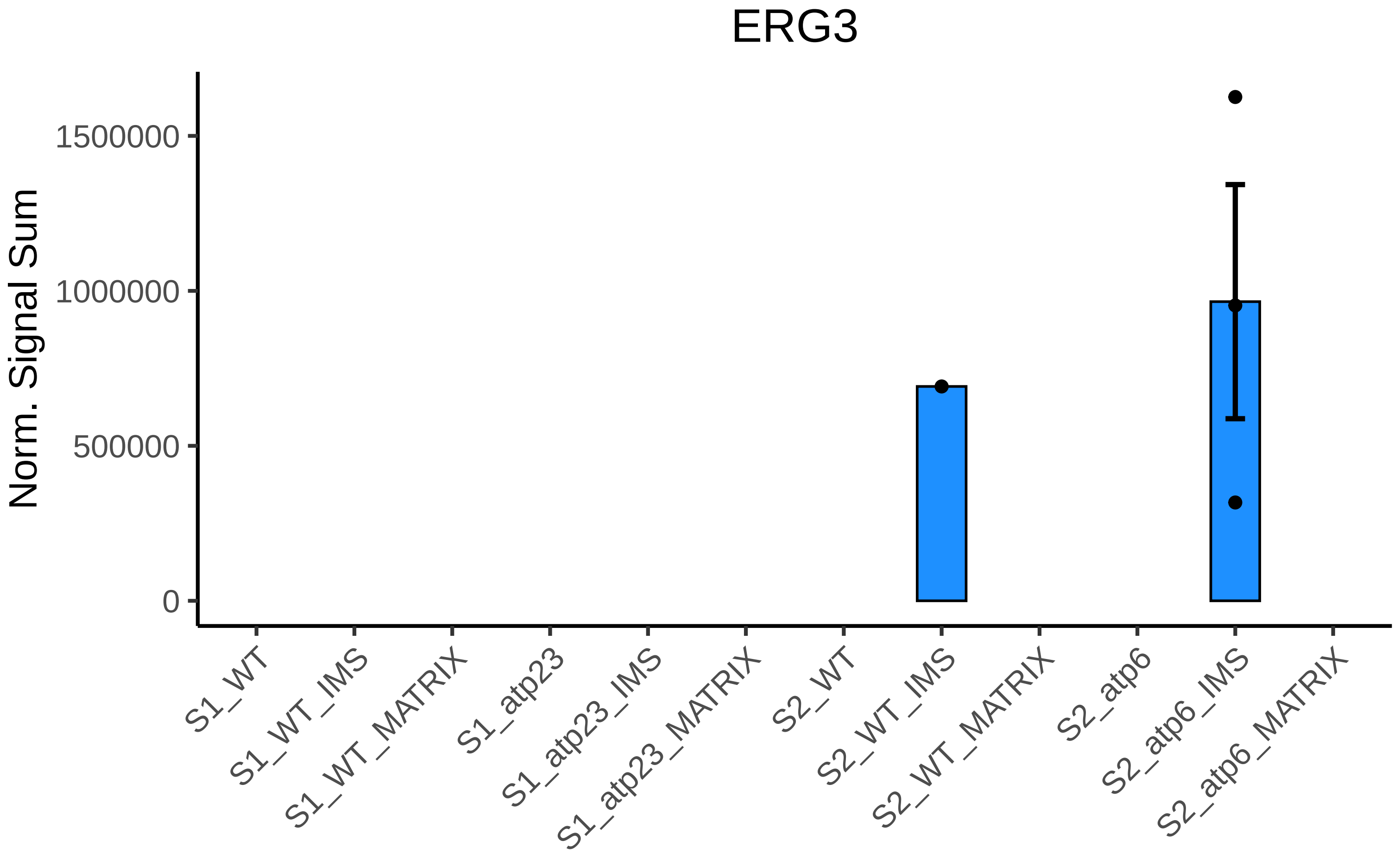

ERG3
Norm. Signal Sum
1500000
1000000
500000
0
S1_WT
S1_WT_IMS
S1_WT_MATRIX
S1_atp23
S1_atp23_IMS
S1_atp23_MATRIX
S2_WT
S2_WT_IMS
S2_WT_MATRIX
S2_atp6
S2_atp6_IMS
S2_atp6_MATRIX

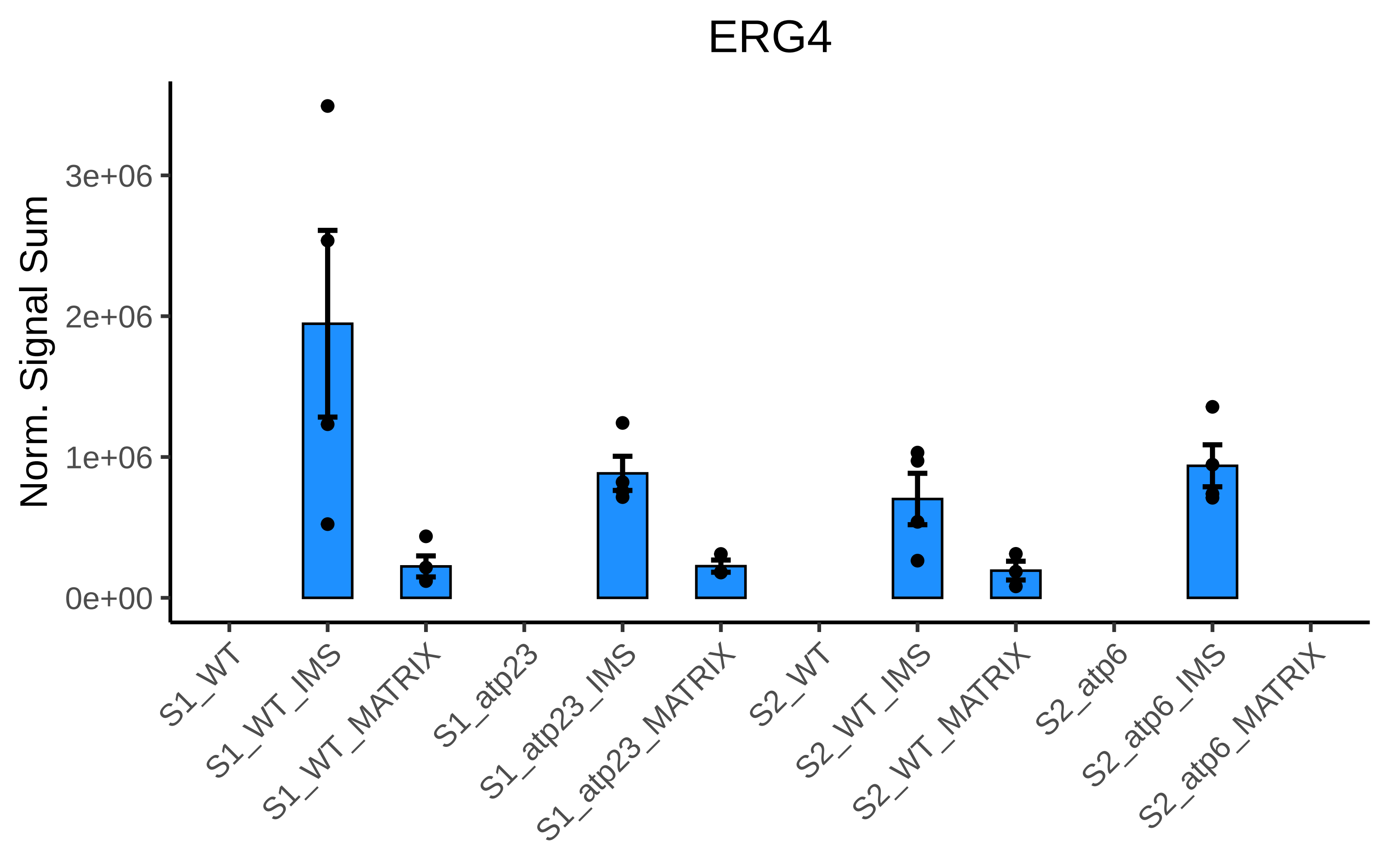

ERG4
Norm. Signal Sum
3e+06
2e+06
1e+06
0e+00
S1_WT
S1_WT_IMS
S1_WT_MATRIX
S1_atp23
S1_atp23_IMS
S1_atp23_MATRIX
S2_WT
S2_WT_IMS
S2_WT_MATRIX
S2_atp6
S2_atp6_IMS
S2_atp6_MATRIX

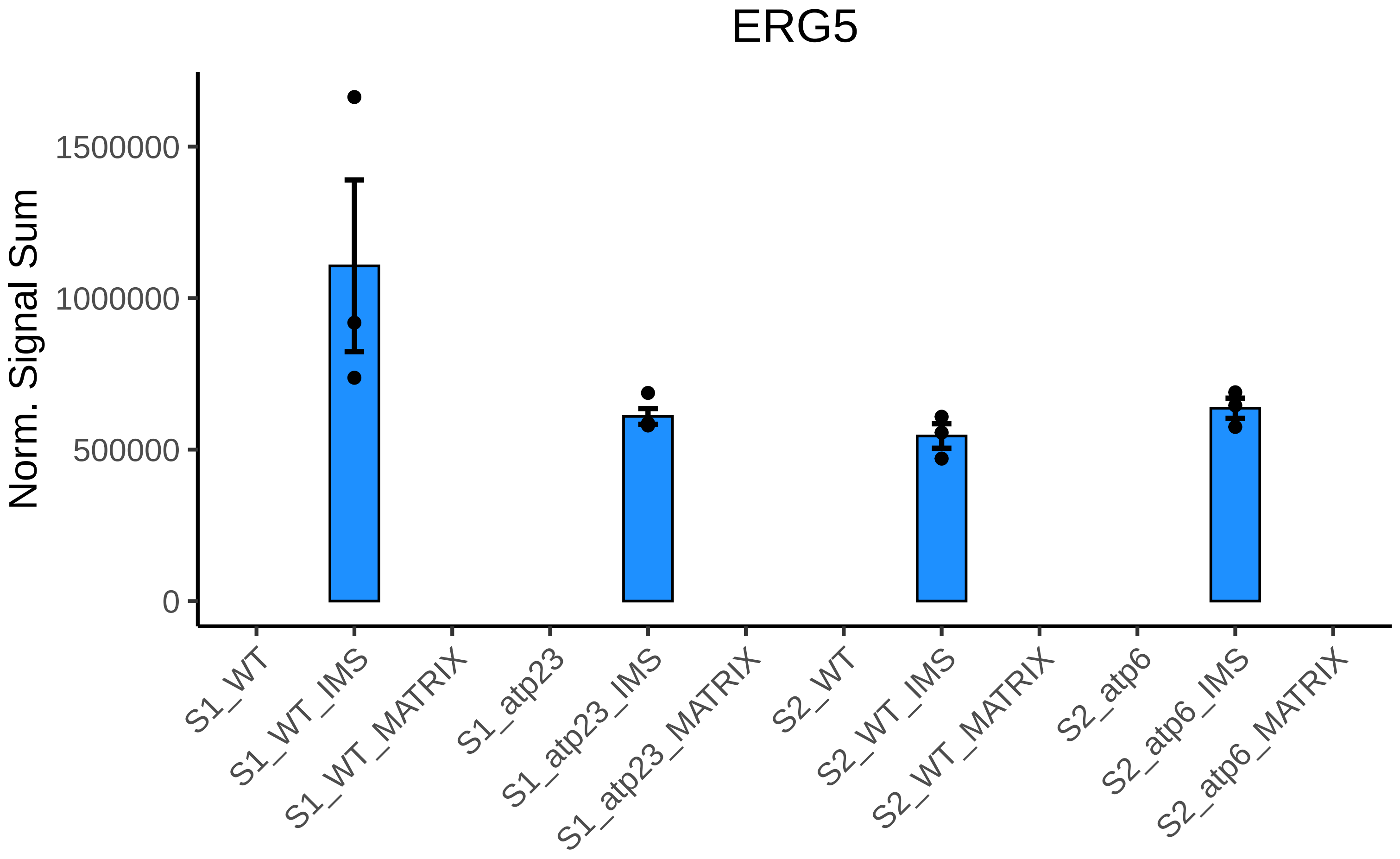

ERG5
Norm. Signal Sum
0
500000
1000000
1500000
S1_WT
S1_WT_IMS
S1_WT_MATRIX
S1_atp23
S1_atp23_IMS
S1_atp23_MATRIX
S2_WT
S2_WT_IMS
S2_WT_MATRIX
S2_atp6
S2_atp6_IMS
S2_atp6_MATRIX

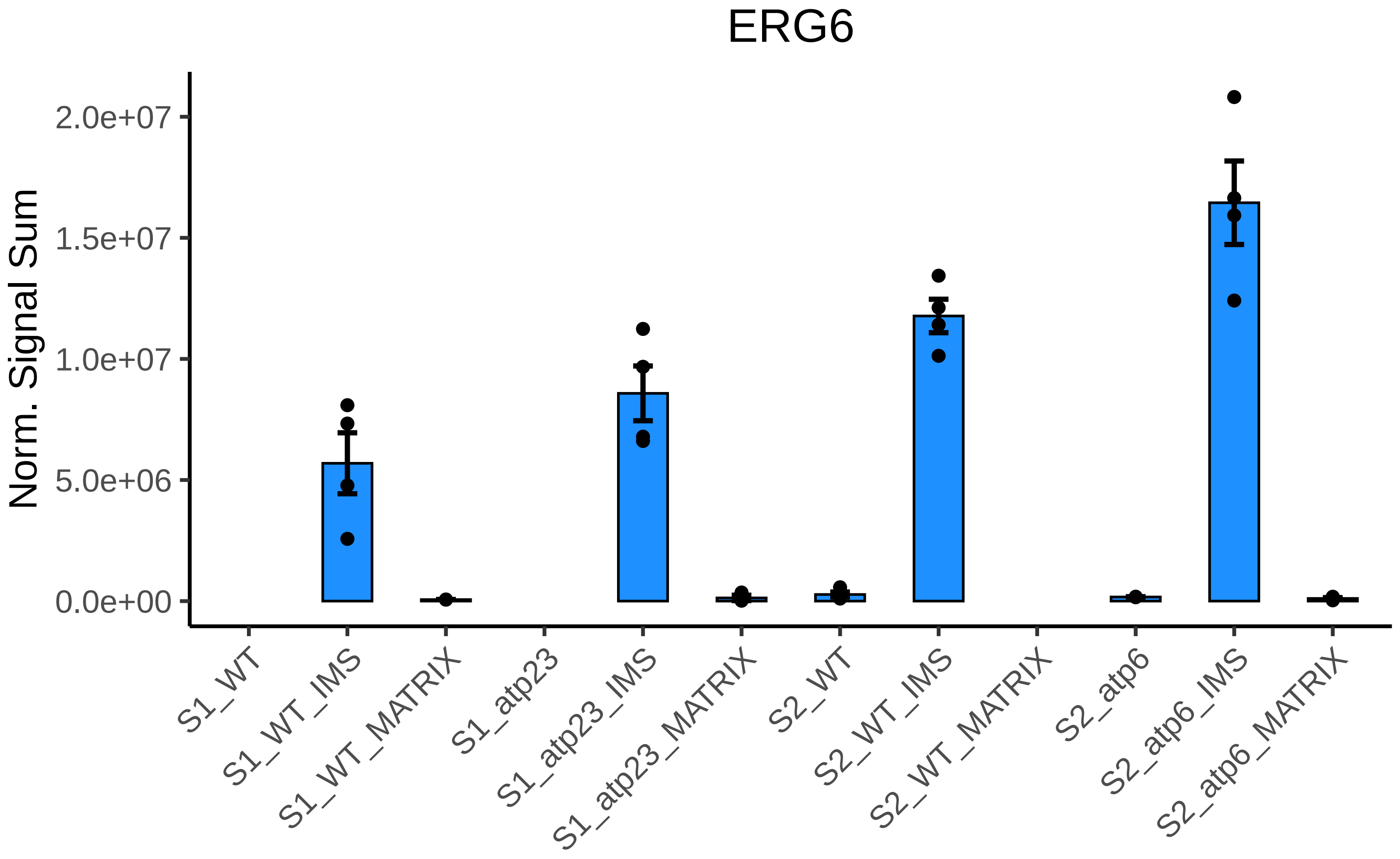

ERG6
Norm. Signal Sum
2.0e+07
1.5e+07
1.0e+07
5.0e+06
0.0e+00
S1_WT
S1_WT_IMS
S1_WT_MATRIX
S1_atp23
S1_atp23_IMS
S1_atp23_MATRIX
S2_WT
S2_WT_IMS
S2_WT_MATRIX
S2_atp6
S2_atp6_IMS
S2_atp6_MATRIX

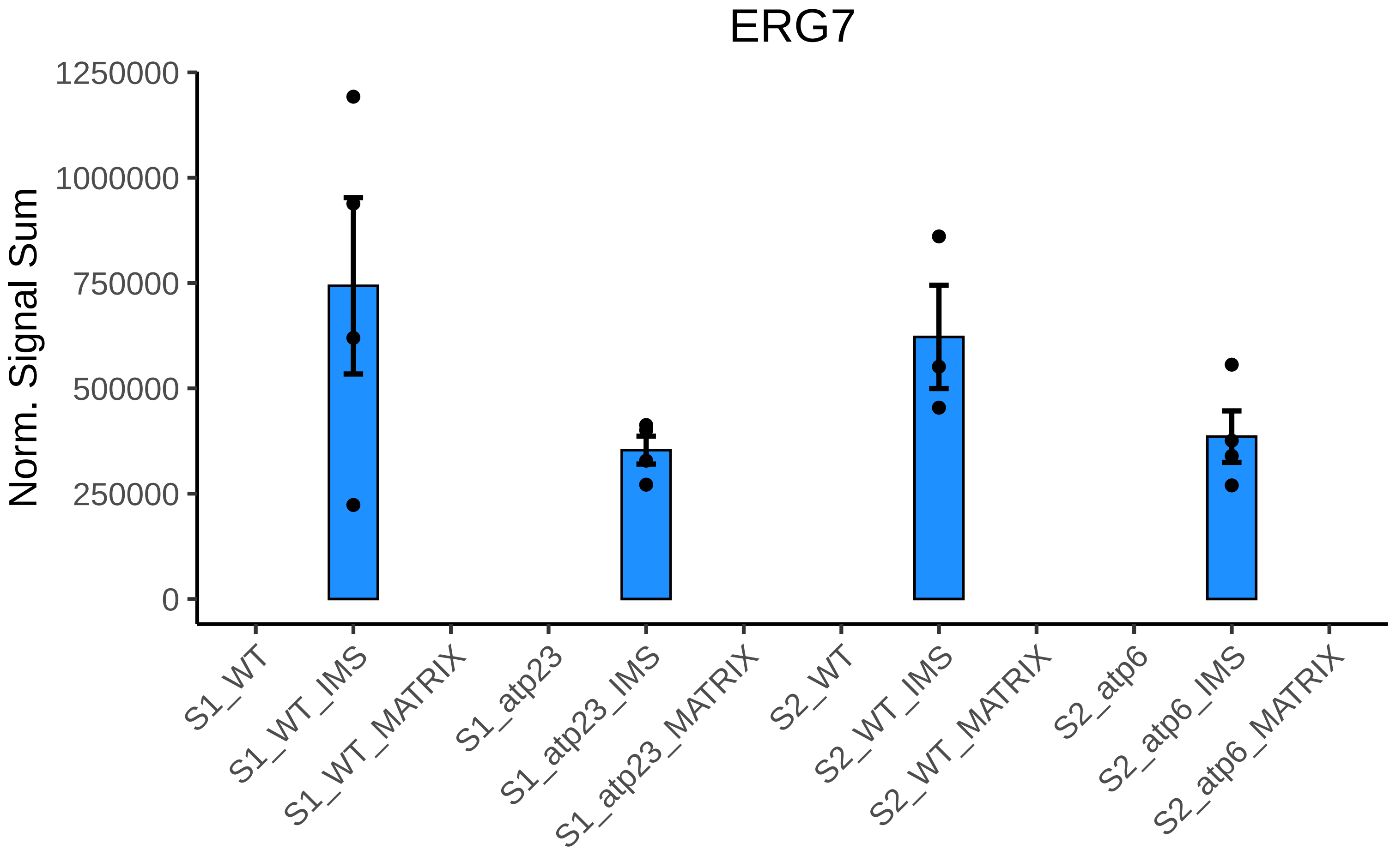

ERG7
Norm. Signal Sum
1250000
1000000
750000
500000
250000
0
S1_WT
S1_WT_IMS
S1_WT_MATRIX
S1_atp23
S1_atp23_IMS
S1_atp23_MATRIX
S2_WT
S2_WT_IMS
S2_WT_MATRIX
S2_atp6
S2_atp6_IMS
S2_atp6_MATRIX

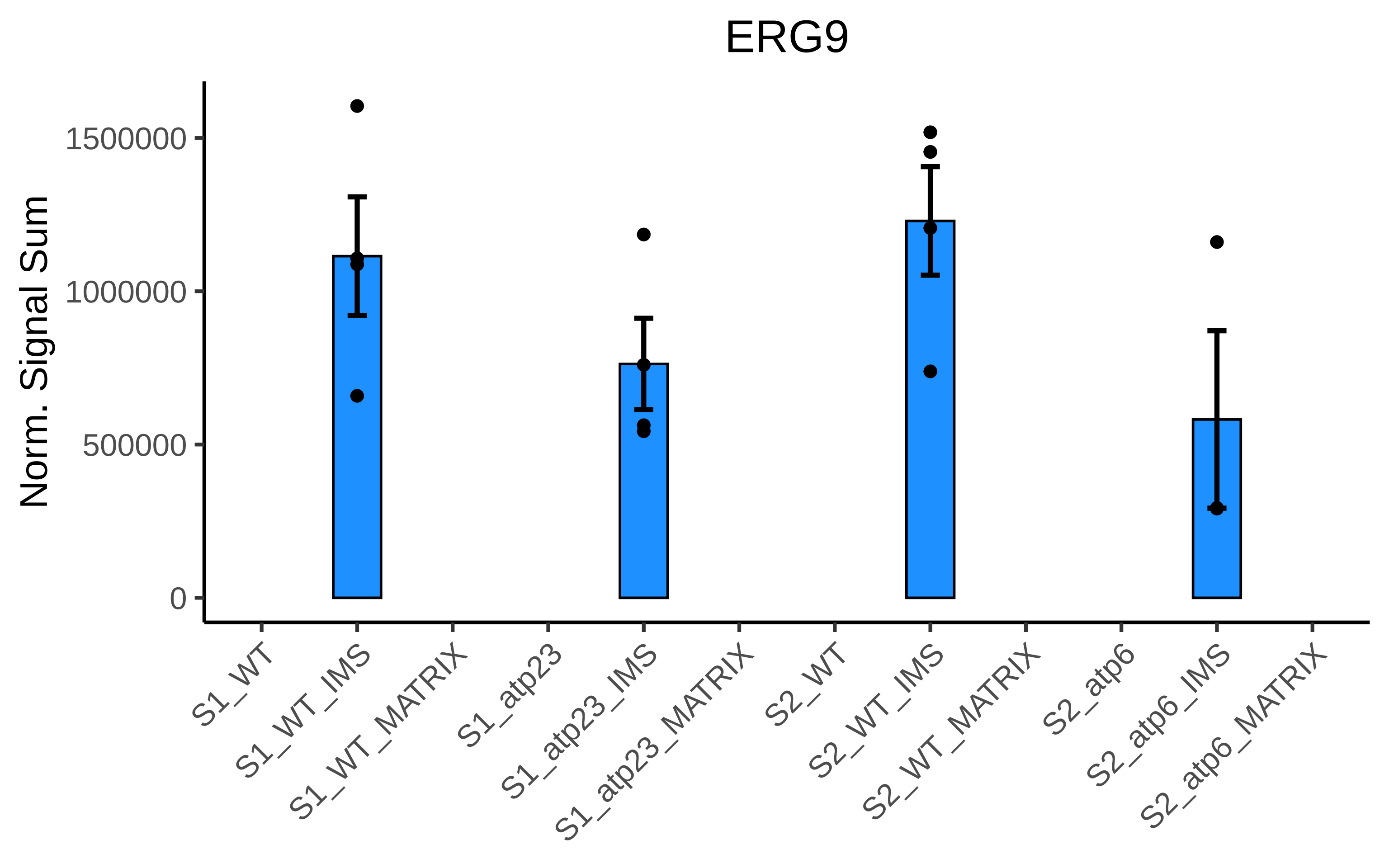

ERG9
Norm. Signal Sum
1500000
1000000
500000
0
S1_WT
S1_WT_IMS
S1_WT_MATRIX
S1_atp23
S1_atp23_IMS
S1_atp23_MATRIX
S2_WT
S2_WT_IMS
S2_WT_MATRIX
S2_atp6
S2_atp6_IMS
S2_atp6_MATRIX

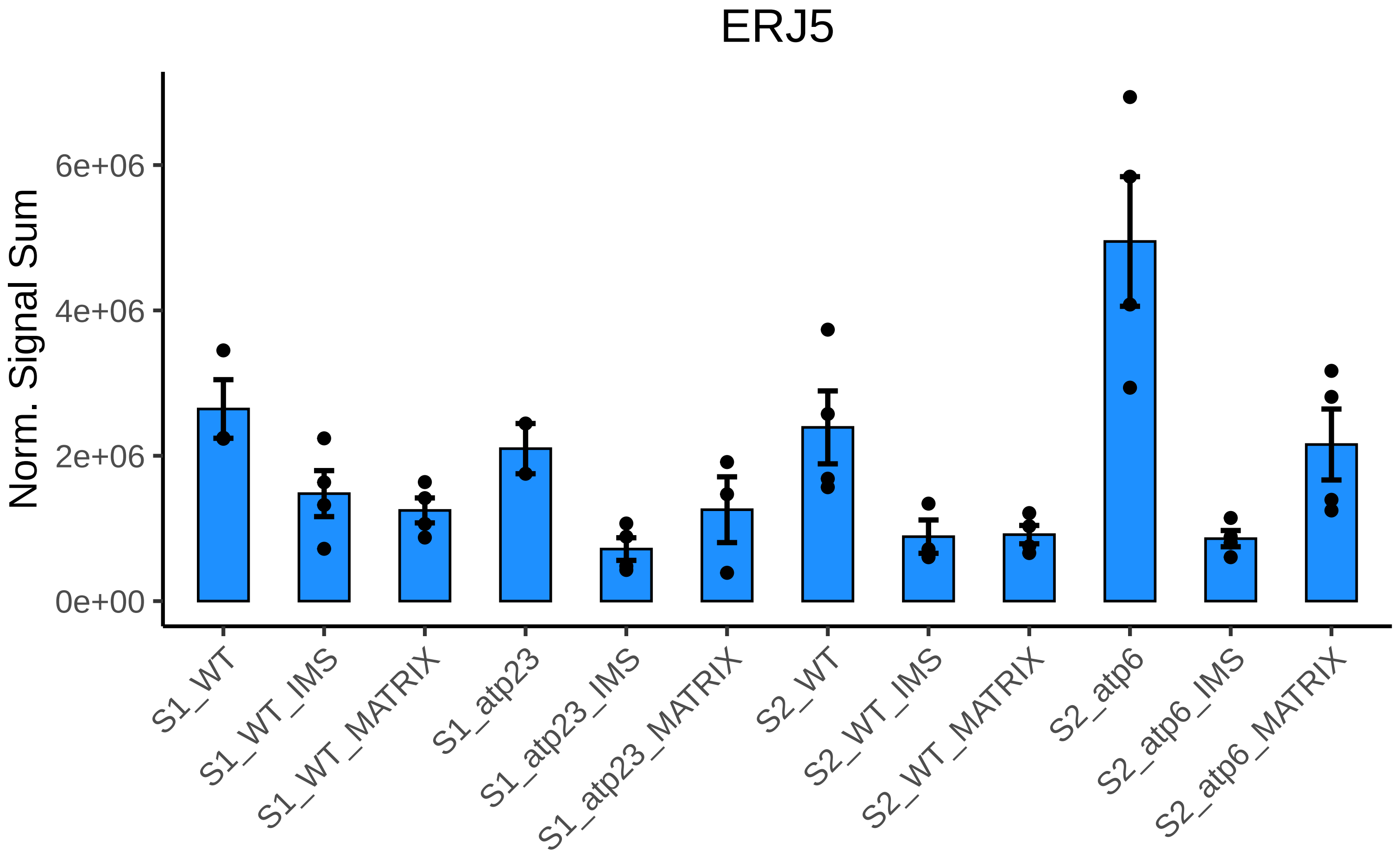
ERJ5
Norm. Signal Sum
0e+00
2e+06
4e+06
6e+06
S1_WT
S1_WT_IMS
S1_WT_MATRIX
S1_atp23
S1_atp23_IMS
S1_atp23_MATRIX
S2_WT
S2_WT_IMS
S2_WT_MATRIX
S2_atp6
S2_atp6_IMS
S2_atp6_MATRIX

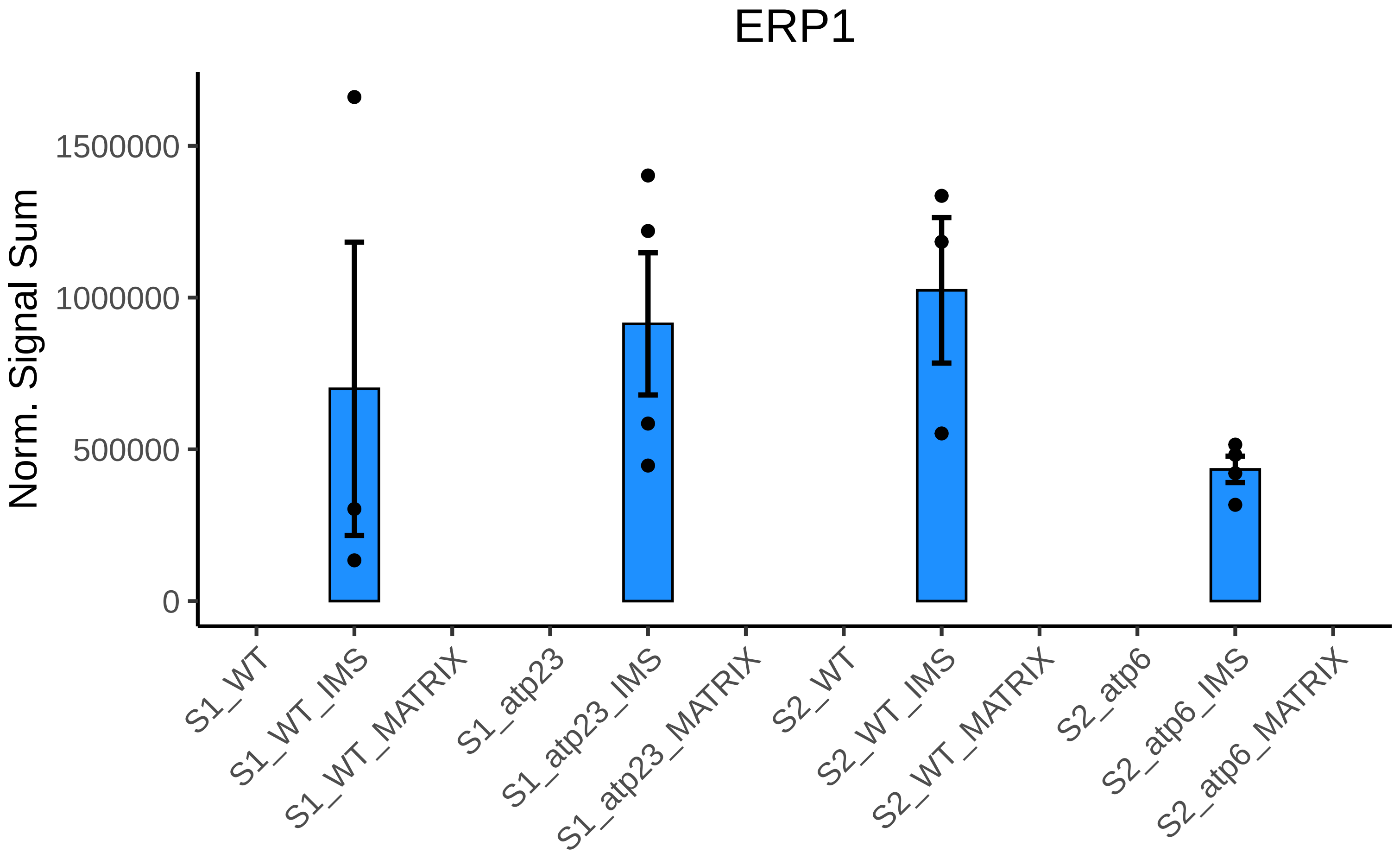
ERP1
Norm. Signal Sum
1500000
1000000
500000
0
S1_WT
S1_WT_IMS
S1_WT_MATRIX
S1_atp23
S1_atp23_IMS
S1_atp23_MATRIX
S2_WT
S2_WT_IMS
S2_WT_MATRIX
S2_atp6
S2_atp6_IMS
S2_atp6_MATRIX

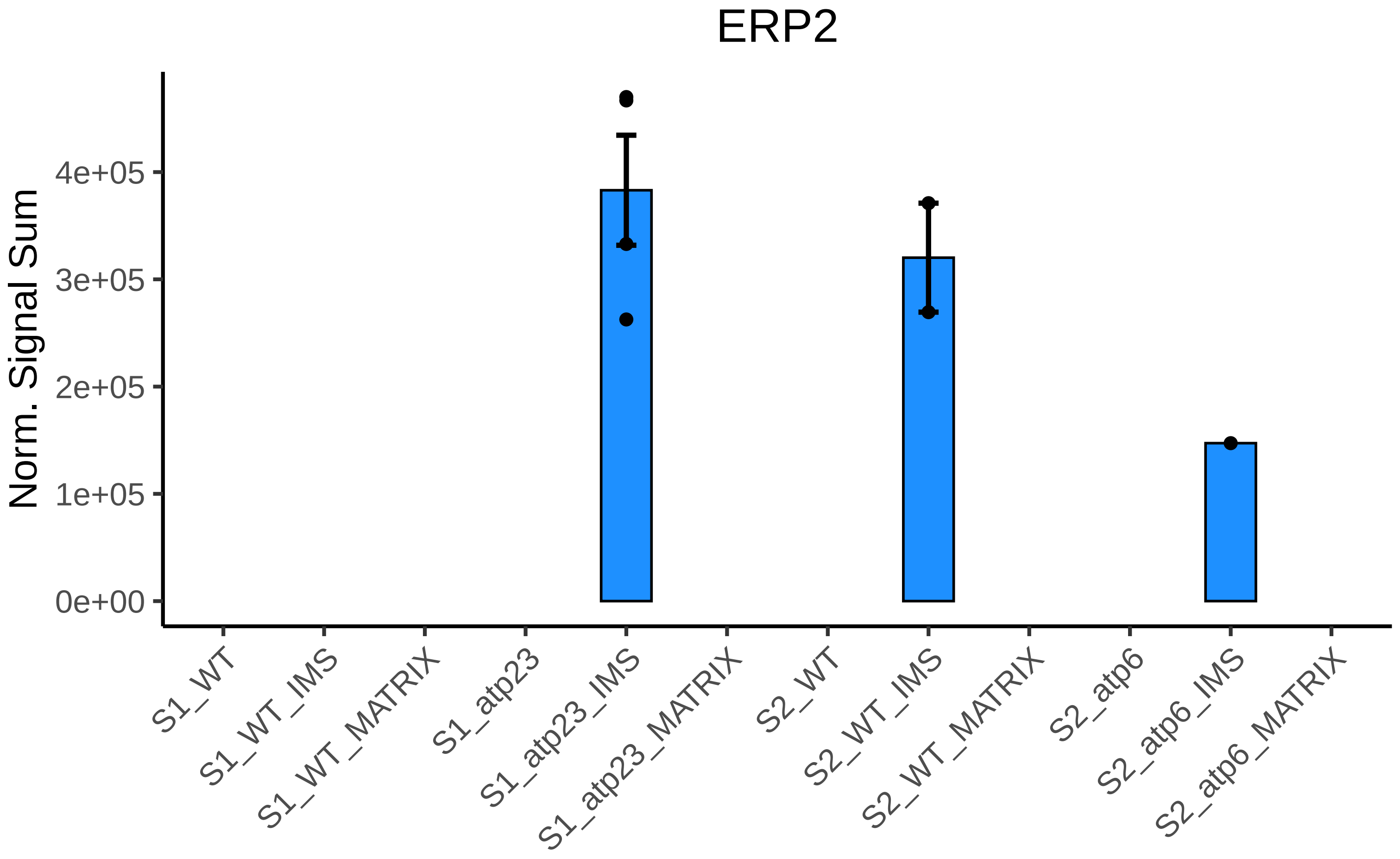
ERP2
Norm. Signal Sum
4e+05
3e+05
2e+05
1e+05
0e+00
S1_WT
S1_WT_IMS
S1_WT_MATRIX
S1_atp23
S1_atp23_IMS
S1_atp23_MATRIX
S2_WT
S2_WT_IMS
S2_WT_MATRIX
S2_atp6
S2_atp6_IMS
S2_atp6_MATRIX

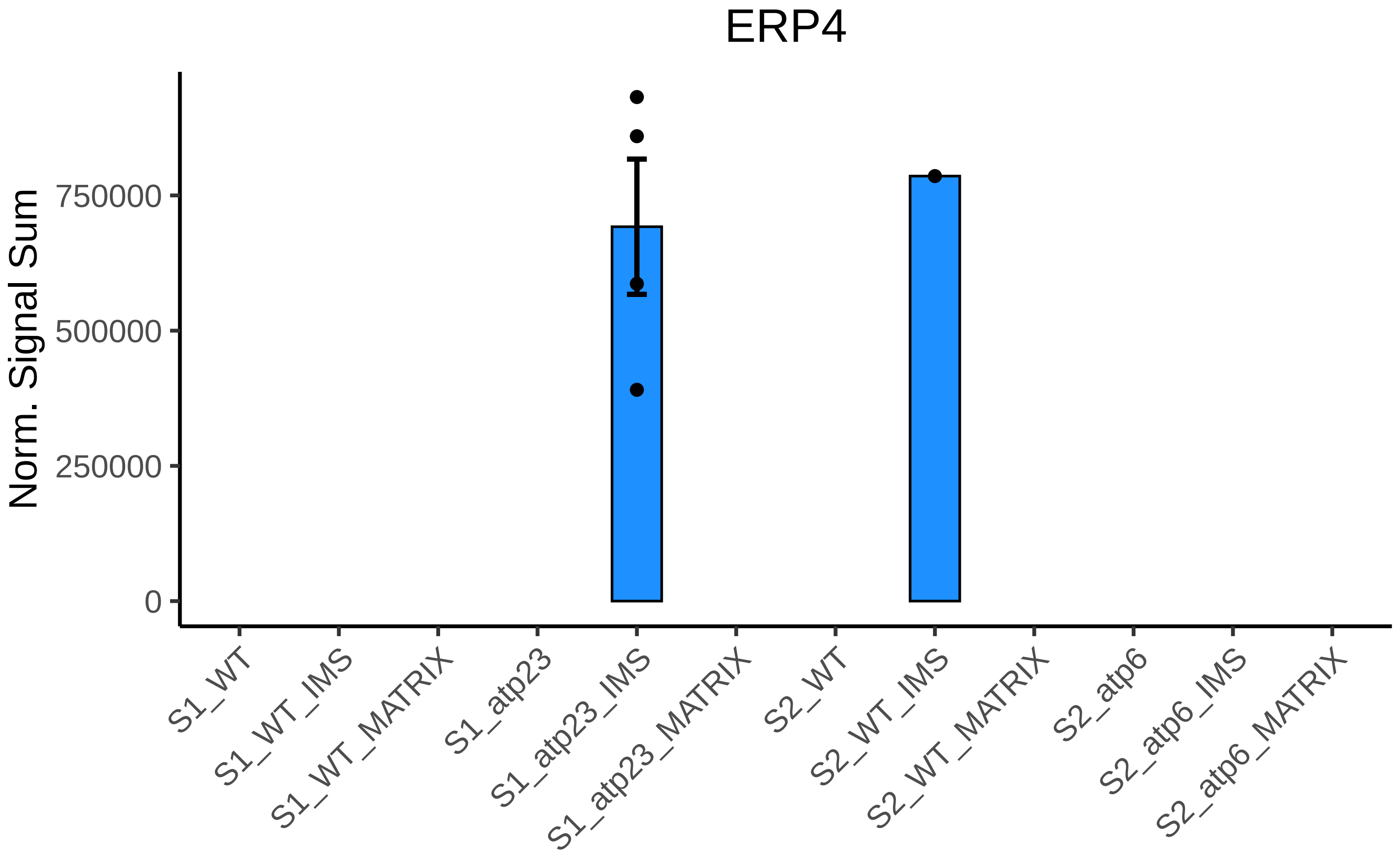

ERP4
Norm. Signal Sum
750000
500000
250000
0
S1_WT
S1_WT_IMS
S1_WT_MATRIX
S1_atp23
S1_atp23_IMS
S1_atp23_MATRIX
S2_WT
S2_WT_IMS
S2_WT_MATRIX
S2_atp6
S2_atp6_IMS
S2_atp6_MATRIX

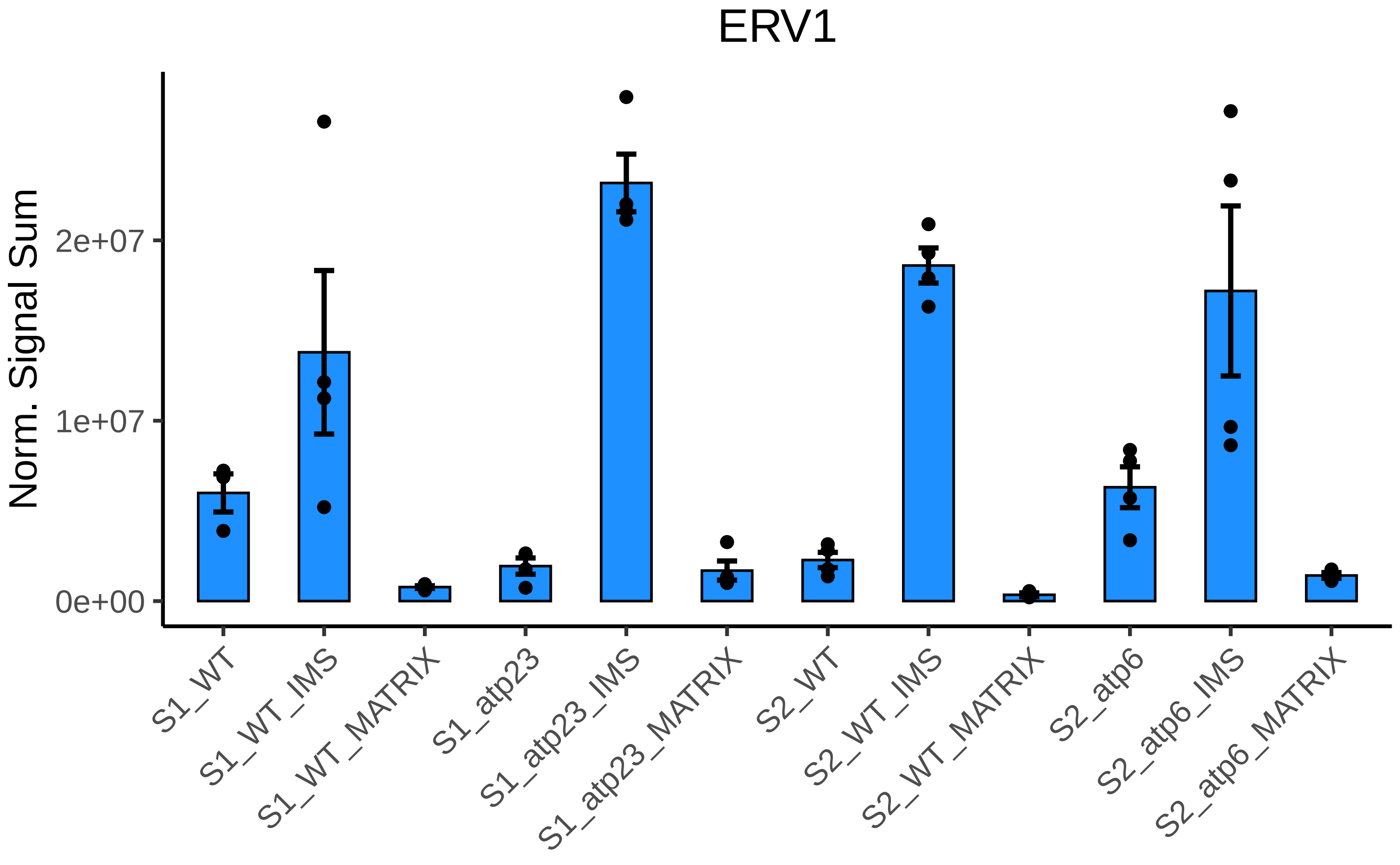

ERV1
Norm. Signal Sum
2e+07
1e+07
0e+00
S1_WT
S1_WT_IMS
S1_WT_MATRIX
S1_atp23
S1_atp23_IMS
S1_atp23_MATRIX
S2_WT
S2_WT_IMS
S2_WT_MATRIX
S2_atp6
S2_atp6_IMS
S2_atp6_MATRIX

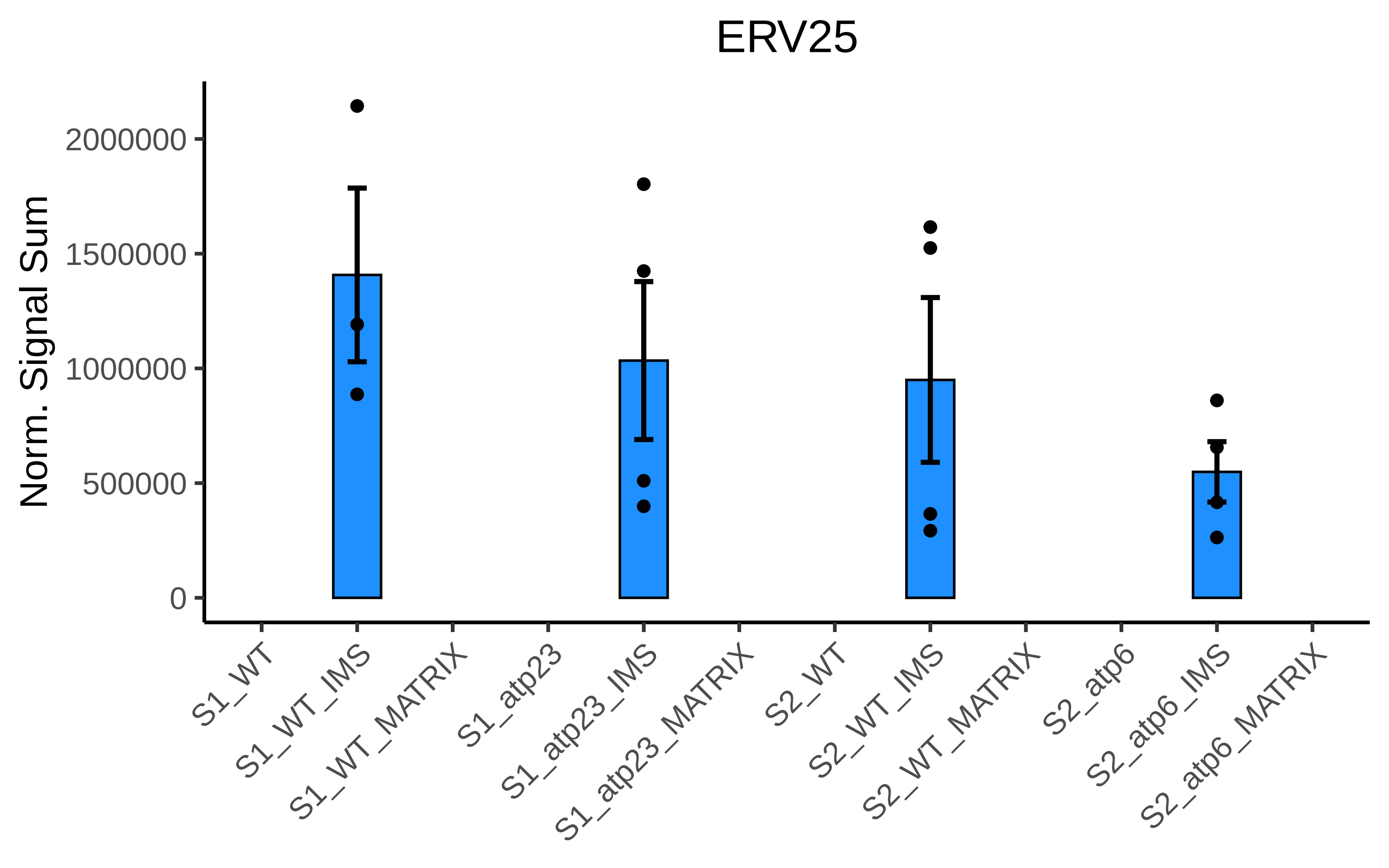
ERV25
Norm. Signal Sum
2000000
1500000
1000000
500000
0
S1_WT
S1_WT_IMS
S1_WT_MATRIX
S1_atp23
S1_atp23_IMS
S1_atp23_MATRIX
S2_WT
S2_WT_IMS
S2_WT_MATRIX
S2_atp6
S2_atp6_IMS
S2_atp6_MATRIX

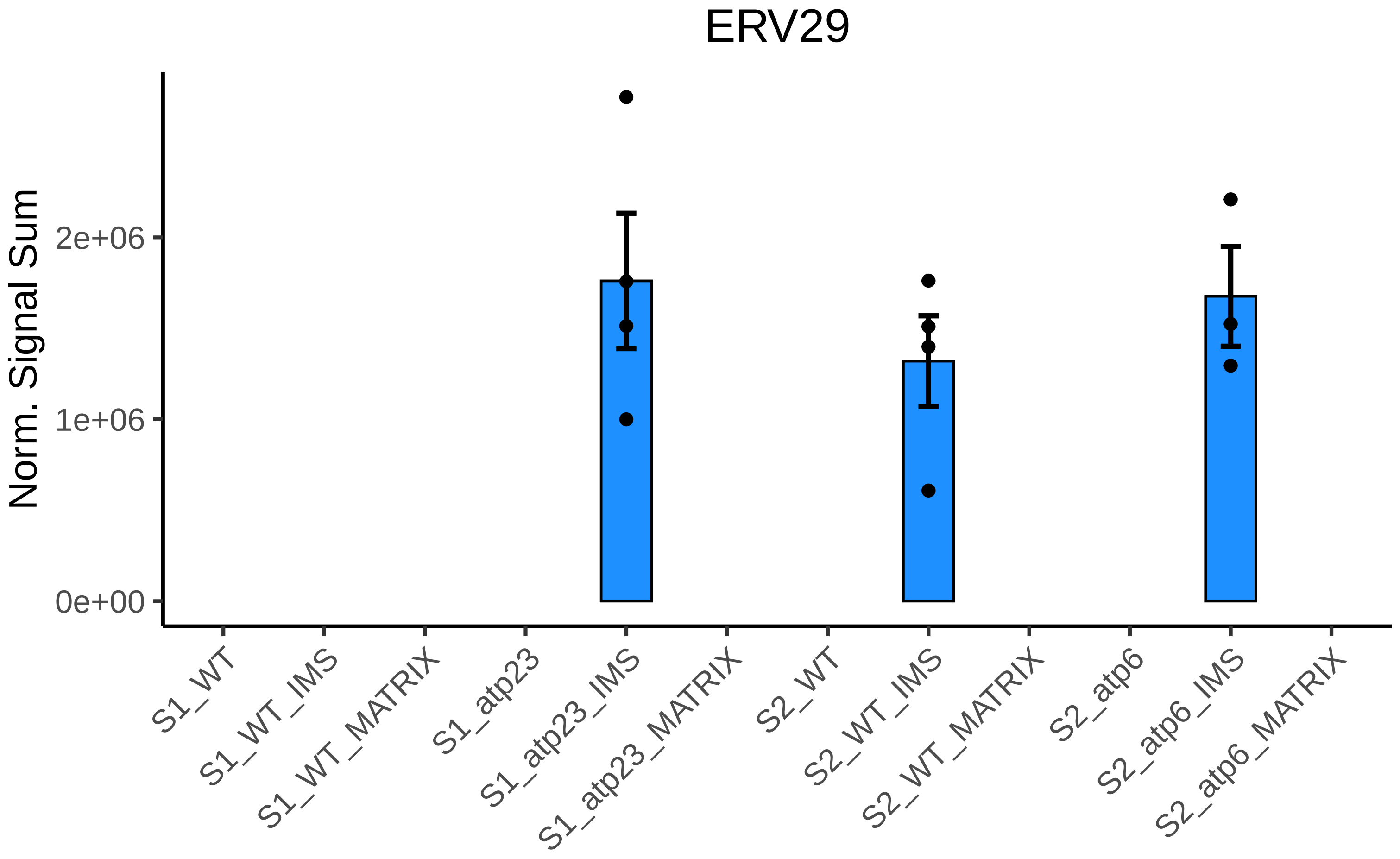

ERV29
Norm. Signal Sum
2e+06
1e+06
0e+00
S1_WT
S1_WT_IMS
S1_WT_MATRIX
S1_atp23
S1_atp23_IMS
S1_atp23_MATRIX
S2_WT
S2_WT_IMS
S2_WT_MATRIX
S2_atp6
S2_atp6_IMS
S2_atp6_MATRIX

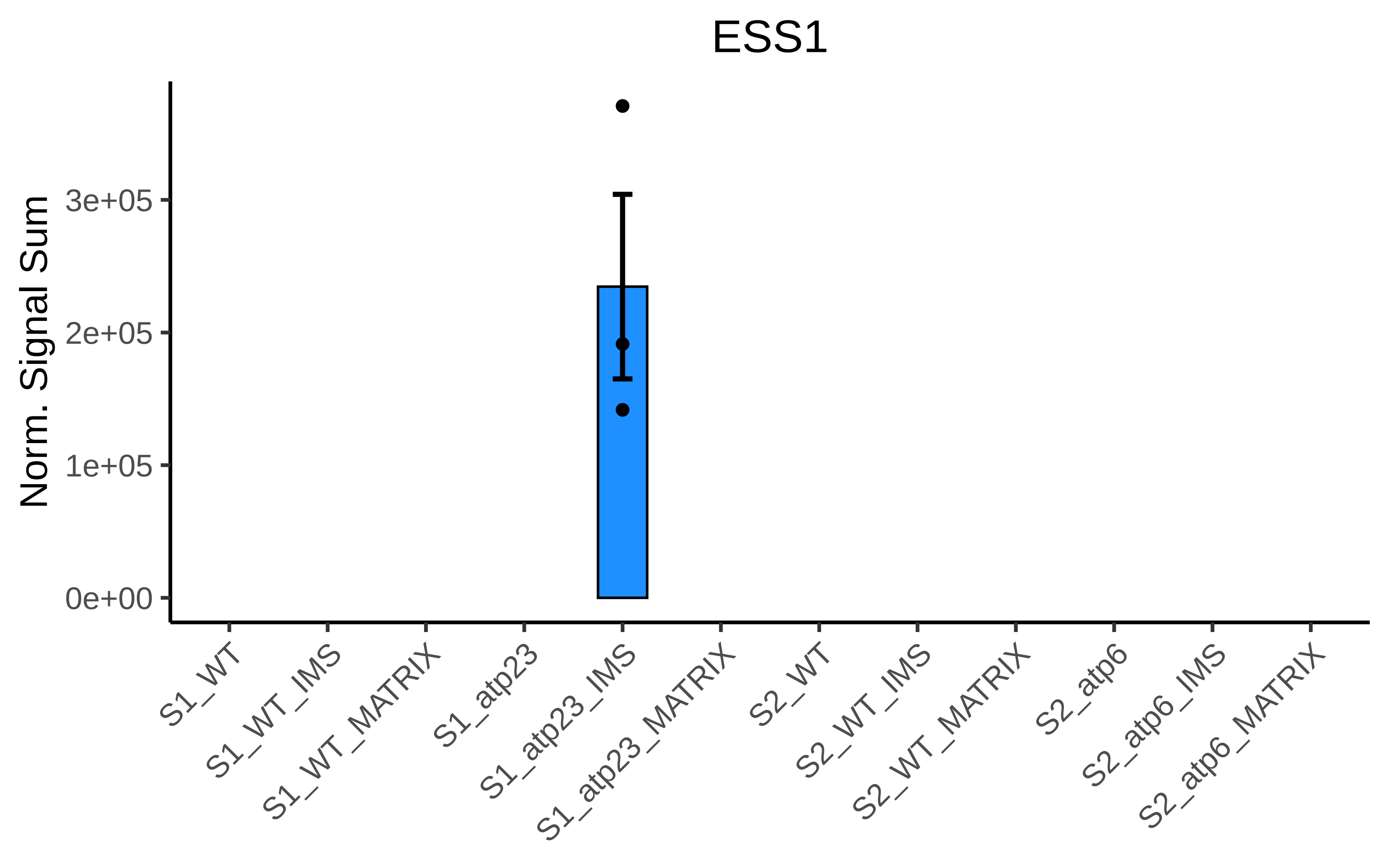
ESS1
Norm. Signal Sum
3e+05
2e+05
1e+05
0e+00
S1_WT
S1_WT_IMS
S1_WT_MATRIX
S1_atp23
S1_atp23_IMS
S1_atp23_MATRIX
S2_WT
S2_WT_IMS
S2_WT_MATRIX
S2_atp6
S2_atp6_IMS
S2_atp6_MATRIX

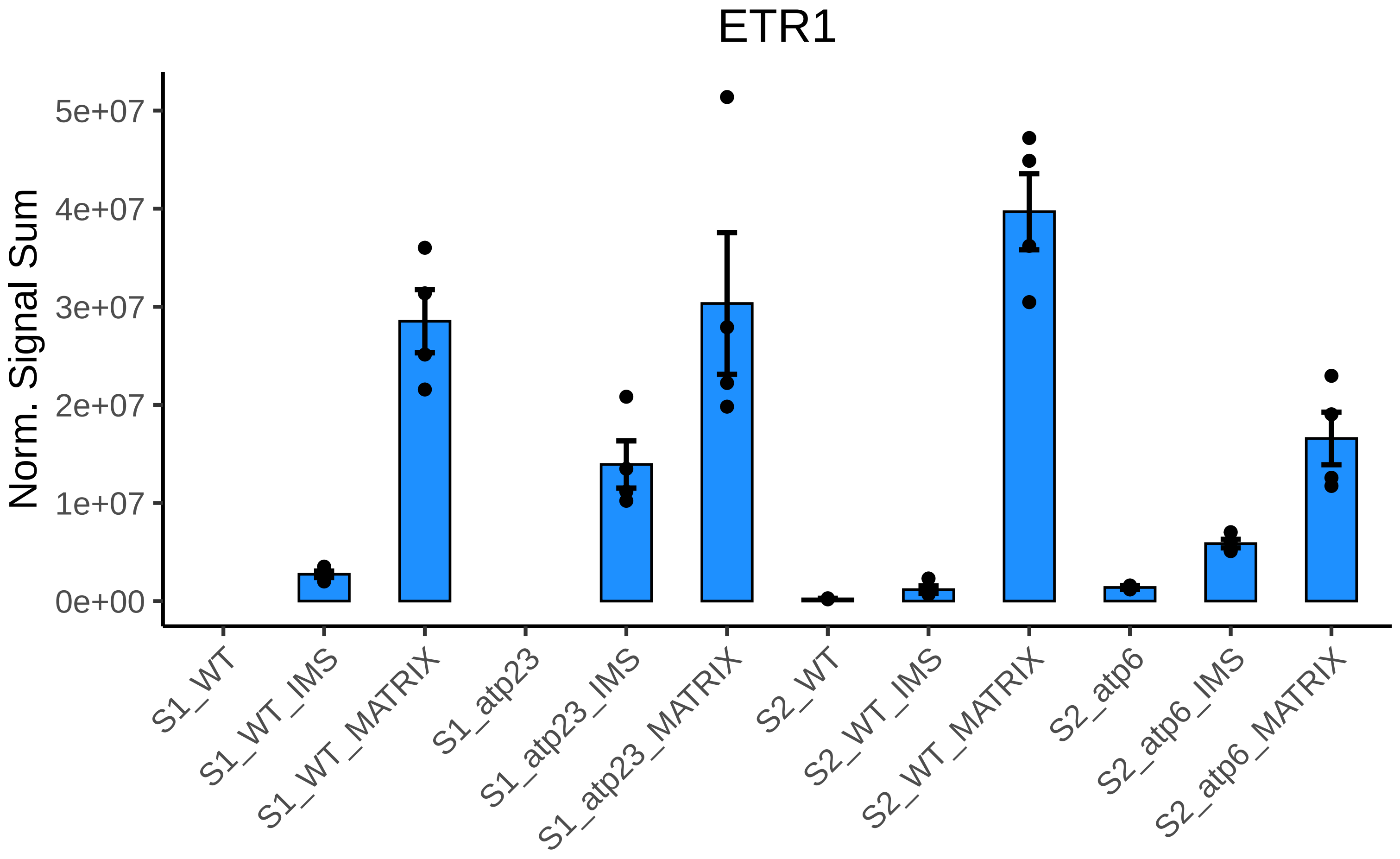
ETR1
Norm. Signal Sum
5e+07
4e+07
3e+07
2e+07
1e+07
0e+00
S1_WT
S1_WT_IMS
S1_WT_MATRIX
S1_atp23
S1_atp23_IMS
S1_atp23_MATRIX
S2_WT
S2_WT_IMS
S2_WT_MATRIX
S2_atp6
S2_atp6_IMS
S2_atp6_MATRIX

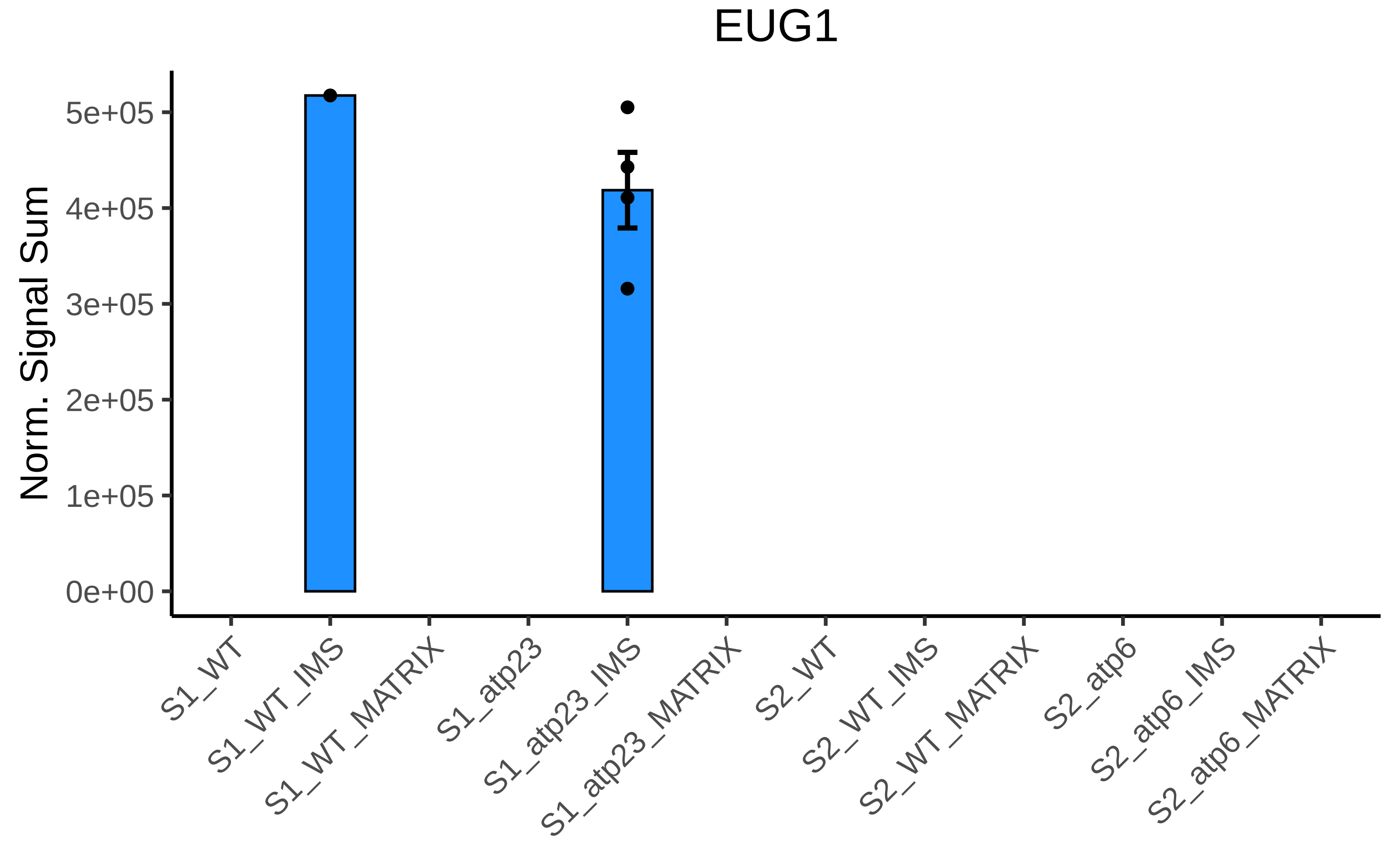

EUG1
Norm. Signal Sum
5e+05
4e+05
3e+05
2e+05
1e+05
0e+00
S1_WT
S1_WT_IMS
S1_WT_MATRIX
S1_atp23
S1_atp23_IMS
S1_atp23_MATRIX
S2_WT
S2_WT_IMS
S2_WT_MATRIX
S2_atp6
S2_atp6_IMS
S2_atp6_MATRIX

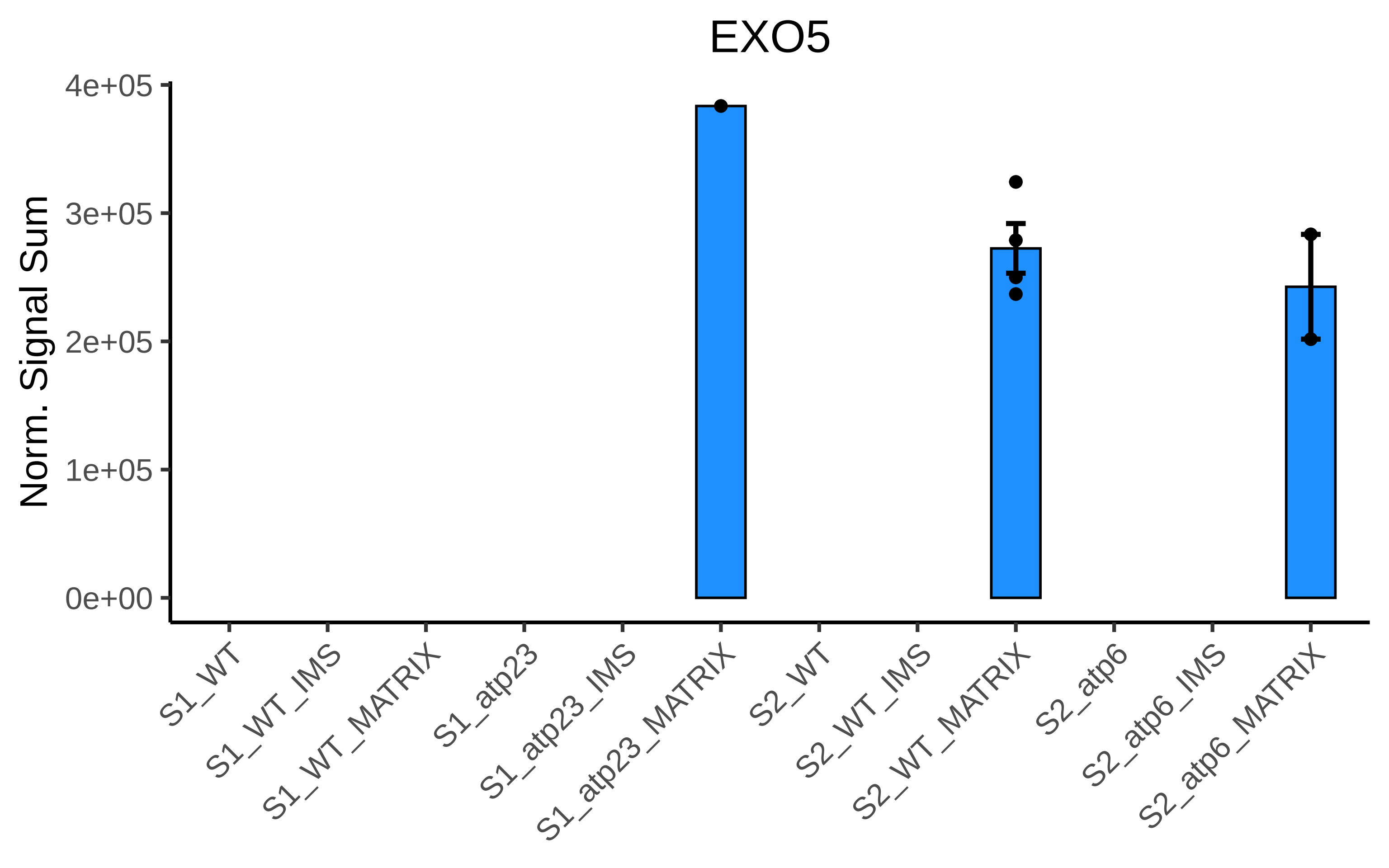
EXO5
Norm. Signal Sum
4e+05
3e+05
2e+05
1e+05
0e+00
S1_WT
S1_WT_IMS
S1_WT_MATRIX
S1_atp23
S1_atp23_IMS
S1_atp23_MATRIX
S2_WT
S2_WT_IMS
S2_WT_MATRIX
S2_atp6
S2_atp6_IMS
S2_atp6_MATRIX

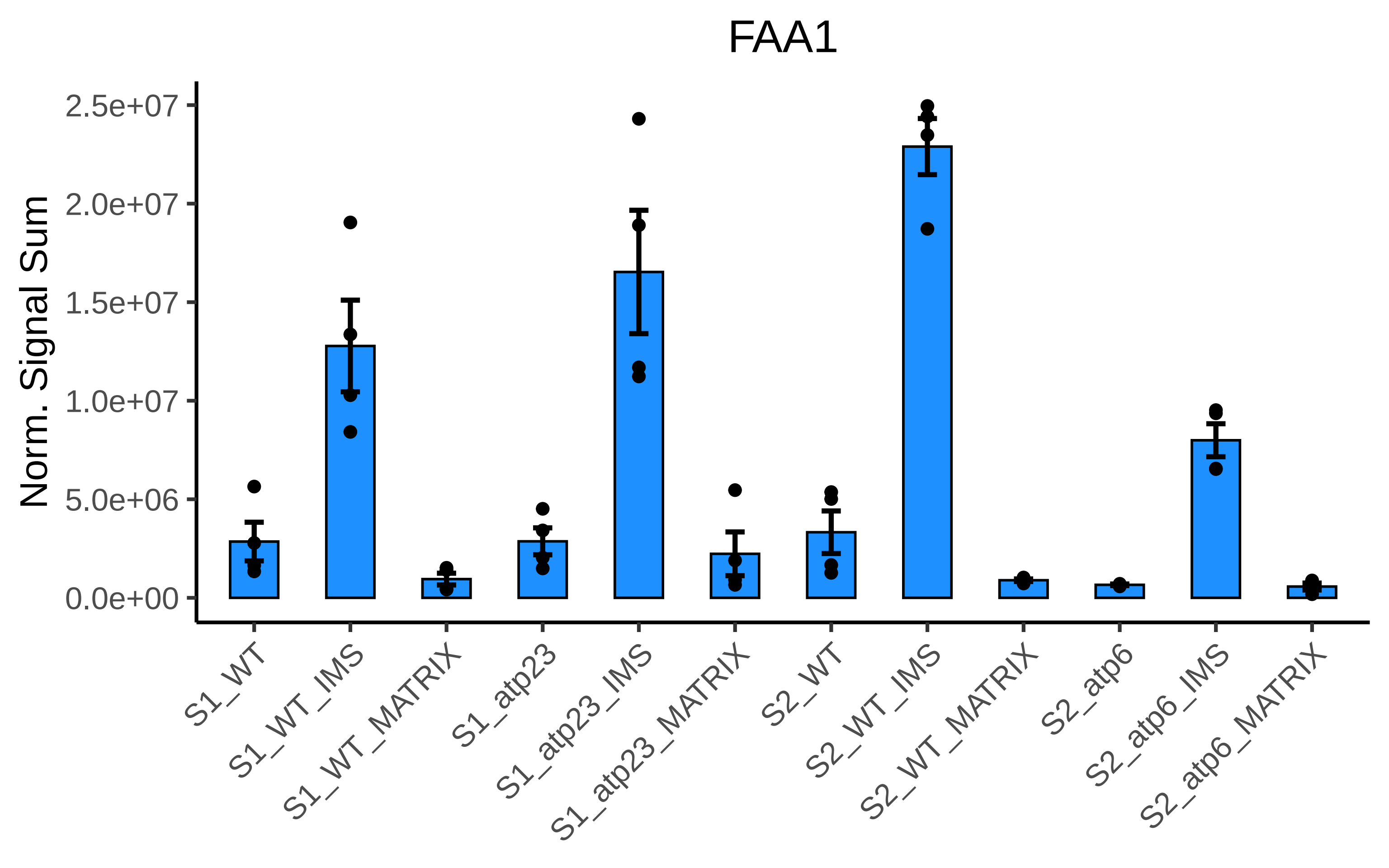
FAA1
Norm. Signal Sum
2.5e+07
2.0e+07
1.5e+07
1.0e+07
5.0e+06
0.0e+00
S1_WT
S1_WT_IMS
S1_WT_MATRIX
S1_atp23
S1_atp23_IMS
S1_atp23_MATRIX
S2_WT
S2_WT_IMS
S2_WT_MATRIX
S2_atp6
S2_atp6_IMS
S2_atp6_MATRIX

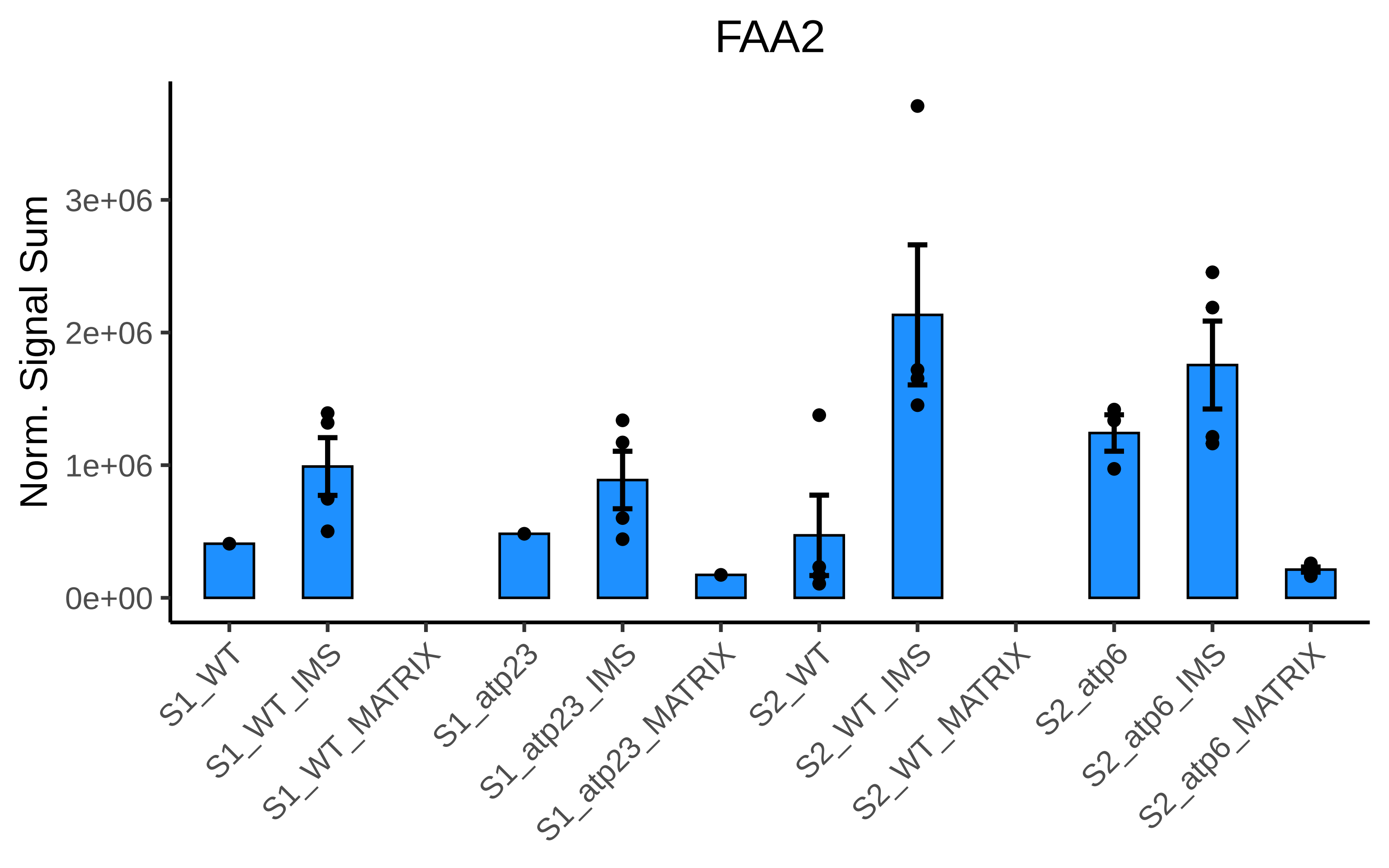

FAA2
Norm. Signal Sum
3e+06
2e+06
1e+06
0e+00
S1_WT
S1_WT_IMS
S1_WT_MATRIX
S1_atp23
S1_atp23_IMS
S1_atp23_MATRIX
S2_WT
S2_WT_IMS
S2_WT_MATRIX
S2_atp6
S2_atp6_IMS
S2_atp6_MATRIX

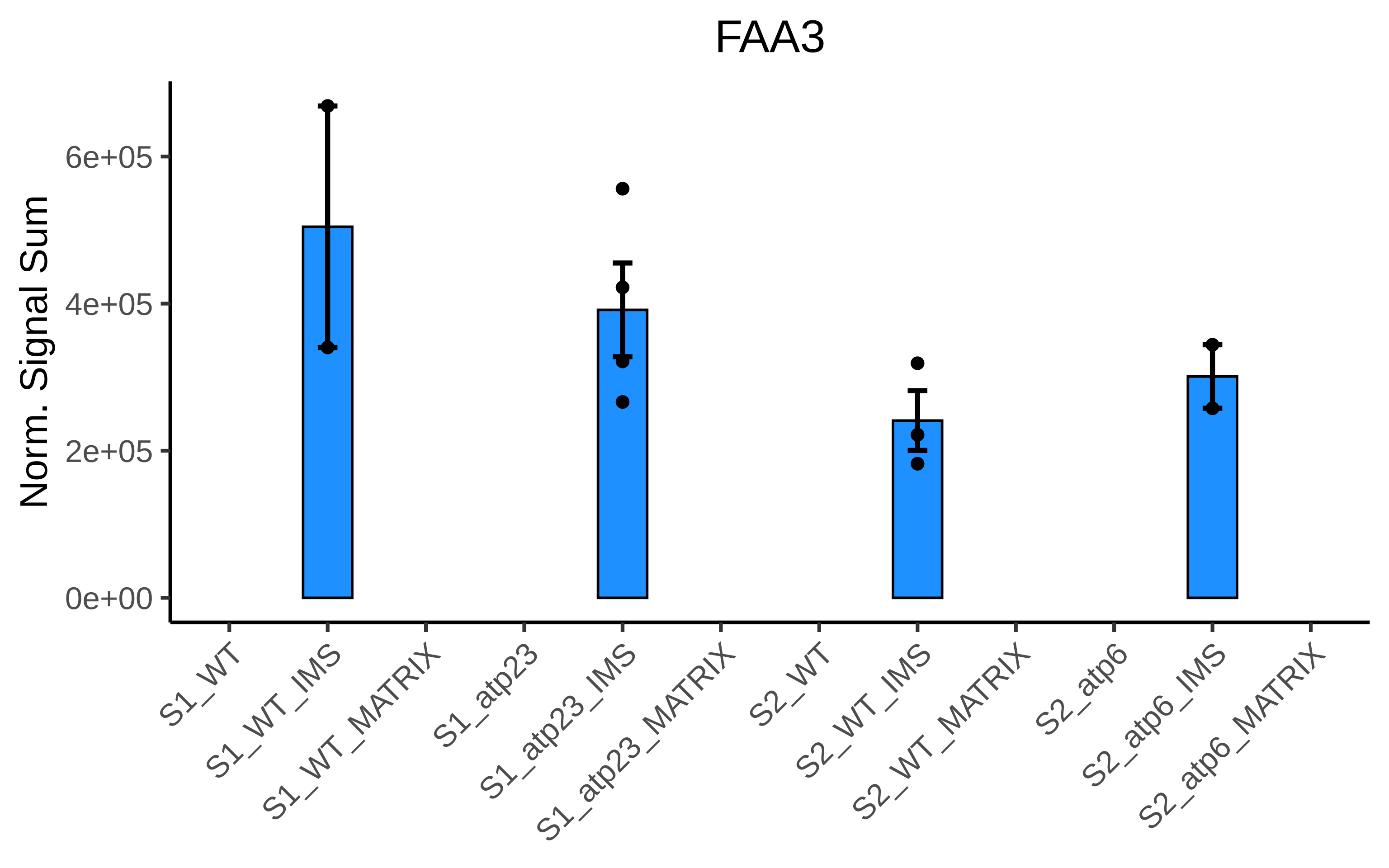
FAA3
Norm. Signal Sum
6e+05
4e+05
2e+05
0e+00
S1_WT
S1_WT_IMS
S1_WT_MATRIX
S1_atp23
S1_atp23_IMS
S1_atp23_MATRIX
S2_WT
S2_WT_IMS
S2_WT_MATRIX
S2_atp6
S2_atp6_IMS
S2_atp6_MATRIX

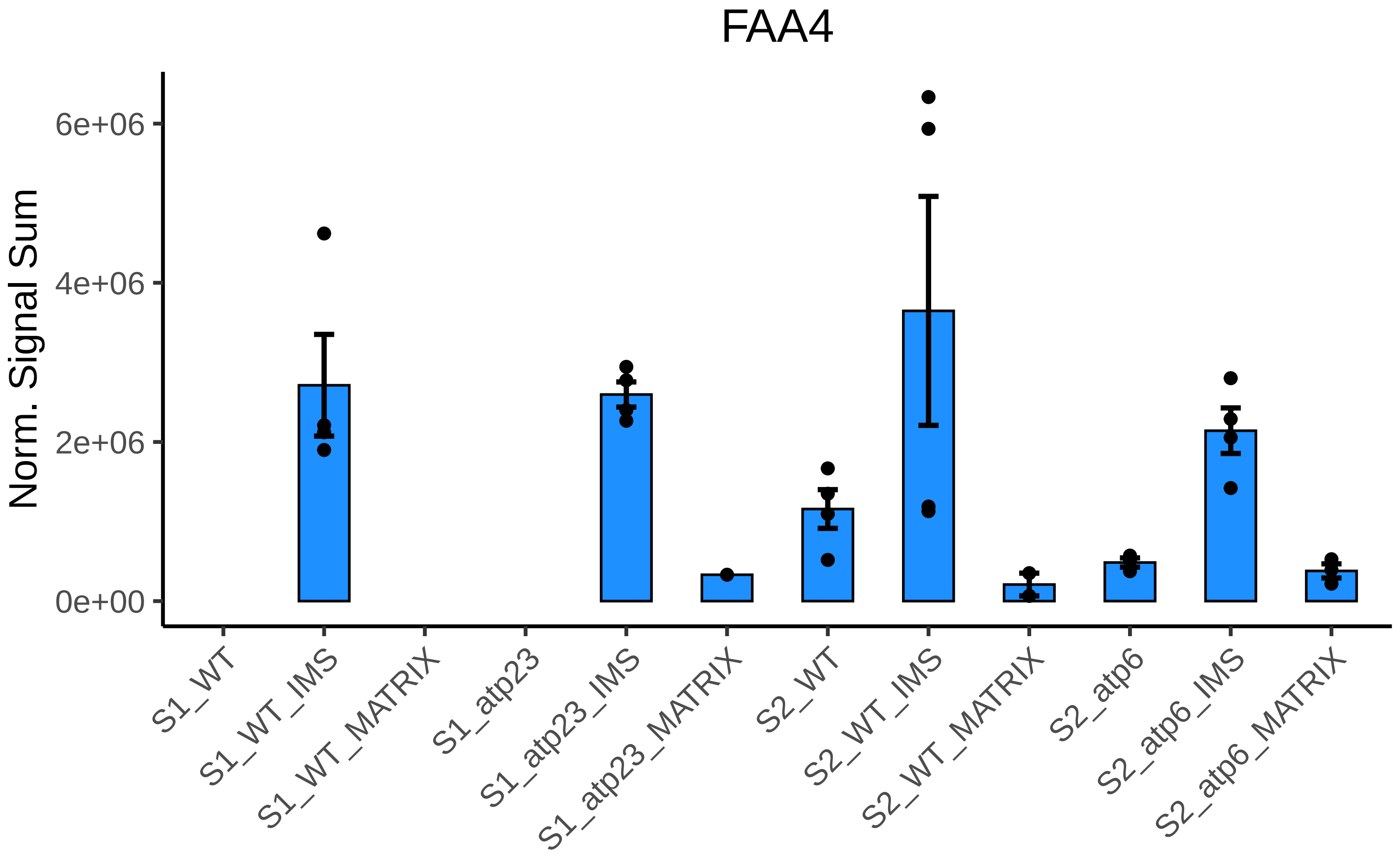

FAA4
Norm. Signal Sum
6e+06
4e+06
2e+06
0e+00
S1_WT
S1_WT_IMS
S1_WT_MATRIX
S1_atp23
S1_atp23_IMS
S1_atp23_MATRIX
S2_WT
S2_WT_IMS
S2_WT_MATRIX
S2_atp6
S2_atp6_IMS
S2_atp6_MATRIX

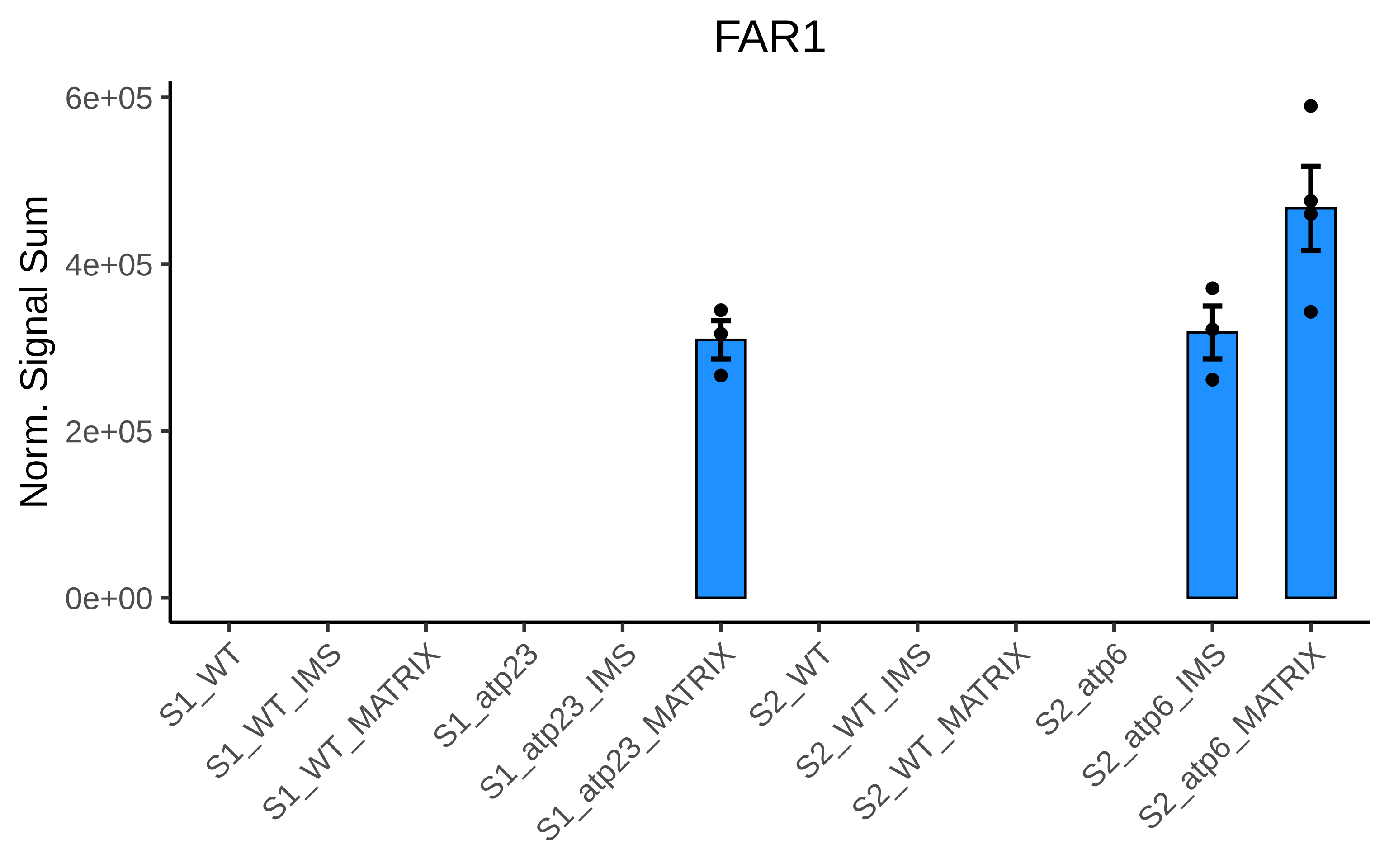

FAR1
Norm. Signal Sum
6e+05
4e+05
2e+05
0e+00
S1_WT
S1_WT_IMS
S1_WT_MATRIX
S1_atp23
S1_atp23_IMS
S1_atp23_MATRIX
S2_WT
S2_WT_IMS
S2_WT_MATRIX
S2_atp6
S2_atp6_IMS
S2_atp6_MATRIX

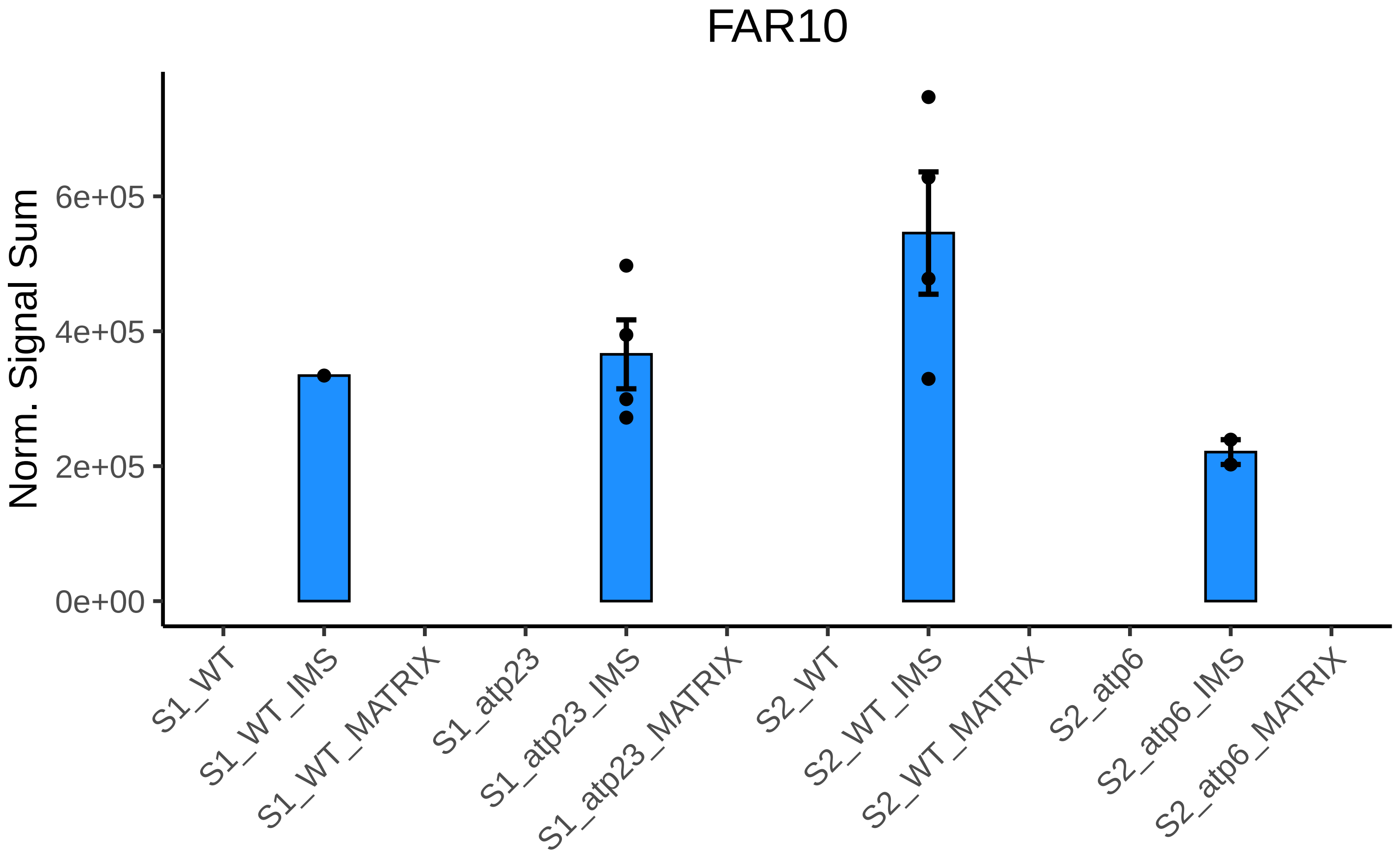
FAR10
Norm. Signal Sum
6e+05
4e+05
2e+05
0e+00
S1_WT
S1_WT_IMS
S1_WT_MATRIX
S1_atp23
S1_atp23_IMS
S1_atp23_MATRIX
S2_WT
S2_WT_IMS
S2_WT_MATRIX
S2_atp6
S2_atp6_IMS
S2_atp6_MATRIX

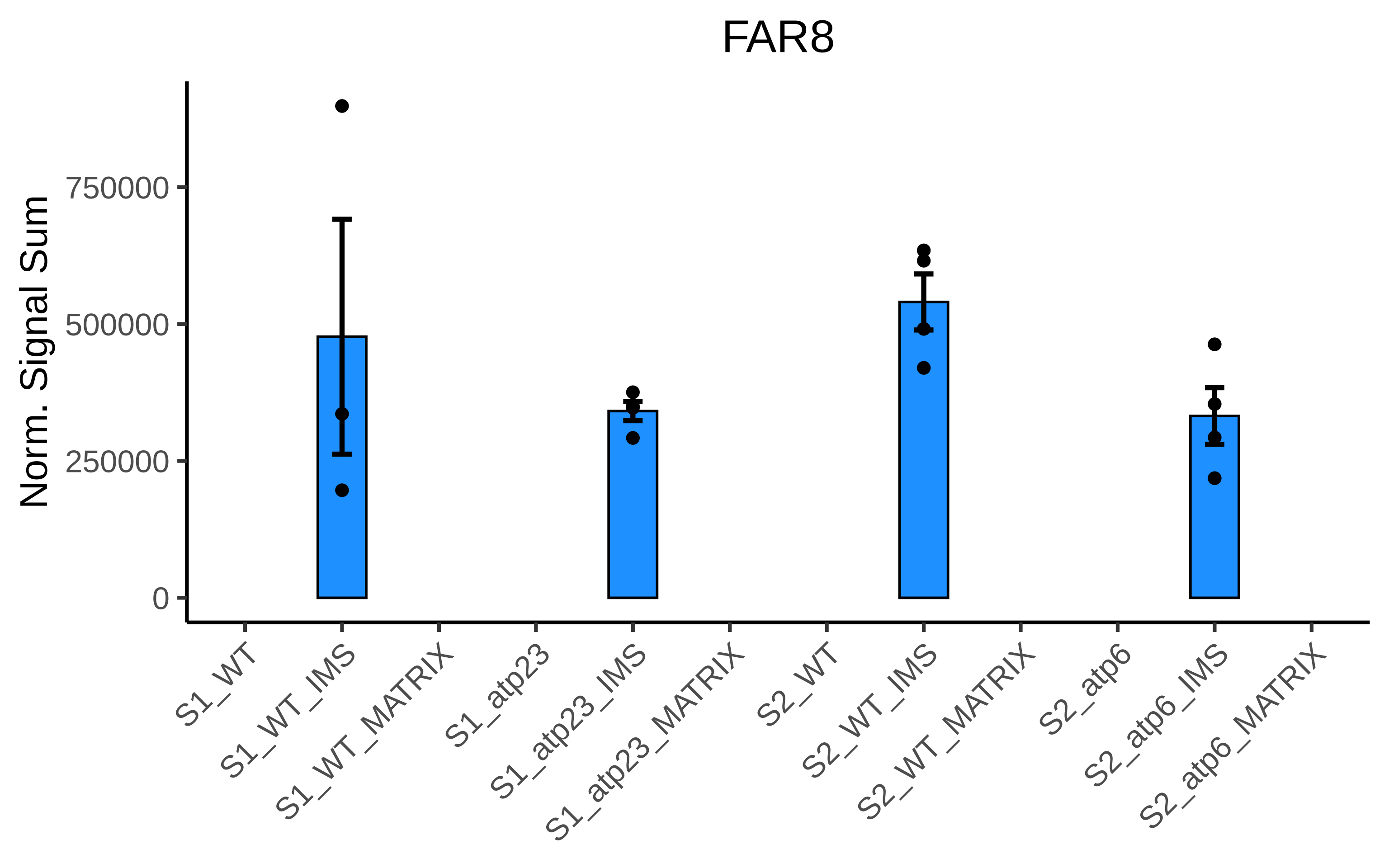

FAR8
Norm. Signal Sum
750000
500000
250000
0
S1_WT
S1_WT_IMS
S1_WT_MATRIX
S1_atp23
S1_atp23_IMS
S1_atp23_MATRIX
S2_WT
S2_WT_IMS
S2_WT_MATRIX
S2_atp6
S2_atp6_IMS
S2_atp6_MATRIX

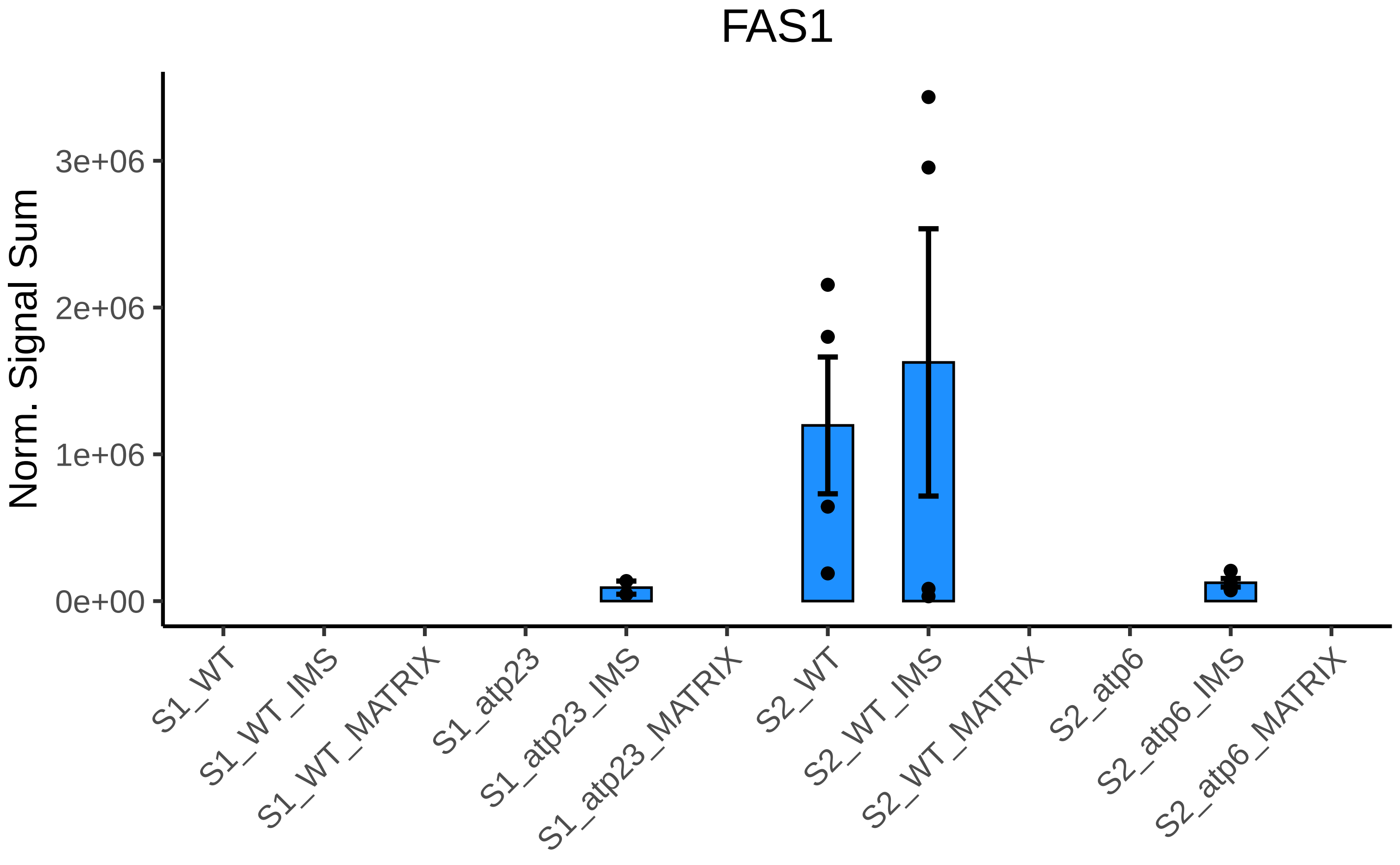
FAS1
Norm. Signal Sum
3e+06
2e+06
1e+06
0e+00
S1_WT
S1_WT_IMS
S1_WT_MATRIX
S1_atp23
S1_atp23_IMS
S1_atp23_MATRIX
S2_WT
S2_WT_IMS
S2_WT_MATRIX
S2_atp6
S2_atp6_IMS
S2_atp6_MATRIX

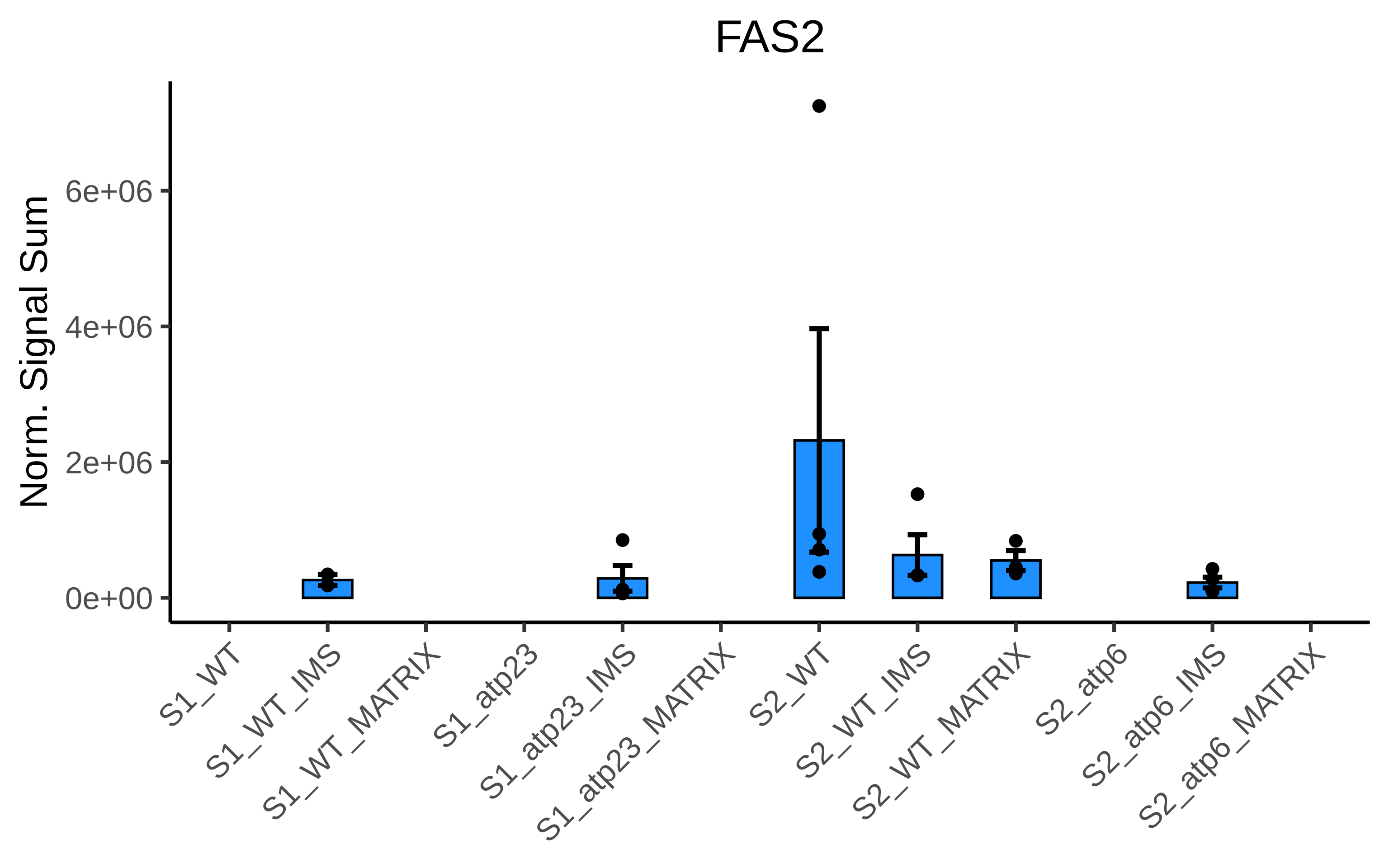

FAS2
Norm. Signal Sum
6e+06
4e+06
2e+06
0e+00
S1_WT
S1_WT_IMS
S1_WT_MATRIX
S1_atp23
S1_atp23_IMS
S1_atp23_MATRIX
S2_WT
S2_WT_IMS
S2_WT_MATRIX
S2_atp6
S2_atp6_IMS
S2_atp6_MATRIX

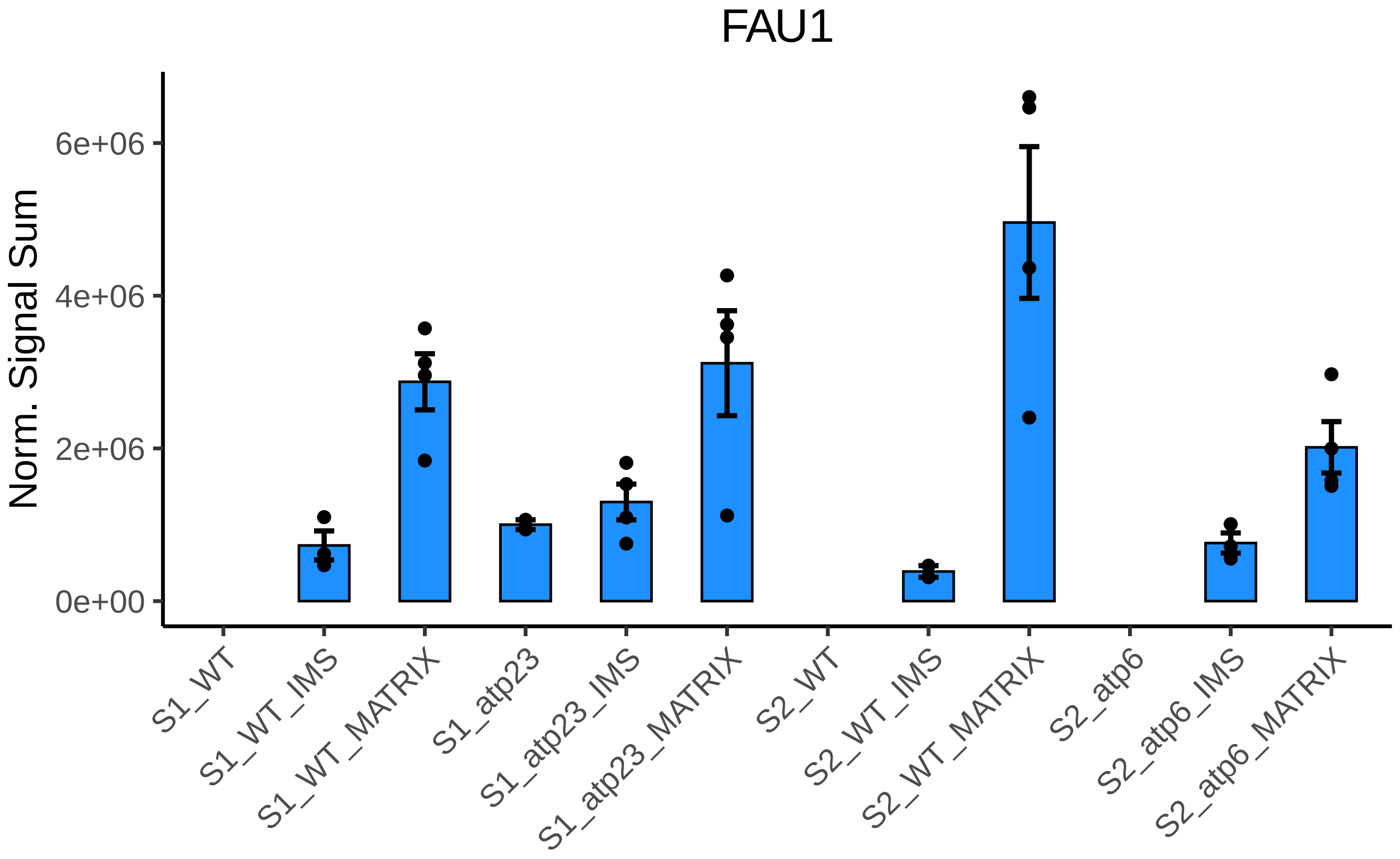

FAU1
Norm. Signal Sum
6e+06
4e+06
2e+06
0e+00
S1_WT
S1_WT_IMS
S1_WT_MATRIX
S1_atp23
S1_atp23_IMS
S1_atp23_MATRIX
S2_WT
S2_WT_IMS
S2_WT_MATRIX
S2_atp6
S2_atp6_IMS
S2_atp6_MATRIX

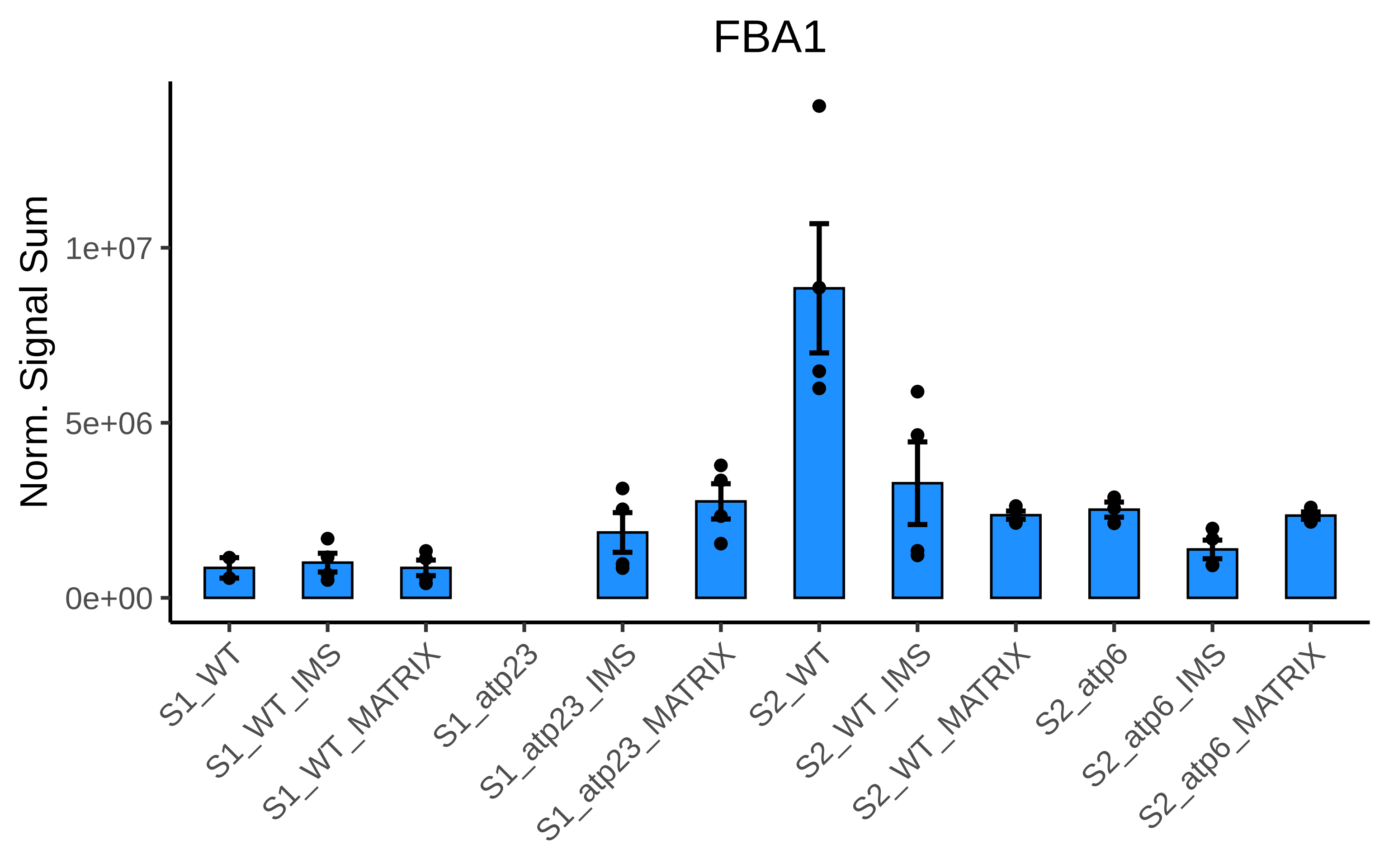
FBA1
Norm. Signal Sum
1e+07
5e+06
0e+00
S1_WT
S1_WT_IMS
S1_WT_MATRIX
S1_atp23
S1_atp23_IMS
S1_atp23_MATRIX
S2_WT
S2_WT_IMS
S2_WT_MATRIX
S2_atp6
S2_atp6_IMS
S2_atp6_MATRIX

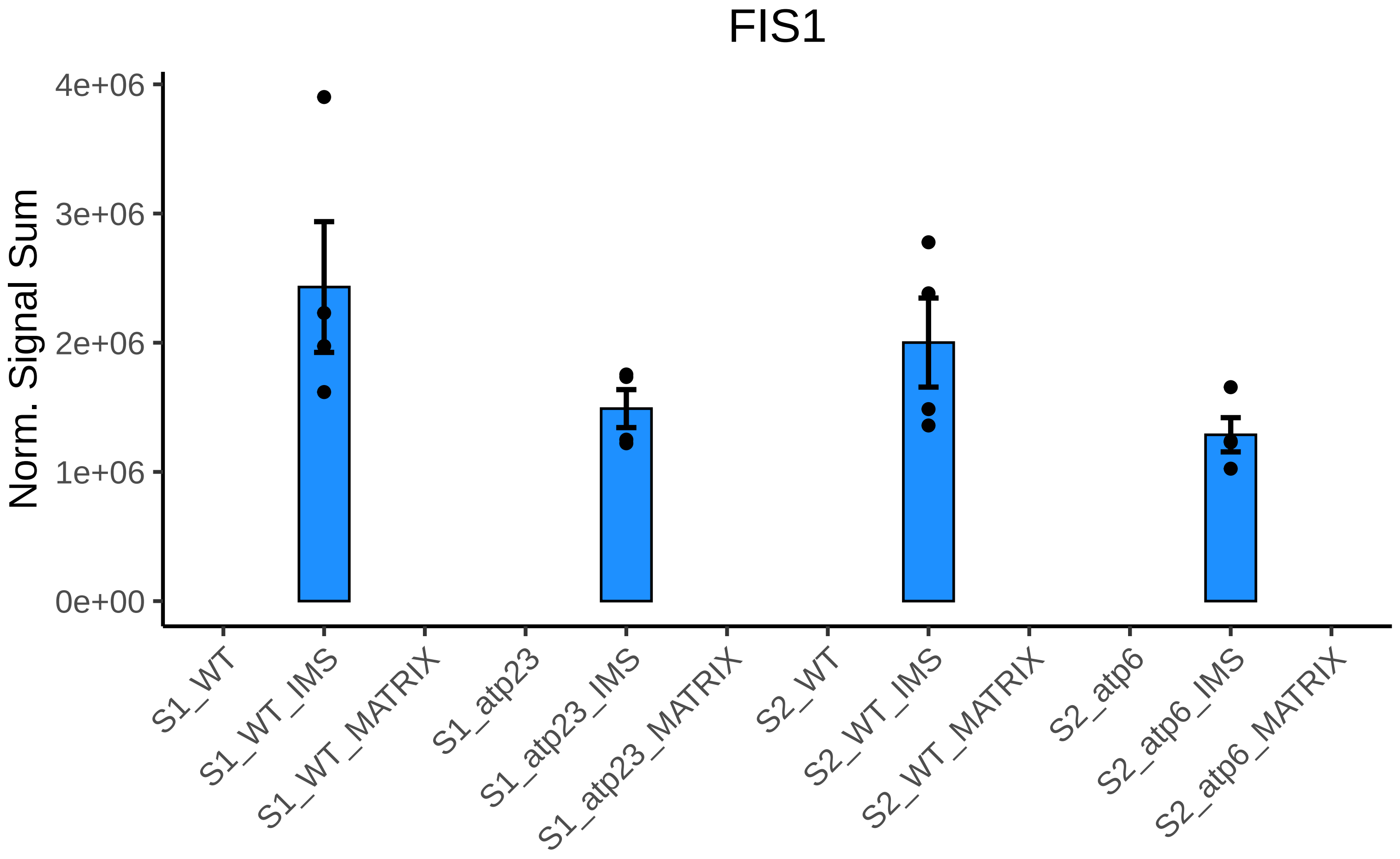
FIS1
Norm. Signal Sum
4e+06
3e+06
2e+06
1e+06
0e+00
S1_WT
S1_WT_IMS
S1_WT_MATRIX
S1_atp23
S1_atp23_IMS
S1_atp23_MATRIX
S2_WT
S2_WT_IMS
S2_WT_MATRIX
S2_atp6
S2_atp6_IMS
S2_atp6_MATRIX

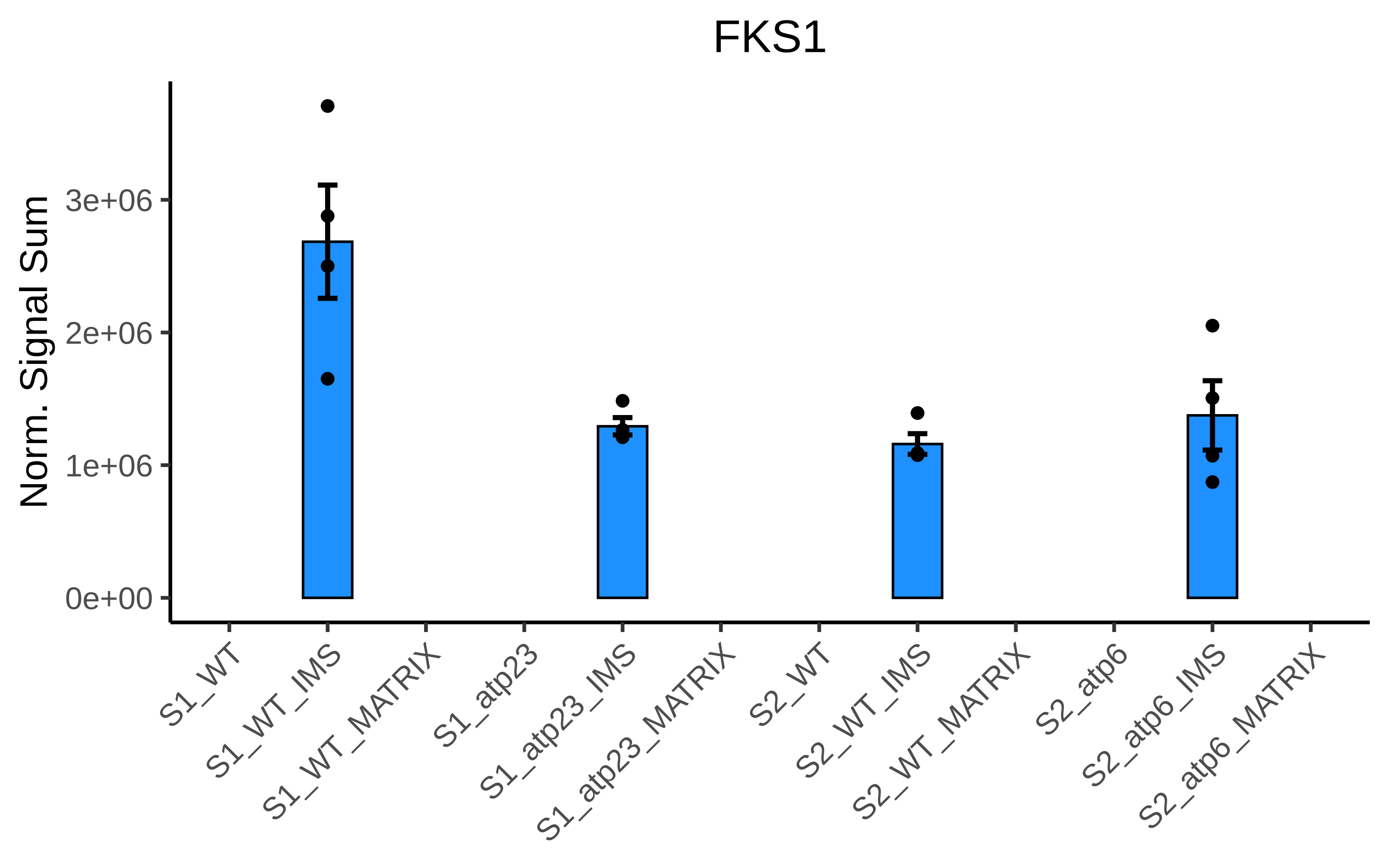

FKS1
Norm. Signal Sum
3e+06
2e+06
1e+06
0e+00
S1_WT
S1_WT_IMS
S1_WT_MATRIX
S1_atp23
S1_atp23_IMS
S1_atp23_MATRIX
S2_WT
S2_WT_IMS
S2_WT_MATRIX
S2_atp6
S2_atp6_IMS
S2_atp6_MATRIX

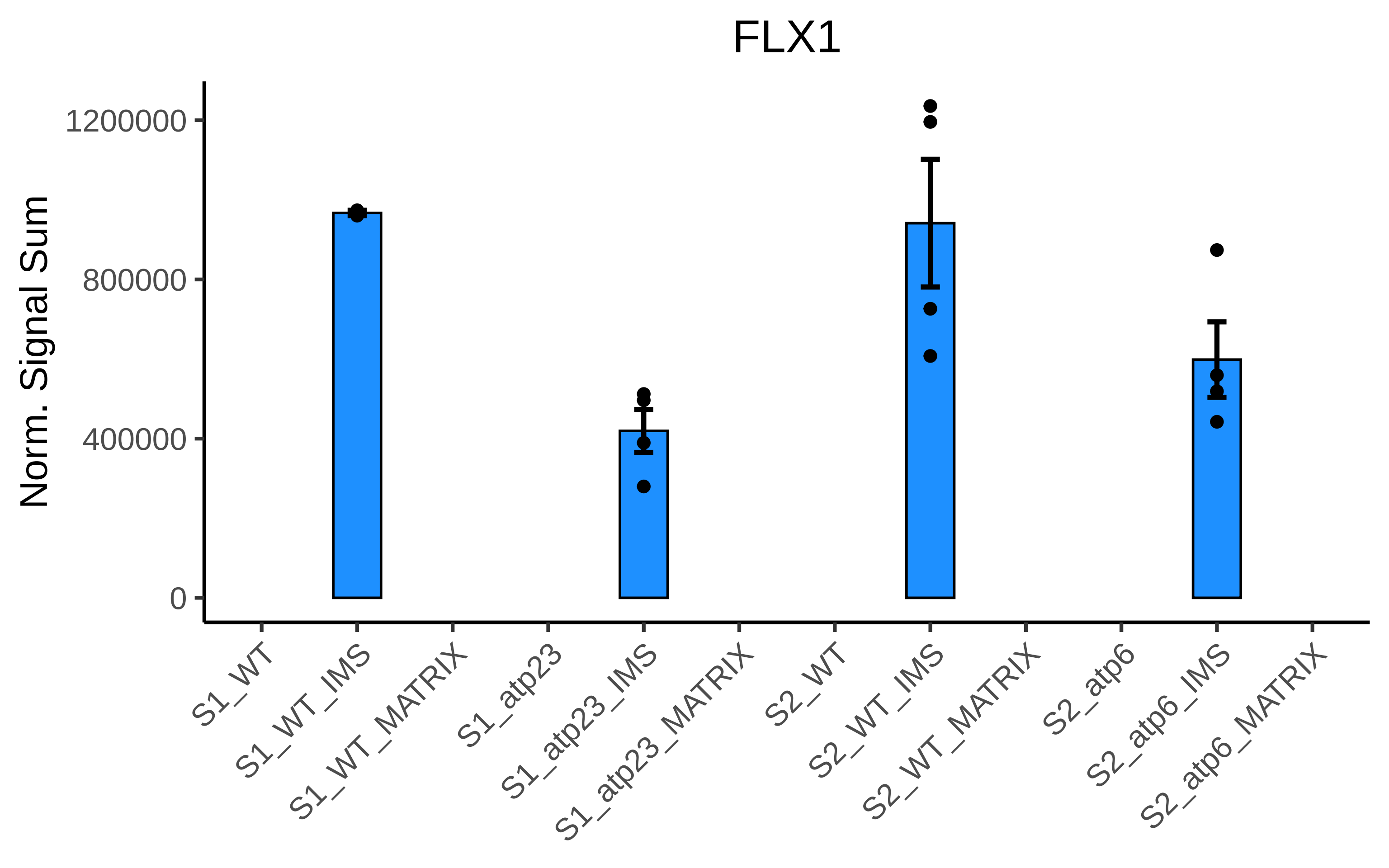

FLX1
Norm. Signal Sum
1200000
800000
400000
0
S1_WT
S1_WT_IMS
S1_WT_MATRIX
S1_atp23
S1_atp23_IMS
S1_atp23_MATRIX
S2_WT
S2_WT_IMS
S2_WT_MATRIX
S2_atp6
S2_atp6_IMS
S2_atp6_MATRIX

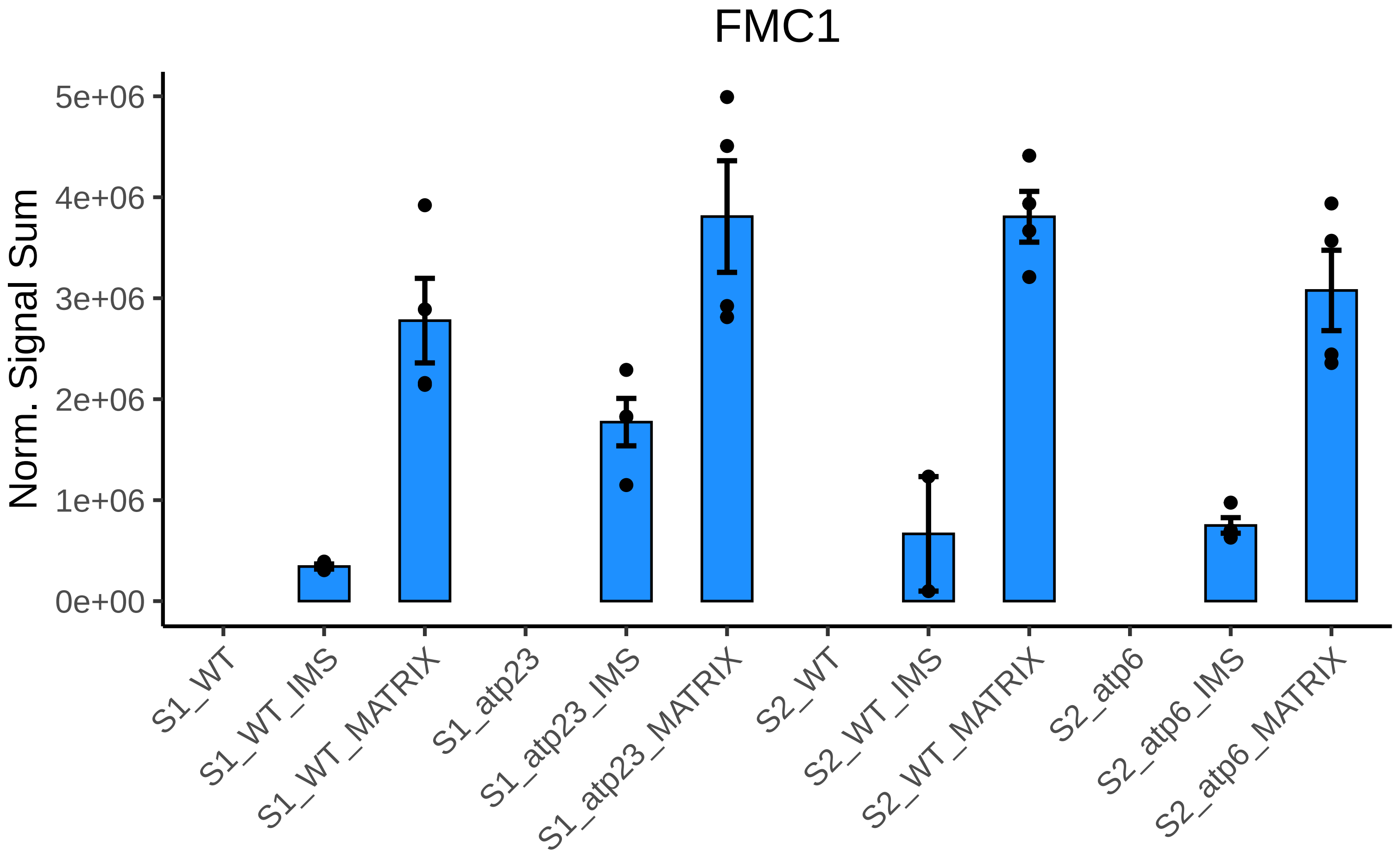
FMC1
Norm. Signal Sum
5e+06
4e+06
3e+06
2e+06
1e+06
0e+00
S1_WT
S1_WT_IMS
S1_WT_MATRIX
S1_atp23
S1_atp23_IMS
S1_atp23_MATRIX
S2_WT
S2_WT_IMS
S2_WT_MATRIX
S2_atp6
S2_atp6_IMS
S2_atp6_MATRIX

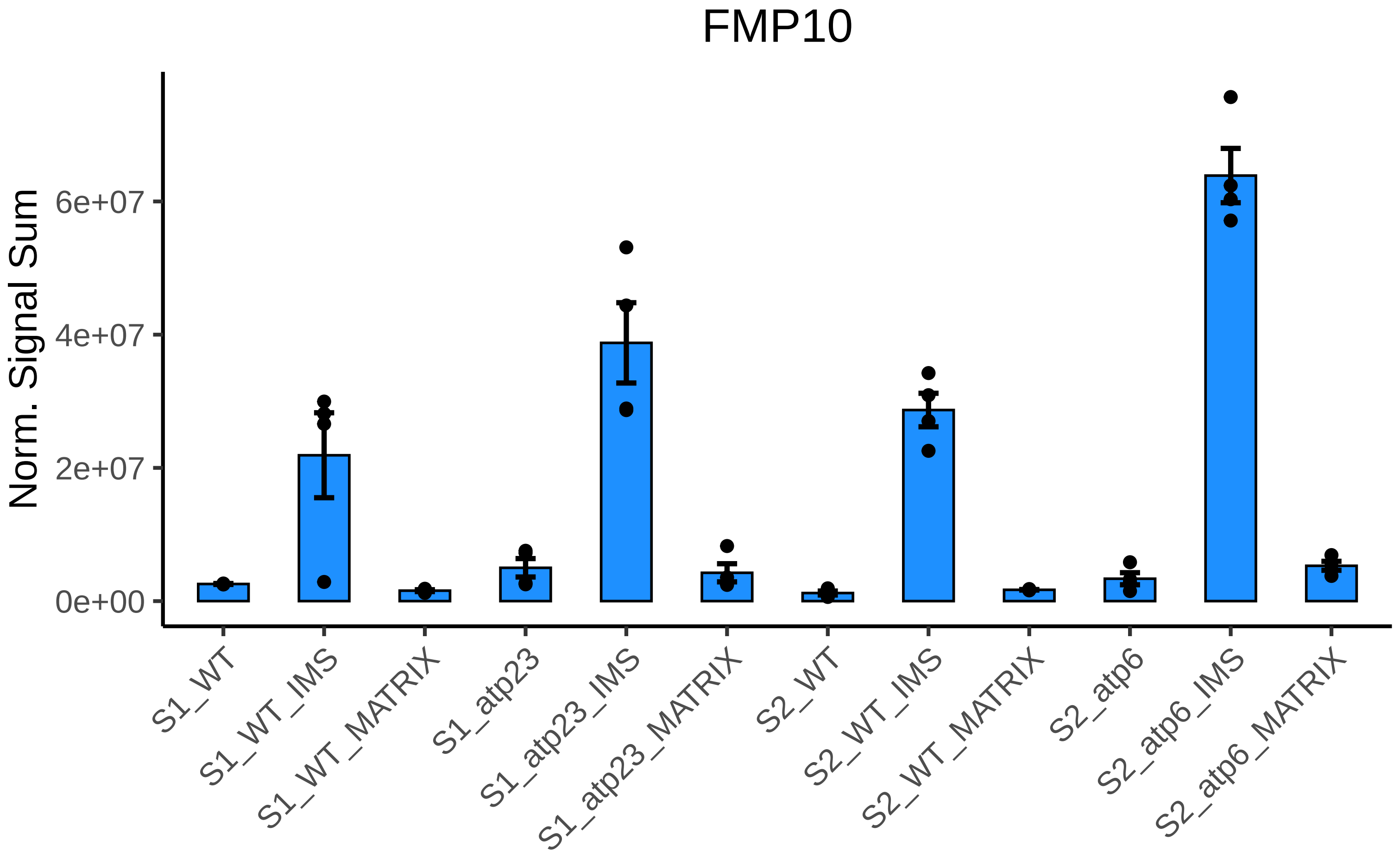
FMP10
Norm. Signal Sum
6e+07
4e+07
2e+07
0e+00
S1_WT
S1_WT_IMS
S1_WT_MATRIX
S1_atp23
S1_atp23_IMS
S1_atp23_MATRIX
S2_WT
S2_WT_IMS
S2_WT_MATRIX
S2_atp6
S2_atp6_IMS
S2_atp6_MATRIX

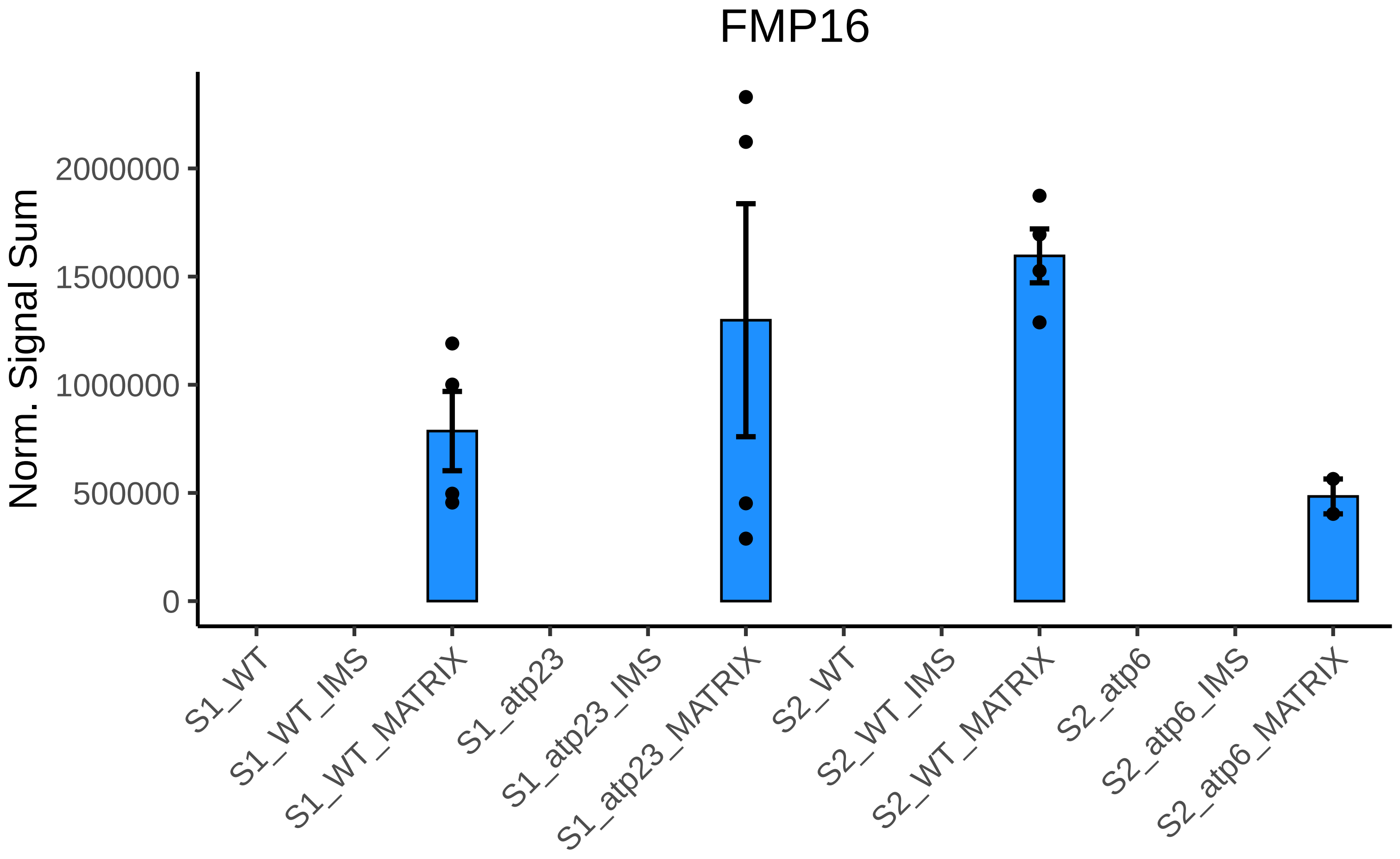
FMP16
Norm. Signal Sum
2000000
1500000
1000000
500000
0
S1_WT
S1_WT_IMS
S1_WT_MATRIX
S1_atp23
S1_atp23_IMS
S1_atp23_MATRIX
S2_WT
S2_WT_IMS
S2_WT_MATRIX
S2_atp6
S2_atp6_IMS
S2_atp6_MATRIX

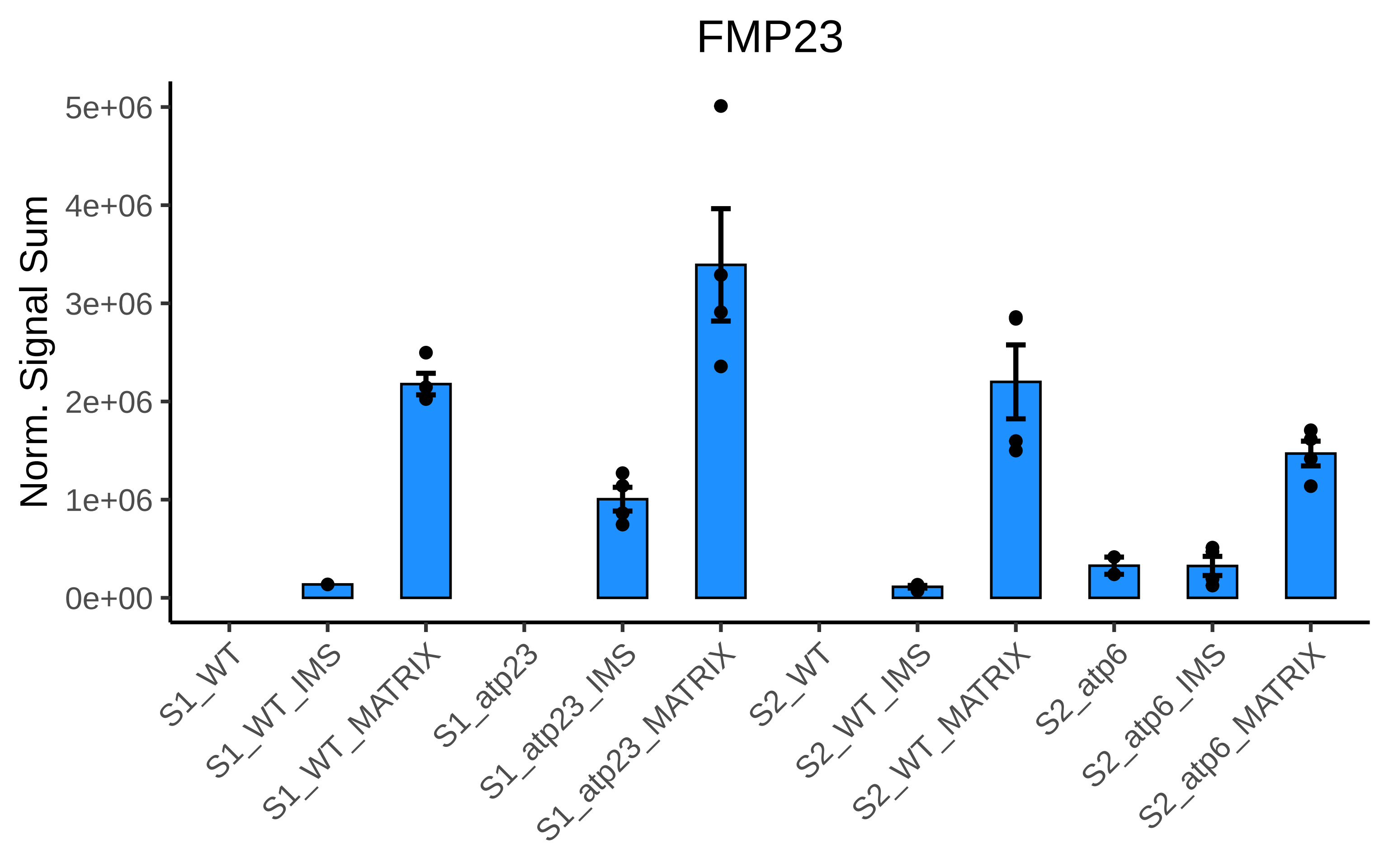
FMP23
Norm. Signal Sum
5e+06
4e+06
3e+06
2e+06
1e+06
0e+00
S1_WT
S1_WT_IMS
S1_WT_MATRIX
S1_atp23
S1_atp23_IMS
S1_atp23_MATRIX
S2_WT
S2_WT_IMS
S2_WT_MATRIX
S2_atp6
S2_atp6_IMS
S2_atp6_MATRIX

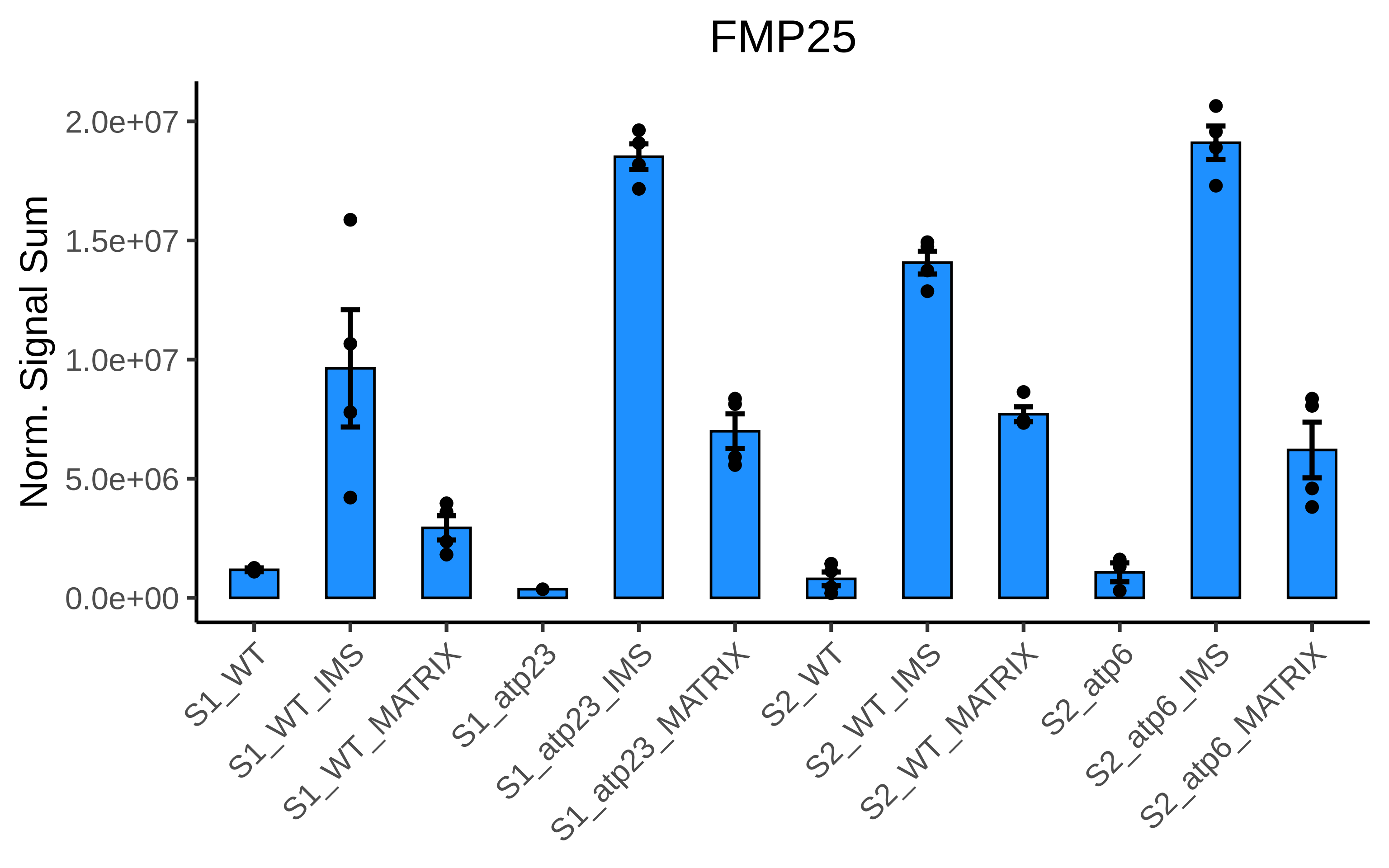

FMP25
Norm. Signal Sum
2.0e+07
1.5e+07
1.0e+07
5.0e+06
0.0e+00
S1_WT
S1_WT_IMS
S1_WT_MATRIX
S1_atp23
S1_atp23_IMS
S1_atp23_MATRIX
S2_WT
S2_WT_IMS
S2_WT_MATRIX
S2_atp6
S2_atp6_IMS
S2_atp6_MATRIX

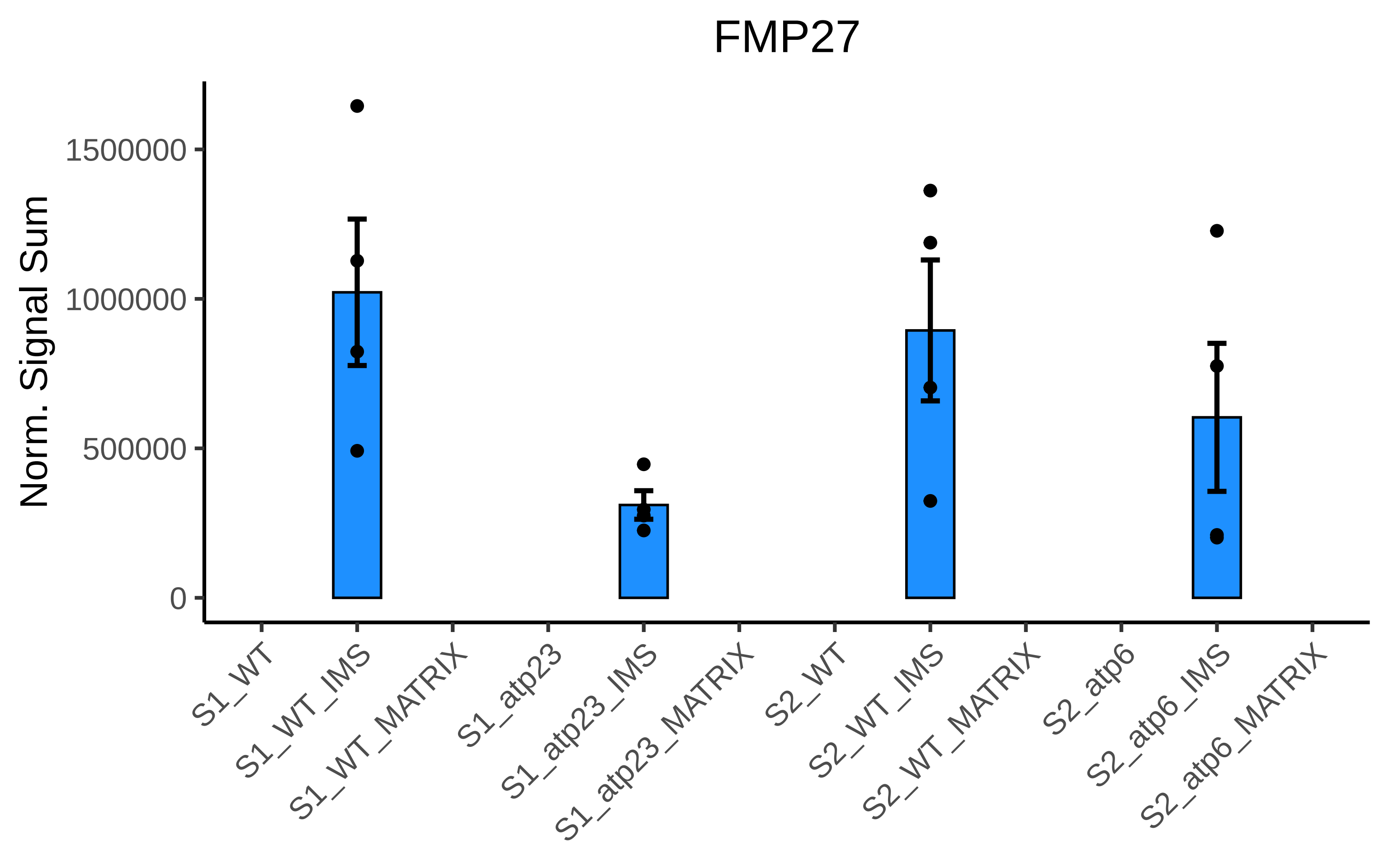
FMP27
Norm. Signal Sum
1500000
1000000
500000
0
S1_WT
S1_WT_IMS
S1_WT_MATRIX
S1_atp23
S1_atp23_IMS
S1_atp23_MATRIX
S2_WT
S2_WT_IMS
S2_WT_MATRIX
S2_atp6
S2_atp6_IMS
S2_atp6_MATRIX

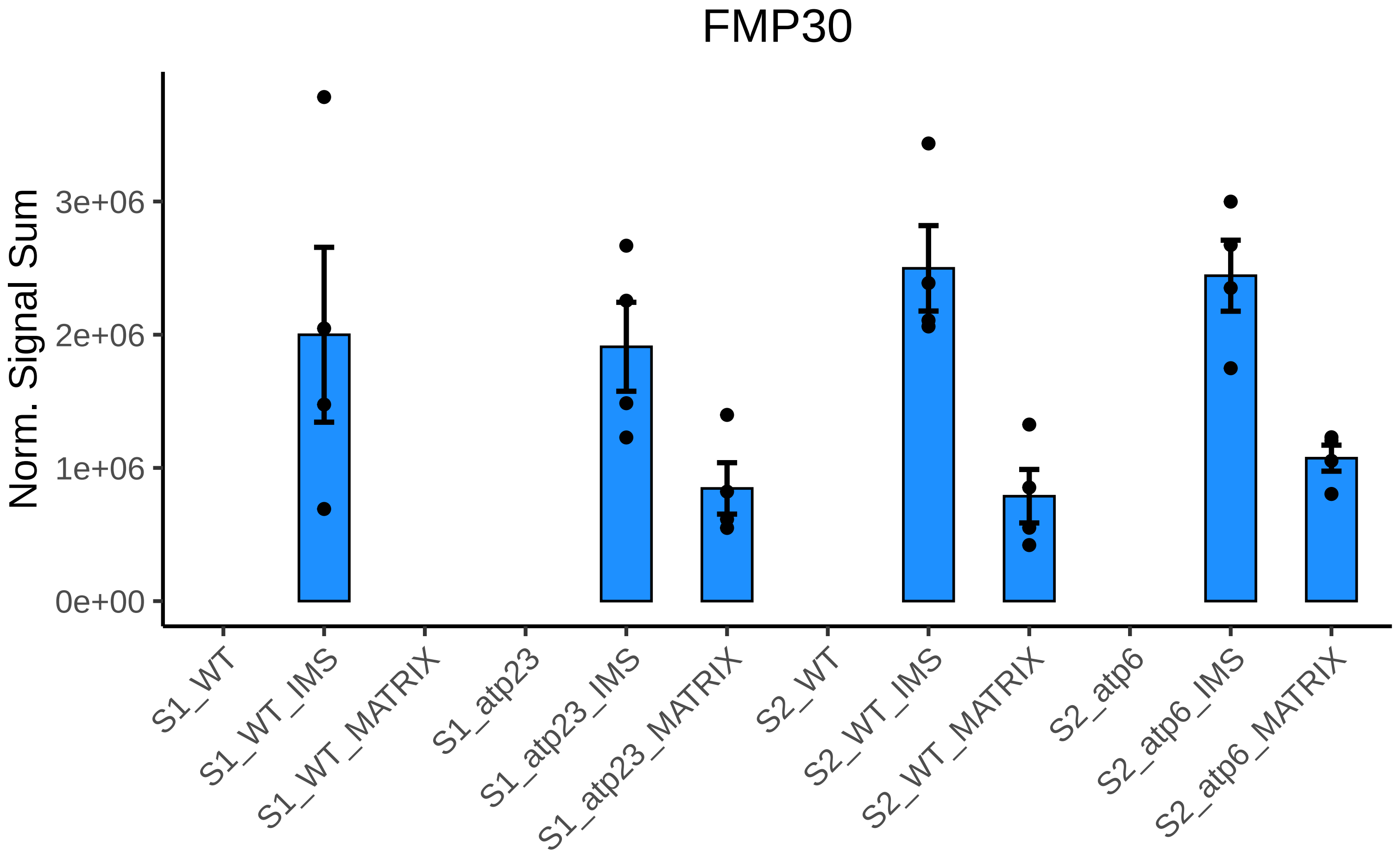
FMP30
Norm. Signal Sum
3e+06
2e+06
1e+06
0e+00
S1_WT
S1_WT_IMS
S1_WT_MATRIX
S1_atp23
S1_atp23_IMS
S1_atp23_MATRIX
S2_WT
S2_WT_IMS
S2_WT_MATRIX
S2_atp6
S2_atp6_IMS
S2_atp6_MATRIX

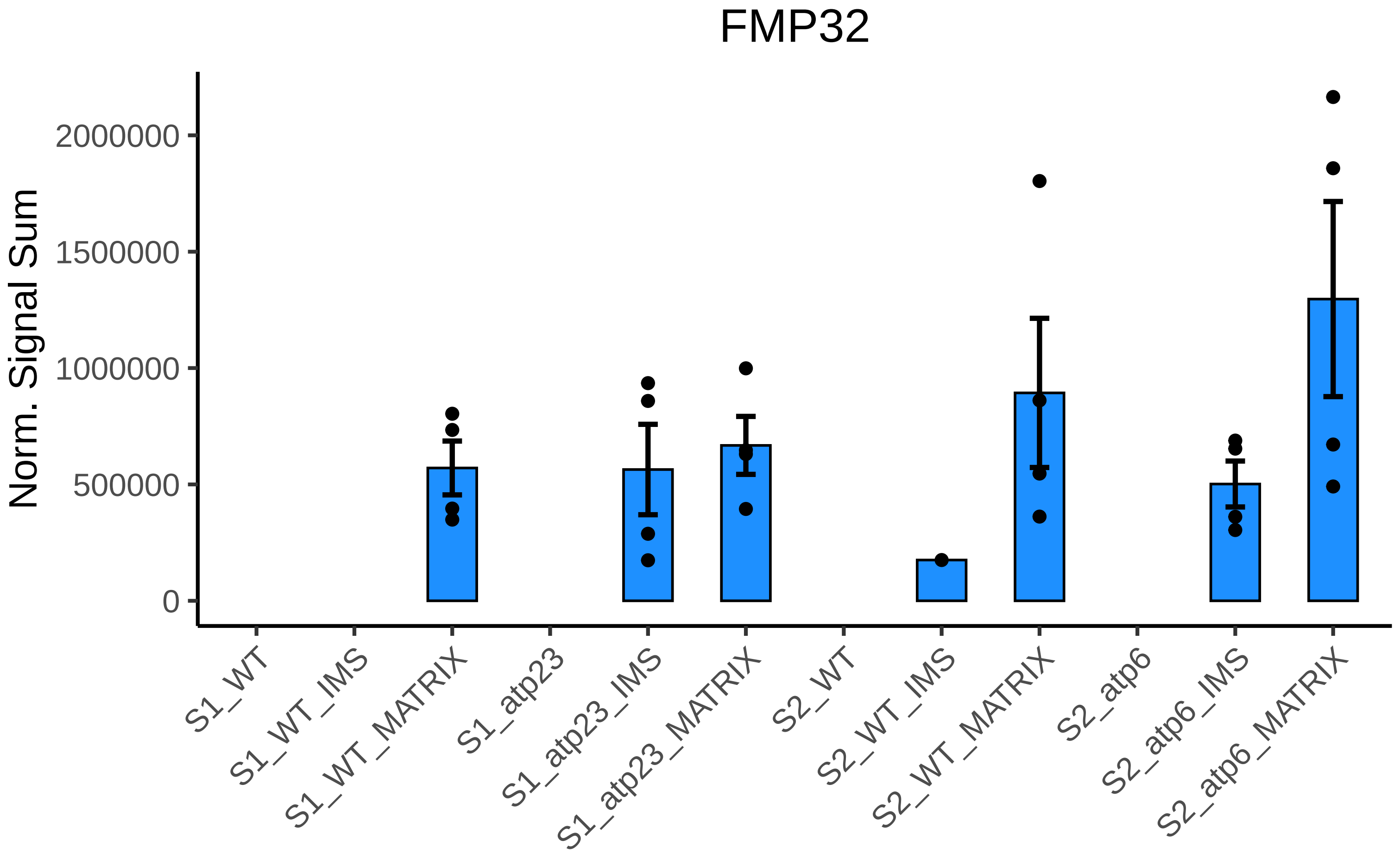
FMP32
Norm. Signal Sum
2000000
1500000
1000000
500000
0
S1_WT
S1_WT_IMS
S1_WT_MATRIX
S1_atp23
S1_atp23_IMS
S1_atp23_MATRIX
S2_WT
S2_WT_IMS
S2_WT_MATRIX
S2_atp6
S2_atp6_IMS
S2_atp6_MATRIX

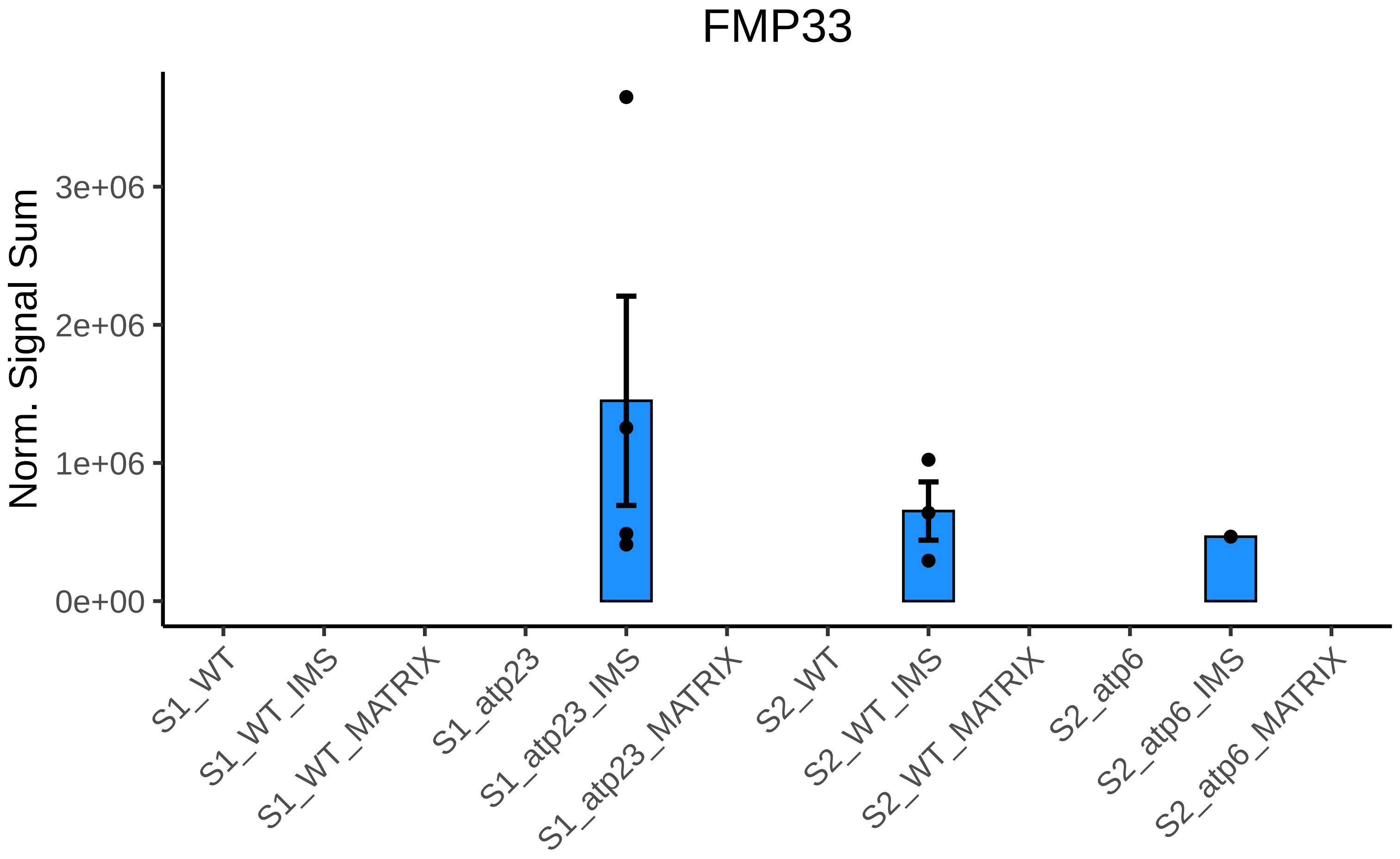
FMP33
Norm. Signal Sum
3e+06
2e+06
1e+06
0e+00
S1_WT
S1_WT_IMS
S1_WT_MATRIX
S1_atp23
S1_atp23_IMS
S1_atp23_MATRIX
S2_WT
S2_WT_IMS
S2_WT_MATRIX
S2_atp6
S2_atp6_IMS
S2_atp6_MATRIX

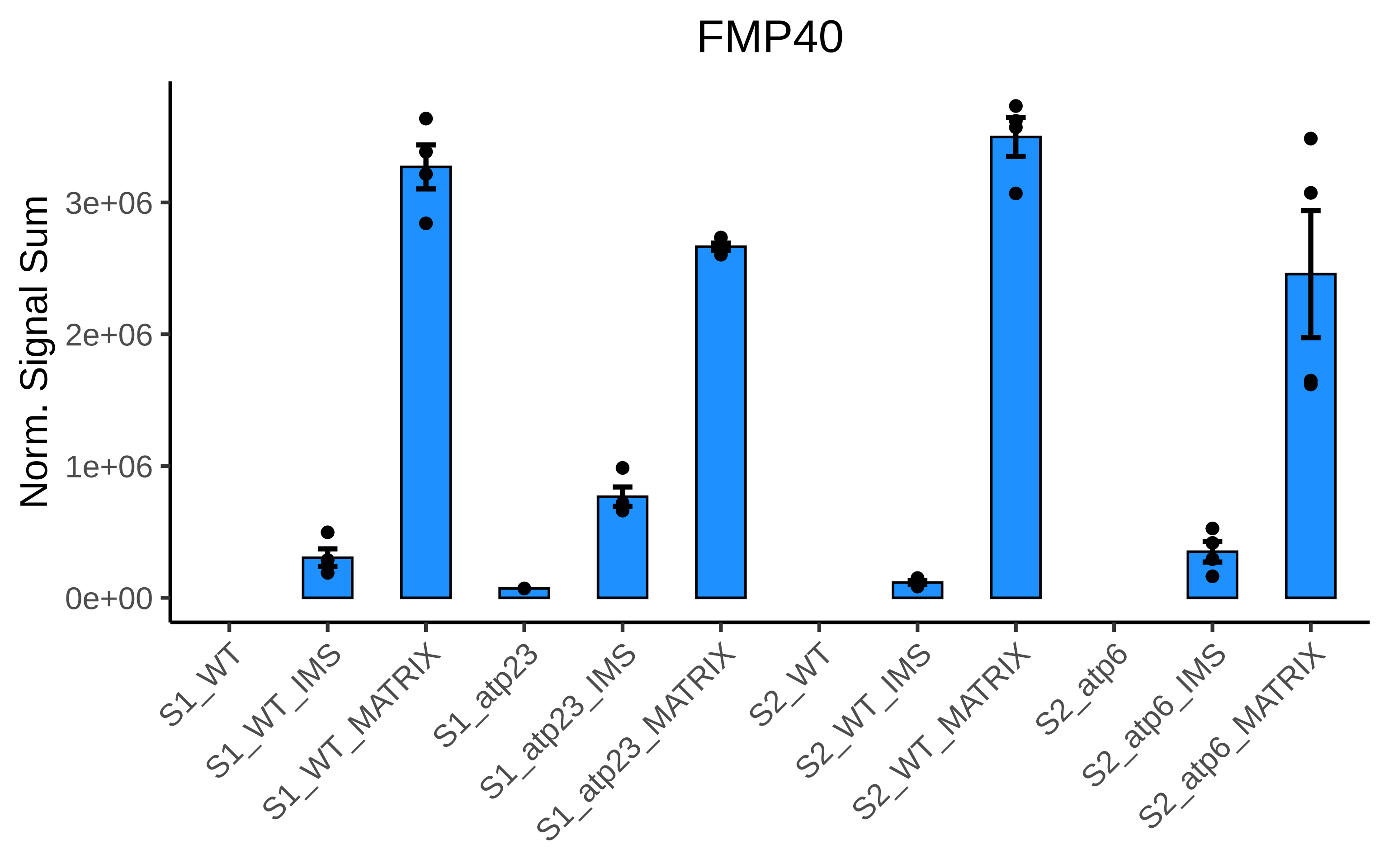

FMP40
Norm. Signal Sum
3e+06
2e+06
1e+06
0e+00
S1_WT
S1_WT_IMS
S1_WT_MATRIX
S1_atp23
S1_atp23_IMS
S1_atp23_MATRIX
S2_WT
S2_WT_IMS
S2_WT_MATRIX
S2_atp6
S2_atp6_IMS
S2_atp6_MATRIX

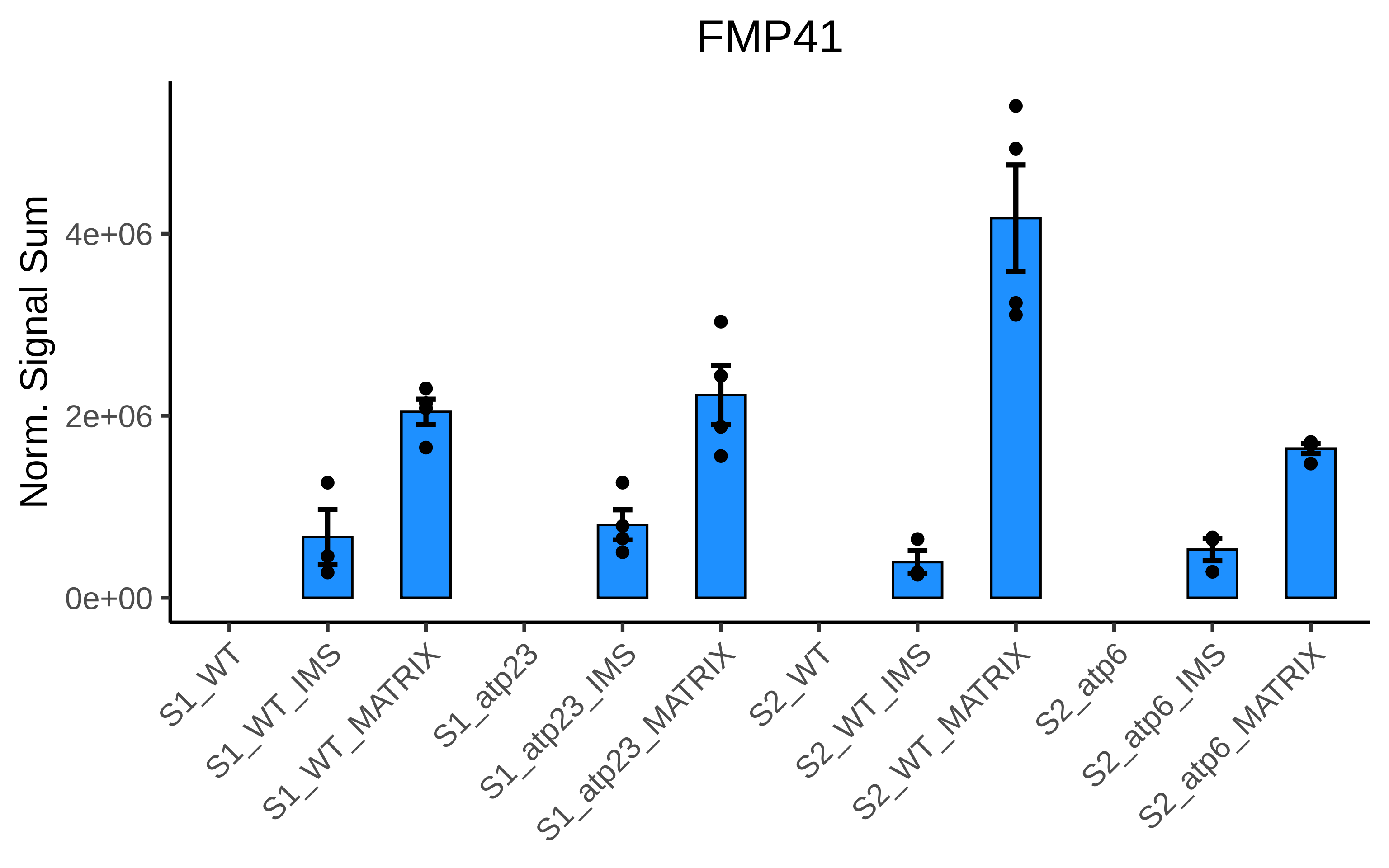

FMP41
Norm. Signal Sum
4e+06
2e+06
0e+00
S1_WT
S1_WT_IMS
S1_WT_MATRIX
S1_atp23
S1_atp23_IMS
S1_atp23_MATRIX
S2_WT
S2_WT_IMS
S2_WT_MATRIX
S2_atp6
S2_atp6_IMS
S2_atp6_MATRIX

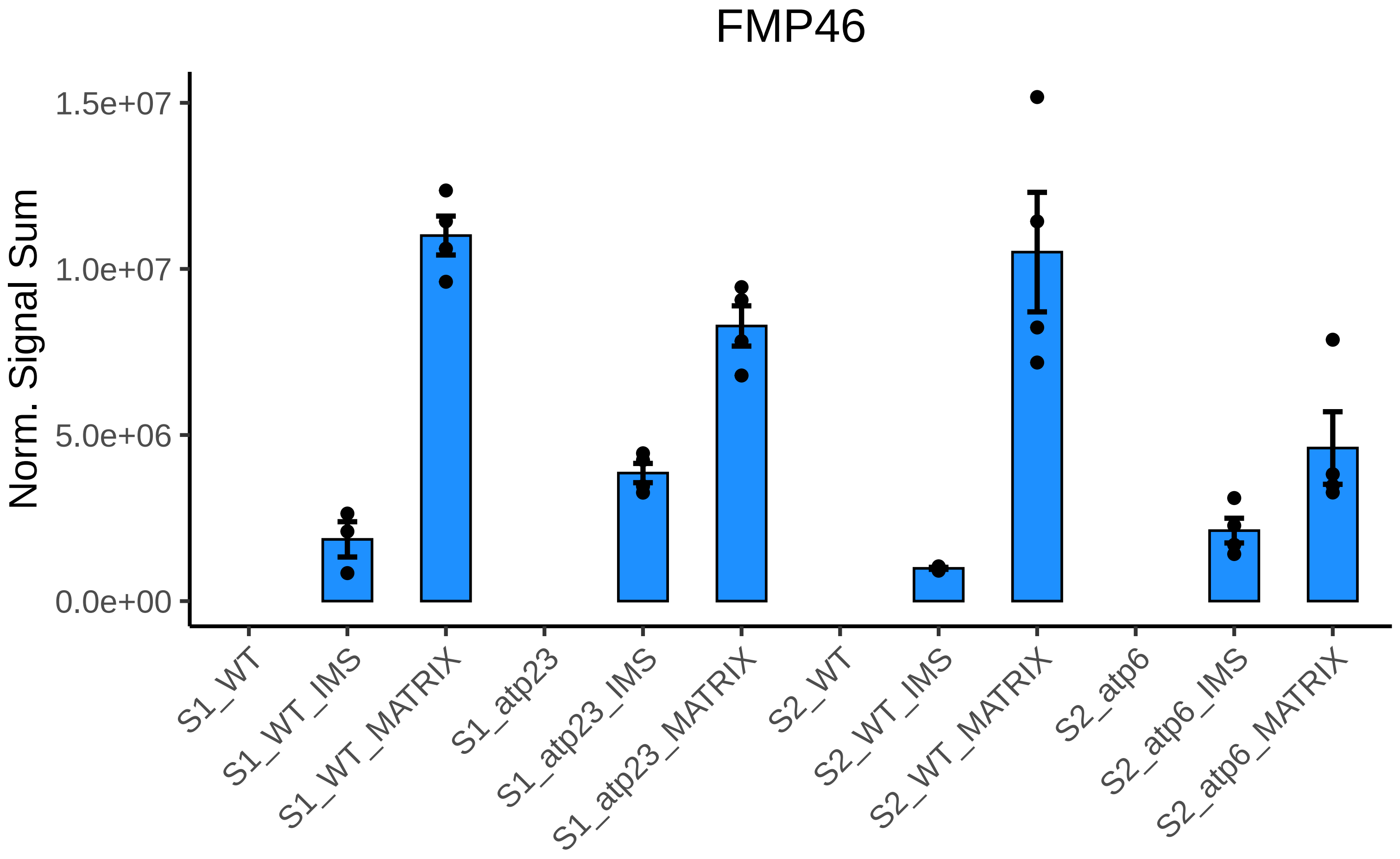
FMP46
Norm. Signal Sum
1.5e+07
1.0e+07
5.0e+06
0.0e+00
S1_WT
S1_WT_IMS
S1_WT_MATRIX
S1_atp23
S1_atp23_IMS
S1_atp23_MATRIX
S2_WT
S2_WT_IMS
S2_WT_MATRIX
S2_atp6
S2_atp6_IMS
S2_atp6_MATRIX

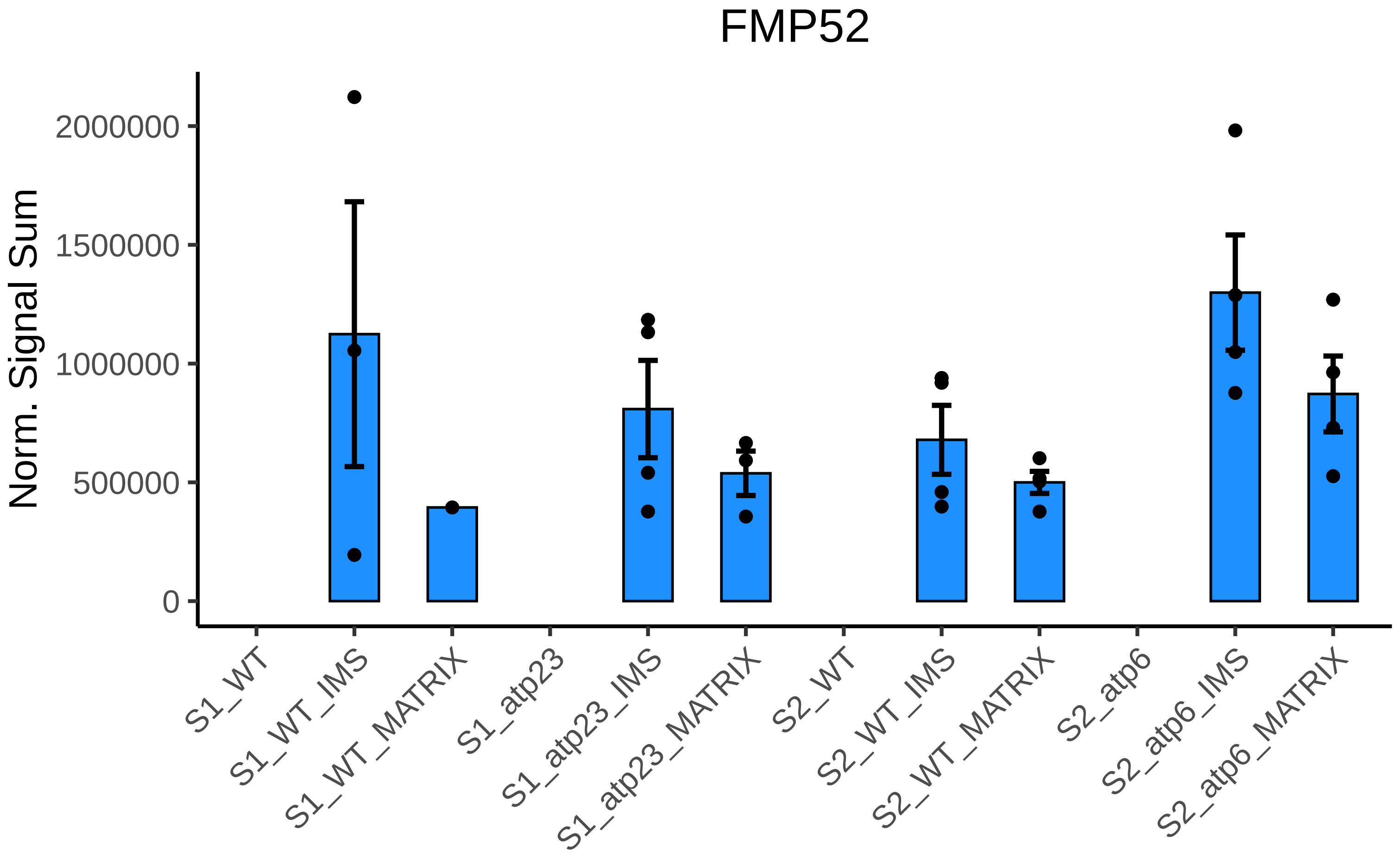

FMP52
Norm. Signal Sum
2000000
1500000
1000000
500000
0
S1_WT
S1_WT_IMS
S1_WT_MATRIX
S1_atp23
S1_atp23_IMS
S1_atp23_MATRIX
S2_WT
S2_WT_IMS
S2_WT_MATRIX
S2_atp6
S2_atp6_IMS
S2_atp6_MATRIX

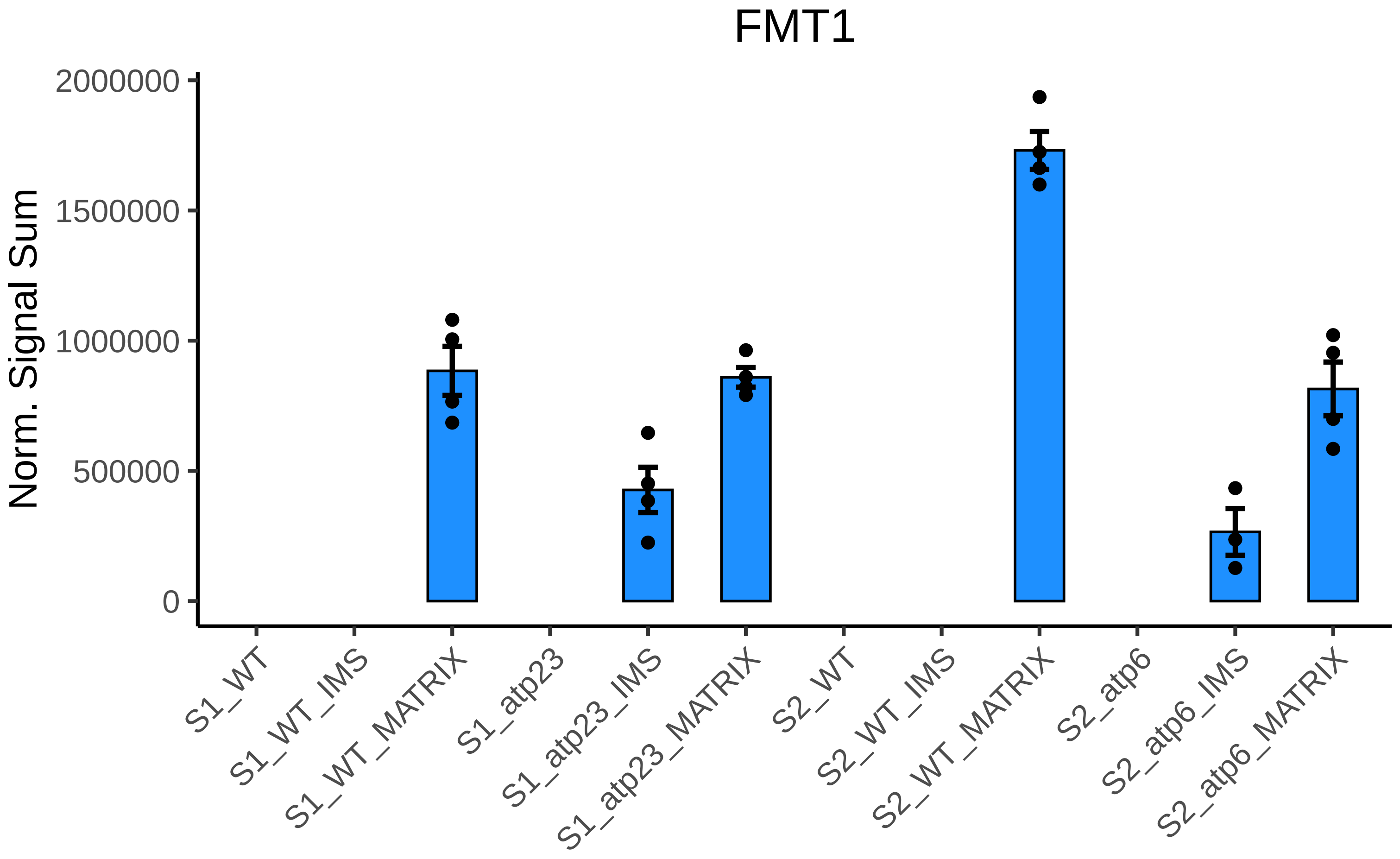

FMT1
Norm. Signal Sum
2000000
1500000
1000000
500000
0
S1_WT
S1_WT_IMS
S1_WT_MATRIX
S1_atp23
S1_atp23_IMS
S1_atp23_MATRIX
S2_WT
S2_WT_IMS
S2_WT_MATRIX
S2_atp6
S2_atp6_IMS
S2_atp6_MATRIX

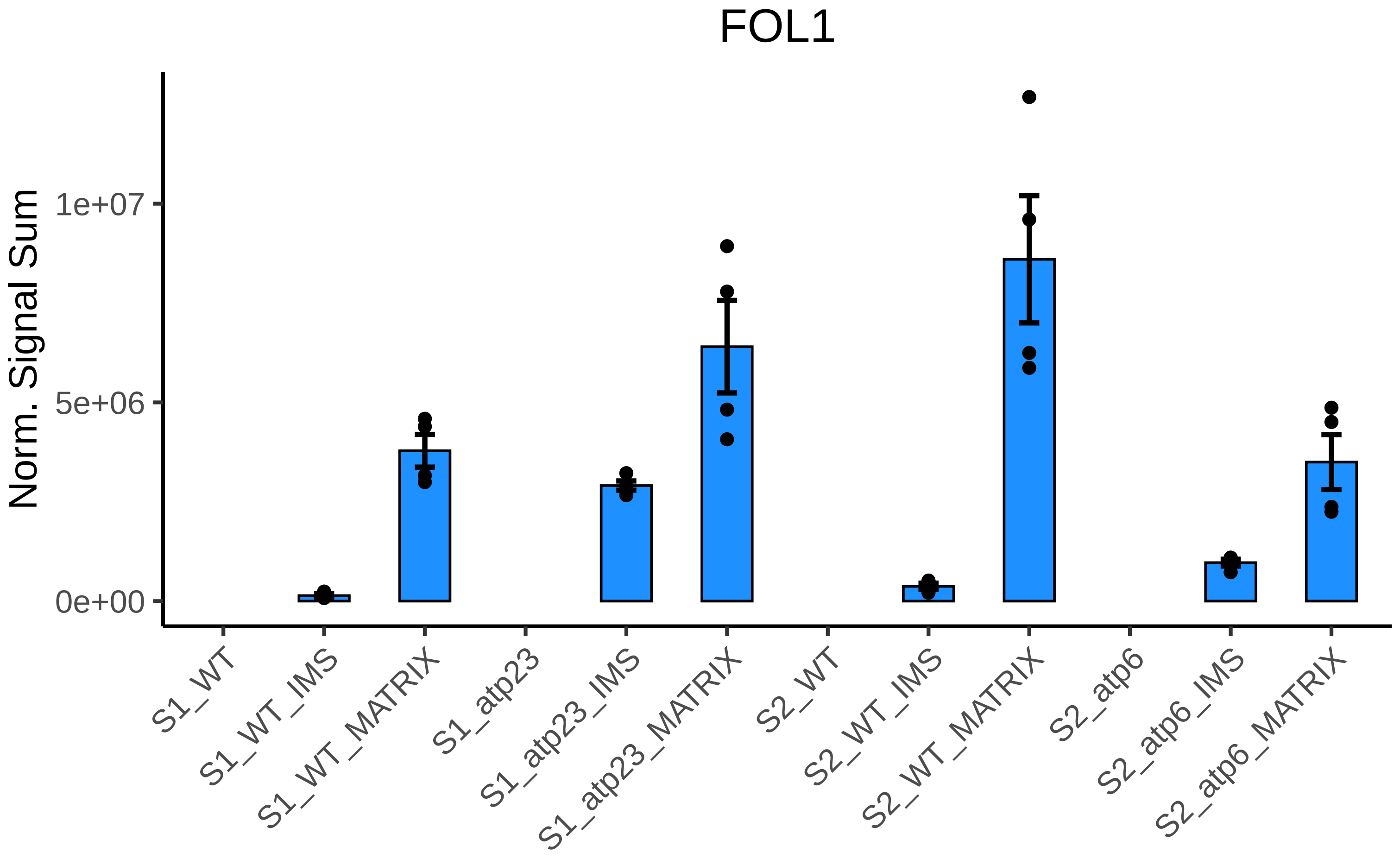

FOL1
Norm. Signal Sum
1e+07
5e+06
0e+00
S1_WT
S1_WT_IMS
S1_WT_MATRIX
S1_atp23
S1_atp23_IMS
S1_atp23_MATRIX
S2_WT
S2_WT_IMS
S2_WT_MATRIX
S2_atp6
S2_atp6_IMS
S2_atp6_MATRIX

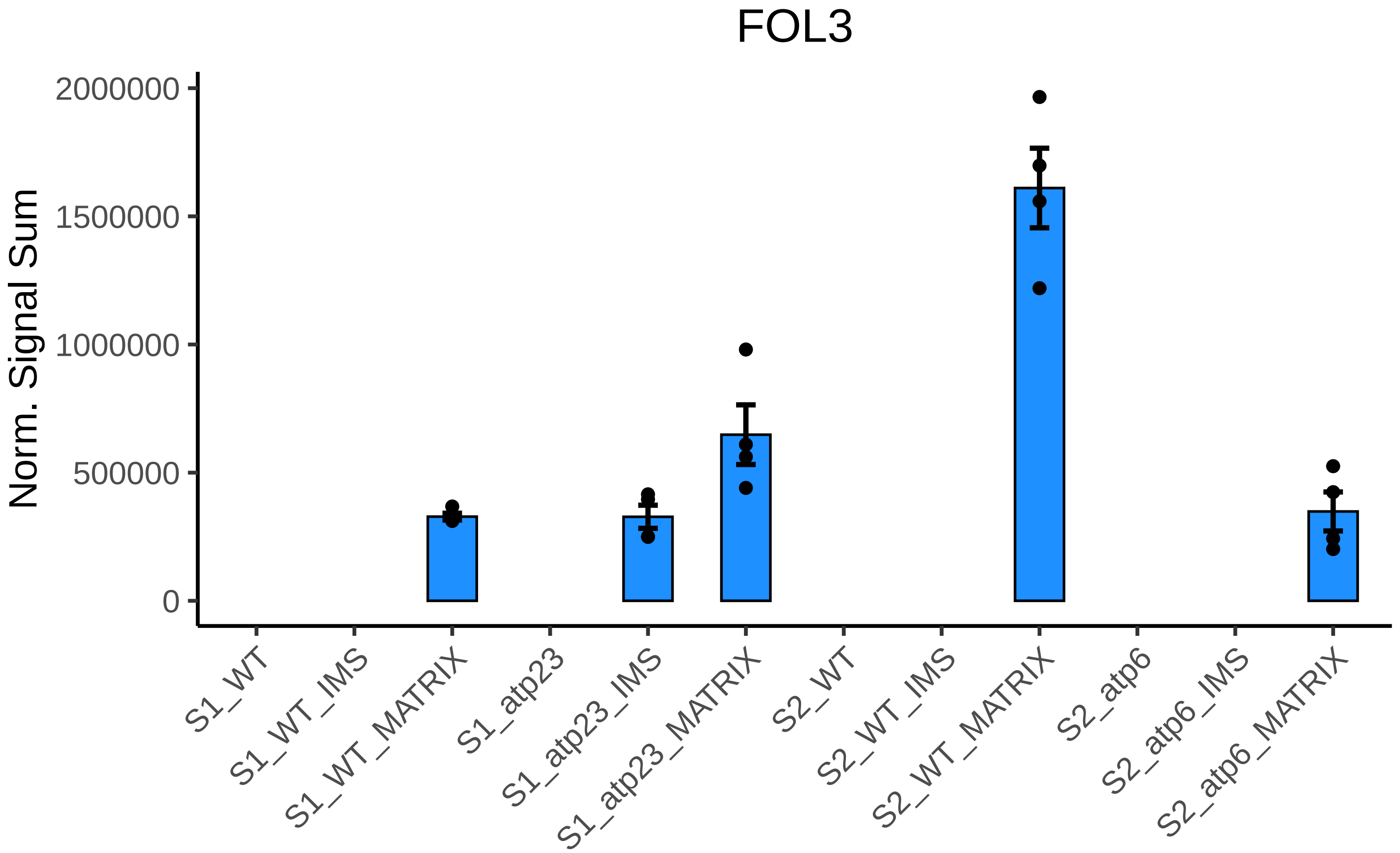

FOL3
Norm. Signal Sum
2000000
1500000
1000000
500000
0
S1_WT
S1_WT_IMS
S1_WT_MATRIX
S1_atp23
S1_atp23_IMS
S1_atp23_MATRIX
S2_WT
S2_WT_IMS
S2_WT_MATRIX
S2_atp6
S2_atp6_IMS
S2_atp6_MATRIX

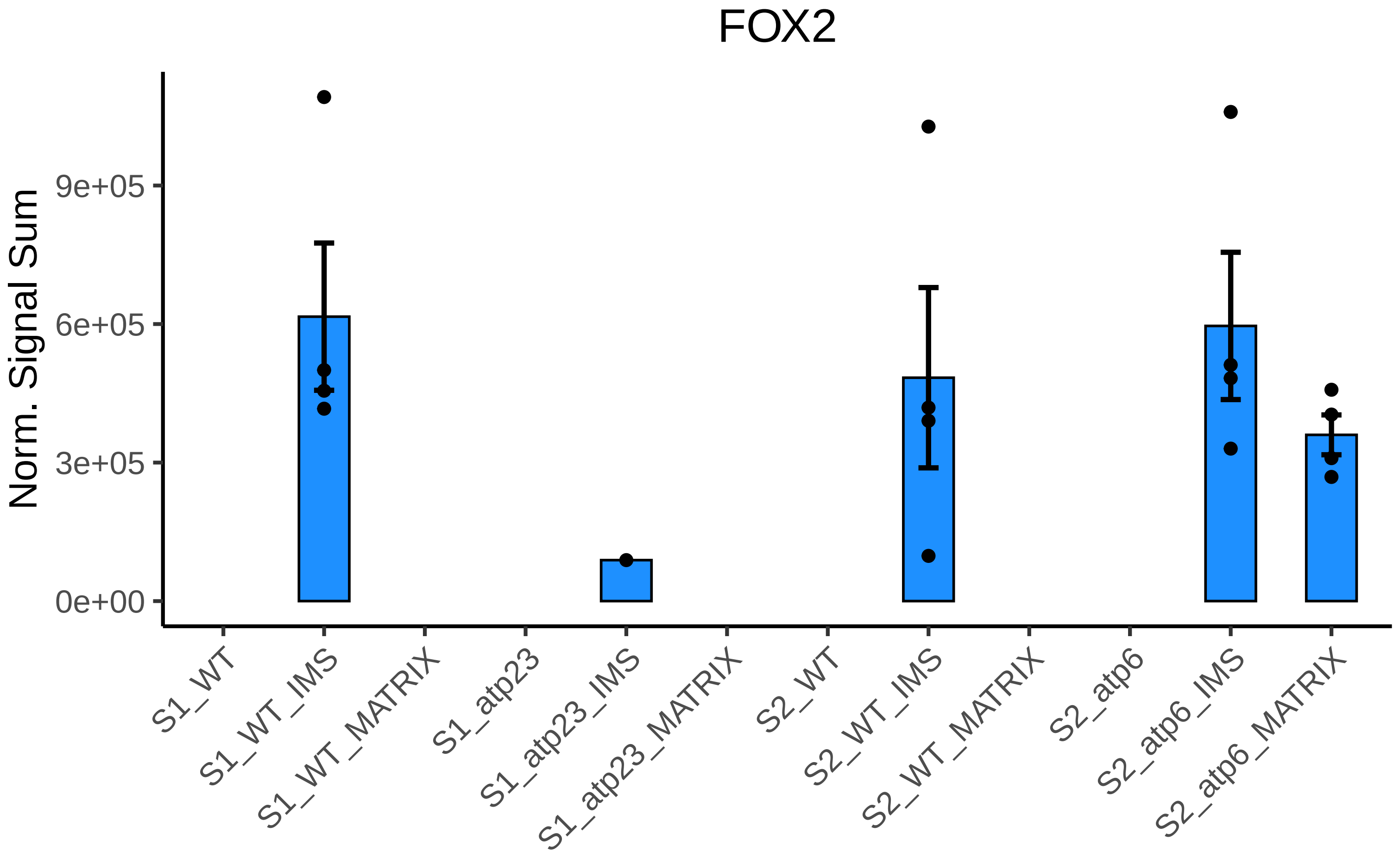

FOX2
Norm. Signal Sum
9e+05
6e+05
3e+05
0e+00
S1_WT
S1_WT_IMS
S1_WT_MATRIX
S1_atp23
S1_atp23_IMS
S1_atp23_MATRIX
S2_WT
S2_WT_IMS
S2_WT_MATRIX
S2_atp6
S2_atp6_IMS
S2_atp6_MATRIX

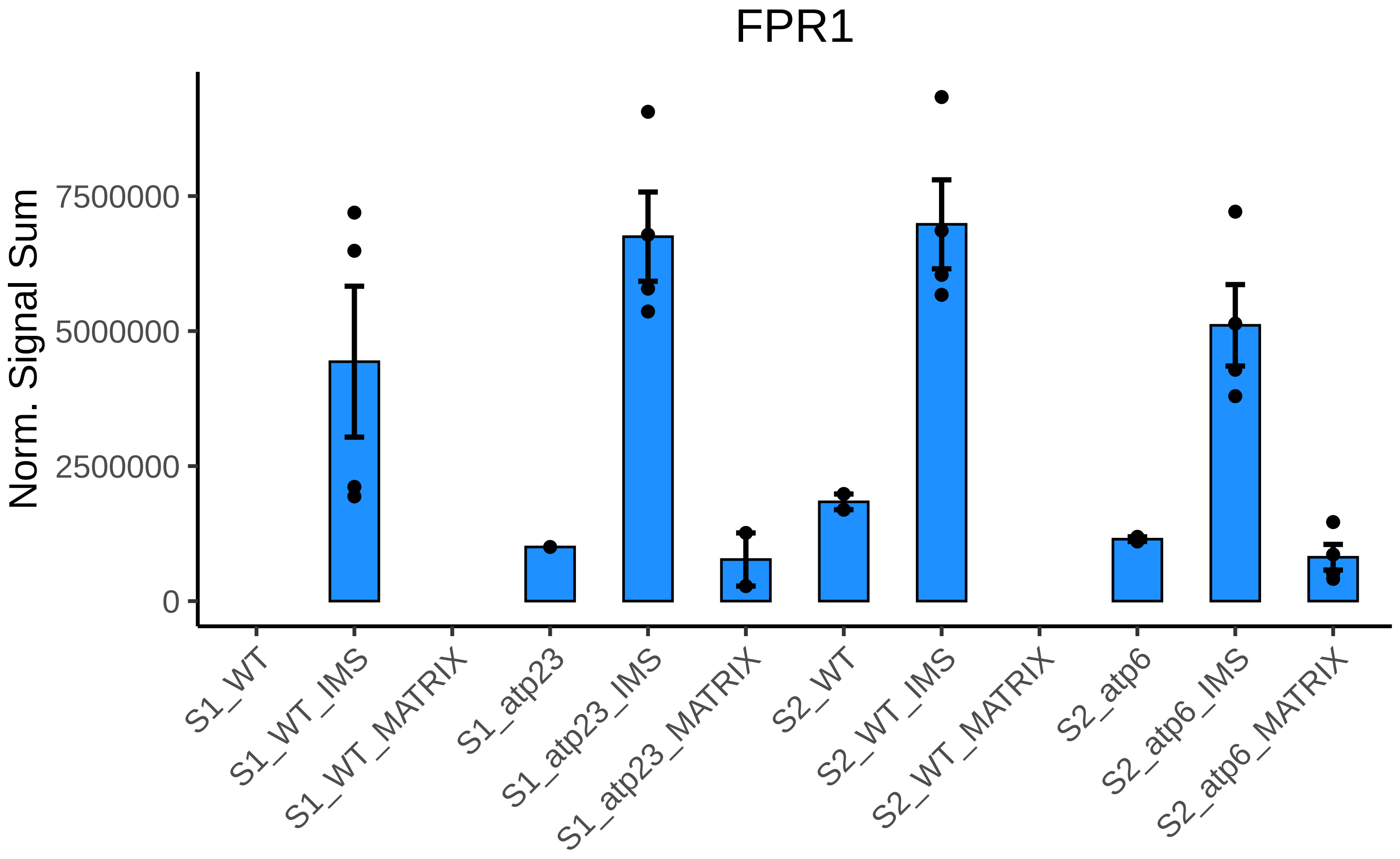

FPR1
Norm. Signal Sum
7500000
5000000
2500000
0
S1_WT
S1_WT_IMS
S1_WT_MATRIX
S1_atp23
S1_atp23_IMS
S1_atp23_MATRIX
S2_WT
S2_WT_IMS
S2_WT_MATRIX
S2_atp6
S2_atp6_IMS
S2_atp6_MATRIX

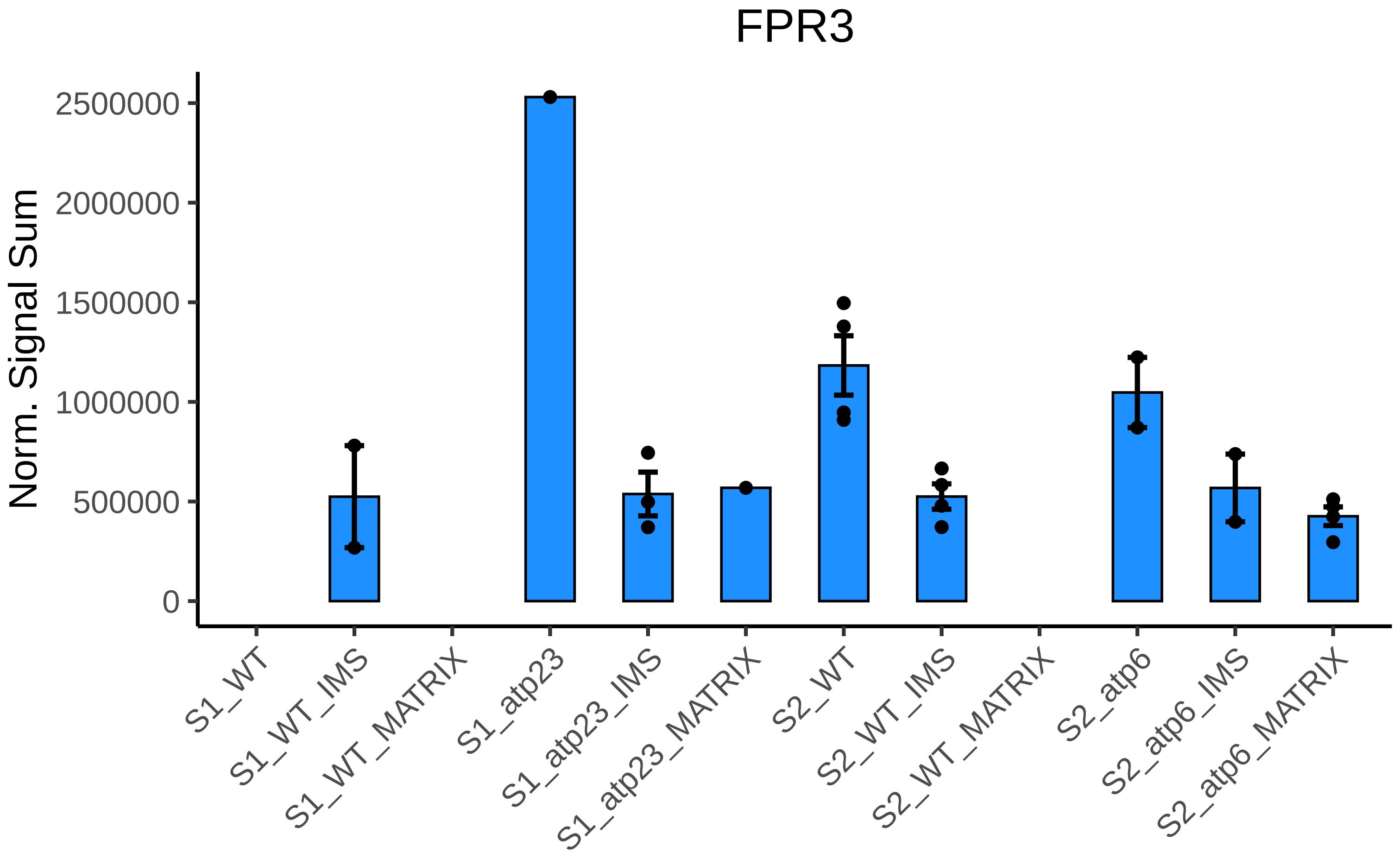

FPR3
Norm. Signal Sum
2500000
2000000
1500000
1000000
500000
0
S1_WT
S1_WT_IMS
S1_WT_MATRIX
S1_atp23
S1_atp23_IMS
S1_atp23_MATRIX
S2_WT
S2_WT_IMS
S2_WT_MATRIX
S2_atp6
S2_atp6_IMS
S2_atp6_MATRIX

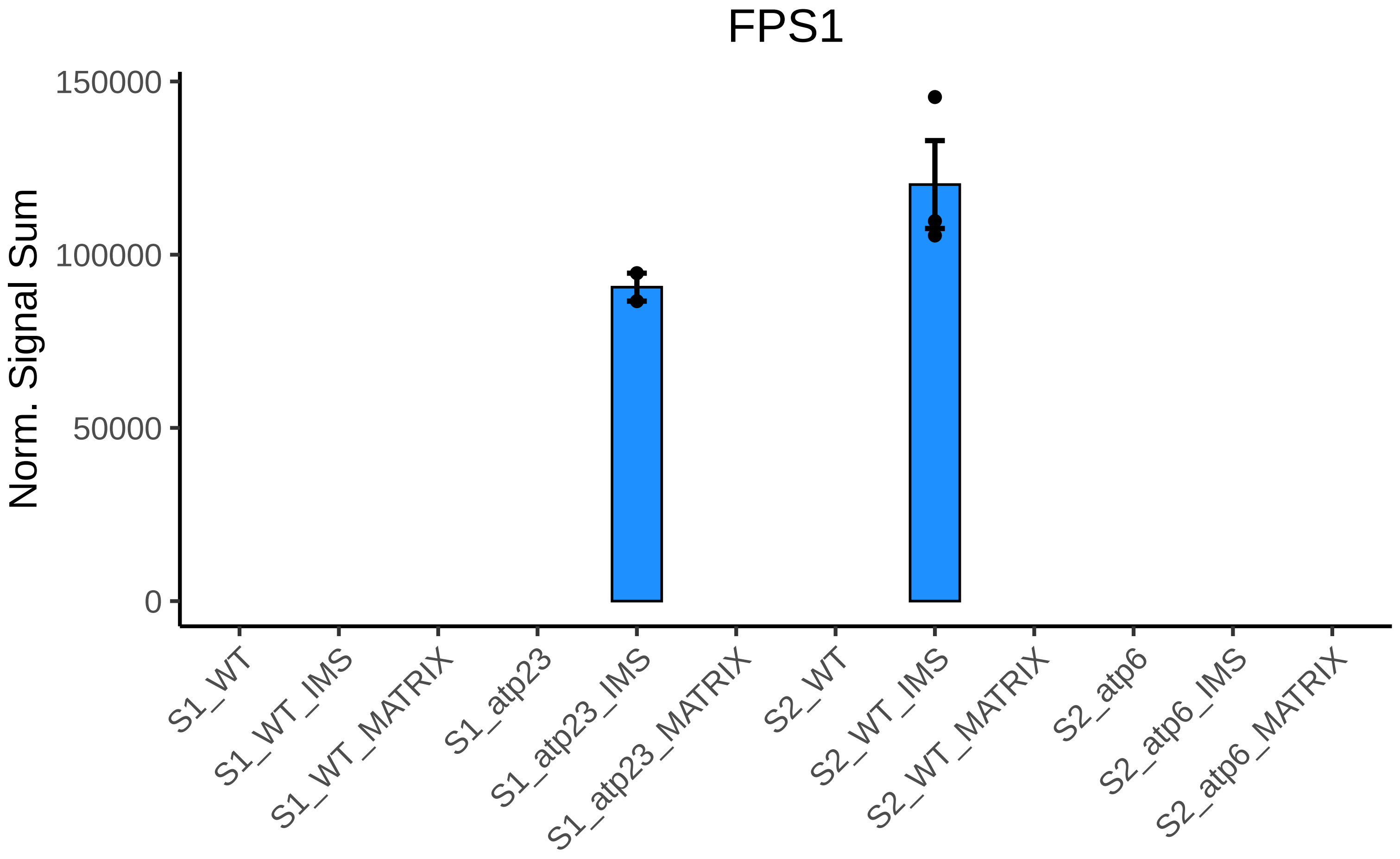
FPS1
Norm. Signal Sum
150000
100000
50000
0
S1_WT
S1_WT_IMS
S1_WT_MATRIX
S1_atp23
S1_atp23_IMS
S1_atp23_MATRIX
S2_WT
S2_WT_IMS
S2_WT_MATRIX
S2_atp6
S2_atp6_IMS
S2_atp6_MATRIX

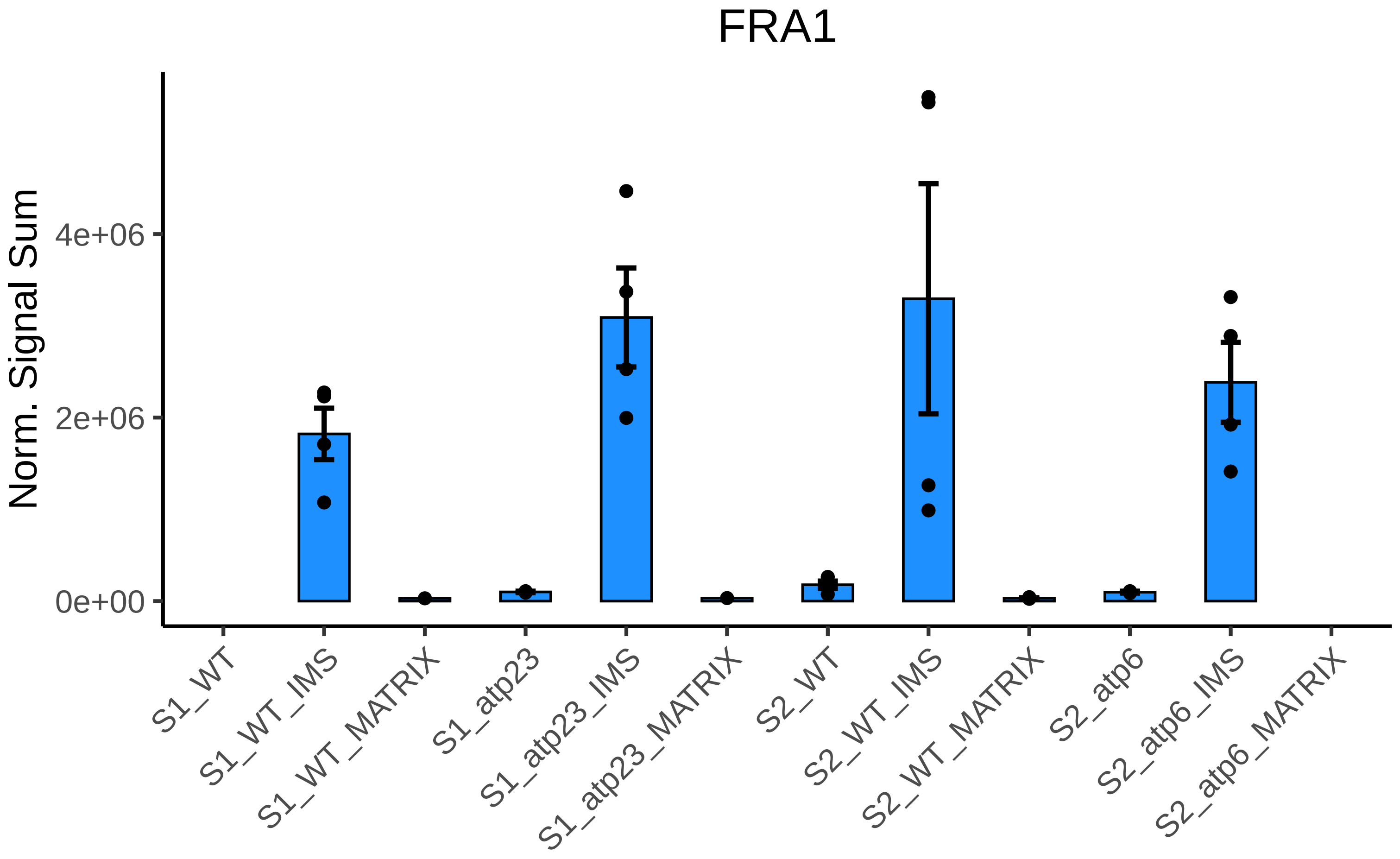
FRA1
Norm. Signal Sum
4e+06
2e+06
0e+00
S1_WT
S1_WT_IMS
S1_WT_MATRIX
S1_atp23
S1_atp23_IMS
S1_atp23_MATRIX
S2_WT
S2_WT_IMS
S2_WT_MATRIX
S2_atp6
S2_atp6_IMS
S2_atp6_MATRIX

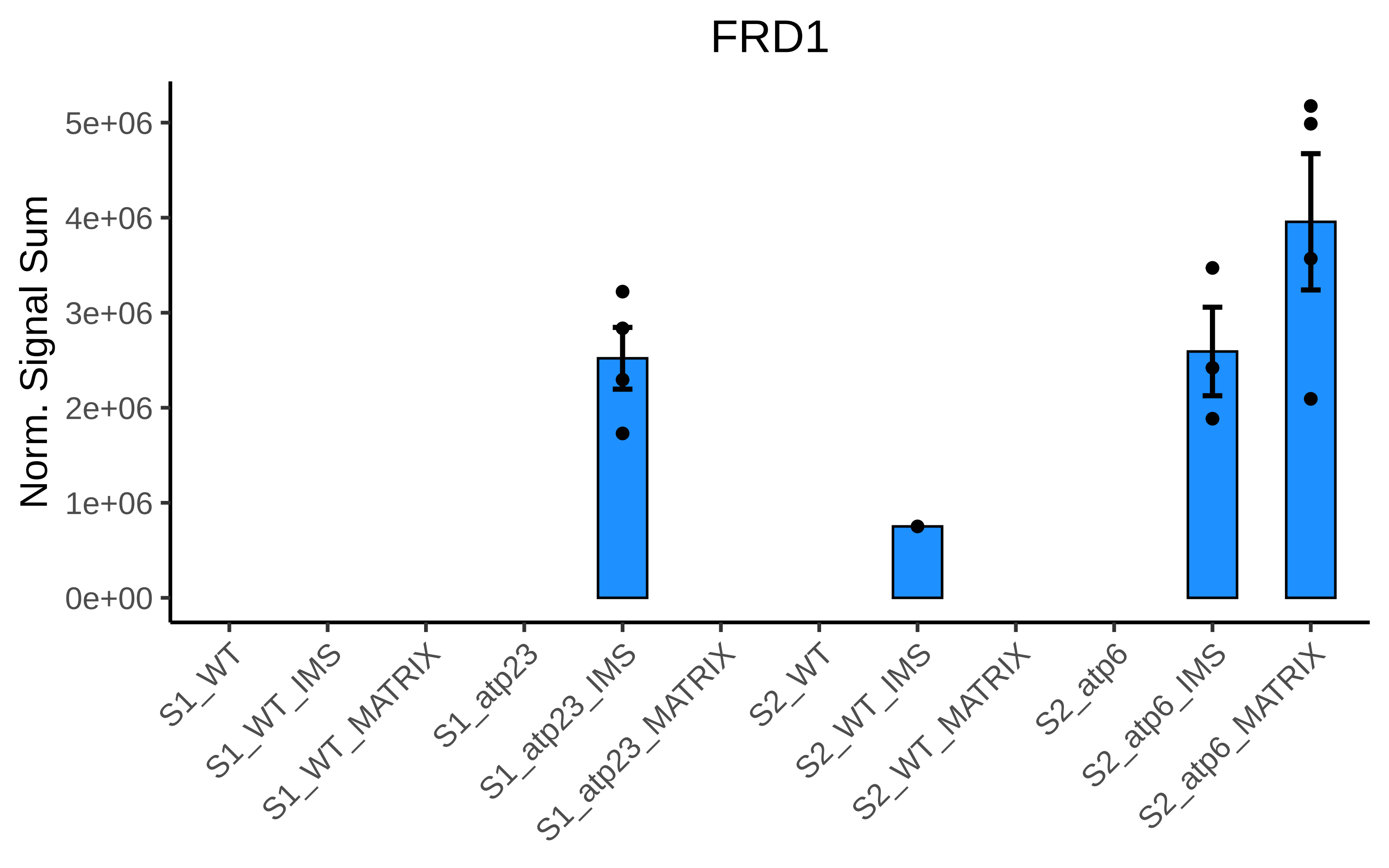

FRD1
Norm. Signal Sum
5e+06
4e+06
3e+06
2e+06
1e+06
0e+00
S1_WT
S1_WT_IMS
S1_WT_MATRIX
S1_atp23
S1_atp23_IMS
S1_atp23_MATRIX
S2_WT
S2_WT_IMS
S2_WT_MATRIX
S2_atp6
S2_atp6_IMS
S2_atp6_MATRIX

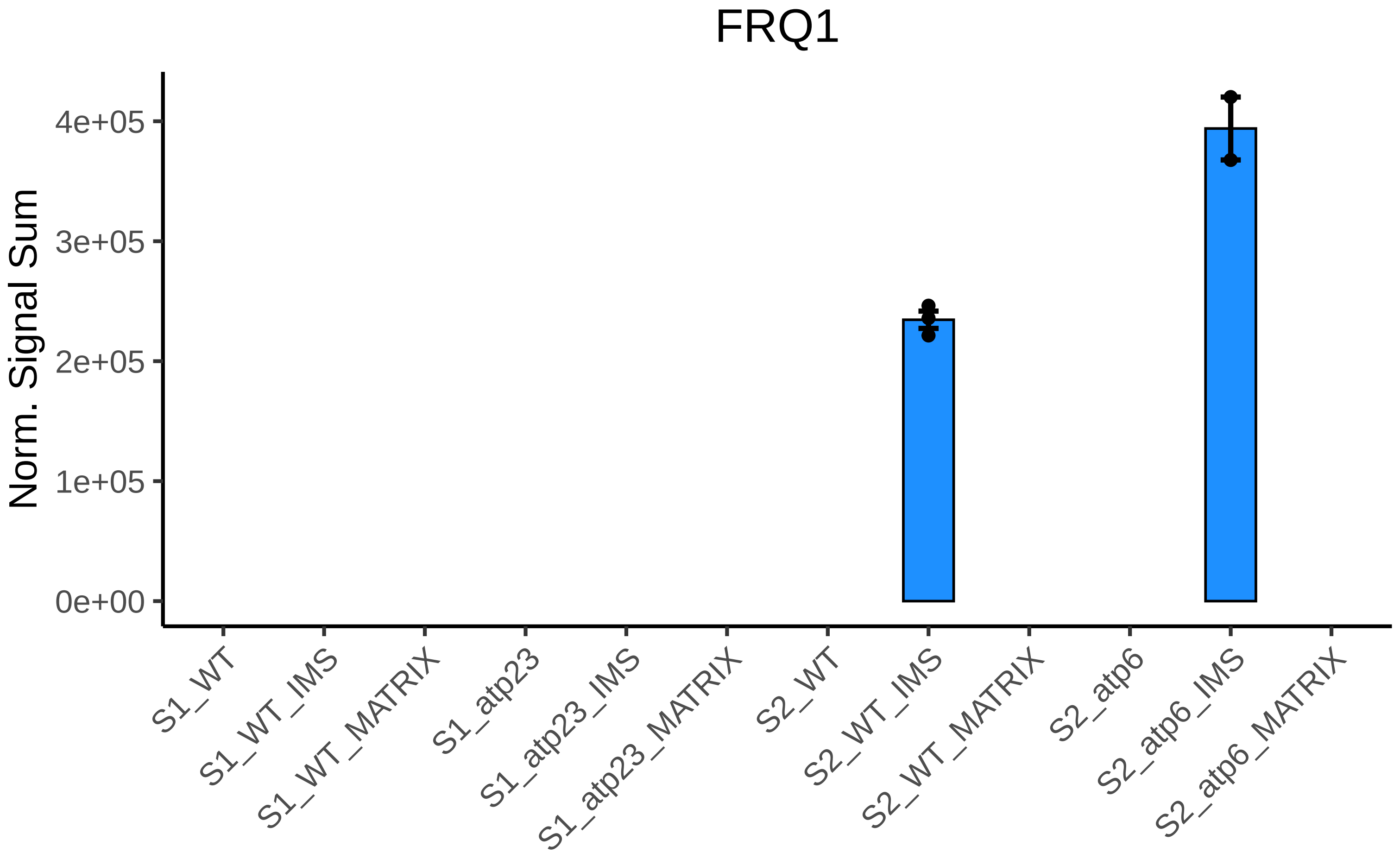

FRQ1
Norm. Signal Sum
4e+05
3e+05
2e+05
1e+05
0e+00
S1_WT
S1_WT_IMS
S1_WT_MATRIX
S1_atp23
S1_atp23_IMS
S1_atp23_MATRIX
S2_WT
S2_WT_IMS
S2_WT_MATRIX
S2_atp6
S2_atp6_IMS
S2_atp6_MATRIX

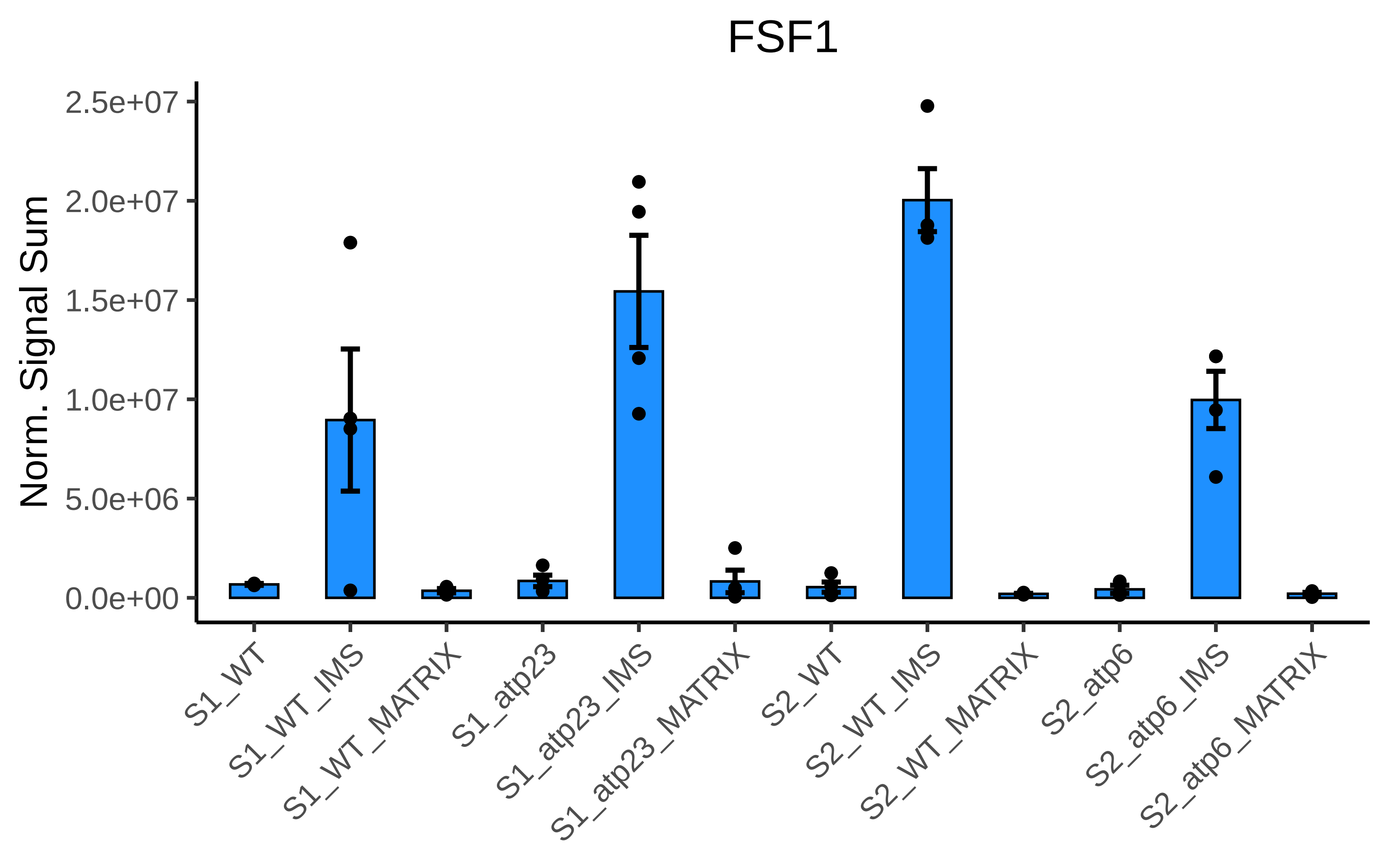

FSF1
Norm. Signal Sum
2.5e+07
2.0e+07
1.5e+07
1.0e+07
5.0e+06
0.0e+00
S1_WT
S1_WT_IMS
S1_WT_MATRIX
S1_atp23
S1_atp23_IMS
S1_atp23_MATRIX
S2_WT
S2_WT_IMS
S2_WT_MATRIX
S2_atp6
S2_atp6_IMS
S2_atp6_MATRIX

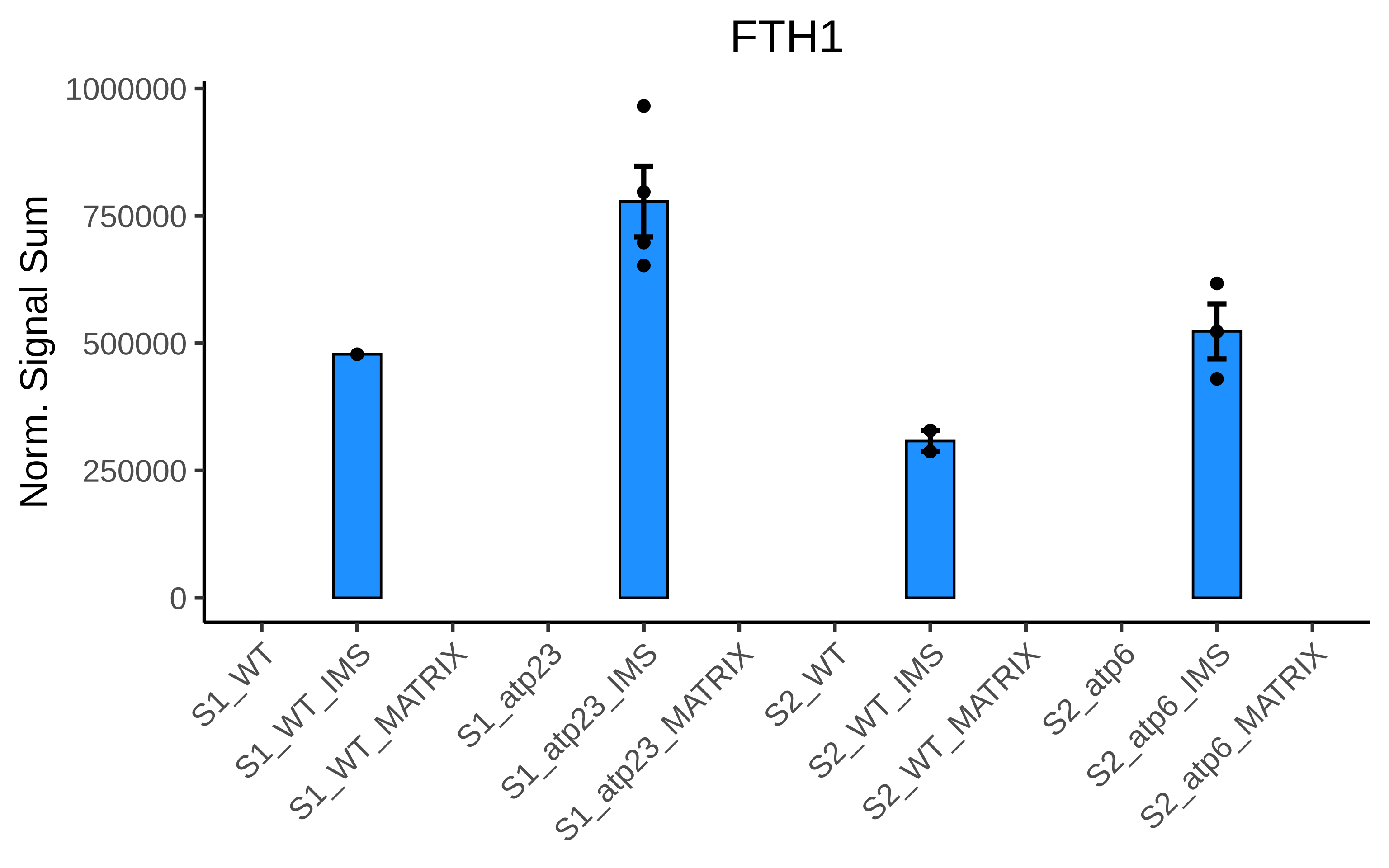
FTH1
Norm. Signal Sum
1000000
750000
500000
250000
0
S1_WT
S1_WT_IMS
S1_WT_MATRIX
S1_atp23
S1_atp23_IMS
S1_atp23_MATRIX
S2_WT
S2_WT_IMS
S2_WT_MATRIX
S2_atp6
S2_atp6_IMS
S2_atp6_MATRIX

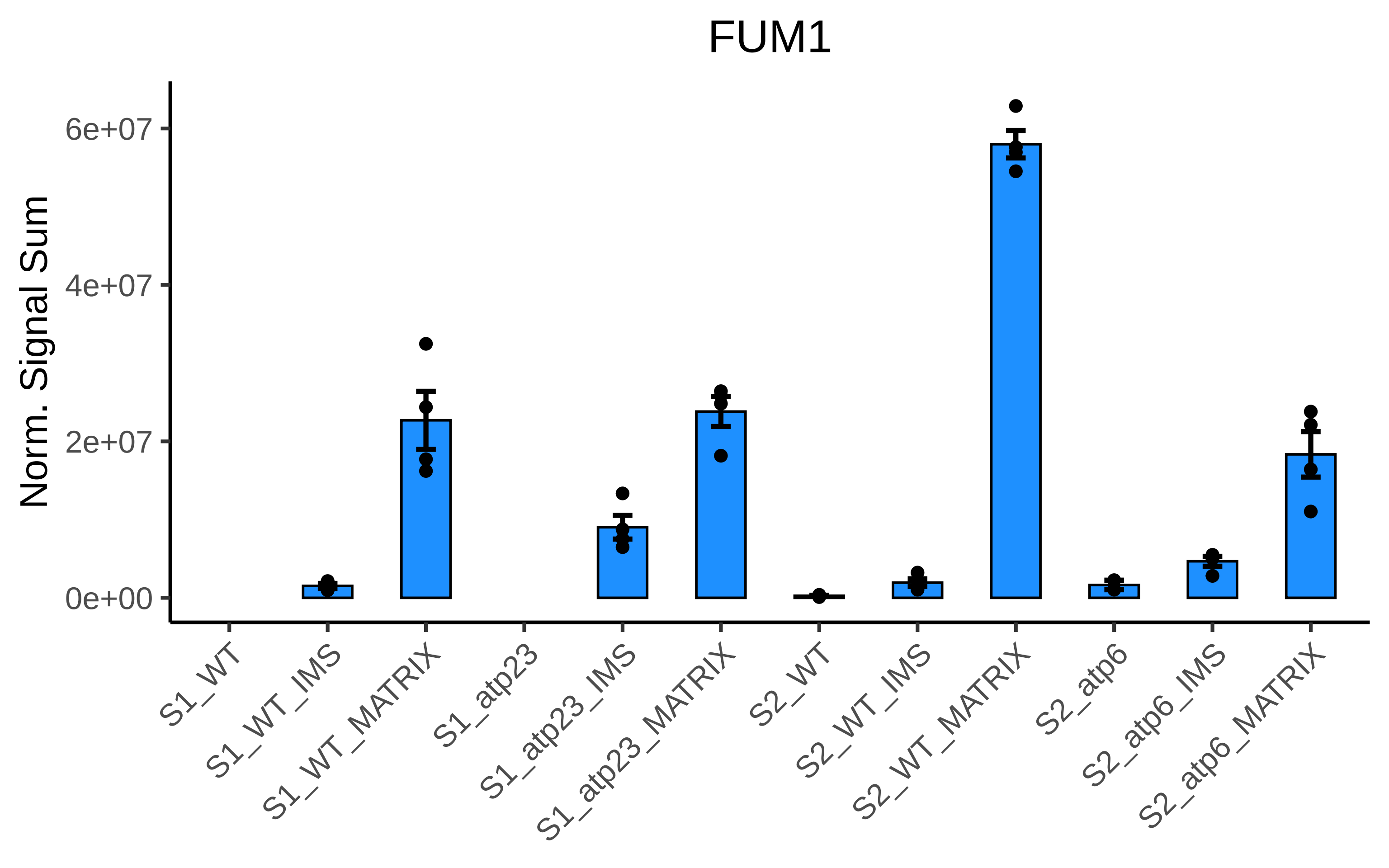

FUM1
Norm. Signal Sum
6e+07
4e+07
2e+07
0e+00
S1_WT
S1_WT_IMS
S1_WT_MATRIX
S1_atp23
S1_atp23_IMS
S1_atp23_MATRIX
S2_WT
S2_WT_IMS
S2_WT_MATRIX
S2_atp6
S2_atp6_IMS
S2_atp6_MATRIX

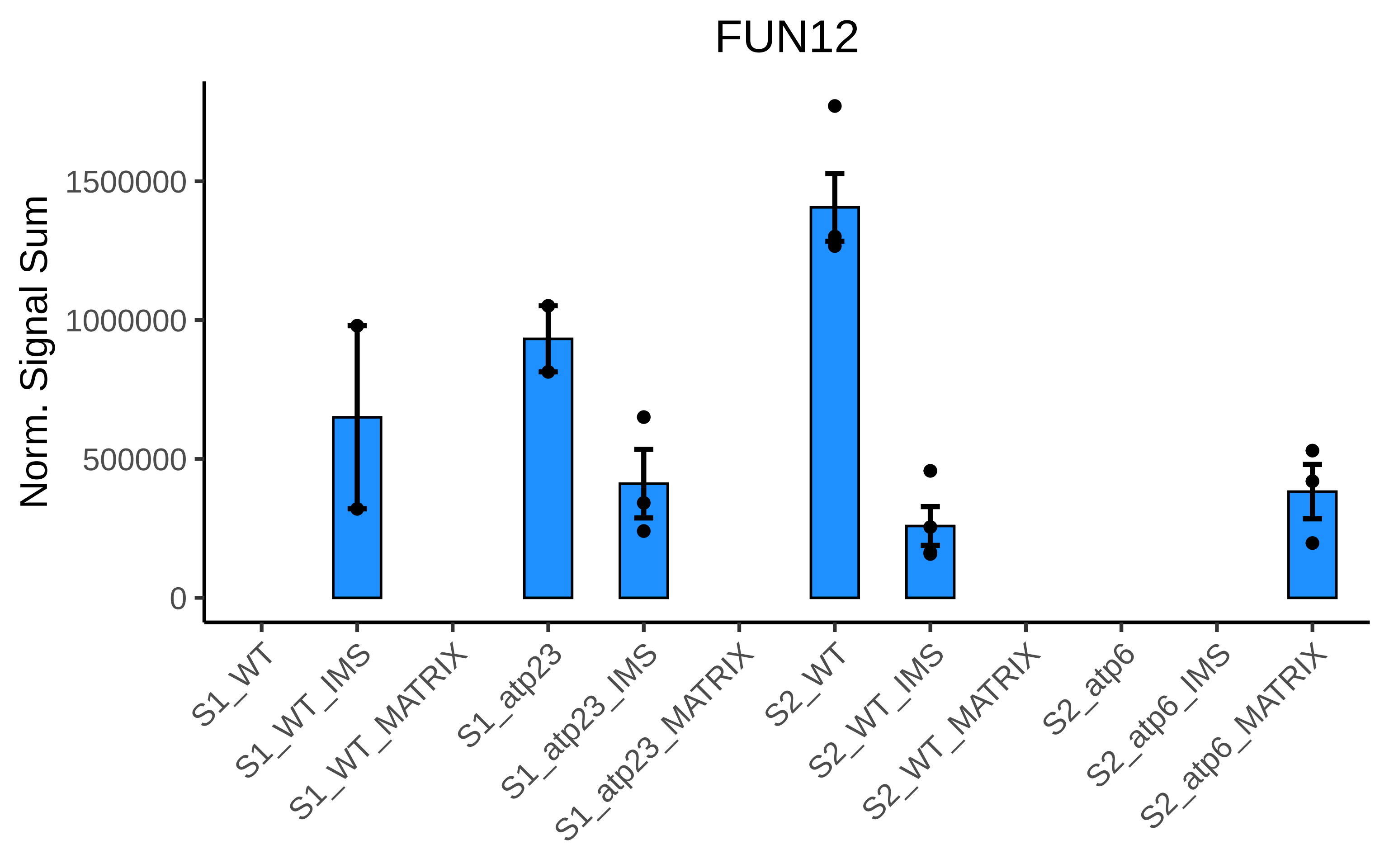

FUN12
Norm. Signal Sum
1500000
1000000
500000
0
S1_WT
S1_WT_IMS
S1_WT_MATRIX
S1_atp23
S1_atp23_IMS
S1_atp23_MATRIX
S2_WT
S2_WT_IMS
S2_WT_MATRIX
S2_atp6
S2_atp6_IMS
S2_atp6_MATRIX

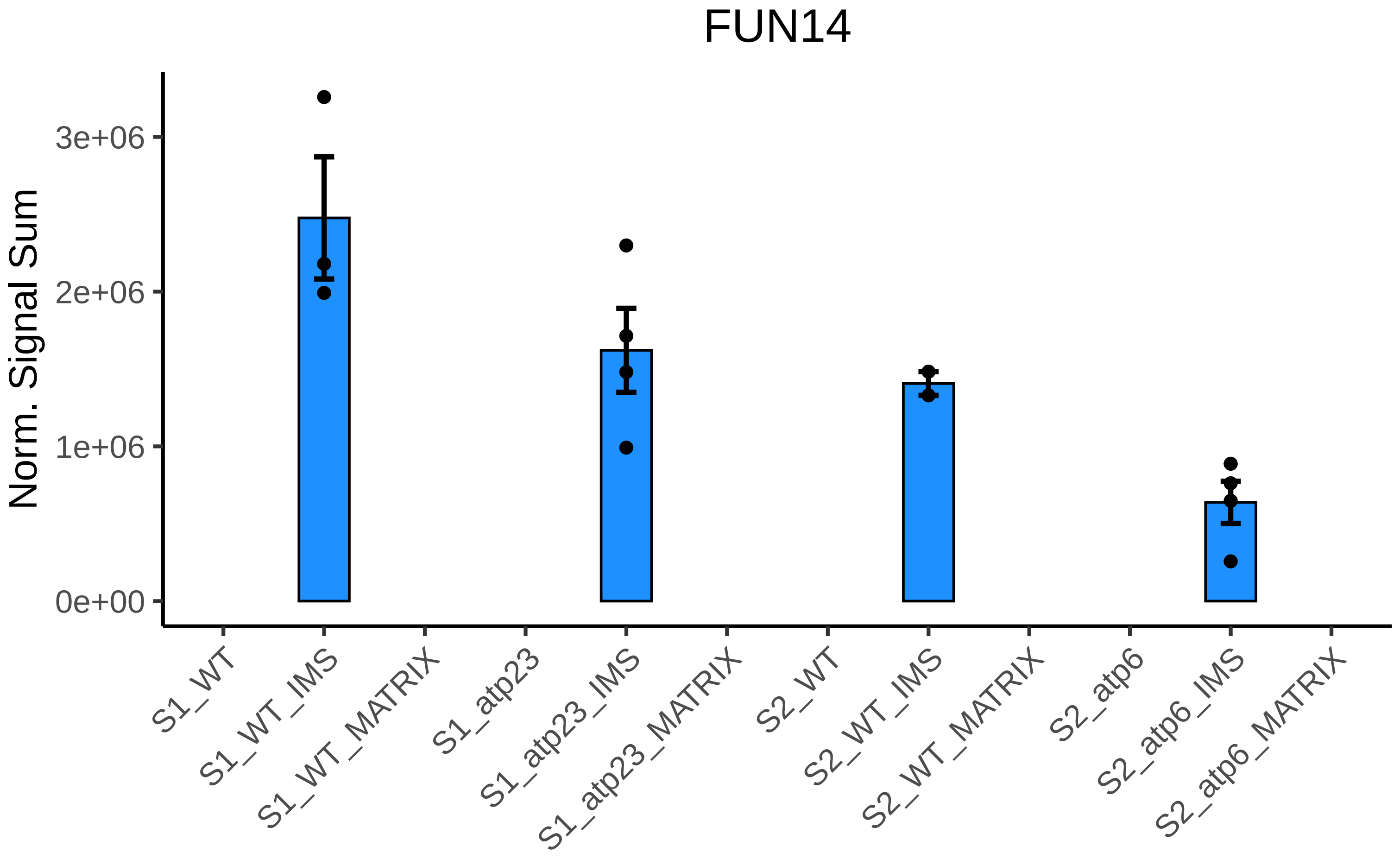

FUN14
Norm. Signal Sum
3e+06
2e+06
1e+06
0e+00
S1_WT
S1_WT_IMS
S1_WT_MATRIX
S1_atp23
S1_atp23_IMS
S1_atp23_MATRIX
S2_WT
S2_WT_IMS
S2_WT_MATRIX
S2_atp6
S2_atp6_IMS
S2_atp6_MATRIX

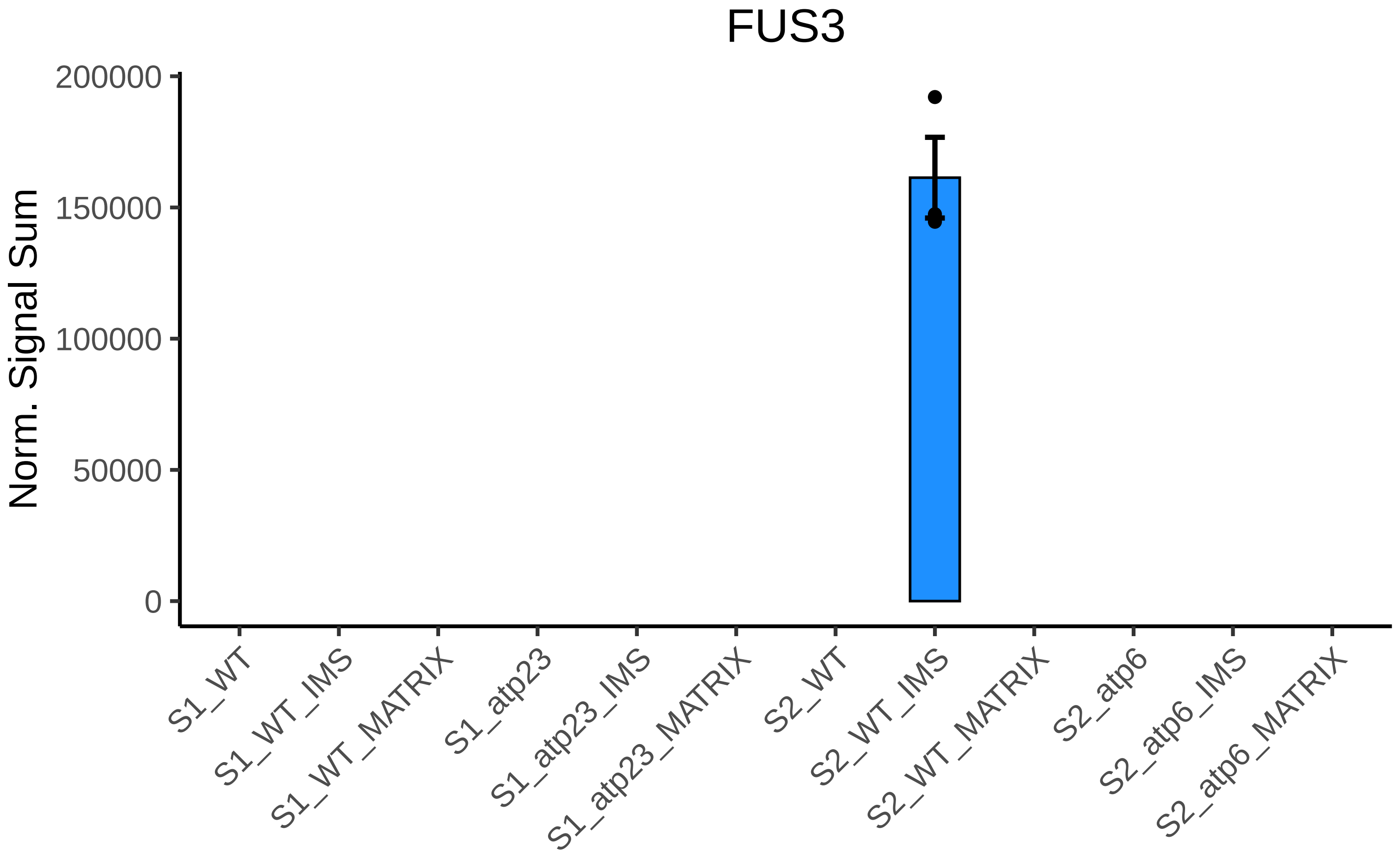

FUS3
Norm. Signal Sum
200000
150000
100000
50000
0
S1_WT
S1_WT_IMS
S1_WT_MATRIX
S1_atp23
S1_atp23_IMS
S1_atp23_MATRIX
S2_WT
S2_WT_IMS
S2_WT_MATRIX
S2_atp6
S2_atp6_IMS
S2_atp6_MATRIX

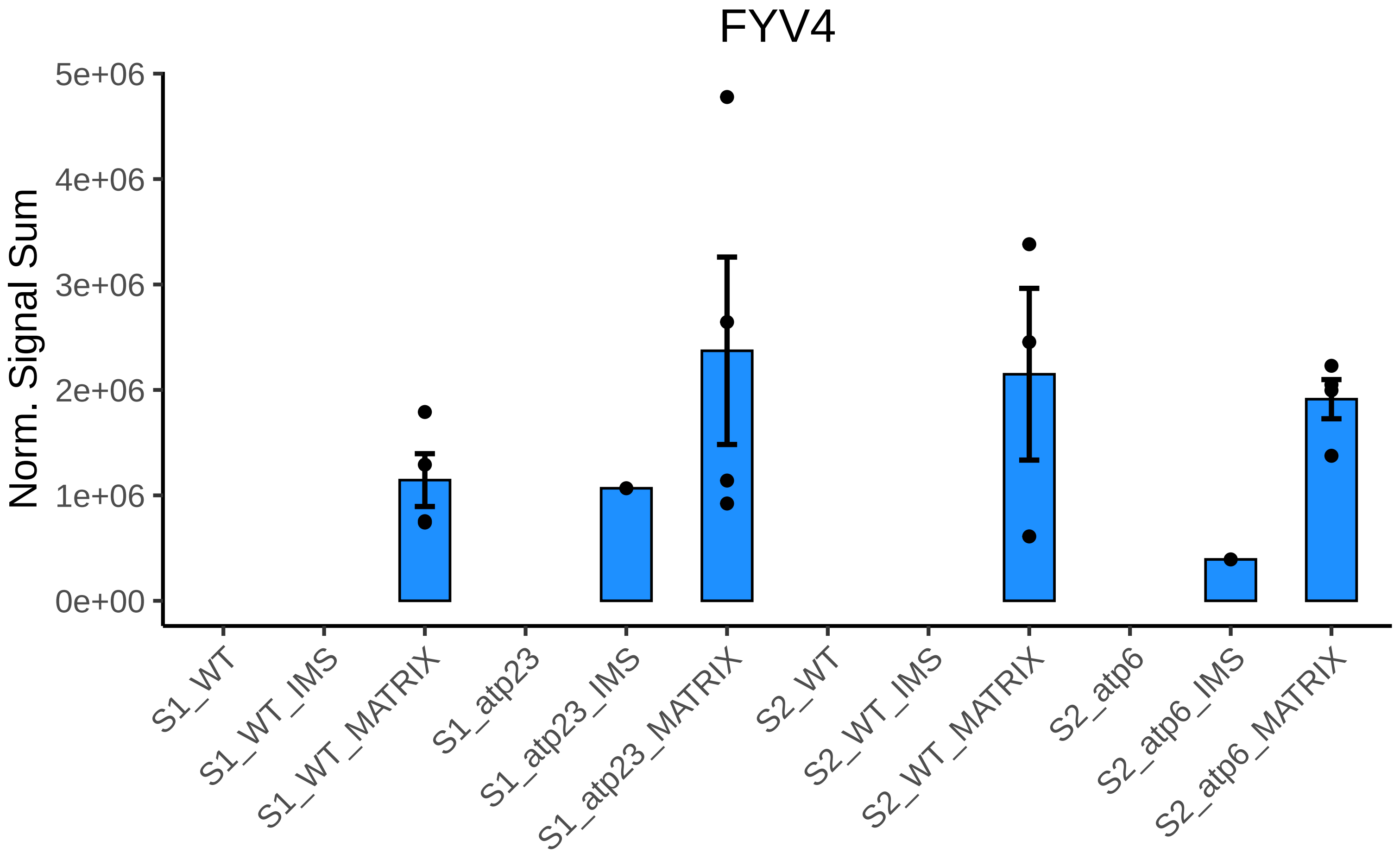

FYV4
Norm. Signal Sum
5e+06
4e+06
3e+06
2e+06
1e+06
0e+00
S1_WT
S1_WT_IMS
S1_WT_MATRIX
S1_atp23
S1_atp23_IMS
S1_atp23_MATRIX
S2_WT
S2_WT_IMS
S2_WT_MATRIX
S2_atp6
S2_atp6_IMS
S2_atp6_MATRIX

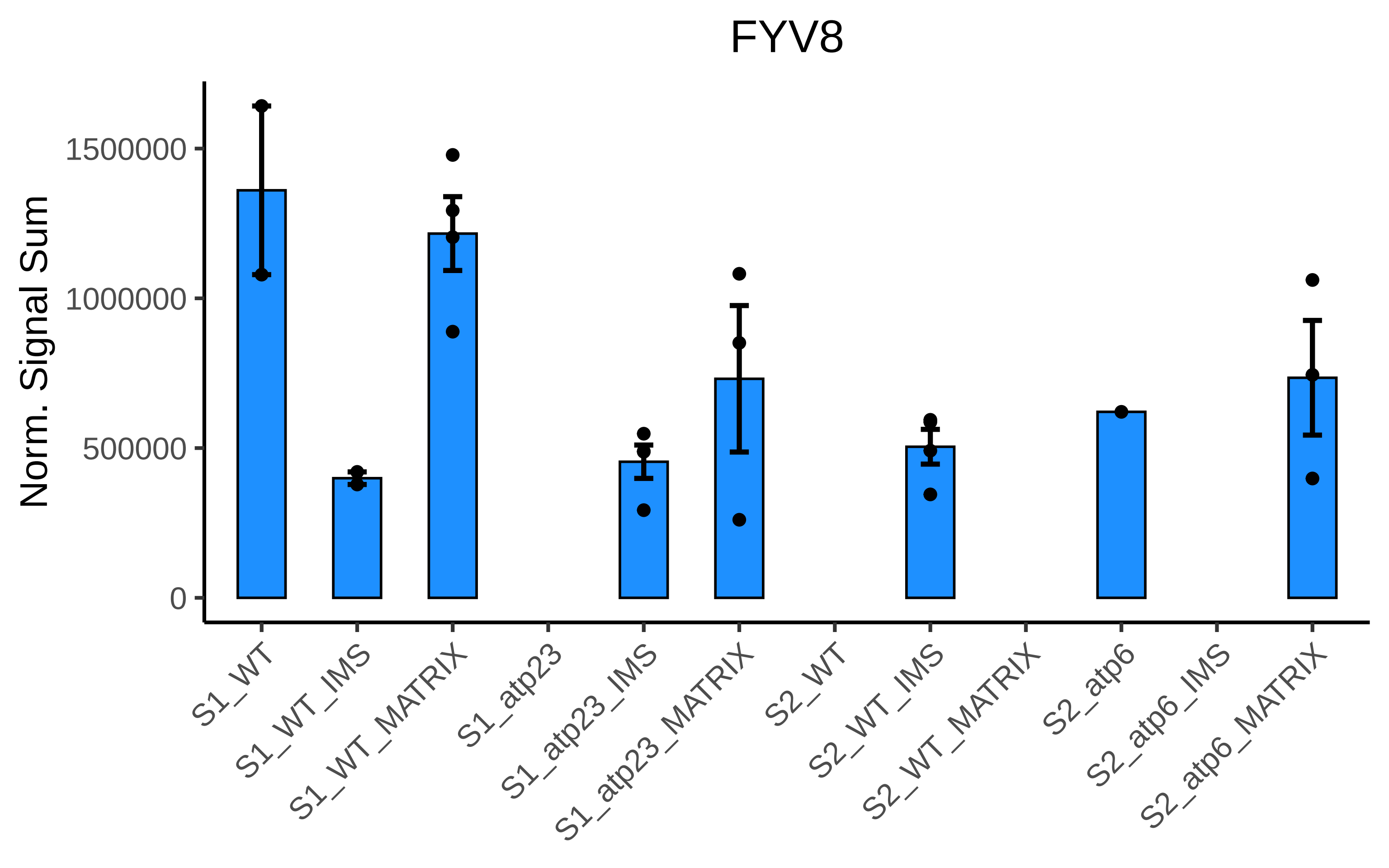
FYV8
Norm. Signal Sum
1500000
1000000
500000
0
S1_WT
S1_WT_IMS
S1_WT_MATRIX
S1_atp23
S1_atp23_IMS
S1_atp23_MATRIX
S2_WT
S2_WT_IMS
S2_WT_MATRIX
S2_atp6
S2_atp6_IMS
S2_atp6_MATRIX

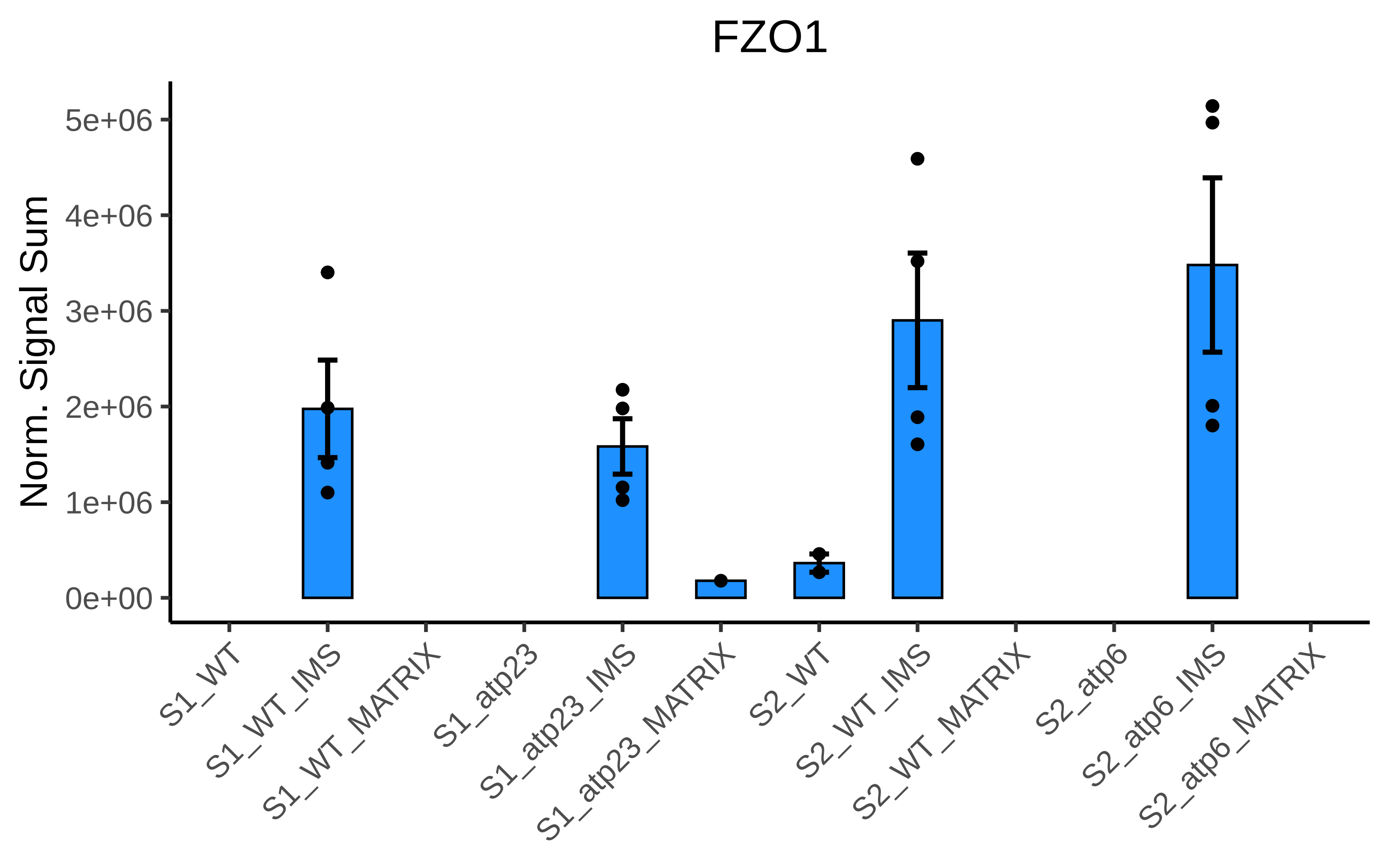
FZO1
Norm. Signal Sum
5e+06
4e+06
3e+06
2e+06
1e+06
0e+00
S1_WT
S1_WT_IMS
S1_WT_MATRIX
S1_atp23
S1_atp23_IMS
S1_atp23_MATRIX
S2_WT
S2_WT_IMS
S2_WT_MATRIX
S2_atp6
S2_atp6_IMS
S2_atp6_MATRIX

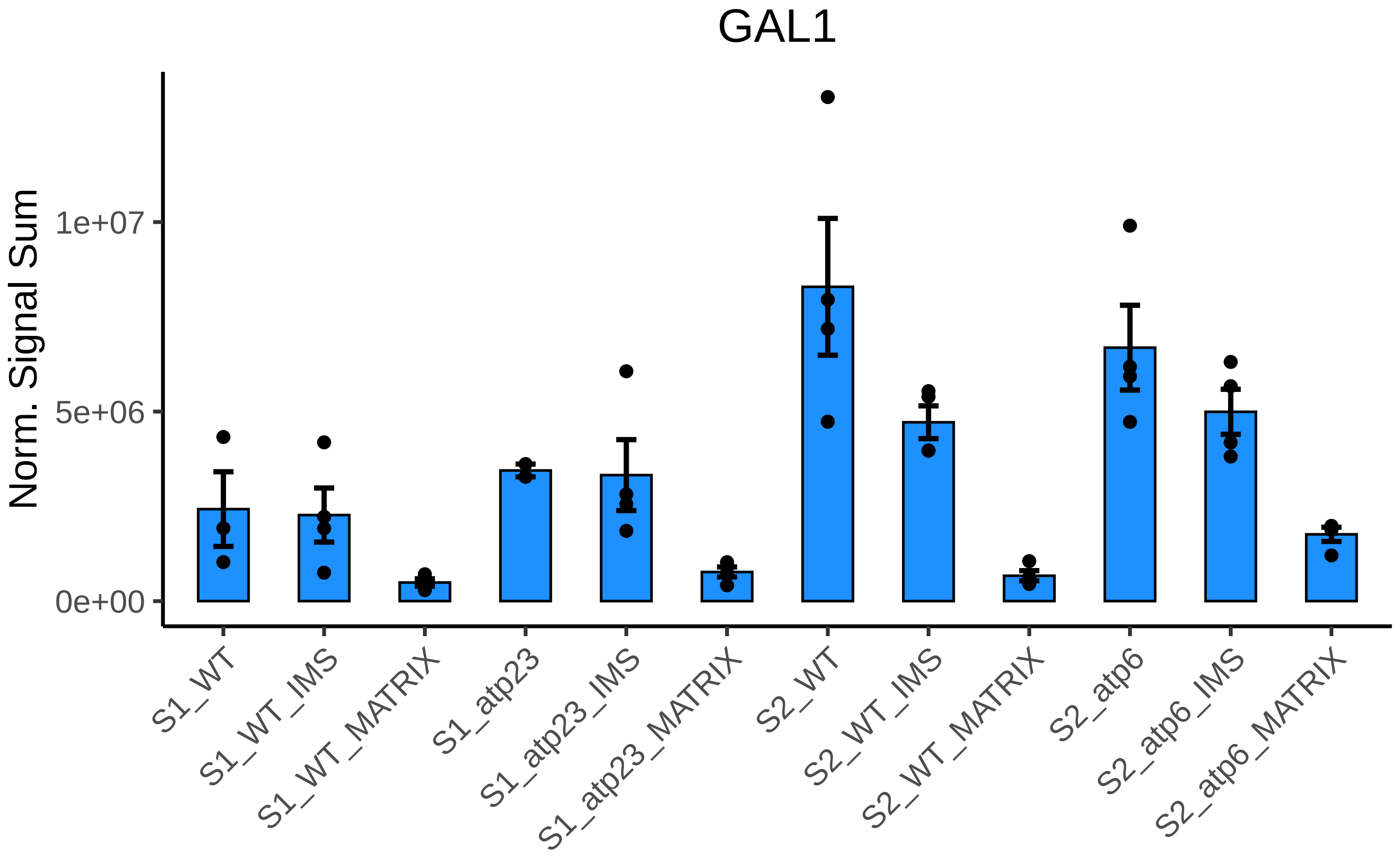
GAL1
Norm. Signal Sum
1e+07
5e+06
0e+00
S1_WT
S1_WT_IMS
S1_WT_MATRIX
S1_atp23
S1_atp23_IMS
S1_atp23_MATRIX
S2_WT
S2_WT_IMS
S2_WT_MATRIX
S2_atp6
S2_atp6_IMS
S2_atp6_MATRIX

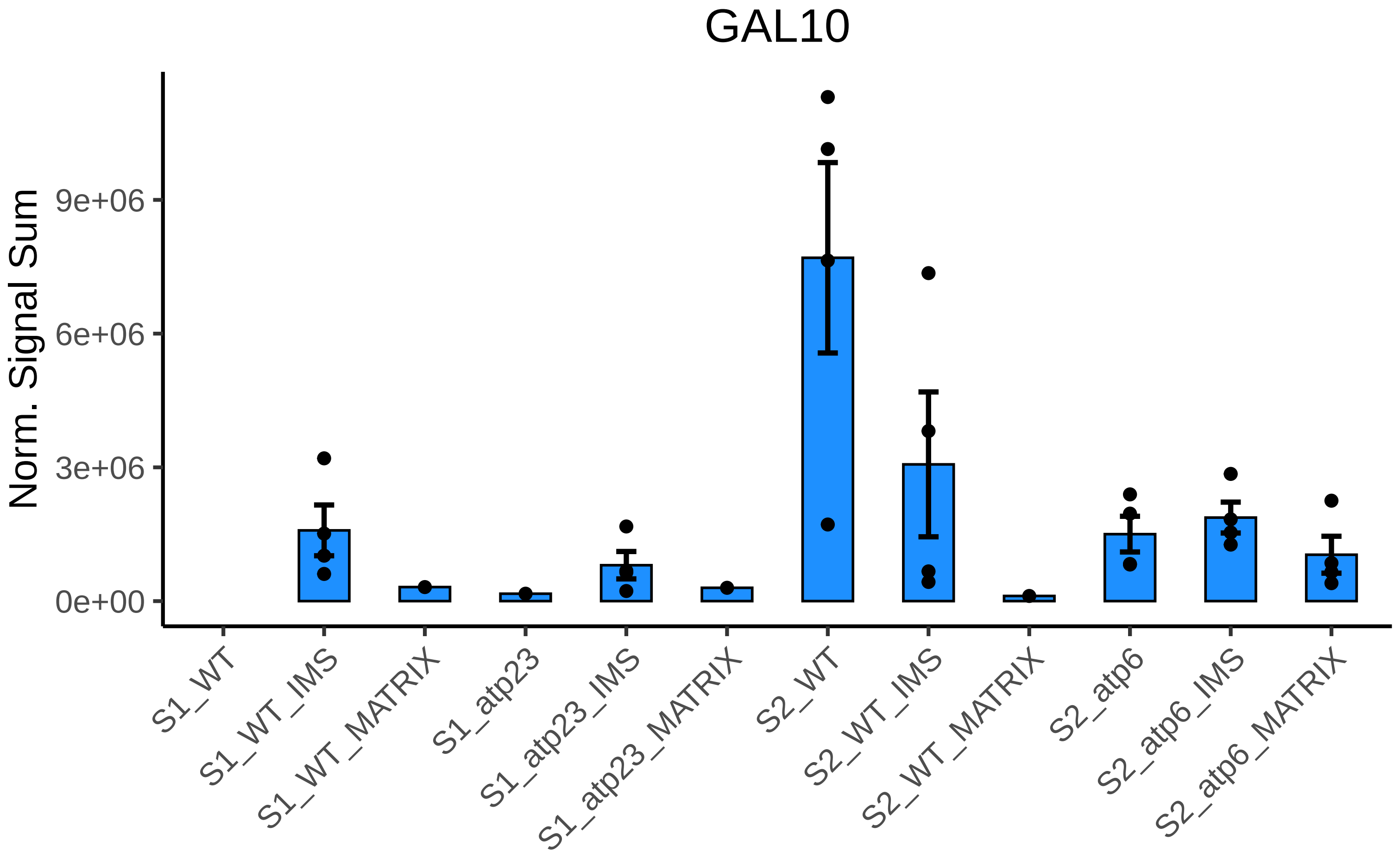
GAL10
Norm. Signal Sum
9e+06
6e+06
3e+06
0e+00
S1_WT
S1_WT_IMS
S1_WT_MATRIX
S1_atp23
S1_atp23_IMS
S1_atp23_MATRIX
S2_WT
S2_WT_IMS
S2_WT_MATRIX
S2_atp6
S2_atp6_IMS
S2_atp6_MATRIX

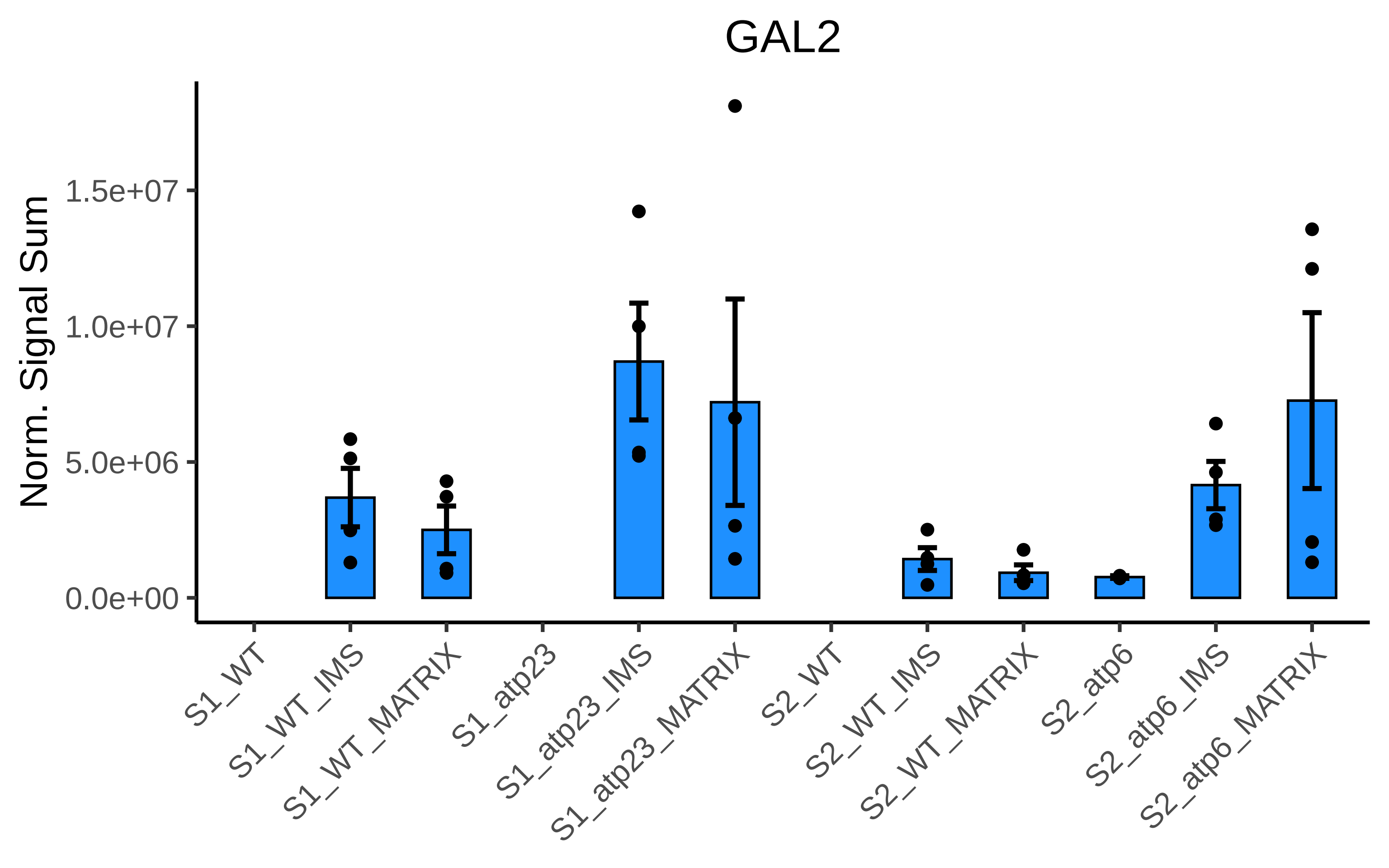
GAL2
Norm. Signal Sum
1.5e+07
1.0e+07
5.0e+06
0.0e+00
S1_WT
S1_WT_IMS
S1_WT_MATRIX
S1_atp23
S1_atp23_IMS
S1_atp23_MATRIX
S2_WT
S2_WT_IMS
S2_WT_MATRIX
S2_atp6
S2_atp6_IMS
S2_atp6_MATRIX

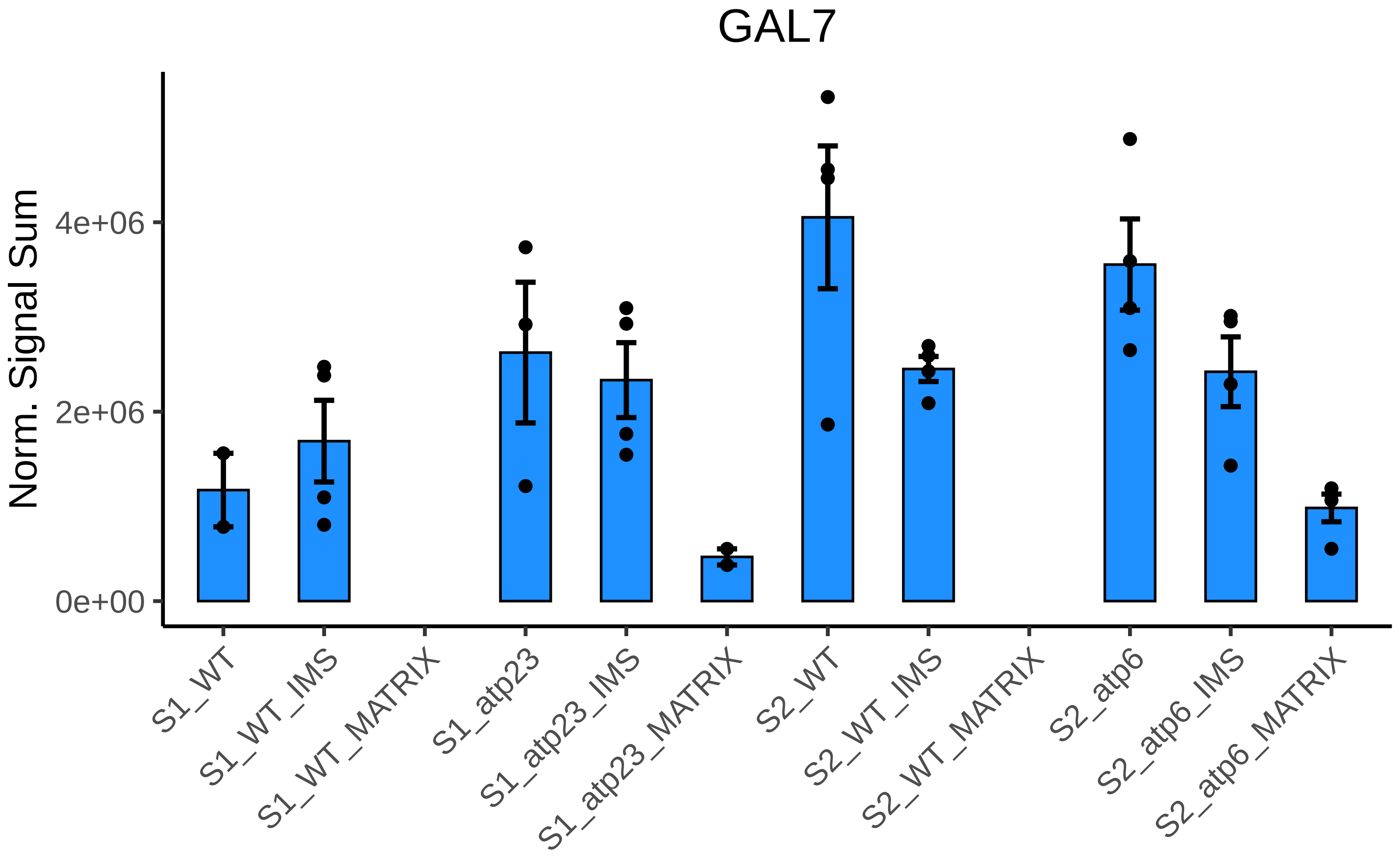
GAL7
Norm. Signal Sum
4e+06
2e+06
0e+00
S1_WT
S1_WT_IMS
S1_WT_MATRIX
S1_atp23
S1_atp23_IMS
S1_atp23_MATRIX
S2_WT
S2_WT_IMS
S2_WT_MATRIX
S2_atp6
S2_atp6_IMS
S2_atp6_MATRIX

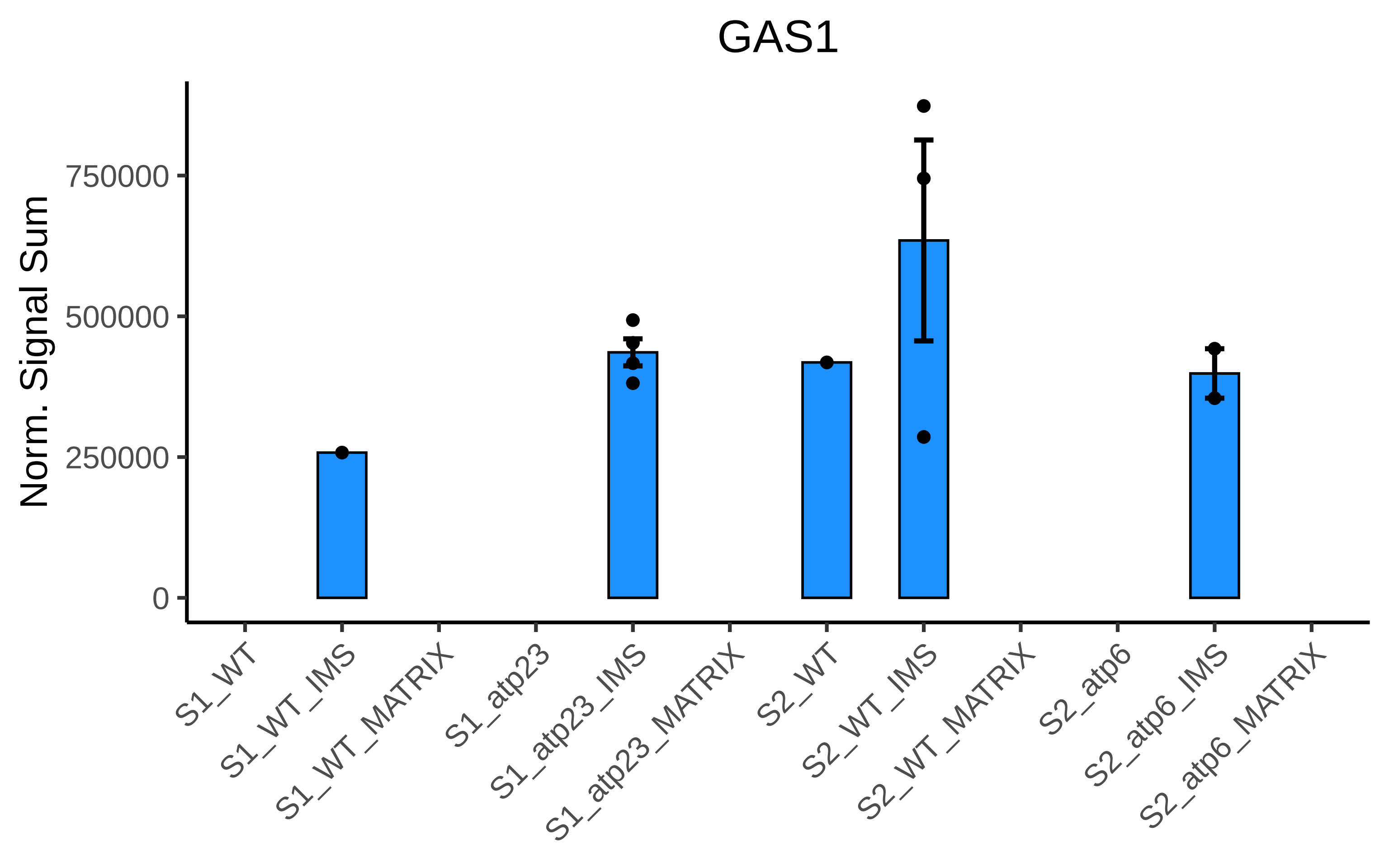

GAS1
Norm. Signal Sum
750000
500000
250000
0
S1_WT
S1_WT_IMS
S1_WT_MATRIX
S1_atp23
S1_atp23_IMS
S1_atp23_MATRIX
S2_WT
S2_WT_IMS
S2_WT_MATRIX
S2_atp6
S2_atp6_IMS
S2_atp6_MATRIX

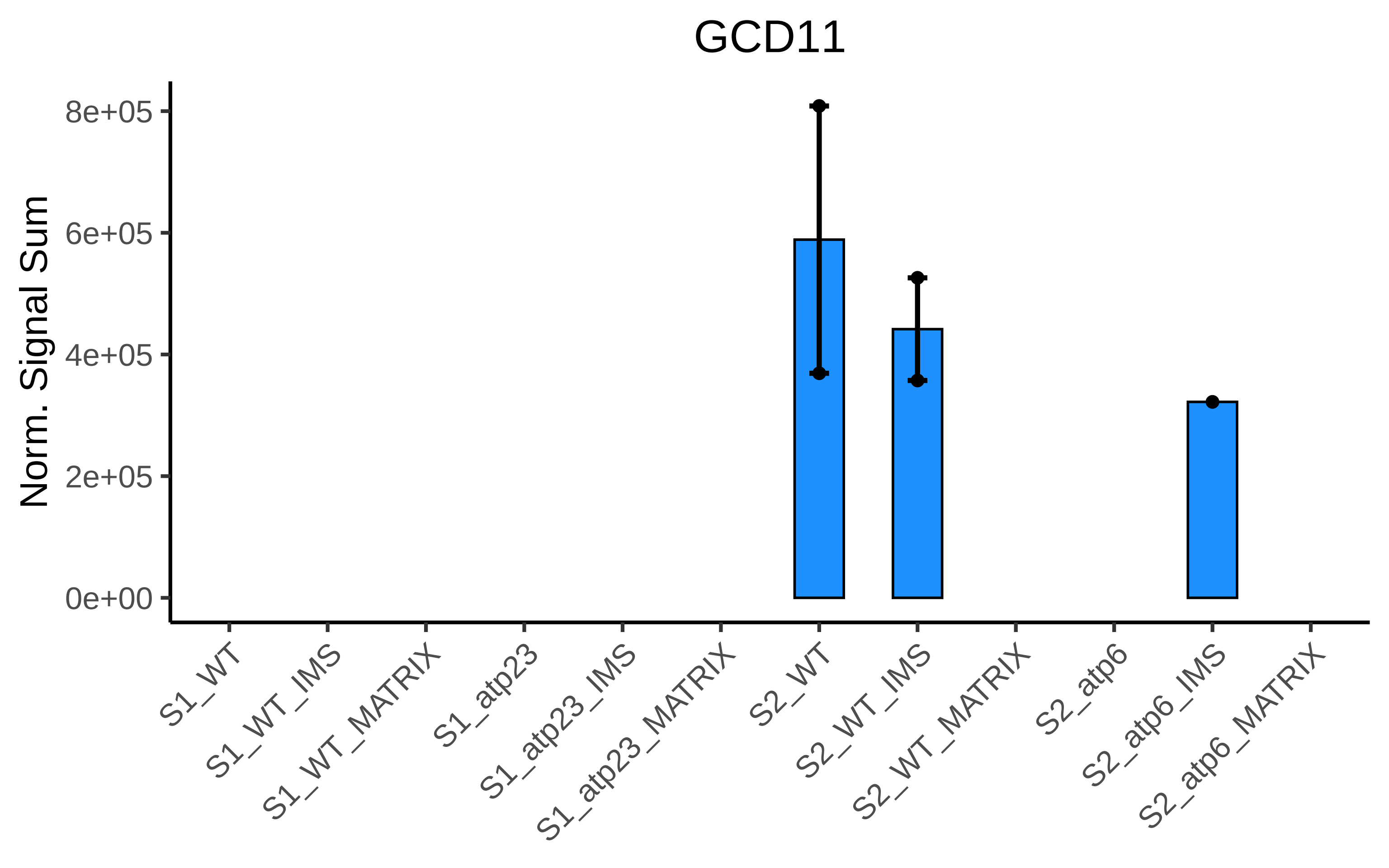
GCD11
Norm. Signal Sum
8e+05
6e+05
4e+05
2e+05
0e+00
S1_WT
S1_WT_IMS
S1_WT_MATRIX
S1_atp23
S1_atp23_IMS
S1_atp23_MATRIX
S2_WT
S2_WT_IMS
S2_WT_MATRIX
S2_atp6
S2_atp6_IMS
S2_atp6_MATRIX

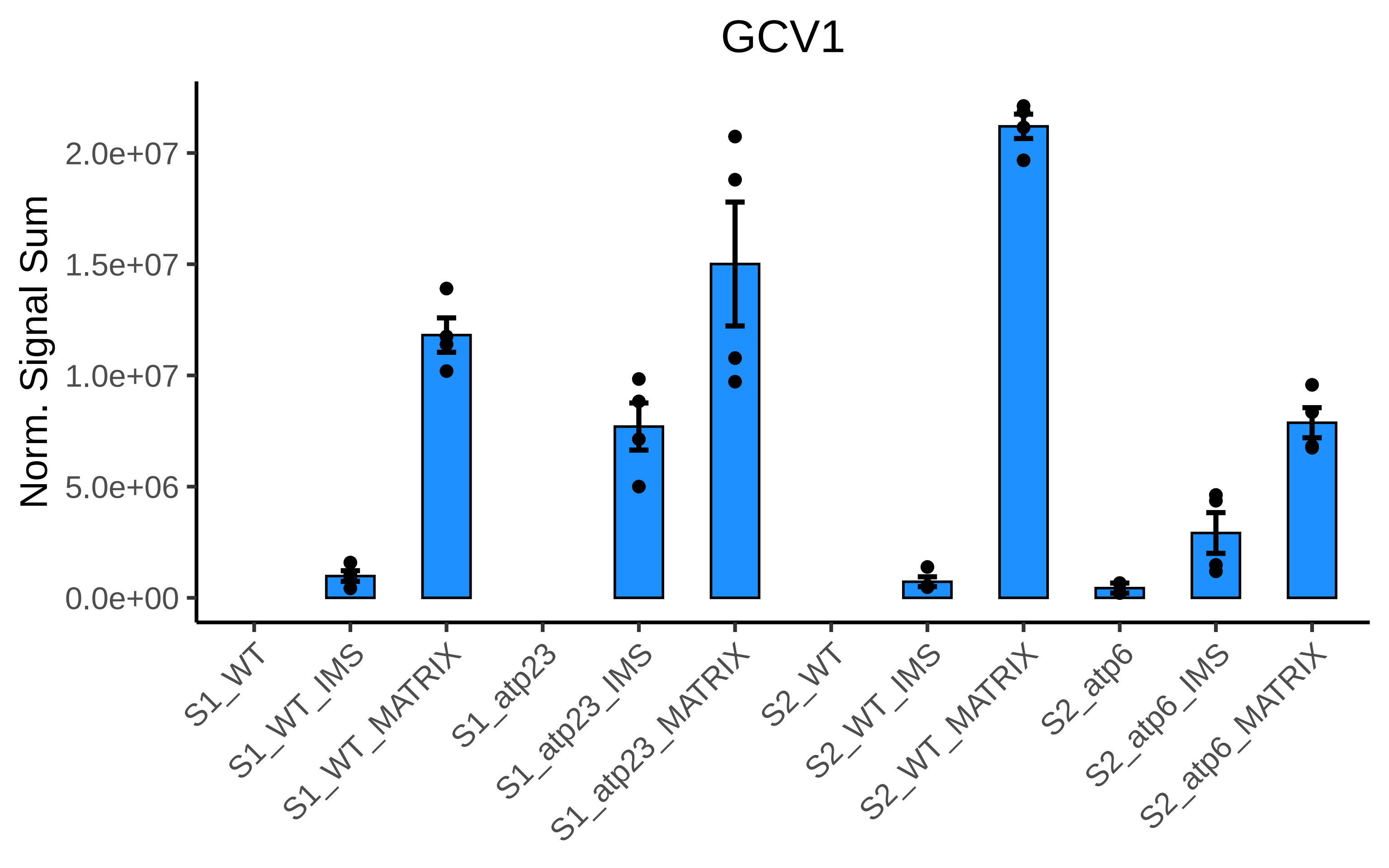
GCV1
Norm. Signal Sum
2.0e+07
1.5e+07
1.0e+07
5.0e+06
0.0e+00
S1_WT
S1_WT_IMS
S1_WT_MATRIX
S1_atp23
S1_atp23_IMS
S1_atp23_MATRIX
S2_WT
S2_WT_IMS
S2_WT_MATRIX
S2_atp6
S2_atp6_IMS
S2_atp6_MATRIX

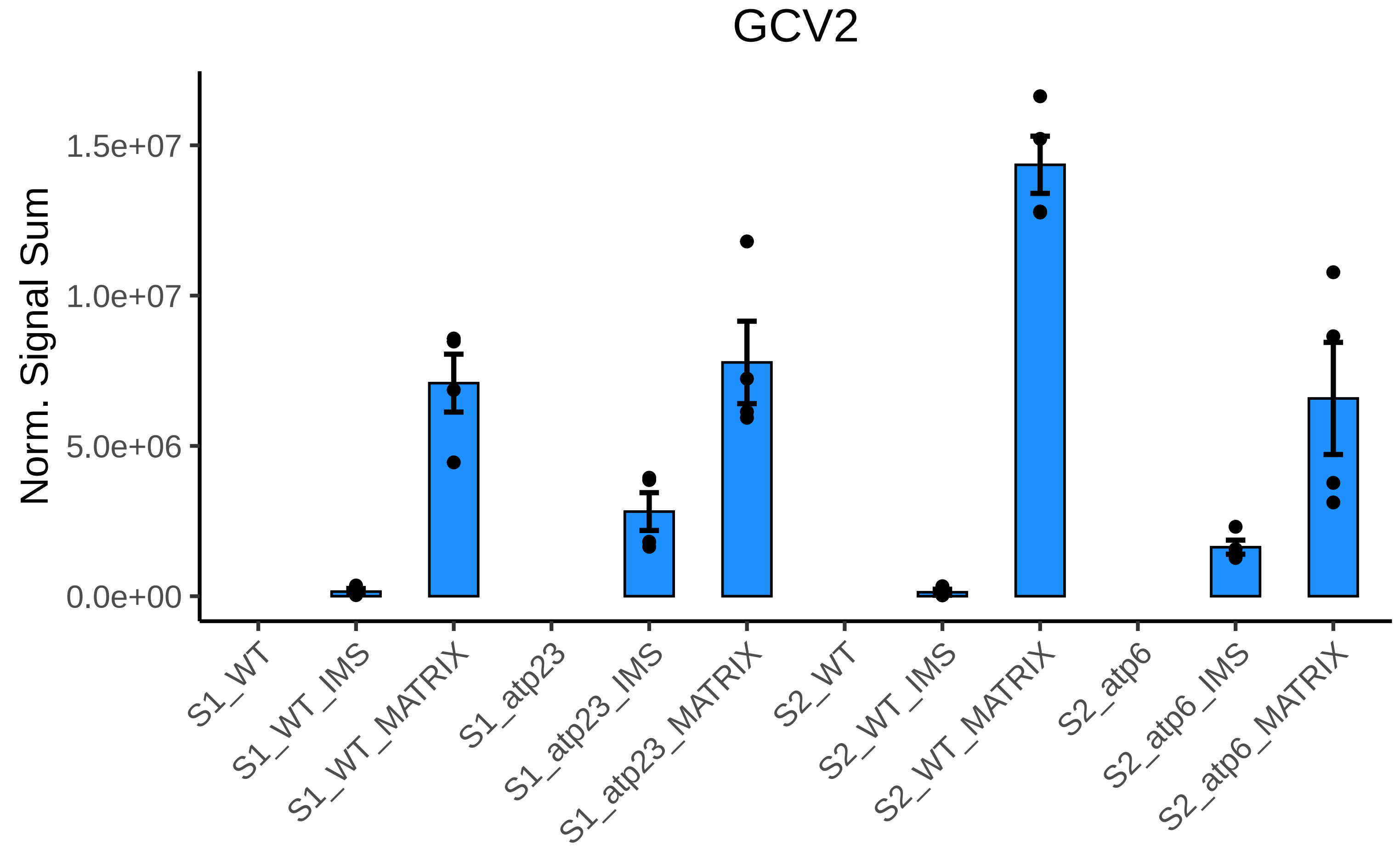
GCV2
Norm. Signal Sum
1.5e+07
1.0e+07
5.0e+06
0.0e+00
S1_WT
S1_WT_IMS
S1_WT_MATRIX
S1_atp23
S1_atp23_IMS
S1_atp23_MATRIX
S2_WT
S2_WT_IMS
S2_WT_MATRIX
S2_atp6
S2_atp6_IMS
S2_atp6_MATRIX

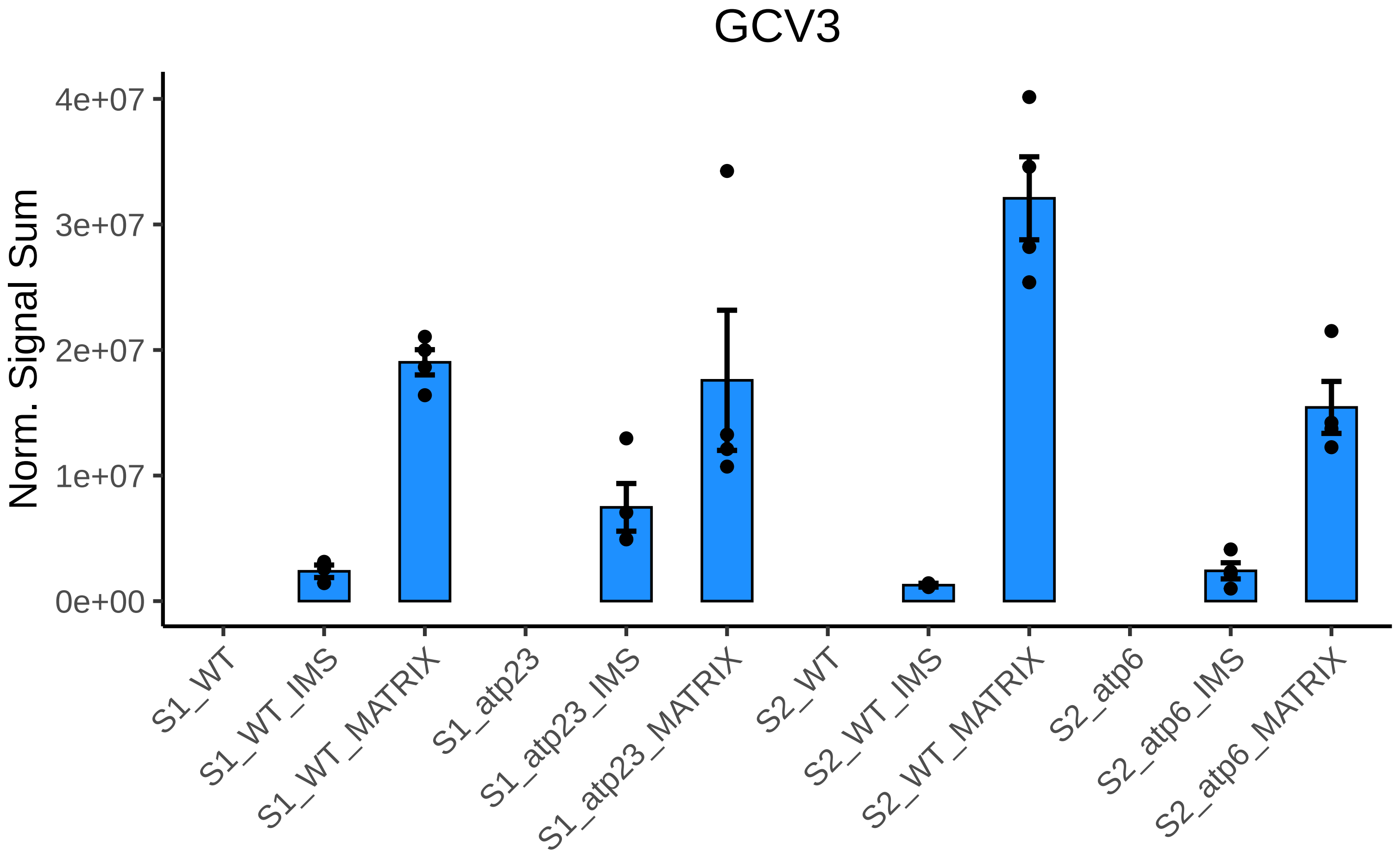
GCV3
Norm. Signal Sum
4e+07
3e+07
2e+07
1e+07
0e+00
S1_WT
S1_WT_IMS
S1_WT_MATRIX
S1_atp23
S1_atp23_IMS
S1_atp23_MATRIX
S2_WT
S2_WT_IMS
S2_WT_MATRIX
S2_atp6
S2_atp6_IMS
S2_atp6_MATRIX

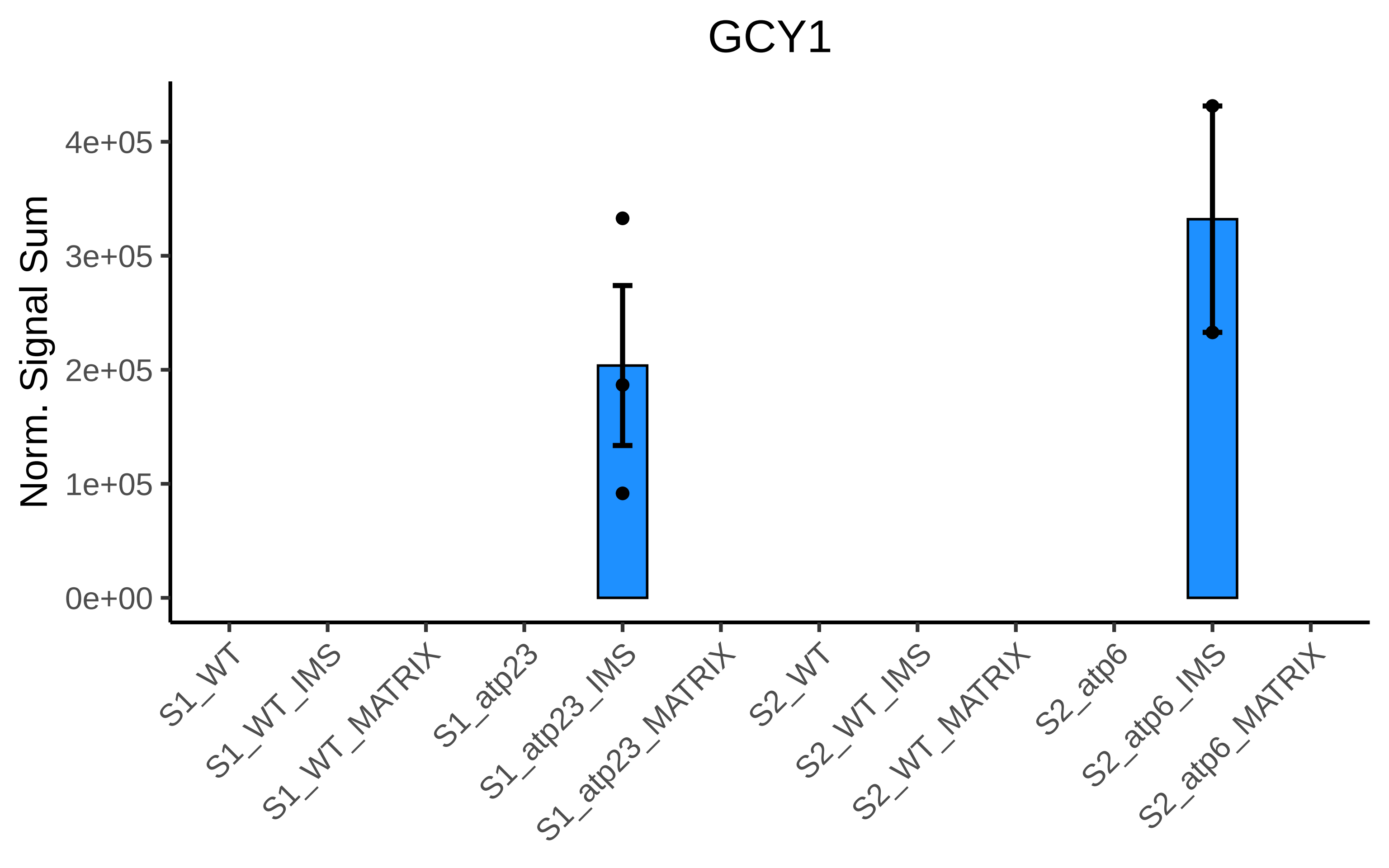
GCY1
Norm. Signal Sum
4e+05
3e+05
2e+05
1e+05
0e+00
S1_WT
S1_WT_IMS
S1_WT_MATRIX
S1_atp23
S1_atp23_IMS
S1_atp23_MATRIX
S2_WT
S2_WT_IMS
S2_WT_MATRIX
S2_atp6
S2_atp6_IMS
S2_atp6_MATRIX

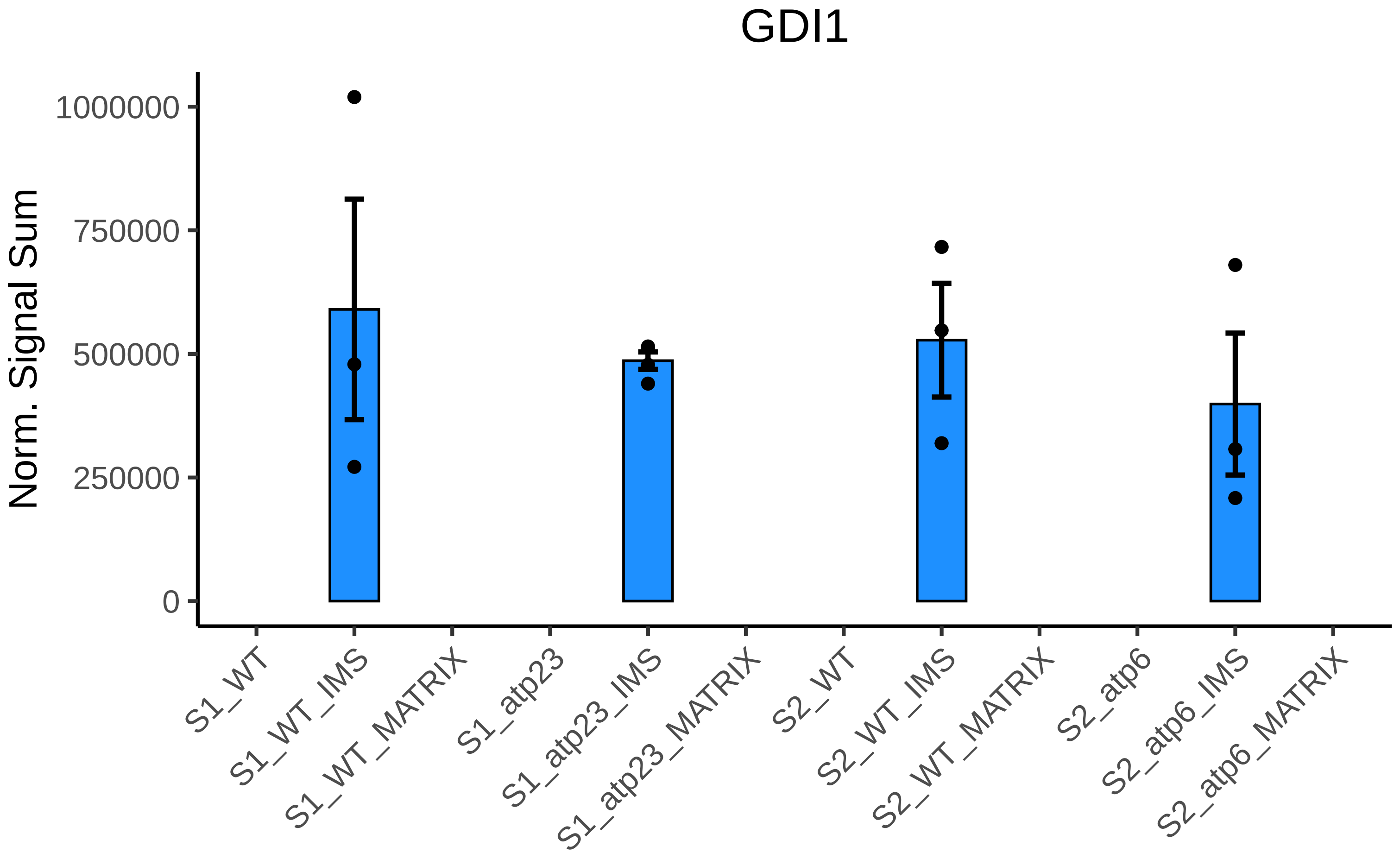

GDI1
Norm. Signal Sum
1000000
750000
500000
250000
0
S1_WT
S1_WT_IMS
S1_WT_MATRIX
S1_atp23
S1_atp23_IMS
S1_atp23_MATRIX
S2_WT
S2_WT_IMS
S2_WT_MATRIX
S2_atp6
S2_atp6_IMS
S2_atp6_MATRIX

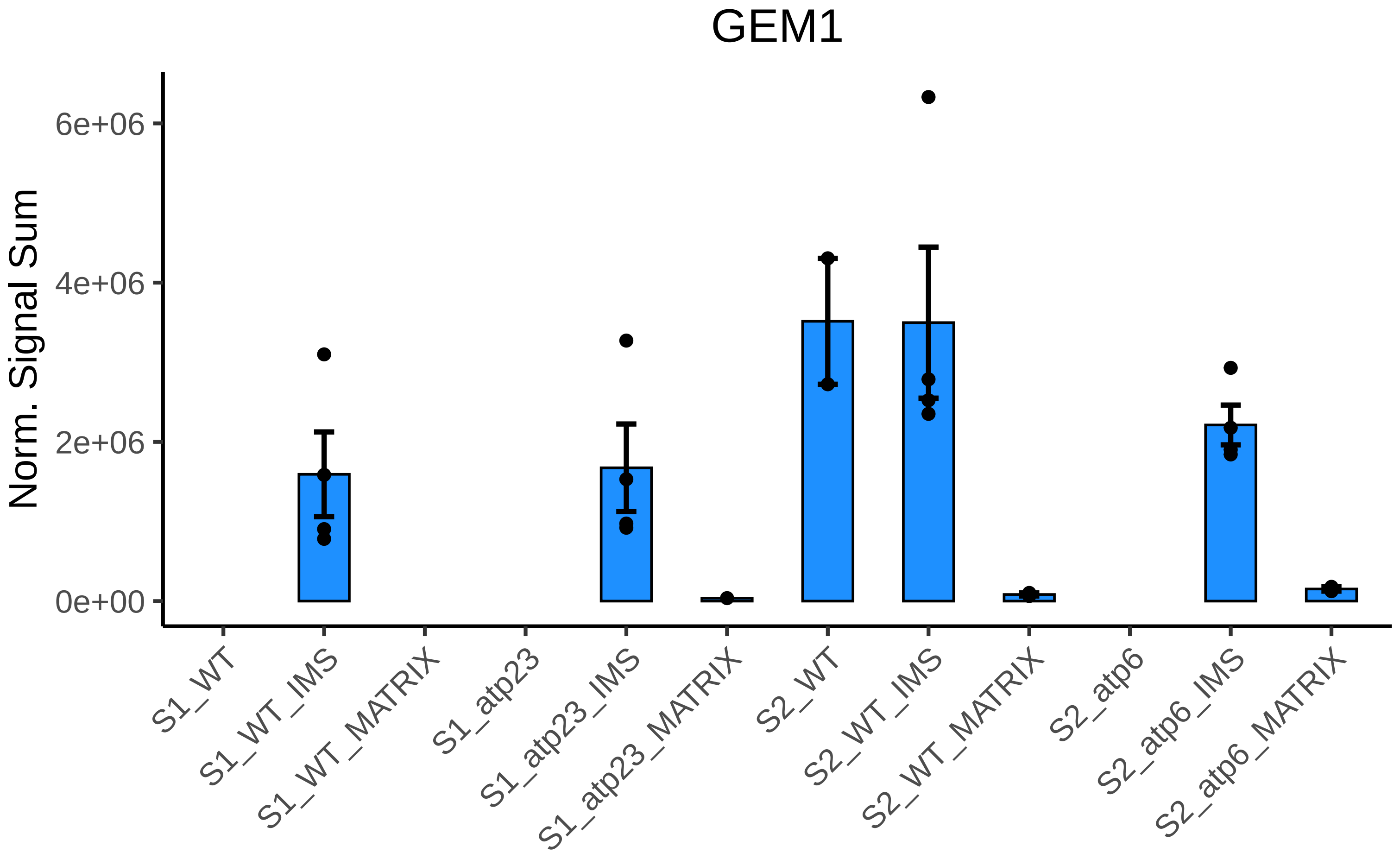

GEM1
Norm. Signal Sum
6e+06
4e+06
2e+06
0e+00
S1_WT
S1_WT_IMS
S1_WT_MATRIX
S1_atp23
S1_atp23_IMS
S1_atp23_MATRIX
S2_WT
S2_WT_IMS
S2_WT_MATRIX
S2_atp6
S2_atp6_IMS
S2_atp6_MATRIX

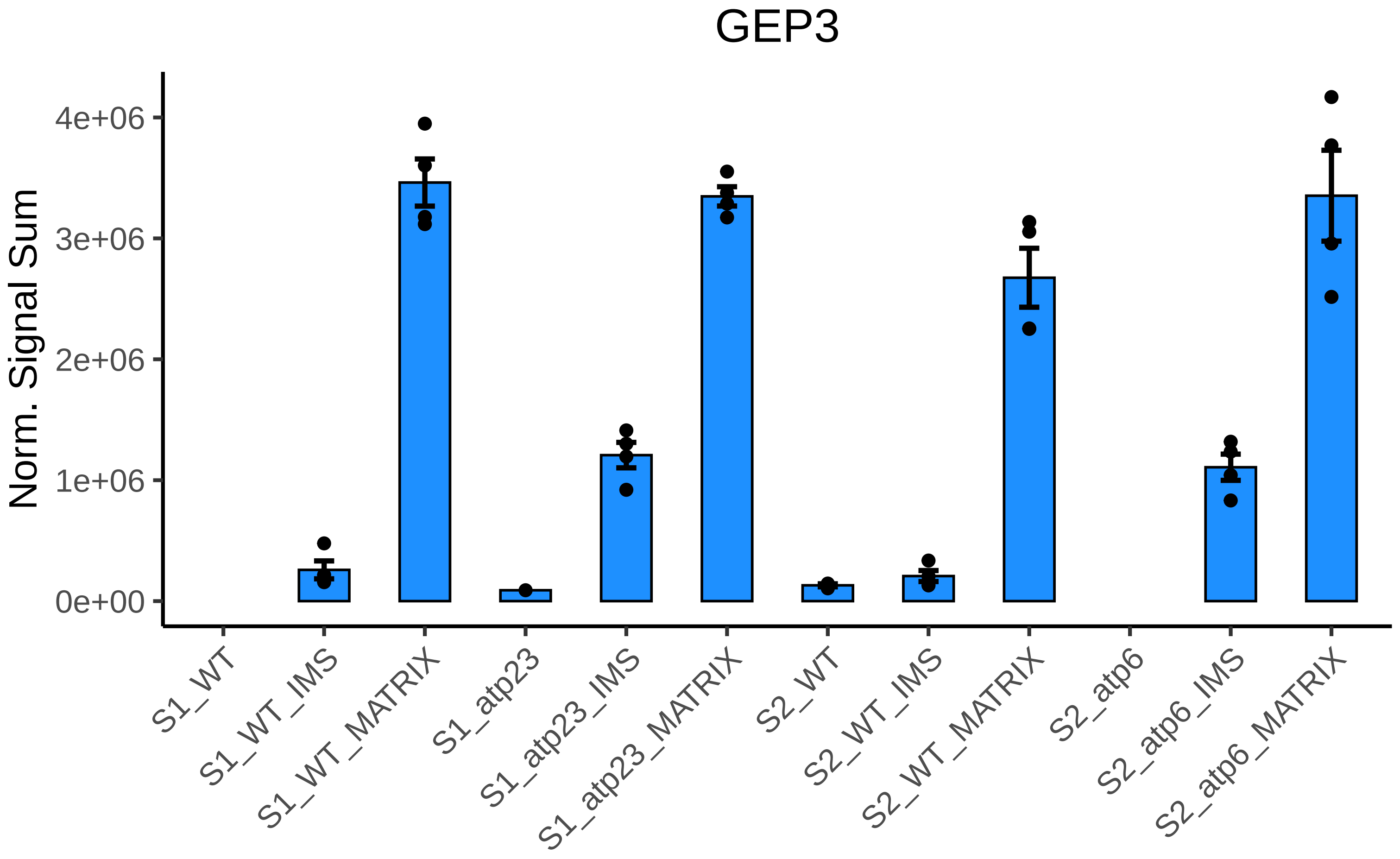

GEP3
Norm. Signal Sum
4e+06
3e+06
2e+06
1e+06
0e+00
S1_WT
S1_WT_IMS
S1_WT_MATRIX
S1_atp23
S1_atp23_IMS
S1_atp23_MATRIX
S2_WT
S2_WT_IMS
S2_WT_MATRIX
S2_atp6
S2_atp6_IMS
S2_atp6_MATRIX

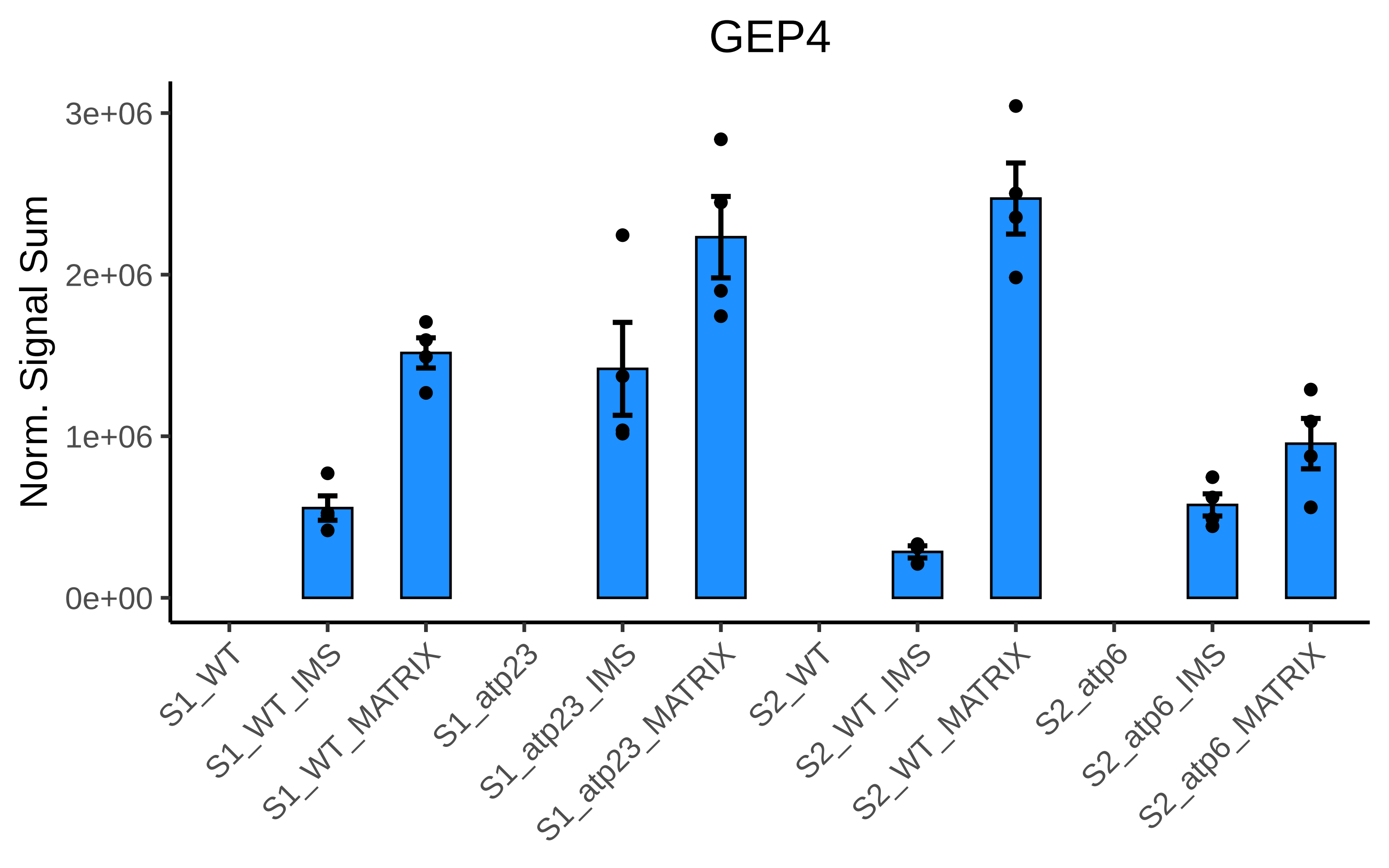
GEP4
Norm. Signal Sum
3e+06
2e+06
1e+06
0e+00
S1_WT
S1_WT_IMS
S1_WT_MATRIX
S1_atp23
S1_atp23_IMS
S1_atp23_MATRIX
S2_WT
S2_WT_IMS
S2_WT_MATRIX
S2_atp6
S2_atp6_IMS
S2_atp6_MATRIX

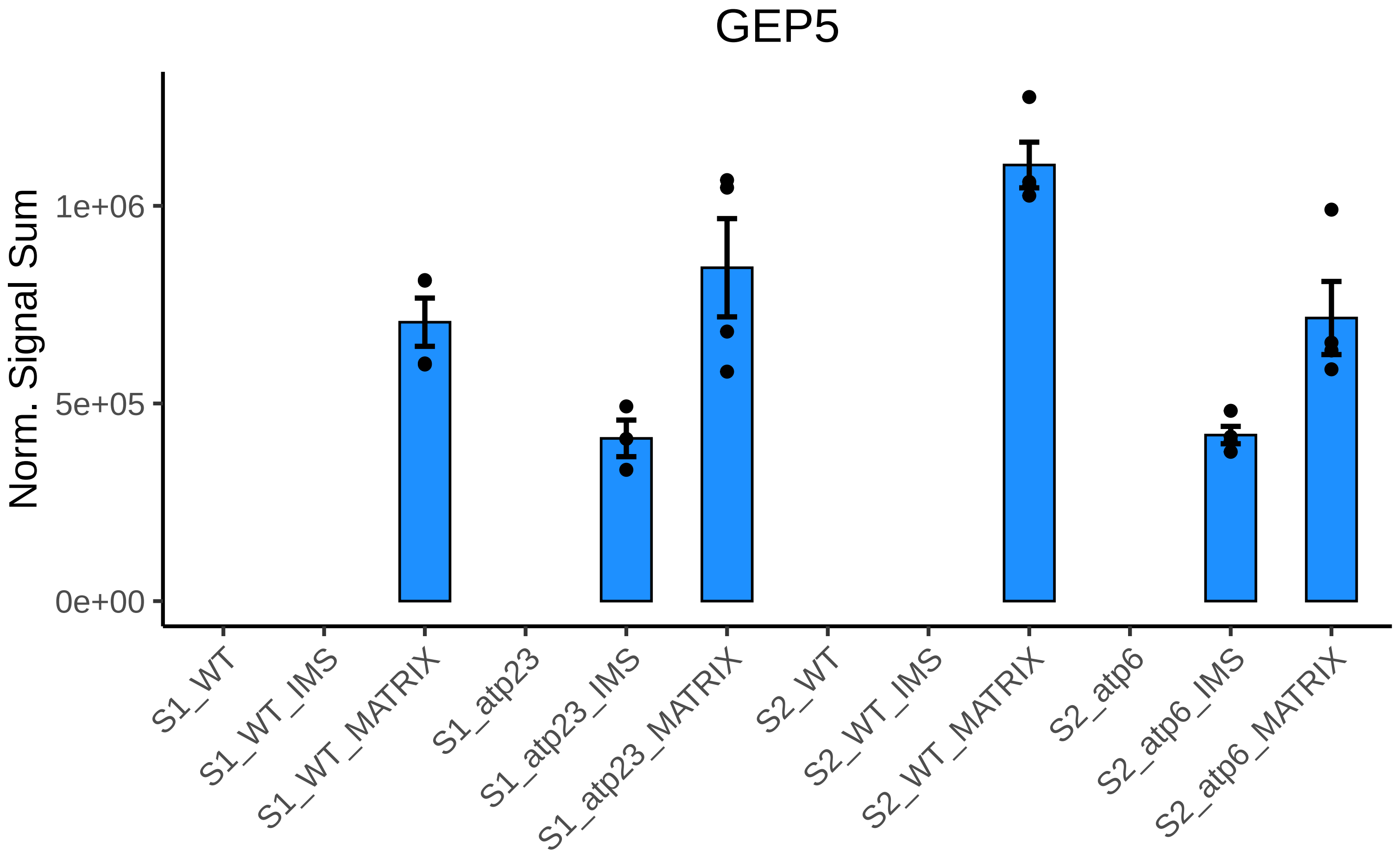

GEP5
Norm. Signal Sum
1e+06
5e+05
0e+00
S1_WT
S1_WT_IMS
S1_WT_MATRIX
S1_atp23
S1_atp23_IMS
S1_atp23_MATRIX
S2_WT
S2_WT_IMS
S2_WT_MATRIX
S2_atp6
S2_atp6_IMS
S2_atp6_MATRIX

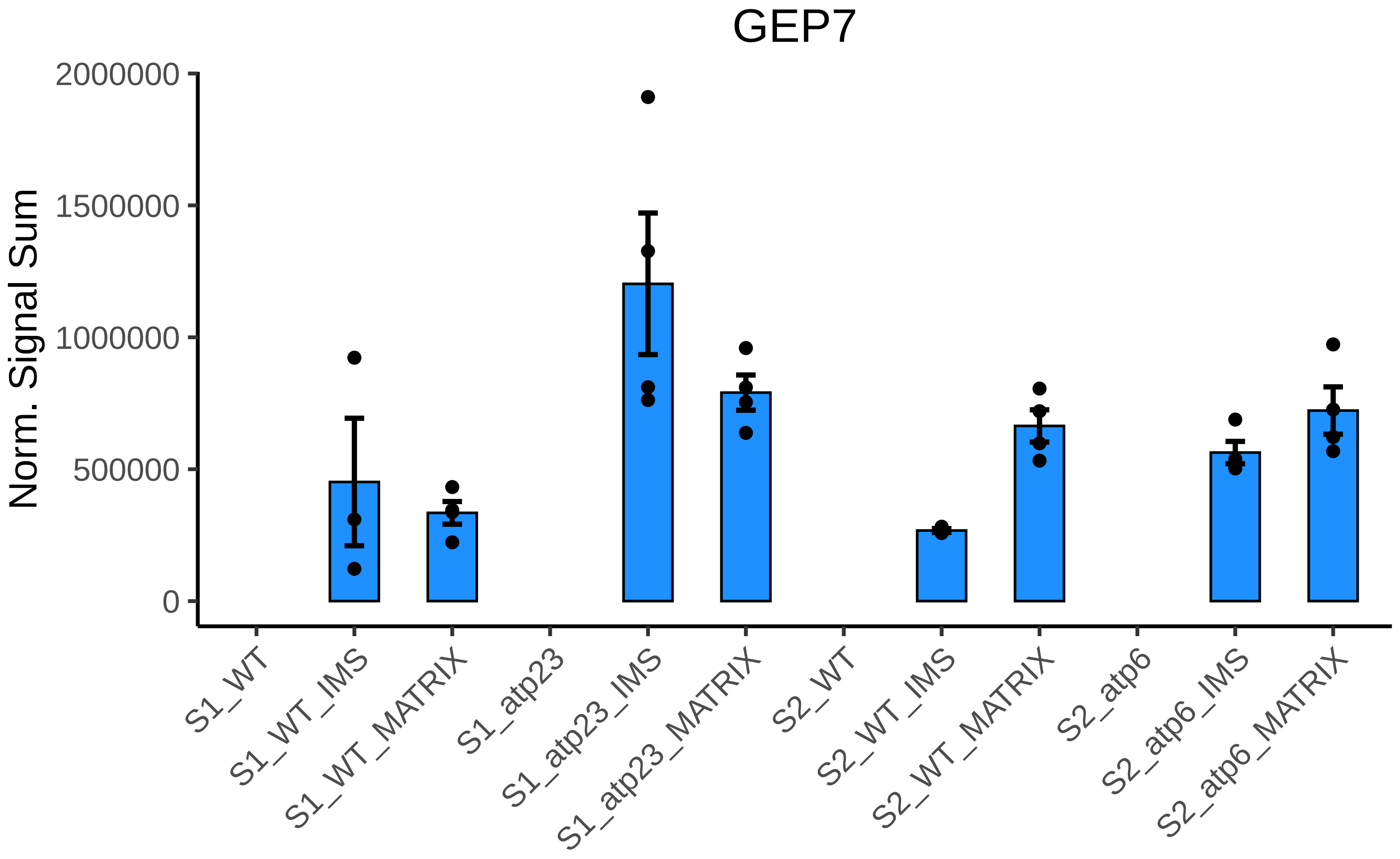

GEP7
Norm. Signal Sum
2000000
1500000
1000000
500000
0
S1_WT
S1_WT_IMS
S1_WT_MATRIX
S1_atp23
S1_atp23_IMS
S1_atp23_MATRIX
S2_WT
S2_WT_IMS
S2_WT_MATRIX
S2_atp6
S2_atp6_IMS
S2_atp6_MATRIX

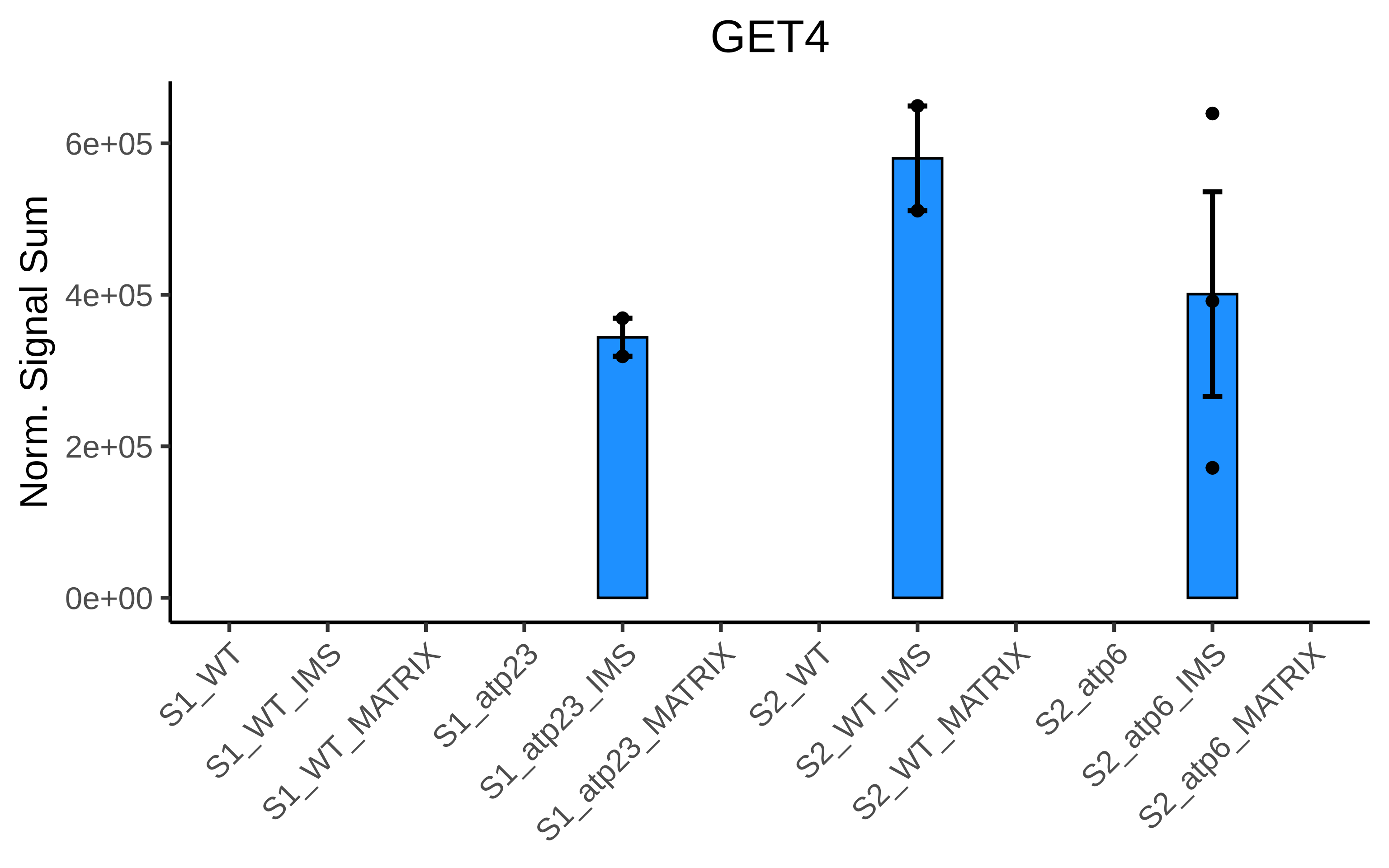
GET4
Norm. Signal Sum
6e+05
4e+05
2e+05
0e+00
S1_WT
S1_WT_IMS
S1_WT_MATRIX
S1_atp23
S1_atp23_IMS
S1_atp23_MATRIX
S2_WT
S2_WT_IMS
S2_WT_MATRIX
S2_atp6
S2_atp6_IMS
S2_atp6_MATRIX

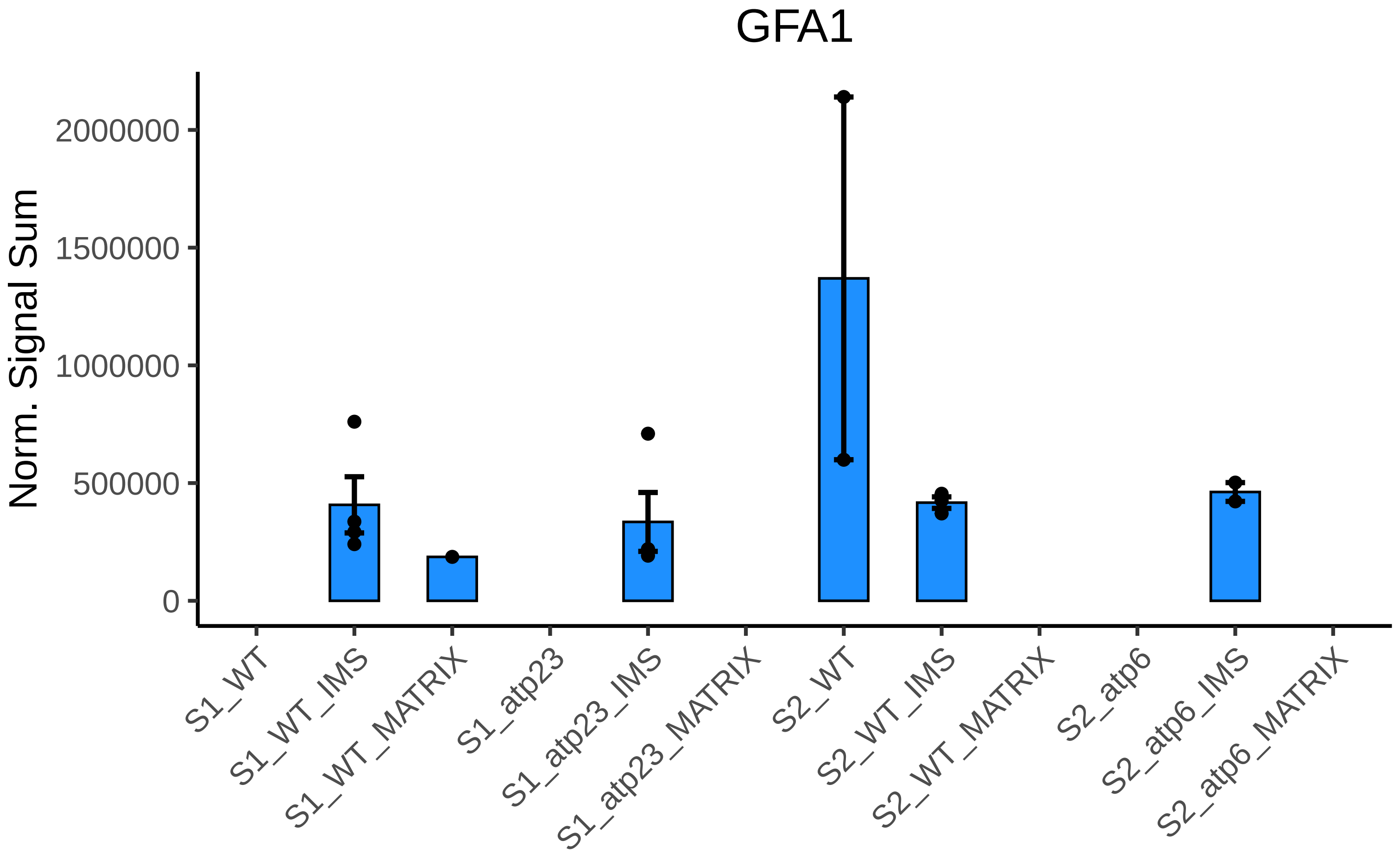
GFA1
Norm. Signal Sum
2000000
1500000
1000000
500000
0
S1_WT
S1_WT_IMS
S1_WT_MATRIX
S1_atp23
S1_atp23_IMS
S1_atp23_MATRIX
S2_WT
S2_WT_IMS
S2_WT_MATRIX
S2_atp6
S2_atp6_IMS
S2_atp6_MATRIX

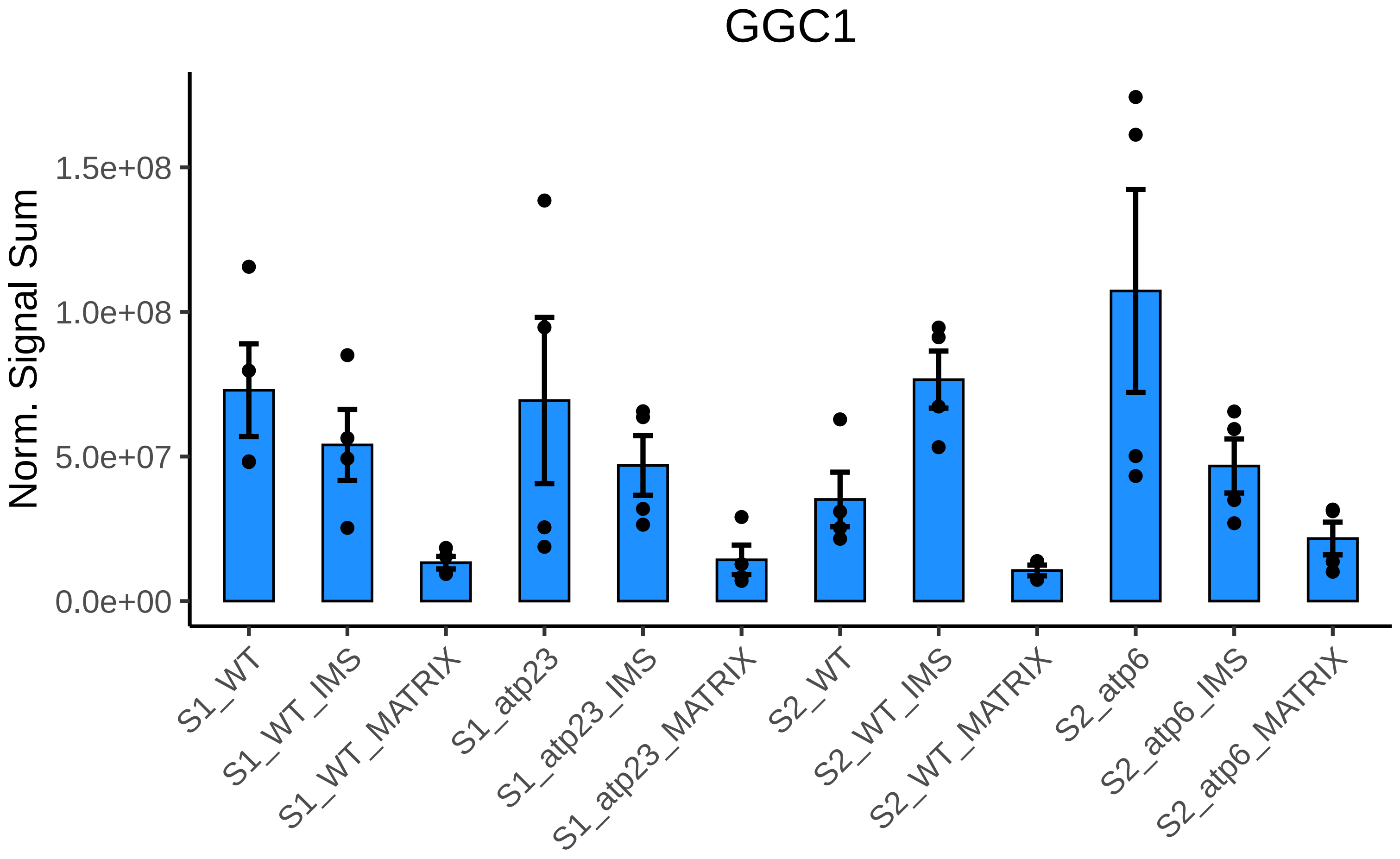
GGC1
Norm. Signal Sum
1.5e+08
1.0e+08
5.0e+07
0.0e+00
S1_WT
S1_WT_IMS
S1_WT_MATRIX
S1_atp23
S1_atp23_IMS
S1_atp23_MATRIX
S2_WT
S2_WT_IMS
S2_WT_MATRIX
S2_atp6
S2_atp6_IMS
S2_atp6_MATRIX

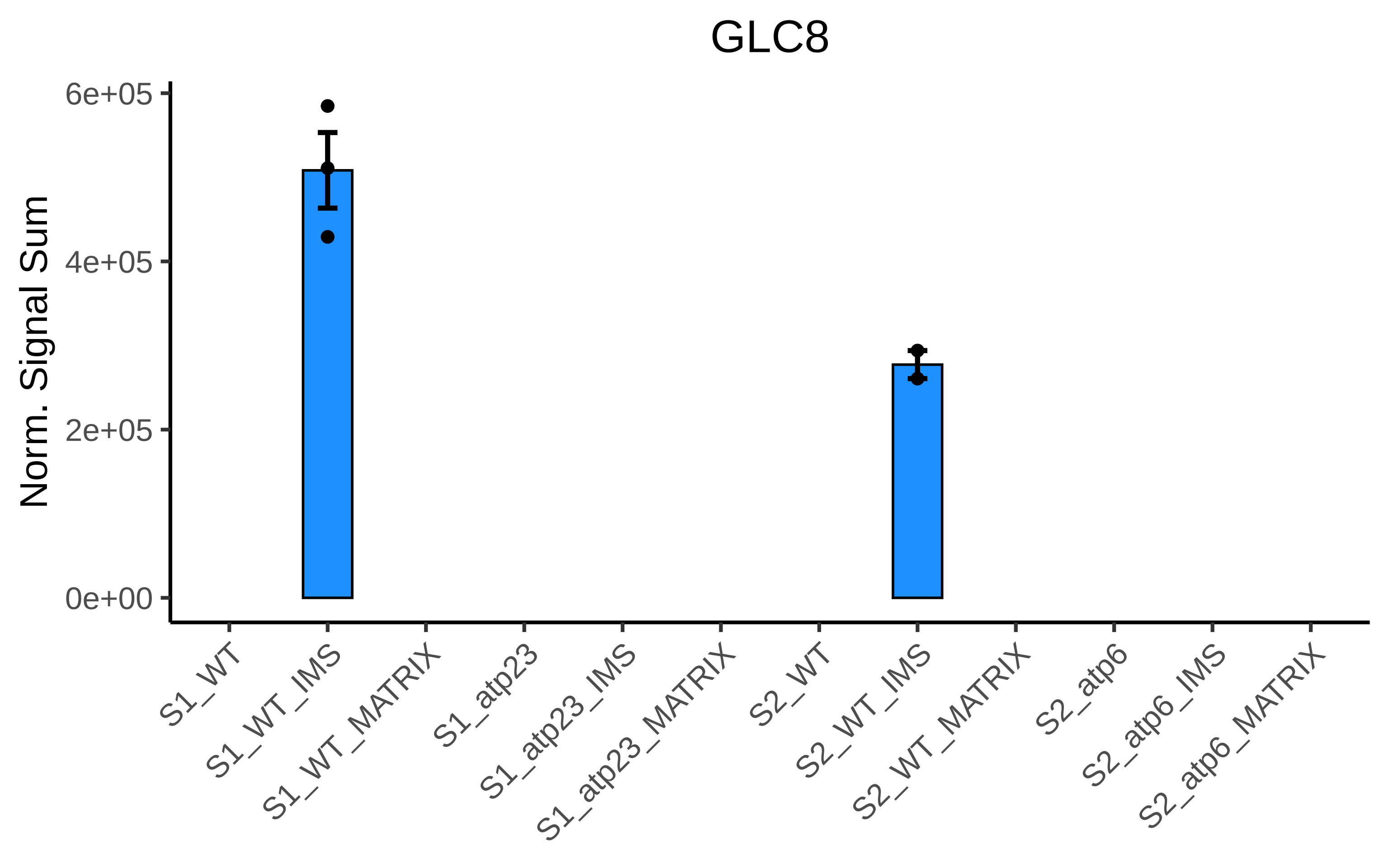

GLC8
Norm. Signal Sum
6e+05
4e+05
2e+05
0e+00
S1_WT
S1_WT_IMS
S1_WT_MATRIX
S1_atp23
S1_atp23_IMS
S1_atp23_MATRIX
S2_WT
S2_WT_IMS
S2_WT_MATRIX
S2_atp6
S2_atp6_IMS
S2_atp6_MATRIX

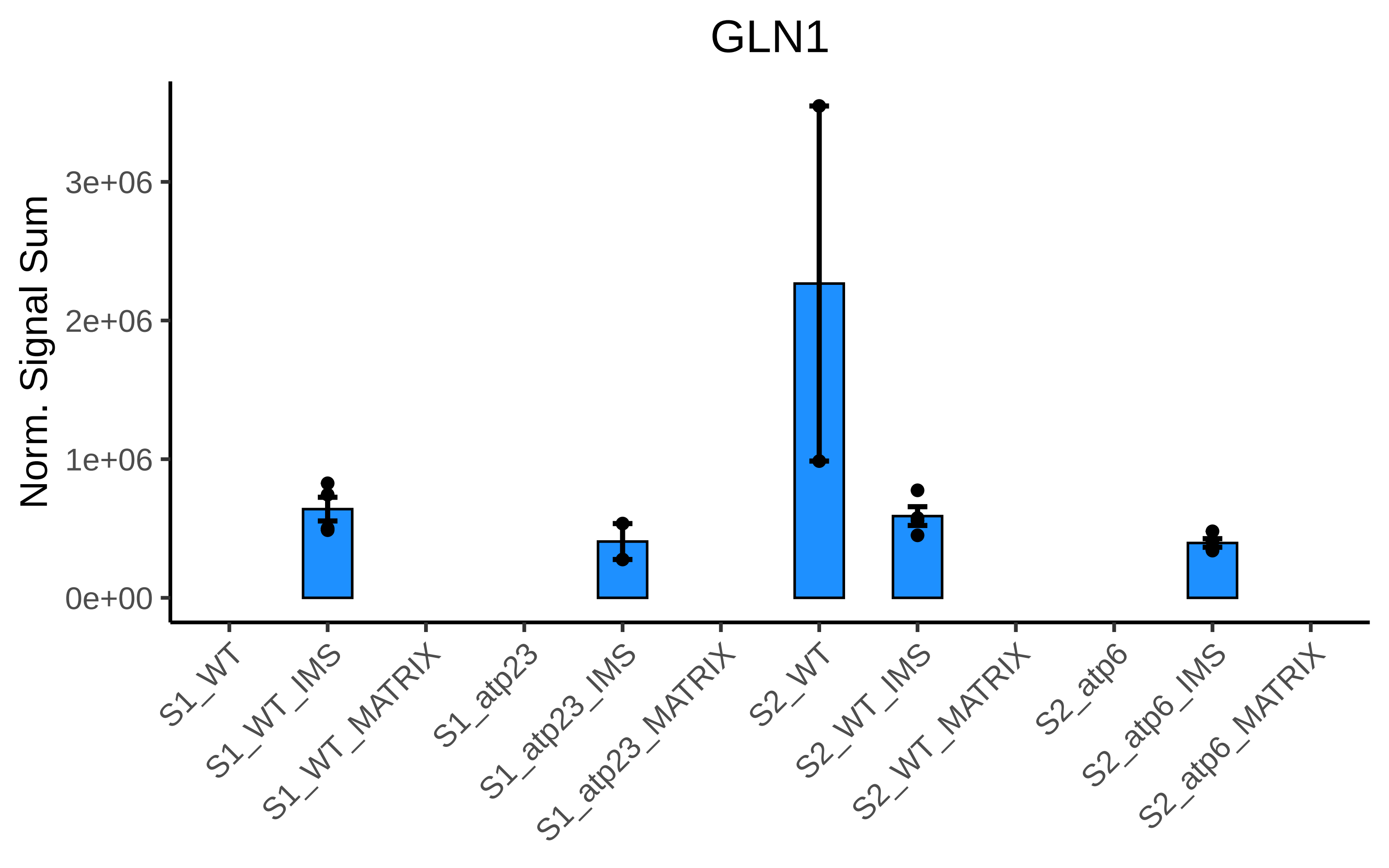
GLN1
Norm. Signal Sum
3e+06
2e+06
1e+06
0e+00
S1_WT
S1_WT_IMS
S1_WT_MATRIX
S1_atp23
S1_atp23_IMS
S1_atp23_MATRIX
S2_WT
S2_WT_IMS
S2_WT_MATRIX
S2_atp6
S2_atp6_IMS
S2_atp6_MATRIX

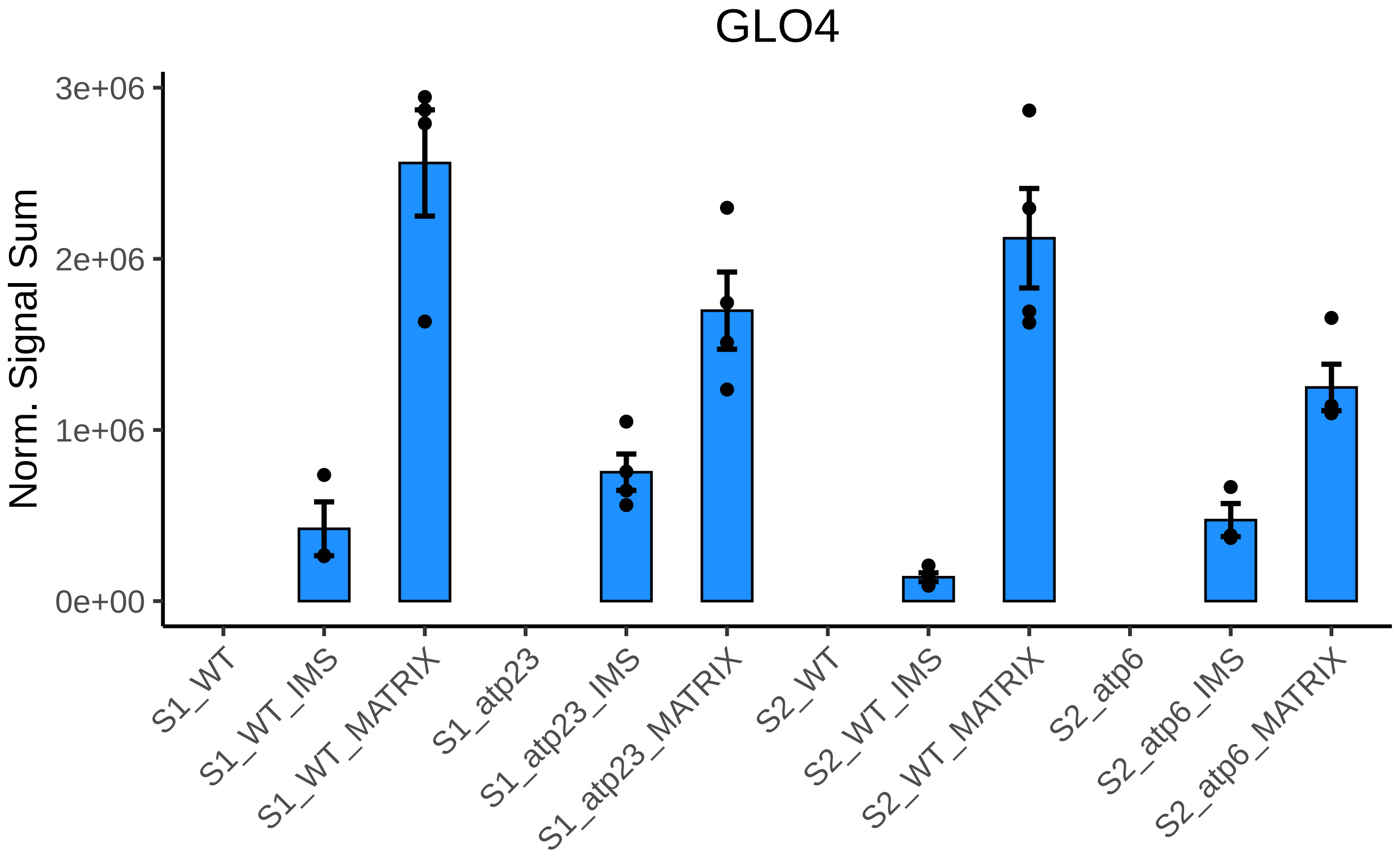

GLO4
Norm. Signal Sum
3e+06
2e+06
1e+06
0e+00
S1_WT
S1_WT_IMS
S1_WT_MATRIX
S1_atp23
S1_atp23_IMS
S1_atp23_MATRIX
S2_WT
S2_WT_IMS
S2_WT_MATRIX
S2_atp6
S2_atp6_IMS
S2_atp6_MATRIX

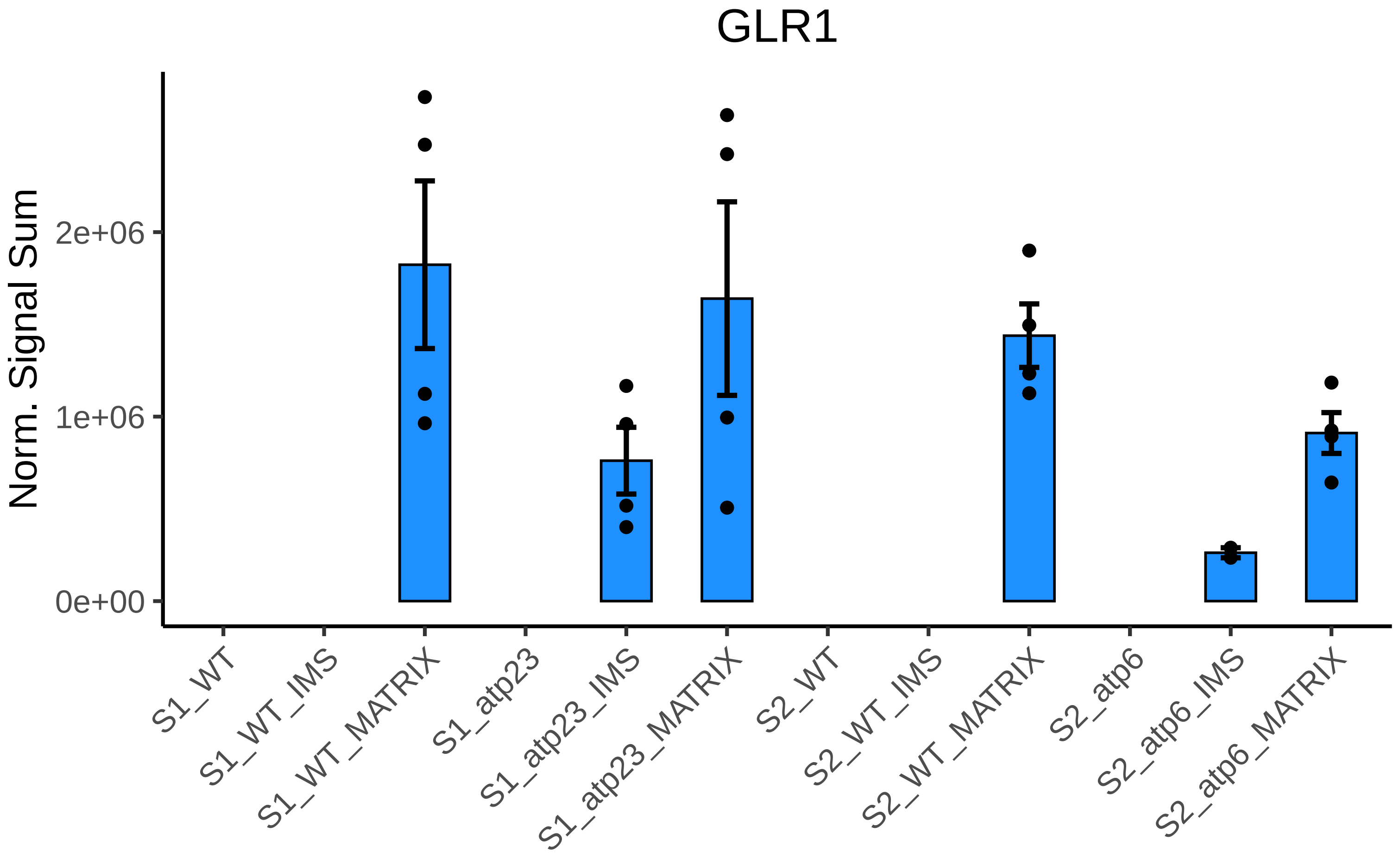
GLR1
Norm. Signal Sum
2e+06
1e+06
0e+00
S1_WT
S1_WT_IMS
S1_WT_MATRIX
S1_atp23
S1_atp23_IMS
S1_atp23_MATRIX
S2_WT
S2_WT_IMS
S2_WT_MATRIX
S2_atp6
S2_atp6_IMS
S2_atp6_MATRIX

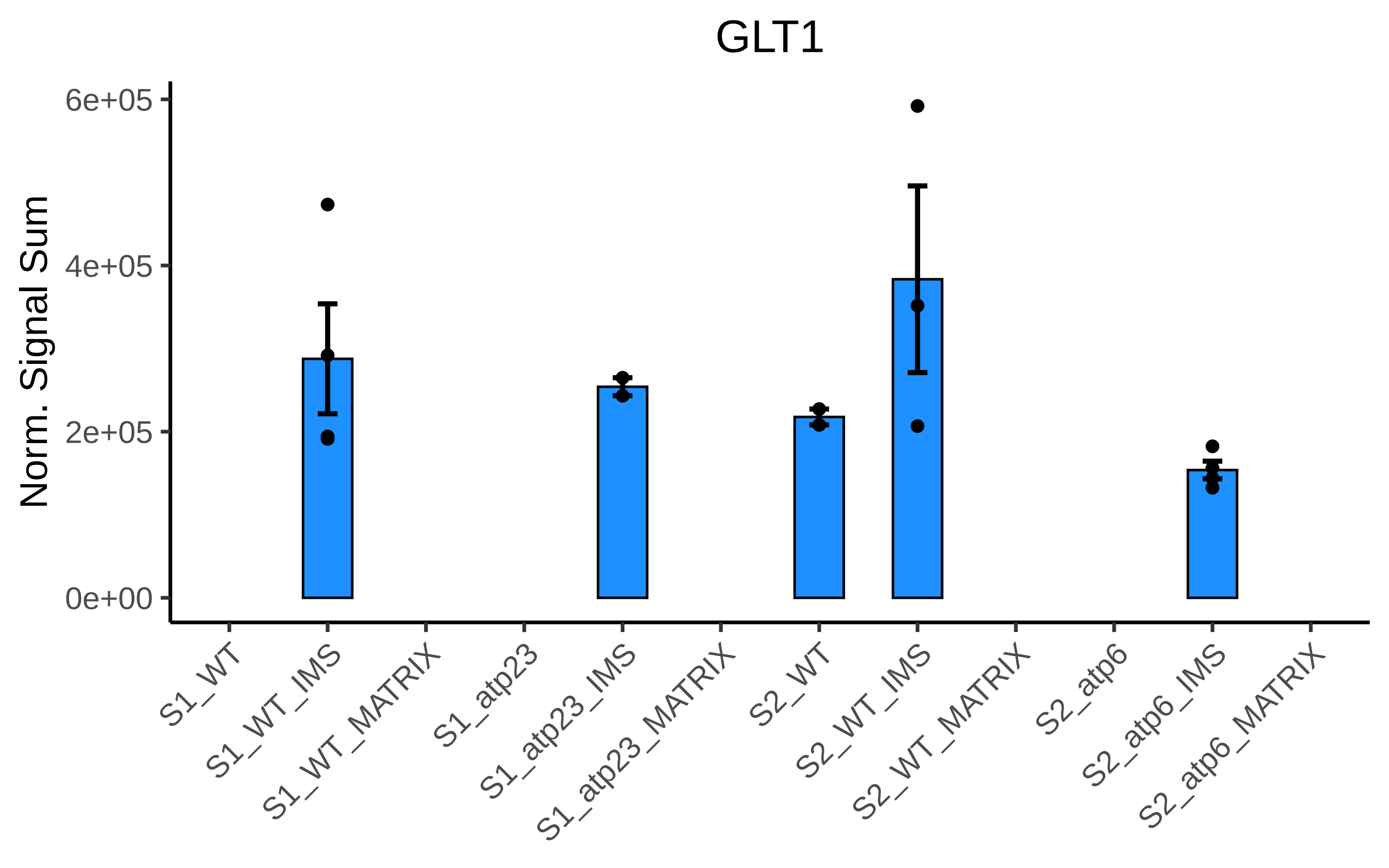

GLT1
Norm. Signal Sum
6e+05
4e+05
2e+05
0e+00
S1_WT
S1_WT_IMS
S1_WT_MATRIX
S1_atp23
S1_atp23_IMS
S1_atp23_MATRIX
S2_WT
S2_WT_IMS
S2_WT_MATRIX
S2_atp6
S2_atp6_IMS
S2_atp6_MATRIX

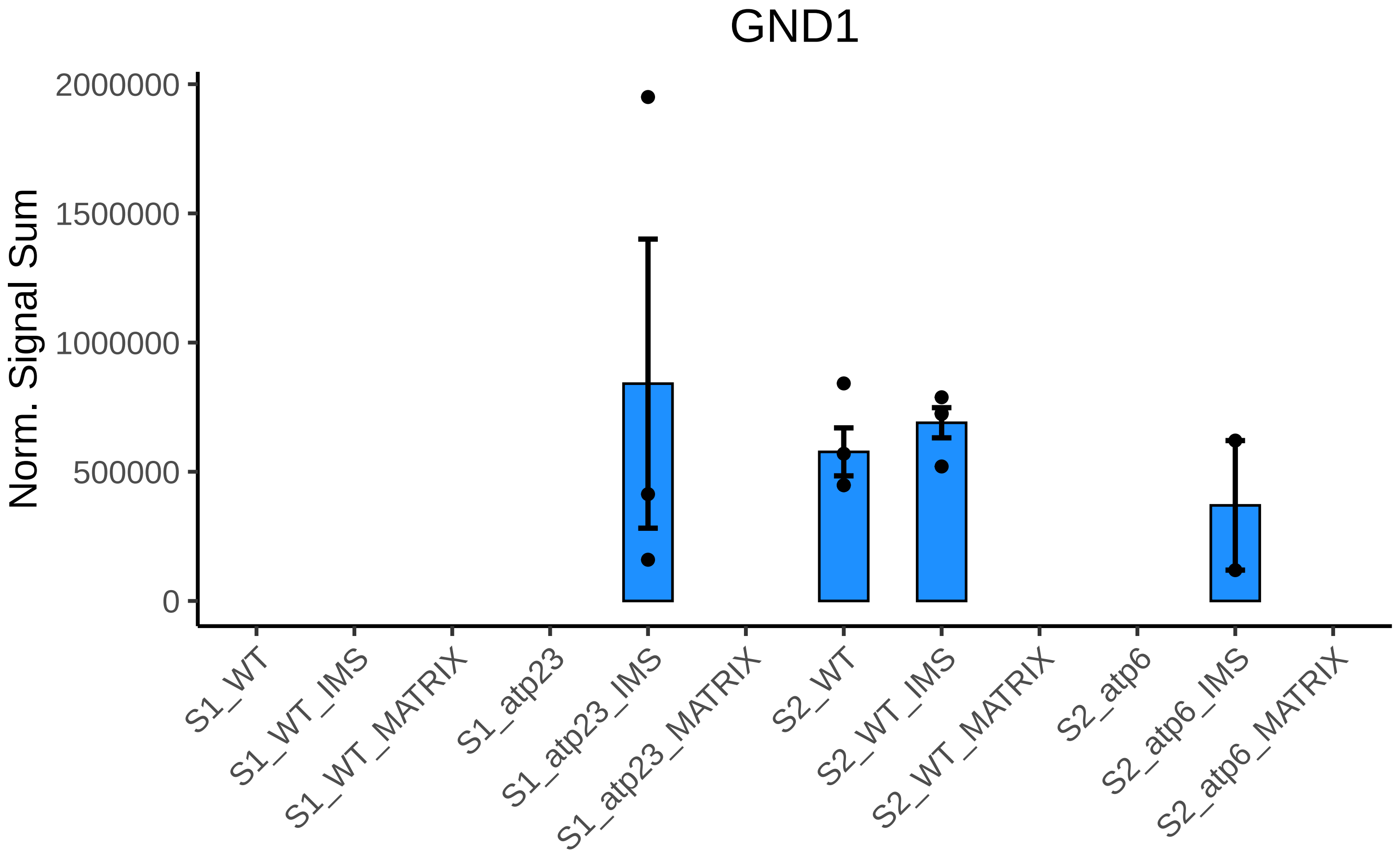

GND1
Norm. Signal Sum
2000000
1500000
1000000
500000
0
S1_WT
S1_WT_IMS
S1_WT_MATRIX
S1_atp23
S1_atp23_IMS
S1_atp23_MATRIX
S2_WT
S2_WT_IMS
S2_WT_MATRIX
S2_atp6
S2_atp6_IMS
S2_atp6_MATRIX

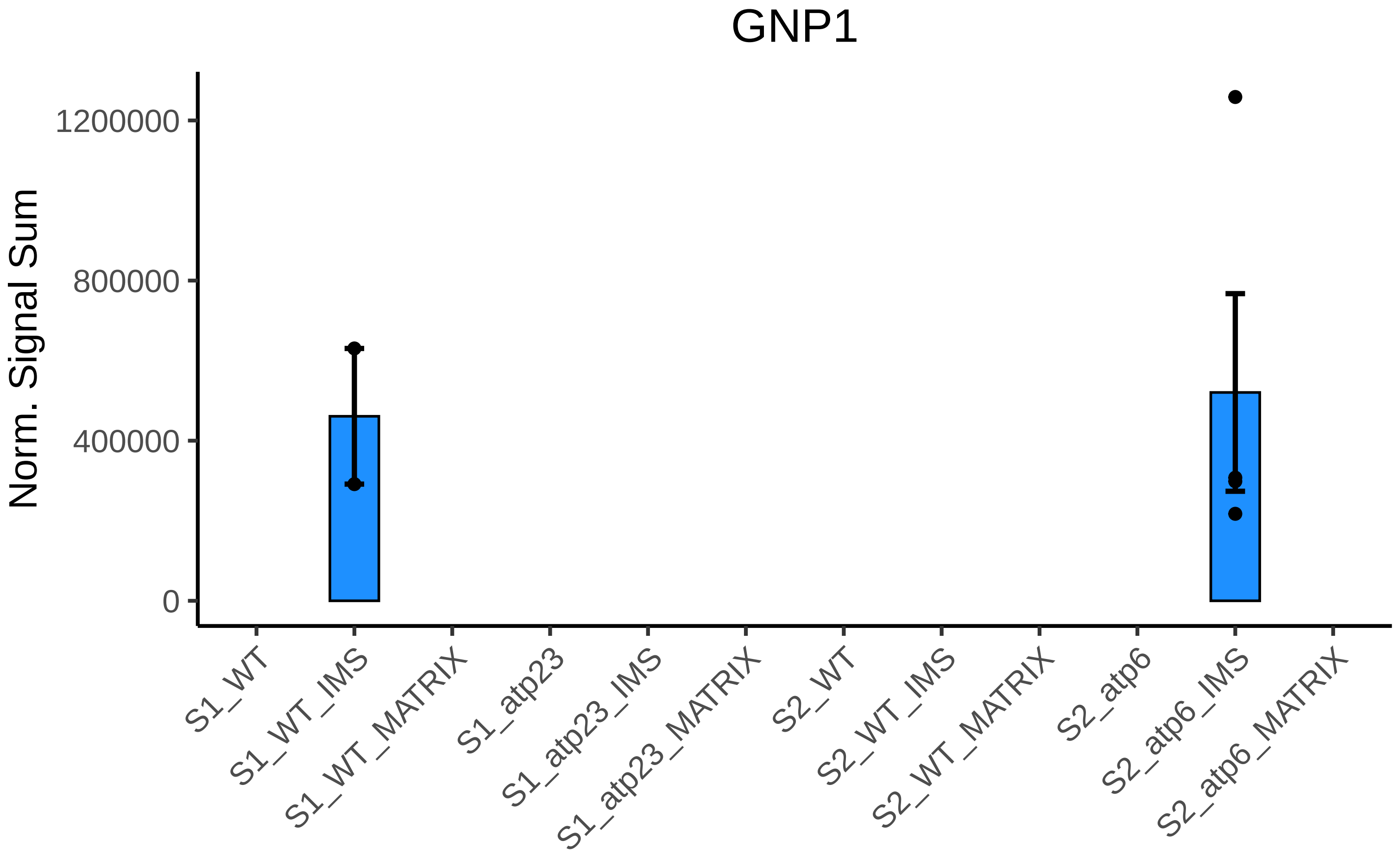
GNP1
Norm. Signal Sum
1200000
800000
400000
0
S1_WT
S1_WT_IMS
S1_WT_MATRIX
S1_atp23
S1_atp23_IMS
S1_atp23_MATRIX
S2_WT
S2_WT_IMS
S2_WT_MATRIX
S2_atp6
S2_atp6_IMS
S2_atp6_MATRIX

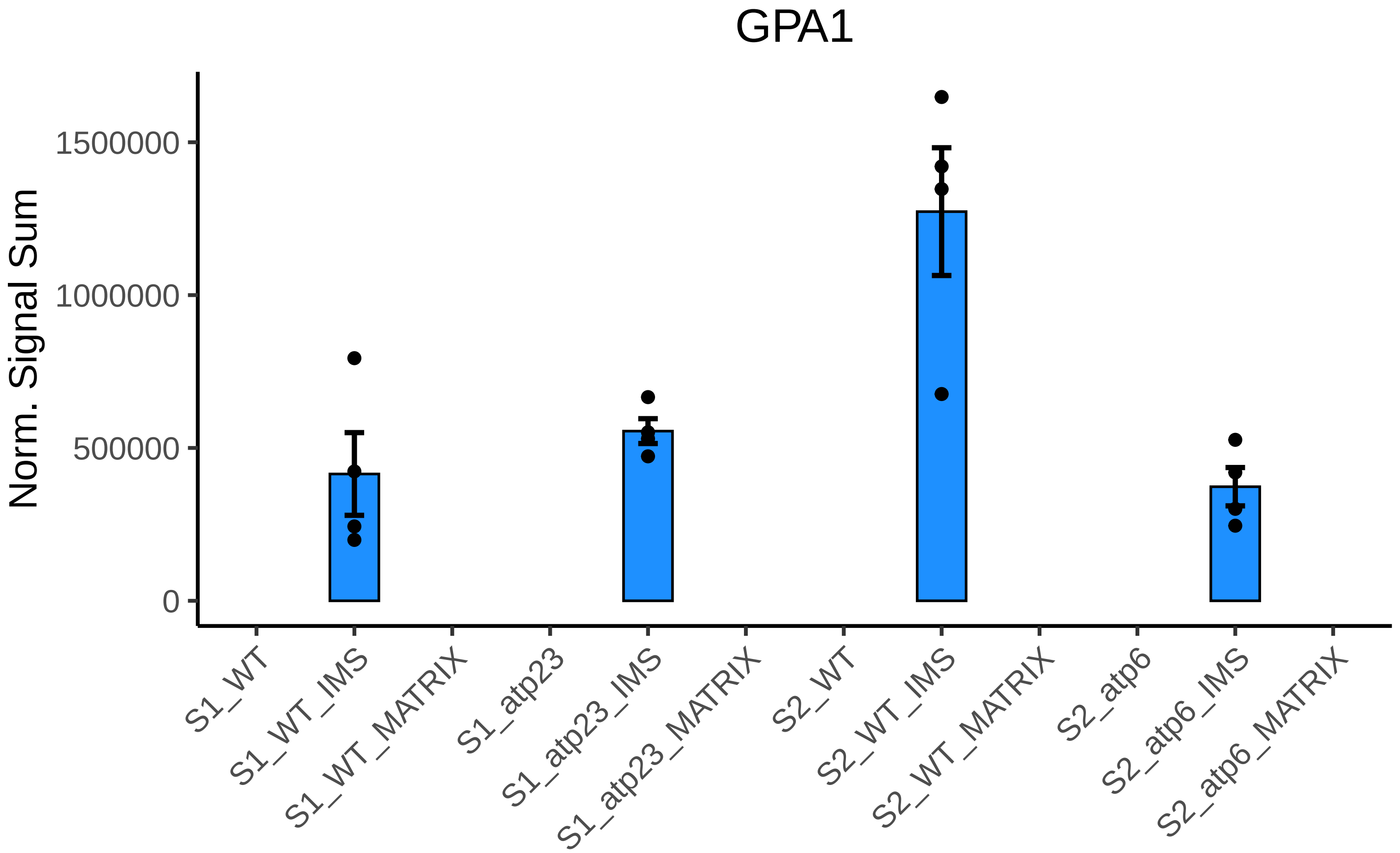
GPA1
Norm. Signal Sum
1500000
1000000
500000
0
S1_WT
S1_WT_IMS
S1_WT_MATRIX
S1_atp23
S1_atp23_IMS
S1_atp23_MATRIX
S2_WT
S2_WT_IMS
S2_WT_MATRIX
S2_atp6
S2_atp6_IMS
S2_atp6_MATRIX

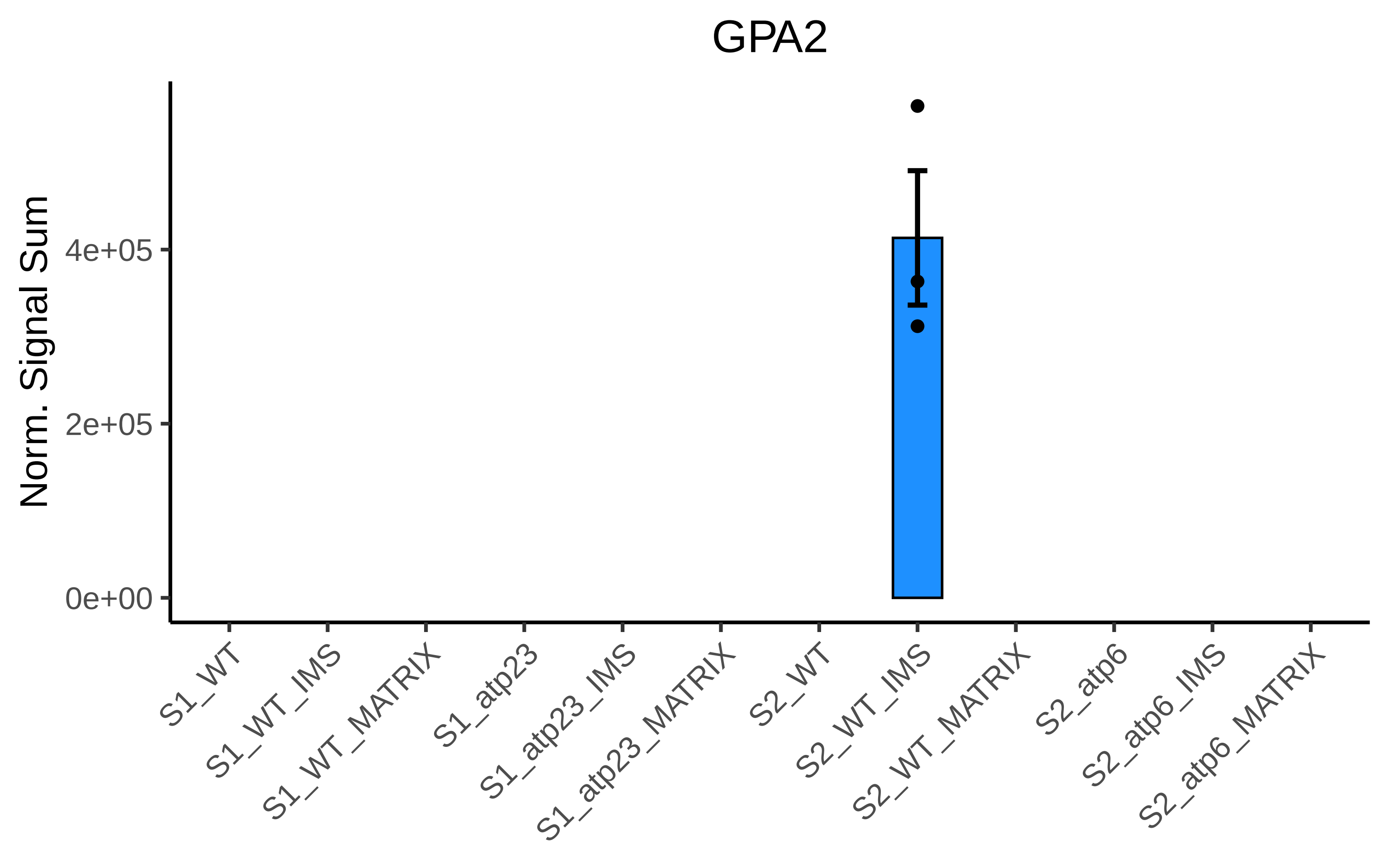
GPA2
Norm. Signal Sum
4e+05
2e+05
0e+00
S1_WT
S1_WT_IMS
S1_WT_MATRIX
S1_atp23
S1_atp23_IMS
S1_atp23_MATRIX
S2_WT
S2_WT_IMS
S2_WT_MATRIX
S2_atp6
S2_atp6_IMS
S2_atp6_MATRIX

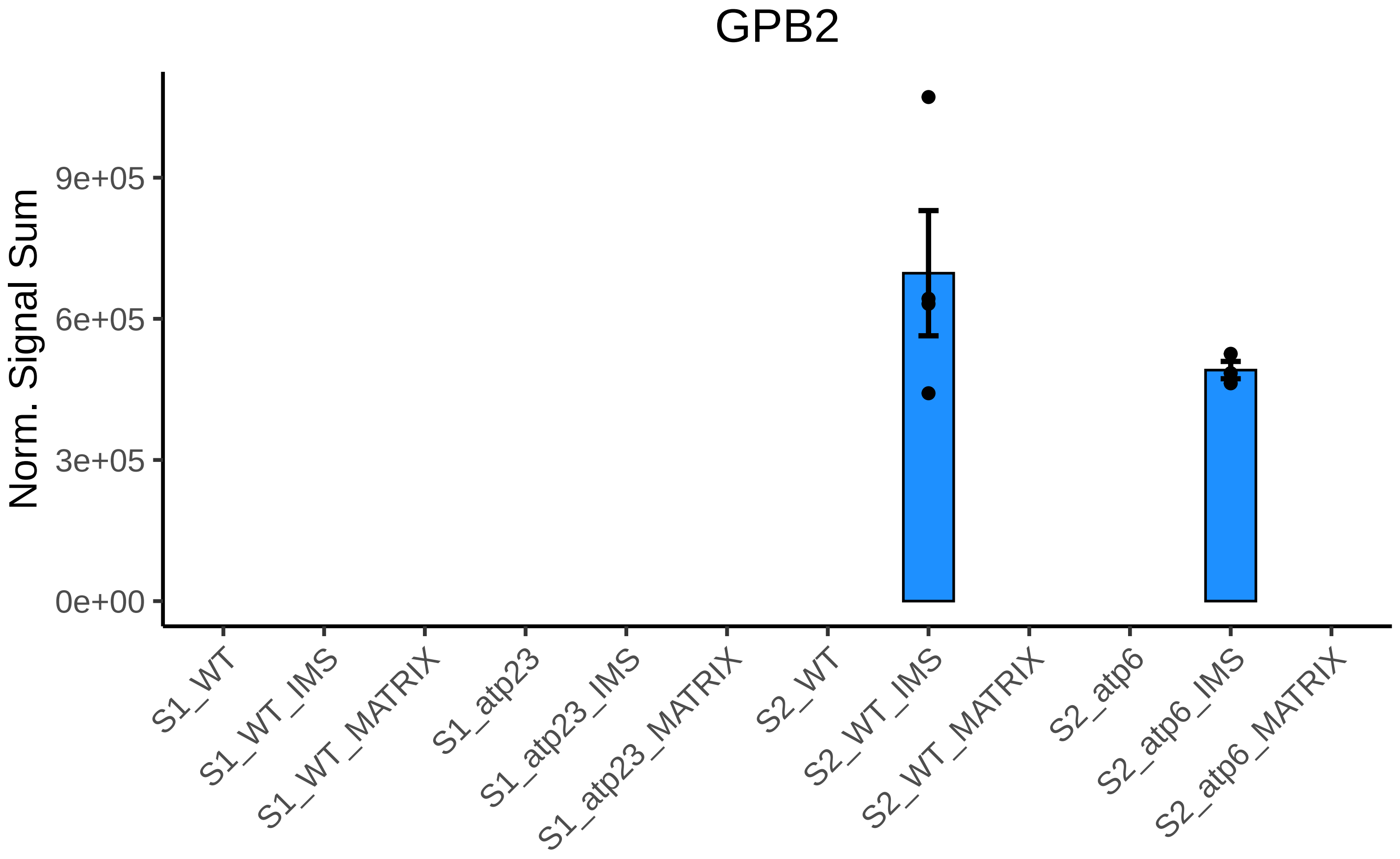

GPB2
Norm. Signal Sum
9e+05
6e+05
3e+05
0e+00
S1_WT
S1_WT_IMS
S1_WT_MATRIX
S1_atp23
S1_atp23_IMS
S1_atp23_MATRIX
S2_WT
S2_WT_IMS
S2_WT_MATRIX
S2_atp6
S2_atp6_IMS
S2_atp6_MATRIX

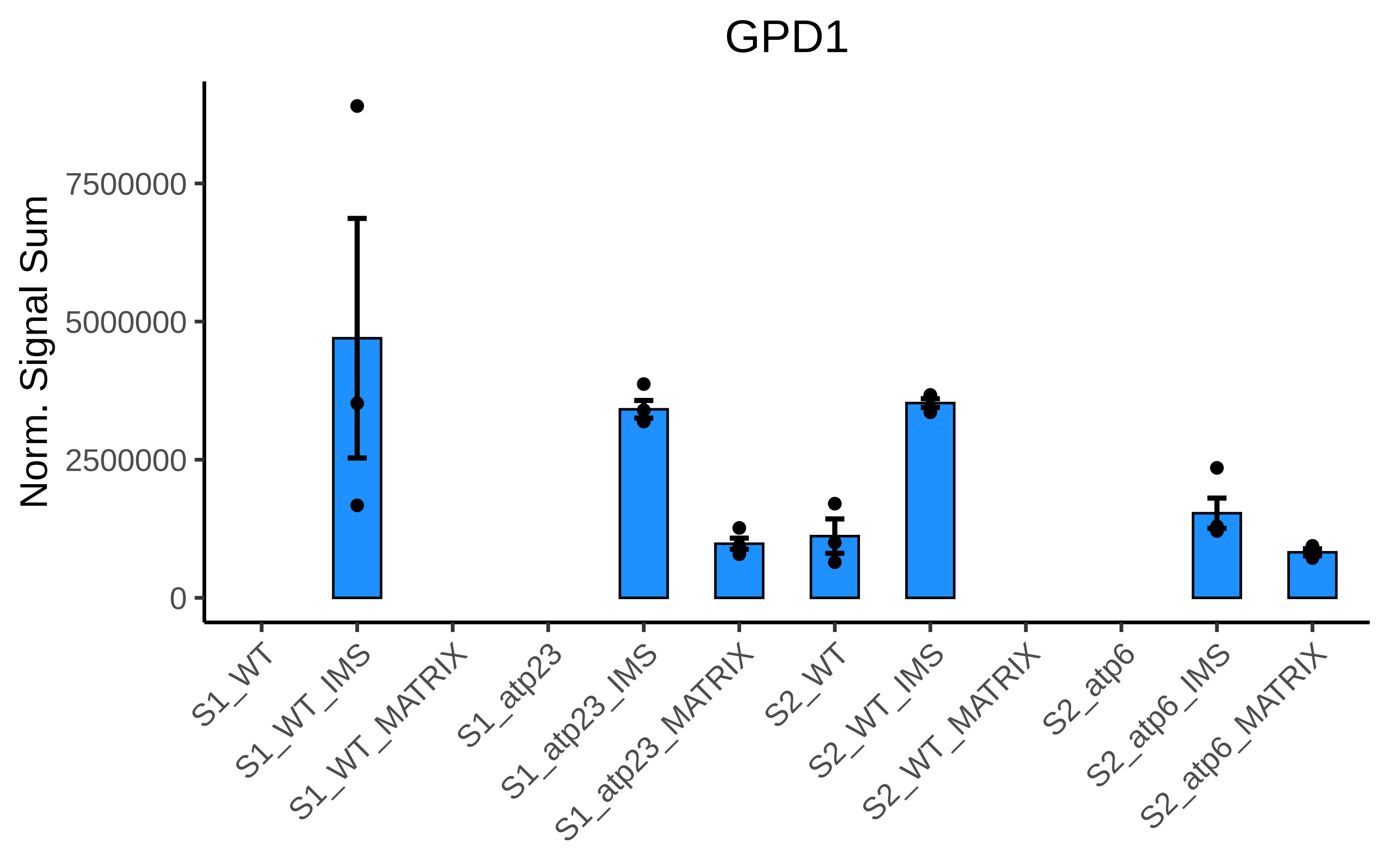

GPD1
Norm. Signal Sum
7500000
5000000
2500000
0
S1_WT
S1_WT_IMS
S1_WT_MATRIX
S1_atp23
S1_atp23_IMS
S1_atp23_MATRIX
S2_WT
S2_WT_IMS
S2_WT_MATRIX
S2_atp6
S2_atp6_IMS
S2_atp6_MATRIX

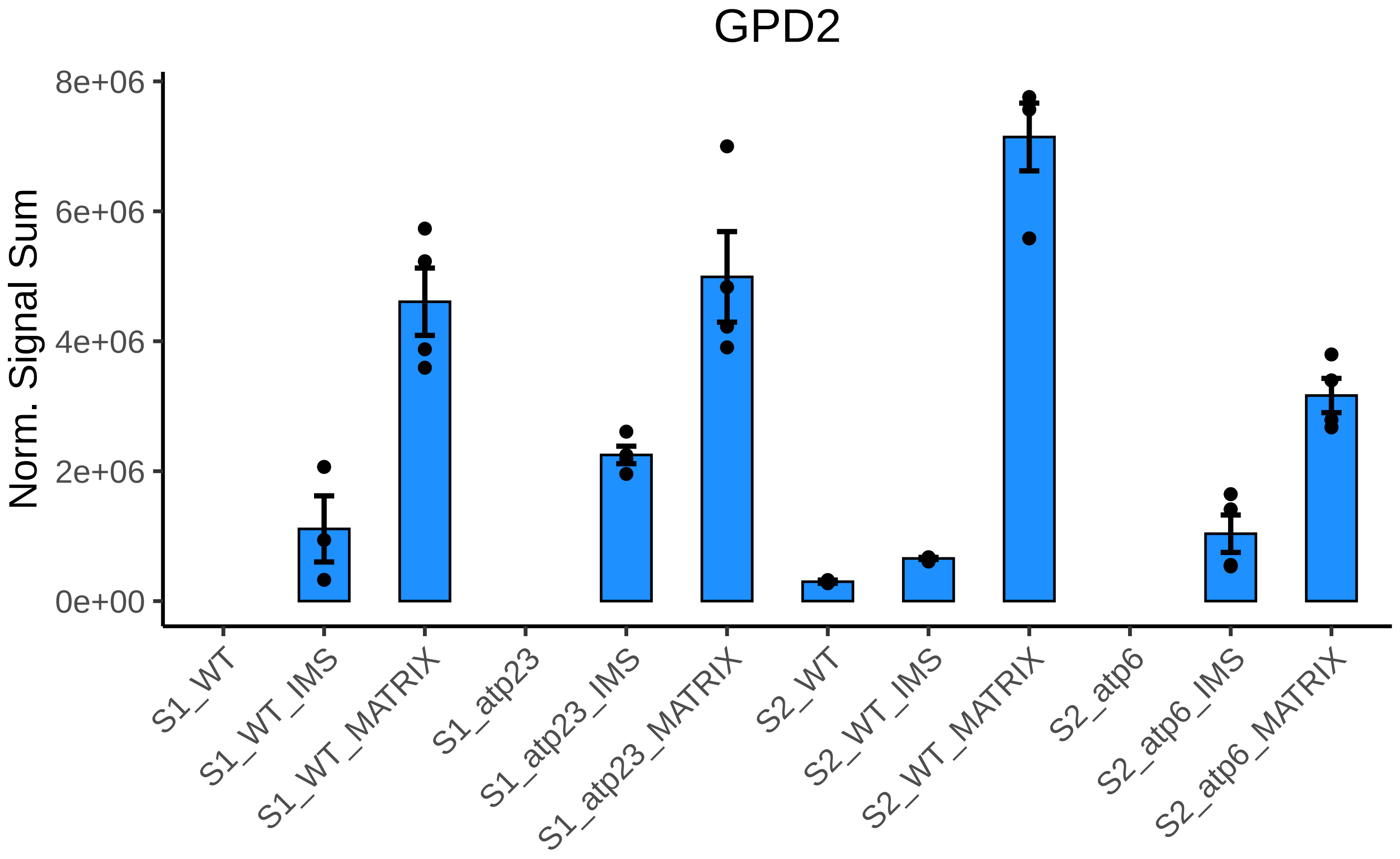
GPD2
Norm. Signal Sum
8e+06
6e+06
4e+06
2e+06
0e+00
S1_WT
S1_WT_IMS
S1_WT_MATRIX
S1_atp23
S1_atp23_IMS
S1_atp23_MATRIX
S2_WT
S2_WT_IMS
S2_WT_MATRIX
S2_atp6
S2_atp6_IMS
S2_atp6_MATRIX

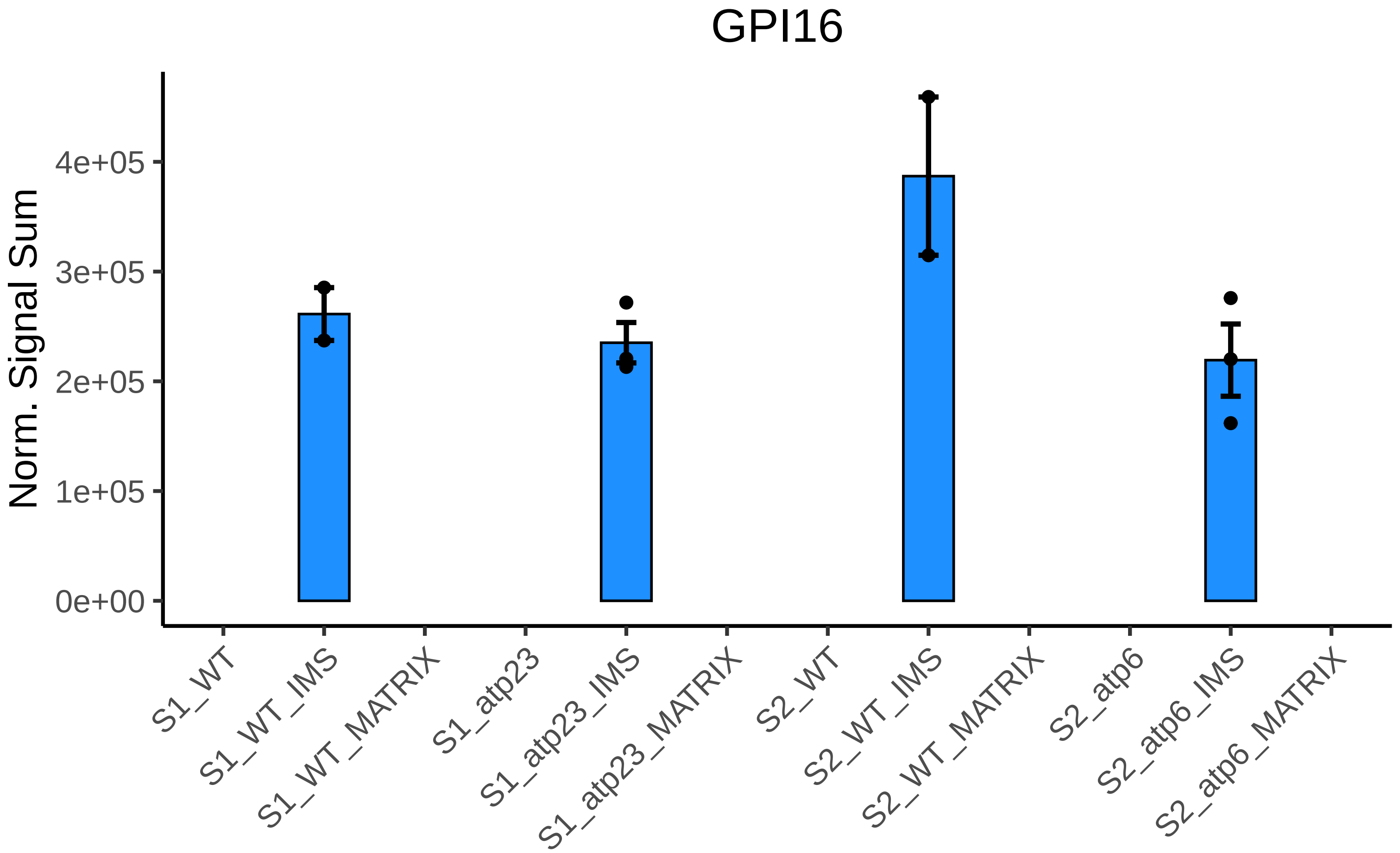

GPI16
Norm. Signal Sum
4e+05
3e+05
2e+05
1e+05
0e+00
S1_WT
S1_WT_IMS
S1_WT_MATRIX
S1_atp23
S1_atp23_IMS
S1_atp23_MATRIX
S2_WT
S2_WT_IMS
S2_WT_MATRIX
S2_atp6
S2_atp6_IMS
S2_atp6_MATRIX

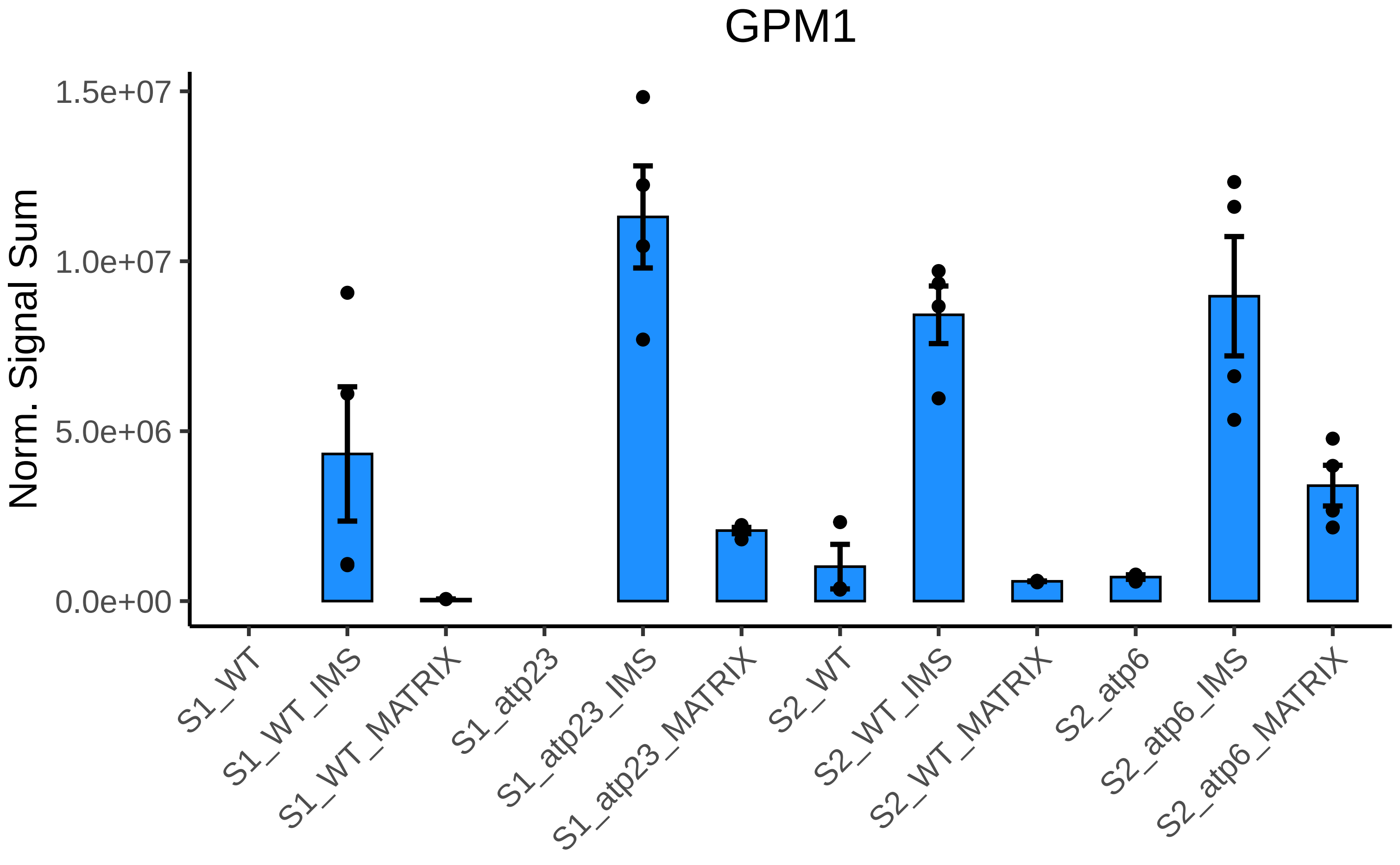
GPM1
Norm. Signal Sum
1.5e+07
1.0e+07
5.0e+06
0.0e+00
S1_WT
S1_WT_IMS
S1_WT_MATRIX
S1_atp23
S1_atp23_IMS
S1_atp23_MATRIX
S2_WT
S2_WT_IMS
S2_WT_MATRIX
S2_atp6
S2_atp6_IMS
S2_atp6_MATRIX

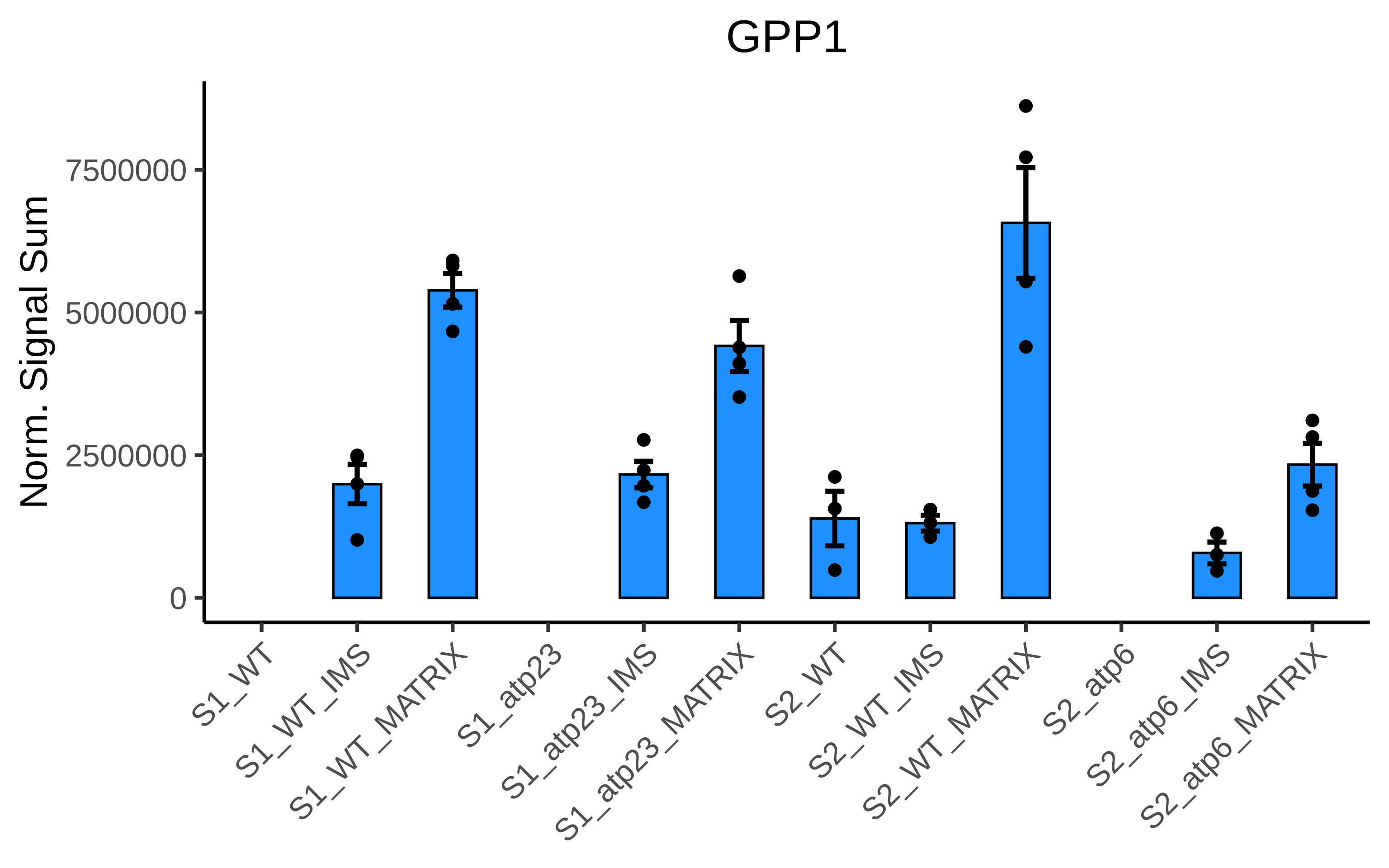

GPP1
Norm. Signal Sum
7500000
5000000
2500000
0
S1_WT
S1_WT_IMS
S1_WT_MATRIX
S1_atp23
S1_atp23_IMS
S1_atp23_MATRIX
S2_WT
S2_WT_IMS
S2_WT_MATRIX
S2_atp6
S2_atp6_IMS
S2_atp6_MATRIX

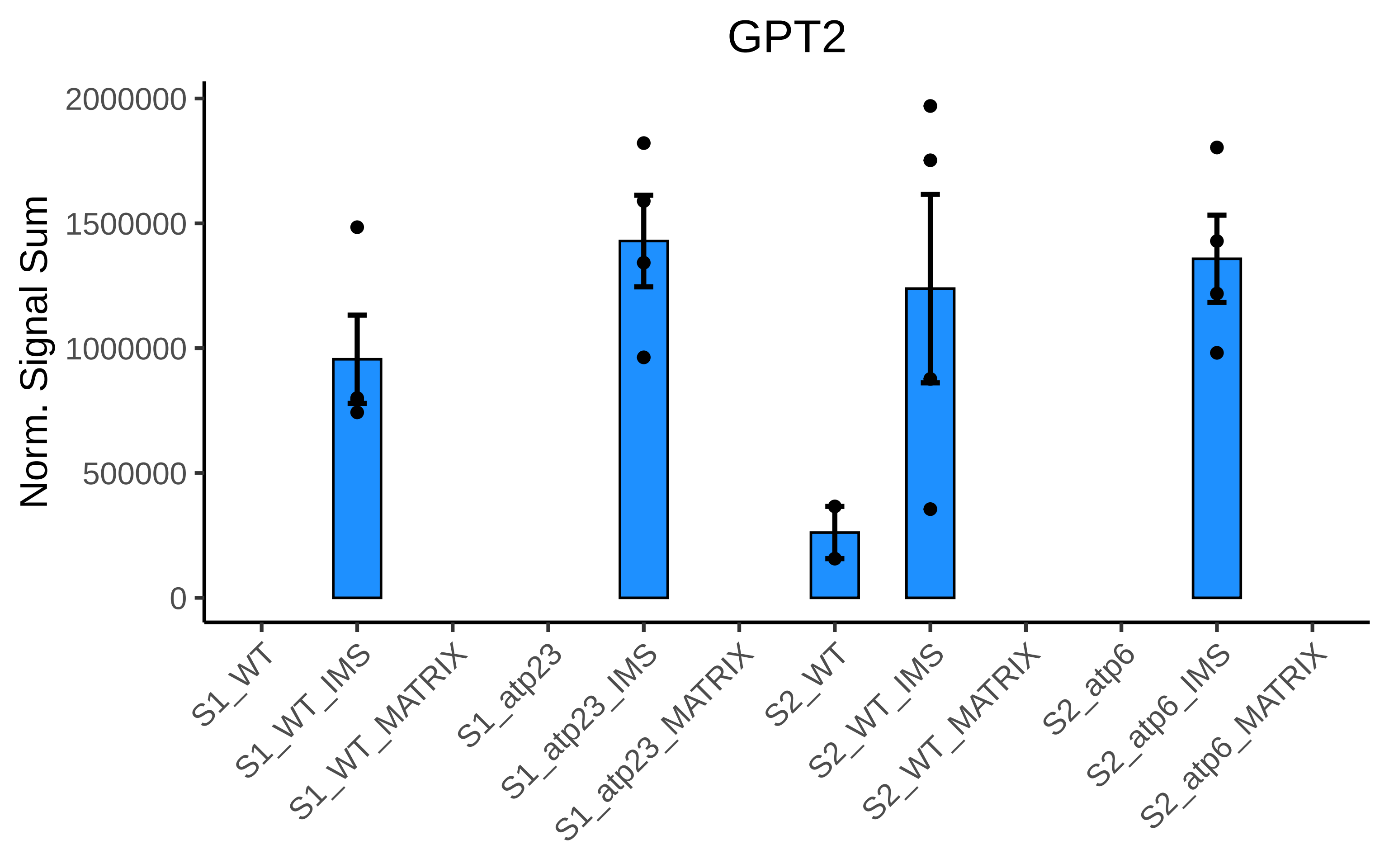
GPT2
Norm. Signal Sum
2000000
1500000
1000000
500000
0
S1_WT
S1_WT_IMS
S1_WT_MATRIX
S1_atp23
S1_atp23_IMS
S1_atp23_MATRIX
S2_WT
S2_WT_IMS
S2_WT_MATRIX
S2_atp6
S2_atp6_IMS
S2_atp6_MATRIX

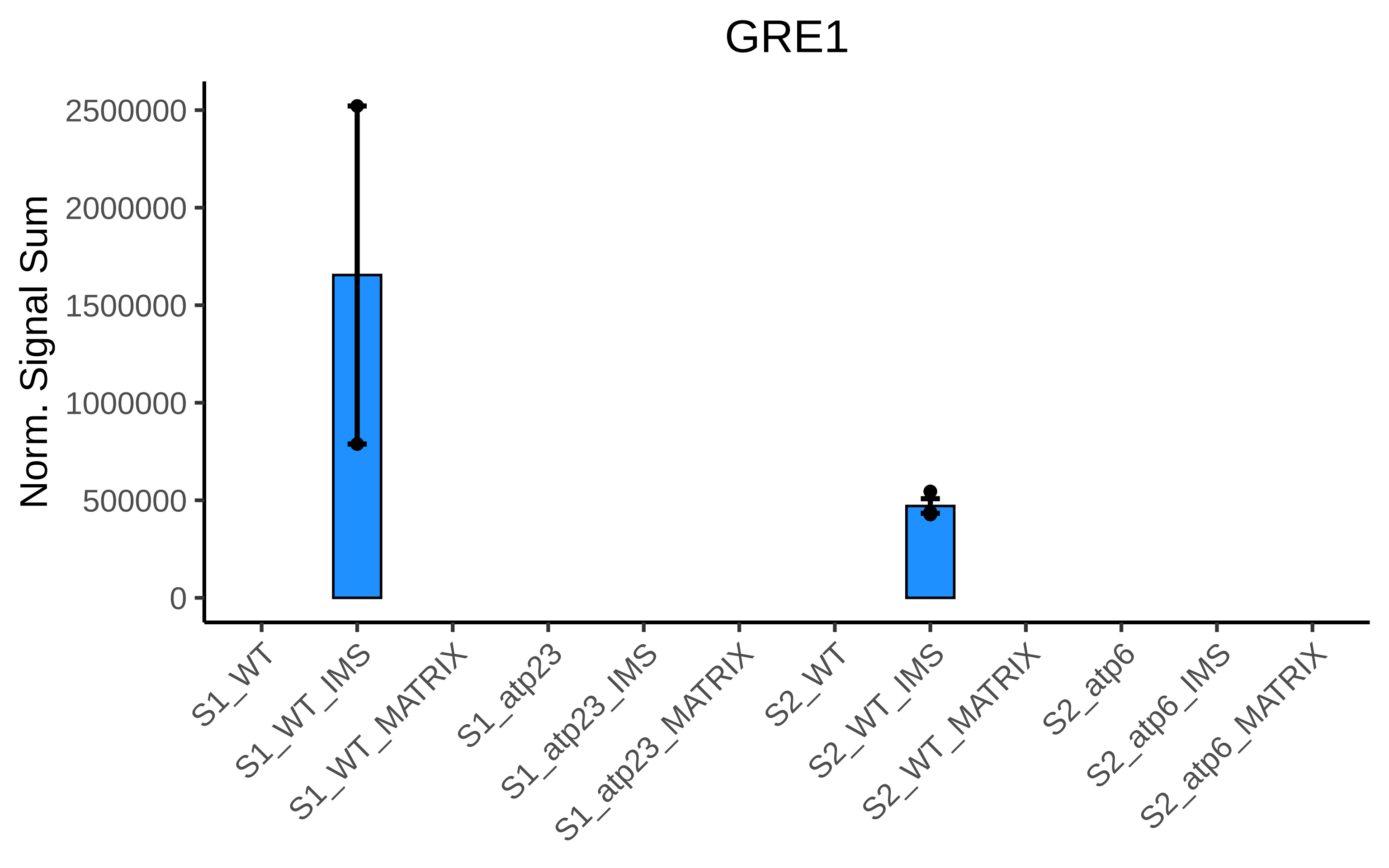
GRE1
Norm. Signal Sum
2500000
2000000
1500000
1000000
500000
0
S1_WT
S1_WT_IMS
S1_WT_MATRIX
S1_atp23
S1_atp23_IMS
S1_atp23_MATRIX
S2_WT
S2_WT_IMS
S2_WT_MATRIX
S2_atp6
S2_atp6_IMS
S2_atp6_MATRIX

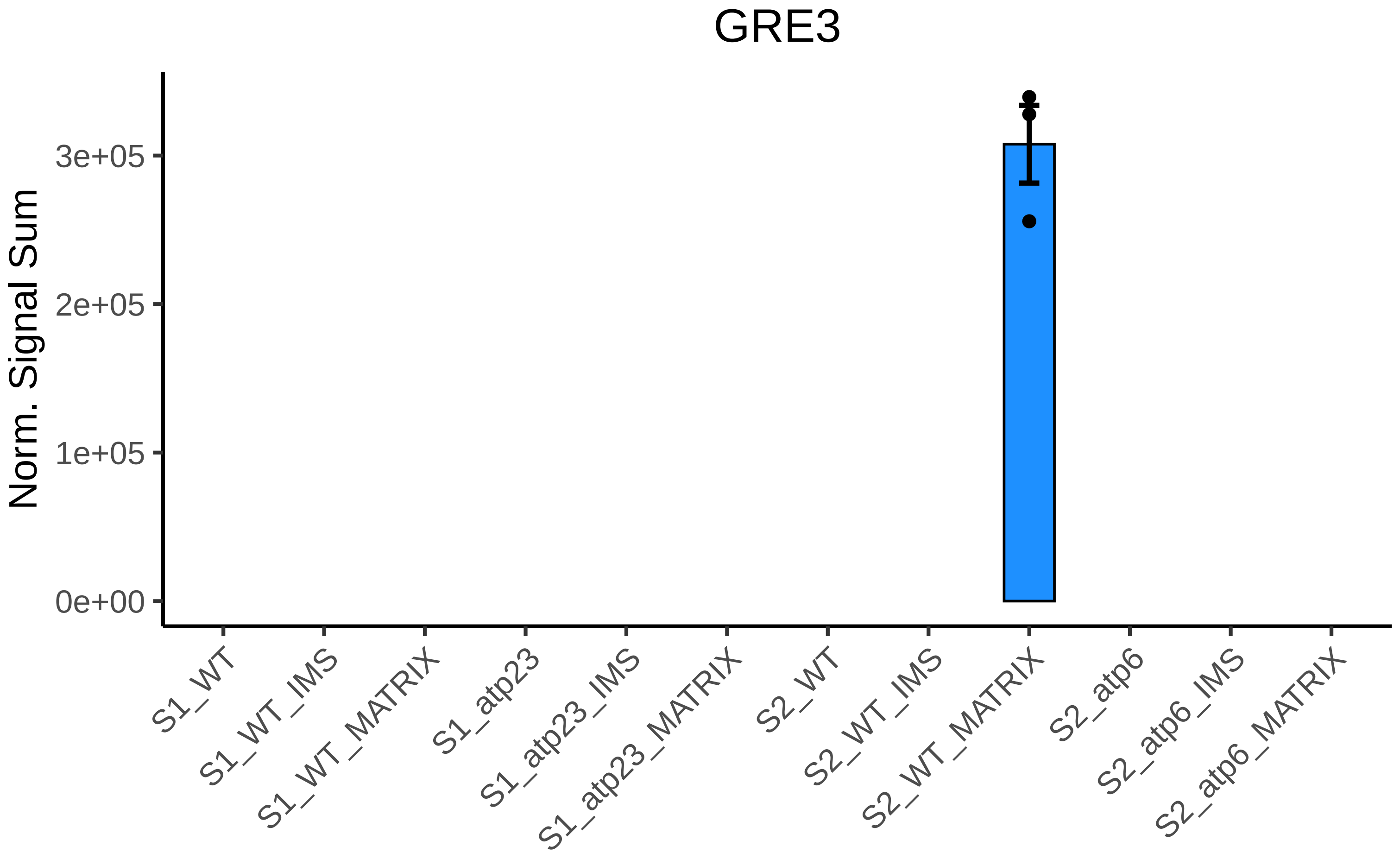
GRE3
Norm. Signal Sum
3e+05
2e+05
1e+05
0e+00
S1_WT
S1_WT_IMS
S1_WT_MATRIX
S1_atp23
S1_atp23_IMS
S1_atp23_MATRIX
S2_WT
S2_WT_IMS
S2_WT_MATRIX
S2_atp6
S2_atp6_IMS
S2_atp6_MATRIX

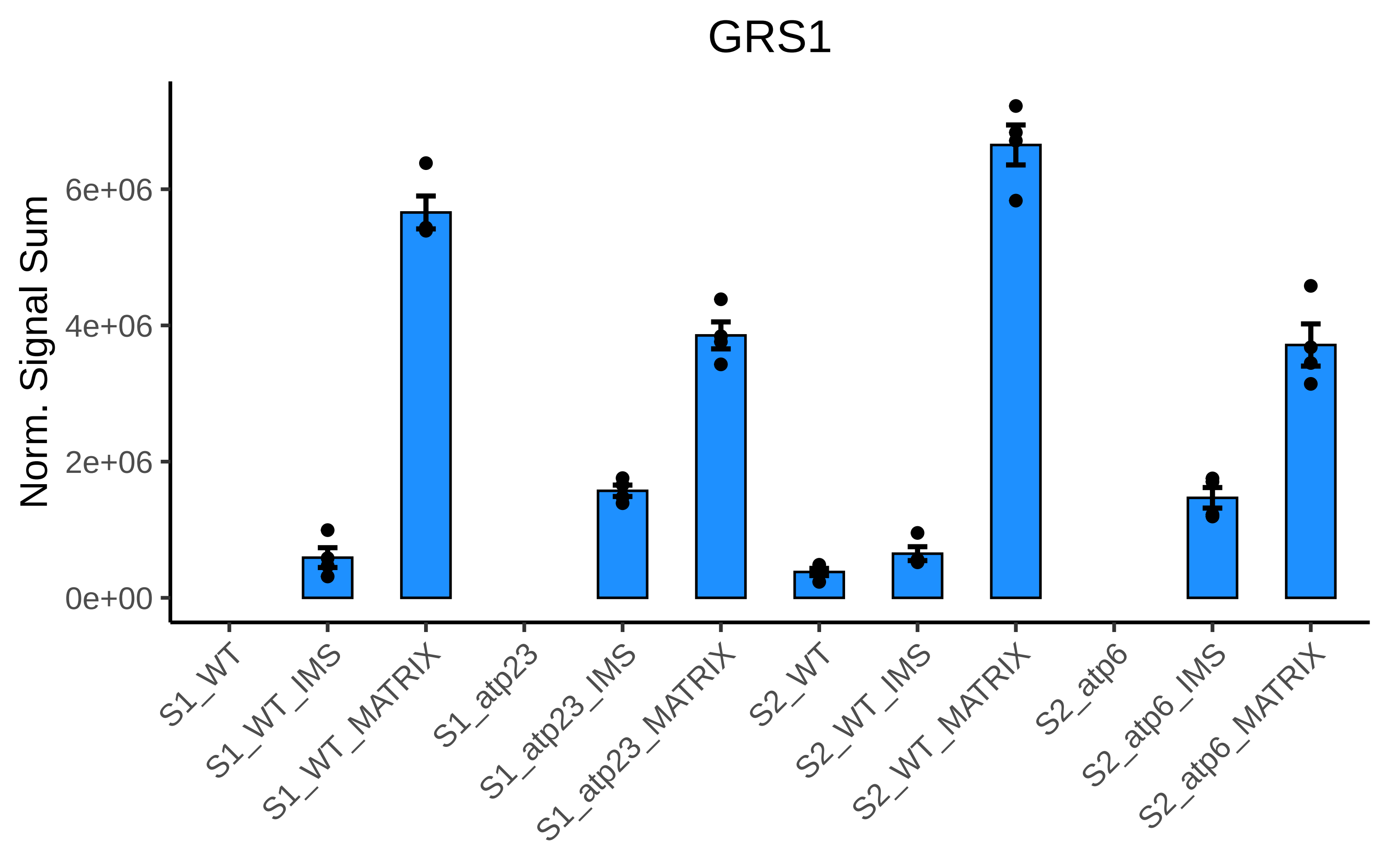

GRS1
Norm. Signal Sum
6e+06
4e+06
2e+06
0e+00
S1_WT
S1_WT_IMS
S1_WT_MATRIX
S1_atp23
S1_atp23_IMS
S1_atp23_MATRIX
S2_WT
S2_WT_IMS
S2_WT_MATRIX
S2_atp6
S2_atp6_IMS
S2_atp6_MATRIX

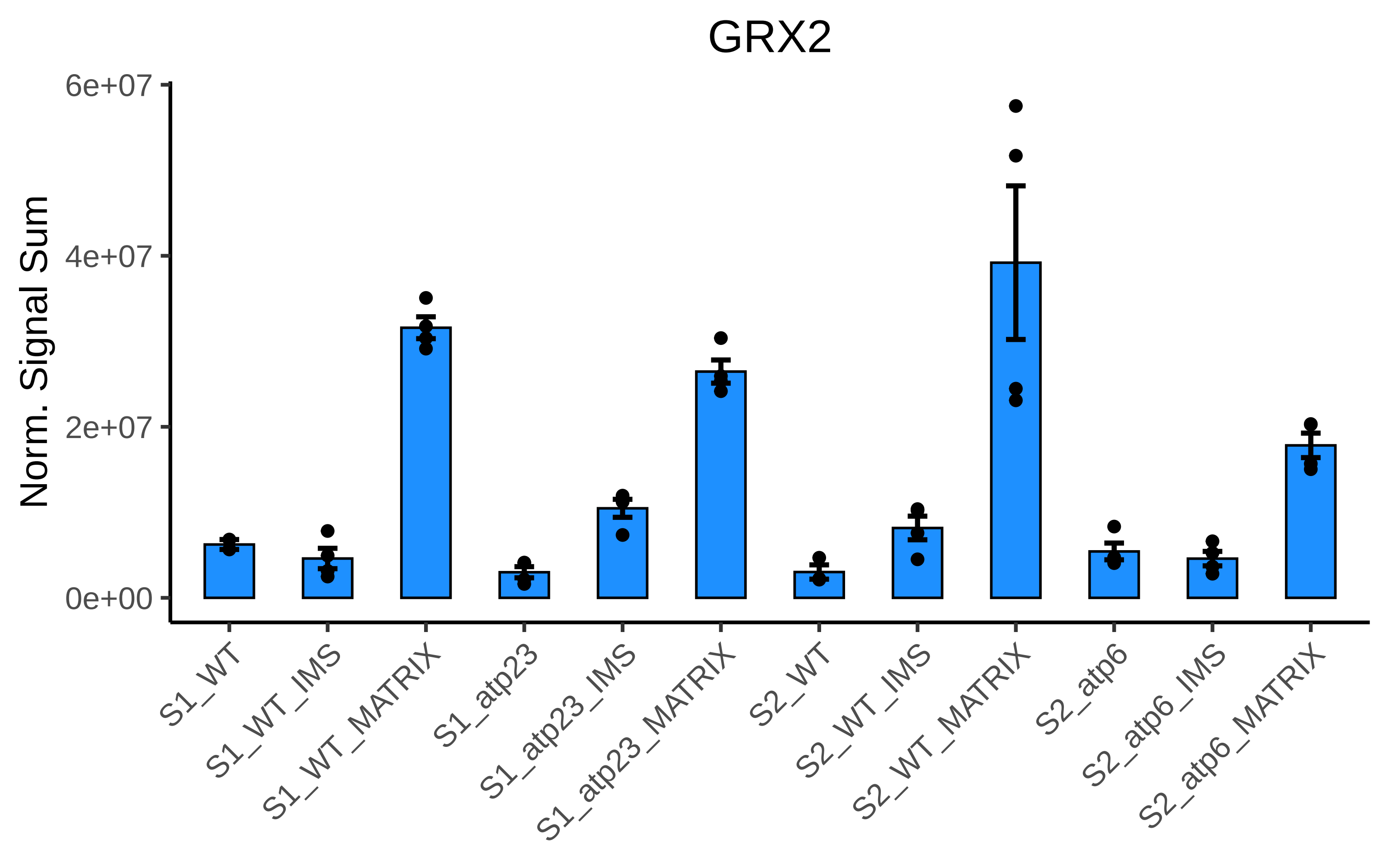

GRX2
Norm. Signal Sum
6e+07
4e+07
2e+07
0e+00
S1_WT
S1_WT_IMS
S1_WT_MATRIX
S1_atp23
S1_atp23_IMS
S1_atp23_MATRIX
S2_WT
S2_WT_IMS
S2_WT_MATRIX
S2_atp6
S2_atp6_IMS
S2_atp6_MATRIX

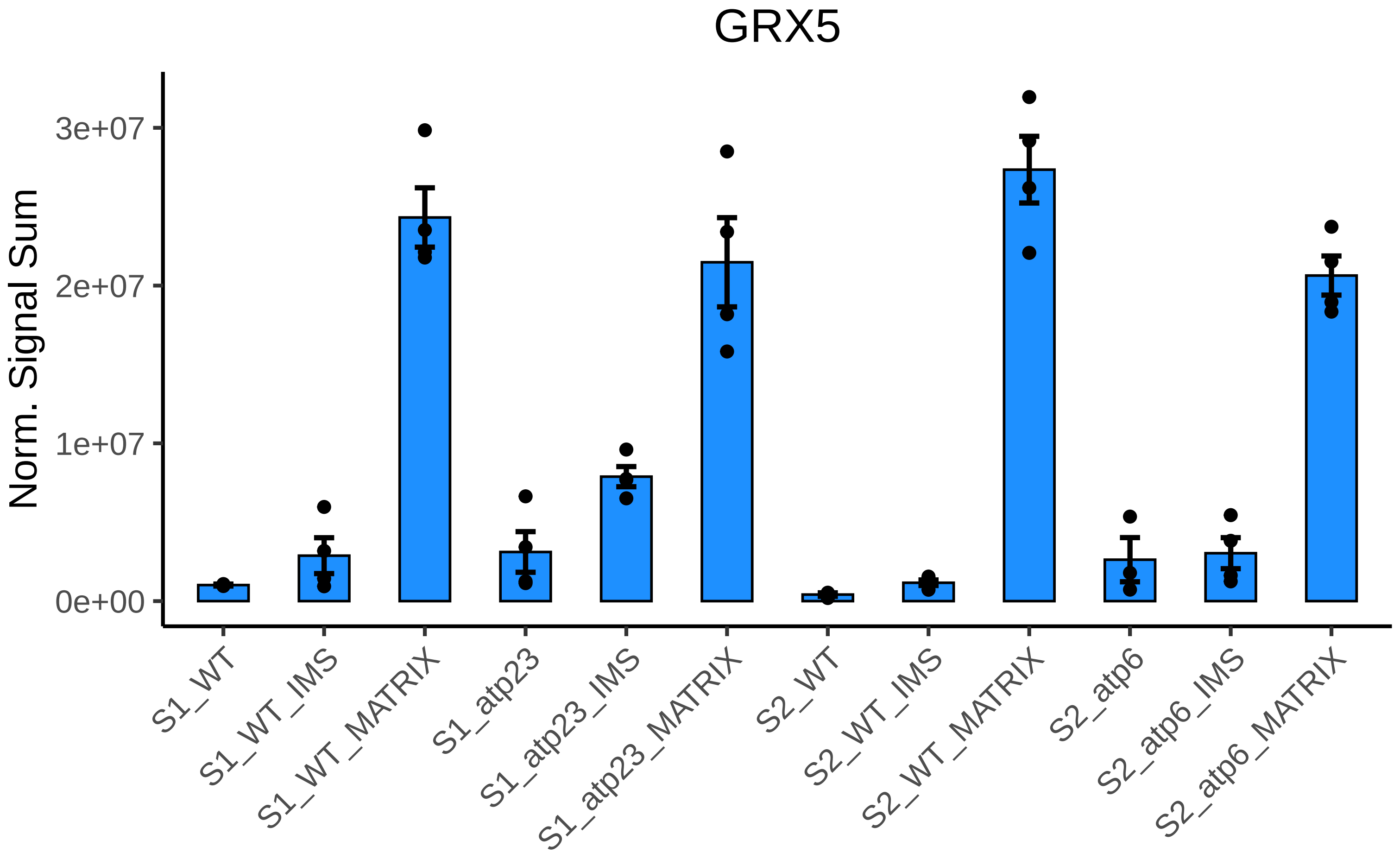
GRX5
Norm. Signal Sum
3e+07
2e+07
1e+07
0e+00
S1_WT
S1_WT_IMS
S1_WT_MATRIX
S1_atp23
S1_atp23_IMS
S1_atp23_MATRIX
S2_WT
S2_WT_IMS
S2_WT_MATRIX
S2_atp6
S2_atp6_IMS
S2_atp6_MATRIX

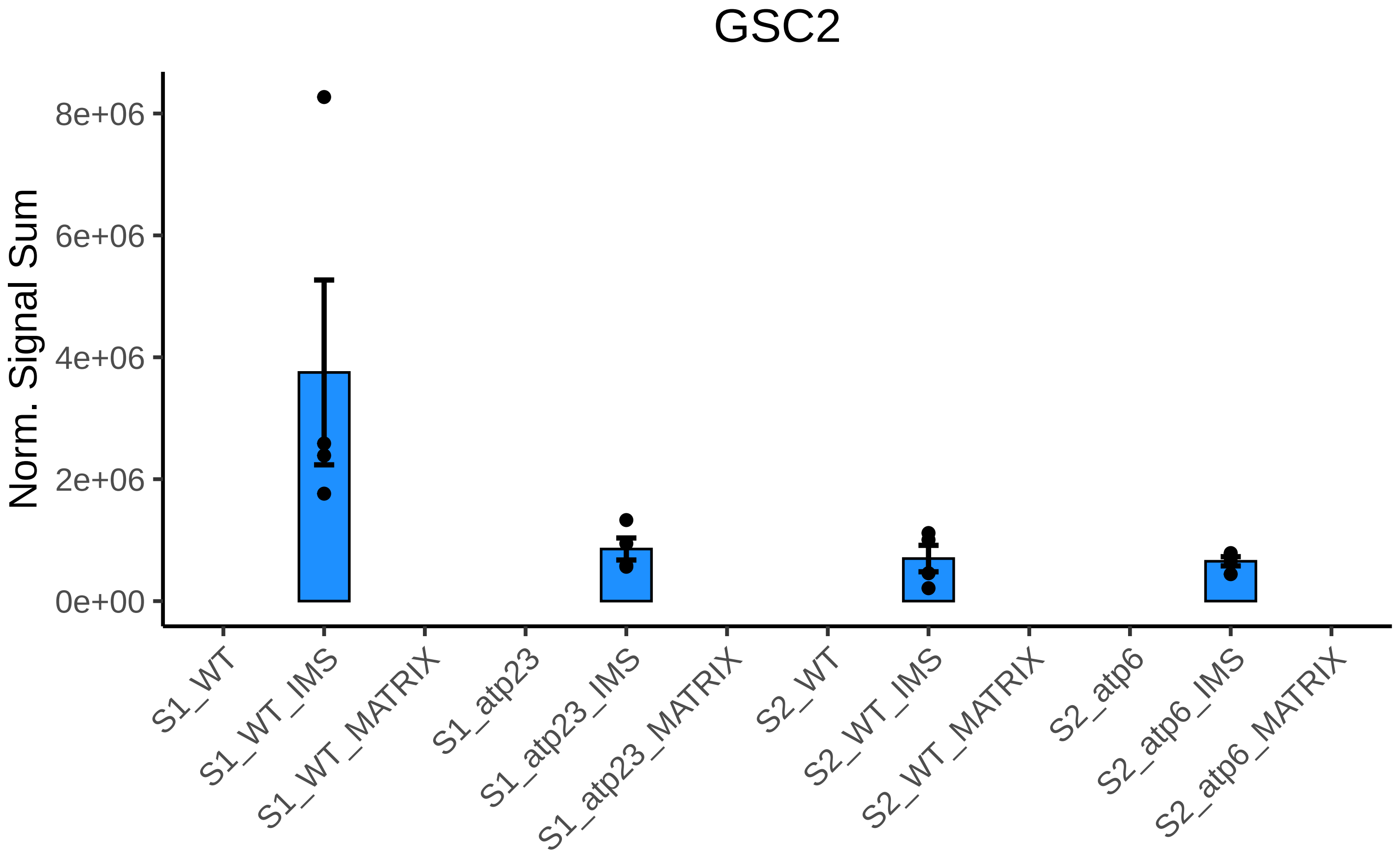
GSC2
Norm. Signal Sum
8e+06
6e+06
4e+06
2e+06
0e+00
S1_WT
S1_WT_IMS
S1_WT_MATRIX
S1_atp23
S1_atp23_IMS
S1_atp23_MATRIX
S2_WT
S2_WT_IMS
S2_WT_MATRIX
S2_atp6
S2_atp6_IMS
S2_atp6_MATRIX

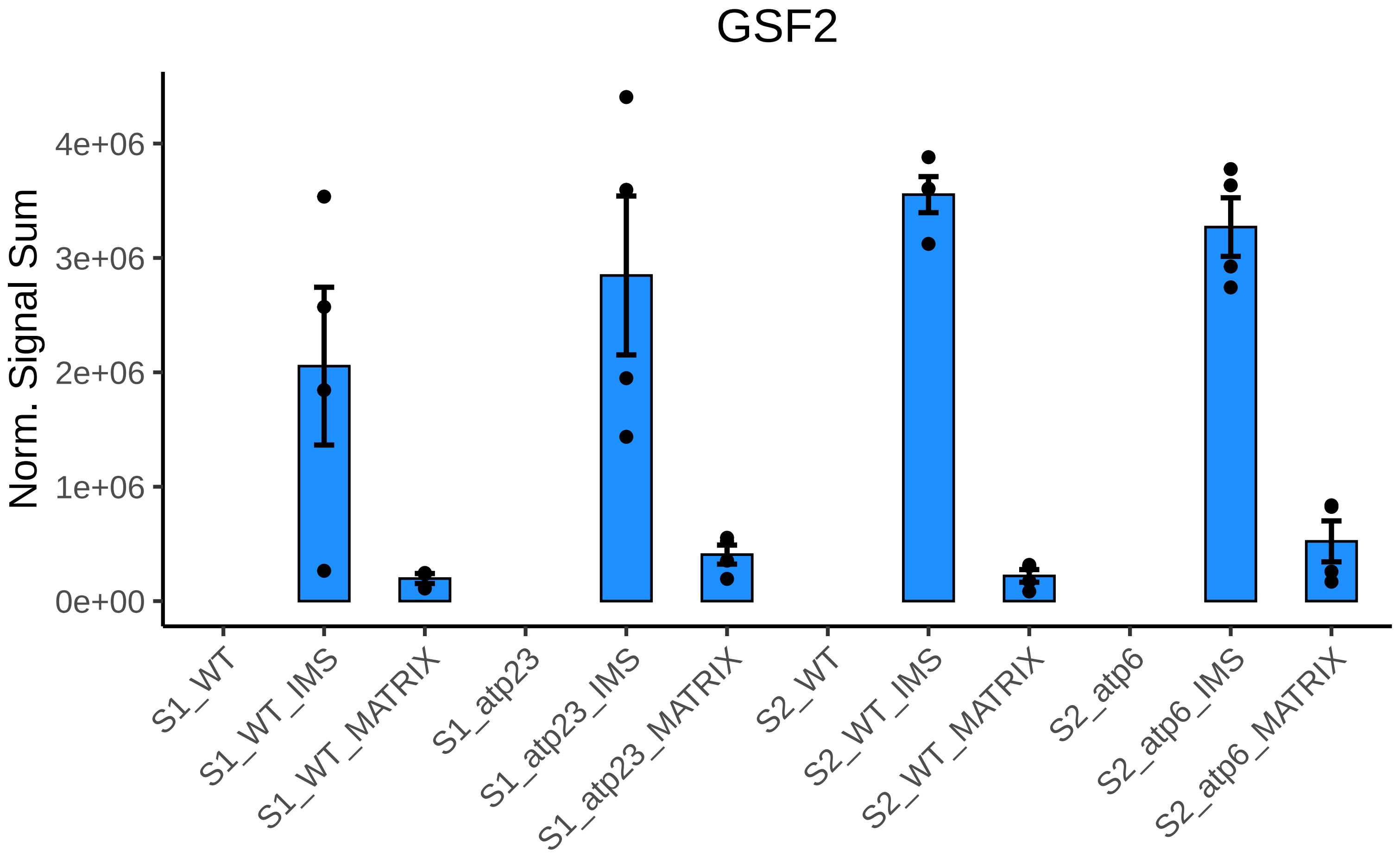

GSF2
Norm. Signal Sum
4e+06
3e+06
2e+06
1e+06
0e+00
S1_WT
S1_WT_IMS
S1_WT_MATRIX
S1_atp23
S1_atp23_IMS
S1_atp23_MATRIX
S2_WT
S2_WT_IMS
S2_WT_MATRIX
S2_atp6
S2_atp6_IMS
S2_atp6_MATRIX

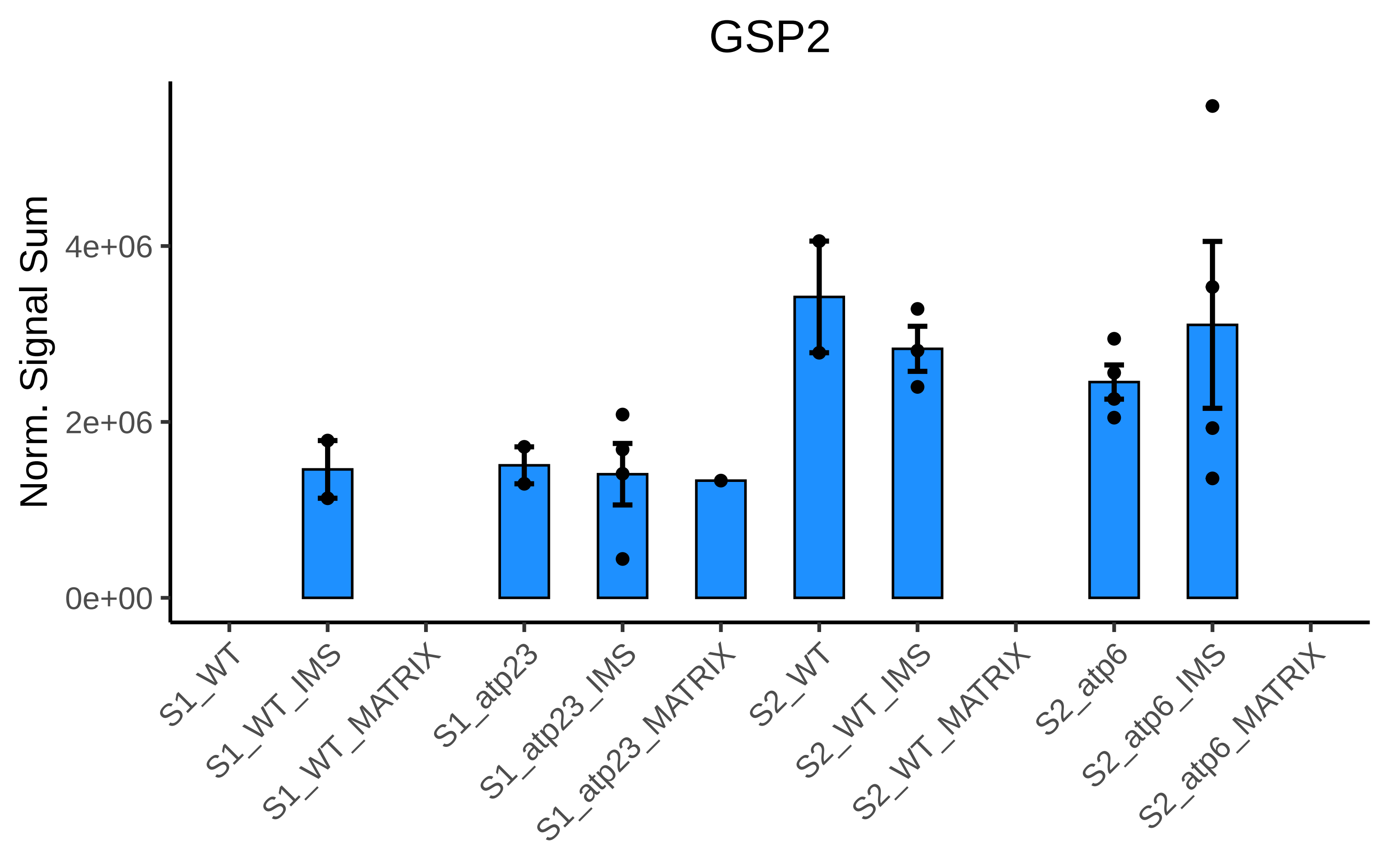
GSP2
Norm. Signal Sum
4e+06
2e+06
0e+00
S1_WT
S1_WT_IMS
S1_WT_MATRIX
S1_atp23
S1_atp23_IMS
S1_atp23_MATRIX
S2_WT
S2_WT_IMS
S2_WT_MATRIX
S2_atp6
S2_atp6_IMS
S2_atp6_MATRIX

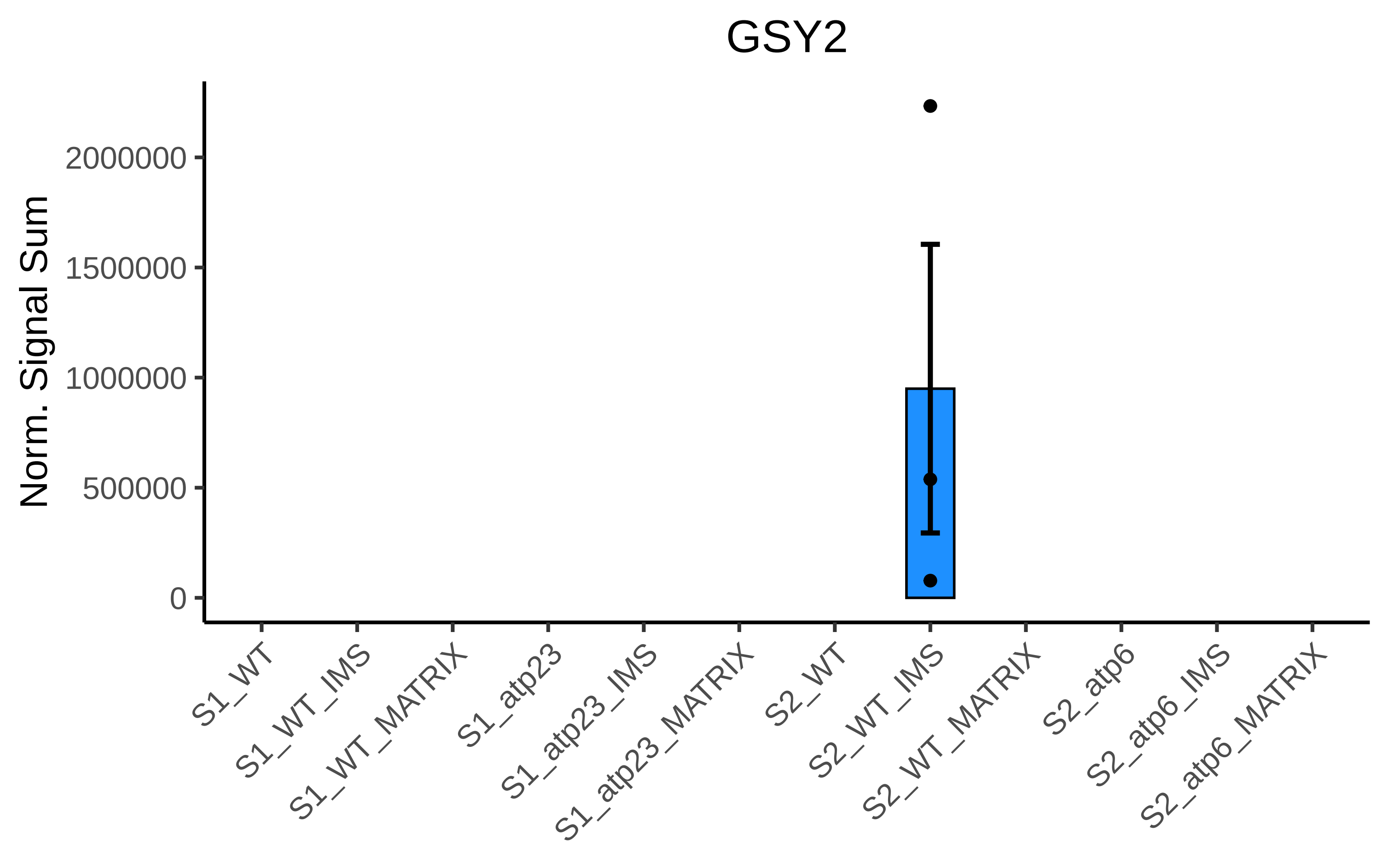
GSY2
Norm. Signal Sum
2000000
1500000
1000000
500000
0
S1_WT
S1_WT_IMS
S1_WT_MATRIX
S1_atp23
S1_atp23_IMS
S1_atp23_MATRIX
S2_WT
S2_WT_IMS
S2_WT_MATRIX
S2_atp6
S2_atp6_IMS
S2_atp6_MATRIX

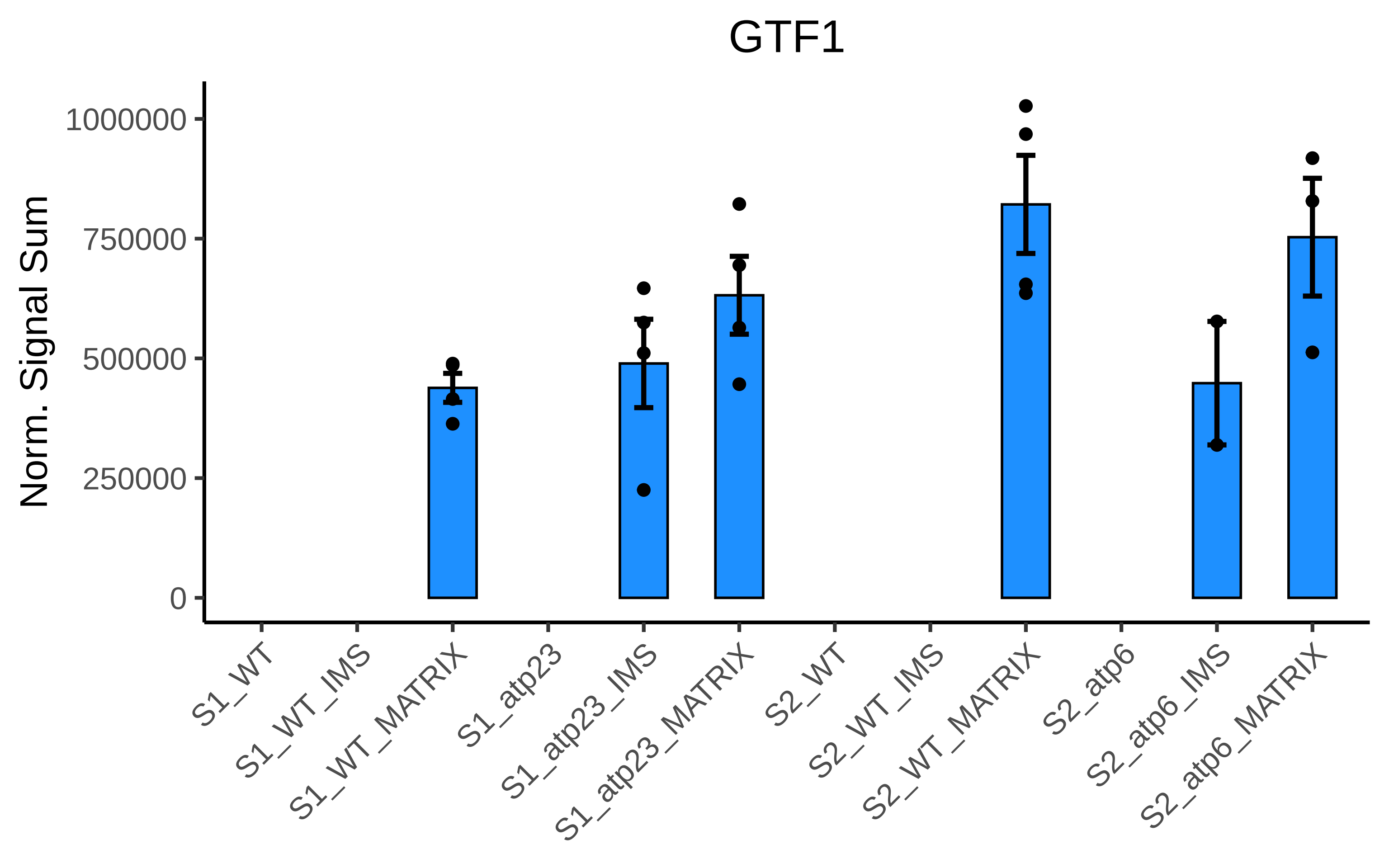

GTF1
Norm. Signal Sum
1000000
750000
500000
250000
0
S1_WT
S1_WT_IMS
S1_WT_MATRIX
S1_atp23
S1_atp23_IMS
S1_atp23_MATRIX
S2_WT
S2_WT_IMS
S2_WT_MATRIX
S2_atp6
S2_atp6_IMS
S2_atp6_MATRIX

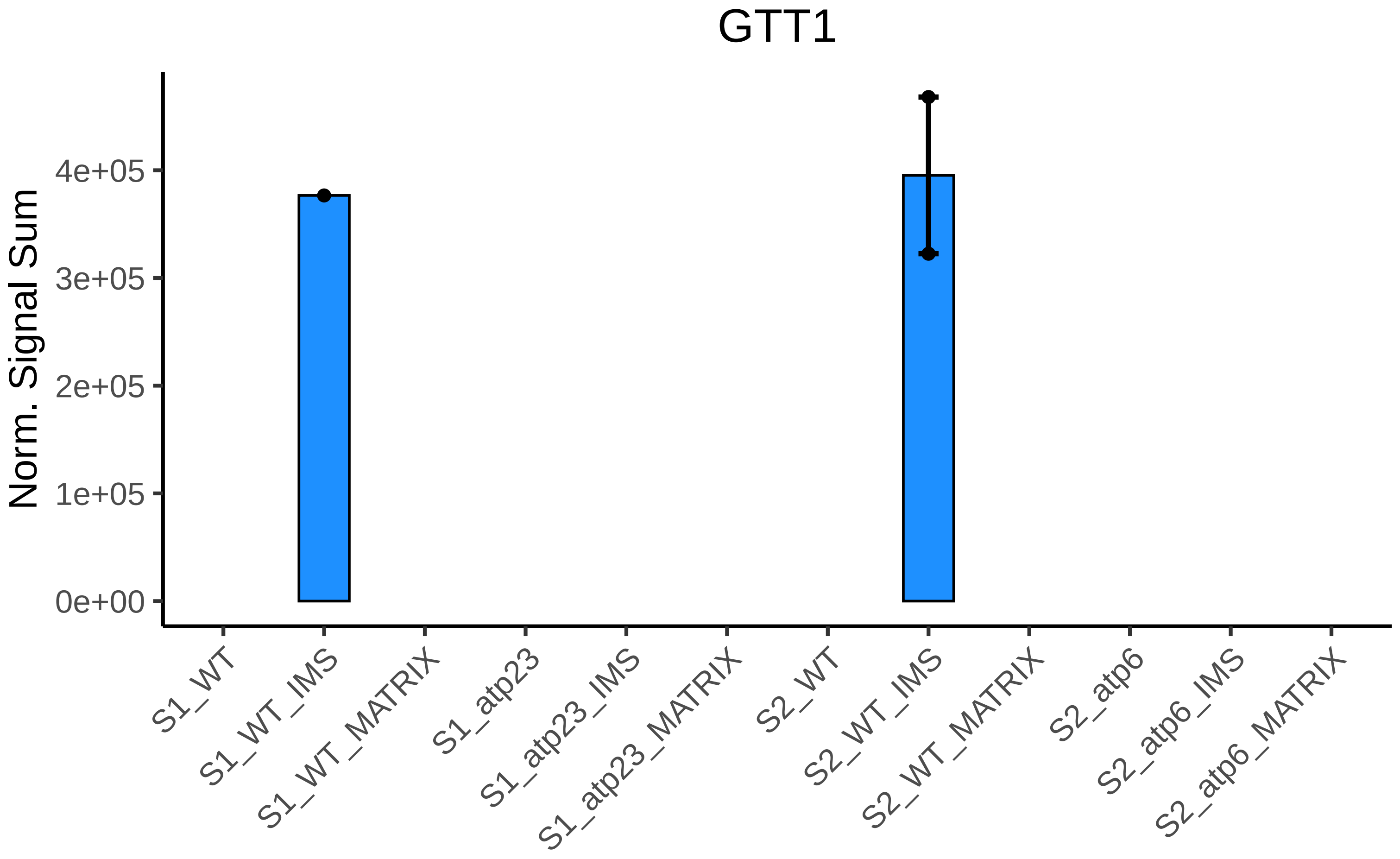
GTT1
Norm. Signal Sum
0e+00
1e+05
2e+05
3e+05
4e+05
S1_WT
S1_WT_IMS
S1_WT_MATRIX
S1_atp23
S1_atp23_IMS
S1_atp23_MATRIX
S2_WT
S2_WT_IMS
S2_WT_MATRIX
S2_atp6
S2_atp6_IMS
S2_atp6_MATRIX

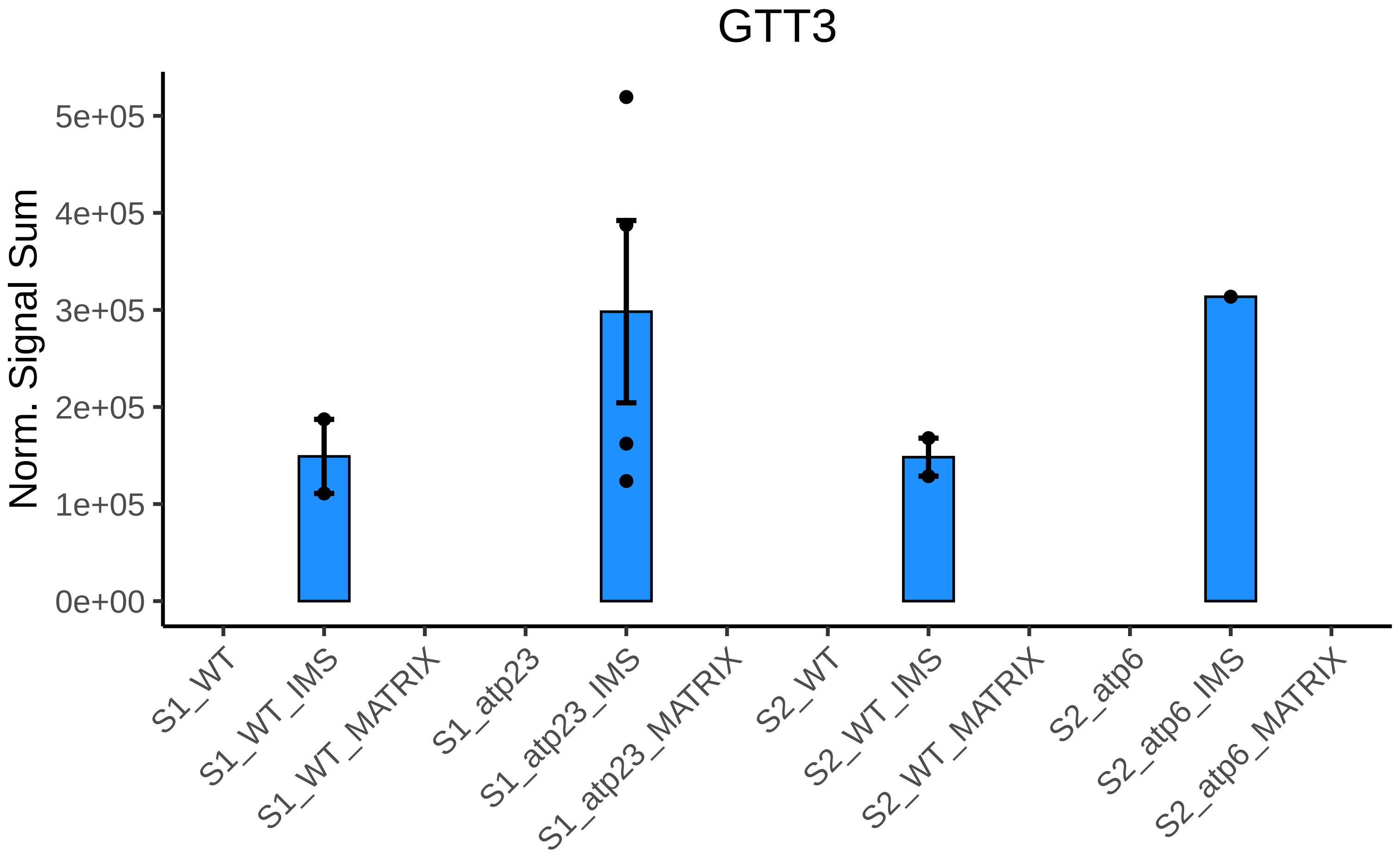
GTT3
Norm. Signal Sum
5e+05
4e+05
3e+05
2e+05
1e+05
0e+00
S1_WT
S1_WT_IMS
S1_WT_MATRIX
S1_atp23
S1_atp23_IMS
S1_atp23_MATRIX
S2_WT
S2_WT_IMS
S2_WT_MATRIX
S2_atp6
S2_atp6_IMS
S2_atp6_MATRIX

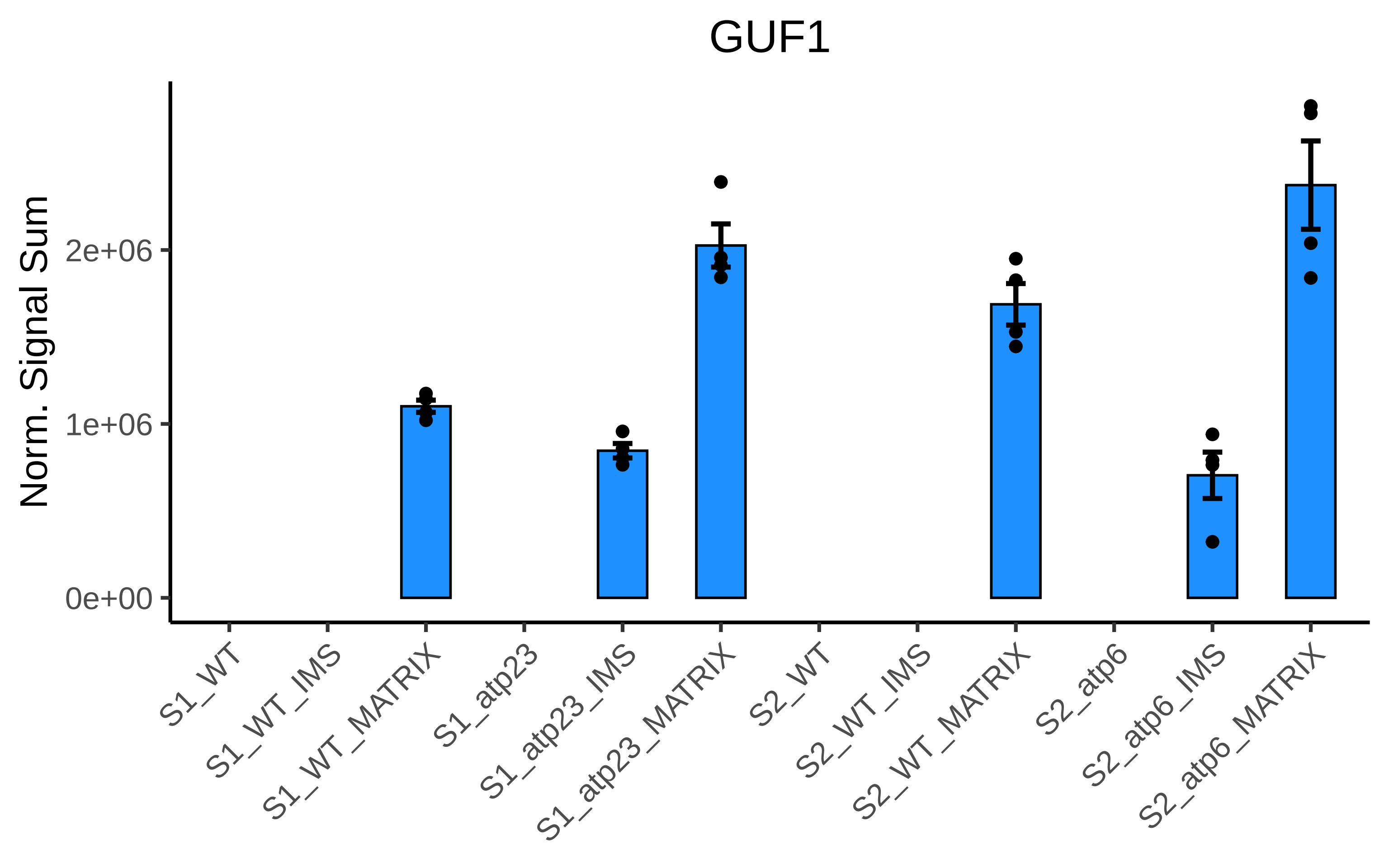
GUF1
Norm. Signal Sum
0e+00
1e+06
2e+06
S1_WT
S1_WT_IMS
S1_WT_MATRIX
S1_atp23
S1_atp23_IMS
S1_atp23_MATRIX
S2_WT
S2_WT_IMS
S2_WT_MATRIX
S2_atp6
S2_atp6_IMS
S2_atp6_MATRIX

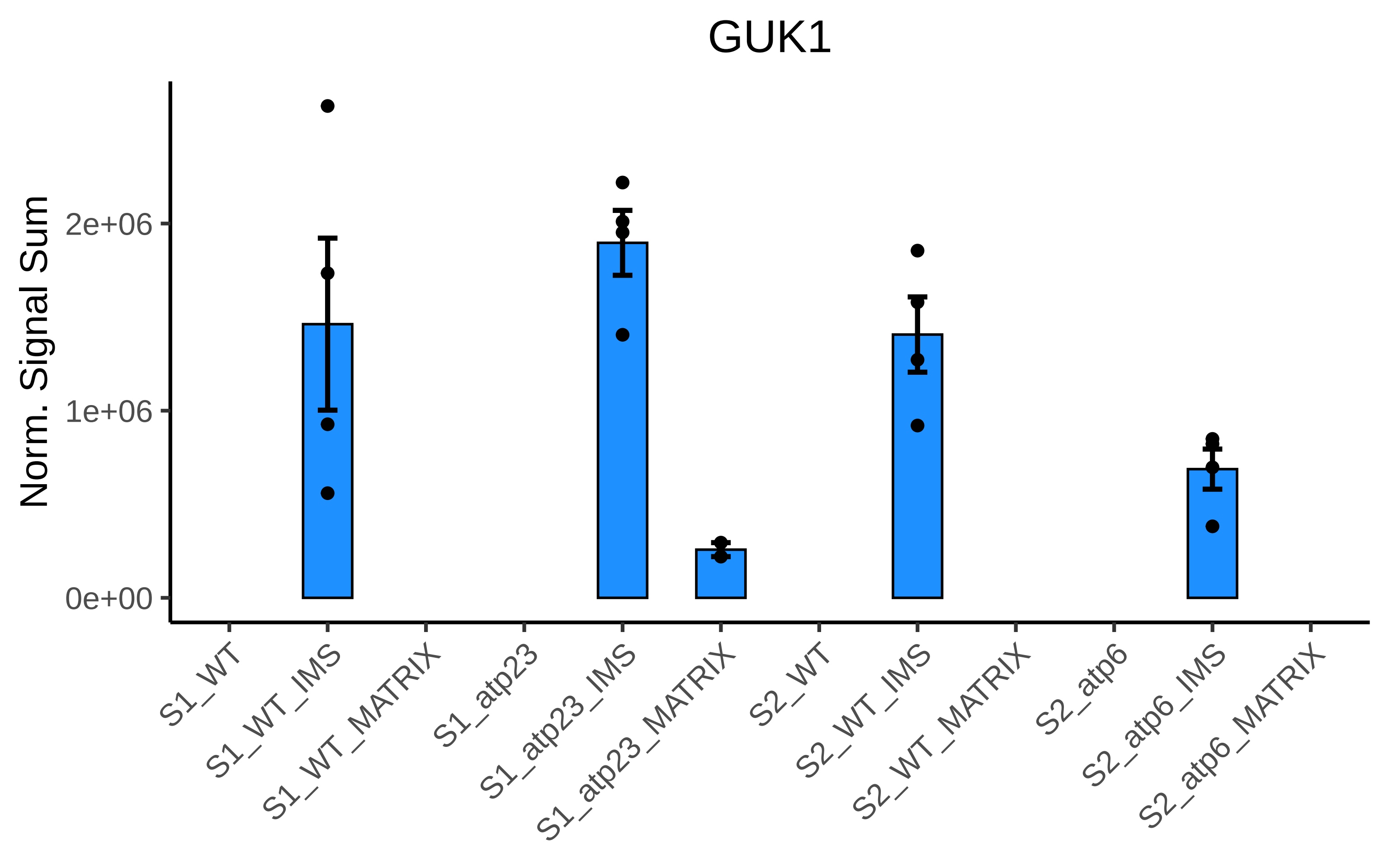
GUK1
Norm. Signal Sum
2e+06
1e+06
0e+00
S1_WT
S1_WT_IMS
S1_WT_MATRIX
S1_atp23
S1_atp23_IMS
S1_atp23_MATRIX
S2_WT
S2_WT_IMS
S2_WT_MATRIX
S2_atp6
S2_atp6_IMS
S2_atp6_MATRIX

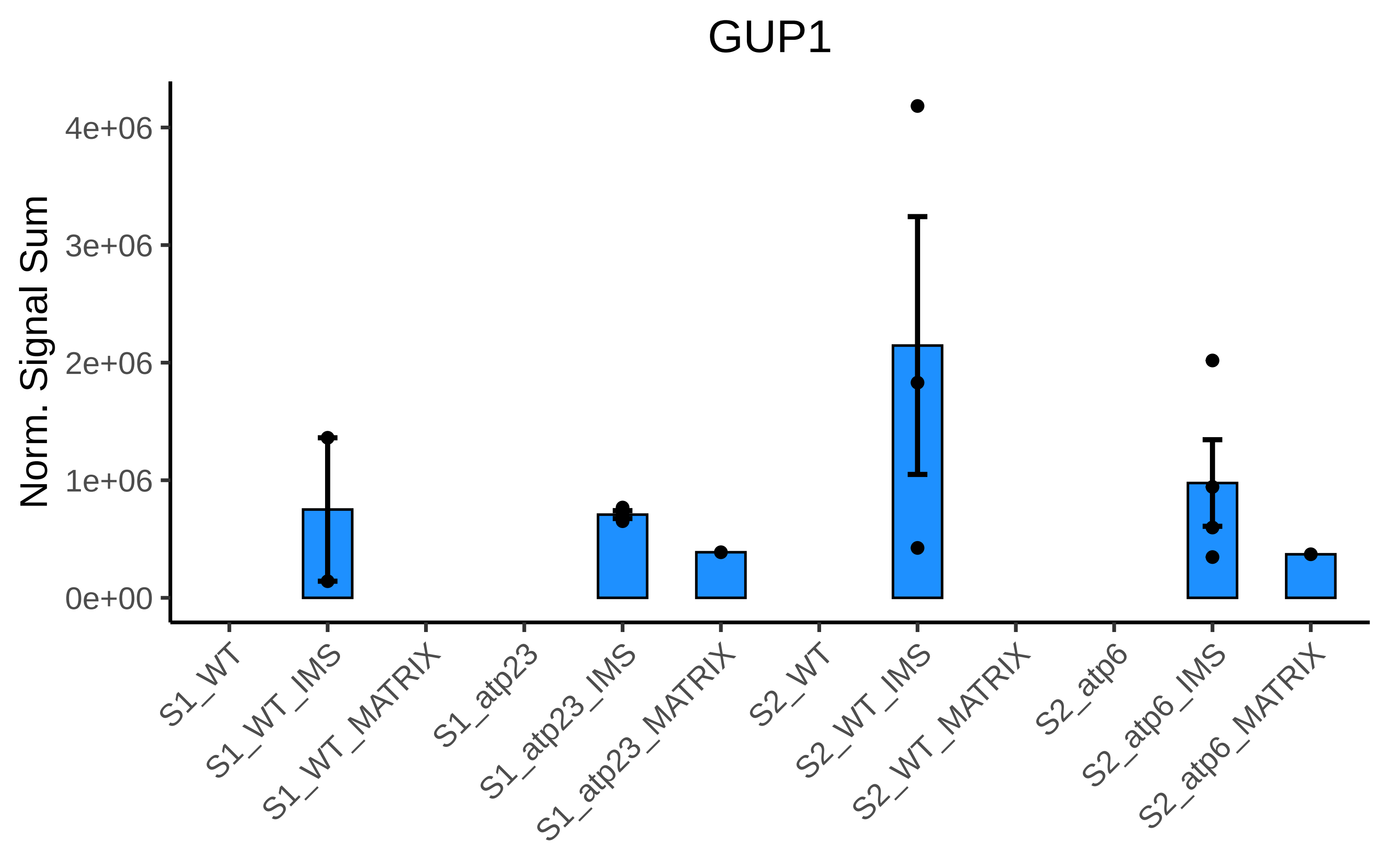

GUP1
Norm. Signal Sum
4e+06
3e+06
2e+06
1e+06
0e+00
S1_WT
S1_WT_IMS
S1_WT_MATRIX
S1_atp23
S1_atp23_IMS
S1_atp23_MATRIX
S2_WT
S2_WT_IMS
S2_WT_MATRIX
S2_atp6
S2_atp6_IMS
S2_atp6_MATRIX

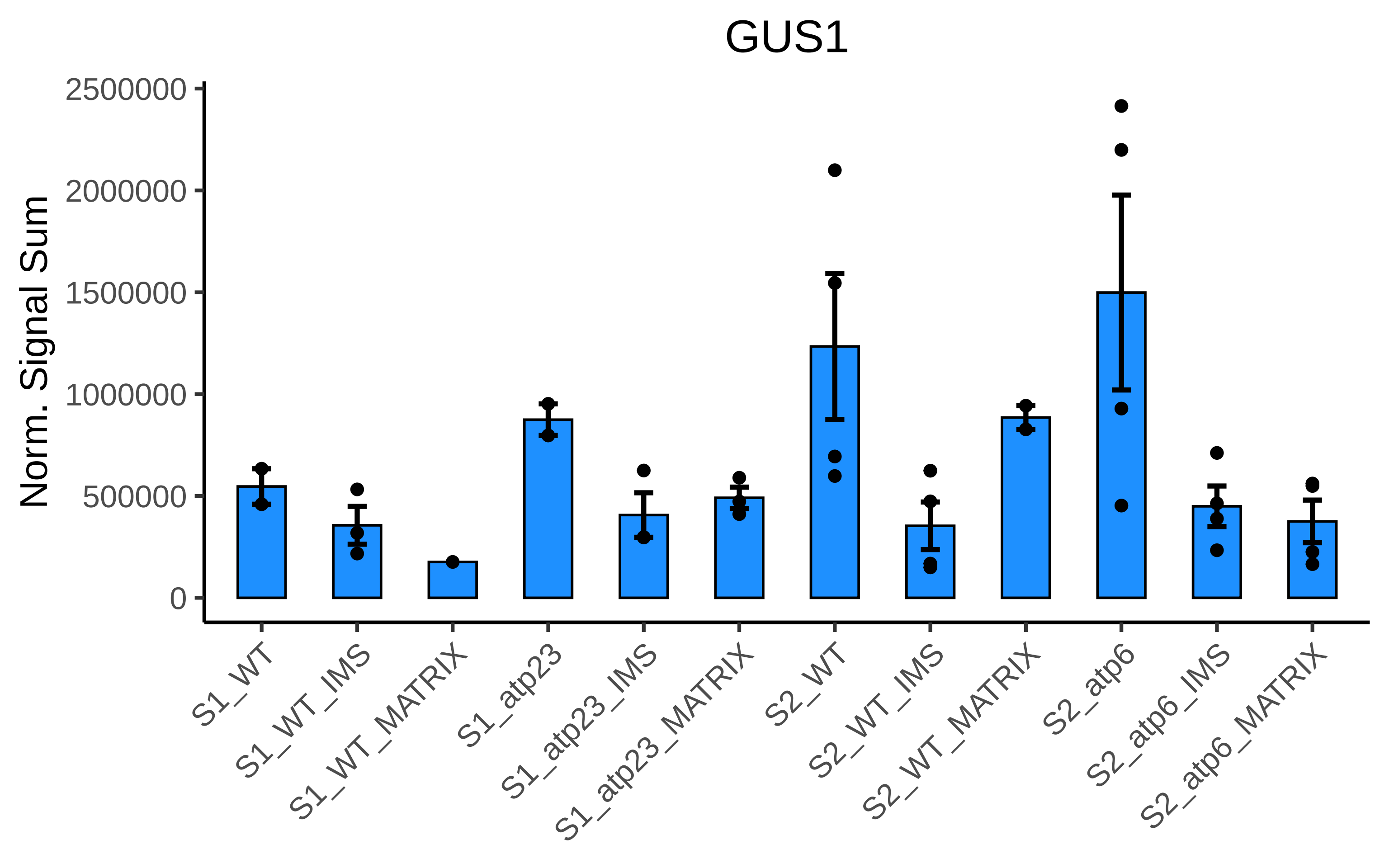
GUS1
Norm. Signal Sum
2500000
2000000
1500000
1000000
500000
0
S1_WT
S1_WT_IMS
S1_WT_MATRIX
S1_atp23
S1_atp23_IMS
S1_atp23_MATRIX
S2_WT
S2_WT_IMS
S2_WT_MATRIX
S2_atp6
S2_atp6_IMS
S2_atp6_MATRIX

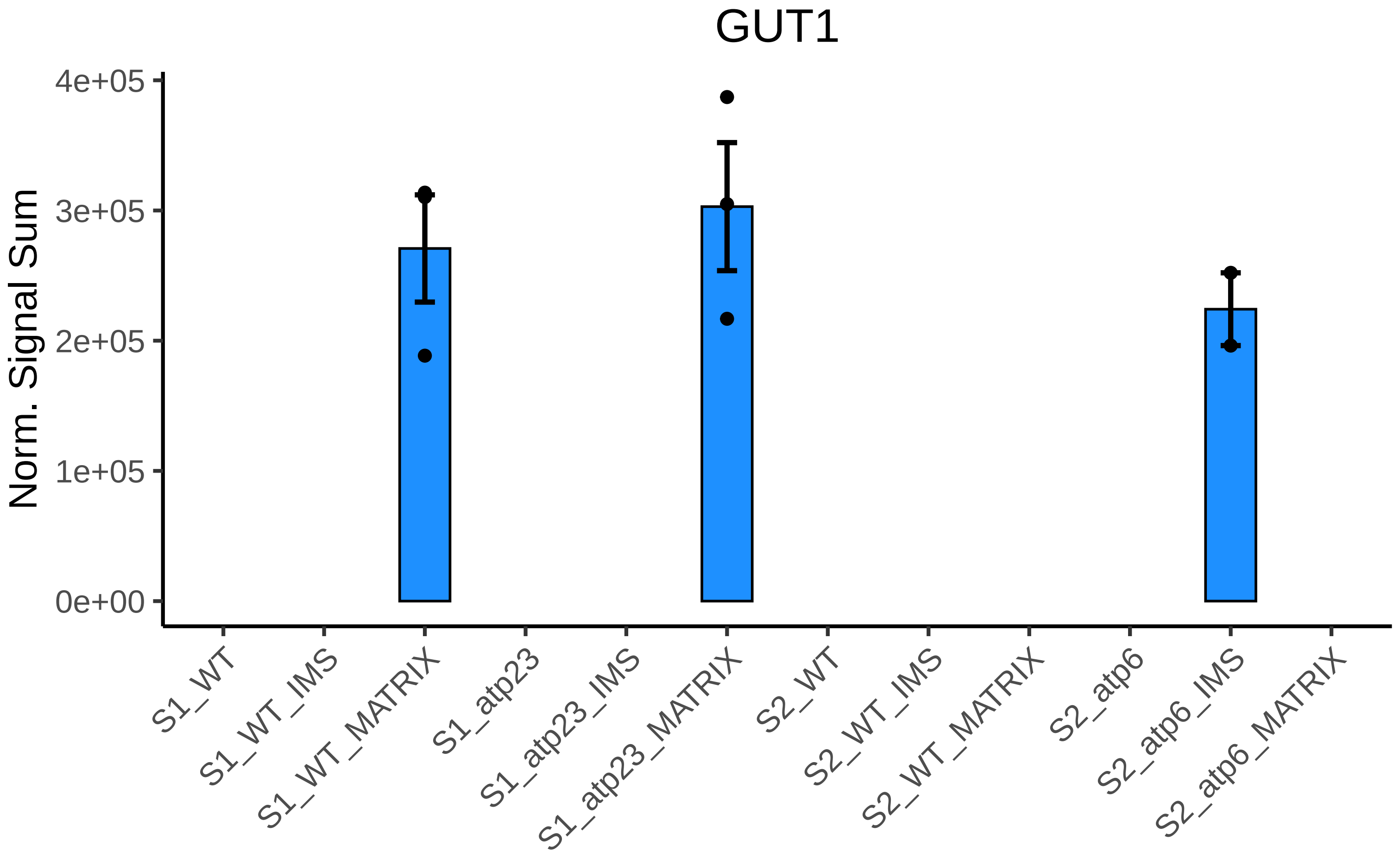
GUT1
Norm. Signal Sum
4e+05
3e+05
2e+05
1e+05
0e+00
S1_WT
S1_WT_IMS
S1_WT_MATRIX
S1_atp23
S1_atp23_IMS
S1_atp23_MATRIX
S2_WT
S2_WT_IMS
S2_WT_MATRIX
S2_atp6
S2_atp6_IMS
S2_atp6_MATRIX

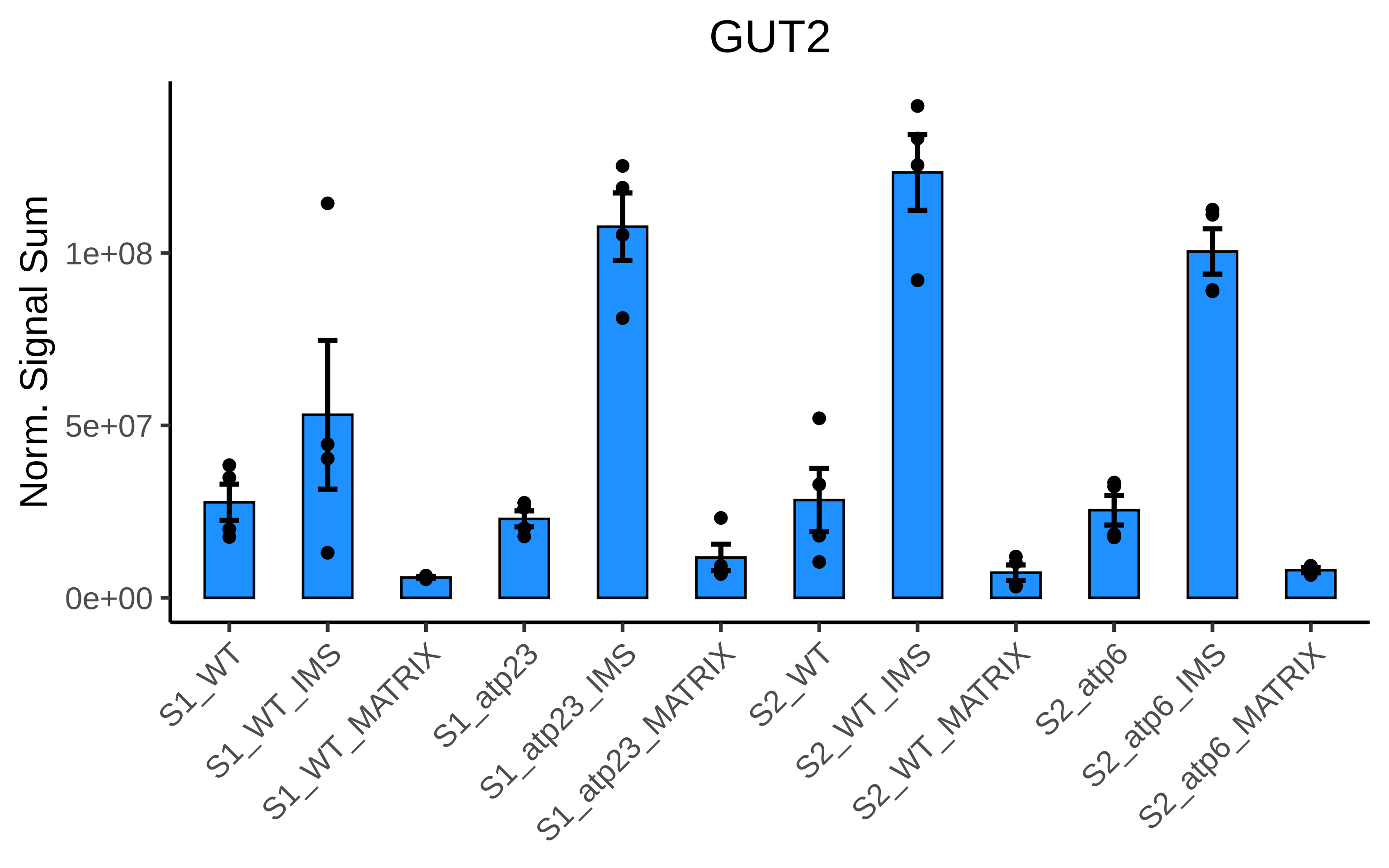

GUT2
Norm. Signal Sum
0e+00
5e+07
1e+08
S1_WT
S1_WT_IMS
S1_WT_MATRIX
S1_atp23
S1_atp23_IMS
S1_atp23_MATRIX
S2_WT
S2_WT_IMS
S2_WT_MATRIX
S2_atp6
S2_atp6_IMS
S2_atp6_MATRIX

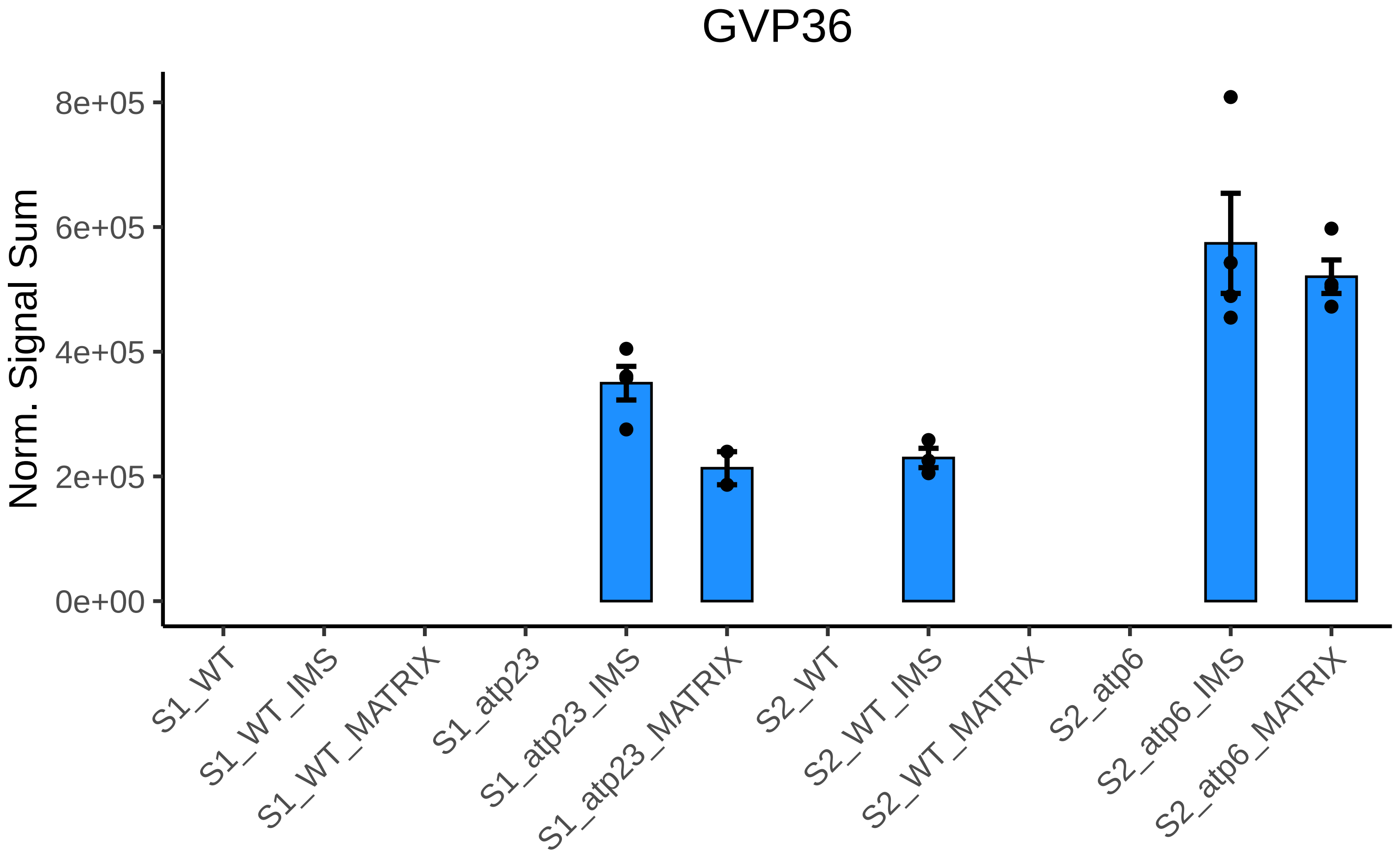
GVP36
Norm. Signal Sum
8e+05
6e+05
4e+05
2e+05
0e+00
S1_WT
S1_WT_IMS
S1_WT_MATRIX
S1_atp23
S1_atp23_IMS
S1_atp23_MATRIX
S2_WT
S2_WT_IMS
S2_WT_MATRIX
S2_atp6
S2_atp6_IMS
S2_atp6_MATRIX

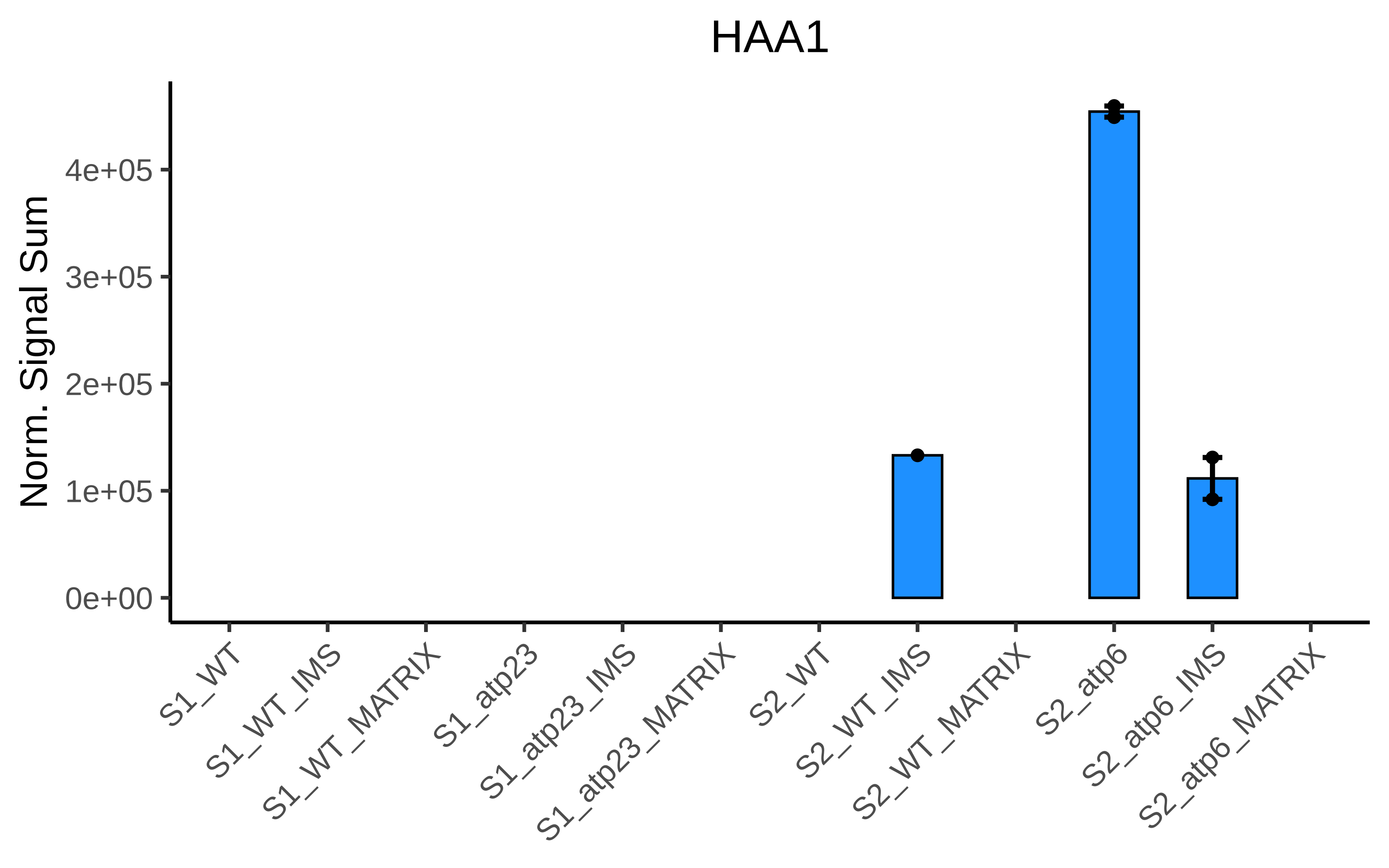
HAA1
Norm. Signal Sum
4e+05
3e+05
2e+05
1e+05
0e+00
S1_WT
S1_WT_IMS
S1_WT_MATRIX
S1_atp23
S1_atp23_IMS
S1_atp23_MATRIX
S2_WT
S2_WT_IMS
S2_WT_MATRIX
S2_atp6
S2_atp6_IMS
S2_atp6_MATRIX

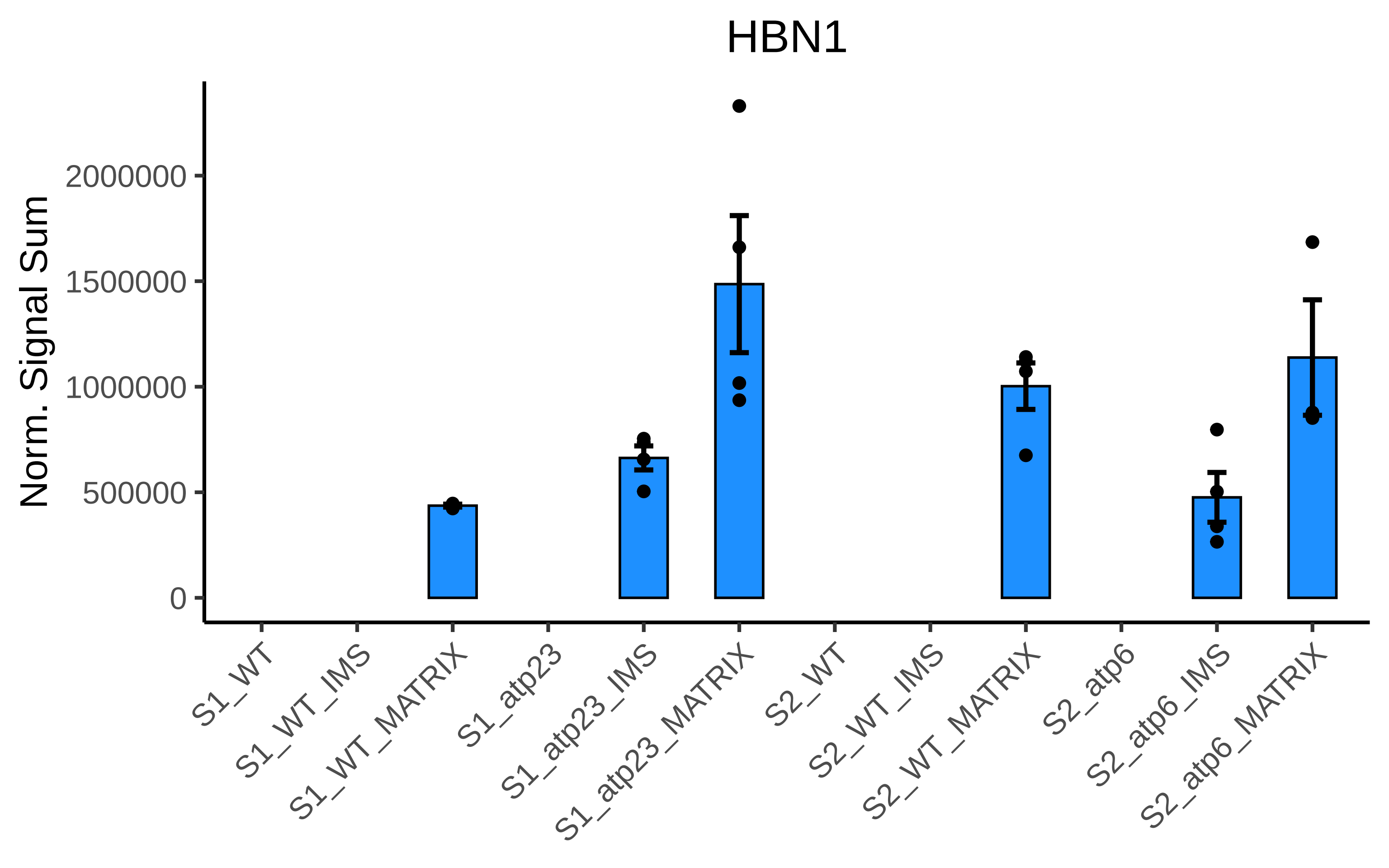

HBN1
Norm. Signal Sum
2000000
1500000
1000000
500000
0
S1_WT
S1_WT_IMS
S1_WT_MATRIX
S1_atp23
S1_atp23_IMS
S1_atp23_MATRIX
S2_WT
S2_WT_IMS
S2_WT_MATRIX
S2_atp6
S2_atp6_IMS
S2_atp6_MATRIX

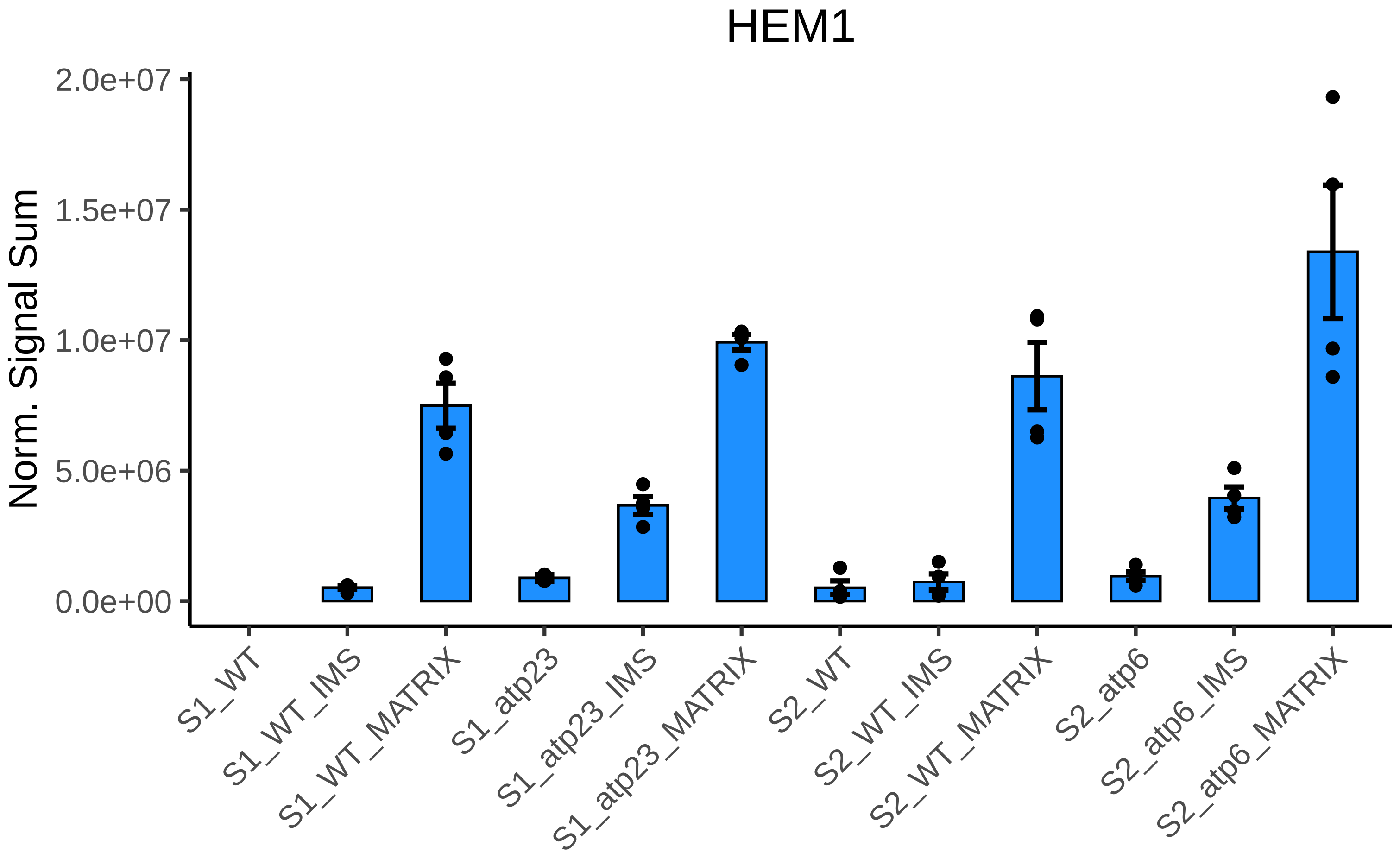
HEM1
Norm. Signal Sum
2.0e+07
1.5e+07
1.0e+07
5.0e+06
0.0e+00
S1_WT
S1_WT_IMS
S1_WT_MATRIX
S1_atp23
S1_atp23_IMS
S1_atp23_MATRIX
S2_WT
S2_WT_IMS
S2_WT_MATRIX
S2_atp6
S2_atp6_IMS
S2_atp6_MATRIX

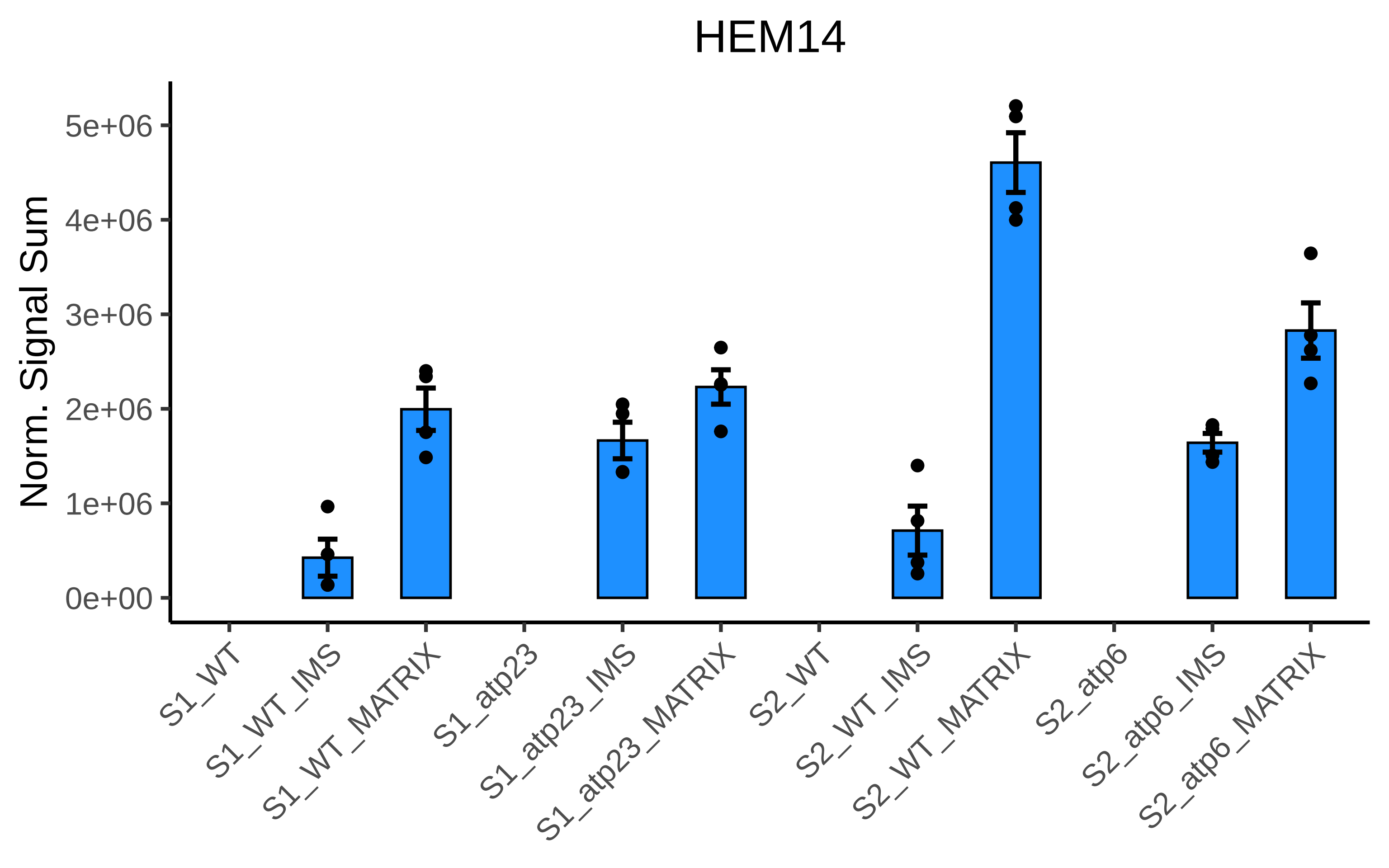
HEM14
Norm. Signal Sum
5e+06
4e+06
3e+06
2e+06
1e+06
0e+00
S1_WT
S1_WT_IMS
S1_WT_MATRIX
S1_atp23
S1_atp23_IMS
S1_atp23_MATRIX
S2_WT
S2_WT_IMS
S2_WT_MATRIX
S2_atp6
S2_atp6_IMS
S2_atp6_MATRIX

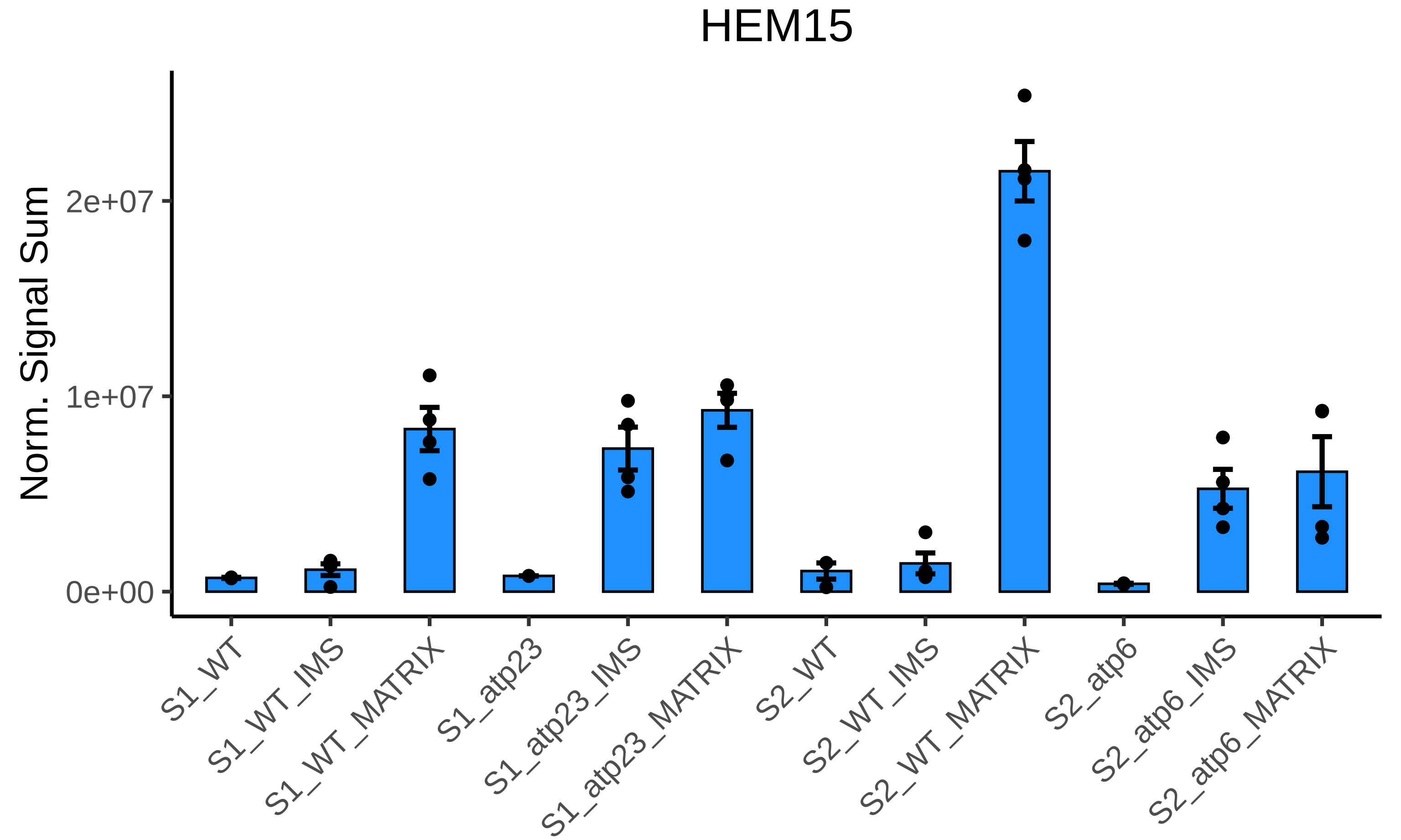

HEM15
Norm. Signal Sum
2e+07
1e+07
0e+00
S1_WT
S1_WT_IMS
S1_WT_MATRIX
S1_atp23
S1_atp23_IMS
S1_atp23_MATRIX
S2_WT
S2_WT_IMS
S2_WT_MATRIX
S2_atp6
S2_atp6_IMS
S2_atp6_MATRIX

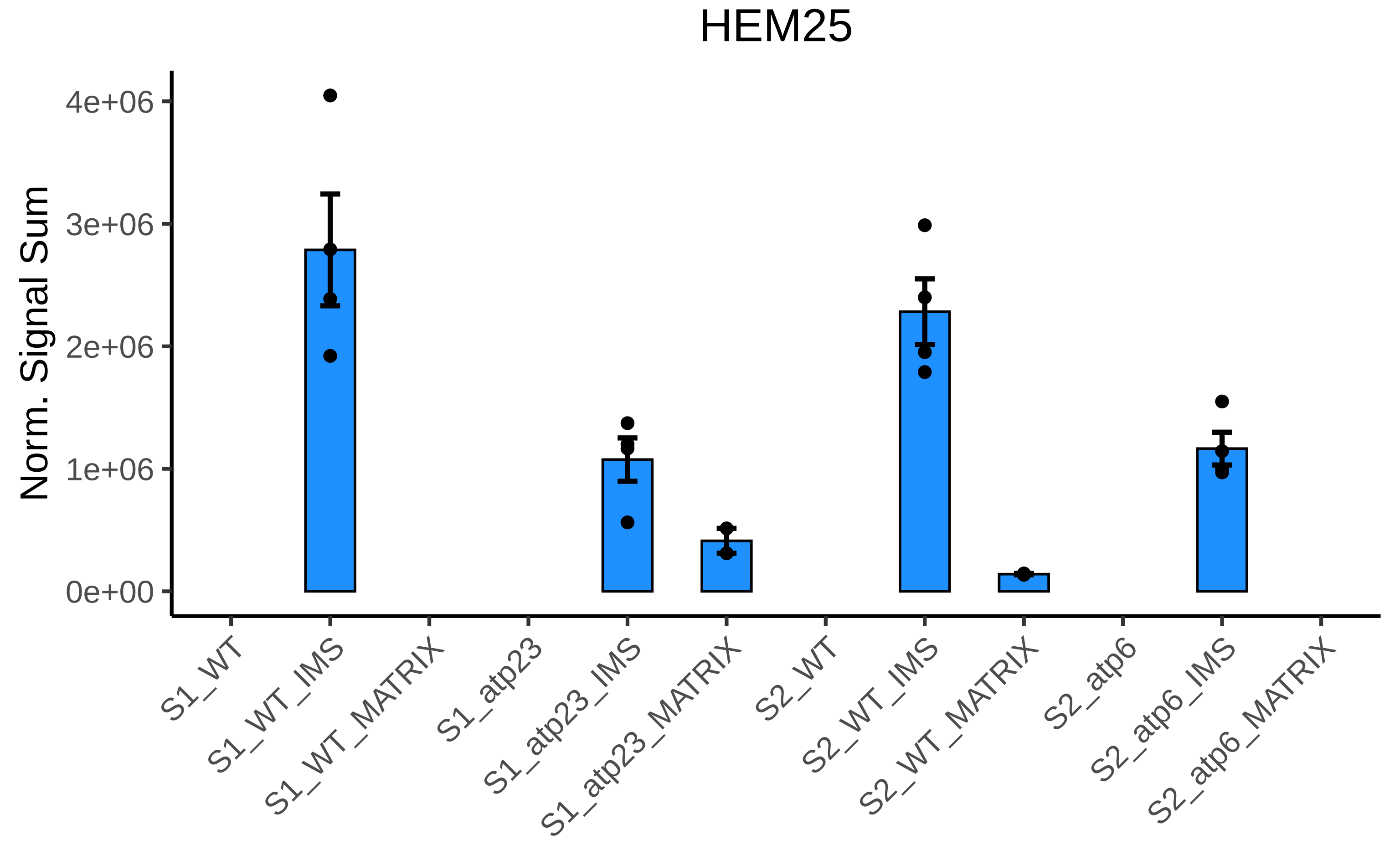

HEM25
Norm. Signal Sum
4e+06
3e+06
2e+06
1e+06
0e+00
S1_WT
S1_WT_IMS
S1_WT_MATRIX
S1_atp23
S1_atp23_IMS
S1_atp23_MATRIX
S2_WT
S2_WT_IMS
S2_WT_MATRIX
S2_atp6
S2_atp6_IMS
S2_atp6_MATRIX

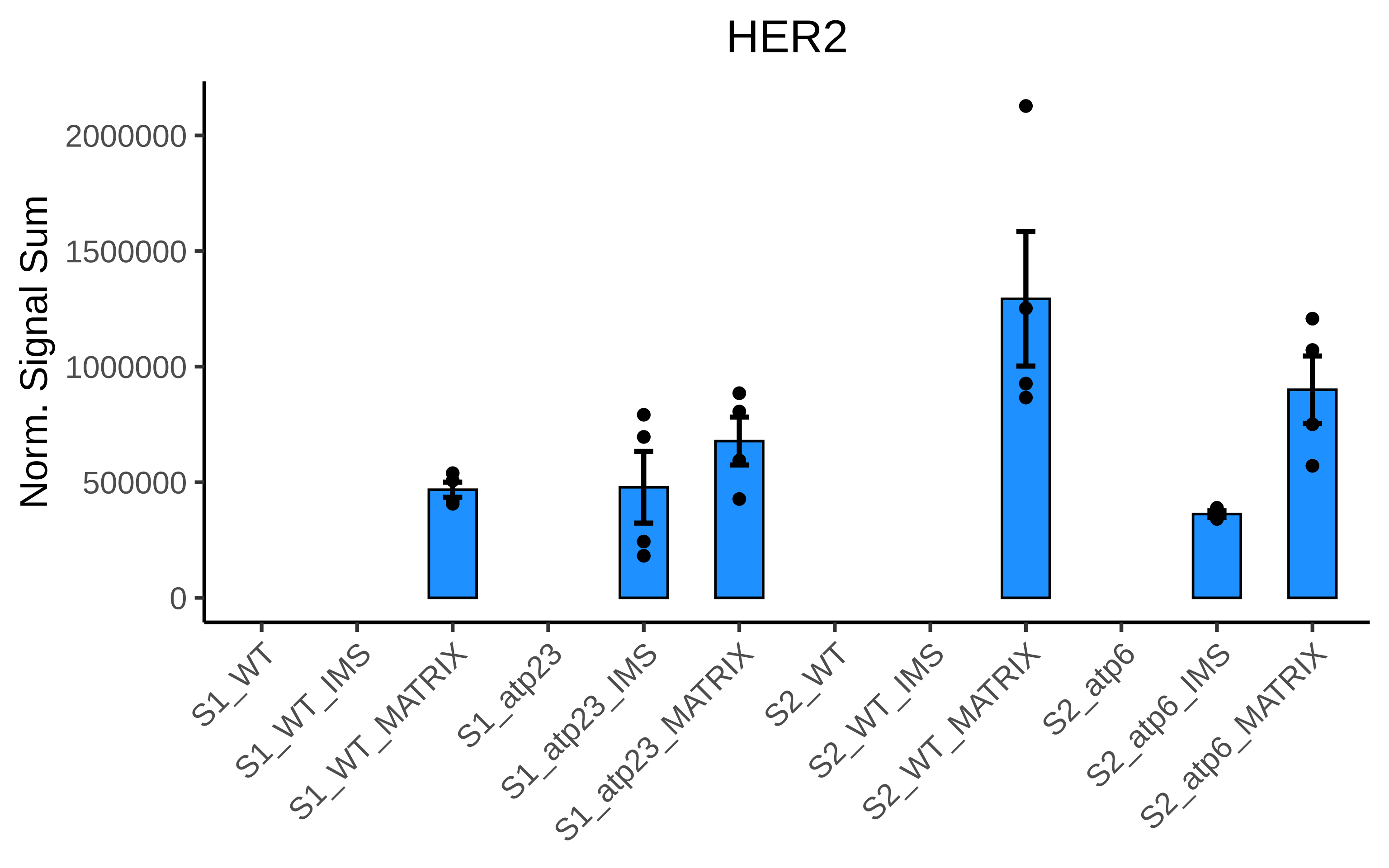

HER2
Norm. Signal Sum
2000000
1500000
1000000
500000
0
S1_WT
S1_WT_IMS
S1_WT_MATRIX
S1_atp23
S1_atp23_IMS
S1_atp23_MATRIX
S2_WT
S2_WT_IMS
S2_WT_MATRIX
S2_atp6
S2_atp6_IMS
S2_atp6_MATRIX

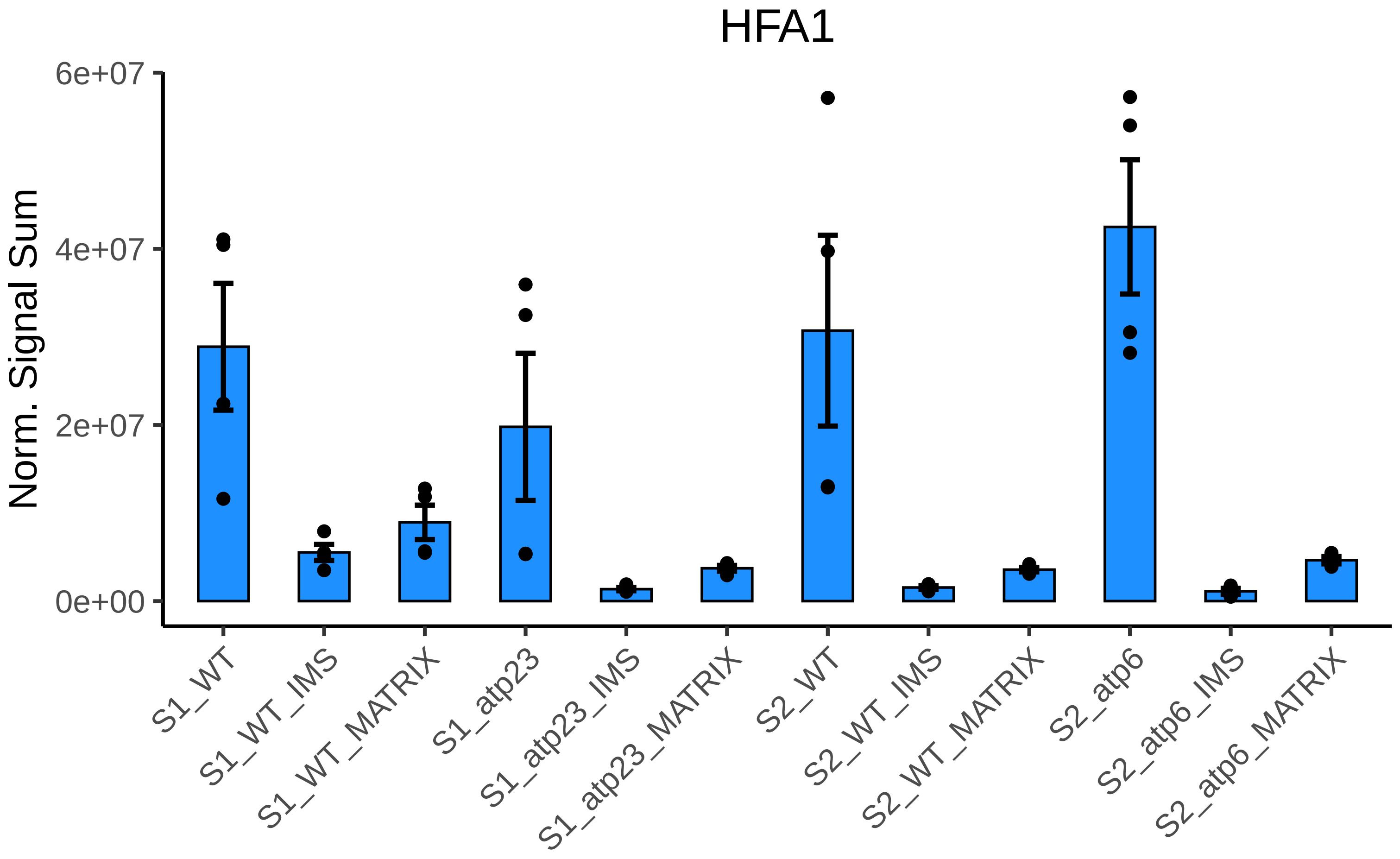
HFA1
Norm. Signal Sum
6e+07
4e+07
2e+07
0e+00
S1_WT
S1_WT_IMS
S1_WT_MATRIX
S1_atp23
S1_atp23_IMS
S1_atp23_MATRIX
S2_WT
S2_WT_IMS
S2_WT_MATRIX
S2_atp6
S2_atp6_IMS
S2_atp6_MATRIX

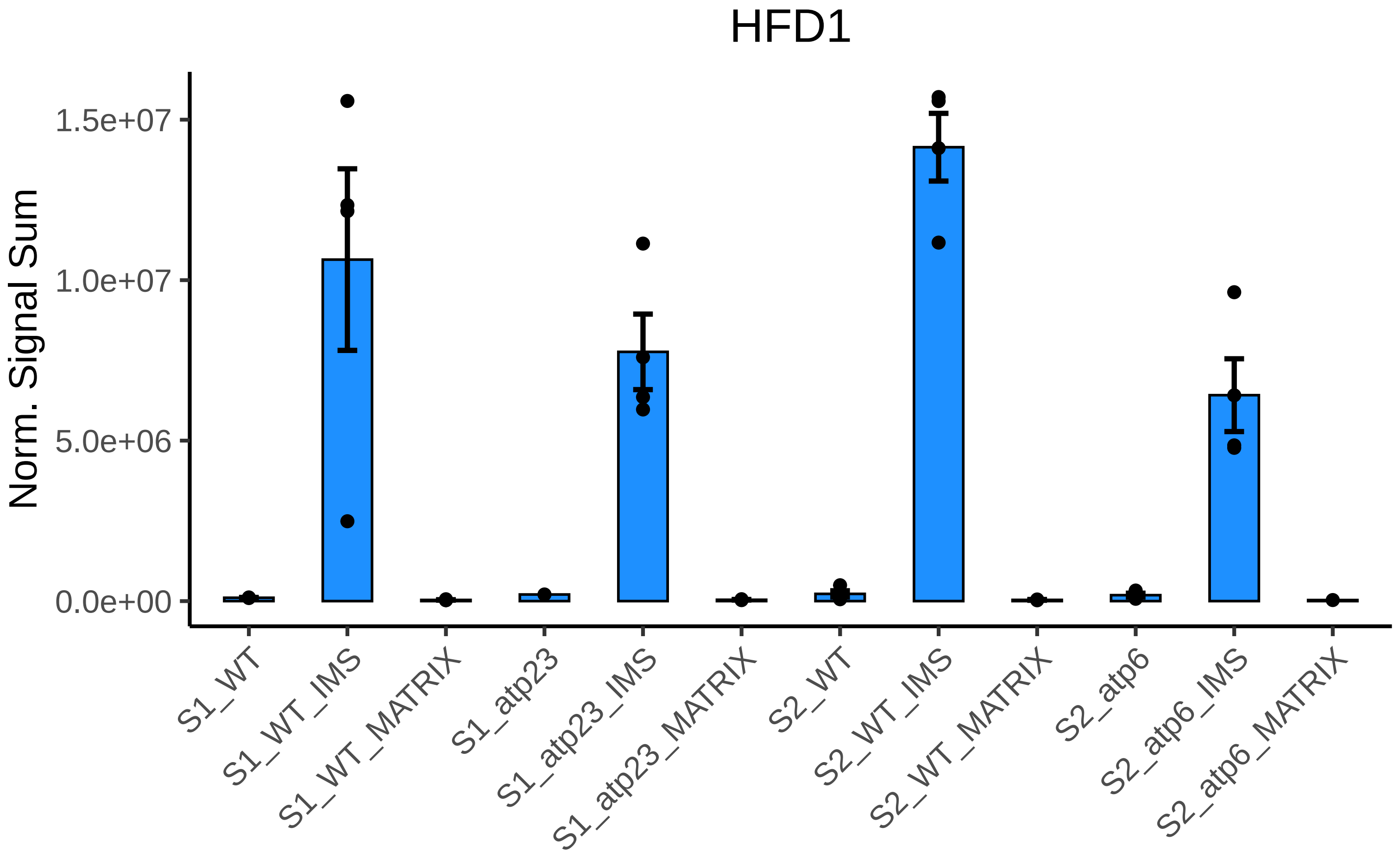

HFD1
Norm. Signal Sum
1.5e+07
1.0e+07
5.0e+06
0.0e+00
S1_WT
S1_WT_IMS
S1_WT_MATRIX
S1_atp23
S1_atp23_IMS
S1_atp23_MATRIX
S2_WT
S2_WT_IMS
S2_WT_MATRIX
S2_atp6
S2_atp6_IMS
S2_atp6_MATRIX

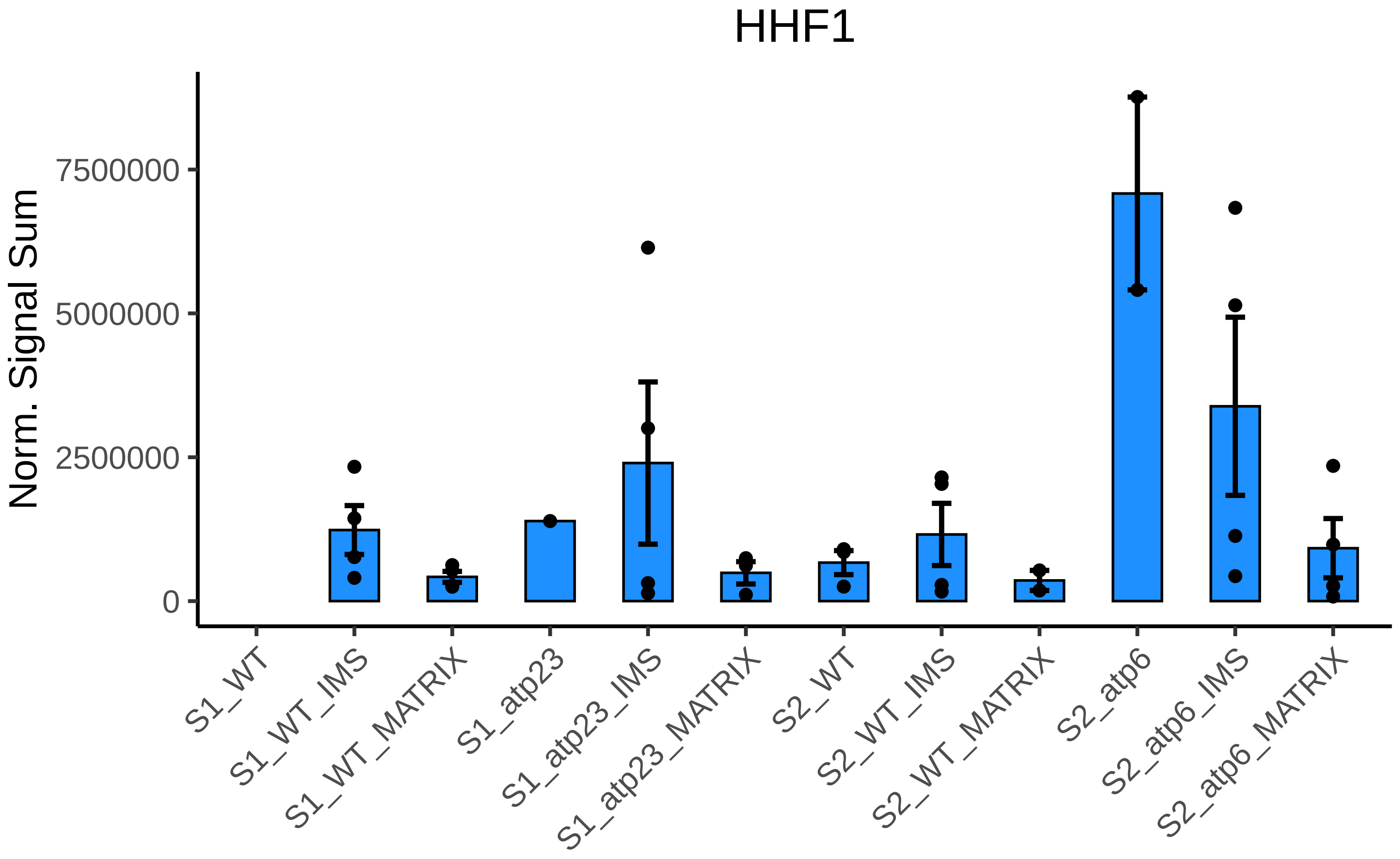
HHF1
Norm. Signal Sum
7500000
5000000
2500000
0
S1_WT
S1_WT_IMS
S1_WT_MATRIX
S1_atp23
S1_atp23_IMS
S1_atp23_MATRIX
S2_WT
S2_WT_IMS
S2_WT_MATRIX
S2_atp6
S2_atp6_IMS
S2_atp6_MATRIX

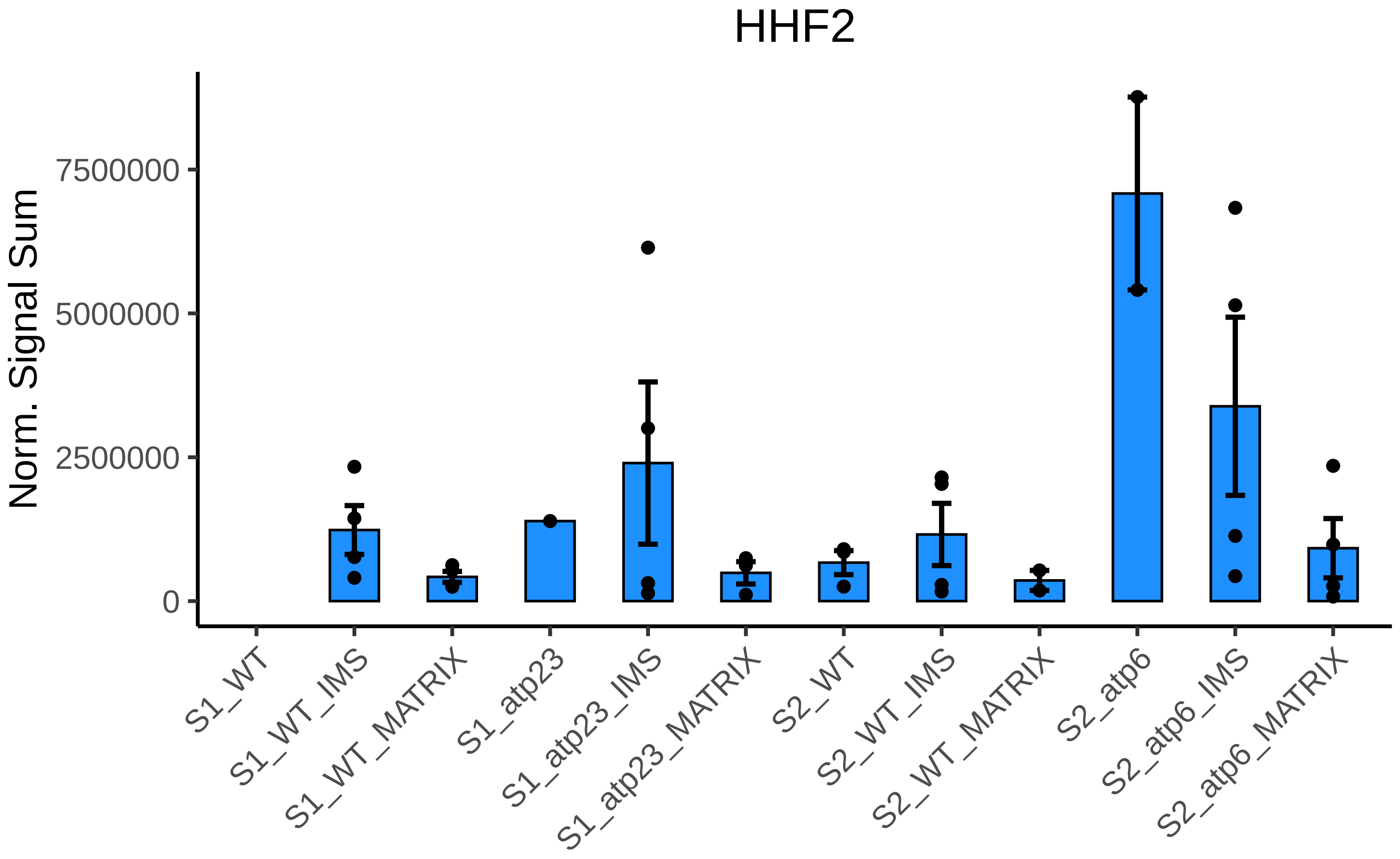

HHF2
Norm. Signal Sum
7500000
5000000
2500000
0
S1_WT
S1_WT_IMS
S1_WT_MATRIX
S1_atp23
S1_atp23_IMS
S1_atp23_MATRIX
S2_WT
S2_WT_IMS
S2_WT_MATRIX
S2_atp6
S2_atp6_IMS
S2_atp6_MATRIX

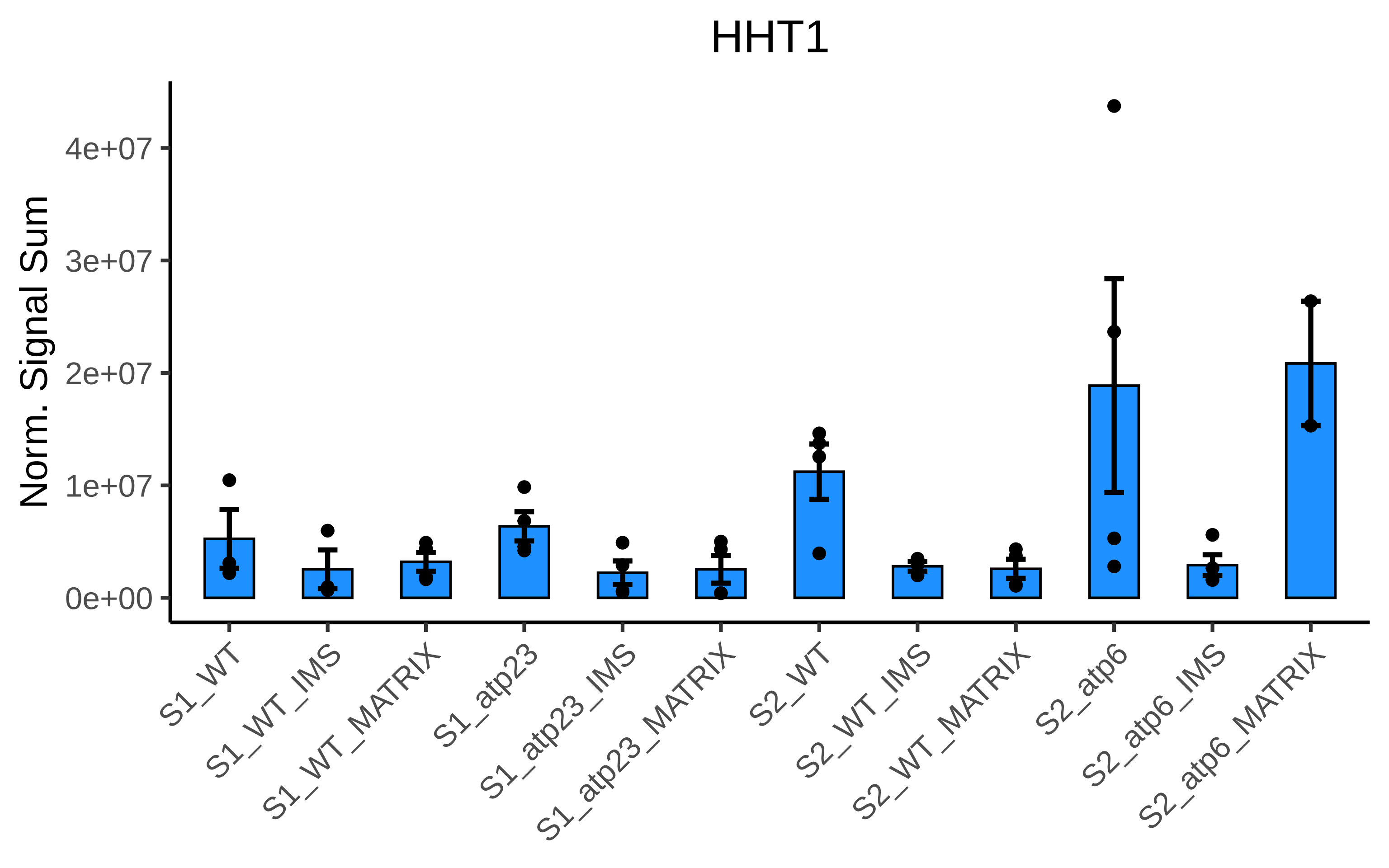
HHT1
Norm. Signal Sum
4e+07
3e+07
2e+07
1e+07
0e+00
S1_WT
S1_WT_IMS
S1_WT_MATRIX
S1_atp23
S1_atp23_IMS
S1_atp23_MATRIX
S2_WT
S2_WT_IMS
S2_WT_MATRIX
S2_atp6
S2_atp6_IMS
S2_atp6_MATRIX

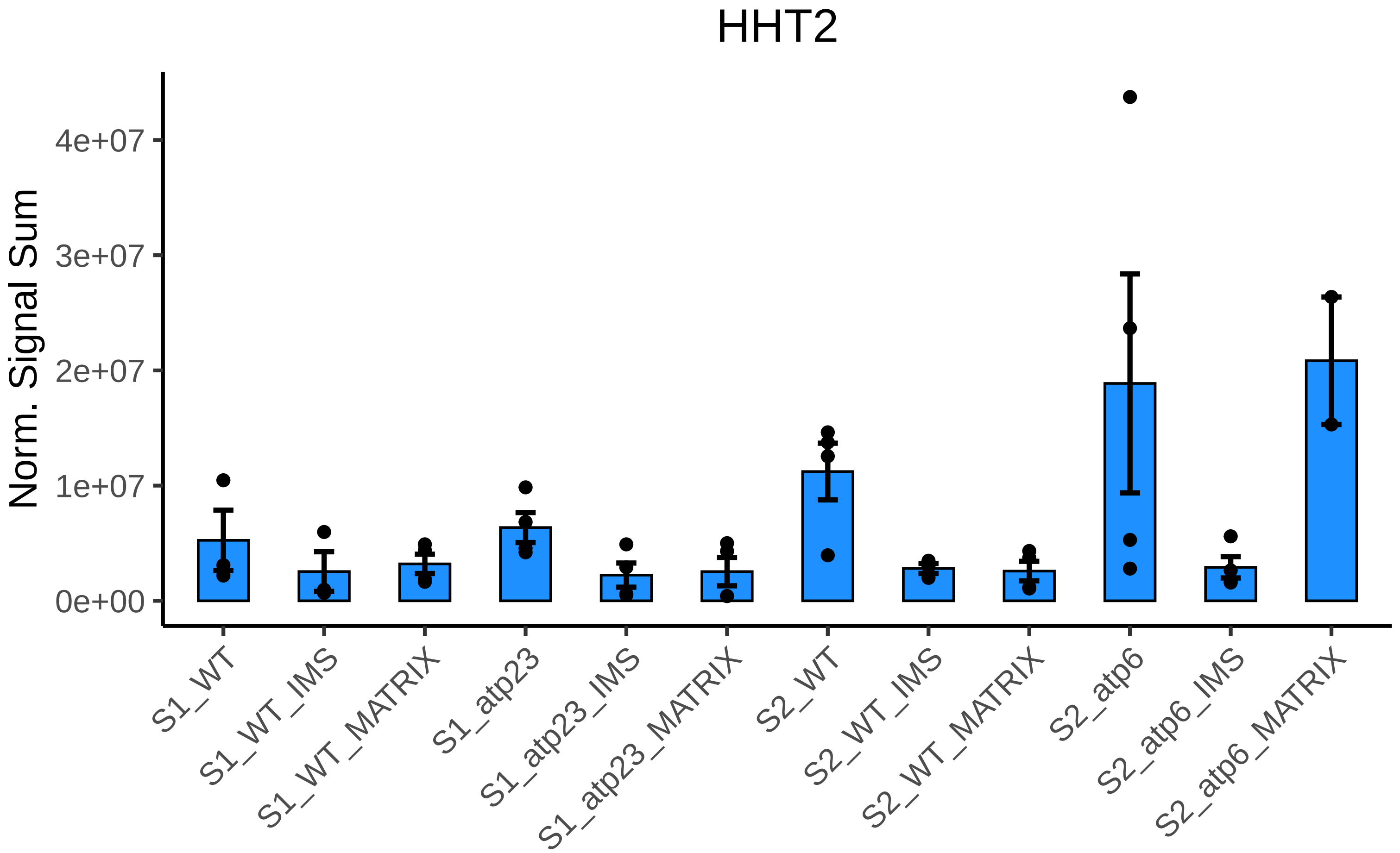

HHT2
Norm. Signal Sum
4e+07
3e+07
2e+07
1e+07
0e+00
S1_WT
S1_WT_IMS
S1_WT_MATRIX
S1_atp23
S1_atp23_IMS
S1_atp23_MATRIX
S2_WT
S2_WT_IMS
S2_WT_MATRIX
S2_atp6
S2_atp6_IMS
S2_atp6_MATRIX

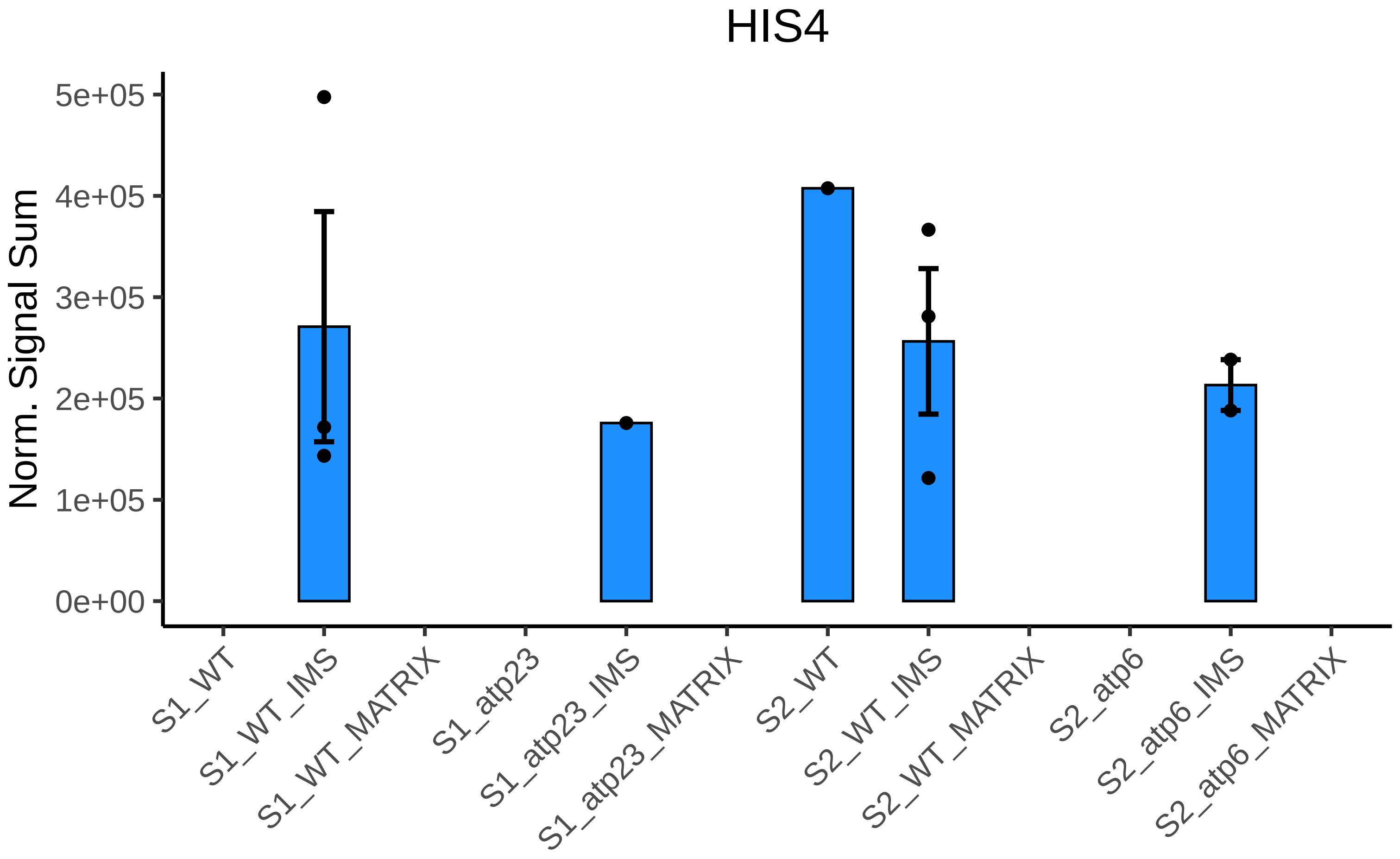

HIS4
Norm. Signal Sum
5e+05
4e+05
3e+05
2e+05
1e+05
0e+00
S1_WT
S1_WT_IMS
S1_WT_MATRIX
S1_atp23
S1_atp23_IMS
S1_atp23_MATRIX
S2_WT
S2_WT_IMS
S2_WT_MATRIX
S2_atp6
S2_atp6_IMS
S2_atp6_MATRIX

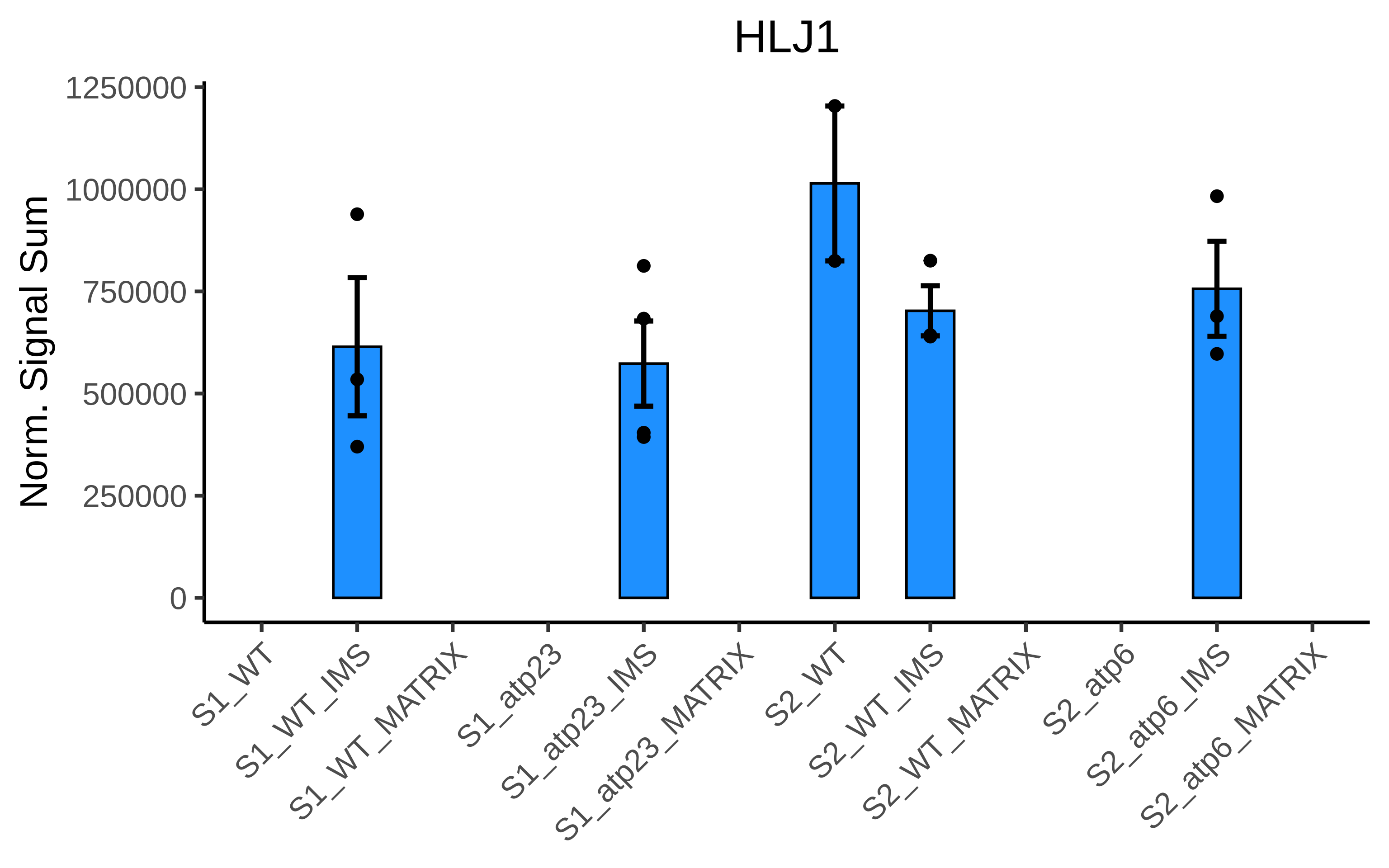
HLJ1
Norm. Signal Sum
1250000
1000000
750000
500000
250000
0
S1_WT
S1_WT_IMS
S1_WT_MATRIX
S1_atp23
S1_atp23_IMS
S1_atp23_MATRIX
S2_WT
S2_WT_IMS
S2_WT_MATRIX
S2_atp6
S2_atp6_IMS
S2_atp6_MATRIX

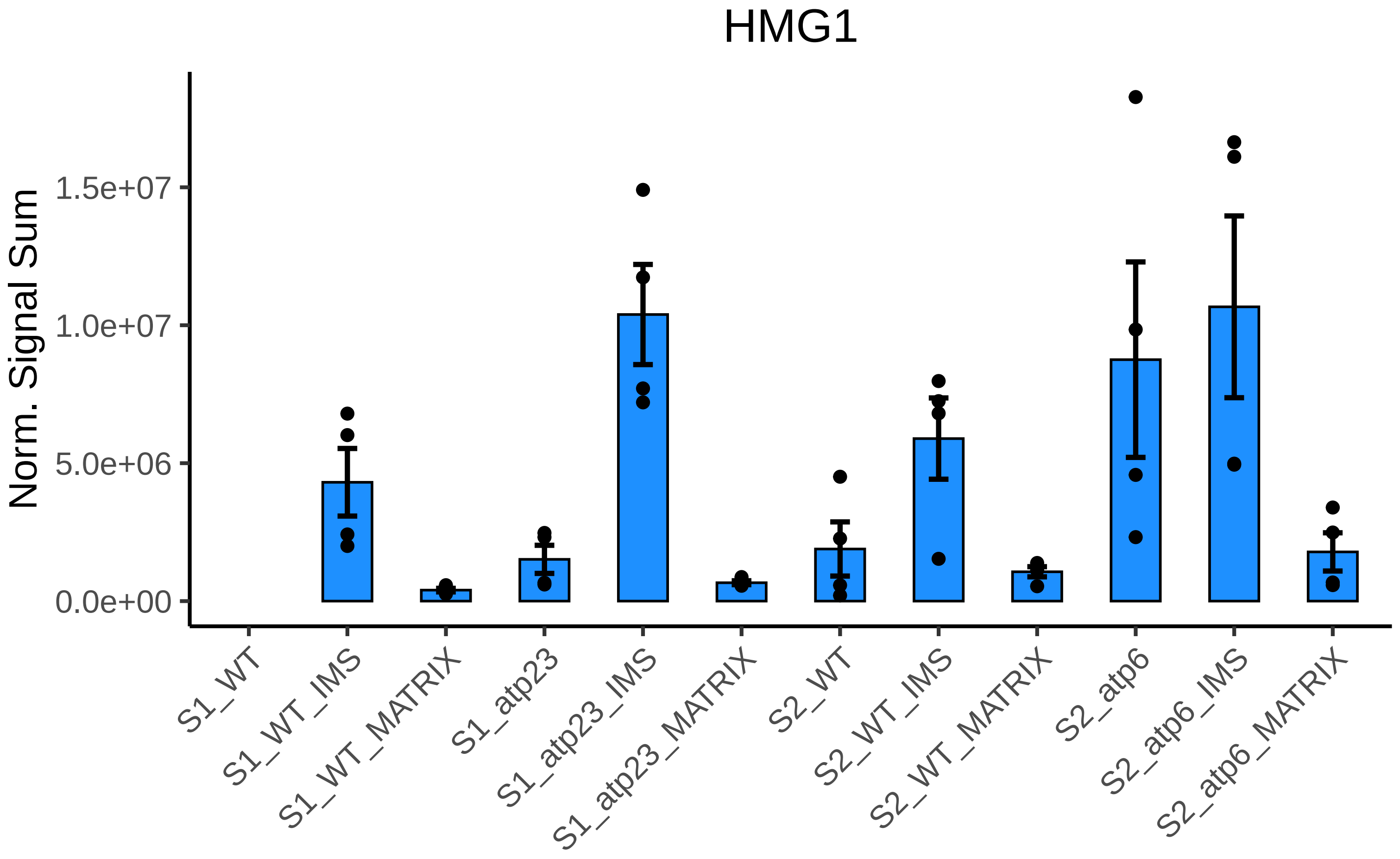

HMG1
Norm. Signal Sum
1.5e+07
1.0e+07
5.0e+06
0.0e+00
S1_WT
S1_WT_IMS
S1_WT_MATRIX
S1_atp23
S1_atp23_IMS
S1_atp23_MATRIX
S2_WT
S2_WT_IMS
S2_WT_MATRIX
S2_atp6
S2_atp6_IMS
S2_atp6_MATRIX

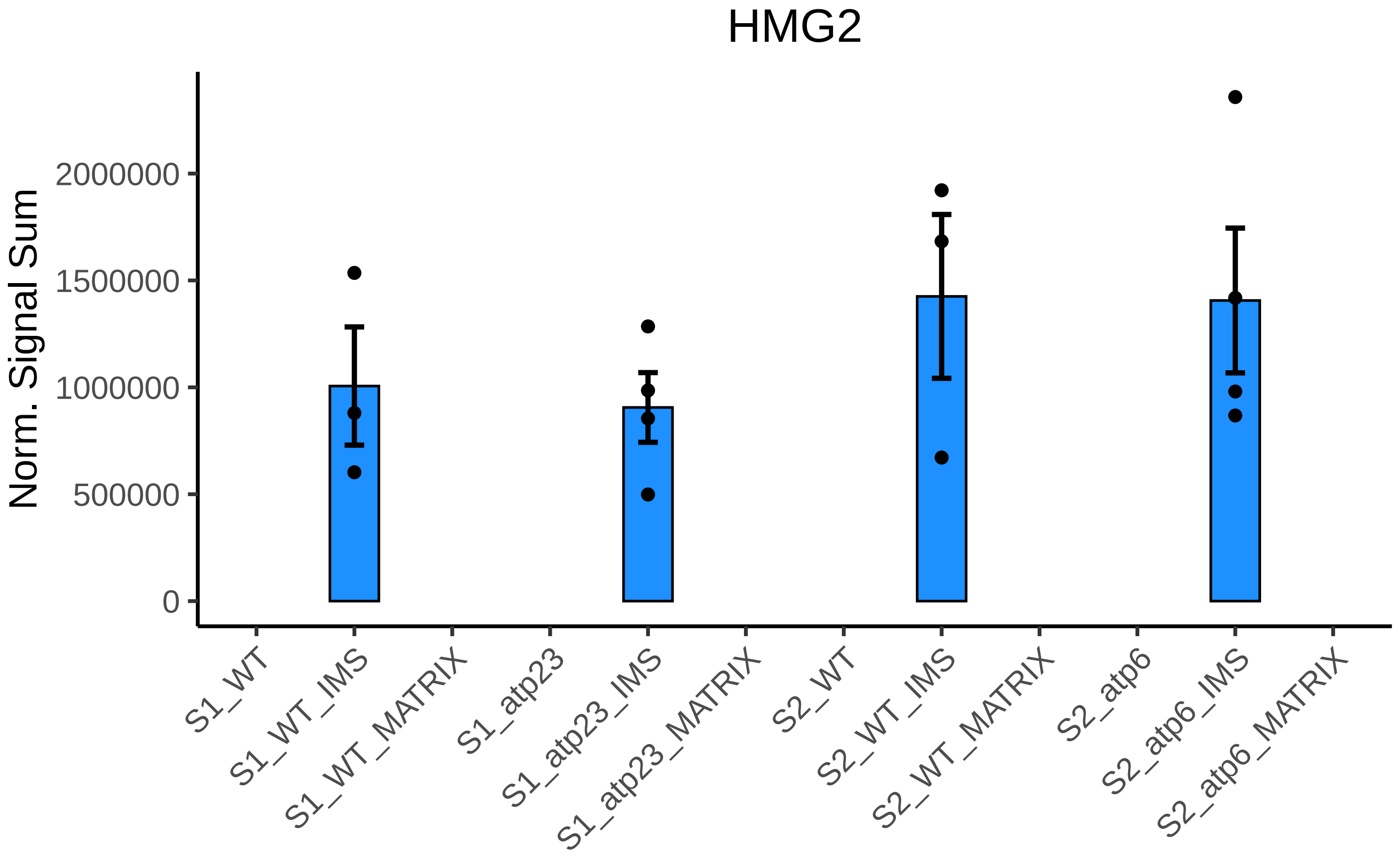

HMG2
Norm. Signal Sum
2000000
1500000
1000000
500000
0
S1_WT
S1_WT_IMS
S1_WT_MATRIX
S1_atp23
S1_atp23_IMS
S1_atp23_MATRIX
S2_WT
S2_WT_IMS
S2_WT_MATRIX
S2_atp6
S2_atp6_IMS
S2_atp6_MATRIX

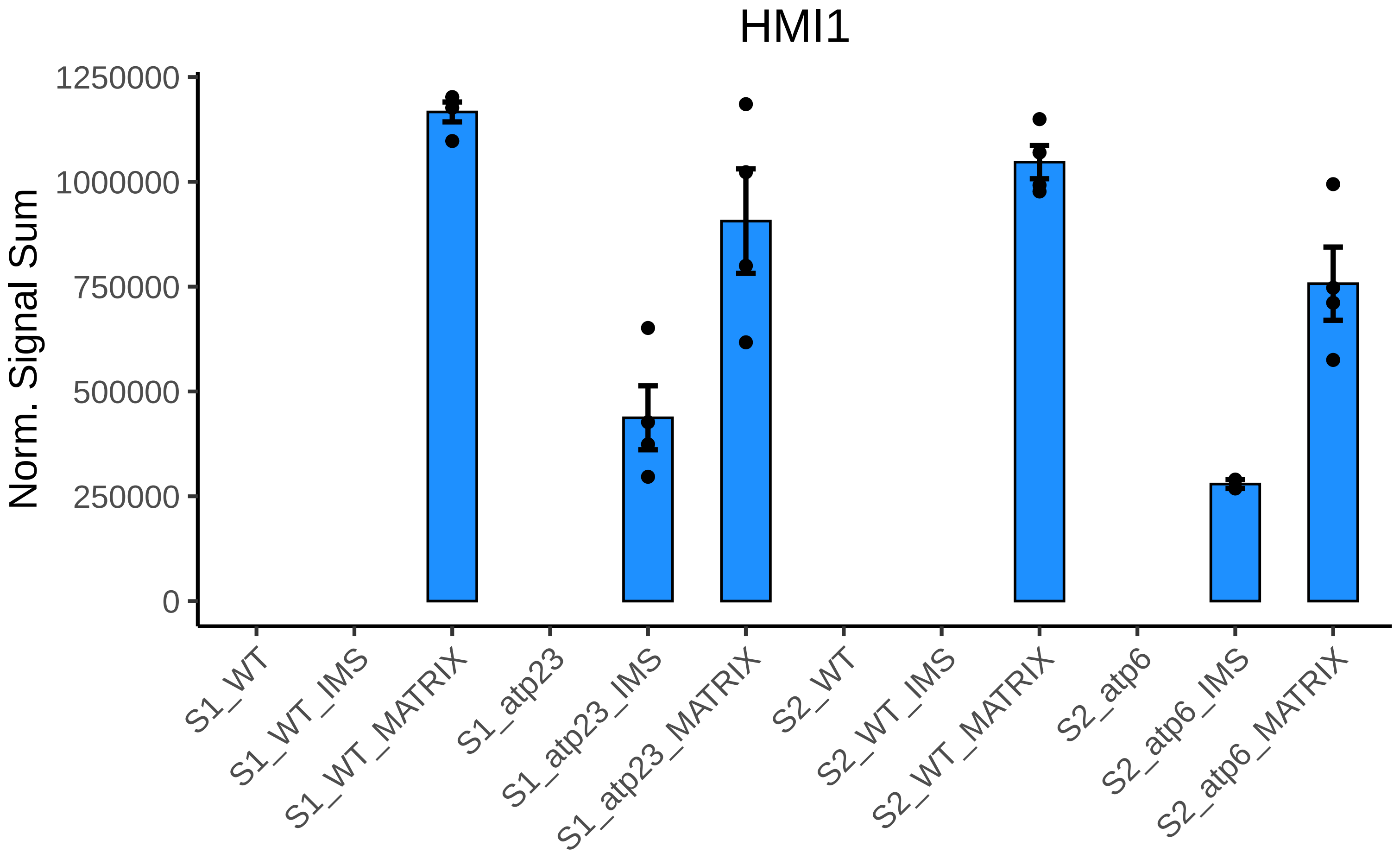

HMI1
Norm. Signal Sum
1250000
1000000
750000
500000
250000
0
S1_WT
S1_WT_IMS
S1_WT_MATRIX
S1_atp23
S1_atp23_IMS
S1_atp23_MATRIX
S2_WT
S2_WT_IMS
S2_WT_MATRIX
S2_atp6
S2_atp6_IMS
S2_atp6_MATRIX

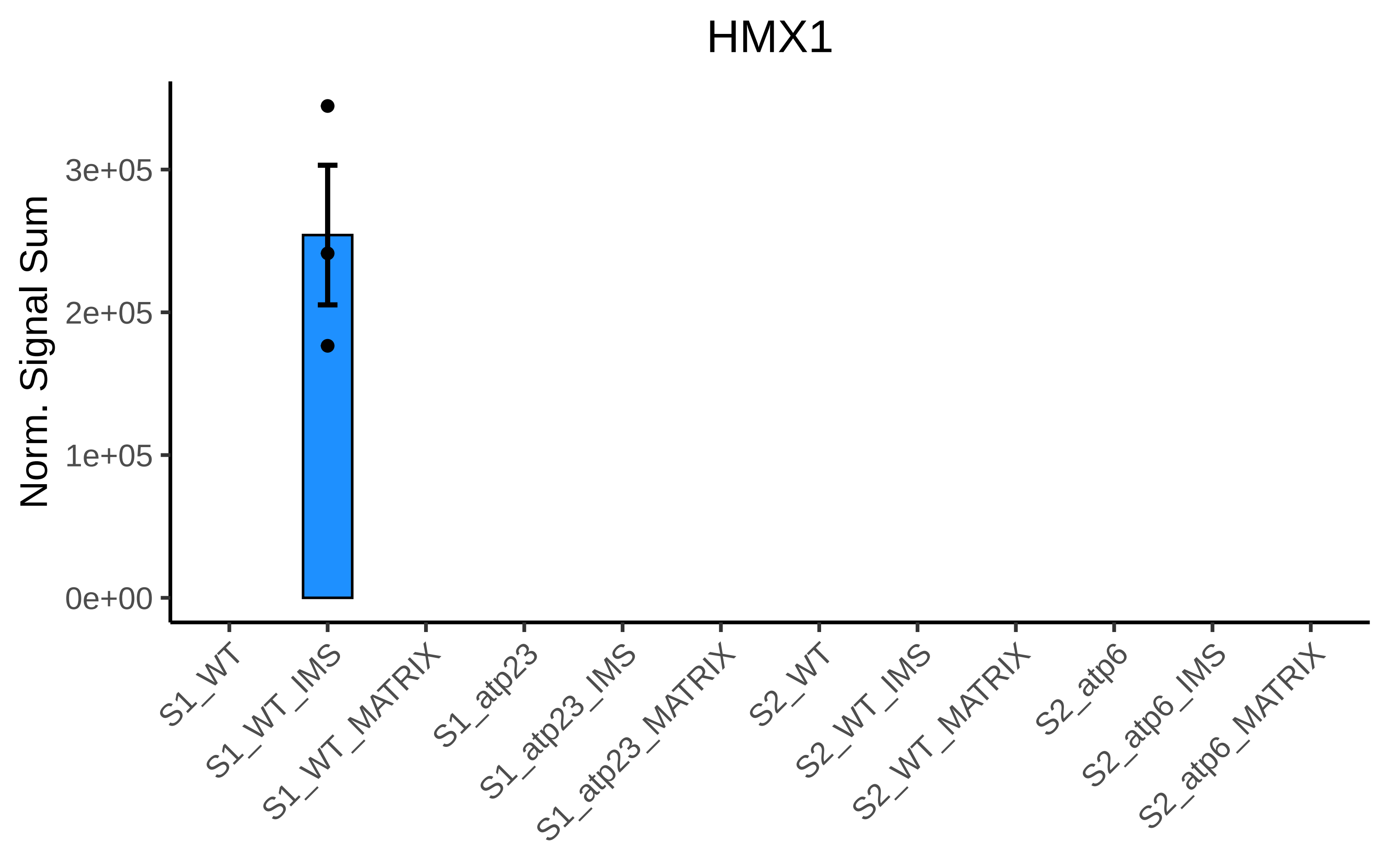

HMX1
Norm. Signal Sum
3e+05
2e+05
1e+05
0e+00
S1_WT
S1_WT_IMS
S1_WT_MATRIX
S1_atp23
S1_atp23_IMS
S1_atp23_MATRIX
S2_WT
S2_WT_IMS
S2_WT_MATRIX
S2_atp6
S2_atp6_IMS
S2_atp6_MATRIX

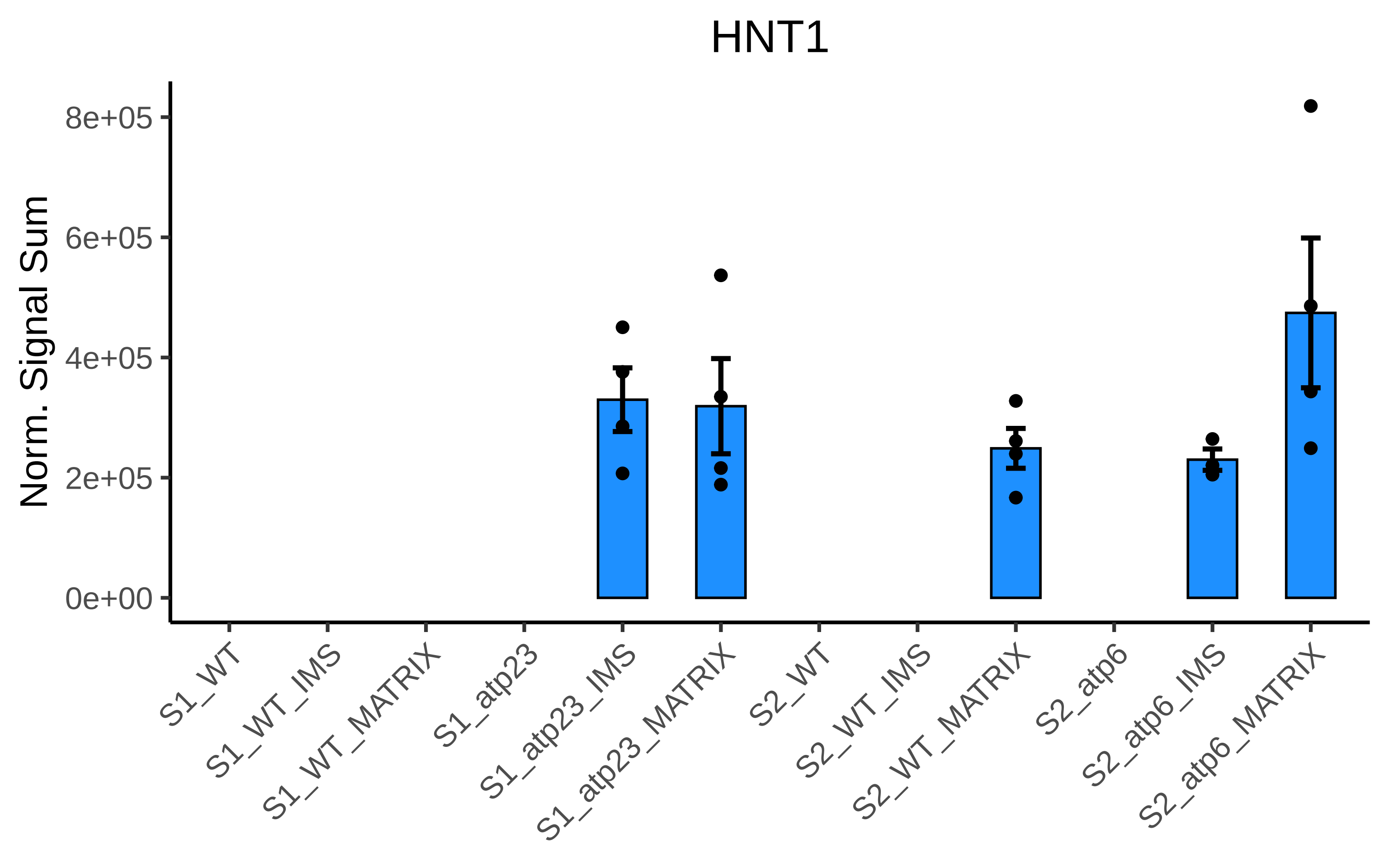
HNT1
Norm. Signal Sum
8e+05
6e+05
4e+05
2e+05
0e+00
S1_WT
S1_WT_IMS
S1_WT_MATRIX
S1_atp23
S1_atp23_IMS
S1_atp23_MATRIX
S2_WT
S2_WT_IMS
S2_WT_MATRIX
S2_atp6
S2_atp6_IMS
S2_atp6_MATRIX

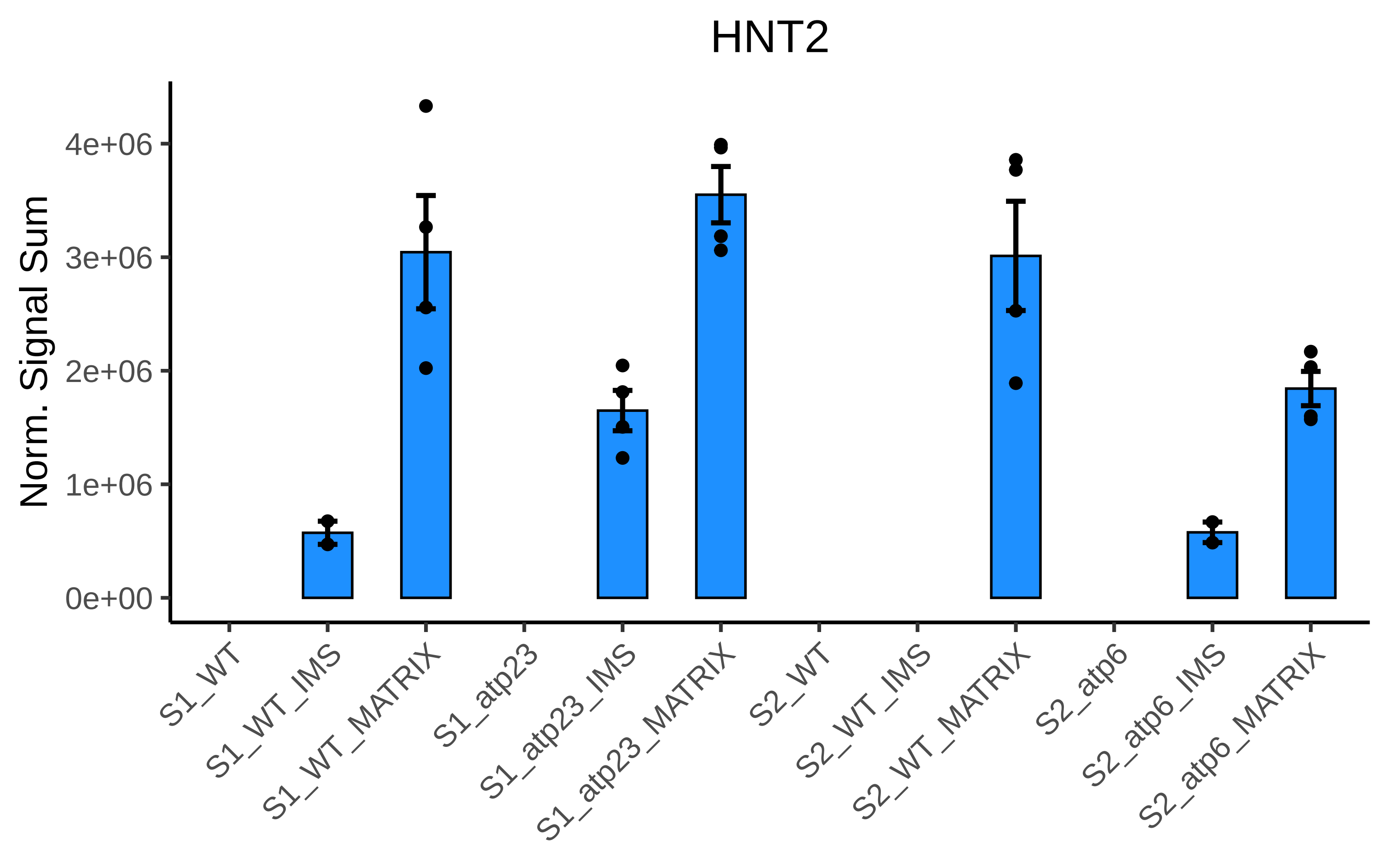
HNT2
Norm. Signal Sum
4e+06
3e+06
2e+06
1e+06
0e+00
S1_WT
S1_WT_IMS
S1_WT_MATRIX
S1_atp23
S1_atp23_IMS
S1_atp23_MATRIX
S2_WT
S2_WT_IMS
S2_WT_MATRIX
S2_atp6
S2_atp6_IMS
S2_atp6_MATRIX

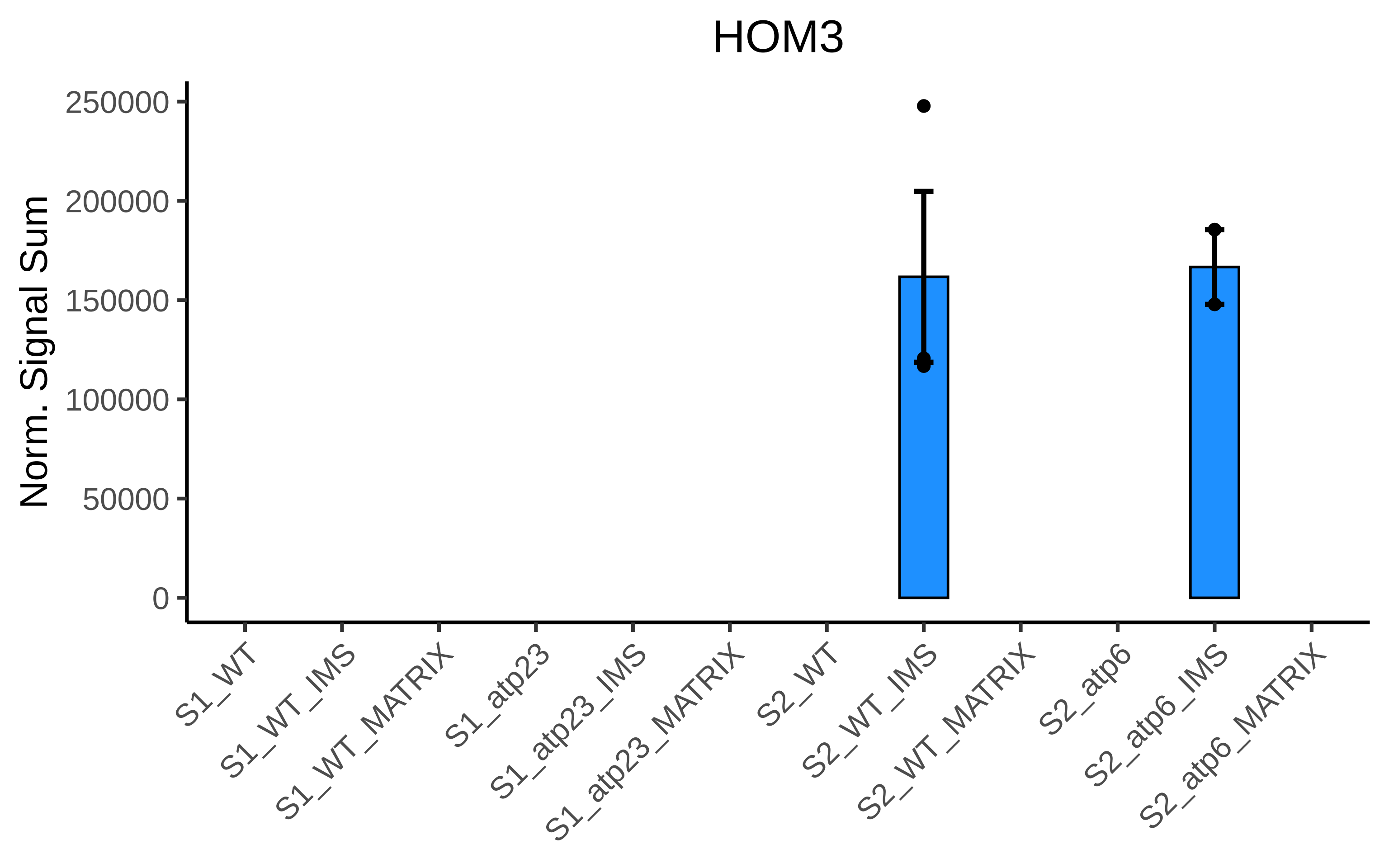
HOM3
Norm. Signal Sum
250000
200000
150000
100000
50000
0
S1_WT
S1_WT_IMS
S1_WT_MATRIX
S1_atp23
S1_atp23_IMS
S1_atp23_MATRIX
S2_WT
S2_WT_IMS
S2_WT_MATRIX
S2_atp6
S2_atp6_IMS
S2_atp6_MATRIX

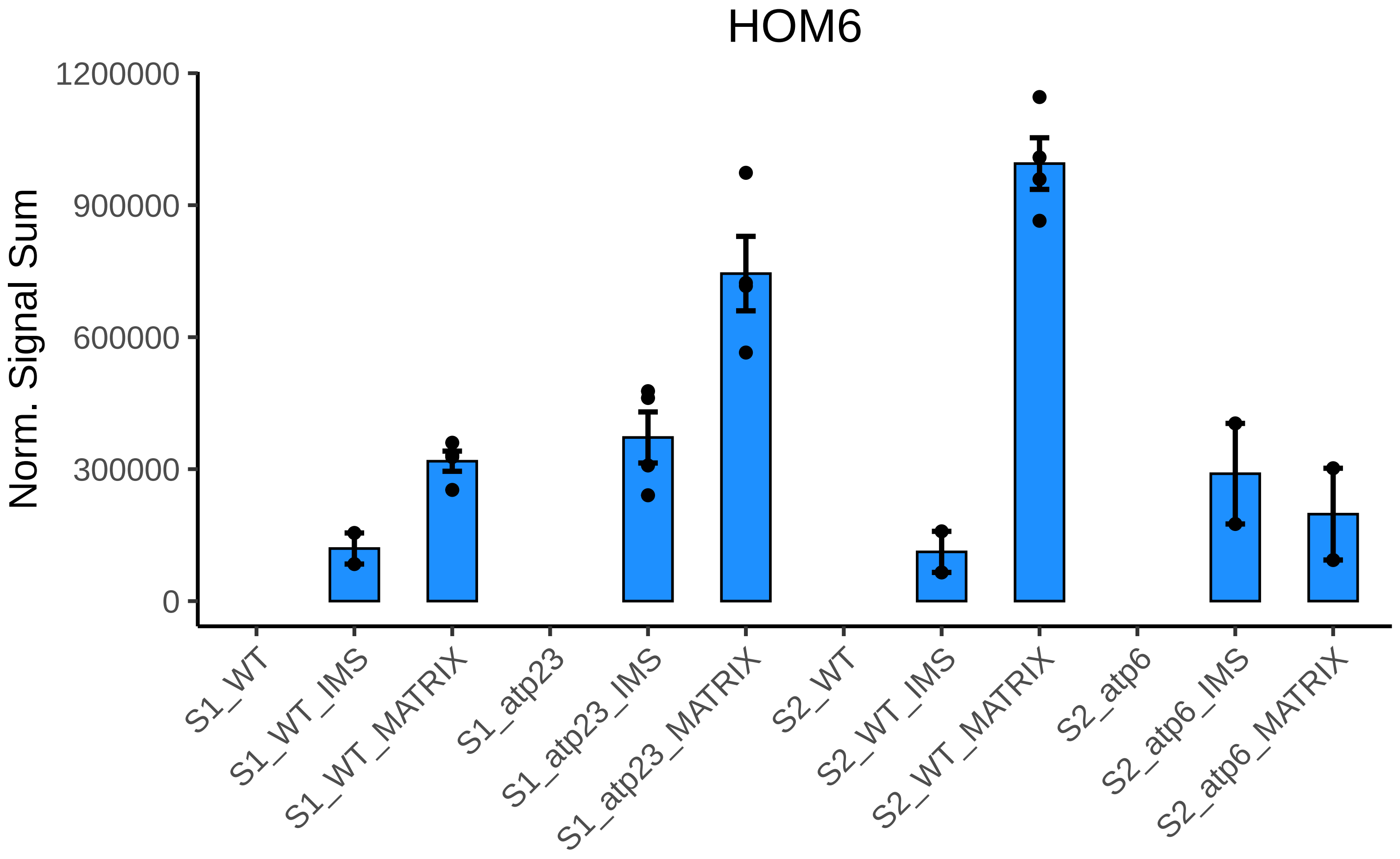
HOM6
Norm. Signal Sum
1200000
900000
600000
300000
0
S1_WT
S1_WT_IMS
S1_WT_MATRIX
S1_atp23
S1_atp23_IMS
S1_atp23_MATRIX
S2_WT
S2_WT_IMS
S2_WT_MATRIX
S2_atp6
S2_atp6_IMS
S2_atp6_MATRIX

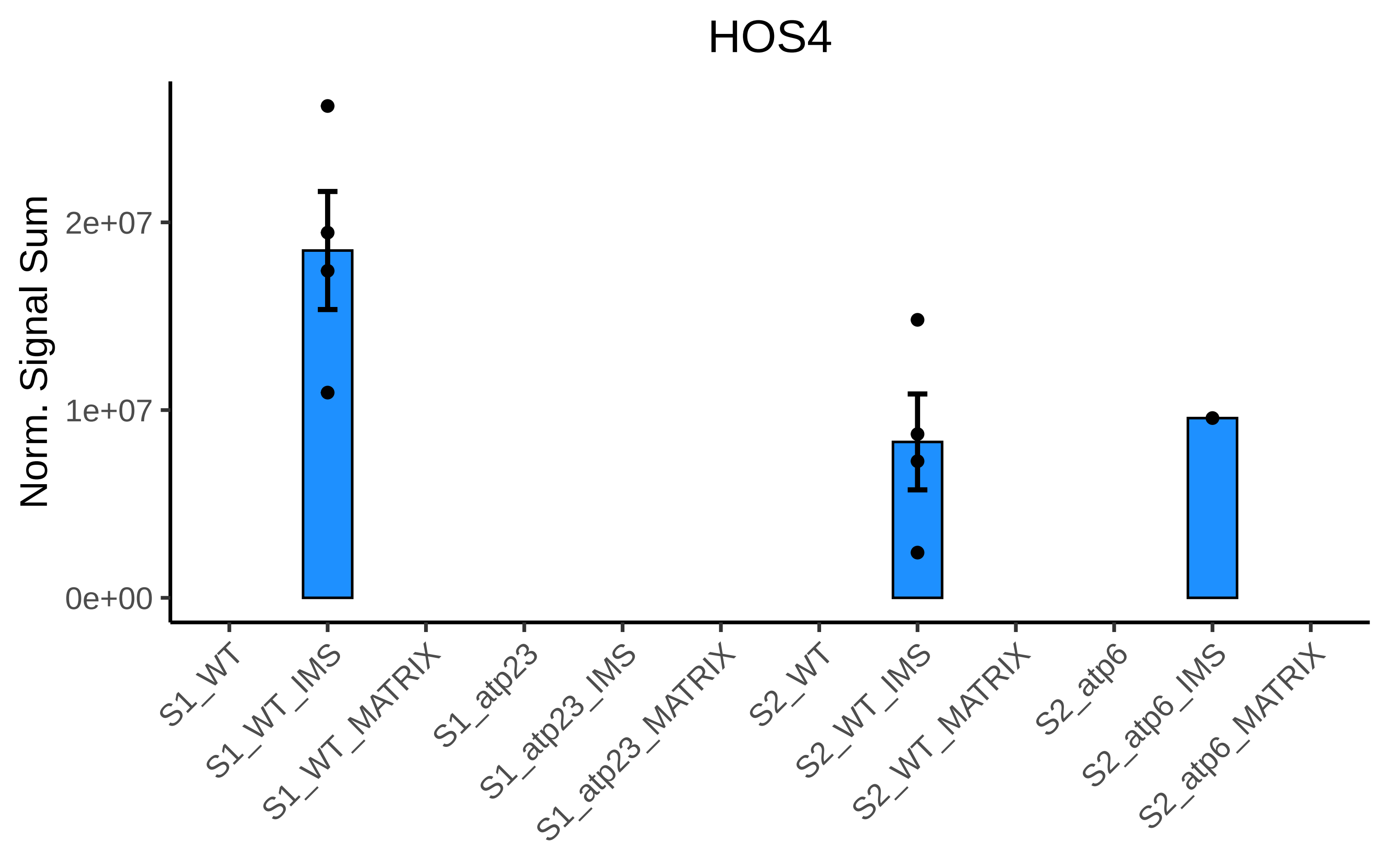
HOS4
Norm. Signal Sum
2e+07
1e+07
0e+00
S1_WT
S1_WT_IMS
S1_WT_MATRIX
S1_atp23
S1_atp23_IMS
S1_atp23_MATRIX
S2_WT
S2_WT_IMS
S2_WT_MATRIX
S2_atp6
S2_atp6_IMS
S2_atp6_MATRIX

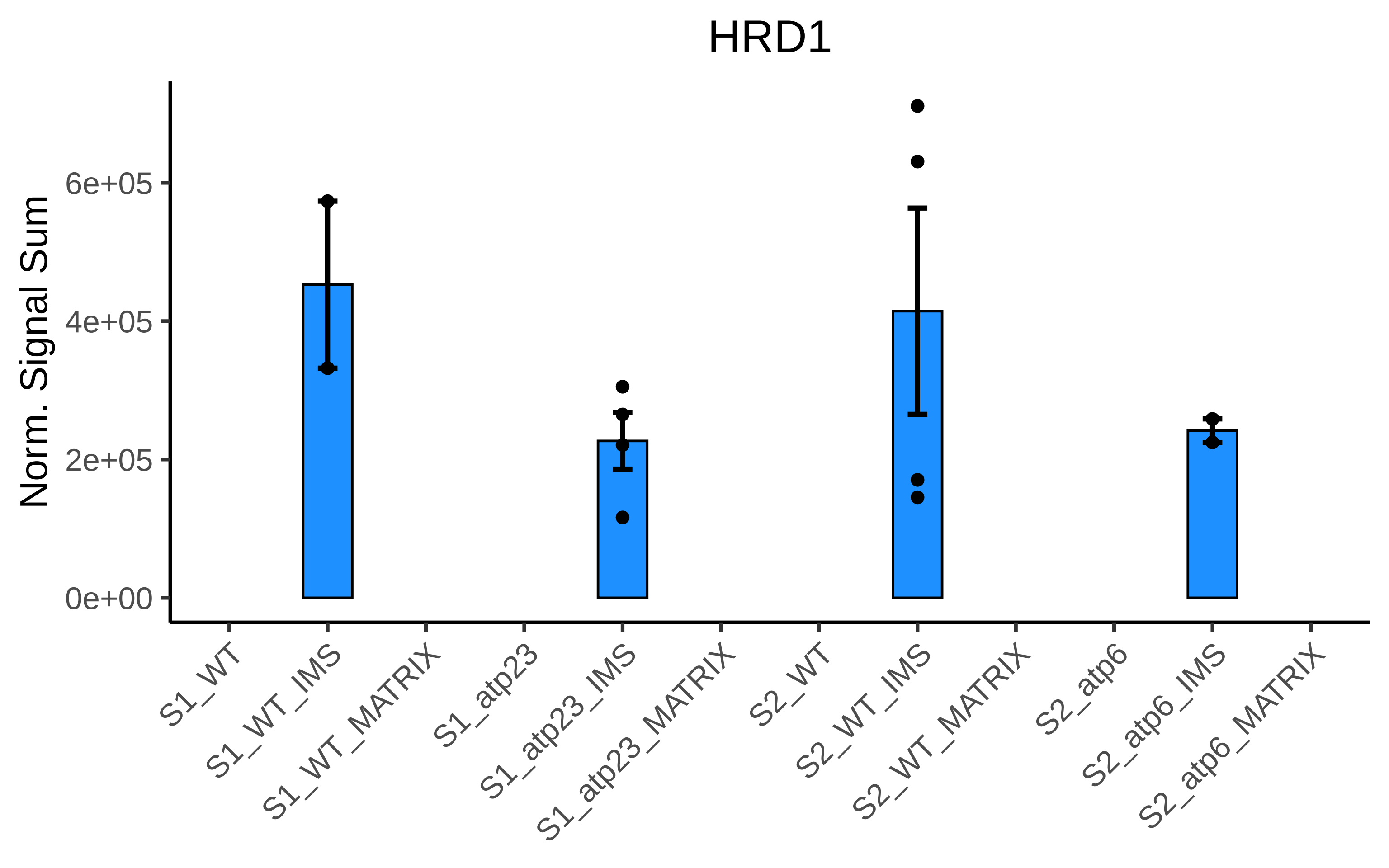
HRD1
Norm. Signal Sum
6e+05
4e+05
2e+05
0e+00
S1_WT
S1_WT_IMS
S1_WT_MATRIX
S1_atp23
S1_atp23_IMS
S1_atp23_MATRIX
S2_WT
S2_WT_IMS
S2_WT_MATRIX
S2_atp6
S2_atp6_IMS
S2_atp6_MATRIX

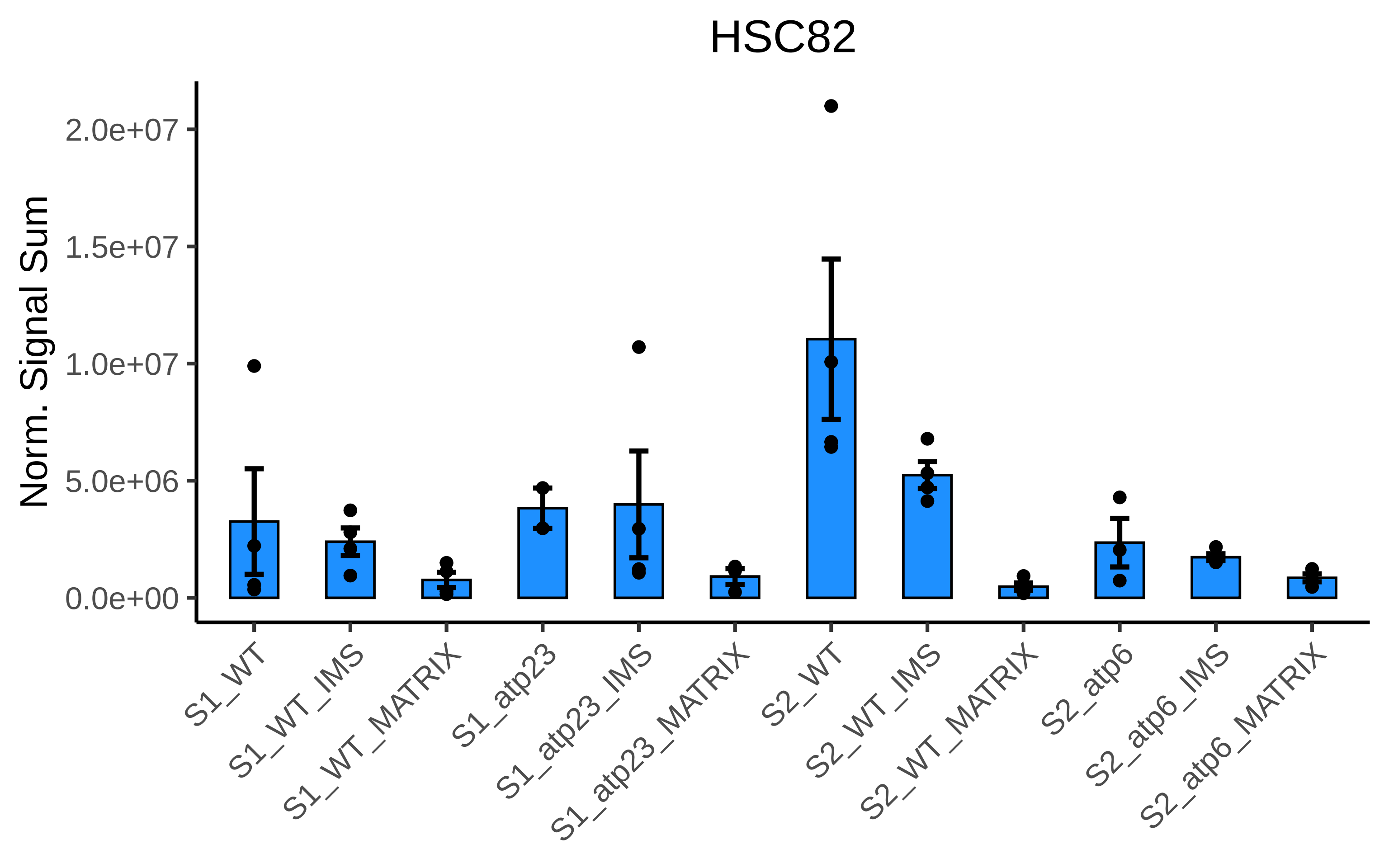
HSC82
Norm. Signal Sum
2.0e+07
1.5e+07
1.0e+07
5.0e+06
0.0e+00
S1_WT
S1_WT_IMS
S1_WT_MATRIX
S1_atp23
S1_atp23_IMS
S1_atp23_MATRIX
S2_WT
S2_WT_IMS
S2_WT_MATRIX
S2_atp6
S2_atp6_IMS
S2_atp6_MATRIX

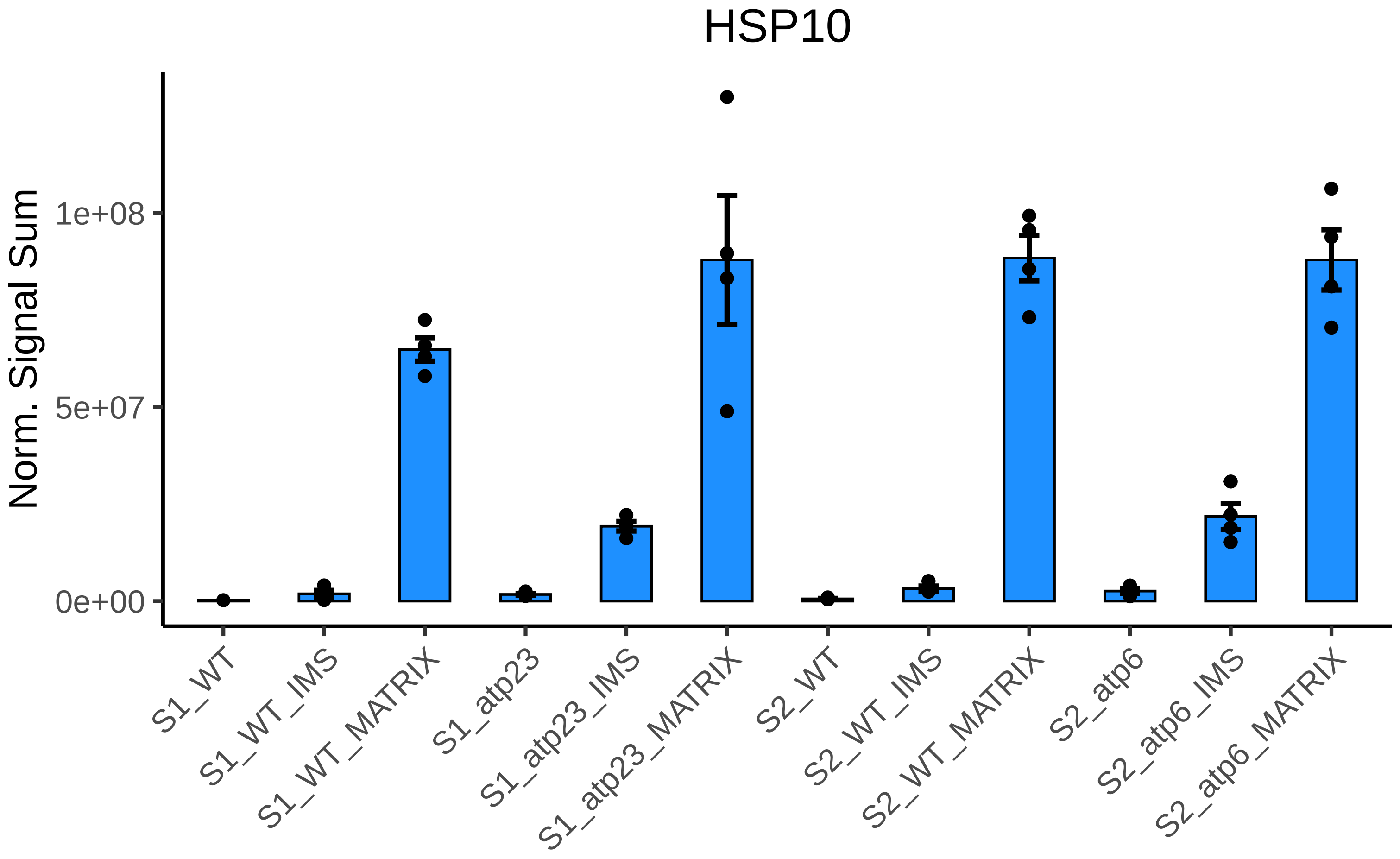
HSP10
Norm. Signal Sum
1e+08
5e+07
0e+00
S1_WT
S1_WT_IMS
S1_WT_MATRIX
S1_atp23
S1_atp23_IMS
S1_atp23_MATRIX
S2_WT
S2_WT_IMS
S2_WT_MATRIX
S2_atp6
S2_atp6_IMS
S2_atp6_MATRIX

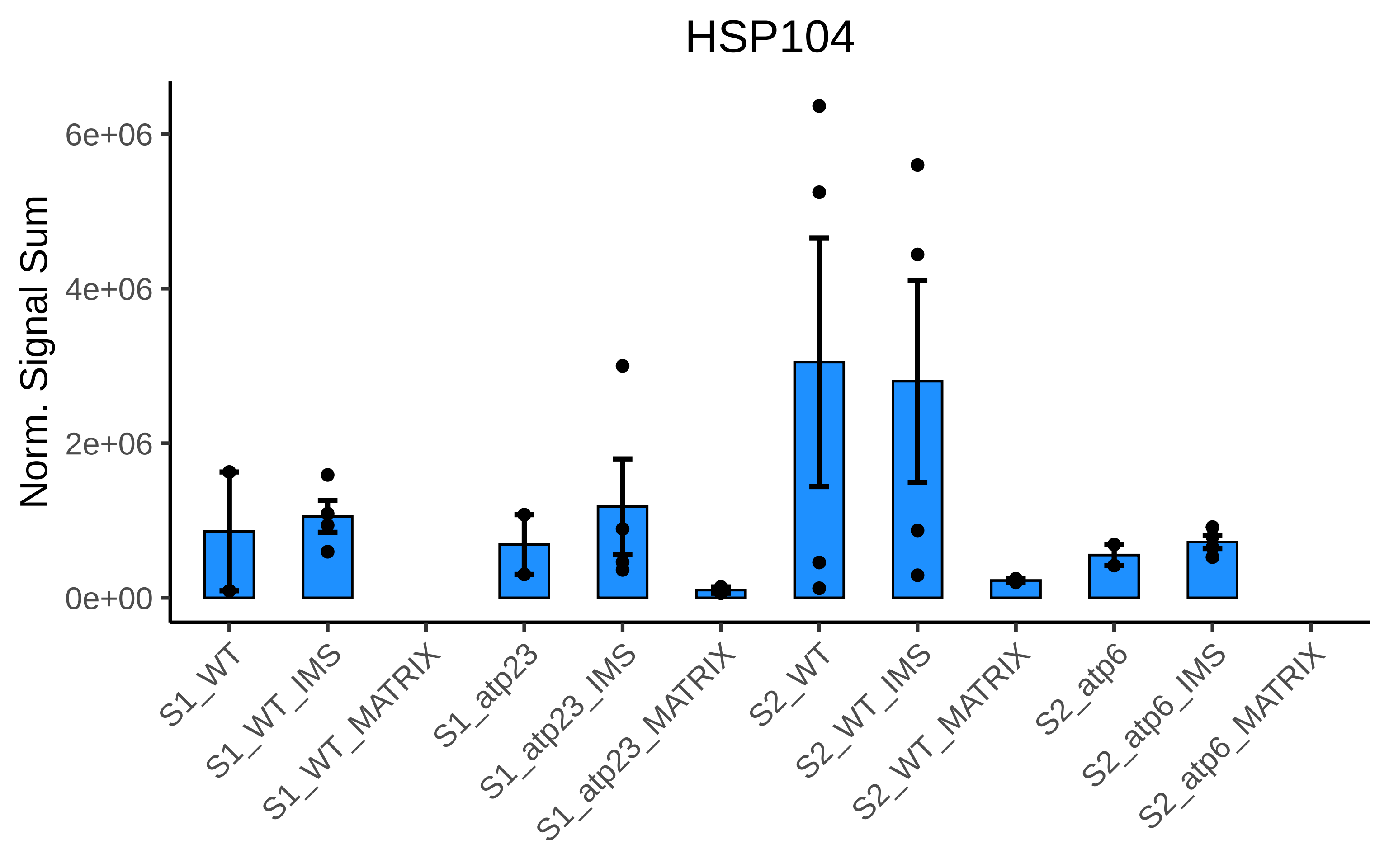
HSP104
Norm. Signal Sum
6e+06
4e+06
2e+06
0e+00
S1_WT
S1_WT_IMS
S1_WT_MATRIX
S1_atp23
S1_atp23_IMS
S1_atp23_MATRIX
S2_WT
S2_WT_IMS
S2_WT_MATRIX
S2_atp6
S2_atp6_IMS
S2_atp6_MATRIX

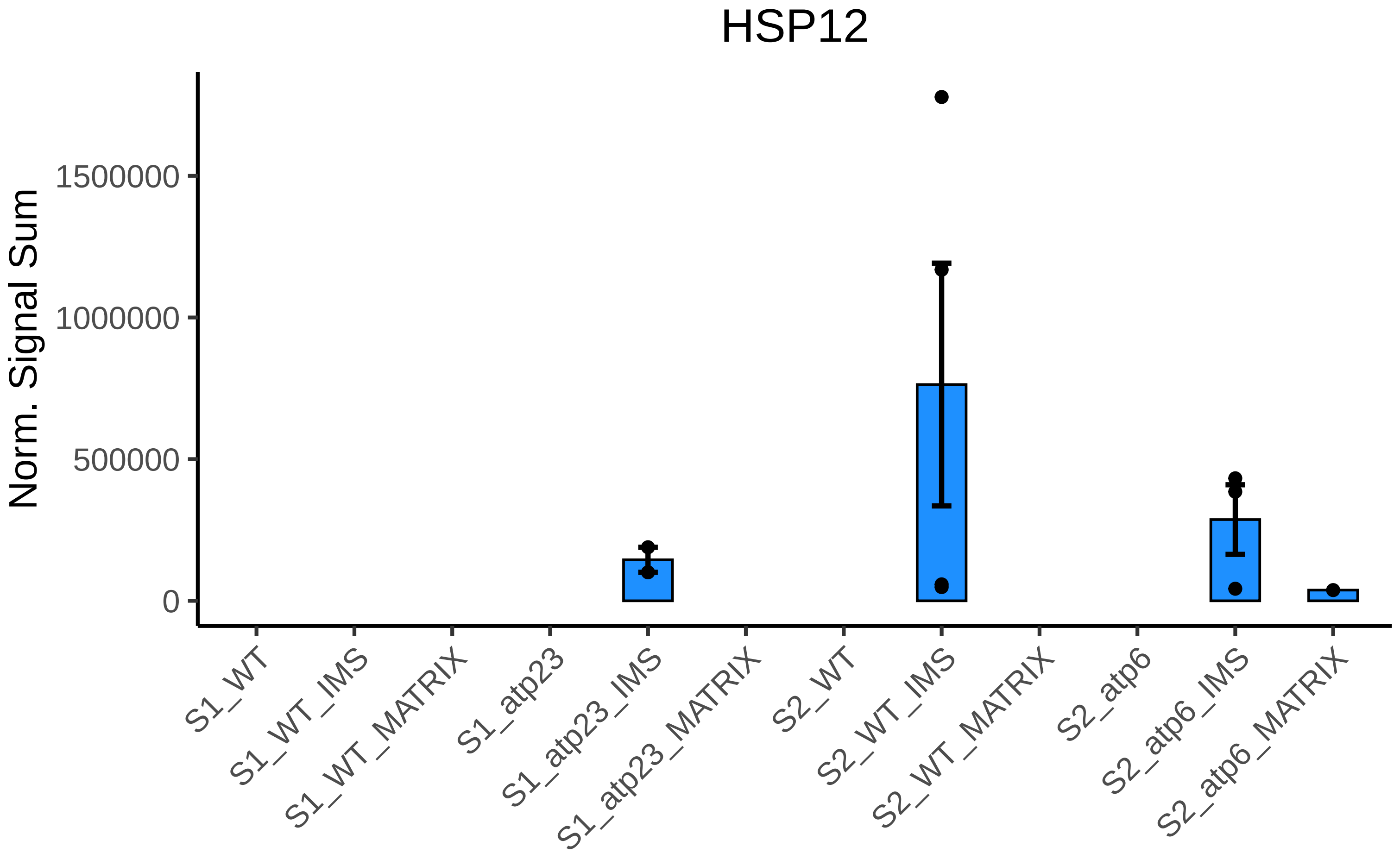

HSP12
Norm. Signal Sum
1500000
1000000
500000
0
S1_WT
S1_WT_IMS
S1_WT_MATRIX
S1_atp23
S1_atp23_IMS
S1_atp23_MATRIX
S2_WT
S2_WT_IMS
S2_WT_MATRIX
S2_atp6
S2_atp6_IMS
S2_atp6_MATRIX

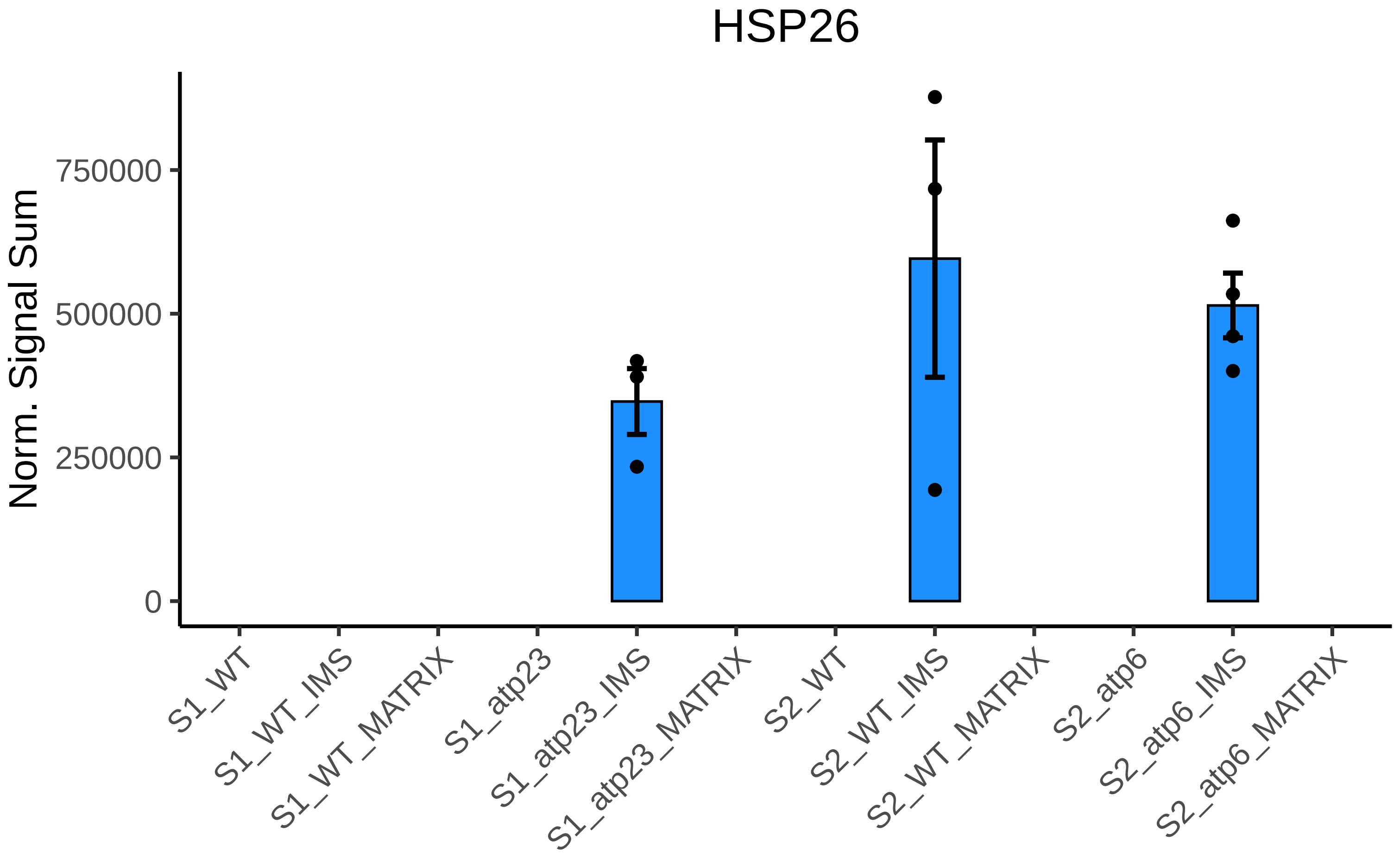
HSP26
Norm. Signal Sum
750000
500000
250000
0
S1_WT
S1_WT_IMS
S1_WT_MATRIX
S1_atp23
S1_atp23_IMS
S1_atp23_MATRIX
S2_WT
S2_WT_IMS
S2_WT_MATRIX
S2_atp6
S2_atp6_IMS
S2_atp6_MATRIX

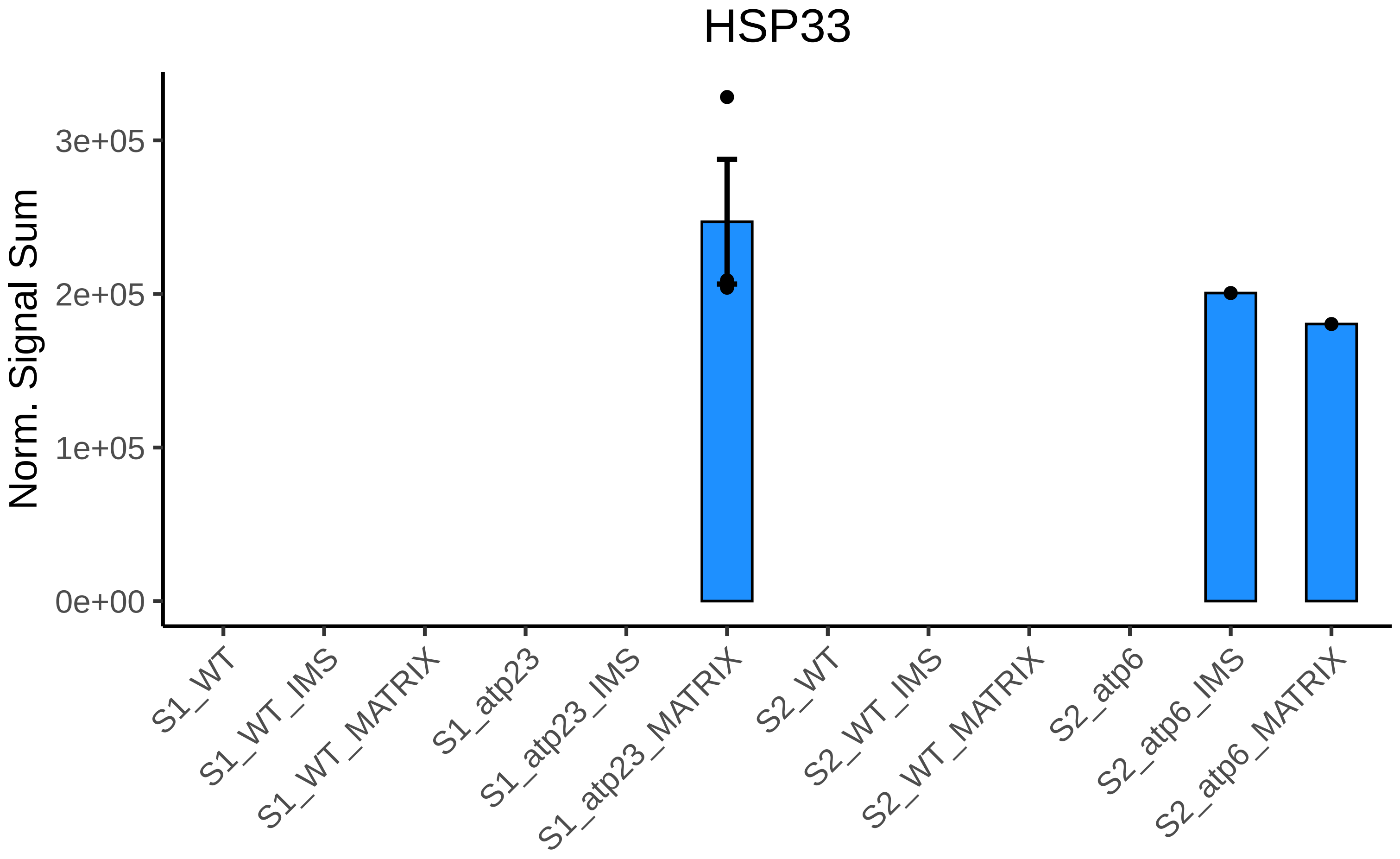
HSP33
Norm. Signal Sum
3e+05
2e+05
1e+05
0e+00
S1_WT
S1_WT_IMS
S1_WT_MATRIX
S1_atp23
S1_atp23_IMS
S1_atp23_MATRIX
S2_WT
S2_WT_IMS
S2_WT_MATRIX
S2_atp6
S2_atp6_IMS
S2_atp6_MATRIX

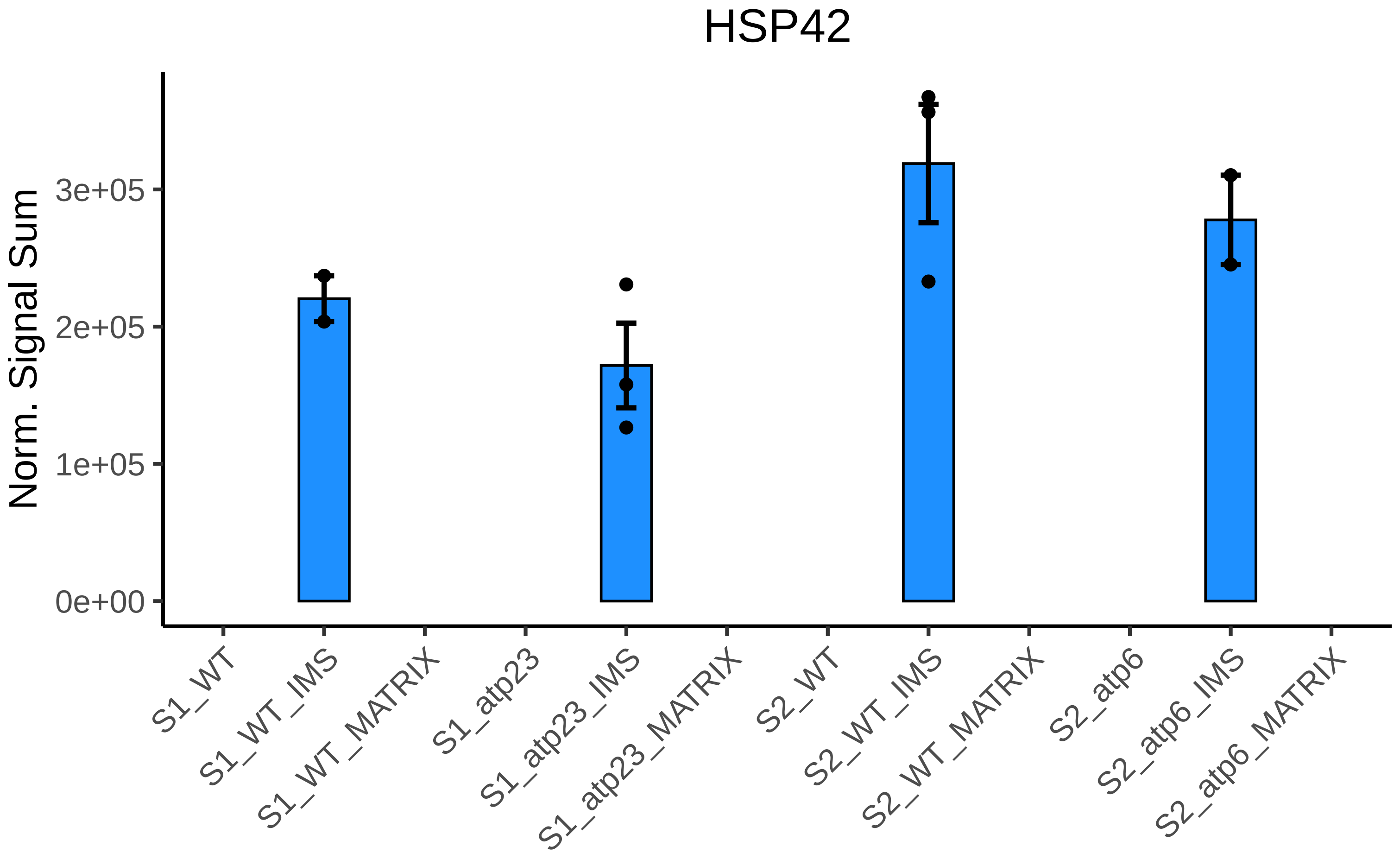

HSP42
Norm. Signal Sum
3e+05
2e+05
1e+05
0e+00
S1_WT
S1_WT_IMS
S1_WT_MATRIX
S1_atp23
S1_atp23_IMS
S1_atp23_MATRIX
S2_WT
S2_WT_IMS
S2_WT_MATRIX
S2_atp6
S2_atp6_IMS
S2_atp6_MATRIX

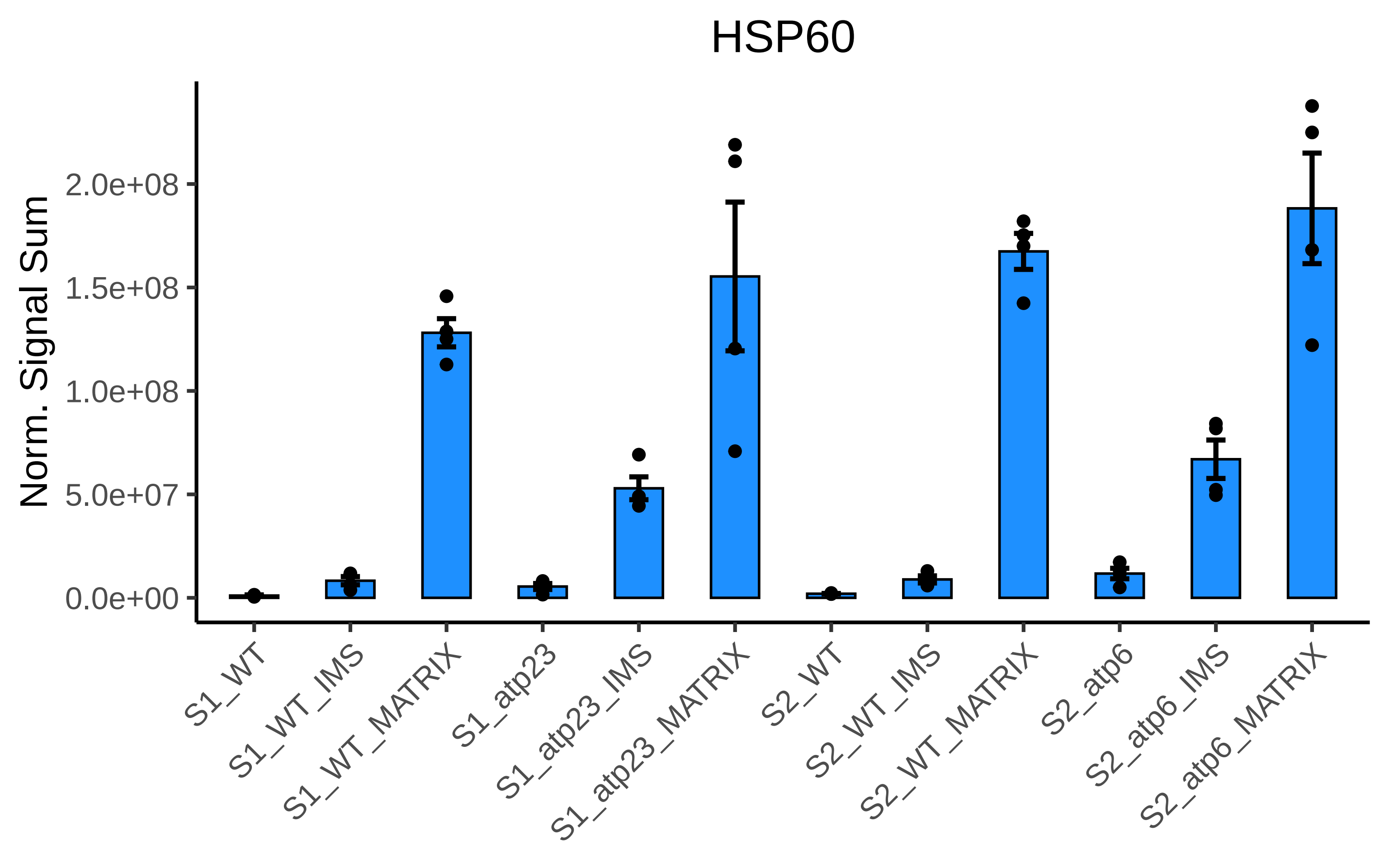
HSP60
Norm. Signal Sum
2.0e+08
1.5e+08
1.0e+08
5.0e+07
0.0e+00
S1_WT
S1_WT_IMS
S1_WT_MATRIX
S1_atp23
S1_atp23_IMS
S1_atp23_MATRIX
S2_WT
S2_WT_IMS
S2_WT_MATRIX
S2_atp6
S2_atp6_IMS
S2_atp6_MATRIX

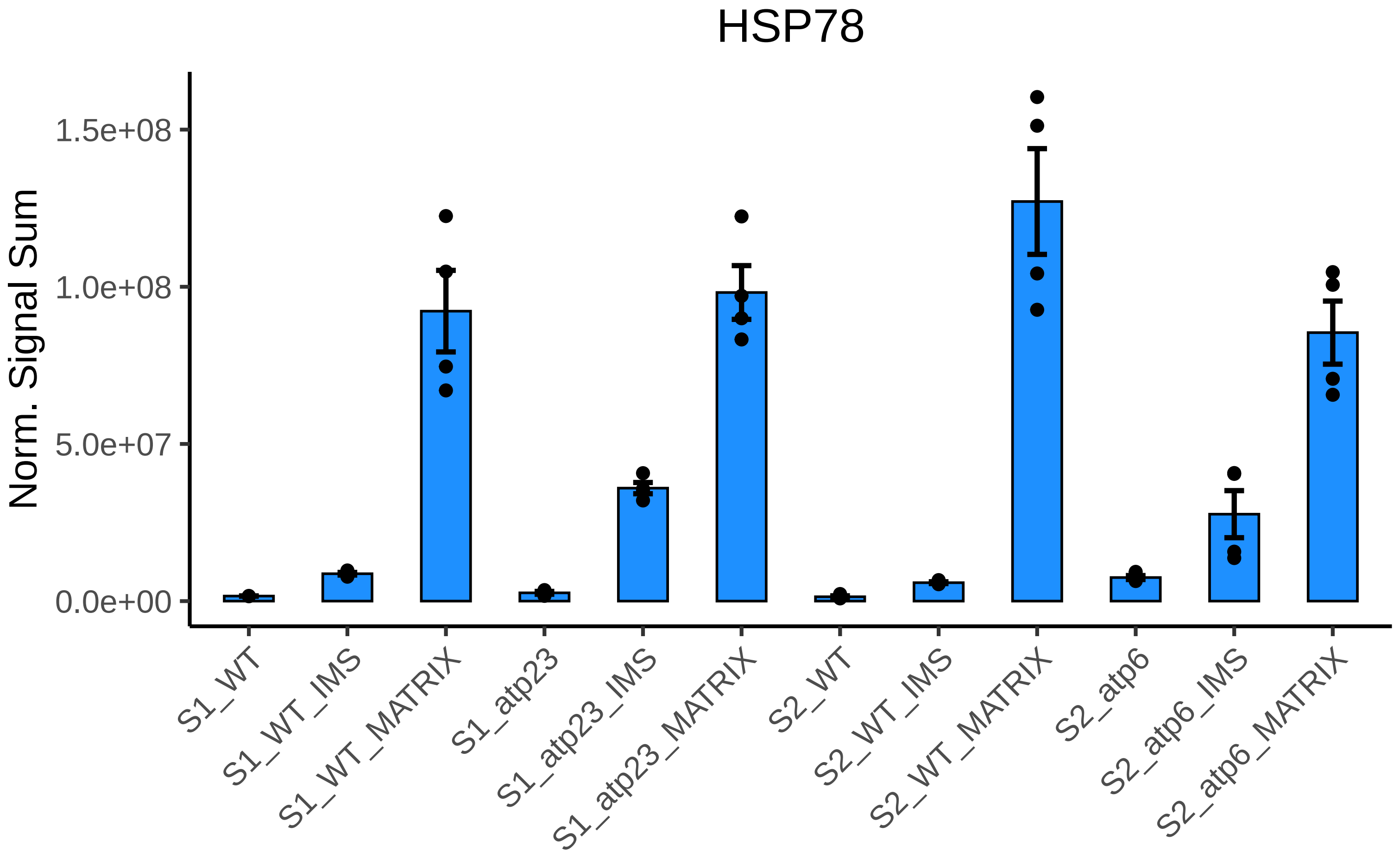

HSP78
Norm. Signal Sum
1.5e+08
1.0e+08
5.0e+07
0.0e+00
S1_WT
S1_WT_IMS
S1_WT_MATRIX
S1_atp23
S1_atp23_IMS
S1_atp23_MATRIX
S2_WT
S2_WT_IMS
S2_WT_MATRIX
S2_atp6
S2_atp6_IMS
S2_atp6_MATRIX

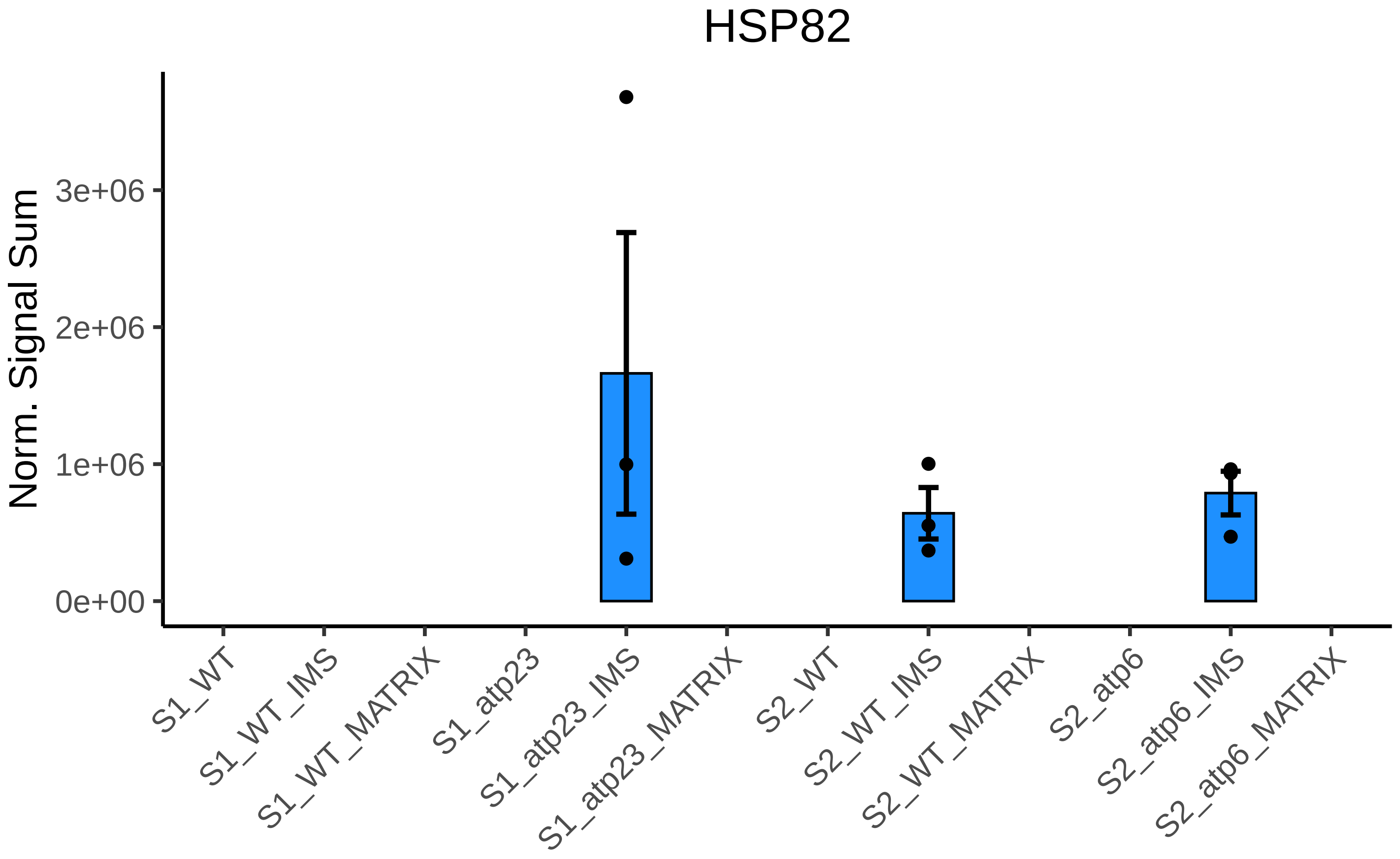

HSP82
Norm. Signal Sum
3e+06
2e+06
1e+06
0e+00
S1_WT
S1_WT_IMS
S1_WT_MATRIX
S1_atp23
S1_atp23_IMS
S1_atp23_MATRIX
S2_WT
S2_WT_IMS
S2_WT_MATRIX
S2_atp6
S2_atp6_IMS
S2_atp6_MATRIX

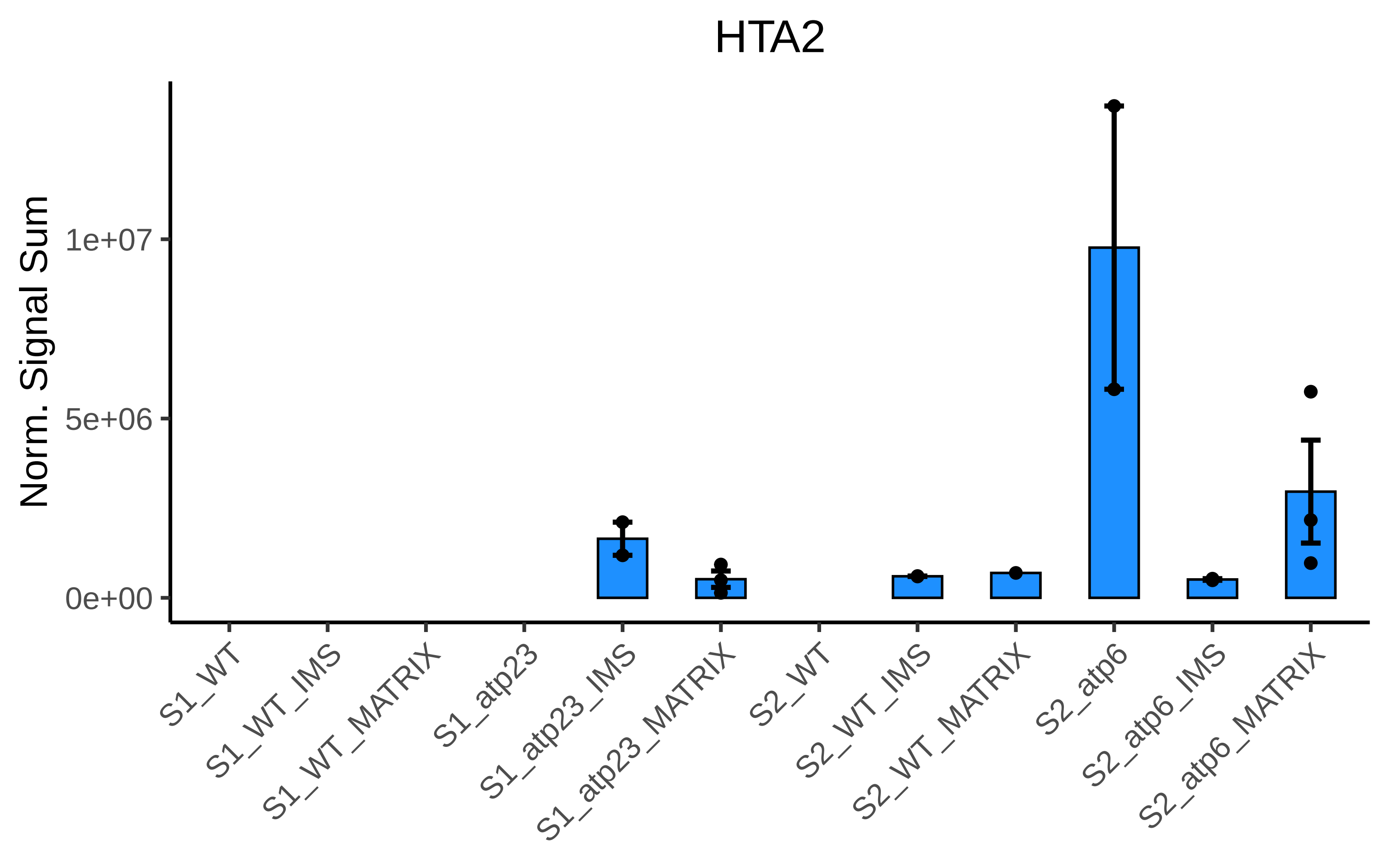
HTA2
Norm. Signal Sum
1e+07
5e+06
0e+00
S1_WT
S1_WT_IMS
S1_WT_MATRIX
S1_atp23
S1_atp23_IMS
S1_atp23_MATRIX
S2_WT
S2_WT_IMS
S2_WT_MATRIX
S2_atp6
S2_atp6_IMS
S2_atp6_MATRIX

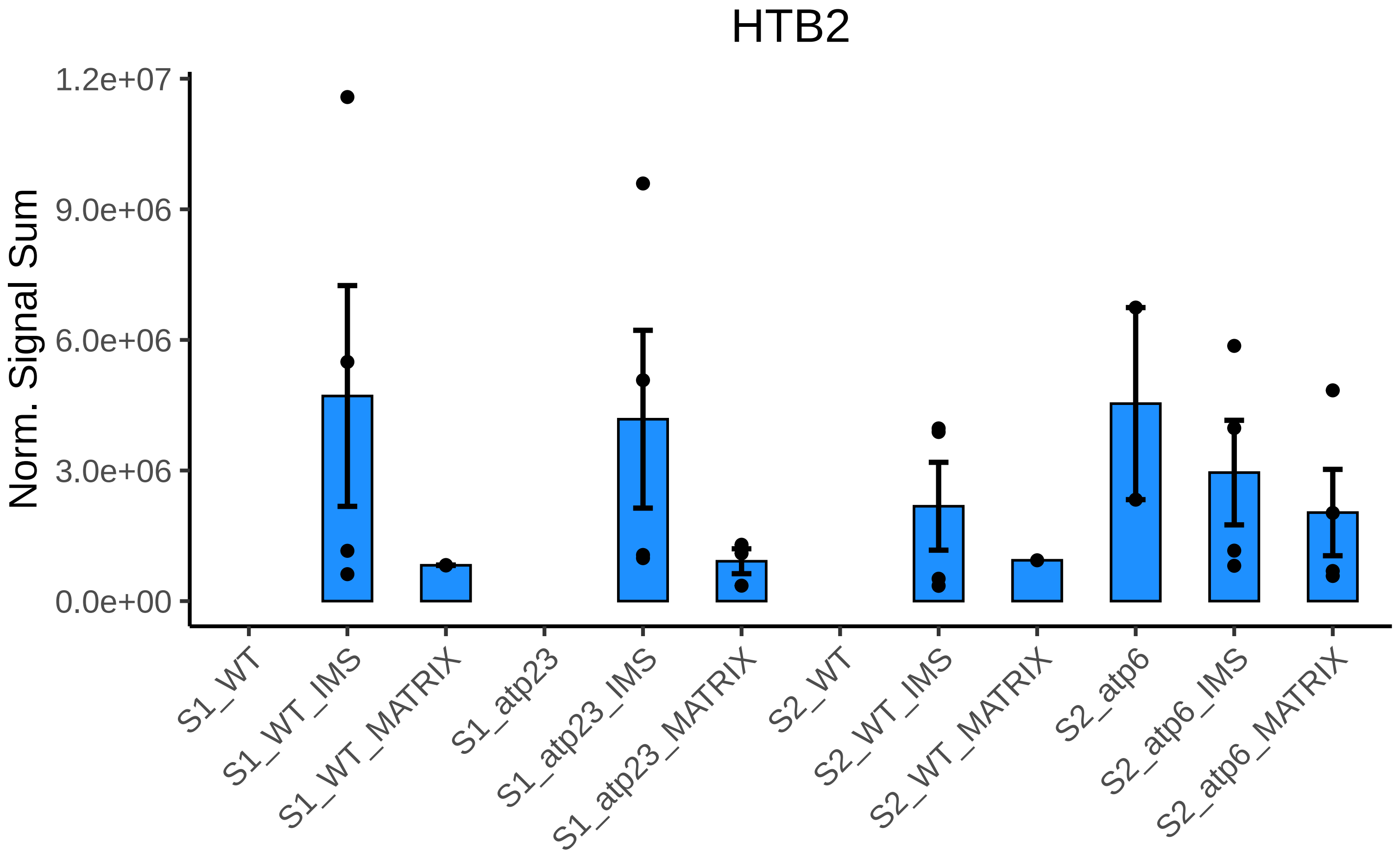

HTB2
Norm. Signal Sum
1.2e+07
9.0e+06
6.0e+06
3.0e+06
0.0e+00
S1_WT
S1_WT_IMS
S1_WT_MATRIX
S1_atp23
S1_atp23_IMS
S1_atp23_MATRIX
S2_WT
S2_WT_IMS
S2_WT_MATRIX
S2_atp6
S2_atp6_IMS
S2_atp6_MATRIX

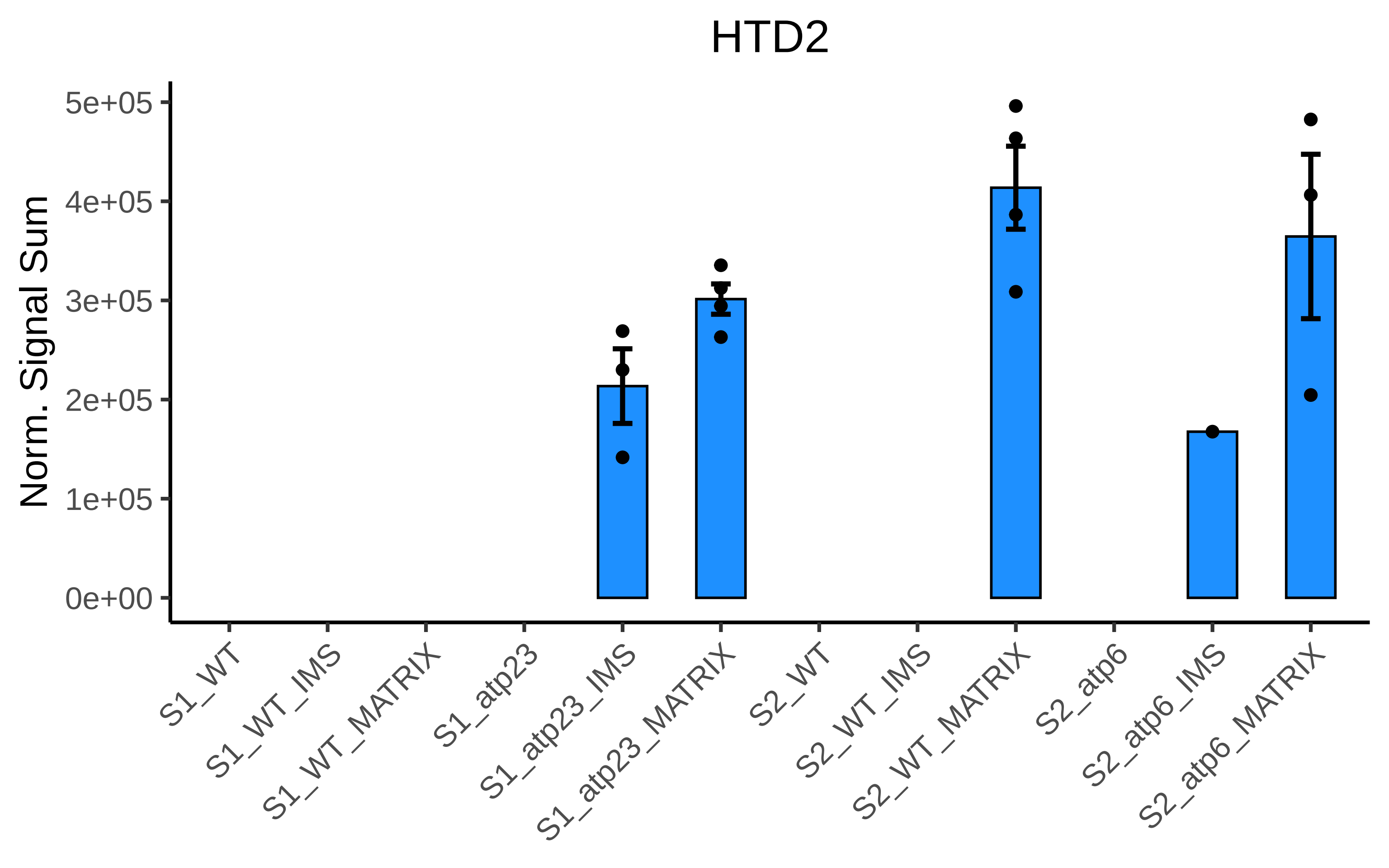
HTD2
Norm. Signal Sum
5e+05
4e+05
3e+05
2e+05
1e+05
0e+00
S1_WT
S1_WT_IMS
S1_WT_MATRIX
S1_atp23
S1_atp23_IMS
S1_atp23_MATRIX
S2_WT
S2_WT_IMS
S2_WT_MATRIX
S2_atp6
S2_atp6_IMS
S2_atp6_MATRIX

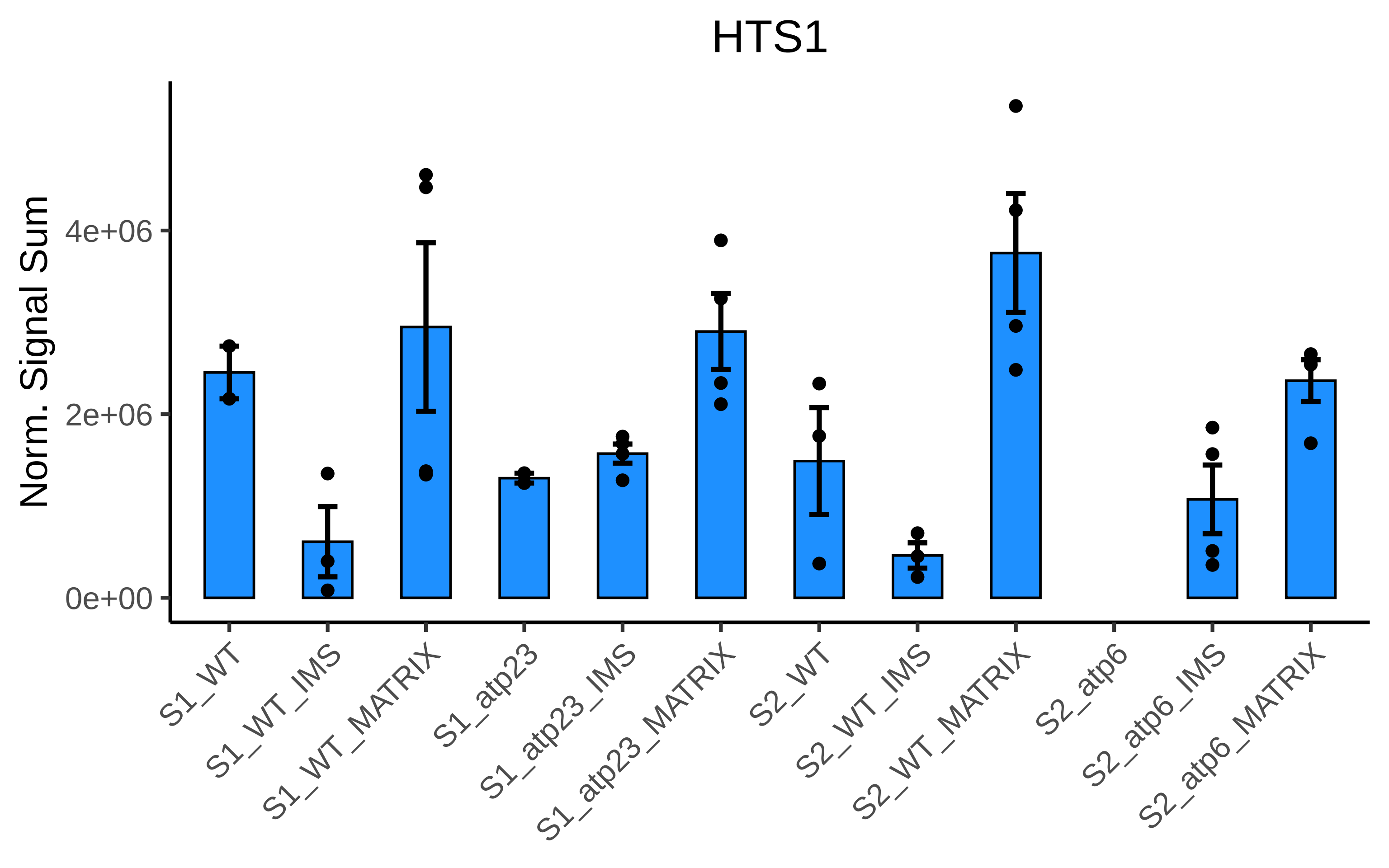
HTS1
Norm. Signal Sum
0e+00
2e+06
4e+06
S1_WT
S1_WT_IMS
S1_WT_MATRIX
S1_atp23
S1_atp23_IMS
S1_atp23_MATRIX
S2_WT
S2_WT_IMS
S2_WT_MATRIX
S2_atp6
S2_atp6_IMS
S2_atp6_MATRIX

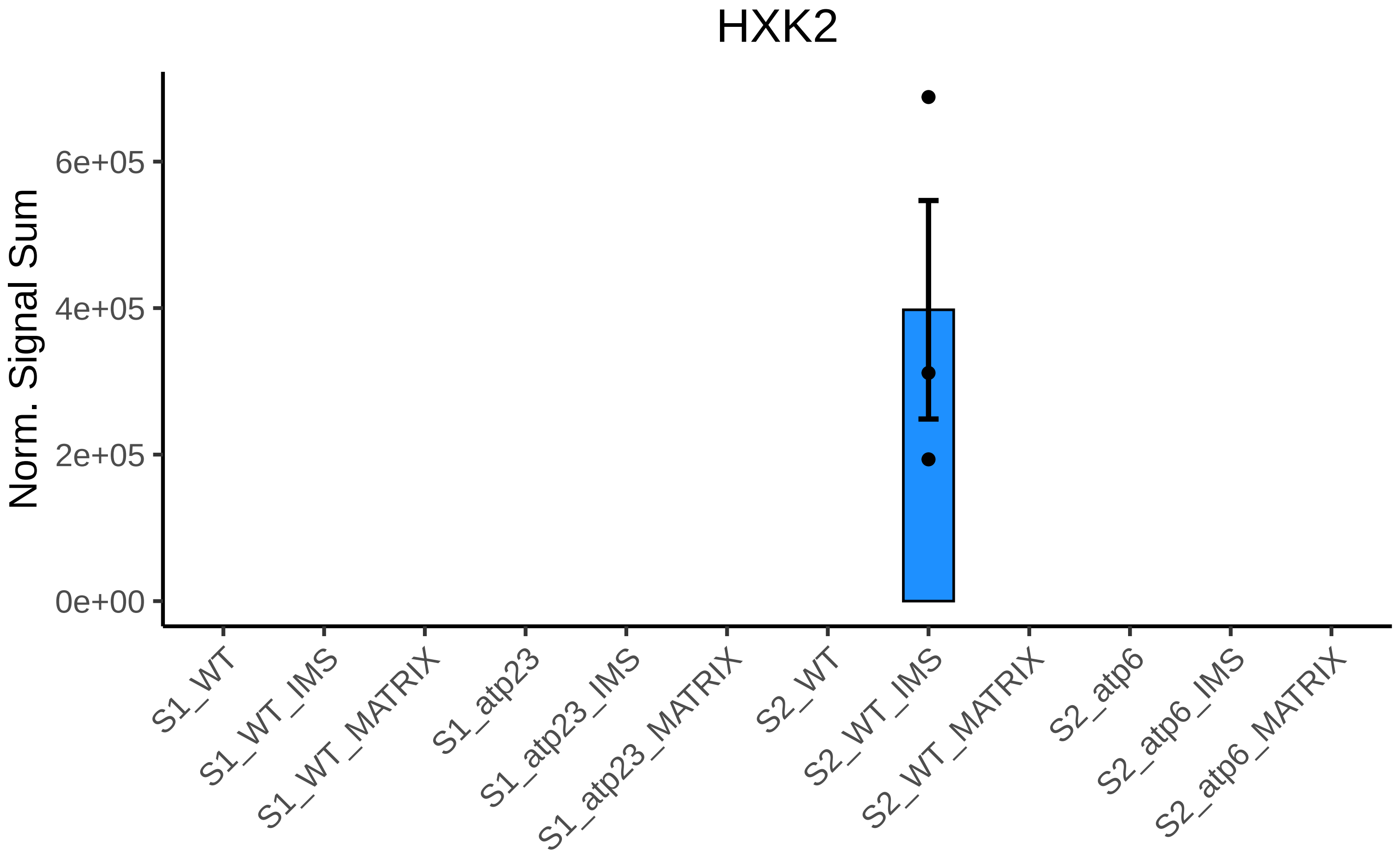
HXK2
Norm. Signal Sum
6e+05
4e+05
2e+05
0e+00
S1_WT
S1_WT_IMS
S1_WT_MATRIX
S1_atp23
S1_atp23_IMS
S1_atp23_MATRIX
S2_WT
S2_WT_IMS
S2_WT_MATRIX
S2_atp6
S2_atp6_IMS
S2_atp6_MATRIX

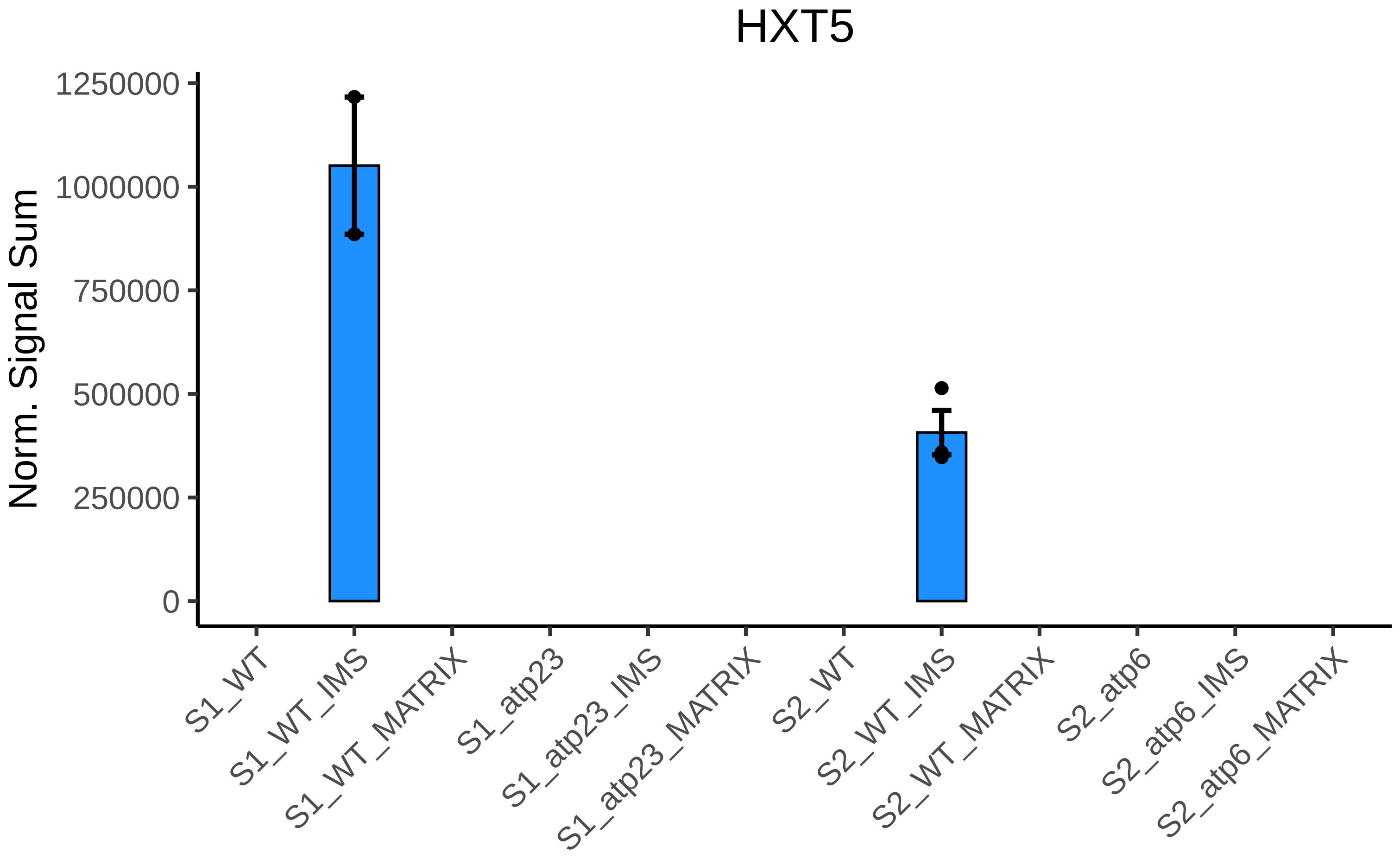
HXT5
Norm. Signal Sum
1250000
1000000
750000
500000
250000
0
S1_WT
S1_WT_IMS
S1_WT_MATRIX
S1_atp23
S1_atp23_IMS
S1_atp23_MATRIX
S2_WT
S2_WT_IMS
S2_WT_MATRIX
S2_atp6
S2_atp6_IMS
S2_atp6_MATRIX

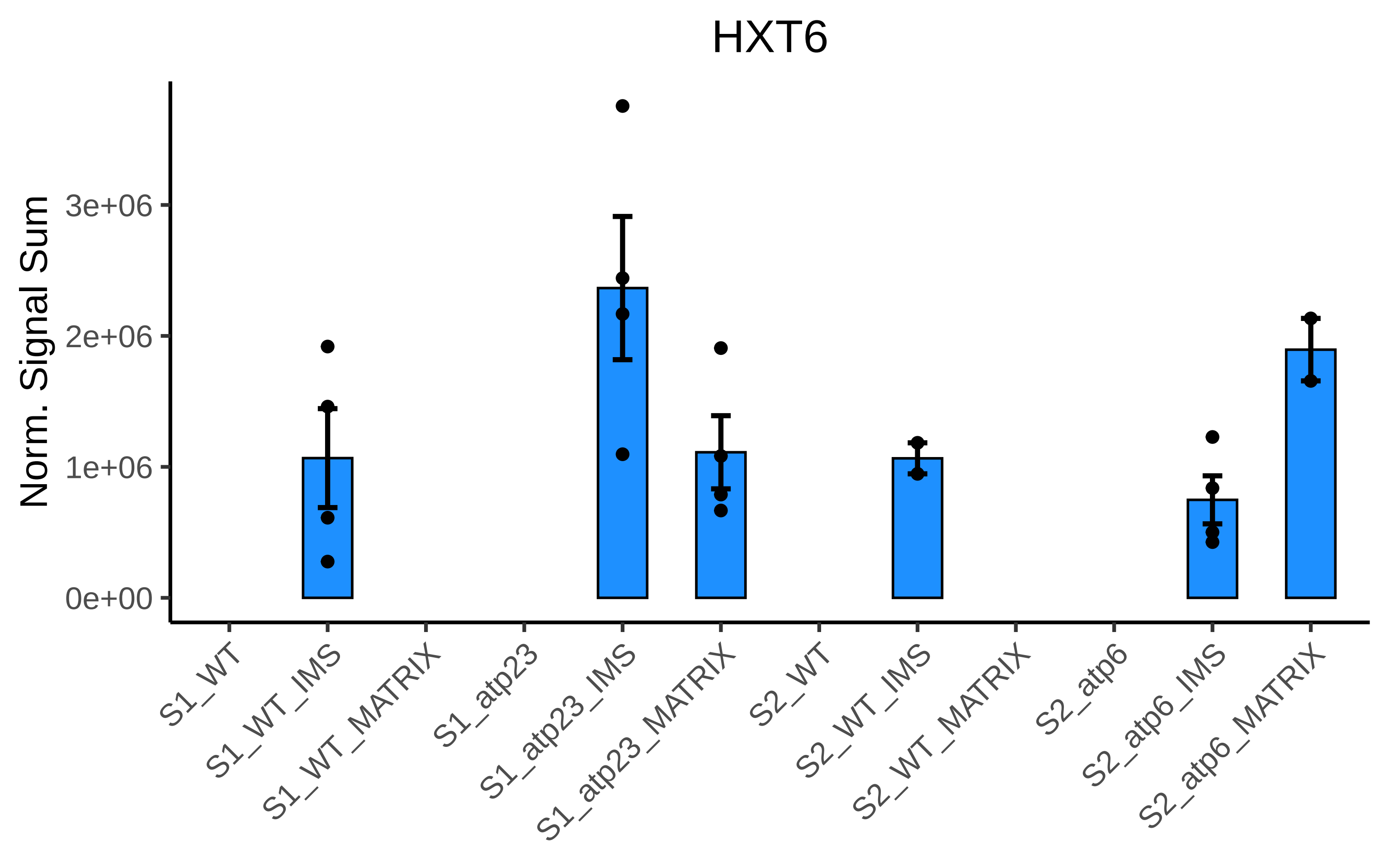
HXT6
Norm. Signal Sum
3e+06
2e+06
1e+06
0e+00
S1_WT
S1_WT_IMS
S1_WT_MATRIX
S1_atp23
S1_atp23_IMS
S1_atp23_MATRIX
S2_WT
S2_WT_IMS
S2_WT_MATRIX
S2_atp6
S2_atp6_IMS
S2_atp6_MATRIX

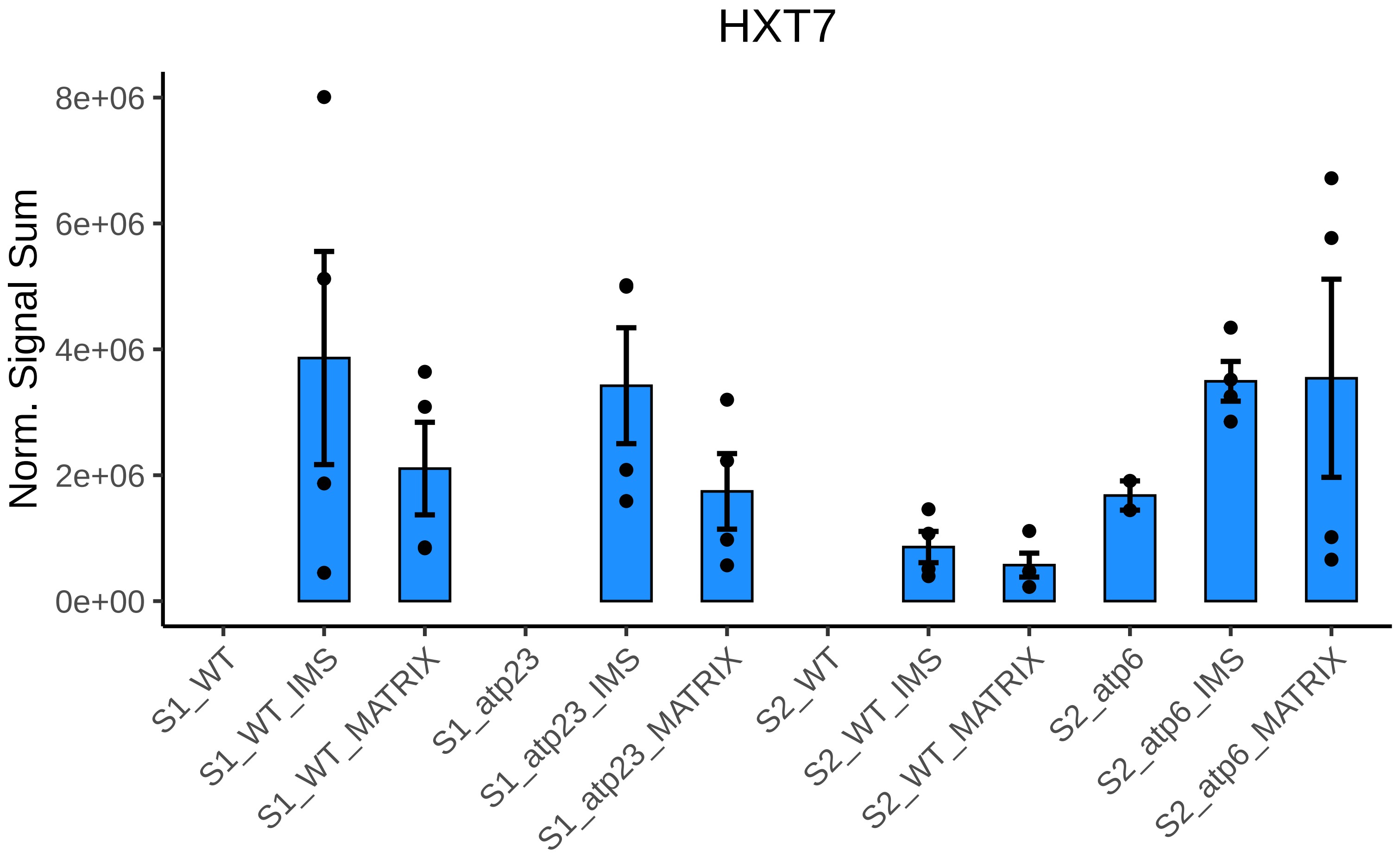

HXT7
Norm. Signal Sum
8e+06
6e+06
4e+06
2e+06
0e+00
S1_WT
S1_WT_IMS
S1_WT_MATRIX
S1_atp23
S1_atp23_IMS
S1_atp23_MATRIX
S2_WT
S2_WT_IMS
S2_WT_MATRIX
S2_atp6
S2_atp6_IMS
S2_atp6_MATRIX

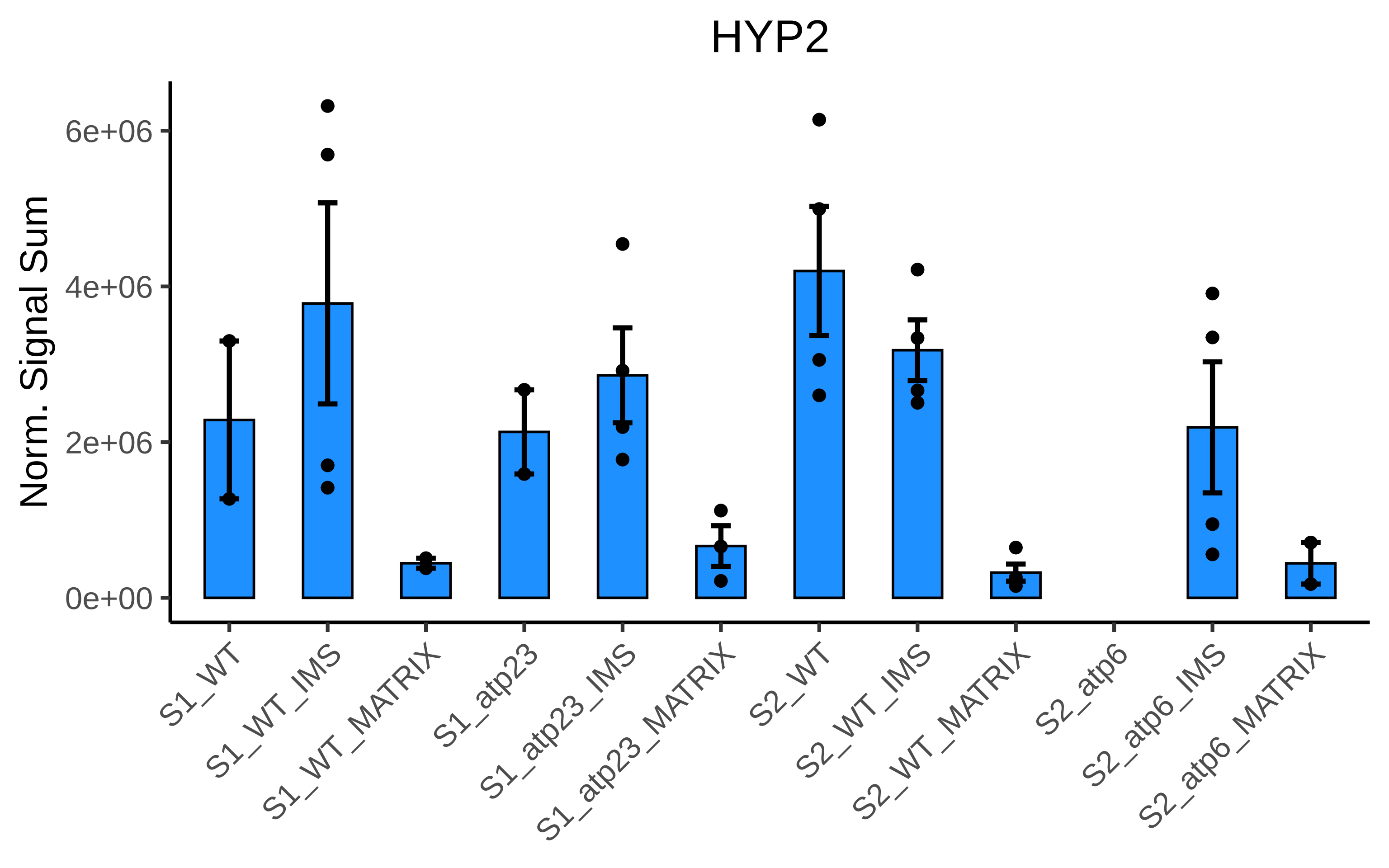
HYP2
Norm. Signal Sum
6e+06
4e+06
2e+06
0e+00
S1_WT
S1_WT_IMS
S1_WT_MATRIX
S1_atp23
S1_atp23_IMS
S1_atp23_MATRIX
S2_WT
S2_WT_IMS
S2_WT_MATRIX
S2_atp6
S2_atp6_IMS
S2_atp6_MATRIX

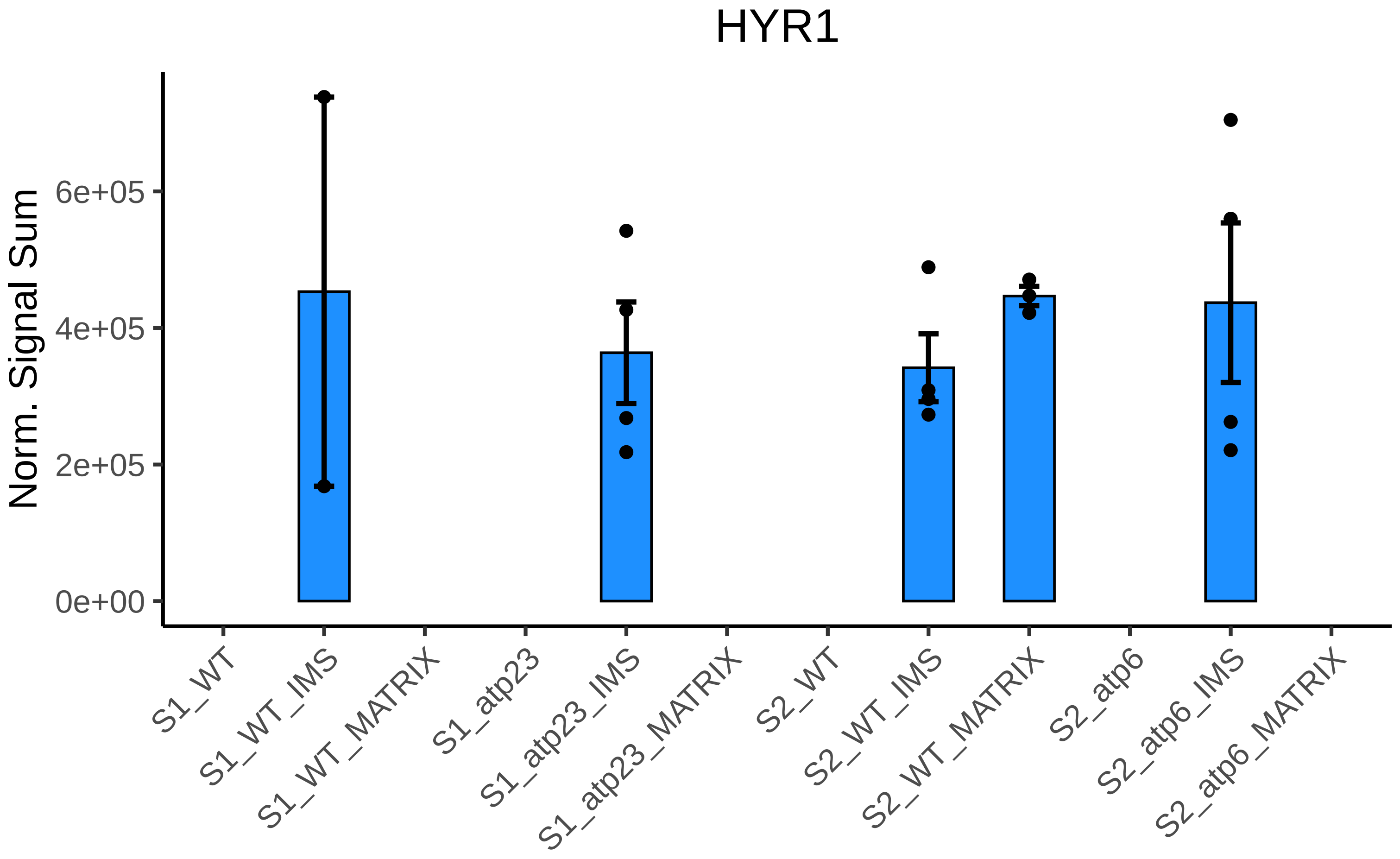

HYR1
Norm. Signal Sum
6e+05
4e+05
2e+05
0e+00
S1_WT
S1_WT_IMS
S1_WT_MATRIX
S1_atp23
S1_atp23_IMS
S1_atp23_MATRIX
S2_WT
S2_WT_IMS
S2_WT_MATRIX
S2_atp6
S2_atp6_IMS
S2_atp6_MATRIX

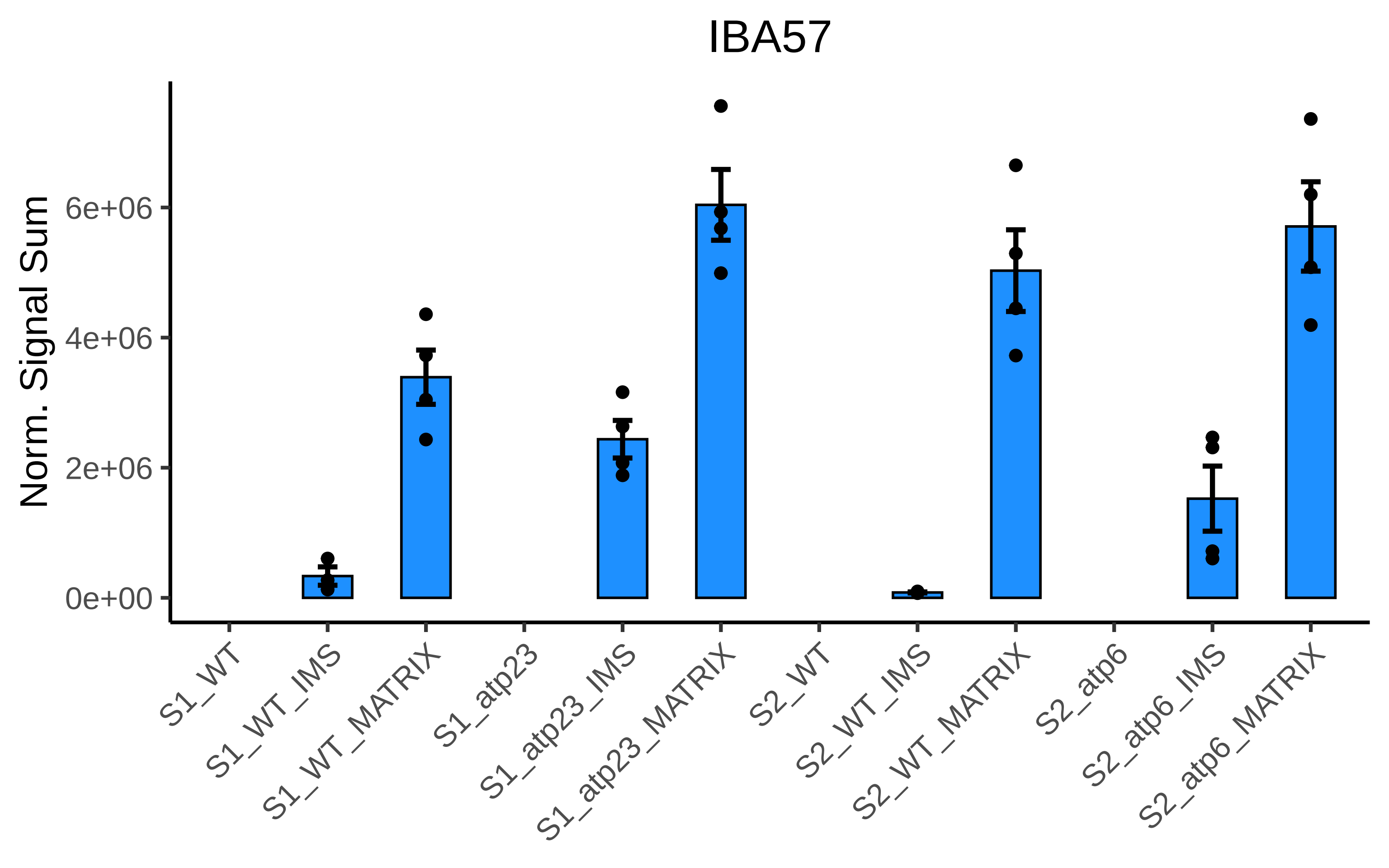
IBA57
Norm. Signal Sum
0e+00
2e+06
4e+06
6e+06
S1_WT
S1_WT_IMS
S1_WT_MATRIX
S1_atp23
S1_atp23_IMS
S1_atp23_MATRIX
S2_WT
S2_WT_IMS
S2_WT_MATRIX
S2_atp6
S2_atp6_IMS
S2_atp6_MATRIX

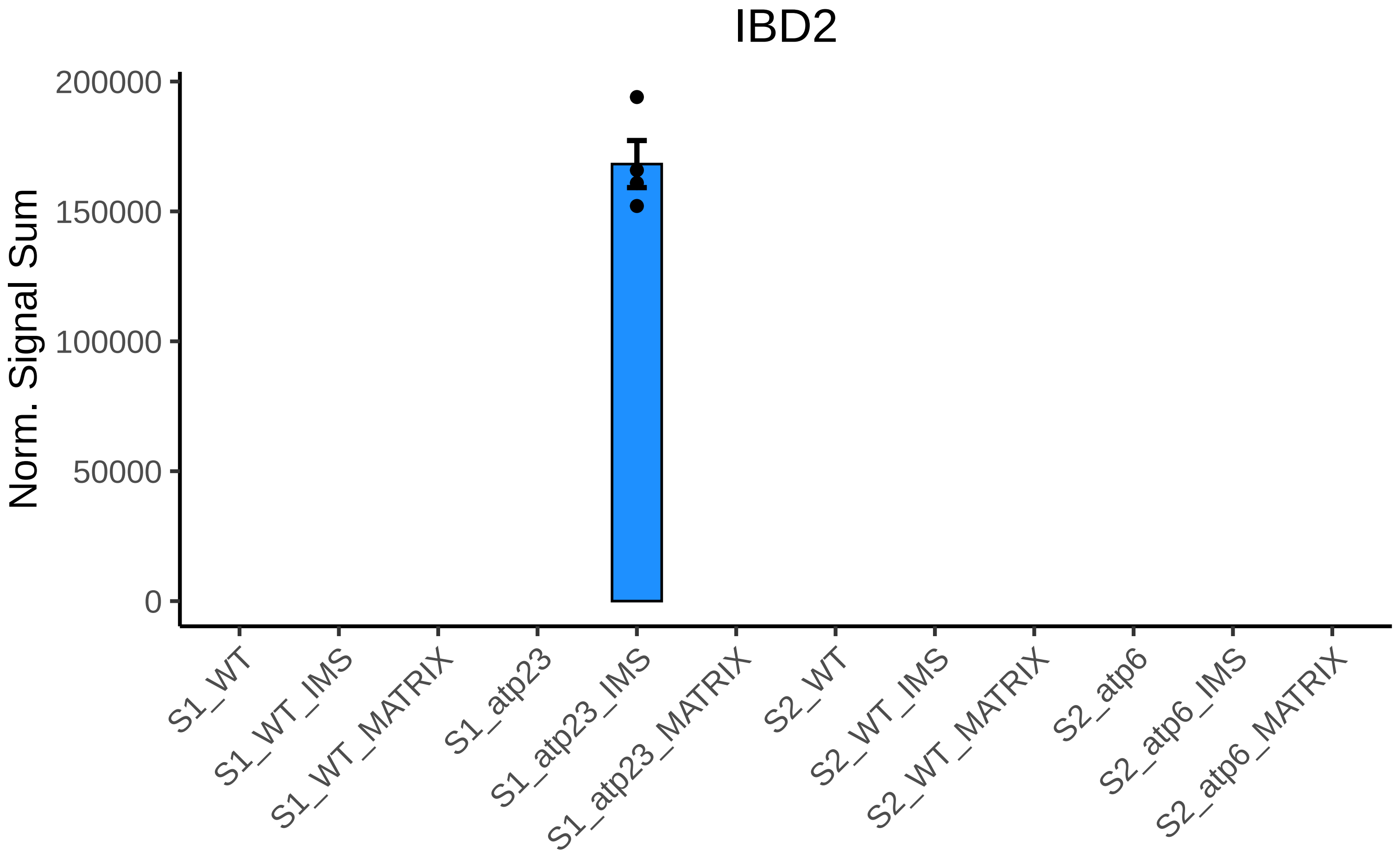

IBD2
Norm. Signal Sum
200000
150000
100000
50000
0
S1_WT
S1_WT_IMS
S1_WT_MATRIX
S1_atp23
S1_atp23_IMS
S1_atp23_MATRIX
S2_WT
S2_WT_IMS
S2_WT_MATRIX
S2_atp6
S2_atp6_IMS
S2_atp6_MATRIX

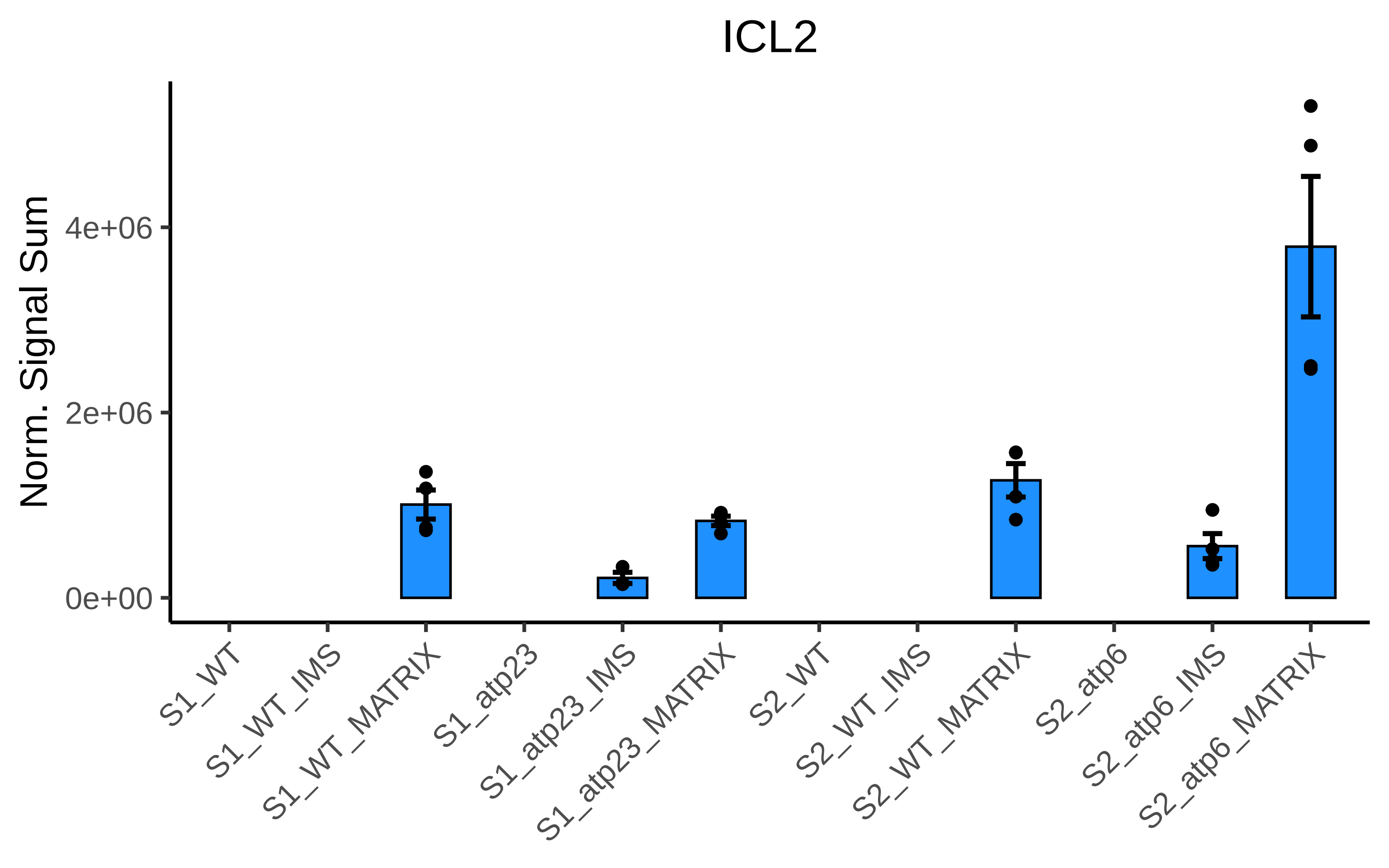
ICL2
Norm. Signal Sum
4e+06
2e+06
0e+00
S1_WT
S1_WT_IMS
S1_WT_MATRIX
S1_atp23
S1_atp23_IMS
S1_atp23_MATRIX
S2_WT
S2_WT_IMS
S2_WT_MATRIX
S2_atp6
S2_atp6_IMS
S2_atp6_MATRIX

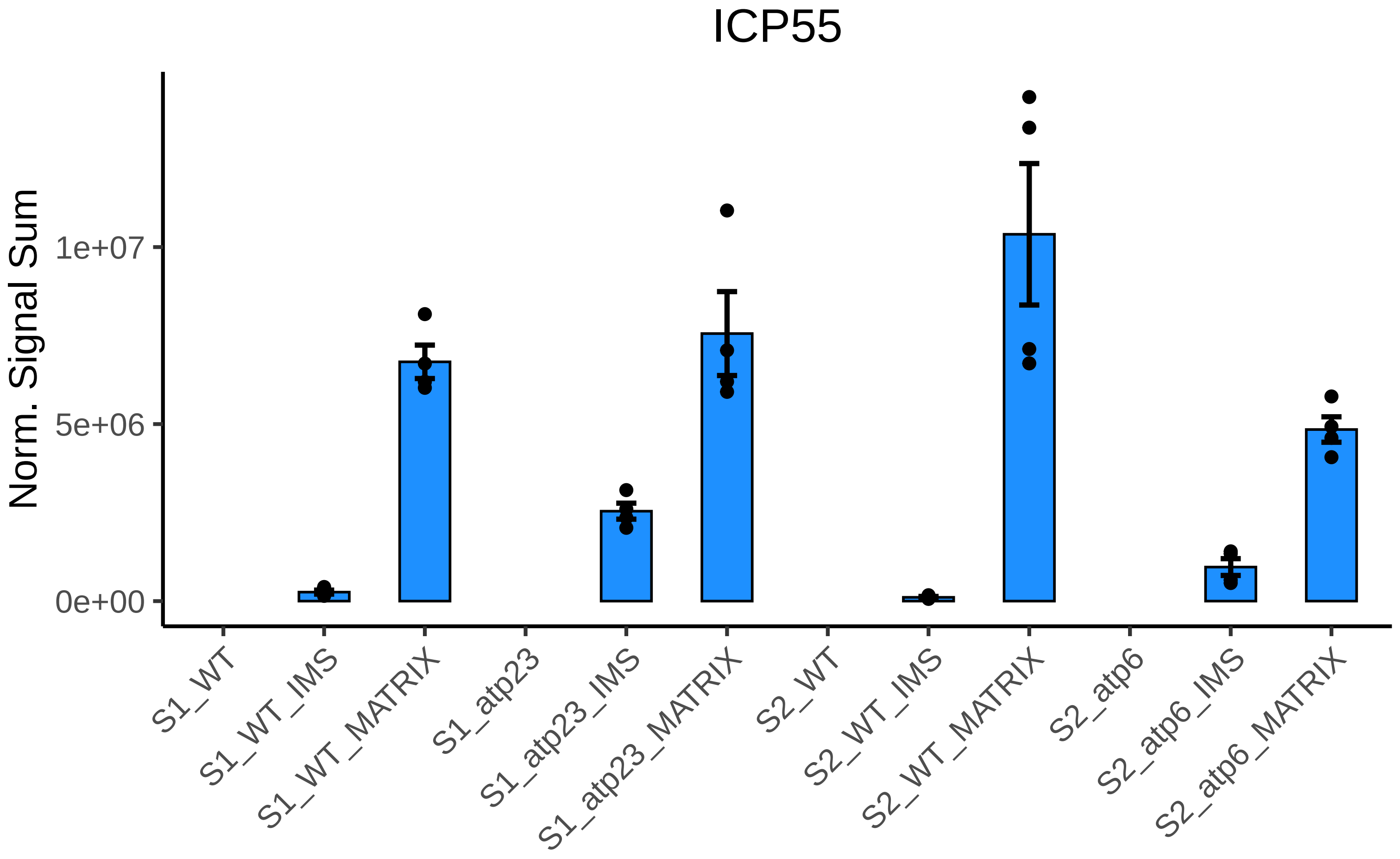
ICP55
Norm. Signal Sum
1e+07
5e+06
0e+00
S1_WT
S1_WT_IMS
S1_WT_MATRIX
S1_atp23
S1_atp23_IMS
S1_atp23_MATRIX
S2_WT
S2_WT_IMS
S2_WT_MATRIX
S2_atp6
S2_atp6_IMS
S2_atp6_MATRIX

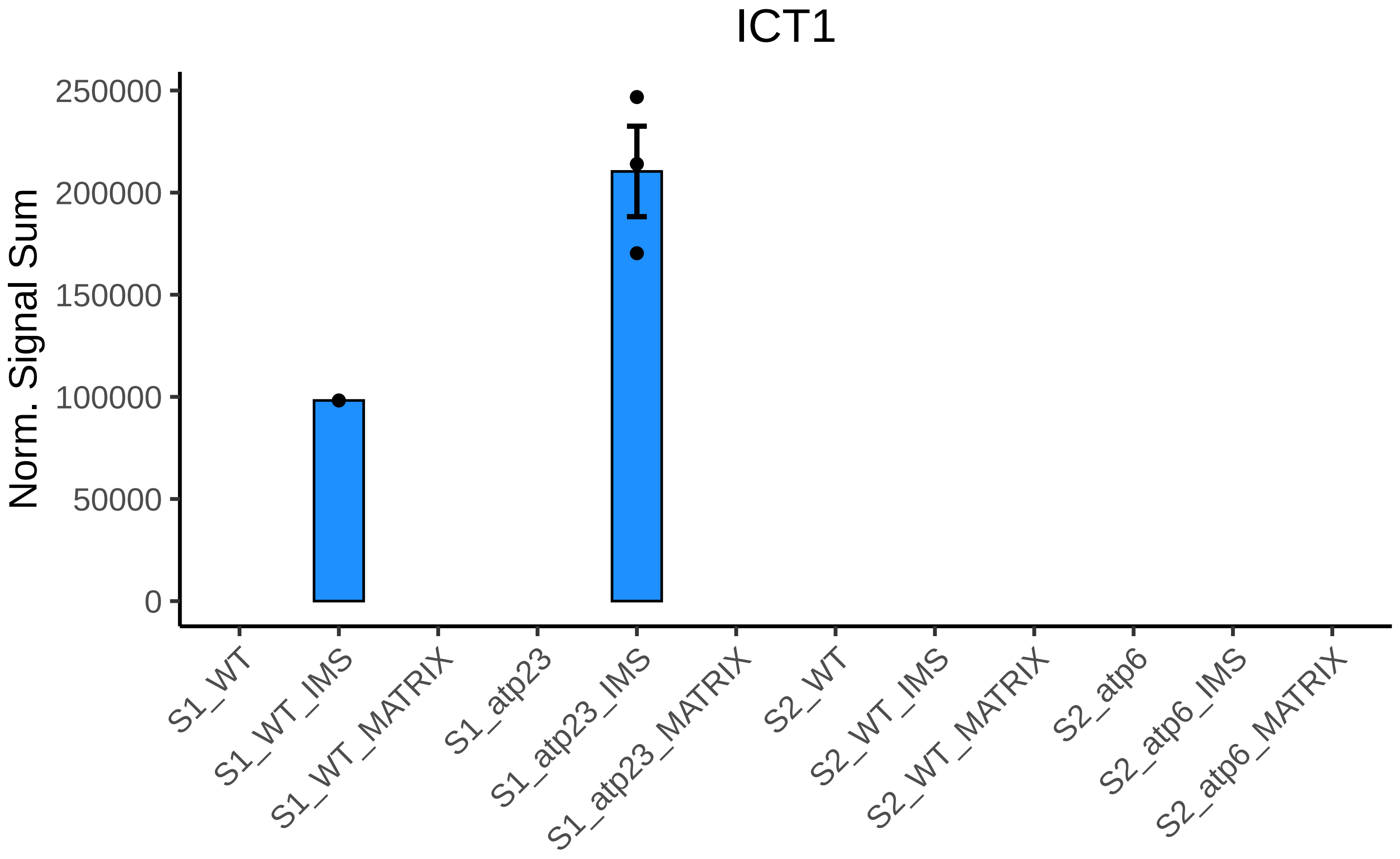

ICT1
Norm. Signal Sum
250000
200000
150000
100000
50000
0
S1_WT
S1_WT_IMS
S1_WT_MATRIX
S1_atp23
S1_atp23_IMS
S1_atp23_MATRIX
S2_WT
S2_WT_IMS
S2_WT_MATRIX
S2_atp6
S2_atp6_IMS
S2_atp6_MATRIX

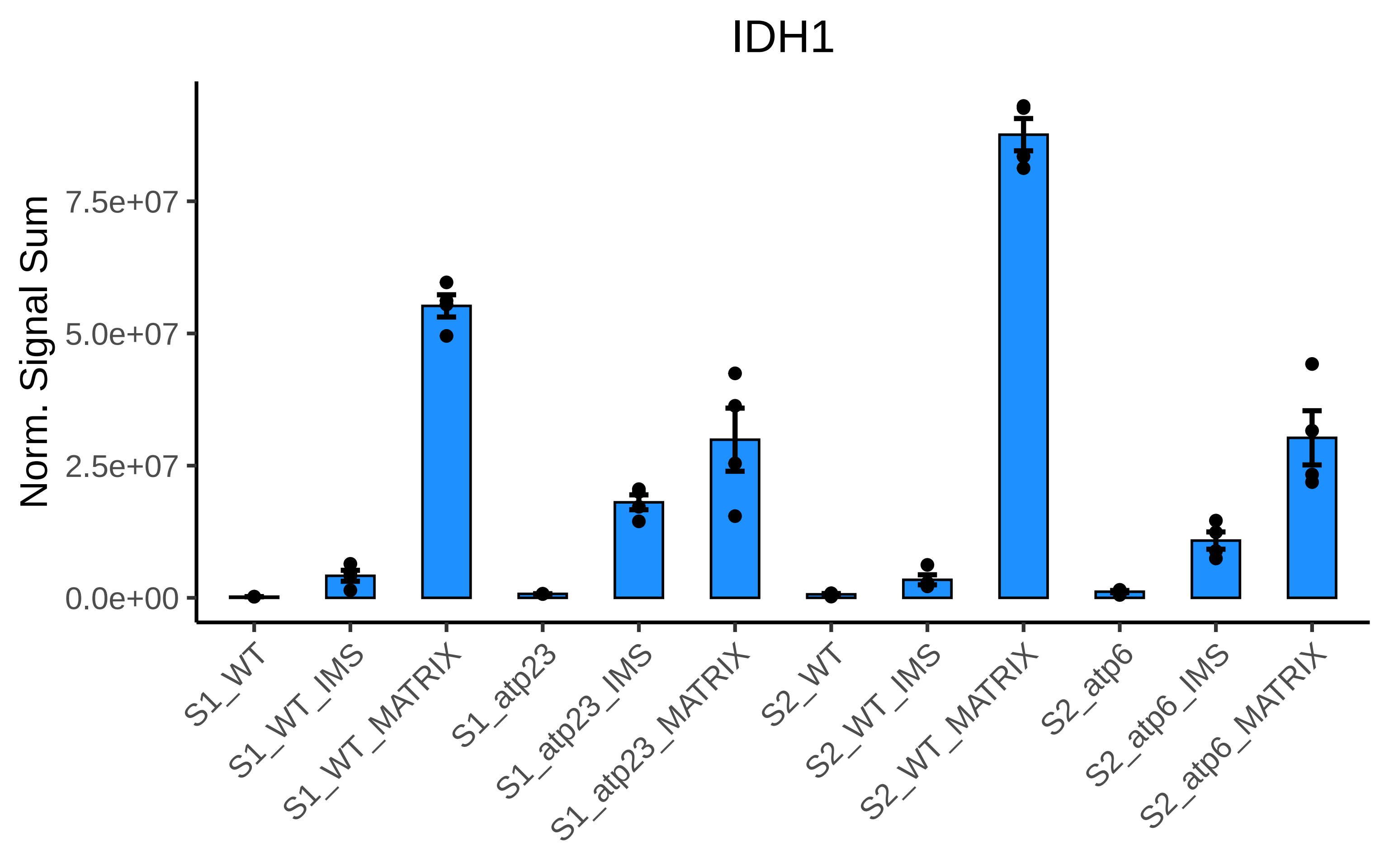

IDH1
Norm. Signal Sum
7.5e+07
5.0e+07
2.5e+07
0.0e+00
S1_WT
S1_WT_IMS
S1_WT_MATRIX
S1_atp23
S1_atp23_IMS
S1_atp23_MATRIX
S2_WT
S2_WT_IMS
S2_WT_MATRIX
S2_atp6
S2_atp6_IMS
S2_atp6_MATRIX

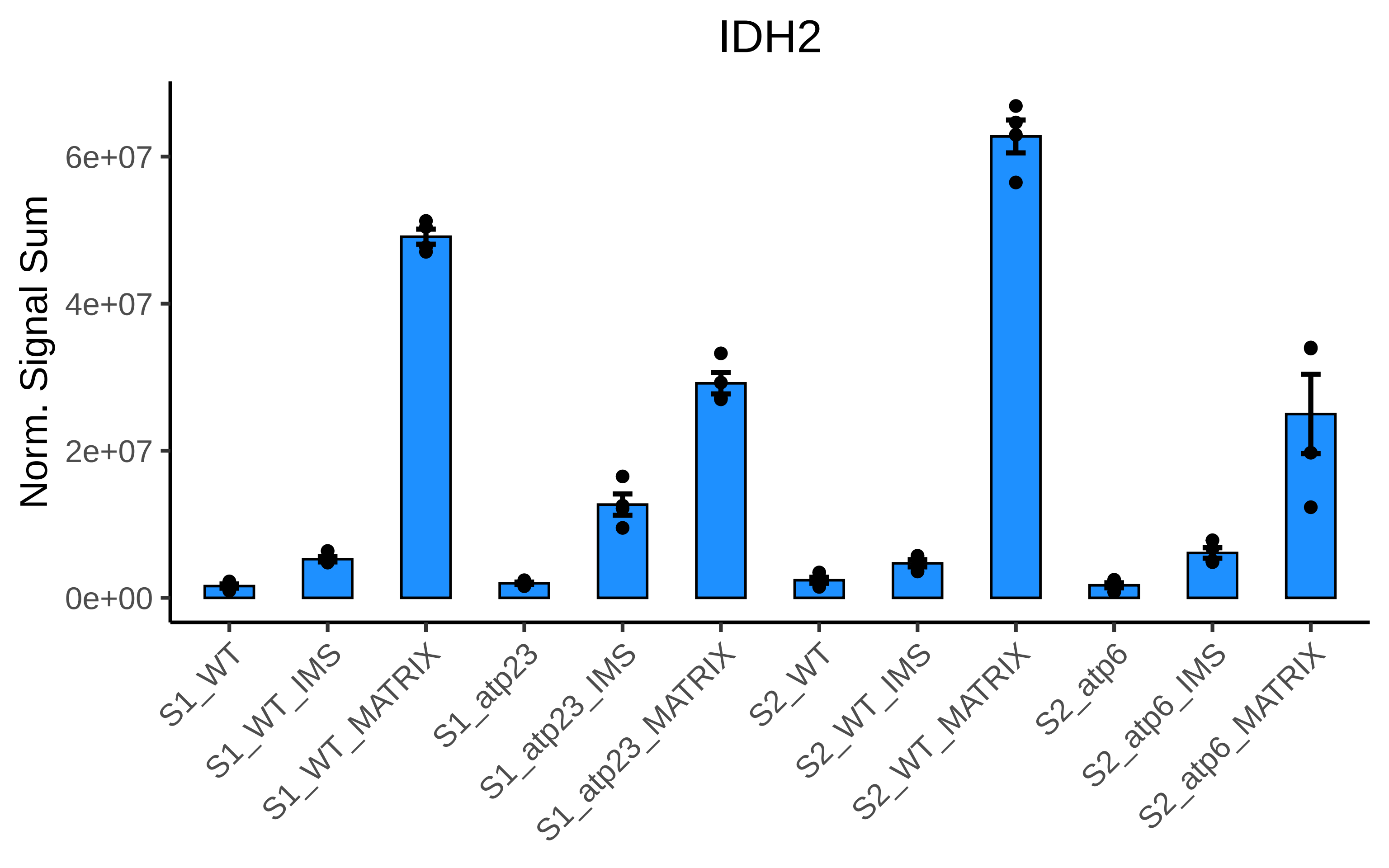

IDH2
Norm. Signal Sum
6e+07
4e+07
2e+07
0e+00
S1_WT
S1_WT_IMS
S1_WT_MATRIX
S1_atp23
S1_atp23_IMS
S1_atp23_MATRIX
S2_WT
S2_WT_IMS
S2_WT_MATRIX
S2_atp6
S2_atp6_IMS
S2_atp6_MATRIX

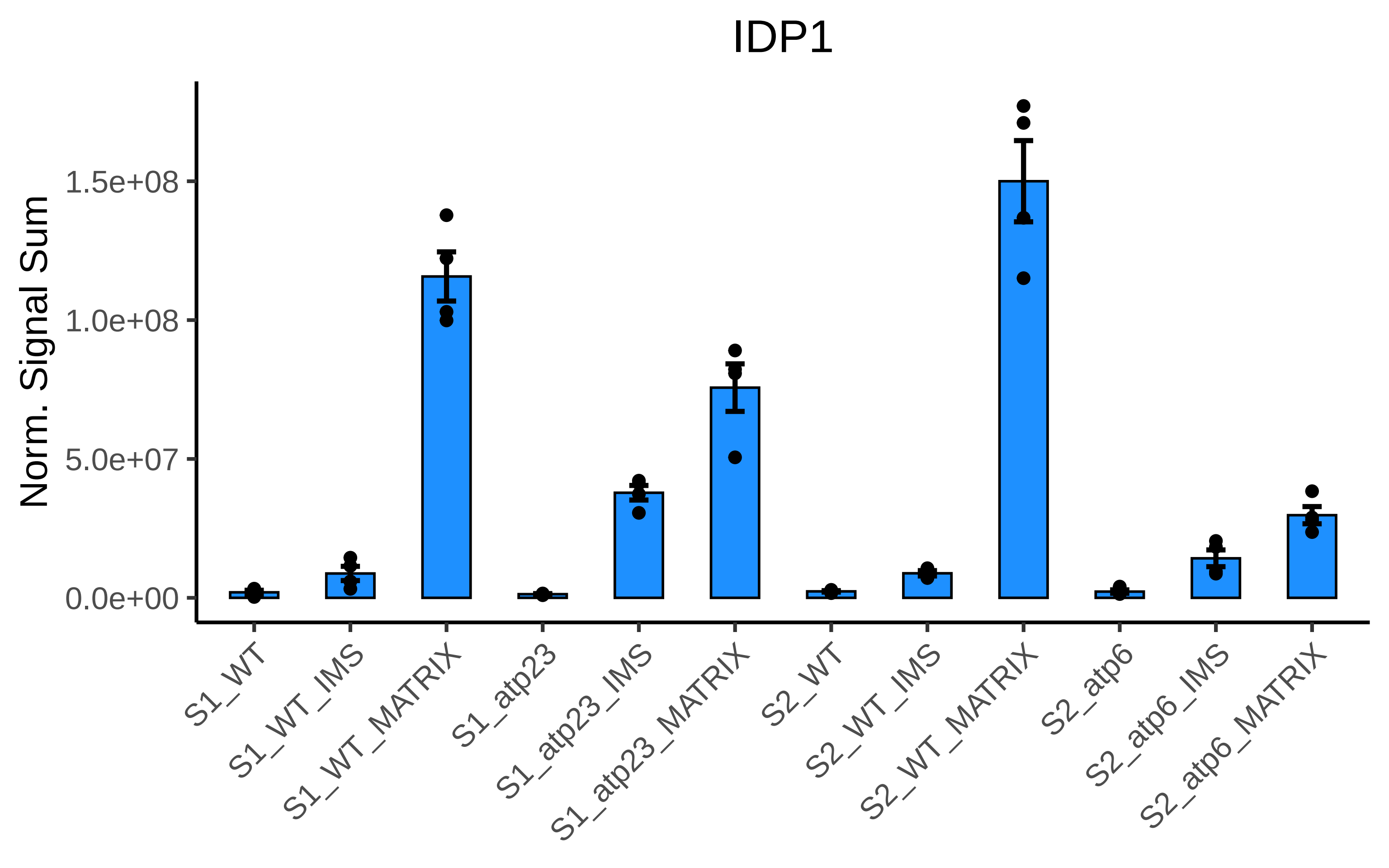

IDP1
Norm. Signal Sum
1.5e+08
1.0e+08
5.0e+07
0.0e+00
S1_WT
S1_WT_IMS
S1_WT_MATRIX
S1_atp23
S1_atp23_IMS
S1_atp23_MATRIX
S2_WT
S2_WT_IMS
S2_WT_MATRIX
S2_atp6
S2_atp6_IMS
S2_atp6_MATRIX

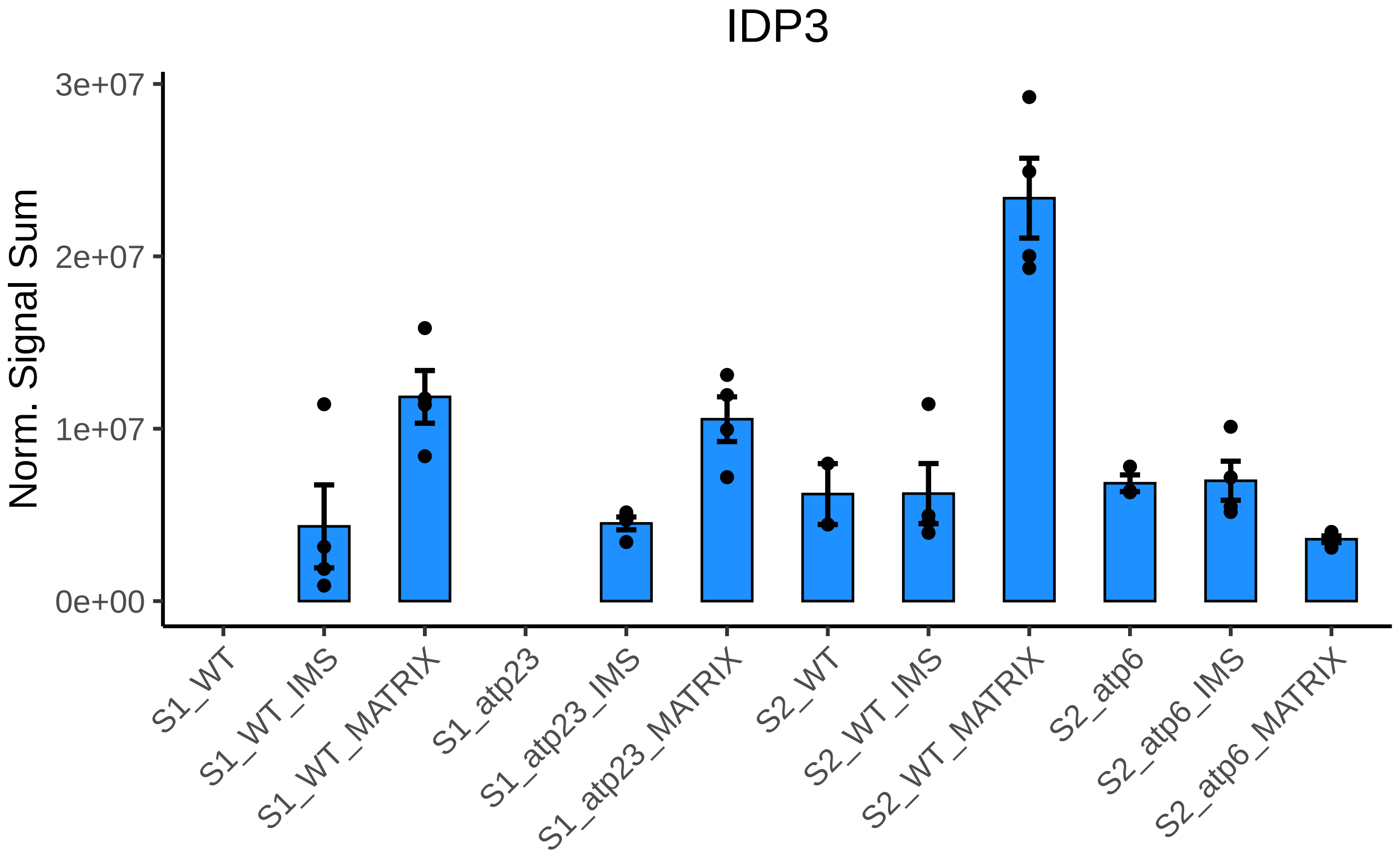

IDP3
Norm. Signal Sum
3e+07
2e+07
1e+07
0e+00
S1_WT
S1_WT_IMS
S1_WT_MATRIX
S1_atp23
S1_atp23_IMS
S1_atp23_MATRIX
S2_WT
S2_WT_IMS
S2_WT_MATRIX
S2_atp6
S2_atp6_IMS
S2_atp6_MATRIX

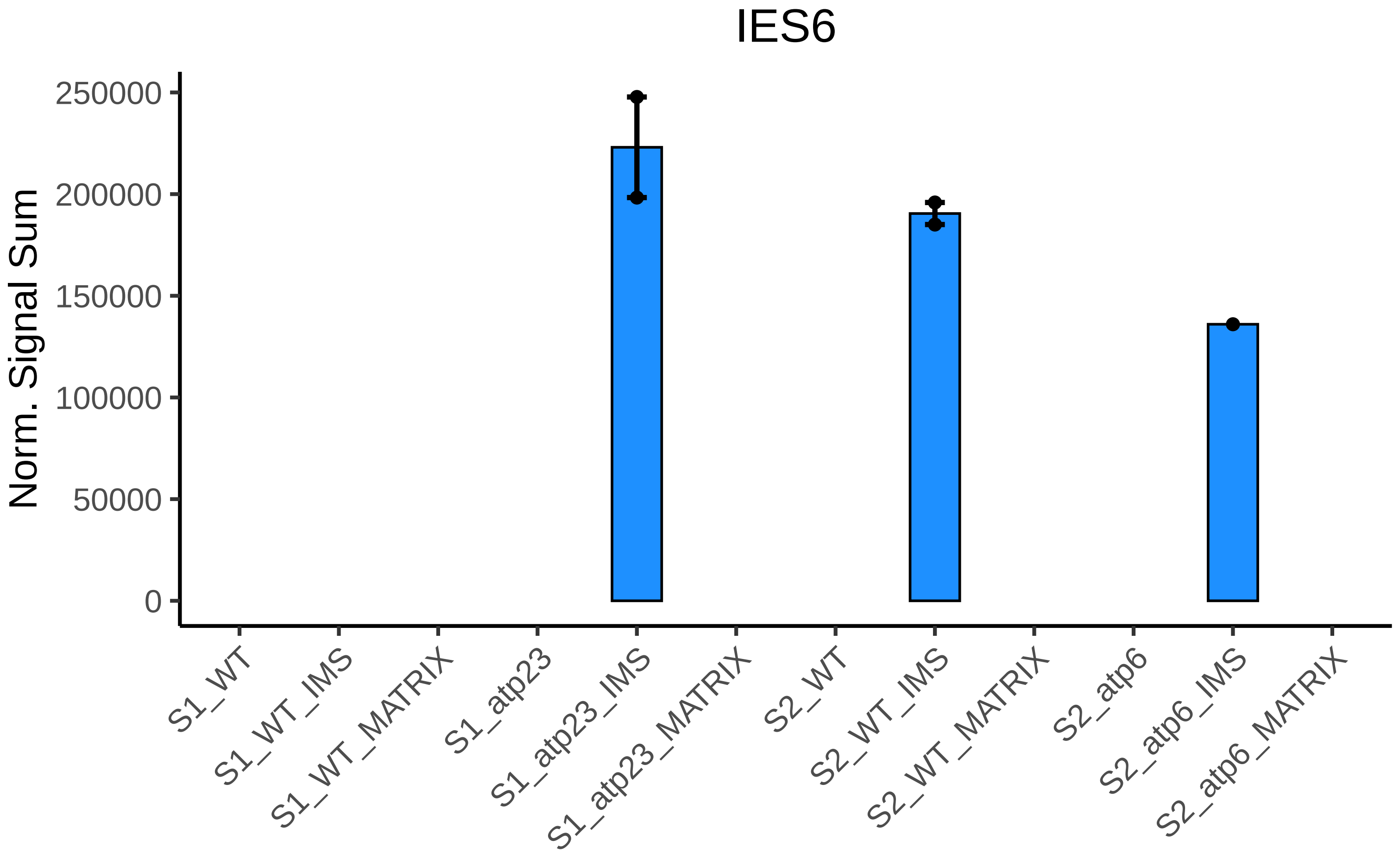
IES6
Norm. Signal Sum
250000
200000
150000
100000
50000
0
S1_WT
S1_WT_IMS
S1_WT_MATRIX
S1_atp23
S1_atp23_IMS
S1_atp23_MATRIX
S2_WT
S2_WT_IMS
S2_WT_MATRIX
S2_atp6
S2_atp6_IMS
S2_atp6_MATRIX

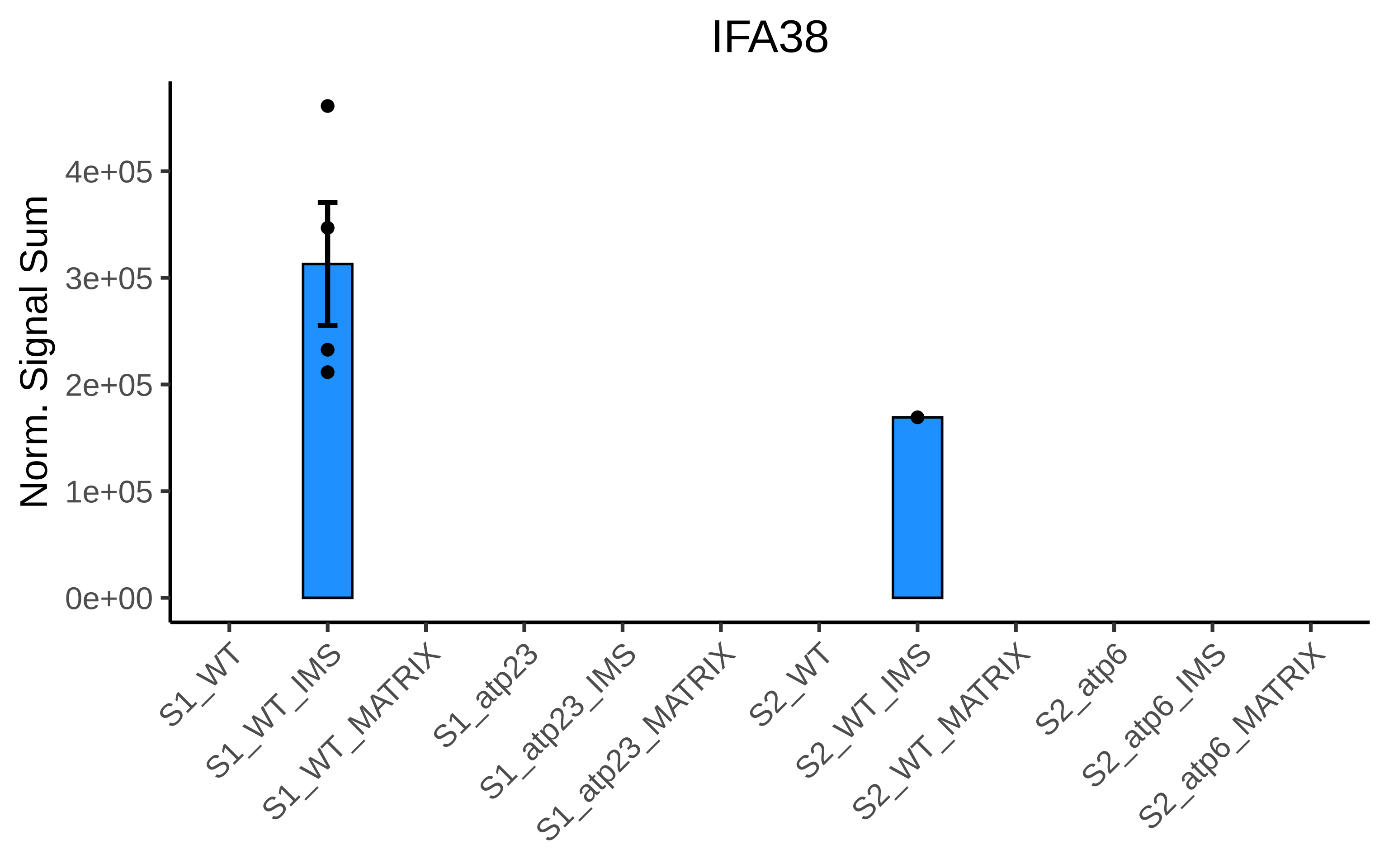
IFA38
Norm. Signal Sum
4e+05
3e+05
2e+05
1e+05
0e+00
S1_WT
S1_WT_IMS
S1_WT_MATRIX
S1_atp23
S1_atp23_IMS
S1_atp23_MATRIX
S2_WT
S2_WT_IMS
S2_WT_MATRIX
S2_atp6
S2_atp6_IMS
S2_atp6_MATRIX

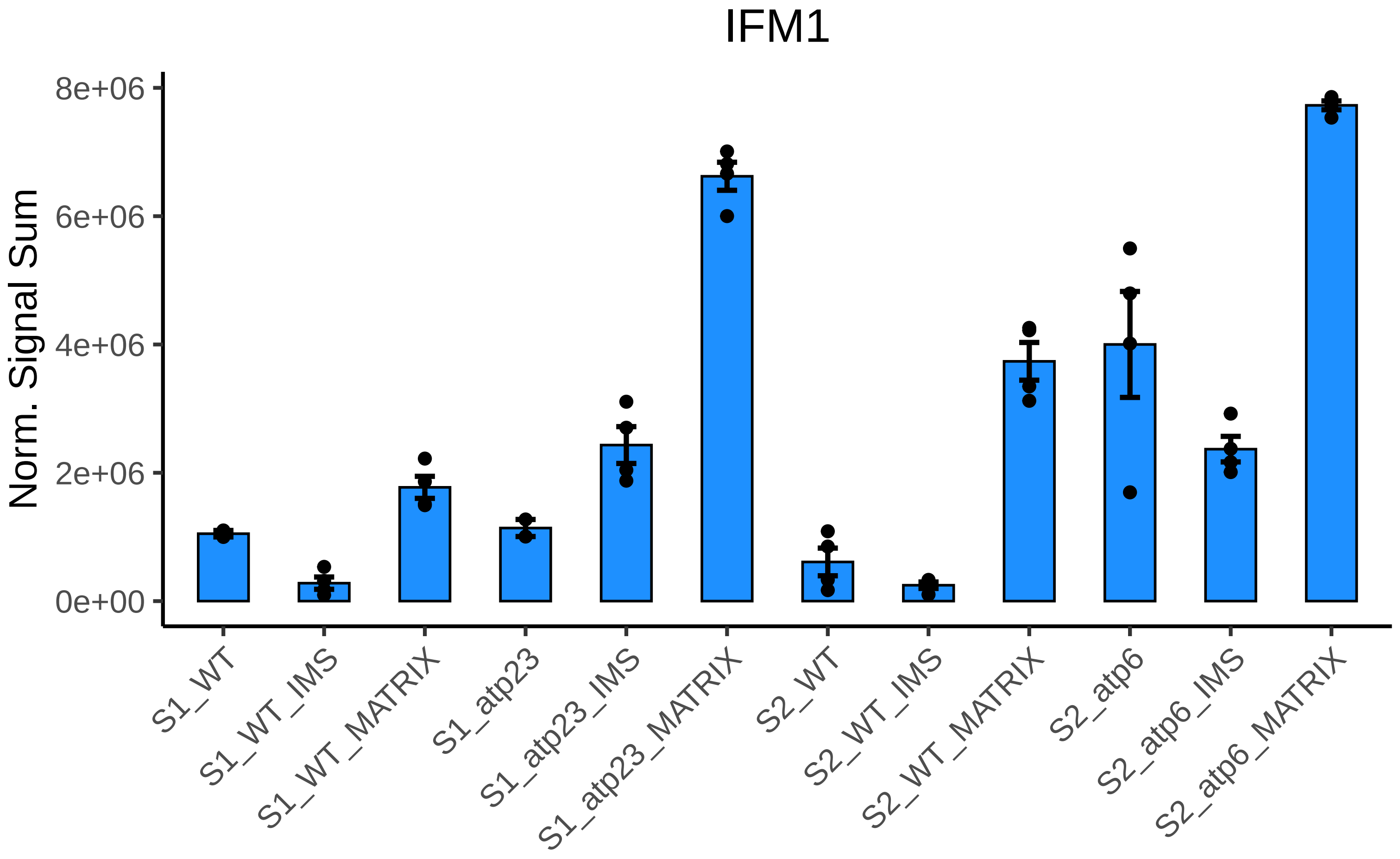
IFM1
Norm. Signal Sum
8e+06
6e+06
4e+06
2e+06
0e+00
S1_WT
S1_WT_IMS
S1_WT_MATRIX
S1_atp23
S1_atp23_IMS
S1_atp23_MATRIX
S2_WT
S2_WT_IMS
S2_WT_MATRIX
S2_atp6
S2_atp6_IMS
S2_atp6_MATRIX

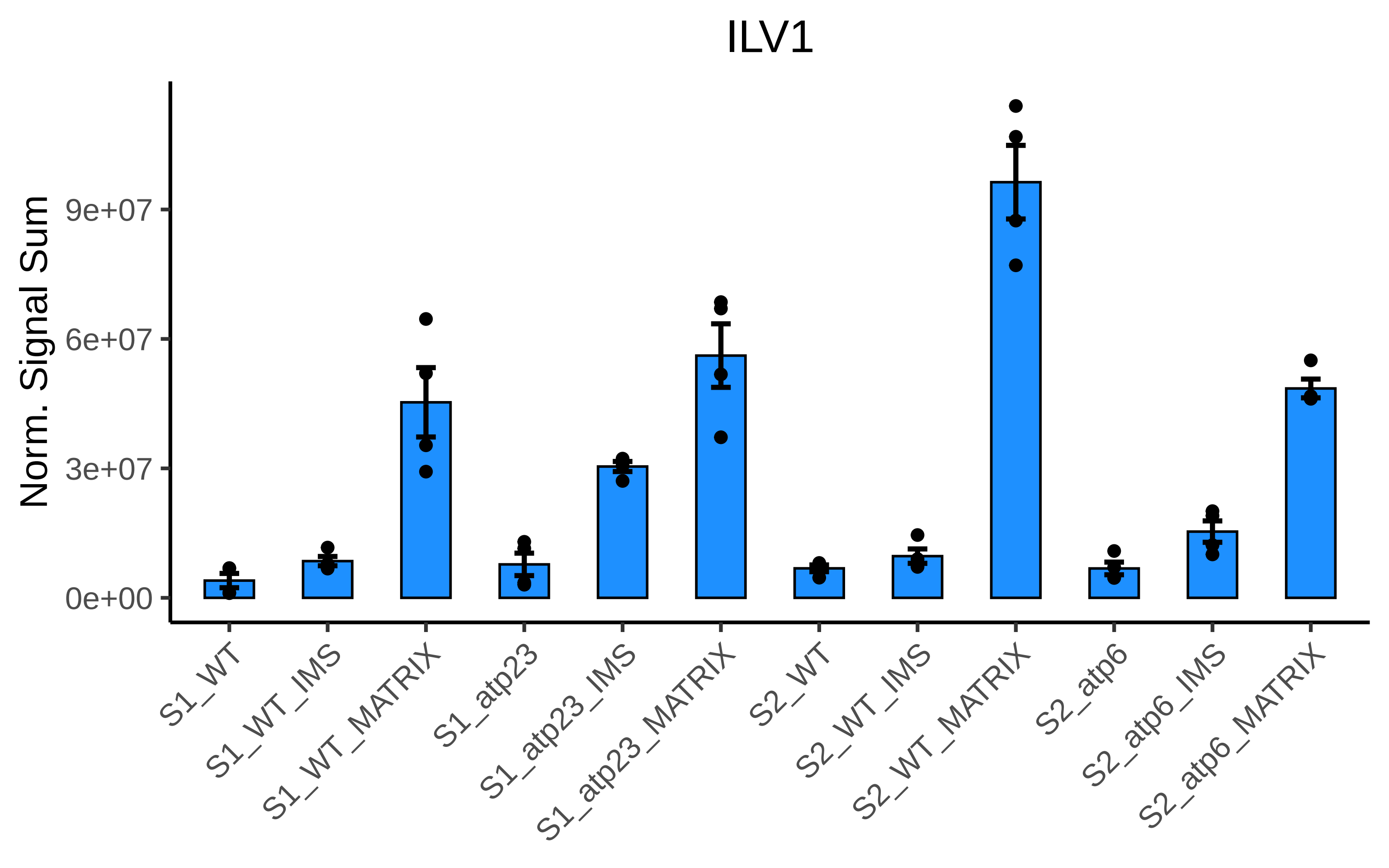
ILV1
Norm. Signal Sum
9e+07
6e+07
3e+07
0e+00
S1_WT
S1_WT_IMS
S1_WT_MATRIX
S1_atp23
S1_atp23_IMS
S1_atp23_MATRIX
S2_WT
S2_WT_IMS
S2_WT_MATRIX
S2_atp6
S2_atp6_IMS
S2_atp6_MATRIX

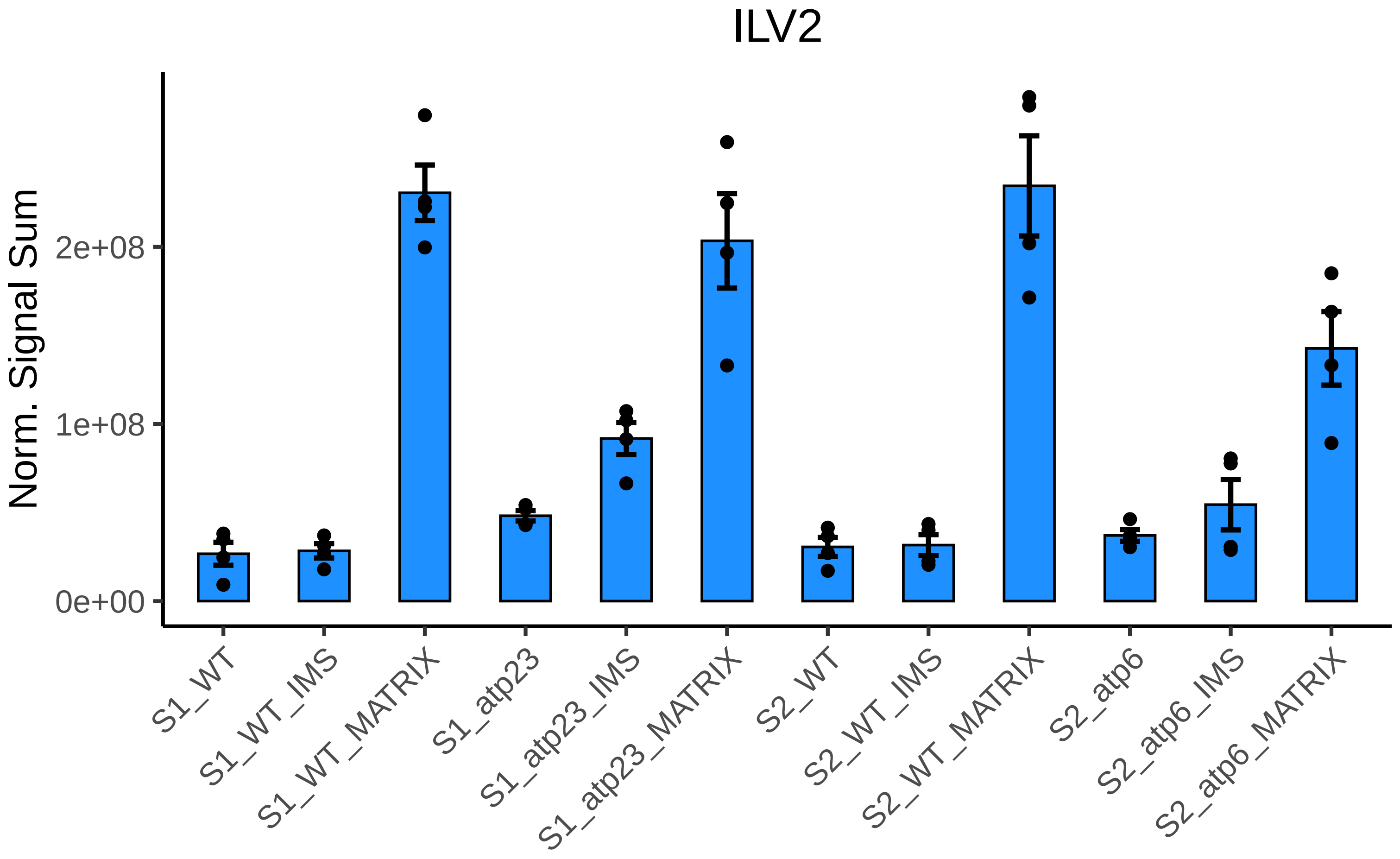
ILV2
Norm. Signal Sum
2e+08
1e+08
0e+00
S1_WT
S1_WT_IMS
S1_WT_MATRIX
S1_atp23
S1_atp23_IMS
S1_atp23_MATRIX
S2_WT
S2_WT_IMS
S2_WT_MATRIX
S2_atp6
S2_atp6_IMS
S2_atp6_MATRIX

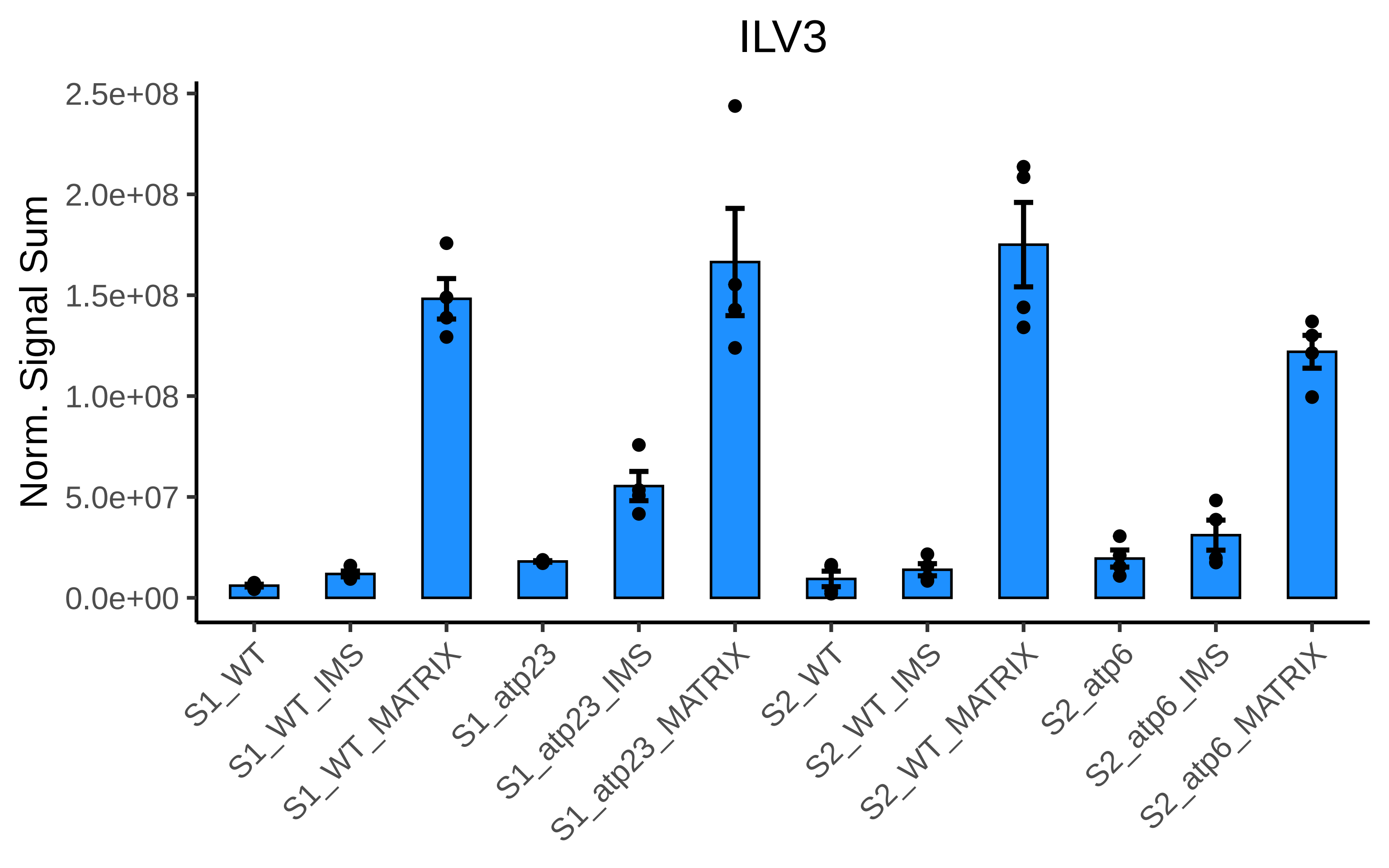
ILV3
Norm. Signal Sum
2.5e+08
2.0e+08
1.5e+08
1.0e+08
5.0e+07
0.0e+00
S1_WT
S1_WT_IMS
S1_WT_MATRIX
S1_atp23
S1_atp23_IMS
S1_atp23_MATRIX
S2_WT
S2_WT_IMS
S2_WT_MATRIX
S2_atp6
S2_atp6_IMS
S2_atp6_MATRIX

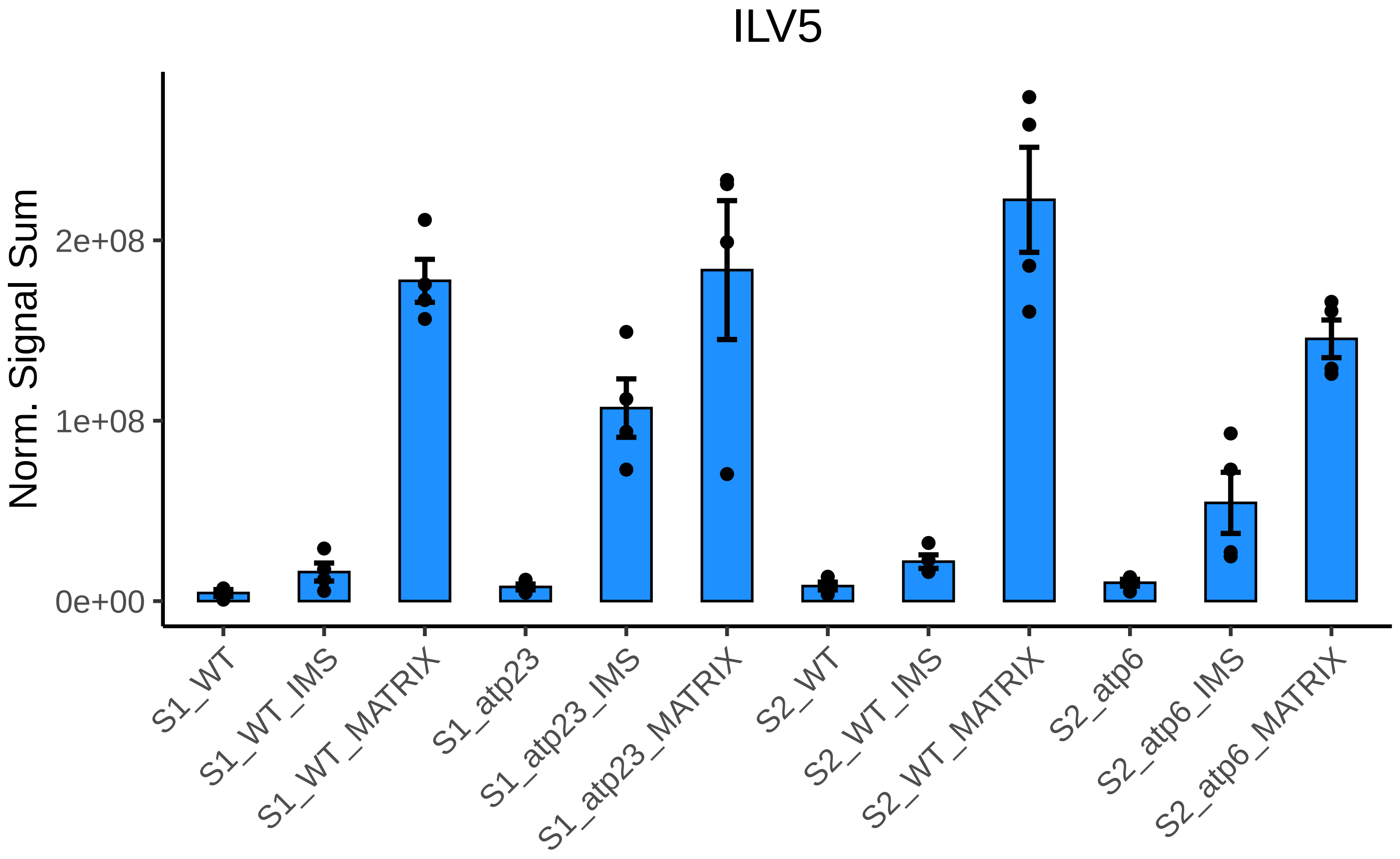

ILV5
Norm. Signal Sum
2e+08
1e+08
0e+00
S1_WT
S1_WT_IMS
S1_WT_MATRIX
S1_atp23
S1_atp23_IMS
S1_atp23_MATRIX
S2_WT
S2_WT_IMS
S2_WT_MATRIX
S2_atp6
S2_atp6_IMS
S2_atp6_MATRIX

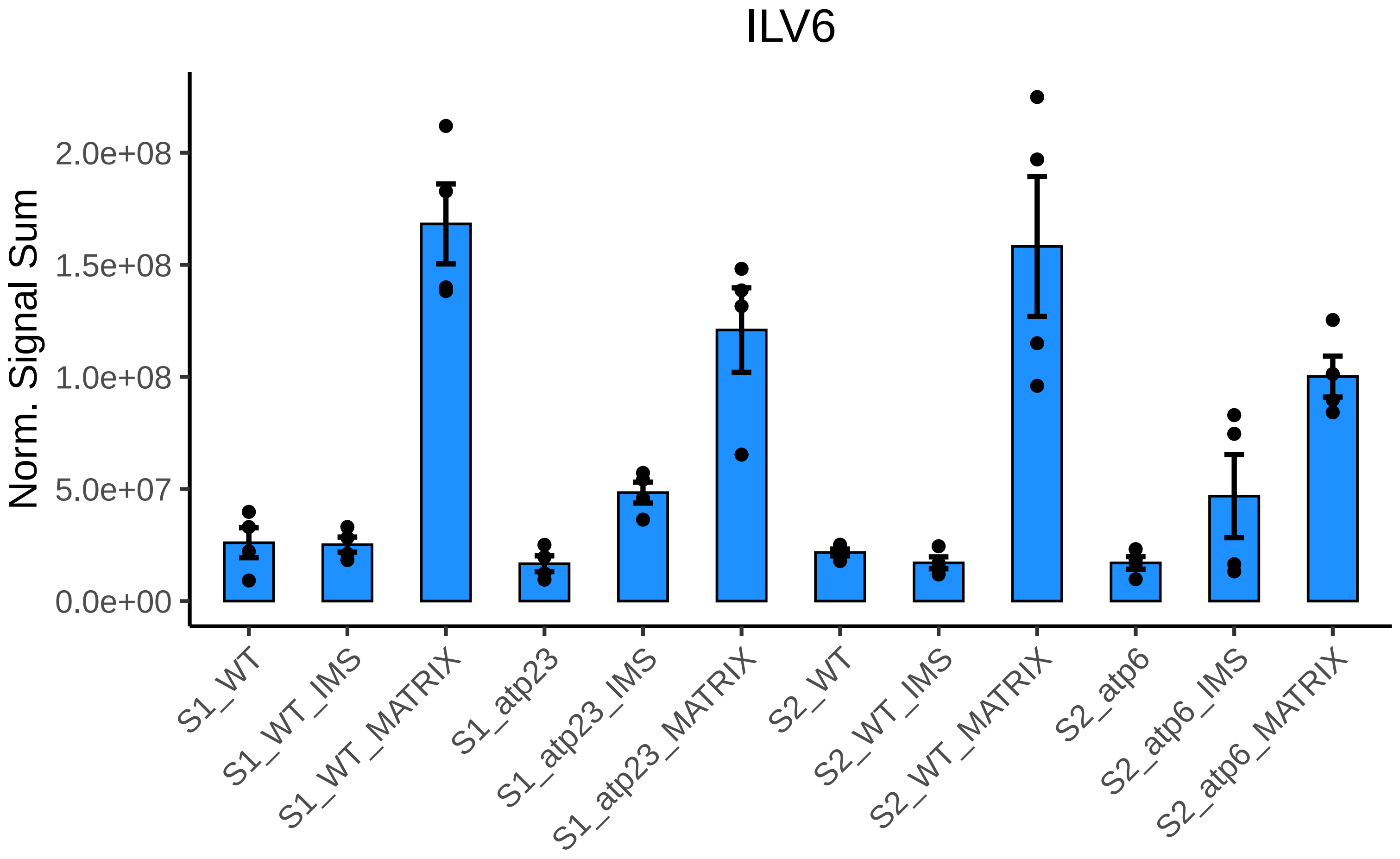
ILV6
Norm. Signal Sum
2.0e+08
1.5e+08
1.0e+08
5.0e+07
0.0e+00
S1_WT
S1_WT_IMS
S1_WT_MATRIX
S1_atp23
S1_atp23_IMS
S1_atp23_MATRIX
S2_WT
S2_WT_IMS
S2_WT_MATRIX
S2_atp6
S2_atp6_IMS
S2_atp6_MATRIX

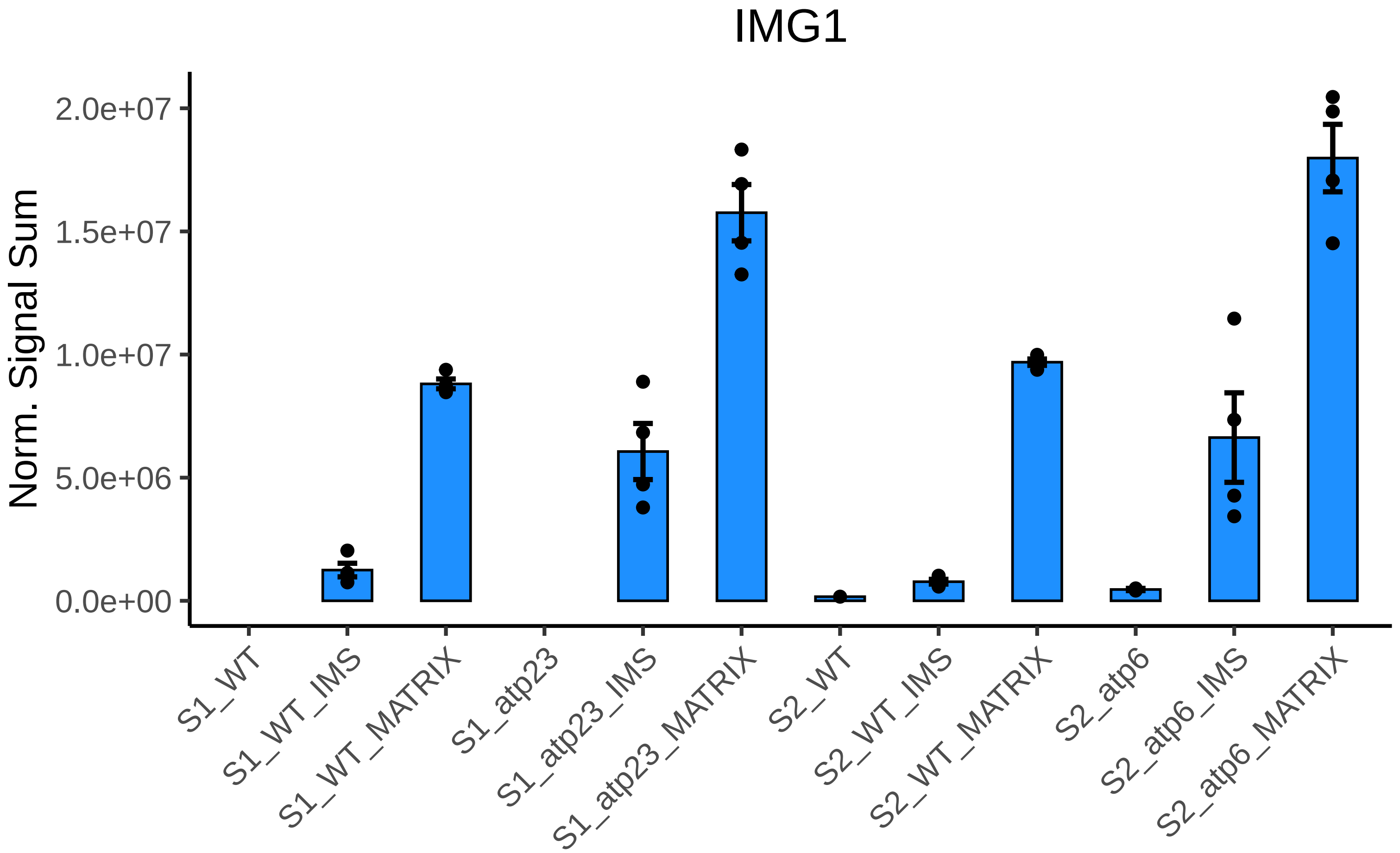
IMG1
Norm. Signal Sum
2.0e+07
1.5e+07
1.0e+07
5.0e+06
0.0e+00
S1_WT
S1_WT_IMS
S1_WT_MATRIX
S1_atp23
S1_atp23_IMS
S1_atp23_MATRIX
S2_WT
S2_WT_IMS
S2_WT_MATRIX
S2_atp6
S2_atp6_IMS
S2_atp6_MATRIX

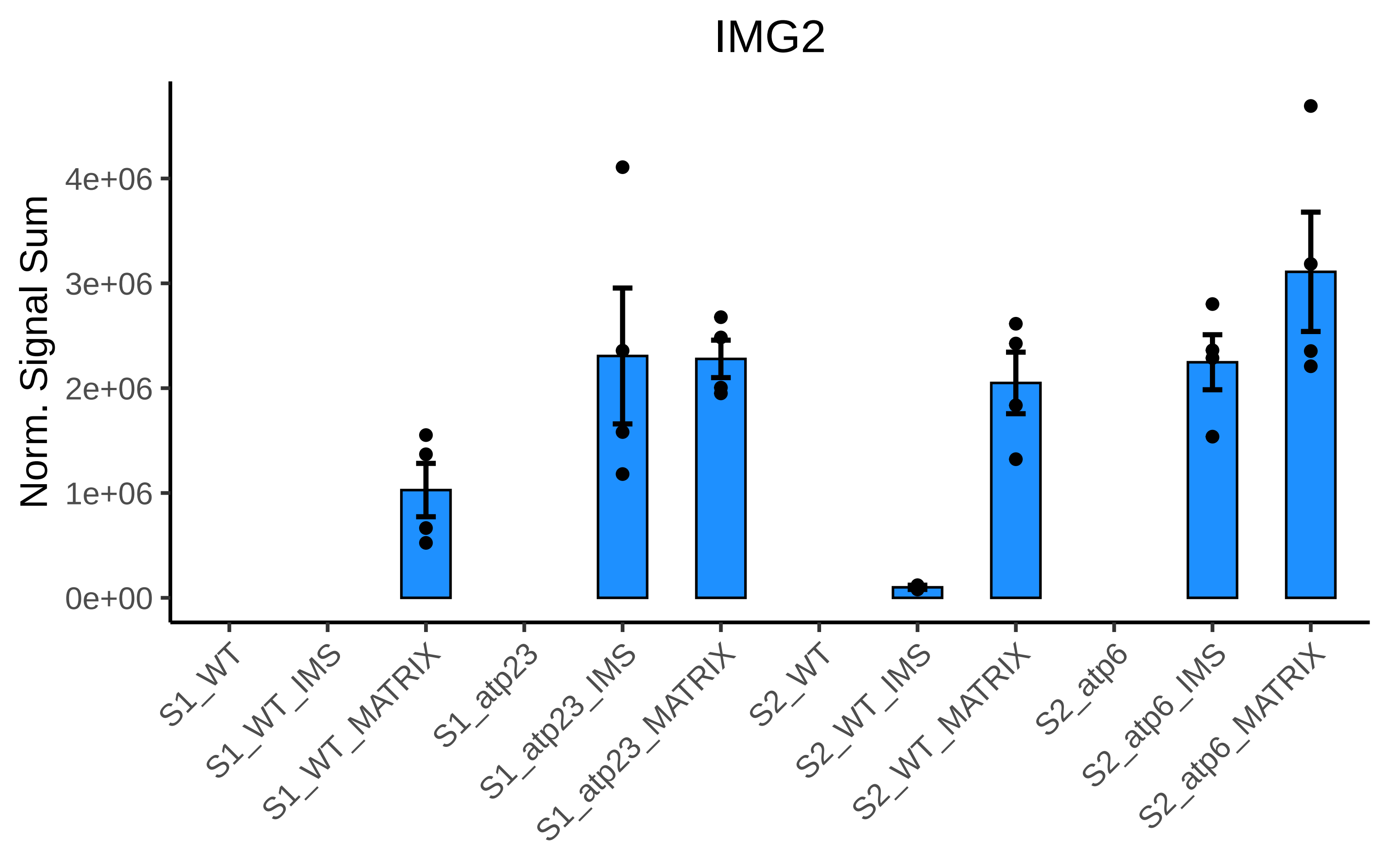
IMG2
Norm. Signal Sum
4e+06
3e+06
2e+06
1e+06
0e+00
S1_WT
S1_WT_IMS
S1_WT_MATRIX
S1_atp23
S1_atp23_IMS
S1_atp23_MATRIX
S2_WT
S2_WT_IMS
S2_WT_MATRIX
S2_atp6
S2_atp6_IMS
S2_atp6_MATRIX

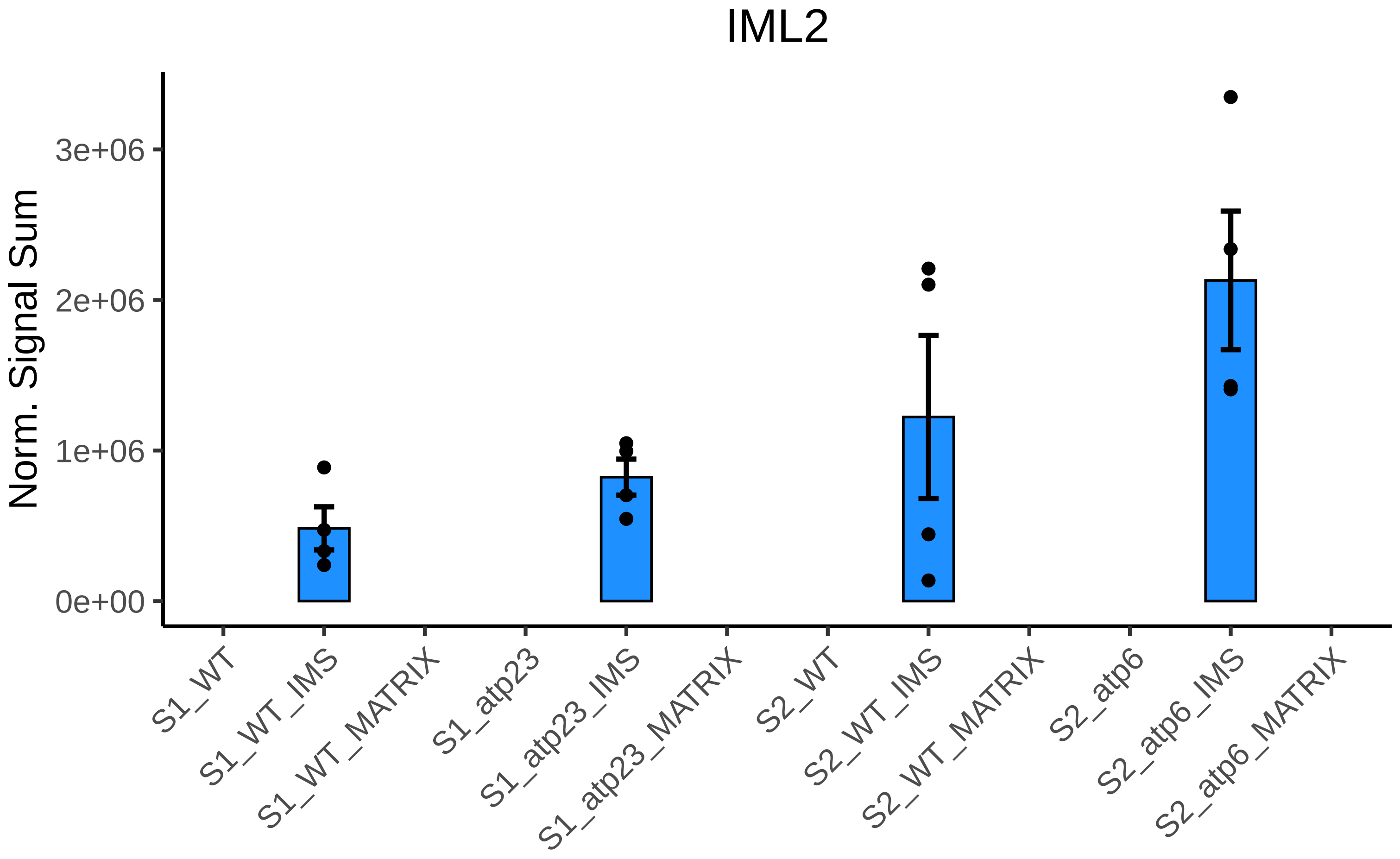
IML2
Norm. Signal Sum
3e+06
2e+06
1e+06
0e+00
S1_WT
S1_WT_IMS
S1_WT_MATRIX
S1_atp23
S1_atp23_IMS
S1_atp23_MATRIX
S2_WT
S2_WT_IMS
S2_WT_MATRIX
S2_atp6
S2_atp6_IMS
S2_atp6_MATRIX

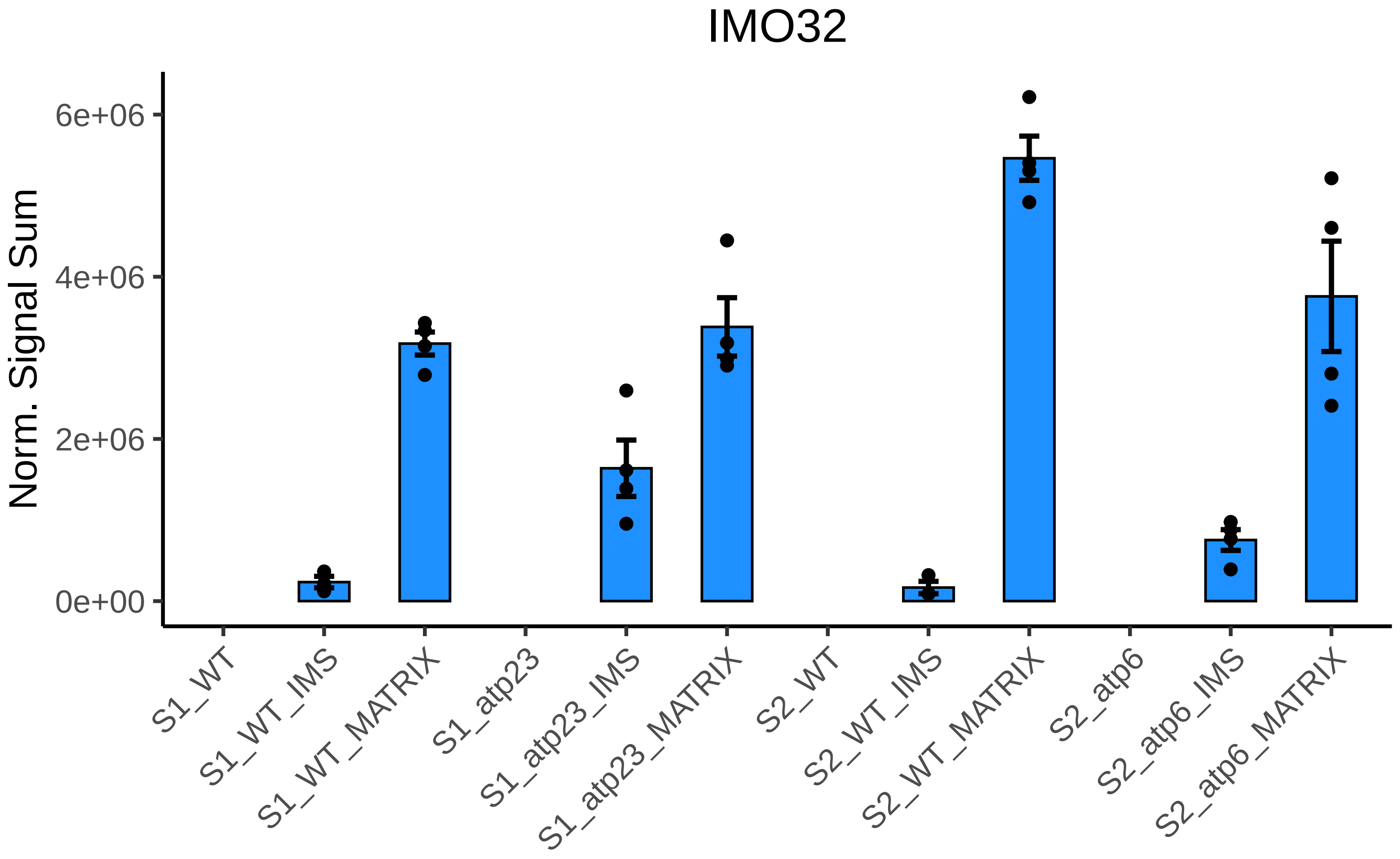

IMO32
Norm. Signal Sum
6e+06
4e+06
2e+06
0e+00
S1_WT
S1_WT_IMS
S1_WT_MATRIX
S1_atp23
S1_atp23_IMS
S1_atp23_MATRIX
S2_WT
S2_WT_IMS
S2_WT_MATRIX
S2_atp6
S2_atp6_IMS
S2_atp6_MATRIX

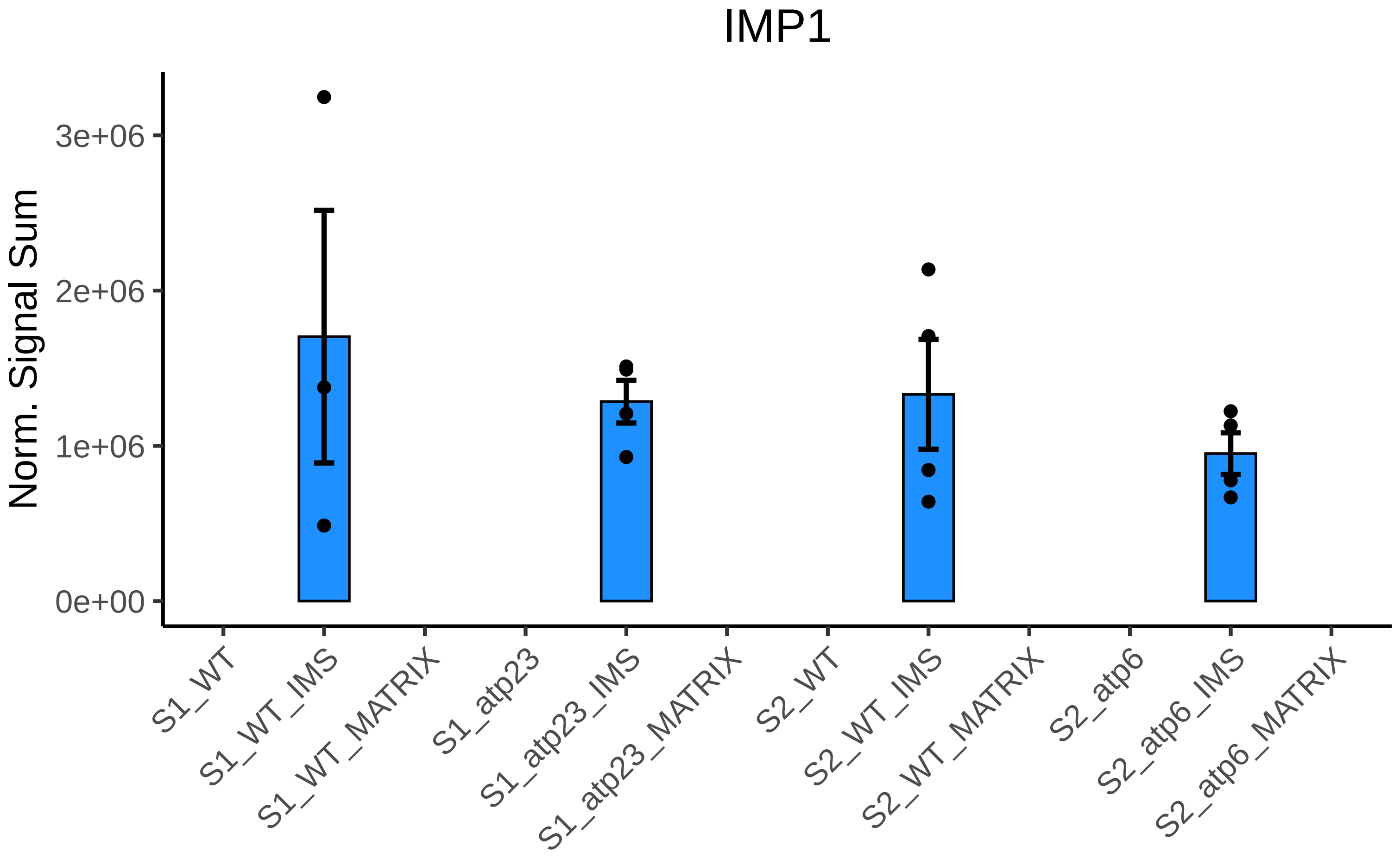

IMP1
Norm. Signal Sum
3e+06
2e+06
1e+06
0e+00
S1_WT
S1_WT_IMS
S1_WT_MATRIX
S1_atp23
S1_atp23_IMS
S1_atp23_MATRIX
S2_WT
S2_WT_IMS
S2_WT_MATRIX
S2_atp6
S2_atp6_IMS
S2_atp6_MATRIX

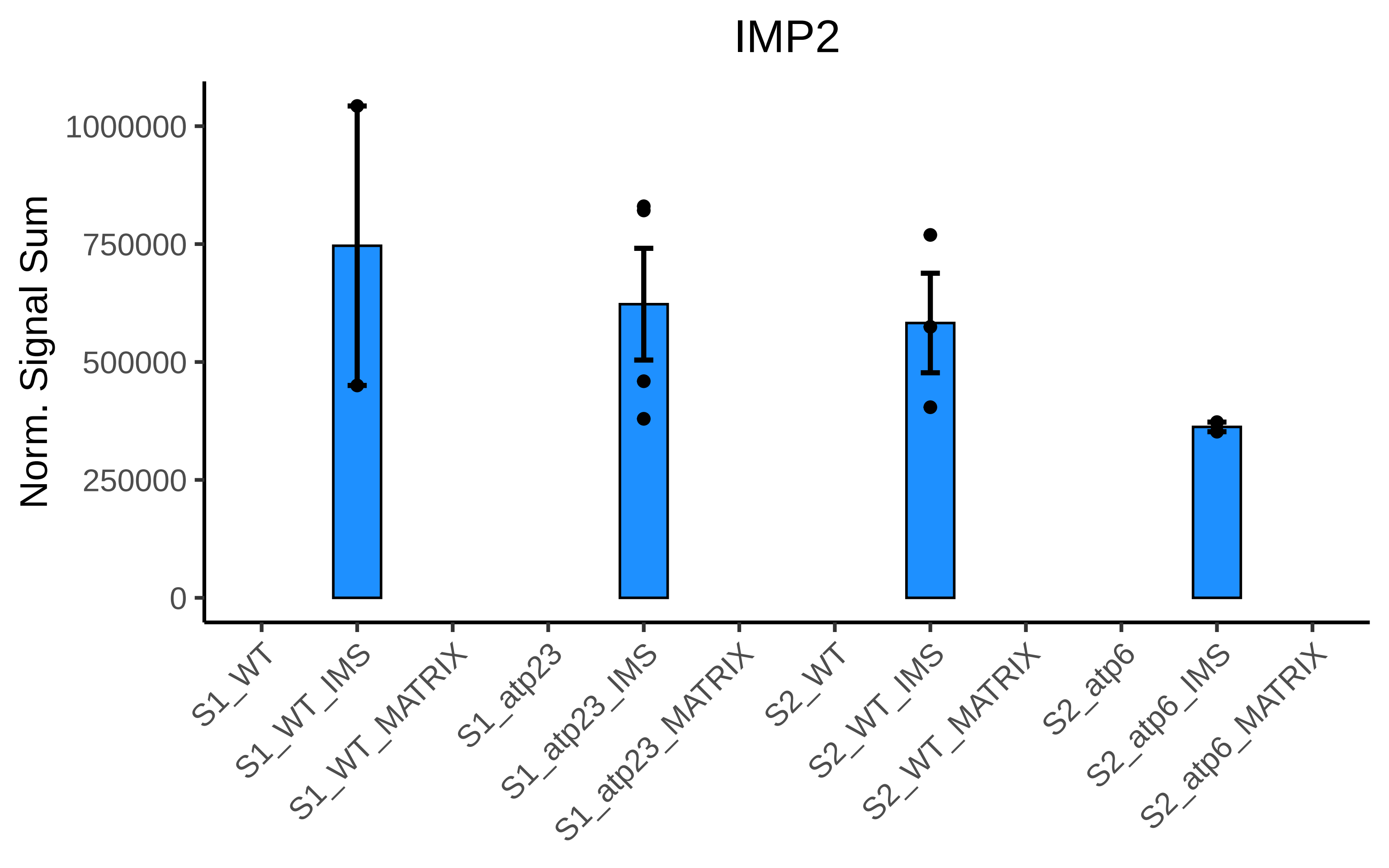

IMP2
Norm. Signal Sum
1000000
750000
500000
250000
0
S1_WT
S1_WT_IMS
S1_WT_MATRIX
S1_atp23
S1_atp23_IMS
S1_atp23_MATRIX
S2_WT
S2_WT_IMS
S2_WT_MATRIX
S2_atp6
S2_atp6_IMS
S2_atp6_MATRIX

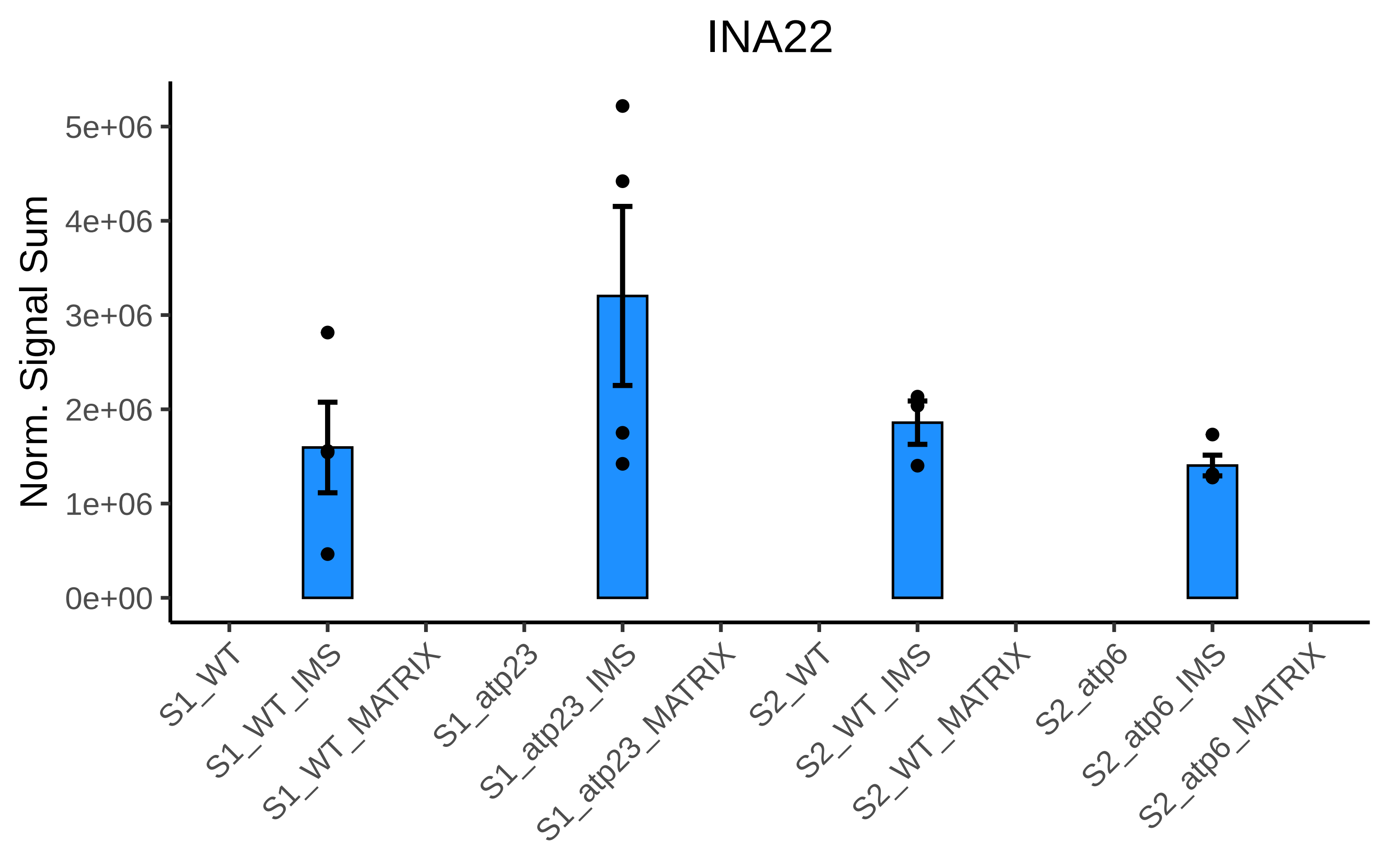
INA22
Norm. Signal Sum
0e+00
1e+06
2e+06
3e+06
4e+06
5e+06
S1_WT
S1_WT_IMS
S1_WT_MATRIX
S1_atp23
S1_atp23_IMS
S1_atp23_MATRIX
S2_WT
S2_WT_IMS
S2_WT_MATRIX
S2_atp6
S2_atp6_IMS
S2_atp6_MATRIX

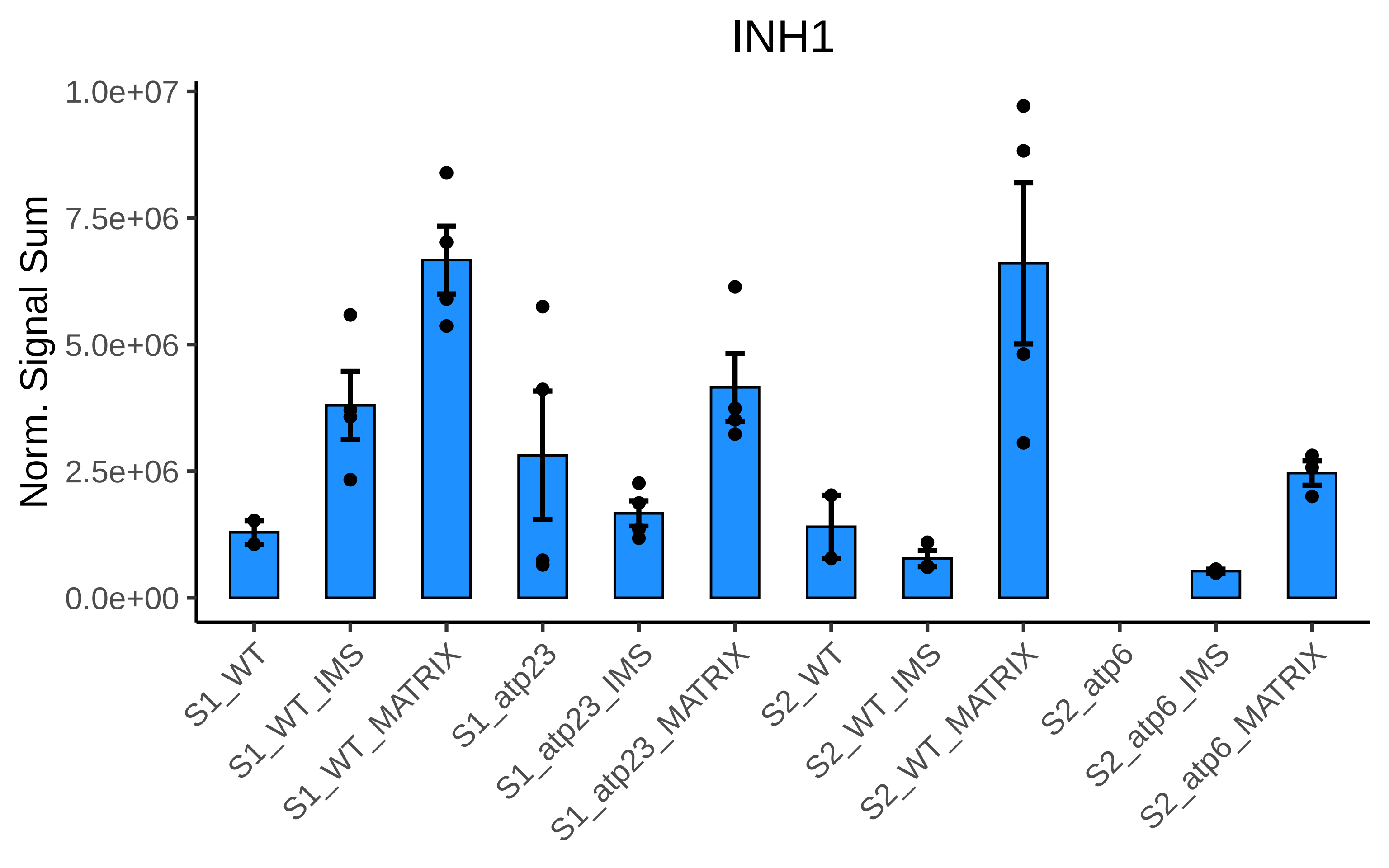

INH1
Norm. Signal Sum
1.0e+07
7.5e+06
5.0e+06
2.5e+06
0.0e+00
S1_WT
S1_WT_IMS
S1_WT_MATRIX
S1_atp23
S1_atp23_IMS
S1_atp23_MATRIX
S2_WT
S2_WT_IMS
S2_WT_MATRIX
S2_atp6
S2_atp6_IMS
S2_atp6_MATRIX

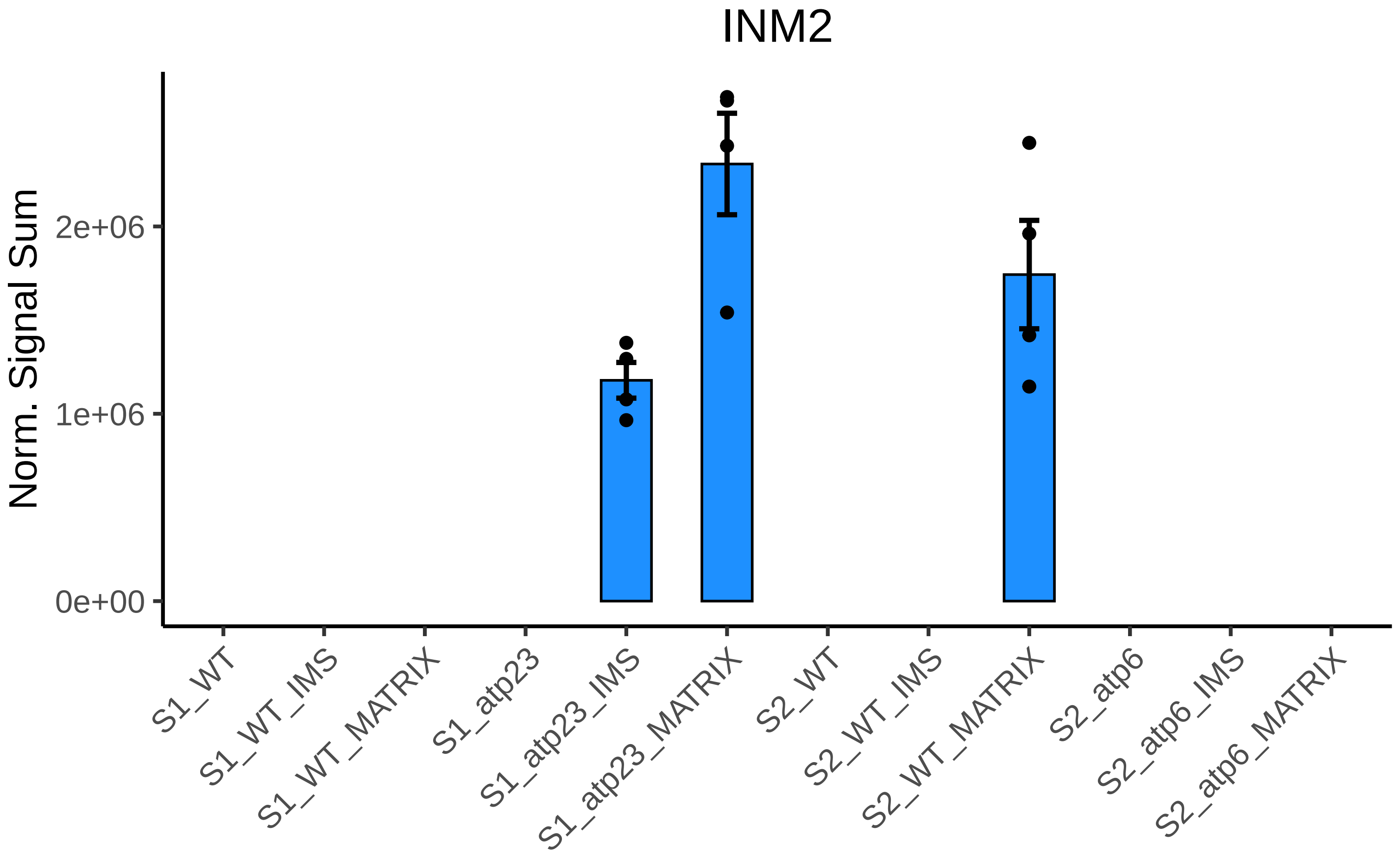

INM2
Norm. Signal Sum
2e+06
1e+06
0e+00
S1_WT
S1_WT_IMS
S1_WT_MATRIX
S1_atp23
S1_atp23_IMS
S1_atp23_MATRIX
S2_WT
S2_WT_IMS
S2_WT_MATRIX
S2_atp6
S2_atp6_IMS
S2_atp6_MATRIX

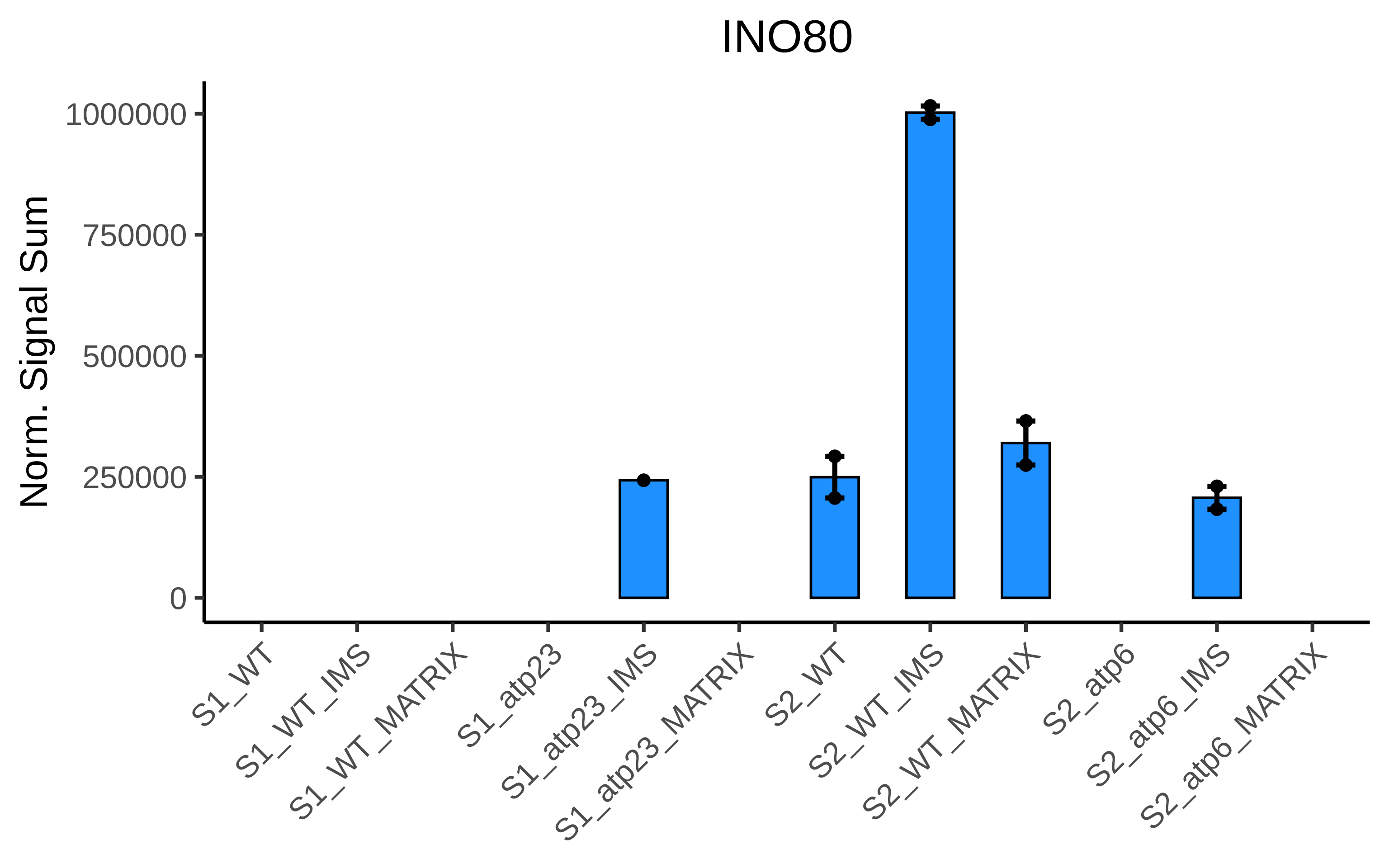

INO80
Norm. Signal Sum
1000000
750000
500000
250000
0
S1_WT
S1_WT_IMS
S1_WT_MATRIX
S1_atp23
S1_atp23_IMS
S1_atp23_MATRIX
S2_WT
S2_WT_IMS
S2_WT_MATRIX
S2_atp6
S2_atp6_IMS
S2_atp6_MATRIX

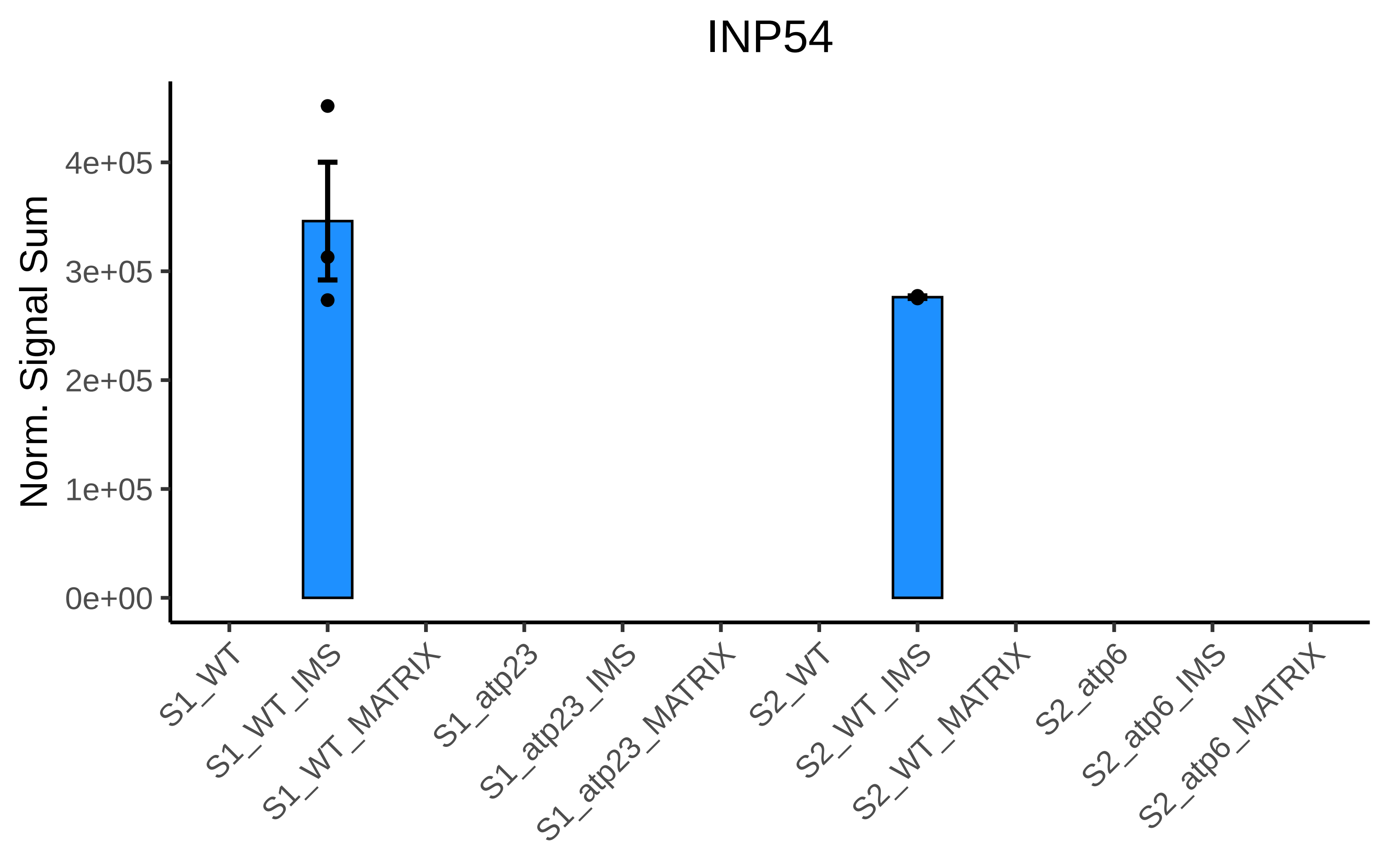

INP54
Norm. Signal Sum
0e+00
1e+05
2e+05
3e+05
4e+05
S1_WT
S1_WT_IMS
S1_WT_MATRIX
S1_atp23
S1_atp23_IMS
S1_atp23_MATRIX
S2_WT
S2_WT_IMS
S2_WT_MATRIX
S2_atp6
S2_atp6_IMS
S2_atp6_MATRIX

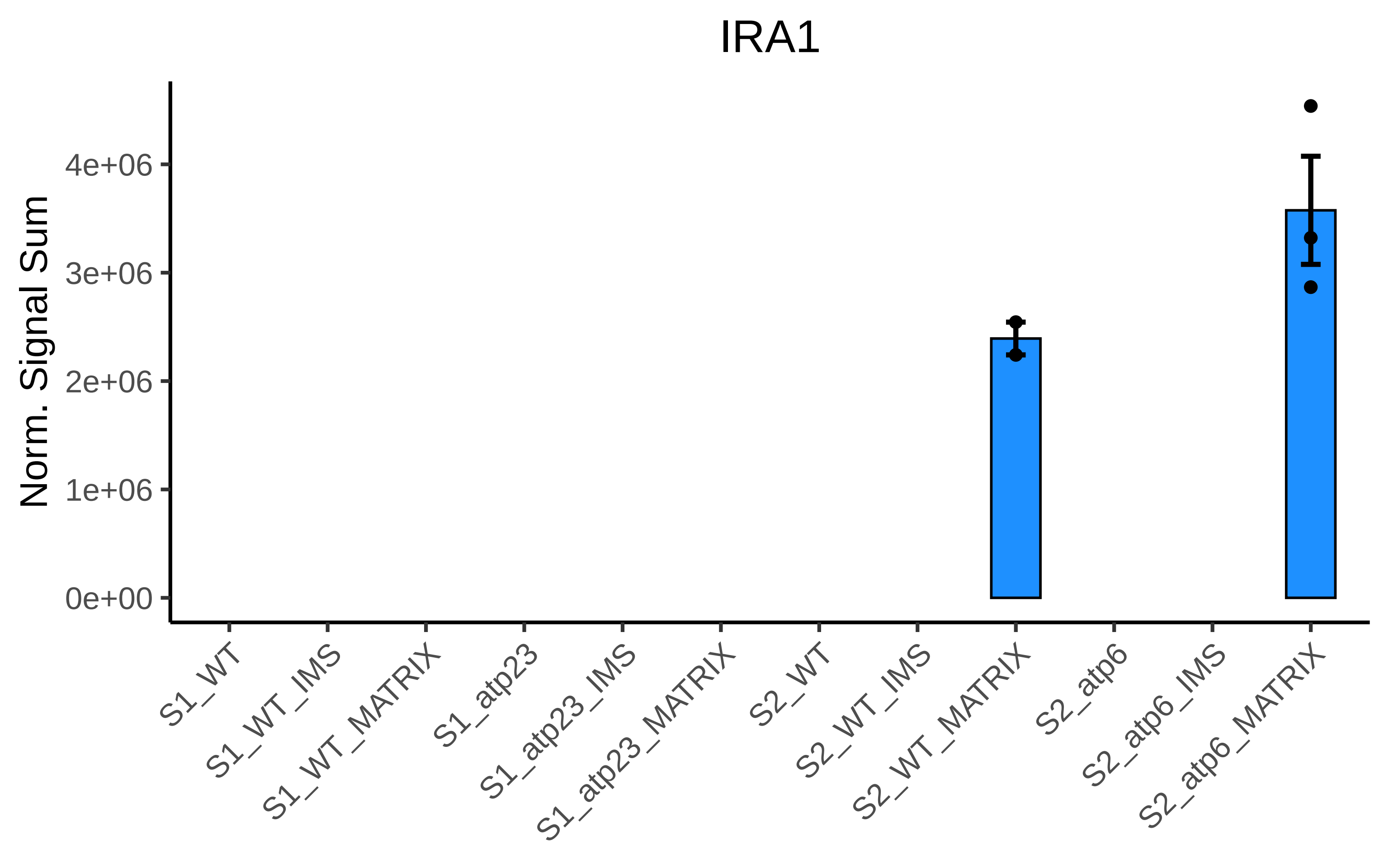
IRA1
Norm. Signal Sum
4e+06
3e+06
2e+06
1e+06
0e+00
S1_WT
S1_WT_IMS
S1_WT_MATRIX
S1_atp23
S1_atp23_IMS
S1_atp23_MATRIX
S2_WT
S2_WT_IMS
S2_WT_MATRIX
S2_atp6
S2_atp6_IMS
S2_atp6_MATRIX

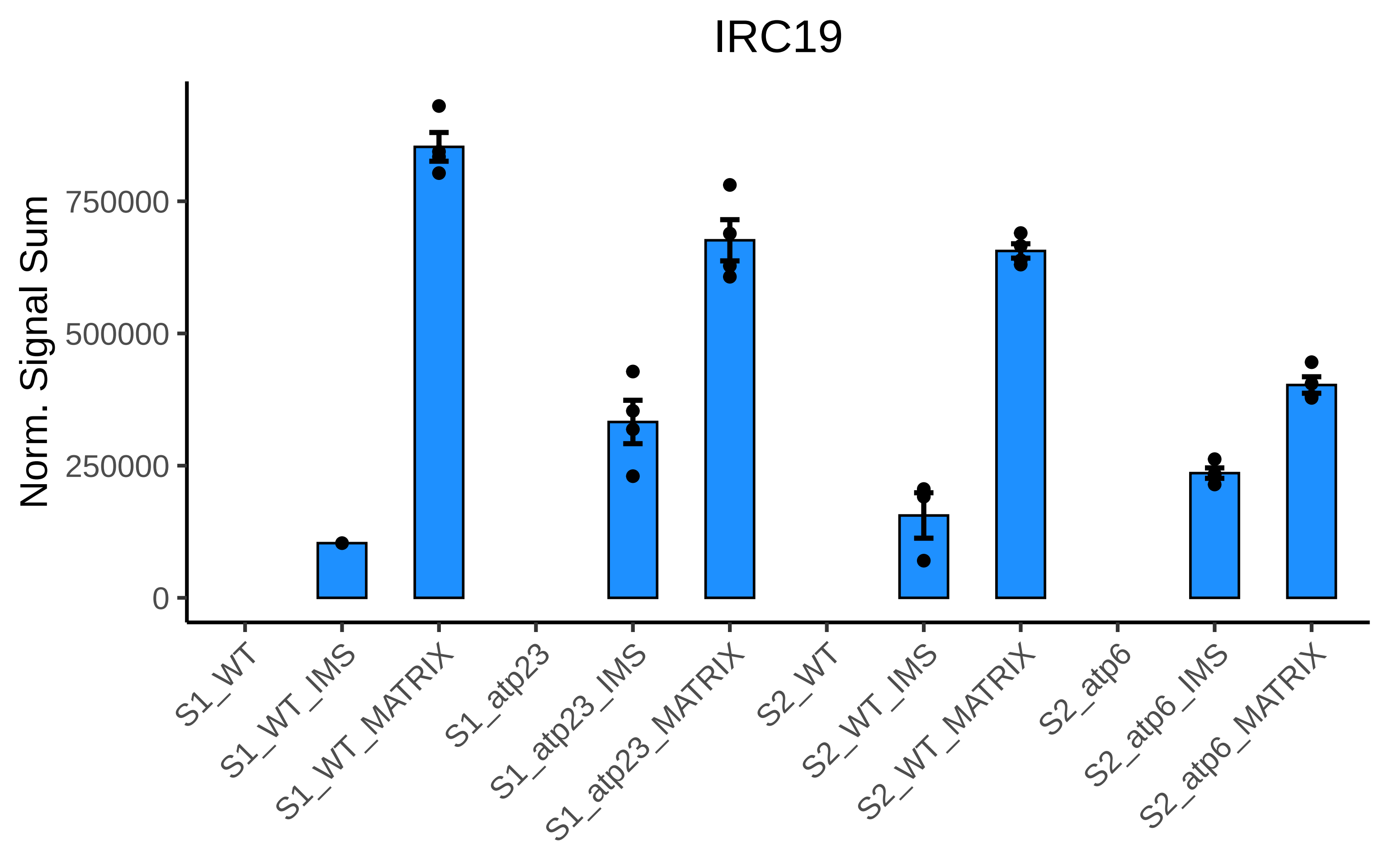

IRC19
Norm. Signal Sum
750000
500000
250000
0
S1_WT
S1_WT_IMS
S1_WT_MATRIX
S1_atp23
S1_atp23_IMS
S1_atp23_MATRIX
S2_WT
S2_WT_IMS
S2_WT_MATRIX
S2_atp6
S2_atp6_IMS
S2_atp6_MATRIX

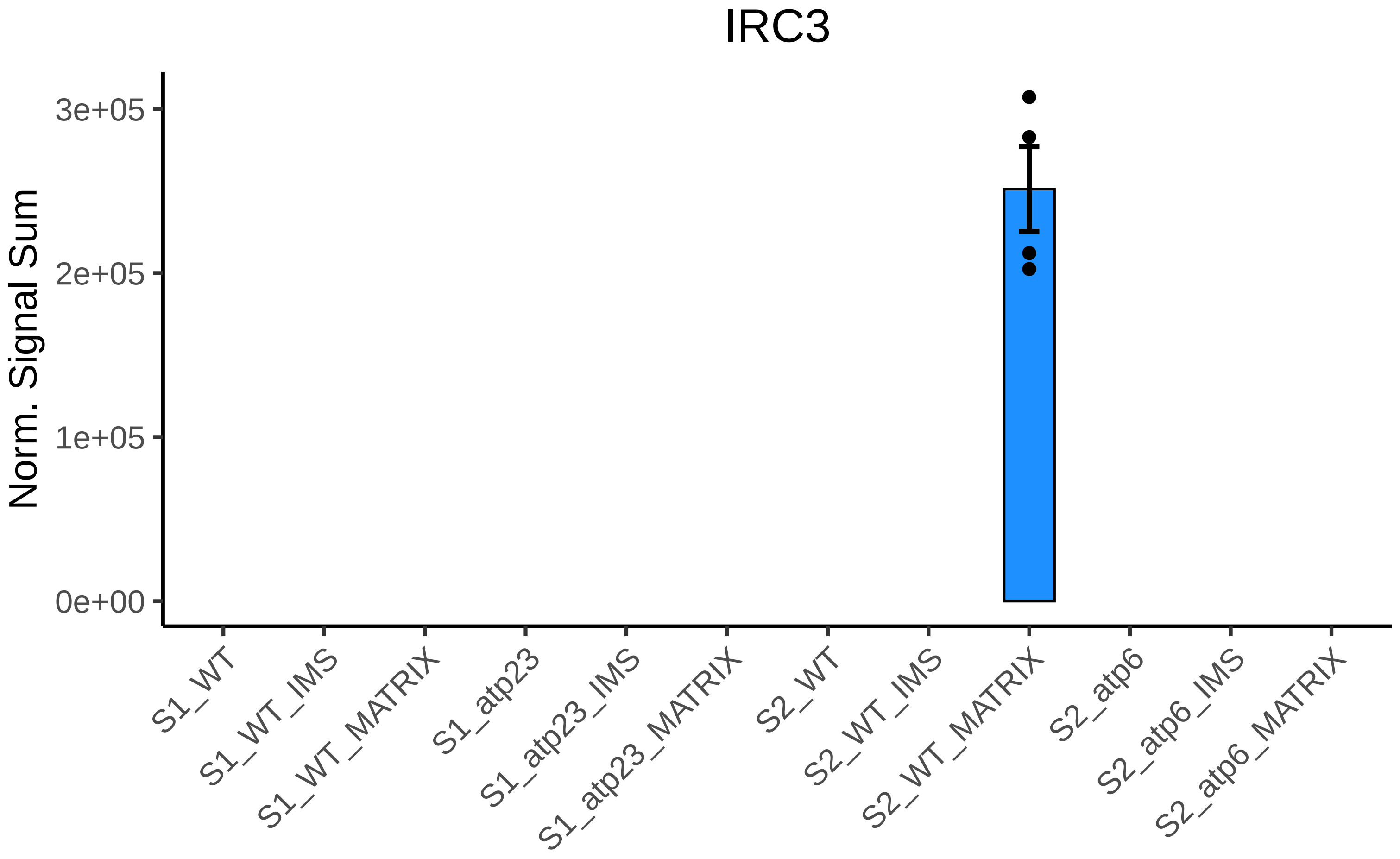

IRC3
Norm. Signal Sum
3e+05
2e+05
1e+05
0e+00
S1_WT
S1_WT_IMS
S1_WT_MATRIX
S1_atp23
S1_atp23_IMS
S1_atp23_MATRIX
S2_WT
S2_WT_IMS
S2_WT_MATRIX
S2_atp6
S2_atp6_IMS
S2_atp6_MATRIX

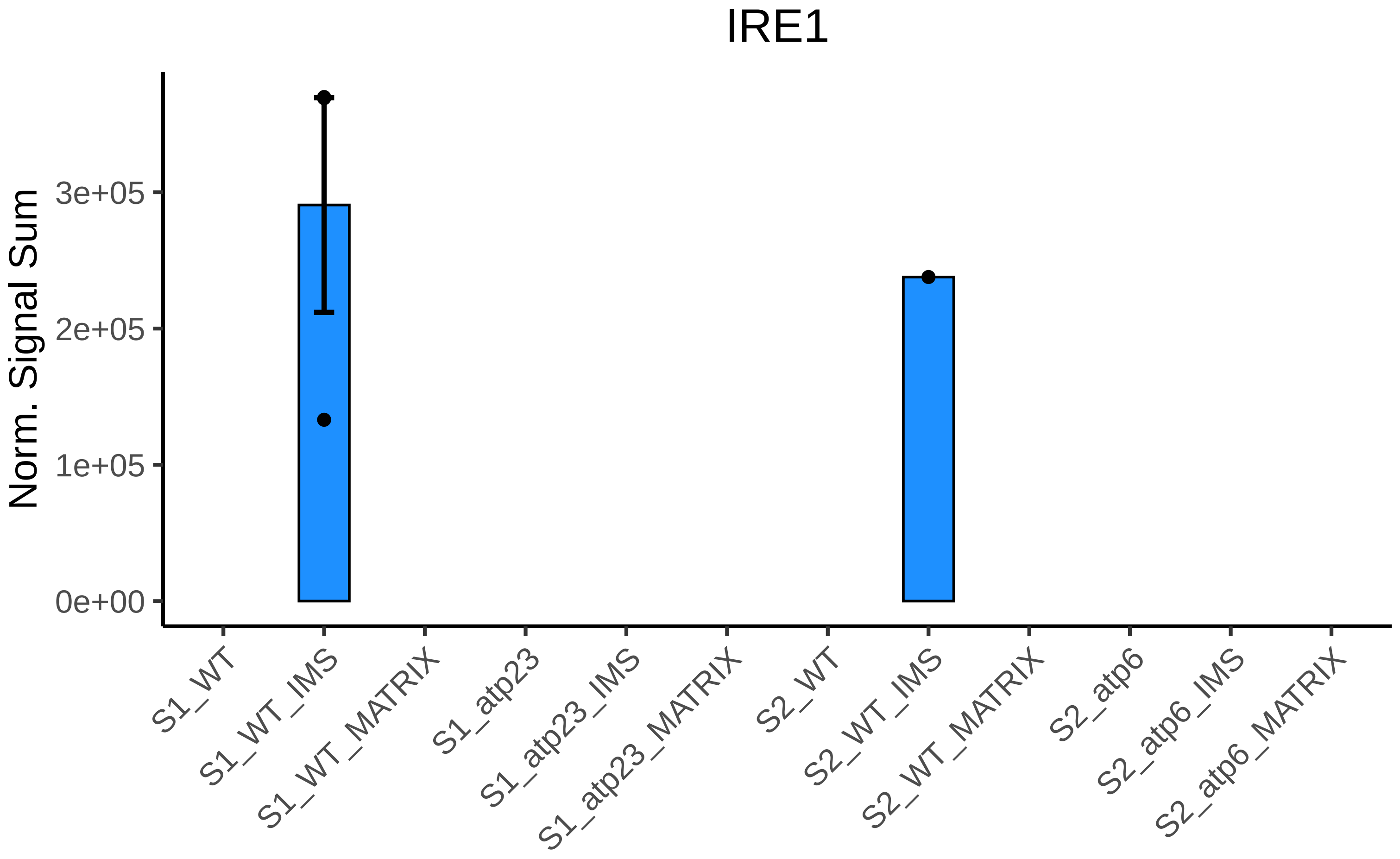

IRE1
Norm. Signal Sum
3e+05
2e+05
1e+05
0e+00
S1_WT
S1_WT_IMS
S1_WT_MATRIX
S1_atp23
S1_atp23_IMS
S1_atp23_MATRIX
S2_WT
S2_WT_IMS
S2_WT_MATRIX
S2_atp6
S2_atp6_IMS
S2_atp6_MATRIX

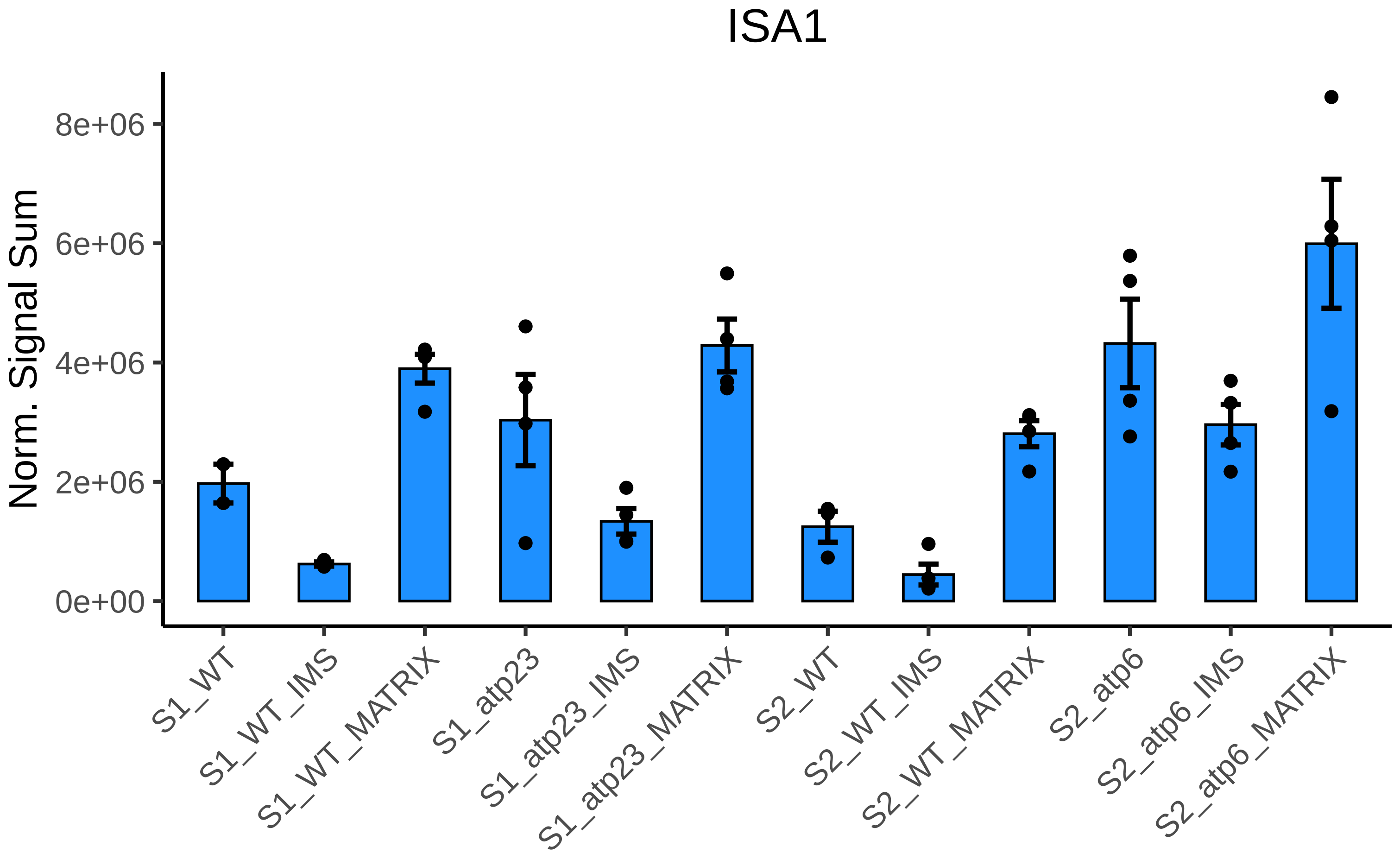
ISA1
Norm. Signal Sum
8e+06
6e+06
4e+06
2e+06
0e+00
S1_WT
S1_WT_IMS
S1_WT_MATRIX
S1_atp23
S1_atp23_IMS
S1_atp23_MATRIX
S2_WT
S2_WT_IMS
S2_WT_MATRIX
S2_atp6
S2_atp6_IMS
S2_atp6_MATRIX

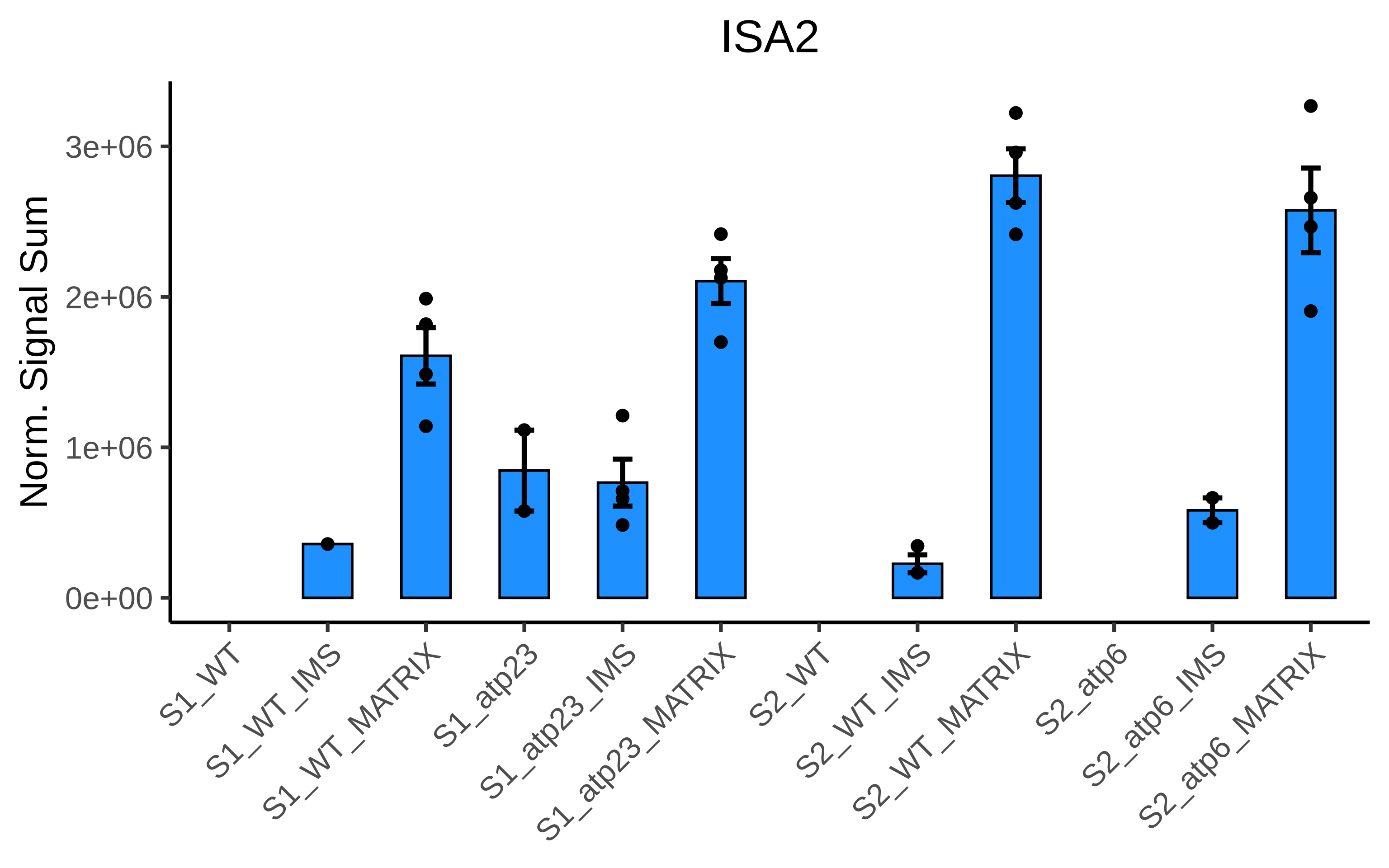
ISA2
Norm. Signal Sum
3e+06
2e+06
1e+06
0e+00
S1_WT
S1_WT_IMS
S1_WT_MATRIX
S1_atp23
S1_atp23_IMS
S1_atp23_MATRIX
S2_WT
S2_WT_IMS
S2_WT_MATRIX
S2_atp6
S2_atp6_IMS
S2_atp6_MATRIX

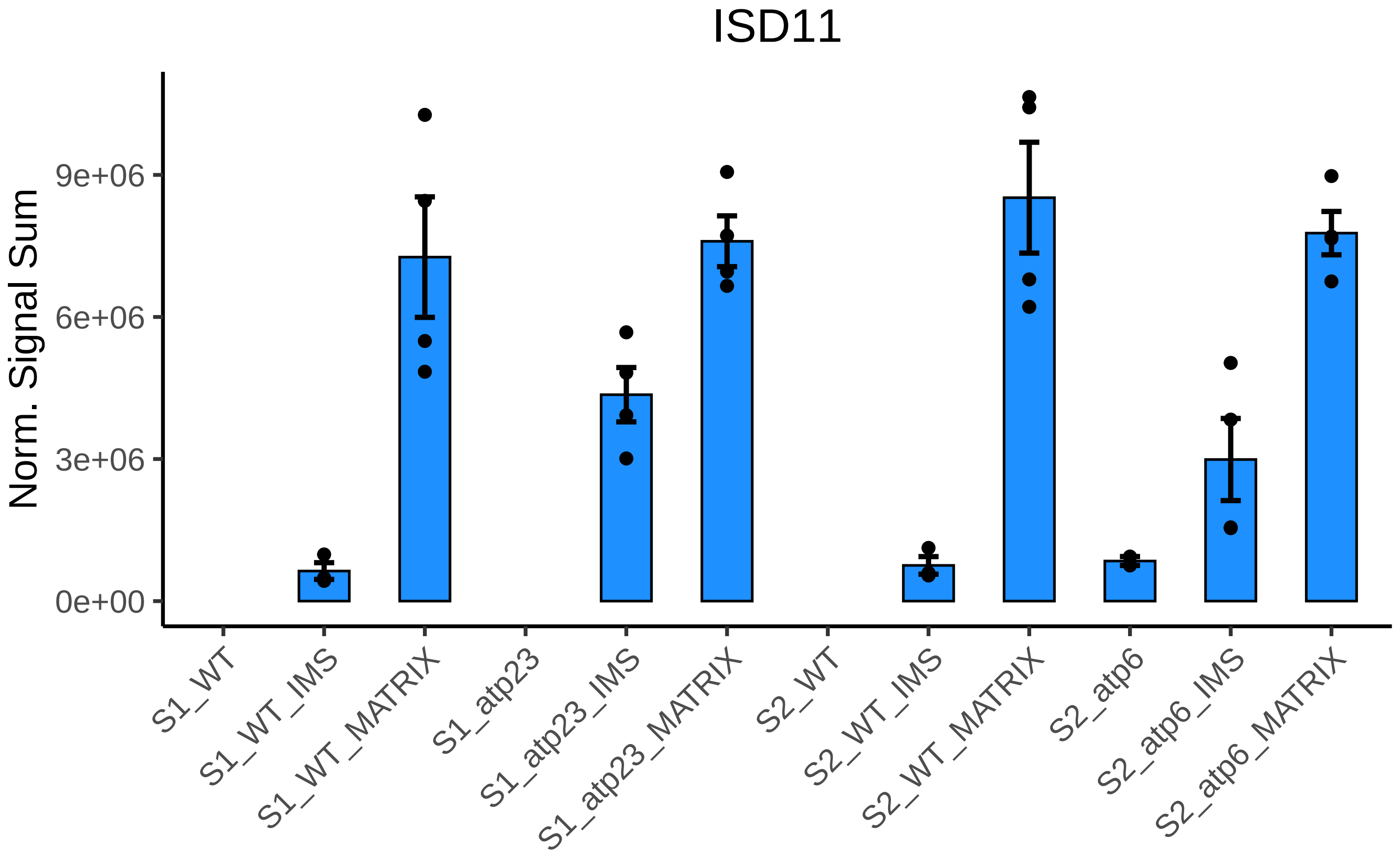
ISD11
Norm. Signal Sum
9e+06
6e+06
3e+06
0e+00
S1_WT
S1_WT_IMS
S1_WT_MATRIX
S1_atp23
S1_atp23_IMS
S1_atp23_MATRIX
S2_WT
S2_WT_IMS
S2_WT_MATRIX
S2_atp6
S2_atp6_IMS
S2_atp6_MATRIX

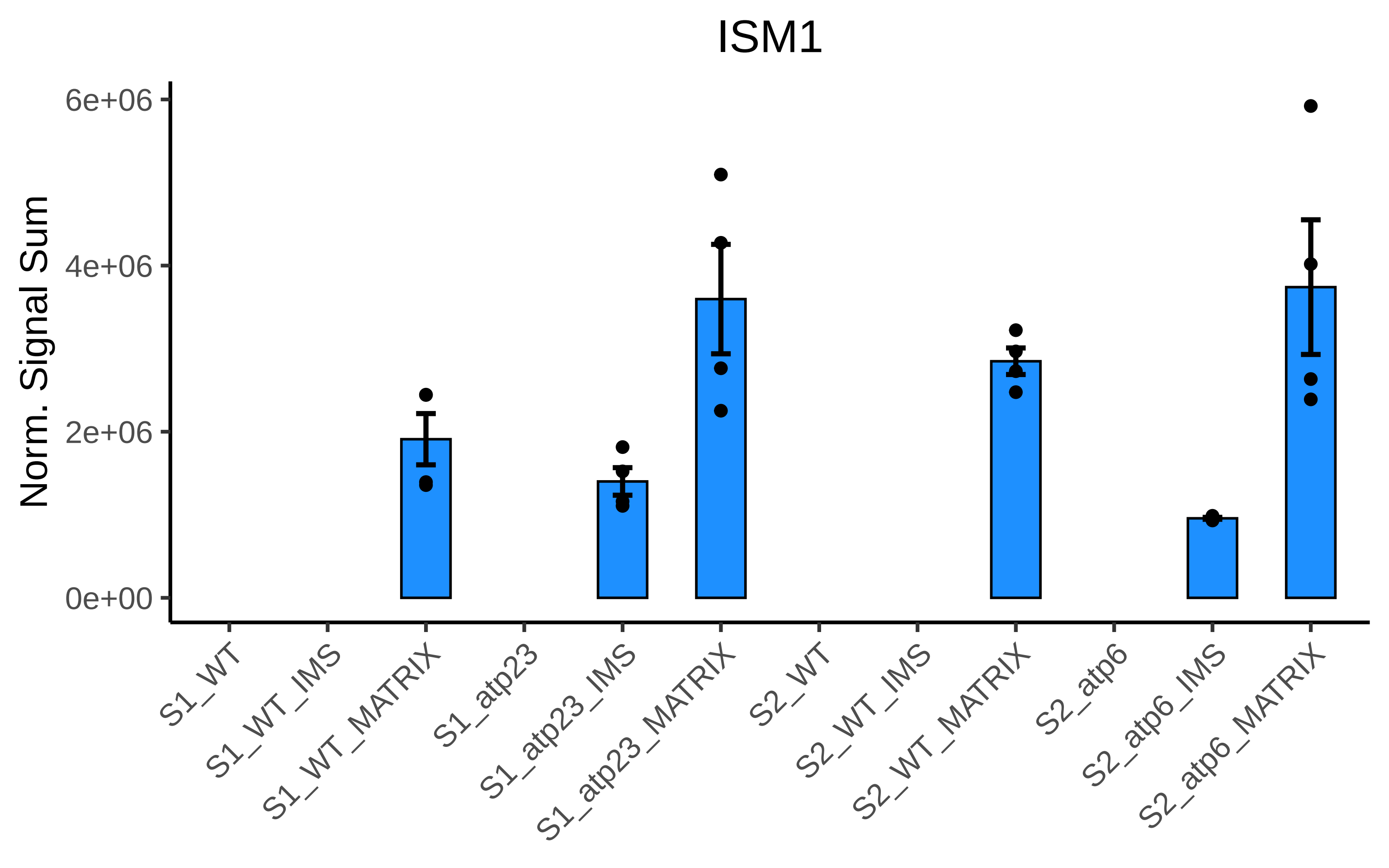

ISM1
Norm. Signal Sum
6e+06
4e+06
2e+06
0e+00
S1_WT
S1_WT_IMS
S1_WT_MATRIX
S1_atp23
S1_atp23_IMS
S1_atp23_MATRIX
S2_WT
S2_WT_IMS
S2_WT_MATRIX
S2_atp6
S2_atp6_IMS
S2_atp6_MATRIX

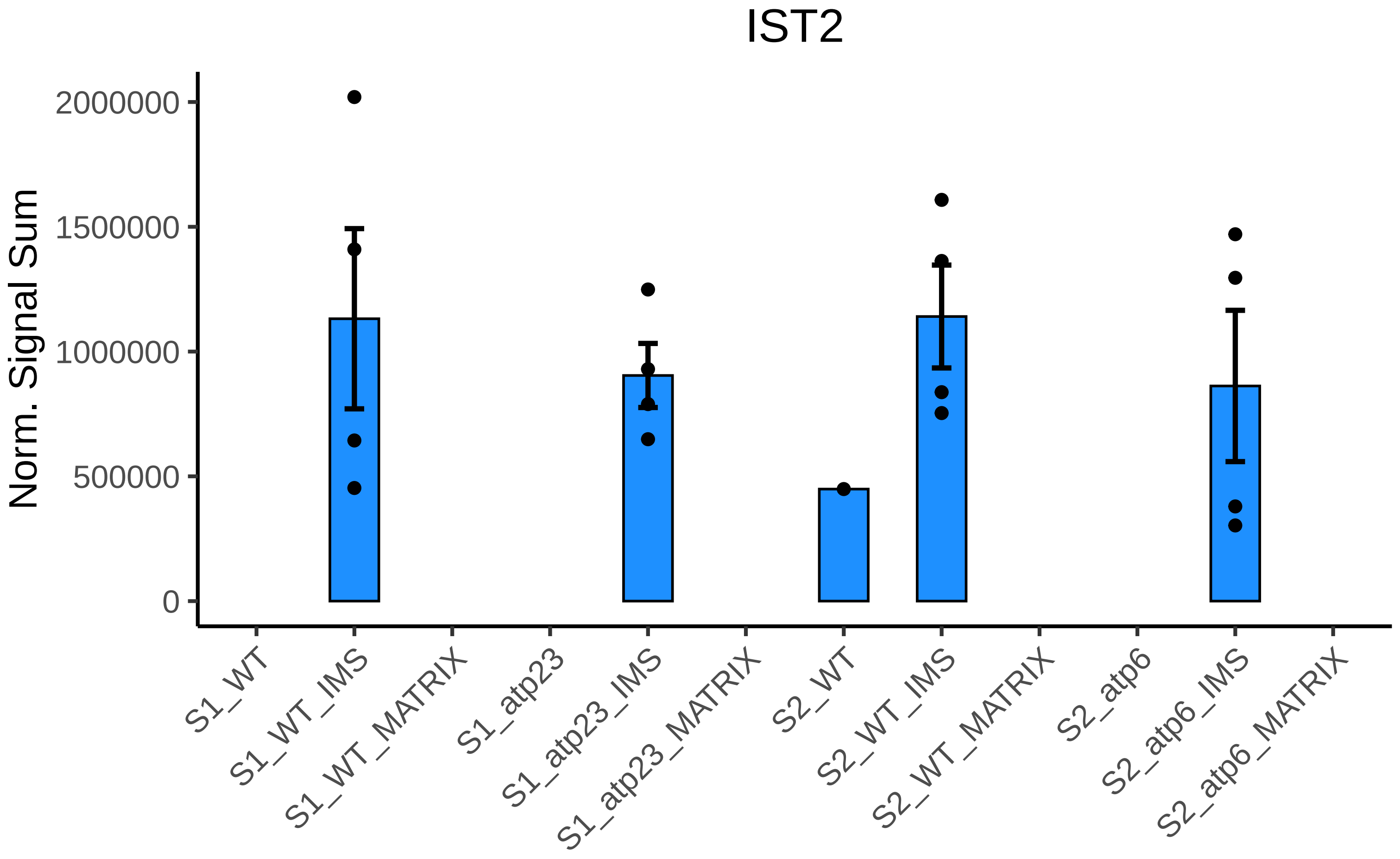
IST2
Norm. Signal Sum
2000000
1500000
1000000
500000
0
S1_WT
S1_WT_IMS
S1_WT_MATRIX
S1_atp23
S1_atp23_IMS
S1_atp23_MATRIX
S2_WT
S2_WT_IMS
S2_WT_MATRIX
S2_atp6
S2_atp6_IMS
S2_atp6_MATRIX

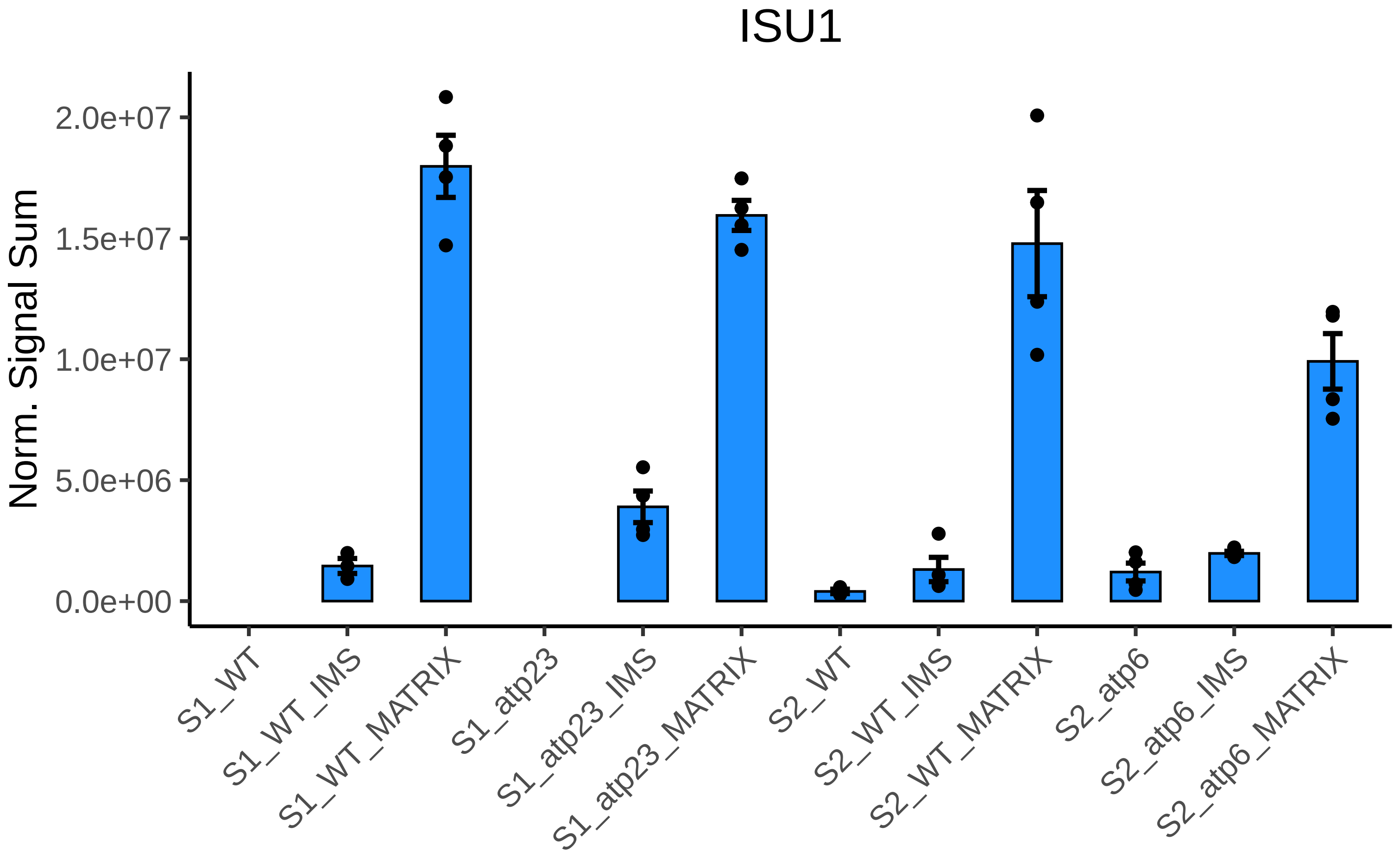

ISU1
Norm. Signal Sum
2.0e+07
1.5e+07
1.0e+07
5.0e+06
0.0e+00
S1_WT
S1_WT_IMS
S1_WT_MATRIX
S1_atp23
S1_atp23_IMS
S1_atp23_MATRIX
S2_WT
S2_WT_IMS
S2_WT_MATRIX
S2_atp6
S2_atp6_IMS
S2_atp6_MATRIX

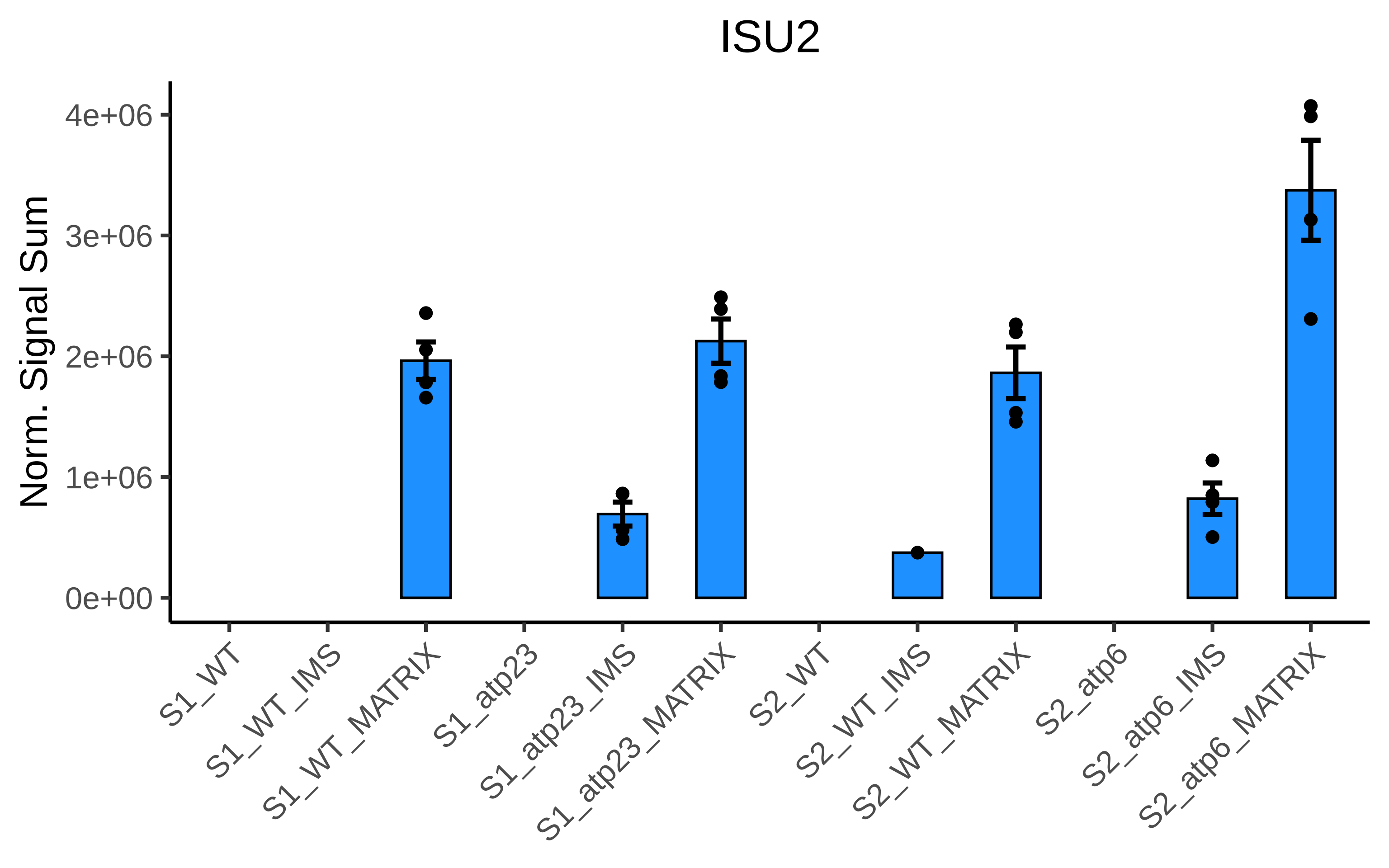
ISU2
Norm. Signal Sum
4e+06
3e+06
2e+06
1e+06
0e+00
S1_WT
S1_WT_IMS
S1_WT_MATRIX
S1_atp23
S1_atp23_IMS
S1_atp23_MATRIX
S2_WT
S2_WT_IMS
S2_WT_MATRIX
S2_atp6
S2_atp6_IMS
S2_atp6_MATRIX

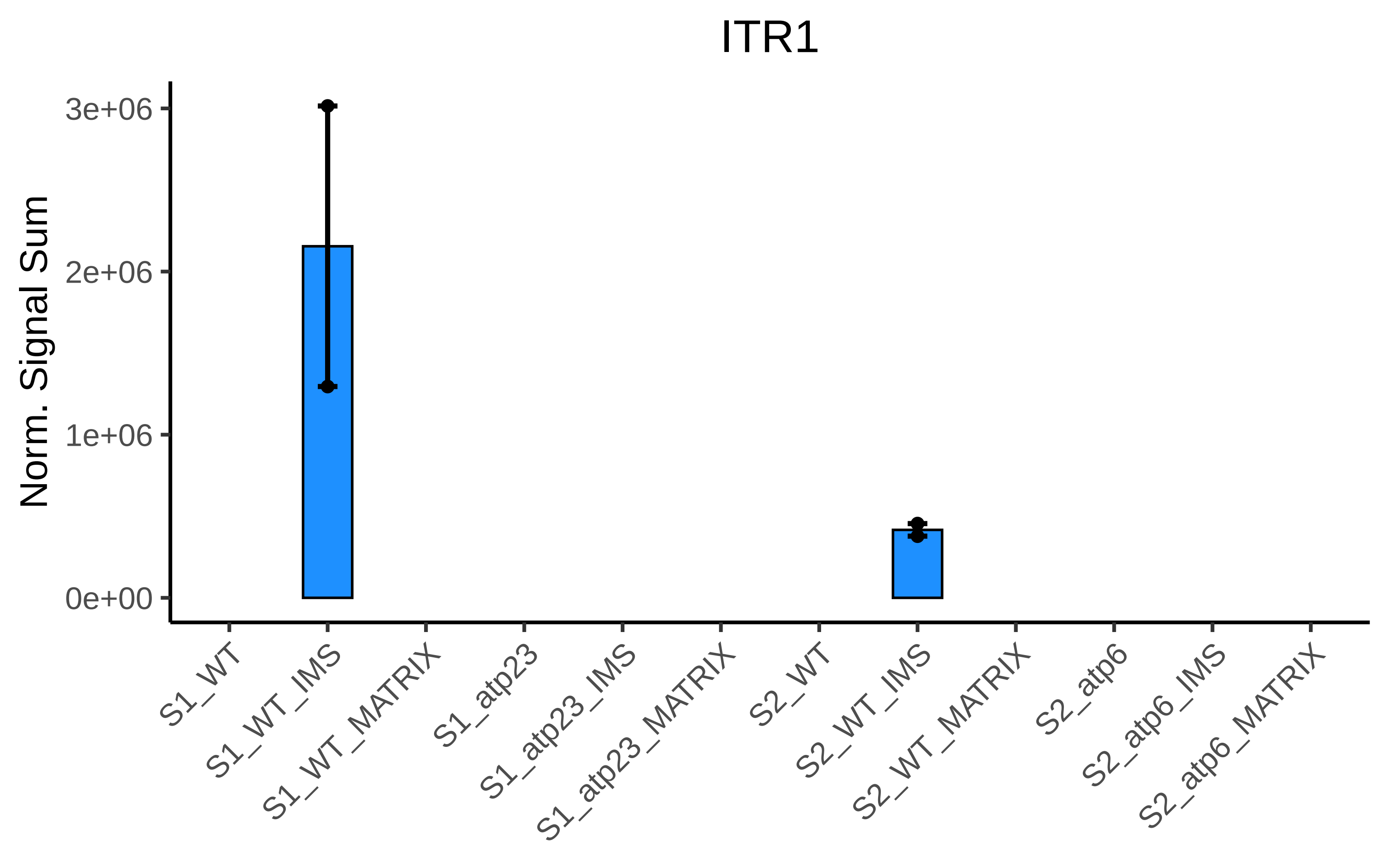
ITR1
Norm. Signal Sum
3e+06
2e+06
1e+06
0e+00
S1_WT
S1_WT_IMS
S1_WT_MATRIX
S1_atp23
S1_atp23_IMS
S1_atp23_MATRIX
S2_WT
S2_WT_IMS
S2_WT_MATRIX
S2_atp6
S2_atp6_IMS
S2_atp6_MATRIX

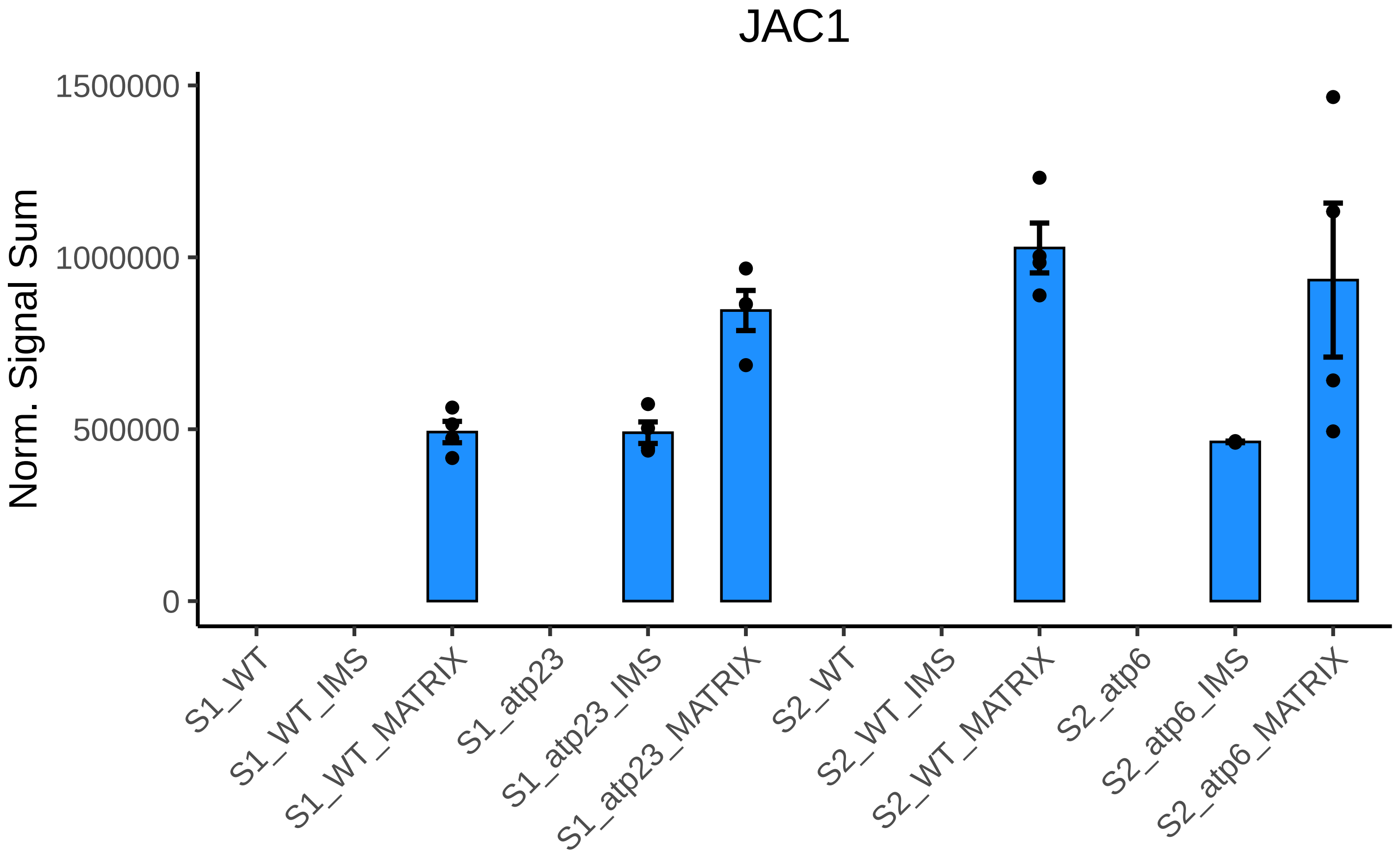

JAC1
Norm. Signal Sum
1500000
1000000
500000
0
S1_WT
S1_WT_IMS
S1_WT_MATRIX
S1_atp23
S1_atp23_IMS
S1_atp23_MATRIX
S2_WT
S2_WT_IMS
S2_WT_MATRIX
S2_atp6
S2_atp6_IMS
S2_atp6_MATRIX

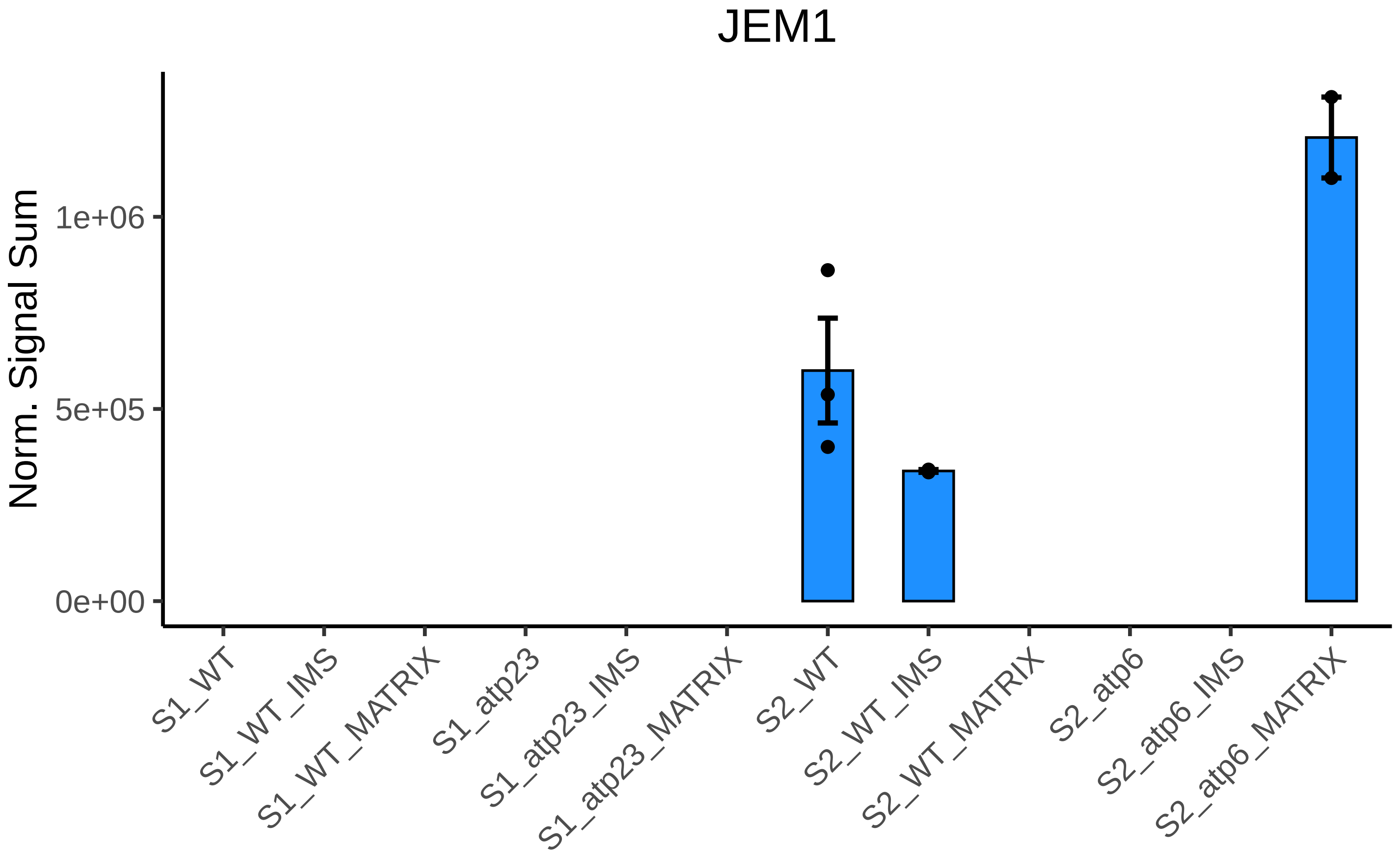

JEM1
Norm. Signal Sum
1e+06
5e+05
0e+00
S1_WT
S1_WT_IMS
S1_WT_MATRIX
S1_atp23
S1_atp23_IMS
S1_atp23_MATRIX
S2_WT
S2_WT_IMS
S2_WT_MATRIX
S2_atp6
S2_atp6_IMS
S2_atp6_MATRIX

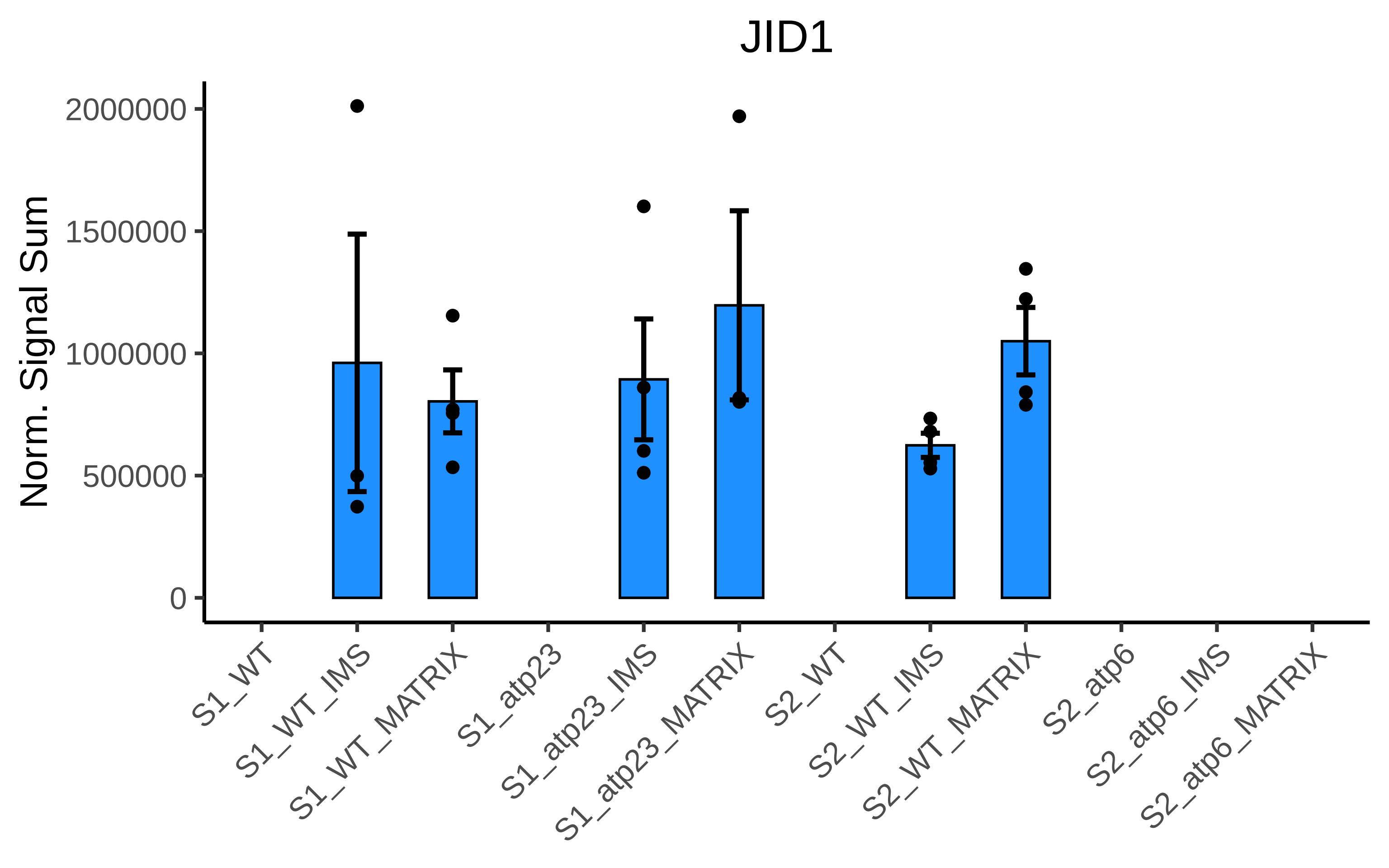
JID1
Norm. Signal Sum
2000000
1500000
1000000
500000
0
S1_WT
S1_WT_IMS
S1_WT_MATRIX
S1_atp23
S1_atp23_IMS
S1_atp23_MATRIX
S2_WT
S2_WT_IMS
S2_WT_MATRIX
S2_atp6
S2_atp6_IMS
S2_atp6_MATRIX

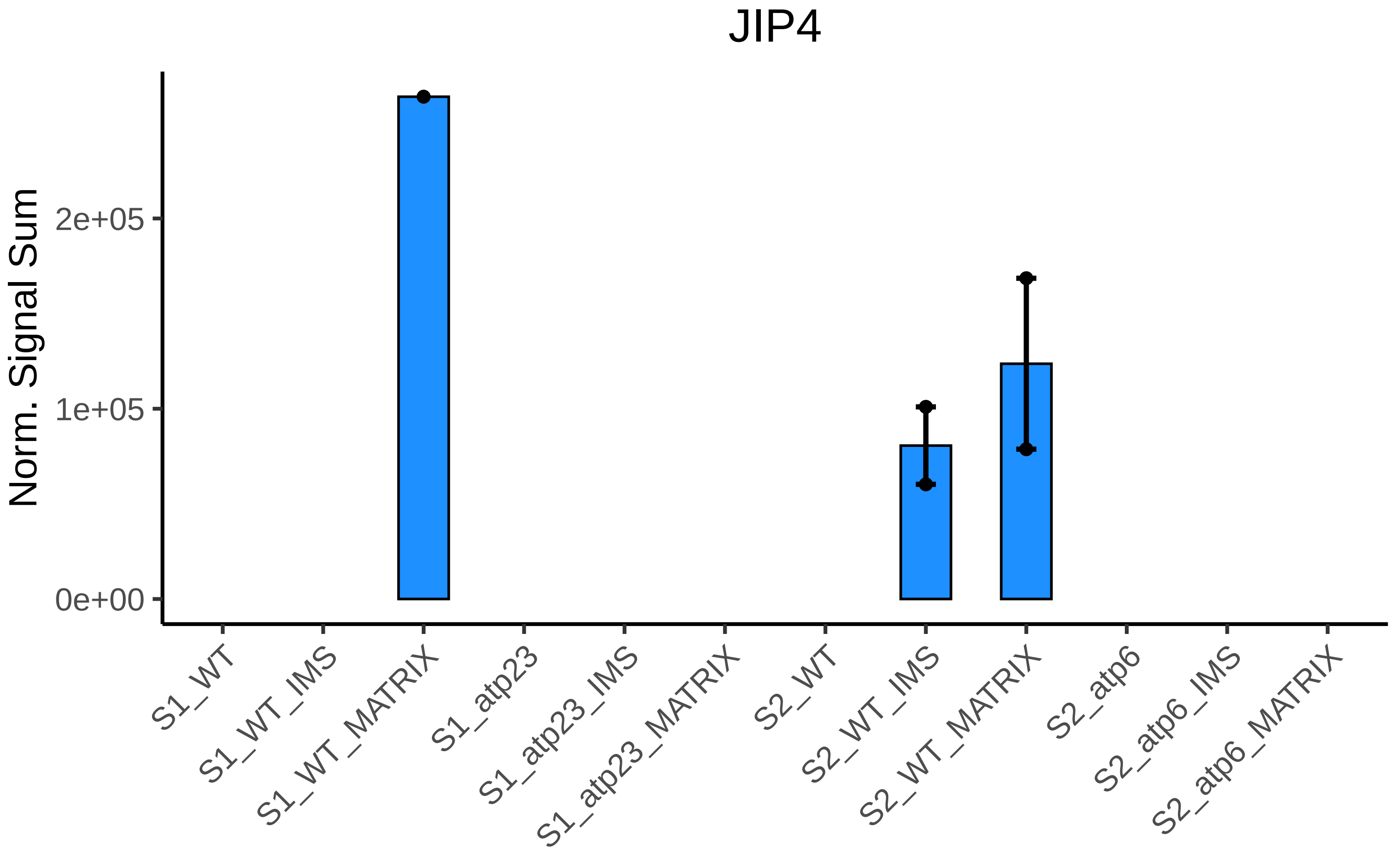
JIP4
Norm. Signal Sum
2e+05
1e+05
0e+00
S1_WT
S1_WT_IMS
S1_WT_MATRIX
S1_atp23
S1_atp23_IMS
S1_atp23_MATRIX
S2_WT
S2_WT_IMS
S2_WT_MATRIX
S2_atp6
S2_atp6_IMS
S2_atp6_MATRIX

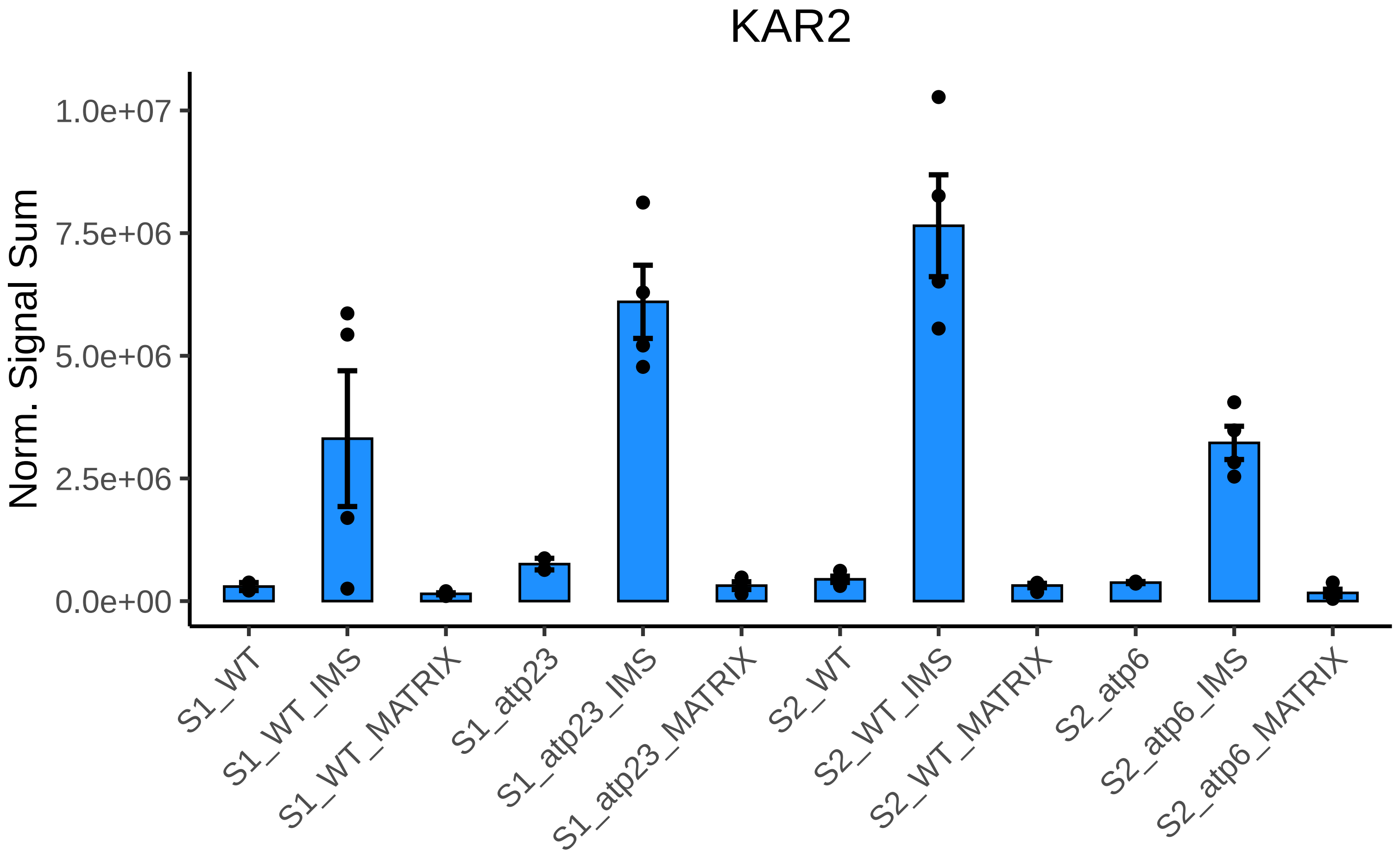

KAR2
Norm. Signal Sum
1.0e+07
7.5e+06
5.0e+06
2.5e+06
0.0e+00
S1_WT
S1_WT_IMS
S1_WT_MATRIX
S1_atp23
S1_atp23_IMS
S1_atp23_MATRIX
S2_WT
S2_WT_IMS
S2_WT_MATRIX
S2_atp6
S2_atp6_IMS
S2_atp6_MATRIX

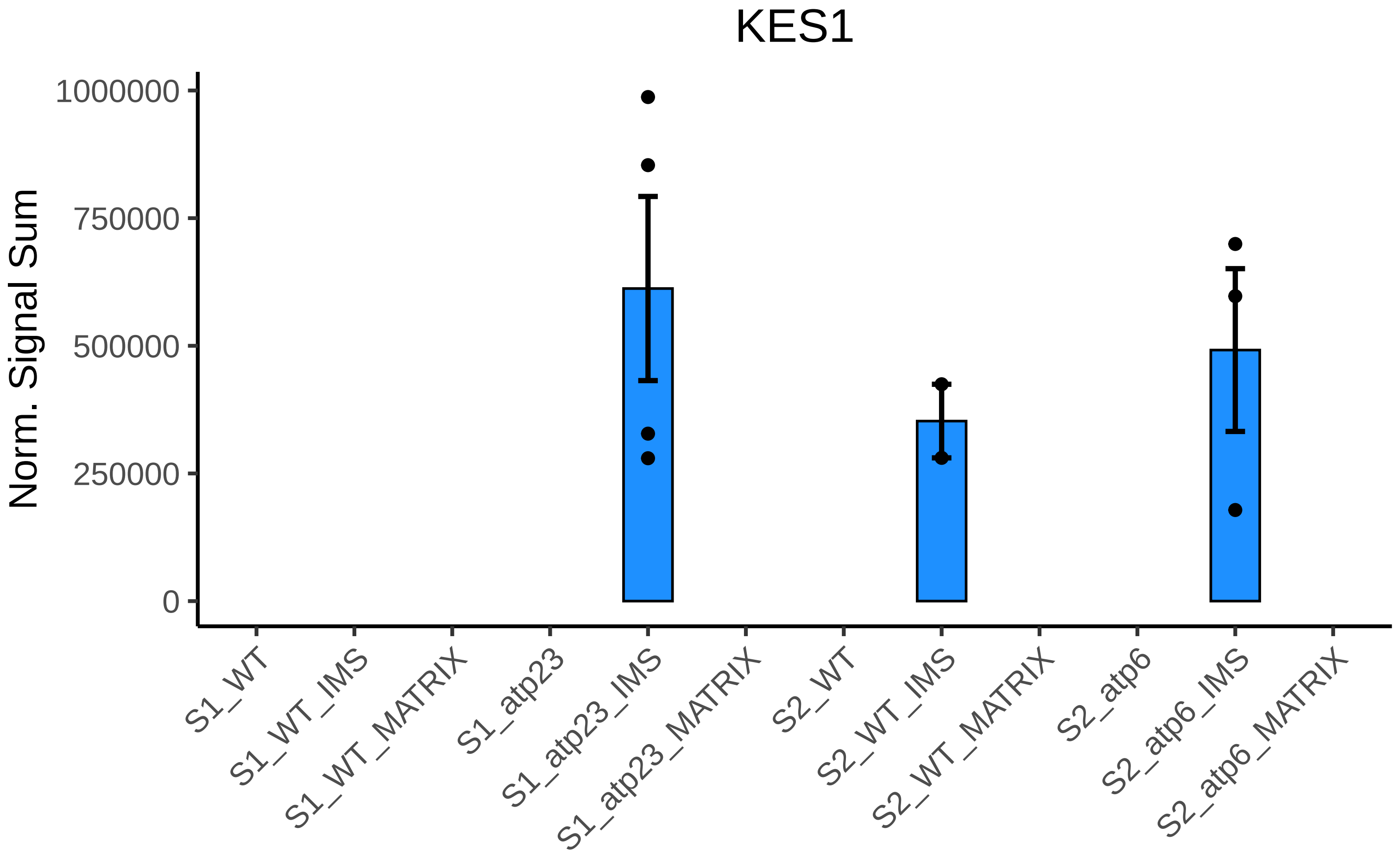
KES1
Norm. Signal Sum
1000000
750000
500000
250000
0
S1_WT
S1_WT_IMS
S1_WT_MATRIX
S1_atp23
S1_atp23_IMS
S1_atp23_MATRIX
S2_WT
S2_WT_IMS
S2_WT_MATRIX
S2_atp6
S2_atp6_IMS
S2_atp6_MATRIX

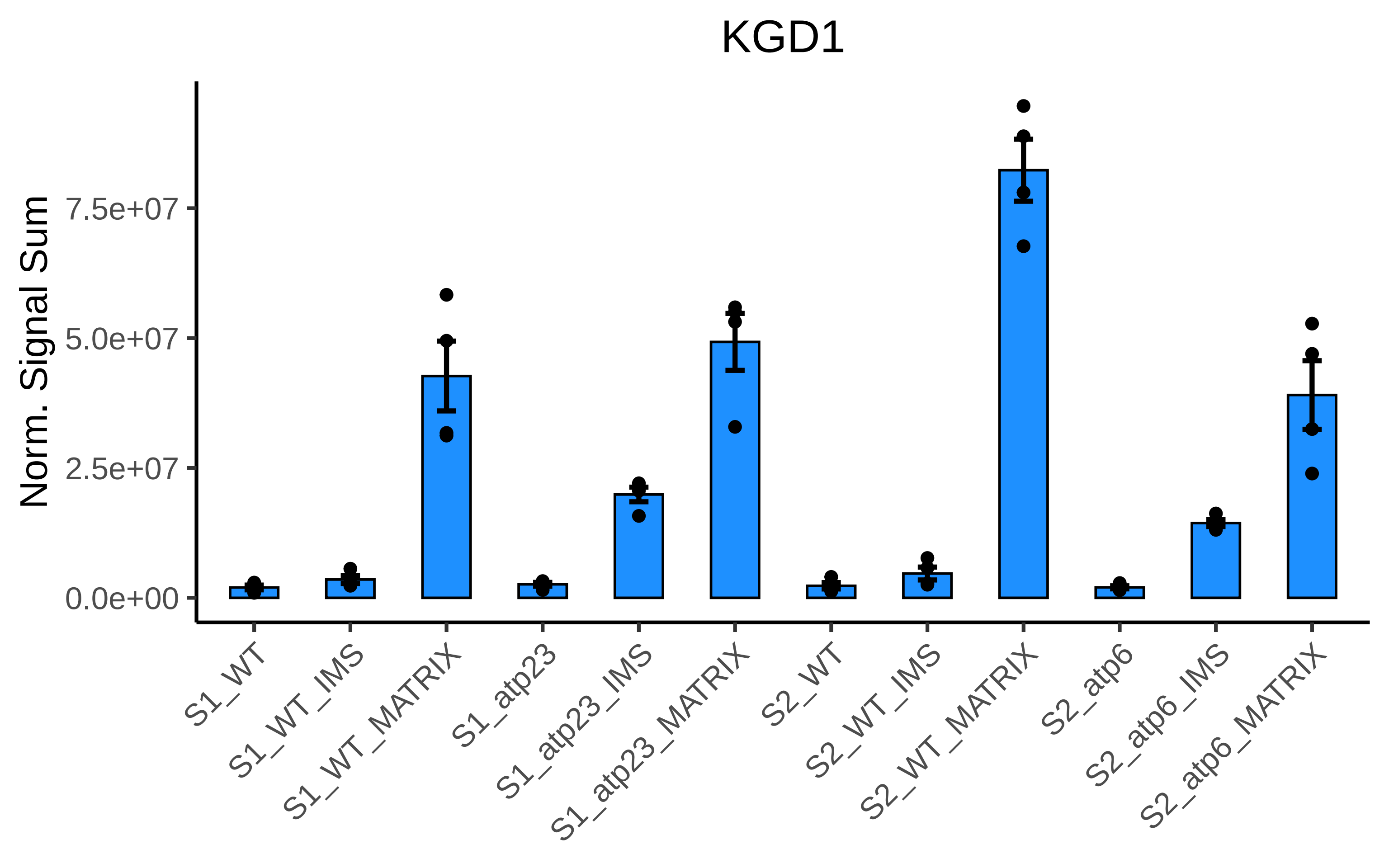
KGD1
Norm. Signal Sum
7.5e+07
5.0e+07
2.5e+07
0.0e+00
S1_WT
S1_WT_IMS
S1_WT_MATRIX
S1_atp23
S1_atp23_IMS
S1_atp23_MATRIX
S2_WT
S2_WT_IMS
S2_WT_MATRIX
S2_atp6
S2_atp6_IMS
S2_atp6_MATRIX

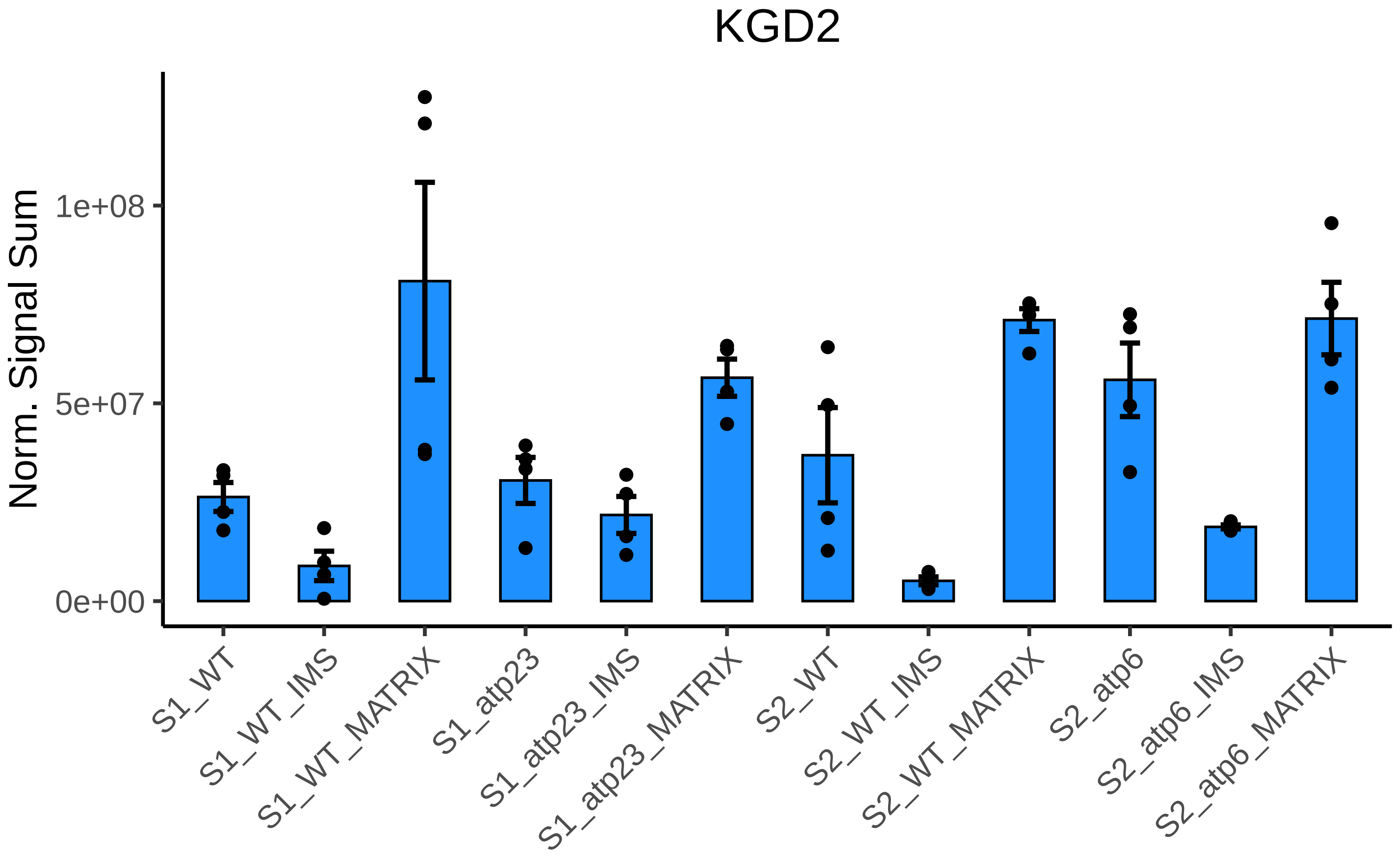

KGD2
Norm. Signal Sum
1e+08
5e+07
0e+00
S1_WT
S1_WT_IMS
S1_WT_MATRIX
S1_atp23
S1_atp23_IMS
S1_atp23_MATRIX
S2_WT
S2_WT_IMS
S2_WT_MATRIX
S2_atp6
S2_atp6_IMS
S2_atp6_MATRIX

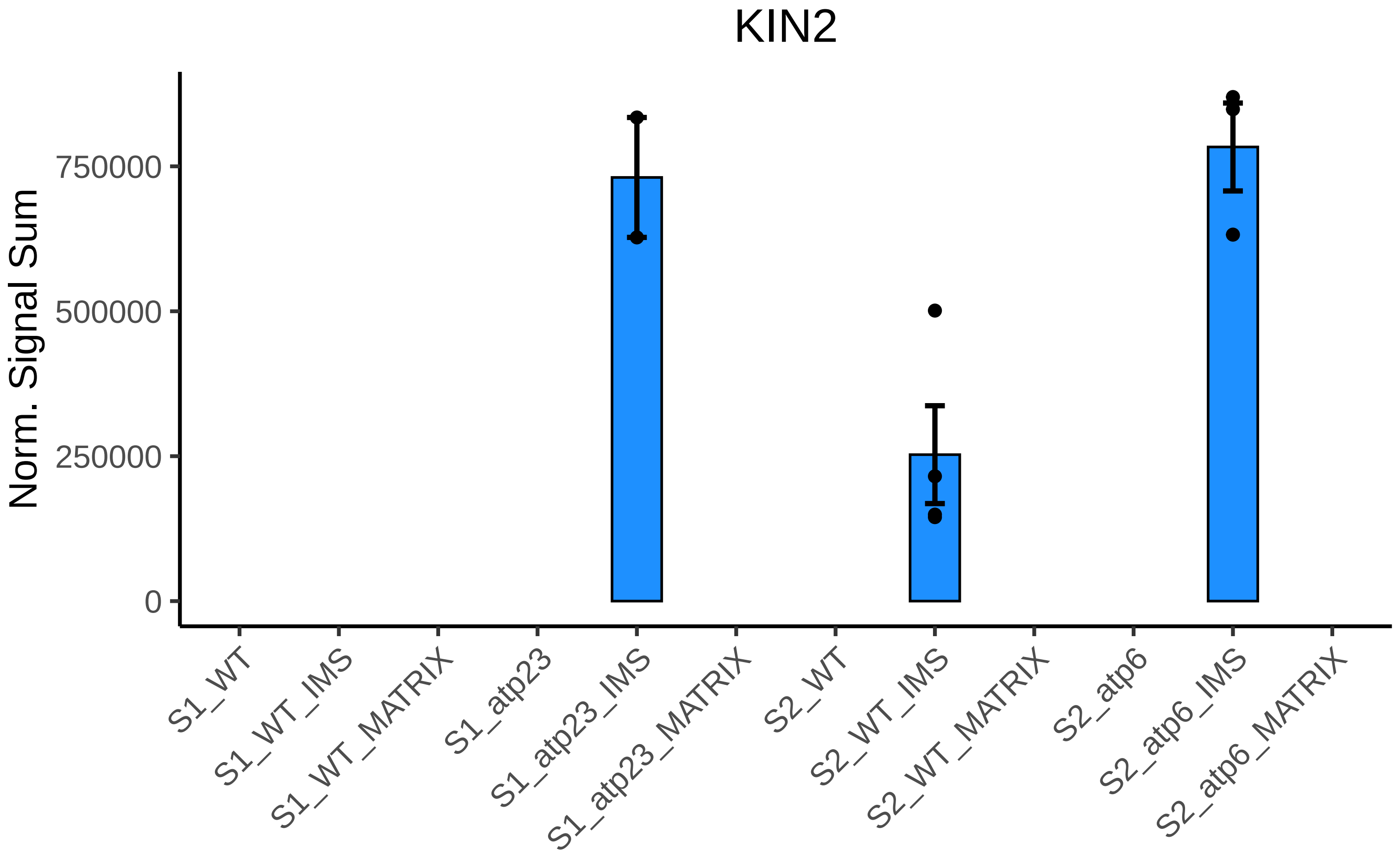

KIN2
Norm. Signal Sum
750000
500000
250000
0
S1_WT
S1_WT_IMS
S1_WT_MATRIX
S1_atp23
S1_atp23_IMS
S1_atp23_MATRIX
S2_WT
S2_WT_IMS
S2_WT_MATRIX
S2_atp6
S2_atp6_IMS
S2_atp6_MATRIX

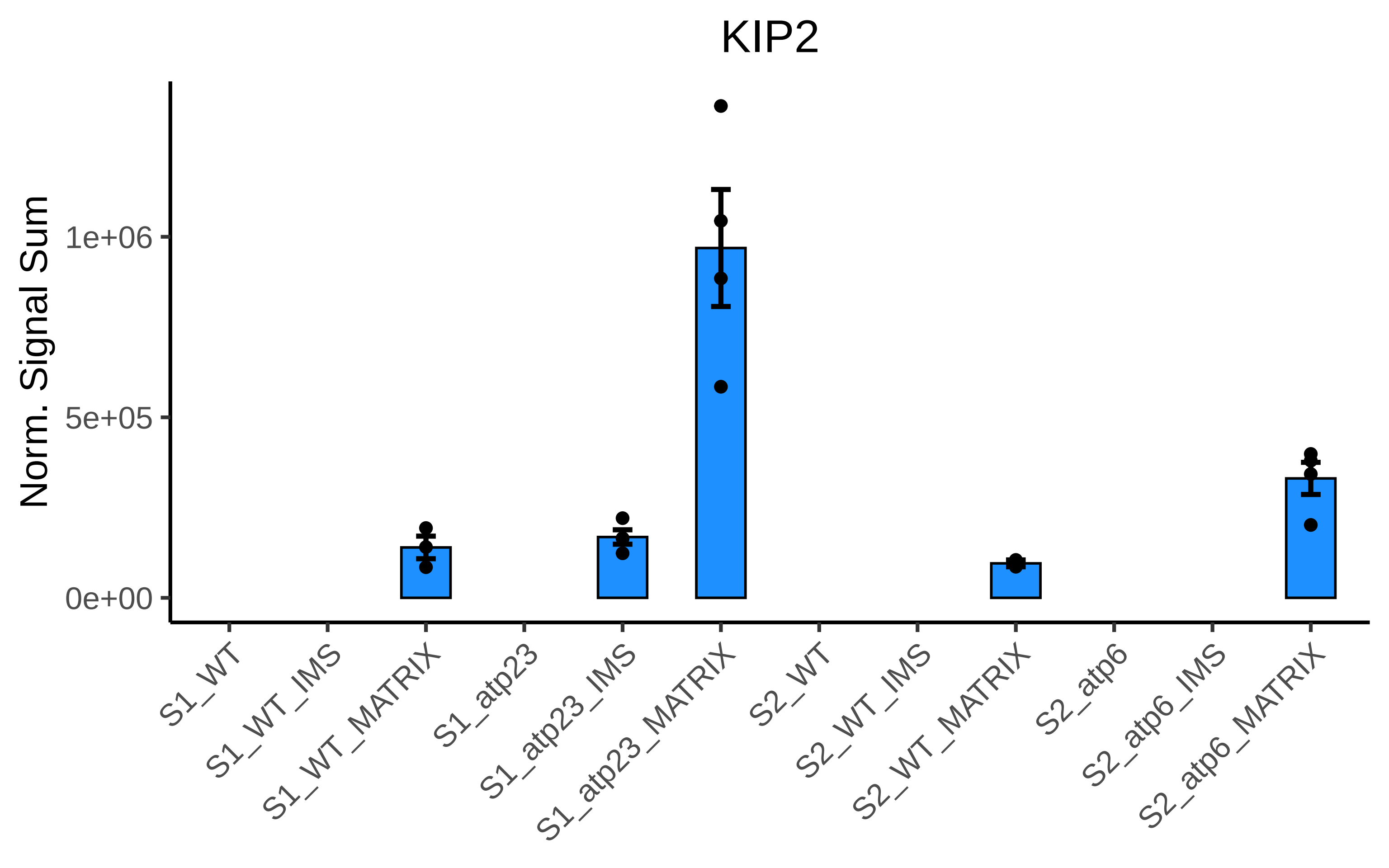
KIP2
Norm. Signal Sum
1e+06
5e+05
0e+00
S1_WT
S1_WT_IMS
S1_WT_MATRIX
S1_atp23
S1_atp23_IMS
S1_atp23_MATRIX
S2_WT
S2_WT_IMS
S2_WT_MATRIX
S2_atp6
S2_atp6_IMS
S2_atp6_MATRIX

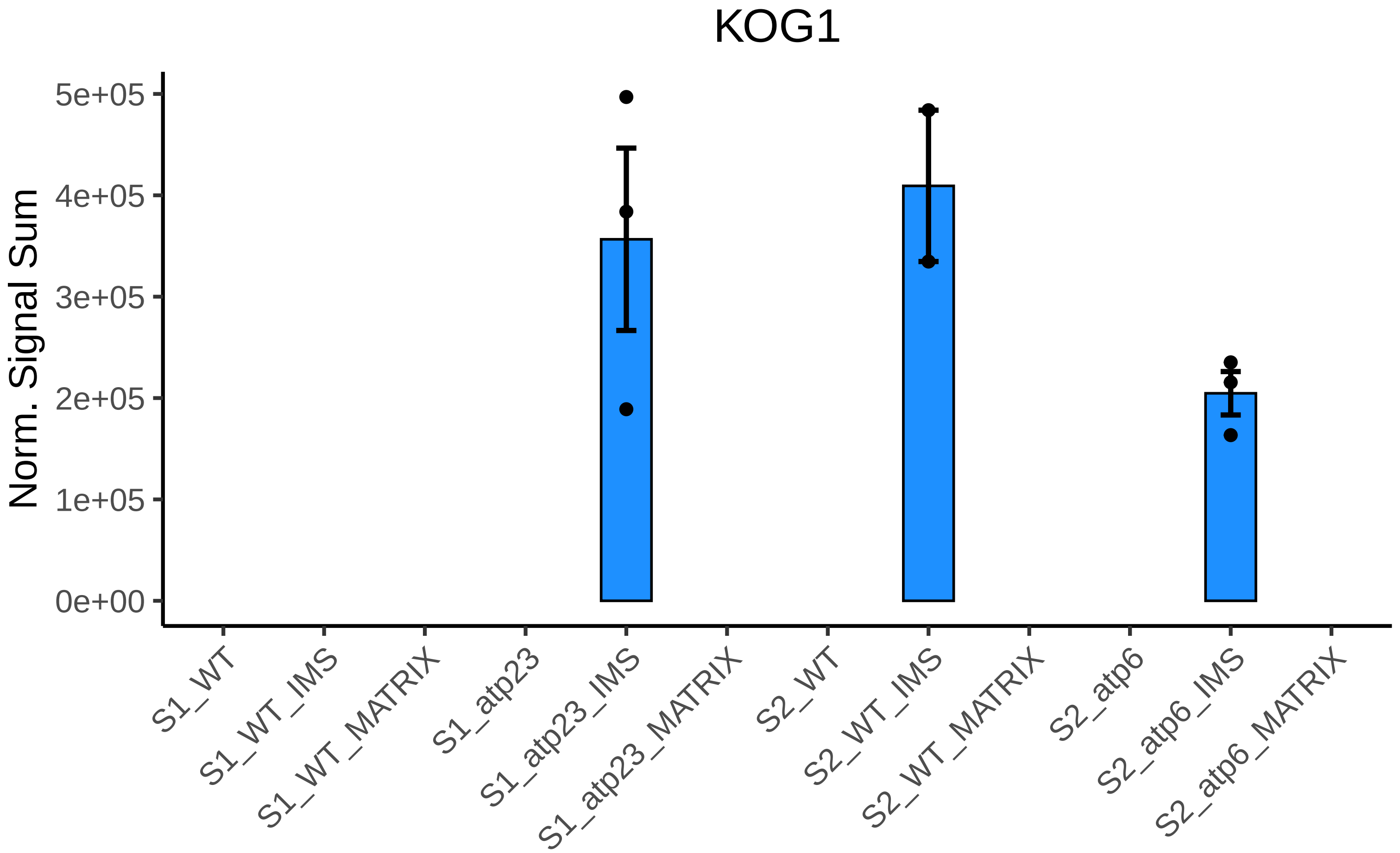

KOG1
Norm. Signal Sum
5e+05
4e+05
3e+05
2e+05
1e+05
0e+00
S1_WT
S1_WT_IMS
S1_WT_MATRIX
S1_atp23
S1_atp23_IMS
S1_atp23_MATRIX
S2_WT
S2_WT_IMS
S2_WT_MATRIX
S2_atp6
S2_atp6_IMS
S2_atp6_MATRIX

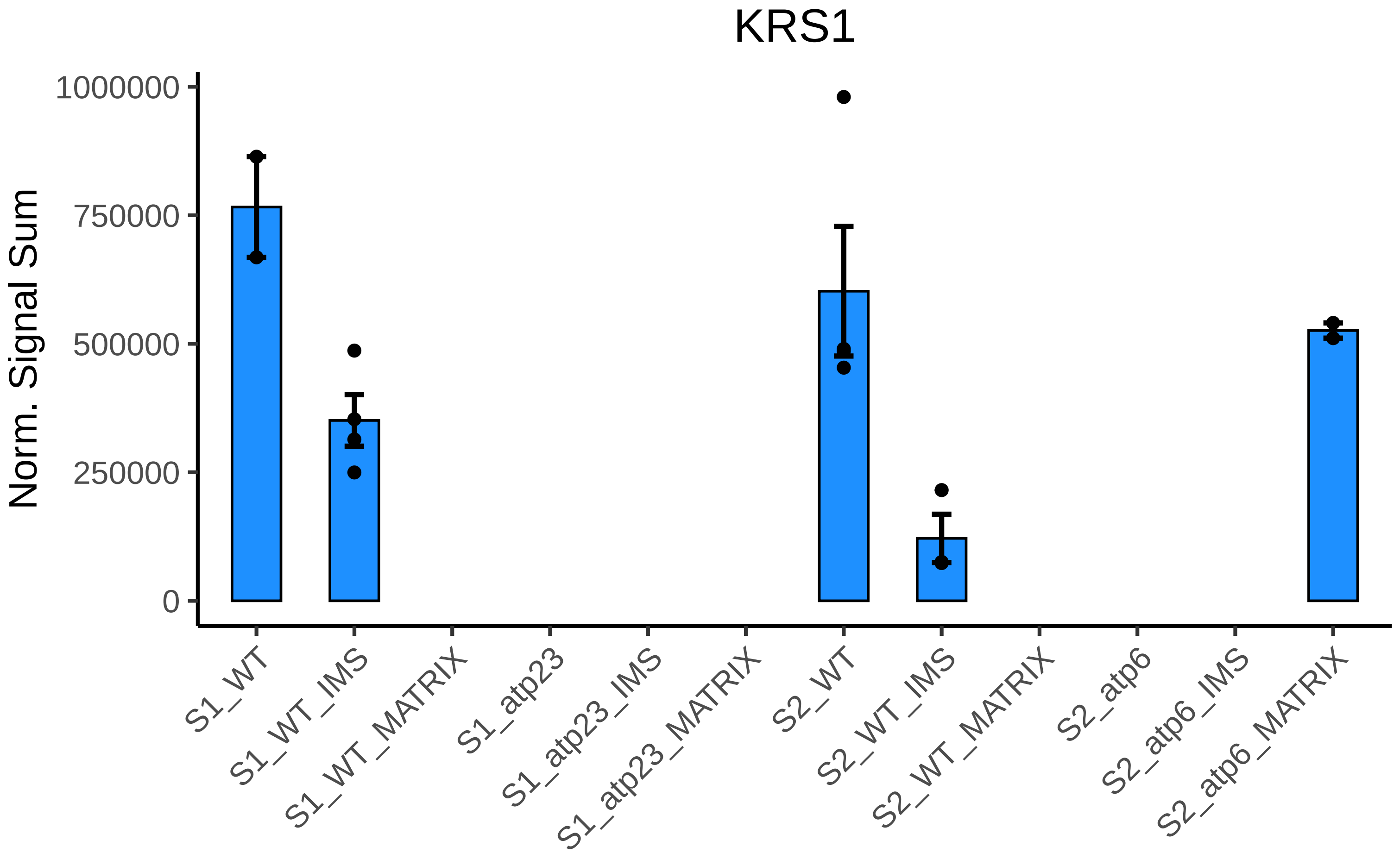
KRS1
Norm. Signal Sum
1000000
750000
500000
250000
0
S1_WT
S1_WT_IMS
S1_WT_MATRIX
S1_atp23
S1_atp23_IMS
S1_atp23_MATRIX
S2_WT
S2_WT_IMS
S2_WT_MATRIX
S2_atp6
S2_atp6_IMS
S2_atp6_MATRIX

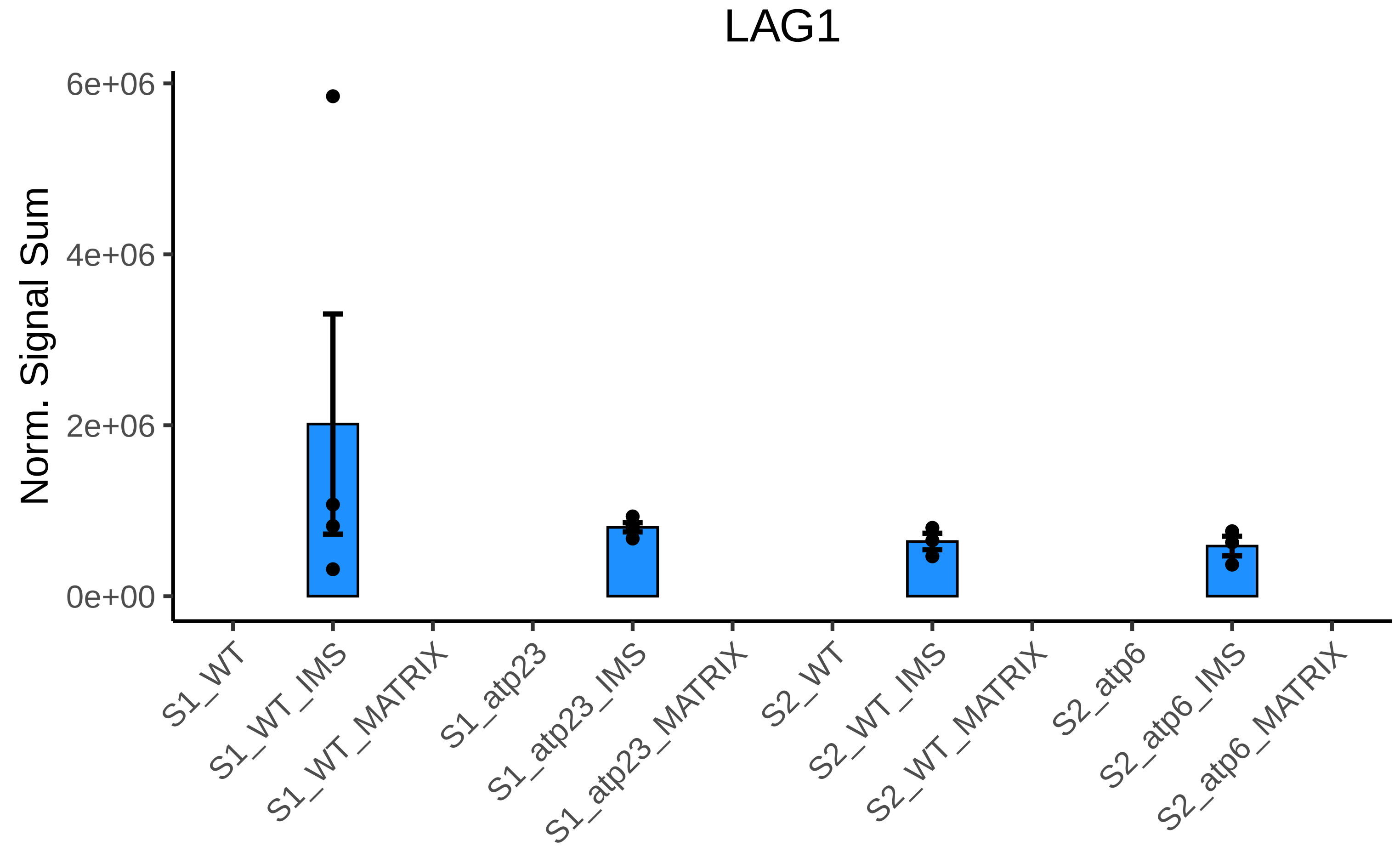

LAG1
Norm. Signal Sum
6e+06
4e+06
2e+06
0e+00
S1_WT
S1_WT_IMS
S1_WT_MATRIX
S1_atp23
S1_atp23_IMS
S1_atp23_MATRIX
S2_WT
S2_WT_IMS
S2_WT_MATRIX
S2_atp6
S2_atp6_IMS
S2_atp6_MATRIX

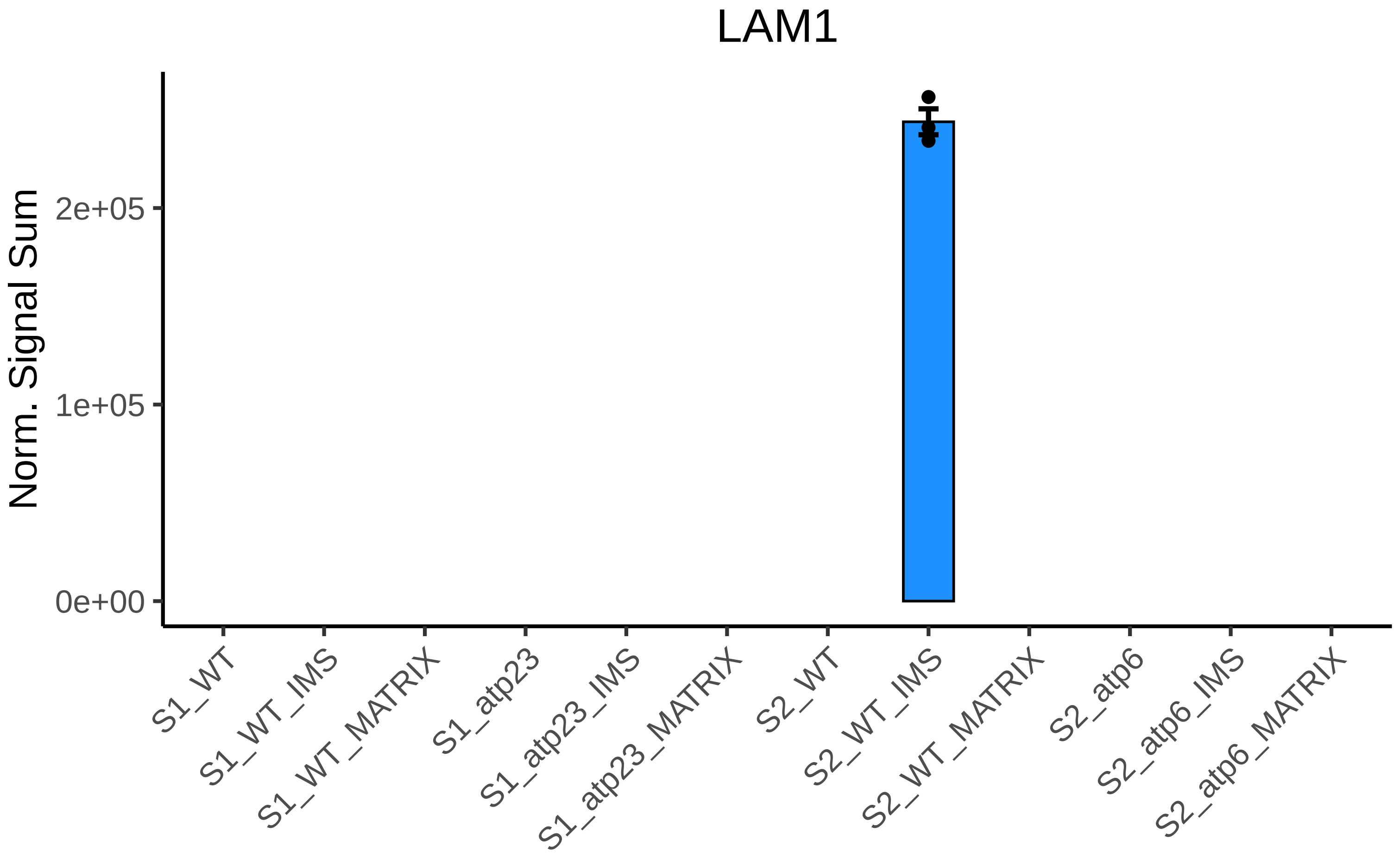
LAM1
Norm. Signal Sum
2e+05
1e+05
0e+00
S1_WT
S1_WT_IMS
S1_WT_MATRIX
S1_atp23
S1_atp23_IMS
S1_atp23_MATRIX
S2_WT
S2_WT_IMS
S2_WT_MATRIX
S2_atp6
S2_atp6_IMS
S2_atp6_MATRIX

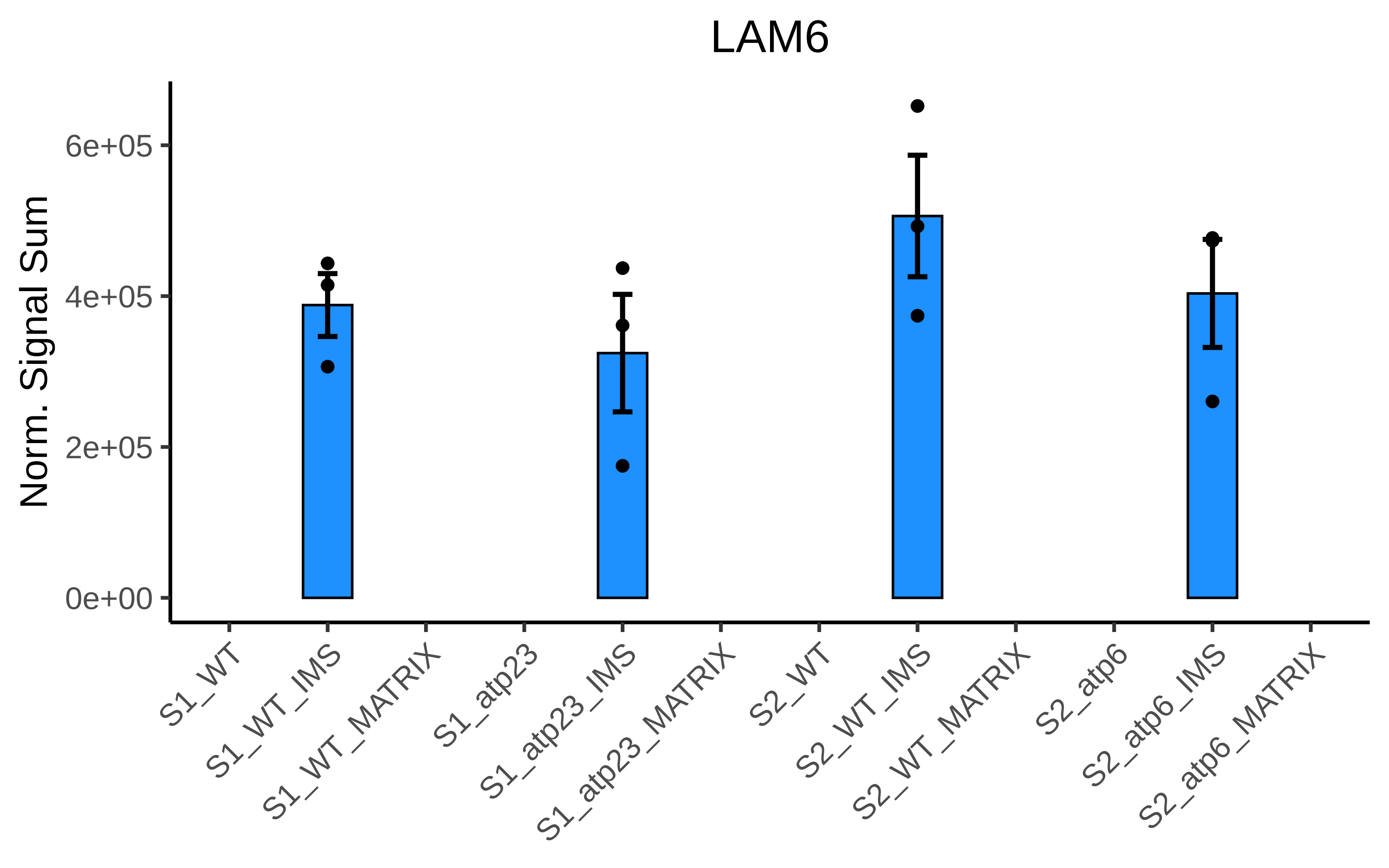

LAM6
Norm. Signal Sum
6e+05
4e+05
2e+05
0e+00
S1_WT
S1_WT_IMS
S1_WT_MATRIX
S1_atp23
S1_atp23_IMS
S1_atp23_MATRIX
S2_WT
S2_WT_IMS
S2_WT_MATRIX
S2_atp6
S2_atp6_IMS
S2_atp6_MATRIX

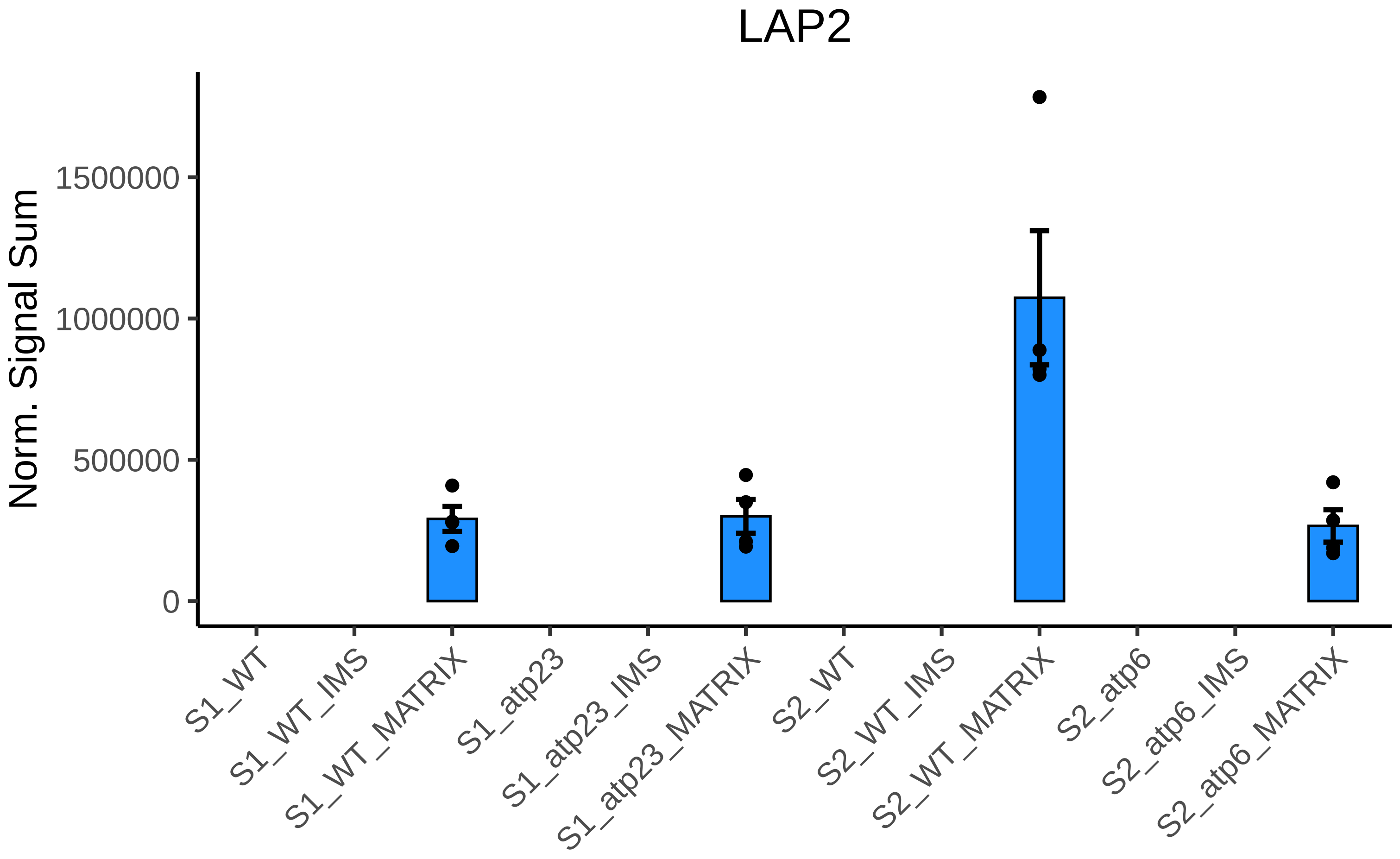

LAP2
Norm. Signal Sum
1500000
1000000
500000
0
S1_WT
S1_WT_IMS
S1_WT_MATRIX
S1_atp23
S1_atp23_IMS
S1_atp23_MATRIX
S2_WT
S2_WT_IMS
S2_WT_MATRIX
S2_atp6
S2_atp6_IMS
S2_atp6_MATRIX

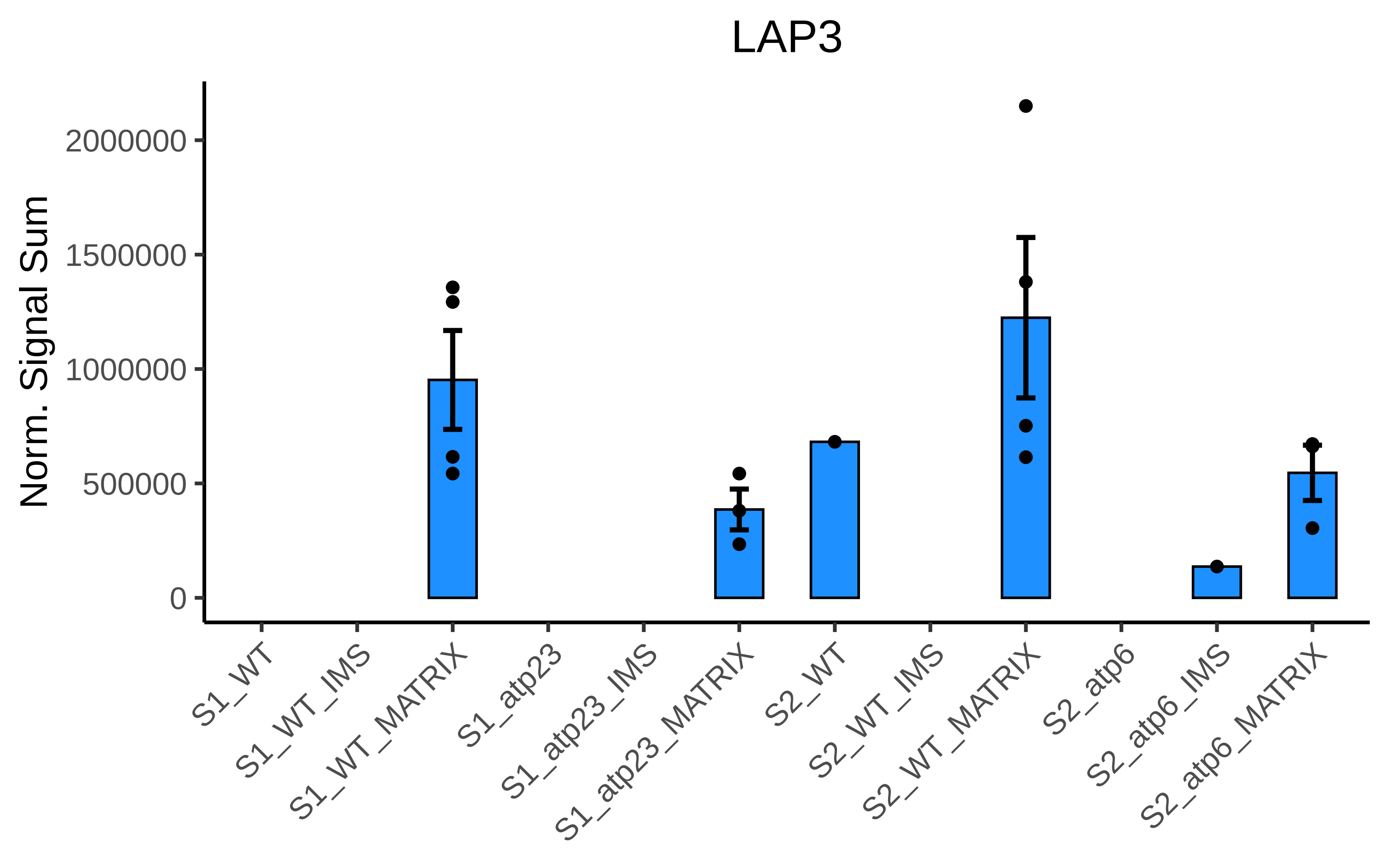

LAP3
Norm. Signal Sum
2000000
1500000
1000000
500000
0
S1_WT
S1_WT_IMS
S1_WT_MATRIX
S1_atp23
S1_atp23_IMS
S1_atp23_MATRIX
S2_WT
S2_WT_IMS
S2_WT_MATRIX
S2_atp6
S2_atp6_IMS
S2_atp6_MATRIX

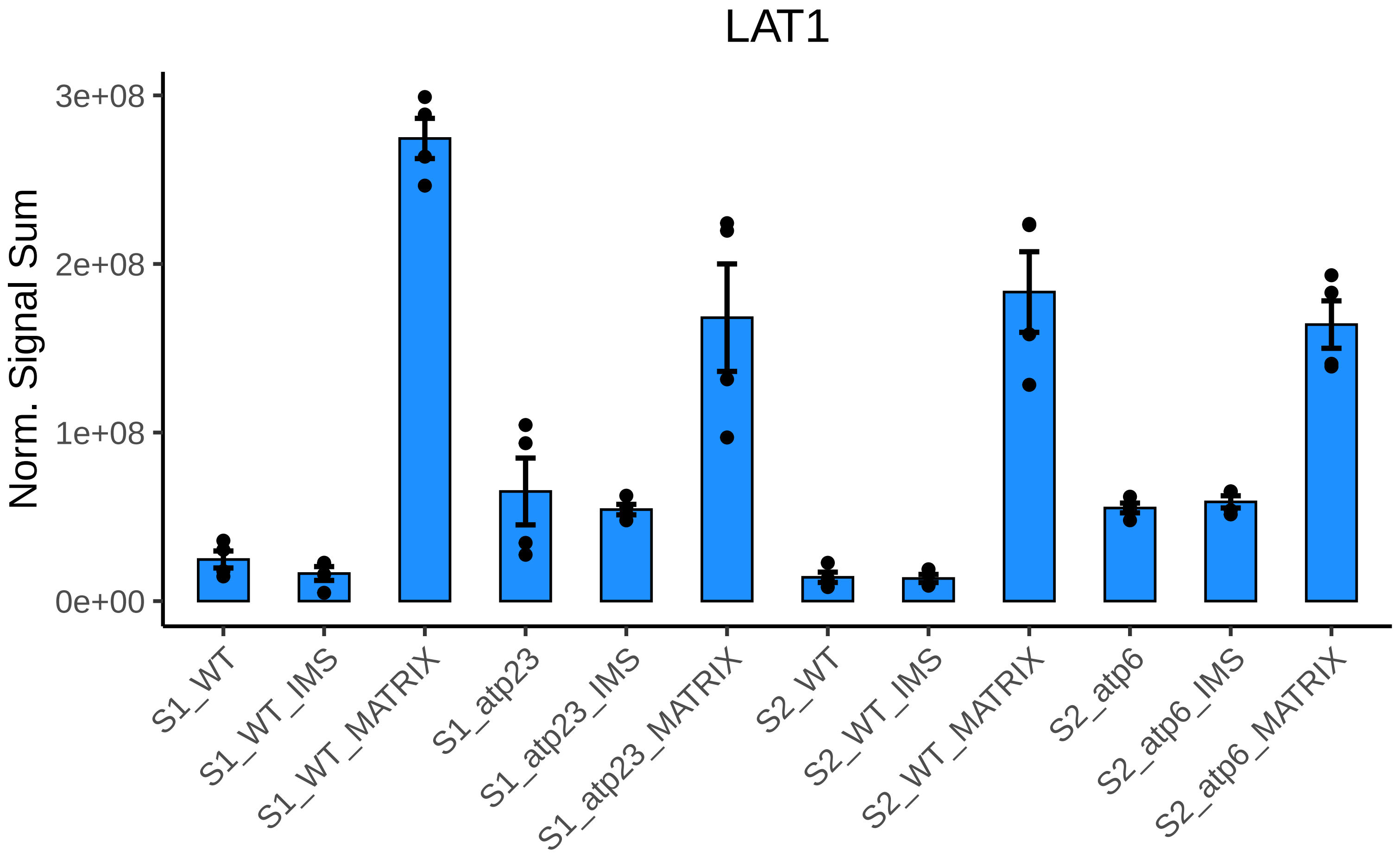
LAT1
Norm. Signal Sum
3e+08
2e+08
1e+08
0e+00
S1_WT
S1_WT_IMS
S1_WT_MATRIX
S1_atp23
S1_atp23_IMS
S1_atp23_MATRIX
S2_WT
S2_WT_IMS
S2_WT_MATRIX
S2_atp6
S2_atp6_IMS
S2_atp6_MATRIX

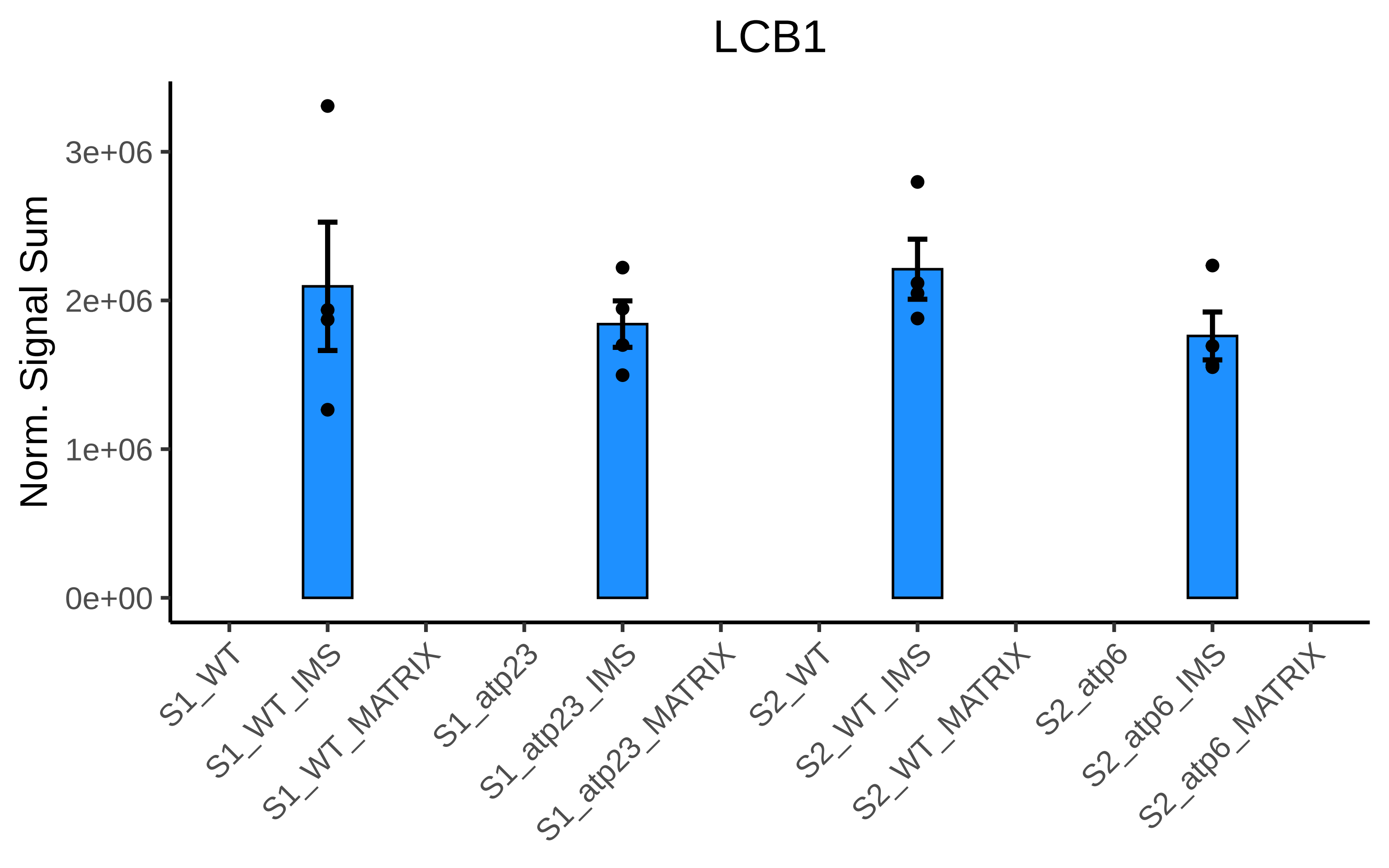
LCB1
Norm. Signal Sum
3e+06
2e+06
1e+06
0e+00
S1_WT
S1_WT_IMS
S1_WT_MATRIX
S1_atp23
S1_atp23_IMS
S1_atp23_MATRIX
S2_WT
S2_WT_IMS
S2_WT_MATRIX
S2_atp6
S2_atp6_IMS
S2_atp6_MATRIX

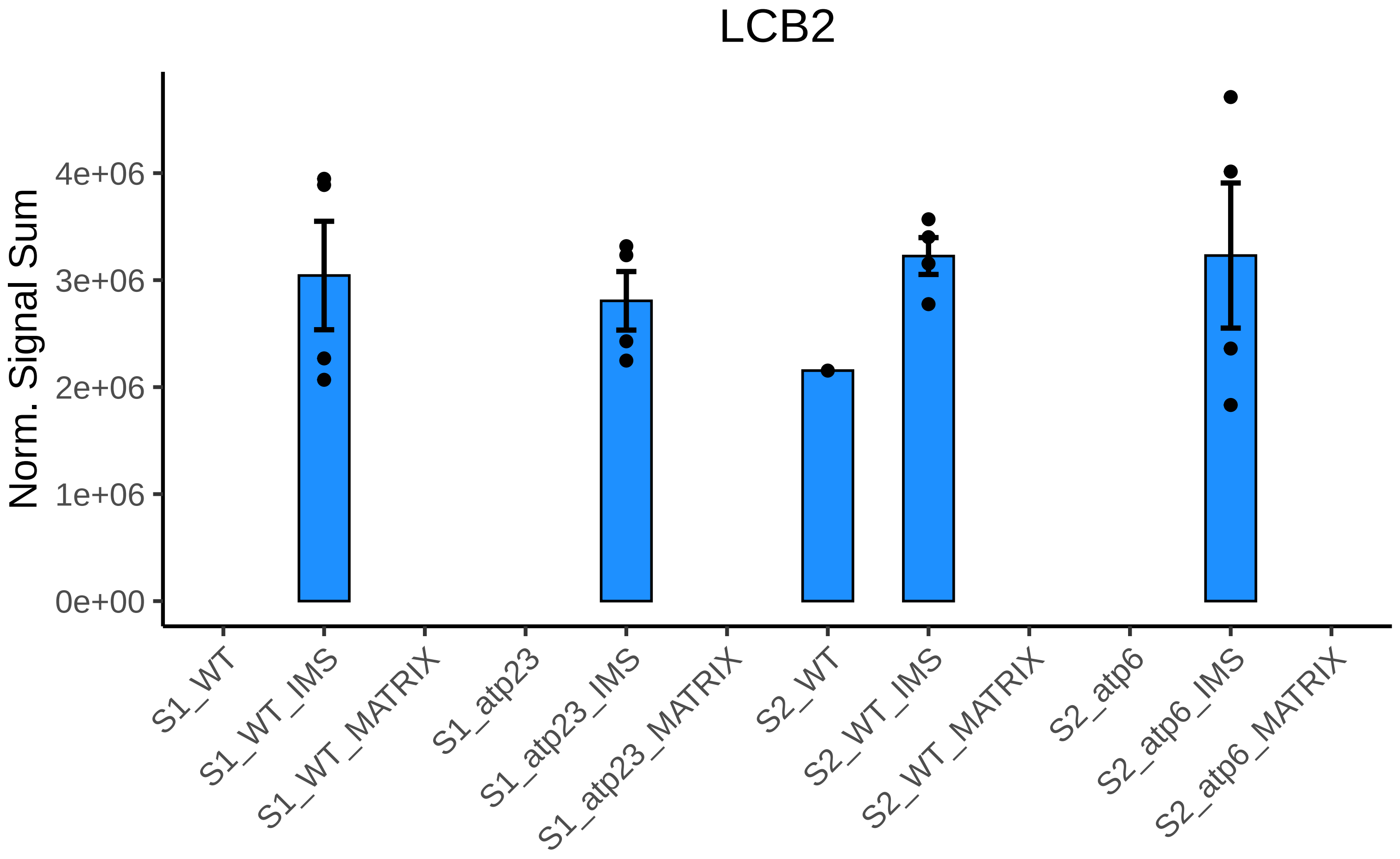

LCB2
Norm. Signal Sum
4e+06
3e+06
2e+06
1e+06
0e+00
S1_WT
S1_WT_IMS
S1_WT_MATRIX
S1_atp23
S1_atp23_IMS
S1_atp23_MATRIX
S2_WT
S2_WT_IMS
S2_WT_MATRIX
S2_atp6
S2_atp6_IMS
S2_atp6_MATRIX

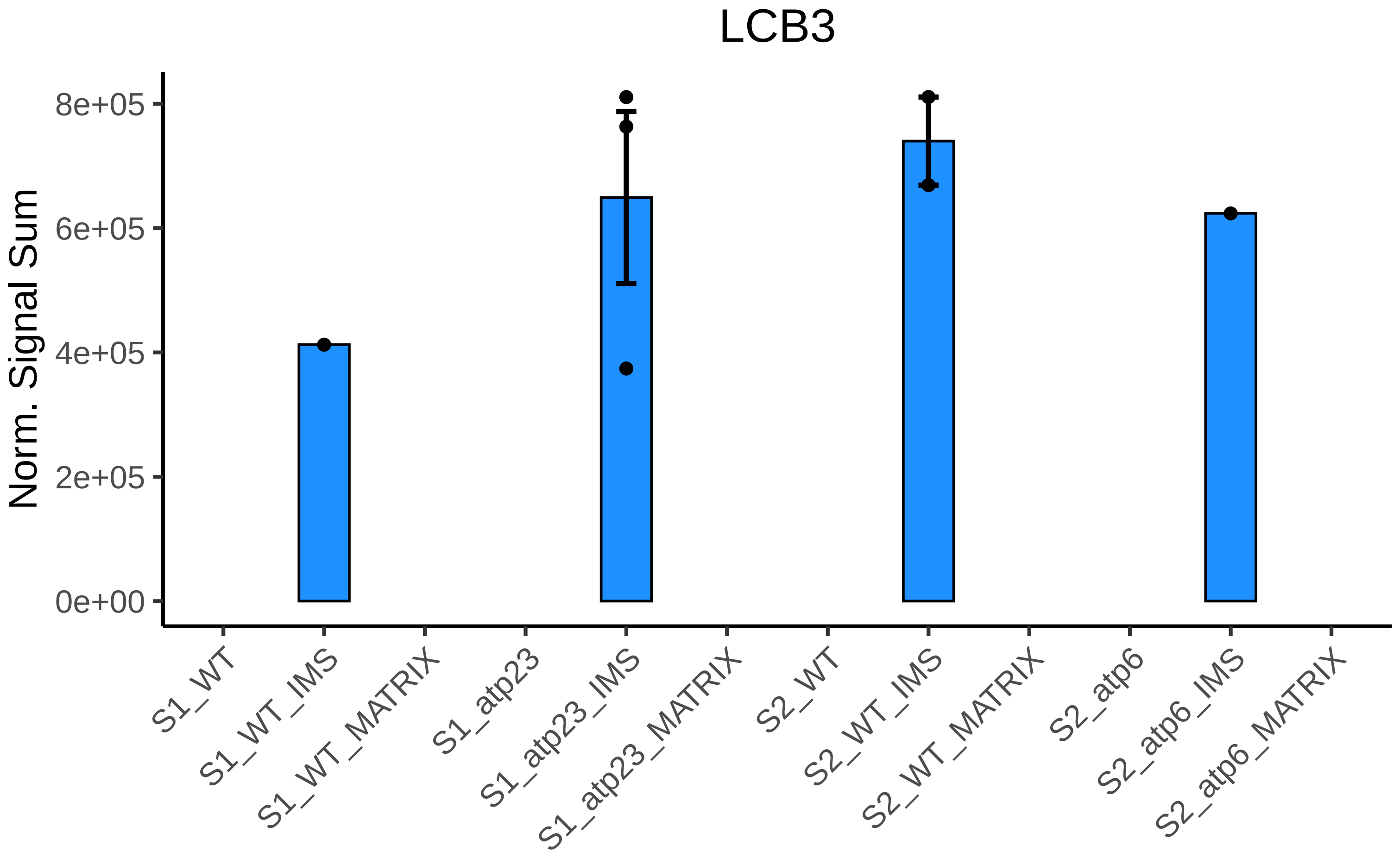

LCB3
Norm. Signal Sum
8e+05
6e+05
4e+05
2e+05
0e+00
S1_WT
S1_WT_IMS
S1_WT_MATRIX
S1_atp23
S1_atp23_IMS
S1_atp23_MATRIX
S2_WT
S2_WT_IMS
S2_WT_MATRIX
S2_atp6
S2_atp6_IMS
S2_atp6_MATRIX

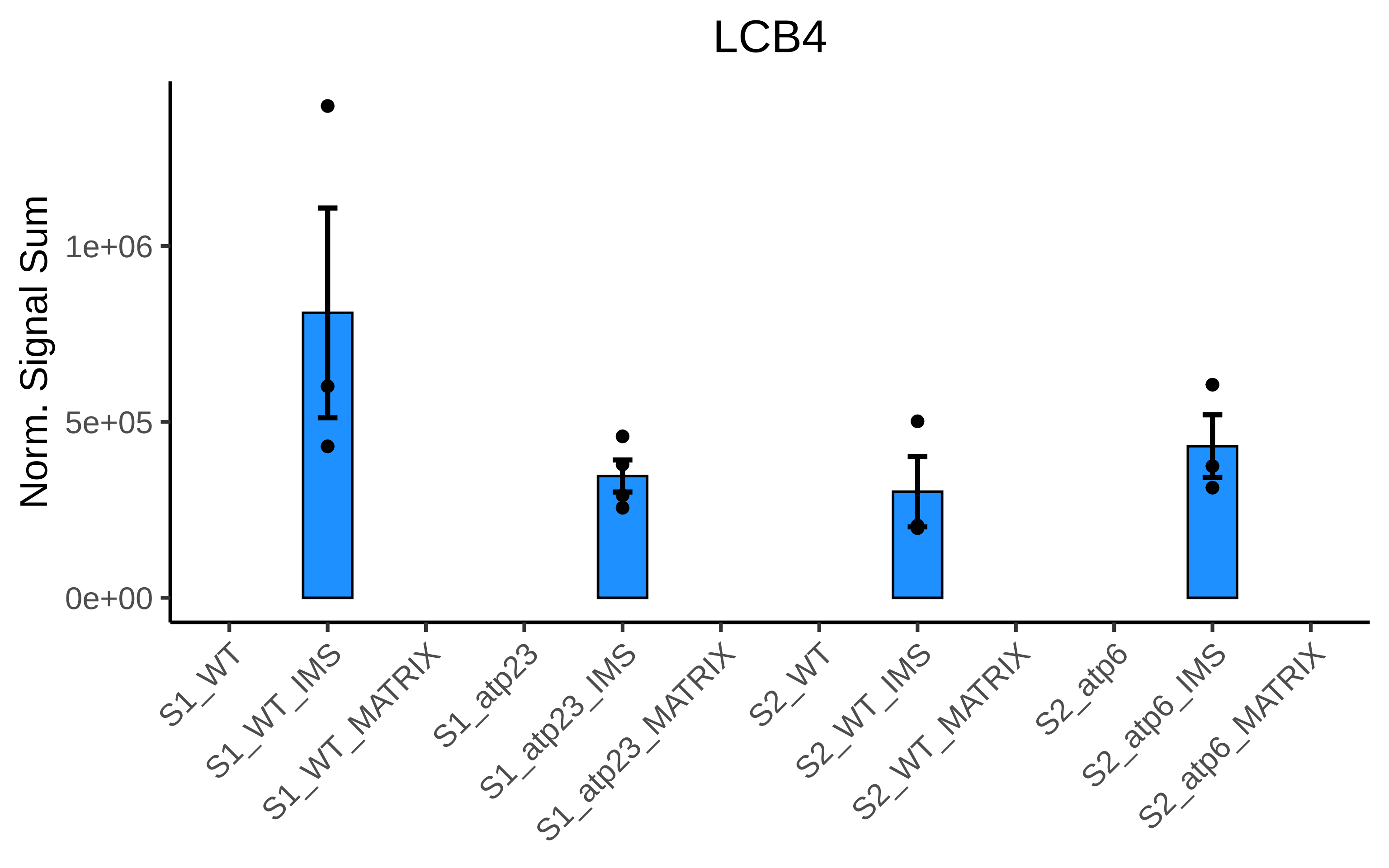

LCB4
Norm. Signal Sum
1e+06
5e+05
0e+00
S1_WT
S1_WT_IMS
S1_WT_MATRIX
S1_atp23
S1_atp23_IMS
S1_atp23_MATRIX
S2_WT
S2_WT_IMS
S2_WT_MATRIX
S2_atp6
S2_atp6_IMS
S2_atp6_MATRIX

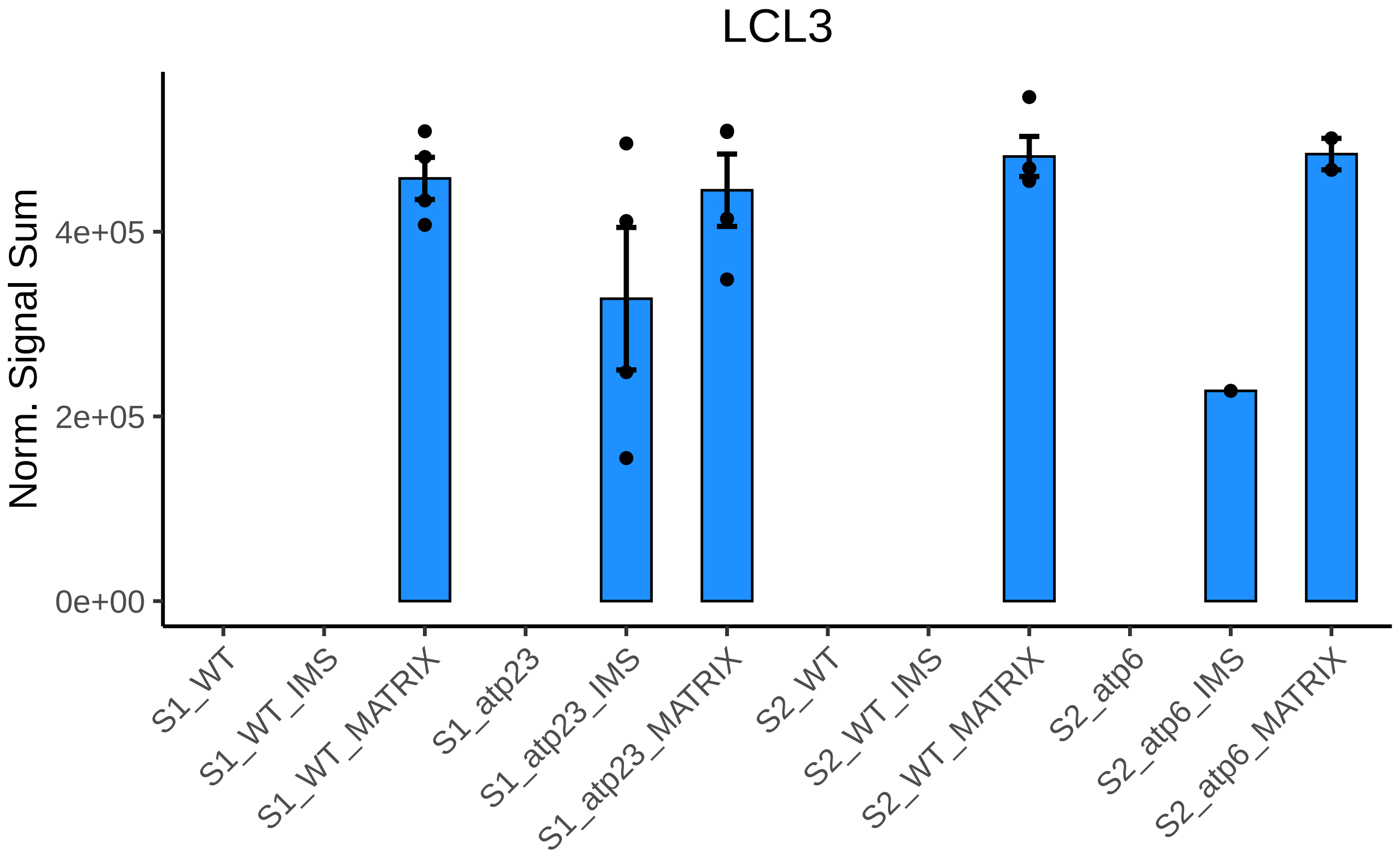

LCL3
Norm. Signal Sum
4e+05
2e+05
0e+00
S1_WT
S1_WT_IMS
S1_WT_MATRIX
S1_atp23
S1_atp23_IMS
S1_atp23_MATRIX
S2_WT
S2_WT_IMS
S2_WT_MATRIX
S2_atp6
S2_atp6_IMS
S2_atp6_MATRIX

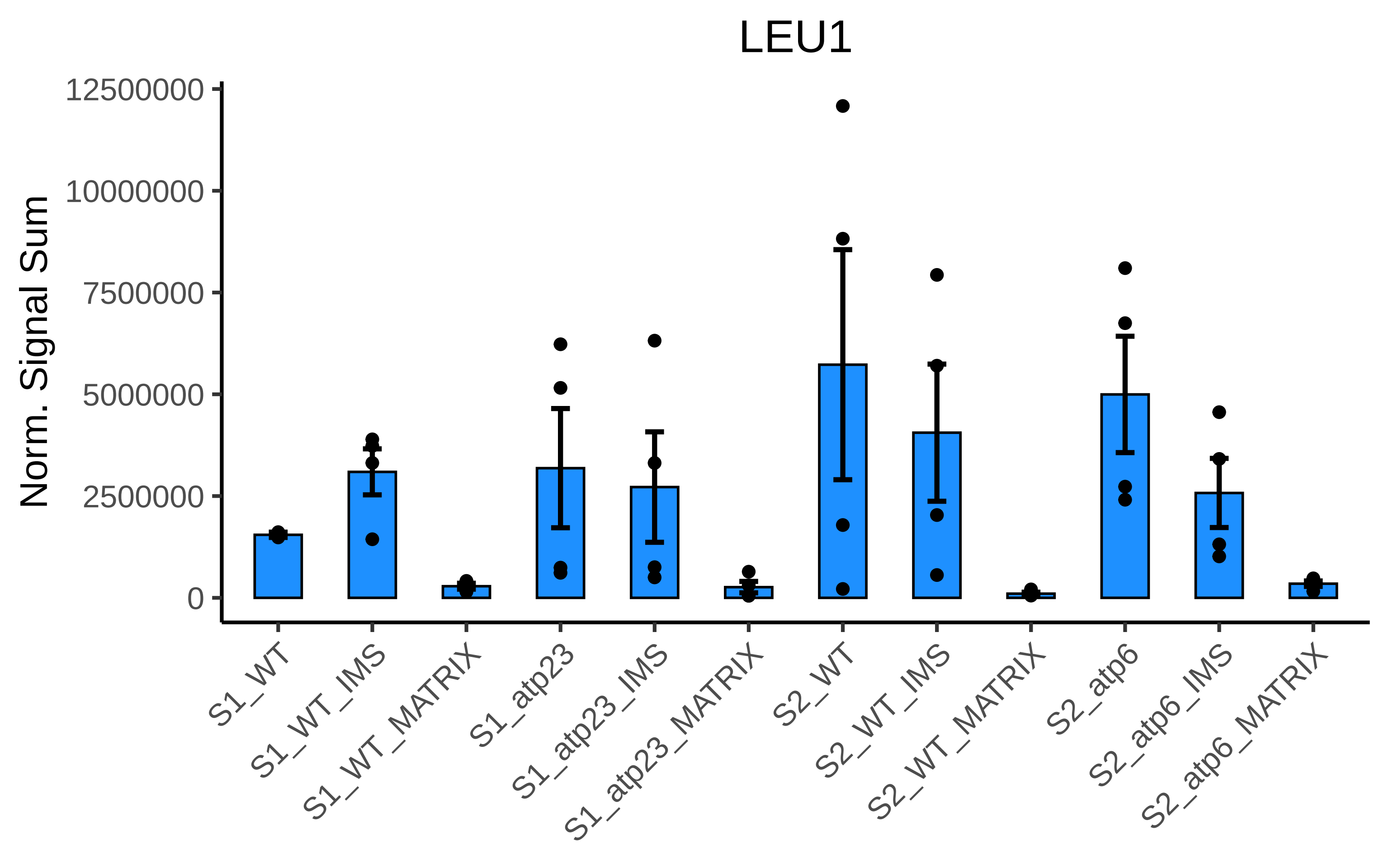

LEU1
Norm. Signal Sum
12500000
10000000
7500000
5000000
2500000
0
S1_WT
S1_WT_IMS
S1_WT_MATRIX
S1_atp23
S1_atp23_IMS
S1_atp23_MATRIX
S2_WT
S2_WT_IMS
S2_WT_MATRIX
S2_atp6
S2_atp6_IMS
S2_atp6_MATRIX

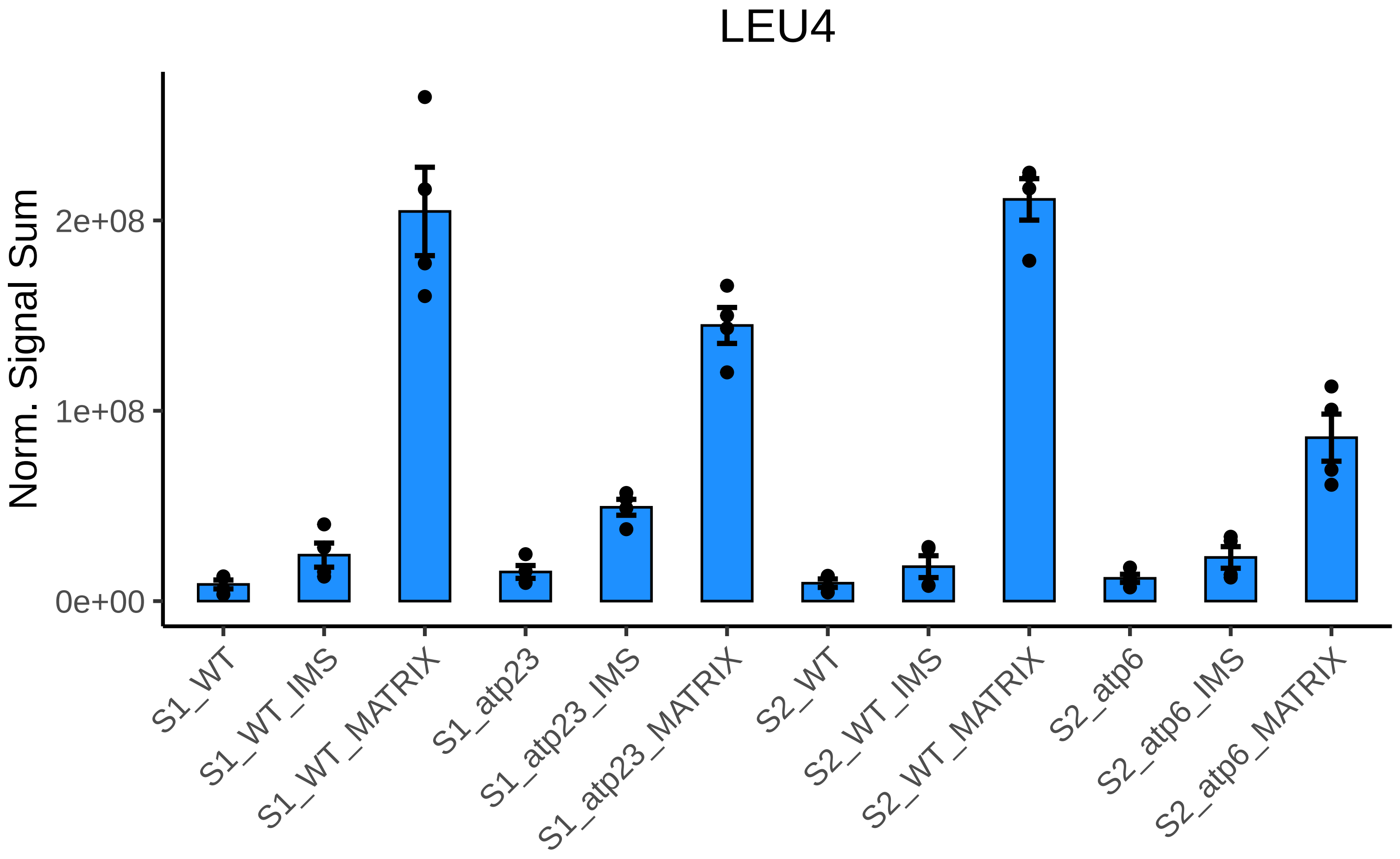
LEU4
Norm. Signal Sum
2e+08
1e+08
0e+00
S1_WT
S1_WT_IMS
S1_WT_MATRIX
S1_atp23
S1_atp23_IMS
S1_atp23_MATRIX
S2_WT
S2_WT_IMS
S2_WT_MATRIX
S2_atp6
S2_atp6_IMS
S2_atp6_MATRIX

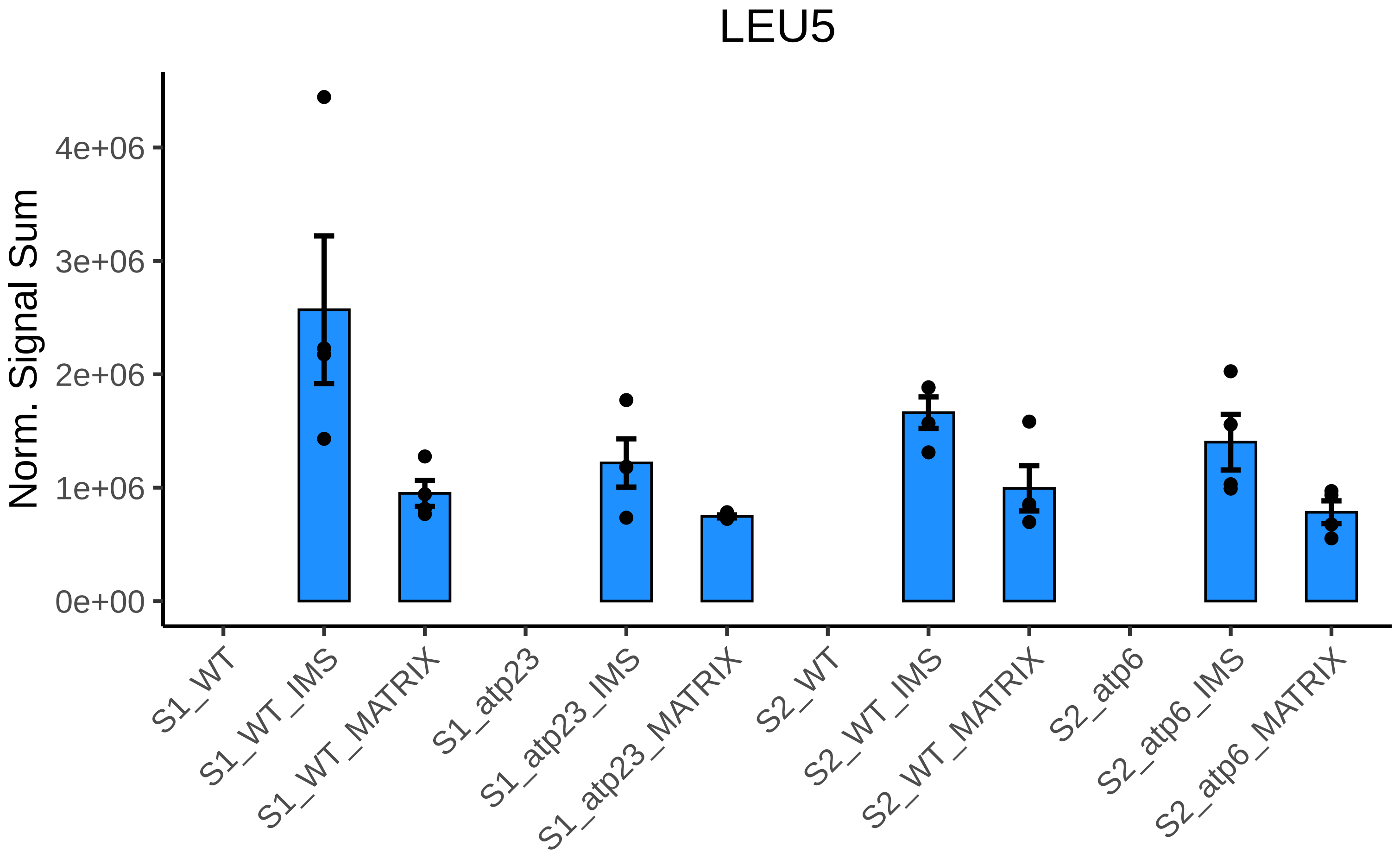
LEU5
Norm. Signal Sum
4e+06
3e+06
2e+06
1e+06
0e+00
S1_WT
S1_WT_IMS
S1_WT_MATRIX
S1_atp23
S1_atp23_IMS
S1_atp23_MATRIX
S2_WT
S2_WT_IMS
S2_WT_MATRIX
S2_atp6
S2_atp6_IMS
S2_atp6_MATRIX

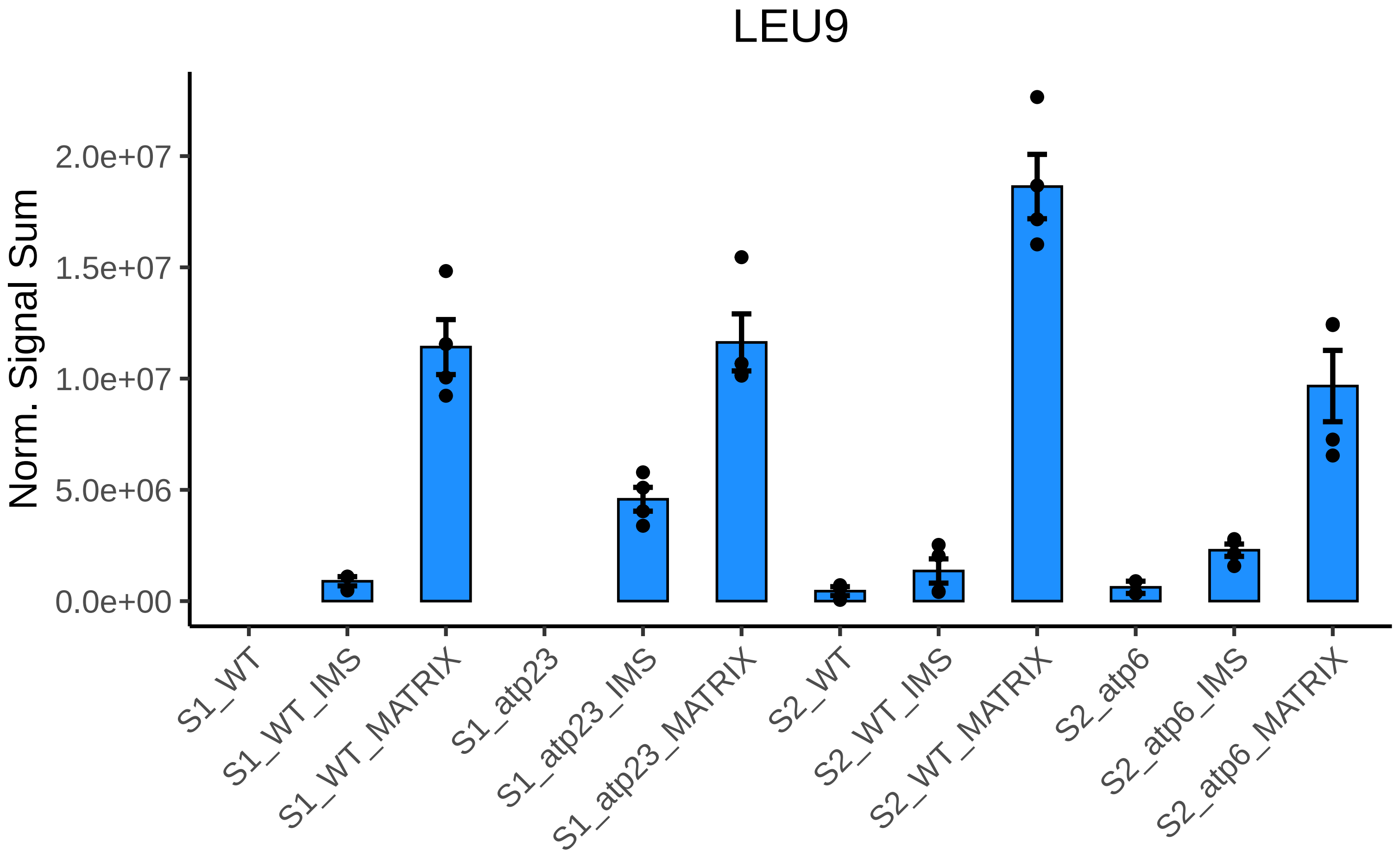

LEU9
Norm. Signal Sum
2.0e+07
1.5e+07
1.0e+07
5.0e+06
0.0e+00
S1_WT
S1_WT_IMS
S1_WT_MATRIX
S1_atp23
S1_atp23_IMS
S1_atp23_MATRIX
S2_WT
S2_WT_IMS
S2_WT_MATRIX
S2_atp6
S2_atp6_IMS
S2_atp6_MATRIX

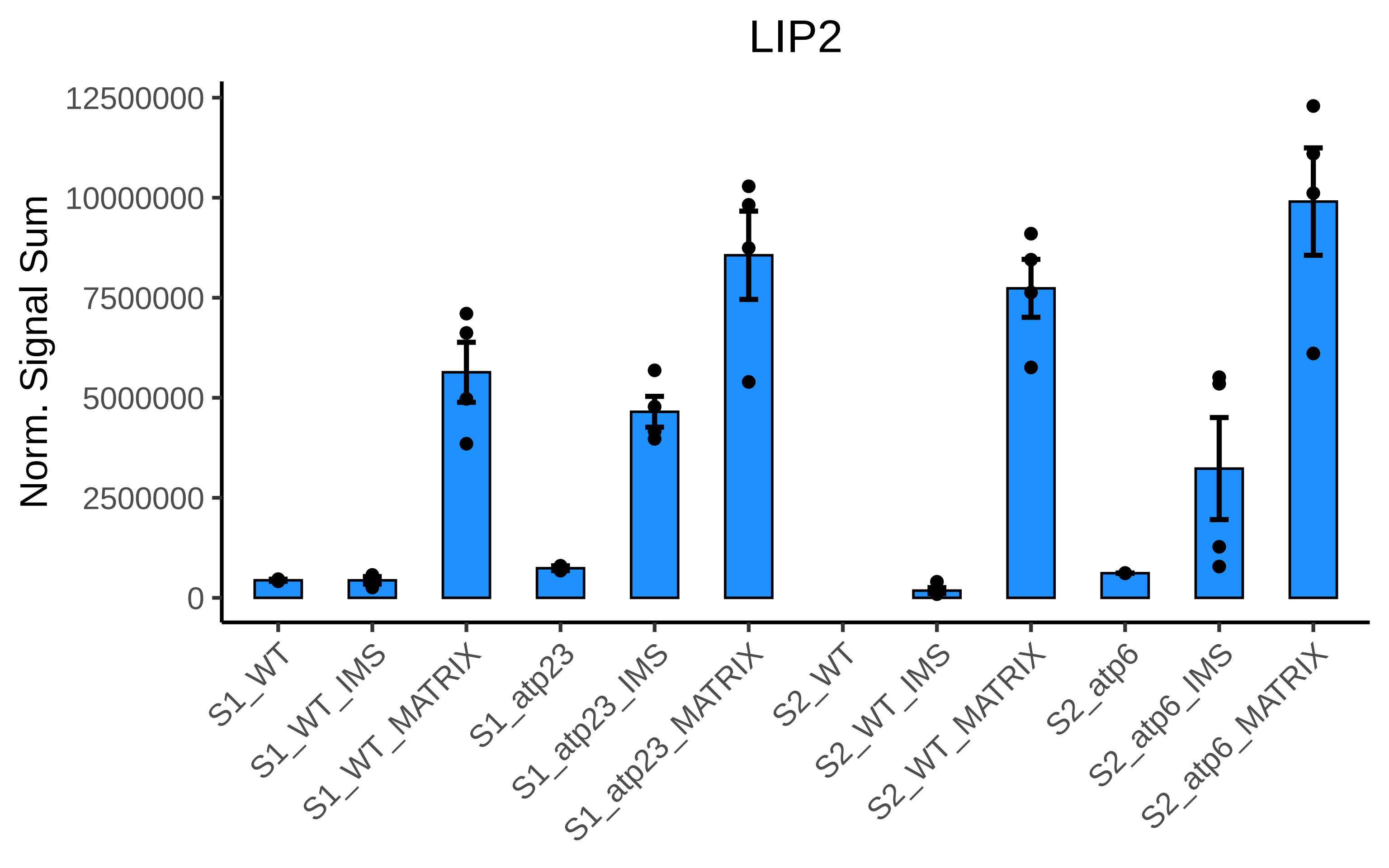
LIP2
Norm. Signal Sum
12500000
10000000
7500000
5000000
2500000
0
S1_WT
S1_WT_IMS
S1_WT_MATRIX
S1_atp23
S1_atp23_IMS
S1_atp23_MATRIX
S2_WT
S2_WT_IMS
S2_WT_MATRIX
S2_atp6
S2_atp6_IMS
S2_atp6_MATRIX

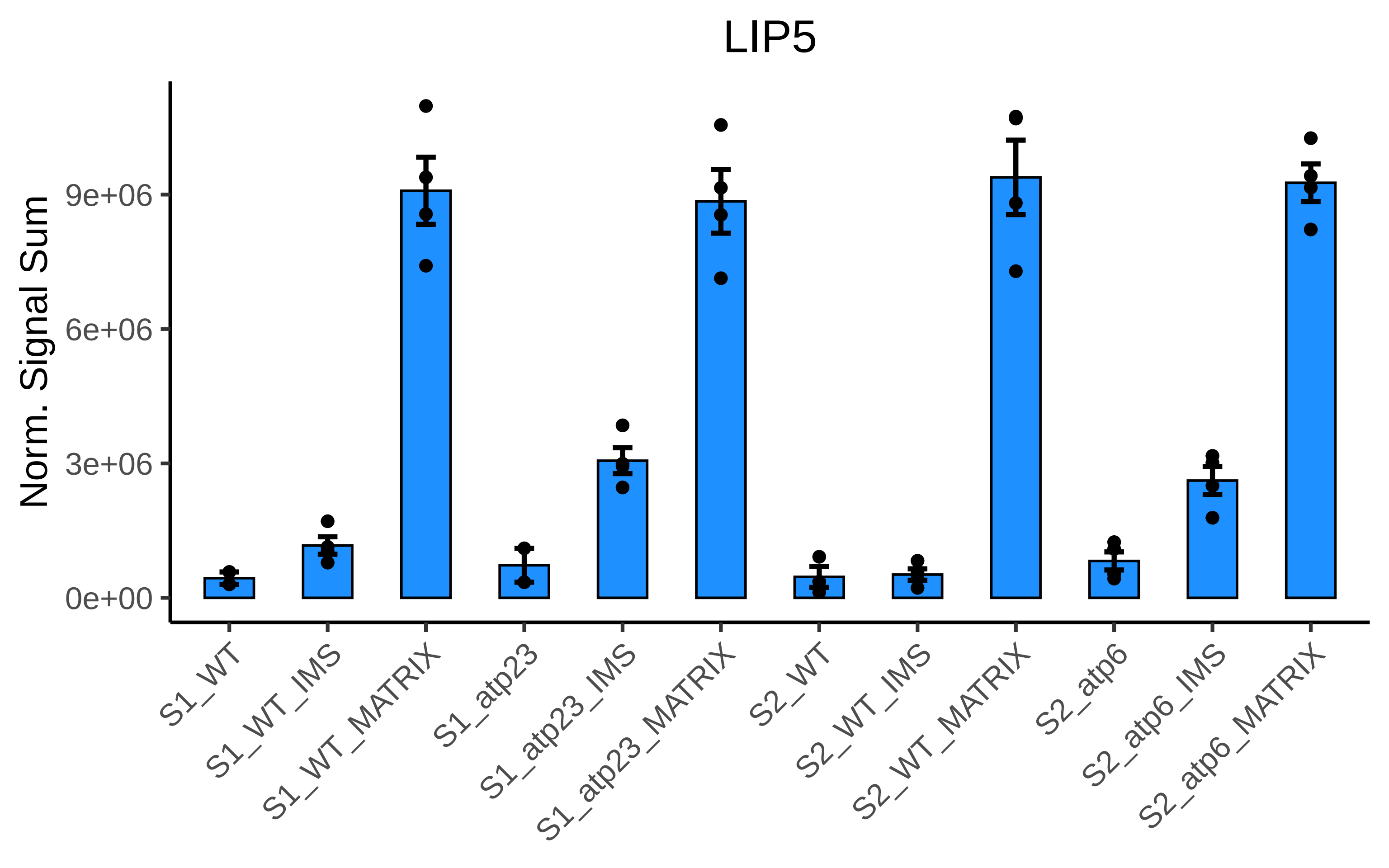
LIP5
Norm. Signal Sum
9e+06
6e+06
3e+06
0e+00
S1_WT
S1_WT_IMS
S1_WT_MATRIX
S1_atp23
S1_atp23_IMS
S1_atp23_MATRIX
S2_WT
S2_WT_IMS
S2_WT_MATRIX
S2_atp6
S2_atp6_IMS
S2_atp6_MATRIX

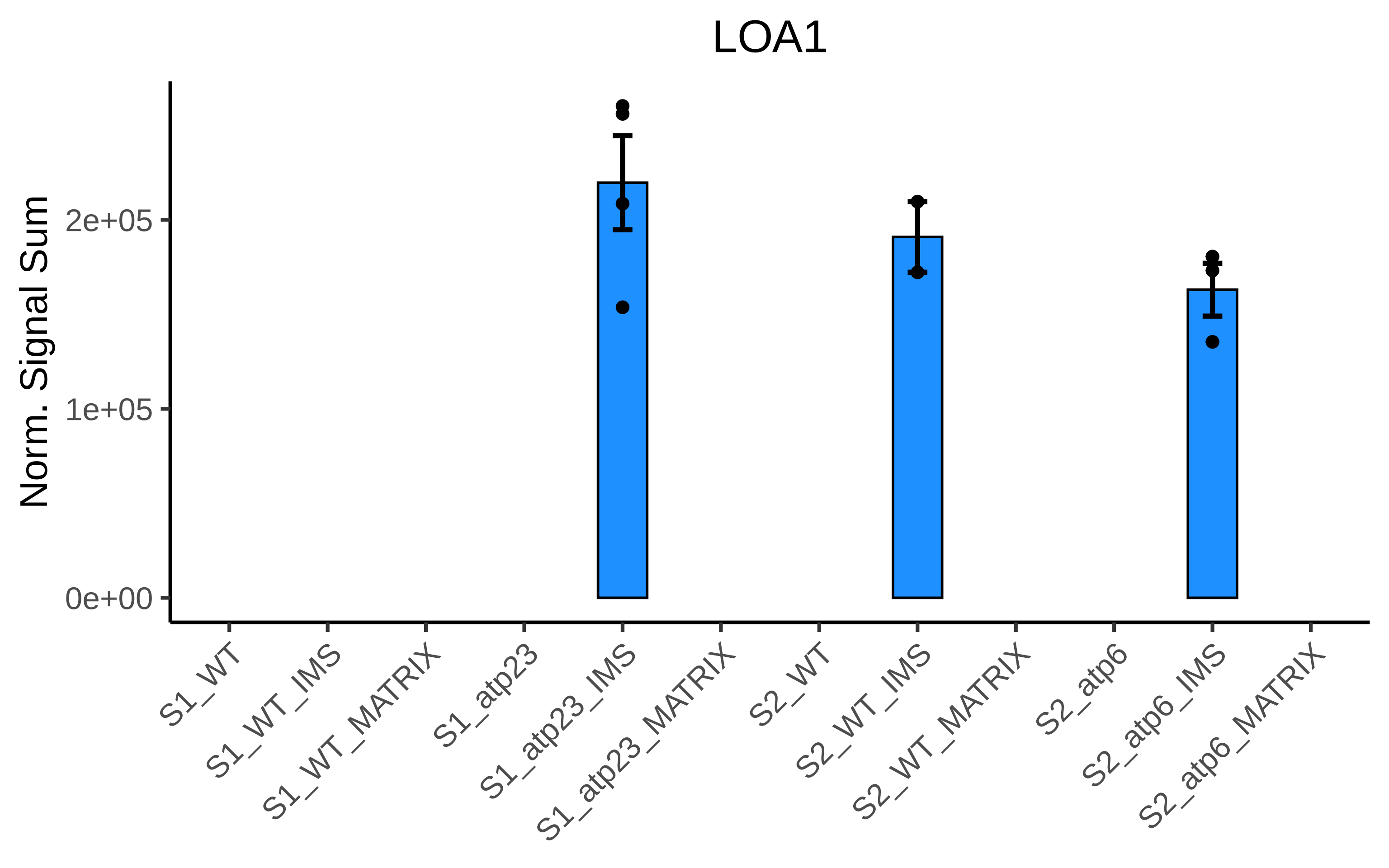

LOA1
Norm. Signal Sum
2e+05
1e+05
0e+00
S1_WT
S1_WT_IMS
S1_WT_MATRIX
S1_atp23
S1_atp23_IMS
S1_atp23_MATRIX
S2_WT
S2_WT_IMS
S2_WT_MATRIX
S2_atp6
S2_atp6_IMS
S2_atp6_MATRIX

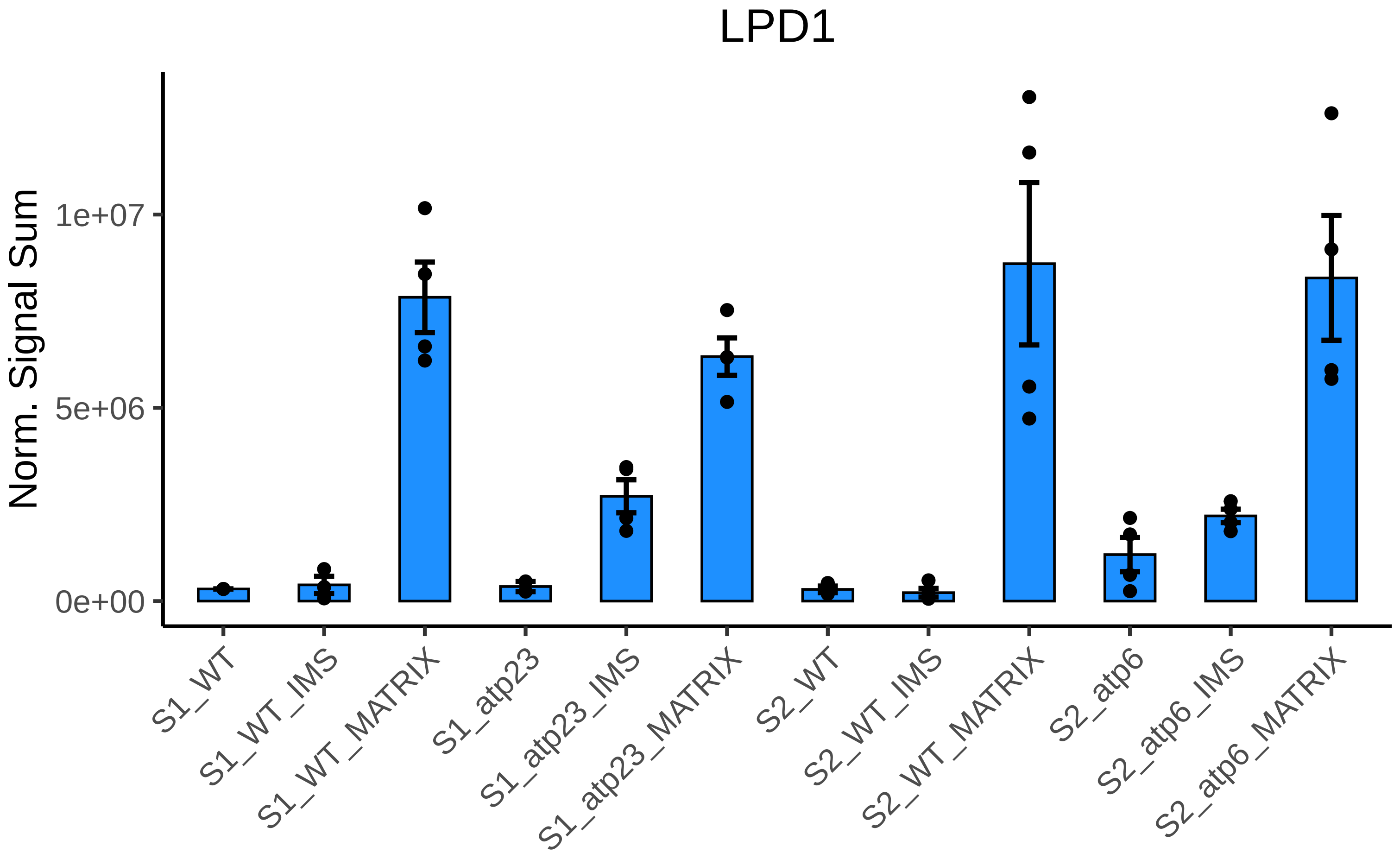
LPD1
Norm. Signal Sum
1e+07
5e+06
0e+00
S1_WT
S1_WT_IMS
S1_WT_MATRIX
S1_atp23
S1_atp23_IMS
S1_atp23_MATRIX
S2_WT
S2_WT_IMS
S2_WT_MATRIX
S2_atp6
S2_atp6_IMS
S2_atp6_MATRIX

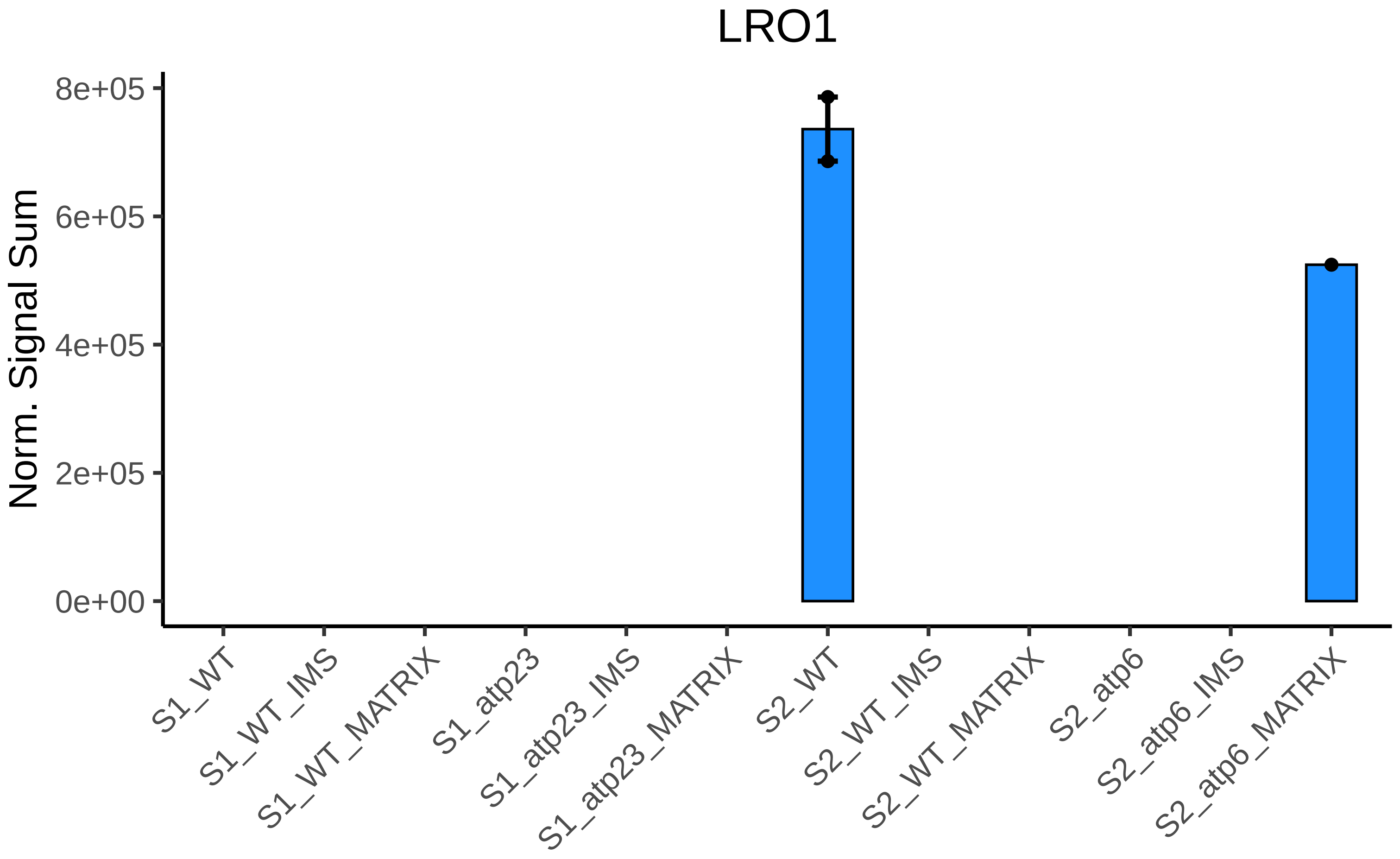

LRO1
Norm. Signal Sum
8e+05
6e+05
4e+05
2e+05
0e+00
S1_WT
S1_WT_IMS
S1_WT_MATRIX
S1_atp23
S1_atp23_IMS
S1_atp23_MATRIX
S2_WT
S2_WT_IMS
S2_WT_MATRIX
S2_atp6
S2_atp6_IMS
S2_atp6_MATRIX

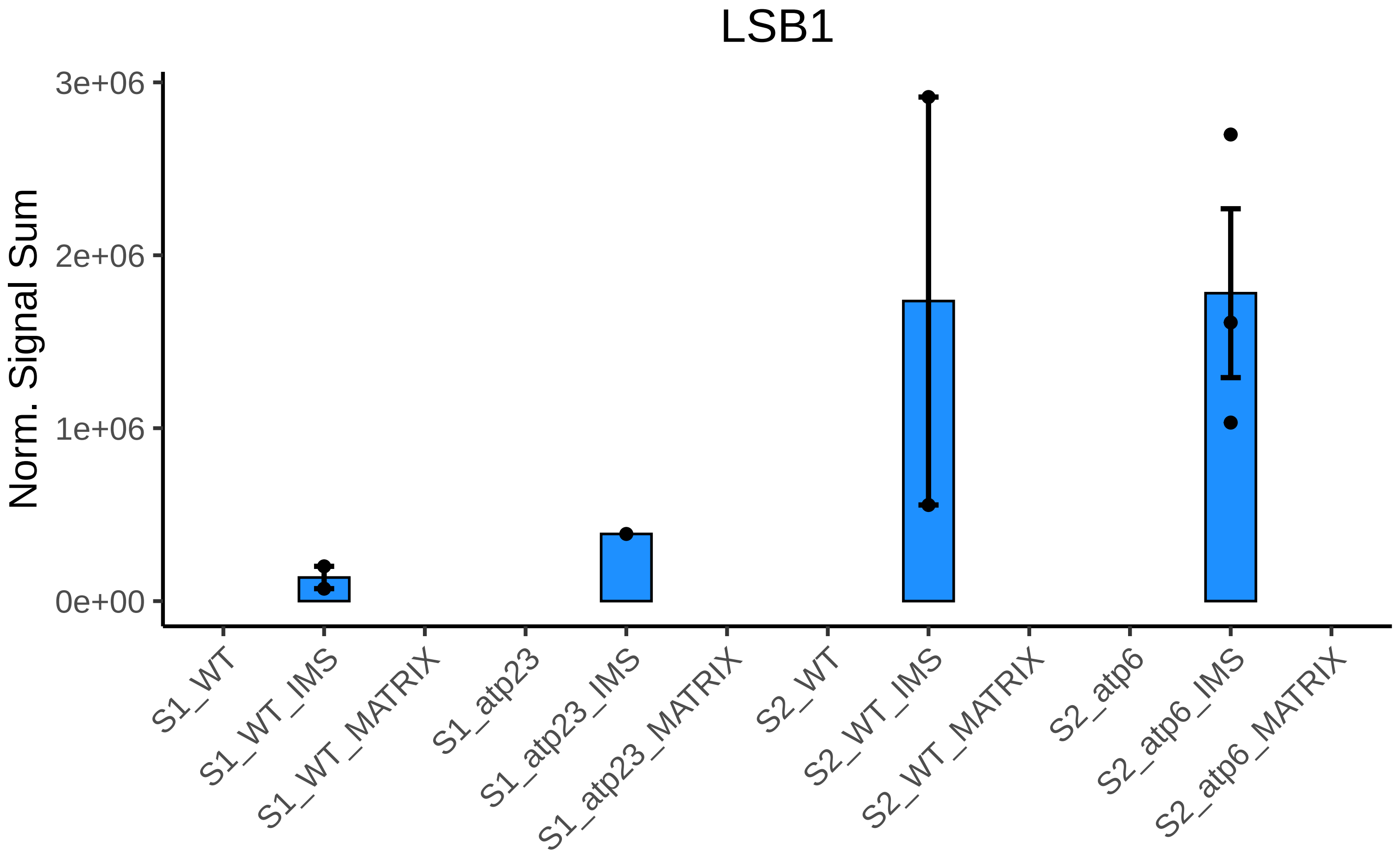

LSB1
Norm. Signal Sum
3e+06
2e+06
1e+06
0e+00
S1_WT
S1_WT_IMS
S1_WT_MATRIX
S1_atp23
S1_atp23_IMS
S1_atp23_MATRIX
S2_WT
S2_WT_IMS
S2_WT_MATRIX
S2_atp6
S2_atp6_IMS
S2_atp6_MATRIX

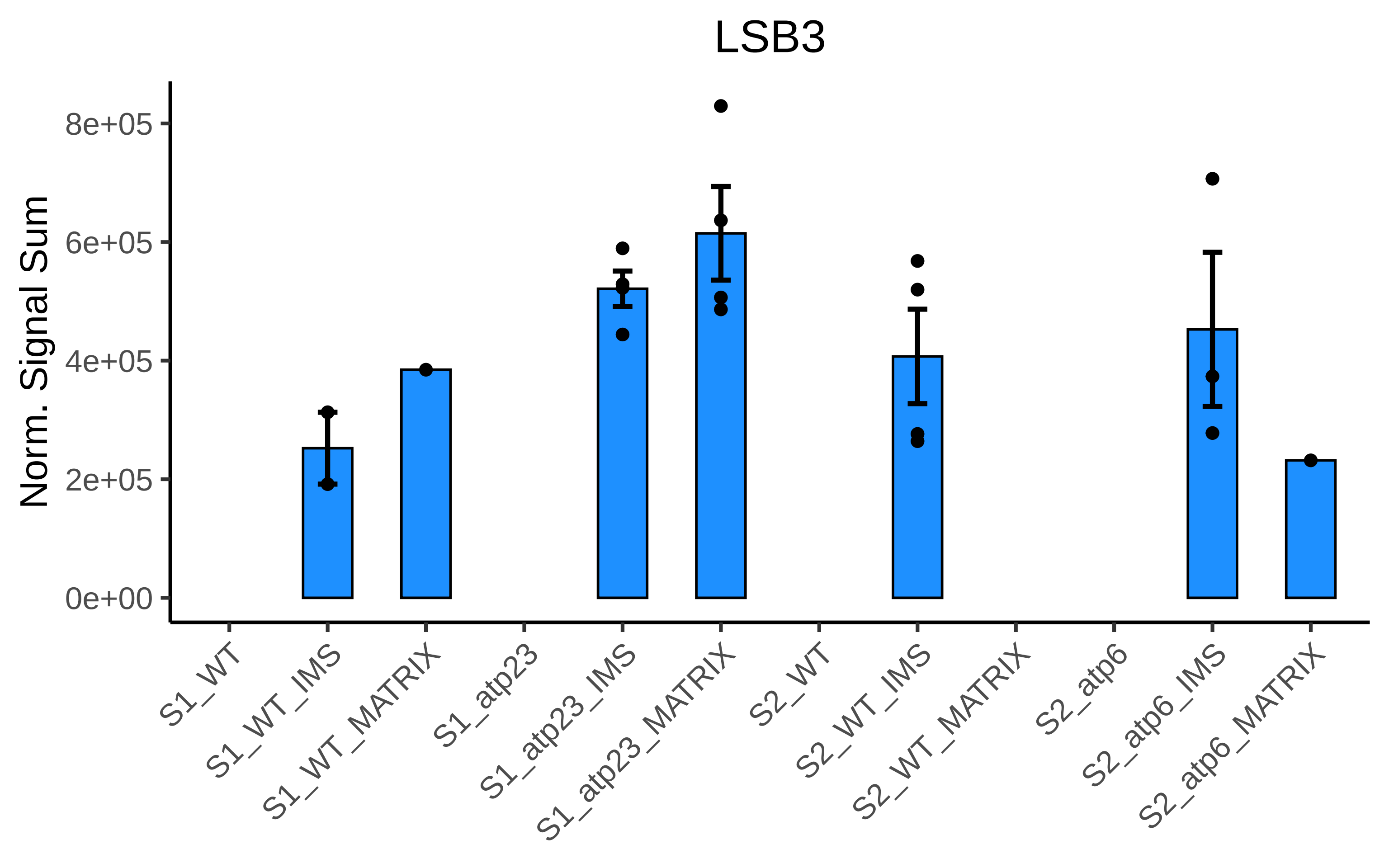
LSB3
Norm. Signal Sum
8e+05
6e+05
4e+05
2e+05
0e+00
S1_WT
S1_WT_IMS
S1_WT_MATRIX
S1_atp23
S1_atp23_IMS
S1_atp23_MATRIX
S2_WT
S2_WT_IMS
S2_WT_MATRIX
S2_atp6
S2_atp6_IMS
S2_atp6_MATRIX

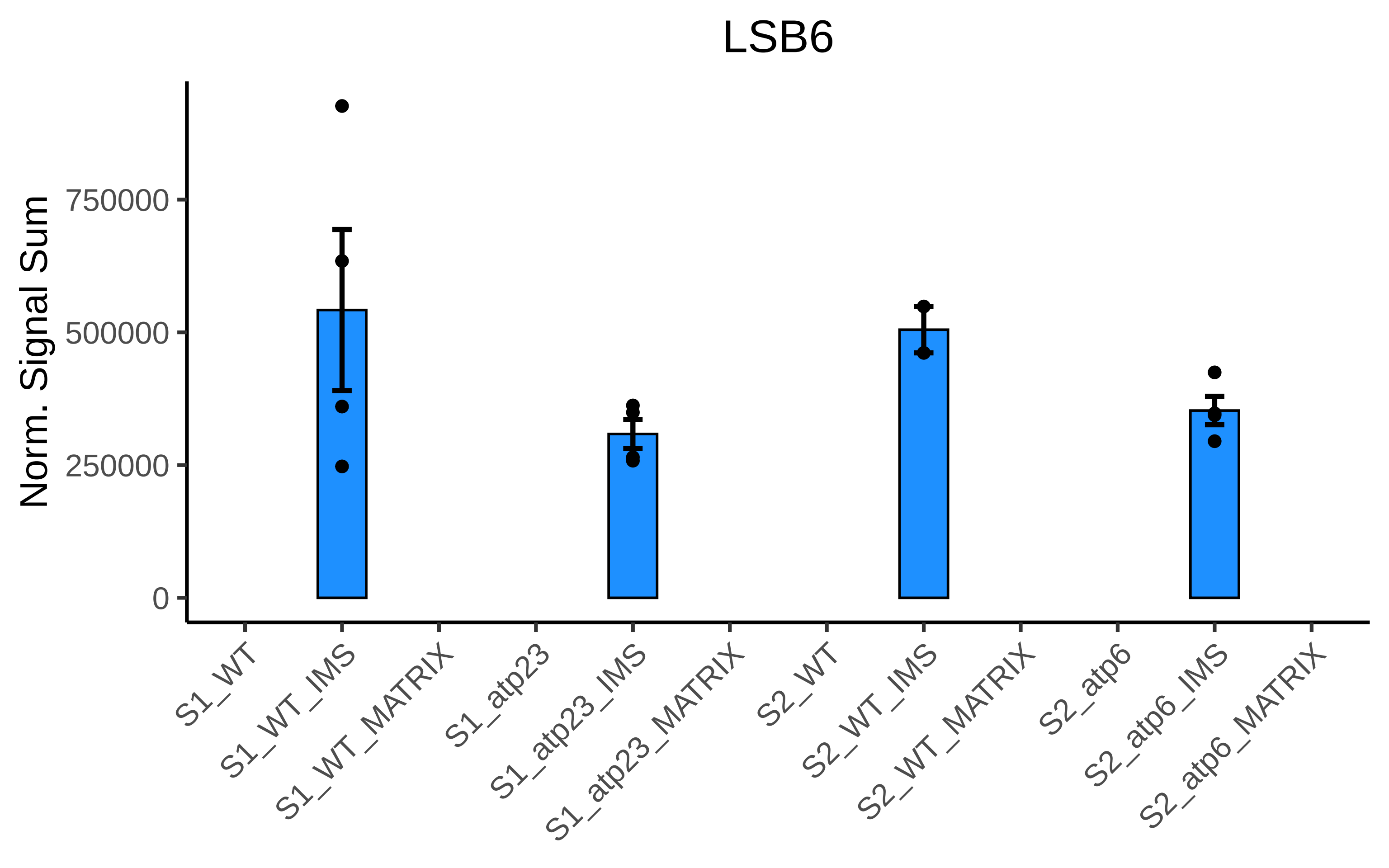
LSB6
Norm. Signal Sum
750000
500000
250000
0
S1_WT
S1_WT_IMS
S1_WT_MATRIX
S1_atp23
S1_atp23_IMS
S1_atp23_MATRIX
S2_WT
S2_WT_IMS
S2_WT_MATRIX
S2_atp6
S2_atp6_IMS
S2_atp6_MATRIX

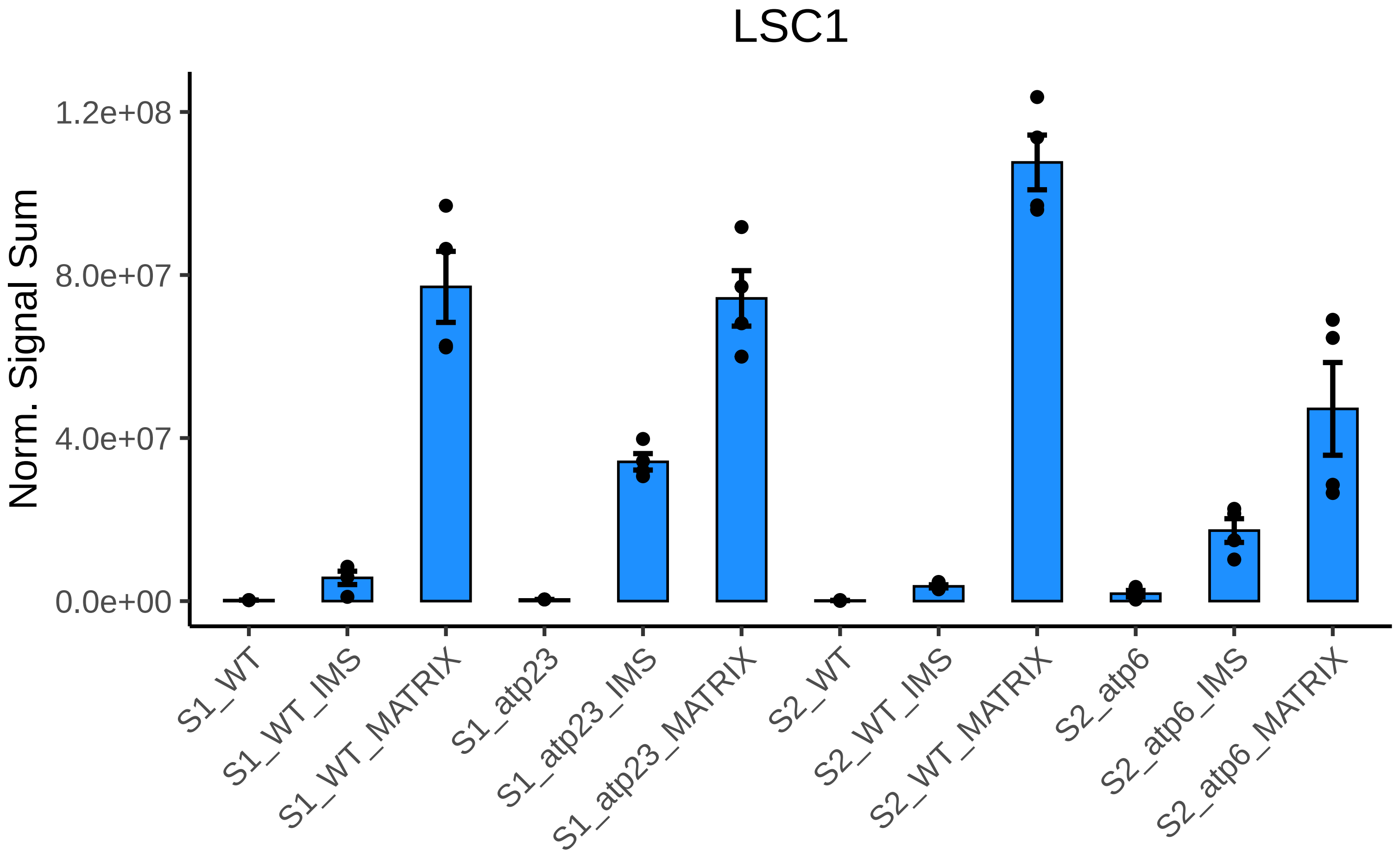

LSC1
Norm. Signal Sum
1.2e+08
8.0e+07
4.0e+07
0.0e+00
S1_WT
S1_WT_IMS
S1_WT_MATRIX
S1_atp23
S1_atp23_IMS
S1_atp23_MATRIX
S2_WT
S2_WT_IMS
S2_WT_MATRIX
S2_atp6
S2_atp6_IMS
S2_atp6_MATRIX

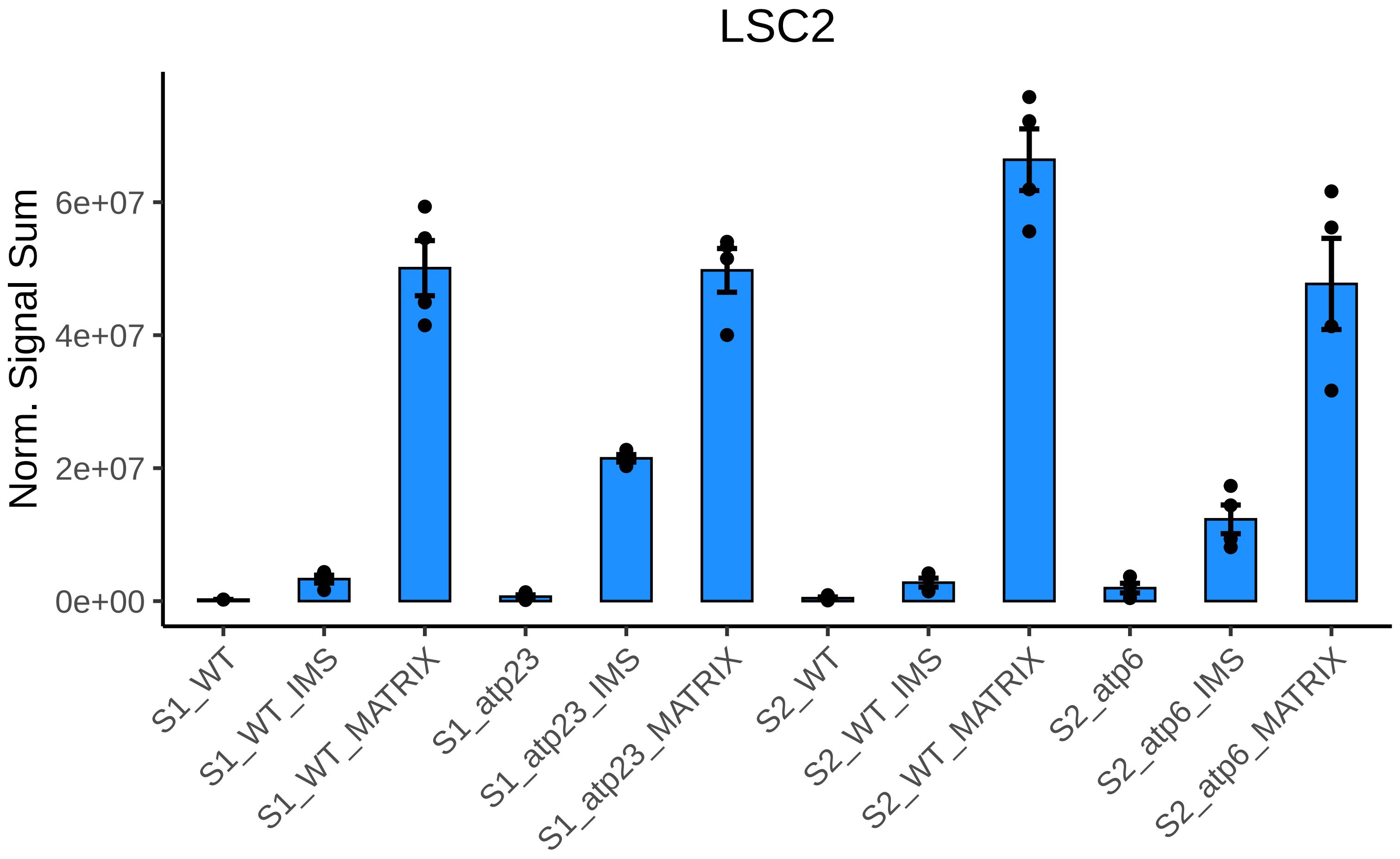
LSC2
Norm. Signal Sum
6e+07
4e+07
2e+07
0e+00
S1_WT
S1_WT_IMS
S1_WT_MATRIX
S1_atp23
S1_atp23_IMS
S1_atp23_MATRIX
S2_WT
S2_WT_IMS
S2_WT_MATRIX
S2_atp6
S2_atp6_IMS
S2_atp6_MATRIX

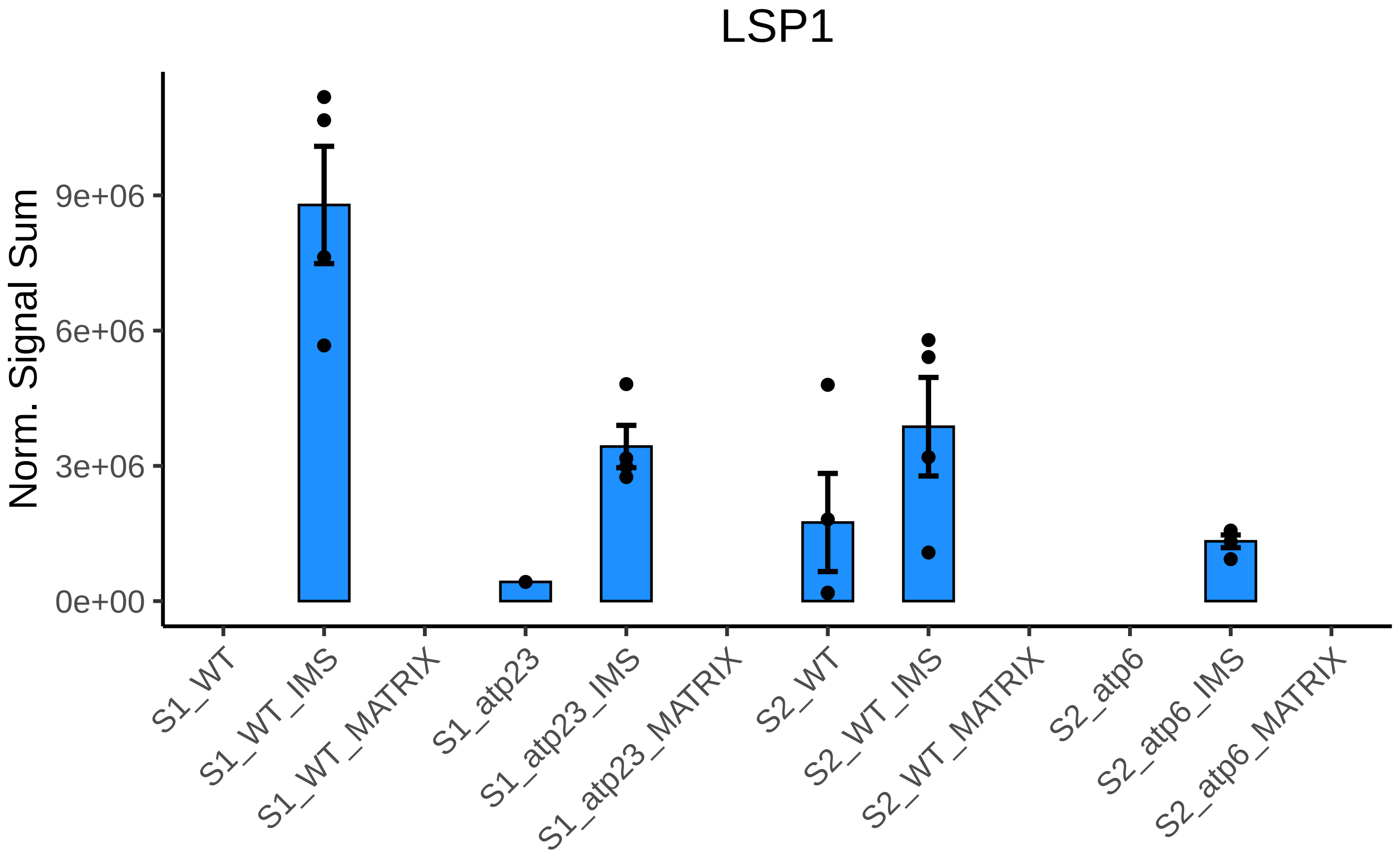

LSP1
Norm. Signal Sum
9e+06
6e+06
3e+06
0e+00
S1_WT
S1_WT_IMS
S1_WT_MATRIX
S1_atp23
S1_atp23_IMS
S1_atp23_MATRIX
S2_WT
S2_WT_IMS
S2_WT_MATRIX
S2_atp6
S2_atp6_IMS
S2_atp6_MATRIX

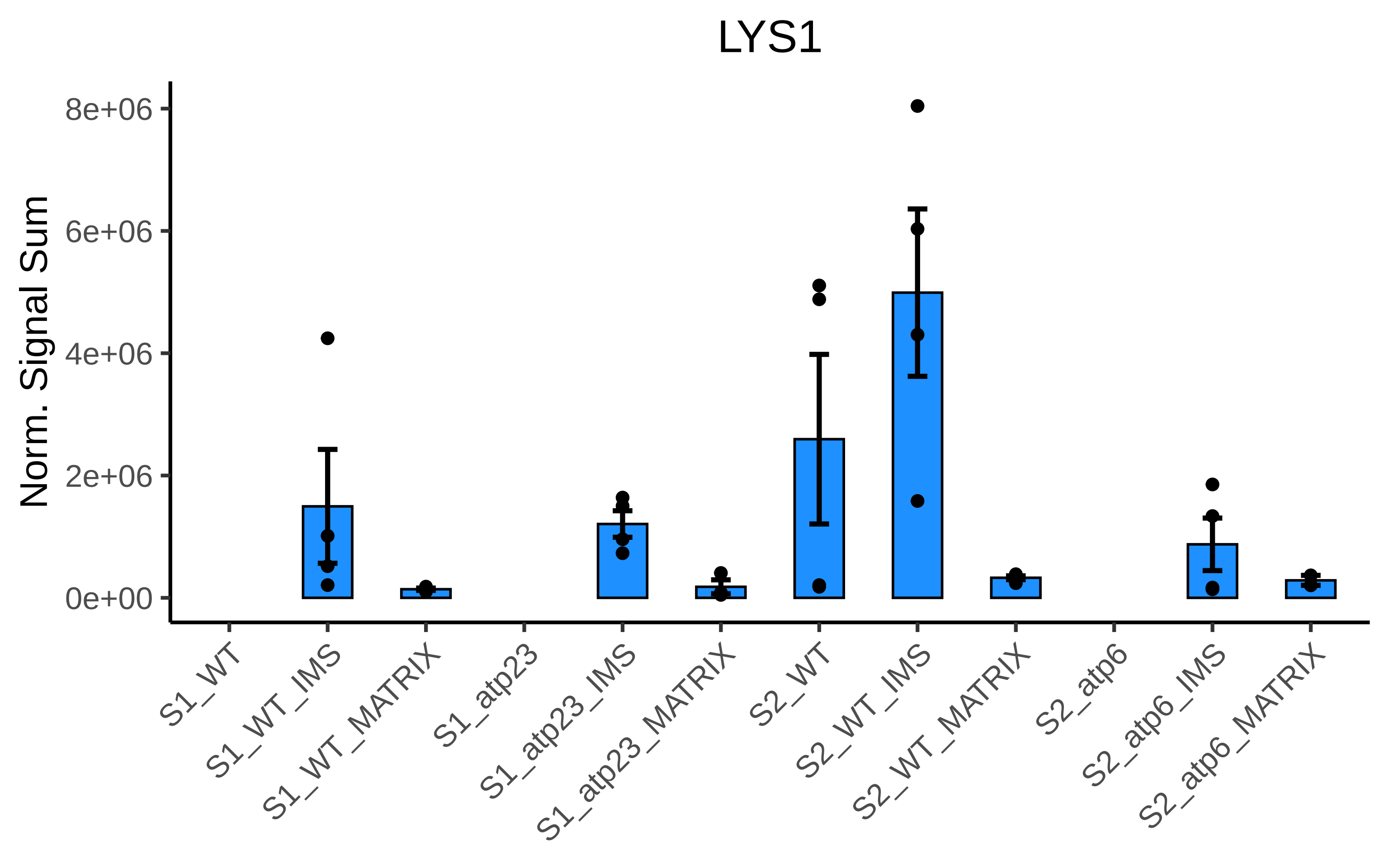

LYS1
Norm. Signal Sum
8e+06
6e+06
4e+06
2e+06
0e+00
S1_WT
S1_WT_IMS
S1_WT_MATRIX
S1_atp23
S1_atp23_IMS
S1_atp23_MATRIX
S2_WT
S2_WT_IMS
S2_WT_MATRIX
S2_atp6
S2_atp6_IMS
S2_atp6_MATRIX

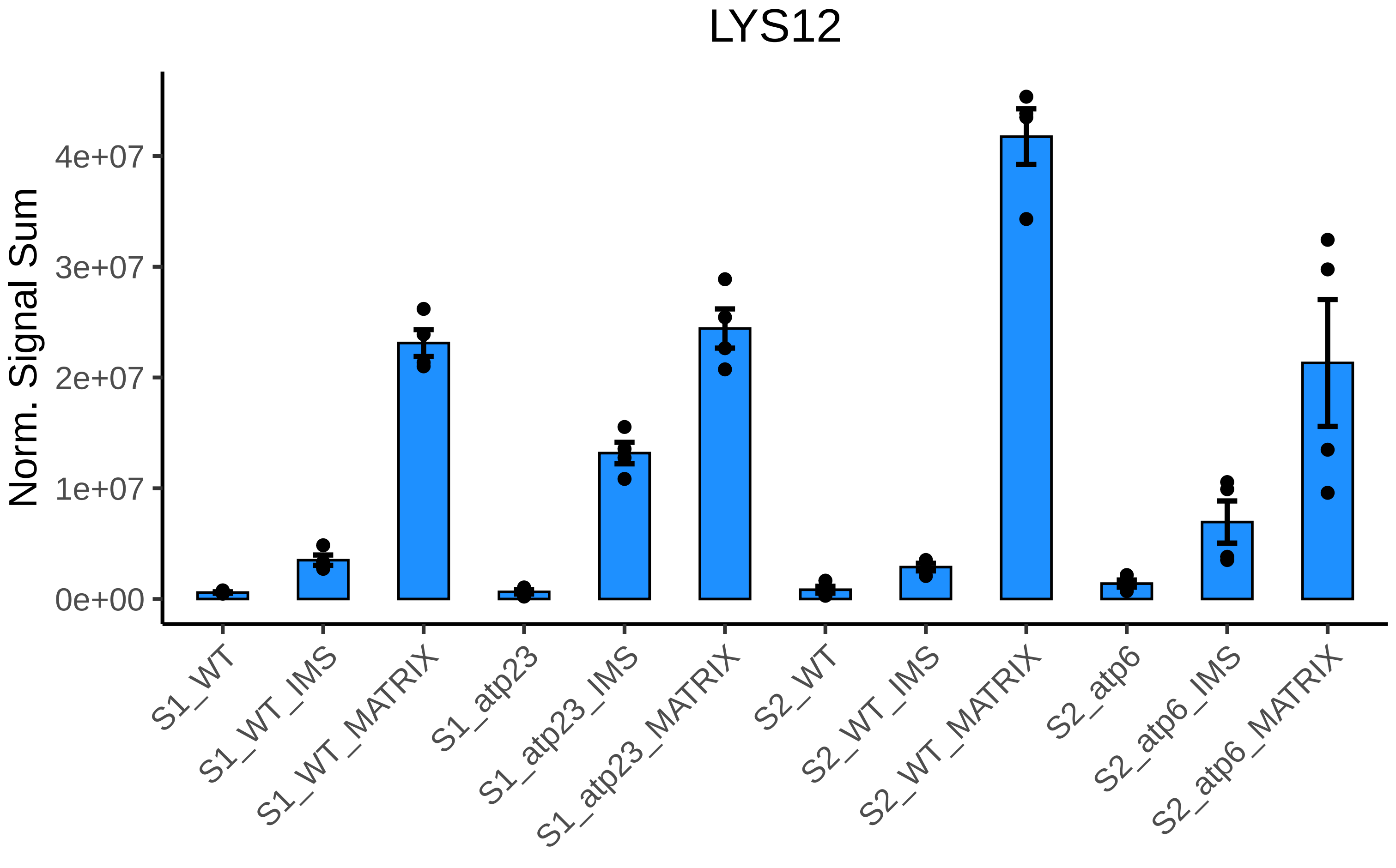
LYS12
Norm. Signal Sum
4e+07
3e+07
2e+07
1e+07
0e+00
S1_WT
S1_WT_IMS
S1_WT_MATRIX
S1_atp23
S1_atp23_IMS
S1_atp23_MATRIX
S2_WT
S2_WT_IMS
S2_WT_MATRIX
S2_atp6
S2_atp6_IMS
S2_atp6_MATRIX

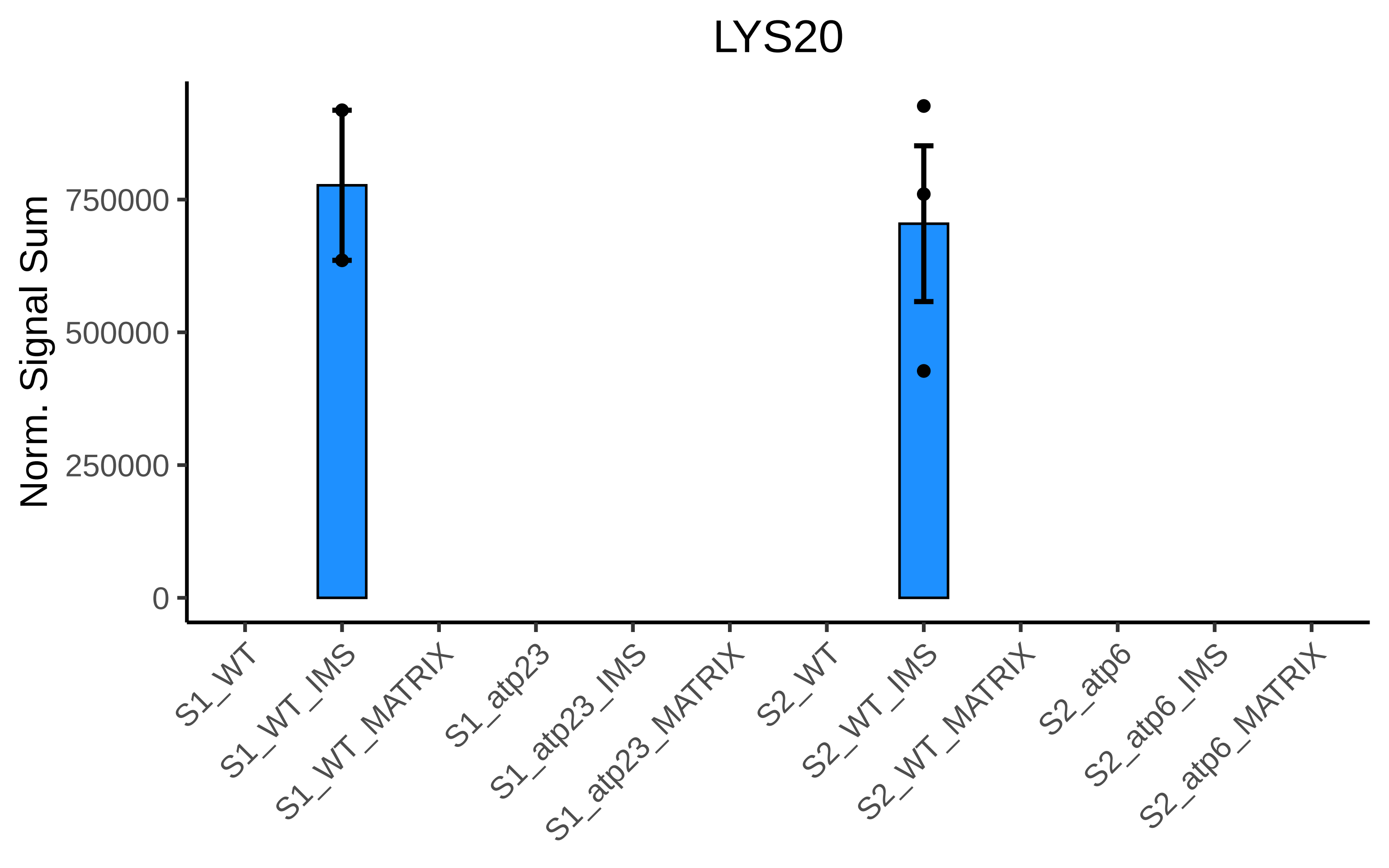

LYS20
Norm. Signal Sum
750000
500000
250000
0
S1_WT
S1_WT_IMS
S1_WT_MATRIX
S1_atp23
S1_atp23_IMS
S1_atp23_MATRIX
S2_WT
S2_WT_IMS
S2_WT_MATRIX
S2_atp6
S2_atp6_IMS
S2_atp6_MATRIX

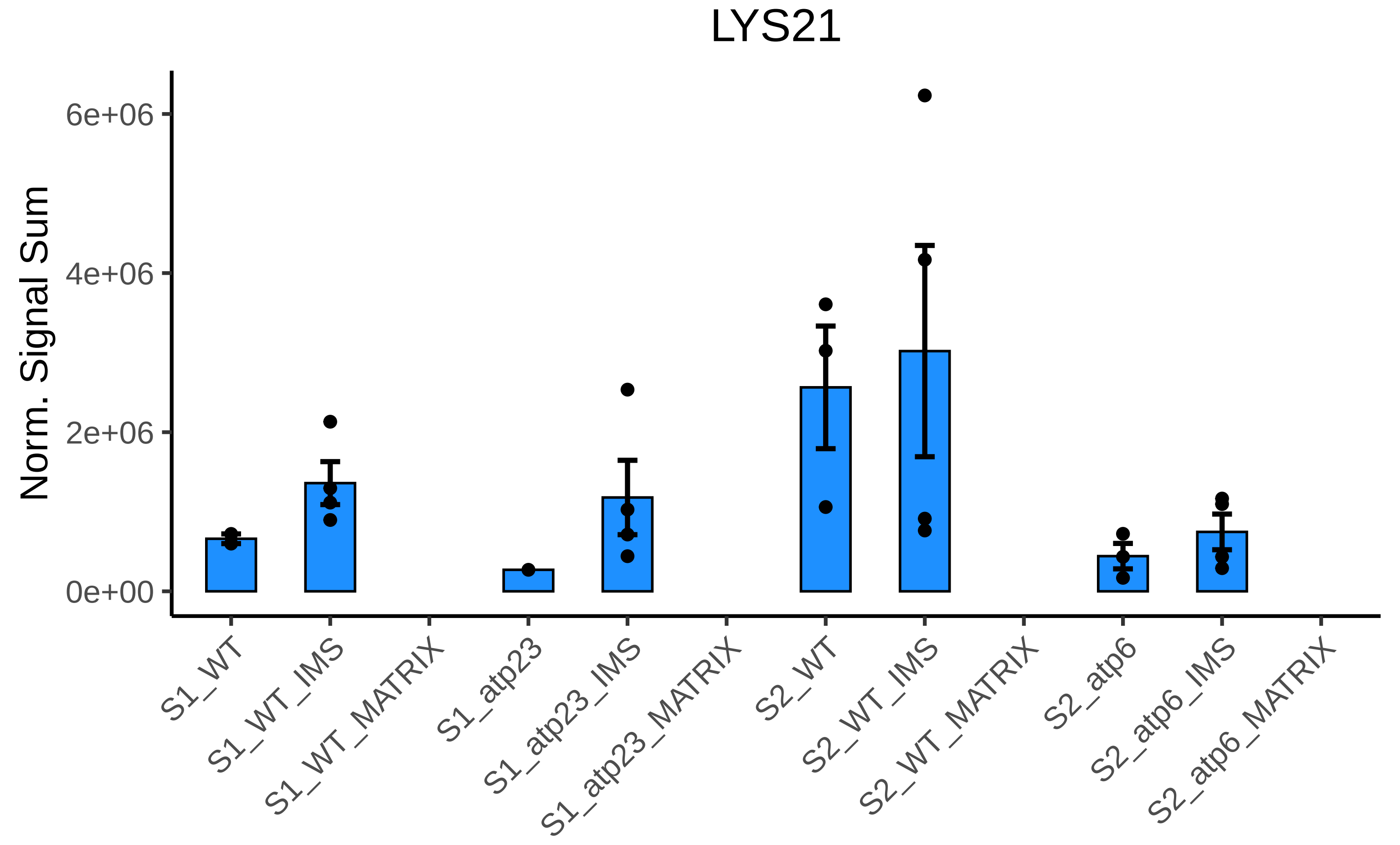

LYS21
Norm. Signal Sum
6e+06
4e+06
2e+06
0e+00
S1_WT
S1_WT_IMS
S1_WT_MATRIX
S1_atp23
S1_atp23_IMS
S1_atp23_MATRIX
S2_WT
S2_WT_IMS
S2_WT_MATRIX
S2_atp6
S2_atp6_IMS
S2_atp6_MATRIX

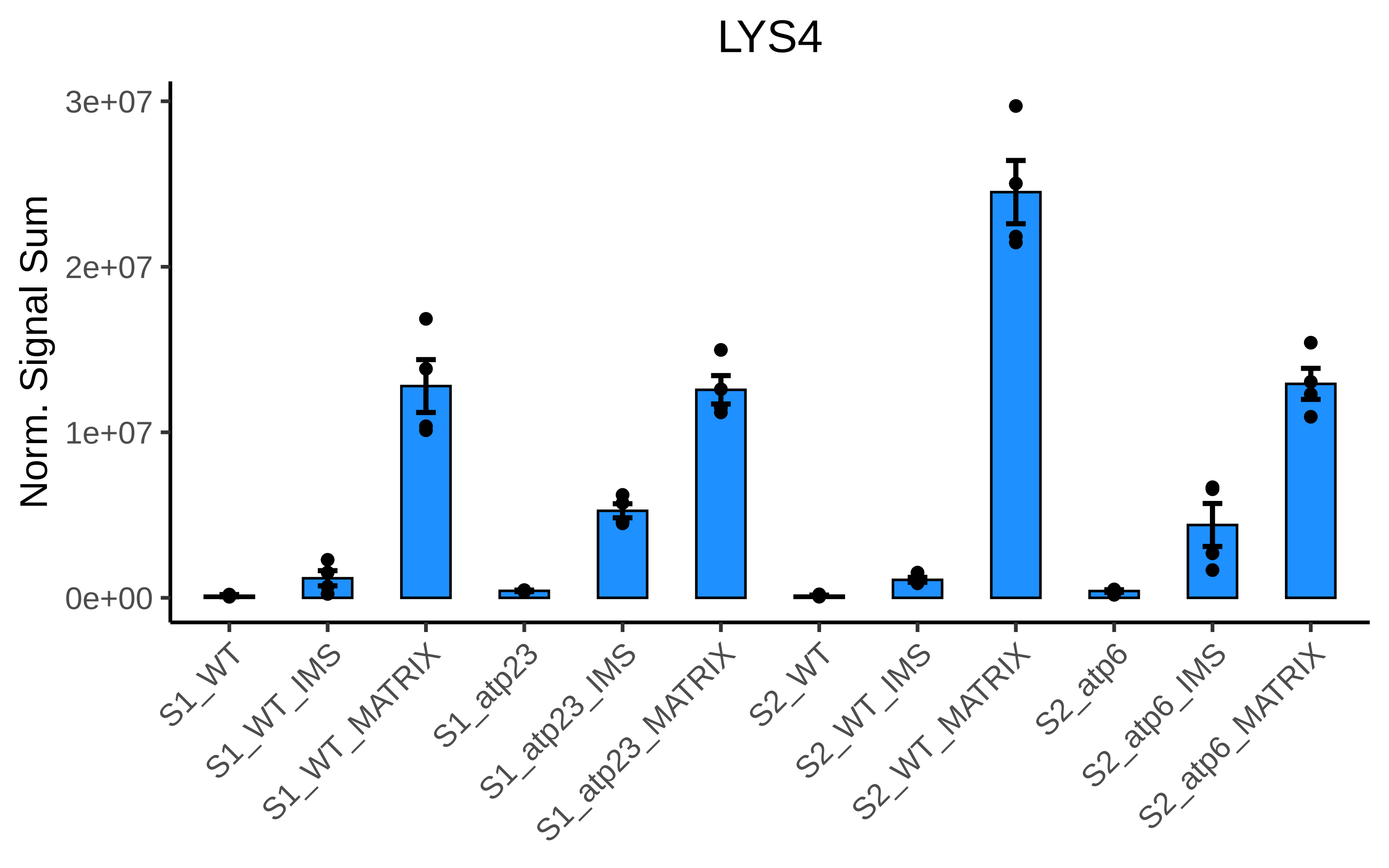

LYS4
Norm. Signal Sum
3e+07
2e+07
1e+07
0e+00
S1_WT
S1_WT_IMS
S1_WT_MATRIX
S1_atp23
S1_atp23_IMS
S1_atp23_MATRIX
S2_WT
S2_WT_IMS
S2_WT_MATRIX
S2_atp6
S2_atp6_IMS
S2_atp6_MATRIX

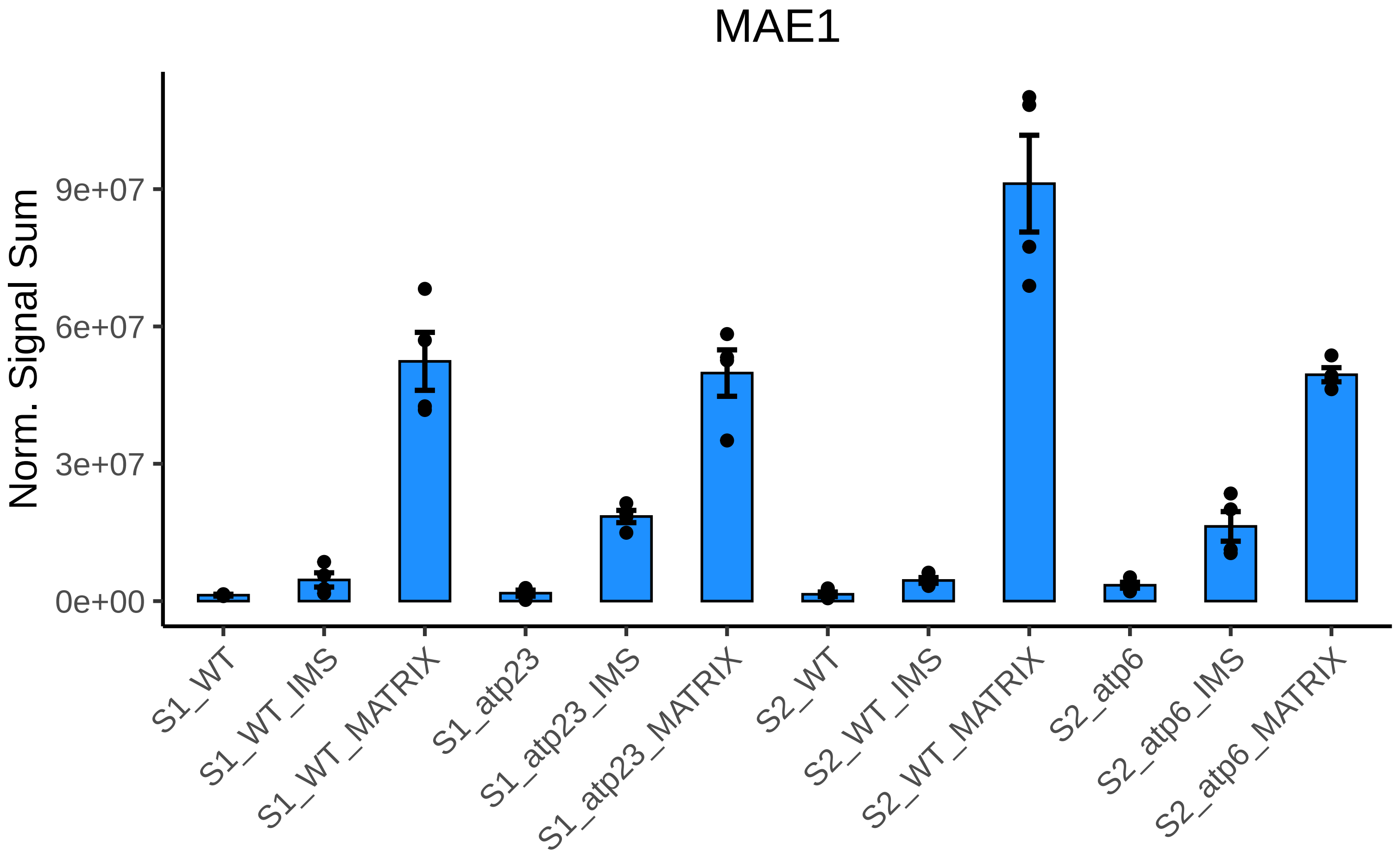

MAE1
Norm. Signal Sum
9e+07
6e+07
3e+07
0e+00
S1_WT
S1_WT_IMS
S1_WT_MATRIX
S1_atp23
S1_atp23_IMS
S1_atp23_MATRIX
S2_WT
S2_WT_IMS
S2_WT_MATRIX
S2_atp6
S2_atp6_IMS
S2_atp6_MATRIX

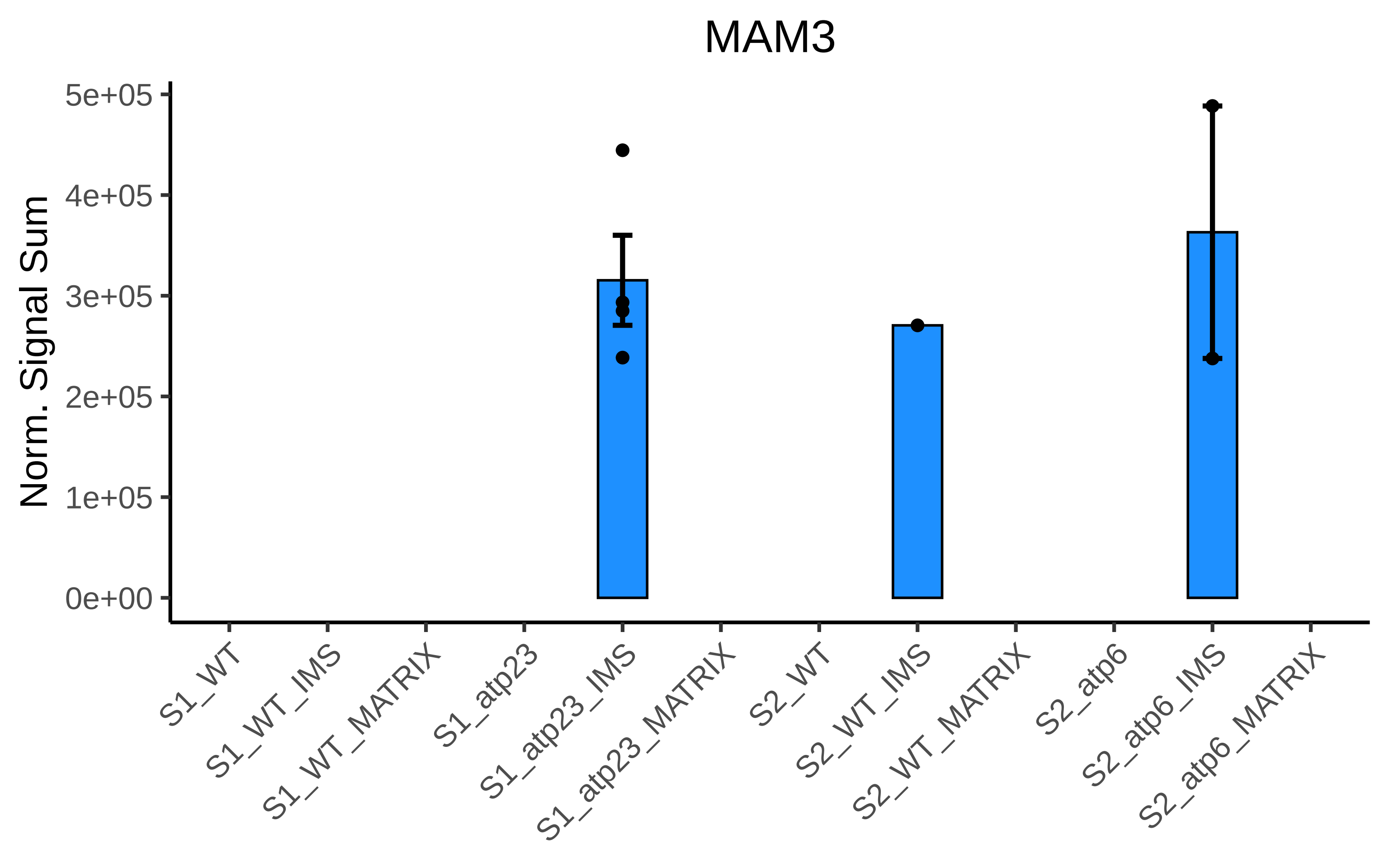
MAM3
Norm. Signal Sum
5e+05
4e+05
3e+05
2e+05
1e+05
0e+00
S1_WT
S1_WT_IMS
S1_WT_MATRIX
S1_atp23
S1_atp23_IMS
S1_atp23_MATRIX
S2_WT
S2_WT_IMS
S2_WT_MATRIX
S2_atp6
S2_atp6_IMS
S2_atp6_MATRIX

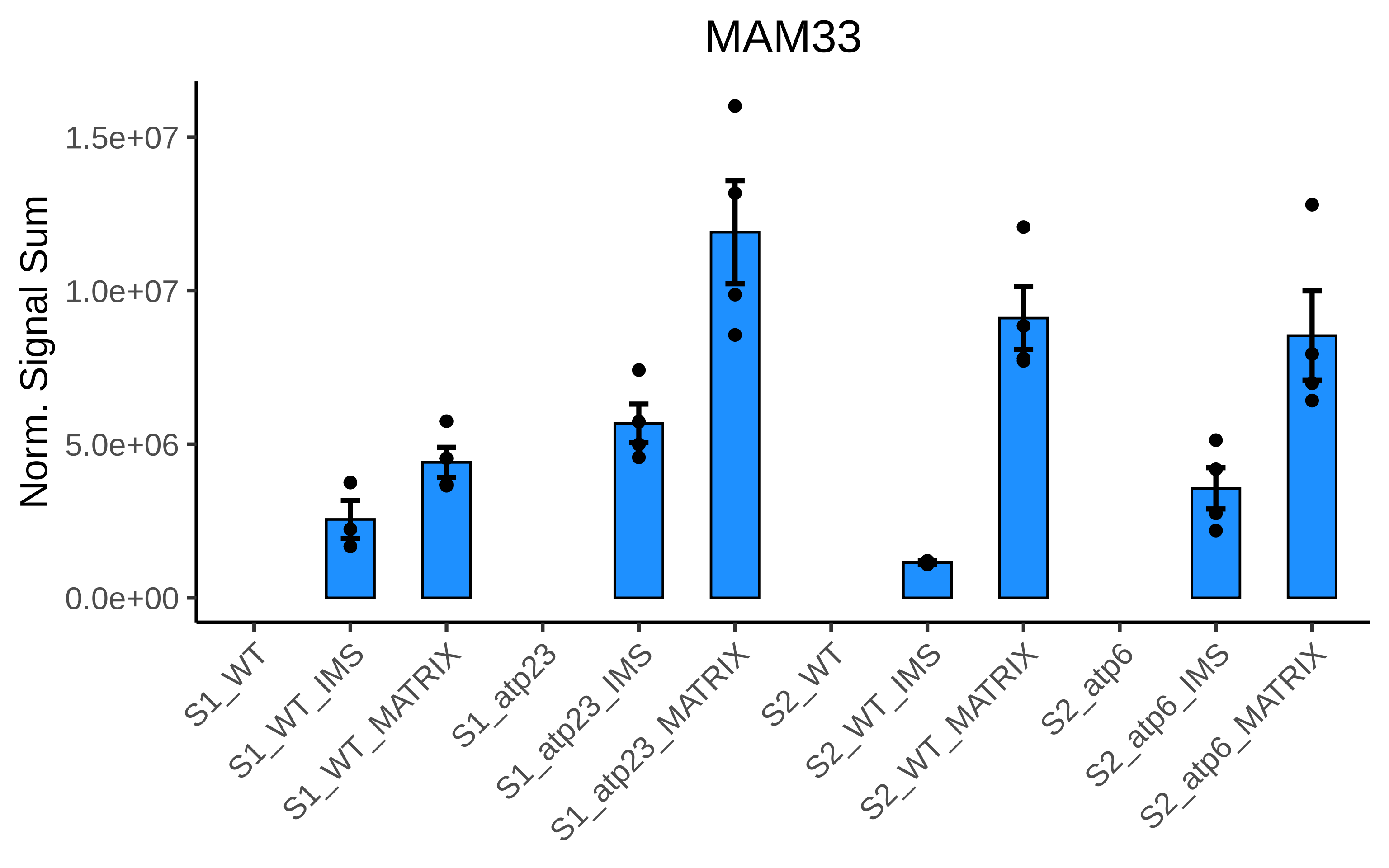

MAM33
Norm. Signal Sum
1.5e+07
1.0e+07
5.0e+06
0.0e+00
S1_WT
S1_WT_IMS
S1_WT_MATRIX
S1_atp23
S1_atp23_IMS
S1_atp23_MATRIX
S2_WT
S2_WT_IMS
S2_WT_MATRIX
S2_atp6
S2_atp6_IMS
S2_atp6_MATRIX

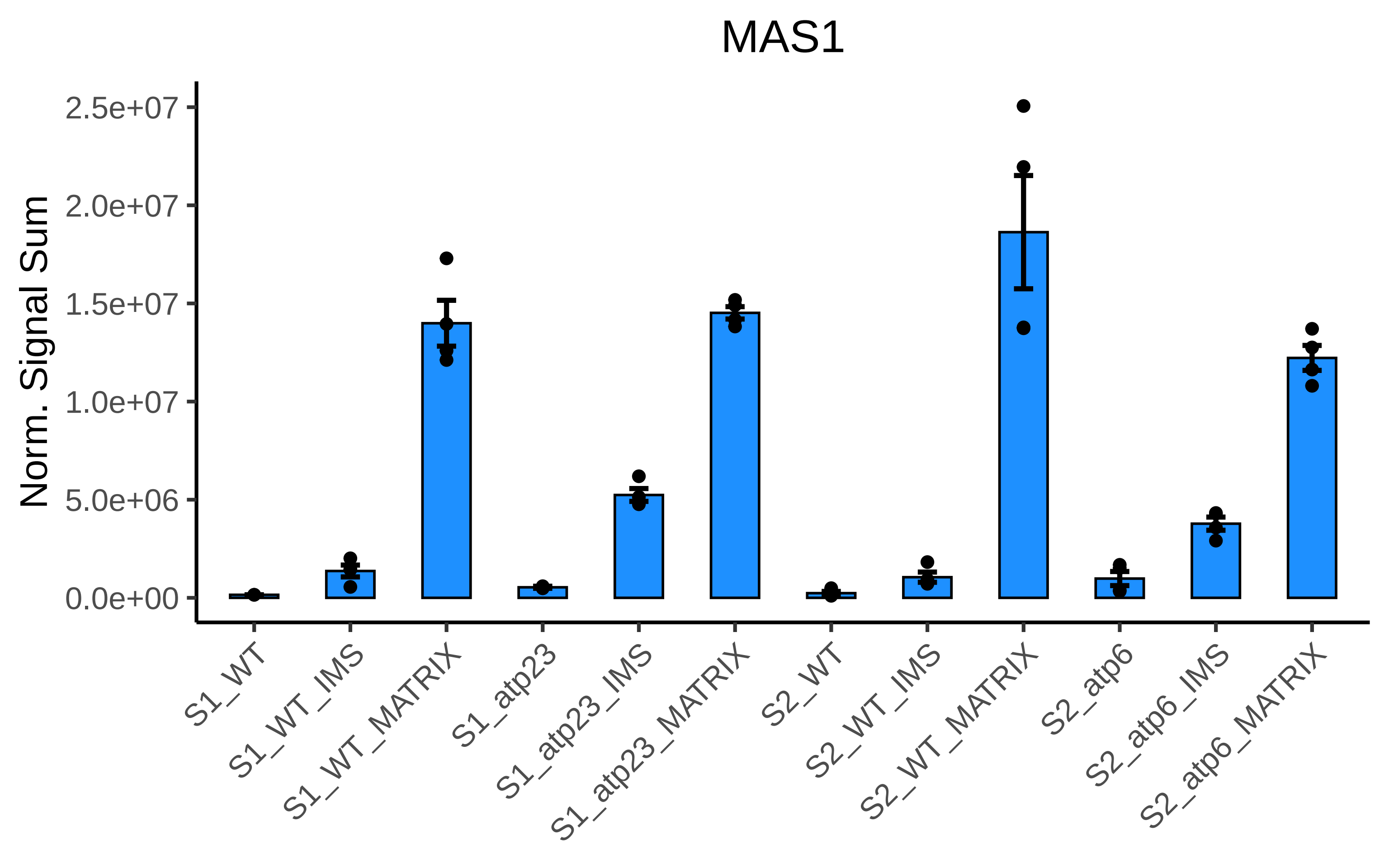

MAS1
Norm. Signal Sum
2.5e+07
2.0e+07
1.5e+07
1.0e+07
5.0e+06
0.0e+00
S1_WT
S1_WT_IMS
S1_WT_MATRIX
S1_atp23
S1_atp23_IMS
S1_atp23_MATRIX
S2_WT
S2_WT_IMS
S2_WT_MATRIX
S2_atp6
S2_atp6_IMS
S2_atp6_MATRIX

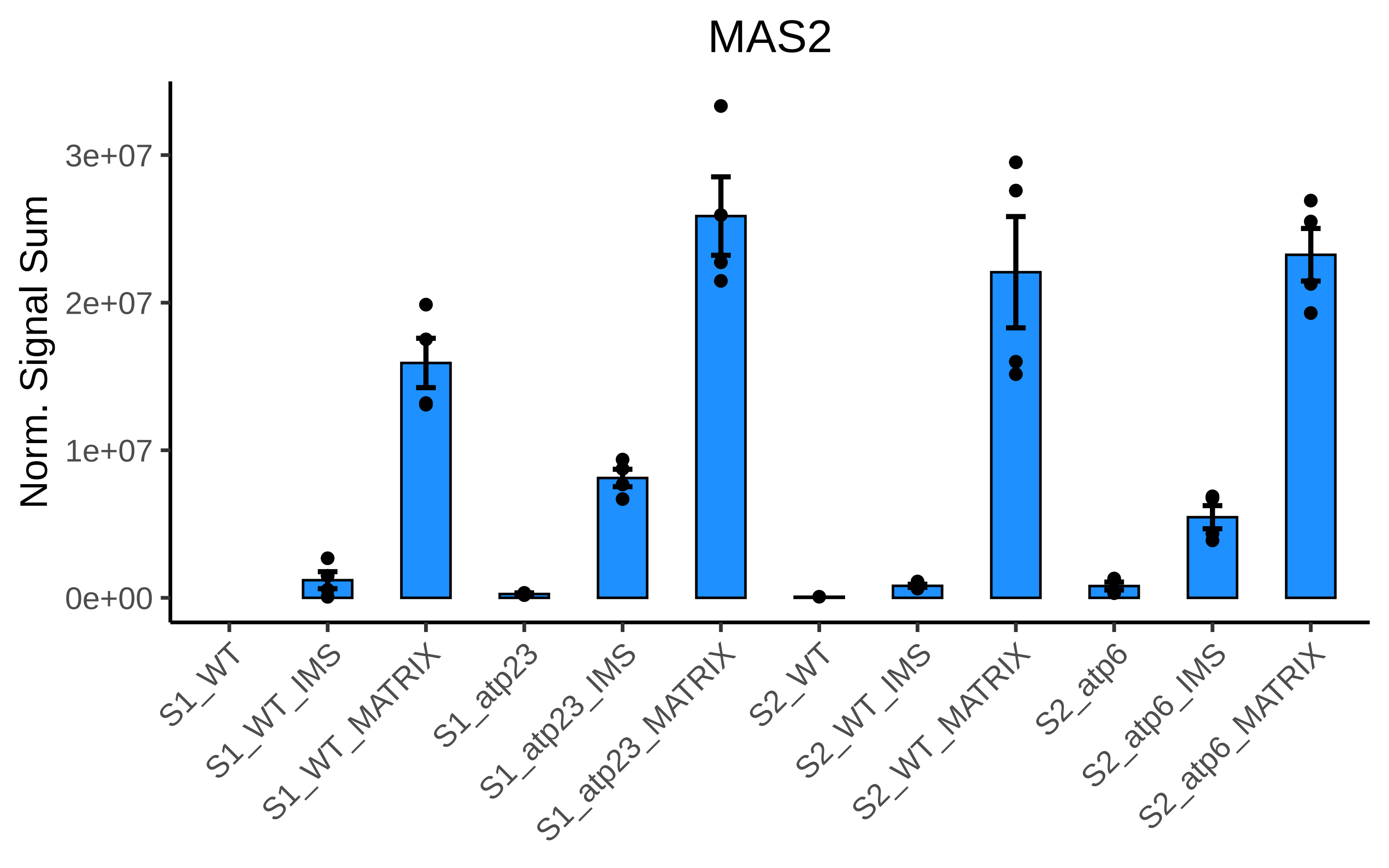
MAS2
Norm. Signal Sum
3e+07
2e+07
1e+07
0e+00
S1_WT
S1_WT_IMS
S1_WT_MATRIX
S1_atp23
S1_atp23_IMS
S1_atp23_MATRIX
S2_WT
S2_WT_IMS
S2_WT_MATRIX
S2_atp6
S2_atp6_IMS
S2_atp6_MATRIX

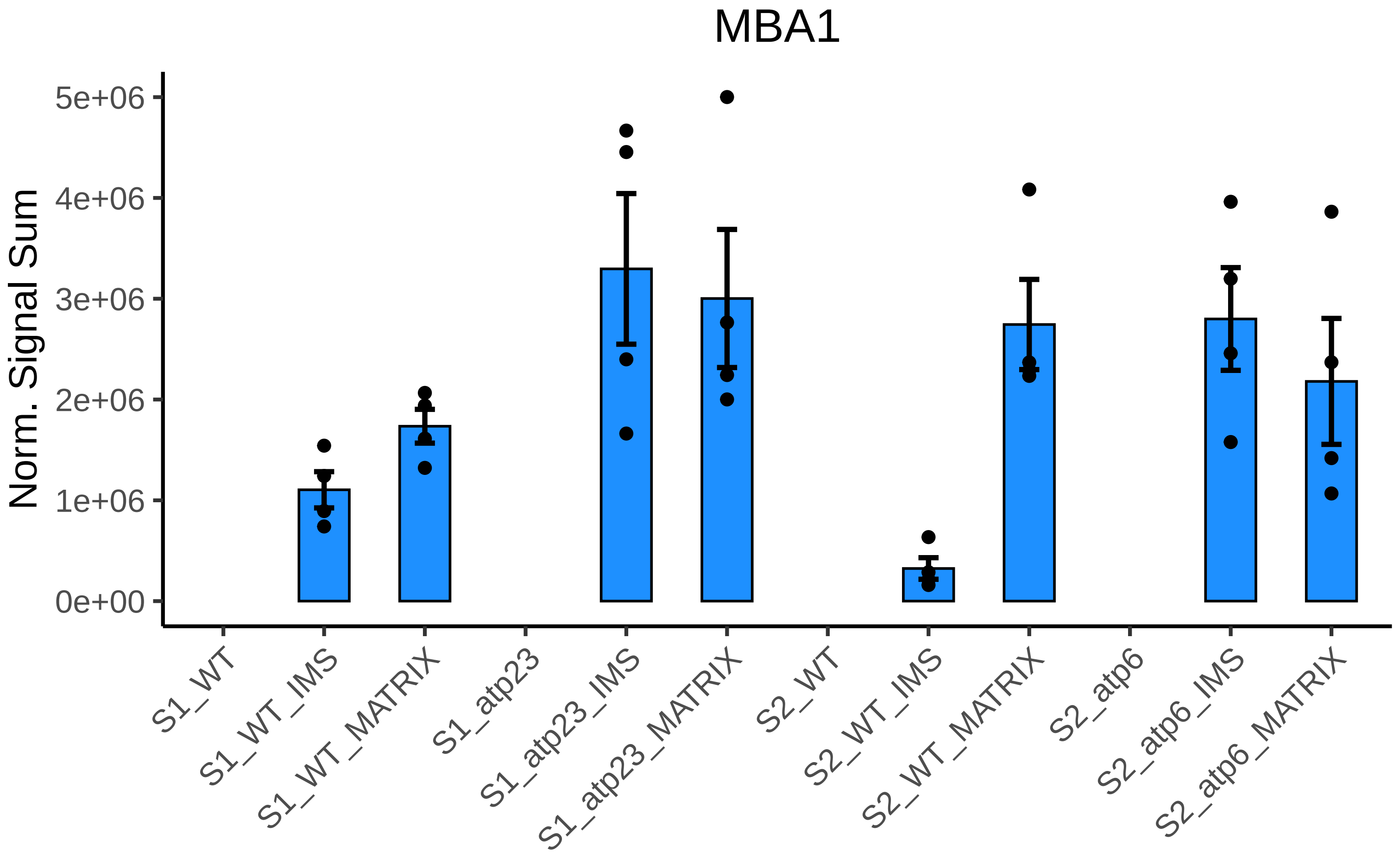

MBA1
Norm. Signal Sum
5e+06
4e+06
3e+06
2e+06
1e+06
0e+00
S1_WT
S1_WT_IMS
S1_WT_MATRIX
S1_atp23
S1_atp23_IMS
S1_atp23_MATRIX
S2_WT
S2_WT_IMS
S2_WT_MATRIX
S2_atp6
S2_atp6_IMS
S2_atp6_MATRIX

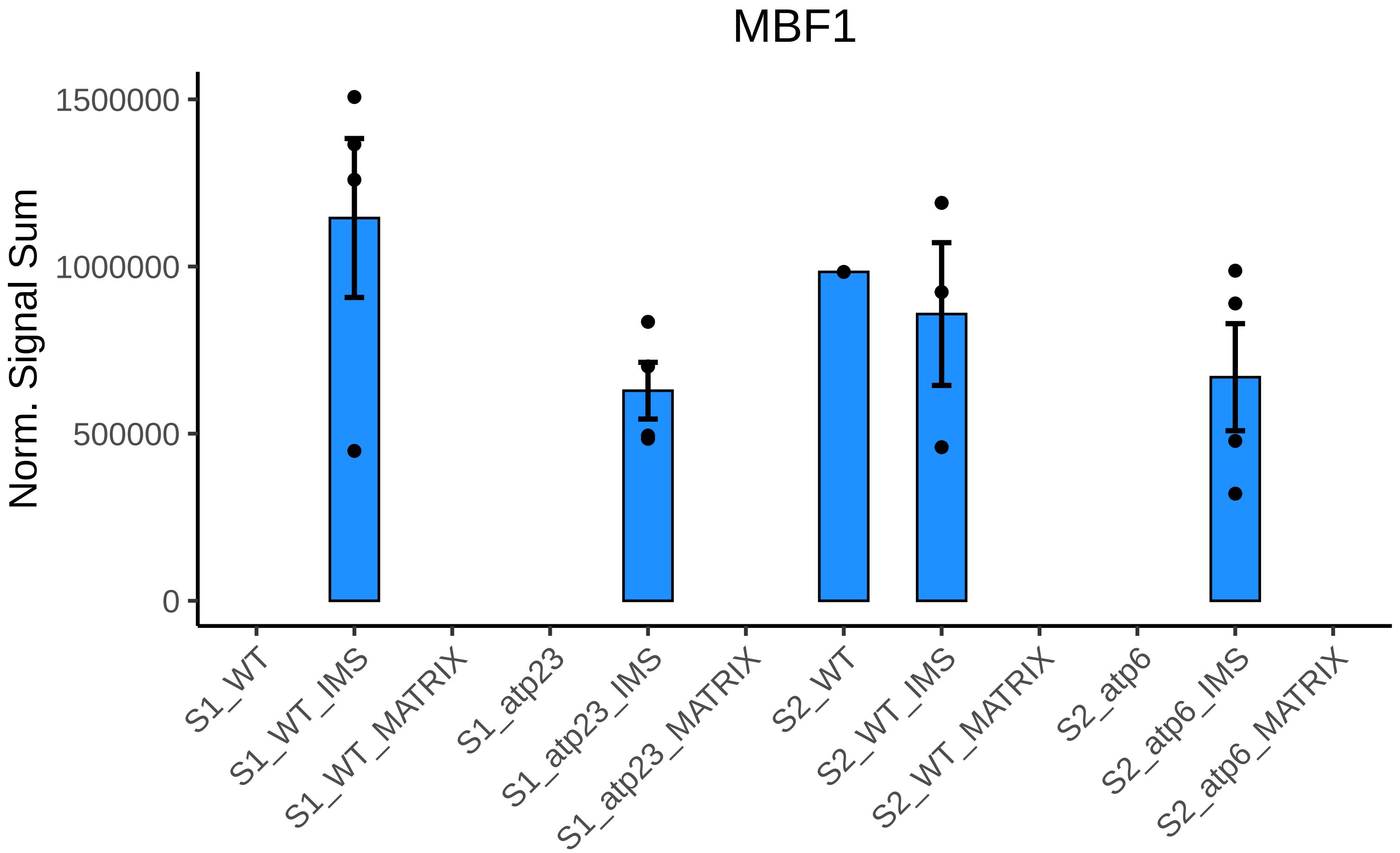

MBF1
Norm. Signal Sum
1500000
1000000
500000
0
S1_WT
S1_WT_IMS
S1_WT_MATRIX
S1_atp23
S1_atp23_IMS
S1_atp23_MATRIX
S2_WT
S2_WT_IMS
S2_WT_MATRIX
S2_atp6
S2_atp6_IMS
S2_atp6_MATRIX

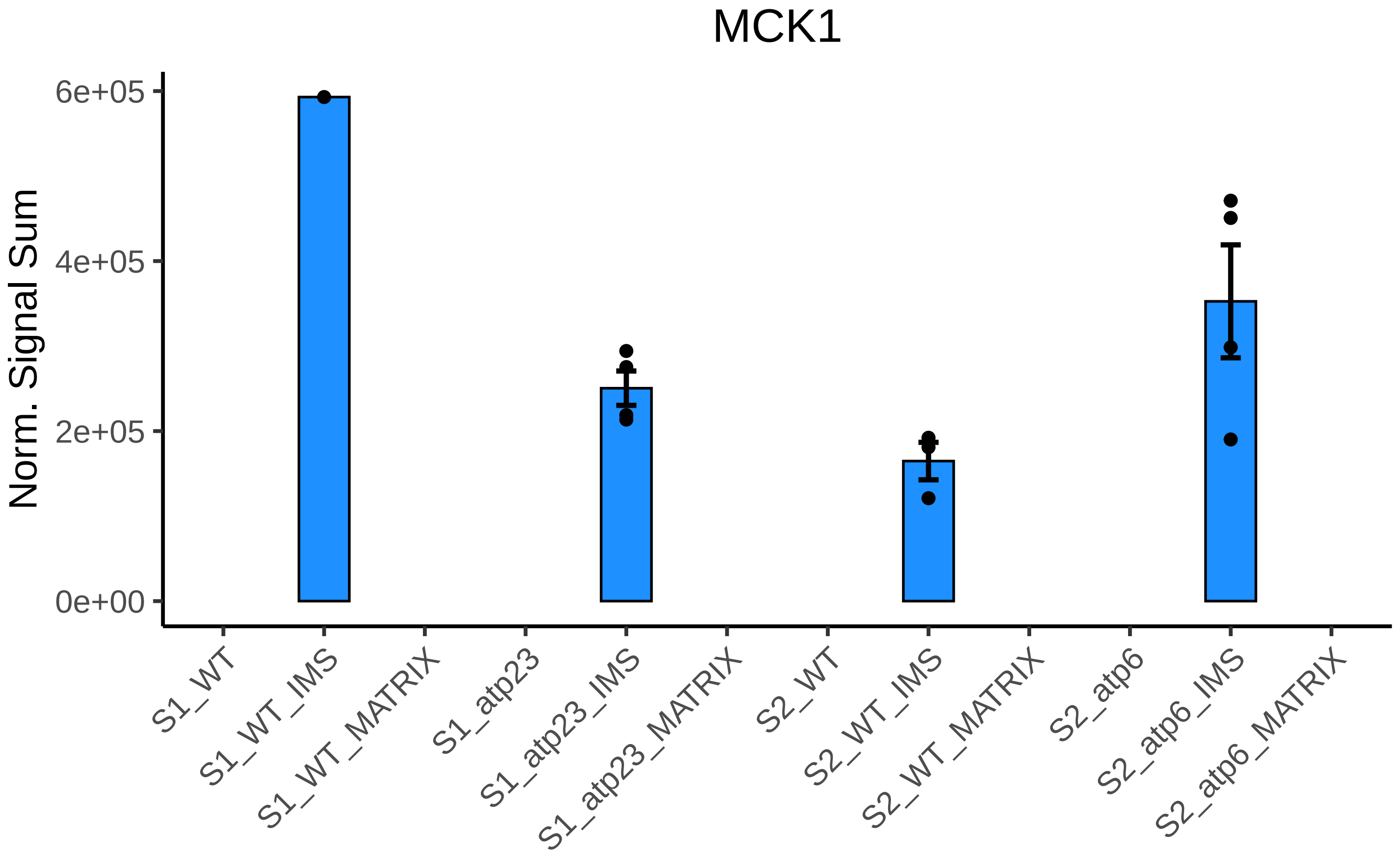

MCK1
Norm. Signal Sum
6e+05
4e+05
2e+05
0e+00
S1_WT
S1_WT_IMS
S1_WT_MATRIX
S1_atp23
S1_atp23_IMS
S1_atp23_MATRIX
S2_WT
S2_WT_IMS
S2_WT_MATRIX
S2_atp6
S2_atp6_IMS
S2_atp6_MATRIX

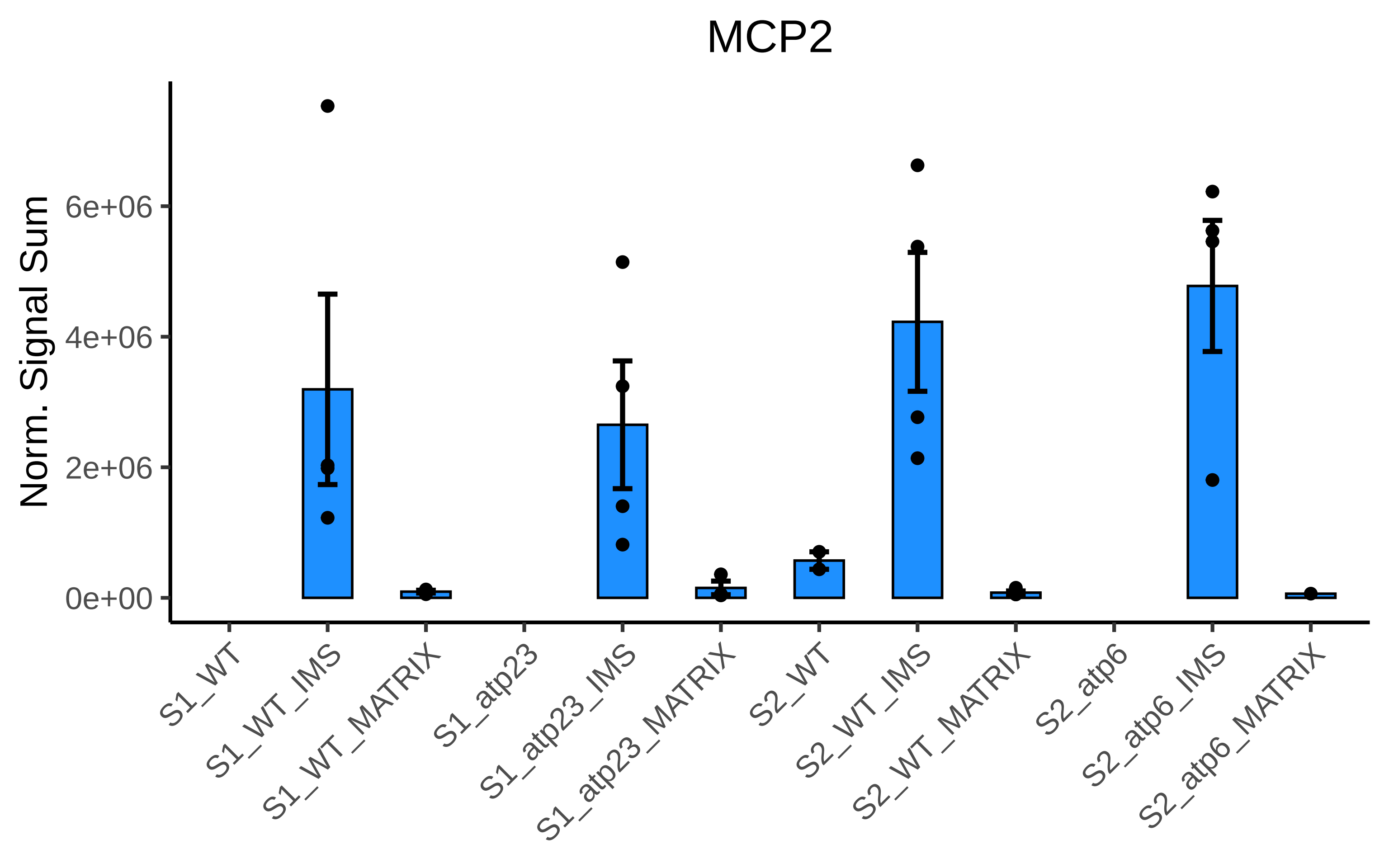
MCP2
Norm. Signal Sum
6e+06
4e+06
2e+06
0e+00
S1_WT
S1_WT_IMS
S1_WT_MATRIX
S1_atp23
S1_atp23_IMS
S1_atp23_MATRIX
S2_WT
S2_WT_IMS
S2_WT_MATRIX
S2_atp6
S2_atp6_IMS
S2_atp6_MATRIX

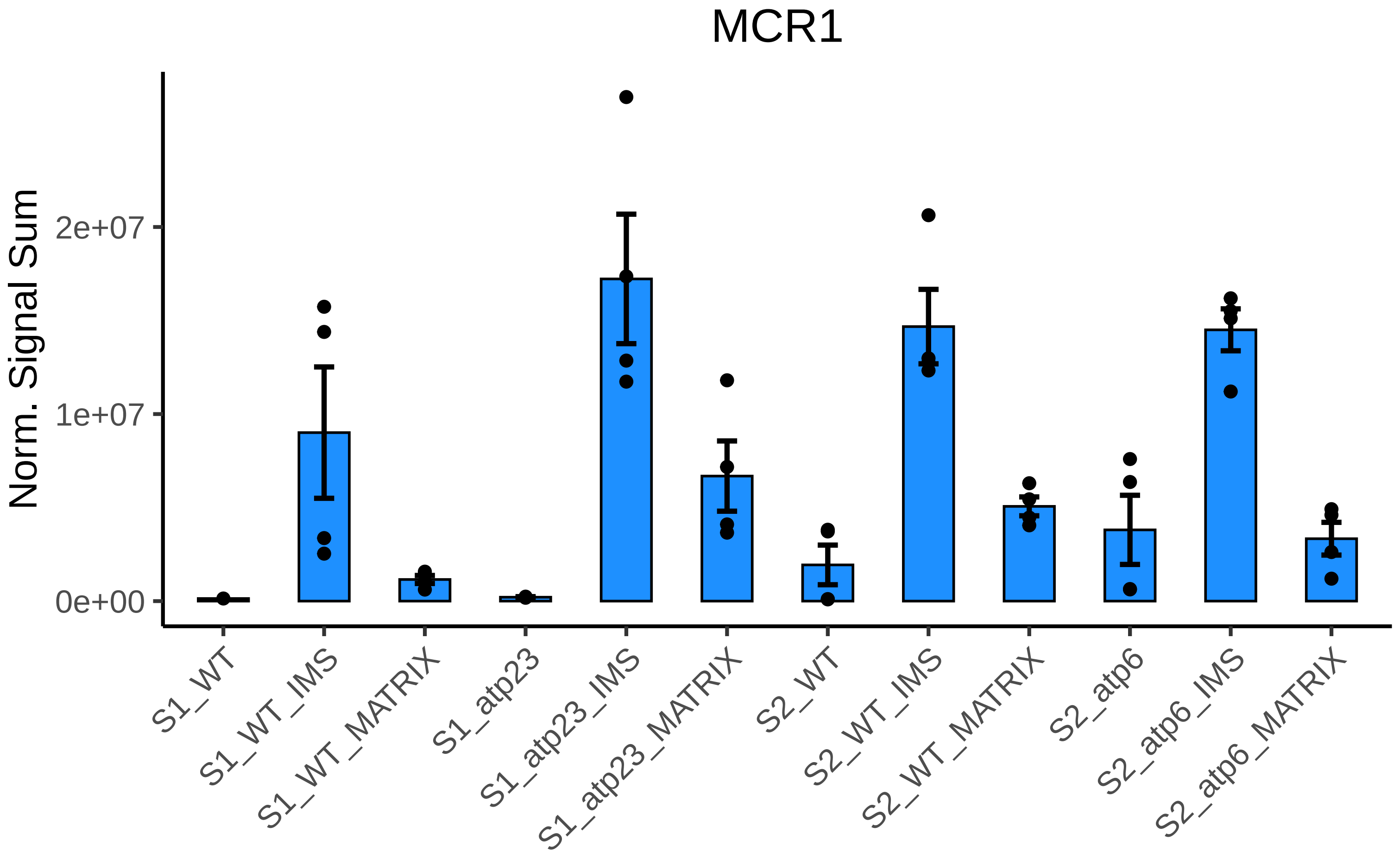

MCR1
Norm. Signal Sum
2e+07
1e+07
0e+00
S1_WT
S1_WT_IMS
S1_WT_MATRIX
S1_atp23
S1_atp23_IMS
S1_atp23_MATRIX
S2_WT
S2_WT_IMS
S2_WT_MATRIX
S2_atp6
S2_atp6_IMS
S2_atp6_MATRIX

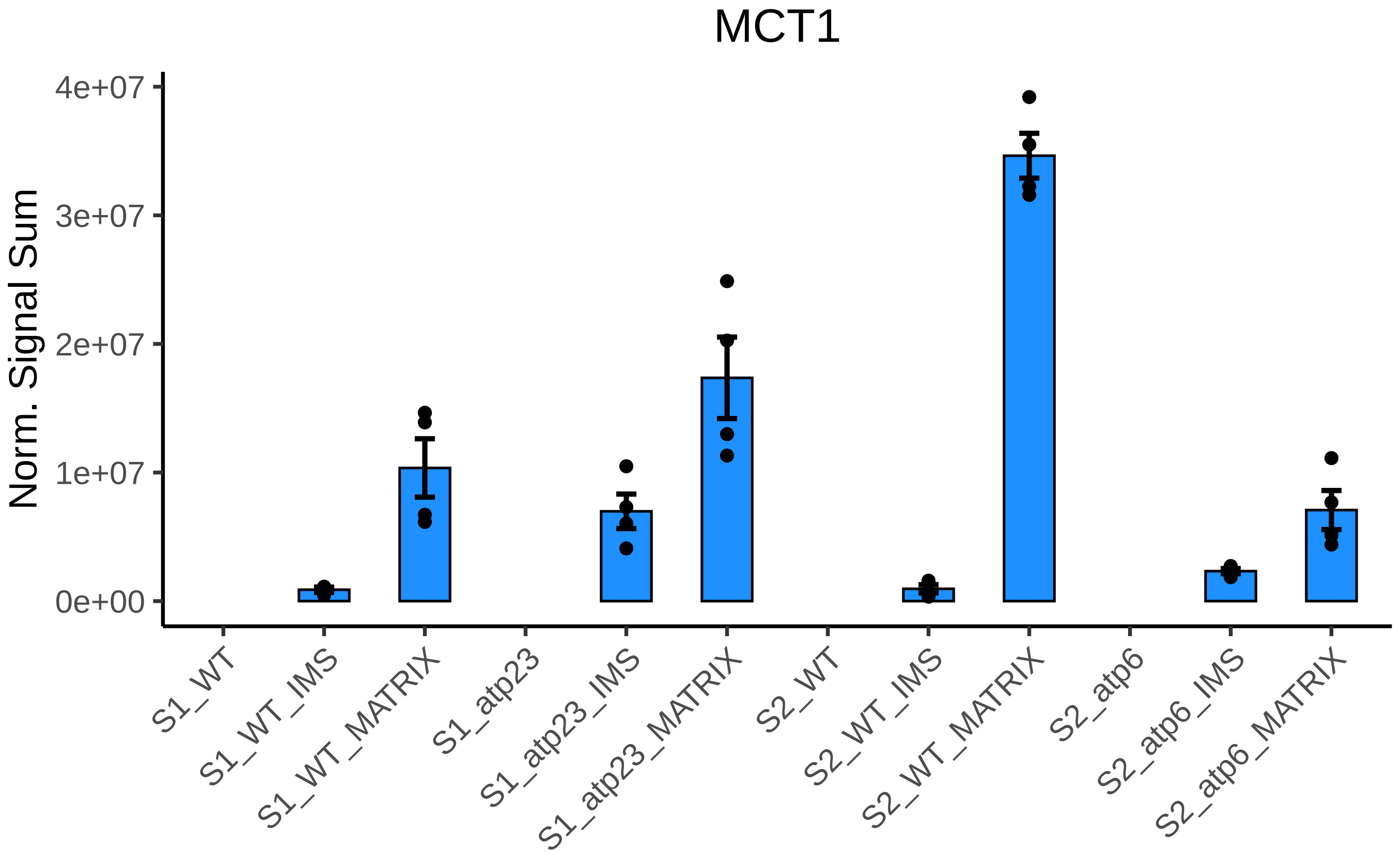

MCT1
Norm. Signal Sum
4e+07
3e+07
2e+07
1e+07
0e+00
S1_WT
S1_WT_IMS
S1_WT_MATRIX
S1_atp23
S1_atp23_IMS
S1_atp23_MATRIX
S2_WT
S2_WT_IMS
S2_WT_MATRIX
S2_atp6
S2_atp6_IMS
S2_atp6_MATRIX

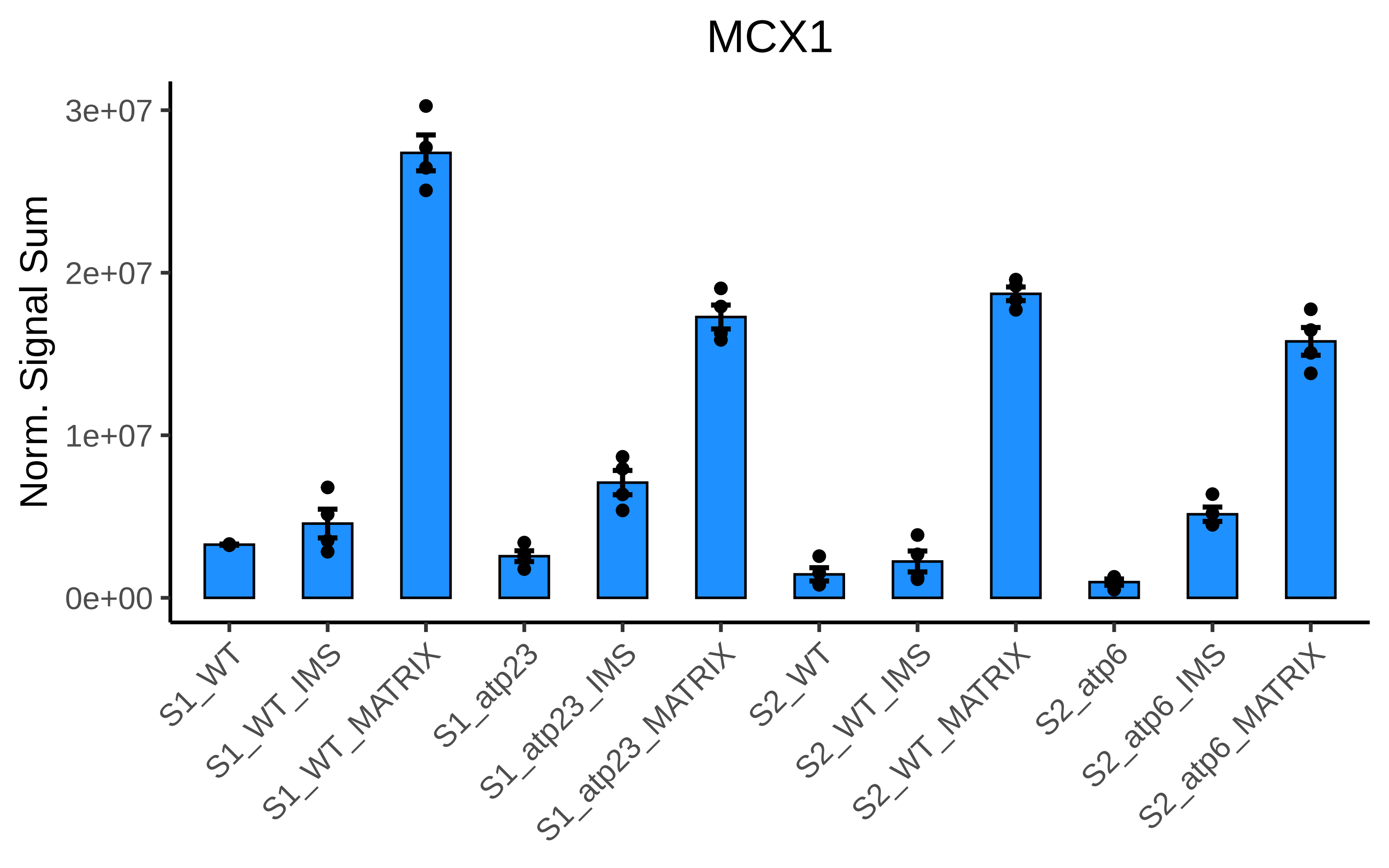
MCX1
Norm. Signal Sum
3e+07
2e+07
1e+07
0e+00
S1_WT
S1_WT_IMS
S1_WT_MATRIX
S1_atp23
S1_atp23_IMS
S1_atp23_MATRIX
S2_WT
S2_WT_IMS
S2_WT_MATRIX
S2_atp6
S2_atp6_IMS
S2_atp6_MATRIX

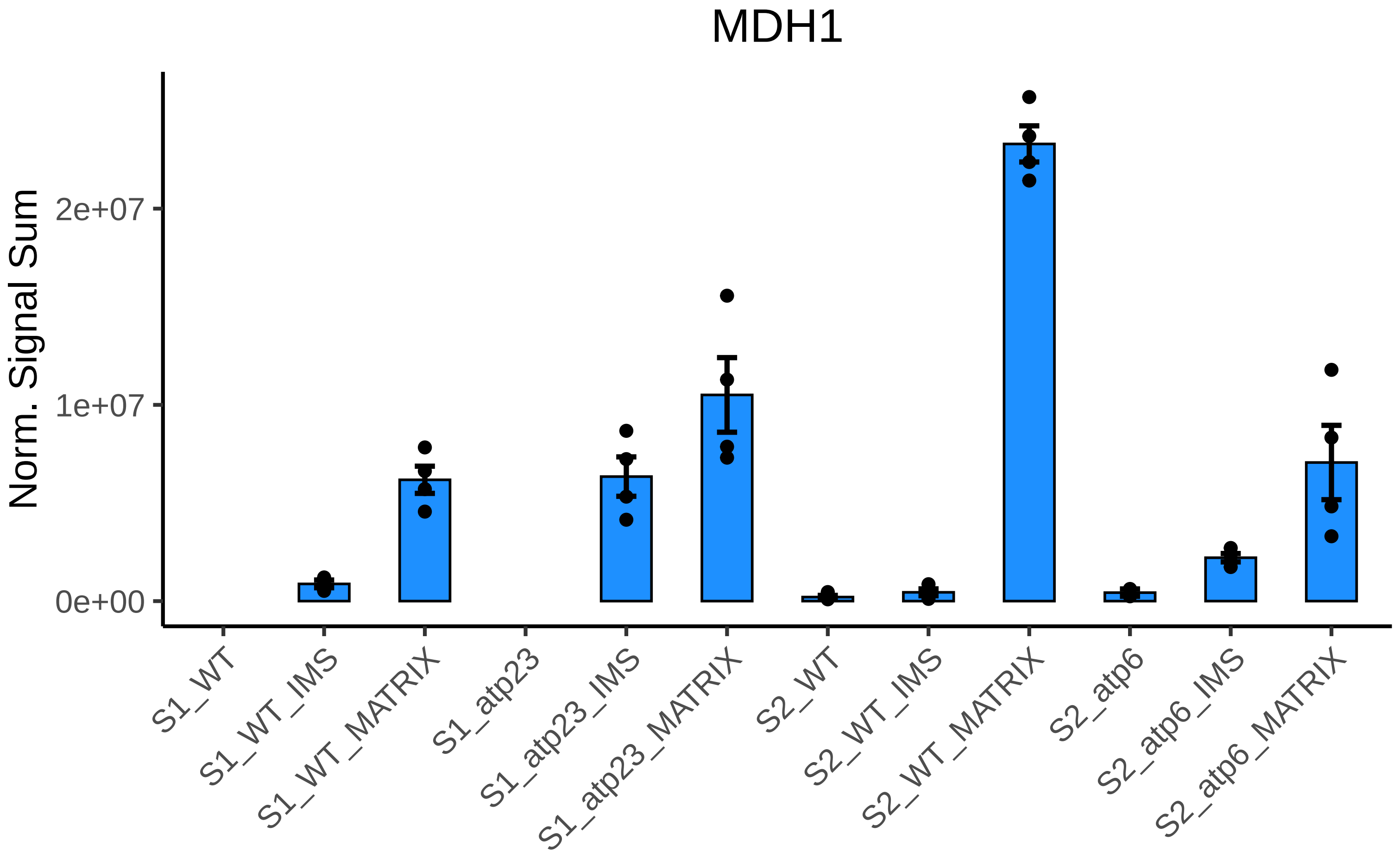

MDH1
Norm. Signal Sum
2e+07
1e+07
0e+00
S1_WT
S1_WT_IMS
S1_WT_MATRIX
S1_atp23
S1_atp23_IMS
S1_atp23_MATRIX
S2_WT
S2_WT_IMS
S2_WT_MATRIX
S2_atp6
S2_atp6_IMS
S2_atp6_MATRIX

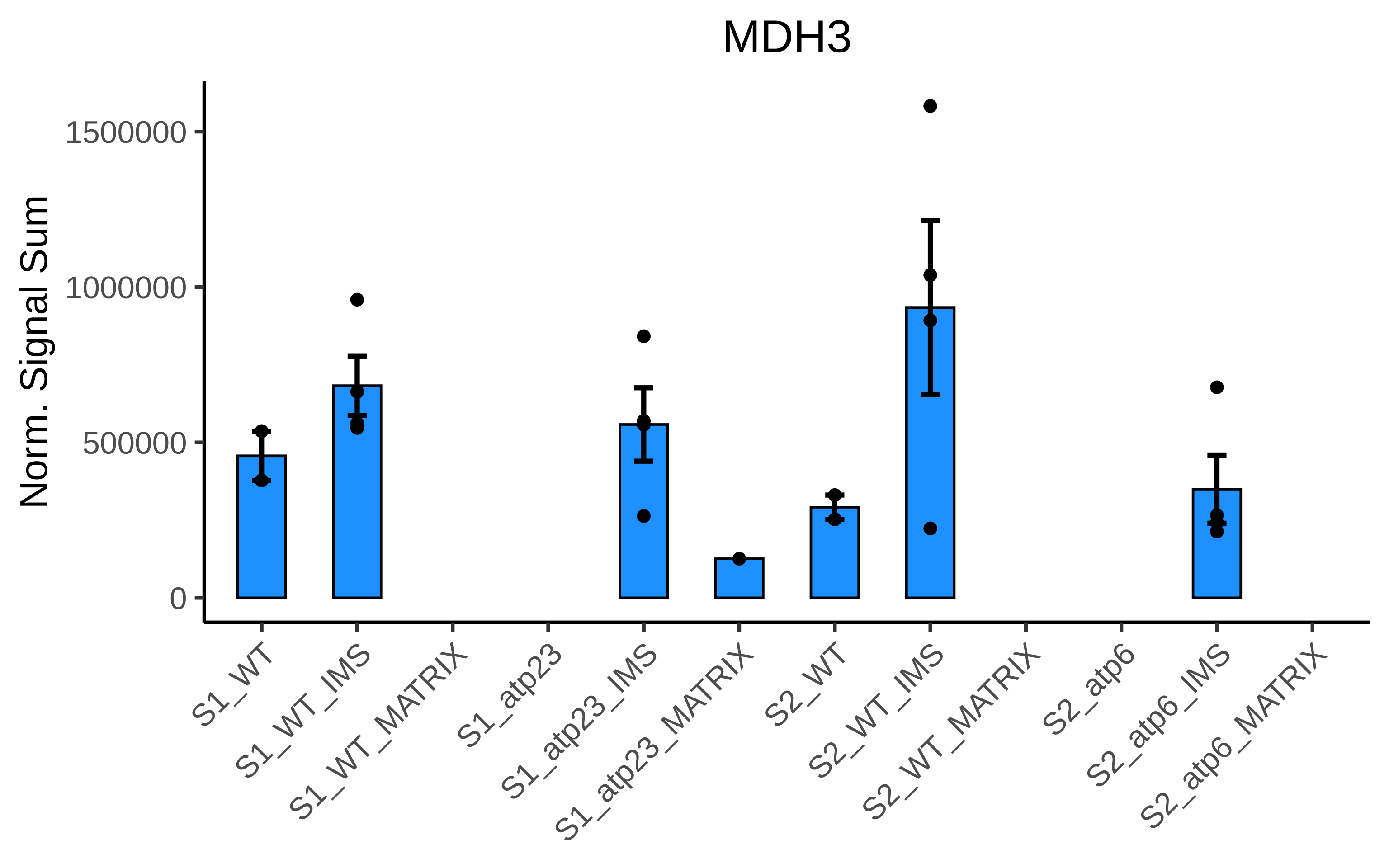

MDH3
Norm. Signal Sum
1500000
1000000
500000
0
S1_WT
S1_WT_IMS
S1_WT_MATRIX
S1_atp23
S1_atp23_IMS
S1_atp23_MATRIX
S2_WT
S2_WT_IMS
S2_WT_MATRIX
S2_atp6
S2_atp6_IMS
S2_atp6_MATRIX

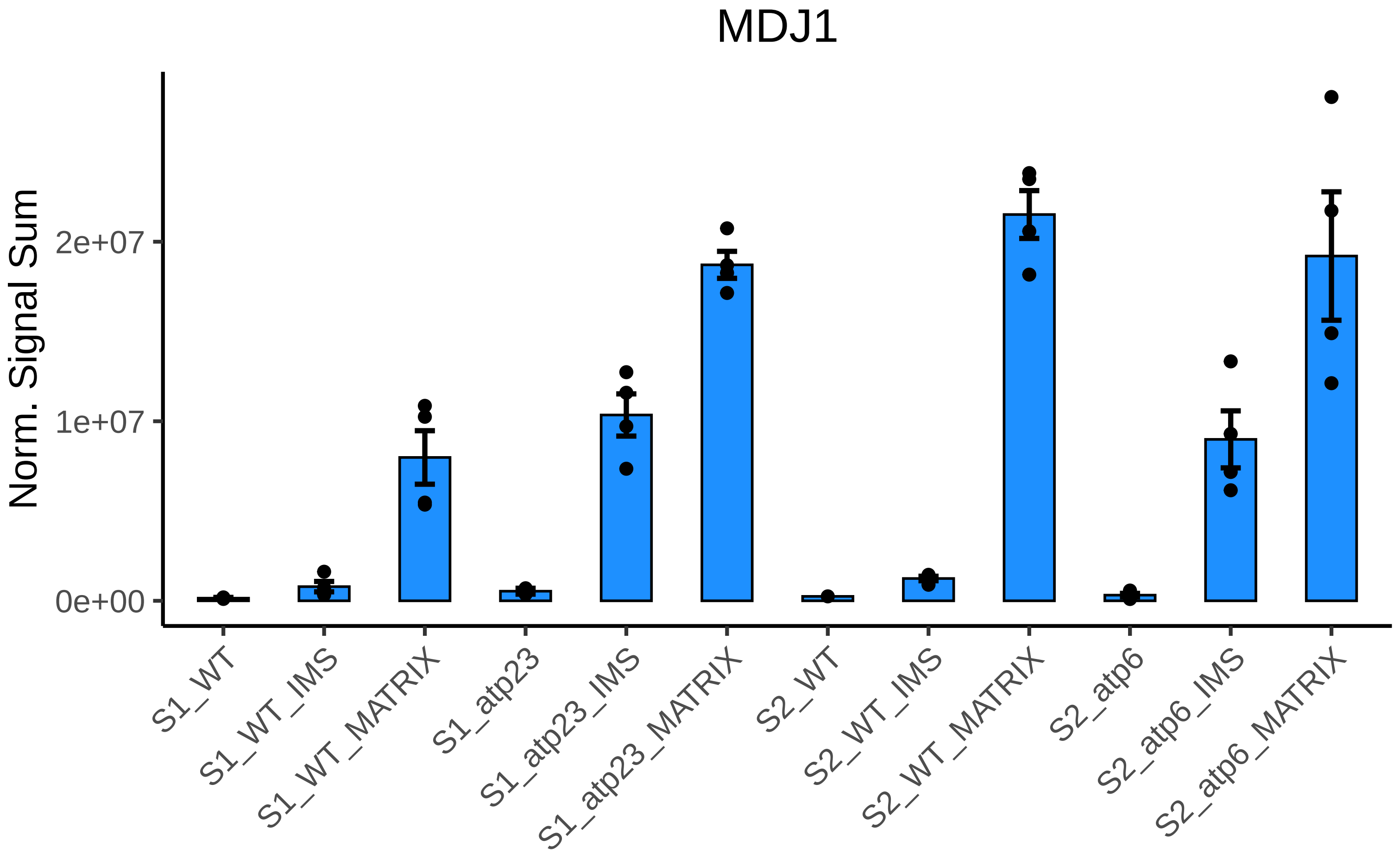
MDJ1
Norm. Signal Sum
0e+00
1e+07
2e+07
S1_WT
S1_WT_IMS
S1_WT_MATRIX
S1_atp23
S1_atp23_IMS
S1_atp23_MATRIX
S2_WT
S2_WT_IMS
S2_WT_MATRIX
S2_atp6
S2_atp6_IMS
S2_atp6_MATRIX

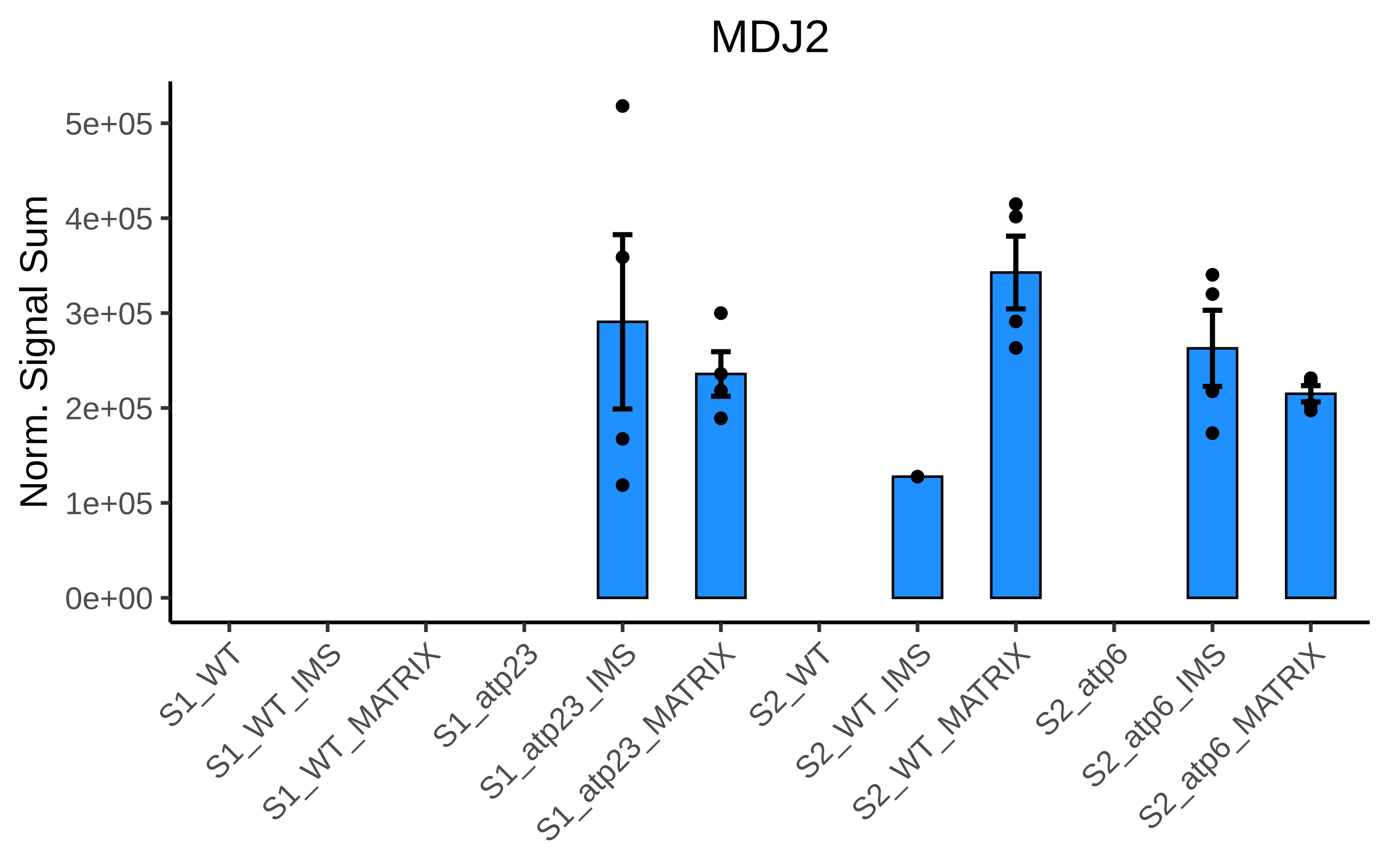
MDJ2
Norm. Signal Sum
5e+05
4e+05
3e+05
2e+05
1e+05
0e+00
S1_WT
S1_WT_IMS
S1_WT_MATRIX
S1_atp23
S1_atp23_IMS
S1_atp23_MATRIX
S2_WT
S2_WT_IMS
S2_WT_MATRIX
S2_atp6
S2_atp6_IMS
S2_atp6_MATRIX

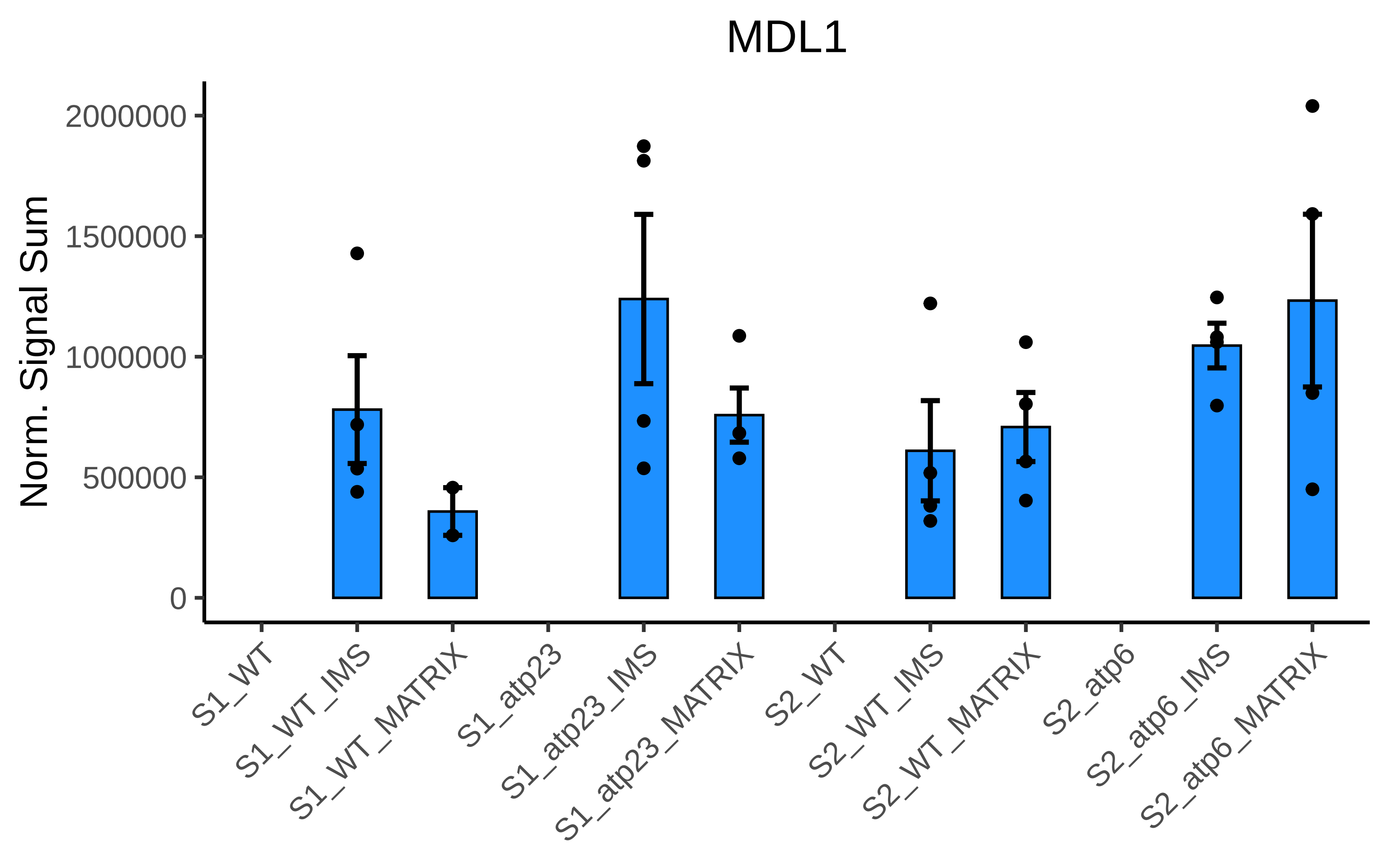

MDL1
Norm. Signal Sum
0
500000
1000000
1500000
2000000
S1_WT
S1_WT_IMS
S1_WT_MATRIX
S1_atp23
S1_atp23_IMS
S1_atp23_MATRIX
S2_WT
S2_WT_IMS
S2_WT_MATRIX
S2_atp6
S2_atp6_IMS
S2_atp6_MATRIX

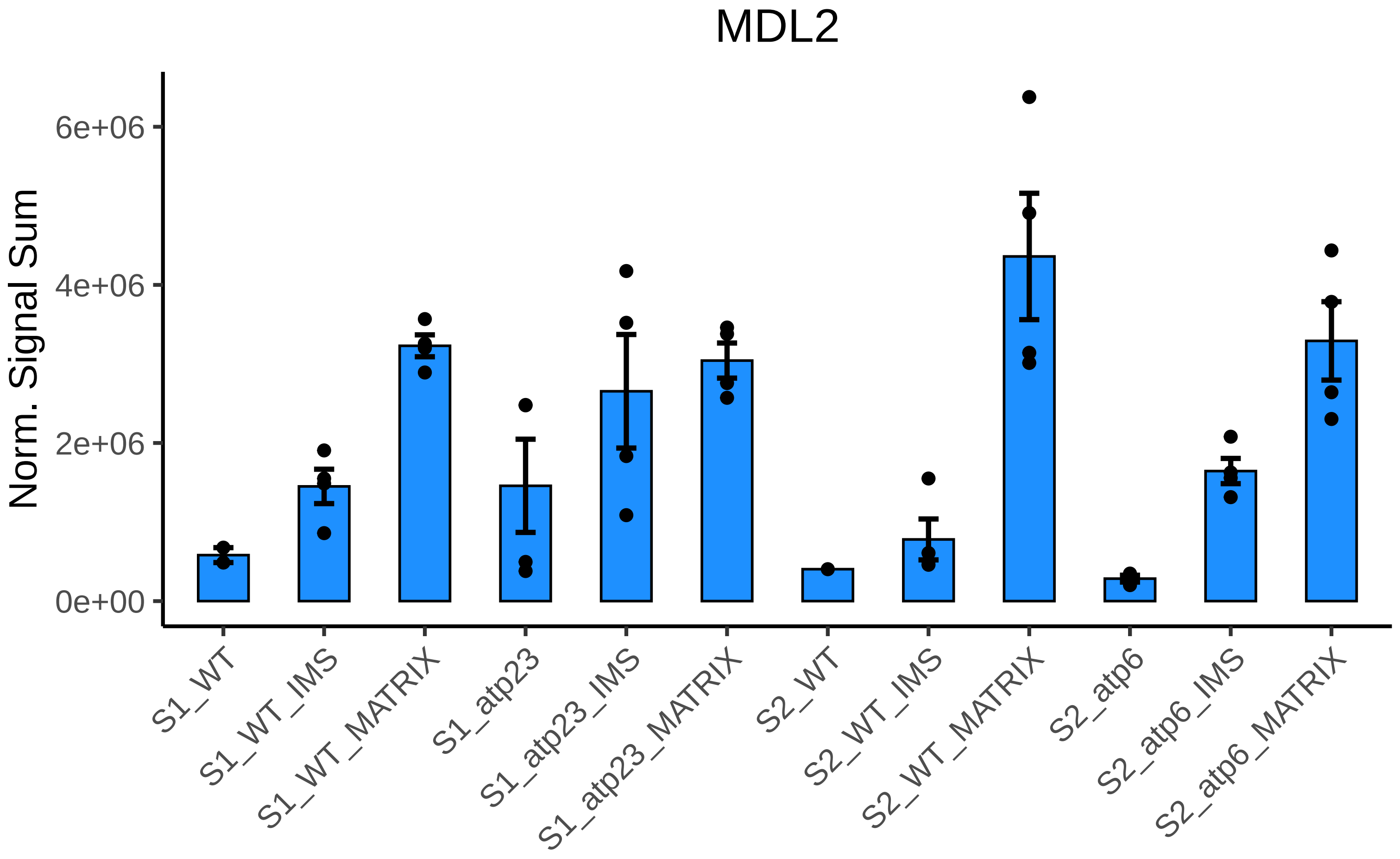
MDL2
Norm. Signal Sum
6e+06
4e+06
2e+06
0e+00
S1_WT
S1_WT_IMS
S1_WT_MATRIX
S1_atp23
S1_atp23_IMS
S1_atp23_MATRIX
S2_WT
S2_WT_IMS
S2_WT_MATRIX
S2_atp6
S2_atp6_IMS
S2_atp6_MATRIX

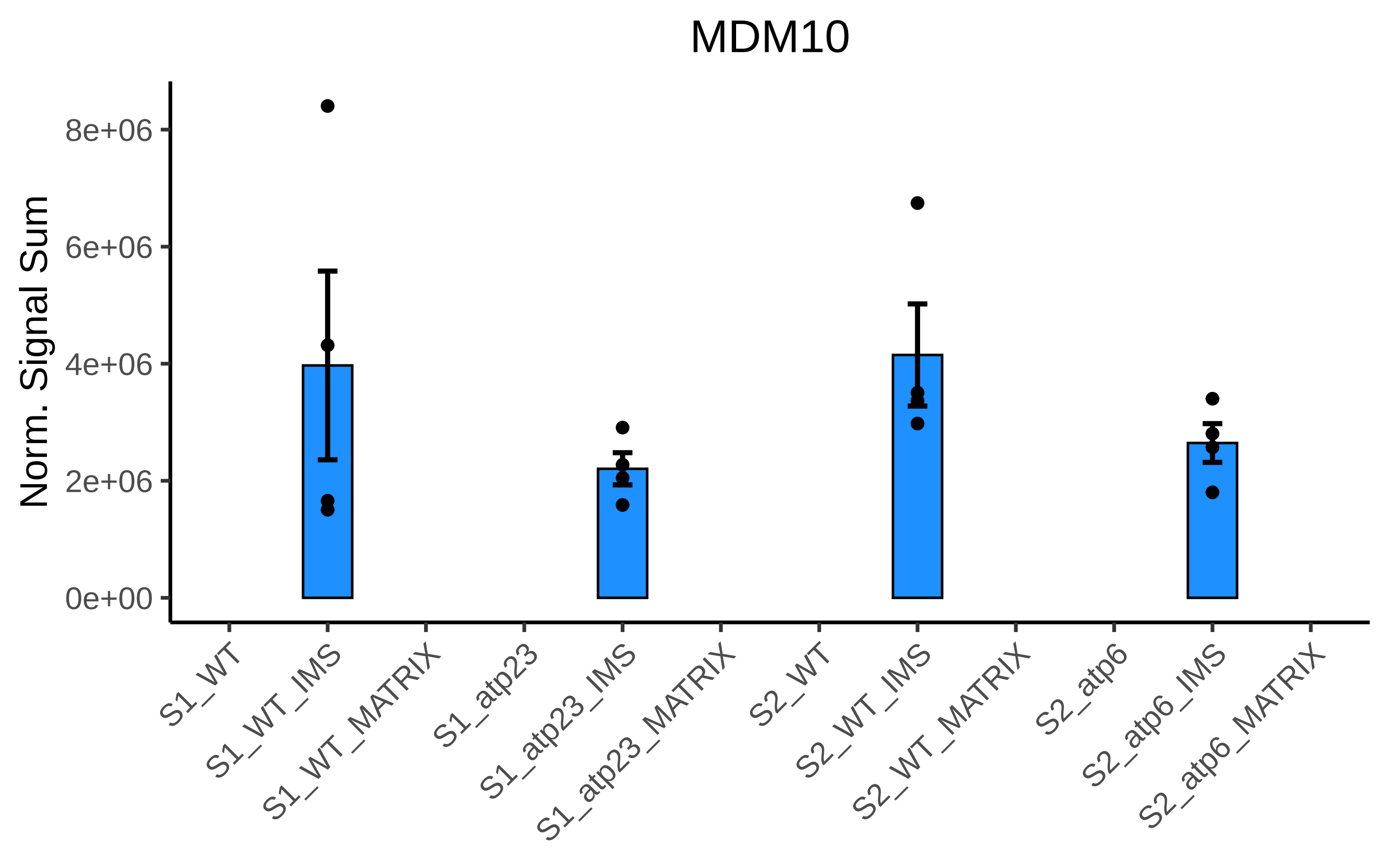
MDM10
Norm. Signal Sum
0e+00
2e+06
4e+06
6e+06
8e+06
S1_WT
S1_WT_IMS
S1_WT_MATRIX
S1_atp23
S1_atp23_IMS
S1_atp23_MATRIX
S2_WT
S2_WT_IMS
S2_WT_MATRIX
S2_atp6
S2_atp6_IMS
S2_atp6_MATRIX

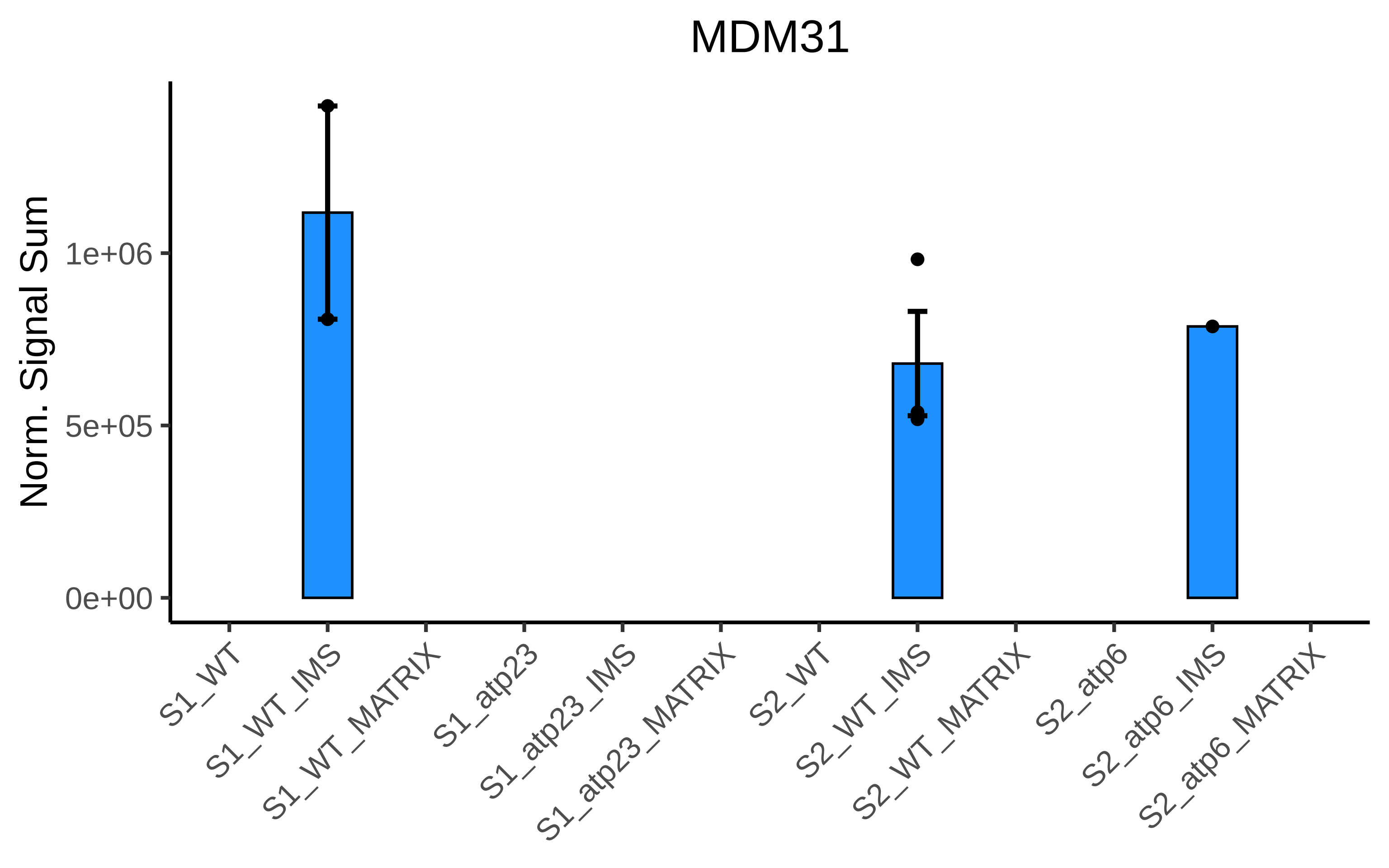

MDM31
Norm. Signal Sum
1e+06
5e+05
0e+00
S1_WT
S1_WT_IMS
S1_WT_MATRIX
S1_atp23
S1_atp23_IMS
S1_atp23_MATRIX
S2_WT
S2_WT_IMS
S2_WT_MATRIX
S2_atp6
S2_atp6_IMS
S2_atp6_MATRIX

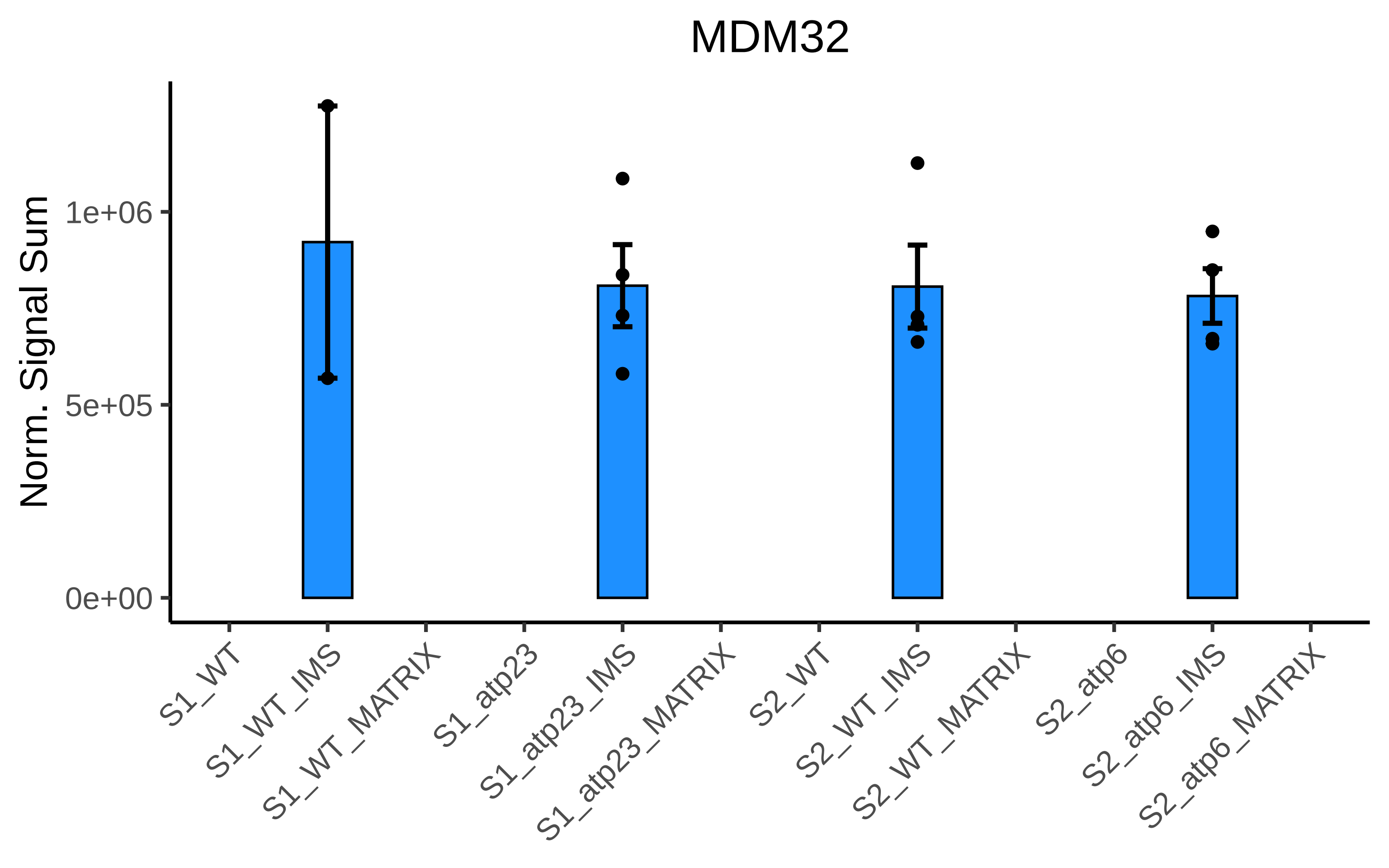

MDM32
Norm. Signal Sum
1e+06
5e+05
0e+00
S1_WT
S1_WT_IMS
S1_WT_MATRIX
S1_atp23
S1_atp23_IMS
S1_atp23_MATRIX
S2_WT
S2_WT_IMS
S2_WT_MATRIX
S2_atp6
S2_atp6_IMS
S2_atp6_MATRIX

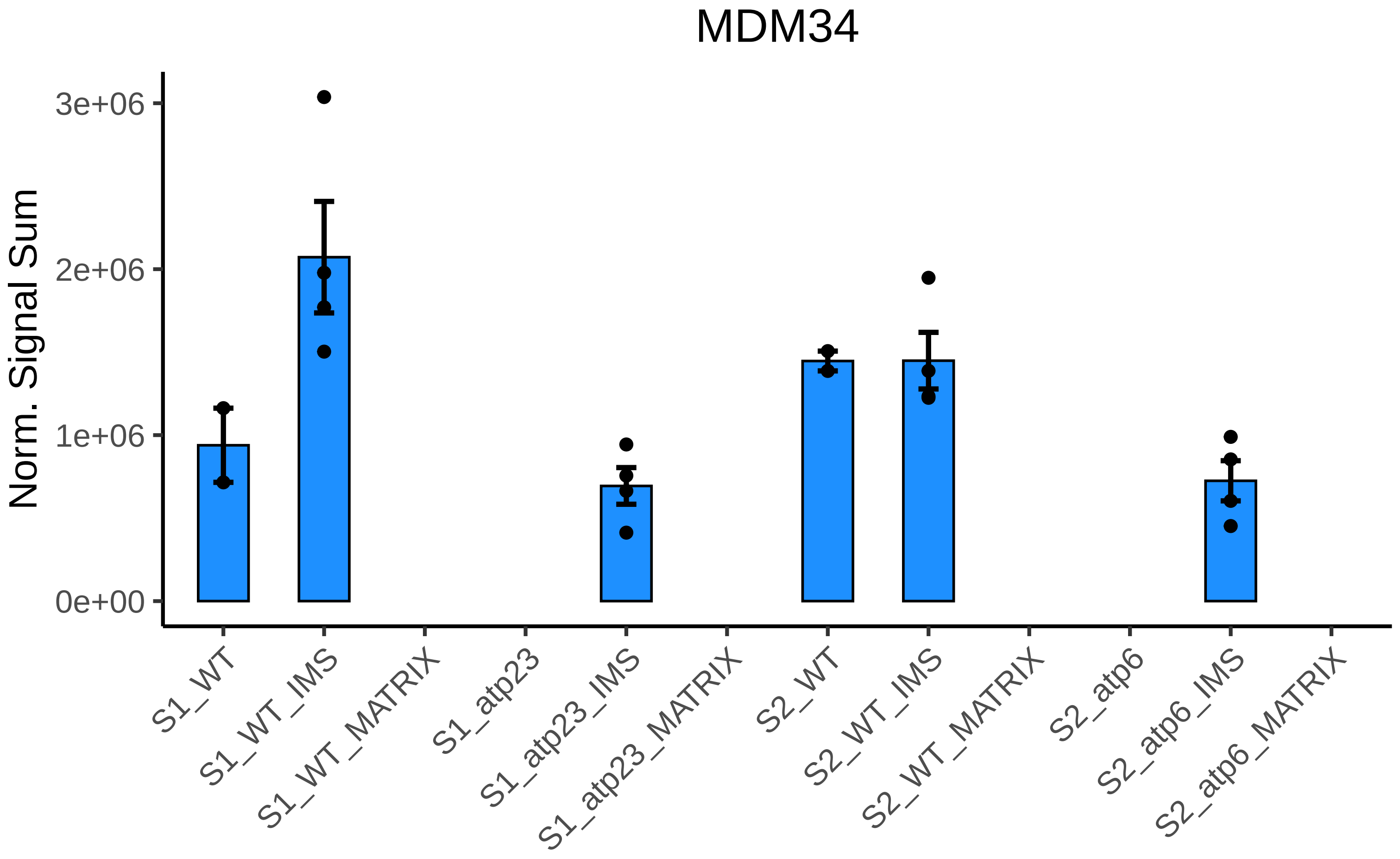
MDM34
Norm. Signal Sum
3e+06
2e+06
1e+06
0e+00
S1_WT
S1_WT_IMS
S1_WT_MATRIX
S1_atp23
S1_atp23_IMS
S1_atp23_MATRIX
S2_WT
S2_WT_IMS
S2_WT_MATRIX
S2_atp6
S2_atp6_IMS
S2_atp6_MATRIX

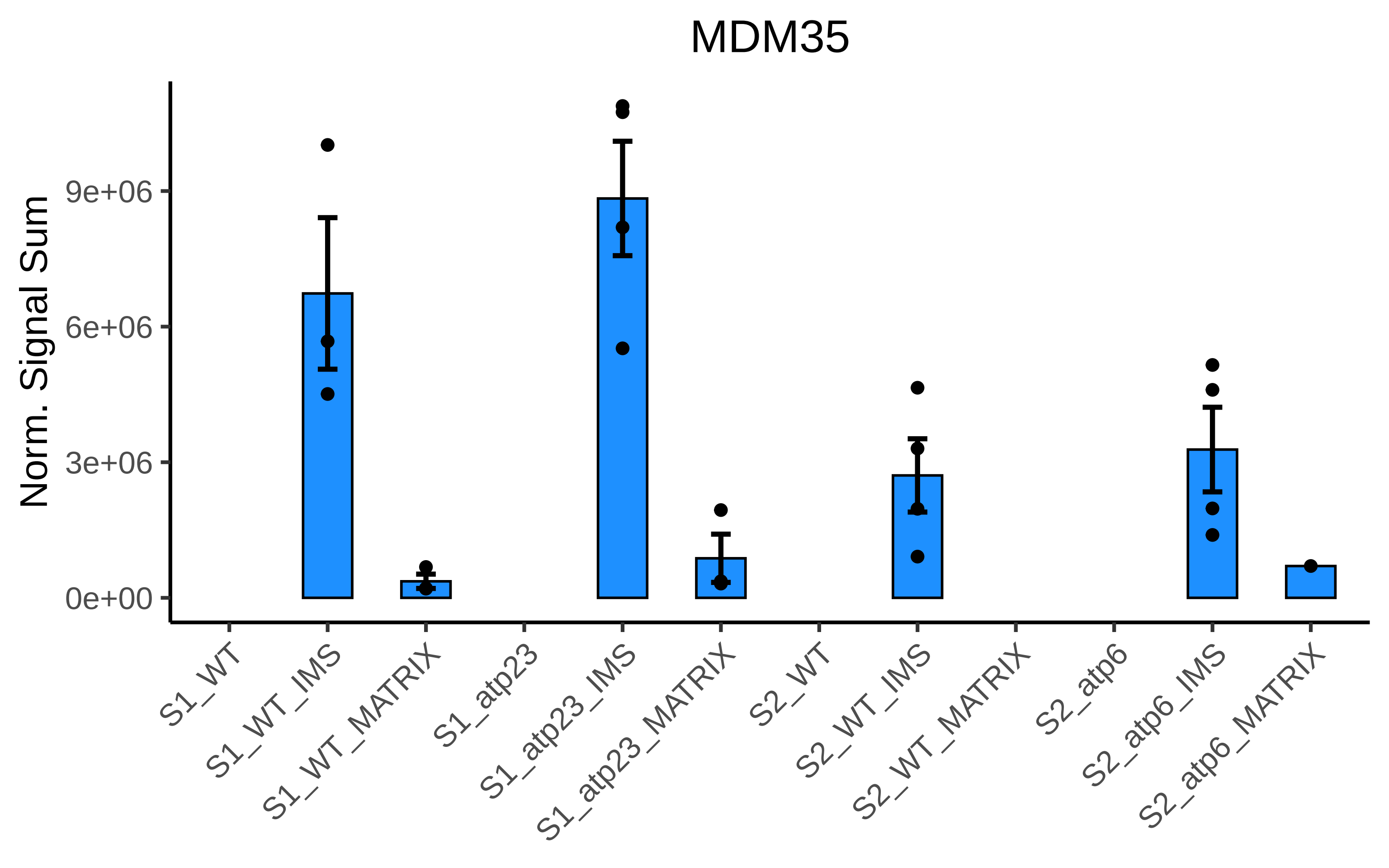
MDM35
Norm. Signal Sum
9e+06
6e+06
3e+06
0e+00
S1_WT
S1_WT_IMS
S1_WT_MATRIX
S1_atp23
S1_atp23_IMS
S1_atp23_MATRIX
S2_WT
S2_WT_IMS
S2_WT_MATRIX
S2_atp6
S2_atp6_IMS
S2_atp6_MATRIX

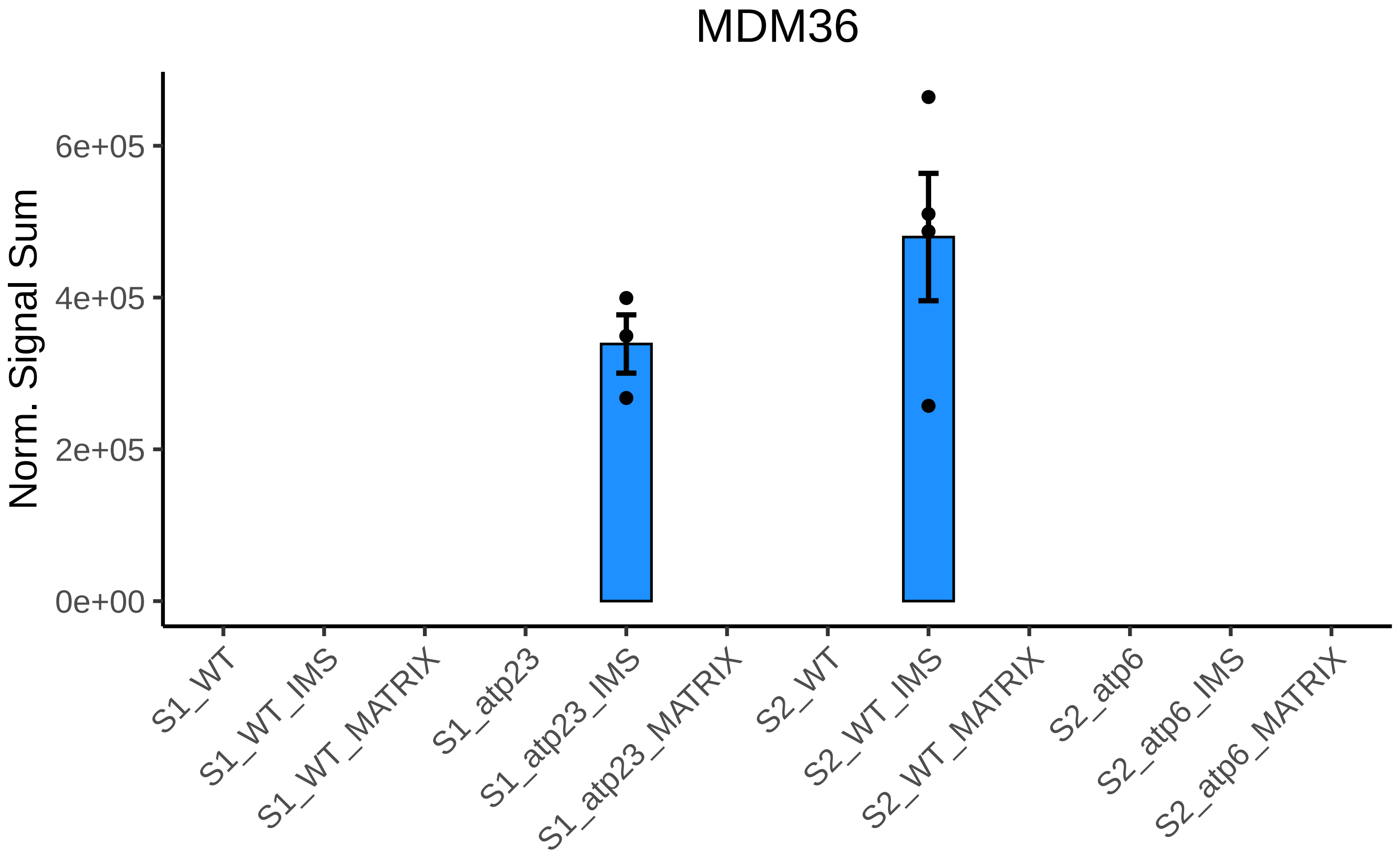
MDM36
Norm. Signal Sum
6e+05
4e+05
2e+05
0e+00
S1_WT
S1_WT_IMS
S1_WT_MATRIX
S1_atp23
S1_atp23_IMS
S1_atp23_MATRIX
S2_WT
S2_WT_IMS
S2_WT_MATRIX
S2_atp6
S2_atp6_IMS
S2_atp6_MATRIX

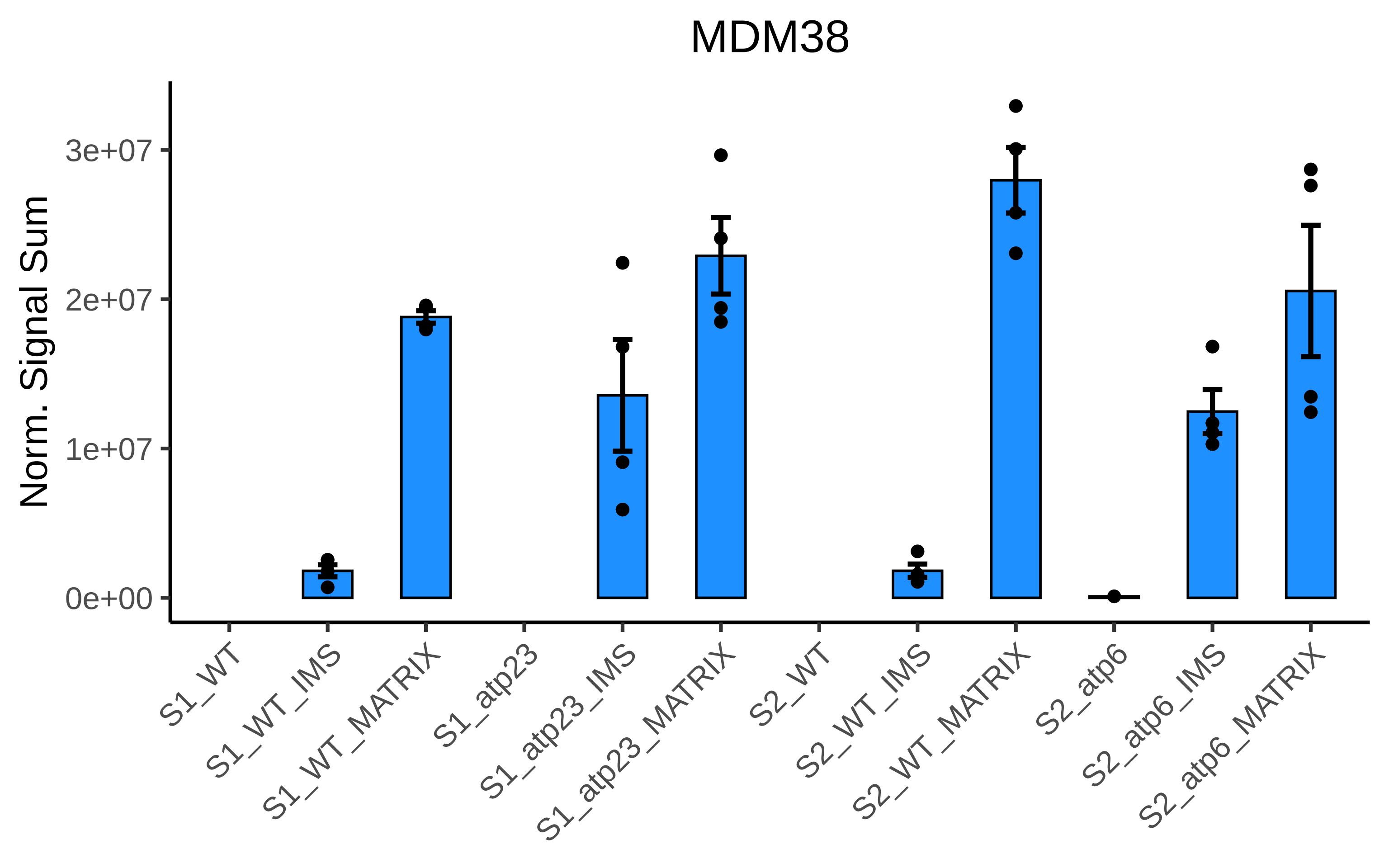
MDM38
Norm. Signal Sum
3e+07
2e+07
1e+07
0e+00
S1_WT
S1_WT_IMS
S1_WT_MATRIX
S1_atp23
S1_atp23_IMS
S1_atp23_MATRIX
S2_WT
S2_WT_IMS
S2_WT_MATRIX
S2_atp6
S2_atp6_IMS
S2_atp6_MATRIX

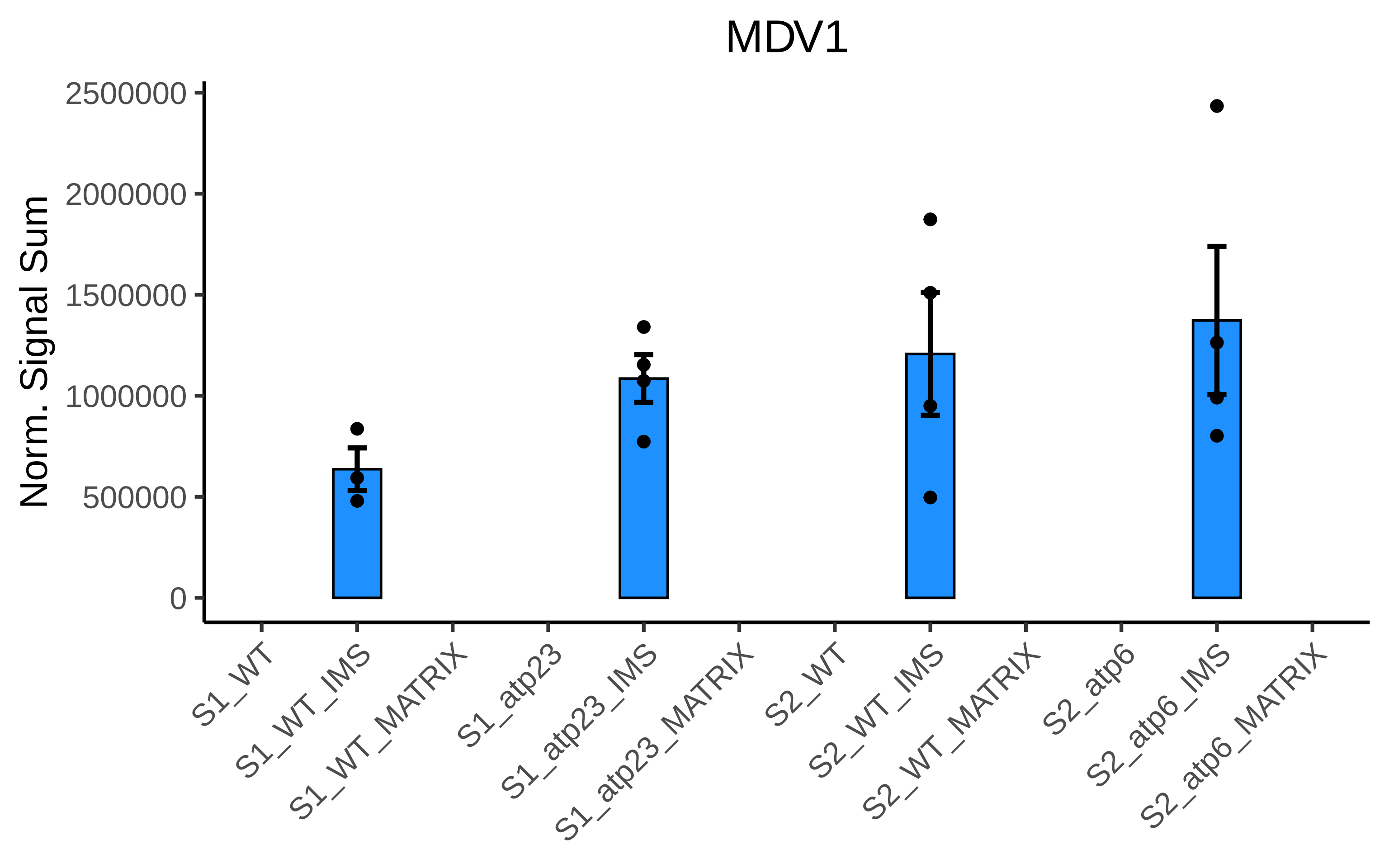
MDV1
Norm. Signal Sum
2500000
2000000
1500000
1000000
500000
0
S1_WT
S1_WT_IMS
S1_WT_MATRIX
S1_atp23
S1_atp23_IMS
S1_atp23_MATRIX
S2_WT
S2_WT_IMS
S2_WT_MATRIX
S2_atp6
S2_atp6_IMS
S2_atp6_MATRIX

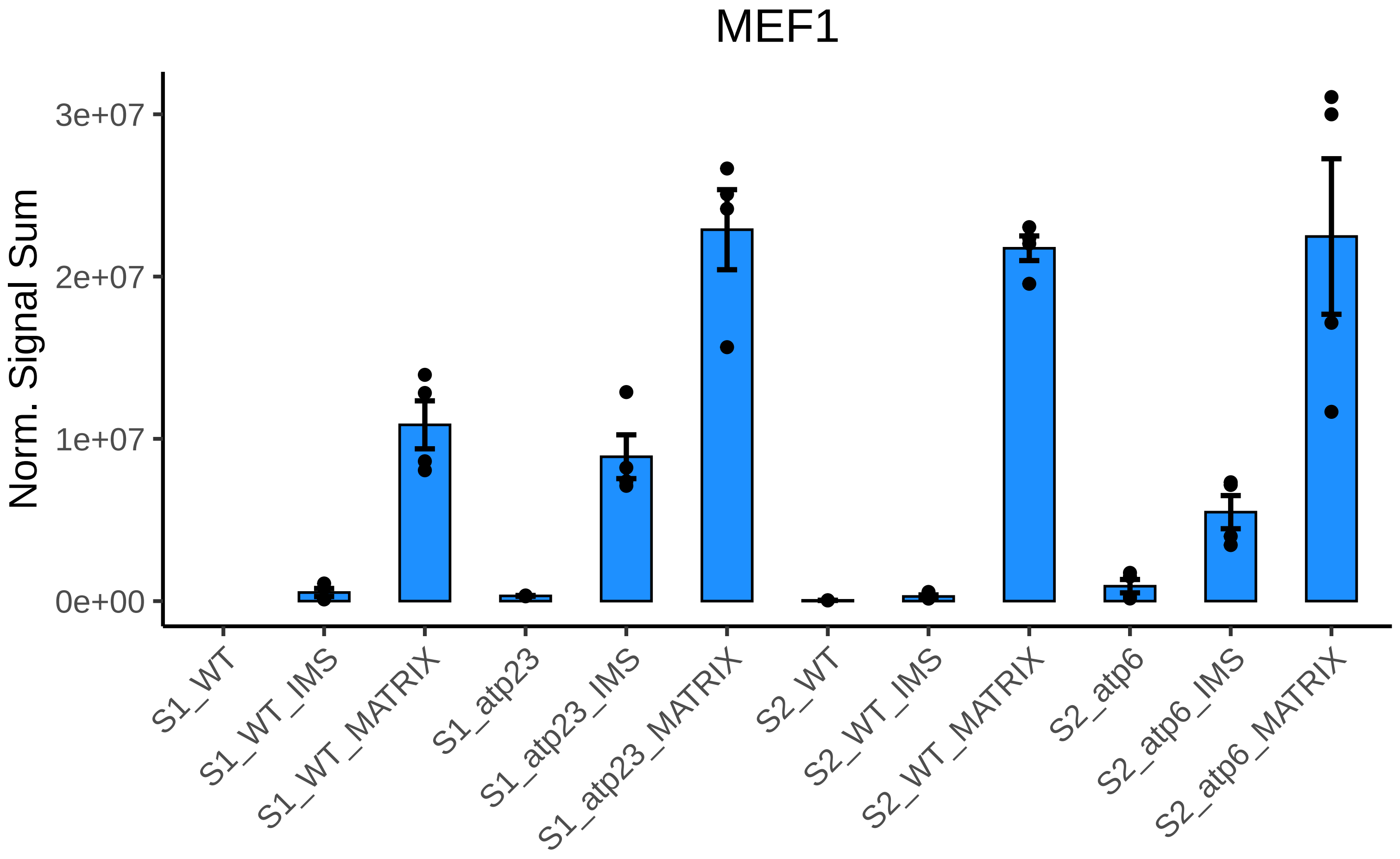

MEF1
Norm. Signal Sum
3e+07
2e+07
1e+07
0e+00
S1_WT
S1_WT_IMS
S1_WT_MATRIX
S1_atp23
S1_atp23_IMS
S1_atp23_MATRIX
S2_WT
S2_WT_IMS
S2_WT_MATRIX
S2_atp6
S2_atp6_IMS
S2_atp6_MATRIX

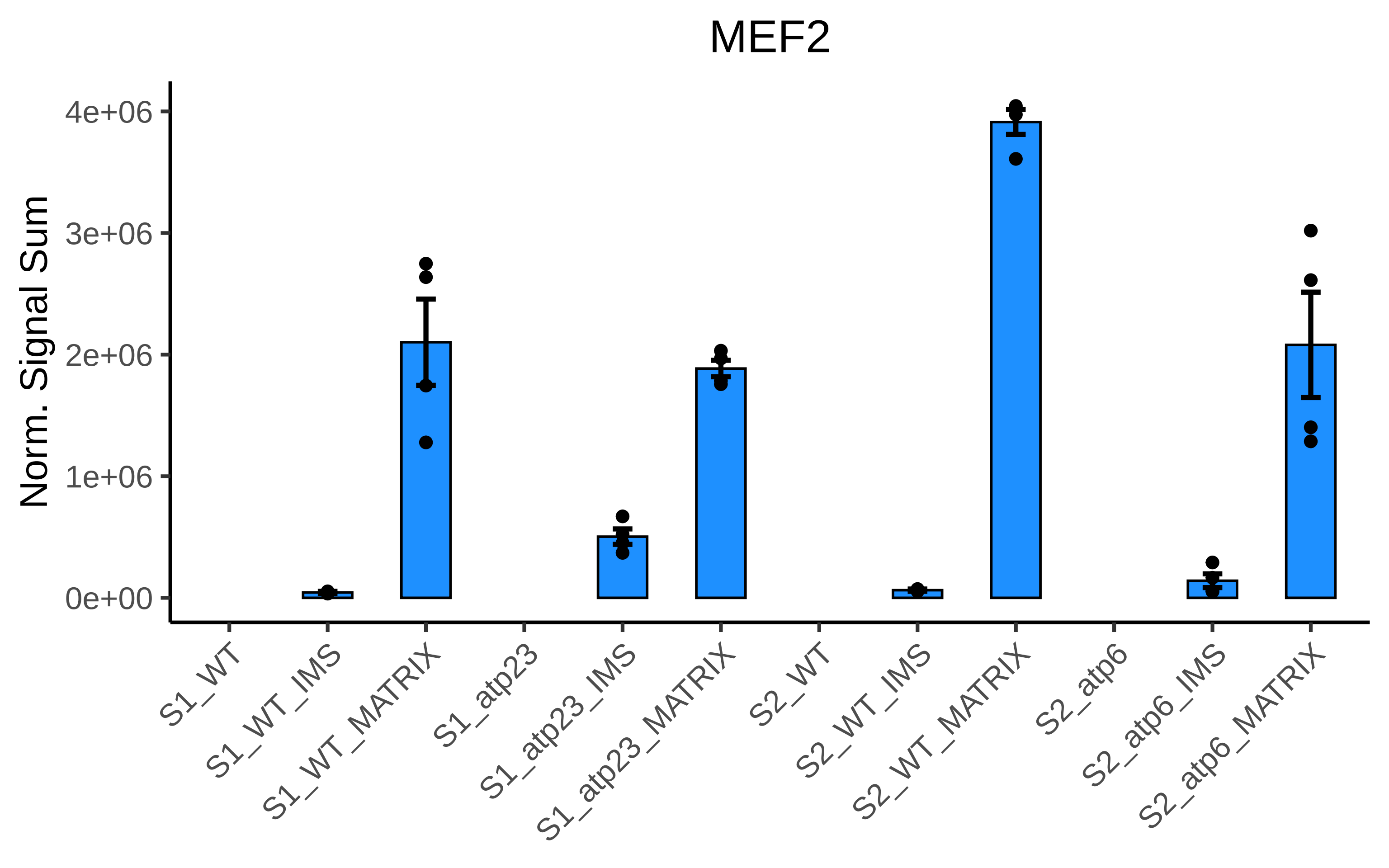
MEF2
Norm. Signal Sum
4e+06
3e+06
2e+06
1e+06
0e+00
S1_WT
S1_WT_IMS
S1_WT_MATRIX
S1_atp23
S1_atp23_IMS
S1_atp23_MATRIX
S2_WT
S2_WT_IMS
S2_WT_MATRIX
S2_atp6
S2_atp6_IMS
S2_atp6_MATRIX

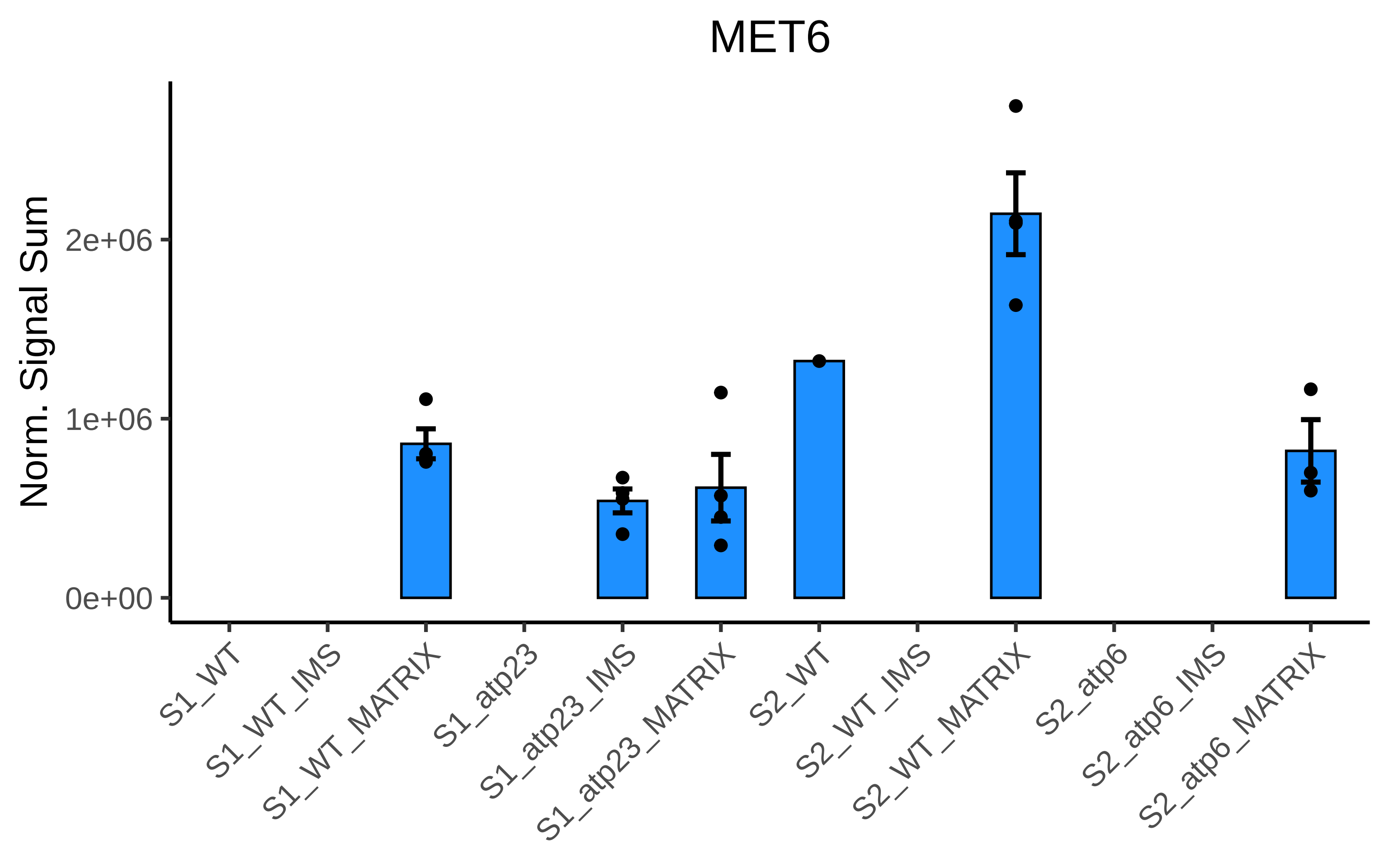

MET6
Norm. Signal Sum
2e+06
1e+06
0e+00
S1_WT
S1_WT_IMS
S1_WT_MATRIX
S1_atp23
S1_atp23_IMS
S1_atp23_MATRIX
S2_WT
S2_WT_IMS
S2_WT_MATRIX
S2_atp6
S2_atp6_IMS
S2_atp6_MATRIX

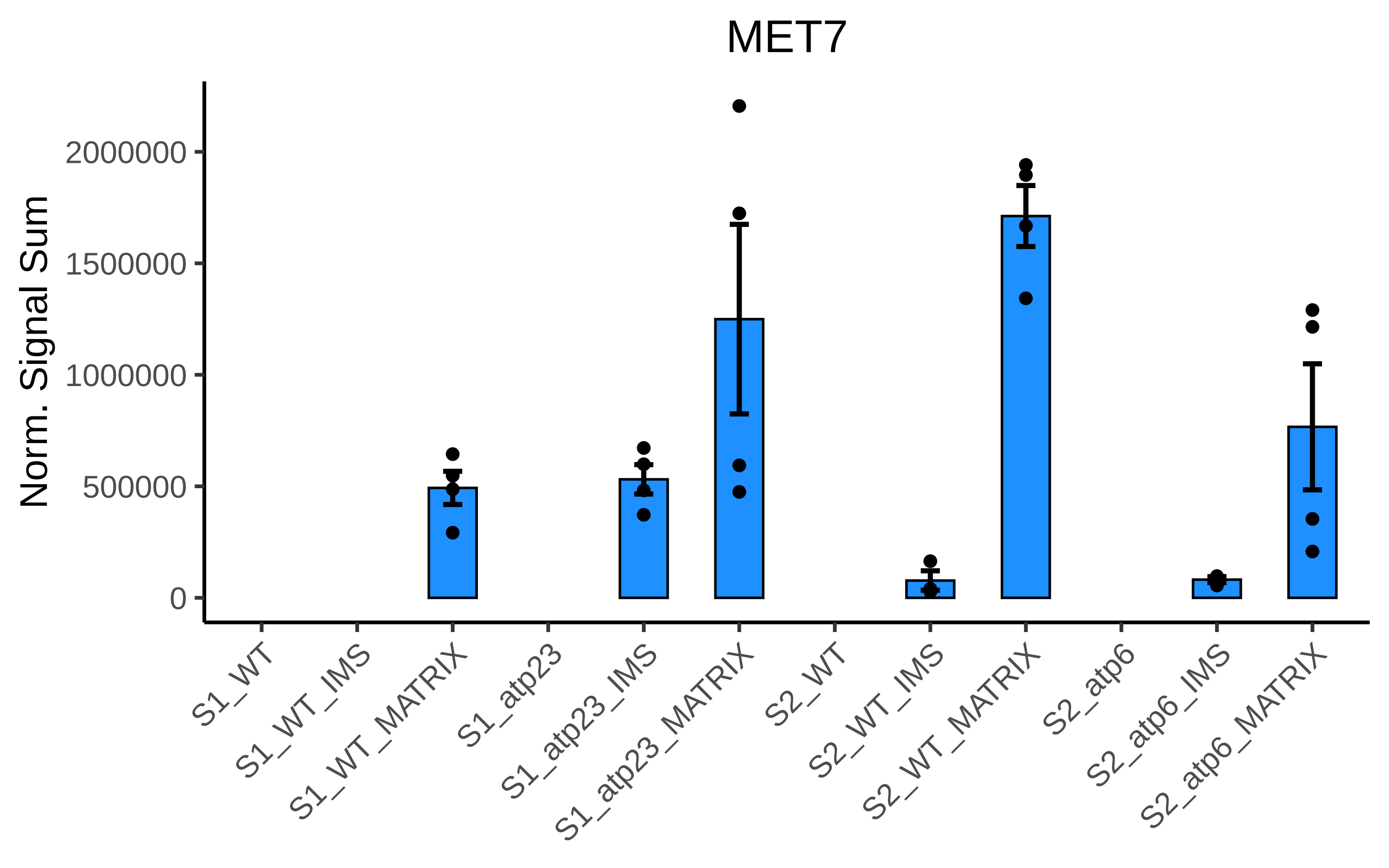

MET7
Norm. Signal Sum
2000000
1500000
1000000
500000
0
S1_WT
S1_WT_IMS
S1_WT_MATRIX
S1_atp23
S1_atp23_IMS
S1_atp23_MATRIX
S2_WT
S2_WT_IMS
S2_WT_MATRIX
S2_atp6
S2_atp6_IMS
S2_atp6_MATRIX

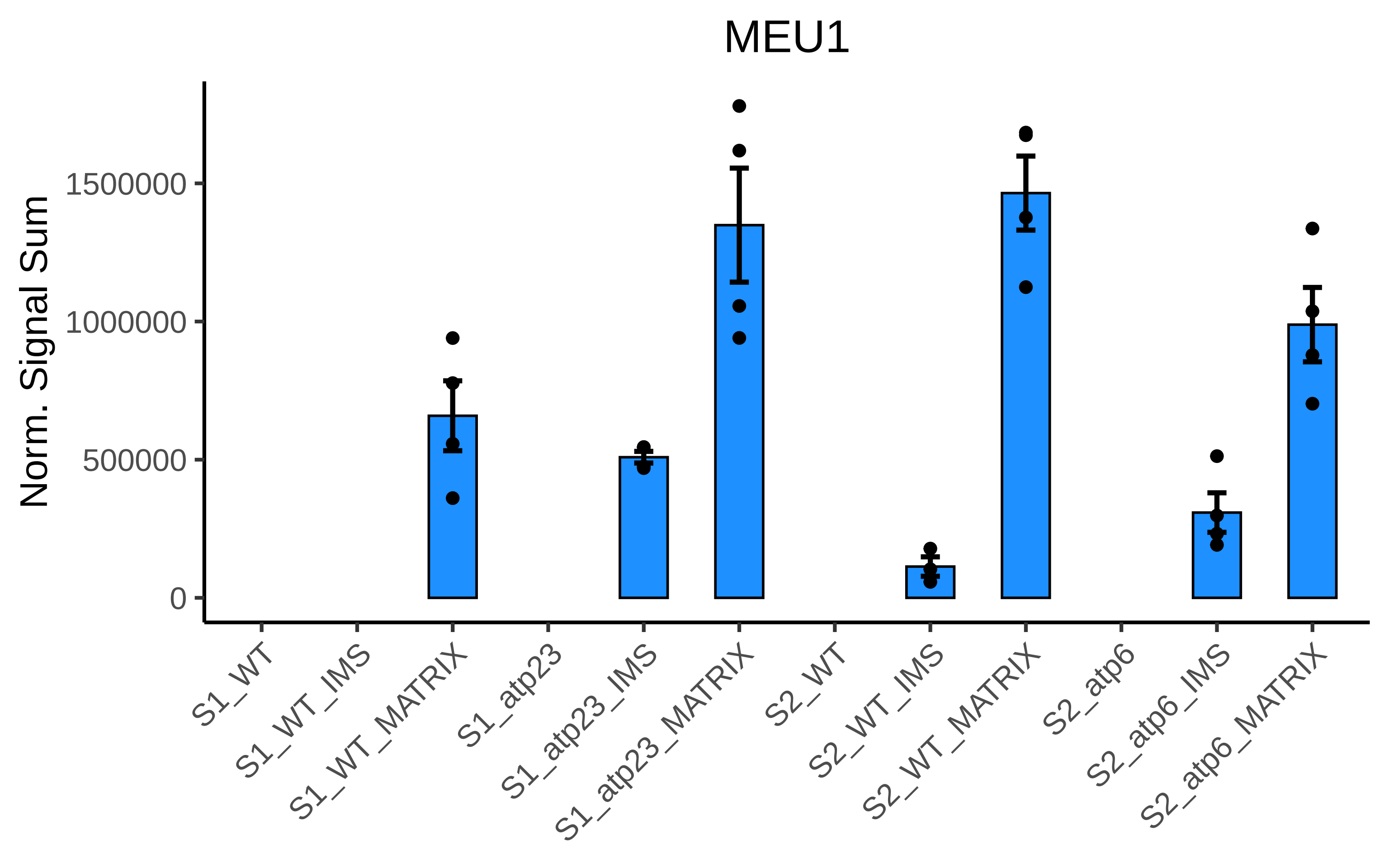

MEU1
Norm. Signal Sum
1500000
1000000
500000
0
S1_WT
S1_WT_IMS
S1_WT_MATRIX
S1_atp23
S1_atp23_IMS
S1_atp23_MATRIX
S2_WT
S2_WT_IMS
S2_WT_MATRIX
S2_atp6
S2_atp6_IMS
S2_atp6_MATRIX

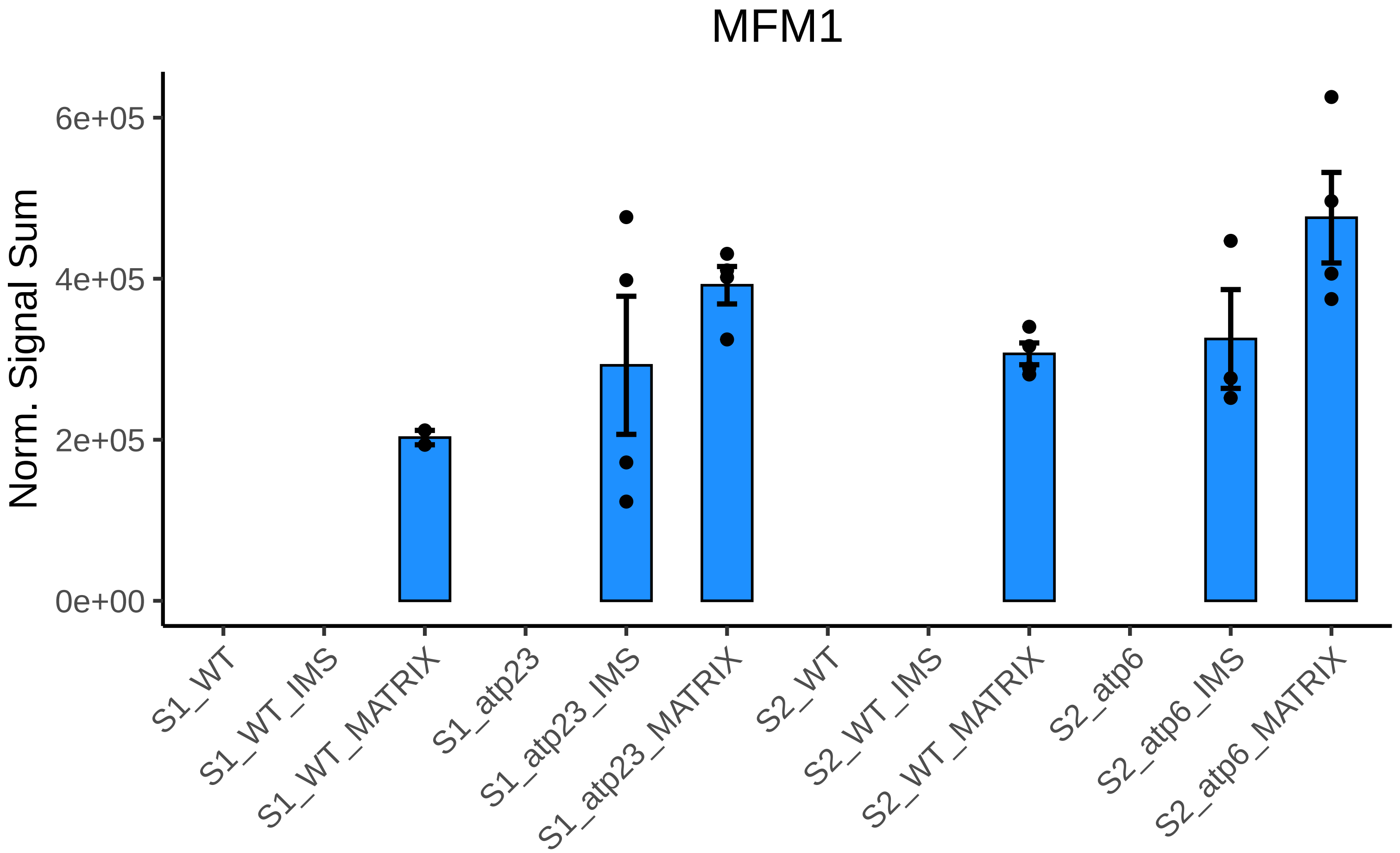
MFM1
Norm. Signal Sum
0e+00
2e+05
4e+05
6e+05
S1_WT
S1_WT_IMS
S1_WT_MATRIX
S1_atp23
S1_atp23_IMS
S1_atp23_MATRIX
S2_WT
S2_WT_IMS
S2_WT_MATRIX
S2_atp6
S2_atp6_IMS
S2_atp6_MATRIX

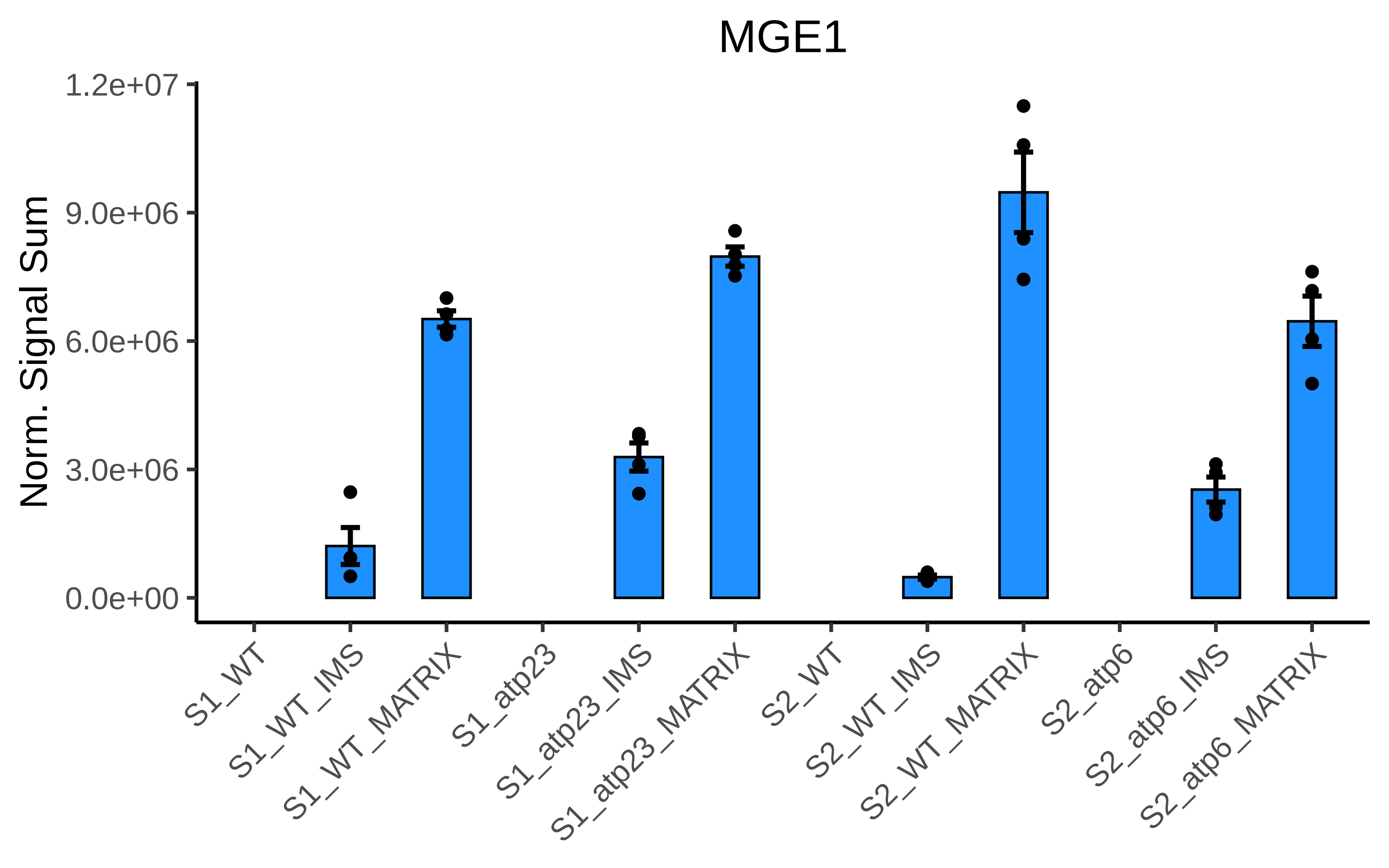

MGE1
Norm. Signal Sum
1.2e+07
9.0e+06
6.0e+06
3.0e+06
0.0e+00
S1_WT
S1_WT_IMS
S1_WT_MATRIX
S1_atp23
S1_atp23_IMS
S1_atp23_MATRIX
S2_WT
S2_WT_IMS
S2_WT_MATRIX
S2_atp6
S2_atp6_IMS
S2_atp6_MATRIX

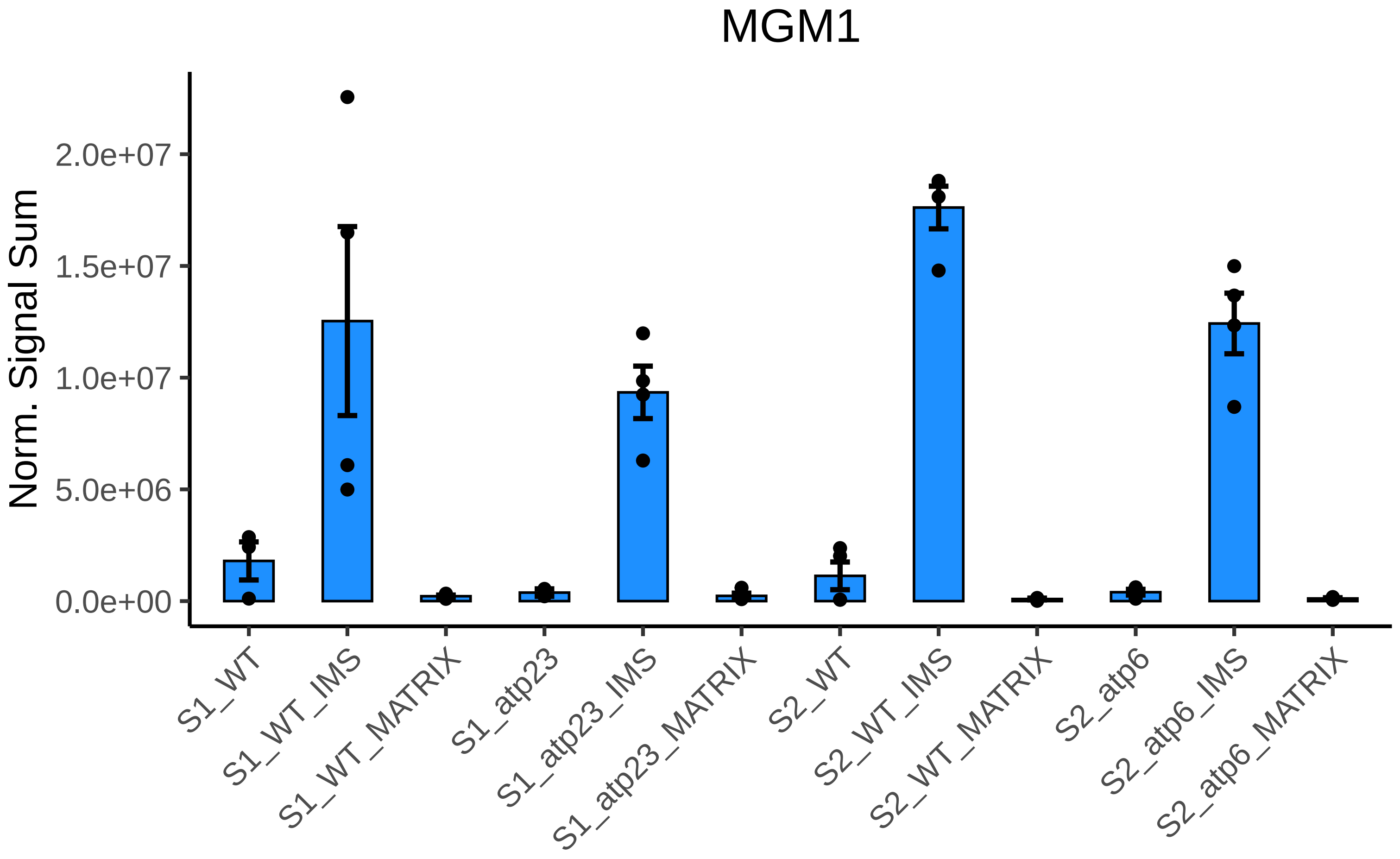
MGM1
Norm. Signal Sum
2.0e+07
1.5e+07
1.0e+07
5.0e+06
0.0e+00
S1_WT
S1_WT_IMS
S1_WT_MATRIX
S1_atp23
S1_atp23_IMS
S1_atp23_MATRIX
S2_WT
S2_WT_IMS
S2_WT_MATRIX
S2_atp6
S2_atp6_IMS
S2_atp6_MATRIX

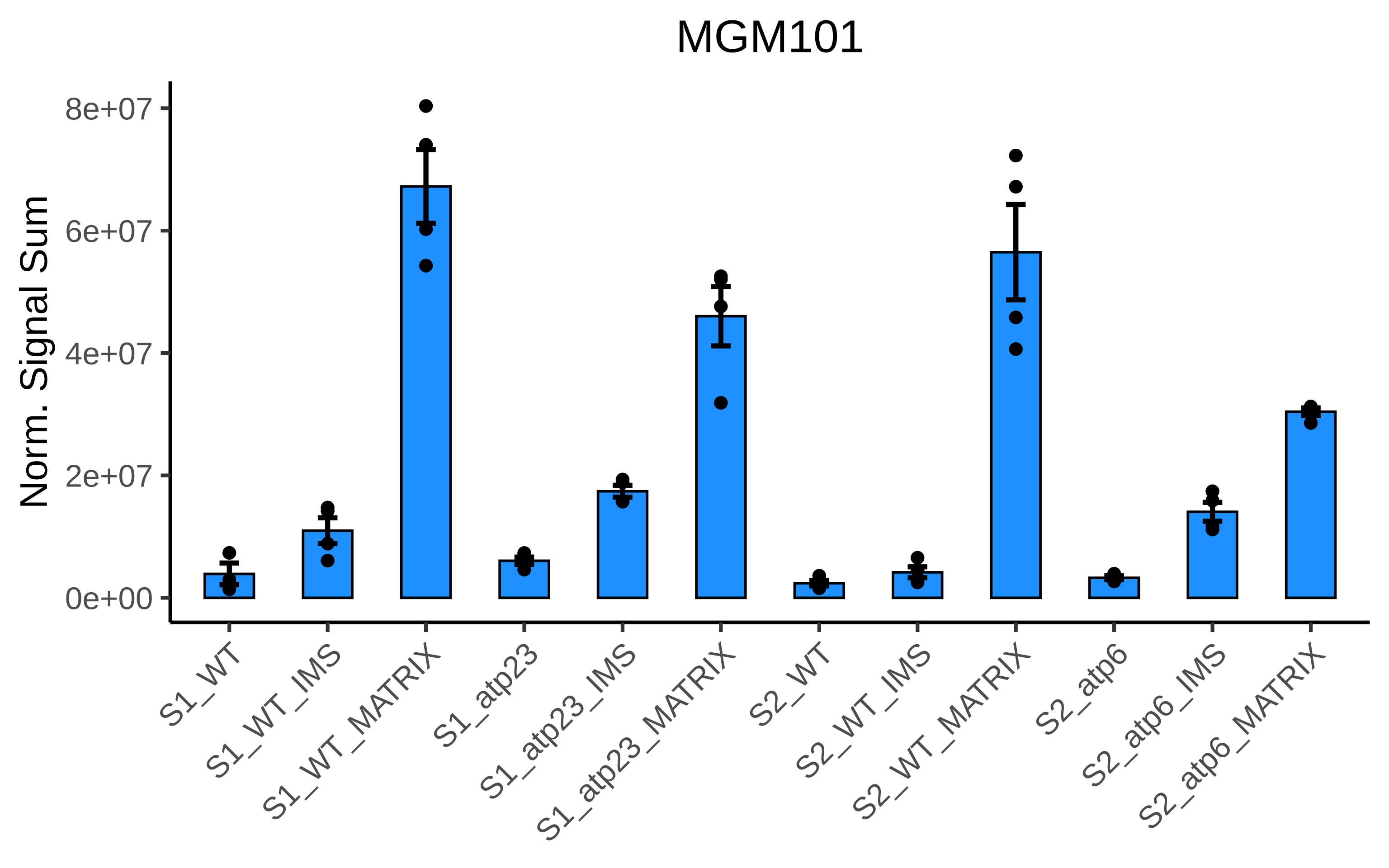

MGM101
Norm. Signal Sum
8e+07
6e+07
4e+07
2e+07
0e+00
S1_WT
S1_WT_IMS
S1_WT_MATRIX
S1_atp23
S1_atp23_IMS
S1_atp23_MATRIX
S2_WT
S2_WT_IMS
S2_WT_MATRIX
S2_atp6
S2_atp6_IMS
S2_atp6_MATRIX

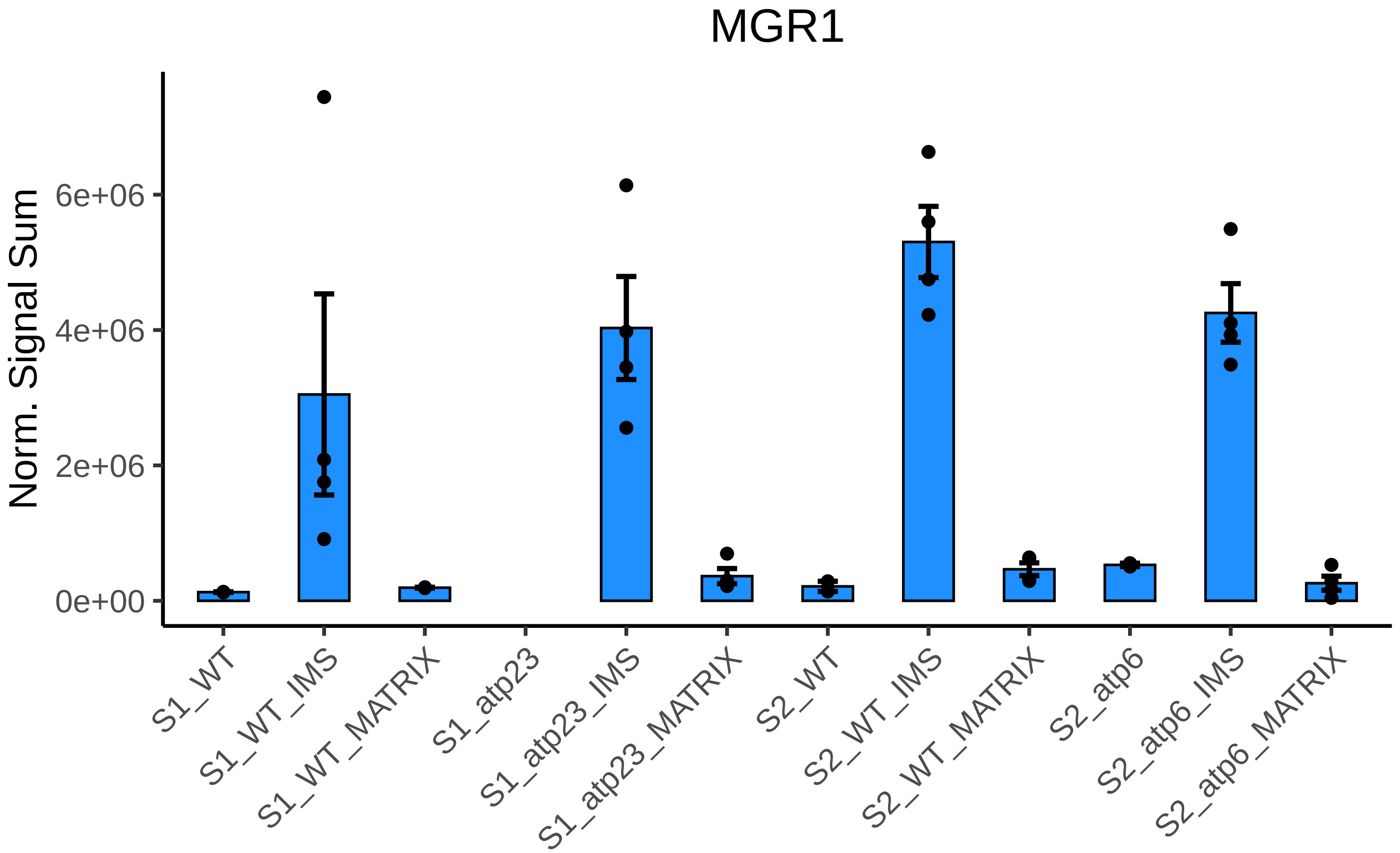
MGR1
Norm. Signal Sum
6e+06
4e+06
2e+06
0e+00
S1_WT
S1_WT_IMS
S1_WT_MATRIX
S1_atp23
S1_atp23_IMS
S1_atp23_MATRIX
S2_WT
S2_WT_IMS
S2_WT_MATRIX
S2_atp6
S2_atp6_IMS
S2_atp6_MATRIX

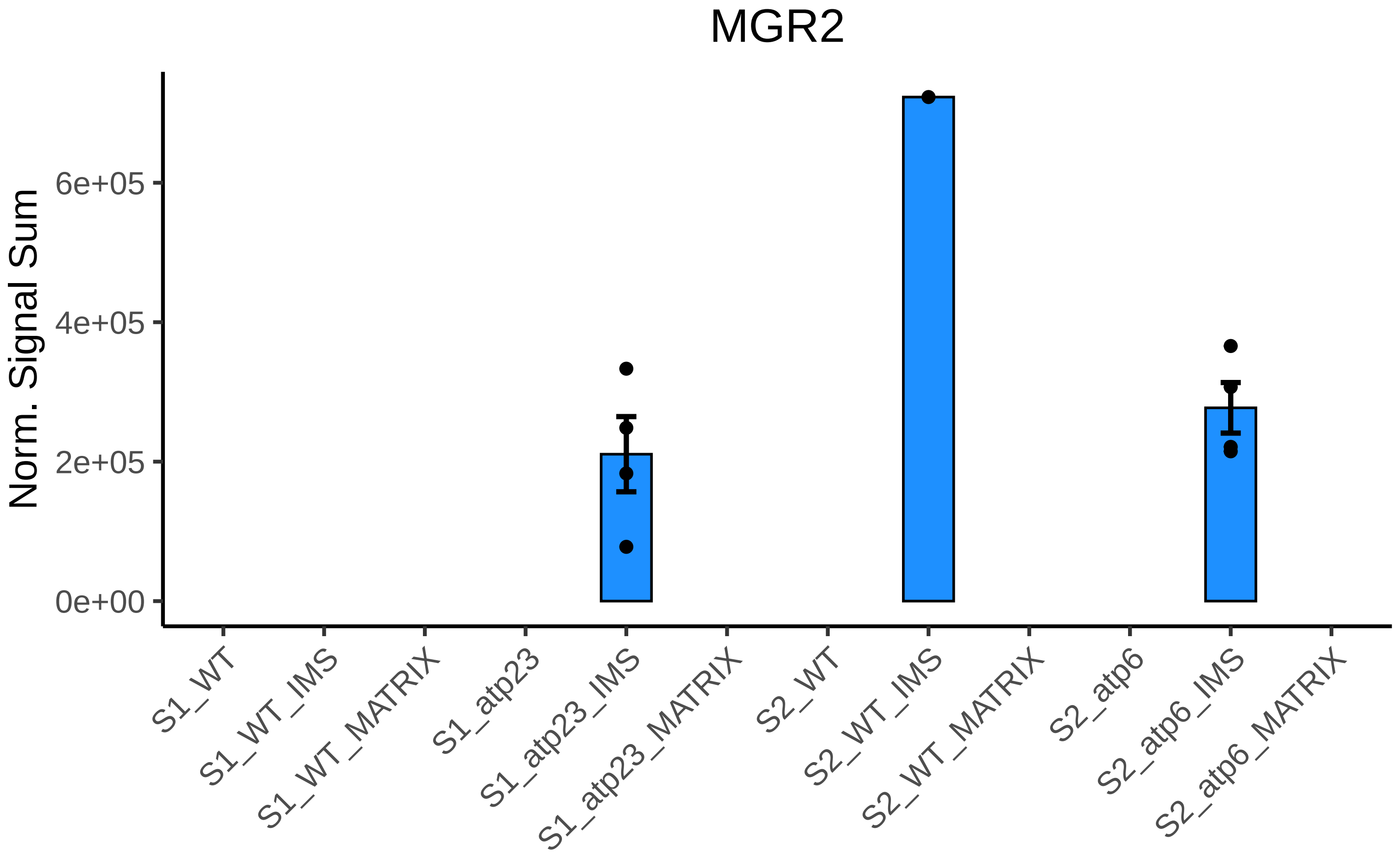

MGR2
Norm. Signal Sum
6e+05
4e+05
2e+05
0e+00
S1_WT
S1_WT_IMS
S1_WT_MATRIX
S1_atp23
S1_atp23_IMS
S1_atp23_MATRIX
S2_WT
S2_WT_IMS
S2_WT_MATRIX
S2_atp6
S2_atp6_IMS
S2_atp6_MATRIX

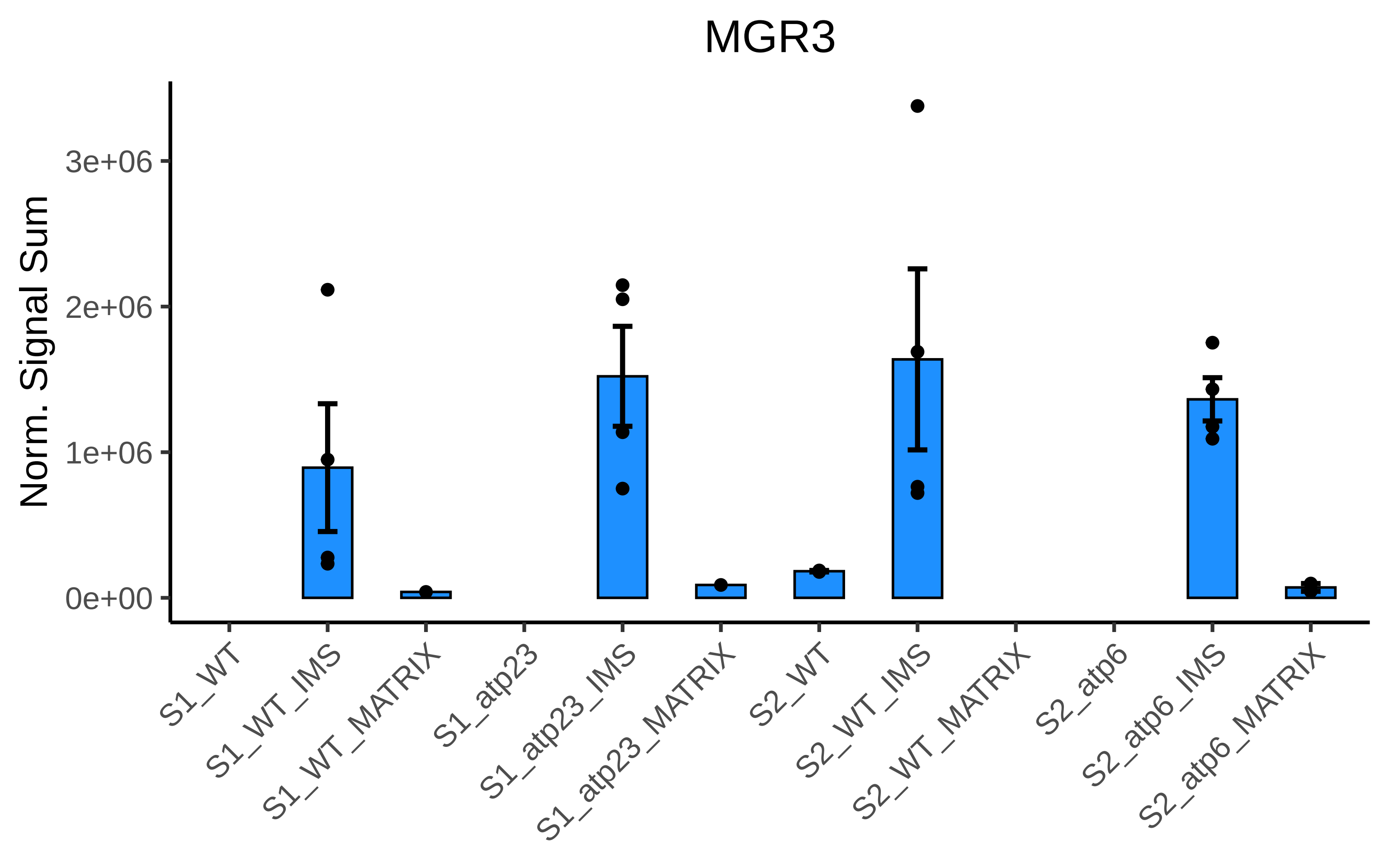
MGR3
Norm. Signal Sum
3e+06
2e+06
1e+06
0e+00
S1_WT
S1_WT_IMS
S1_WT_MATRIX
S1_atp23
S1_atp23_IMS
S1_atp23_MATRIX
S2_WT
S2_WT_IMS
S2_WT_MATRIX
S2_atp6
S2_atp6_IMS
S2_atp6_MATRIX

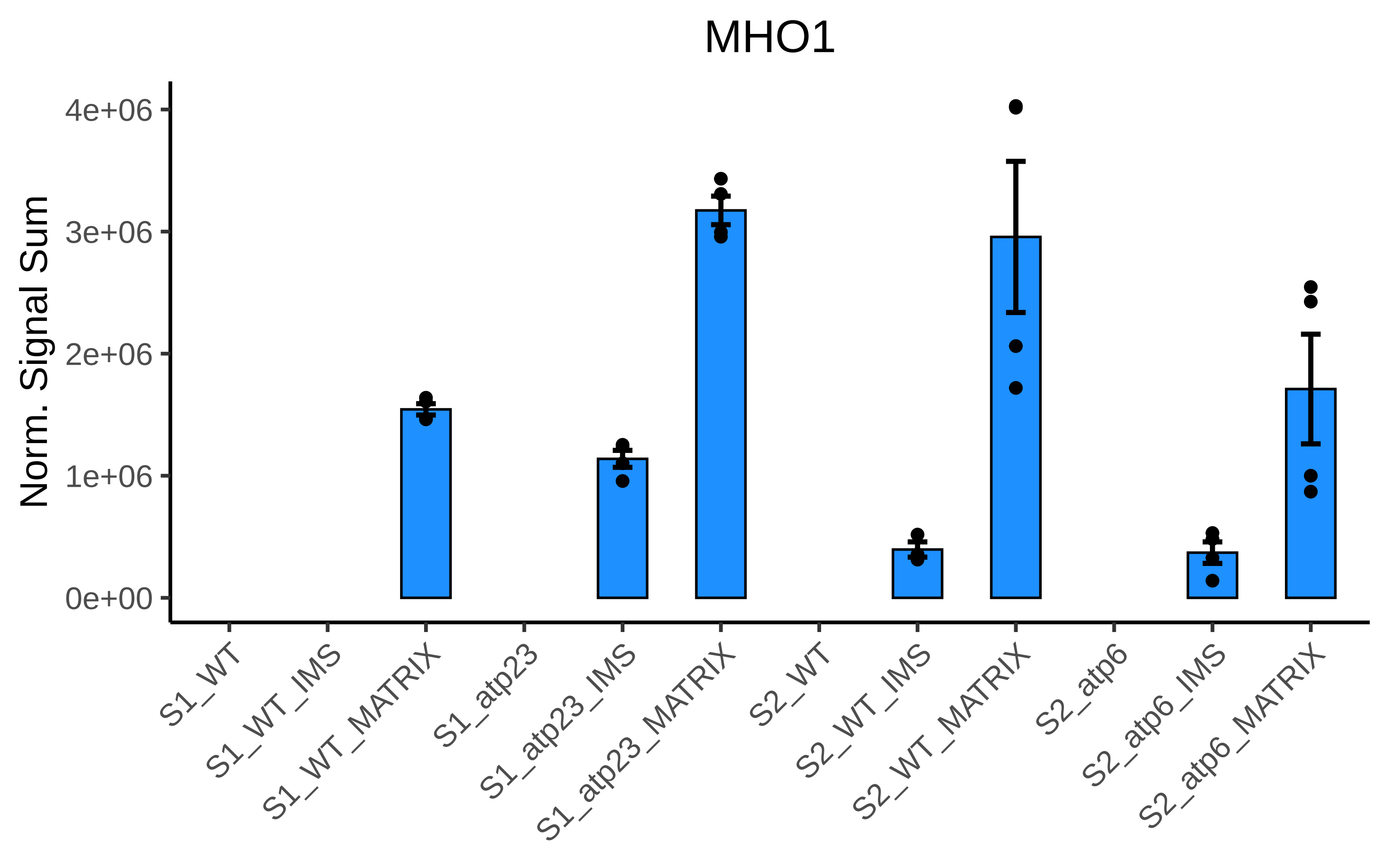

MHO1
Norm. Signal Sum
4e+06
3e+06
2e+06
1e+06
0e+00
S1_WT
S1_WT_IMS
S1_WT_MATRIX
S1_atp23
S1_atp23_IMS
S1_atp23_MATRIX
S2_WT
S2_WT_IMS
S2_WT_MATRIX
S2_atp6
S2_atp6_IMS
S2_atp6_MATRIX

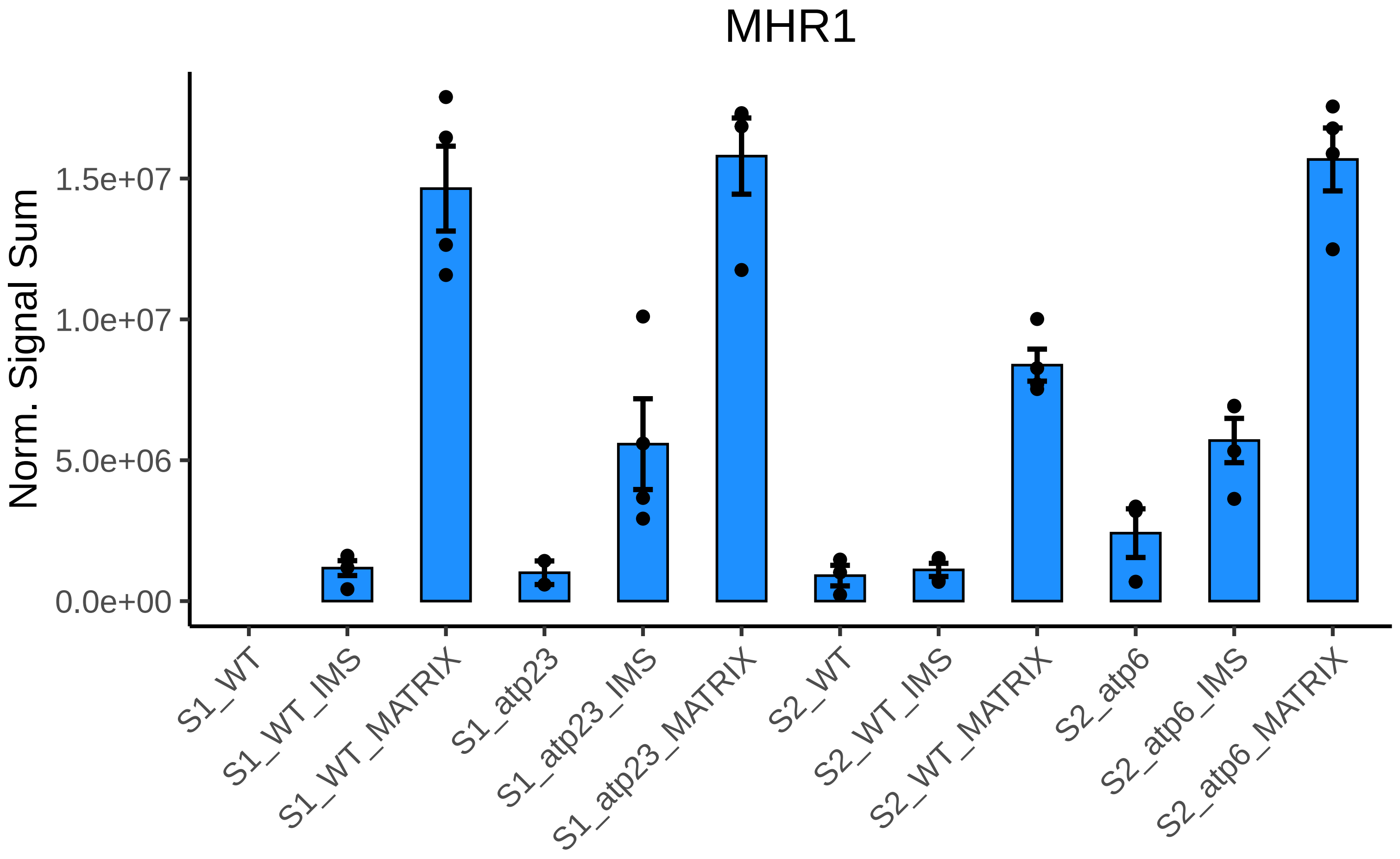

MHR1
Norm. Signal Sum
1.5e+07
1.0e+07
5.0e+06
0.0e+00
S1_WT
S1_WT_IMS
S1_WT_MATRIX
S1_atp23
S1_atp23_IMS
S1_atp23_MATRIX
S2_WT
S2_WT_IMS
S2_WT_MATRIX
S2_atp6
S2_atp6_IMS
S2_atp6_MATRIX

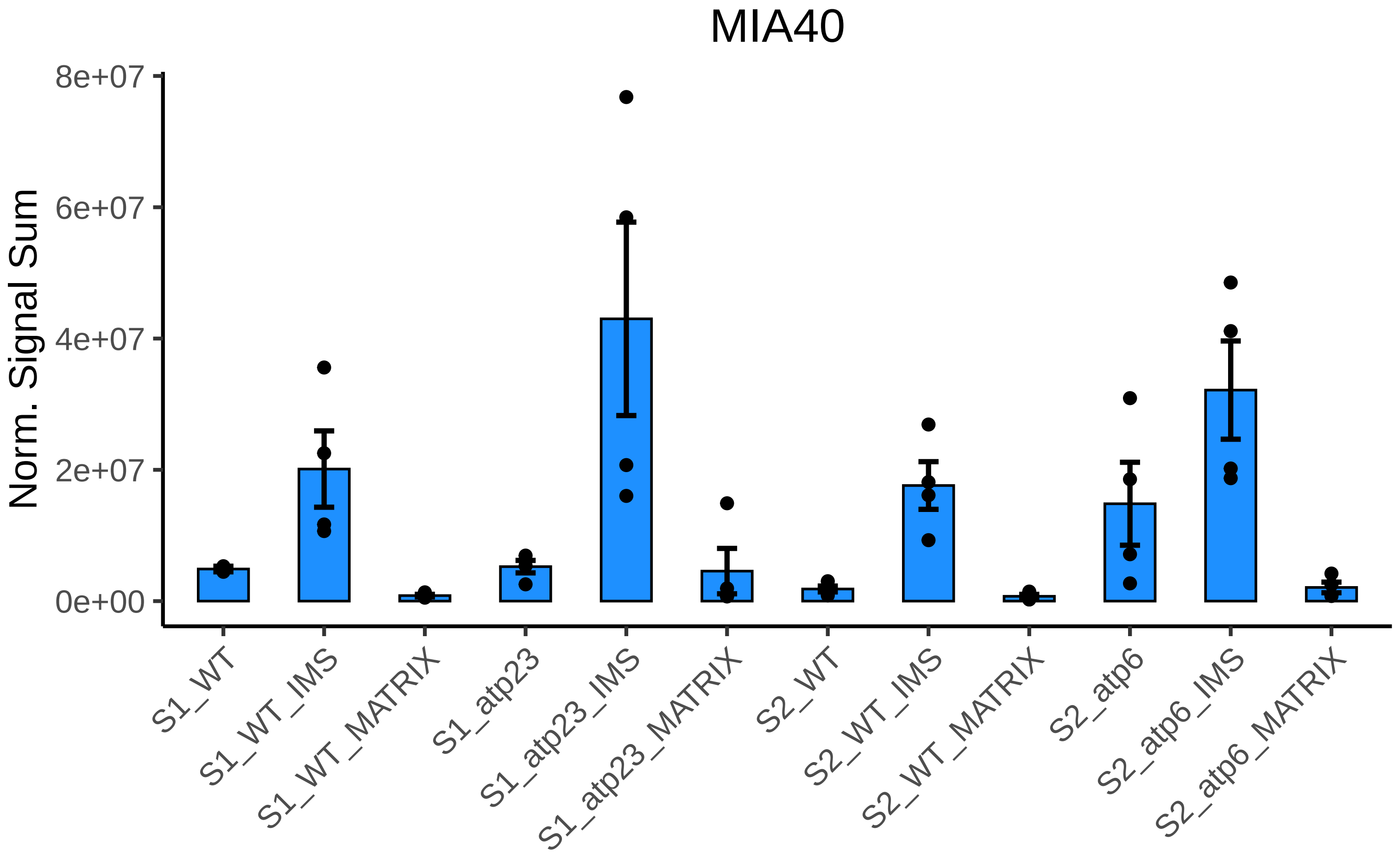

MIA40
Norm. Signal Sum
8e+07
6e+07
4e+07
2e+07
0e+00
S1_WT
S1_WT_IMS
S1_WT_MATRIX
S1_atp23
S1_atp23_IMS
S1_atp23_MATRIX
S2_WT
S2_WT_IMS
S2_WT_MATRIX
S2_atp6
S2_atp6_IMS
S2_atp6_MATRIX

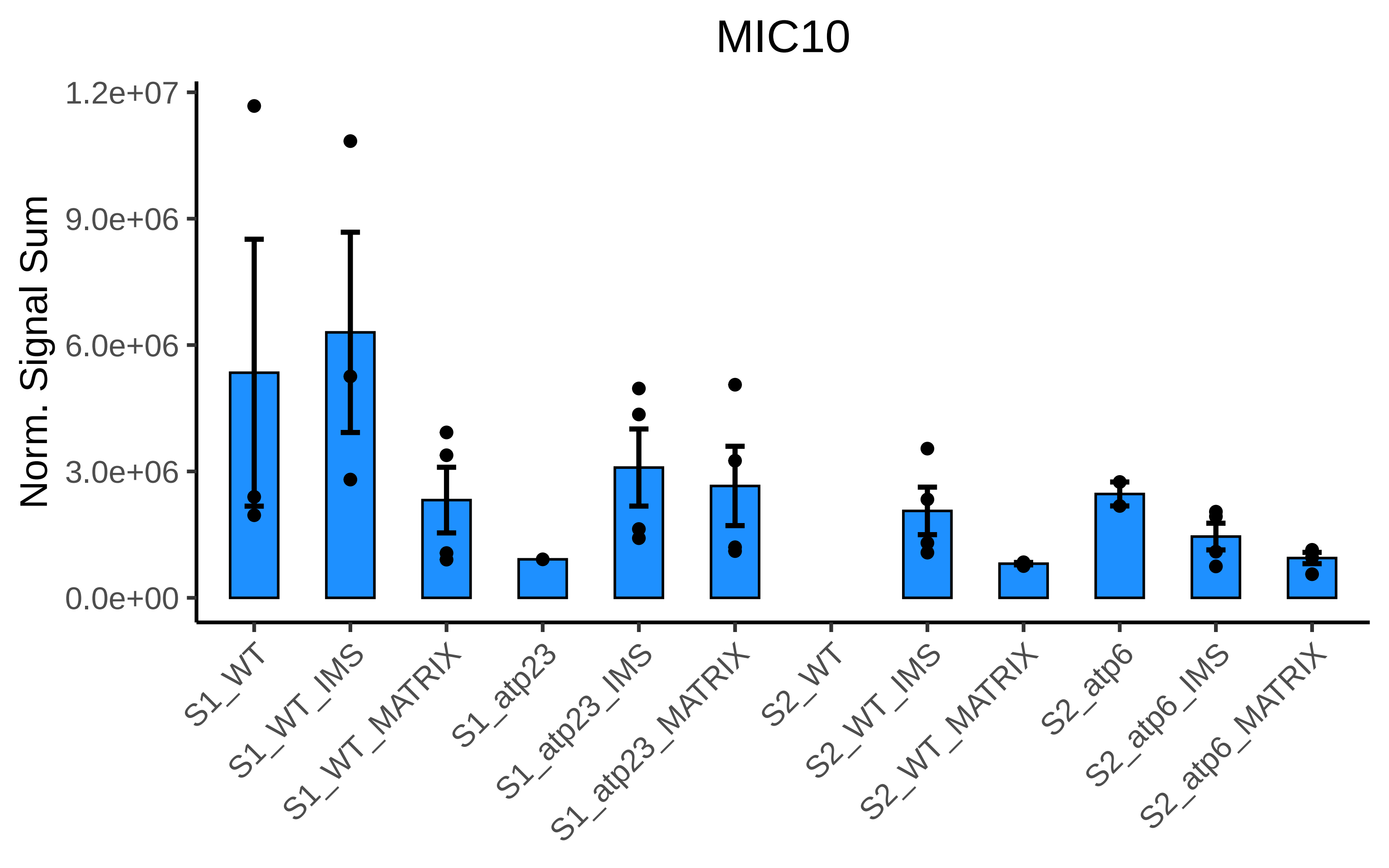

MIC10
Norm. Signal Sum
1.2e+07
9.0e+06
6.0e+06
3.0e+06
0.0e+00
S1_WT
S1_WT_IMS
S1_WT_MATRIX
S1_atp23
S1_atp23_IMS
S1_atp23_MATRIX
S2_WT
S2_WT_IMS
S2_WT_MATRIX
S2_atp6
S2_atp6_IMS
S2_atp6_MATRIX

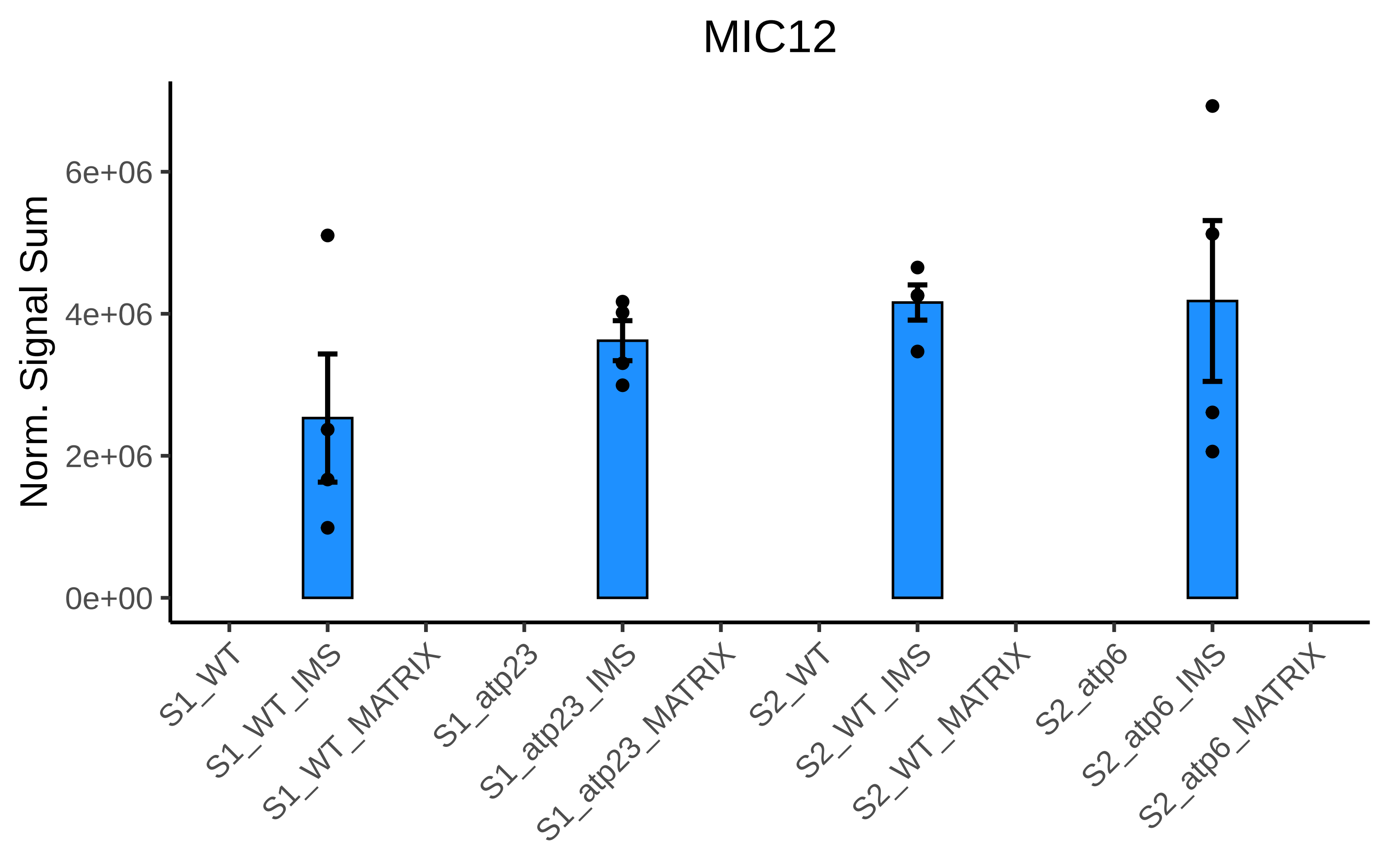

MIC12
Norm. Signal Sum
6e+06
4e+06
2e+06
0e+00
S1_WT
S1_WT_IMS
S1_WT_MATRIX
S1_atp23
S1_atp23_IMS
S1_atp23_MATRIX
S2_WT
S2_WT_IMS
S2_WT_MATRIX
S2_atp6
S2_atp6_IMS
S2_atp6_MATRIX

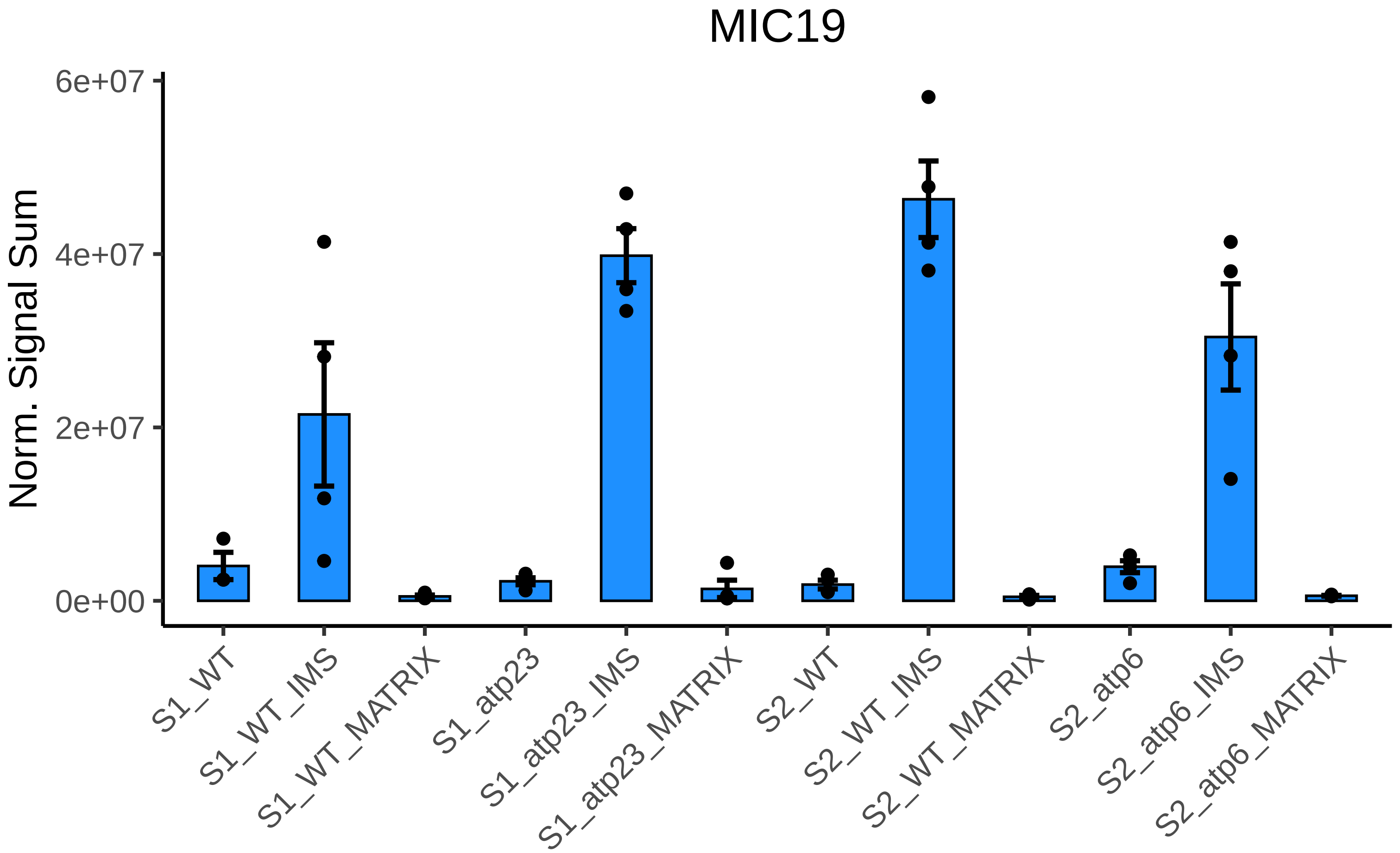

MIC19
Norm. Signal Sum
6e+07
4e+07
2e+07
0e+00
S1_WT
S1_WT_IMS
S1_WT_MATRIX
S1_atp23
S1_atp23_IMS
S1_atp23_MATRIX
S2_WT
S2_WT_IMS
S2_WT_MATRIX
S2_atp6
S2_atp6_IMS
S2_atp6_MATRIX

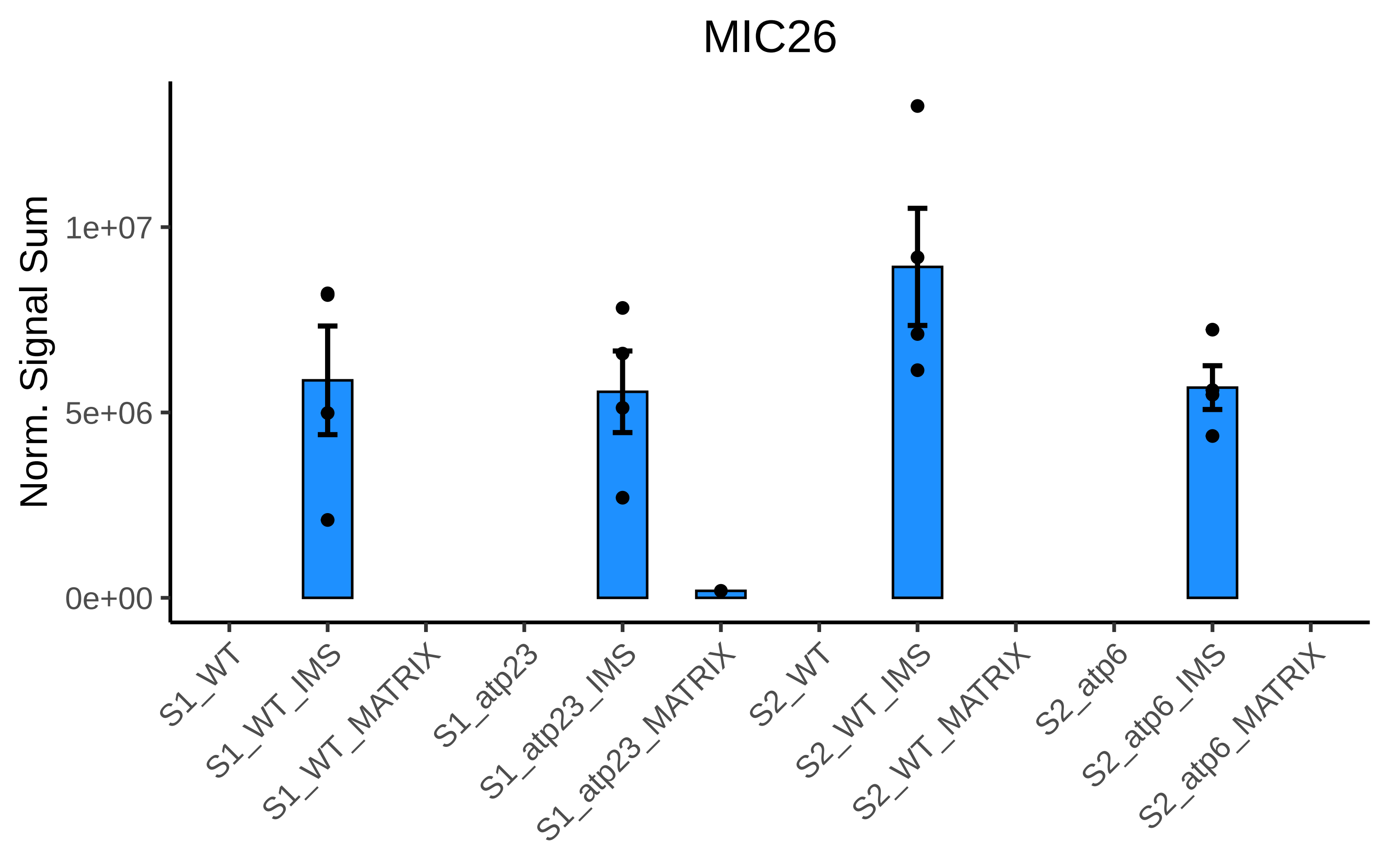

MIC26
Norm. Signal Sum
1e+07
5e+06
0e+00
S1_WT
S1_WT_IMS
S1_WT_MATRIX
S1_atp23
S1_atp23_IMS
S1_atp23_MATRIX
S2_WT
S2_WT_IMS
S2_WT_MATRIX
S2_atp6
S2_atp6_IMS
S2_atp6_MATRIX

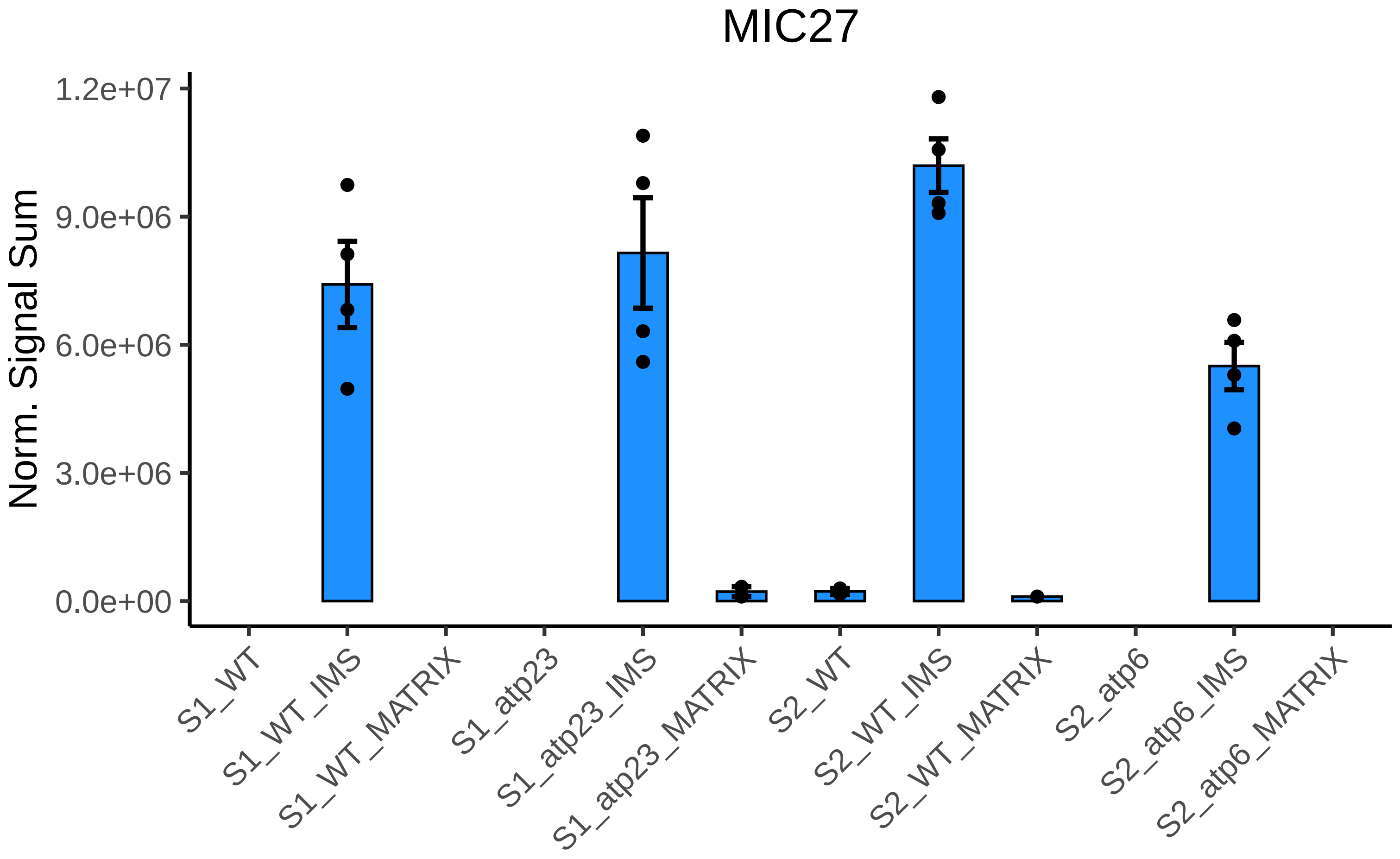

MIC27
Norm. Signal Sum
1.2e+07
9.0e+06
6.0e+06
3.0e+06
0.0e+00
S1_WT
S1_WT_IMS
S1_WT_MATRIX
S1_atp23
S1_atp23_IMS
S1_atp23_MATRIX
S2_WT
S2_WT_IMS
S2_WT_MATRIX
S2_atp6
S2_atp6_IMS
S2_atp6_MATRIX

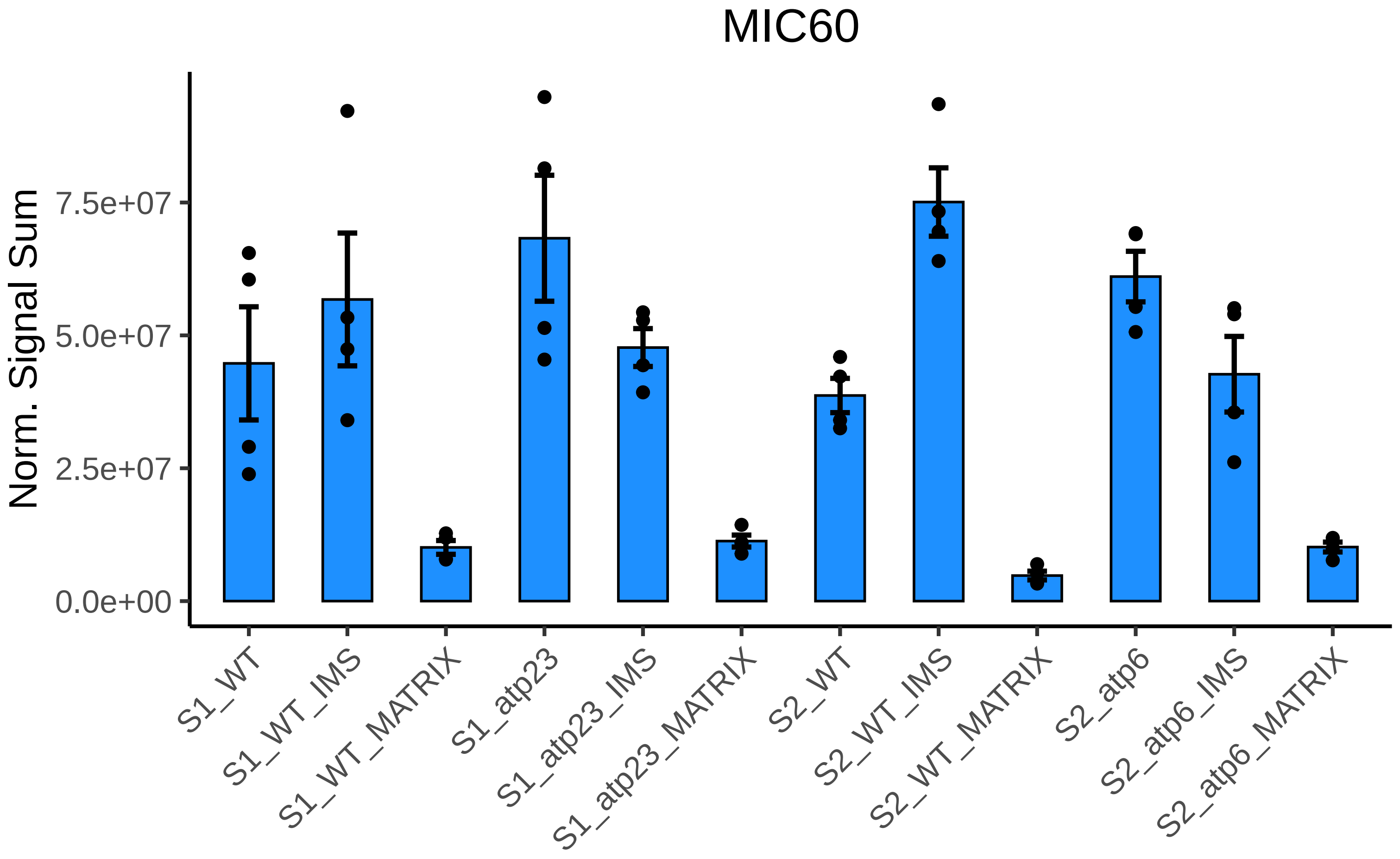

MIC60
Norm. Signal Sum
7.5e+07
5.0e+07
2.5e+07
0.0e+00
S1_WT
S1_WT_IMS
S1_WT_MATRIX
S1_atp23
S1_atp23_IMS
S1_atp23_MATRIX
S2_WT
S2_WT_IMS
S2_WT_MATRIX
S2_atp6
S2_atp6_IMS
S2_atp6_MATRIX

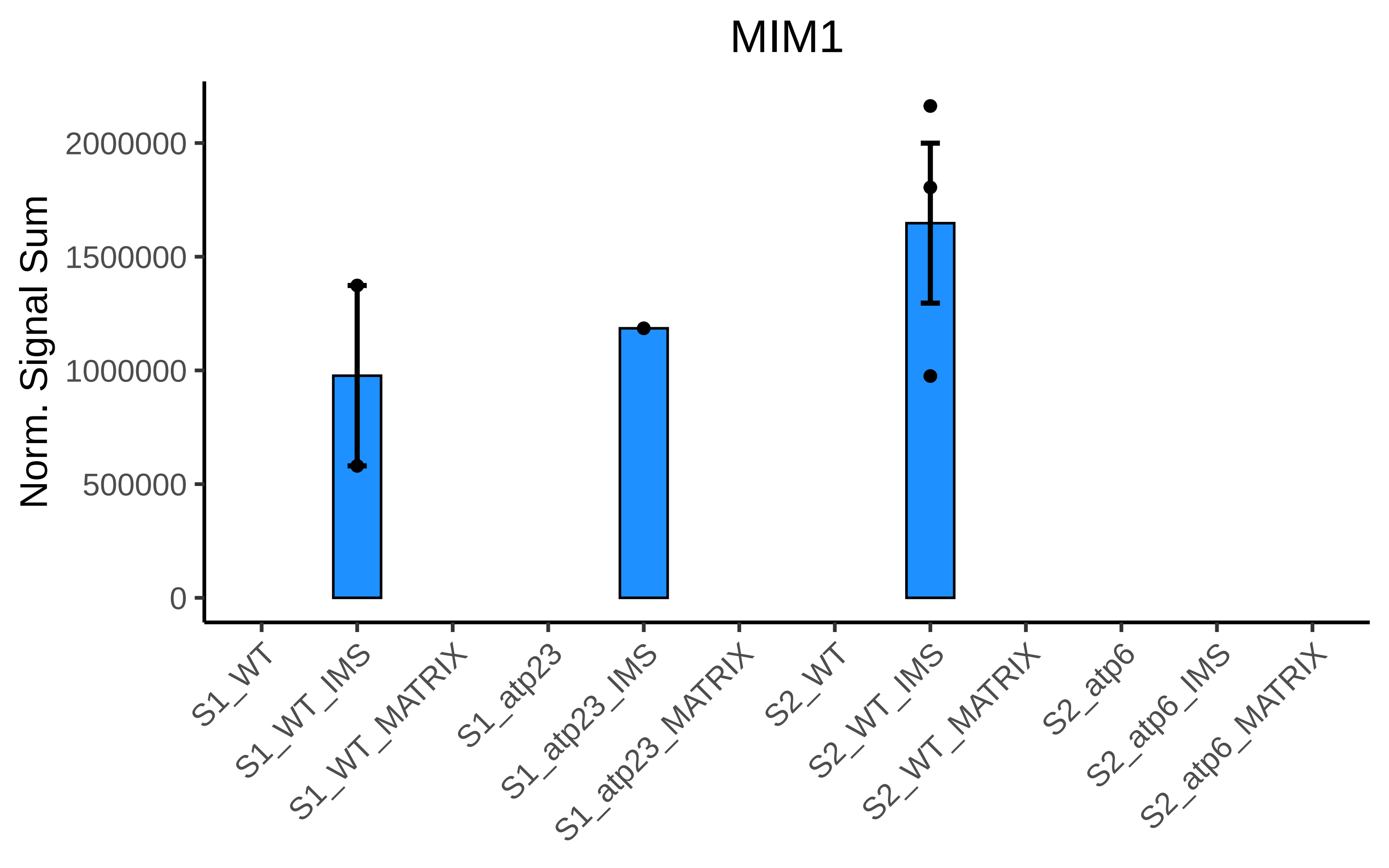
MIM1
Norm. Signal Sum
2000000
1500000
1000000
500000
0
S1_WT
S1_WT_IMS
S1_WT_MATRIX
S1_atp23
S1_atp23_IMS
S1_atp23_MATRIX
S2_WT
S2_WT_IMS
S2_WT_MATRIX
S2_atp6
S2_atp6_IMS
S2_atp6_MATRIX

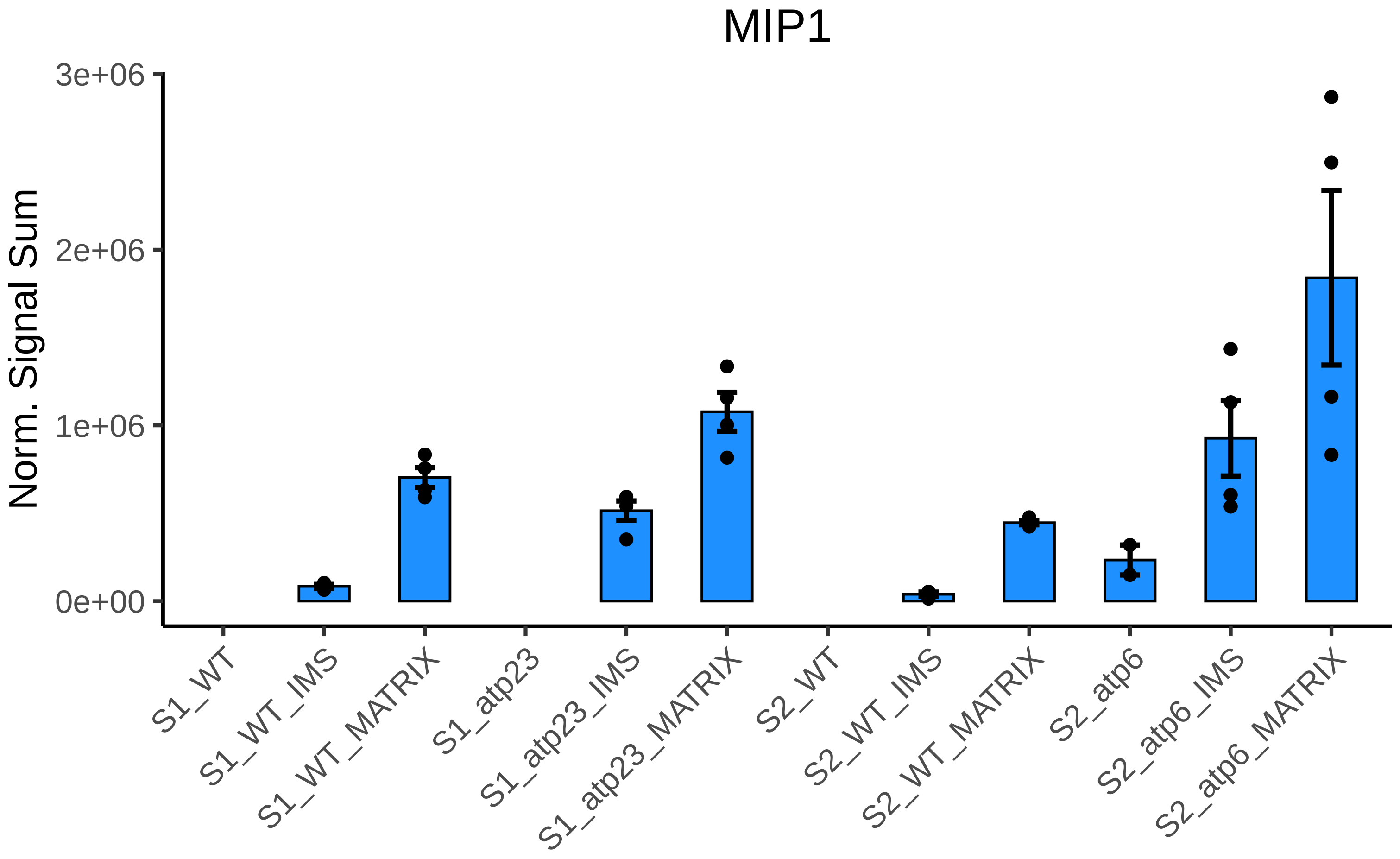

MIP1
Norm. Signal Sum
3e+06
2e+06
1e+06
0e+00
S1_WT
S1_WT_IMS
S1_WT_MATRIX
S1_atp23
S1_atp23_IMS
S1_atp23_MATRIX
S2_WT
S2_WT_IMS
S2_WT_MATRIX
S2_atp6
S2_atp6_IMS
S2_atp6_MATRIX

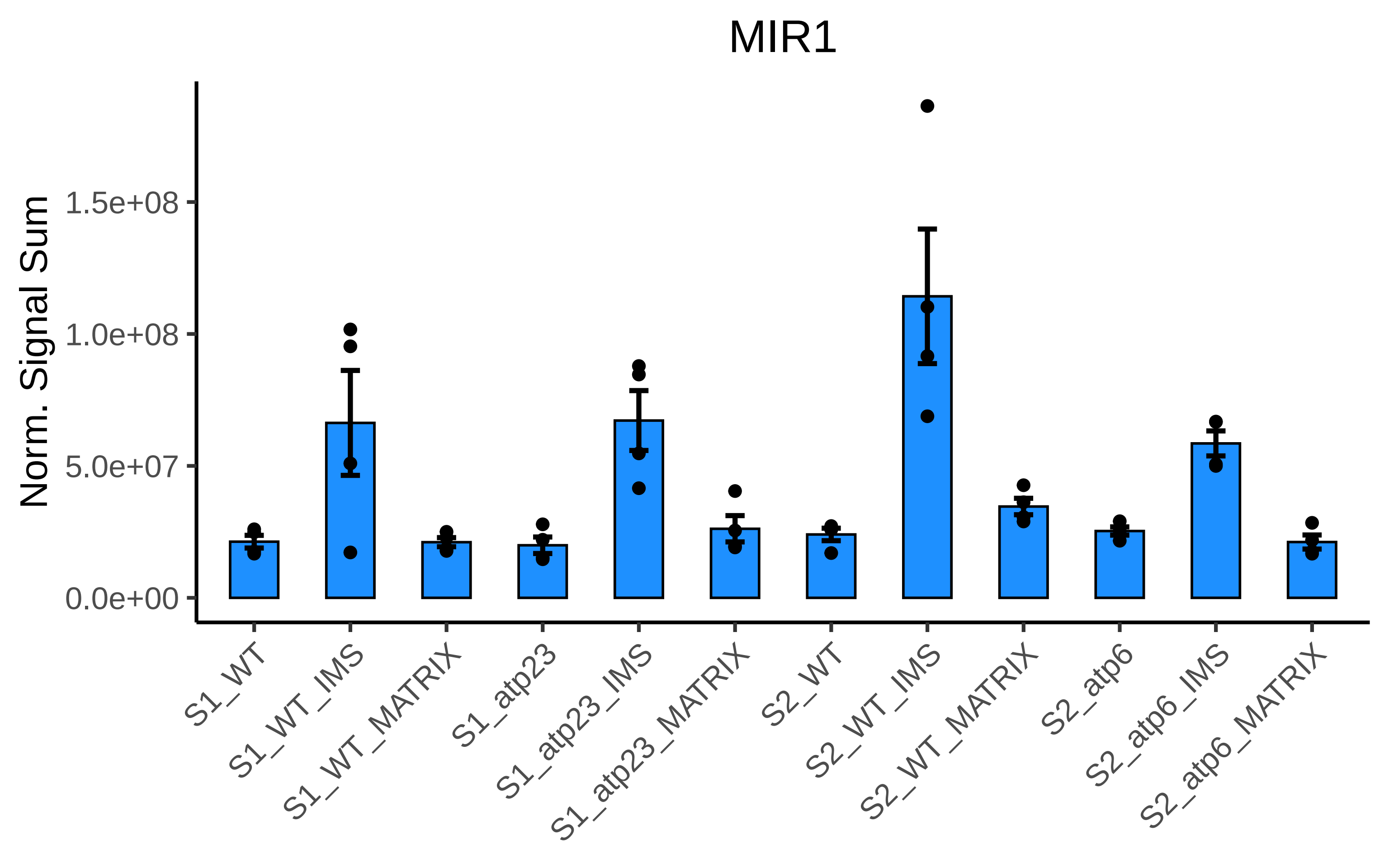

MIR1
Norm. Signal Sum
1.5e+08
1.0e+08
5.0e+07
0.0e+00
S1_WT
S1_WT_IMS
S1_WT_MATRIX
S1_atp23
S1_atp23_IMS
S1_atp23_MATRIX
S2_WT
S2_WT_IMS
S2_WT_MATRIX
S2_atp6
S2_atp6_IMS
S2_atp6_MATRIX

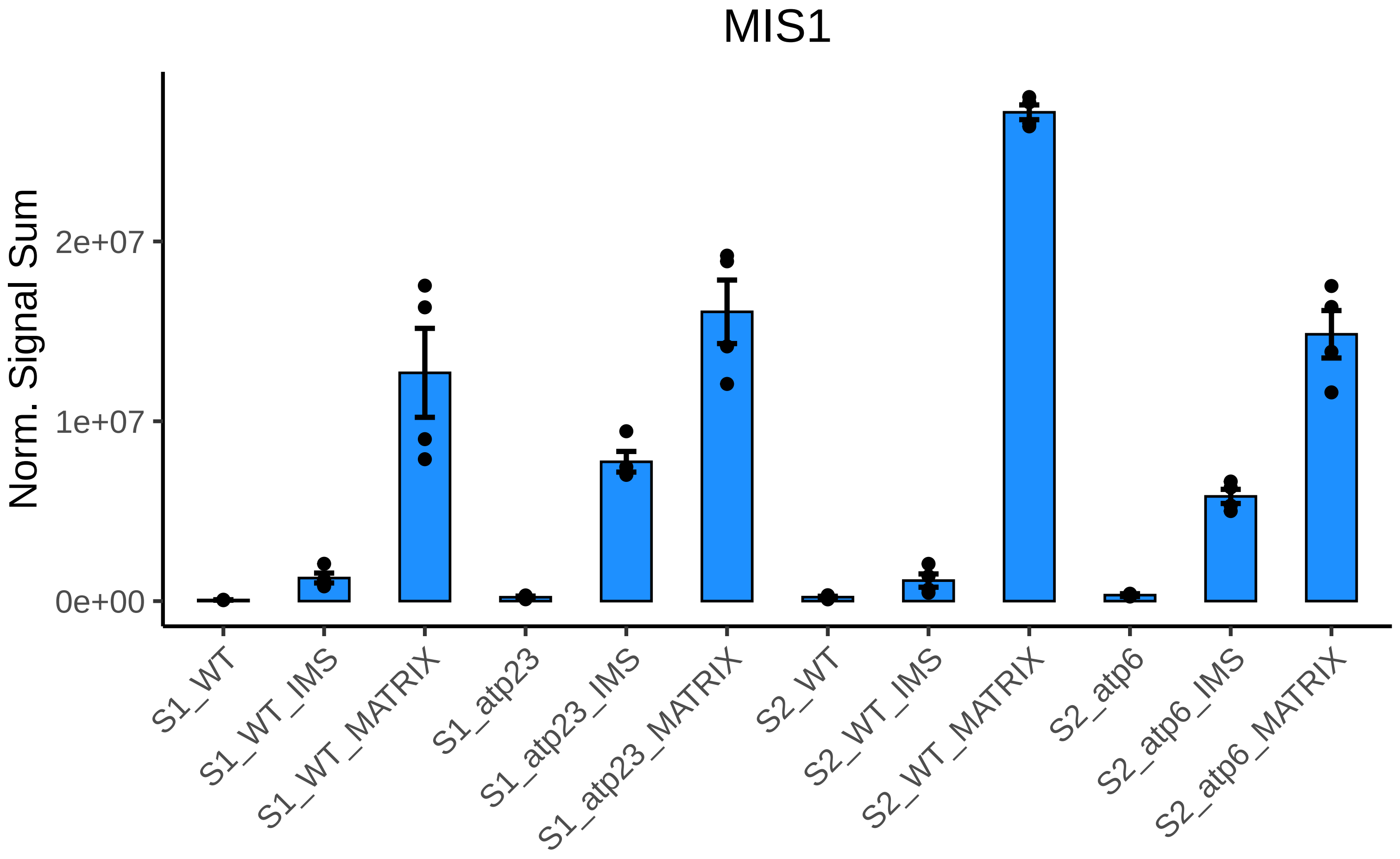
MIS1
Norm. Signal Sum
2e+07
1e+07
0e+00
S1_WT
S1_WT_IMS
S1_WT_MATRIX
S1_atp23
S1_atp23_IMS
S1_atp23_MATRIX
S2_WT
S2_WT_IMS
S2_WT_MATRIX
S2_atp6
S2_atp6_IMS
S2_atp6_MATRIX

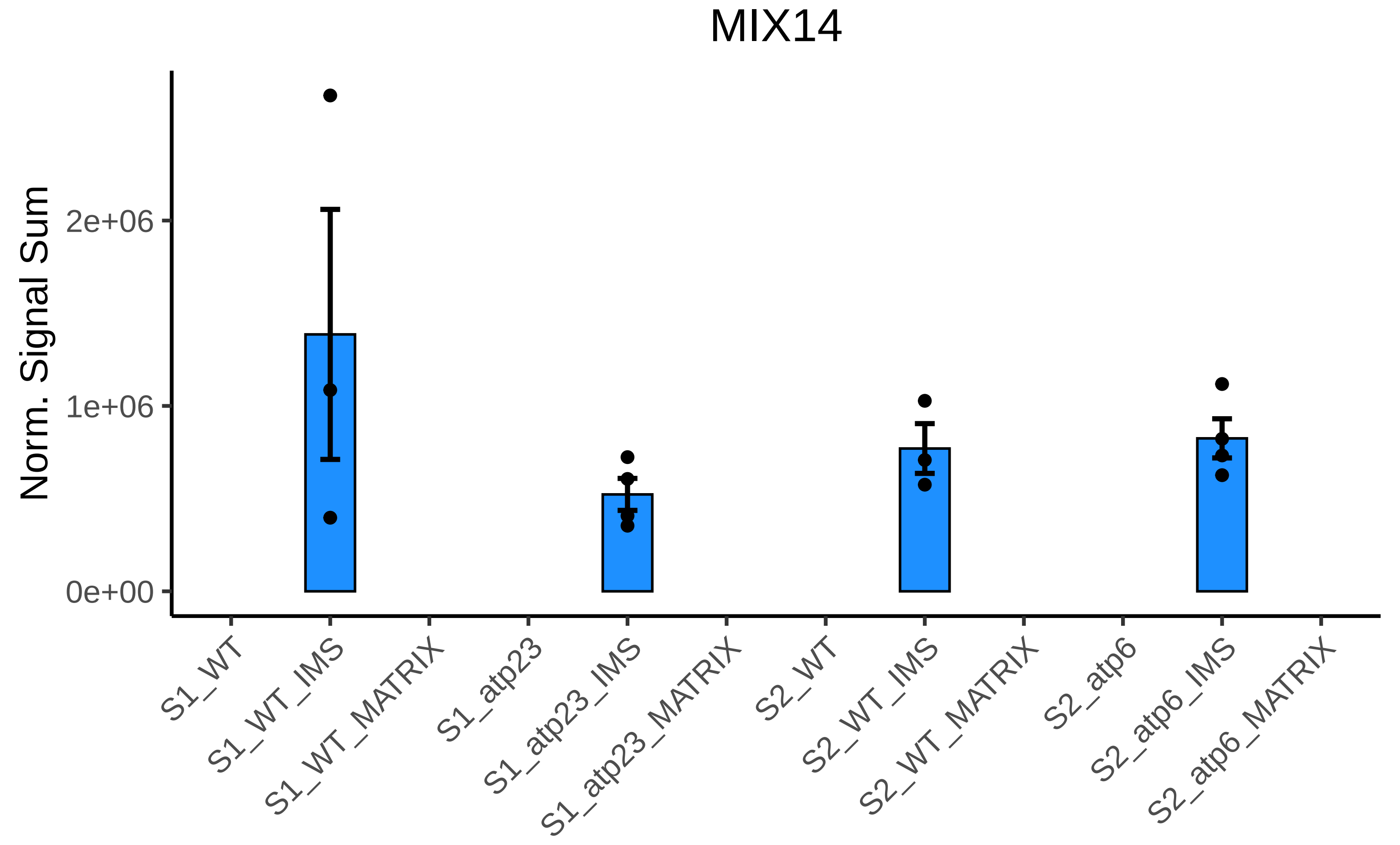

MIX14
Norm. Signal Sum
2e+06
1e+06
0e+00
S1_WT
S1_WT_IMS
S1_WT_MATRIX
S1_atp23
S1_atp23_IMS
S1_atp23_MATRIX
S2_WT
S2_WT_IMS
S2_WT_MATRIX
S2_atp6
S2_atp6_IMS
S2_atp6_MATRIX

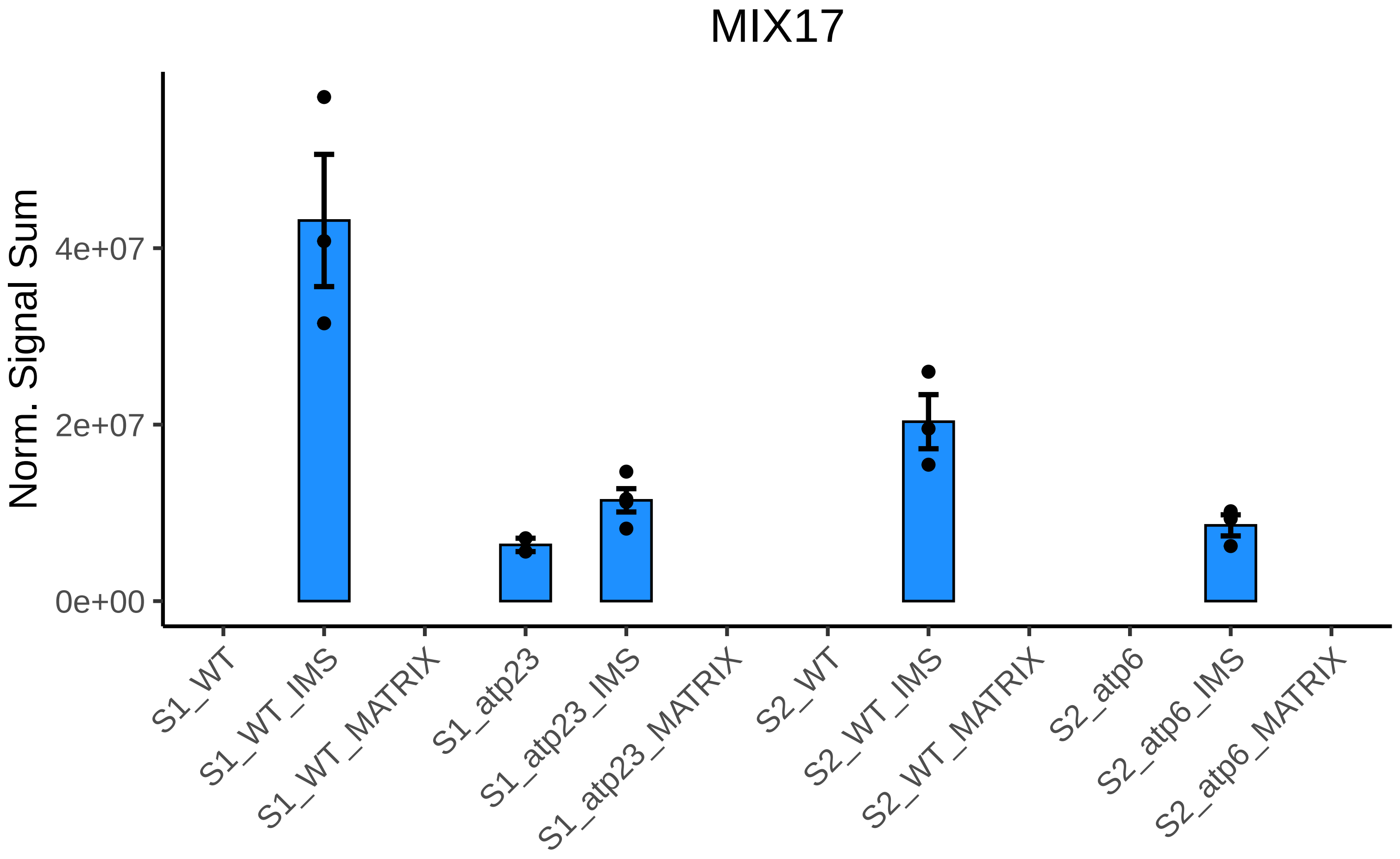
MIX17
Norm. Signal Sum
4e+07
2e+07
0e+00
S1_WT
S1_WT_IMS
S1_WT_MATRIX
S1_atp23
S1_atp23_IMS
S1_atp23_MATRIX
S2_WT
S2_WT_IMS
S2_WT_MATRIX
S2_atp6
S2_atp6_IMS
S2_atp6_MATRIX

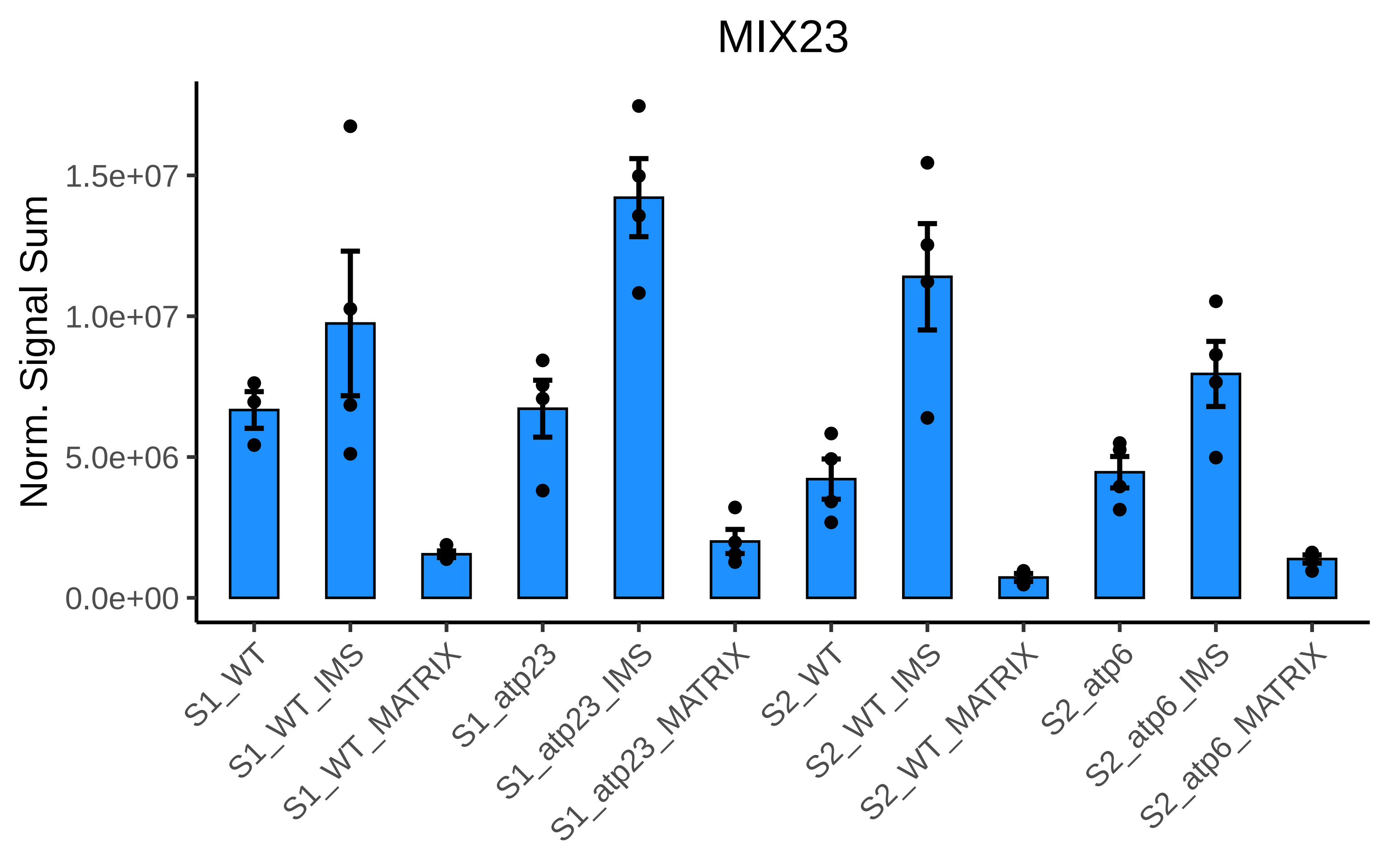

MIX23
Norm. Signal Sum
1.5e+07
1.0e+07
5.0e+06
0.0e+00
S1_WT
S1_WT_IMS
S1_WT_MATRIX
S1_atp23
S1_atp23_IMS
S1_atp23_MATRIX
S2_WT
S2_WT_IMS
S2_WT_MATRIX
S2_atp6
S2_atp6_IMS
S2_atp6_MATRIX

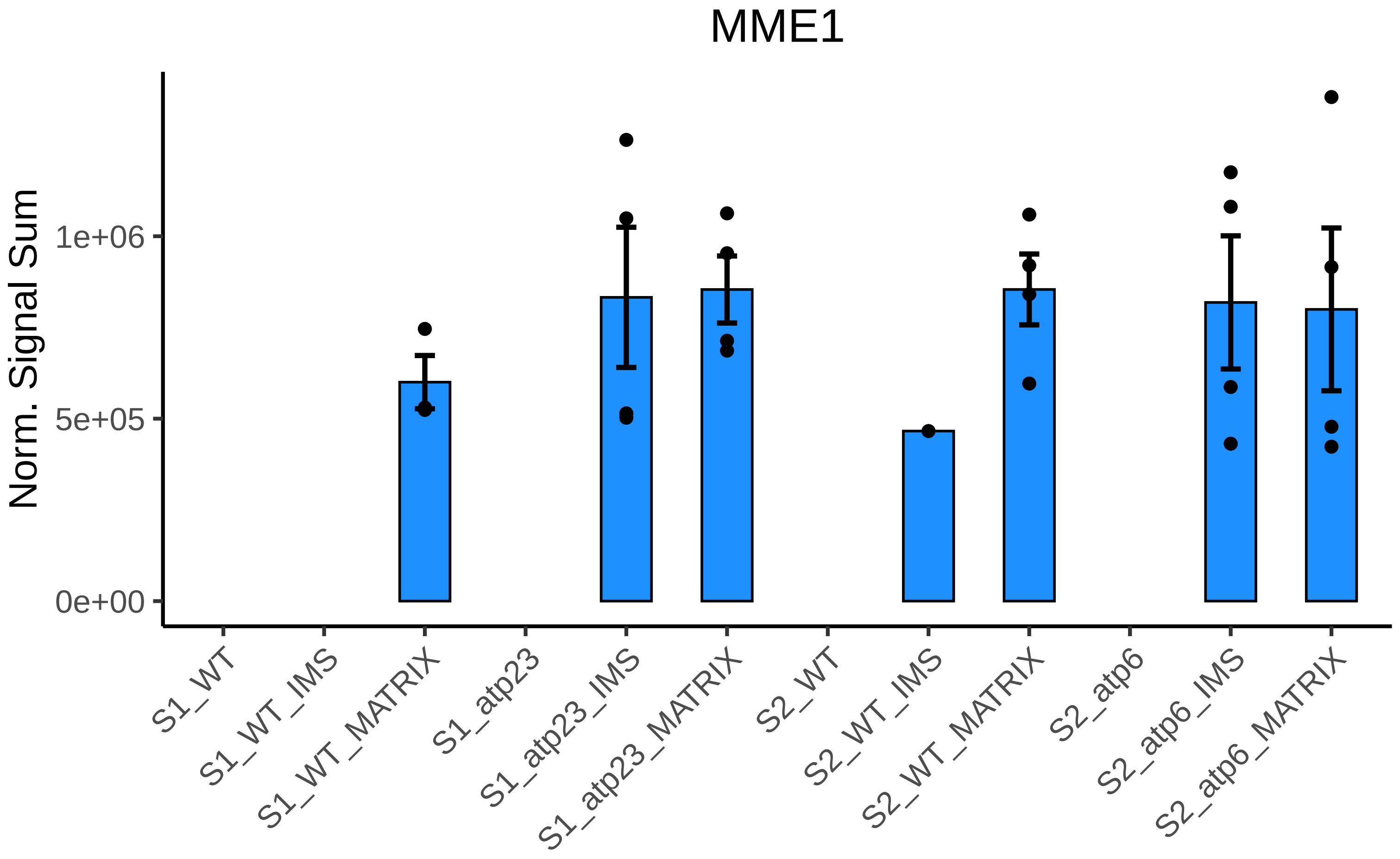

MME1
Norm. Signal Sum
1e+06
5e+05
0e+00
S1_WT
S1_WT_IMS
S1_WT_MATRIX
S1_atp23
S1_atp23_IMS
S1_atp23_MATRIX
S2_WT
S2_WT_IMS
S2_WT_MATRIX
S2_atp6
S2_atp6_IMS
S2_atp6_MATRIX

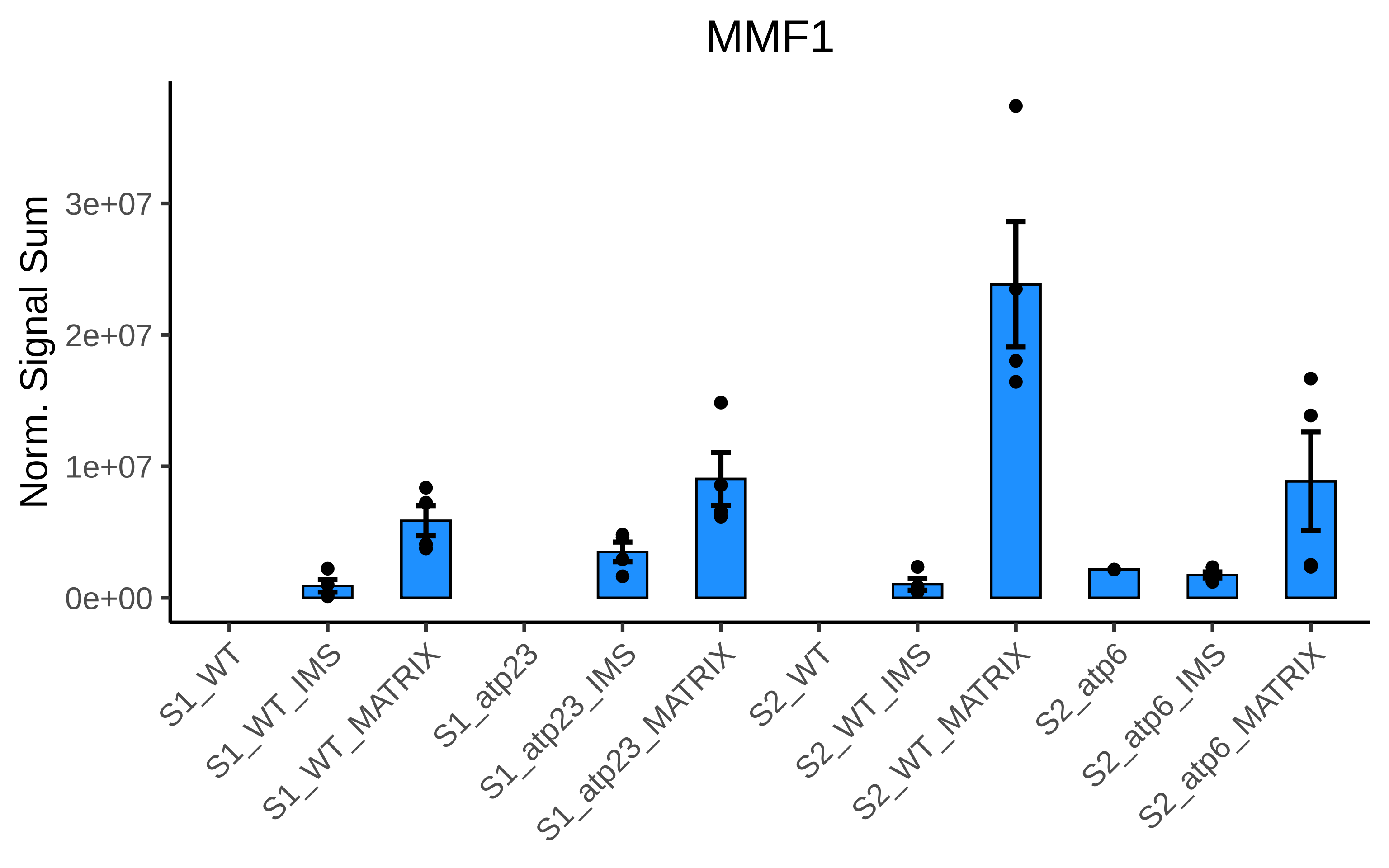
MMF1
Norm. Signal Sum
3e+07
2e+07
1e+07
0e+00
S1_WT
S1_WT_IMS
S1_WT_MATRIX
S1_atp23
S1_atp23_IMS
S1_atp23_MATRIX
S2_WT
S2_WT_IMS
S2_WT_MATRIX
S2_atp6
S2_atp6_IMS
S2_atp6_MATRIX

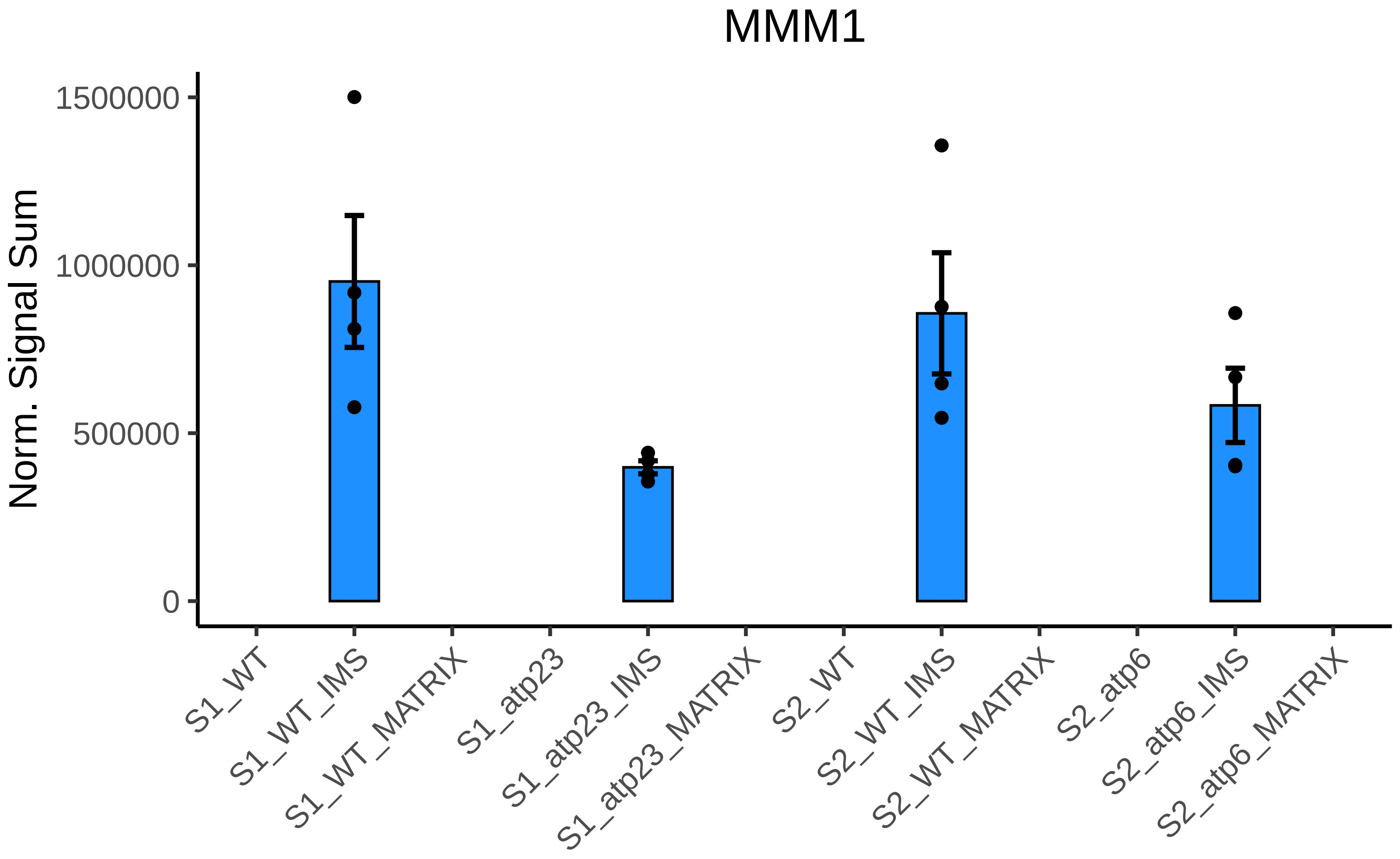
MMM1
Norm. Signal Sum
1500000
1000000
500000
0
S1_WT
S1_WT_IMS
S1_WT_MATRIX
S1_atp23
S1_atp23_IMS
S1_atp23_MATRIX
S2_WT
S2_WT_IMS
S2_WT_MATRIX
S2_atp6
S2_atp6_IMS
S2_atp6_MATRIX

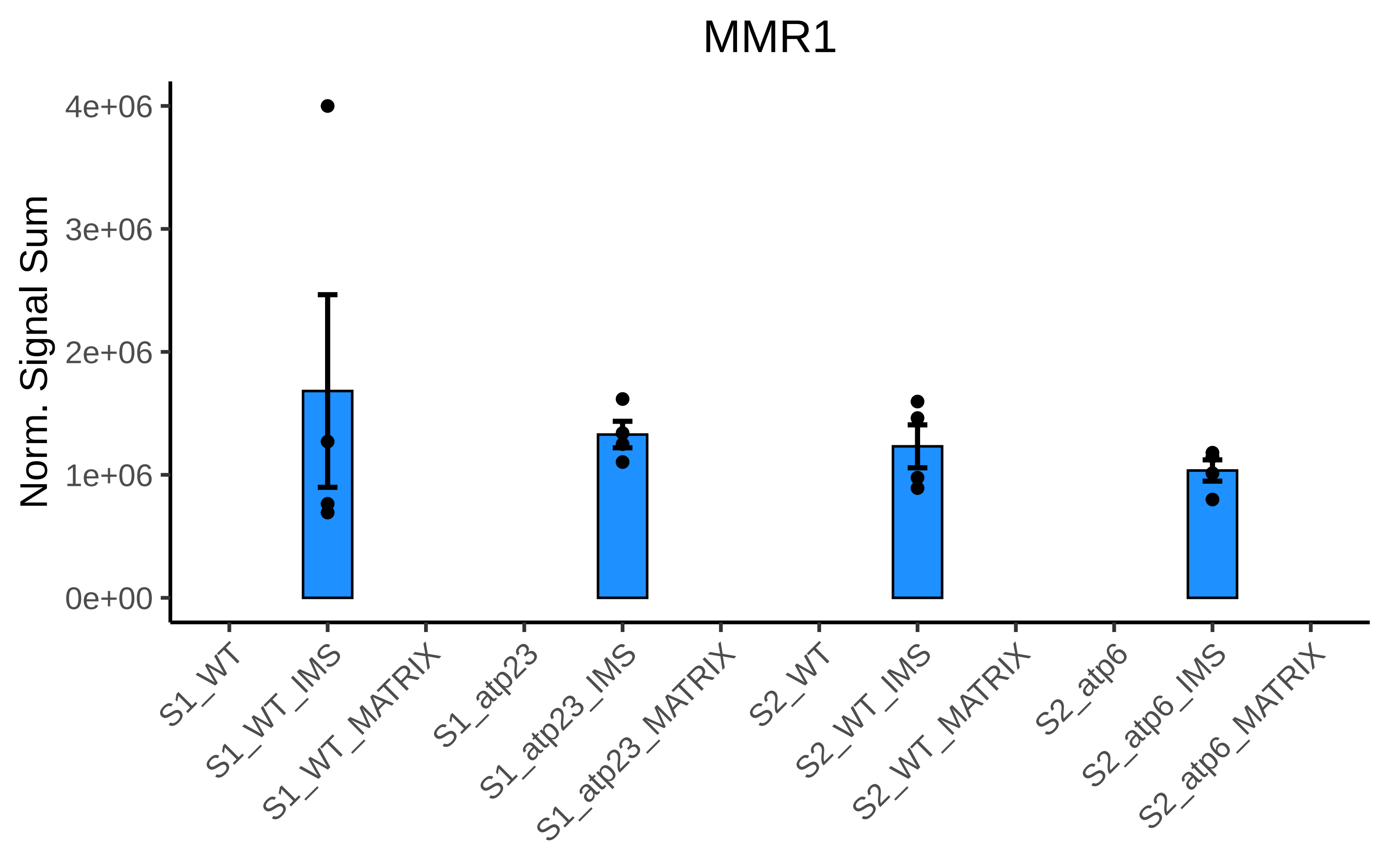
MMR1
Norm. Signal Sum
4e+06
3e+06
2e+06
1e+06
0e+00
S1_WT
S1_WT_IMS
S1_WT_MATRIX
S1_atp23
S1_atp23_IMS
S1_atp23_MATRIX
S2_WT
S2_WT_IMS
S2_WT_MATRIX
S2_atp6
S2_atp6_IMS
S2_atp6_MATRIX

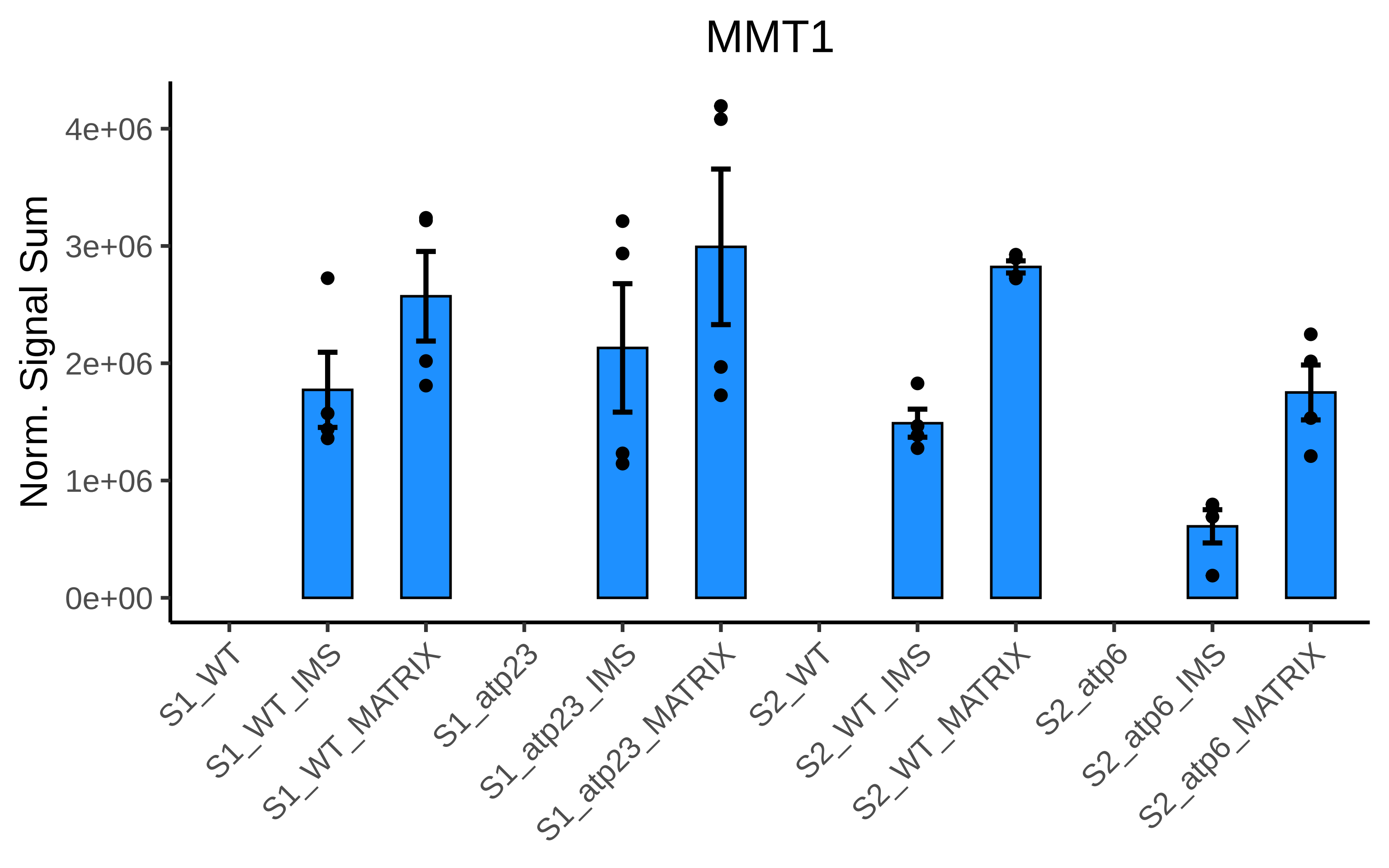
MMT1
Norm. Signal Sum
4e+06
3e+06
2e+06
1e+06
0e+00
S1_WT
S1_WT_IMS
S1_WT_MATRIX
S1_atp23
S1_atp23_IMS
S1_atp23_MATRIX
S2_WT
S2_WT_IMS
S2_WT_MATRIX
S2_atp6
S2_atp6_IMS
S2_atp6_MATRIX

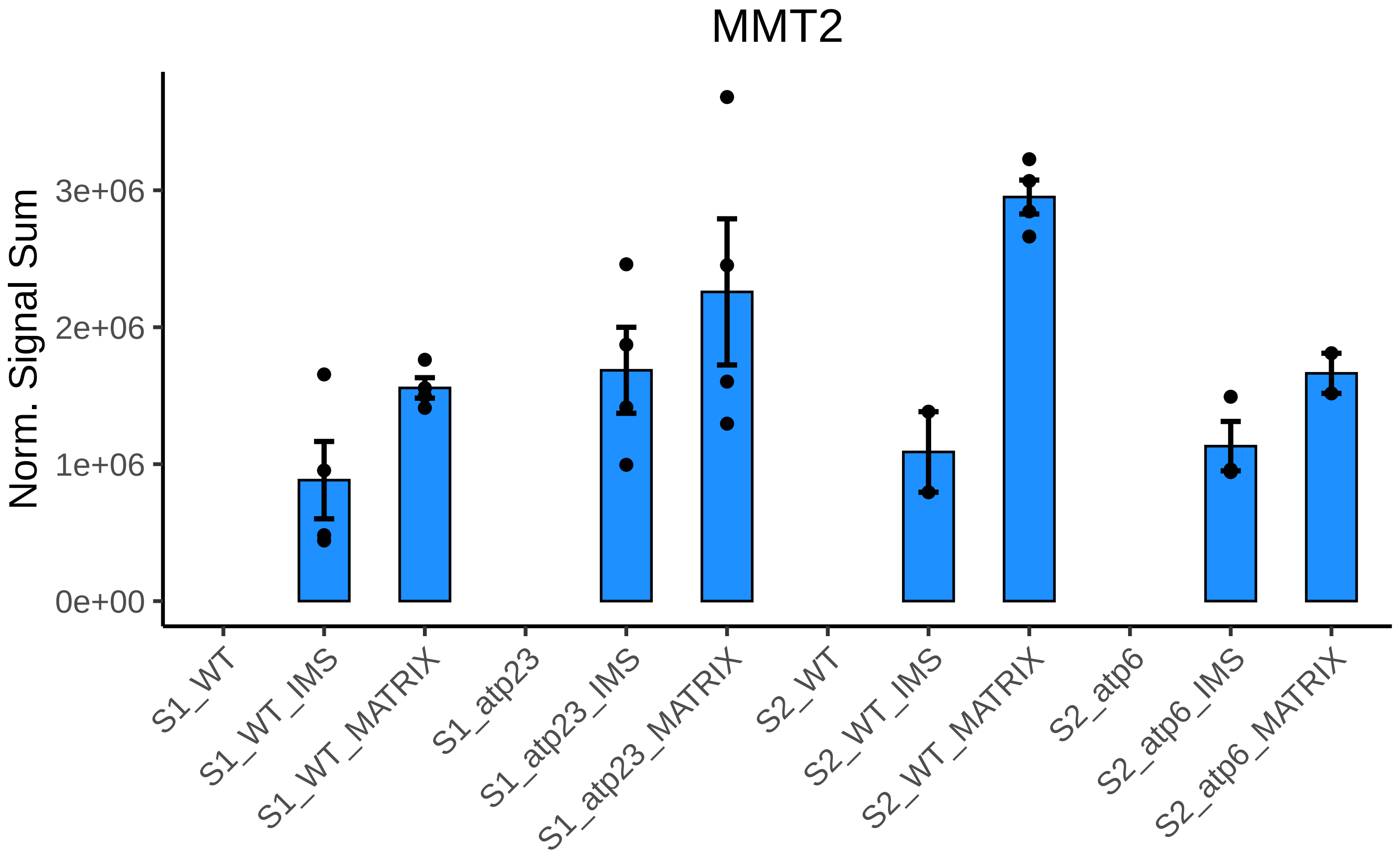

MMT2
Norm. Signal Sum
3e+06
2e+06
1e+06
0e+00
S1_WT
S1_WT_IMS
S1_WT_MATRIX
S1_atp23
S1_atp23_IMS
S1_atp23_MATRIX
S2_WT
S2_WT_IMS
S2_WT_MATRIX
S2_atp6
S2_atp6_IMS
S2_atp6_MATRIX

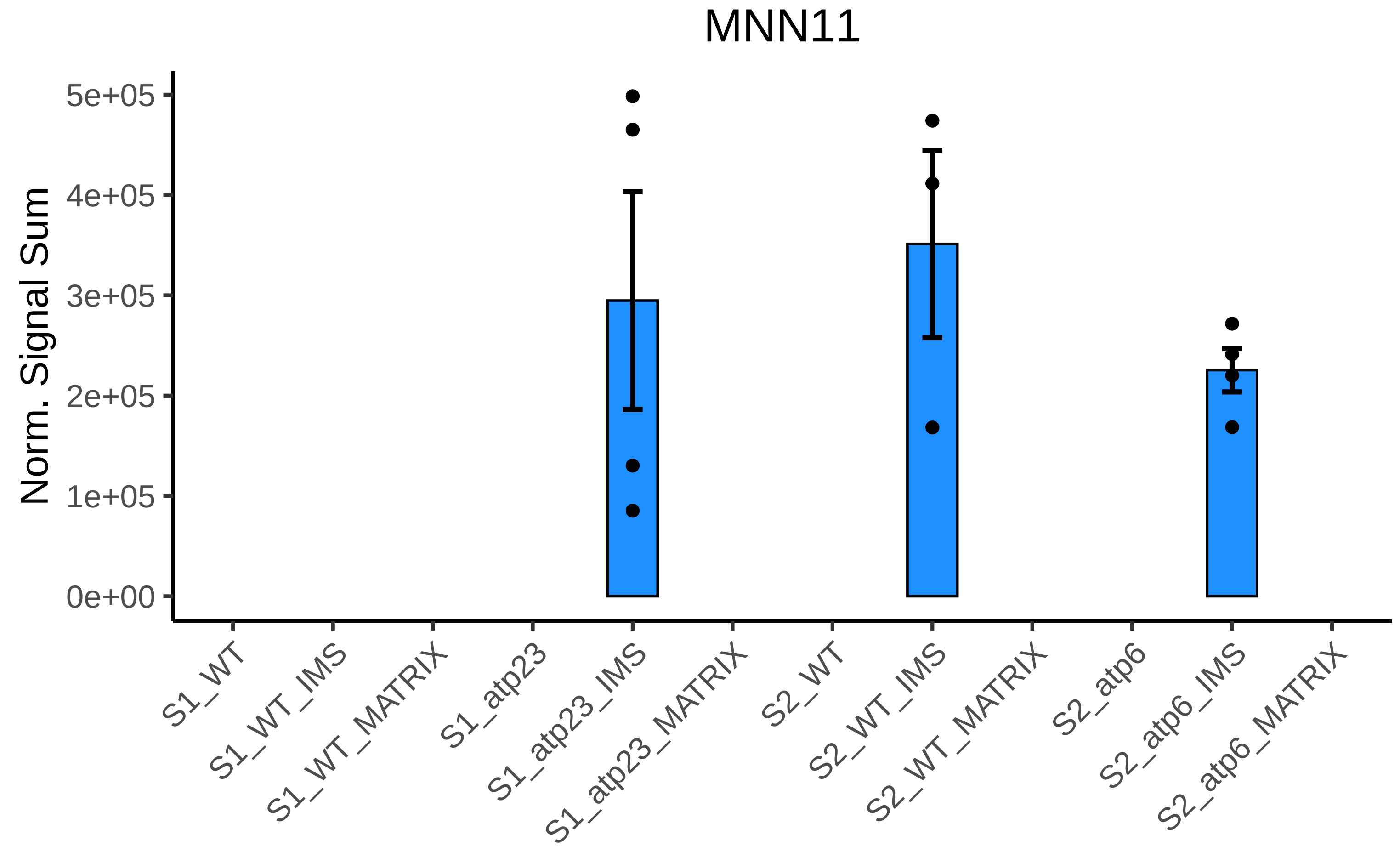

MNN11
Norm. Signal Sum
5e+05
4e+05
3e+05
2e+05
1e+05
0e+00
S1_WT
S1_WT_IMS
S1_WT_MATRIX
S1_atp23
S1_atp23_IMS
S1_atp23_MATRIX
S2_WT
S2_WT_IMS
S2_WT_MATRIX
S2_atp6
S2_atp6_IMS
S2_atp6_MATRIX

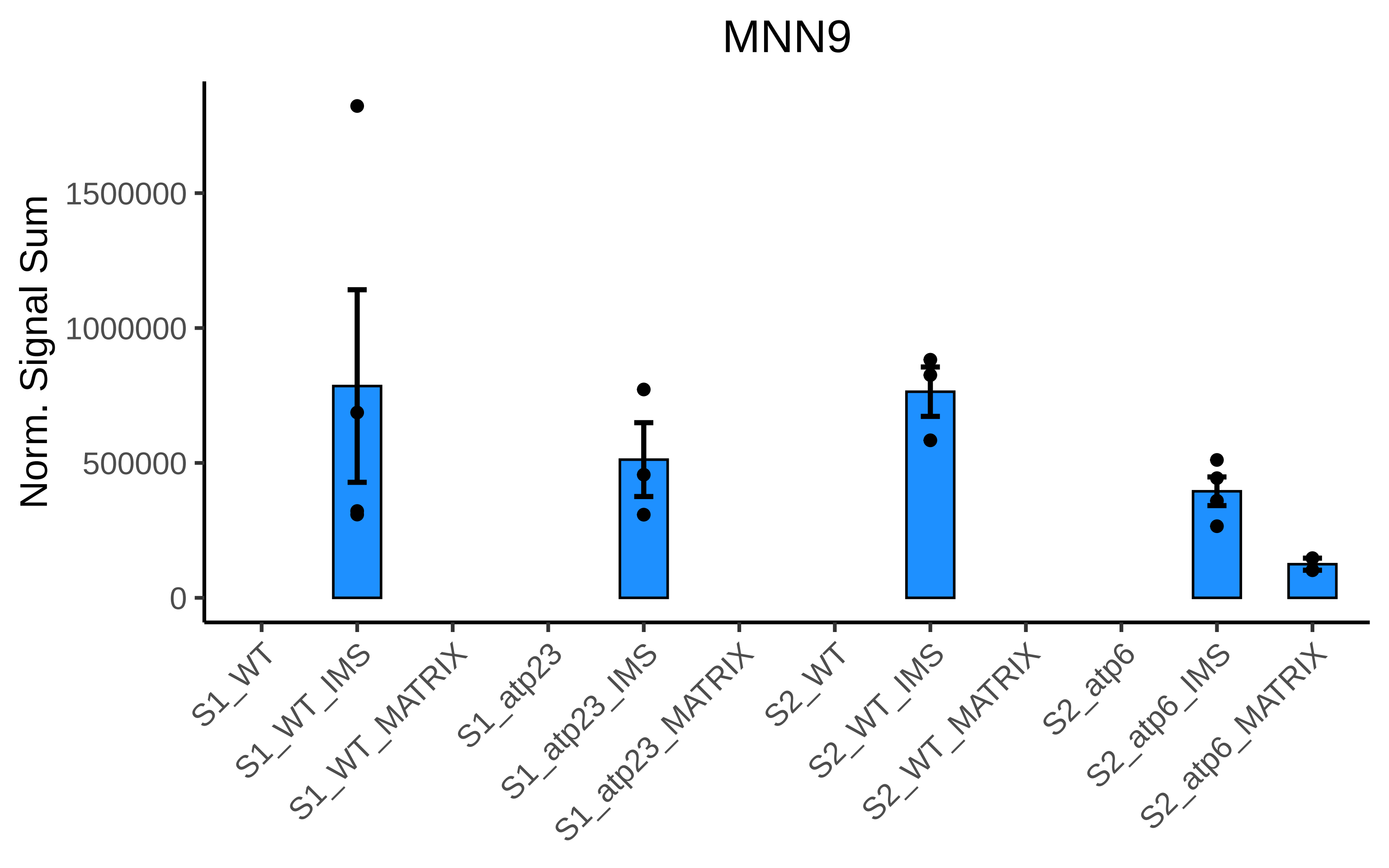
MNN9
Norm. Signal Sum
1500000
1000000
500000
0
S1_WT
S1_WT_IMS
S1_WT_MATRIX
S1_atp23
S1_atp23_IMS
S1_atp23_MATRIX
S2_WT
S2_WT_IMS
S2_WT_MATRIX
S2_atp6
S2_atp6_IMS
S2_atp6_MATRIX

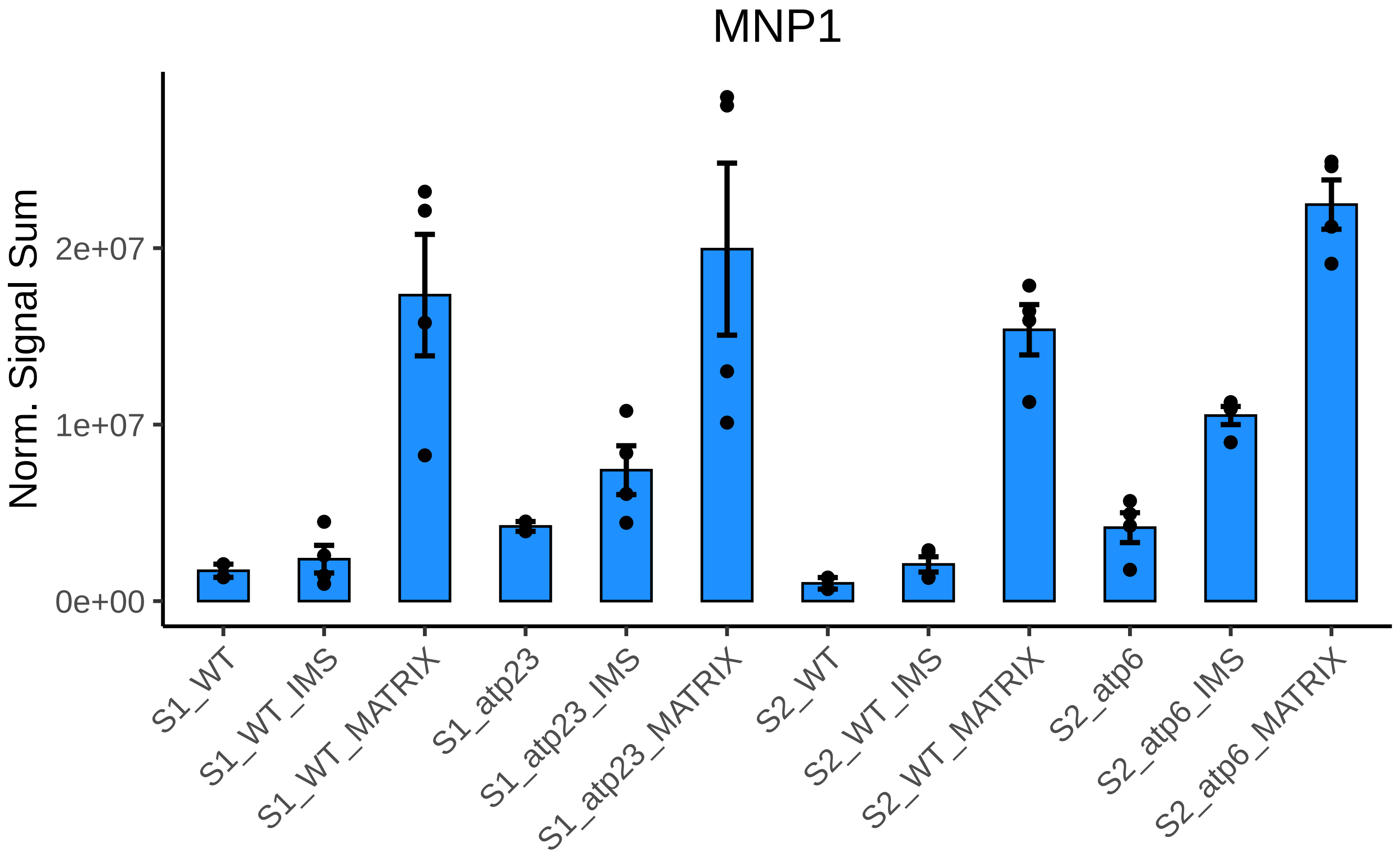
MNP1
Norm. Signal Sum
2e+07
1e+07
0e+00
S1_WT
S1_WT_IMS
S1_WT_MATRIX
S1_atp23
S1_atp23_IMS
S1_atp23_MATRIX
S2_WT
S2_WT_IMS
S2_WT_MATRIX
S2_atp6
S2_atp6_IMS
S2_atp6_MATRIX

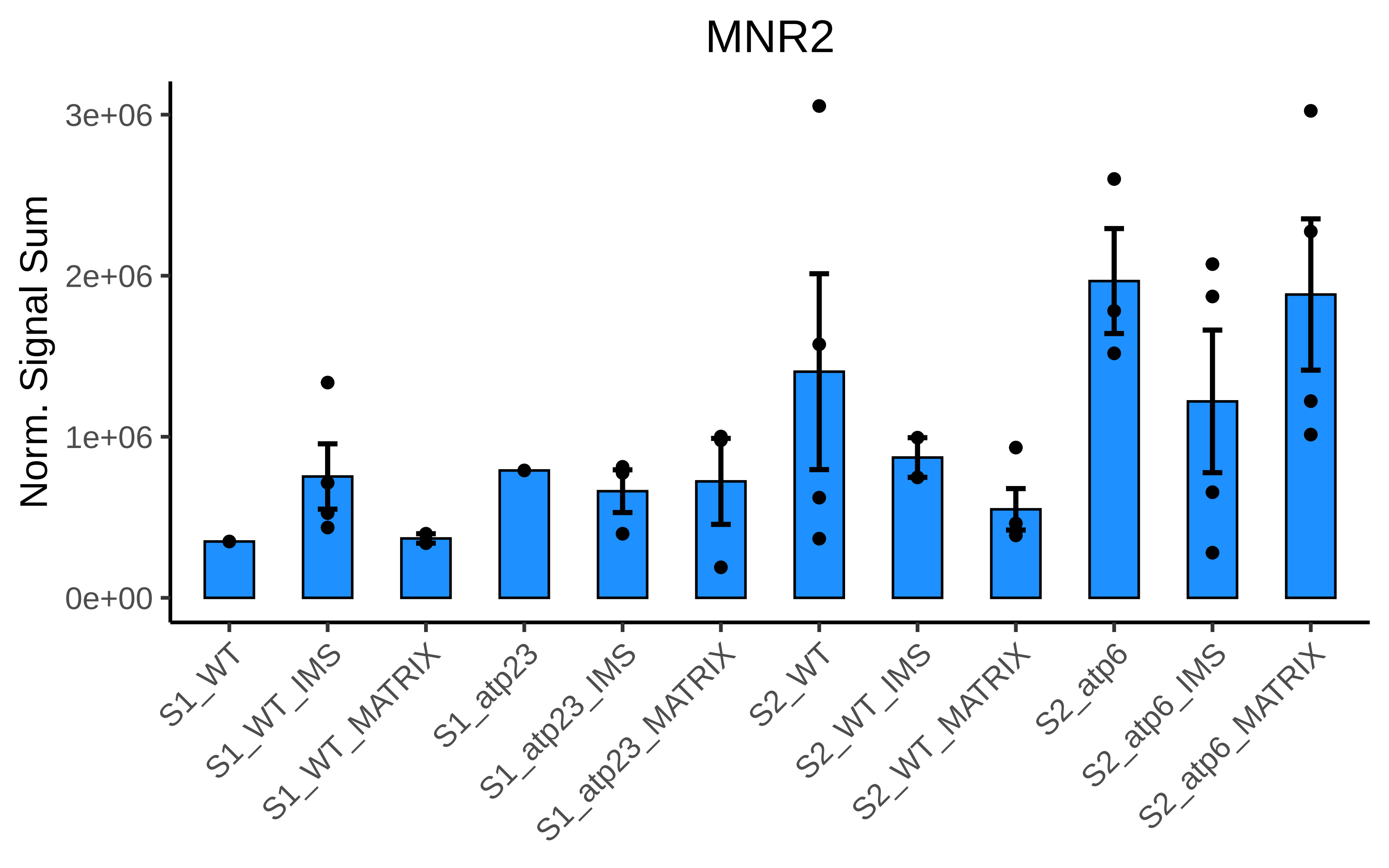
MNR2
Norm. Signal Sum
3e+06
2e+06
1e+06
0e+00
S1_WT
S1_WT_IMS
S1_WT_MATRIX
S1_atp23
S1_atp23_IMS
S1_atp23_MATRIX
S2_WT
S2_WT_IMS
S2_WT_MATRIX
S2_atp6
S2_atp6_IMS
S2_atp6_MATRIX

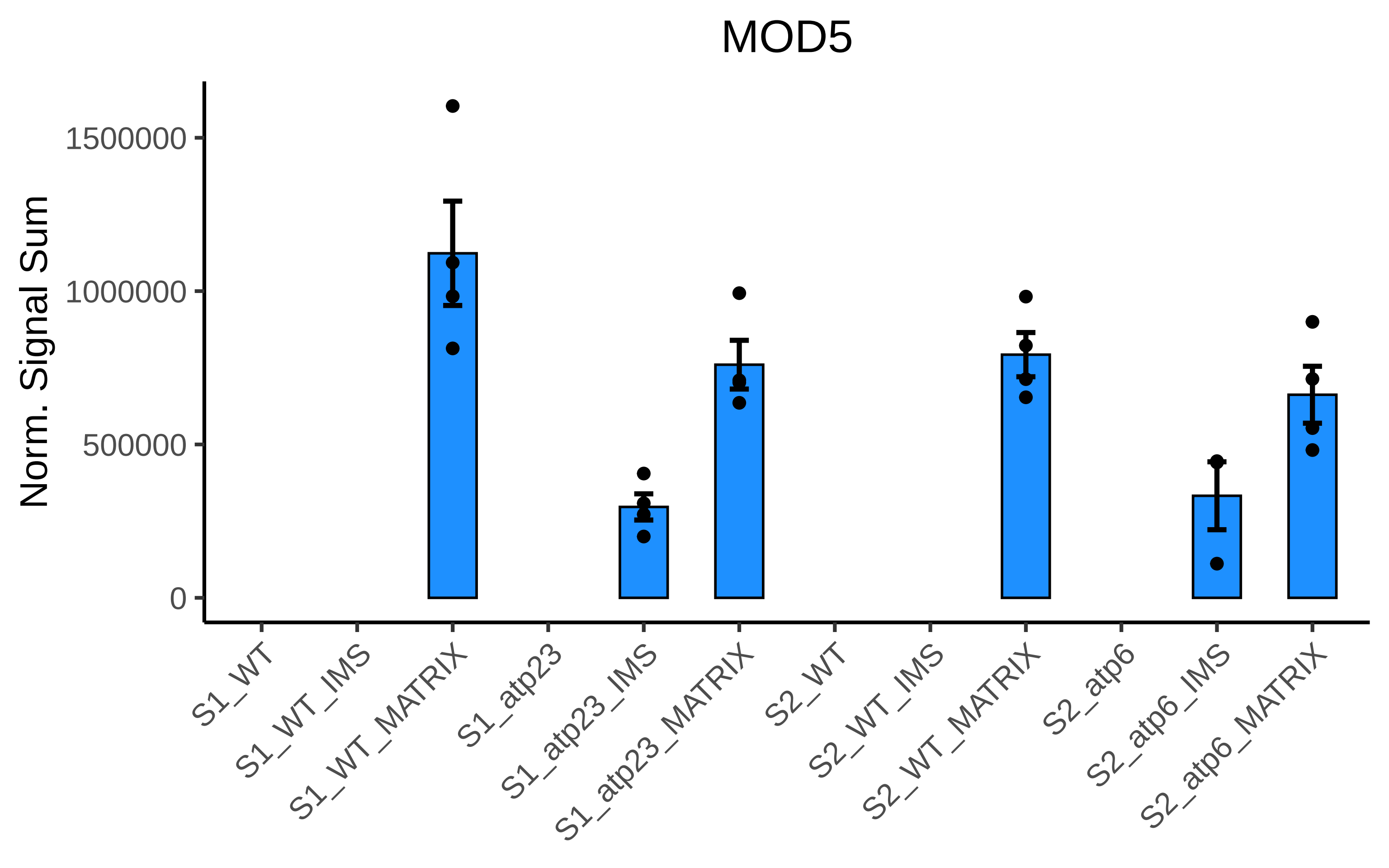

MOD5
Norm. Signal Sum
1500000
1000000
500000
0
S1_WT
S1_WT_IMS
S1_WT_MATRIX
S1_atp23
S1_atp23_IMS
S1_atp23_MATRIX
S2_WT
S2_WT_IMS
S2_WT_MATRIX
S2_atp6
S2_atp6_IMS
S2_atp6_MATRIX

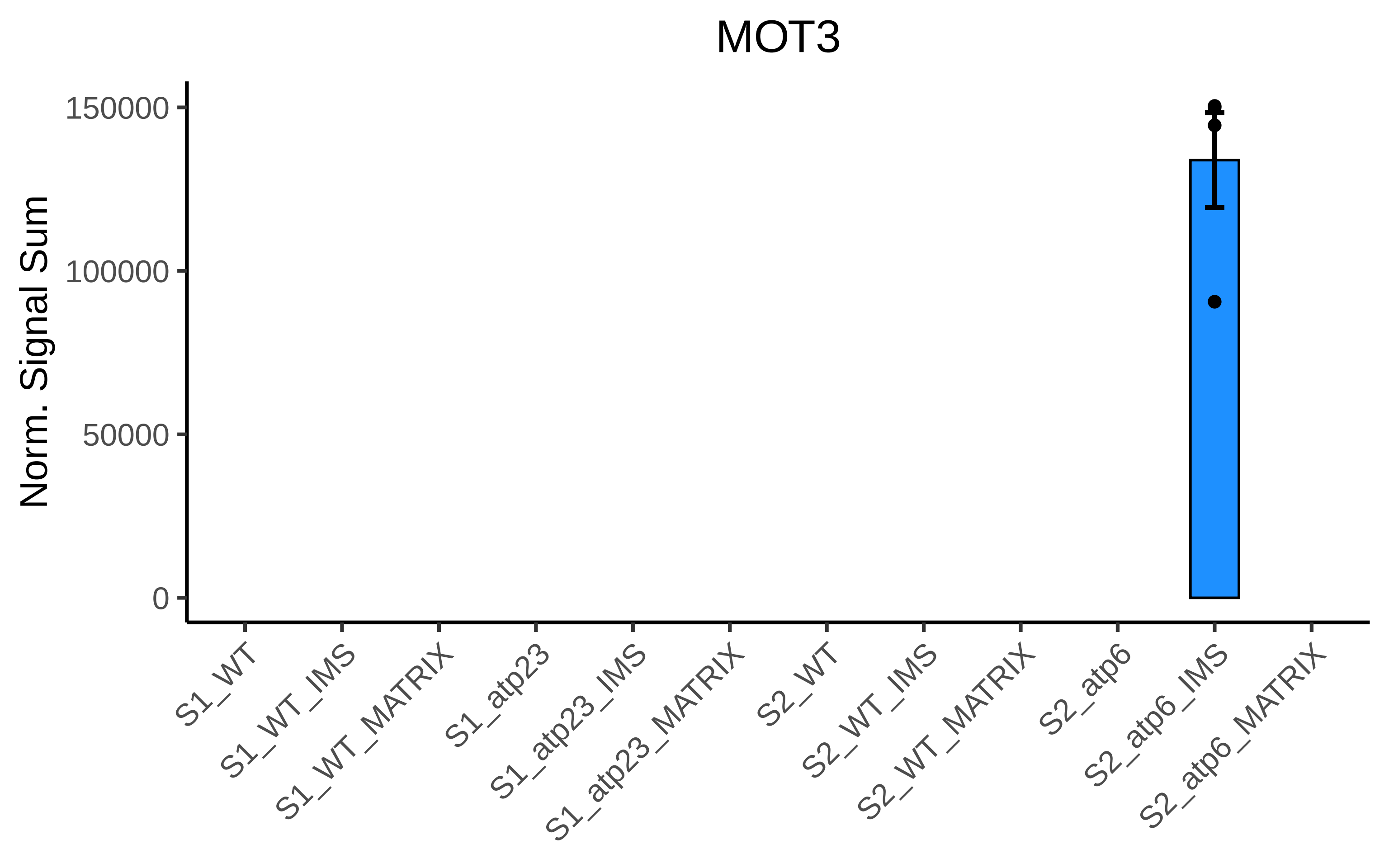
MOT3
Norm. Signal Sum
150000
100000
50000
0
S1_WT
S1_WT_IMS
S1_WT_MATRIX
S1_atp23
S1_atp23_IMS
S1_atp23_MATRIX
S2_WT
S2_WT_IMS
S2_WT_MATRIX
S2_atp6
S2_atp6_IMS
S2_atp6_MATRIX

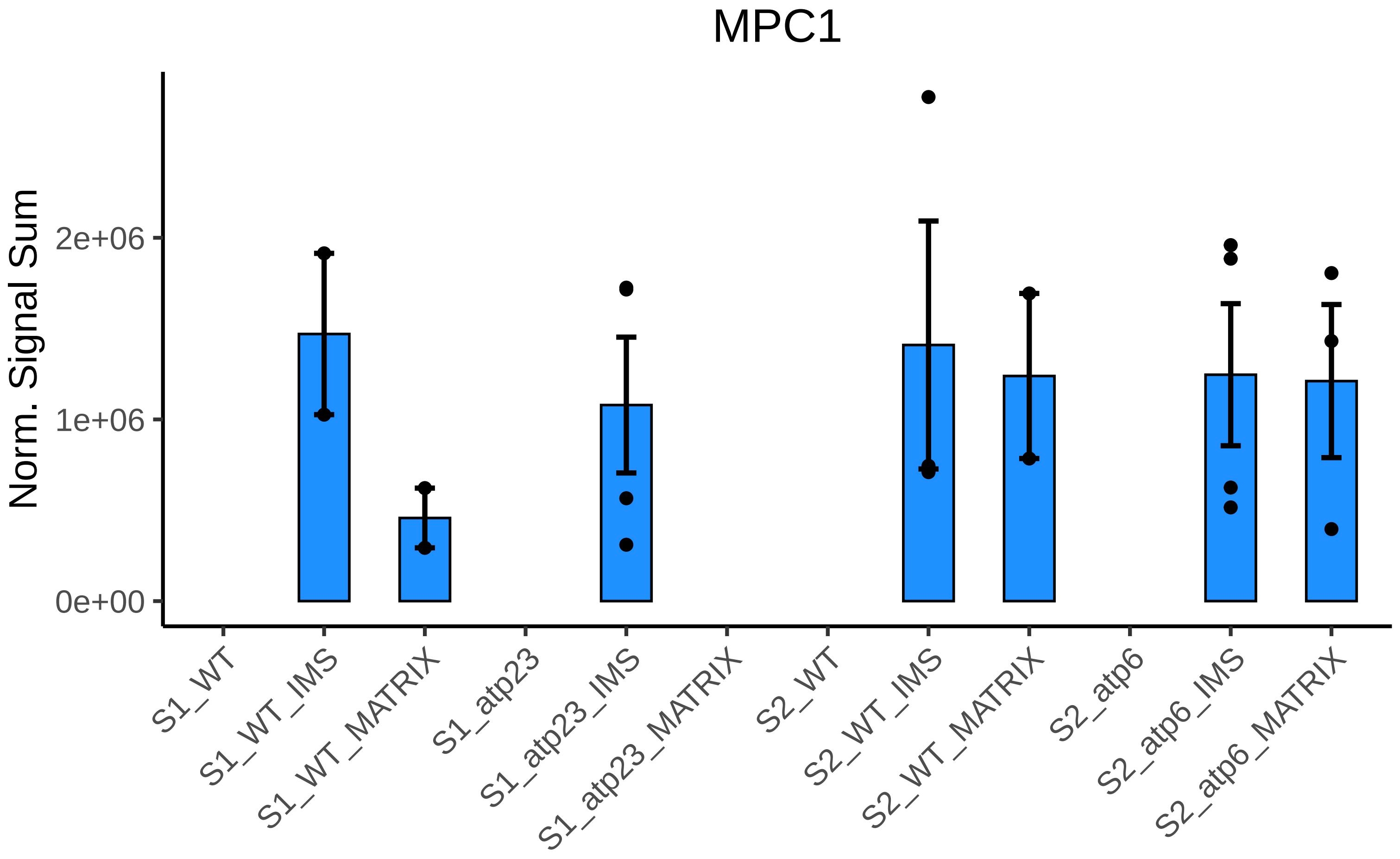
MPC1
Norm. Signal Sum
2e+06
1e+06
0e+00
S1_WT
S1_WT_IMS
S1_WT_MATRIX
S1_atp23
S1_atp23_IMS
S1_atp23_MATRIX
S2_WT
S2_WT_IMS
S2_WT_MATRIX
S2_atp6
S2_atp6_IMS
S2_atp6_MATRIX

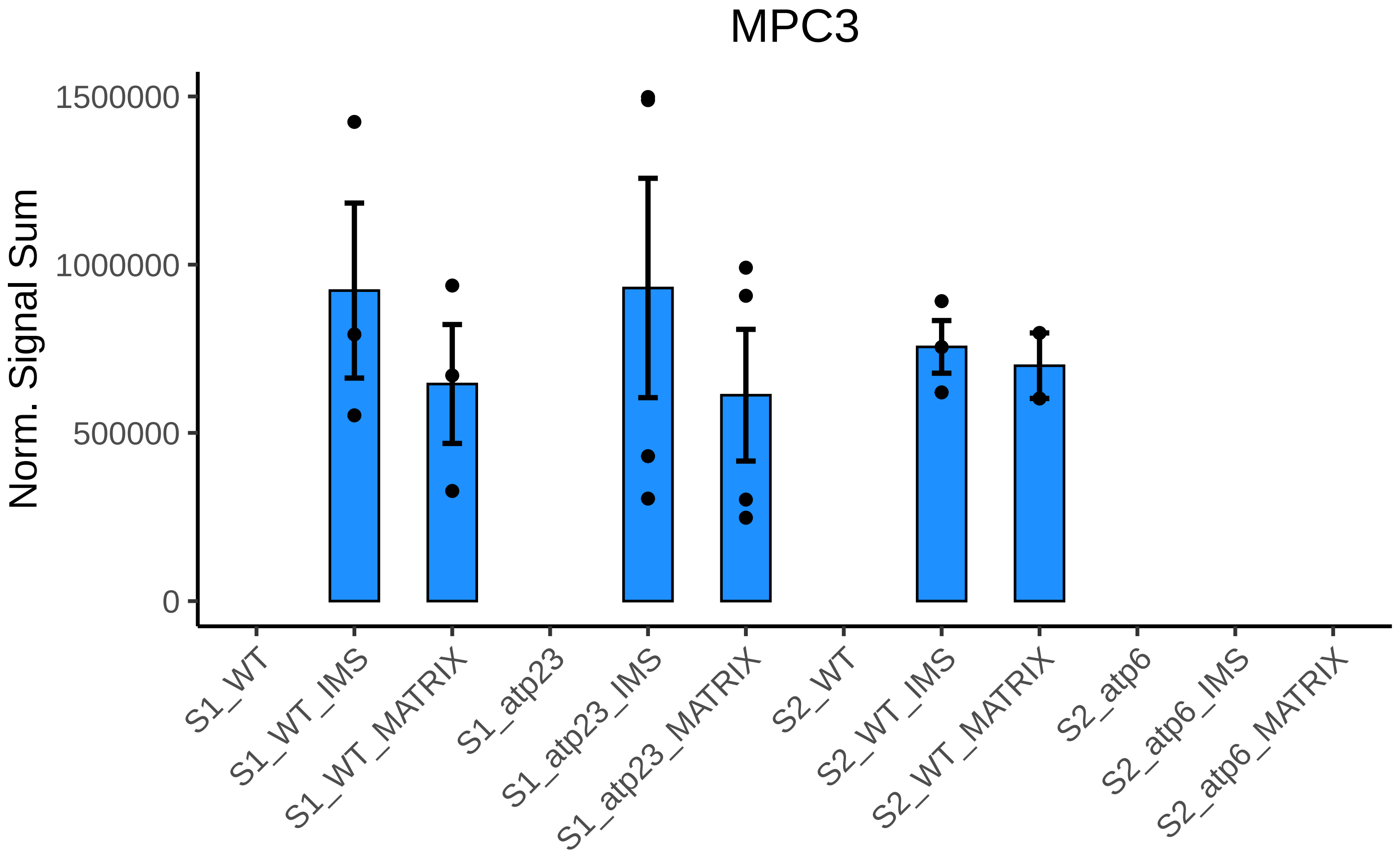
MPC3
Norm. Signal Sum
1500000
1000000
500000
0
S1_WT
S1_WT_IMS
S1_WT_MATRIX
S1_atp23
S1_atp23_IMS
S1_atp23_MATRIX
S2_WT
S2_WT_IMS
S2_WT_MATRIX
S2_atp6
S2_atp6_IMS
S2_atp6_MATRIX

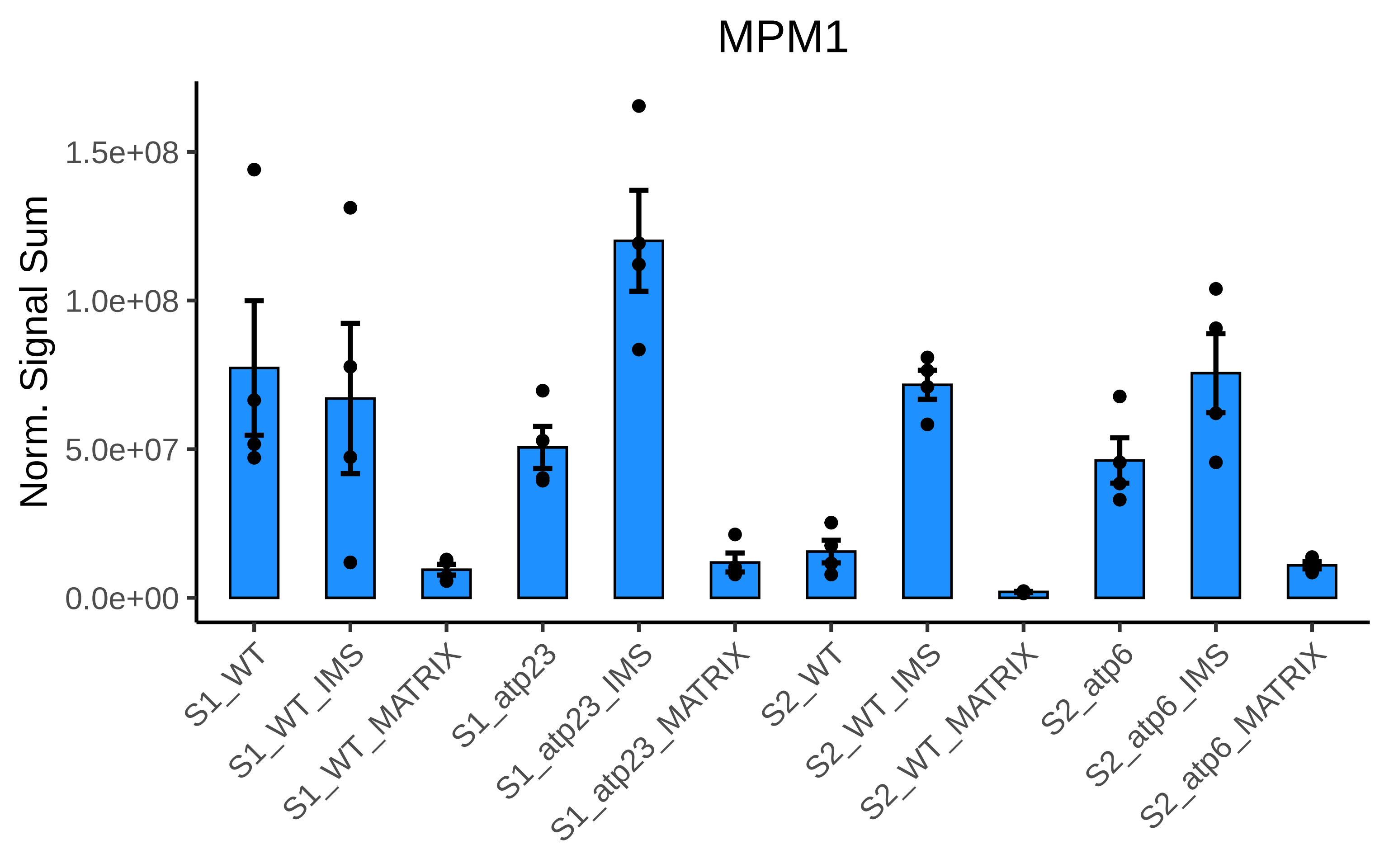

MPM1
Norm. Signal Sum
1.5e+08
1.0e+08
5.0e+07
0.0e+00
S1_WT
S1_WT_IMS
S1_WT_MATRIX
S1_atp23
S1_atp23_IMS
S1_atp23_MATRIX
S2_WT
S2_WT_IMS
S2_WT_MATRIX
S2_atp6
S2_atp6_IMS
S2_atp6_MATRIX

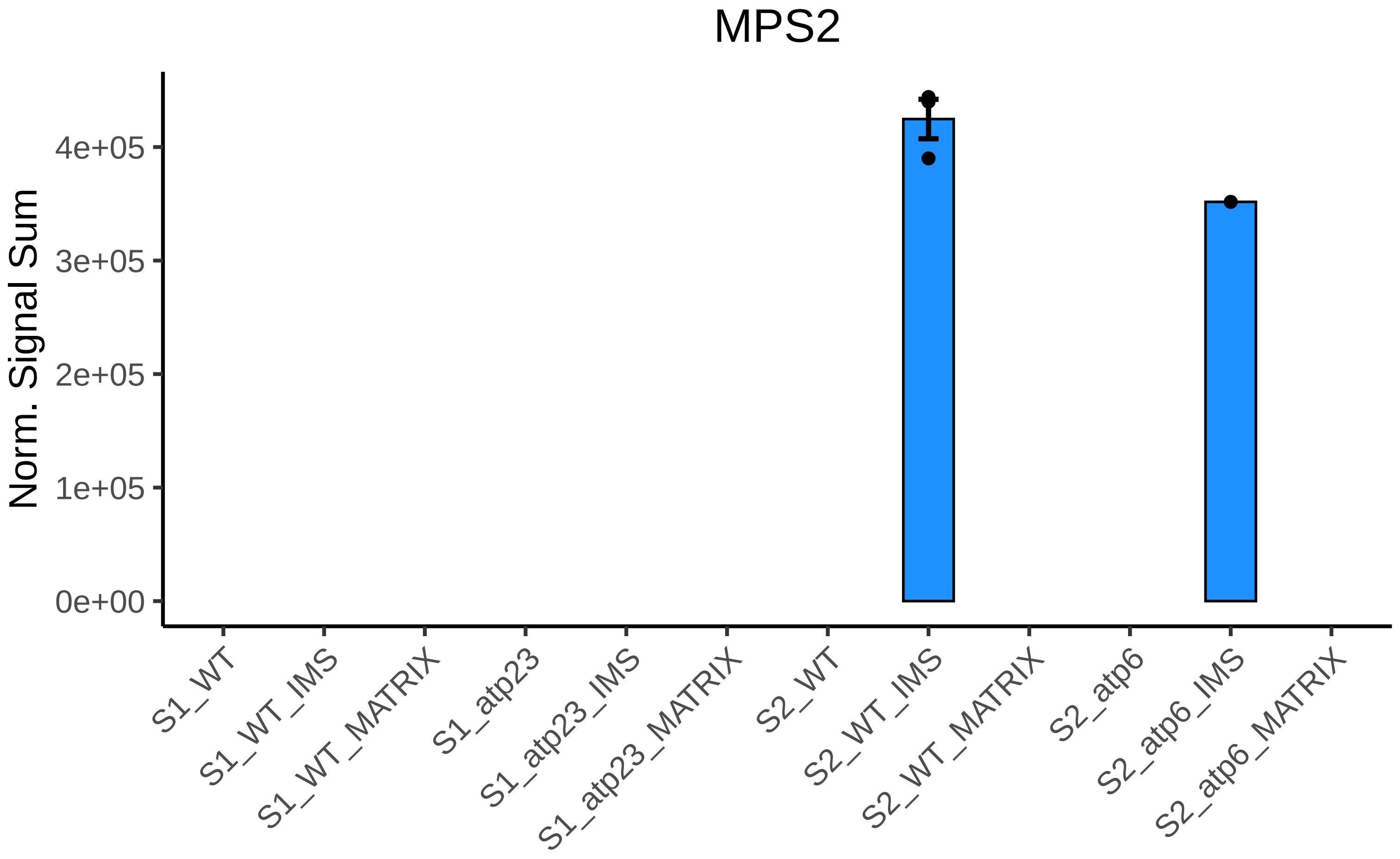
MPS2
Norm. Signal Sum
4e+05
3e+05
2e+05
1e+05
0e+00
S1_WT
S1_WT_IMS
S1_WT_MATRIX
S1_atp23
S1_atp23_IMS
S1_atp23_MATRIX
S2_WT
S2_WT_IMS
S2_WT_MATRIX
S2_atp6
S2_atp6_IMS
S2_atp6_MATRIX

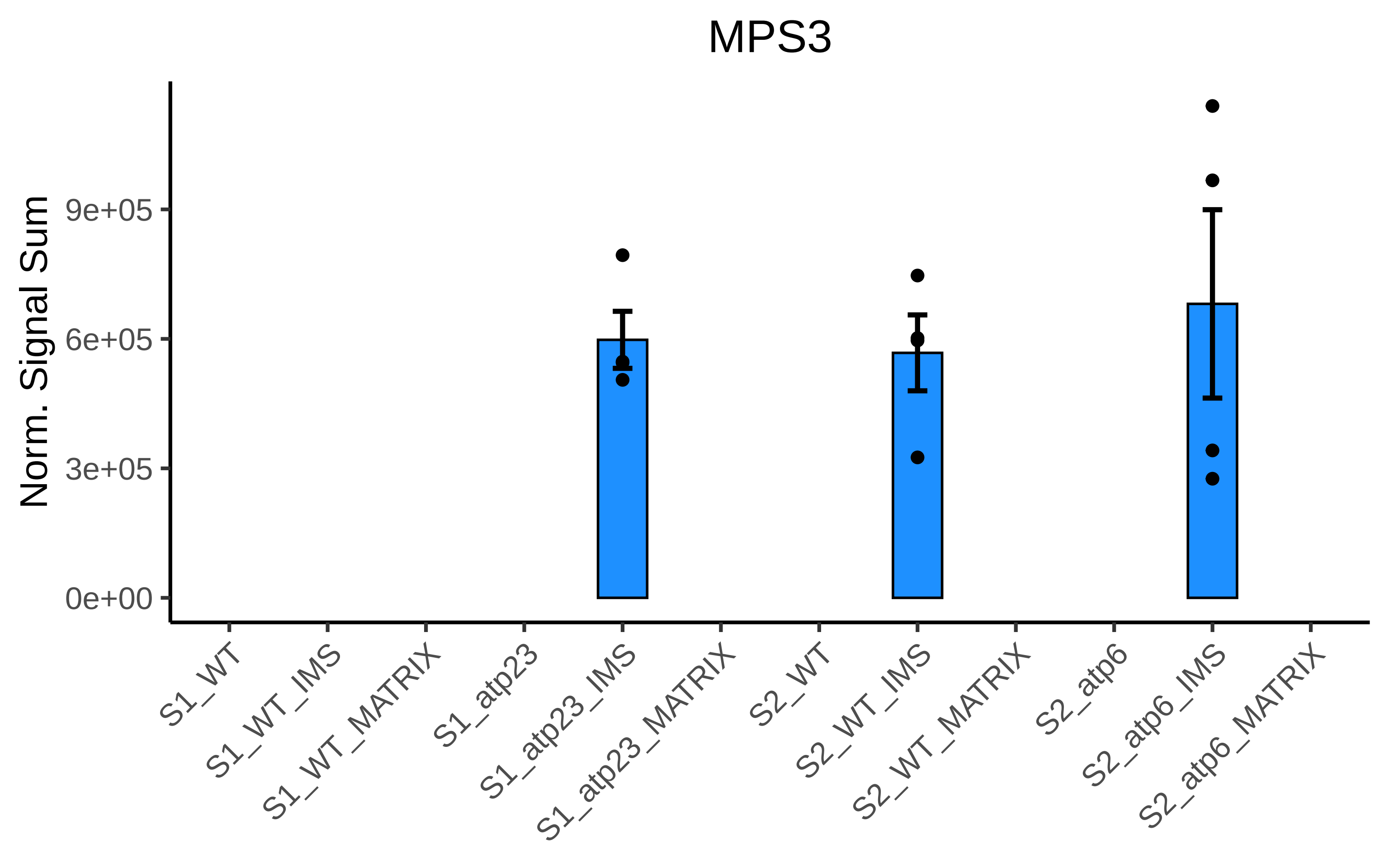
MPS3
Norm. Signal Sum
9e+05
6e+05
3e+05
0e+00
S1_WT
S1_WT_IMS
S1_WT_MATRIX
S1_atp23
S1_atp23_IMS
S1_atp23_MATRIX
S2_WT
S2_WT_IMS
S2_WT_MATRIX
S2_atp6
S2_atp6_IMS
S2_atp6_MATRIX

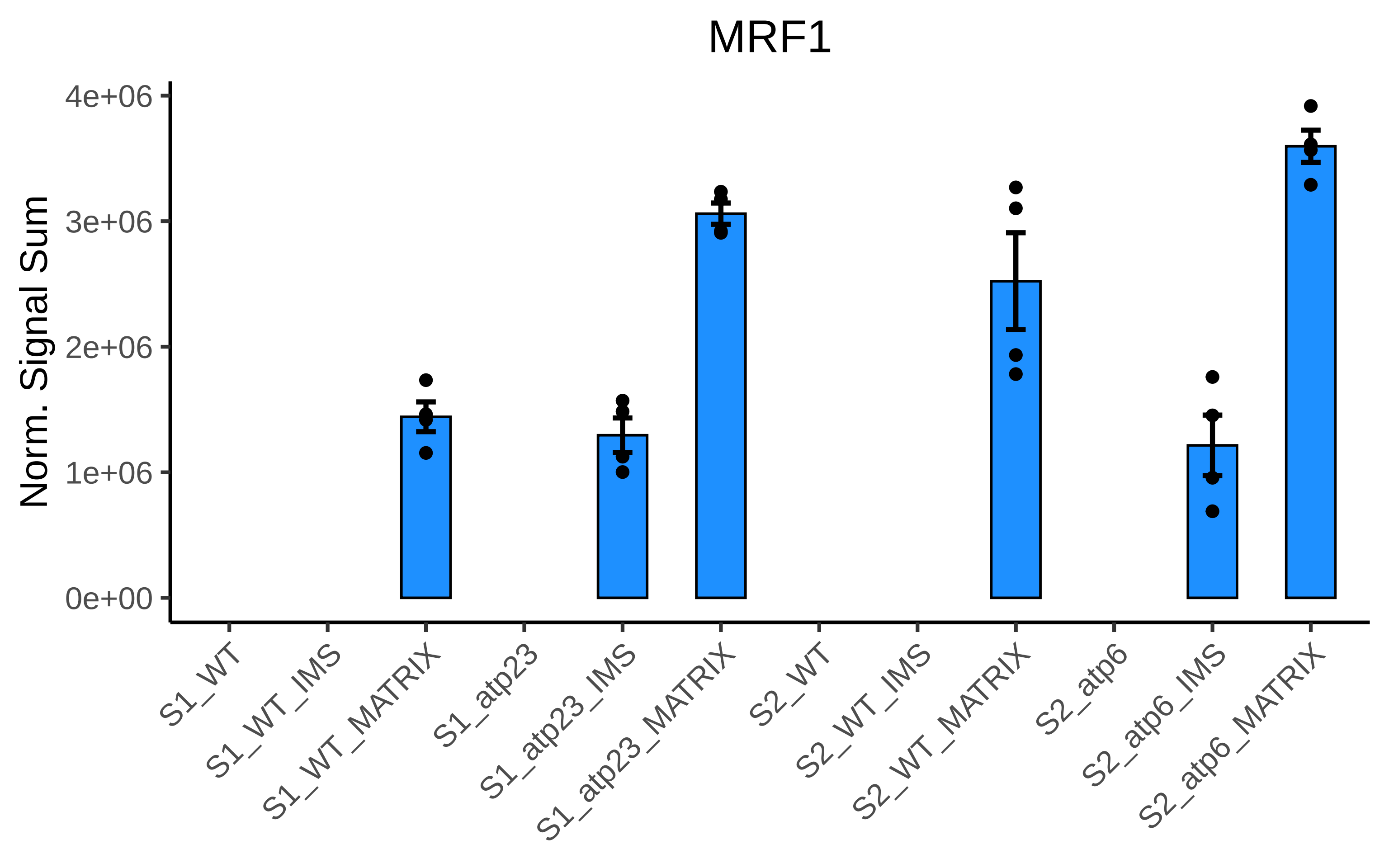

MRF1
Norm. Signal Sum
4e+06
3e+06
2e+06
1e+06
0e+00
S1_WT
S1_WT_IMS
S1_WT_MATRIX
S1_atp23
S1_atp23_IMS
S1_atp23_MATRIX
S2_WT
S2_WT_IMS
S2_WT_MATRIX
S2_atp6
S2_atp6_IMS
S2_atp6_MATRIX

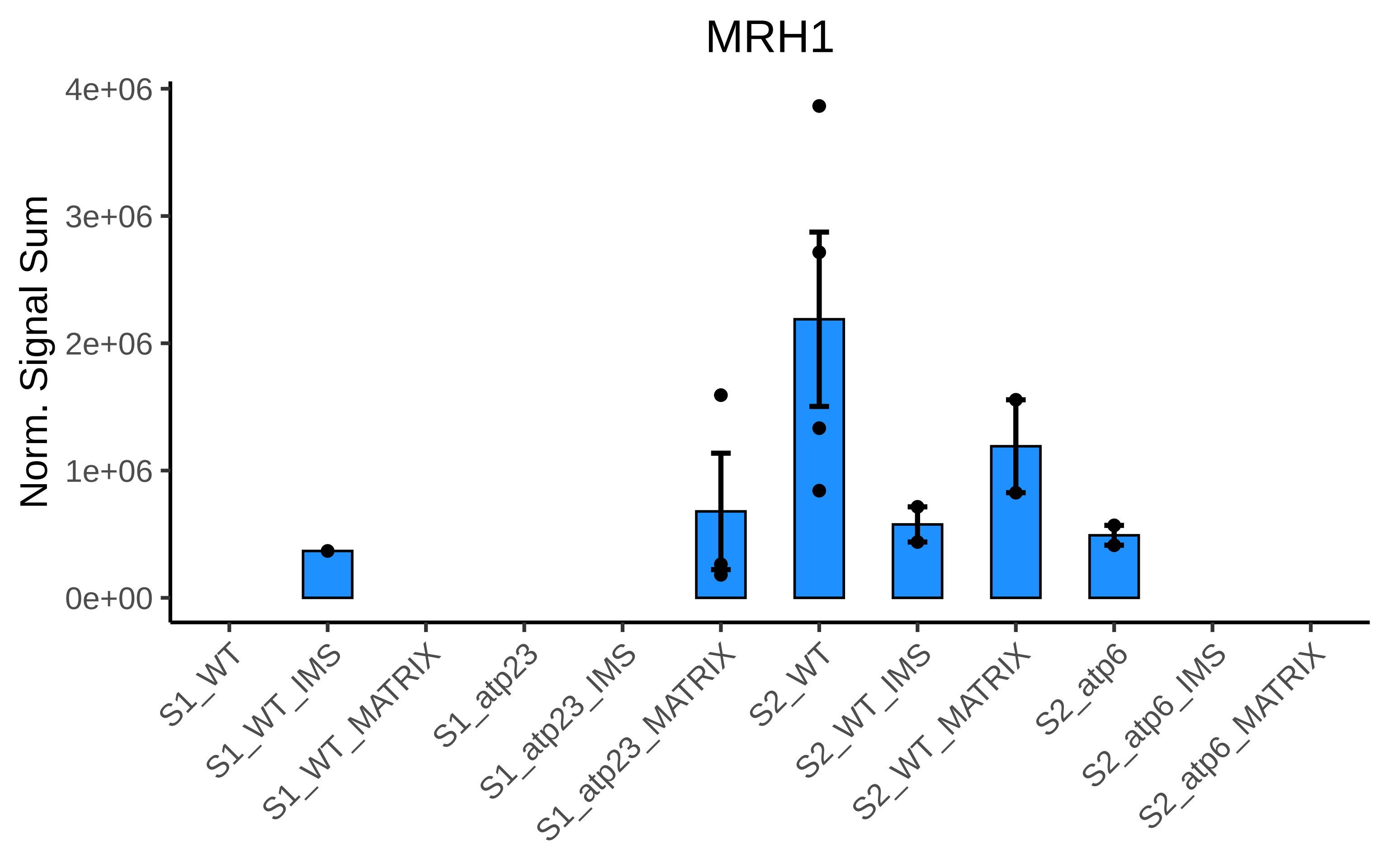

MRH1
Norm. Signal Sum
4e+06
3e+06
2e+06
1e+06
0e+00
S1_WT
S1_WT_IMS
S1_WT_MATRIX
S1_atp23
S1_atp23_IMS
S1_atp23_MATRIX
S2_WT
S2_WT_IMS
S2_WT_MATRIX
S2_atp6
S2_atp6_IMS
S2_atp6_MATRIX

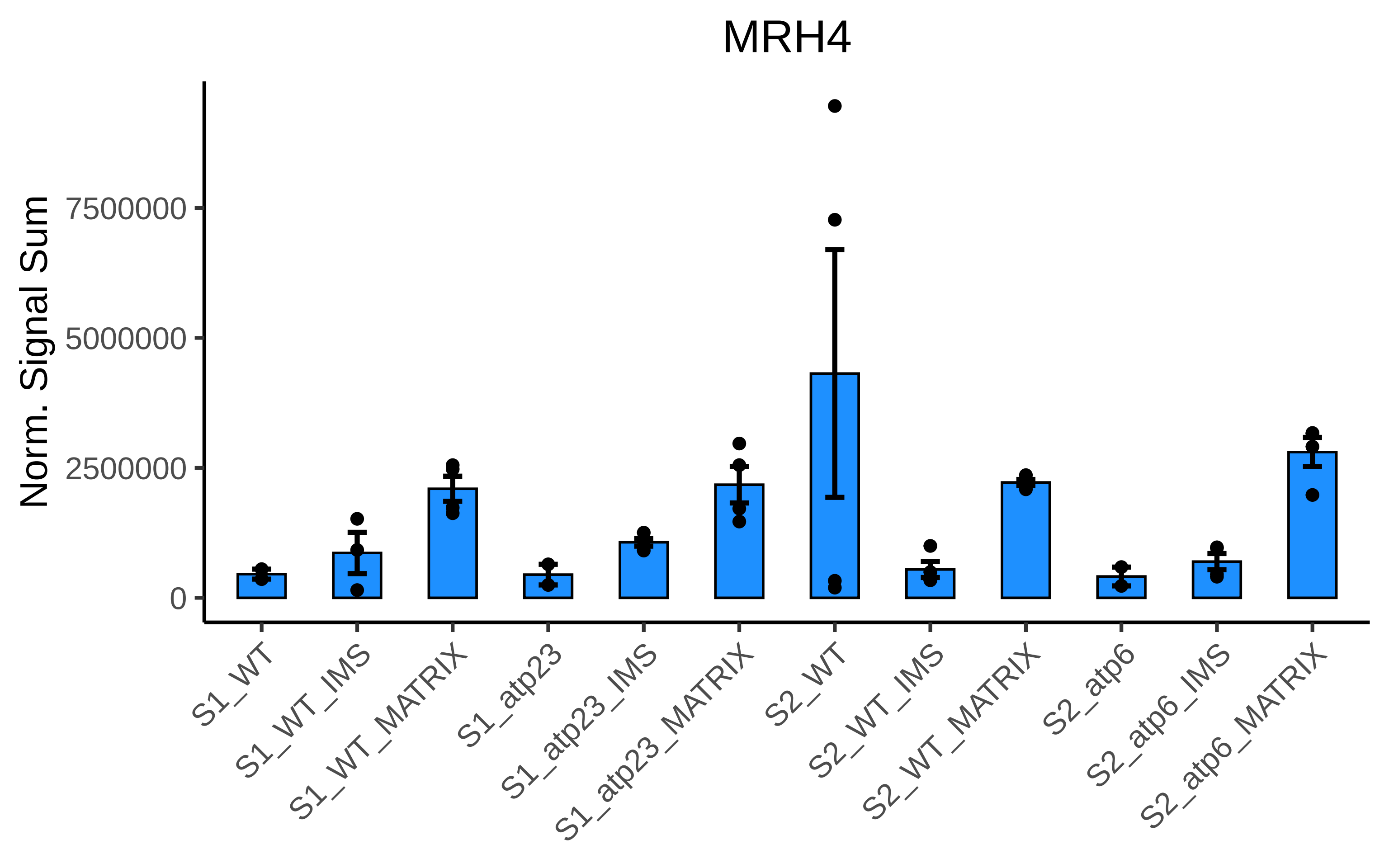

MRH4
Norm. Signal Sum
7500000
5000000
2500000
0
S1_WT
S1_WT_IMS
S1_WT_MATRIX
S1_atp23
S1_atp23_IMS
S1_atp23_MATRIX
S2_WT
S2_WT_IMS
S2_WT_MATRIX
S2_atp6
S2_atp6_IMS
S2_atp6_MATRIX

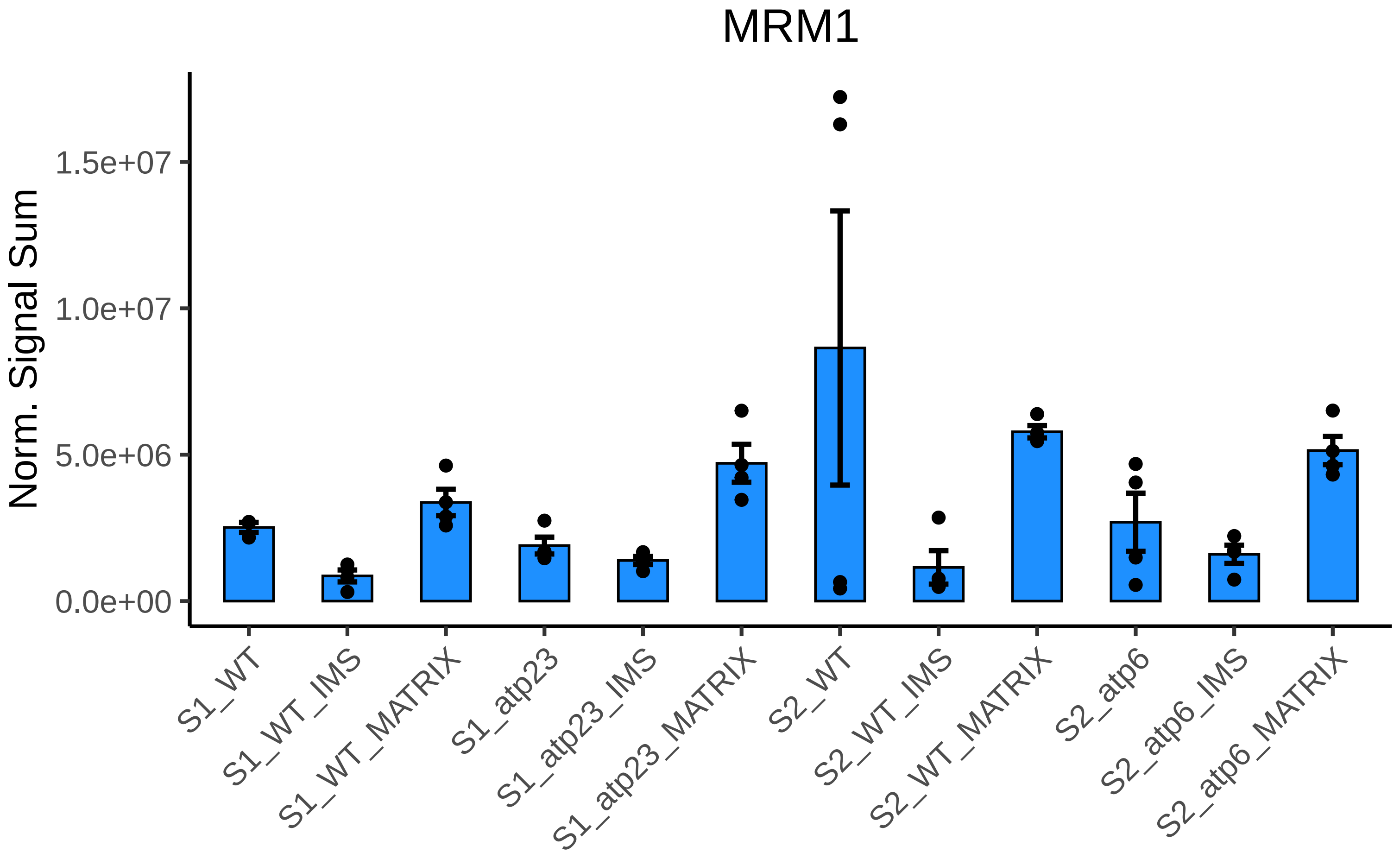

MRM1
Norm. Signal Sum
1.5e+07
1.0e+07
5.0e+06
0.0e+00
S1_WT
S1_WT_IMS
S1_WT_MATRIX
S1_atp23
S1_atp23_IMS
S1_atp23_MATRIX
S2_WT
S2_WT_IMS
S2_WT_MATRIX
S2_atp6
S2_atp6_IMS
S2_atp6_MATRIX

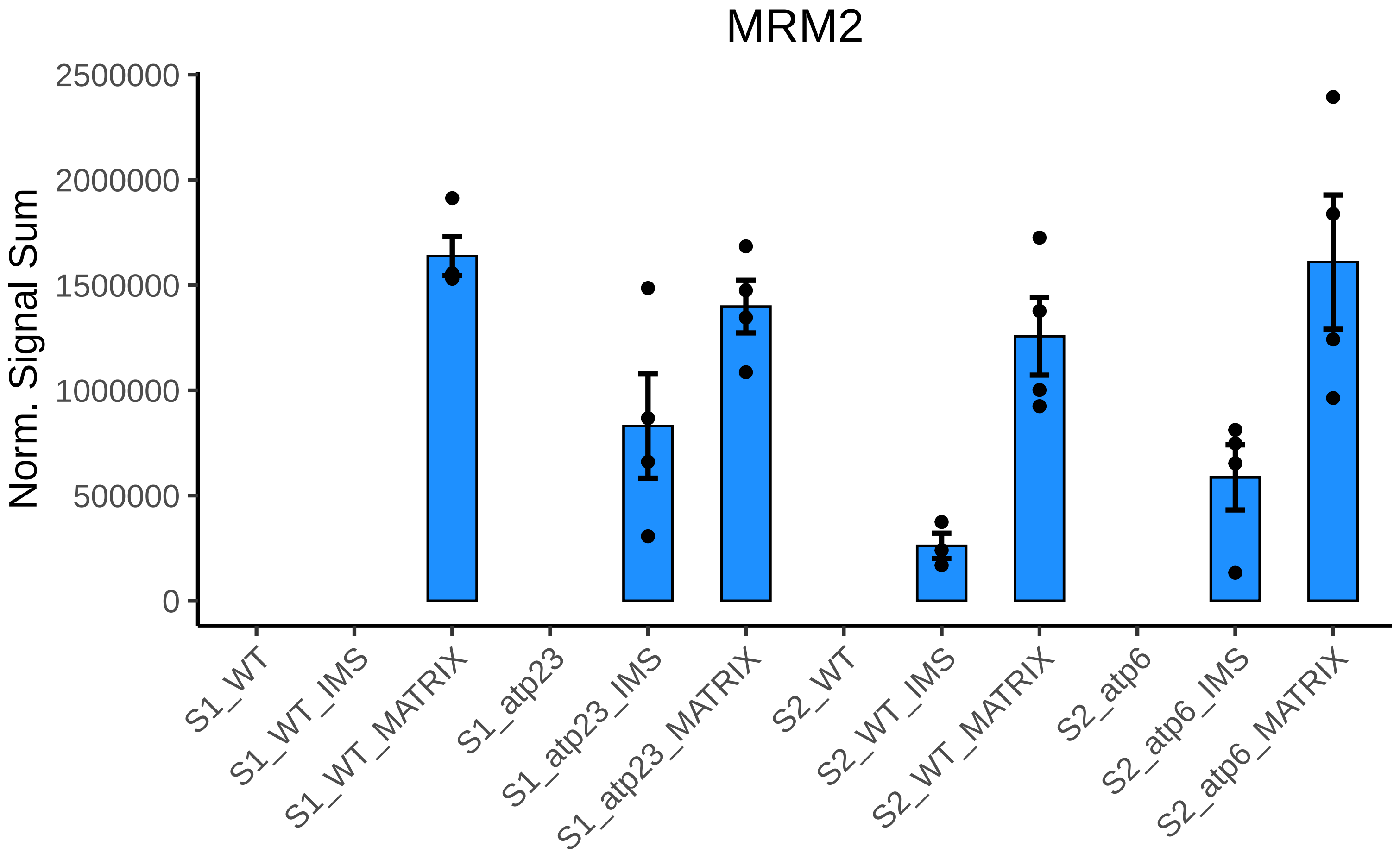
MRM2
Norm. Signal Sum
2500000
2000000
1500000
1000000
500000
0
S1_WT
S1_WT_IMS
S1_WT_MATRIX
S1_atp23
S1_atp23_IMS
S1_atp23_MATRIX
S2_WT
S2_WT_IMS
S2_WT_MATRIX
S2_atp6
S2_atp6_IMS
S2_atp6_MATRIX

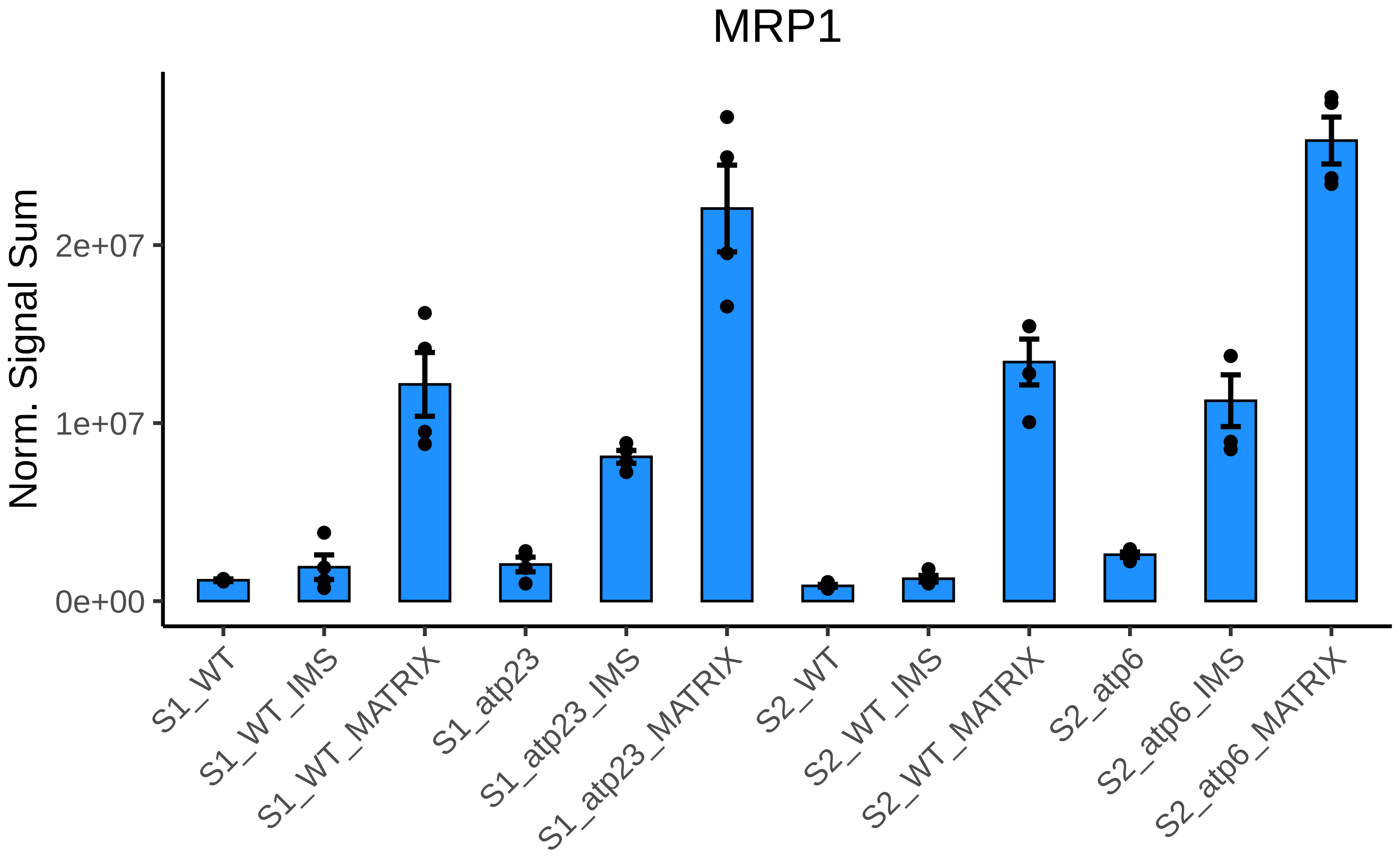

MRP1
Norm. Signal Sum
2e+07
1e+07
0e+00
S1_WT
S1_WT_IMS
S1_WT_MATRIX
S1_atp23
S1_atp23_IMS
S1_atp23_MATRIX
S2_WT
S2_WT_IMS
S2_WT_MATRIX
S2_atp6
S2_atp6_IMS
S2_atp6_MATRIX

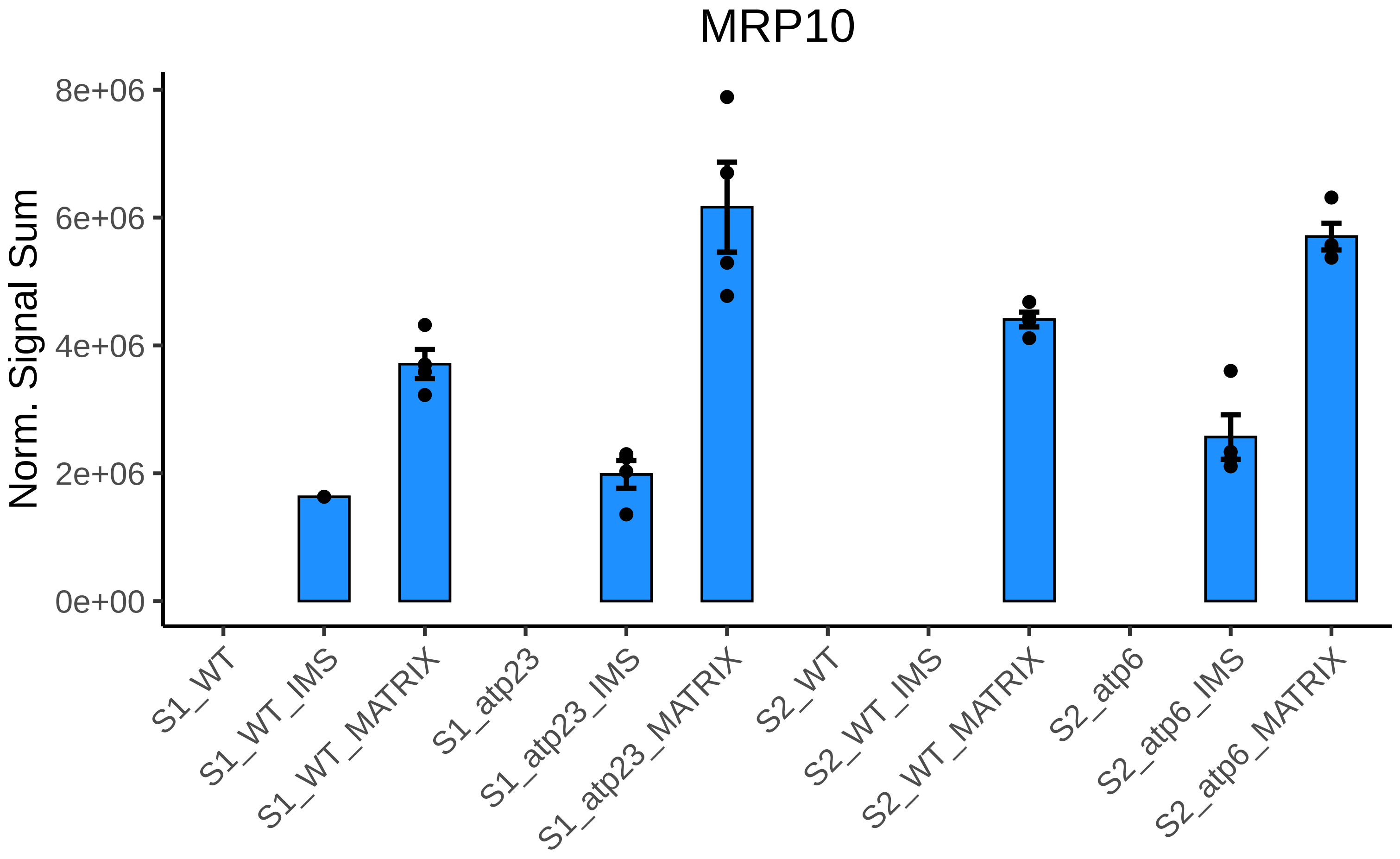

MRP10
Norm. Signal Sum
8e+06
6e+06
4e+06
2e+06
0e+00
S1_WT
S1_WT_IMS
S1_WT_MATRIX
S1_atp23
S1_atp23_IMS
S1_atp23_MATRIX
S2_WT
S2_WT_IMS
S2_WT_MATRIX
S2_atp6
S2_atp6_IMS
S2_atp6_MATRIX

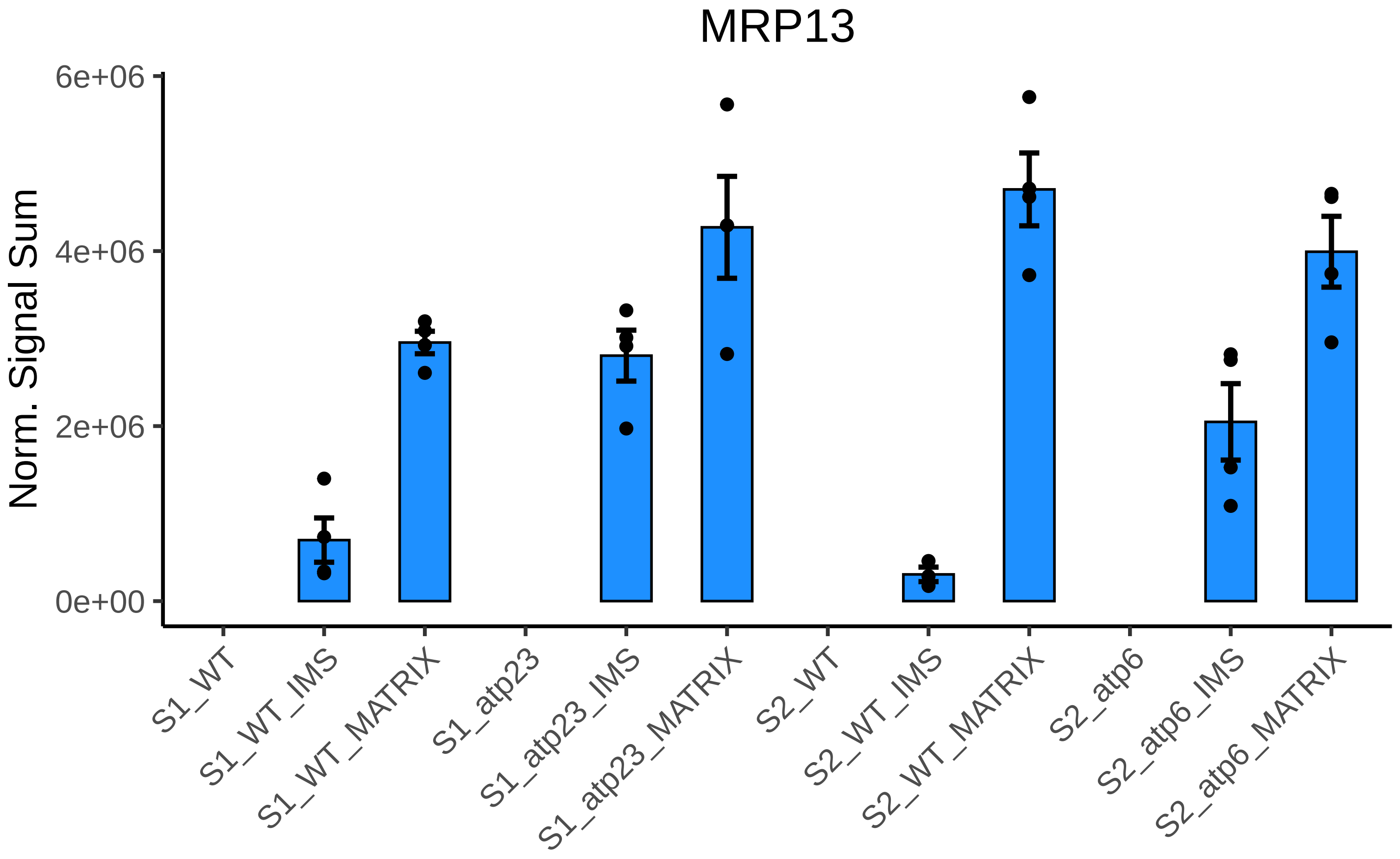

MRP13
Norm. Signal Sum
6e+06
4e+06
2e+06
0e+00
S1_WT
S1_WT_IMS
S1_WT_MATRIX
S1_atp23
S1_atp23_IMS
S1_atp23_MATRIX
S2_WT
S2_WT_IMS
S2_WT_MATRIX
S2_atp6
S2_atp6_IMS
S2_atp6_MATRIX

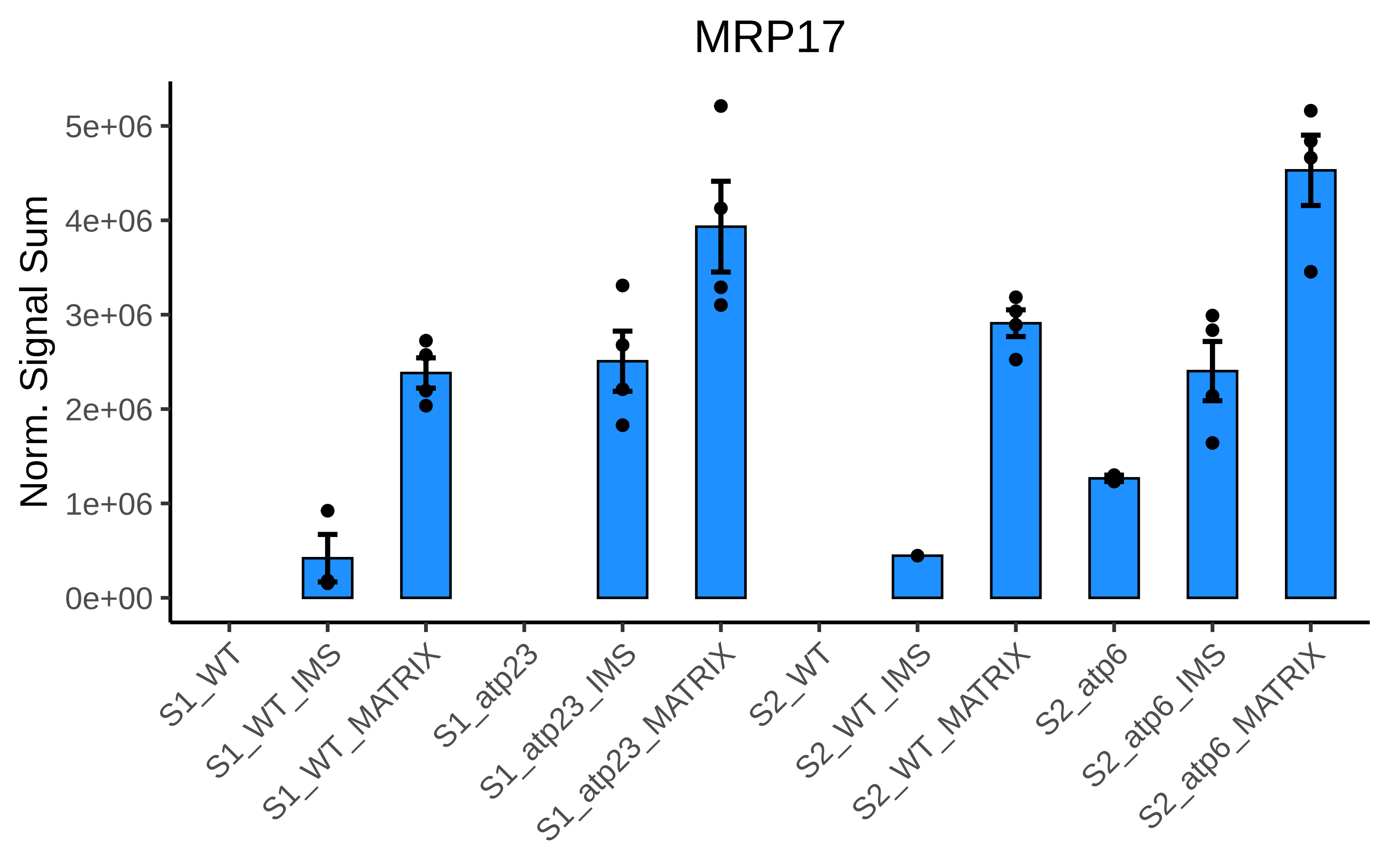

MRP17
Norm. Signal Sum
5e+06
4e+06
3e+06
2e+06
1e+06
0e+00
S1_WT
S1_WT_IMS
S1_WT_MATRIX
S1_atp23
S1_atp23_IMS
S1_atp23_MATRIX
S2_WT
S2_WT_IMS
S2_WT_MATRIX
S2_atp6
S2_atp6_IMS
S2_atp6_MATRIX

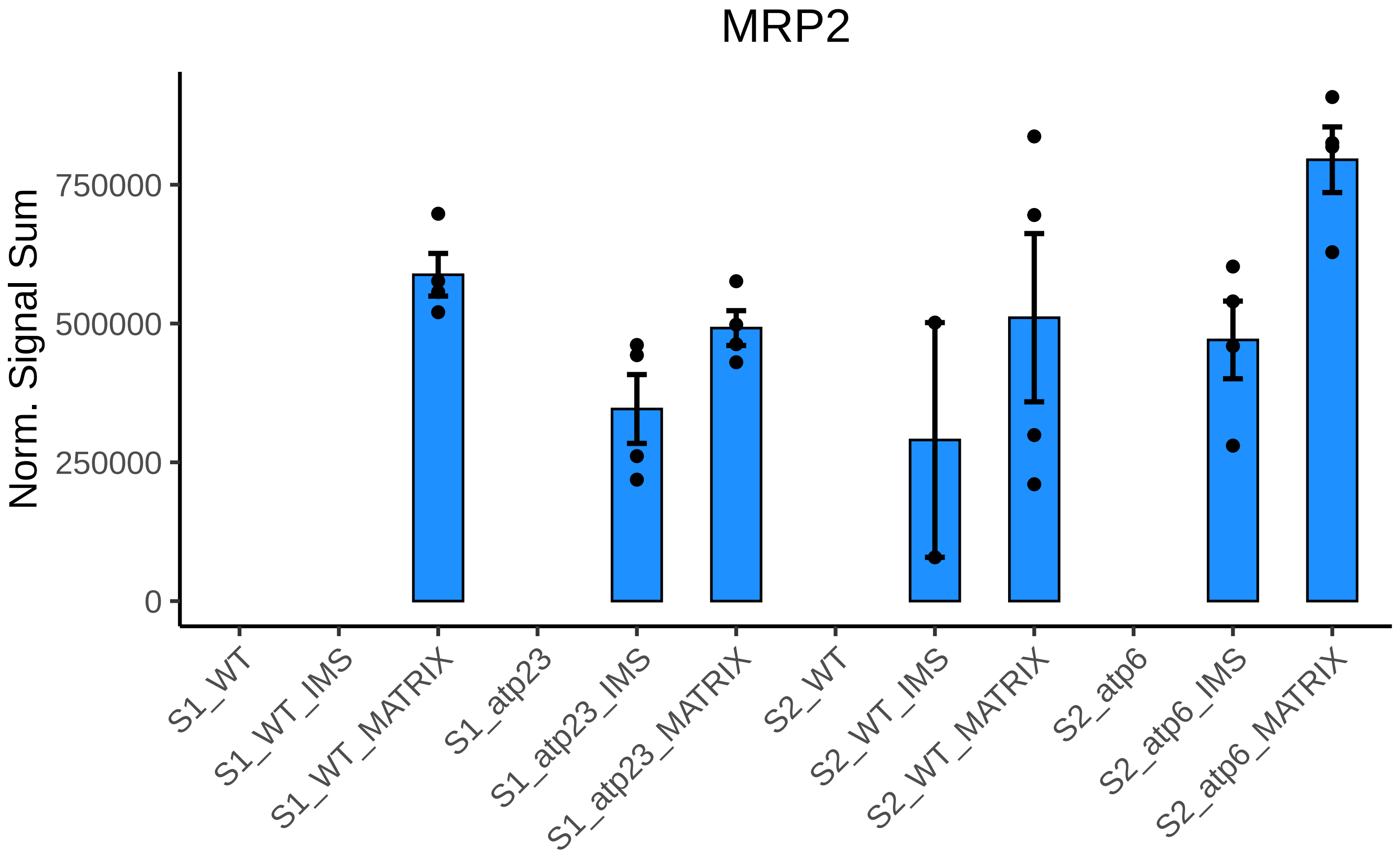
MRP2
Norm. Signal Sum
750000
500000
250000
0
S1_WT
S1_WT_IMS
S1_WT_MATRIX
S1_atp23
S1_atp23_IMS
S1_atp23_MATRIX
S2_WT
S2_WT_IMS
S2_WT_MATRIX
S2_atp6
S2_atp6_IMS
S2_atp6_MATRIX

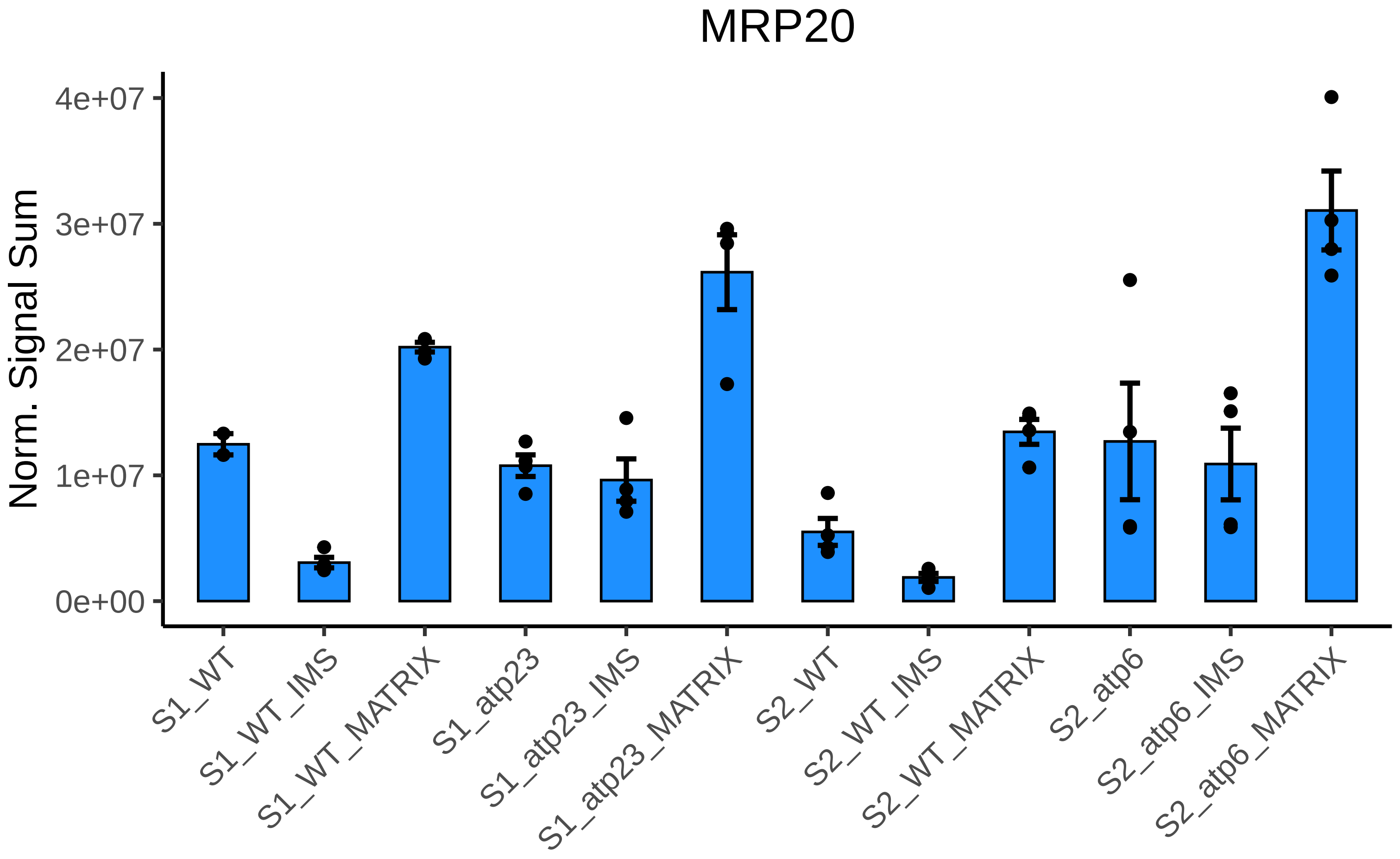

MRP20
Norm. Signal Sum
4e+07
3e+07
2e+07
1e+07
0e+00
S1_WT
S1_WT_IMS
S1_WT_MATRIX
S1_atp23
S1_atp23_IMS
S1_atp23_MATRIX
S2_WT
S2_WT_IMS
S2_WT_MATRIX
S2_atp6
S2_atp6_IMS
S2_atp6_MATRIX

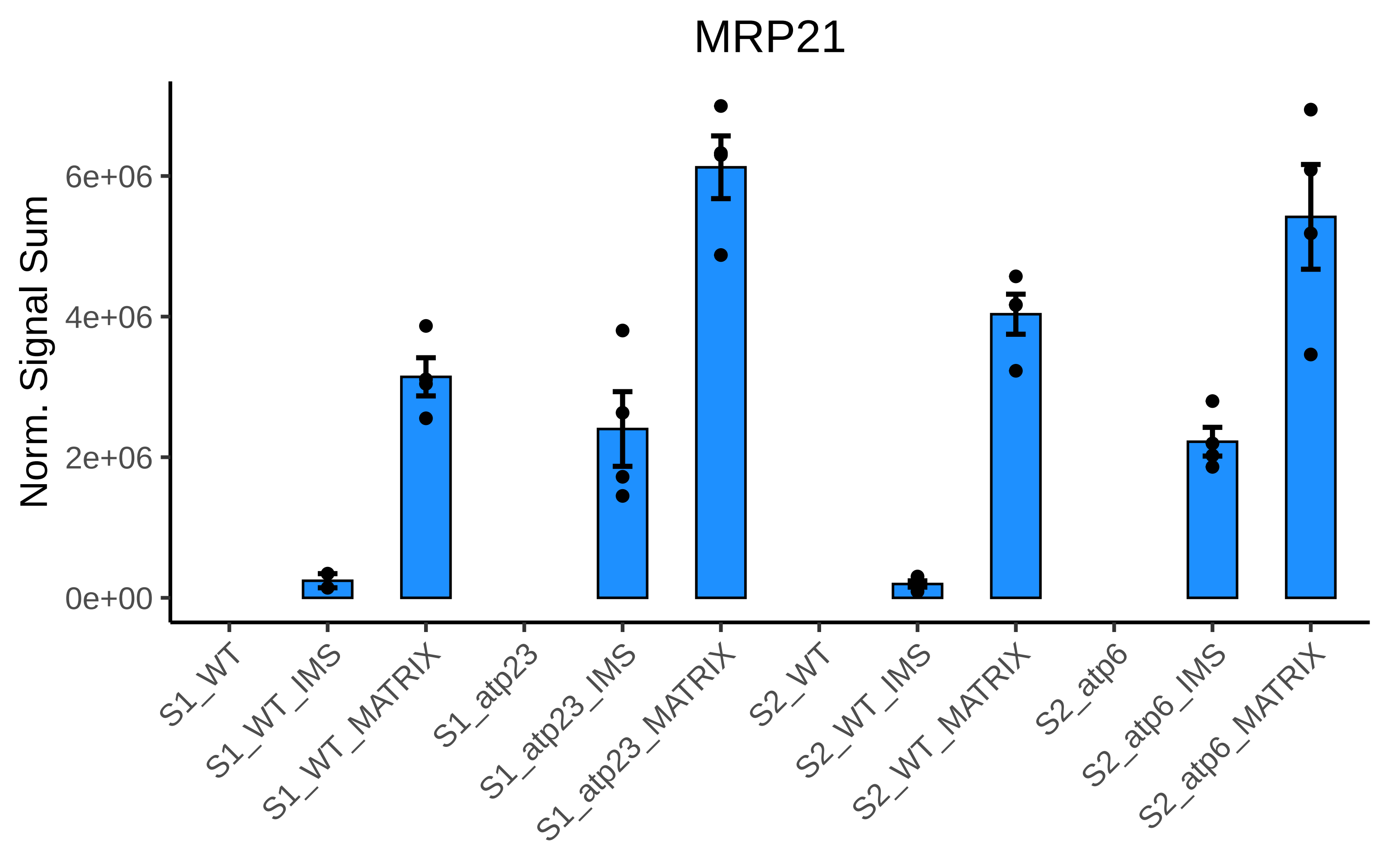
MRP21
Norm. Signal Sum
6e+06
4e+06
2e+06
0e+00
S1_WT
S1_WT_IMS
S1_WT_MATRIX
S1_atp23
S1_atp23_IMS
S1_atp23_MATRIX
S2_WT
S2_WT_IMS
S2_WT_MATRIX
S2_atp6
S2_atp6_IMS
S2_atp6_MATRIX

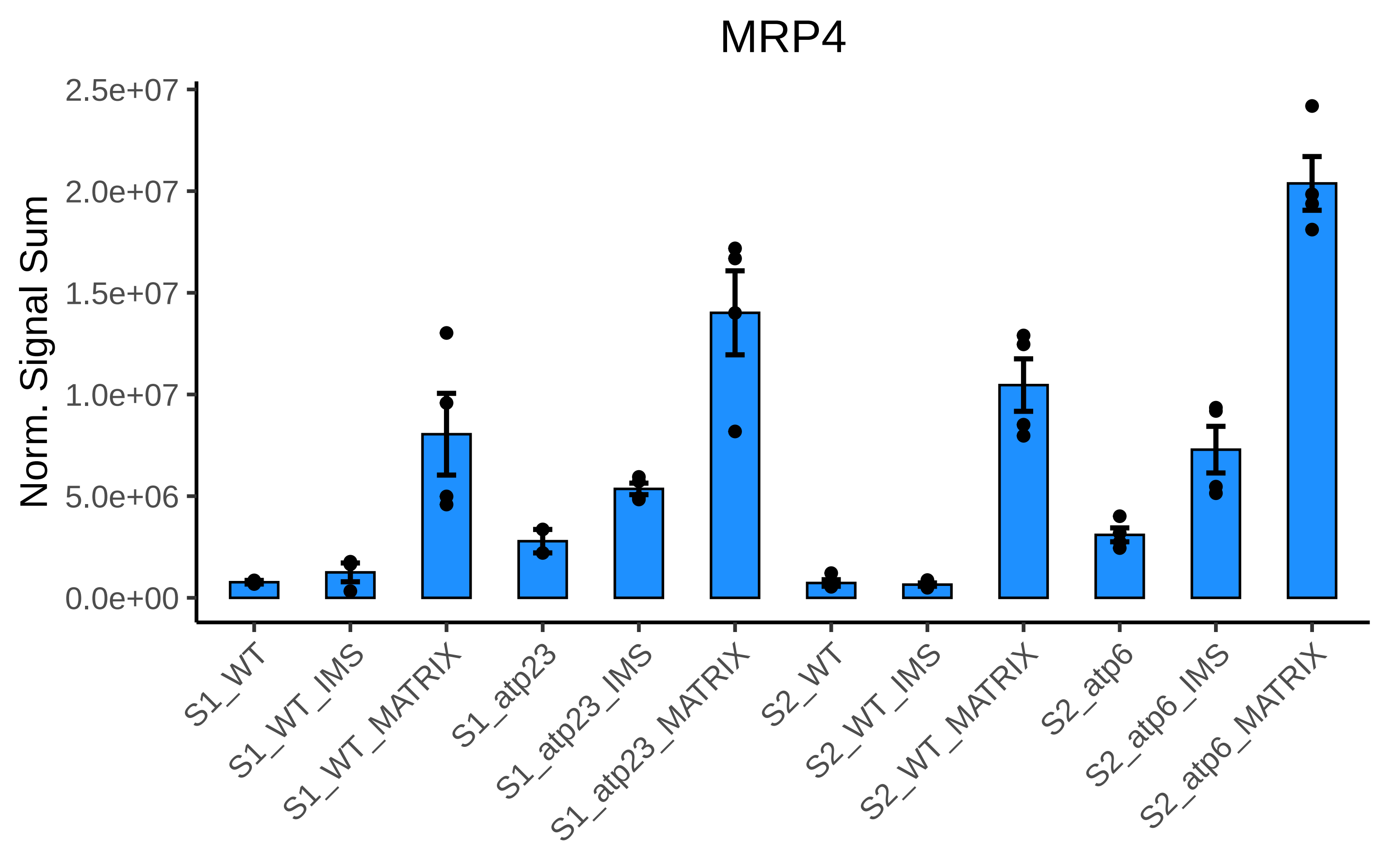

MRP4
Norm. Signal Sum
2.5e+07
2.0e+07
1.5e+07
1.0e+07
5.0e+06
0.0e+00
S1_WT
S1_WT_IMS
S1_WT_MATRIX
S1_atp23
S1_atp23_IMS
S1_atp23_MATRIX
S2_WT
S2_WT_IMS
S2_WT_MATRIX
S2_atp6
S2_atp6_IMS
S2_atp6_MATRIX

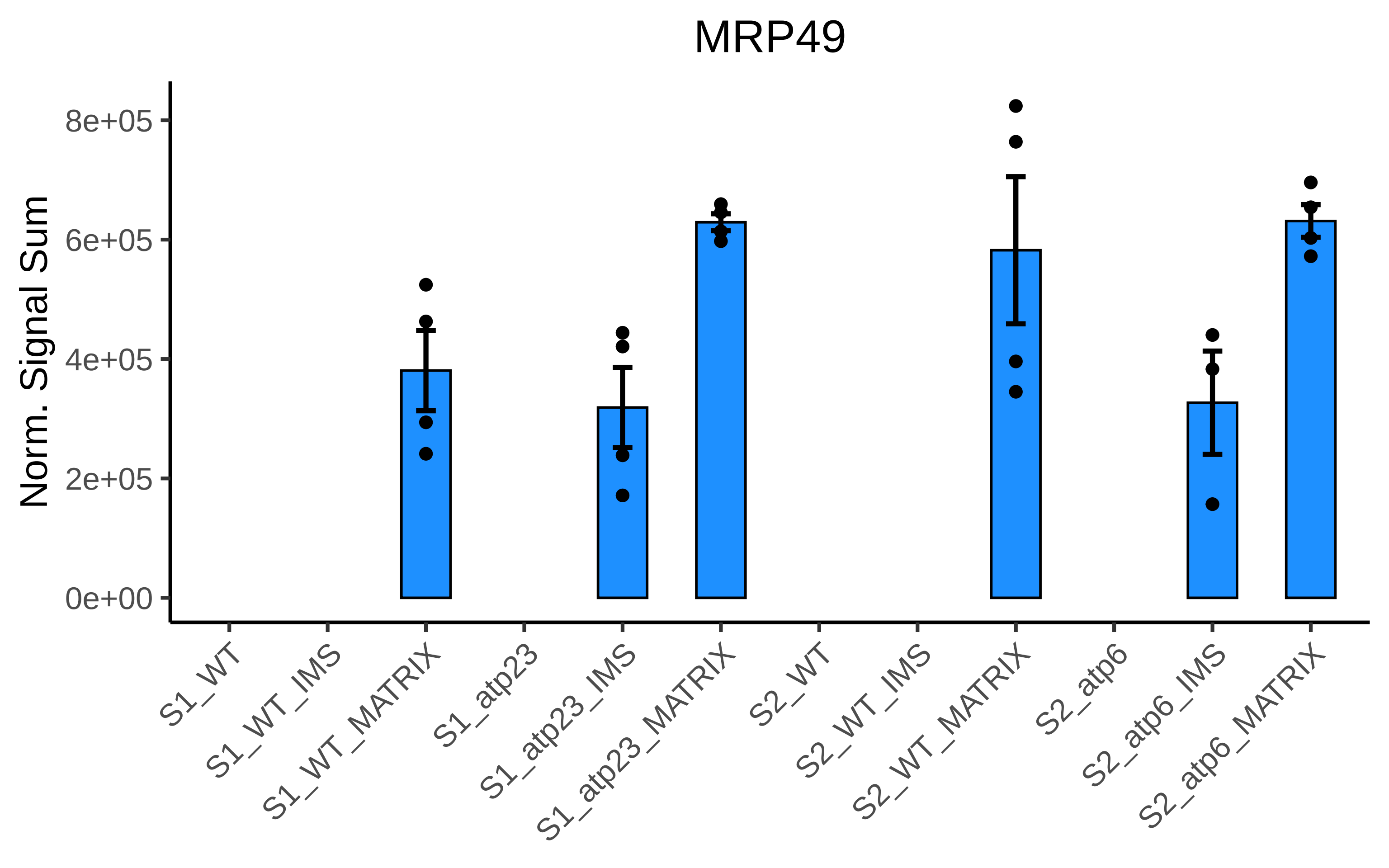
MRP49
Norm. Signal Sum
8e+05
6e+05
4e+05
2e+05
0e+00
S1_WT
S1_WT_IMS
S1_WT_MATRIX
S1_atp23
S1_atp23_IMS
S1_atp23_MATRIX
S2_WT
S2_WT_IMS
S2_WT_MATRIX
S2_atp6
S2_atp6_IMS
S2_atp6_MATRIX

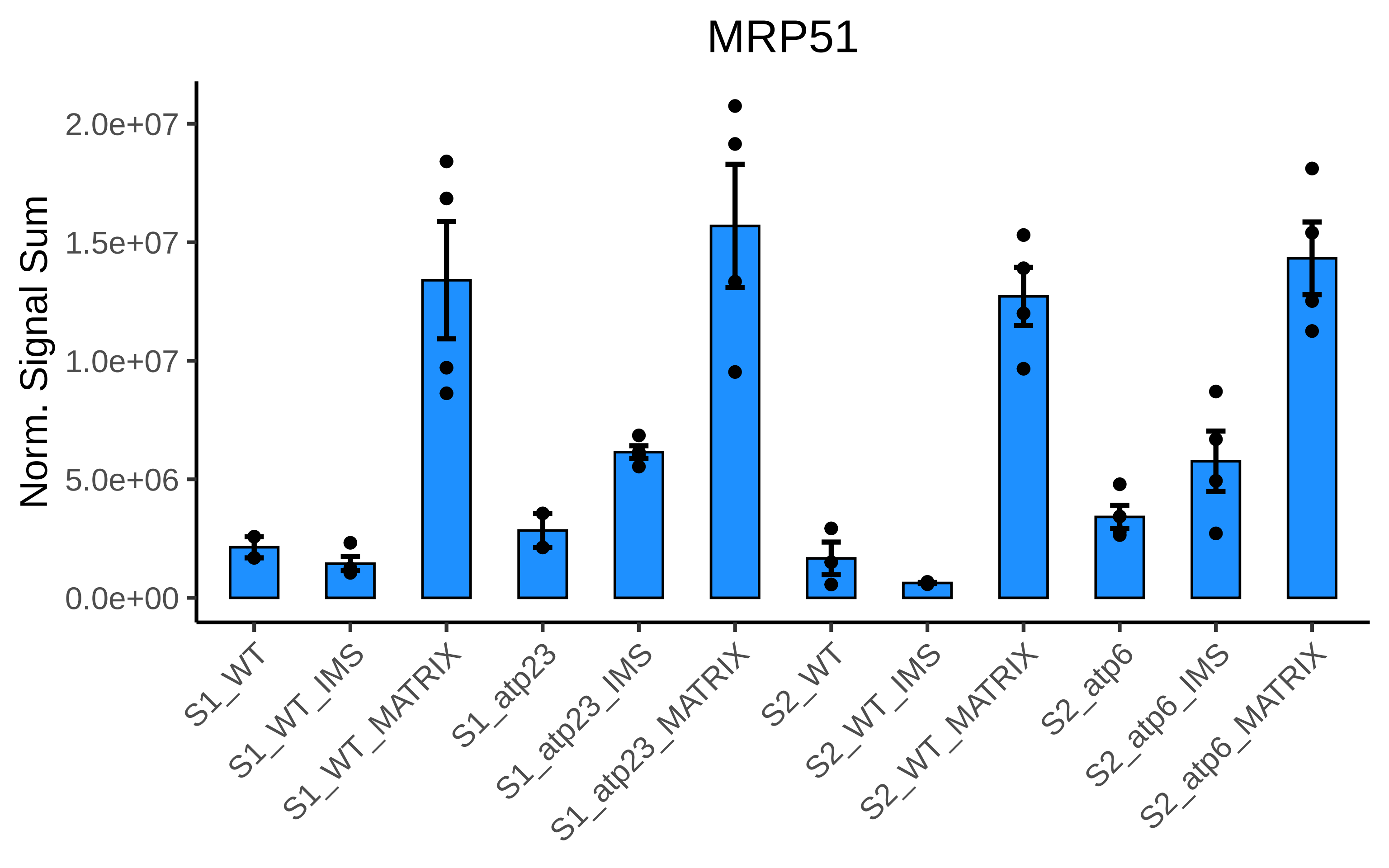
MRP51
Norm. Signal Sum
2.0e+07
1.5e+07
1.0e+07
5.0e+06
0.0e+00
S1_WT
S1_WT_IMS
S1_WT_MATRIX
S1_atp23
S1_atp23_IMS
S1_atp23_MATRIX
S2_WT
S2_WT_IMS
S2_WT_MATRIX
S2_atp6
S2_atp6_IMS
S2_atp6_MATRIX

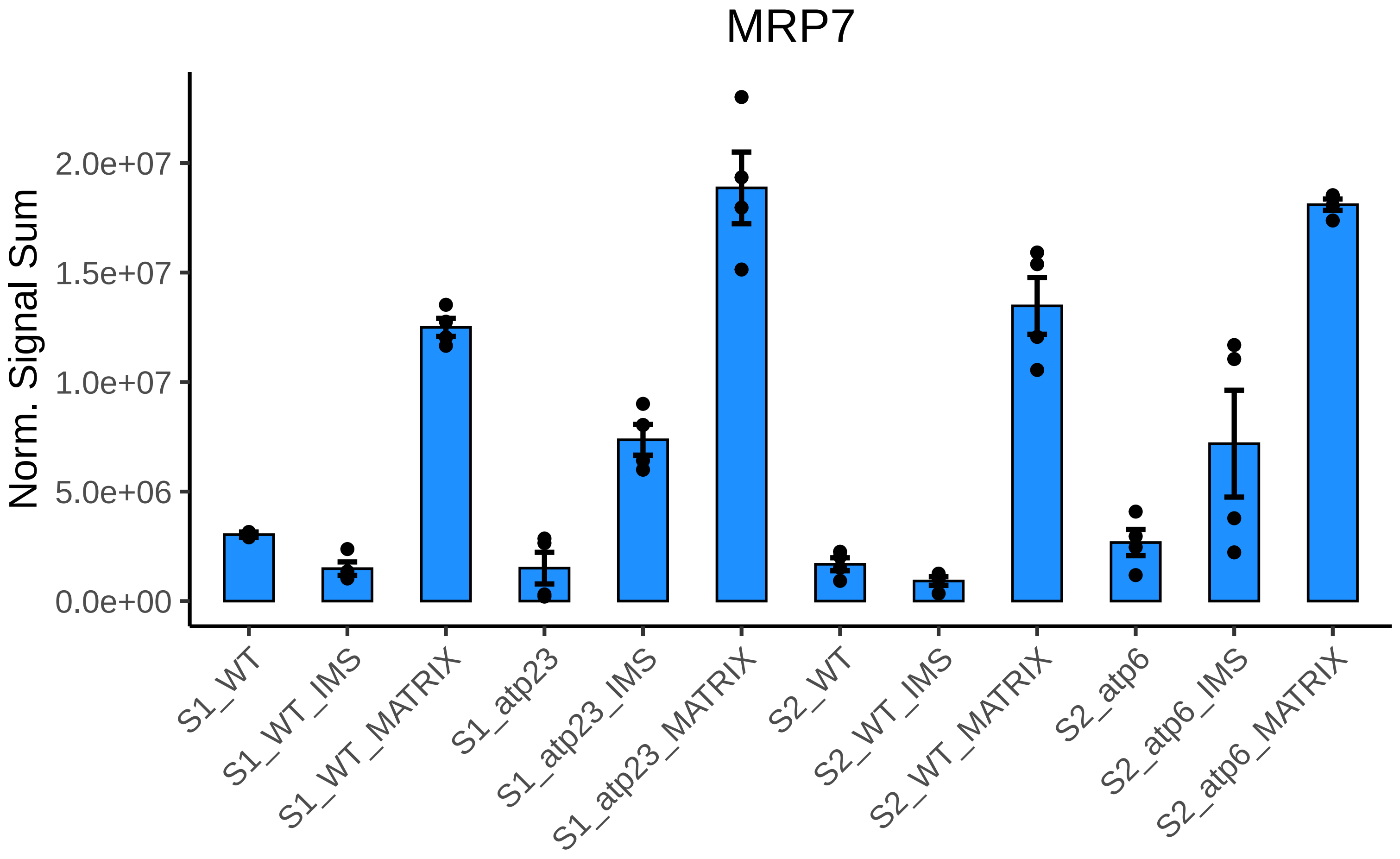
MRP7
Norm. Signal Sum
2.0e+07
1.5e+07
1.0e+07
5.0e+06
0.0e+00
S1_WT
S1_WT_IMS
S1_WT_MATRIX
S1_atp23
S1_atp23_IMS
S1_atp23_MATRIX
S2_WT
S2_WT_IMS
S2_WT_MATRIX
S2_atp6
S2_atp6_IMS
S2_atp6_MATRIX

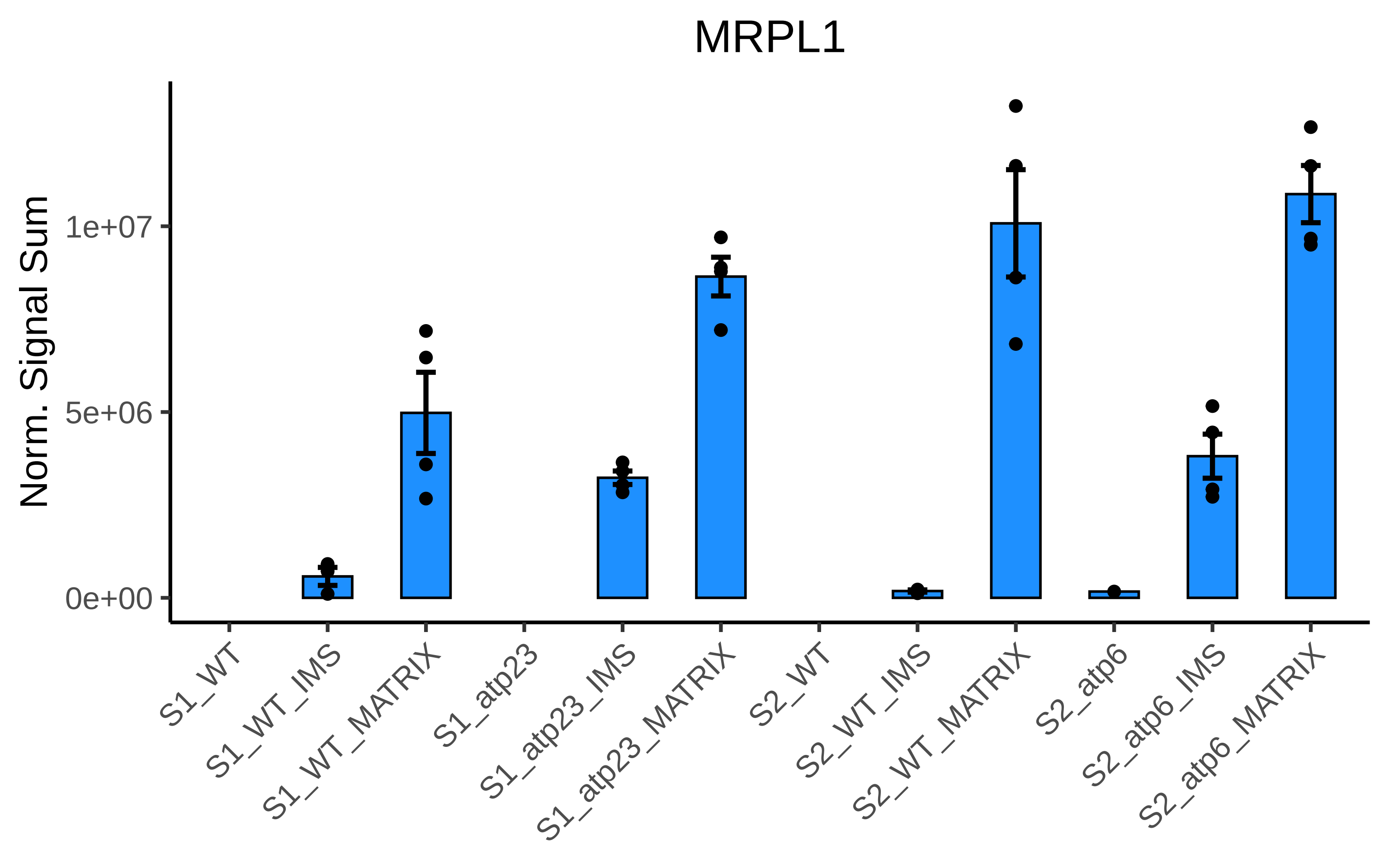
MRPL1
Norm. Signal Sum
1e+07
5e+06
0e+00
S1_WT
S1_WT_IMS
S1_WT_MATRIX
S1_atp23
S1_atp23_IMS
S1_atp23_MATRIX
S2_WT
S2_WT_IMS
S2_WT_MATRIX
S2_atp6
S2_atp6_IMS
S2_atp6_MATRIX

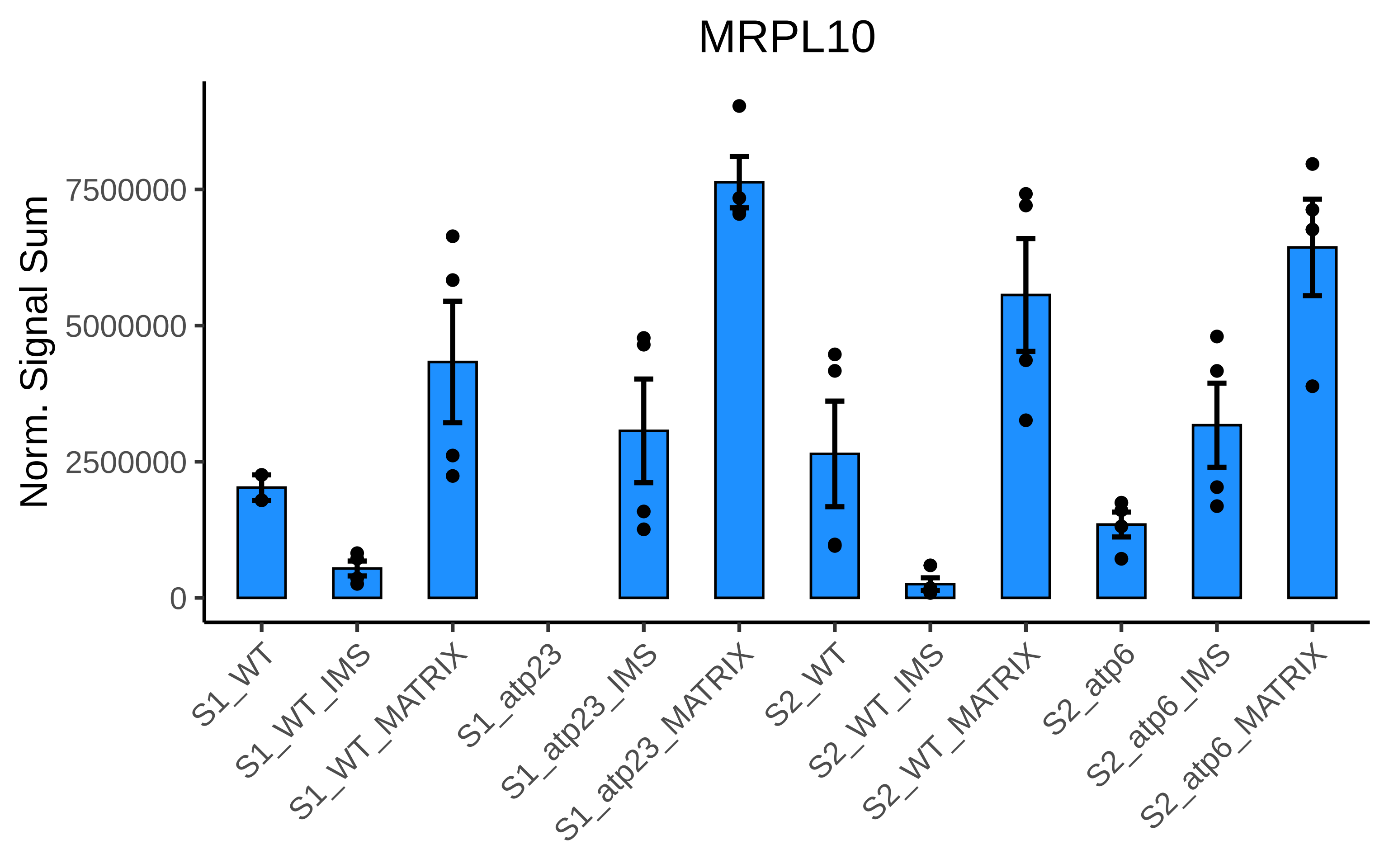
MRPL10
Norm. Signal Sum
7500000
5000000
2500000
0
S1_WT
S1_WT_IMS
S1_WT_MATRIX
S1_atp23
S1_atp23_IMS
S1_atp23_MATRIX
S2_WT
S2_WT_IMS
S2_WT_MATRIX
S2_atp6
S2_atp6_IMS
S2_atp6_MATRIX

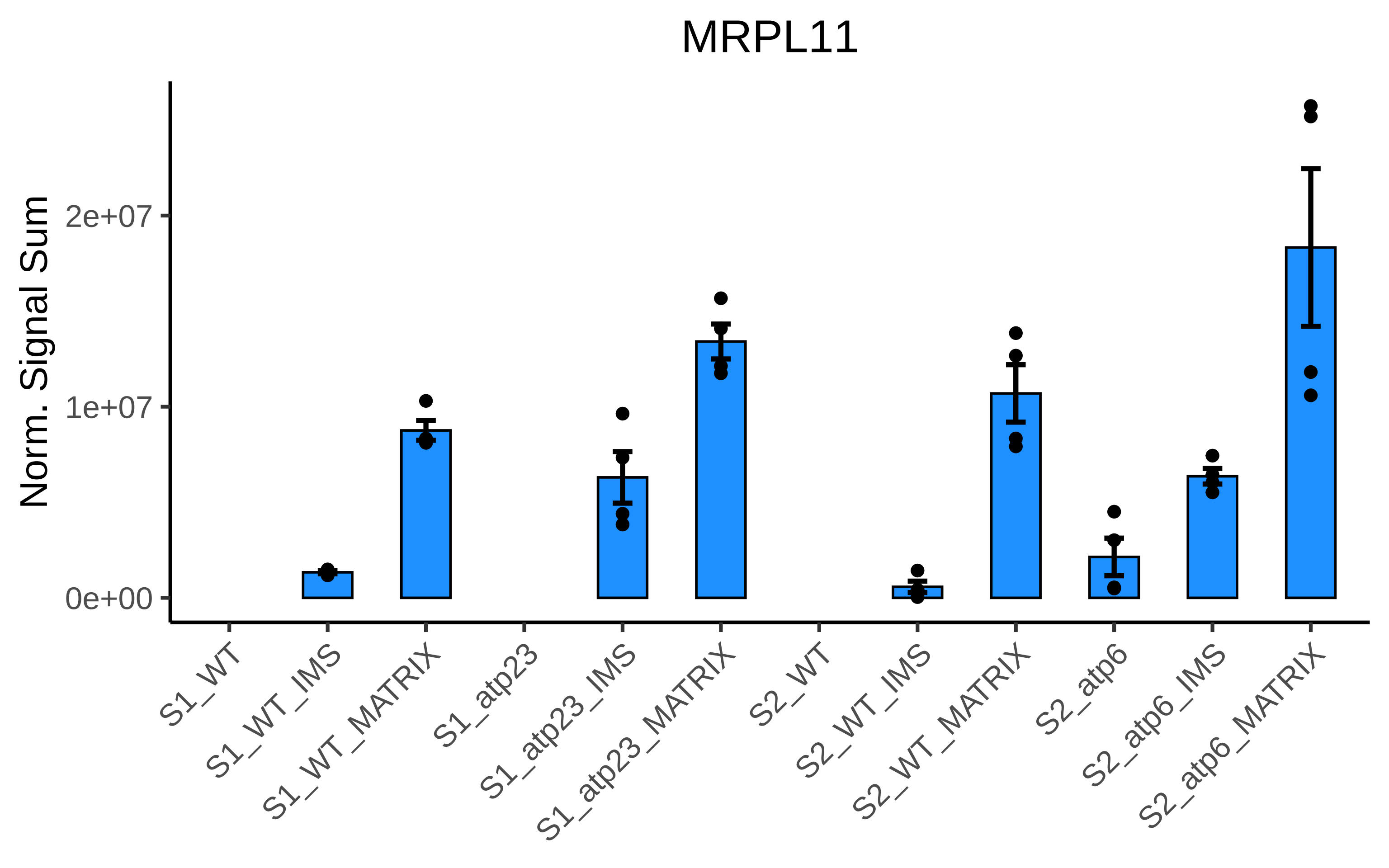

MRPL11
Norm. Signal Sum
2e+07
1e+07
0e+00
S1_WT
S1_WT_IMS
S1_WT_MATRIX
S1_atp23
S1_atp23_IMS
S1_atp23_MATRIX
S2_WT
S2_WT_IMS
S2_WT_MATRIX
S2_atp6
S2_atp6_IMS
S2_atp6_MATRIX

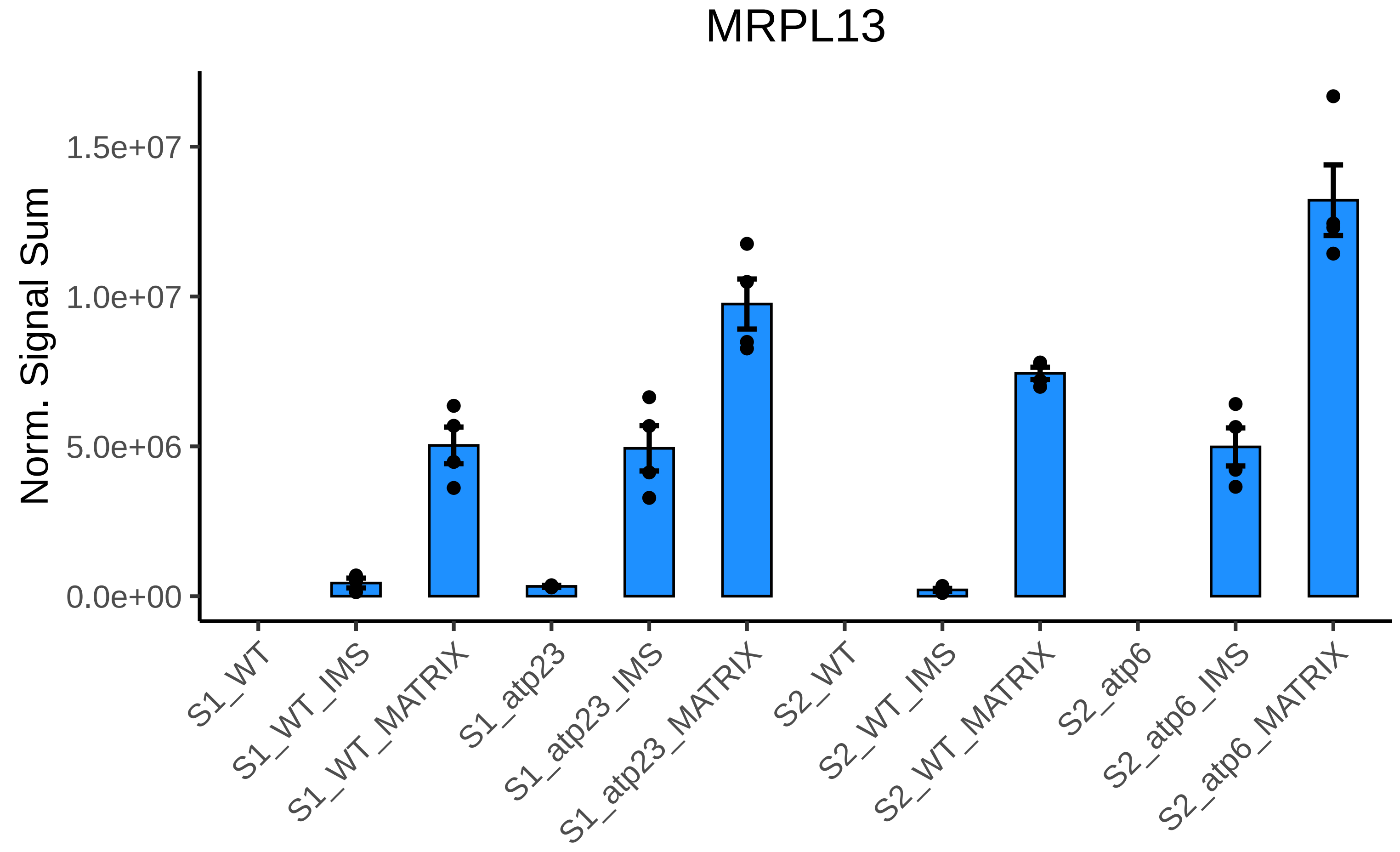

MRPL13
Norm. Signal Sum
1.5e+07
1.0e+07
5.0e+06
0.0e+00
S1_WT
S1_WT_IMS
S1_WT_MATRIX
S1_atp23
S1_atp23_IMS
S1_atp23_MATRIX
S2_WT
S2_WT_IMS
S2_WT_MATRIX
S2_atp6
S2_atp6_IMS
S2_atp6_MATRIX

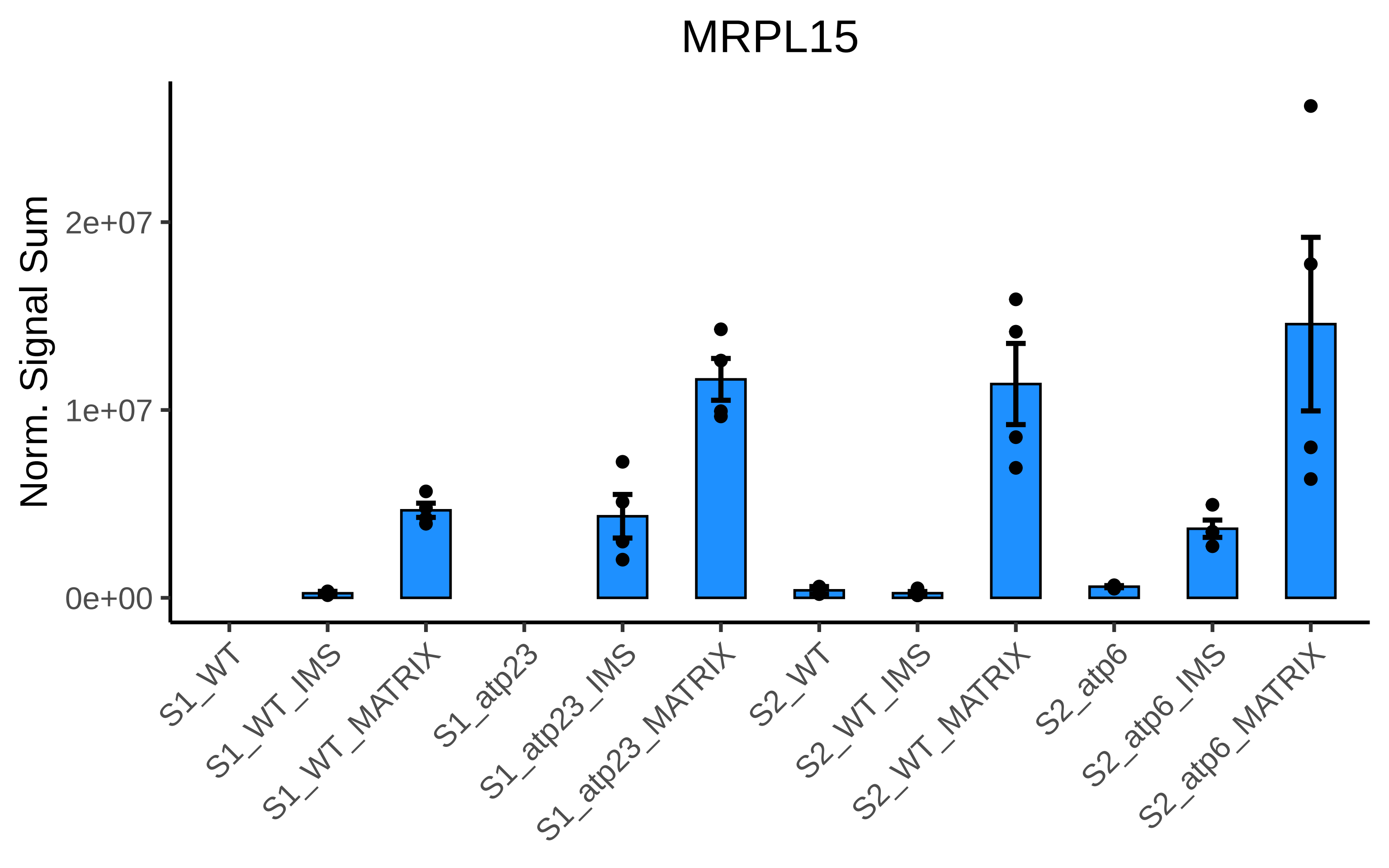
MRPL15
Norm. Signal Sum
2e+07
1e+07
0e+00
S1_WT
S1_WT_IMS
S1_WT_MATRIX
S1_atp23
S1_atp23_IMS
S1_atp23_MATRIX
S2_WT
S2_WT_IMS
S2_WT_MATRIX
S2_atp6
S2_atp6_IMS
S2_atp6_MATRIX

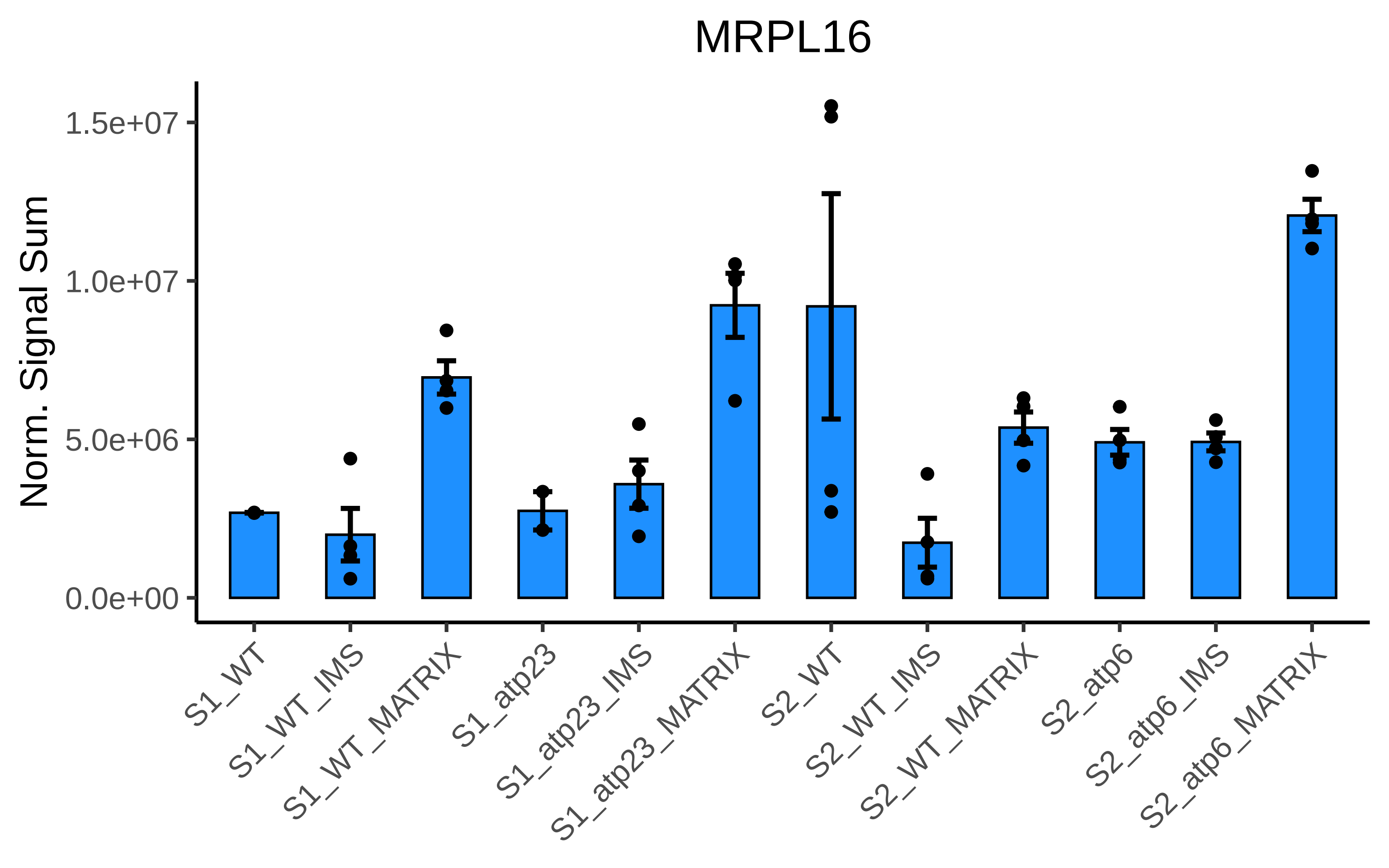
MRPL16
Norm. Signal Sum
1.5e+07
1.0e+07
5.0e+06
0.0e+00
S1_WT
S1_WT_IMS
S1_WT_MATRIX
S1_atp23
S1_atp23_IMS
S1_atp23_MATRIX
S2_WT
S2_WT_IMS
S2_WT_MATRIX
S2_atp6
S2_atp6_IMS
S2_atp6_MATRIX

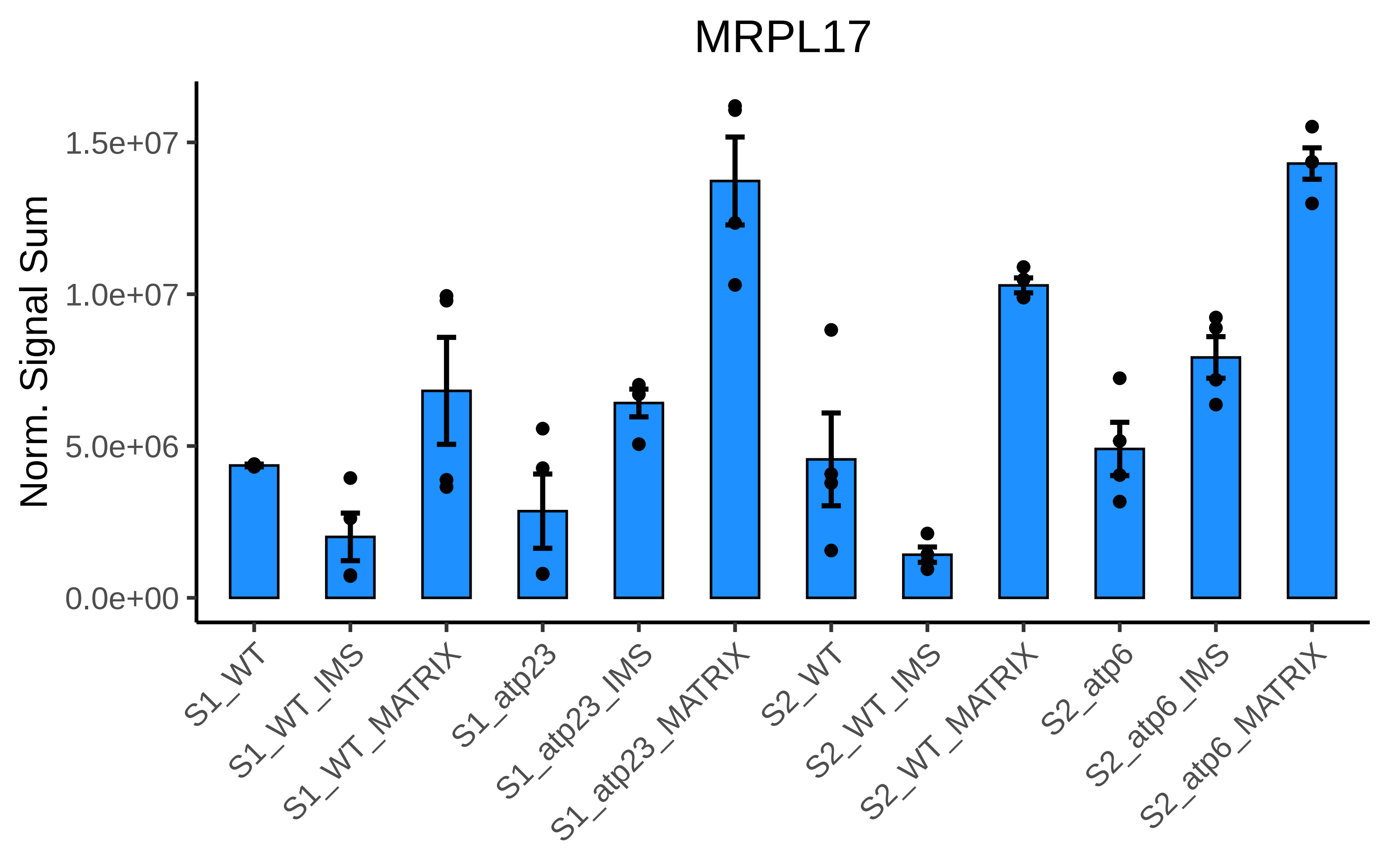
MRPL17
Norm. Signal Sum
0.0e+00
5.0e+06
1.0e+07
1.5e+07
S1_WT
S1_WT_IMS
S1_WT_MATRIX
S1_atp23
S1_atp23_IMS
S1_atp23_MATRIX
S2_WT
S2_WT_IMS
S2_WT_MATRIX
S2_atp6
S2_atp6_IMS
S2_atp6_MATRIX

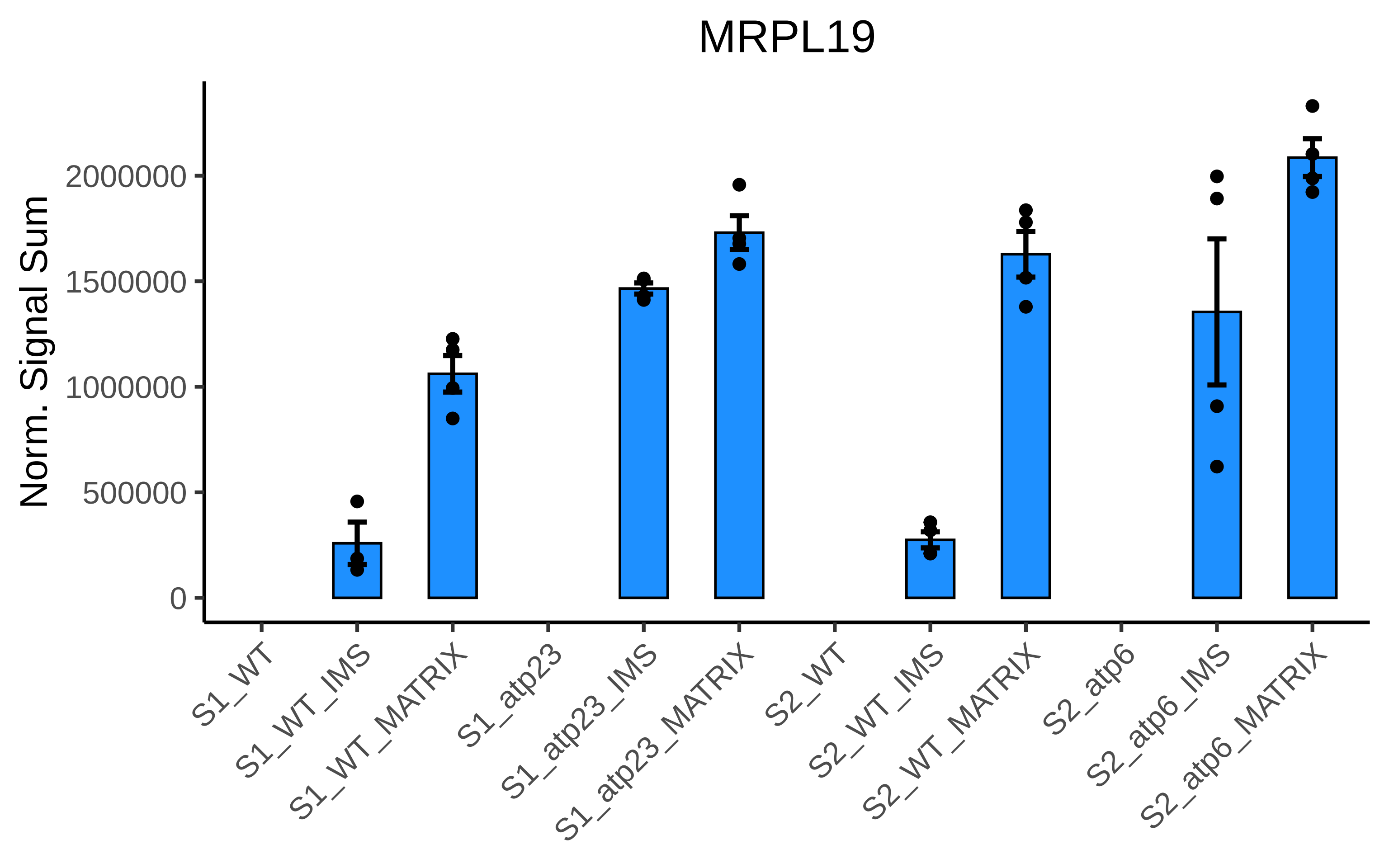
MRPL19
Norm. Signal Sum
2000000
1500000
1000000
500000
0
S1_WT
S1_WT_IMS
S1_WT_MATRIX
S1_atp23
S1_atp23_IMS
S1_atp23_MATRIX
S2_WT
S2_WT_IMS
S2_WT_MATRIX
S2_atp6
S2_atp6_IMS
S2_atp6_MATRIX

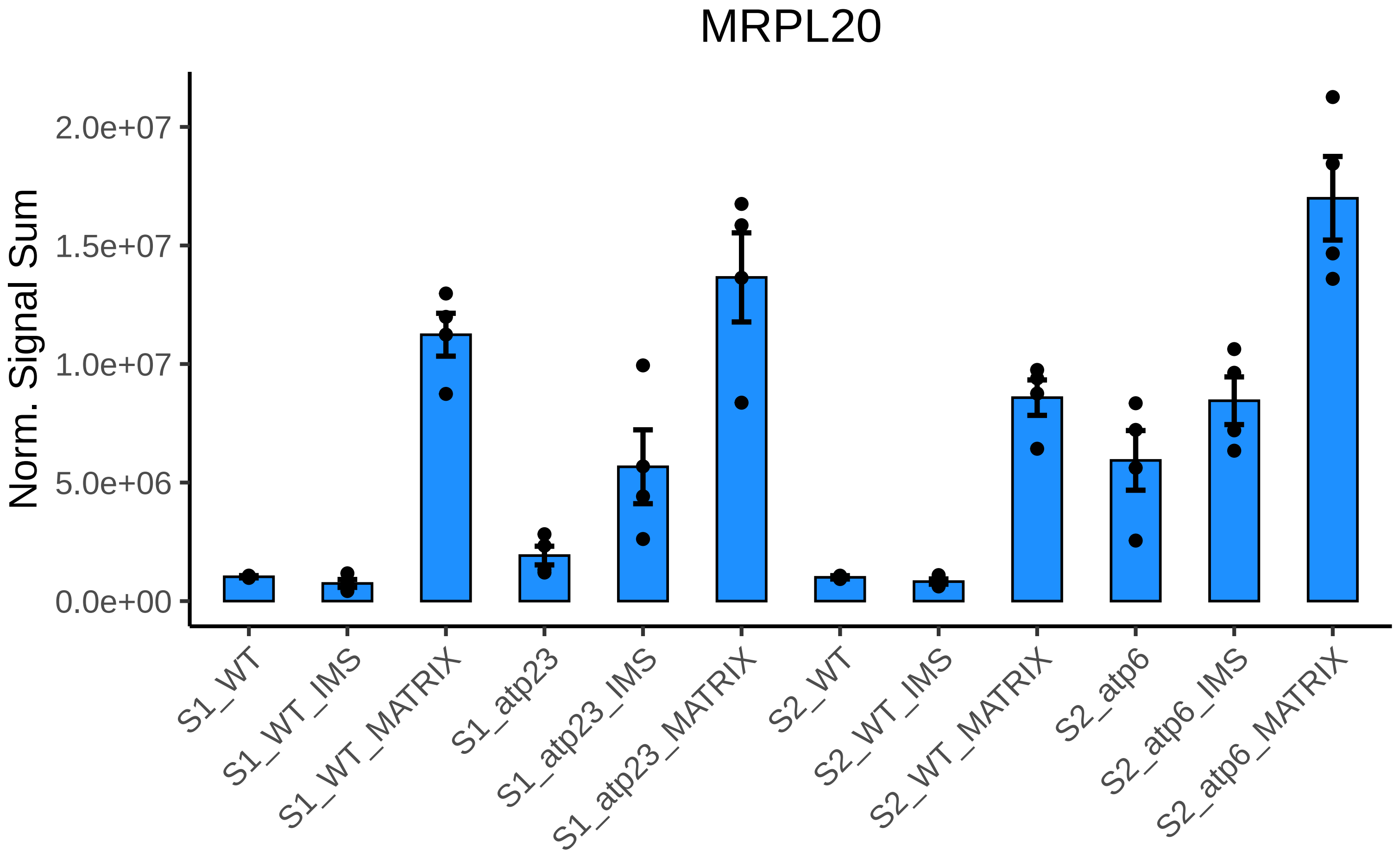

MRPL20
Norm. Signal Sum
2.0e+07
1.5e+07
1.0e+07
5.0e+06
0.0e+00
S1_WT
S1_WT_IMS
S1_WT_MATRIX
S1_atp23
S1_atp23_IMS
S1_atp23_MATRIX
S2_WT
S2_WT_IMS
S2_WT_MATRIX
S2_atp6
S2_atp6_IMS
S2_atp6_MATRIX

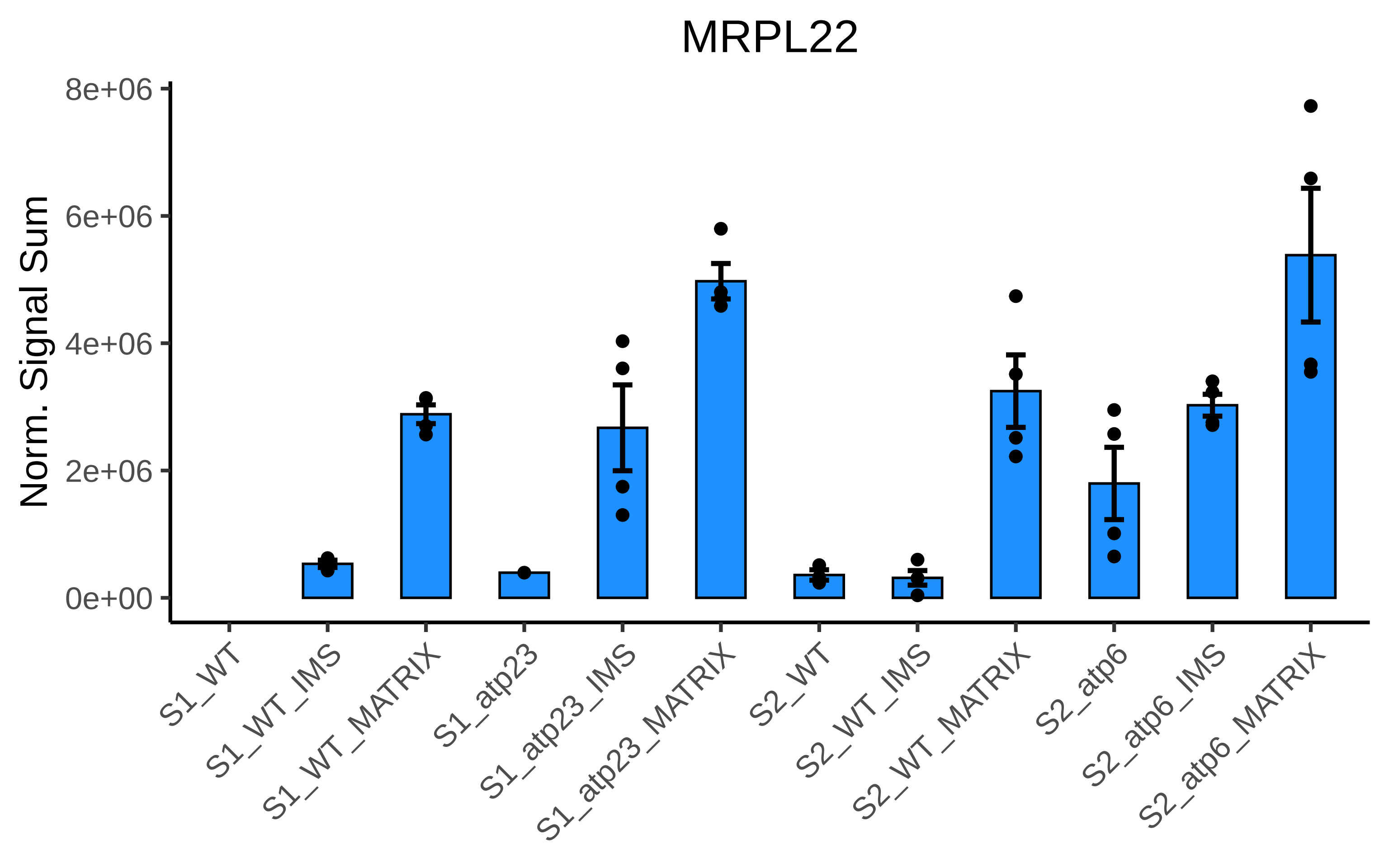

MRPL22
Norm. Signal Sum
8e+06
6e+06
4e+06
2e+06
0e+00
S1_WT
S1_WT_IMS
S1_WT_MATRIX
S1_atp23
S1_atp23_IMS
S1_atp23_MATRIX
S2_WT
S2_WT_IMS
S2_WT_MATRIX
S2_atp6
S2_atp6_IMS
S2_atp6_MATRIX

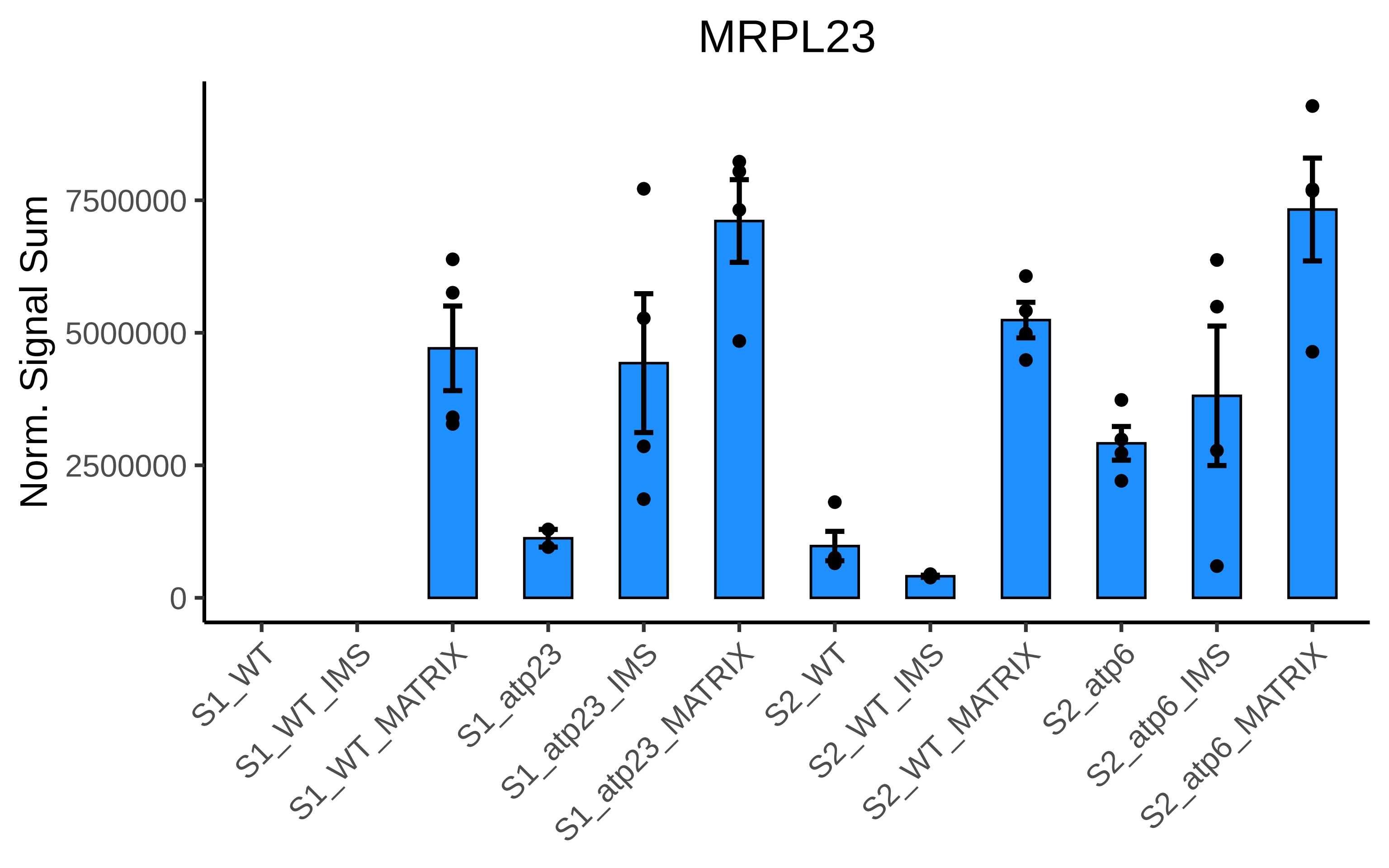

MRPL23
Norm. Signal Sum
7500000
5000000
2500000
0
S1_WT
S1_WT_IMS
S1_WT_MATRIX
S1_atp23
S1_atp23_IMS
S1_atp23_MATRIX
S2_WT
S2_WT_IMS
S2_WT_MATRIX
S2_atp6
S2_atp6_IMS
S2_atp6_MATRIX

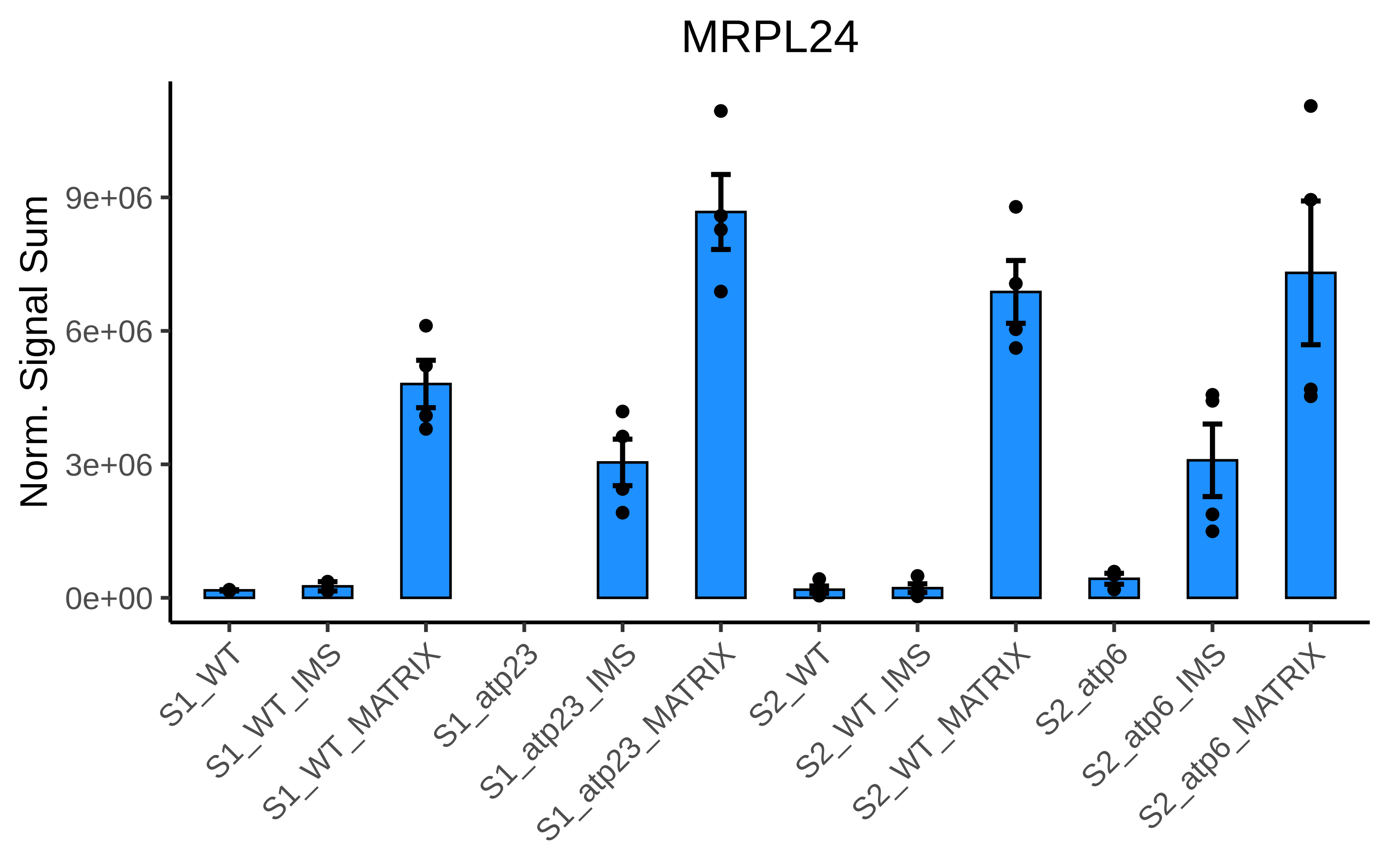
MRPL24
Norm. Signal Sum
9e+06
6e+06
3e+06
0e+00
S1_WT
S1_WT_IMS
S1_WT_MATRIX
S1_atp23
S1_atp23_IMS
S1_atp23_MATRIX
S2_WT
S2_WT_IMS
S2_WT_MATRIX
S2_atp6
S2_atp6_IMS
S2_atp6_MATRIX

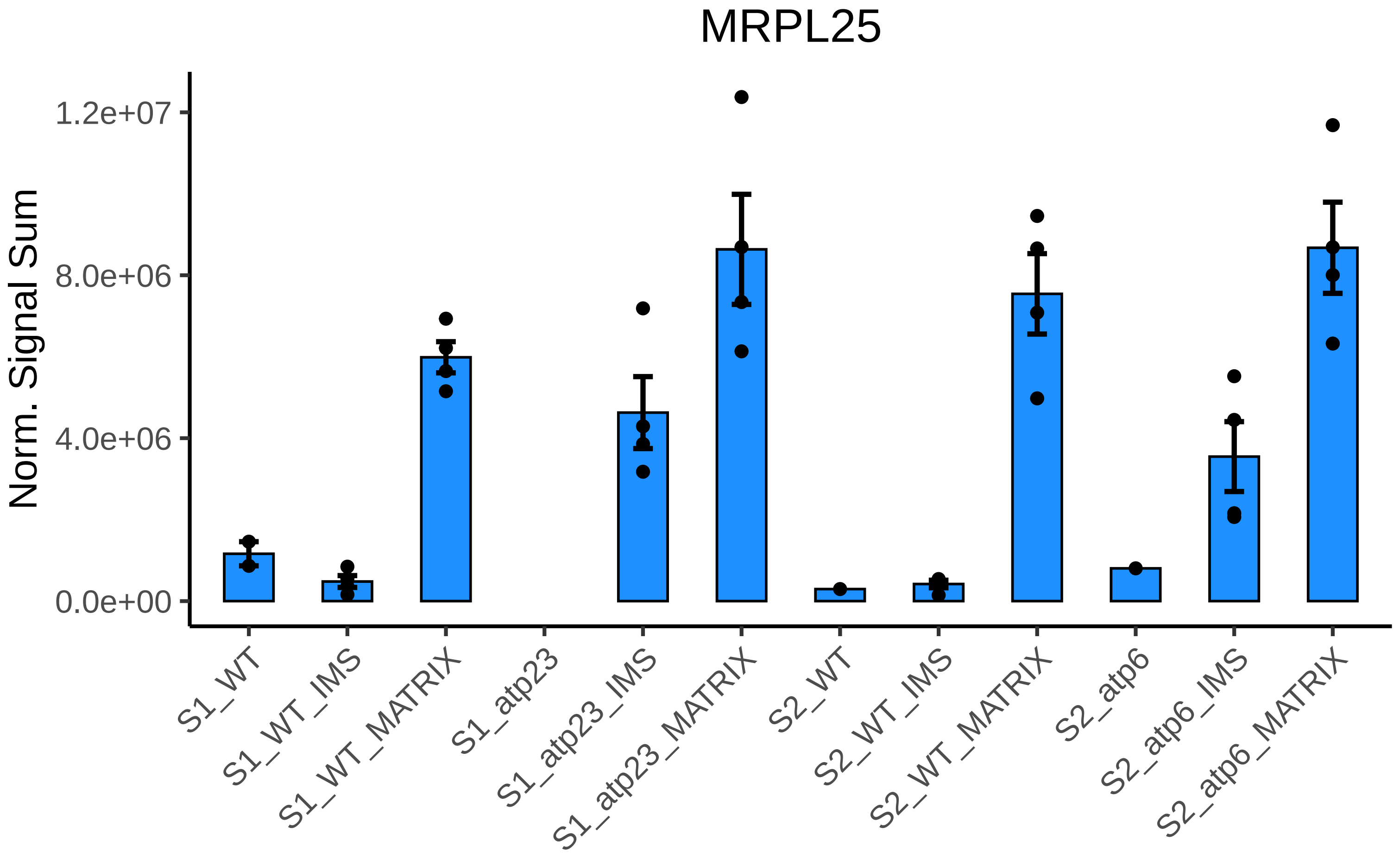

MRPL25
Norm. Signal Sum
1.2e+07
8.0e+06
4.0e+06
0.0e+00
S1_WT
S1_WT_IMS
S1_WT_MATRIX
S1_atp23
S1_atp23_IMS
S1_atp23_MATRIX
S2_WT
S2_WT_IMS
S2_WT_MATRIX
S2_atp6
S2_atp6_IMS
S2_atp6_MATRIX

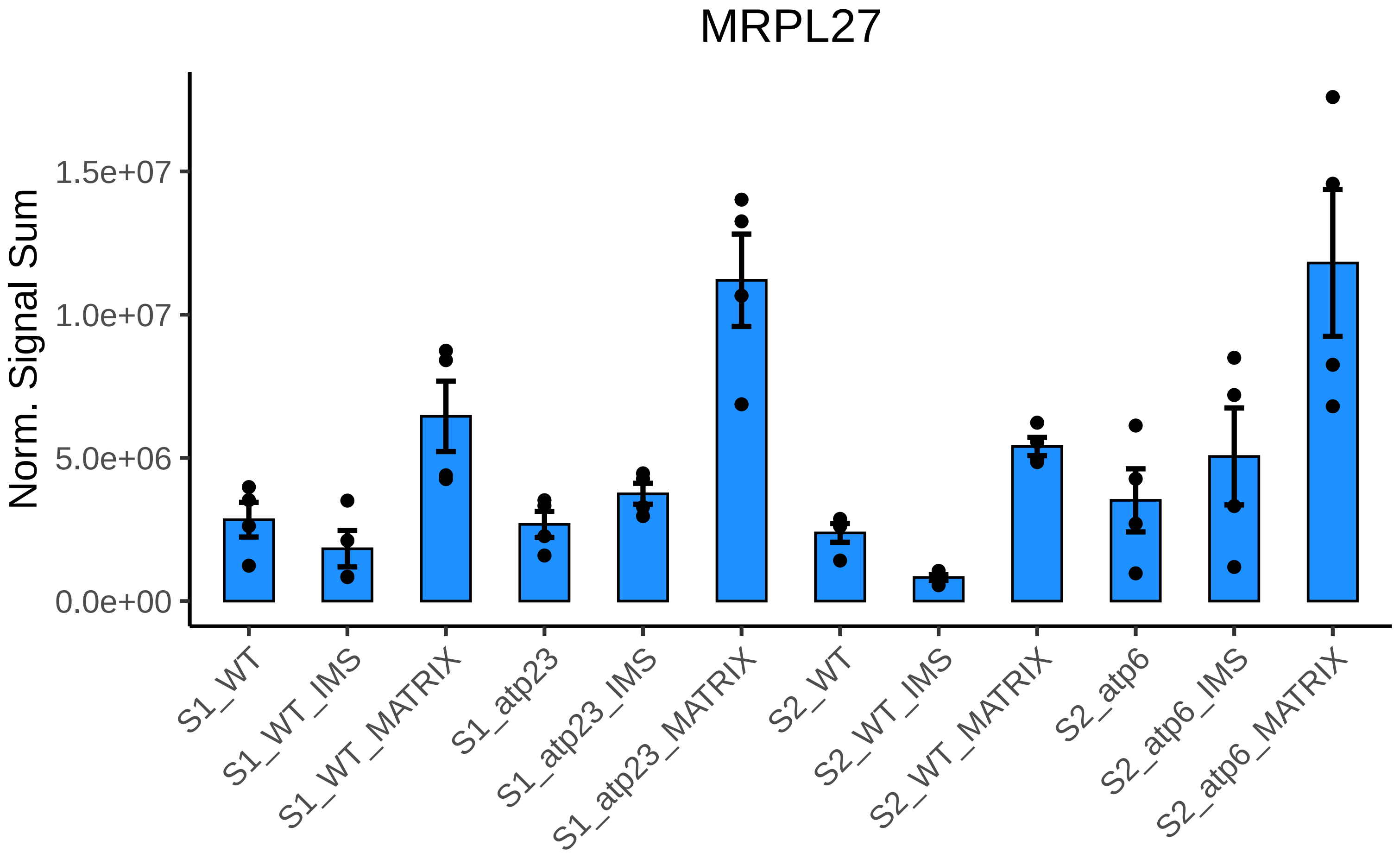
MRPL27
Norm. Signal Sum
0.0e+00
5.0e+06
1.0e+07
1.5e+07
S1_WT
S1_WT_IMS
S1_WT_MATRIX
S1_atp23
S1_atp23_IMS
S1_atp23_MATRIX
S2_WT
S2_WT_IMS
S2_WT_MATRIX
S2_atp6
S2_atp6_IMS
S2_atp6_MATRIX

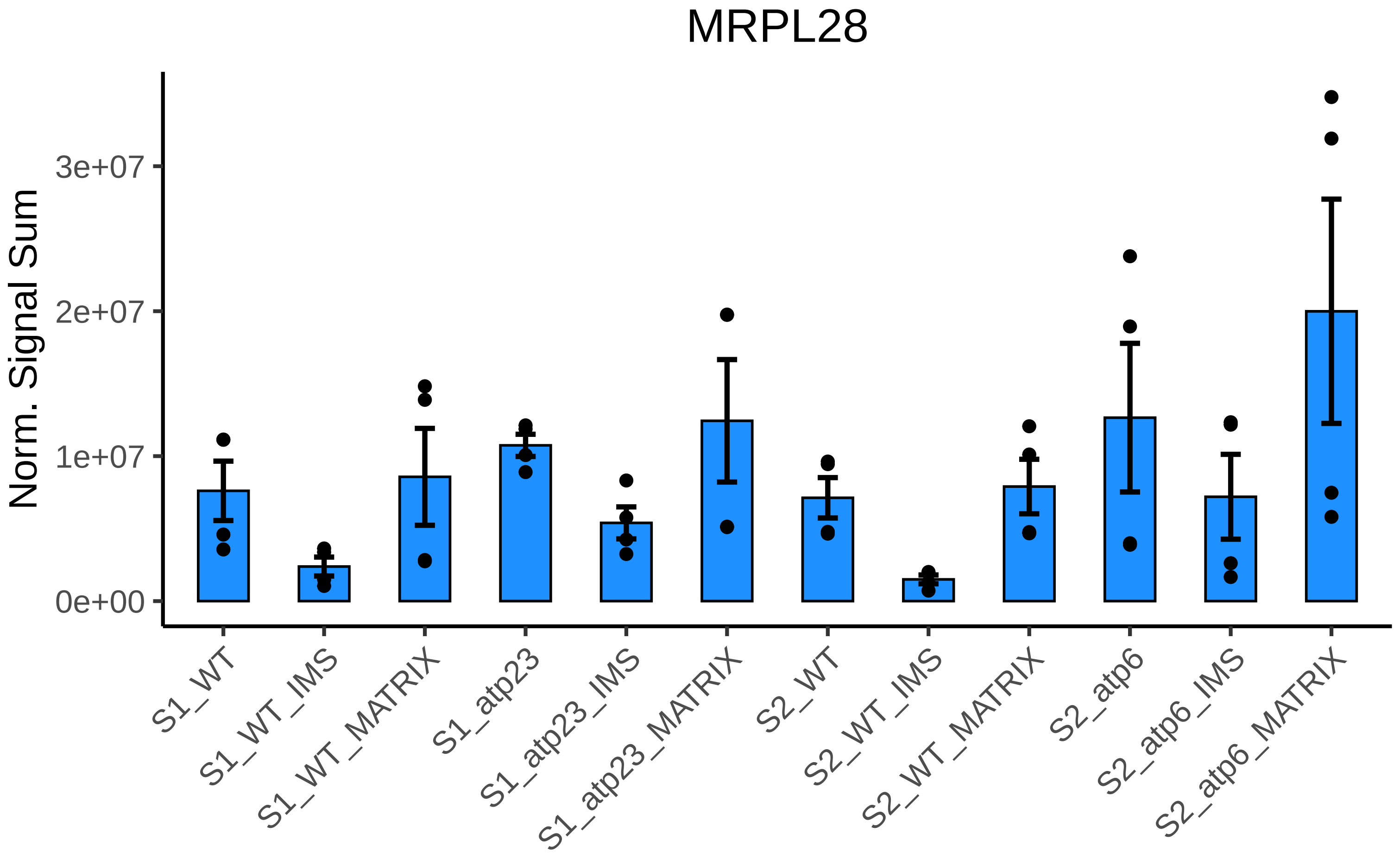
MRPL28
Norm. Signal Sum
3e+07
2e+07
1e+07
0e+00
S1_WT
S1_WT_IMS
S1_WT_MATRIX
S1_atp23
S1_atp23_IMS
S1_atp23_MATRIX
S2_WT
S2_WT_IMS
S2_WT_MATRIX
S2_atp6
S2_atp6_IMS
S2_atp6_MATRIX

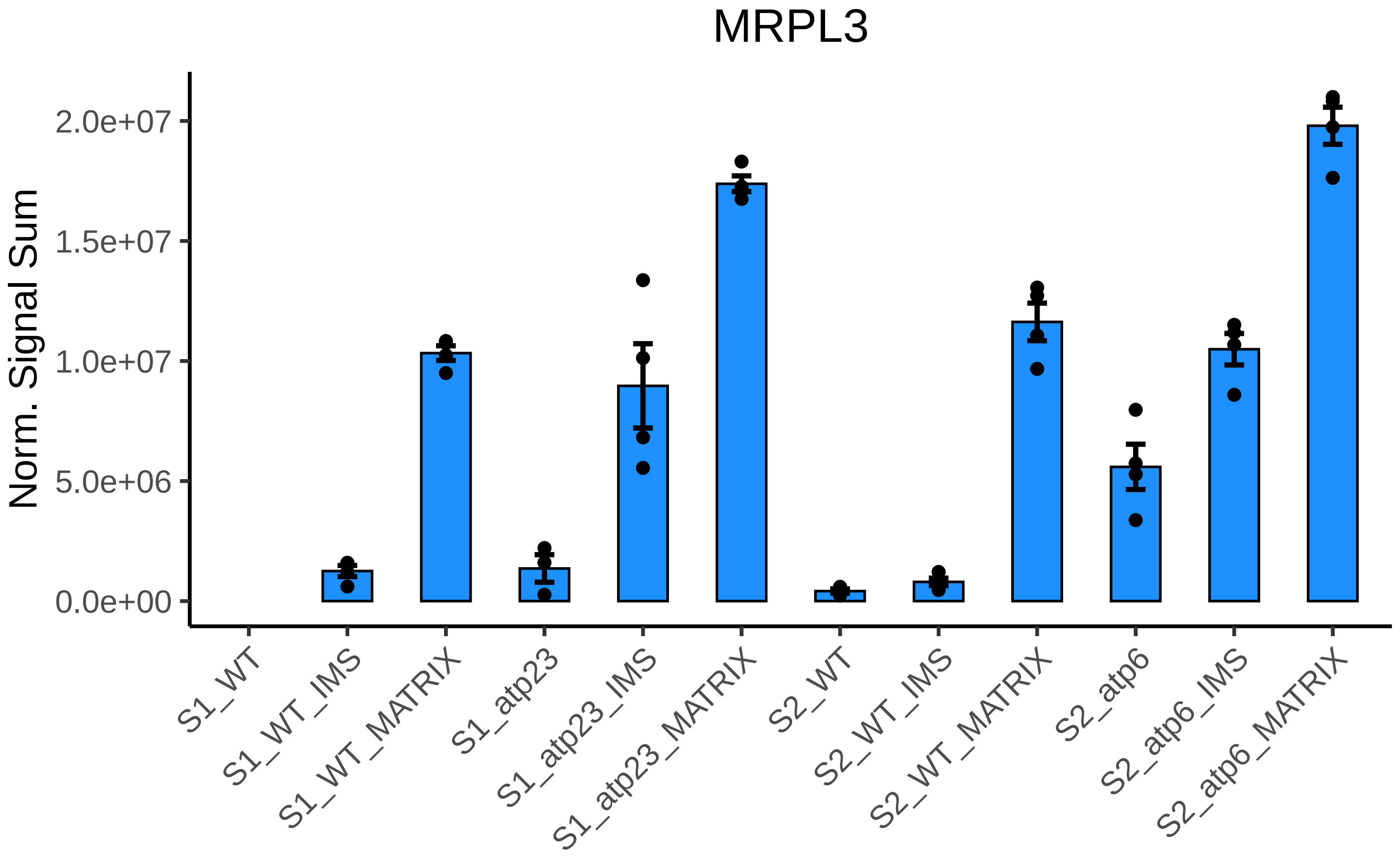

MRPL3
Norm. Signal Sum
2.0e+07
1.5e+07
1.0e+07
5.0e+06
0.0e+00
S1_WT
S1_WT_IMS
S1_WT_MATRIX
S1_atp23
S1_atp23_IMS
S1_atp23_MATRIX
S2_WT
S2_WT_IMS
S2_WT_MATRIX
S2_atp6
S2_atp6_IMS
S2_atp6_MATRIX

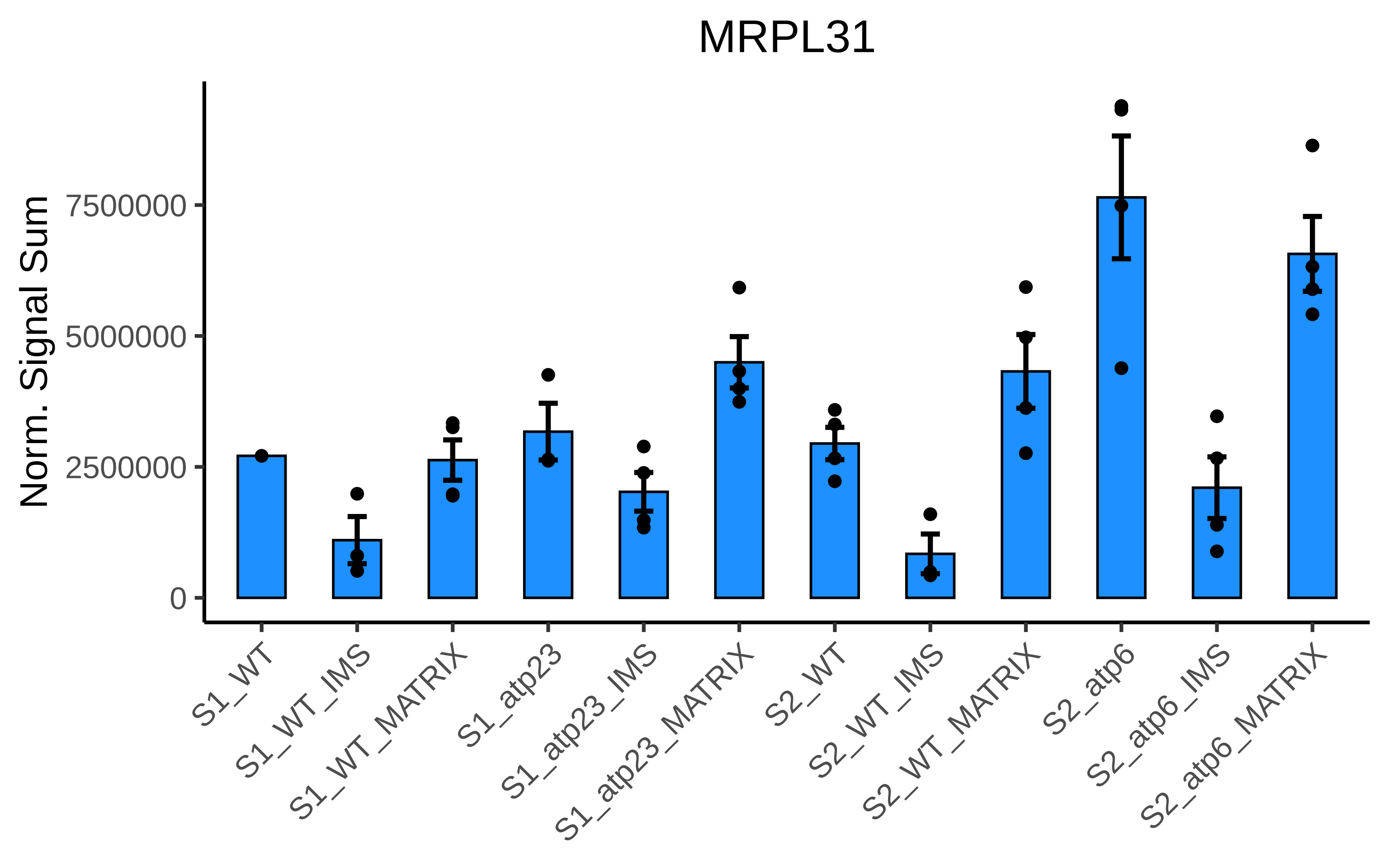
MRPL31
Norm. Signal Sum
7500000
5000000
2500000
0
S1_WT
S1_WT_IMS
S1_WT_MATRIX
S1_atp23
S1_atp23_IMS
S1_atp23_MATRIX
S2_WT
S2_WT_IMS
S2_WT_MATRIX
S2_atp6
S2_atp6_IMS
S2_atp6_MATRIX

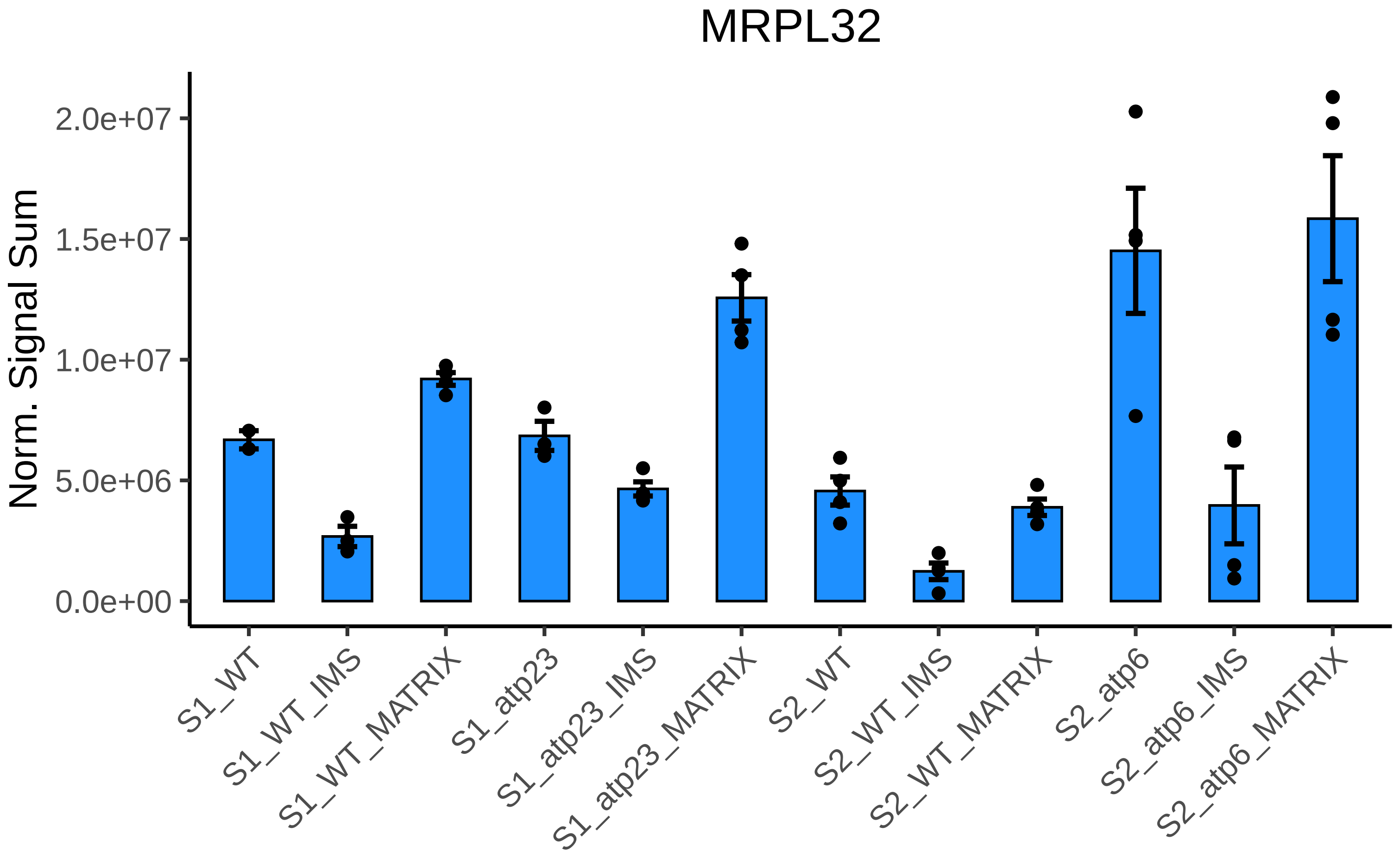

MRPL32
Norm. Signal Sum
2.0e+07
1.5e+07
1.0e+07
5.0e+06
0.0e+00
S1_WT
S1_WT_IMS
S1_WT_MATRIX
S1_atp23
S1_atp23_IMS
S1_atp23_MATRIX
S2_WT
S2_WT_IMS
S2_WT_MATRIX
S2_atp6
S2_atp6_IMS
S2_atp6_MATRIX

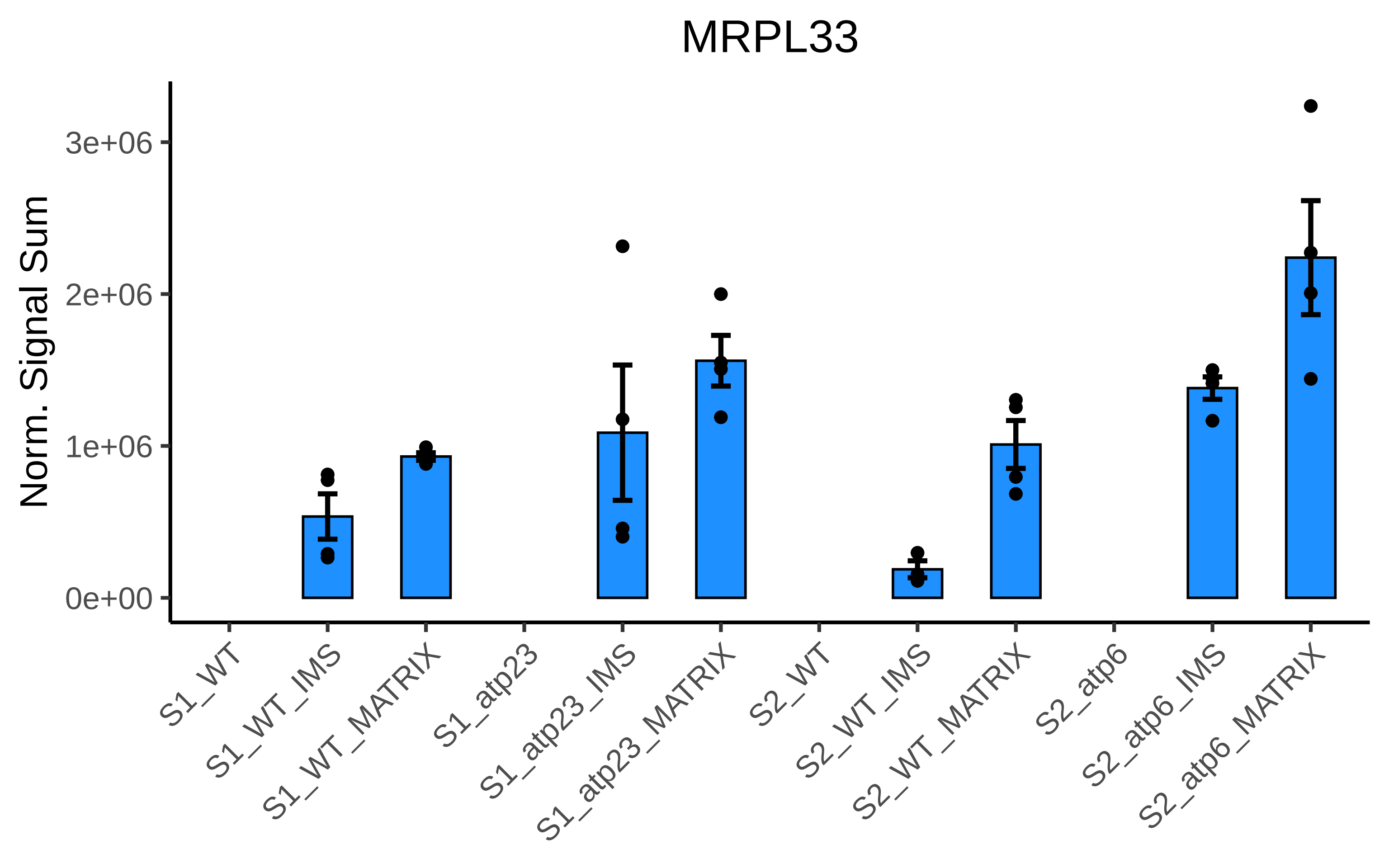
MRPL33
Norm. Signal Sum
3e+06
2e+06
1e+06
0e+00
S1_WT
S1_WT_IMS
S1_WT_MATRIX
S1_atp23
S1_atp23_IMS
S1_atp23_MATRIX
S2_WT
S2_WT_IMS
S2_WT_MATRIX
S2_atp6
S2_atp6_IMS
S2_atp6_MATRIX

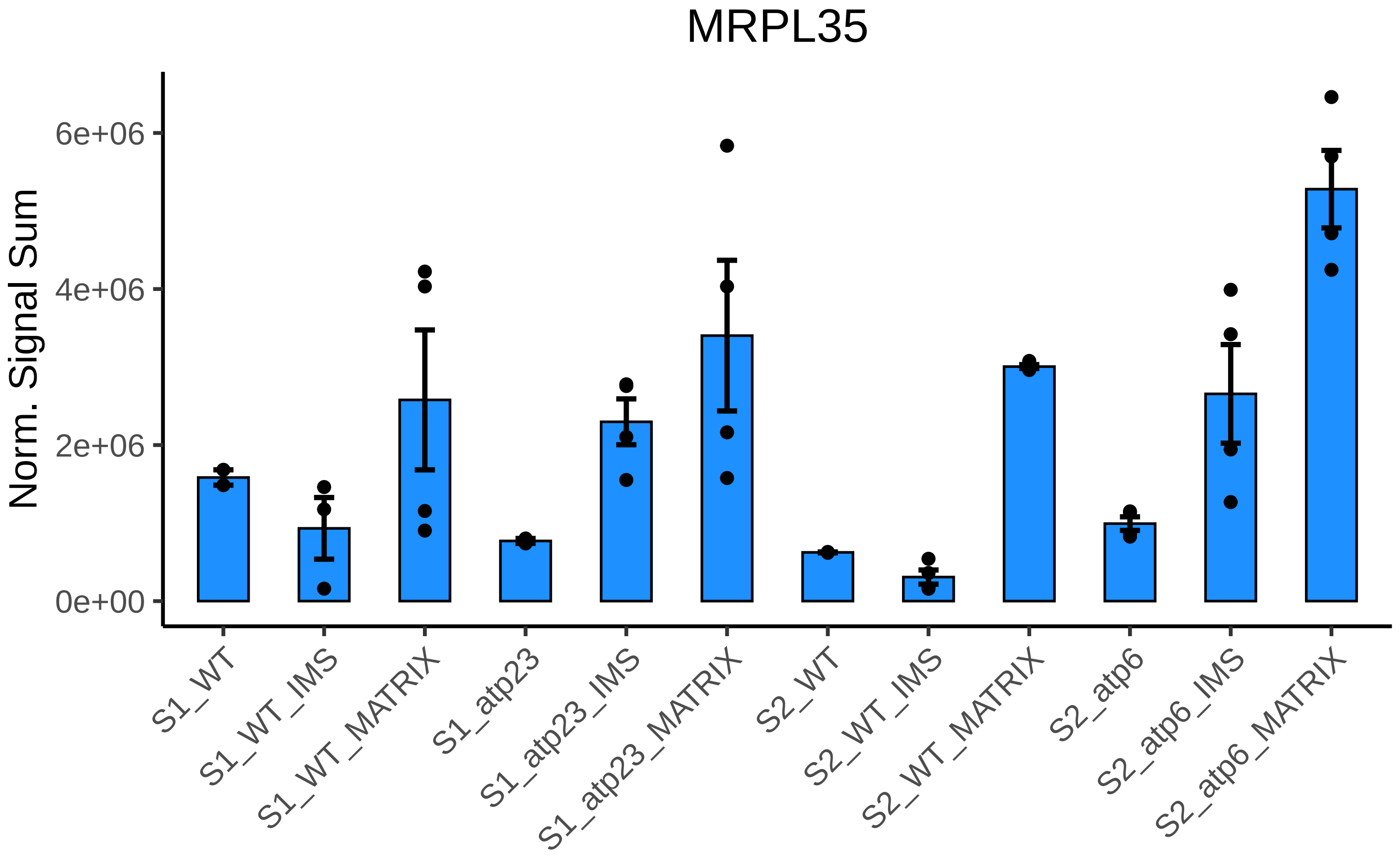

MRPL35
Norm. Signal Sum
6e+06
4e+06
2e+06
0e+00
S1_WT
S1_WT_IMS
S1_WT_MATRIX
S1_atp23
S1_atp23_IMS
S1_atp23_MATRIX
S2_WT
S2_WT_IMS
S2_WT_MATRIX
S2_atp6
S2_atp6_IMS
S2_atp6_MATRIX

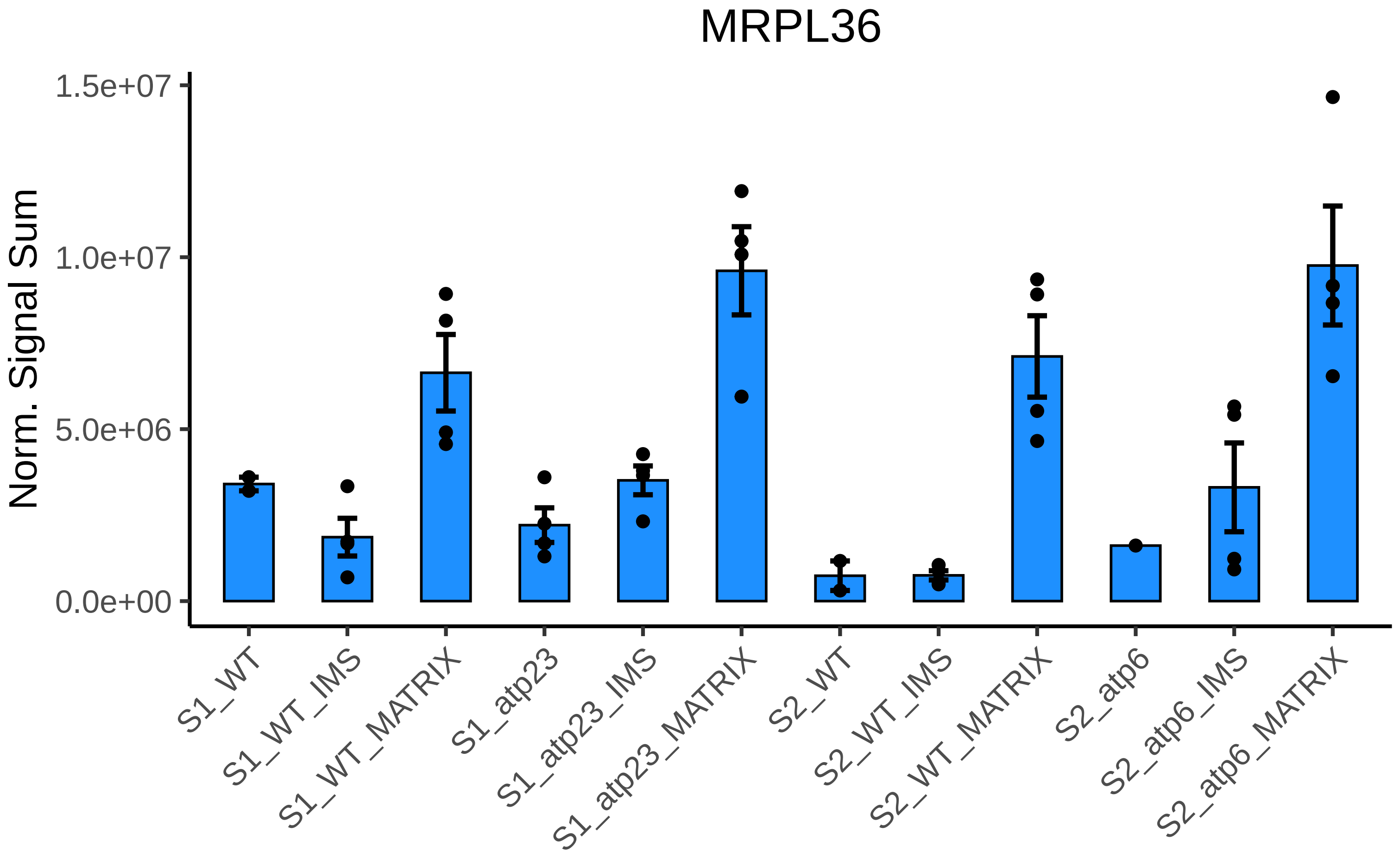
MRPL36
Norm. Signal Sum
1.5e+07
1.0e+07
5.0e+06
0.0e+00
S1_WT
S1_WT_IMS
S1_WT_MATRIX
S1_atp23
S1_atp23_IMS
S1_atp23_MATRIX
S2_WT
S2_WT_IMS
S2_WT_MATRIX
S2_atp6
S2_atp6_IMS
S2_atp6_MATRIX

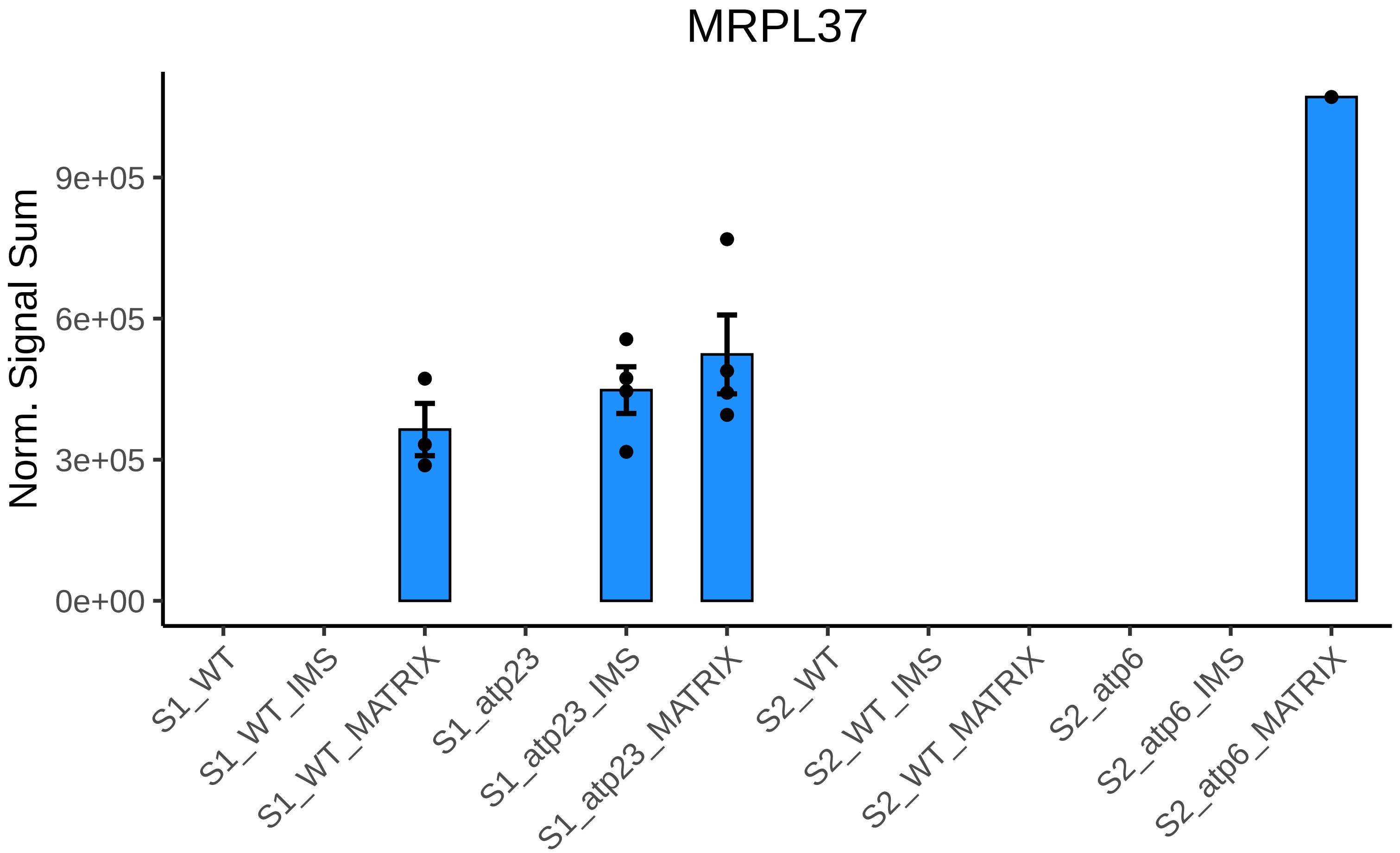

MRPL37
Norm. Signal Sum
9e+05
6e+05
3e+05
0e+00
S1_WT
S1_WT_IMS
S1_WT_MATRIX
S1_atp23
S1_atp23_IMS
S1_atp23_MATRIX
S2_WT
S2_WT_IMS
S2_WT_MATRIX
S2_atp6
S2_atp6_IMS
S2_atp6_MATRIX

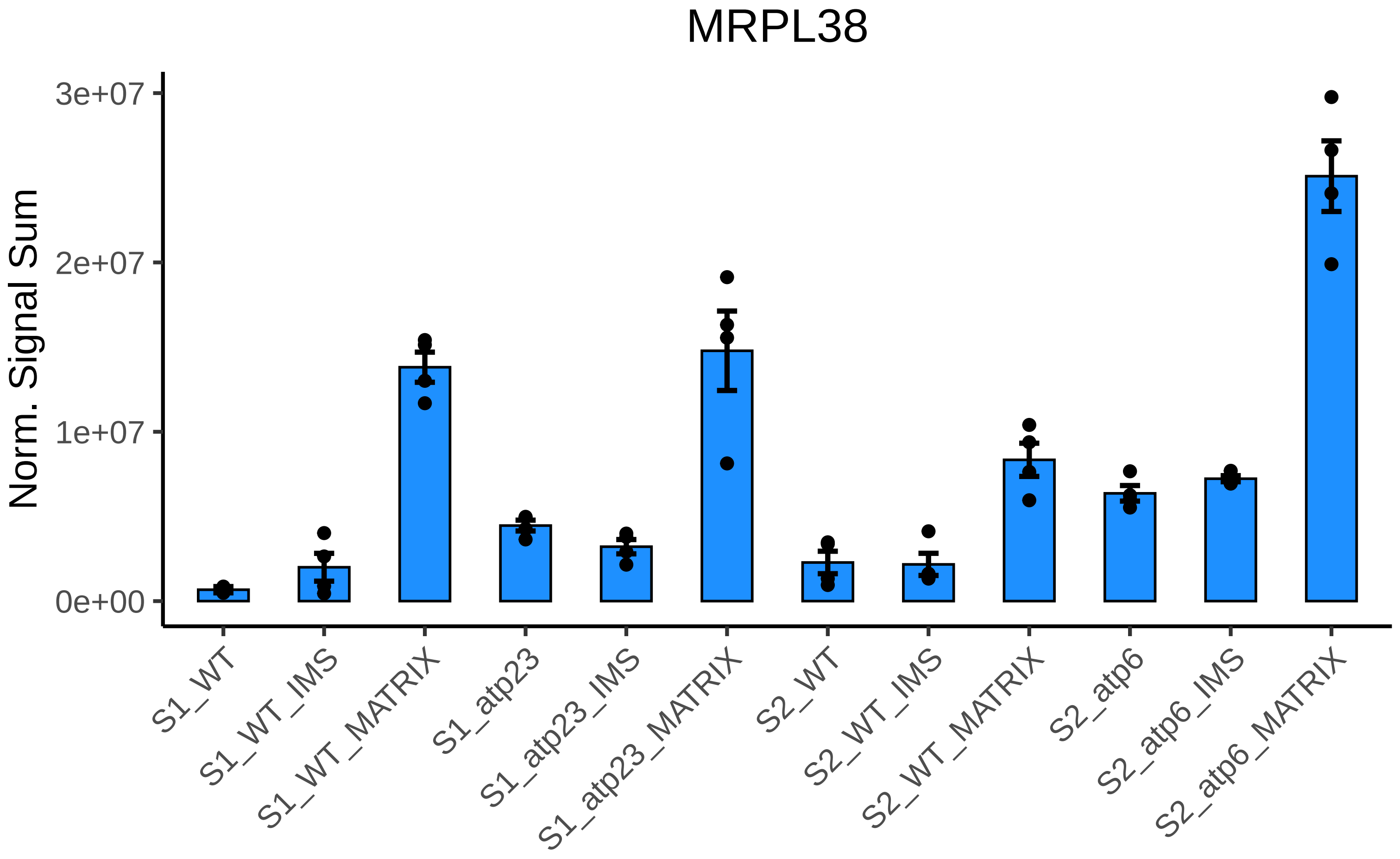

MRPL38
Norm. Signal Sum
3e+07
2e+07
1e+07
0e+00
S1_WT
S1_WT_IMS
S1_WT_MATRIX
S1_atp23
S1_atp23_IMS
S1_atp23_MATRIX
S2_WT
S2_WT_IMS
S2_WT_MATRIX
S2_atp6
S2_atp6_IMS
S2_atp6_MATRIX

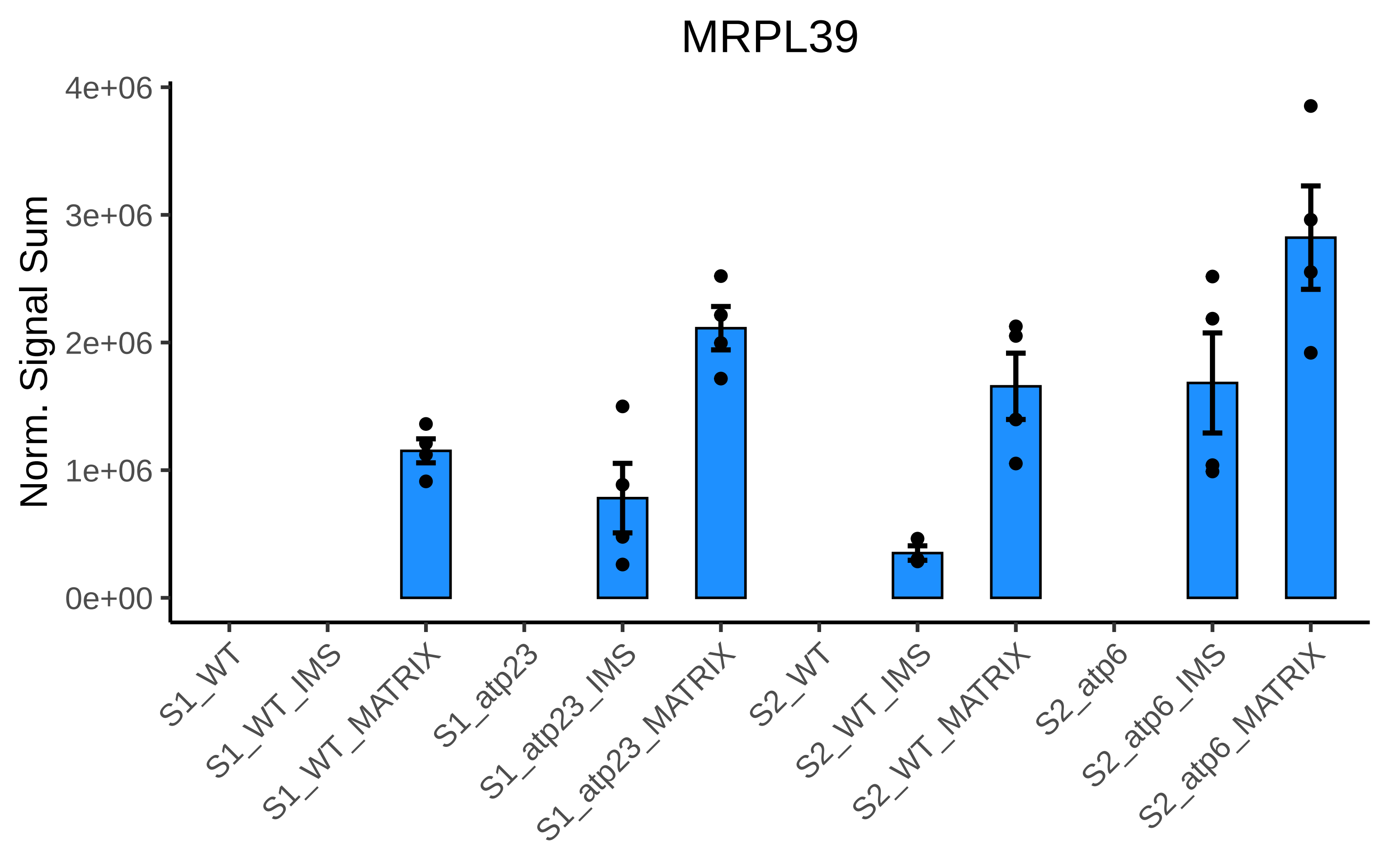

MRPL39
Norm. Signal Sum
4e+06
3e+06
2e+06
1e+06
0e+00
S1_WT
S1_WT_IMS
S1_WT_MATRIX
S1_atp23
S1_atp23_IMS
S1_atp23_MATRIX
S2_WT
S2_WT_IMS
S2_WT_MATRIX
S2_atp6
S2_atp6_IMS
S2_atp6_MATRIX

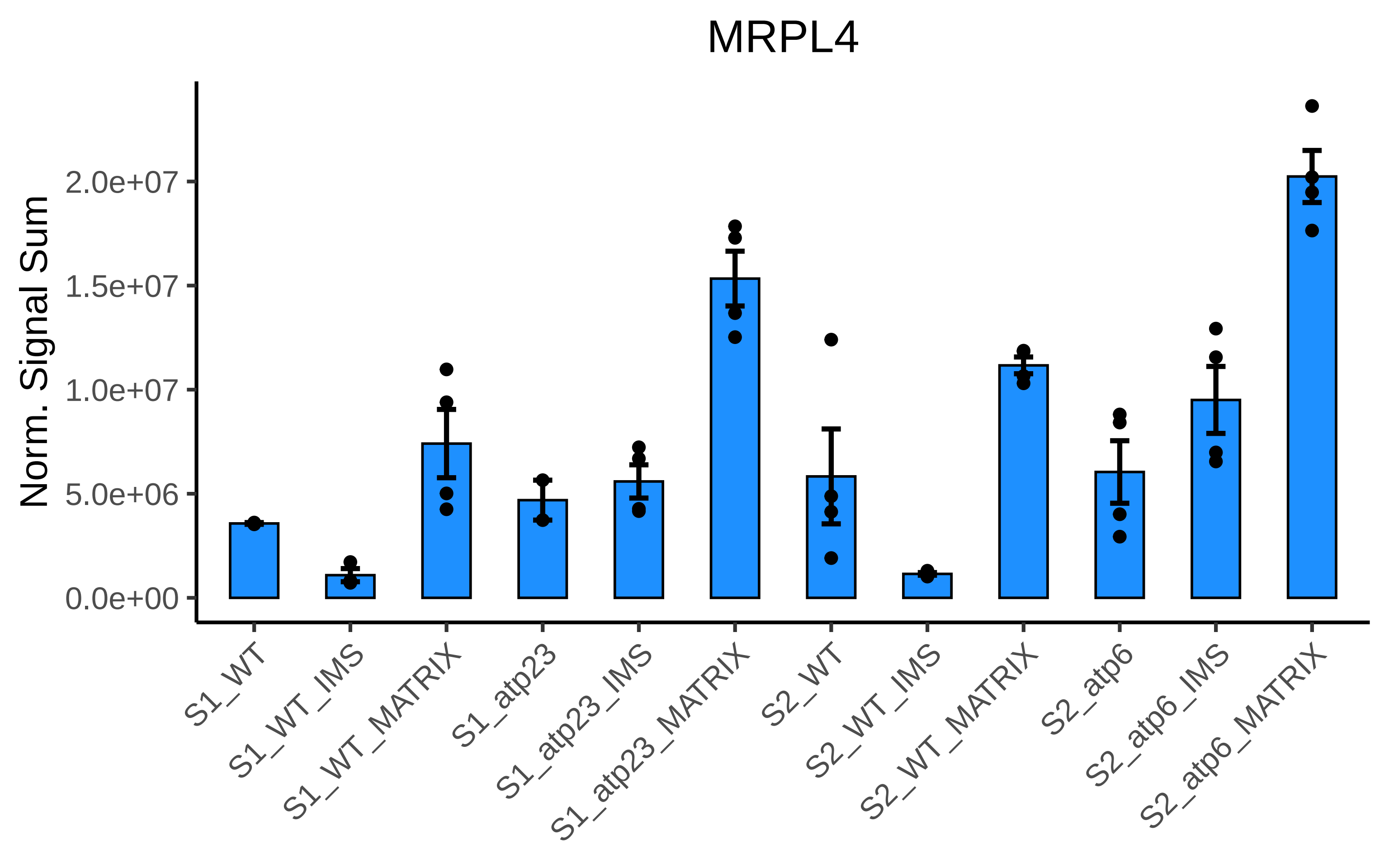
MRPL4
Norm. Signal Sum
2.0e+07
1.5e+07
1.0e+07
5.0e+06
0.0e+00
S1_WT
S1_WT_IMS
S1_WT_MATRIX
S1_atp23
S1_atp23_IMS
S1_atp23_MATRIX
S2_WT
S2_WT_IMS
S2_WT_MATRIX
S2_atp6
S2_atp6_IMS
S2_atp6_MATRIX

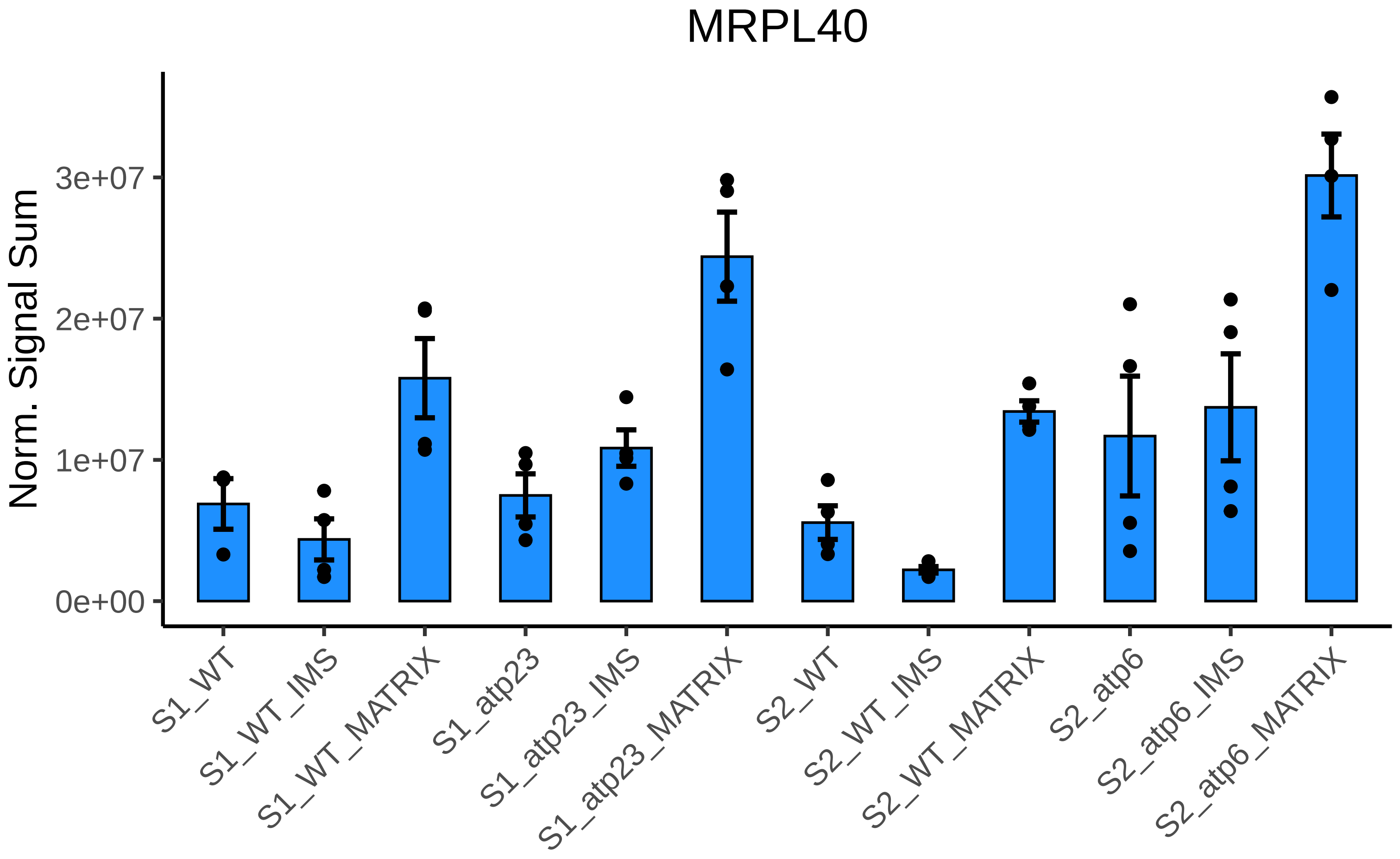
MRPL40
Norm. Signal Sum
3e+07
2e+07
1e+07
0e+00
S1_WT
S1_WT_IMS
S1_WT_MATRIX
S1_atp23
S1_atp23_IMS
S1_atp23_MATRIX
S2_WT
S2_WT_IMS
S2_WT_MATRIX
S2_atp6
S2_atp6_IMS
S2_atp6_MATRIX

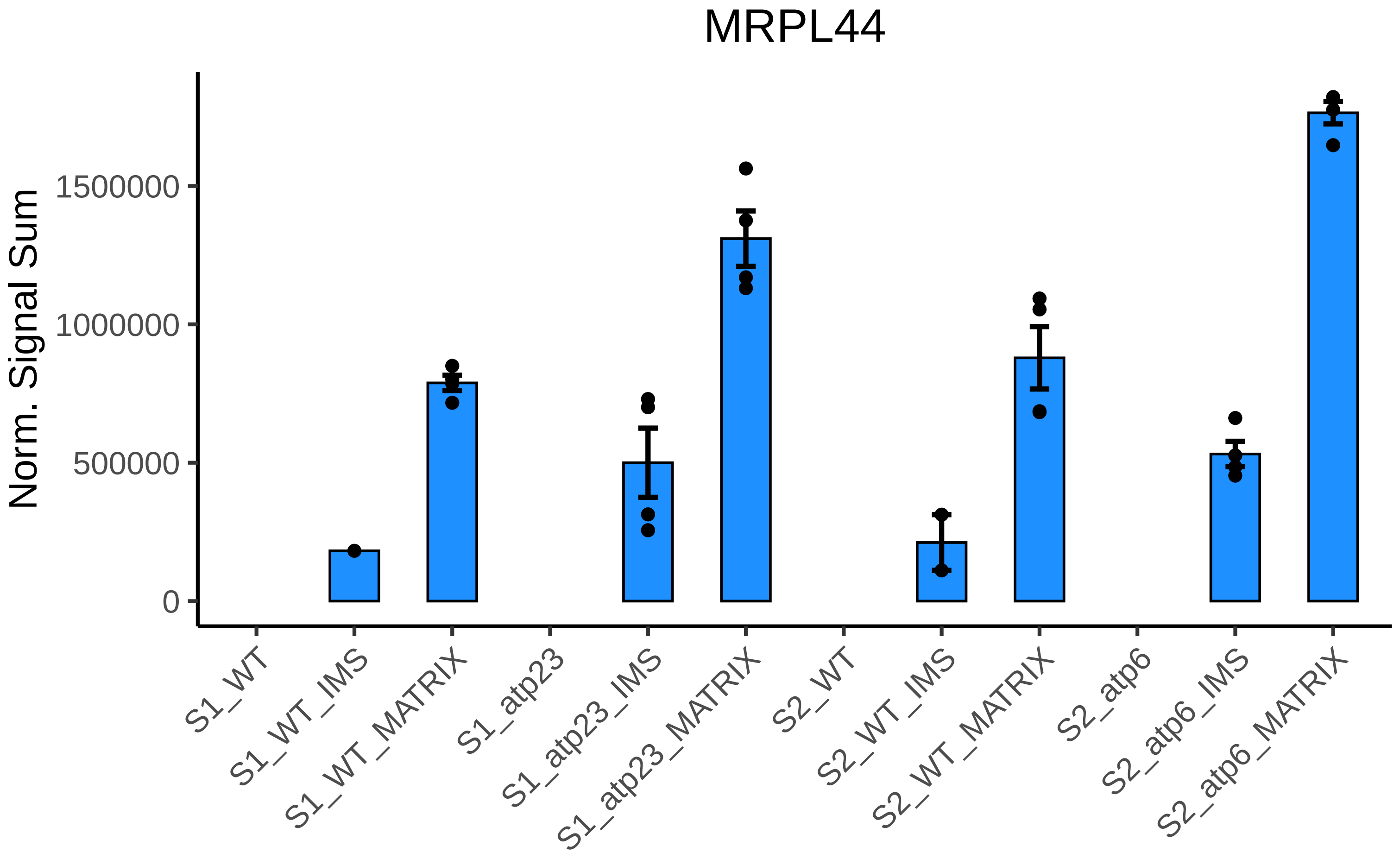
MRPL44
Norm. Signal Sum
0
500000
1000000
1500000
S1_WT
S1_WT_IMS
S1_WT_MATRIX
S1_atp23
S1_atp23_IMS
S1_atp23_MATRIX
S2_WT
S2_WT_IMS
S2_WT_MATRIX
S2_atp6
S2_atp6_IMS
S2_atp6_MATRIX

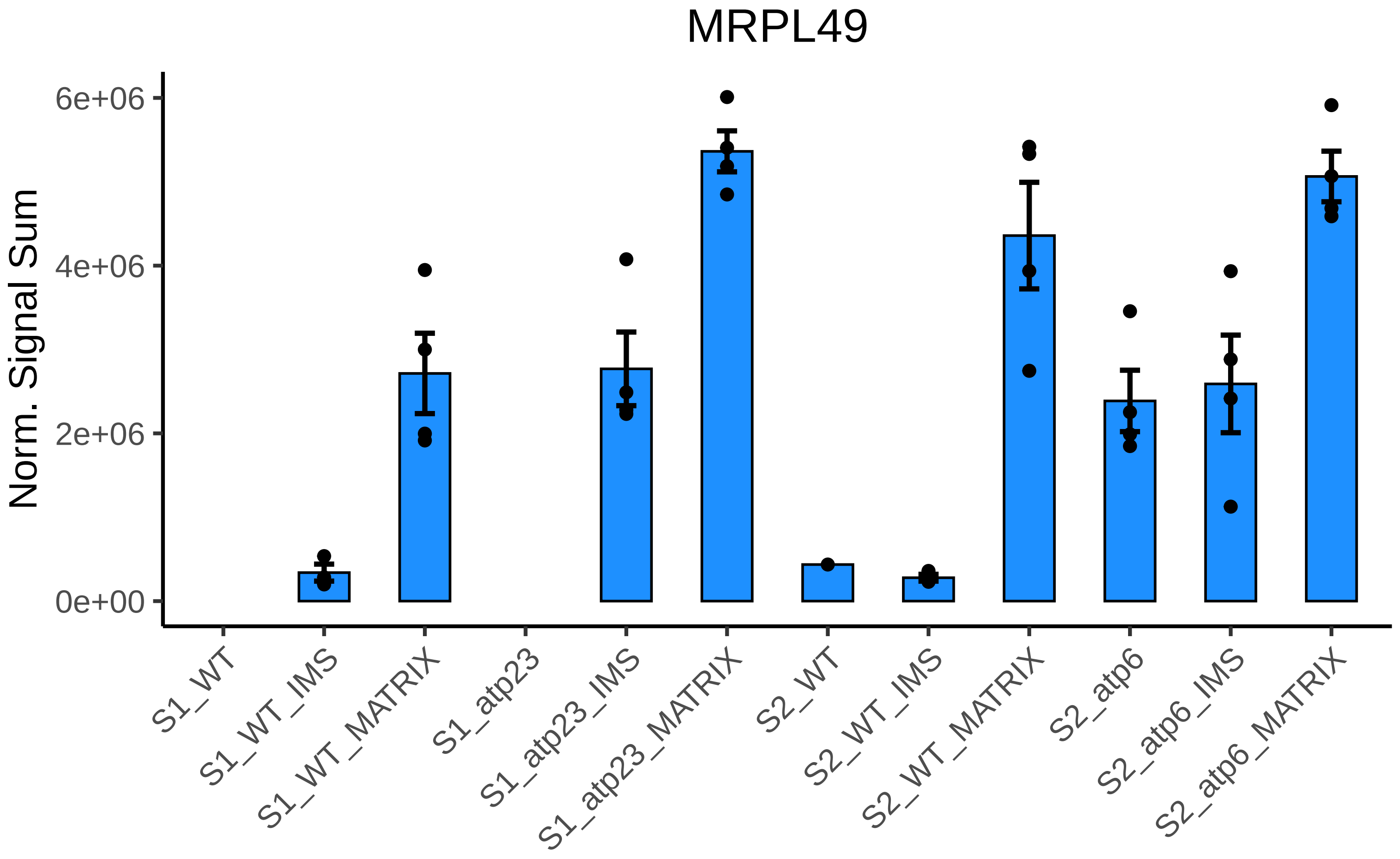
MRPL49
Norm. Signal Sum
6e+06
4e+06
2e+06
0e+00
S1_WT
S1_WT_IMS
S1_WT_MATRIX
S1_atp23
S1_atp23_IMS
S1_atp23_MATRIX
S2_WT
S2_WT_IMS
S2_WT_MATRIX
S2_atp6
S2_atp6_IMS
S2_atp6_MATRIX

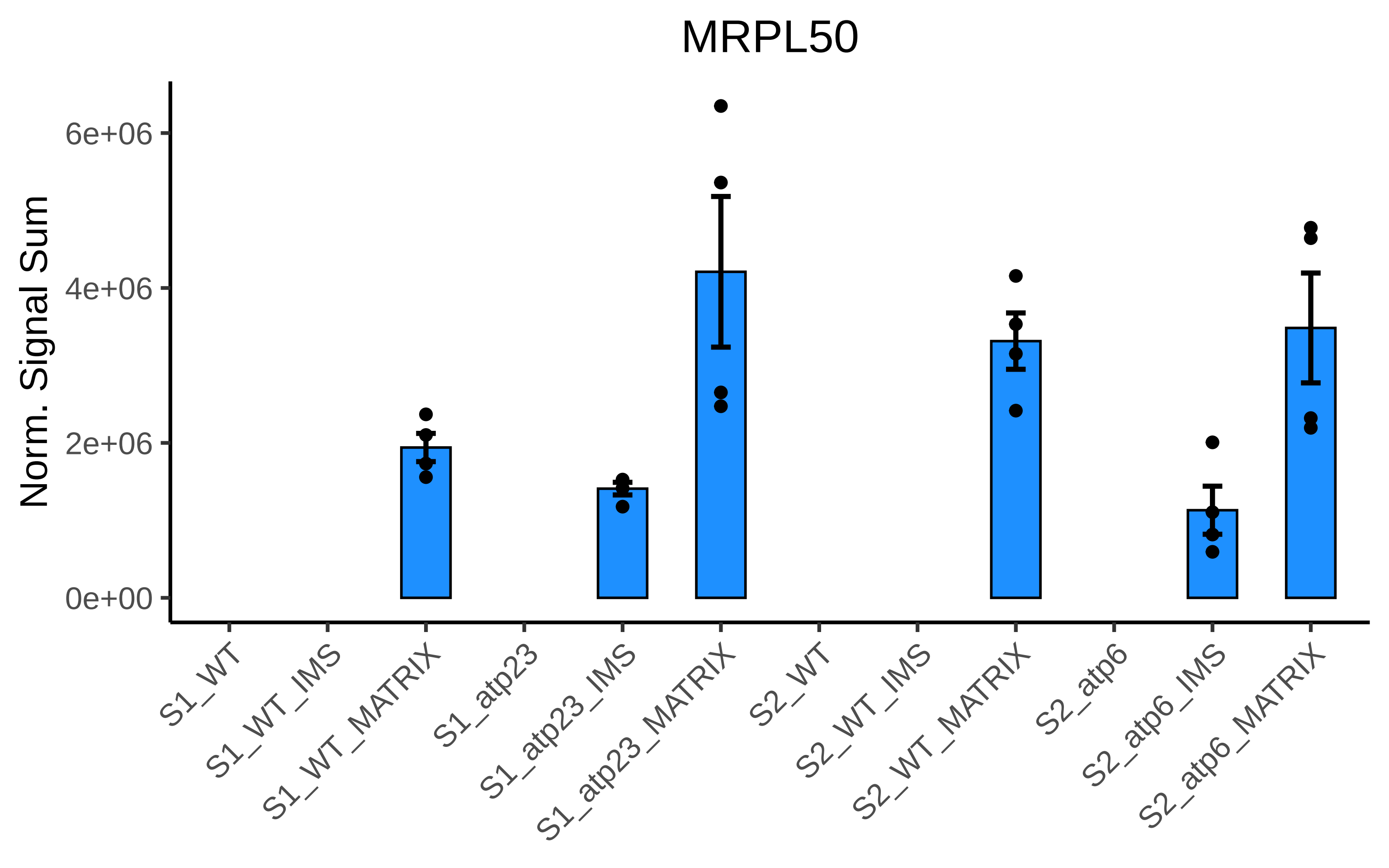
MRPL50
Norm. Signal Sum
6e+06
4e+06
2e+06
0e+00
S1_WT
S1_WT_IMS
S1_WT_MATRIX
S1_atp23
S1_atp23_IMS
S1_atp23_MATRIX
S2_WT
S2_WT_IMS
S2_WT_MATRIX
S2_atp6
S2_atp6_IMS
S2_atp6_MATRIX

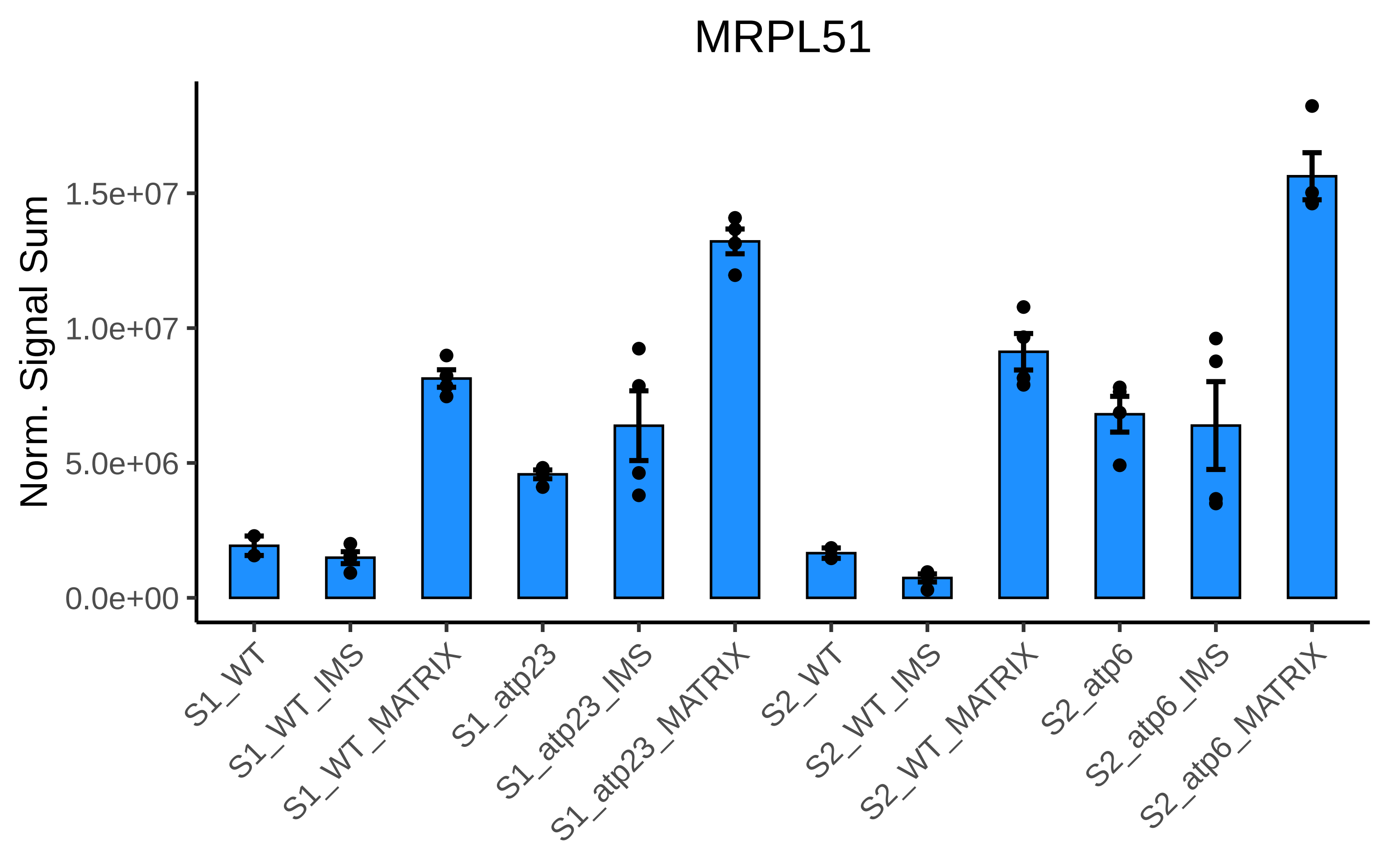
MRPL51
Norm. Signal Sum
1.5e+07
1.0e+07
5.0e+06
0.0e+00
S1_WT
S1_WT_IMS
S1_WT_MATRIX
S1_atp23
S1_atp23_IMS
S1_atp23_MATRIX
S2_WT
S2_WT_IMS
S2_WT_MATRIX
S2_atp6
S2_atp6_IMS
S2_atp6_MATRIX

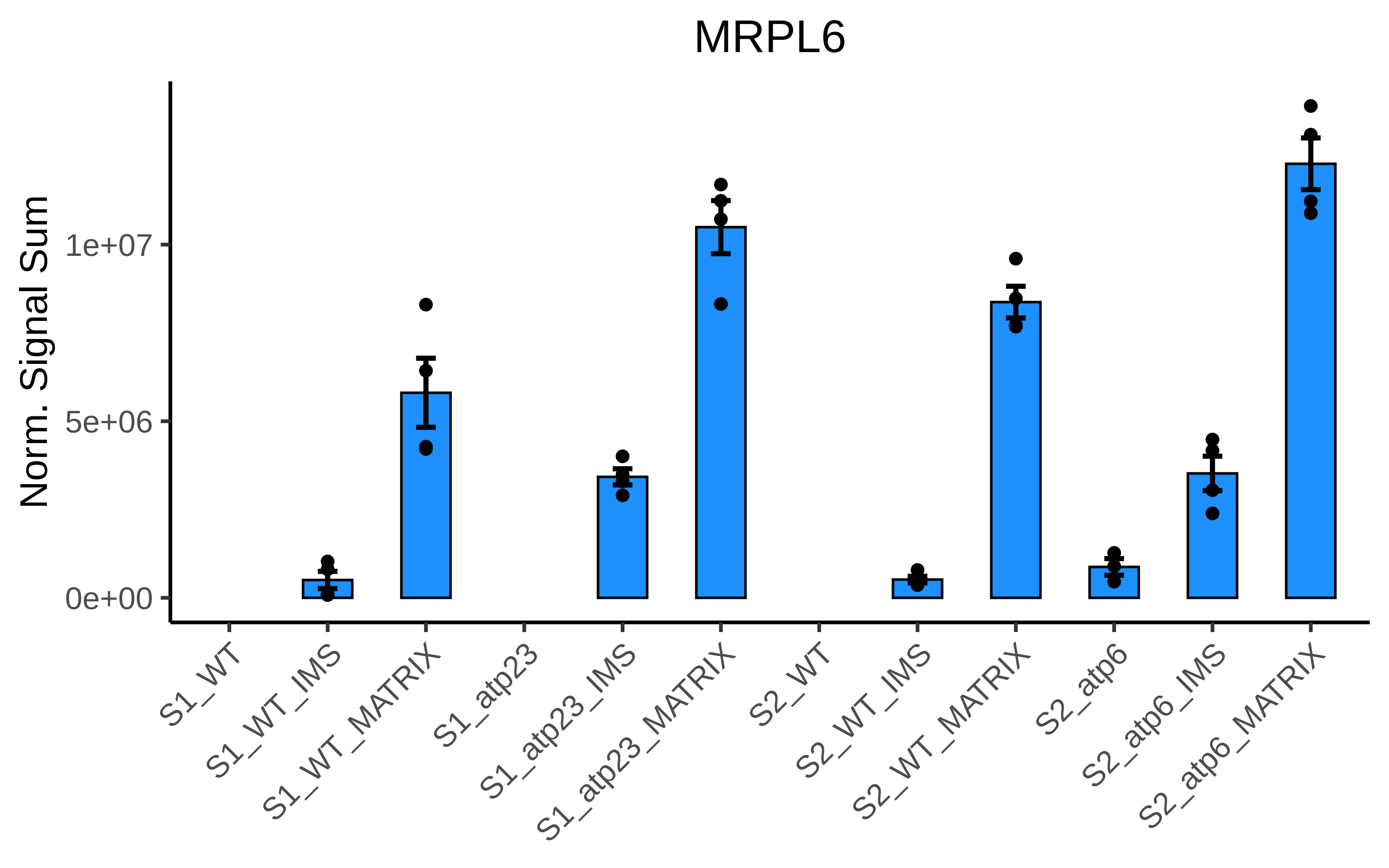
MRPL6
Norm. Signal Sum
1e+07
5e+06
0e+00
S1_WT
S1_WT_IMS
S1_WT_MATRIX
S1_atp23
S1_atp23_IMS
S1_atp23_MATRIX
S2_WT
S2_WT_IMS
S2_WT_MATRIX
S2_atp6
S2_atp6_IMS
S2_atp6_MATRIX

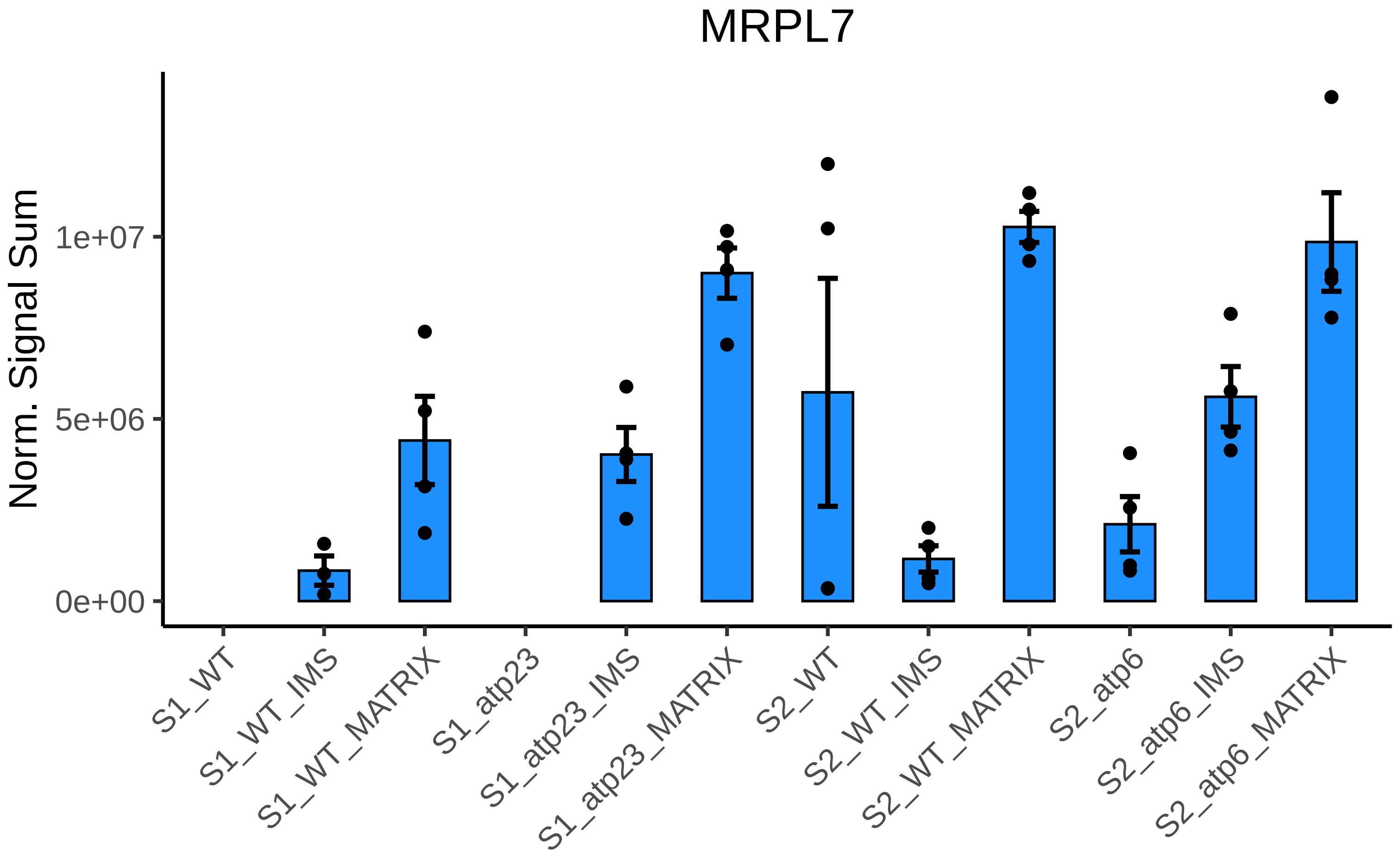
MRPL7
Norm. Signal Sum
1e+07
5e+06
0e+00
S1_WT
S1_WT_IMS
S1_WT_MATRIX
S1_atp23
S1_atp23_IMS
S1_atp23_MATRIX
S2_WT
S2_WT_IMS
S2_WT_MATRIX
S2_atp6
S2_atp6_IMS
S2_atp6_MATRIX

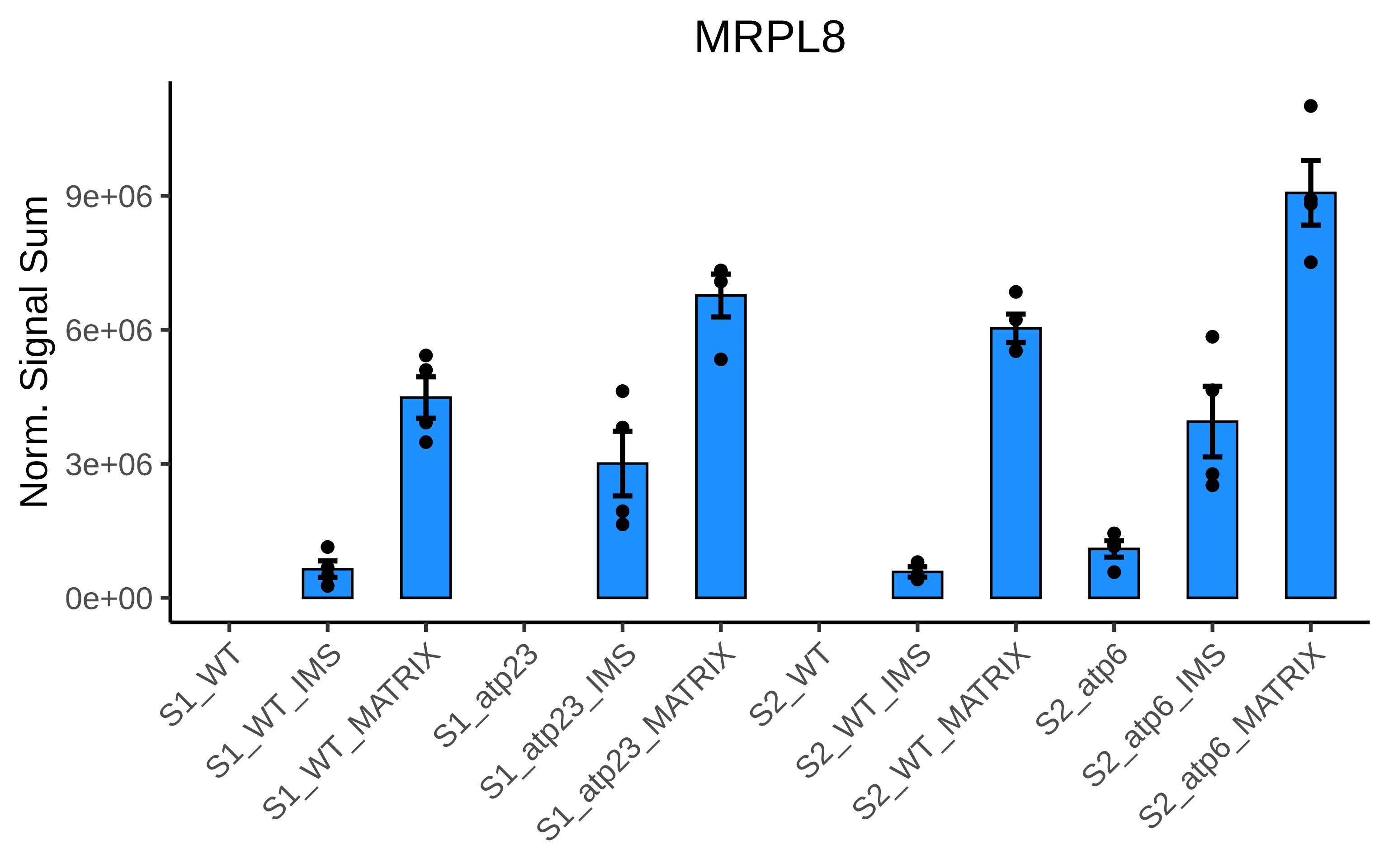
MRPL8
Norm. Signal Sum
9e+06
6e+06
3e+06
0e+00
S1_WT
S1_WT_IMS
S1_WT_MATRIX
S1_atp23
S1_atp23_IMS
S1_atp23_MATRIX
S2_WT
S2_WT_IMS
S2_WT_MATRIX
S2_atp6
S2_atp6_IMS
S2_atp6_MATRIX

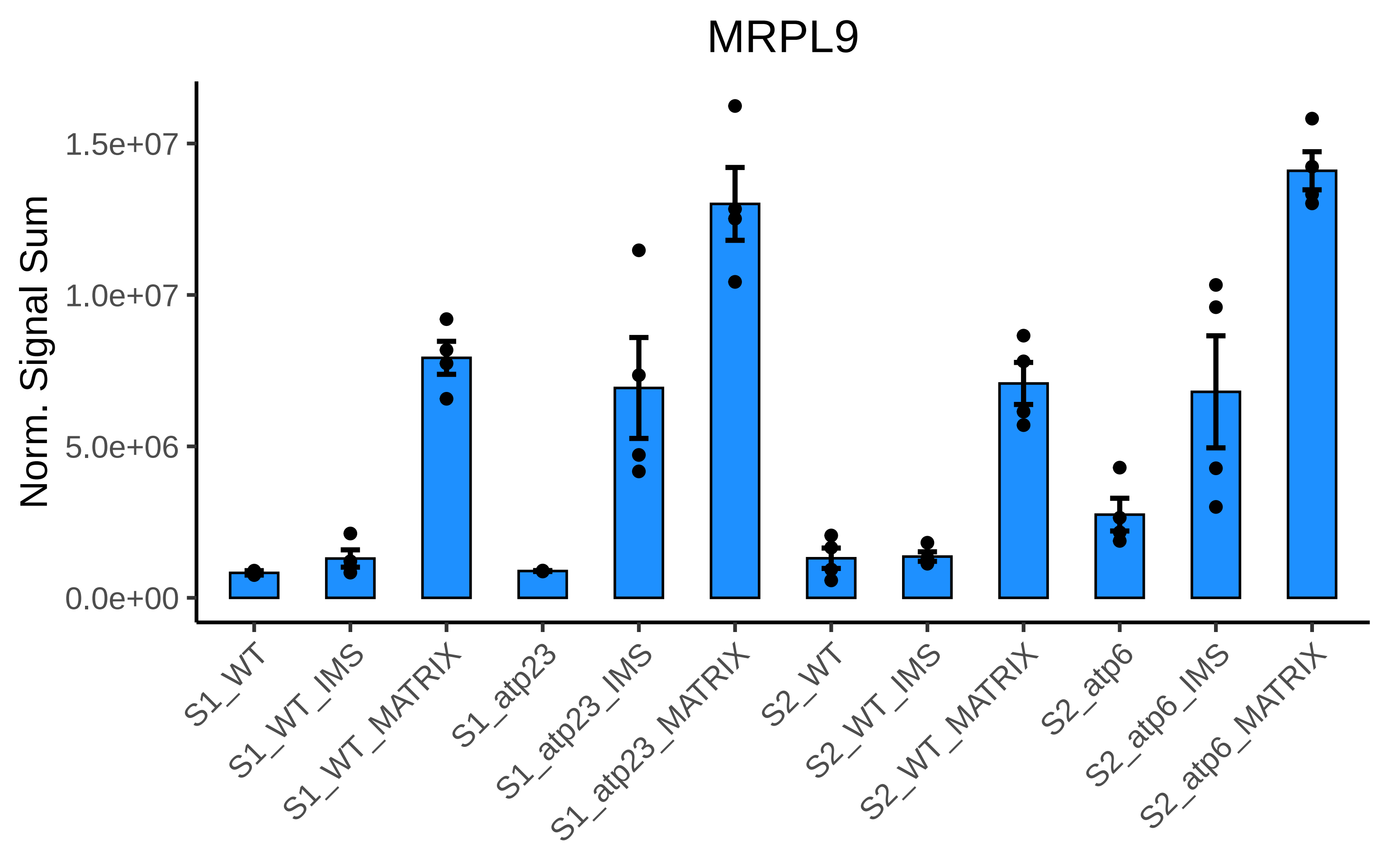
MRPL9
Norm. Signal Sum
1.5e+07
1.0e+07
5.0e+06
0.0e+00
S1_WT
S1_WT_IMS
S1_WT_MATRIX
S1_atp23
S1_atp23_IMS
S1_atp23_MATRIX
S2_WT
S2_WT_IMS
S2_WT_MATRIX
S2_atp6
S2_atp6_IMS
S2_atp6_MATRIX

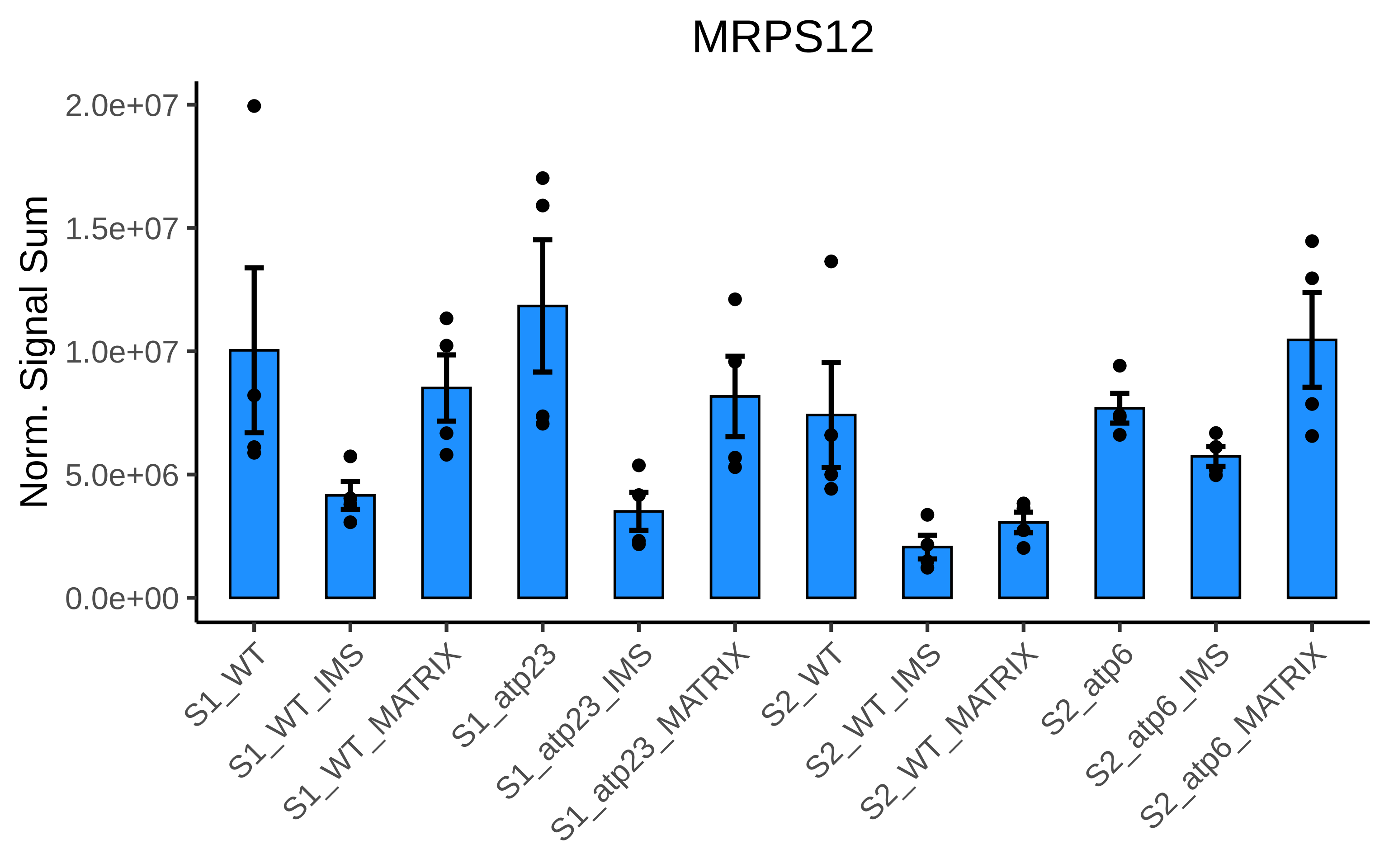
MRPS12
Norm. Signal Sum
2.0e+07
1.5e+07
1.0e+07
5.0e+06
0.0e+00
S1_WT
S1_WT_IMS
S1_WT_MATRIX
S1_atp23
S1_atp23_IMS
S1_atp23_MATRIX
S2_WT
S2_WT_IMS
S2_WT_MATRIX
S2_atp6
S2_atp6_IMS
S2_atp6_MATRIX

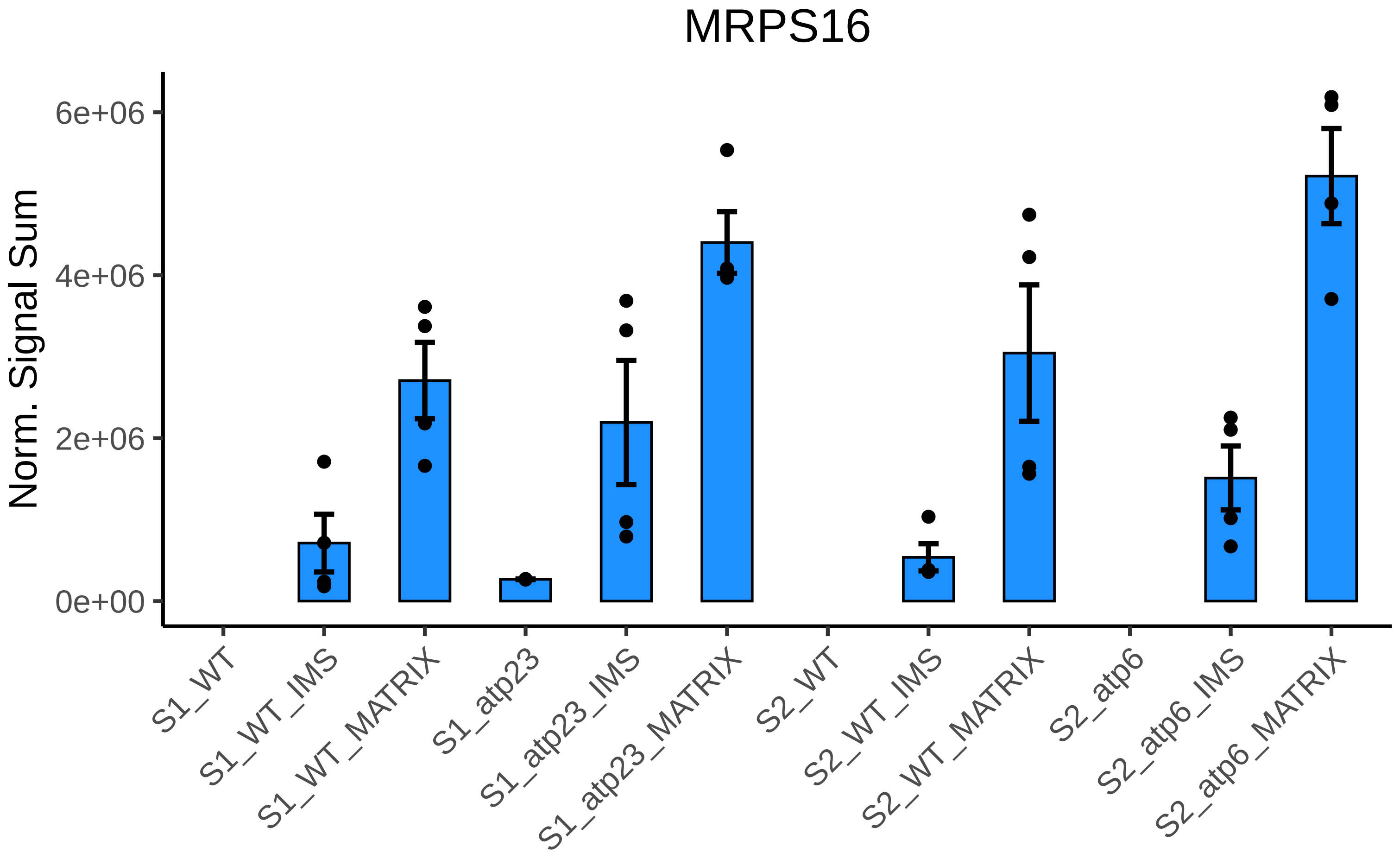
MRPS16
Norm. Signal Sum
6e+06
4e+06
2e+06
0e+00
S1_WT
S1_WT_IMS
S1_WT_MATRIX
S1_atp23
S1_atp23_IMS
S1_atp23_MATRIX
S2_WT
S2_WT_IMS
S2_WT_MATRIX
S2_atp6
S2_atp6_IMS
S2_atp6_MATRIX

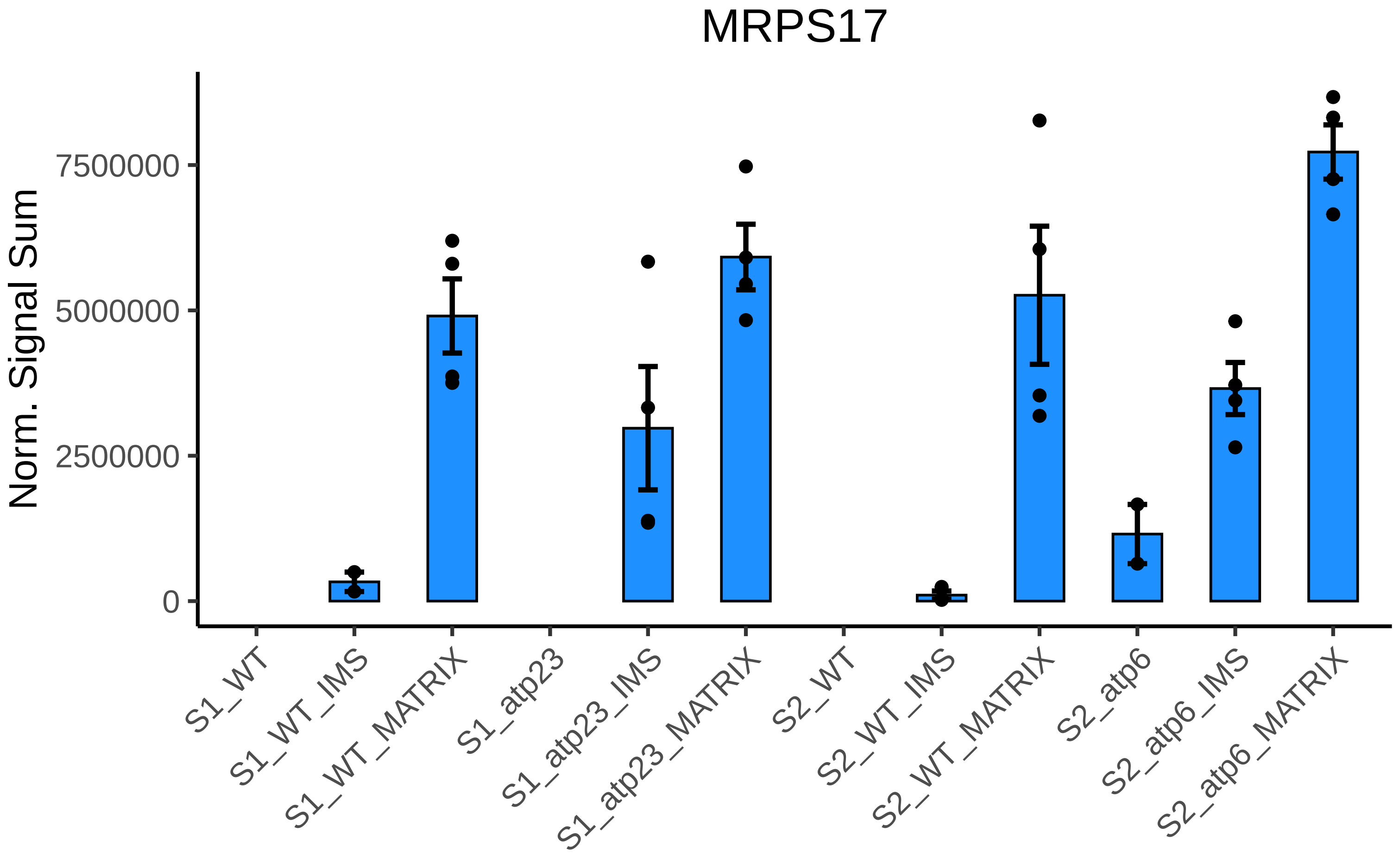

MRPS17
Norm. Signal Sum
7500000
5000000
2500000
0
S1_WT
S1_WT_IMS
S1_WT_MATRIX
S1_atp23
S1_atp23_IMS
S1_atp23_MATRIX
S2_WT
S2_WT_IMS
S2_WT_MATRIX
S2_atp6
S2_atp6_IMS
S2_atp6_MATRIX

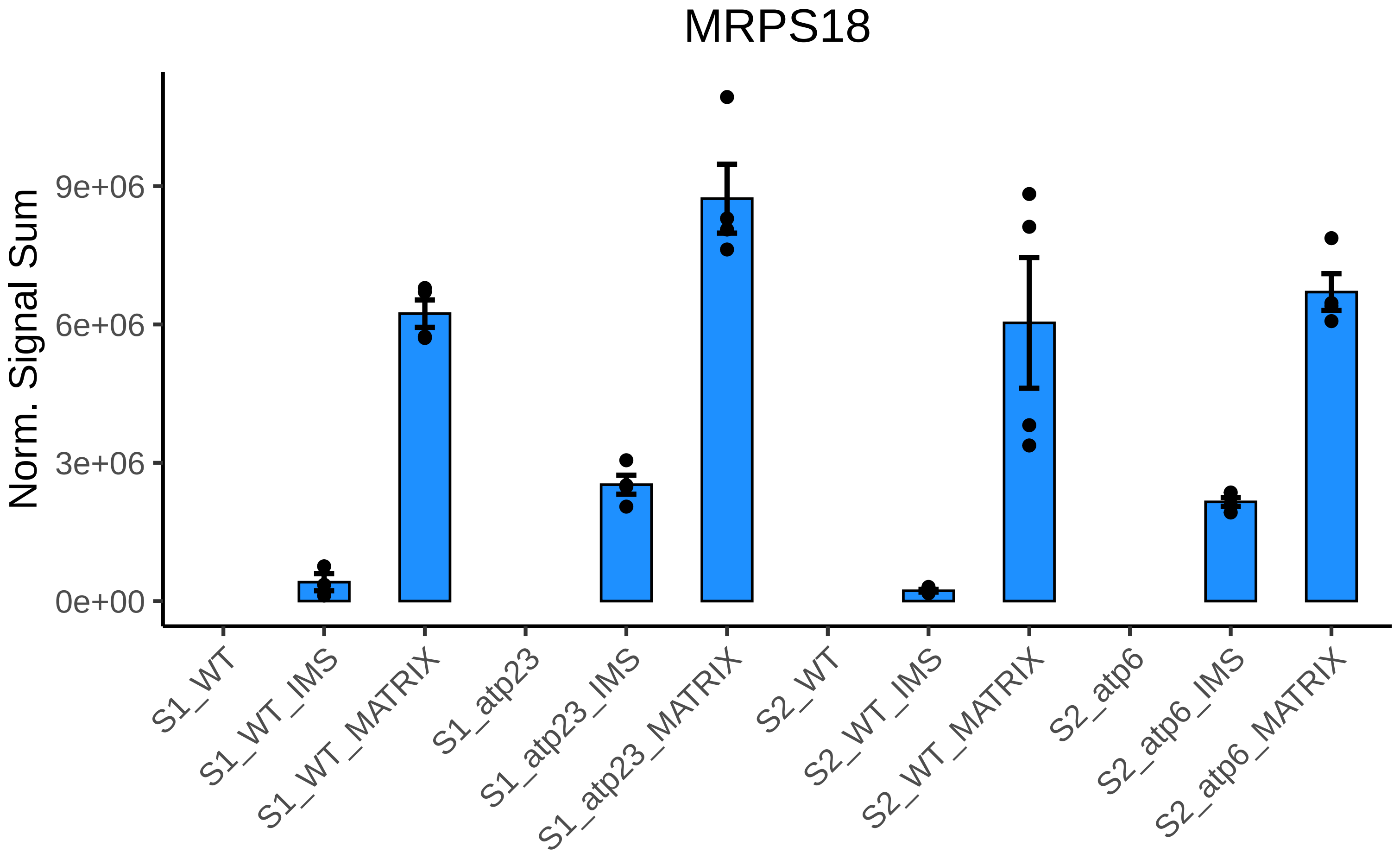

MRPS18
Norm. Signal Sum
9e+06
6e+06
3e+06
0e+00
S1_WT
S1_WT_IMS
S1_WT_MATRIX
S1_atp23
S1_atp23_IMS
S1_atp23_MATRIX
S2_WT
S2_WT_IMS
S2_WT_MATRIX
S2_atp6
S2_atp6_IMS
S2_atp6_MATRIX

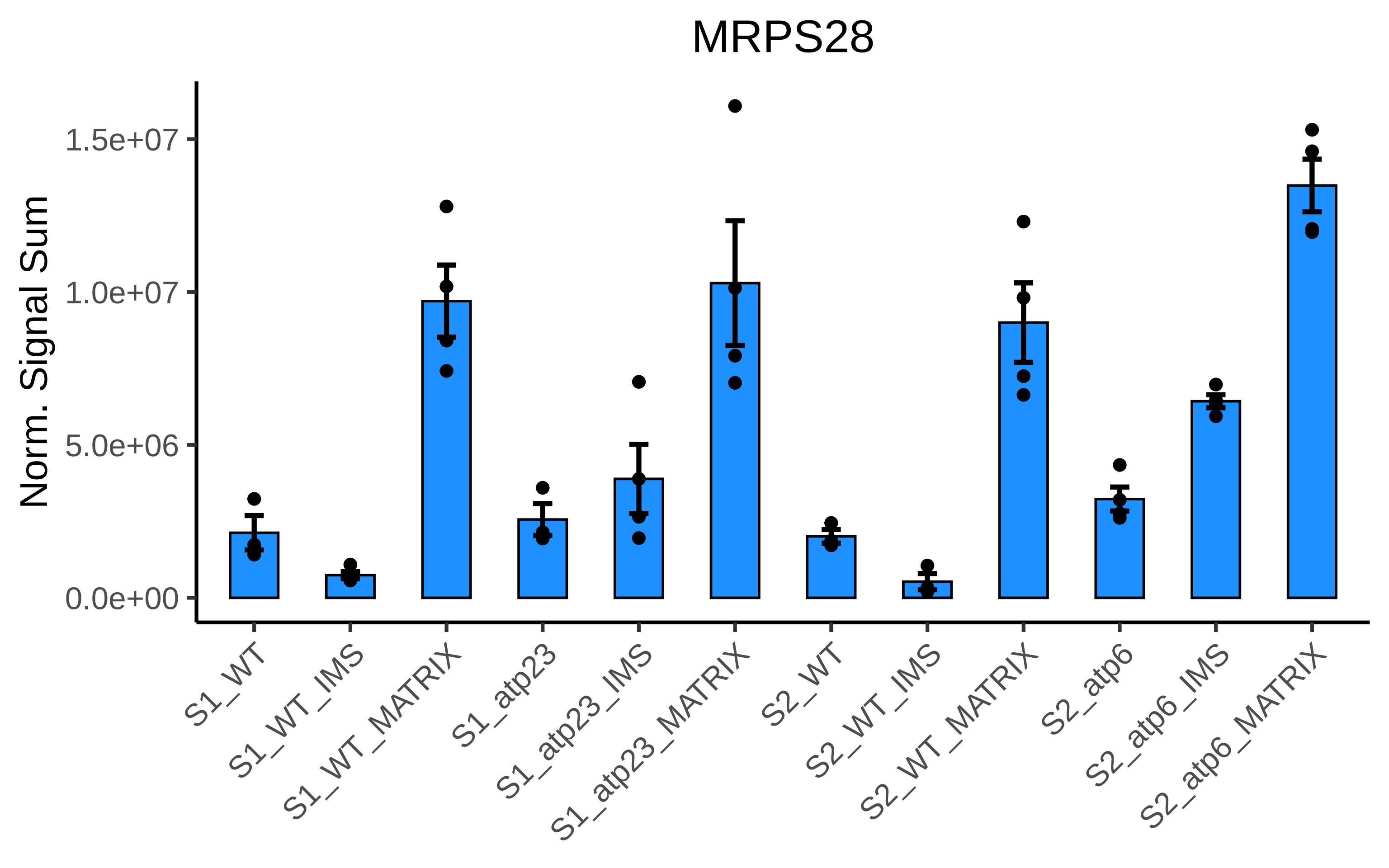
MRPS28
Norm. Signal Sum
1.5e+07
1.0e+07
5.0e+06
0.0e+00
S1_WT
S1_WT_IMS
S1_WT_MATRIX
S1_atp23
S1_atp23_IMS
S1_atp23_MATRIX
S2_WT
S2_WT_IMS
S2_WT_MATRIX
S2_atp6
S2_atp6_IMS
S2_atp6_MATRIX

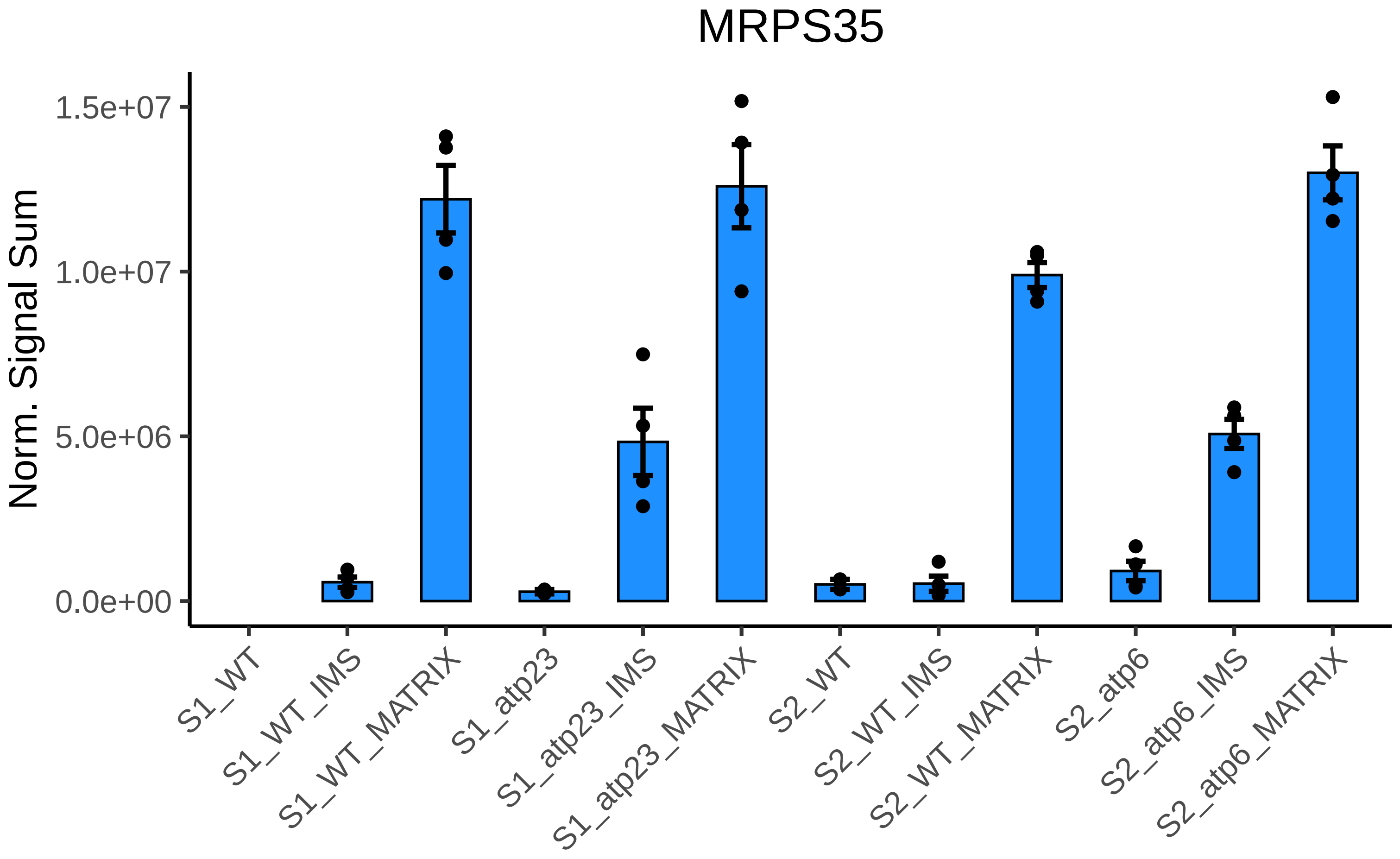

MRPS35
Norm. Signal Sum
1.5e+07
1.0e+07
5.0e+06
0.0e+00
S1_WT
S1_WT_IMS
S1_WT_MATRIX
S1_atp23
S1_atp23_IMS
S1_atp23_MATRIX
S2_WT
S2_WT_IMS
S2_WT_MATRIX
S2_atp6
S2_atp6_IMS
S2_atp6_MATRIX

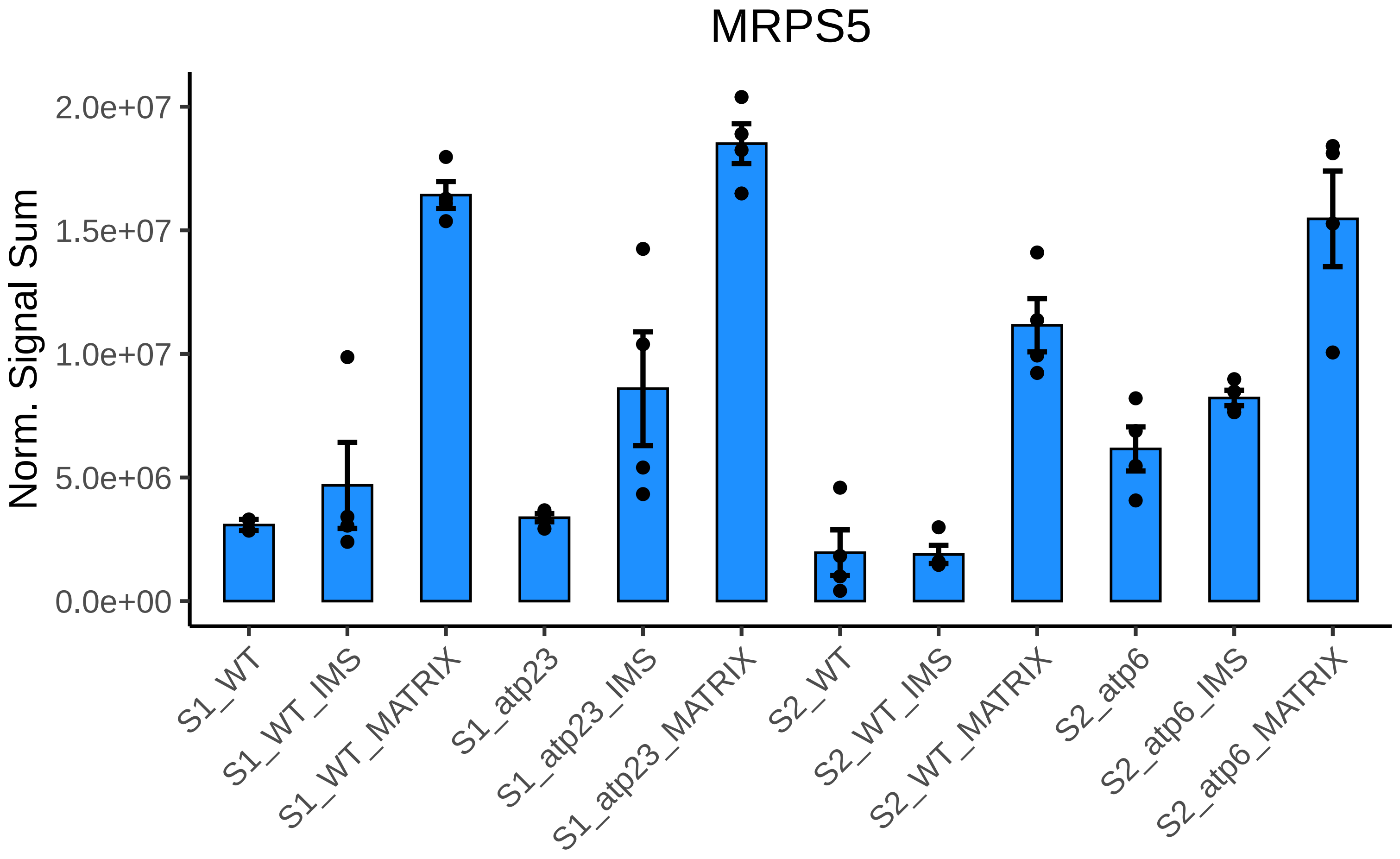
MRPS5
Norm. Signal Sum
2.0e+07
1.5e+07
1.0e+07
5.0e+06
0.0e+00
S1_WT
S1_WT_IMS
S1_WT_MATRIX
S1_atp23
S1_atp23_IMS
S1_atp23_MATRIX
S2_WT
S2_WT_IMS
S2_WT_MATRIX
S2_atp6
S2_atp6_IMS
S2_atp6_MATRIX

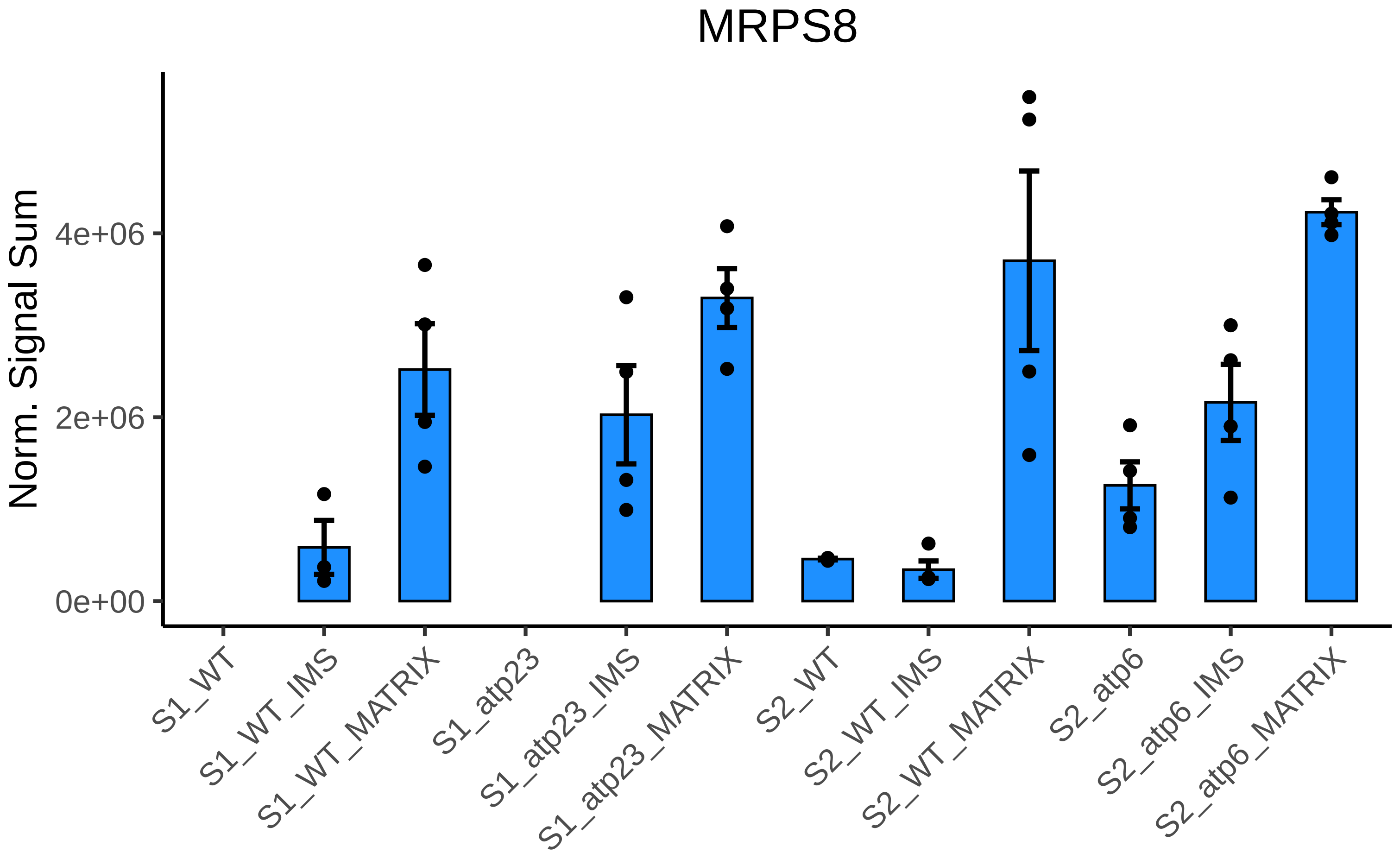

MRPS8
Norm. Signal Sum
4e+06
2e+06
0e+00
S1_WT
S1_WT_IMS
S1_WT_MATRIX
S1_atp23
S1_atp23_IMS
S1_atp23_MATRIX
S2_WT
S2_WT_IMS
S2_WT_MATRIX
S2_atp6
S2_atp6_IMS
S2_atp6_MATRIX

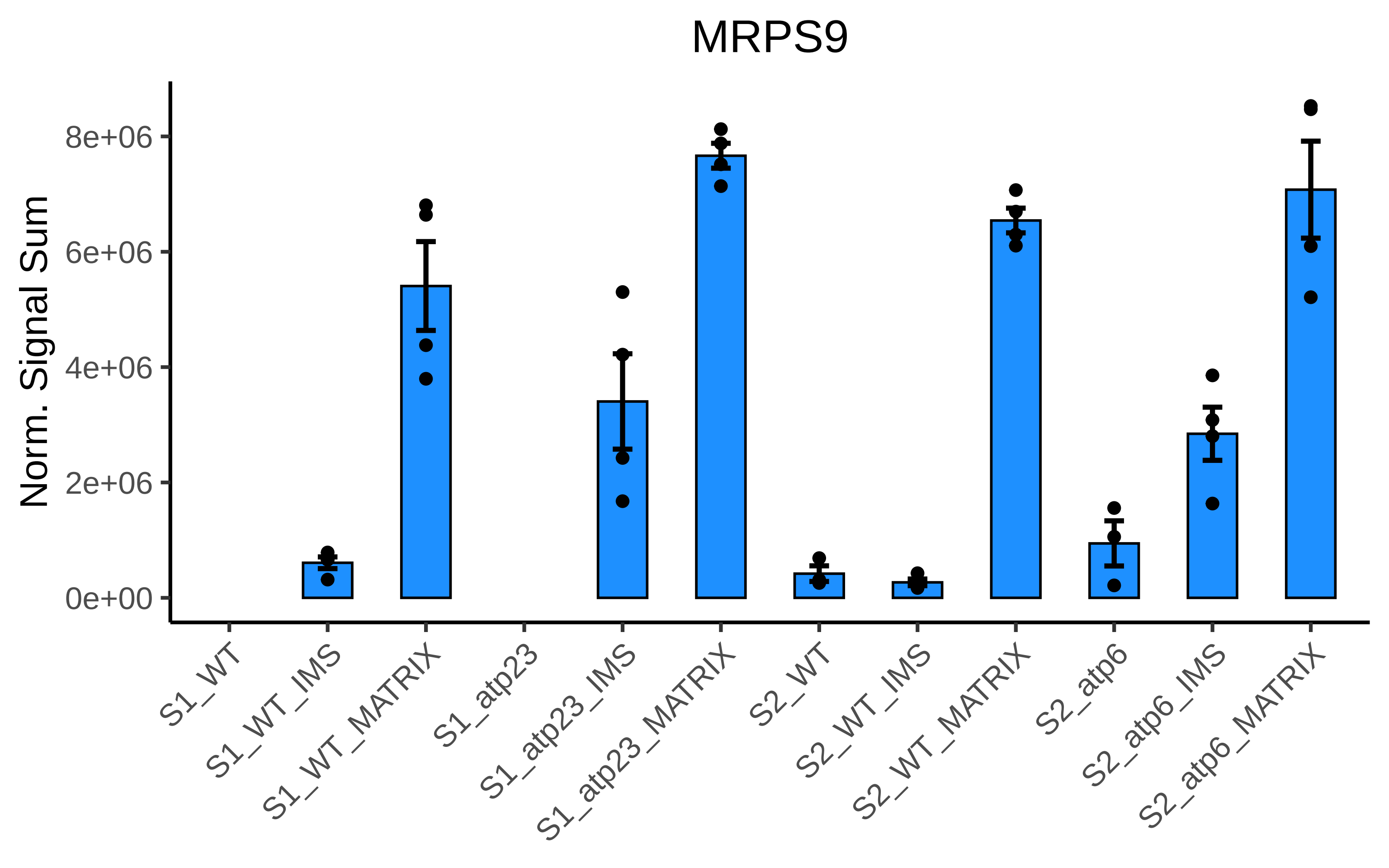
MRPS9
Norm. Signal Sum
8e+06
6e+06
4e+06
2e+06
0e+00
S1_WT
S1_WT_IMS
S1_WT_MATRIX
S1_atp23
S1_atp23_IMS
S1_atp23_MATRIX
S2_WT
S2_WT_IMS
S2_WT_MATRIX
S2_atp6
S2_atp6_IMS
S2_atp6_MATRIX

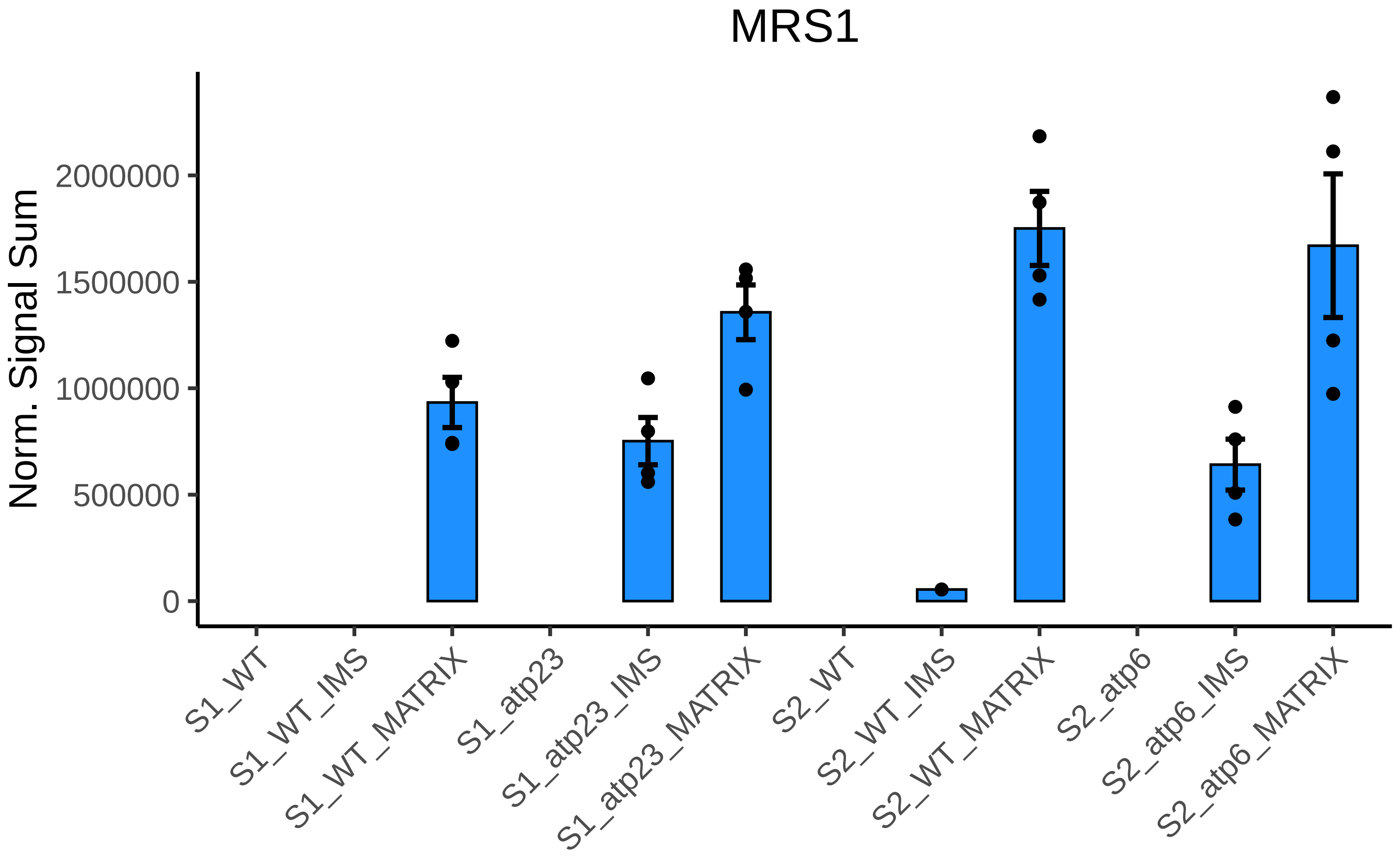
MRS1
Norm. Signal Sum
2000000
1500000
1000000
500000
0
S1_WT
S1_WT_IMS
S1_WT_MATRIX
S1_atp23
S1_atp23_IMS
S1_atp23_MATRIX
S2_WT
S2_WT_IMS
S2_WT_MATRIX
S2_atp6
S2_atp6_IMS
S2_atp6_MATRIX

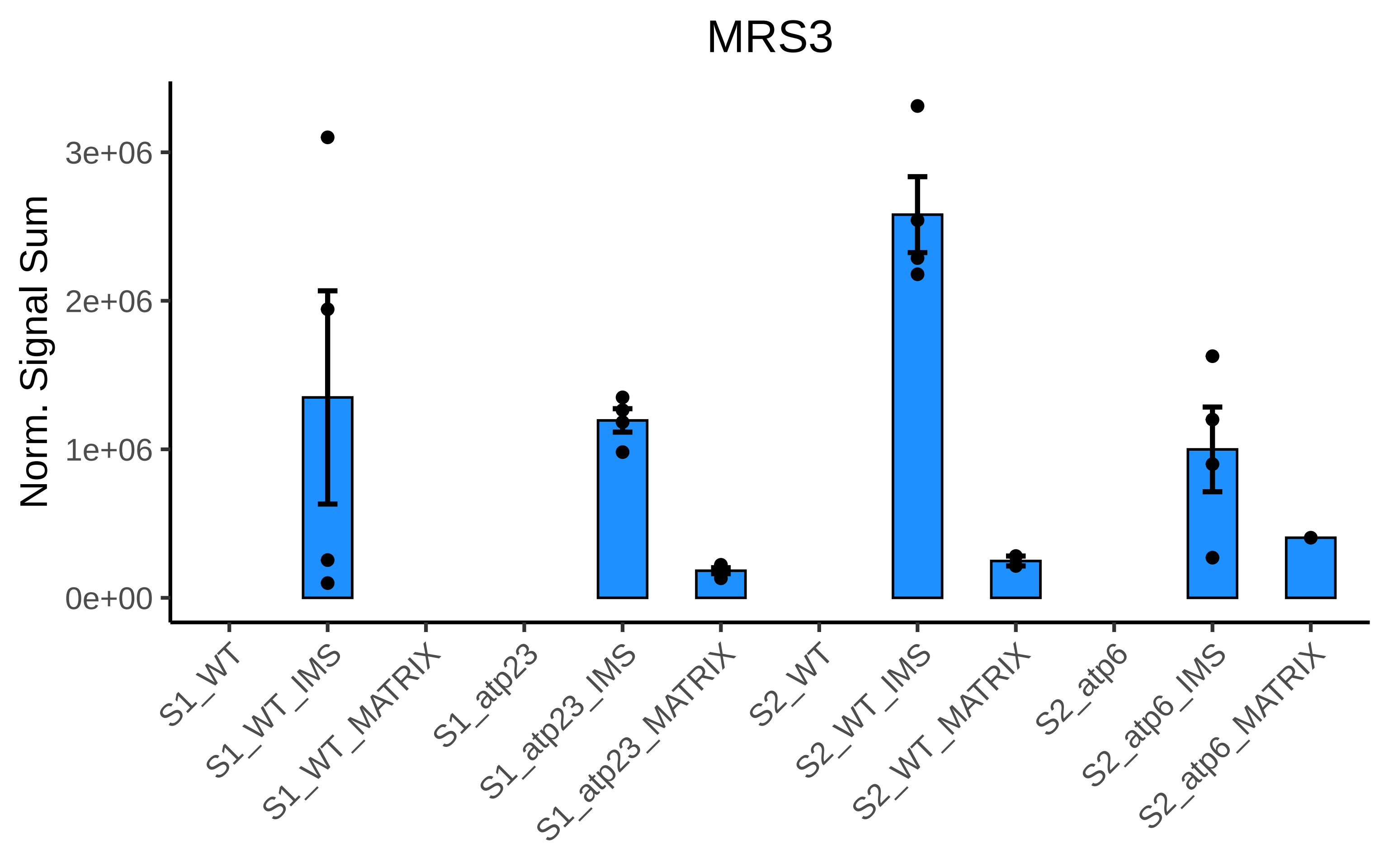

MRS3
Norm. Signal Sum
3e+06
2e+06
1e+06
0e+00
S1_WT
S1_WT_IMS
S1_WT_MATRIX
S1_atp23
S1_atp23_IMS
S1_atp23_MATRIX
S2_WT
S2_WT_IMS
S2_WT_MATRIX
S2_atp6
S2_atp6_IMS
S2_atp6_MATRIX

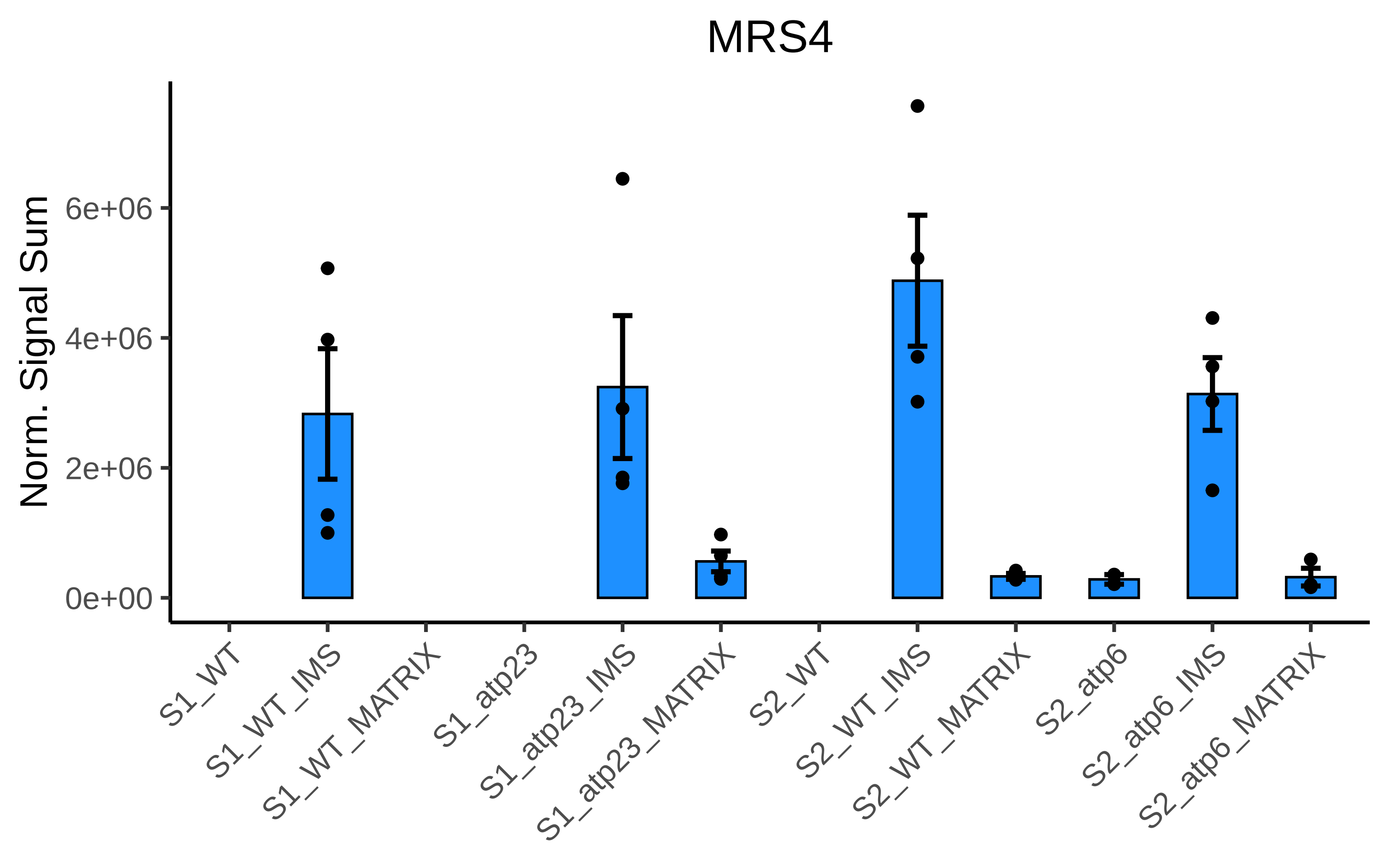

MRS4
Norm. Signal Sum
6e+06
4e+06
2e+06
0e+00
S1_WT
S1_WT_IMS
S1_WT_MATRIX
S1_atp23
S1_atp23_IMS
S1_atp23_MATRIX
S2_WT
S2_WT_IMS
S2_WT_MATRIX
S2_atp6
S2_atp6_IMS
S2_atp6_MATRIX

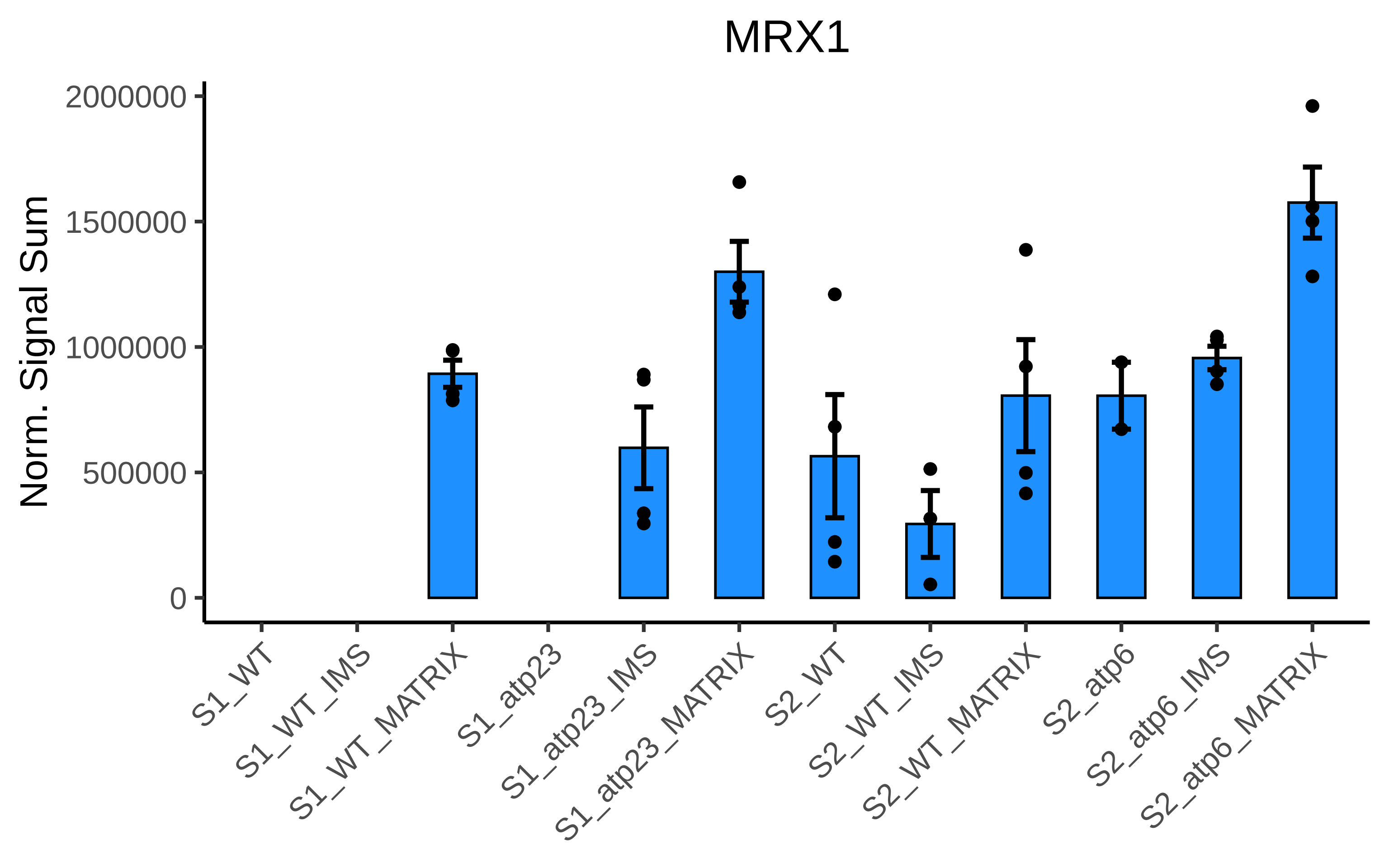

MRX1
Norm. Signal Sum
2000000
1500000
1000000
500000
0
S1_WT
S1_WT_IMS
S1_WT_MATRIX
S1_atp23
S1_atp23_IMS
S1_atp23_MATRIX
S2_WT
S2_WT_IMS
S2_WT_MATRIX
S2_atp6
S2_atp6_IMS
S2_atp6_MATRIX

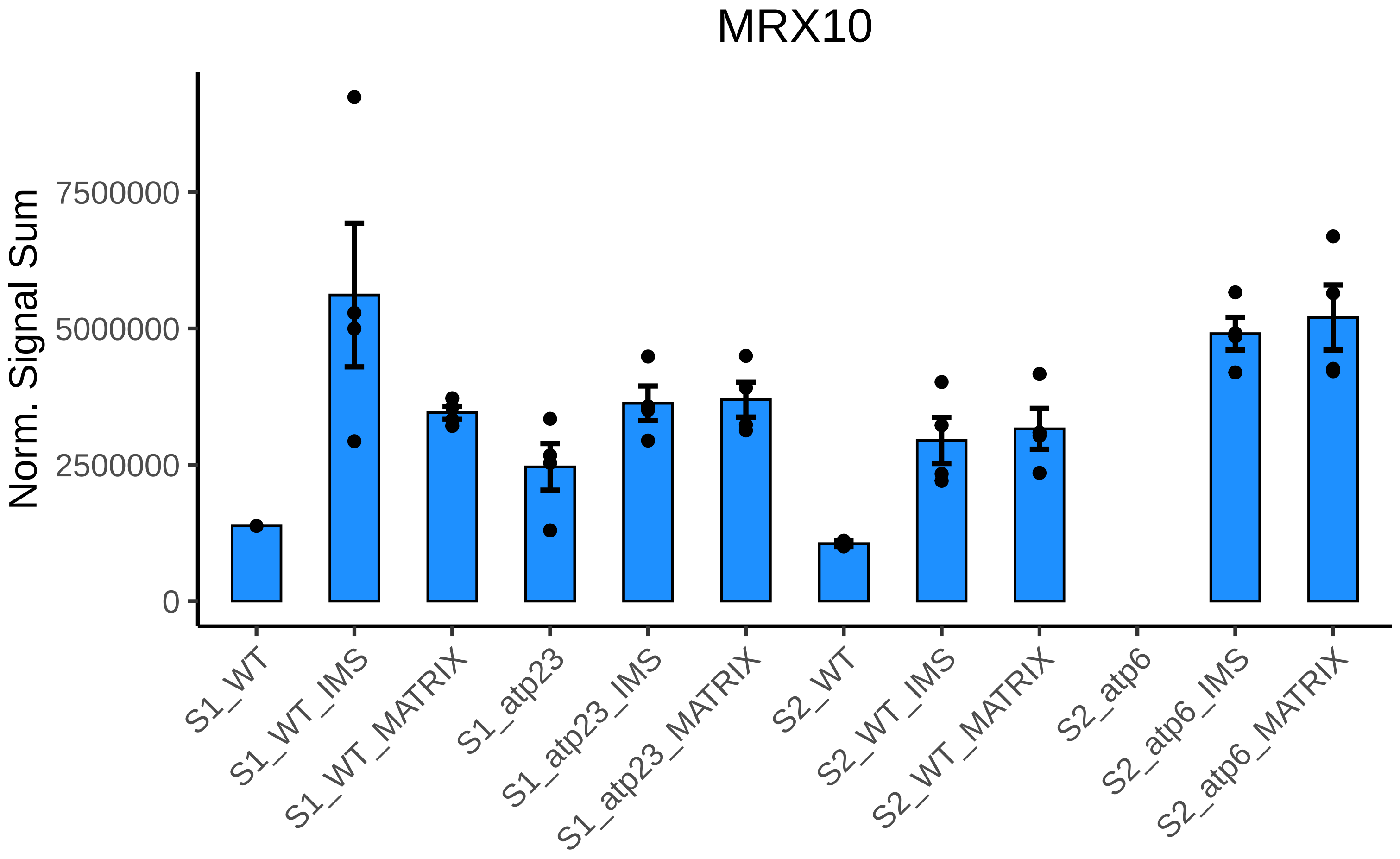

MRX10
Norm. Signal Sum
7500000
5000000
2500000
0
S1_WT
S1_WT_IMS
S1_WT_MATRIX
S1_atp23
S1_atp23_IMS
S1_atp23_MATRIX
S2_WT
S2_WT_IMS
S2_WT_MATRIX
S2_atp6
S2_atp6_IMS
S2_atp6_MATRIX

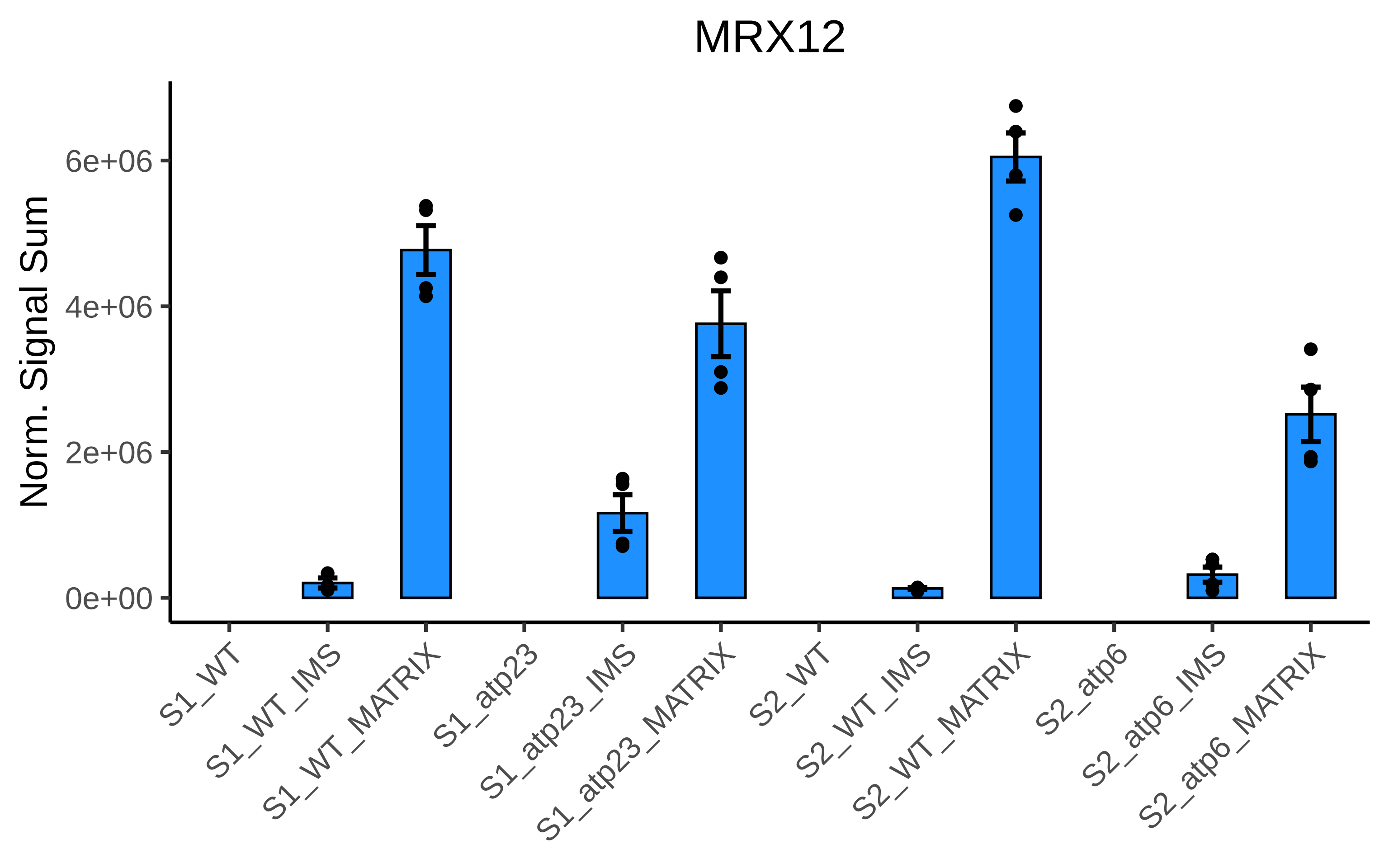
MRX12
Norm. Signal Sum
0e+00
2e+06
4e+06
6e+06
S1_WT
S1_WT_IMS
S1_WT_MATRIX
S1_atp23
S1_atp23_IMS
S1_atp23_MATRIX
S2_WT
S2_WT_IMS
S2_WT_MATRIX
S2_atp6
S2_atp6_IMS
S2_atp6_MATRIX

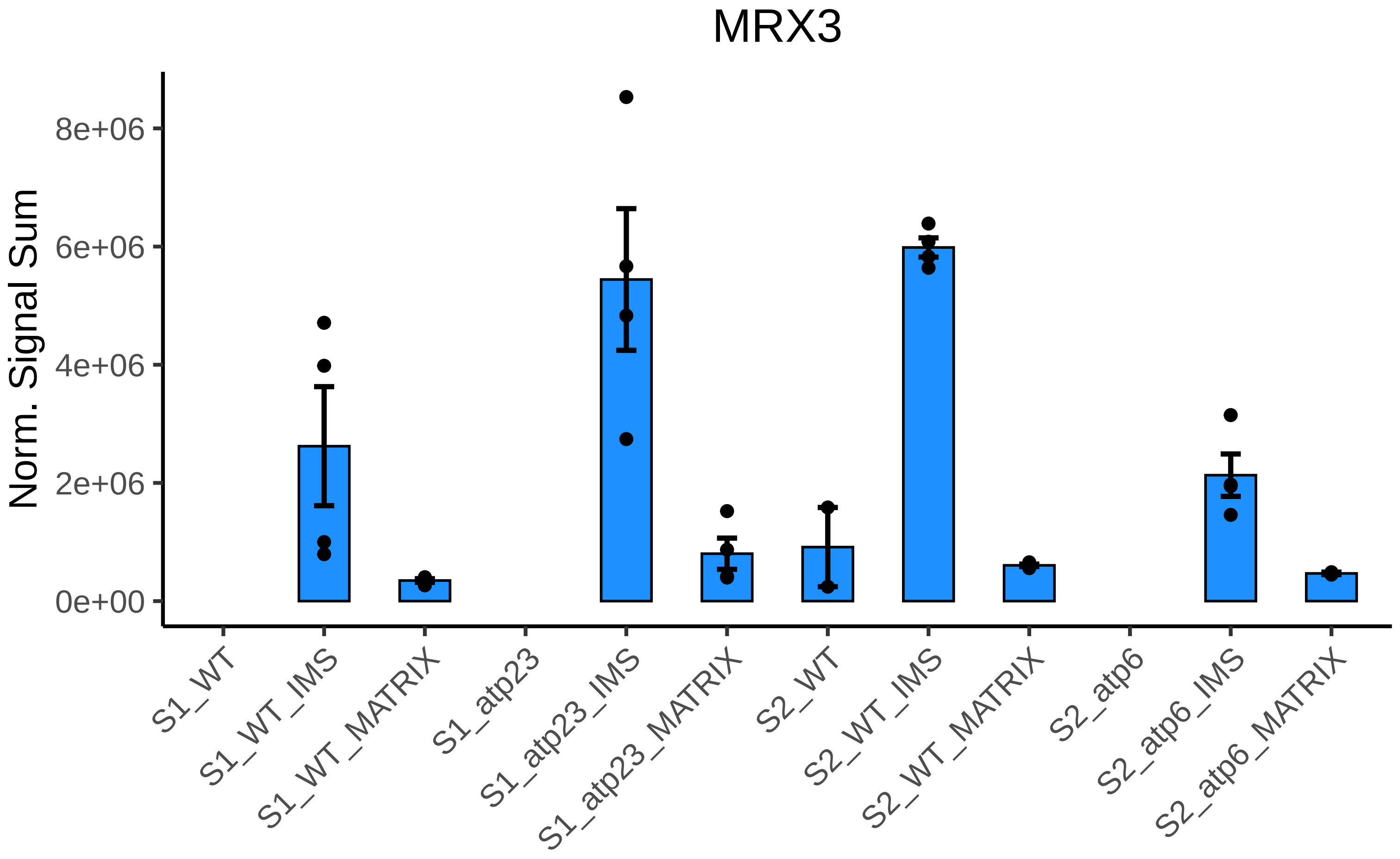

MRX3
Norm. Signal Sum
8e+06
6e+06
4e+06
2e+06
0e+00
S1_WT
S1_WT_IMS
S1_WT_MATRIX
S1_atp23
S1_atp23_IMS
S1_atp23_MATRIX
S2_WT
S2_WT_IMS
S2_WT_MATRIX
S2_atp6
S2_atp6_IMS
S2_atp6_MATRIX

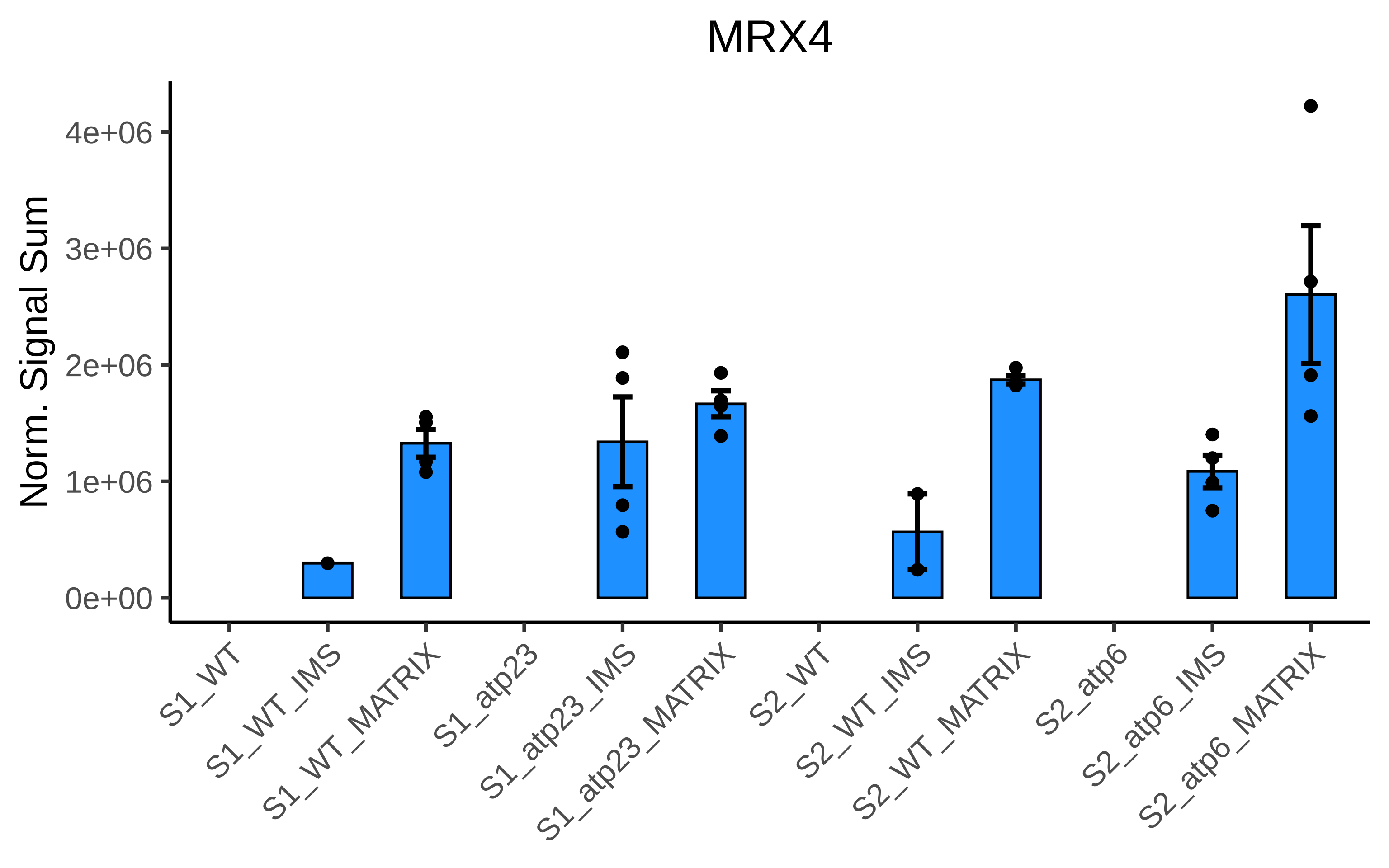

MRX4
Norm. Signal Sum
4e+06
3e+06
2e+06
1e+06
0e+00
S1_WT
S1_WT_IMS
S1_WT_MATRIX
S1_atp23
S1_atp23_IMS
S1_atp23_MATRIX
S2_WT
S2_WT_IMS
S2_WT_MATRIX
S2_atp6
S2_atp6_IMS
S2_atp6_MATRIX

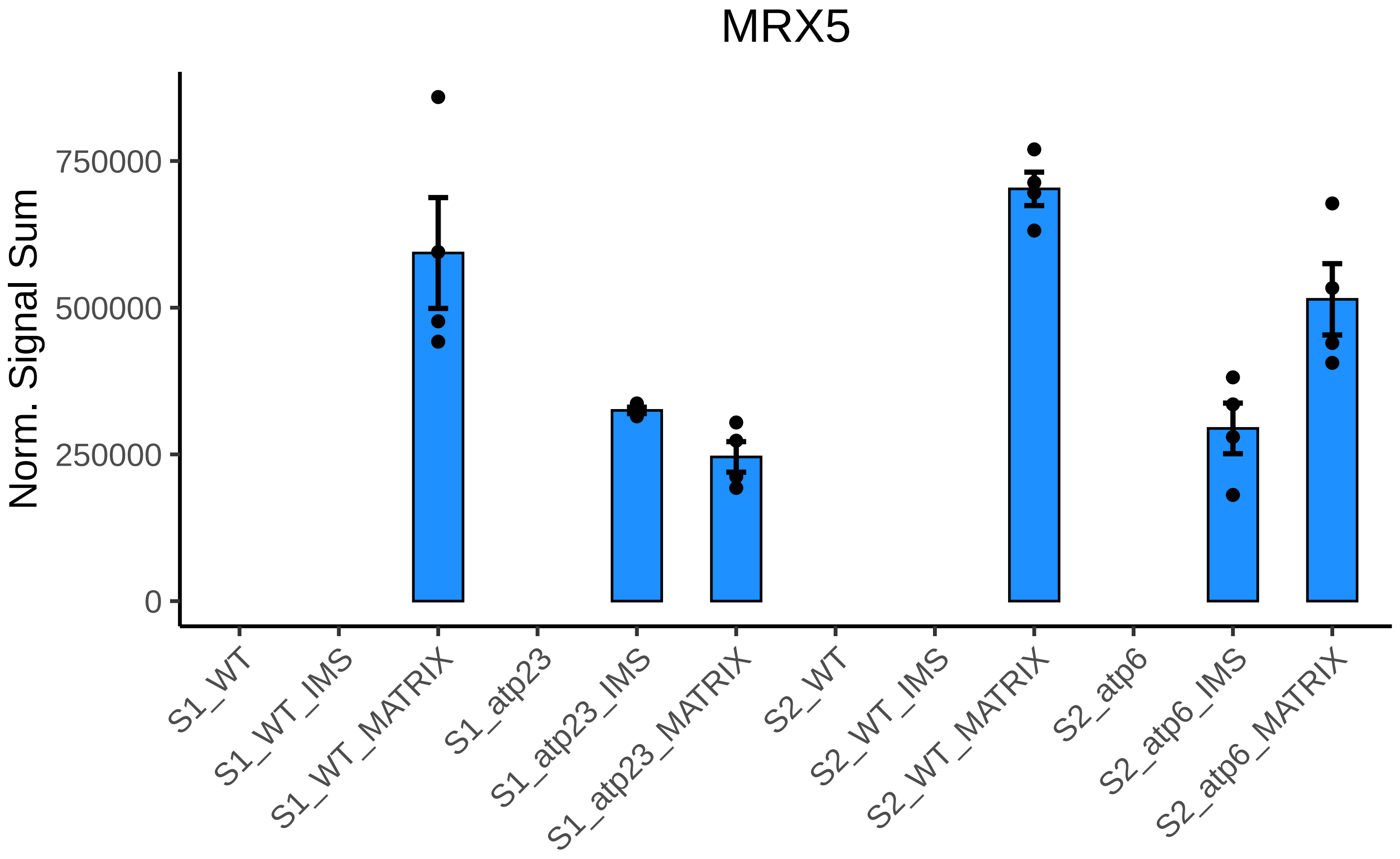

MRX5
Norm. Signal Sum
750000
500000
250000
0
S1_WT
S1_WT_IMS
S1_WT_MATRIX
S1_atp23
S1_atp23_IMS
S1_atp23_MATRIX
S2_WT
S2_WT_IMS
S2_WT_MATRIX
S2_atp6
S2_atp6_IMS
S2_atp6_MATRIX

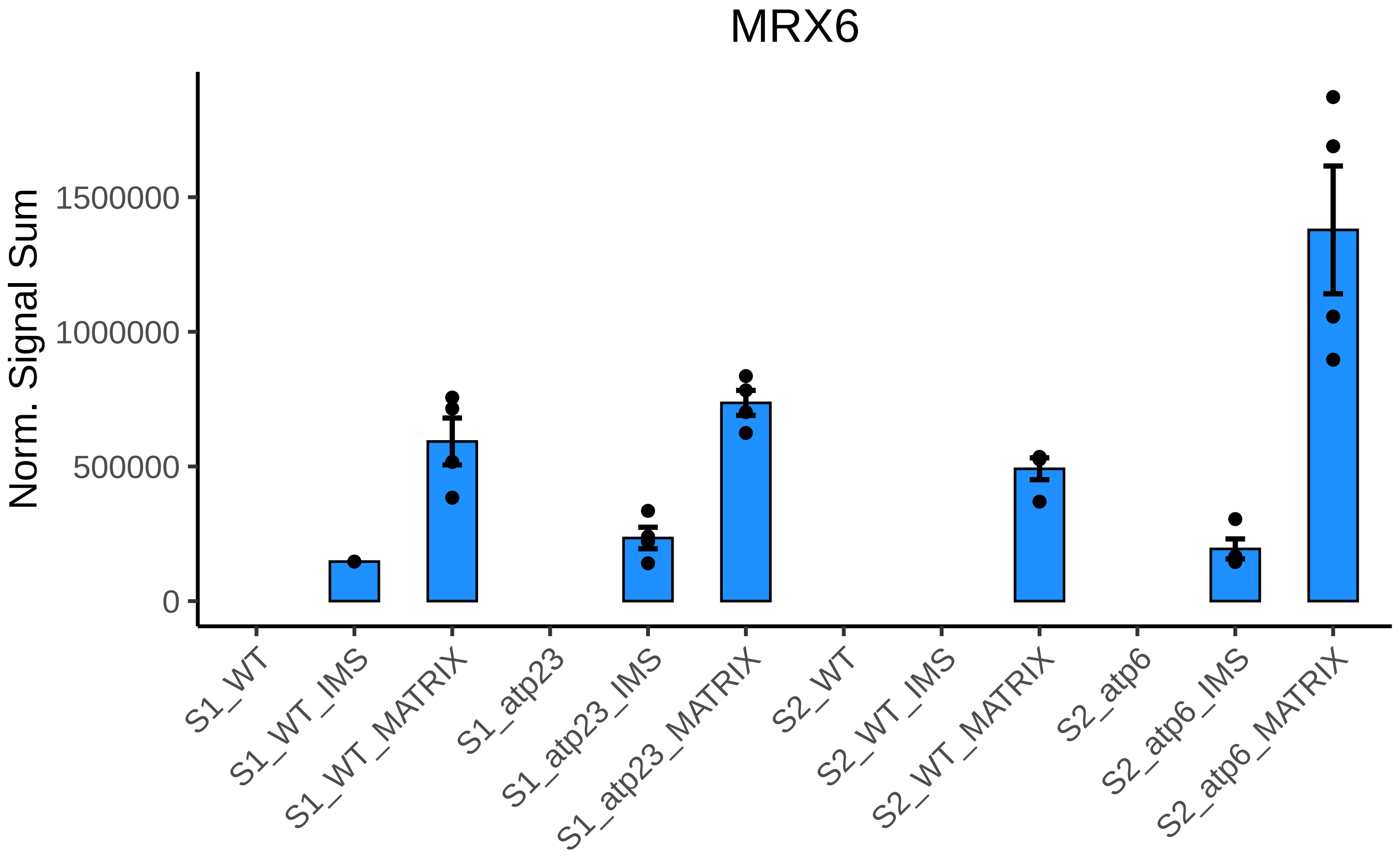
MRX6
Norm. Signal Sum
1500000
1000000
500000
0
S1_WT
S1_WT_IMS
S1_WT_MATRIX
S1_atp23
S1_atp23_IMS
S1_atp23_MATRIX
S2_WT
S2_WT_IMS
S2_WT_MATRIX
S2_atp6
S2_atp6_IMS
S2_atp6_MATRIX

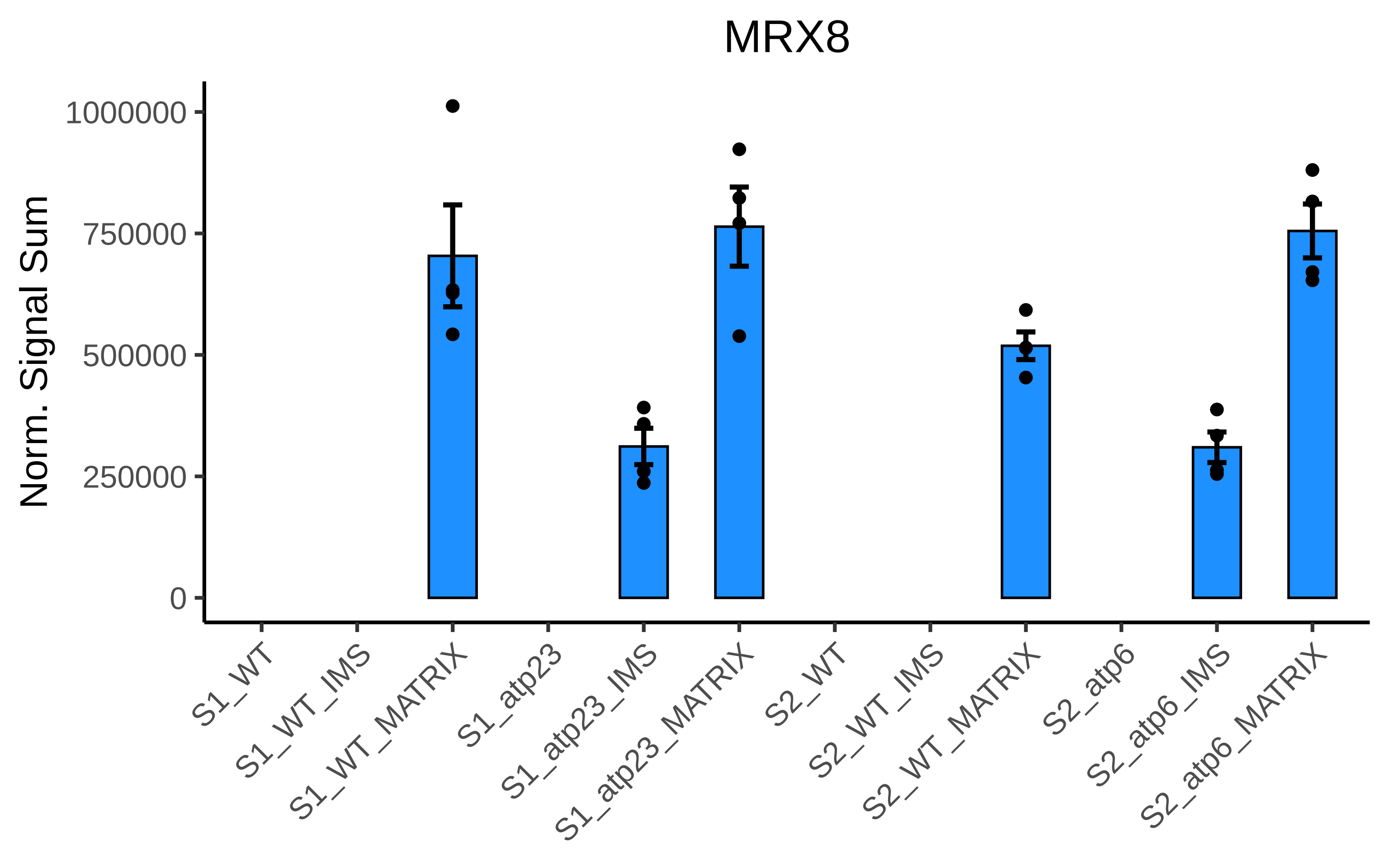
MRX8
Norm. Signal Sum
1000000
750000
500000
250000
0
S1_WT
S1_WT_IMS
S1_WT_MATRIX
S1_atp23
S1_atp23_IMS
S1_atp23_MATRIX
S2_WT
S2_WT_IMS
S2_WT_MATRIX
S2_atp6
S2_atp6_IMS
S2_atp6_MATRIX

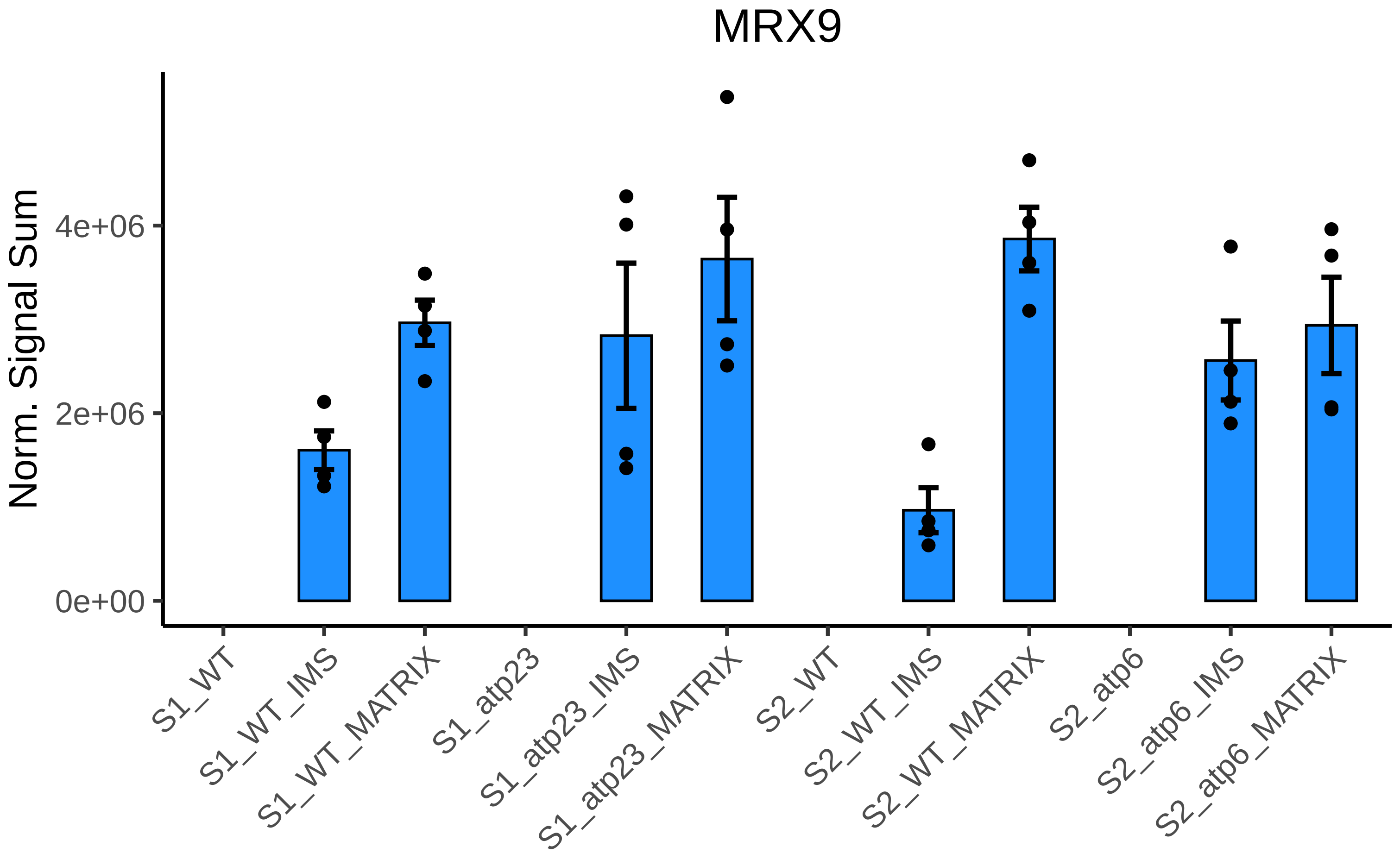
MRX9
Norm. Signal Sum
4e+06
2e+06
0e+00
S1_WT
S1_WT_IMS
S1_WT_MATRIX
S1_atp23
S1_atp23_IMS
S1_atp23_MATRIX
S2_WT
S2_WT_IMS
S2_WT_MATRIX
S2_atp6
S2_atp6_IMS
S2_atp6_MATRIX

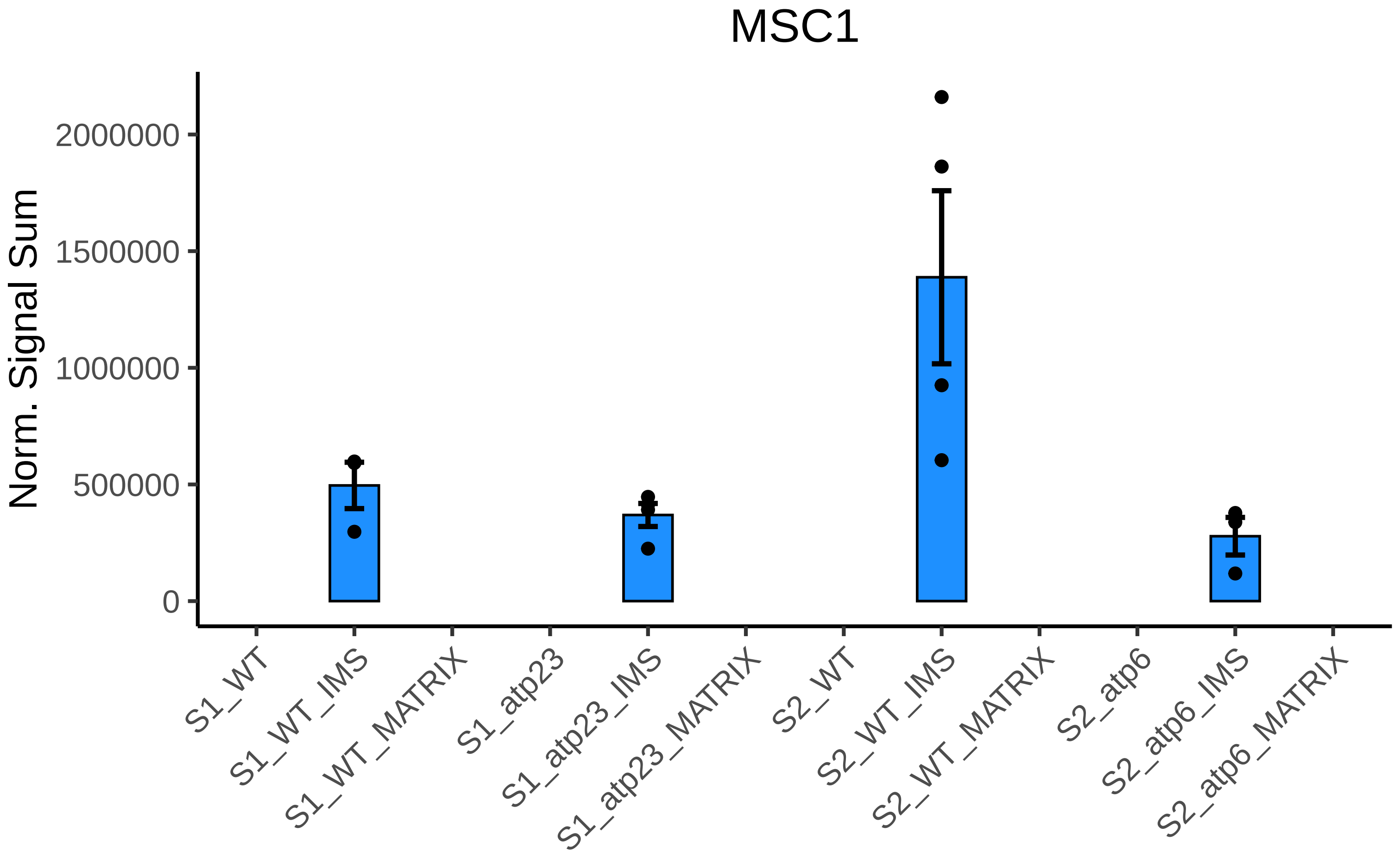

MSC1
Norm. Signal Sum
2000000
1500000
1000000
500000
0
S1_WT
S1_WT_IMS
S1_WT_MATRIX
S1_atp23
S1_atp23_IMS
S1_atp23_MATRIX
S2_WT
S2_WT_IMS
S2_WT_MATRIX
S2_atp6
S2_atp6_IMS
S2_atp6_MATRIX

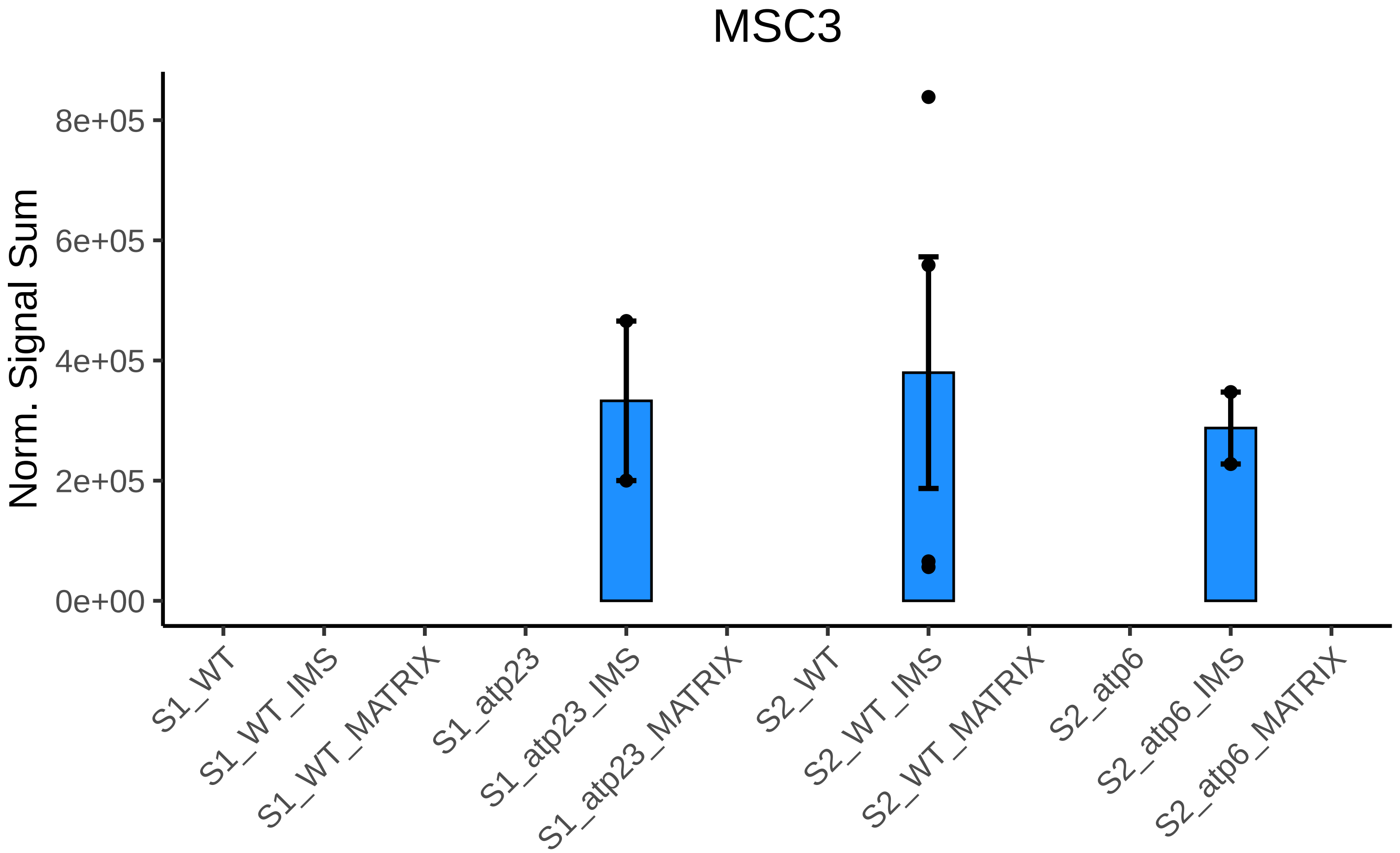
MSC3
Norm. Signal Sum
8e+05
6e+05
4e+05
2e+05
0e+00
S1_WT
S1_WT_IMS
S1_WT_MATRIX
S1_atp23
S1_atp23_IMS
S1_atp23_MATRIX
S2_WT
S2_WT_IMS
S2_WT_MATRIX
S2_atp6
S2_atp6_IMS
S2_atp6_MATRIX

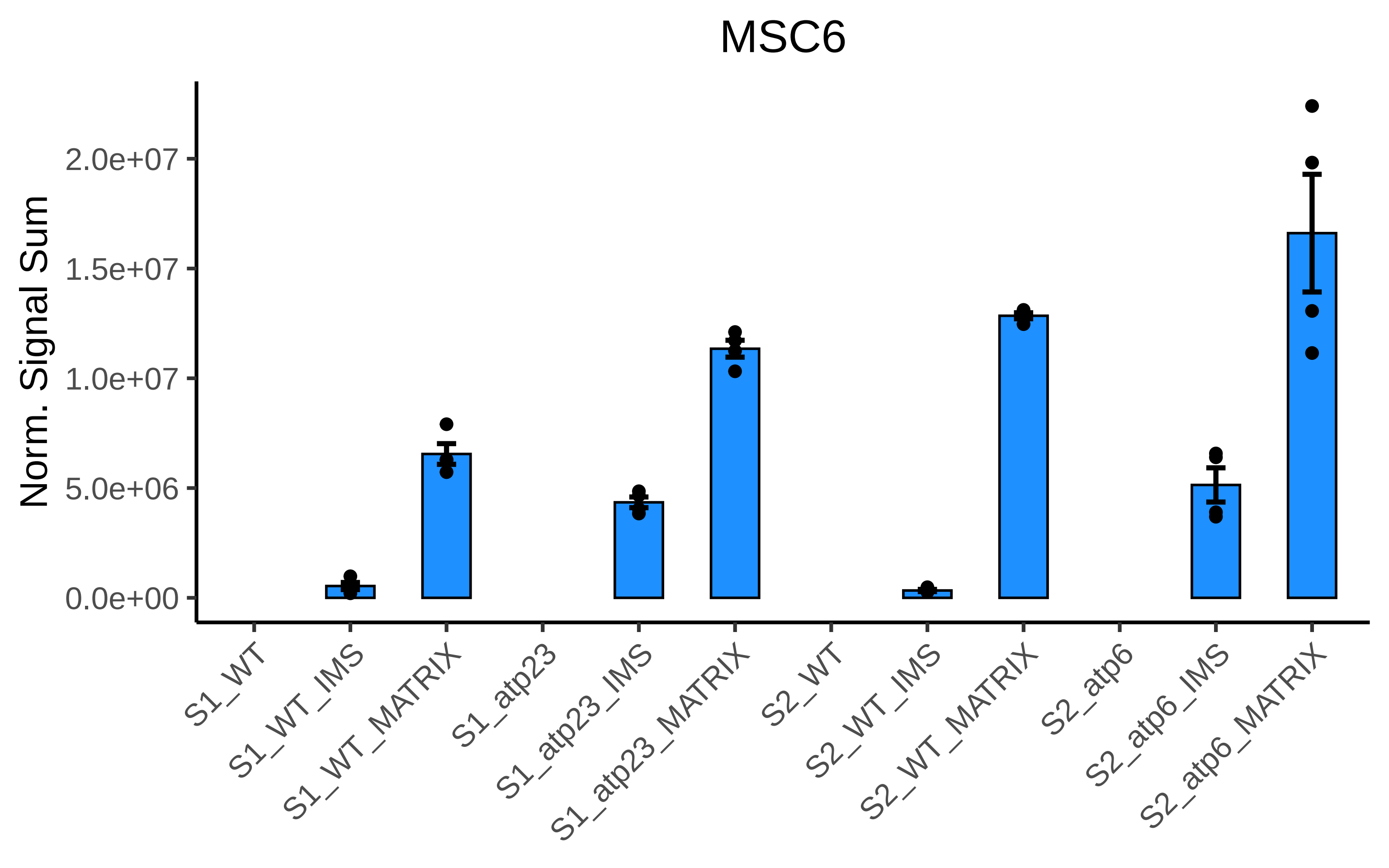

MSC6
Norm. Signal Sum
2.0e+07
1.5e+07
1.0e+07
5.0e+06
0.0e+00
S1_WT
S1_WT_IMS
S1_WT_MATRIX
S1_atp23
S1_atp23_IMS
S1_atp23_MATRIX
S2_WT
S2_WT_IMS
S2_WT_MATRIX
S2_atp6
S2_atp6_IMS
S2_atp6_MATRIX

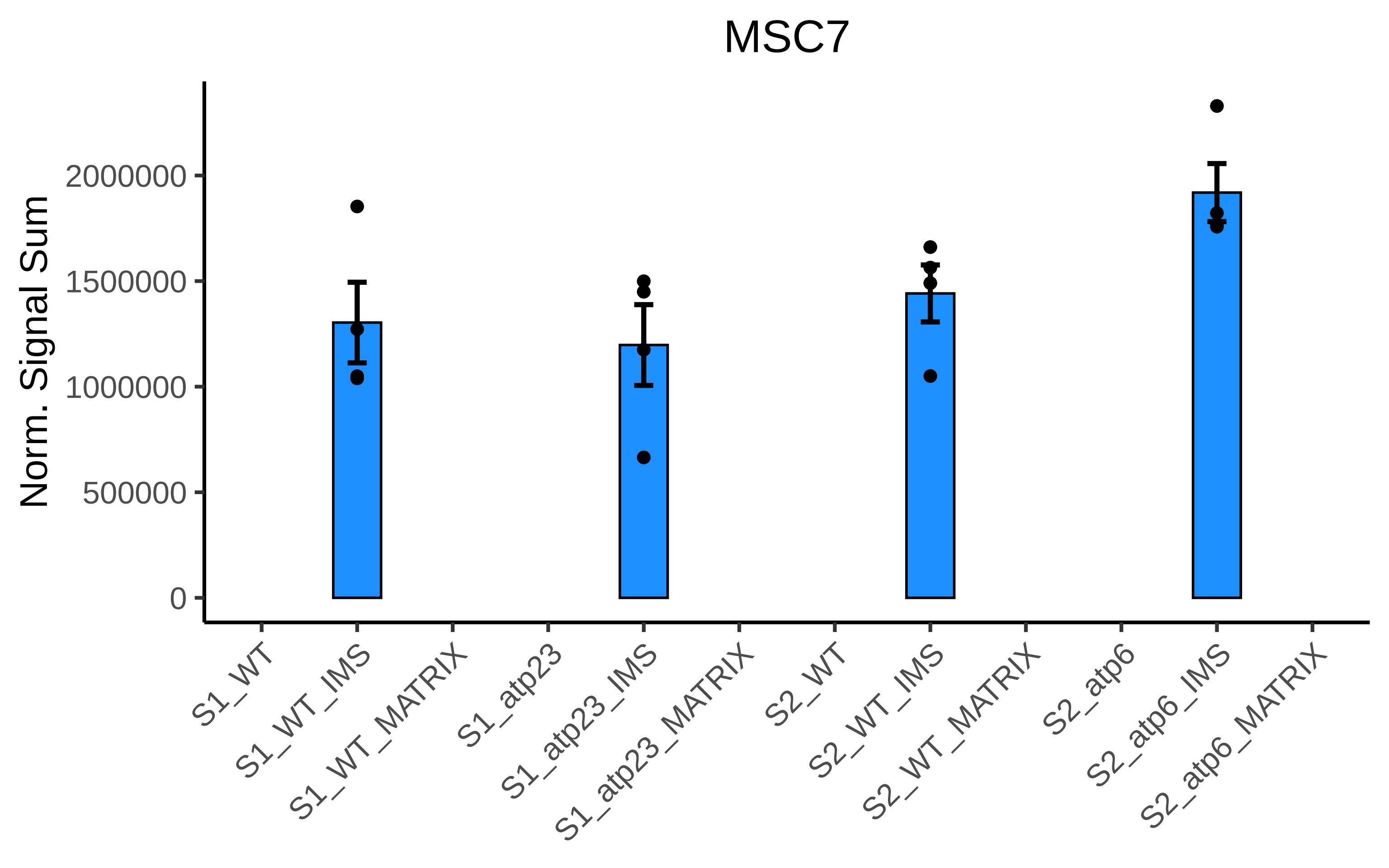
MSC7
Norm. Signal Sum
2000000
1500000
1000000
500000
0
S1_WT
S1_WT_IMS
S1_WT_MATRIX
S1_atp23
S1_atp23_IMS
S1_atp23_MATRIX
S2_WT
S2_WT_IMS
S2_WT_MATRIX
S2_atp6
S2_atp6_IMS
S2_atp6_MATRIX

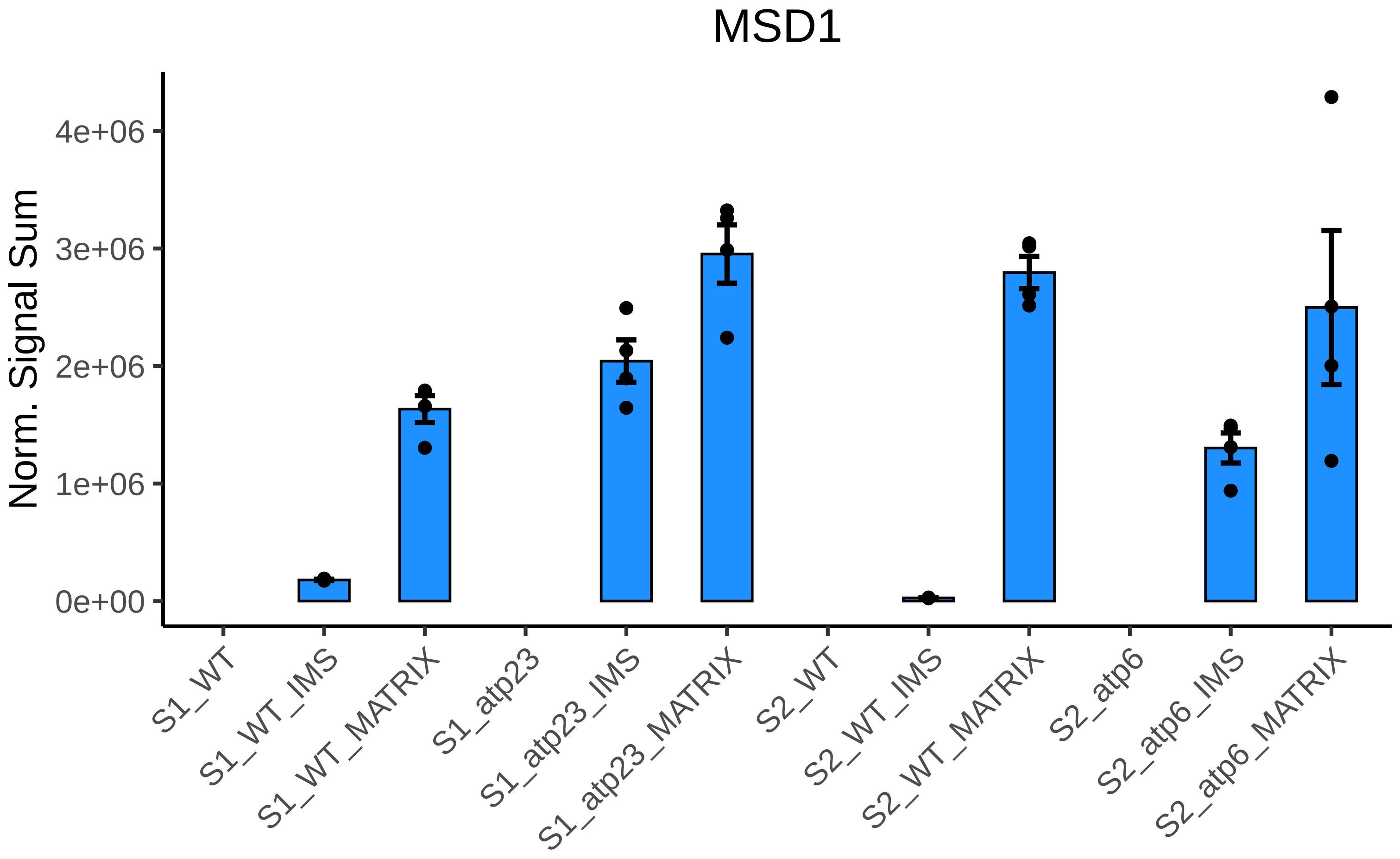
MSD1
Norm. Signal Sum
4e+06
3e+06
2e+06
1e+06
0e+00
S1_WT
S1_WT_IMS
S1_WT_MATRIX
S1_atp23
S1_atp23_IMS
S1_atp23_MATRIX
S2_WT
S2_WT_IMS
S2_WT_MATRIX
S2_atp6
S2_atp6_IMS
S2_atp6_MATRIX

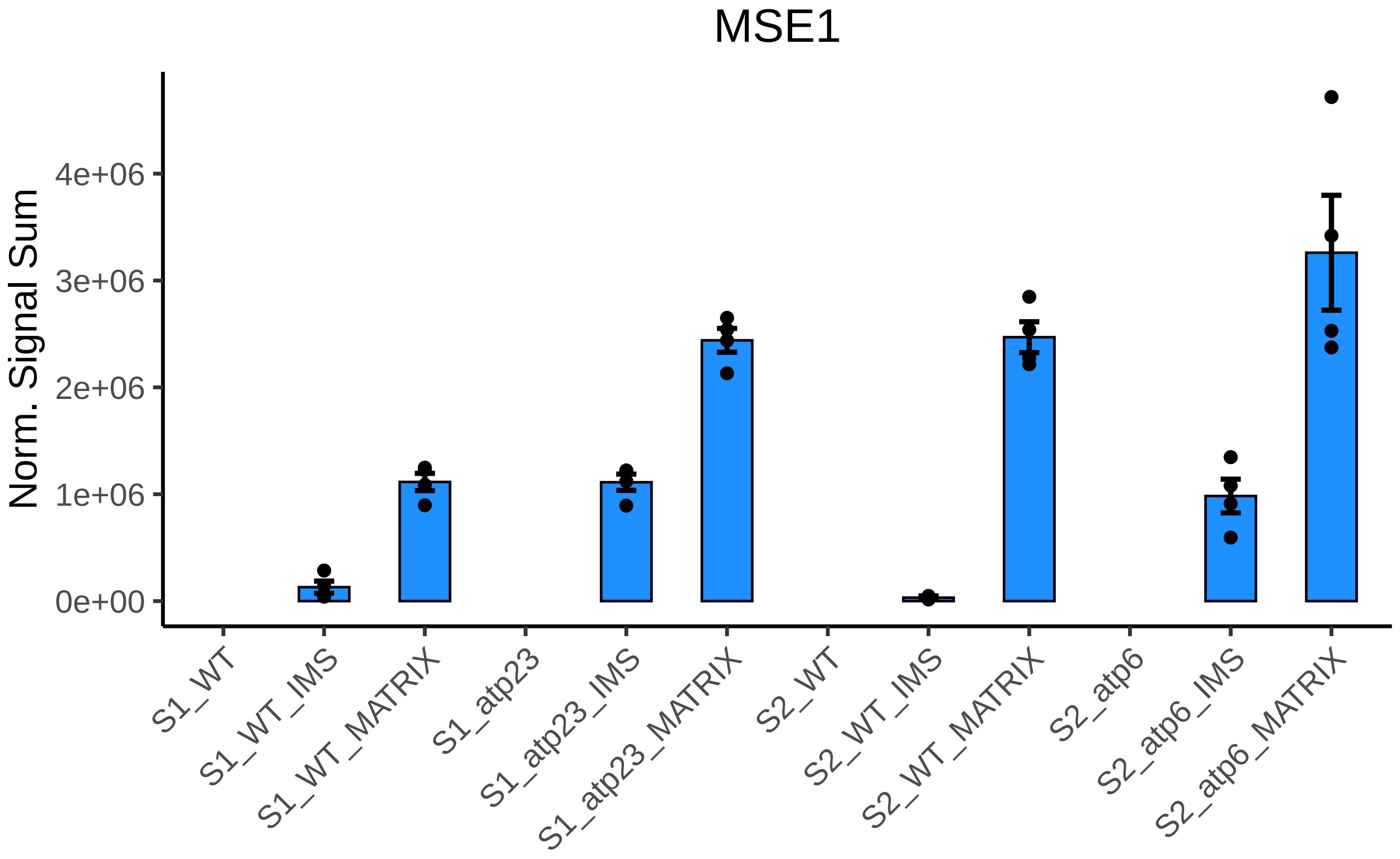

MSE1
Norm. Signal Sum
4e+06
3e+06
2e+06
1e+06
0e+00
S1_WT
S1_WT_IMS
S1_WT_MATRIX
S1_atp23
S1_atp23_IMS
S1_atp23_MATRIX
S2_WT
S2_WT_IMS
S2_WT_MATRIX
S2_atp6
S2_atp6_IMS
S2_atp6_MATRIX

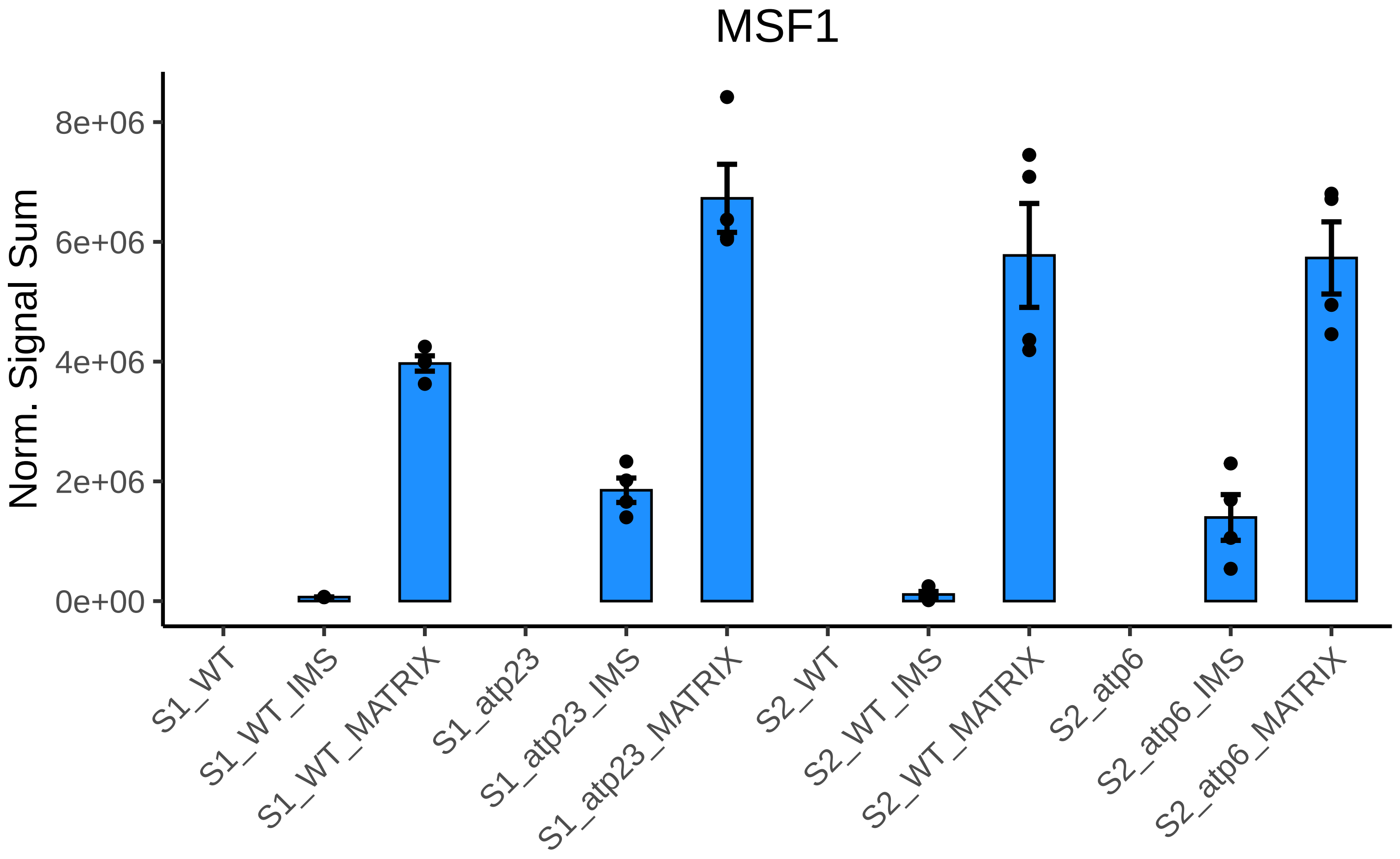
MSF1
Norm. Signal Sum
8e+06
6e+06
4e+06
2e+06
0e+00
S1_WT
S1_WT_IMS
S1_WT_MATRIX
S1_atp23
S1_atp23_IMS
S1_atp23_MATRIX
S2_WT
S2_WT_IMS
S2_WT_MATRIX
S2_atp6
S2_atp6_IMS
S2_atp6_MATRIX

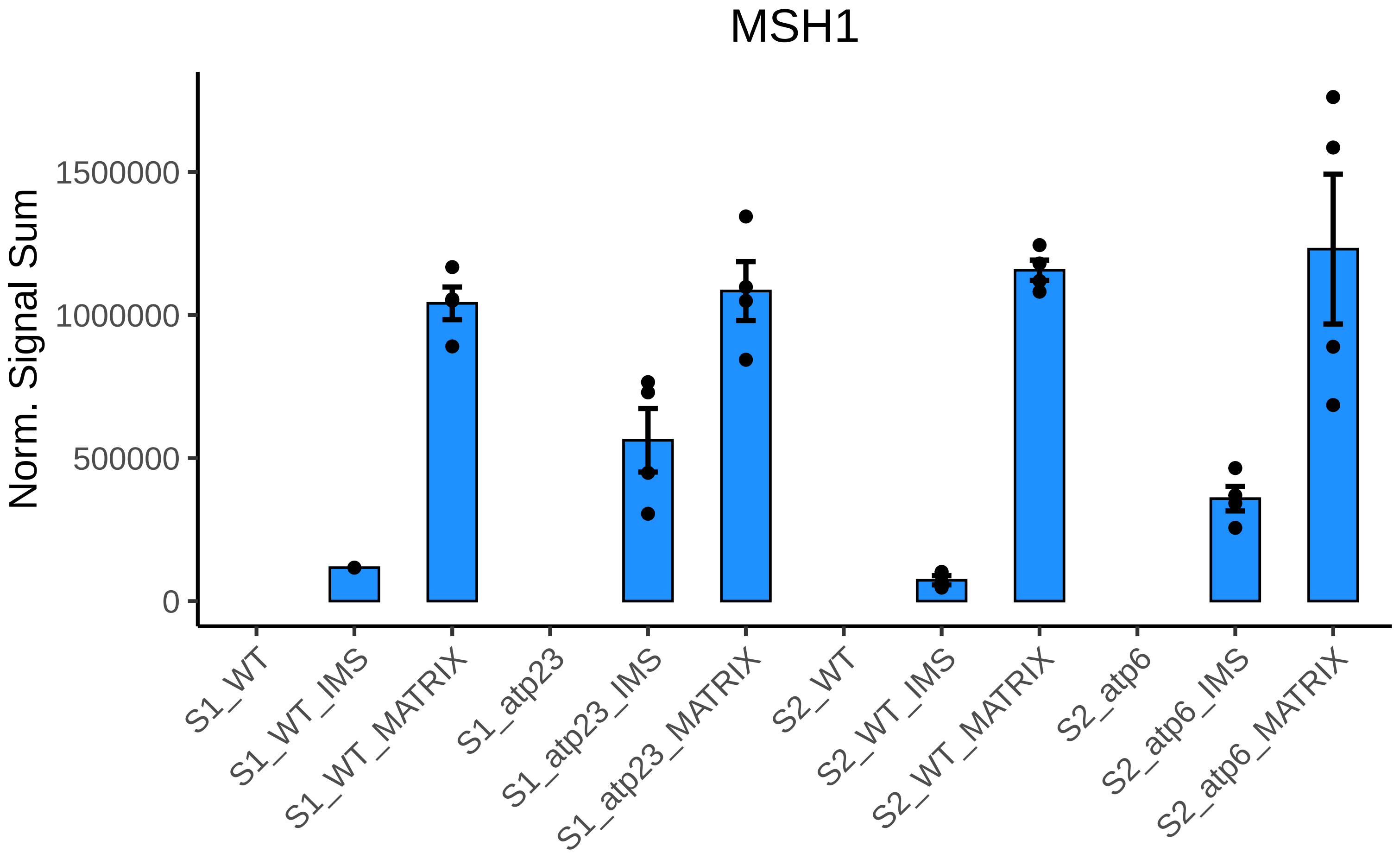
MSH1
Norm. Signal Sum
1500000
1000000
500000
0
S1_WT
S1_WT_IMS
S1_WT_MATRIX
S1_atp23
S1_atp23_IMS
S1_atp23_MATRIX
S2_WT
S2_WT_IMS
S2_WT_MATRIX
S2_atp6
S2_atp6_IMS
S2_atp6_MATRIX

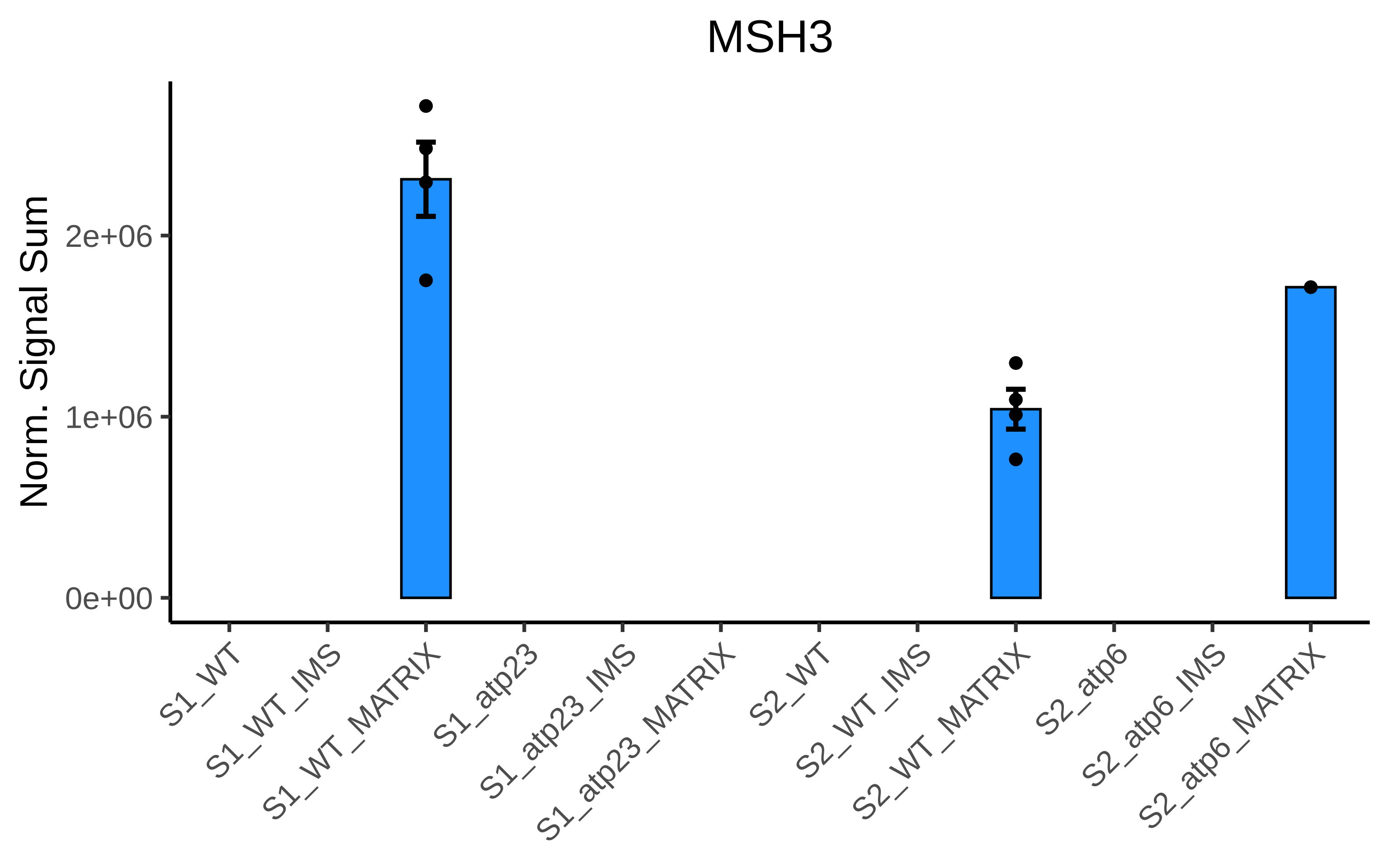

MSH3
Norm. Signal Sum
2e+06
1e+06
0e+00
S1_WT
S1_WT_IMS
S1_WT_MATRIX
S1_atp23
S1_atp23_IMS
S1_atp23_MATRIX
S2_WT
S2_WT_IMS
S2_WT_MATRIX
S2_atp6
S2_atp6_IMS
S2_atp6_MATRIX

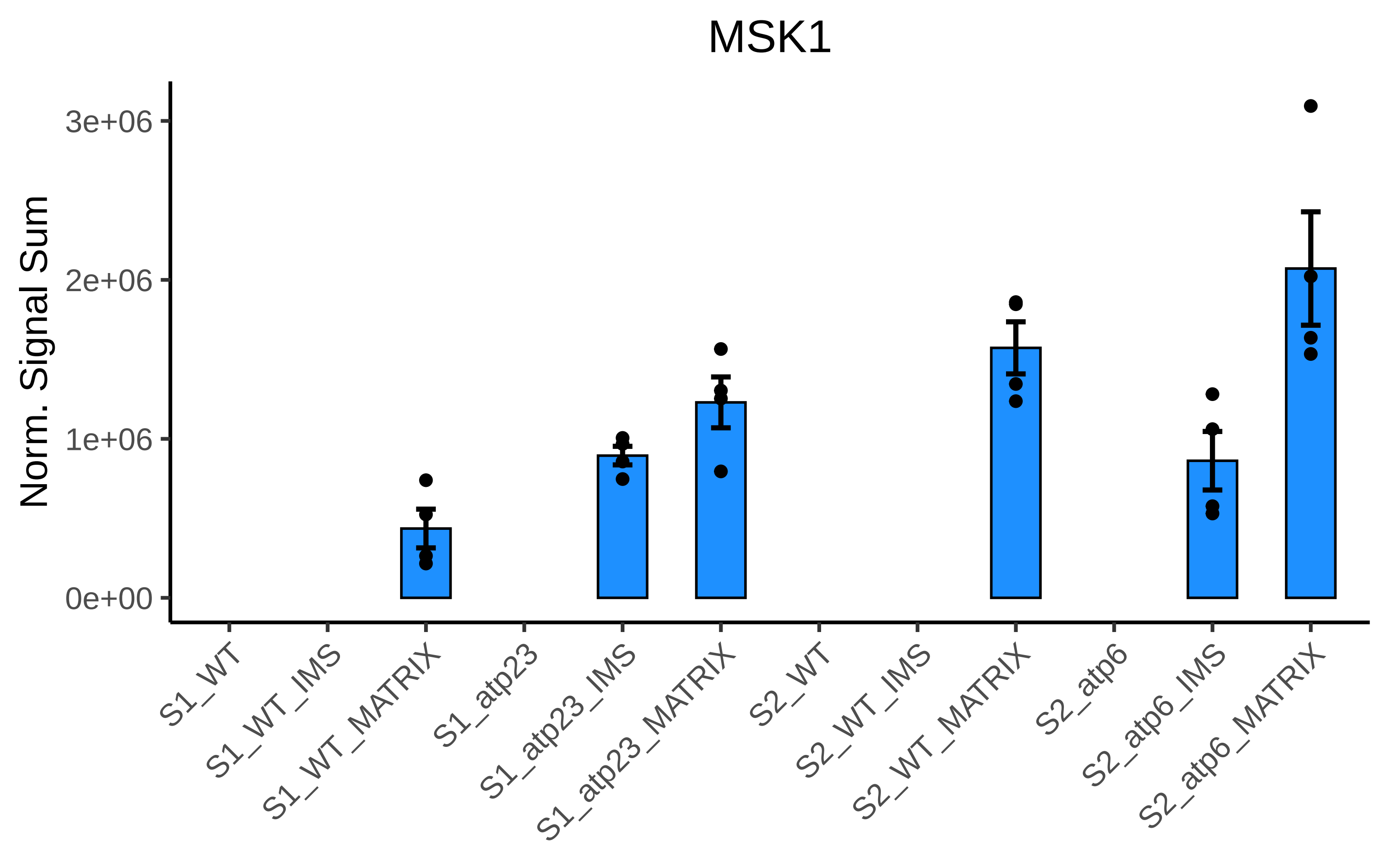

MSK1
Norm. Signal Sum
3e+06
2e+06
1e+06
0e+00
S1_WT
S1_WT_IMS
S1_WT_MATRIX
S1_atp23
S1_atp23_IMS
S1_atp23_MATRIX
S2_WT
S2_WT_IMS
S2_WT_MATRIX
S2_atp6
S2_atp6_IMS
S2_atp6_MATRIX

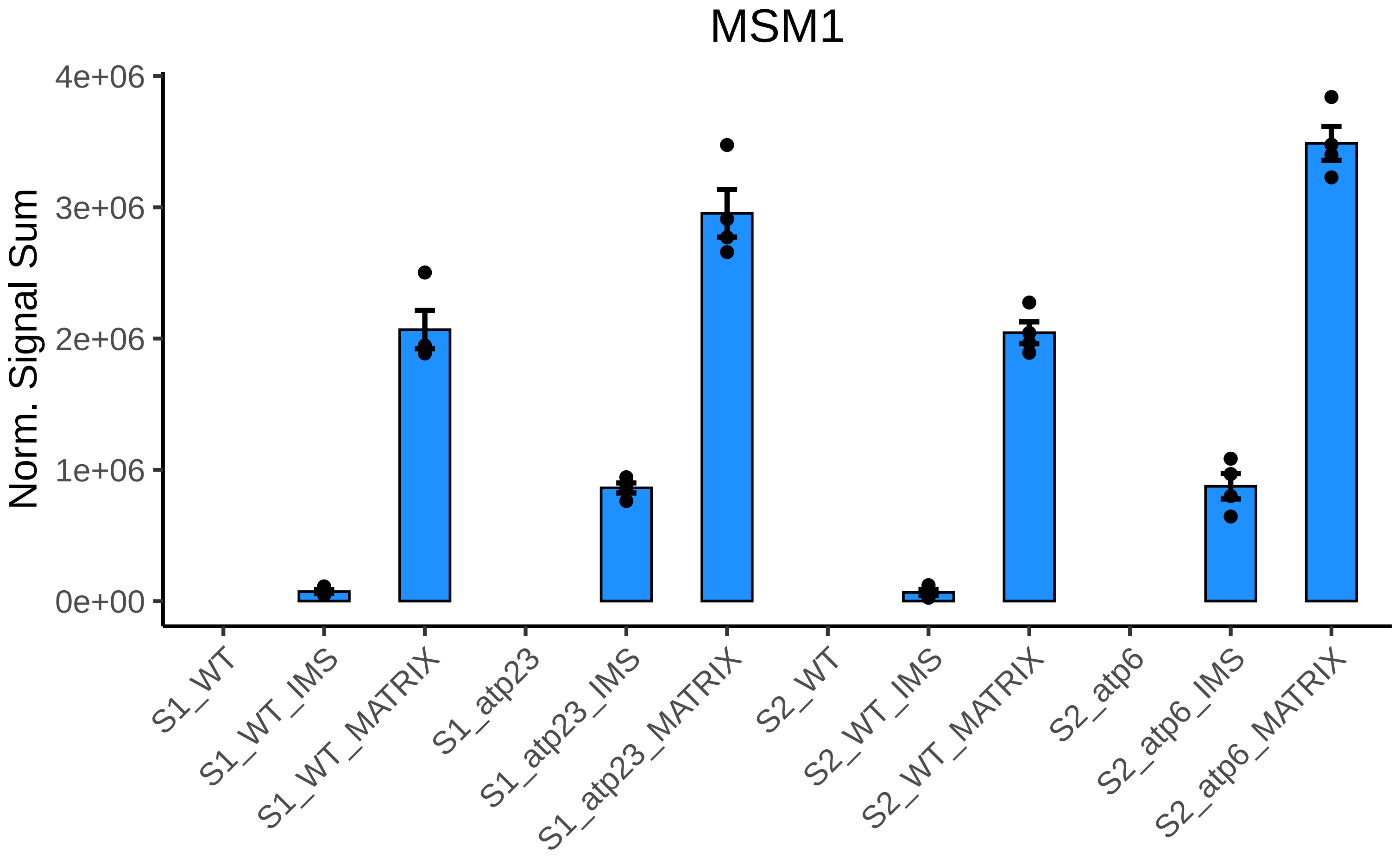

MSM1
Norm. Signal Sum
4e+06
3e+06
2e+06
1e+06
0e+00
S1_WT
S1_WT_IMS
S1_WT_MATRIX
S1_atp23
S1_atp23_IMS
S1_atp23_MATRIX
S2_WT
S2_WT_IMS
S2_WT_MATRIX
S2_atp6
S2_atp6_IMS
S2_atp6_MATRIX

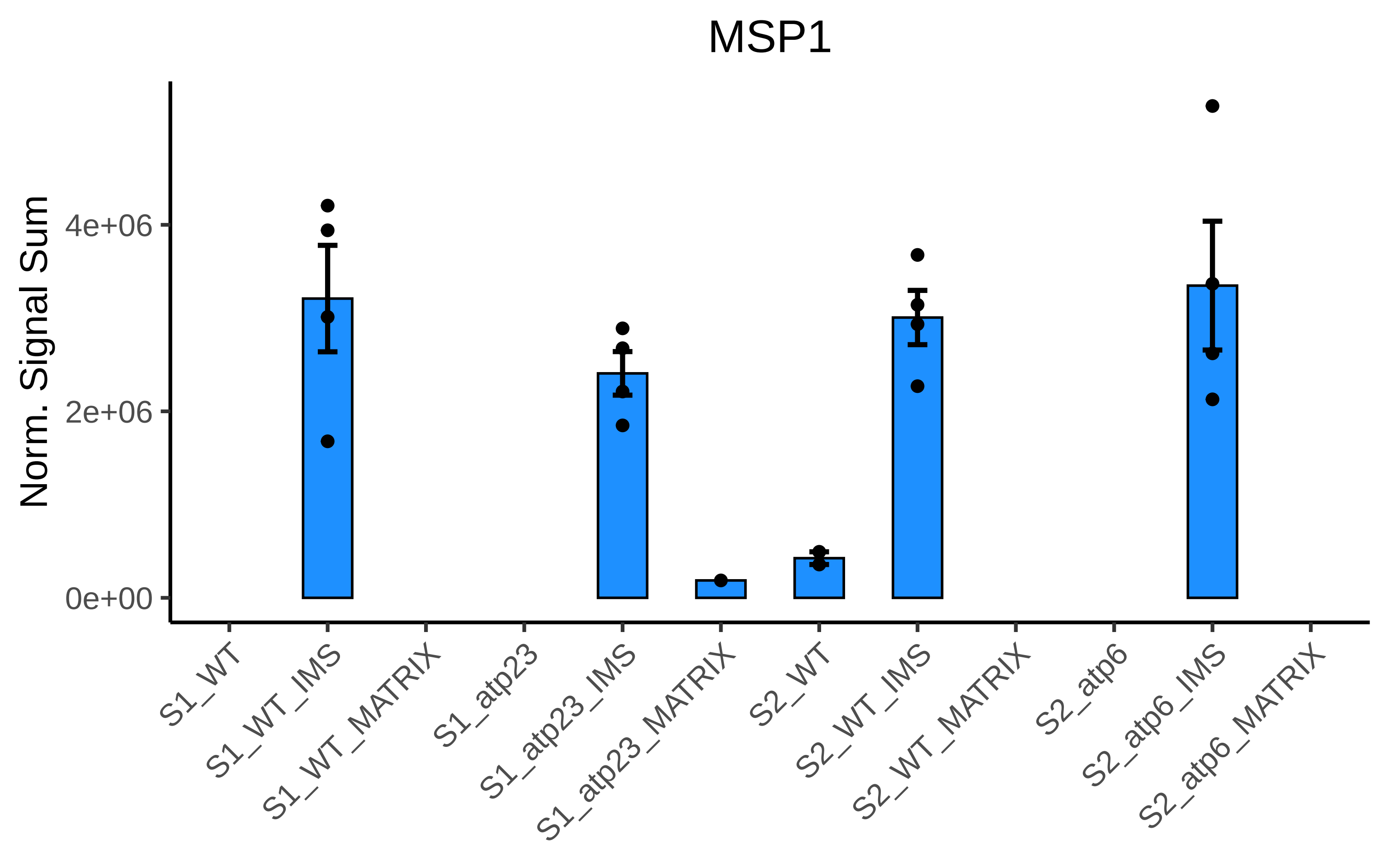
MSP1
Norm. Signal Sum
4e+06
2e+06
0e+00
S1_WT
S1_WT_IMS
S1_WT_MATRIX
S1_atp23
S1_atp23_IMS
S1_atp23_MATRIX
S2_WT
S2_WT_IMS
S2_WT_MATRIX
S2_atp6
S2_atp6_IMS
S2_atp6_MATRIX

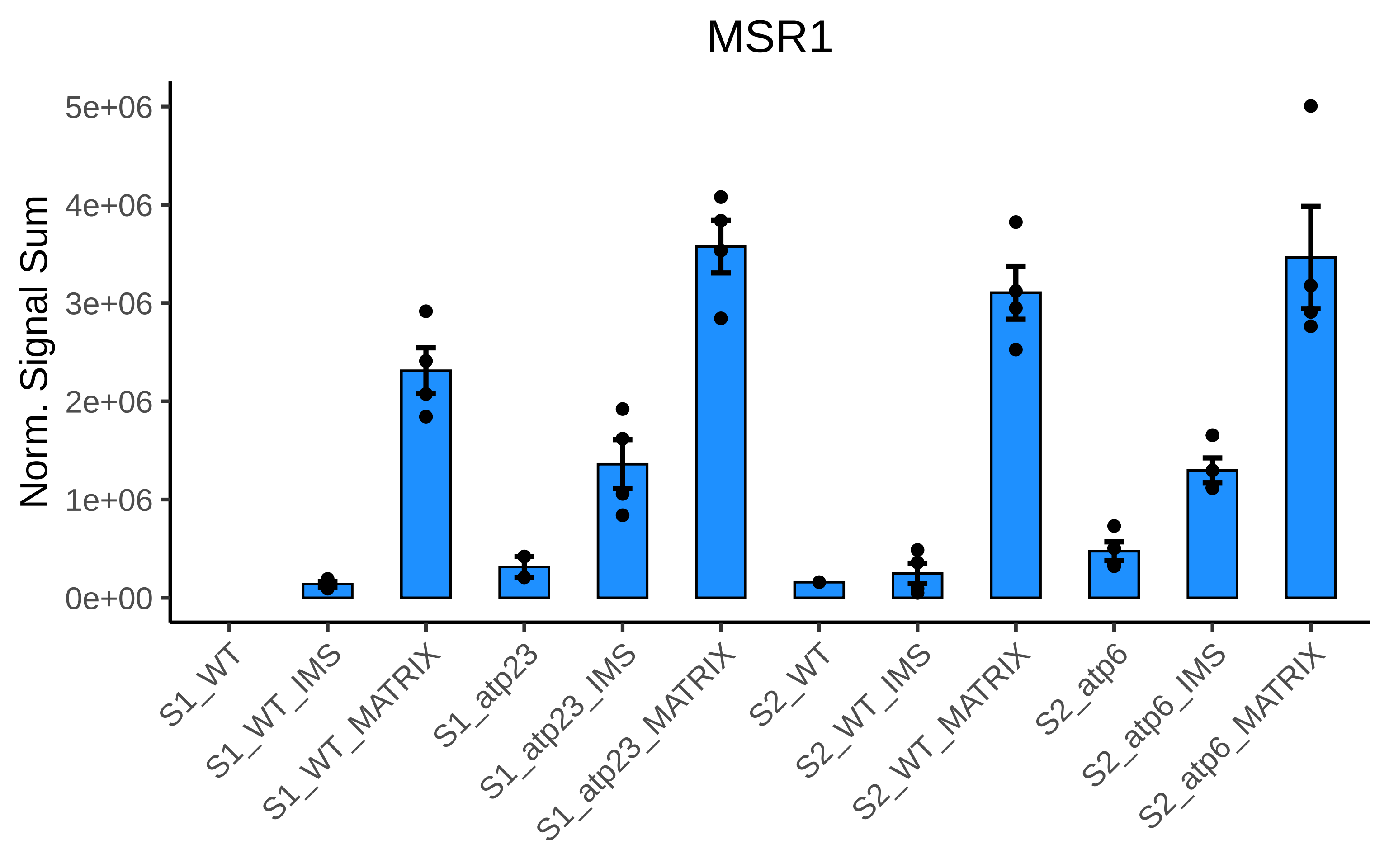
MSR1
Norm. Signal Sum
5e+06
4e+06
3e+06
2e+06
1e+06
0e+00
S1_WT
S1_WT_IMS
S1_WT_MATRIX
S1_atp23
S1_atp23_IMS
S1_atp23_MATRIX
S2_WT
S2_WT_IMS
S2_WT_MATRIX
S2_atp6
S2_atp6_IMS
S2_atp6_MATRIX

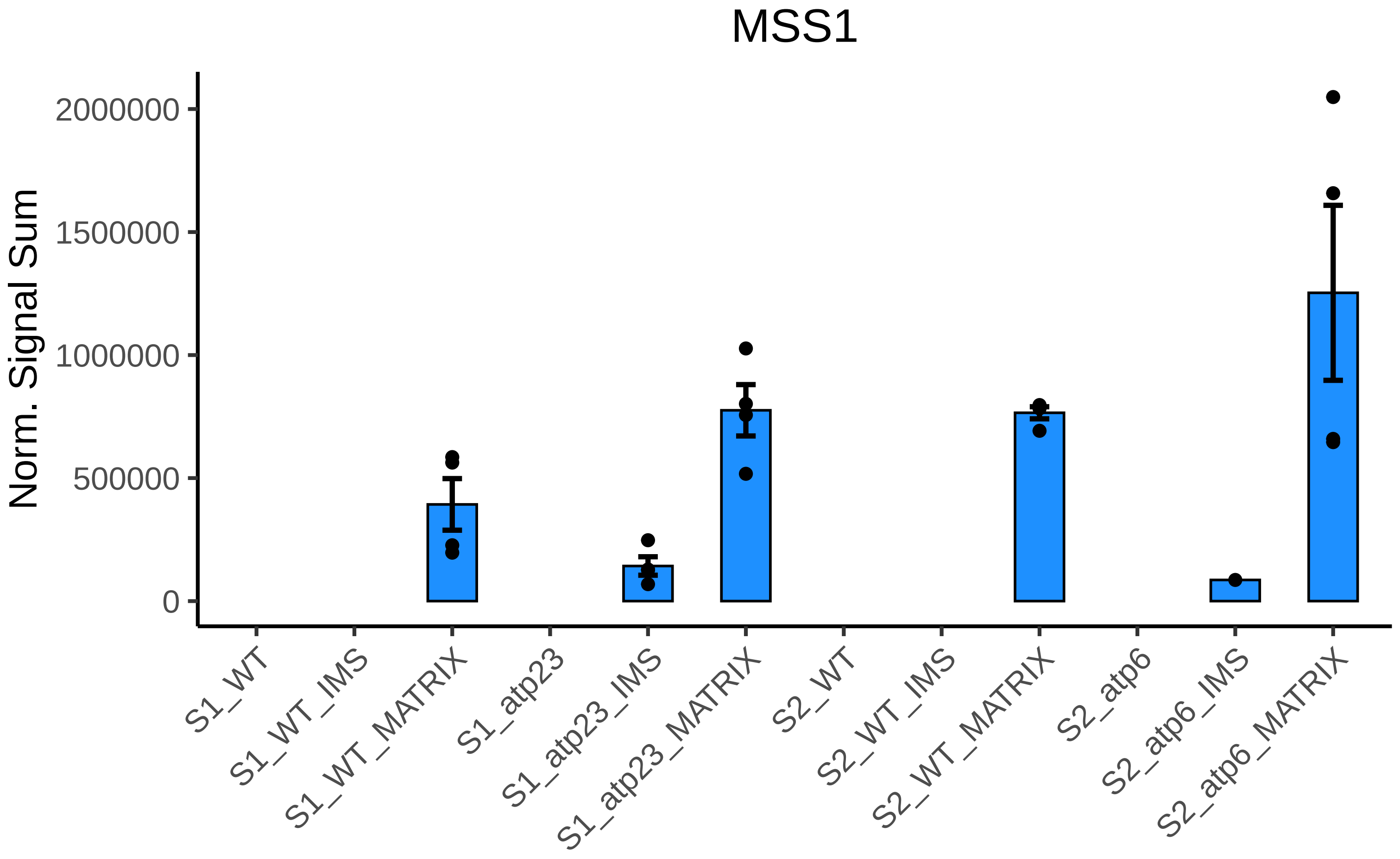

MSS1
Norm. Signal Sum
2000000
1500000
1000000
500000
0
S1_WT
S1_WT_IMS
S1_WT_MATRIX
S1_atp23
S1_atp23_IMS
S1_atp23_MATRIX
S2_WT
S2_WT_IMS
S2_WT_MATRIX
S2_atp6
S2_atp6_IMS
S2_atp6_MATRIX

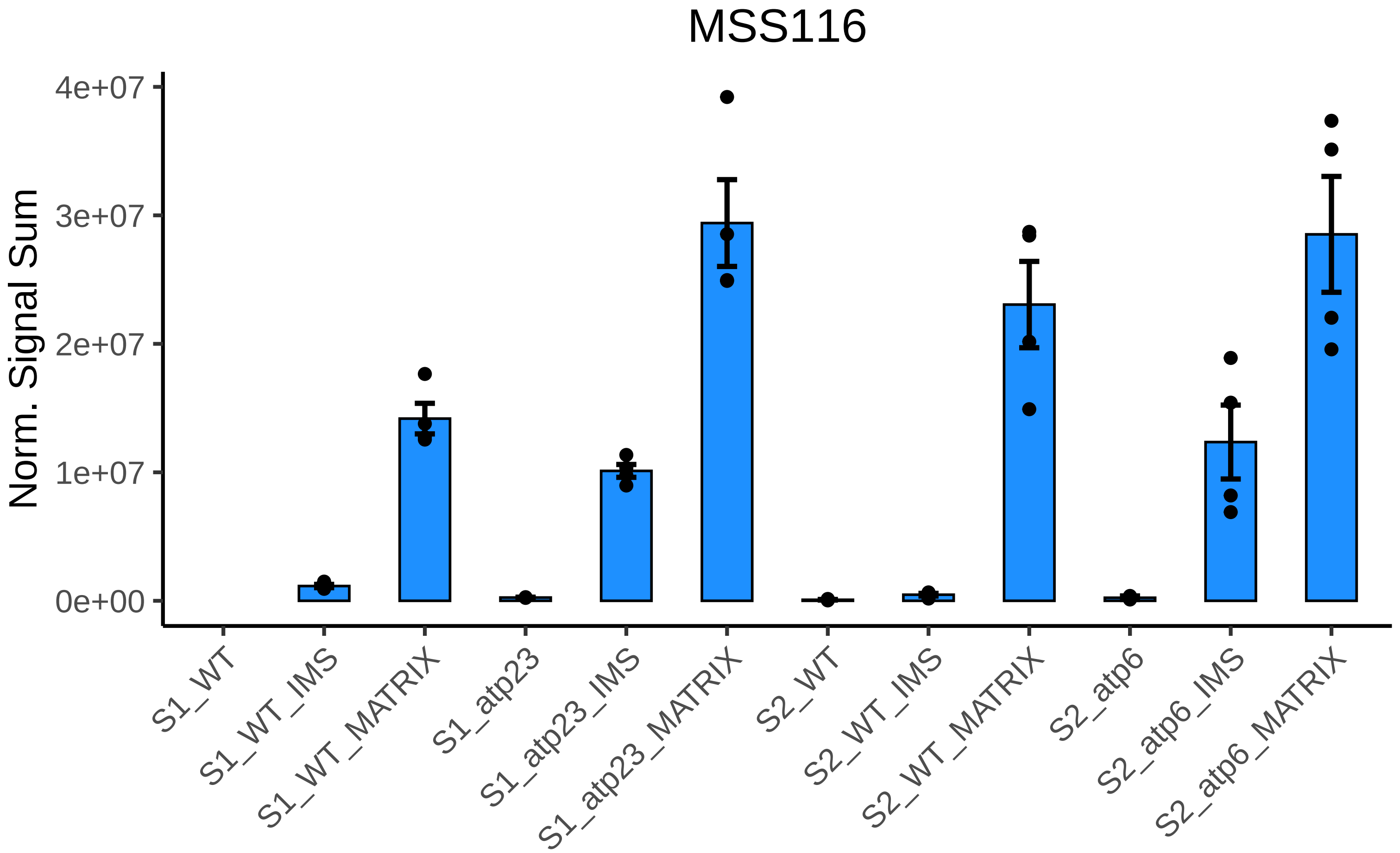
MSS116
Norm. Signal Sum
4e+07
3e+07
2e+07
1e+07
0e+00
S1_WT
S1_WT_IMS
S1_WT_MATRIX
S1_atp23
S1_atp23_IMS
S1_atp23_MATRIX
S2_WT
S2_WT_IMS
S2_WT_MATRIX
S2_atp6
S2_atp6_IMS
S2_atp6_MATRIX

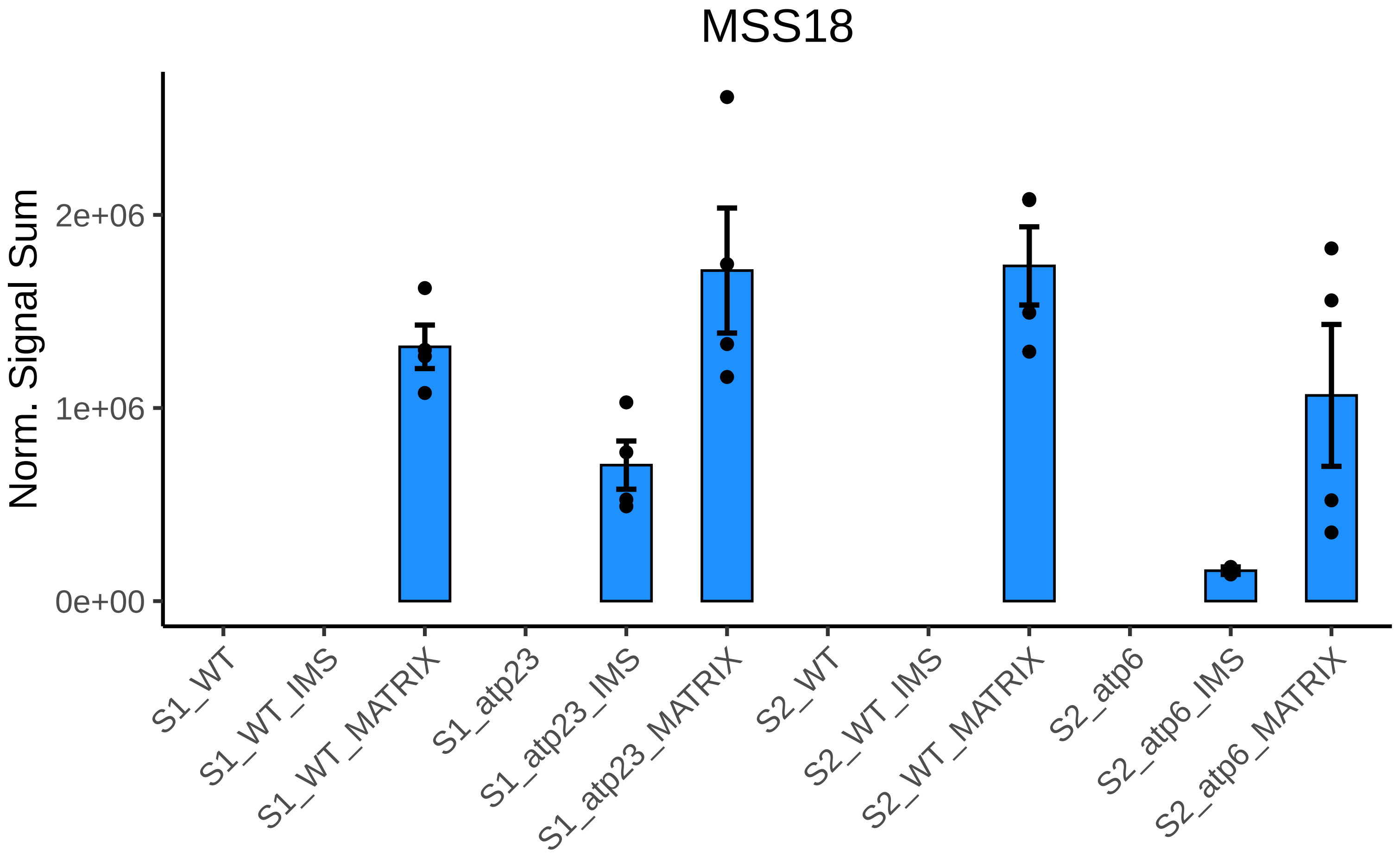

MSS18
Norm. Signal Sum
2e+06
1e+06
0e+00
S1_WT
S1_WT_IMS
S1_WT_MATRIX
S1_atp23
S1_atp23_IMS
S1_atp23_MATRIX
S2_WT
S2_WT_IMS
S2_WT_MATRIX
S2_atp6
S2_atp6_IMS
S2_atp6_MATRIX

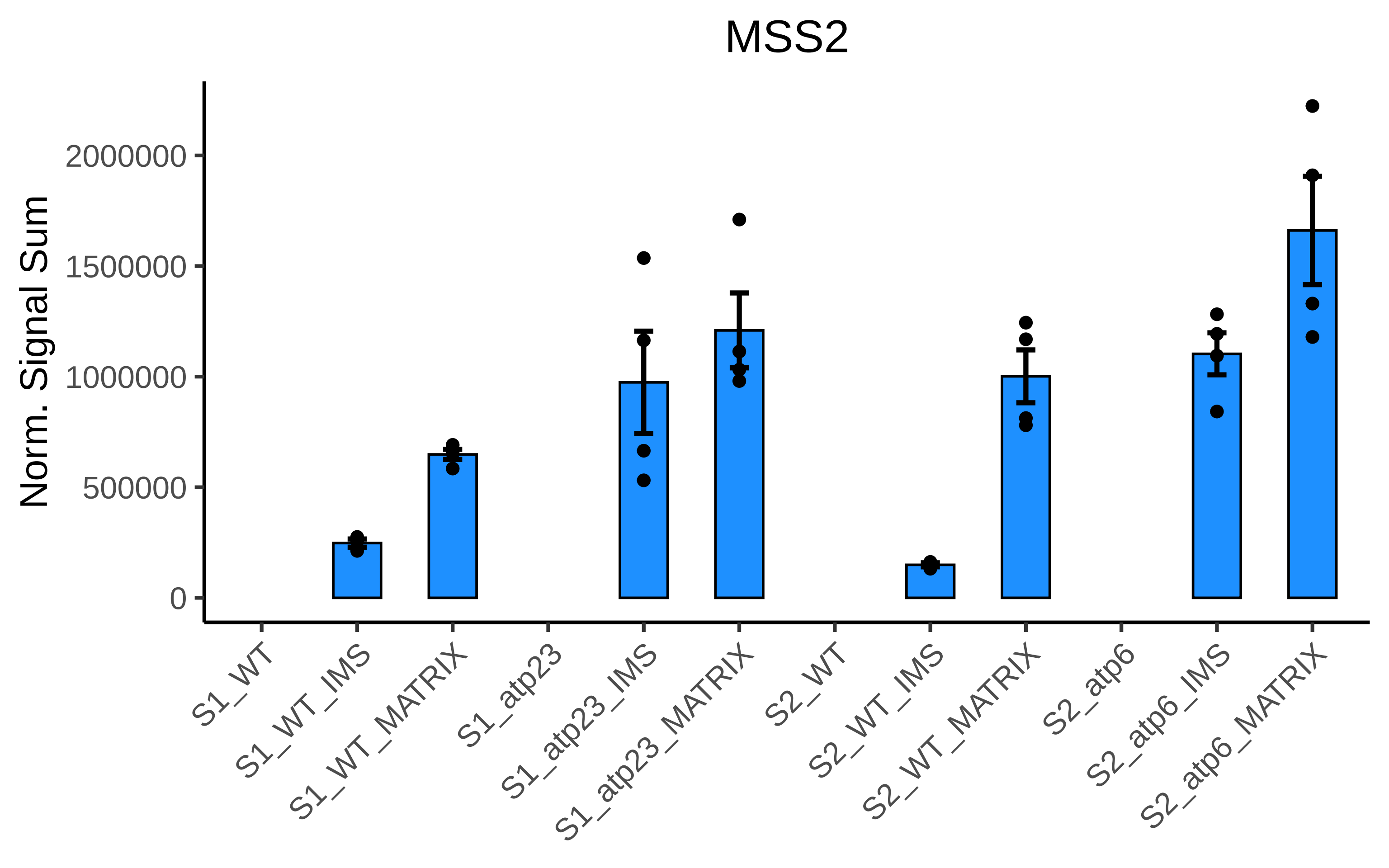
MSS2
Norm. Signal Sum
2000000
1500000
1000000
500000
0
S1_WT
S1_WT_IMS
S1_WT_MATRIX
S1_atp23
S1_atp23_IMS
S1_atp23_MATRIX
S2_WT
S2_WT_IMS
S2_WT_MATRIX
S2_atp6
S2_atp6_IMS
S2_atp6_MATRIX

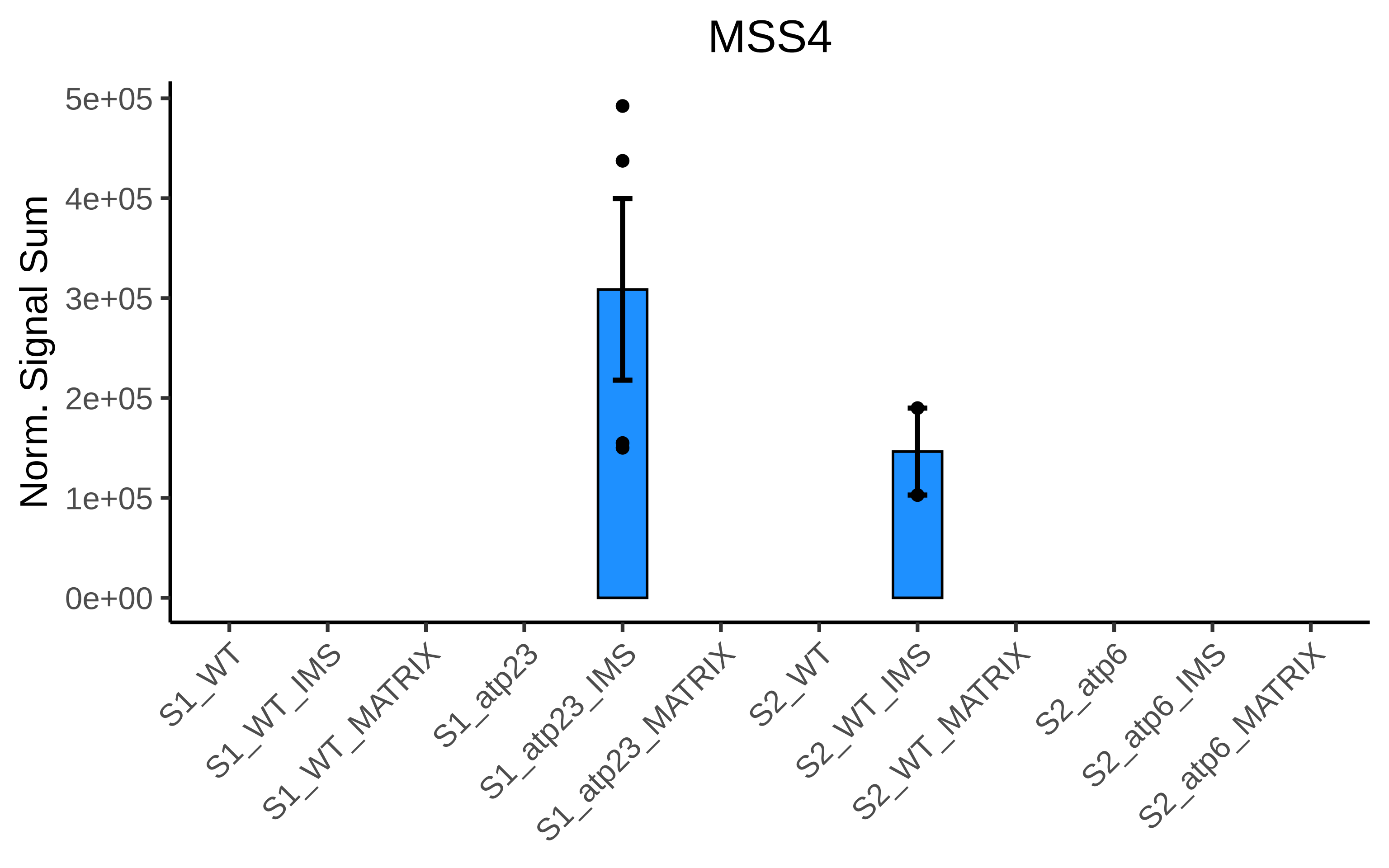

MSS4
Norm. Signal Sum
5e+05
4e+05
3e+05
2e+05
1e+05
0e+00
S1_WT
S1_WT_IMS
S1_WT_MATRIX
S1_atp23
S1_atp23_IMS
S1_atp23_MATRIX
S2_WT
S2_WT_IMS
S2_WT_MATRIX
S2_atp6
S2_atp6_IMS
S2_atp6_MATRIX

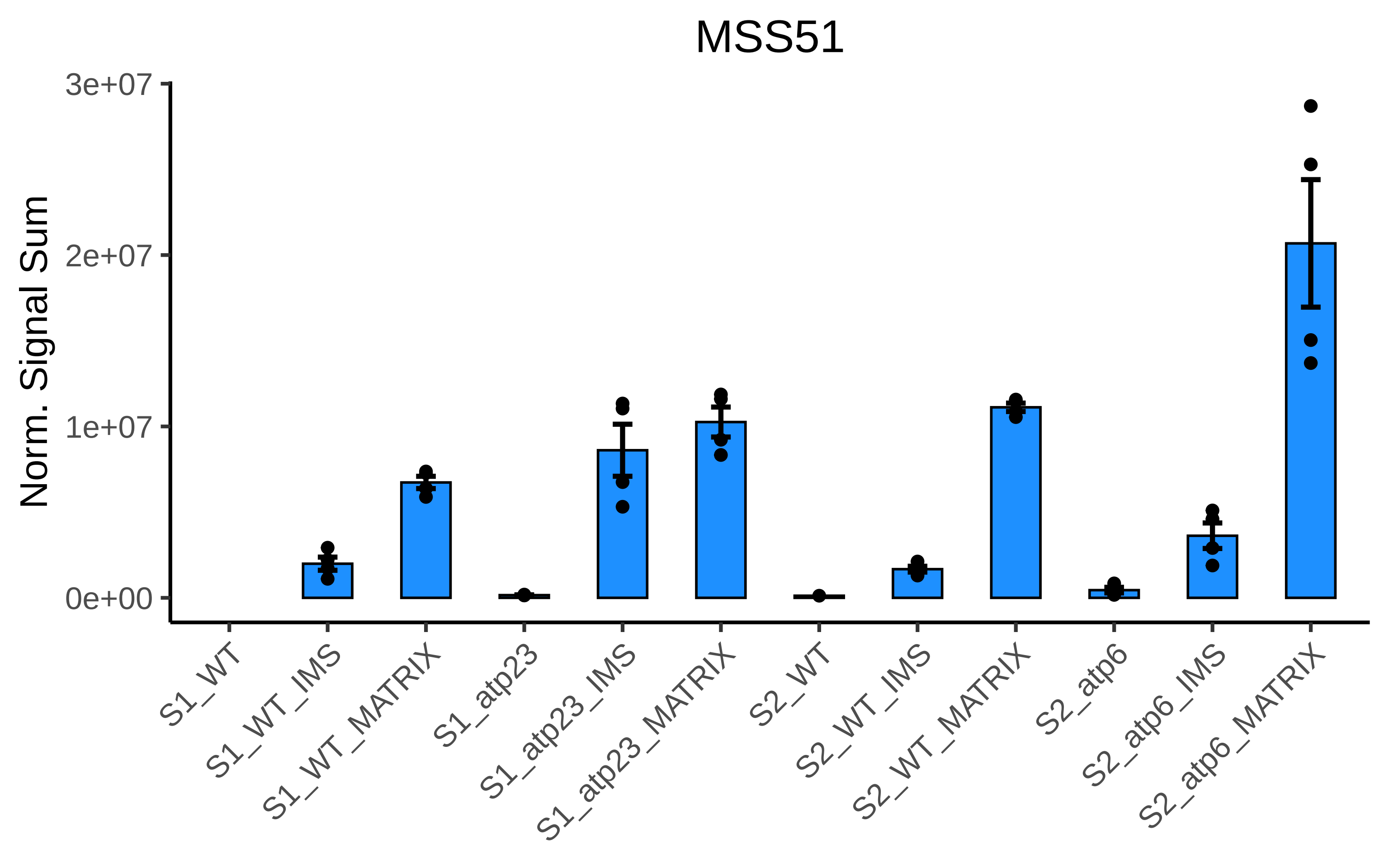
MSS51
Norm. Signal Sum
3e+07
2e+07
1e+07
0e+00
S1_WT
S1_WT_IMS
S1_WT_MATRIX
S1_atp23
S1_atp23_IMS
S1_atp23_MATRIX
S2_WT
S2_WT_IMS
S2_WT_MATRIX
S2_atp6
S2_atp6_IMS
S2_atp6_MATRIX

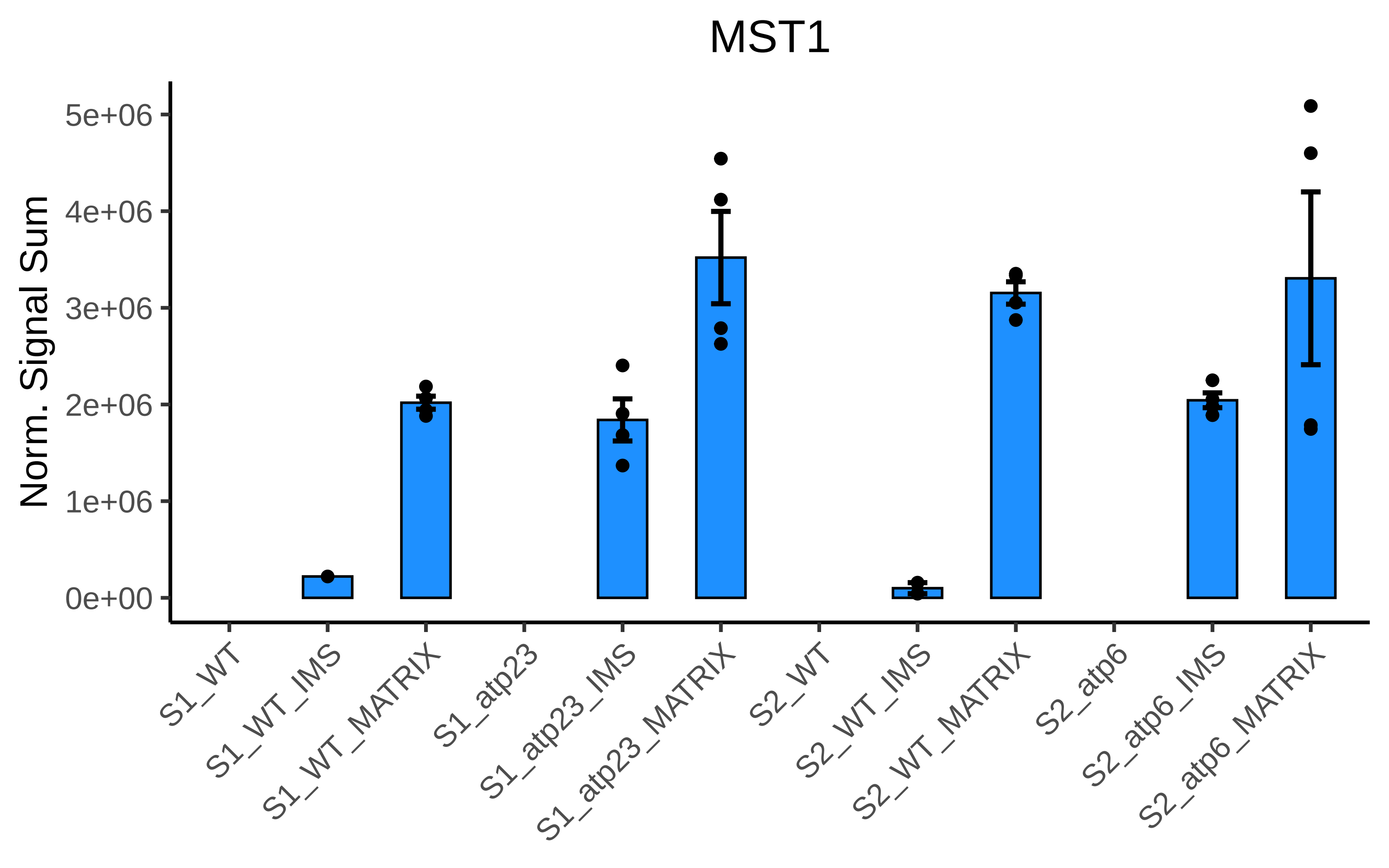

MST1
Norm. Signal Sum
5e+06
4e+06
3e+06
2e+06
1e+06
0e+00
S1_WT
S1_WT_IMS
S1_WT_MATRIX
S1_atp23
S1_atp23_IMS
S1_atp23_MATRIX
S2_WT
S2_WT_IMS
S2_WT_MATRIX
S2_atp6
S2_atp6_IMS
S2_atp6_MATRIX

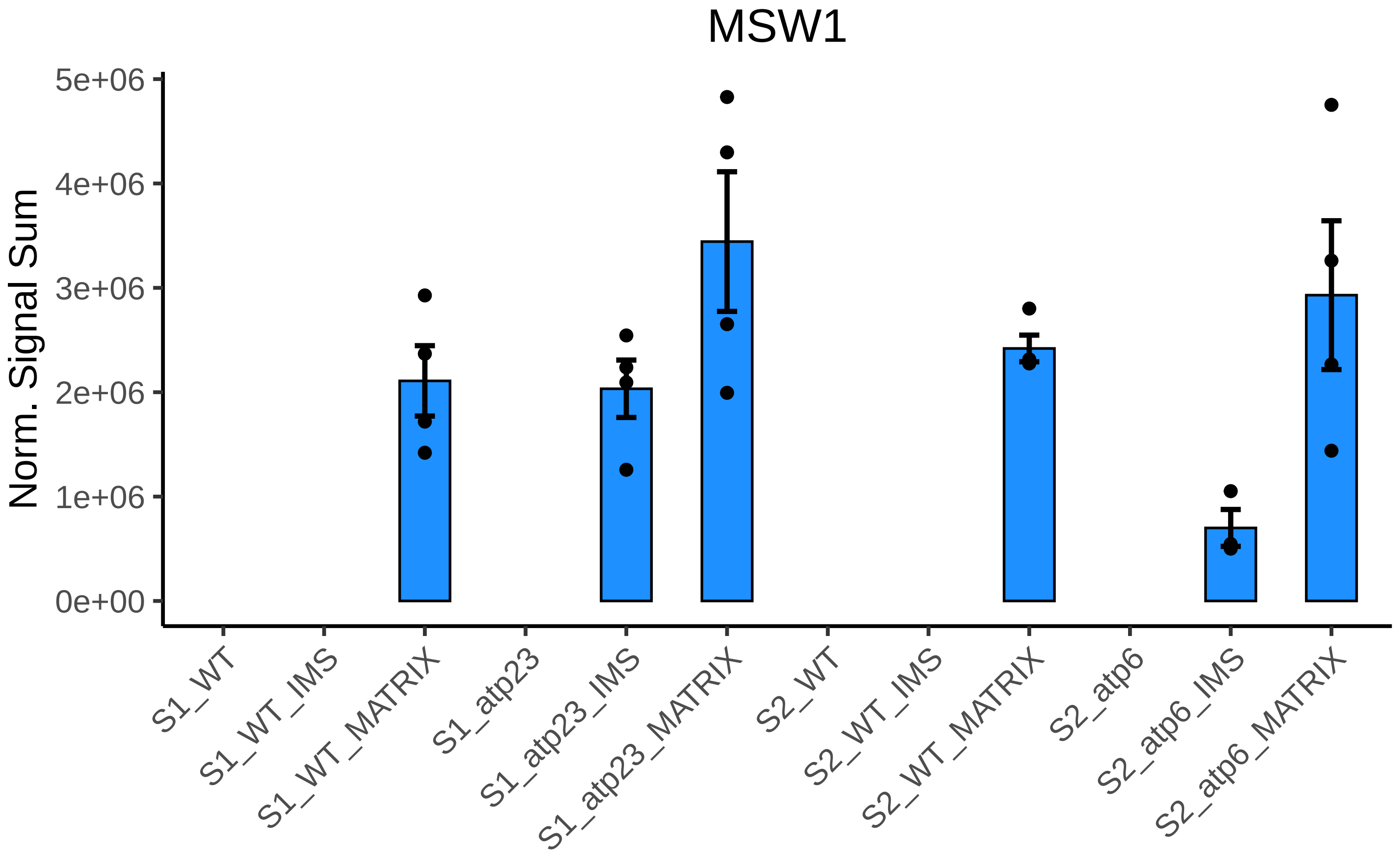

MSW1
Norm. Signal Sum
5e+06
4e+06
3e+06
2e+06
1e+06
0e+00
S1_WT
S1_WT_IMS
S1_WT_MATRIX
S1_atp23
S1_atp23_IMS
S1_atp23_MATRIX
S2_WT
S2_WT_IMS
S2_WT_MATRIX
S2_atp6
S2_atp6_IMS
S2_atp6_MATRIX

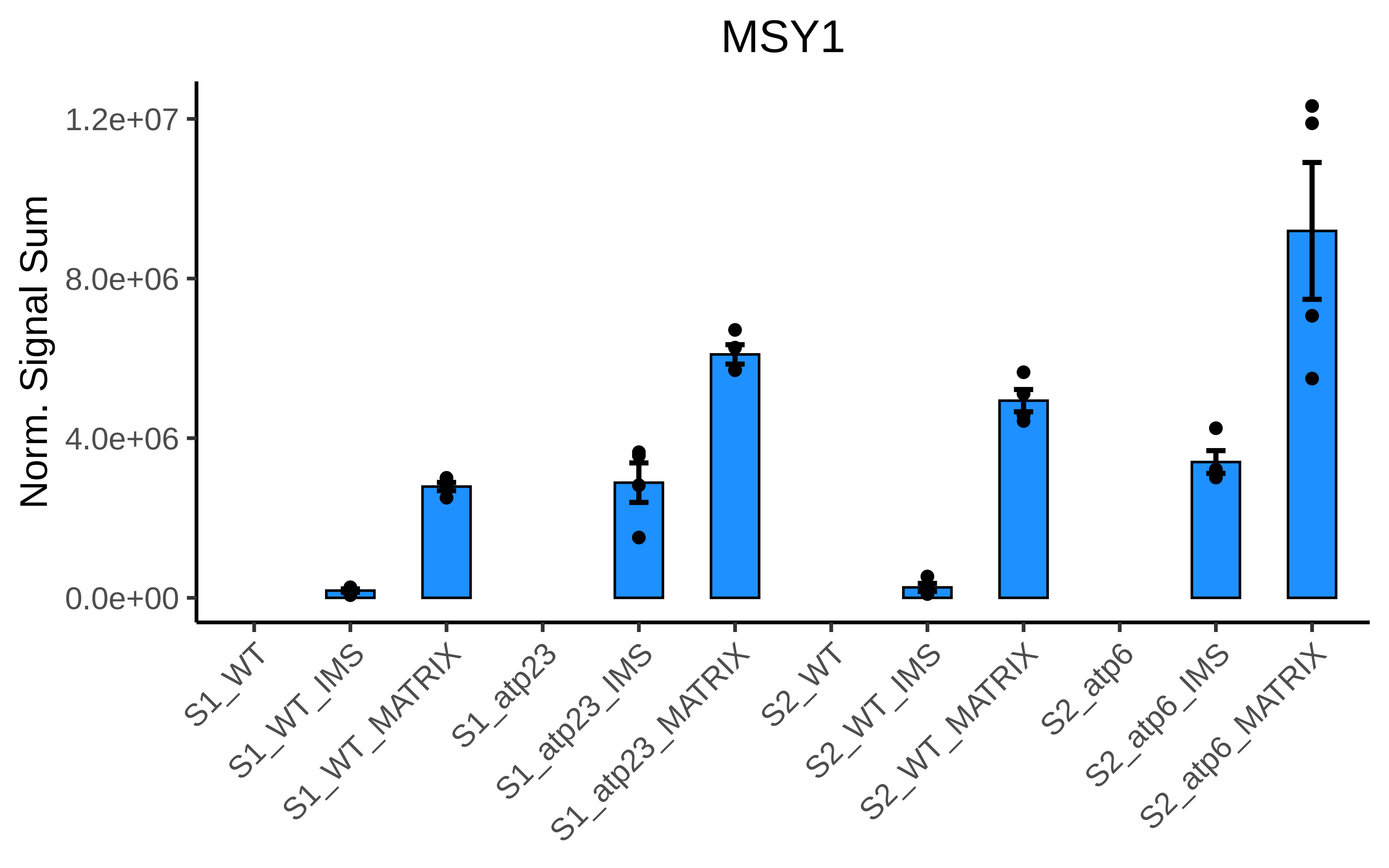
MSY1
Norm. Signal Sum
1.2e+07
8.0e+06
4.0e+06
0.0e+00
S1_WT
S1_WT_IMS
S1_WT_MATRIX
S1_atp23
S1_atp23_IMS
S1_atp23_MATRIX
S2_WT
S2_WT_IMS
S2_WT_MATRIX
S2_atp6
S2_atp6_IMS
S2_atp6_MATRIX

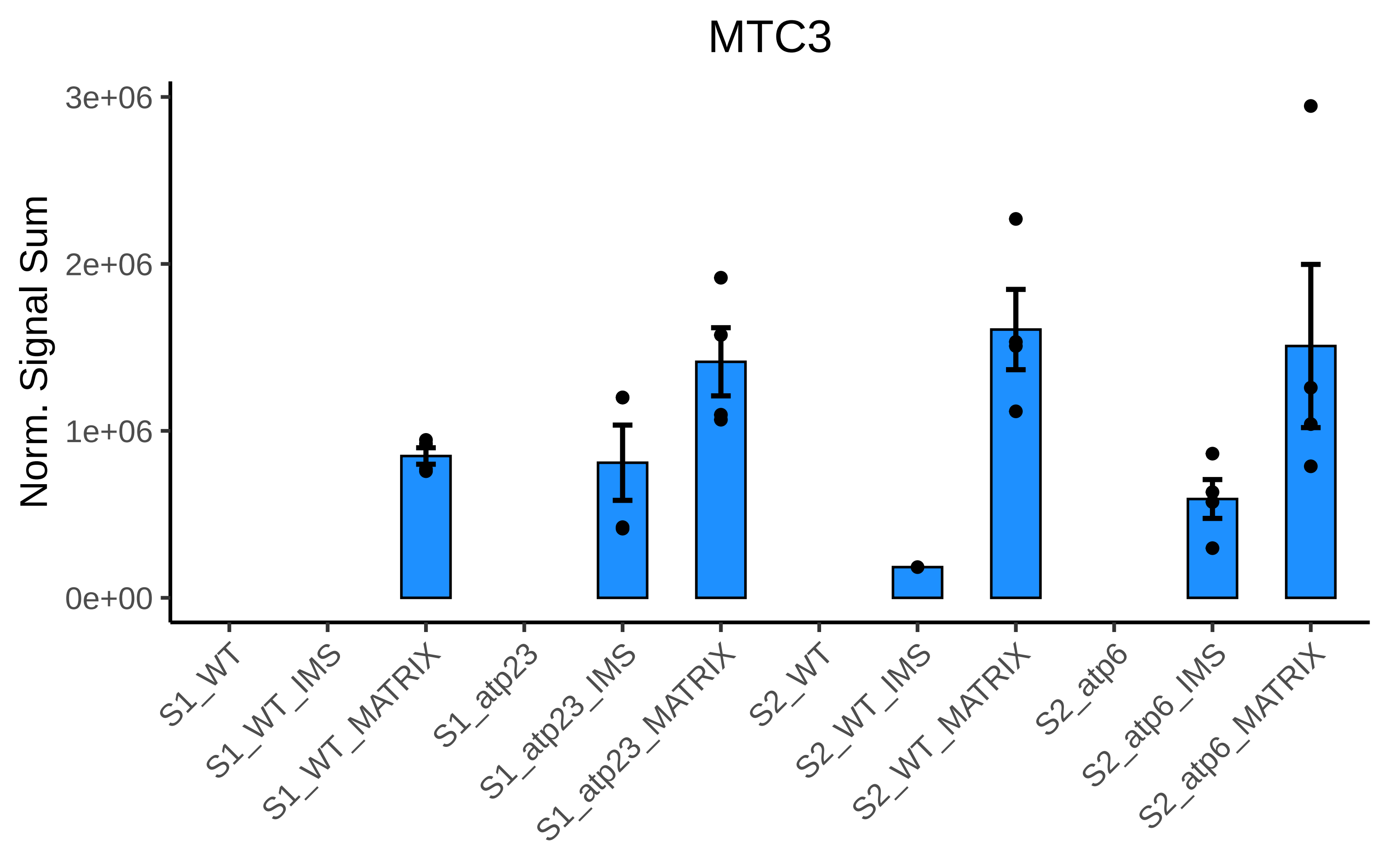
MTC3
Norm. Signal Sum
3e+06
2e+06
1e+06
0e+00
S1_WT
S1_WT_IMS
S1_WT_MATRIX
S1_atp23
S1_atp23_IMS
S1_atp23_MATRIX
S2_WT
S2_WT_IMS
S2_WT_MATRIX
S2_atp6
S2_atp6_IMS
S2_atp6_MATRIX

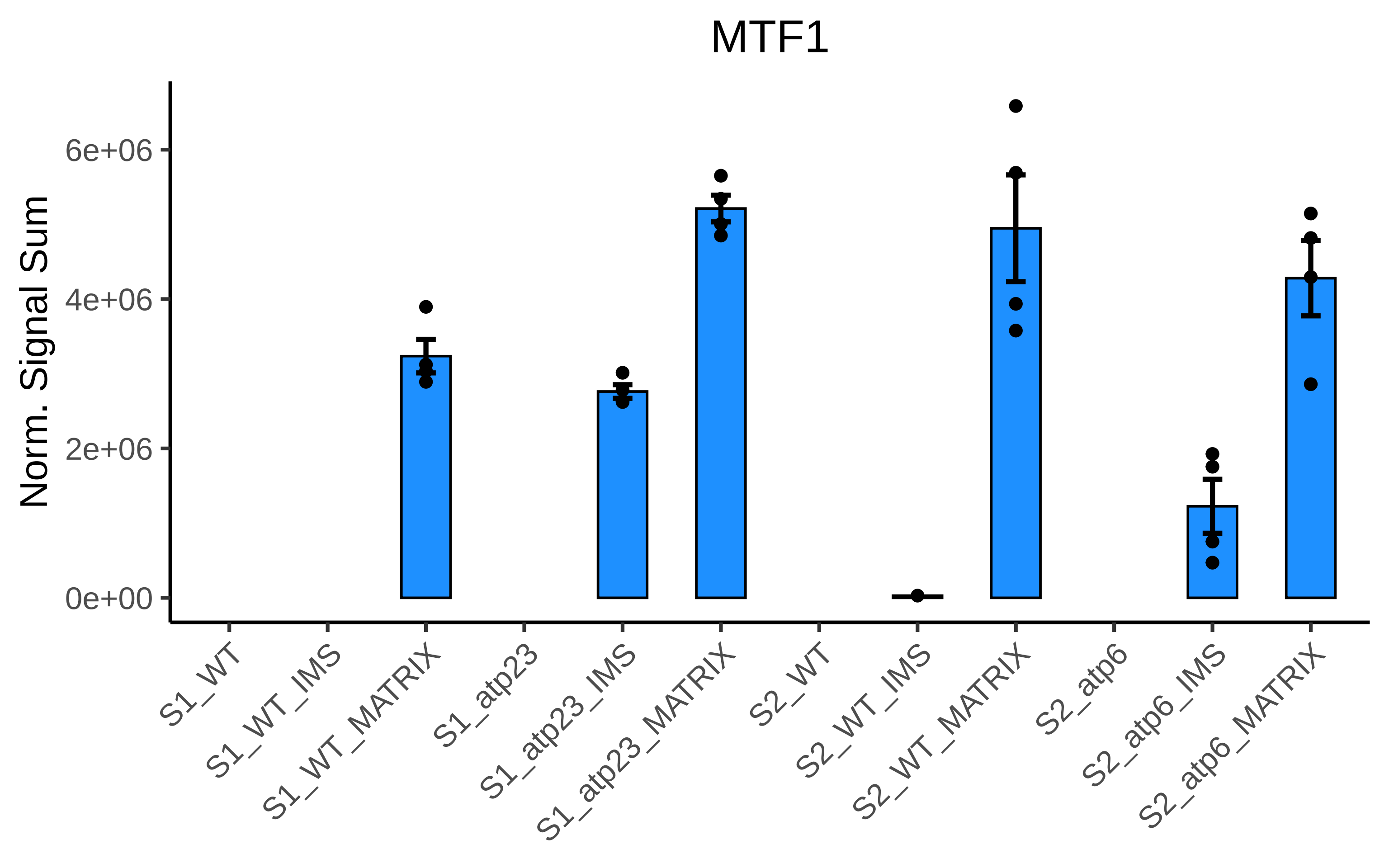
MTF1
Norm. Signal Sum
6e+06
4e+06
2e+06
0e+00
S1_WT
S1_WT_IMS
S1_WT_MATRIX
S1_atp23
S1_atp23_IMS
S1_atp23_MATRIX
S2_WT
S2_WT_IMS
S2_WT_MATRIX
S2_atp6
S2_atp6_IMS
S2_atp6_MATRIX

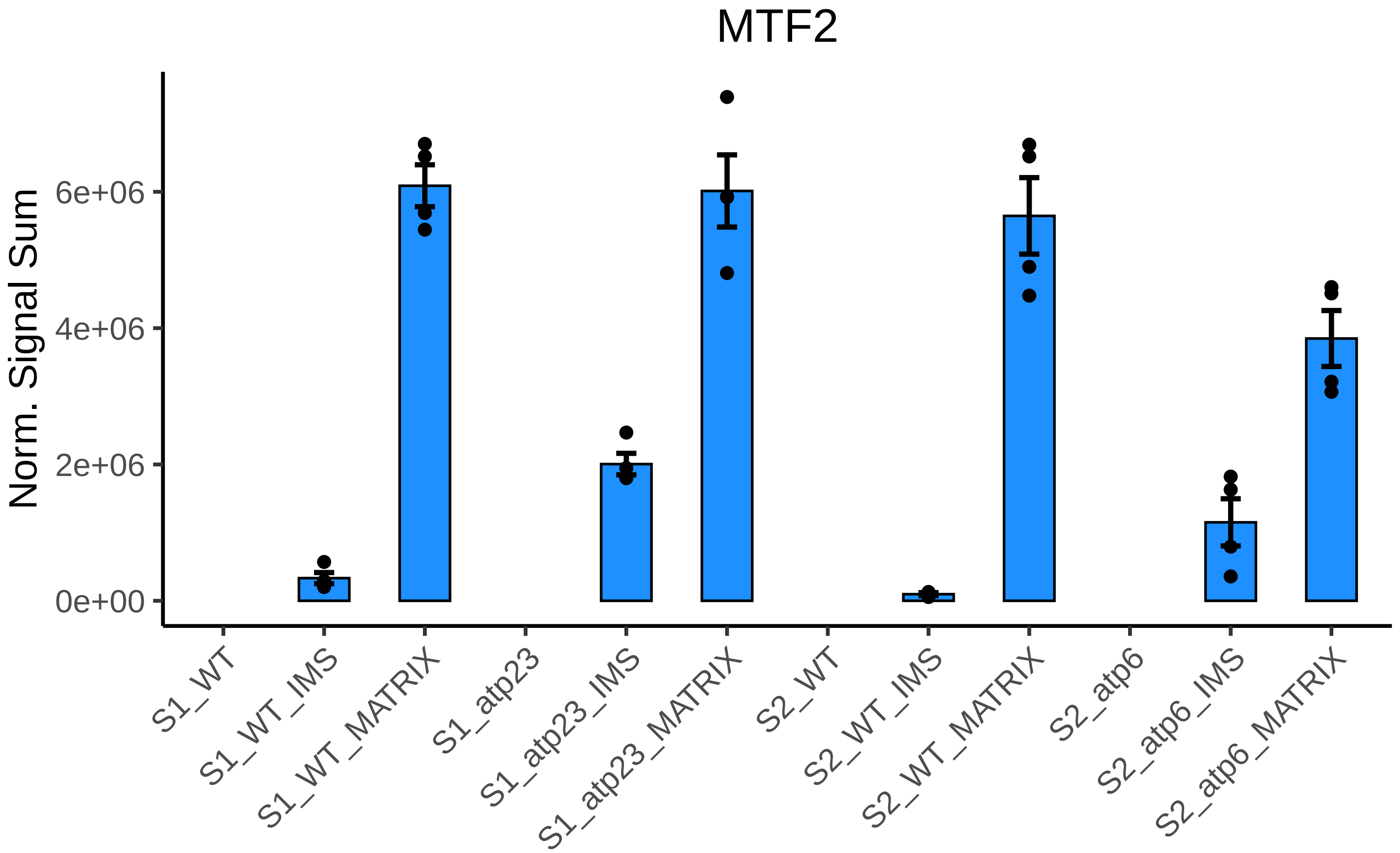

MTF2
Norm. Signal Sum
6e+06
4e+06
2e+06
0e+00
S1_WT
S1_WT_IMS
S1_WT_MATRIX
S1_atp23
S1_atp23_IMS
S1_atp23_MATRIX
S2_WT
S2_WT_IMS
S2_WT_MATRIX
S2_atp6
S2_atp6_IMS
S2_atp6_MATRIX

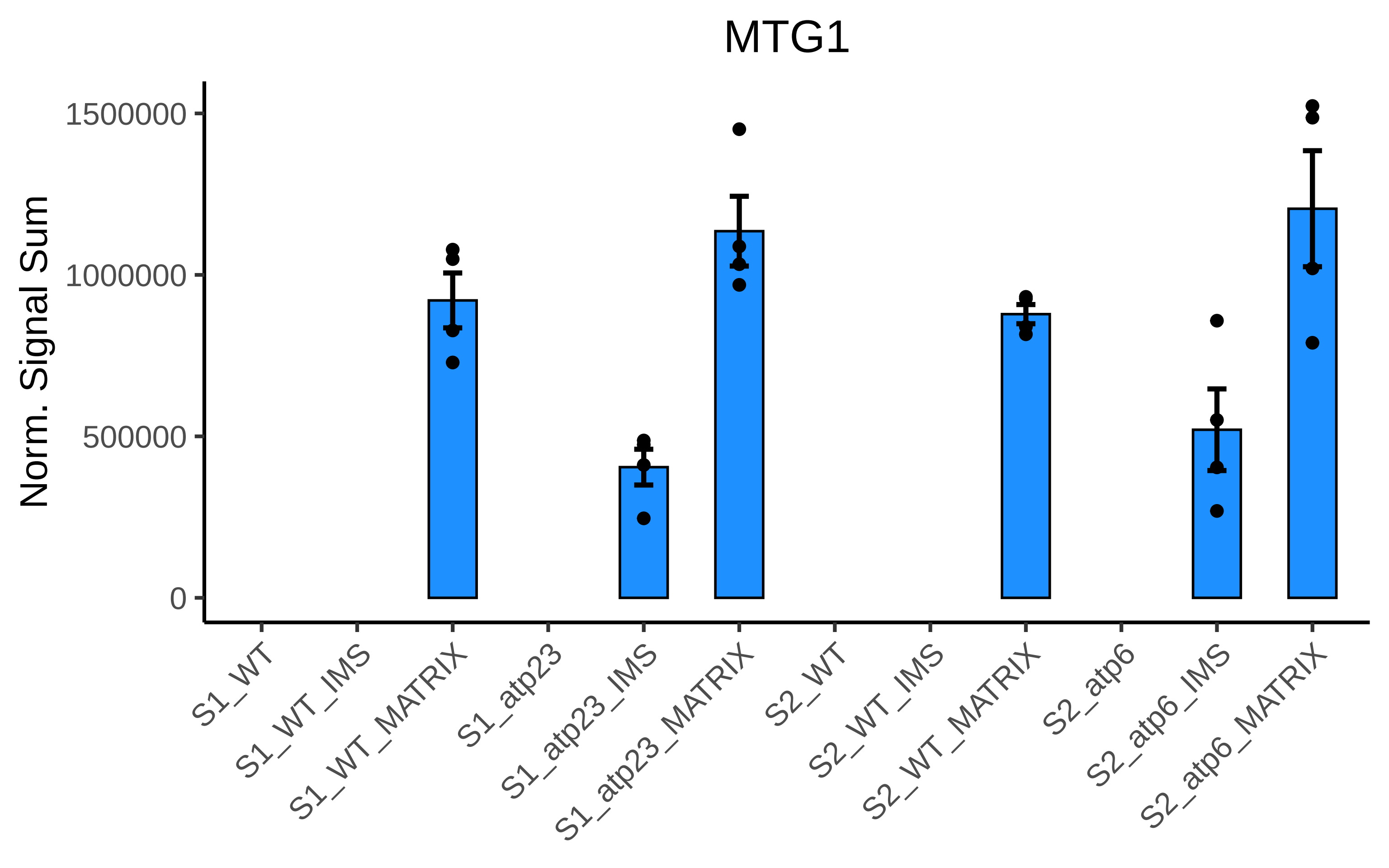
MTG1
Norm. Signal Sum
1500000
1000000
500000
0
S1_WT
S1_WT_IMS
S1_WT_MATRIX
S1_atp23
S1_atp23_IMS
S1_atp23_MATRIX
S2_WT
S2_WT_IMS
S2_WT_MATRIX
S2_atp6
S2_atp6_IMS
S2_atp6_MATRIX

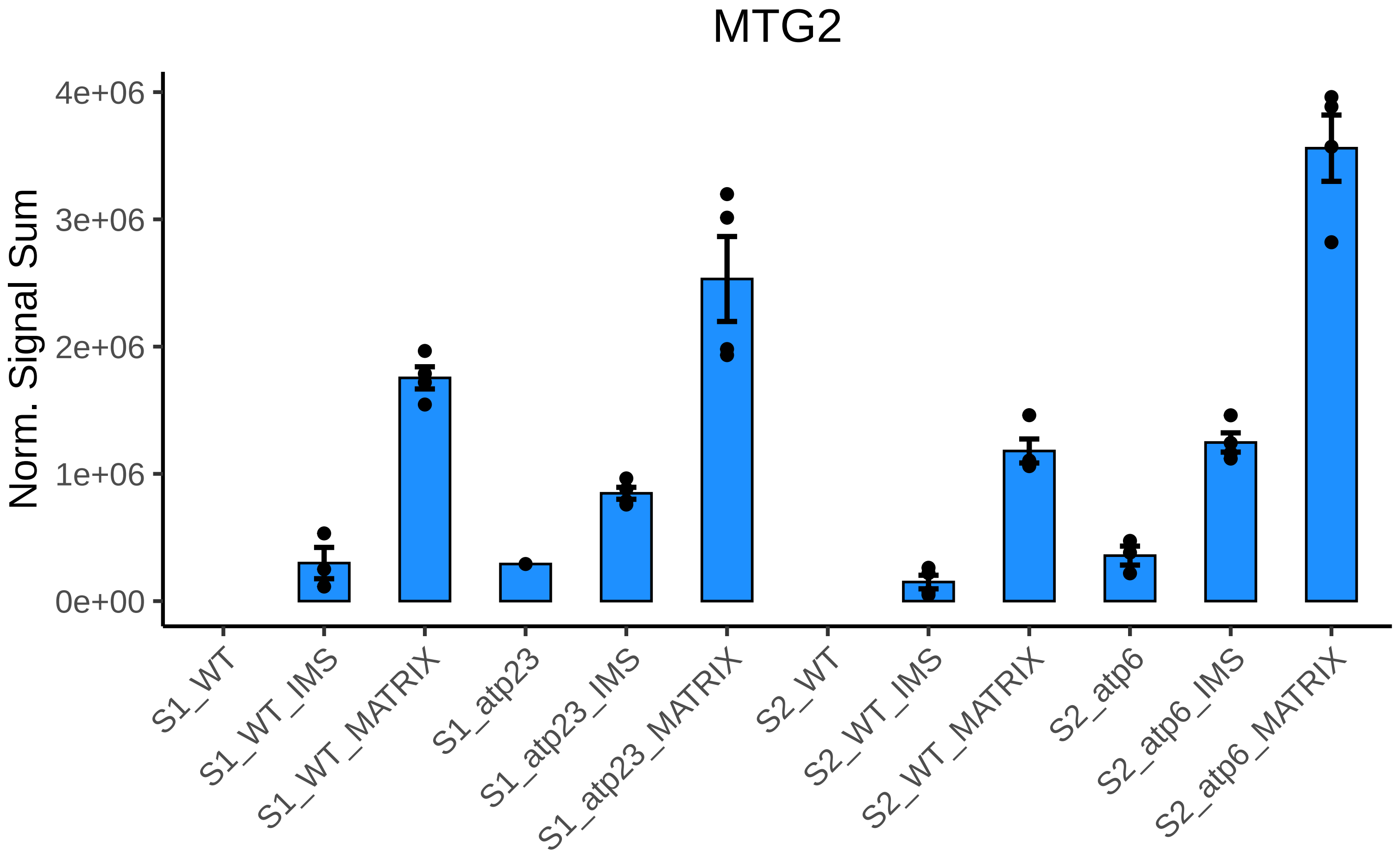

MTG2
Norm. Signal Sum
4e+06
3e+06
2e+06
1e+06
0e+00
S1_WT
S1_WT_IMS
S1_WT_MATRIX
S1_atp23
S1_atp23_IMS
S1_atp23_MATRIX
S2_WT
S2_WT_IMS
S2_WT_MATRIX
S2_atp6
S2_atp6_IMS
S2_atp6_MATRIX

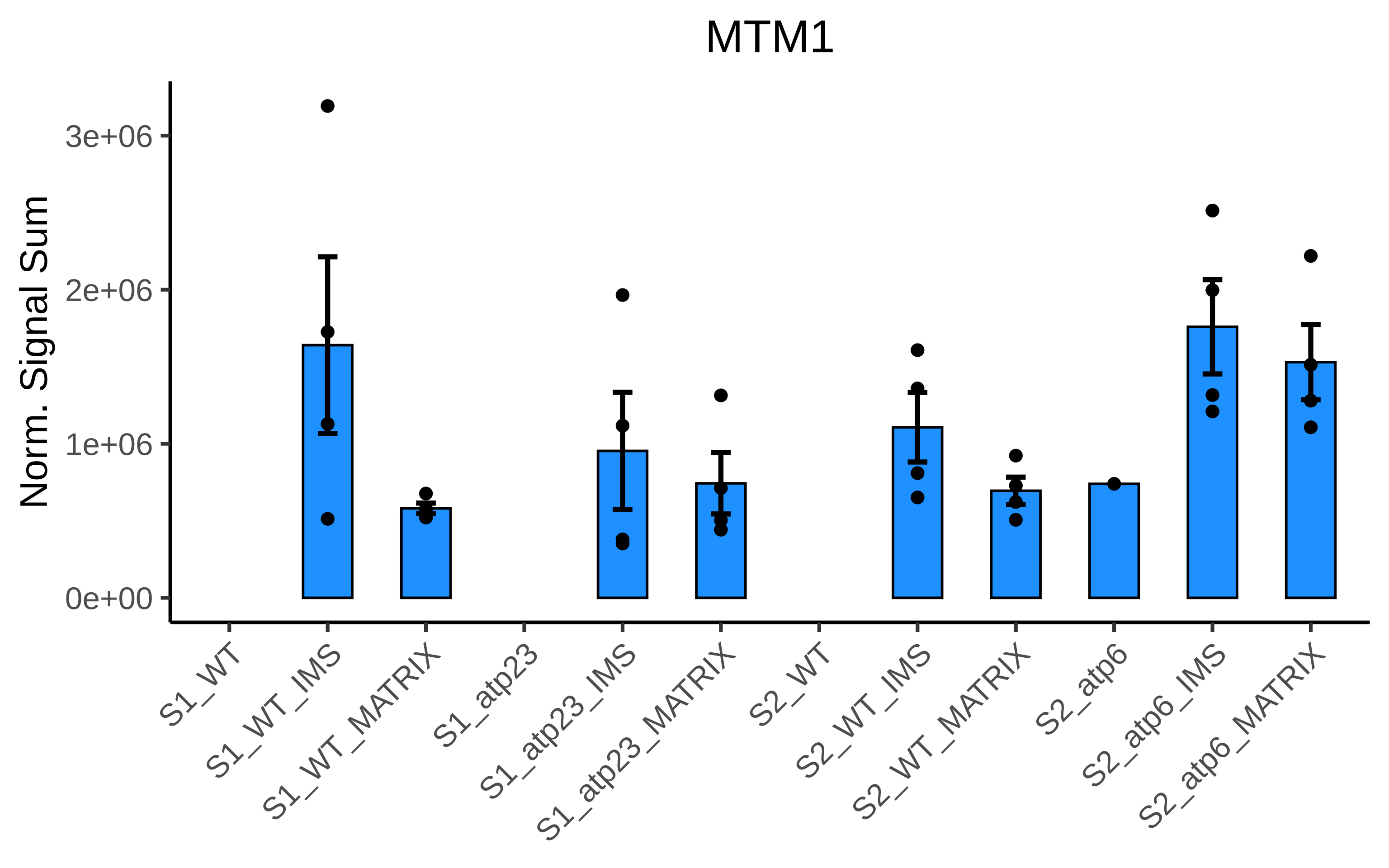
MTM1
Norm. Signal Sum
3e+06
2e+06
1e+06
0e+00
S1_WT
S1_WT_IMS
S1_WT_MATRIX
S1_atp23
S1_atp23_IMS
S1_atp23_MATRIX
S2_WT
S2_WT_IMS
S2_WT_MATRIX
S2_atp6
S2_atp6_IMS
S2_atp6_MATRIX

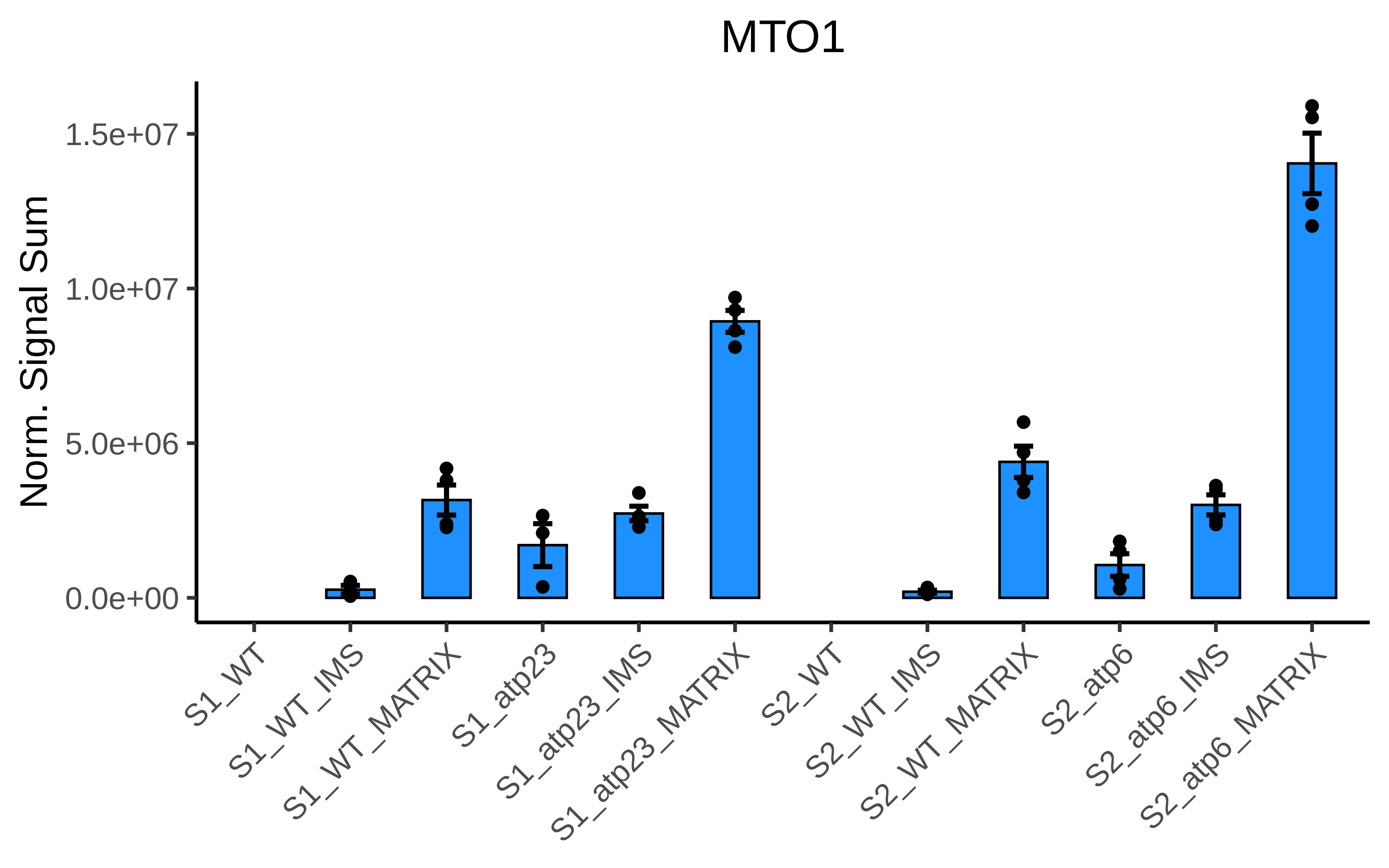
MTO1
Norm. Signal Sum
1.5e+07
1.0e+07
5.0e+06
0.0e+00
S1_WT
S1_WT_IMS
S1_WT_MATRIX
S1_atp23
S1_atp23_IMS
S1_atp23_MATRIX
S2_WT
S2_WT_IMS
S2_WT_MATRIX
S2_atp6
S2_atp6_IMS
S2_atp6_MATRIX

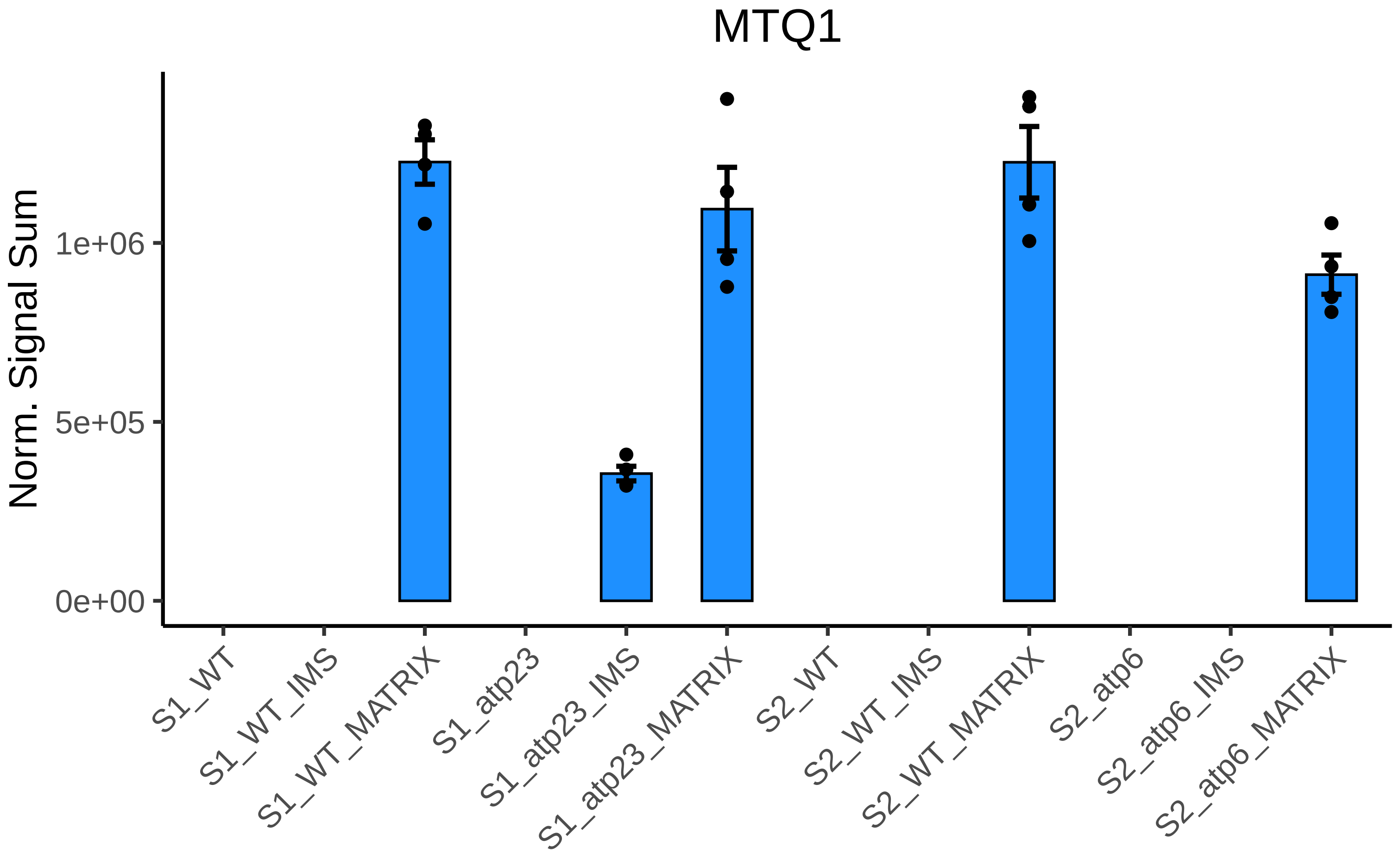
MTQ1
Norm. Signal Sum
1e+06
5e+05
0e+00
S1_WT
S1_WT_IMS
S1_WT_MATRIX
S1_atp23
S1_atp23_IMS
S1_atp23_MATRIX
S2_WT
S2_WT_IMS
S2_WT_MATRIX
S2_atp6
S2_atp6_IMS
S2_atp6_MATRIX

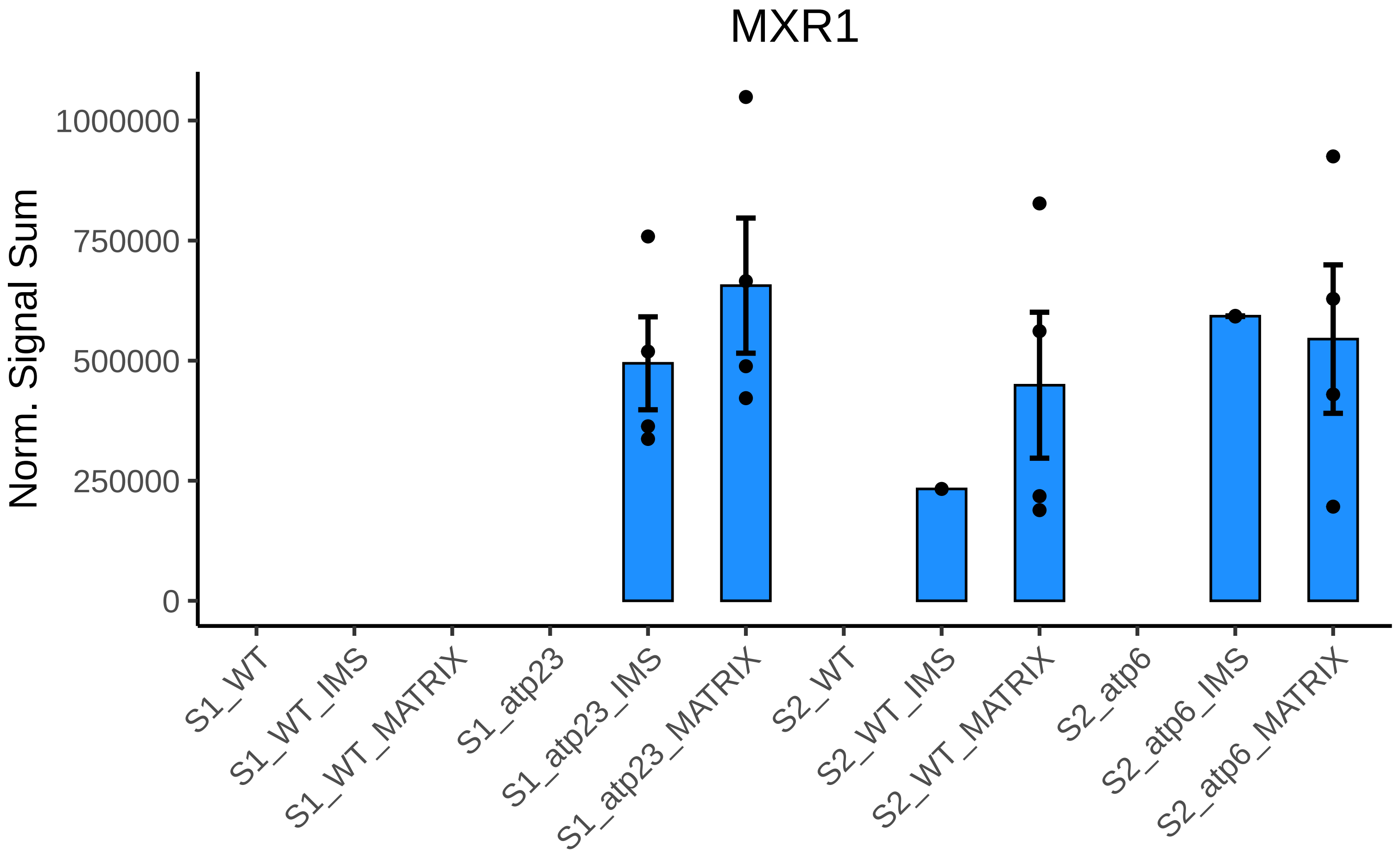
MXR1
Norm. Signal Sum
1000000
750000
500000
250000
0
S1_WT
S1_WT_IMS
S1_WT_MATRIX
S1_atp23
S1_atp23_IMS
S1_atp23_MATRIX
S2_WT
S2_WT_IMS
S2_WT_MATRIX
S2_atp6
S2_atp6_IMS
S2_atp6_MATRIX

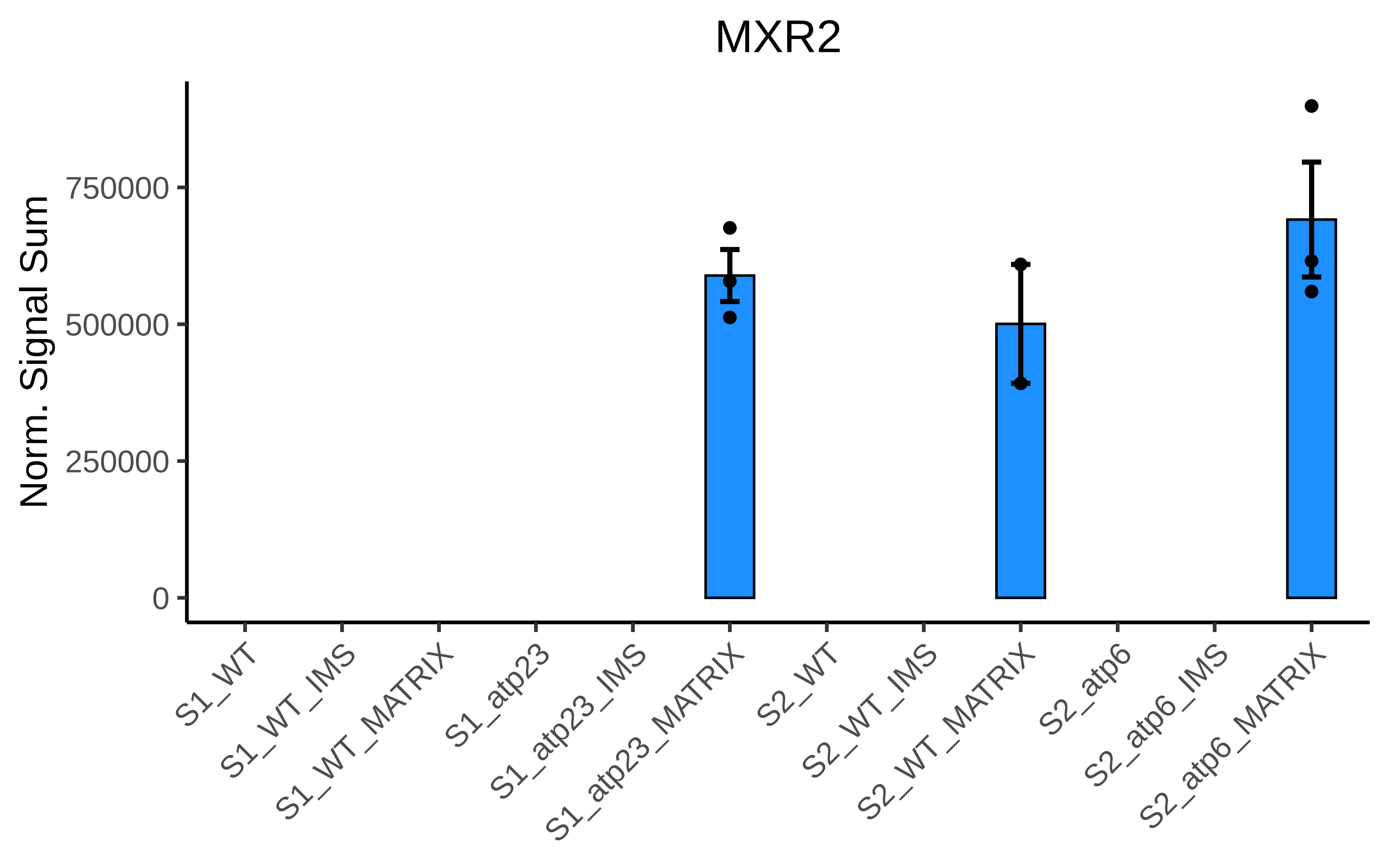

MXR2
Norm. Signal Sum
750000
500000
250000
0
S1_WT
S1_WT_IMS
S1_WT_MATRIX
S1_atp23
S1_atp23_IMS
S1_atp23_MATRIX
S2_WT
S2_WT_IMS
S2_WT_MATRIX
S2_atp6
S2_atp6_IMS
S2_atp6_MATRIX

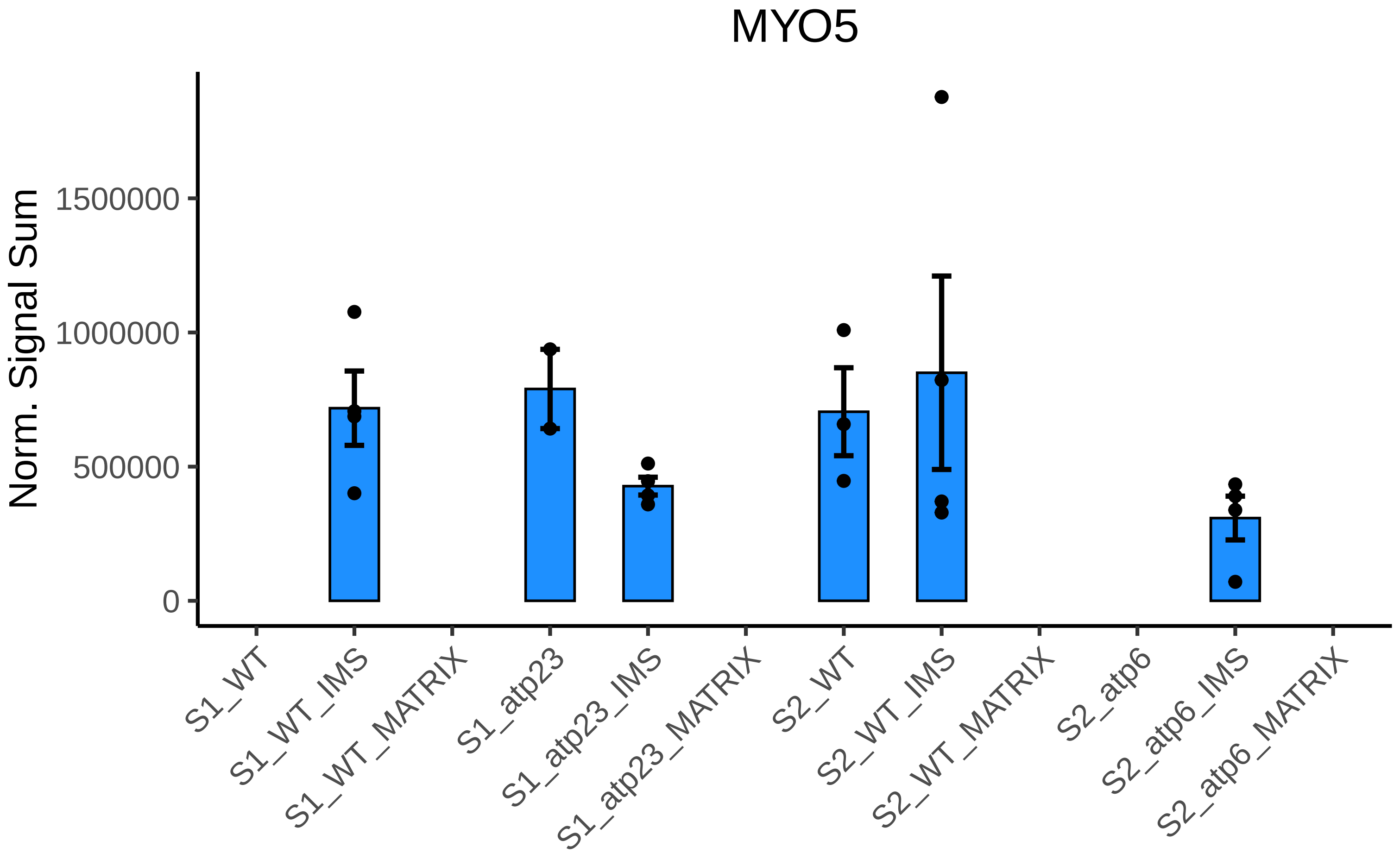

MYO5
Norm. Signal Sum
1500000
1000000
500000
0
S1_WT
S1_WT_IMS
S1_WT_MATRIX
S1_atp23
S1_atp23_IMS
S1_atp23_MATRIX
S2_WT
S2_WT_IMS
S2_WT_MATRIX
S2_atp6
S2_atp6_IMS
S2_atp6_MATRIX

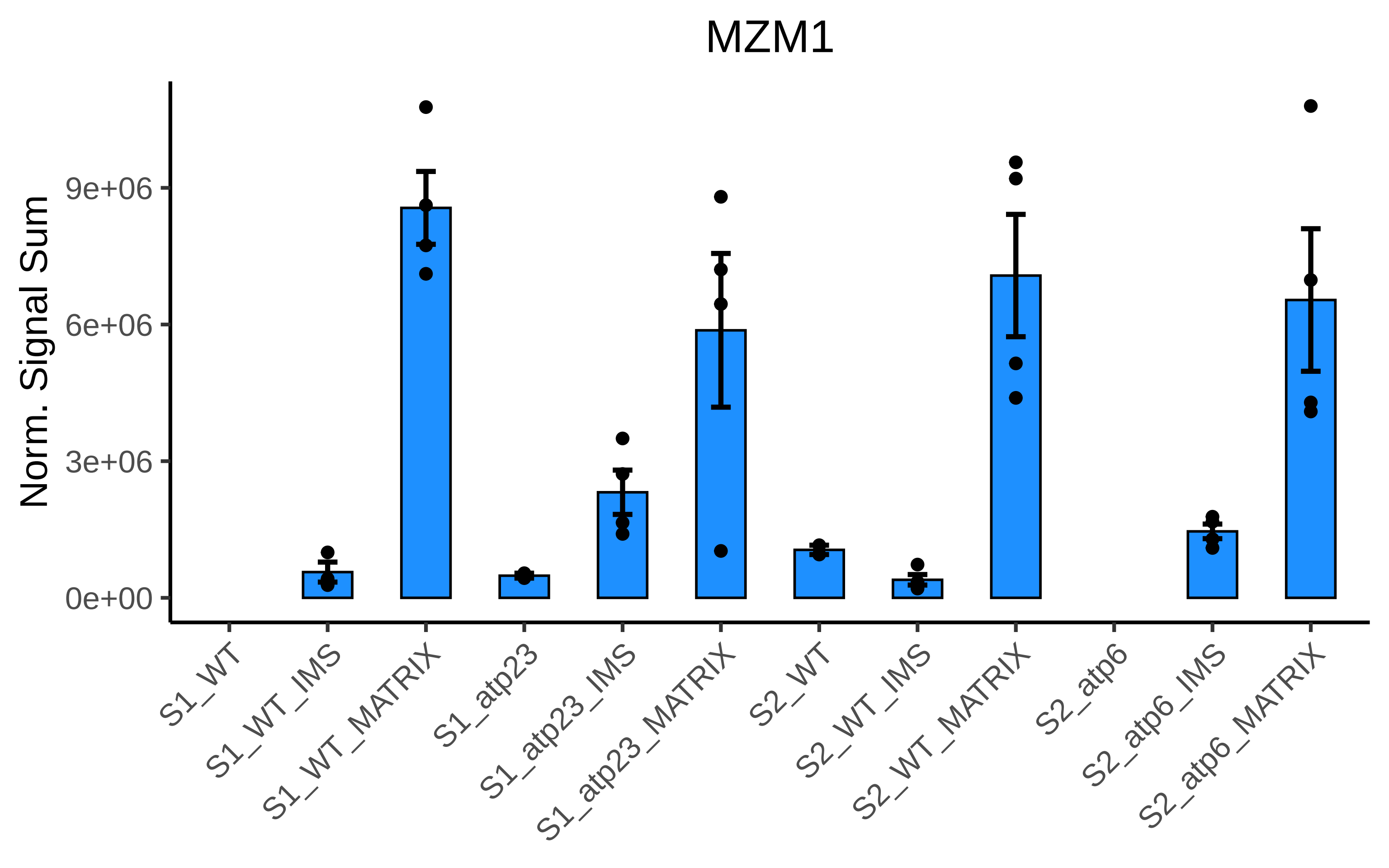

MZM1
Norm. Signal Sum
9e+06
6e+06
3e+06
0e+00
S1_WT
S1_WT_IMS
S1_WT_MATRIX
S1_atp23
S1_atp23_IMS
S1_atp23_MATRIX
S2_WT
S2_WT_IMS
S2_WT_MATRIX
S2_atp6
S2_atp6_IMS
S2_atp6_MATRIX

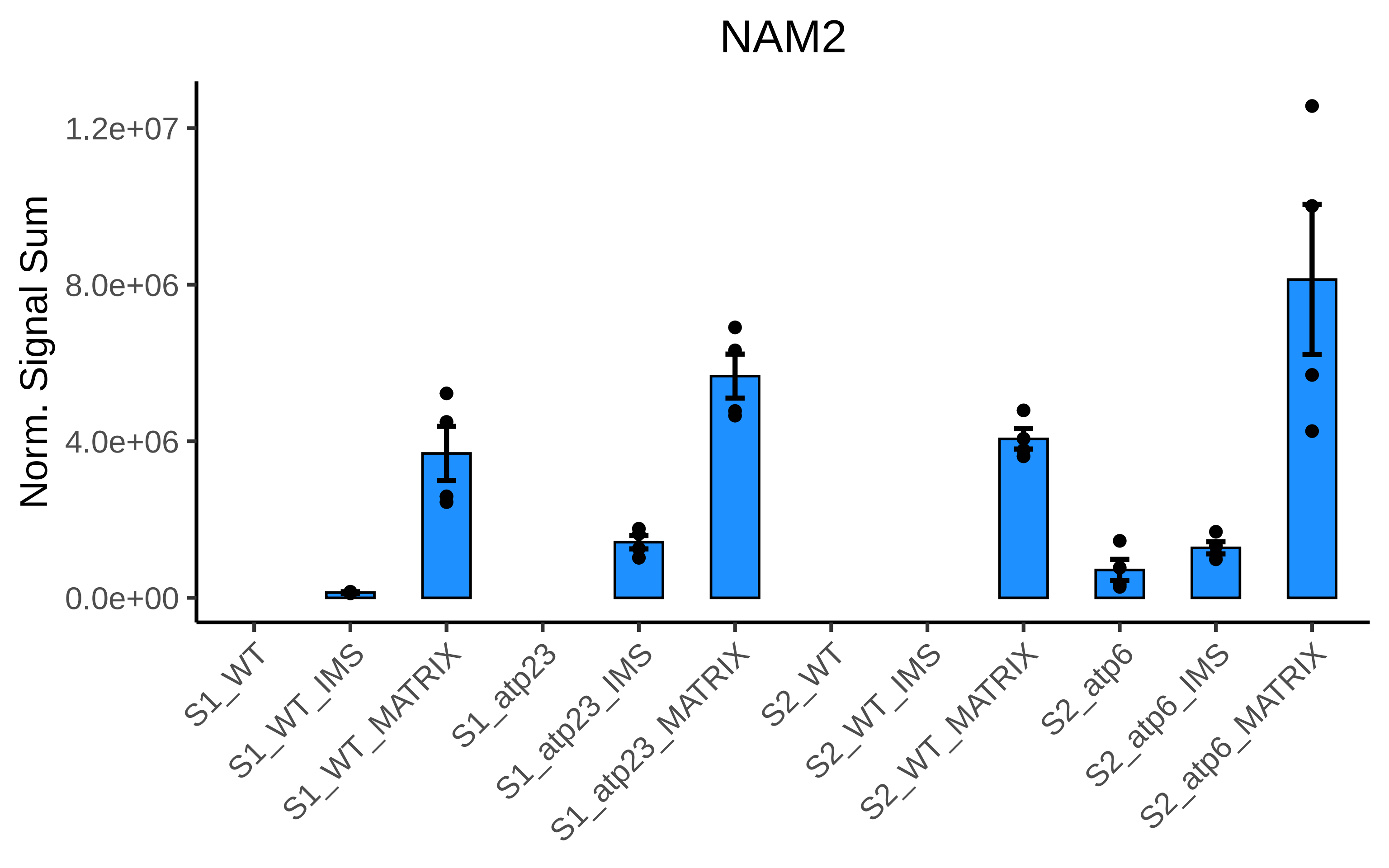
NAM2
Norm. Signal Sum
1.2e+07
8.0e+06
4.0e+06
0.0e+00
S1_WT
S1_WT_IMS
S1_WT_MATRIX
S1_atp23
S1_atp23_IMS
S1_atp23_MATRIX
S2_WT
S2_WT_IMS
S2_WT_MATRIX
S2_atp6
S2_atp6_IMS
S2_atp6_MATRIX

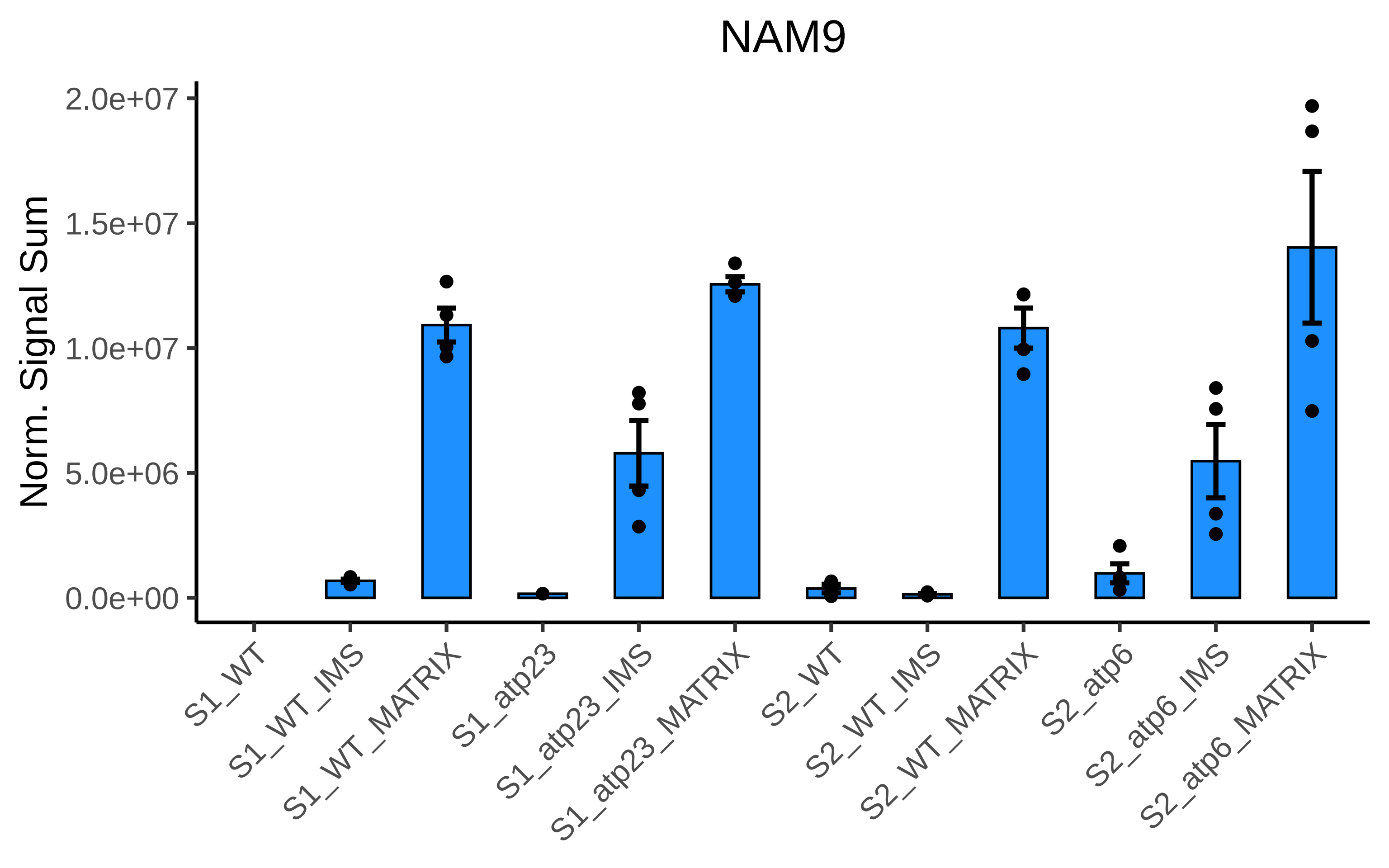

NAM9
Norm. Signal Sum
2.0e+07
1.5e+07
1.0e+07
5.0e+06
0.0e+00
S1_WT
S1_WT_IMS
S1_WT_MATRIX
S1_atp23
S1_atp23_IMS
S1_atp23_MATRIX
S2_WT
S2_WT_IMS
S2_WT_MATRIX
S2_atp6
S2_atp6_IMS
S2_atp6_MATRIX

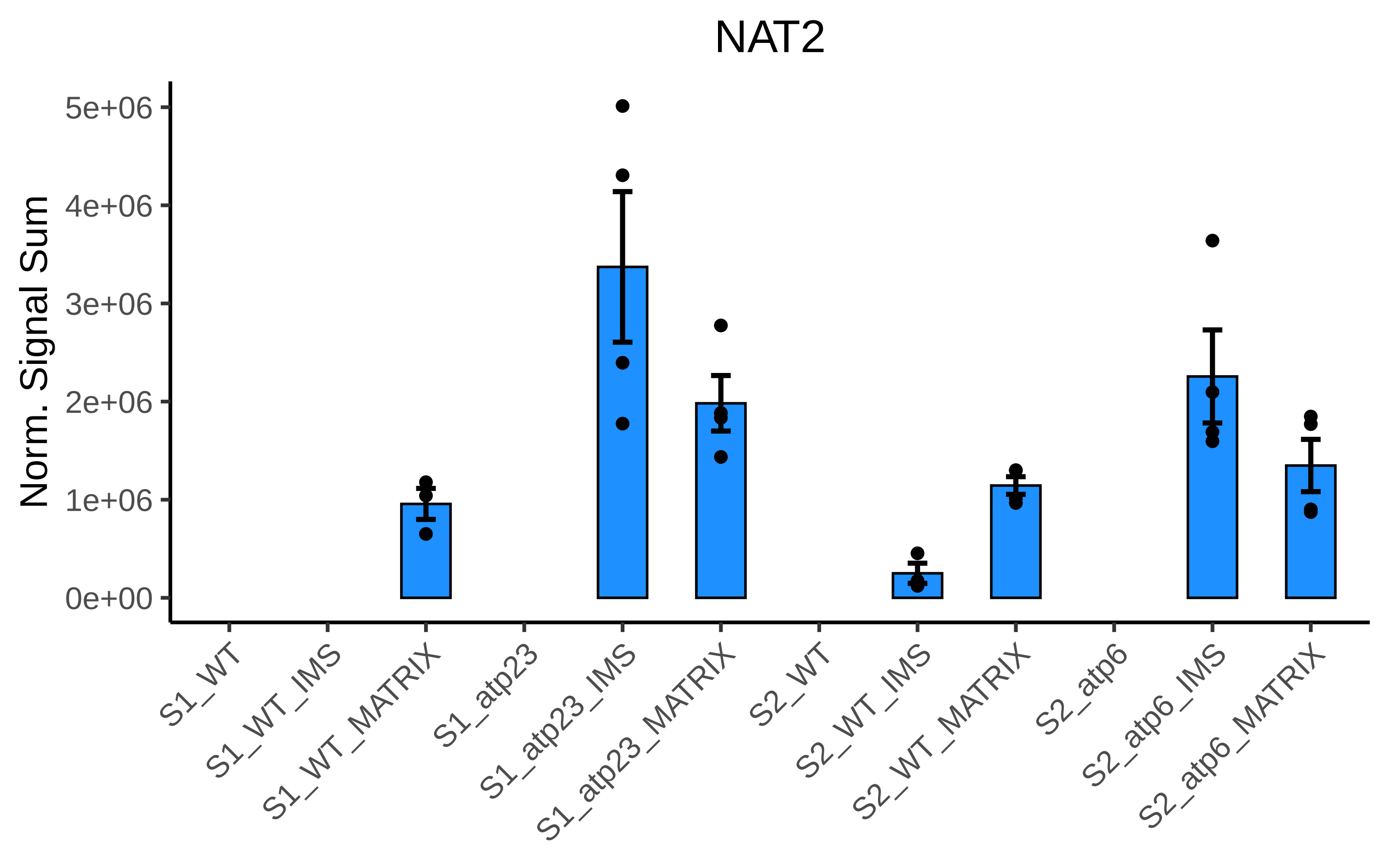
NAT2
Norm. Signal Sum
5e+06
4e+06
3e+06
2e+06
1e+06
0e+00
S1_WT
S1_WT_IMS
S1_WT_MATRIX
S1_atp23
S1_atp23_IMS
S1_atp23_MATRIX
S2_WT
S2_WT_IMS
S2_WT_MATRIX
S2_atp6
S2_atp6_IMS
S2_atp6_MATRIX

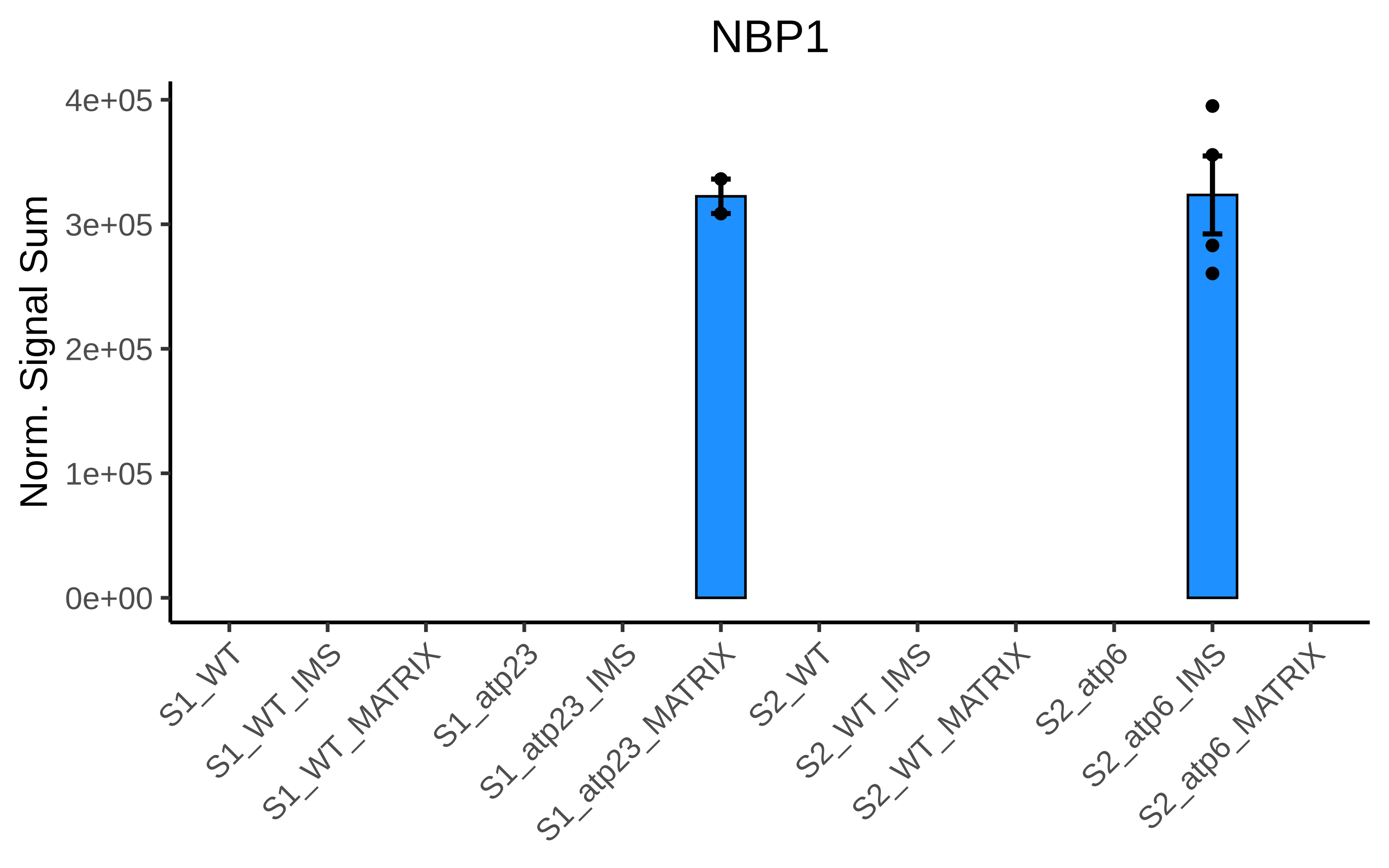

NBP1
Norm. Signal Sum
4e+05
3e+05
2e+05
1e+05
0e+00
S1_WT
S1_WT_IMS
S1_WT_MATRIX
S1_atp23
S1_atp23_IMS
S1_atp23_MATRIX
S2_WT
S2_WT_IMS
S2_WT_MATRIX
S2_atp6
S2_atp6_IMS
S2_atp6_MATRIX

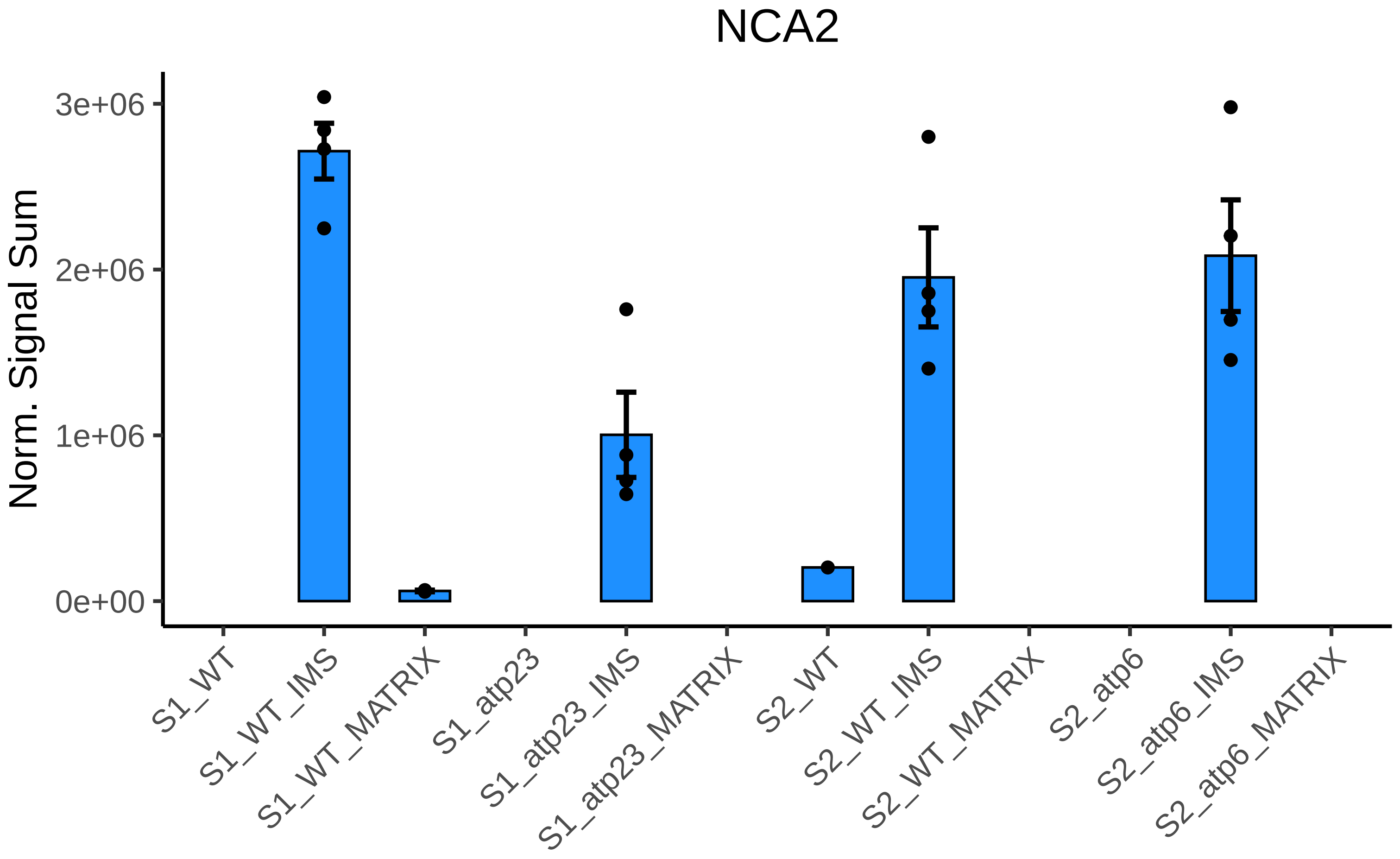

NCA2
Norm. Signal Sum
3e+06
2e+06
1e+06
0e+00
S1_WT
S1_WT_IMS
S1_WT_MATRIX
S1_atp23
S1_atp23_IMS
S1_atp23_MATRIX
S2_WT
S2_WT_IMS
S2_WT_MATRIX
S2_atp6
S2_atp6_IMS
S2_atp6_MATRIX

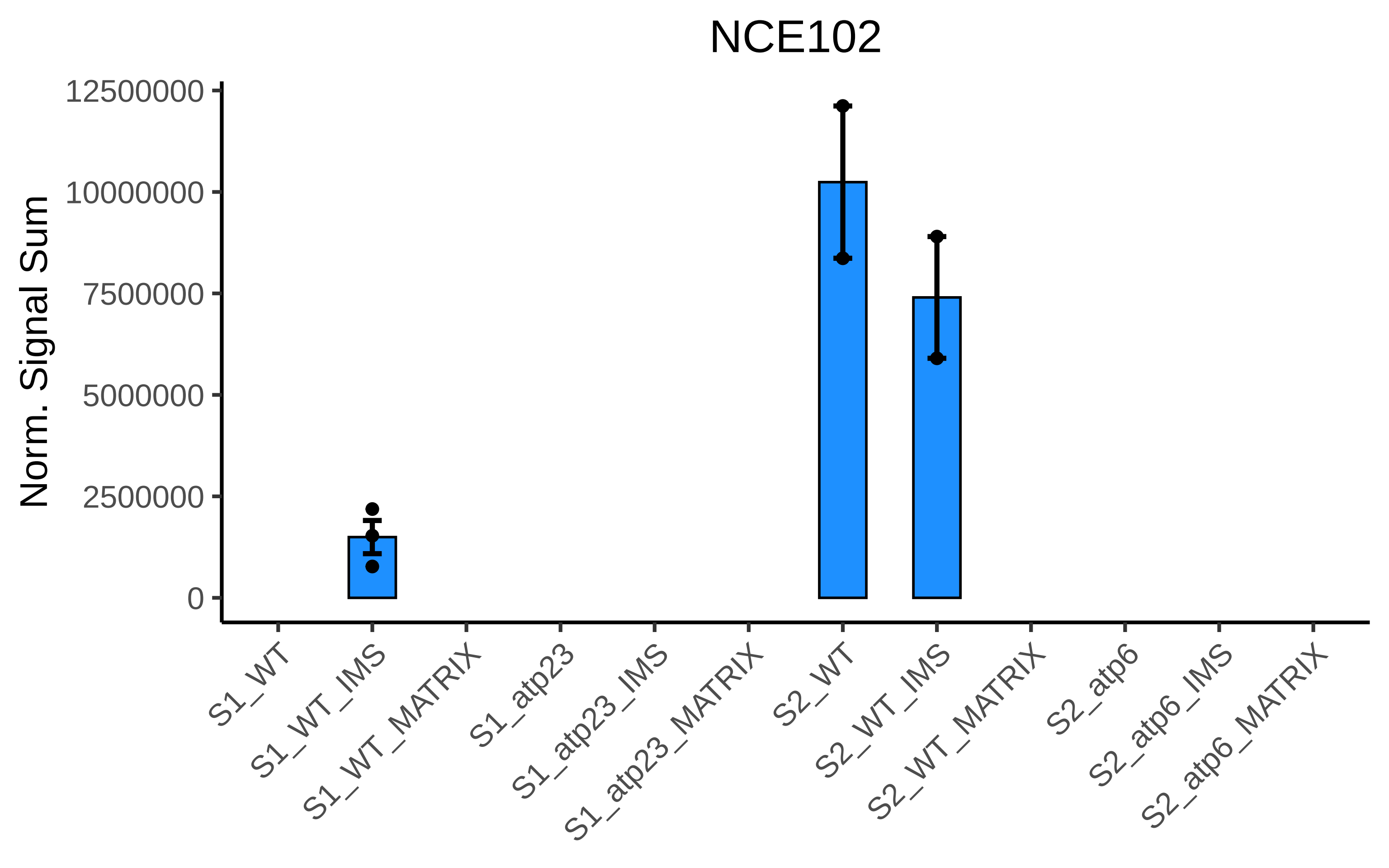

NCE102
Norm. Signal Sum
12500000
10000000
7500000
5000000
2500000
0
S1_WT
S1_WT_IMS
S1_WT_MATRIX
S1_atp23
S1_atp23_IMS
S1_atp23_MATRIX
S2_WT
S2_WT_IMS
S2_WT_MATRIX
S2_atp6
S2_atp6_IMS
S2_atp6_MATRIX

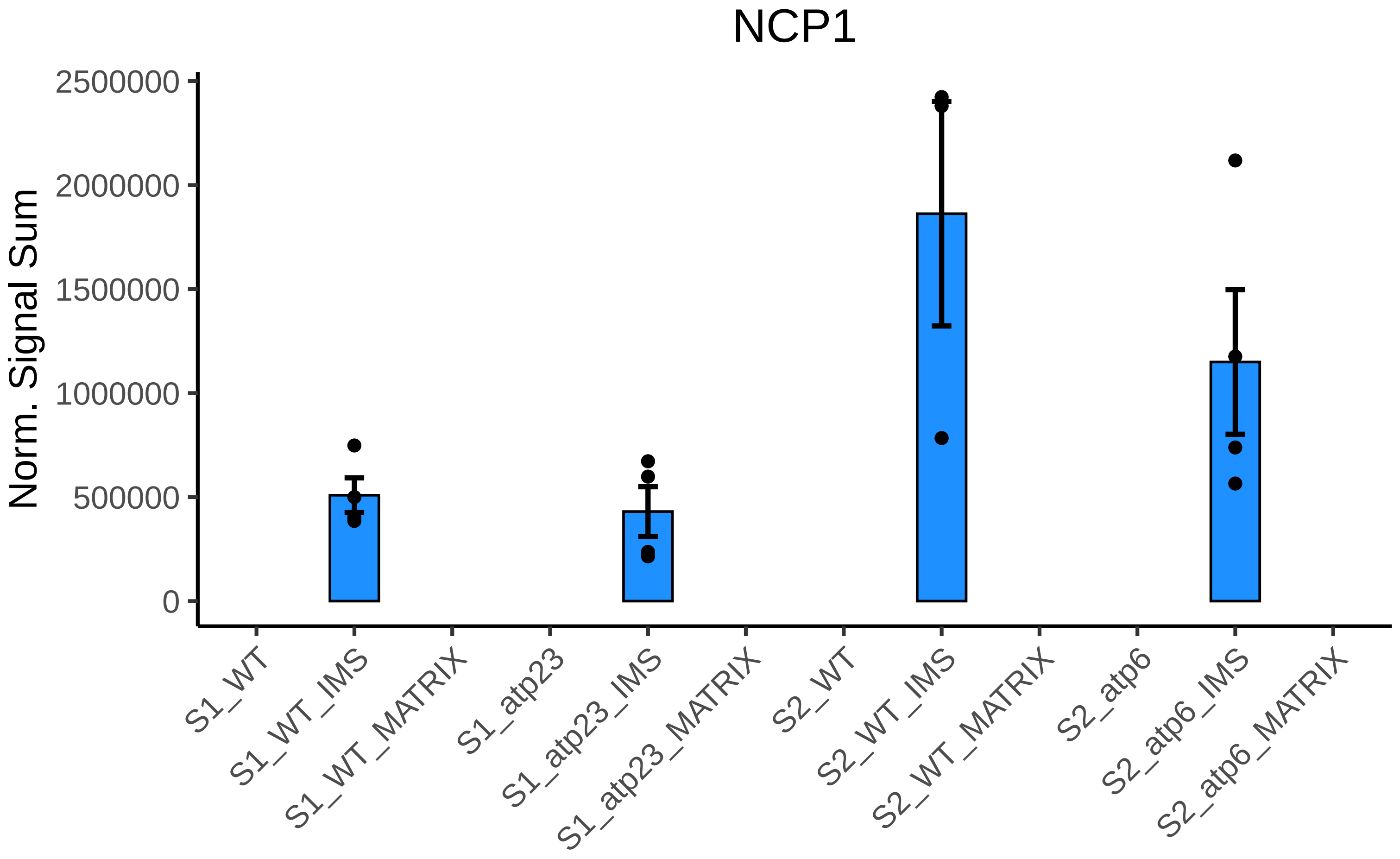
NCP1
Norm. Signal Sum
2500000
2000000
1500000
1000000
500000
0
S1_WT
S1_WT_IMS
S1_WT_MATRIX
S1_atp23
S1_atp23_IMS
S1_atp23_MATRIX
S2_WT
S2_WT_IMS
S2_WT_MATRIX
S2_atp6
S2_atp6_IMS
S2_atp6_MATRIX

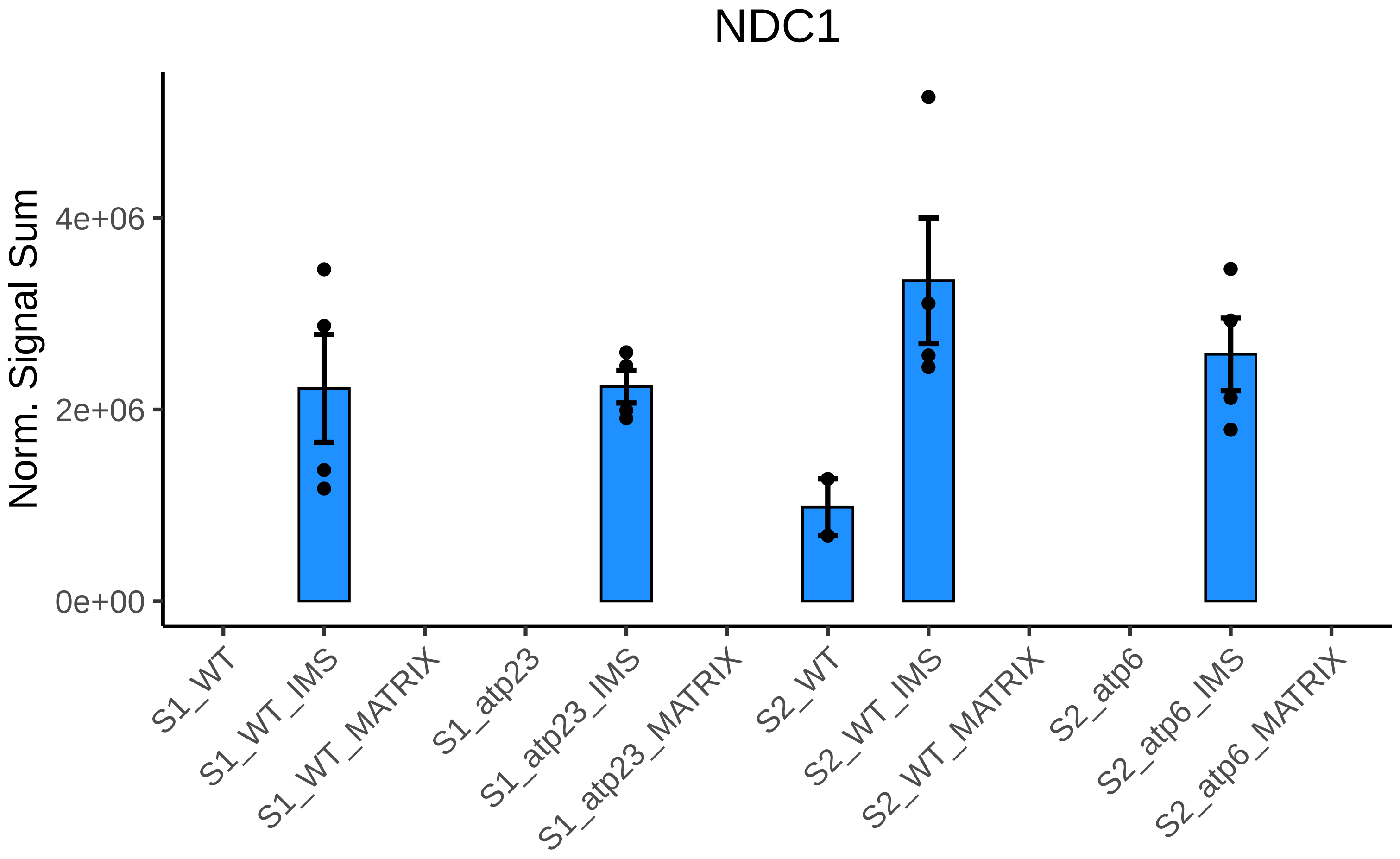
NDC1
Norm. Signal Sum
4e+06
2e+06
0e+00
S1_WT
S1_WT_IMS
S1_WT_MATRIX
S1_atp23
S1_atp23_IMS
S1_atp23_MATRIX
S2_WT
S2_WT_IMS
S2_WT_MATRIX
S2_atp6
S2_atp6_IMS
S2_atp6_MATRIX

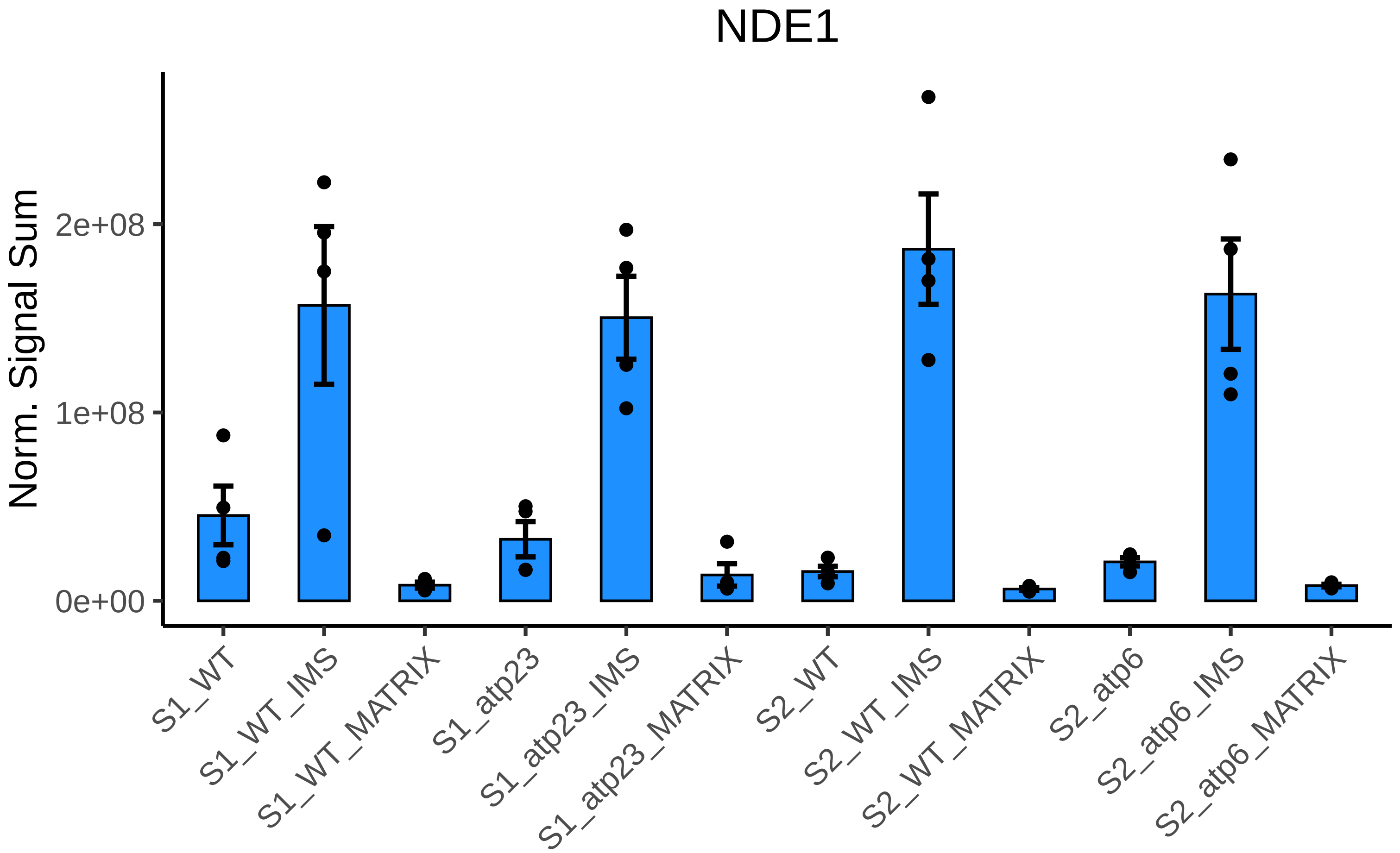
NDE1
Norm. Signal Sum
2e+08
1e+08
0e+00
S1_WT
S1_WT_IMS
S1_WT_MATRIX
S1_atp23
S1_atp23_IMS
S1_atp23_MATRIX
S2_WT
S2_WT_IMS
S2_WT_MATRIX
S2_atp6
S2_atp6_IMS
S2_atp6_MATRIX

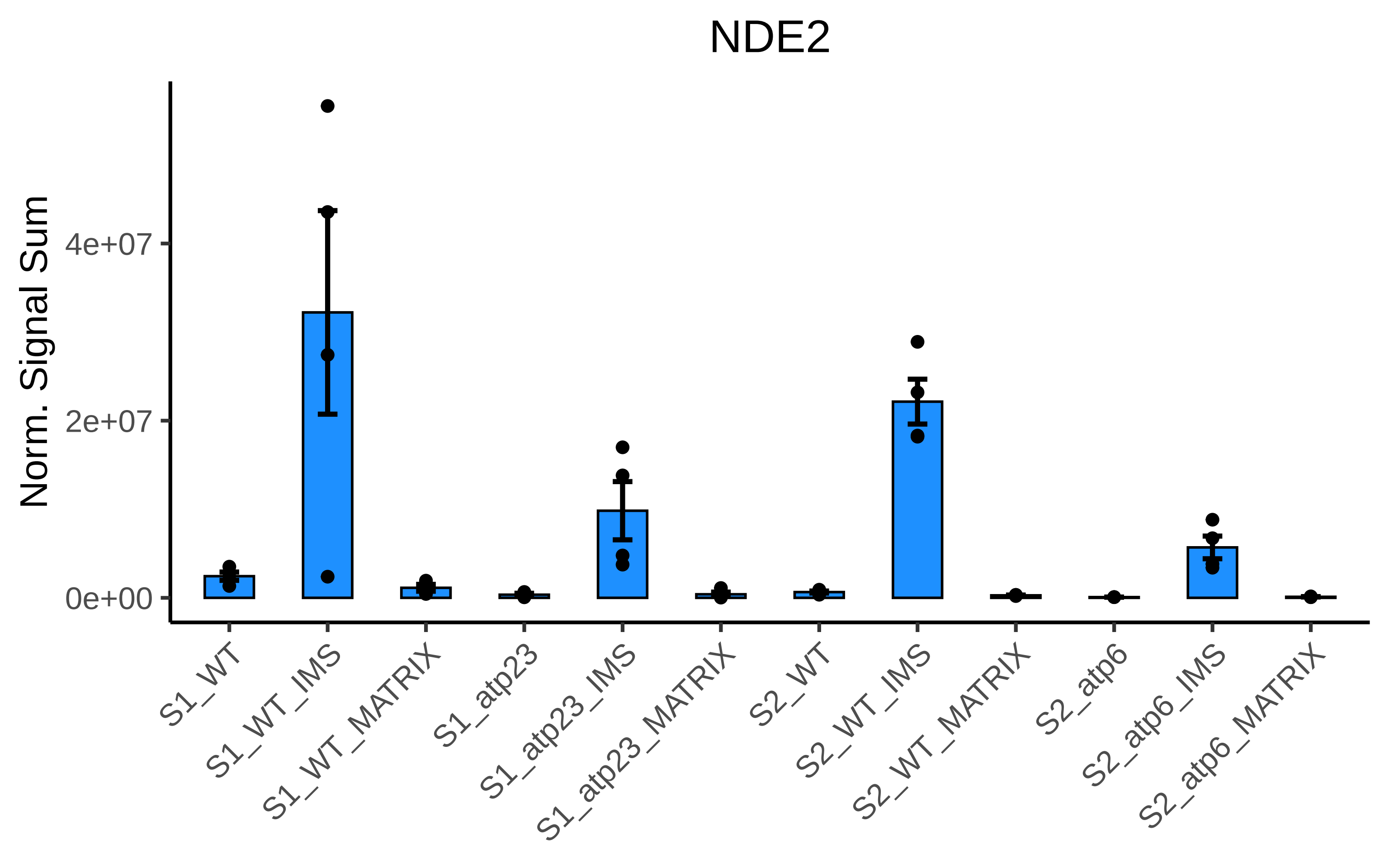
NDE2
Norm. Signal Sum
4e+07
2e+07
0e+00
S1_WT
S1_WT_IMS
S1_WT_MATRIX
S1_atp23
S1_atp23_IMS
S1_atp23_MATRIX
S2_WT
S2_WT_IMS
S2_WT_MATRIX
S2_atp6
S2_atp6_IMS
S2_atp6_MATRIX

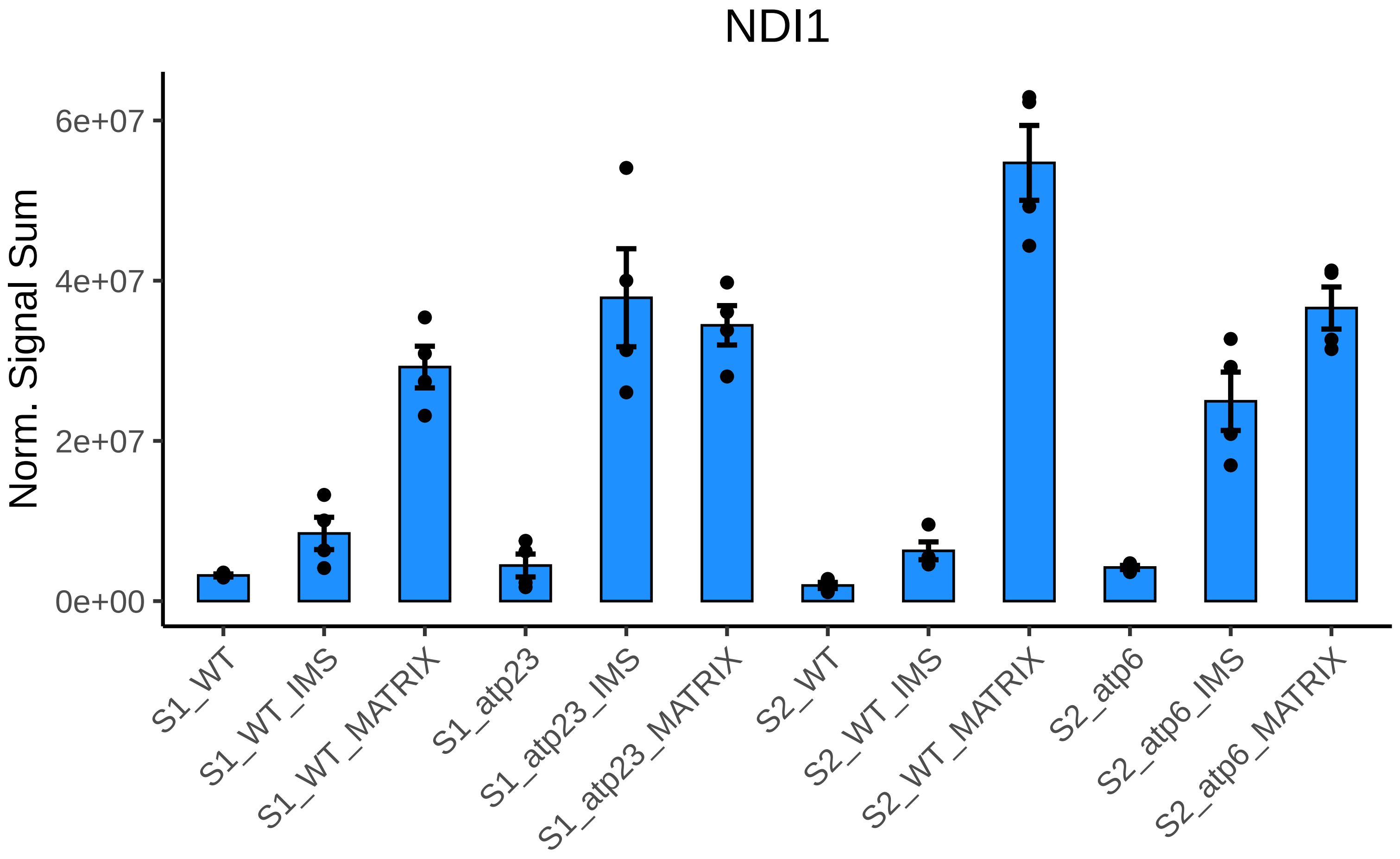
NDI1
Norm. Signal Sum
6e+07
4e+07
2e+07
0e+00
S1_WT
S1_WT_IMS
S1_WT_MATRIX
S1_atp23
S1_atp23_IMS
S1_atp23_MATRIX
S2_WT
S2_WT_IMS
S2_WT_MATRIX
S2_atp6
S2_atp6_IMS
S2_atp6_MATRIX

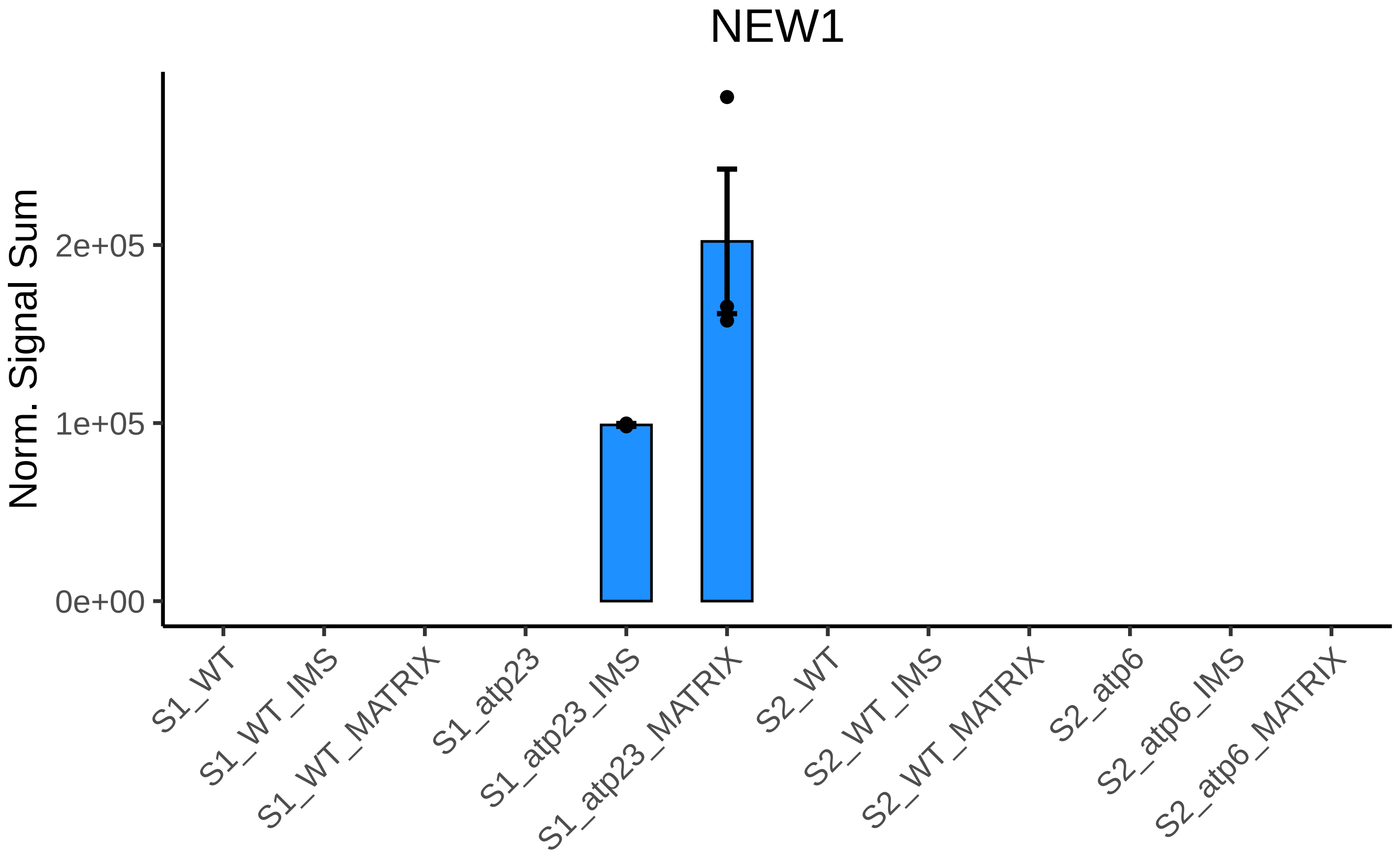

NEW1
Norm. Signal Sum
2e+05
1e+05
0e+00
S1_WT
S1_WT_IMS
S1_WT_MATRIX
S1_atp23
S1_atp23_IMS
S1_atp23_MATRIX
S2_WT
S2_WT_IMS
S2_WT_MATRIX
S2_atp6
S2_atp6_IMS
S2_atp6_MATRIX

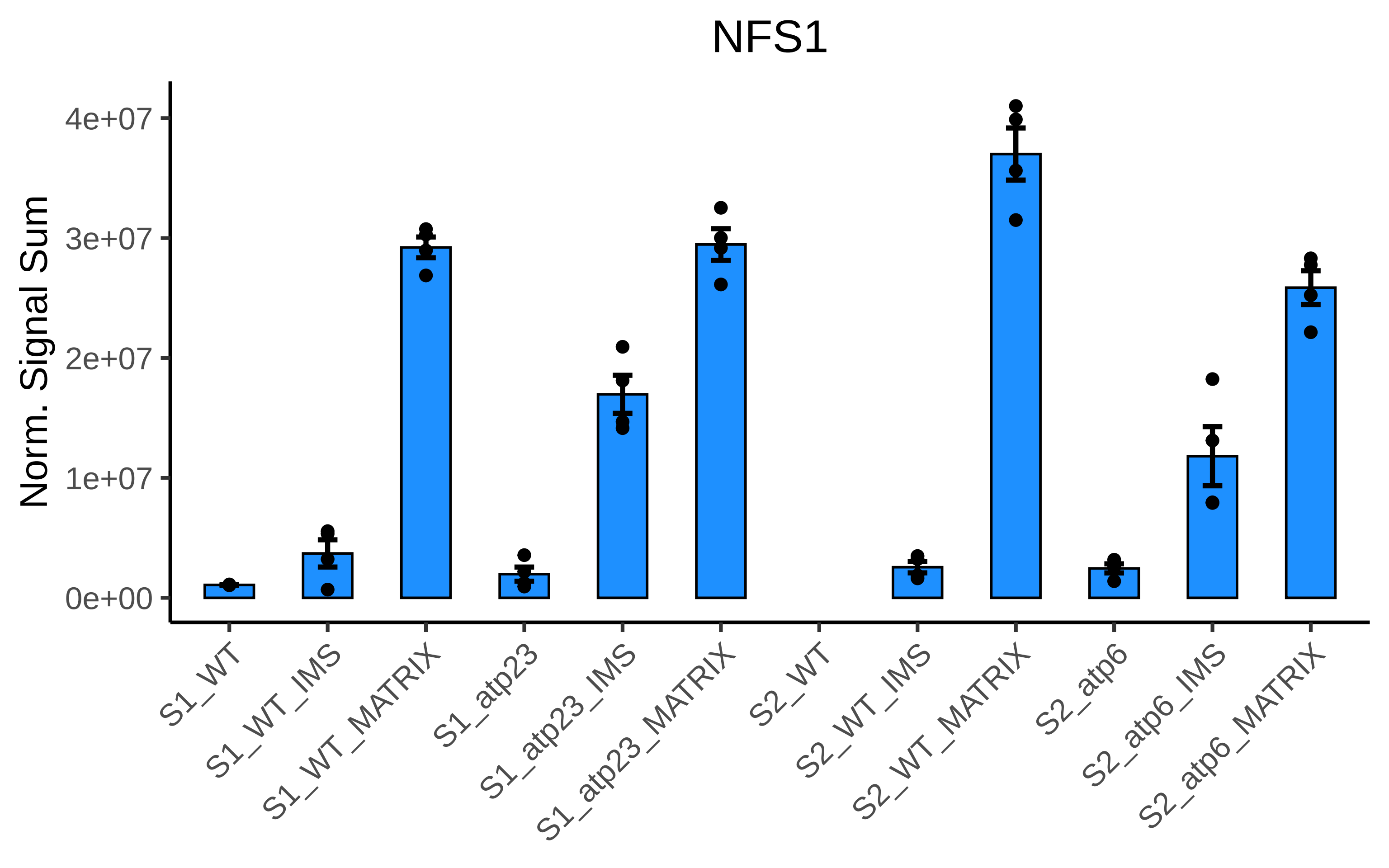
NFS1
Norm. Signal Sum
4e+07
3e+07
2e+07
1e+07
0e+00
S1_WT
S1_WT_IMS
S1_WT_MATRIX
S1_atp23
S1_atp23_IMS
S1_atp23_MATRIX
S2_WT
S2_WT_IMS
S2_WT_MATRIX
S2_atp6
S2_atp6_IMS
S2_atp6_MATRIX

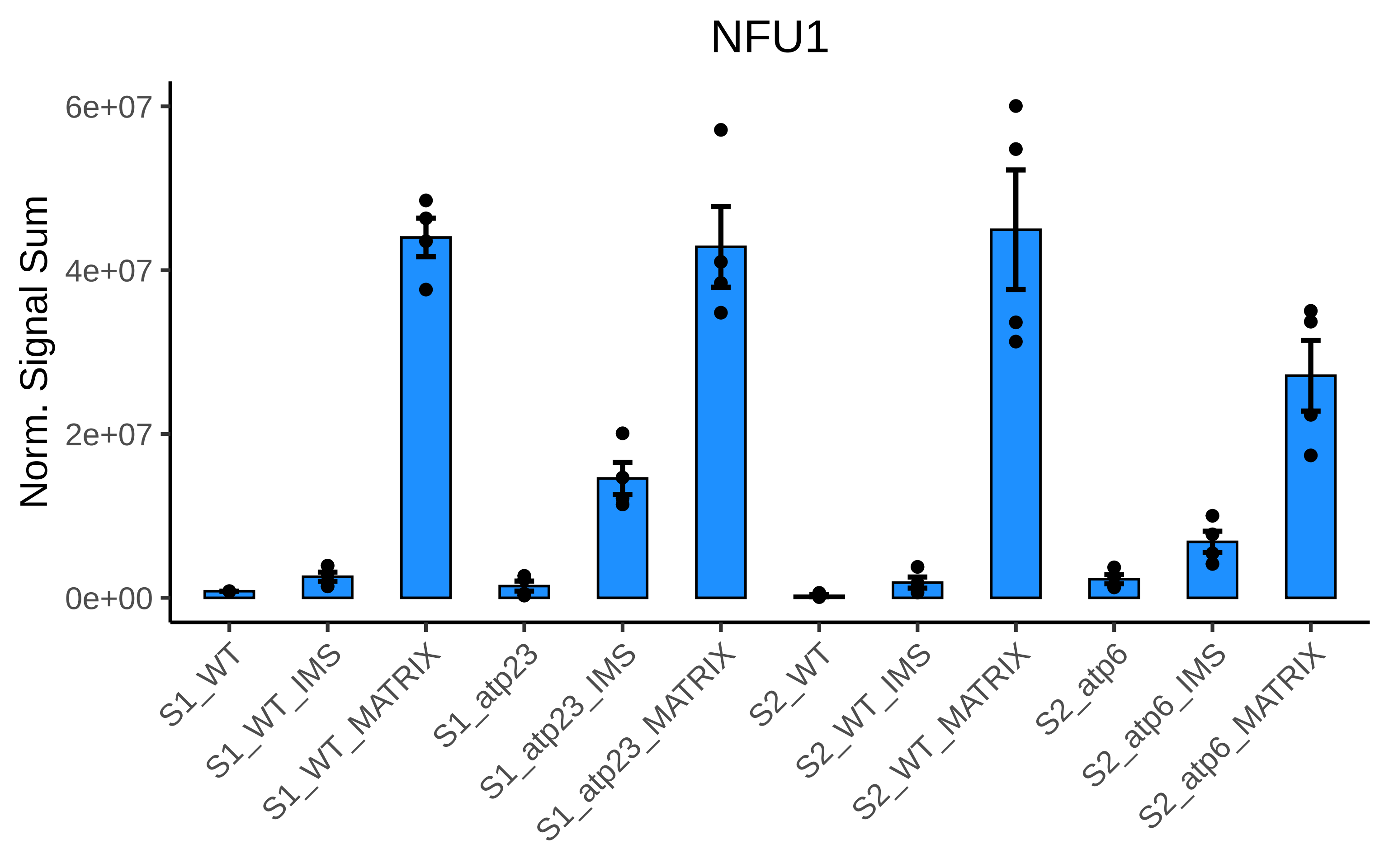
NFU1
Norm. Signal Sum
6e+07
4e+07
2e+07
0e+00
S1_WT
S1_WT_IMS
S1_WT_MATRIX
S1_atp23
S1_atp23_IMS
S1_atp23_MATRIX
S2_WT
S2_WT_IMS
S2_WT_MATRIX
S2_atp6
S2_atp6_IMS
S2_atp6_MATRIX

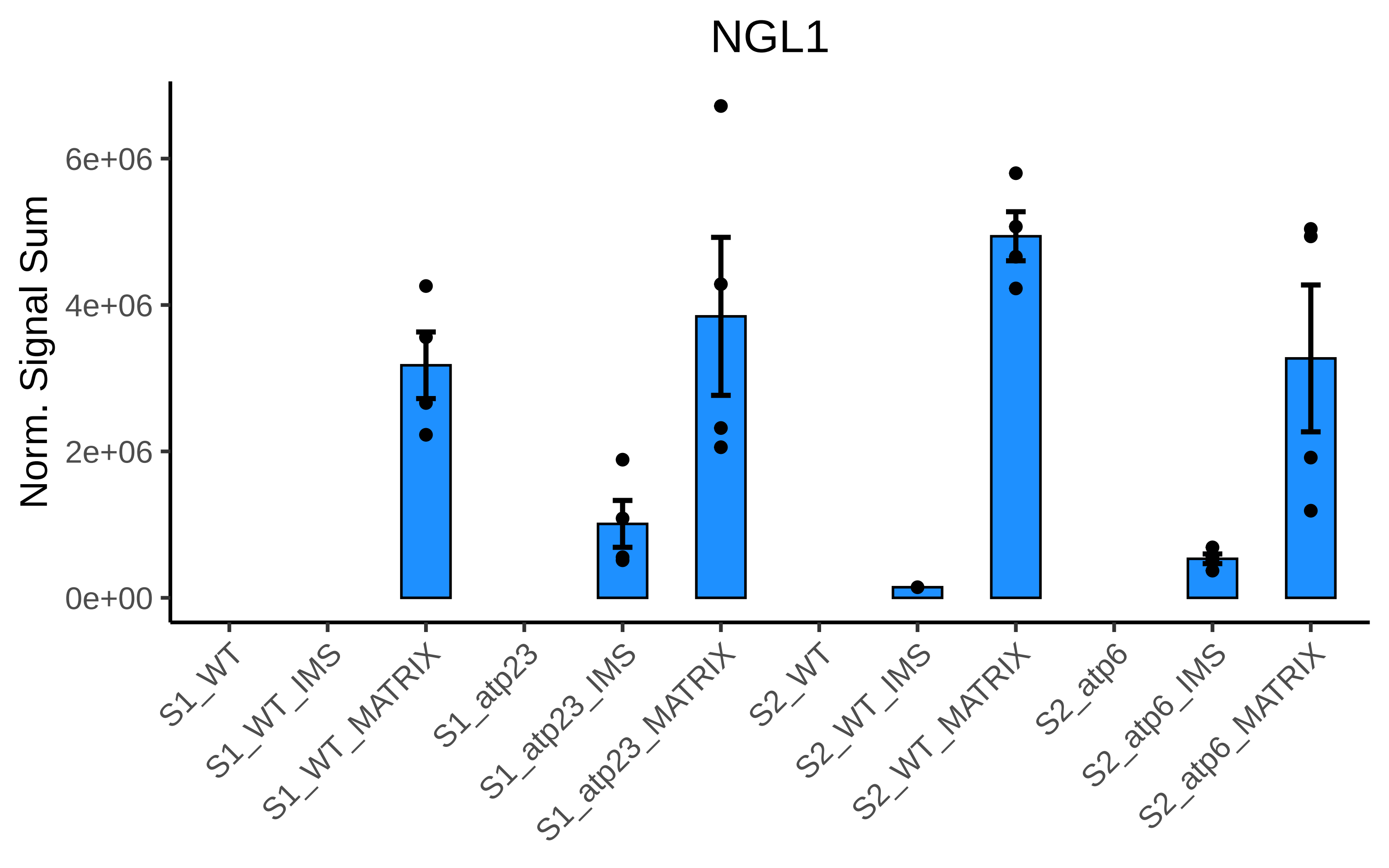

NGL1
Norm. Signal Sum
6e+06
4e+06
2e+06
0e+00
S1_WT
S1_WT_IMS
S1_WT_MATRIX
S1_atp23
S1_atp23_IMS
S1_atp23_MATRIX
S2_WT
S2_WT_IMS
S2_WT_MATRIX
S2_atp6
S2_atp6_IMS
S2_atp6_MATRIX

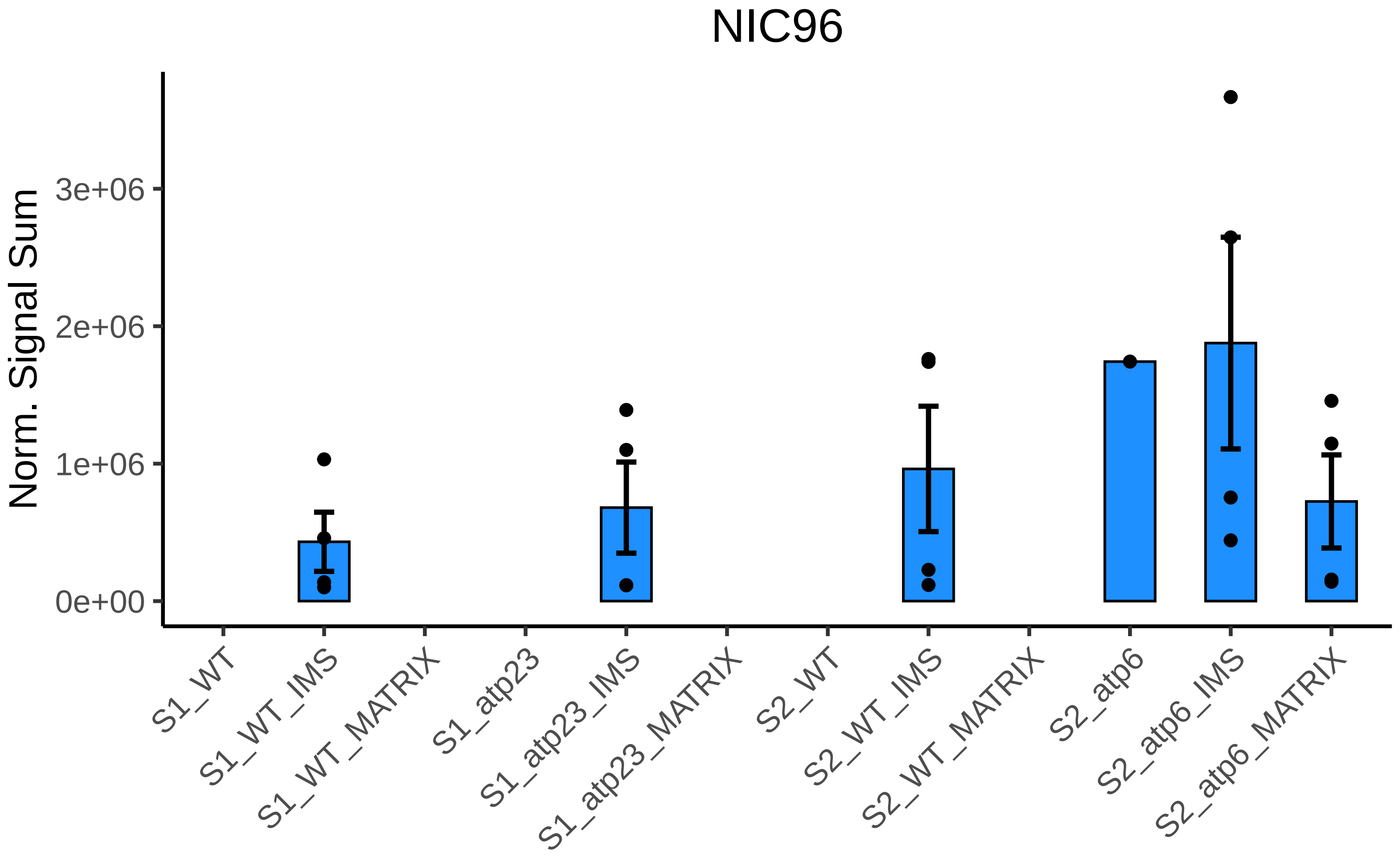
NIC96
Norm. Signal Sum
3e+06
2e+06
1e+06
0e+00
S1_WT
S1_WT_IMS
S1_WT_MATRIX
S1_atp23
S1_atp23_IMS
S1_atp23_MATRIX
S2_WT
S2_WT_IMS
S2_WT_MATRIX
S2_atp6
S2_atp6_IMS
S2_atp6_MATRIX

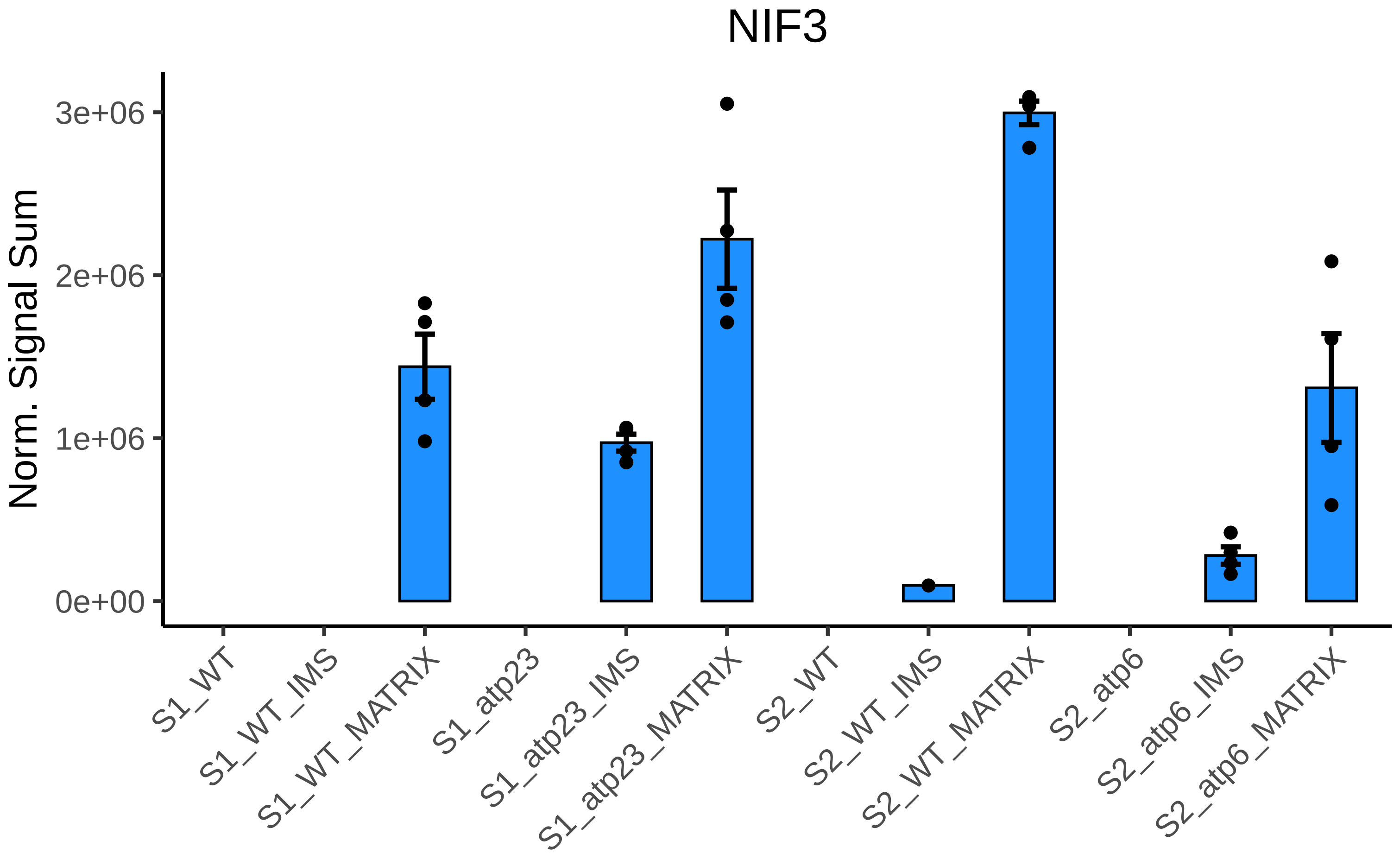
NIF3
Norm. Signal Sum
3e+06
2e+06
1e+06
0e+00
S1_WT
S1_WT_IMS
S1_WT_MATRIX
S1_atp23
S1_atp23_IMS
S1_atp23_MATRIX
S2_WT
S2_WT_IMS
S2_WT_MATRIX
S2_atp6
S2_atp6_IMS
S2_atp6_MATRIX

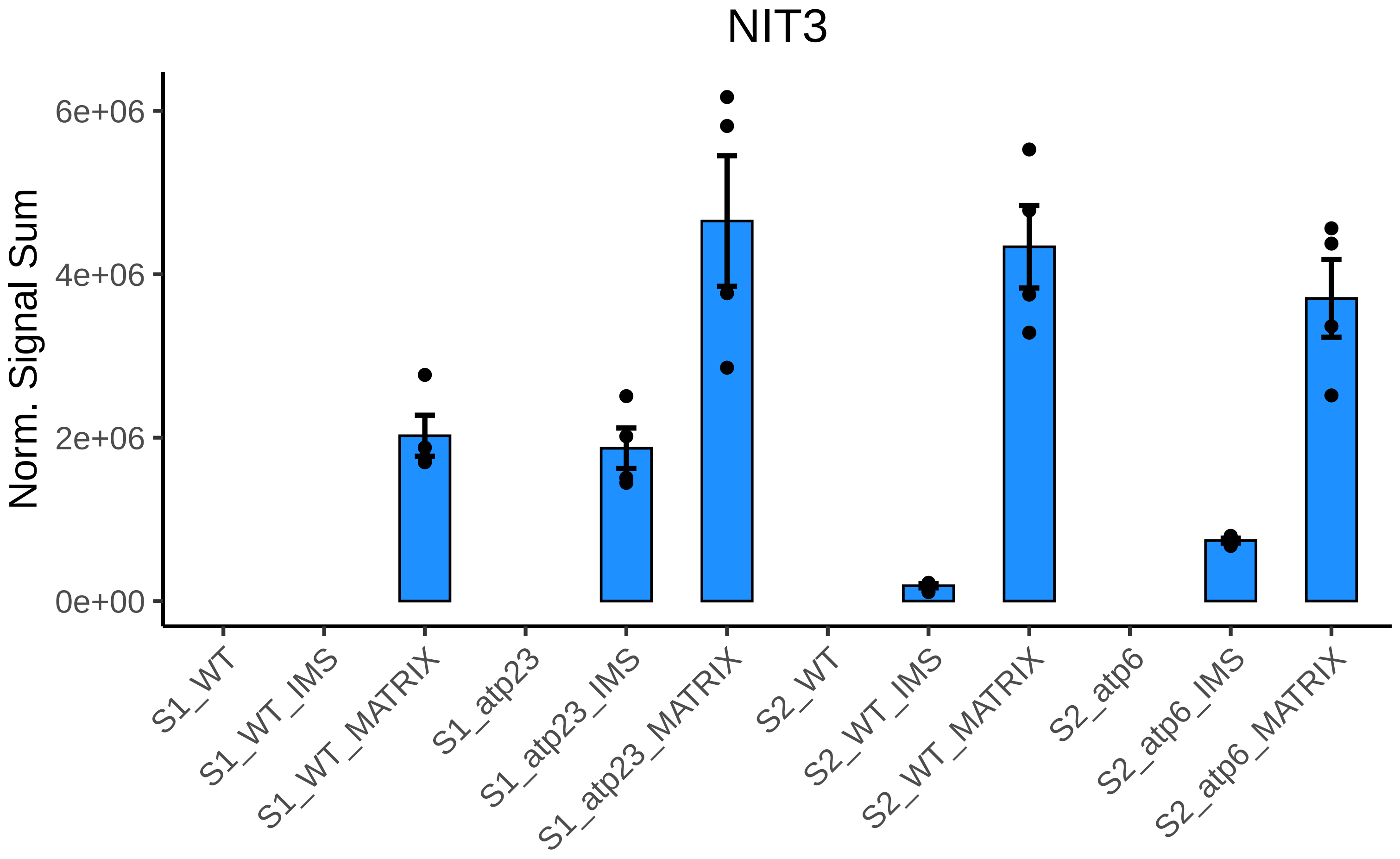
NIT3
Norm. Signal Sum
6e+06
4e+06
2e+06
0e+00
S1_WT
S1_WT_IMS
S1_WT_MATRIX
S1_atp23
S1_atp23_IMS
S1_atp23_MATRIX
S2_WT
S2_WT_IMS
S2_WT_MATRIX
S2_atp6
S2_atp6_IMS
S2_atp6_MATRIX

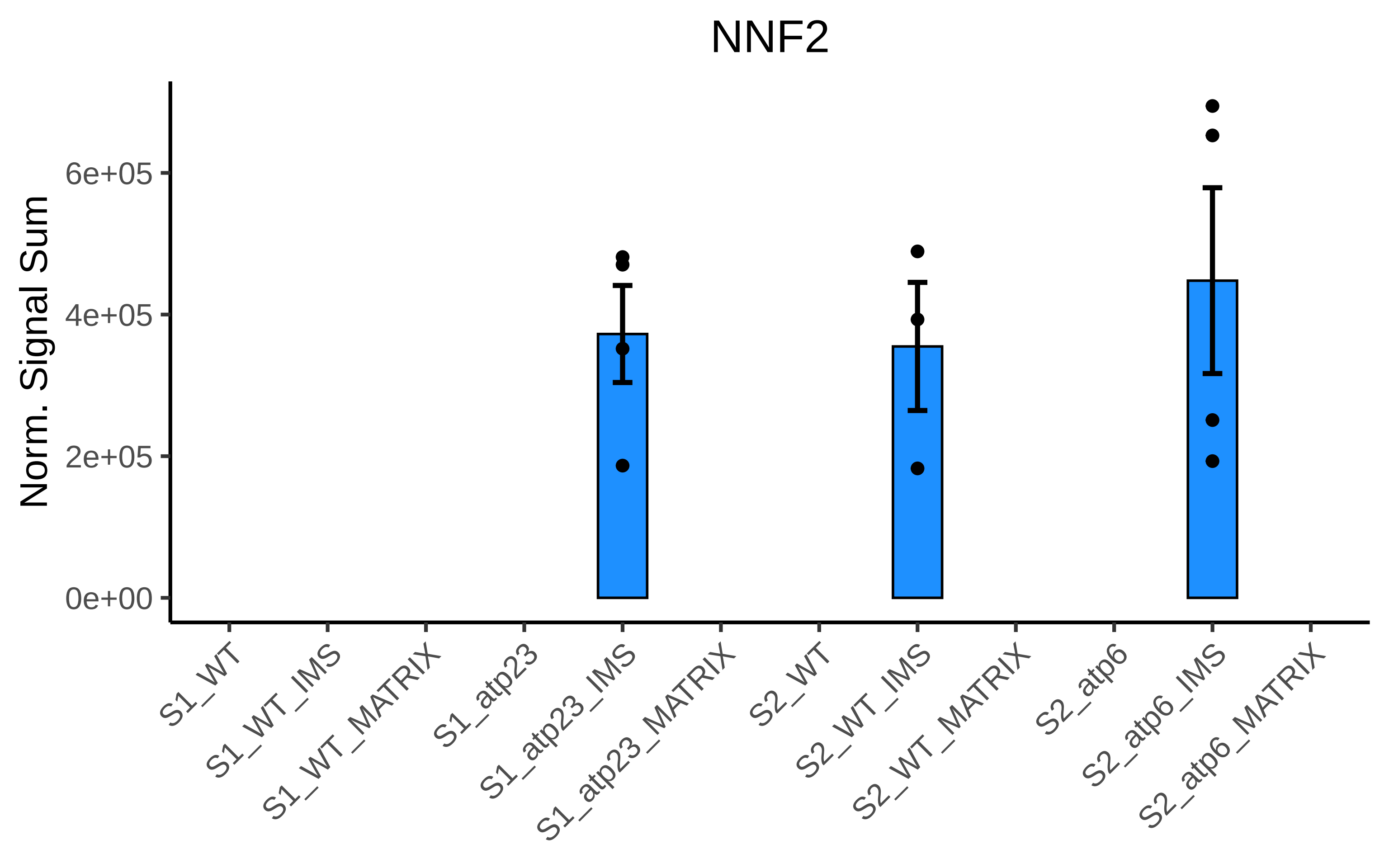

NNF2
Norm. Signal Sum
0e+00
2e+05
4e+05
6e+05
S1_WT
S1_WT_IMS
S1_WT_MATRIX
S1_atp23
S1_atp23_IMS
S1_atp23_MATRIX
S2_WT
S2_WT_IMS
S2_WT_MATRIX
S2_atp6
S2_atp6_IMS
S2_atp6_MATRIX

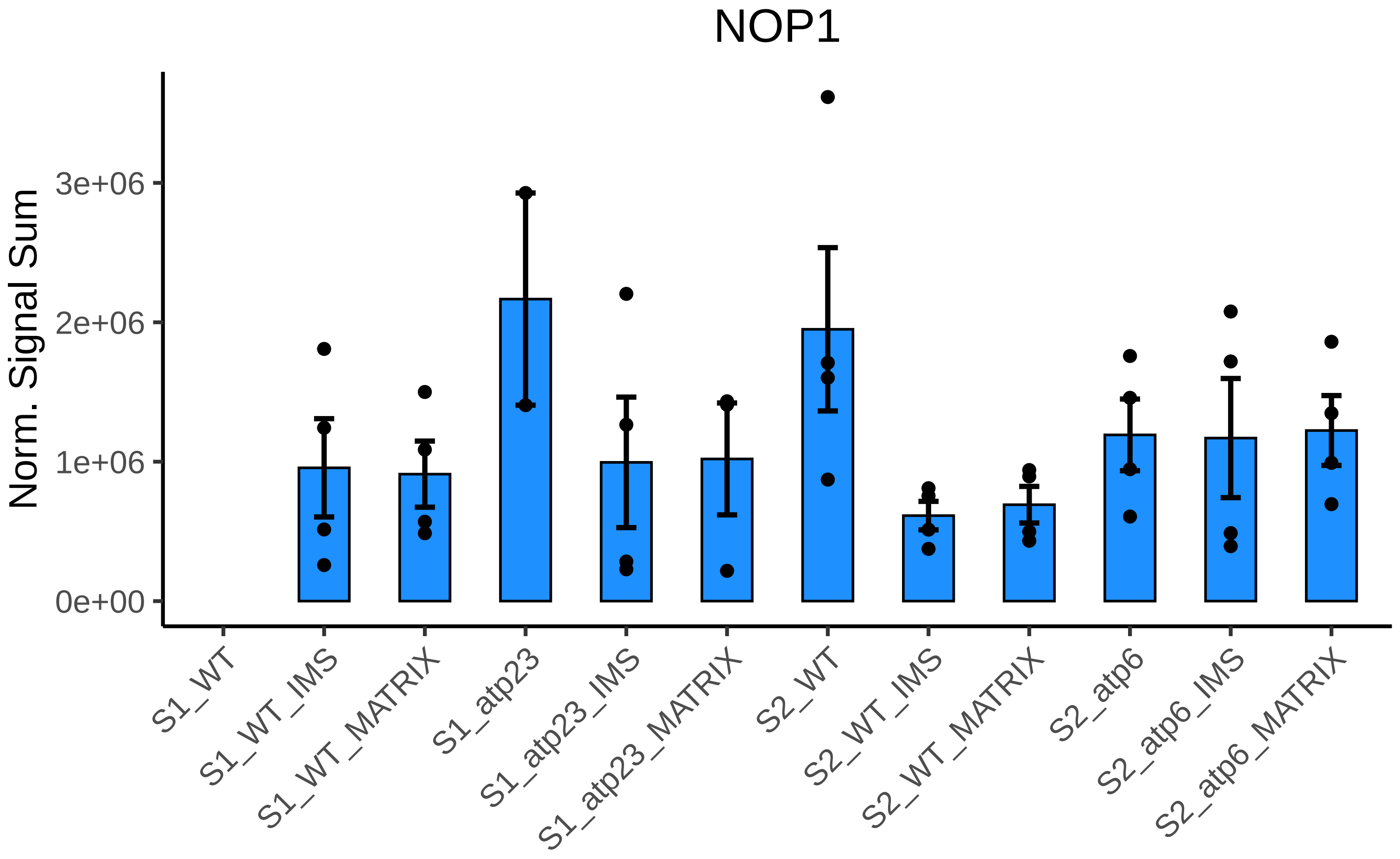

NOP1
Norm. Signal Sum
3e+06
2e+06
1e+06
0e+00
S1_WT
S1_WT_IMS
S1_WT_MATRIX
S1_atp23
S1_atp23_IMS
S1_atp23_MATRIX
S2_WT
S2_WT_IMS
S2_WT_MATRIX
S2_atp6
S2_atp6_IMS
S2_atp6_MATRIX

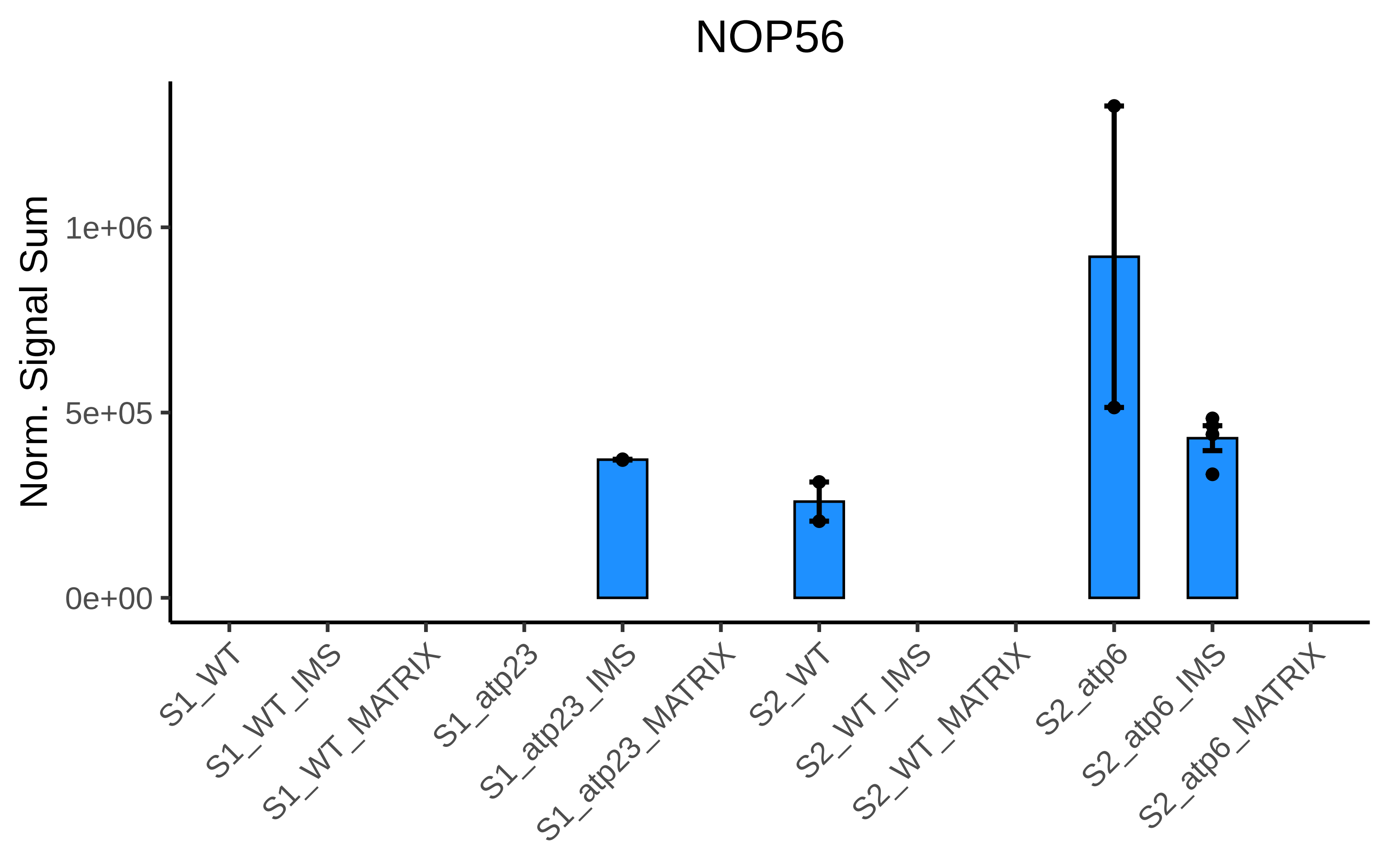
NOP56
Norm. Signal Sum
1e+06
5e+05
0e+00
S1_WT
S1_WT_IMS
S1_WT_MATRIX
S1_atp23
S1_atp23_IMS
S1_atp23_MATRIX
S2_WT
S2_WT_IMS
S2_WT_MATRIX
S2_atp6
S2_atp6_IMS
S2_atp6_MATRIX

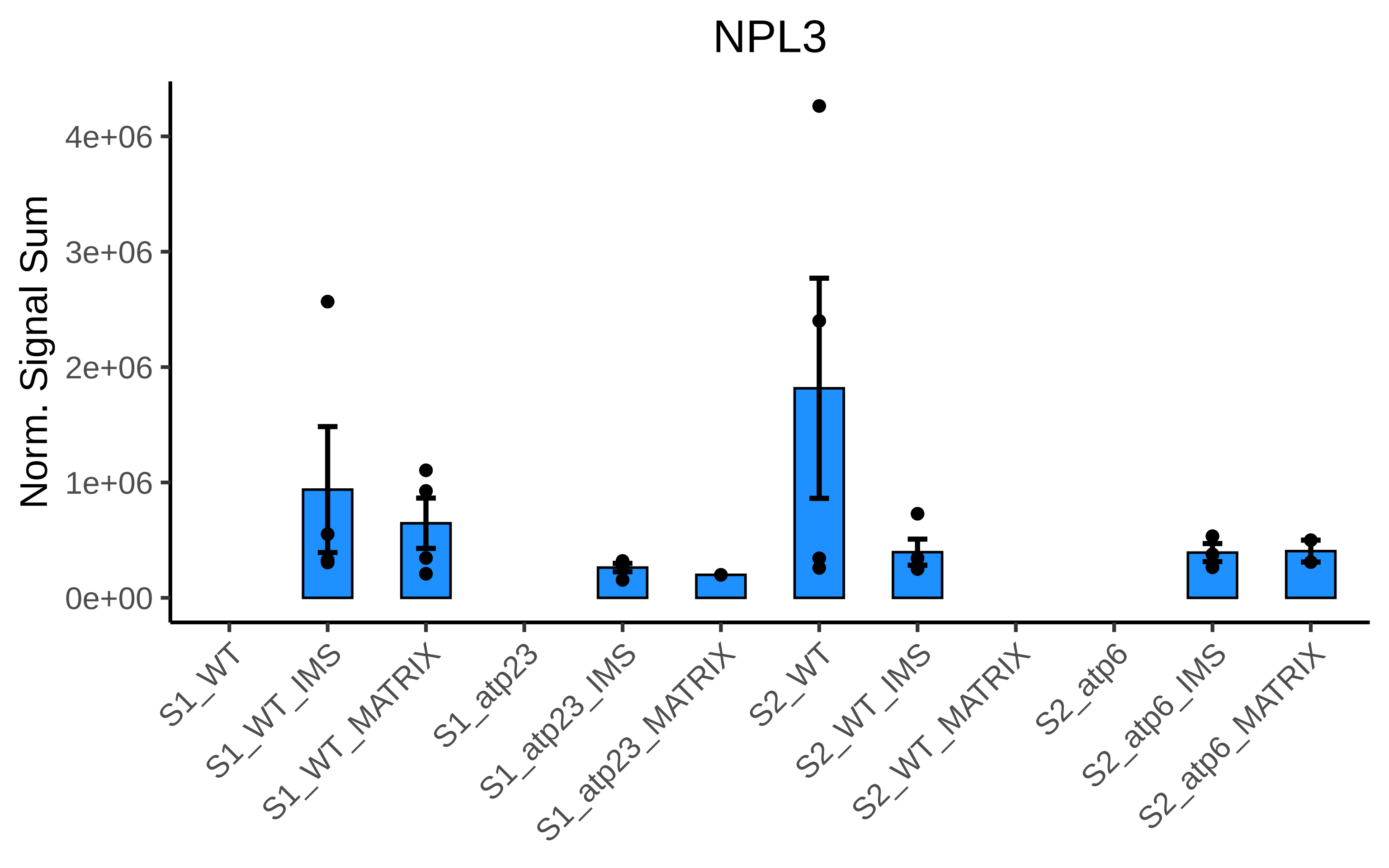
NPL3
Norm. Signal Sum
4e+06
3e+06
2e+06
1e+06
0e+00
S1_WT
S1_WT_IMS
S1_WT_MATRIX
S1_atp23
S1_atp23_IMS
S1_atp23_MATRIX
S2_WT
S2_WT_IMS
S2_WT_MATRIX
S2_atp6
S2_atp6_IMS
S2_atp6_MATRIX

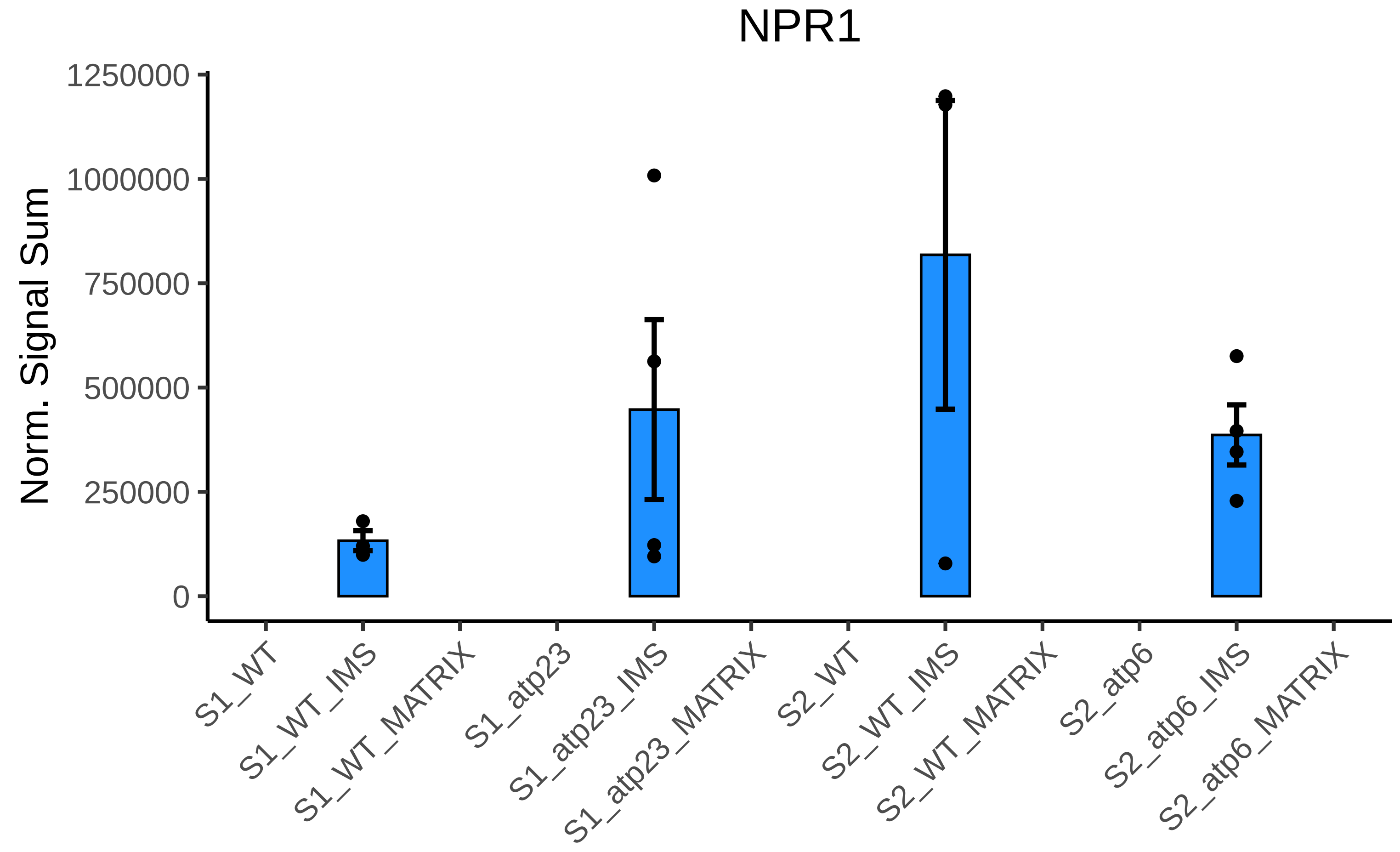
NPR1
Norm. Signal Sum
1250000
1000000
750000
500000
250000
0
S1_WT
S1_WT_IMS
S1_WT_MATRIX
S1_atp23
S1_atp23_IMS
S1_atp23_MATRIX
S2_WT
S2_WT_IMS
S2_WT_MATRIX
S2_atp6
S2_atp6_IMS
S2_atp6_MATRIX

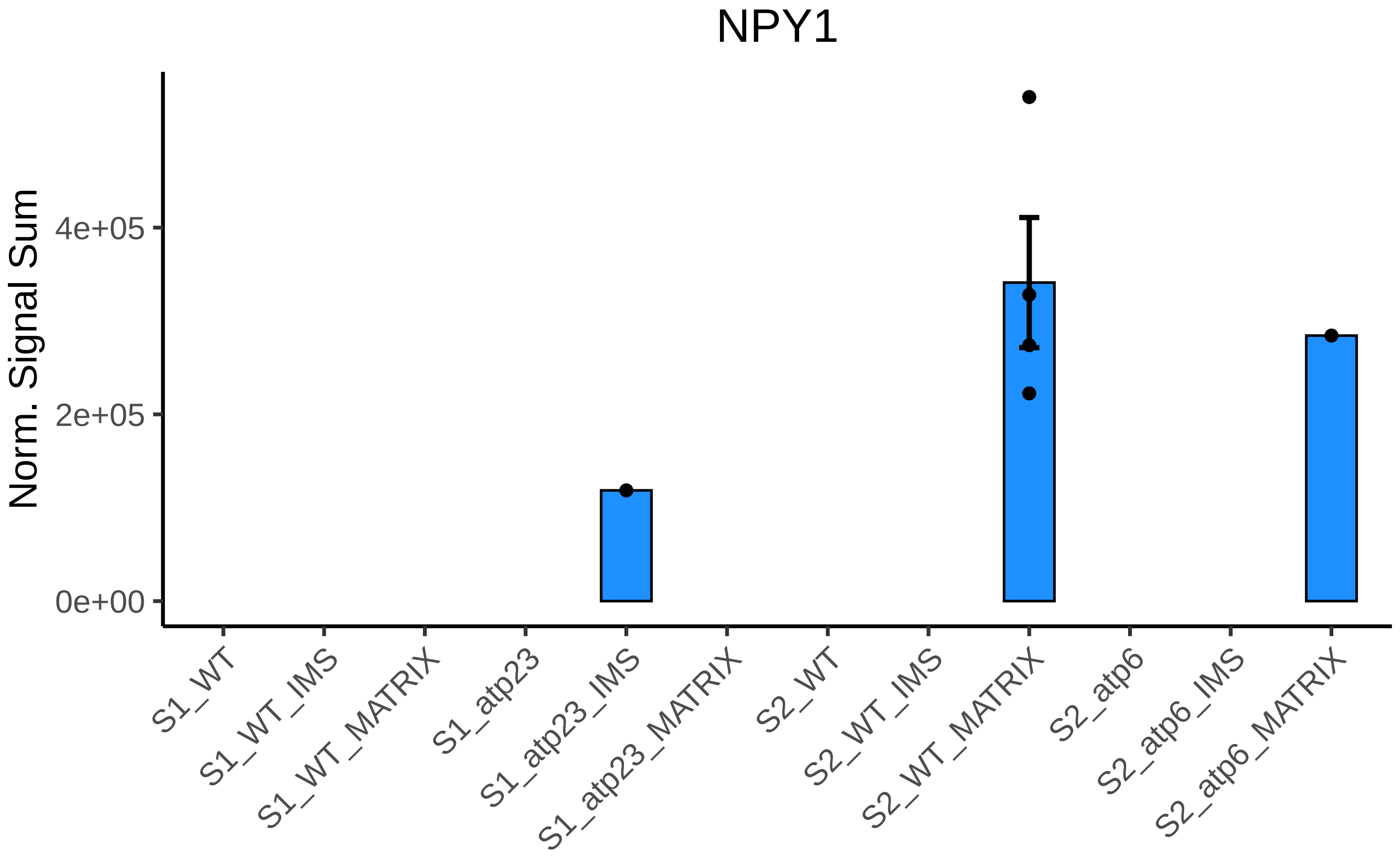
NPY1
Norm. Signal Sum
4e+05
2e+05
0e+00
S1_WT
S1_WT_IMS
S1_WT_MATRIX
S1_atp23
S1_atp23_IMS
S1_atp23_MATRIX
S2_WT
S2_WT_IMS
S2_WT_MATRIX
S2_atp6
S2_atp6_IMS
S2_atp6_MATRIX

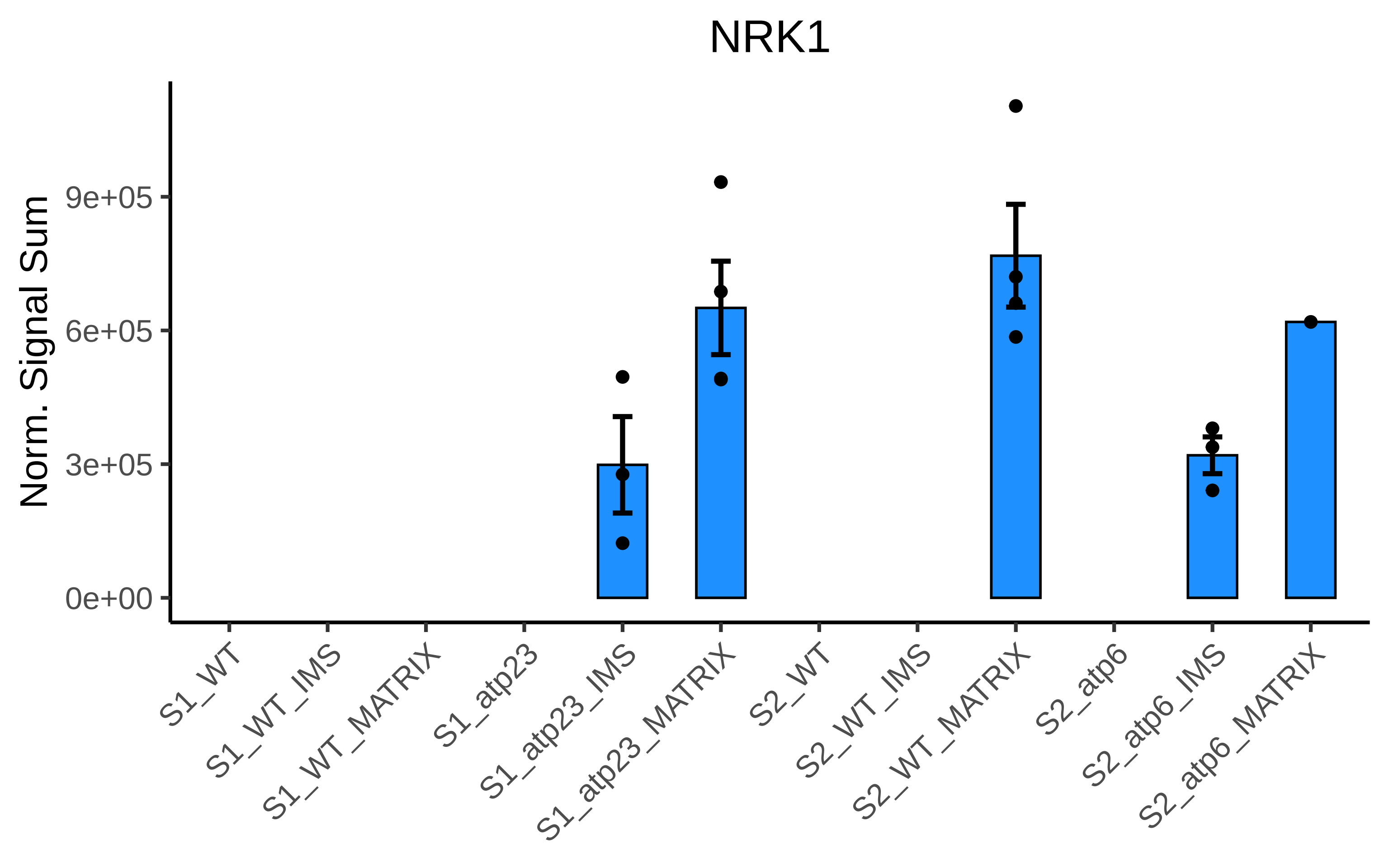
NRK1
Norm. Signal Sum
9e+05
6e+05
3e+05
0e+00
S1_WT
S1_WT_IMS
S1_WT_MATRIX
S1_atp23
S1_atp23_IMS
S1_atp23_MATRIX
S2_WT
S2_WT_IMS
S2_WT_MATRIX
S2_atp6
S2_atp6_IMS
S2_atp6_MATRIX

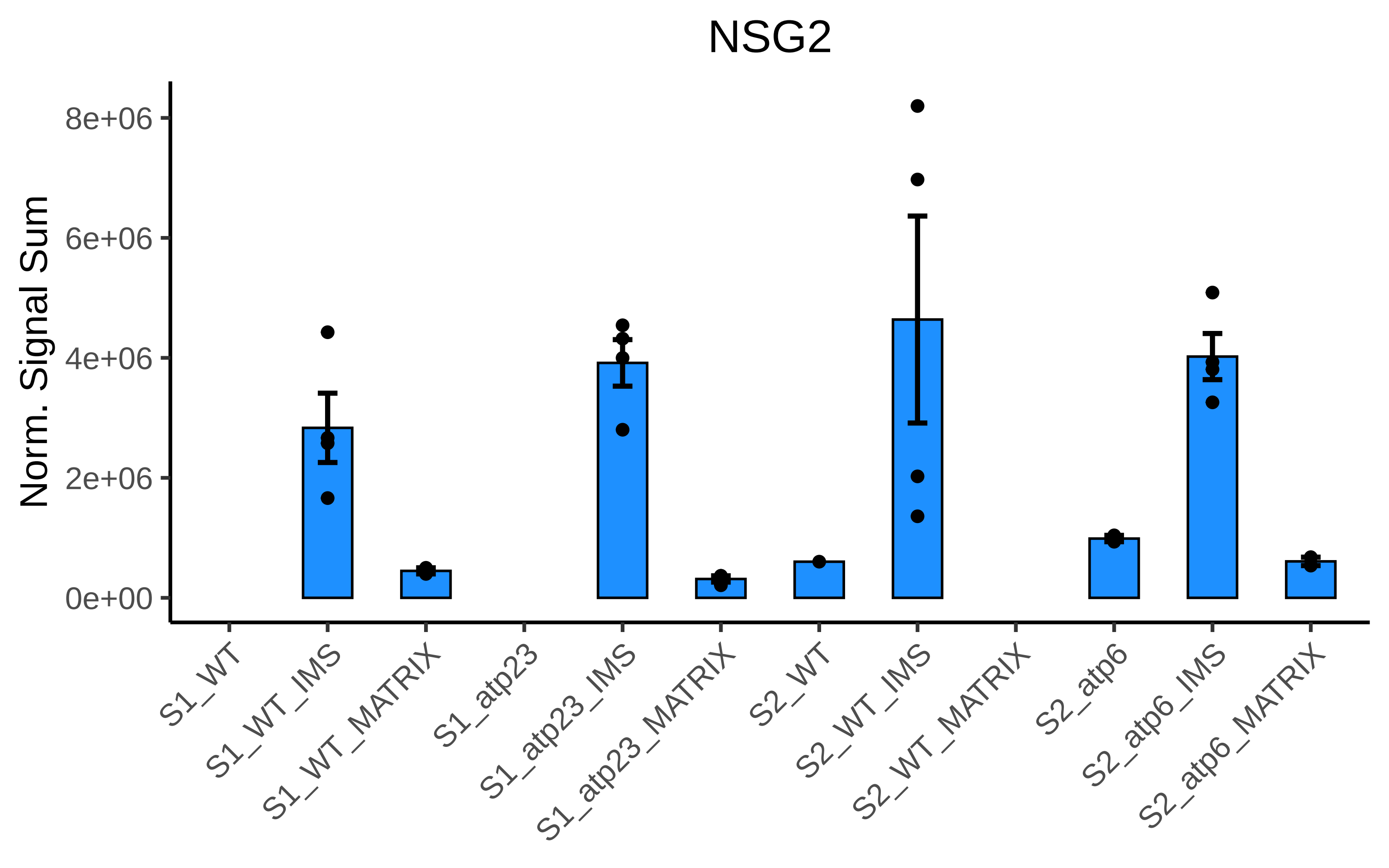
NSG2
Norm. Signal Sum
8e+06
6e+06
4e+06
2e+06
0e+00
S1_WT
S1_WT_IMS
S1_WT_MATRIX
S1_atp23
S1_atp23_IMS
S1_atp23_MATRIX
S2_WT
S2_WT_IMS
S2_WT_MATRIX
S2_atp6
S2_atp6_IMS
S2_atp6_MATRIX

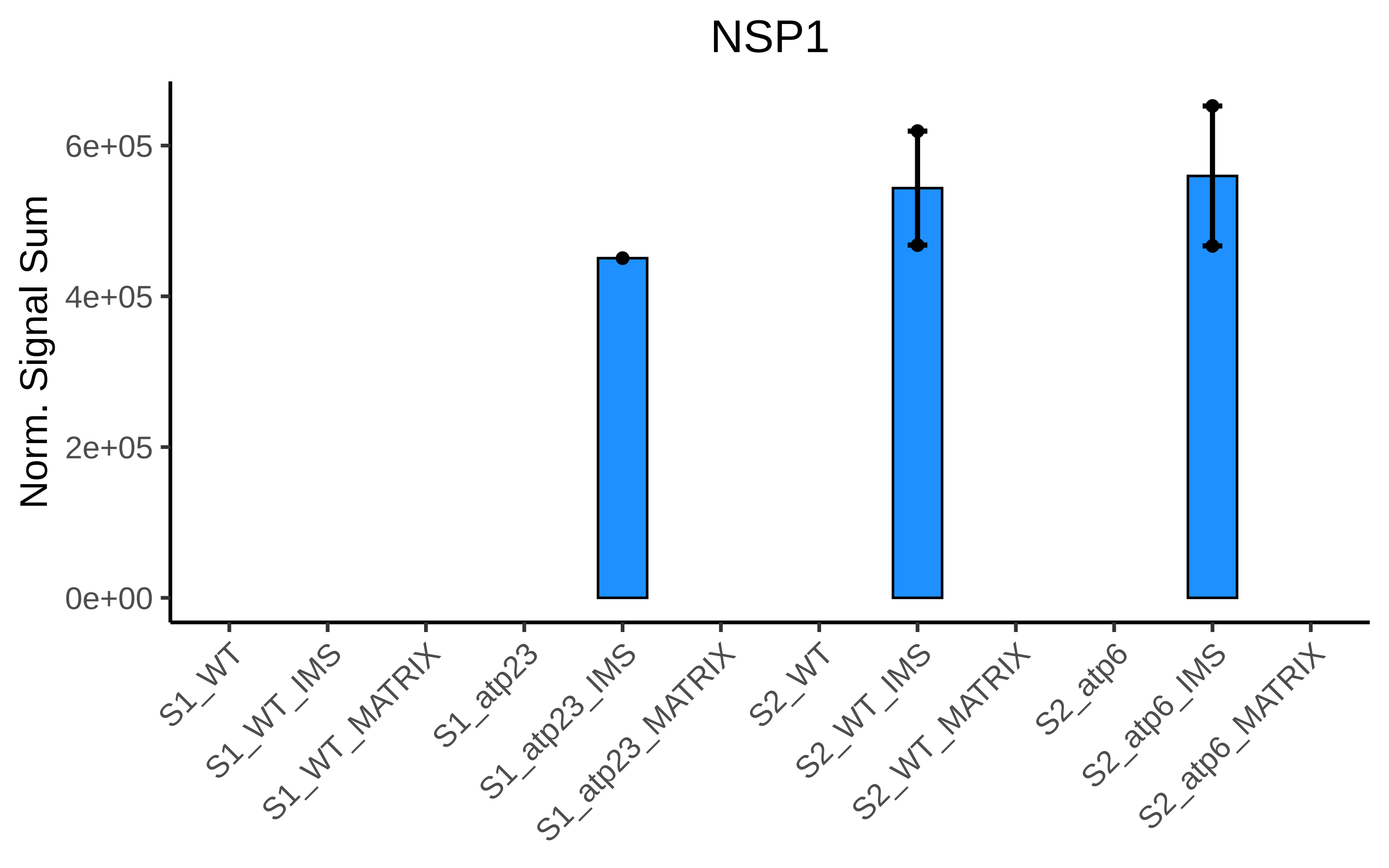

NSP1
Norm. Signal Sum
6e+05
4e+05
2e+05
0e+00
S1_WT
S1_WT_IMS
S1_WT_MATRIX
S1_atp23
S1_atp23_IMS
S1_atp23_MATRIX
S2_WT
S2_WT_IMS
S2_WT_MATRIX
S2_atp6
S2_atp6_IMS
S2_atp6_MATRIX

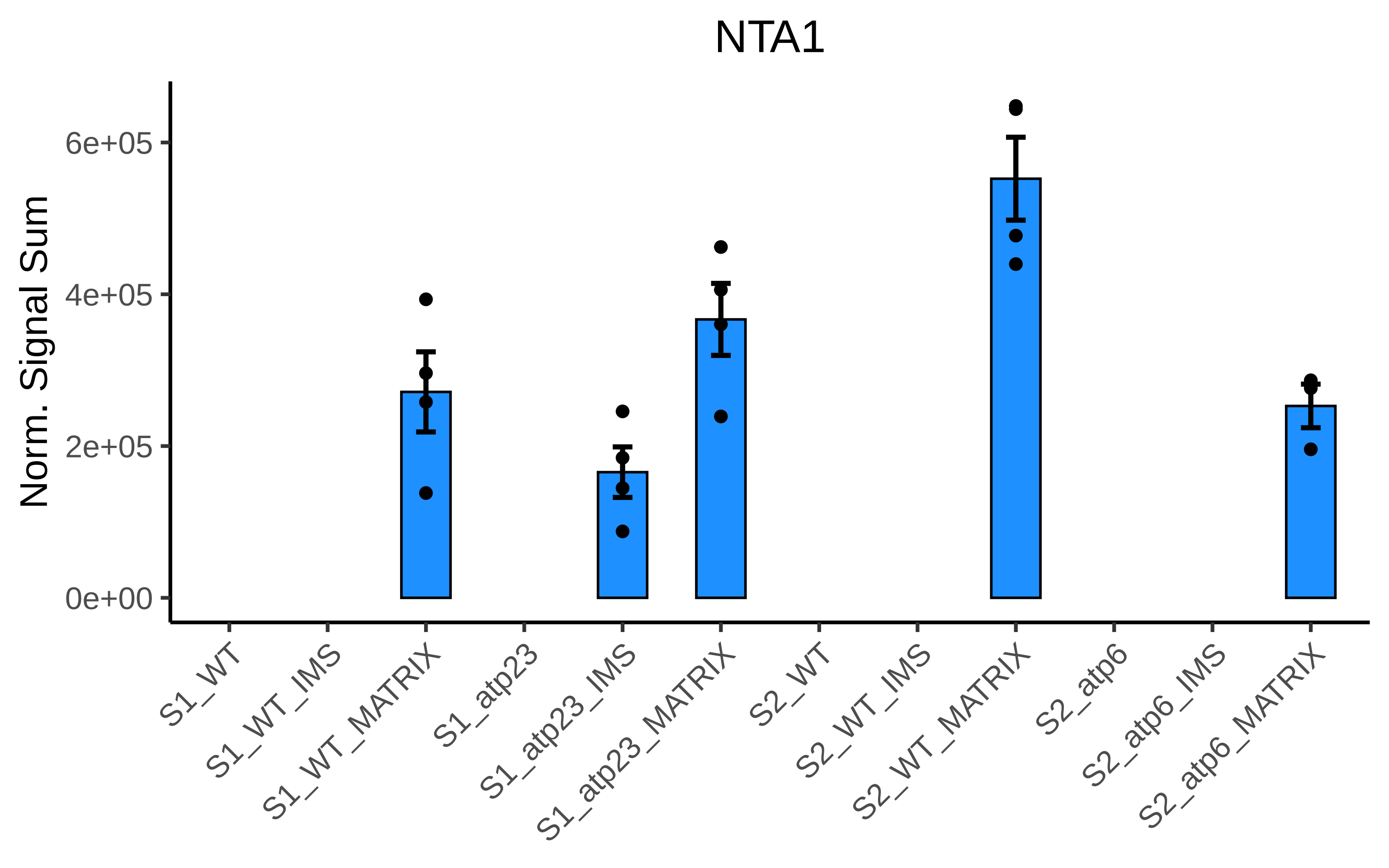

NTA1
Norm. Signal Sum
6e+05
4e+05
2e+05
0e+00
S1_WT
S1_WT_IMS
S1_WT_MATRIX
S1_atp23
S1_atp23_IMS
S1_atp23_MATRIX
S2_WT
S2_WT_IMS
S2_WT_MATRIX
S2_atp6
S2_atp6_IMS
S2_atp6_MATRIX

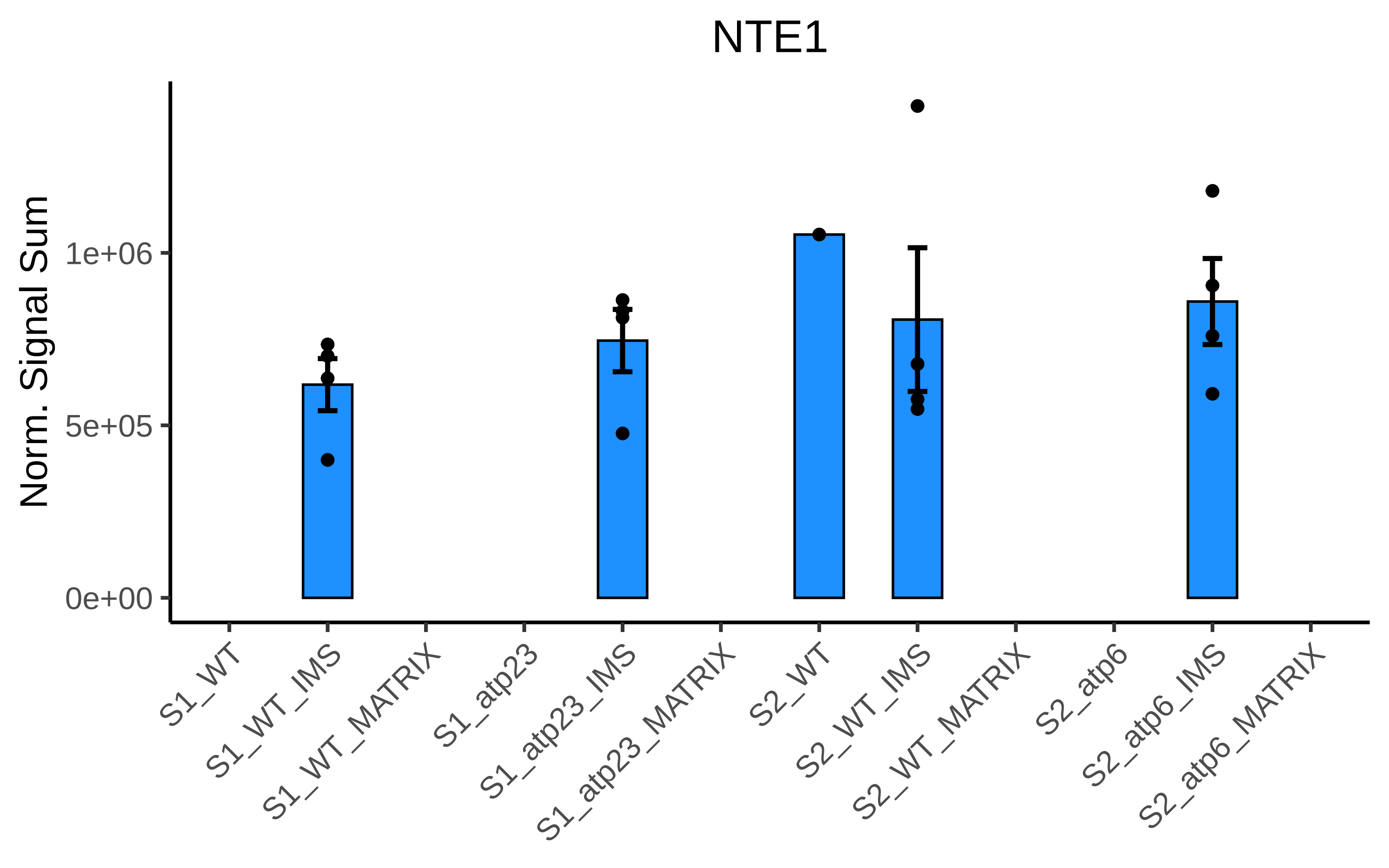
NTE1
Norm. Signal Sum
0e+00
5e+05
1e+06
S1_WT
S1_WT_IMS
S1_WT_MATRIX
S1_atp23
S1_atp23_IMS
S1_atp23_MATRIX
S2_WT
S2_WT_IMS
S2_WT_MATRIX
S2_atp6
S2_atp6_IMS
S2_atp6_MATRIX

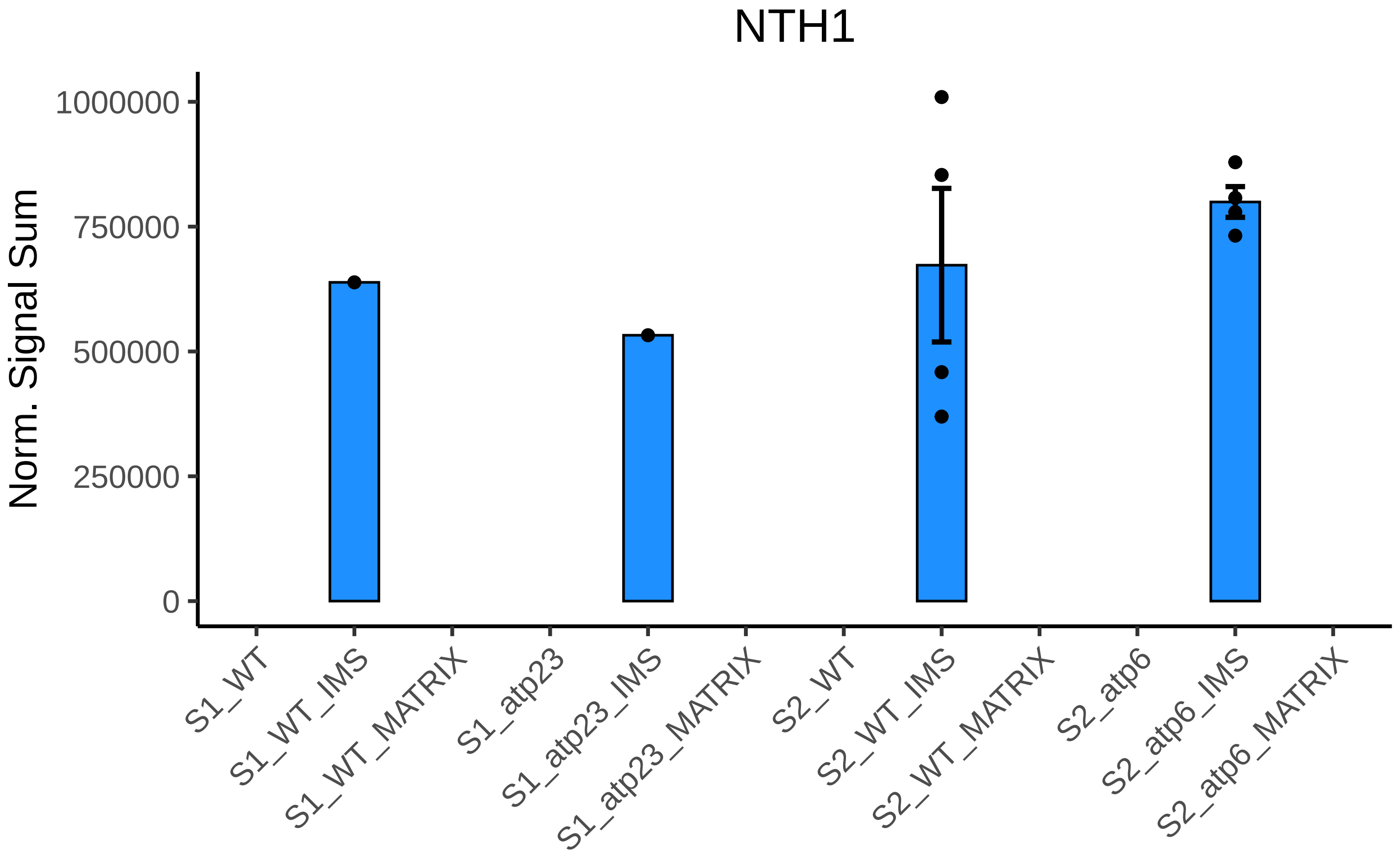
NTH1
Norm. Signal Sum
1000000
750000
500000
250000
0
S1_WT
S1_WT_IMS
S1_WT_MATRIX
S1_atp23
S1_atp23_IMS
S1_atp23_MATRIX
S2_WT
S2_WT_IMS
S2_WT_MATRIX
S2_atp6
S2_atp6_IMS
S2_atp6_MATRIX

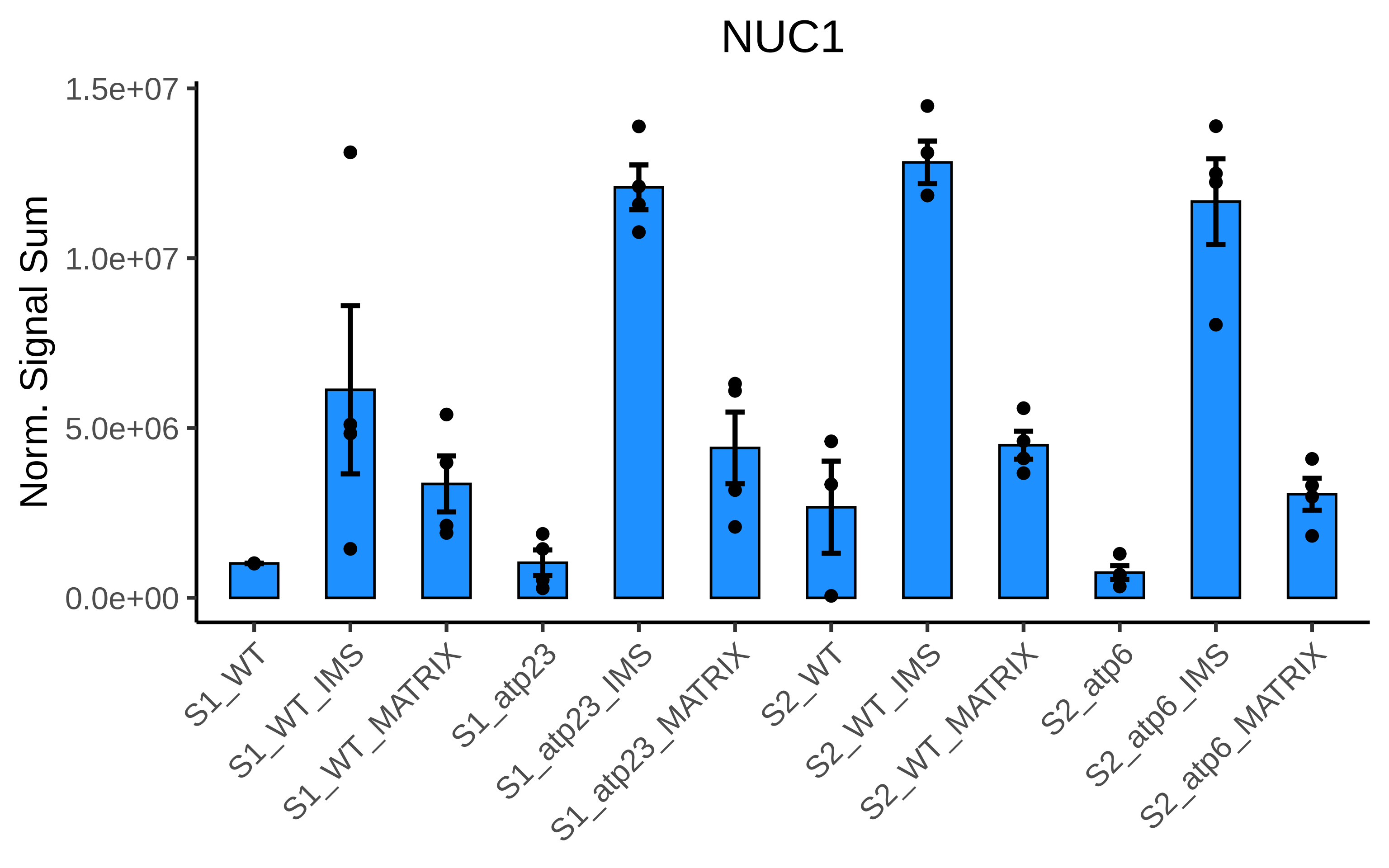

NUC1
Norm. Signal Sum
1.5e+07
1.0e+07
5.0e+06
0.0e+00
S1_WT
S1_WT_IMS
S1_WT_MATRIX
S1_atp23
S1_atp23_IMS
S1_atp23_MATRIX
S2_WT
S2_WT_IMS
S2_WT_MATRIX
S2_atp6
S2_atp6_IMS
S2_atp6_MATRIX

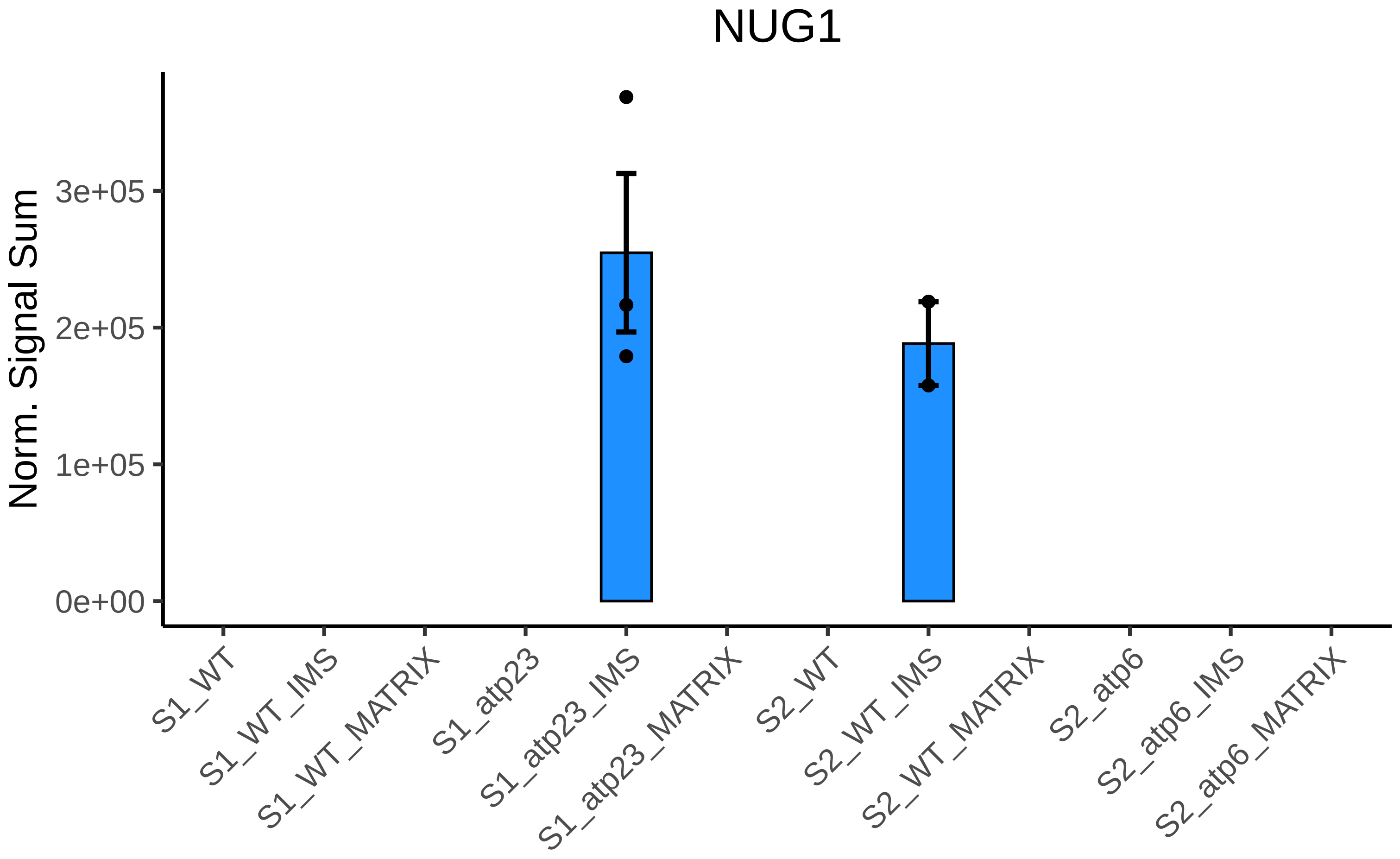
NUG1
Norm. Signal Sum
3e+05
2e+05
1e+05
0e+00
S1_WT
S1_WT_IMS
S1_WT_MATRIX
S1_atp23
S1_atp23_IMS
S1_atp23_MATRIX
S2_WT
S2_WT_IMS
S2_WT_MATRIX
S2_atp6
S2_atp6_IMS
S2_atp6_MATRIX

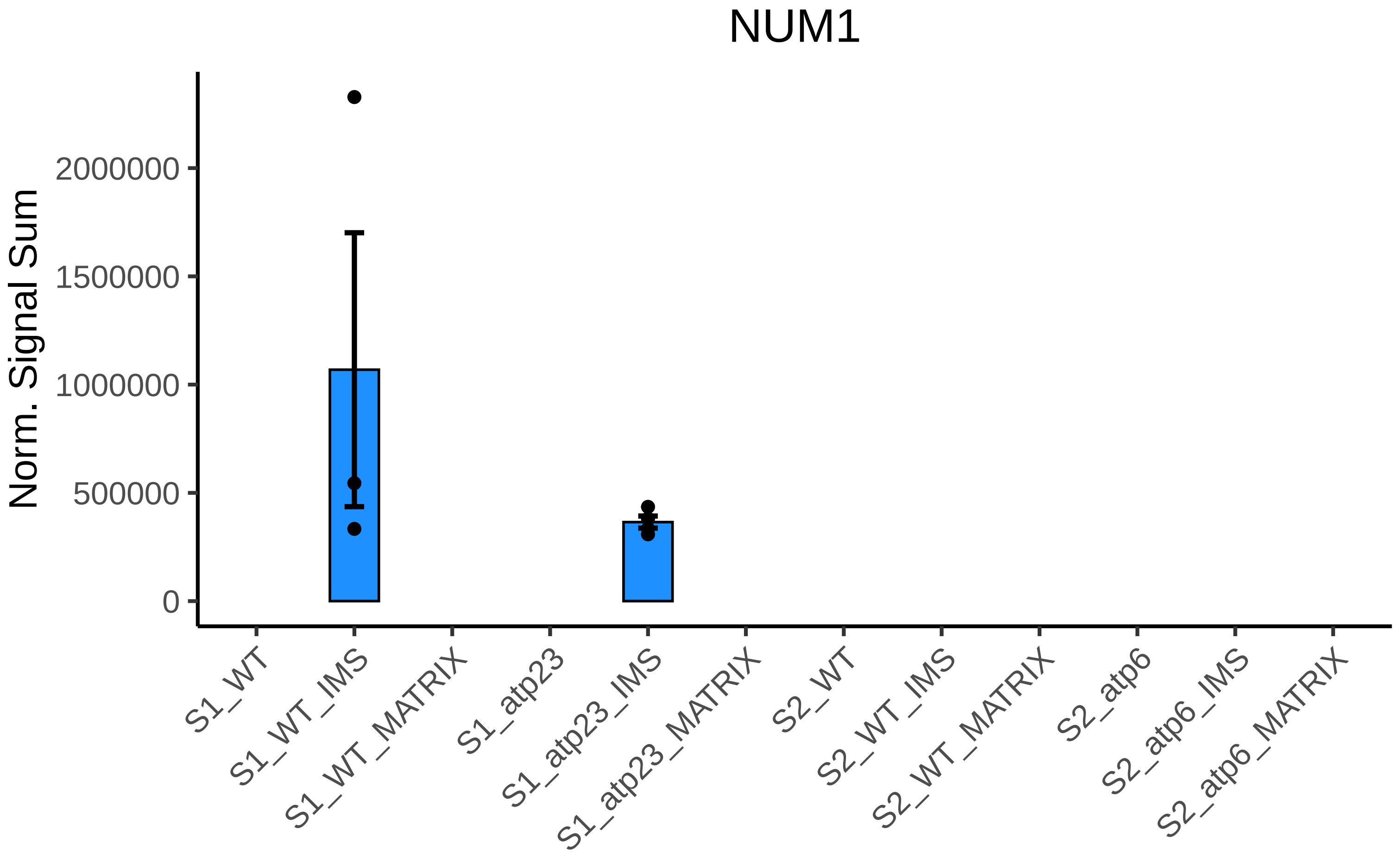

NUM1
Norm. Signal Sum
2000000
1500000
1000000
500000
0
S1_WT
S1_WT_IMS
S1_WT_MATRIX
S1_atp23
S1_atp23_IMS
S1_atp23_MATRIX
S2_WT
S2_WT_IMS
S2_WT_MATRIX
S2_atp6
S2_atp6_IMS
S2_atp6_MATRIX

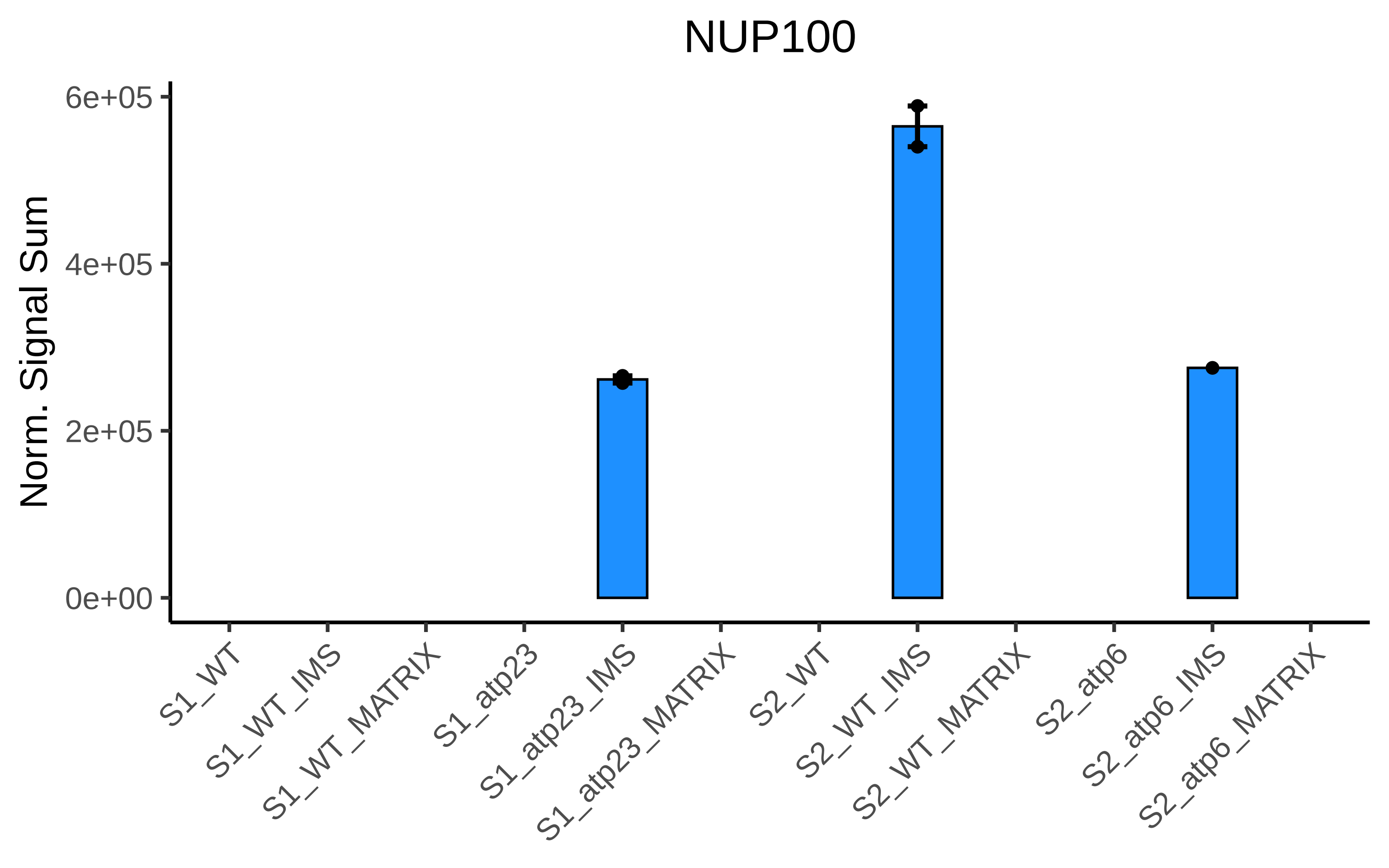
NUP100
Norm. Signal Sum
6e+05
4e+05
2e+05
0e+00
S1_WT
S1_WT_IMS
S1_WT_MATRIX
S1_atp23
S1_atp23_IMS
S1_atp23_MATRIX
S2_WT
S2_WT_IMS
S2_WT_MATRIX
S2_atp6
S2_atp6_IMS
S2_atp6_MATRIX

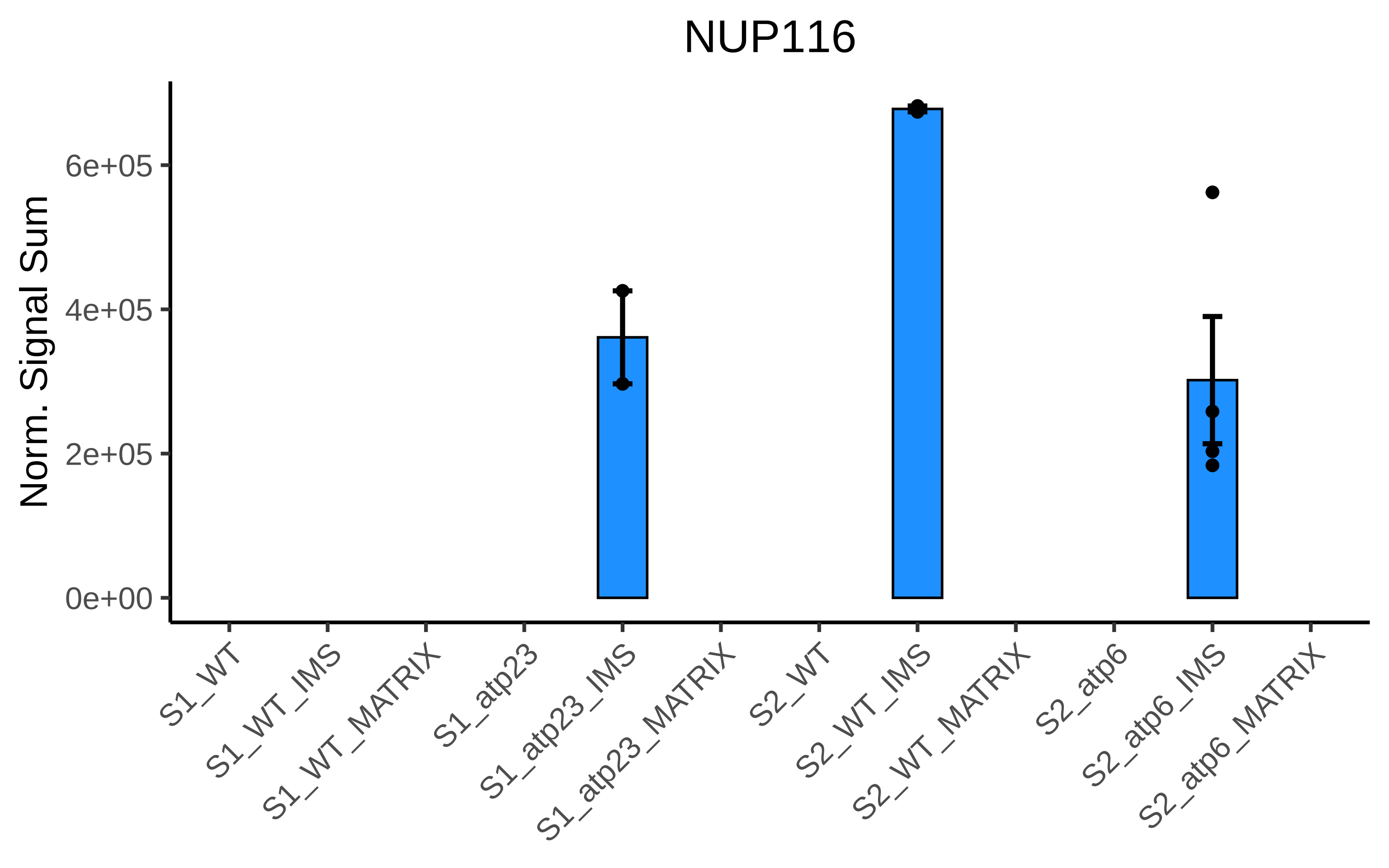

NUP116
Norm. Signal Sum
6e+05
4e+05
2e+05
0e+00
S1_WT
S1_WT_IMS
S1_WT_MATRIX
S1_atp23
S1_atp23_IMS
S1_atp23_MATRIX
S2_WT
S2_WT_IMS
S2_WT_MATRIX
S2_atp6
S2_atp6_IMS
S2_atp6_MATRIX

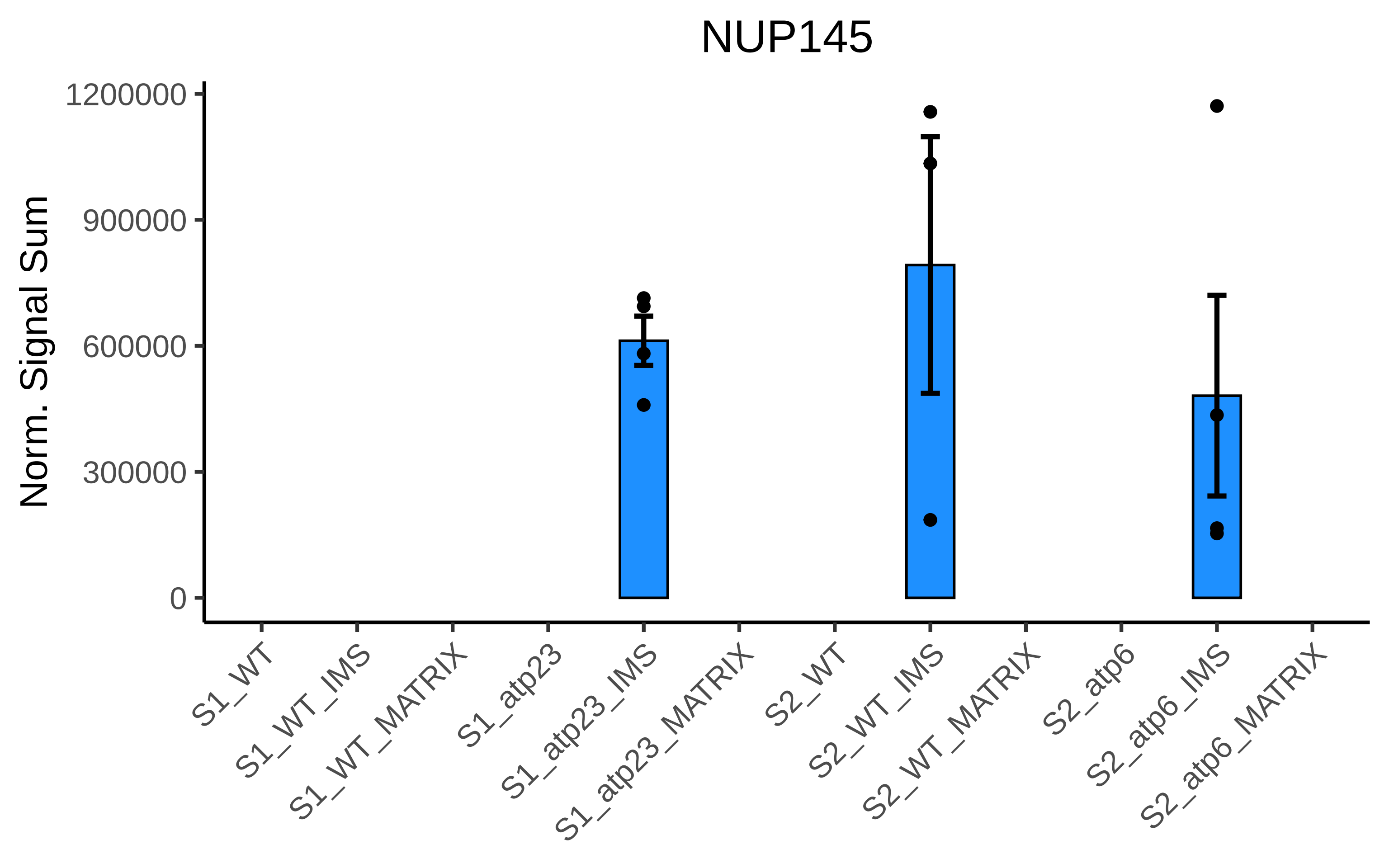

NUP145
Norm. Signal Sum
1200000
900000
600000
300000
0
S1_WT
S1_WT_IMS
S1_WT_MATRIX
S1_atp23
S1_atp23_IMS
S1_atp23_MATRIX
S2_WT
S2_WT_IMS
S2_WT_MATRIX
S2_atp6
S2_atp6_IMS
S2_atp6_MATRIX

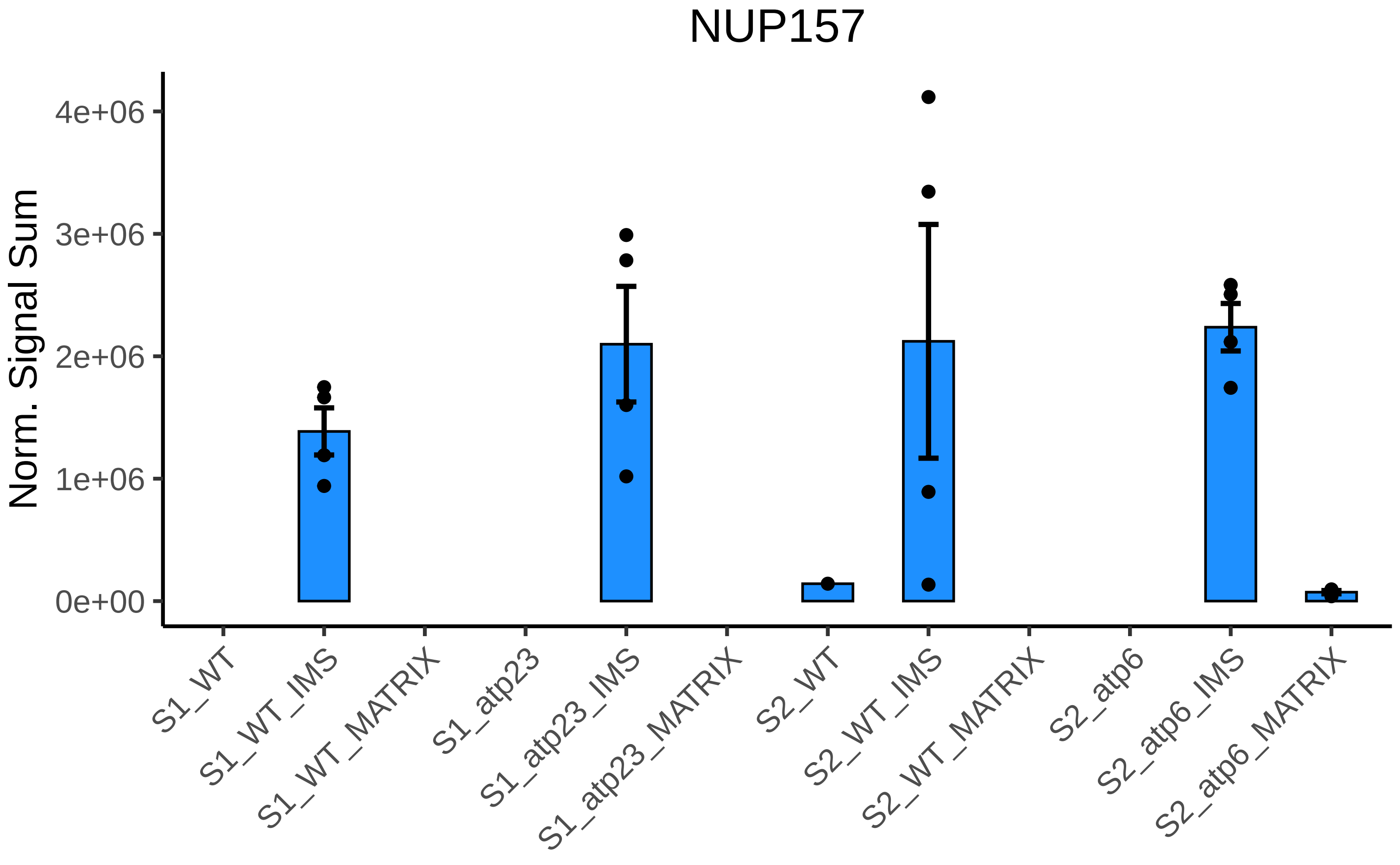
NUP157
Norm. Signal Sum
4e+06
3e+06
2e+06
1e+06
0e+00
S1_WT
S1_WT_IMS
S1_WT_MATRIX
S1_atp23
S1_atp23_IMS
S1_atp23_MATRIX
S2_WT
S2_WT_IMS
S2_WT_MATRIX
S2_atp6
S2_atp6_IMS
S2_atp6_MATRIX

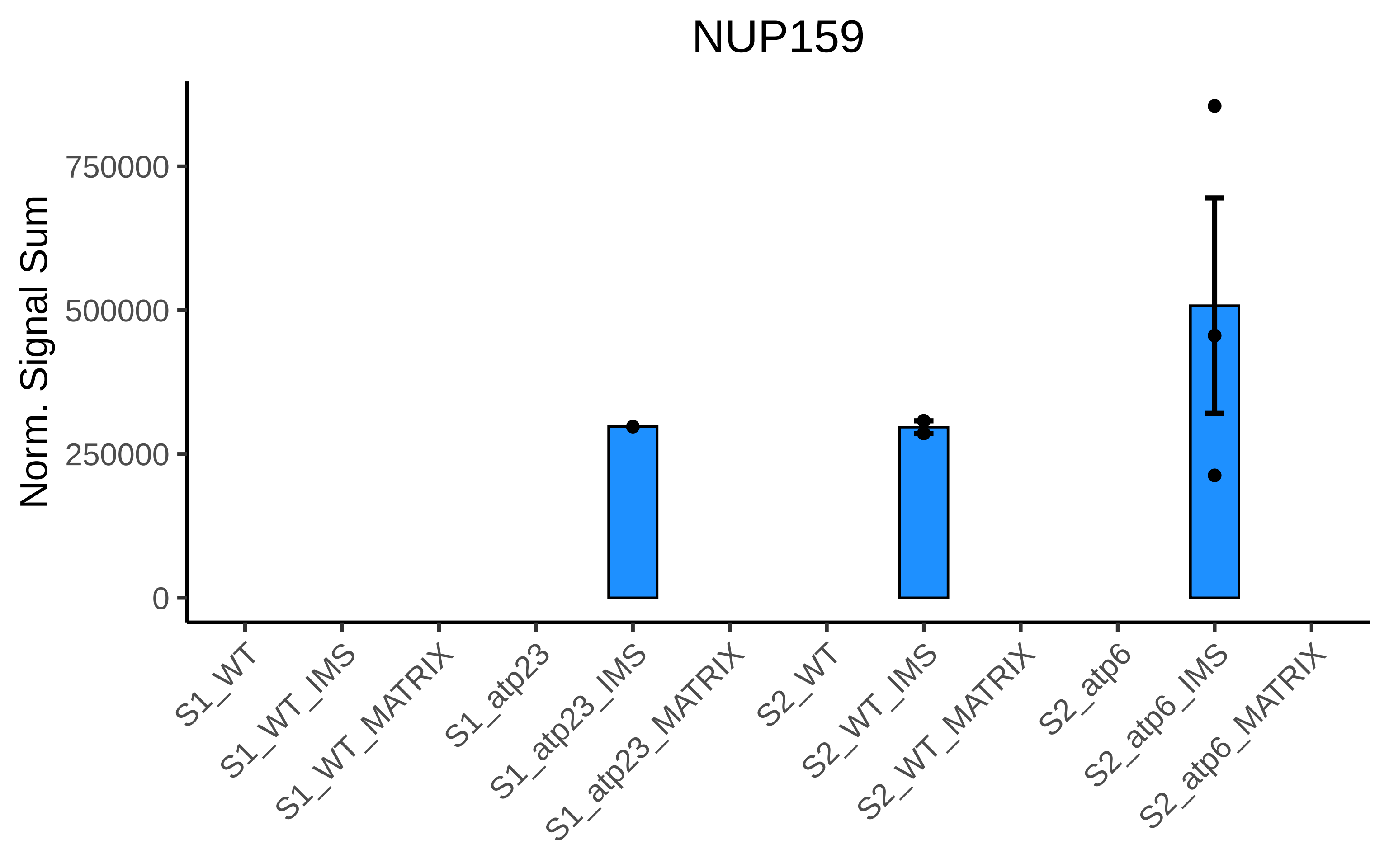

NUP159
Norm. Signal Sum
750000
500000
250000
0
S1_WT
S1_WT_IMS
S1_WT_MATRIX
S1_atp23
S1_atp23_IMS
S1_atp23_MATRIX
S2_WT
S2_WT_IMS
S2_WT_MATRIX
S2_atp6
S2_atp6_IMS
S2_atp6_MATRIX

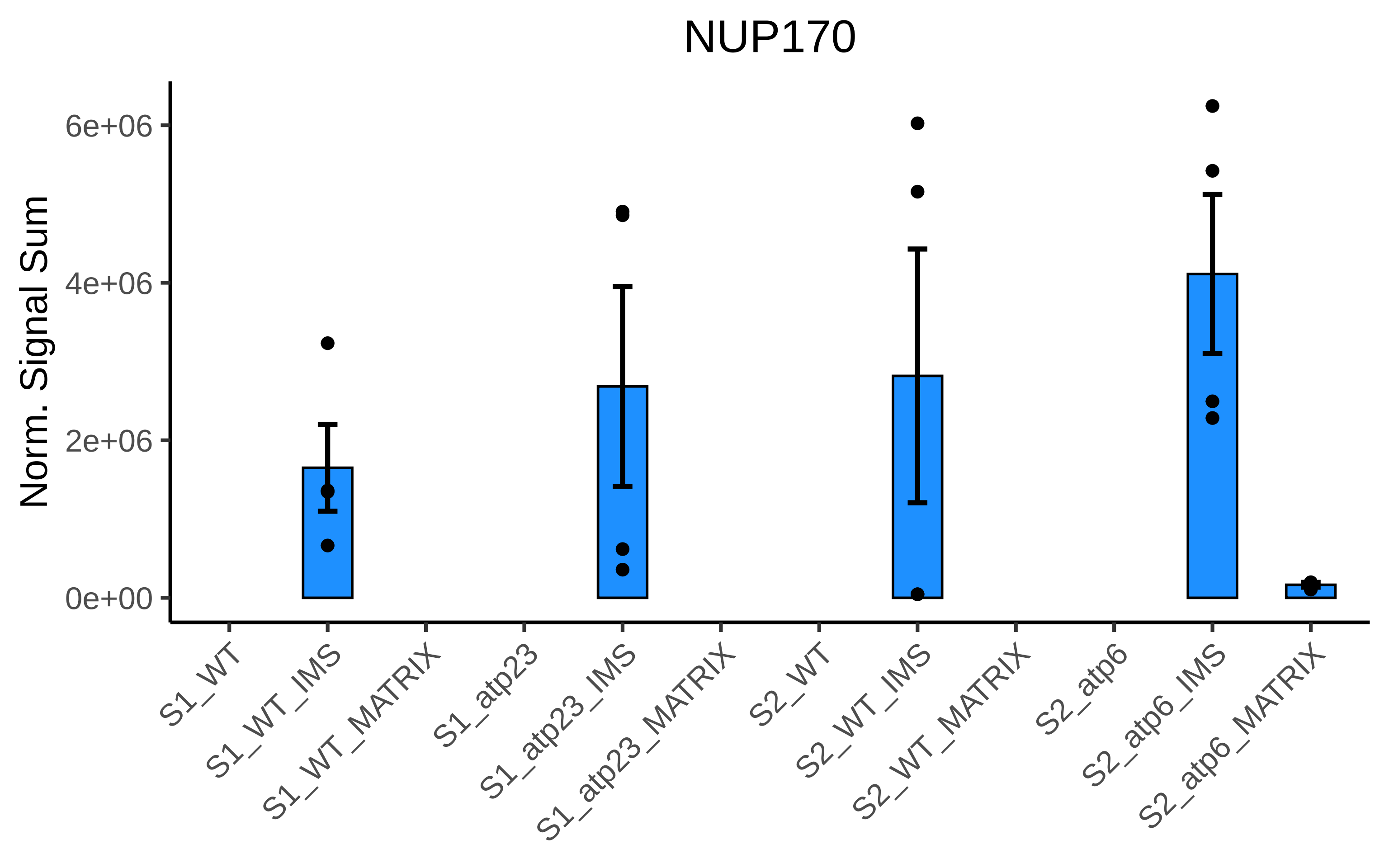
NUP170
Norm. Signal Sum
6e+06
4e+06
2e+06
0e+00
S1_WT
S1_WT_IMS
S1_WT_MATRIX
S1_atp23
S1_atp23_IMS
S1_atp23_MATRIX
S2_WT
S2_WT_IMS
S2_WT_MATRIX
S2_atp6
S2_atp6_IMS
S2_atp6_MATRIX

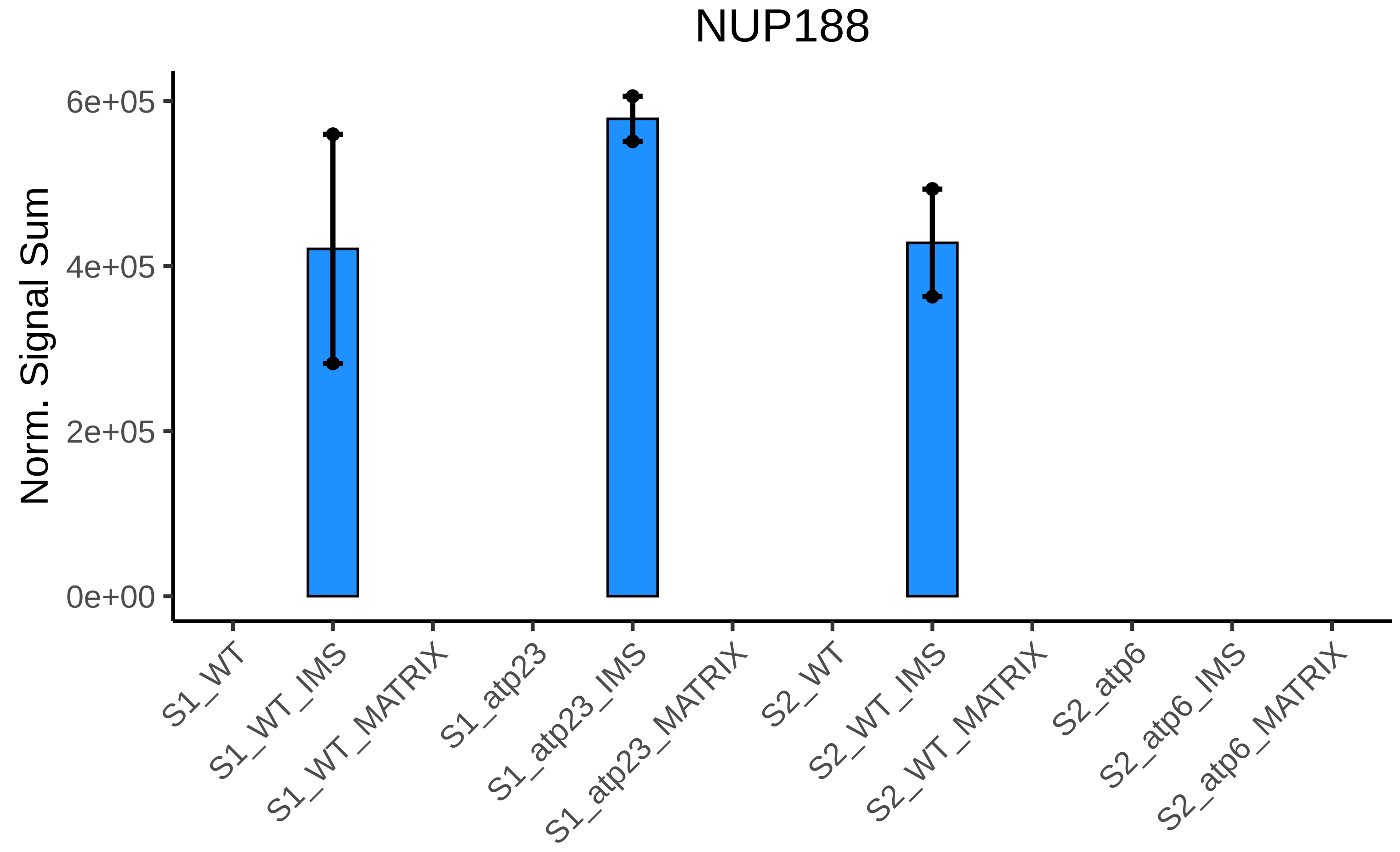

NUP188
Norm. Signal Sum
6e+05
4e+05
2e+05
0e+00
S1_WT
S1_WT_IMS
S1_WT_MATRIX
S1_atp23
S1_atp23_IMS
S1_atp23_MATRIX
S2_WT
S2_WT_IMS
S2_WT_MATRIX
S2_atp6
S2_atp6_IMS
S2_atp6_MATRIX

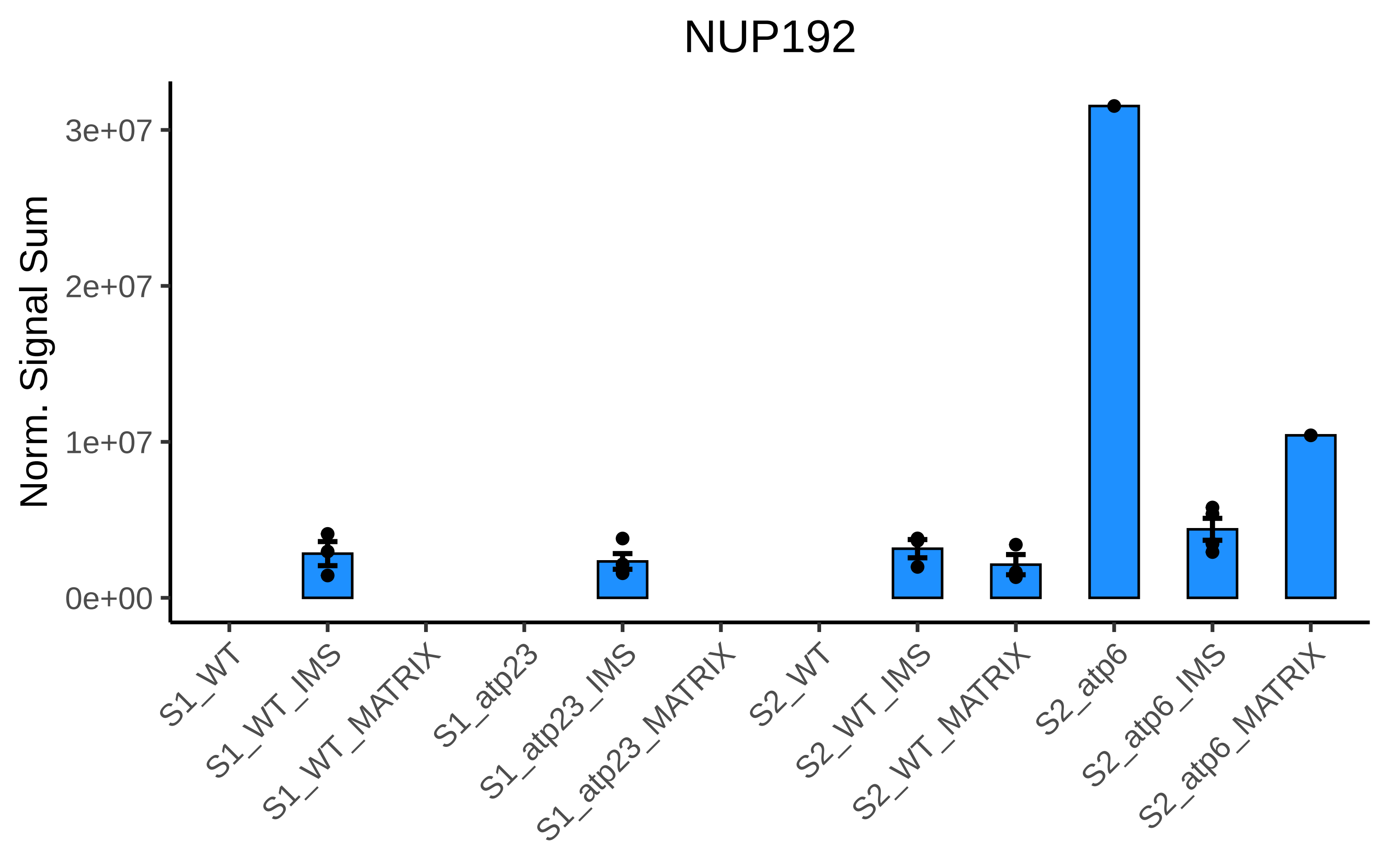
NUP192
Norm. Signal Sum
3e+07
2e+07
1e+07
0e+00
S1_WT
S1_WT_IMS
S1_WT_MATRIX
S1_atp23
S1_atp23_IMS
S1_atp23_MATRIX
S2_WT
S2_WT_IMS
S2_WT_MATRIX
S2_atp6
S2_atp6_IMS
S2_atp6_MATRIX

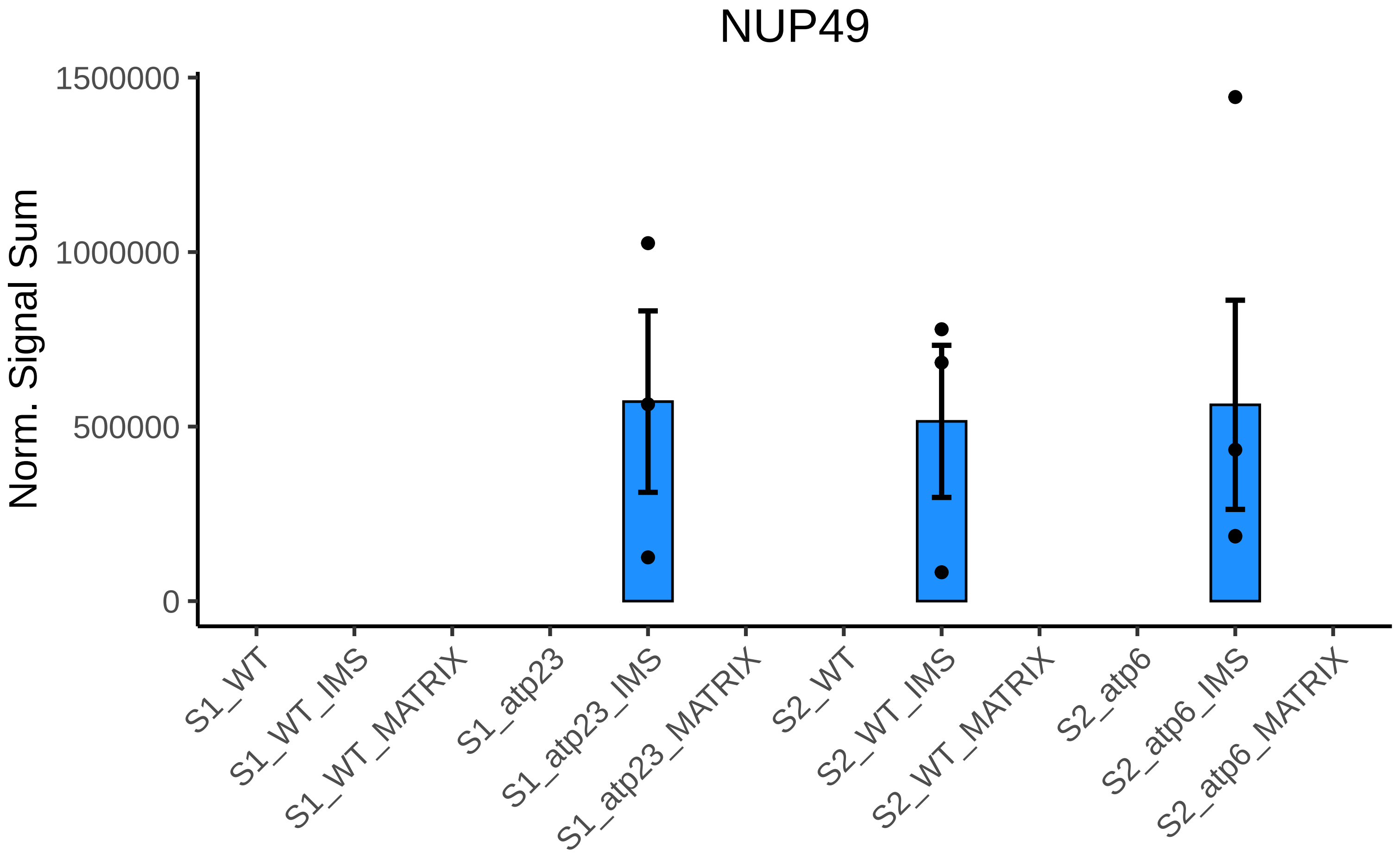
NUP49
Norm. Signal Sum
1500000
1000000
500000
0
S1_WT
S1_WT_IMS
S1_WT_MATRIX
S1_atp23
S1_atp23_IMS
S1_atp23_MATRIX
S2_WT
S2_WT_IMS
S2_WT_MATRIX
S2_atp6
S2_atp6_IMS
S2_atp6_MATRIX

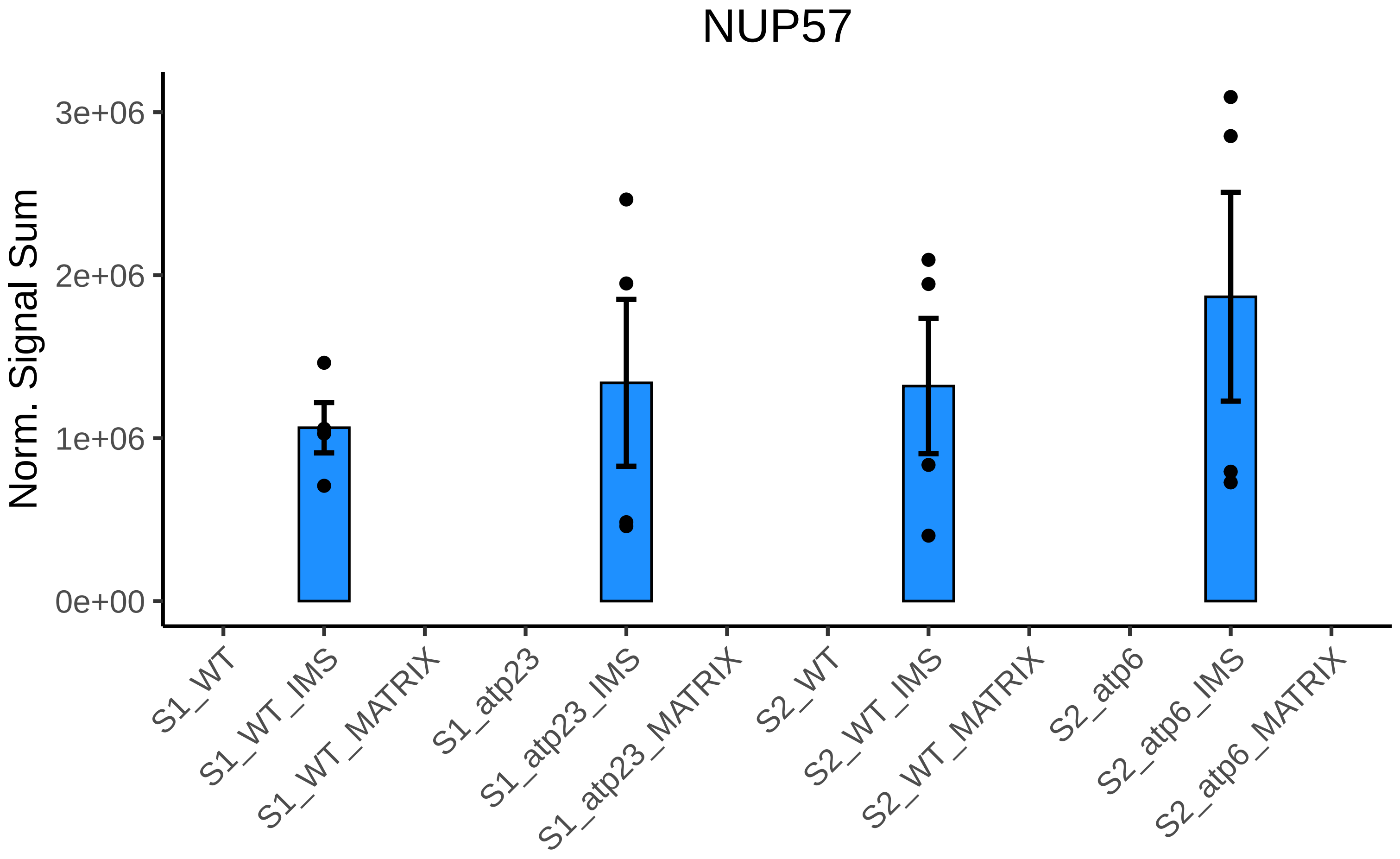

NUP57
Norm. Signal Sum
3e+06
2e+06
1e+06
0e+00
S1_WT
S1_WT_IMS
S1_WT_MATRIX
S1_atp23
S1_atp23_IMS
S1_atp23_MATRIX
S2_WT
S2_WT_IMS
S2_WT_MATRIX
S2_atp6
S2_atp6_IMS
S2_atp6_MATRIX

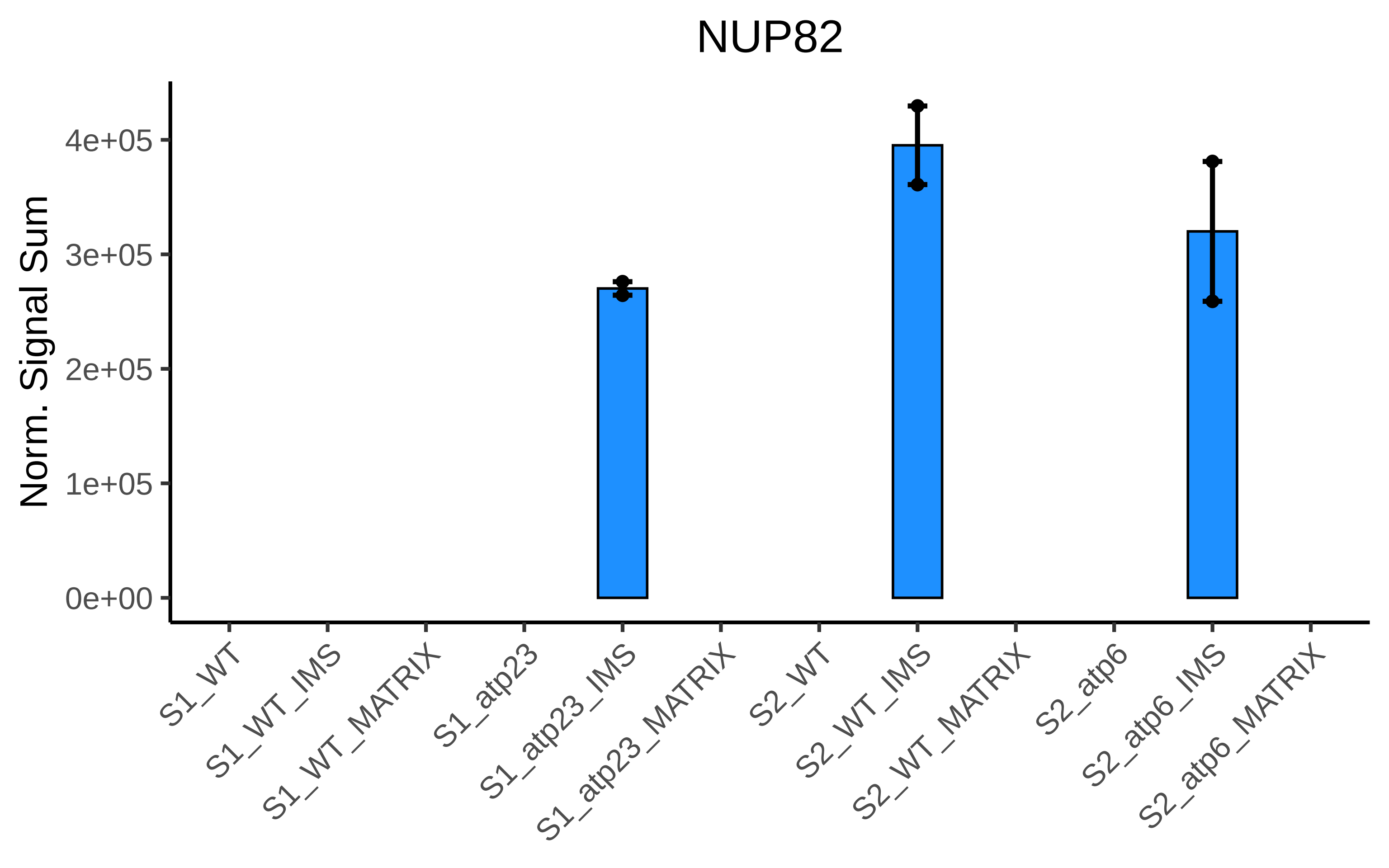
NUP82
Norm. Signal Sum
0e+00
1e+05
2e+05
3e+05
4e+05
S1_WT
S1_WT_IMS
S1_WT_MATRIX
S1_atp23
S1_atp23_IMS
S1_atp23_MATRIX
S2_WT
S2_WT_IMS
S2_WT_MATRIX
S2_atp6
S2_atp6_IMS
S2_atp6_MATRIX

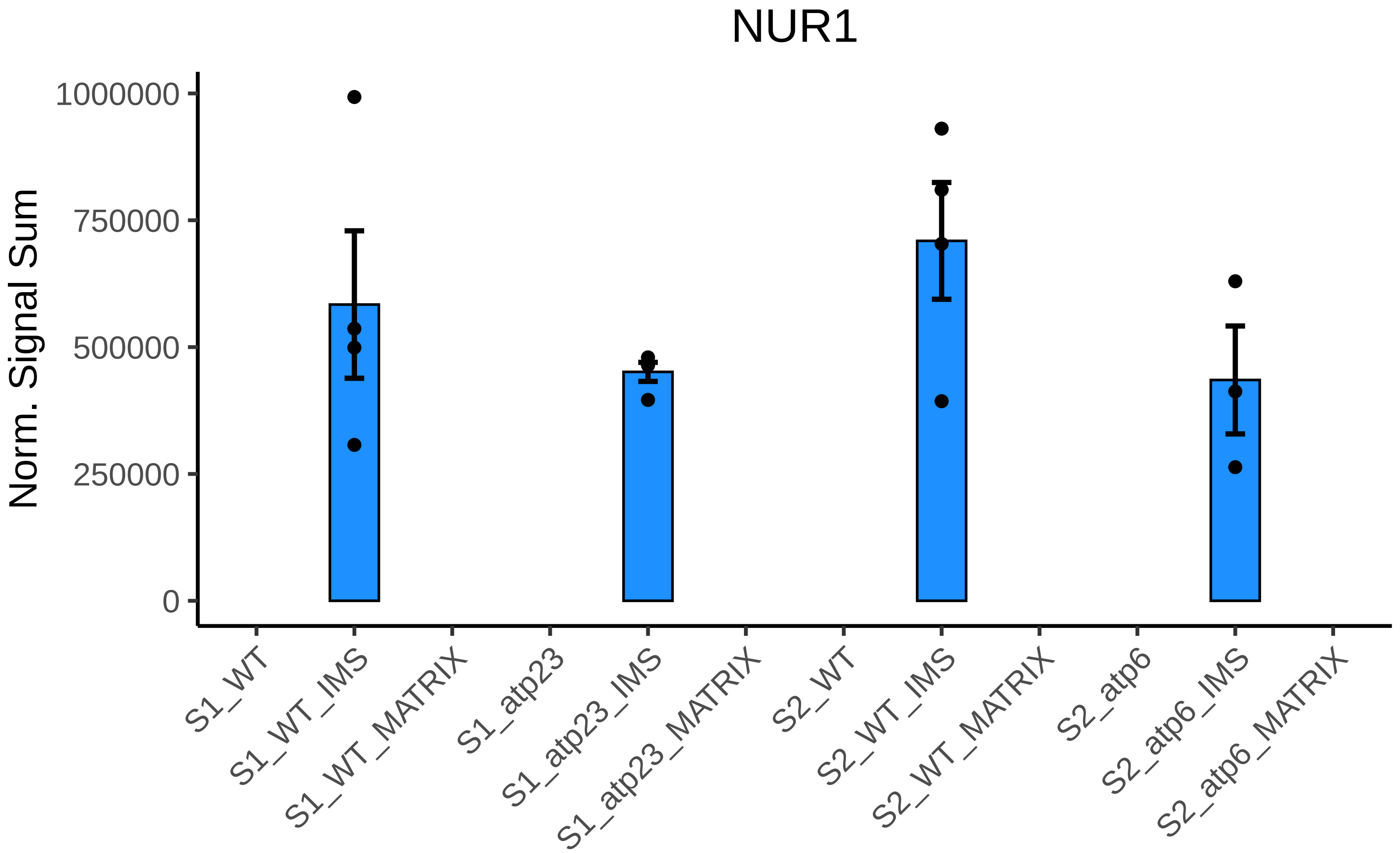
NUR1
Norm. Signal Sum
1000000
750000
500000
250000
0
S1_WT
S1_WT_IMS
S1_WT_MATRIX
S1_atp23
S1_atp23_IMS
S1_atp23_MATRIX
S2_WT
S2_WT_IMS
S2_WT_MATRIX
S2_atp6
S2_atp6_IMS
S2_atp6_MATRIX

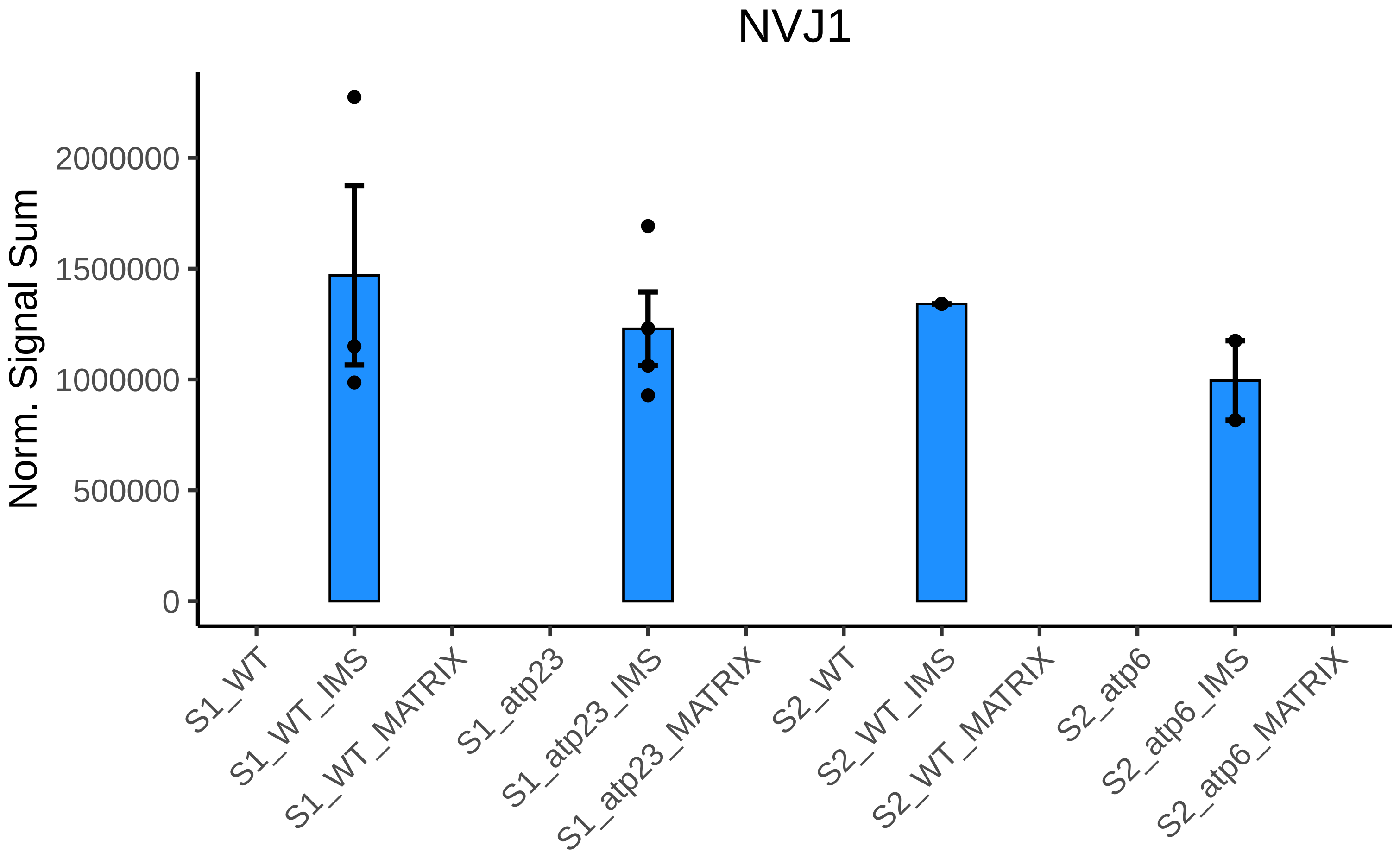
NVJ1
Norm. Signal Sum
2000000
1500000
1000000
500000
0
S1_WT
S1_WT_IMS
S1_WT_MATRIX
S1_atp23
S1_atp23_IMS
S1_atp23_MATRIX
S2_WT
S2_WT_IMS
S2_WT_MATRIX
S2_atp6
S2_atp6_IMS
S2_atp6_MATRIX

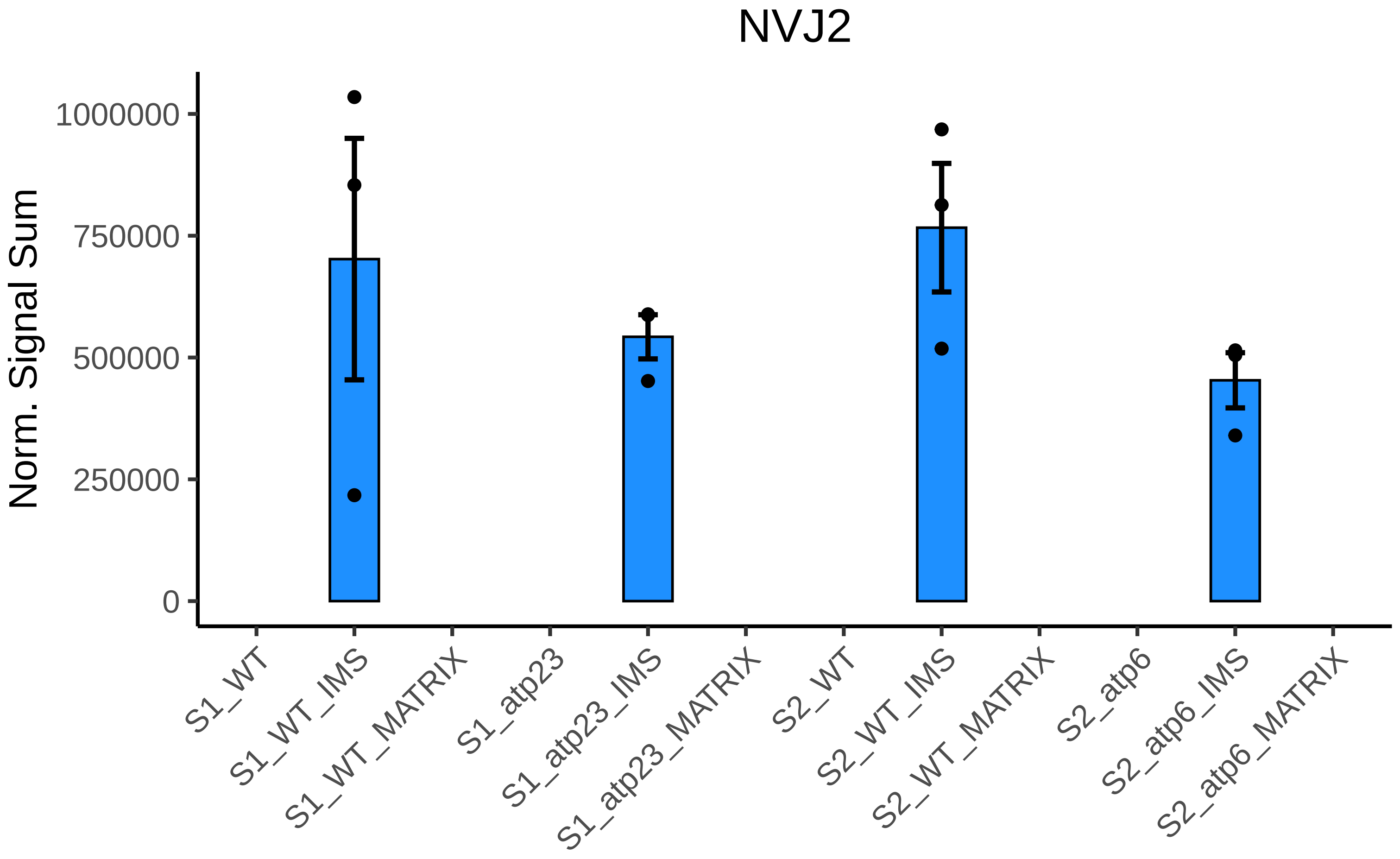

NVJ2
Norm. Signal Sum
1000000
750000
500000
250000
0
S1_WT
S1_WT_IMS
S1_WT_MATRIX
S1_atp23
S1_atp23_IMS
S1_atp23_MATRIX
S2_WT
S2_WT_IMS
S2_WT_MATRIX
S2_atp6
S2_atp6_IMS
S2_atp6_MATRIX

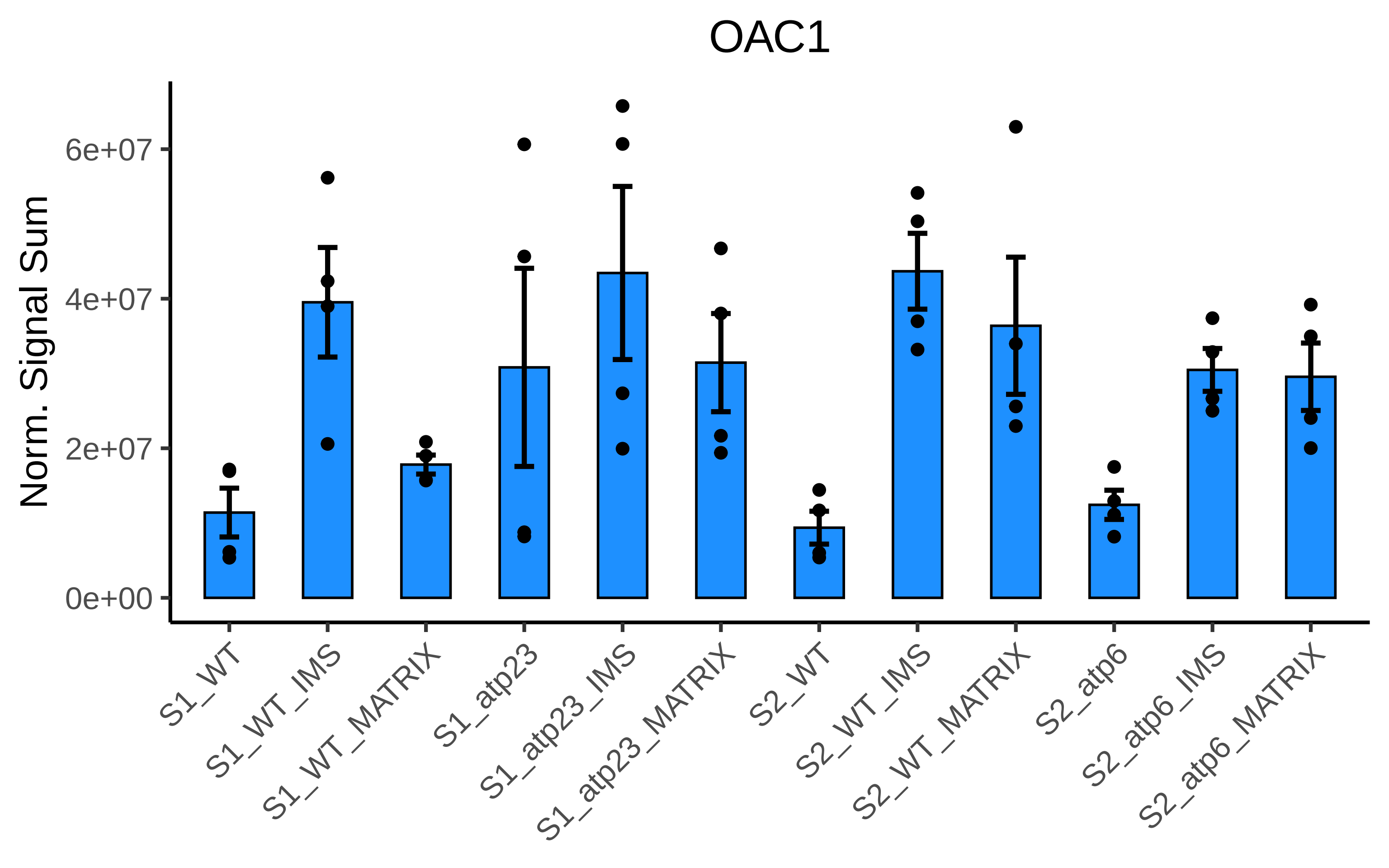

OAC1
Norm. Signal Sum
6e+07
4e+07
2e+07
0e+00
S1_WT
S1_WT_IMS
S1_WT_MATRIX
S1_atp23
S1_atp23_IMS
S1_atp23_MATRIX
S2_WT
S2_WT_IMS
S2_WT_MATRIX
S2_atp6
S2_atp6_IMS
S2_atp6_MATRIX

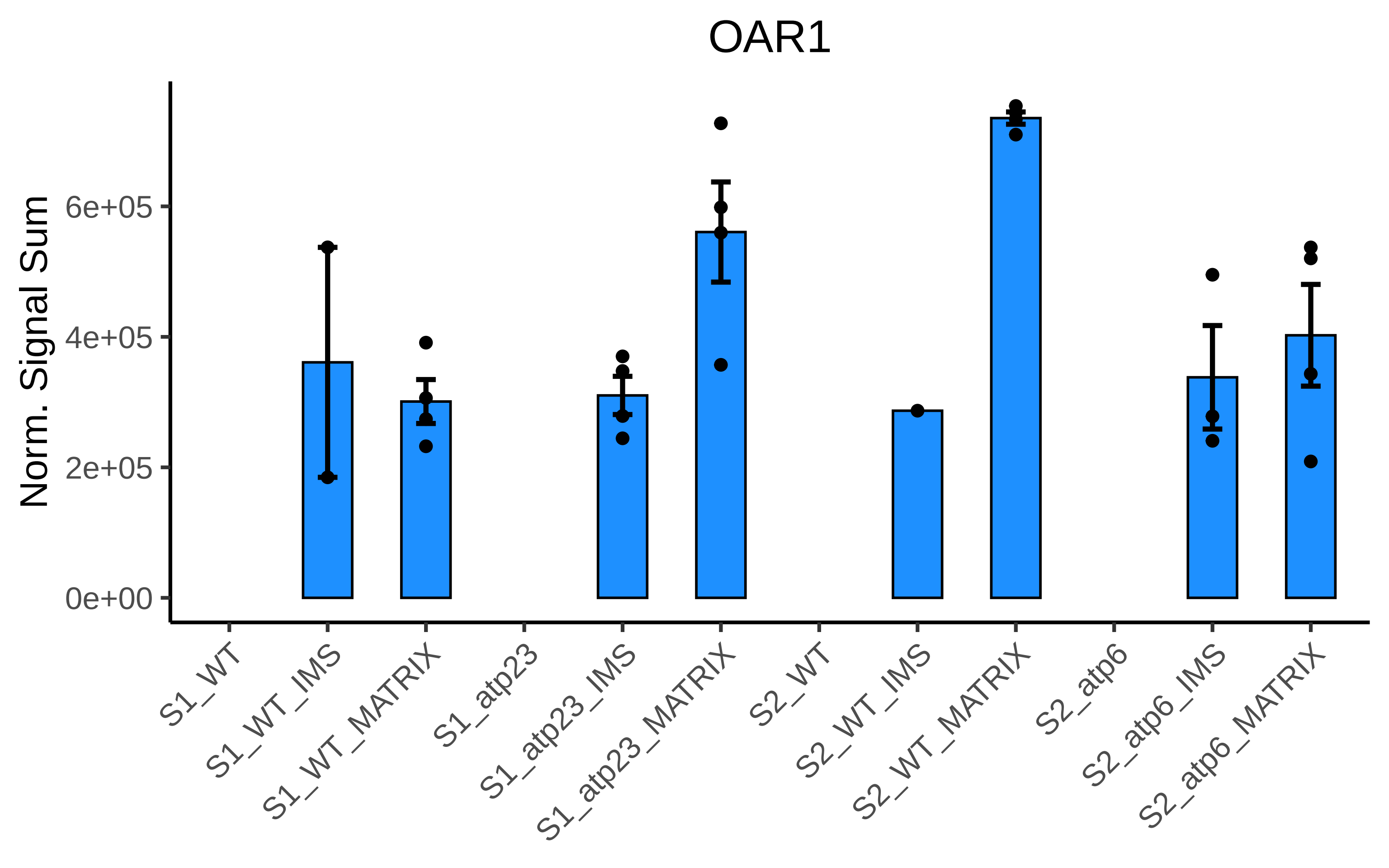

OAR1
Norm. Signal Sum
0e+00
2e+05
4e+05
6e+05
S1_WT
S1_WT_IMS
S1_WT_MATRIX
S1_atp23
S1_atp23_IMS
S1_atp23_MATRIX
S2_WT
S2_WT_IMS
S2_WT_MATRIX
S2_atp6
S2_atp6_IMS
S2_atp6_MATRIX

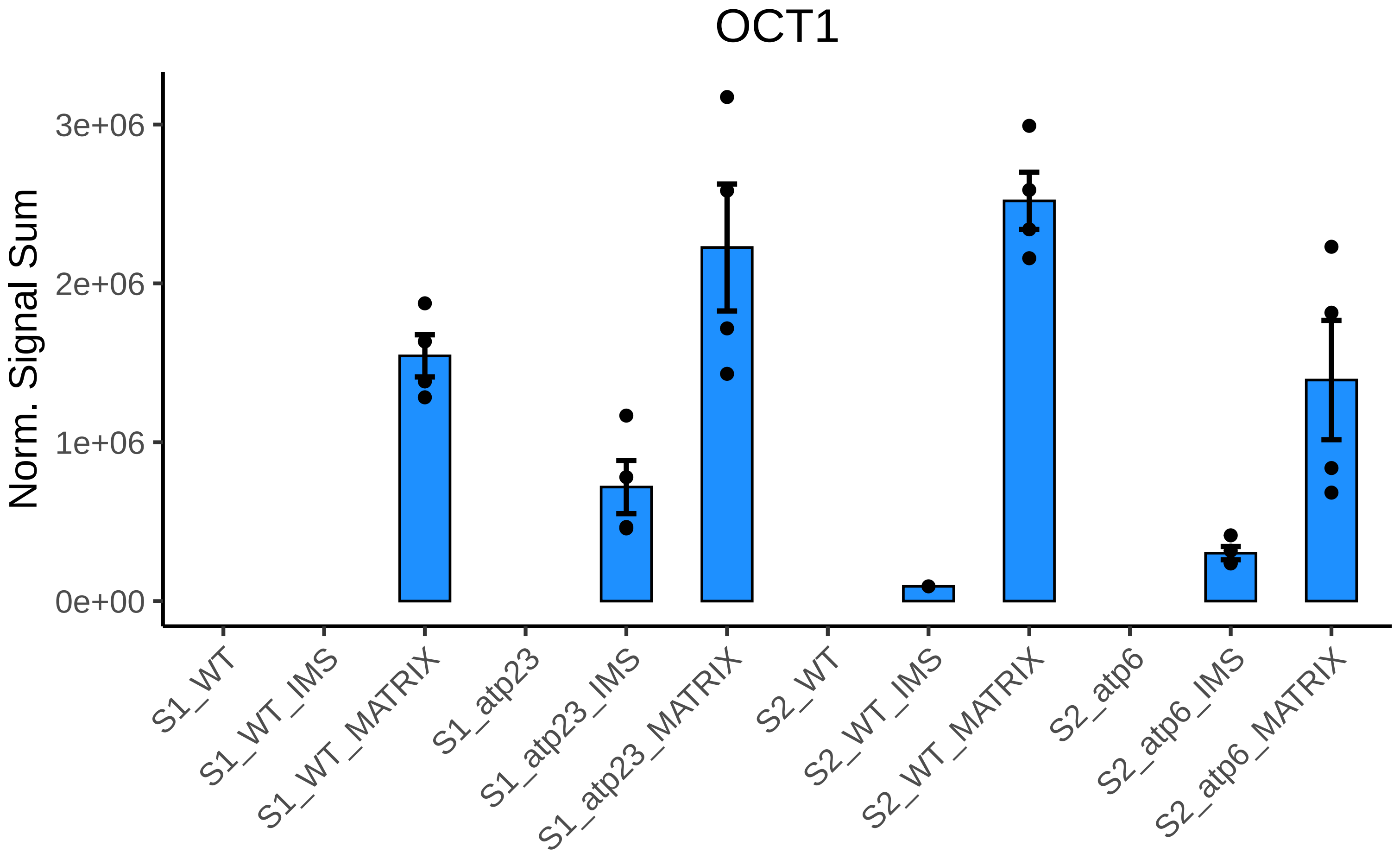

OCT1
Norm. Signal Sum
3e+06
2e+06
1e+06
0e+00
S1_WT
S1_WT_IMS
S1_WT_MATRIX
S1_atp23
S1_atp23_IMS
S1_atp23_MATRIX
S2_WT
S2_WT_IMS
S2_WT_MATRIX
S2_atp6
S2_atp6_IMS
S2_atp6_MATRIX

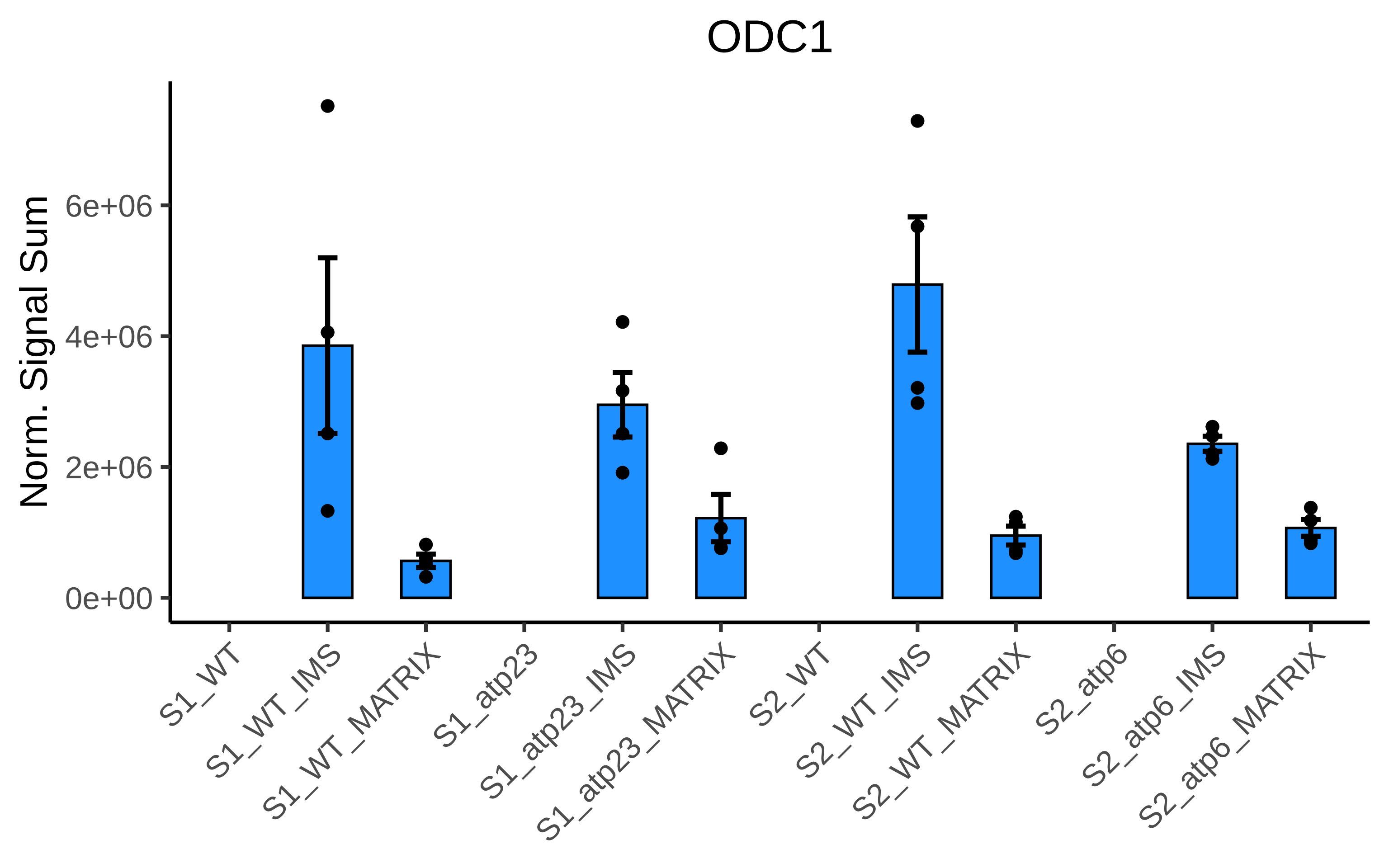
ODC1
Norm. Signal Sum
6e+06
4e+06
2e+06
0e+00
S1_WT
S1_WT_IMS
S1_WT_MATRIX
S1_atp23
S1_atp23_IMS
S1_atp23_MATRIX
S2_WT
S2_WT_IMS
S2_WT_MATRIX
S2_atp6
S2_atp6_IMS
S2_atp6_MATRIX

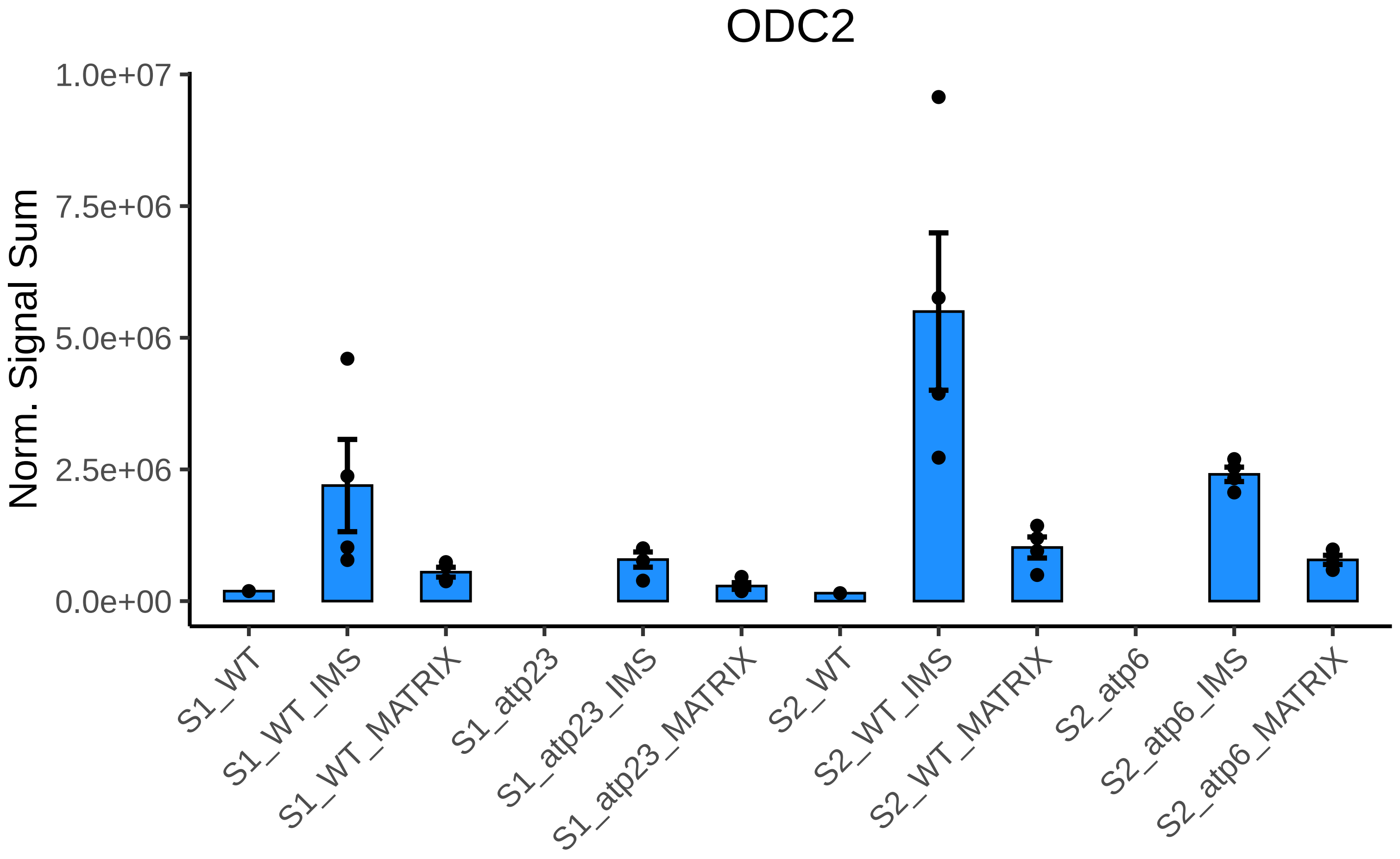
ODC2
Norm. Signal Sum
1.0e+07
7.5e+06
5.0e+06
2.5e+06
0.0e+00
S1_WT
S1_WT_IMS
S1_WT_MATRIX
S1_atp23
S1_atp23_IMS
S1_atp23_MATRIX
S2_WT
S2_WT_IMS
S2_WT_MATRIX
S2_atp6
S2_atp6_IMS
S2_atp6_MATRIX

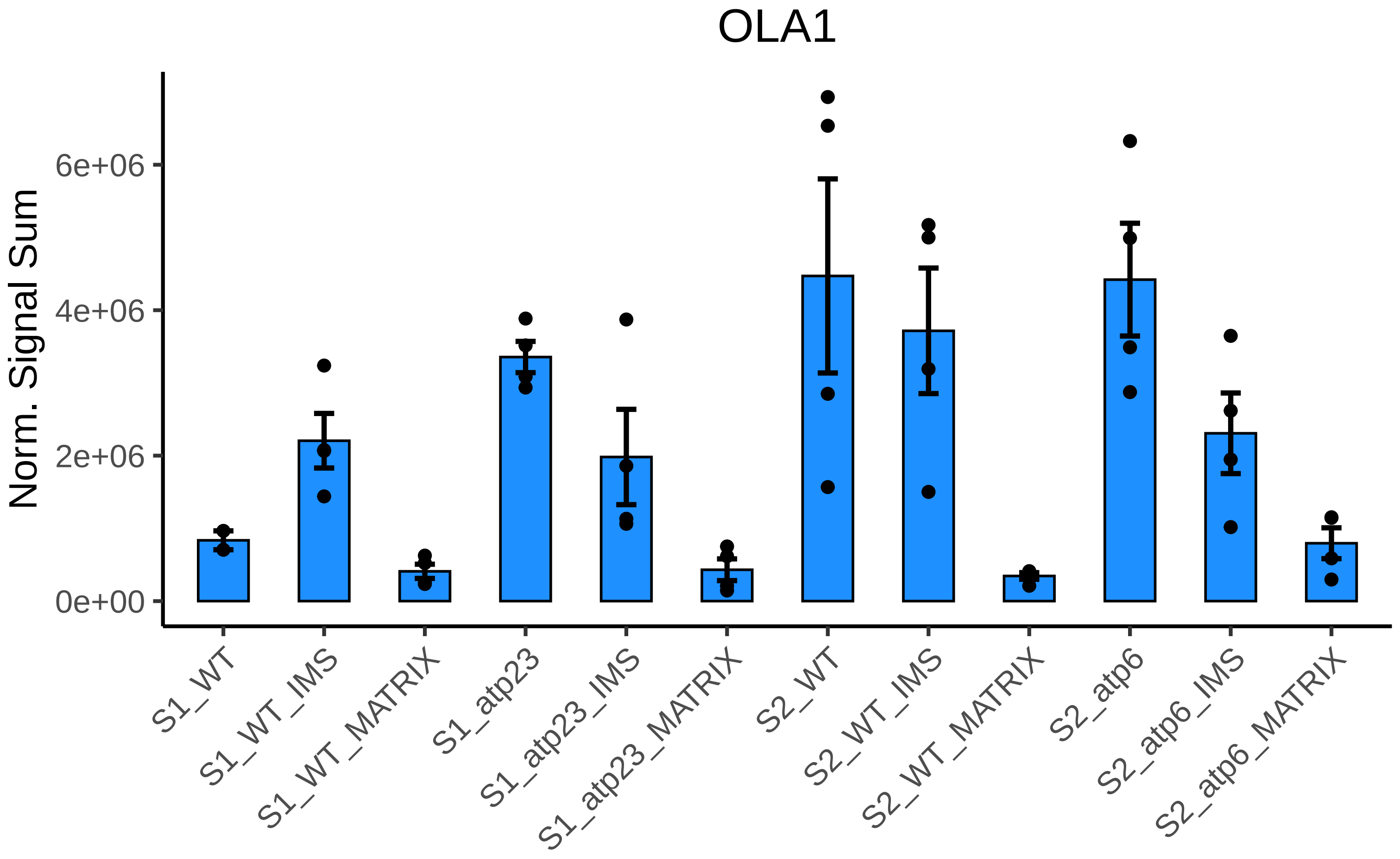

OLA1
Norm. Signal Sum
6e+06
4e+06
2e+06
0e+00
S1_WT
S1_WT_IMS
S1_WT_MATRIX
S1_atp23
S1_atp23_IMS
S1_atp23_MATRIX
S2_WT
S2_WT_IMS
S2_WT_MATRIX
S2_atp6
S2_atp6_IMS
S2_atp6_MATRIX

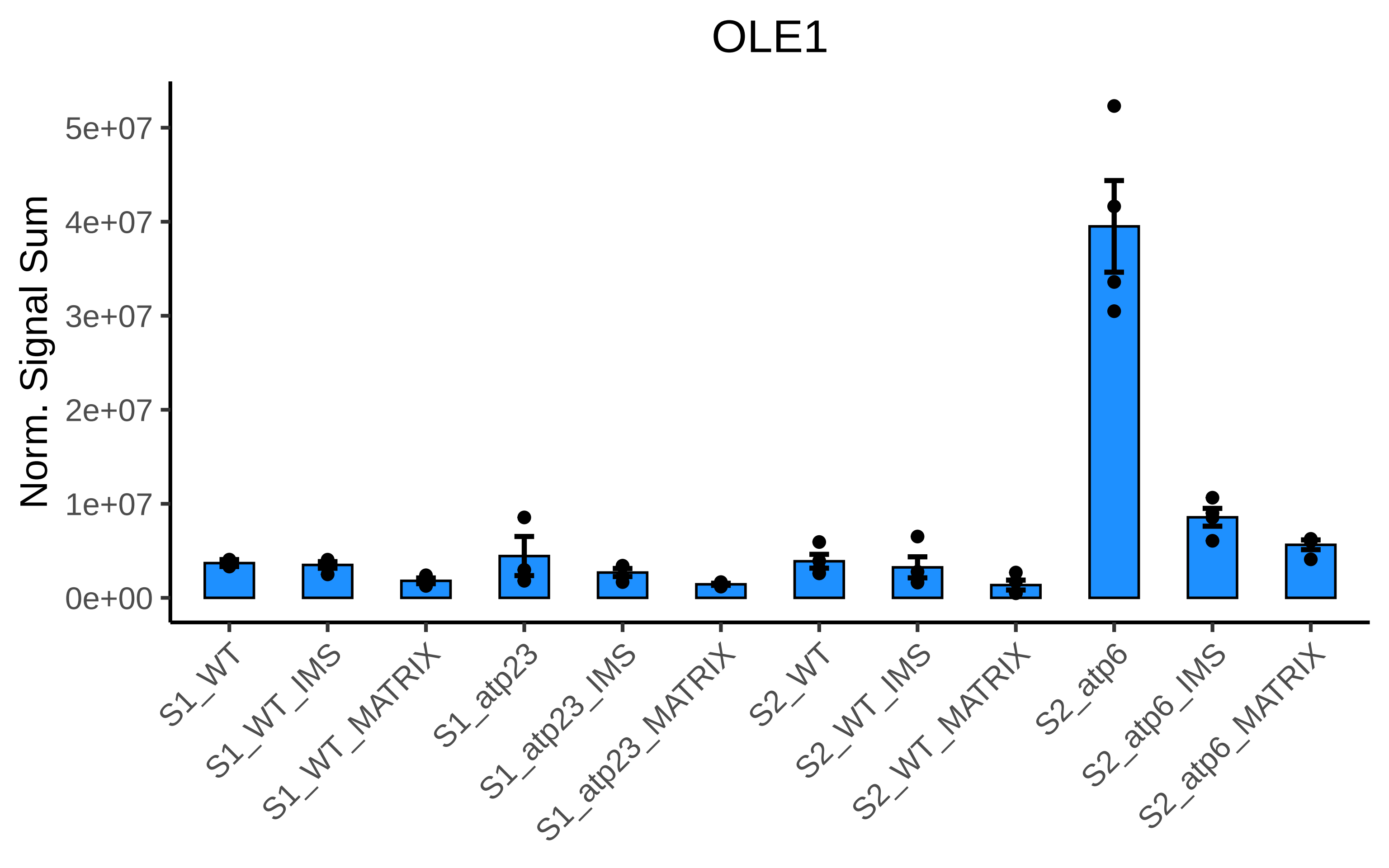

OLE1
Norm. Signal Sum
5e+07
4e+07
3e+07
2e+07
1e+07
0e+00
S1_WT
S1_WT_IMS
S1_WT_MATRIX
S1_atp23
S1_atp23_IMS
S1_atp23_MATRIX
S2_WT
S2_WT_IMS
S2_WT_MATRIX
S2_atp6
S2_atp6_IMS
S2_atp6_MATRIX

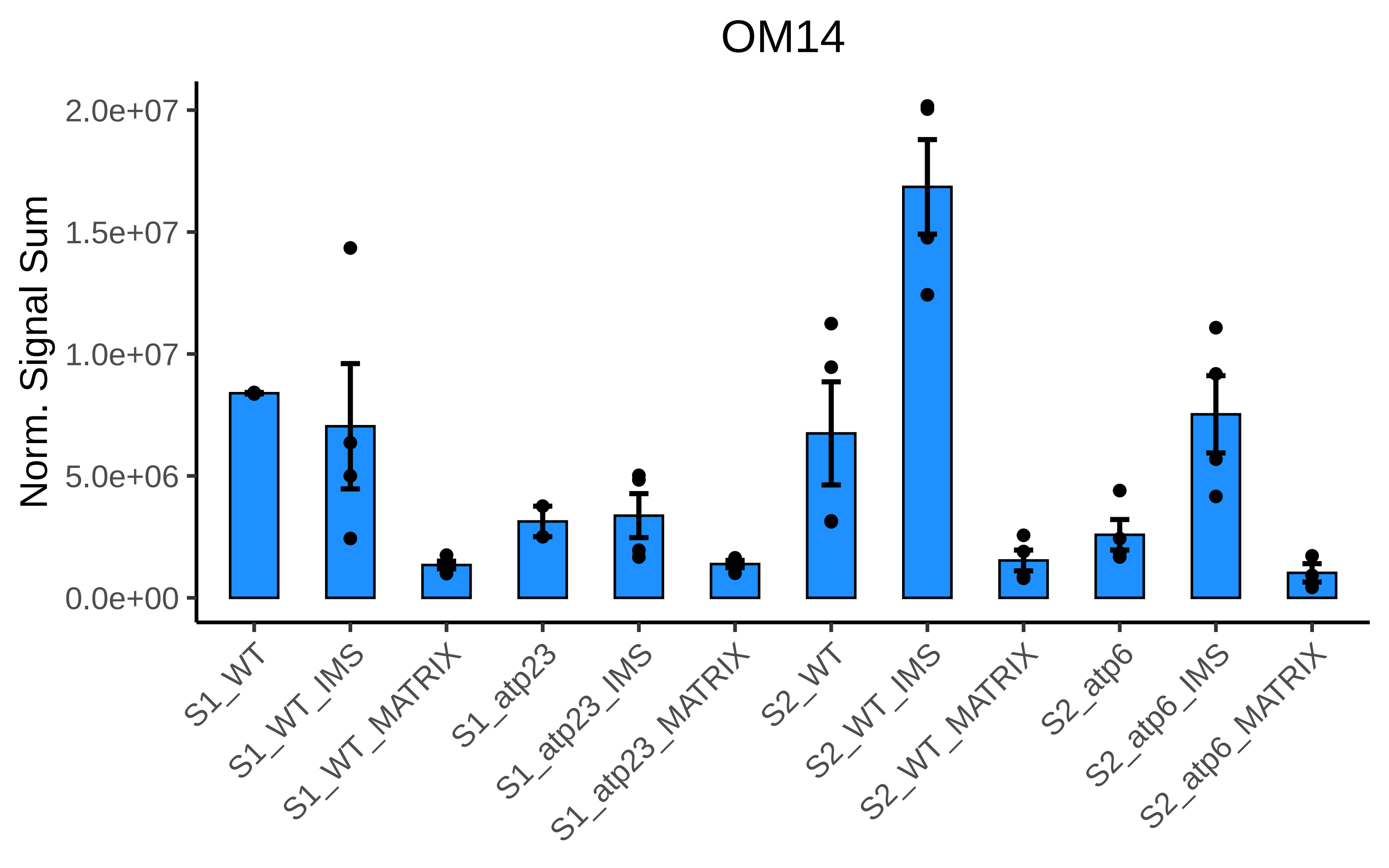

OM14
Norm. Signal Sum
2.0e+07
1.5e+07
1.0e+07
5.0e+06
0.0e+00
S1_WT
S1_WT_IMS
S1_WT_MATRIX
S1_atp23
S1_atp23_IMS
S1_atp23_MATRIX
S2_WT
S2_WT_IMS
S2_WT_MATRIX
S2_atp6
S2_atp6_IMS
S2_atp6_MATRIX

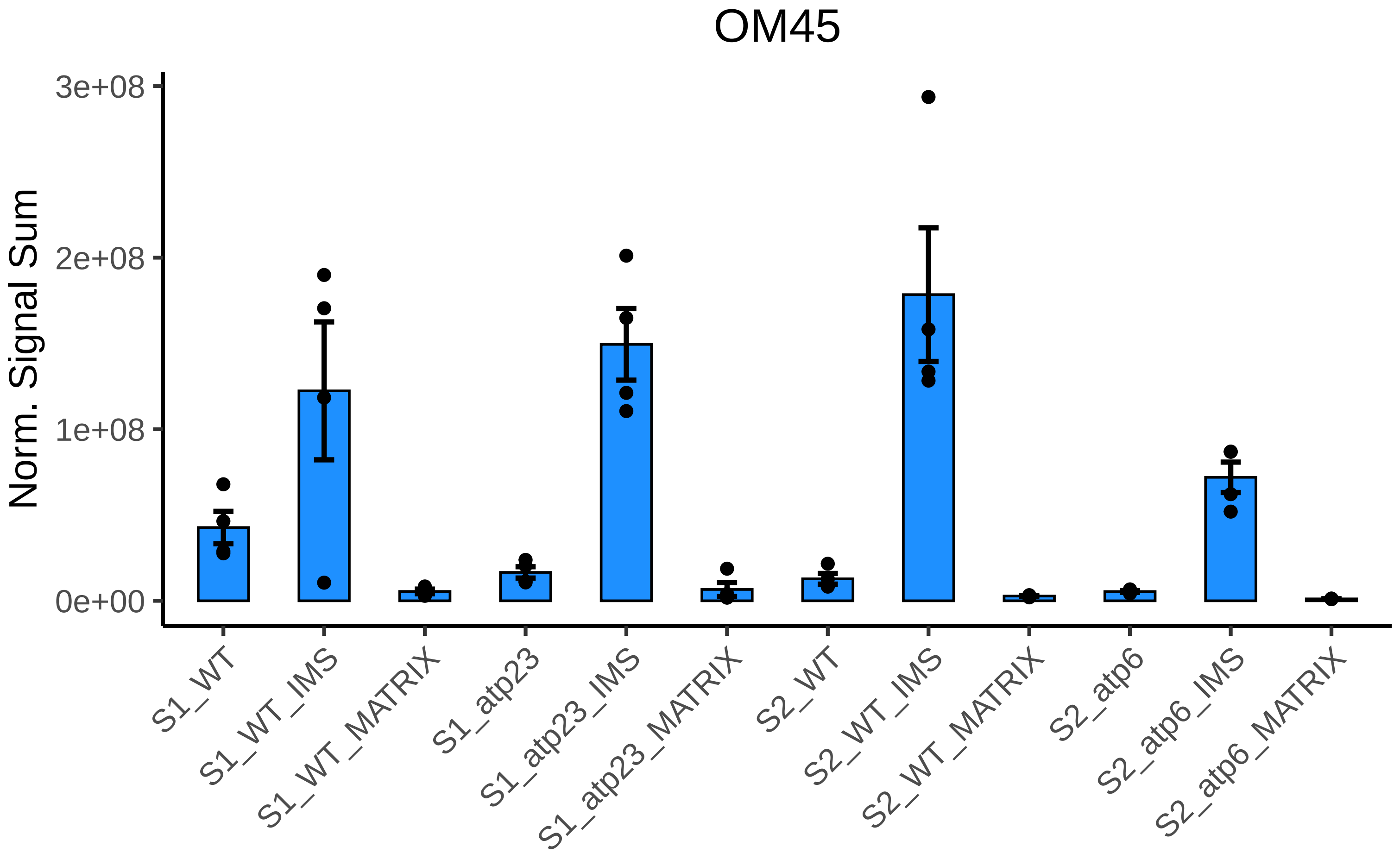
OM45
Norm. Signal Sum
3e+08
2e+08
1e+08
0e+00
S1_WT
S1_WT_IMS
S1_WT_MATRIX
S1_atp23
S1_atp23_IMS
S1_atp23_MATRIX
S2_WT
S2_WT_IMS
S2_WT_MATRIX
S2_atp6
S2_atp6_IMS
S2_atp6_MATRIX

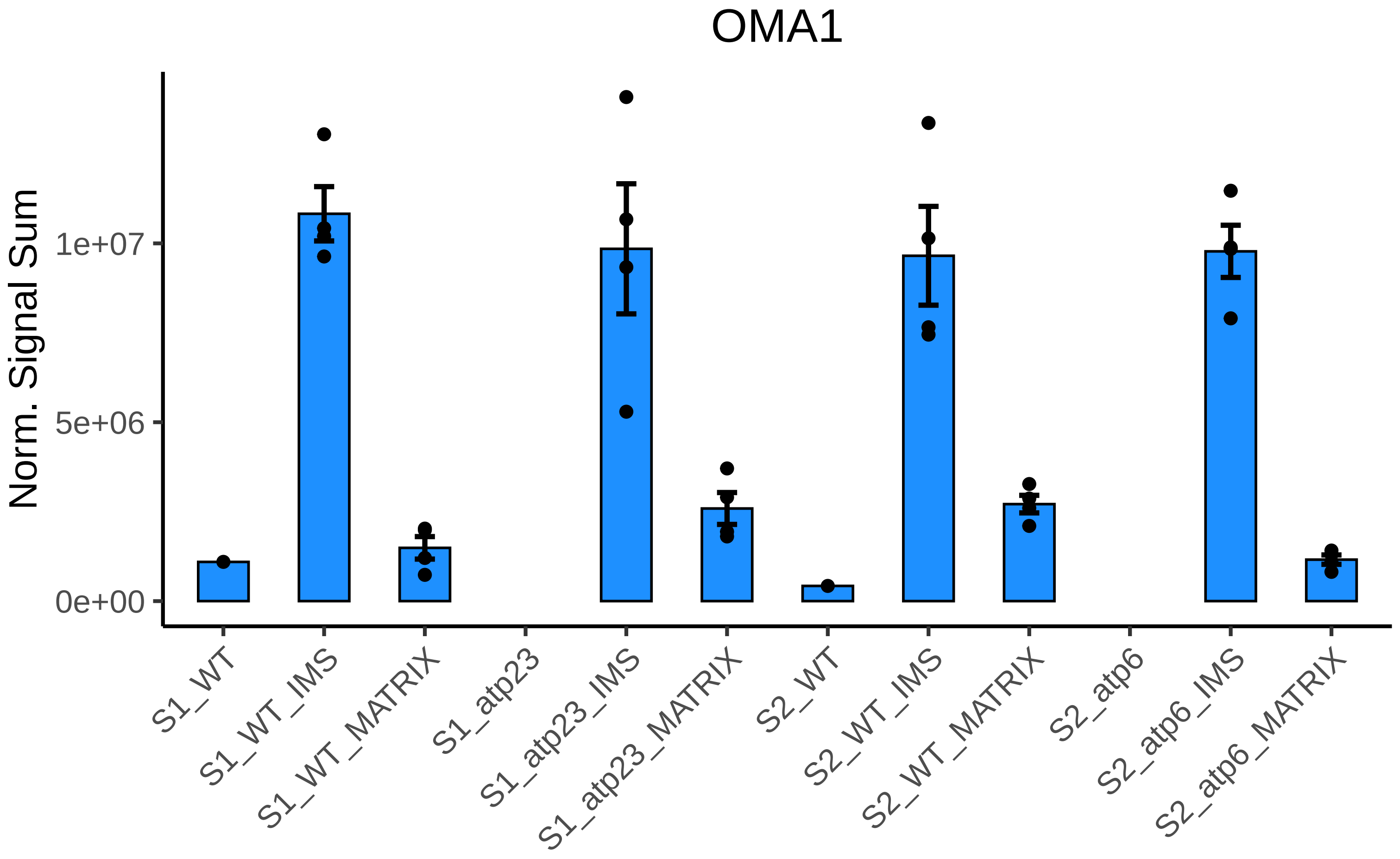
OMA1
Norm. Signal Sum
1e+07
5e+06
0e+00
S1_WT
S1_WT_IMS
S1_WT_MATRIX
S1_atp23
S1_atp23_IMS
S1_atp23_MATRIX
S2_WT
S2_WT_IMS
S2_WT_MATRIX
S2_atp6
S2_atp6_IMS
S2_atp6_MATRIX

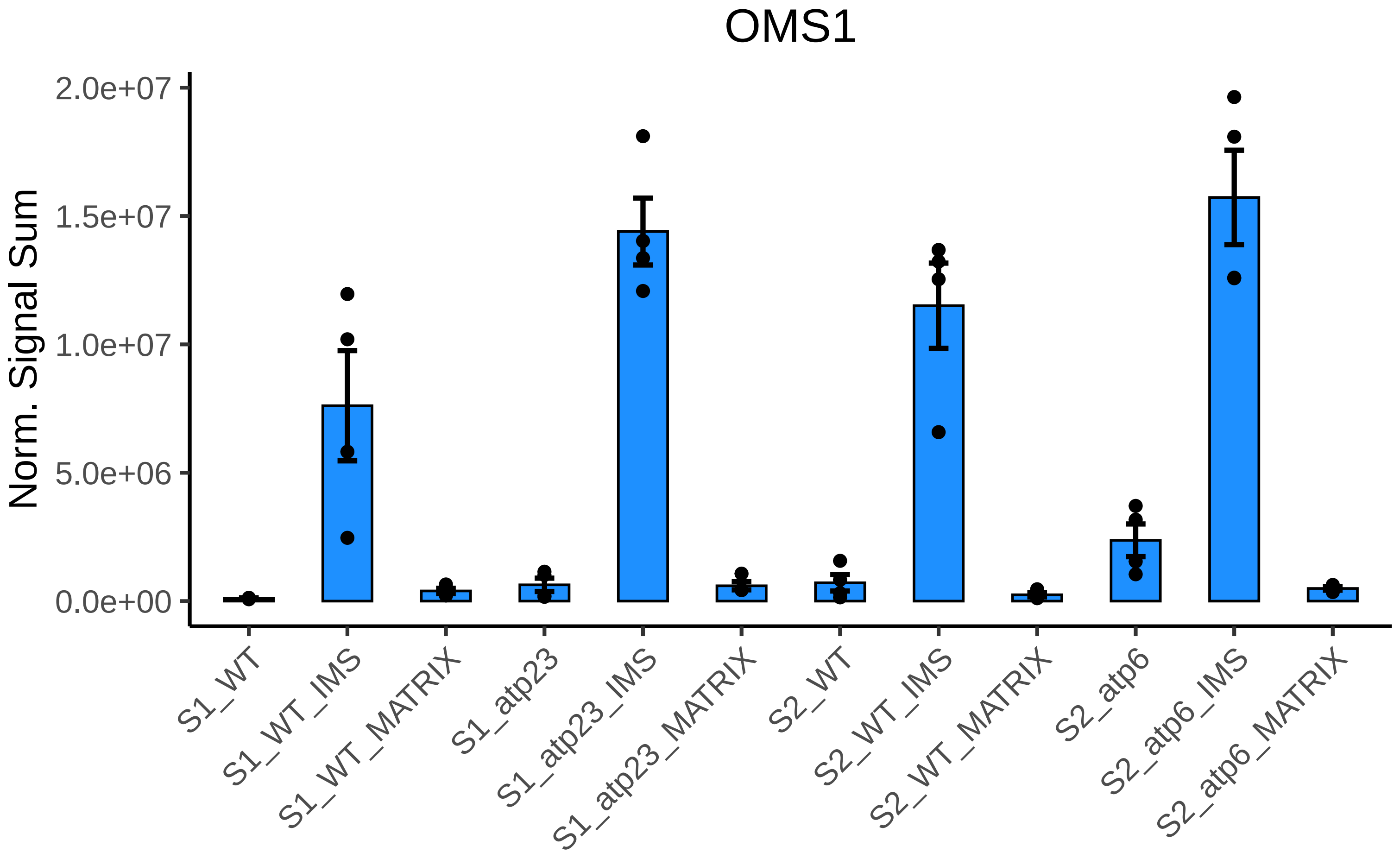
OMS1
Norm. Signal Sum
2.0e+07
1.5e+07
1.0e+07
5.0e+06
0.0e+00
S1_WT
S1_WT_IMS
S1_WT_MATRIX
S1_atp23
S1_atp23_IMS
S1_atp23_MATRIX
S2_WT
S2_WT_IMS
S2_WT_MATRIX
S2_atp6
S2_atp6_IMS
S2_atp6_MATRIX

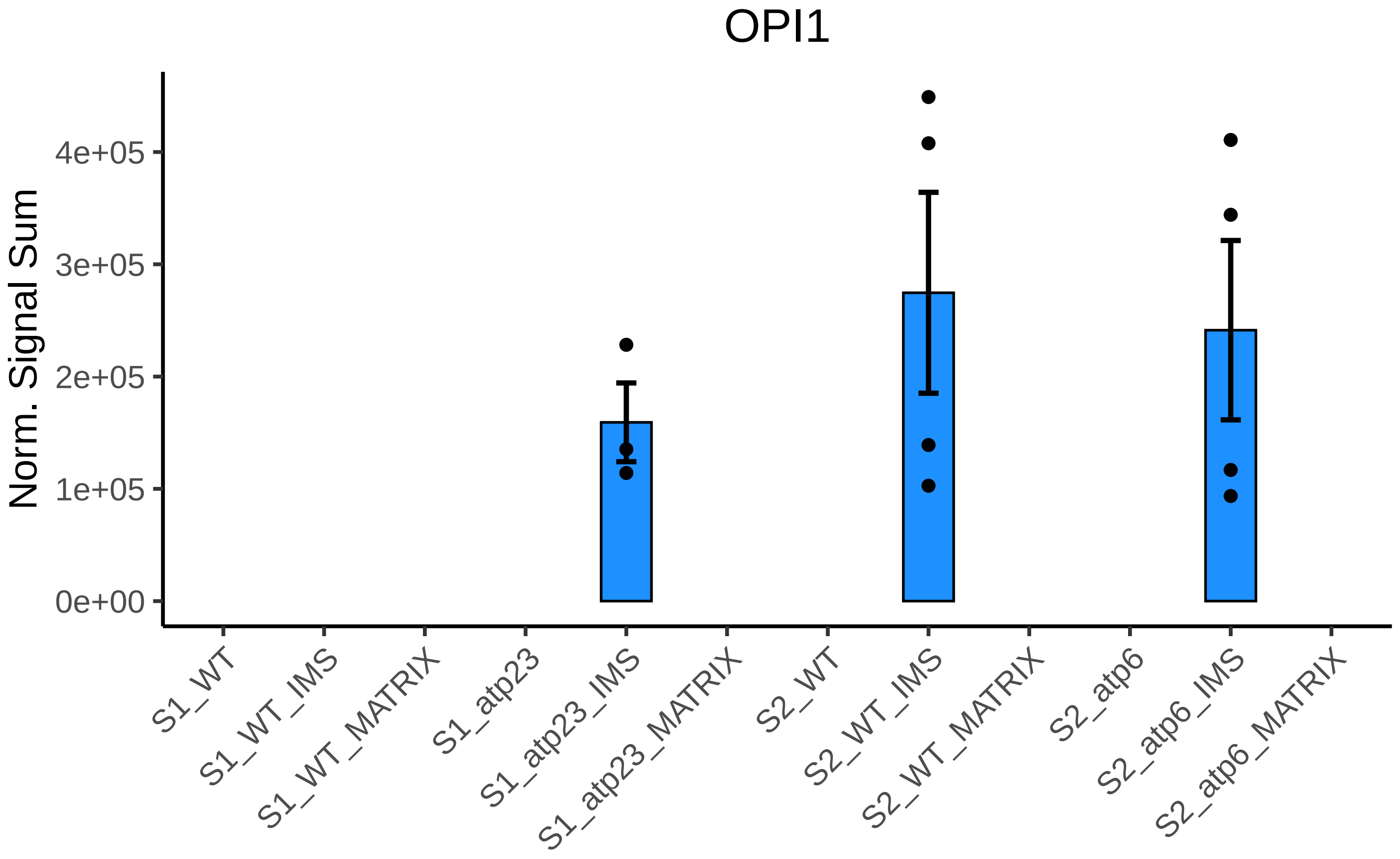

OPI1
Norm. Signal Sum
4e+05
3e+05
2e+05
1e+05
0e+00
S1_WT
S1_WT_IMS
S1_WT_MATRIX
S1_atp23
S1_atp23_IMS
S1_atp23_MATRIX
S2_WT
S2_WT_IMS
S2_WT_MATRIX
S2_atp6
S2_atp6_IMS
S2_atp6_MATRIX

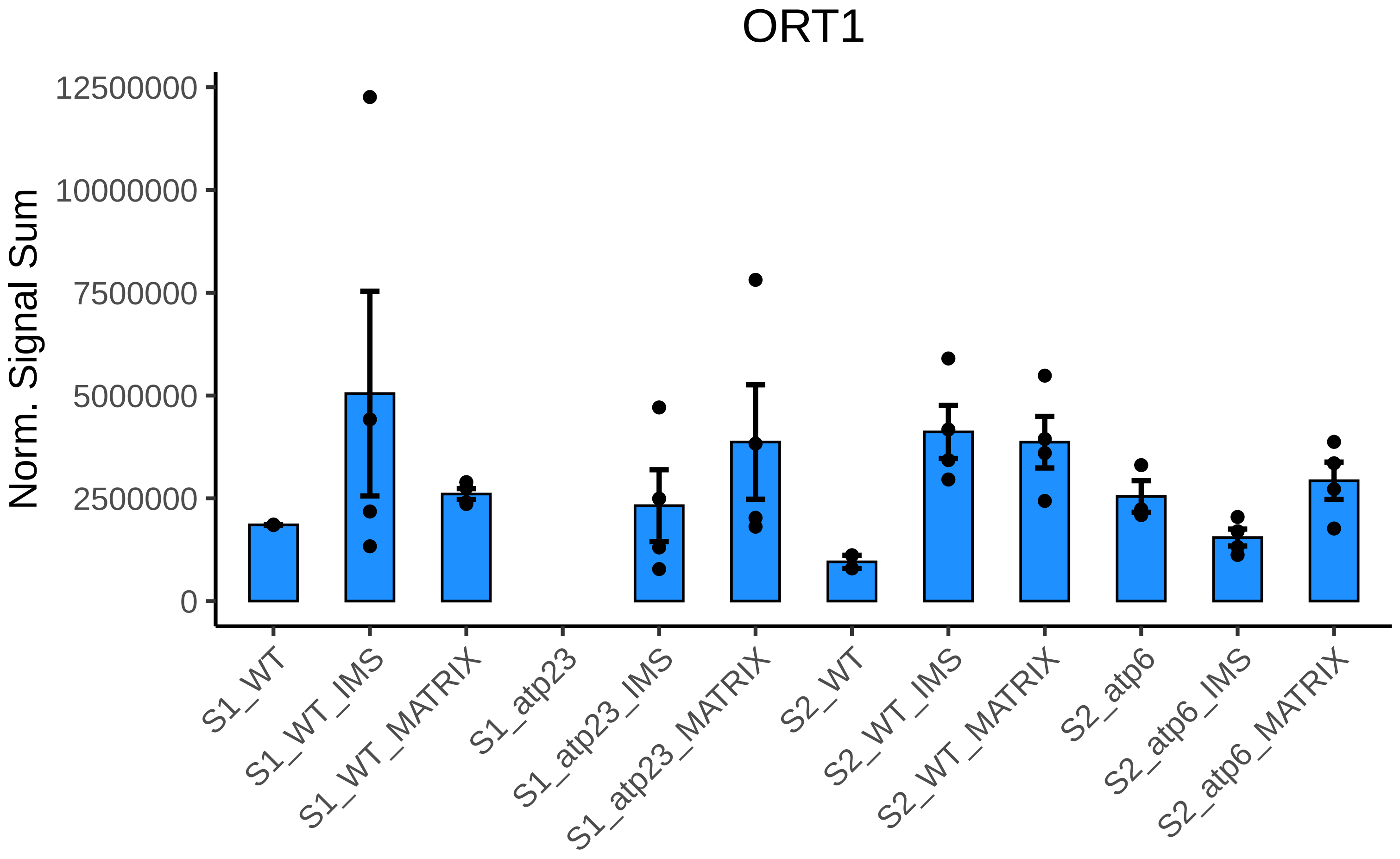

ORT1
Norm. Signal Sum
12500000
10000000
7500000
5000000
2500000
0
S1_WT
S1_WT_IMS
S1_WT_MATRIX
S1_atp23
S1_atp23_IMS
S1_atp23_MATRIX
S2_WT
S2_WT_IMS
S2_WT_MATRIX
S2_atp6
S2_atp6_IMS
S2_atp6_MATRIX

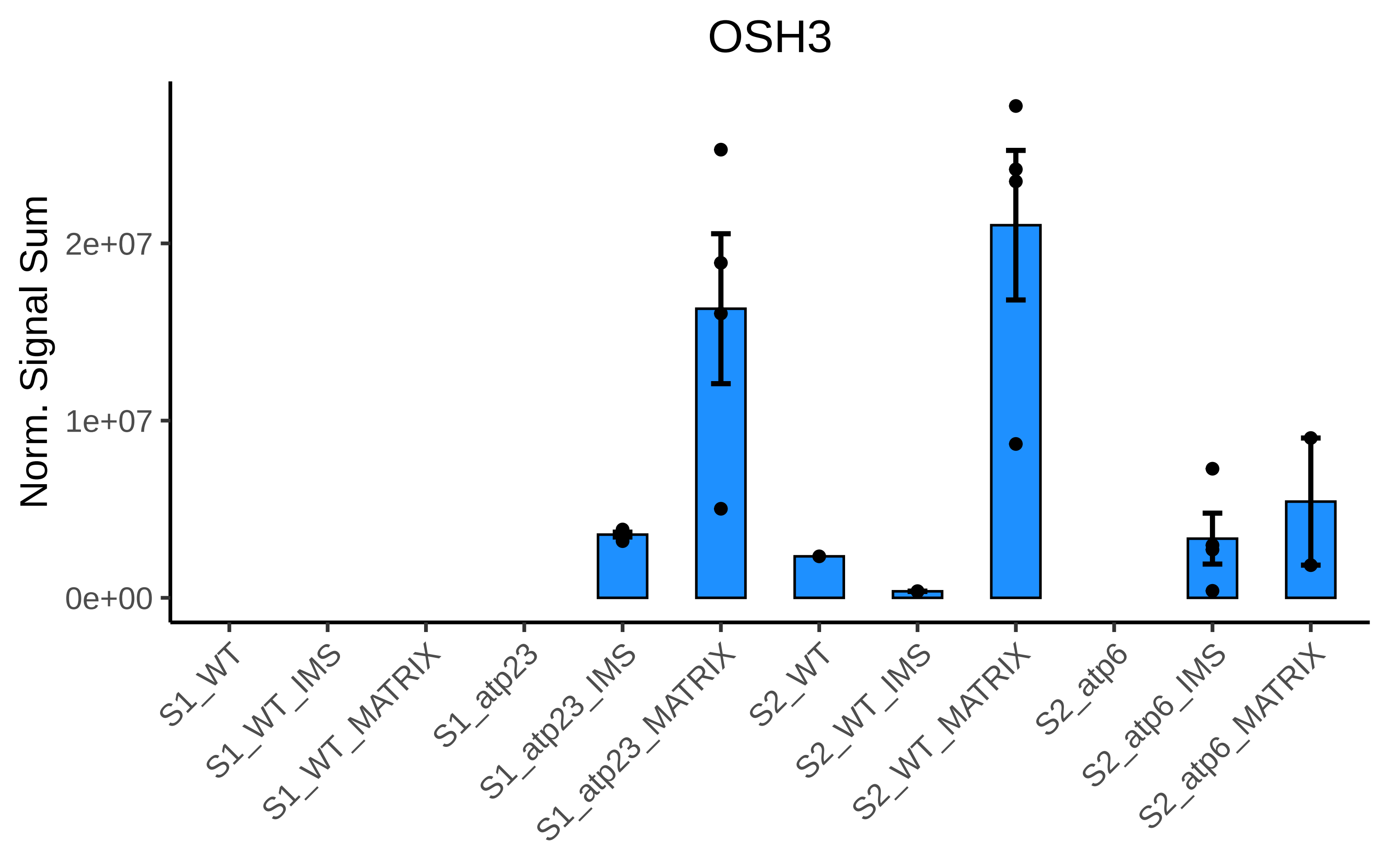
OSH3
Norm. Signal Sum
2e+07
1e+07
0e+00
S1_WT
S1_WT_IMS
S1_WT_MATRIX
S1_atp23
S1_atp23_IMS
S1_atp23_MATRIX
S2_WT
S2_WT_IMS
S2_WT_MATRIX
S2_atp6
S2_atp6_IMS
S2_atp6_MATRIX

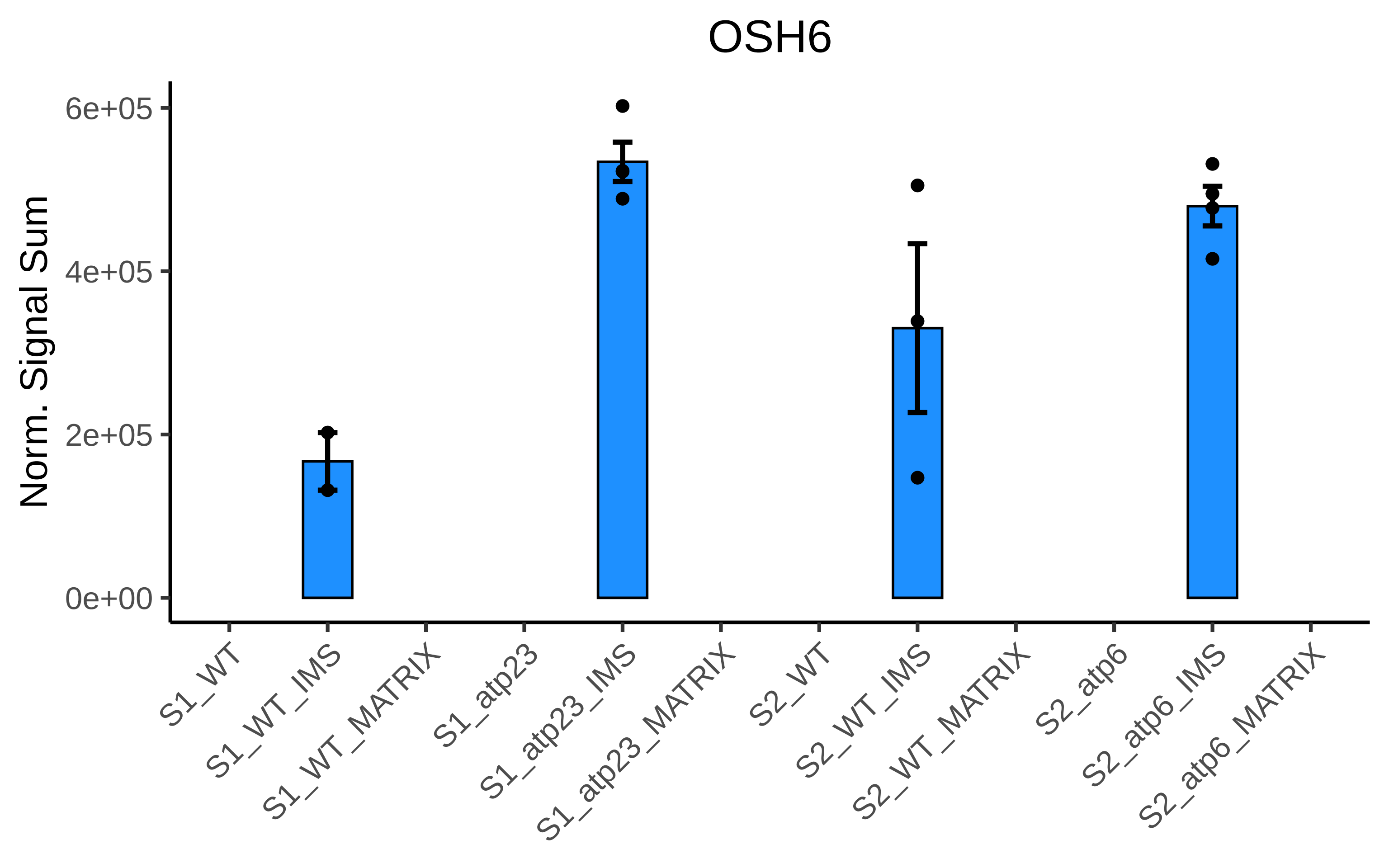

OSH6
Norm. Signal Sum
6e+05
4e+05
2e+05
0e+00
S1_WT
S1_WT_IMS
S1_WT_MATRIX
S1_atp23
S1_atp23_IMS
S1_atp23_MATRIX
S2_WT
S2_WT_IMS
S2_WT_MATRIX
S2_atp6
S2_atp6_IMS
S2_atp6_MATRIX

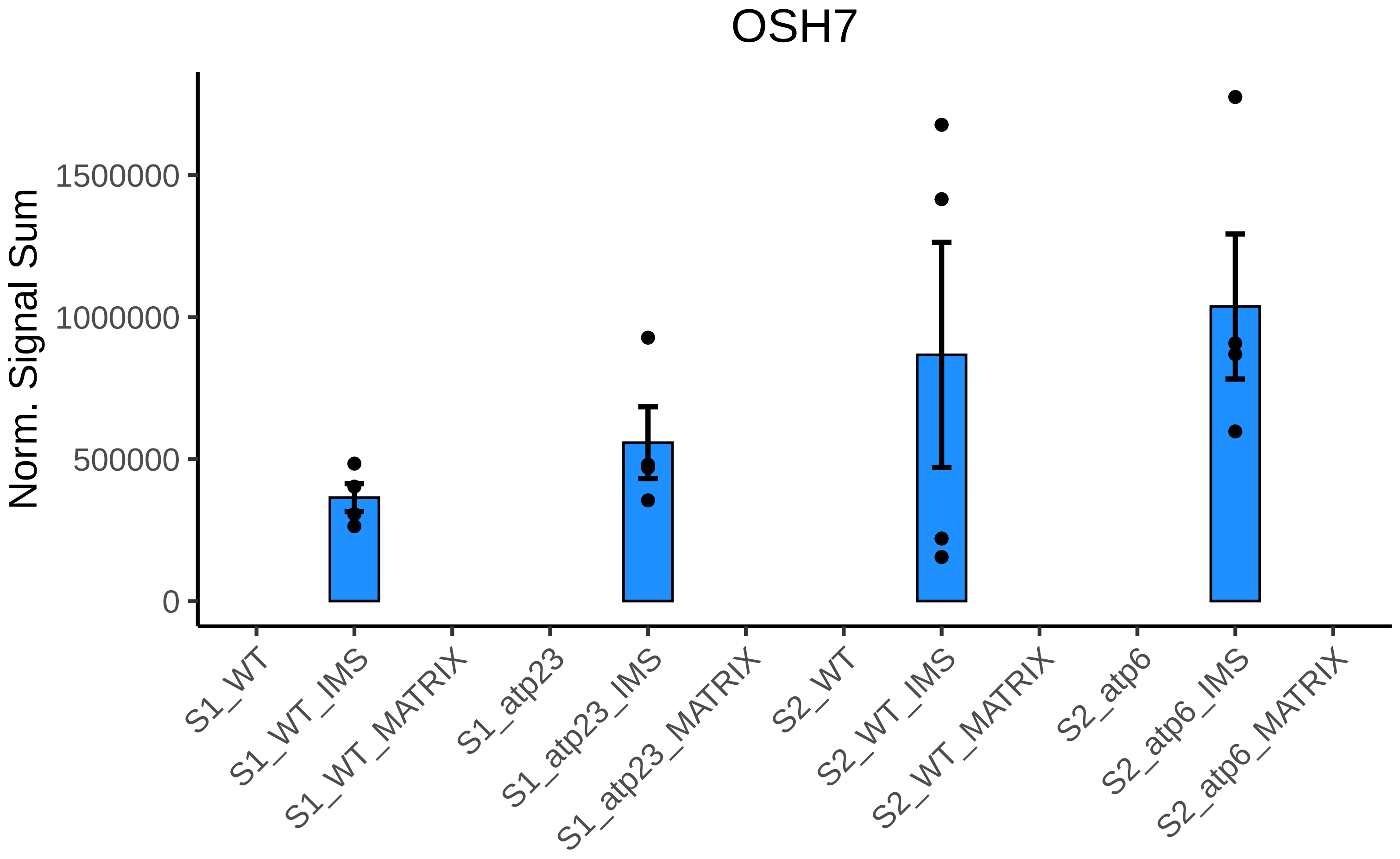
OSH7
Norm. Signal Sum
1500000
1000000
500000
0
S1_WT
S1_WT_IMS
S1_WT_MATRIX
S1_atp23
S1_atp23_IMS
S1_atp23_MATRIX
S2_WT
S2_WT_IMS
S2_WT_MATRIX
S2_atp6
S2_atp6_IMS
S2_atp6_MATRIX

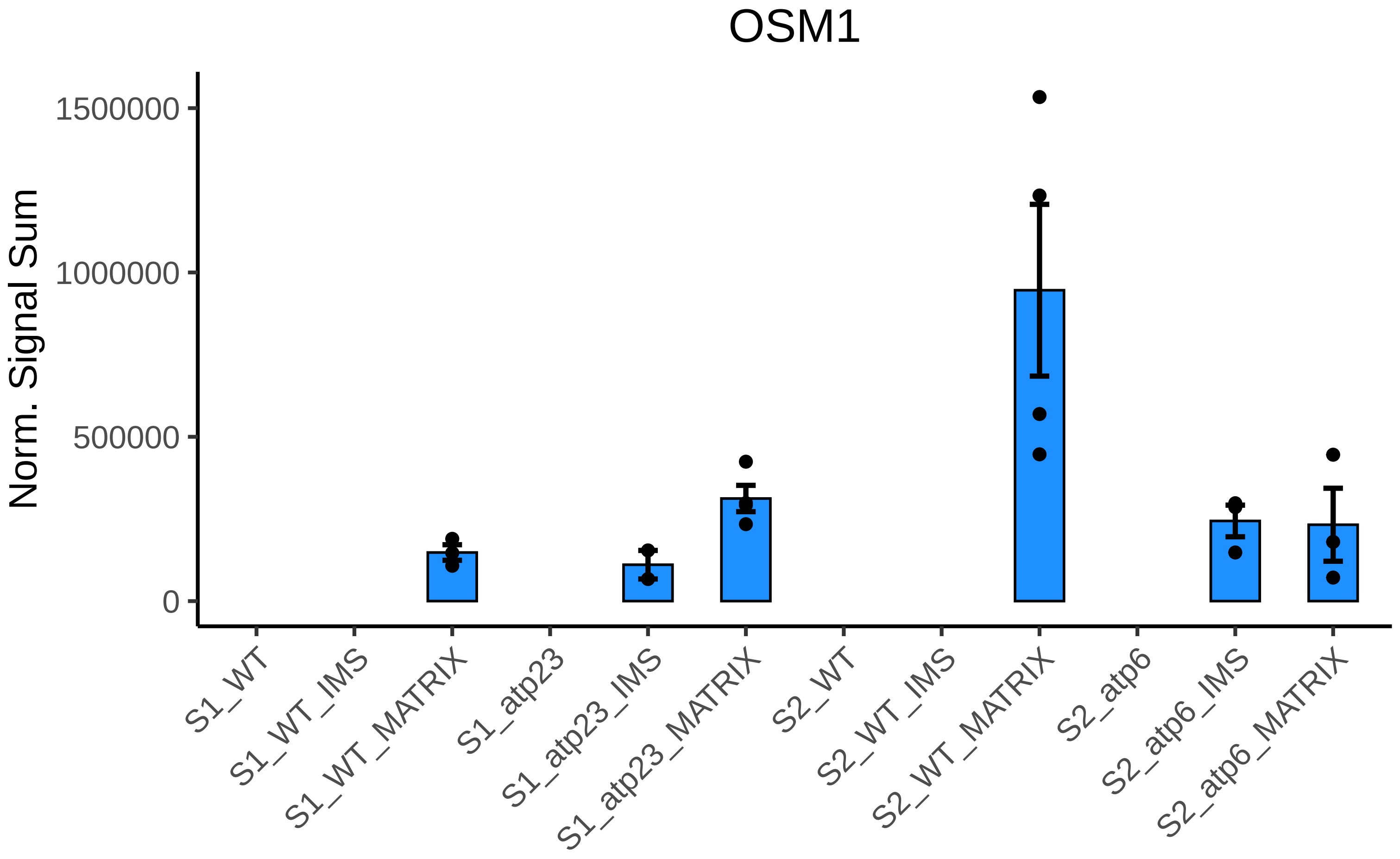
OSM1
Norm. Signal Sum
1500000
1000000
500000
0
S1_WT
S1_WT_IMS
S1_WT_MATRIX
S1_atp23
S1_atp23_IMS
S1_atp23_MATRIX
S2_WT
S2_WT_IMS
S2_WT_MATRIX
S2_atp6
S2_atp6_IMS
S2_atp6_MATRIX

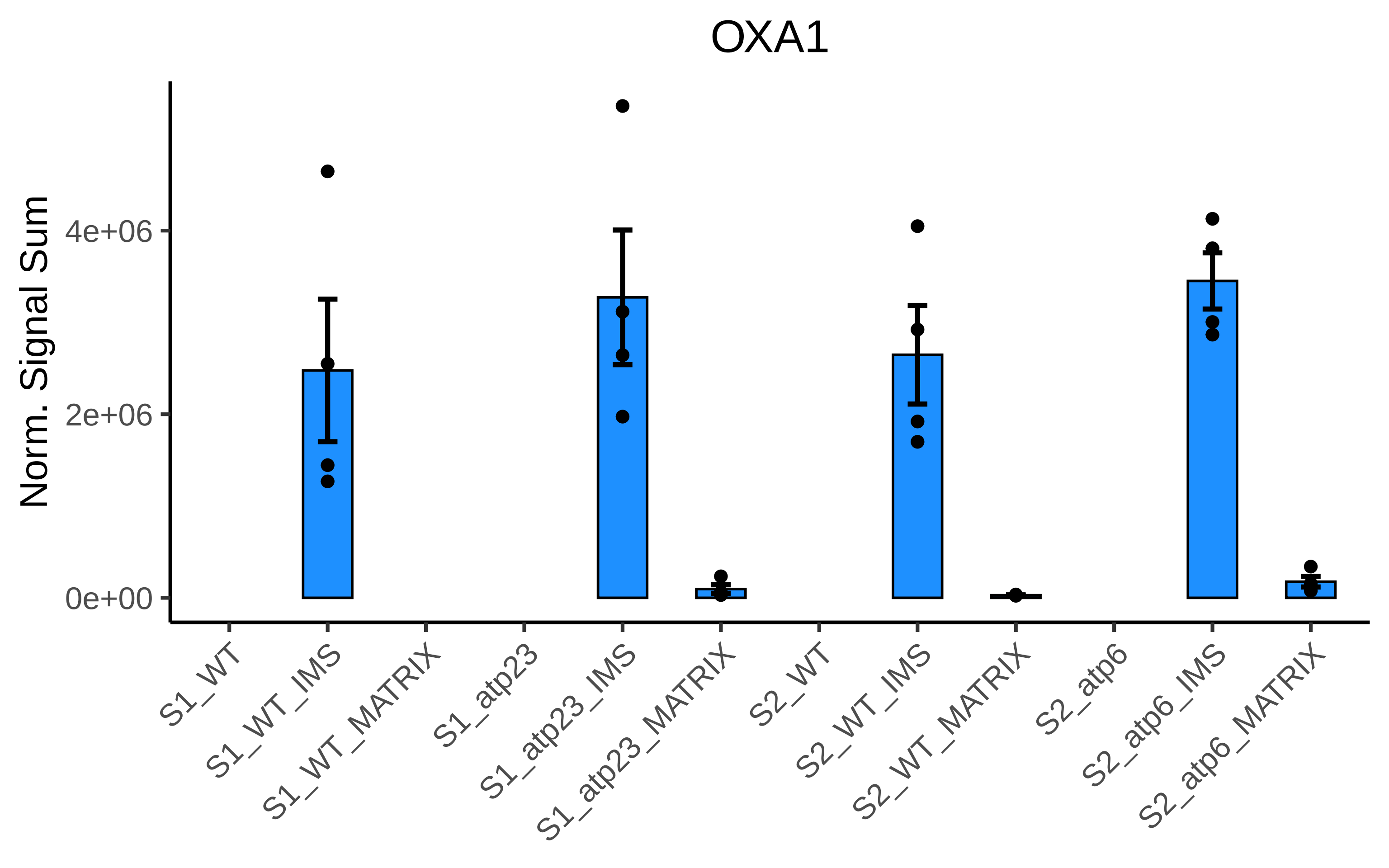
OXA1
Norm. Signal Sum
4e+06
2e+06
0e+00
S1_WT
S1_WT_IMS
S1_WT_MATRIX
S1_atp23
S1_atp23_IMS
S1_atp23_MATRIX
S2_WT
S2_WT_IMS
S2_WT_MATRIX
S2_atp6
S2_atp6_IMS
S2_atp6_MATRIX

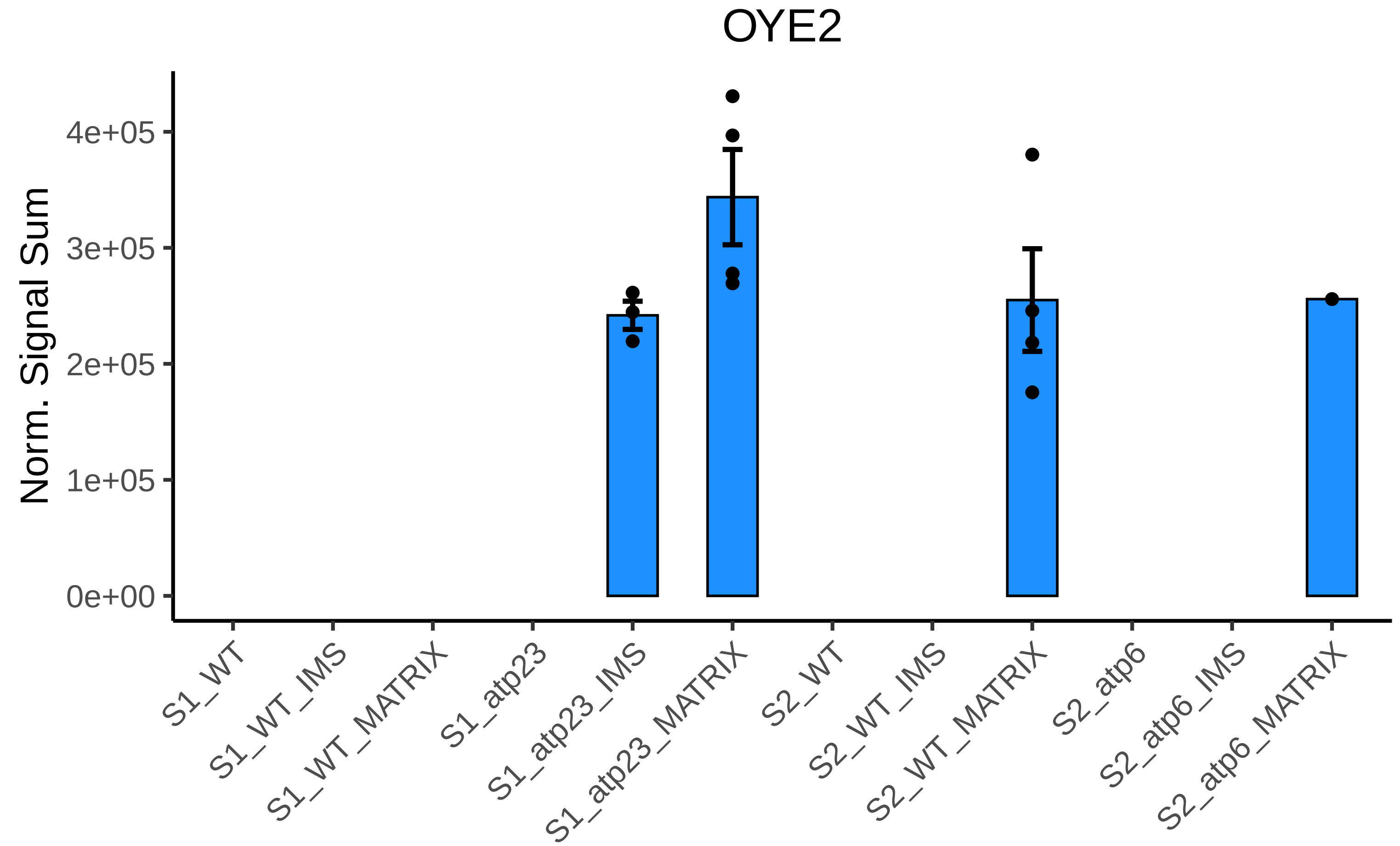
OYE2
Norm. Signal Sum
4e+05
3e+05
2e+05
1e+05
0e+00
S1_WT
S1_WT_IMS
S1_WT_MATRIX
S1_atp23
S1_atp23_IMS
S1_atp23_MATRIX
S2_WT
S2_WT_IMS
S2_WT_MATRIX
S2_atp6
S2_atp6_IMS
S2_atp6_MATRIX

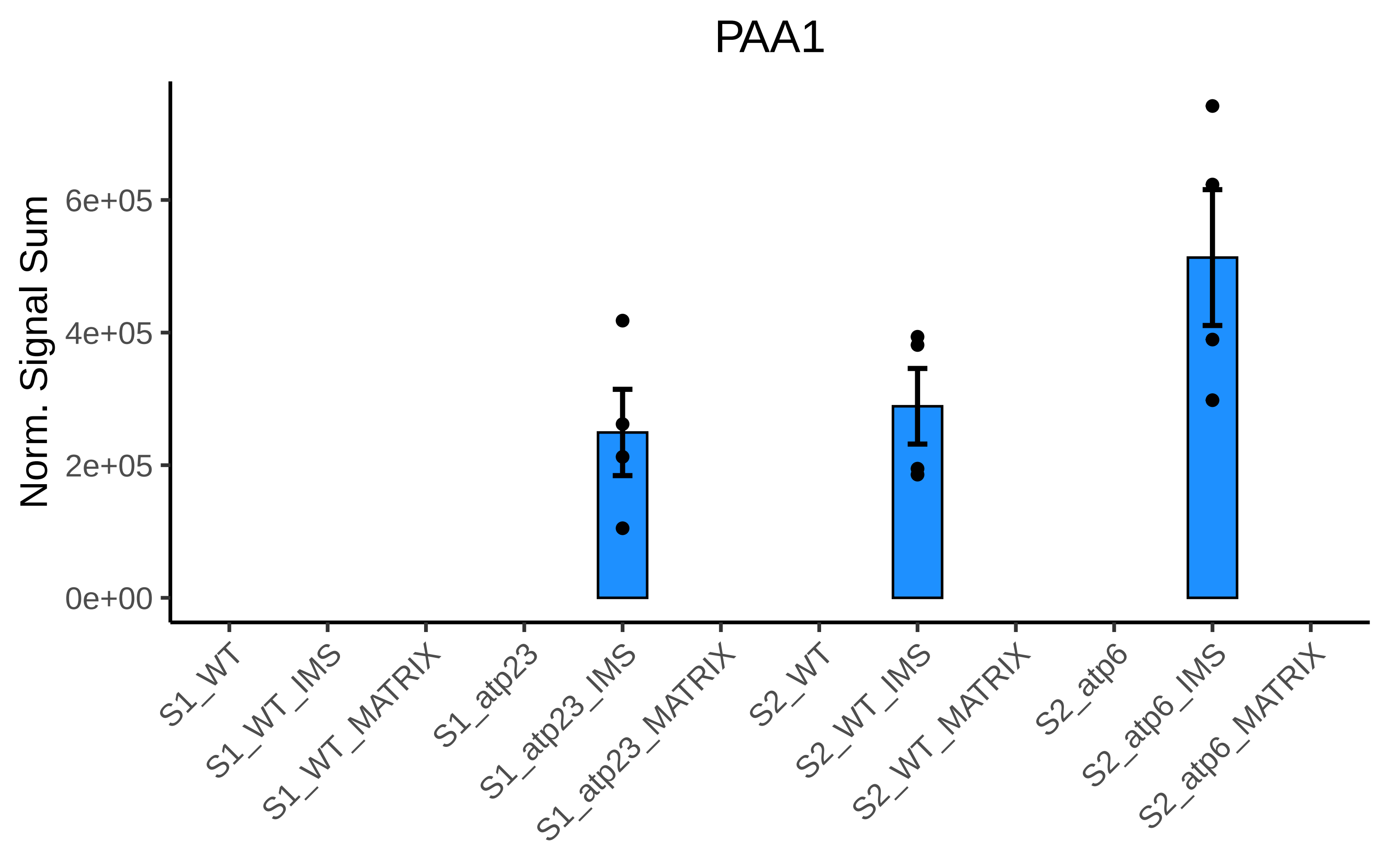
PAA1
Norm. Signal Sum
0e+00
2e+05
4e+05
6e+05
S1_WT
S1_WT_IMS
S1_WT_MATRIX
S1_atp23
S1_atp23_IMS
S1_atp23_MATRIX
S2_WT
S2_WT_IMS
S2_WT_MATRIX
S2_atp6
S2_atp6_IMS
S2_atp6_MATRIX

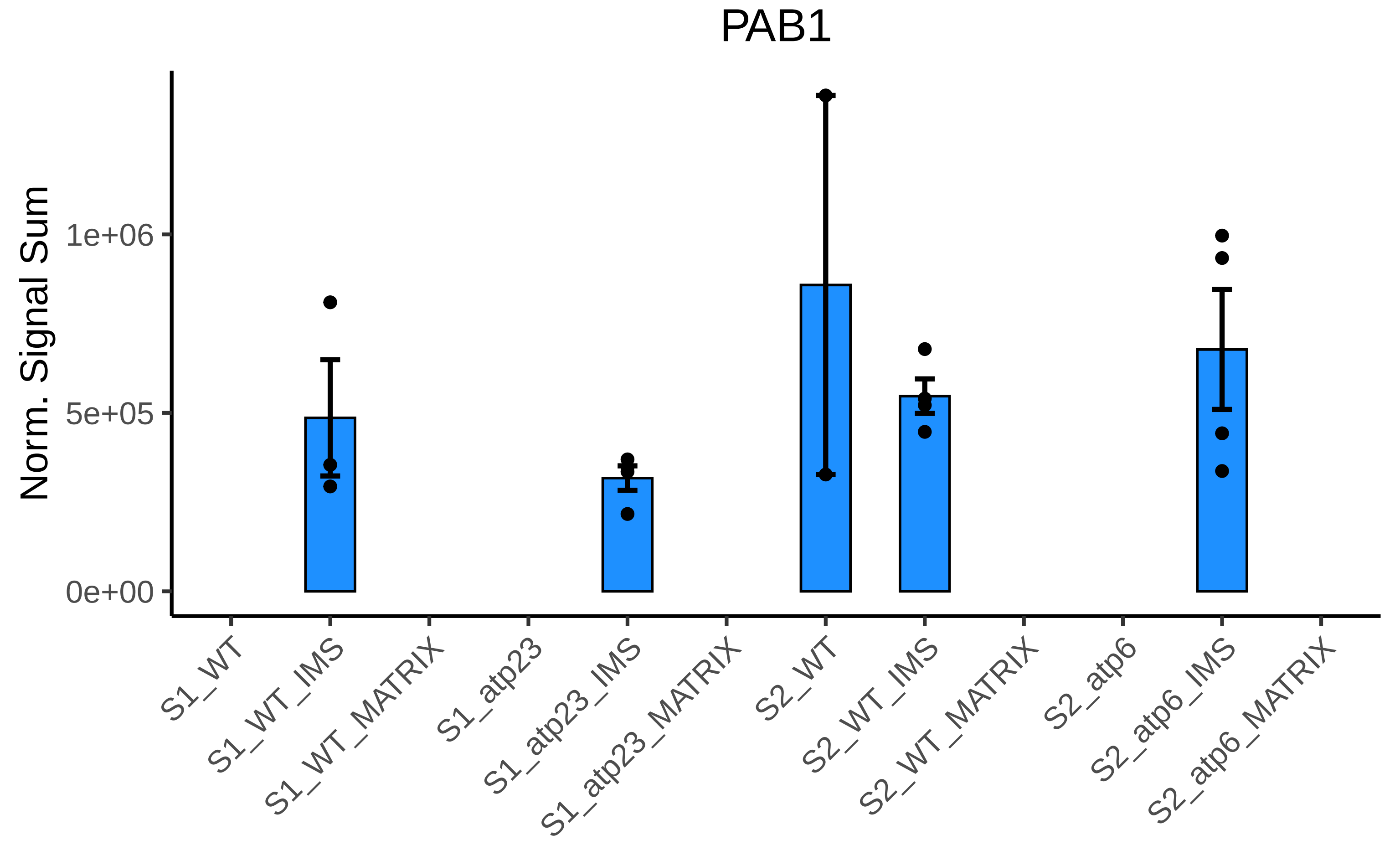
PAB1
Norm. Signal Sum
1e+06
5e+05
0e+00
S1_WT
S1_WT_IMS
S1_WT_MATRIX
S1_atp23
S1_atp23_IMS
S1_atp23_MATRIX
S2_WT
S2_WT_IMS
S2_WT_MATRIX
S2_atp6
S2_atp6_IMS
S2_atp6_MATRIX

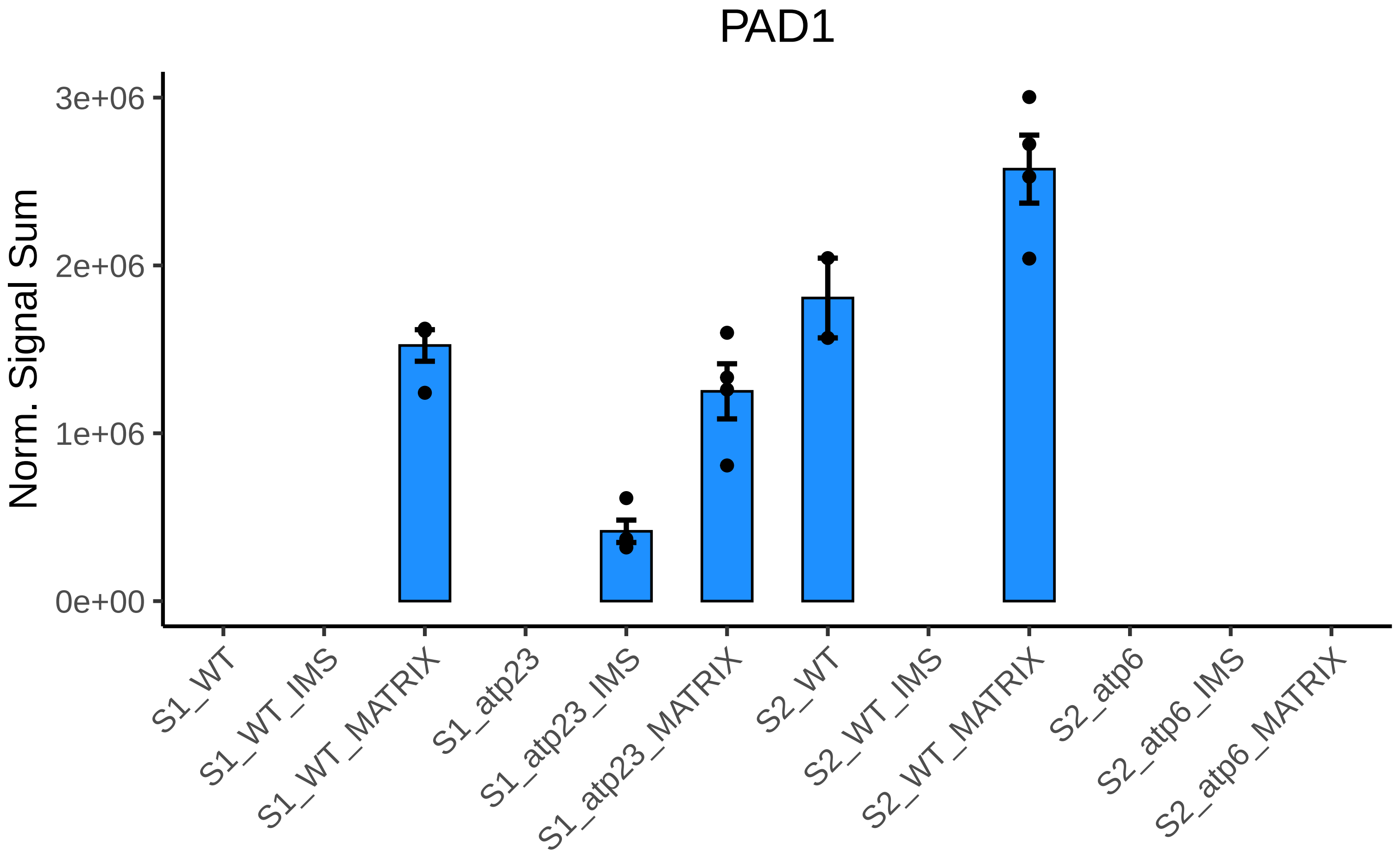
PAD1
Norm. Signal Sum
3e+06
2e+06
1e+06
0e+00
S1_WT
S1_WT_IMS
S1_WT_MATRIX
S1_atp23
S1_atp23_IMS
S1_atp23_MATRIX
S2_WT
S2_WT_IMS
S2_WT_MATRIX
S2_atp6
S2_atp6_IMS
S2_atp6_MATRIX

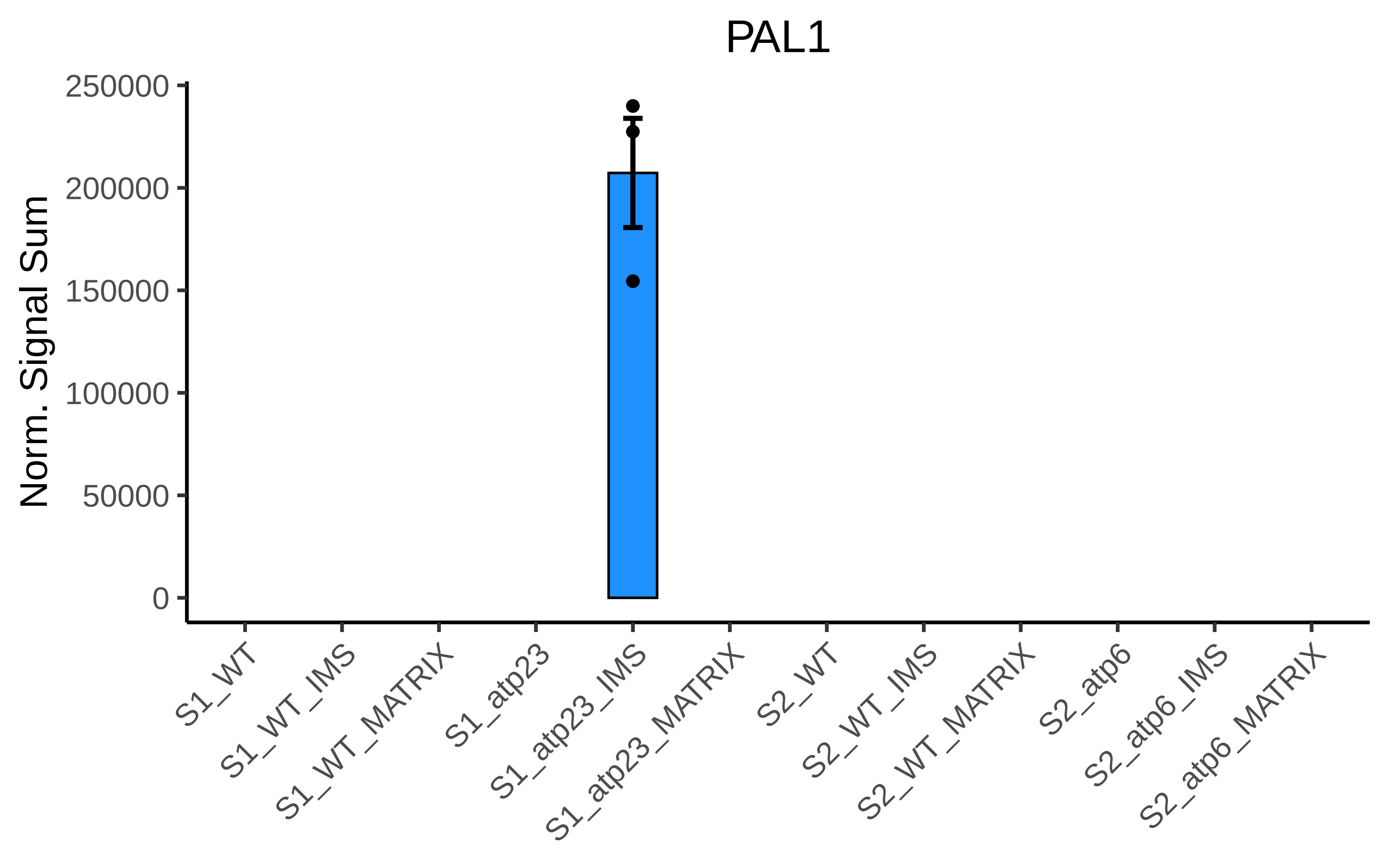

PAL1
Norm. Signal Sum
250000
200000
150000
100000
50000
0
S1_WT
S1_WT_IMS
S1_WT_MATRIX
S1_atp23
S1_atp23_IMS
S1_atp23_MATRIX
S2_WT
S2_WT_IMS
S2_WT_MATRIX
S2_atp6
S2_atp6_IMS
S2_atp6_MATRIX

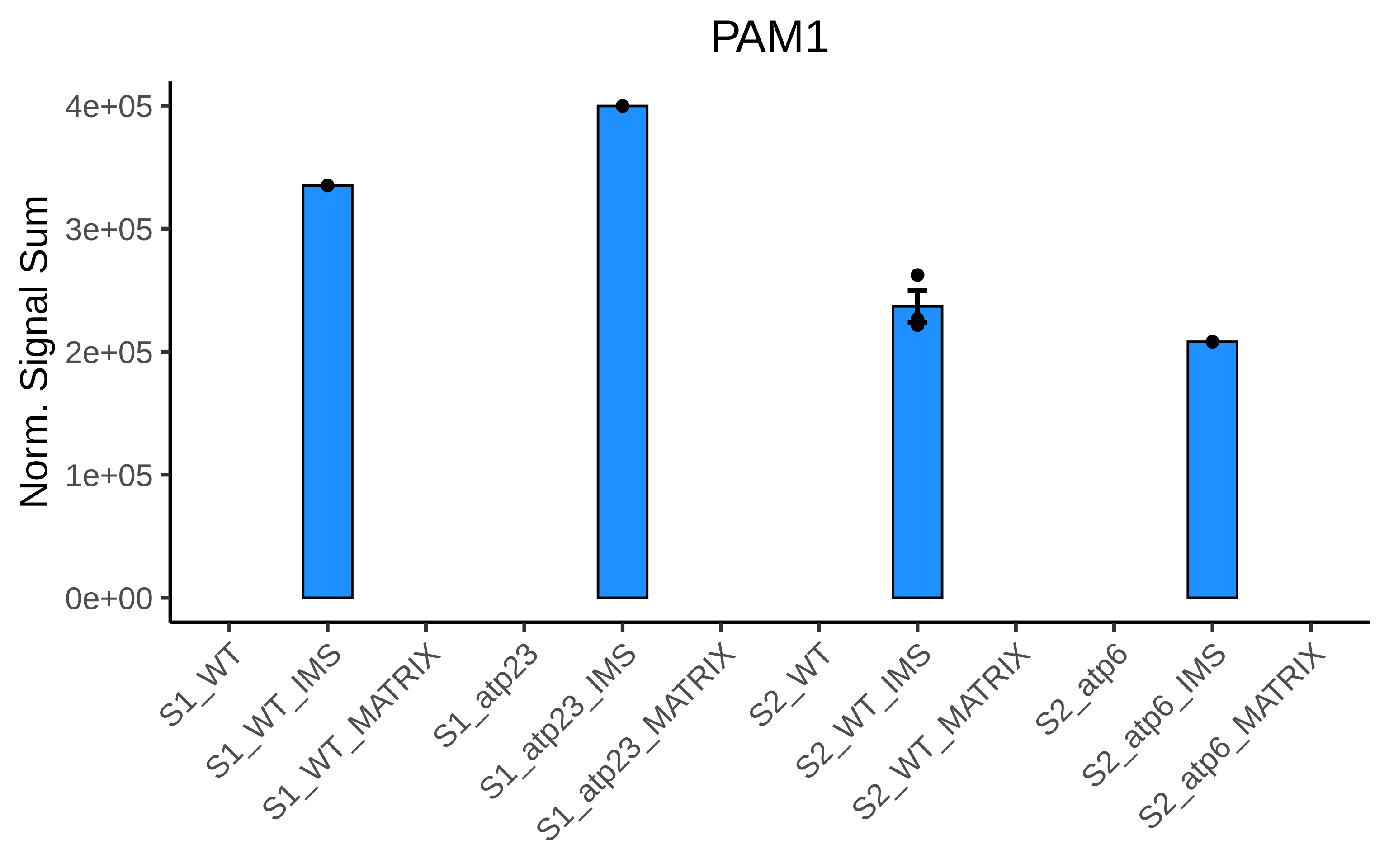
PAM1
Norm. Signal Sum
4e+05
3e+05
2e+05
1e+05
0e+00
S1_WT
S1_WT_IMS
S1_WT_MATRIX
S1_atp23
S1_atp23_IMS
S1_atp23_MATRIX
S2_WT
S2_WT_IMS
S2_WT_MATRIX
S2_atp6
S2_atp6_IMS
S2_atp6_MATRIX

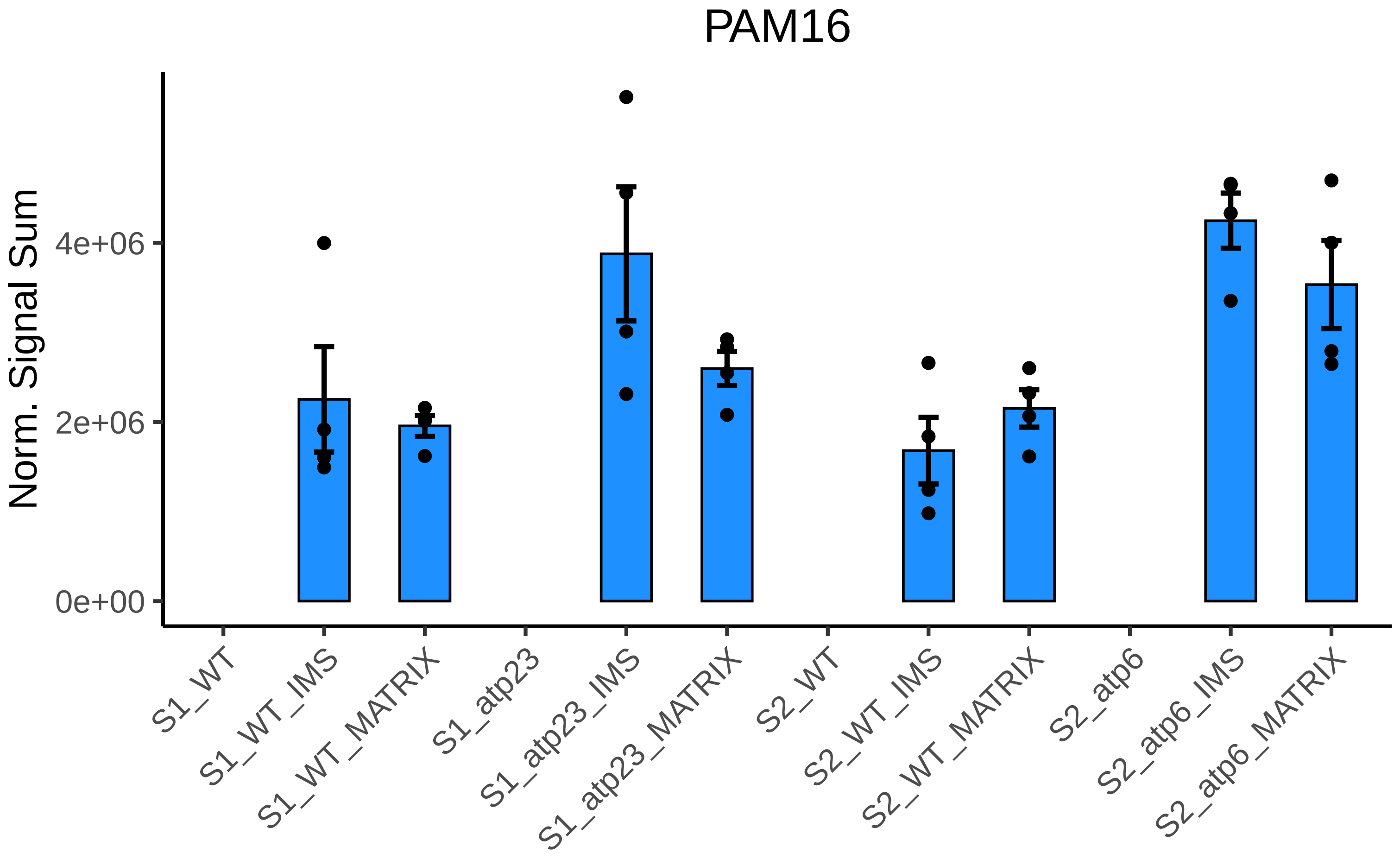

PAM16
Norm. Signal Sum
0e+00
2e+06
4e+06
S1_WT
S1_WT_IMS
S1_WT_MATRIX
S1_atp23
S1_atp23_IMS
S1_atp23_MATRIX
S2_WT
S2_WT_IMS
S2_WT_MATRIX
S2_atp6
S2_atp6_IMS
S2_atp6_MATRIX

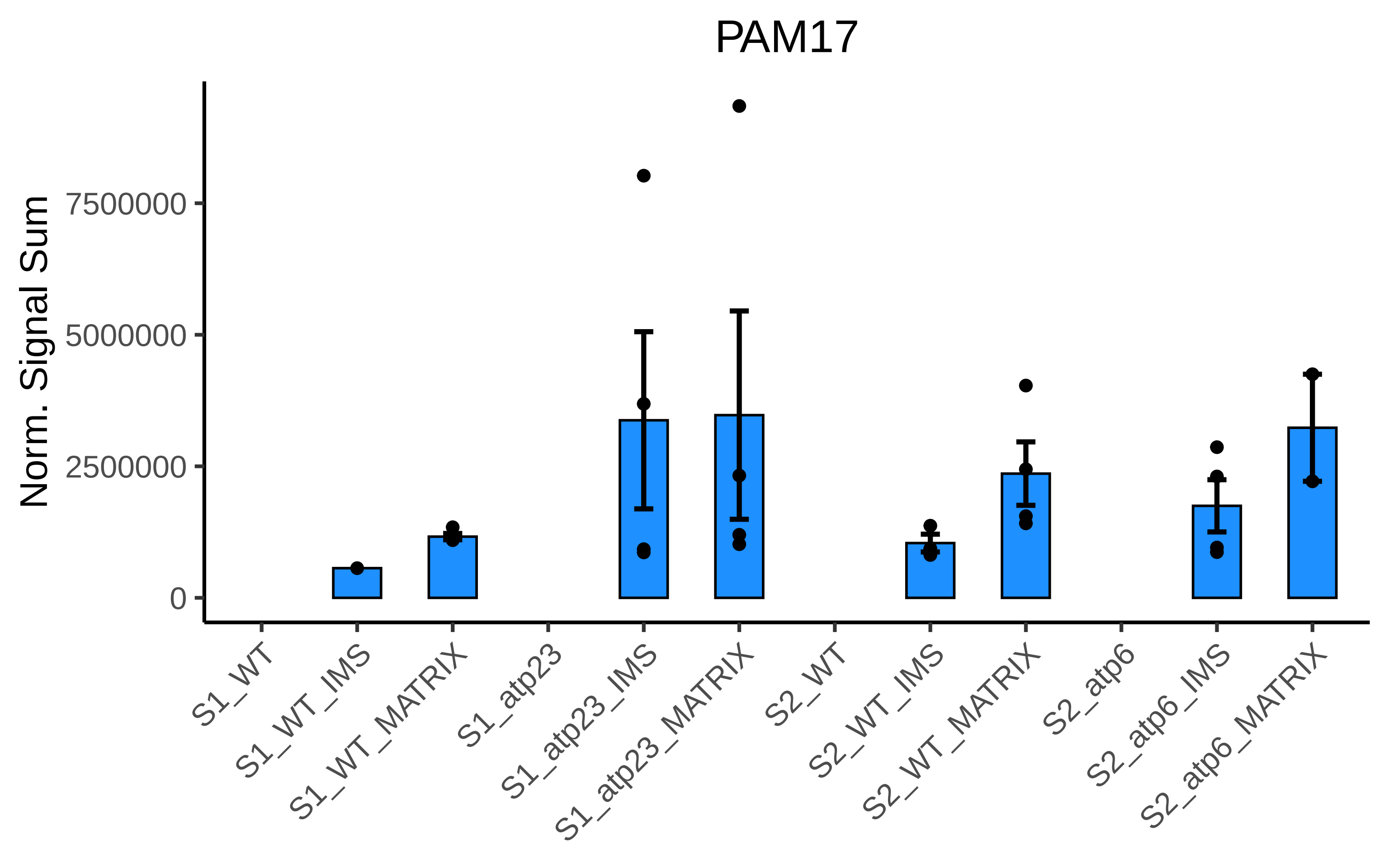
PAM17
Norm. Signal Sum
7500000
5000000
2500000
0
S1_WT
S1_WT_IMS
S1_WT_MATRIX
S1_atp23
S1_atp23_IMS
S1_atp23_MATRIX
S2_WT
S2_WT_IMS
S2_WT_MATRIX
S2_atp6
S2_atp6_IMS
S2_atp6_MATRIX

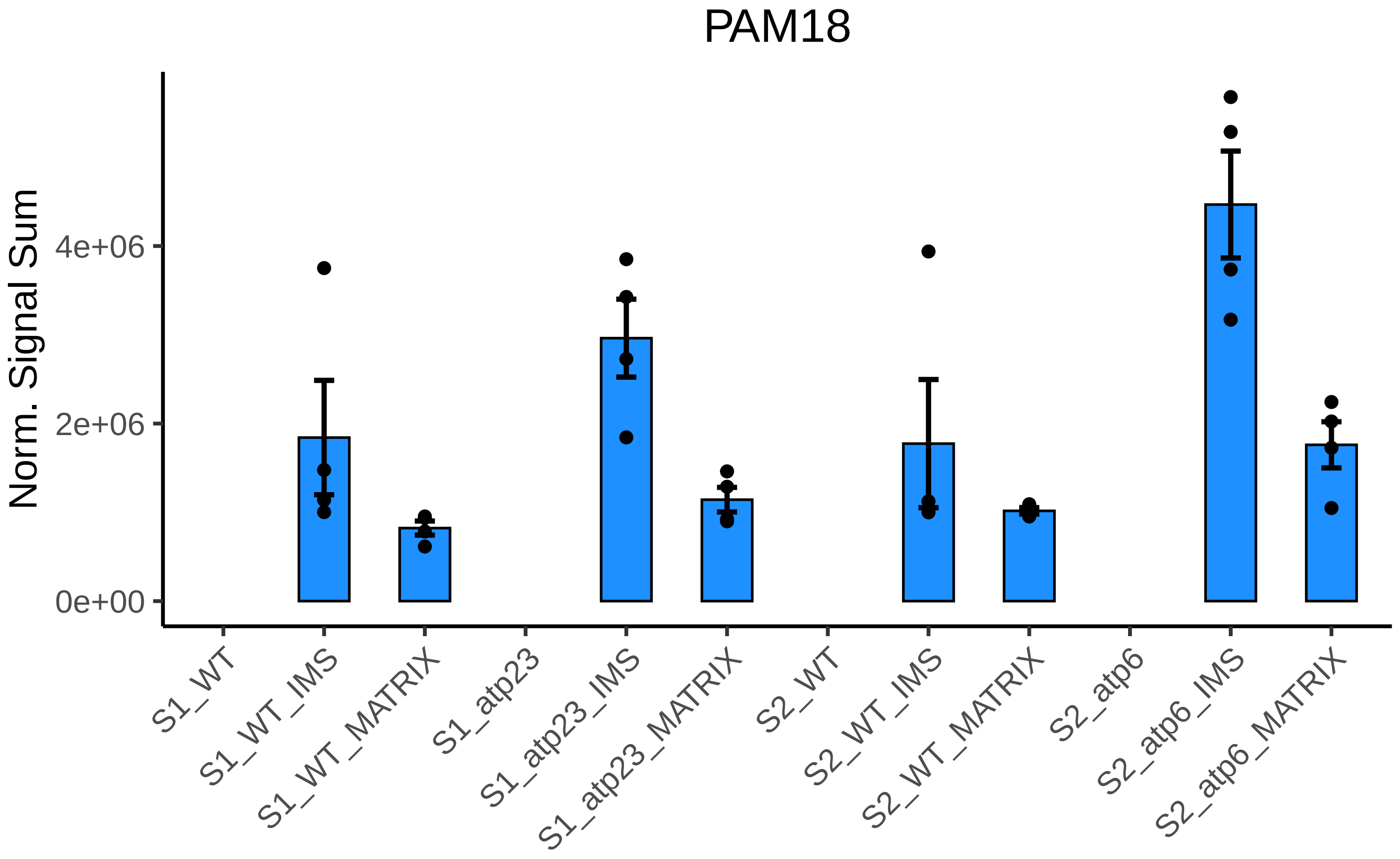

PAM18
Norm. Signal Sum
0e+00
2e+06
4e+06
S1_WT
S1_WT_IMS
S1_WT_MATRIX
S1_atp23
S1_atp23_IMS
S1_atp23_MATRIX
S2_WT
S2_WT_IMS
S2_WT_MATRIX
S2_atp6
S2_atp6_IMS
S2_atp6_MATRIX

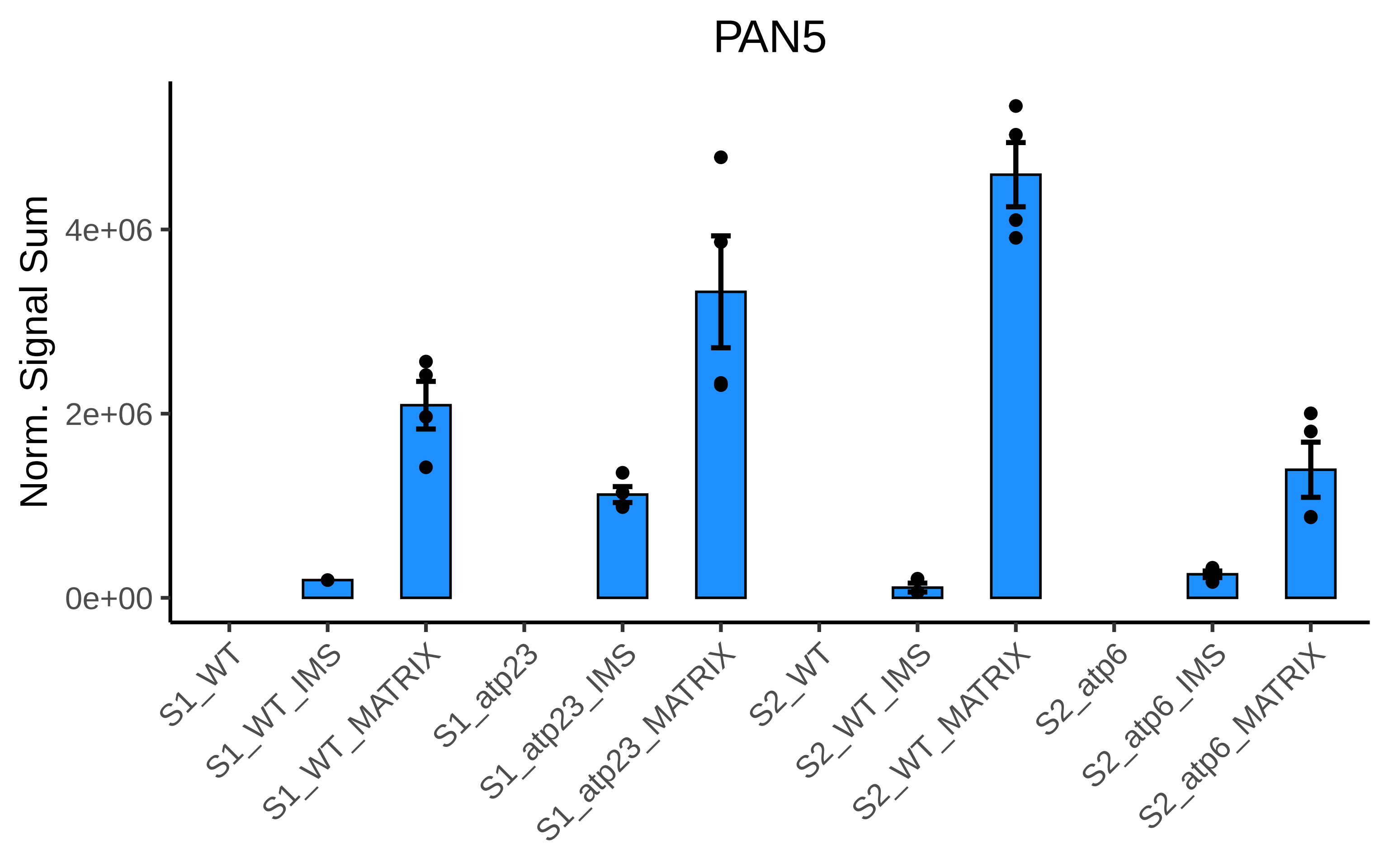
PAN5
Norm. Signal Sum
4e+06
2e+06
0e+00
S1_WT
S1_WT_IMS
S1_WT_MATRIX
S1_atp23
S1_atp23_IMS
S1_atp23_MATRIX
S2_WT
S2_WT_IMS
S2_WT_MATRIX
S2_atp6
S2_atp6_IMS
S2_atp6_MATRIX

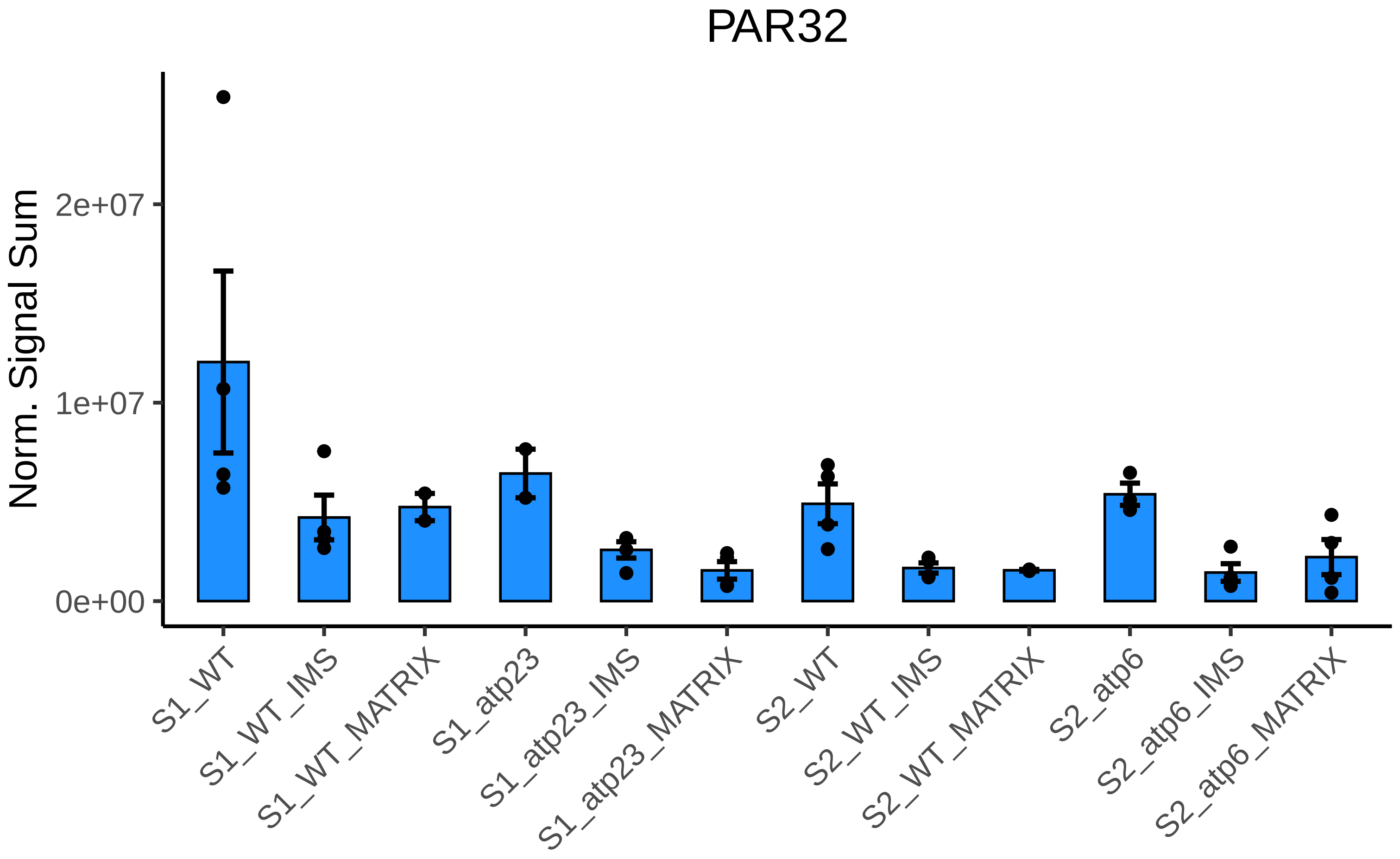
PAR32
Norm. Signal Sum
2e+07
1e+07
0e+00
S1_WT
S1_WT_IMS
S1_WT_MATRIX
S1_atp23
S1_atp23_IMS
S1_atp23_MATRIX
S2_WT
S2_WT_IMS
S2_WT_MATRIX
S2_atp6
S2_atp6_IMS
S2_atp6_MATRIX

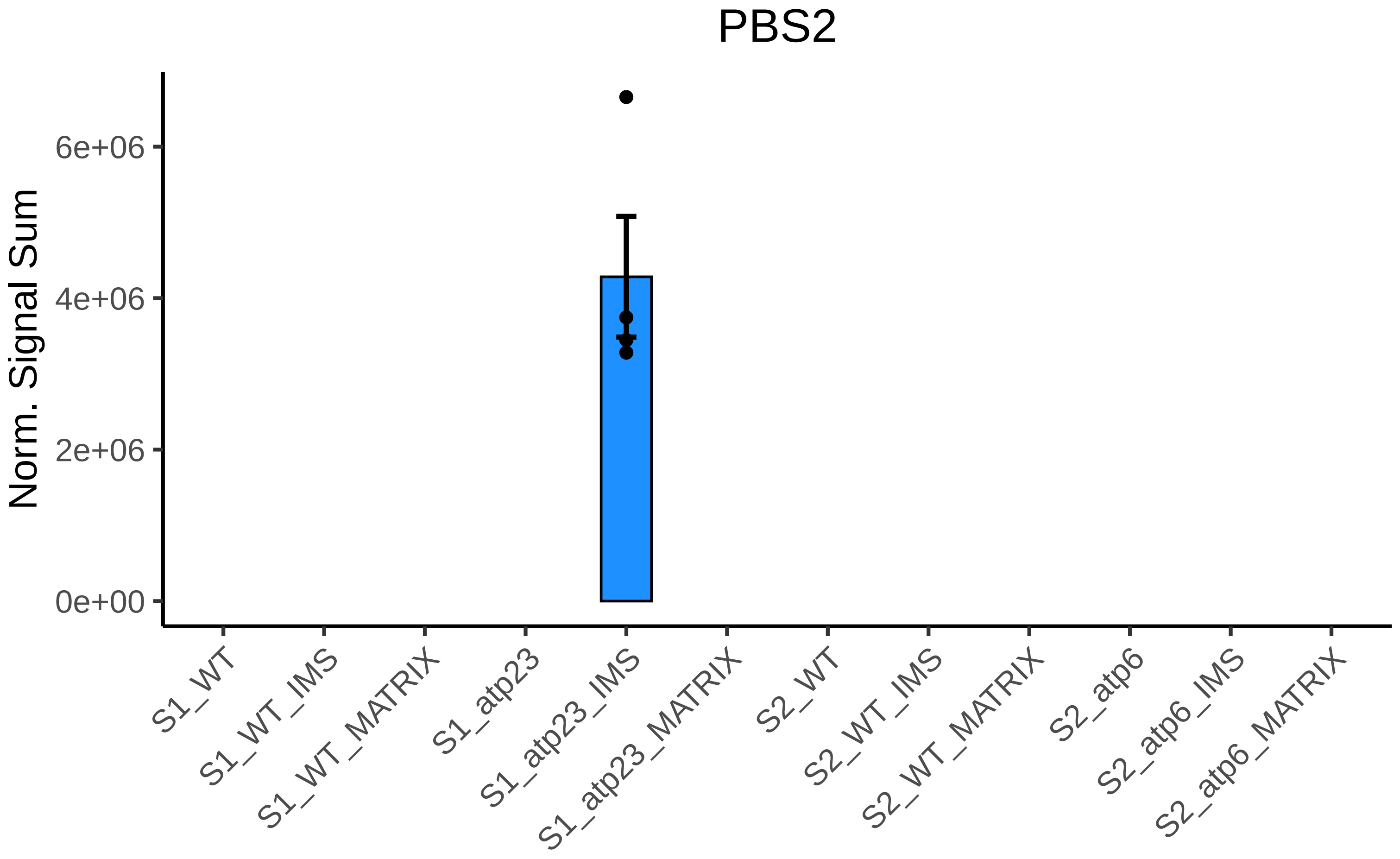
PBS2
Norm. Signal Sum
6e+06
4e+06
2e+06
0e+00
S1_WT
S1_WT_IMS
S1_WT_MATRIX
S1_atp23
S1_atp23_IMS
S1_atp23_MATRIX
S2_WT
S2_WT_IMS
S2_WT_MATRIX
S2_atp6
S2_atp6_IMS
S2_atp6_MATRIX

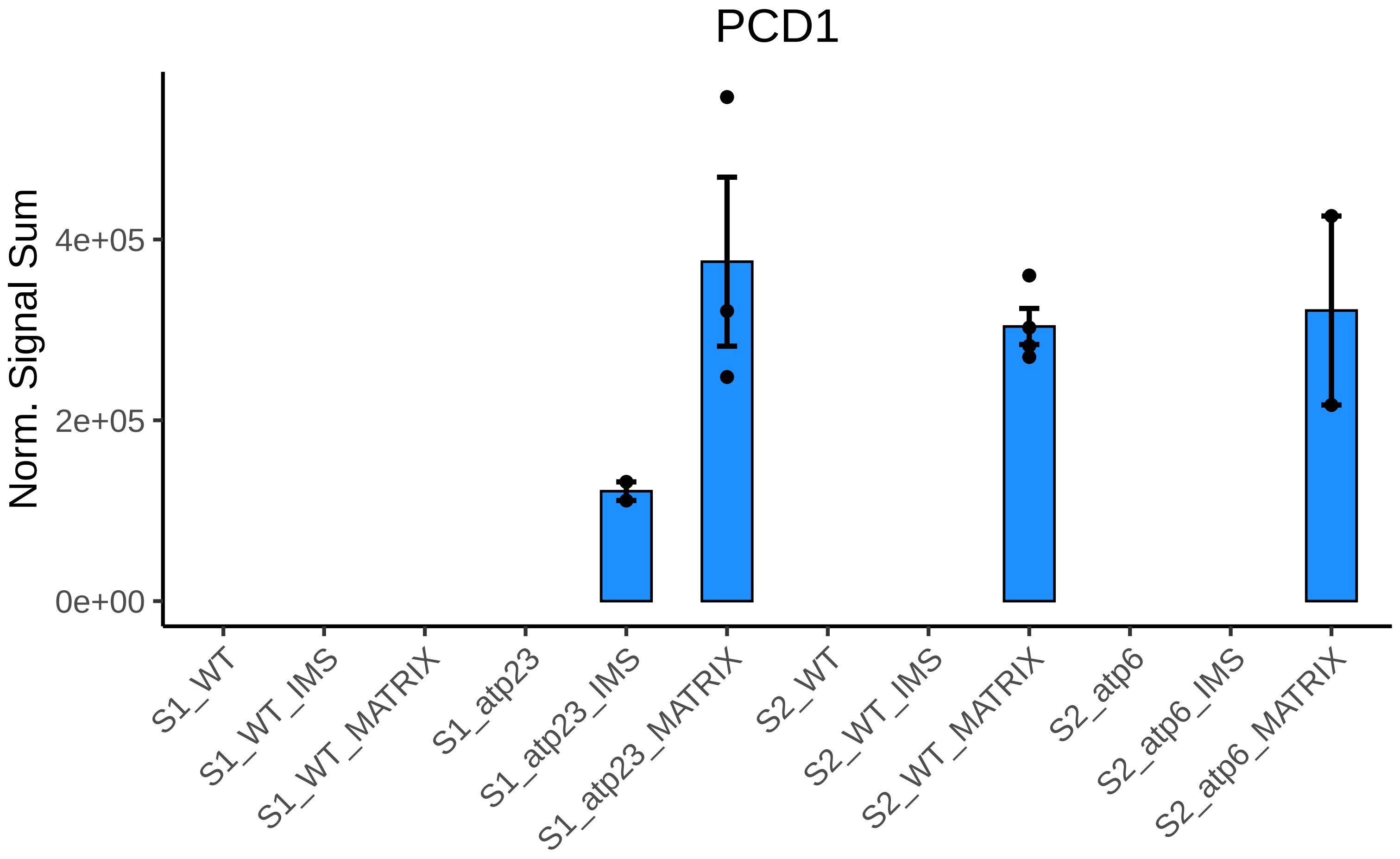
PCD1
Norm. Signal Sum
4e+05
2e+05
0e+00
S1_WT
S1_WT_IMS
S1_WT_MATRIX
S1_atp23
S1_atp23_IMS
S1_atp23_MATRIX
S2_WT
S2_WT_IMS
S2_WT_MATRIX
S2_atp6
S2_atp6_IMS
S2_atp6_MATRIX

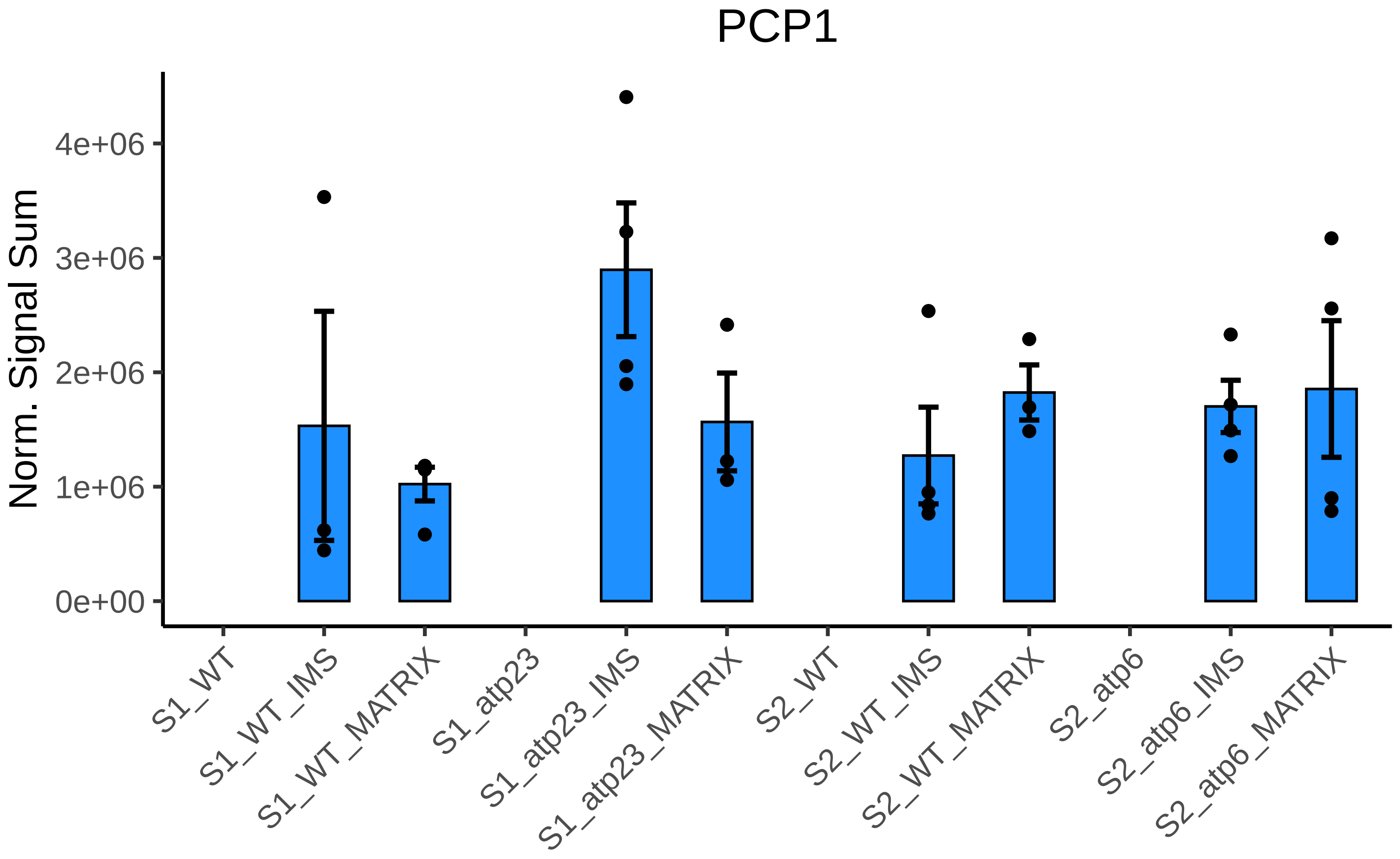
PCP1
Norm. Signal Sum
4e+06
3e+06
2e+06
1e+06
0e+00
S1_WT
S1_WT_IMS
S1_WT_MATRIX
S1_atp23
S1_atp23_IMS
S1_atp23_MATRIX
S2_WT
S2_WT_IMS
S2_WT_MATRIX
S2_atp6
S2_atp6_IMS
S2_atp6_MATRIX

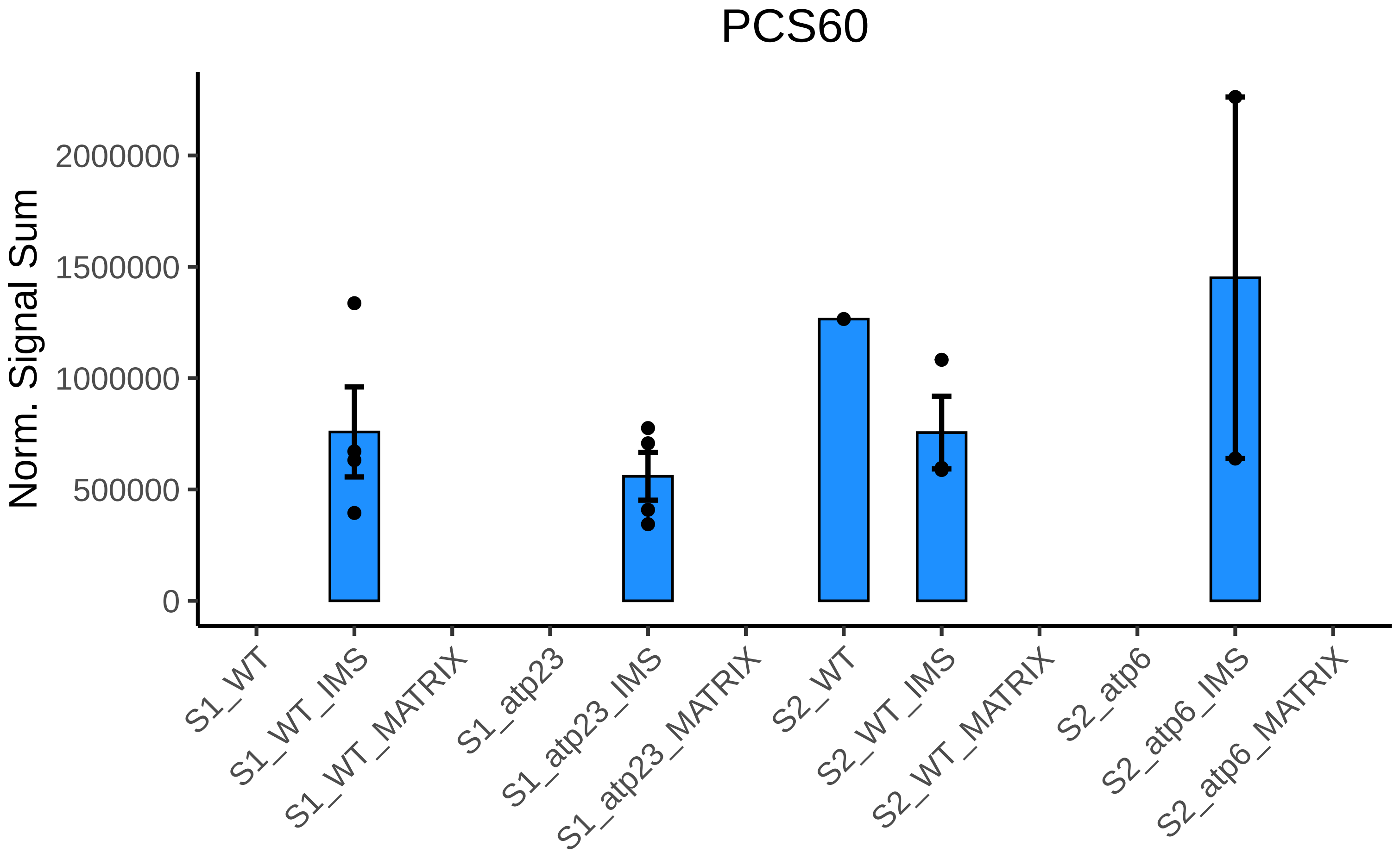
PCS60
Norm. Signal Sum
0
500000
1000000
1500000
2000000
S1_WT
S1_WT_IMS
S1_WT_MATRIX
S1_atp23
S1_atp23_IMS
S1_atp23_MATRIX
S2_WT
S2_WT_IMS
S2_WT_MATRIX
S2_atp6
S2_atp6_IMS
S2_atp6_MATRIX

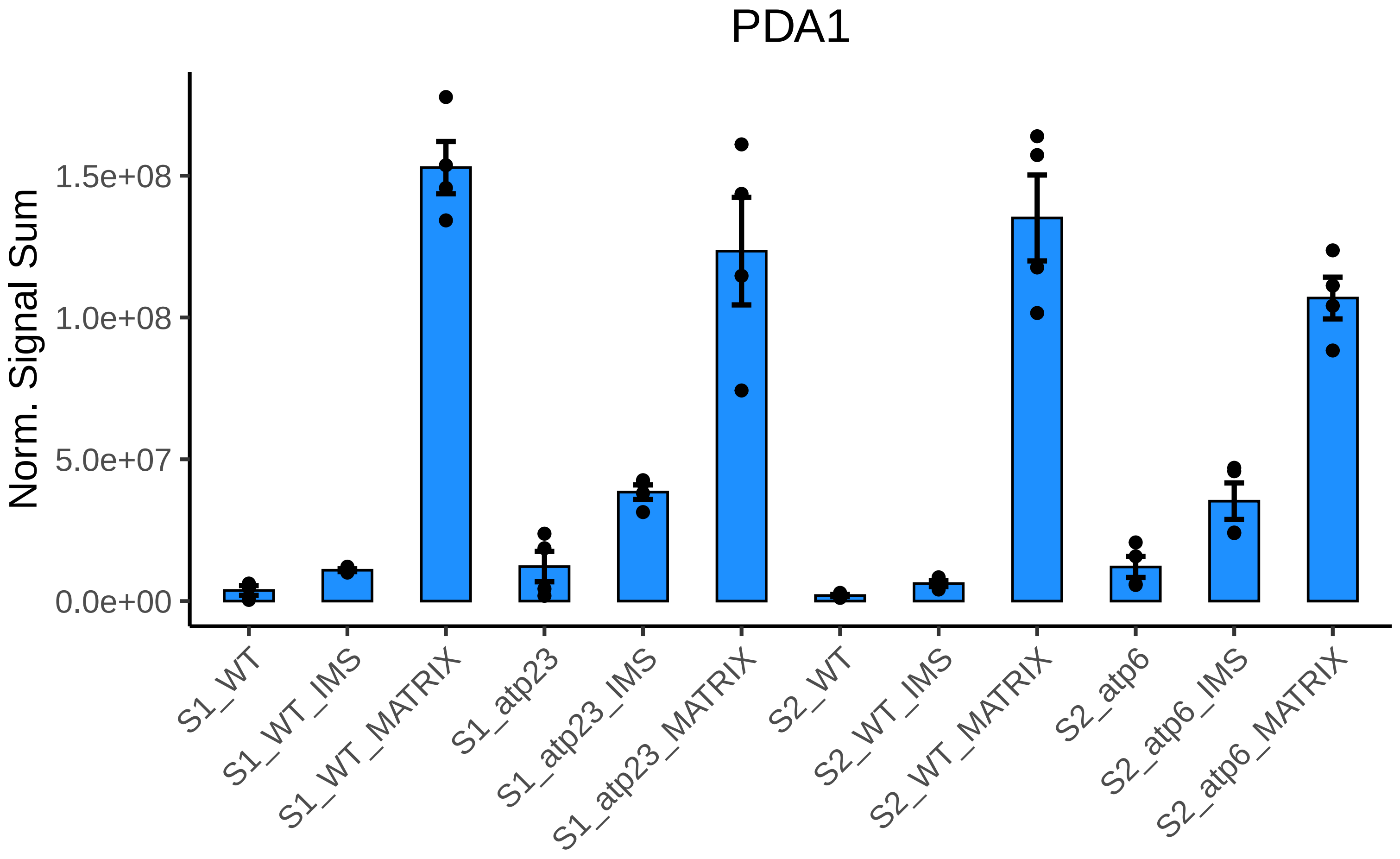

PDA1
Norm. Signal Sum
1.5e+08
1.0e+08
5.0e+07
0.0e+00
S1_WT
S1_WT_IMS
S1_WT_MATRIX
S1_atp23
S1_atp23_IMS
S1_atp23_MATRIX
S2_WT
S2_WT_IMS
S2_WT_MATRIX
S2_atp6
S2_atp6_IMS
S2_atp6_MATRIX

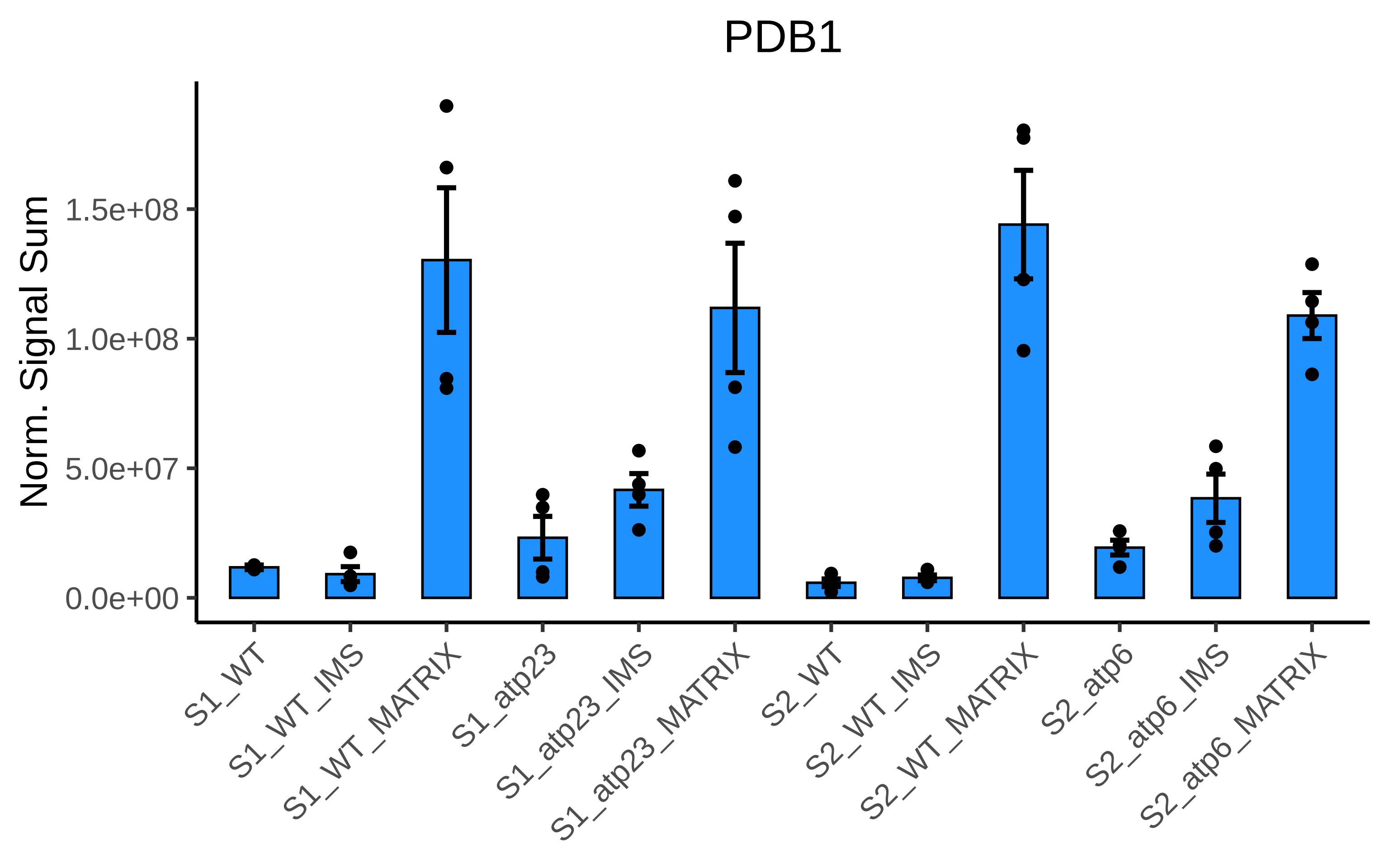

PDB1
Norm. Signal Sum
1.5e+08
1.0e+08
5.0e+07
0.0e+00
S1_WT
S1_WT_IMS
S1_WT_MATRIX
S1_atp23
S1_atp23_IMS
S1_atp23_MATRIX
S2_WT
S2_WT_IMS
S2_WT_MATRIX
S2_atp6
S2_atp6_IMS
S2_atp6_MATRIX

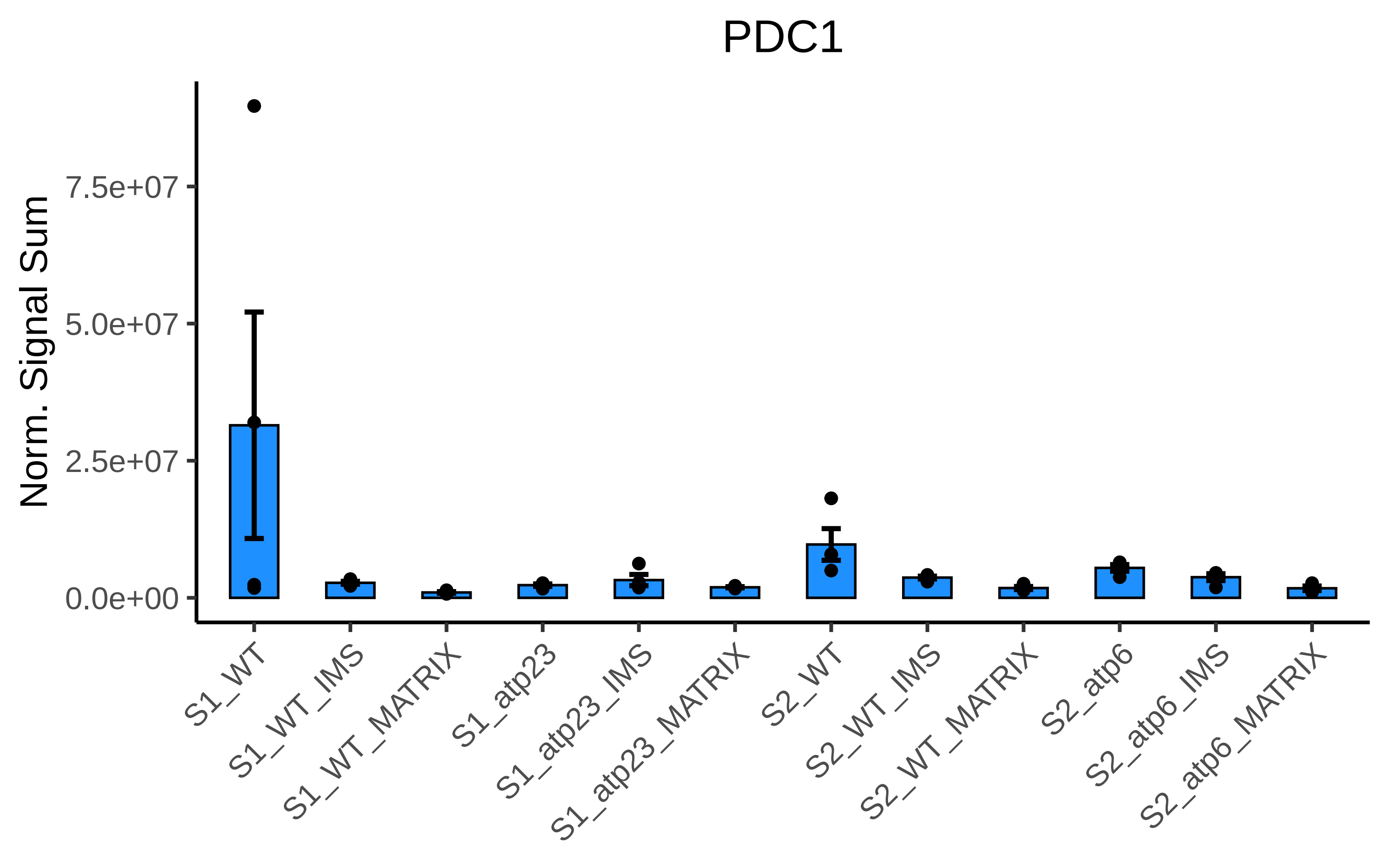
PDC1
Norm. Signal Sum
7.5e+07
5.0e+07
2.5e+07
0.0e+00
S1_WT
S1_WT_IMS
S1_WT_MATRIX
S1_atp23
S1_atp23_IMS
S1_atp23_MATRIX
S2_WT
S2_WT_IMS
S2_WT_MATRIX
S2_atp6
S2_atp6_IMS
S2_atp6_MATRIX

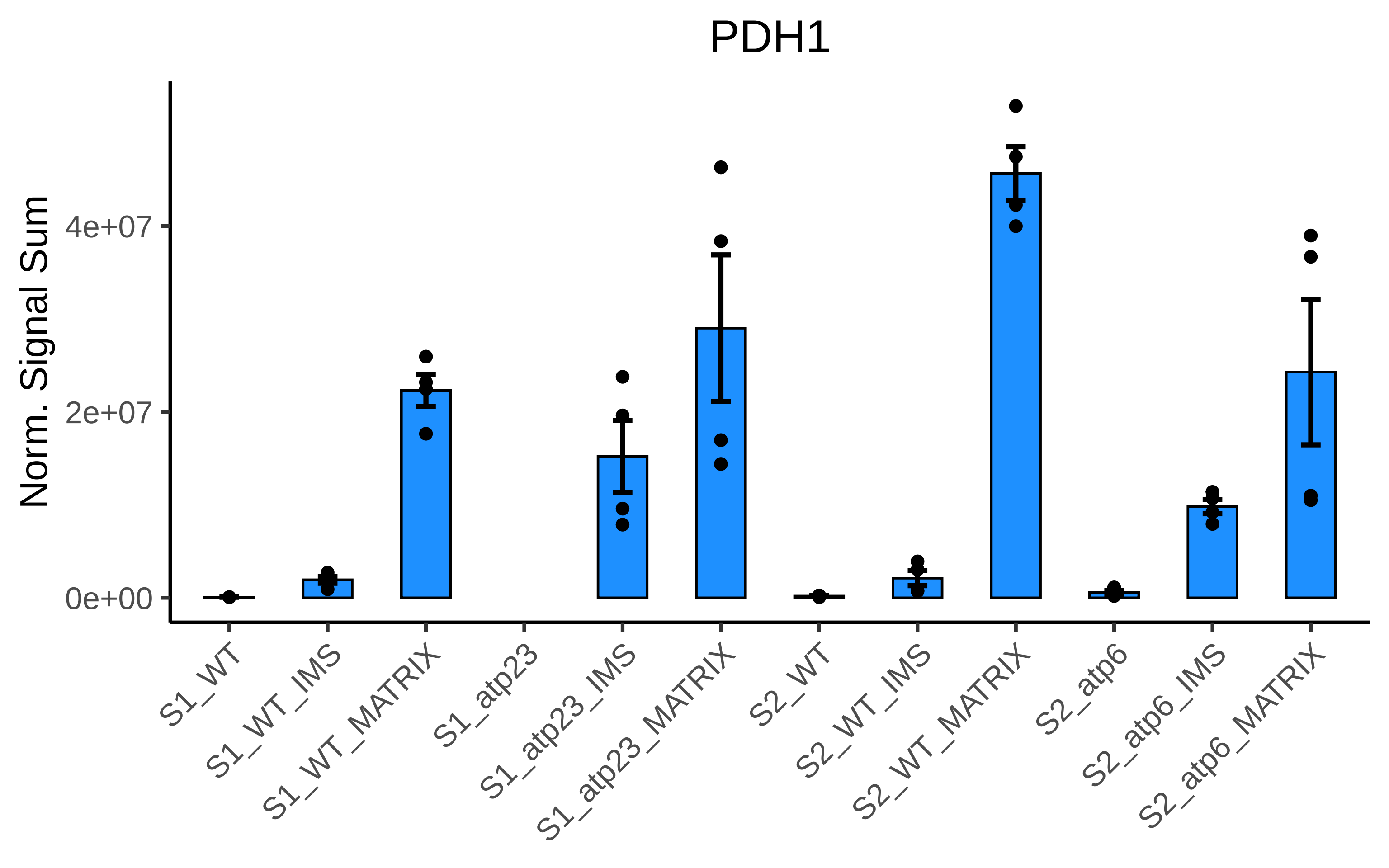

PDH1
Norm. Signal Sum
4e+07
2e+07
0e+00
S1_WT
S1_WT_IMS
S1_WT_MATRIX
S1_atp23
S1_atp23_IMS
S1_atp23_MATRIX
S2_WT
S2_WT_IMS
S2_WT_MATRIX
S2_atp6
S2_atp6_IMS
S2_atp6_MATRIX

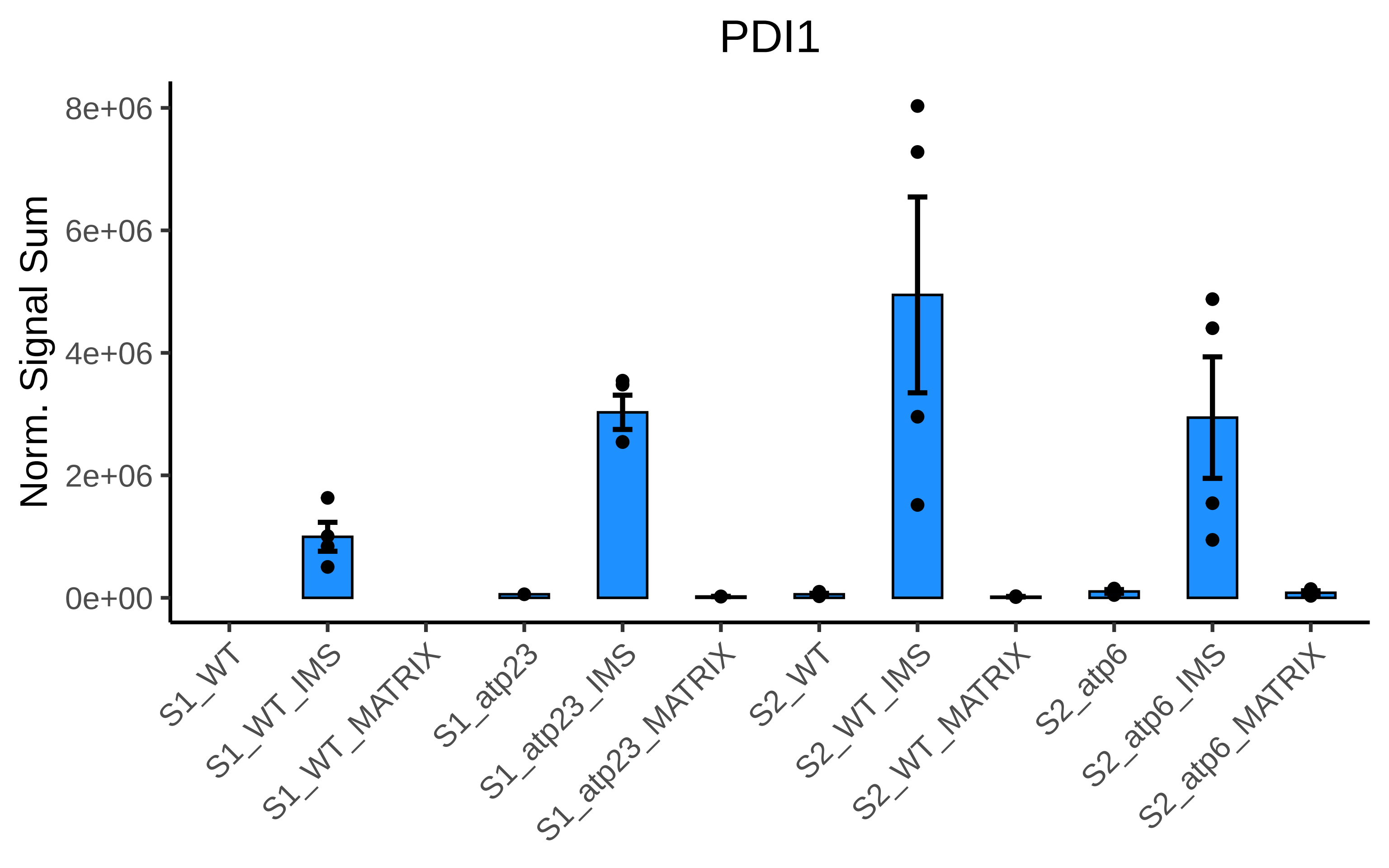

PDI1
Norm. Signal Sum
8e+06
6e+06
4e+06
2e+06
0e+00
S1_WT
S1_WT_IMS
S1_WT_MATRIX
S1_atp23
S1_atp23_IMS
S1_atp23_MATRIX
S2_WT
S2_WT_IMS
S2_WT_MATRIX
S2_atp6
S2_atp6_IMS
S2_atp6_MATRIX

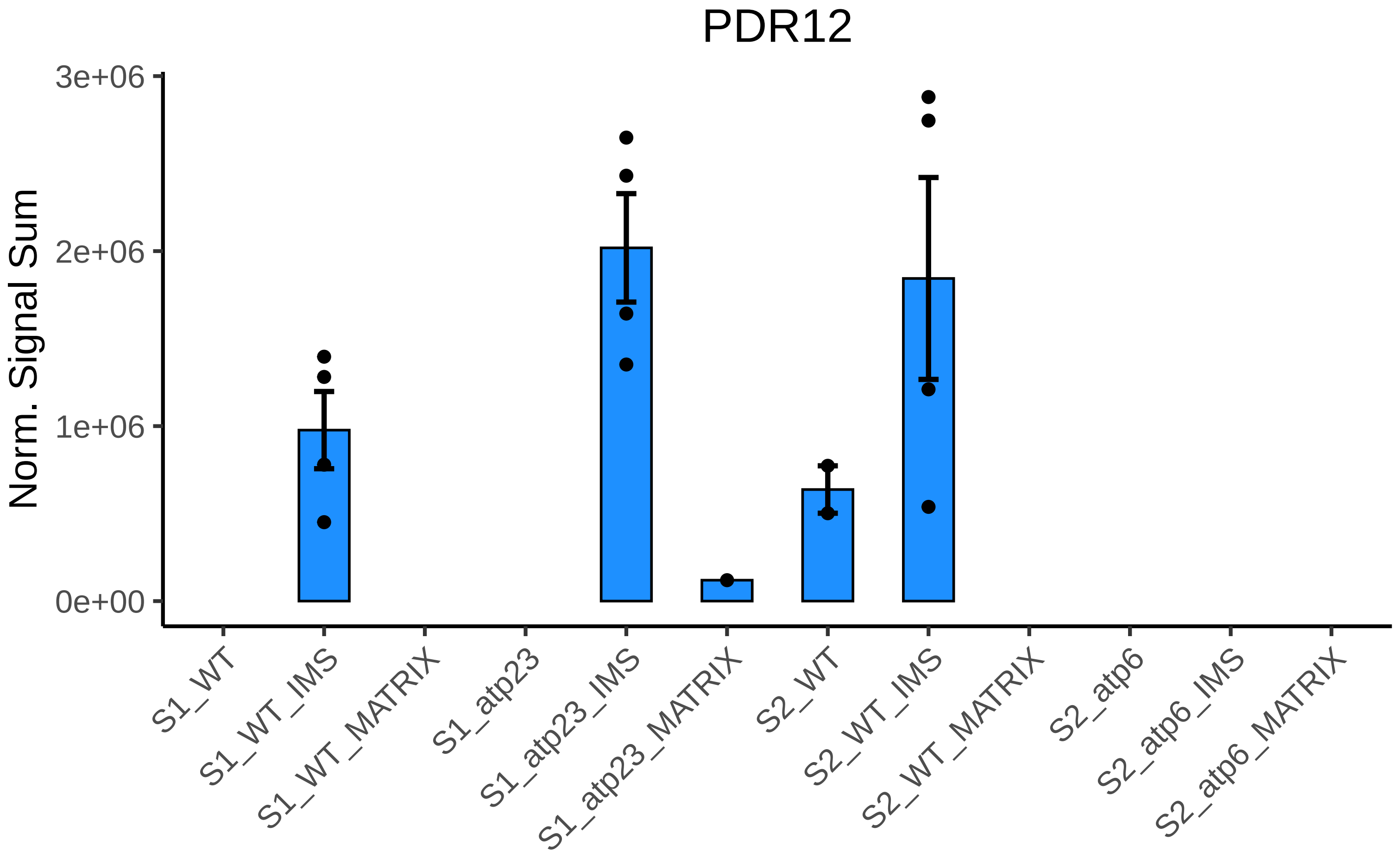
PDR12
Norm. Signal Sum
3e+06
2e+06
1e+06
0e+00
S1_WT
S1_WT_IMS
S1_WT_MATRIX
S1_atp23
S1_atp23_IMS
S1_atp23_MATRIX
S2_WT
S2_WT_IMS
S2_WT_MATRIX
S2_atp6
S2_atp6_IMS
S2_atp6_MATRIX

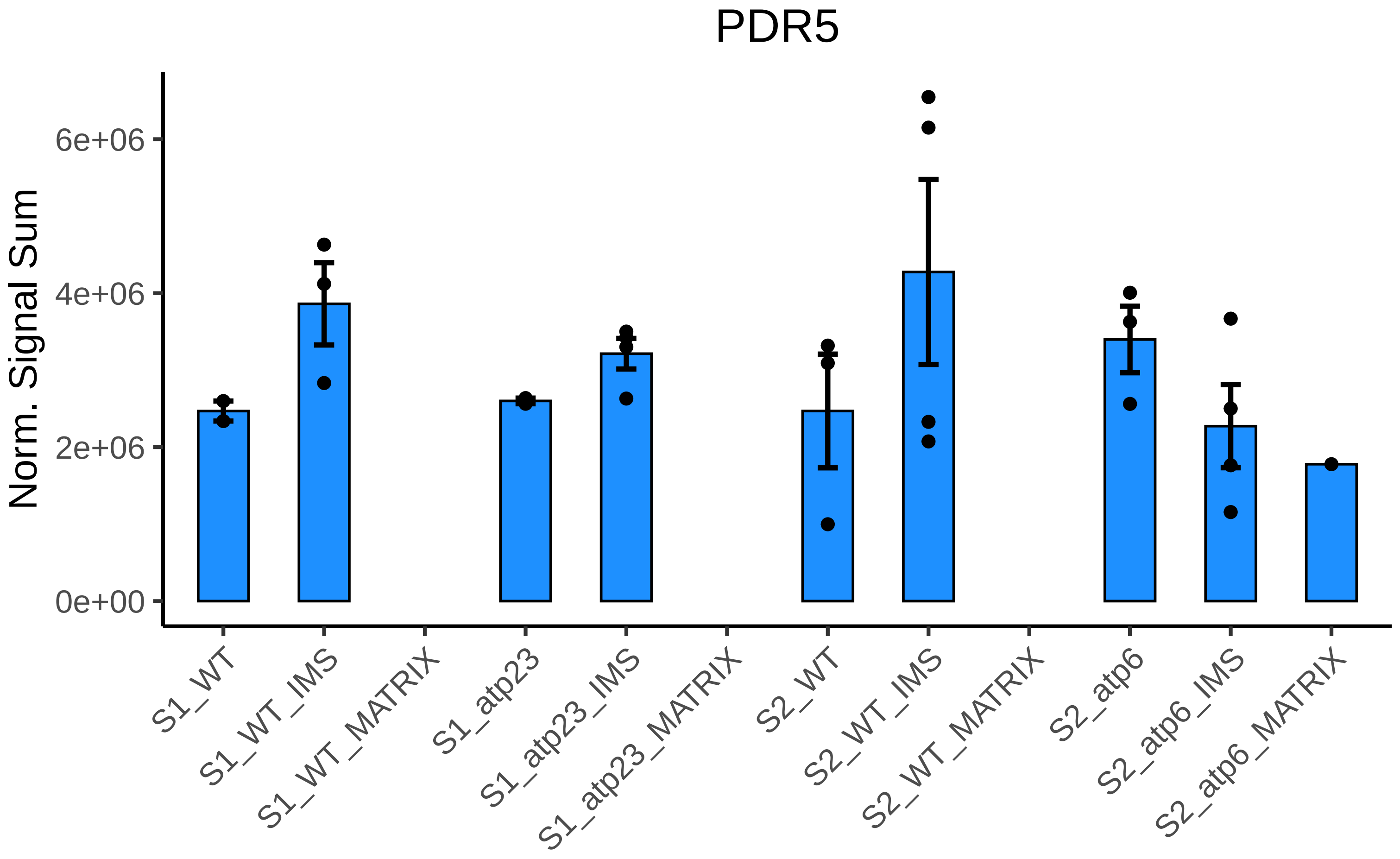

PDR5
Norm. Signal Sum
6e+06
4e+06
2e+06
0e+00
S1_WT
S1_WT_IMS
S1_WT_MATRIX
S1_atp23
S1_atp23_IMS
S1_atp23_MATRIX
S2_WT
S2_WT_IMS
S2_WT_MATRIX
S2_atp6
S2_atp6_IMS
S2_atp6_MATRIX

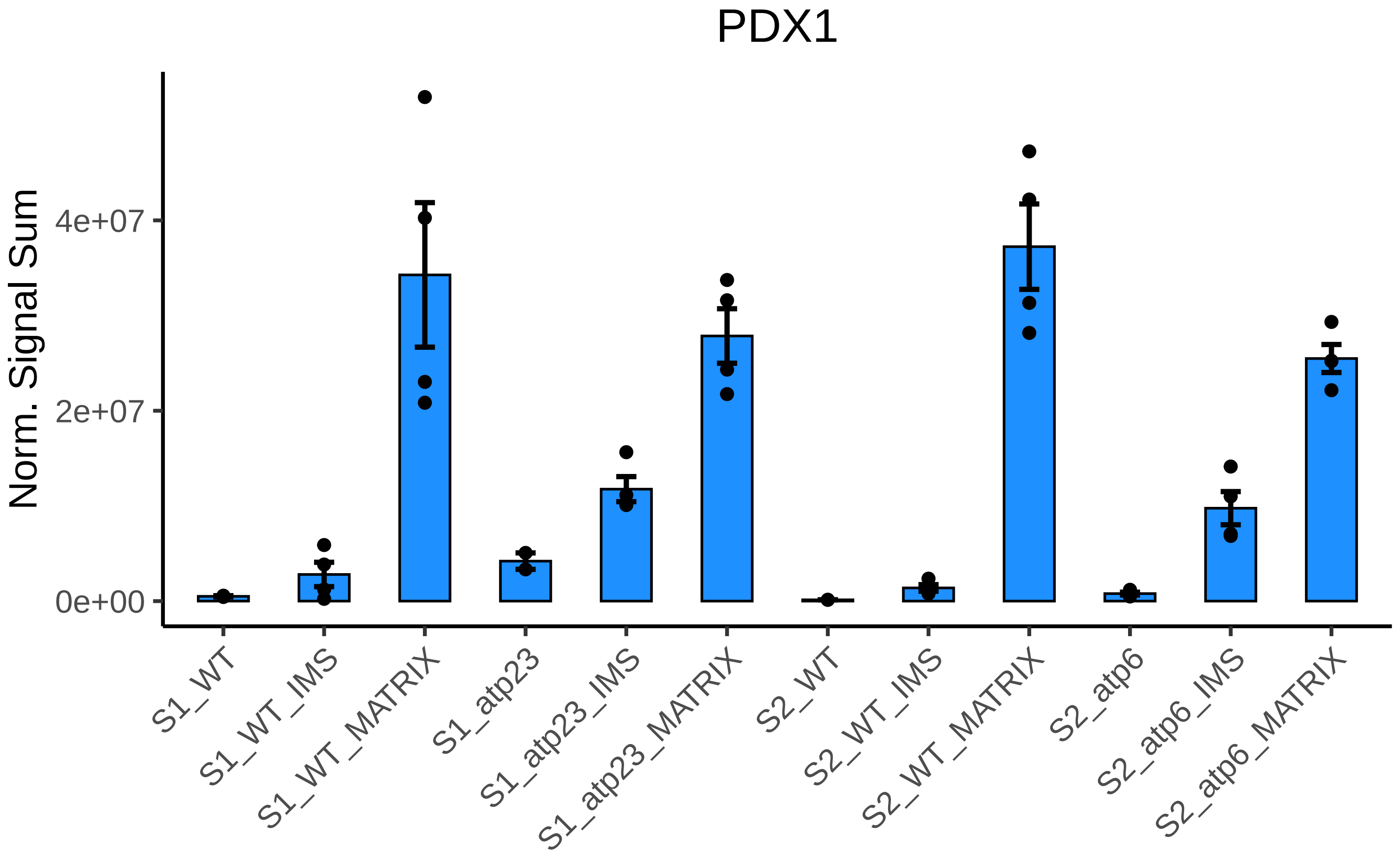

PDX1
Norm. Signal Sum
4e+07
2e+07
0e+00
S1_WT
S1_WT_IMS
S1_WT_MATRIX
S1_atp23
S1_atp23_IMS
S1_atp23_MATRIX
S2_WT
S2_WT_IMS
S2_WT_MATRIX
S2_atp6
S2_atp6_IMS
S2_atp6_MATRIX

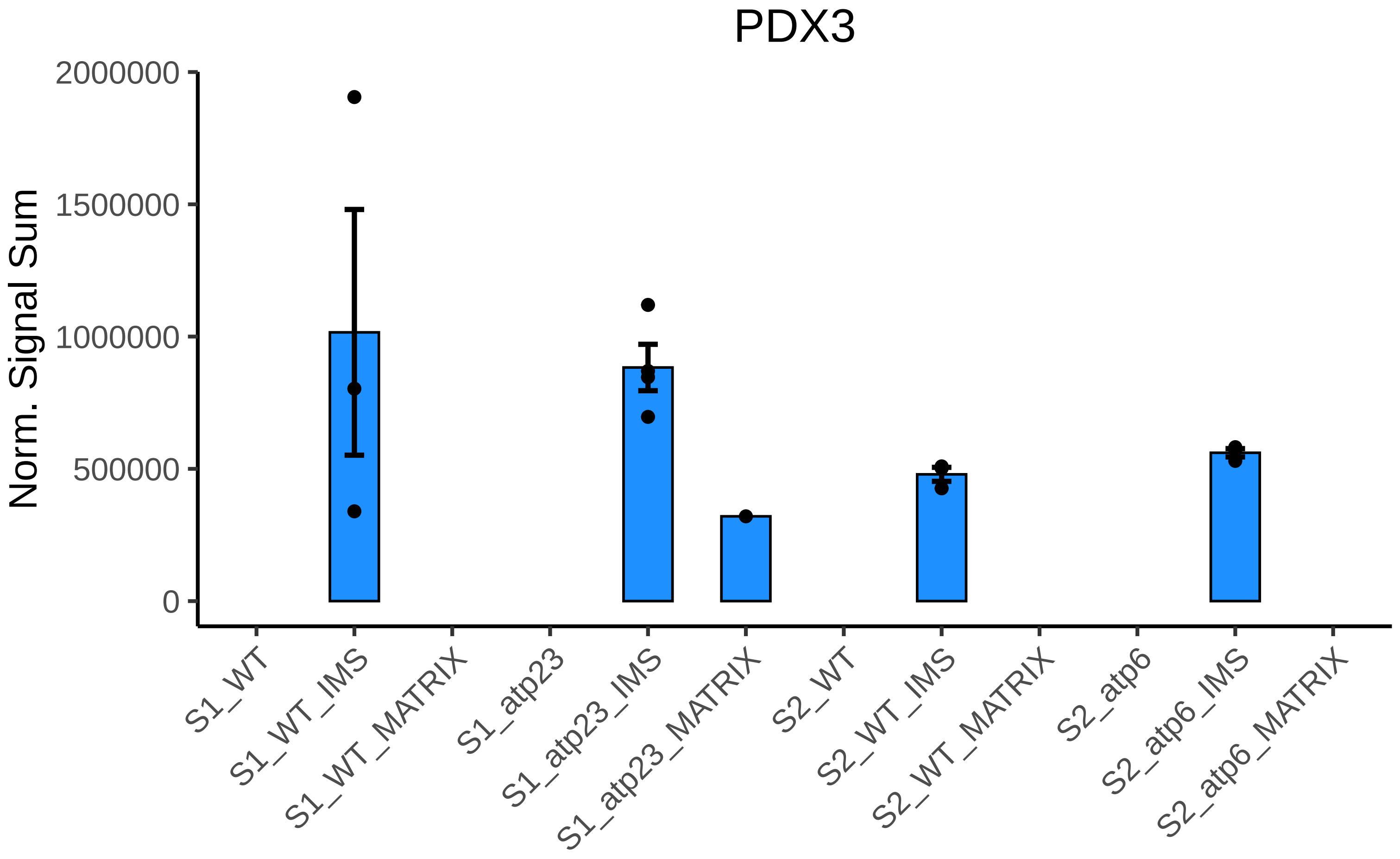

PDX3
Norm. Signal Sum
2000000
1500000
1000000
500000
0
S1_WT
S1_WT_IMS
S1_WT_MATRIX
S1_atp23
S1_atp23_IMS
S1_atp23_MATRIX
S2_WT
S2_WT_IMS
S2_WT_MATRIX
S2_atp6
S2_atp6_IMS
S2_atp6_MATRIX

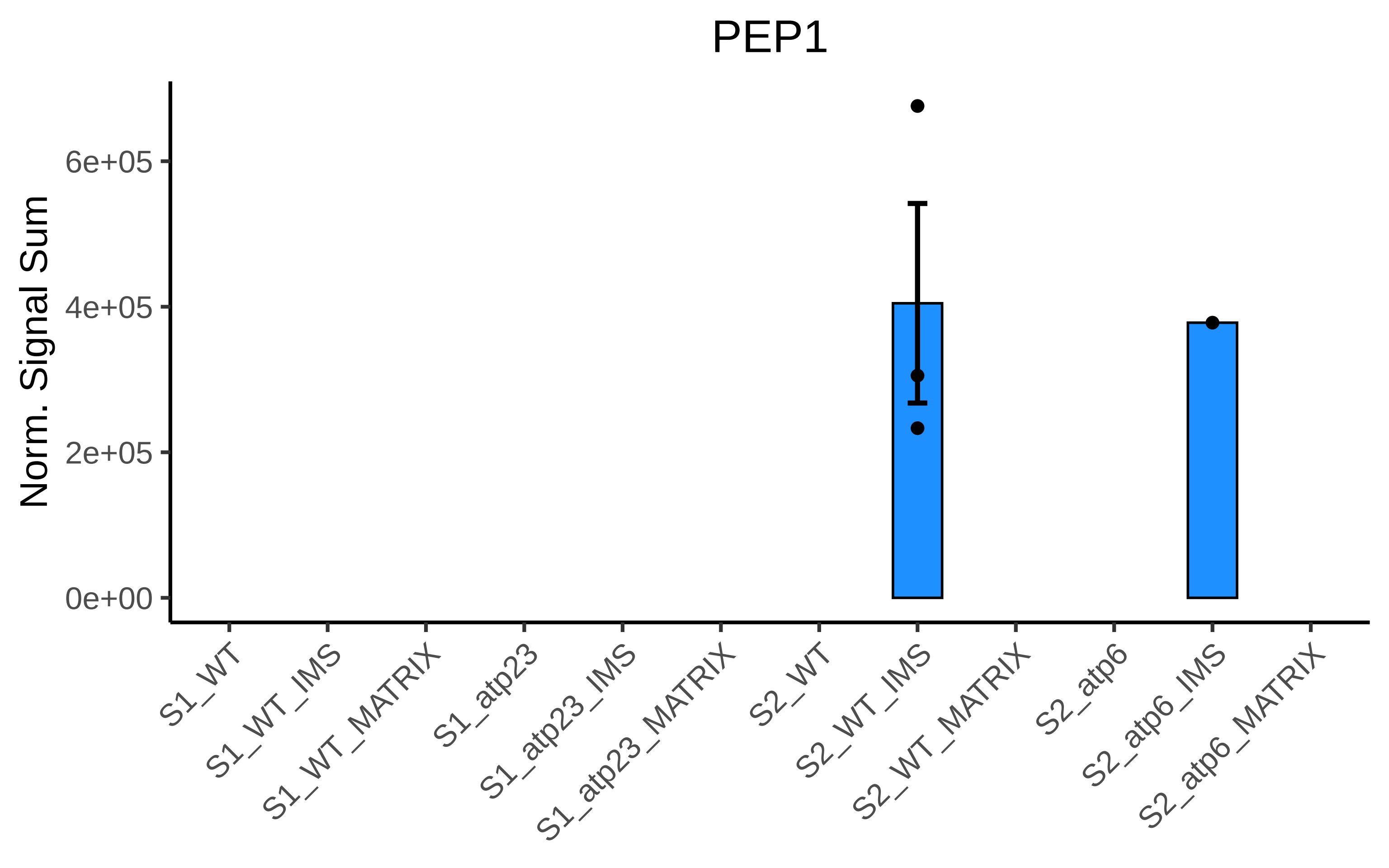
PEP1
Norm. Signal Sum
6e+05
4e+05
2e+05
0e+00
S1_WT
S1_WT_IMS
S1_WT_MATRIX
S1_atp23
S1_atp23_IMS
S1_atp23_MATRIX
S2_WT
S2_WT_IMS
S2_WT_MATRIX
S2_atp6
S2_atp6_IMS
S2_atp6_MATRIX

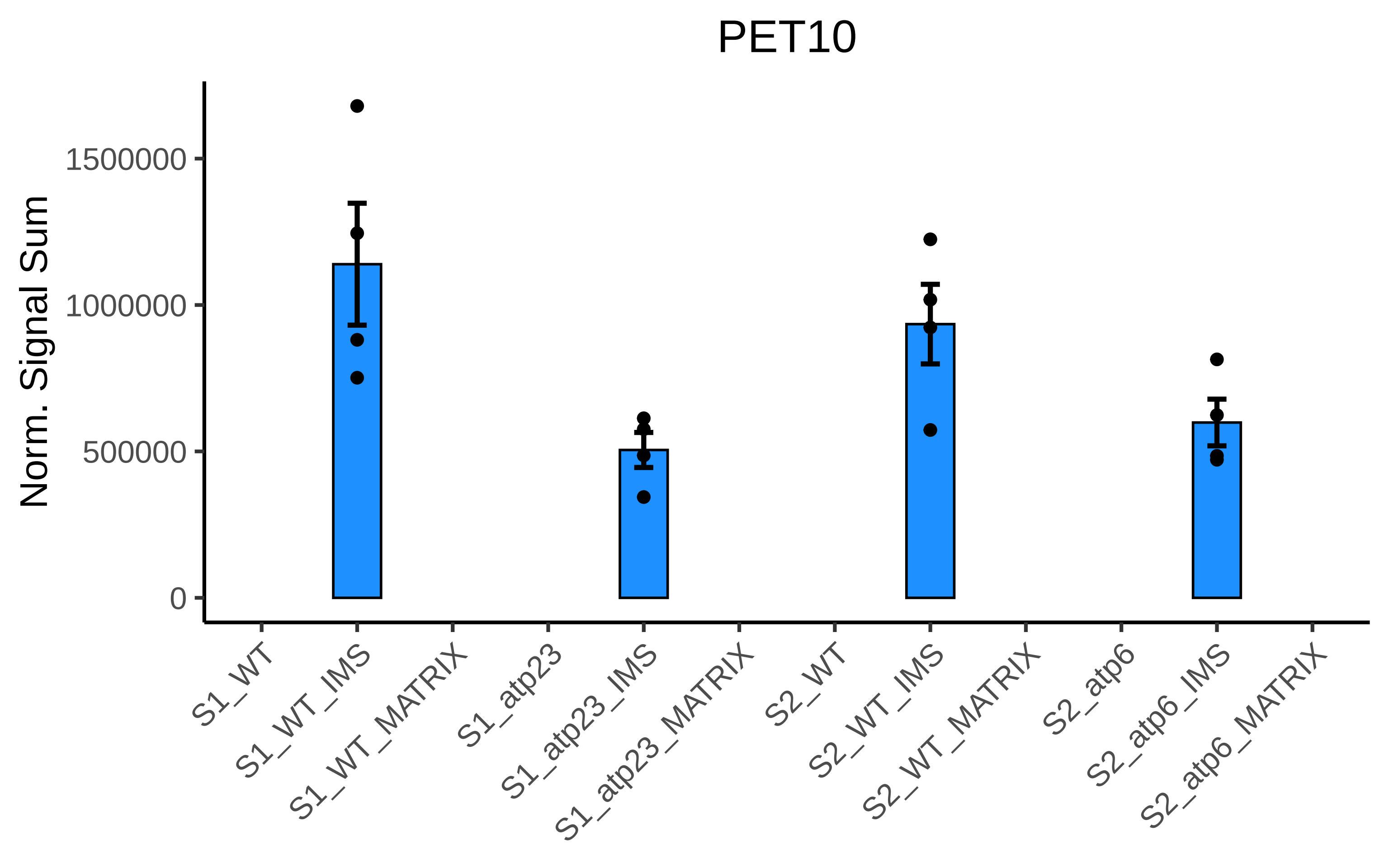
PET10
Norm. Signal Sum
1500000
1000000
500000
0
S1_WT
S1_WT_IMS
S1_WT_MATRIX
S1_atp23
S1_atp23_IMS
S1_atp23_MATRIX
S2_WT
S2_WT_IMS
S2_WT_MATRIX
S2_atp6
S2_atp6_IMS
S2_atp6_MATRIX

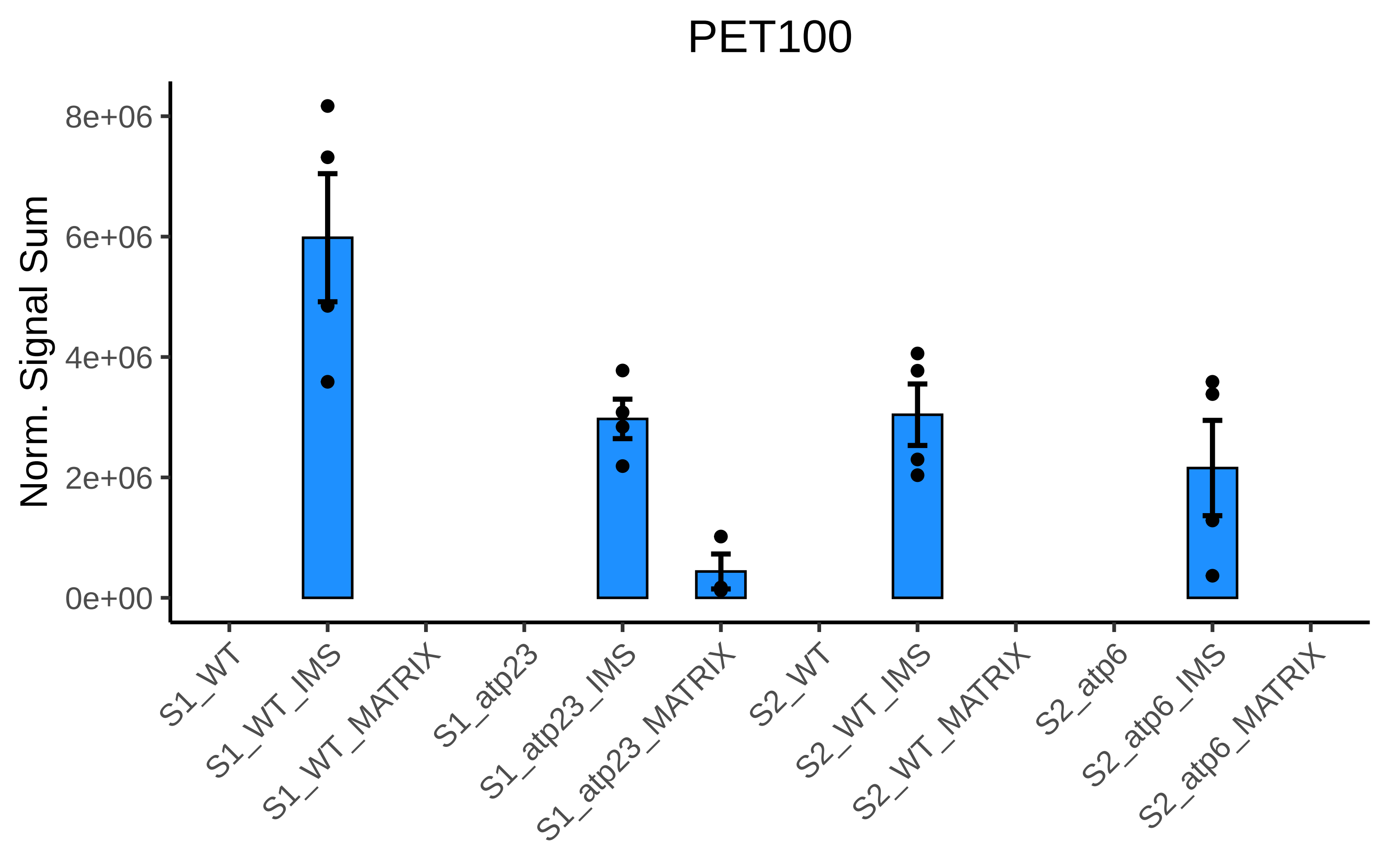

PET100
Norm. Signal Sum
8e+06
6e+06
4e+06
2e+06
0e+00
S1_WT
S1_WT_IMS
S1_WT_MATRIX
S1_atp23
S1_atp23_IMS
S1_atp23_MATRIX
S2_WT
S2_WT_IMS
S2_WT_MATRIX
S2_atp6
S2_atp6_IMS
S2_atp6_MATRIX

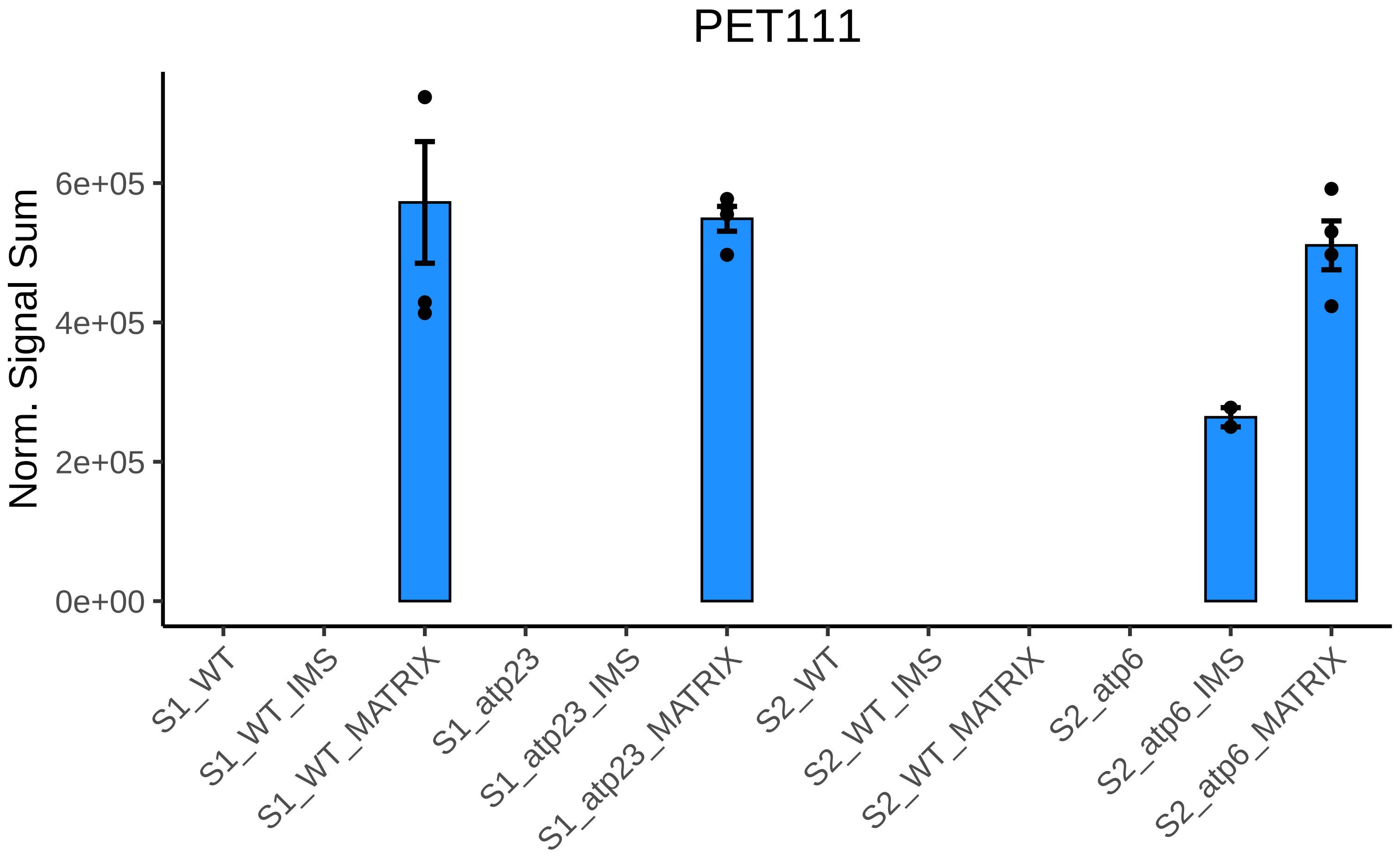

PET111
Norm. Signal Sum
0e+00
2e+05
4e+05
6e+05
S1_WT
S1_WT_IMS
S1_WT_MATRIX
S1_atp23
S1_atp23_IMS
S1_atp23_MATRIX
S2_WT
S2_WT_IMS
S2_WT_MATRIX
S2_atp6
S2_atp6_IMS
S2_atp6_MATRIX

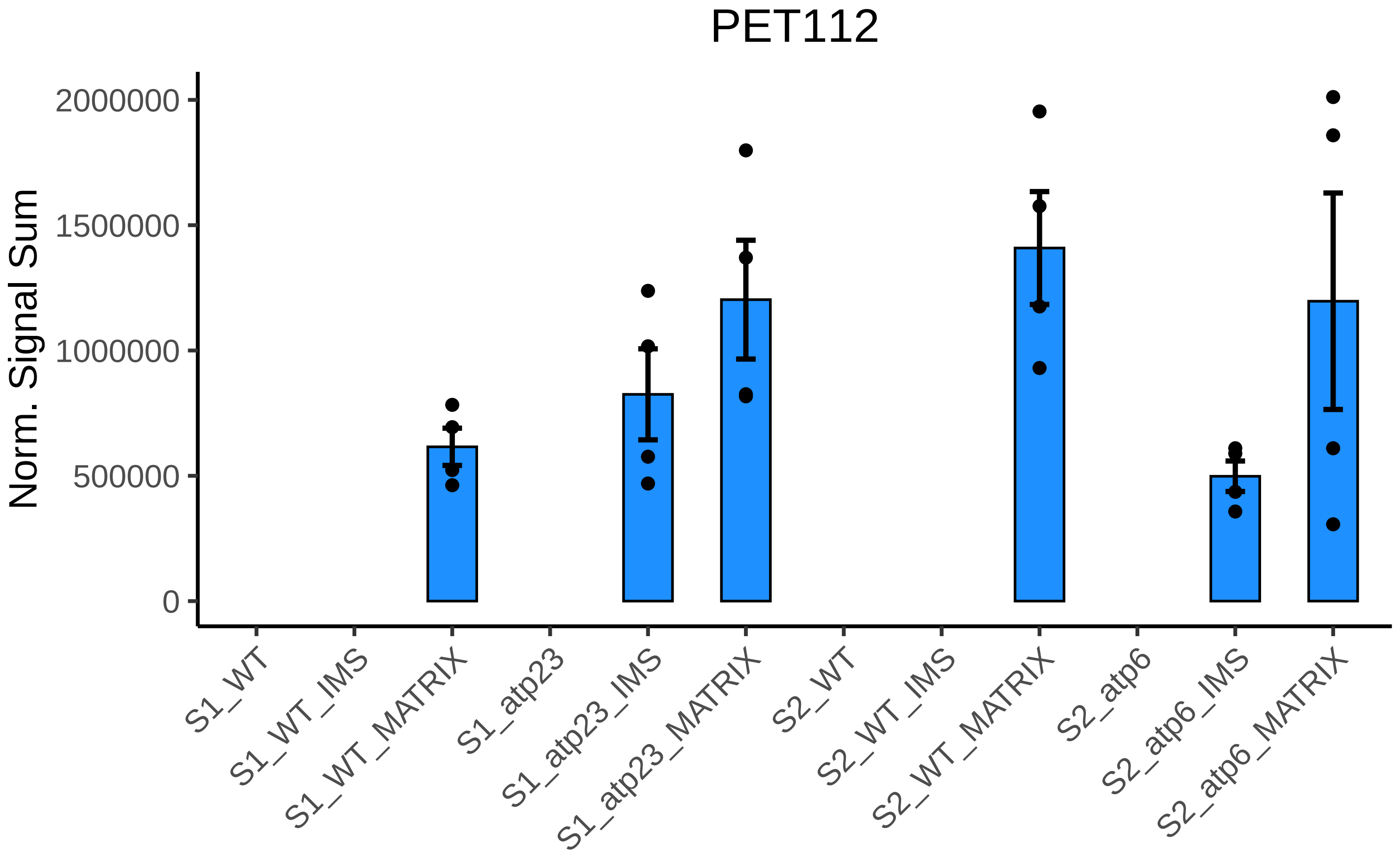
PET112
Norm. Signal Sum
2000000
1500000
1000000
500000
0
S1_WT
S1_WT_IMS
S1_WT_MATRIX
S1_atp23
S1_atp23_IMS
S1_atp23_MATRIX
S2_WT
S2_WT_IMS
S2_WT_MATRIX
S2_atp6
S2_atp6_IMS
S2_atp6_MATRIX

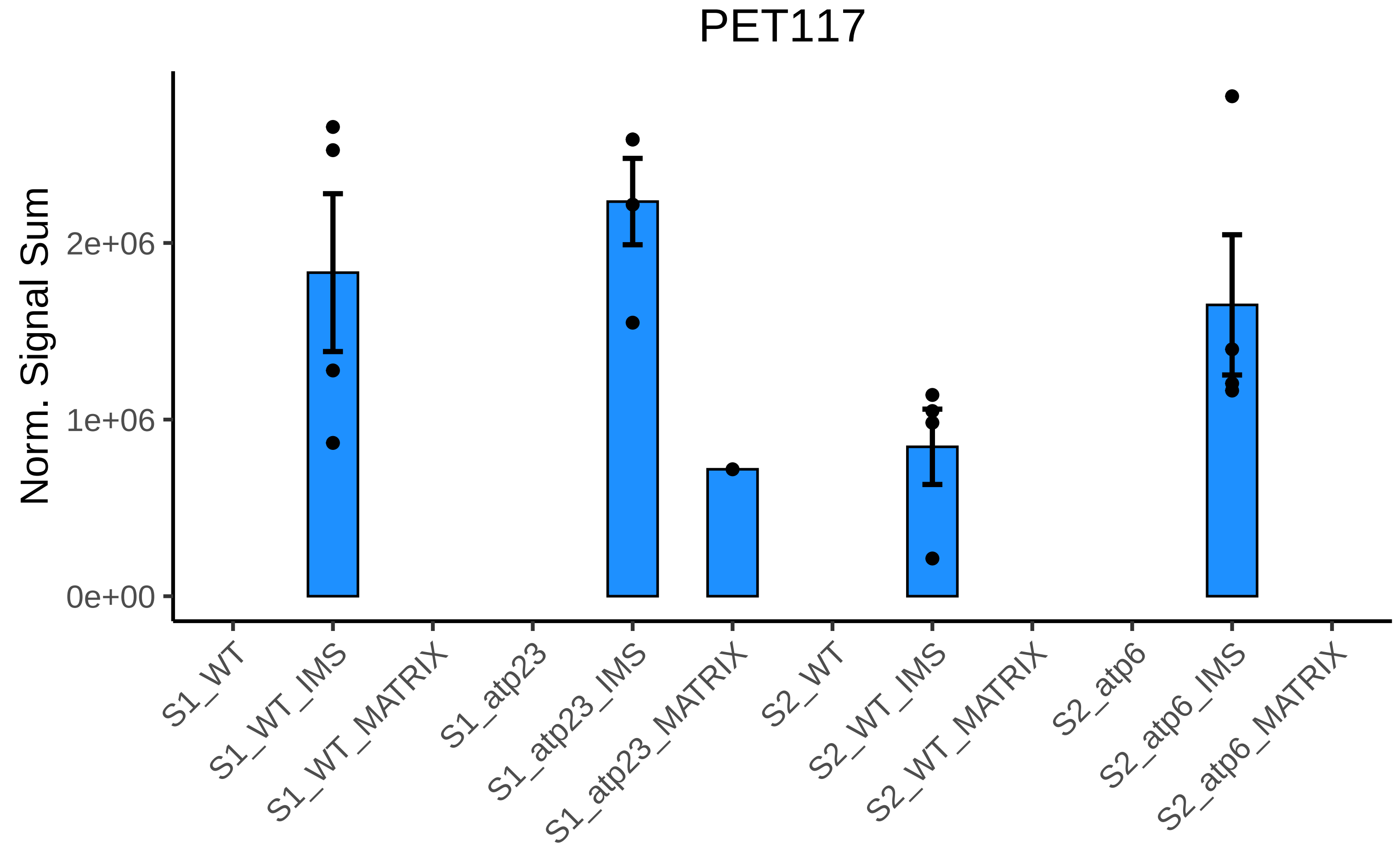

PET117
Norm. Signal Sum
2e+06
1e+06
0e+00
S1_WT
S1_WT_IMS
S1_WT_MATRIX
S1_atp23
S1_atp23_IMS
S1_atp23_MATRIX
S2_WT
S2_WT_IMS
S2_WT_MATRIX
S2_atp6
S2_atp6_IMS
S2_atp6_MATRIX

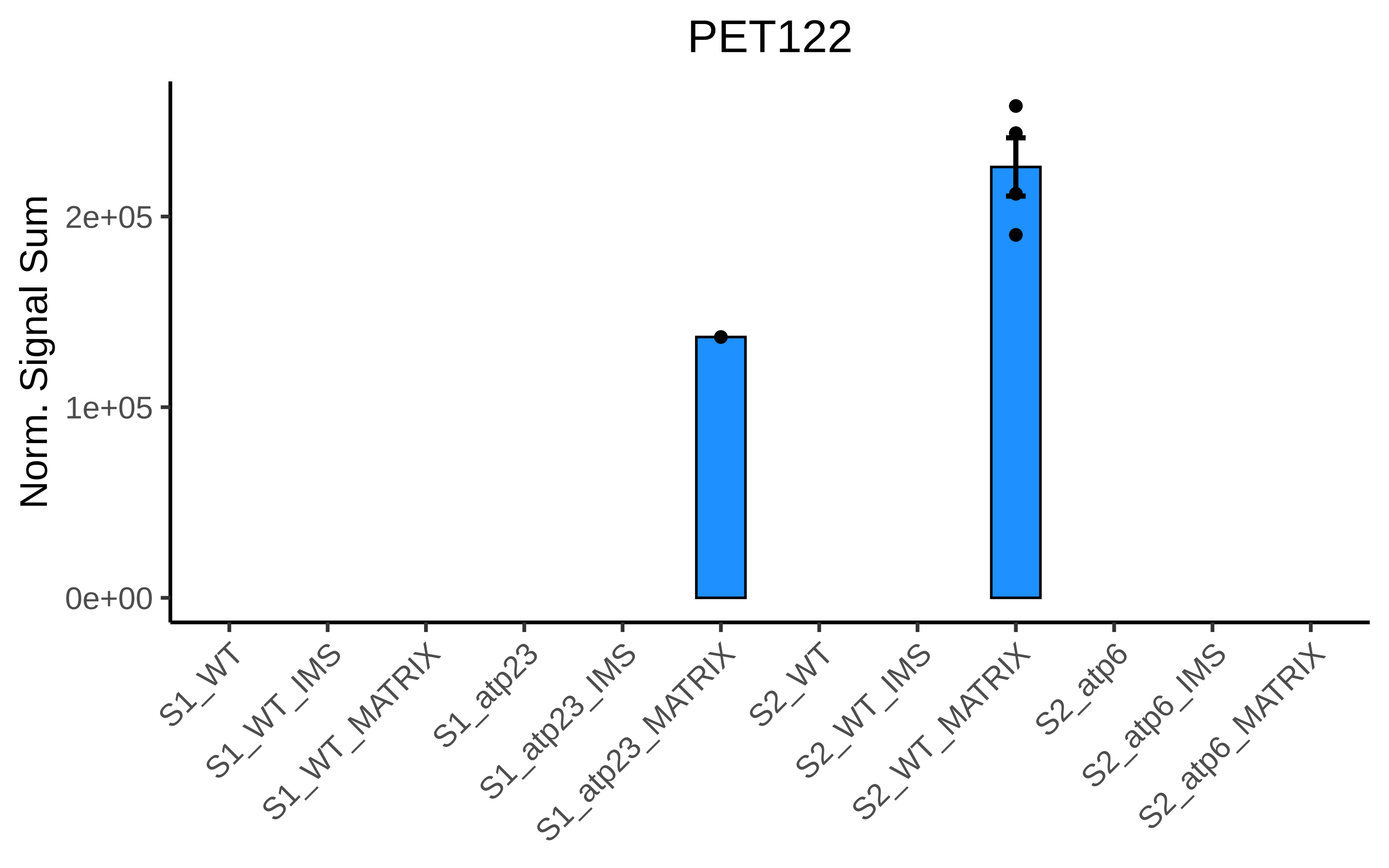
PET122
Norm. Signal Sum
0e+00
1e+05
2e+05
S1_WT
S1_WT_IMS
S1_WT_MATRIX
S1_atp23
S1_atp23_IMS
S1_atp23_MATRIX
S2_WT
S2_WT_IMS
S2_WT_MATRIX
S2_atp6
S2_atp6_IMS
S2_atp6_MATRIX

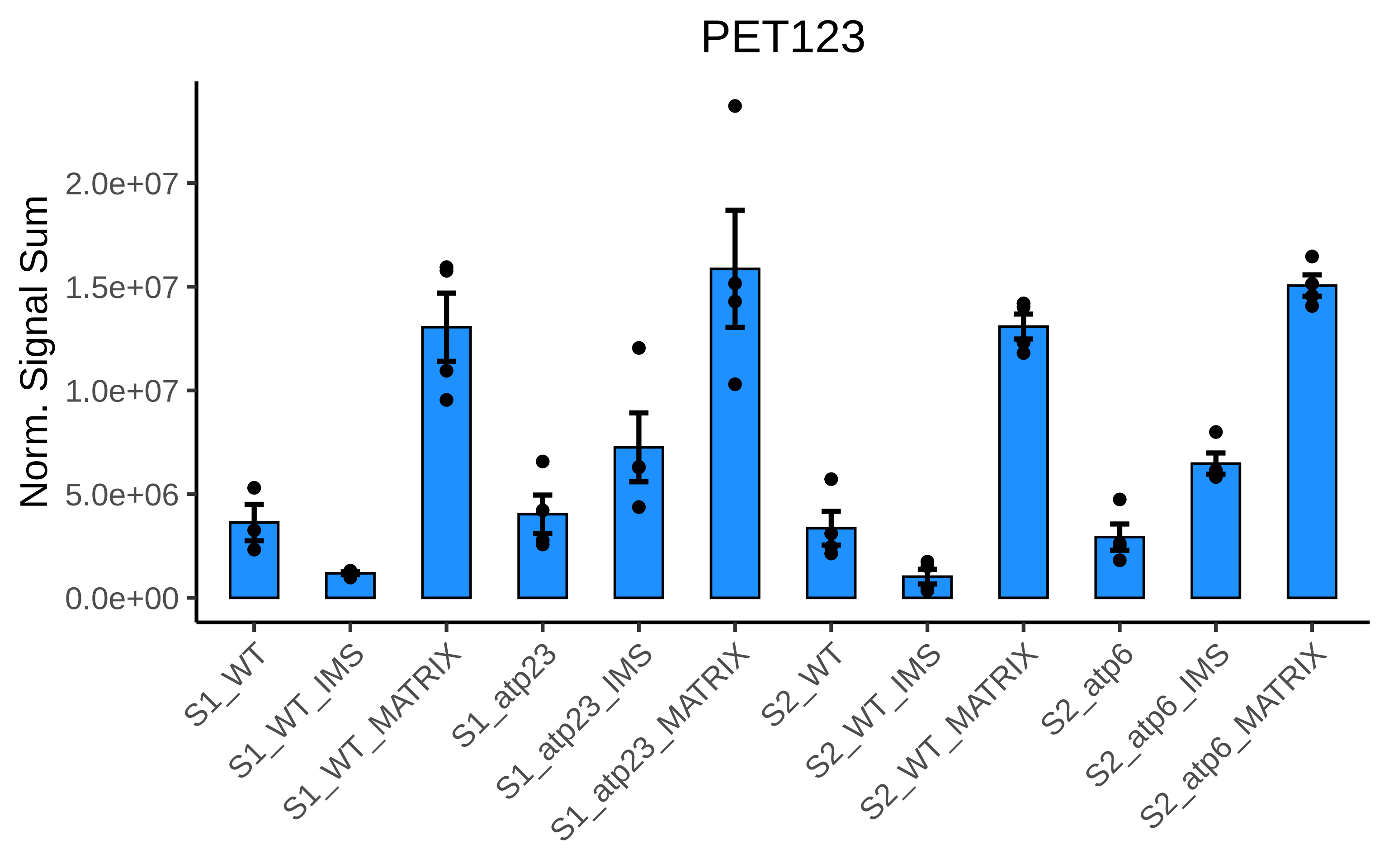
PET123
Norm. Signal Sum
2.0e+07
1.5e+07
1.0e+07
5.0e+06
0.0e+00
S1_WT
S1_WT_IMS
S1_WT_MATRIX
S1_atp23
S1_atp23_IMS
S1_atp23_MATRIX
S2_WT
S2_WT_IMS
S2_WT_MATRIX
S2_atp6
S2_atp6_IMS
S2_atp6_MATRIX

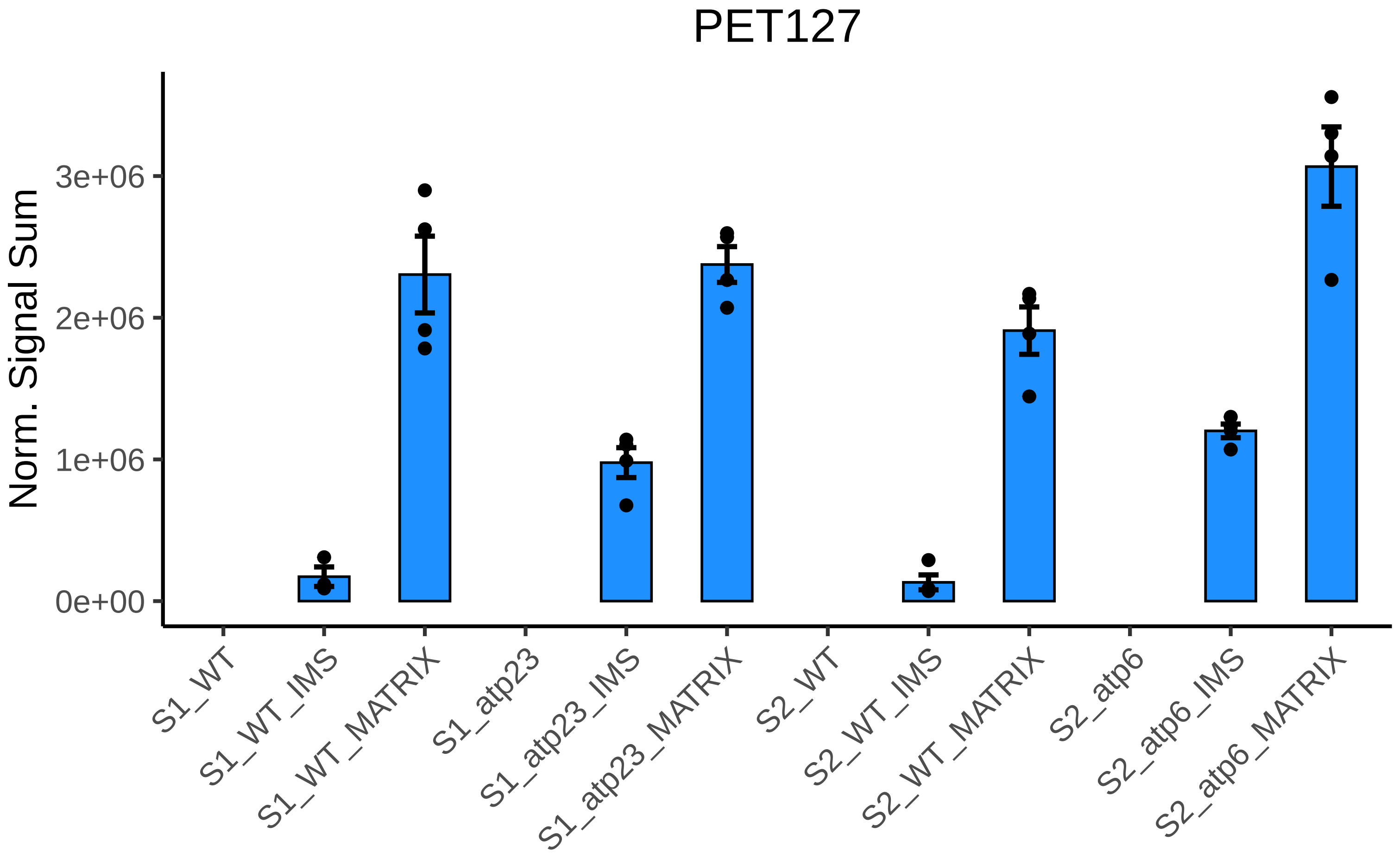

PET127
Norm. Signal Sum
3e+06
2e+06
1e+06
0e+00
S1_WT
S1_WT_IMS
S1_WT_MATRIX
S1_atp23
S1_atp23_IMS
S1_atp23_MATRIX
S2_WT
S2_WT_IMS
S2_WT_MATRIX
S2_atp6
S2_atp6_IMS
S2_atp6_MATRIX

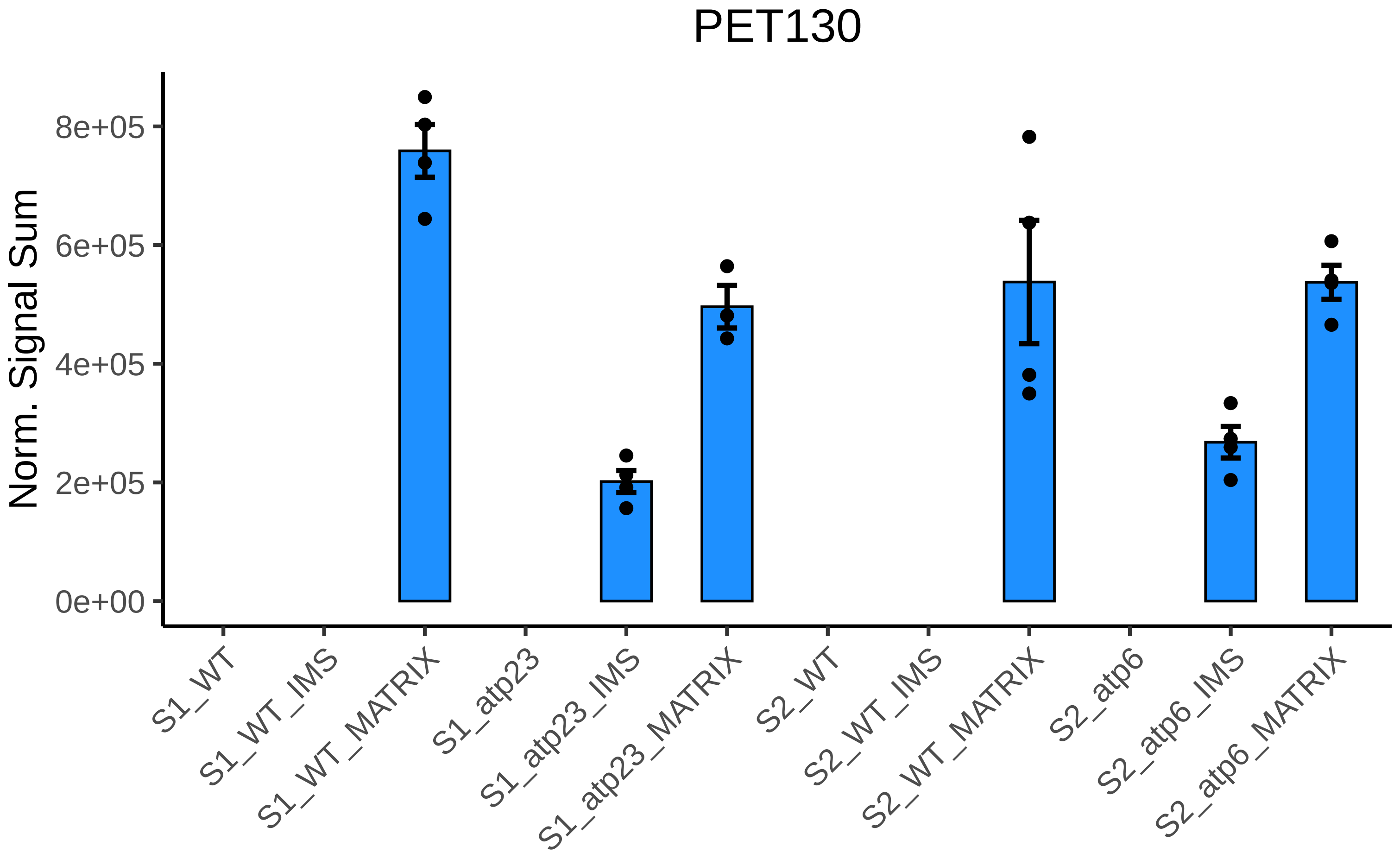

PET130
Norm. Signal Sum
8e+05
6e+05
4e+05
2e+05
0e+00
S1_WT
S1_WT_IMS
S1_WT_MATRIX
S1_atp23
S1_atp23_IMS
S1_atp23_MATRIX
S2_WT
S2_WT_IMS
S2_WT_MATRIX
S2_atp6
S2_atp6_IMS
S2_atp6_MATRIX

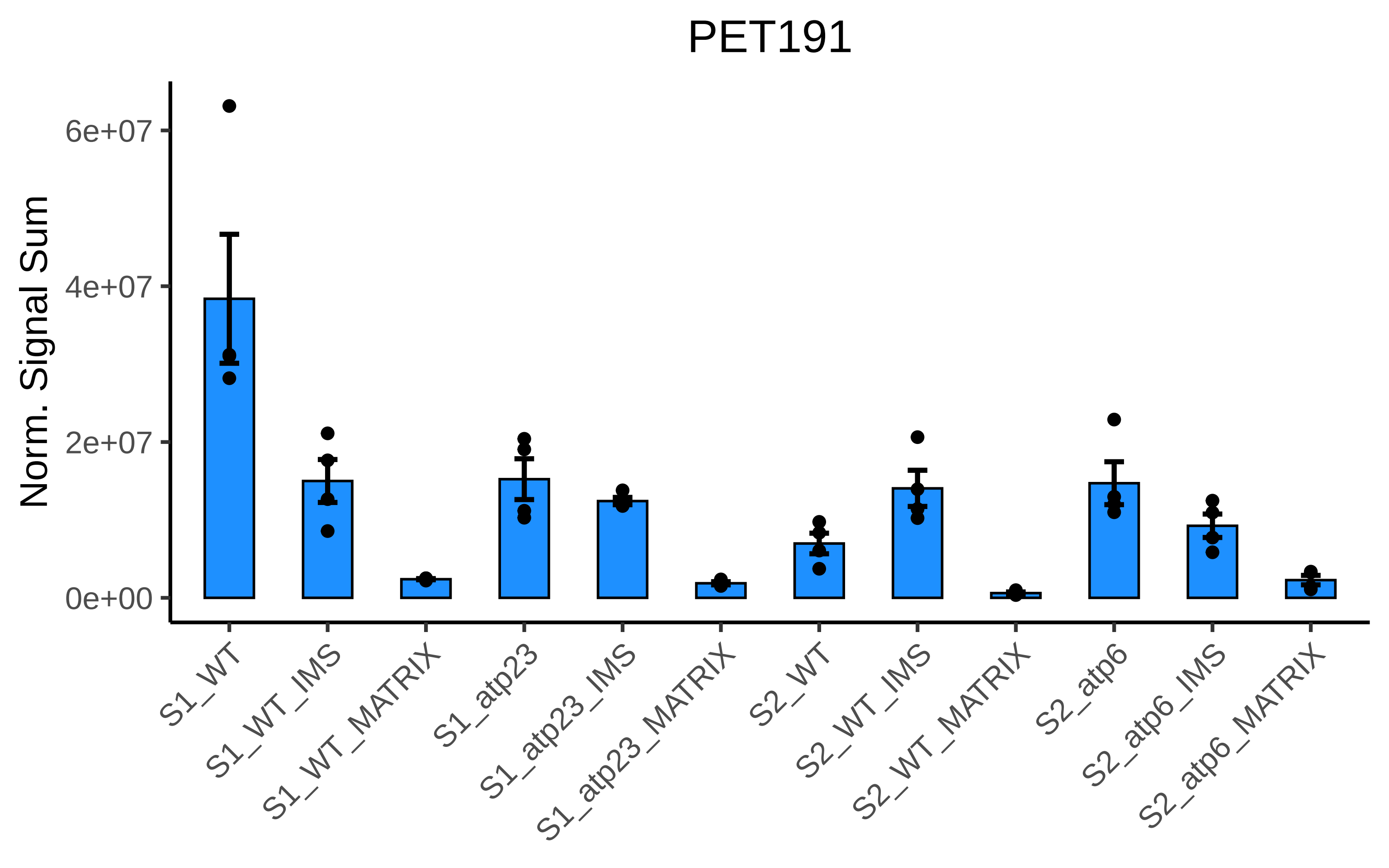
PET191
Norm. Signal Sum
6e+07
4e+07
2e+07
0e+00
S1_WT
S1_WT_IMS
S1_WT_MATRIX
S1_atp23
S1_atp23_IMS
S1_atp23_MATRIX
S2_WT
S2_WT_IMS
S2_WT_MATRIX
S2_atp6
S2_atp6_IMS
S2_atp6_MATRIX

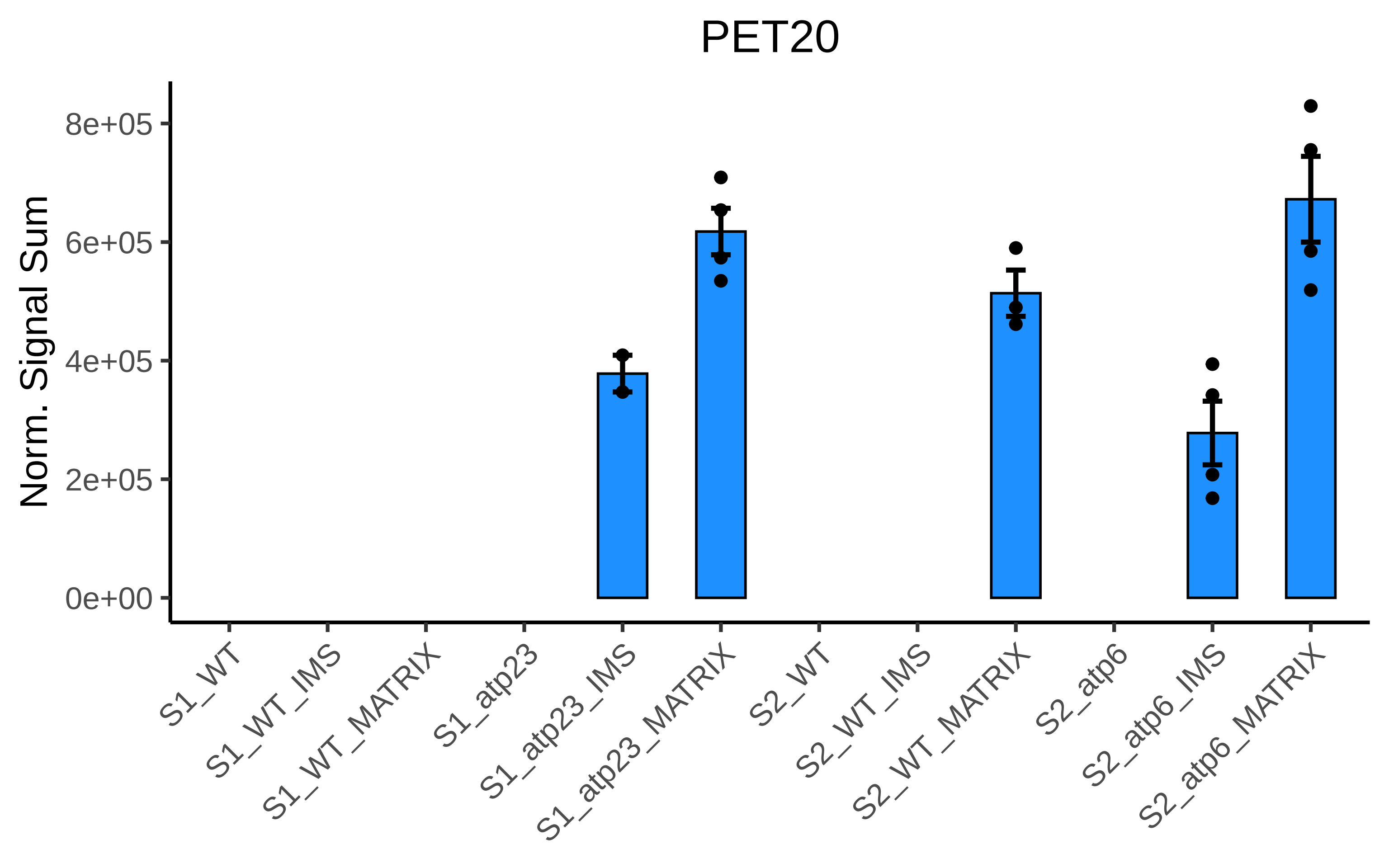
PET20
Norm. Signal Sum
8e+05
6e+05
4e+05
2e+05
0e+00
S1_WT
S1_WT_IMS
S1_WT_MATRIX
S1_atp23
S1_atp23_IMS
S1_atp23_MATRIX
S2_WT
S2_WT_IMS
S2_WT_MATRIX
S2_atp6
S2_atp6_IMS
S2_atp6_MATRIX

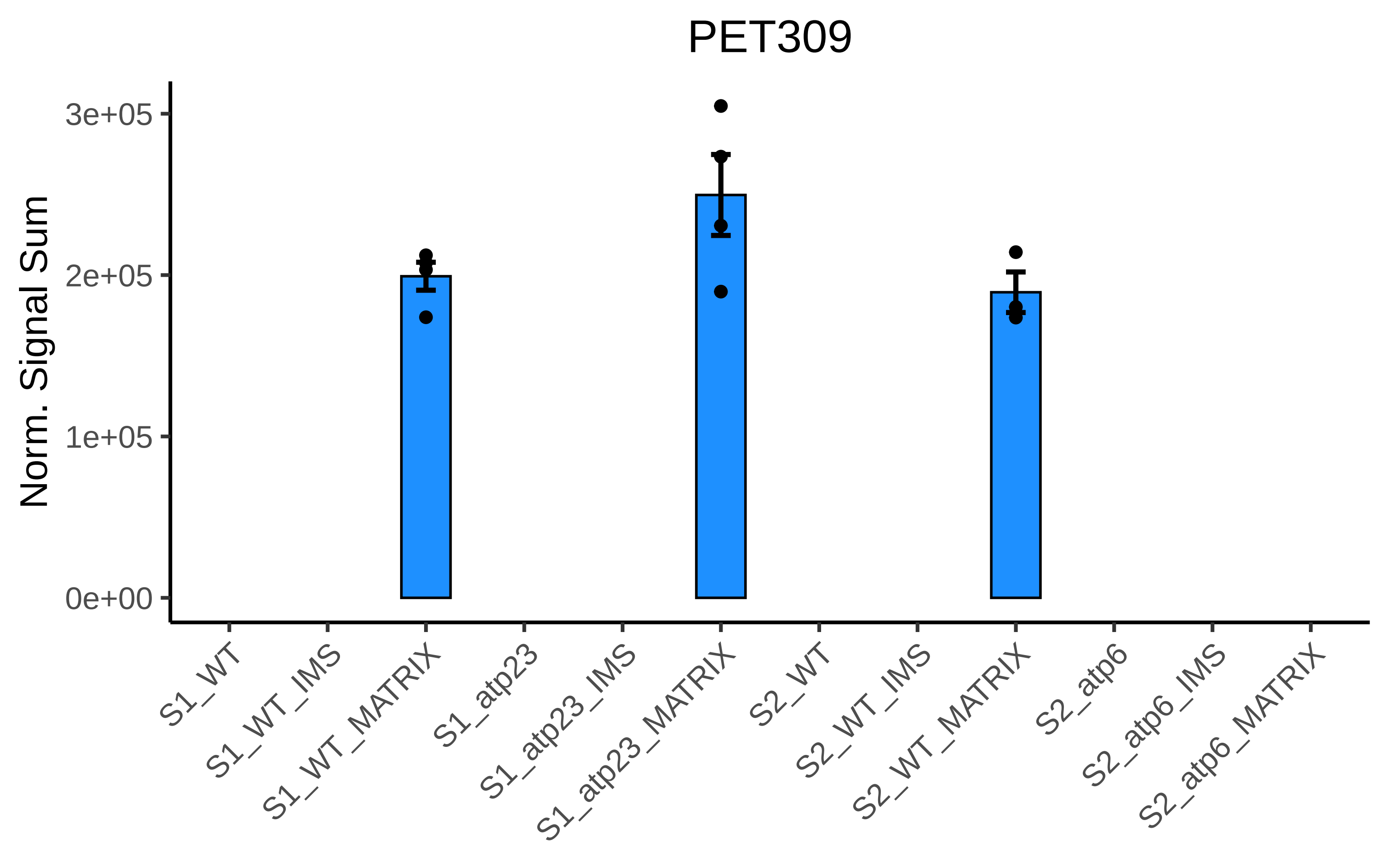
PET309
Norm. Signal Sum
3e+05
2e+05
1e+05
0e+00
S1_WT
S1_WT_IMS
S1_WT_MATRIX
S1_atp23
S1_atp23_IMS
S1_atp23_MATRIX
S2_WT
S2_WT_IMS
S2_WT_MATRIX
S2_atp6
S2_atp6_IMS
S2_atp6_MATRIX

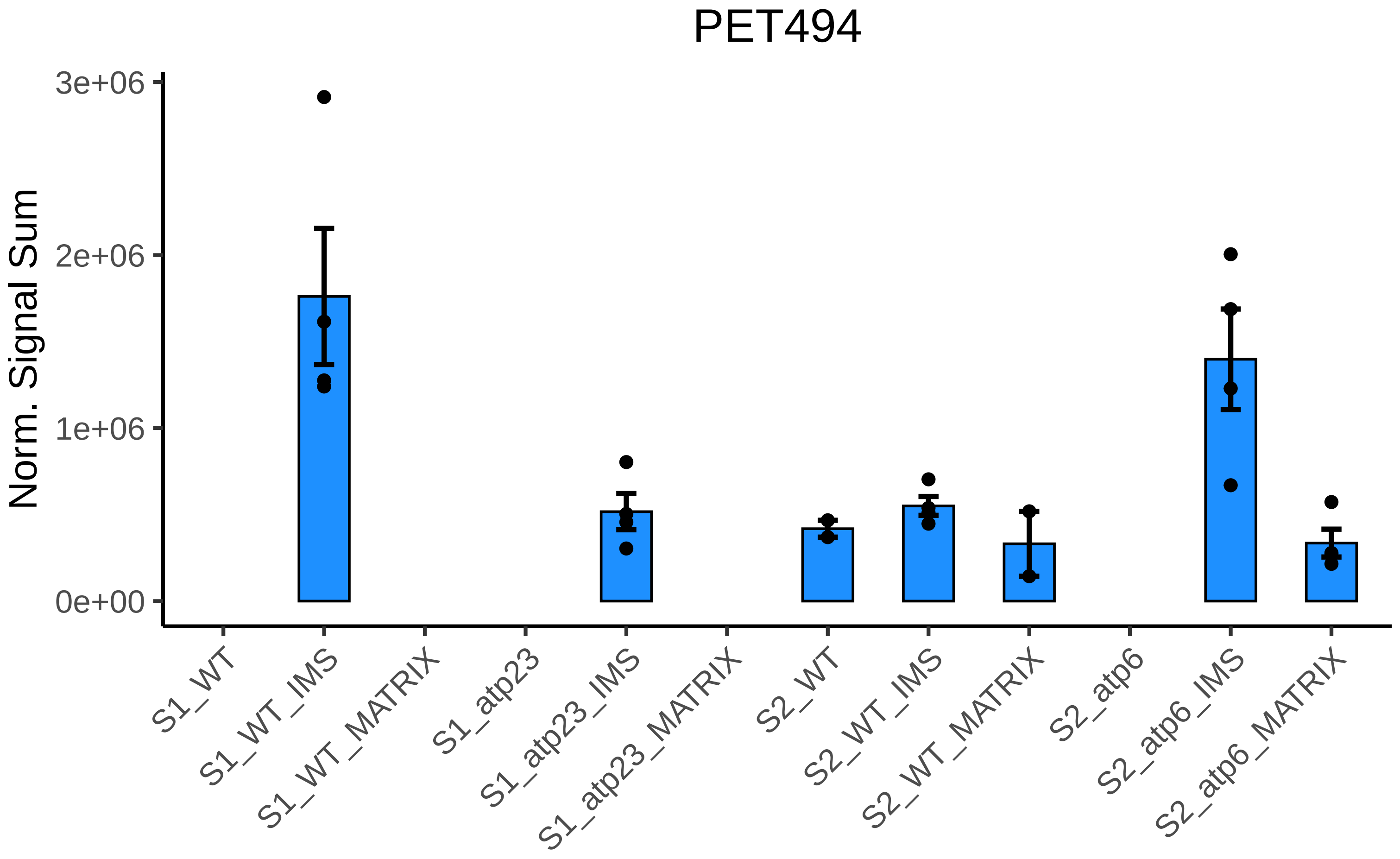

PET494
Norm. Signal Sum
3e+06
2e+06
1e+06
0e+00
S1_WT
S1_WT_IMS
S1_WT_MATRIX
S1_atp23
S1_atp23_IMS
S1_atp23_MATRIX
S2_WT
S2_WT_IMS
S2_WT_MATRIX
S2_atp6
S2_atp6_IMS
S2_atp6_MATRIX

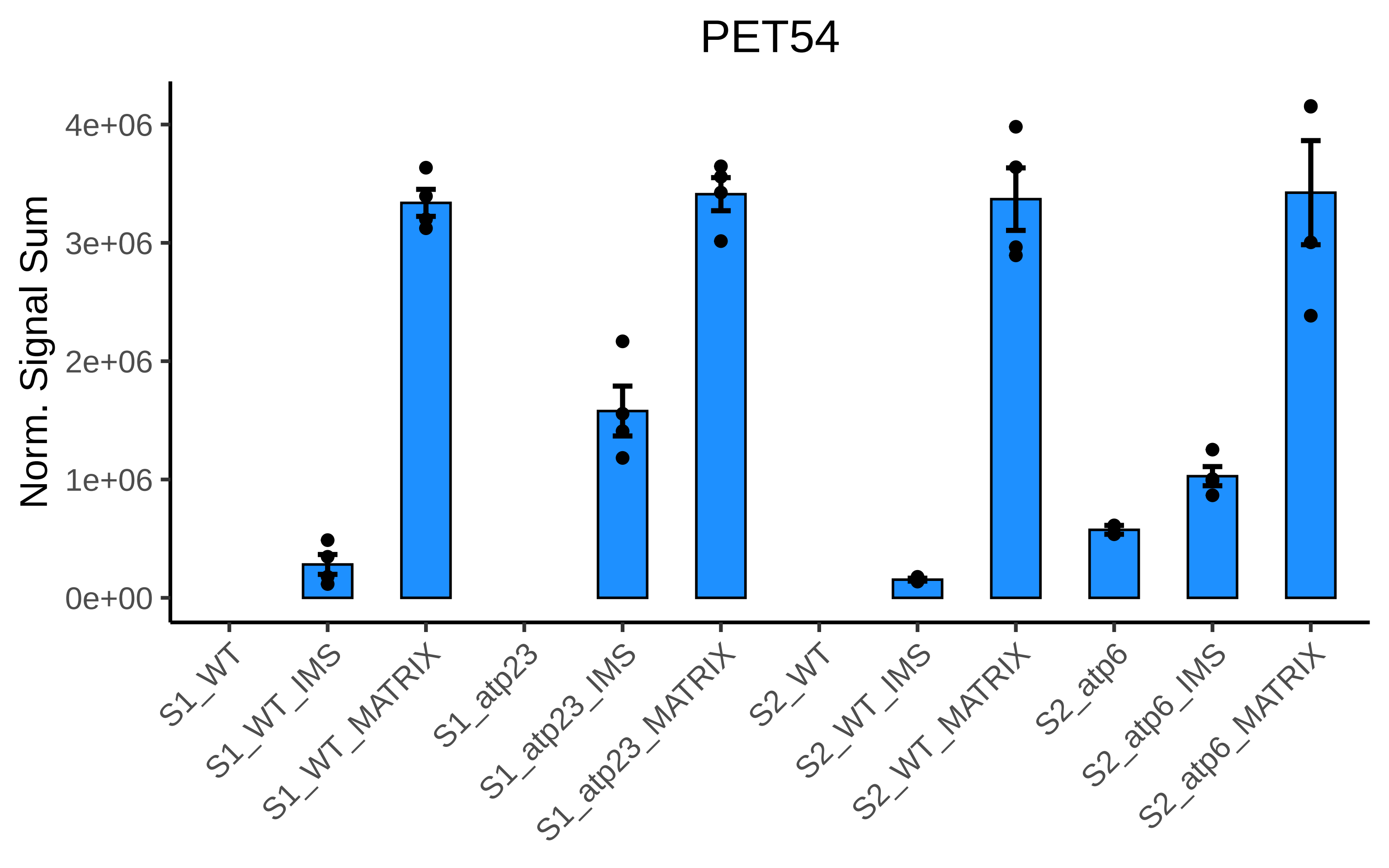
PET54
Norm. Signal Sum
4e+06
3e+06
2e+06
1e+06
0e+00
S1_WT
S1_WT_IMS
S1_WT_MATRIX
S1_atp23
S1_atp23_IMS
S1_atp23_MATRIX
S2_WT
S2_WT_IMS
S2_WT_MATRIX
S2_atp6
S2_atp6_IMS
S2_atp6_MATRIX

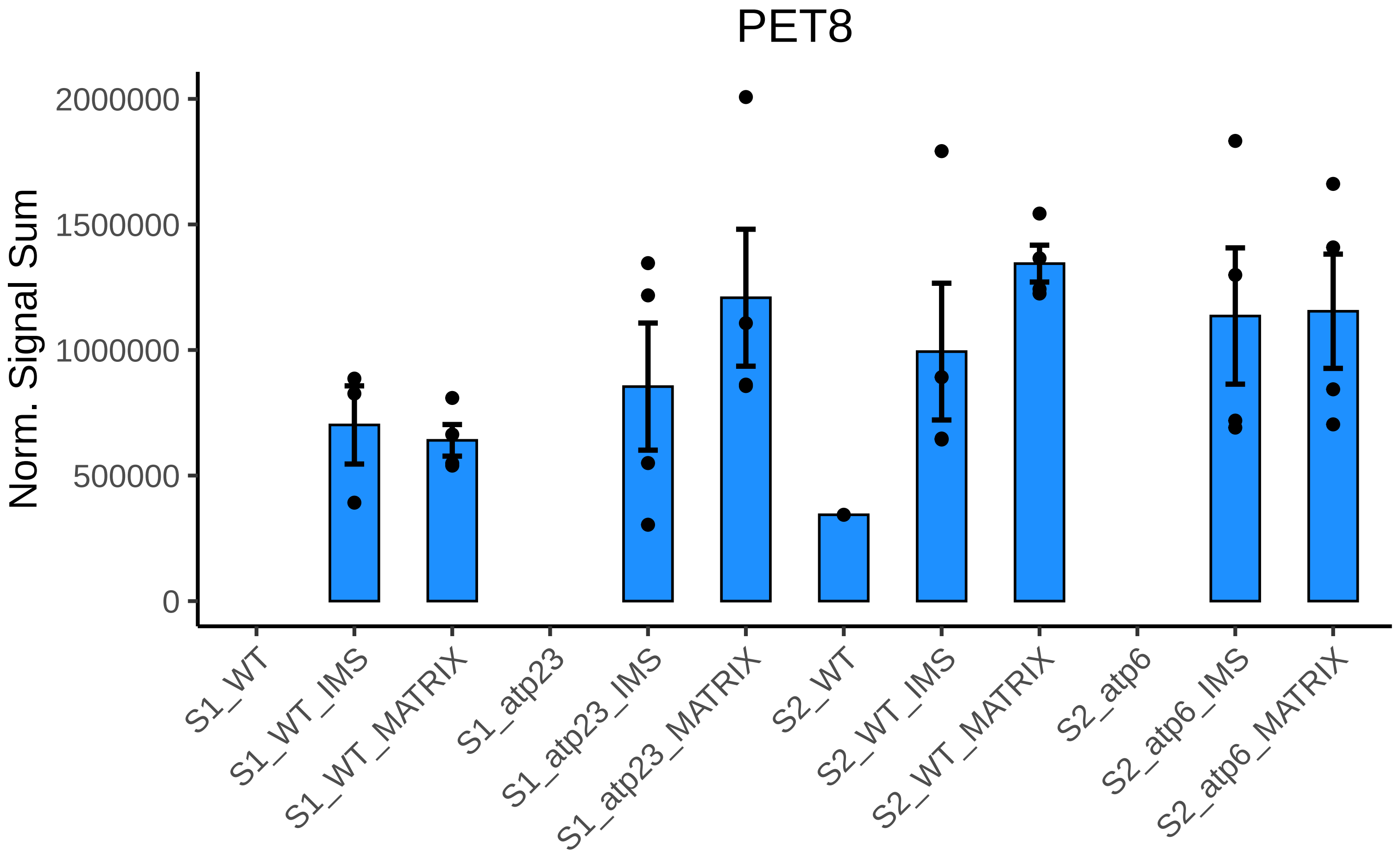

PET8
Norm. Signal Sum
2000000
1500000
1000000
500000
0
S1_WT
S1_WT_IMS
S1_WT_MATRIX
S1_atp23
S1_atp23_IMS
S1_atp23_MATRIX
S2_WT
S2_WT_IMS
S2_WT_MATRIX
S2_atp6
S2_atp6_IMS
S2_atp6_MATRIX

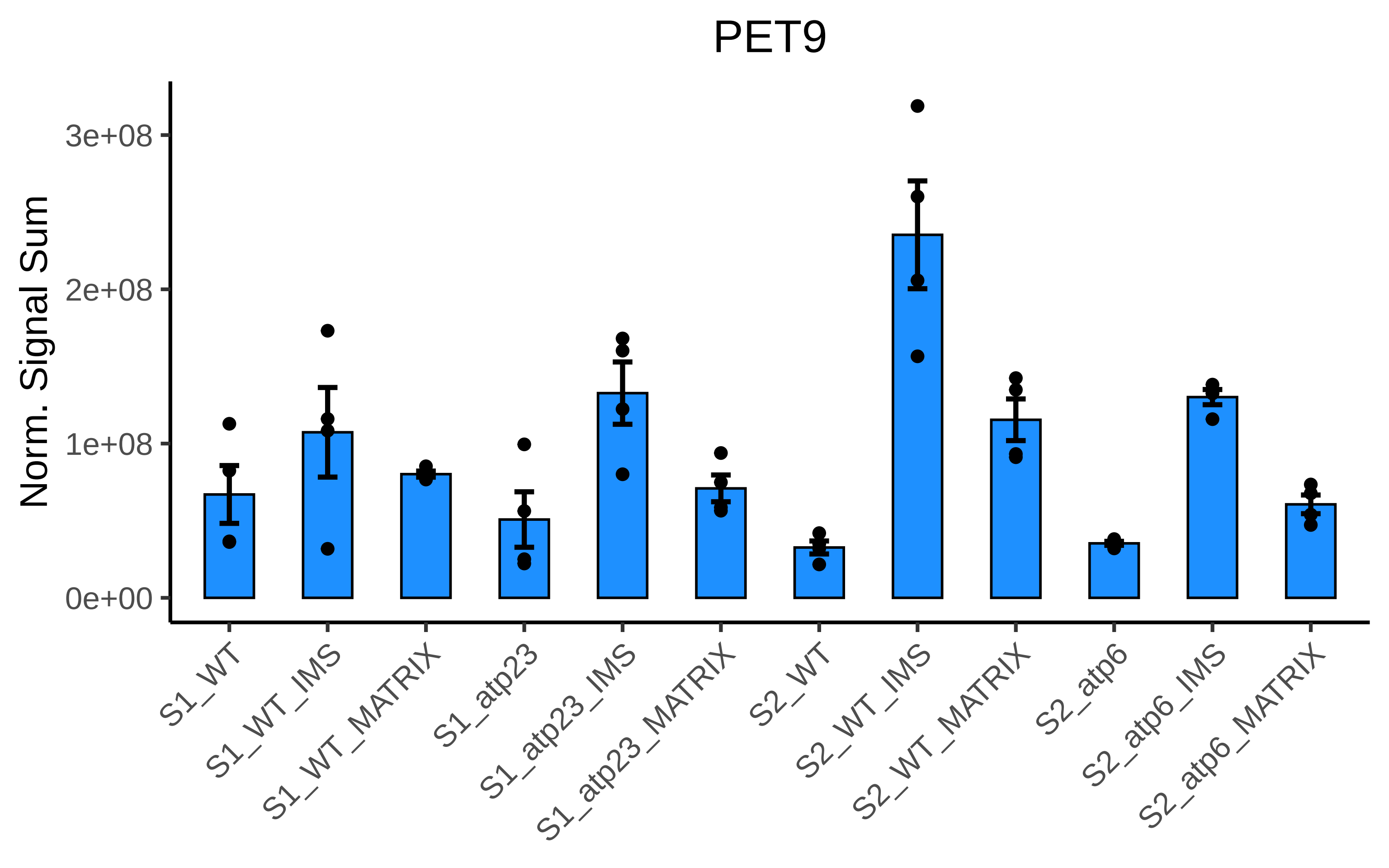
PET9
Norm. Signal Sum
3e+08
2e+08
1e+08
0e+00
S1_WT
S1_WT_IMS
S1_WT_MATRIX
S1_atp23
S1_atp23_IMS
S1_atp23_MATRIX
S2_WT
S2_WT_IMS
S2_WT_MATRIX
S2_atp6
S2_atp6_IMS
S2_atp6_MATRIX

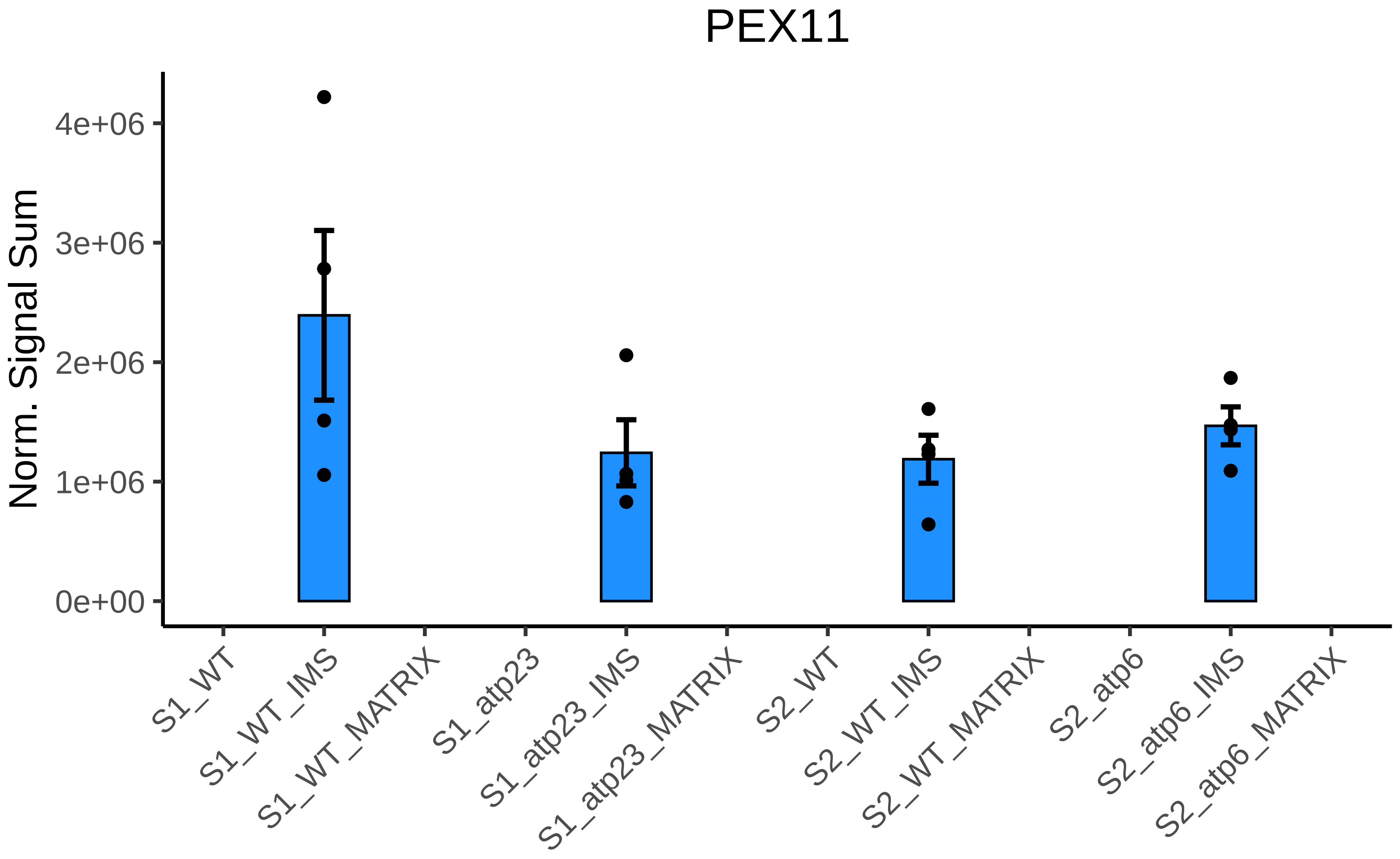
PEX11
Norm. Signal Sum
4e+06
3e+06
2e+06
1e+06
0e+00
S1_WT
S1_WT_IMS
S1_WT_MATRIX
S1_atp23
S1_atp23_IMS
S1_atp23_MATRIX
S2_WT
S2_WT_IMS
S2_WT_MATRIX
S2_atp6
S2_atp6_IMS
S2_atp6_MATRIX

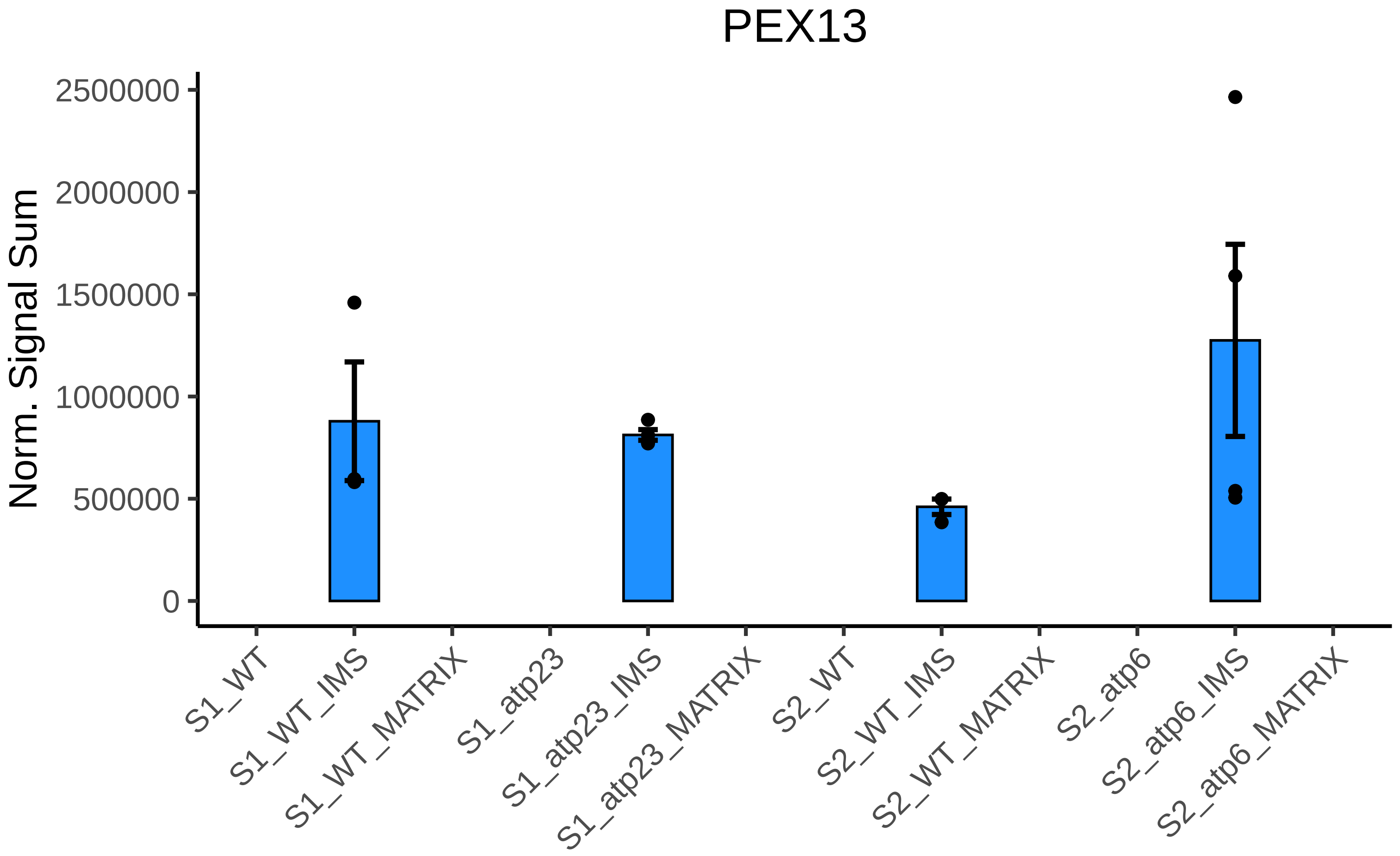

PEX13
Norm. Signal Sum
2500000
2000000
1500000
1000000
500000
0
S1_WT
S1_WT_IMS
S1_WT_MATRIX
S1_atp23
S1_atp23_IMS
S1_atp23_MATRIX
S2_WT
S2_WT_IMS
S2_WT_MATRIX
S2_atp6
S2_atp6_IMS
S2_atp6_MATRIX

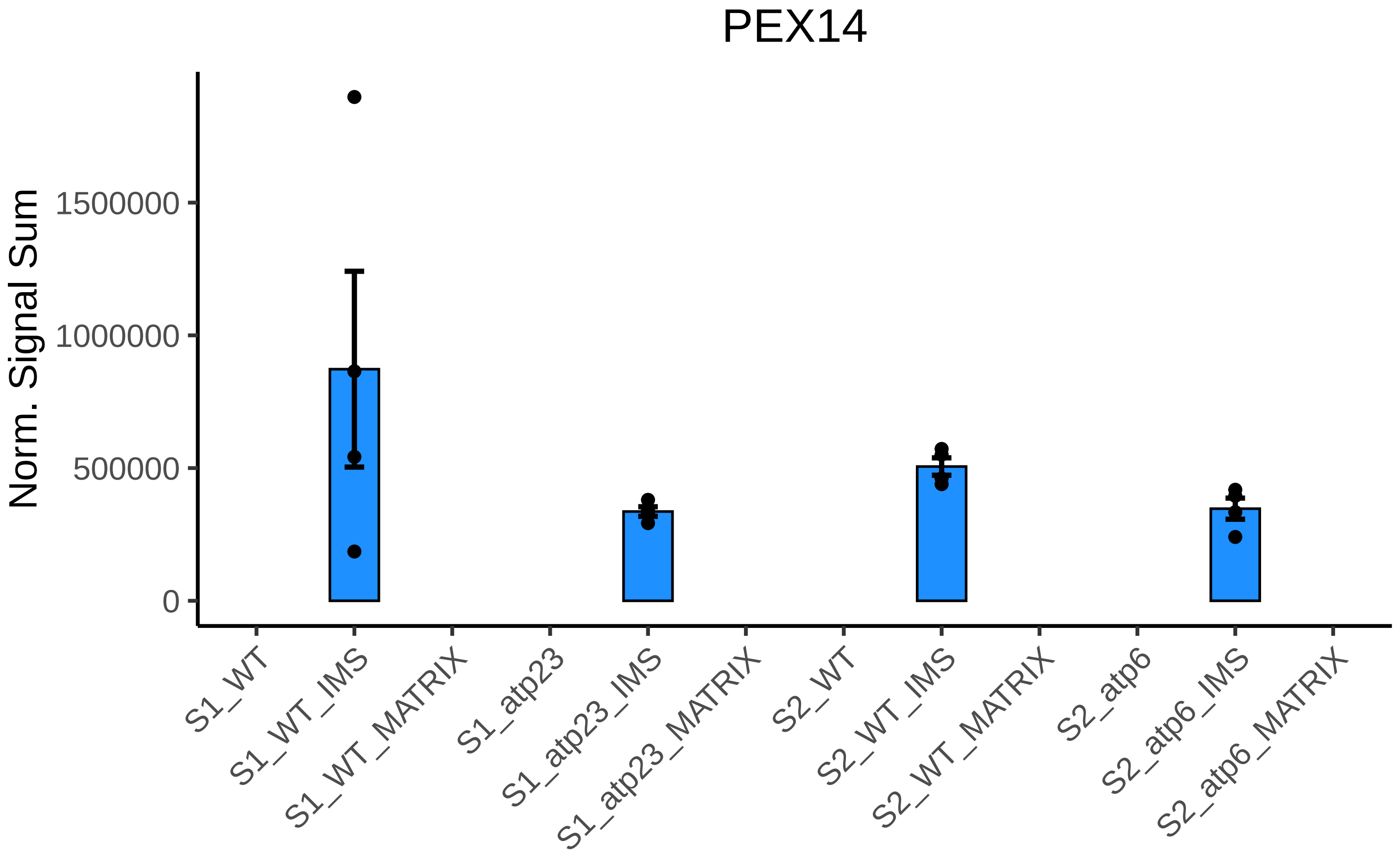
PEX14
Norm. Signal Sum
1500000
1000000
500000
0
S1_WT
S1_WT_IMS
S1_WT_MATRIX
S1_atp23
S1_atp23_IMS
S1_atp23_MATRIX
S2_WT
S2_WT_IMS
S2_WT_MATRIX
S2_atp6
S2_atp6_IMS
S2_atp6_MATRIX

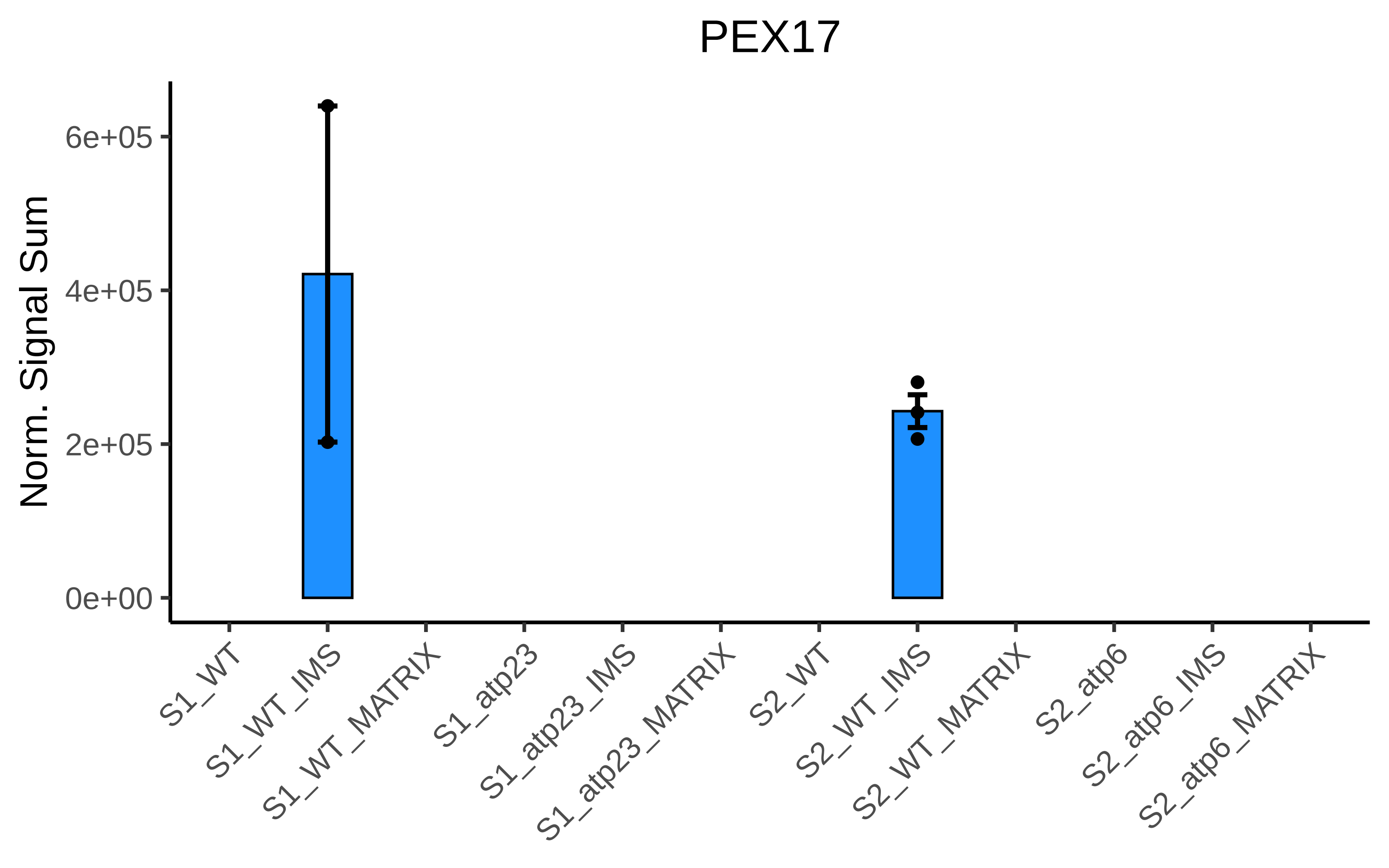
PEX17
Norm. Signal Sum
6e+05
4e+05
2e+05
0e+00
S1_WT
S1_WT_IMS
S1_WT_MATRIX
S1_atp23
S1_atp23_IMS
S1_atp23_MATRIX
S2_WT
S2_WT_IMS
S2_WT_MATRIX
S2_atp6
S2_atp6_IMS
S2_atp6_MATRIX

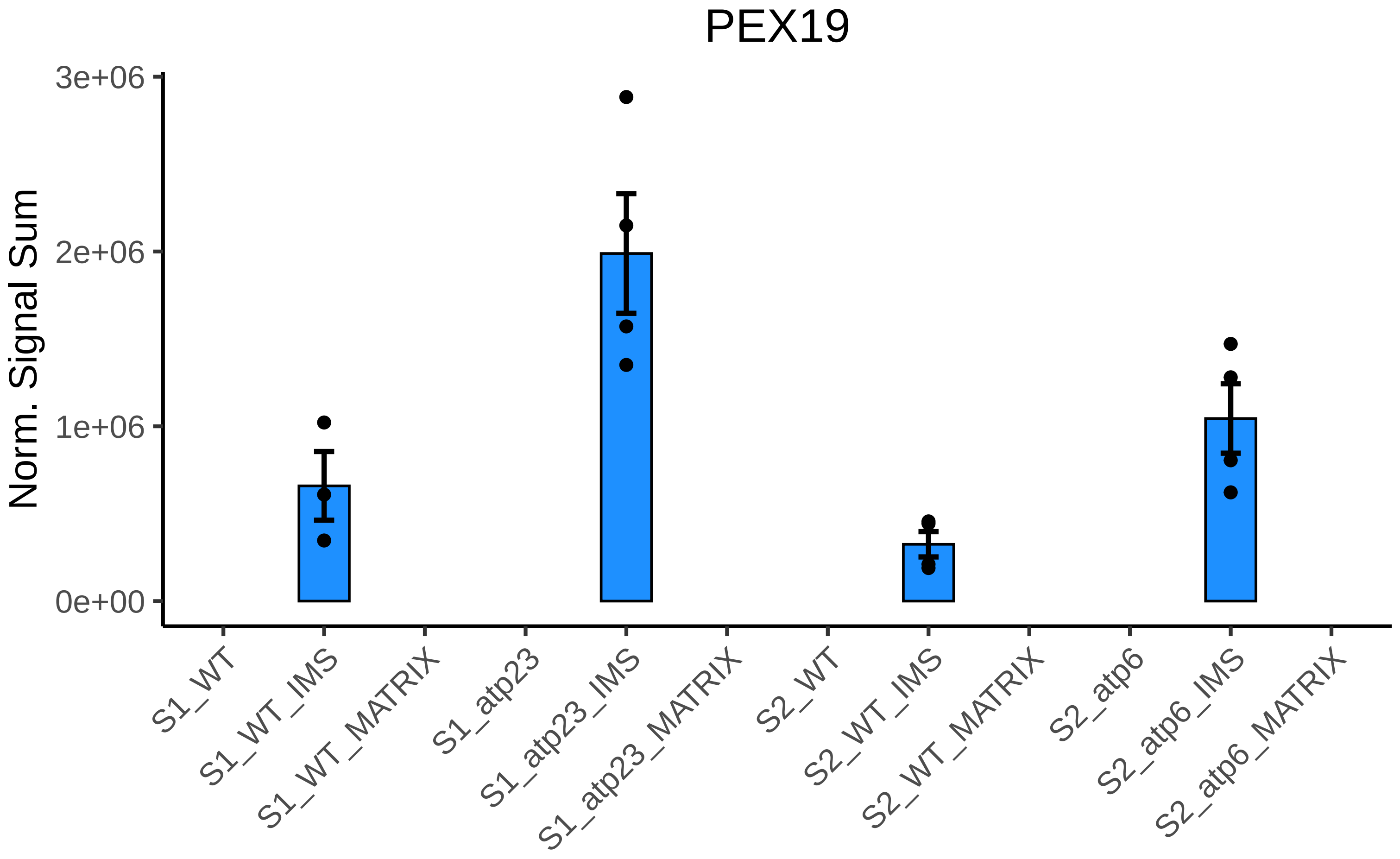
PEX19
Norm. Signal Sum
3e+06
2e+06
1e+06
0e+00
S1_WT
S1_WT_IMS
S1_WT_MATRIX
S1_atp23
S1_atp23_IMS
S1_atp23_MATRIX
S2_WT
S2_WT_IMS
S2_WT_MATRIX
S2_atp6
S2_atp6_IMS
S2_atp6_MATRIX

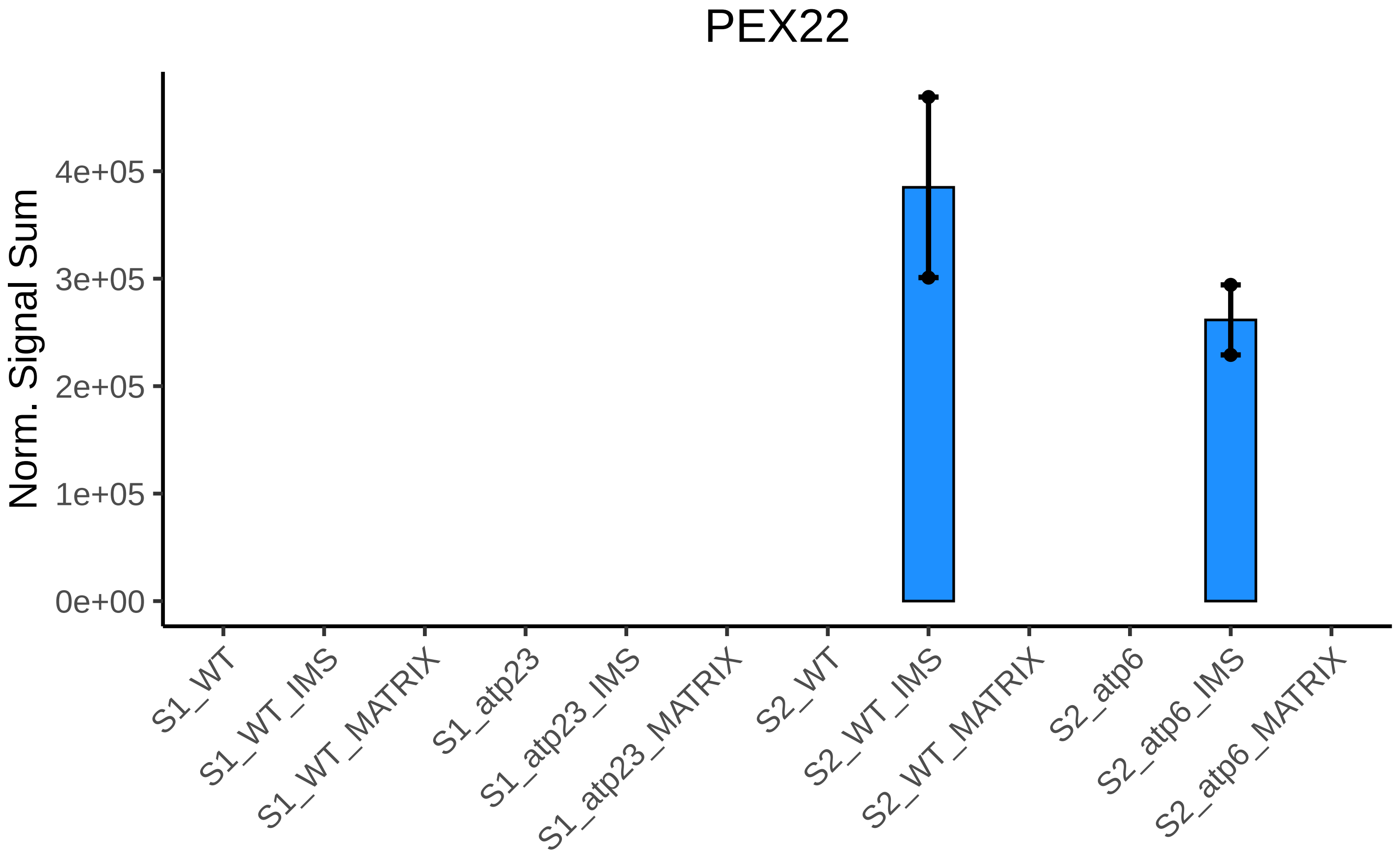
PEX22
Norm. Signal Sum
4e+05
3e+05
2e+05
1e+05
0e+00
S1_WT
S1_WT_IMS
S1_WT_MATRIX
S1_atp23
S1_atp23_IMS
S1_atp23_MATRIX
S2_WT
S2_WT_IMS
S2_WT_MATRIX
S2_atp6
S2_atp6_IMS
S2_atp6_MATRIX

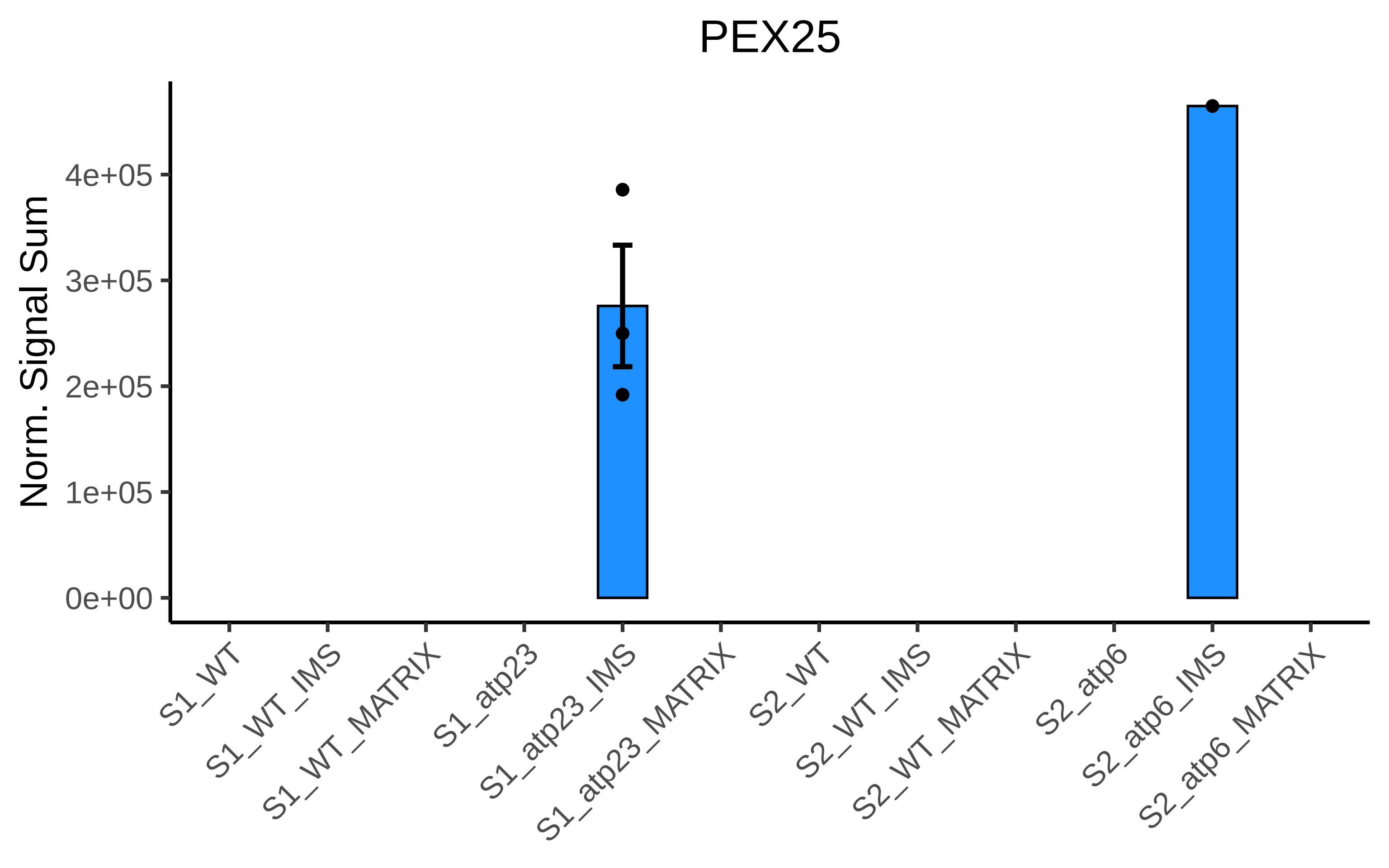
PEX25
Norm. Signal Sum
0e+00
1e+05
2e+05
3e+05
4e+05
S1_WT
S1_WT_IMS
S1_WT_MATRIX
S1_atp23
S1_atp23_IMS
S1_atp23_MATRIX
S2_WT
S2_WT_IMS
S2_WT_MATRIX
S2_atp6
S2_atp6_IMS
S2_atp6_MATRIX

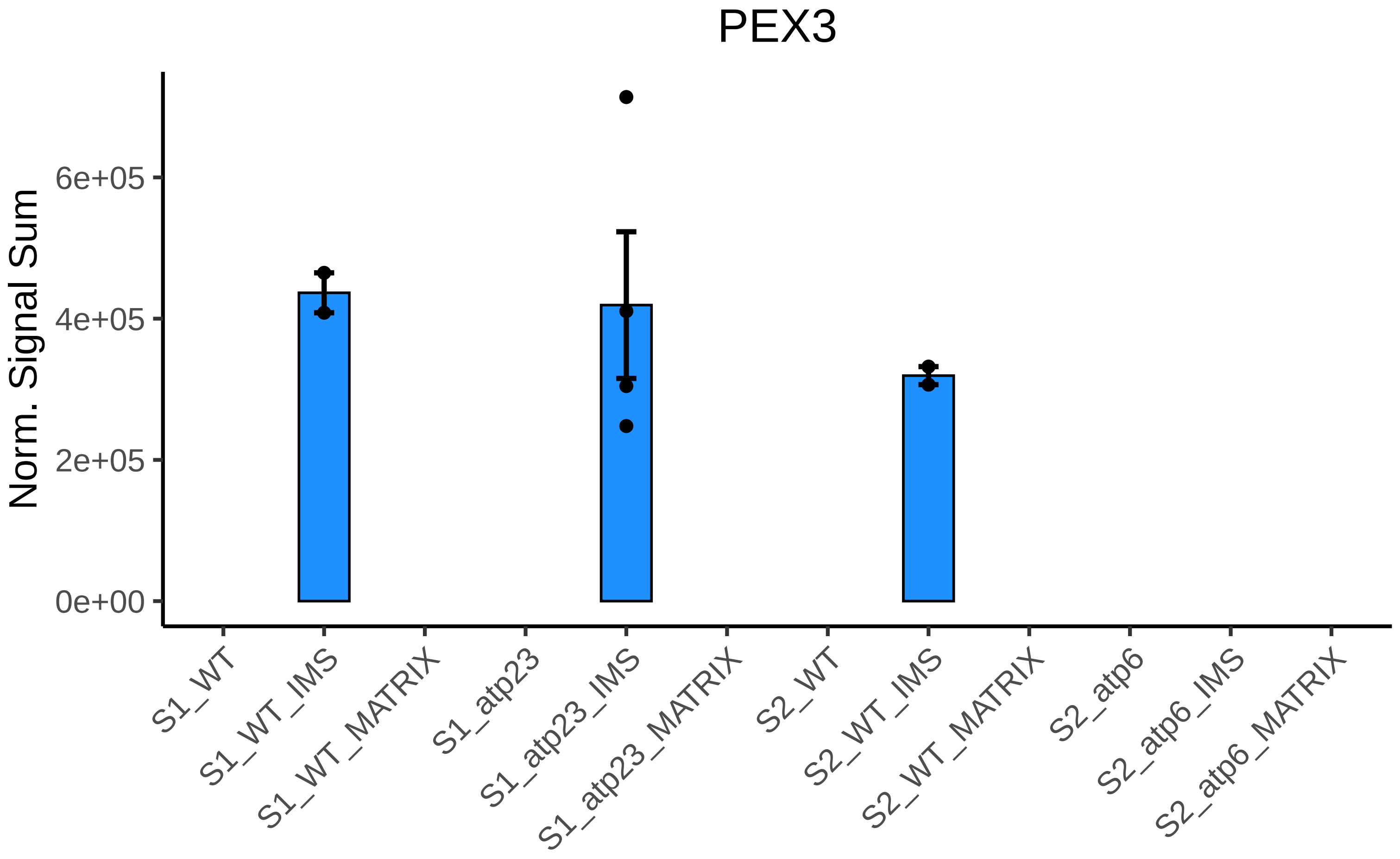

PEX3
Norm. Signal Sum
6e+05
4e+05
2e+05
0e+00
S1_WT
S1_WT_IMS
S1_WT_MATRIX
S1_atp23
S1_atp23_IMS
S1_atp23_MATRIX
S2_WT
S2_WT_IMS
S2_WT_MATRIX
S2_atp6
S2_atp6_IMS
S2_atp6_MATRIX

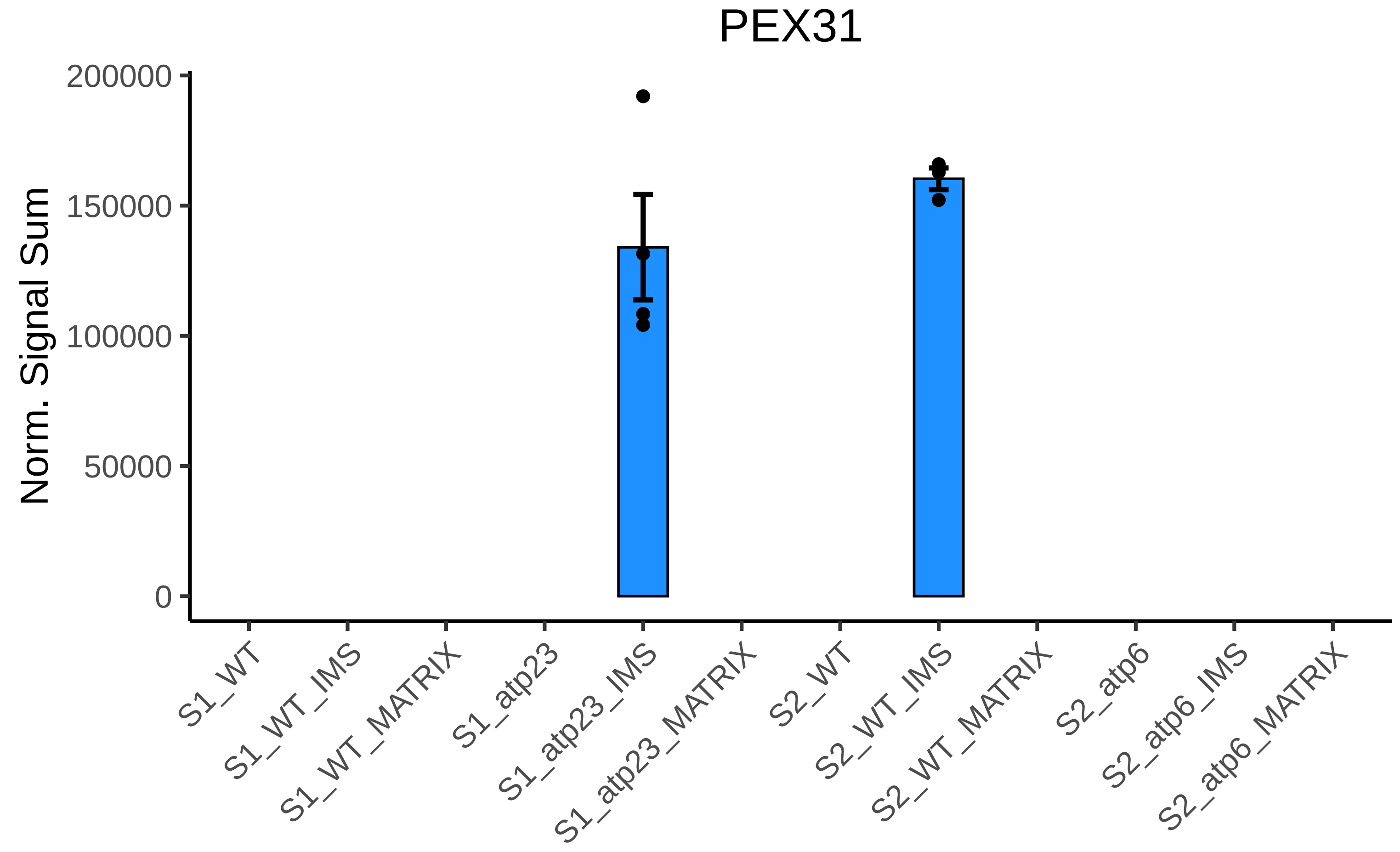
PEX31
Norm. Signal Sum
200000
150000
100000
50000
0
S1_WT
S1_WT_IMS
S1_WT_MATRIX
S1_atp23
S1_atp23_IMS
S1_atp23_MATRIX
S2_WT
S2_WT_IMS
S2_WT_MATRIX
S2_atp6
S2_atp6_IMS
S2_atp6_MATRIX

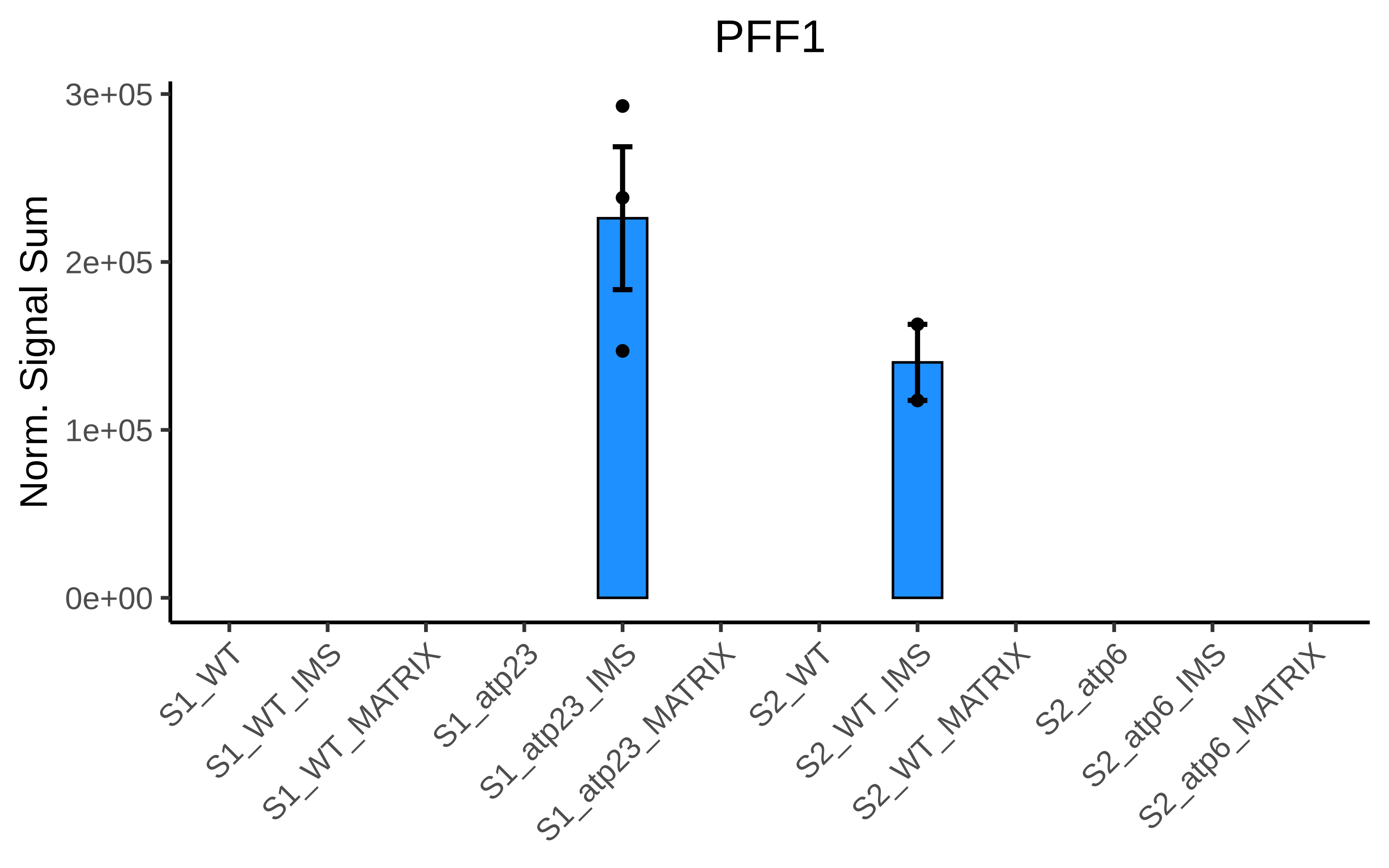
PFF1
Norm. Signal Sum
3e+05
2e+05
1e+05
0e+00
S1_WT
S1_WT_IMS
S1_WT_MATRIX
S1_atp23
S1_atp23_IMS
S1_atp23_MATRIX
S2_WT
S2_WT_IMS
S2_WT_MATRIX
S2_atp6
S2_atp6_IMS
S2_atp6_MATRIX

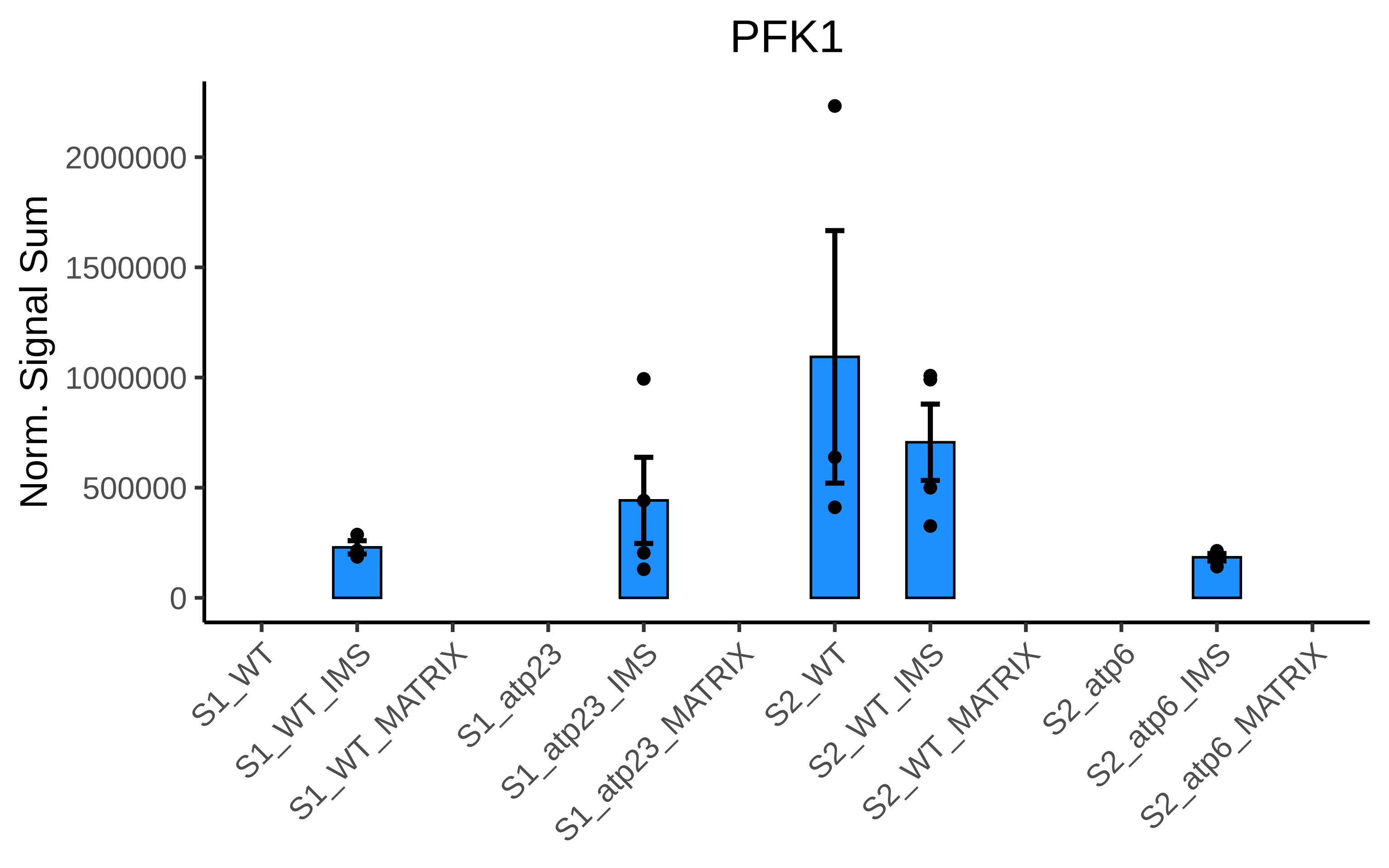

PFK1
Norm. Signal Sum
2000000
1500000
1000000
500000
0
S1_WT
S1_WT_IMS
S1_WT_MATRIX
S1_atp23
S1_atp23_IMS
S1_atp23_MATRIX
S2_WT
S2_WT_IMS
S2_WT_MATRIX
S2_atp6
S2_atp6_IMS
S2_atp6_MATRIX

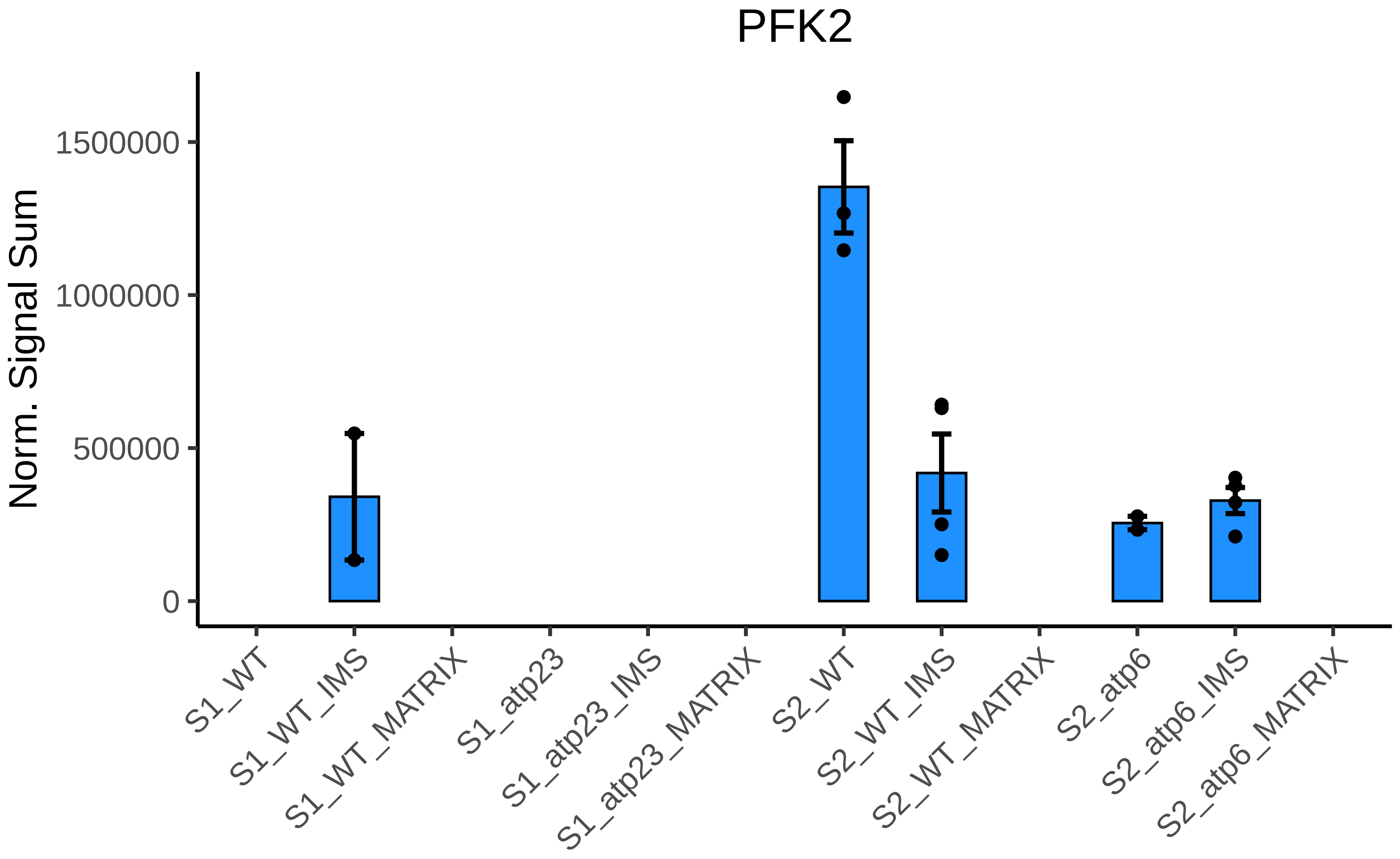
PFK2
Norm. Signal Sum
1500000
1000000
500000
0
S1_WT
S1_WT_IMS
S1_WT_MATRIX
S1_atp23
S1_atp23_IMS
S1_atp23_MATRIX
S2_WT
S2_WT_IMS
S2_WT_MATRIX
S2_atp6
S2_atp6_IMS
S2_atp6_MATRIX

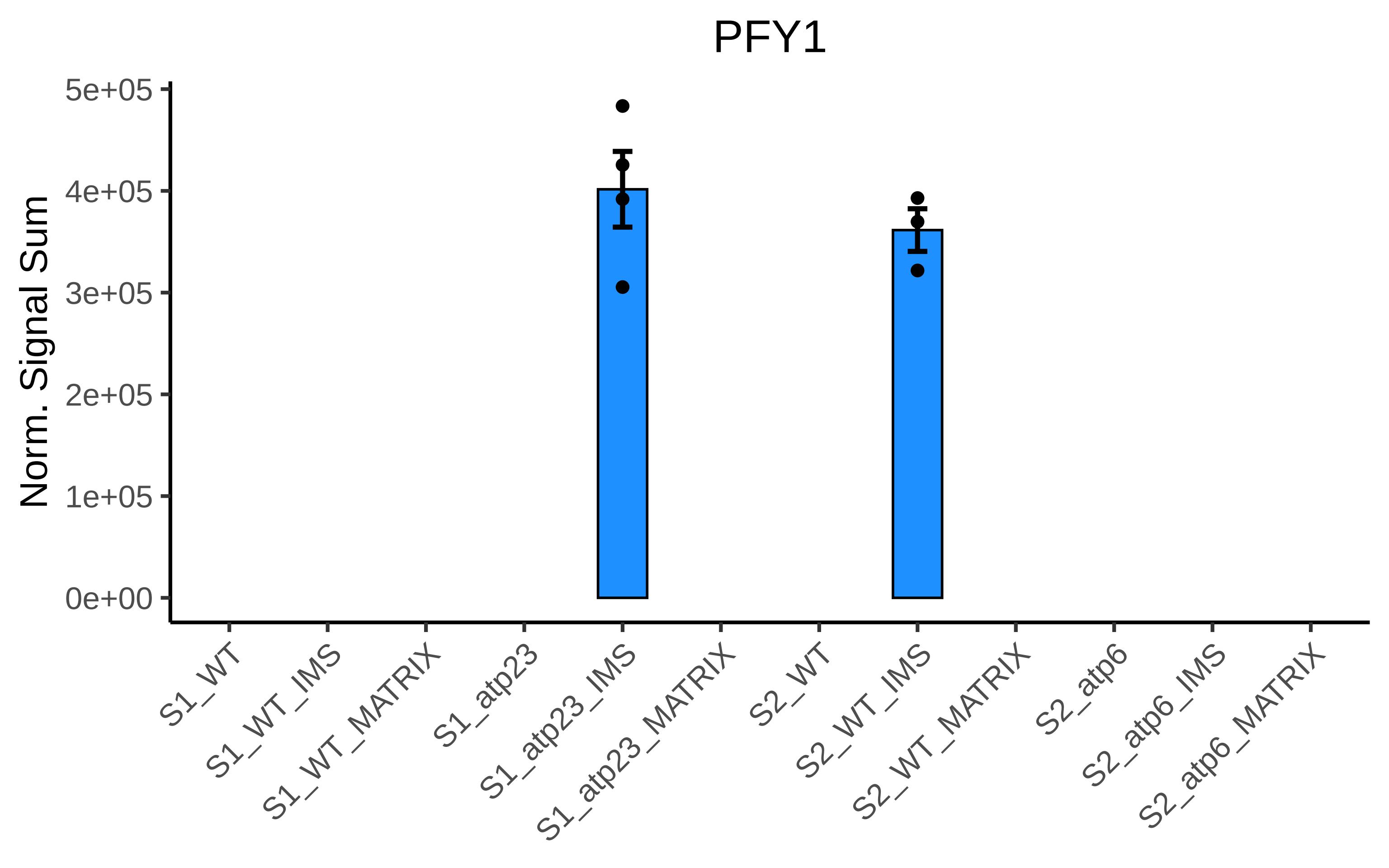

PFY1
Norm. Signal Sum
5e+05
4e+05
3e+05
2e+05
1e+05
0e+00
S1_WT
S1_WT_IMS
S1_WT_MATRIX
S1_atp23
S1_atp23_IMS
S1_atp23_MATRIX
S2_WT
S2_WT_IMS
S2_WT_MATRIX
S2_atp6
S2_atp6_IMS
S2_atp6_MATRIX

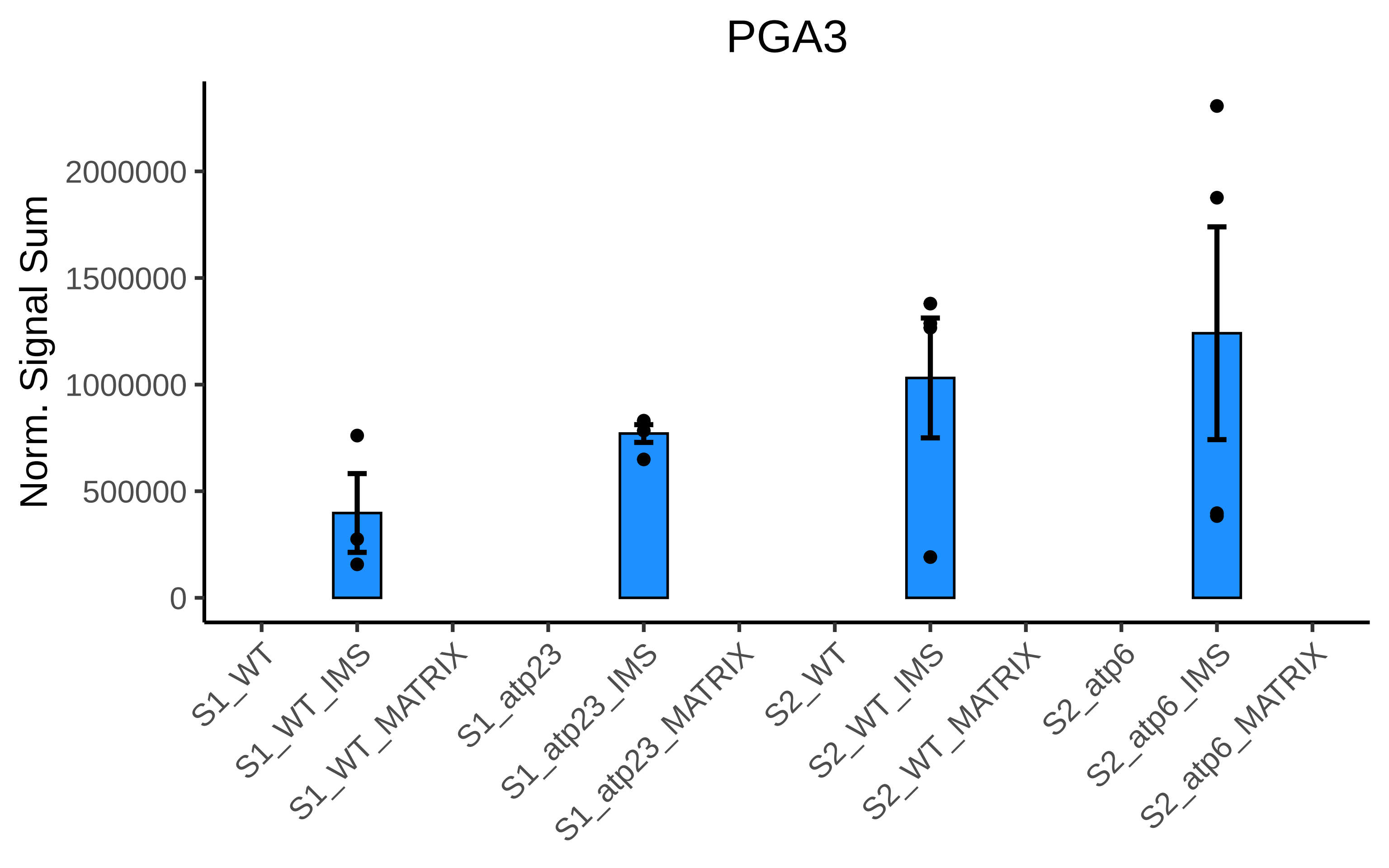
PGA3
Norm. Signal Sum
0
500000
1000000
1500000
2000000
S1_WT
S1_WT_IMS
S1_WT_MATRIX
S1_atp23
S1_atp23_IMS
S1_atp23_MATRIX
S2_WT
S2_WT_IMS
S2_WT_MATRIX
S2_atp6
S2_atp6_IMS
S2_atp6_MATRIX

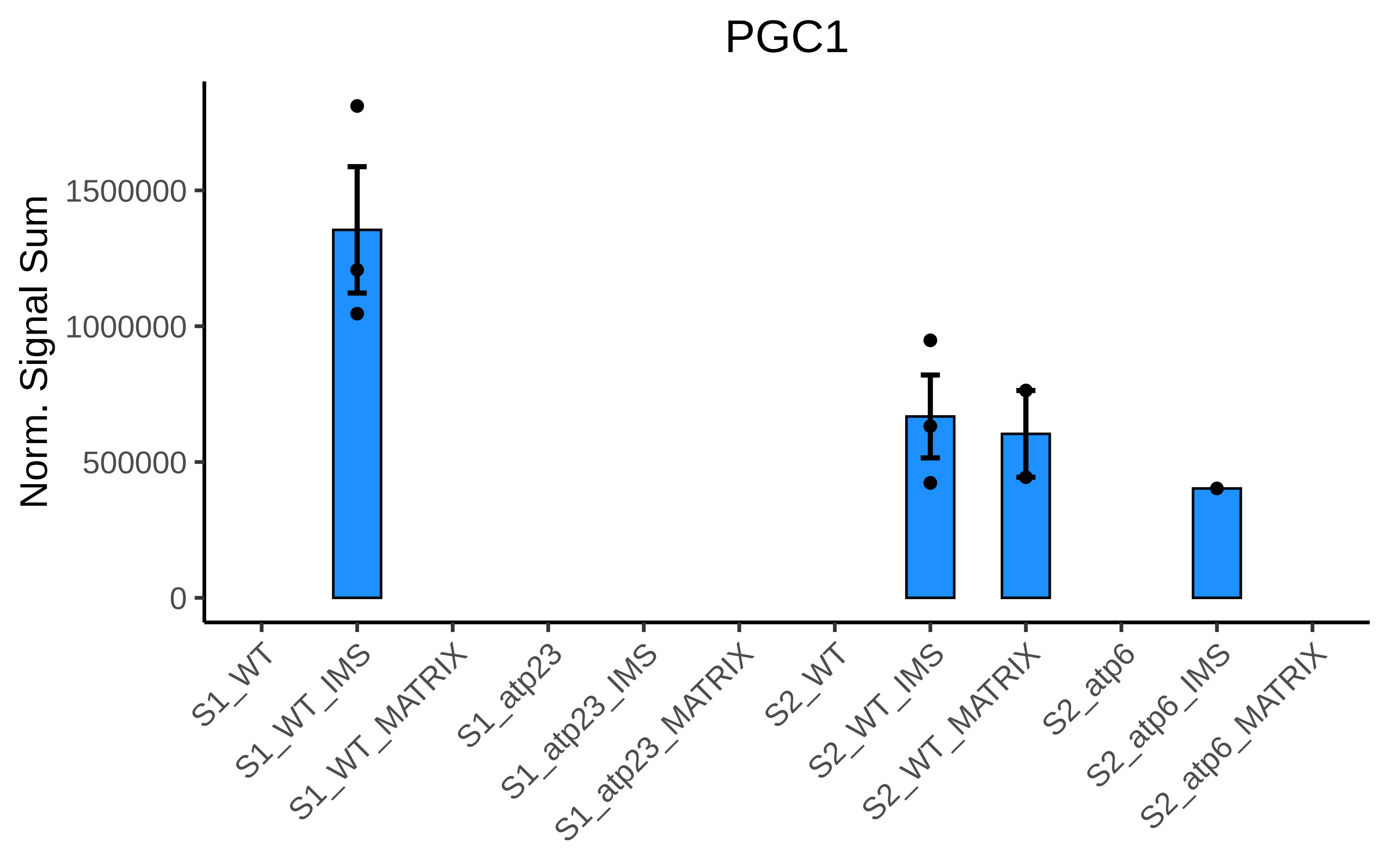
PGC1
Norm. Signal Sum
0
500000
1000000
1500000
S1_WT
S1_WT_IMS
S1_WT_MATRIX
S1_atp23
S1_atp23_IMS
S1_atp23_MATRIX
S2_WT
S2_WT_IMS
S2_WT_MATRIX
S2_atp6
S2_atp6_IMS
S2_atp6_MATRIX

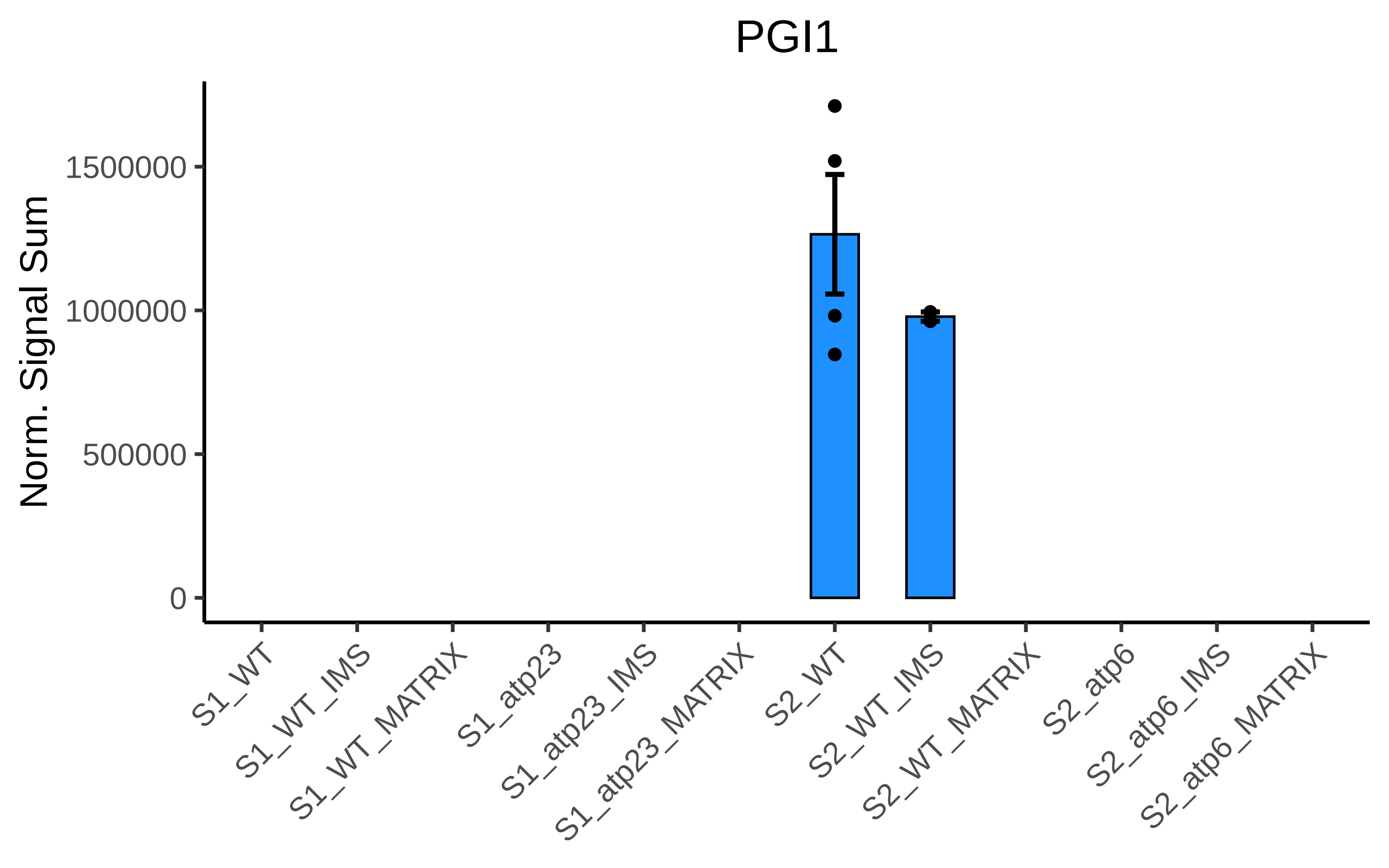
PGI1
Norm. Signal Sum
1500000
1000000
500000
0
S1_WT
S1_WT_IMS
S1_WT_MATRIX
S1_atp23
S1_atp23_IMS
S1_atp23_MATRIX
S2_WT
S2_WT_IMS
S2_WT_MATRIX
S2_atp6
S2_atp6_IMS
S2_atp6_MATRIX

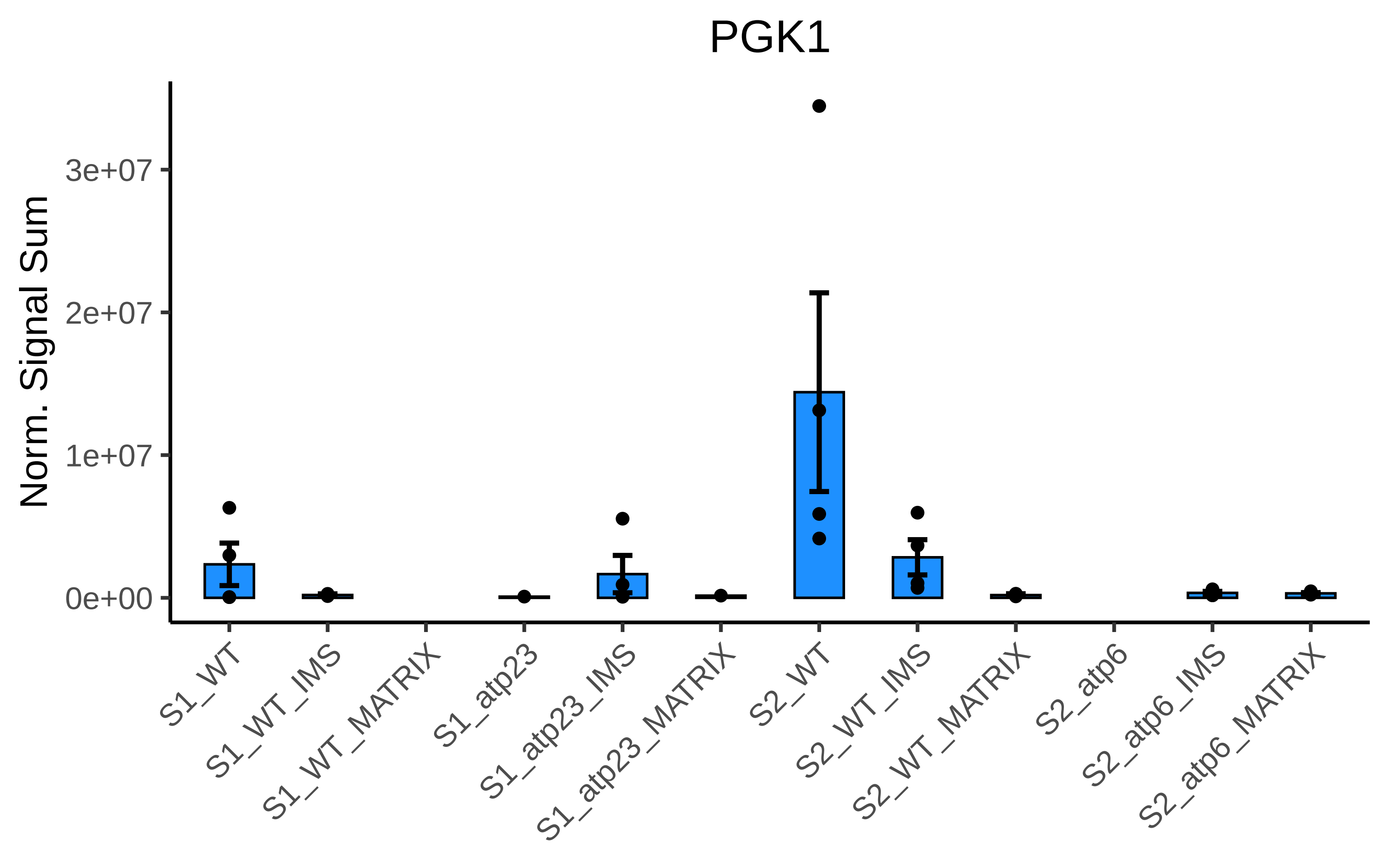
PGK1
Norm. Signal Sum
3e+07
2e+07
1e+07
0e+00
S1_WT
S1_WT_IMS
S1_WT_MATRIX
S1_atp23
S1_atp23_IMS
S1_atp23_MATRIX
S2_WT
S2_WT_IMS
S2_WT_MATRIX
S2_atp6
S2_atp6_IMS
S2_atp6_MATRIX

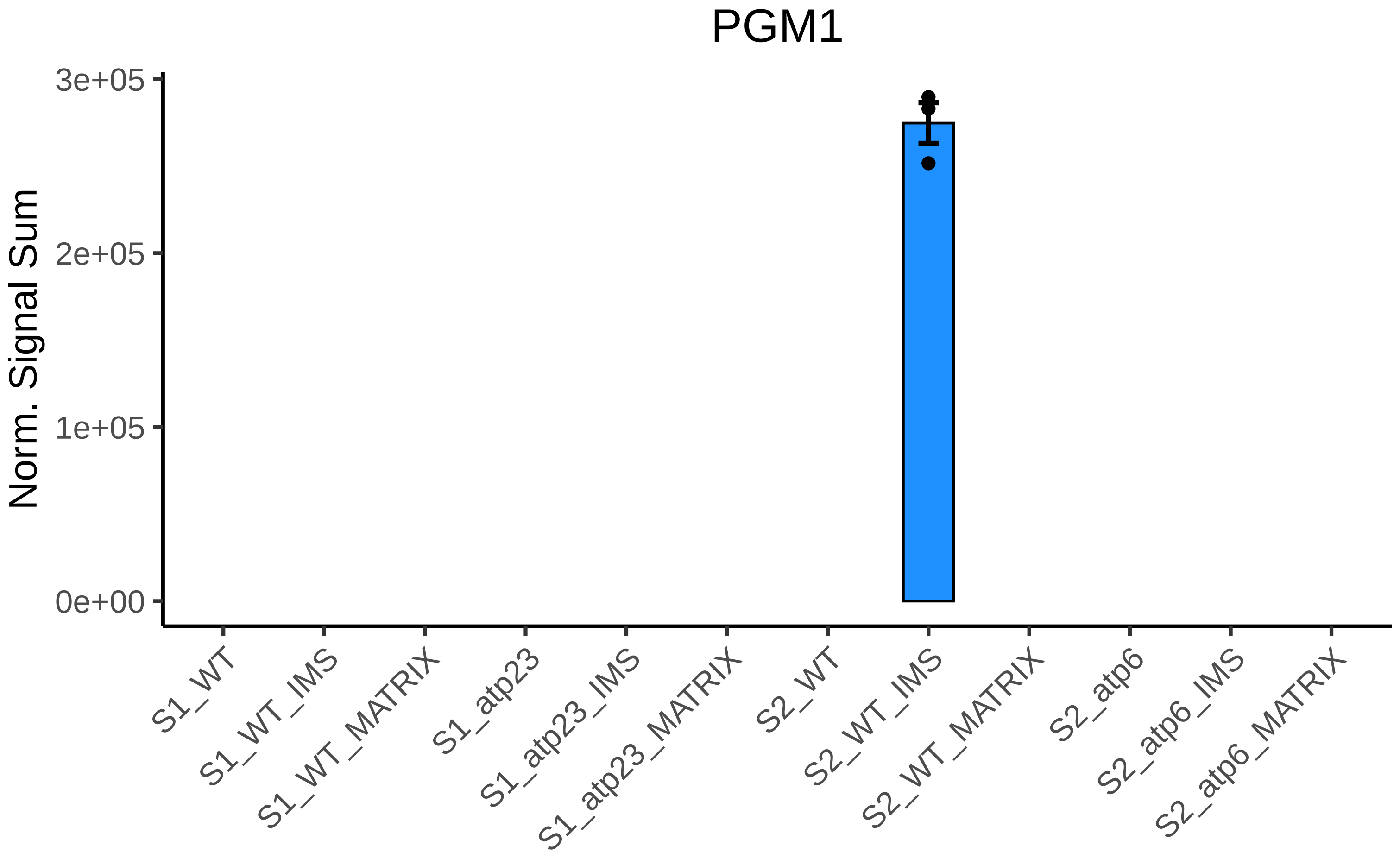
PGM1
Norm. Signal Sum
3e+05
2e+05
1e+05
0e+00
S1_WT
S1_WT_IMS
S1_WT_MATRIX
S1_atp23
S1_atp23_IMS
S1_atp23_MATRIX
S2_WT
S2_WT_IMS
S2_WT_MATRIX
S2_atp6
S2_atp6_IMS
S2_atp6_MATRIX

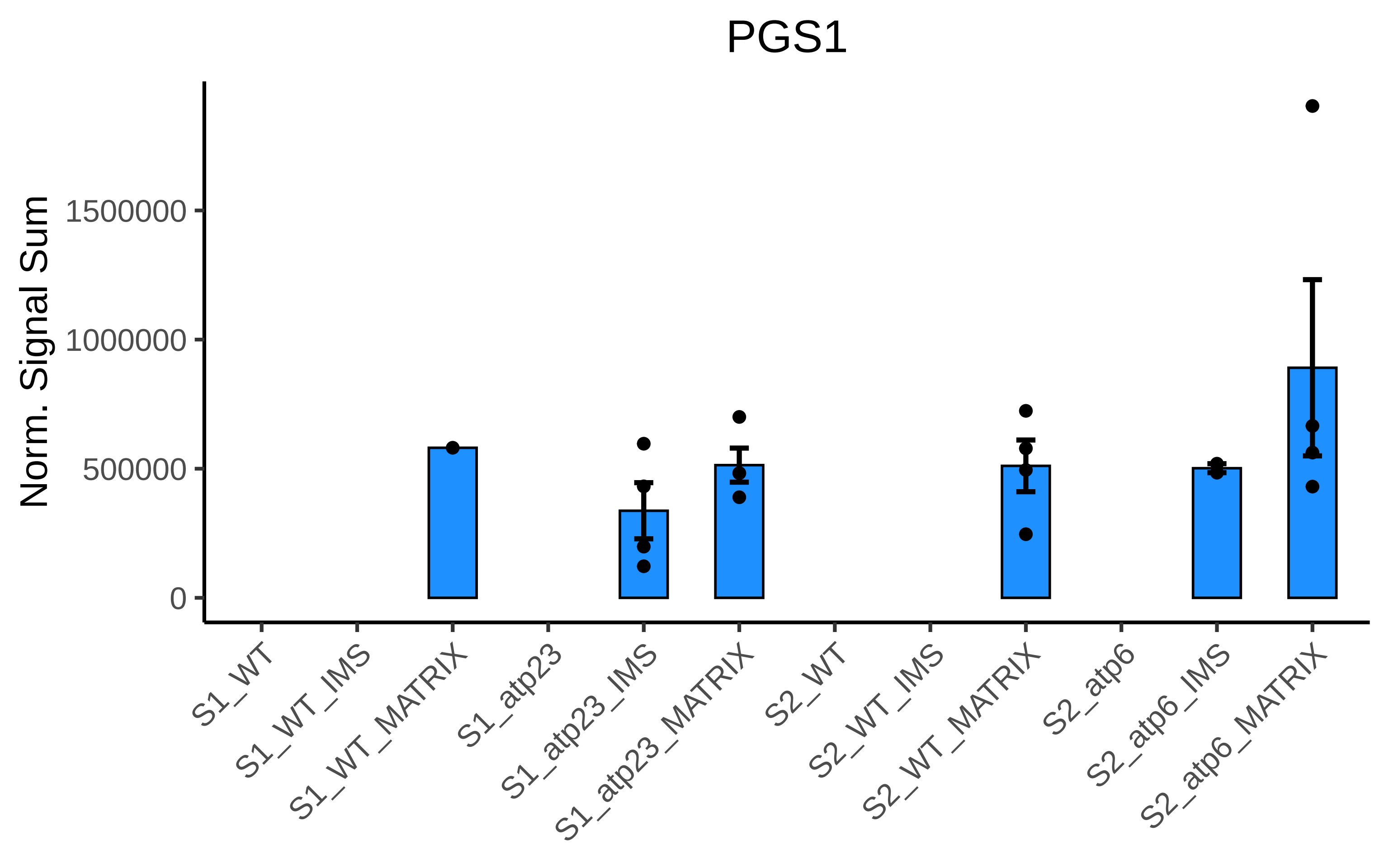
PGS1
Norm. Signal Sum
1500000
1000000
500000
0
S1_WT
S1_WT_IMS
S1_WT_MATRIX
S1_atp23
S1_atp23_IMS
S1_atp23_MATRIX
S2_WT
S2_WT_IMS
S2_WT_MATRIX
S2_atp6
S2_atp6_IMS
S2_atp6_MATRIX

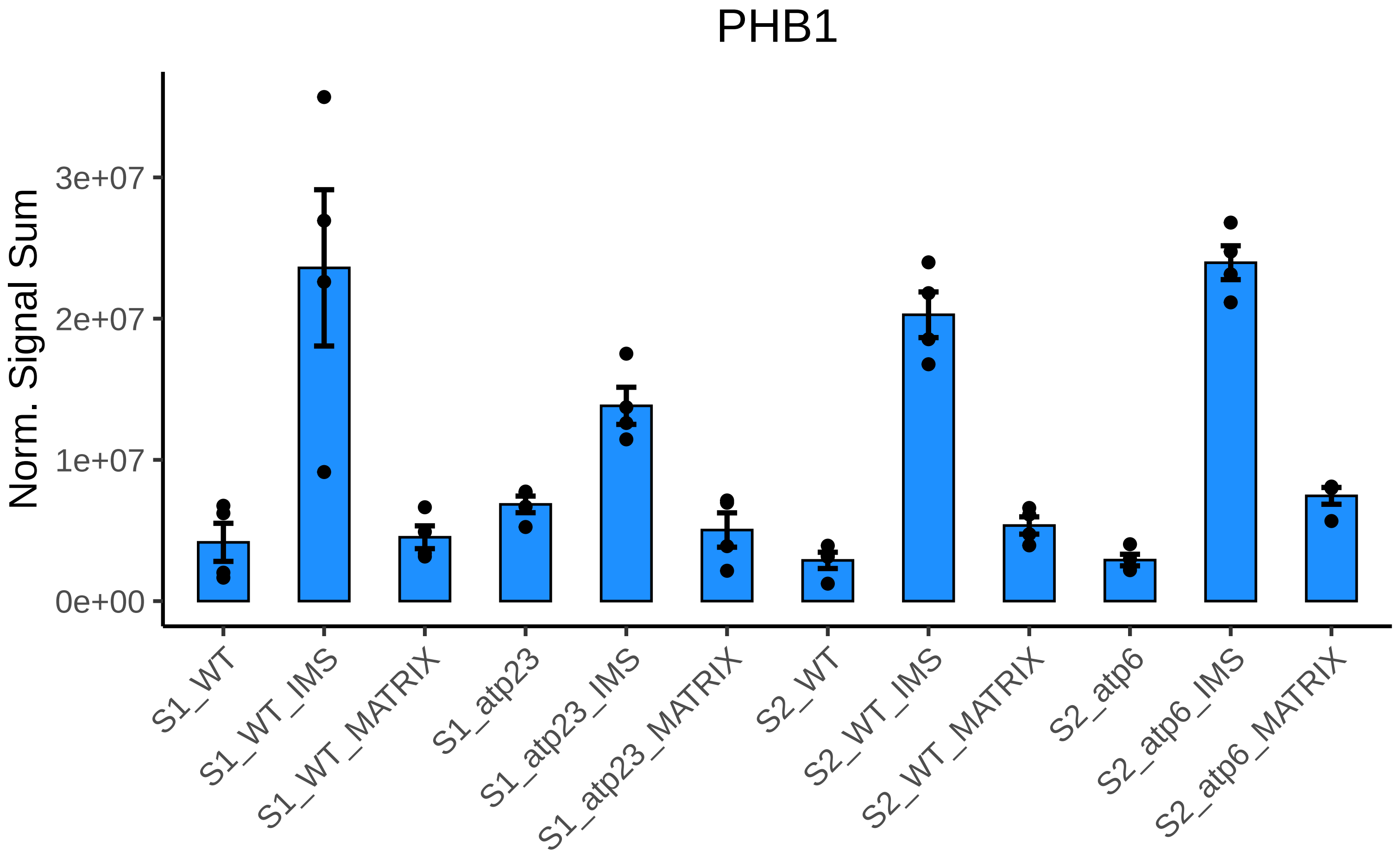

PHB1
Norm. Signal Sum
3e+07
2e+07
1e+07
0e+00
S1_WT
S1_WT_IMS
S1_WT_MATRIX
S1_atp23
S1_atp23_IMS
S1_atp23_MATRIX
S2_WT
S2_WT_IMS
S2_WT_MATRIX
S2_atp6
S2_atp6_IMS
S2_atp6_MATRIX

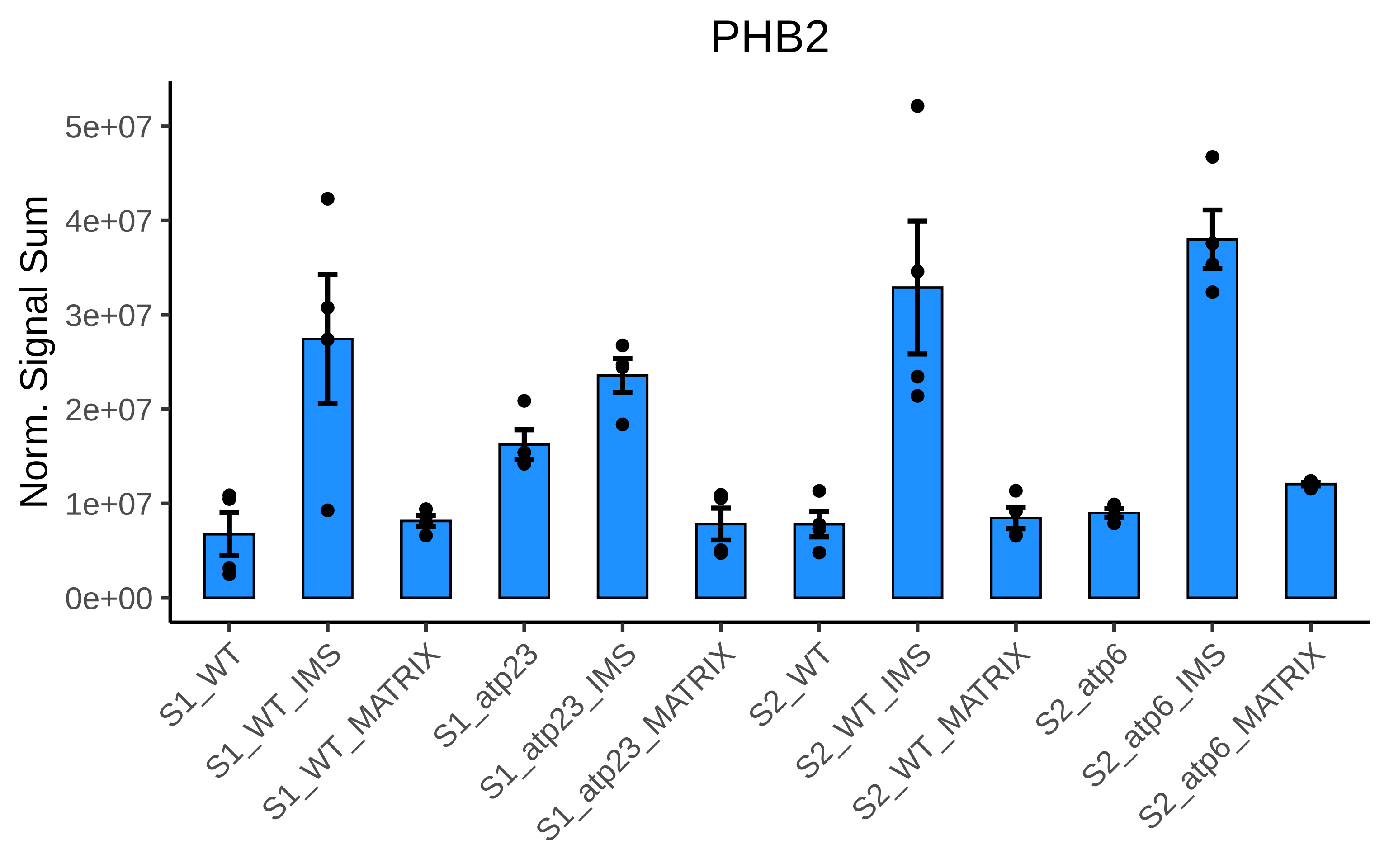

PHB2
Norm. Signal Sum
5e+07
4e+07
3e+07
2e+07
1e+07
0e+00
S1_WT
S1_WT_IMS
S1_WT_MATRIX
S1_atp23
S1_atp23_IMS
S1_atp23_MATRIX
S2_WT
S2_WT_IMS
S2_WT_MATRIX
S2_atp6
S2_atp6_IMS
S2_atp6_MATRIX

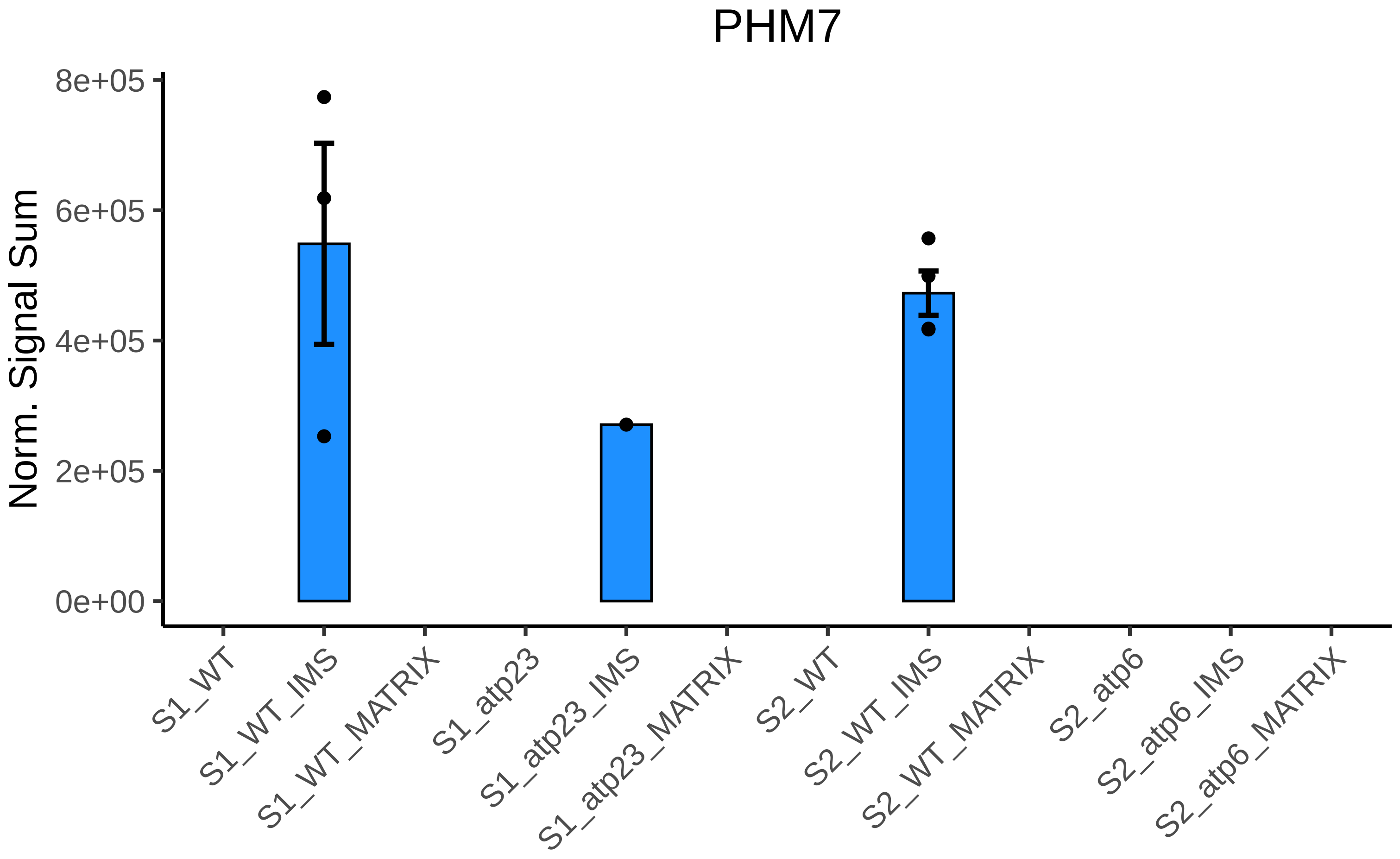
PHM7
Norm. Signal Sum
8e+05
6e+05
4e+05
2e+05
0e+00
S1_WT
S1_WT_IMS
S1_WT_MATRIX
S1_atp23
S1_atp23_IMS
S1_atp23_MATRIX
S2_WT
S2_WT_IMS
S2_WT_MATRIX
S2_atp6
S2_atp6_IMS
S2_atp6_MATRIX

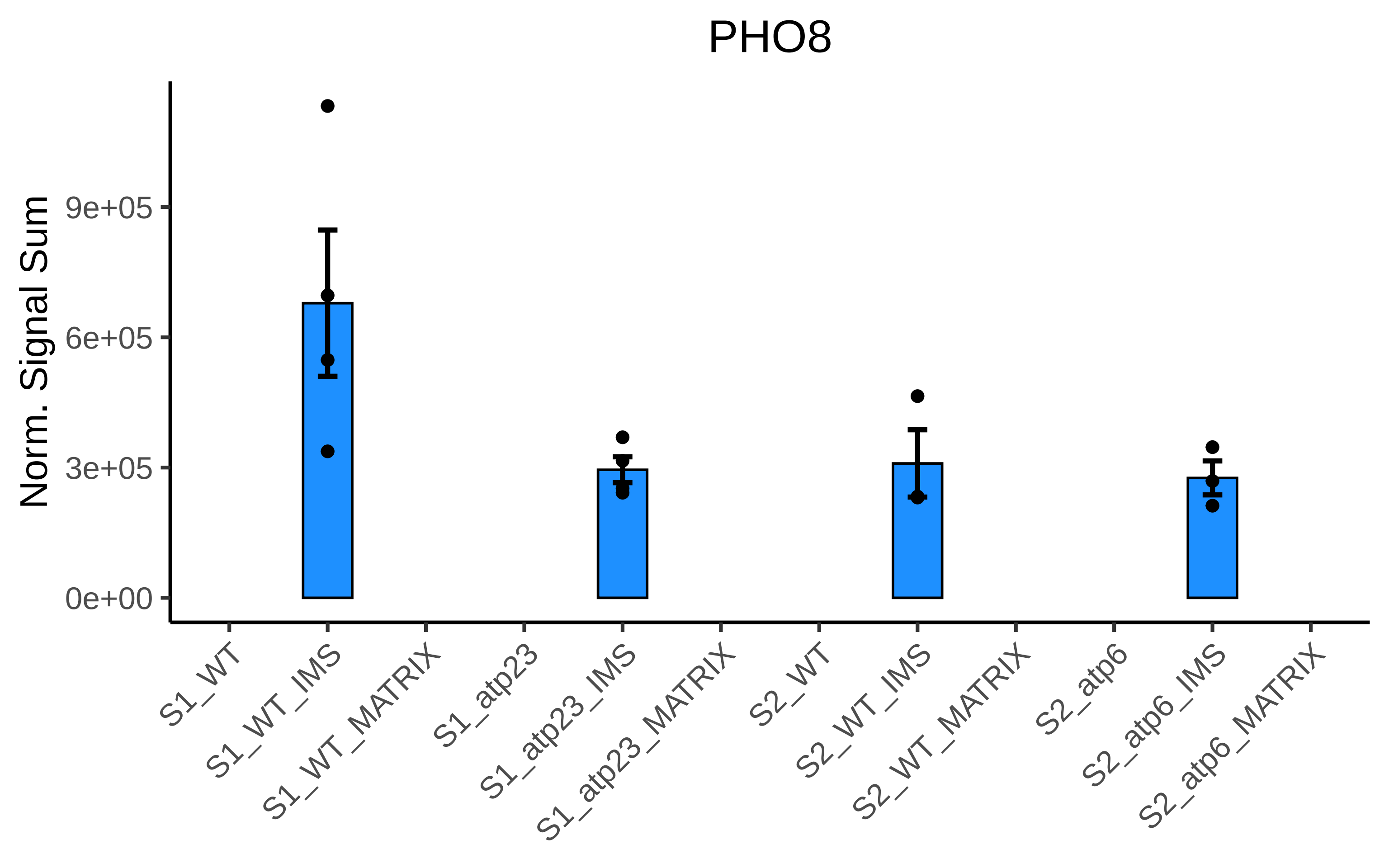
PHO8
Norm. Signal Sum
0e+00
3e+05
6e+05
9e+05
S1_WT
S1_WT_IMS
S1_WT_MATRIX
S1_atp23
S1_atp23_IMS
S1_atp23_MATRIX
S2_WT
S2_WT_IMS
S2_WT_MATRIX
S2_atp6
S2_atp6_IMS
S2_atp6_MATRIX

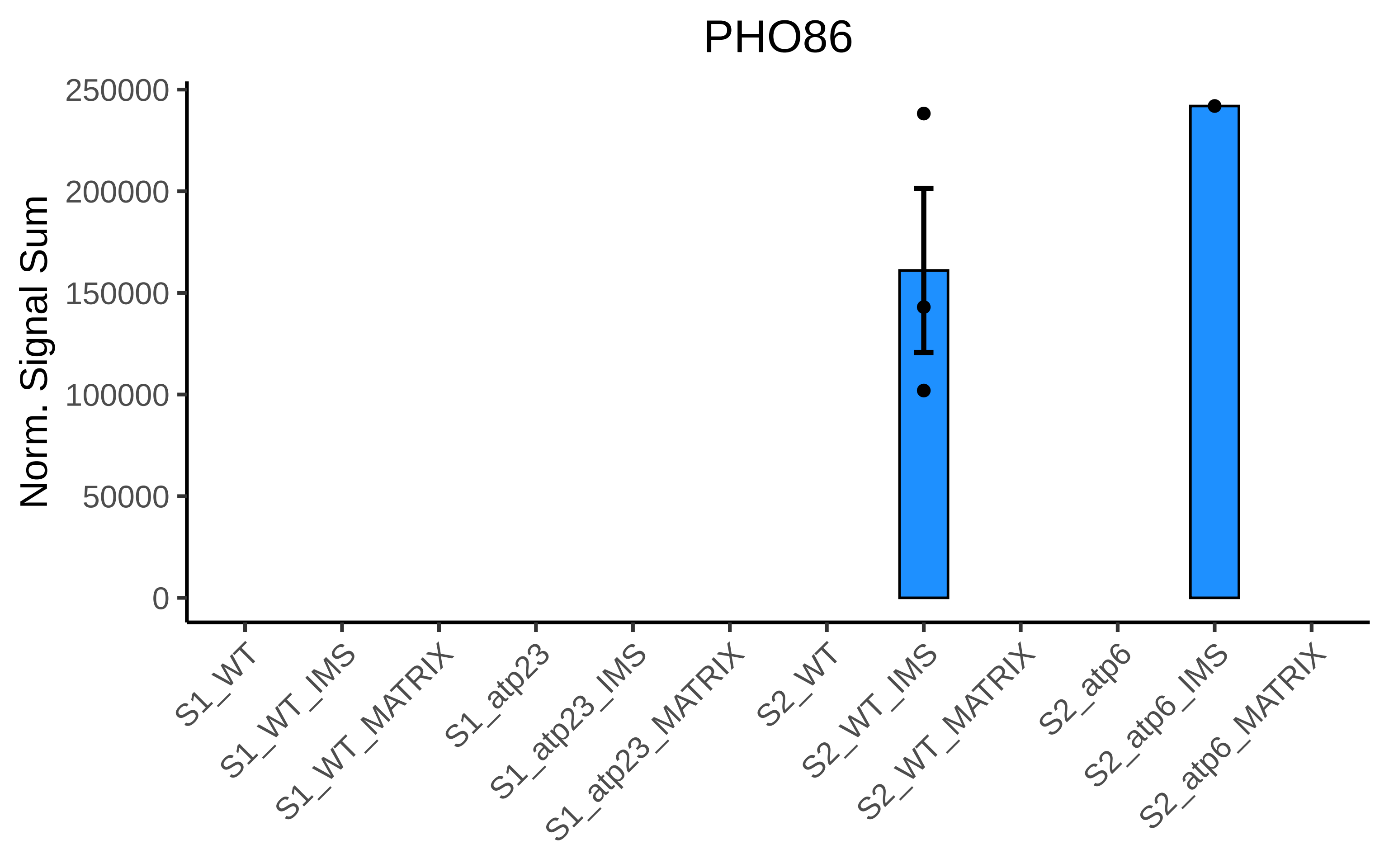
PHO86
Norm. Signal Sum
250000
200000
150000
100000
50000
0
S1_WT
S1_WT_IMS
S1_WT_MATRIX
S1_atp23
S1_atp23_IMS
S1_atp23_MATRIX
S2_WT
S2_WT_IMS
S2_WT_MATRIX
S2_atp6
S2_atp6_IMS
S2_atp6_MATRIX

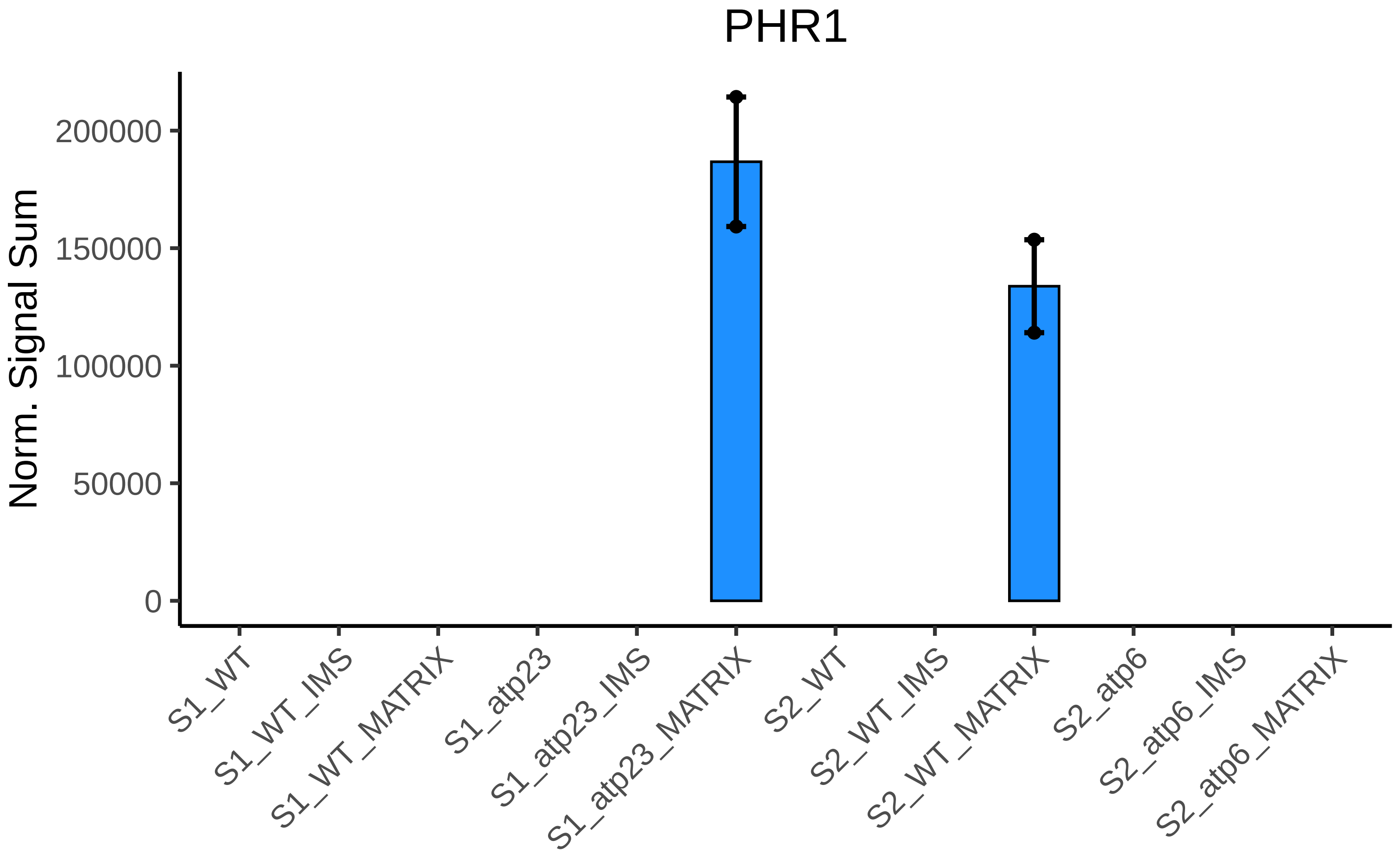

PHR1
Norm. Signal Sum
200000
150000
100000
50000
0
S1_WT
S1_WT_IMS
S1_WT_MATRIX
S1_atp23
S1_atp23_IMS
S1_atp23_MATRIX
S2_WT
S2_WT_IMS
S2_WT_MATRIX
S2_atp6
S2_atp6_IMS
S2_atp6_MATRIX

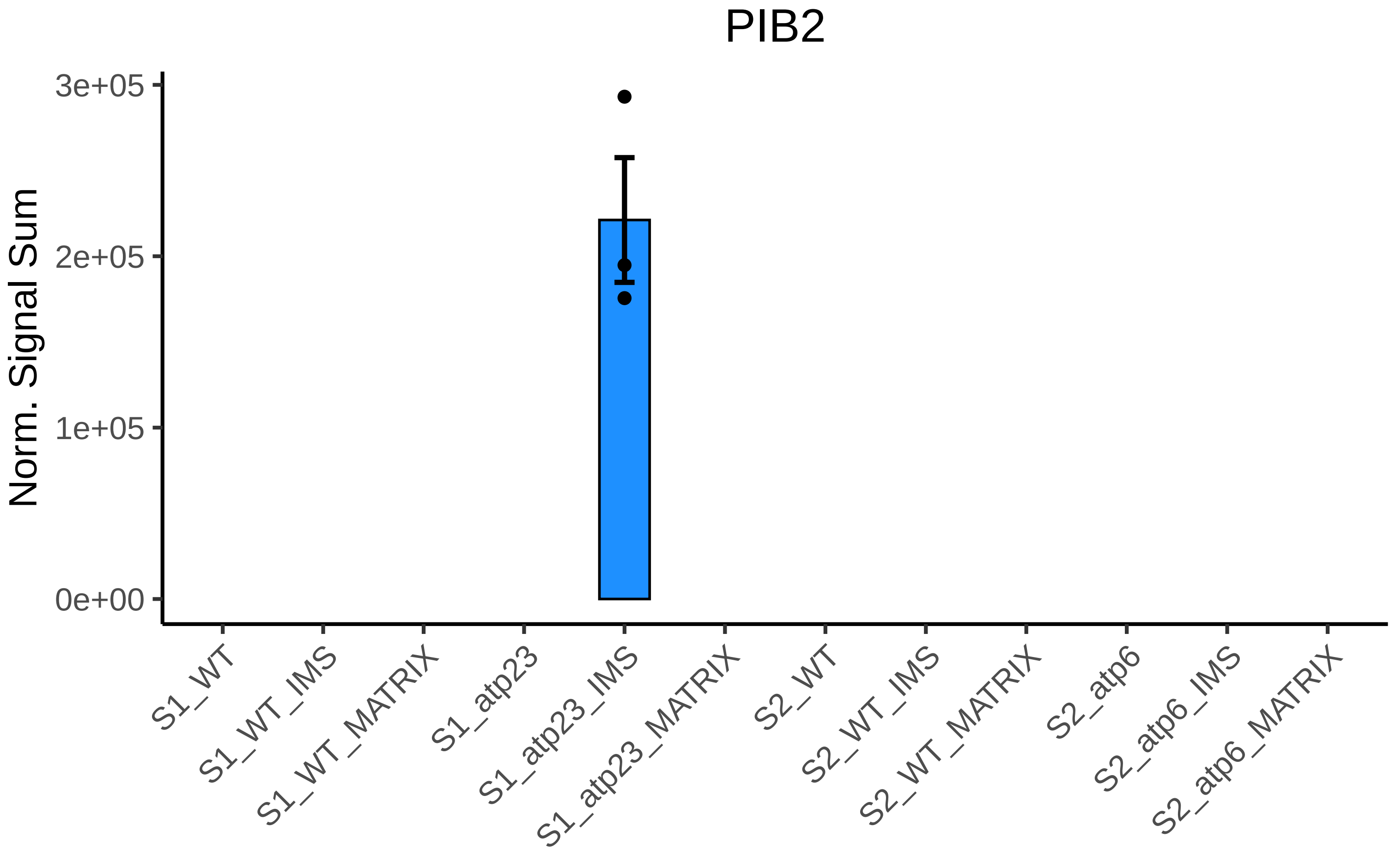

PIB2
Norm. Signal Sum
3e+05
2e+05
1e+05
0e+00
S1_WT
S1_WT_IMS
S1_WT_MATRIX
S1_atp23
S1_atp23_IMS
S1_atp23_MATRIX
S2_WT
S2_WT_IMS
S2_WT_MATRIX
S2_atp6
S2_atp6_IMS
S2_atp6_MATRIX

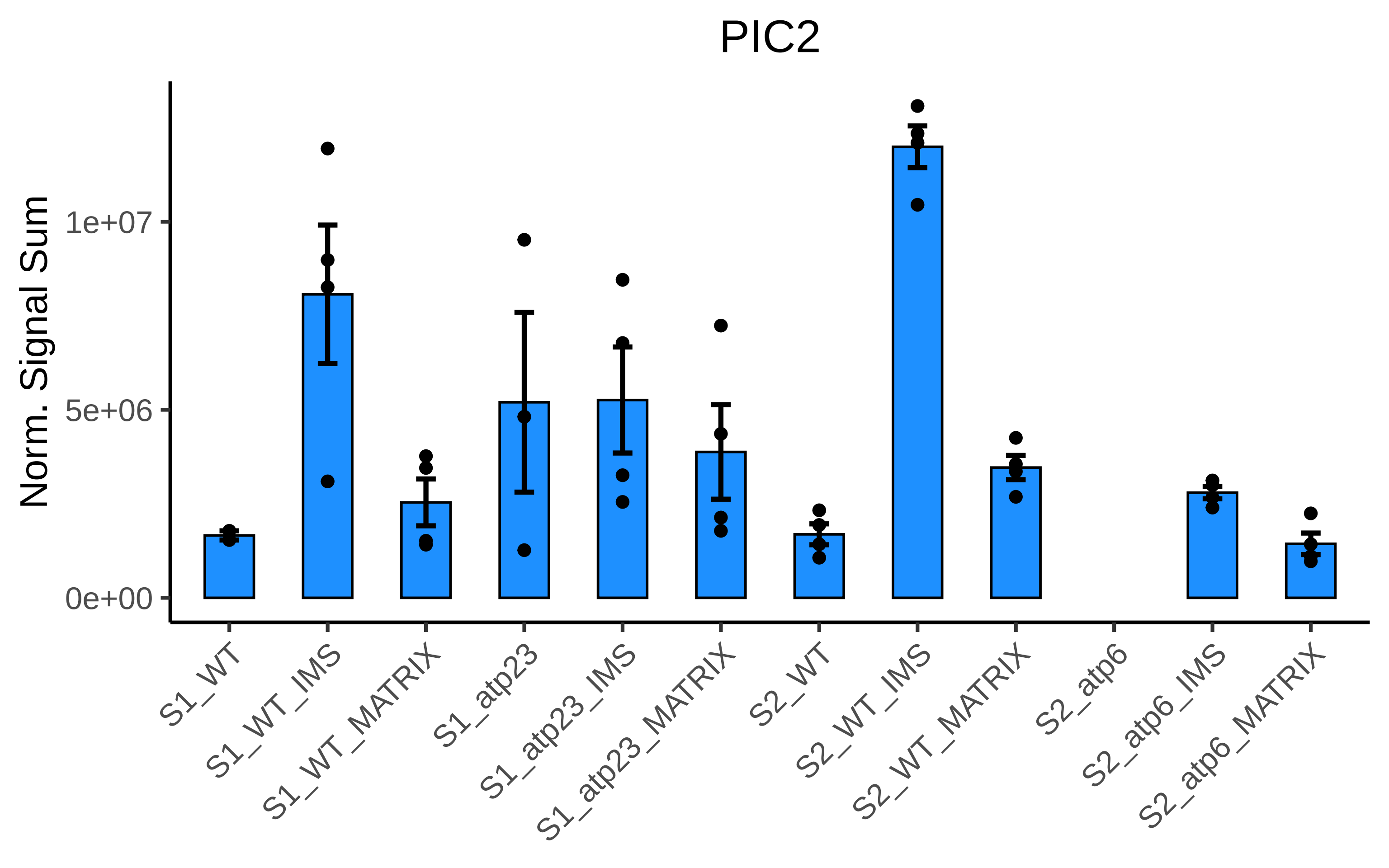
PIC2
Norm. Signal Sum
1e+07
5e+06
0e+00
S1_WT
S1_WT_IMS
S1_WT_MATRIX
S1_atp23
S1_atp23_IMS
S1_atp23_MATRIX
S2_WT
S2_WT_IMS
S2_WT_MATRIX
S2_atp6
S2_atp6_IMS
S2_atp6_MATRIX

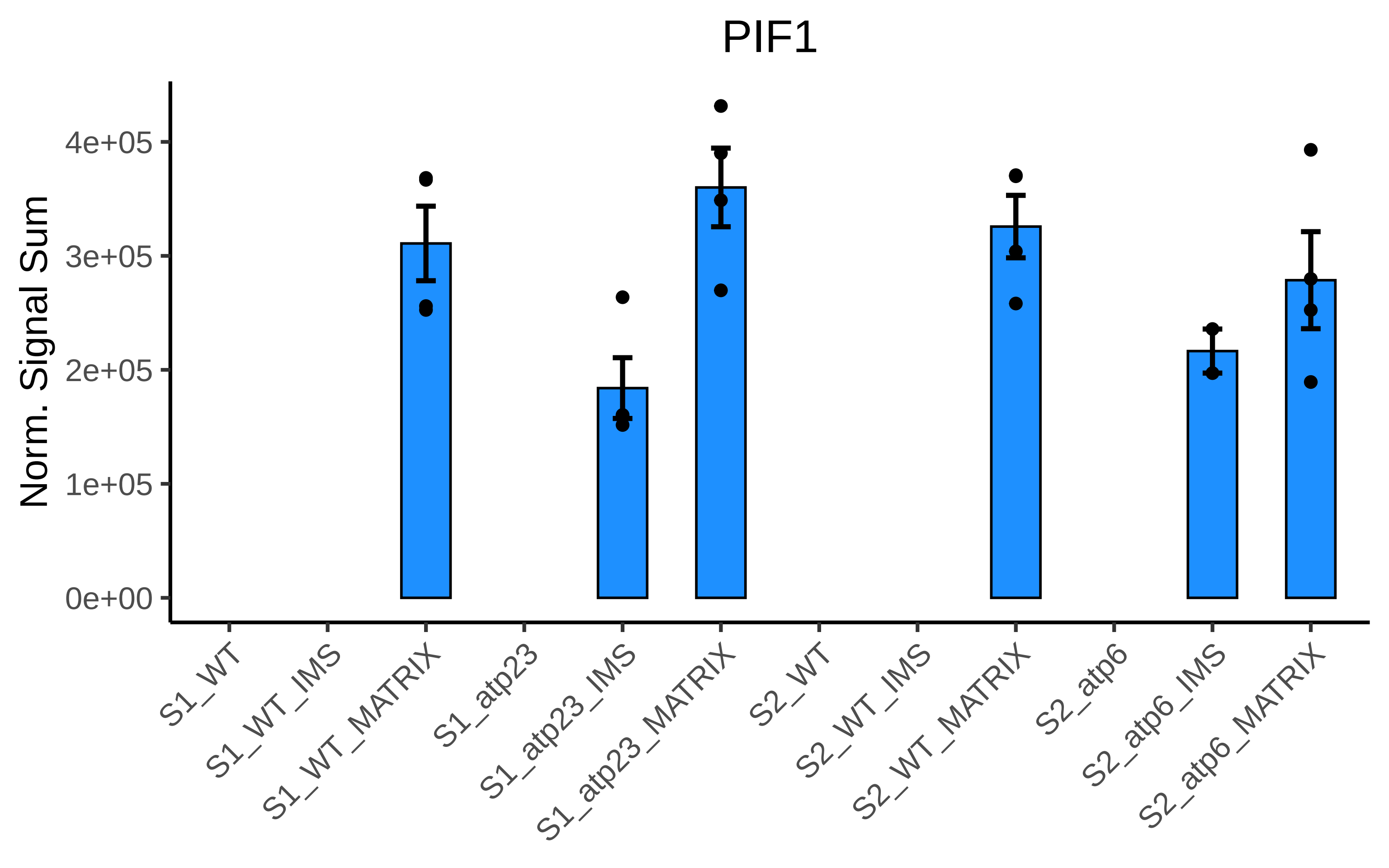
PIF1
Norm. Signal Sum
4e+05
3e+05
2e+05
1e+05
0e+00
S1_WT
S1_WT_IMS
S1_WT_MATRIX
S1_atp23
S1_atp23_IMS
S1_atp23_MATRIX
S2_WT
S2_WT_IMS
S2_WT_MATRIX
S2_atp6
S2_atp6_IMS
S2_atp6_MATRIX

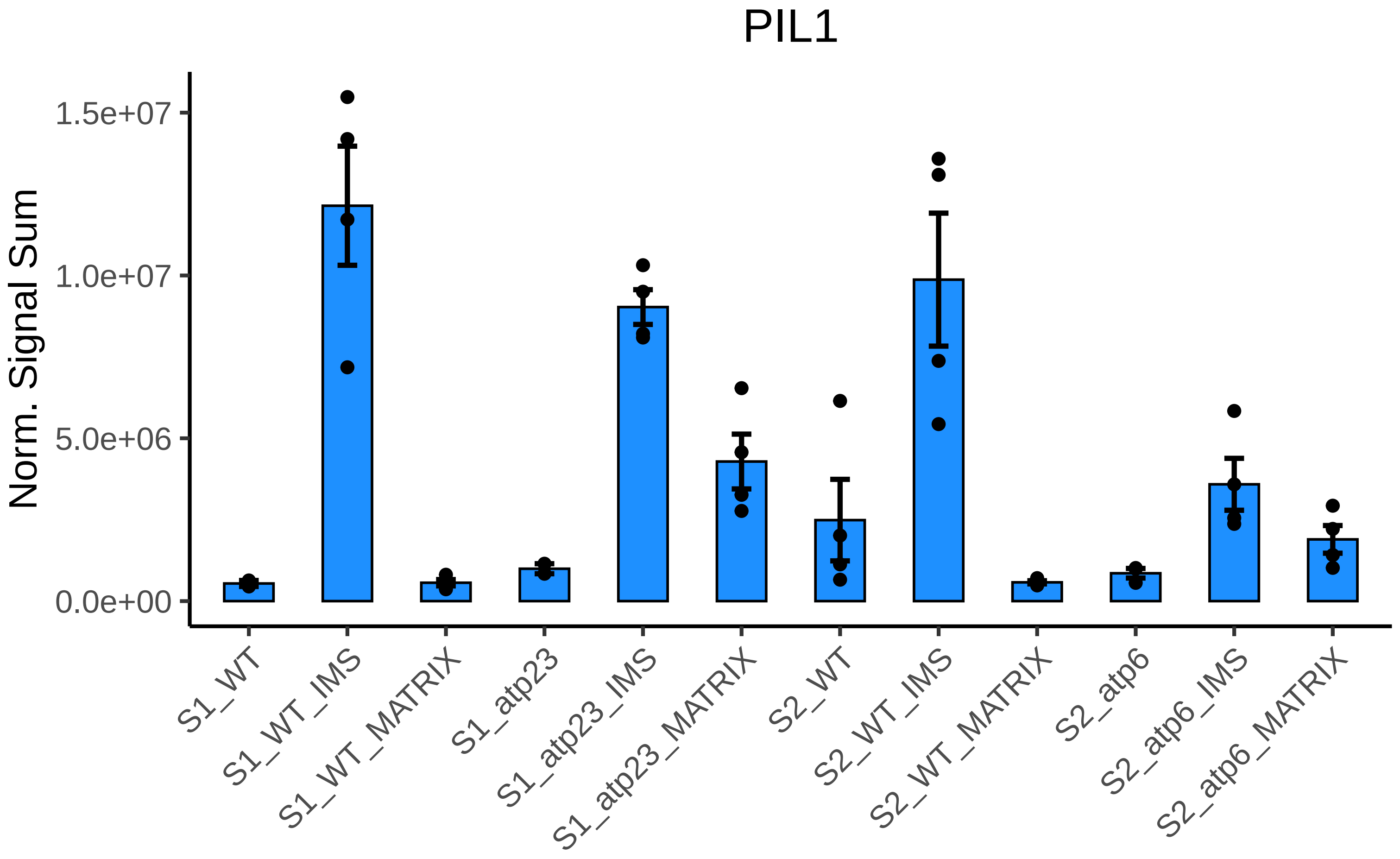
PIL1
Norm. Signal Sum
1.5e+07
1.0e+07
5.0e+06
0.0e+00
S1_WT
S1_WT_IMS
S1_WT_MATRIX
S1_atp23
S1_atp23_IMS
S1_atp23_MATRIX
S2_WT
S2_WT_IMS
S2_WT_MATRIX
S2_atp6
S2_atp6_IMS
S2_atp6_MATRIX

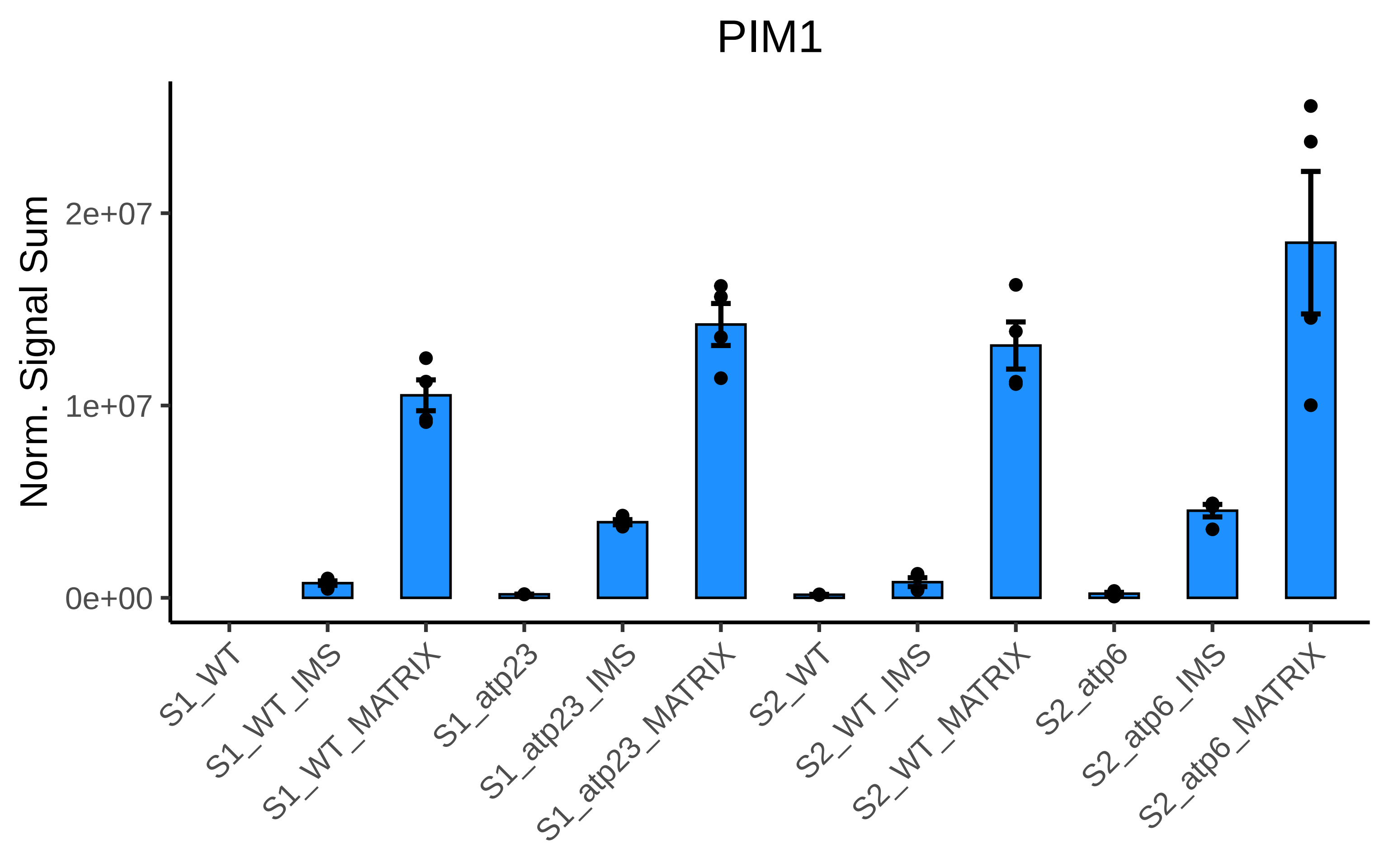

PIM1
Norm. Signal Sum
2e+07
1e+07
0e+00
S1_WT
S1_WT_IMS
S1_WT_MATRIX
S1_atp23
S1_atp23_IMS
S1_atp23_MATRIX
S2_WT
S2_WT_IMS
S2_WT_MATRIX
S2_atp6
S2_atp6_IMS
S2_atp6_MATRIX

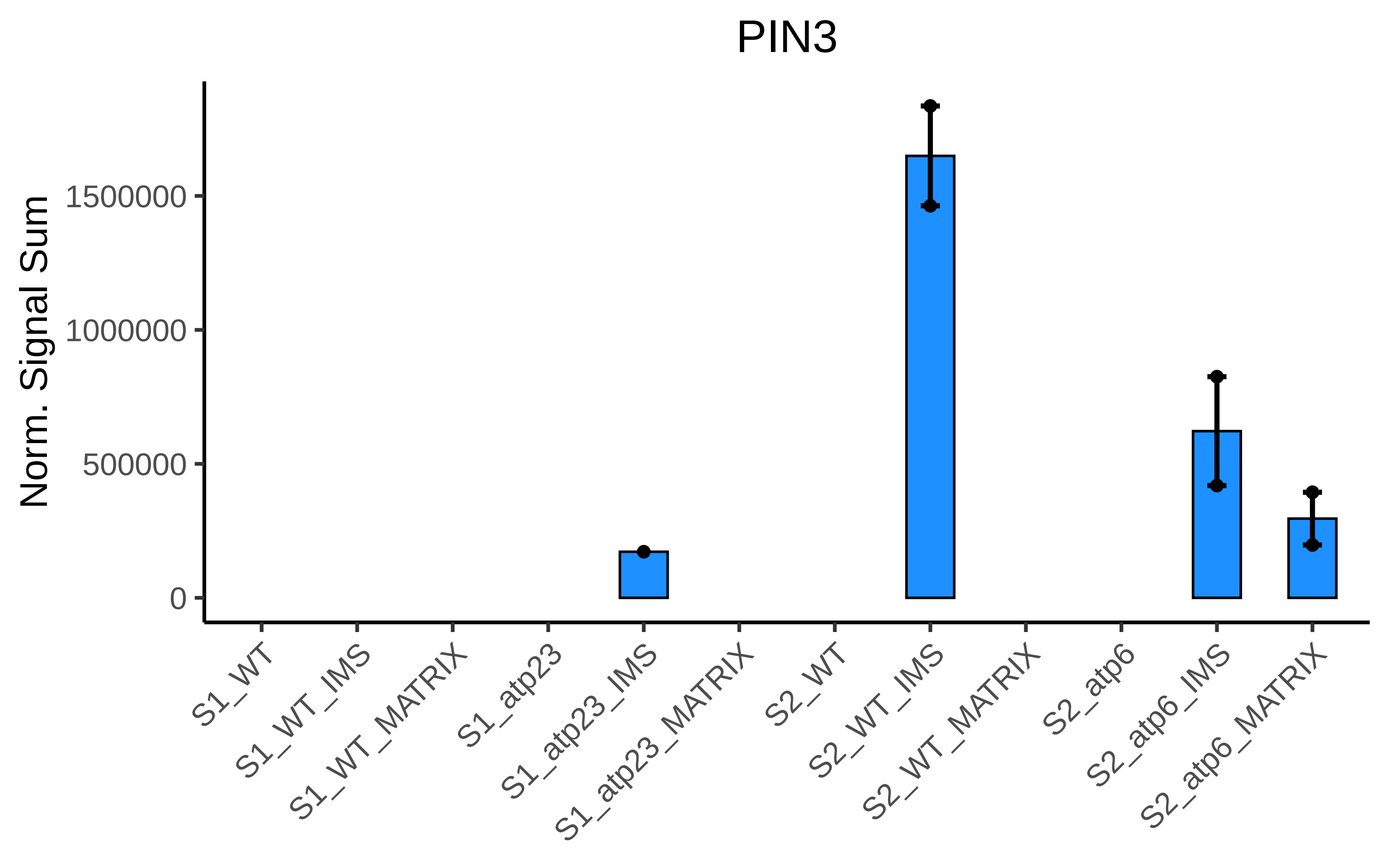

PIN3
Norm. Signal Sum
1500000
1000000
500000
0
S1_WT
S1_WT_IMS
S1_WT_MATRIX
S1_atp23
S1_atp23_IMS
S1_atp23_MATRIX
S2_WT
S2_WT_IMS
S2_WT_MATRIX
S2_atp6
S2_atp6_IMS
S2_atp6_MATRIX

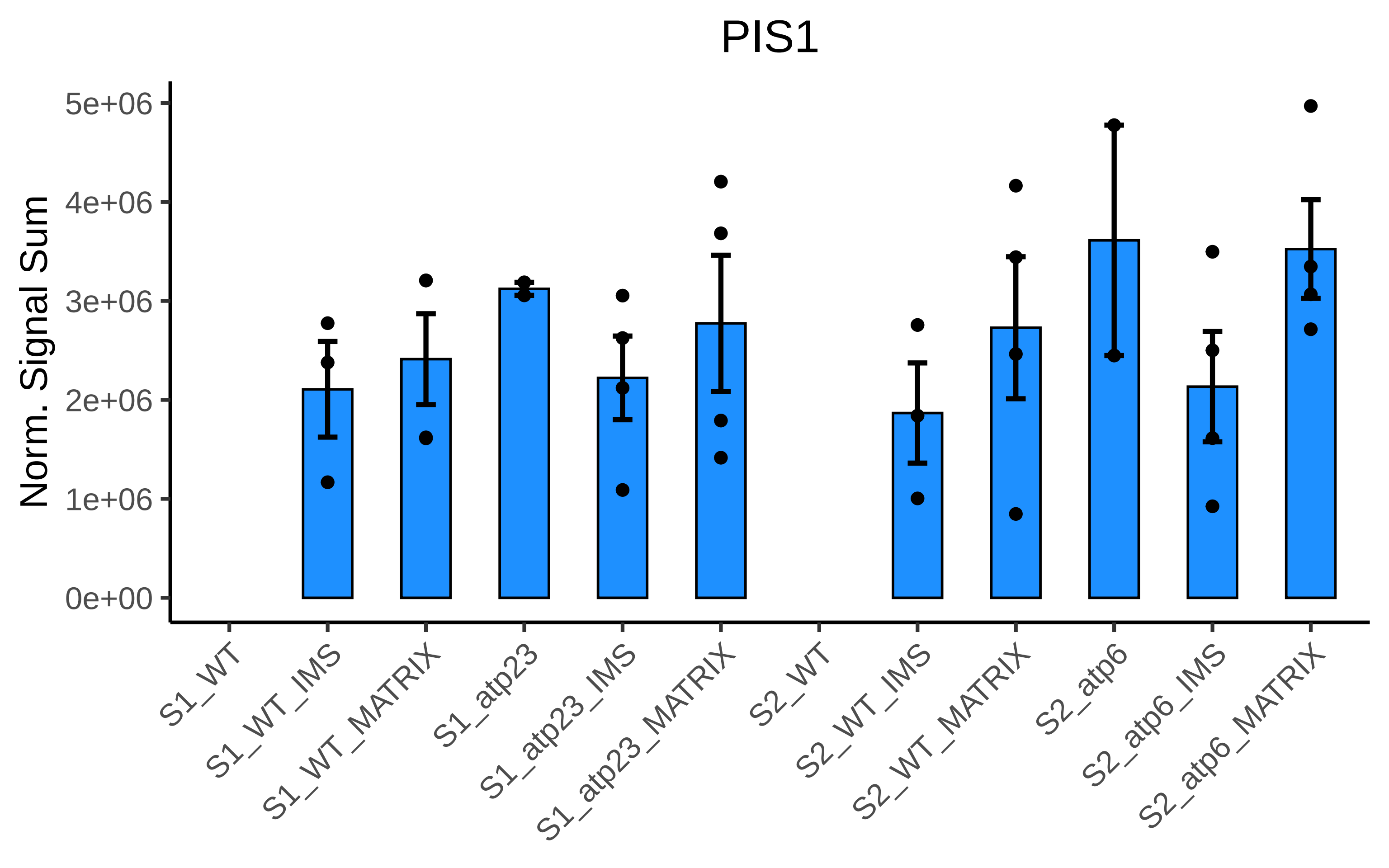

PIS1
Norm. Signal Sum
5e+06
4e+06
3e+06
2e+06
1e+06
0e+00
S1_WT
S1_WT_IMS
S1_WT_MATRIX
S1_atp23
S1_atp23_IMS
S1_atp23_MATRIX
S2_WT
S2_WT_IMS
S2_WT_MATRIX
S2_atp6
S2_atp6_IMS
S2_atp6_MATRIX

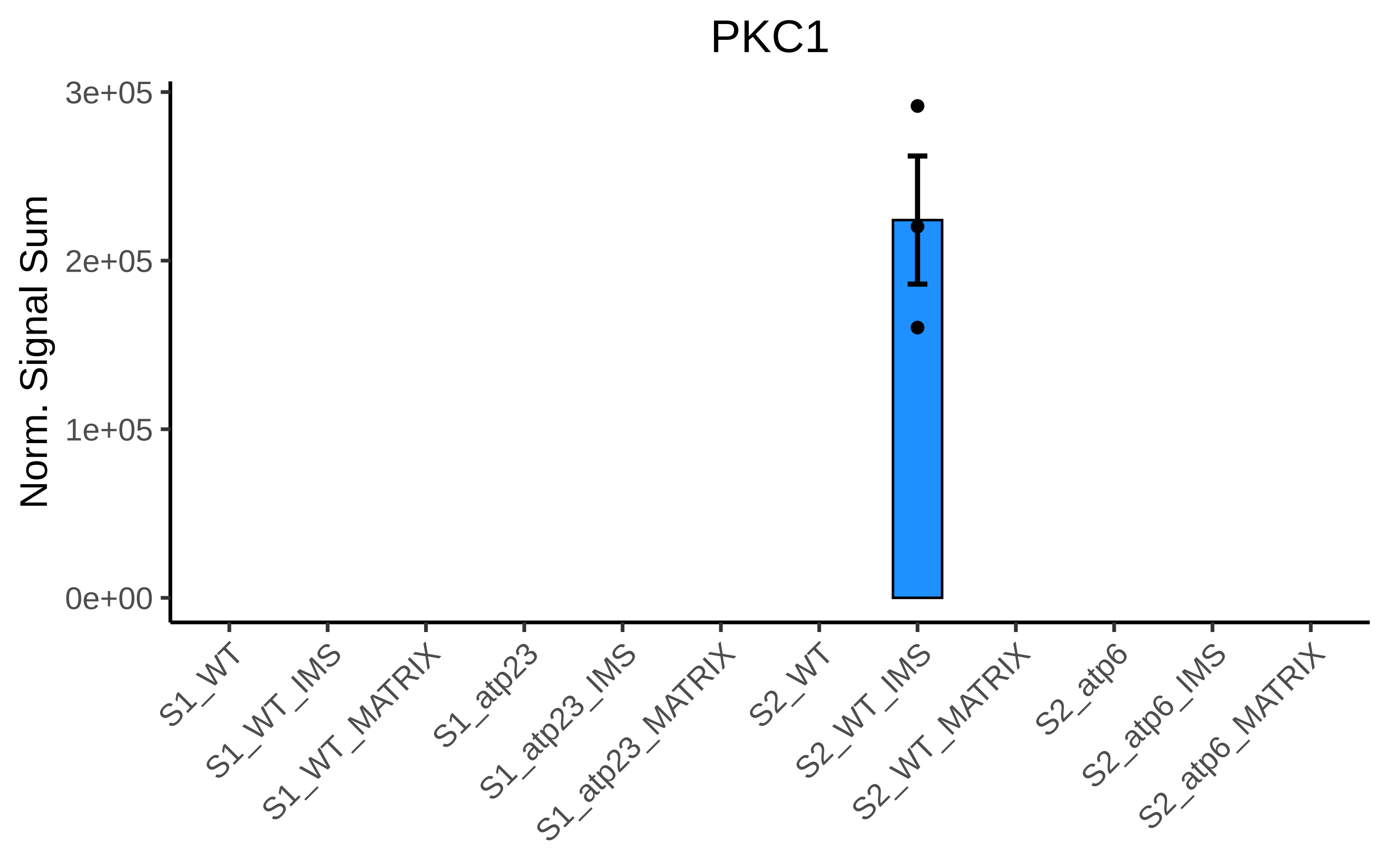

PKC1
Norm. Signal Sum
3e+05
2e+05
1e+05
0e+00
S1_WT
S1_WT_IMS
S1_WT_MATRIX
S1_atp23
S1_atp23_IMS
S1_atp23_MATRIX
S2_WT
S2_WT_IMS
S2_WT_MATRIX
S2_atp6
S2_atp6_IMS
S2_atp6_MATRIX

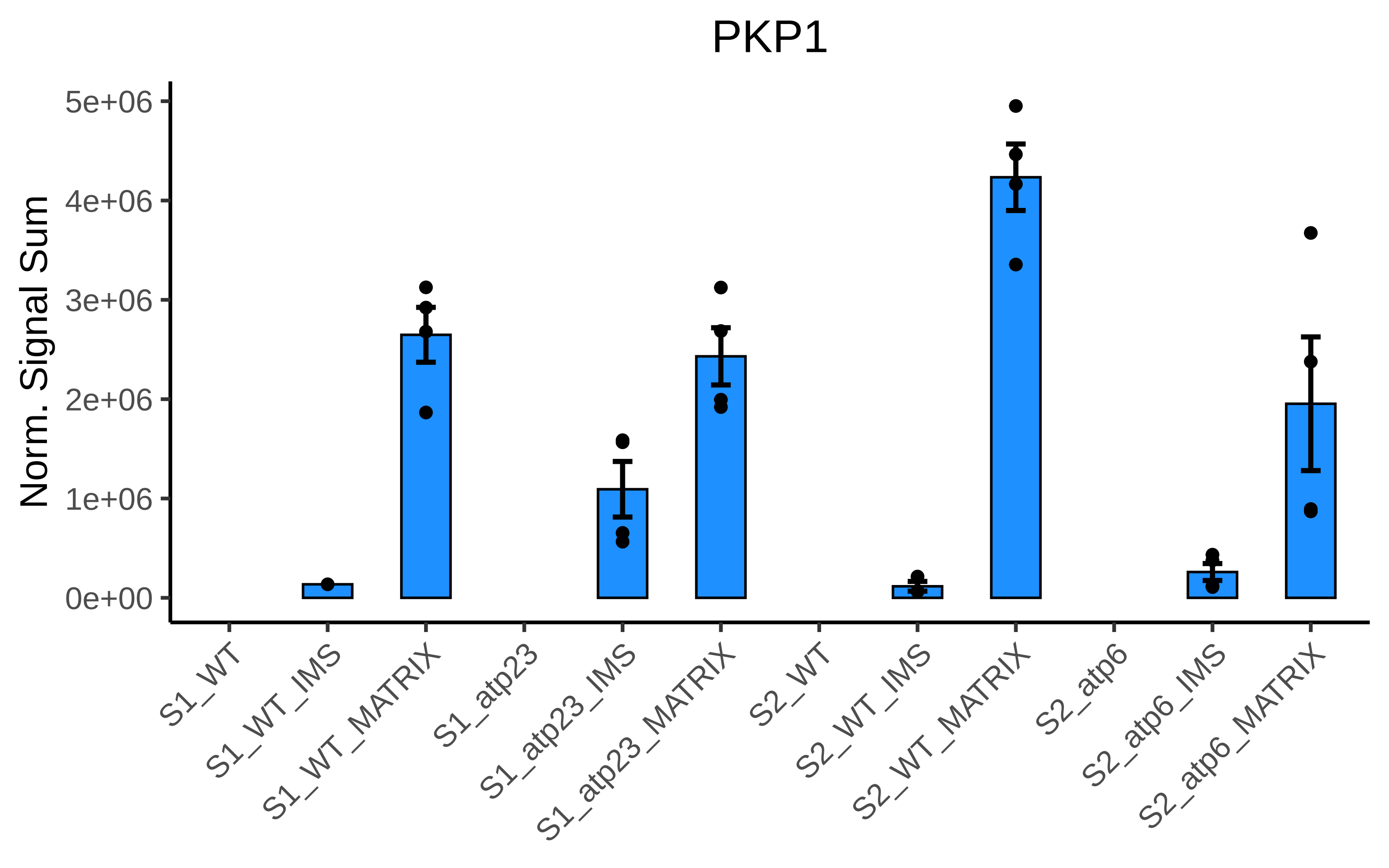

PKP1
Norm. Signal Sum
5e+06
4e+06
3e+06
2e+06
1e+06
0e+00
S1_WT
S1_WT_IMS
S1_WT_MATRIX
S1_atp23
S1_atp23_IMS
S1_atp23_MATRIX
S2_WT
S2_WT_IMS
S2_WT_MATRIX
S2_atp6
S2_atp6_IMS
S2_atp6_MATRIX

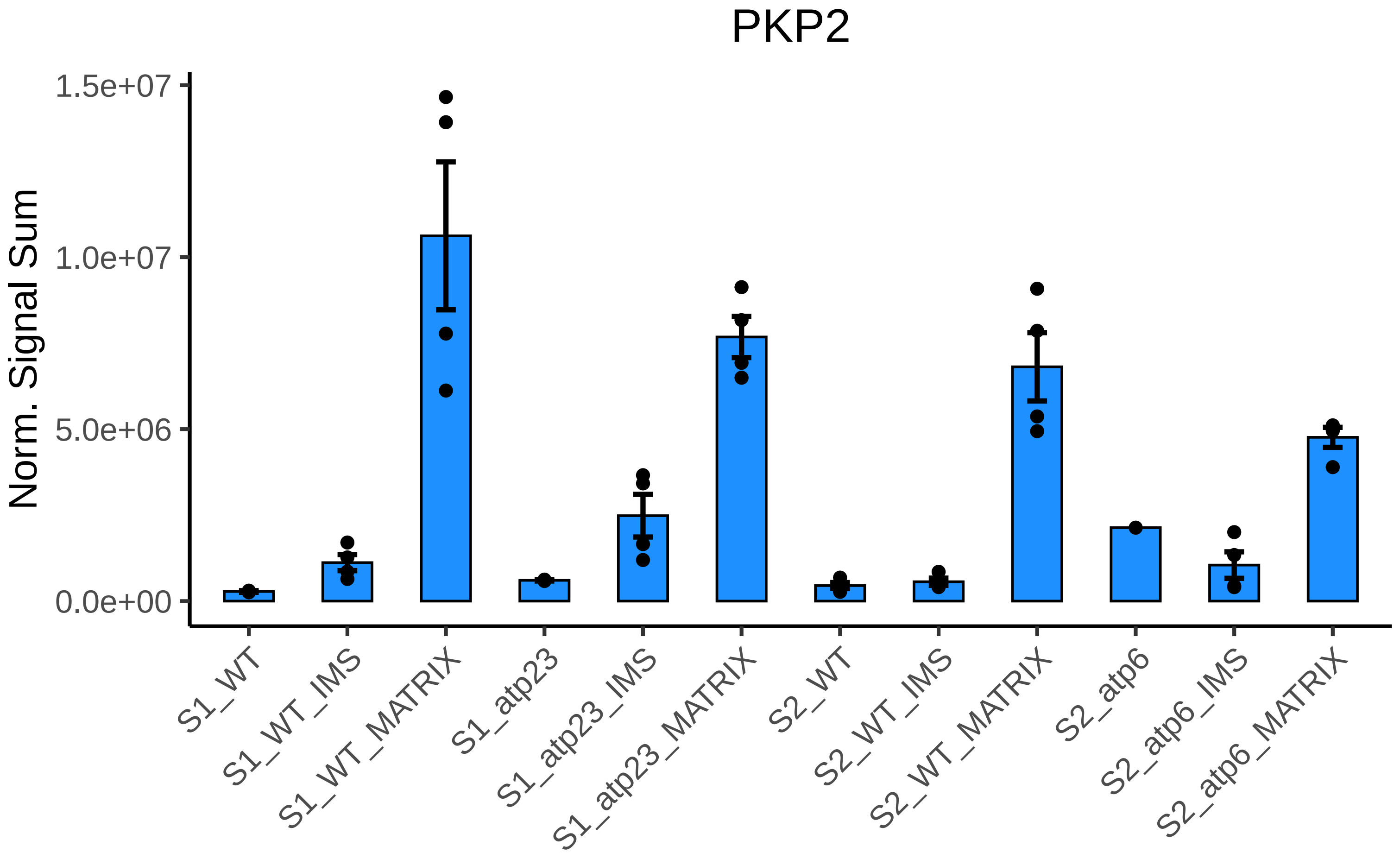
PKP2
Norm. Signal Sum
1.5e+07
1.0e+07
5.0e+06
0.0e+00
S1_WT
S1_WT_IMS
S1_WT_MATRIX
S1_atp23
S1_atp23_IMS
S1_atp23_MATRIX
S2_WT
S2_WT_IMS
S2_WT_MATRIX
S2_atp6
S2_atp6_IMS
S2_atp6_MATRIX

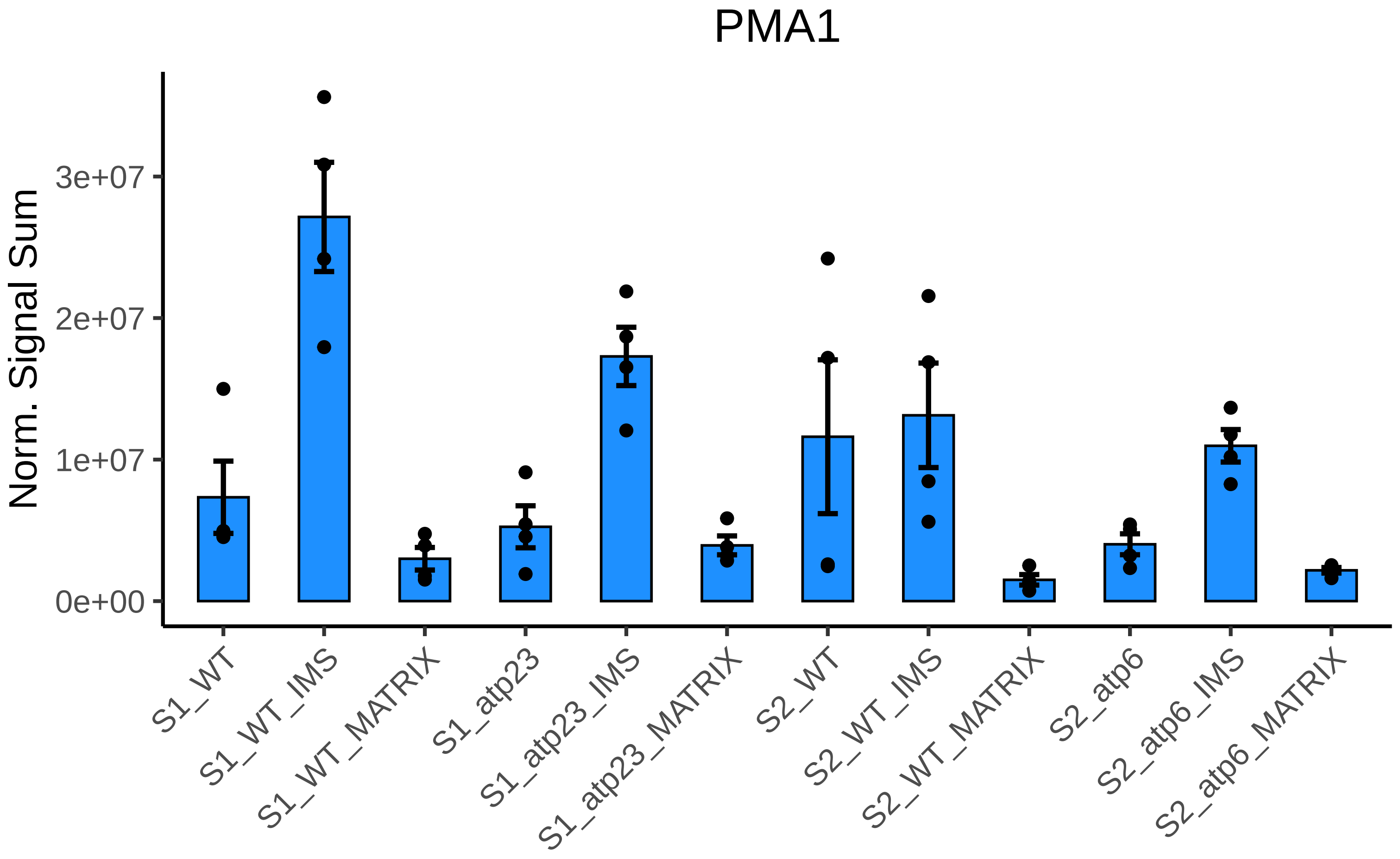

PMA1
Norm. Signal Sum
3e+07
2e+07
1e+07
0e+00
S1_WT
S1_WT_IMS
S1_WT_MATRIX
S1_atp23
S1_atp23_IMS
S1_atp23_MATRIX
S2_WT
S2_WT_IMS
S2_WT_MATRIX
S2_atp6
S2_atp6_IMS
S2_atp6_MATRIX

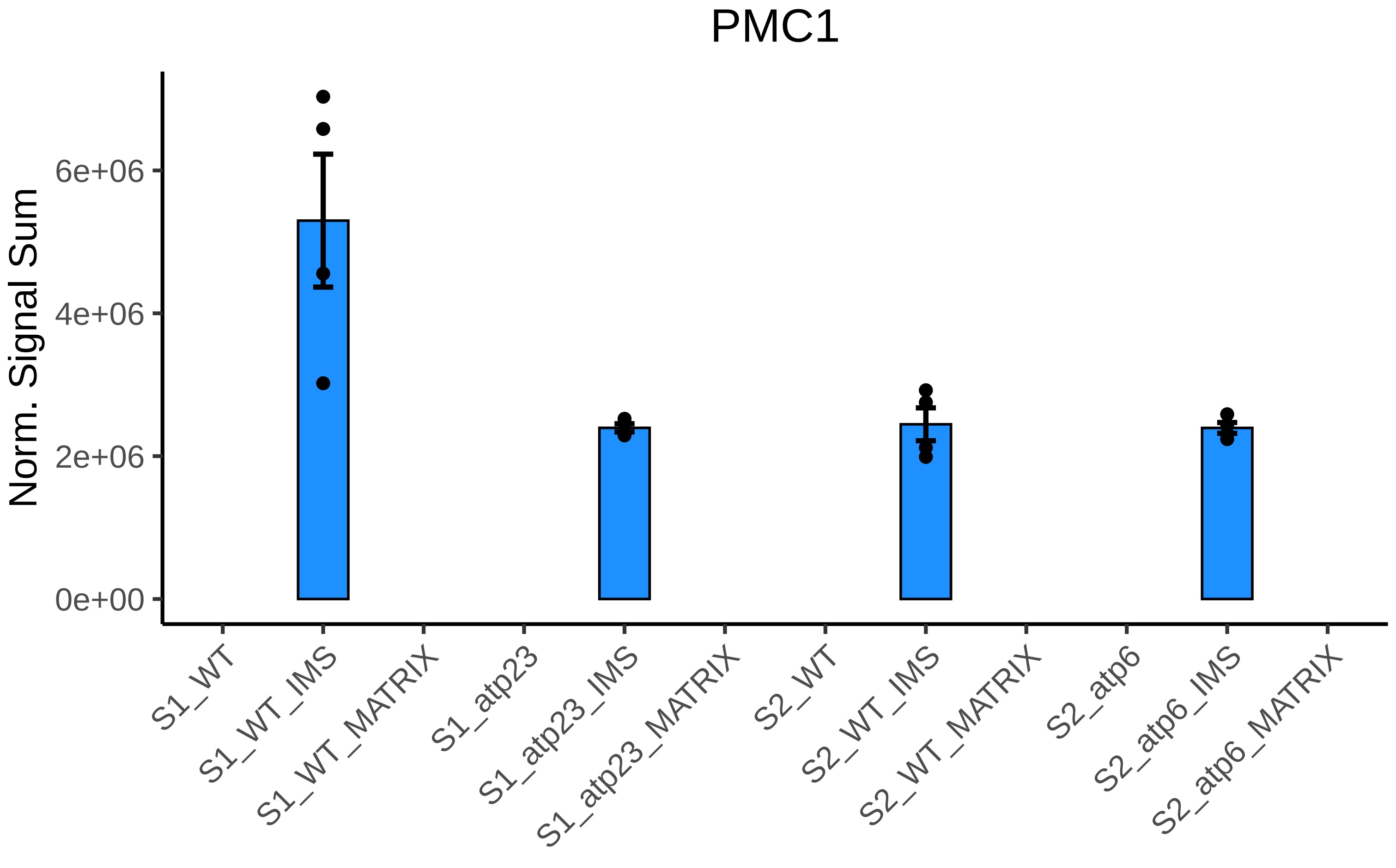

PMC1
Norm. Signal Sum
6e+06
4e+06
2e+06
0e+00
S1_WT
S1_WT_IMS
S1_WT_MATRIX
S1_atp23
S1_atp23_IMS
S1_atp23_MATRIX
S2_WT
S2_WT_IMS
S2_WT_MATRIX
S2_atp6
S2_atp6_IMS
S2_atp6_MATRIX

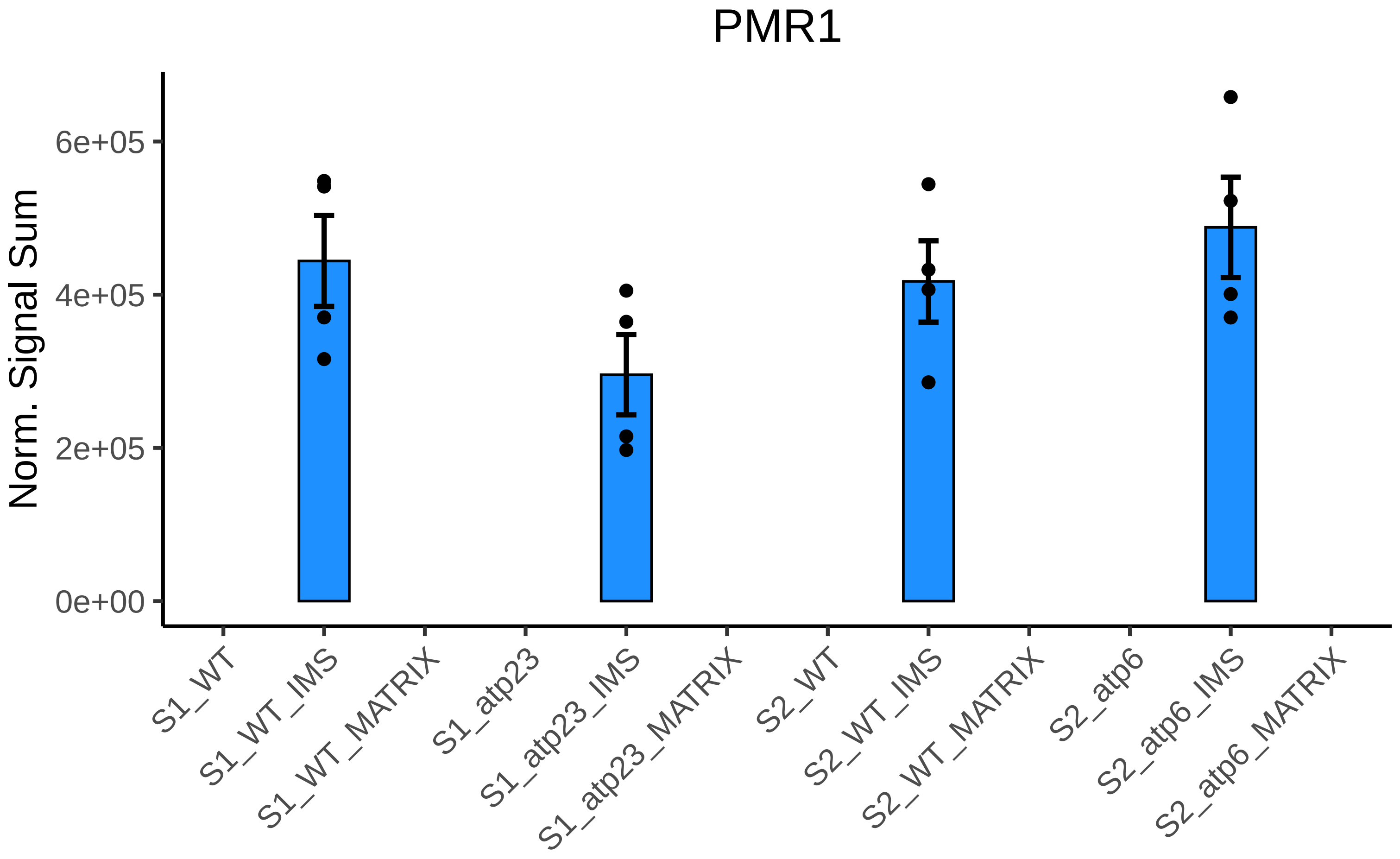
PMR1
Norm. Signal Sum
6e+05
4e+05
2e+05
0e+00
S1_WT
S1_WT_IMS
S1_WT_MATRIX
S1_atp23
S1_atp23_IMS
S1_atp23_MATRIX
S2_WT
S2_WT_IMS
S2_WT_MATRIX
S2_atp6
S2_atp6_IMS
S2_atp6_MATRIX

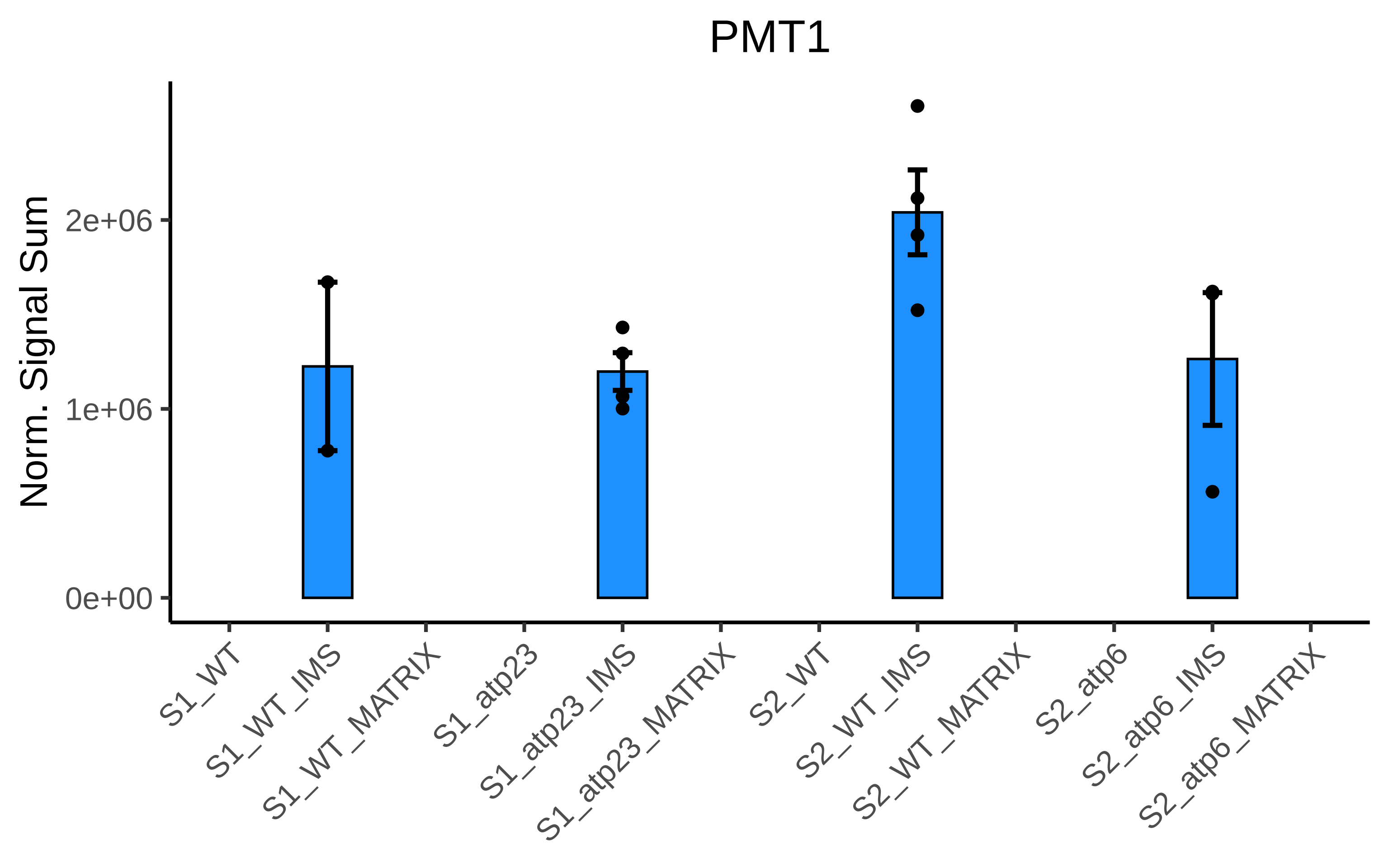

PMT1
Norm. Signal Sum
2e+06
1e+06
0e+00
S1_WT
S1_WT_IMS
S1_WT_MATRIX
S1_atp23
S1_atp23_IMS
S1_atp23_MATRIX
S2_WT
S2_WT_IMS
S2_WT_MATRIX
S2_atp6
S2_atp6_IMS
S2_atp6_MATRIX

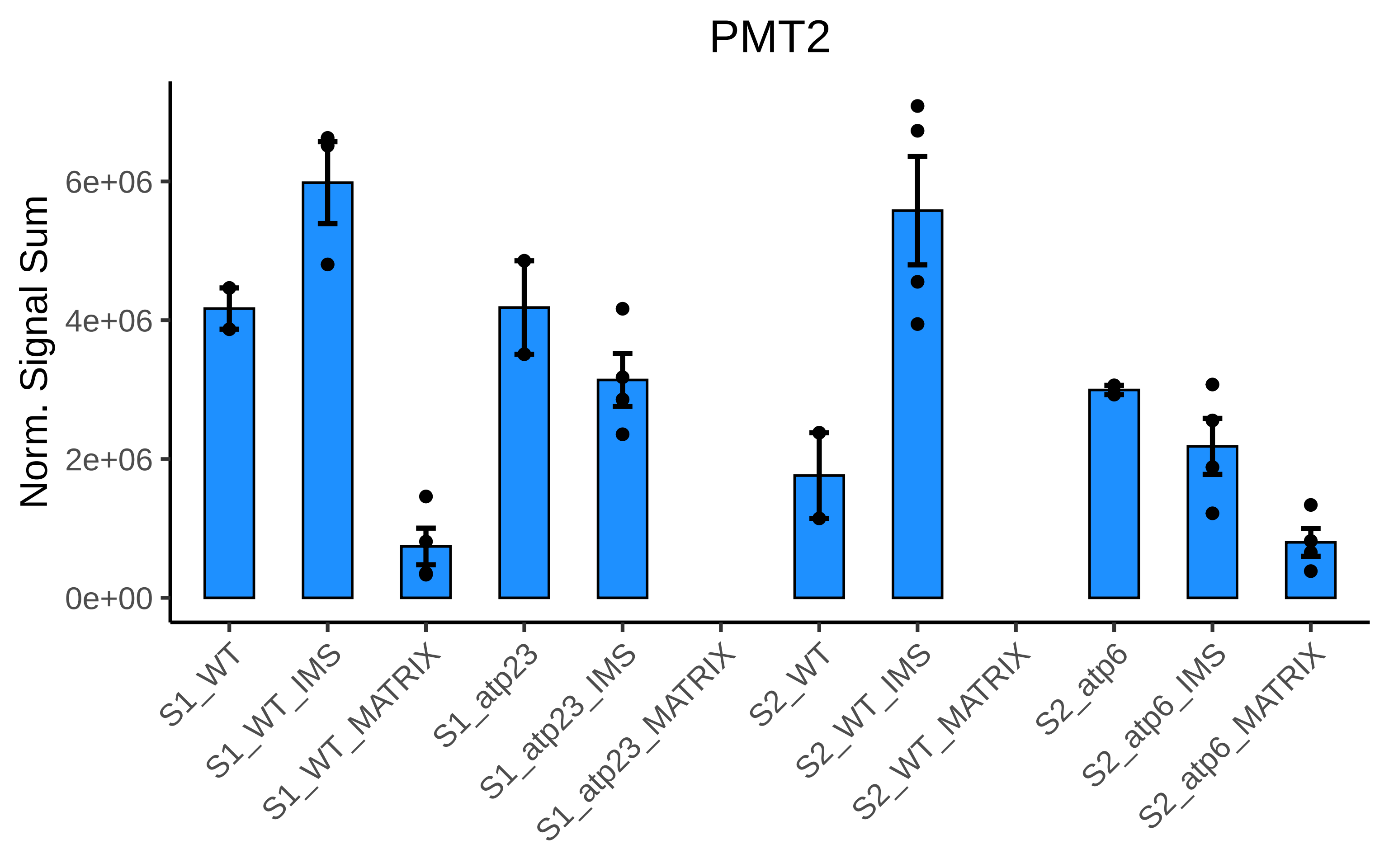
PMT2
Norm. Signal Sum
0e+00
2e+06
4e+06
6e+06
S1_WT
S1_WT_IMS
S1_WT_MATRIX
S1_atp23
S1_atp23_IMS
S1_atp23_MATRIX
S2_WT
S2_WT_IMS
S2_WT_MATRIX
S2_atp6
S2_atp6_IMS
S2_atp6_MATRIX

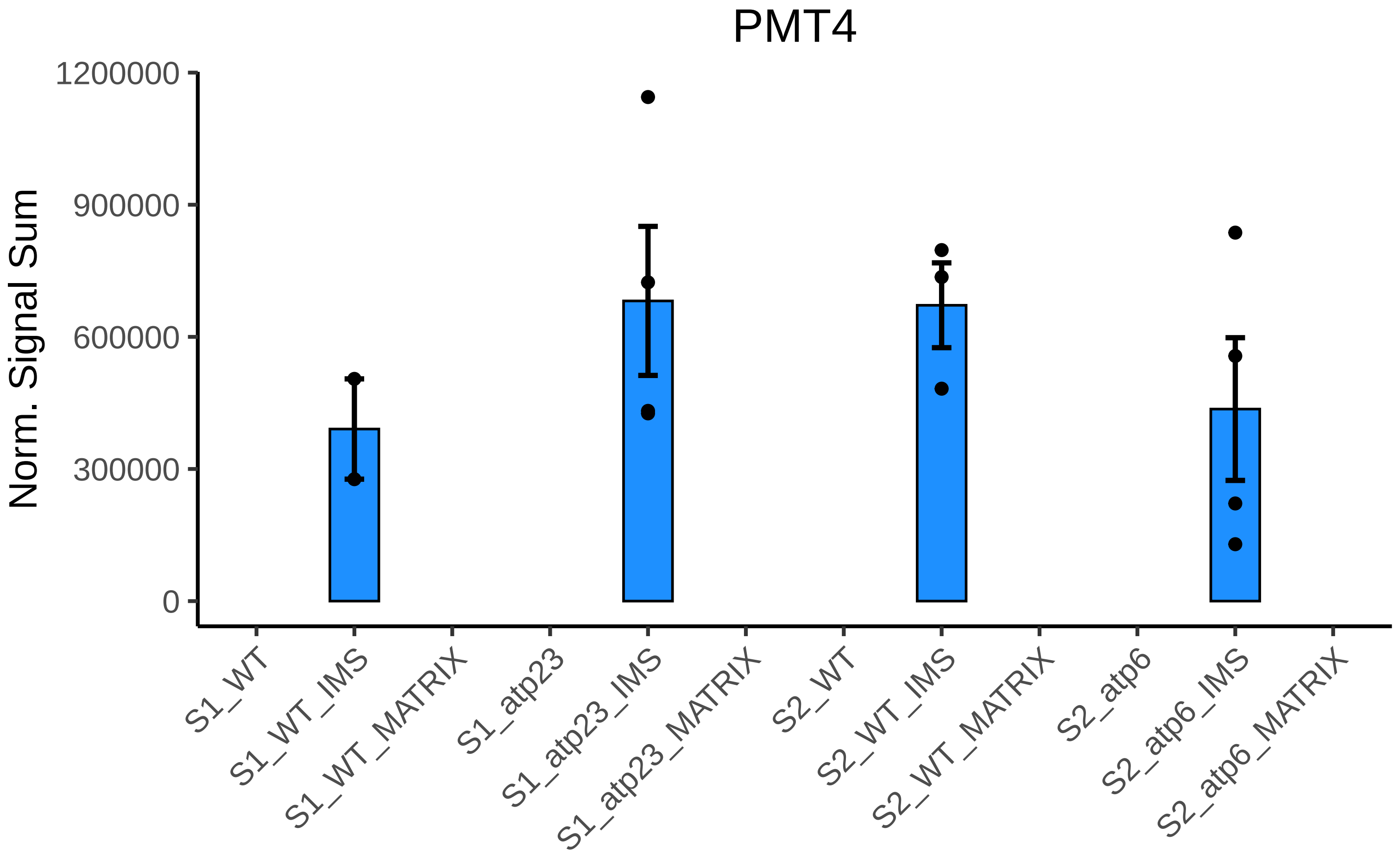
PMT4
Norm. Signal Sum
1200000
900000
600000
300000
0
S1_WT
S1_WT_IMS
S1_WT_MATRIX
S1_atp23
S1_atp23_IMS
S1_atp23_MATRIX
S2_WT
S2_WT_IMS
S2_WT_MATRIX
S2_atp6
S2_atp6_IMS
S2_atp6_MATRIX

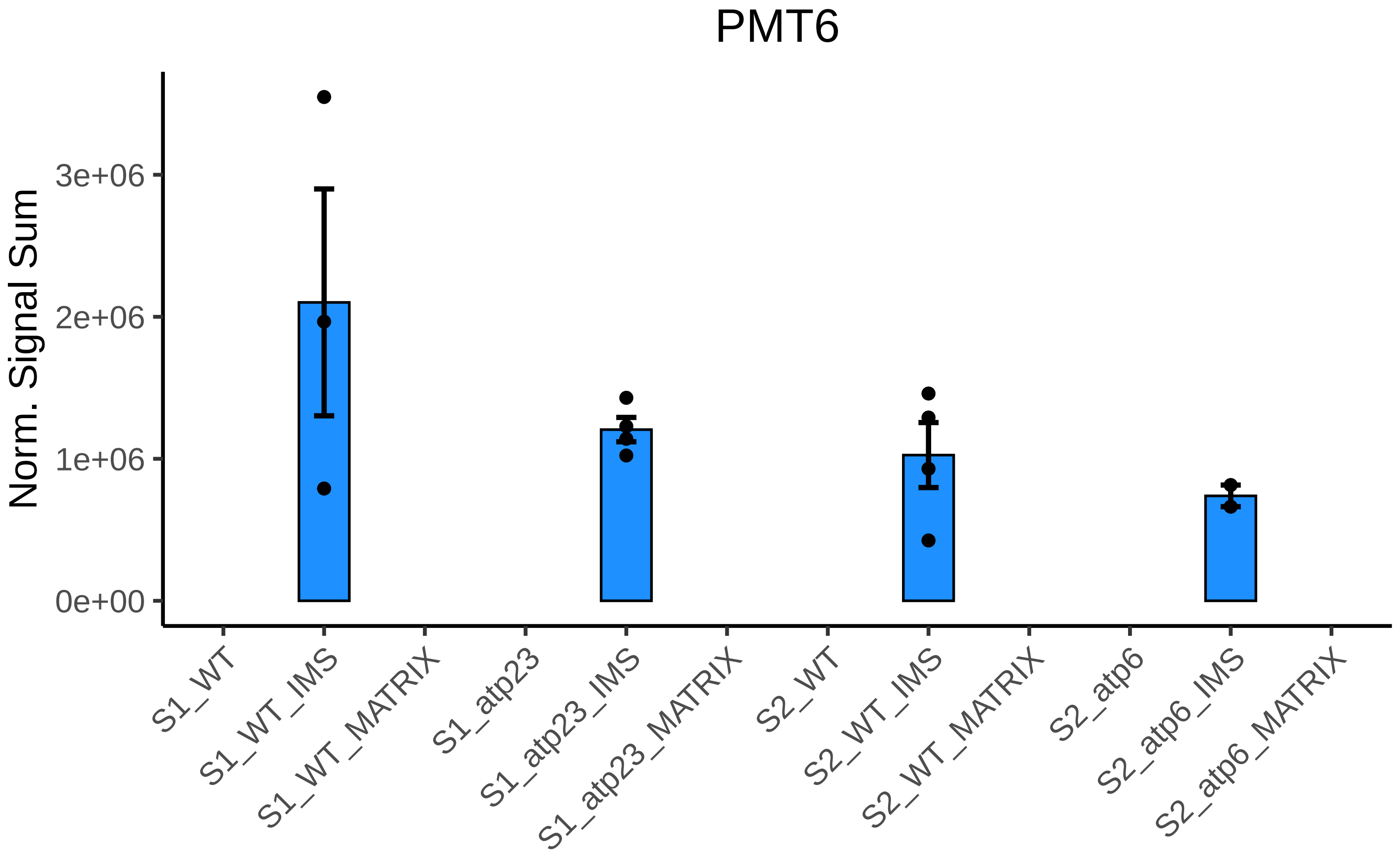

PMT6
Norm. Signal Sum
3e+06
2e+06
1e+06
0e+00
S1_WT
S1_WT_IMS
S1_WT_MATRIX
S1_atp23
S1_atp23_IMS
S1_atp23_MATRIX
S2_WT
S2_WT_IMS
S2_WT_MATRIX
S2_atp6
S2_atp6_IMS
S2_atp6_MATRIX

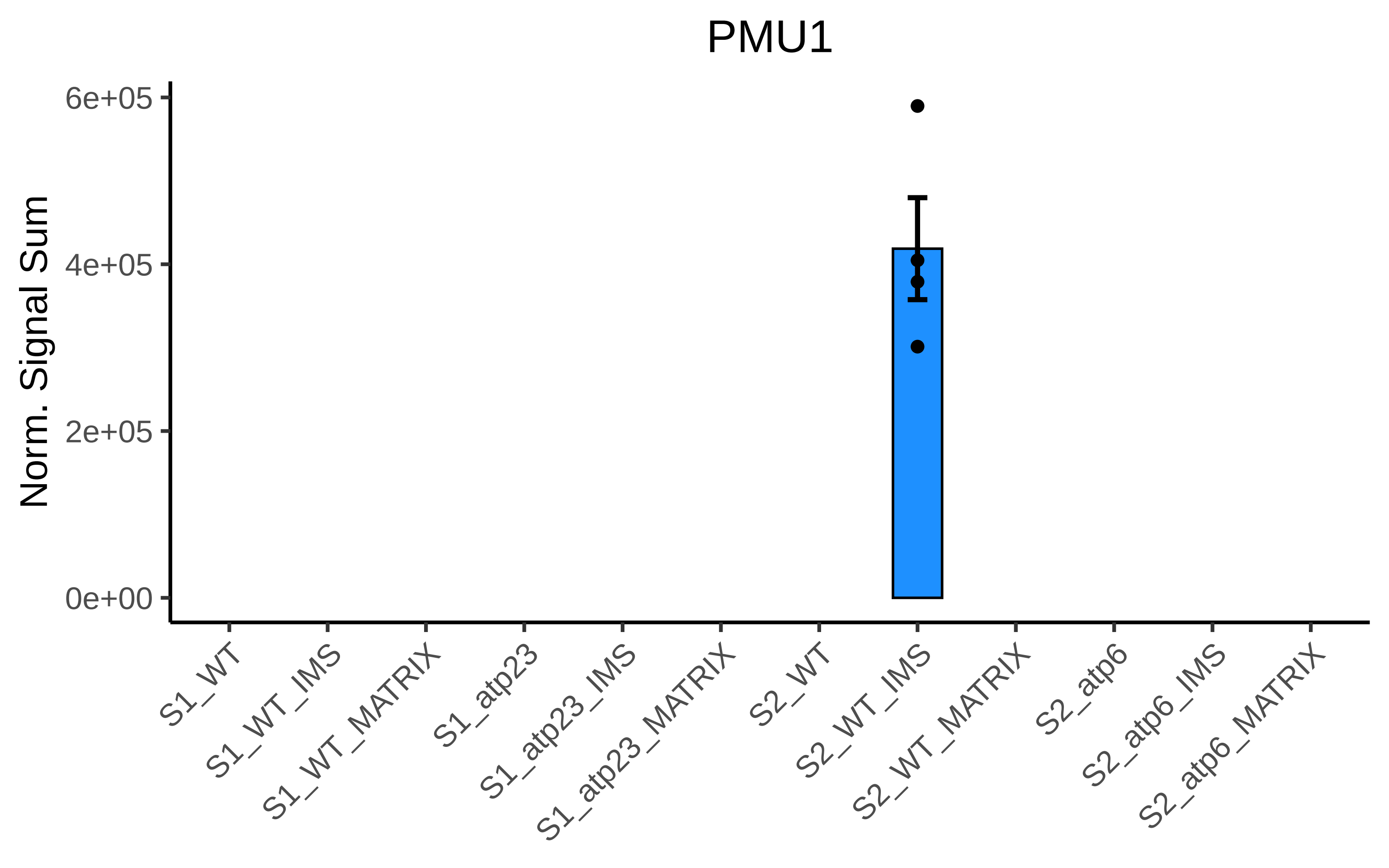
PMU1
Norm. Signal Sum
6e+05
4e+05
2e+05
0e+00
S1_WT
S1_WT_IMS
S1_WT_MATRIX
S1_atp23
S1_atp23_IMS
S1_atp23_MATRIX
S2_WT
S2_WT_IMS
S2_WT_MATRIX
S2_atp6
S2_atp6_IMS
S2_atp6_MATRIX

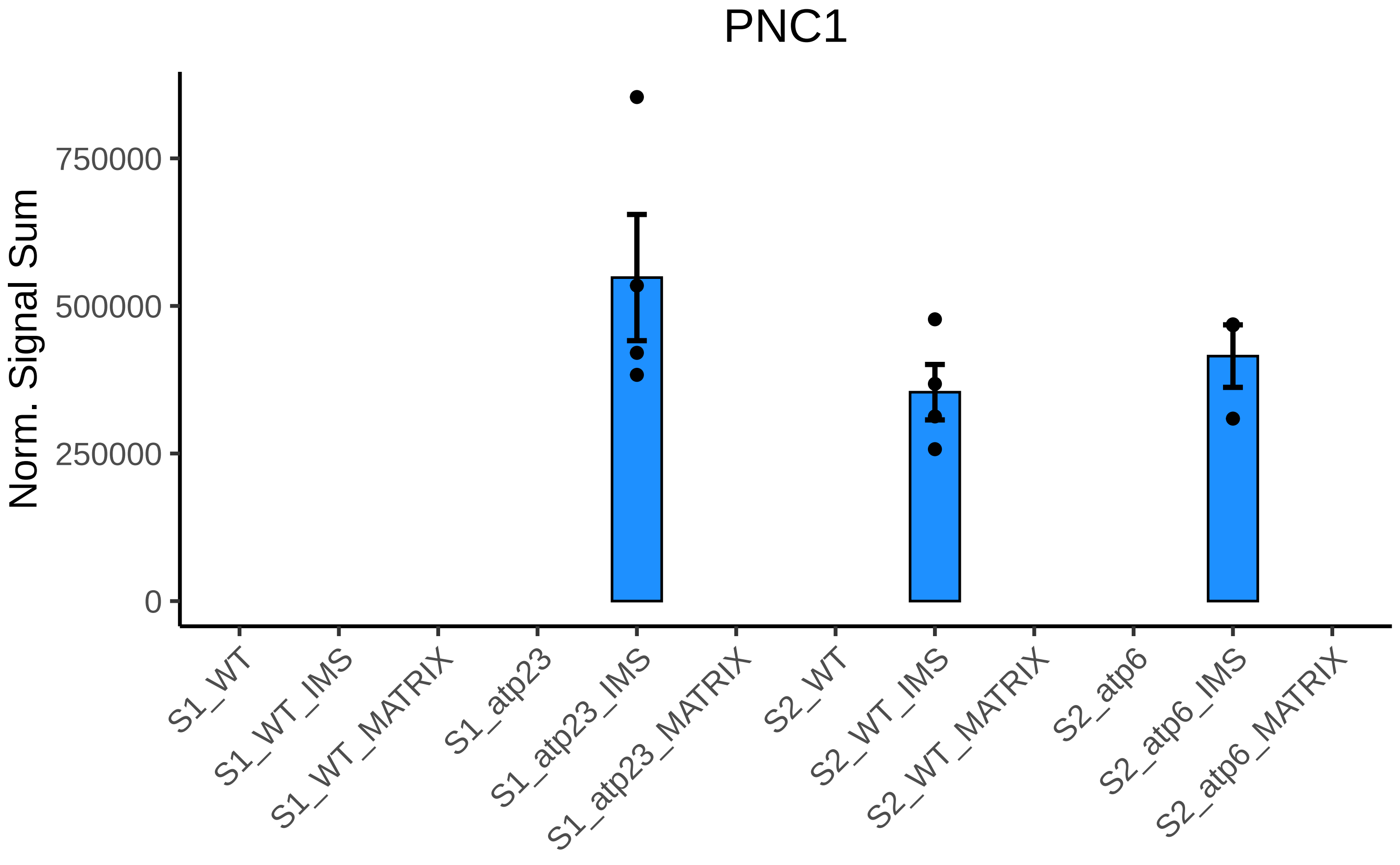

PNC1
Norm. Signal Sum
750000
500000
250000
0
S1_WT
S1_WT_IMS
S1_WT_MATRIX
S1_atp23
S1_atp23_IMS
S1_atp23_MATRIX
S2_WT
S2_WT_IMS
S2_WT_MATRIX
S2_atp6
S2_atp6_IMS
S2_atp6_MATRIX

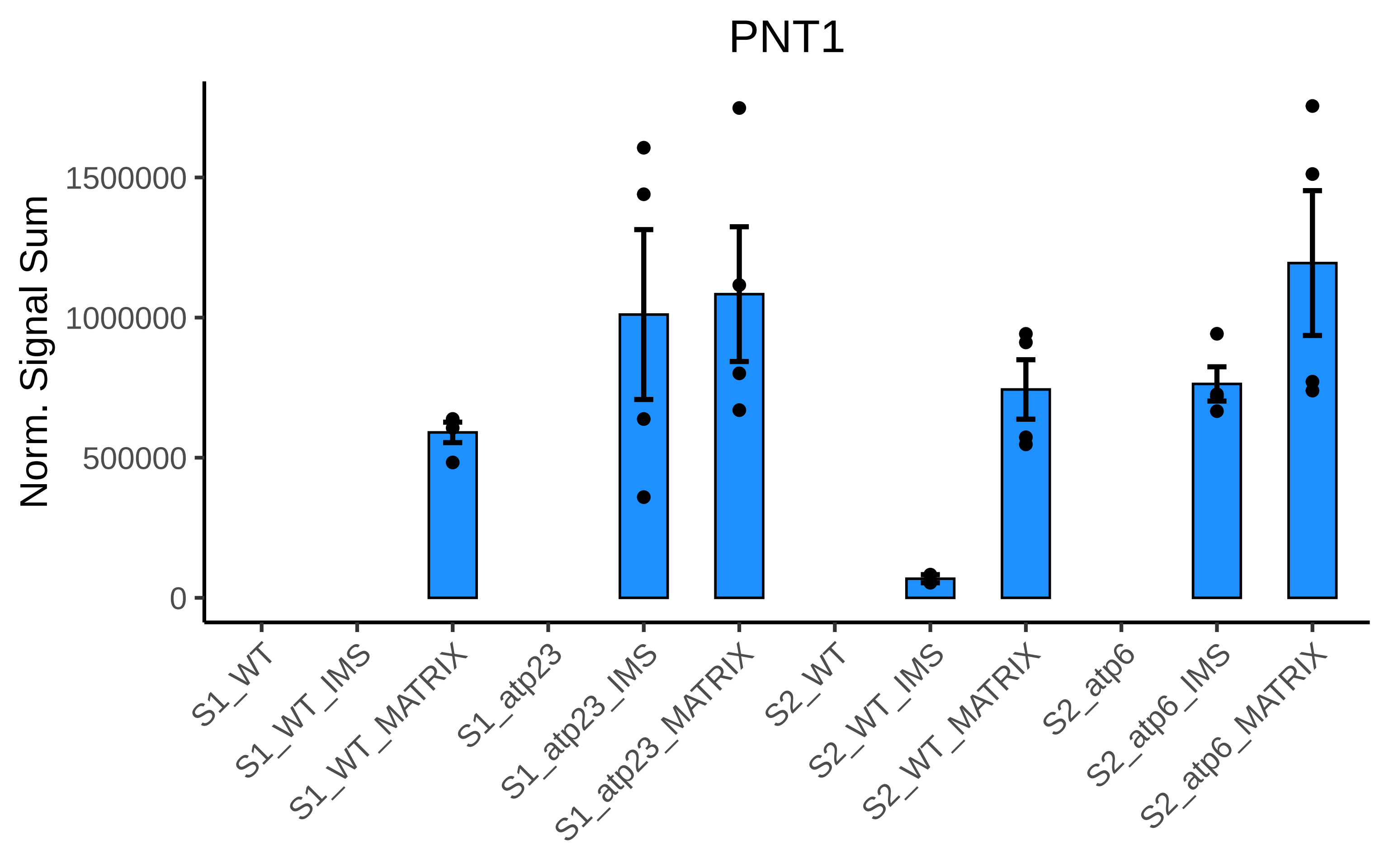

PNT1
Norm. Signal Sum
1500000
1000000
500000
0
S1_WT
S1_WT_IMS
S1_WT_MATRIX
S1_atp23
S1_atp23_IMS
S1_atp23_MATRIX
S2_WT
S2_WT_IMS
S2_WT_MATRIX
S2_atp6
S2_atp6_IMS
S2_atp6_MATRIX

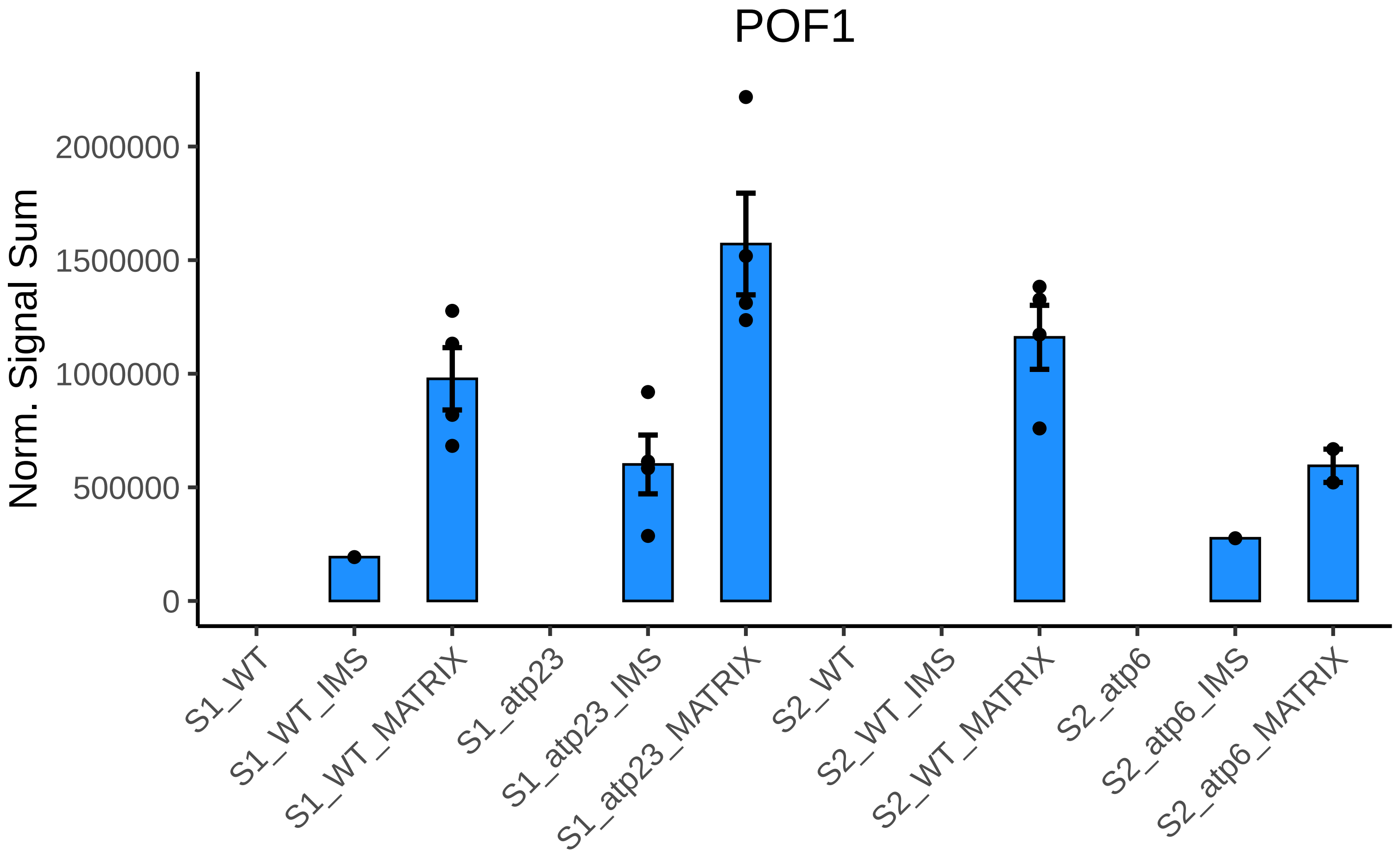

POF1
Norm. Signal Sum
0
500000
1000000
1500000
2000000
S1_WT
S1_WT_IMS
S1_WT_MATRIX
S1_atp23
S1_atp23_IMS
S1_atp23_MATRIX
S2_WT
S2_WT_IMS
S2_WT_MATRIX
S2_atp6
S2_atp6_IMS
S2_atp6_MATRIX

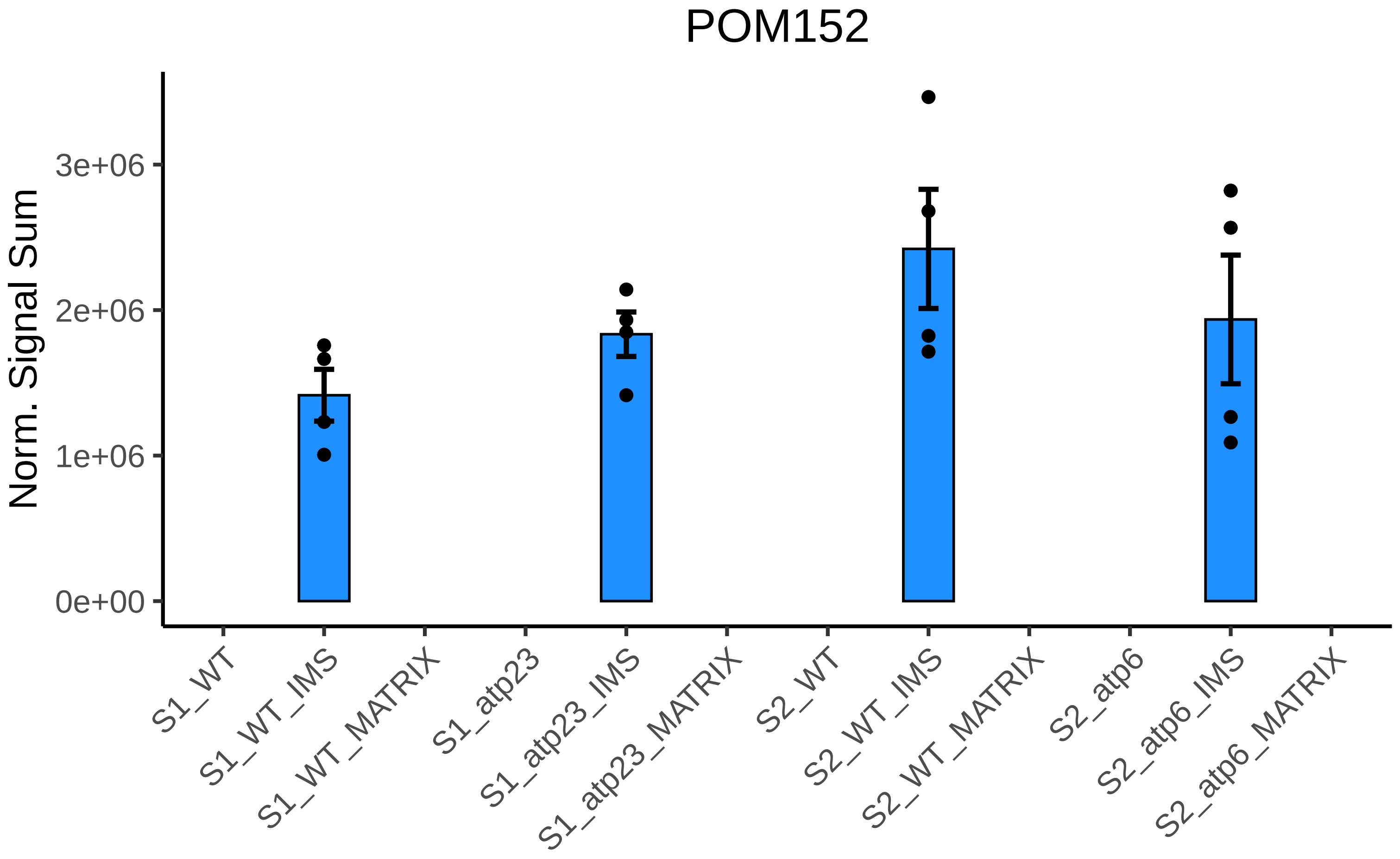
POM152
Norm. Signal Sum
3e+06
2e+06
1e+06
0e+00
S1_WT
S1_WT_IMS
S1_WT_MATRIX
S1_atp23
S1_atp23_IMS
S1_atp23_MATRIX
S2_WT
S2_WT_IMS
S2_WT_MATRIX
S2_atp6
S2_atp6_IMS
S2_atp6_MATRIX

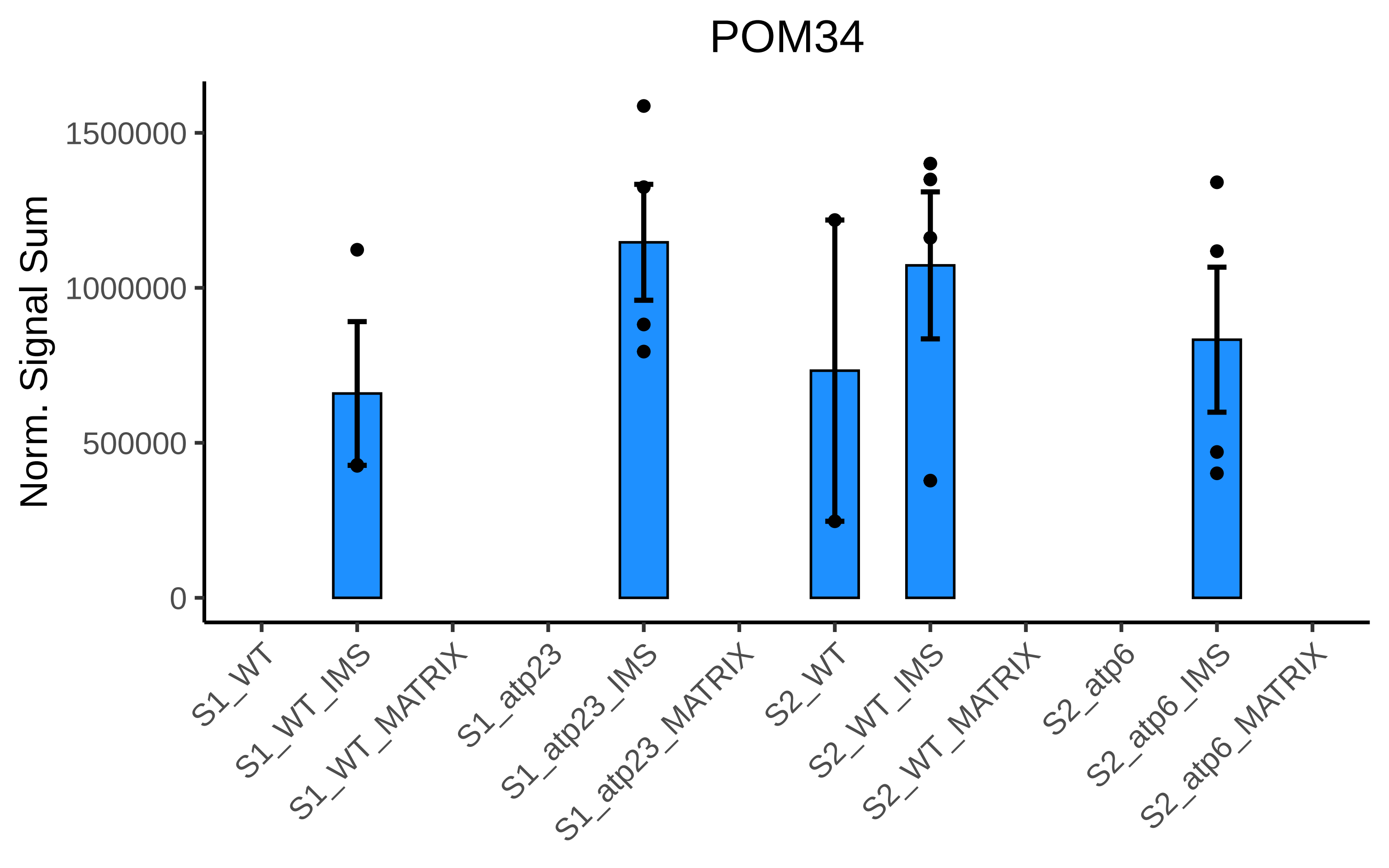

POM34
Norm. Signal Sum
1500000
1000000
500000
0
S1_WT
S1_WT_IMS
S1_WT_MATRIX
S1_atp23
S1_atp23_IMS
S1_atp23_MATRIX
S2_WT
S2_WT_IMS
S2_WT_MATRIX
S2_atp6
S2_atp6_IMS
S2_atp6_MATRIX

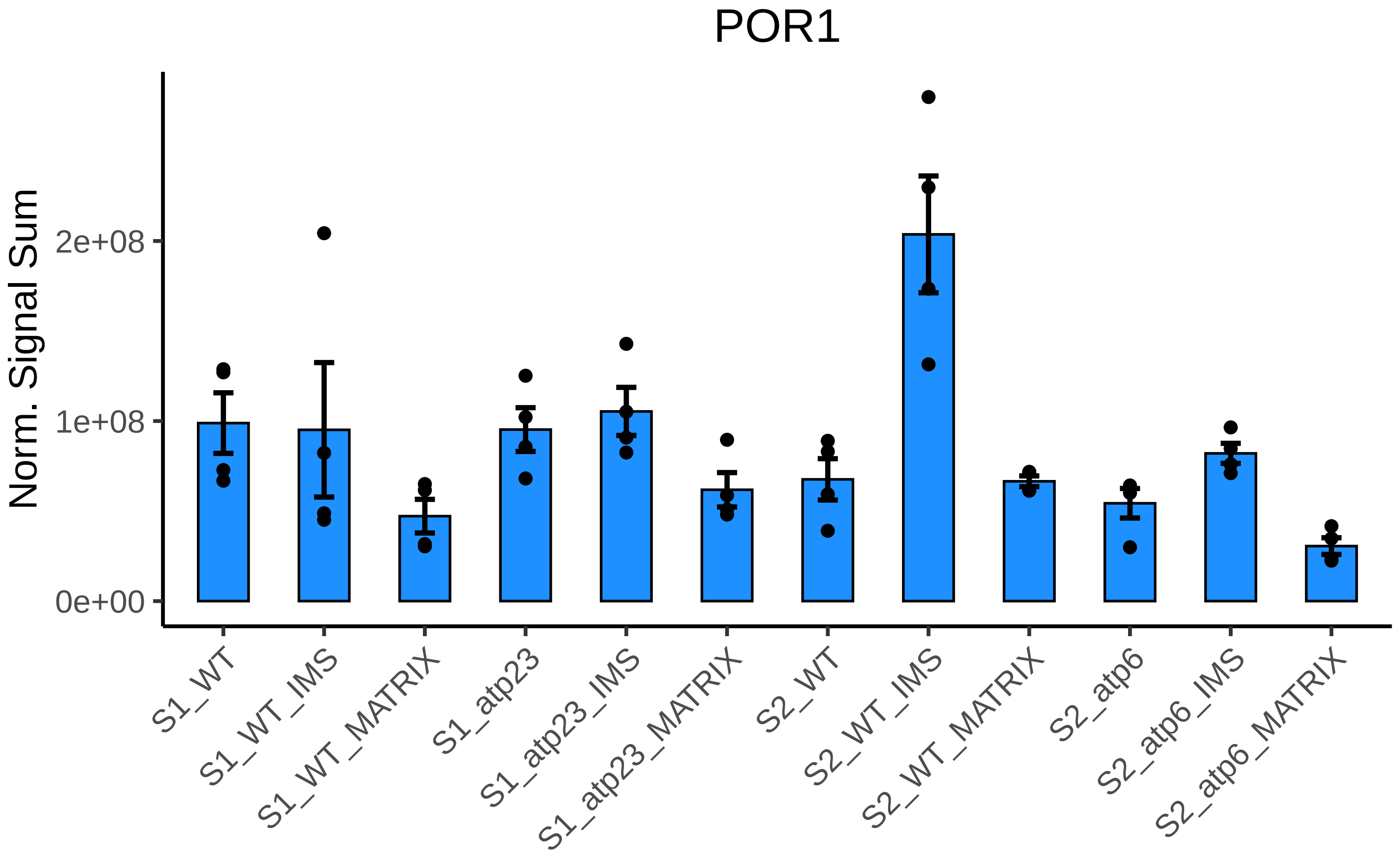
POR1
Norm. Signal Sum
2e+08
1e+08
0e+00
S1_WT
S1_WT_IMS
S1_WT_MATRIX
S1_atp23
S1_atp23_IMS
S1_atp23_MATRIX
S2_WT
S2_WT_IMS
S2_WT_MATRIX
S2_atp6
S2_atp6_IMS
S2_atp6_MATRIX

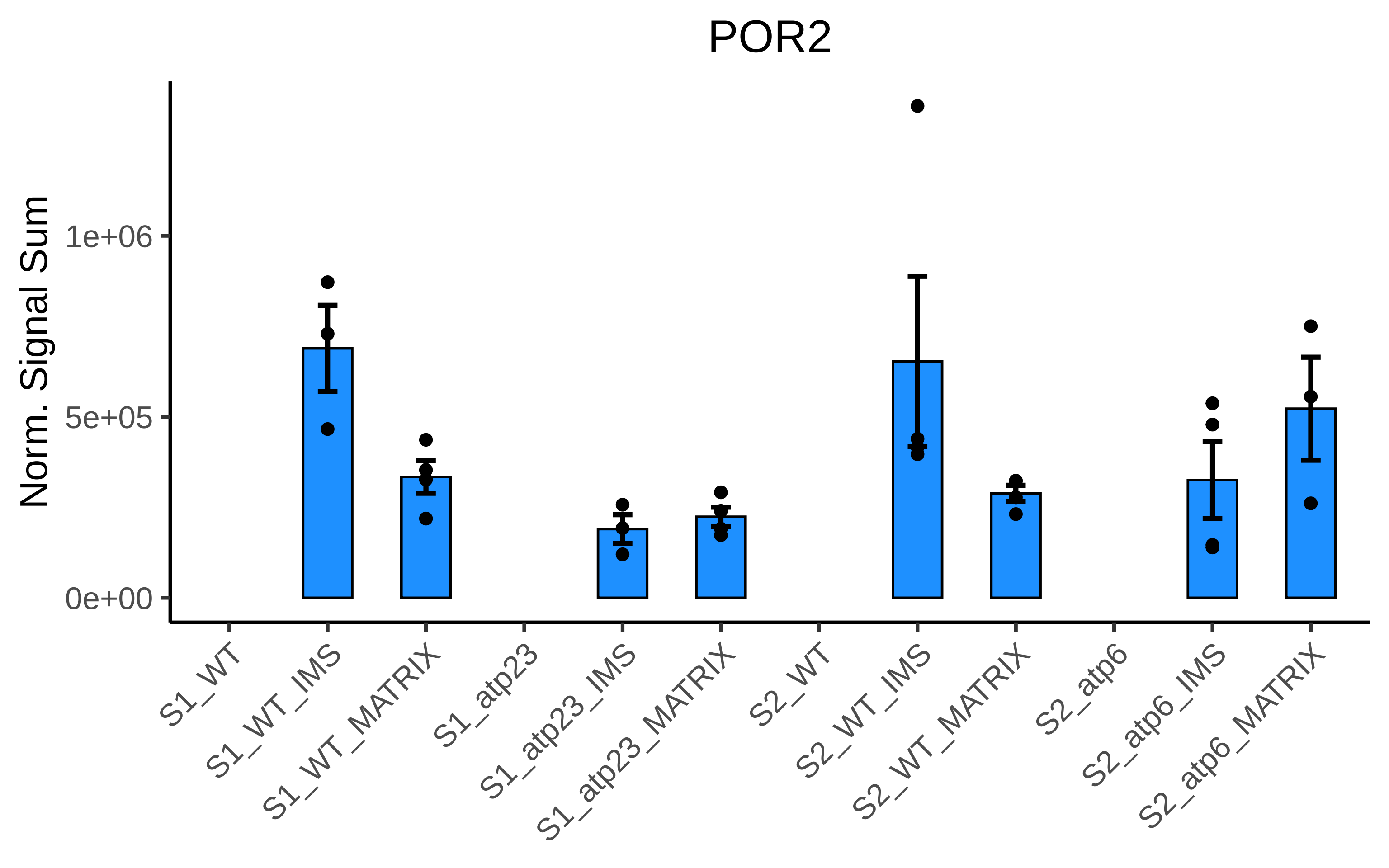
POR2
Norm. Signal Sum
1e+06
5e+05
0e+00
S1_WT
S1_WT_IMS
S1_WT_MATRIX
S1_atp23
S1_atp23_IMS
S1_atp23_MATRIX
S2_WT
S2_WT_IMS
S2_WT_MATRIX
S2_atp6
S2_atp6_IMS
S2_atp6_MATRIX

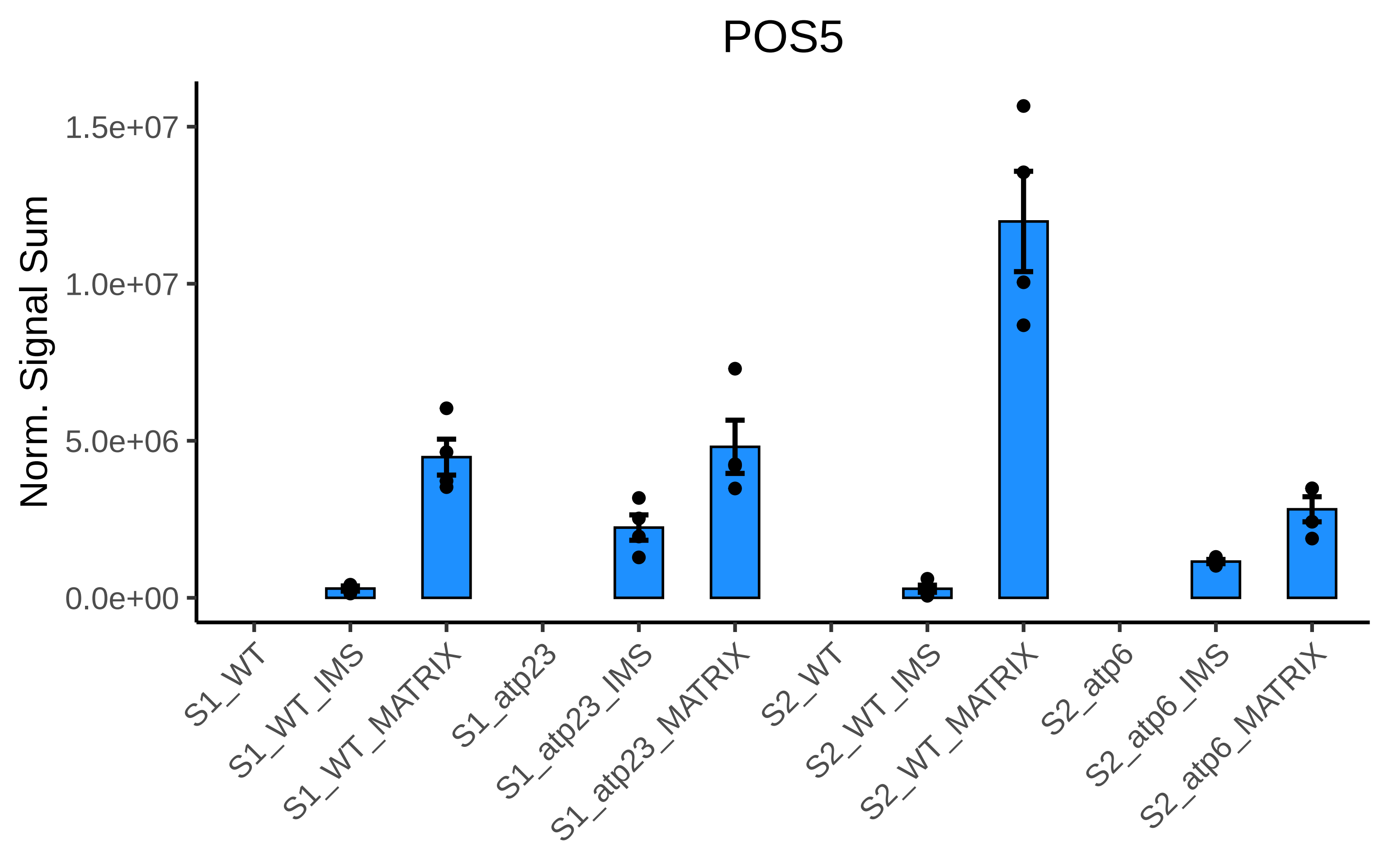

POS5
Norm. Signal Sum
1.5e+07
1.0e+07
5.0e+06
0.0e+00
S1_WT
S1_WT_IMS
S1_WT_MATRIX
S1_atp23
S1_atp23_IMS
S1_atp23_MATRIX
S2_WT
S2_WT_IMS
S2_WT_MATRIX
S2_atp6
S2_atp6_IMS
S2_atp6_MATRIX

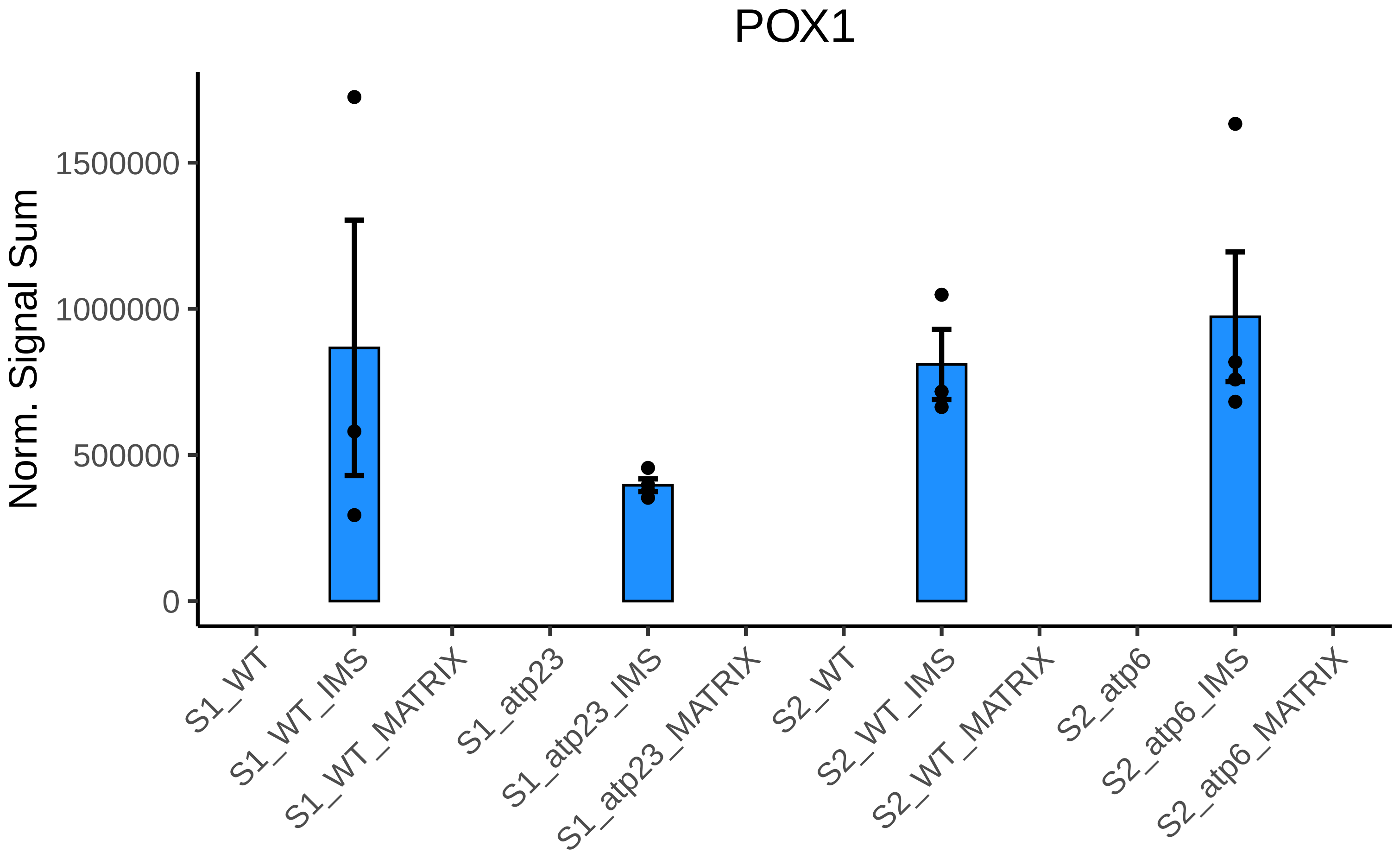
POX1
Norm. Signal Sum
1500000
1000000
500000
0
S1_WT
S1_WT_IMS
S1_WT_MATRIX
S1_atp23
S1_atp23_IMS
S1_atp23_MATRIX
S2_WT
S2_WT_IMS
S2_WT_MATRIX
S2_atp6
S2_atp6_IMS
S2_atp6_MATRIX

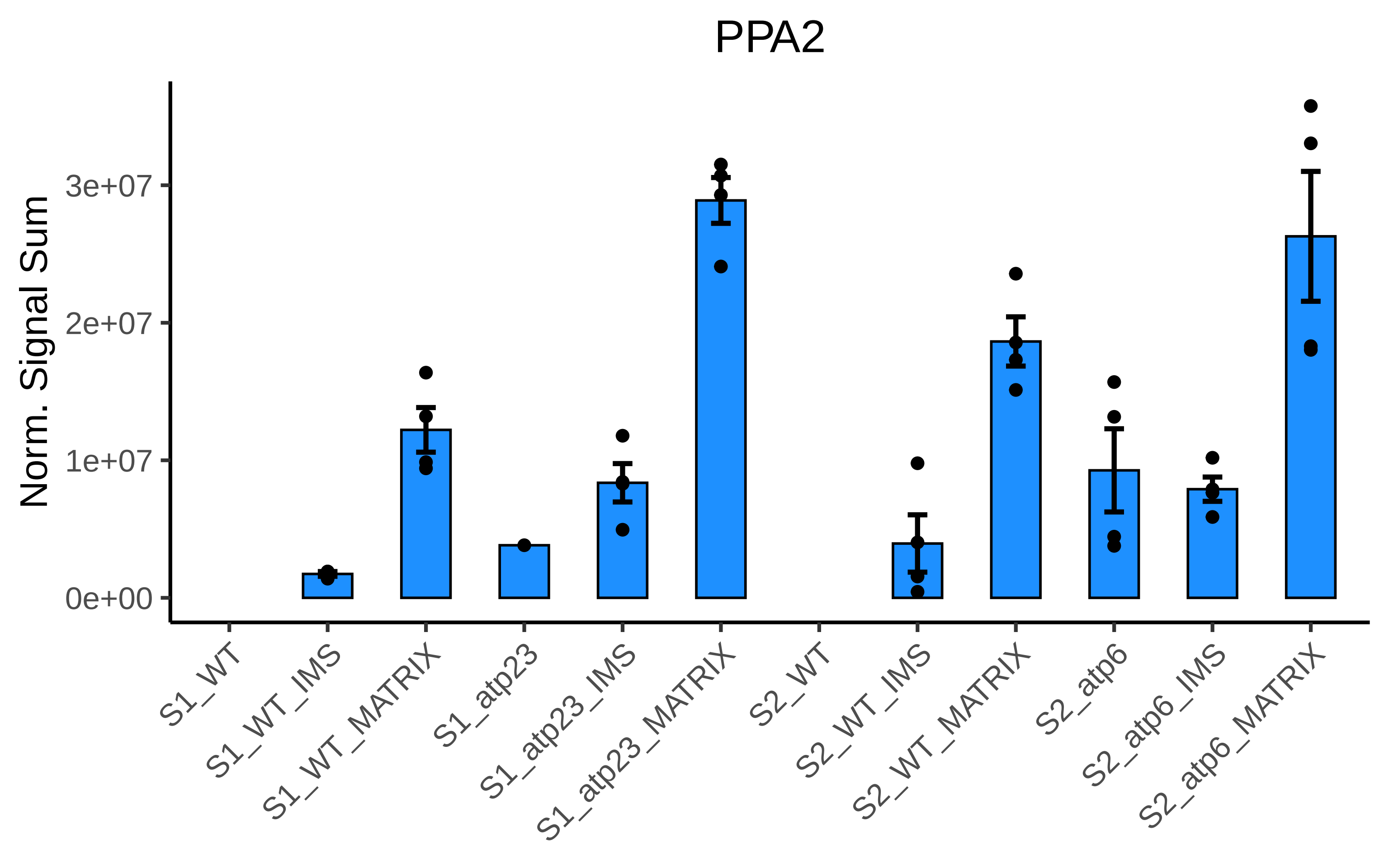
PPA2
Norm. Signal Sum
0e+00
1e+07
2e+07
3e+07
S1_WT
S1_WT_IMS
S1_WT_MATRIX
S1_atp23
S1_atp23_IMS
S1_atp23_MATRIX
S2_WT
S2_WT_IMS
S2_WT_MATRIX
S2_atp6
S2_atp6_IMS
S2_atp6_MATRIX

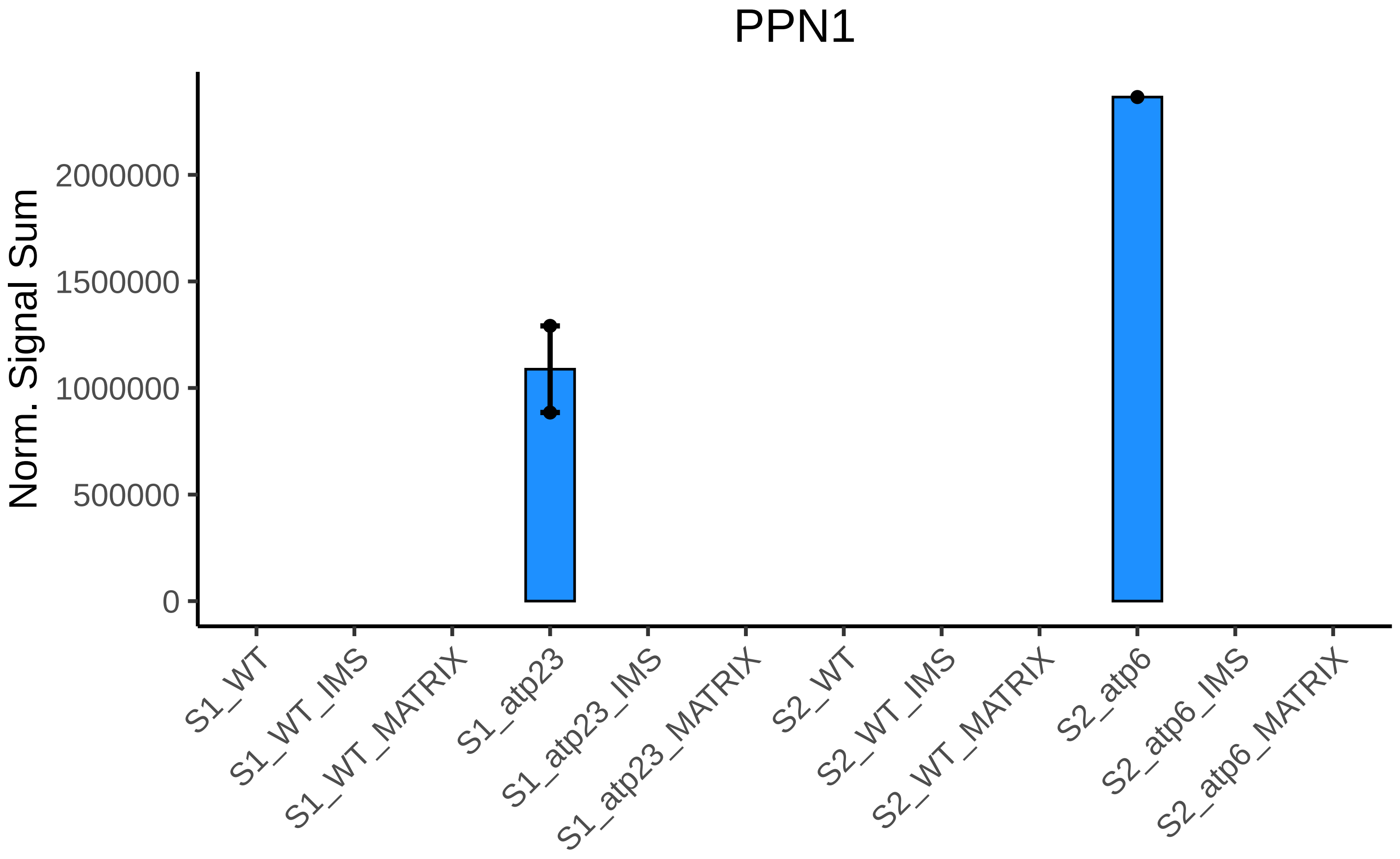
PPN1
Norm. Signal Sum
2000000
1500000
1000000
500000
0
S1_WT
S1_WT_IMS
S1_WT_MATRIX
S1_atp23
S1_atp23_IMS
S1_atp23_MATRIX
S2_WT
S2_WT_IMS
S2_WT_MATRIX
S2_atp6
S2_atp6_IMS
S2_atp6_MATRIX

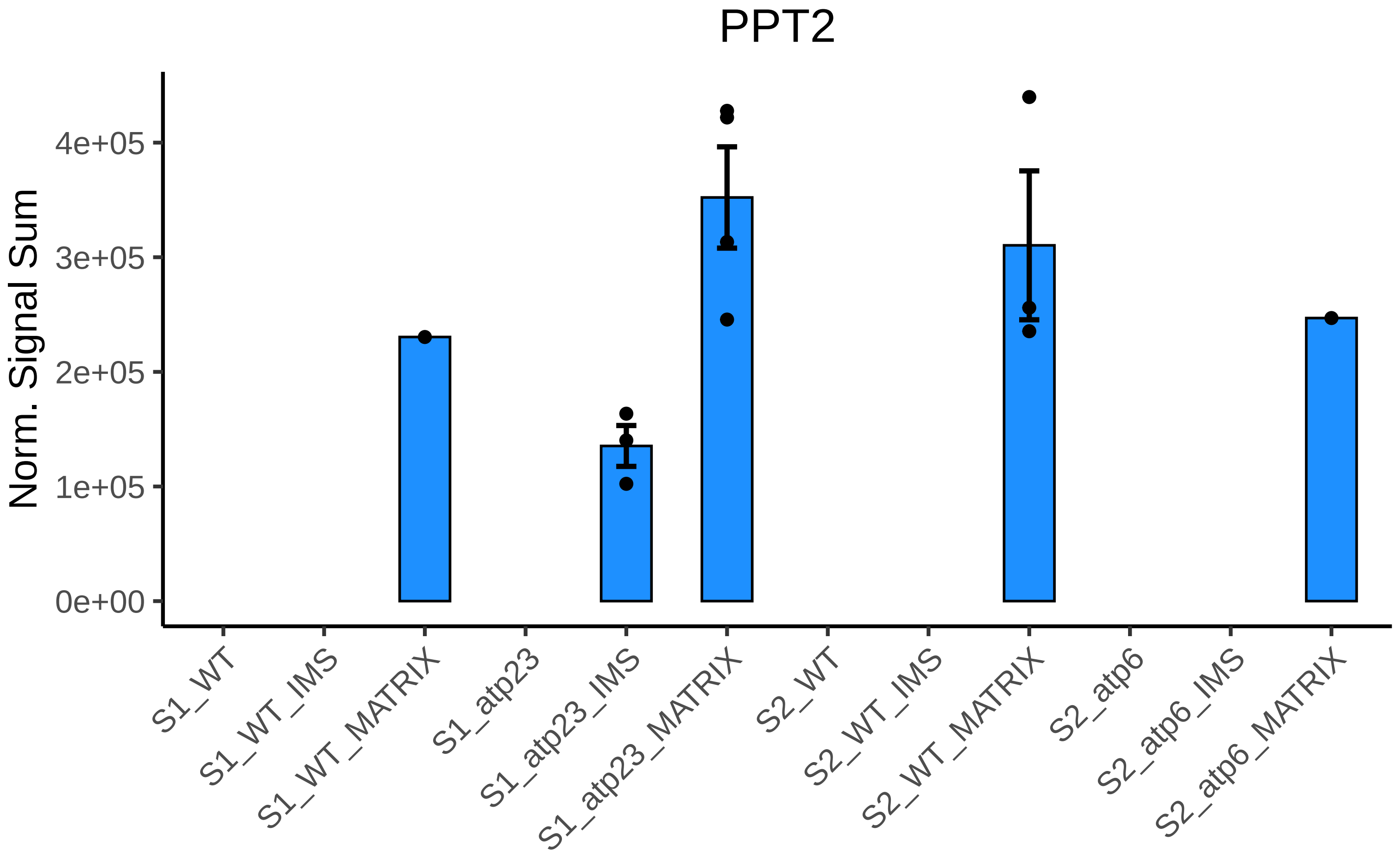

PPT2
Norm. Signal Sum
4e+05
3e+05
2e+05
1e+05
0e+00
S1_WT
S1_WT_IMS
S1_WT_MATRIX
S1_atp23
S1_atp23_IMS
S1_atp23_MATRIX
S2_WT
S2_WT_IMS
S2_WT_MATRIX
S2_atp6
S2_atp6_IMS
S2_atp6_MATRIX

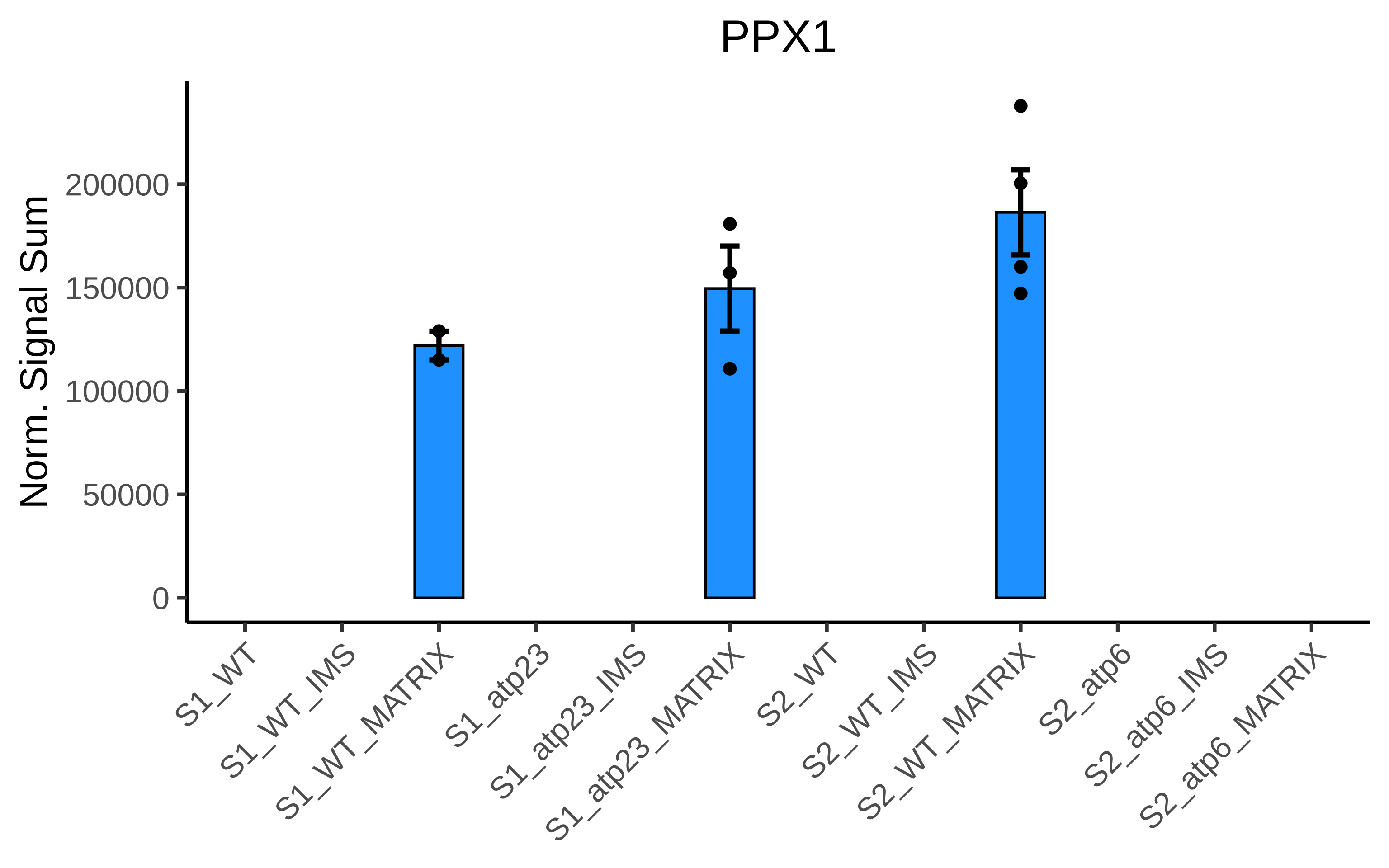
PPX1
Norm. Signal Sum
0
50000
100000
150000
200000
S1_WT
S1_WT_IMS
S1_WT_MATRIX
S1_atp23
S1_atp23_IMS
S1_atp23_MATRIX
S2_WT
S2_WT_IMS
S2_WT_MATRIX
S2_atp6
S2_atp6_IMS
S2_atp6_MATRIX

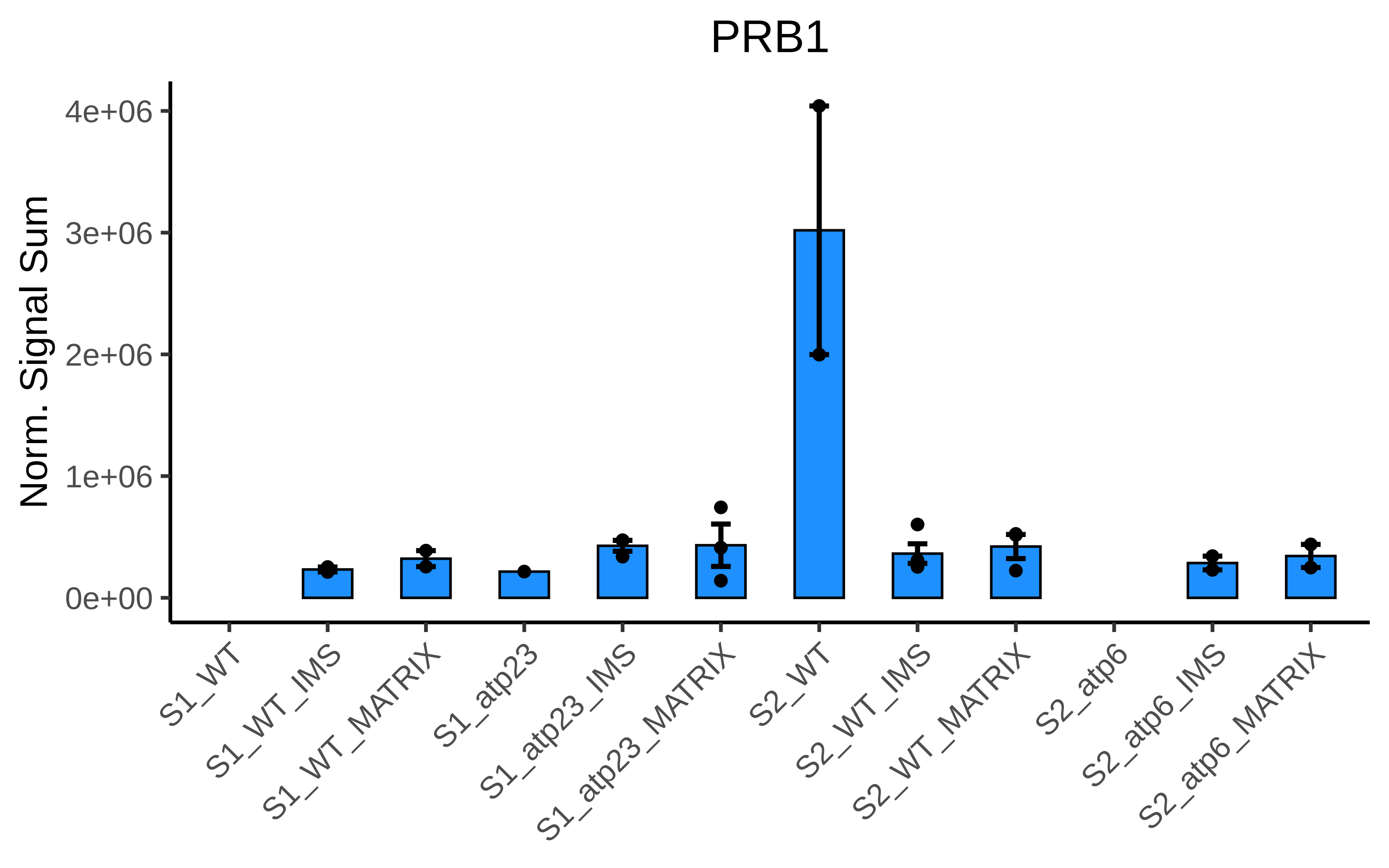
PRB1
Norm. Signal Sum
4e+06
3e+06
2e+06
1e+06
0e+00
S1_WT
S1_WT_IMS
S1_WT_MATRIX
S1_atp23
S1_atp23_IMS
S1_atp23_MATRIX
S2_WT
S2_WT_IMS
S2_WT_MATRIX
S2_atp6
S2_atp6_IMS
S2_atp6_MATRIX

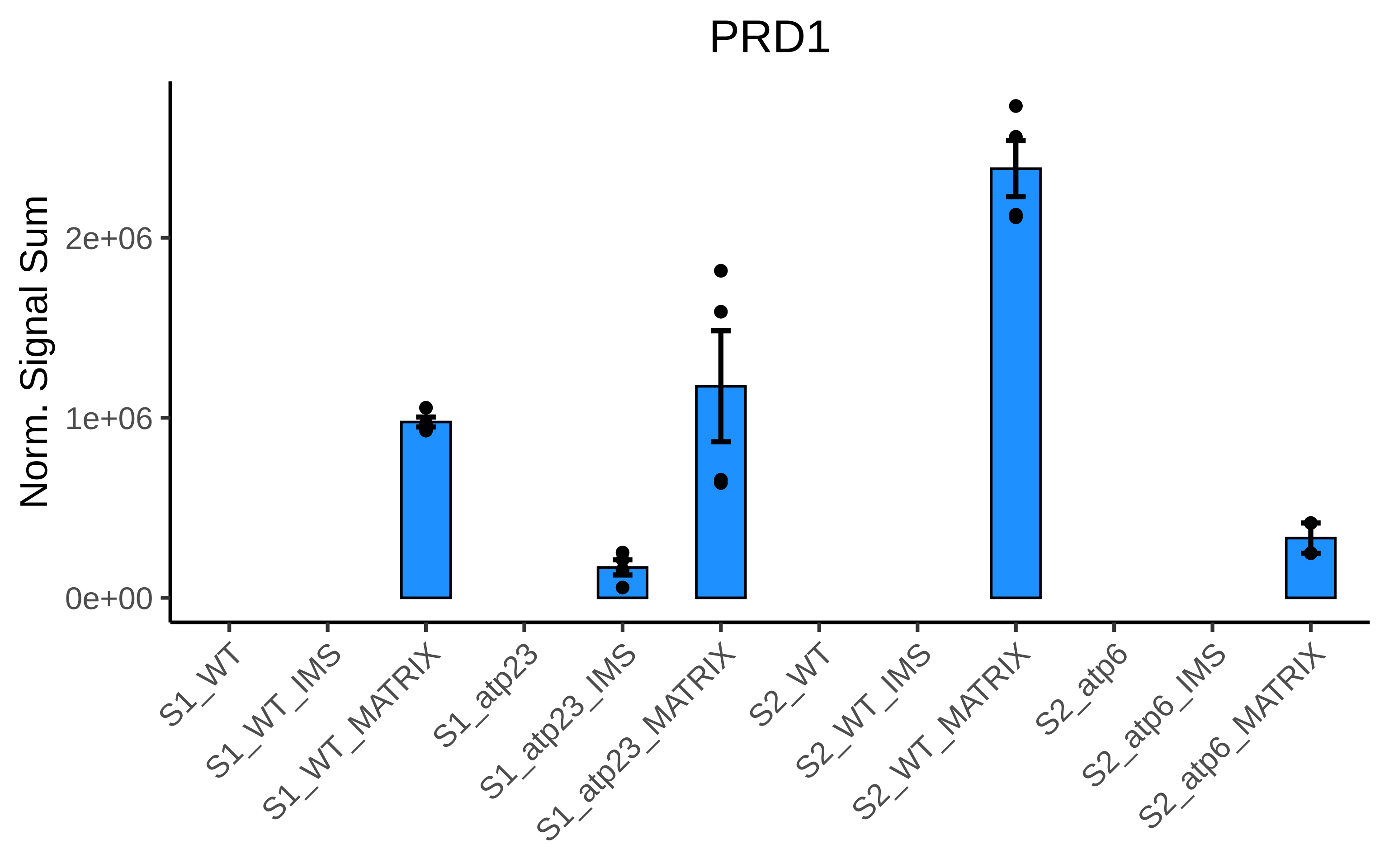
PRD1
Norm. Signal Sum
0e+00
1e+06
2e+06
S1_WT
S1_WT_IMS
S1_WT_MATRIX
S1_atp23
S1_atp23_IMS
S1_atp23_MATRIX
S2_WT
S2_WT_IMS
S2_WT_MATRIX
S2_atp6
S2_atp6_IMS
S2_atp6_MATRIX

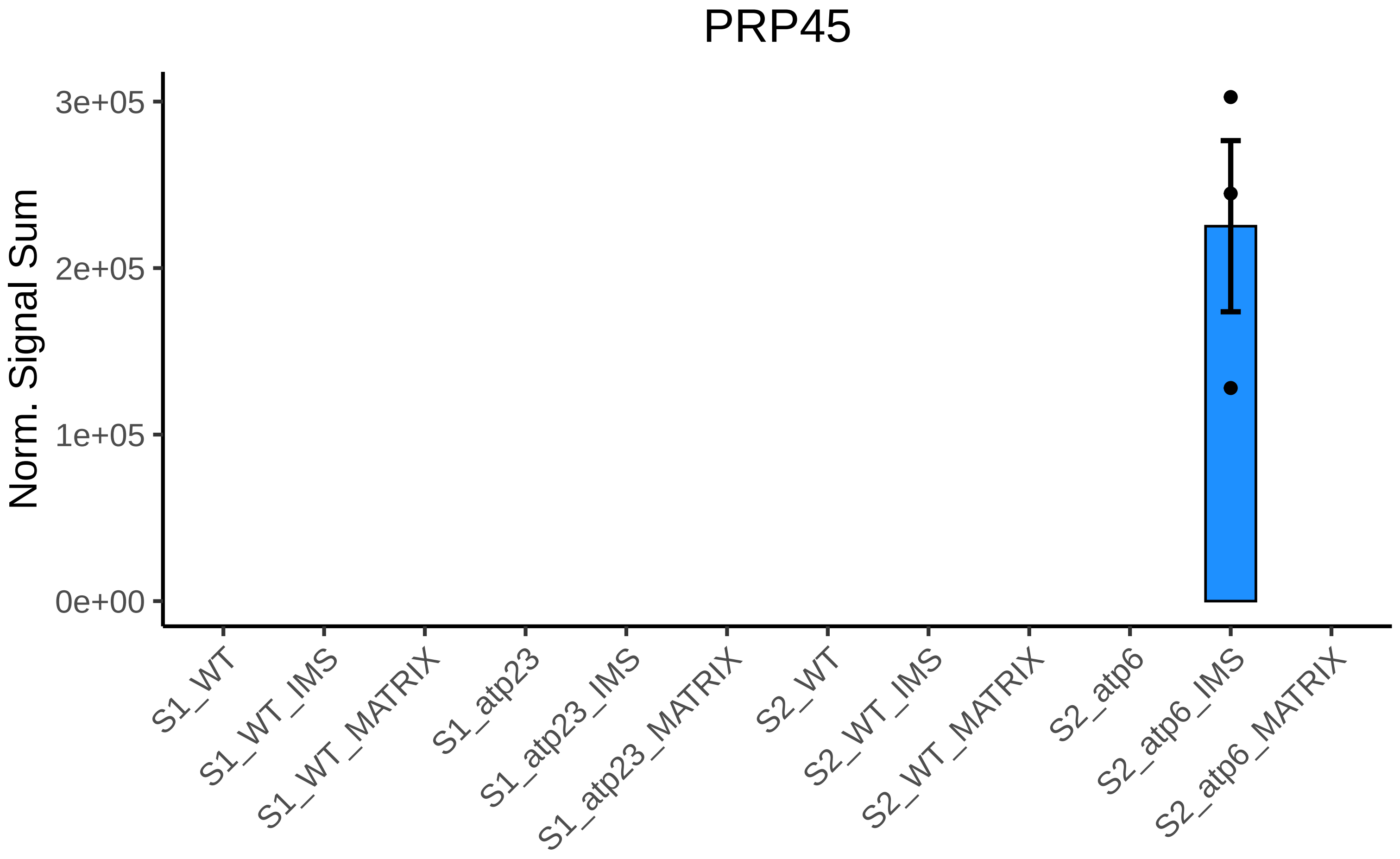
PRP45
Norm. Signal Sum
3e+05
2e+05
1e+05
0e+00
S1_WT
S1_WT_IMS
S1_WT_MATRIX
S1_atp23
S1_atp23_IMS
S1_atp23_MATRIX
S2_WT
S2_WT_IMS
S2_WT_MATRIX
S2_atp6
S2_atp6_IMS
S2_atp6_MATRIX

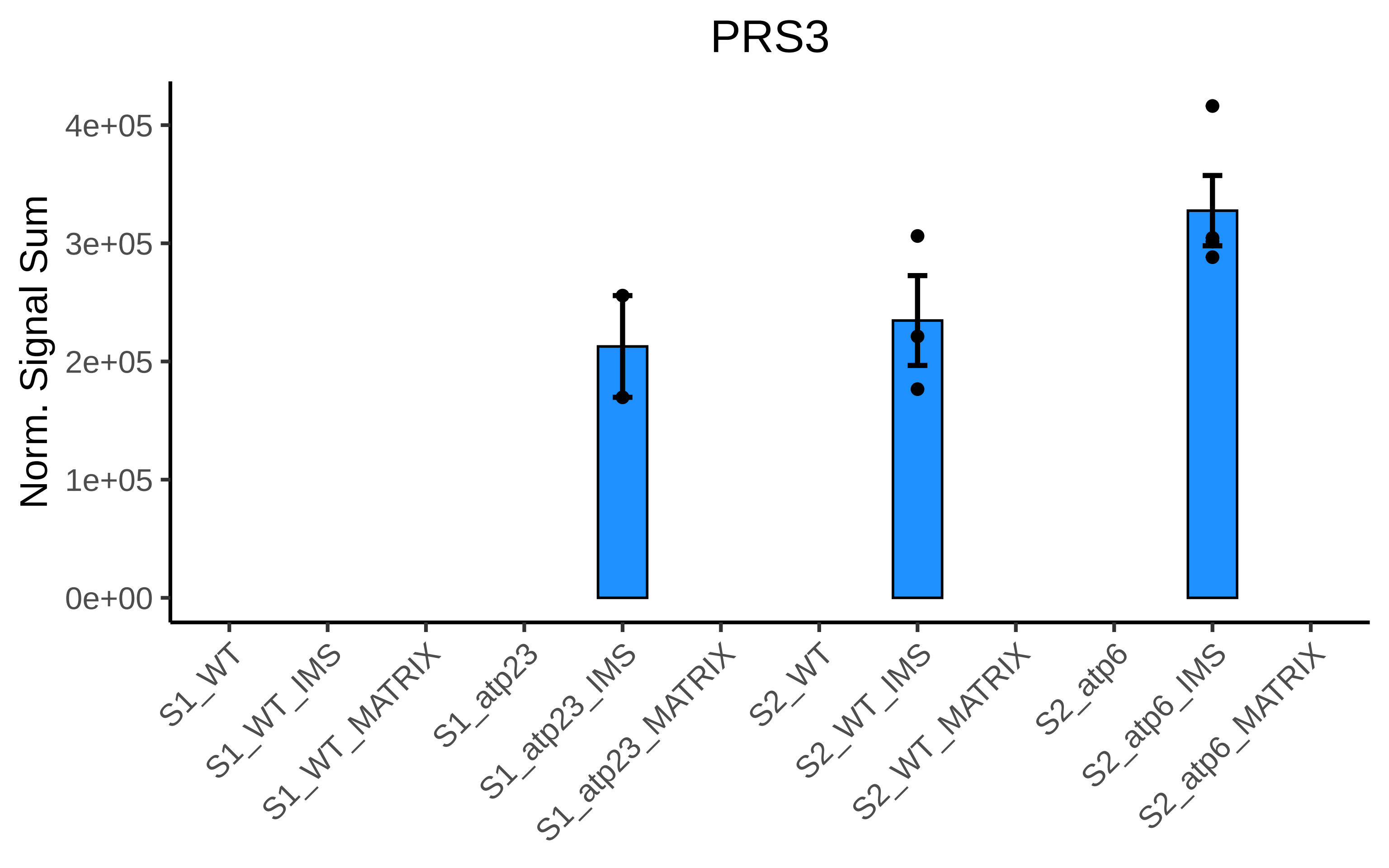
PRS3
Norm. Signal Sum
4e+05
3e+05
2e+05
1e+05
0e+00
S1_WT
S1_WT_IMS
S1_WT_MATRIX
S1_atp23
S1_atp23_IMS
S1_atp23_MATRIX
S2_WT
S2_WT_IMS
S2_WT_MATRIX
S2_atp6
S2_atp6_IMS
S2_atp6_MATRIX

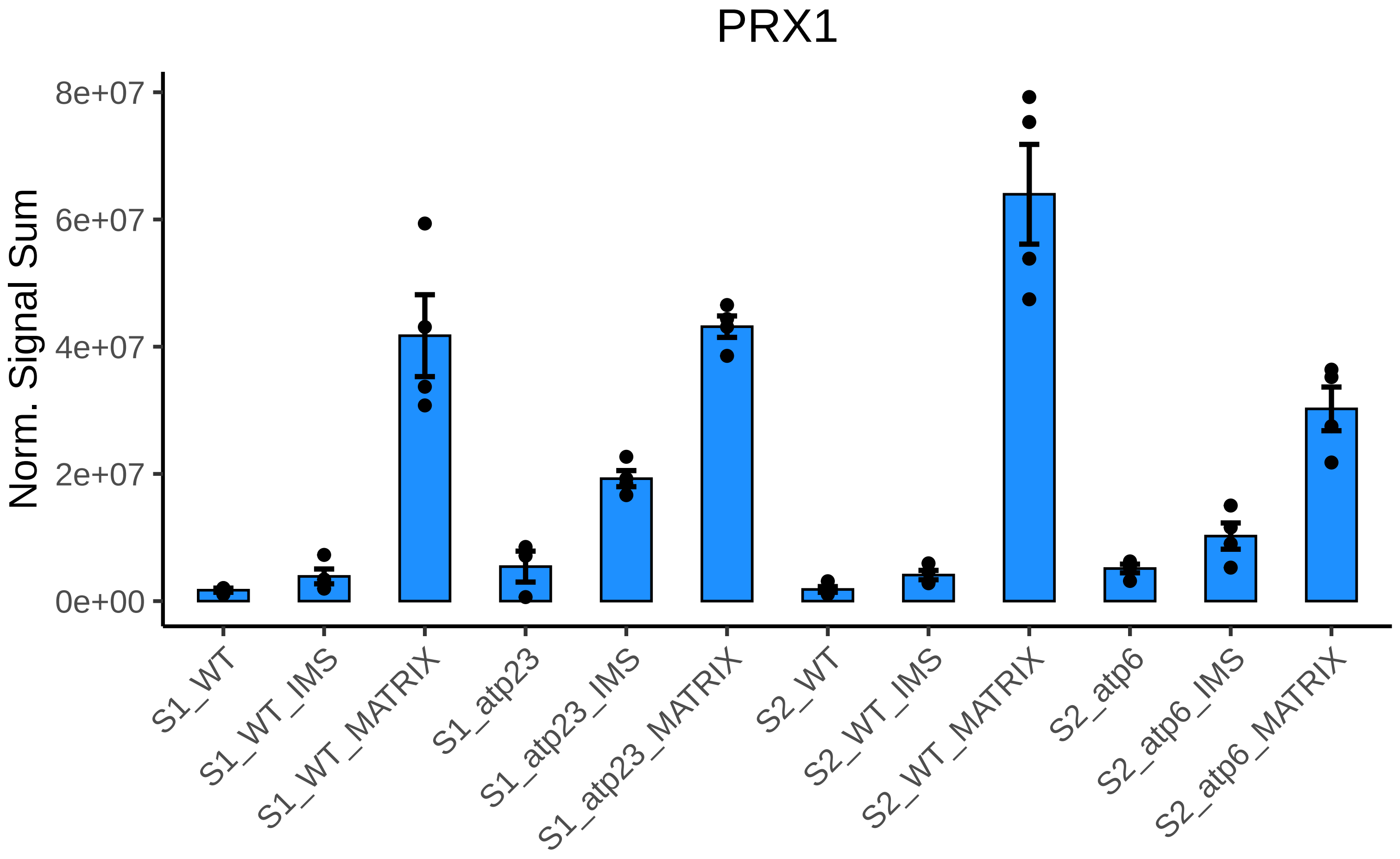
PRX1
Norm. Signal Sum
8e+07
6e+07
4e+07
2e+07
0e+00
S1_WT
S1_WT_IMS
S1_WT_MATRIX
S1_atp23
S1_atp23_IMS
S1_atp23_MATRIX
S2_WT
S2_WT_IMS
S2_WT_MATRIX
S2_atp6
S2_atp6_IMS
S2_atp6_MATRIX

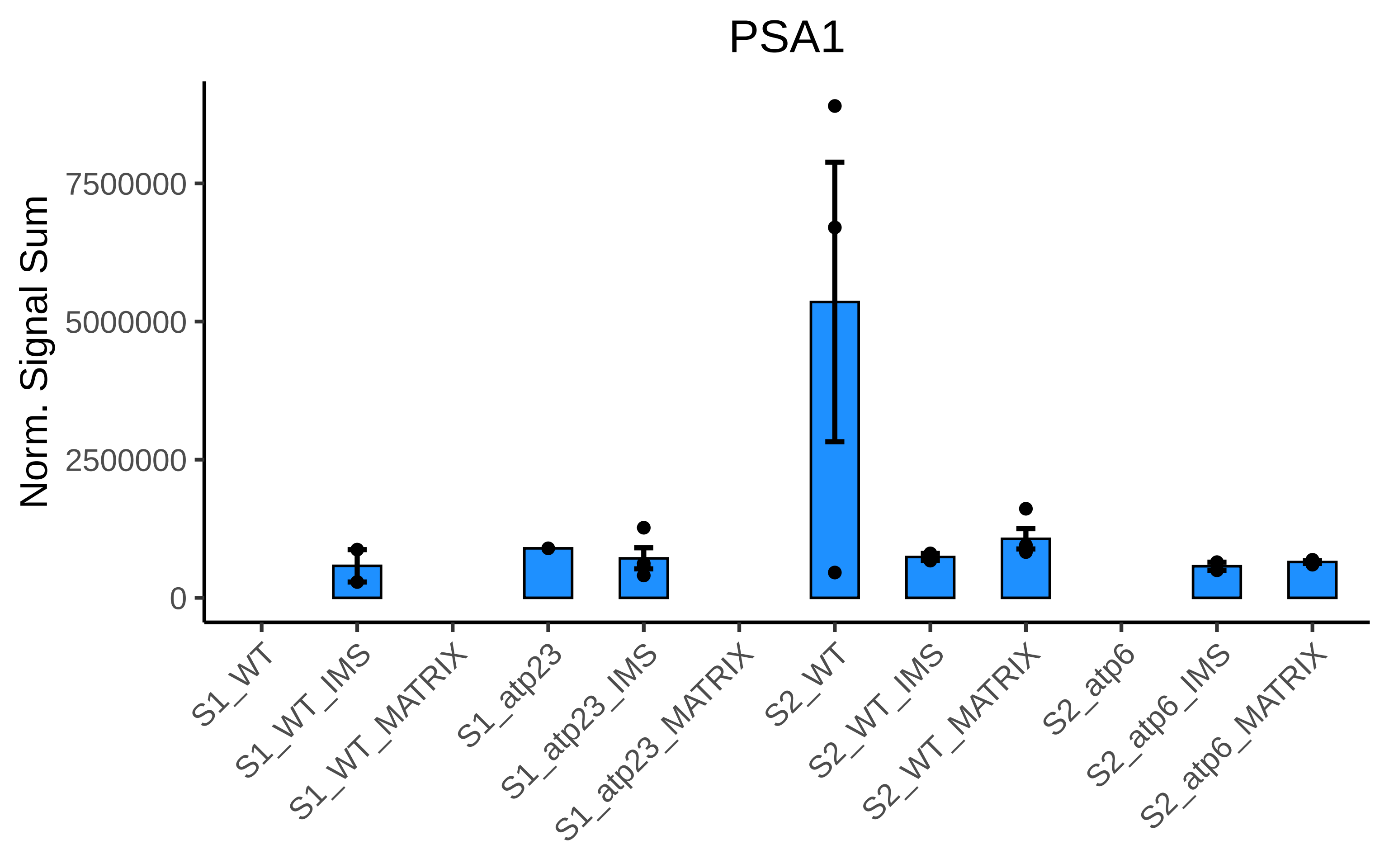
PSA1
Norm. Signal Sum
7500000
5000000
2500000
0
S1_WT
S1_WT_IMS
S1_WT_MATRIX
S1_atp23
S1_atp23_IMS
S1_atp23_MATRIX
S2_WT
S2_WT_IMS
S2_WT_MATRIX
S2_atp6
S2_atp6_IMS
S2_atp6_MATRIX

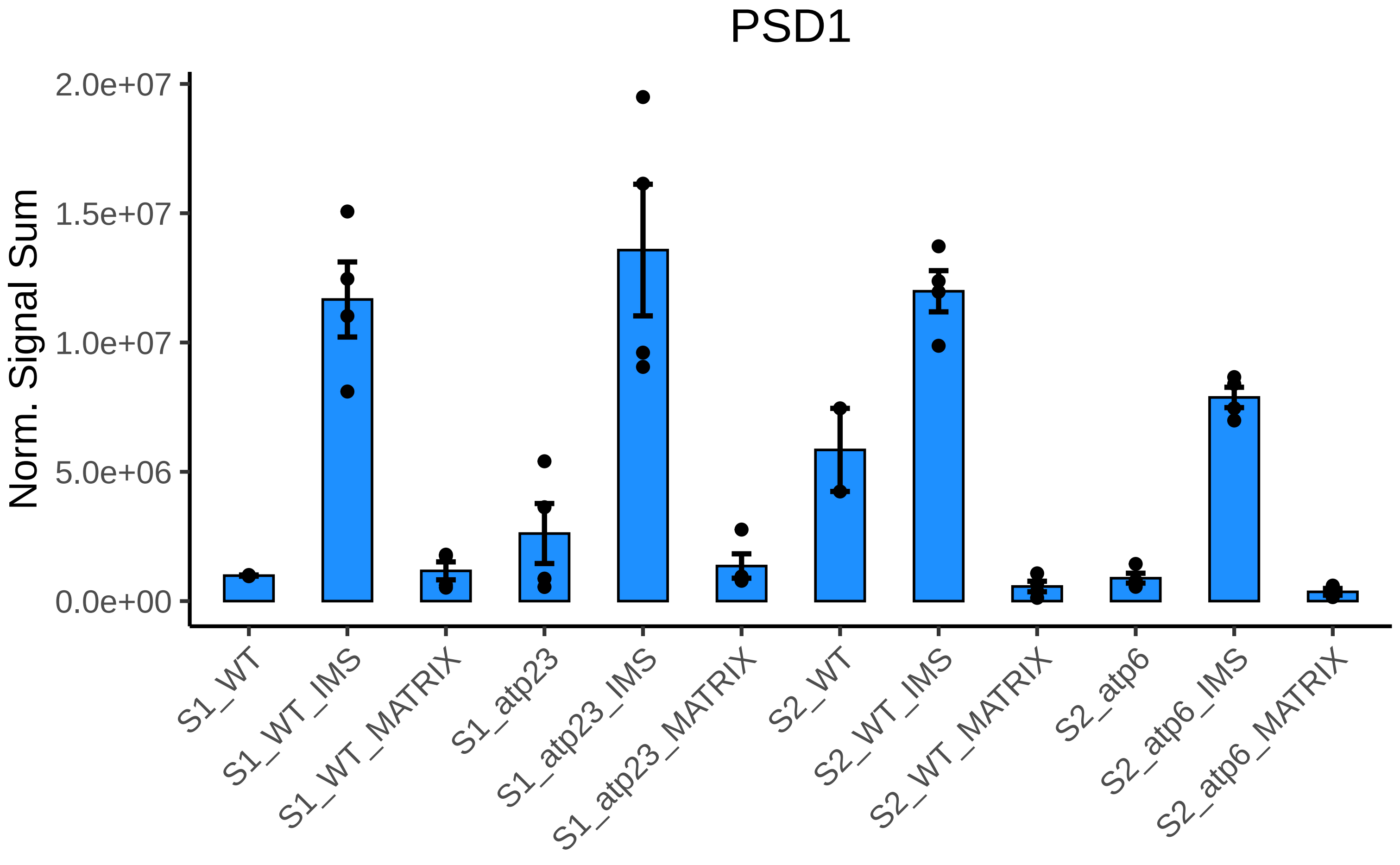

PSD1
Norm. Signal Sum
2.0e+07
1.5e+07
1.0e+07
5.0e+06
0.0e+00
S1_WT
S1_WT_IMS
S1_WT_MATRIX
S1_atp23
S1_atp23_IMS
S1_atp23_MATRIX
S2_WT
S2_WT_IMS
S2_WT_MATRIX
S2_atp6
S2_atp6_IMS
S2_atp6_MATRIX

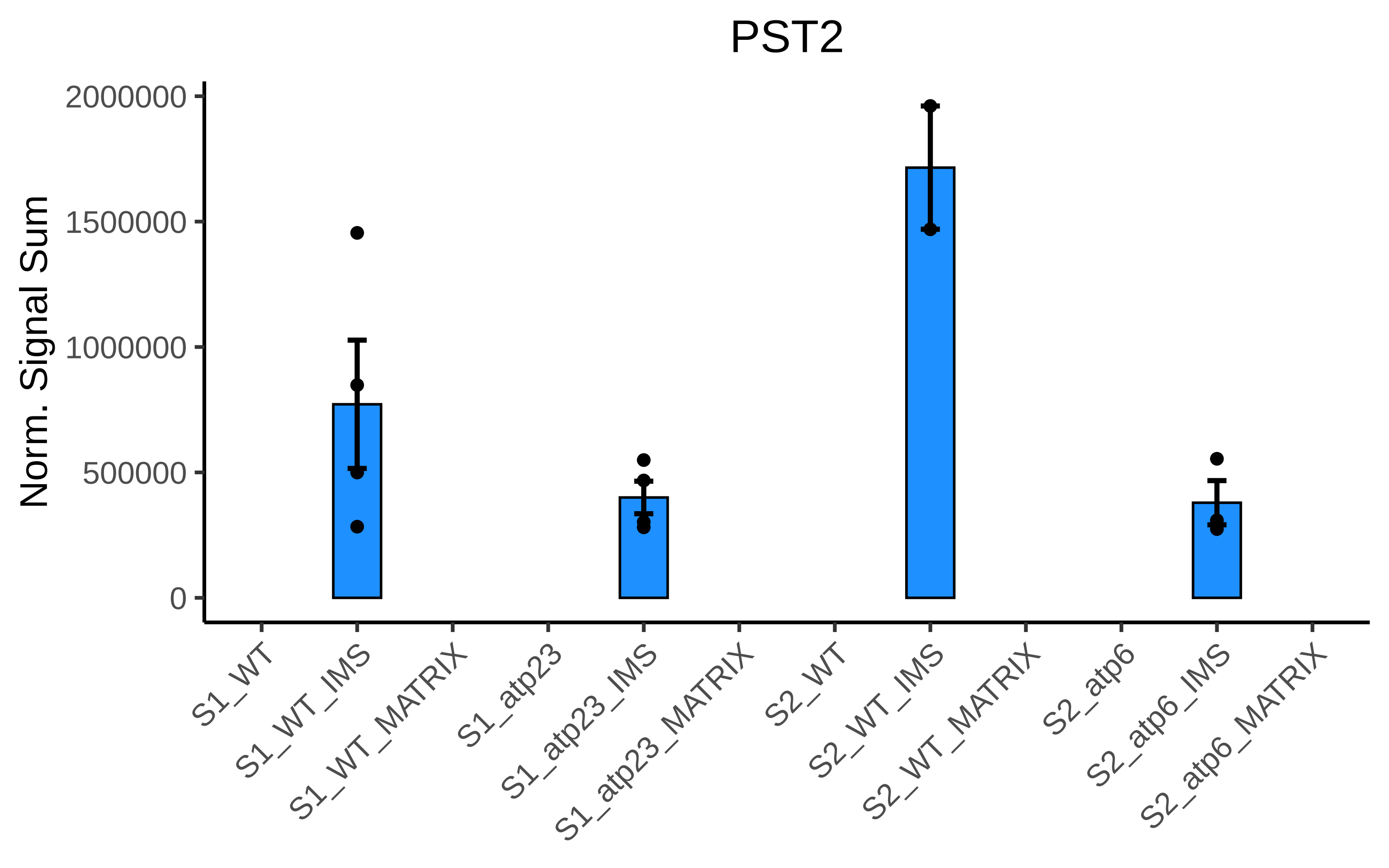

PST2
Norm. Signal Sum
2000000
1500000
1000000
500000
0
S1_WT
S1_WT_IMS
S1_WT_MATRIX
S1_atp23
S1_atp23_IMS
S1_atp23_MATRIX
S2_WT
S2_WT_IMS
S2_WT_MATRIX
S2_atp6
S2_atp6_IMS
S2_atp6_MATRIX

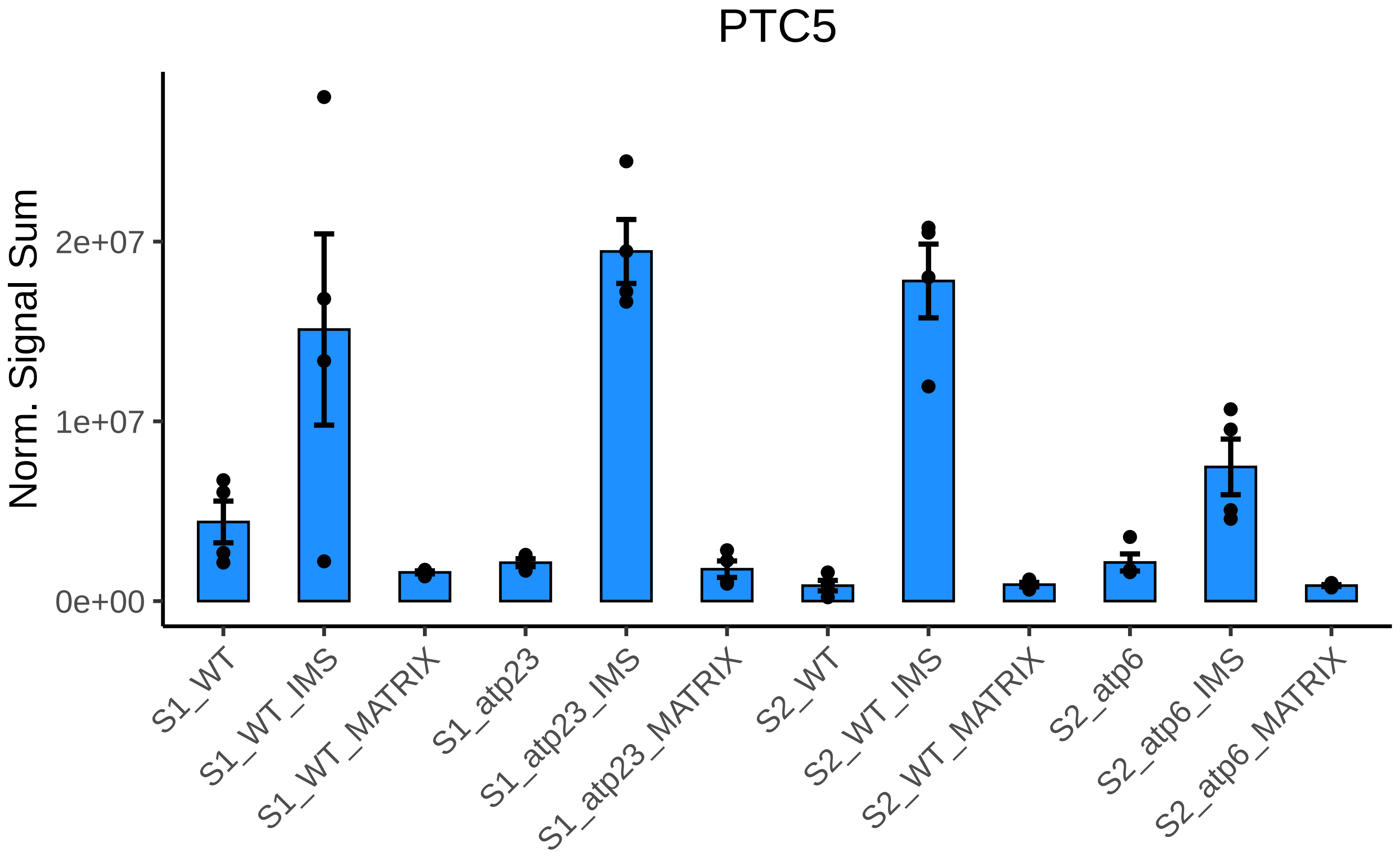
PTC5
Norm. Signal Sum
2e+07
1e+07
0e+00
S1_WT
S1_WT_IMS
S1_WT_MATRIX
S1_atp23
S1_atp23_IMS
S1_atp23_MATRIX
S2_WT
S2_WT_IMS
S2_WT_MATRIX
S2_atp6
S2_atp6_IMS
S2_atp6_MATRIX

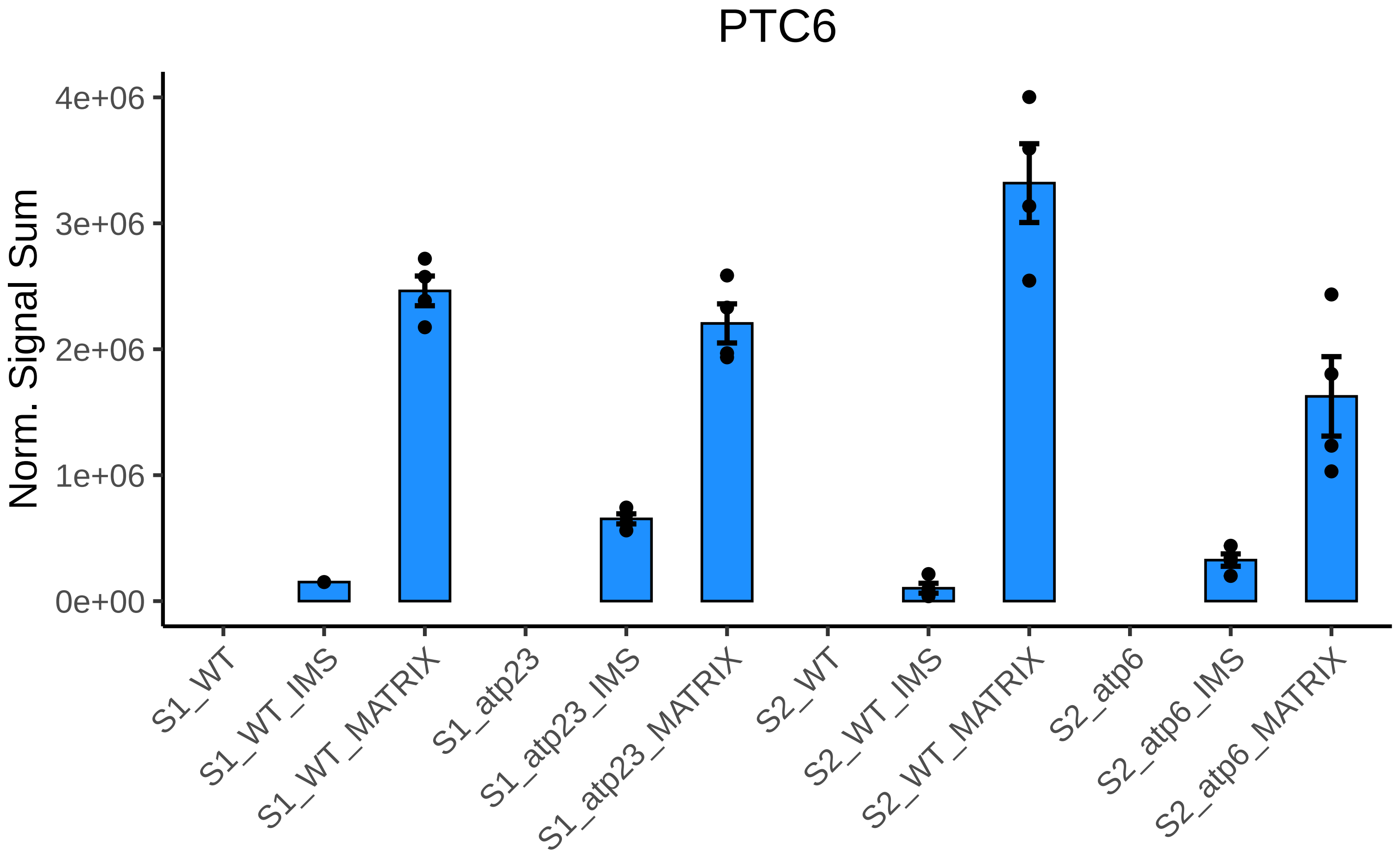
PTC6
Norm. Signal Sum
4e+06
3e+06
2e+06
1e+06
0e+00
S1_WT
S1_WT_IMS
S1_WT_MATRIX
S1_atp23
S1_atp23_IMS
S1_atp23_MATRIX
S2_WT
S2_WT_IMS
S2_WT_MATRIX
S2_atp6
S2_atp6_IMS
S2_atp6_MATRIX

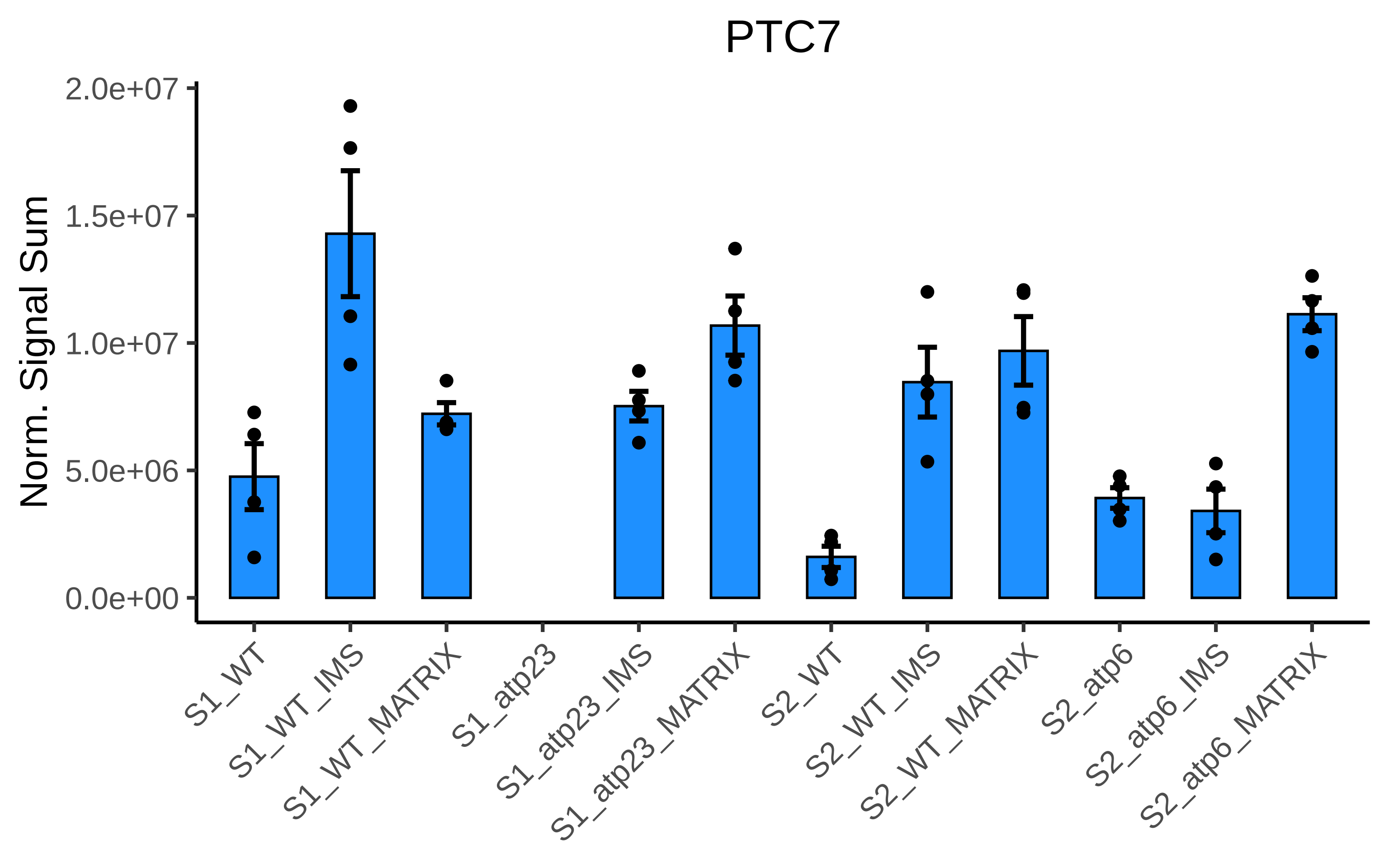

PTC7
Norm. Signal Sum
2.0e+07
1.5e+07
1.0e+07
5.0e+06
0.0e+00
S1_WT
S1_WT_IMS
S1_WT_MATRIX
S1_atp23
S1_atp23_IMS
S1_atp23_MATRIX
S2_WT
S2_WT_IMS
S2_WT_MATRIX
S2_atp6
S2_atp6_IMS
S2_atp6_MATRIX

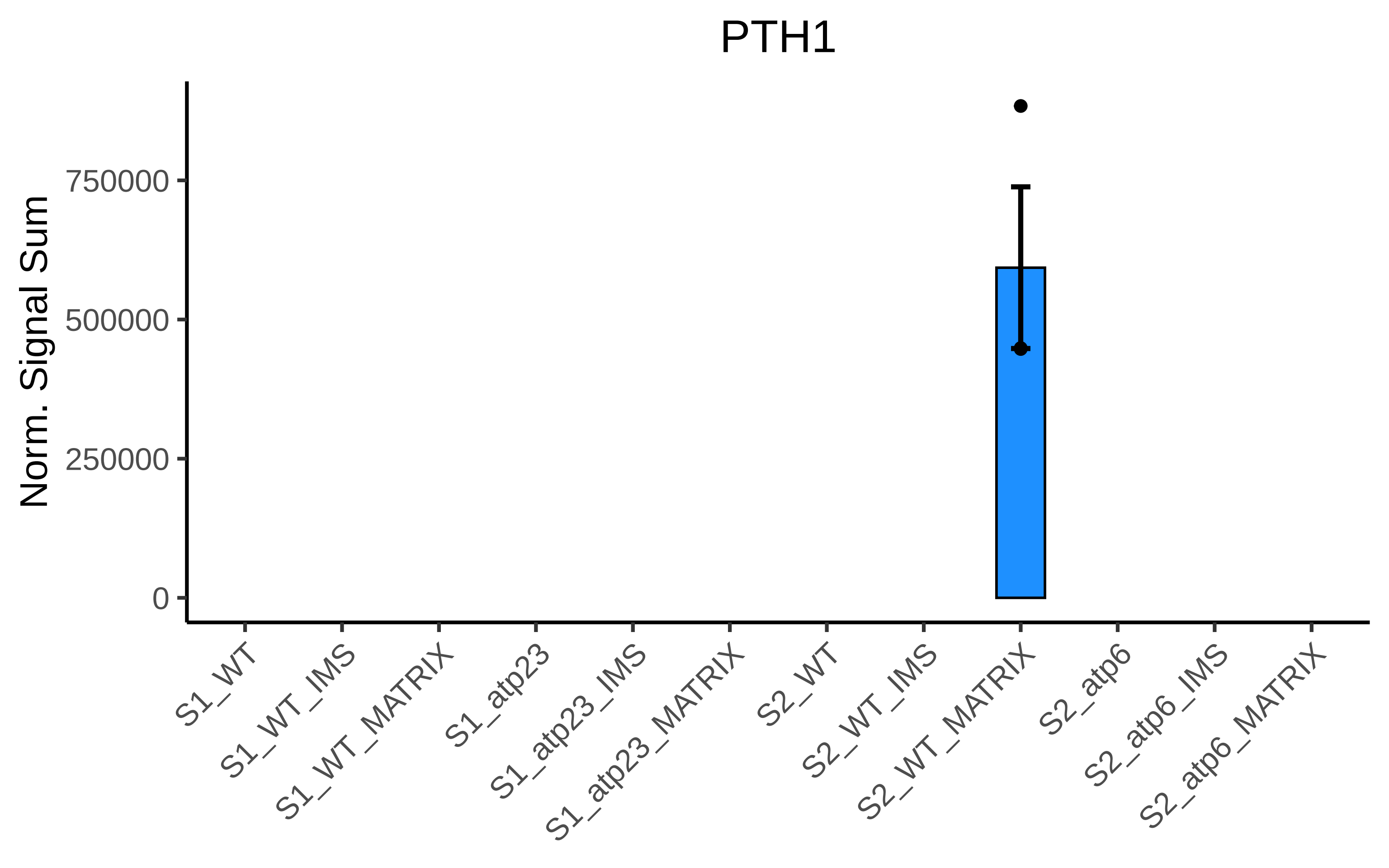
PTH1
Norm. Signal Sum
750000
500000
250000
0
S1_WT
S1_WT_IMS
S1_WT_MATRIX
S1_atp23
S1_atp23_IMS
S1_atp23_MATRIX
S2_WT
S2_WT_IMS
S2_WT_MATRIX
S2_atp6
S2_atp6_IMS
S2_atp6_MATRIX

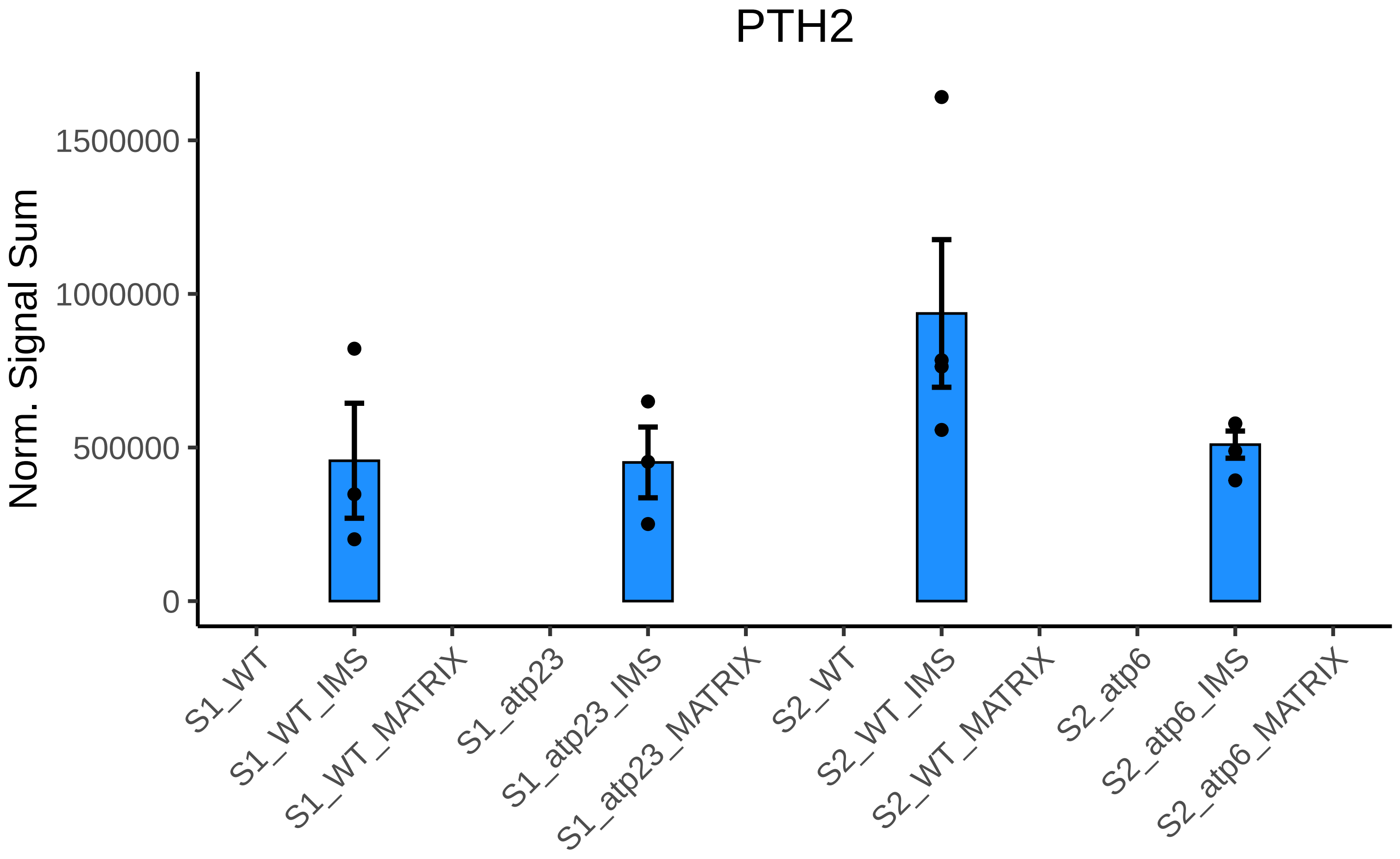

PTH2
Norm. Signal Sum
1500000
1000000
500000
0
S1_WT
S1_WT_IMS
S1_WT_MATRIX
S1_atp23
S1_atp23_IMS
S1_atp23_MATRIX
S2_WT
S2_WT_IMS
S2_WT_MATRIX
S2_atp6
S2_atp6_IMS
S2_atp6_MATRIX

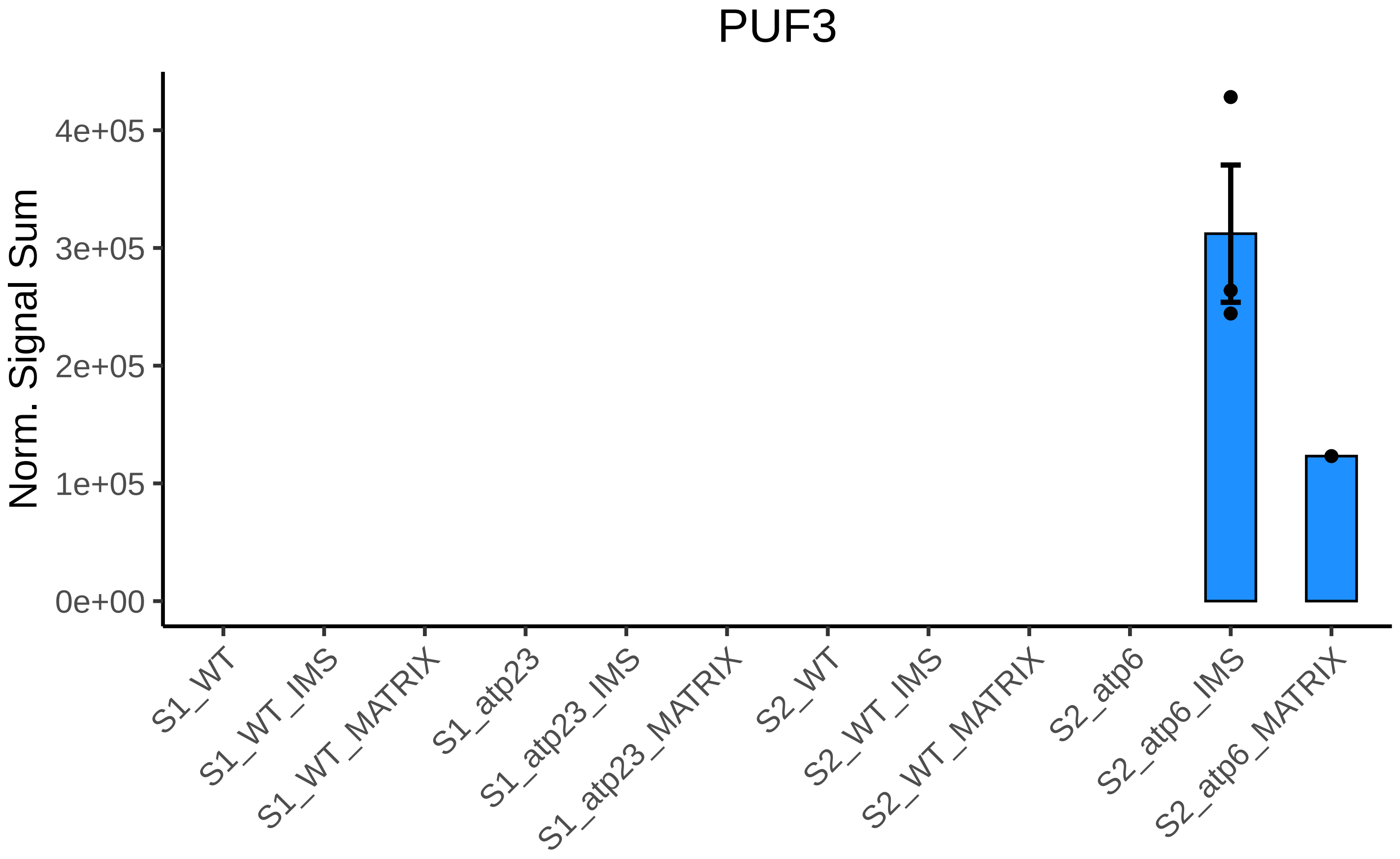

PUF3
Norm. Signal Sum
4e+05
3e+05
2e+05
1e+05
0e+00
S1_WT
S1_WT_IMS
S1_WT_MATRIX
S1_atp23
S1_atp23_IMS
S1_atp23_MATRIX
S2_WT
S2_WT_IMS
S2_WT_MATRIX
S2_atp6
S2_atp6_IMS
S2_atp6_MATRIX

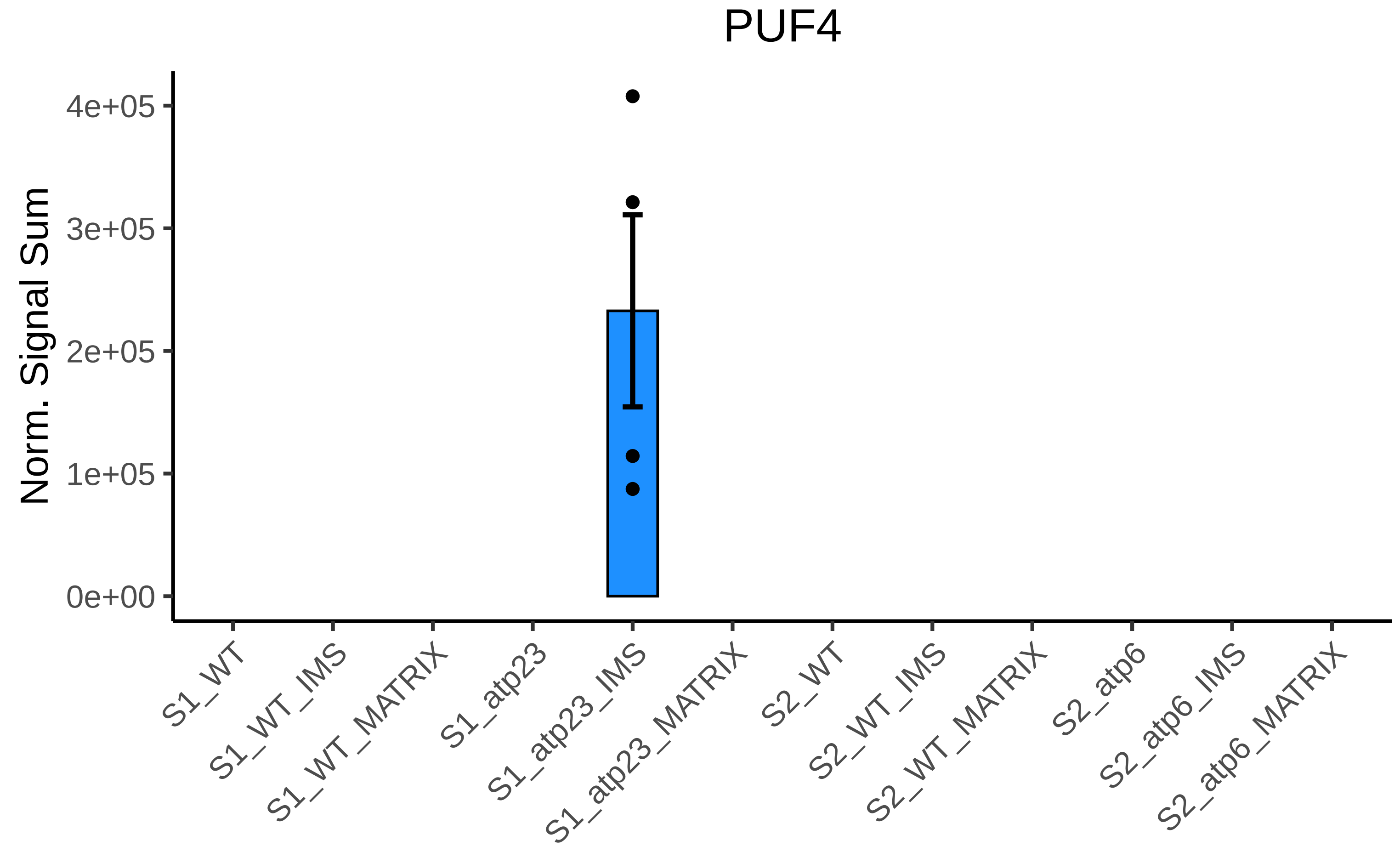

PUF4
Norm. Signal Sum
4e+05
3e+05
2e+05
1e+05
0e+00
S1_WT
S1_WT_IMS
S1_WT_MATRIX
S1_atp23
S1_atp23_IMS
S1_atp23_MATRIX
S2_WT
S2_WT_IMS
S2_WT_MATRIX
S2_atp6
S2_atp6_IMS
S2_atp6_MATRIX

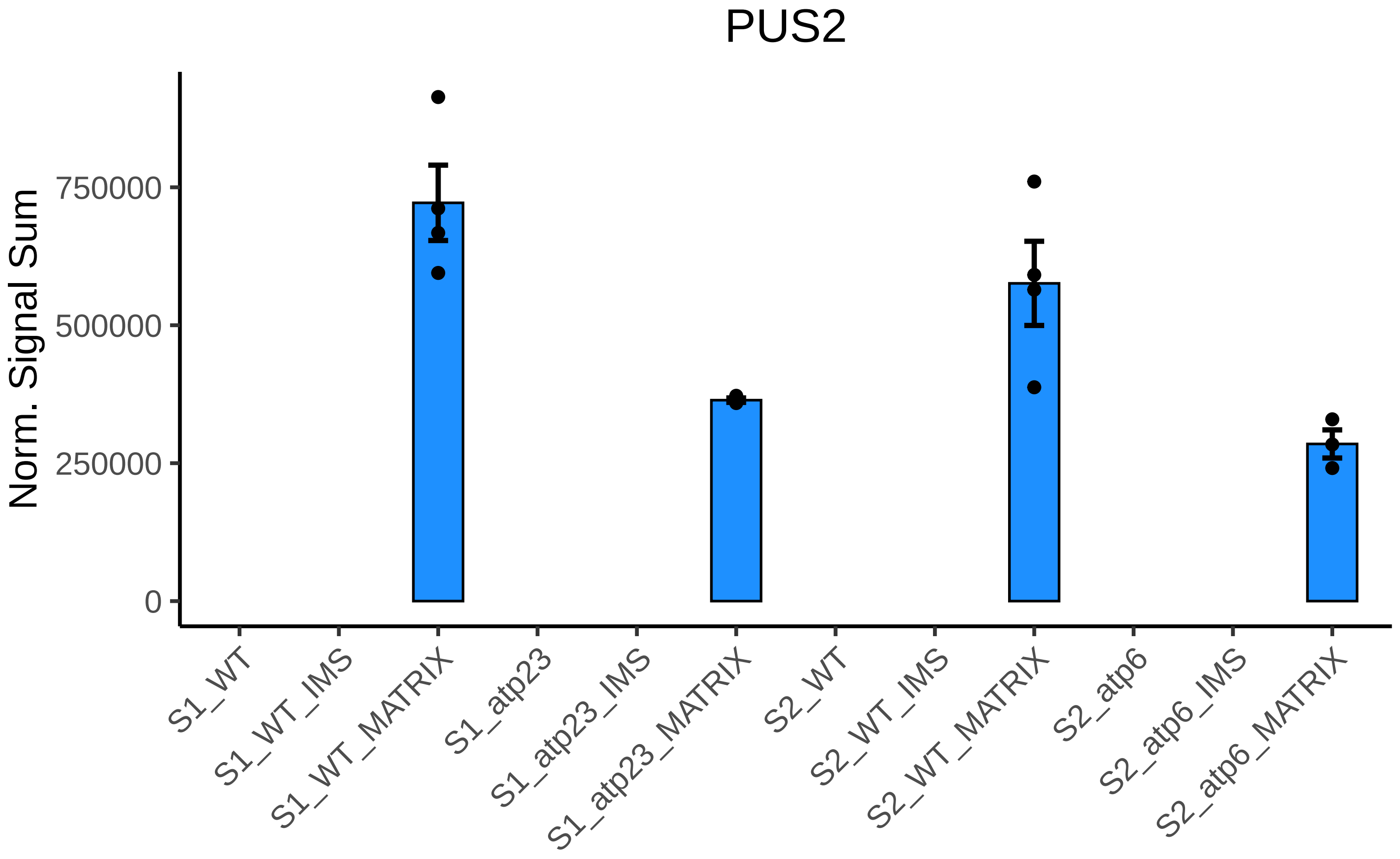

PUS2
Norm. Signal Sum
750000
500000
250000
0
S1_WT
S1_WT_IMS
S1_WT_MATRIX
S1_atp23
S1_atp23_IMS
S1_atp23_MATRIX
S2_WT
S2_WT_IMS
S2_WT_MATRIX
S2_atp6
S2_atp6_IMS
S2_atp6_MATRIX

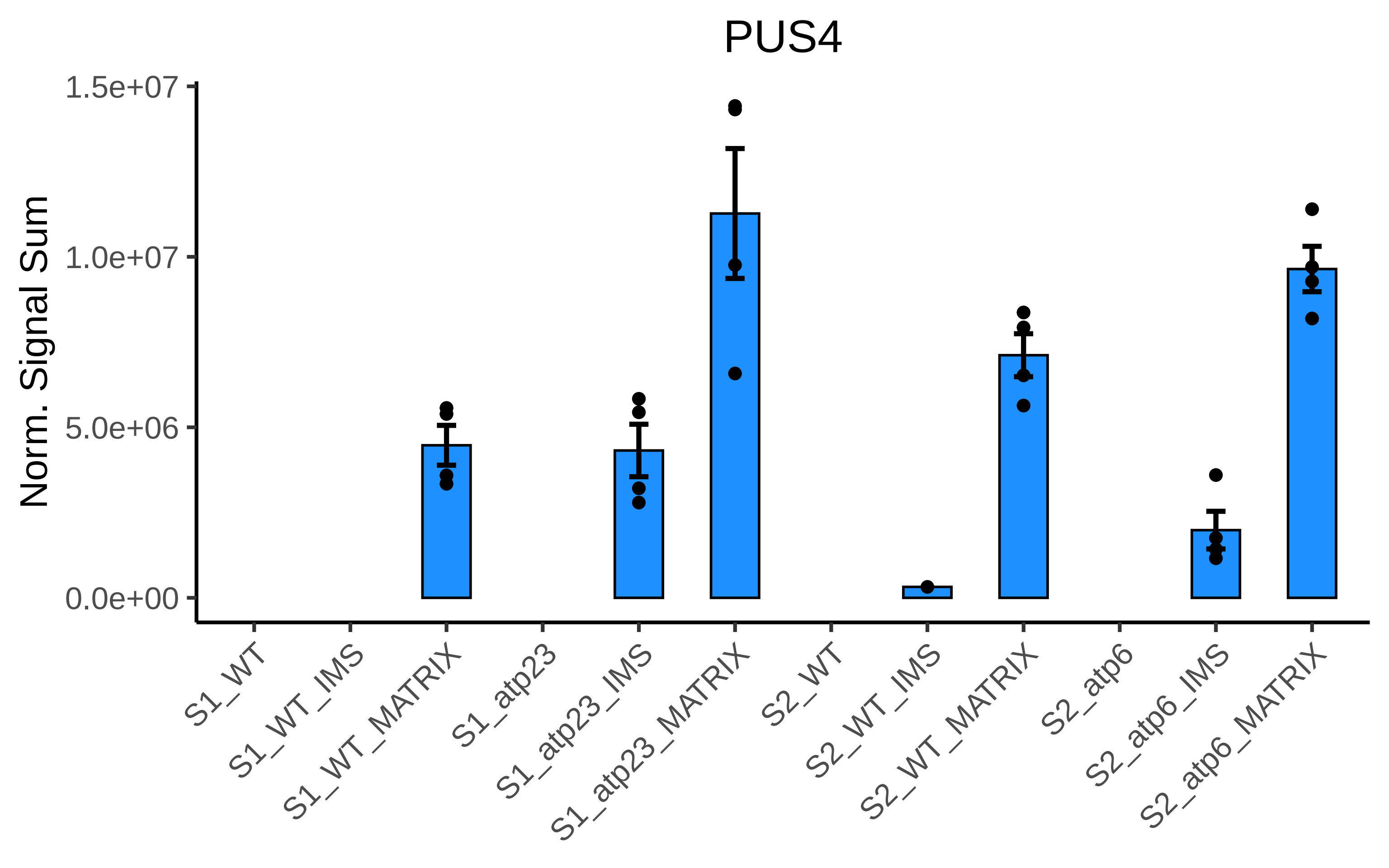
PUS4
Norm. Signal Sum
1.5e+07
1.0e+07
5.0e+06
0.0e+00
S1_WT
S1_WT_IMS
S1_WT_MATRIX
S1_atp23
S1_atp23_IMS
S1_atp23_MATRIX
S2_WT
S2_WT_IMS
S2_WT_MATRIX
S2_atp6
S2_atp6_IMS
S2_atp6_MATRIX

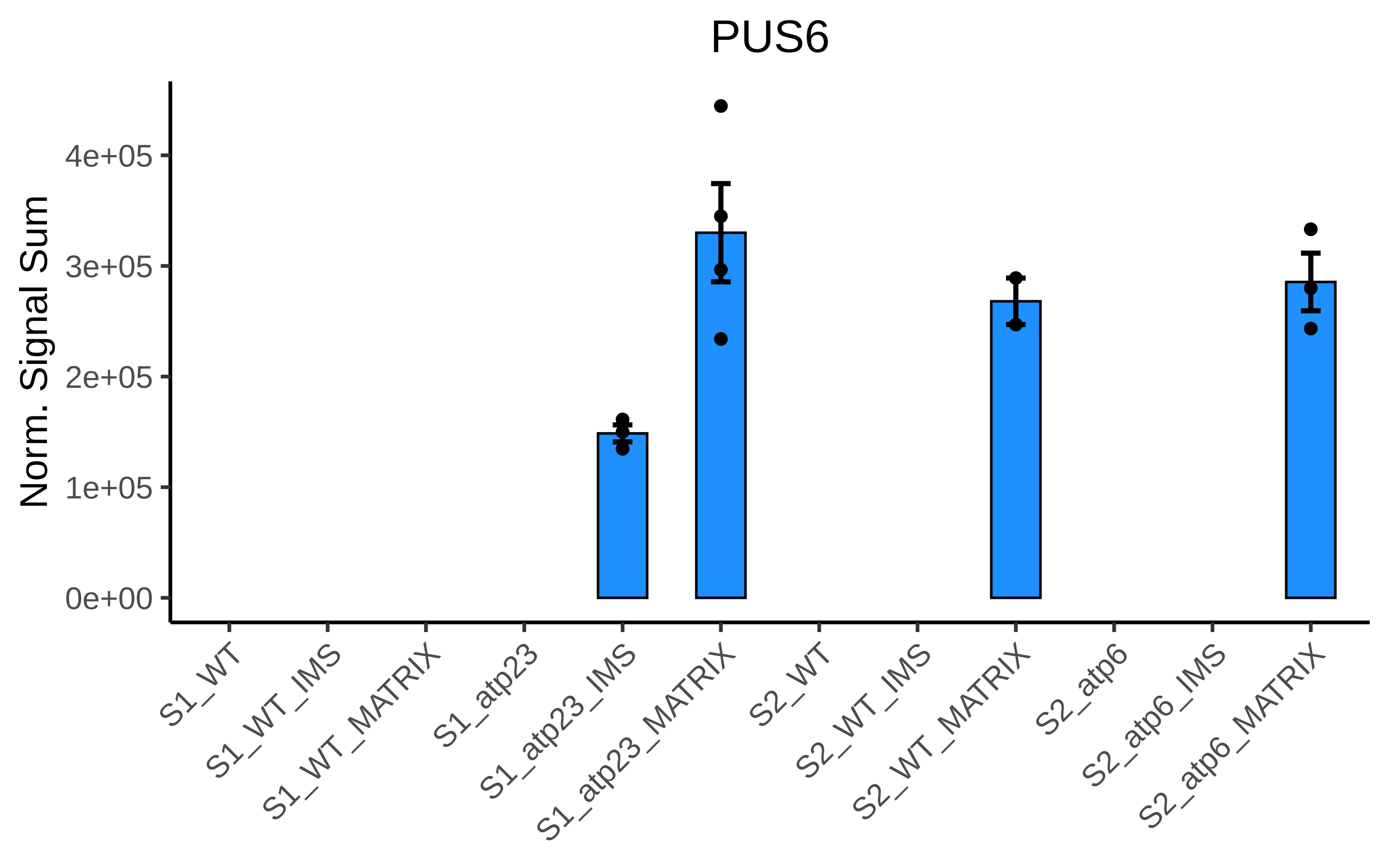
PUS6
Norm. Signal Sum
4e+05
3e+05
2e+05
1e+05
0e+00
S1_WT
S1_WT_IMS
S1_WT_MATRIX
S1_atp23
S1_atp23_IMS
S1_atp23_MATRIX
S2_WT
S2_WT_IMS
S2_WT_MATRIX
S2_atp6
S2_atp6_IMS
S2_atp6_MATRIX

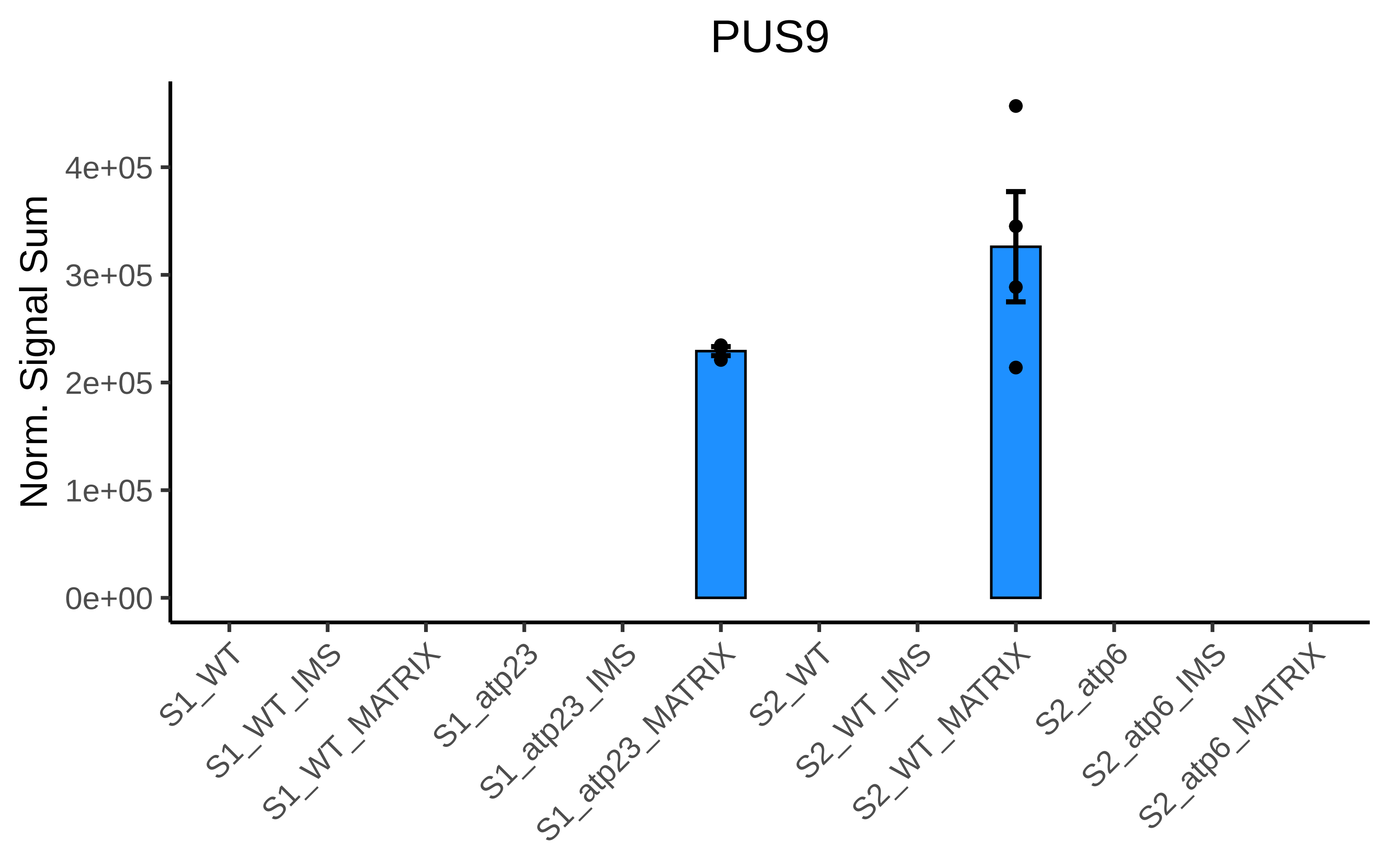
PUS9
Norm. Signal Sum
4e+05
3e+05
2e+05
1e+05
0e+00
S1_WT
S1_WT_IMS
S1_WT_MATRIX
S1_atp23
S1_atp23_IMS
S1_atp23_MATRIX
S2_WT
S2_WT_IMS
S2_WT_MATRIX
S2_atp6
S2_atp6_IMS
S2_atp6_MATRIX

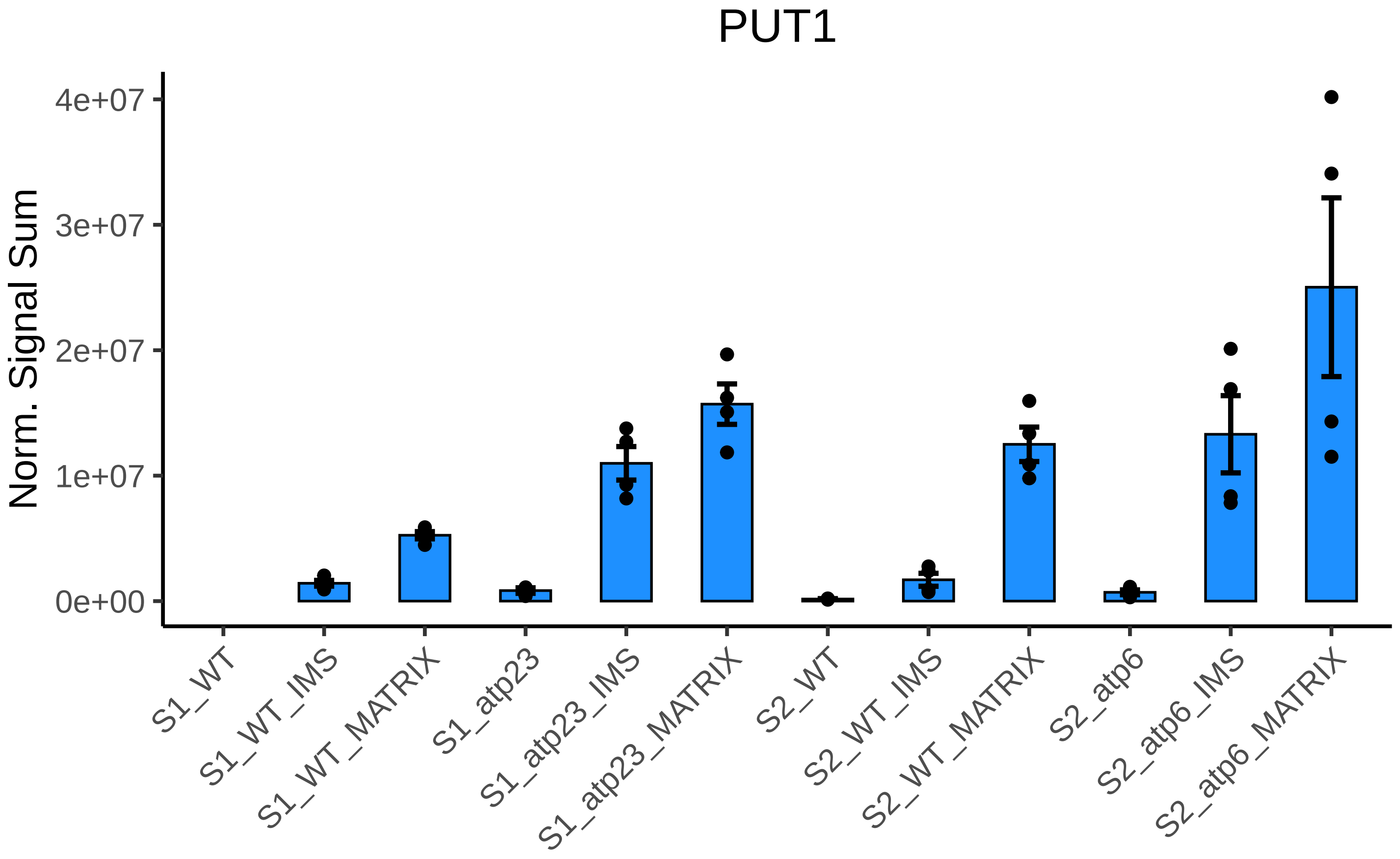

PUT1
Norm. Signal Sum
4e+07
3e+07
2e+07
1e+07
0e+00
S1_WT
S1_WT_IMS
S1_WT_MATRIX
S1_atp23
S1_atp23_IMS
S1_atp23_MATRIX
S2_WT
S2_WT_IMS
S2_WT_MATRIX
S2_atp6
S2_atp6_IMS
S2_atp6_MATRIX

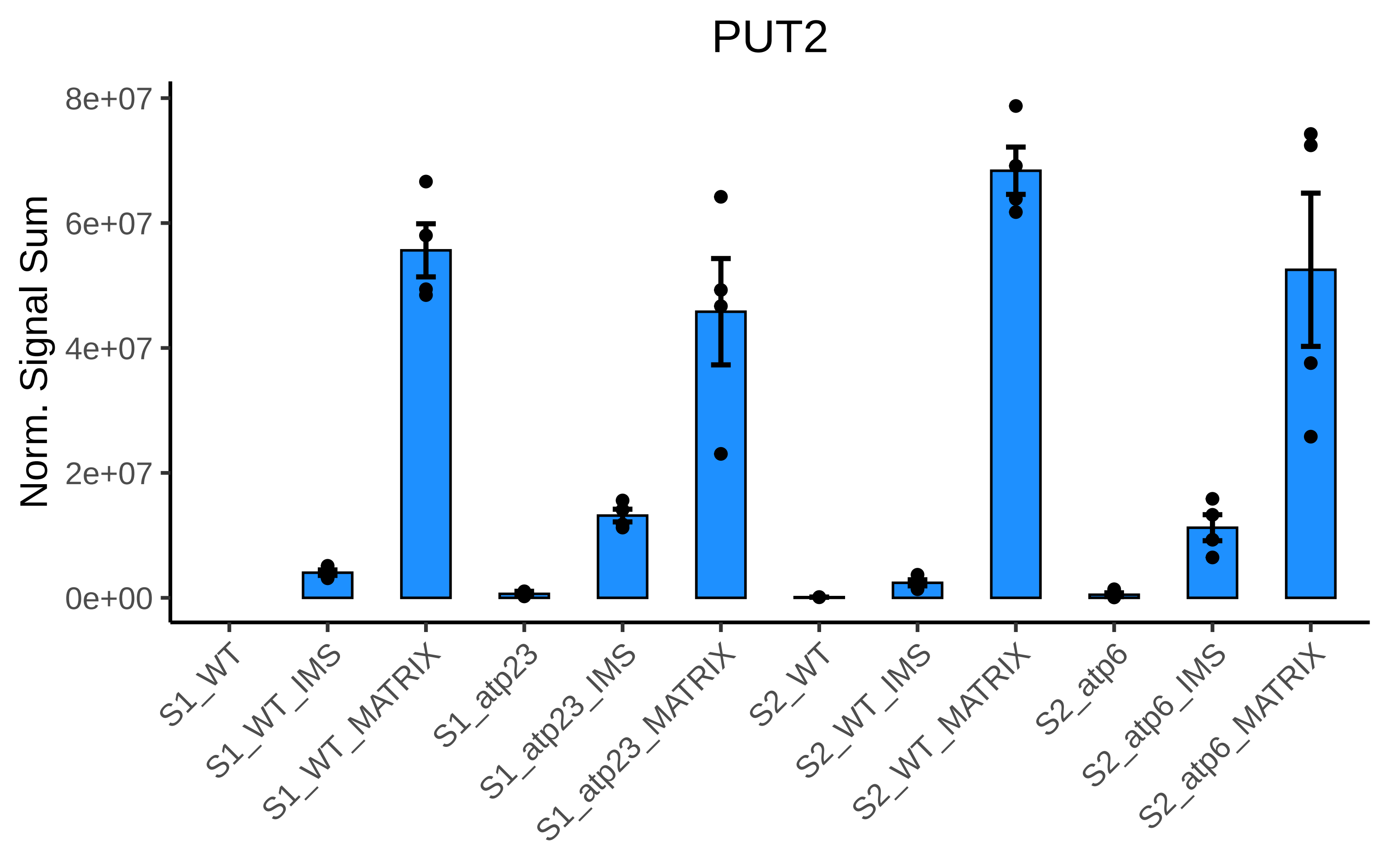

PUT2
Norm. Signal Sum
8e+07
6e+07
4e+07
2e+07
0e+00
S1_WT
S1_WT_IMS
S1_WT_MATRIX
S1_atp23
S1_atp23_IMS
S1_atp23_MATRIX
S2_WT
S2_WT_IMS
S2_WT_MATRIX
S2_atp6
S2_atp6_IMS
S2_atp6_MATRIX

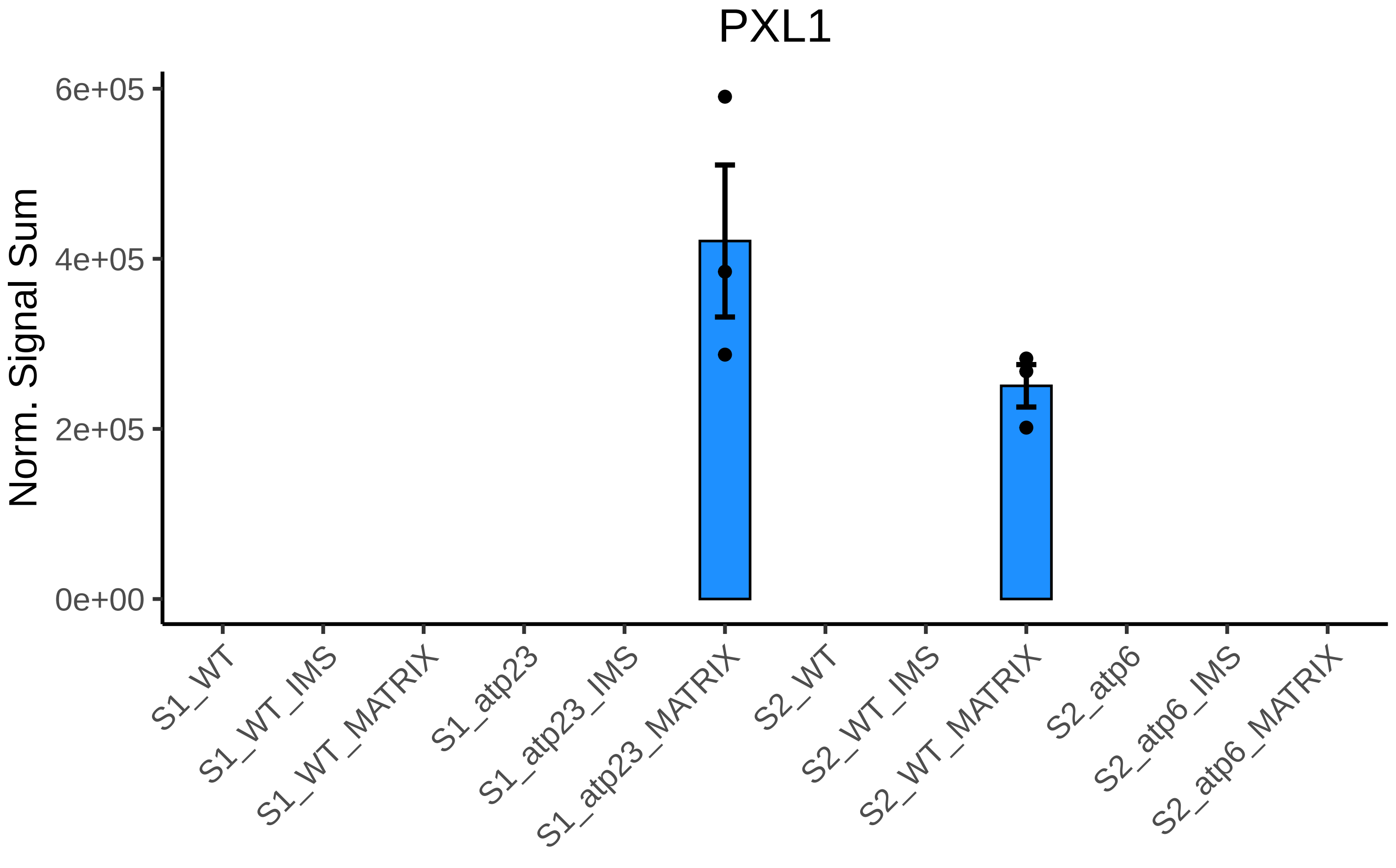

PXL1
Norm. Signal Sum
6e+05
4e+05
2e+05
0e+00
S1_WT
S1_WT_IMS
S1_WT_MATRIX
S1_atp23
S1_atp23_IMS
S1_atp23_MATRIX
S2_WT
S2_WT_IMS
S2_WT_MATRIX
S2_atp6
S2_atp6_IMS
S2_atp6_MATRIX

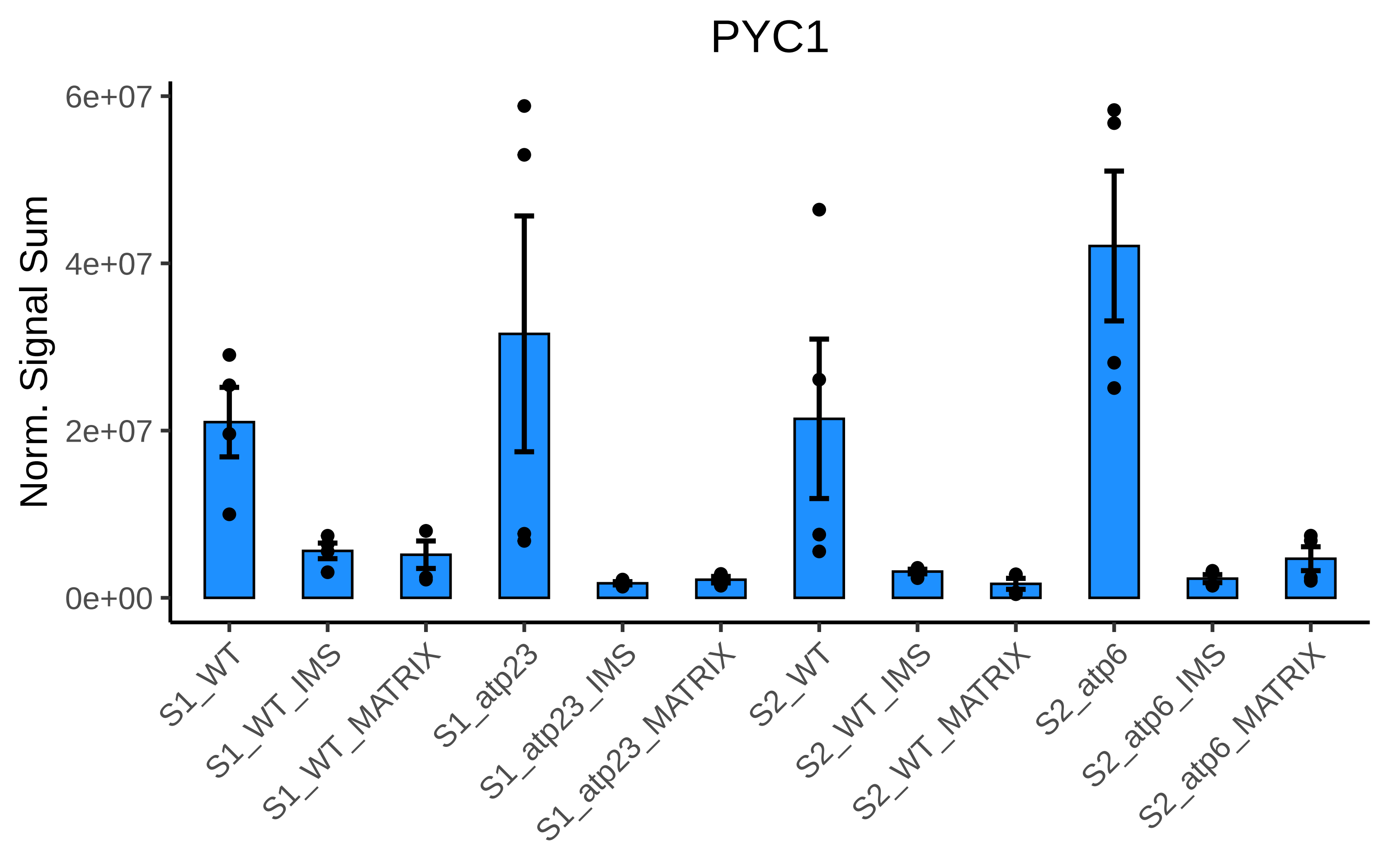
PYC1
Norm. Signal Sum
6e+07
4e+07
2e+07
0e+00
S1_WT
S1_WT_IMS
S1_WT_MATRIX
S1_atp23
S1_atp23_IMS
S1_atp23_MATRIX
S2_WT
S2_WT_IMS
S2_WT_MATRIX
S2_atp6
S2_atp6_IMS
S2_atp6_MATRIX

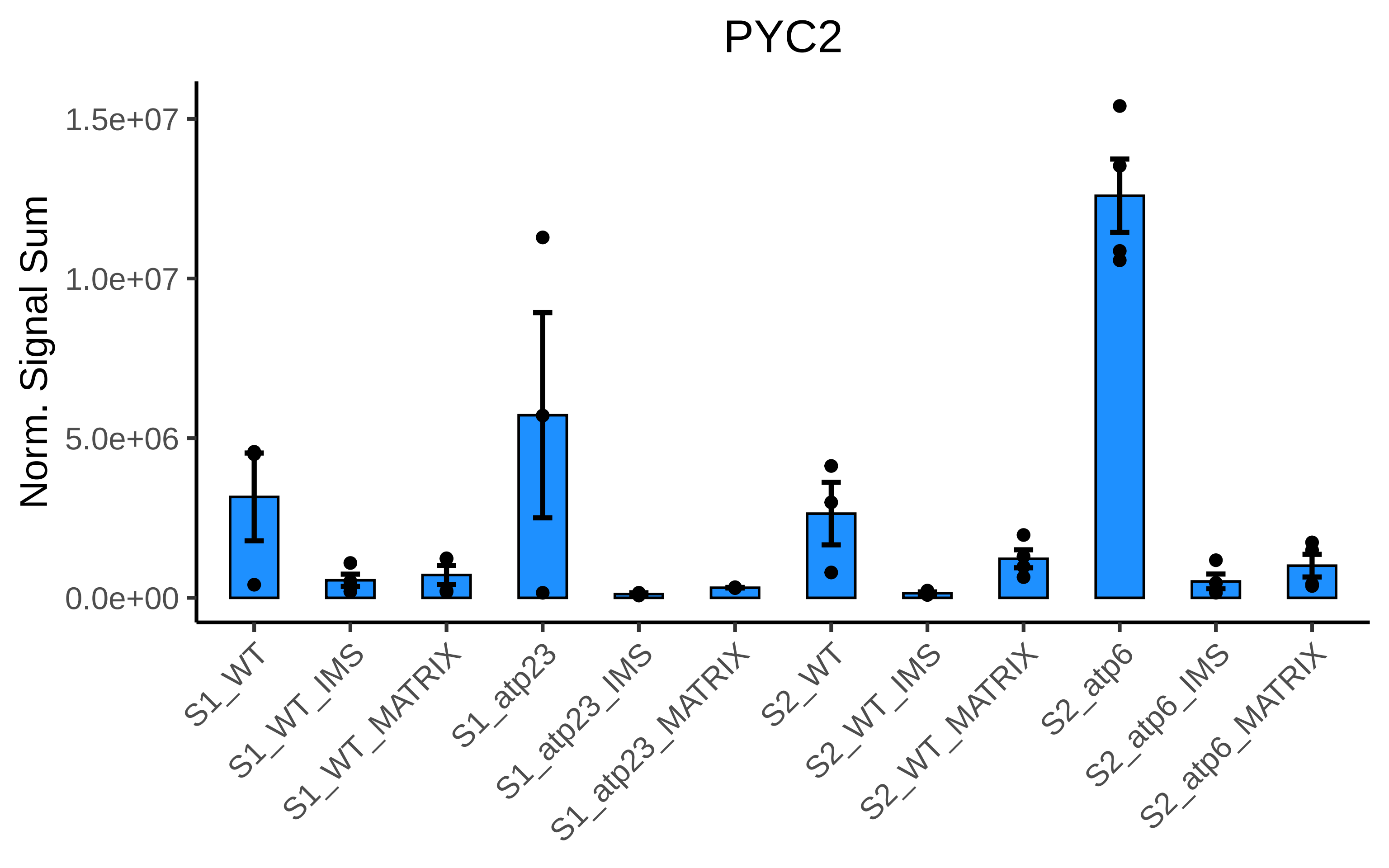

PYC2
Norm. Signal Sum
1.5e+07
1.0e+07
5.0e+06
0.0e+00
S1_WT
S1_WT_IMS
S1_WT_MATRIX
S1_atp23
S1_atp23_IMS
S1_atp23_MATRIX
S2_WT
S2_WT_IMS
S2_WT_MATRIX
S2_atp6
S2_atp6_IMS
S2_atp6_MATRIX

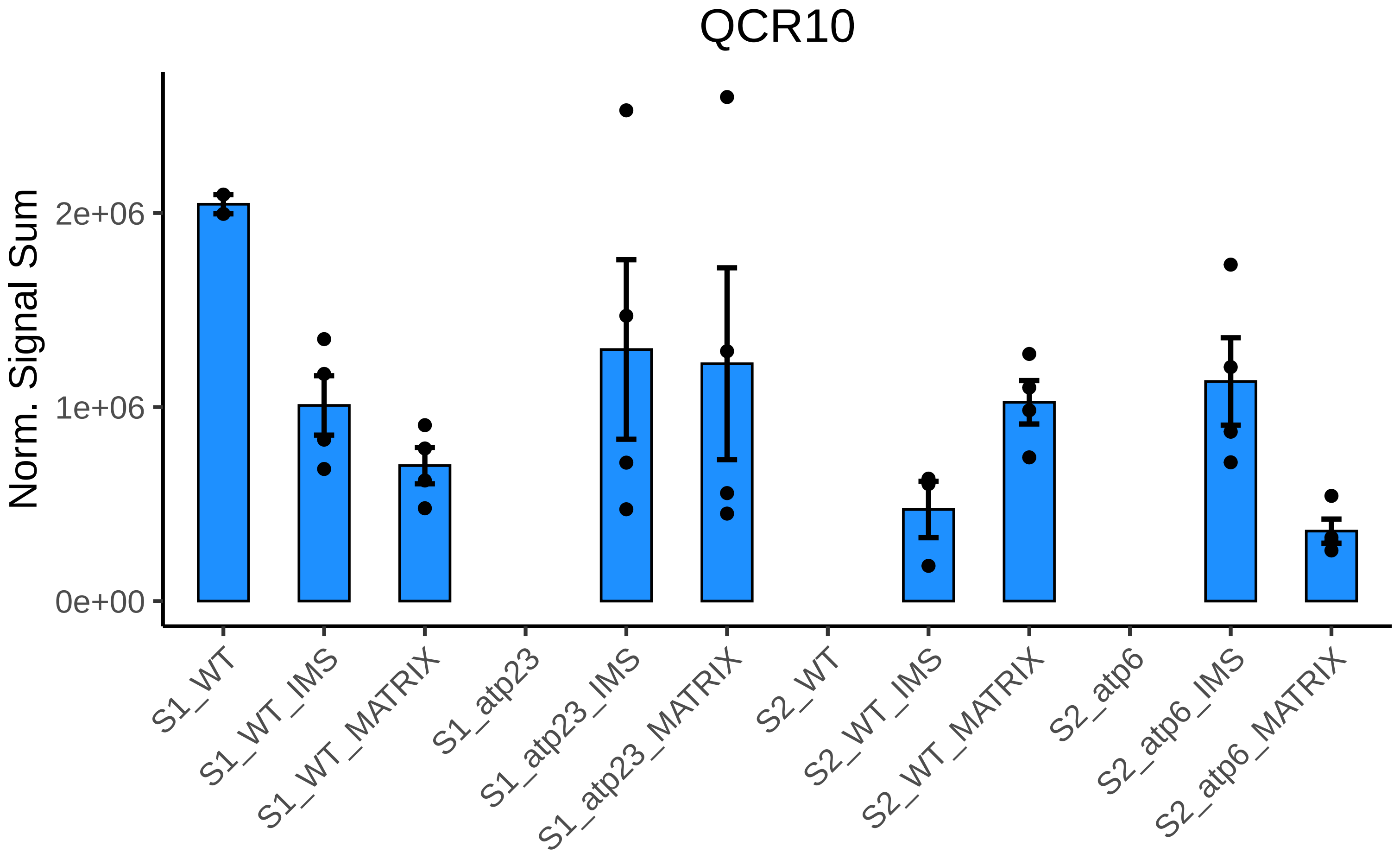
QCR10
Norm. Signal Sum
2e+06
1e+06
0e+00
S1_WT
S1_WT_IMS
S1_WT_MATRIX
S1_atp23
S1_atp23_IMS
S1_atp23_MATRIX
S2_WT
S2_WT_IMS
S2_WT_MATRIX
S2_atp6
S2_atp6_IMS
S2_atp6_MATRIX

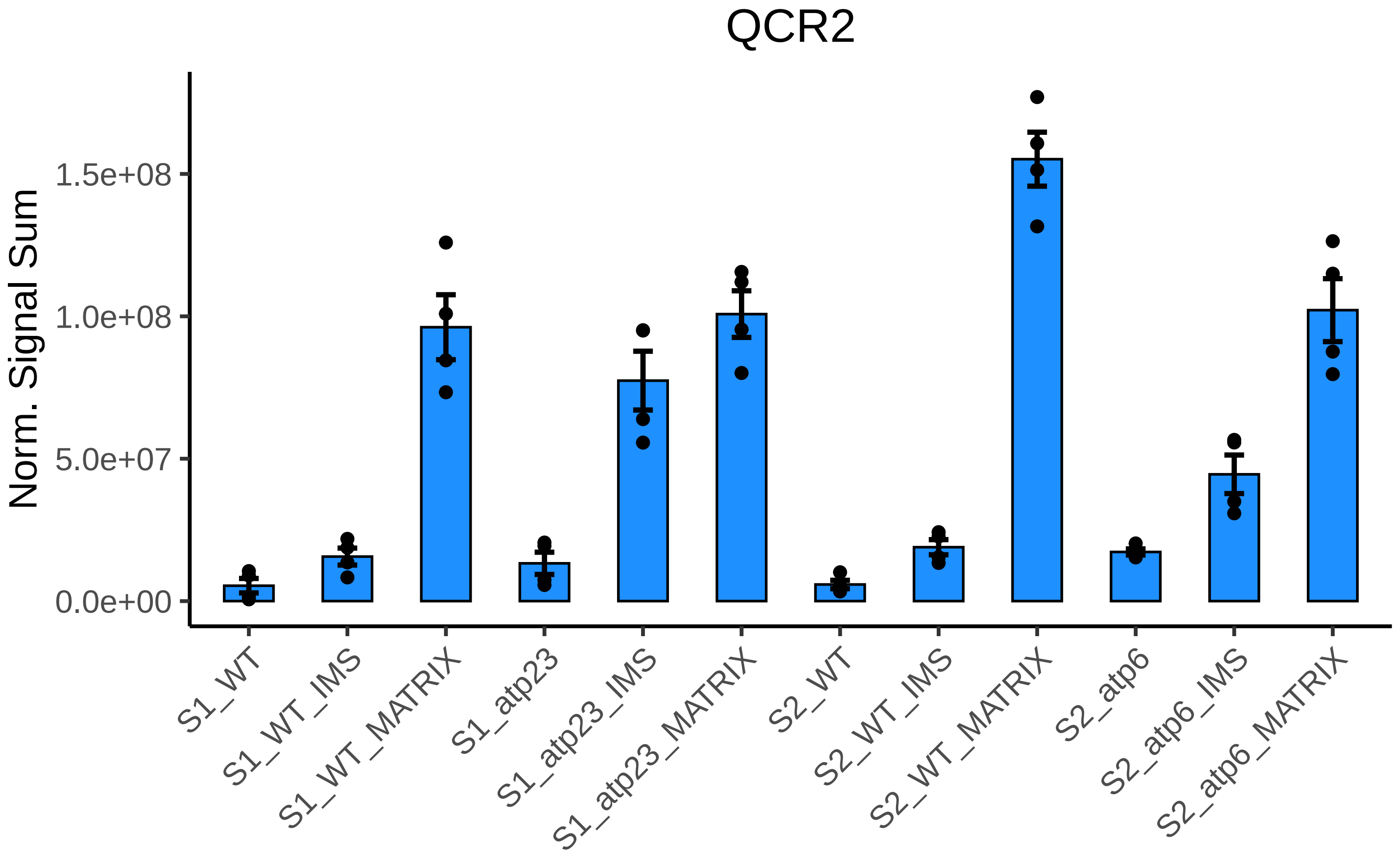
QCR2
Norm. Signal Sum
1.5e+08
1.0e+08
5.0e+07
0.0e+00
S1_WT
S1_WT_IMS
S1_WT_MATRIX
S1_atp23
S1_atp23_IMS
S1_atp23_MATRIX
S2_WT
S2_WT_IMS
S2_WT_MATRIX
S2_atp6
S2_atp6_IMS
S2_atp6_MATRIX

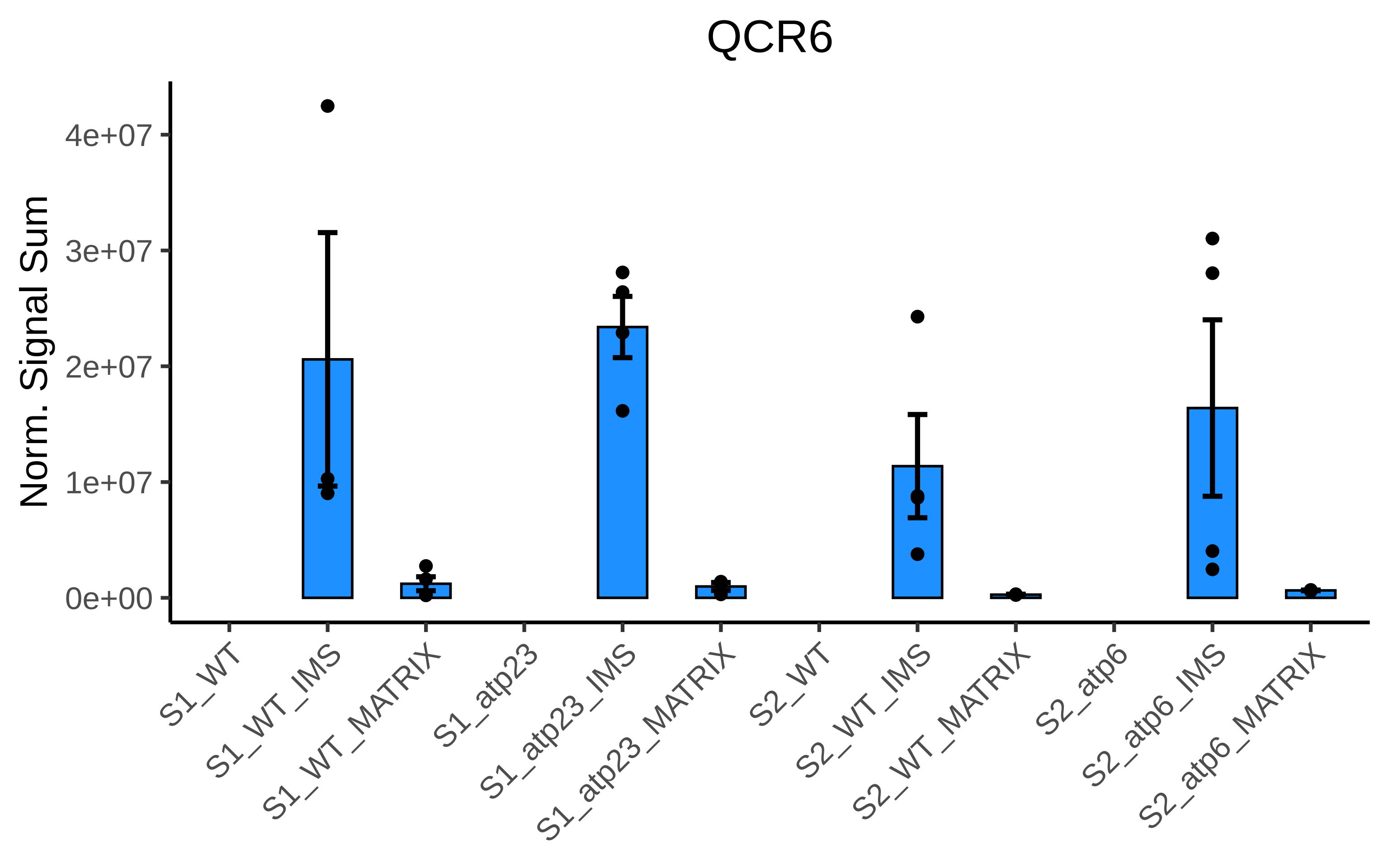
QCR6
Norm. Signal Sum
4e+07
3e+07
2e+07
1e+07
0e+00
S1_WT
S1_WT_IMS
S1_WT_MATRIX
S1_atp23
S1_atp23_IMS
S1_atp23_MATRIX
S2_WT
S2_WT_IMS
S2_WT_MATRIX
S2_atp6
S2_atp6_IMS
S2_atp6_MATRIX

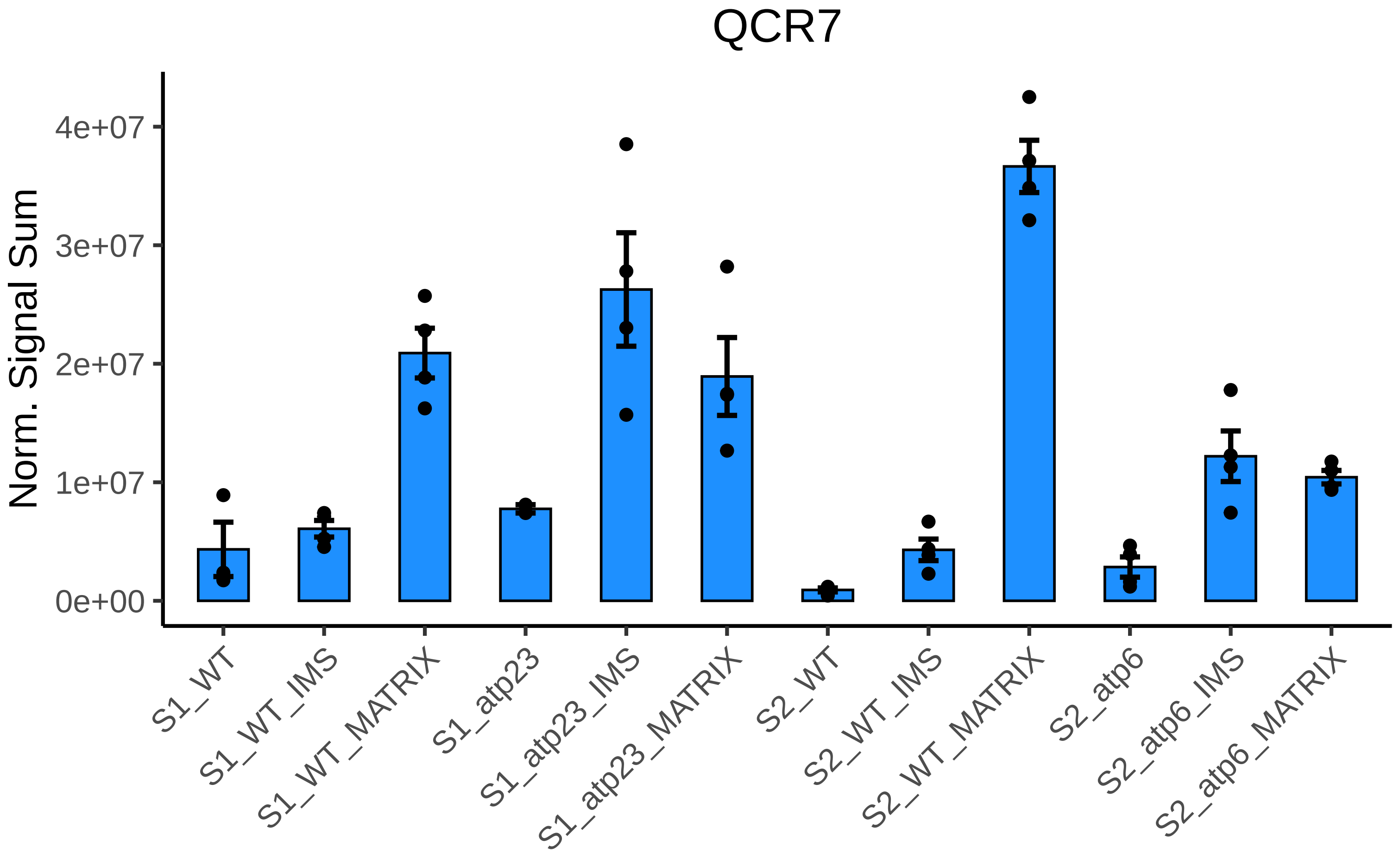

QCR7
Norm. Signal Sum
4e+07
3e+07
2e+07
1e+07
0e+00
S1_WT
S1_WT_IMS
S1_WT_MATRIX
S1_atp23
S1_atp23_IMS
S1_atp23_MATRIX
S2_WT
S2_WT_IMS
S2_WT_MATRIX
S2_atp6
S2_atp6_IMS
S2_atp6_MATRIX

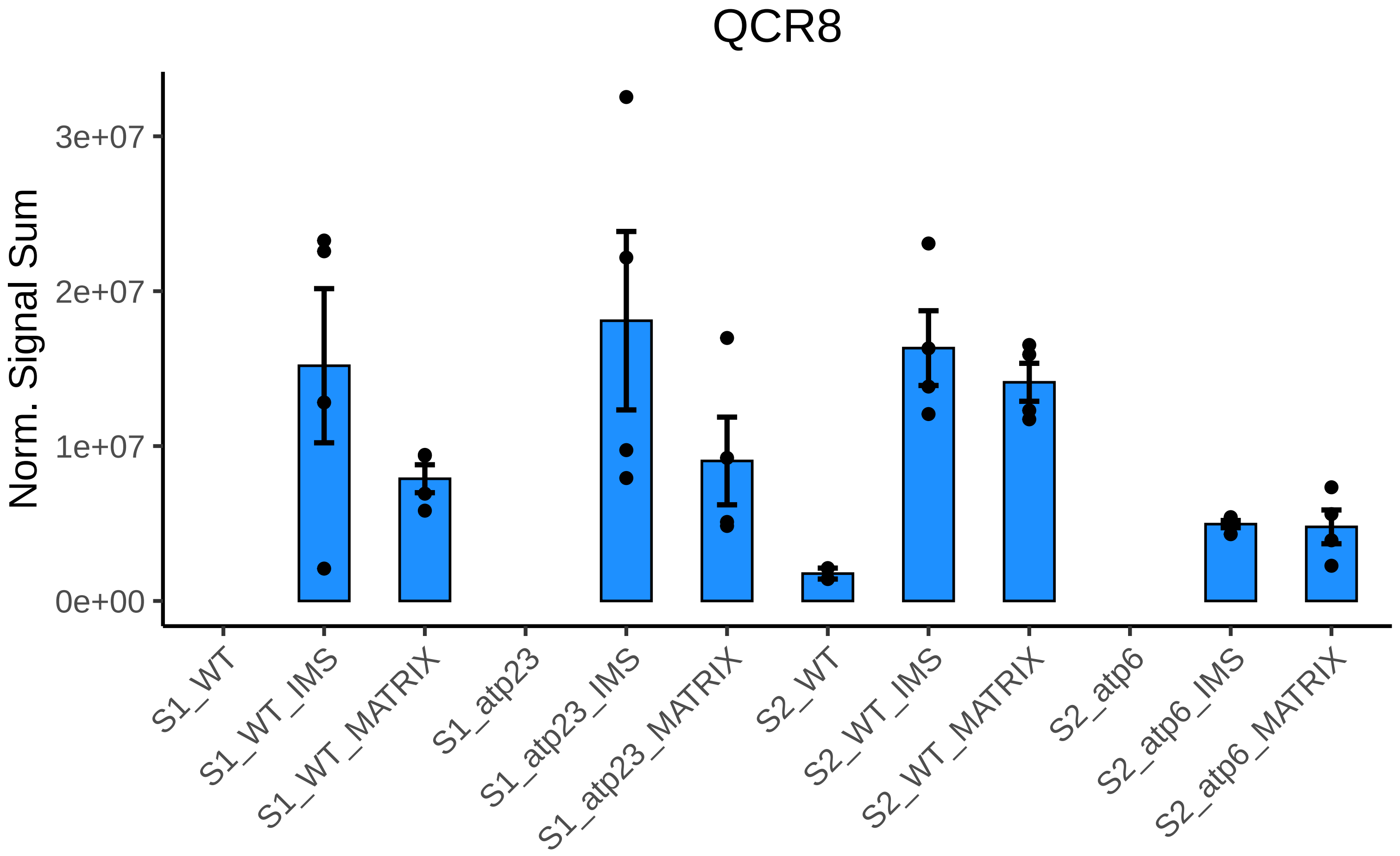
QCR8
Norm. Signal Sum
3e+07
2e+07
1e+07
0e+00
S1_WT
S1_WT_IMS
S1_WT_MATRIX
S1_atp23
S1_atp23_IMS
S1_atp23_MATRIX
S2_WT
S2_WT_IMS
S2_WT_MATRIX
S2_atp6
S2_atp6_IMS
S2_atp6_MATRIX

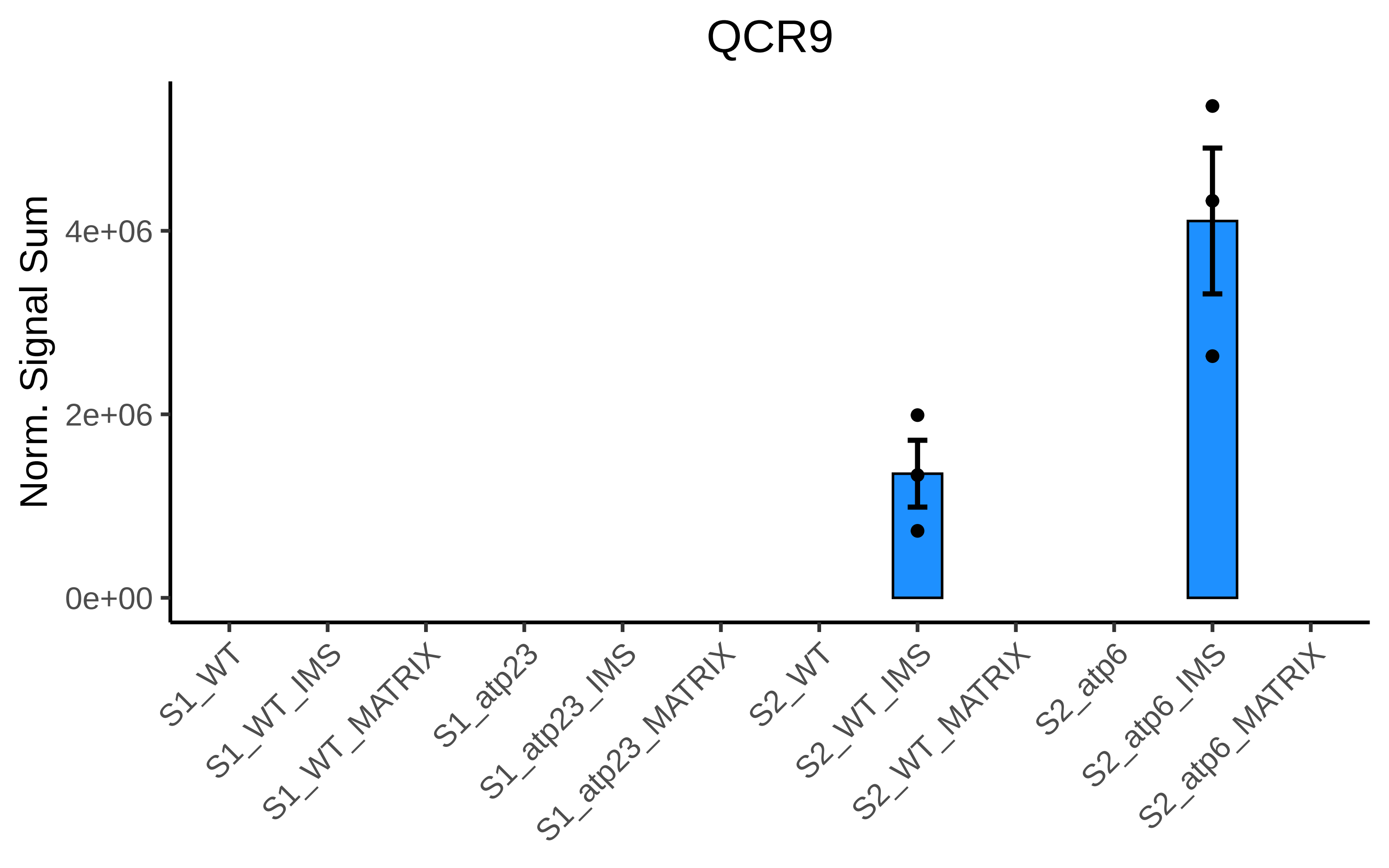

QCR9
Norm. Signal Sum
4e+06
2e+06
0e+00
S1_WT
S1_WT_IMS
S1_WT_MATRIX
S1_atp23
S1_atp23_IMS
S1_atp23_MATRIX
S2_WT
S2_WT_IMS
S2_WT_MATRIX
S2_atp6
S2_atp6_IMS
S2_atp6_MATRIX

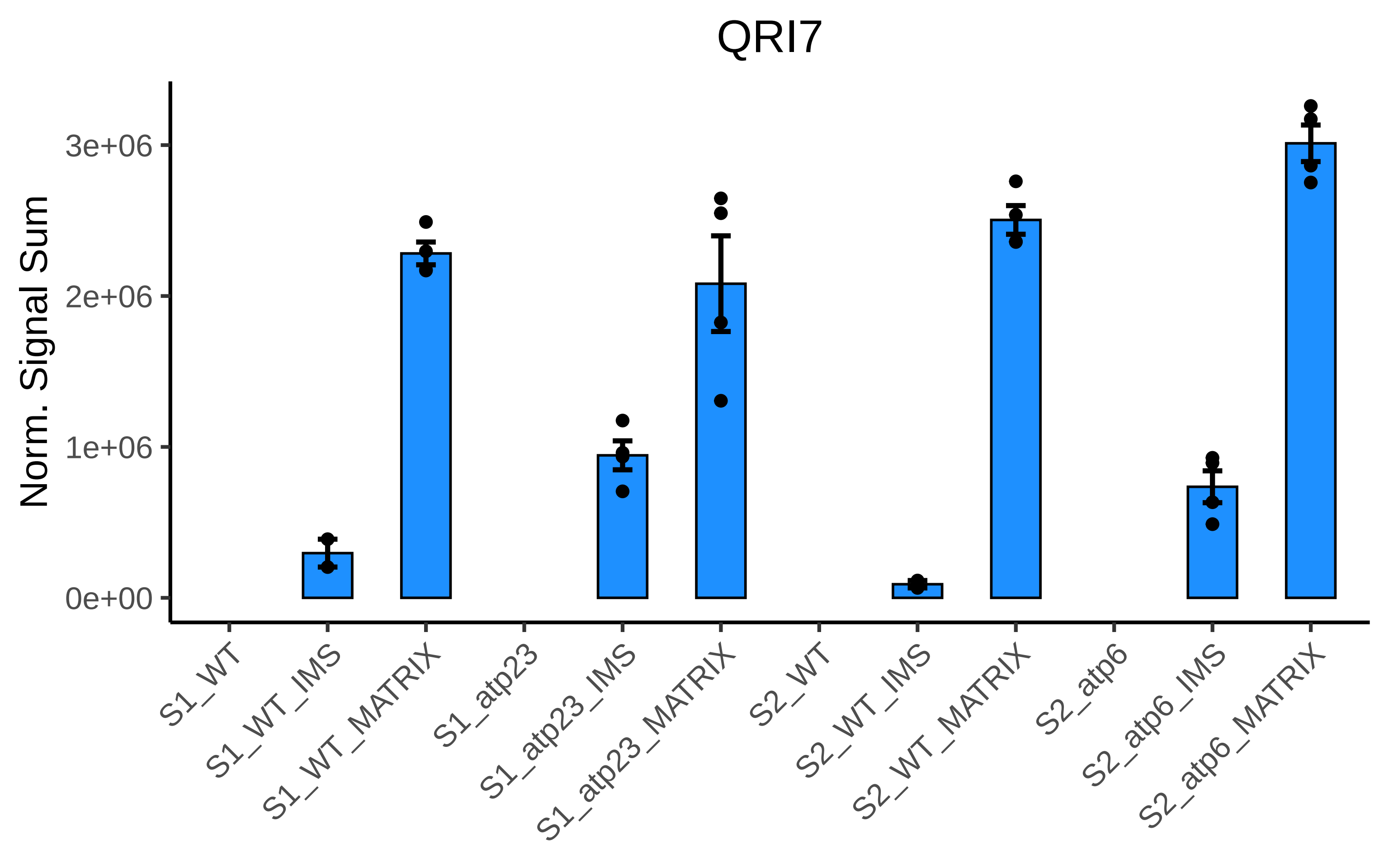
QRI7
Norm. Signal Sum
3e+06
2e+06
1e+06
0e+00
S1_WT
S1_WT_IMS
S1_WT_MATRIX
S1_atp23
S1_atp23_IMS
S1_atp23_MATRIX
S2_WT
S2_WT_IMS
S2_WT_MATRIX
S2_atp6
S2_atp6_IMS
S2_atp6_MATRIX

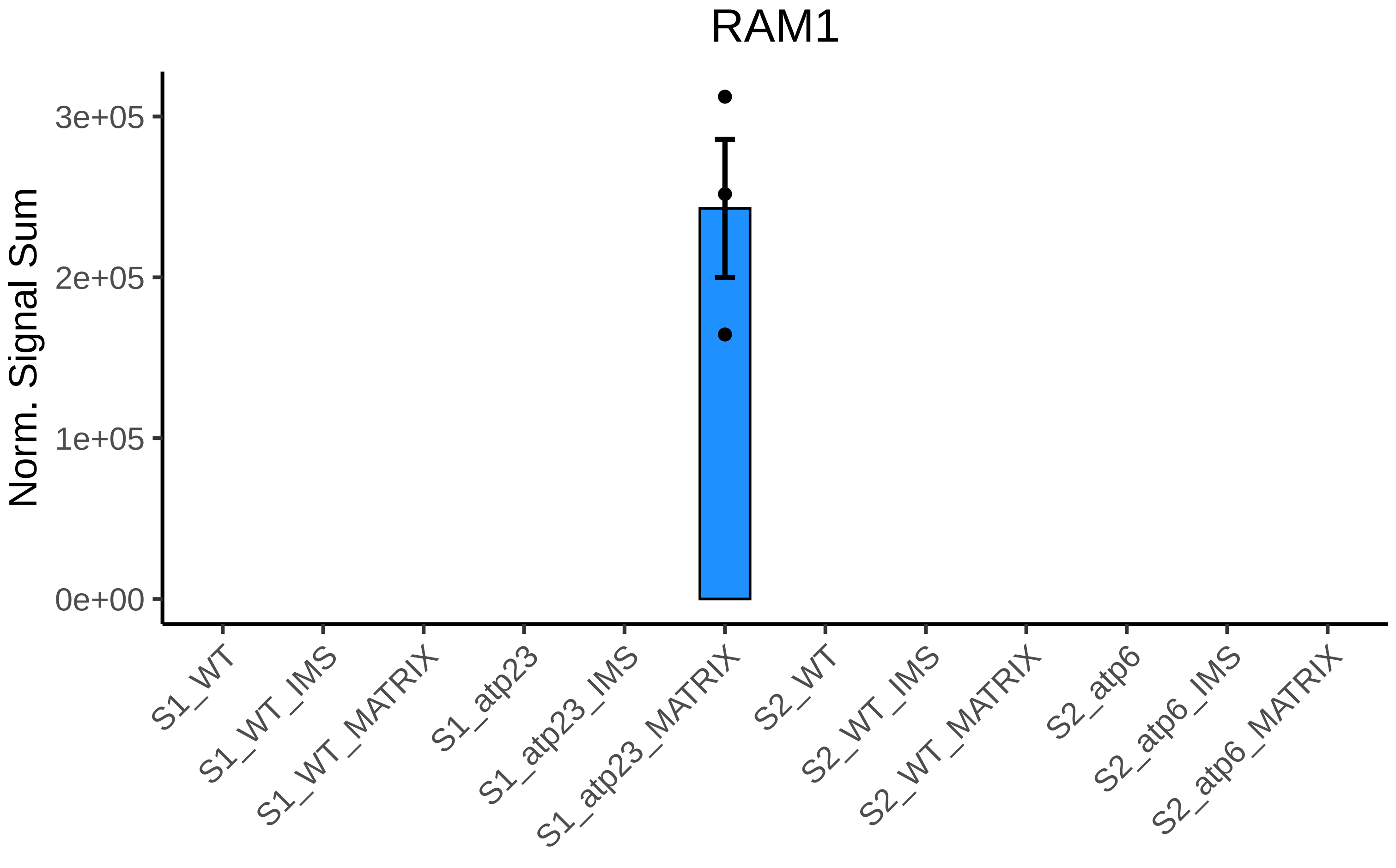

RAM1
Norm. Signal Sum
3e+05
2e+05
1e+05
0e+00
S1_WT
S1_WT_IMS
S1_WT_MATRIX
S1_atp23
S1_atp23_IMS
S1_atp23_MATRIX
S2_WT
S2_WT_IMS
S2_WT_MATRIX
S2_atp6
S2_atp6_IMS
S2_atp6_MATRIX

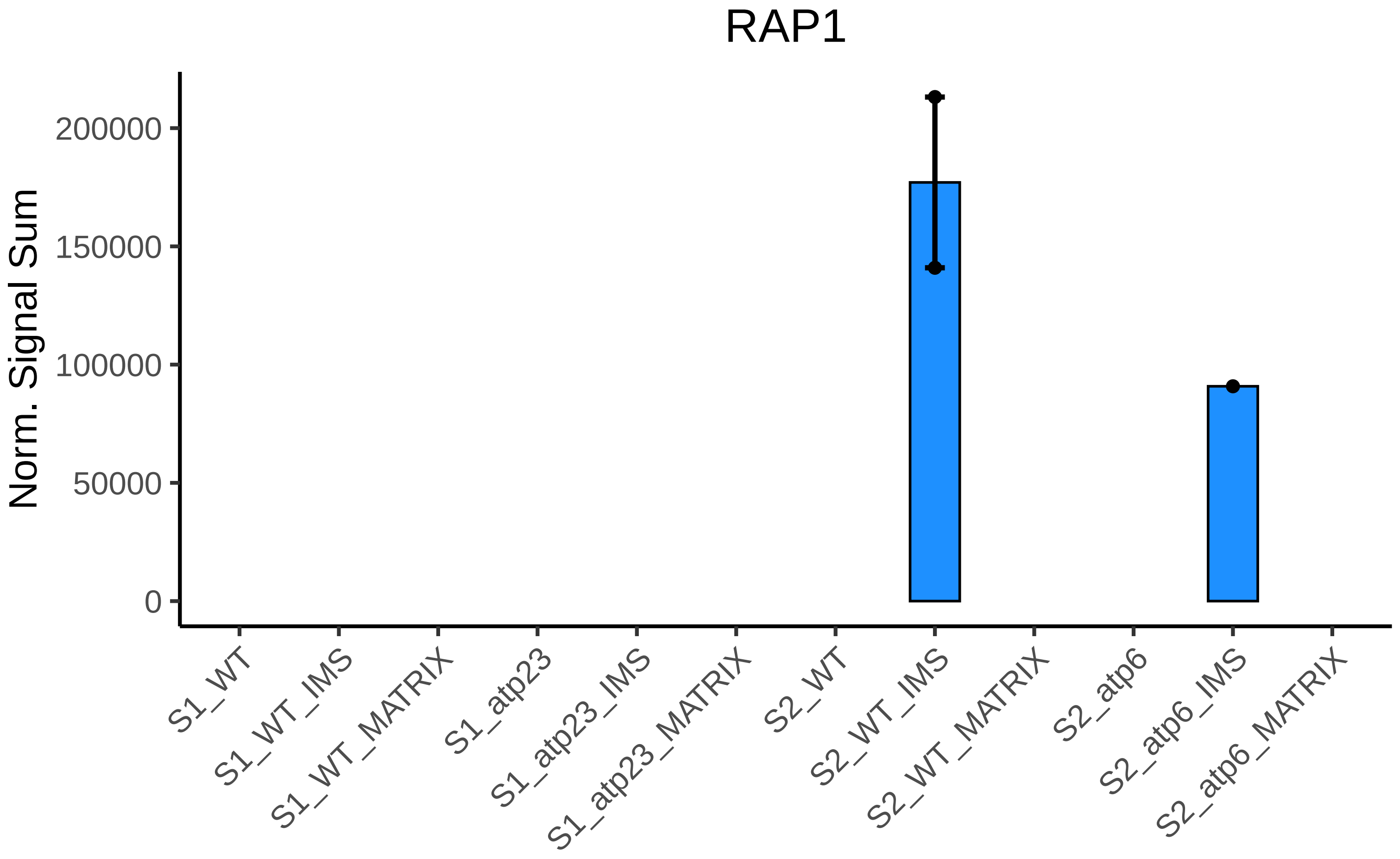

RAP1
Norm. Signal Sum
200000
150000
100000
50000
0
S1_WT
S1_WT_IMS
S1_WT_MATRIX
S1_atp23
S1_atp23_IMS
S1_atp23_MATRIX
S2_WT
S2_WT_IMS
S2_WT_MATRIX
S2_atp6
S2_atp6_IMS
S2_atp6_MATRIX

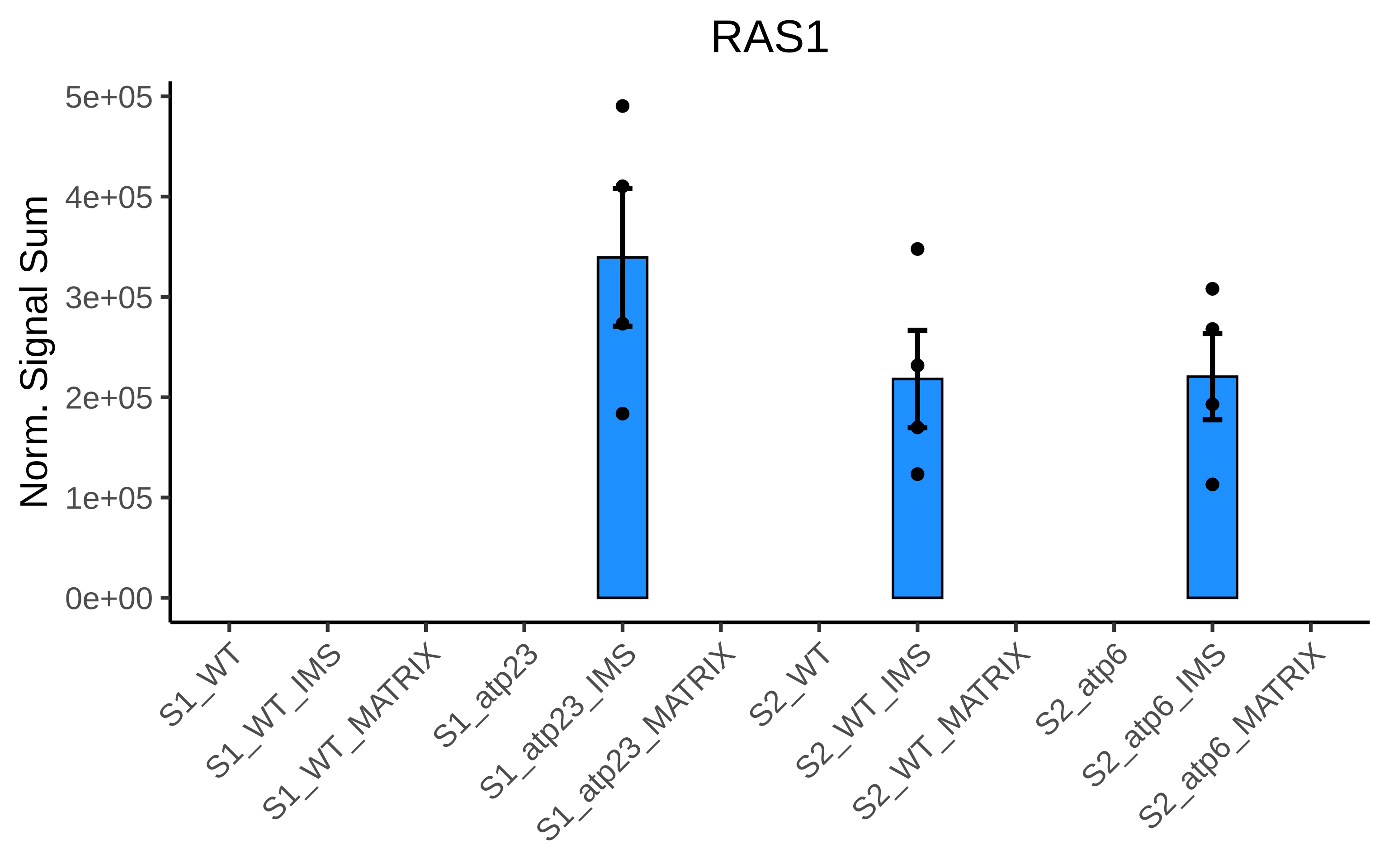

RAS1
Norm. Signal Sum
5e+05
4e+05
3e+05
2e+05
1e+05
0e+00
S1_WT
S1_WT_IMS
S1_WT_MATRIX
S1_atp23
S1_atp23_IMS
S1_atp23_MATRIX
S2_WT
S2_WT_IMS
S2_WT_MATRIX
S2_atp6
S2_atp6_IMS
S2_atp6_MATRIX

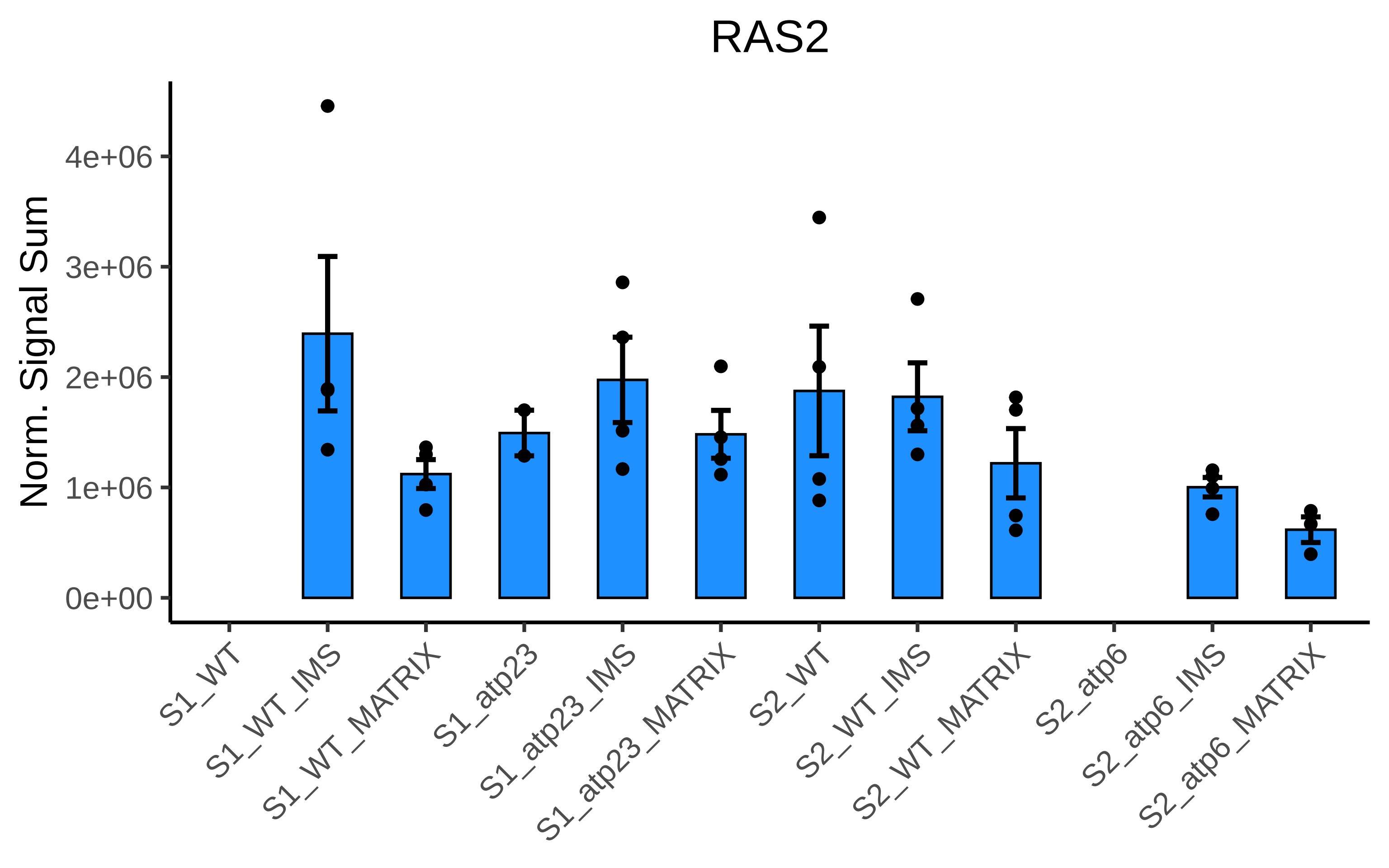

RAS2
Norm. Signal Sum
4e+06
3e+06
2e+06
1e+06
0e+00
S1_WT
S1_WT_IMS
S1_WT_MATRIX
S1_atp23
S1_atp23_IMS
S1_atp23_MATRIX
S2_WT
S2_WT_IMS
S2_WT_MATRIX
S2_atp6
S2_atp6_IMS
S2_atp6_MATRIX

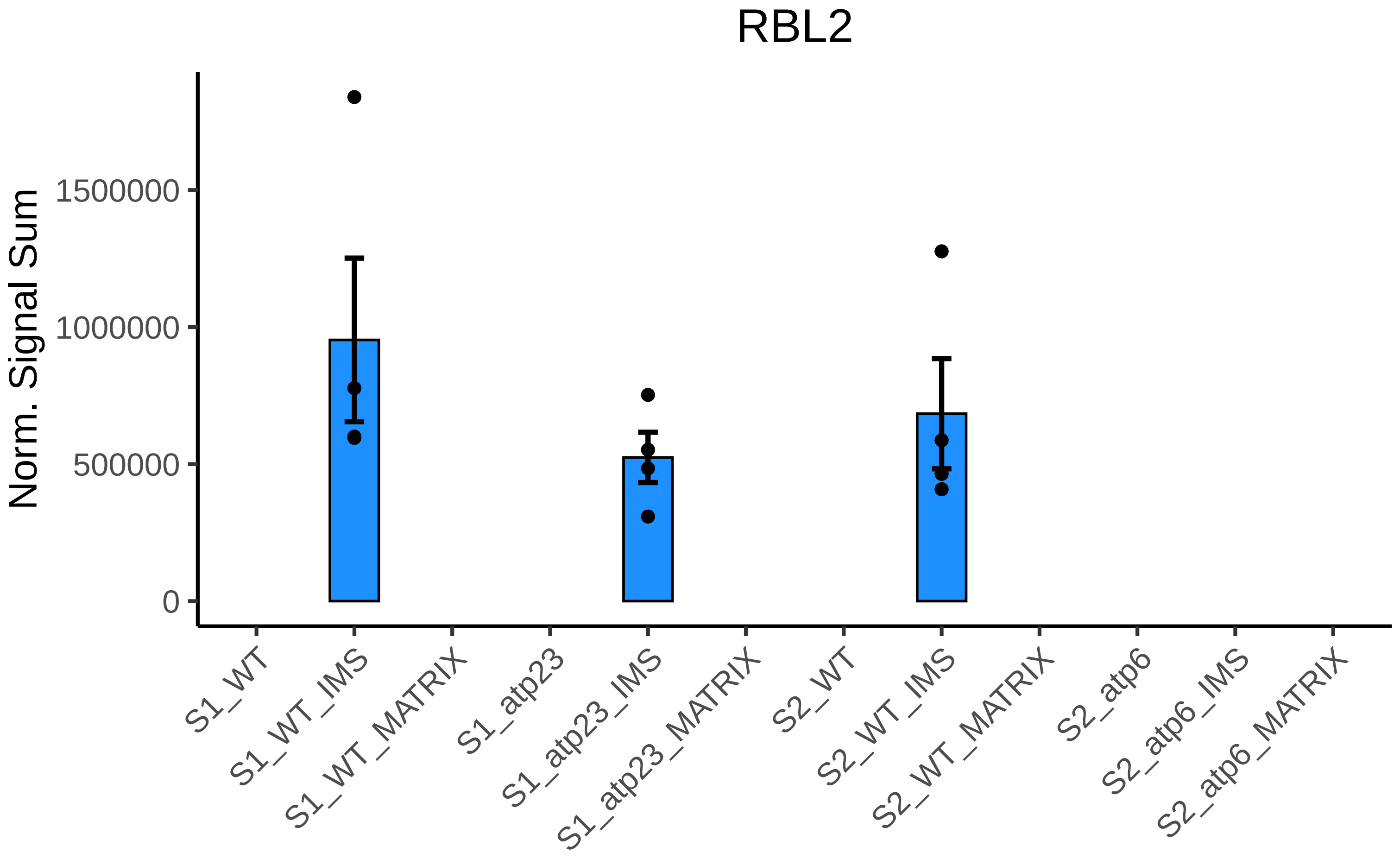
RBL2
Norm. Signal Sum
1500000
1000000
500000
0
S1_WT
S1_WT_IMS
S1_WT_MATRIX
S1_atp23
S1_atp23_IMS
S1_atp23_MATRIX
S2_WT
S2_WT_IMS
S2_WT_MATRIX
S2_atp6
S2_atp6_IMS
S2_atp6_MATRIX

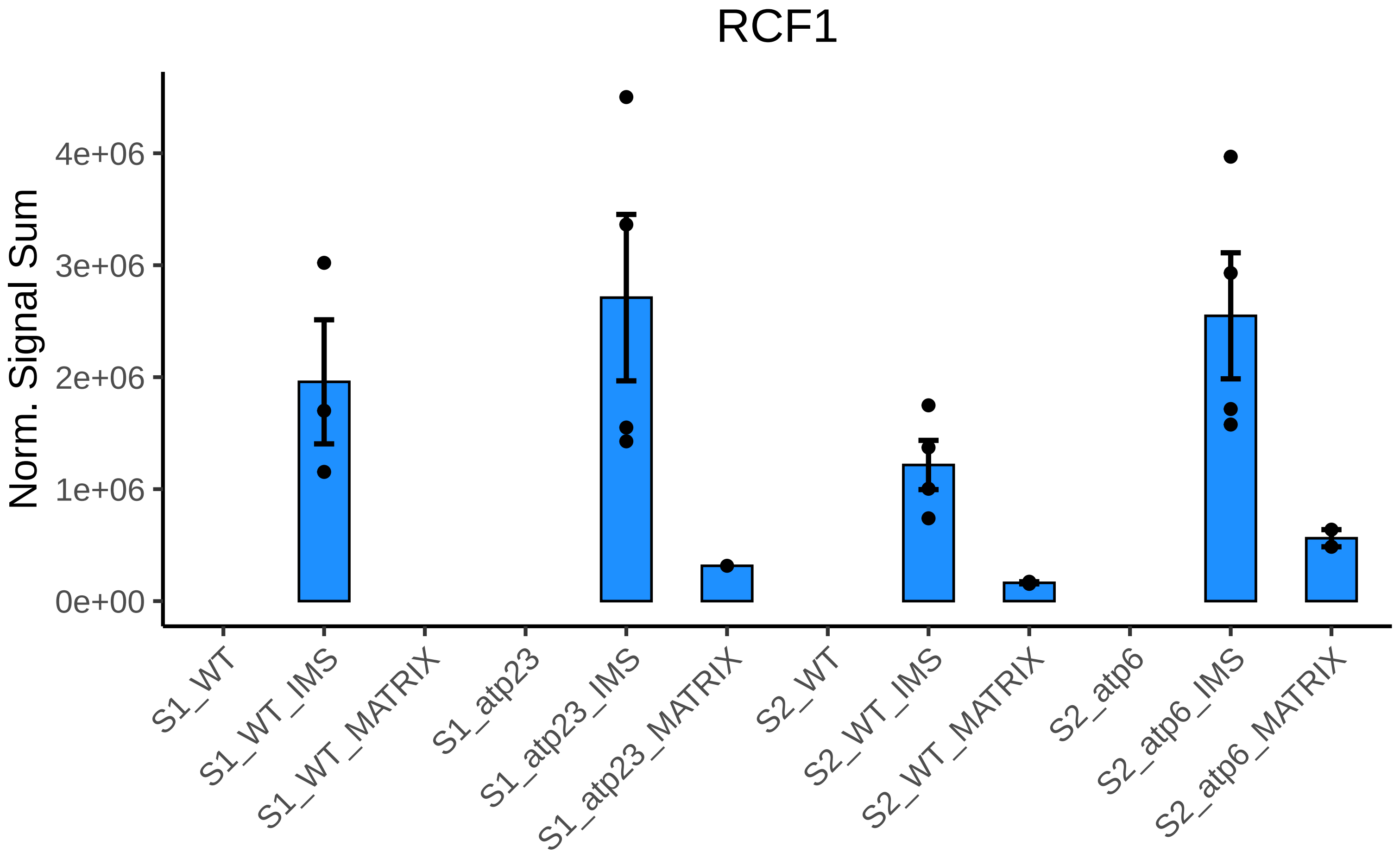
RCF1
Norm. Signal Sum
4e+06
3e+06
2e+06
1e+06
0e+00
S1_WT
S1_WT_IMS
S1_WT_MATRIX
S1_atp23
S1_atp23_IMS
S1_atp23_MATRIX
S2_WT
S2_WT_IMS
S2_WT_MATRIX
S2_atp6
S2_atp6_IMS
S2_atp6_MATRIX

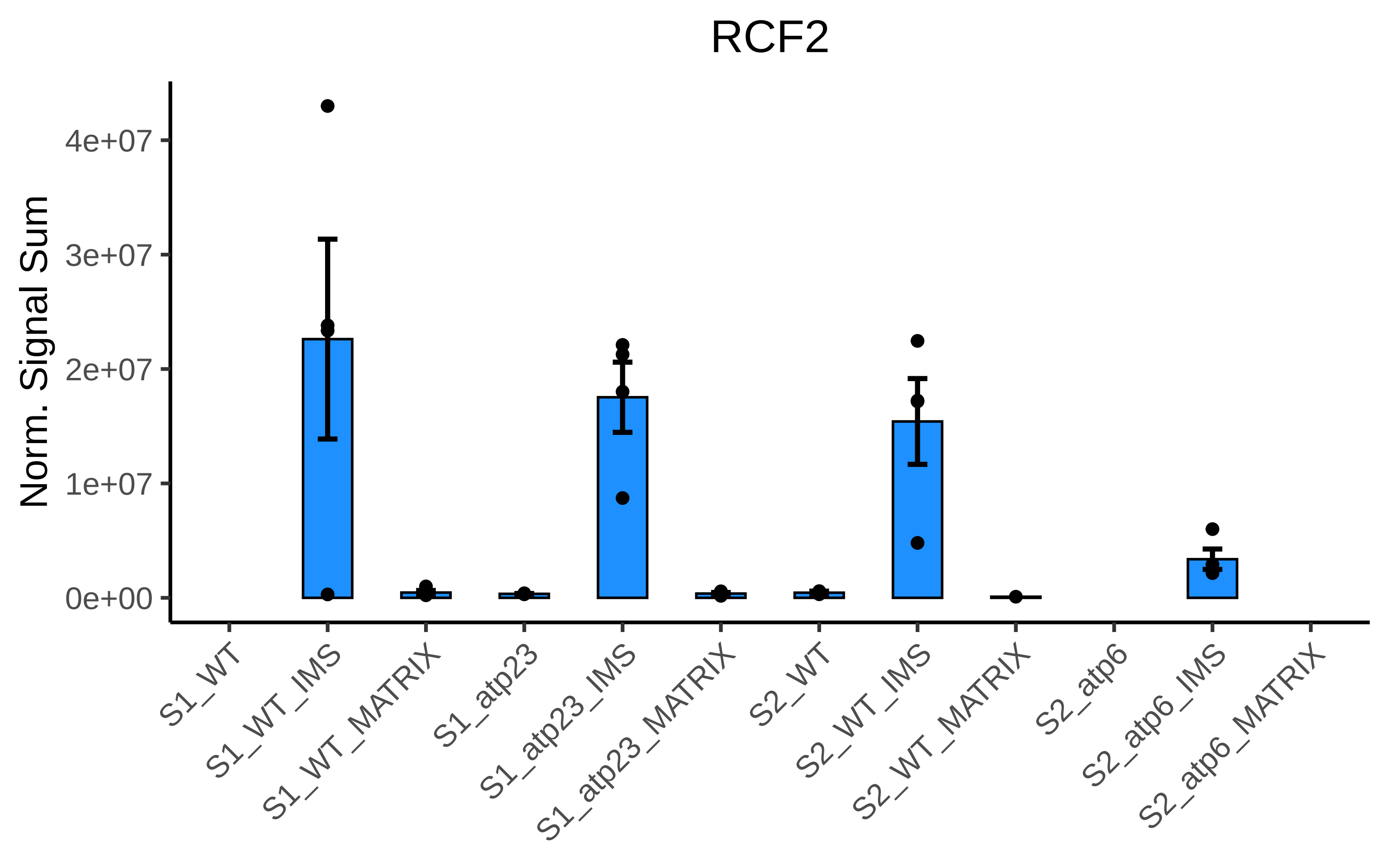
RCF2
Norm. Signal Sum
4e+07
3e+07
2e+07
1e+07
0e+00
S1_WT
S1_WT_IMS
S1_WT_MATRIX
S1_atp23
S1_atp23_IMS
S1_atp23_MATRIX
S2_WT
S2_WT_IMS
S2_WT_MATRIX
S2_atp6
S2_atp6_IMS
S2_atp6_MATRIX

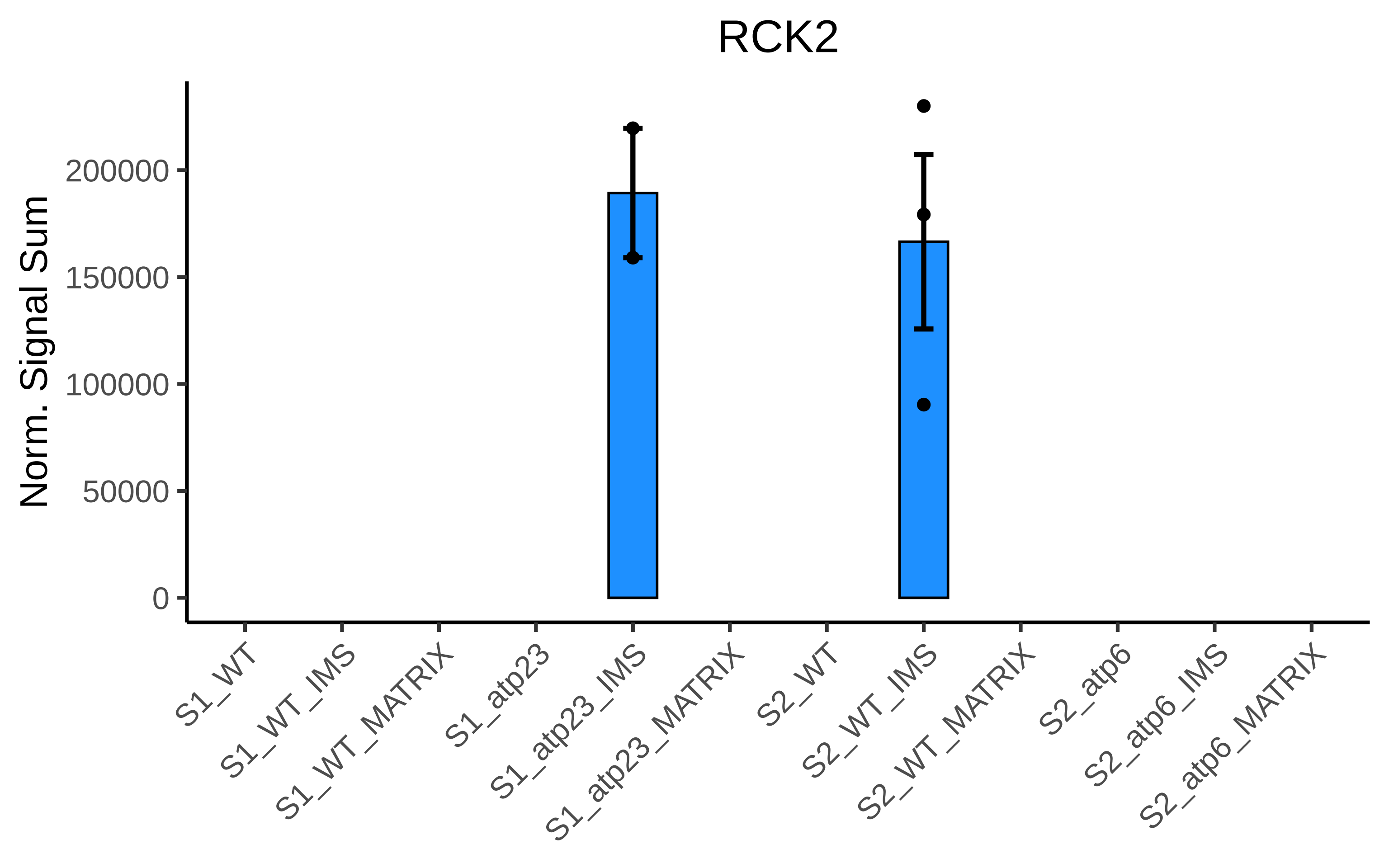

RCK2
Norm. Signal Sum
200000
150000
100000
50000
0
S1_WT
S1_WT_IMS
S1_WT_MATRIX
S1_atp23
S1_atp23_IMS
S1_atp23_MATRIX
S2_WT
S2_WT_IMS
S2_WT_MATRIX
S2_atp6
S2_atp6_IMS
S2_atp6_MATRIX

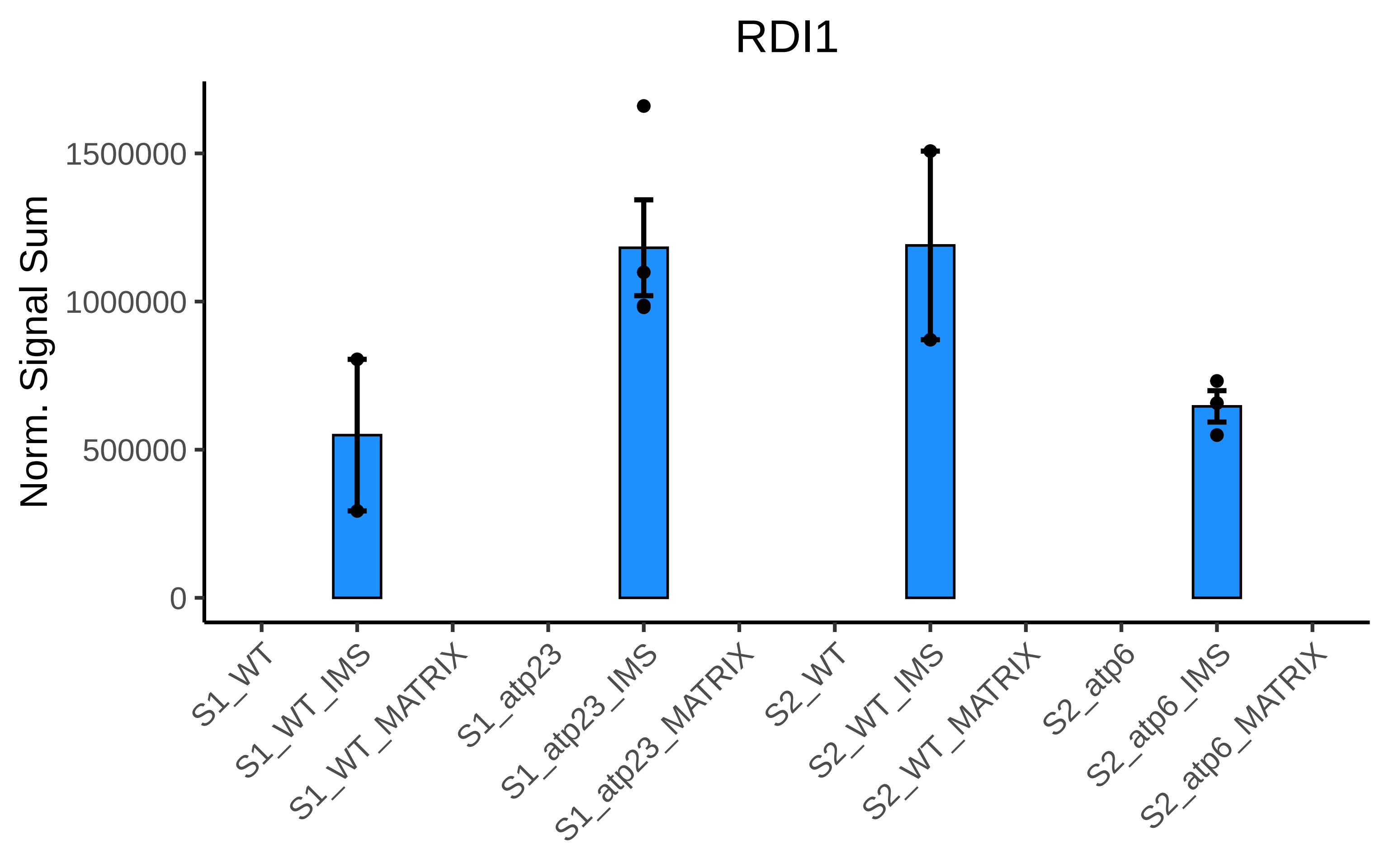
RDI1
Norm. Signal Sum
1500000
1000000
500000
0
S1_WT
S1_WT_IMS
S1_WT_MATRIX
S1_atp23
S1_atp23_IMS
S1_atp23_MATRIX
S2_WT
S2_WT_IMS
S2_WT_MATRIX
S2_atp6
S2_atp6_IMS
S2_atp6_MATRIX

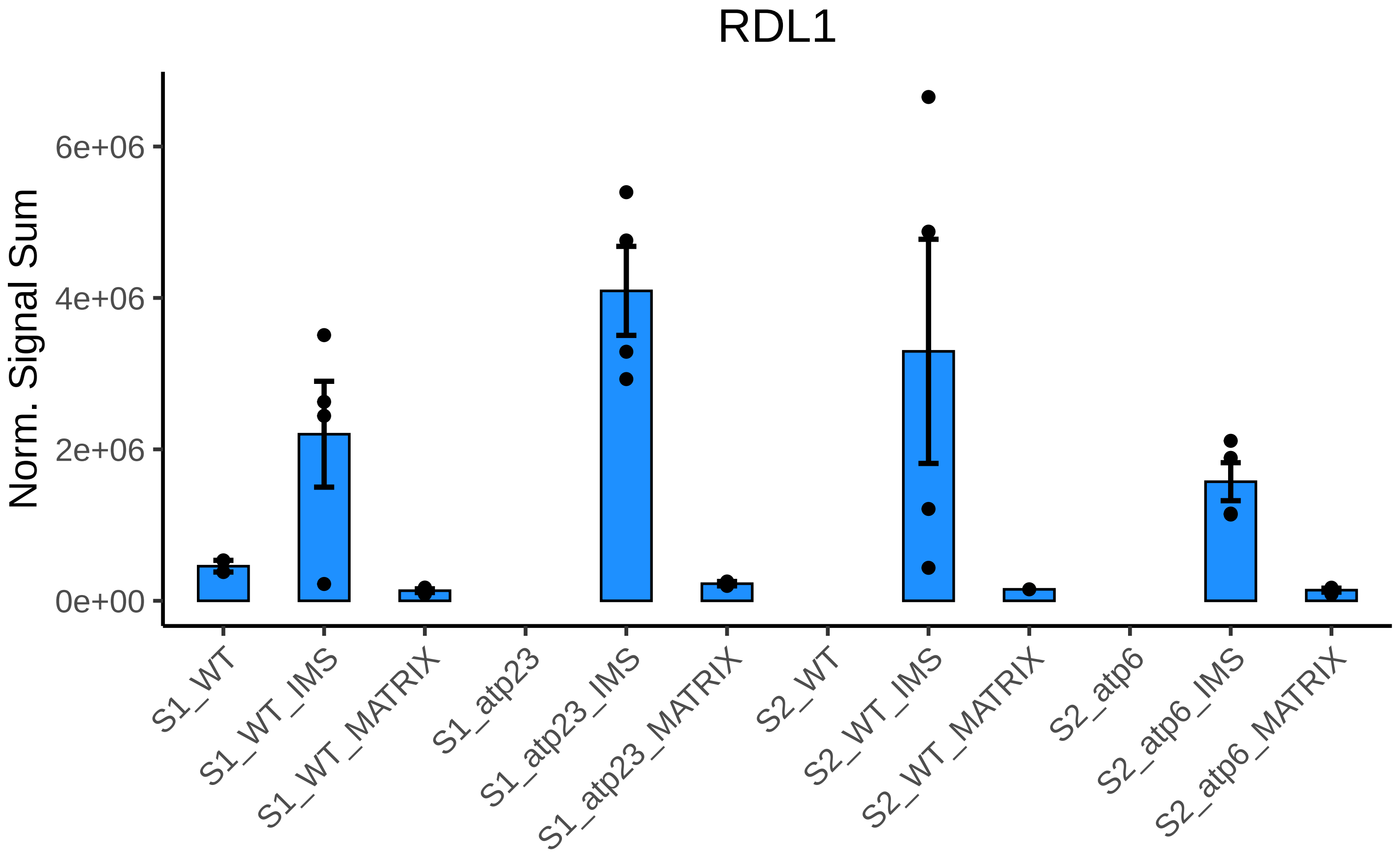
RDL1
Norm. Signal Sum
6e+06
4e+06
2e+06
0e+00
S1_WT
S1_WT_IMS
S1_WT_MATRIX
S1_atp23
S1_atp23_IMS
S1_atp23_MATRIX
S2_WT
S2_WT_IMS
S2_WT_MATRIX
S2_atp6
S2_atp6_IMS
S2_atp6_MATRIX

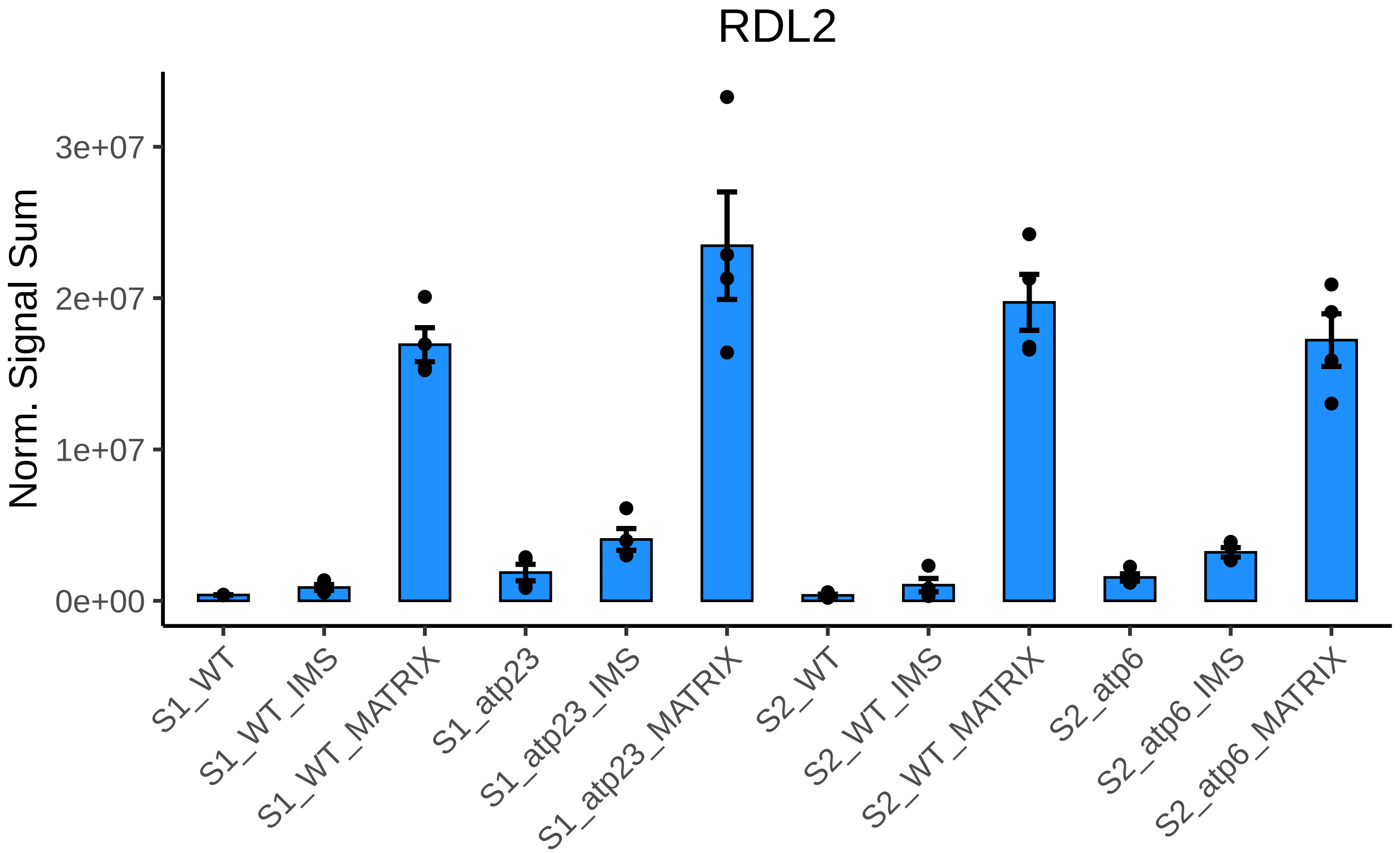
RDL2
Norm. Signal Sum
3e+07
2e+07
1e+07
0e+00
S1_WT
S1_WT_IMS
S1_WT_MATRIX
S1_atp23
S1_atp23_IMS
S1_atp23_MATRIX
S2_WT
S2_WT_IMS
S2_WT_MATRIX
S2_atp6
S2_atp6_IMS
S2_atp6_MATRIX

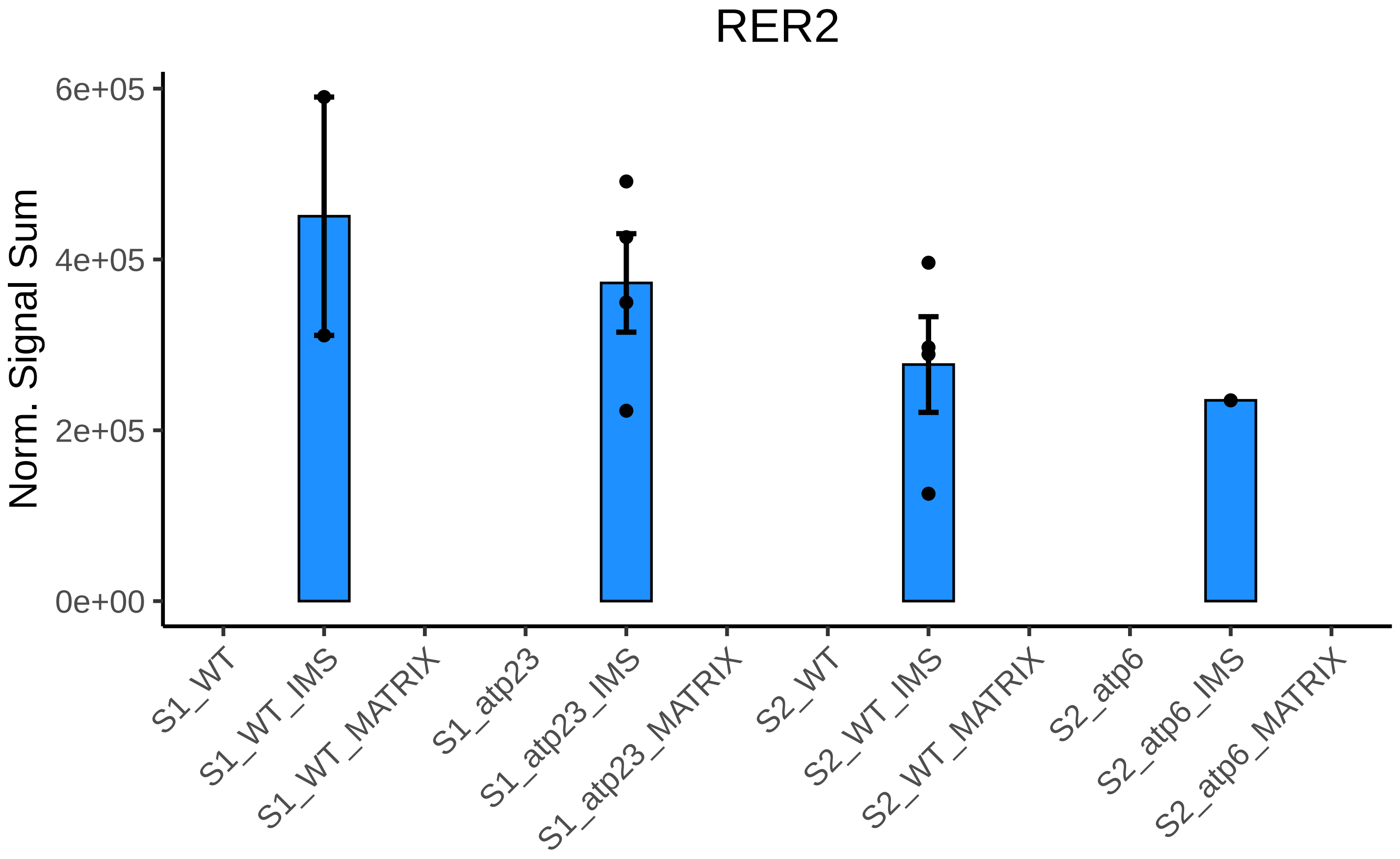
RER2
Norm. Signal Sum
6e+05
4e+05
2e+05
0e+00
S1_WT
S1_WT_IMS
S1_WT_MATRIX
S1_atp23
S1_atp23_IMS
S1_atp23_MATRIX
S2_WT
S2_WT_IMS
S2_WT_MATRIX
S2_atp6
S2_atp6_IMS
S2_atp6_MATRIX

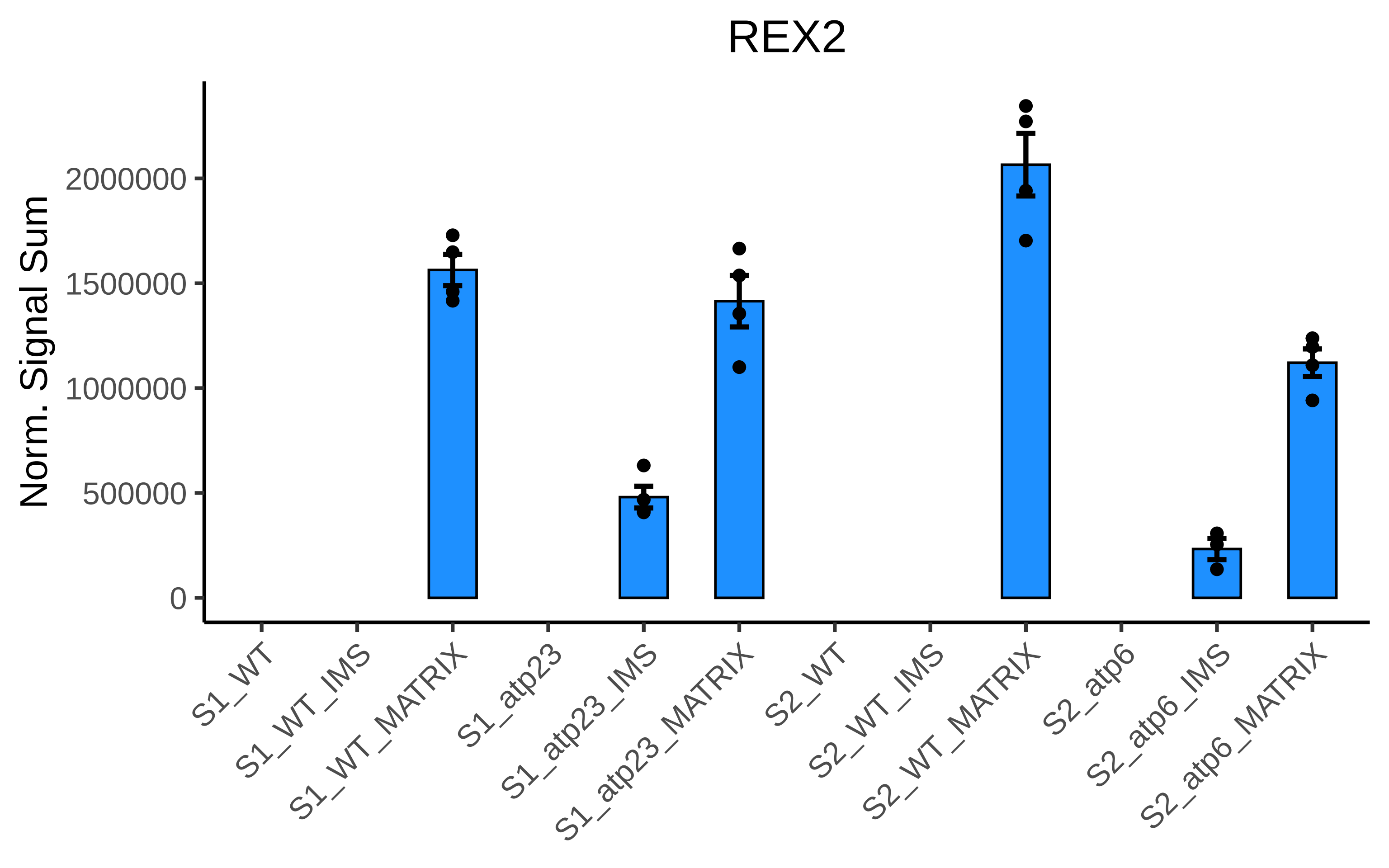
REX2
Norm. Signal Sum
0
500000
1000000
1500000
2000000
S1_WT
S1_WT_IMS
S1_WT_MATRIX
S1_atp23
S1_atp23_IMS
S1_atp23_MATRIX
S2_WT
S2_WT_IMS
S2_WT_MATRIX
S2_atp6
S2_atp6_IMS
S2_atp6_MATRIX

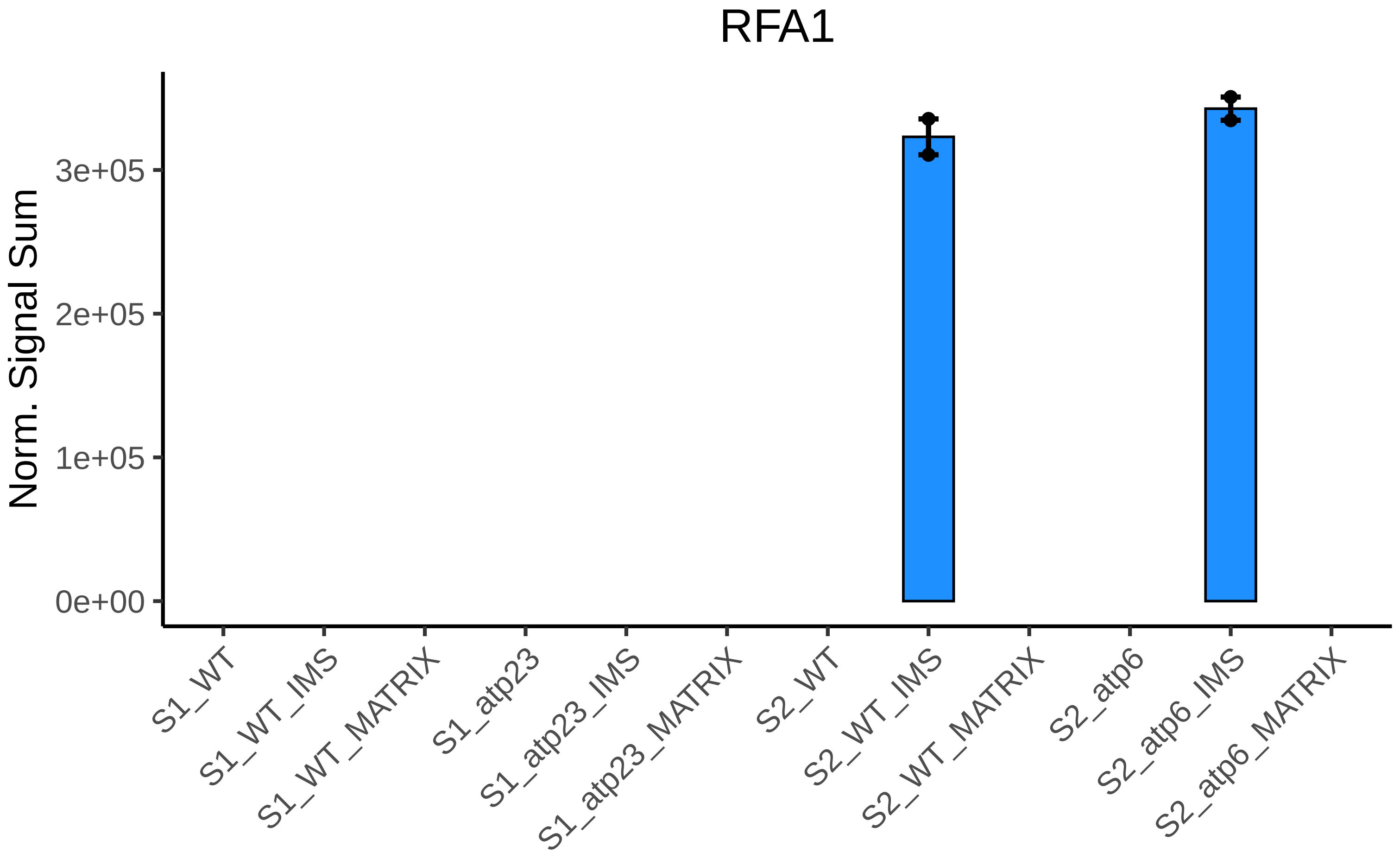

RFA1
Norm. Signal Sum
3e+05
2e+05
1e+05
0e+00
S1_WT
S1_WT_IMS
S1_WT_MATRIX
S1_atp23
S1_atp23_IMS
S1_atp23_MATRIX
S2_WT
S2_WT_IMS
S2_WT_MATRIX
S2_atp6
S2_atp6_IMS
S2_atp6_MATRIX

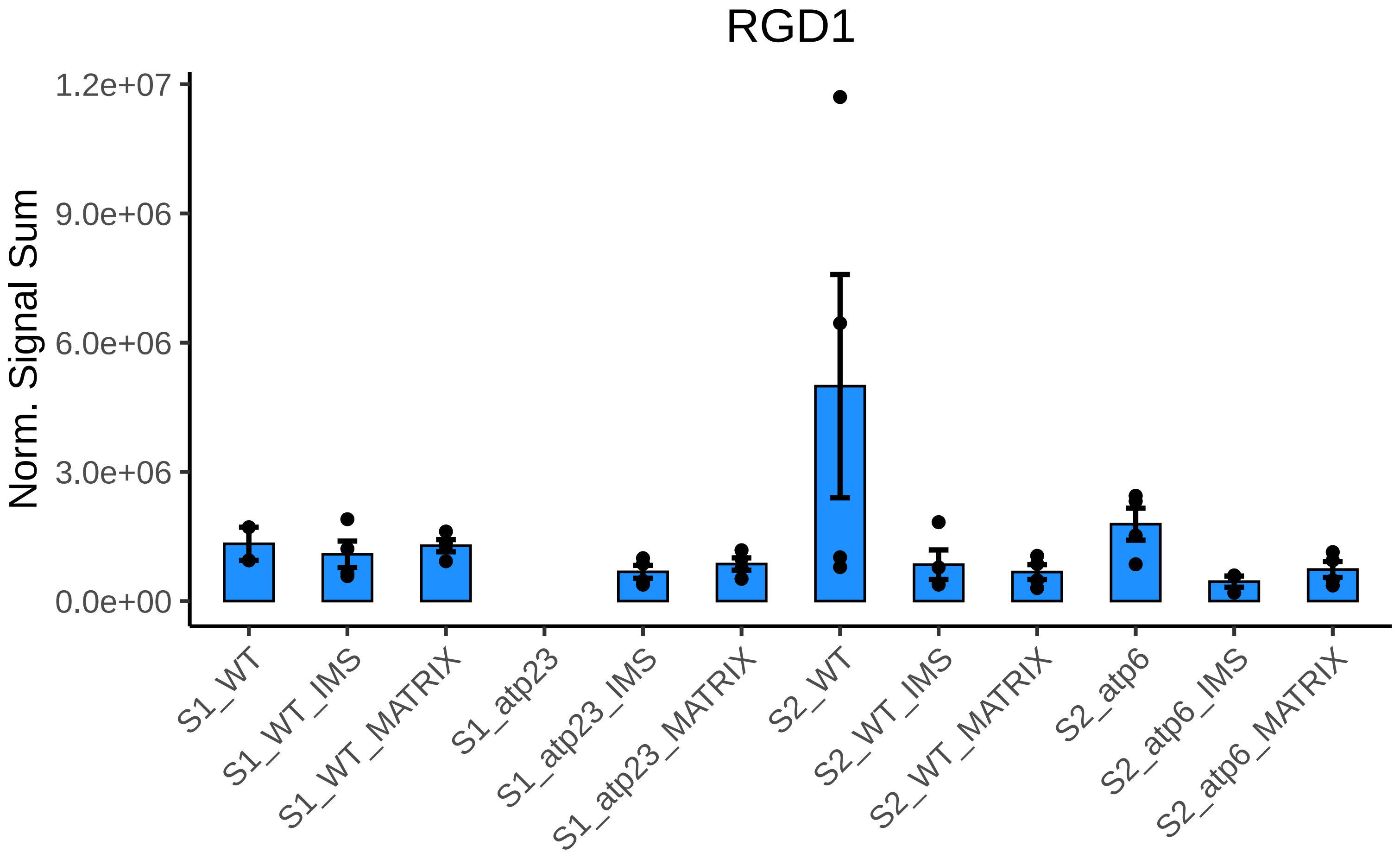

RGD1
Norm. Signal Sum
1.2e+07
9.0e+06
6.0e+06
3.0e+06
0.0e+00
S1_WT
S1_WT_IMS
S1_WT_MATRIX
S1_atp23
S1_atp23_IMS
S1_atp23_MATRIX
S2_WT
S2_WT_IMS
S2_WT_MATRIX
S2_atp6
S2_atp6_IMS
S2_atp6_MATRIX

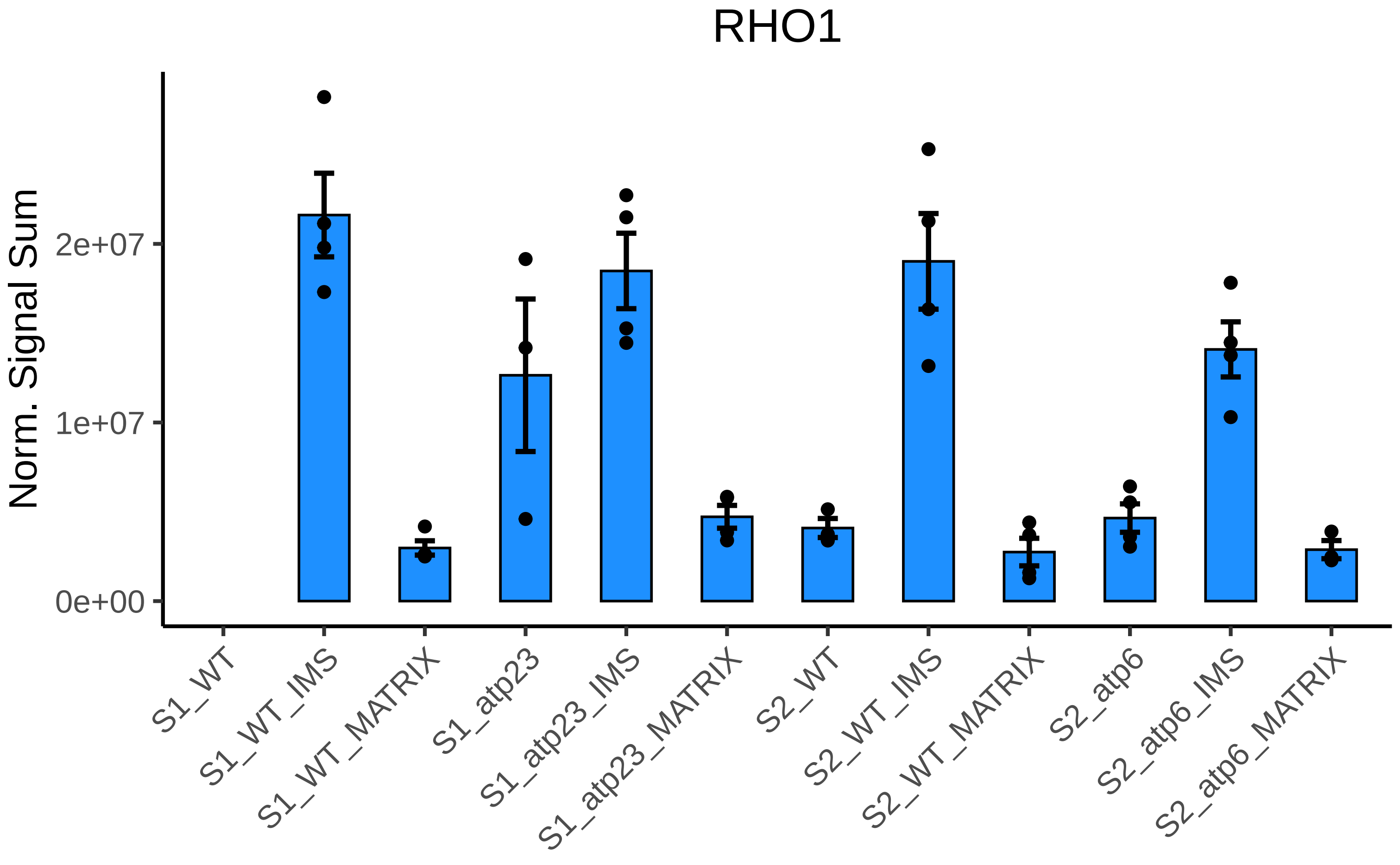
RHO1
Norm. Signal Sum
2e+07
1e+07
0e+00
S1_WT
S1_WT_IMS
S1_WT_MATRIX
S1_atp23
S1_atp23_IMS
S1_atp23_MATRIX
S2_WT
S2_WT_IMS
S2_WT_MATRIX
S2_atp6
S2_atp6_IMS
S2_atp6_MATRIX

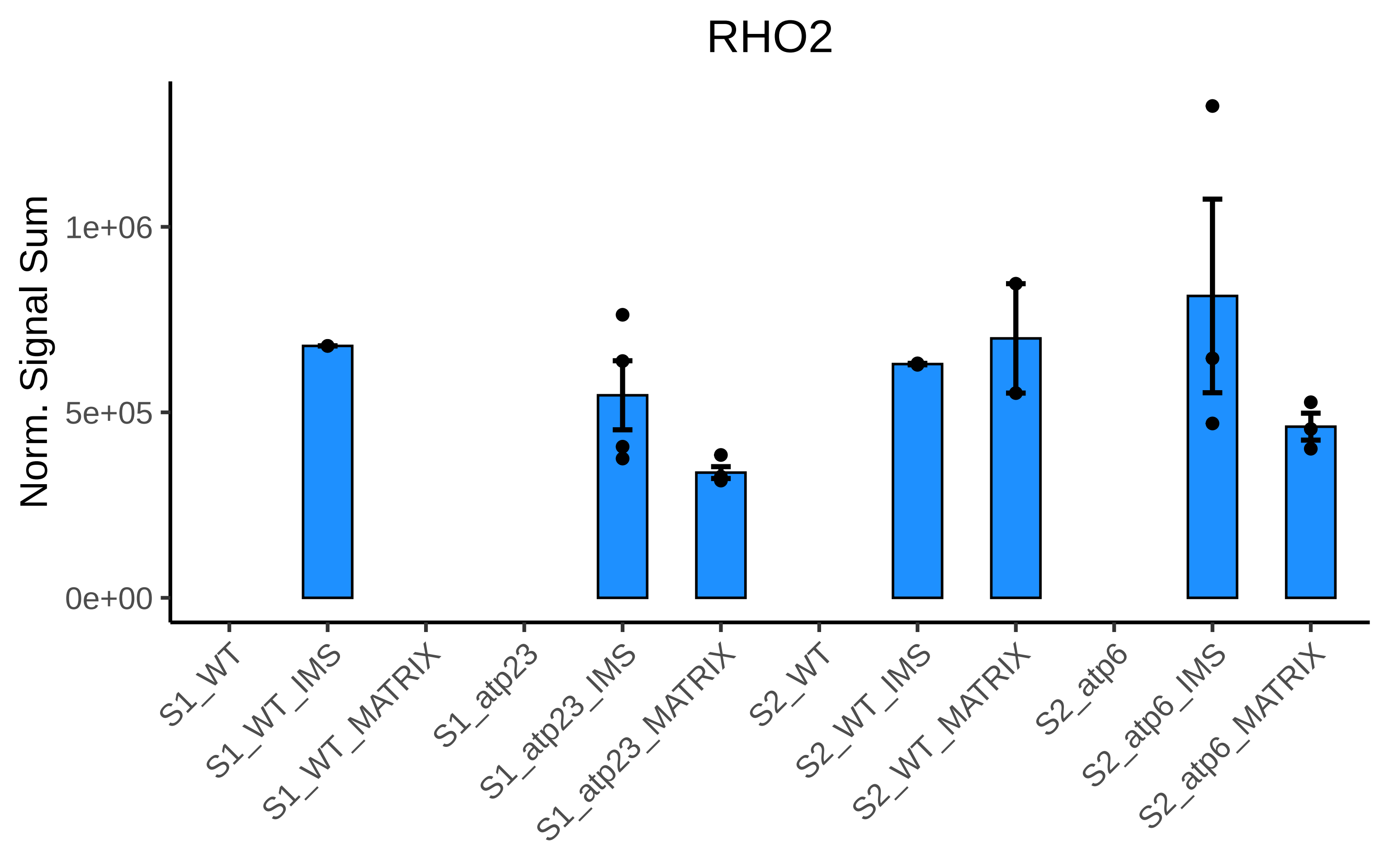

RHO2
Norm. Signal Sum
1e+06
5e+05
0e+00
S1_WT
S1_WT_IMS
S1_WT_MATRIX
S1_atp23
S1_atp23_IMS
S1_atp23_MATRIX
S2_WT
S2_WT_IMS
S2_WT_MATRIX
S2_atp6
S2_atp6_IMS
S2_atp6_MATRIX

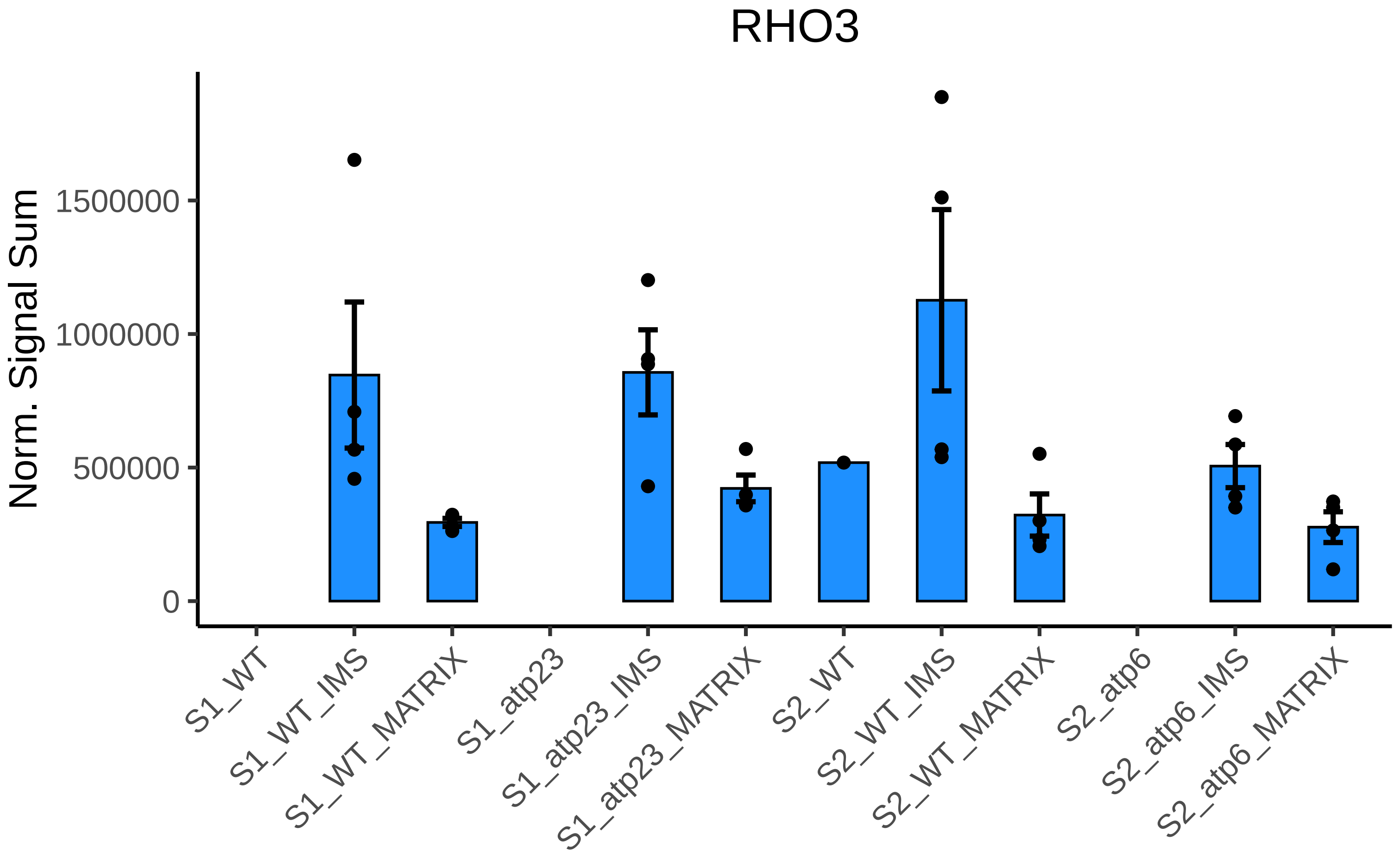

RHO3
Norm. Signal Sum
1500000
1000000
500000
0
S1_WT
S1_WT_IMS
S1_WT_MATRIX
S1_atp23
S1_atp23_IMS
S1_atp23_MATRIX
S2_WT
S2_WT_IMS
S2_WT_MATRIX
S2_atp6
S2_atp6_IMS
S2_atp6_MATRIX

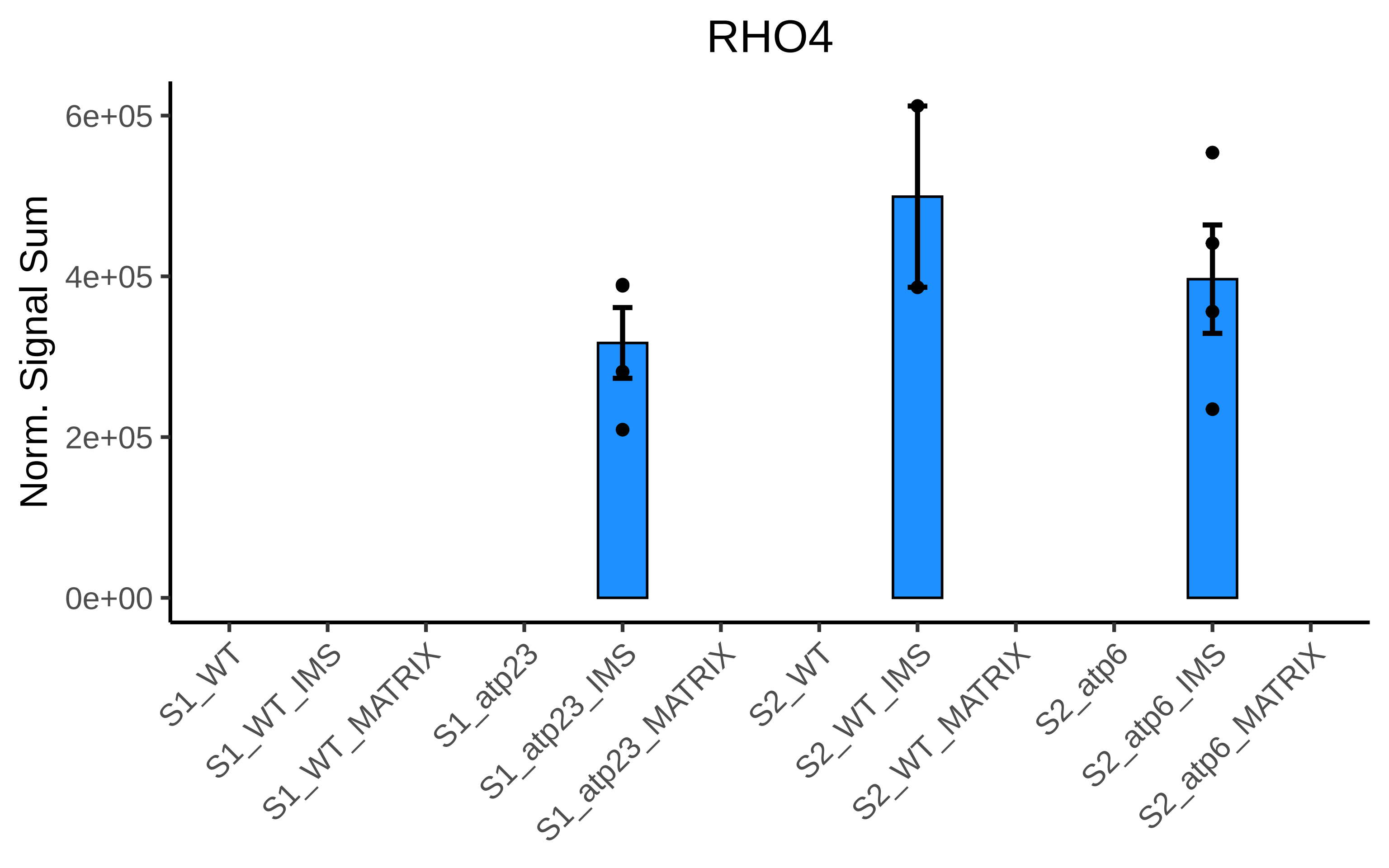
RHO4
Norm. Signal Sum
6e+05
4e+05
2e+05
0e+00
S1_WT
S1_WT_IMS
S1_WT_MATRIX
S1_atp23
S1_atp23_IMS
S1_atp23_MATRIX
S2_WT
S2_WT_IMS
S2_WT_MATRIX
S2_atp6
S2_atp6_IMS
S2_atp6_MATRIX

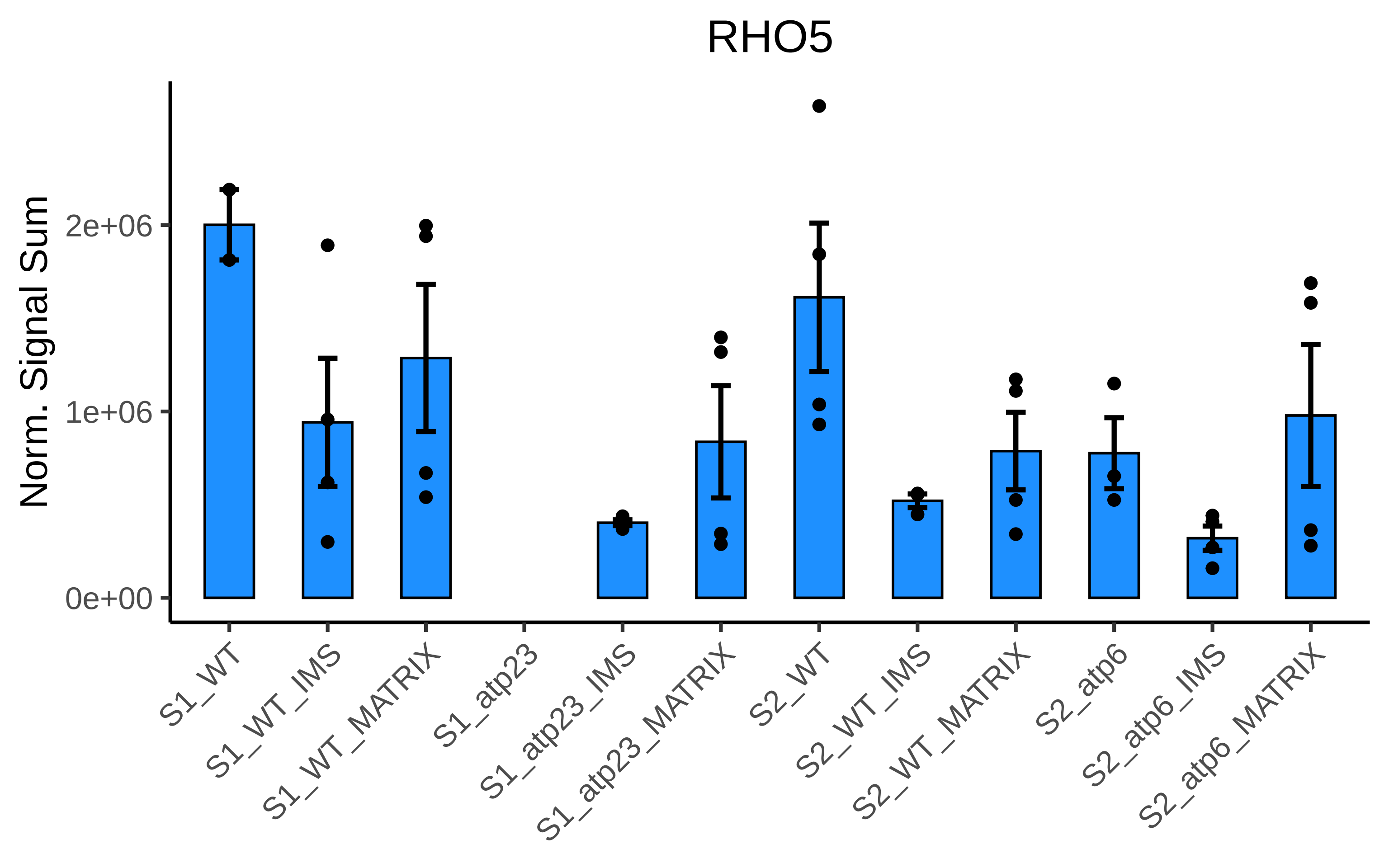
RHO5
Norm. Signal Sum
2e+06
1e+06
0e+00
S1_WT
S1_WT_IMS
S1_WT_MATRIX
S1_atp23
S1_atp23_IMS
S1_atp23_MATRIX
S2_WT
S2_WT_IMS
S2_WT_MATRIX
S2_atp6
S2_atp6_IMS
S2_atp6_MATRIX

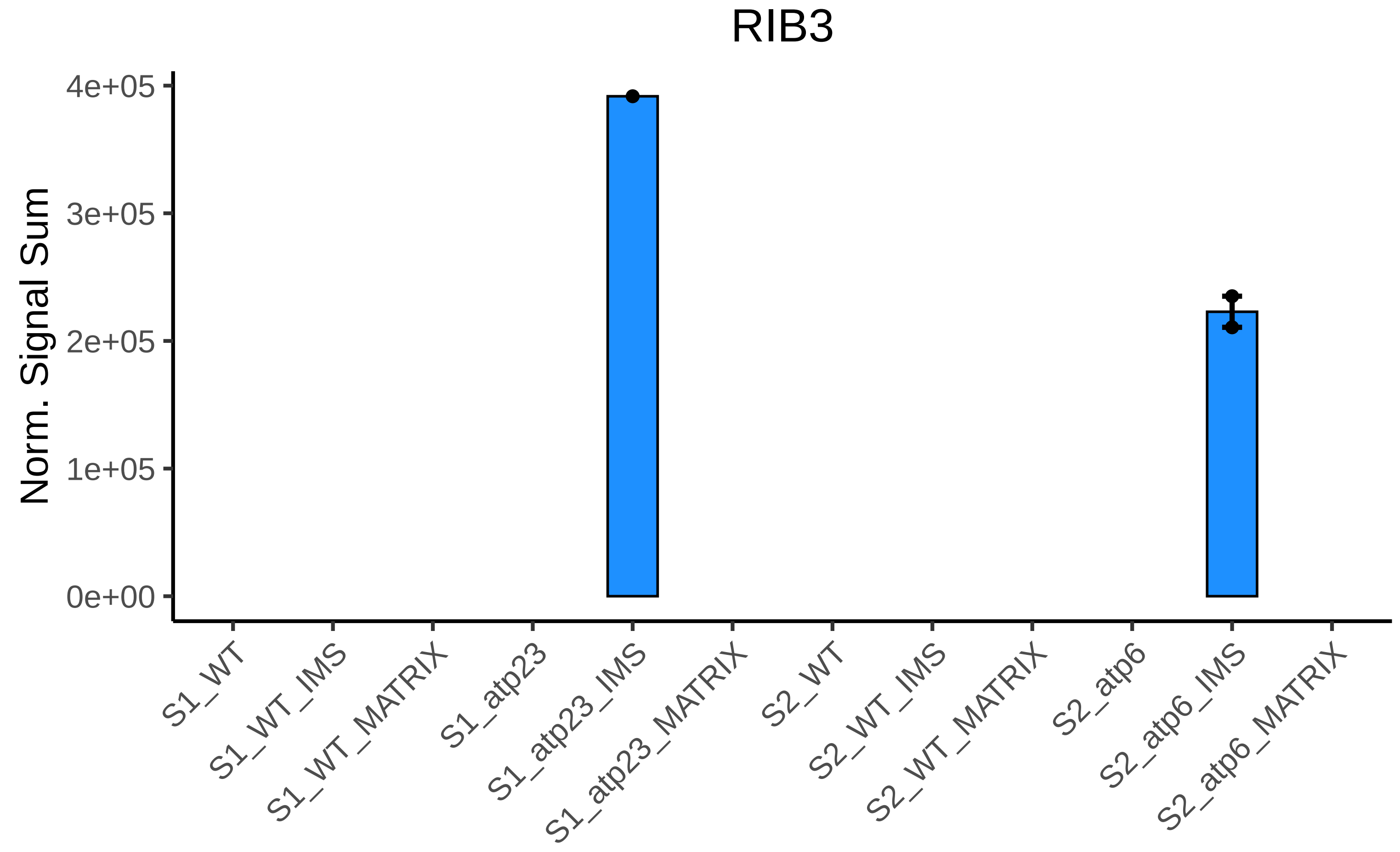
RIB3
Norm. Signal Sum
4e+05
3e+05
2e+05
1e+05
0e+00
S1_WT
S1_WT_IMS
S1_WT_MATRIX
S1_atp23
S1_atp23_IMS
S1_atp23_MATRIX
S2_WT
S2_WT_IMS
S2_WT_MATRIX
S2_atp6
S2_atp6_IMS
S2_atp6_MATRIX

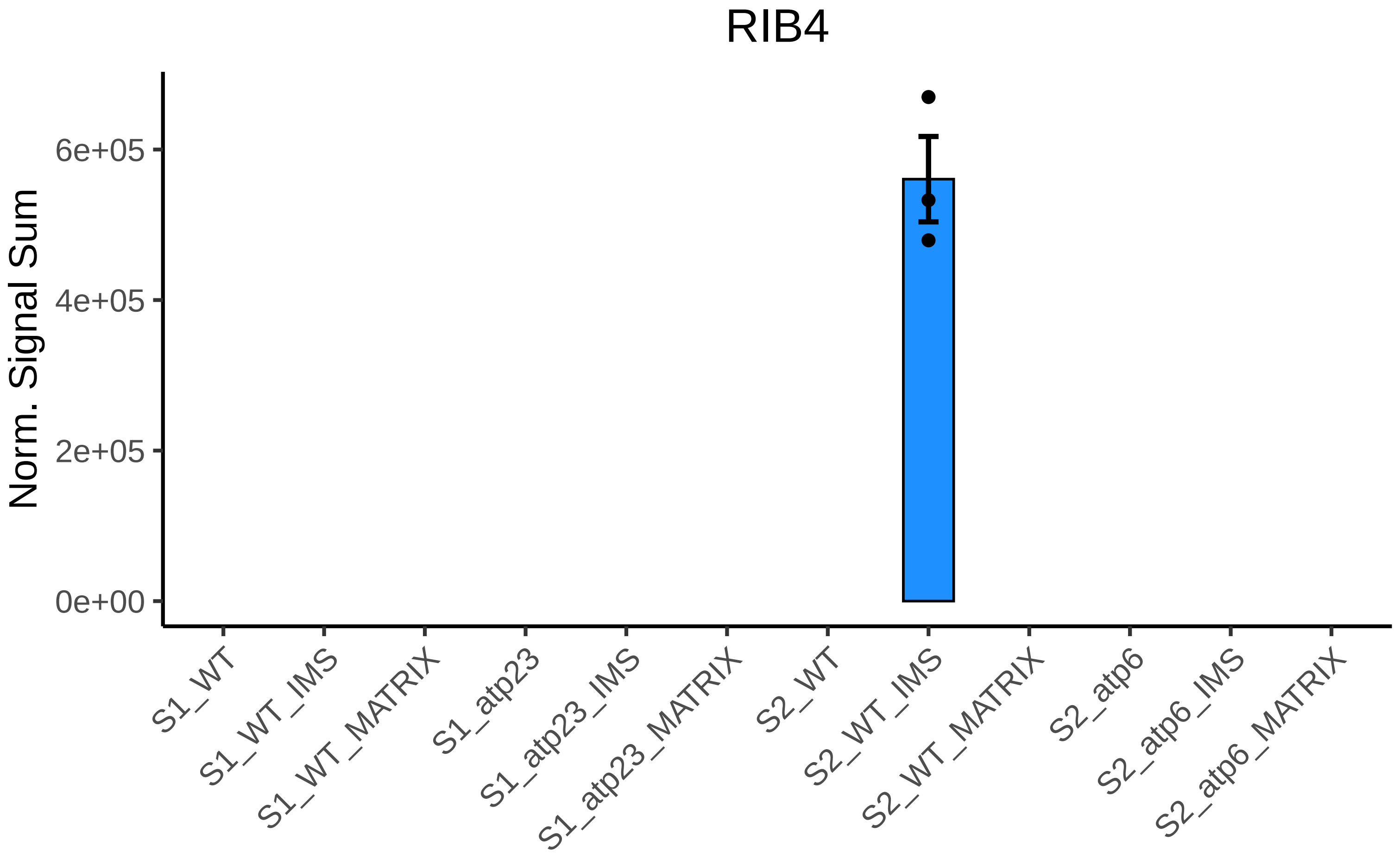

RIB4
Norm. Signal Sum
6e+05
4e+05
2e+05
0e+00
S1_WT
S1_WT_IMS
S1_WT_MATRIX
S1_atp23
S1_atp23_IMS
S1_atp23_MATRIX
S2_WT
S2_WT_IMS
S2_WT_MATRIX
S2_atp6
S2_atp6_IMS
S2_atp6_MATRIX

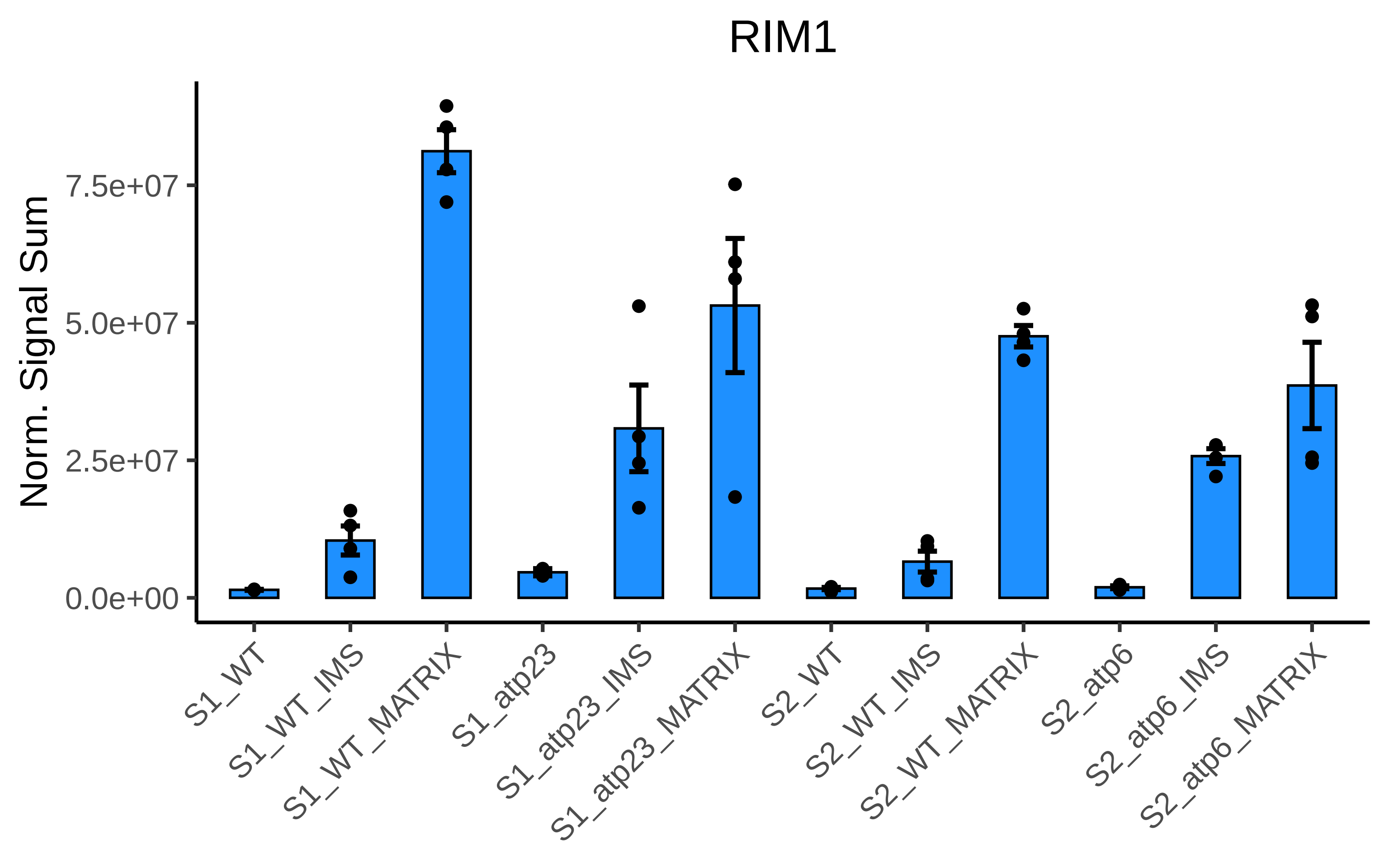

RIM1
Norm. Signal Sum
7.5e+07
5.0e+07
2.5e+07
0.0e+00
S1_WT
S1_WT_IMS
S1_WT_MATRIX
S1_atp23
S1_atp23_IMS
S1_atp23_MATRIX
S2_WT
S2_WT_IMS
S2_WT_MATRIX
S2_atp6
S2_atp6_IMS
S2_atp6_MATRIX

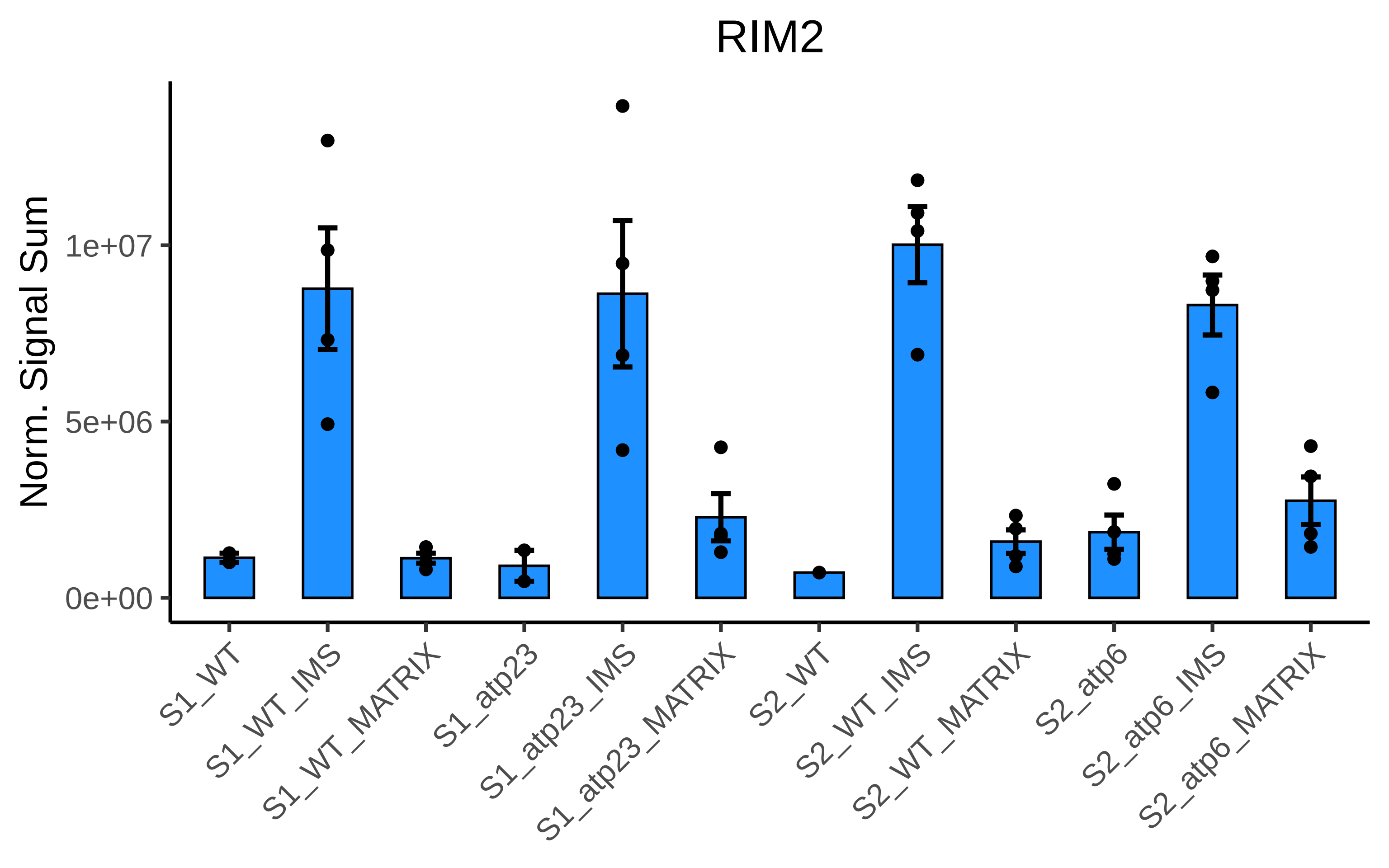
RIM2
Norm. Signal Sum
1e+07
5e+06
0e+00
S1_WT
S1_WT_IMS
S1_WT_MATRIX
S1_atp23
S1_atp23_IMS
S1_atp23_MATRIX
S2_WT
S2_WT_IMS
S2_WT_MATRIX
S2_atp6
S2_atp6_IMS
S2_atp6_MATRIX

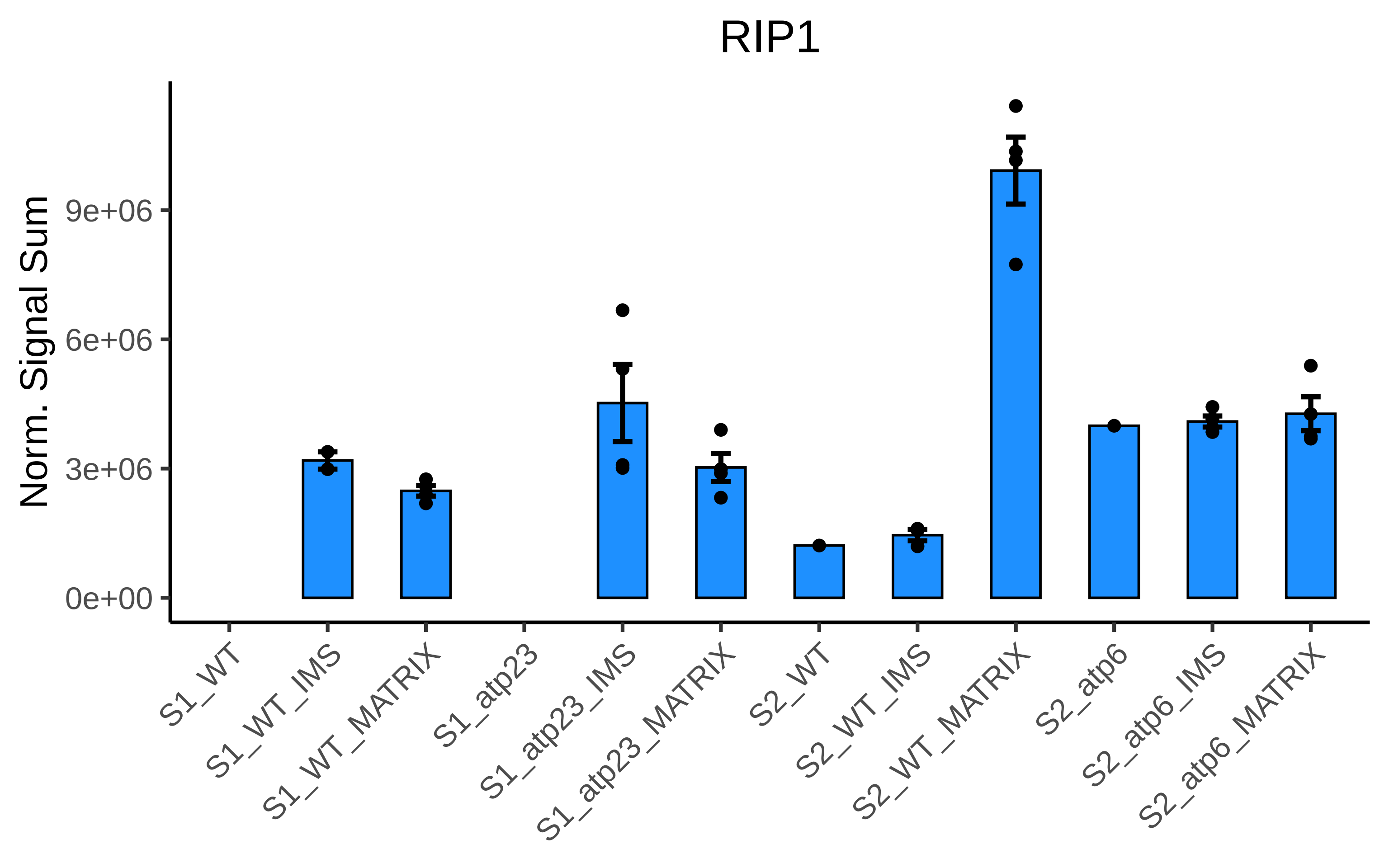
RIP1
Norm. Signal Sum
9e+06
6e+06
3e+06
0e+00
S1_WT
S1_WT_IMS
S1_WT_MATRIX
S1_atp23
S1_atp23_IMS
S1_atp23_MATRIX
S2_WT
S2_WT_IMS
S2_WT_MATRIX
S2_atp6
S2_atp6_IMS
S2_atp6_MATRIX

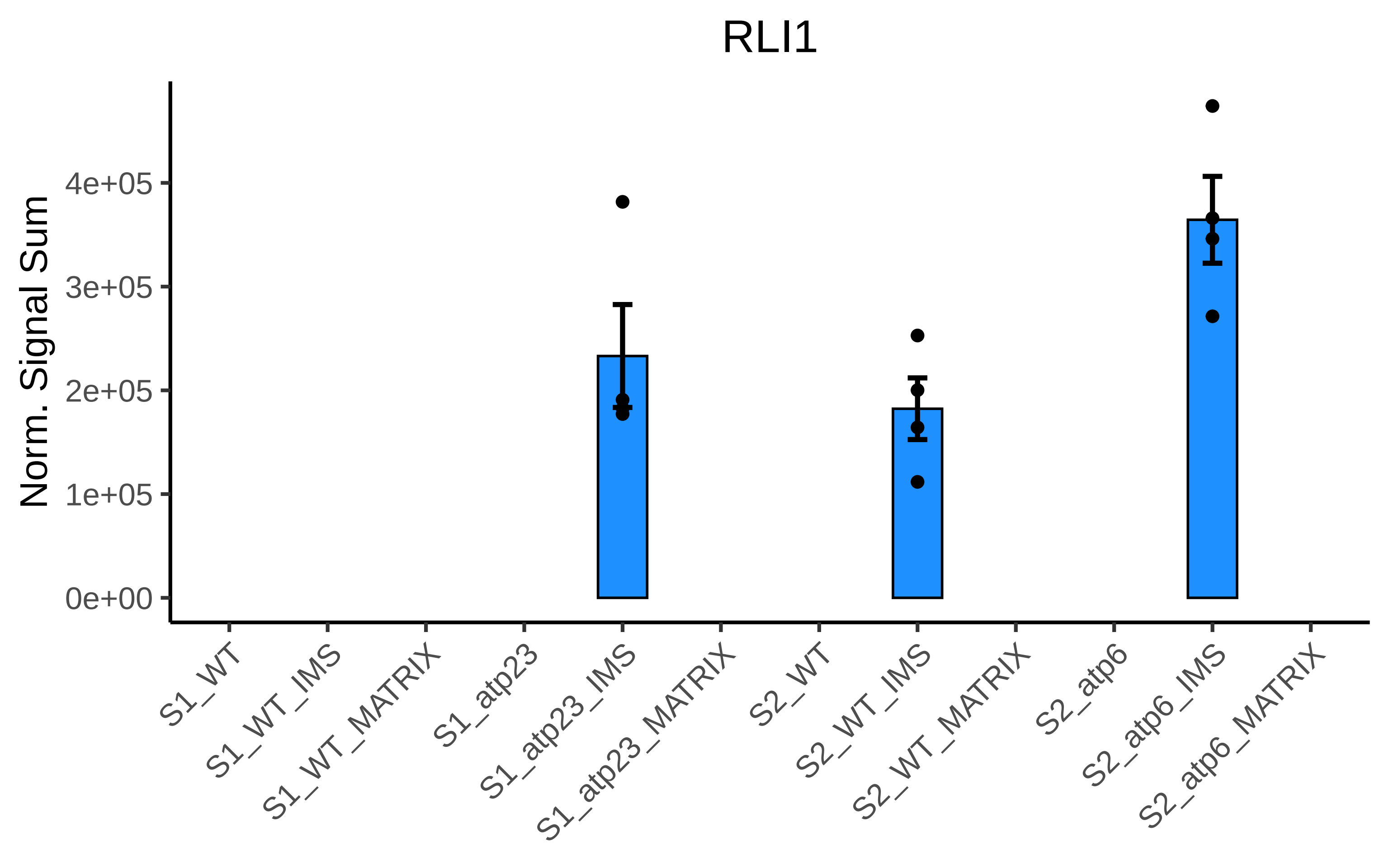

RLI1
Norm. Signal Sum
4e+05
3e+05
2e+05
1e+05
0e+00
S1_WT
S1_WT_IMS
S1_WT_MATRIX
S1_atp23
S1_atp23_IMS
S1_atp23_MATRIX
S2_WT
S2_WT_IMS
S2_WT_MATRIX
S2_atp6
S2_atp6_IMS
S2_atp6_MATRIX

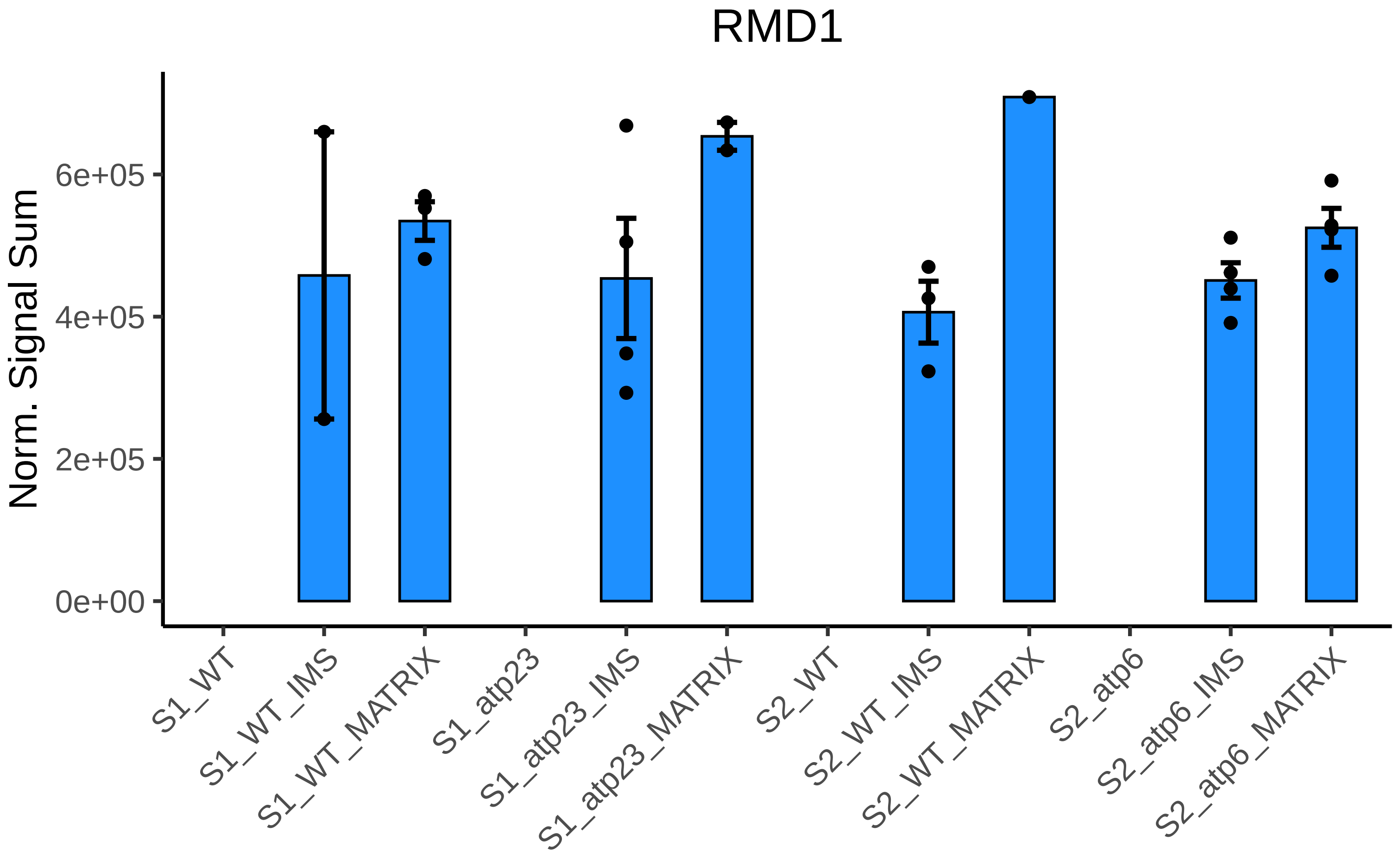

RMD1
Norm. Signal Sum
6e+05
4e+05
2e+05
0e+00
S1_WT
S1_WT_IMS
S1_WT_MATRIX
S1_atp23
S1_atp23_IMS
S1_atp23_MATRIX
S2_WT
S2_WT_IMS
S2_WT_MATRIX
S2_atp6
S2_atp6_IMS
S2_atp6_MATRIX

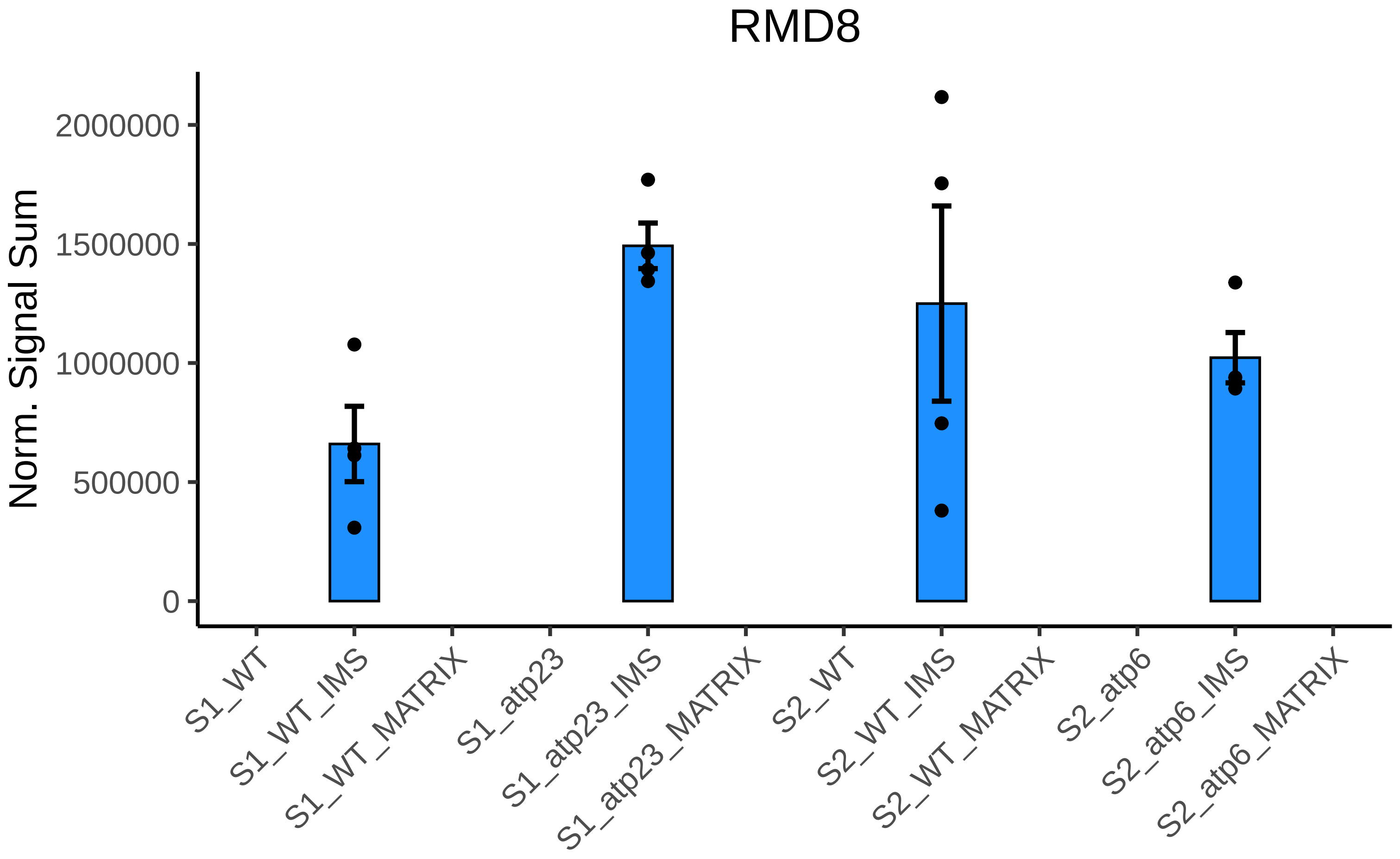

RMD8
Norm. Signal Sum
2000000
1500000
1000000
500000
0
S1_WT
S1_WT_IMS
S1_WT_MATRIX
S1_atp23
S1_atp23_IMS
S1_atp23_MATRIX
S2_WT
S2_WT_IMS
S2_WT_MATRIX
S2_atp6
S2_atp6_IMS
S2_atp6_MATRIX

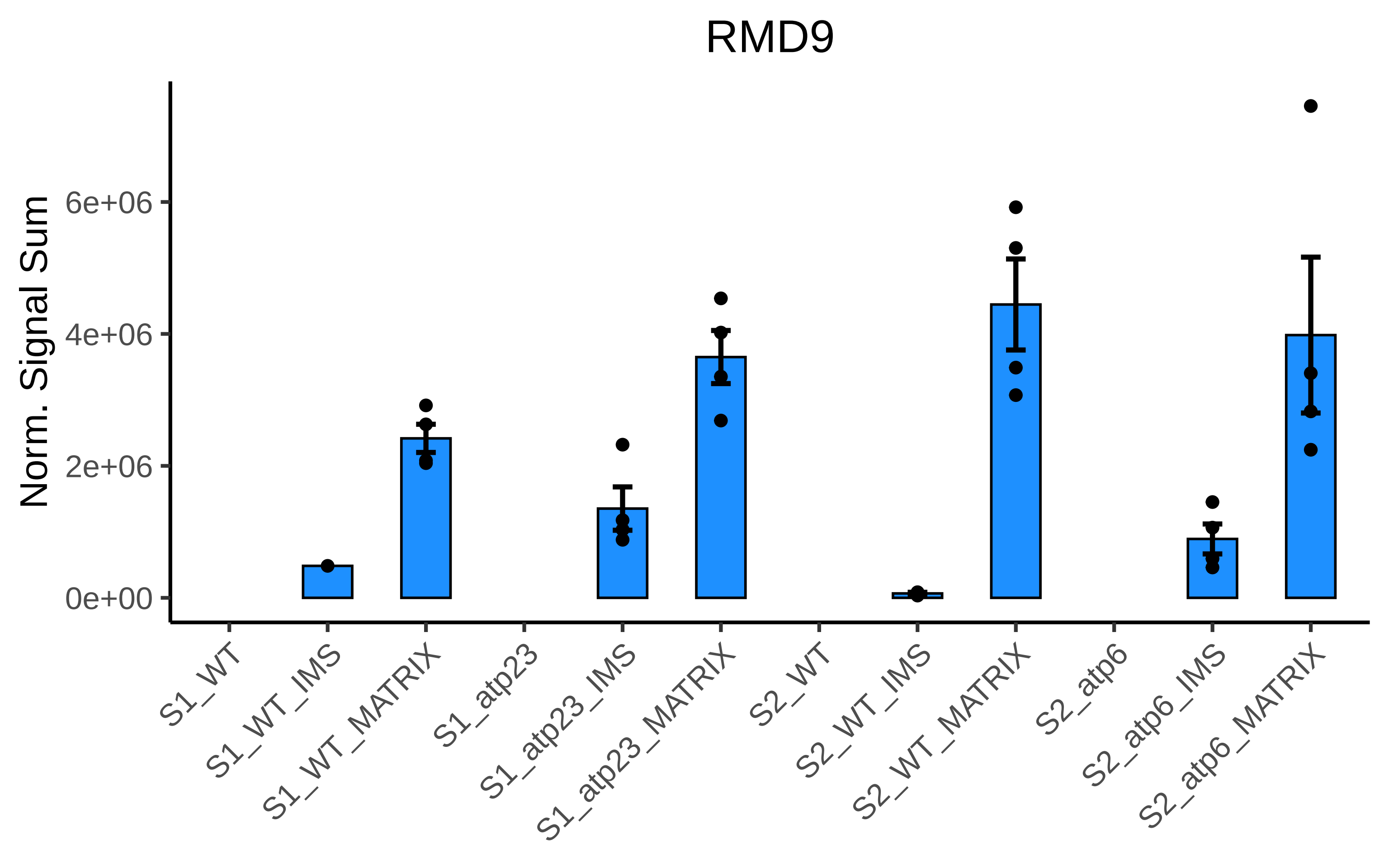

RMD9
Norm. Signal Sum
6e+06
4e+06
2e+06
0e+00
S1_WT
S1_WT_IMS
S1_WT_MATRIX
S1_atp23
S1_atp23_IMS
S1_atp23_MATRIX
S2_WT
S2_WT_IMS
S2_WT_MATRIX
S2_atp6
S2_atp6_IMS
S2_atp6_MATRIX

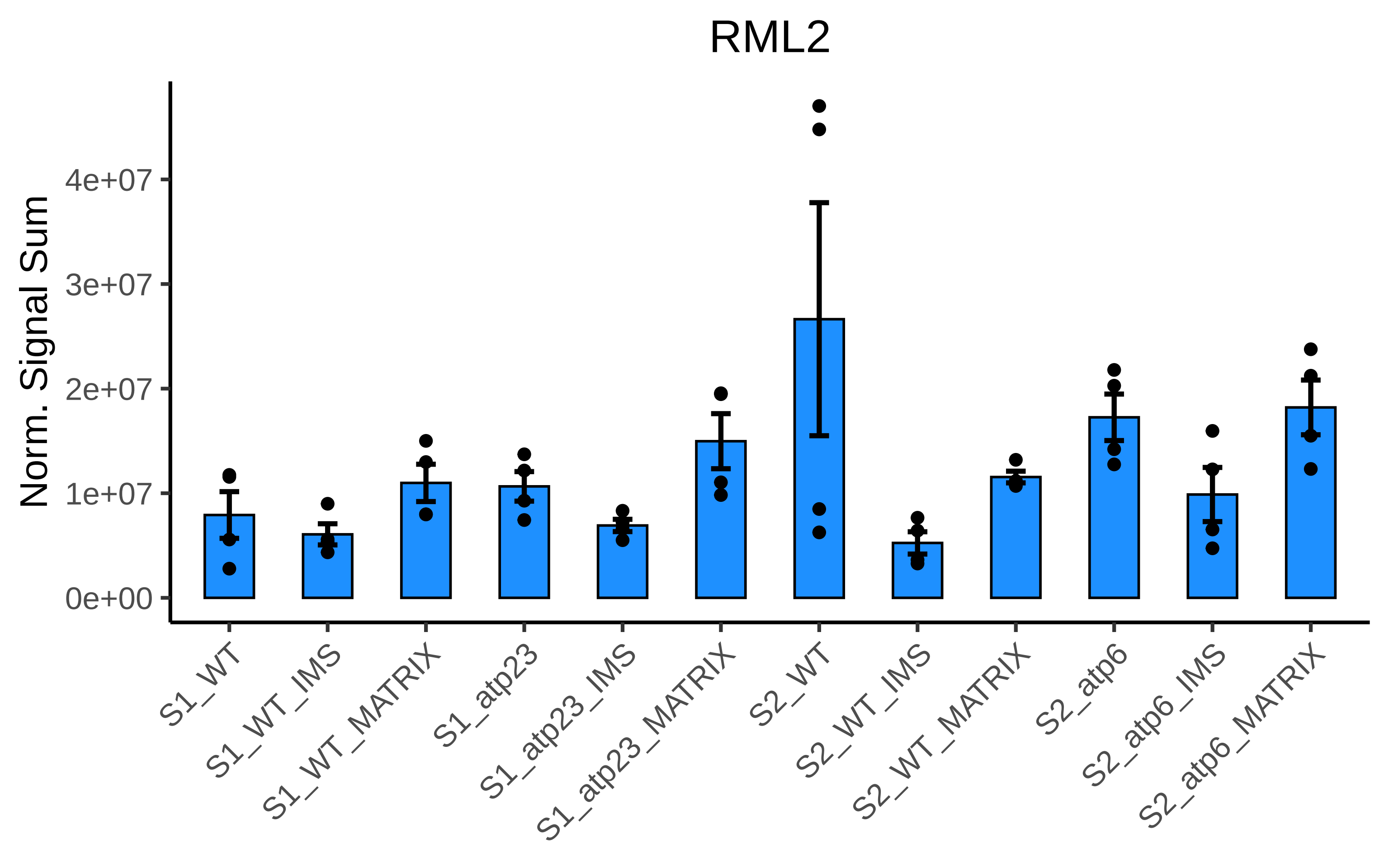

RML2
Norm. Signal Sum
0e+00
1e+07
2e+07
3e+07
4e+07
S1_WT
S1_WT_IMS
S1_WT_MATRIX
S1_atp23
S1_atp23_IMS
S1_atp23_MATRIX
S2_WT
S2_WT_IMS
S2_WT_MATRIX
S2_atp6
S2_atp6_IMS
S2_atp6_MATRIX

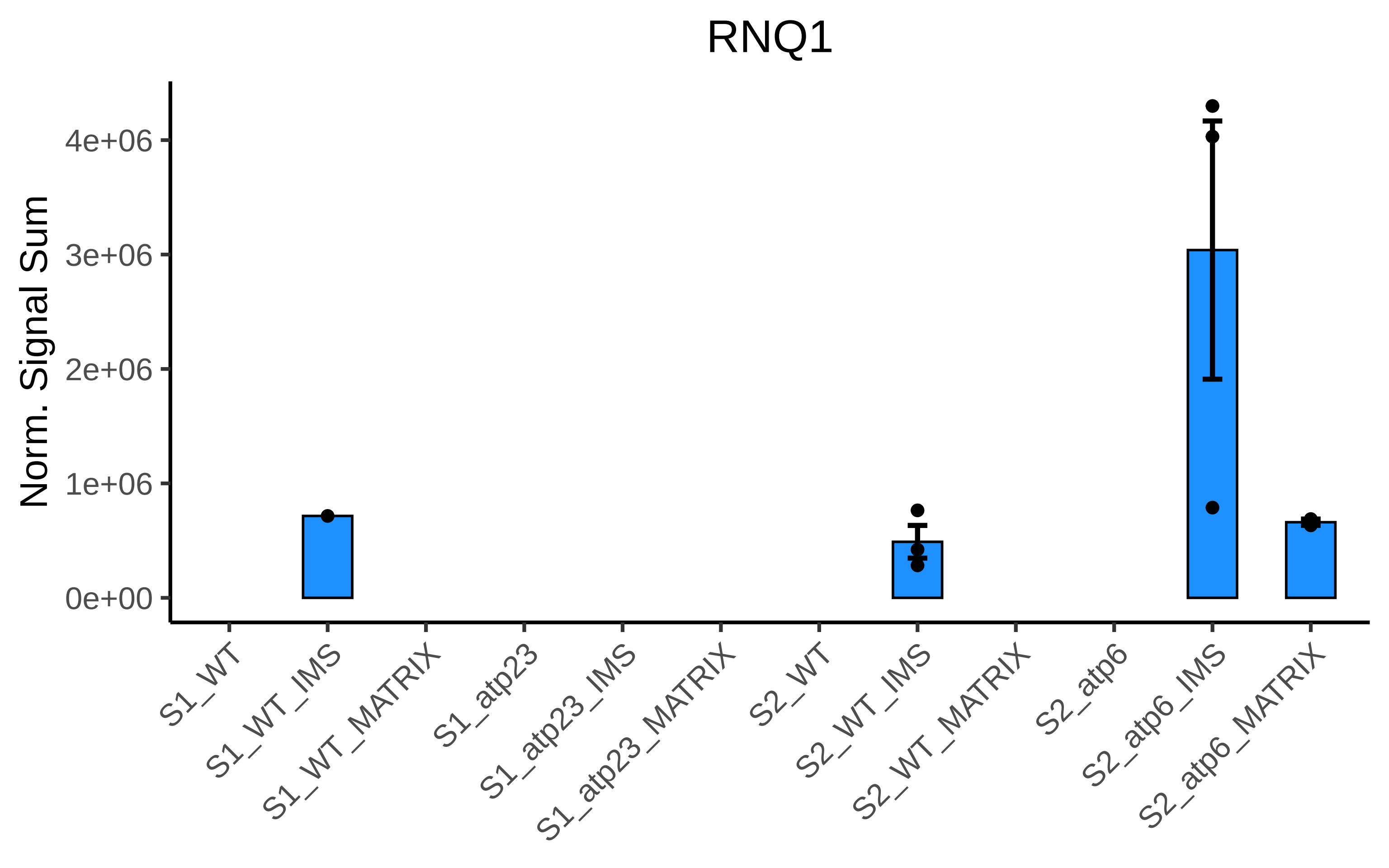
RNQ1
Norm. Signal Sum
0e+00
1e+06
2e+06
3e+06
4e+06
S1_WT
S1_WT_IMS
S1_WT_MATRIX
S1_atp23
S1_atp23_IMS
S1_atp23_MATRIX
S2_WT
S2_WT_IMS
S2_WT_MATRIX
S2_atp6
S2_atp6_IMS
S2_atp6_MATRIX

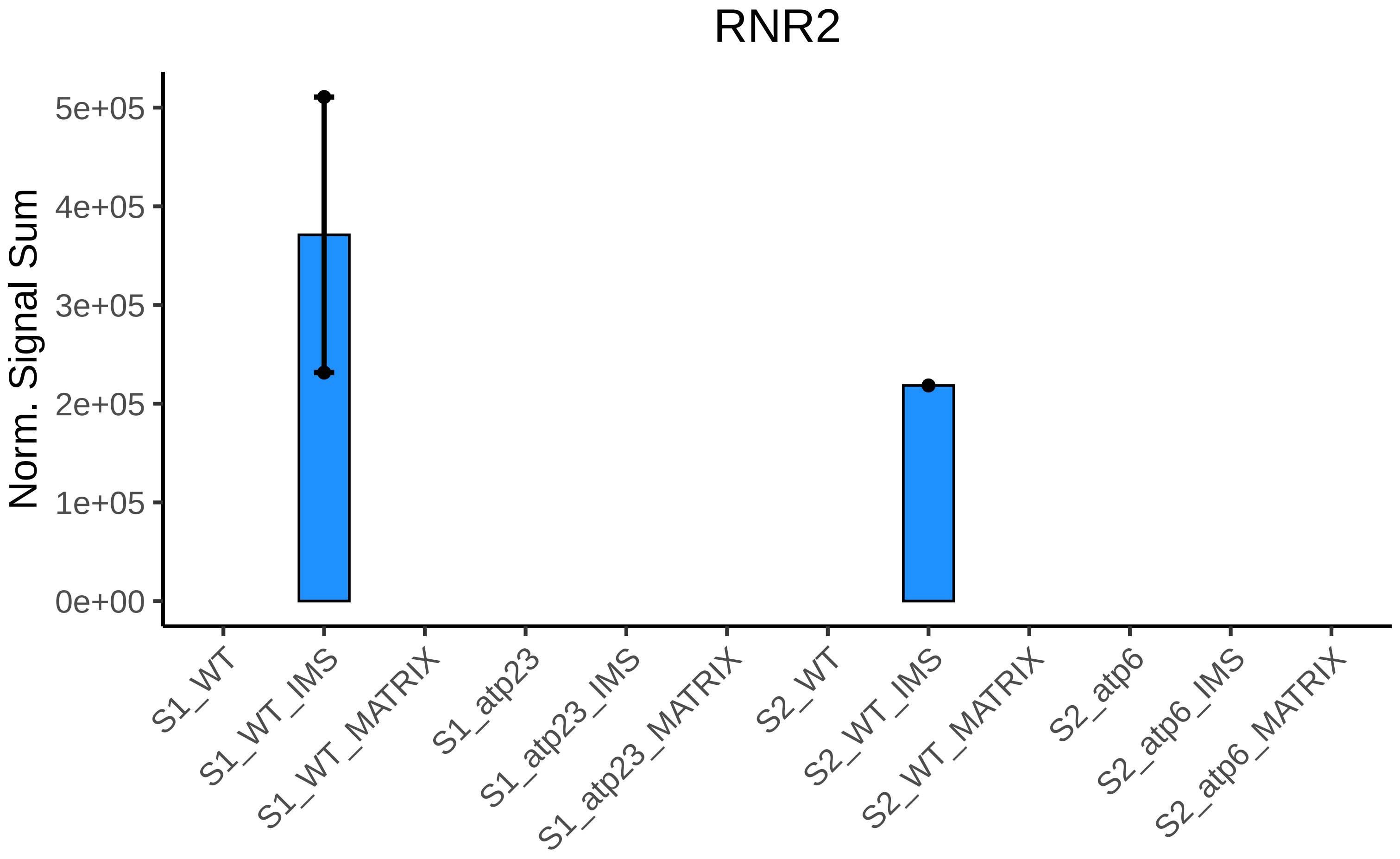

RNR2
Norm. Signal Sum
5e+05
4e+05
3e+05
2e+05
1e+05
0e+00
S1_WT
S1_WT_IMS
S1_WT_MATRIX
S1_atp23
S1_atp23_IMS
S1_atp23_MATRIX
S2_WT
S2_WT_IMS
S2_WT_MATRIX
S2_atp6
S2_atp6_IMS
S2_atp6_MATRIX

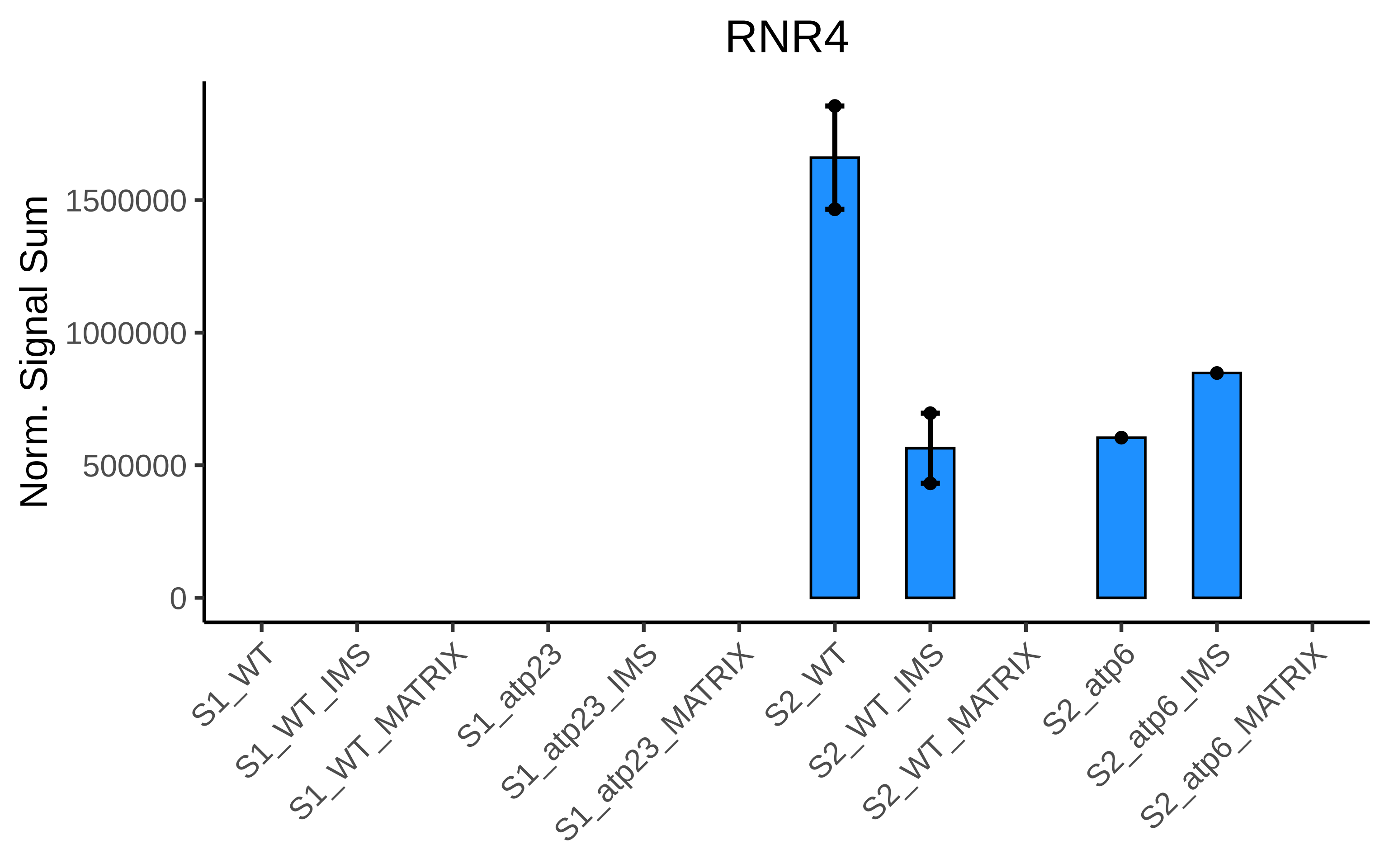
RNR4
Norm. Signal Sum
1500000
1000000
500000
0
S1_WT
S1_WT_IMS
S1_WT_MATRIX
S1_atp23
S1_atp23_IMS
S1_atp23_MATRIX
S2_WT
S2_WT_IMS
S2_WT_MATRIX
S2_atp6
S2_atp6_IMS
S2_atp6_MATRIX

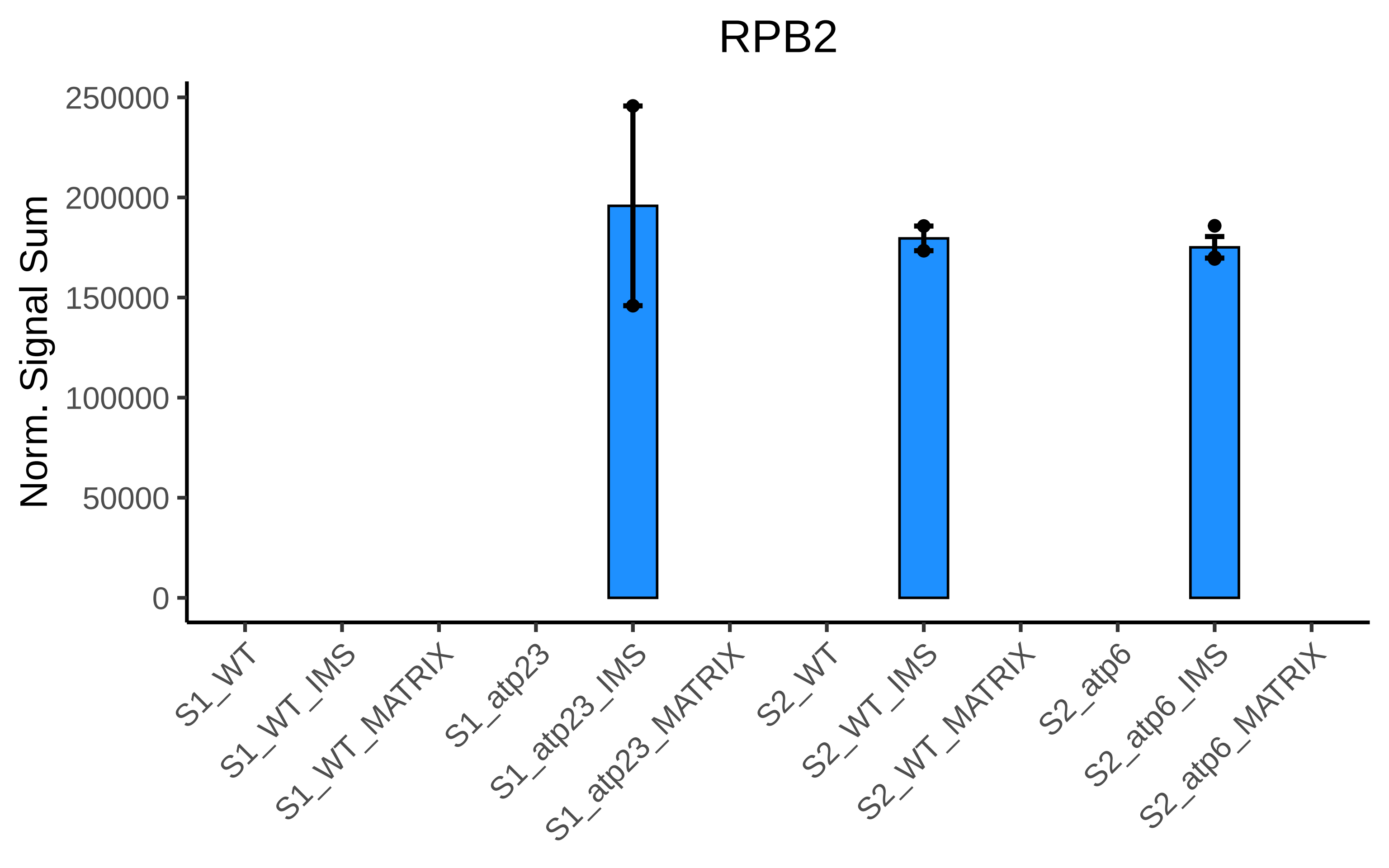
RPB2
Norm. Signal Sum
250000
200000
150000
100000
50000
0
S1_WT
S1_WT_IMS
S1_WT_MATRIX
S1_atp23
S1_atp23_IMS
S1_atp23_MATRIX
S2_WT
S2_WT_IMS
S2_WT_MATRIX
S2_atp6
S2_atp6_IMS
S2_atp6_MATRIX

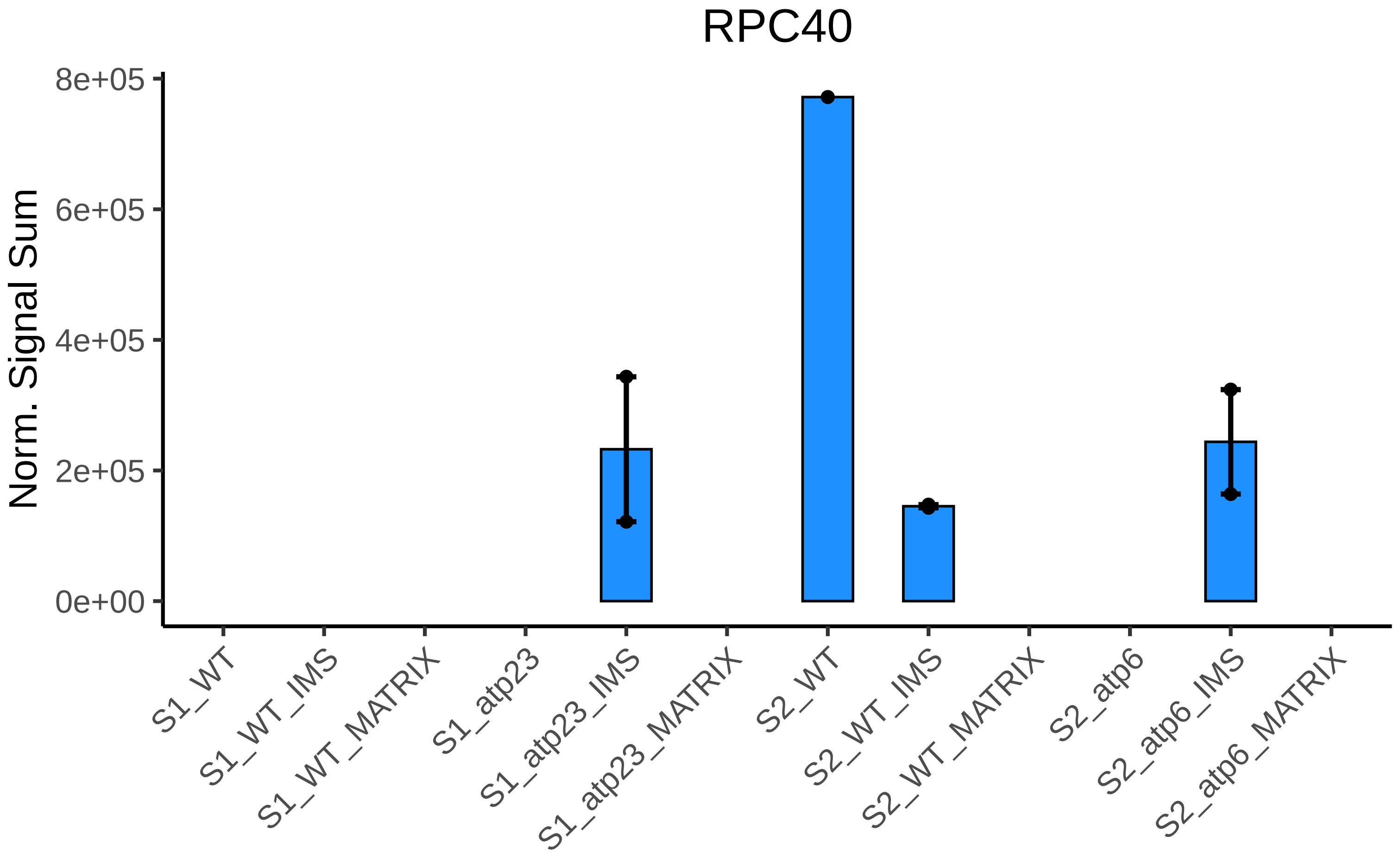

RPC40
Norm. Signal Sum
8e+05
6e+05
4e+05
2e+05
0e+00
S1_WT
S1_WT_IMS
S1_WT_MATRIX
S1_atp23
S1_atp23_IMS
S1_atp23_MATRIX
S2_WT
S2_WT_IMS
S2_WT_MATRIX
S2_atp6
S2_atp6_IMS
S2_atp6_MATRIX

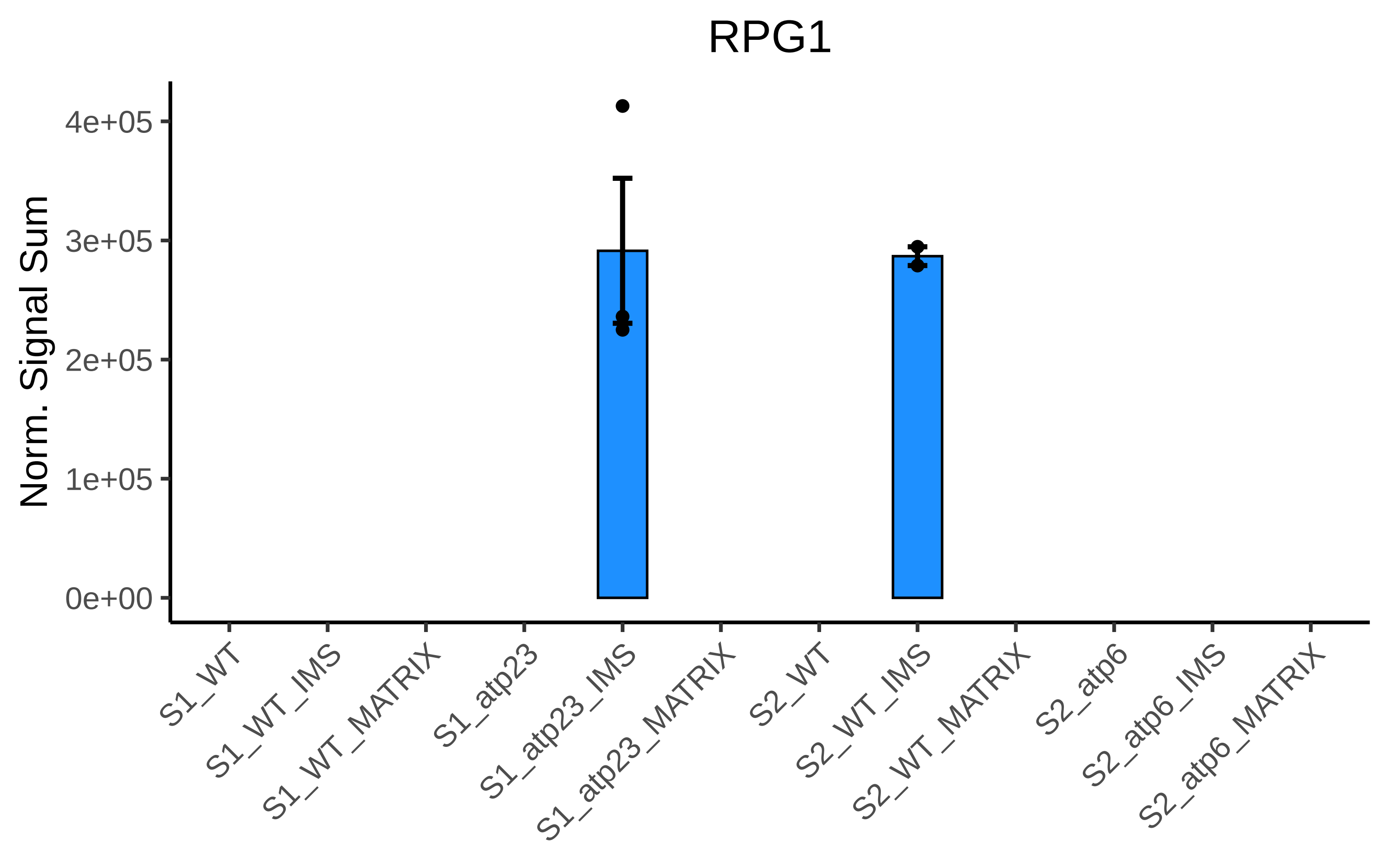

RPG1
Norm. Signal Sum
4e+05
3e+05
2e+05
1e+05
0e+00
S1_WT
S1_WT_IMS
S1_WT_MATRIX
S1_atp23
S1_atp23_IMS
S1_atp23_MATRIX
S2_WT
S2_WT_IMS
S2_WT_MATRIX
S2_atp6
S2_atp6_IMS
S2_atp6_MATRIX

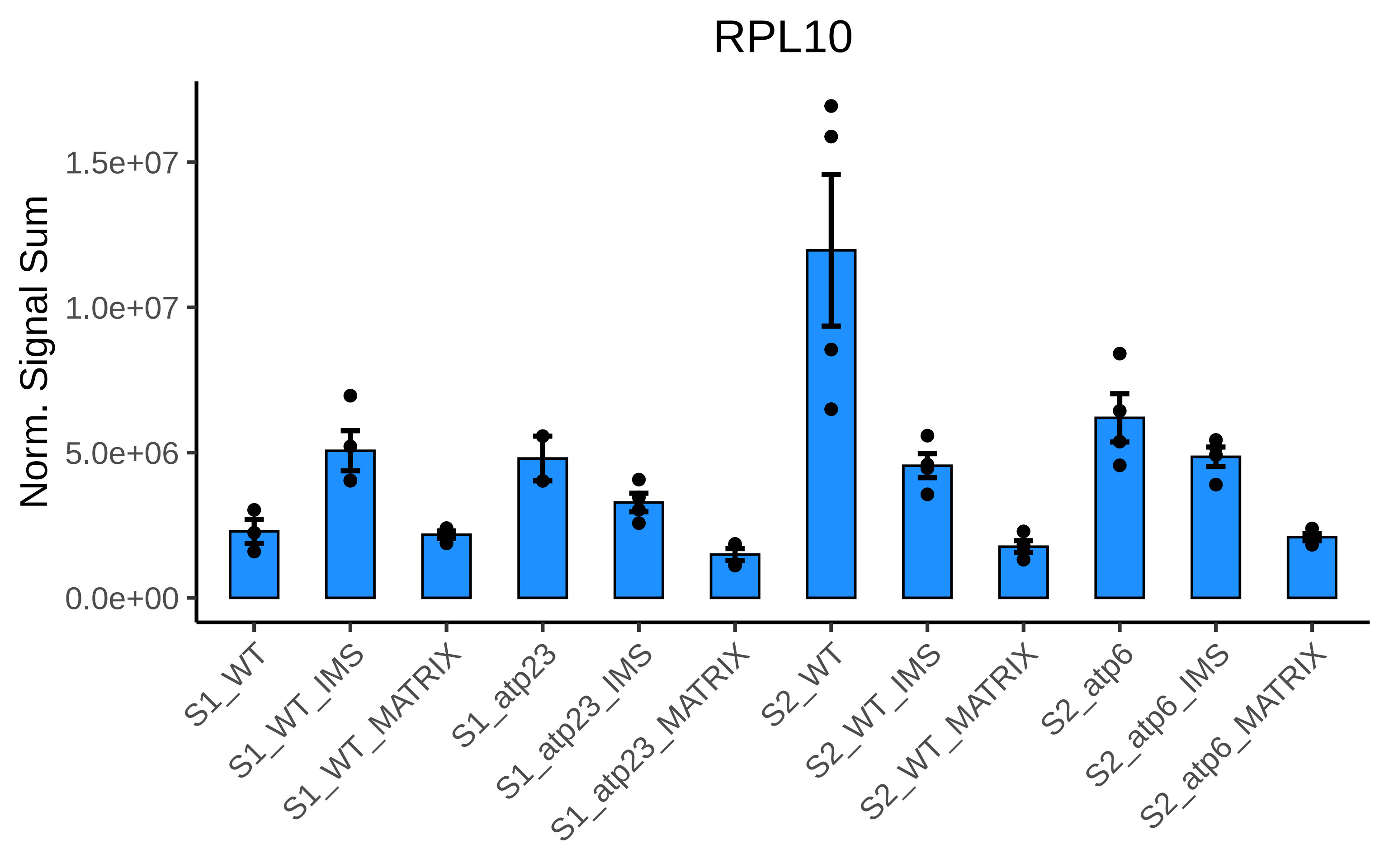
RPL10
Norm. Signal Sum
1.5e+07
1.0e+07
5.0e+06
0.0e+00
S1_WT
S1_WT_IMS
S1_WT_MATRIX
S1_atp23
S1_atp23_IMS
S1_atp23_MATRIX
S2_WT
S2_WT_IMS
S2_WT_MATRIX
S2_atp6
S2_atp6_IMS
S2_atp6_MATRIX

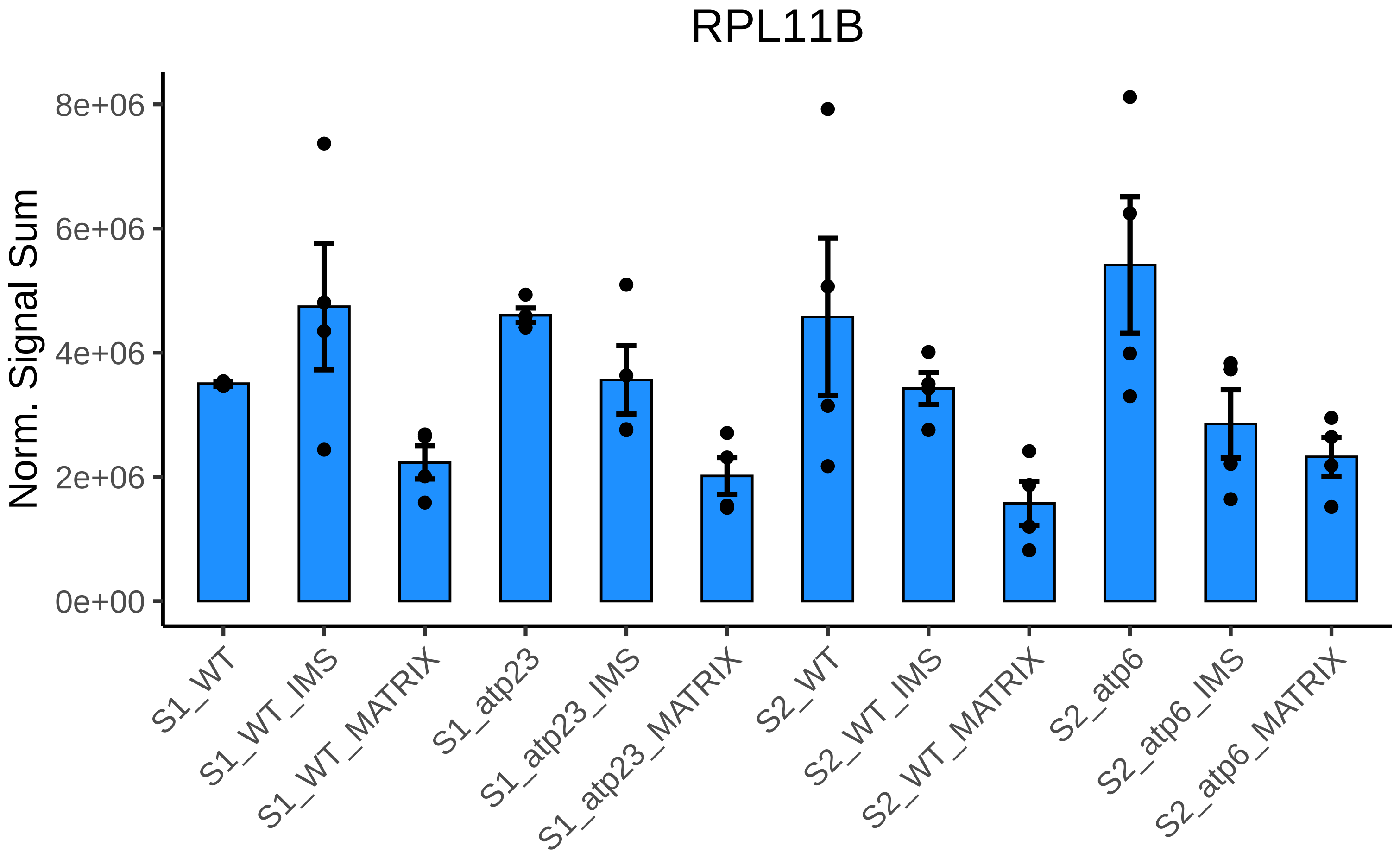
RPL11B
Norm. Signal Sum
8e+06
6e+06
4e+06
2e+06
0e+00
S1_WT
S1_WT_IMS
S1_WT_MATRIX
S1_atp23
S1_atp23_IMS
S1_atp23_MATRIX
S2_WT
S2_WT_IMS
S2_WT_MATRIX
S2_atp6
S2_atp6_IMS
S2_atp6_MATRIX

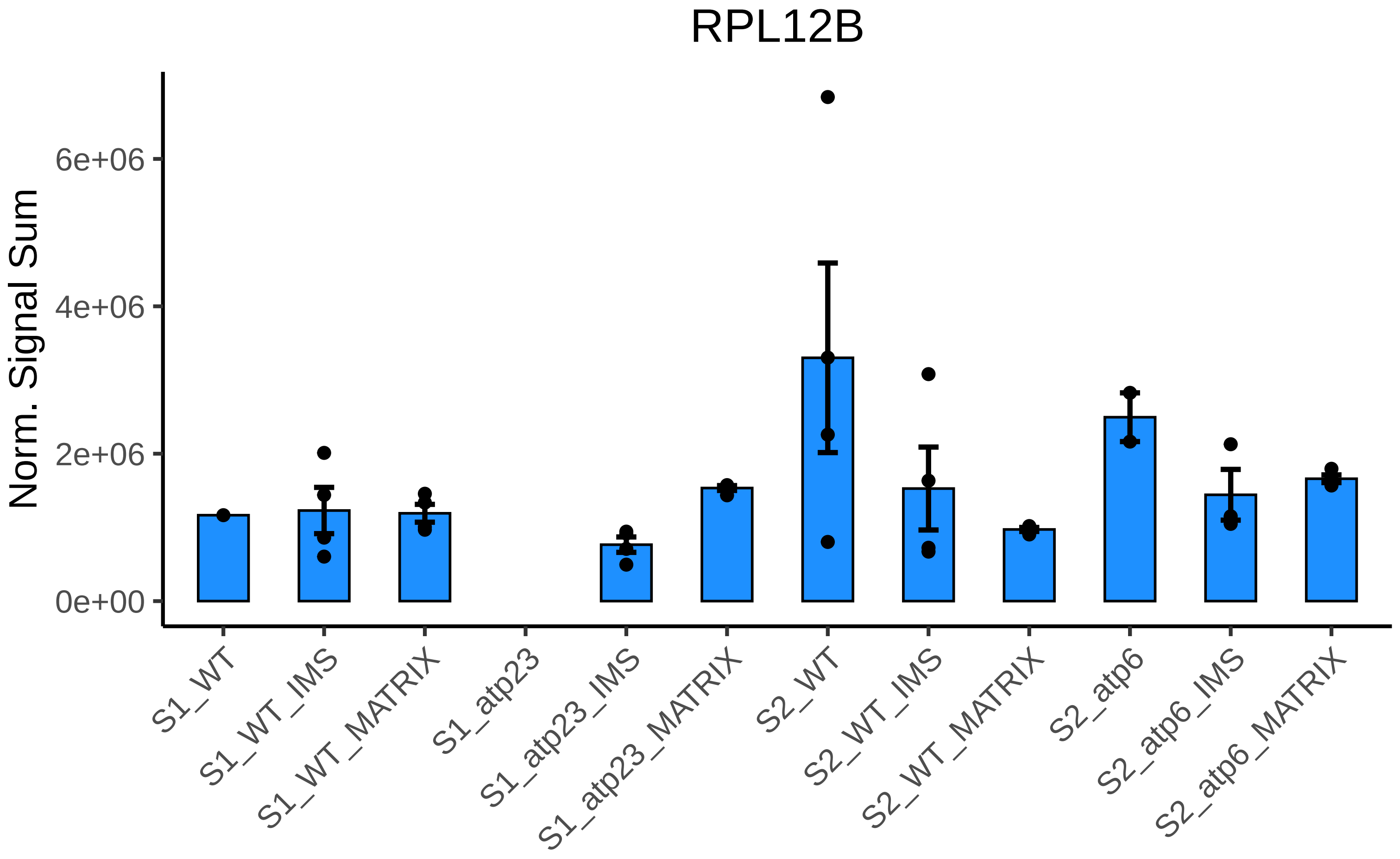

RPL12B
Norm. Signal Sum
6e+06
4e+06
2e+06
0e+00
S1_WT
S1_WT_IMS
S1_WT_MATRIX
S1_atp23
S1_atp23_IMS
S1_atp23_MATRIX
S2_WT
S2_WT_IMS
S2_WT_MATRIX
S2_atp6
S2_atp6_IMS
S2_atp6_MATRIX

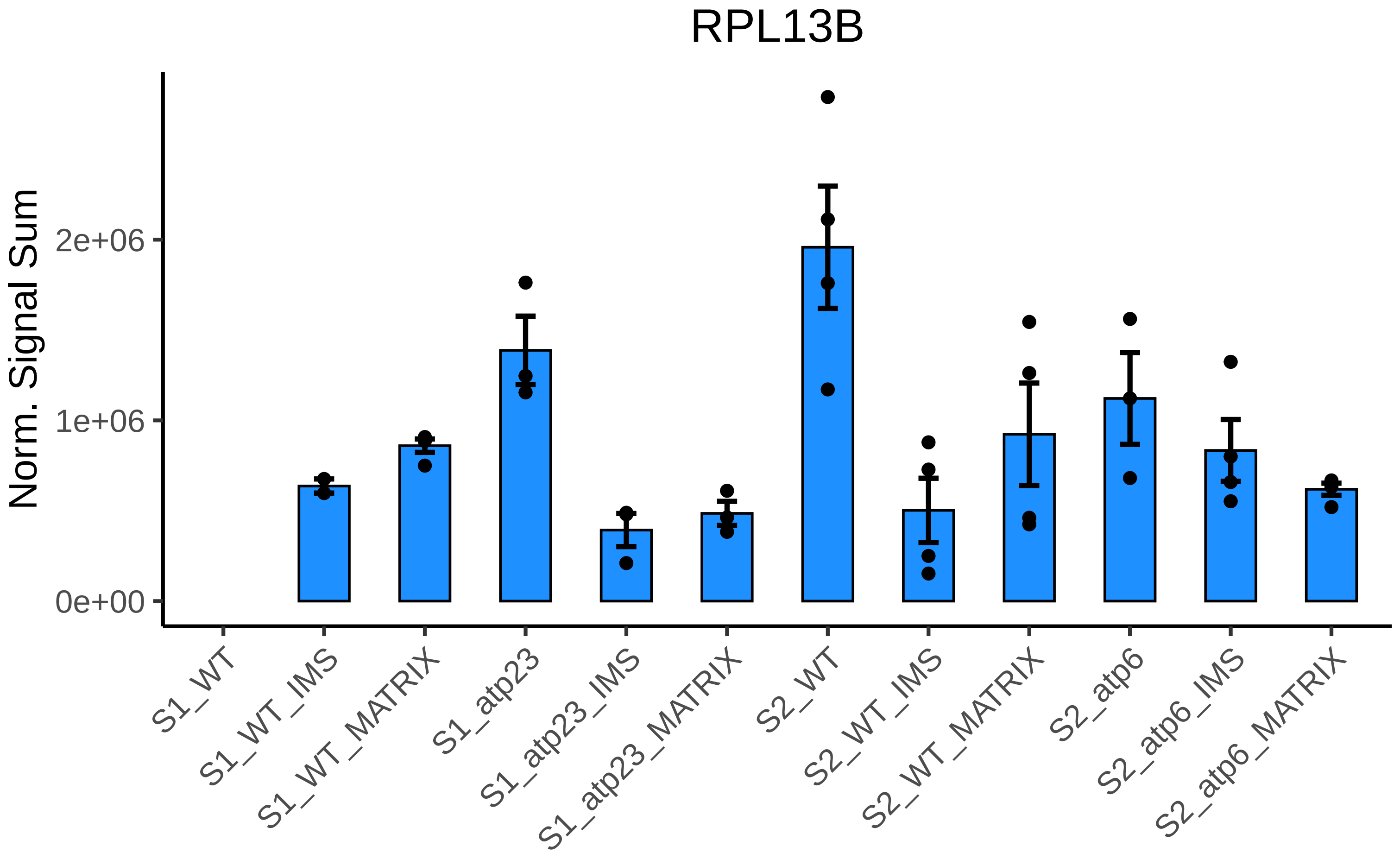

RPL13B
Norm. Signal Sum
2e+06
1e+06
0e+00
S1_WT
S1_WT_IMS
S1_WT_MATRIX
S1_atp23
S1_atp23_IMS
S1_atp23_MATRIX
S2_WT
S2_WT_IMS
S2_WT_MATRIX
S2_atp6
S2_atp6_IMS
S2_atp6_MATRIX

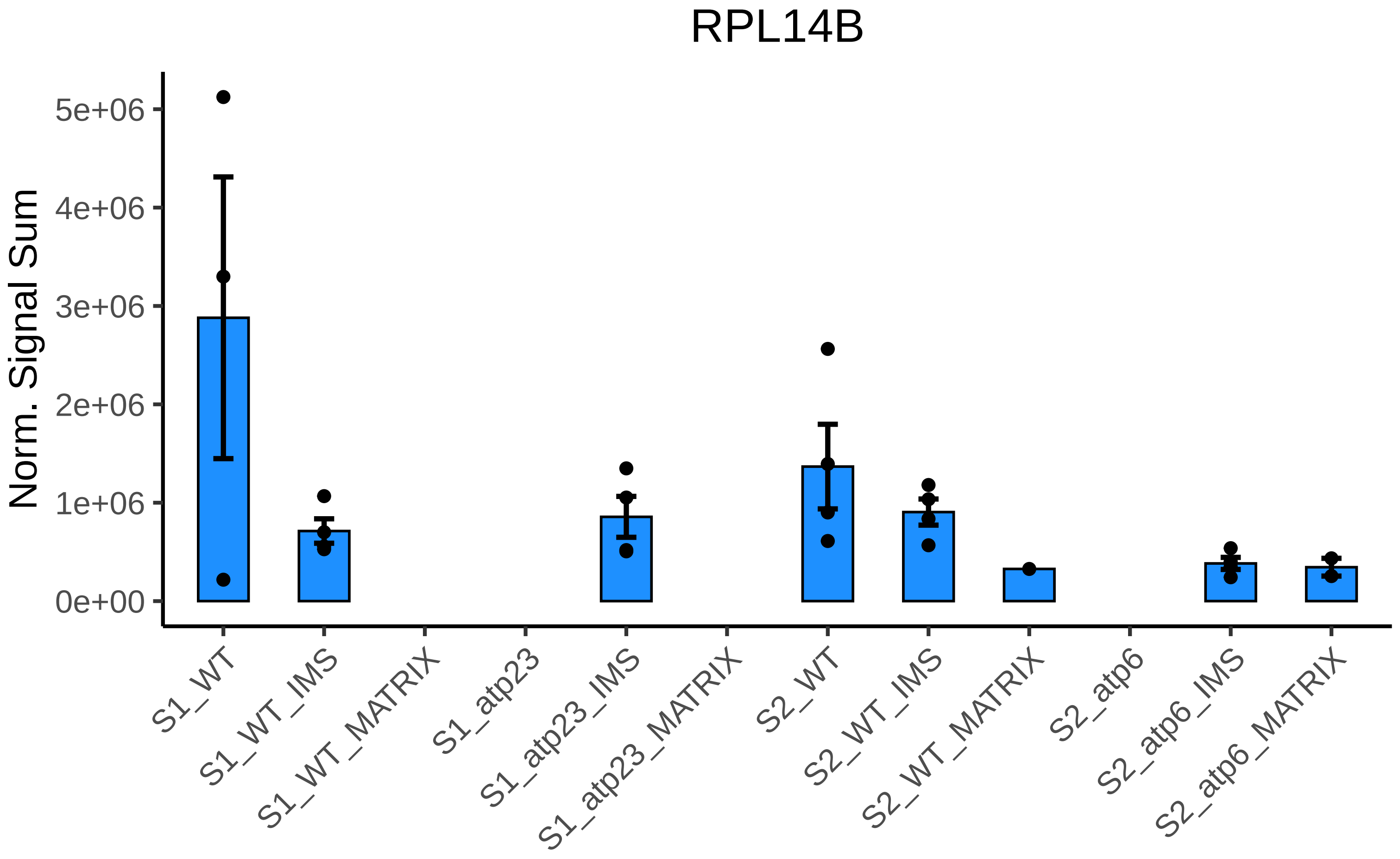
RPL14B
Norm. Signal Sum
5e+06
4e+06
3e+06
2e+06
1e+06
0e+00
S1_WT
S1_WT_IMS
S1_WT_MATRIX
S1_atp23
S1_atp23_IMS
S1_atp23_MATRIX
S2_WT
S2_WT_IMS
S2_WT_MATRIX
S2_atp6
S2_atp6_IMS
S2_atp6_MATRIX

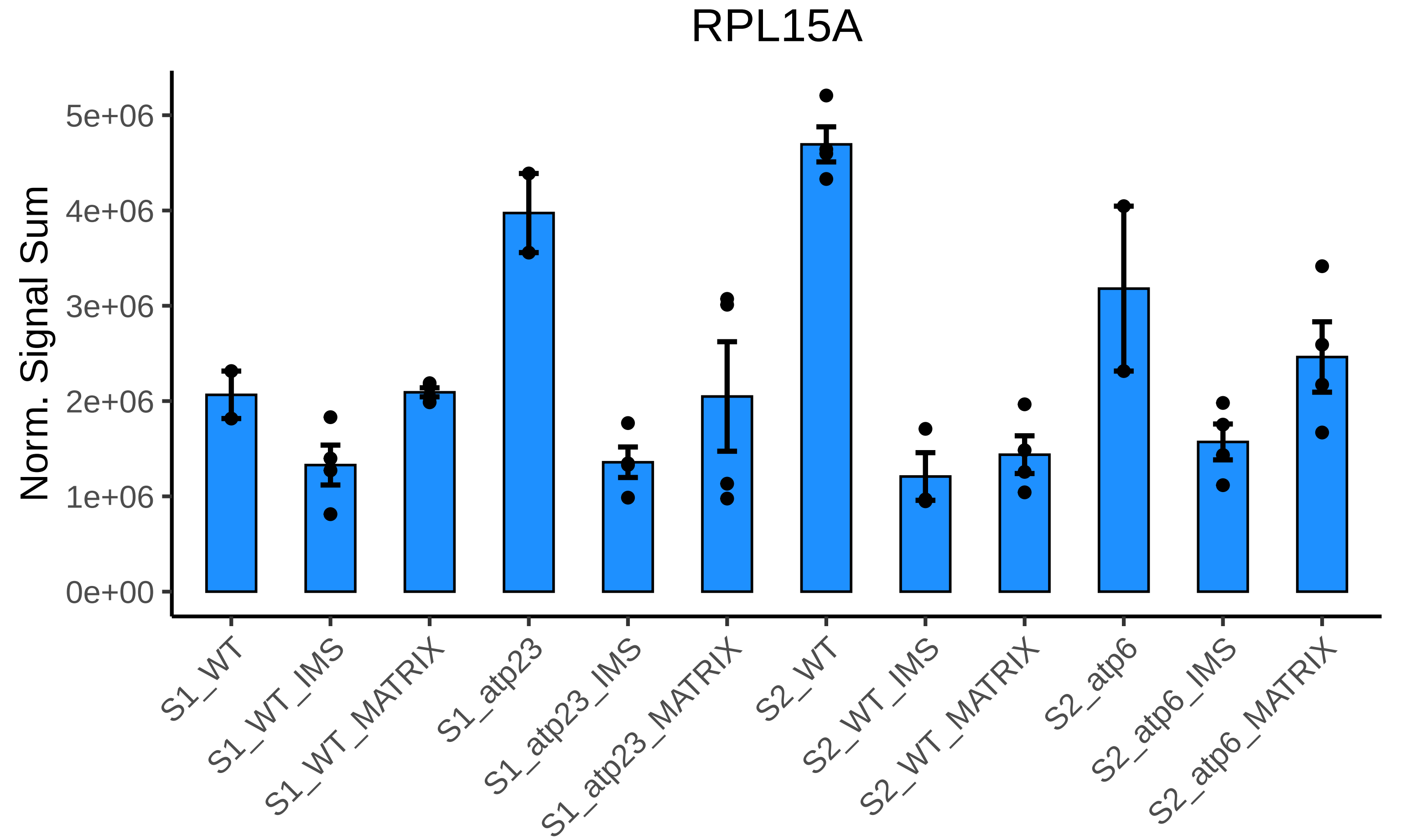

RPL15A
Norm. Signal Sum
5e+06
4e+06
3e+06
2e+06
1e+06
0e+00
S1_WT
S1_WT_IMS
S1_WT_MATRIX
S1_atp23
S1_atp23_IMS
S1_atp23_MATRIX
S2_WT
S2_WT_IMS
S2_WT_MATRIX
S2_atp6
S2_atp6_IMS
S2_atp6_MATRIX

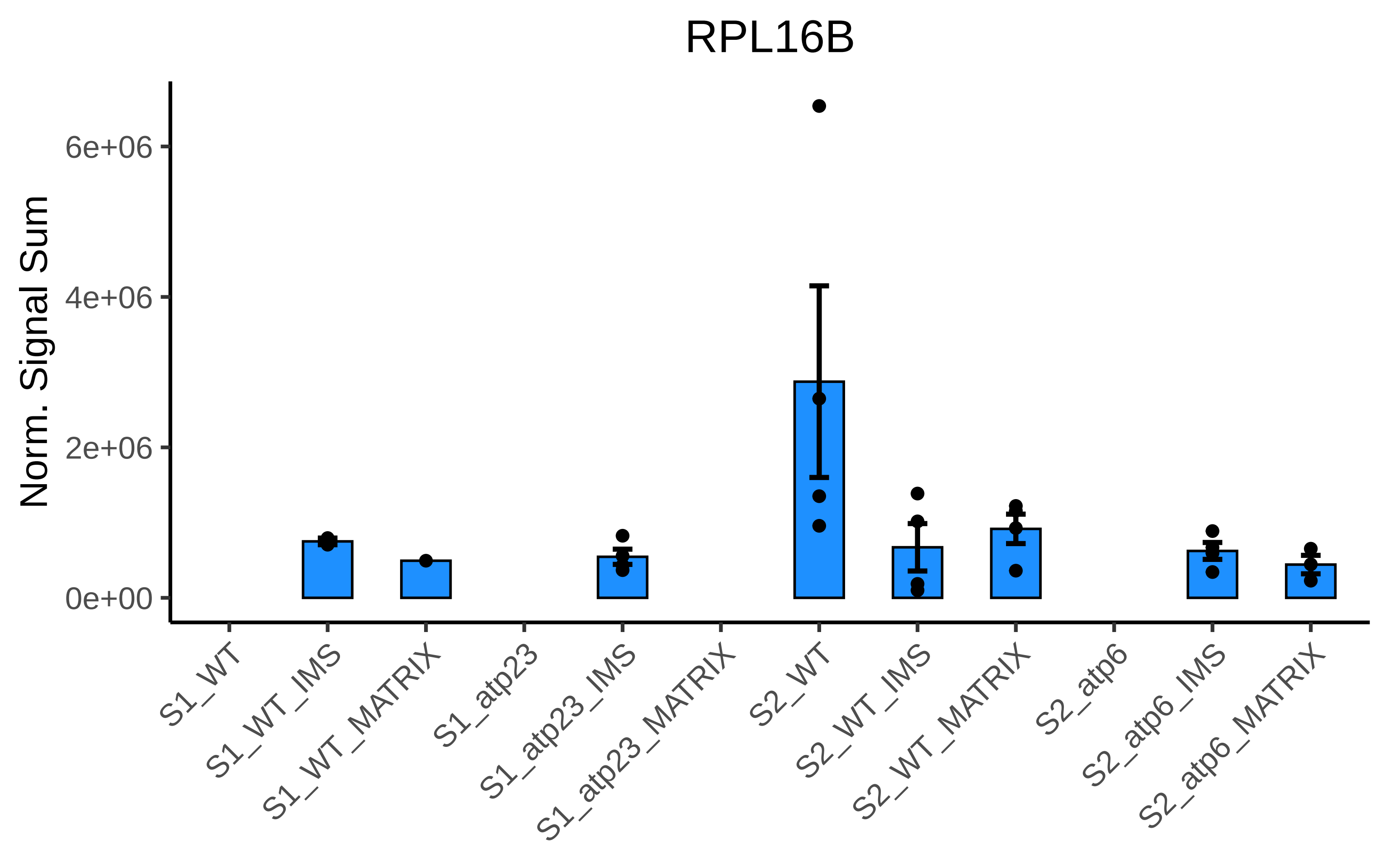

RPL16B
Norm. Signal Sum
6e+06
4e+06
2e+06
0e+00
S1_WT
S1_WT_IMS
S1_WT_MATRIX
S1_atp23
S1_atp23_IMS
S1_atp23_MATRIX
S2_WT
S2_WT_IMS
S2_WT_MATRIX
S2_atp6
S2_atp6_IMS
S2_atp6_MATRIX

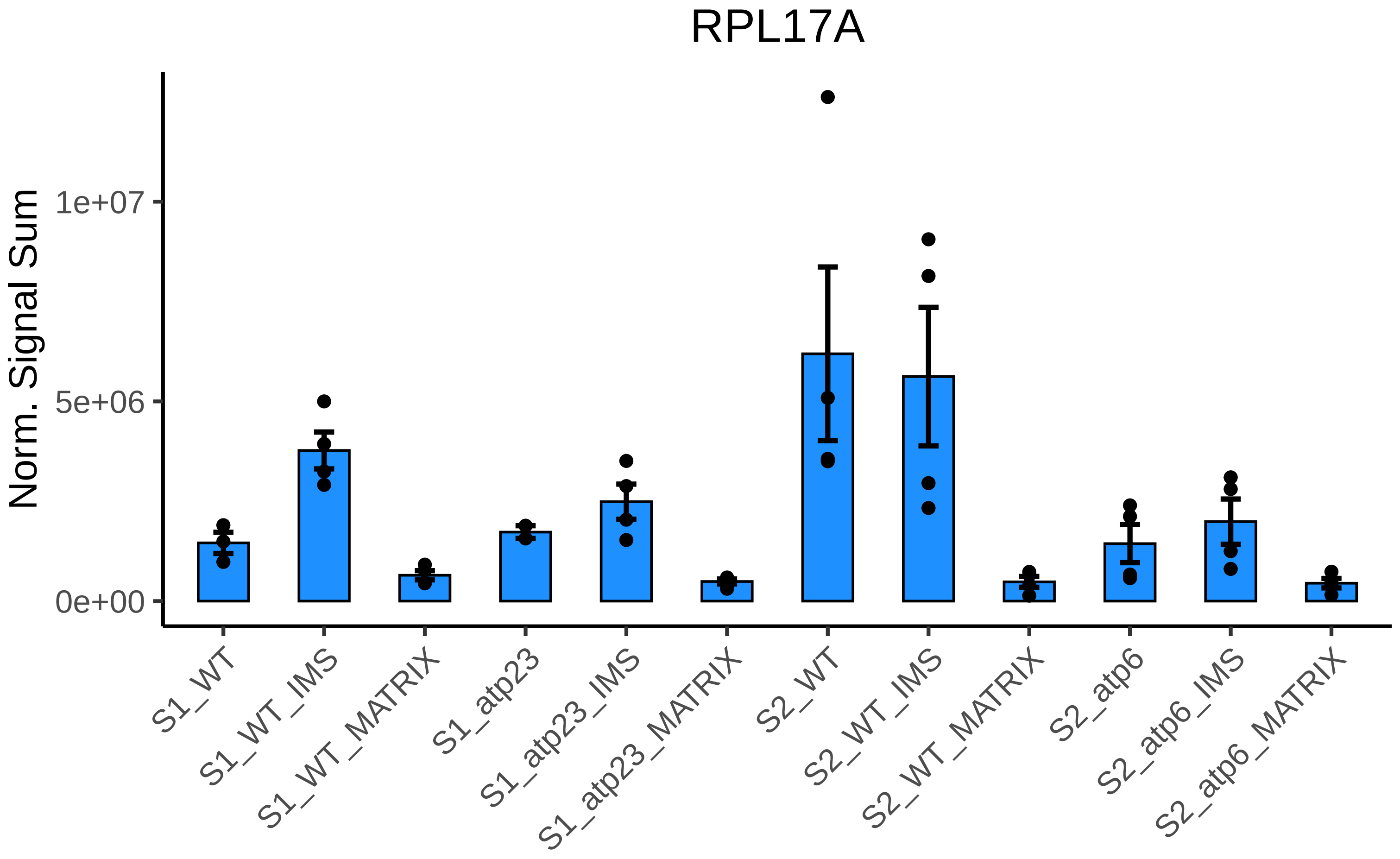

RPL17A
Norm. Signal Sum
1e+07
5e+06
0e+00
S1_WT
S1_WT_IMS
S1_WT_MATRIX
S1_atp23
S1_atp23_IMS
S1_atp23_MATRIX
S2_WT
S2_WT_IMS
S2_WT_MATRIX
S2_atp6
S2_atp6_IMS
S2_atp6_MATRIX

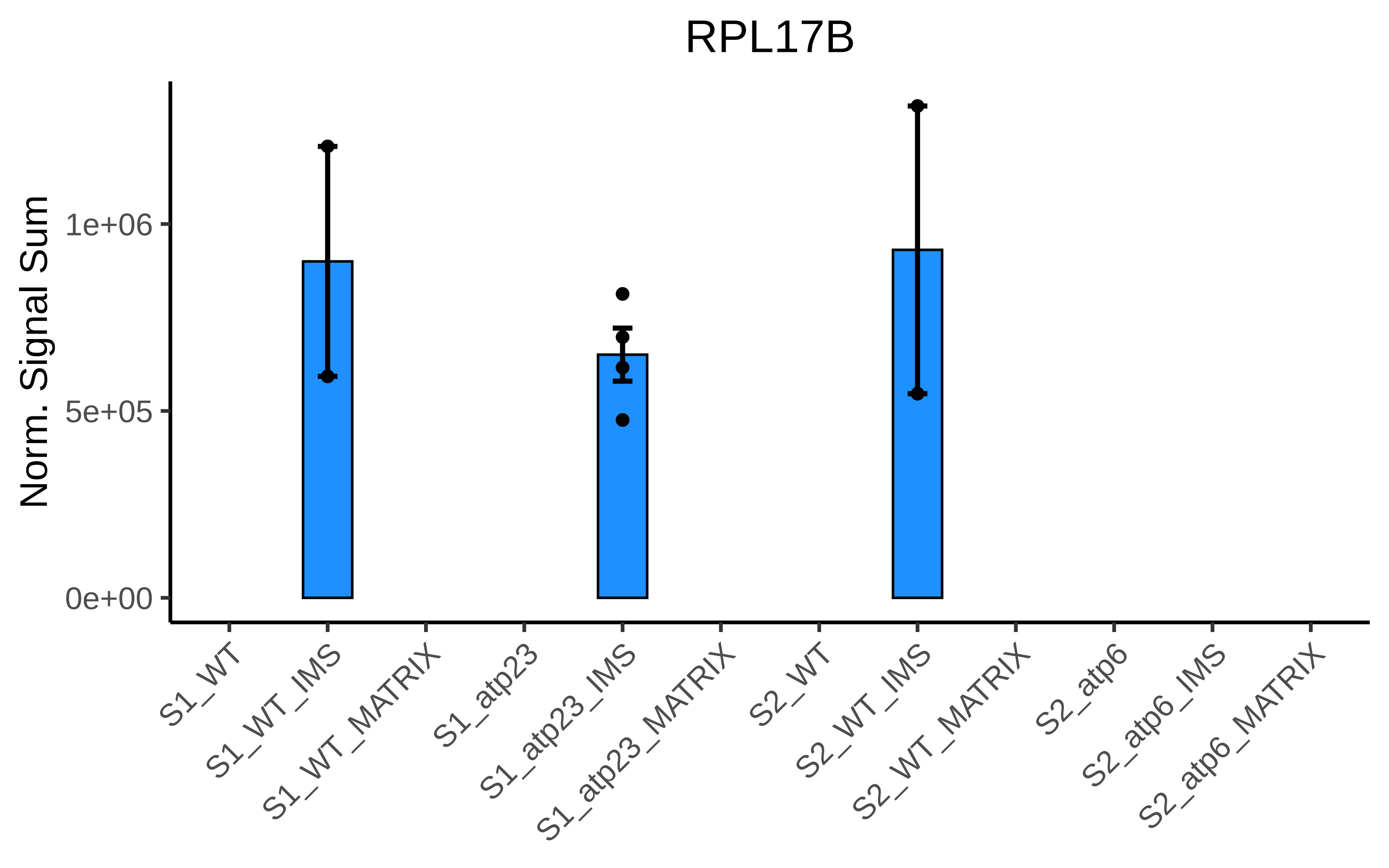

RPL17B
Norm. Signal Sum
1e+06
5e+05
0e+00
S1_WT
S1_WT_IMS
S1_WT_MATRIX
S1_atp23
S1_atp23_IMS
S1_atp23_MATRIX
S2_WT
S2_WT_IMS
S2_WT_MATRIX
S2_atp6
S2_atp6_IMS
S2_atp6_MATRIX

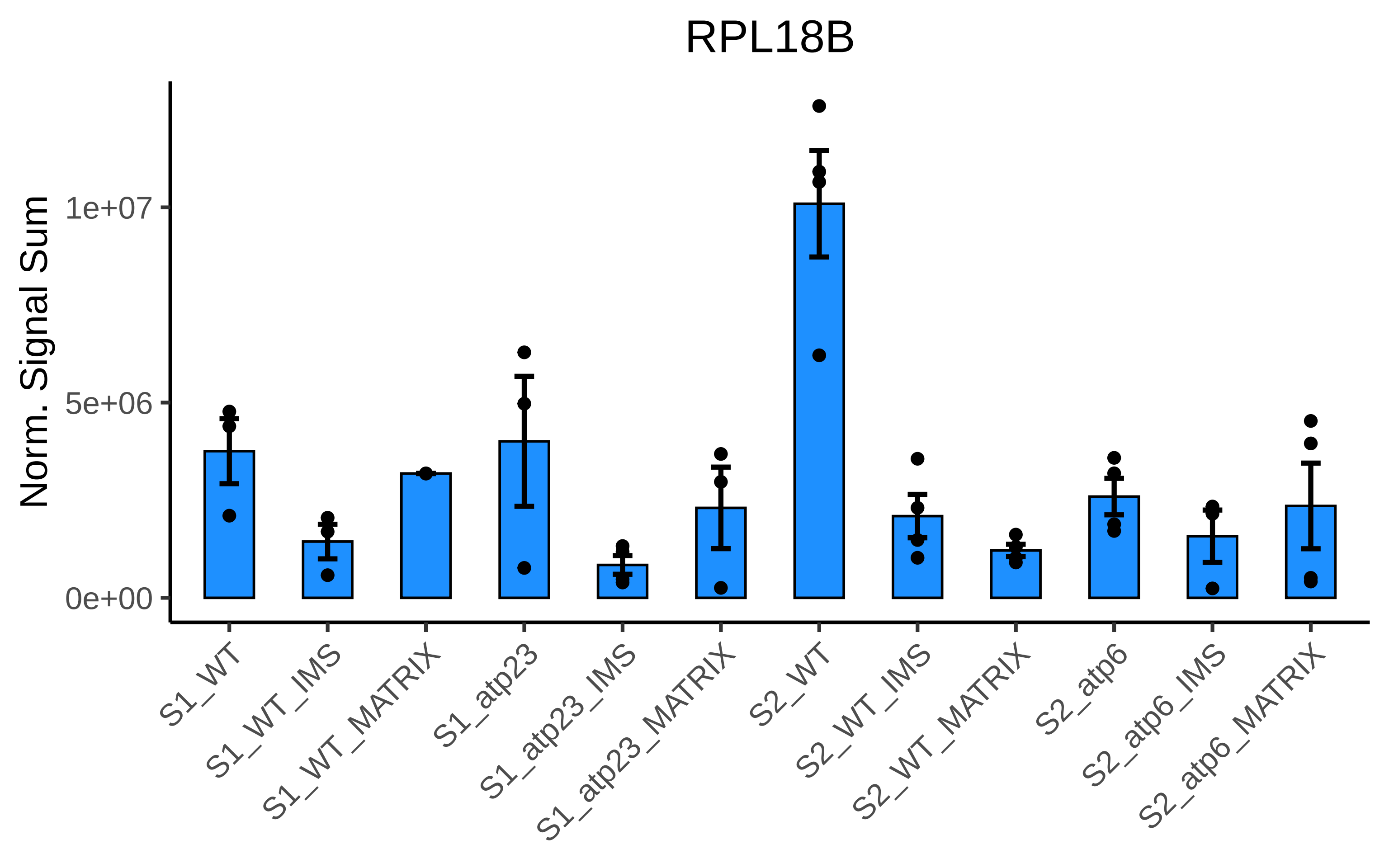
RPL18B
Norm. Signal Sum
1e+07
5e+06
0e+00
S1_WT
S1_WT_IMS
S1_WT_MATRIX
S1_atp23
S1_atp23_IMS
S1_atp23_MATRIX
S2_WT
S2_WT_IMS
S2_WT_MATRIX
S2_atp6
S2_atp6_IMS
S2_atp6_MATRIX

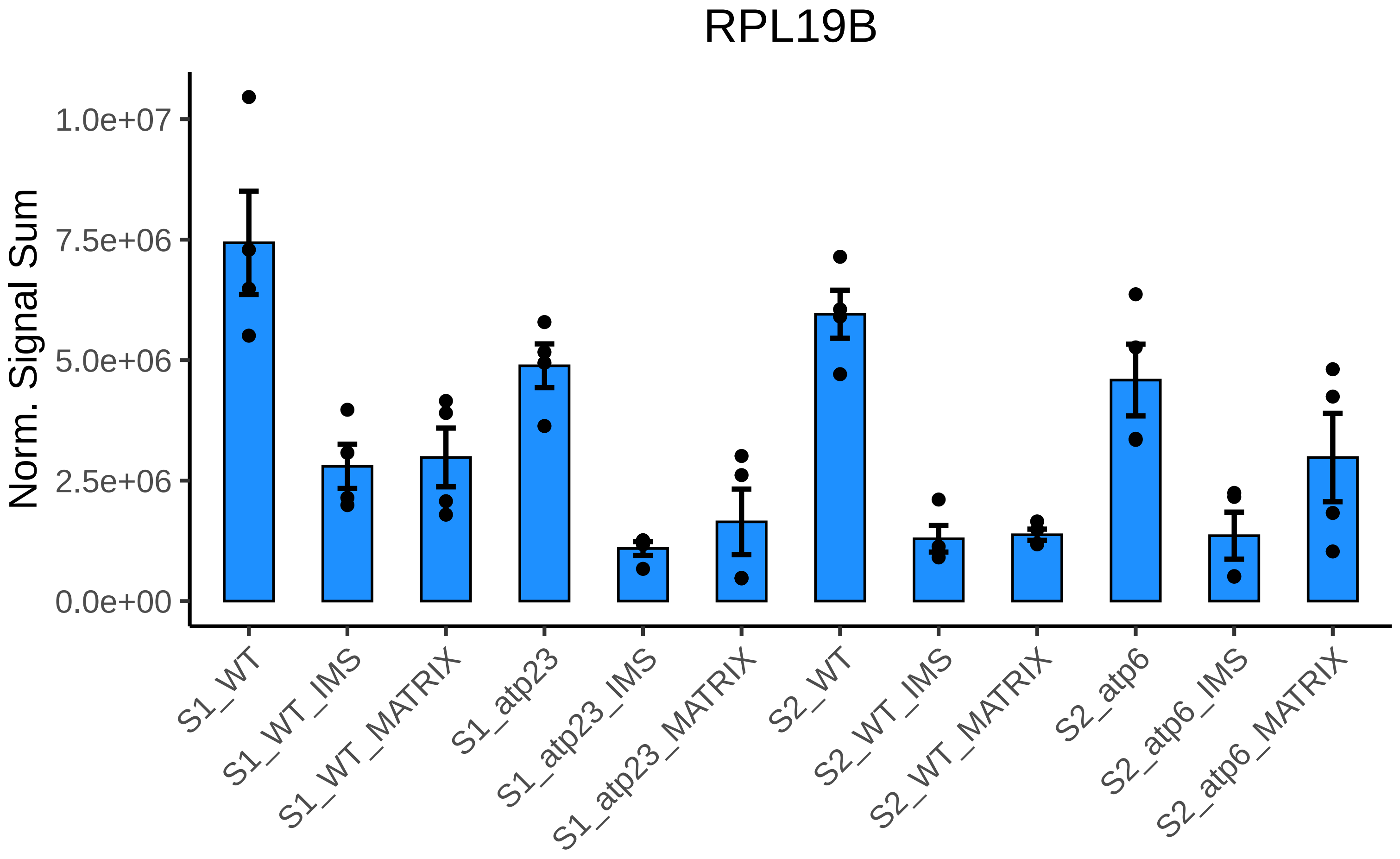
RPL19B
Norm. Signal Sum
1.0e+07
7.5e+06
5.0e+06
2.5e+06
0.0e+00
S1_WT
S1_WT_IMS
S1_WT_MATRIX
S1_atp23
S1_atp23_IMS
S1_atp23_MATRIX
S2_WT
S2_WT_IMS
S2_WT_MATRIX
S2_atp6
S2_atp6_IMS
S2_atp6_MATRIX

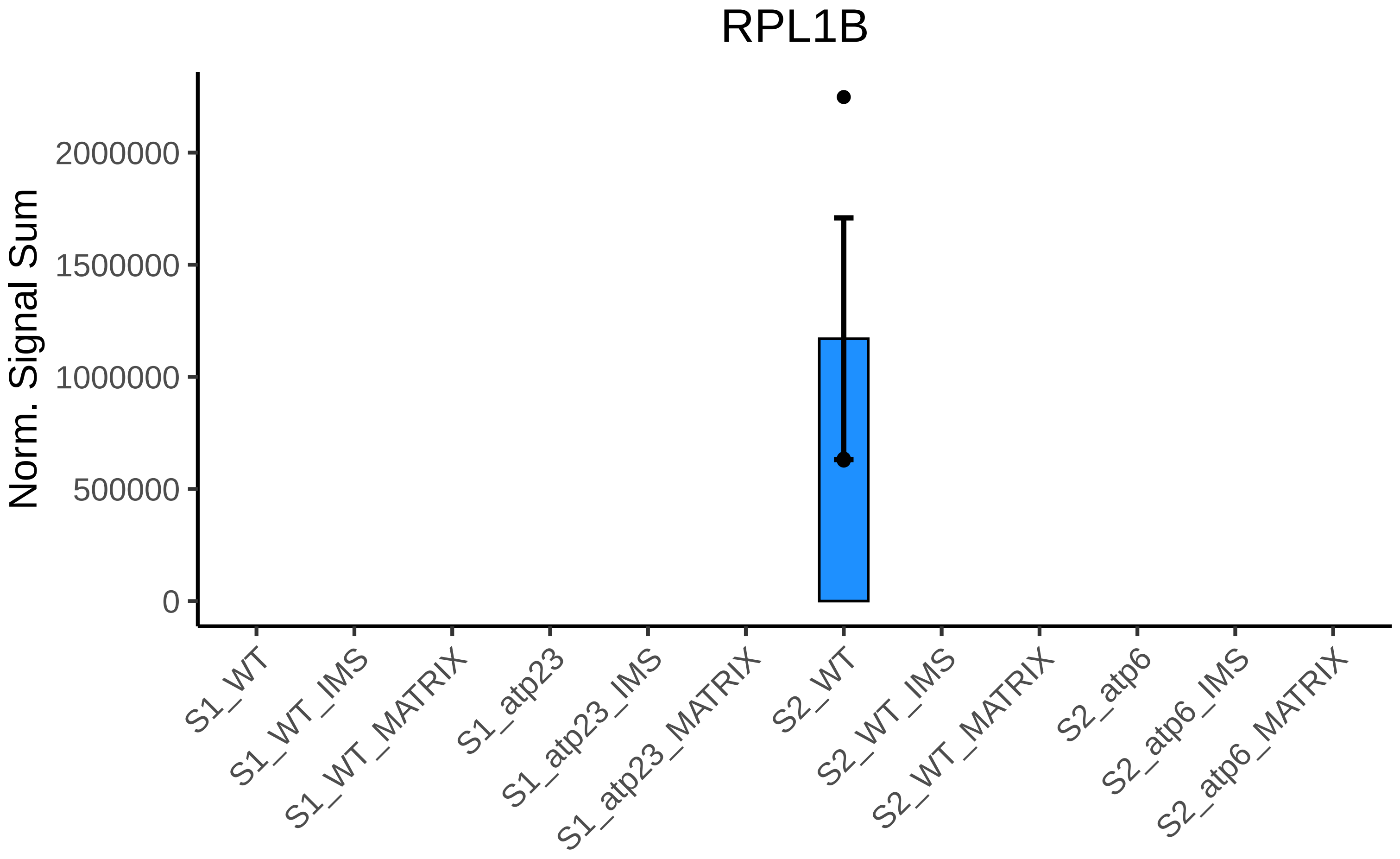
RPL1B
Norm. Signal Sum
2000000
1500000
1000000
500000
0
S1_WT
S1_WT_IMS
S1_WT_MATRIX
S1_atp23
S1_atp23_IMS
S1_atp23_MATRIX
S2_WT
S2_WT_IMS
S2_WT_MATRIX
S2_atp6
S2_atp6_IMS
S2_atp6_MATRIX

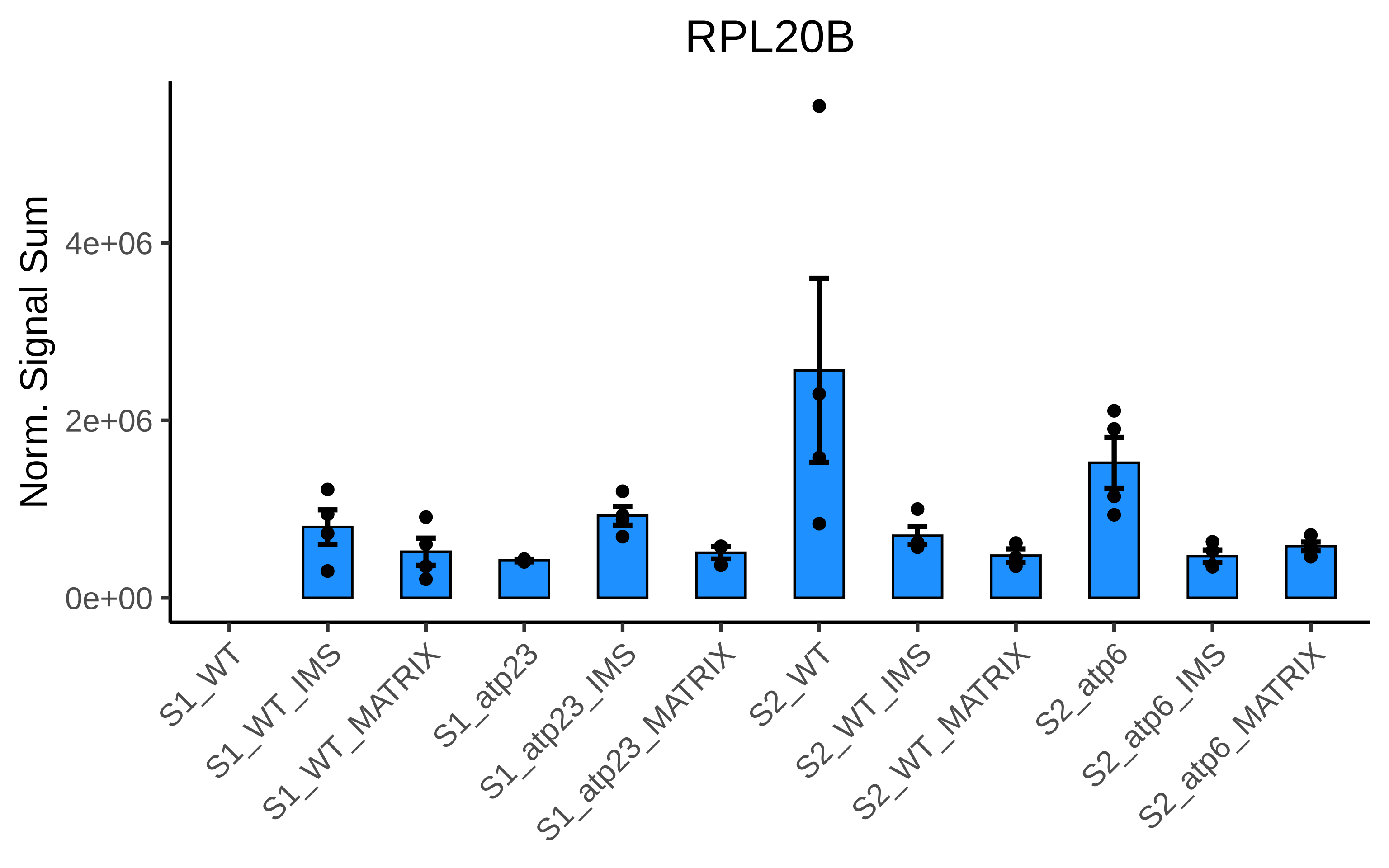

RPL20B
Norm. Signal Sum
4e+06
2e+06
0e+00
S1_WT
S1_WT_IMS
S1_WT_MATRIX
S1_atp23
S1_atp23_IMS
S1_atp23_MATRIX
S2_WT
S2_WT_IMS
S2_WT_MATRIX
S2_atp6
S2_atp6_IMS
S2_atp6_MATRIX

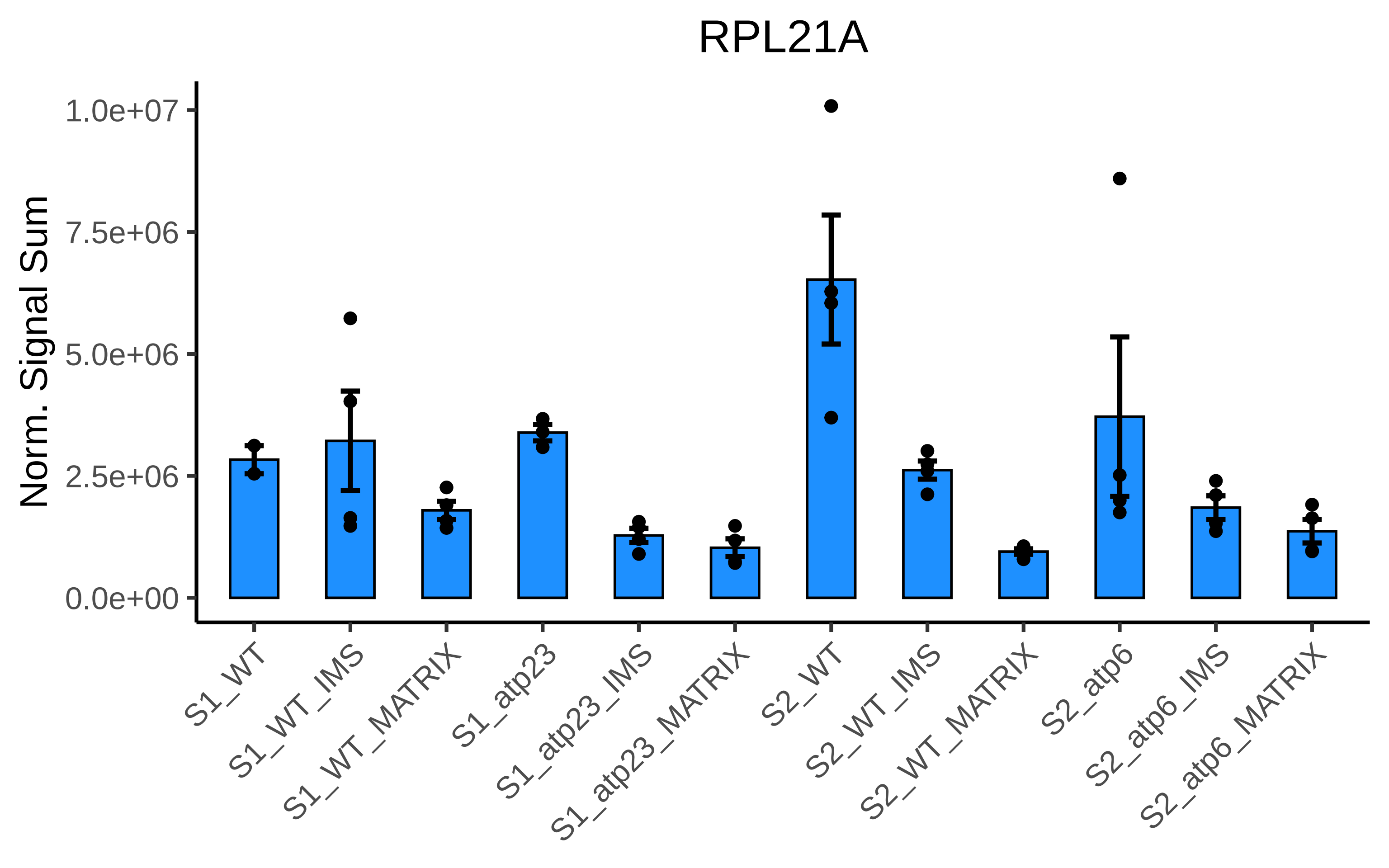

RPL21A
Norm. Signal Sum
1.0e+07
7.5e+06
5.0e+06
2.5e+06
0.0e+00
S1_WT
S1_WT_IMS
S1_WT_MATRIX
S1_atp23
S1_atp23_IMS
S1_atp23_MATRIX
S2_WT
S2_WT_IMS
S2_WT_MATRIX
S2_atp6
S2_atp6_IMS
S2_atp6_MATRIX

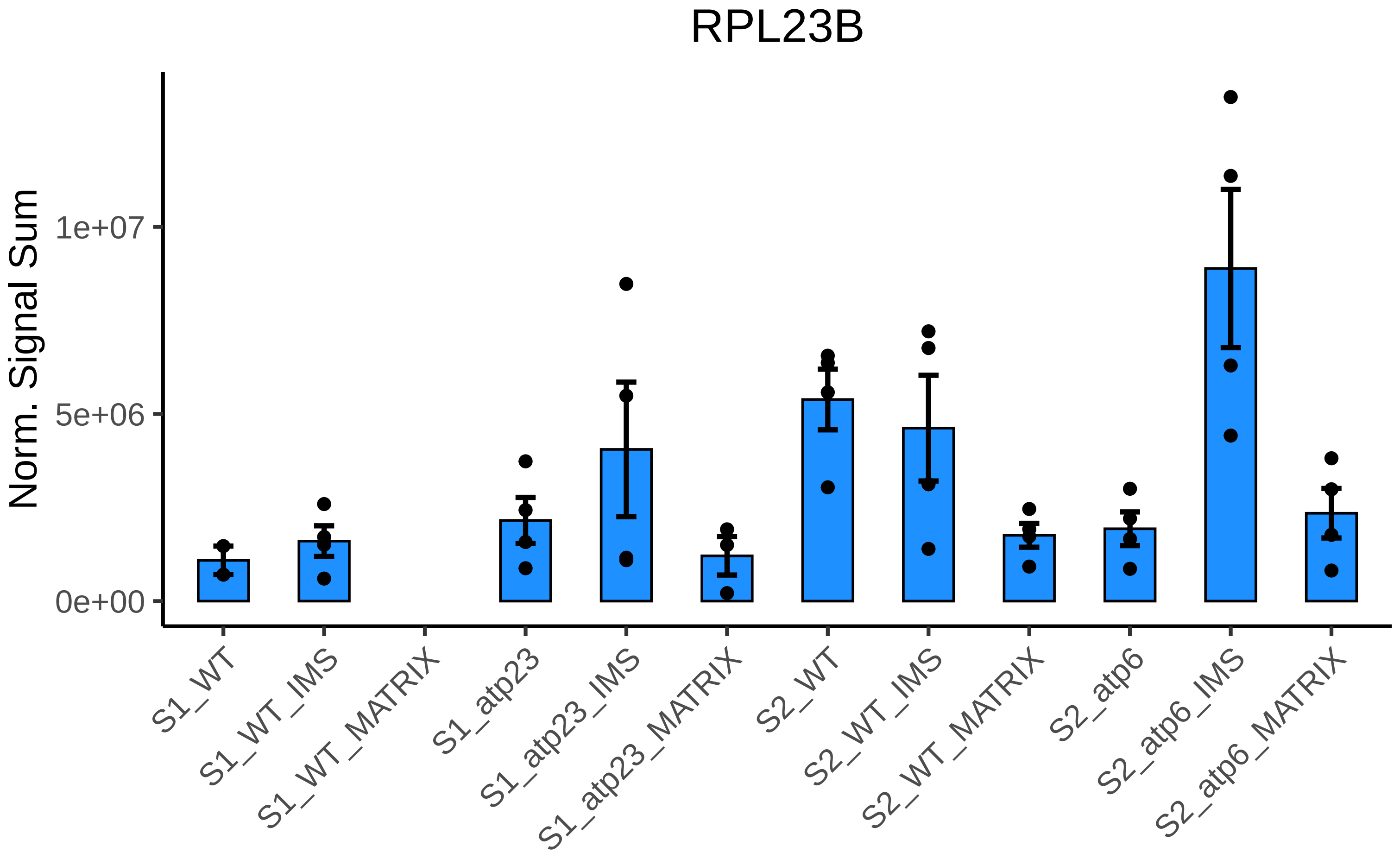
RPL23B
Norm. Signal Sum
1e+07
5e+06
0e+00
S1_WT
S1_WT_IMS
S1_WT_MATRIX
S1_atp23
S1_atp23_IMS
S1_atp23_MATRIX
S2_WT
S2_WT_IMS
S2_WT_MATRIX
S2_atp6
S2_atp6_IMS
S2_atp6_MATRIX

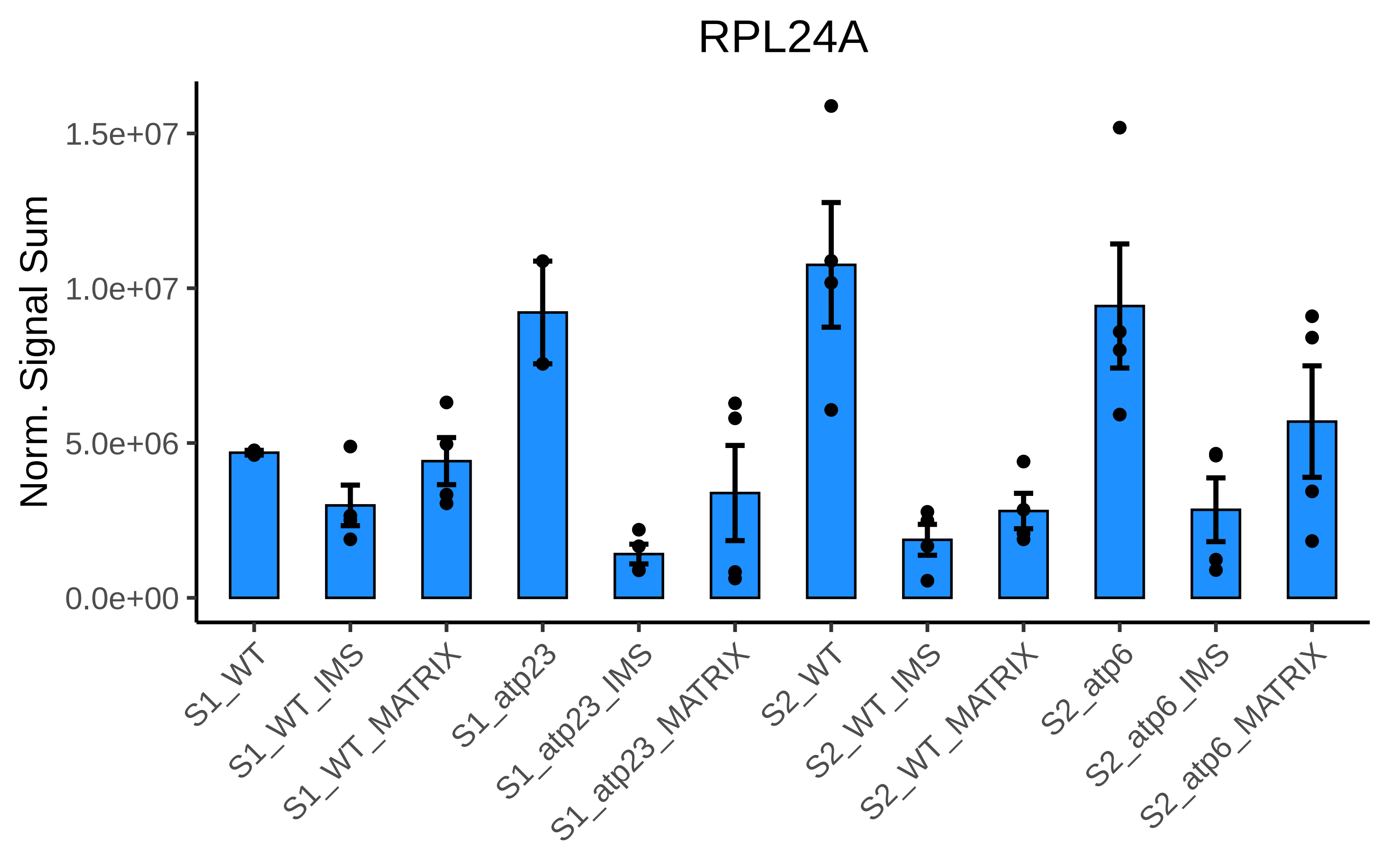
RPL24A
Norm. Signal Sum
1.5e+07
1.0e+07
5.0e+06
0.0e+00
S1_WT
S1_WT_IMS
S1_WT_MATRIX
S1_atp23
S1_atp23_IMS
S1_atp23_MATRIX
S2_WT
S2_WT_IMS
S2_WT_MATRIX
S2_atp6
S2_atp6_IMS
S2_atp6_MATRIX

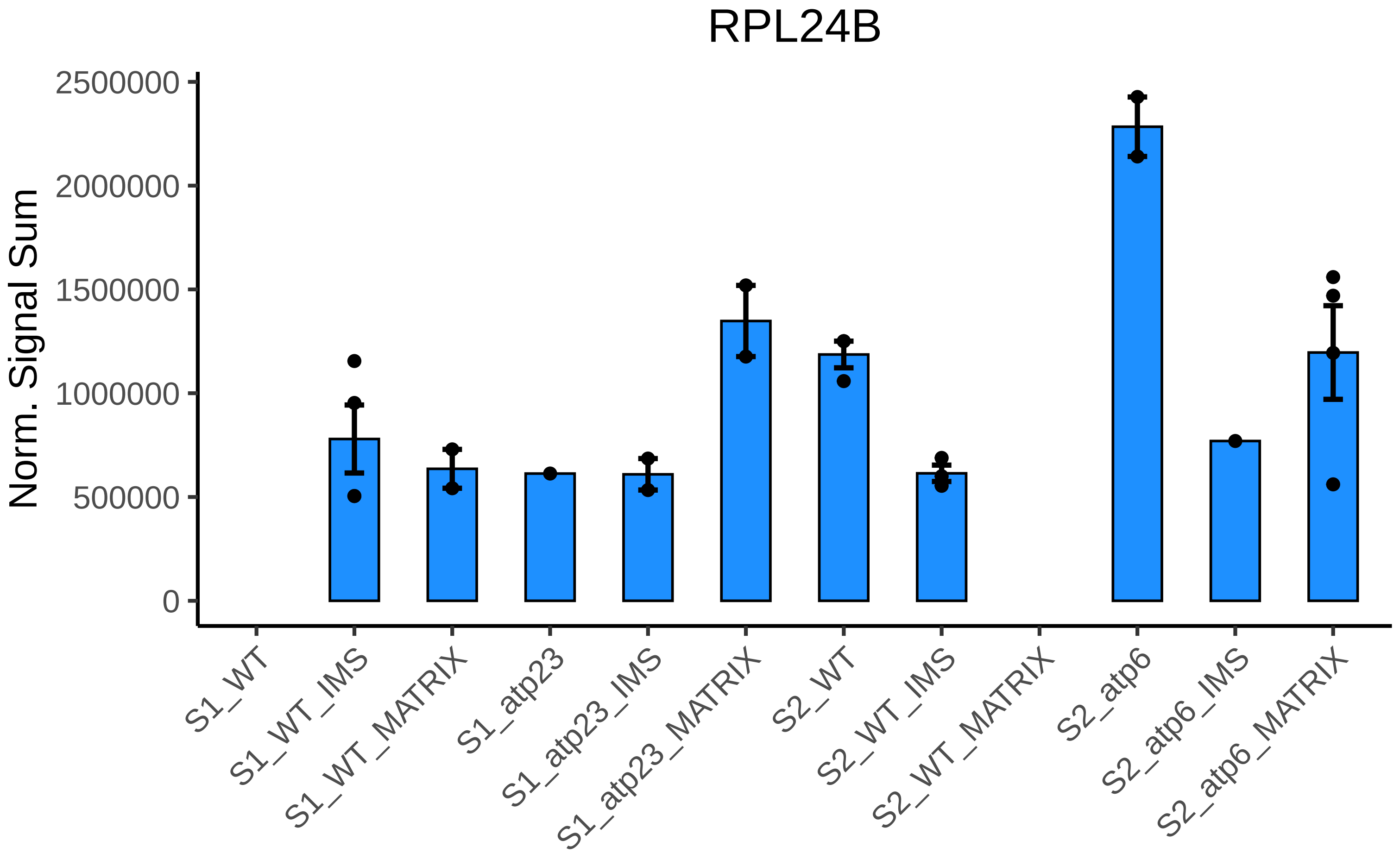
RPL24B
Norm. Signal Sum
2500000
2000000
1500000
1000000
500000
0
S1_WT
S1_WT_IMS
S1_WT_MATRIX
S1_atp23
S1_atp23_IMS
S1_atp23_MATRIX
S2_WT
S2_WT_IMS
S2_WT_MATRIX
S2_atp6
S2_atp6_IMS
S2_atp6_MATRIX

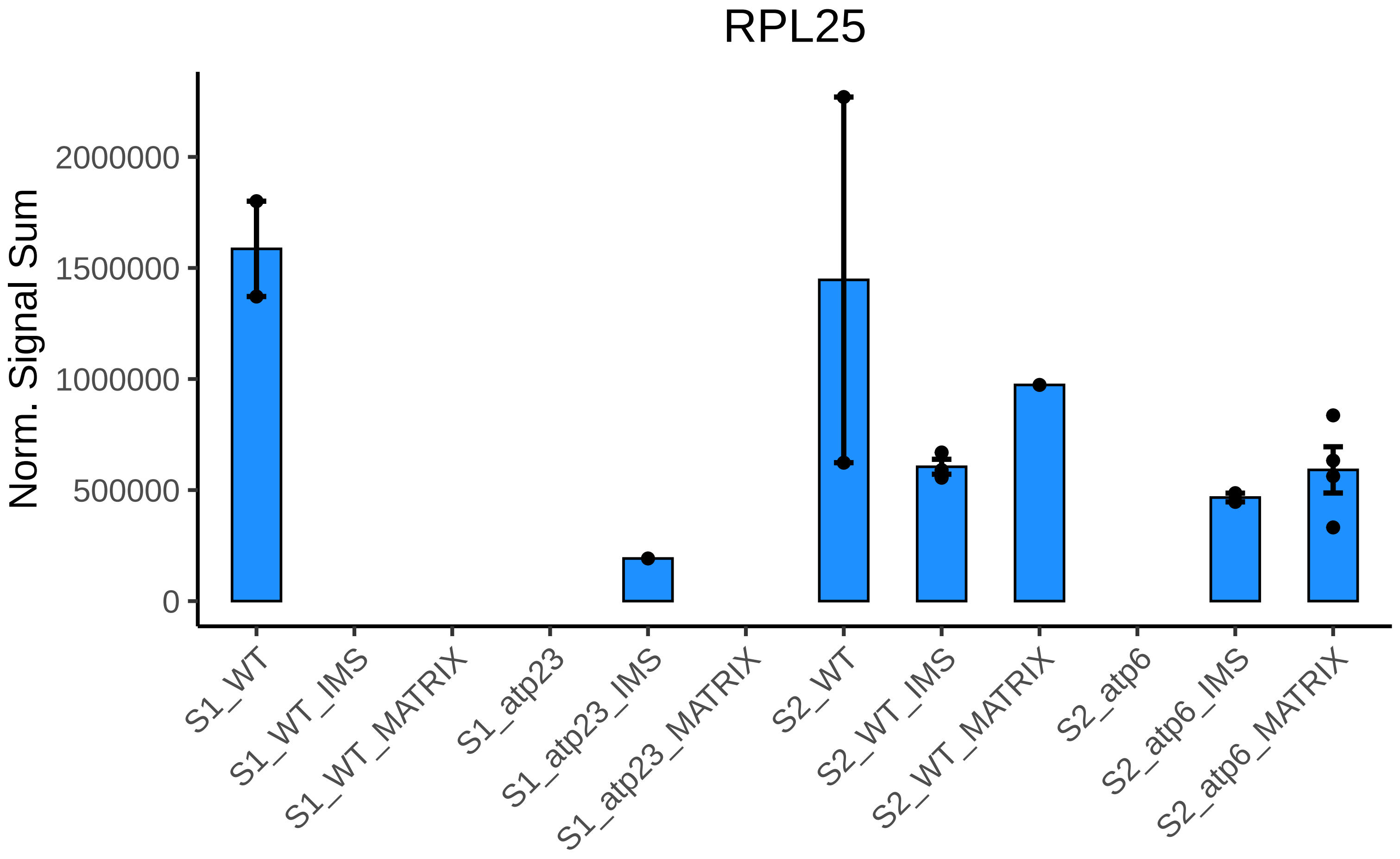

RPL25
Norm. Signal Sum
2000000
1500000
1000000
500000
0
S1_WT
S1_WT_IMS
S1_WT_MATRIX
S1_atp23
S1_atp23_IMS
S1_atp23_MATRIX
S2_WT
S2_WT_IMS
S2_WT_MATRIX
S2_atp6
S2_atp6_IMS
S2_atp6_MATRIX

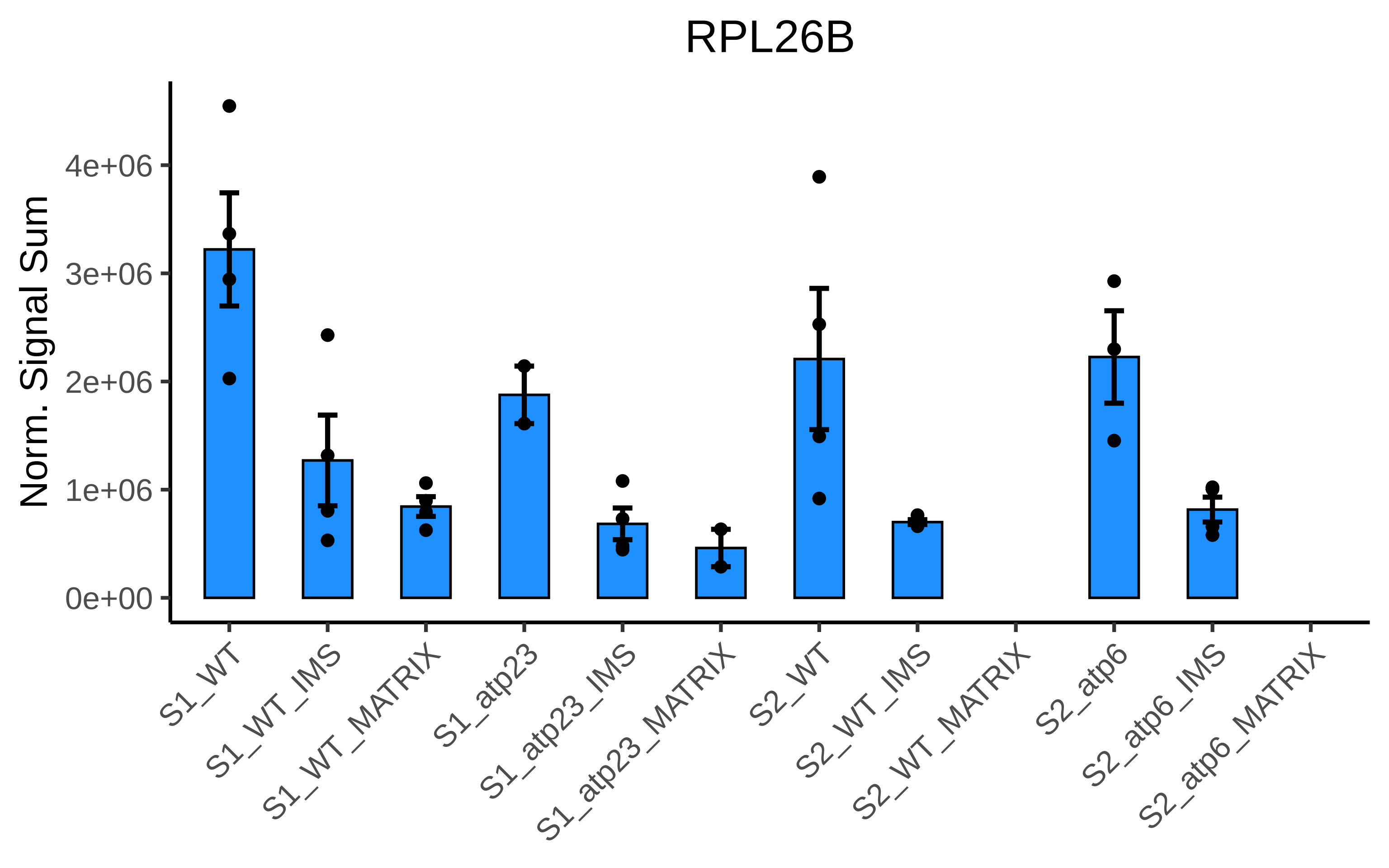
RPL26B
Norm. Signal Sum
4e+06
3e+06
2e+06
1e+06
0e+00
S1_WT
S1_WT_IMS
S1_WT_MATRIX
S1_atp23
S1_atp23_IMS
S1_atp23_MATRIX
S2_WT
S2_WT_IMS
S2_WT_MATRIX
S2_atp6
S2_atp6_IMS
S2_atp6_MATRIX

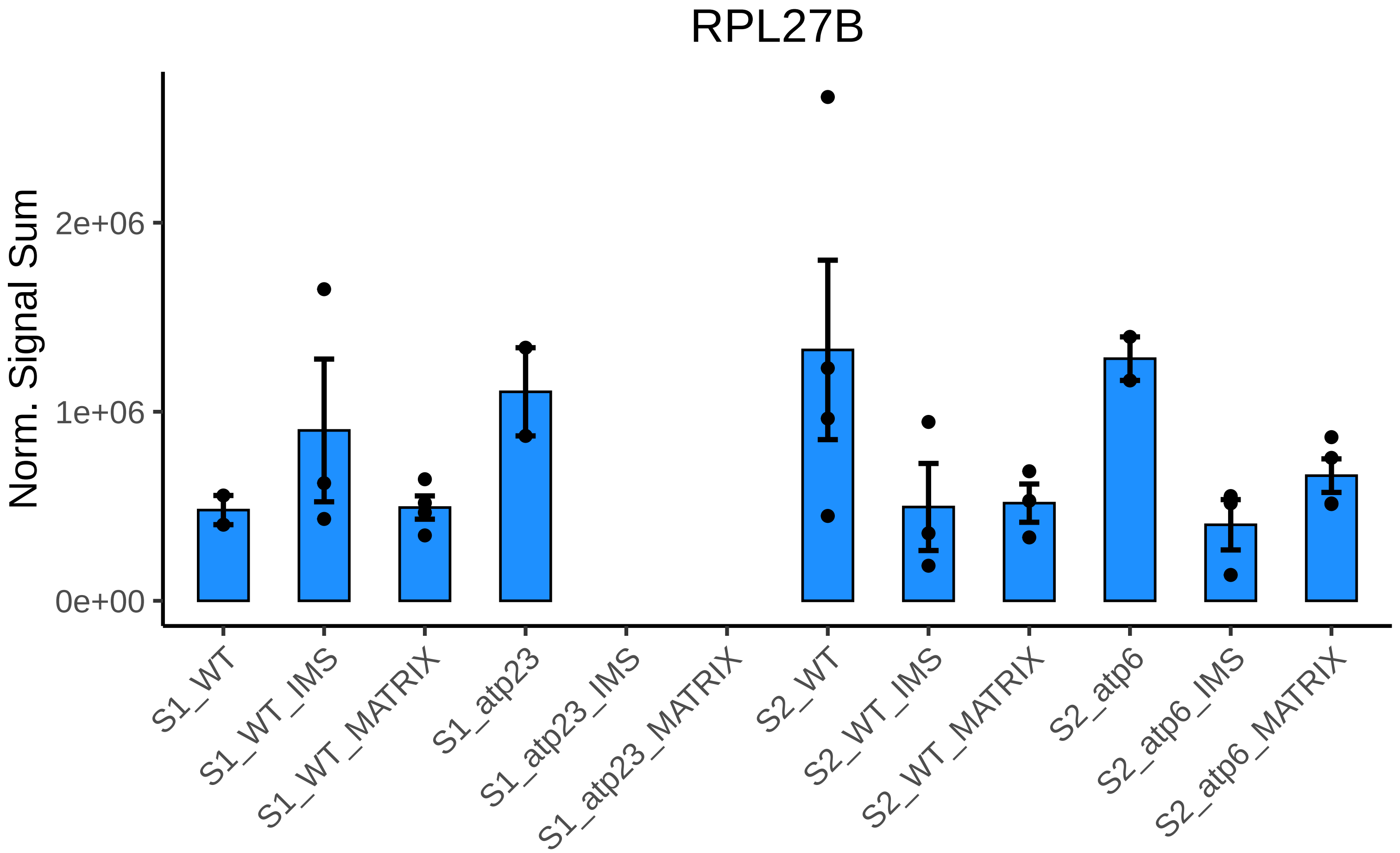

RPL27B
Norm. Signal Sum
2e+06
1e+06
0e+00
S1_WT
S1_WT_IMS
S1_WT_MATRIX
S1_atp23
S1_atp23_IMS
S1_atp23_MATRIX
S2_WT
S2_WT_IMS
S2_WT_MATRIX
S2_atp6
S2_atp6_IMS
S2_atp6_MATRIX

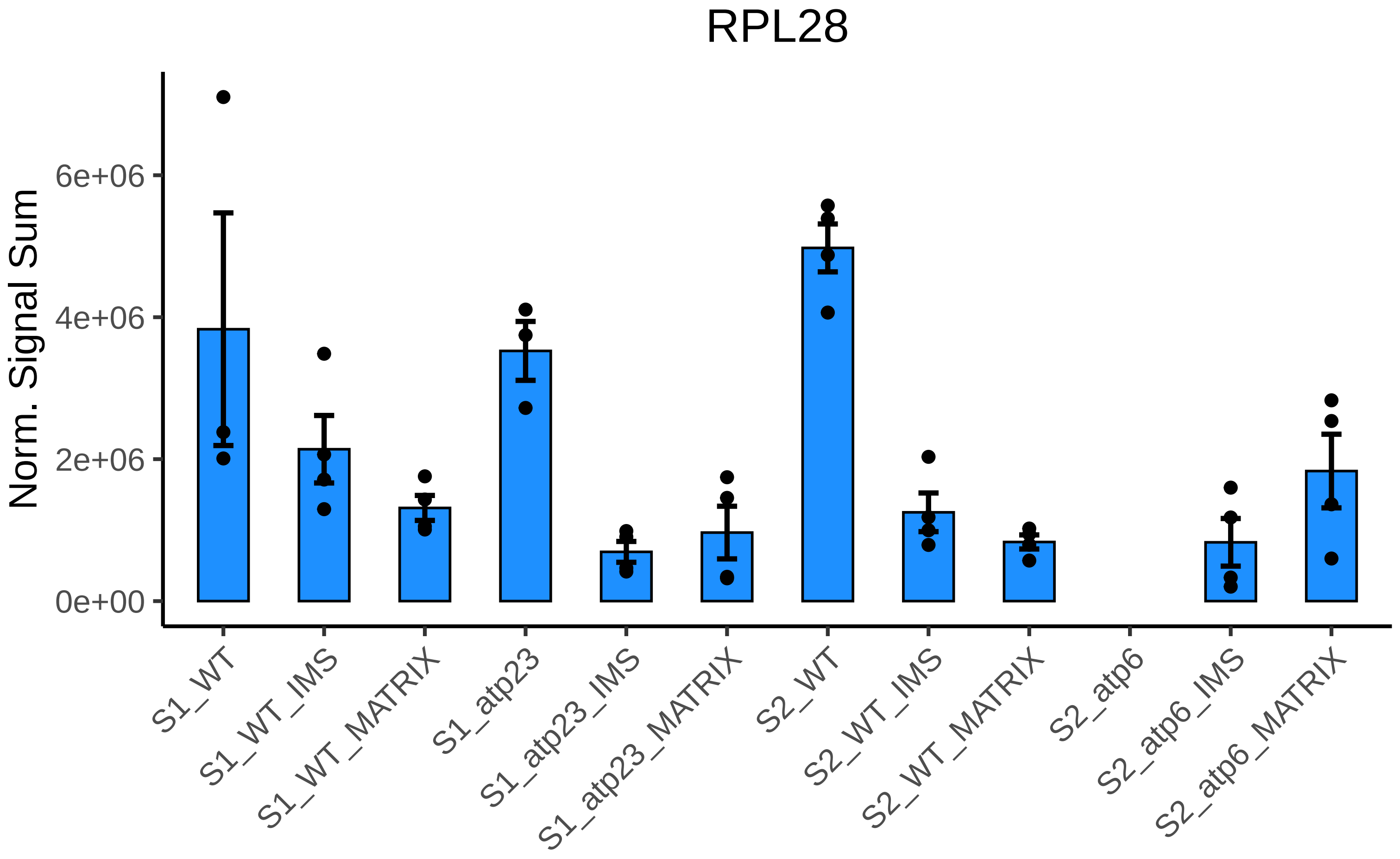

RPL28
Norm. Signal Sum
6e+06
4e+06
2e+06
0e+00
S1_WT
S1_WT_IMS
S1_WT_MATRIX
S1_atp23
S1_atp23_IMS
S1_atp23_MATRIX
S2_WT
S2_WT_IMS
S2_WT_MATRIX
S2_atp6
S2_atp6_IMS
S2_atp6_MATRIX

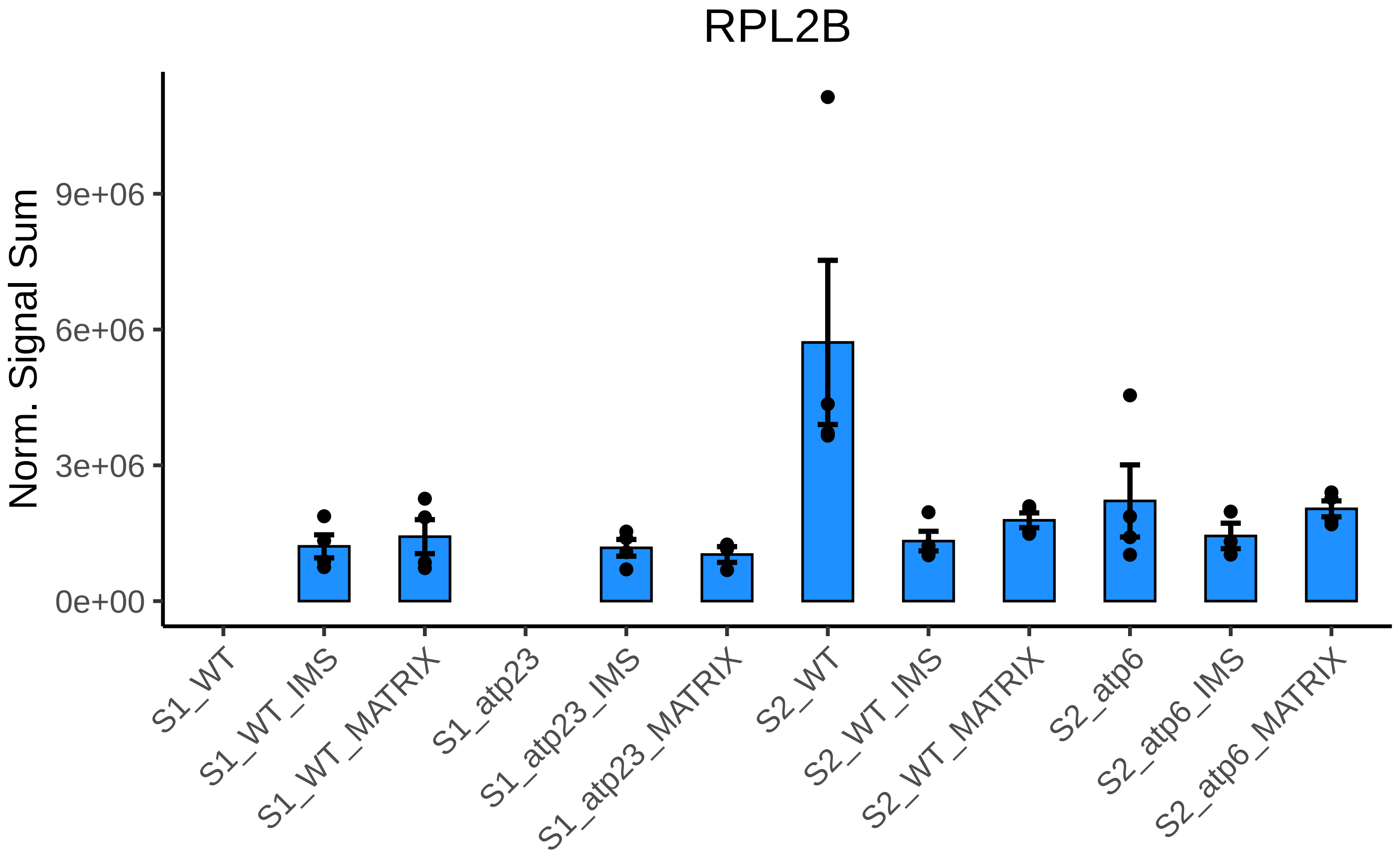

RPL2B
Norm. Signal Sum
9e+06
6e+06
3e+06
0e+00
S1_WT
S1_WT_IMS
S1_WT_MATRIX
S1_atp23
S1_atp23_IMS
S1_atp23_MATRIX
S2_WT
S2_WT_IMS
S2_WT_MATRIX
S2_atp6
S2_atp6_IMS
S2_atp6_MATRIX

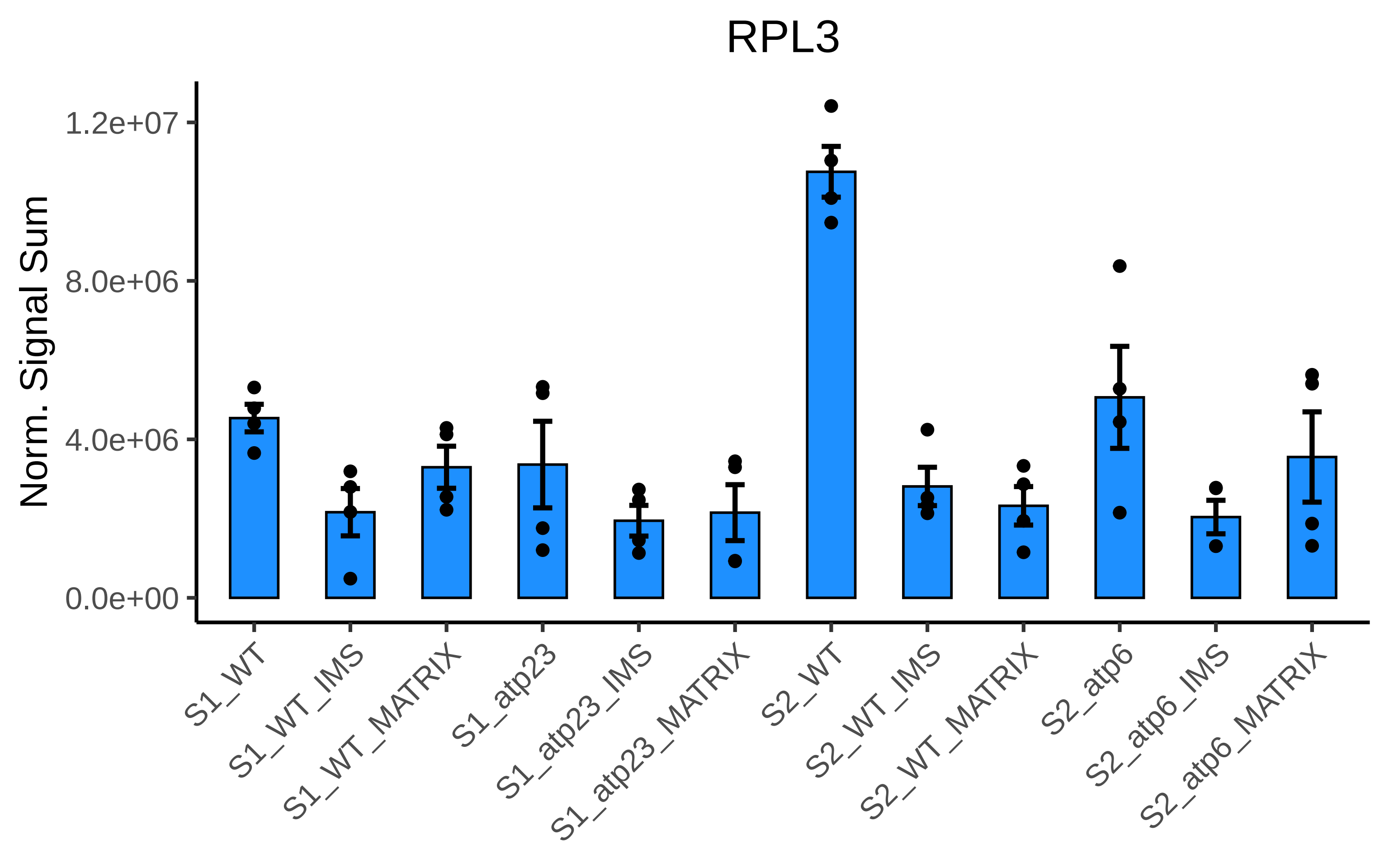

RPL3
Norm. Signal Sum
1.2e+07
8.0e+06
4.0e+06
0.0e+00
S1_WT
S1_WT_IMS
S1_WT_MATRIX
S1_atp23
S1_atp23_IMS
S1_atp23_MATRIX
S2_WT
S2_WT_IMS
S2_WT_MATRIX
S2_atp6
S2_atp6_IMS
S2_atp6_MATRIX

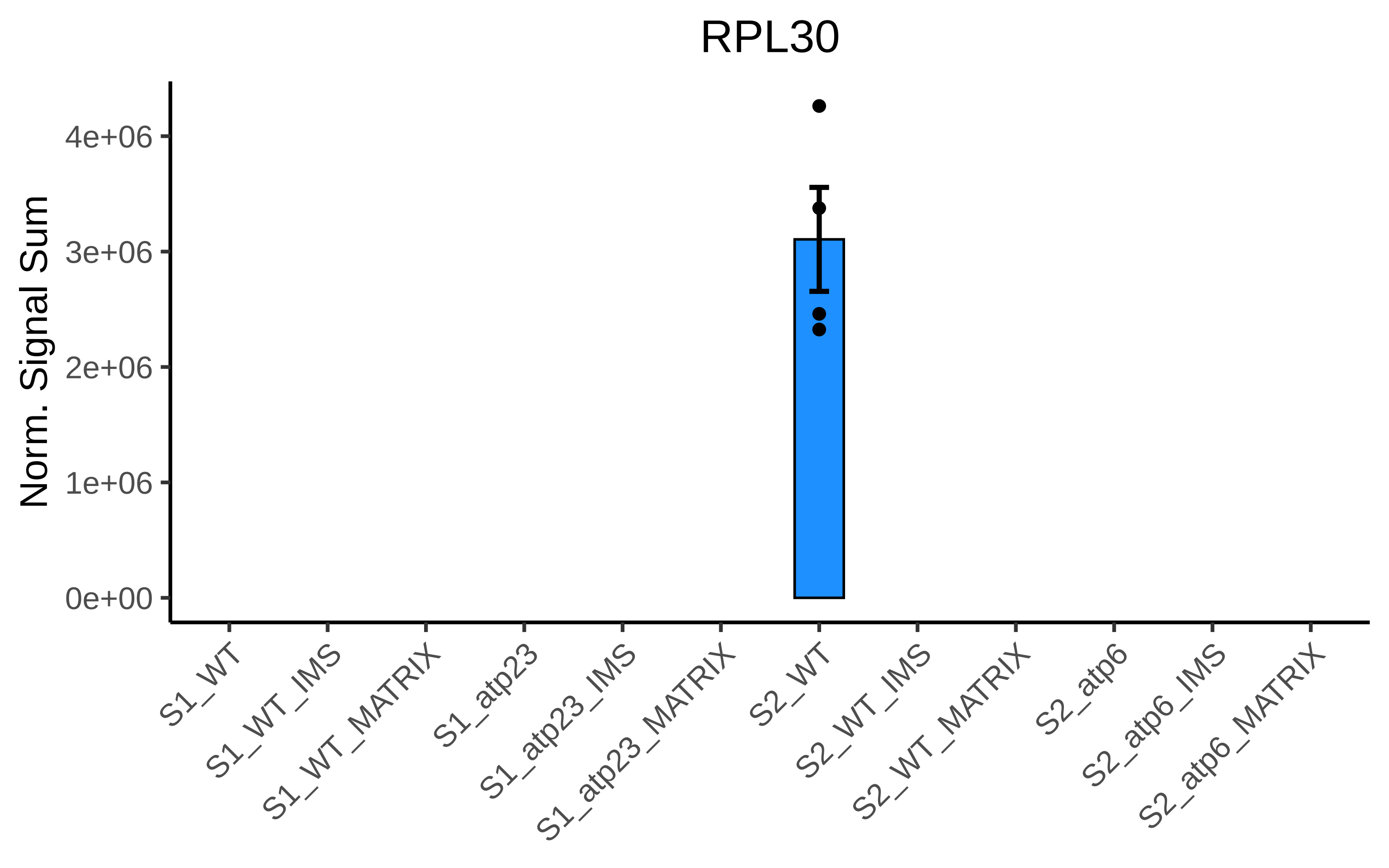

RPL30
Norm. Signal Sum
4e+06
3e+06
2e+06
1e+06
0e+00
S1_WT
S1_WT_IMS
S1_WT_MATRIX
S1_atp23
S1_atp23_IMS
S1_atp23_MATRIX
S2_WT
S2_WT_IMS
S2_WT_MATRIX
S2_atp6
S2_atp6_IMS
S2_atp6_MATRIX

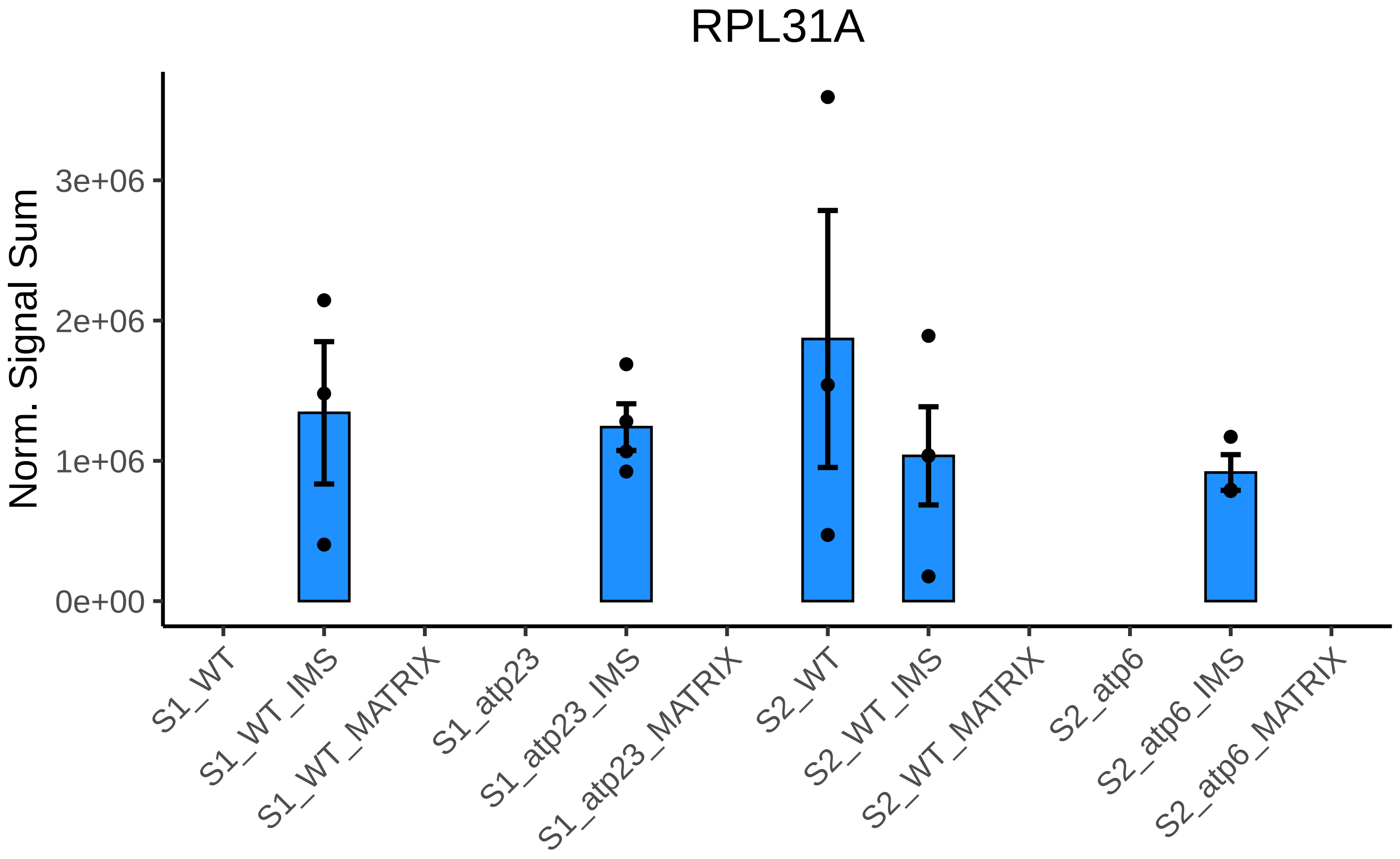
RPL31A
Norm. Signal Sum
3e+06
2e+06
1e+06
0e+00
S1_WT
S1_WT_IMS
S1_WT_MATRIX
S1_atp23
S1_atp23_IMS
S1_atp23_MATRIX
S2_WT
S2_WT_IMS
S2_WT_MATRIX
S2_atp6
S2_atp6_IMS
S2_atp6_MATRIX

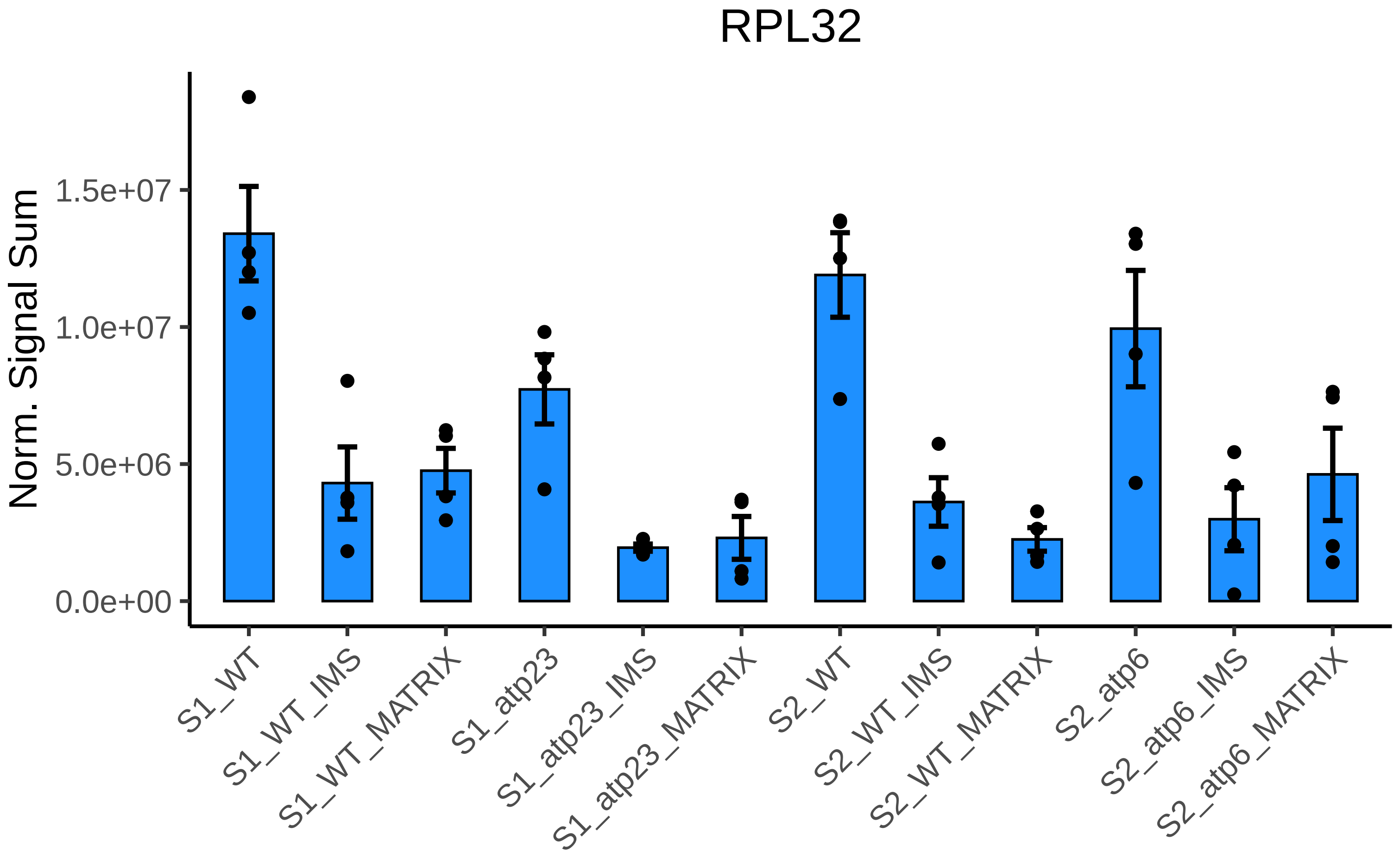

RPL32
Norm. Signal Sum
1.5e+07
1.0e+07
5.0e+06
0.0e+00
S1_WT
S1_WT_IMS
S1_WT_MATRIX
S1_atp23
S1_atp23_IMS
S1_atp23_MATRIX
S2_WT
S2_WT_IMS
S2_WT_MATRIX
S2_atp6
S2_atp6_IMS
S2_atp6_MATRIX

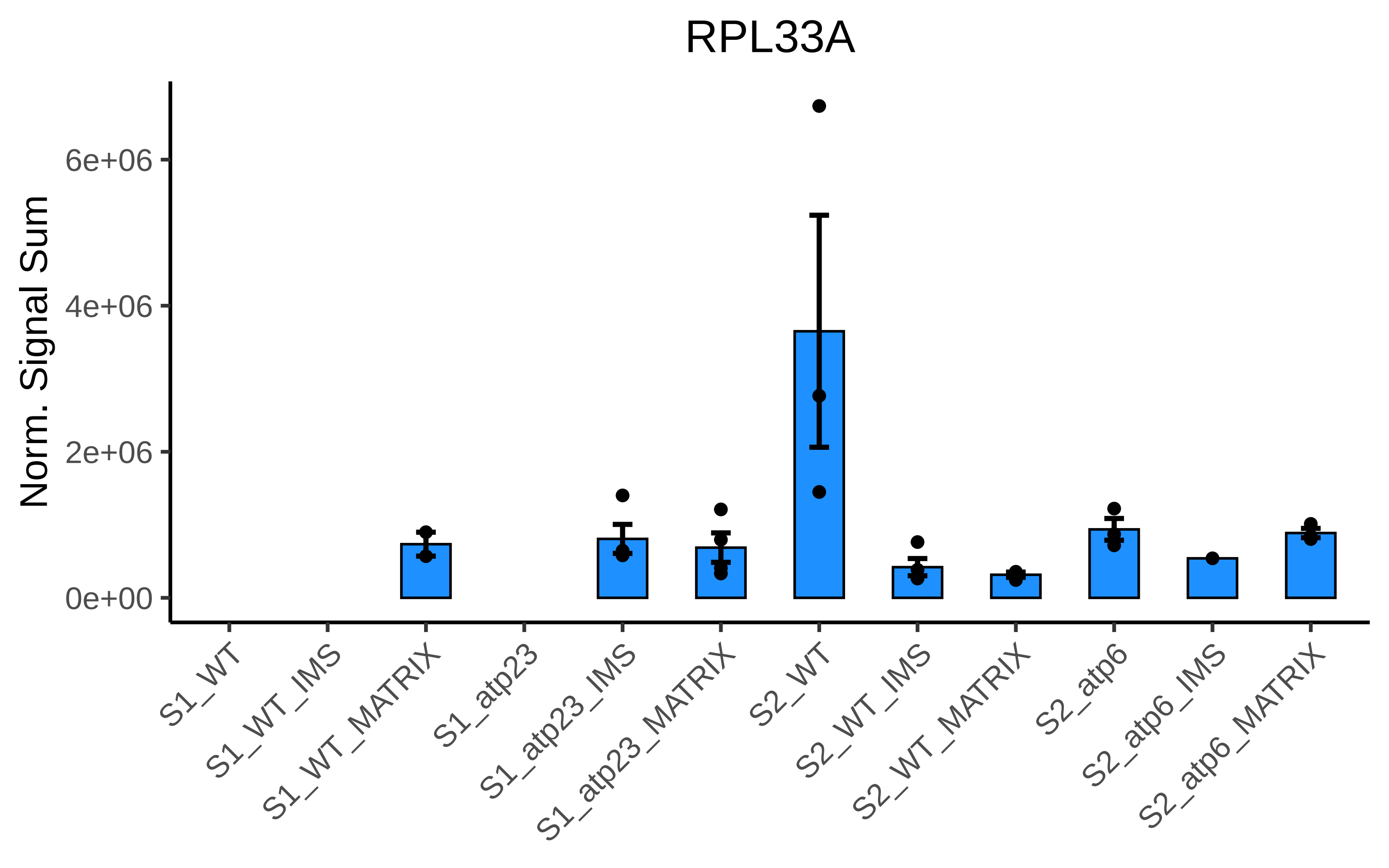

RPL33A
Norm. Signal Sum
6e+06
4e+06
2e+06
0e+00
S1_WT
S1_WT_IMS
S1_WT_MATRIX
S1_atp23
S1_atp23_IMS
S1_atp23_MATRIX
S2_WT
S2_WT_IMS
S2_WT_MATRIX
S2_atp6
S2_atp6_IMS
S2_atp6_MATRIX

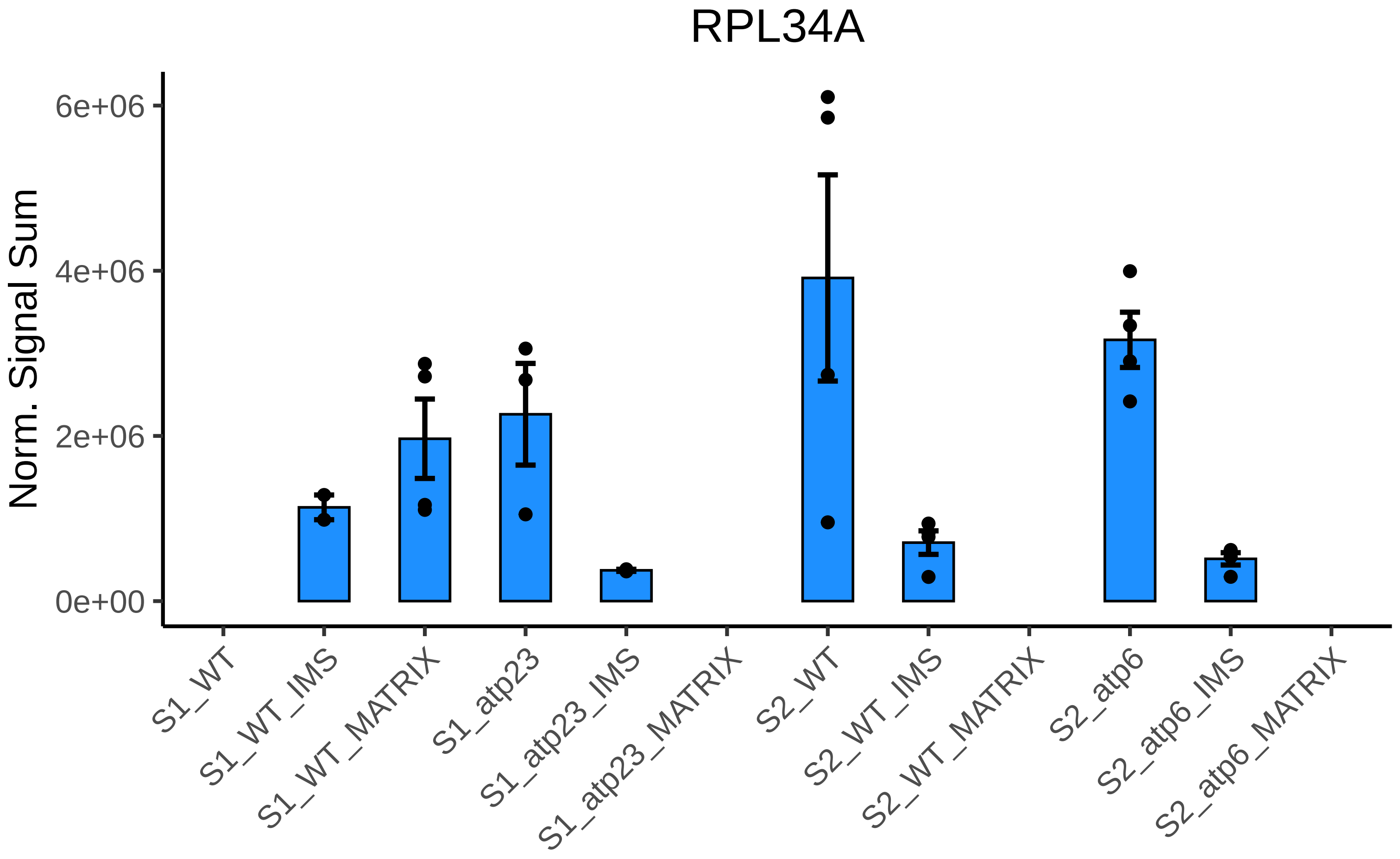
RPL34A
Norm. Signal Sum
6e+06
4e+06
2e+06
0e+00
S1_WT
S1_WT_IMS
S1_WT_MATRIX
S1_atp23
S1_atp23_IMS
S1_atp23_MATRIX
S2_WT
S2_WT_IMS
S2_WT_MATRIX
S2_atp6
S2_atp6_IMS
S2_atp6_MATRIX

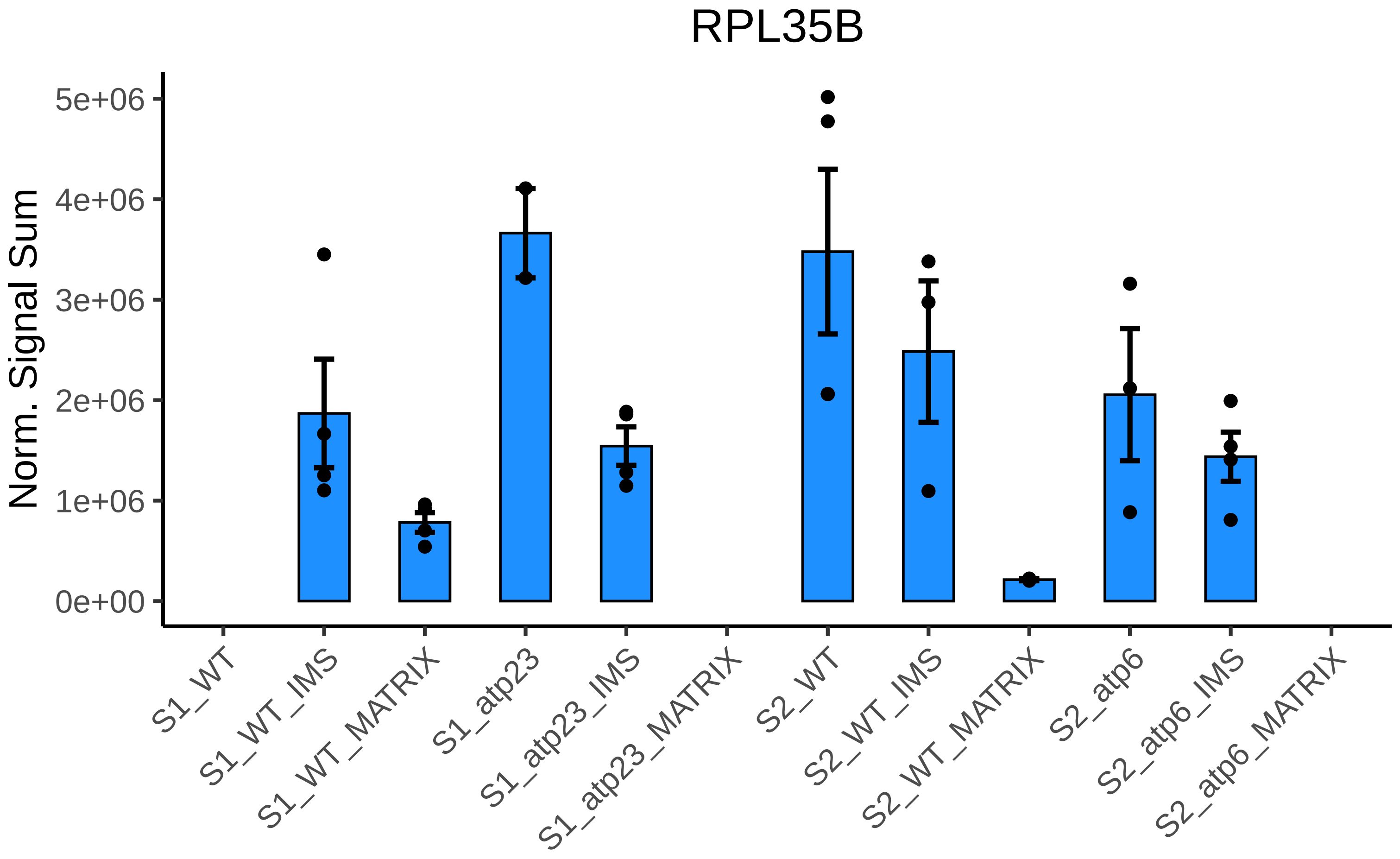

RPL35B
Norm. Signal Sum
5e+06
4e+06
3e+06
2e+06
1e+06
0e+00
S1_WT
S1_WT_IMS
S1_WT_MATRIX
S1_atp23
S1_atp23_IMS
S1_atp23_MATRIX
S2_WT
S2_WT_IMS
S2_WT_MATRIX
S2_atp6
S2_atp6_IMS
S2_atp6_MATRIX

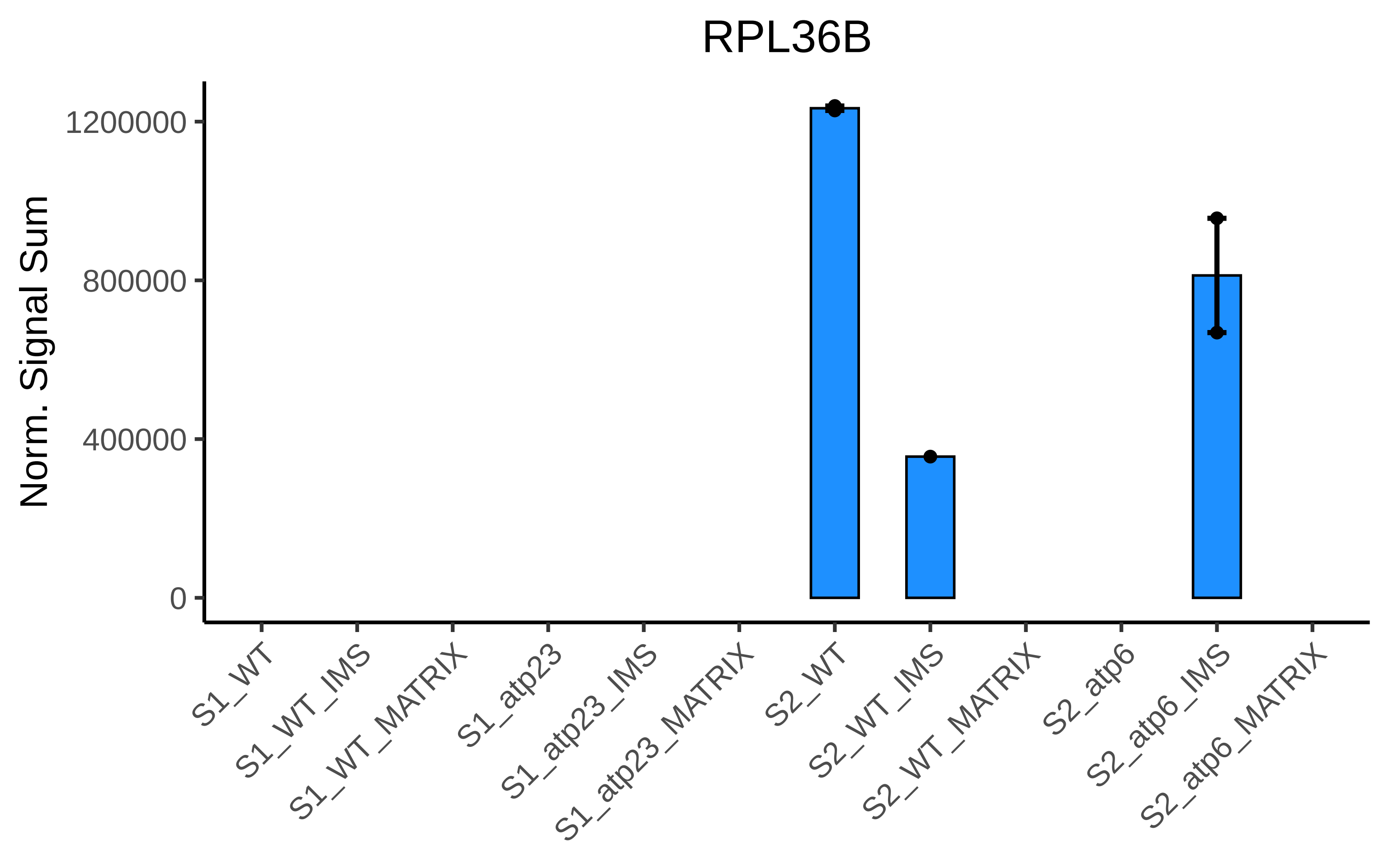
RPL36B
Norm. Signal Sum
1200000
800000
400000
0
S1_WT
S1_WT_IMS
S1_WT_MATRIX
S1_atp23
S1_atp23_IMS
S1_atp23_MATRIX
S2_WT
S2_WT_IMS
S2_WT_MATRIX
S2_atp6
S2_atp6_IMS
S2_atp6_MATRIX

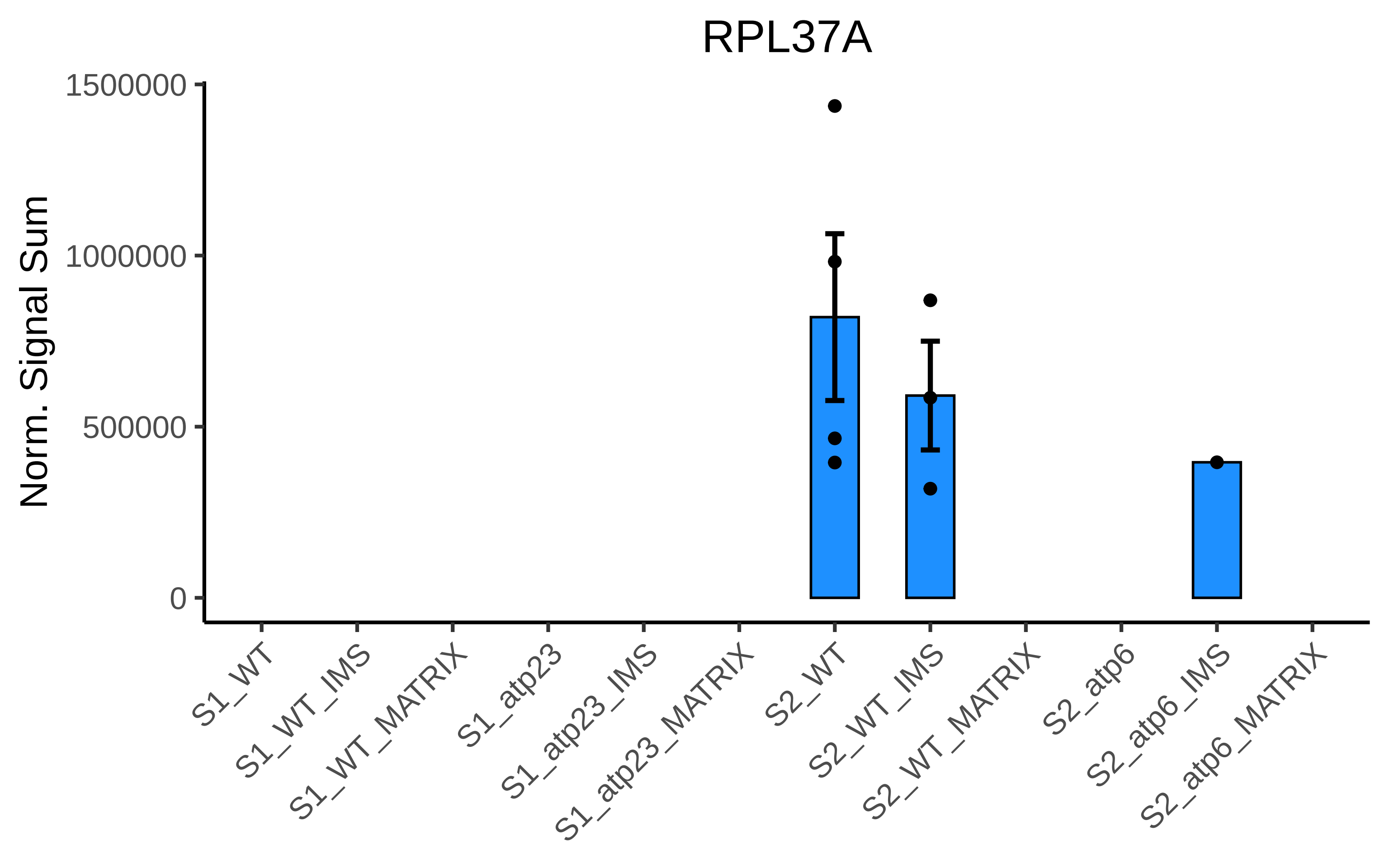
RPL37A
Norm. Signal Sum
1500000
1000000
500000
0
S1_WT
S1_WT_IMS
S1_WT_MATRIX
S1_atp23
S1_atp23_IMS
S1_atp23_MATRIX
S2_WT
S2_WT_IMS
S2_WT_MATRIX
S2_atp6
S2_atp6_IMS
S2_atp6_MATRIX

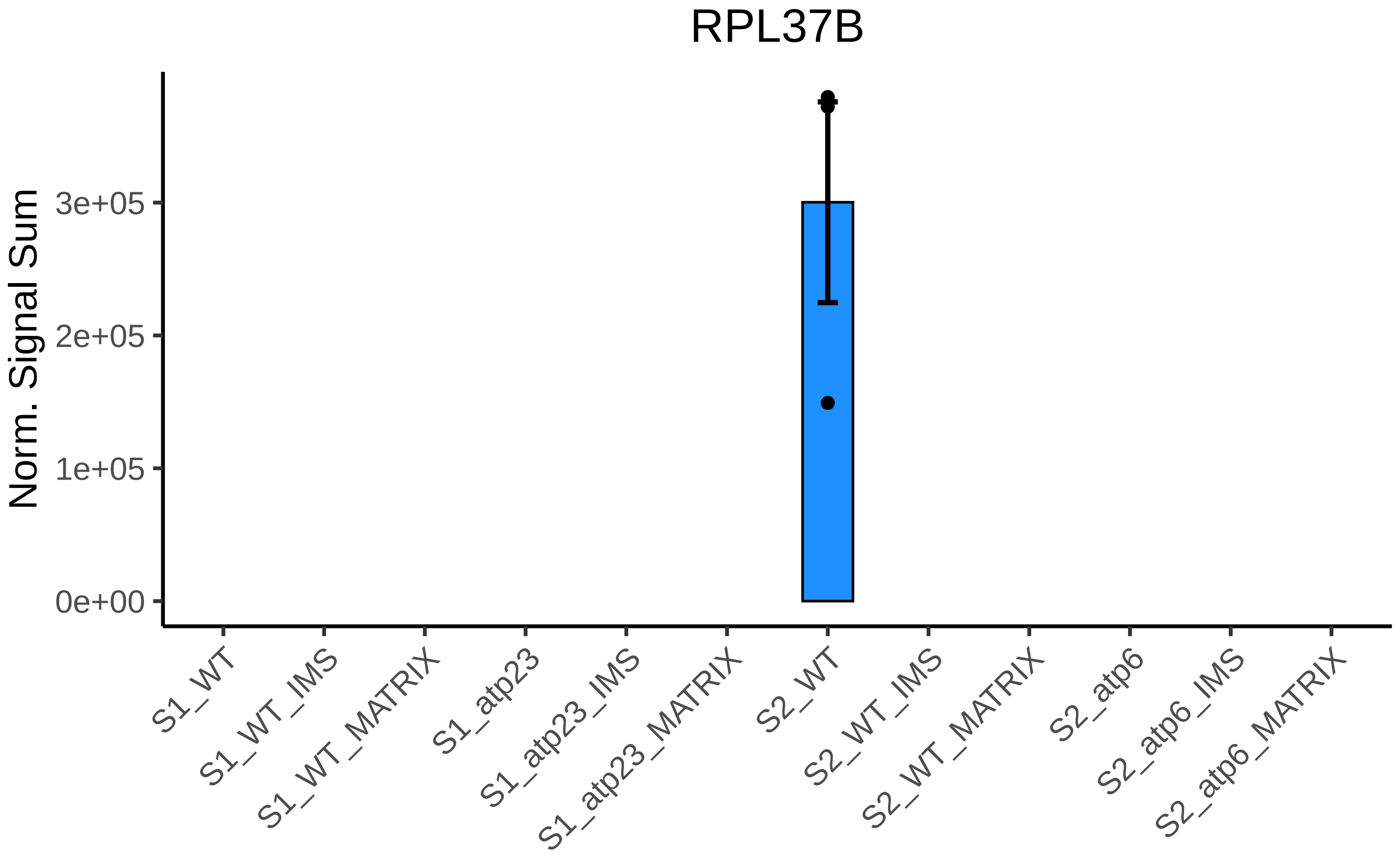
RPL37B
Norm. Signal Sum
3e+05
2e+05
1e+05
0e+00
S1_WT
S1_WT_IMS
S1_WT_MATRIX
S1_atp23
S1_atp23_IMS
S1_atp23_MATRIX
S2_WT
S2_WT_IMS
S2_WT_MATRIX
S2_atp6
S2_atp6_IMS
S2_atp6_MATRIX

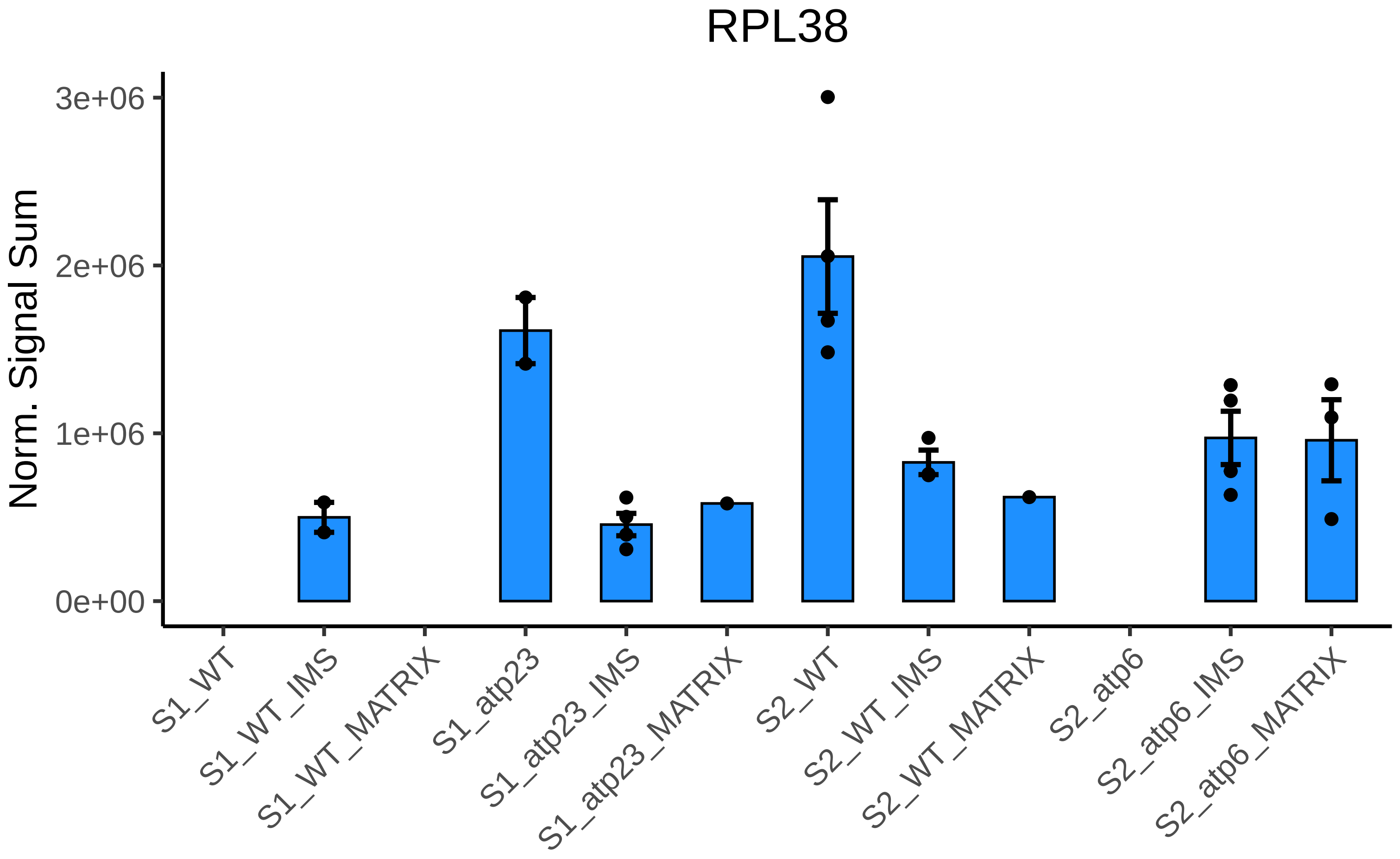
RPL38
Norm. Signal Sum
3e+06
2e+06
1e+06
0e+00
S1_WT
S1_WT_IMS
S1_WT_MATRIX
S1_atp23
S1_atp23_IMS
S1_atp23_MATRIX
S2_WT
S2_WT_IMS
S2_WT_MATRIX
S2_atp6
S2_atp6_IMS
S2_atp6_MATRIX

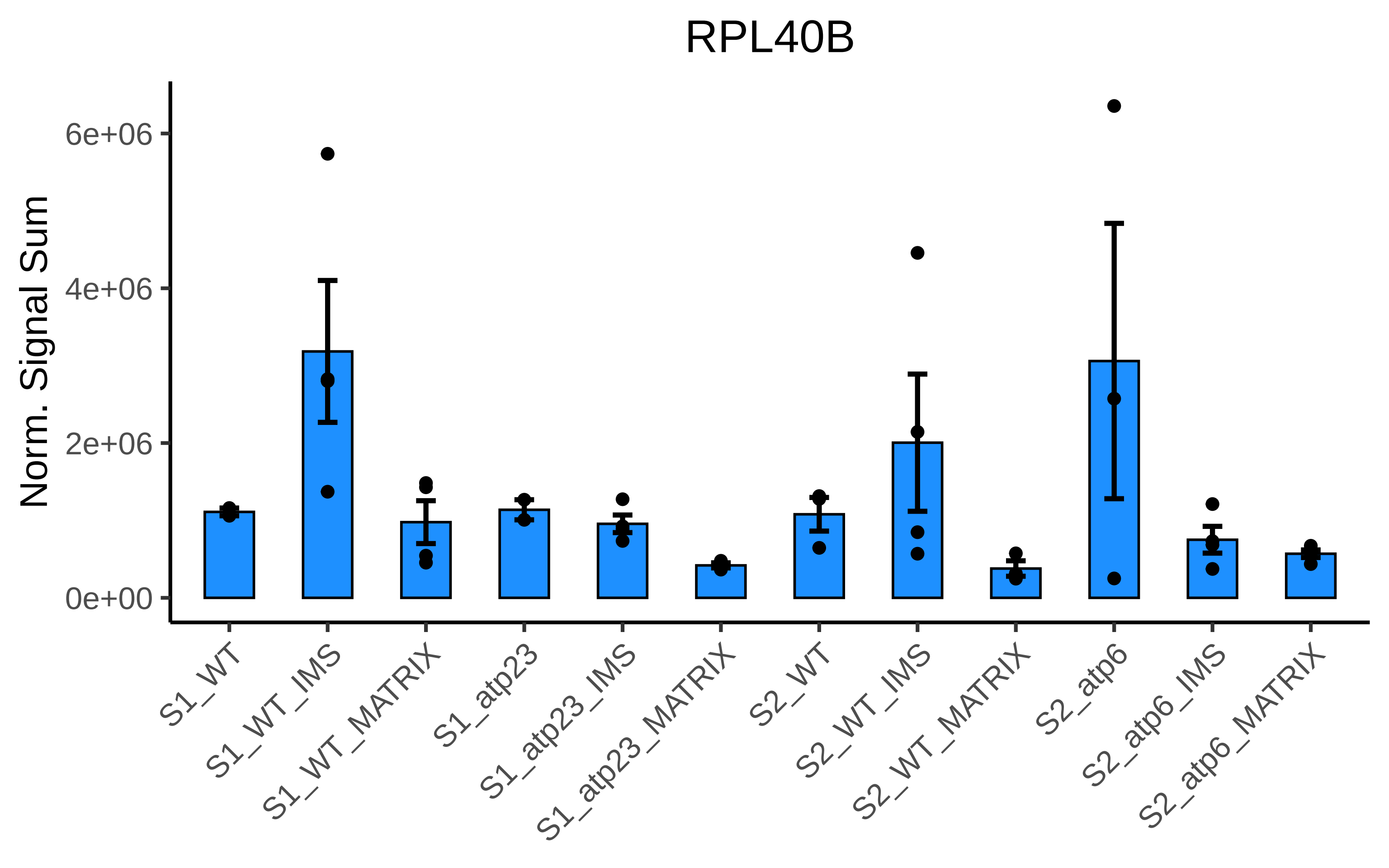
RPL40B
Norm. Signal Sum
6e+06
4e+06
2e+06
0e+00
S1_WT
S1_WT_IMS
S1_WT_MATRIX
S1_atp23
S1_atp23_IMS
S1_atp23_MATRIX
S2_WT
S2_WT_IMS
S2_WT_MATRIX
S2_atp6
S2_atp6_IMS
S2_atp6_MATRIX

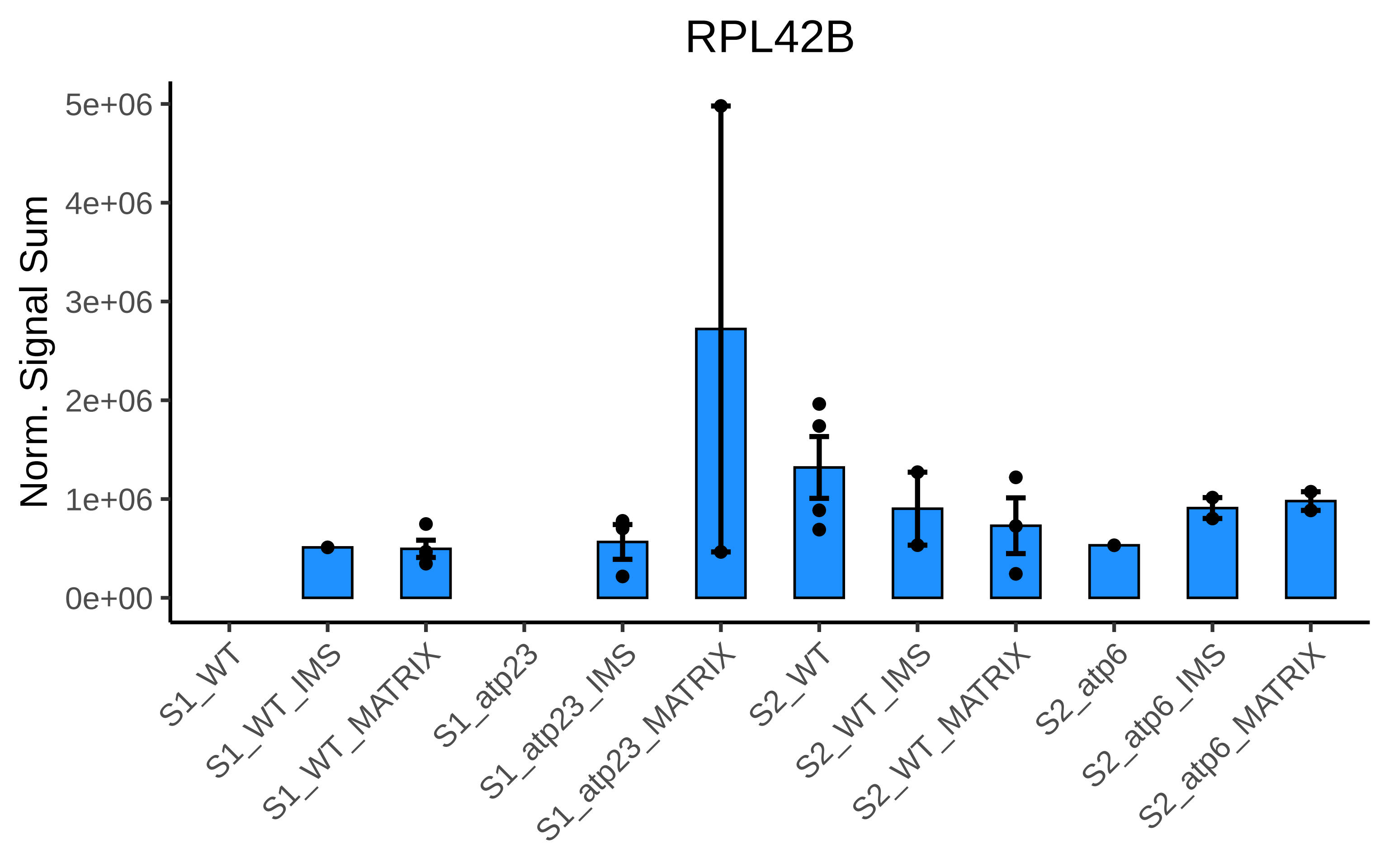
RPL42B
Norm. Signal Sum
5e+06
4e+06
3e+06
2e+06
1e+06
0e+00
S1_WT
S1_WT_IMS
S1_WT_MATRIX
S1_atp23
S1_atp23_IMS
S1_atp23_MATRIX
S2_WT
S2_WT_IMS
S2_WT_MATRIX
S2_atp6
S2_atp6_IMS
S2_atp6_MATRIX

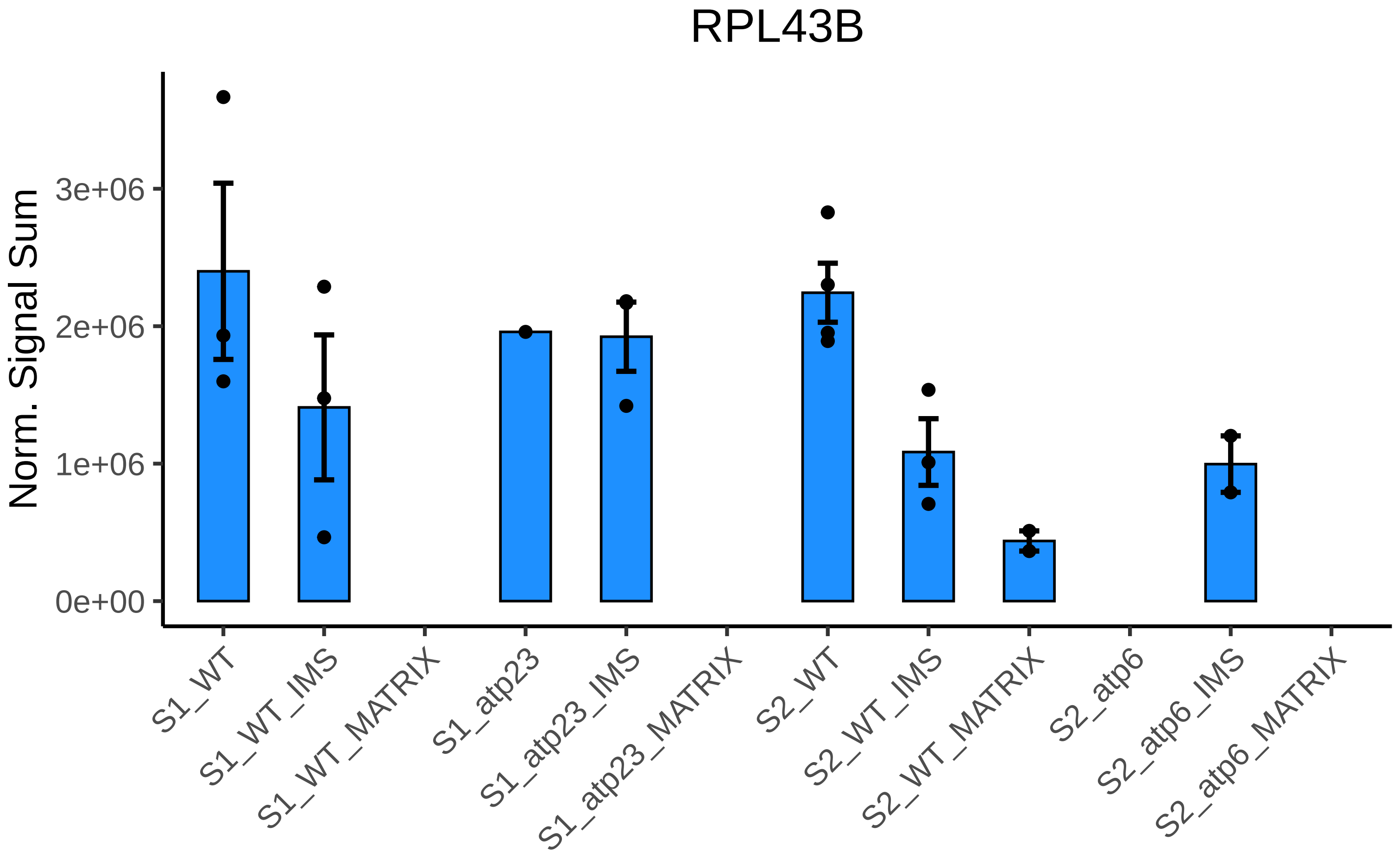

RPL43B
Norm. Signal Sum
3e+06
2e+06
1e+06
0e+00
S1_WT
S1_WT_IMS
S1_WT_MATRIX
S1_atp23
S1_atp23_IMS
S1_atp23_MATRIX
S2_WT
S2_WT_IMS
S2_WT_MATRIX
S2_atp6
S2_atp6_IMS
S2_atp6_MATRIX

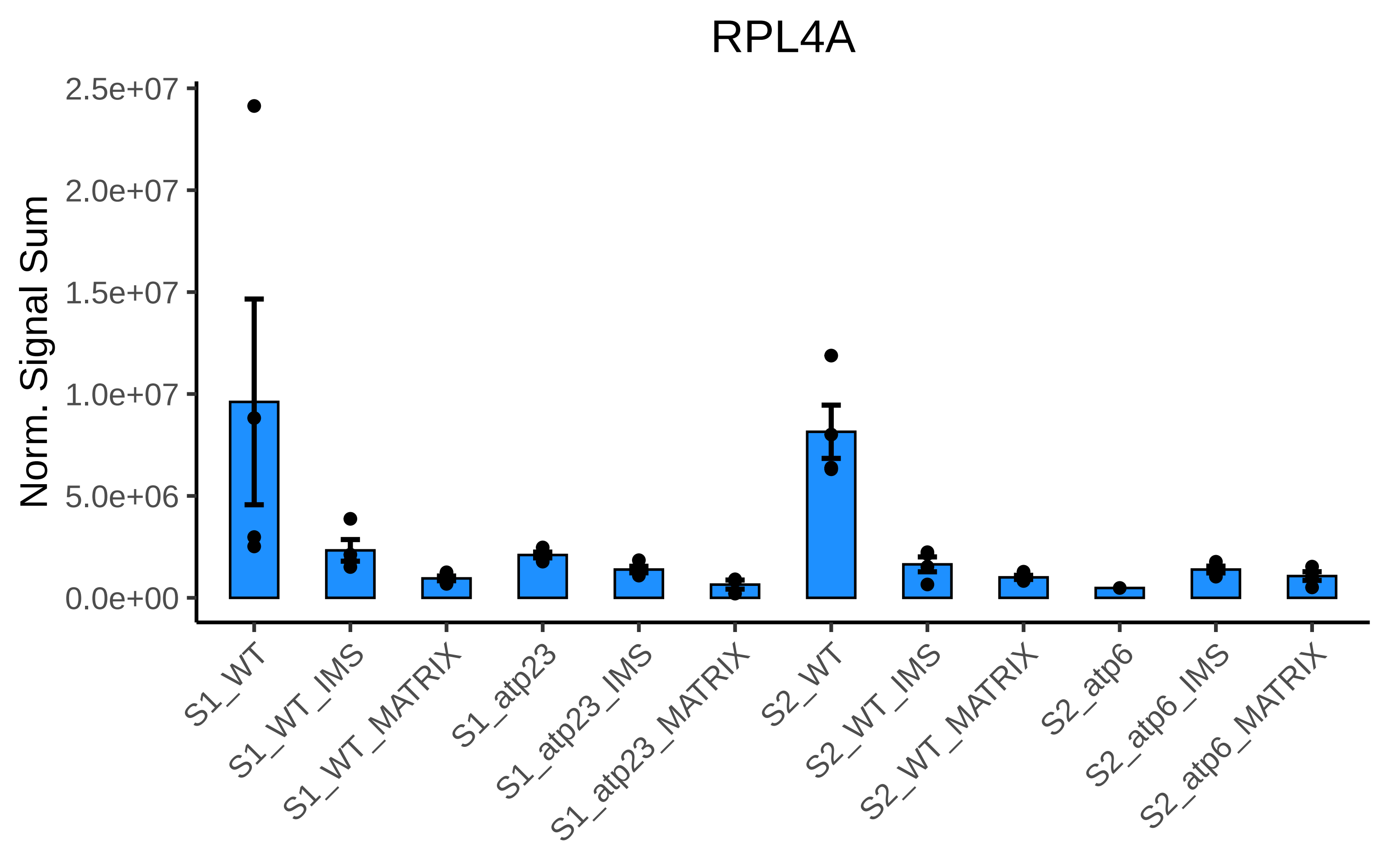
RPL4A
Norm. Signal Sum
2.5e+07
2.0e+07
1.5e+07
1.0e+07
5.0e+06
0.0e+00
S1_WT
S1_WT_IMS
S1_WT_MATRIX
S1_atp23
S1_atp23_IMS
S1_atp23_MATRIX
S2_WT
S2_WT_IMS
S2_WT_MATRIX
S2_atp6
S2_atp6_IMS
S2_atp6_MATRIX

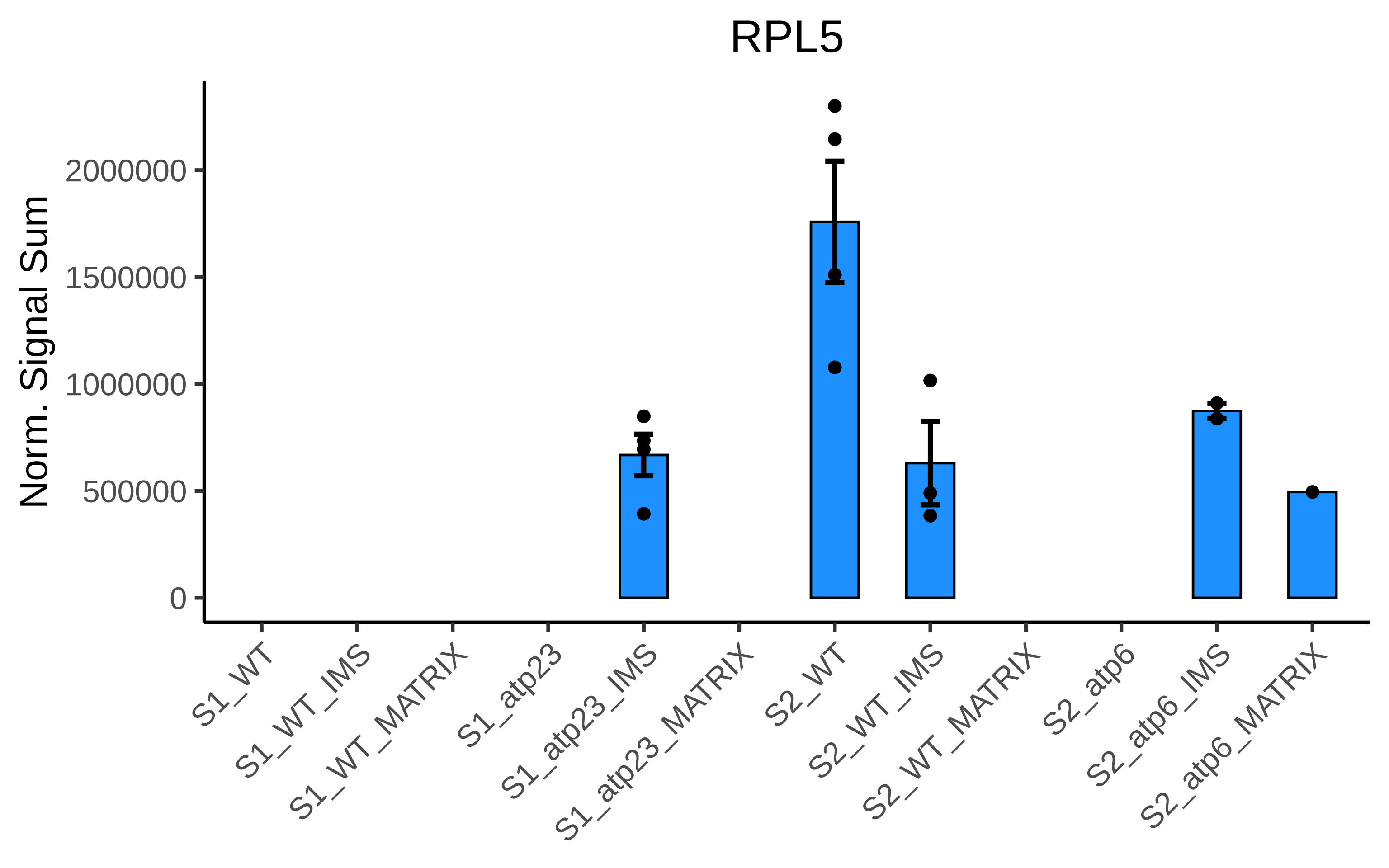

RPL5
Norm. Signal Sum
2000000
1500000
1000000
500000
0
S1_WT
S1_WT_IMS
S1_WT_MATRIX
S1_atp23
S1_atp23_IMS
S1_atp23_MATRIX
S2_WT
S2_WT_IMS
S2_WT_MATRIX
S2_atp6
S2_atp6_IMS
S2_atp6_MATRIX

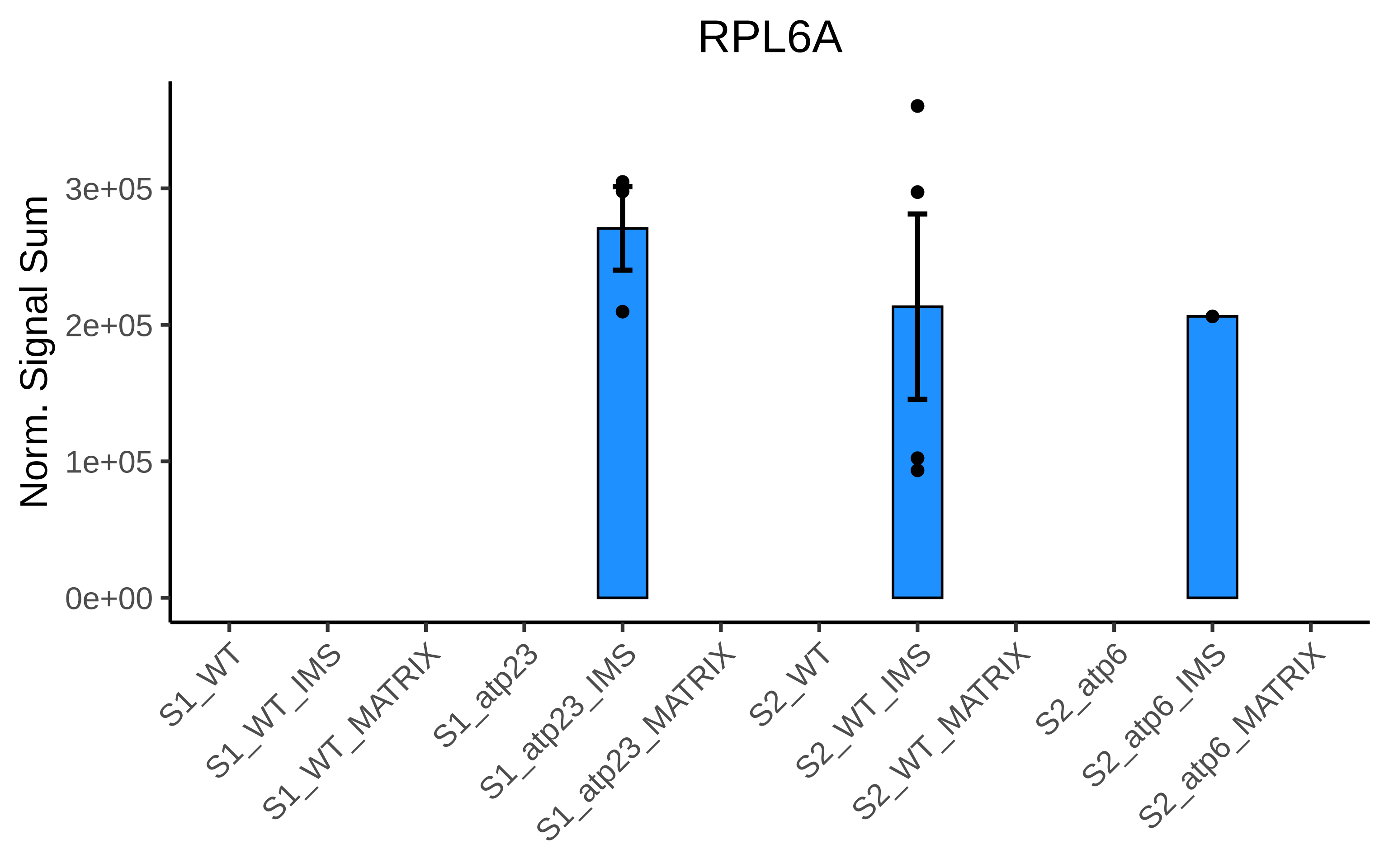

RPL6A
Norm. Signal Sum
3e+05
2e+05
1e+05
0e+00
S1_WT
S1_WT_IMS
S1_WT_MATRIX
S1_atp23
S1_atp23_IMS
S1_atp23_MATRIX
S2_WT
S2_WT_IMS
S2_WT_MATRIX
S2_atp6
S2_atp6_IMS
S2_atp6_MATRIX

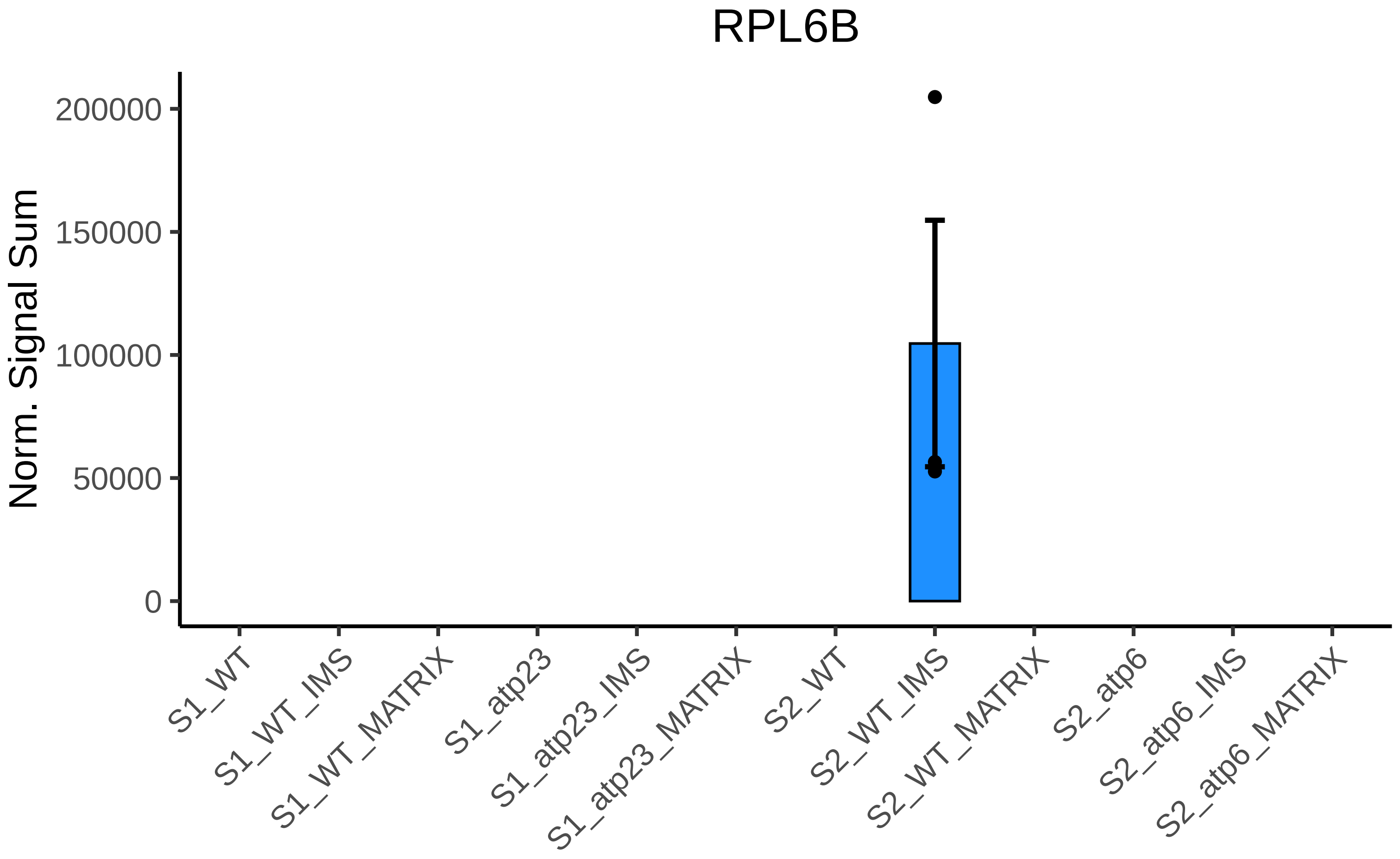

RPL6B
Norm. Signal Sum
200000
150000
100000
50000
0
S1_WT
S1_WT_IMS
S1_WT_MATRIX
S1_atp23
S1_atp23_IMS
S1_atp23_MATRIX
S2_WT
S2_WT_IMS
S2_WT_MATRIX
S2_atp6
S2_atp6_IMS
S2_atp6_MATRIX

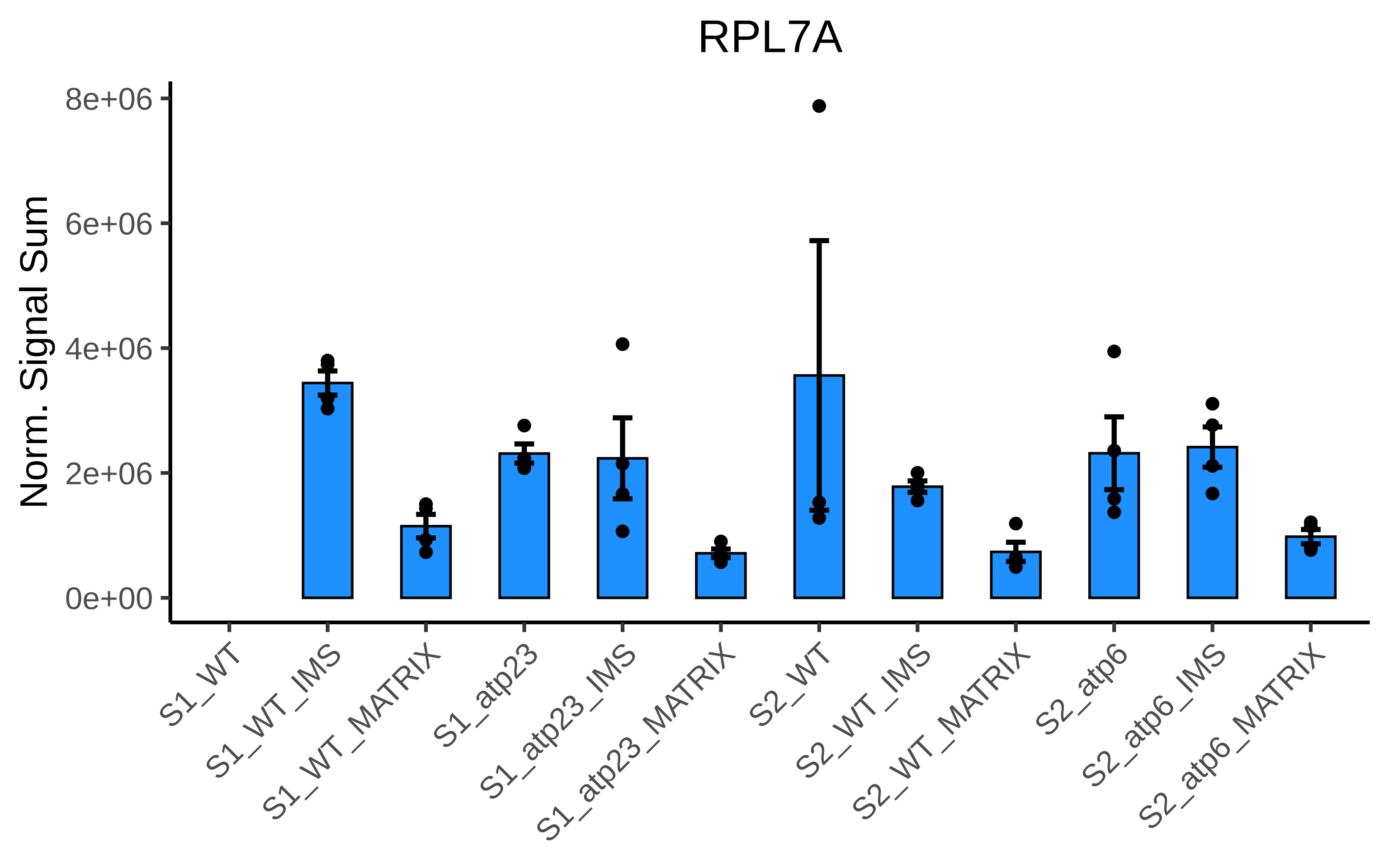

RPL7A
Norm. Signal Sum
8e+06
6e+06
4e+06
2e+06
0e+00
S1_WT
S1_WT_IMS
S1_WT_MATRIX
S1_atp23
S1_atp23_IMS
S1_atp23_MATRIX
S2_WT
S2_WT_IMS
S2_WT_MATRIX
S2_atp6
S2_atp6_IMS
S2_atp6_MATRIX

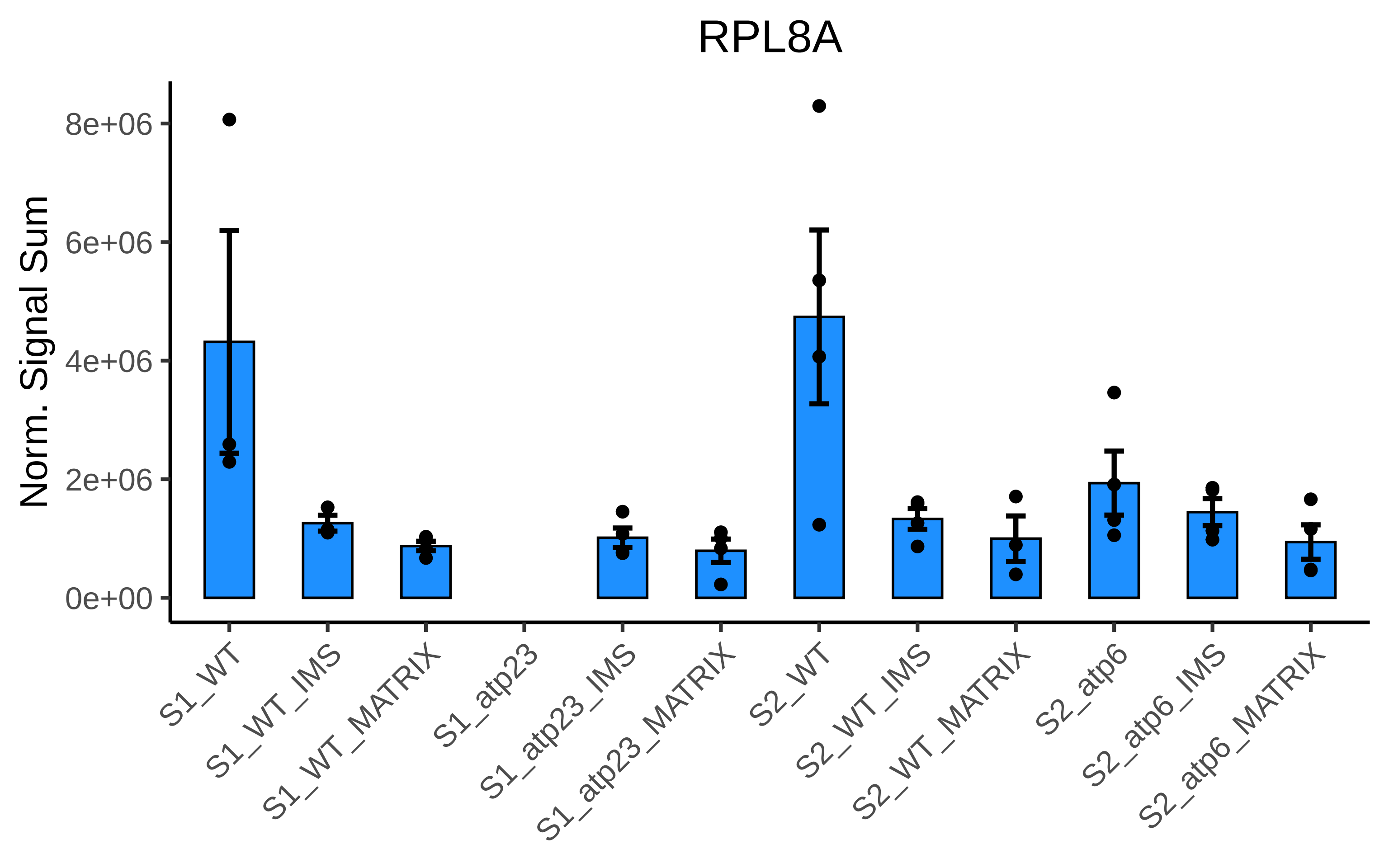
RPL8A
Norm. Signal Sum
8e+06
6e+06
4e+06
2e+06
0e+00
S1_WT
S1_WT_IMS
S1_WT_MATRIX
S1_atp23
S1_atp23_IMS
S1_atp23_MATRIX
S2_WT
S2_WT_IMS
S2_WT_MATRIX
S2_atp6
S2_atp6_IMS
S2_atp6_MATRIX

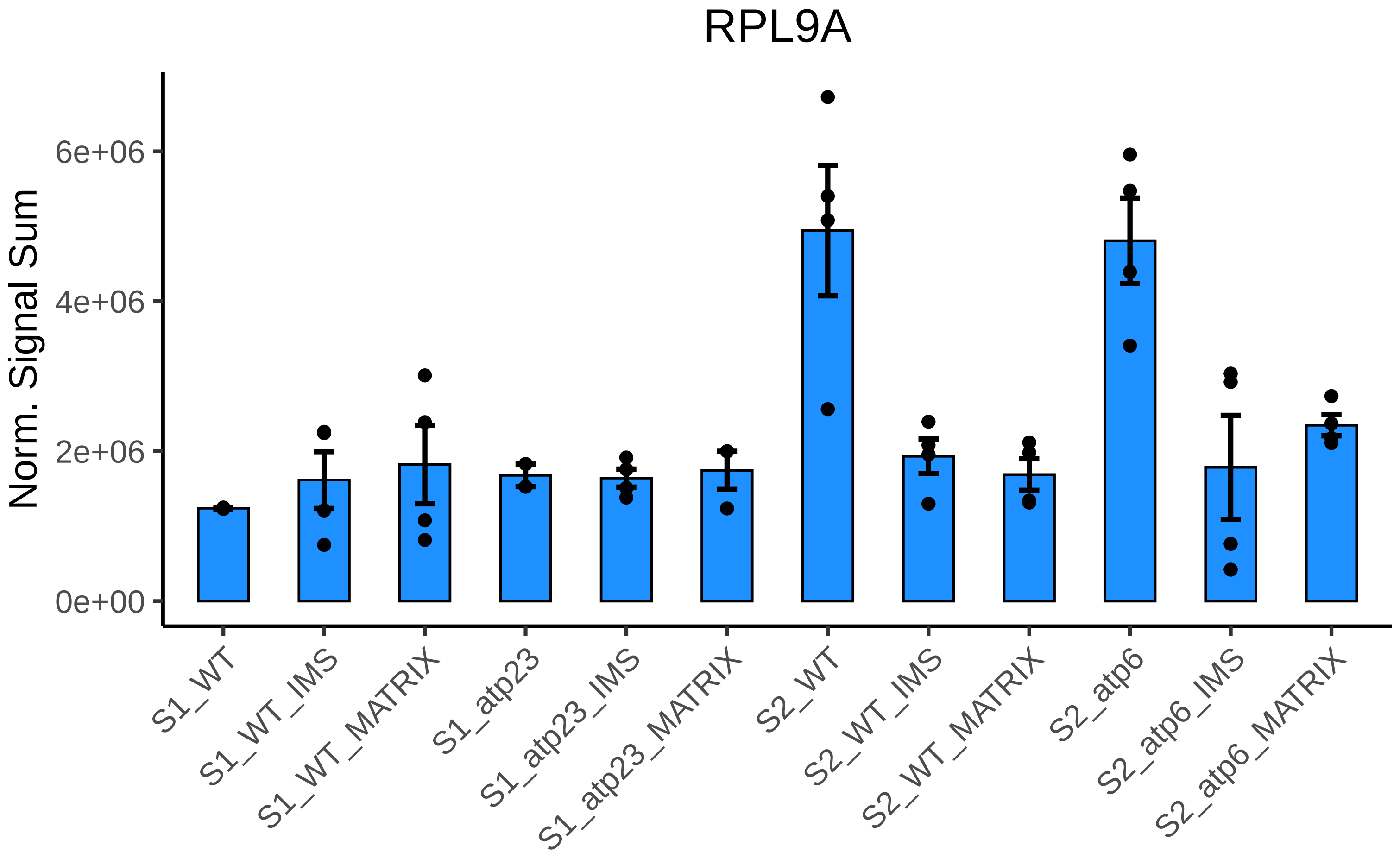
RPL9A
Norm. Signal Sum
6e+06
4e+06
2e+06
0e+00
S1_WT
S1_WT_IMS
S1_WT_MATRIX
S1_atp23
S1_atp23_IMS
S1_atp23_MATRIX
S2_WT
S2_WT_IMS
S2_WT_MATRIX
S2_atp6
S2_atp6_IMS
S2_atp6_MATRIX

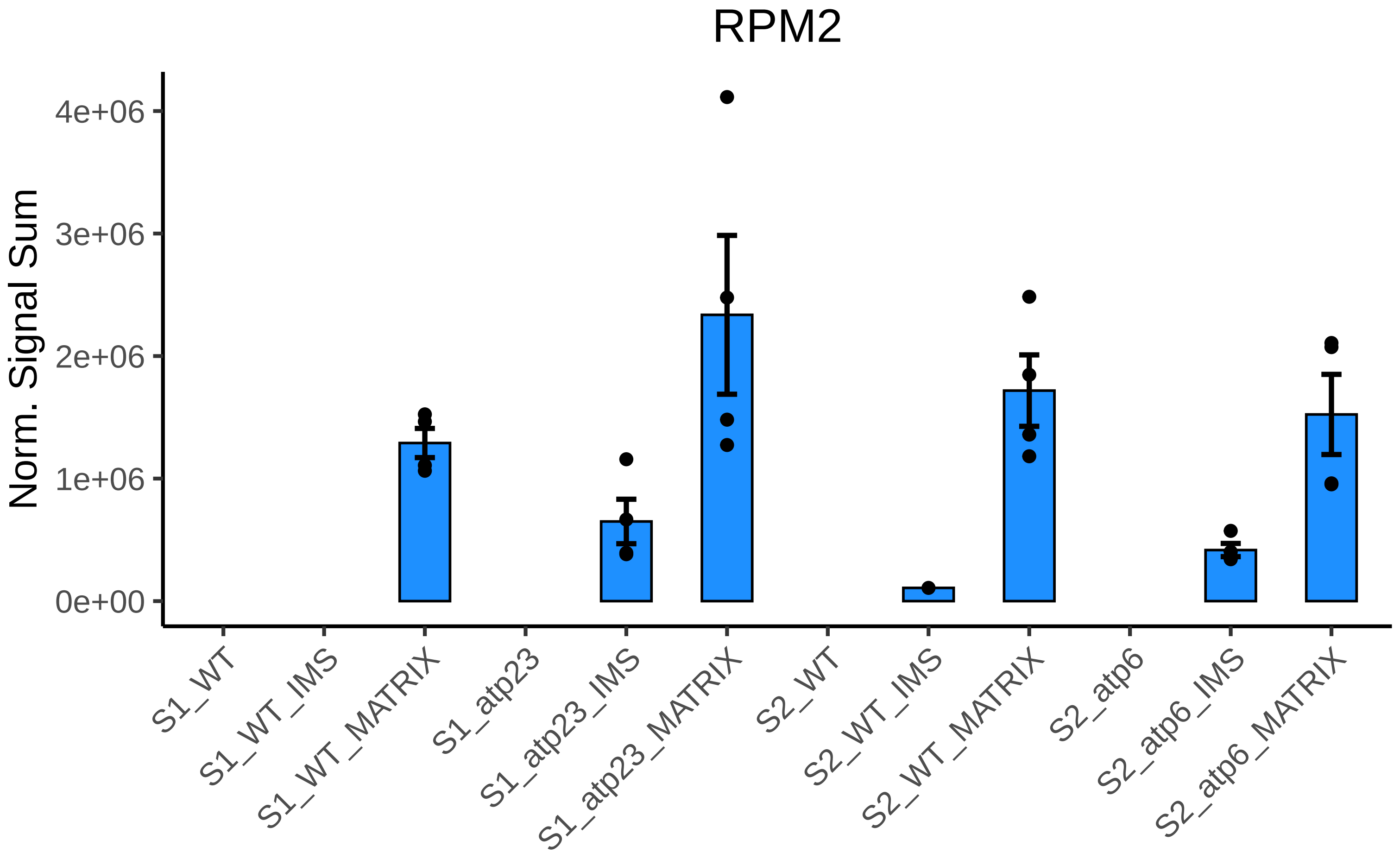

RPM2
Norm. Signal Sum
4e+06
3e+06
2e+06
1e+06
0e+00
S1_WT
S1_WT_IMS
S1_WT_MATRIX
S1_atp23
S1_atp23_IMS
S1_atp23_MATRIX
S2_WT
S2_WT_IMS
S2_WT_MATRIX
S2_atp6
S2_atp6_IMS
S2_atp6_MATRIX

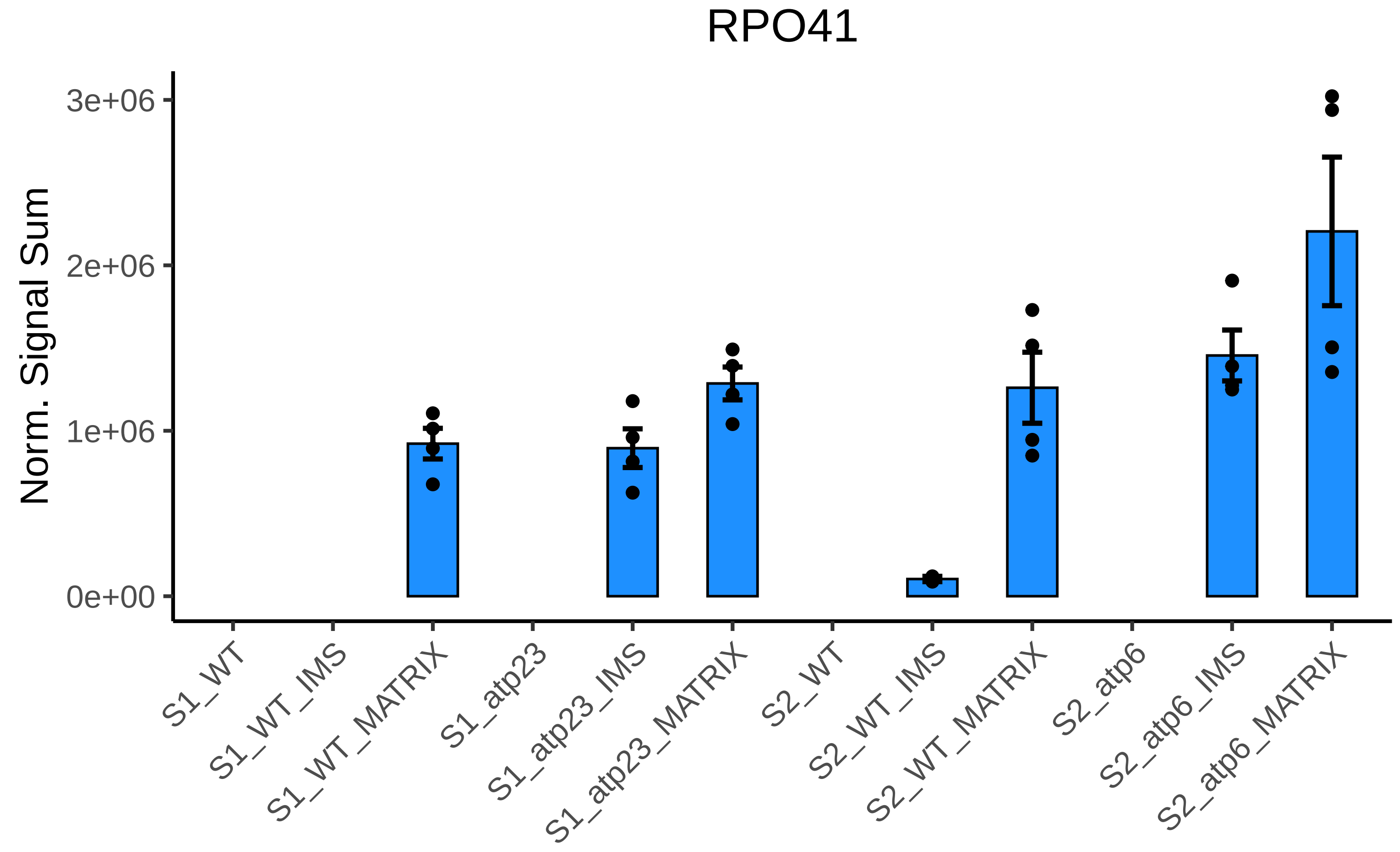

RPO41
Norm. Signal Sum
3e+06
2e+06
1e+06
0e+00
S1_WT
S1_WT_IMS
S1_WT_MATRIX
S1_atp23
S1_atp23_IMS
S1_atp23_MATRIX
S2_WT
S2_WT_IMS
S2_WT_MATRIX
S2_atp6
S2_atp6_IMS
S2_atp6_MATRIX

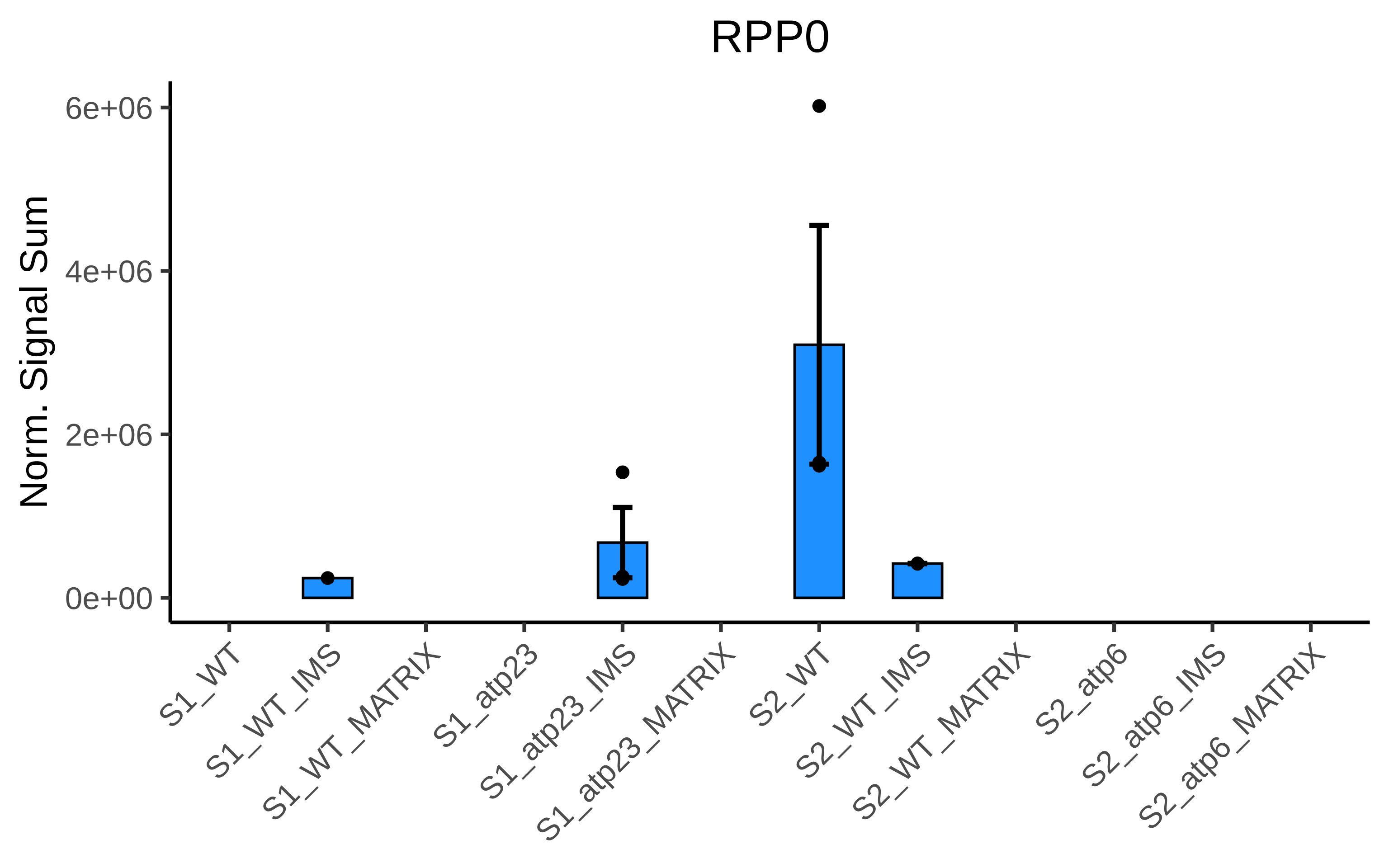
RPP0
Norm. Signal Sum
6e+06
4e+06
2e+06
0e+00
S1_WT
S1_WT_IMS
S1_WT_MATRIX
S1_atp23
S1_atp23_IMS
S1_atp23_MATRIX
S2_WT
S2_WT_IMS
S2_WT_MATRIX
S2_atp6
S2_atp6_IMS
S2_atp6_MATRIX

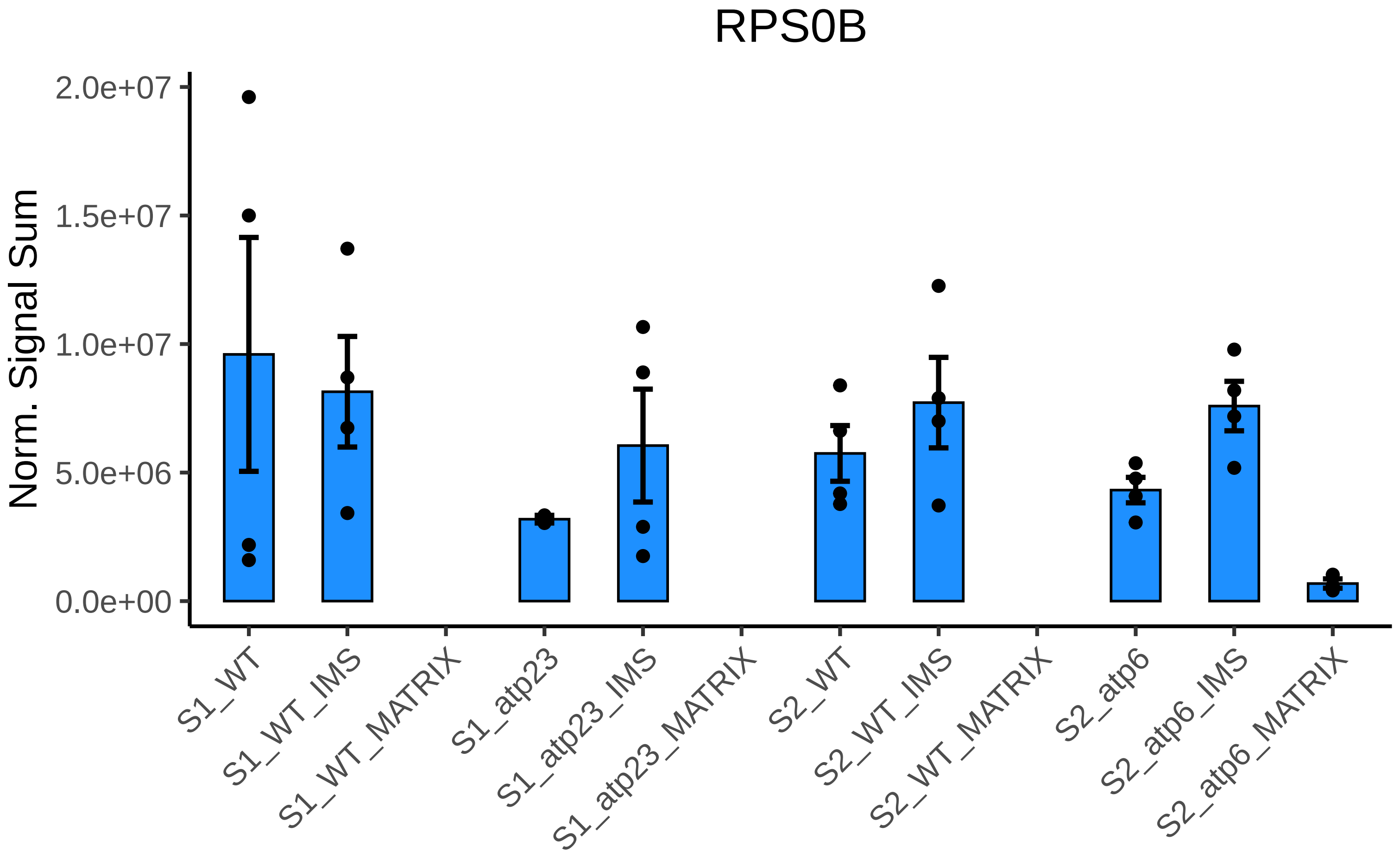
RPS0B
Norm. Signal Sum
2.0e+07
1.5e+07
1.0e+07
5.0e+06
0.0e+00
S1_WT
S1_WT_IMS
S1_WT_MATRIX
S1_atp23
S1_atp23_IMS
S1_atp23_MATRIX
S2_WT
S2_WT_IMS
S2_WT_MATRIX
S2_atp6
S2_atp6_IMS
S2_atp6_MATRIX

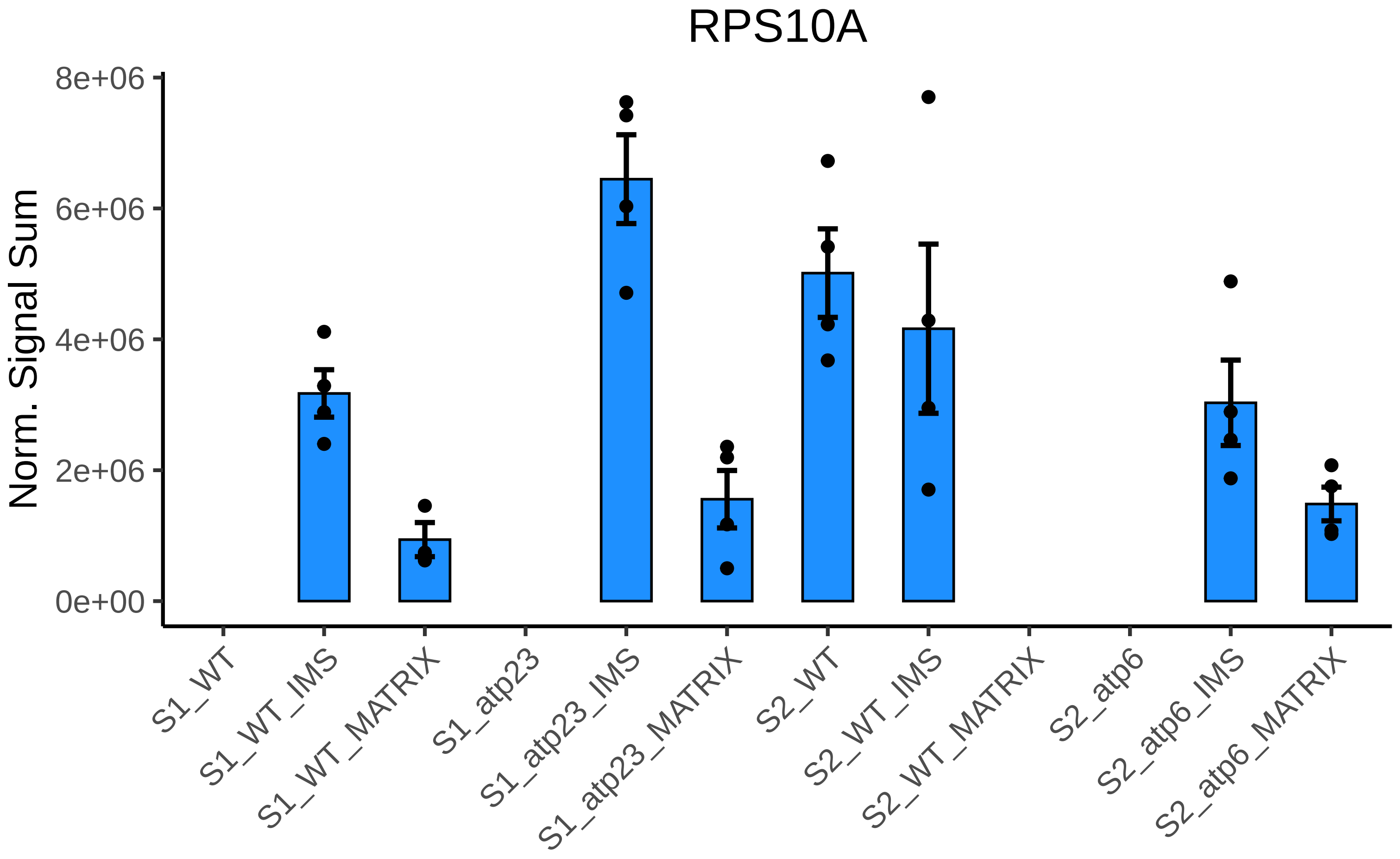

RPS10A
Norm. Signal Sum
8e+06
6e+06
4e+06
2e+06
0e+00
S1_WT
S1_WT_IMS
S1_WT_MATRIX
S1_atp23
S1_atp23_IMS
S1_atp23_MATRIX
S2_WT
S2_WT_IMS
S2_WT_MATRIX
S2_atp6
S2_atp6_IMS
S2_atp6_MATRIX

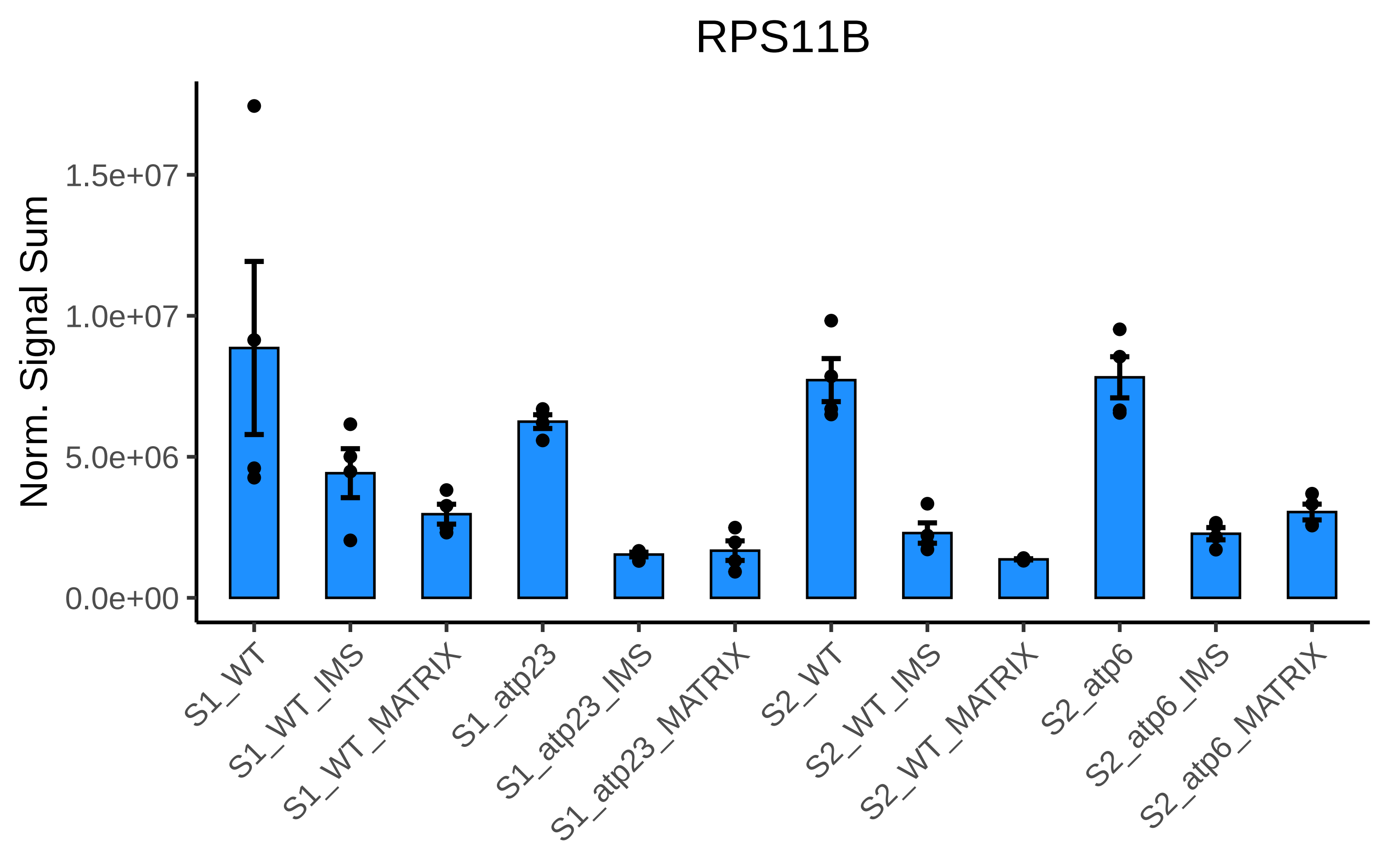
RPS11B
Norm. Signal Sum
1.5e+07
1.0e+07
5.0e+06
0.0e+00
S1_WT
S1_WT_IMS
S1_WT_MATRIX
S1_atp23
S1_atp23_IMS
S1_atp23_MATRIX
S2_WT
S2_WT_IMS
S2_WT_MATRIX
S2_atp6
S2_atp6_IMS
S2_atp6_MATRIX

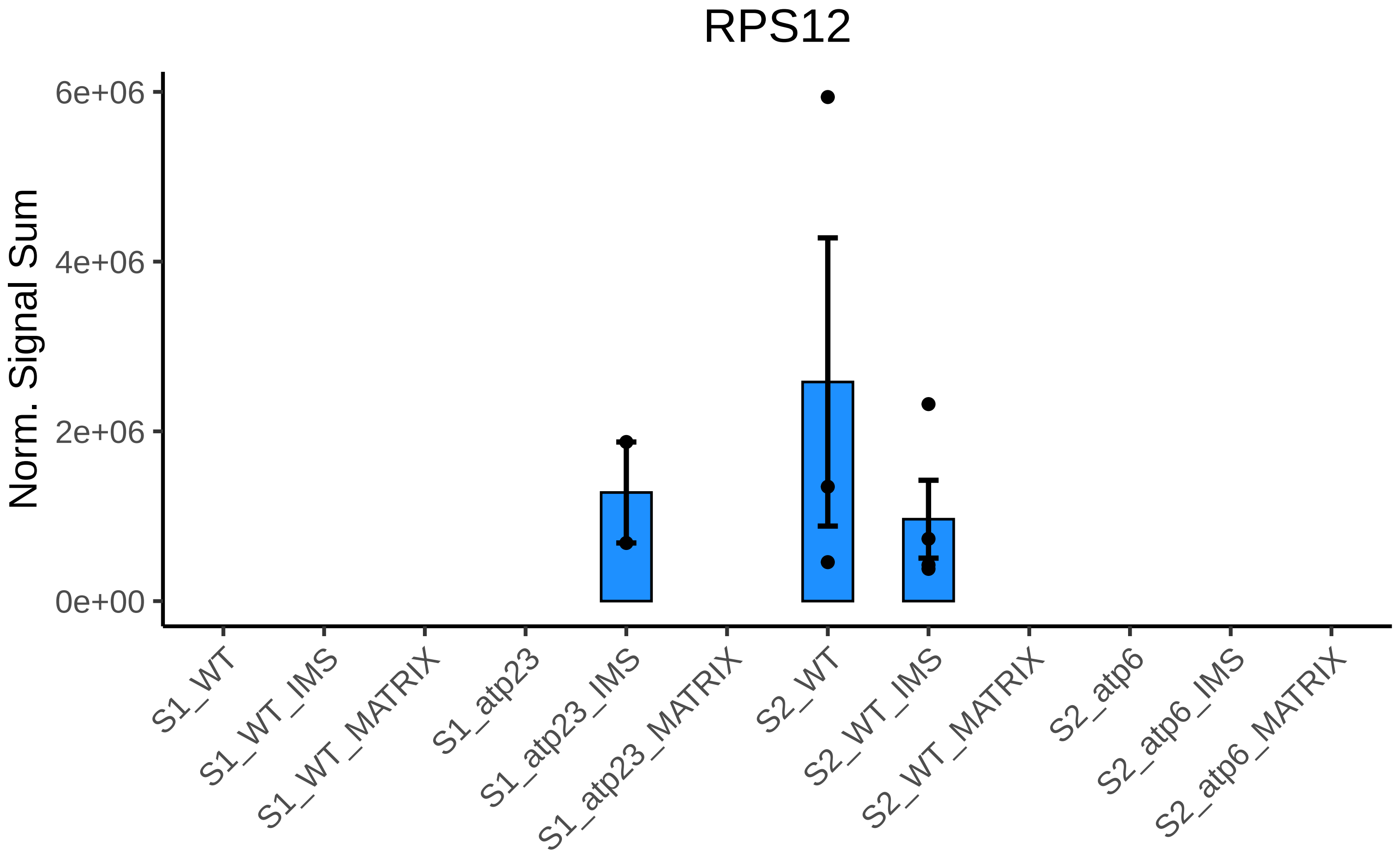

RPS12
Norm. Signal Sum
6e+06
4e+06
2e+06
0e+00
S1_WT
S1_WT_IMS
S1_WT_MATRIX
S1_atp23
S1_atp23_IMS
S1_atp23_MATRIX
S2_WT
S2_WT_IMS
S2_WT_MATRIX
S2_atp6
S2_atp6_IMS
S2_atp6_MATRIX

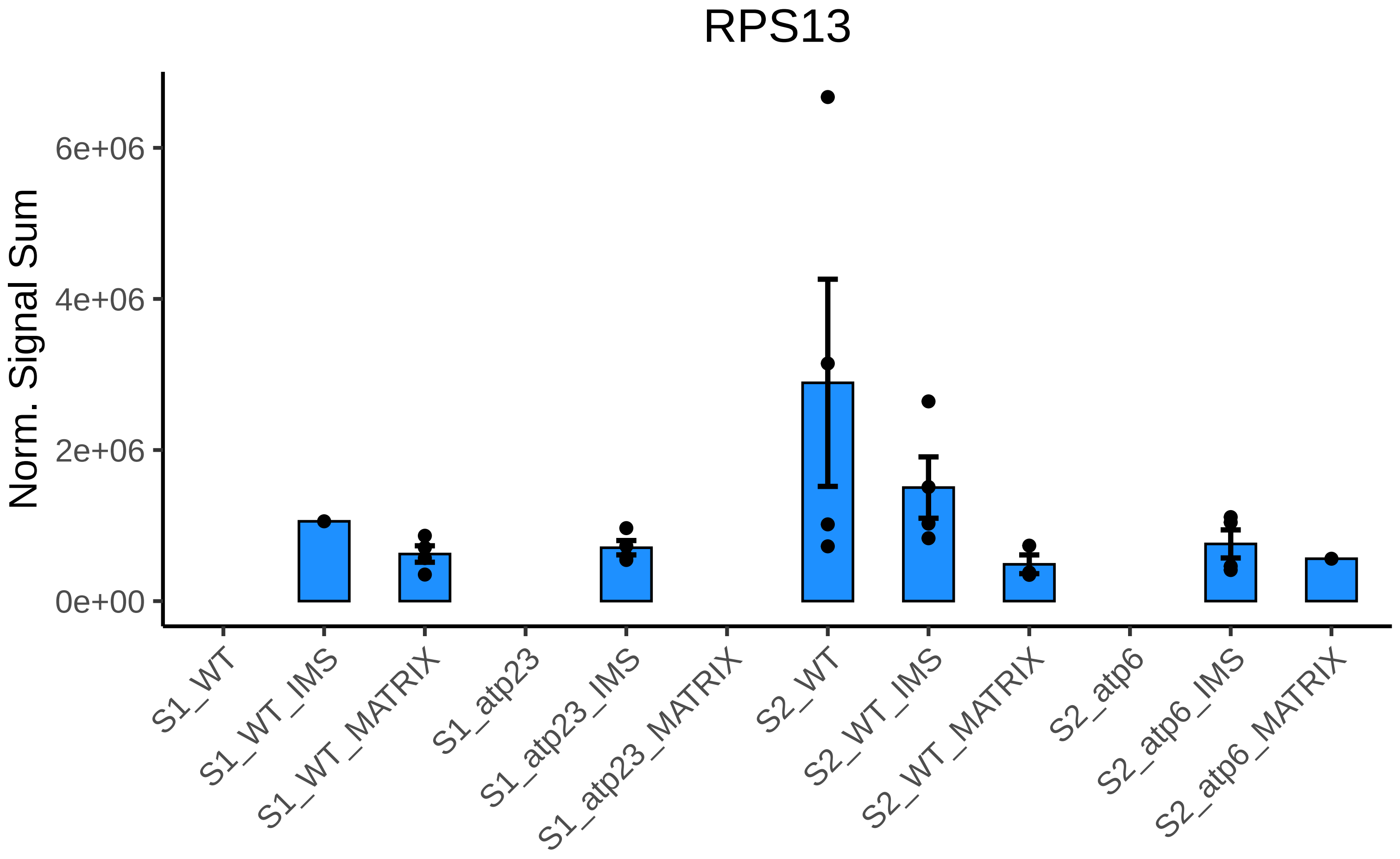
RPS13
Norm. Signal Sum
6e+06
4e+06
2e+06
0e+00
S1_WT
S1_WT_IMS
S1_WT_MATRIX
S1_atp23
S1_atp23_IMS
S1_atp23_MATRIX
S2_WT
S2_WT_IMS
S2_WT_MATRIX
S2_atp6
S2_atp6_IMS
S2_atp6_MATRIX

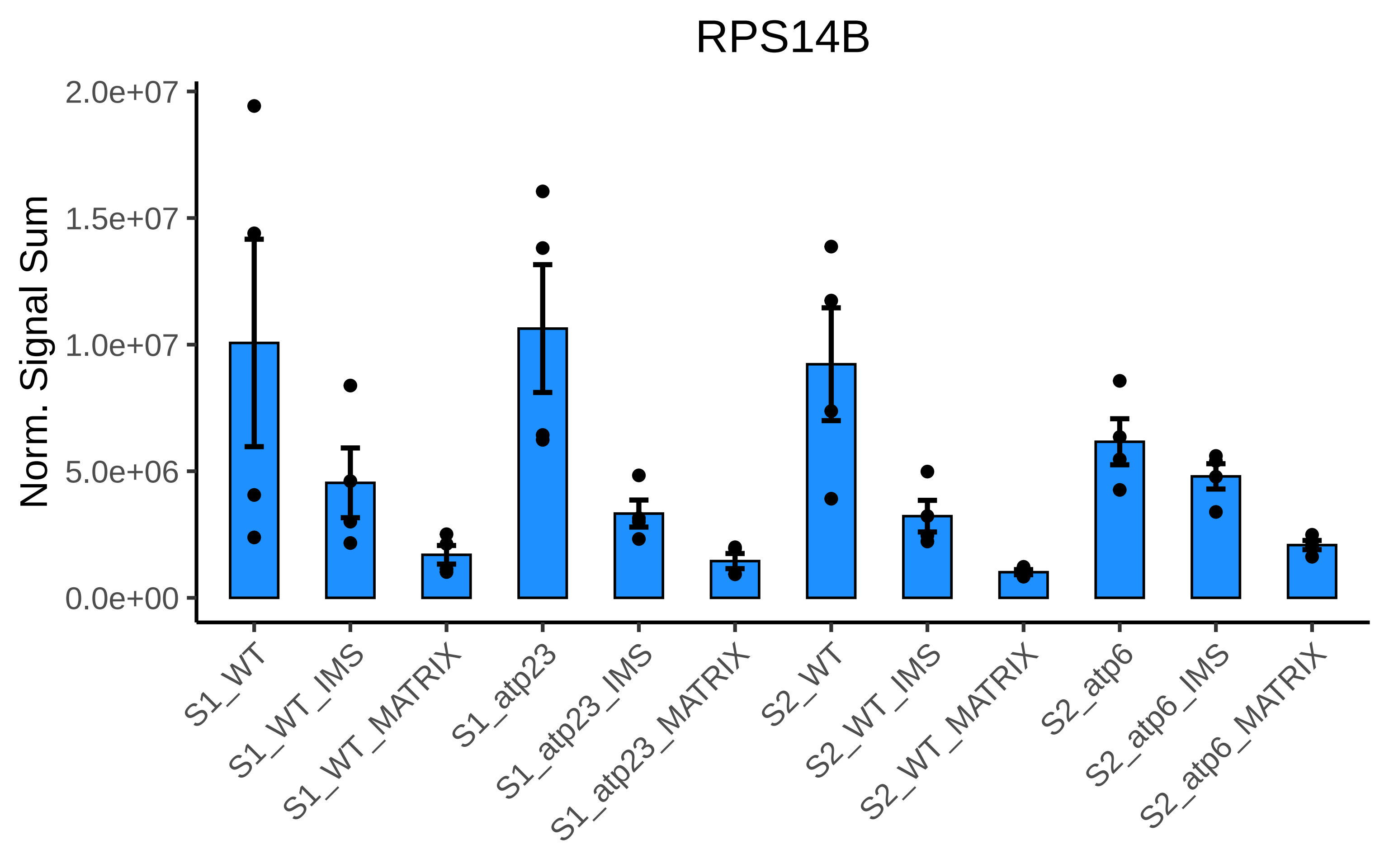
RPS14B
Norm. Signal Sum
2.0e+07
1.5e+07
1.0e+07
5.0e+06
0.0e+00
S1_WT
S1_WT_IMS
S1_WT_MATRIX
S1_atp23
S1_atp23_IMS
S1_atp23_MATRIX
S2_WT
S2_WT_IMS
S2_WT_MATRIX
S2_atp6
S2_atp6_IMS
S2_atp6_MATRIX

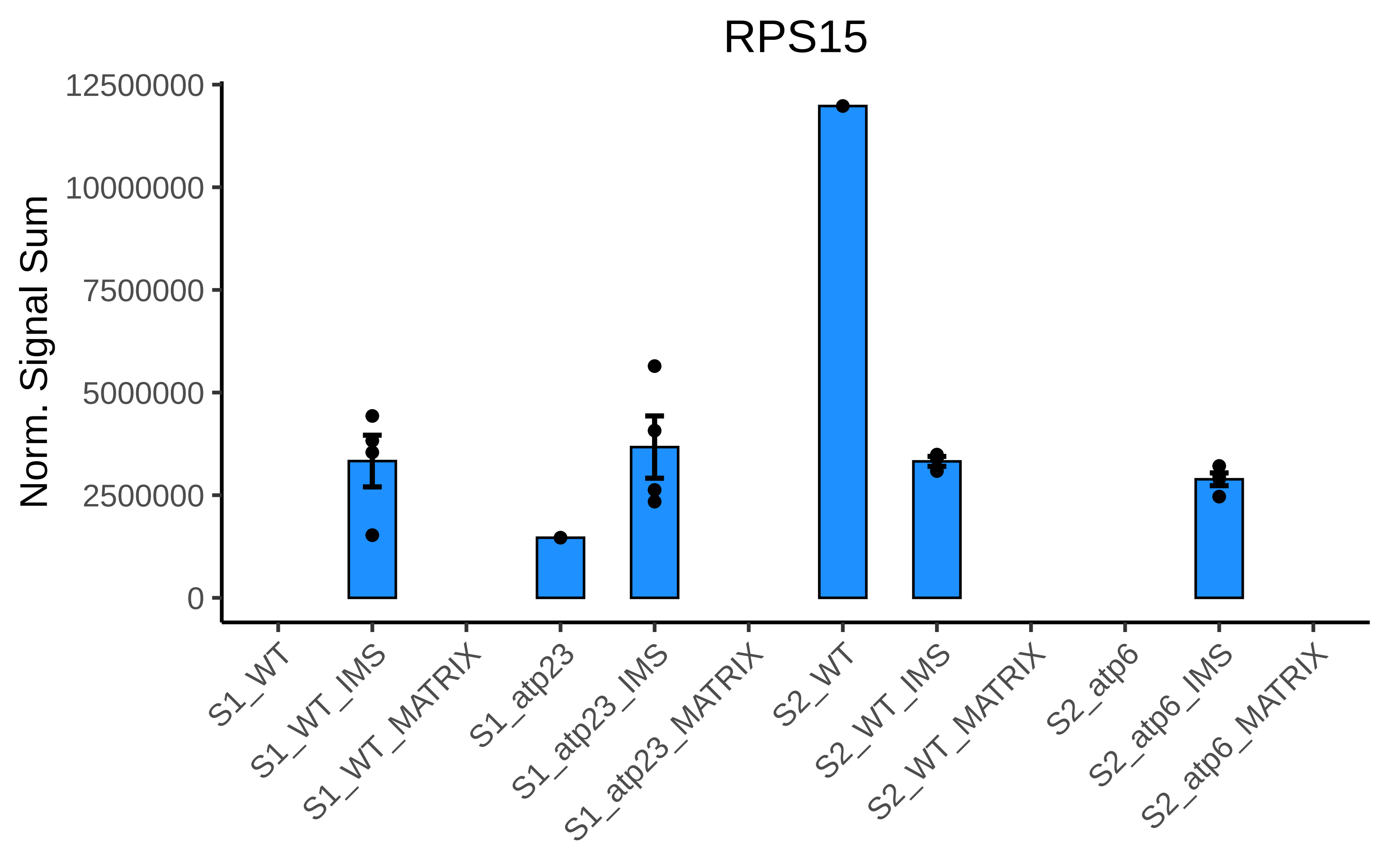
RPS15
Norm. Signal Sum
12500000
10000000
7500000
5000000
2500000
0
S1_WT
S1_WT_IMS
S1_WT_MATRIX
S1_atp23
S1_atp23_IMS
S1_atp23_MATRIX
S2_WT
S2_WT_IMS
S2_WT_MATRIX
S2_atp6
S2_atp6_IMS
S2_atp6_MATRIX

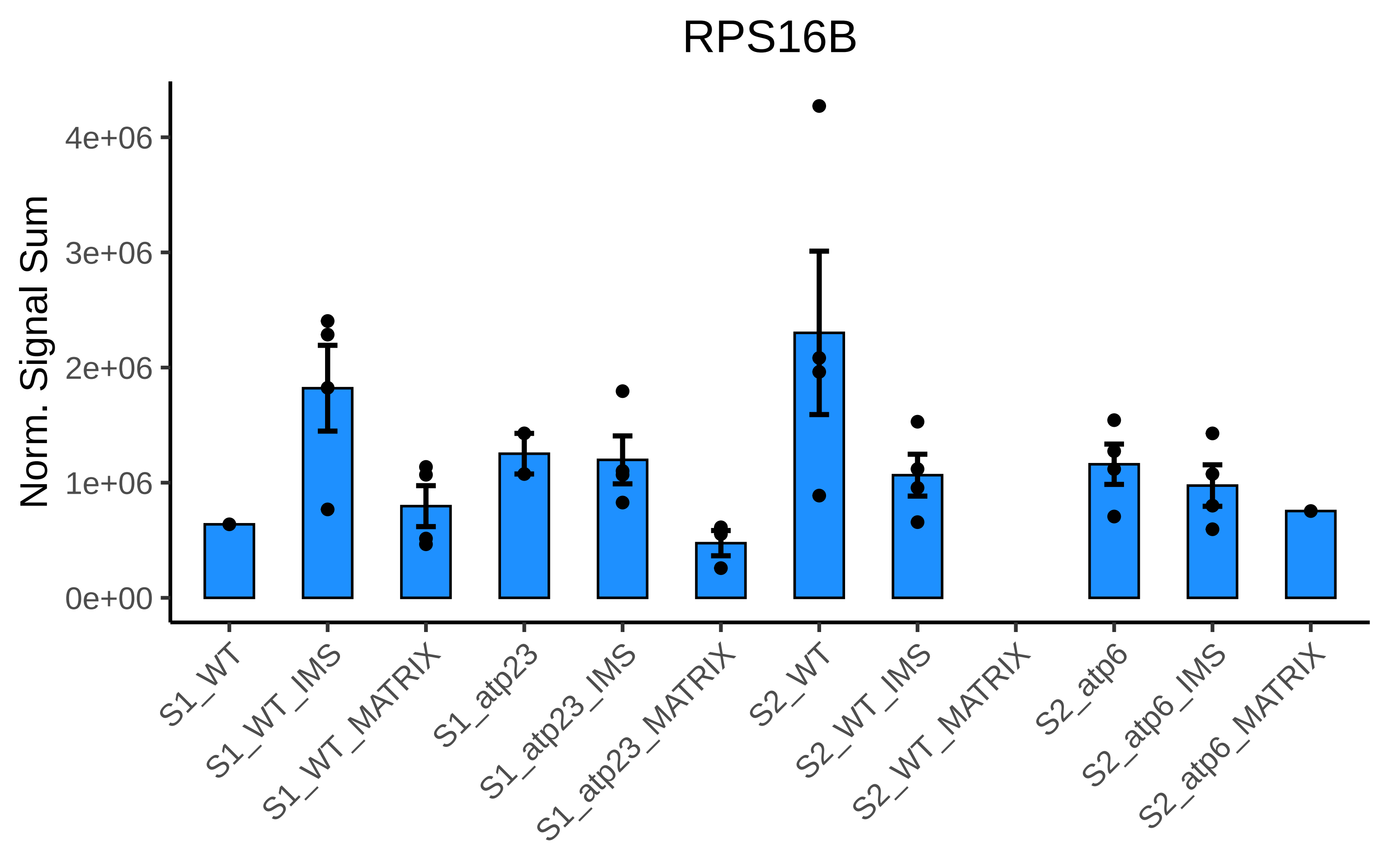

RPS16B
Norm. Signal Sum
4e+06
3e+06
2e+06
1e+06
0e+00
S1_WT
S1_WT_IMS
S1_WT_MATRIX
S1_atp23
S1_atp23_IMS
S1_atp23_MATRIX
S2_WT
S2_WT_IMS
S2_WT_MATRIX
S2_atp6
S2_atp6_IMS
S2_atp6_MATRIX

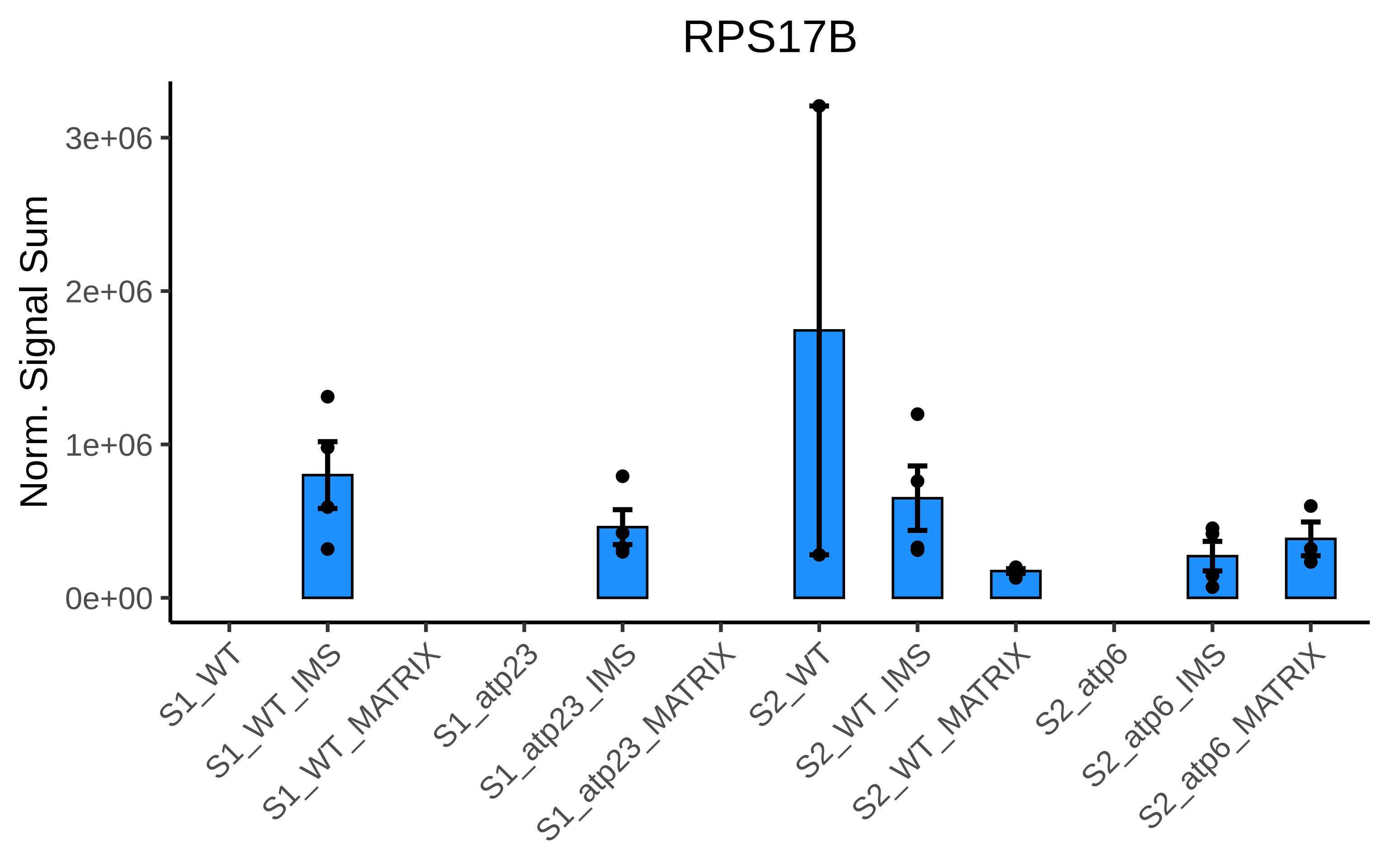
RPS17B
Norm. Signal Sum
3e+06
2e+06
1e+06
0e+00
S1_WT
S1_WT_IMS
S1_WT_MATRIX
S1_atp23
S1_atp23_IMS
S1_atp23_MATRIX
S2_WT
S2_WT_IMS
S2_WT_MATRIX
S2_atp6
S2_atp6_IMS
S2_atp6_MATRIX

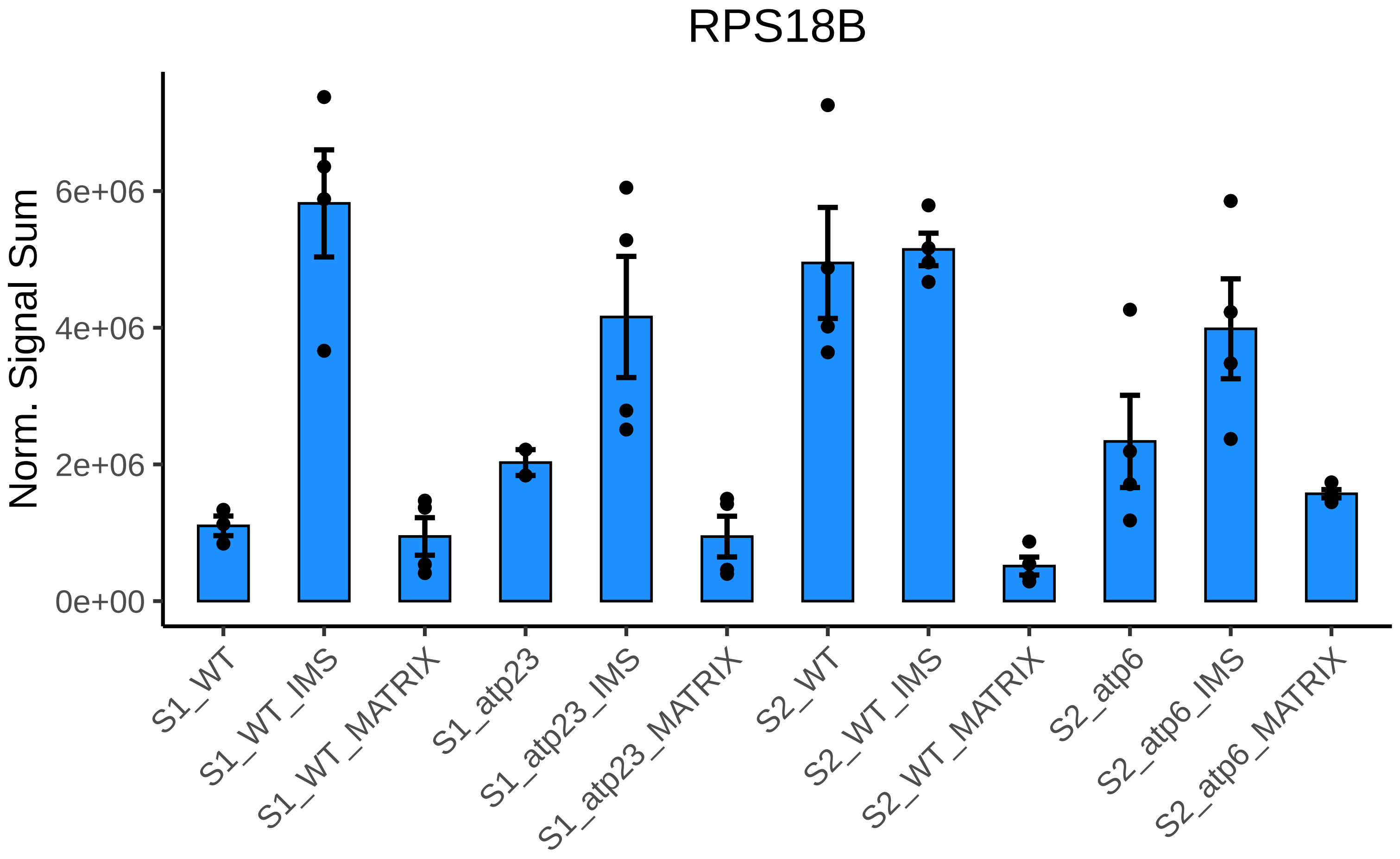

RPS18B
Norm. Signal Sum
6e+06
4e+06
2e+06
0e+00
S1_WT
S1_WT_IMS
S1_WT_MATRIX
S1_atp23
S1_atp23_IMS
S1_atp23_MATRIX
S2_WT
S2_WT_IMS
S2_WT_MATRIX
S2_atp6
S2_atp6_IMS
S2_atp6_MATRIX

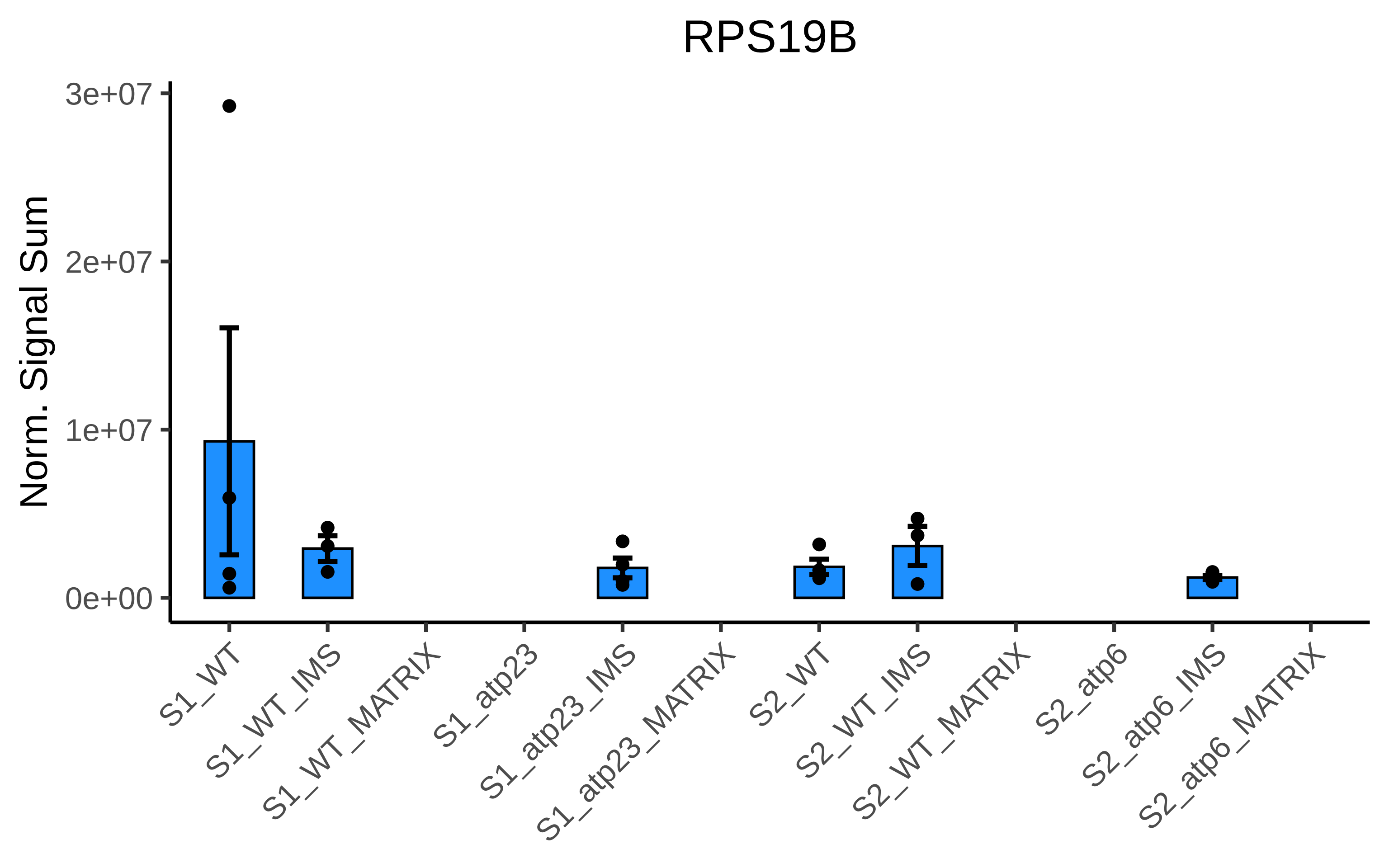
RPS19B
Norm. Signal Sum
3e+07
2e+07
1e+07
0e+00
S1_WT
S1_WT_IMS
S1_WT_MATRIX
S1_atp23
S1_atp23_IMS
S1_atp23_MATRIX
S2_WT
S2_WT_IMS
S2_WT_MATRIX
S2_atp6
S2_atp6_IMS
S2_atp6_MATRIX

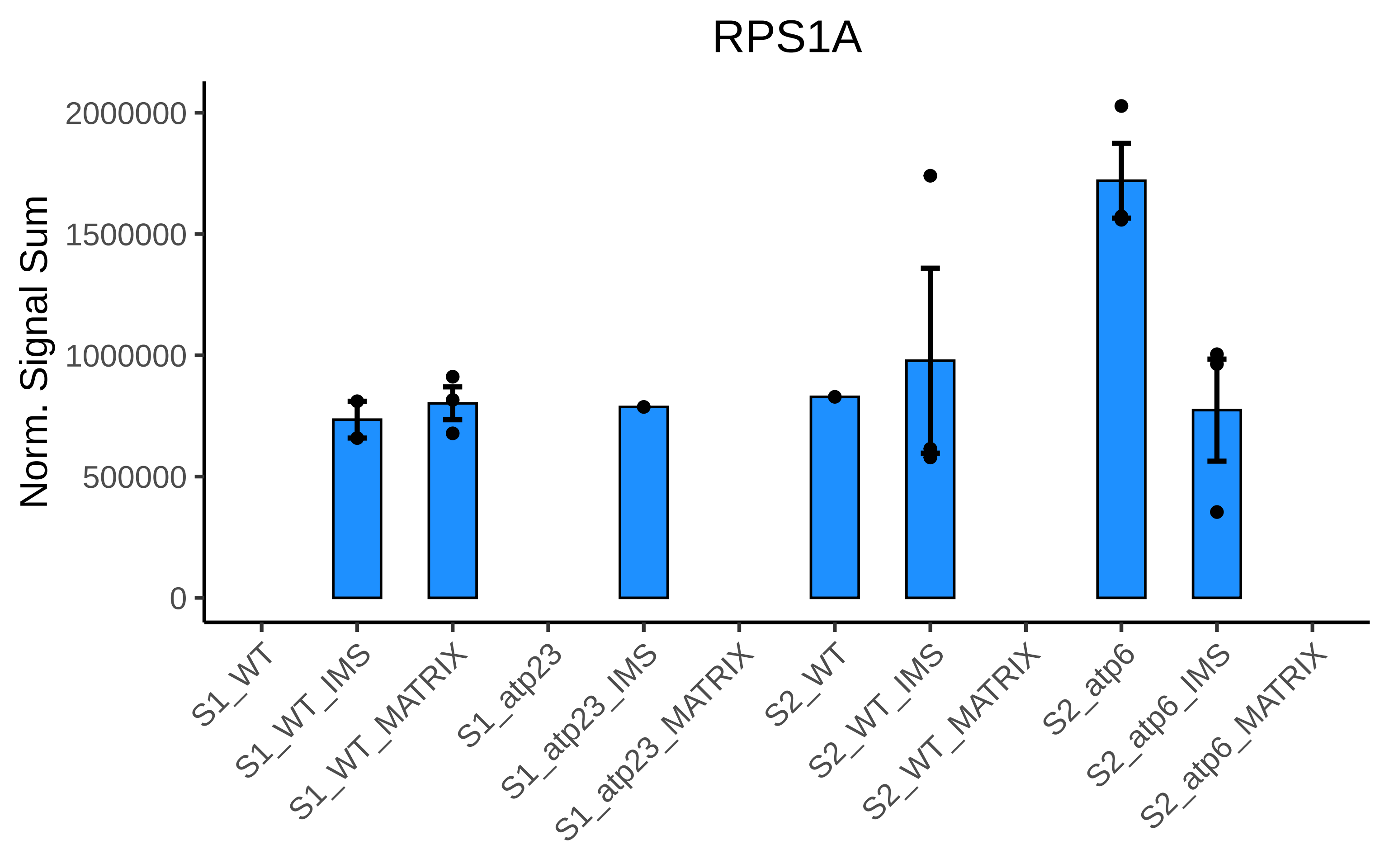

RPS1A
Norm. Signal Sum
2000000
1500000
1000000
500000
0
S1_WT
S1_WT_IMS
S1_WT_MATRIX
S1_atp23
S1_atp23_IMS
S1_atp23_MATRIX
S2_WT
S2_WT_IMS
S2_WT_MATRIX
S2_atp6
S2_atp6_IMS
S2_atp6_MATRIX

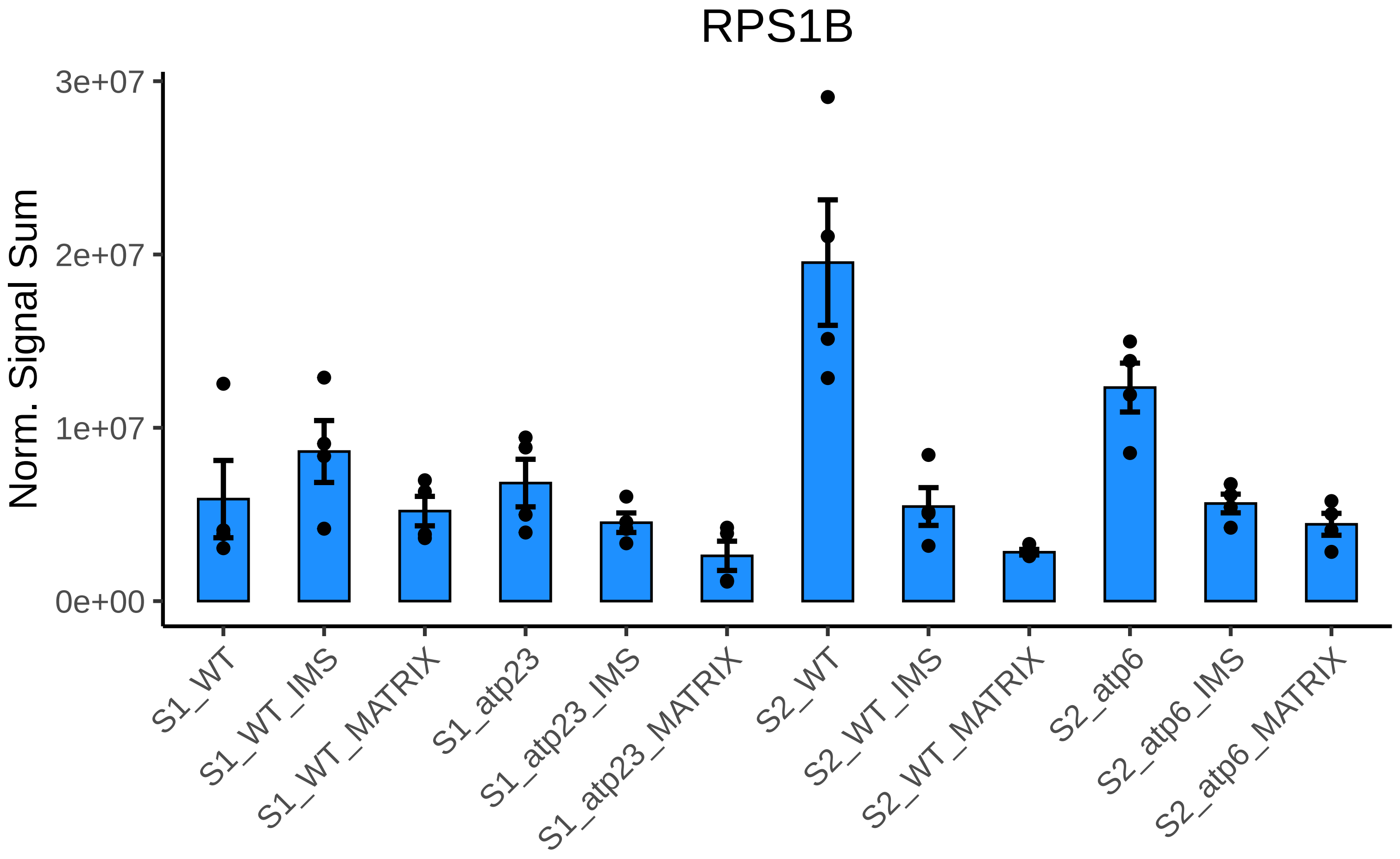

RPS1B
Norm. Signal Sum
3e+07
2e+07
1e+07
0e+00
S1_WT
S1_WT_IMS
S1_WT_MATRIX
S1_atp23
S1_atp23_IMS
S1_atp23_MATRIX
S2_WT
S2_WT_IMS
S2_WT_MATRIX
S2_atp6
S2_atp6_IMS
S2_atp6_MATRIX

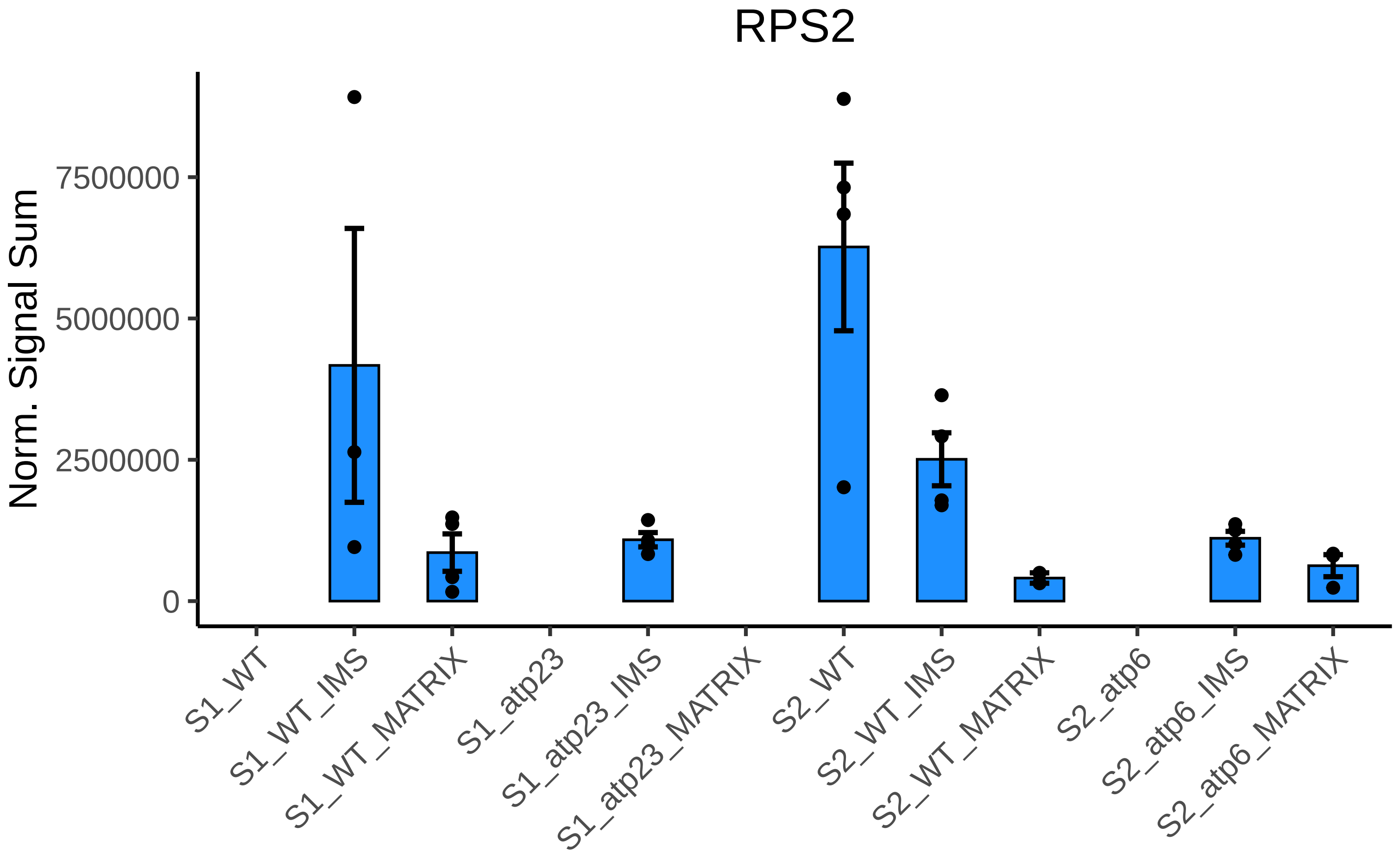

RPS2
Norm. Signal Sum
7500000
5000000
2500000
0
S1_WT
S1_WT_IMS
S1_WT_MATRIX
S1_atp23
S1_atp23_IMS
S1_atp23_MATRIX
S2_WT
S2_WT_IMS
S2_WT_MATRIX
S2_atp6
S2_atp6_IMS
S2_atp6_MATRIX

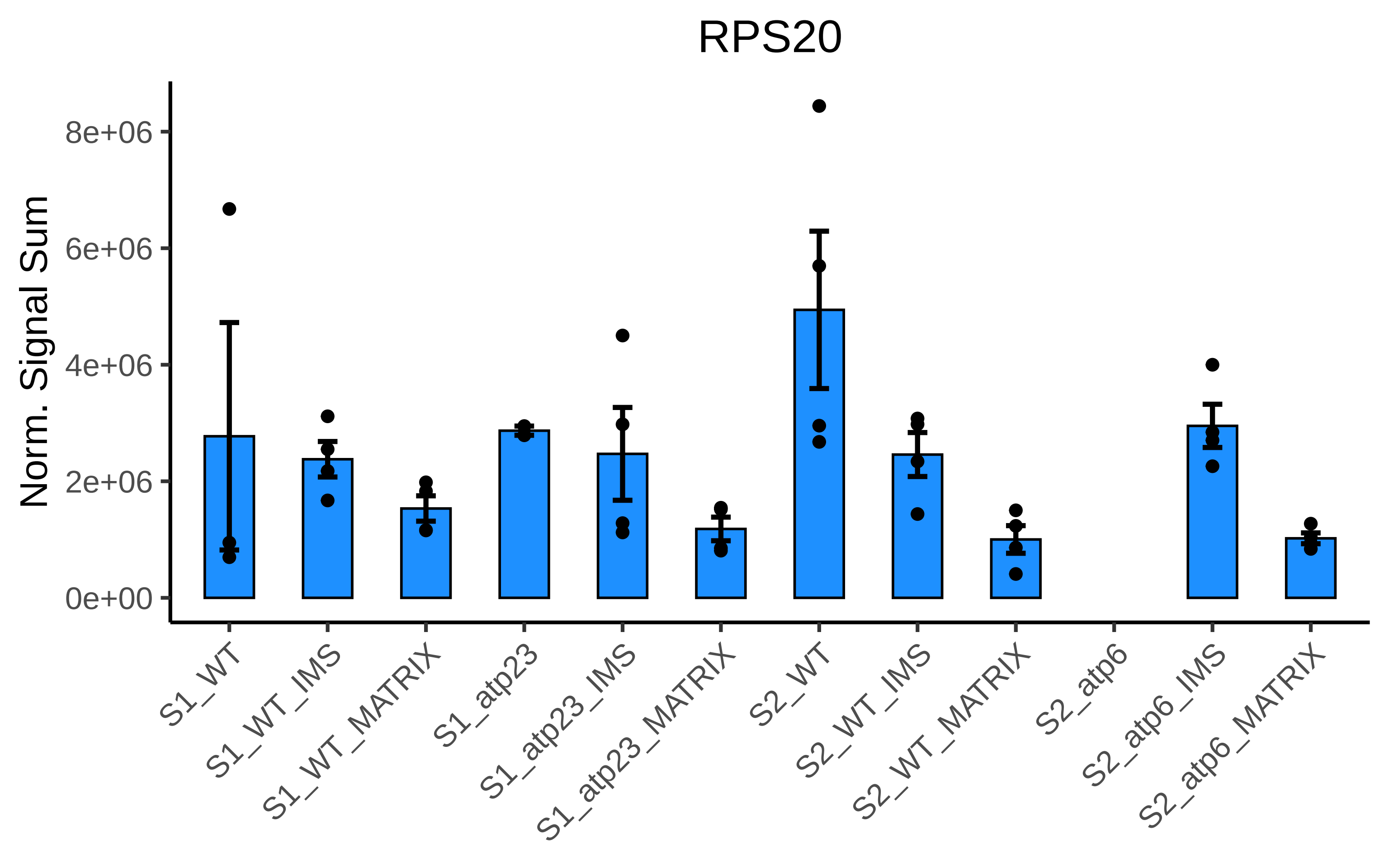

RPS20
Norm. Signal Sum
8e+06
6e+06
4e+06
2e+06
0e+00
S1_WT
S1_WT_IMS
S1_WT_MATRIX
S1_atp23
S1_atp23_IMS
S1_atp23_MATRIX
S2_WT
S2_WT_IMS
S2_WT_MATRIX
S2_atp6
S2_atp6_IMS
S2_atp6_MATRIX

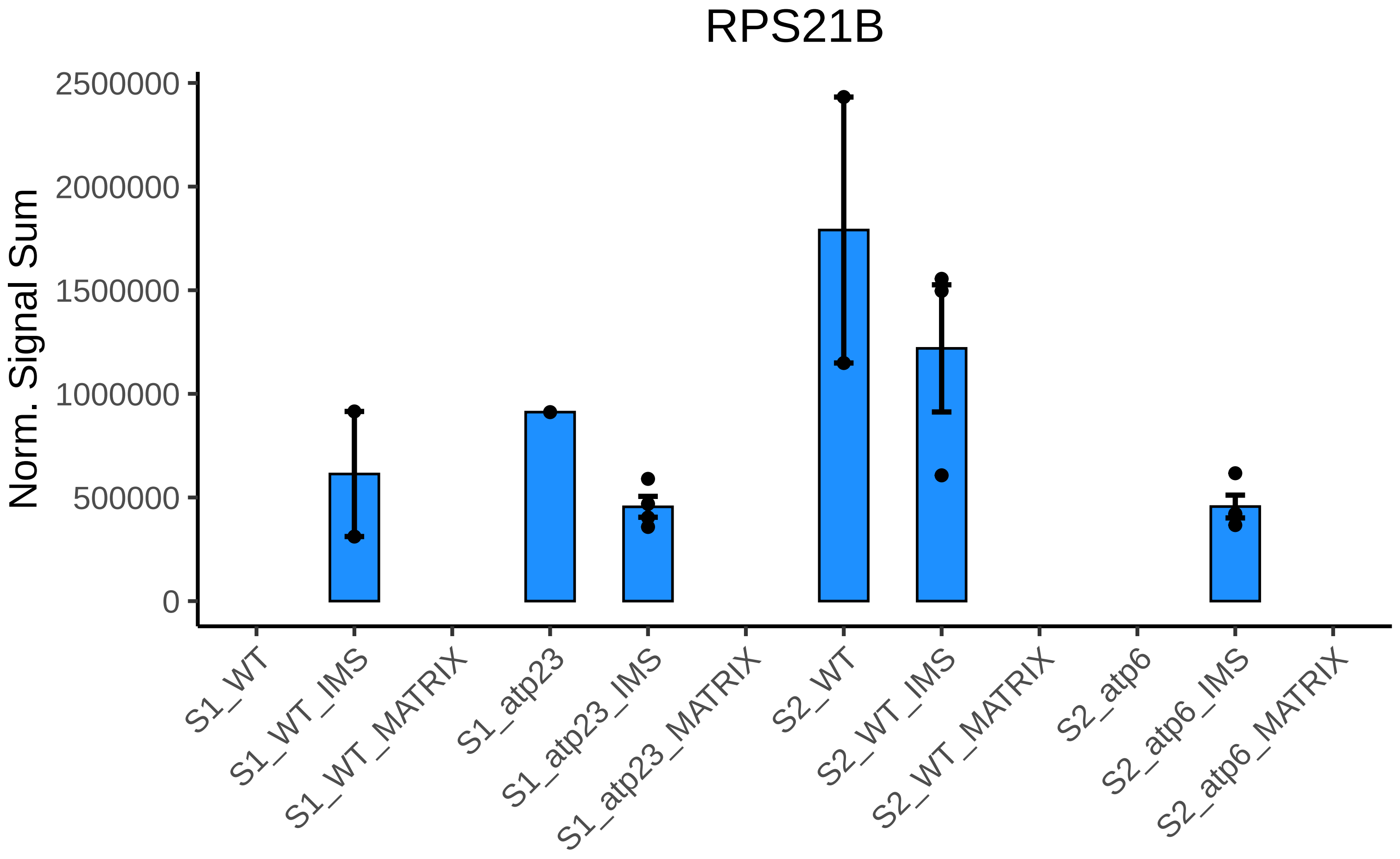

RPS21B
Norm. Signal Sum
2500000
2000000
1500000
1000000
500000
0
S1_WT
S1_WT_IMS
S1_WT_MATRIX
S1_atp23
S1_atp23_IMS
S1_atp23_MATRIX
S2_WT
S2_WT_IMS
S2_WT_MATRIX
S2_atp6
S2_atp6_IMS
S2_atp6_MATRIX

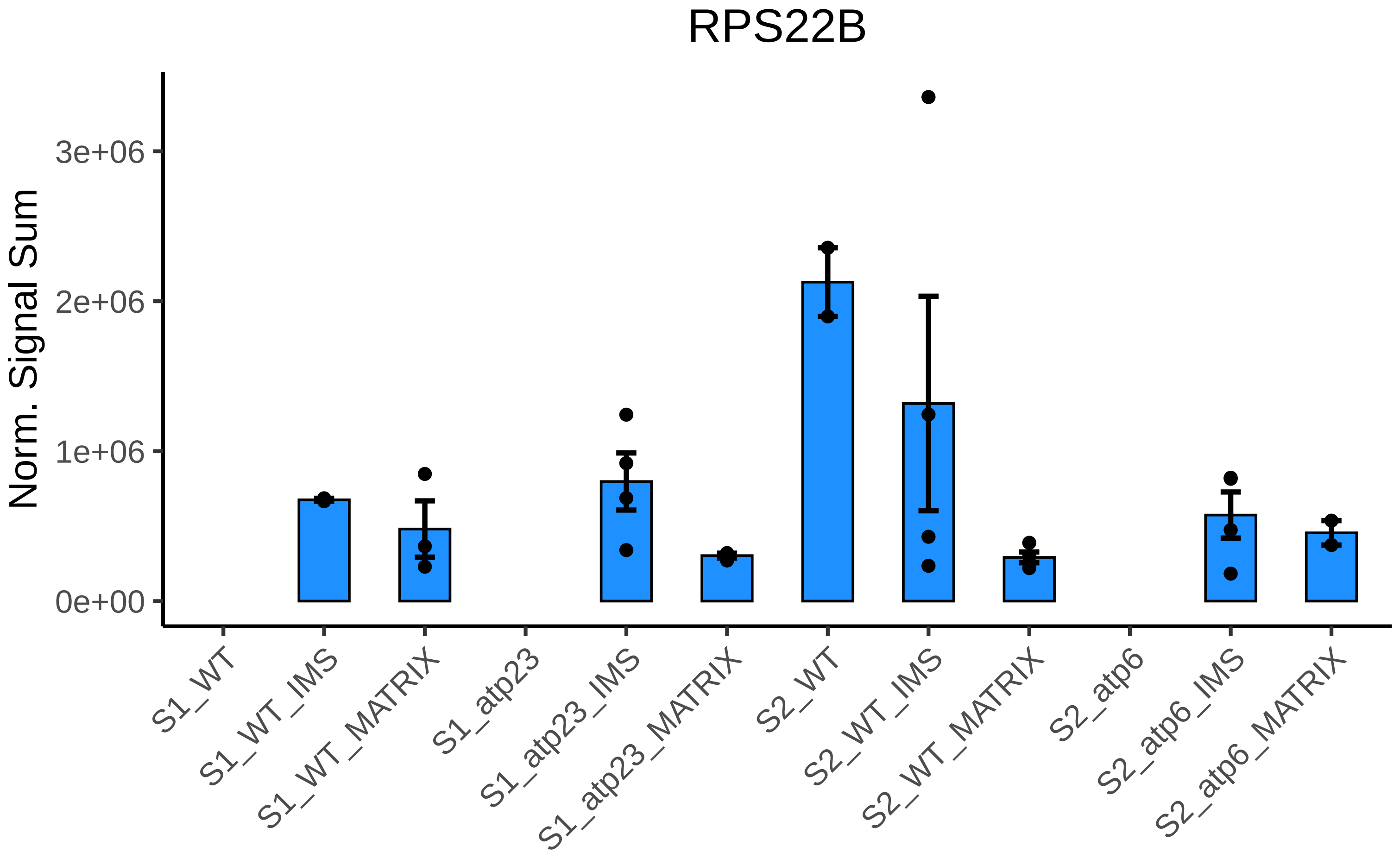

RPS22B
Norm. Signal Sum
3e+06
2e+06
1e+06
0e+00
S1_WT
S1_WT_IMS
S1_WT_MATRIX
S1_atp23
S1_atp23_IMS
S1_atp23_MATRIX
S2_WT
S2_WT_IMS
S2_WT_MATRIX
S2_atp6
S2_atp6_IMS
S2_atp6_MATRIX

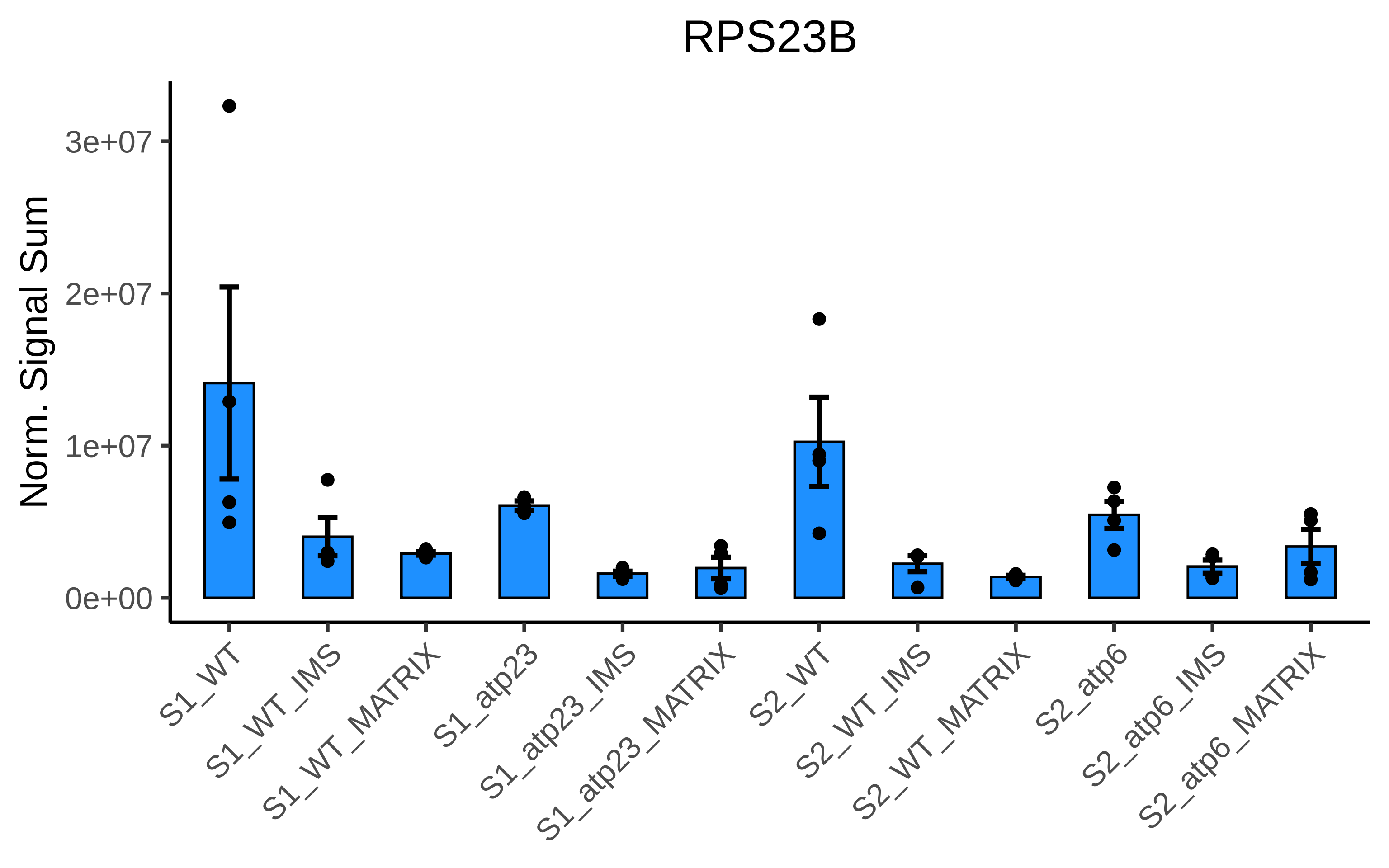
RPS23B
Norm. Signal Sum
3e+07
2e+07
1e+07
0e+00
S1_WT
S1_WT_IMS
S1_WT_MATRIX
S1_atp23
S1_atp23_IMS
S1_atp23_MATRIX
S2_WT
S2_WT_IMS
S2_WT_MATRIX
S2_atp6
S2_atp6_IMS
S2_atp6_MATRIX

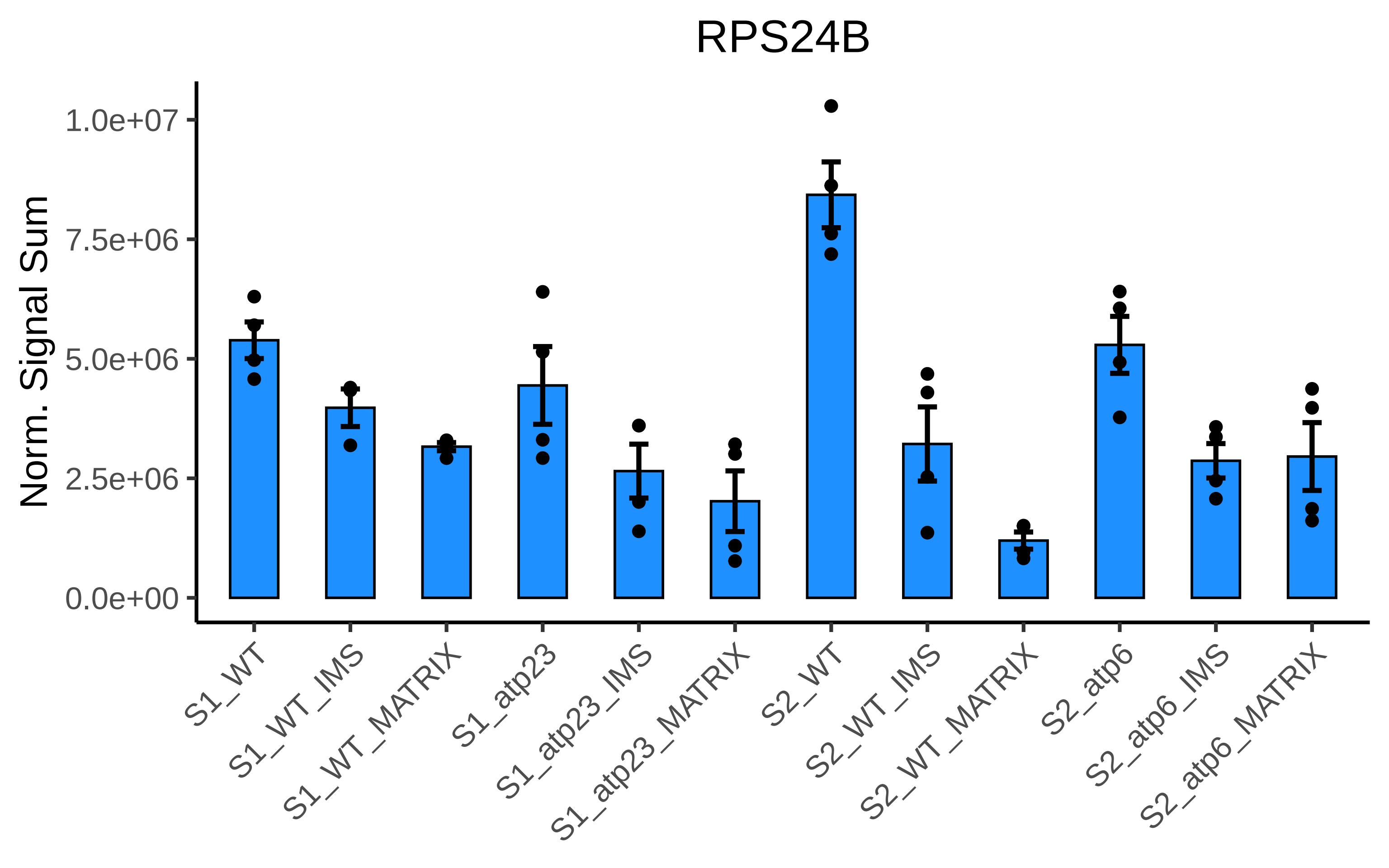

RPS24B
Norm. Signal Sum
1.0e+07
7.5e+06
5.0e+06
2.5e+06
0.0e+00
S1_WT
S1_WT_IMS
S1_WT_MATRIX
S1_atp23
S1_atp23_IMS
S1_atp23_MATRIX
S2_WT
S2_WT_IMS
S2_WT_MATRIX
S2_atp6
S2_atp6_IMS
S2_atp6_MATRIX

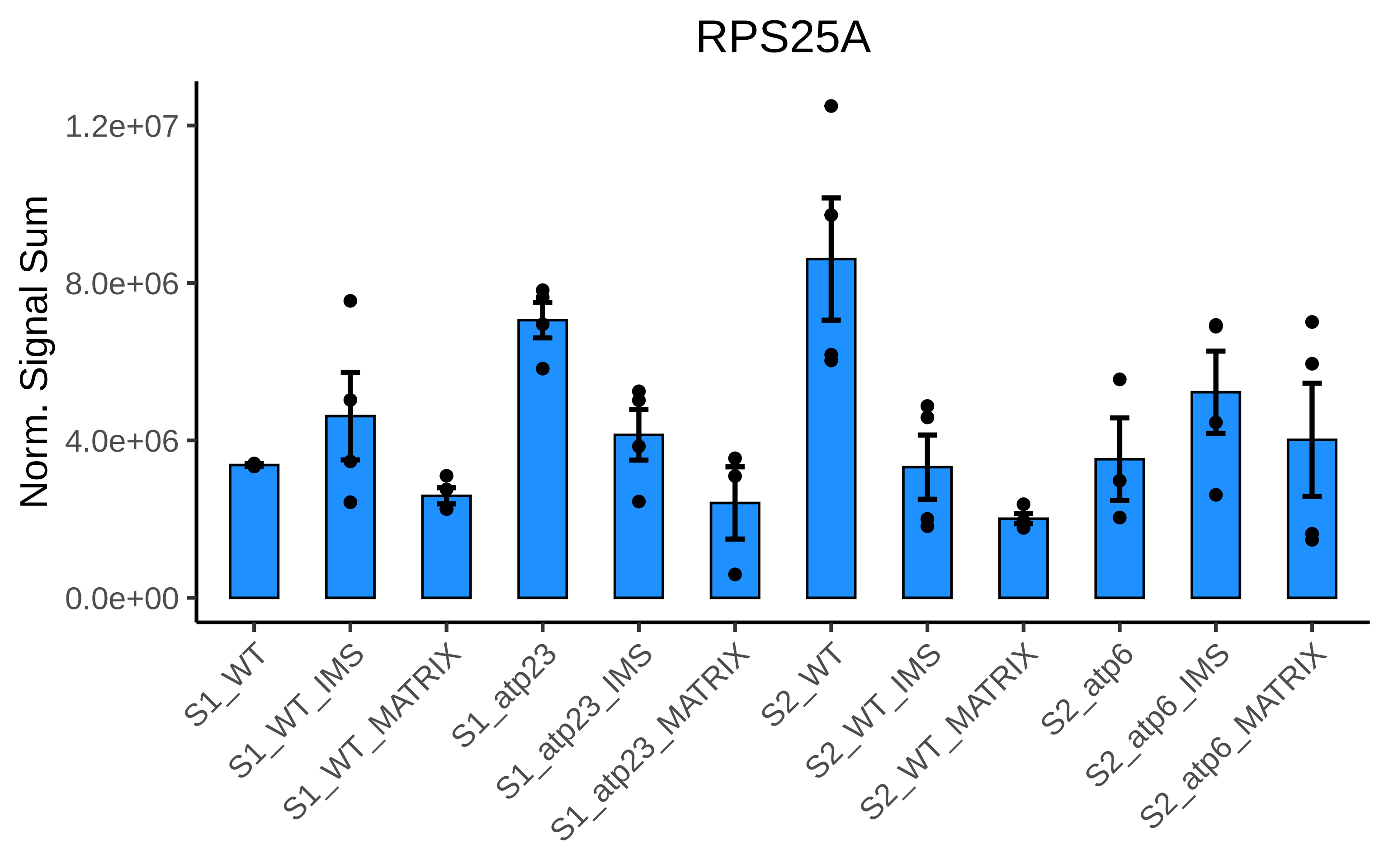
RPS25A
Norm. Signal Sum
1.2e+07
8.0e+06
4.0e+06
0.0e+00
S1_WT
S1_WT_IMS
S1_WT_MATRIX
S1_atp23
S1_atp23_IMS
S1_atp23_MATRIX
S2_WT
S2_WT_IMS
S2_WT_MATRIX
S2_atp6
S2_atp6_IMS
S2_atp6_MATRIX

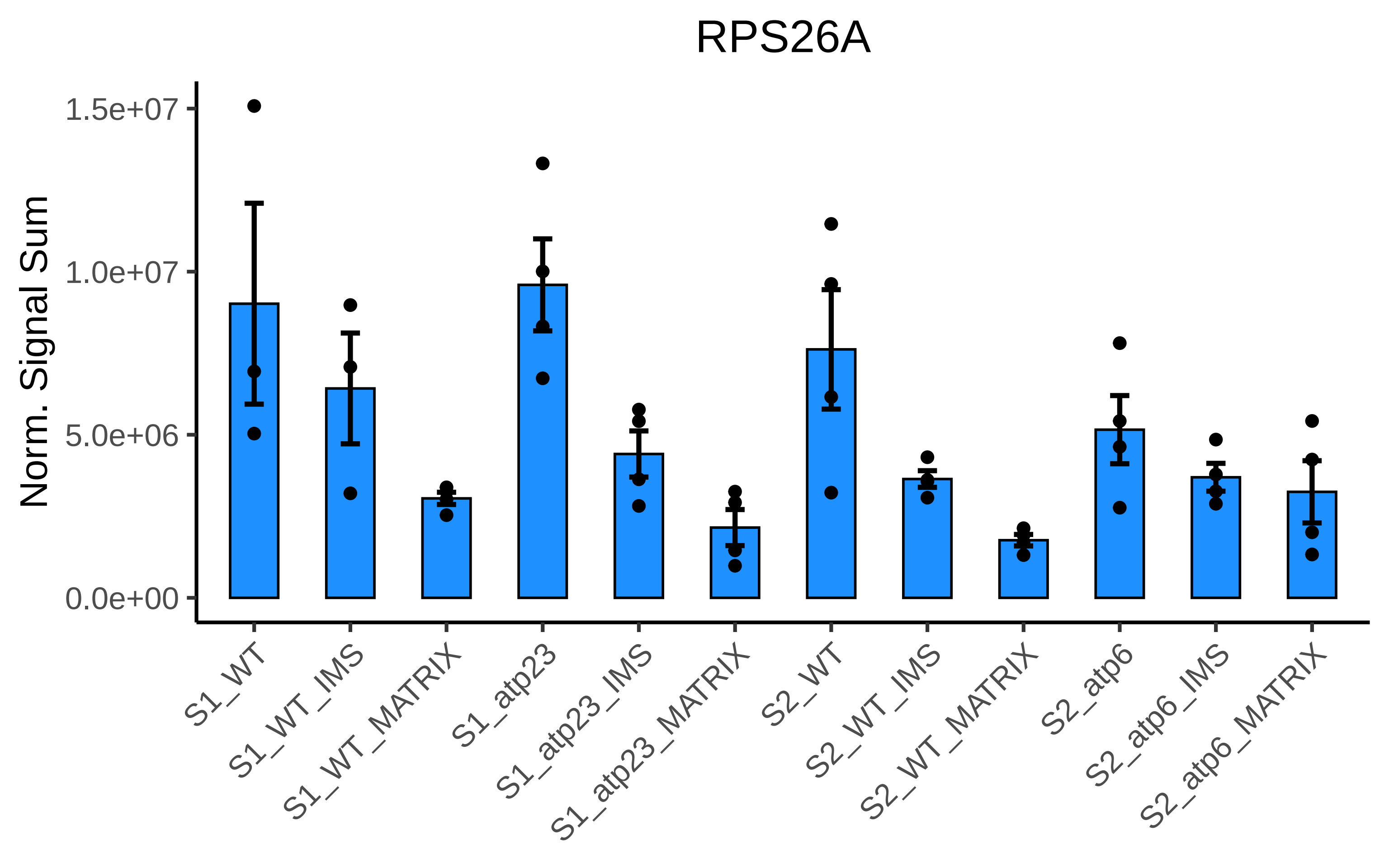
RPS26A
Norm. Signal Sum
1.5e+07
1.0e+07
5.0e+06
0.0e+00
S1_WT
S1_WT_IMS
S1_WT_MATRIX
S1_atp23
S1_atp23_IMS
S1_atp23_MATRIX
S2_WT
S2_WT_IMS
S2_WT_MATRIX
S2_atp6
S2_atp6_IMS
S2_atp6_MATRIX

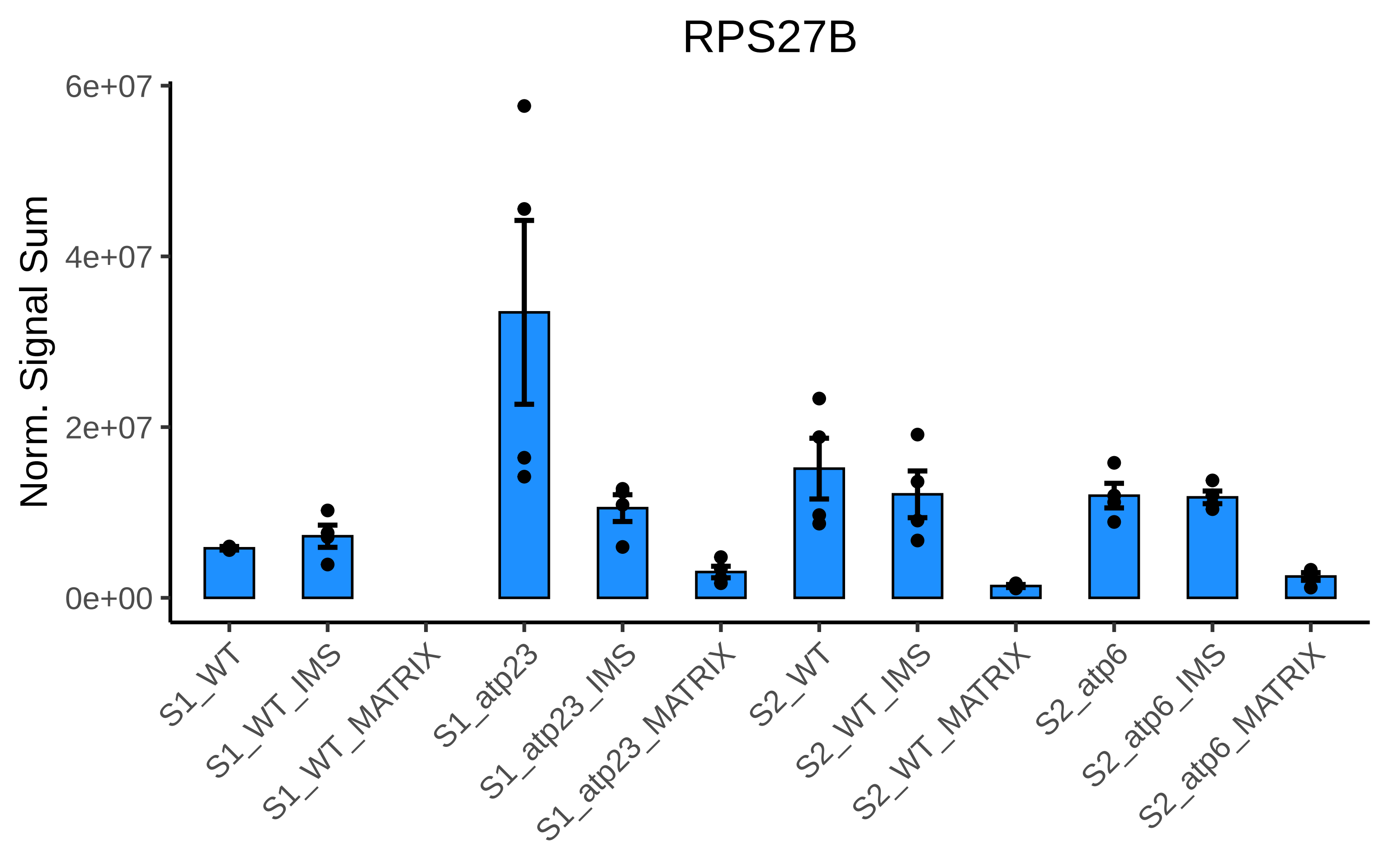
RPS27B
Norm. Signal Sum
6e+07
4e+07
2e+07
0e+00
S1_WT
S1_WT_IMS
S1_WT_MATRIX
S1_atp23
S1_atp23_IMS
S1_atp23_MATRIX
S2_WT
S2_WT_IMS
S2_WT_MATRIX
S2_atp6
S2_atp6_IMS
S2_atp6_MATRIX

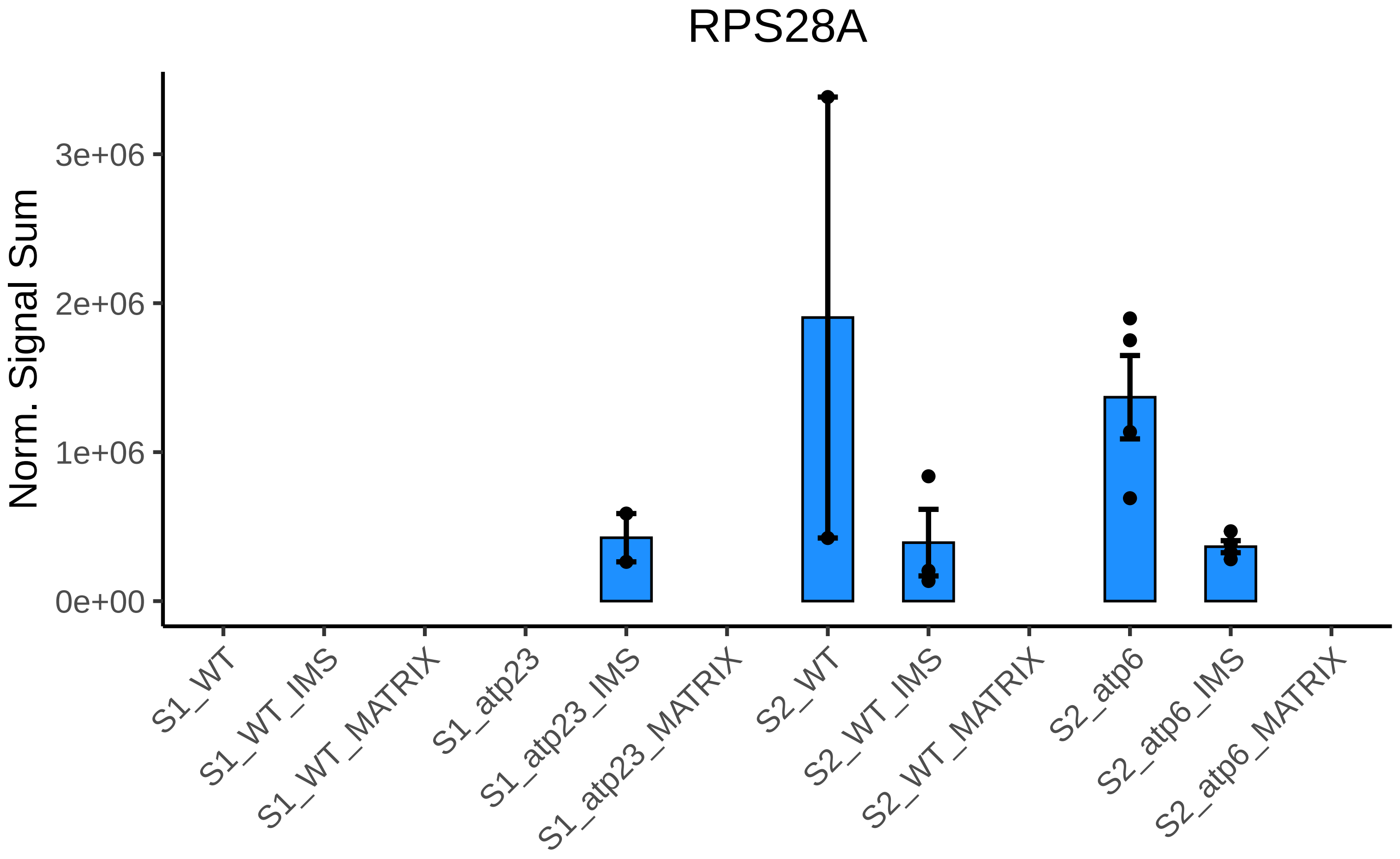

RPS28A
Norm. Signal Sum
3e+06
2e+06
1e+06
0e+00
S1_WT
S1_WT_IMS
S1_WT_MATRIX
S1_atp23
S1_atp23_IMS
S1_atp23_MATRIX
S2_WT
S2_WT_IMS
S2_WT_MATRIX
S2_atp6
S2_atp6_IMS
S2_atp6_MATRIX

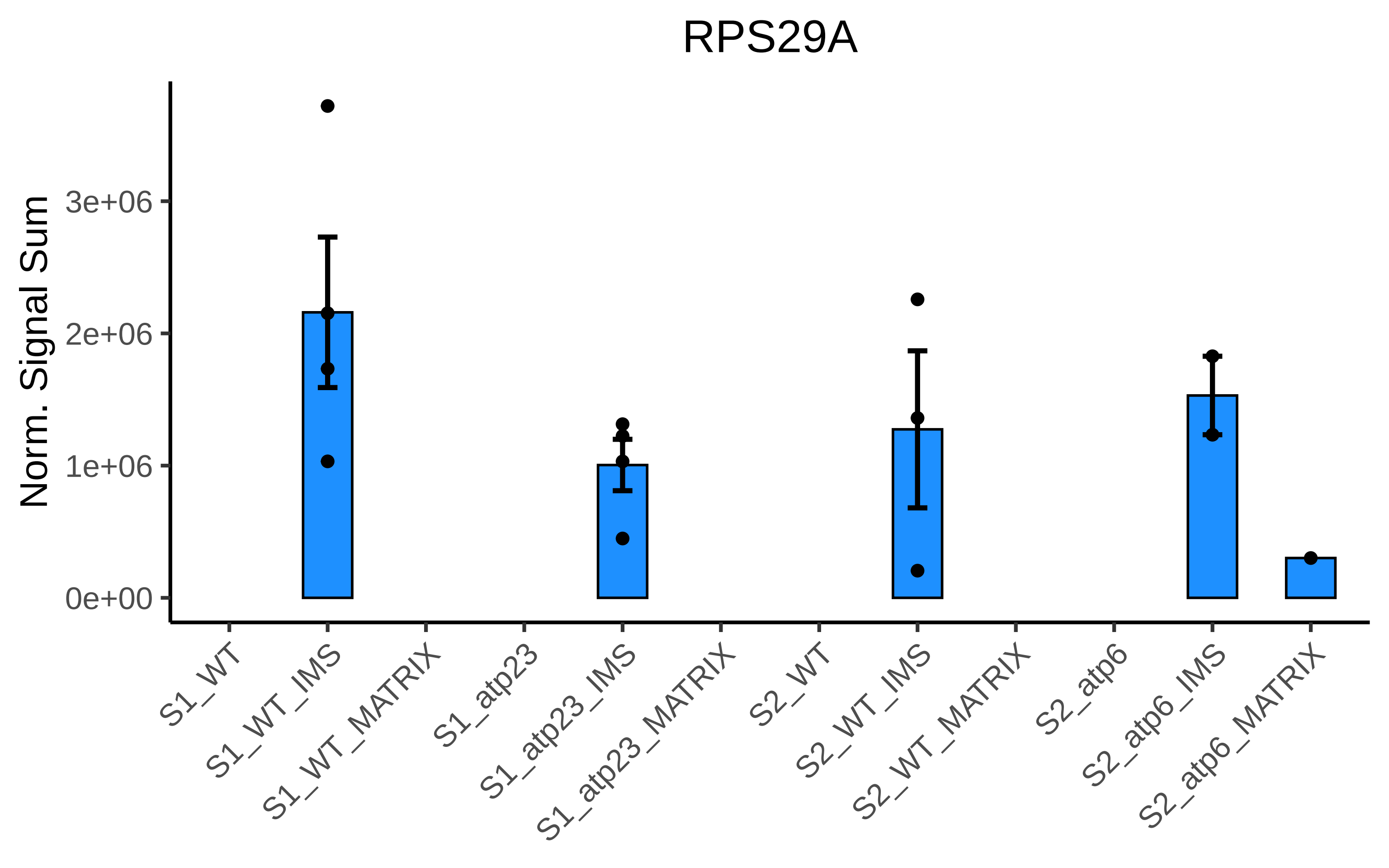
RPS29A
Norm. Signal Sum
3e+06
2e+06
1e+06
0e+00
S1_WT
S1_WT_IMS
S1_WT_MATRIX
S1_atp23
S1_atp23_IMS
S1_atp23_MATRIX
S2_WT
S2_WT_IMS
S2_WT_MATRIX
S2_atp6
S2_atp6_IMS
S2_atp6_MATRIX

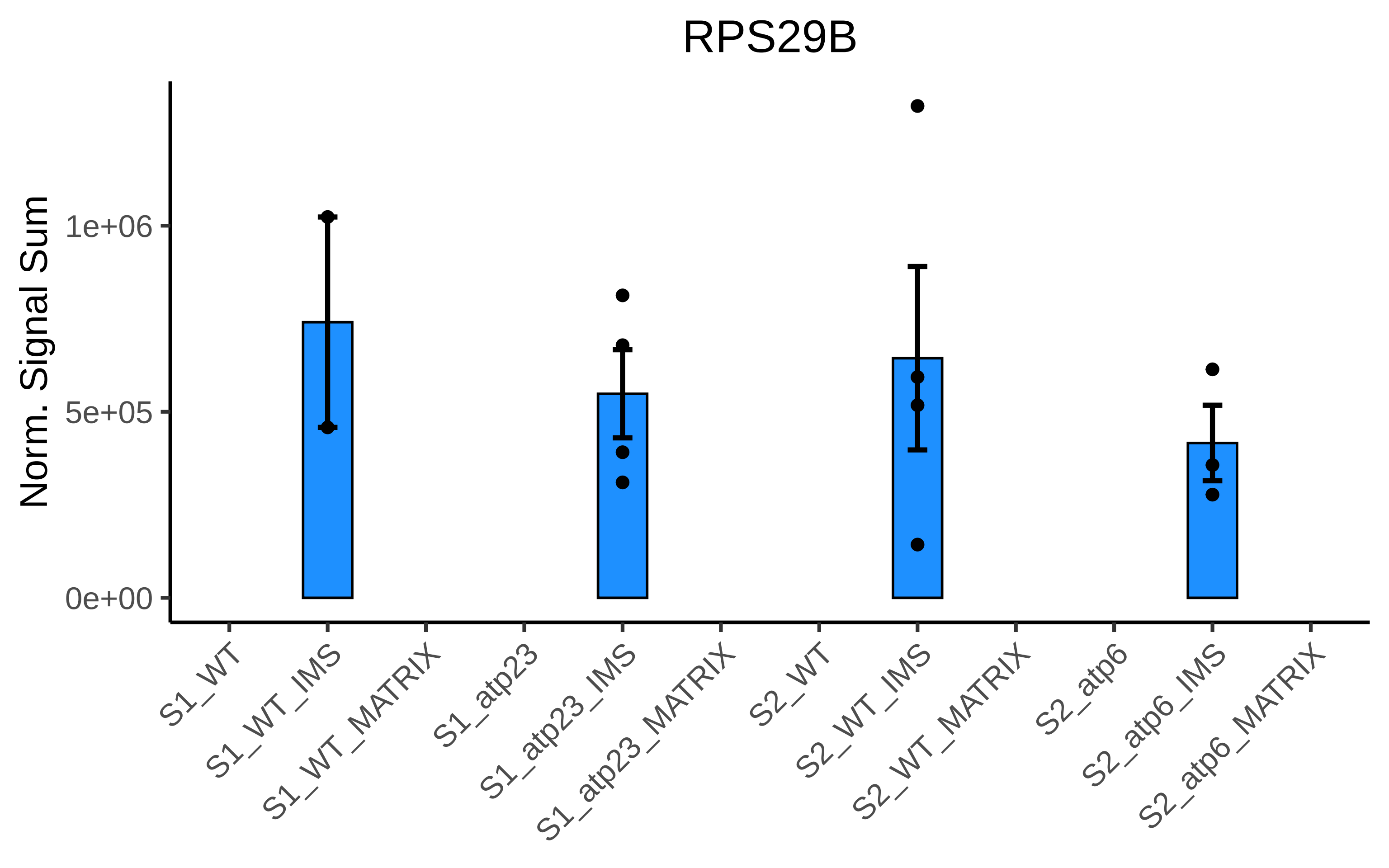

RPS29B
Norm. Signal Sum
1e+06
5e+05
0e+00
S1_WT
S1_WT_IMS
S1_WT_MATRIX
S1_atp23
S1_atp23_IMS
S1_atp23_MATRIX
S2_WT
S2_WT_IMS
S2_WT_MATRIX
S2_atp6
S2_atp6_IMS
S2_atp6_MATRIX

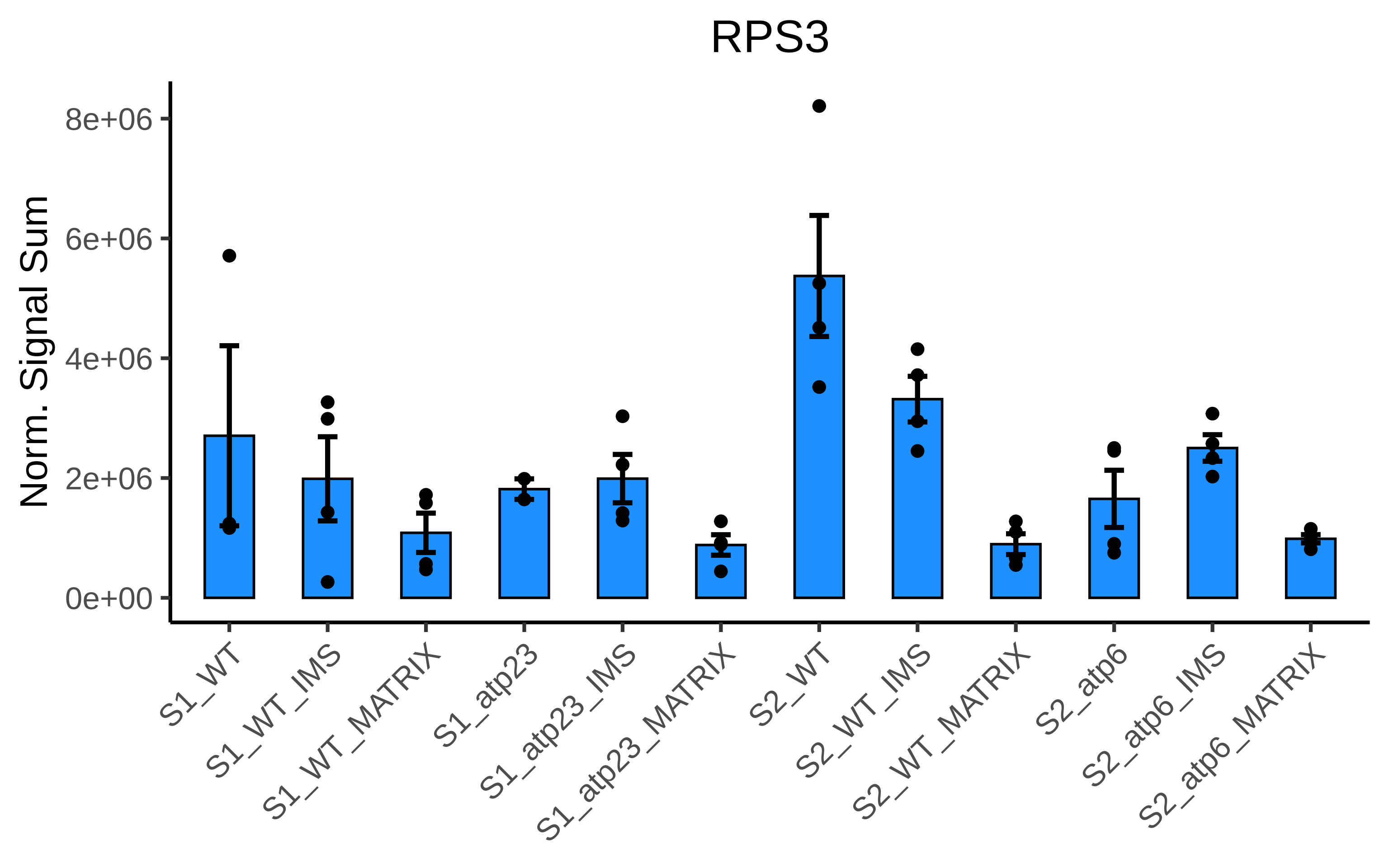

RPS3
Norm. Signal Sum
8e+06
6e+06
4e+06
2e+06
0e+00
S1_WT
S1_WT_IMS
S1_WT_MATRIX
S1_atp23
S1_atp23_IMS
S1_atp23_MATRIX
S2_WT
S2_WT_IMS
S2_WT_MATRIX
S2_atp6
S2_atp6_IMS
S2_atp6_MATRIX

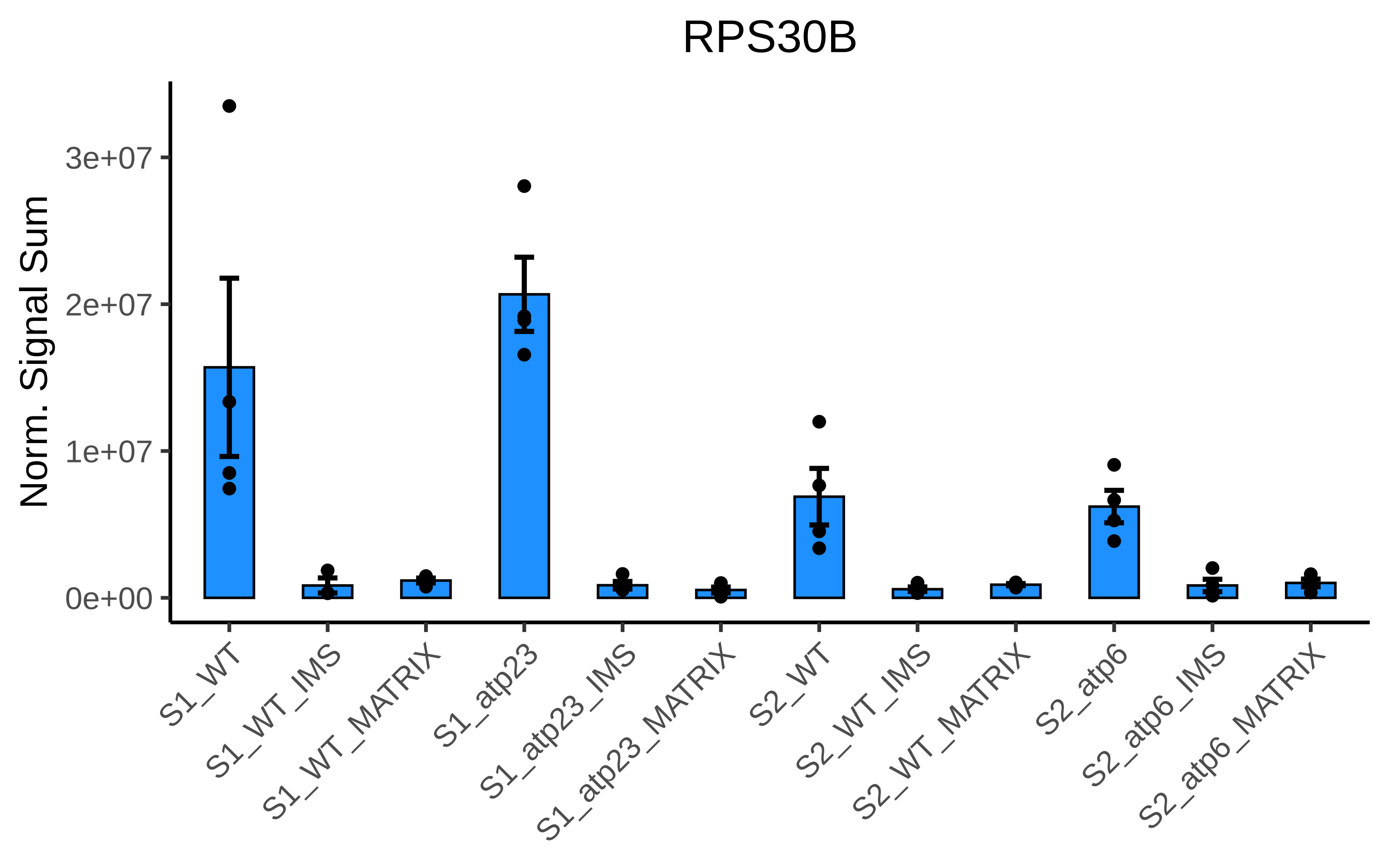
RPS30B
Norm. Signal Sum
3e+07
2e+07
1e+07
0e+00
S1_WT
S1_WT_IMS
S1_WT_MATRIX
S1_atp23
S1_atp23_IMS
S1_atp23_MATRIX
S2_WT
S2_WT_IMS
S2_WT_MATRIX
S2_atp6
S2_atp6_IMS
S2_atp6_MATRIX

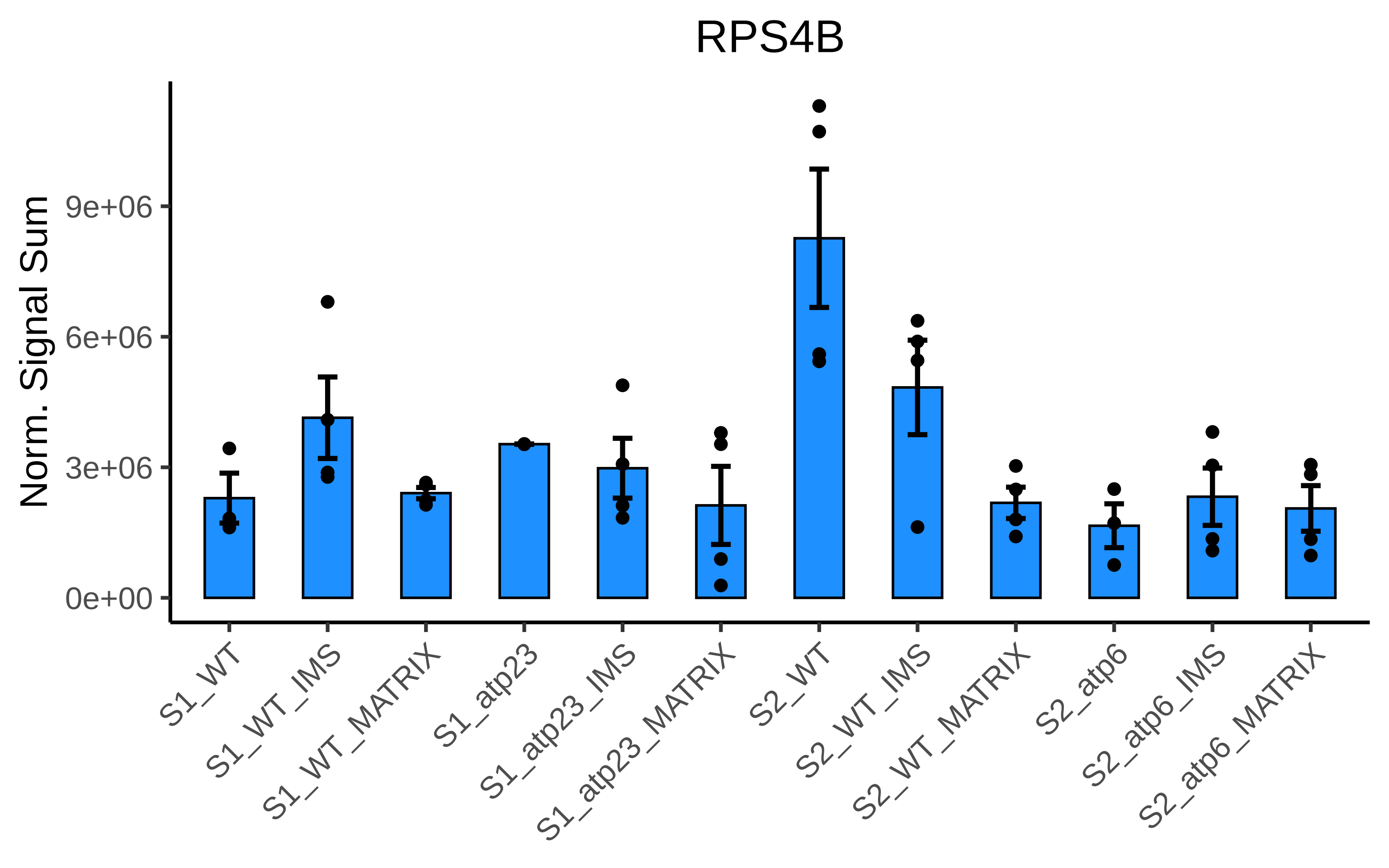

RPS4B
Norm. Signal Sum
9e+06
6e+06
3e+06
0e+00
S1_WT
S1_WT_IMS
S1_WT_MATRIX
S1_atp23
S1_atp23_IMS
S1_atp23_MATRIX
S2_WT
S2_WT_IMS
S2_WT_MATRIX
S2_atp6
S2_atp6_IMS
S2_atp6_MATRIX

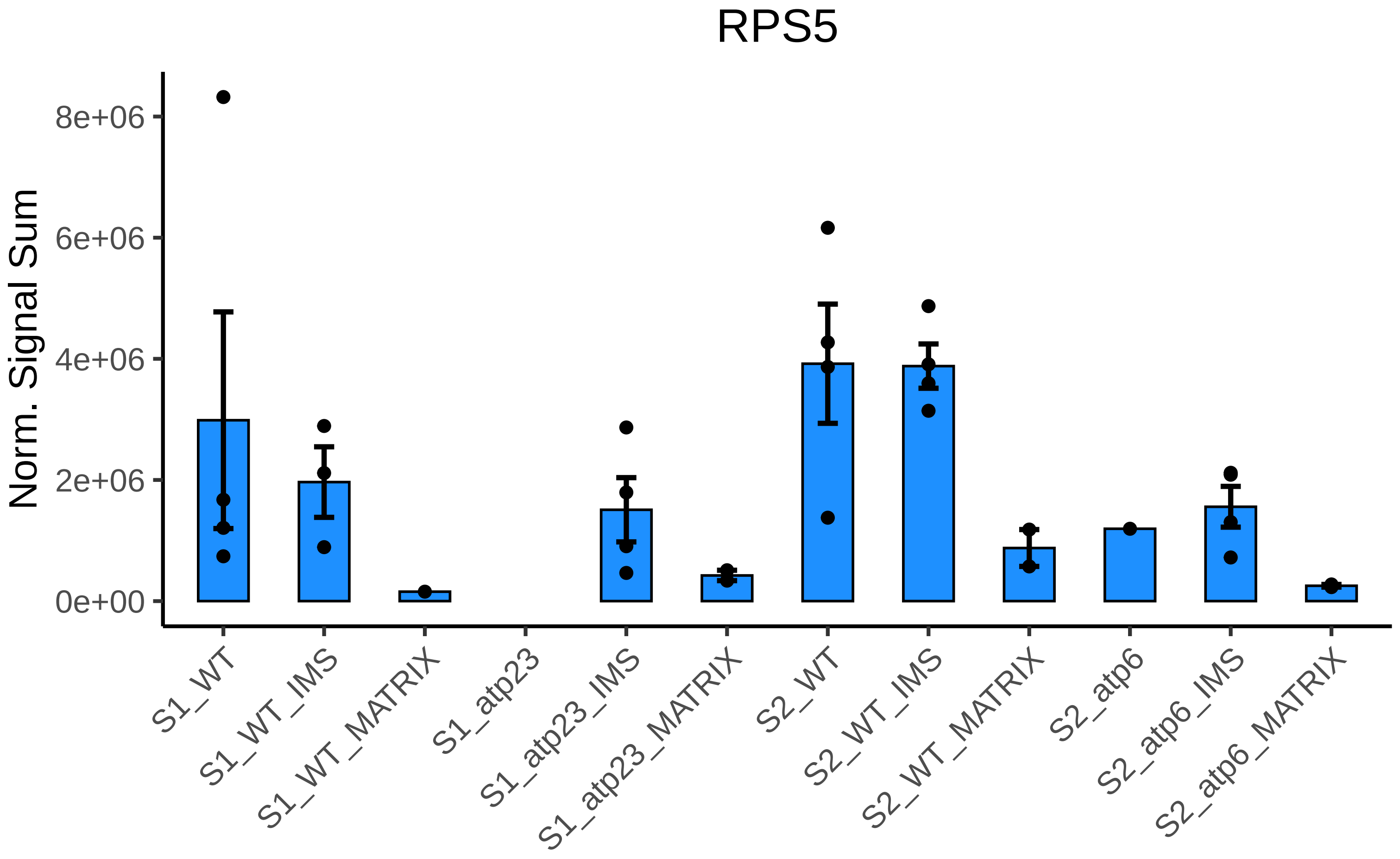
RPS5
Norm. Signal Sum
8e+06
6e+06
4e+06
2e+06
0e+00
S1_WT
S1_WT_IMS
S1_WT_MATRIX
S1_atp23
S1_atp23_IMS
S1_atp23_MATRIX
S2_WT
S2_WT_IMS
S2_WT_MATRIX
S2_atp6
S2_atp6_IMS
S2_atp6_MATRIX

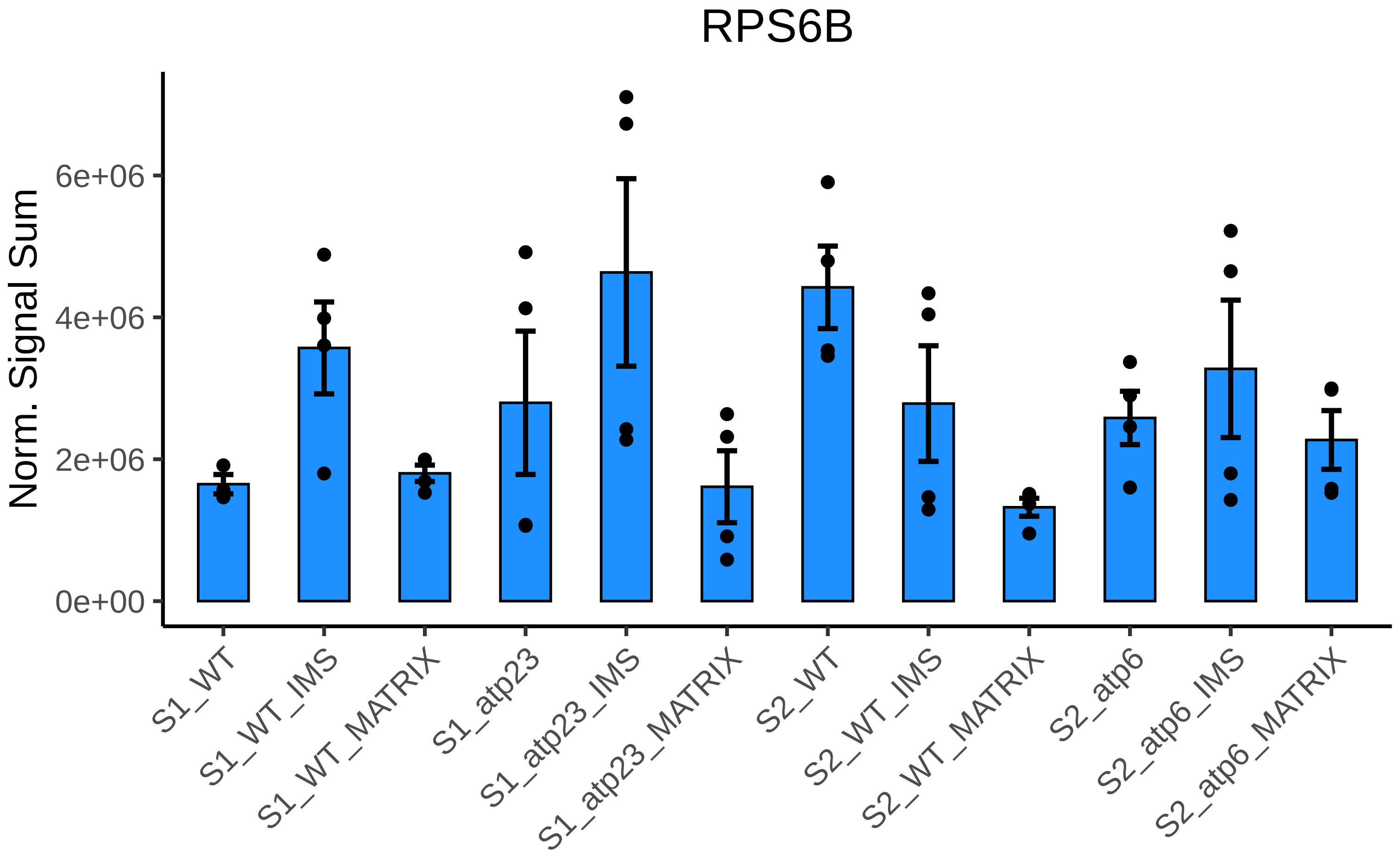
RPS6B
Norm. Signal Sum
6e+06
4e+06
2e+06
0e+00
S1_WT
S1_WT_IMS
S1_WT_MATRIX
S1_atp23
S1_atp23_IMS
S1_atp23_MATRIX
S2_WT
S2_WT_IMS
S2_WT_MATRIX
S2_atp6
S2_atp6_IMS
S2_atp6_MATRIX

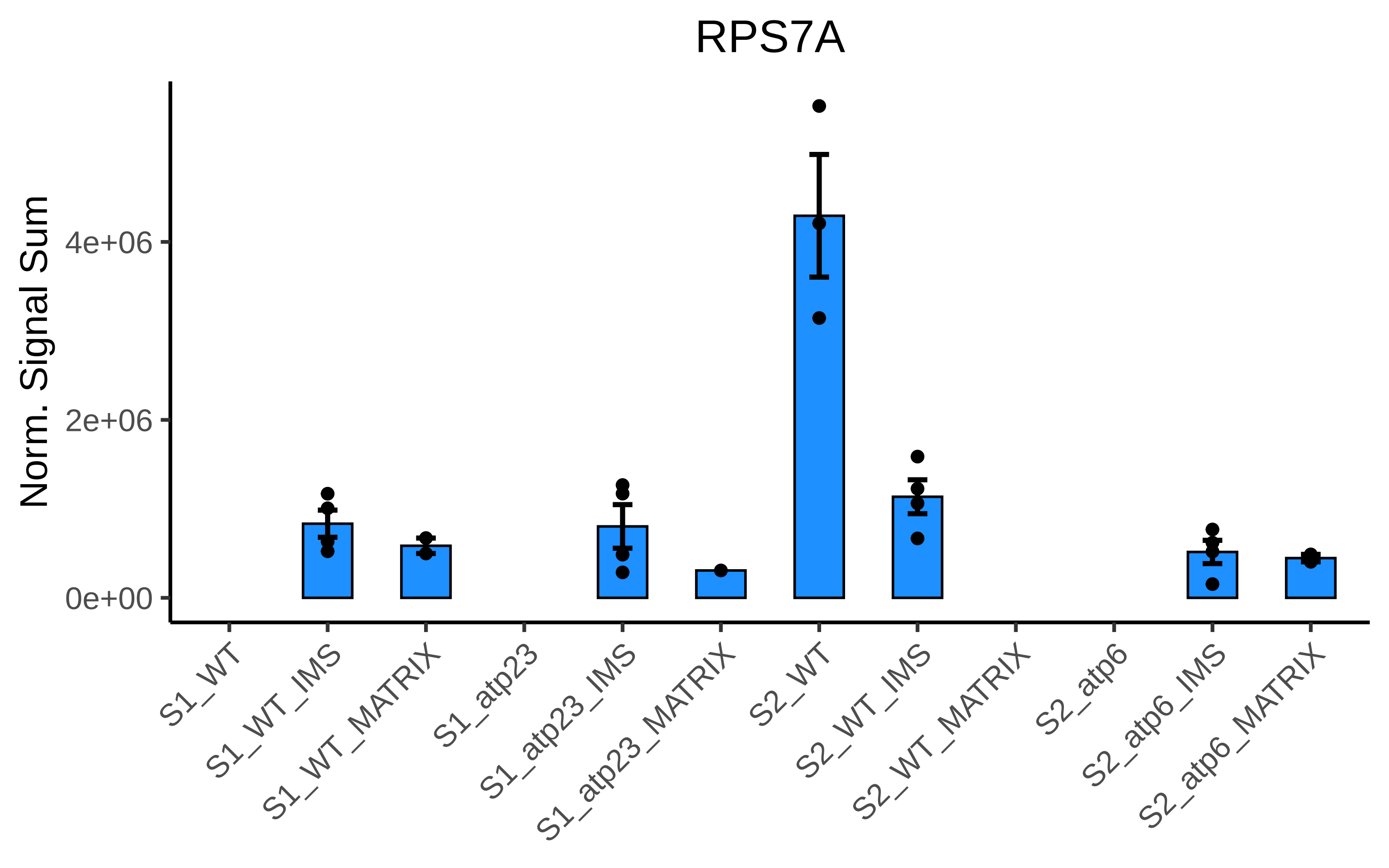

RPS7A
Norm. Signal Sum
4e+06
2e+06
0e+00
S1_WT
S1_WT_IMS
S1_WT_MATRIX
S1_atp23
S1_atp23_IMS
S1_atp23_MATRIX
S2_WT
S2_WT_IMS
S2_WT_MATRIX
S2_atp6
S2_atp6_IMS
S2_atp6_MATRIX

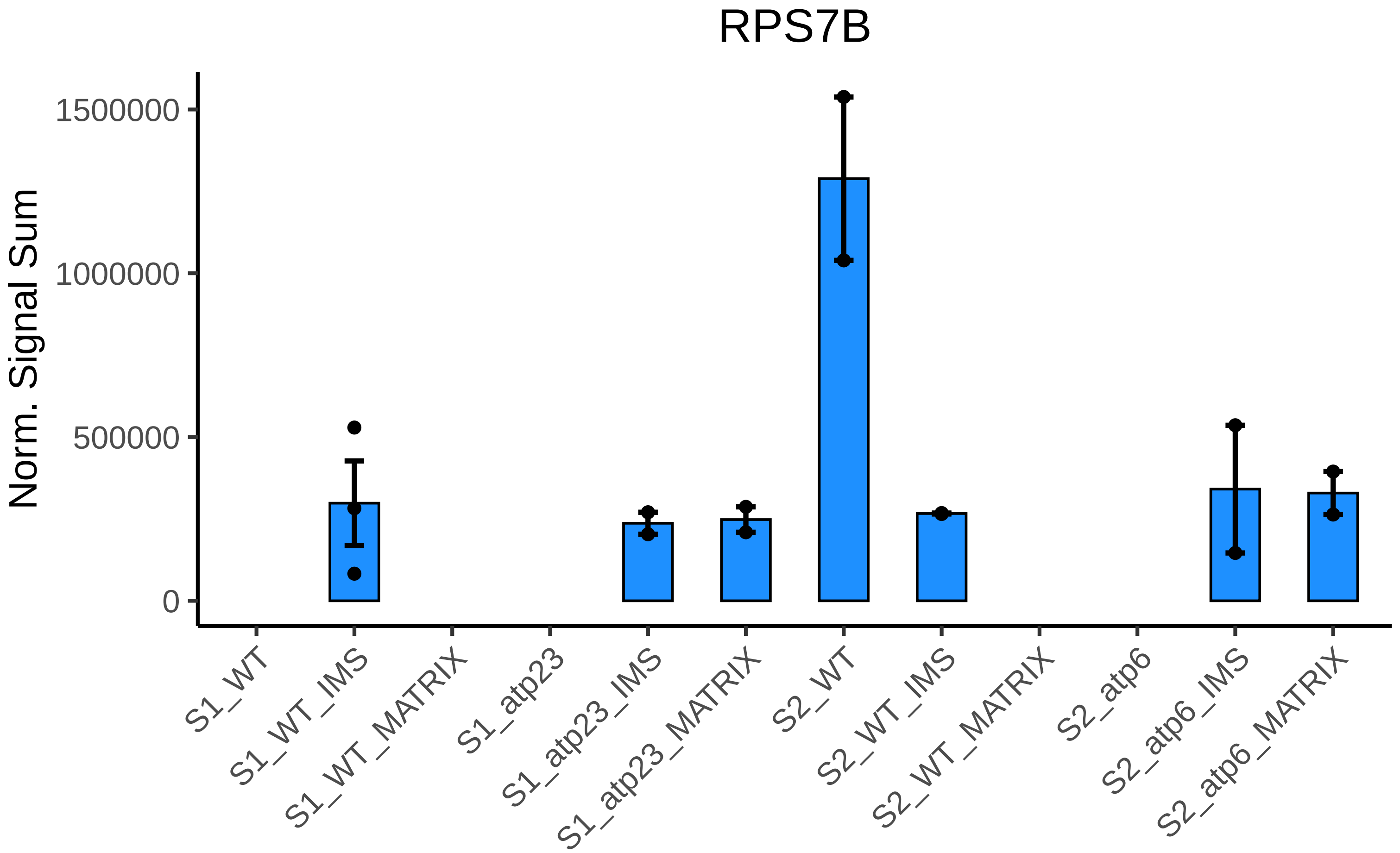
RPS7B
Norm. Signal Sum
1500000
1000000
500000
0
S1_WT
S1_WT_IMS
S1_WT_MATRIX
S1_atp23
S1_atp23_IMS
S1_atp23_MATRIX
S2_WT
S2_WT_IMS
S2_WT_MATRIX
S2_atp6
S2_atp6_IMS
S2_atp6_MATRIX

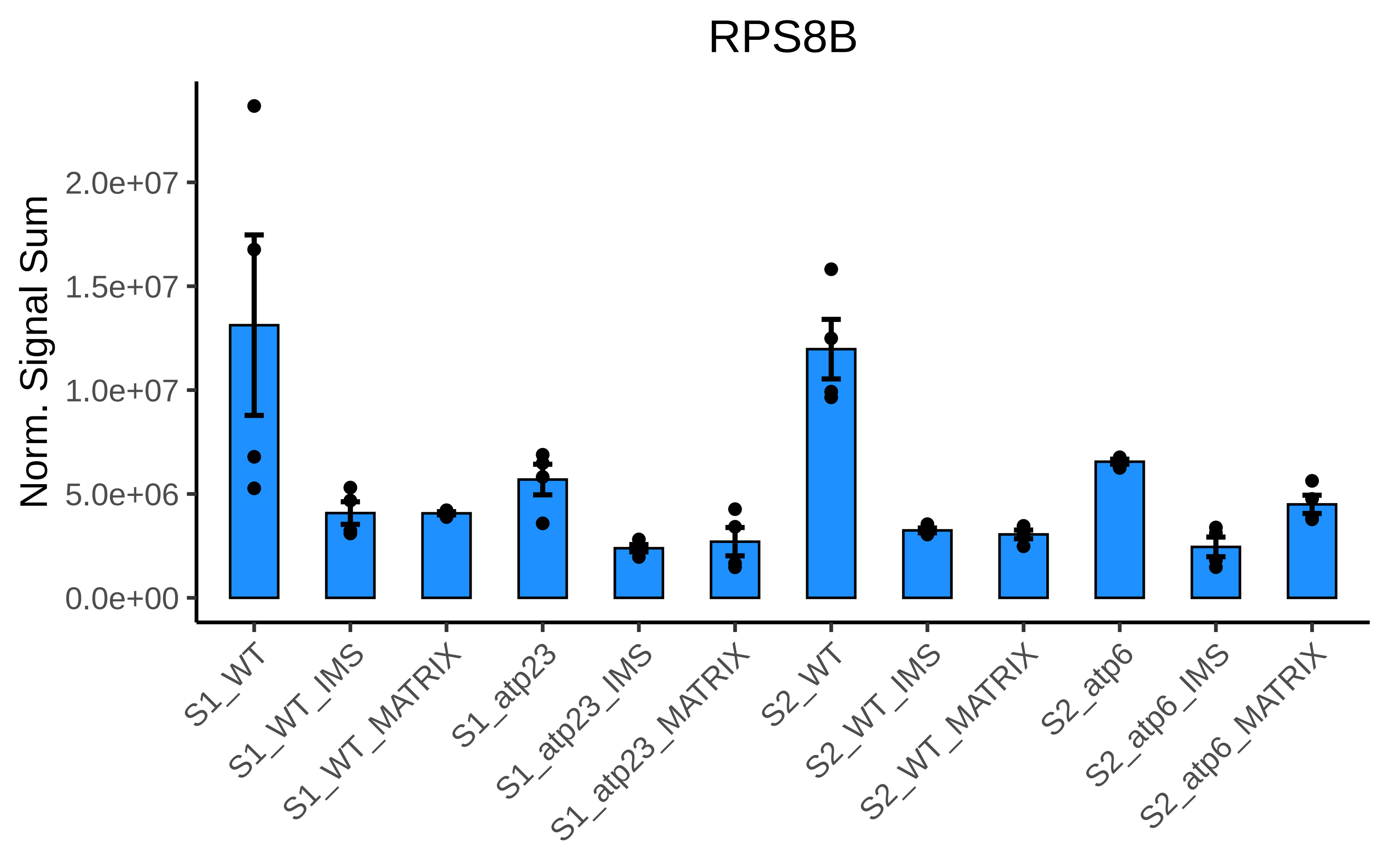
RPS8B
Norm. Signal Sum
2.0e+07
1.5e+07
1.0e+07
5.0e+06
0.0e+00
S1_WT
S1_WT_IMS
S1_WT_MATRIX
S1_atp23
S1_atp23_IMS
S1_atp23_MATRIX
S2_WT
S2_WT_IMS
S2_WT_MATRIX
S2_atp6
S2_atp6_IMS
S2_atp6_MATRIX

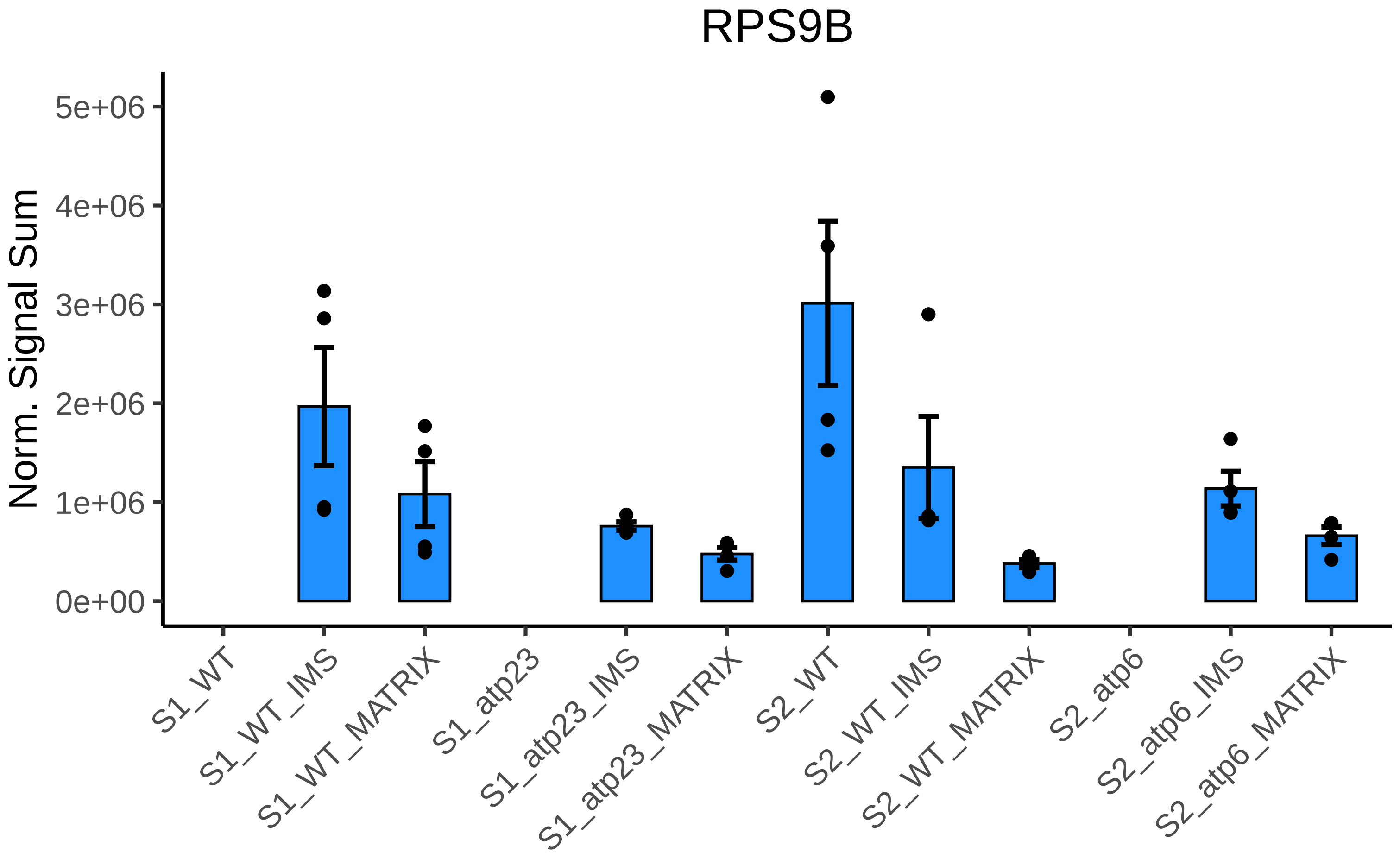

RPS9B
Norm. Signal Sum
5e+06
4e+06
3e+06
2e+06
1e+06
0e+00
S1_WT
S1_WT_IMS
S1_WT_MATRIX
S1_atp23
S1_atp23_IMS
S1_atp23_MATRIX
S2_WT
S2_WT_IMS
S2_WT_MATRIX
S2_atp6
S2_atp6_IMS
S2_atp6_MATRIX

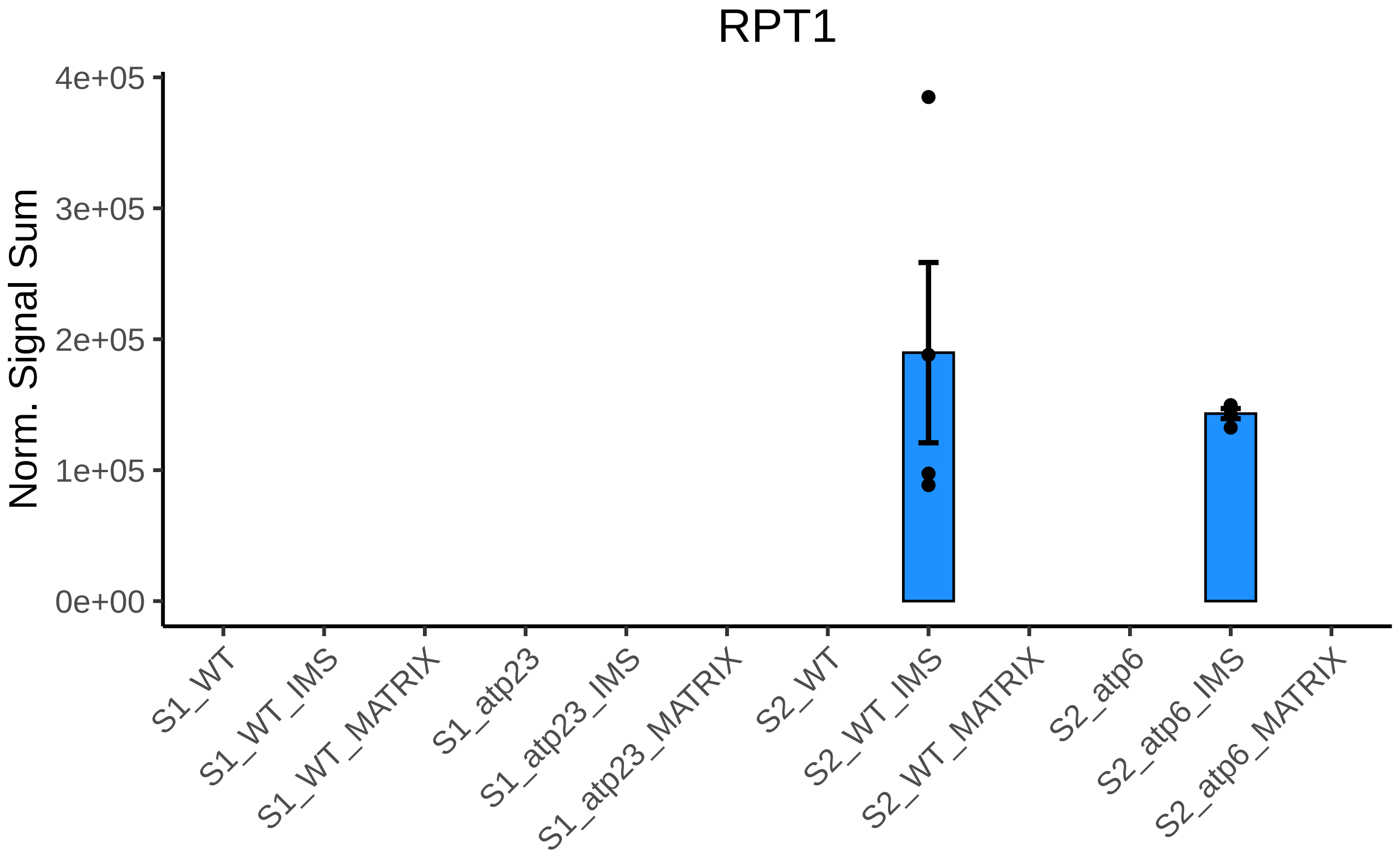

RPT1
Norm. Signal Sum
4e+05
3e+05
2e+05
1e+05
0e+00
S1_WT
S1_WT_IMS
S1_WT_MATRIX
S1_atp23
S1_atp23_IMS
S1_atp23_MATRIX
S2_WT
S2_WT_IMS
S2_WT_MATRIX
S2_atp6
S2_atp6_IMS
S2_atp6_MATRIX

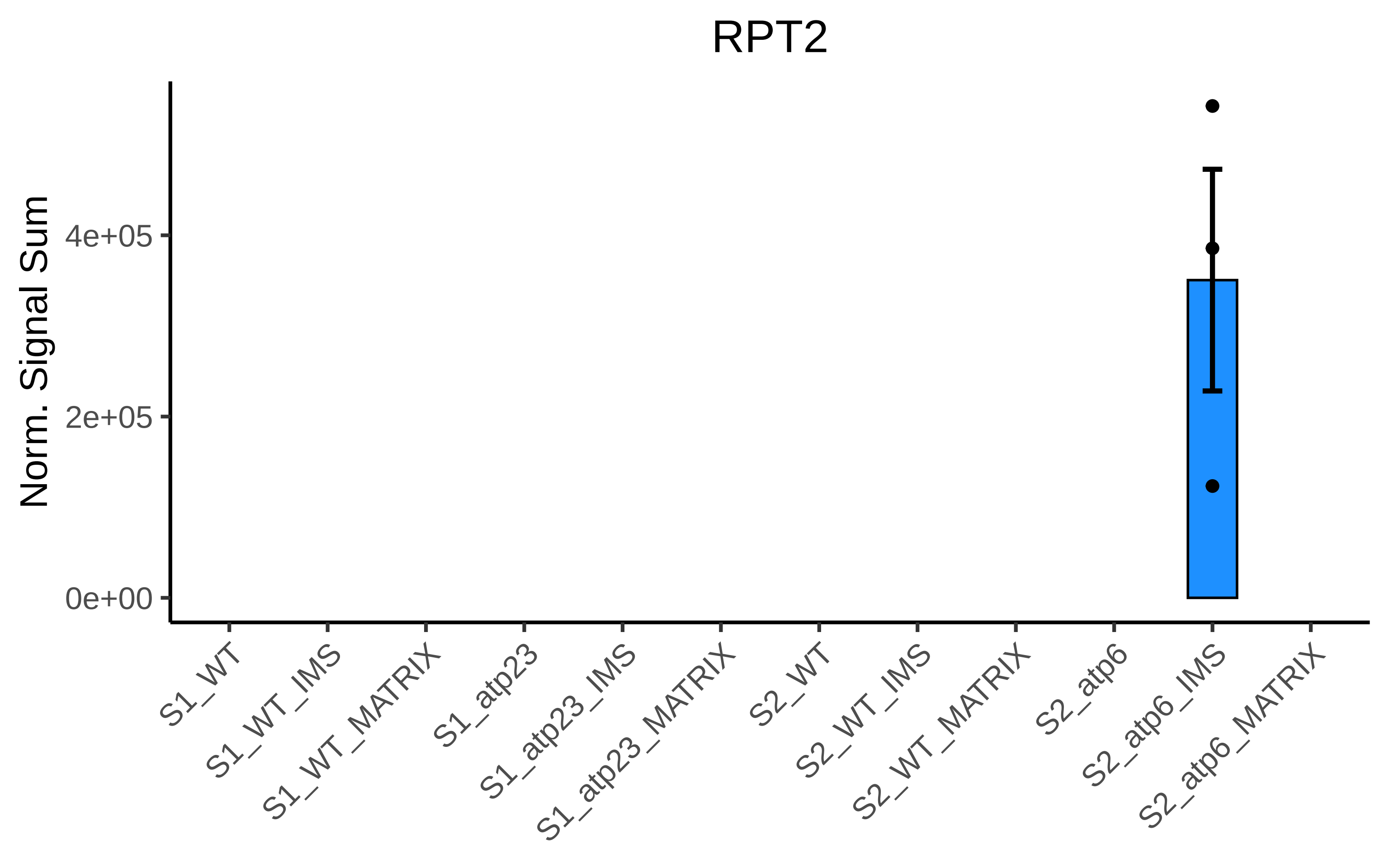
RPT2
Norm. Signal Sum
4e+05
2e+05
0e+00
S1_WT
S1_WT_IMS
S1_WT_MATRIX
S1_atp23
S1_atp23_IMS
S1_atp23_MATRIX
S2_WT
S2_WT_IMS
S2_WT_MATRIX
S2_atp6
S2_atp6_IMS
S2_atp6_MATRIX

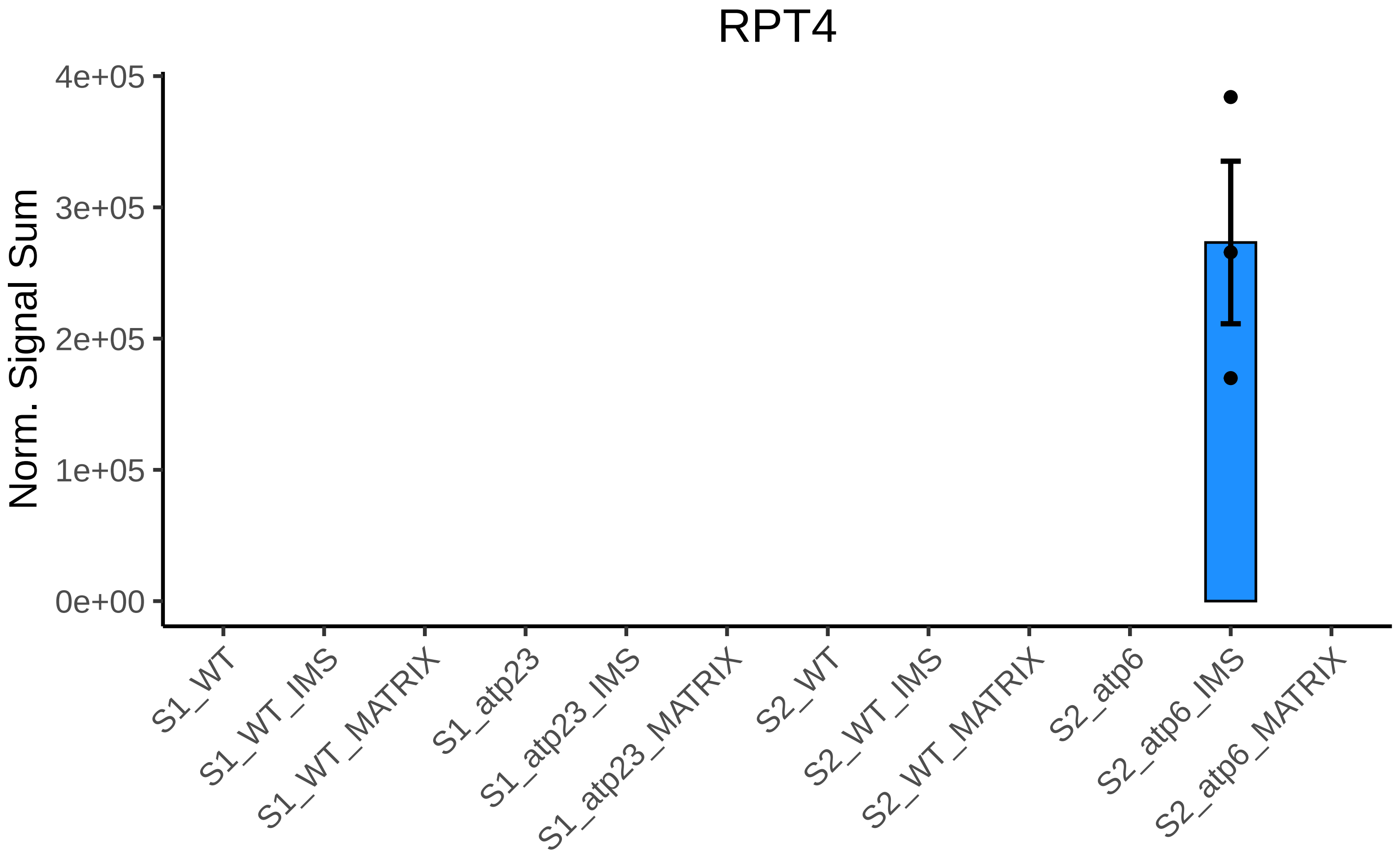
RPT4
Norm. Signal Sum
4e+05
3e+05
2e+05
1e+05
0e+00
S1_WT
S1_WT_IMS
S1_WT_MATRIX
S1_atp23
S1_atp23_IMS
S1_atp23_MATRIX
S2_WT
S2_WT_IMS
S2_WT_MATRIX
S2_atp6
S2_atp6_IMS
S2_atp6_MATRIX

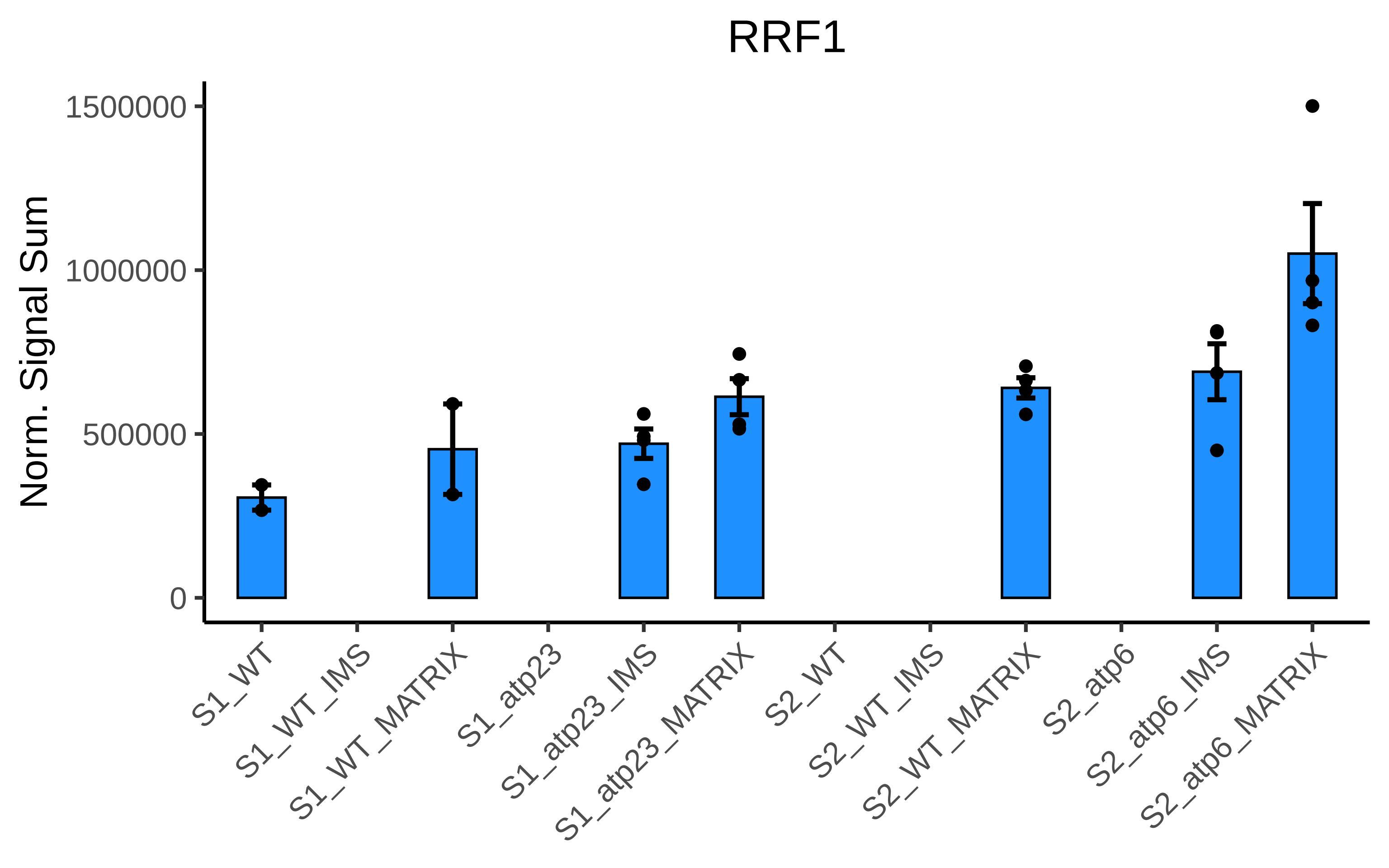

RRF1
Norm. Signal Sum
1500000
1000000
500000
0
S1_WT
S1_WT_IMS
S1_WT_MATRIX
S1_atp23
S1_atp23_IMS
S1_atp23_MATRIX
S2_WT
S2_WT_IMS
S2_WT_MATRIX
S2_atp6
S2_atp6_IMS
S2_atp6_MATRIX

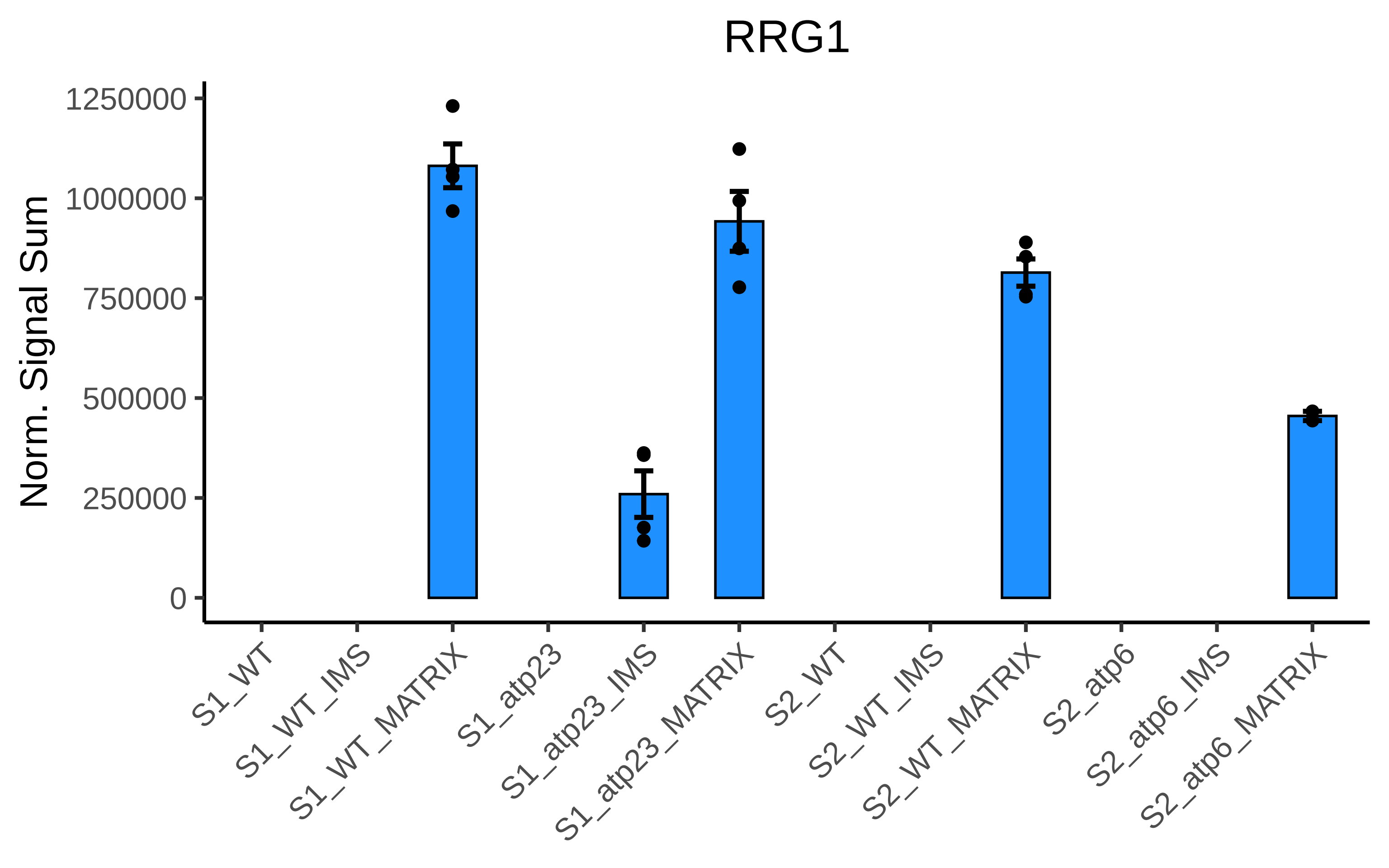

RRG1
Norm. Signal Sum
1250000
1000000
750000
500000
250000
0
S1_WT
S1_WT_IMS
S1_WT_MATRIX
S1_atp23
S1_atp23_IMS
S1_atp23_MATRIX
S2_WT
S2_WT_IMS
S2_WT_MATRIX
S2_atp6
S2_atp6_IMS
S2_atp6_MATRIX

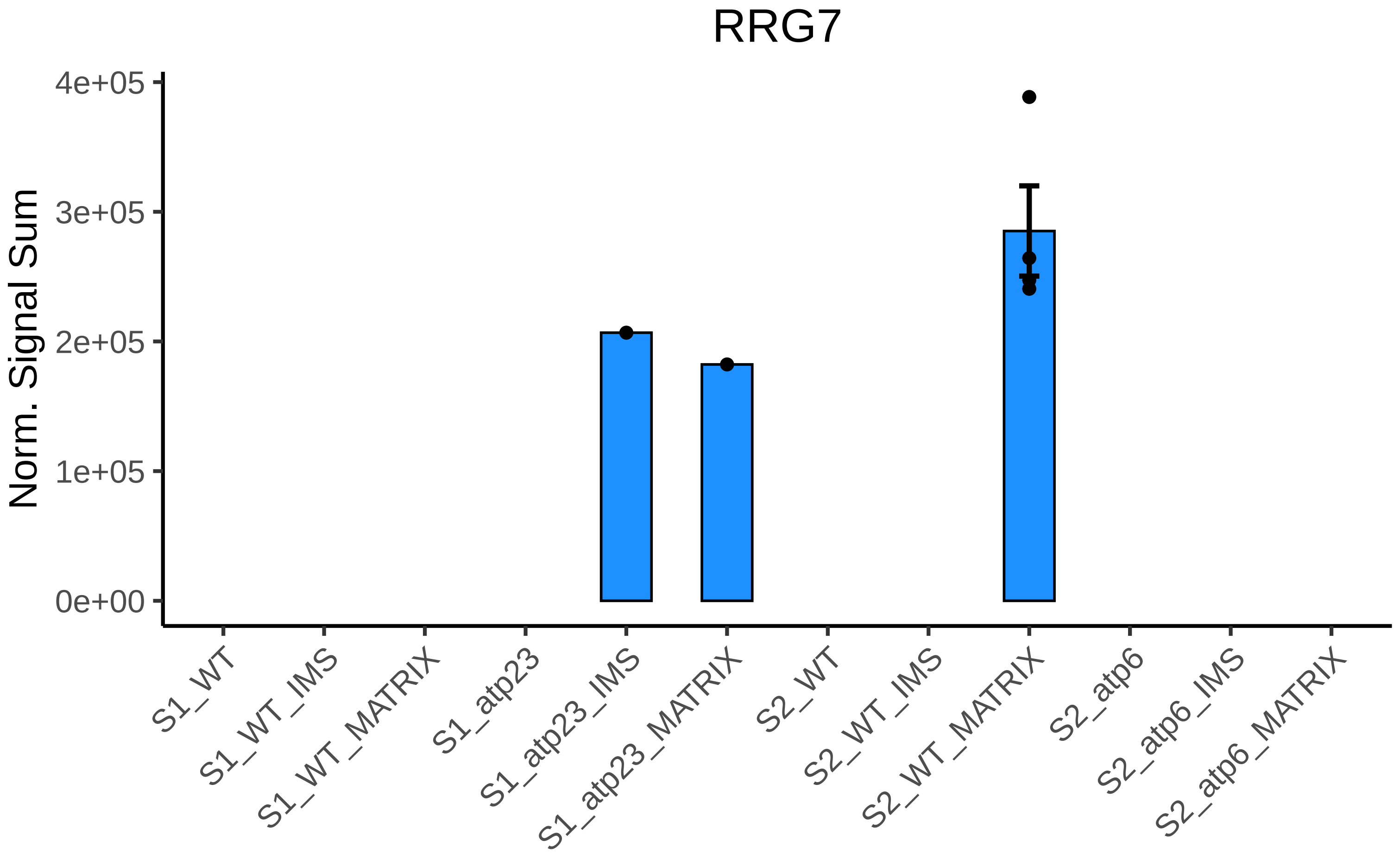

RRG7
Norm. Signal Sum
4e+05
3e+05
2e+05
1e+05
0e+00
S1_WT
S1_WT_IMS
S1_WT_MATRIX
S1_atp23
S1_atp23_IMS
S1_atp23_MATRIX
S2_WT
S2_WT_IMS
S2_WT_MATRIX
S2_atp6
S2_atp6_IMS
S2_atp6_MATRIX

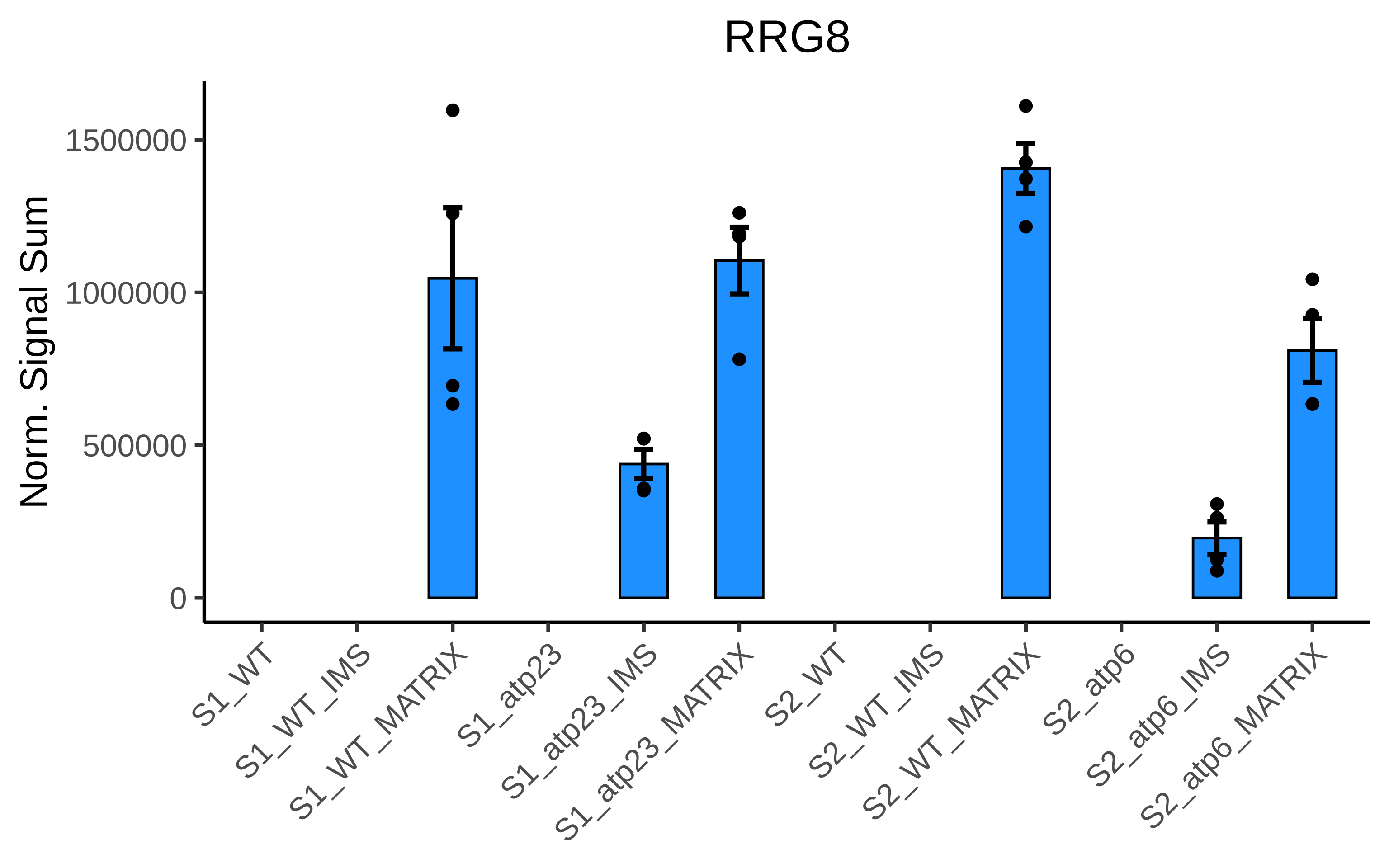

RRG8
Norm. Signal Sum
1500000
1000000
500000
0
S1_WT
S1_WT_IMS
S1_WT_MATRIX
S1_atp23
S1_atp23_IMS
S1_atp23_MATRIX
S2_WT
S2_WT_IMS
S2_WT_MATRIX
S2_atp6
S2_atp6_IMS
S2_atp6_MATRIX

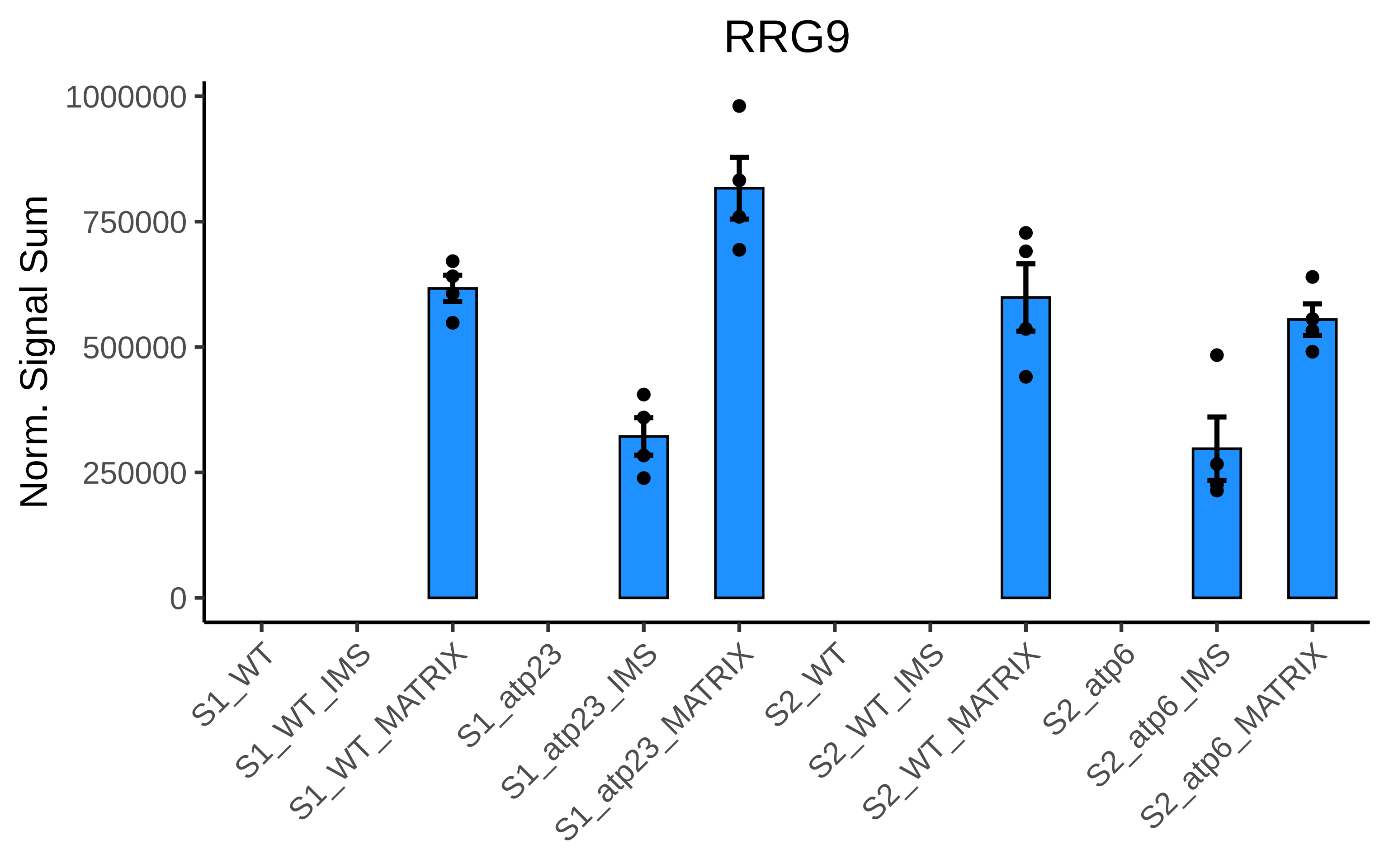
RRG9
Norm. Signal Sum
1000000
750000
500000
250000
0
S1_WT
S1_WT_IMS
S1_WT_MATRIX
S1_atp23
S1_atp23_IMS
S1_atp23_MATRIX
S2_WT
S2_WT_IMS
S2_WT_MATRIX
S2_atp6
S2_atp6_IMS
S2_atp6_MATRIX

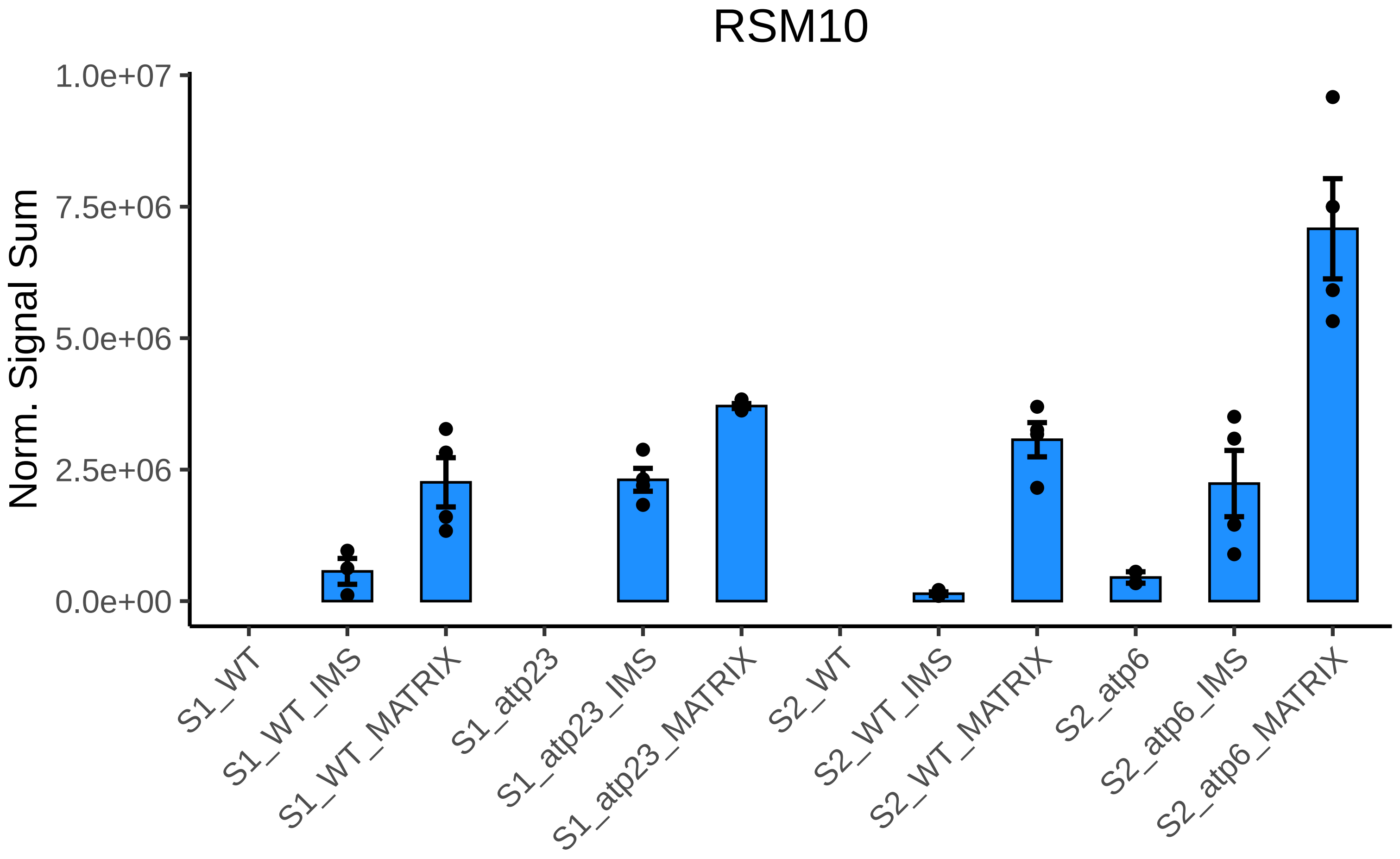
RSM10
Norm. Signal Sum
1.0e+07
7.5e+06
5.0e+06
2.5e+06
0.0e+00
S1_WT
S1_WT_IMS
S1_WT_MATRIX
S1_atp23
S1_atp23_IMS
S1_atp23_MATRIX
S2_WT
S2_WT_IMS
S2_WT_MATRIX
S2_atp6
S2_atp6_IMS
S2_atp6_MATRIX

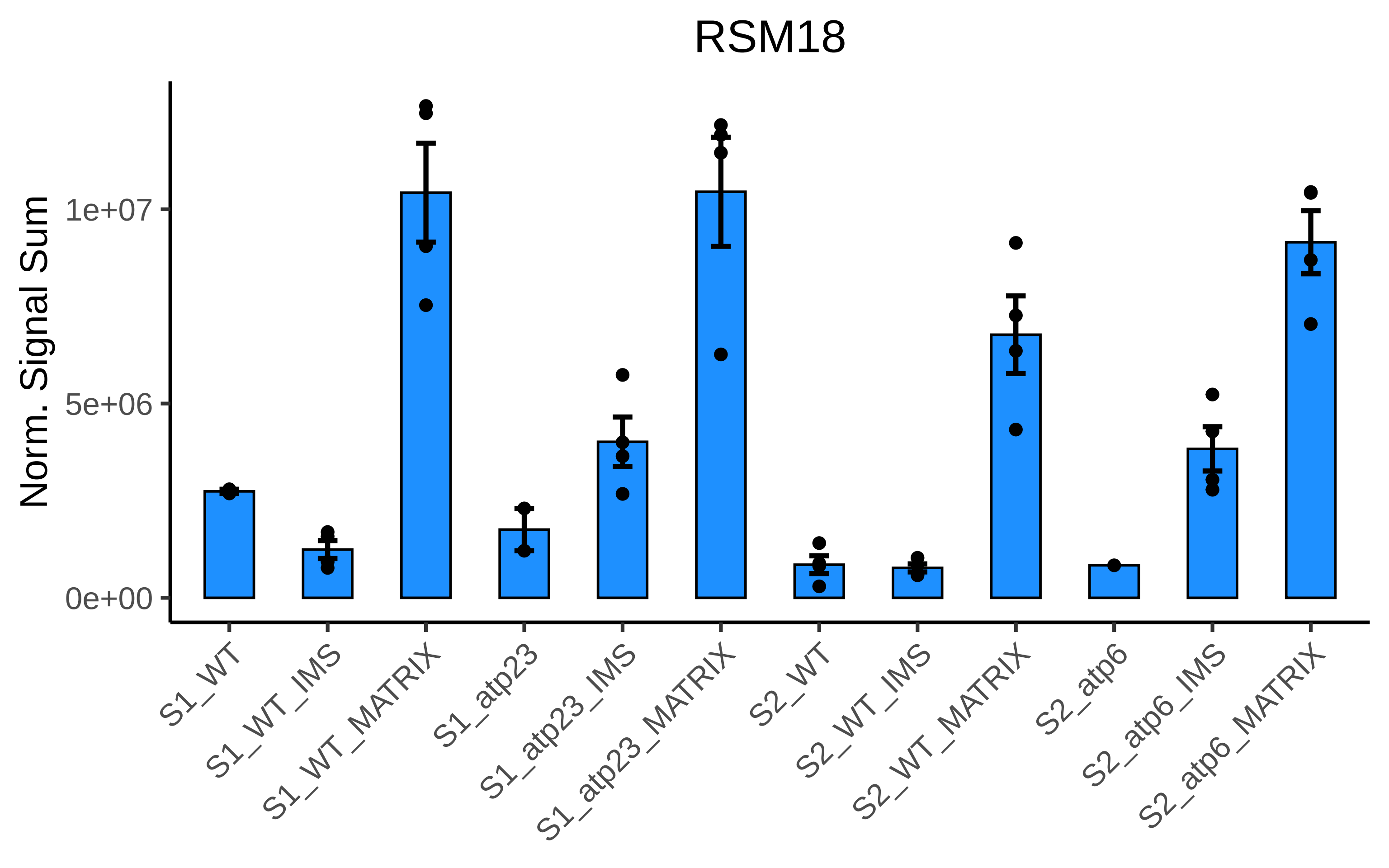
RSM18
Norm. Signal Sum
1e+07
5e+06
0e+00
S1_WT
S1_WT_IMS
S1_WT_MATRIX
S1_atp23
S1_atp23_IMS
S1_atp23_MATRIX
S2_WT
S2_WT_IMS
S2_WT_MATRIX
S2_atp6
S2_atp6_IMS
S2_atp6_MATRIX

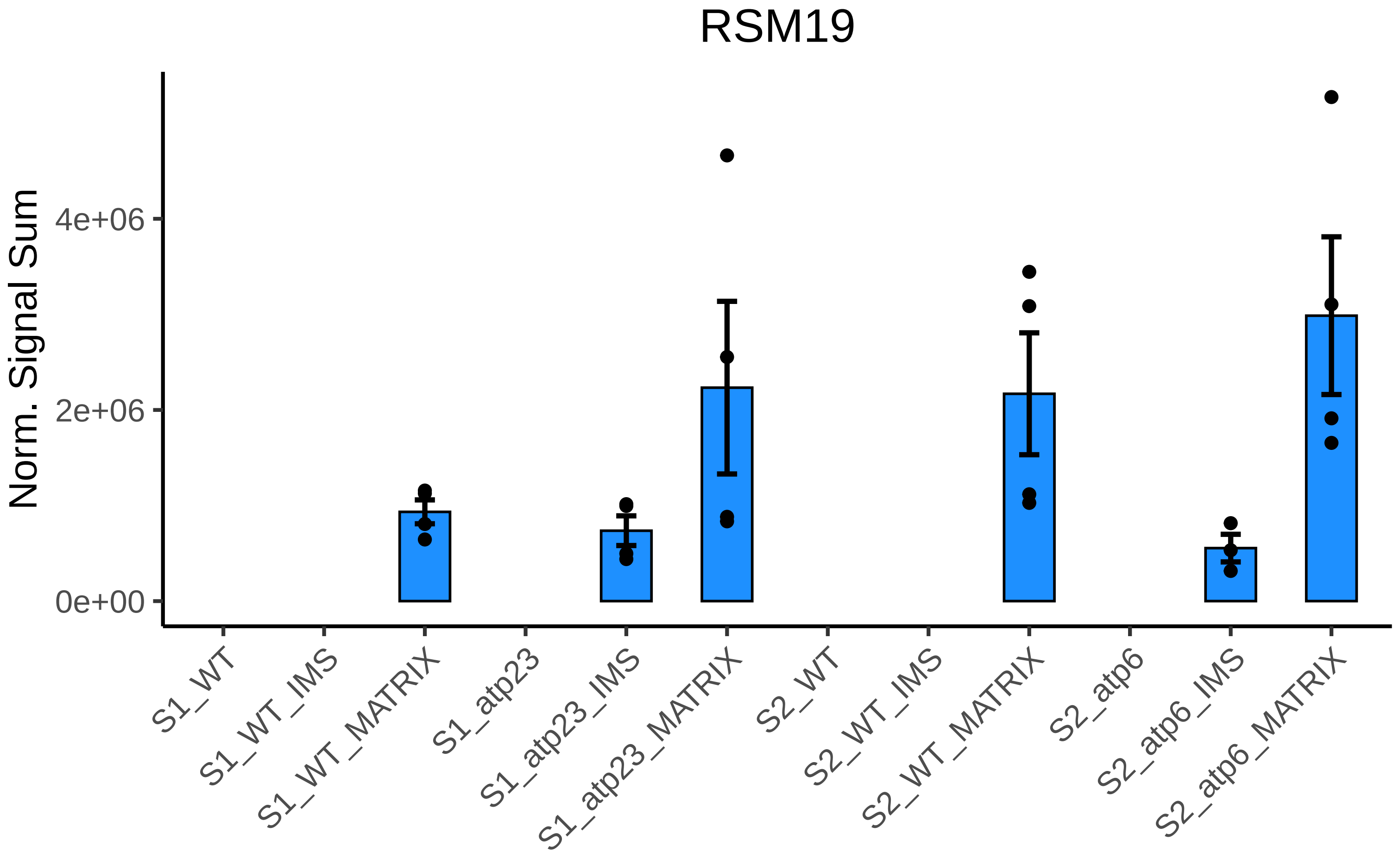

RSM19
Norm. Signal Sum
4e+06
2e+06
0e+00
S1_WT
S1_WT_IMS
S1_WT_MATRIX
S1_atp23
S1_atp23_IMS
S1_atp23_MATRIX
S2_WT
S2_WT_IMS
S2_WT_MATRIX
S2_atp6
S2_atp6_IMS
S2_atp6_MATRIX

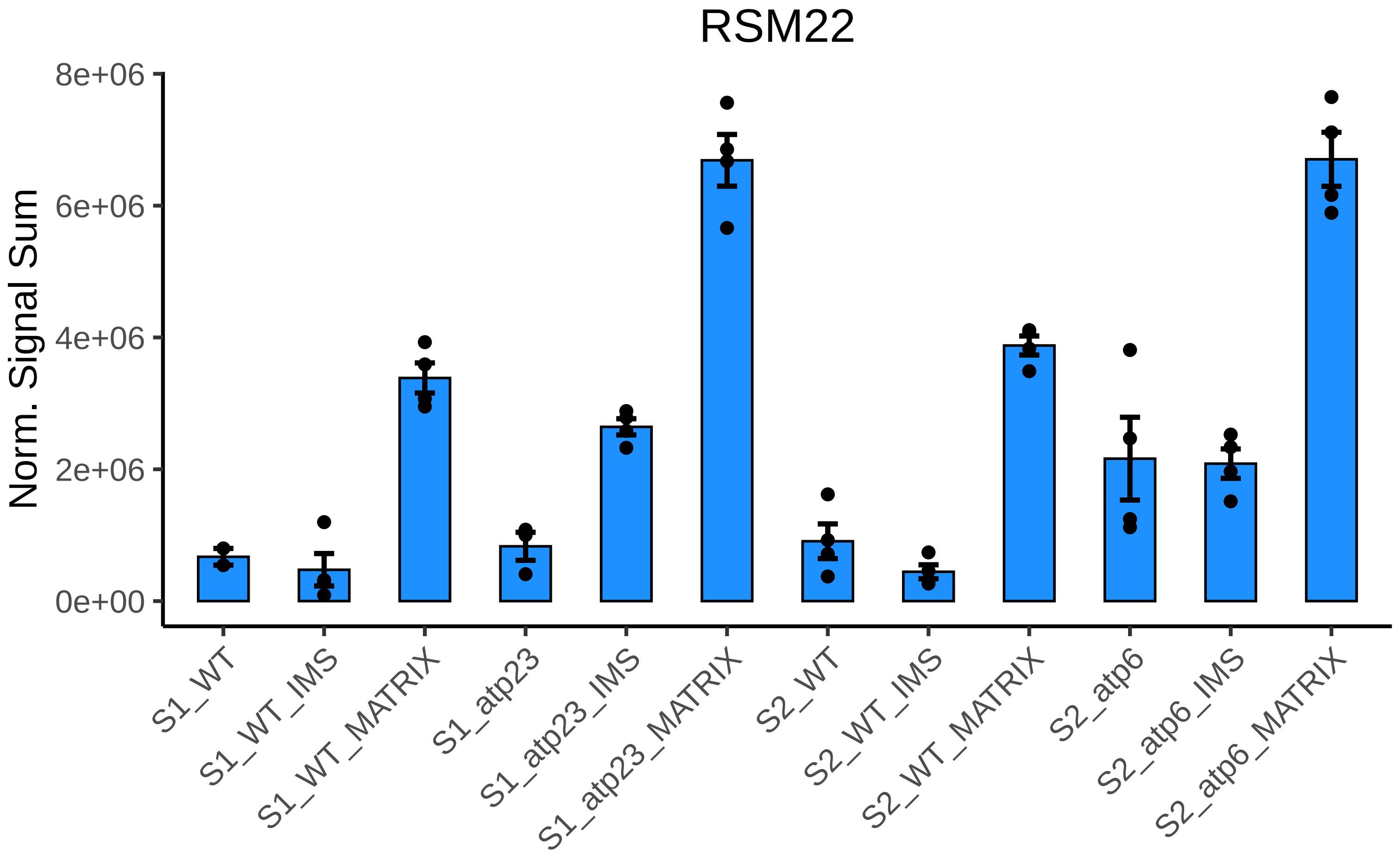
RSM22
Norm. Signal Sum
8e+06
6e+06
4e+06
2e+06
0e+00
S1_WT
S1_WT_IMS
S1_WT_MATRIX
S1_atp23
S1_atp23_IMS
S1_atp23_MATRIX
S2_WT
S2_WT_IMS
S2_WT_MATRIX
S2_atp6
S2_atp6_IMS
S2_atp6_MATRIX

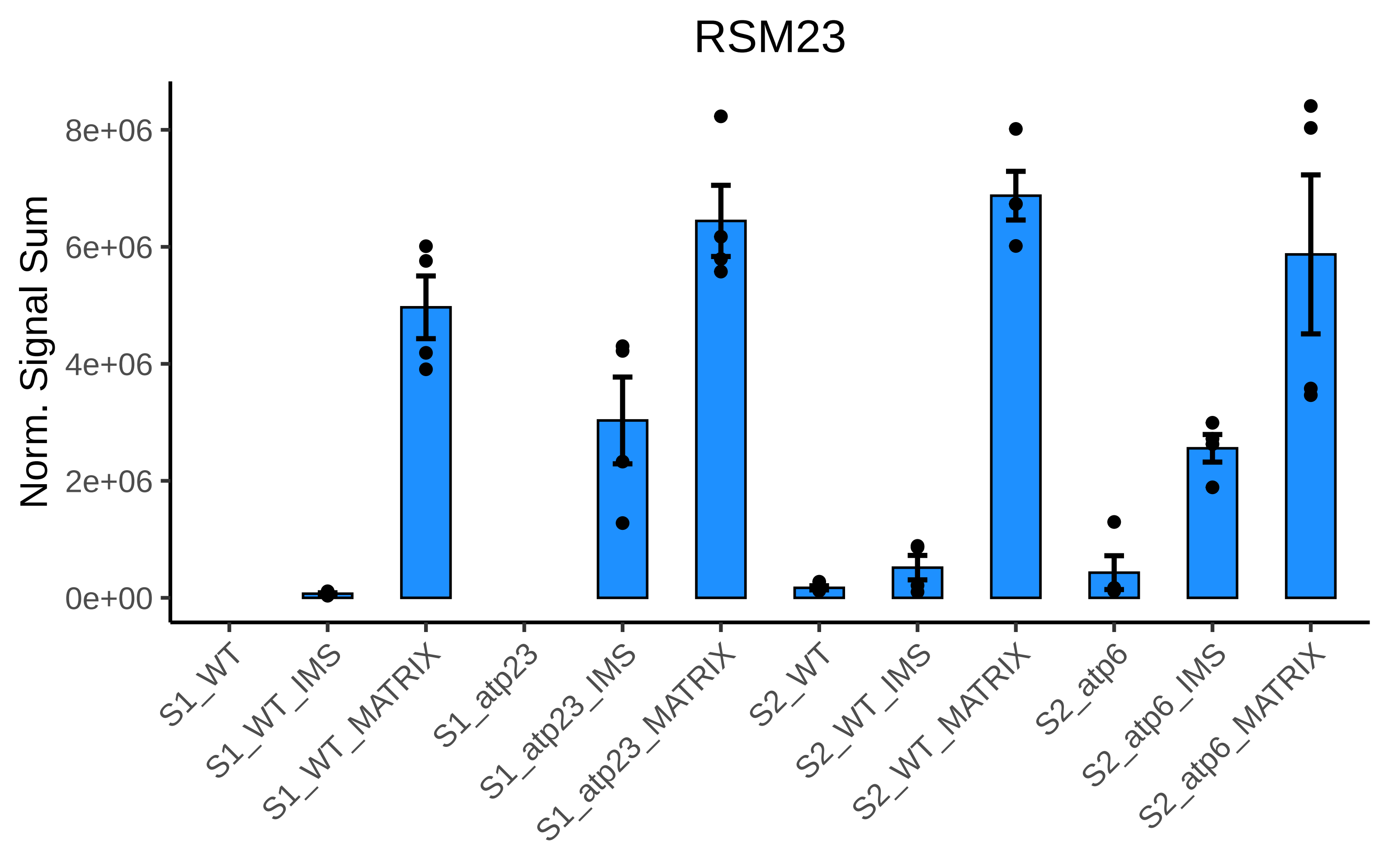
RSM23
Norm. Signal Sum
0e+00
2e+06
4e+06
6e+06
8e+06
S1_WT
S1_WT_IMS
S1_WT_MATRIX
S1_atp23
S1_atp23_IMS
S1_atp23_MATRIX
S2_WT
S2_WT_IMS
S2_WT_MATRIX
S2_atp6
S2_atp6_IMS
S2_atp6_MATRIX

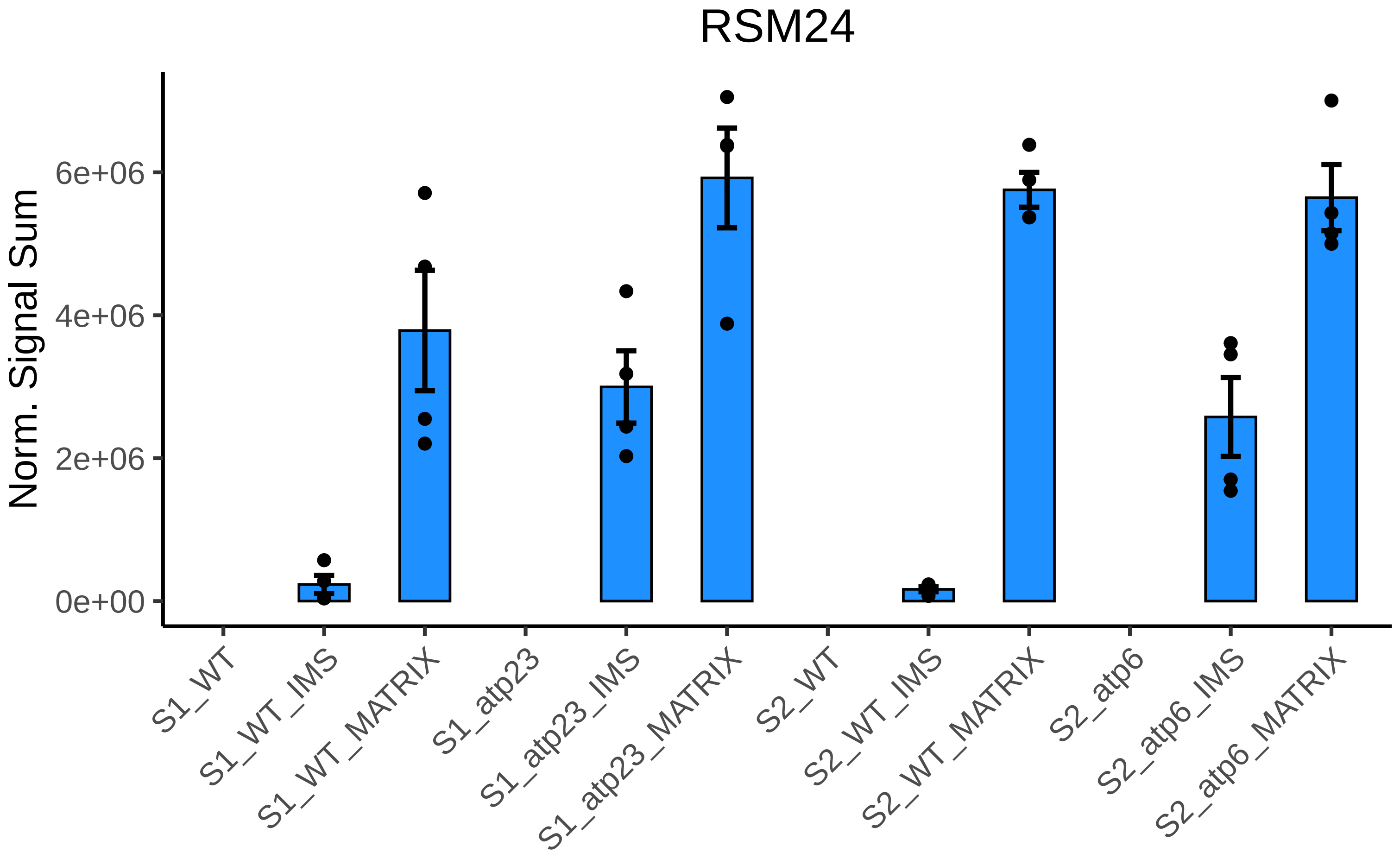
RSM24
Norm. Signal Sum
6e+06
4e+06
2e+06
0e+00
S1_WT
S1_WT_IMS
S1_WT_MATRIX
S1_atp23
S1_atp23_IMS
S1_atp23_MATRIX
S2_WT
S2_WT_IMS
S2_WT_MATRIX
S2_atp6
S2_atp6_IMS
S2_atp6_MATRIX

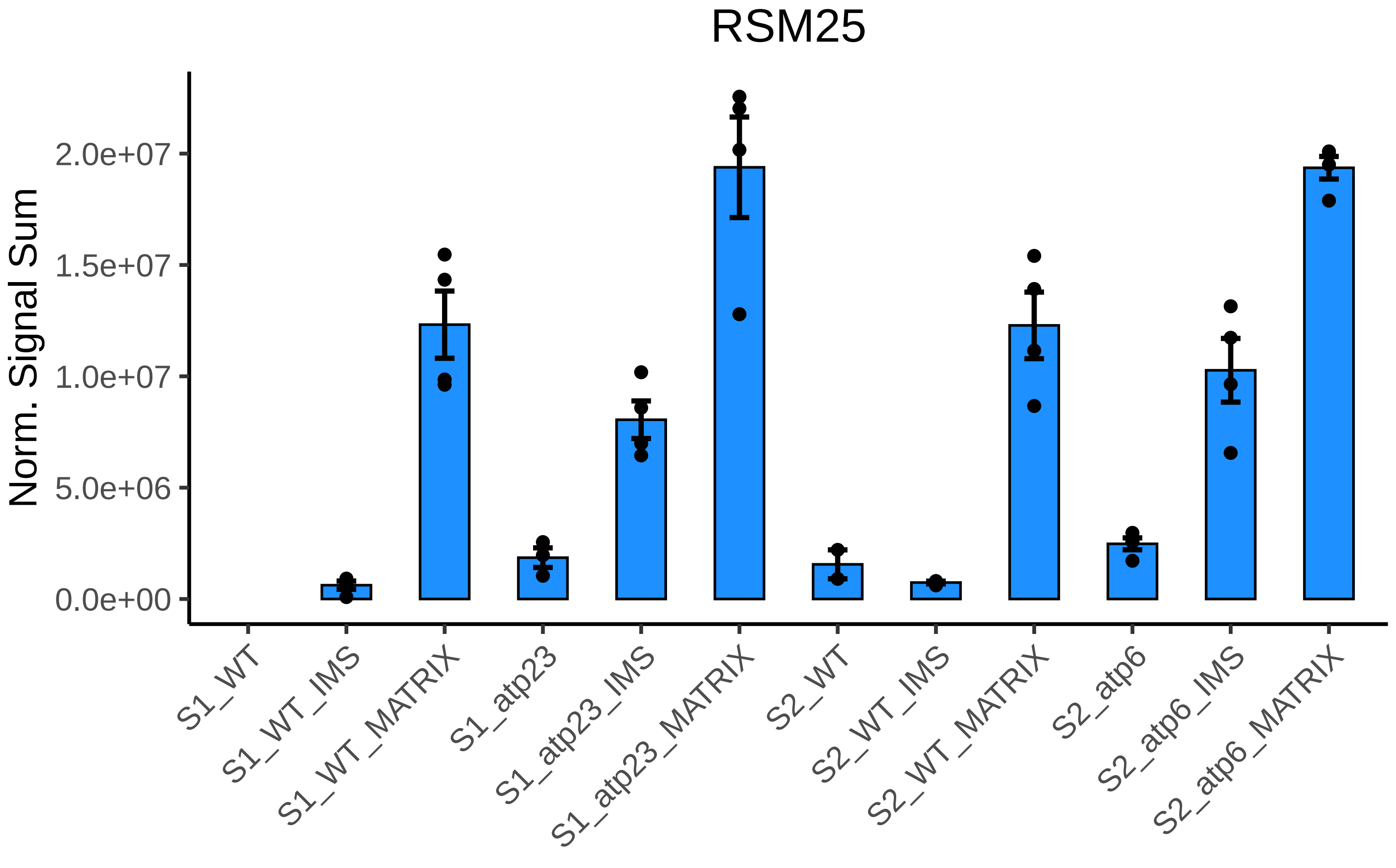
RSM25
Norm. Signal Sum
0.0e+00
5.0e+06
1.0e+07
1.5e+07
2.0e+07
S1_WT
S1_WT_IMS
S1_WT_MATRIX
S1_atp23
S1_atp23_IMS
S1_atp23_MATRIX
S2_WT
S2_WT_IMS
S2_WT_MATRIX
S2_atp6
S2_atp6_IMS
S2_atp6_MATRIX

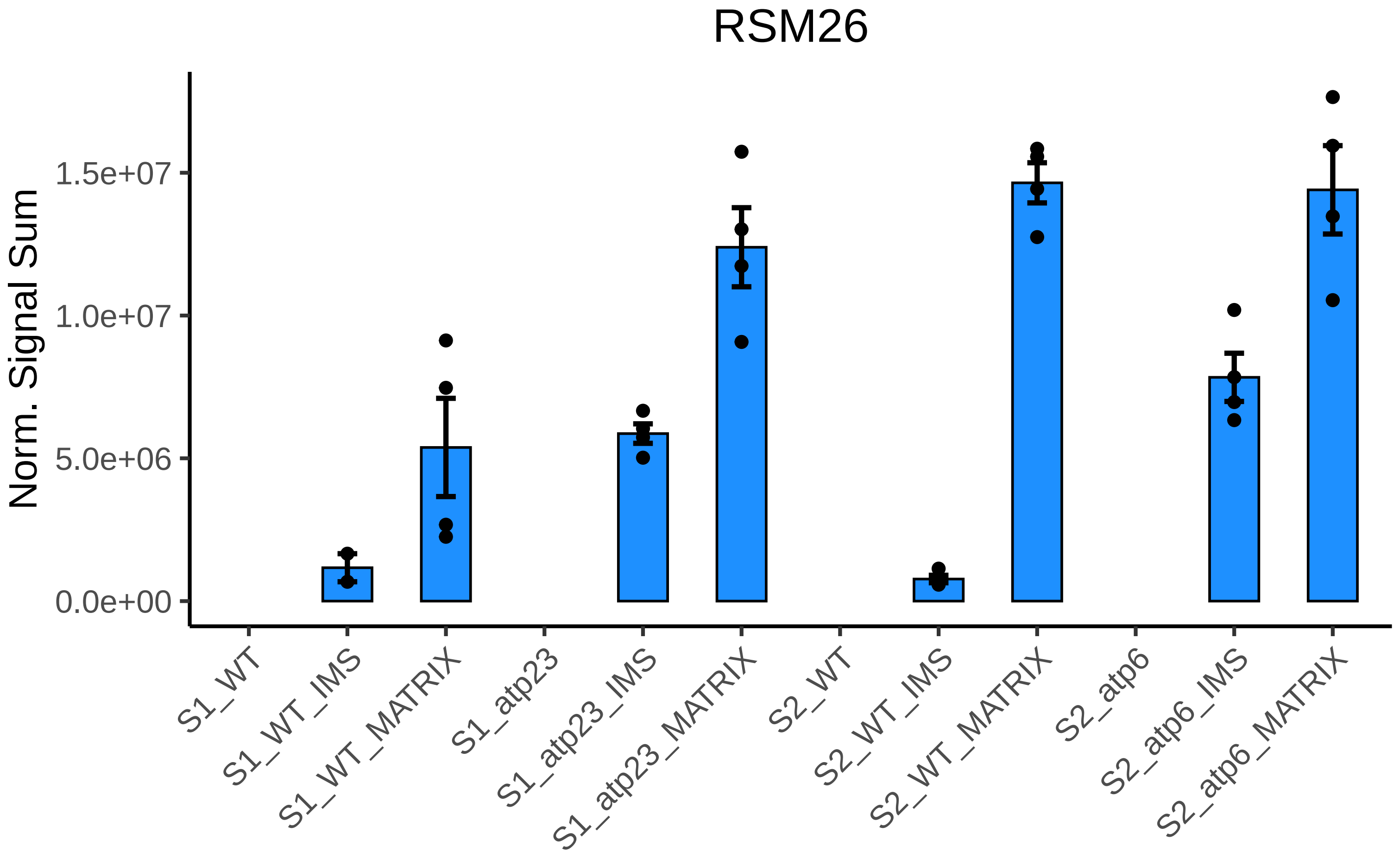

RSM26
Norm. Signal Sum
1.5e+07
1.0e+07
5.0e+06
0.0e+00
S1_WT
S1_WT_IMS
S1_WT_MATRIX
S1_atp23
S1_atp23_IMS
S1_atp23_MATRIX
S2_WT
S2_WT_IMS
S2_WT_MATRIX
S2_atp6
S2_atp6_IMS
S2_atp6_MATRIX

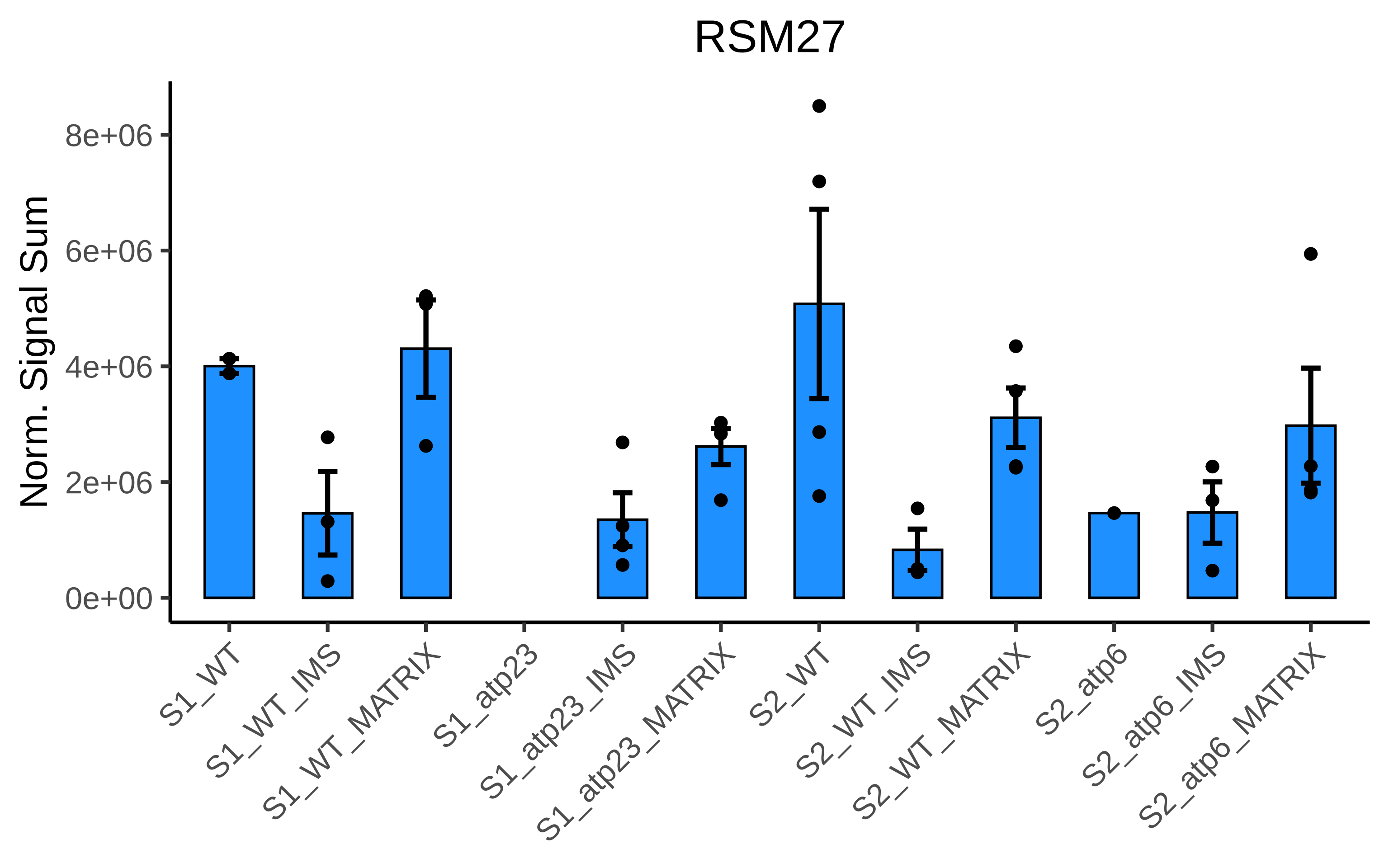
RSM27
Norm. Signal Sum
8e+06
6e+06
4e+06
2e+06
0e+00
S1_WT
S1_WT_IMS
S1_WT_MATRIX
S1_atp23
S1_atp23_IMS
S1_atp23_MATRIX
S2_WT
S2_WT_IMS
S2_WT_MATRIX
S2_atp6
S2_atp6_IMS
S2_atp6_MATRIX

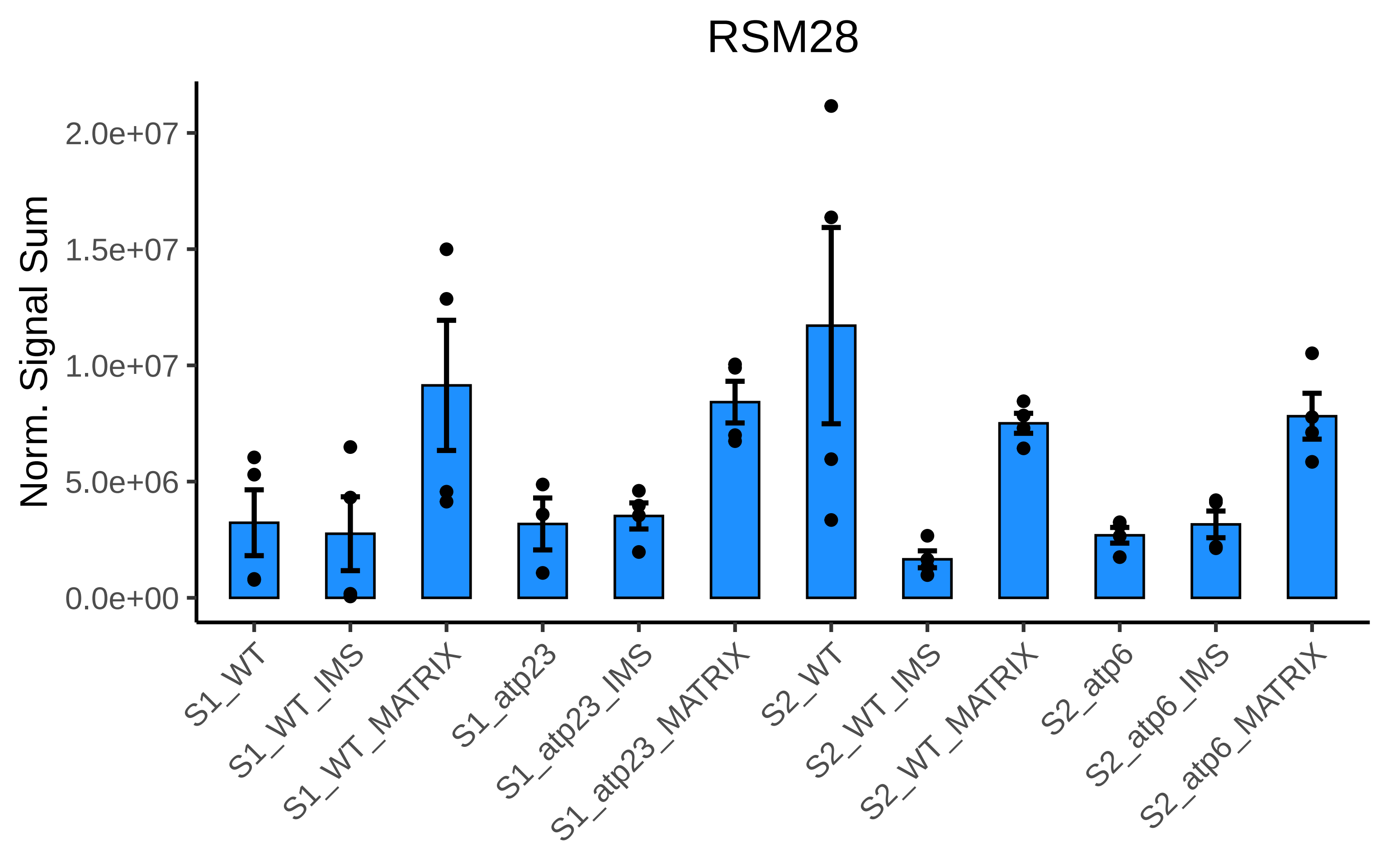

RSM28
Norm. Signal Sum
2.0e+07
1.5e+07
1.0e+07
5.0e+06
0.0e+00
S1_WT
S1_WT_IMS
S1_WT_MATRIX
S1_atp23
S1_atp23_IMS
S1_atp23_MATRIX
S2_WT
S2_WT_IMS
S2_WT_MATRIX
S2_atp6
S2_atp6_IMS
S2_atp6_MATRIX

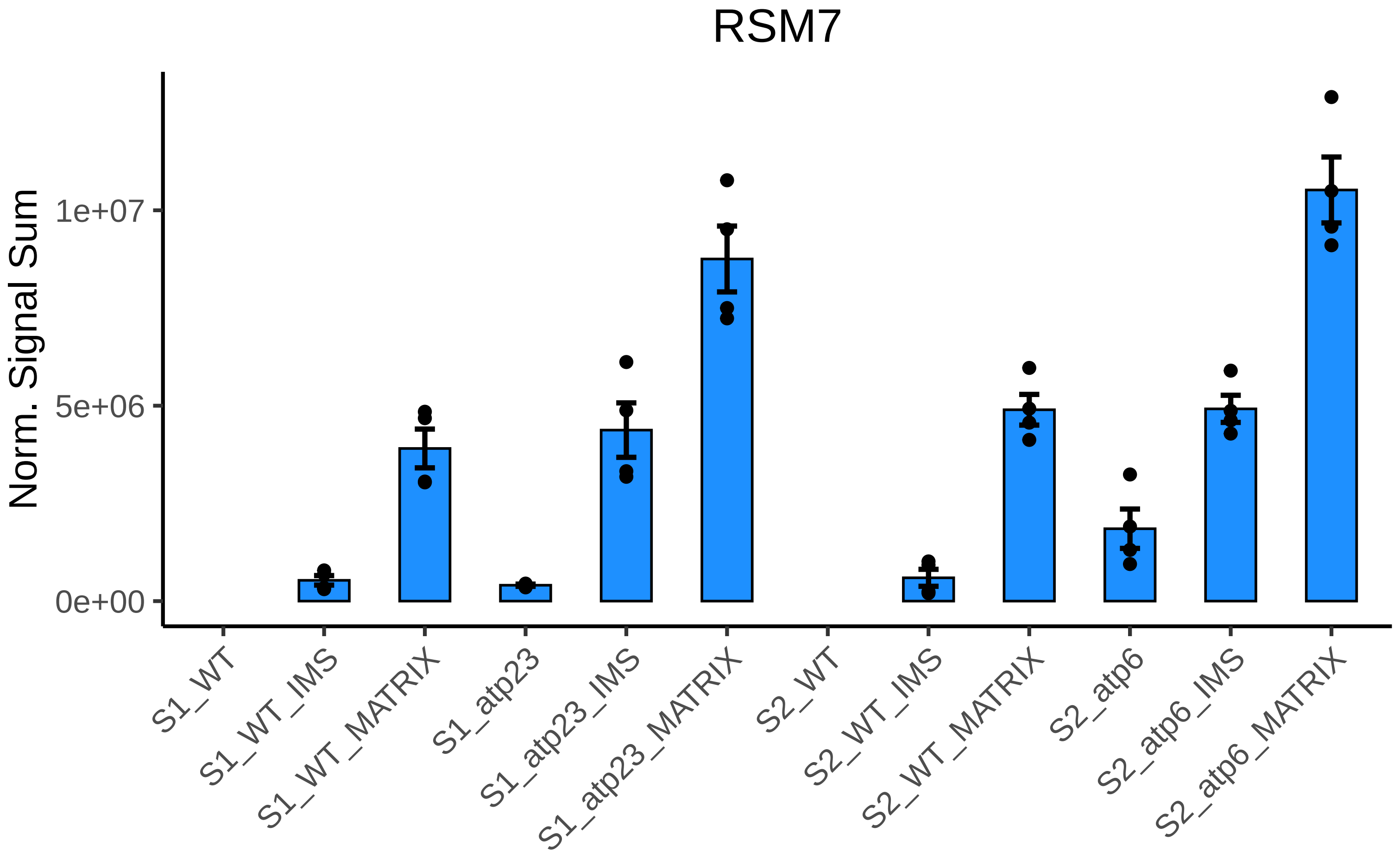
RSM7
Norm. Signal Sum
1e+07
5e+06
0e+00
S1_WT
S1_WT_IMS
S1_WT_MATRIX
S1_atp23
S1_atp23_IMS
S1_atp23_MATRIX
S2_WT
S2_WT_IMS
S2_WT_MATRIX
S2_atp6
S2_atp6_IMS
S2_atp6_MATRIX

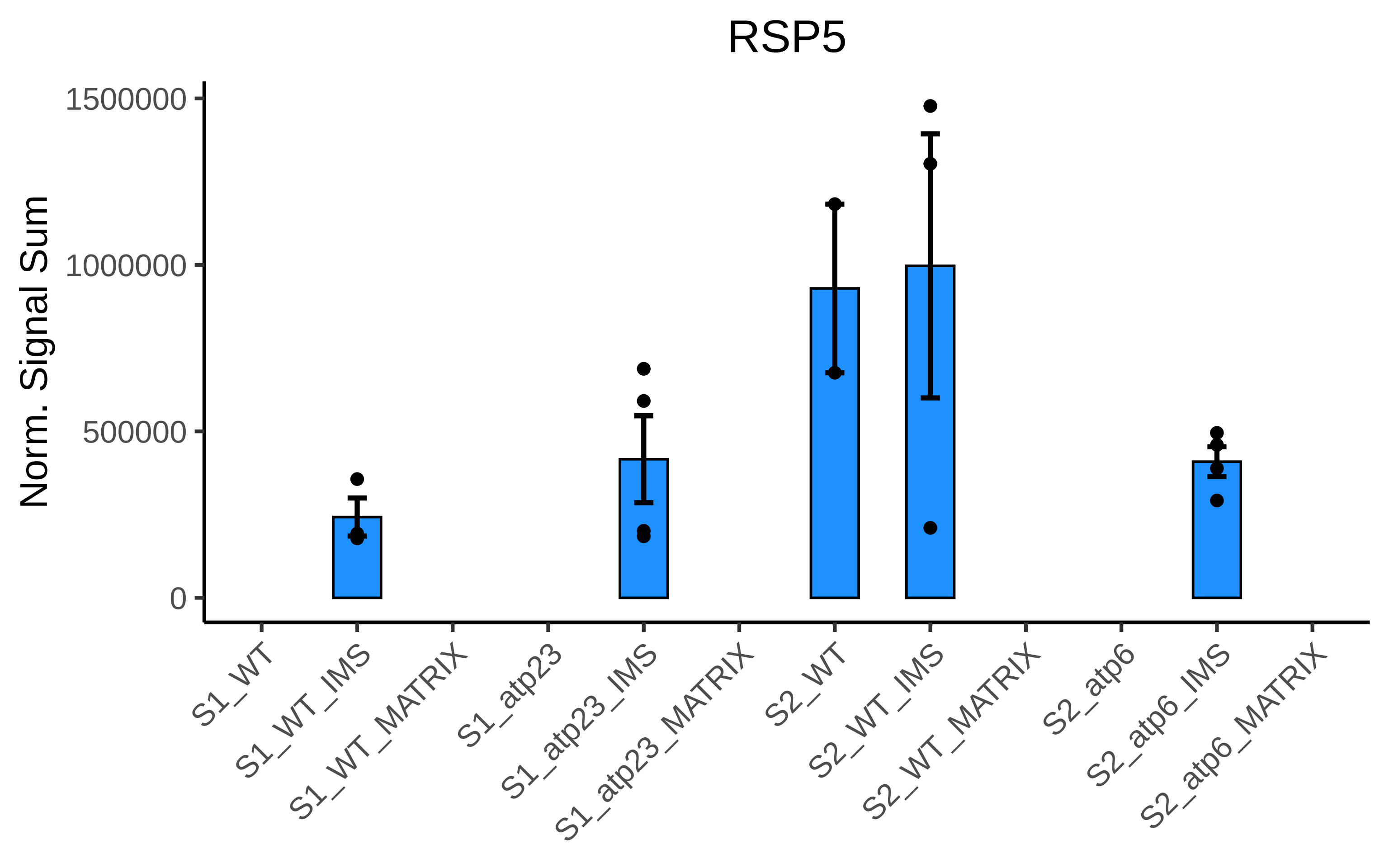
RSP5
Norm. Signal Sum
1500000
1000000
500000
0
S1_WT
S1_WT_IMS
S1_WT_MATRIX
S1_atp23
S1_atp23_IMS
S1_atp23_MATRIX
S2_WT
S2_WT_IMS
S2_WT_MATRIX
S2_atp6
S2_atp6_IMS
S2_atp6_MATRIX

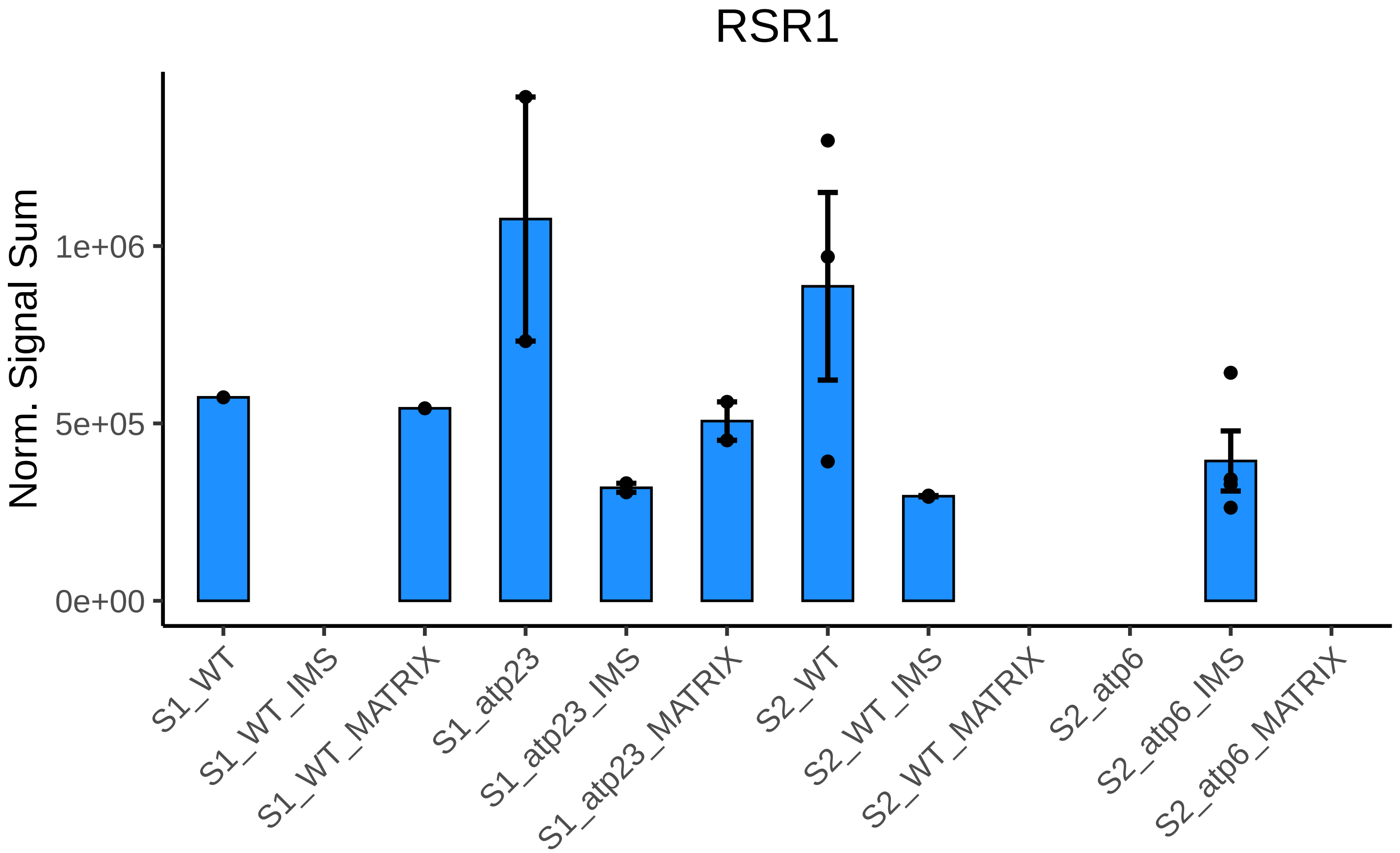
RSR1
Norm. Signal Sum
1e+06
5e+05
0e+00
S1_WT
S1_WT_IMS
S1_WT_MATRIX
S1_atp23
S1_atp23_IMS
S1_atp23_MATRIX
S2_WT
S2_WT_IMS
S2_WT_MATRIX
S2_atp6
S2_atp6_IMS
S2_atp6_MATRIX

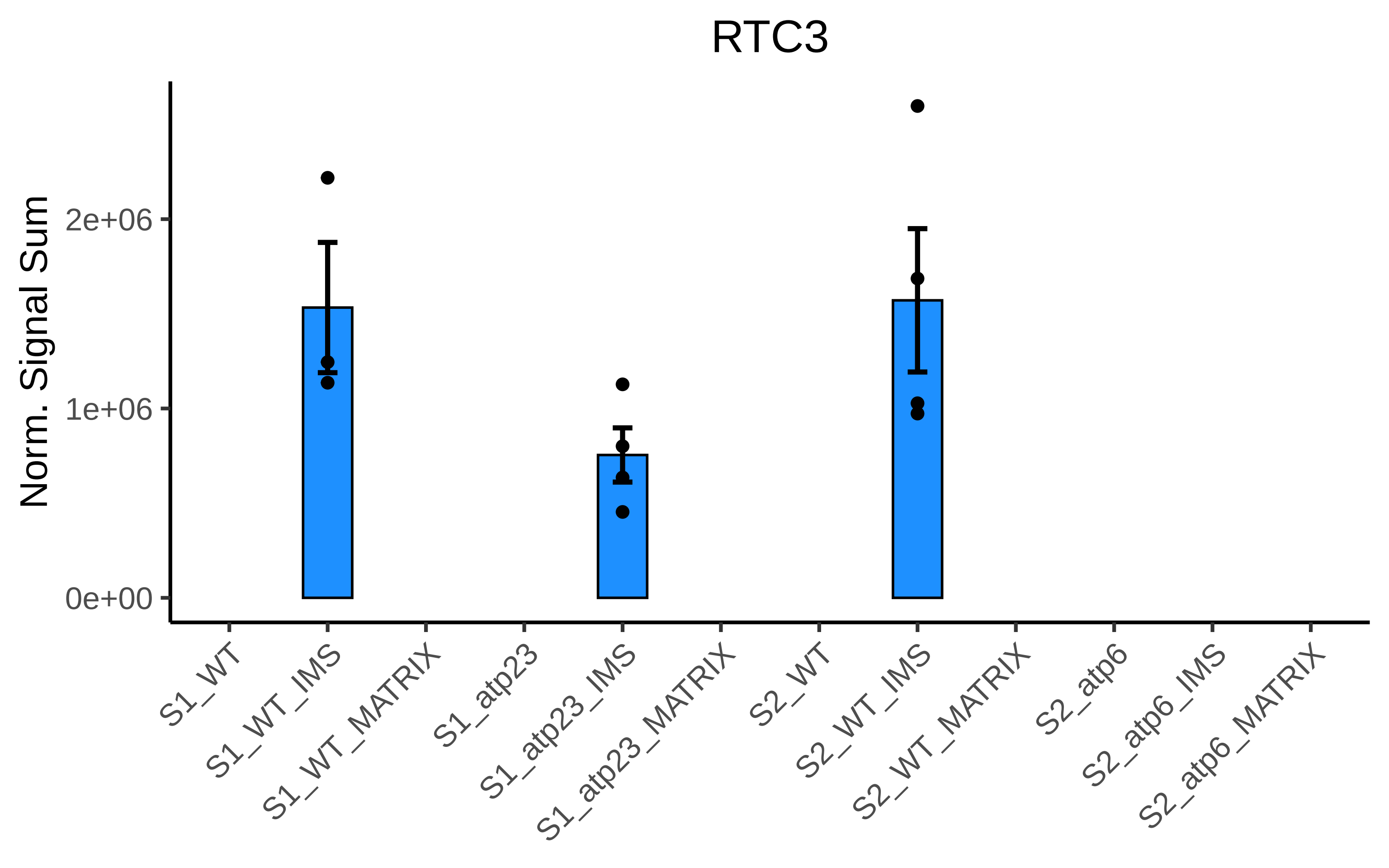
RTC3
Norm. Signal Sum
2e+06
1e+06
0e+00
S1_WT
S1_WT_IMS
S1_WT_MATRIX
S1_atp23
S1_atp23_IMS
S1_atp23_MATRIX
S2_WT
S2_WT_IMS
S2_WT_MATRIX
S2_atp6
S2_atp6_IMS
S2_atp6_MATRIX

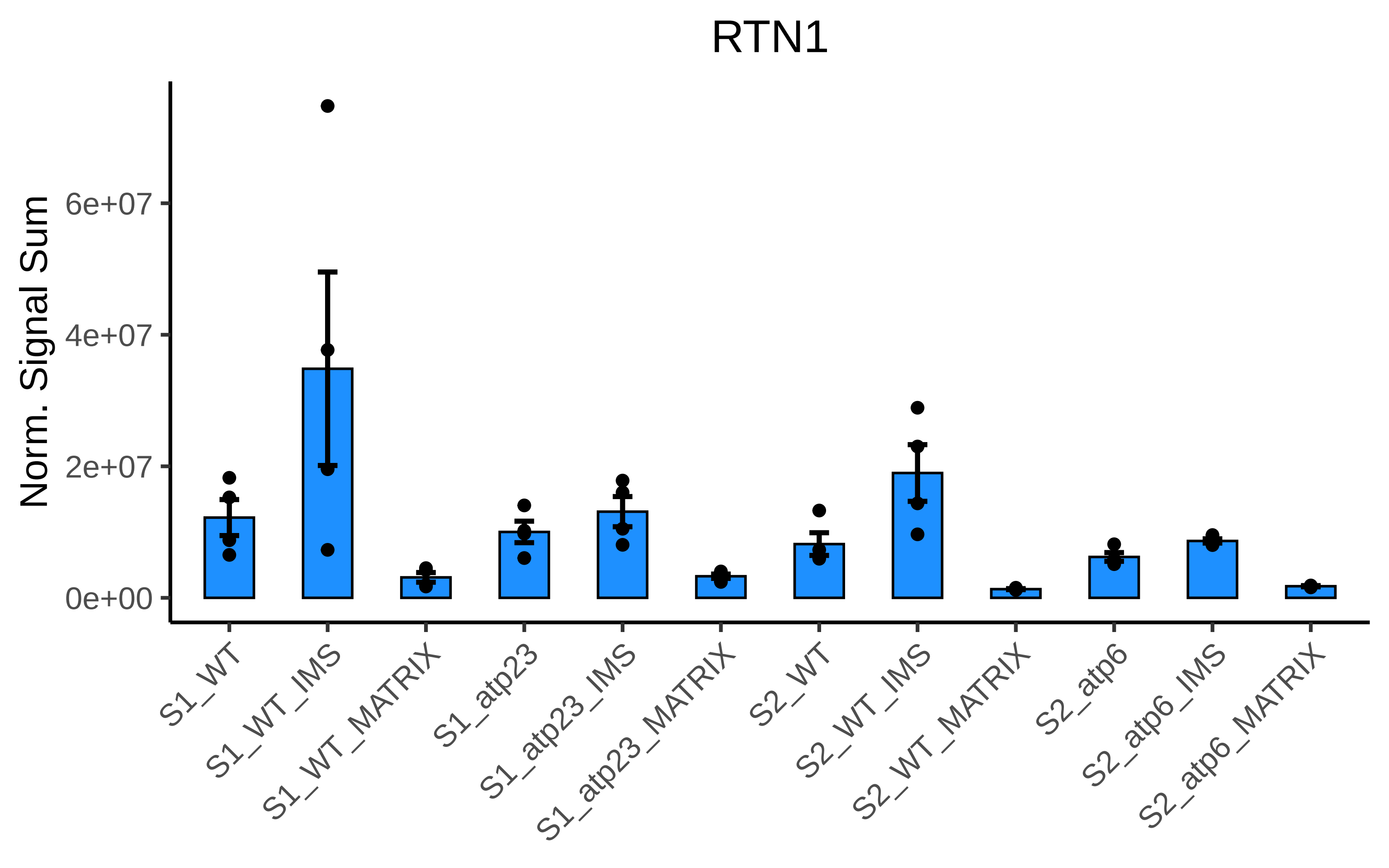
RTN1
Norm. Signal Sum
6e+07
4e+07
2e+07
0e+00
S1_WT
S1_WT_IMS
S1_WT_MATRIX
S1_atp23
S1_atp23_IMS
S1_atp23_MATRIX
S2_WT
S2_WT_IMS
S2_WT_MATRIX
S2_atp6
S2_atp6_IMS
S2_atp6_MATRIX

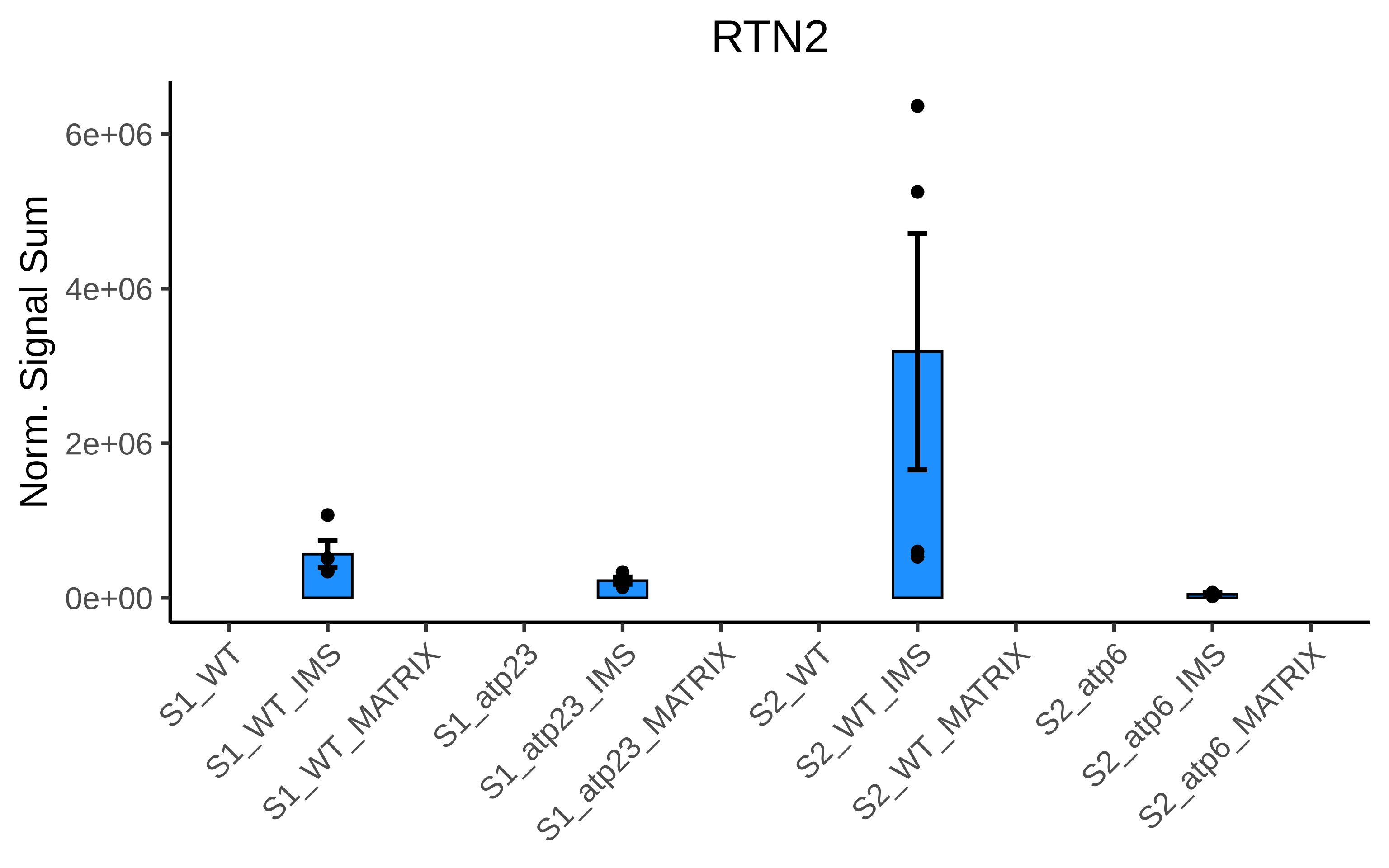

RTN2
Norm. Signal Sum
6e+06
4e+06
2e+06
0e+00
S1_WT
S1_WT_IMS
S1_WT_MATRIX
S1_atp23
S1_atp23_IMS
S1_atp23_MATRIX
S2_WT
S2_WT_IMS
S2_WT_MATRIX
S2_atp6
S2_atp6_IMS
S2_atp6_MATRIX

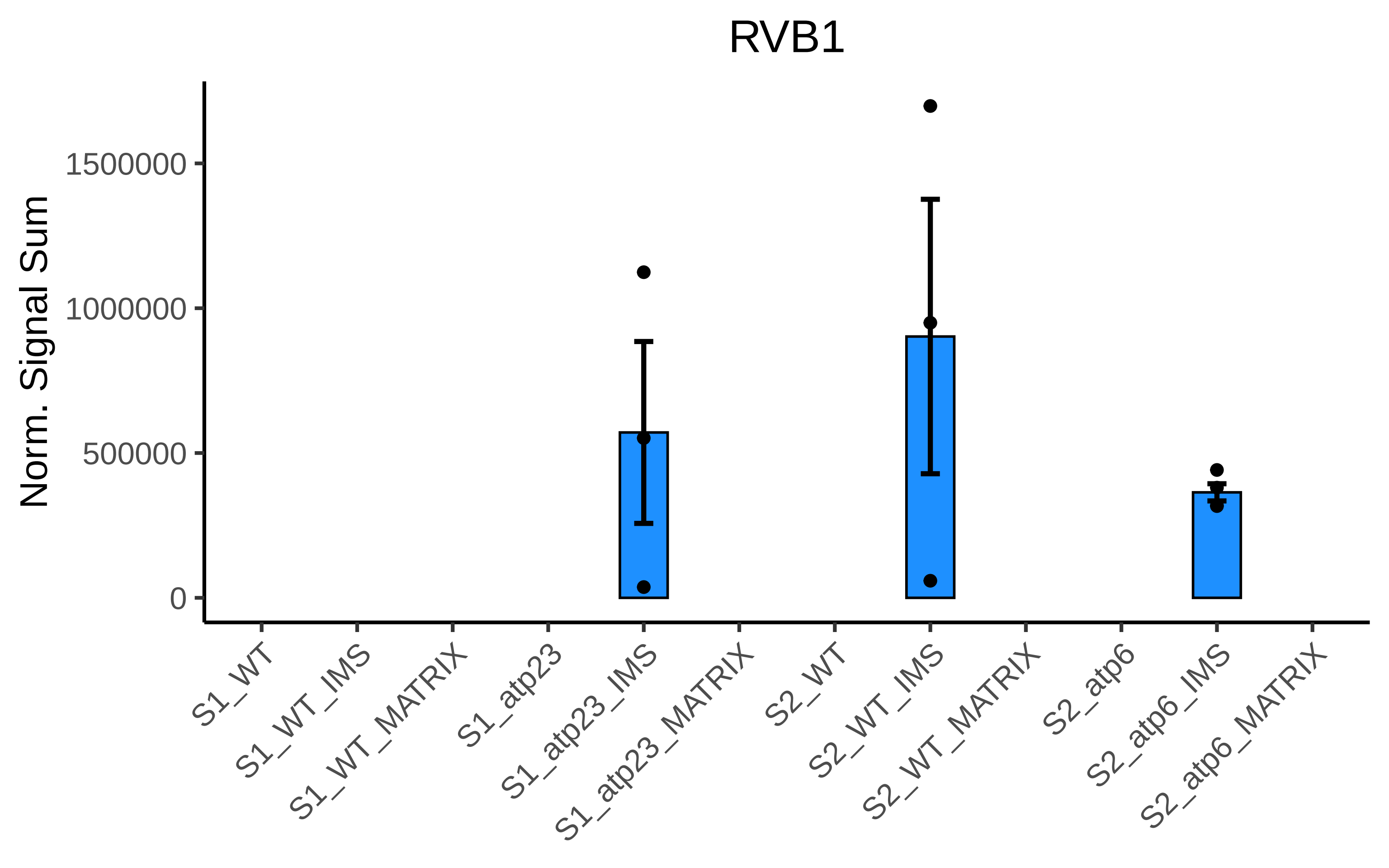
RVB1
Norm. Signal Sum
1500000
1000000
500000
0
S1_WT
S1_WT_IMS
S1_WT_MATRIX
S1_atp23
S1_atp23_IMS
S1_atp23_MATRIX
S2_WT
S2_WT_IMS
S2_WT_MATRIX
S2_atp6
S2_atp6_IMS
S2_atp6_MATRIX

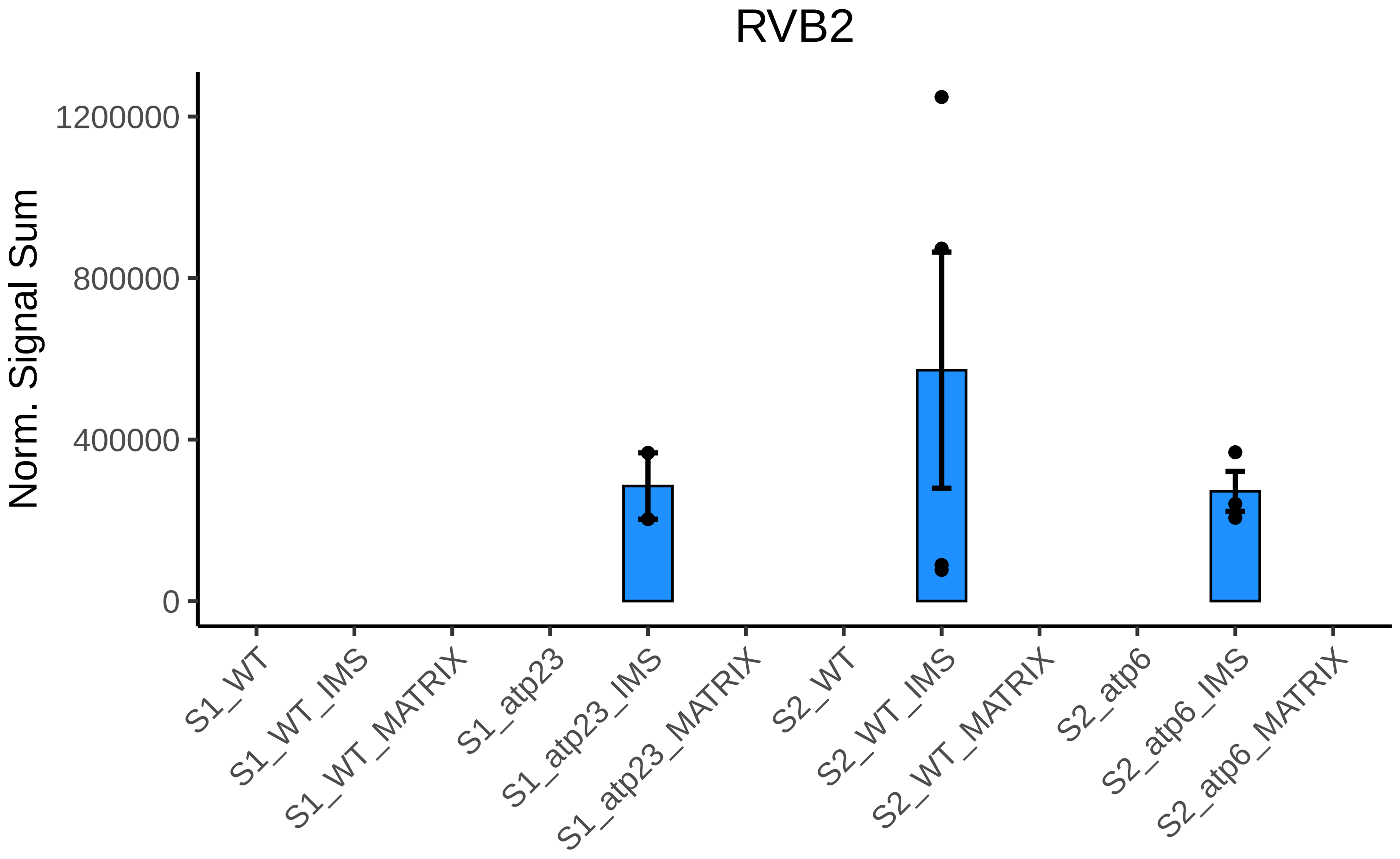

RVB2
Norm. Signal Sum
1200000
800000
400000
0
S1_WT
S1_WT_IMS
S1_WT_MATRIX
S1_atp23
S1_atp23_IMS
S1_atp23_MATRIX
S2_WT
S2_WT_IMS
S2_WT_MATRIX
S2_atp6
S2_atp6_IMS
S2_atp6_MATRIX

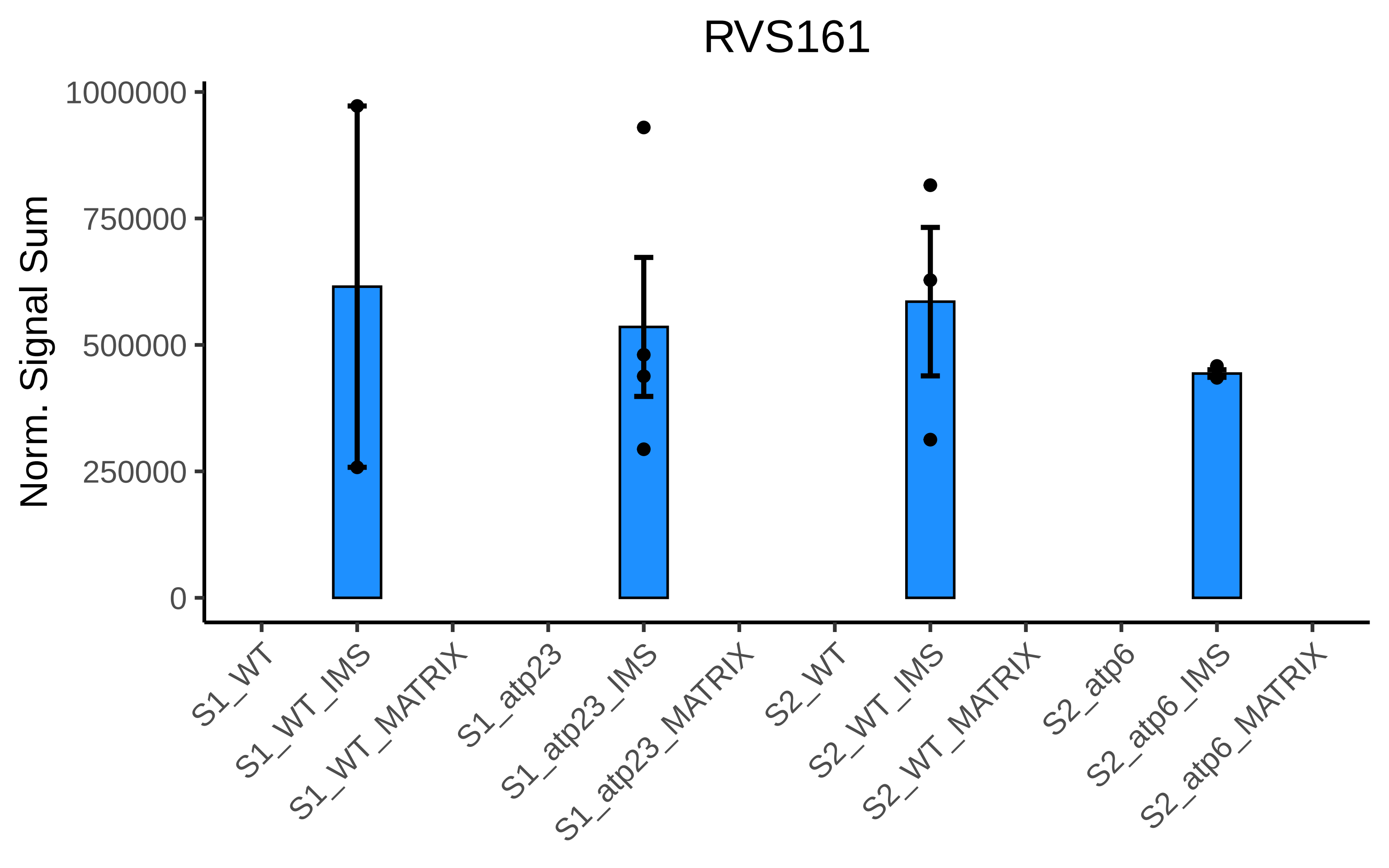

RVS161
Norm. Signal Sum
1000000
750000
500000
250000
0
S1_WT
S1_WT_IMS
S1_WT_MATRIX
S1_atp23
S1_atp23_IMS
S1_atp23_MATRIX
S2_WT
S2_WT_IMS
S2_WT_MATRIX
S2_atp6
S2_atp6_IMS
S2_atp6_MATRIX

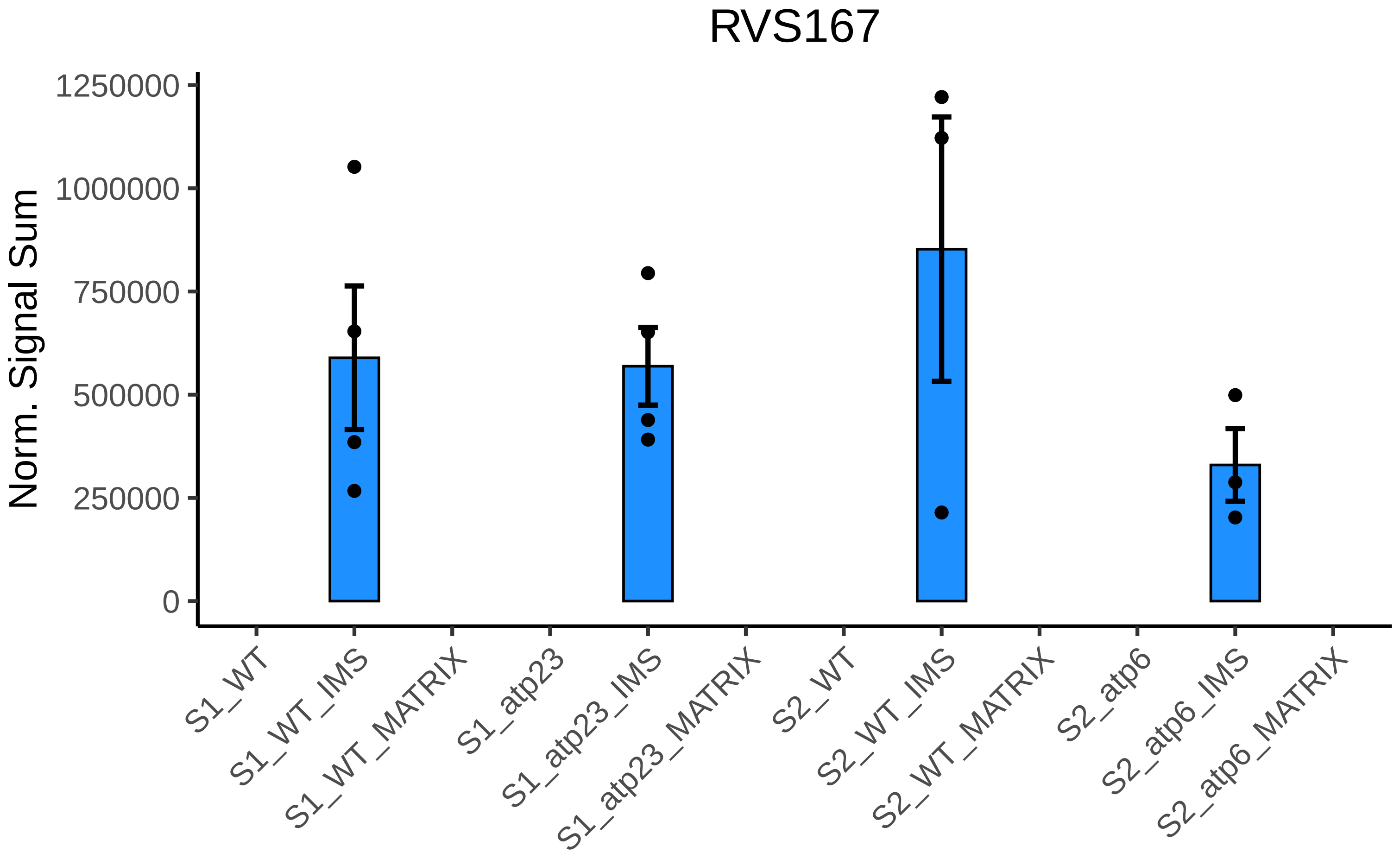

RVS167
Norm. Signal Sum
1250000
1000000
750000
500000
250000
0
S1_WT
S1_WT_IMS
S1_WT_MATRIX
S1_atp23
S1_atp23_IMS
S1_atp23_MATRIX
S2_WT
S2_WT_IMS
S2_WT_MATRIX
S2_atp6
S2_atp6_IMS
S2_atp6_MATRIX

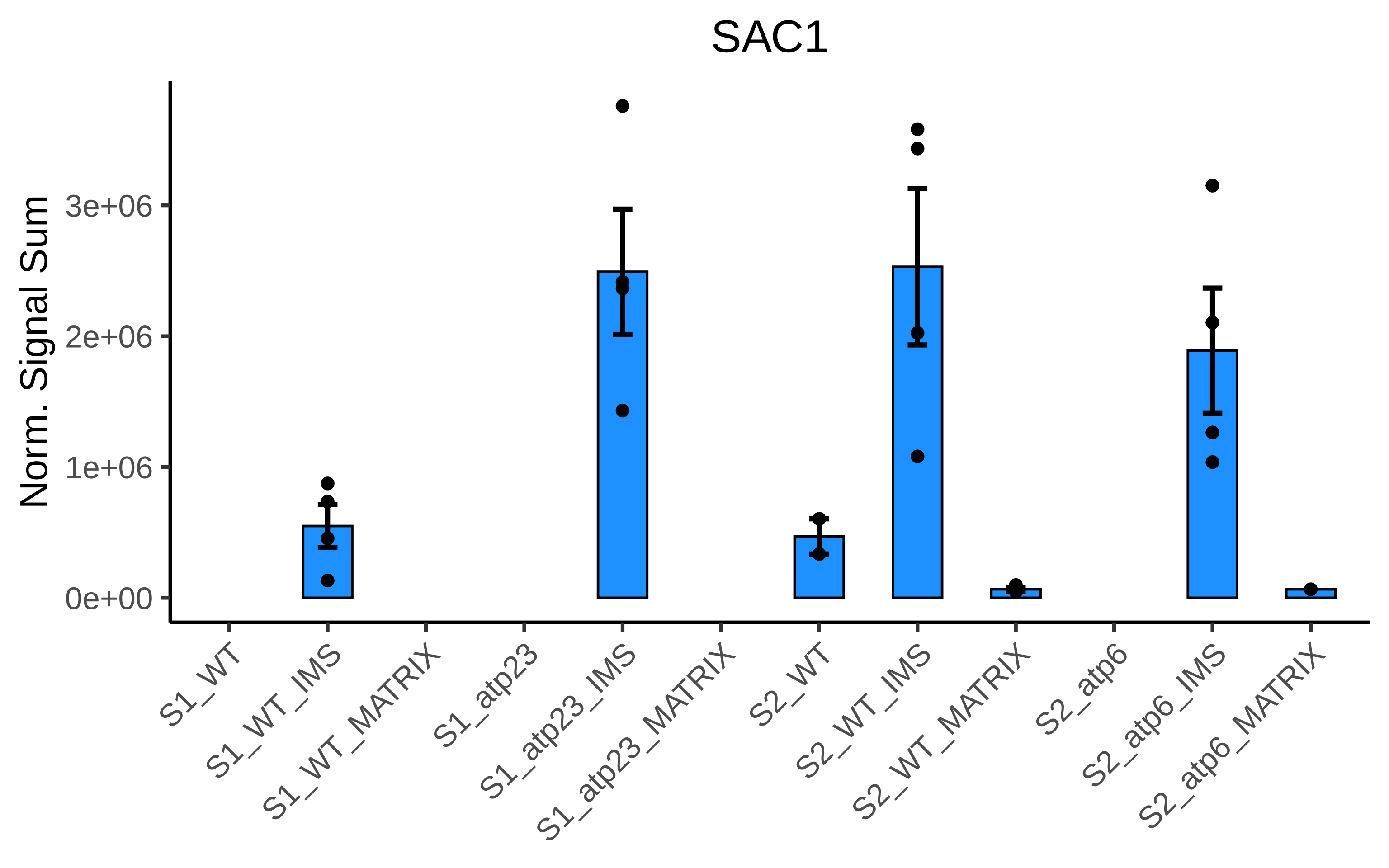
SAC1
Norm. Signal Sum
3e+06
2e+06
1e+06
0e+00
S1_WT
S1_WT_IMS
S1_WT_MATRIX
S1_atp23
S1_atp23_IMS
S1_atp23_MATRIX
S2_WT
S2_WT_IMS
S2_WT_MATRIX
S2_atp6
S2_atp6_IMS
S2_atp6_MATRIX

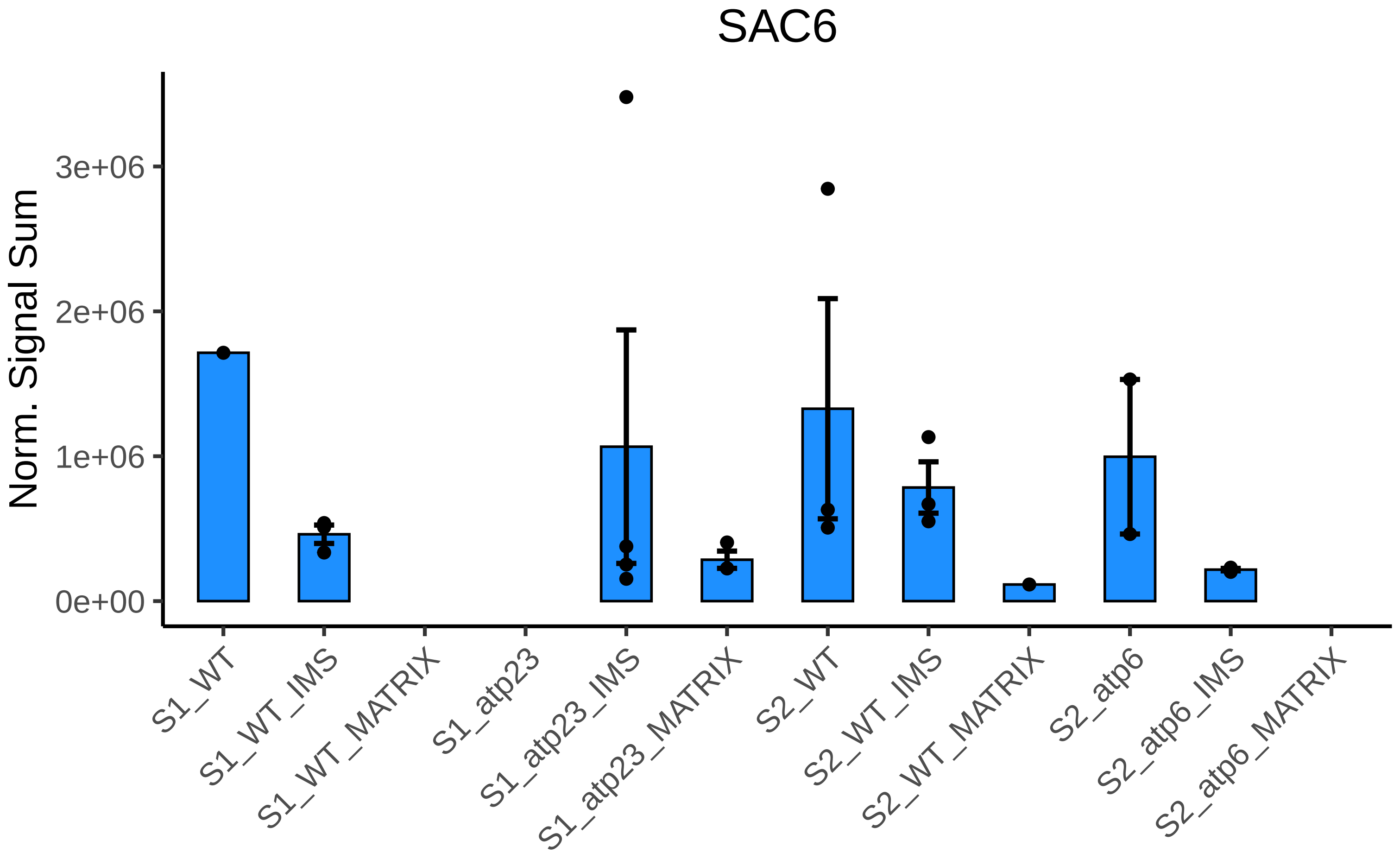

SAC6
Norm. Signal Sum
3e+06
2e+06
1e+06
0e+00
S1_WT
S1_WT_IMS
S1_WT_MATRIX
S1_atp23
S1_atp23_IMS
S1_atp23_MATRIX
S2_WT
S2_WT_IMS
S2_WT_MATRIX
S2_atp6
S2_atp6_IMS
S2_atp6_MATRIX

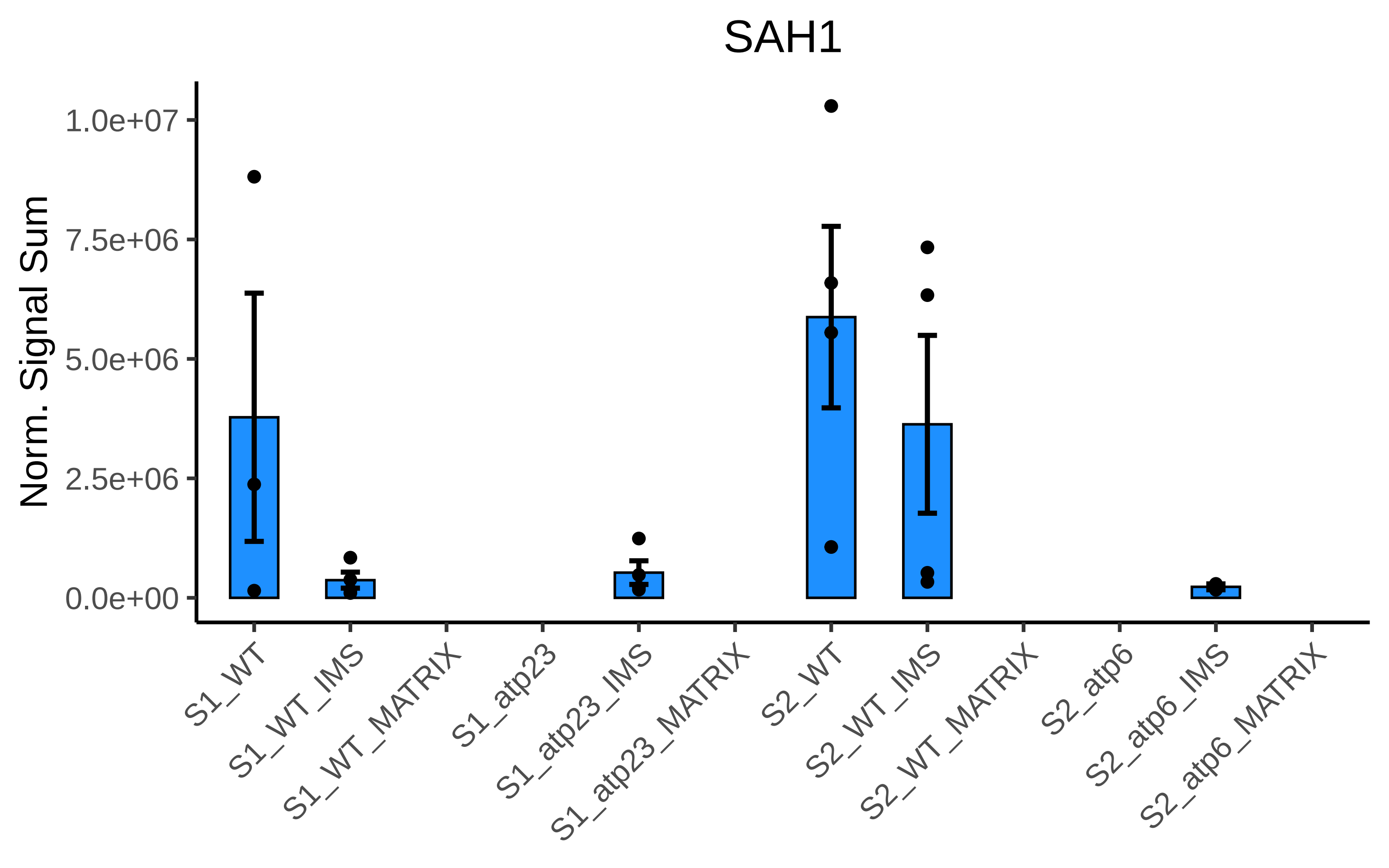
SAH1
Norm. Signal Sum
1.0e+07
7.5e+06
5.0e+06
2.5e+06
0.0e+00
S1_WT
S1_WT_IMS
S1_WT_MATRIX
S1_atp23
S1_atp23_IMS
S1_atp23_MATRIX
S2_WT
S2_WT_IMS
S2_WT_MATRIX
S2_atp6
S2_atp6_IMS
S2_atp6_MATRIX

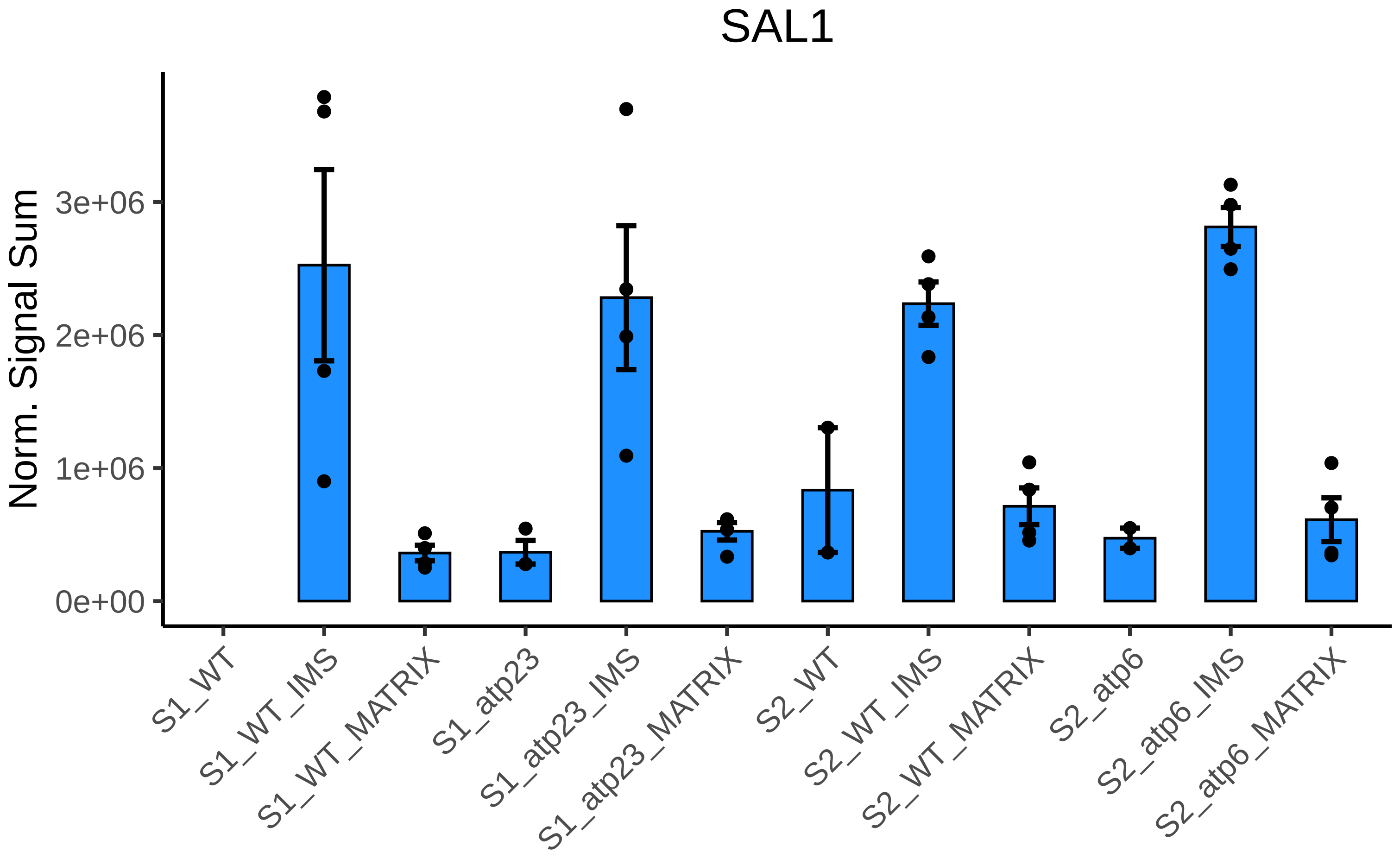
SAL1
Norm. Signal Sum
3e+06
2e+06
1e+06
0e+00
S1_WT
S1_WT_IMS
S1_WT_MATRIX
S1_atp23
S1_atp23_IMS
S1_atp23_MATRIX
S2_WT
S2_WT_IMS
S2_WT_MATRIX
S2_atp6
S2_atp6_IMS
S2_atp6_MATRIX

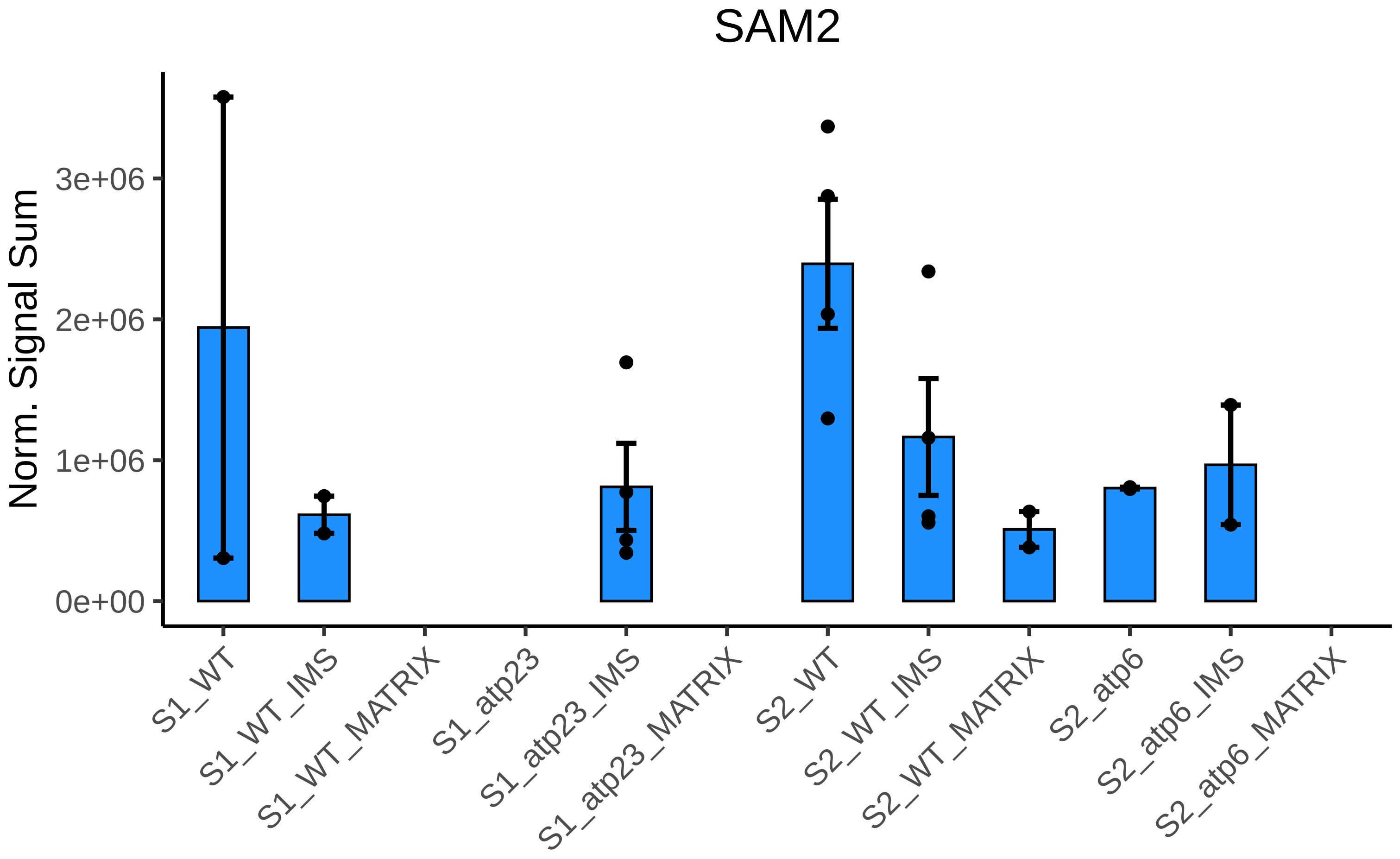
SAM2
Norm. Signal Sum
3e+06
2e+06
1e+06
0e+00
S1_WT
S1_WT_IMS
S1_WT_MATRIX
S1_atp23
S1_atp23_IMS
S1_atp23_MATRIX
S2_WT
S2_WT_IMS
S2_WT_MATRIX
S2_atp6
S2_atp6_IMS
S2_atp6_MATRIX

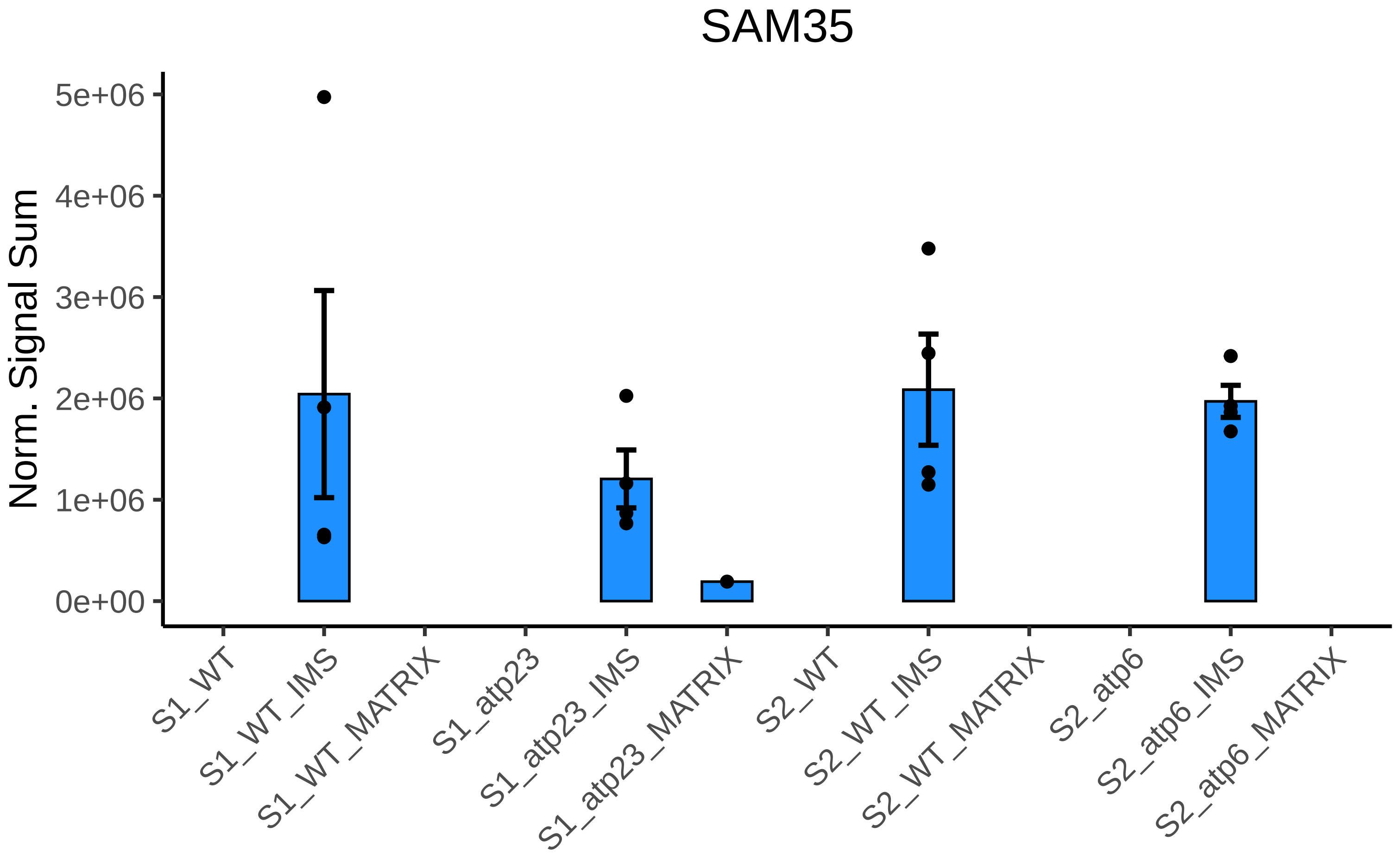
SAM35
Norm. Signal Sum
5e+06
4e+06
3e+06
2e+06
1e+06
0e+00
S1_WT
S1_WT_IMS
S1_WT_MATRIX
S1_atp23
S1_atp23_IMS
S1_atp23_MATRIX
S2_WT
S2_WT_IMS
S2_WT_MATRIX
S2_atp6
S2_atp6_IMS
S2_atp6_MATRIX

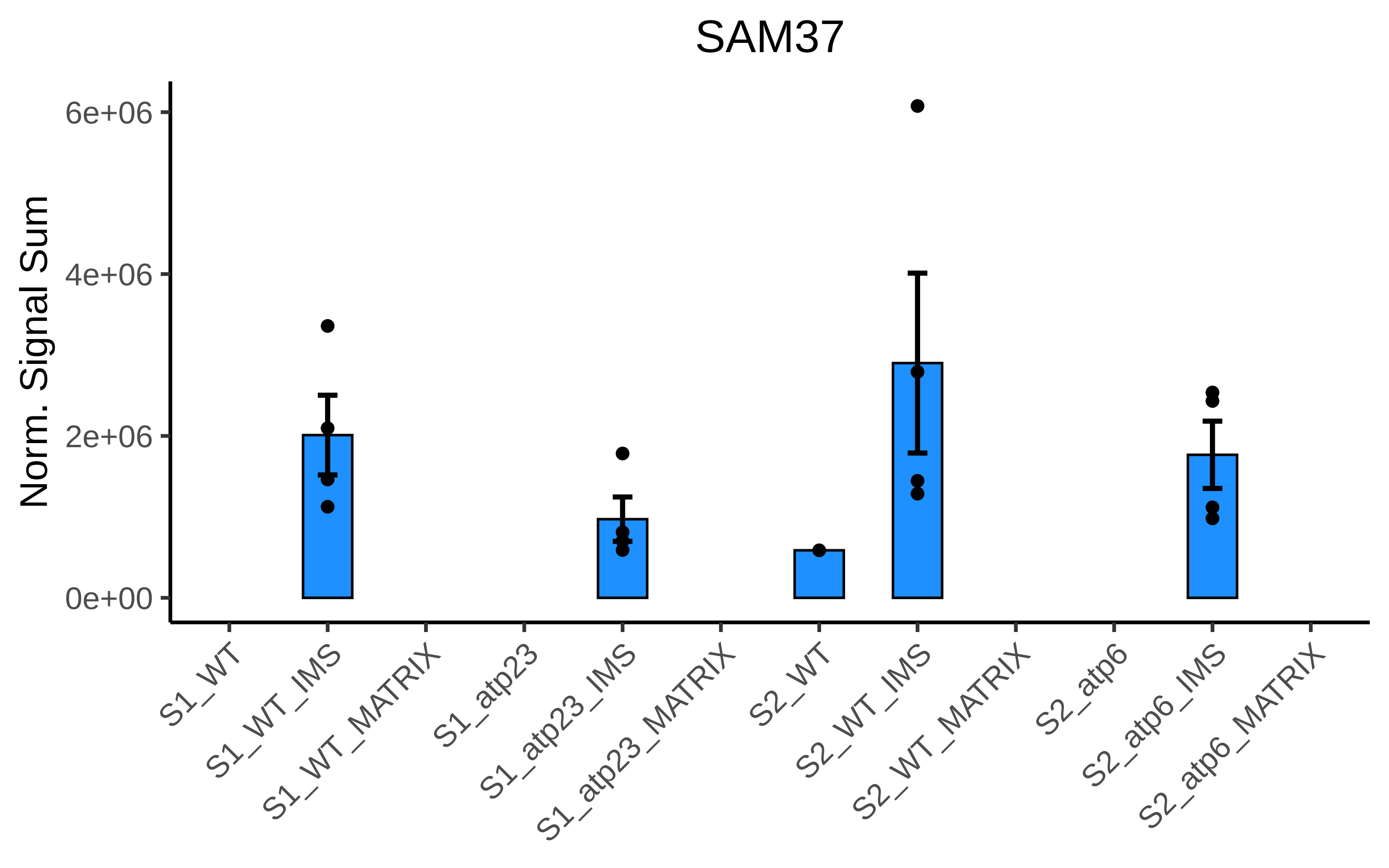

SAM37
Norm. Signal Sum
6e+06
4e+06
2e+06
0e+00
S1_WT
S1_WT_IMS
S1_WT_MATRIX
S1_atp23
S1_atp23_IMS
S1_atp23_MATRIX
S2_WT
S2_WT_IMS
S2_WT_MATRIX
S2_atp6
S2_atp6_IMS
S2_atp6_MATRIX

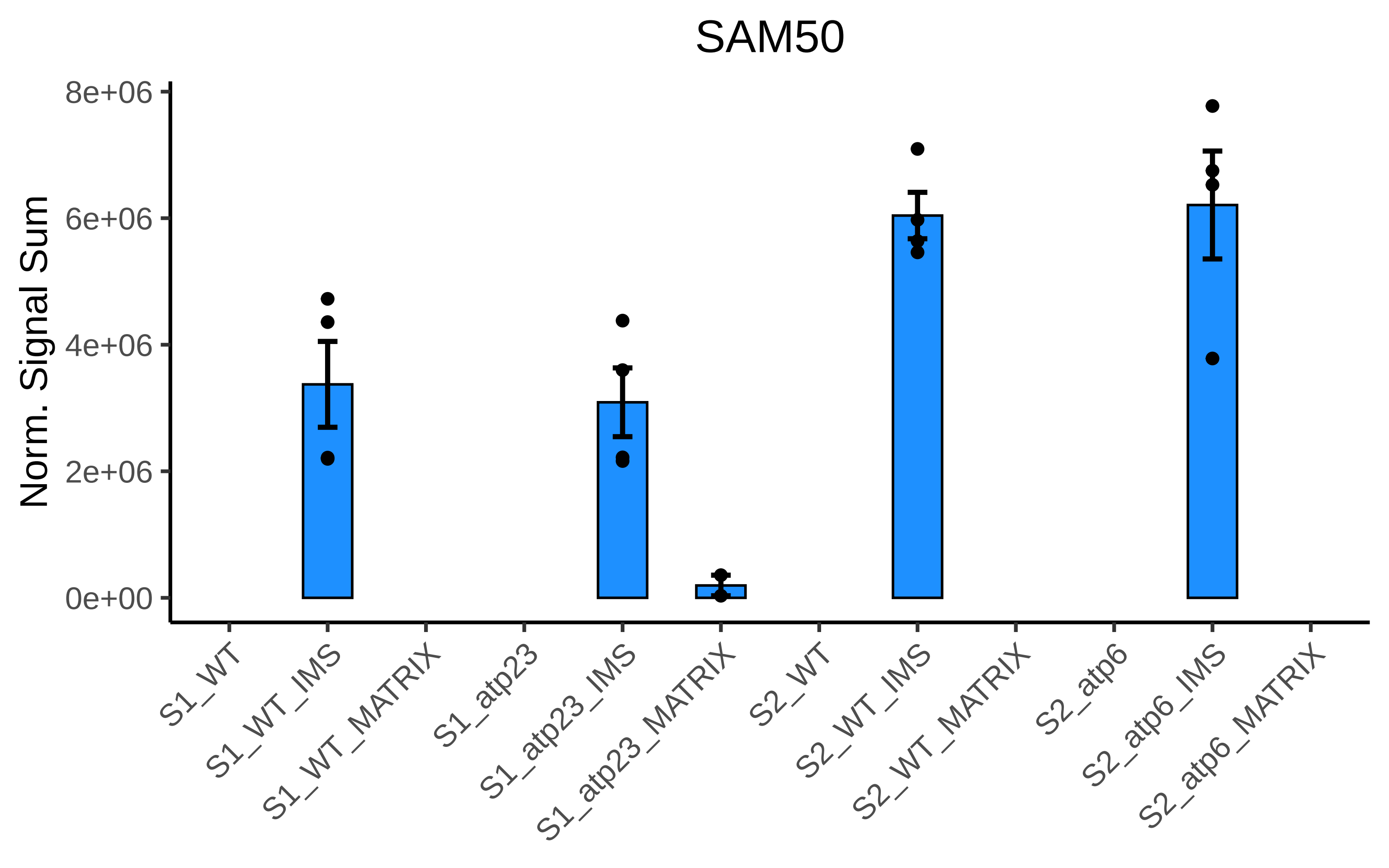

SAM50
Norm. Signal Sum
8e+06
6e+06
4e+06
2e+06
0e+00
S1_WT
S1_WT_IMS
S1_WT_MATRIX
S1_atp23
S1_atp23_IMS
S1_atp23_MATRIX
S2_WT
S2_WT_IMS
S2_WT_MATRIX
S2_atp6
S2_atp6_IMS
S2_atp6_MATRIX

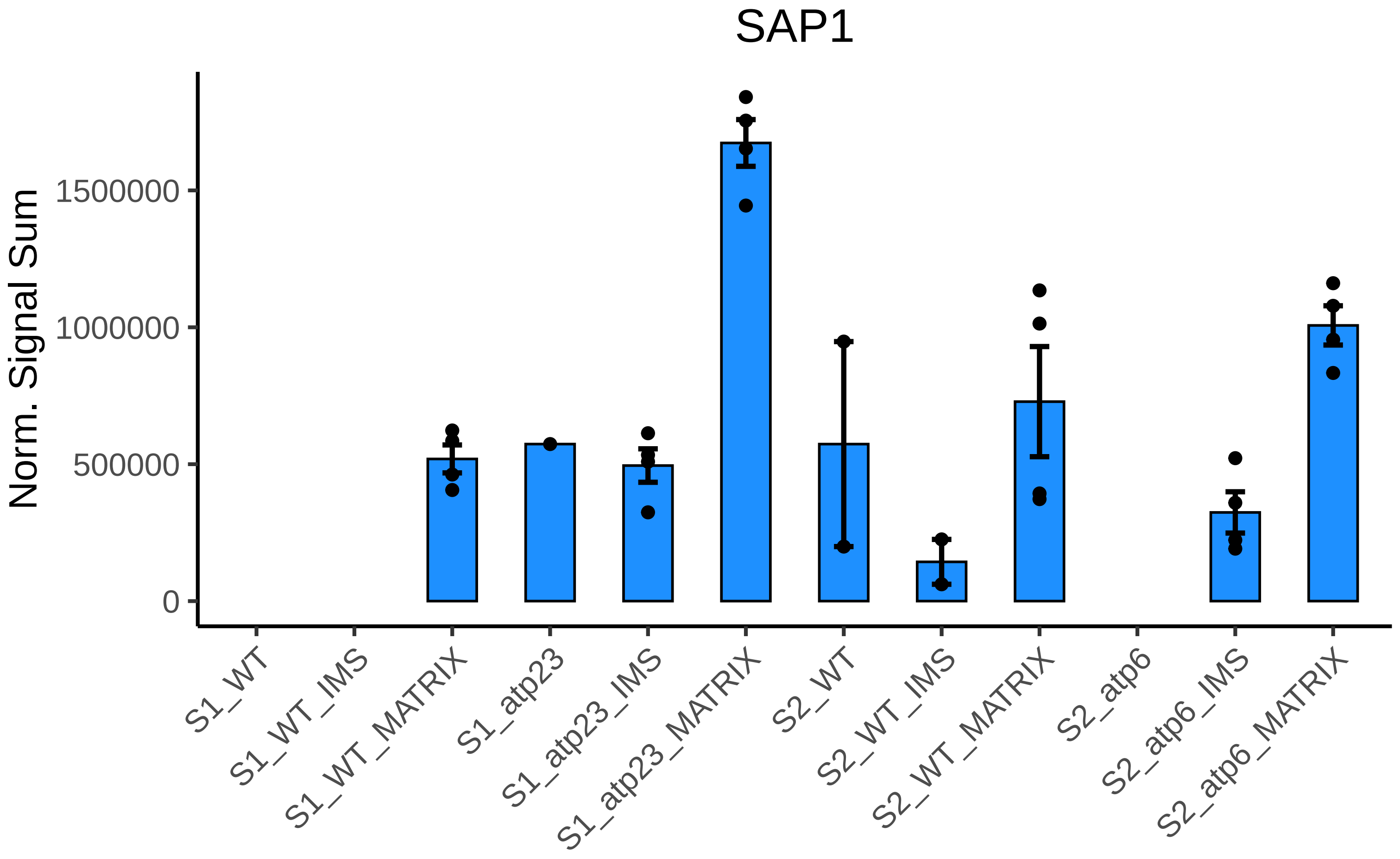

SAP1
Norm. Signal Sum
0
500000
1000000
1500000
S1_WT
S1_WT_IMS
S1_WT_MATRIX
S1_atp23
S1_atp23_IMS
S1_atp23_MATRIX
S2_WT
S2_WT_IMS
S2_WT_MATRIX
S2_atp6
S2_atp6_IMS
S2_atp6_MATRIX

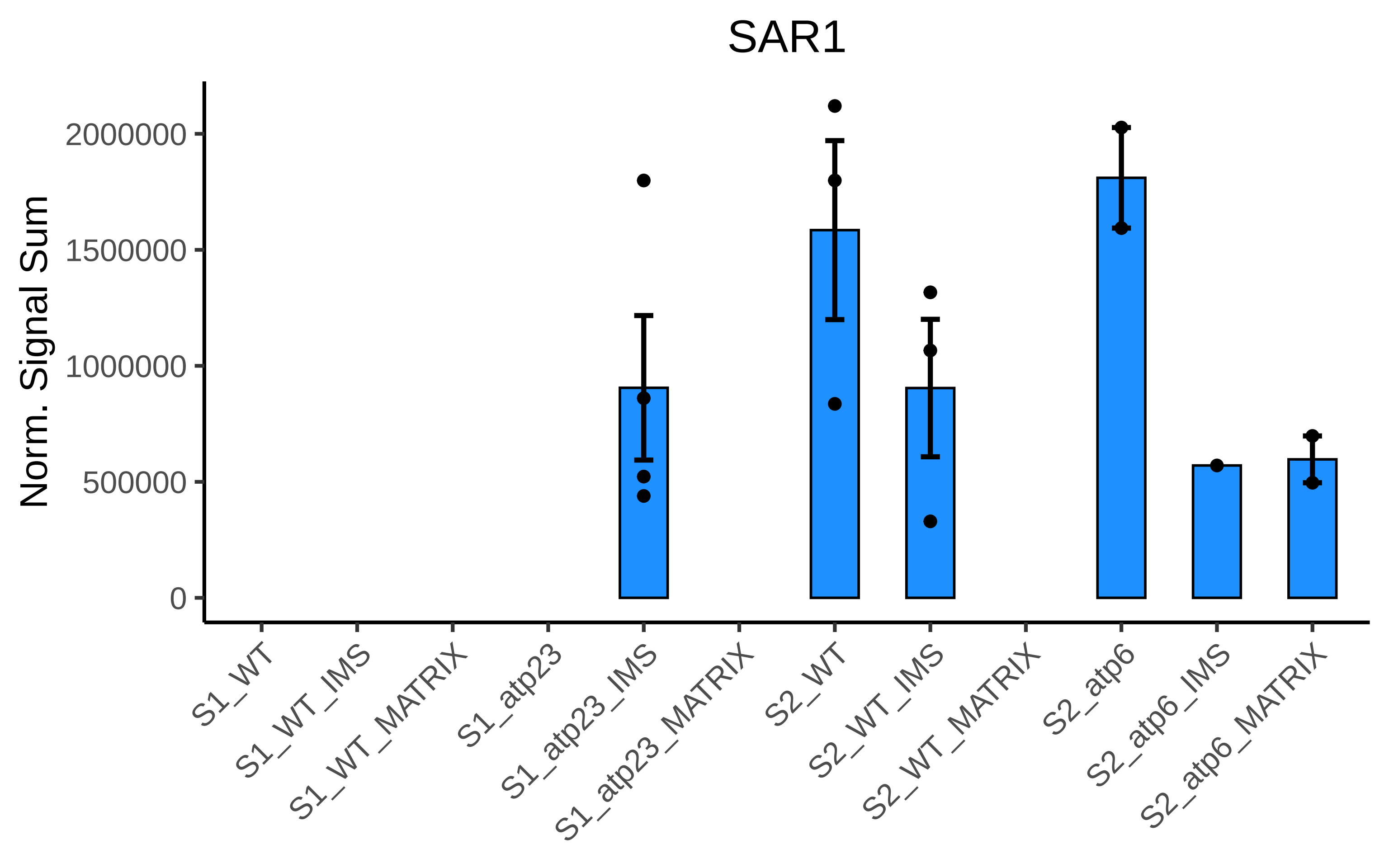

SAR1
Norm. Signal Sum
2000000
1500000
1000000
500000
0
S1_WT
S1_WT_IMS
S1_WT_MATRIX
S1_atp23
S1_atp23_IMS
S1_atp23_MATRIX
S2_WT
S2_WT_IMS
S2_WT_MATRIX
S2_atp6
S2_atp6_IMS
S2_atp6_MATRIX

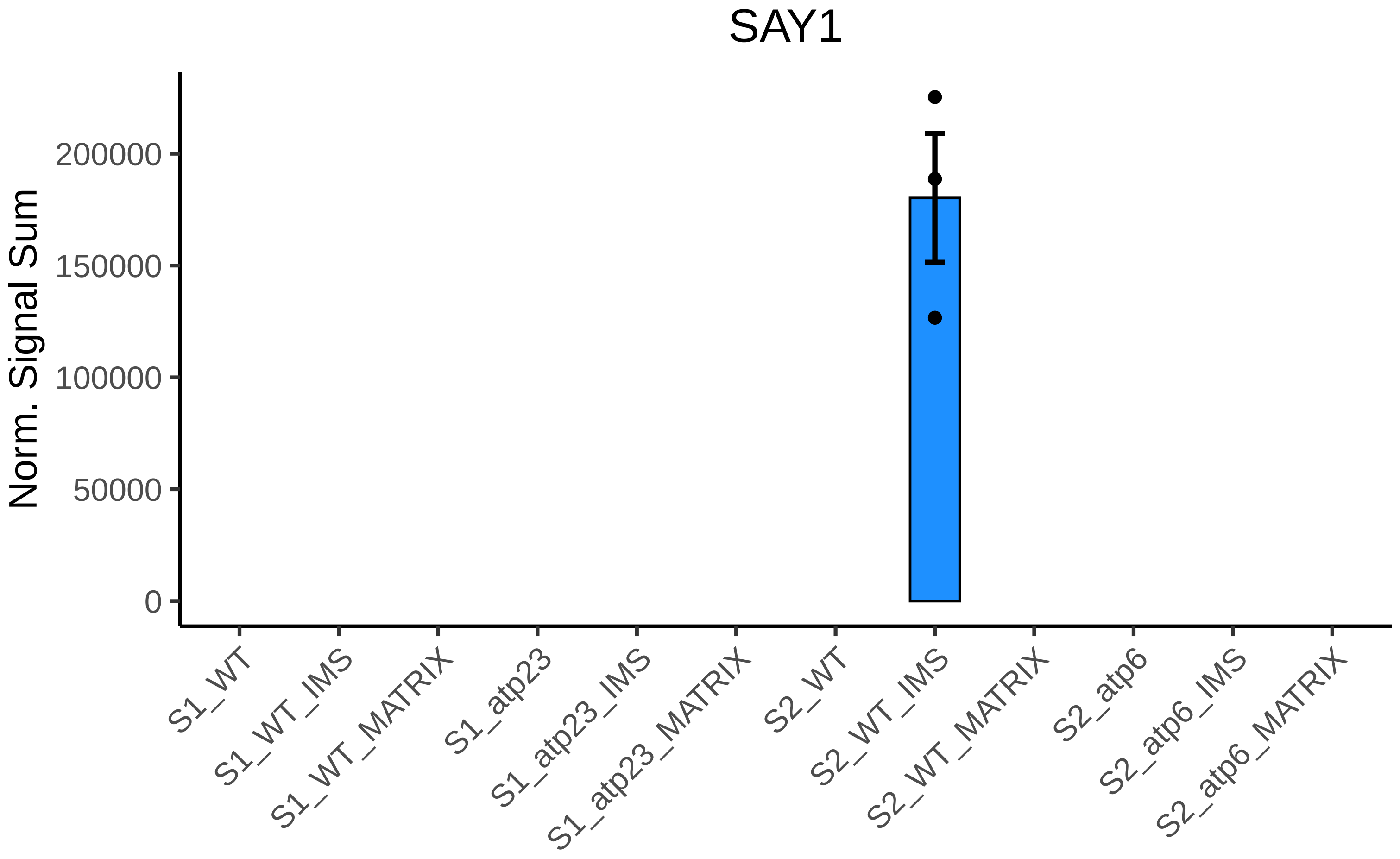

SAY1
Norm. Signal Sum
200000
150000
100000
50000
0
S1_WT
S1_WT_IMS
S1_WT_MATRIX
S1_atp23
S1_atp23_IMS
S1_atp23_MATRIX
S2_WT
S2_WT_IMS
S2_WT_MATRIX
S2_atp6
S2_atp6_IMS
S2_atp6_MATRIX

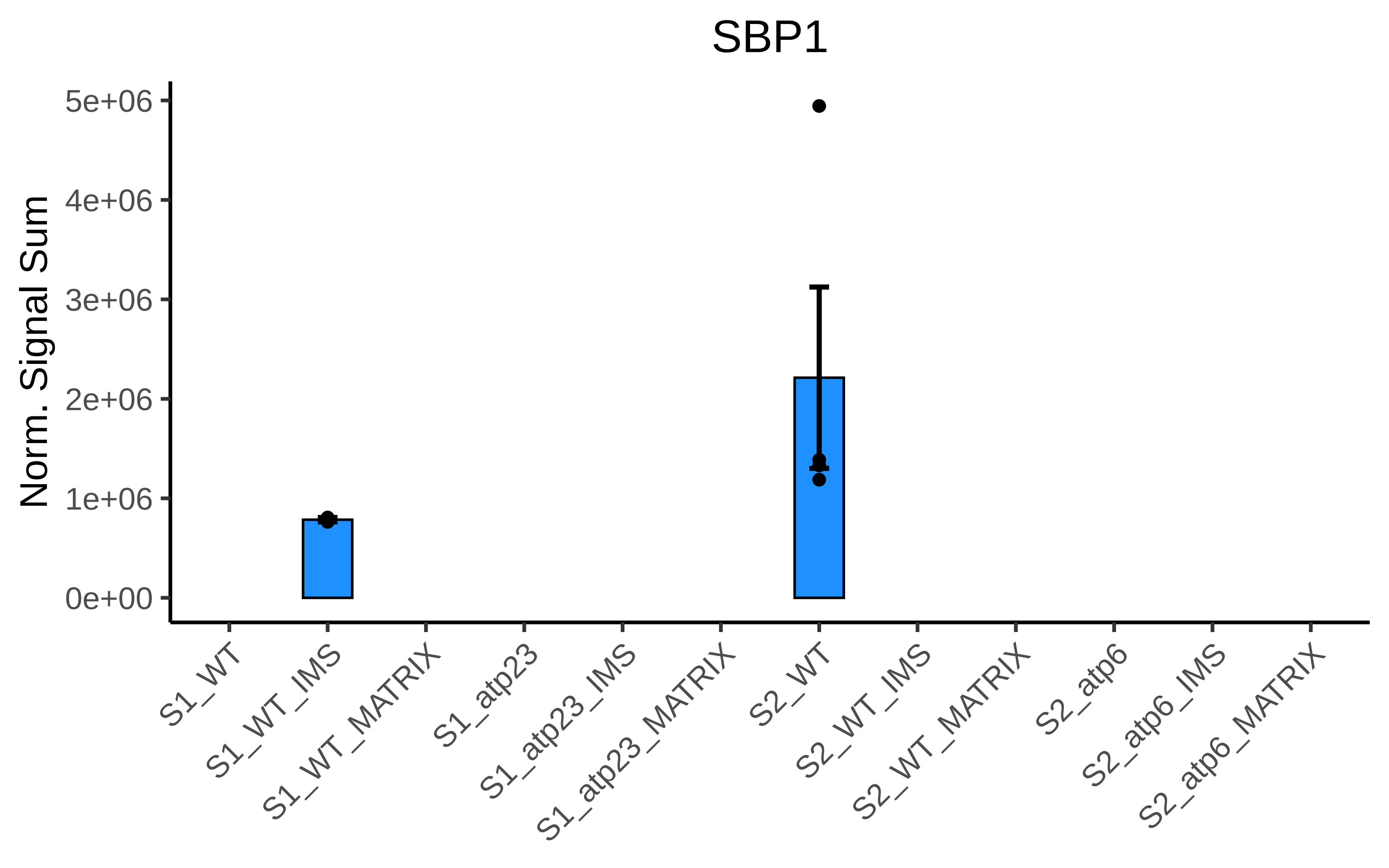

SBP1
Norm. Signal Sum
5e+06
4e+06
3e+06
2e+06
1e+06
0e+00
S1_WT
S1_WT_IMS
S1_WT_MATRIX
S1_atp23
S1_atp23_IMS
S1_atp23_MATRIX
S2_WT
S2_WT_IMS
S2_WT_MATRIX
S2_atp6
S2_atp6_IMS
S2_atp6_MATRIX

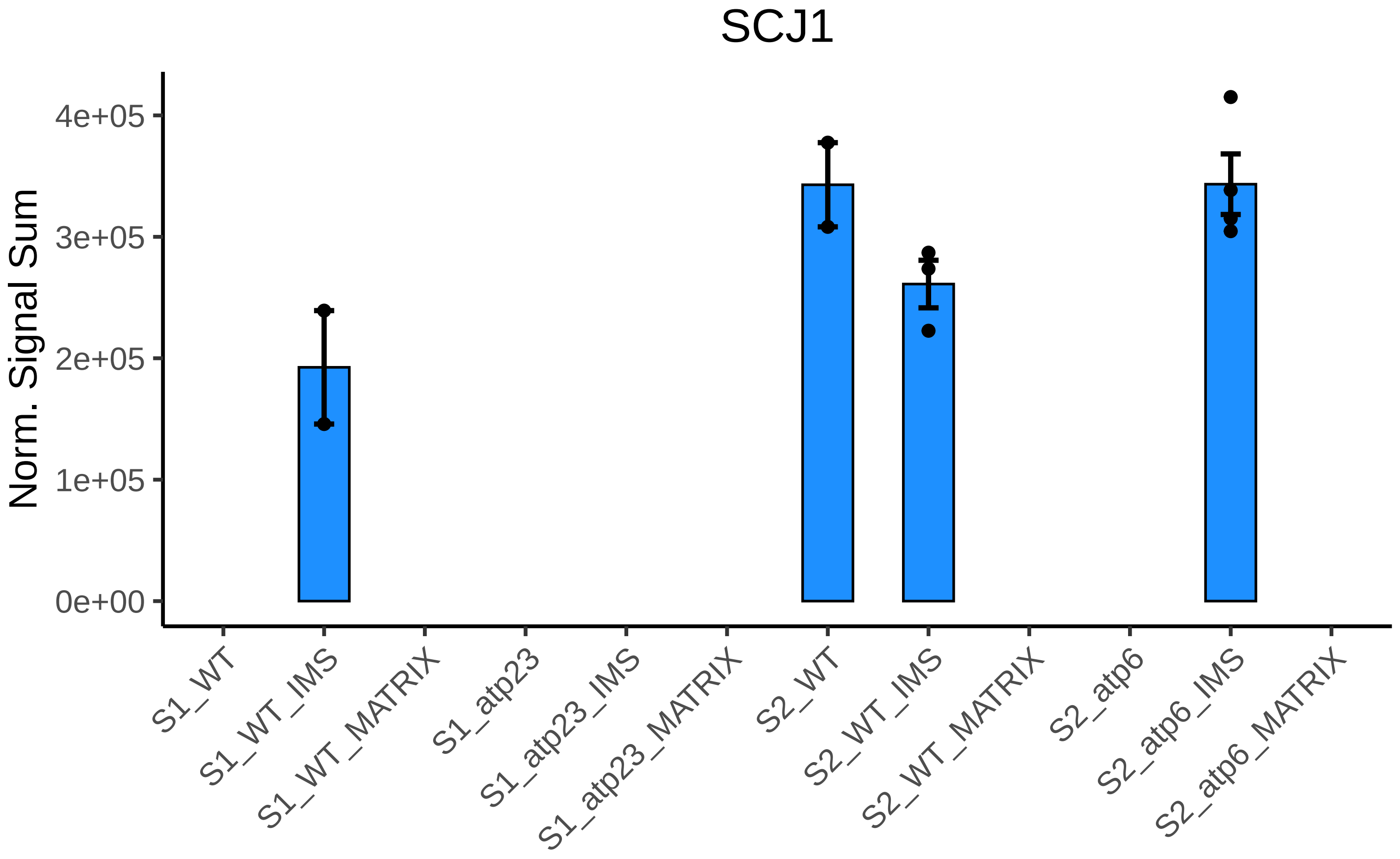
SCJ1
Norm. Signal Sum
4e+05
3e+05
2e+05
1e+05
0e+00
S1_WT
S1_WT_IMS
S1_WT_MATRIX
S1_atp23
S1_atp23_IMS
S1_atp23_MATRIX
S2_WT
S2_WT_IMS
S2_WT_MATRIX
S2_atp6
S2_atp6_IMS
S2_atp6_MATRIX

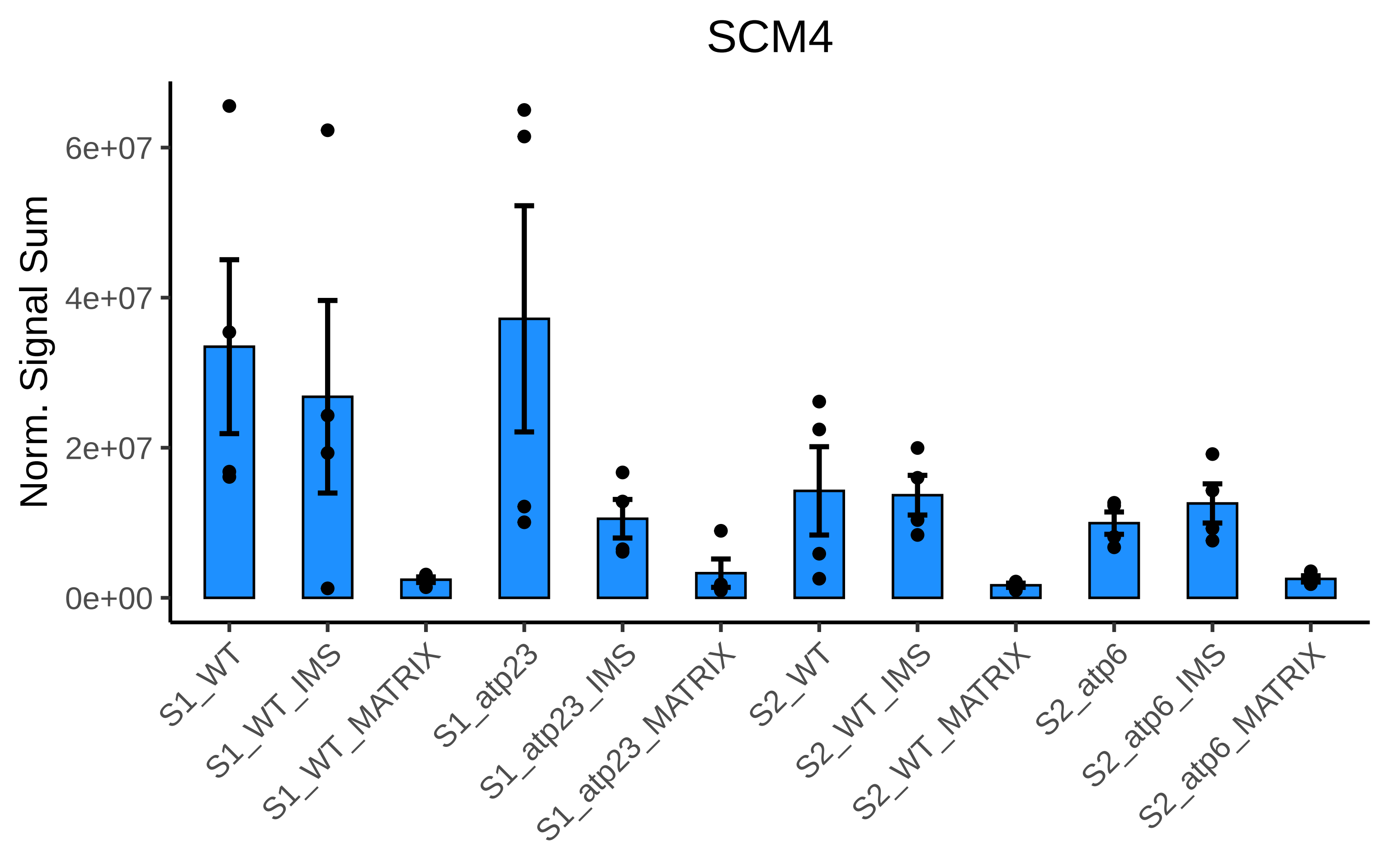

SCM4
Norm. Signal Sum
6e+07
4e+07
2e+07
0e+00
S1_WT
S1_WT_IMS
S1_WT_MATRIX
S1_atp23
S1_atp23_IMS
S1_atp23_MATRIX
S2_WT
S2_WT_IMS
S2_WT_MATRIX
S2_atp6
S2_atp6_IMS
S2_atp6_MATRIX

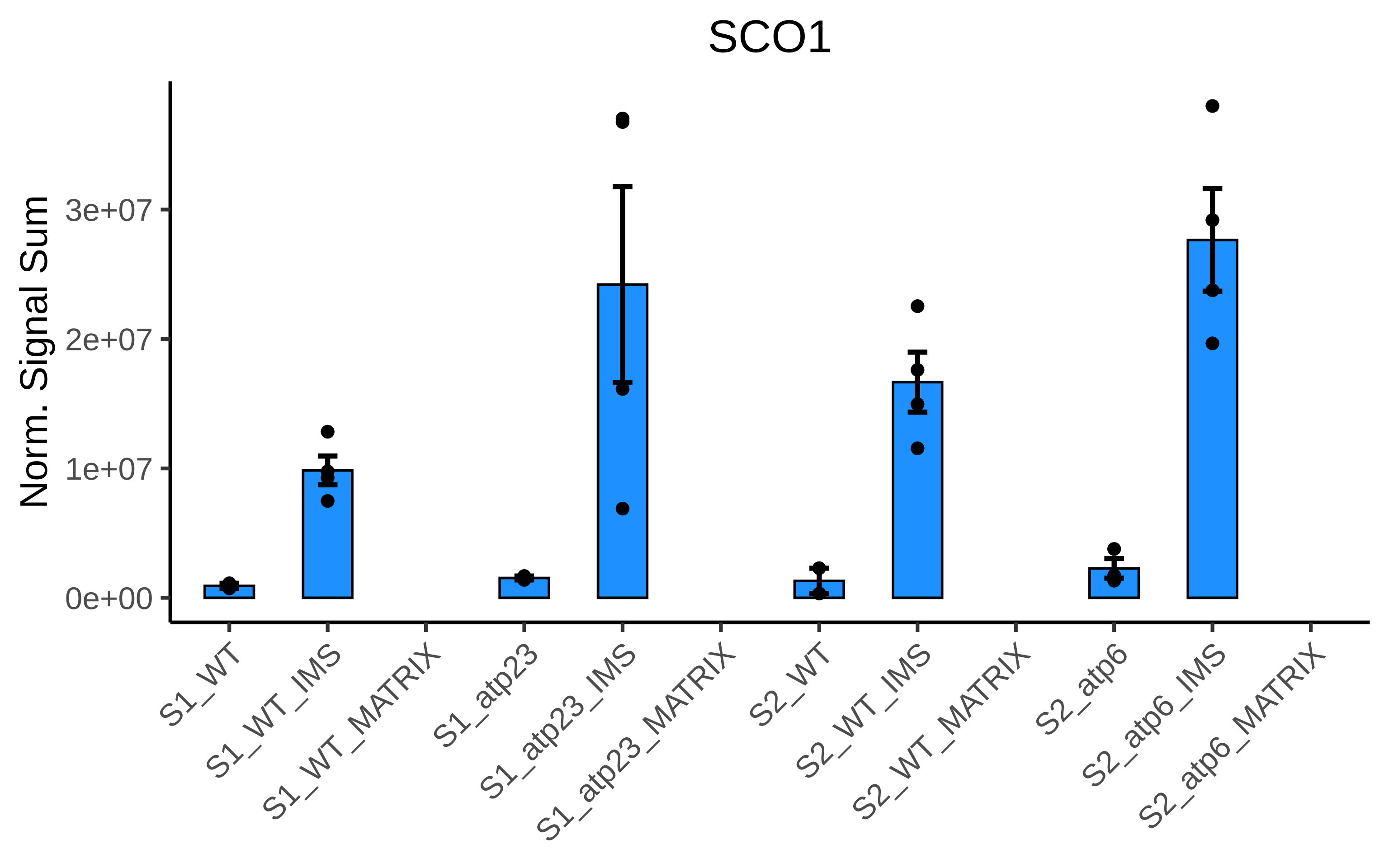

SCO1
Norm. Signal Sum
3e+07
2e+07
1e+07
0e+00
S1_WT
S1_WT_IMS
S1_WT_MATRIX
S1_atp23
S1_atp23_IMS
S1_atp23_MATRIX
S2_WT
S2_WT_IMS
S2_WT_MATRIX
S2_atp6
S2_atp6_IMS
S2_atp6_MATRIX

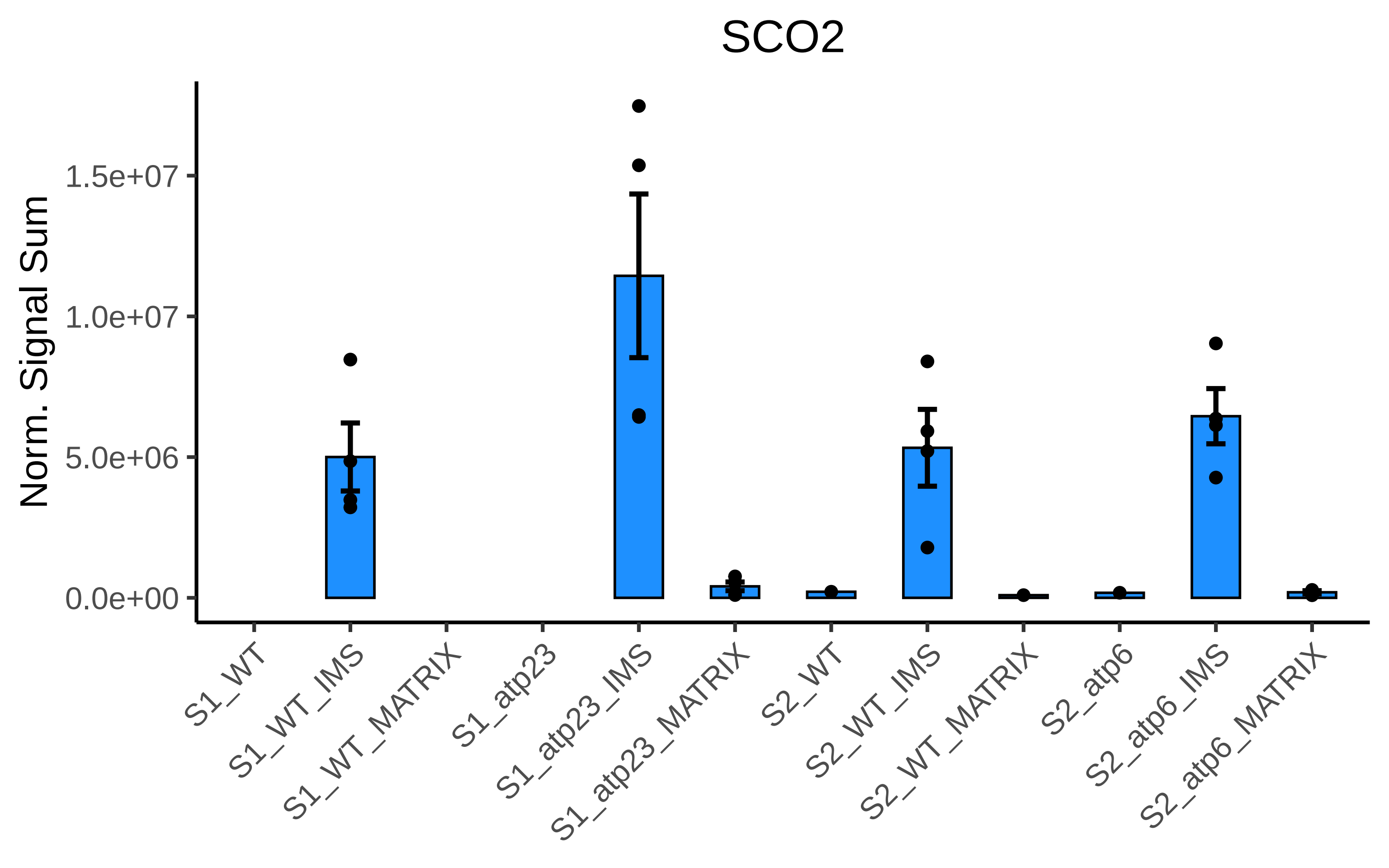
SCO2
Norm. Signal Sum
1.5e+07
1.0e+07
5.0e+06
0.0e+00
S1_WT
S1_WT_IMS
S1_WT_MATRIX
S1_atp23
S1_atp23_IMS
S1_atp23_MATRIX
S2_WT
S2_WT_IMS
S2_WT_MATRIX
S2_atp6
S2_atp6_IMS
S2_atp6_MATRIX

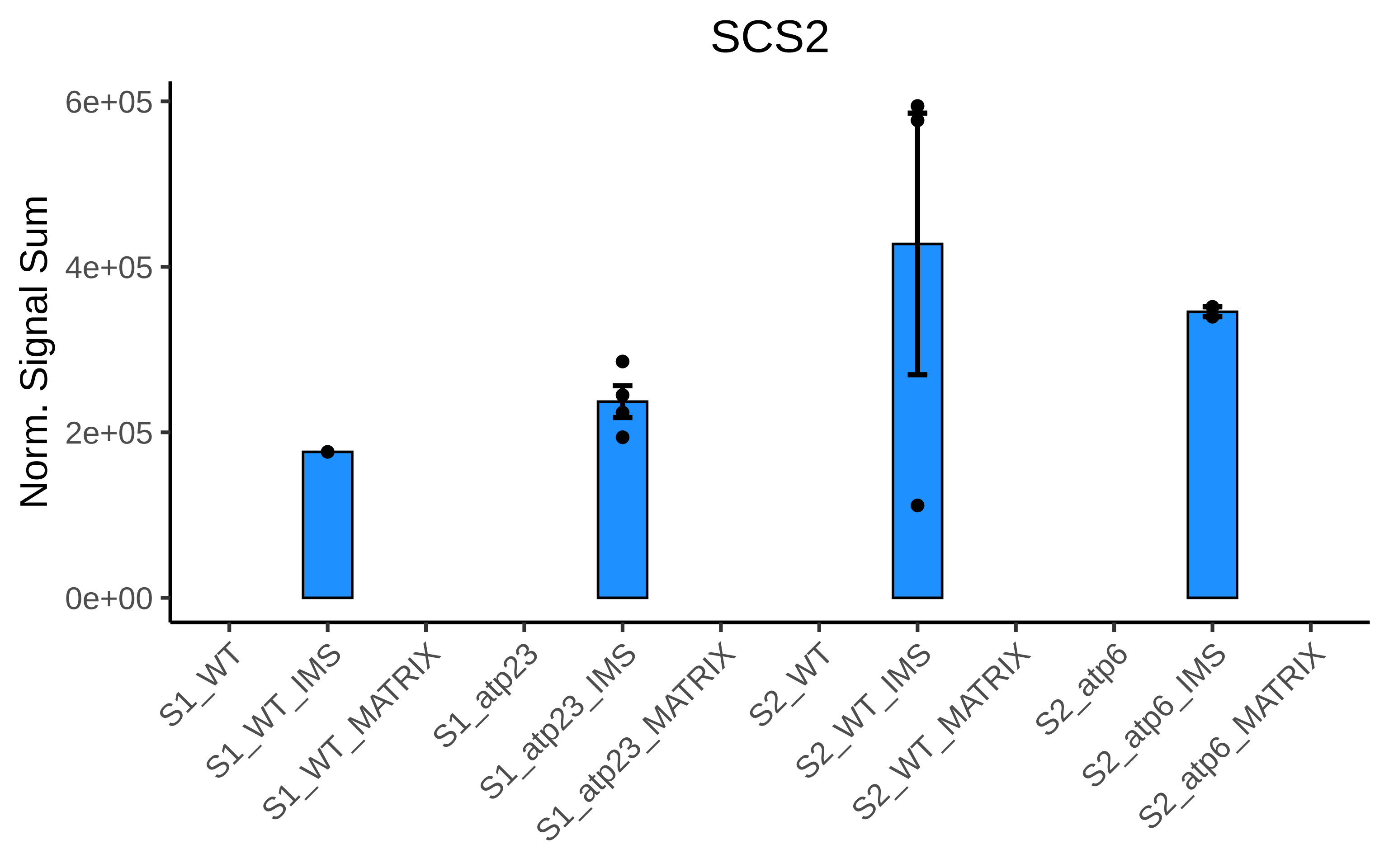

SCS2
Norm. Signal Sum
6e+05
4e+05
2e+05
0e+00
S1_WT
S1_WT_IMS
S1_WT_MATRIX
S1_atp23
S1_atp23_IMS
S1_atp23_MATRIX
S2_WT
S2_WT_IMS
S2_WT_MATRIX
S2_atp6
S2_atp6_IMS
S2_atp6_MATRIX

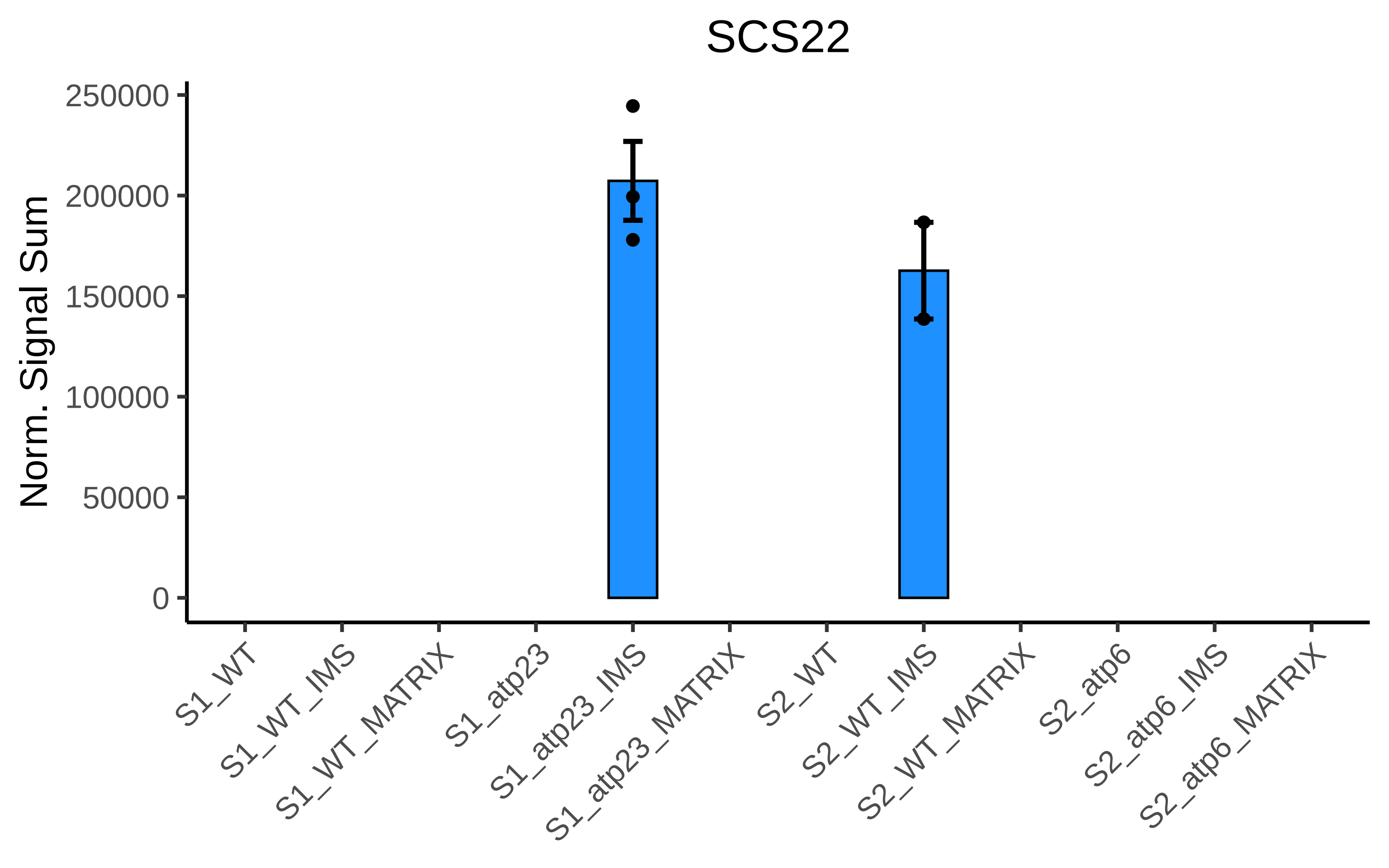

SCS22
Norm. Signal Sum
250000
200000
150000
100000
50000
0
S1_WT
S1_WT_IMS
S1_WT_MATRIX
S1_atp23
S1_atp23_IMS
S1_atp23_MATRIX
S2_WT
S2_WT_IMS
S2_WT_MATRIX
S2_atp6
S2_atp6_IMS
S2_atp6_MATRIX

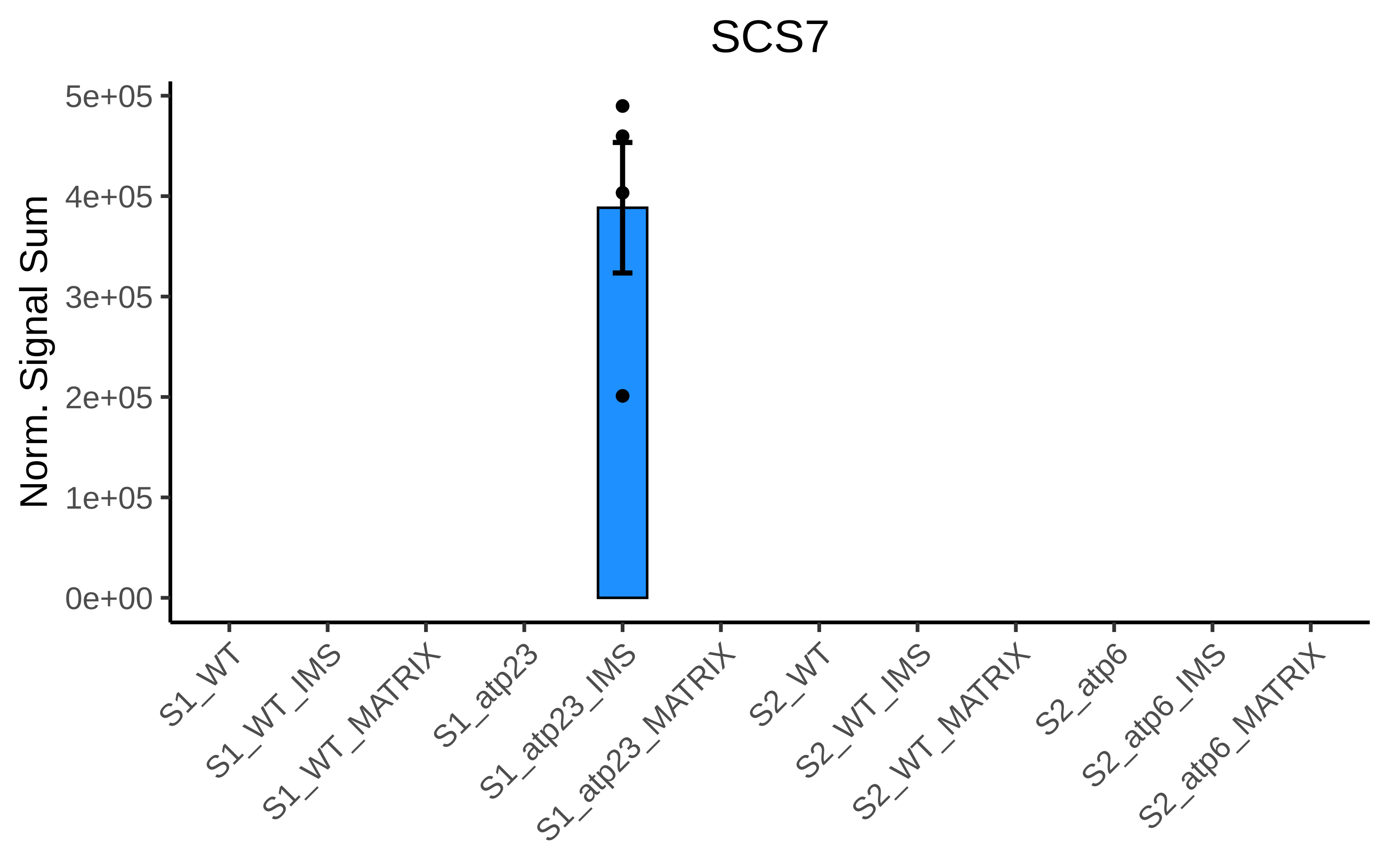

SCS7
Norm. Signal Sum
5e+05
4e+05
3e+05
2e+05
1e+05
0e+00
S1_WT
S1_WT_IMS
S1_WT_MATRIX
S1_atp23
S1_atp23_IMS
S1_atp23_MATRIX
S2_WT
S2_WT_IMS
S2_WT_MATRIX
S2_atp6
S2_atp6_IMS
S2_atp6_MATRIX

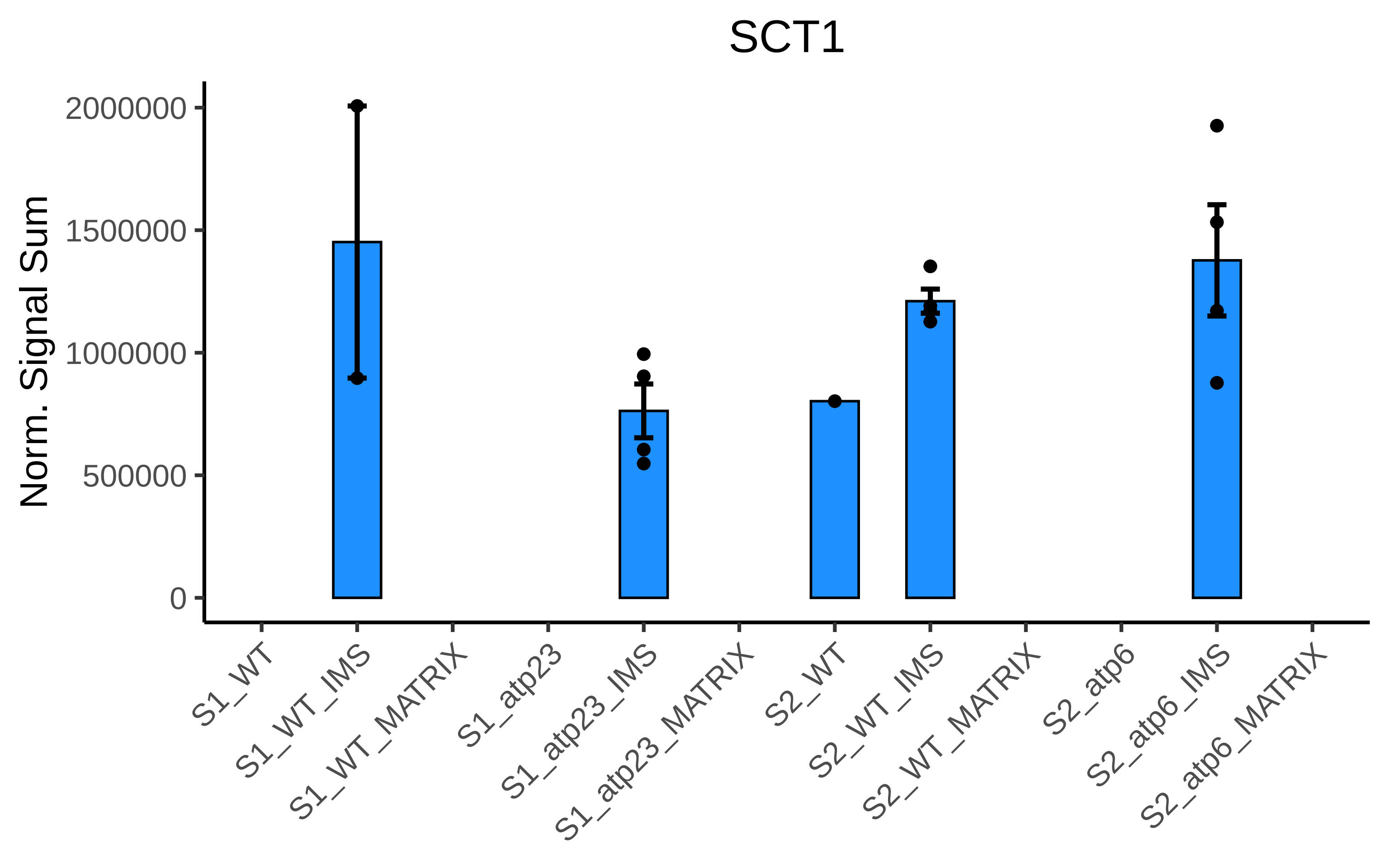
SCT1
Norm. Signal Sum
2000000
1500000
1000000
500000
0
S1_WT
S1_WT_IMS
S1_WT_MATRIX
S1_atp23
S1_atp23_IMS
S1_atp23_MATRIX
S2_WT
S2_WT_IMS
S2_WT_MATRIX
S2_atp6
S2_atp6_IMS
S2_atp6_MATRIX

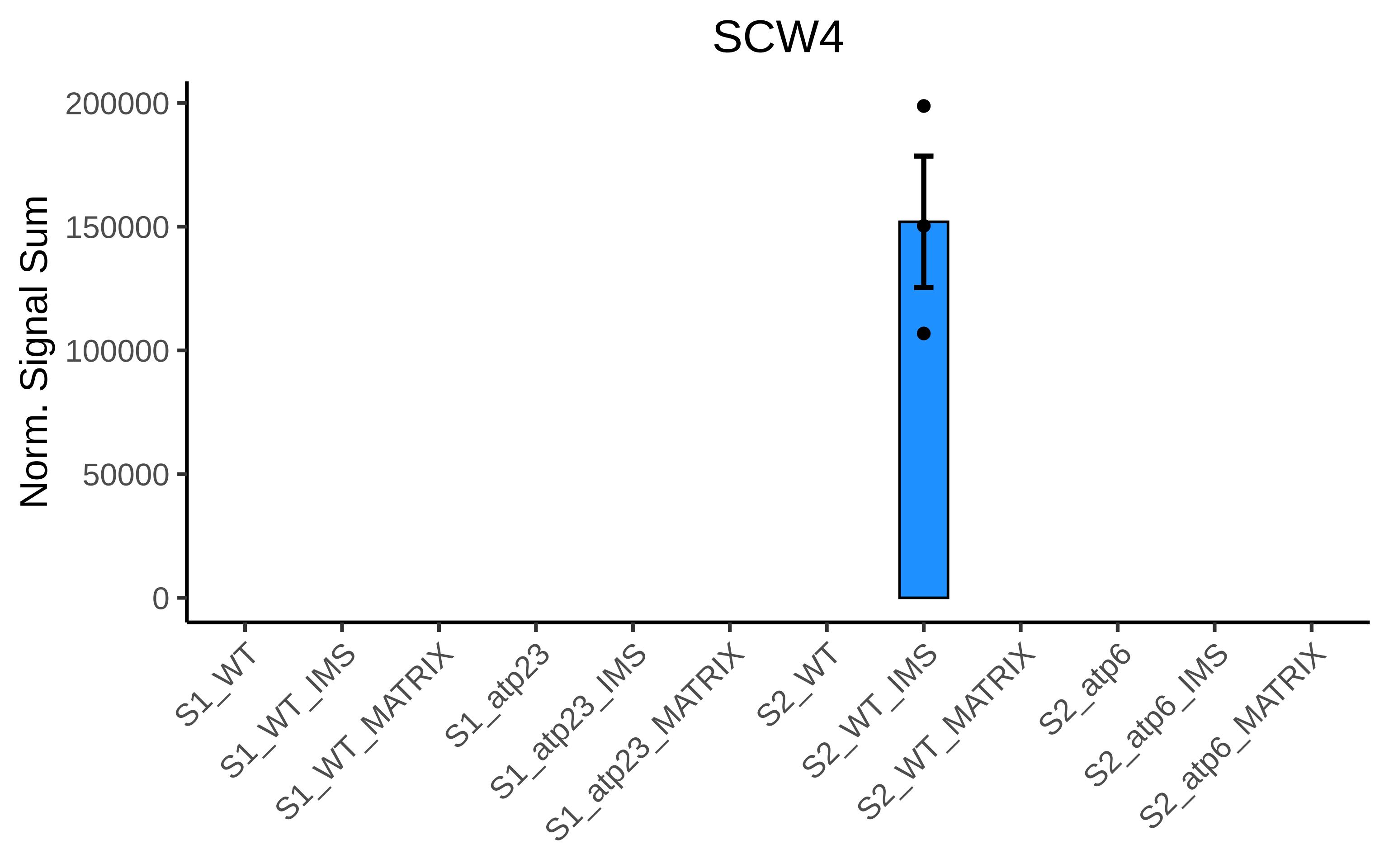

SCW4
Norm. Signal Sum
200000
150000
100000
50000
0
S1_WT
S1_WT_IMS
S1_WT_MATRIX
S1_atp23
S1_atp23_IMS
S1_atp23_MATRIX
S2_WT
S2_WT_IMS
S2_WT_MATRIX
S2_atp6
S2_atp6_IMS
S2_atp6_MATRIX

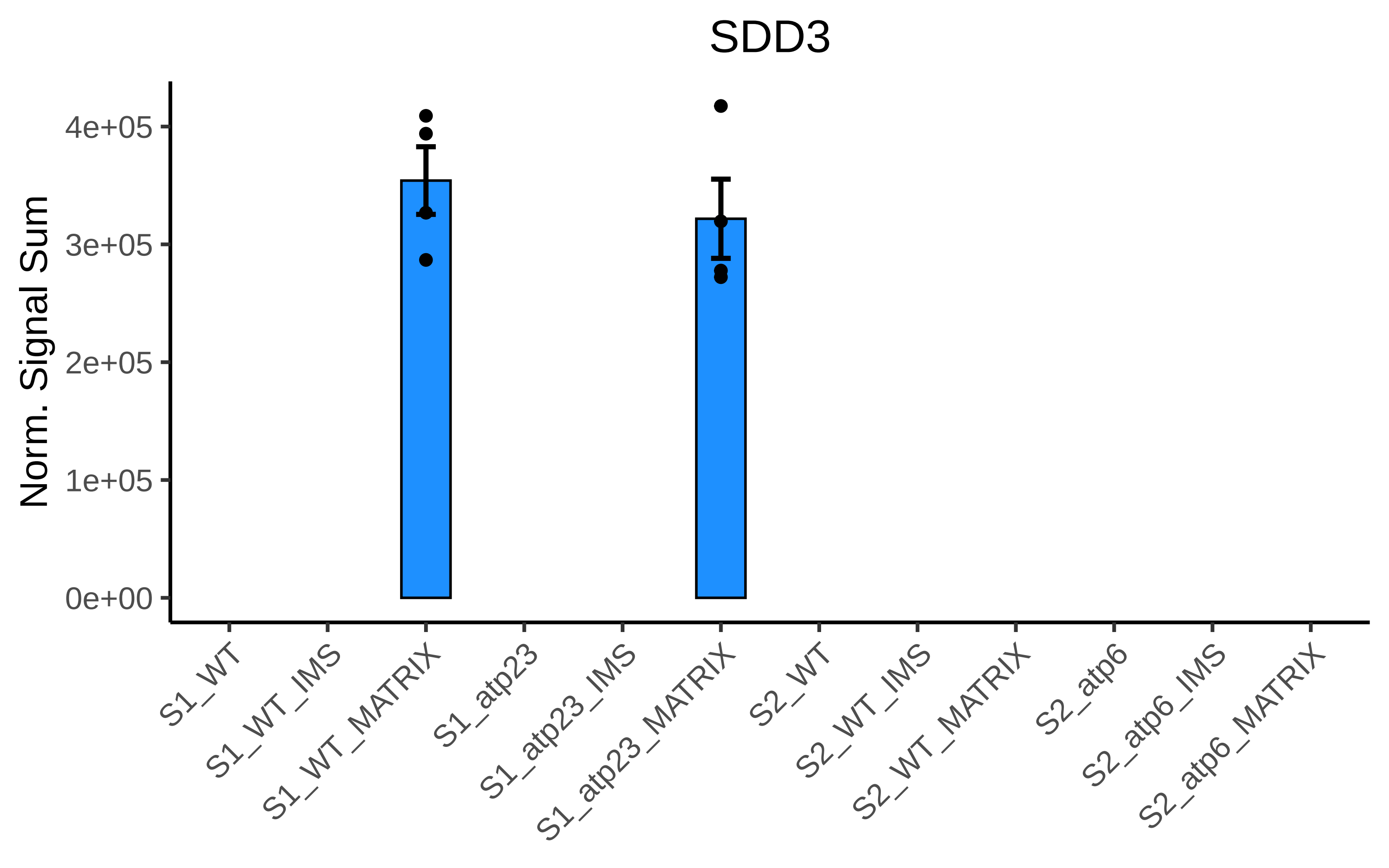

SDD3
Norm. Signal Sum
4e+05
3e+05
2e+05
1e+05
0e+00
S1_WT
S1_WT_IMS
S1_WT_MATRIX
S1_atp23
S1_atp23_IMS
S1_atp23_MATRIX
S2_WT
S2_WT_IMS
S2_WT_MATRIX
S2_atp6
S2_atp6_IMS
S2_atp6_MATRIX

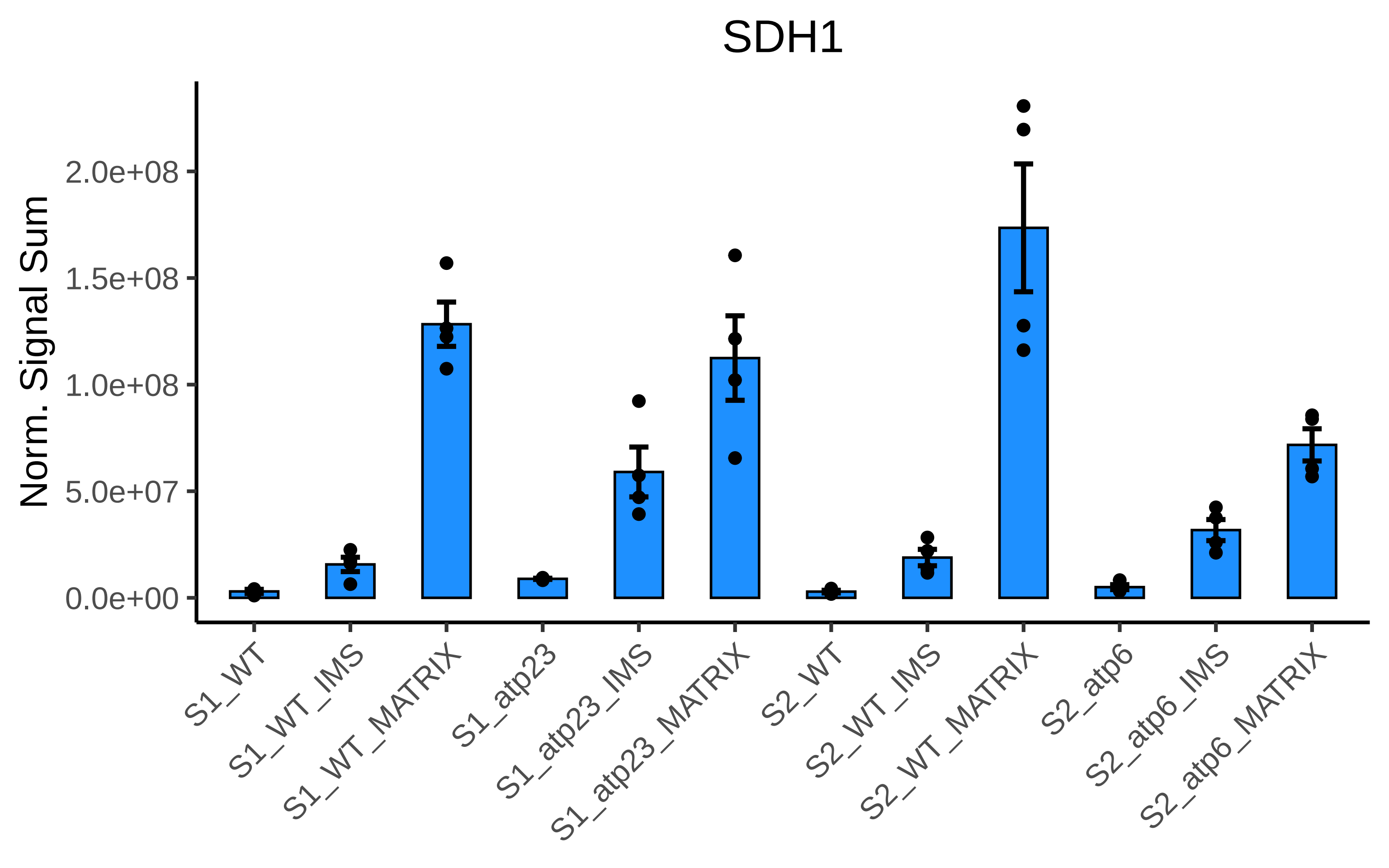
SDH1
Norm. Signal Sum
2.0e+08
1.5e+08
1.0e+08
5.0e+07
0.0e+00
S1_WT
S1_WT_IMS
S1_WT_MATRIX
S1_atp23
S1_atp23_IMS
S1_atp23_MATRIX
S2_WT
S2_WT_IMS
S2_WT_MATRIX
S2_atp6
S2_atp6_IMS
S2_atp6_MATRIX

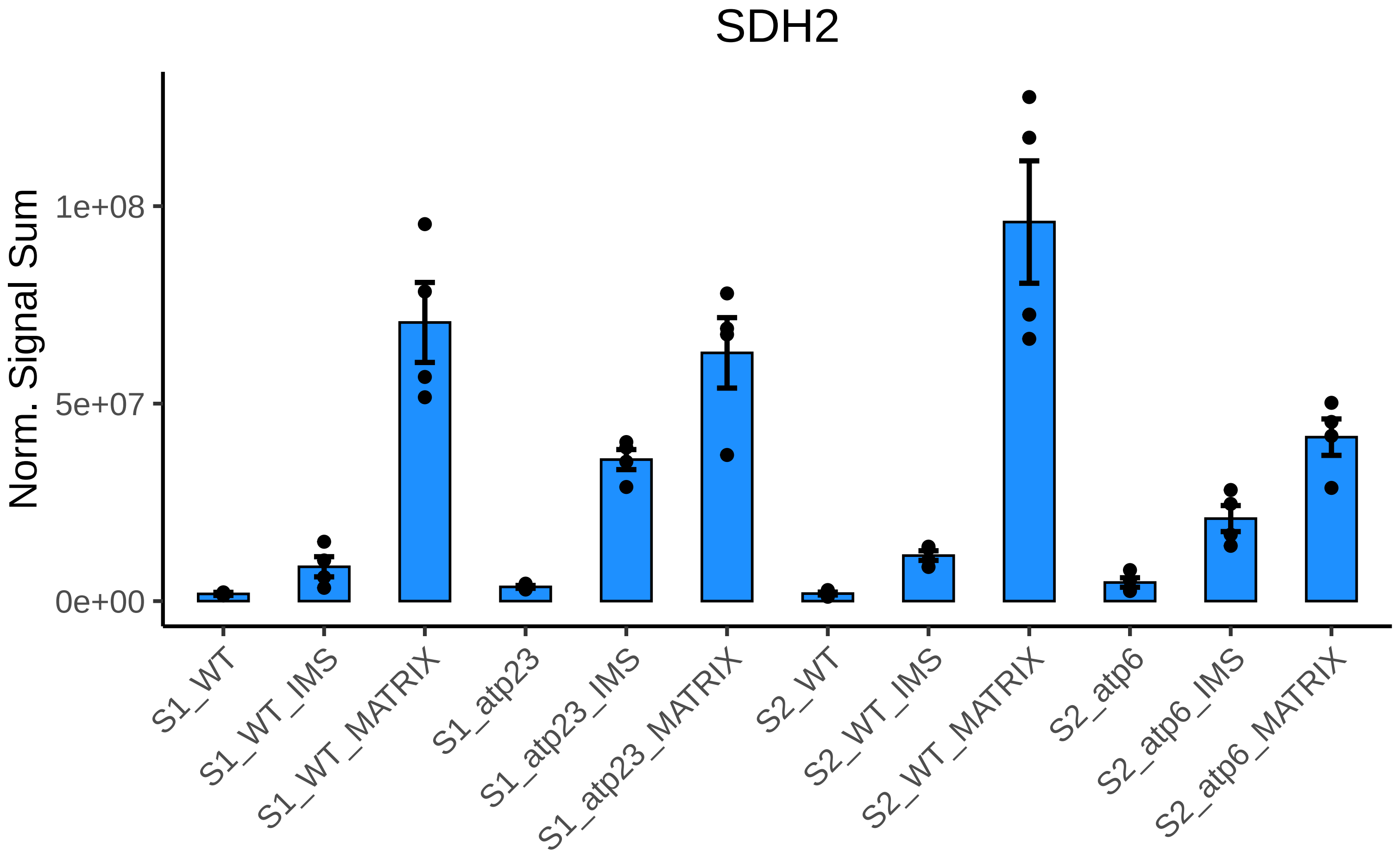
SDH2
Norm. Signal Sum
1e+08
5e+07
0e+00
S1_WT
S1_WT_IMS
S1_WT_MATRIX
S1_atp23
S1_atp23_IMS
S1_atp23_MATRIX
S2_WT
S2_WT_IMS
S2_WT_MATRIX
S2_atp6
S2_atp6_IMS
S2_atp6_MATRIX

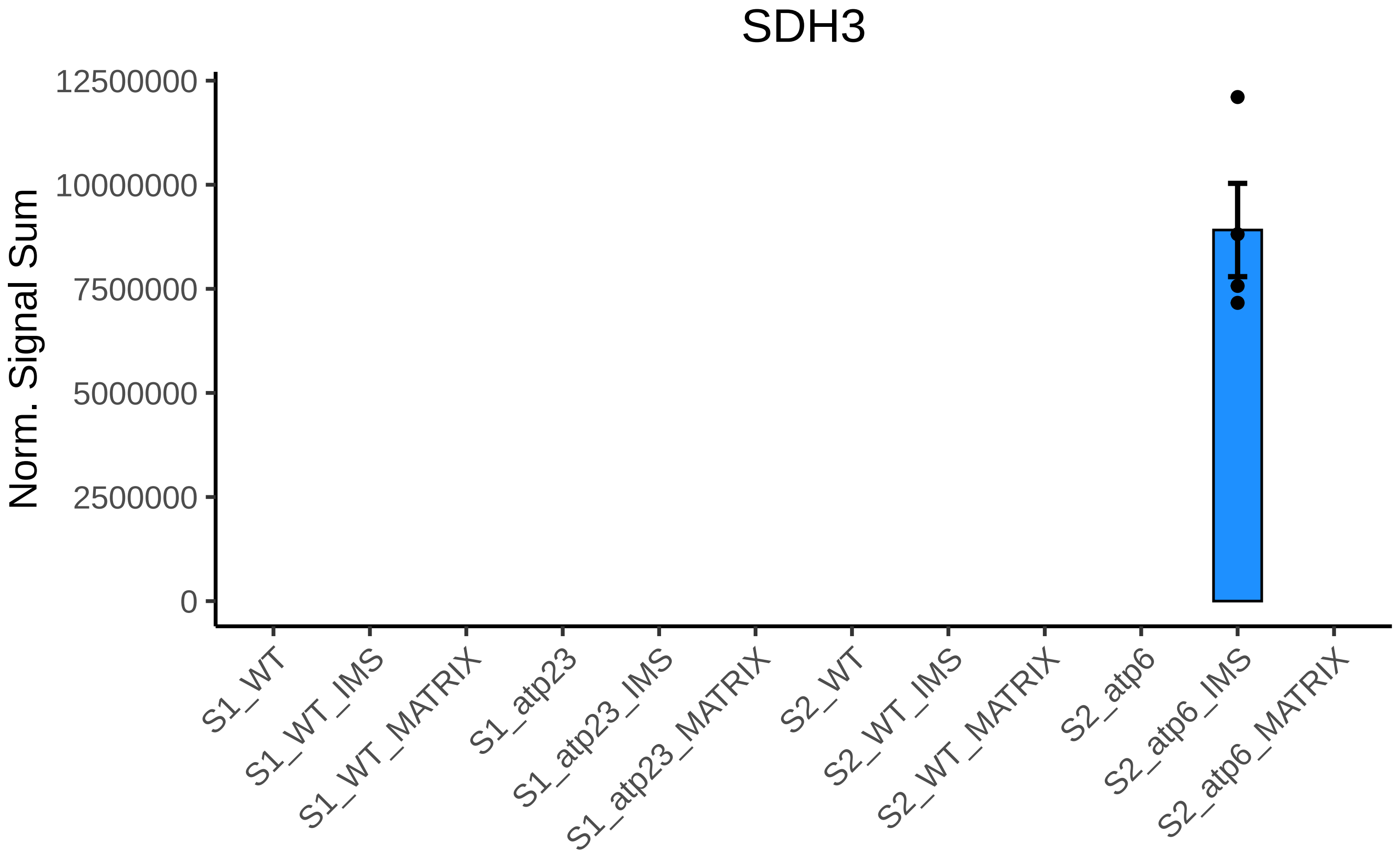

SDH3
Norm. Signal Sum
12500000
10000000
7500000
5000000
2500000
0
S1_WT
S1_WT_IMS
S1_WT_MATRIX
S1_atp23
S1_atp23_IMS
S1_atp23_MATRIX
S2_WT
S2_WT_IMS
S2_WT_MATRIX
S2_atp6
S2_atp6_IMS
S2_atp6_MATRIX

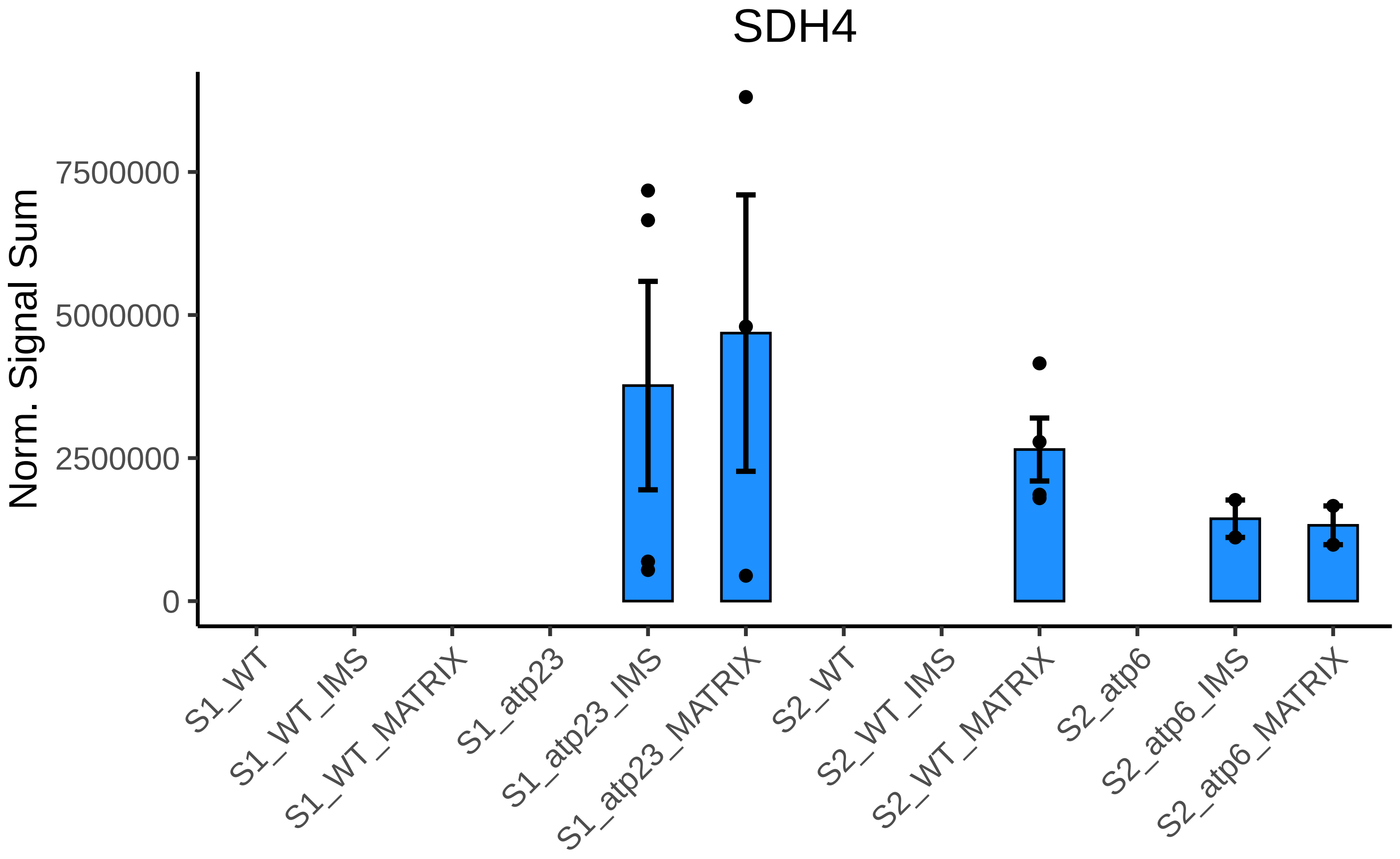
SDH4
Norm. Signal Sum
7500000
5000000
2500000
0
S1_WT
S1_WT_IMS
S1_WT_MATRIX
S1_atp23
S1_atp23_IMS
S1_atp23_MATRIX
S2_WT
S2_WT_IMS
S2_WT_MATRIX
S2_atp6
S2_atp6_IMS
S2_atp6_MATRIX

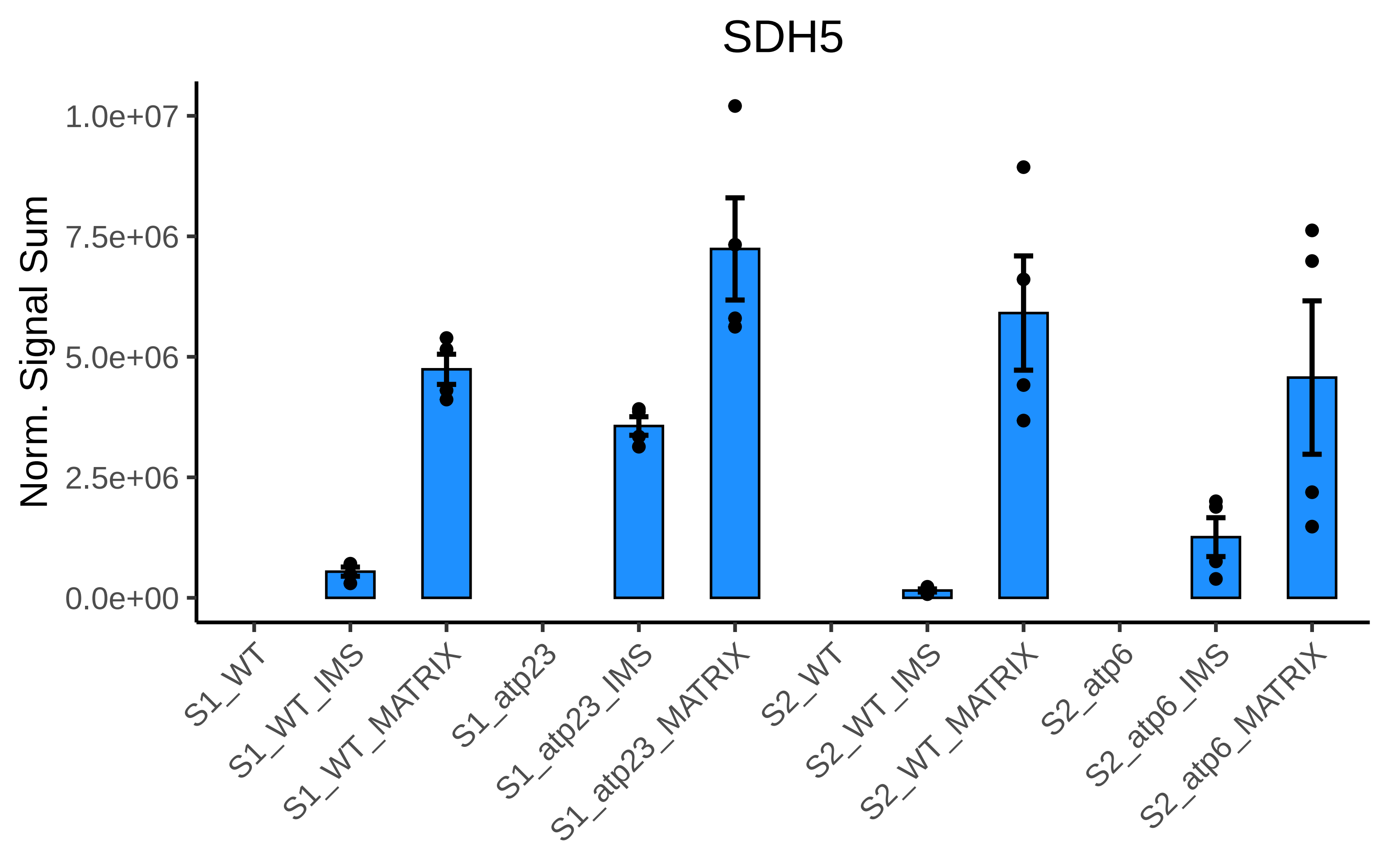
SDH5
Norm. Signal Sum
1.0e+07
7.5e+06
5.0e+06
2.5e+06
0.0e+00
S1_WT
S1_WT_IMS
S1_WT_MATRIX
S1_atp23
S1_atp23_IMS
S1_atp23_MATRIX
S2_WT
S2_WT_IMS
S2_WT_MATRIX
S2_atp6
S2_atp6_IMS
S2_atp6_MATRIX

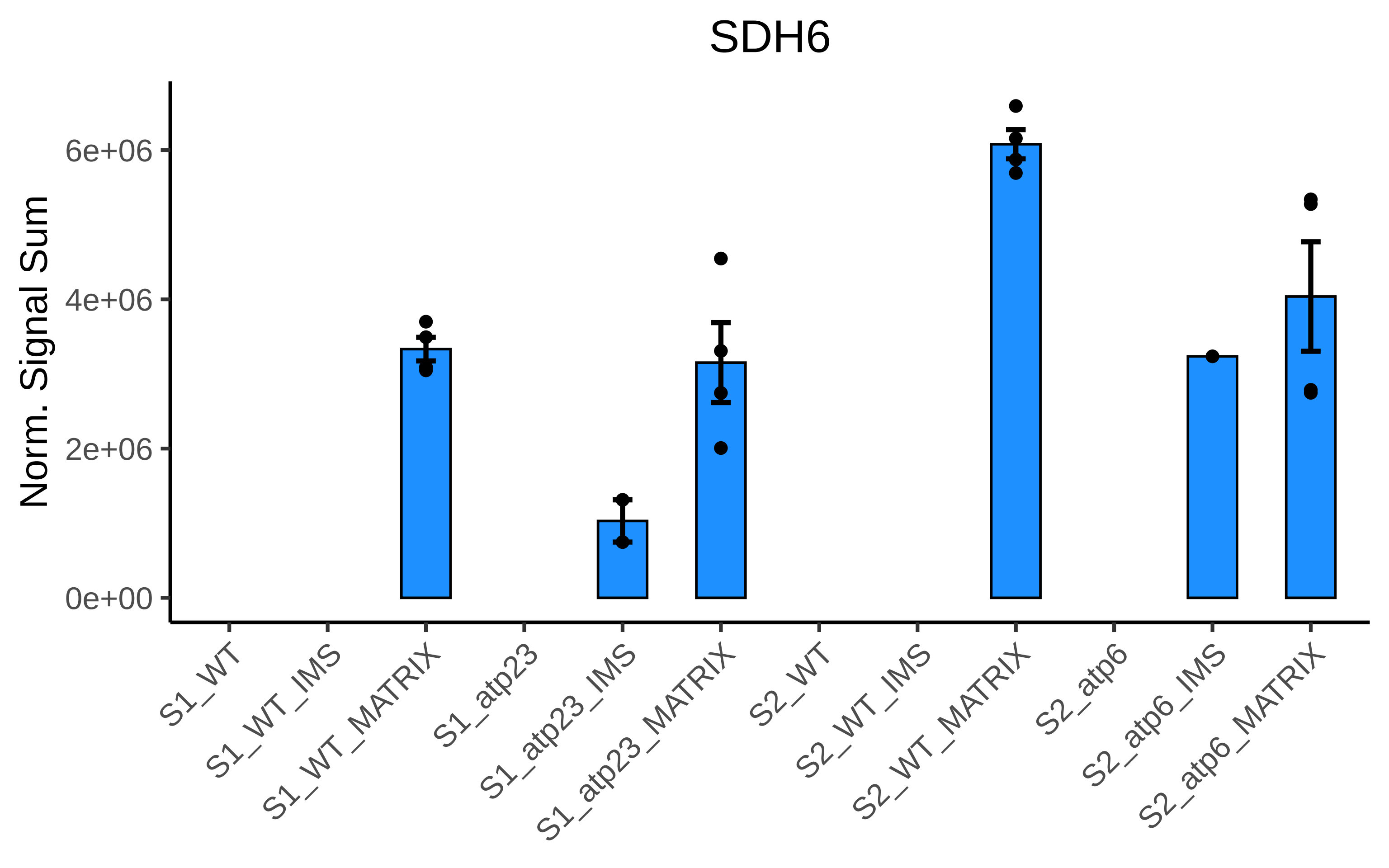
SDH6
Norm. Signal Sum
6e+06
4e+06
2e+06
0e+00
S1_WT
S1_WT_IMS
S1_WT_MATRIX
S1_atp23
S1_atp23_IMS
S1_atp23_MATRIX
S2_WT
S2_WT_IMS
S2_WT_MATRIX
S2_atp6
S2_atp6_IMS
S2_atp6_MATRIX

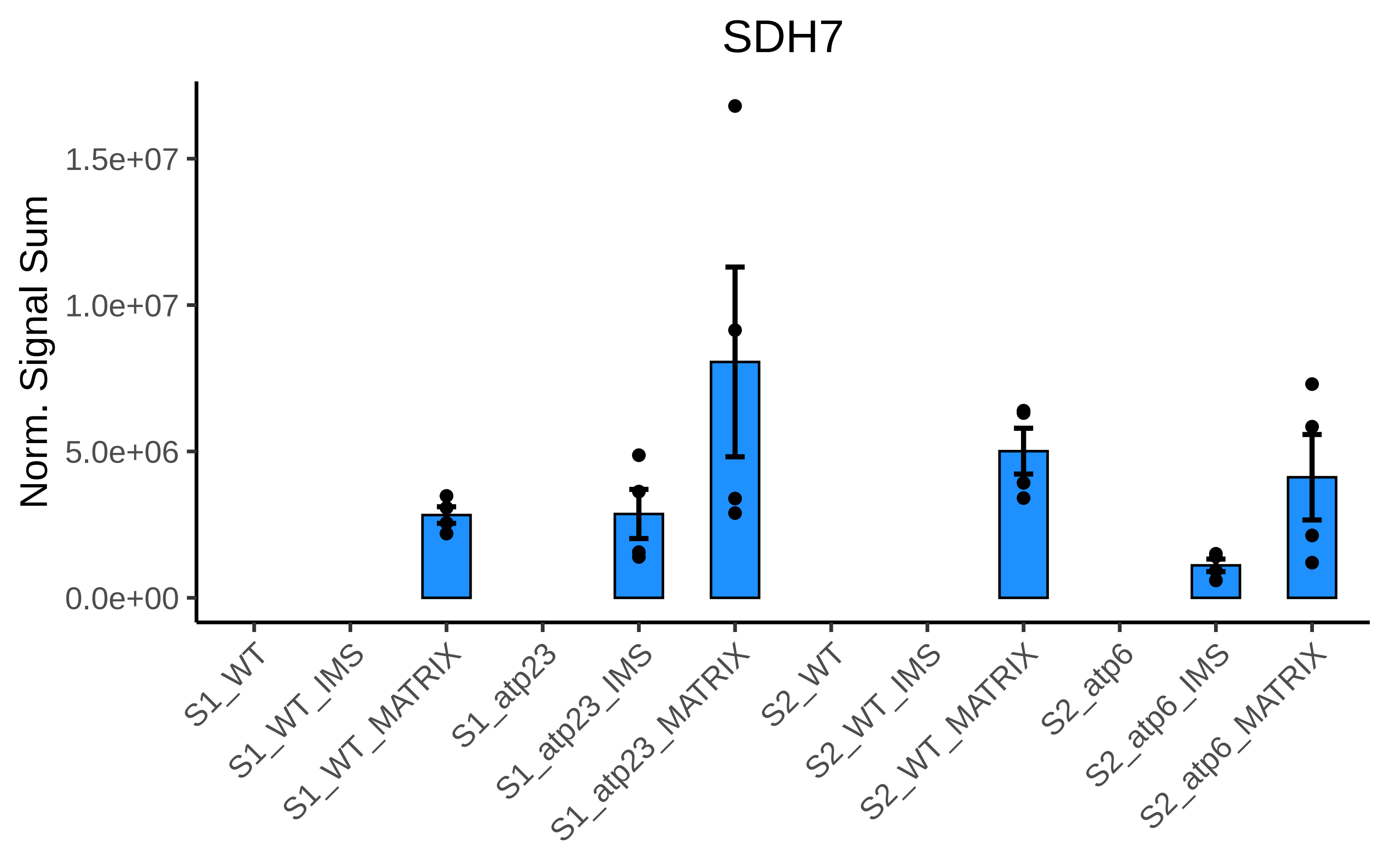

SDH7
Norm. Signal Sum
1.5e+07
1.0e+07
5.0e+06
0.0e+00
S1_WT
S1_WT_IMS
S1_WT_MATRIX
S1_atp23
S1_atp23_IMS
S1_atp23_MATRIX
S2_WT
S2_WT_IMS
S2_WT_MATRIX
S2_atp6
S2_atp6_IMS
S2_atp6_MATRIX

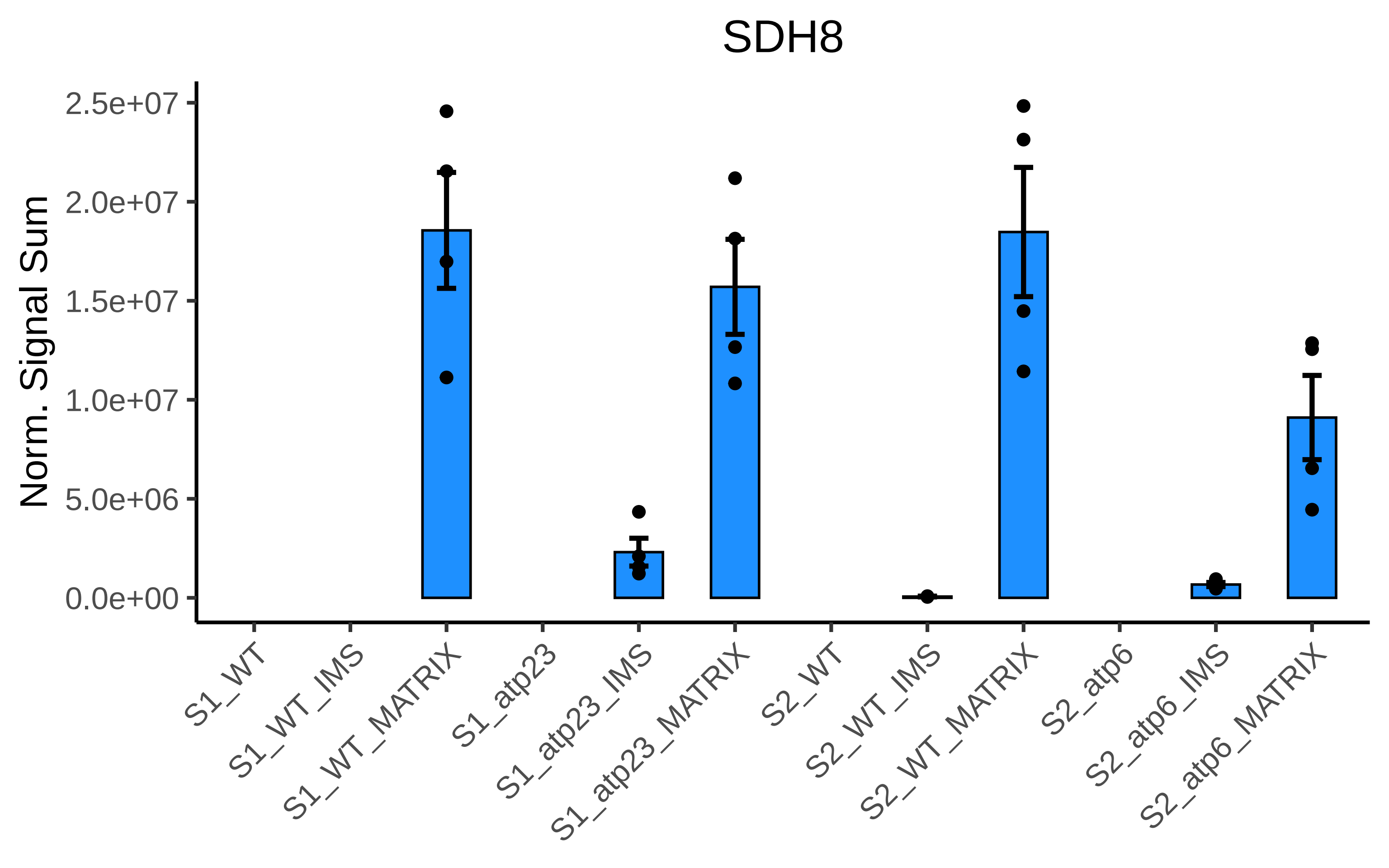

SDH8
Norm. Signal Sum
2.5e+07
2.0e+07
1.5e+07
1.0e+07
5.0e+06
0.0e+00
S1_WT
S1_WT_IMS
S1_WT_MATRIX
S1_atp23
S1_atp23_IMS
S1_atp23_MATRIX
S2_WT
S2_WT_IMS
S2_WT_MATRIX
S2_atp6
S2_atp6_IMS
S2_atp6_MATRIX

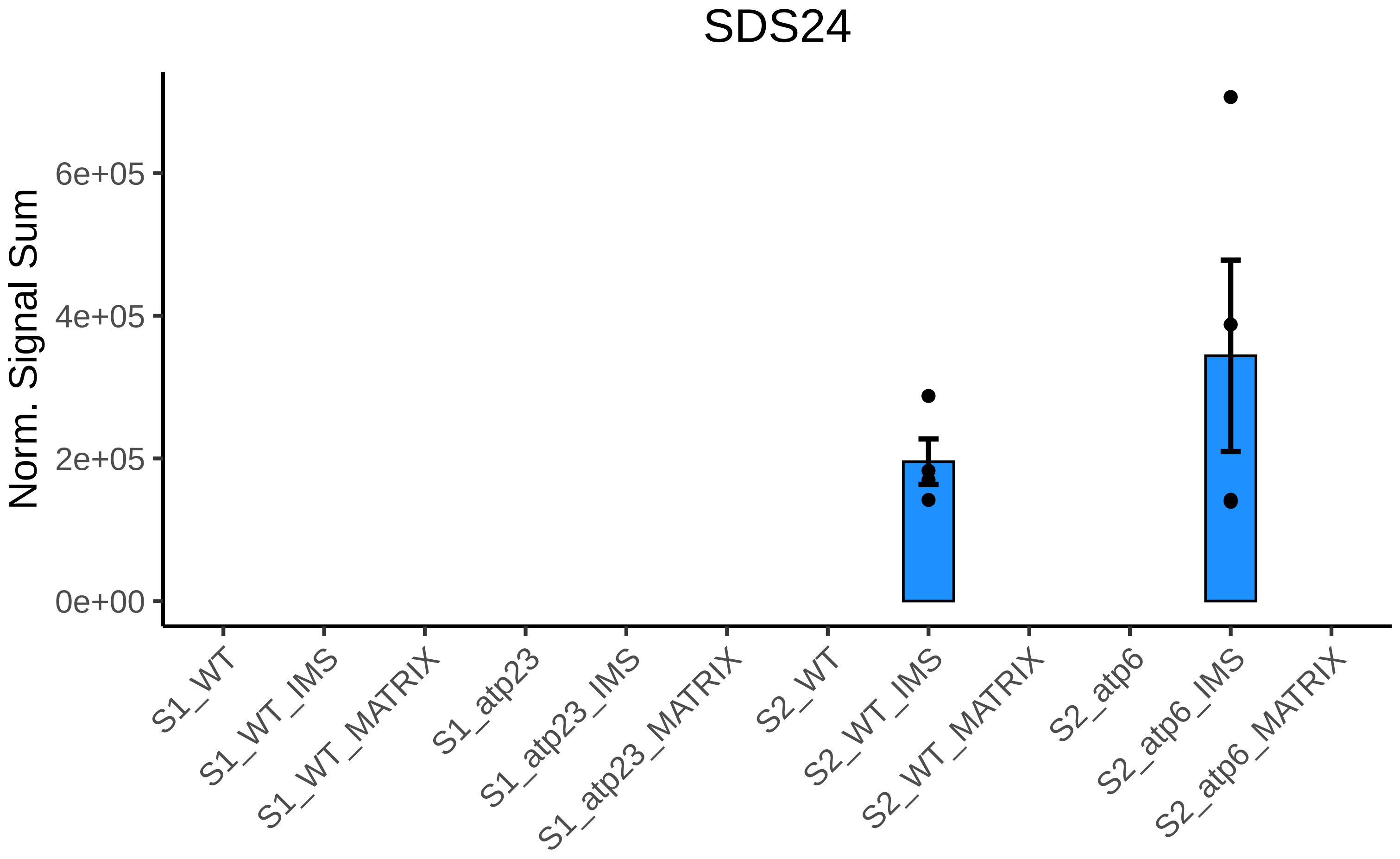
SDS24
Norm. Signal Sum
0e+00
2e+05
4e+05
6e+05
S1_WT
S1_WT_IMS
S1_WT_MATRIX
S1_atp23
S1_atp23_IMS
S1_atp23_MATRIX
S2_WT
S2_WT_IMS
S2_WT_MATRIX
S2_atp6
S2_atp6_IMS
S2_atp6_MATRIX

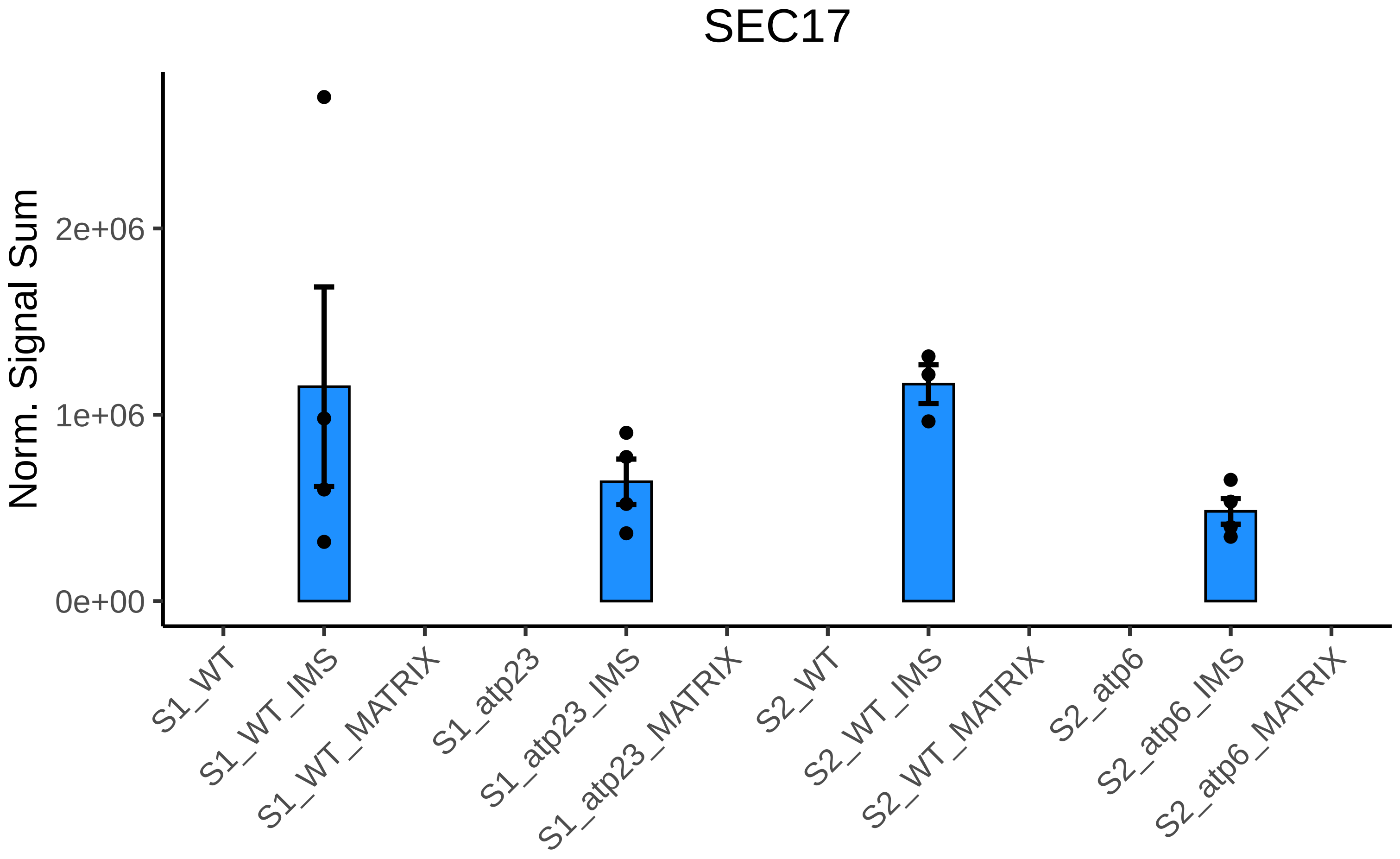

SEC17
Norm. Signal Sum
2e+06
1e+06
0e+00
S1_WT
S1_WT_IMS
S1_WT_MATRIX
S1_atp23
S1_atp23_IMS
S1_atp23_MATRIX
S2_WT
S2_WT_IMS
S2_WT_MATRIX
S2_atp6
S2_atp6_IMS
S2_atp6_MATRIX

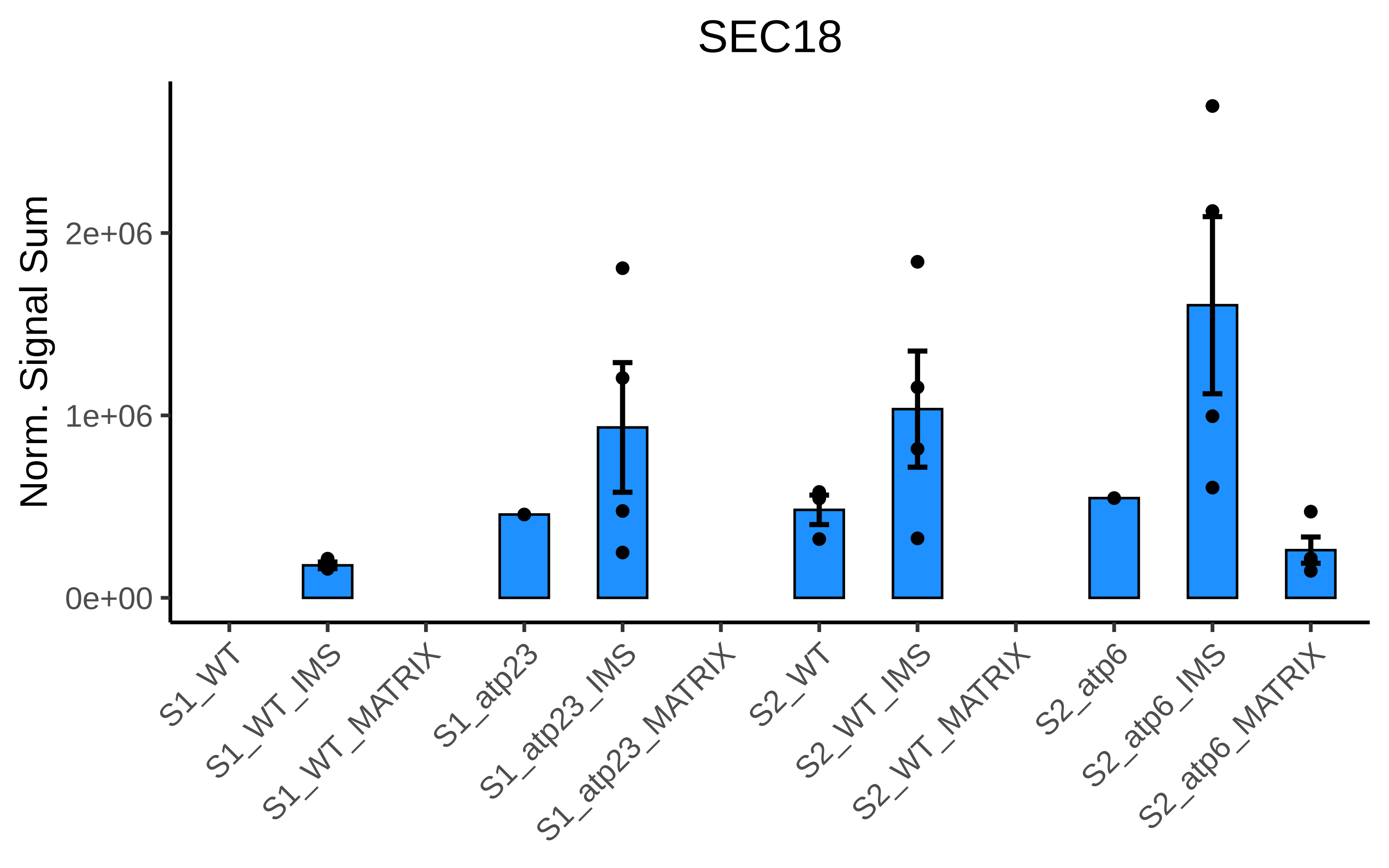

SEC18
Norm. Signal Sum
2e+06
1e+06
0e+00
S1_WT
S1_WT_IMS
S1_WT_MATRIX
S1_atp23
S1_atp23_IMS
S1_atp23_MATRIX
S2_WT
S2_WT_IMS
S2_WT_MATRIX
S2_atp6
S2_atp6_IMS
S2_atp6_MATRIX

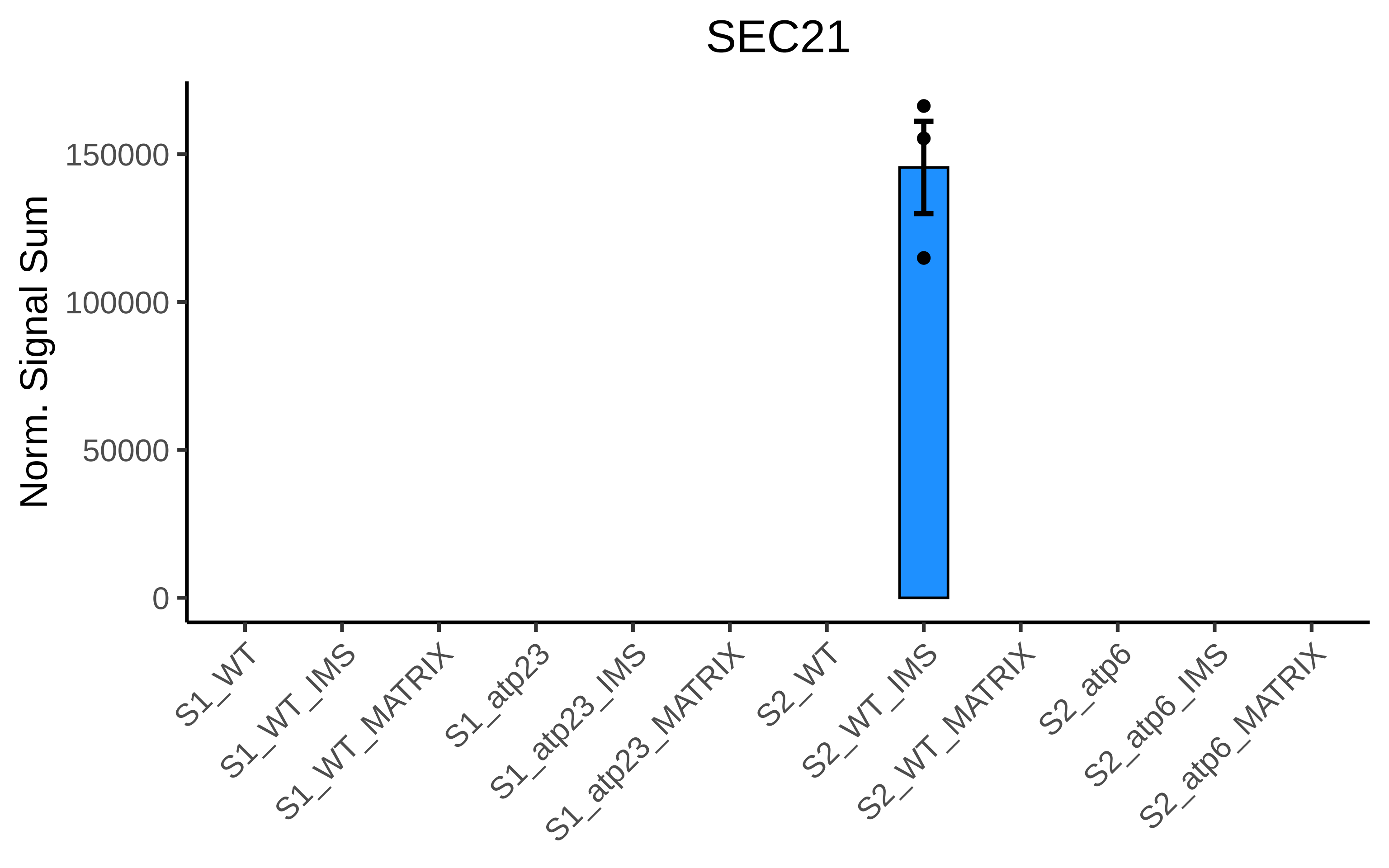

SEC21
Norm. Signal Sum
150000
100000
50000
0
S1_WT
S1_WT_IMS
S1_WT_MATRIX
S1_atp23
S1_atp23_IMS
S1_atp23_MATRIX
S2_WT
S2_WT_IMS
S2_WT_MATRIX
S2_atp6
S2_atp6_IMS
S2_atp6_MATRIX

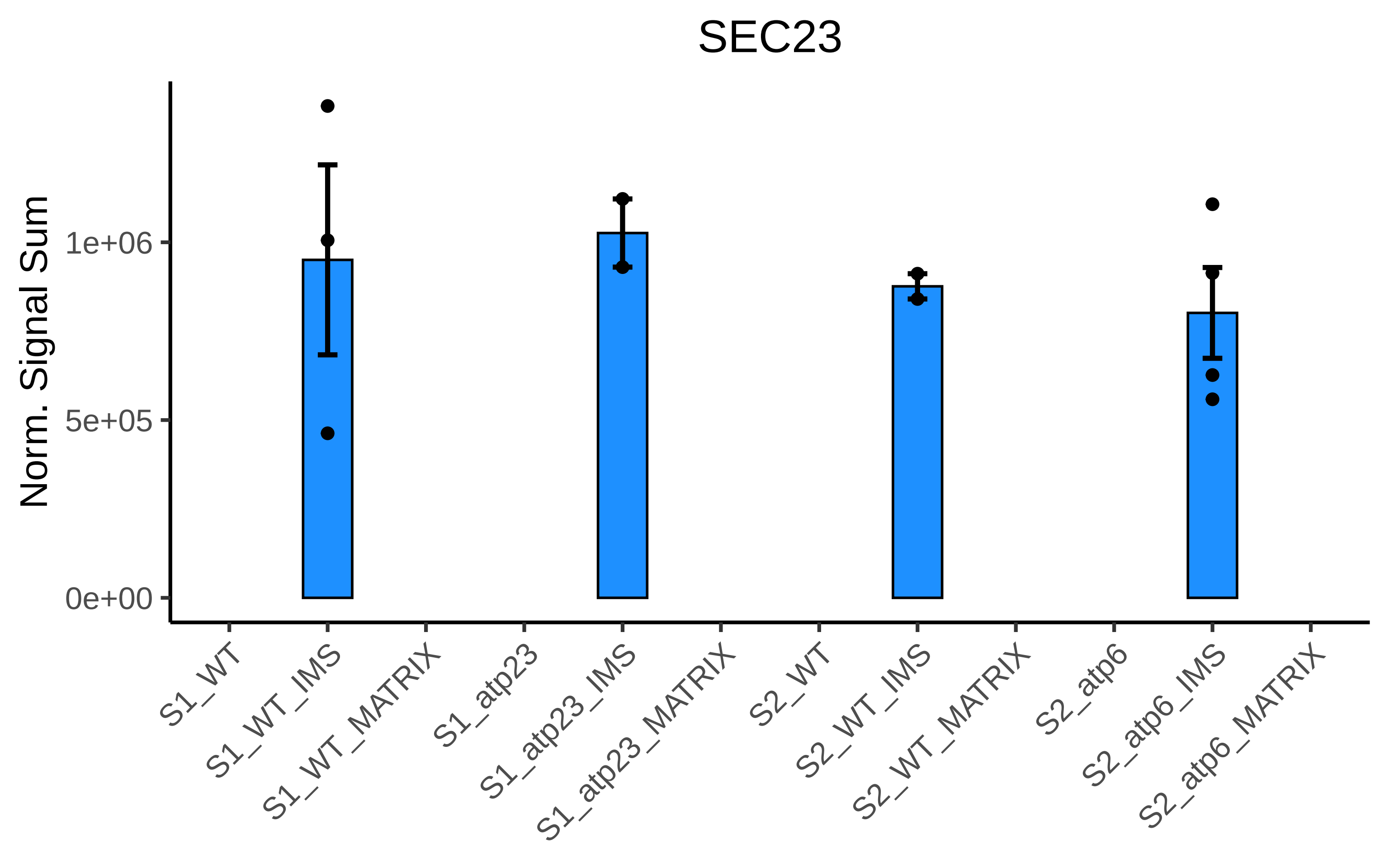
SEC23
Norm. Signal Sum
1e+06
5e+05
0e+00
S1_WT
S1_WT_IMS
S1_WT_MATRIX
S1_atp23
S1_atp23_IMS
S1_atp23_MATRIX
S2_WT
S2_WT_IMS
S2_WT_MATRIX
S2_atp6
S2_atp6_IMS
S2_atp6_MATRIX

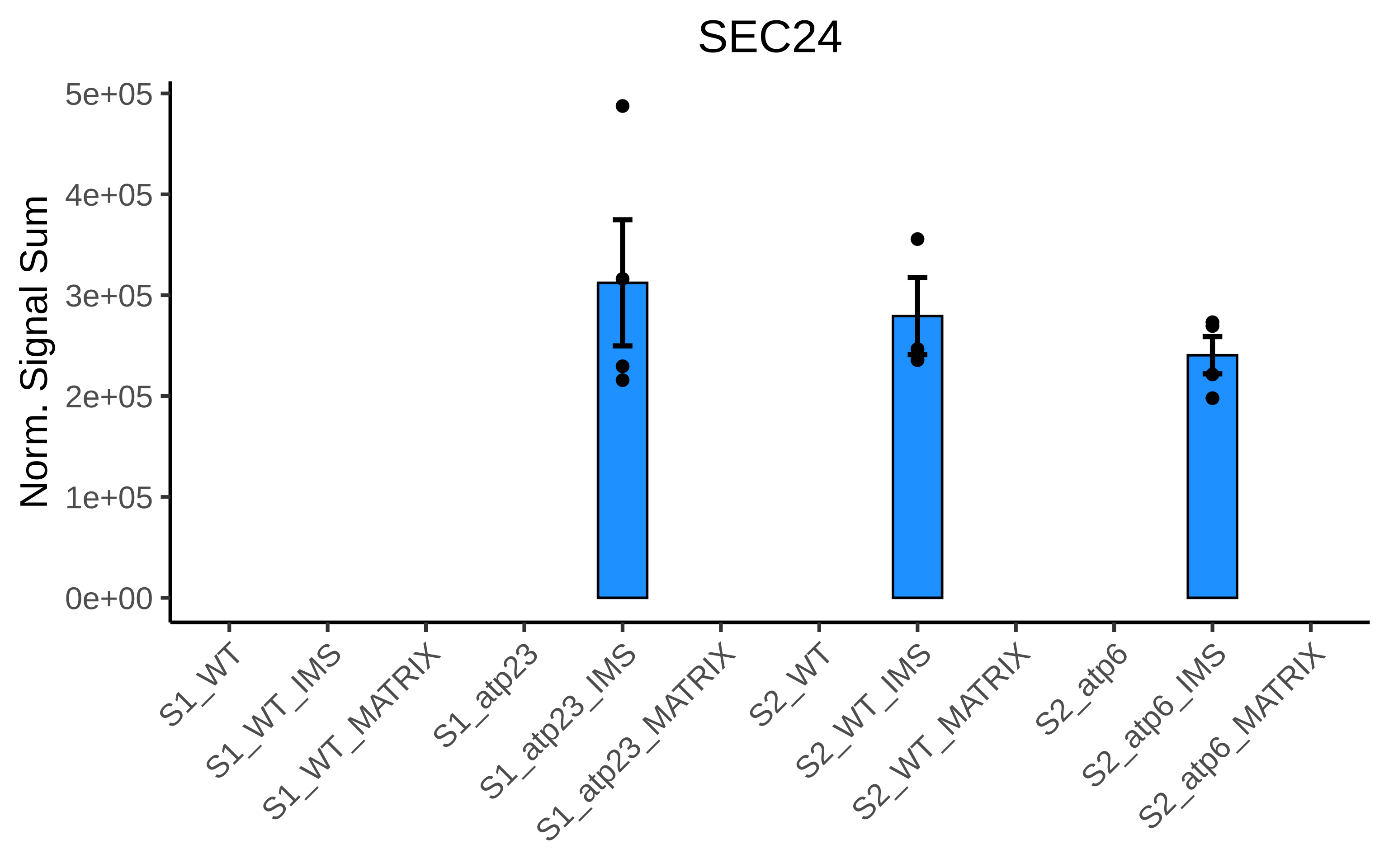
SEC24
Norm. Signal Sum
5e+05
4e+05
3e+05
2e+05
1e+05
0e+00
S1_WT
S1_WT_IMS
S1_WT_MATRIX
S1_atp23
S1_atp23_IMS
S1_atp23_MATRIX
S2_WT
S2_WT_IMS
S2_WT_MATRIX
S2_atp6
S2_atp6_IMS
S2_atp6_MATRIX

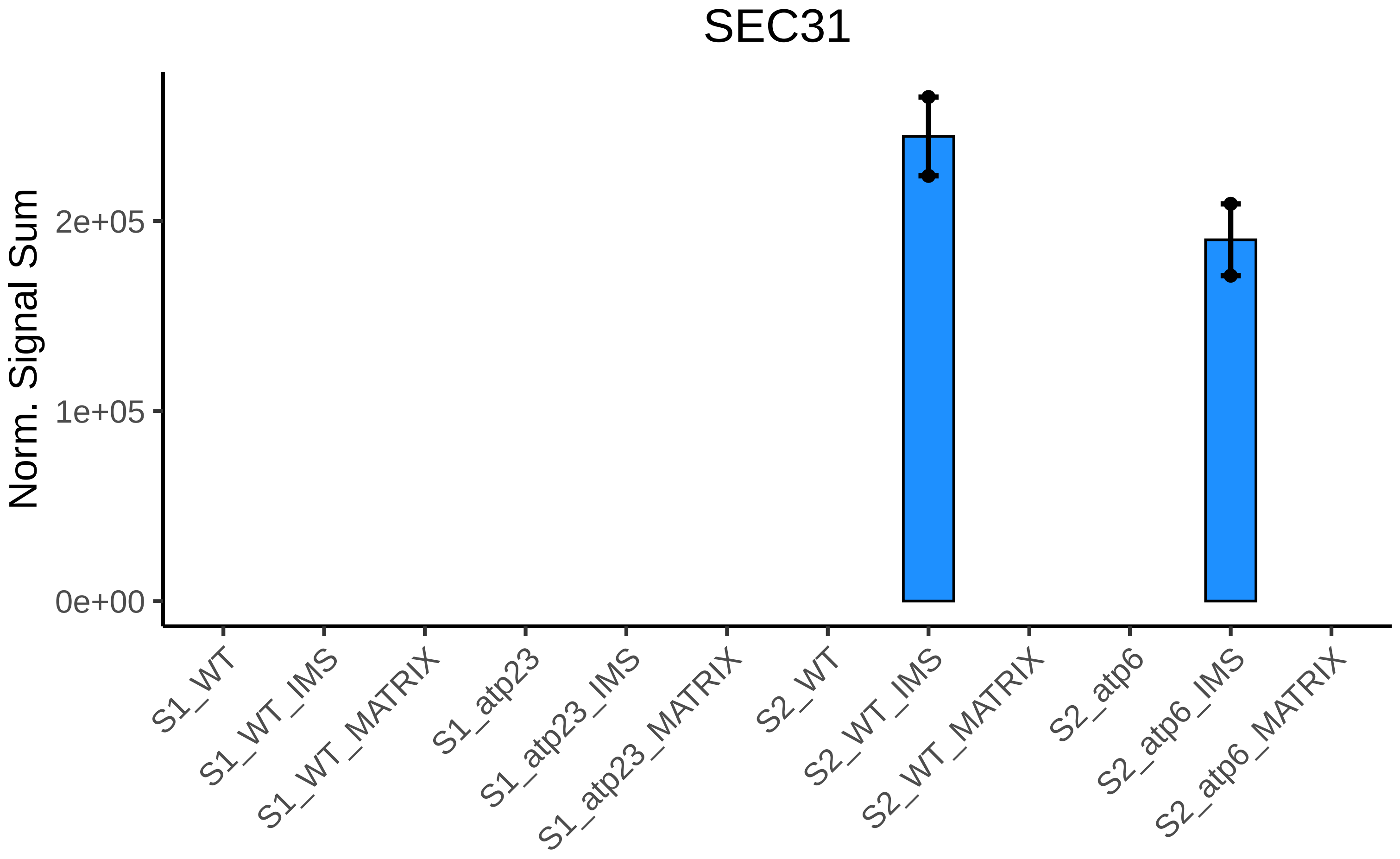

SEC31
Norm. Signal Sum
0e+00
1e+05
2e+05
S1_WT
S1_WT_IMS
S1_WT_MATRIX
S1_atp23
S1_atp23_IMS
S1_atp23_MATRIX
S2_WT
S2_WT_IMS
S2_WT_MATRIX
S2_atp6
S2_atp6_IMS
S2_atp6_MATRIX

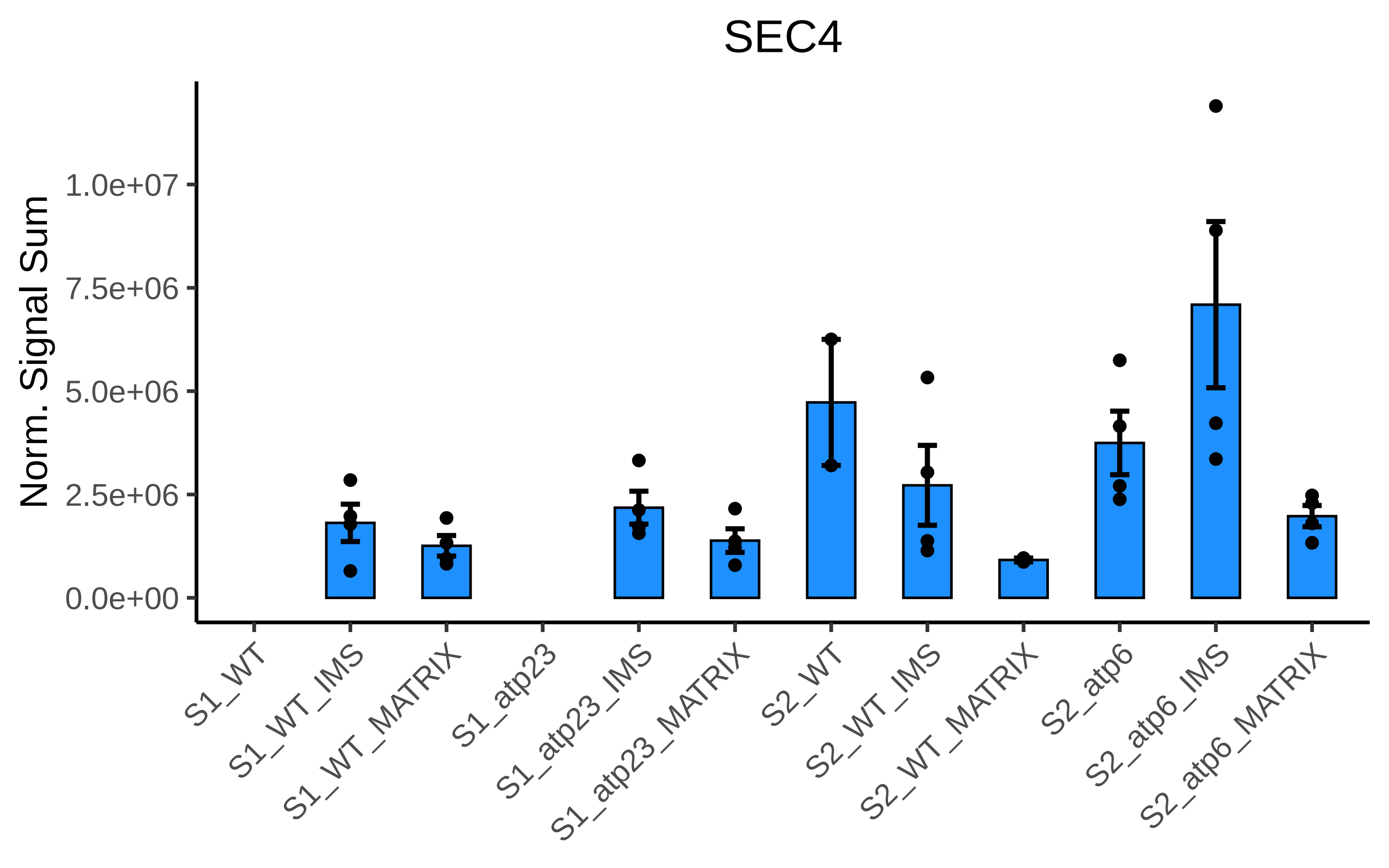
SEC4
Norm. Signal Sum
1.0e+07
7.5e+06
5.0e+06
2.5e+06
0.0e+00
S1_WT
S1_WT_IMS
S1_WT_MATRIX
S1_atp23
S1_atp23_IMS
S1_atp23_MATRIX
S2_WT
S2_WT_IMS
S2_WT_MATRIX
S2_atp6
S2_atp6_IMS
S2_atp6_MATRIX

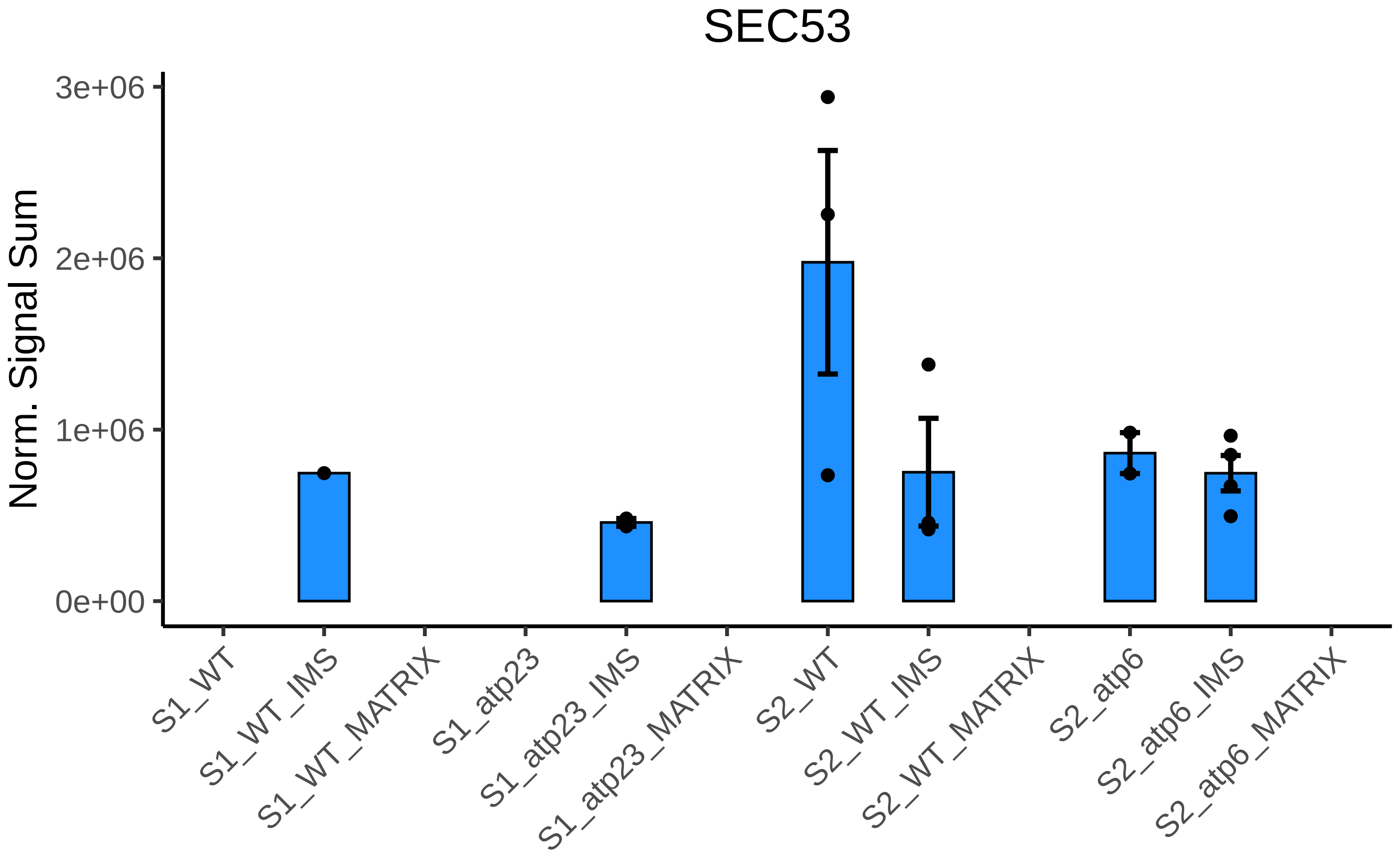

SEC53
Norm. Signal Sum
3e+06
2e+06
1e+06
0e+00
S1_WT
S1_WT_IMS
S1_WT_MATRIX
S1_atp23
S1_atp23_IMS
S1_atp23_MATRIX
S2_WT
S2_WT_IMS
S2_WT_MATRIX
S2_atp6
S2_atp6_IMS
S2_atp6_MATRIX

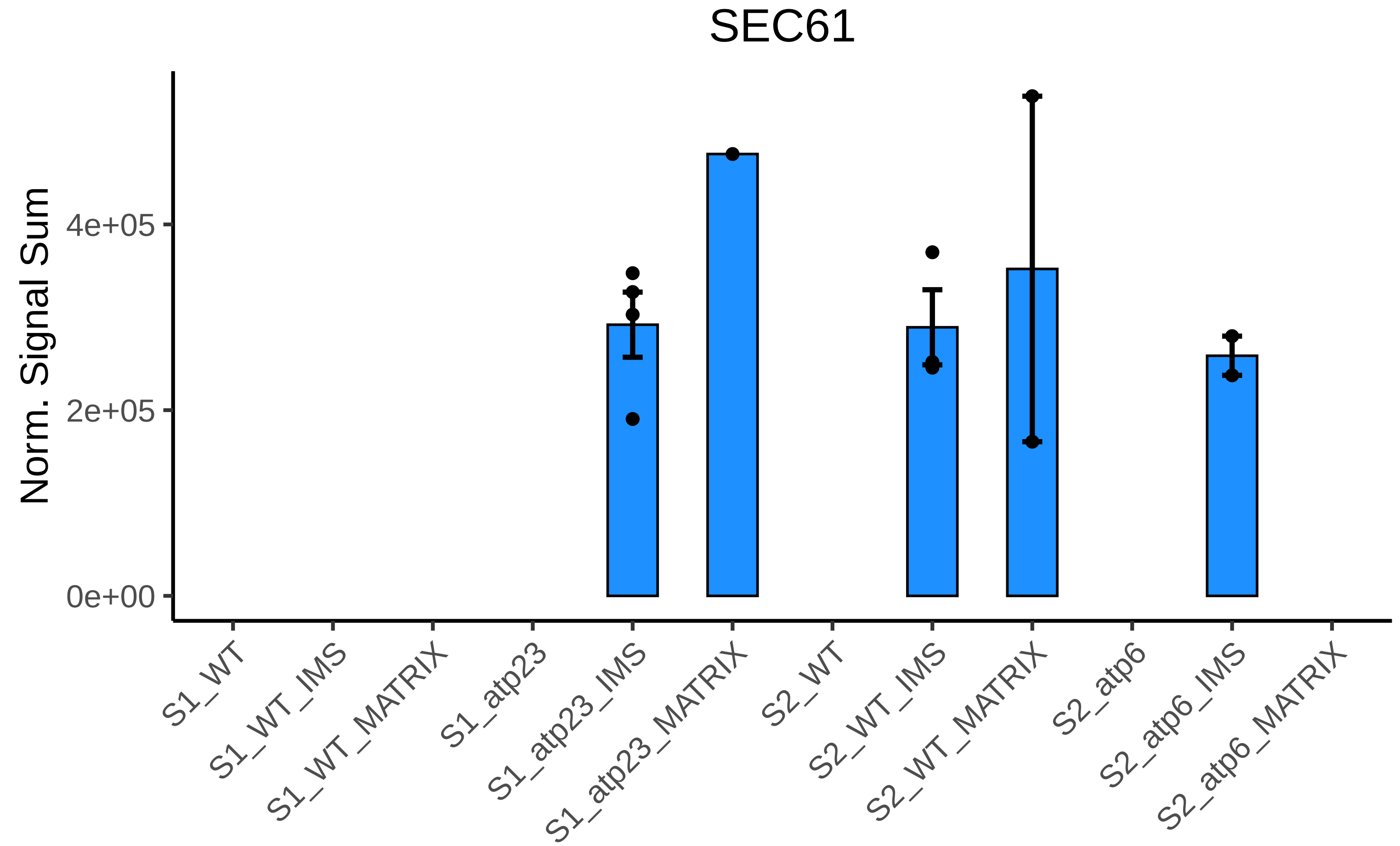

SEC61
Norm. Signal Sum
4e+05
2e+05
0e+00
S1_WT
S1_WT_IMS
S1_WT_MATRIX
S1_atp23
S1_atp23_IMS
S1_atp23_MATRIX
S2_WT
S2_WT_IMS
S2_WT_MATRIX
S2_atp6
S2_atp6_IMS
S2_atp6_MATRIX

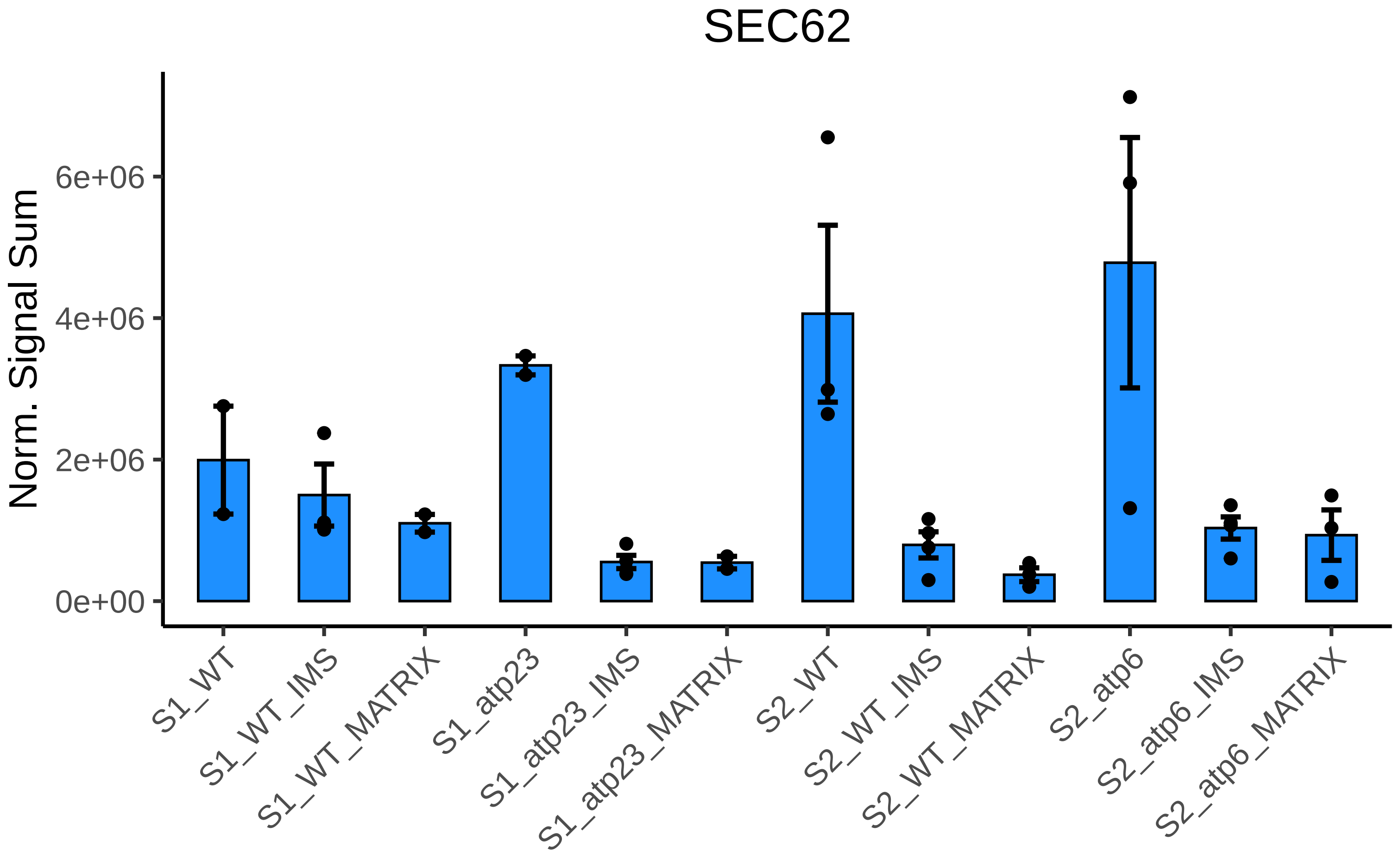

SEC62
Norm. Signal Sum
0e+00
2e+06
4e+06
6e+06
S1_WT
S1_WT_IMS
S1_WT_MATRIX
S1_atp23
S1_atp23_IMS
S1_atp23_MATRIX
S2_WT
S2_WT_IMS
S2_WT_MATRIX
S2_atp6
S2_atp6_IMS
S2_atp6_MATRIX

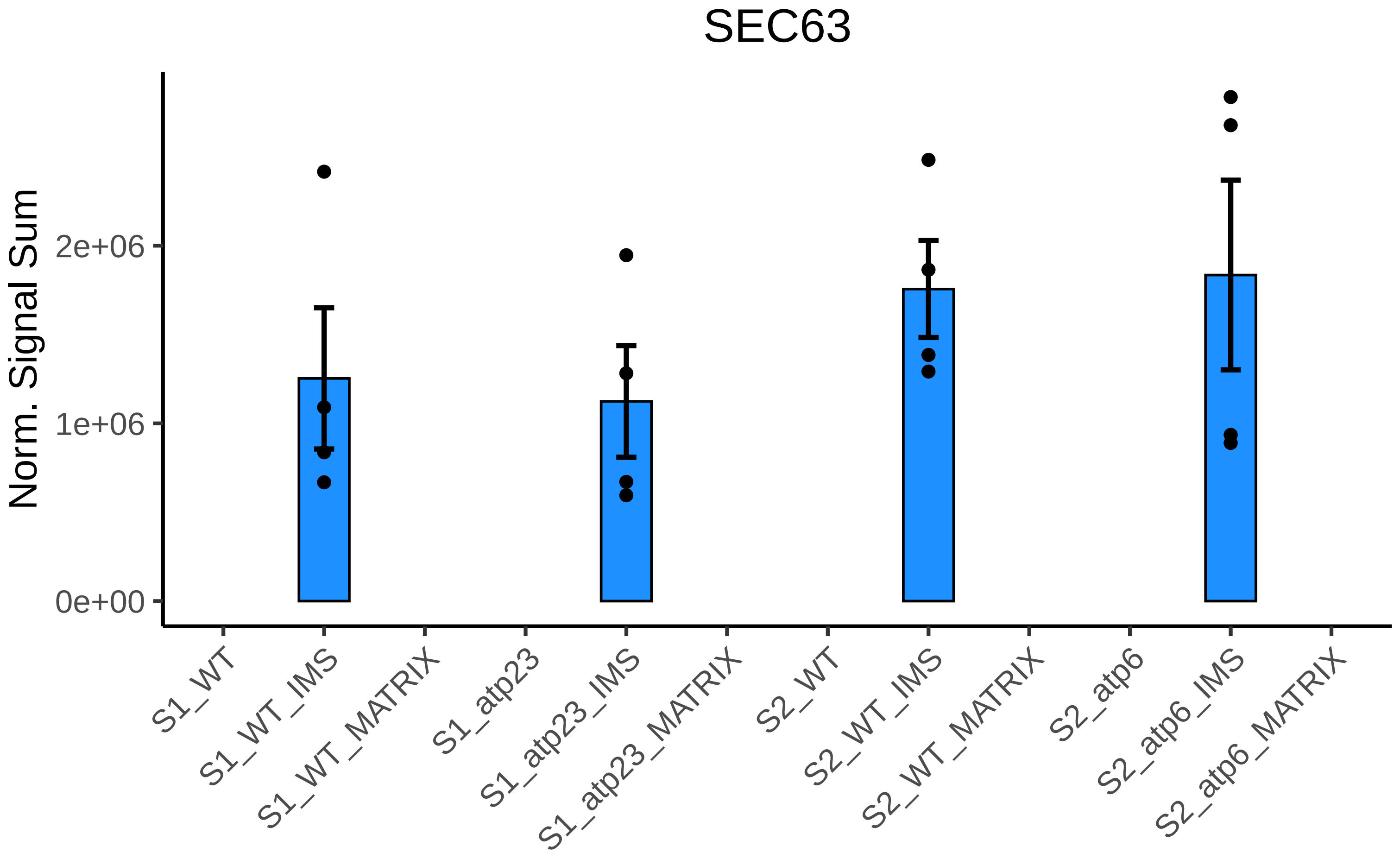

SEC63
Norm. Signal Sum
0e+00
1e+06
2e+06
S1_WT
S1_WT_IMS
S1_WT_MATRIX
S1_atp23
S1_atp23_IMS
S1_atp23_MATRIX
S2_WT
S2_WT_IMS
S2_WT_MATRIX
S2_atp6
S2_atp6_IMS
S2_atp6_MATRIX

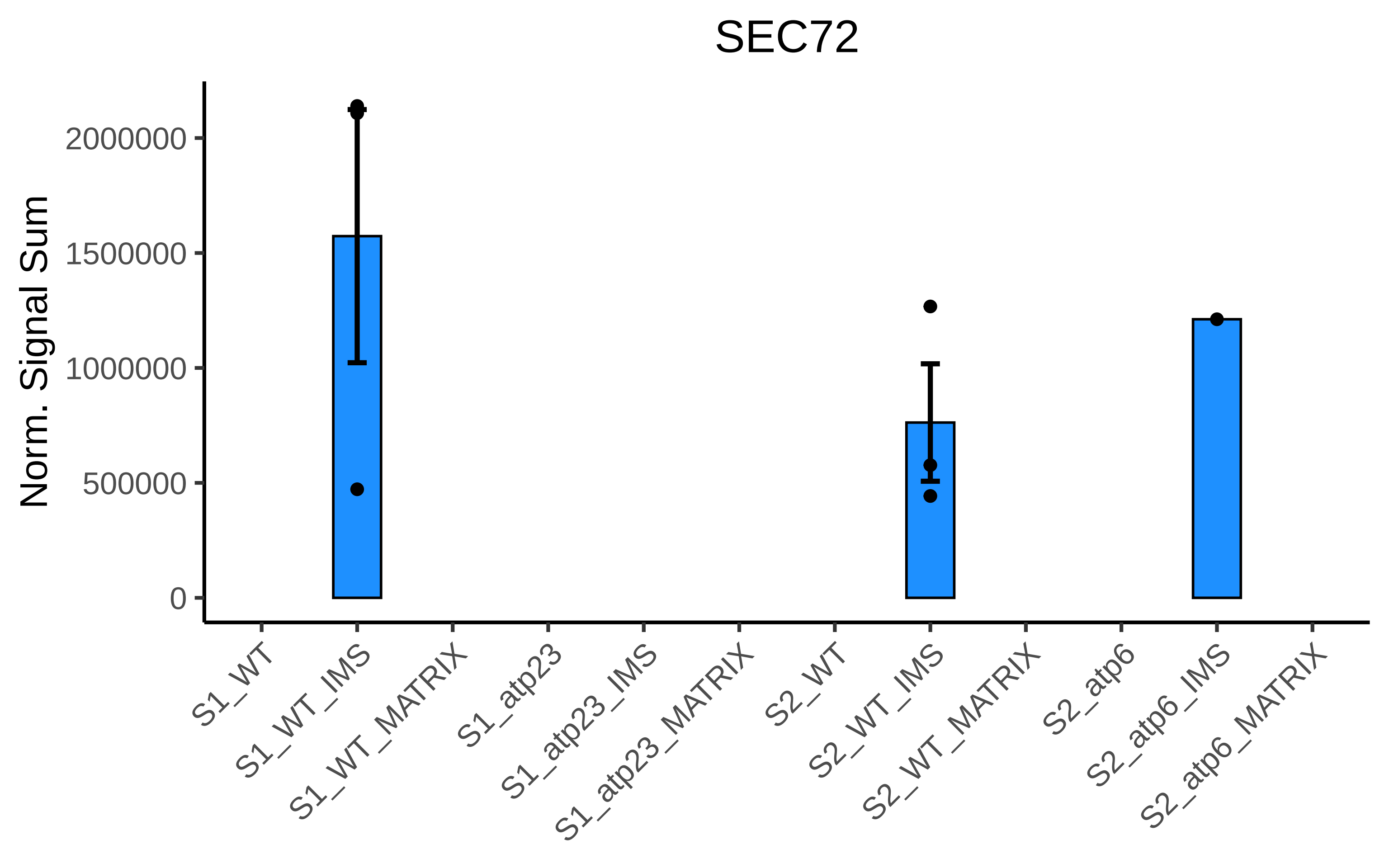

SEC72
Norm. Signal Sum
2000000
1500000
1000000
500000
0
S1_WT
S1_WT_IMS
S1_WT_MATRIX
S1_atp23
S1_atp23_IMS
S1_atp23_MATRIX
S2_WT
S2_WT_IMS
S2_WT_MATRIX
S2_atp6
S2_atp6_IMS
S2_atp6_MATRIX

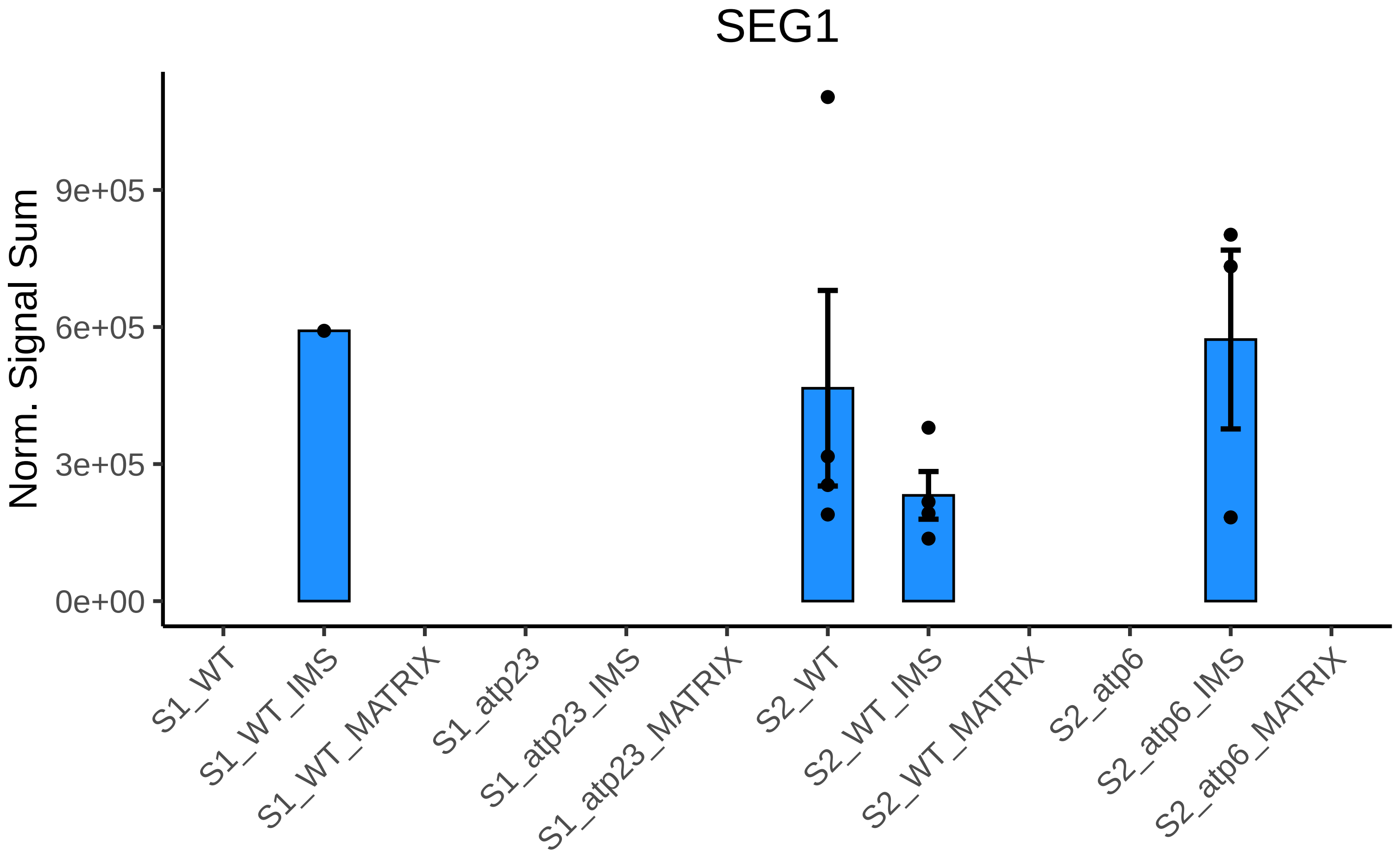

SEG1
Norm. Signal Sum
9e+05
6e+05
3e+05
0e+00
S1_WT
S1_WT_IMS
S1_WT_MATRIX
S1_atp23
S1_atp23_IMS
S1_atp23_MATRIX
S2_WT
S2_WT_IMS
S2_WT_MATRIX
S2_atp6
S2_atp6_IMS
S2_atp6_MATRIX

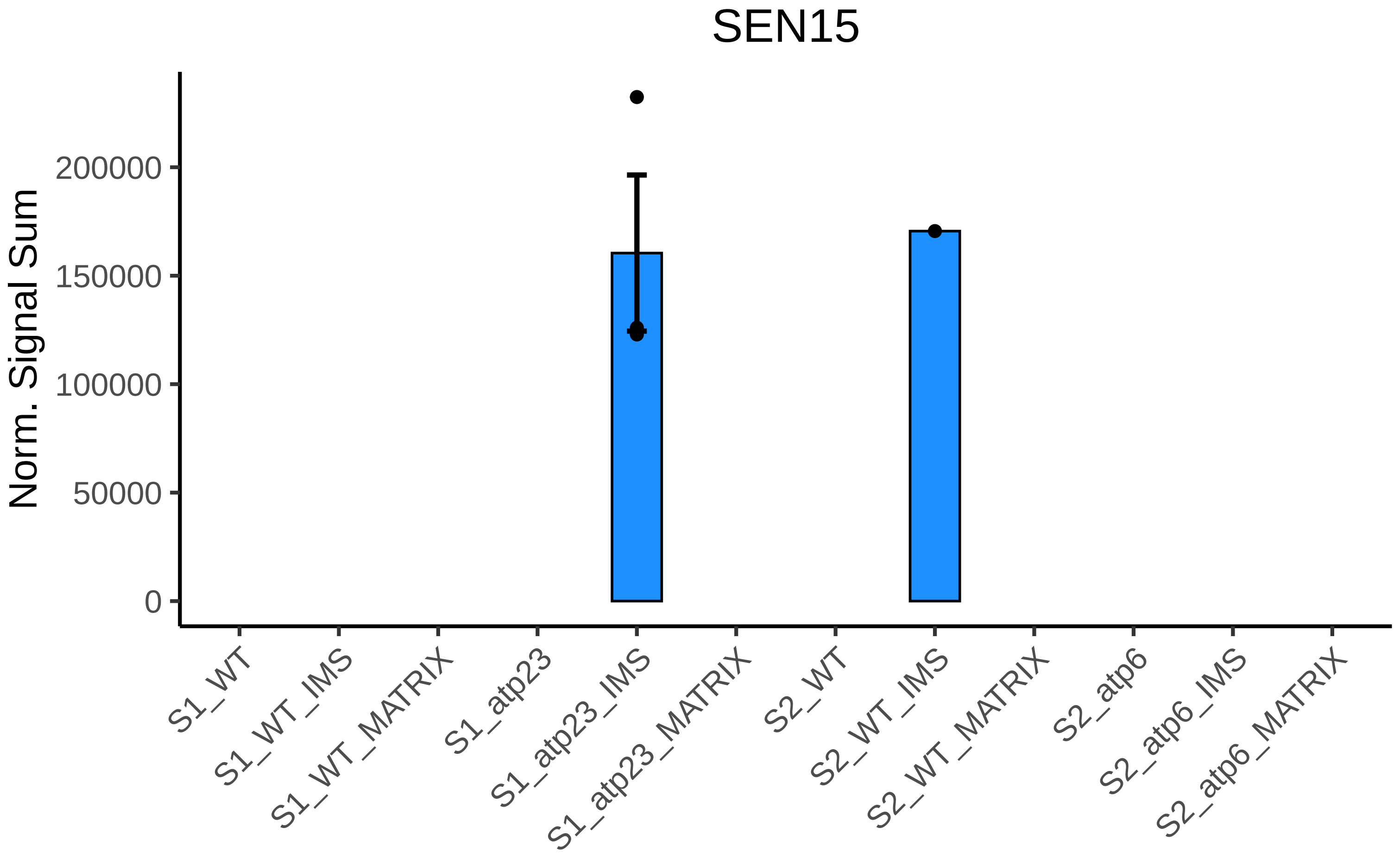

SEN15
Norm. Signal Sum
200000
150000
100000
50000
0
S1_WT
S1_WT_IMS
S1_WT_MATRIX
S1_atp23
S1_atp23_IMS
S1_atp23_MATRIX
S2_WT
S2_WT_IMS
S2_WT_MATRIX
S2_atp6
S2_atp6_IMS
S2_atp6_MATRIX

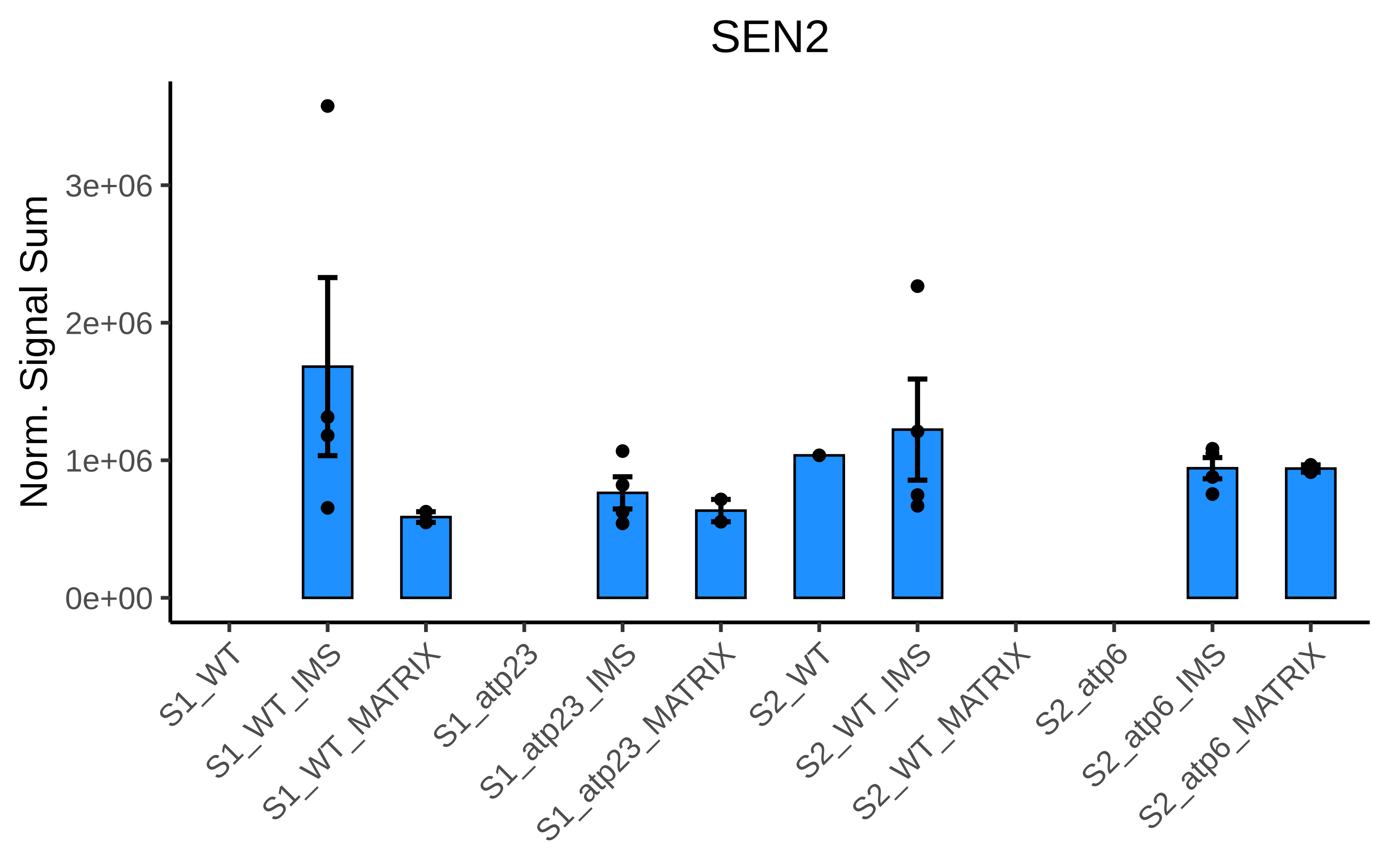
SEN2
Norm. Signal Sum
3e+06
2e+06
1e+06
0e+00
S1_WT
S1_WT_IMS
S1_WT_MATRIX
S1_atp23
S1_atp23_IMS
S1_atp23_MATRIX
S2_WT
S2_WT_IMS
S2_WT_MATRIX
S2_atp6
S2_atp6_IMS
S2_atp6_MATRIX

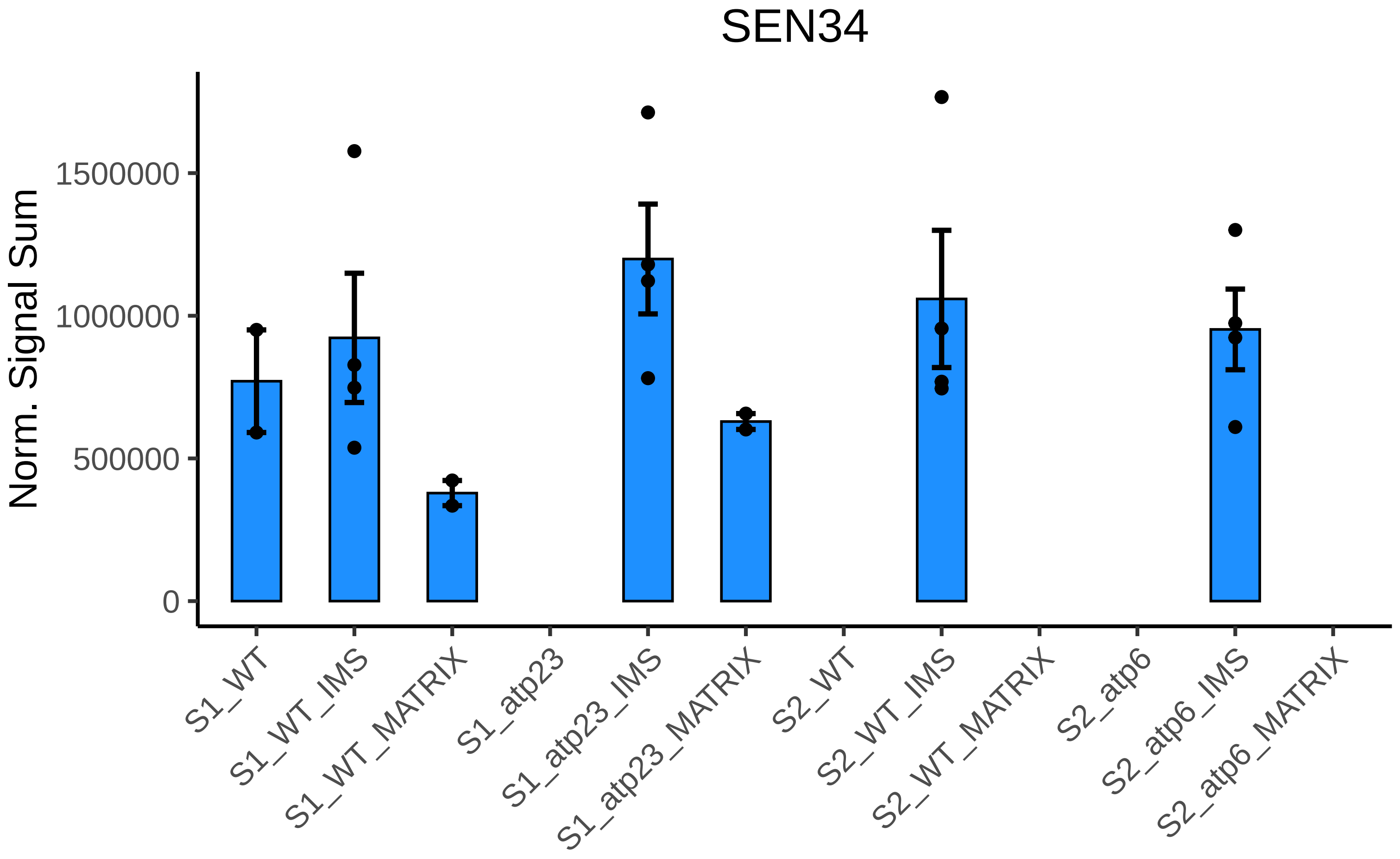
SEN34
Norm. Signal Sum
1500000
1000000
500000
0
S1_WT
S1_WT_IMS
S1_WT_MATRIX
S1_atp23
S1_atp23_IMS
S1_atp23_MATRIX
S2_WT
S2_WT_IMS
S2_WT_MATRIX
S2_atp6
S2_atp6_IMS
S2_atp6_MATRIX

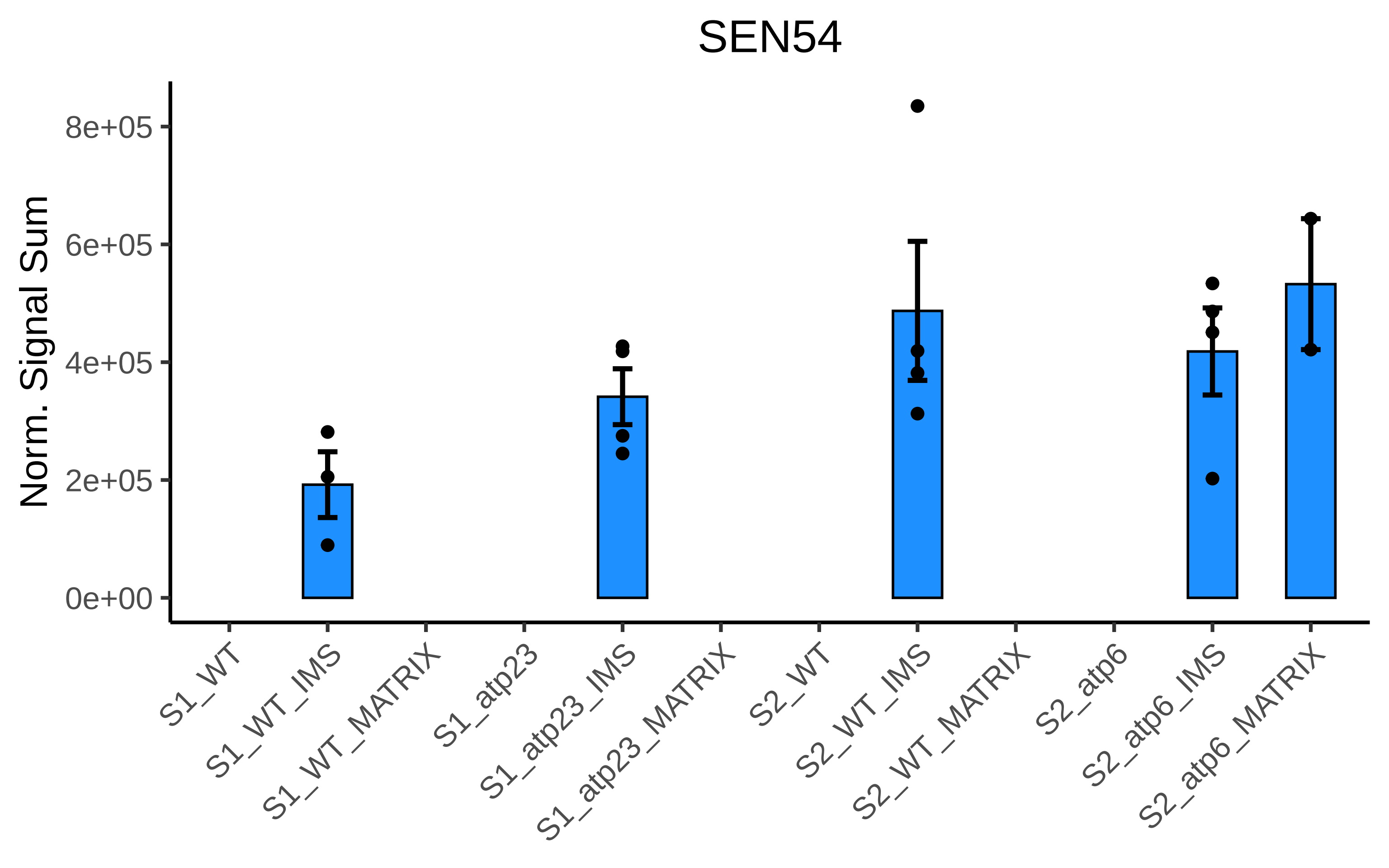
SEN54
Norm. Signal Sum
8e+05
6e+05
4e+05
2e+05
0e+00
S1_WT
S1_WT_IMS
S1_WT_MATRIX
S1_atp23
S1_atp23_IMS
S1_atp23_MATRIX
S2_WT
S2_WT_IMS
S2_WT_MATRIX
S2_atp6
S2_atp6_IMS
S2_atp6_MATRIX

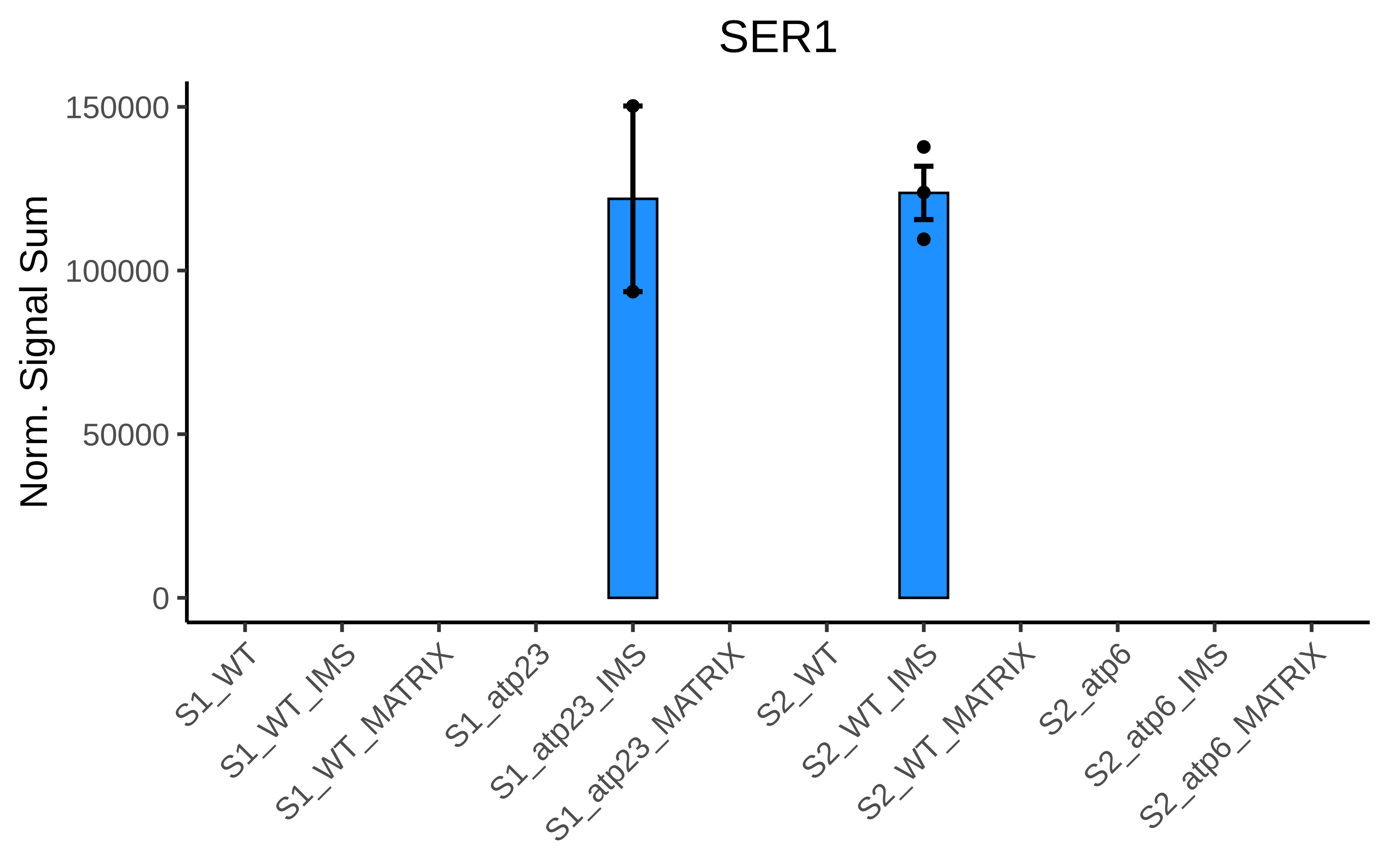
SER1
Norm. Signal Sum
150000
100000
50000
0
S1_WT
S1_WT_IMS
S1_WT_MATRIX
S1_atp23
S1_atp23_IMS
S1_atp23_MATRIX
S2_WT
S2_WT_IMS
S2_WT_MATRIX
S2_atp6
S2_atp6_IMS
S2_atp6_MATRIX

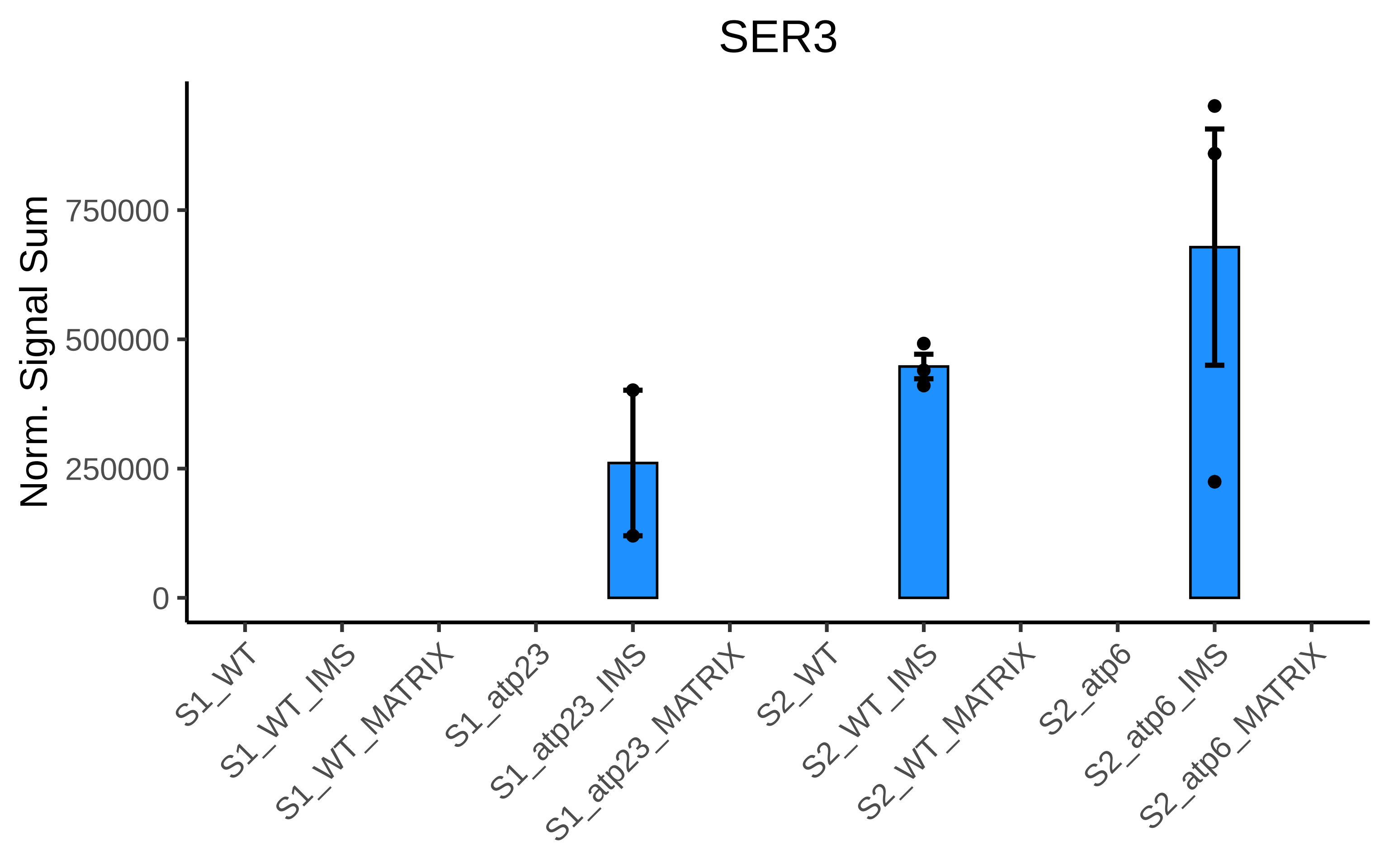
SER3
Norm. Signal Sum
750000
500000
250000
0
S1_WT
S1_WT_IMS
S1_WT_MATRIX
S1_atp23
S1_atp23_IMS
S1_atp23_MATRIX
S2_WT
S2_WT_IMS
S2_WT_MATRIX
S2_atp6
S2_atp6_IMS
S2_atp6_MATRIX

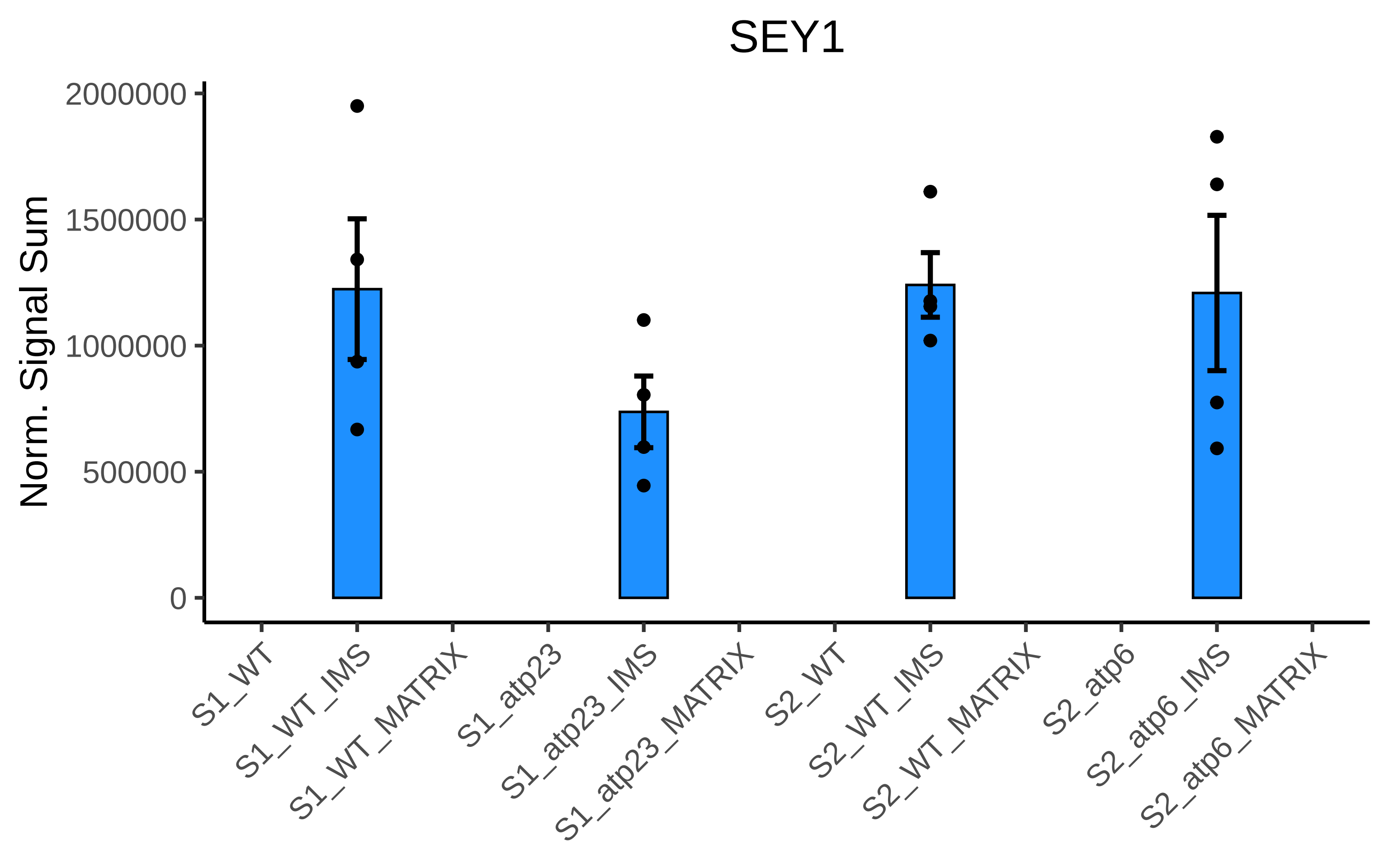

SEY1
Norm. Signal Sum
2000000
1500000
1000000
500000
0
S1_WT
S1_WT_IMS
S1_WT_MATRIX
S1_atp23
S1_atp23_IMS
S1_atp23_MATRIX
S2_WT
S2_WT_IMS
S2_WT_MATRIX
S2_atp6
S2_atp6_IMS
S2_atp6_MATRIX

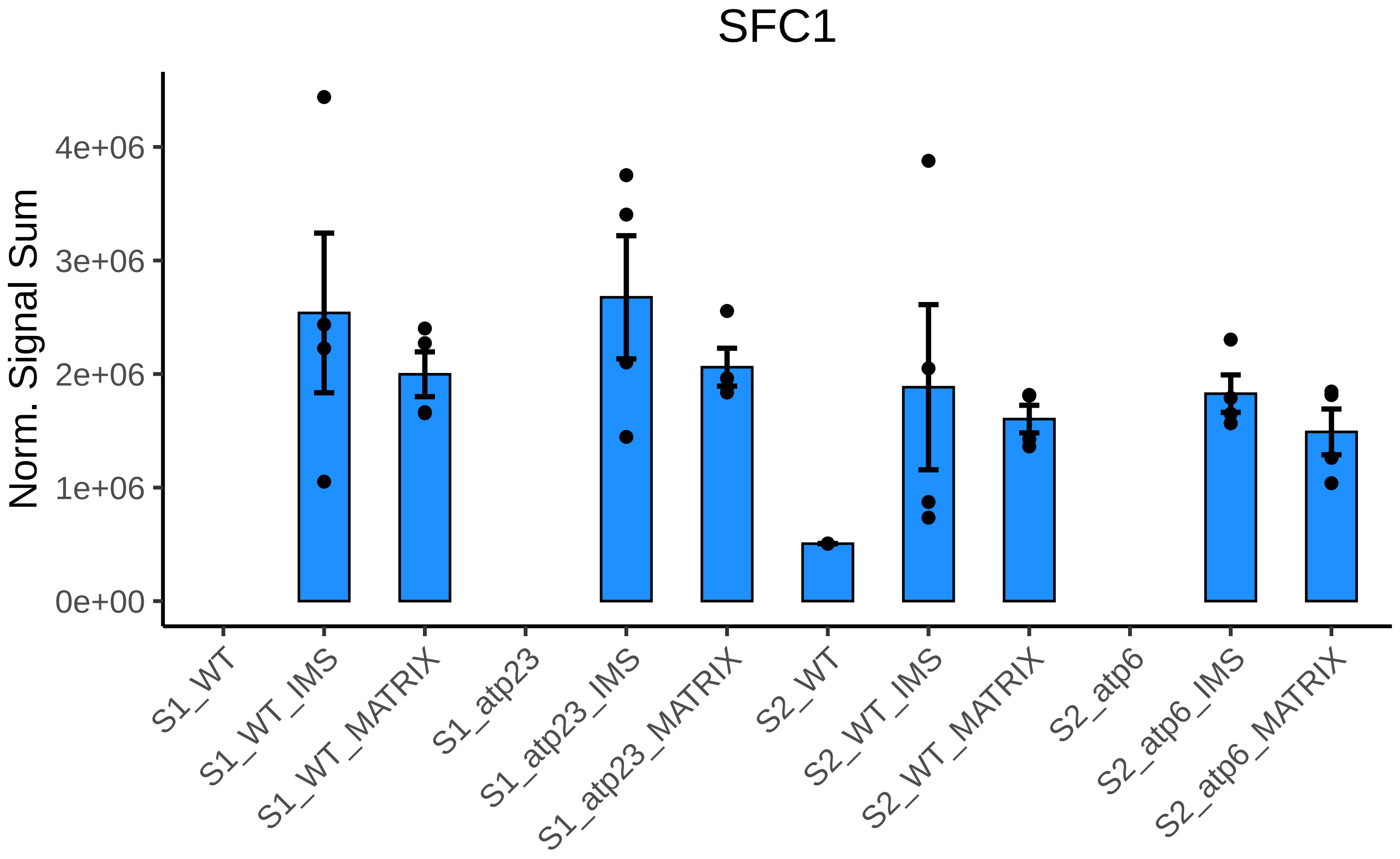

SFC1
Norm. Signal Sum
0e+00
1e+06
2e+06
3e+06
4e+06
S1_WT
S1_WT_IMS
S1_WT_MATRIX
S1_atp23
S1_atp23_IMS
S1_atp23_MATRIX
S2_WT
S2_WT_IMS
S2_WT_MATRIX
S2_atp6
S2_atp6_IMS
S2_atp6_MATRIX

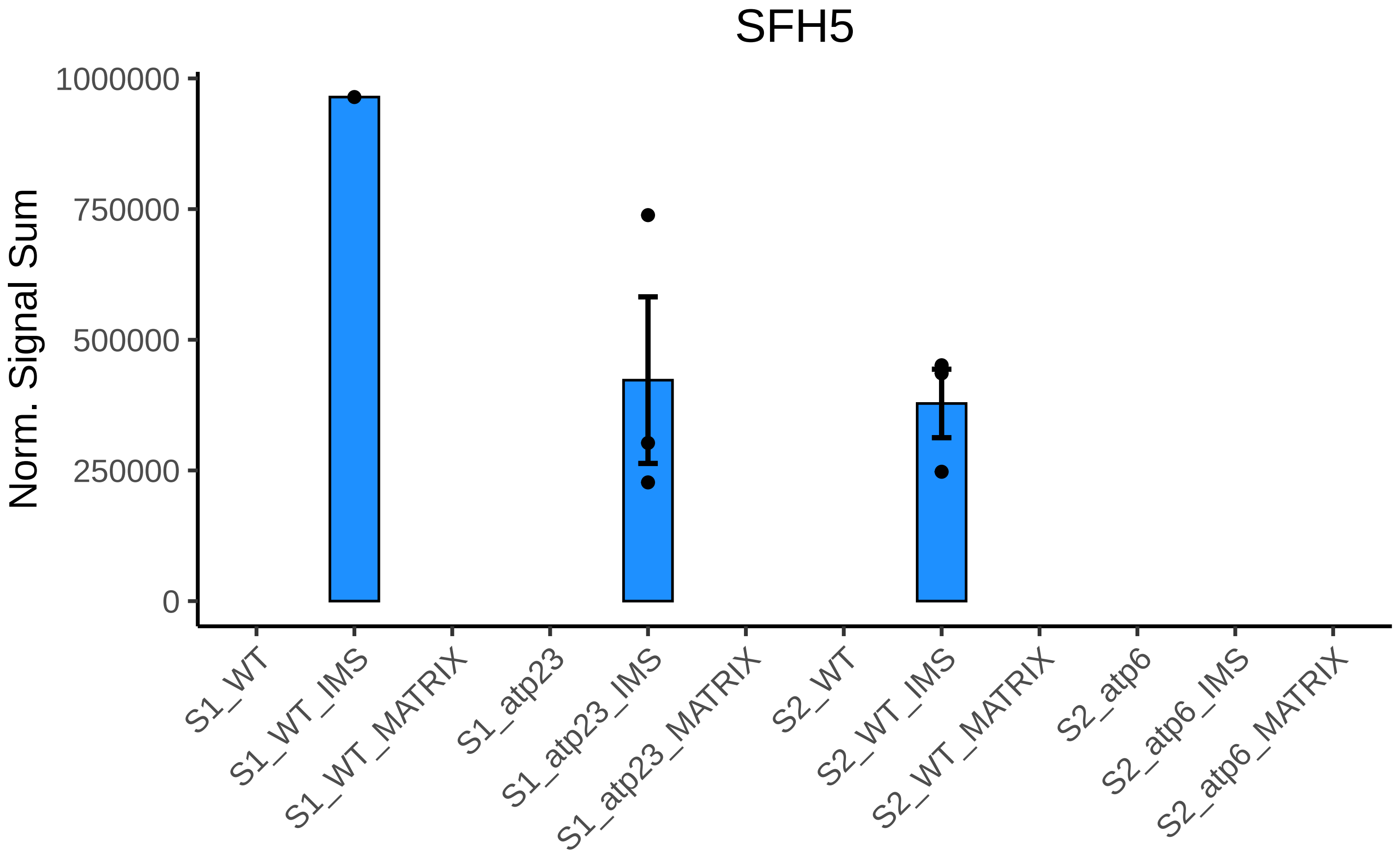

SFH5
Norm. Signal Sum
1000000
750000
500000
250000
0
S1_WT
S1_WT_IMS
S1_WT_MATRIX
S1_atp23
S1_atp23_IMS
S1_atp23_MATRIX
S2_WT
S2_WT_IMS
S2_WT_MATRIX
S2_atp6
S2_atp6_IMS
S2_atp6_MATRIX

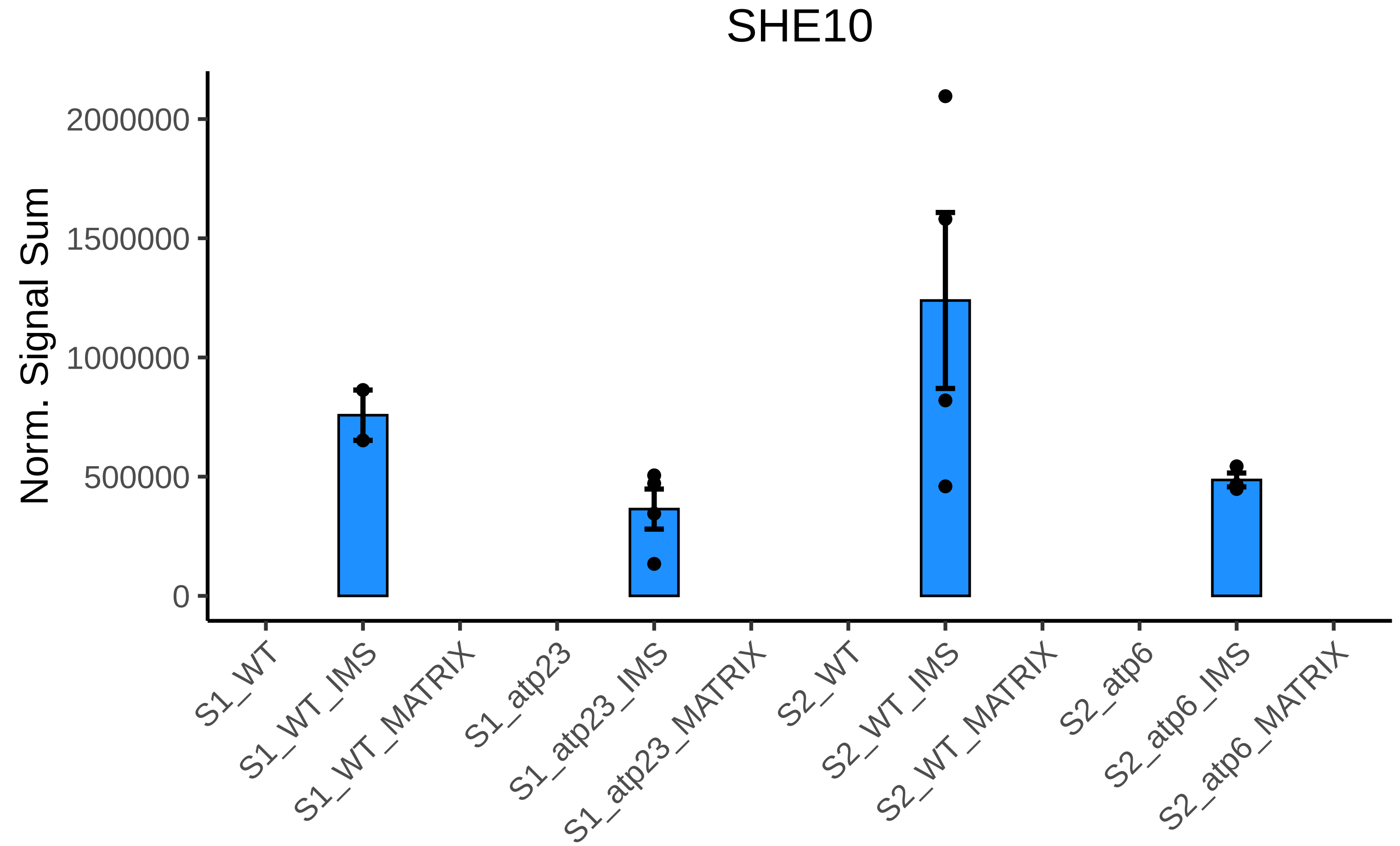

SHE10
Norm. Signal Sum
2000000
1500000
1000000
500000
0
S1_WT
S1_WT_IMS
S1_WT_MATRIX
S1_atp23
S1_atp23_IMS
S1_atp23_MATRIX
S2_WT
S2_WT_IMS
S2_WT_MATRIX
S2_atp6
S2_atp6_IMS
S2_atp6_MATRIX

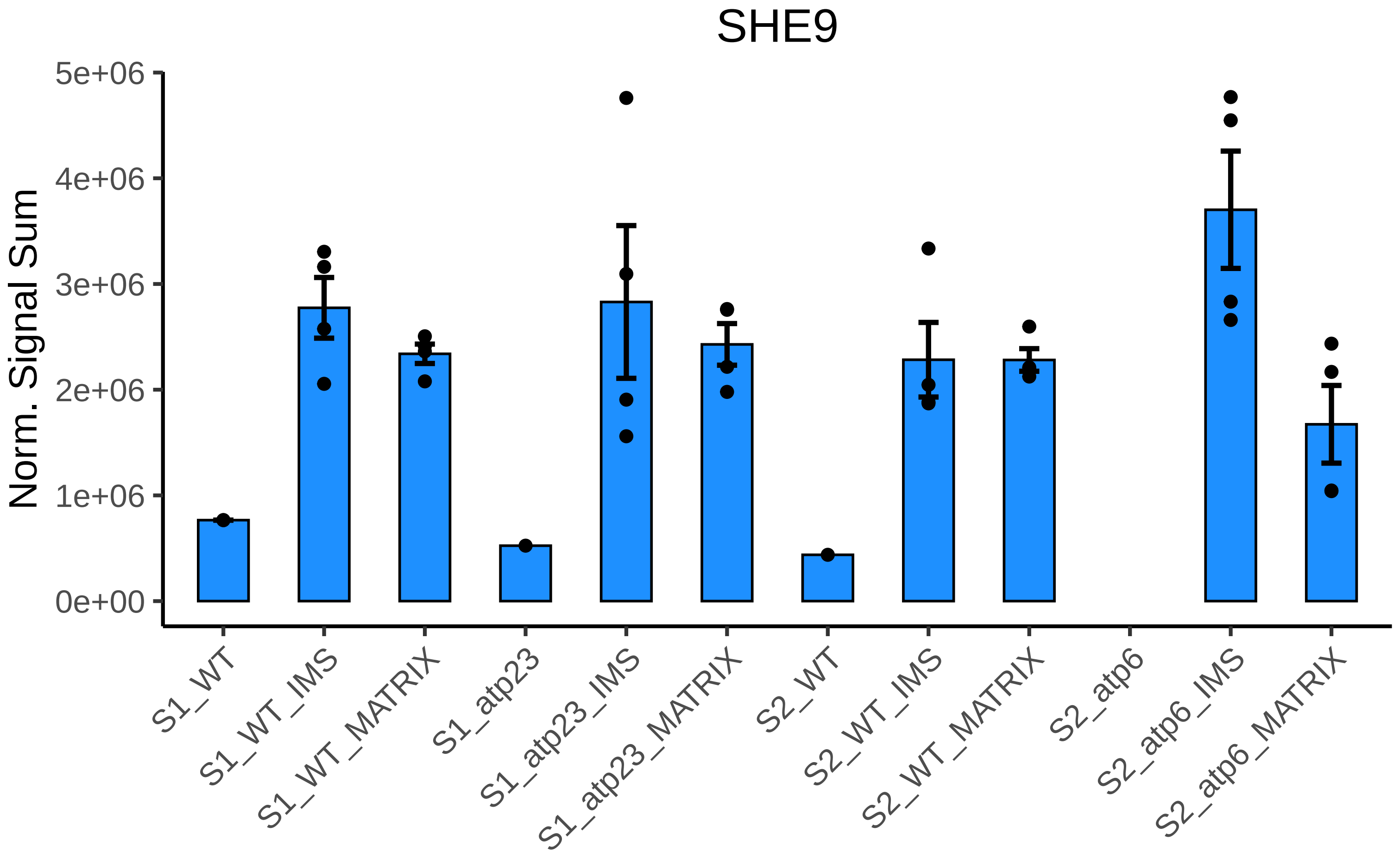
SHE9
Norm. Signal Sum
5e+06
4e+06
3e+06
2e+06
1e+06
0e+00
S1_WT
S1_WT_IMS
S1_WT_MATRIX
S1_atp23
S1_atp23_IMS
S1_atp23_MATRIX
S2_WT
S2_WT_IMS
S2_WT_MATRIX
S2_atp6
S2_atp6_IMS
S2_atp6_MATRIX

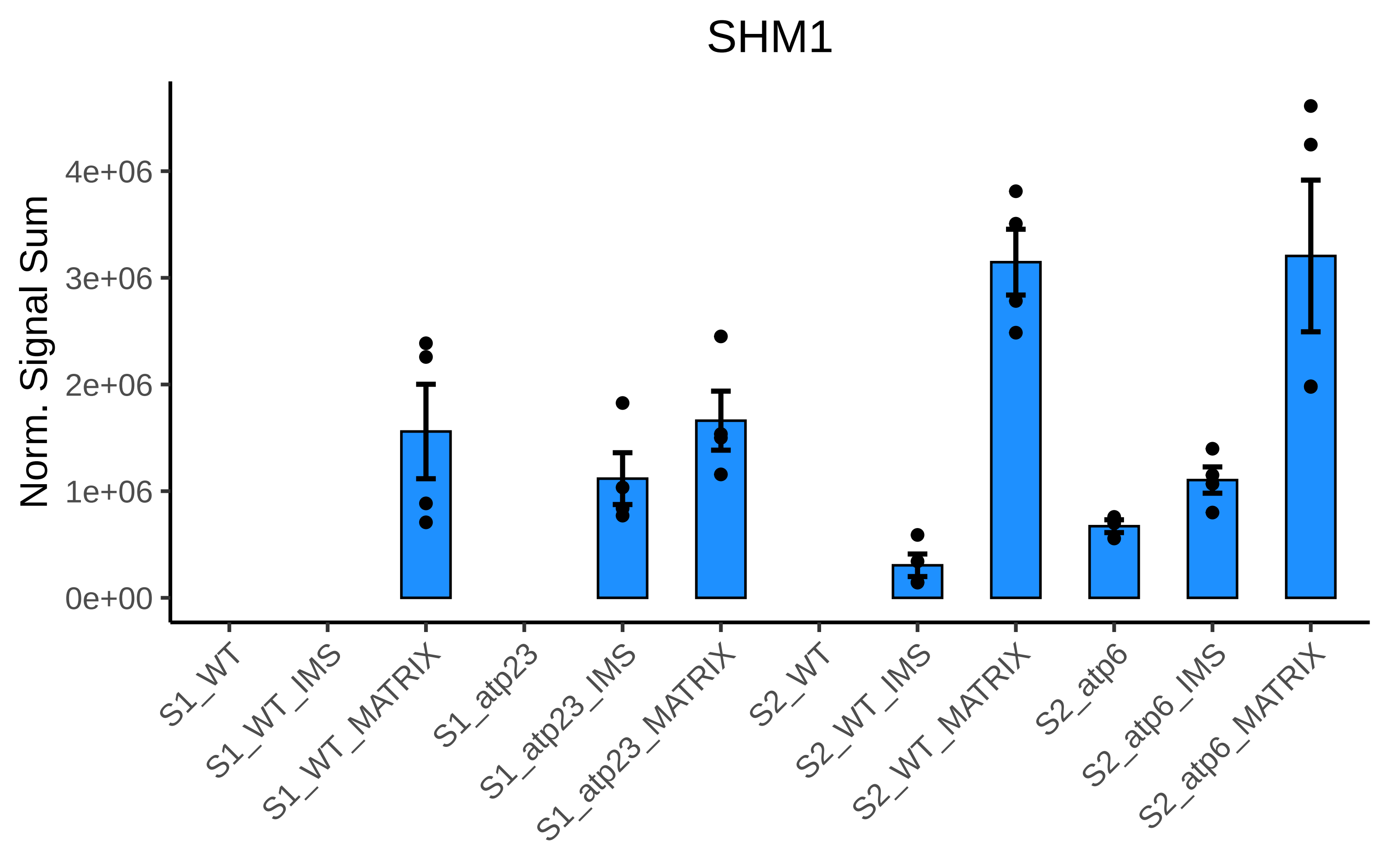
SHM1
Norm. Signal Sum
4e+06
3e+06
2e+06
1e+06
0e+00
S1_WT
S1_WT_IMS
S1_WT_MATRIX
S1_atp23
S1_atp23_IMS
S1_atp23_MATRIX
S2_WT
S2_WT_IMS
S2_WT_MATRIX
S2_atp6
S2_atp6_IMS
S2_atp6_MATRIX

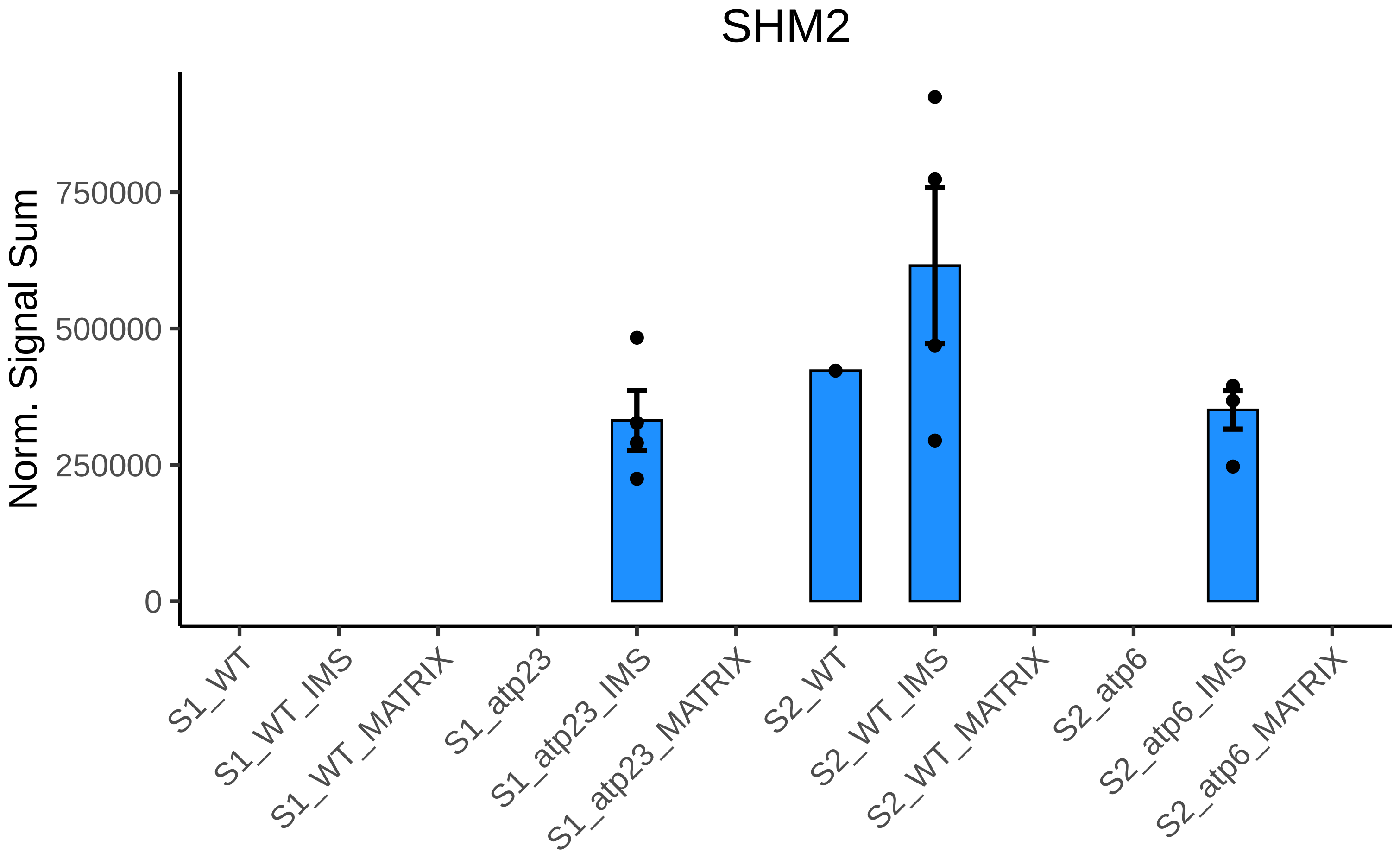
SHM2
Norm. Signal Sum
750000
500000
250000
0
S1_WT
S1_WT_IMS
S1_WT_MATRIX
S1_atp23
S1_atp23_IMS
S1_atp23_MATRIX
S2_WT
S2_WT_IMS
S2_WT_MATRIX
S2_atp6
S2_atp6_IMS
S2_atp6_MATRIX

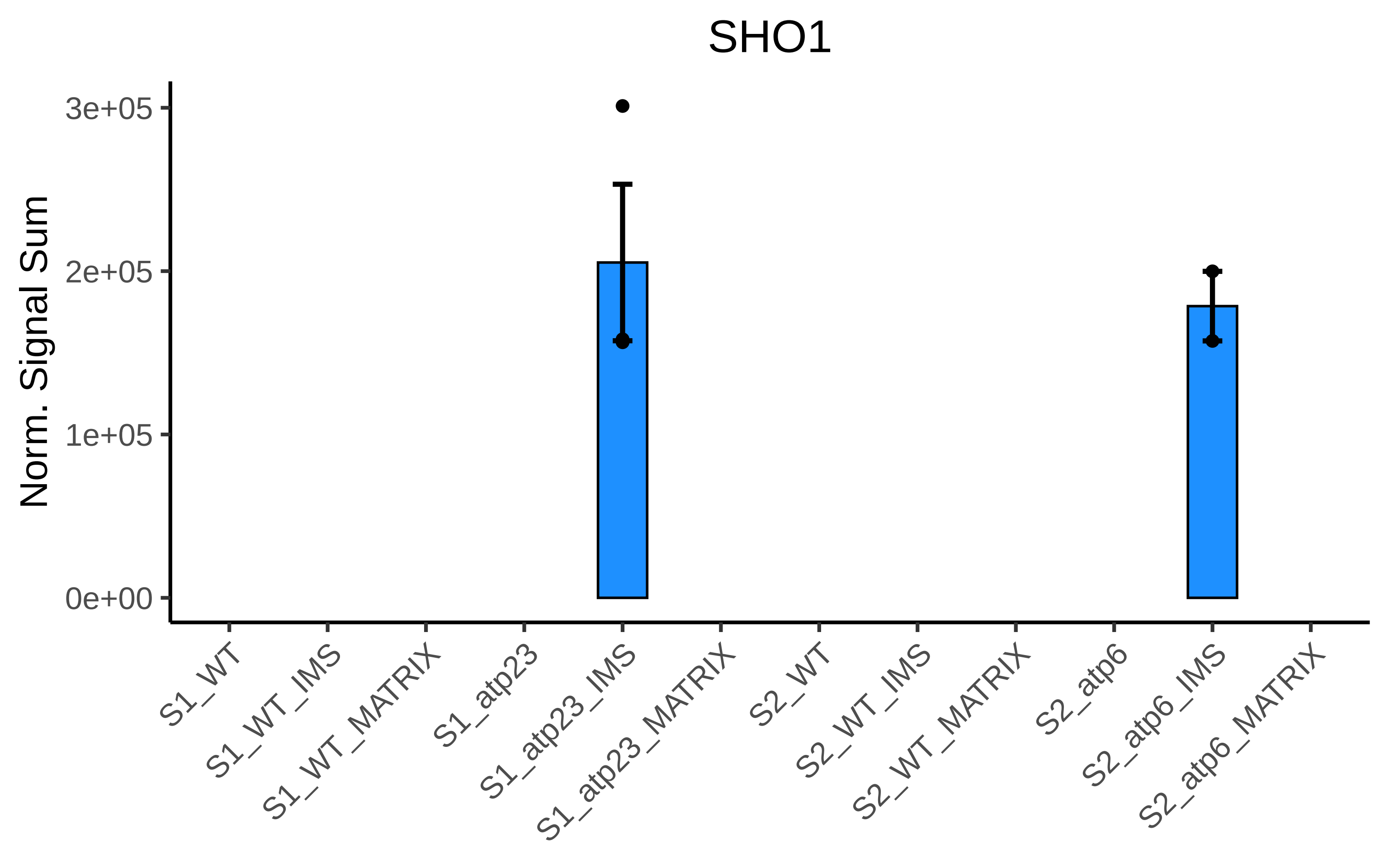
SHO1
Norm. Signal Sum
3e+05
2e+05
1e+05
0e+00
S1_WT
S1_WT_IMS
S1_WT_MATRIX
S1_atp23
S1_atp23_IMS
S1_atp23_MATRIX
S2_WT
S2_WT_IMS
S2_WT_MATRIX
S2_atp6
S2_atp6_IMS
S2_atp6_MATRIX

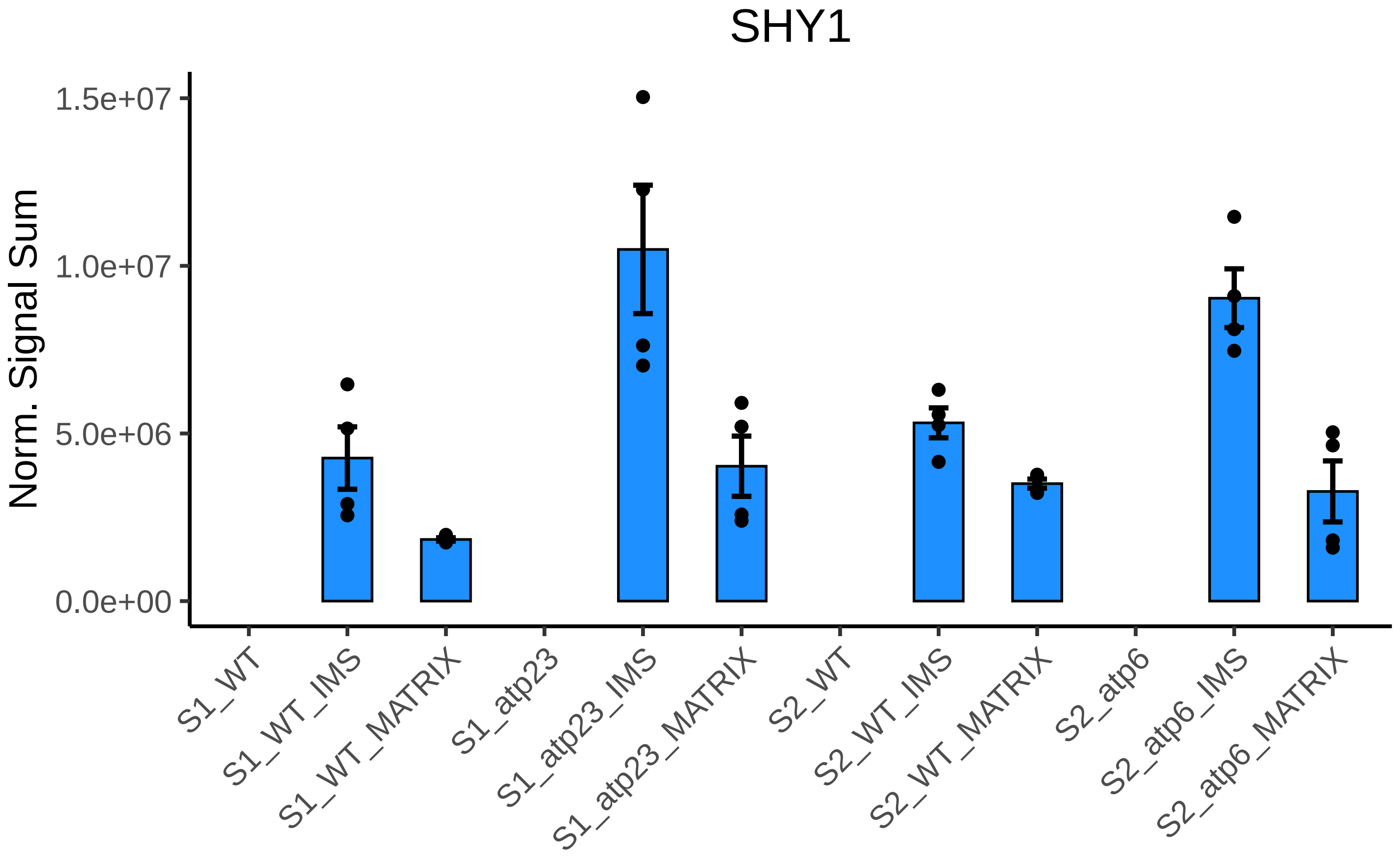
SHY1
Norm. Signal Sum
1.5e+07
1.0e+07
5.0e+06
0.0e+00
S1_WT
S1_WT_IMS
S1_WT_MATRIX
S1_atp23
S1_atp23_IMS
S1_atp23_MATRIX
S2_WT
S2_WT_IMS
S2_WT_MATRIX
S2_atp6
S2_atp6_IMS
S2_atp6_MATRIX

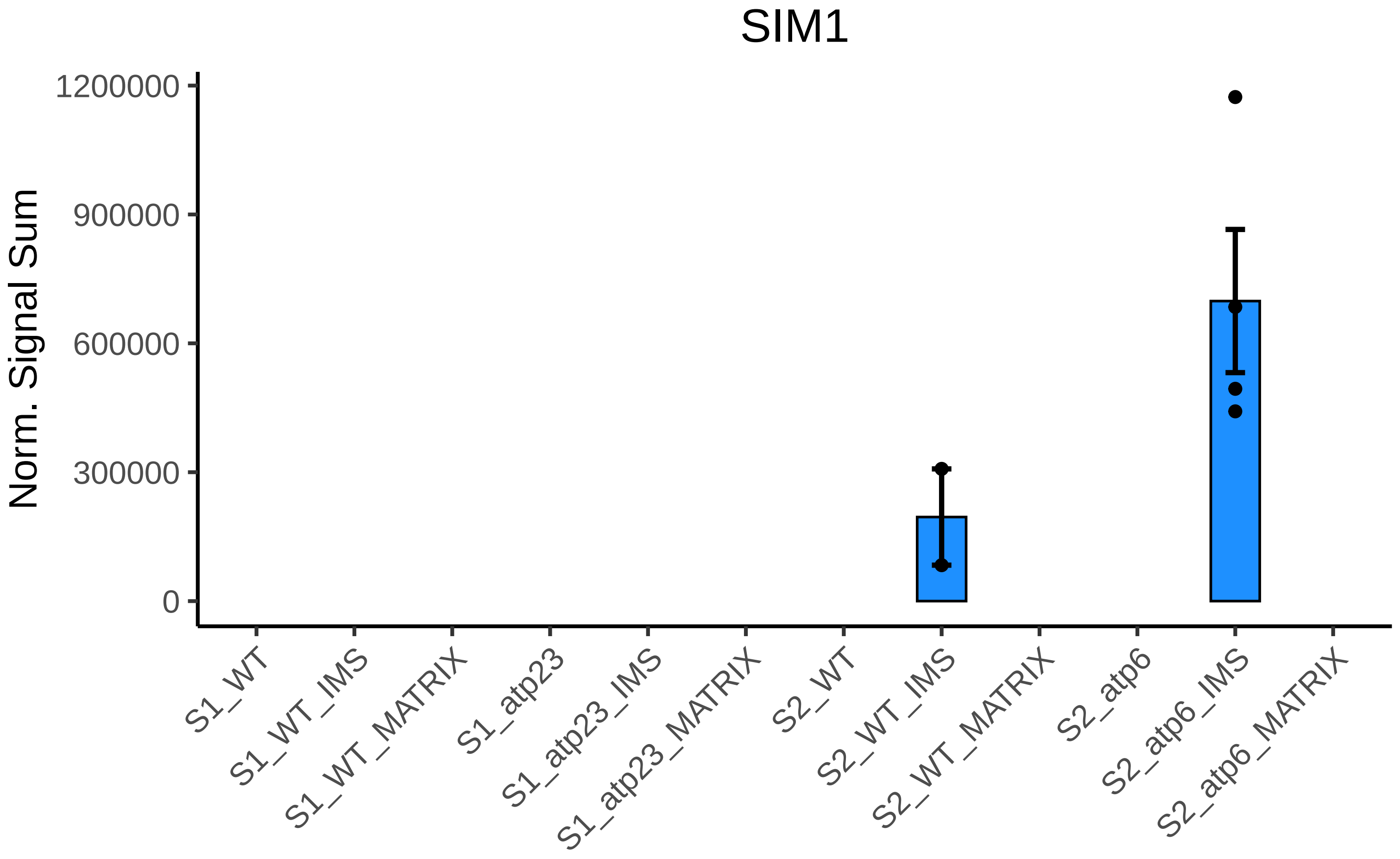

SIM1
Norm. Signal Sum
1200000
900000
600000
300000
0
S1_WT
S1_WT_IMS
S1_WT_MATRIX
S1_atp23
S1_atp23_IMS
S1_atp23_MATRIX
S2_WT
S2_WT_IMS
S2_WT_MATRIX
S2_atp6
S2_atp6_IMS
S2_atp6_MATRIX

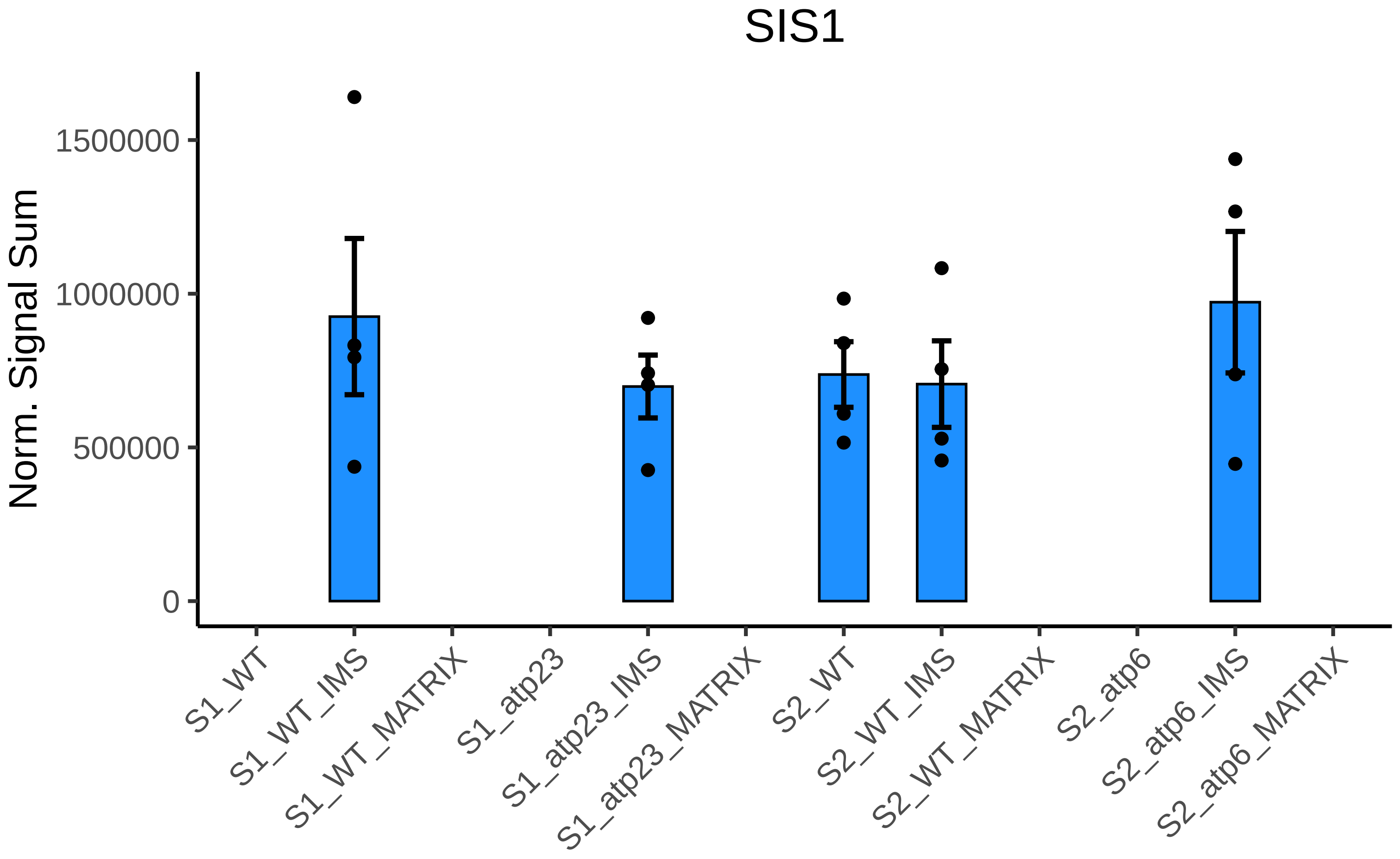
SIS1
Norm. Signal Sum
1500000
1000000
500000
0
S1_WT
S1_WT_IMS
S1_WT_MATRIX
S1_atp23
S1_atp23_IMS
S1_atp23_MATRIX
S2_WT
S2_WT_IMS
S2_WT_MATRIX
S2_atp6
S2_atp6_IMS
S2_atp6_MATRIX

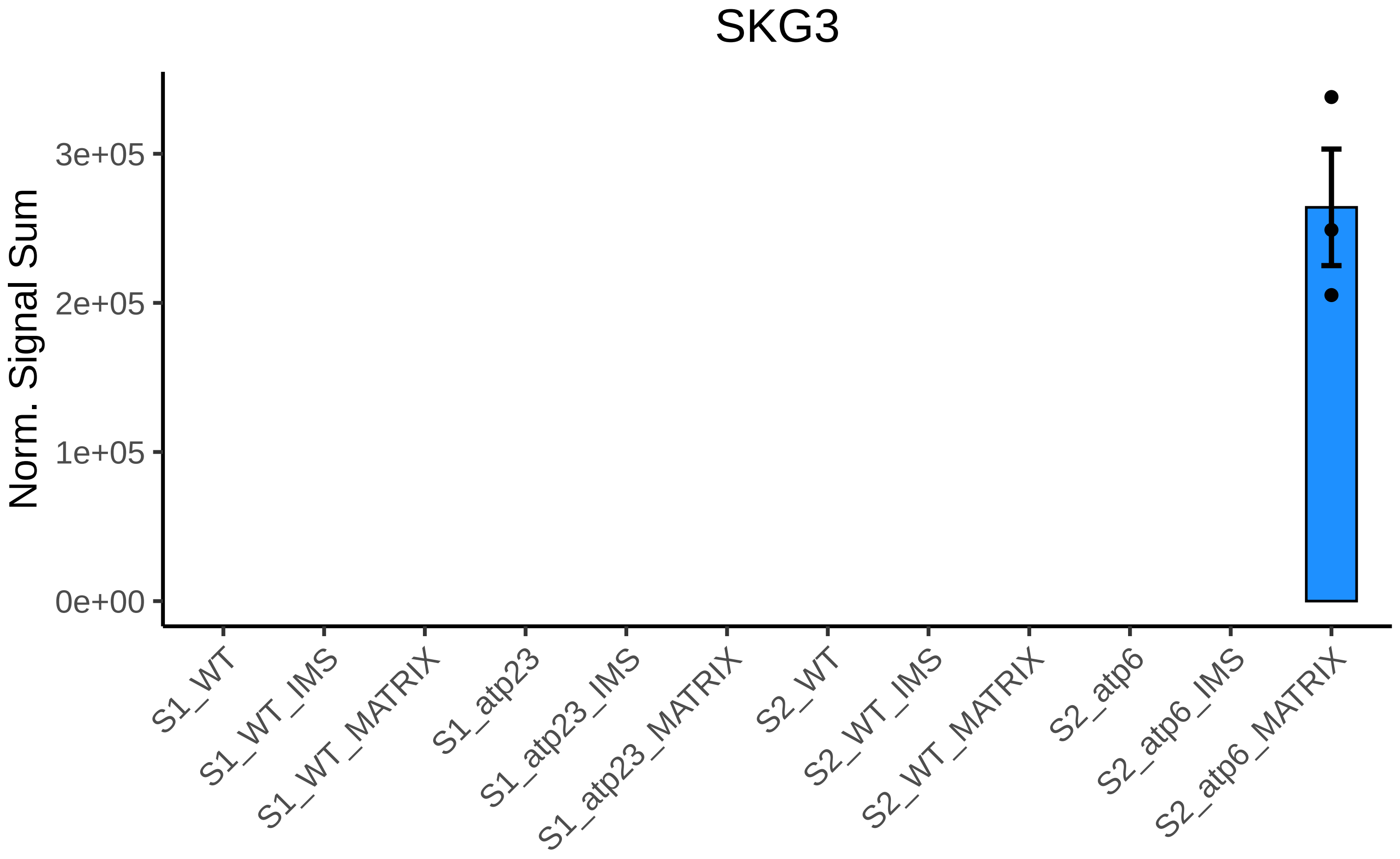
SKG3
Norm. Signal Sum
3e+05
2e+05
1e+05
0e+00
S1_WT
S1_WT_IMS
S1_WT_MATRIX
S1_atp23
S1_atp23_IMS
S1_atp23_MATRIX
S2_WT
S2_WT_IMS
S2_WT_MATRIX
S2_atp6
S2_atp6_IMS
S2_atp6_MATRIX

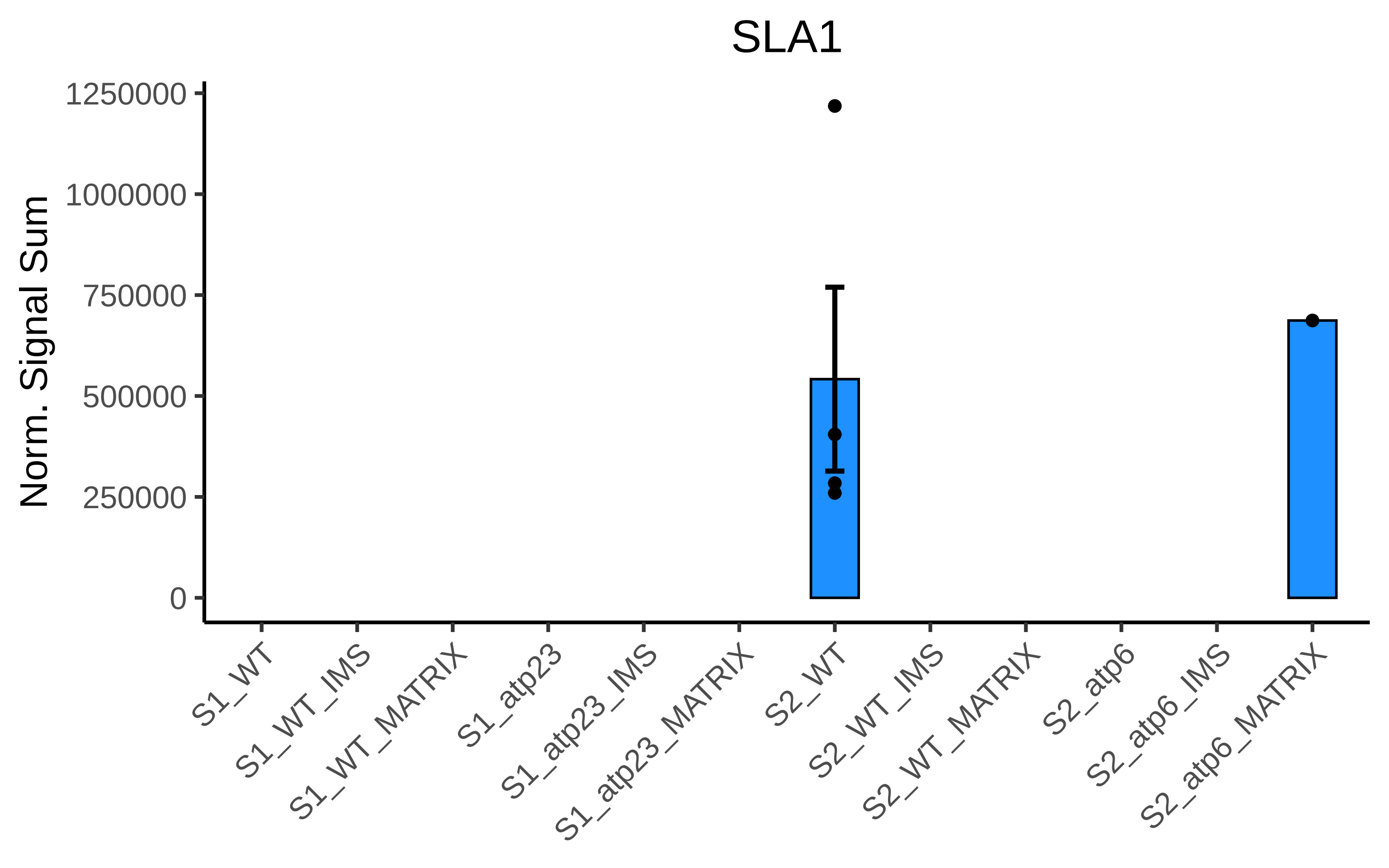

SLA1
Norm. Signal Sum
1250000
1000000
750000
500000
250000
0
S1_WT
S1_WT_IMS
S1_WT_MATRIX
S1_atp23
S1_atp23_IMS
S1_atp23_MATRIX
S2_WT
S2_WT_IMS
S2_WT_MATRIX
S2_atp6
S2_atp6_IMS
S2_atp6_MATRIX

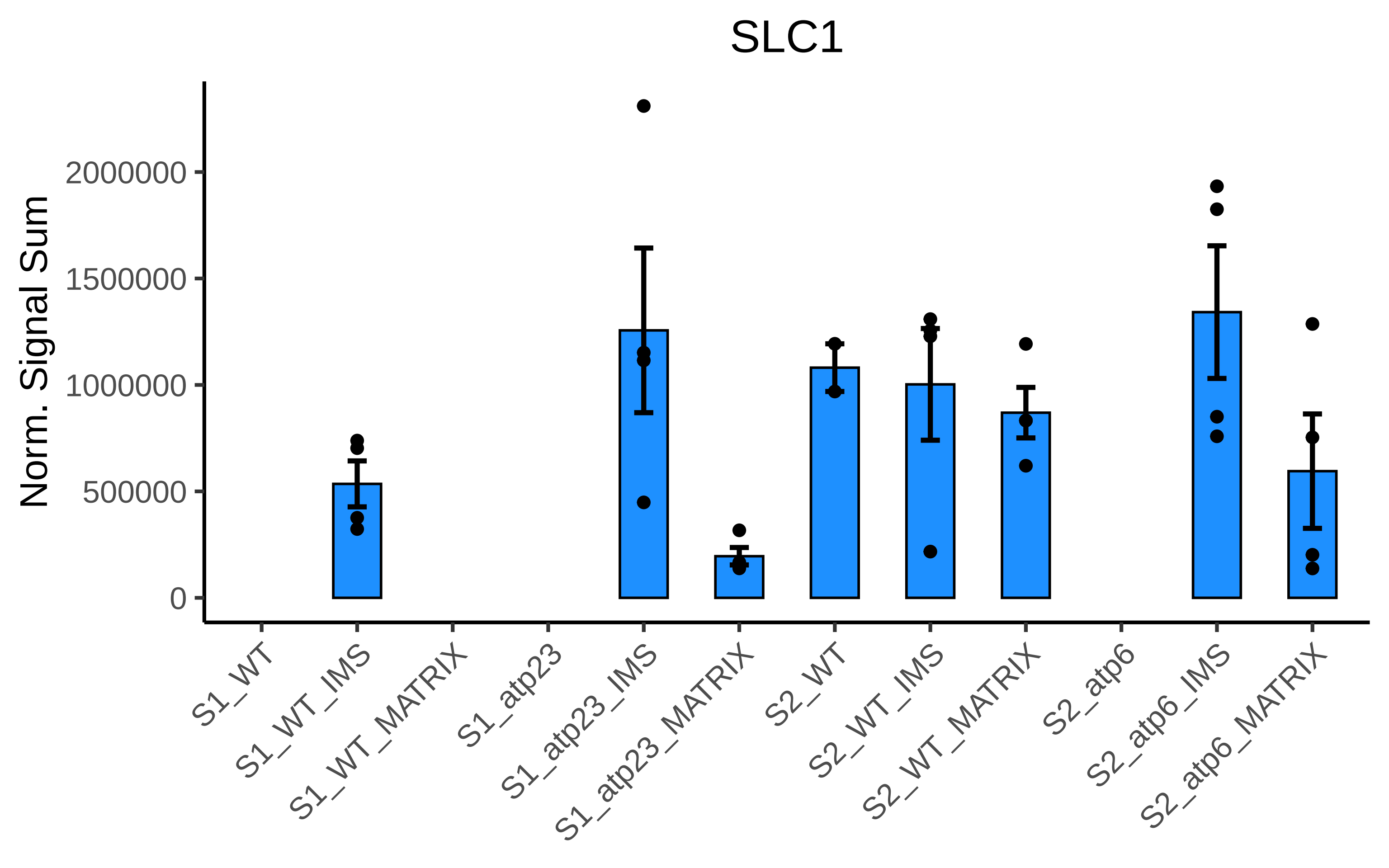

SLC1
Norm. Signal Sum
0
500000
1000000
1500000
2000000
S1_WT
S1_WT_IMS
S1_WT_MATRIX
S1_atp23
S1_atp23_IMS
S1_atp23_MATRIX
S2_WT
S2_WT_IMS
S2_WT_MATRIX
S2_atp6
S2_atp6_IMS
S2_atp6_MATRIX

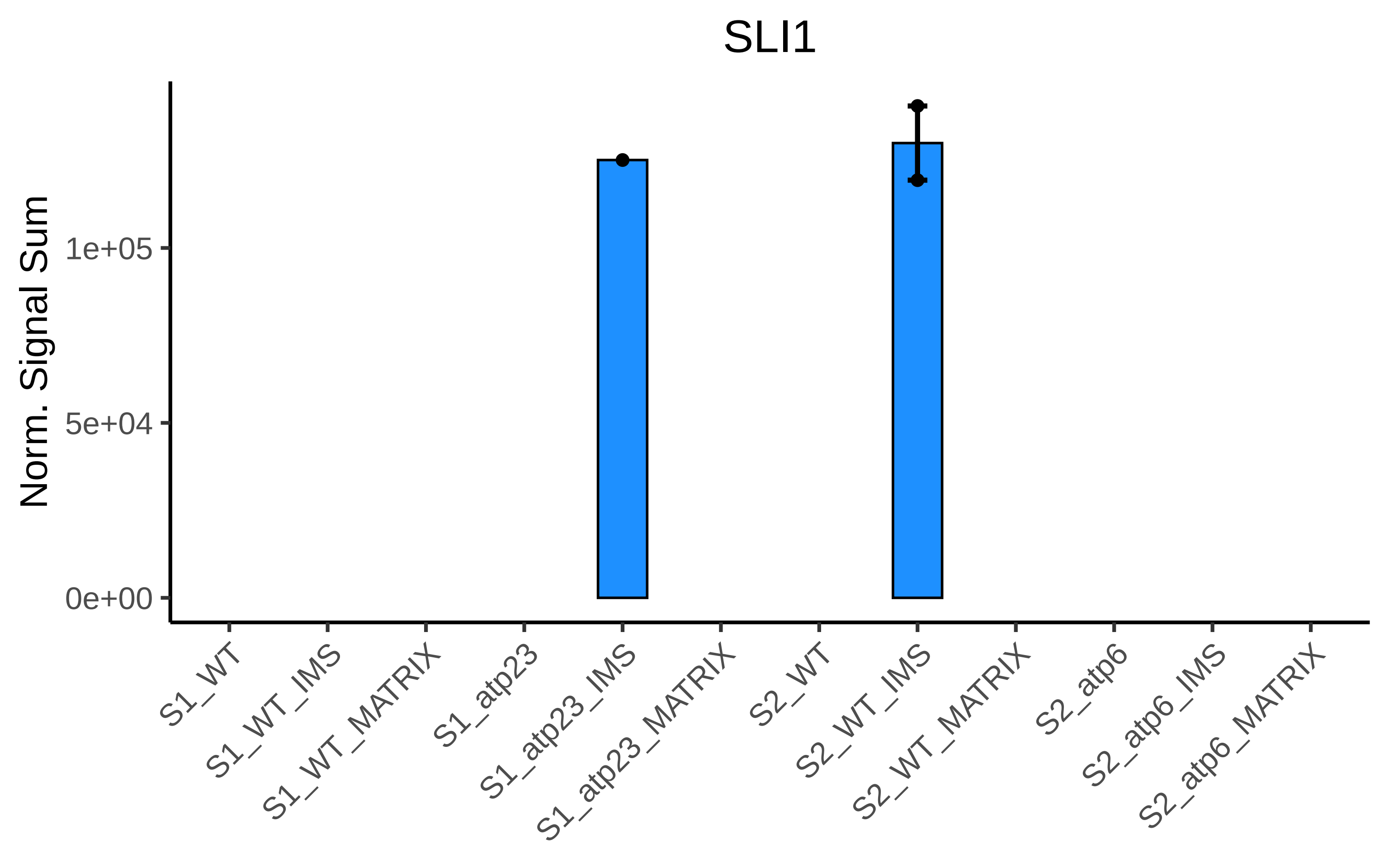

SLI1
Norm. Signal Sum
1e+05
5e+04
0e+00
S1_WT
S1_WT_IMS
S1_WT_MATRIX
S1_atp23
S1_atp23_IMS
S1_atp23_MATRIX
S2_WT
S2_WT_IMS
S2_WT_MATRIX
S2_atp6
S2_atp6_IMS
S2_atp6_MATRIX

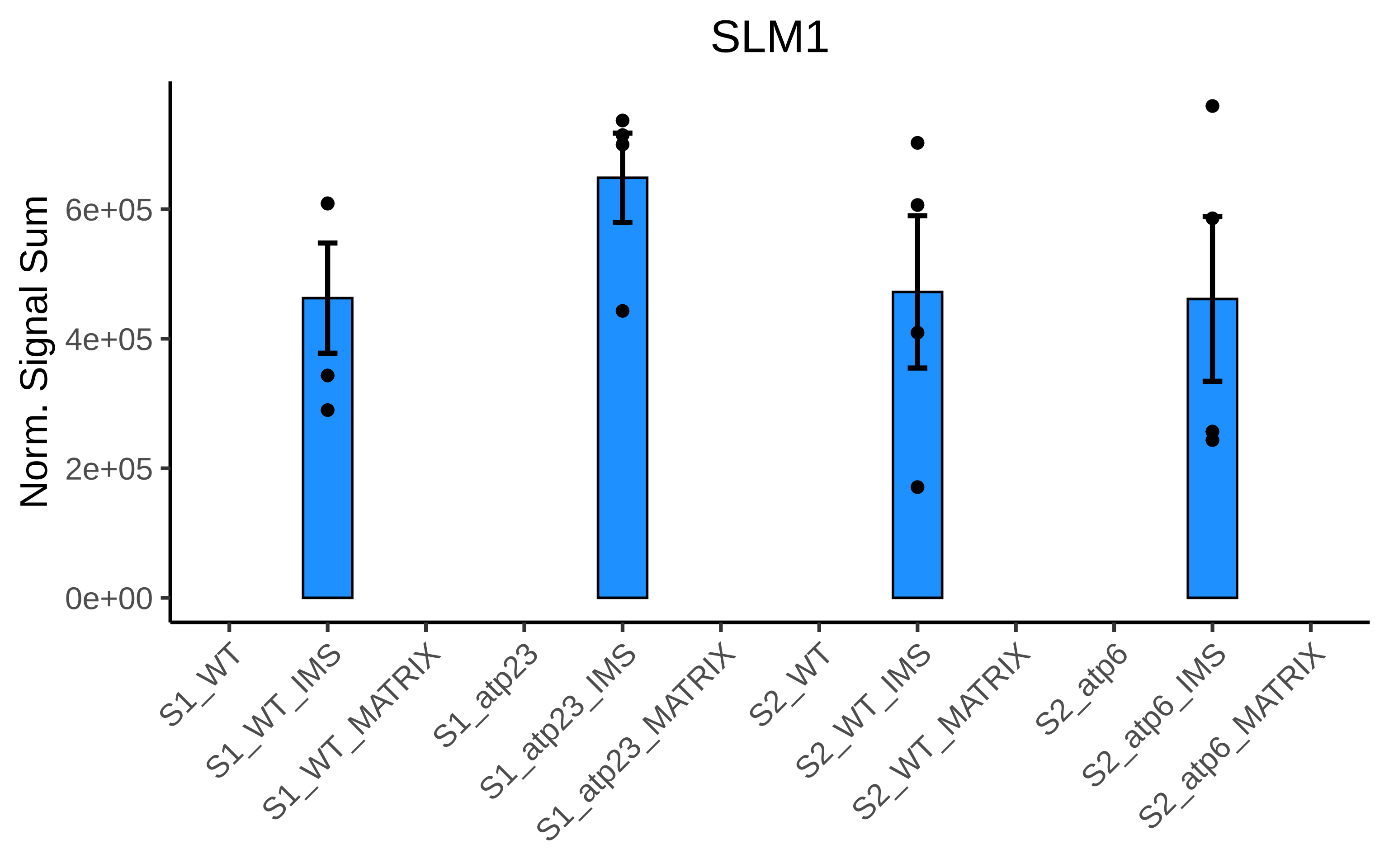

SLM1
Norm. Signal Sum
6e+05
4e+05
2e+05
0e+00
S1_WT
S1_WT_IMS
S1_WT_MATRIX
S1_atp23
S1_atp23_IMS
S1_atp23_MATRIX
S2_WT
S2_WT_IMS
S2_WT_MATRIX
S2_atp6
S2_atp6_IMS
S2_atp6_MATRIX

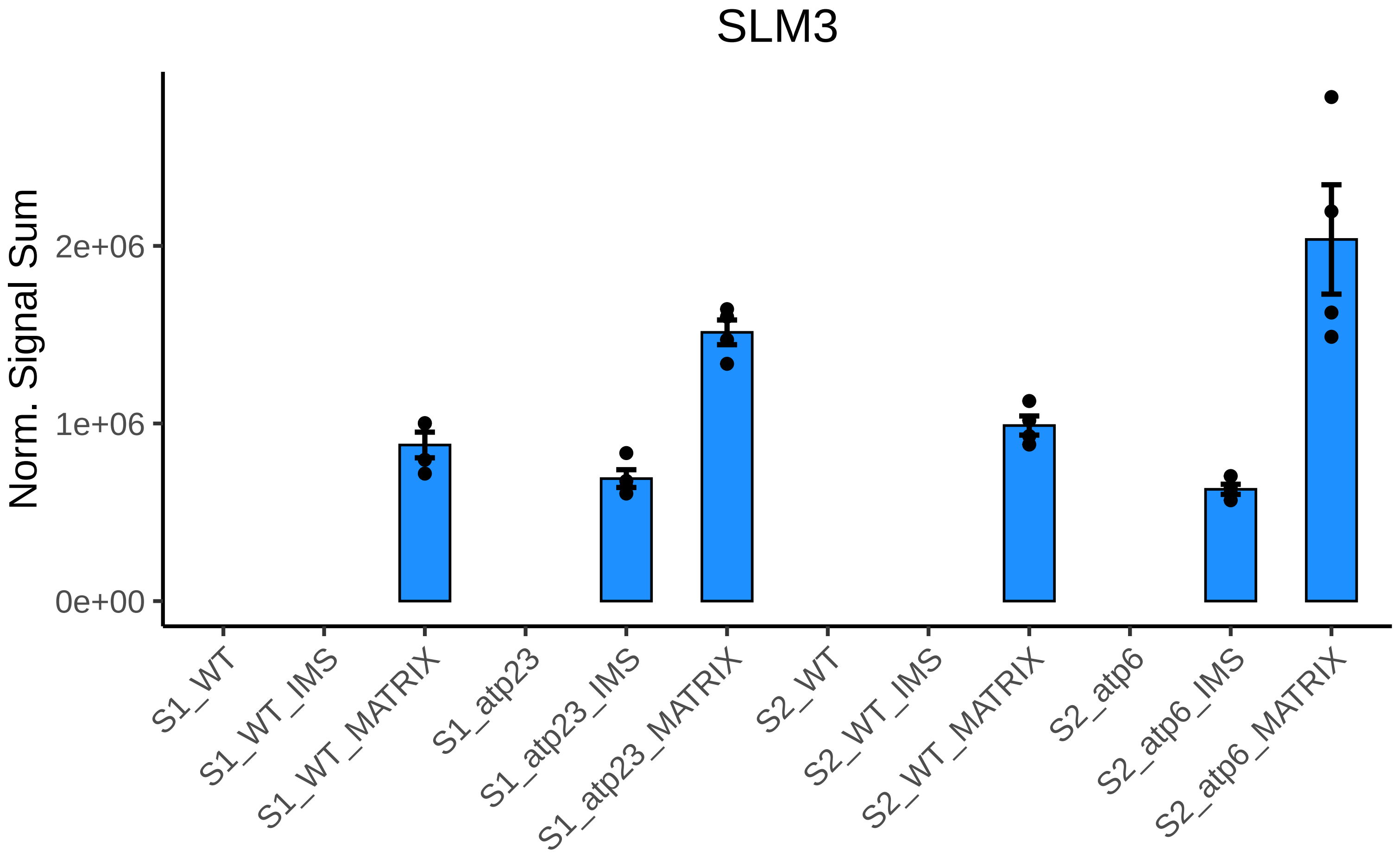

SLM3
Norm. Signal Sum
2e+06
1e+06
0e+00
S1_WT
S1_WT_IMS
S1_WT_MATRIX
S1_atp23
S1_atp23_IMS
S1_atp23_MATRIX
S2_WT
S2_WT_IMS
S2_WT_MATRIX
S2_atp6
S2_atp6_IMS
S2_atp6_MATRIX

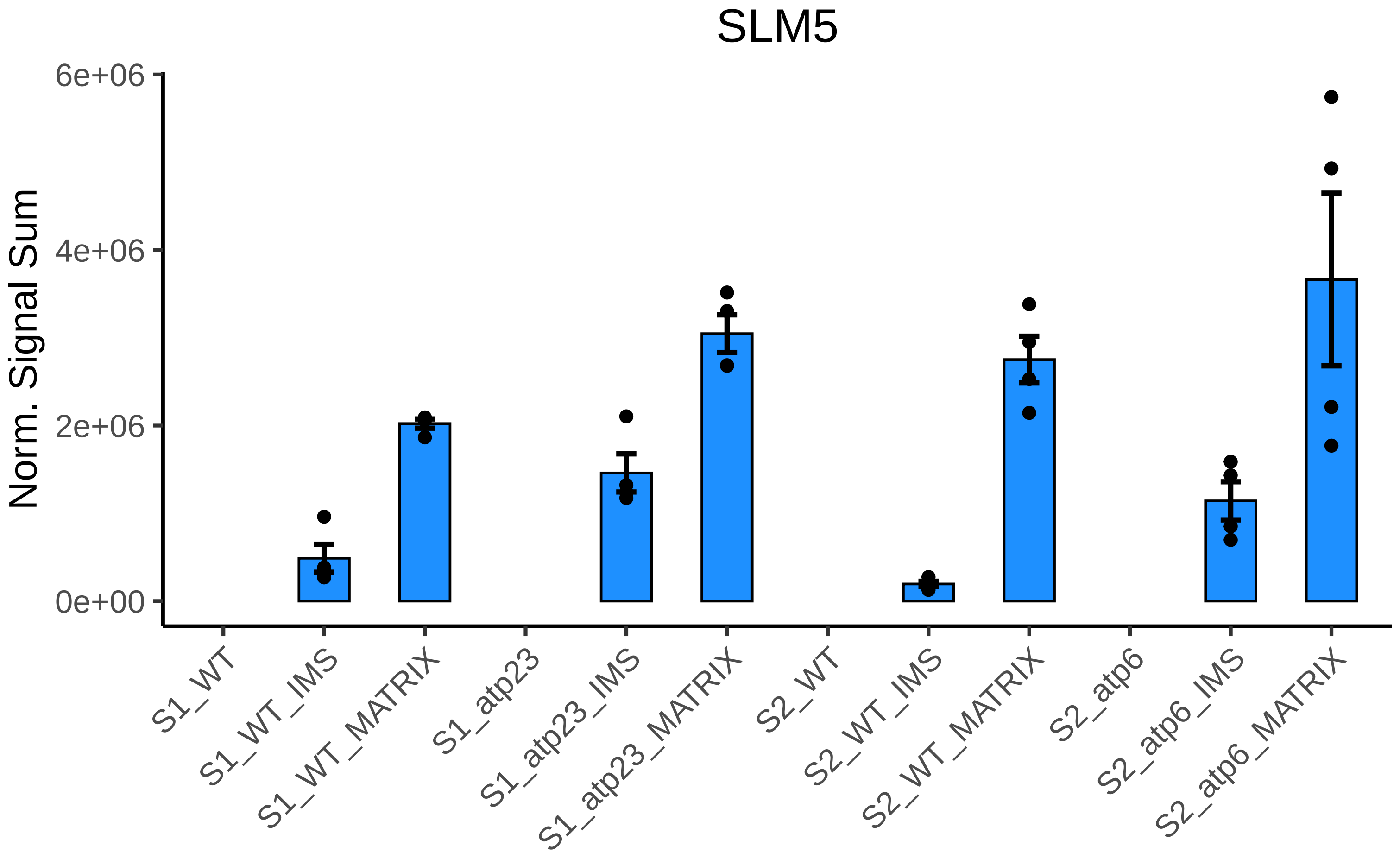

SLM5
Norm. Signal Sum
6e+06
4e+06
2e+06
0e+00
S1_WT
S1_WT_IMS
S1_WT_MATRIX
S1_atp23
S1_atp23_IMS
S1_atp23_MATRIX
S2_WT
S2_WT_IMS
S2_WT_MATRIX
S2_atp6
S2_atp6_IMS
S2_atp6_MATRIX

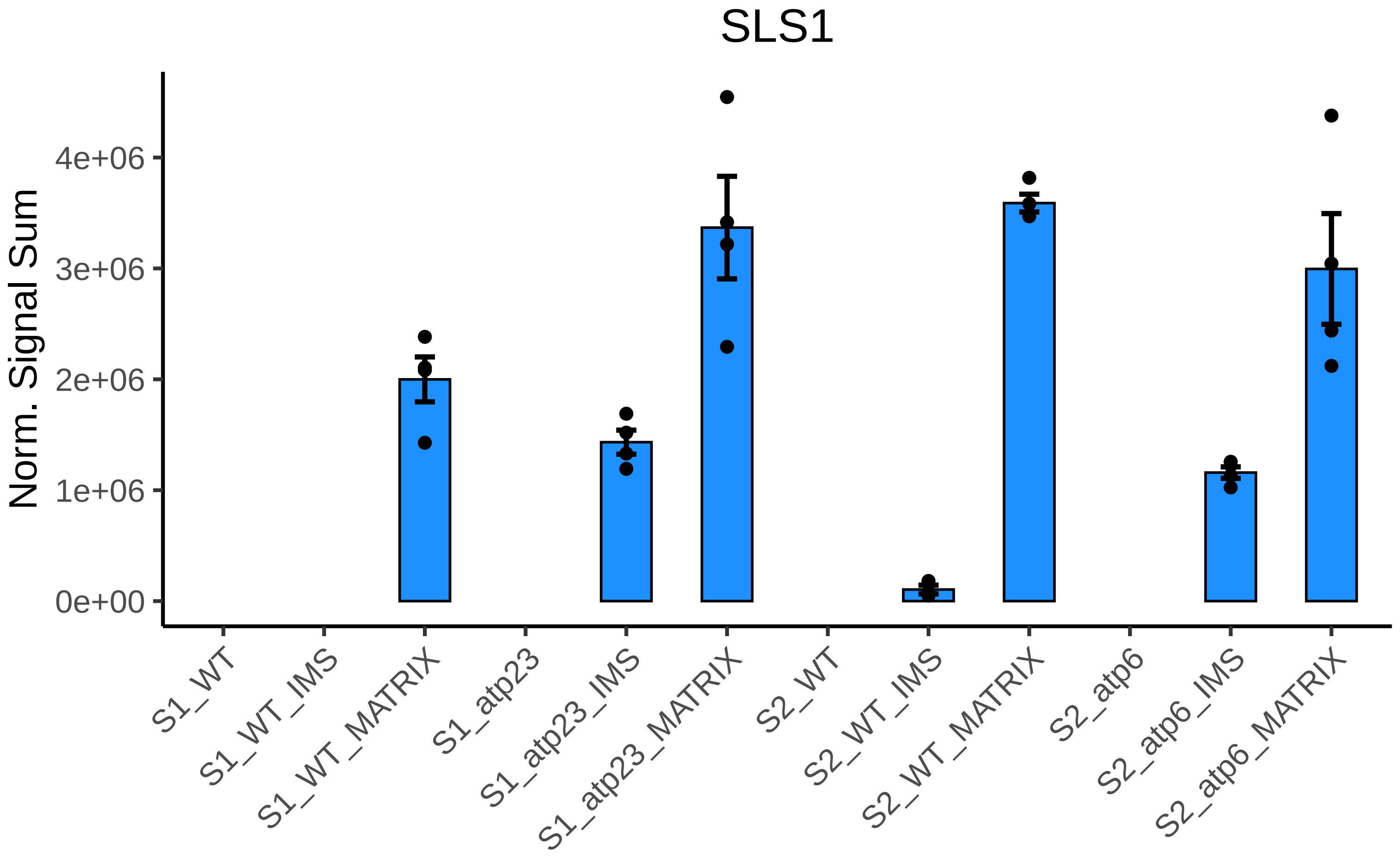

SLS1
Norm. Signal Sum
4e+06
3e+06
2e+06
1e+06
0e+00
S1_WT
S1_WT_IMS
S1_WT_MATRIX
S1_atp23
S1_atp23_IMS
S1_atp23_MATRIX
S2_WT
S2_WT_IMS
S2_WT_MATRIX
S2_atp6
S2_atp6_IMS
S2_atp6_MATRIX

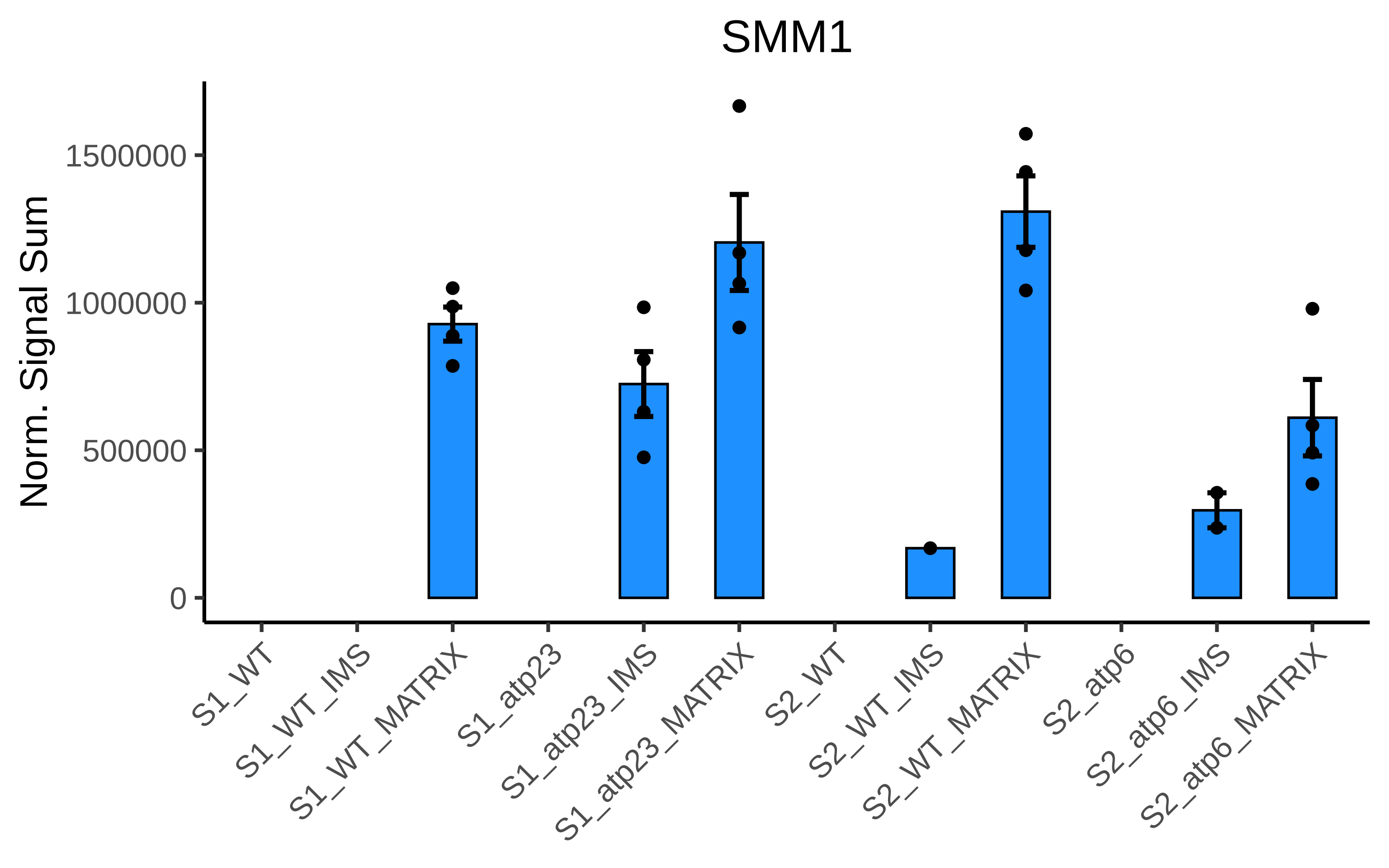

SMM1
Norm. Signal Sum
1500000
1000000
500000
0
S1_WT
S1_WT_IMS
S1_WT_MATRIX
S1_atp23
S1_atp23_IMS
S1_atp23_MATRIX
S2_WT
S2_WT_IMS
S2_WT_MATRIX
S2_atp6
S2_atp6_IMS
S2_atp6_MATRIX

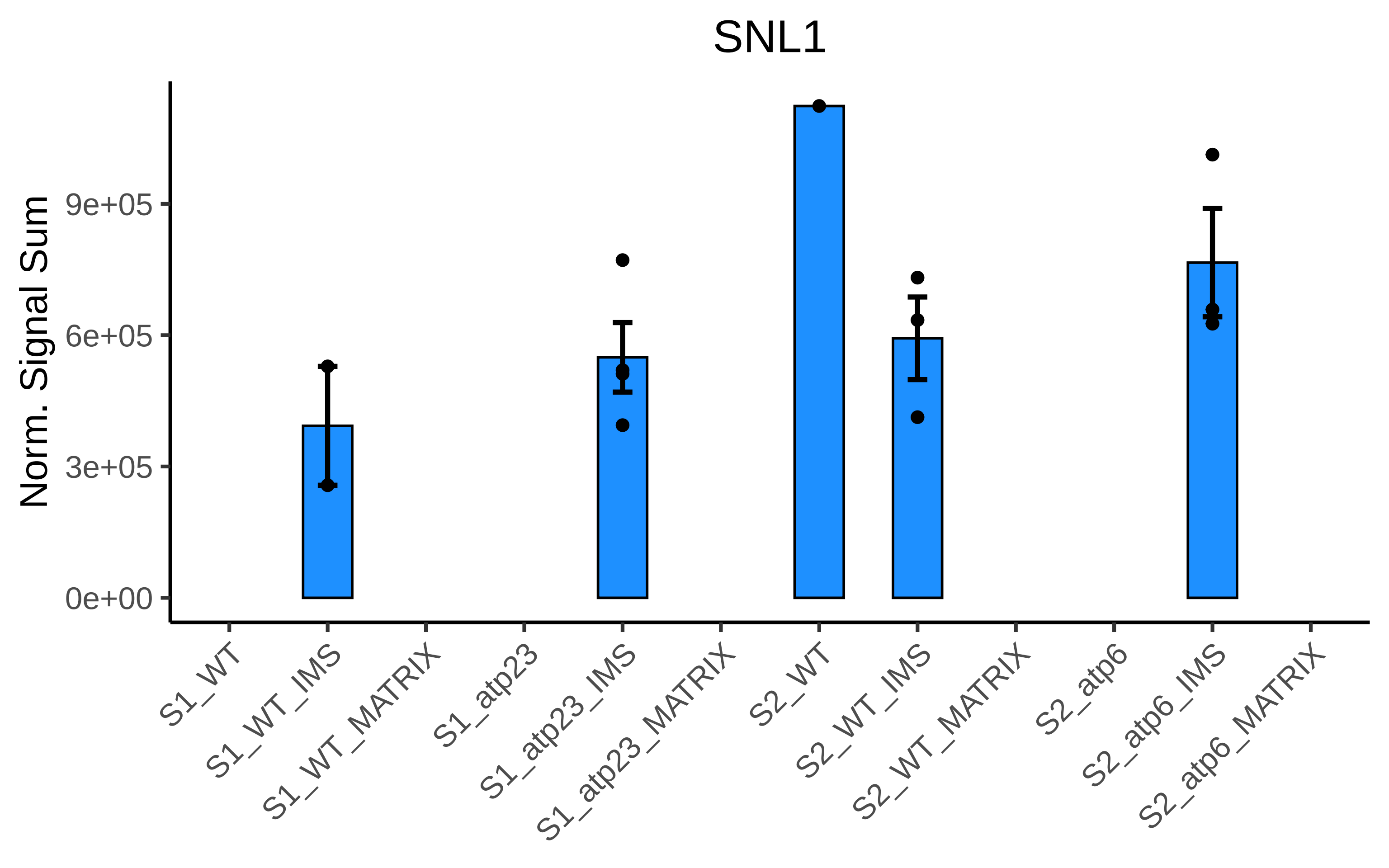
SNL1
Norm. Signal Sum
0e+00
3e+05
6e+05
9e+05
S1_WT
S1_WT_IMS
S1_WT_MATRIX
S1_atp23
S1_atp23_IMS
S1_atp23_MATRIX
S2_WT
S2_WT_IMS
S2_WT_MATRIX
S2_atp6
S2_atp6_IMS
S2_atp6_MATRIX

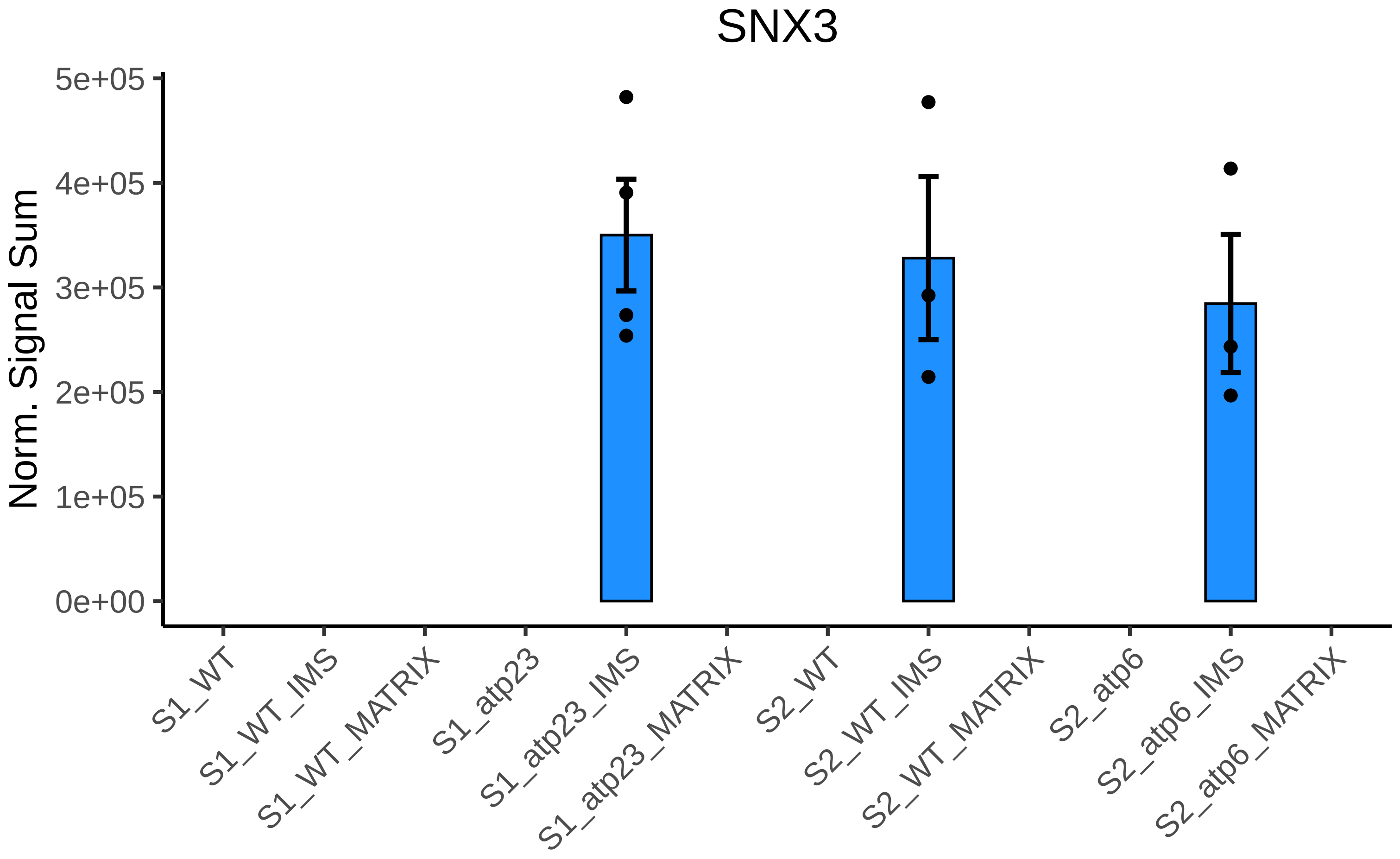
SNX3
Norm. Signal Sum
5e+05
4e+05
3e+05
2e+05
1e+05
0e+00
S1_WT
S1_WT_IMS
S1_WT_MATRIX
S1_atp23
S1_atp23_IMS
S1_atp23_MATRIX
S2_WT
S2_WT_IMS
S2_WT_MATRIX
S2_atp6
S2_atp6_IMS
S2_atp6_MATRIX

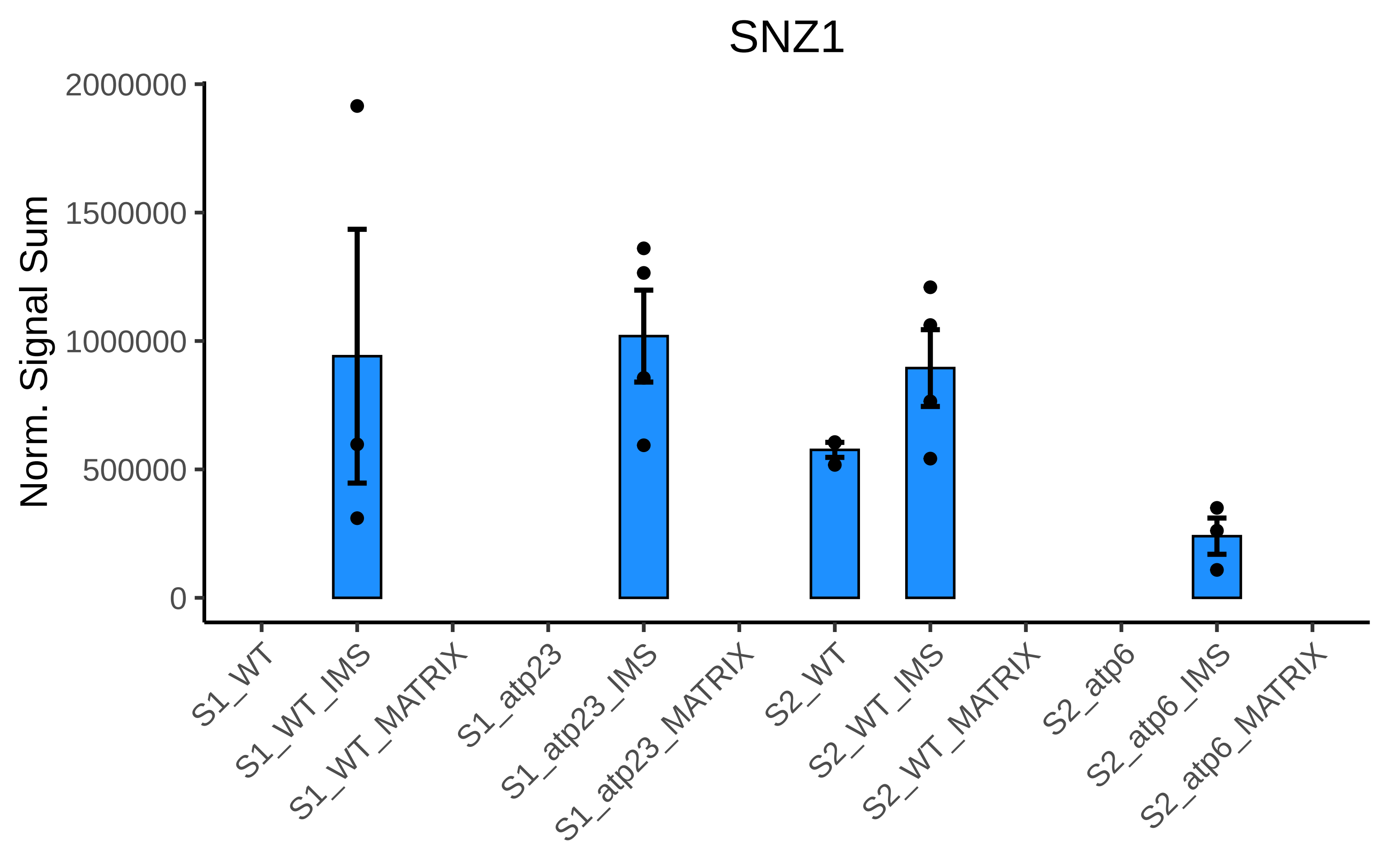

SNZ1
Norm. Signal Sum
2000000
1500000
1000000
500000
0
S1_WT
S1_WT_IMS
S1_WT_MATRIX
S1_atp23
S1_atp23_IMS
S1_atp23_MATRIX
S2_WT
S2_WT_IMS
S2_WT_MATRIX
S2_atp6
S2_atp6_IMS
S2_atp6_MATRIX

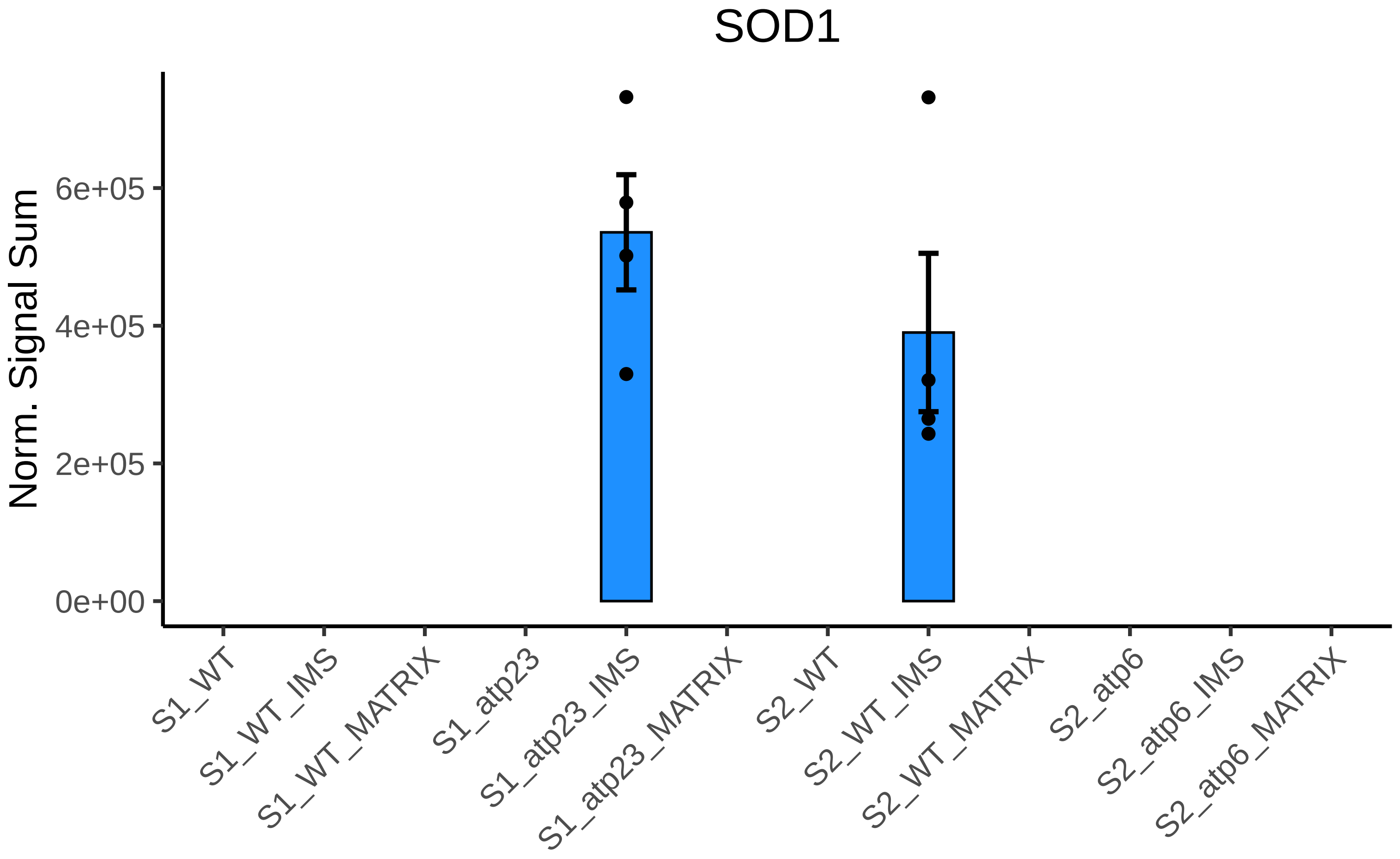

SOD1
Norm. Signal Sum
0e+00
2e+05
4e+05
6e+05
S1_WT
S1_WT_IMS
S1_WT_MATRIX
S1_atp23
S1_atp23_IMS
S1_atp23_MATRIX
S2_WT
S2_WT_IMS
S2_WT_MATRIX
S2_atp6
S2_atp6_IMS
S2_atp6_MATRIX

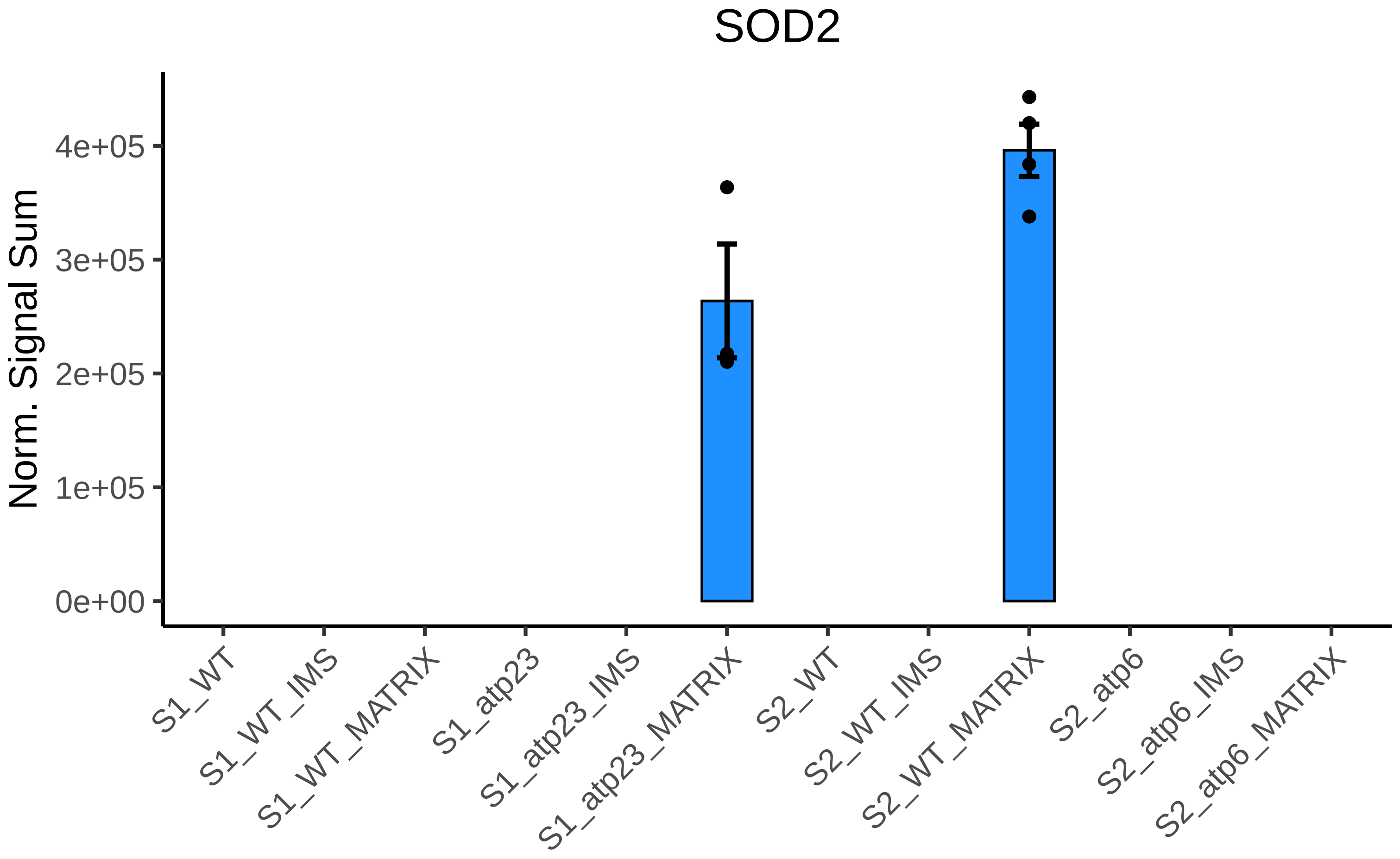

SOD2
Norm. Signal Sum
0e+00
1e+05
2e+05
3e+05
4e+05
S1_WT
S1_WT_IMS
S1_WT_MATRIX
S1_atp23
S1_atp23_IMS
S1_atp23_MATRIX
S2_WT
S2_WT_IMS
S2_WT_MATRIX
S2_atp6
S2_atp6_IMS
S2_atp6_MATRIX

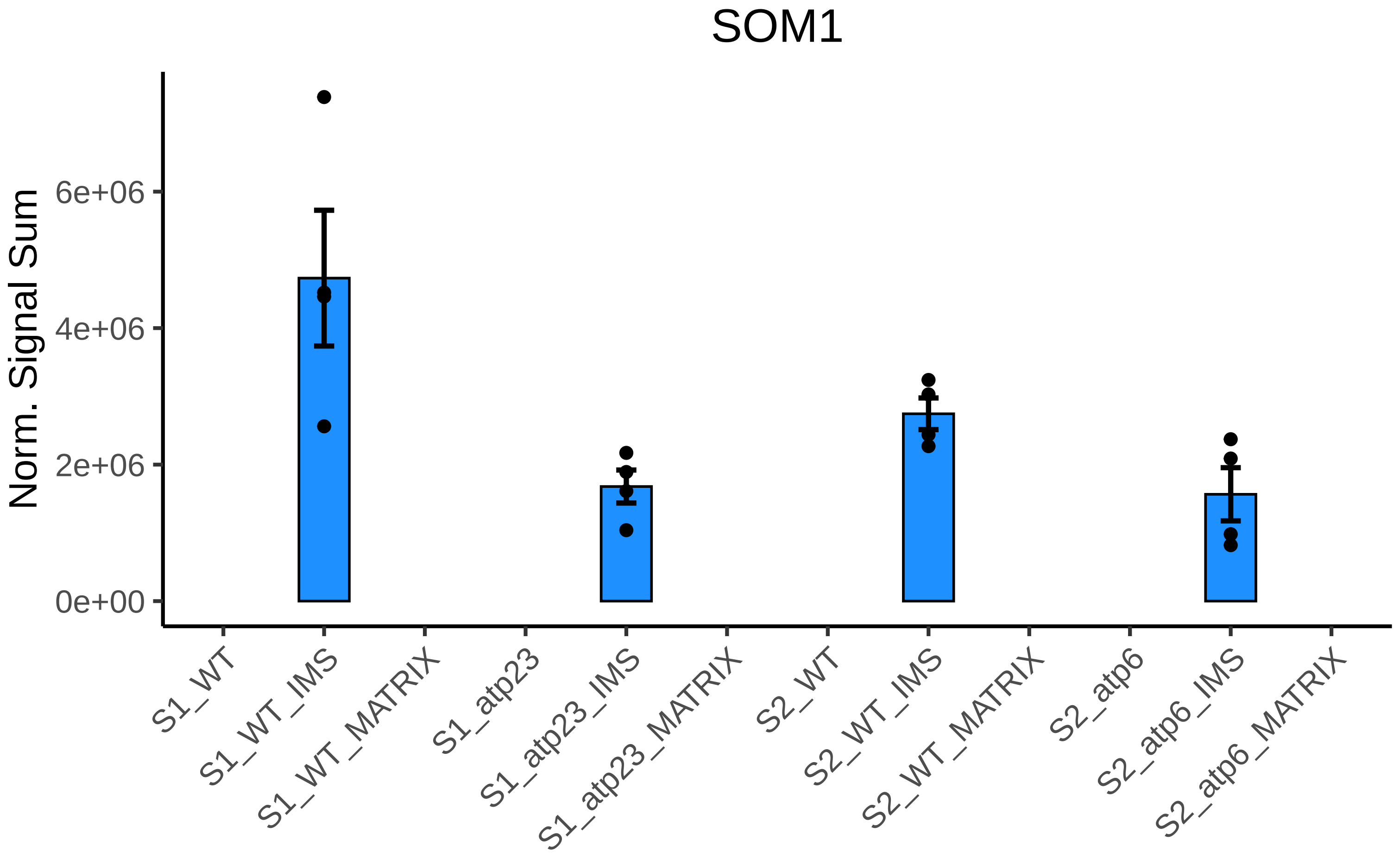

SOM1
Norm. Signal Sum
6e+06
4e+06
2e+06
0e+00
S1_WT
S1_WT_IMS
S1_WT_MATRIX
S1_atp23
S1_atp23_IMS
S1_atp23_MATRIX
S2_WT
S2_WT_IMS
S2_WT_MATRIX
S2_atp6
S2_atp6_IMS
S2_atp6_MATRIX

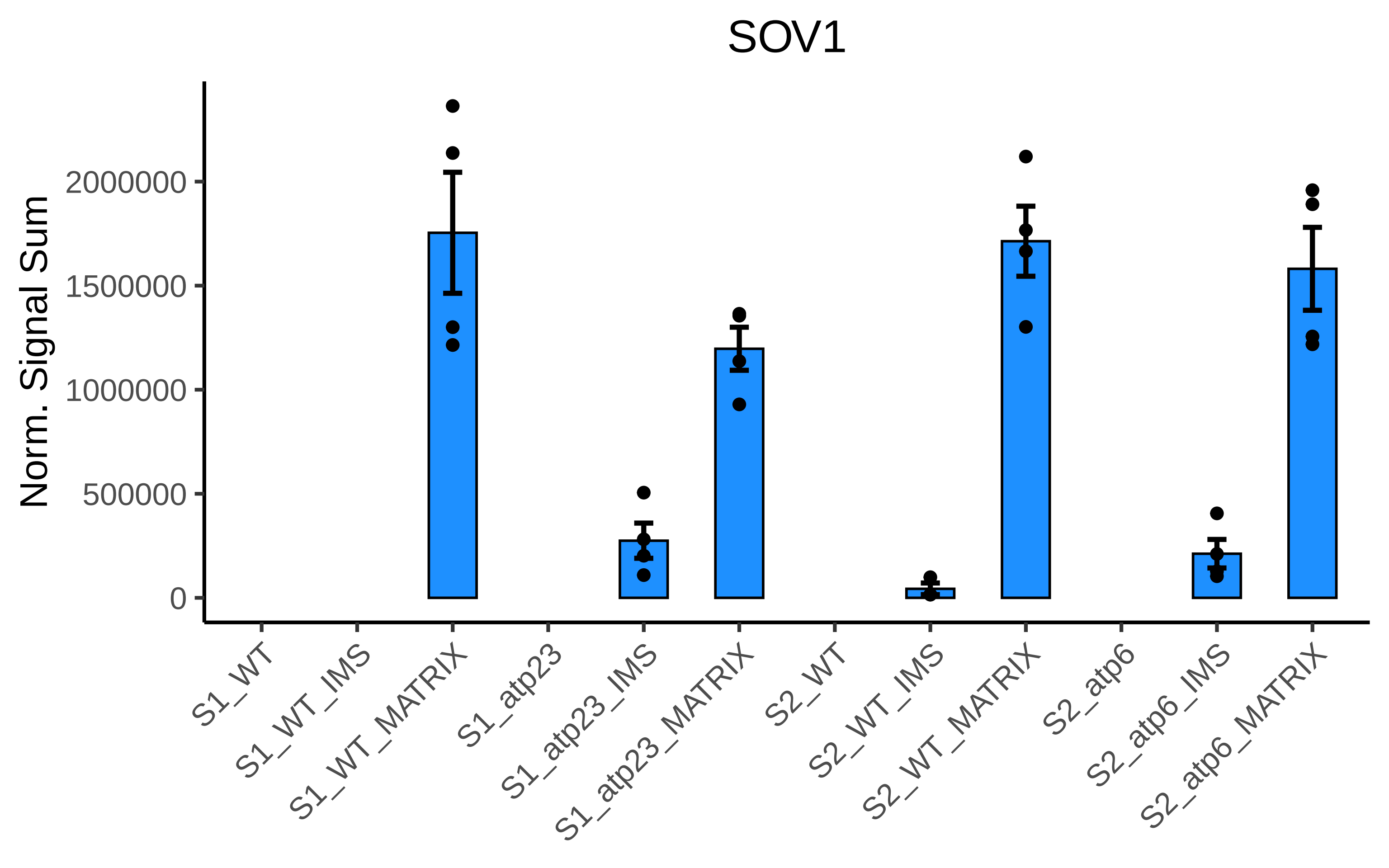
SOV1
Norm. Signal Sum
2000000
1500000
1000000
500000
0
S1_WT
S1_WT_IMS
S1_WT_MATRIX
S1_atp23
S1_atp23_IMS
S1_atp23_MATRIX
S2_WT
S2_WT_IMS
S2_WT_MATRIX
S2_atp6
S2_atp6_IMS
S2_atp6_MATRIX

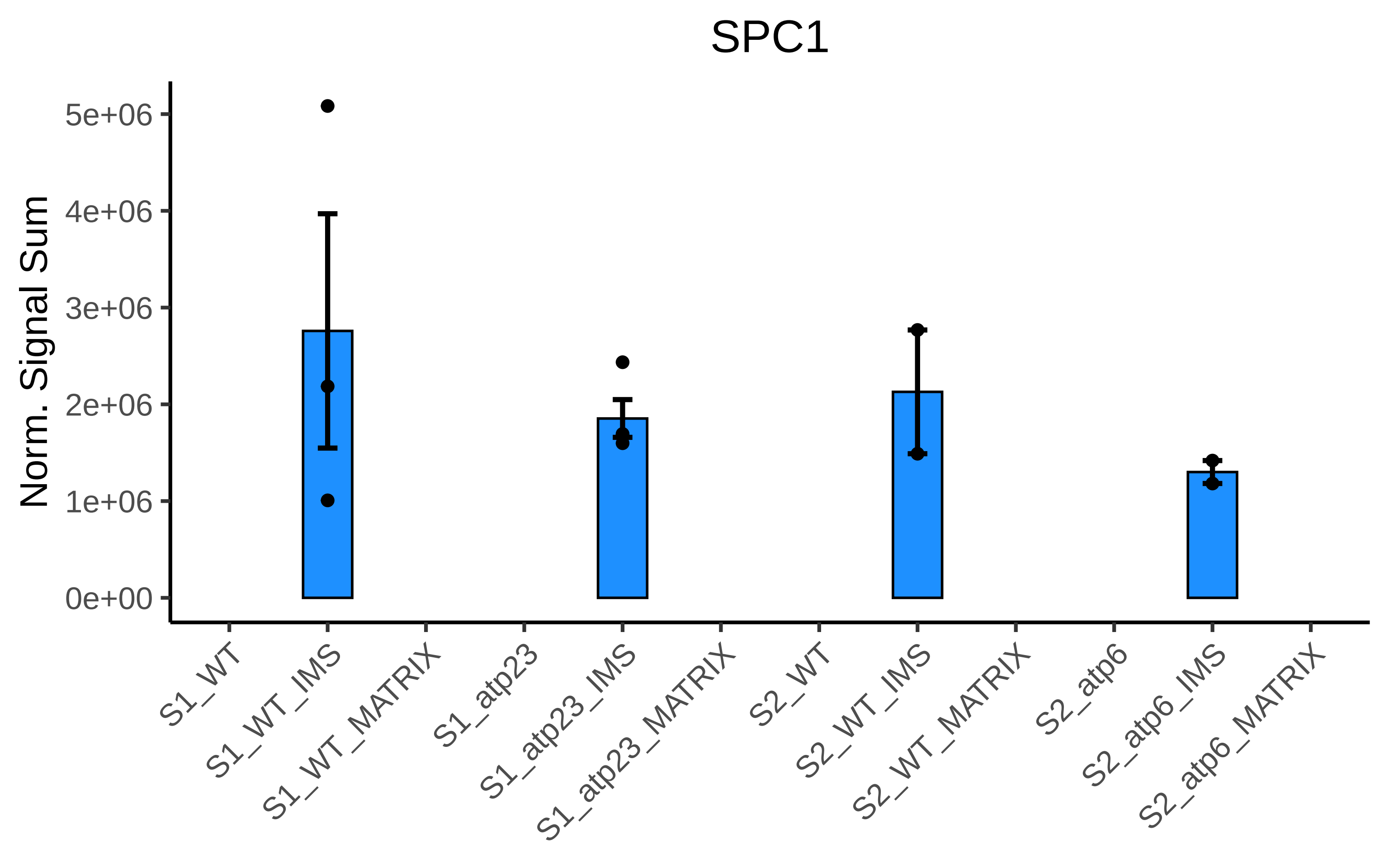
SPC1
Norm. Signal Sum
5e+06
4e+06
3e+06
2e+06
1e+06
0e+00
S1_WT
S1_WT_IMS
S1_WT_MATRIX
S1_atp23
S1_atp23_IMS
S1_atp23_MATRIX
S2_WT
S2_WT_IMS
S2_WT_MATRIX
S2_atp6
S2_atp6_IMS
S2_atp6_MATRIX

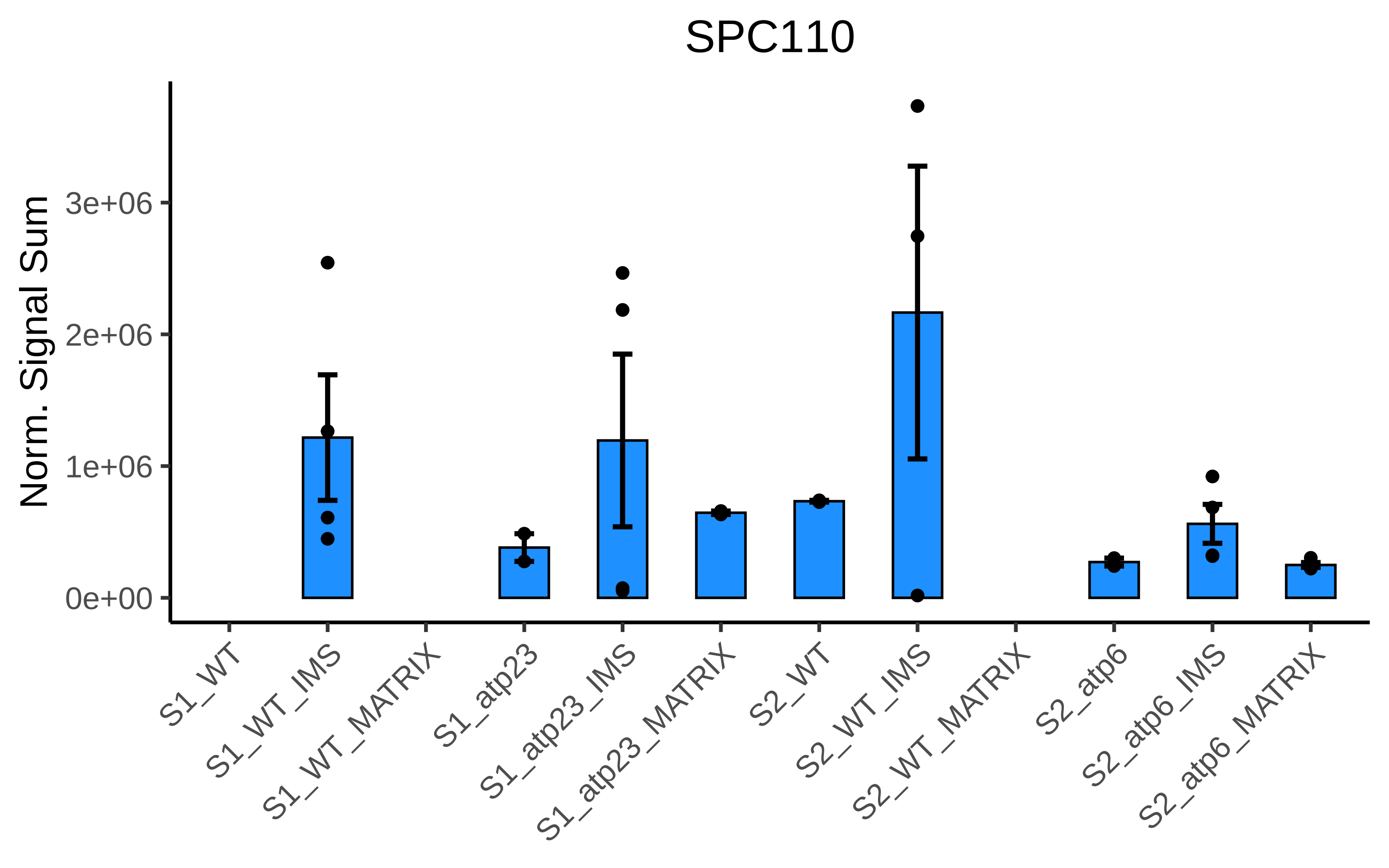
SPC110
Norm. Signal Sum
0e+00
1e+06
2e+06
3e+06
S1_WT
S1_WT_IMS
S1_WT_MATRIX
S1_atp23
S1_atp23_IMS
S1_atp23_MATRIX
S2_WT
S2_WT_IMS
S2_WT_MATRIX
S2_atp6
S2_atp6_IMS
S2_atp6_MATRIX

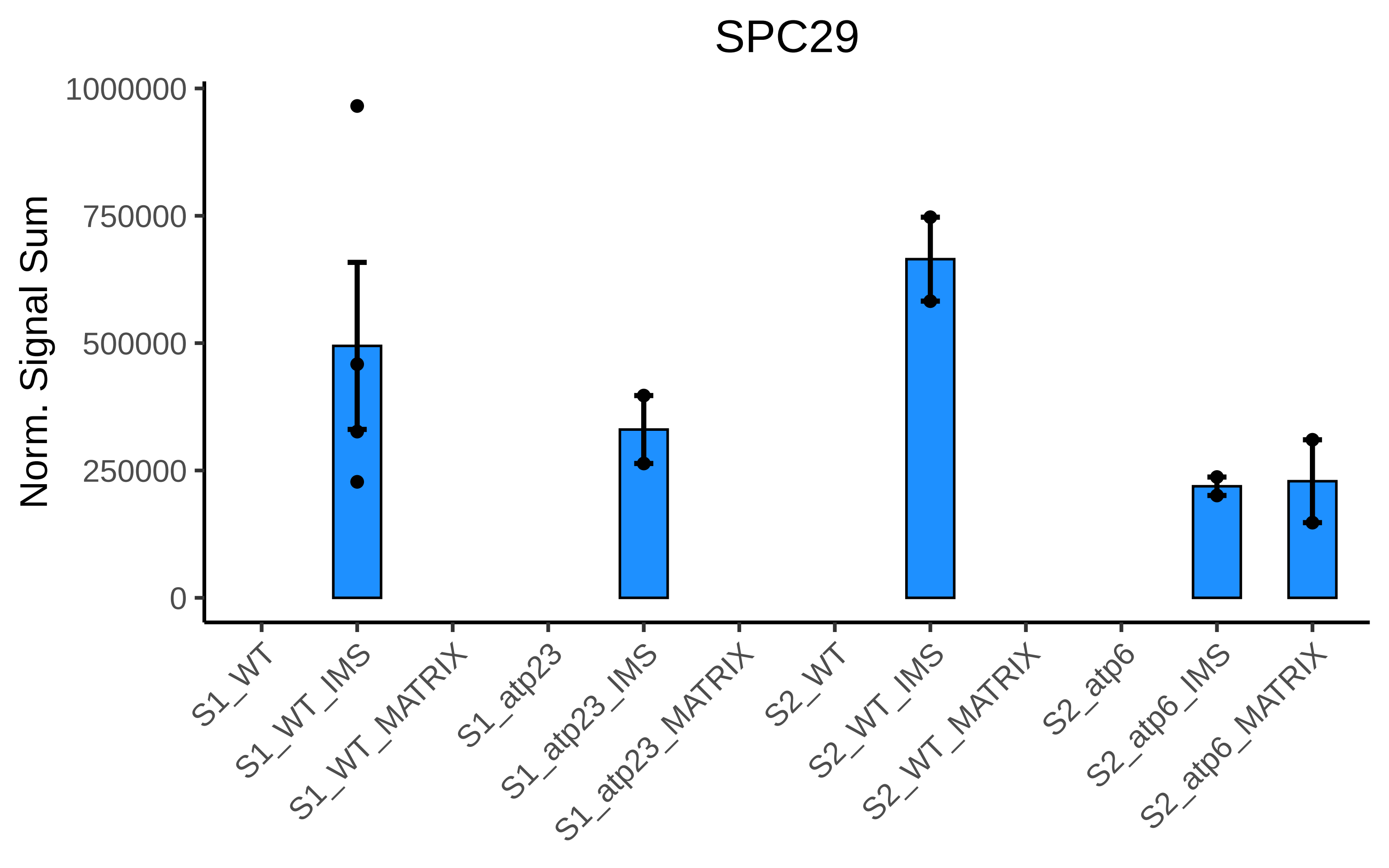

SPC29
Norm. Signal Sum
1000000
750000
500000
250000
0
S1_WT
S1_WT_IMS
S1_WT_MATRIX
S1_atp23
S1_atp23_IMS
S1_atp23_MATRIX
S2_WT
S2_WT_IMS
S2_WT_MATRIX
S2_atp6
S2_atp6_IMS
S2_atp6_MATRIX

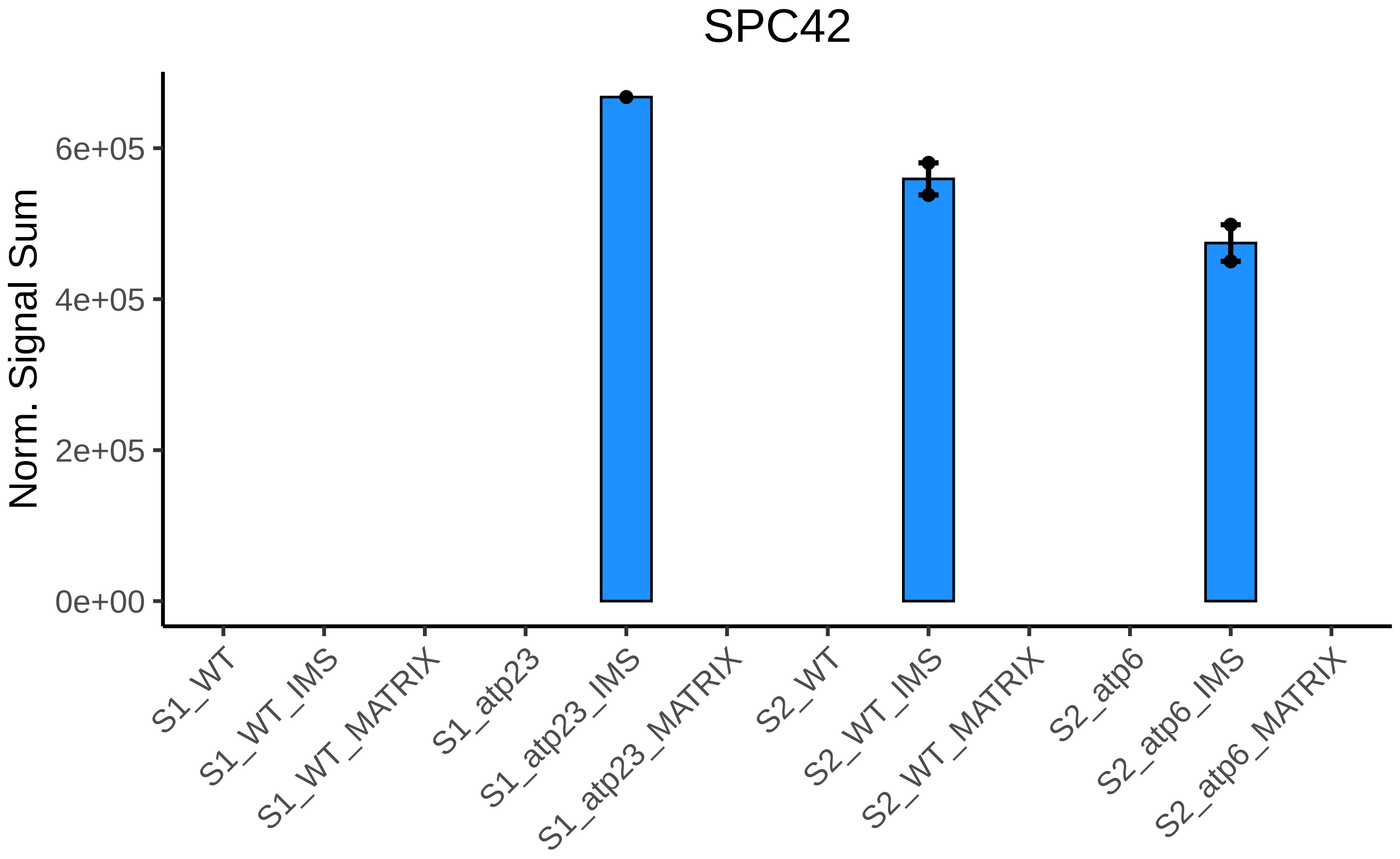

SPC42
Norm. Signal Sum
6e+05
4e+05
2e+05
0e+00
S1_WT
S1_WT_IMS
S1_WT_MATRIX
S1_atp23
S1_atp23_IMS
S1_atp23_MATRIX
S2_WT
S2_WT_IMS
S2_WT_MATRIX
S2_atp6
S2_atp6_IMS
S2_atp6_MATRIX

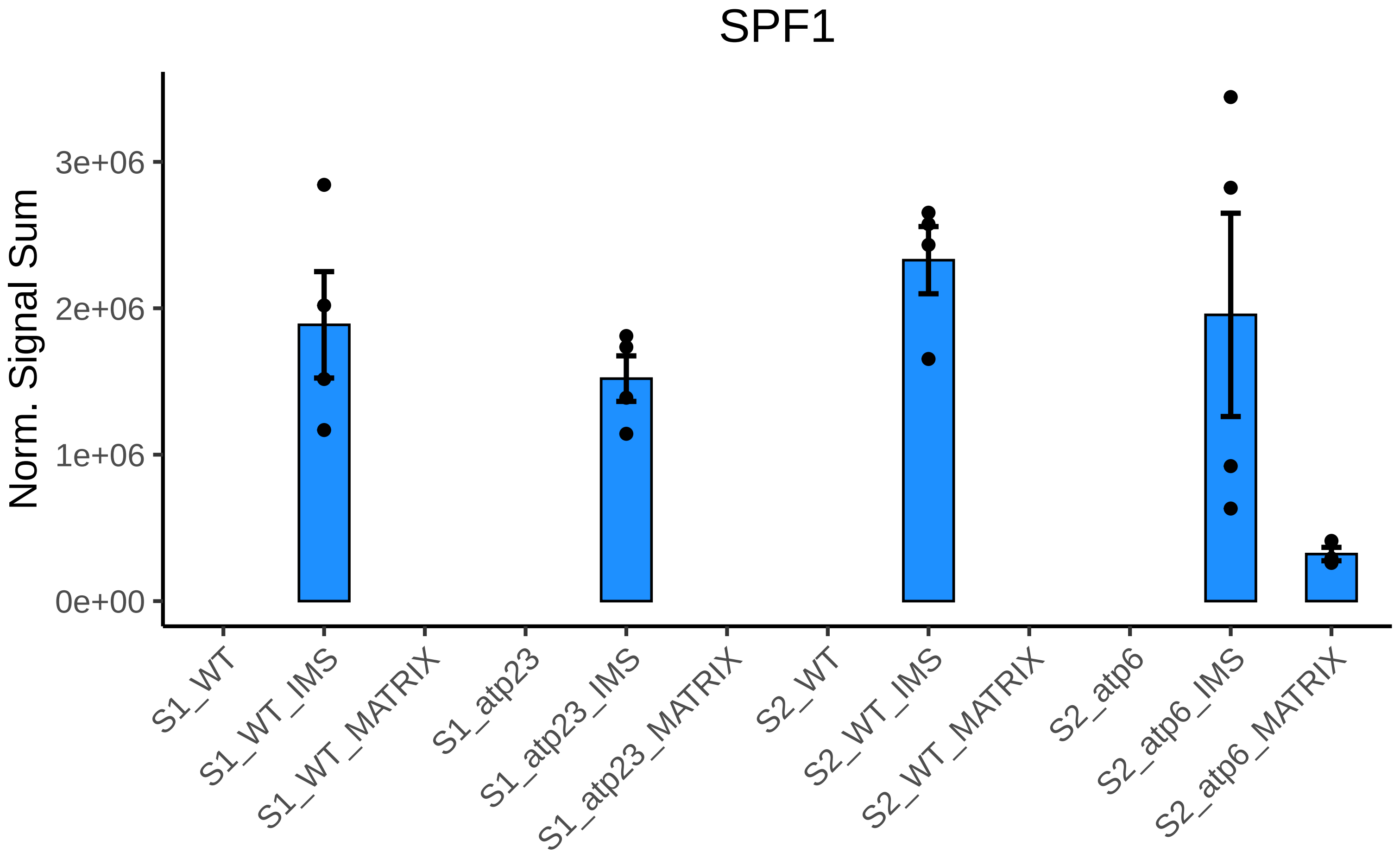

SPF1
Norm. Signal Sum
3e+06
2e+06
1e+06
0e+00
S1_WT
S1_WT_IMS
S1_WT_MATRIX
S1_atp23
S1_atp23_IMS
S1_atp23_MATRIX
S2_WT
S2_WT_IMS
S2_WT_MATRIX
S2_atp6
S2_atp6_IMS
S2_atp6_MATRIX

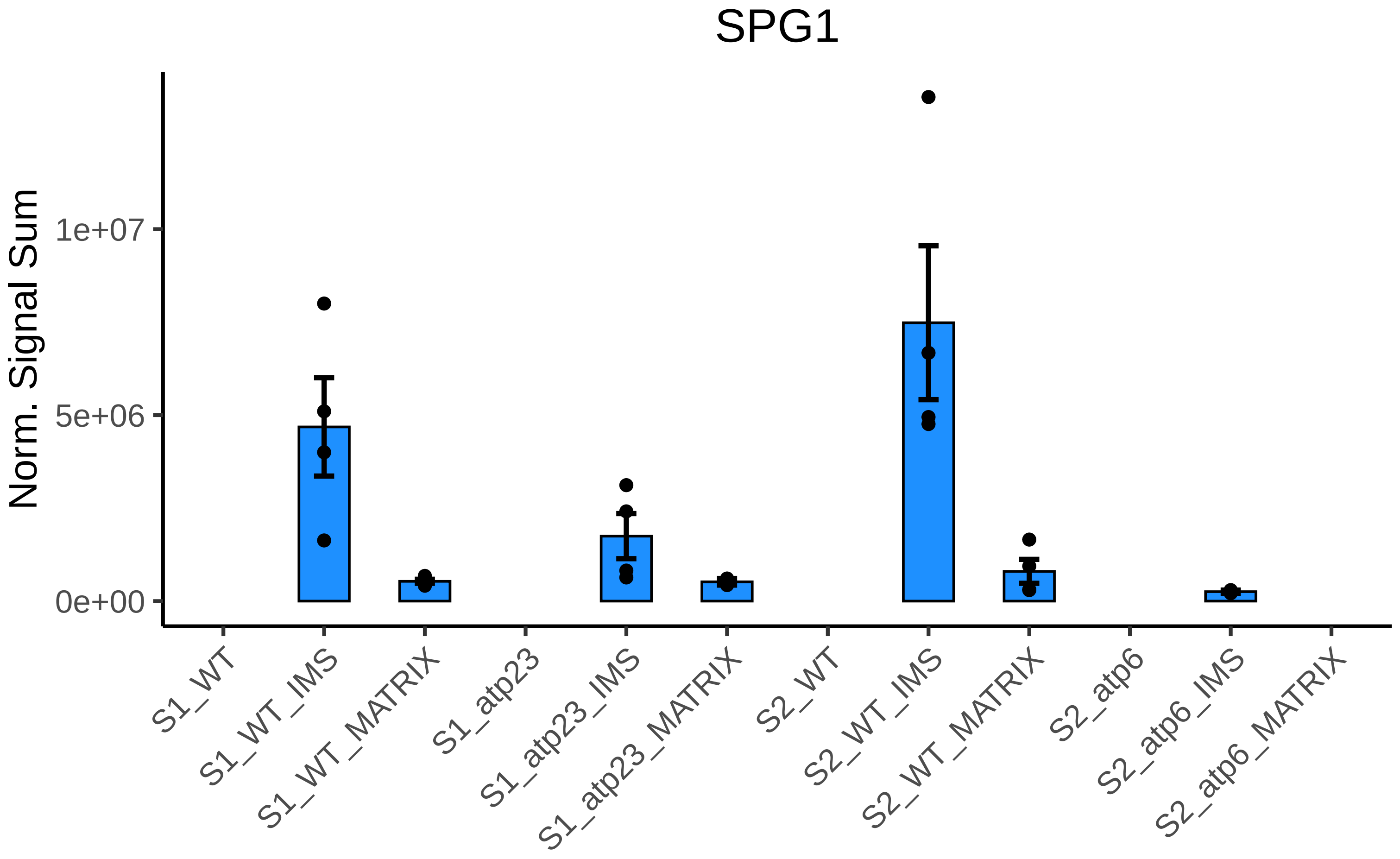
SPG1
Norm. Signal Sum
1e+07
5e+06
0e+00
S1_WT
S1_WT_IMS
S1_WT_MATRIX
S1_atp23
S1_atp23_IMS
S1_atp23_MATRIX
S2_WT
S2_WT_IMS
S2_WT_MATRIX
S2_atp6
S2_atp6_IMS
S2_atp6_MATRIX

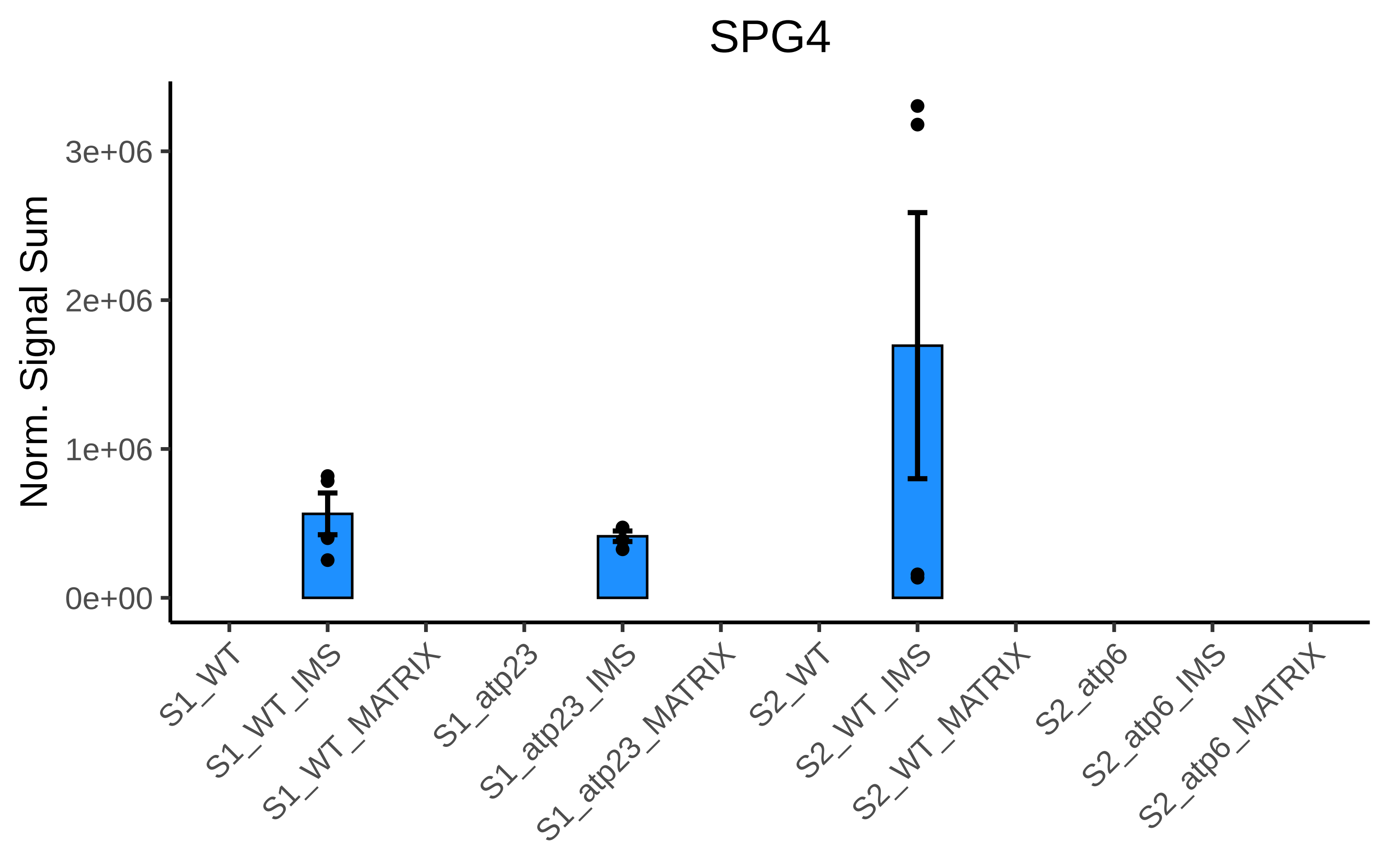
SPG4
Norm. Signal Sum
3e+06
2e+06
1e+06
0e+00
S1_WT
S1_WT_IMS
S1_WT_MATRIX
S1_atp23
S1_atp23_IMS
S1_atp23_MATRIX
S2_WT
S2_WT_IMS
S2_WT_MATRIX
S2_atp6
S2_atp6_IMS
S2_atp6_MATRIX

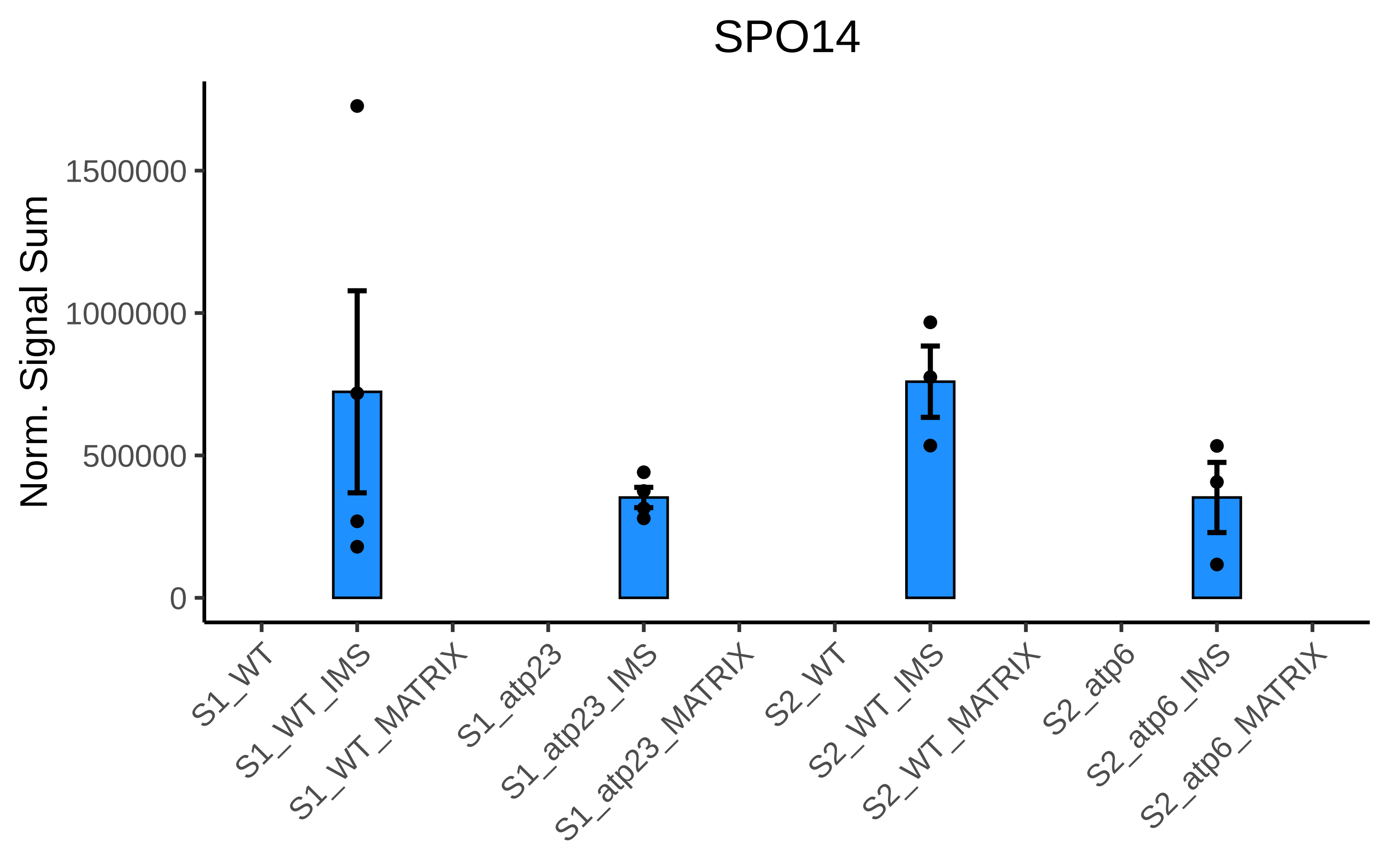

SPO14
Norm. Signal Sum
1500000
1000000
500000
0
S1_WT
S1_WT_IMS
S1_WT_MATRIX
S1_atp23
S1_atp23_IMS
S1_atp23_MATRIX
S2_WT
S2_WT_IMS
S2_WT_MATRIX
S2_atp6
S2_atp6_IMS
S2_atp6_MATRIX

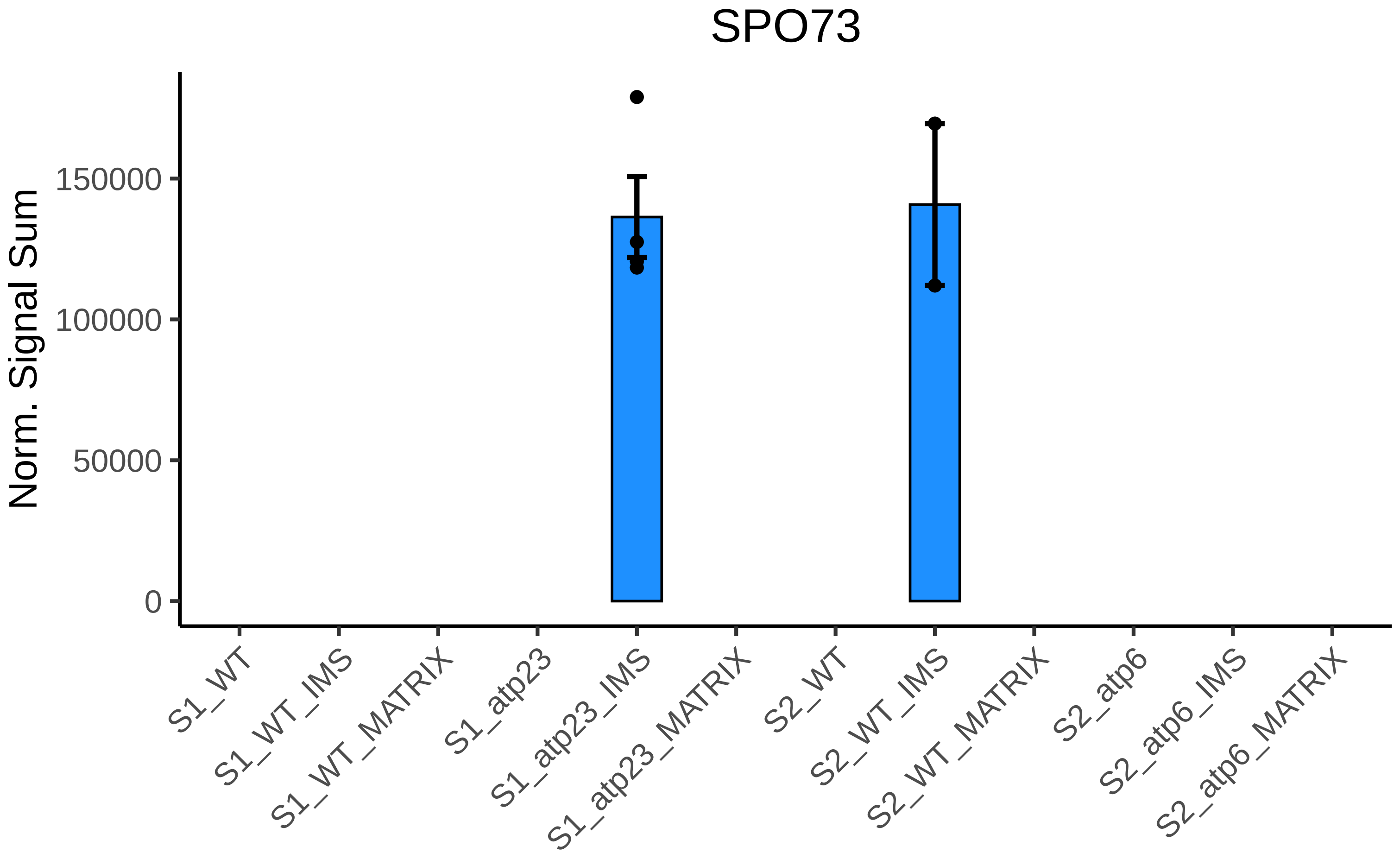

SPO73
Norm. Signal Sum
150000
100000
50000
0
S1_WT
S1_WT_IMS
S1_WT_MATRIX
S1_atp23
S1_atp23_IMS
S1_atp23_MATRIX
S2_WT
S2_WT_IMS
S2_WT_MATRIX
S2_atp6
S2_atp6_IMS
S2_atp6_MATRIX

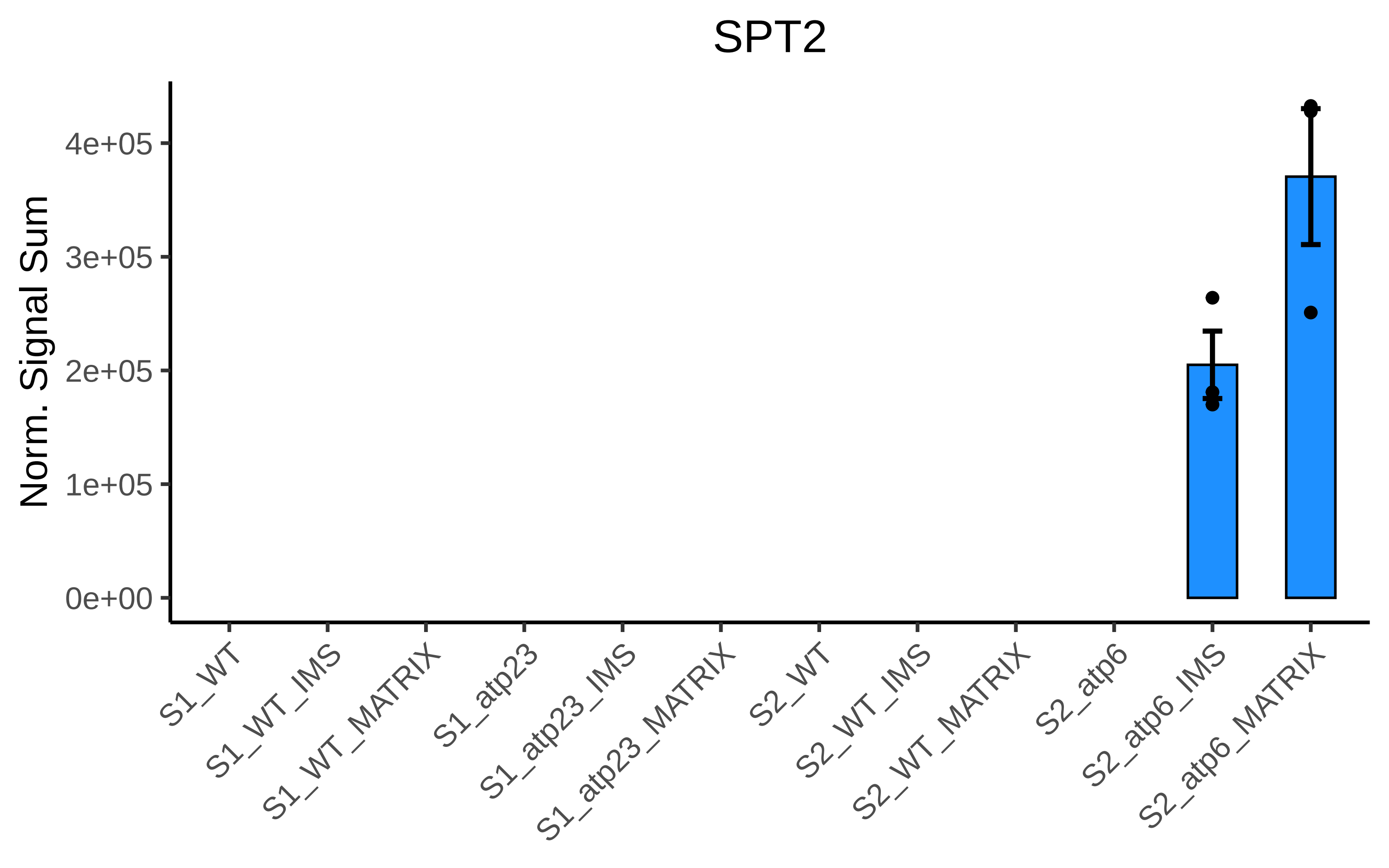

SPT2
Norm. Signal Sum
4e+05
3e+05
2e+05
1e+05
0e+00
S1_WT
S1_WT_IMS
S1_WT_MATRIX
S1_atp23
S1_atp23_IMS
S1_atp23_MATRIX
S2_WT
S2_WT_IMS
S2_WT_MATRIX
S2_atp6
S2_atp6_IMS
S2_atp6_MATRIX

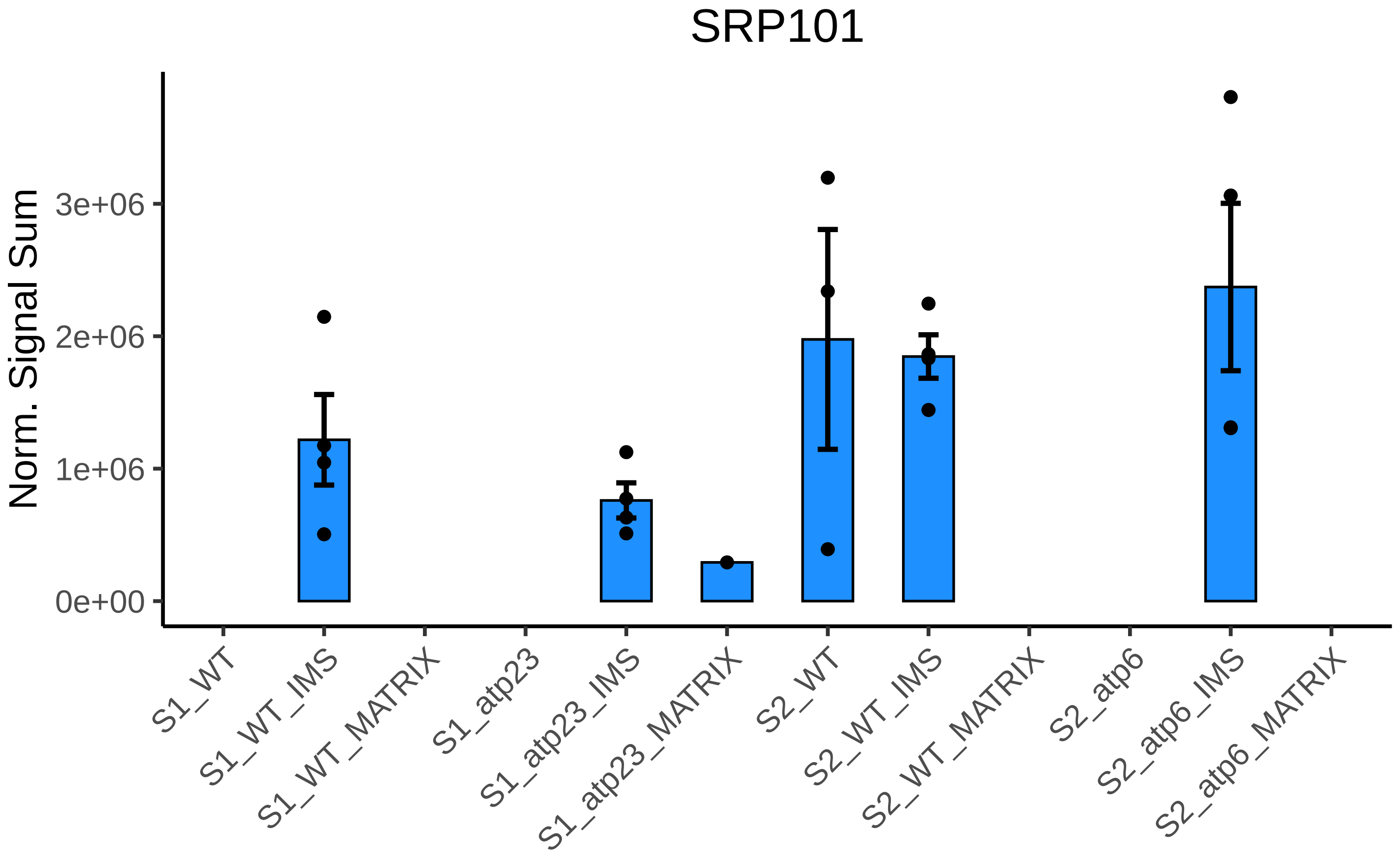

SRP101
Norm. Signal Sum
3e+06
2e+06
1e+06
0e+00
S1_WT
S1_WT_IMS
S1_WT_MATRIX
S1_atp23
S1_atp23_IMS
S1_atp23_MATRIX
S2_WT
S2_WT_IMS
S2_WT_MATRIX
S2_atp6
S2_atp6_IMS
S2_atp6_MATRIX

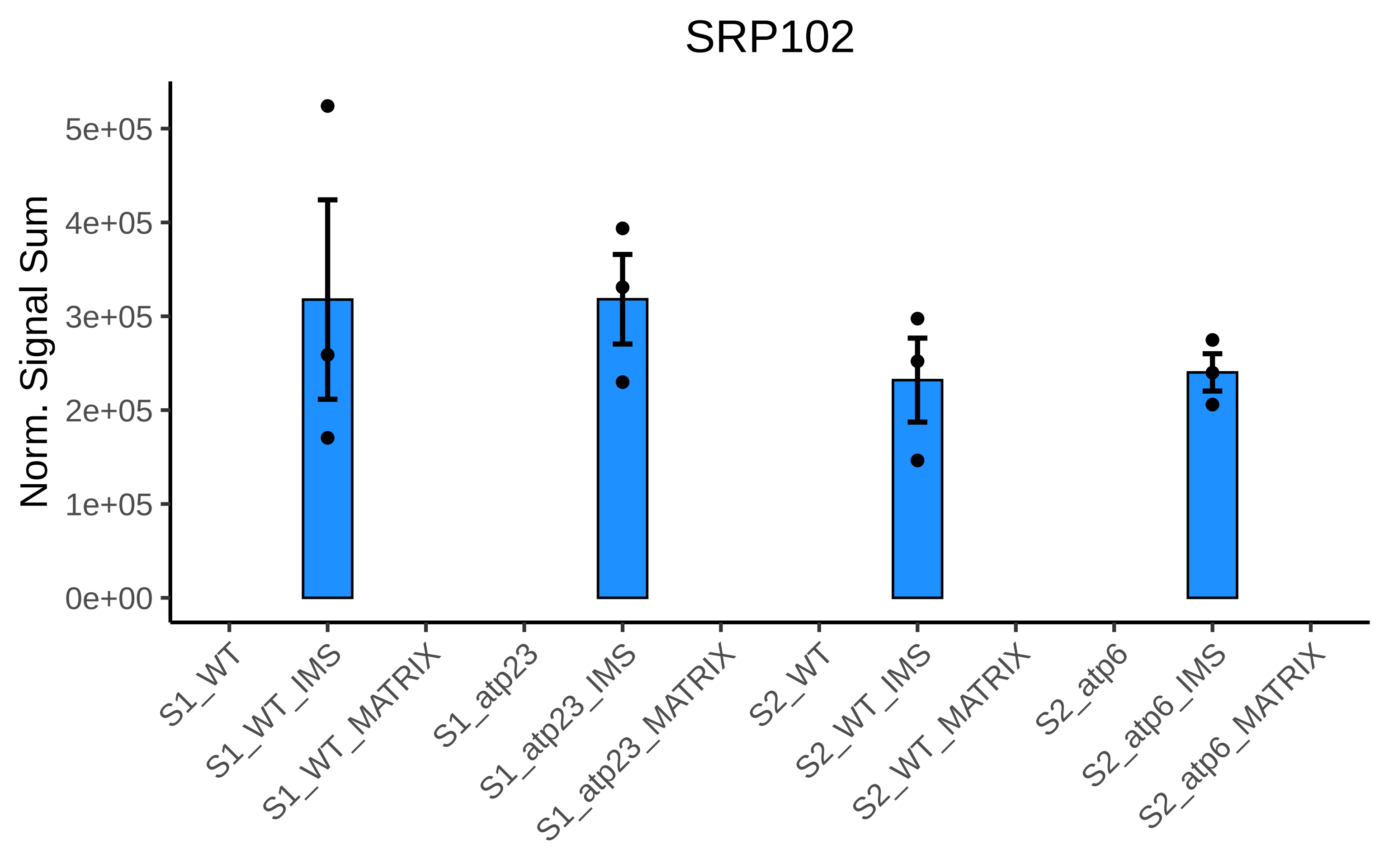
SRP102
Norm. Signal Sum
0e+00
1e+05
2e+05
3e+05
4e+05
5e+05
S1_WT
S1_WT_IMS
S1_WT_MATRIX
S1_atp23
S1_atp23_IMS
S1_atp23_MATRIX
S2_WT
S2_WT_IMS
S2_WT_MATRIX
S2_atp6
S2_atp6_IMS
S2_atp6_MATRIX

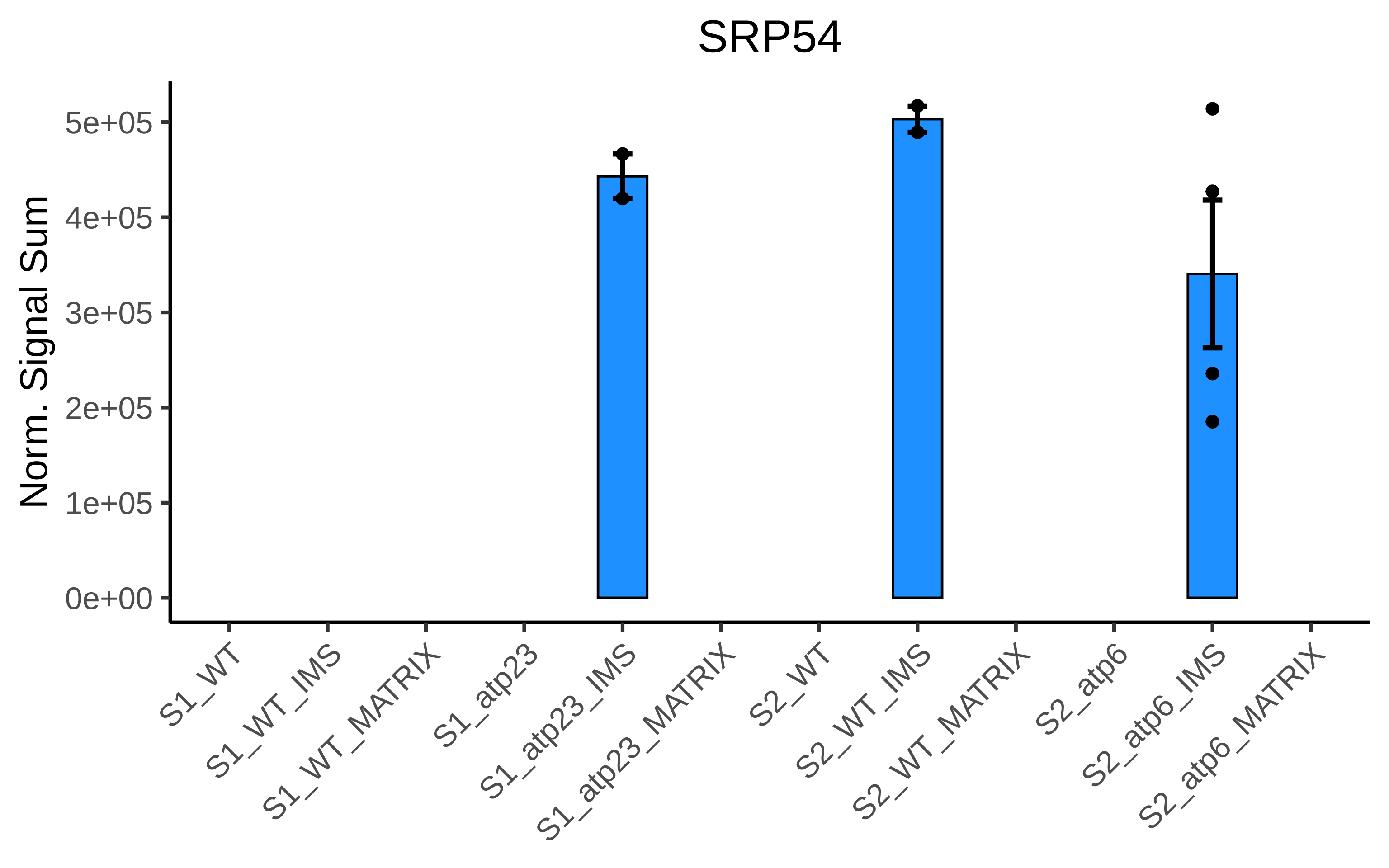
SRP54
Norm. Signal Sum
5e+05
4e+05
3e+05
2e+05
1e+05
0e+00
S1_WT
S1_WT_IMS
S1_WT_MATRIX
S1_atp23
S1_atp23_IMS
S1_atp23_MATRIX
S2_WT
S2_WT_IMS
S2_WT_MATRIX
S2_atp6
S2_atp6_IMS
S2_atp6_MATRIX

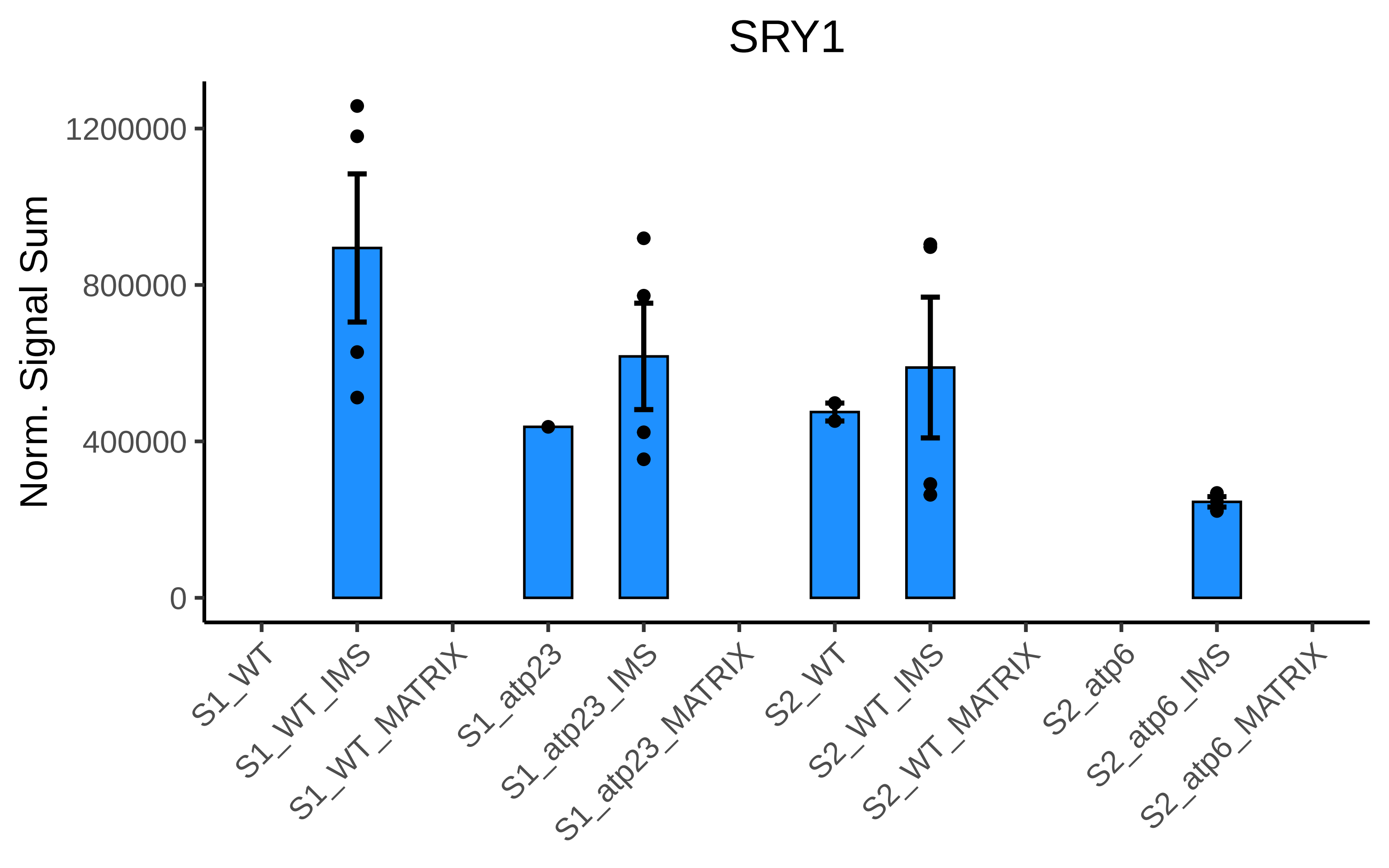

SRY1
Norm. Signal Sum
1200000
800000
400000
0
S1_WT
S1_WT_IMS
S1_WT_MATRIX
S1_atp23
S1_atp23_IMS
S1_atp23_MATRIX
S2_WT
S2_WT_IMS
S2_WT_MATRIX
S2_atp6
S2_atp6_IMS
S2_atp6_MATRIX

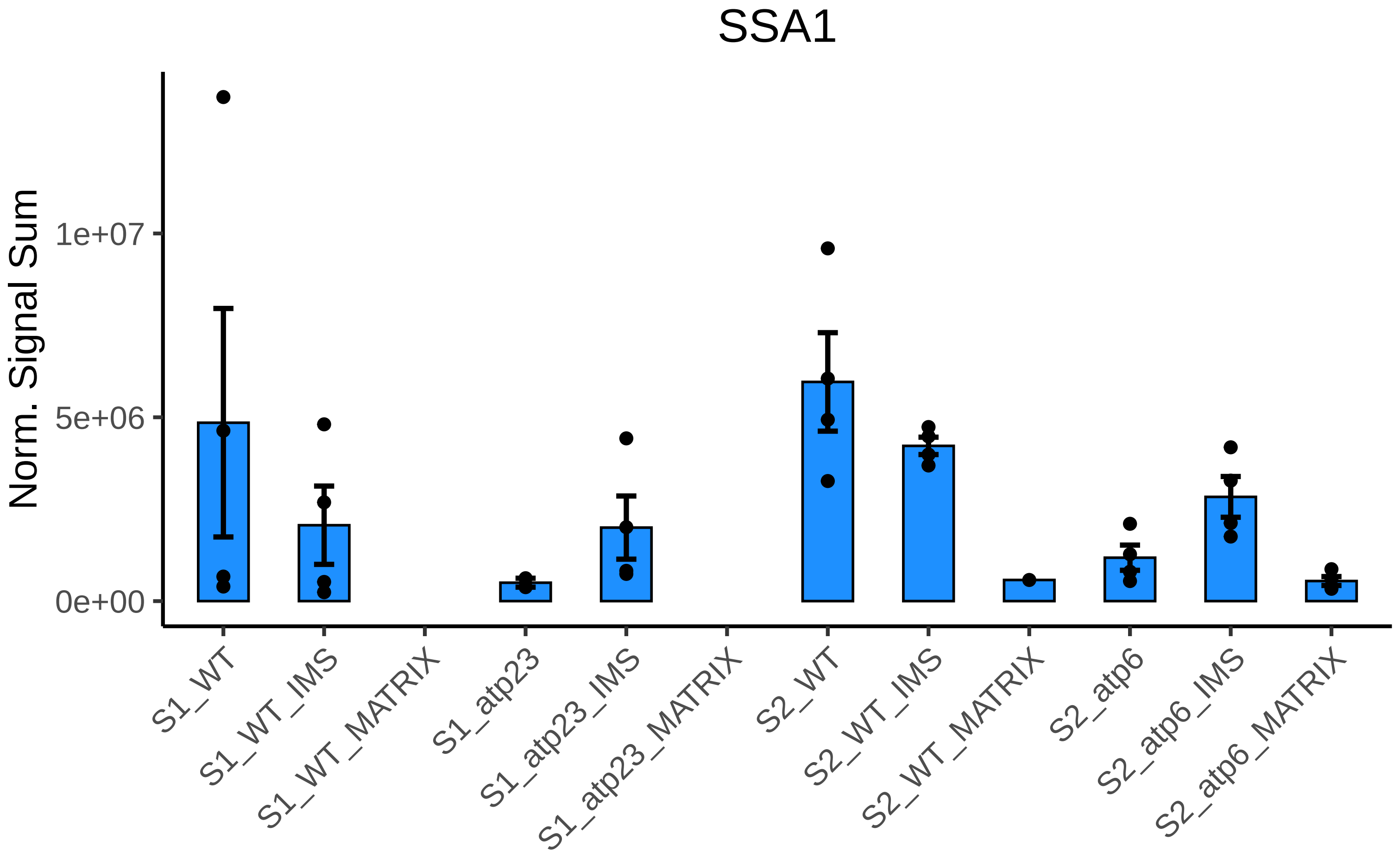
SSA1
Norm. Signal Sum
1e+07
5e+06
0e+00
S1_WT
S1_WT_IMS
S1_WT_MATRIX
S1_atp23
S1_atp23_IMS
S1_atp23_MATRIX
S2_WT
S2_WT_IMS
S2_WT_MATRIX
S2_atp6
S2_atp6_IMS
S2_atp6_MATRIX

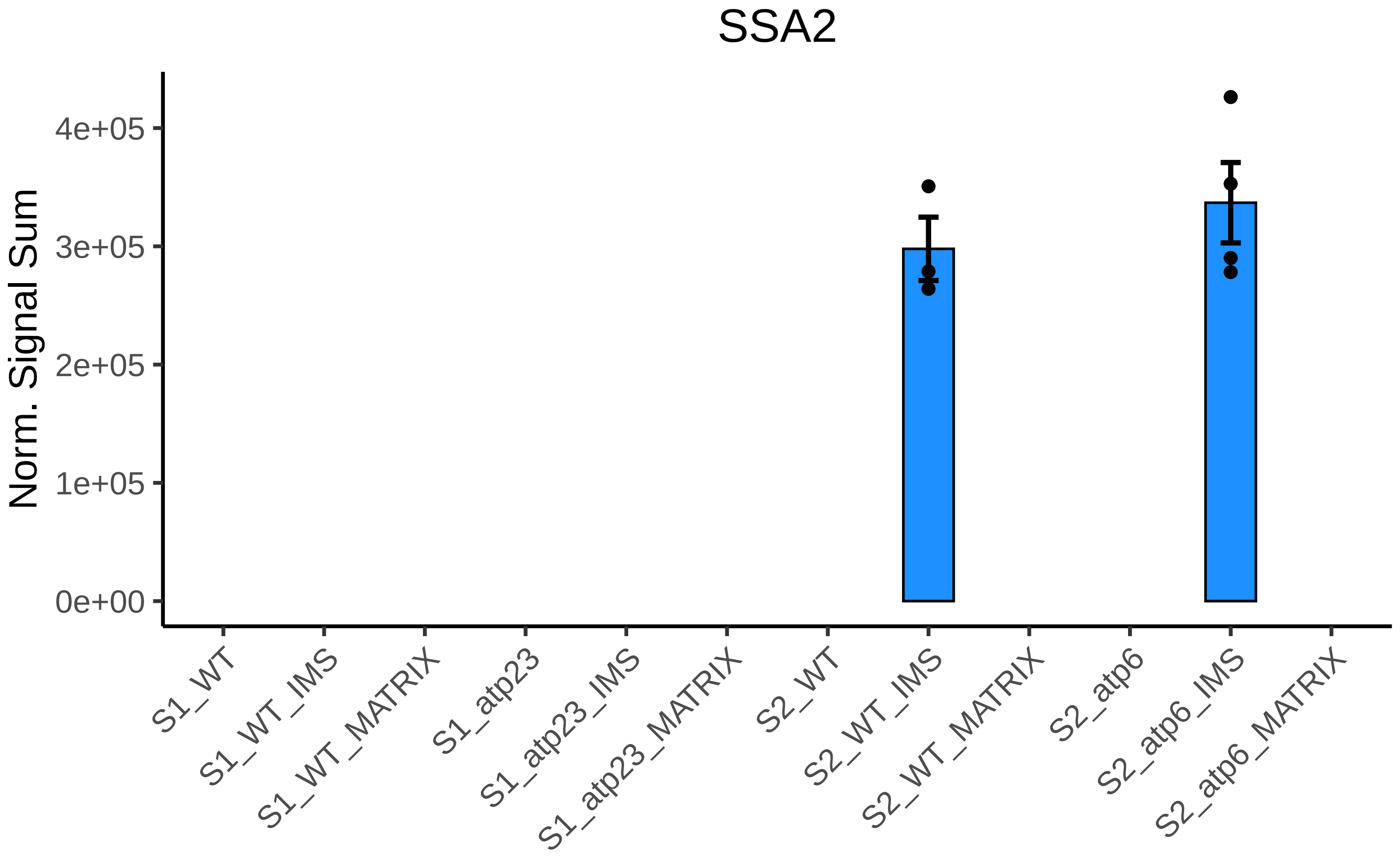
SSA2
Norm. Signal Sum
4e+05
3e+05
2e+05
1e+05
0e+00
S1_WT
S1_WT_IMS
S1_WT_MATRIX
S1_atp23
S1_atp23_IMS
S1_atp23_MATRIX
S2_WT
S2_WT_IMS
S2_WT_MATRIX
S2_atp6
S2_atp6_IMS
S2_atp6_MATRIX

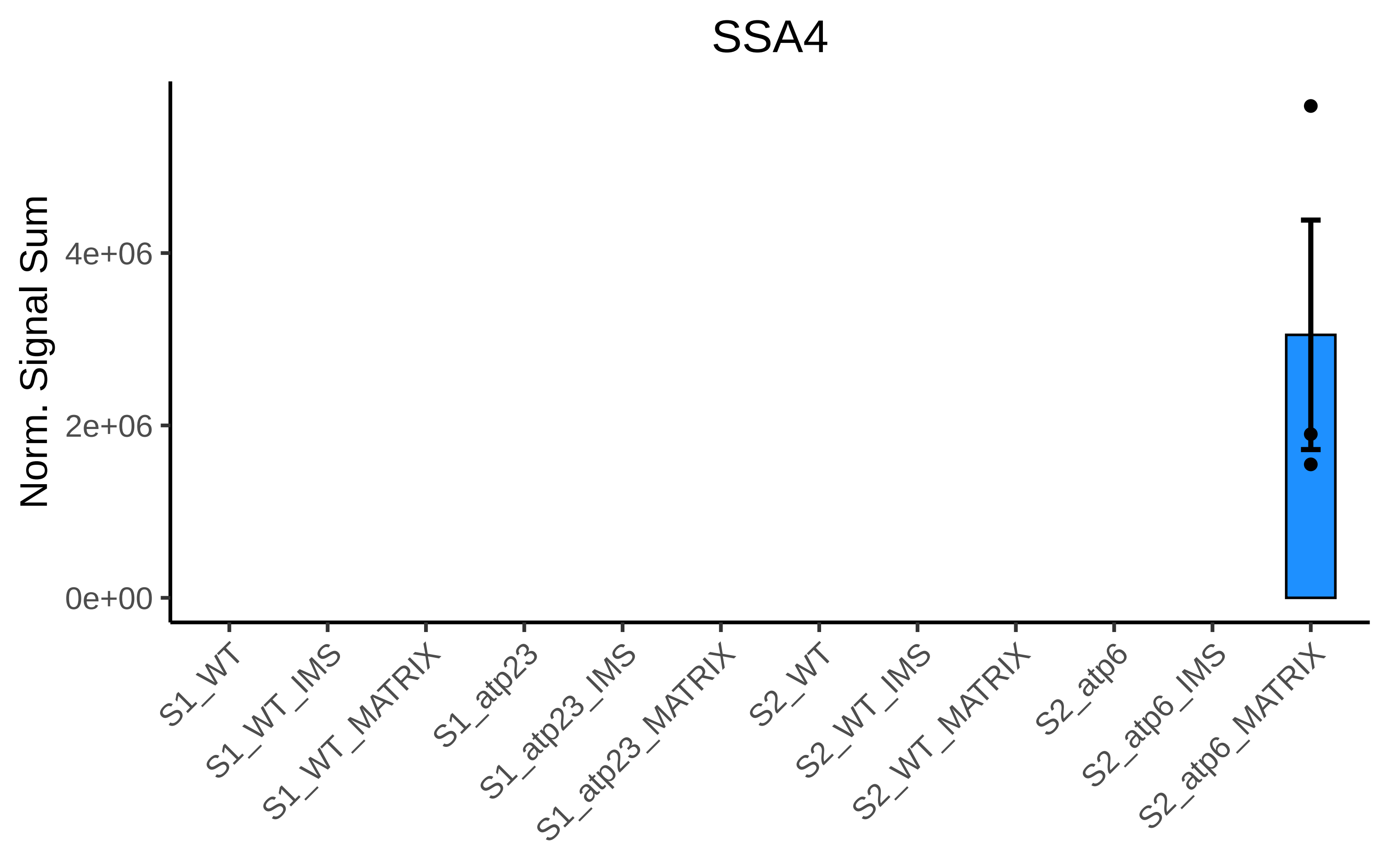
SSA4
Norm. Signal Sum
4e+06
2e+06
0e+00
S1_WT
S1_WT_IMS
S1_WT_MATRIX
S1_atp23
S1_atp23_IMS
S1_atp23_MATRIX
S2_WT
S2_WT_IMS
S2_WT_MATRIX
S2_atp6
S2_atp6_IMS
S2_atp6_MATRIX

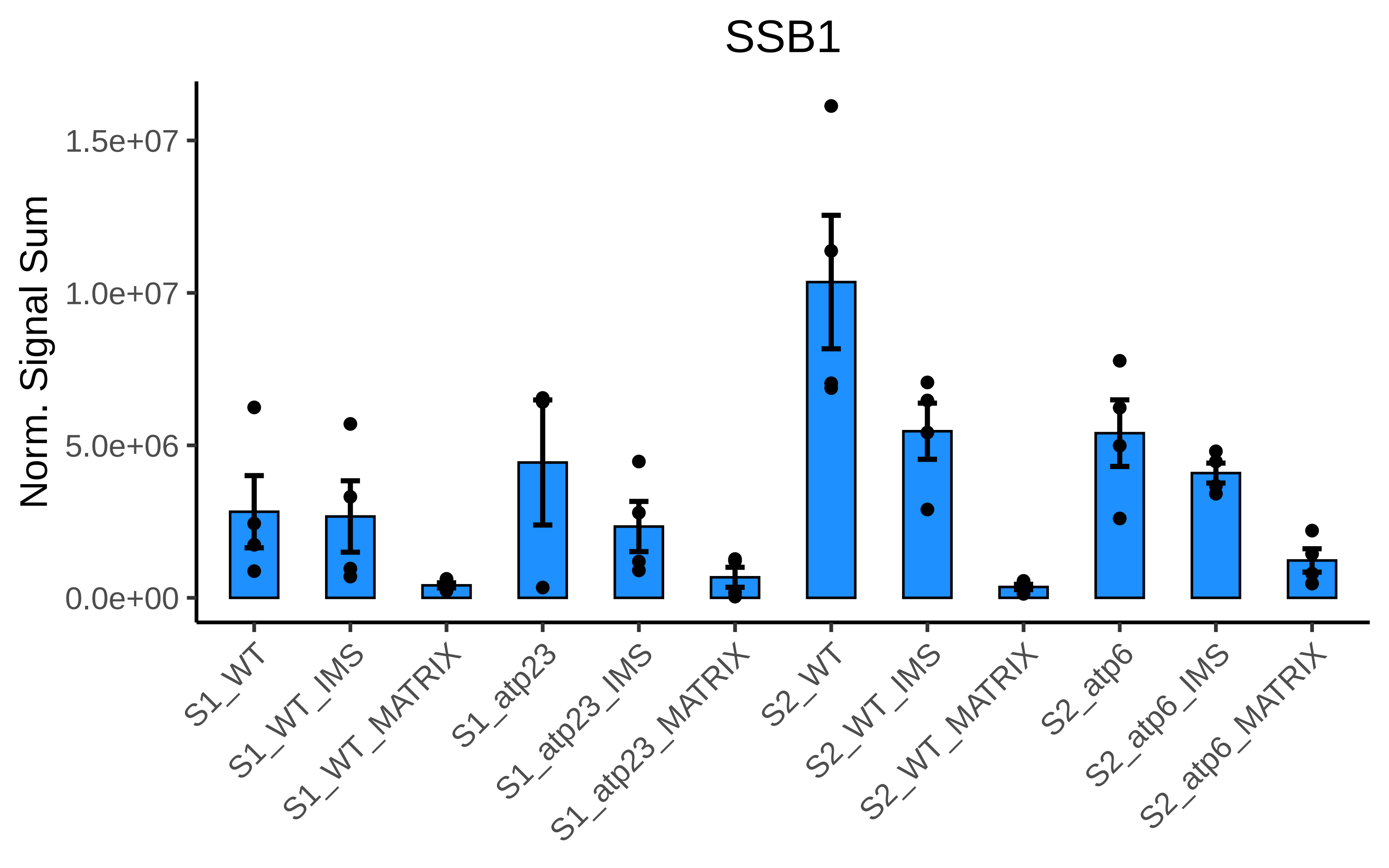

SSB1
Norm. Signal Sum
1.5e+07
1.0e+07
5.0e+06
0.0e+00
S1_WT
S1_WT_IMS
S1_WT_MATRIX
S1_atp23
S1_atp23_IMS
S1_atp23_MATRIX
S2_WT
S2_WT_IMS
S2_WT_MATRIX
S2_atp6
S2_atp6_IMS
S2_atp6_MATRIX

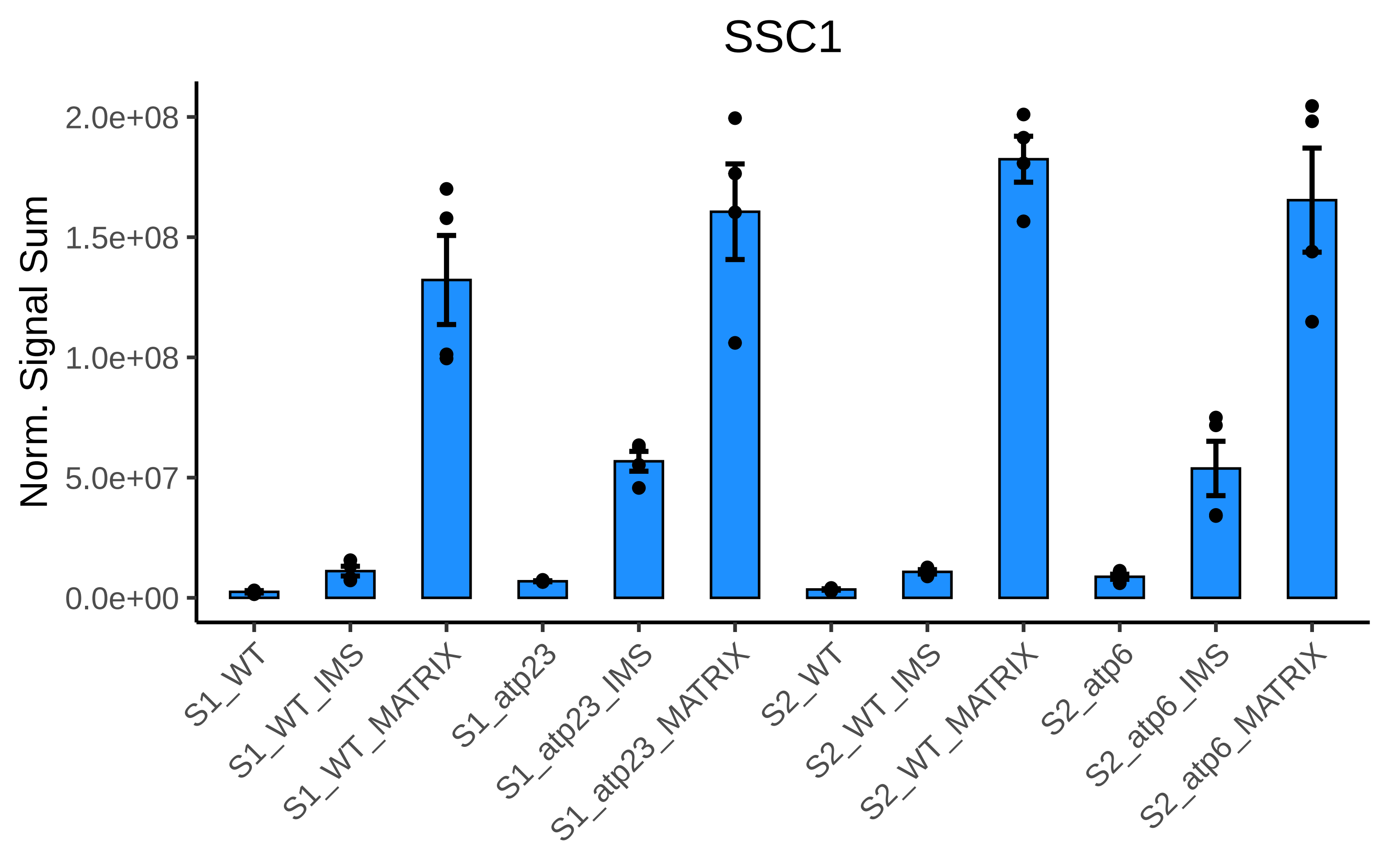

SSC1
Norm. Signal Sum
2.0e+08
1.5e+08
1.0e+08
5.0e+07
0.0e+00
S1_WT
S1_WT_IMS
S1_WT_MATRIX
S1_atp23
S1_atp23_IMS
S1_atp23_MATRIX
S2_WT
S2_WT_IMS
S2_WT_MATRIX
S2_atp6
S2_atp6_IMS
S2_atp6_MATRIX

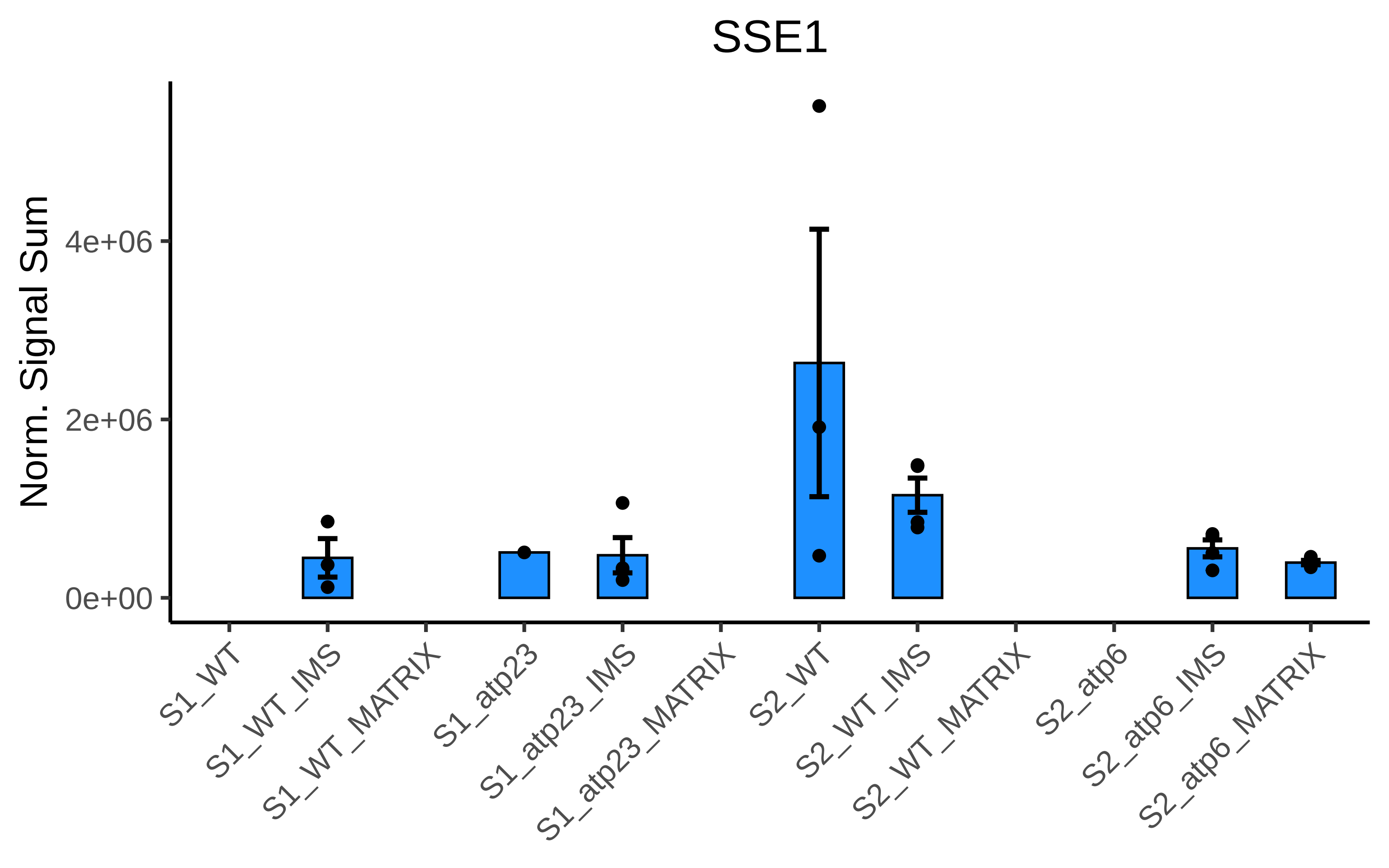

SSE1
Norm. Signal Sum
4e+06
2e+06
0e+00
S1_WT
S1_WT_IMS
S1_WT_MATRIX
S1_atp23
S1_atp23_IMS
S1_atp23_MATRIX
S2_WT
S2_WT_IMS
S2_WT_MATRIX
S2_atp6
S2_atp6_IMS
S2_atp6_MATRIX

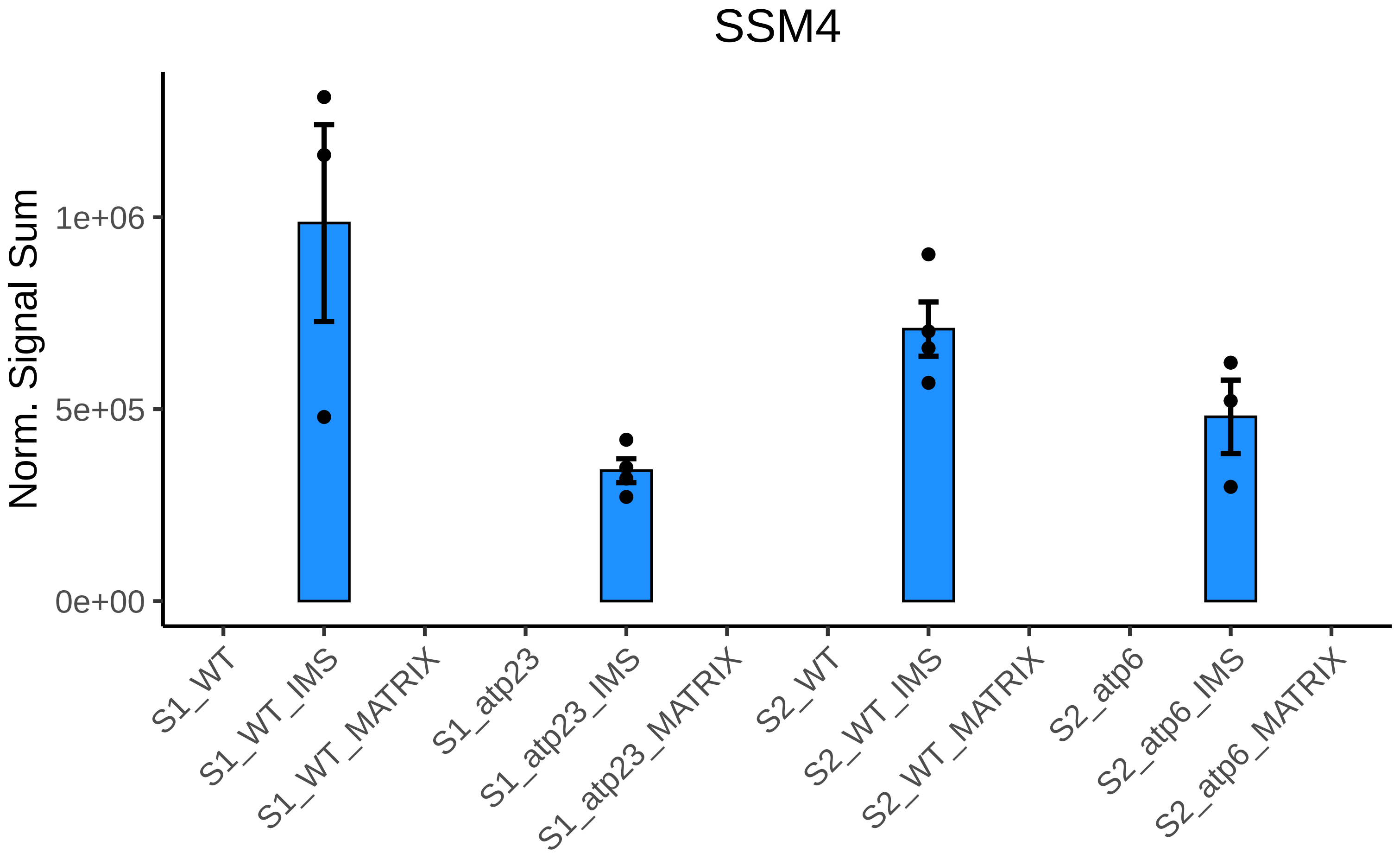
SSM4
Norm. Signal Sum
1e+06
5e+05
0e+00
S1_WT
S1_WT_IMS
S1_WT_MATRIX
S1_atp23
S1_atp23_IMS
S1_atp23_MATRIX
S2_WT
S2_WT_IMS
S2_WT_MATRIX
S2_atp6
S2_atp6_IMS
S2_atp6_MATRIX

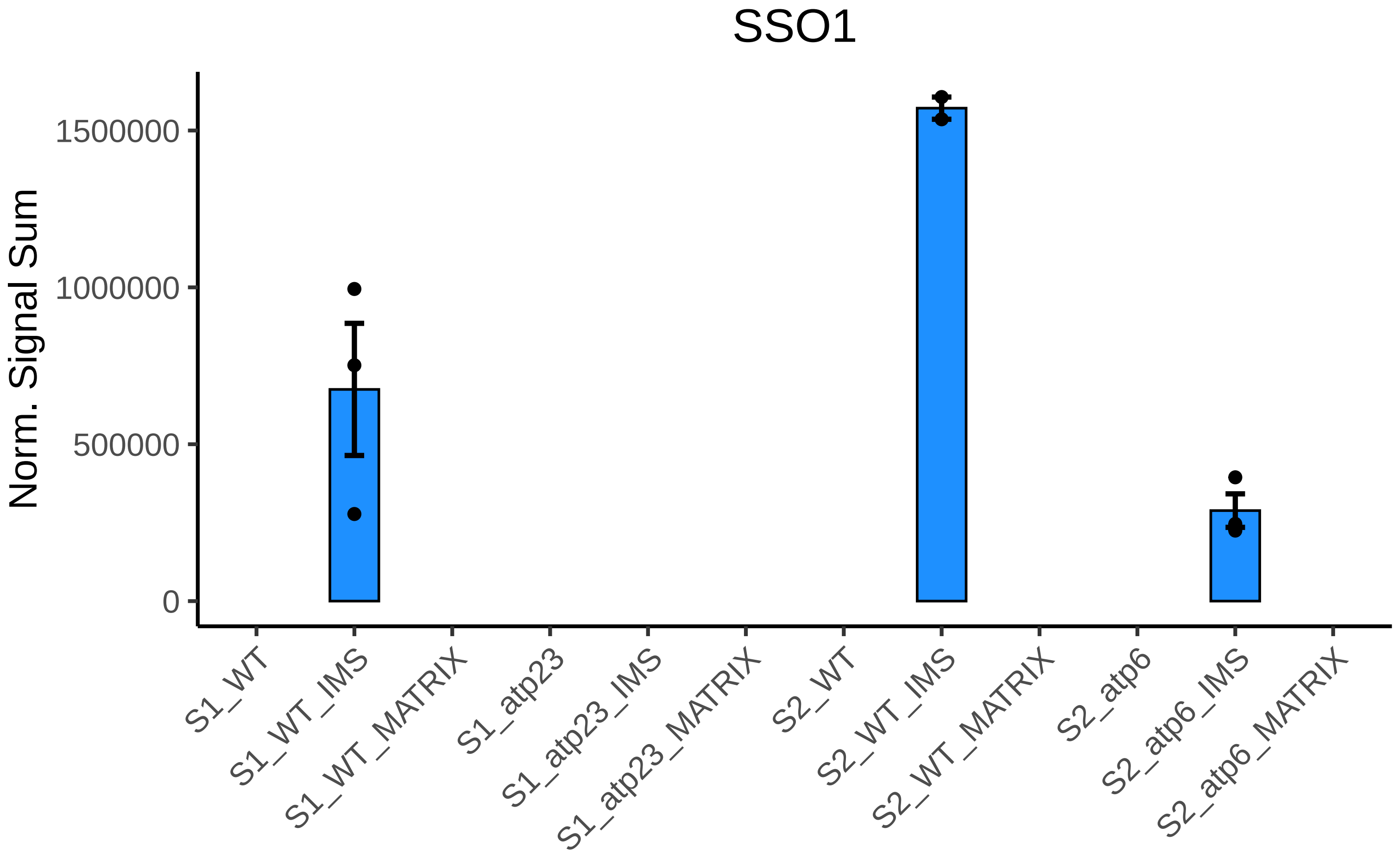

SSO1
Norm. Signal Sum
1500000
1000000
500000
0
S1_WT
S1_WT_IMS
S1_WT_MATRIX
S1_atp23
S1_atp23_IMS
S1_atp23_MATRIX
S2_WT
S2_WT_IMS
S2_WT_MATRIX
S2_atp6
S2_atp6_IMS
S2_atp6_MATRIX

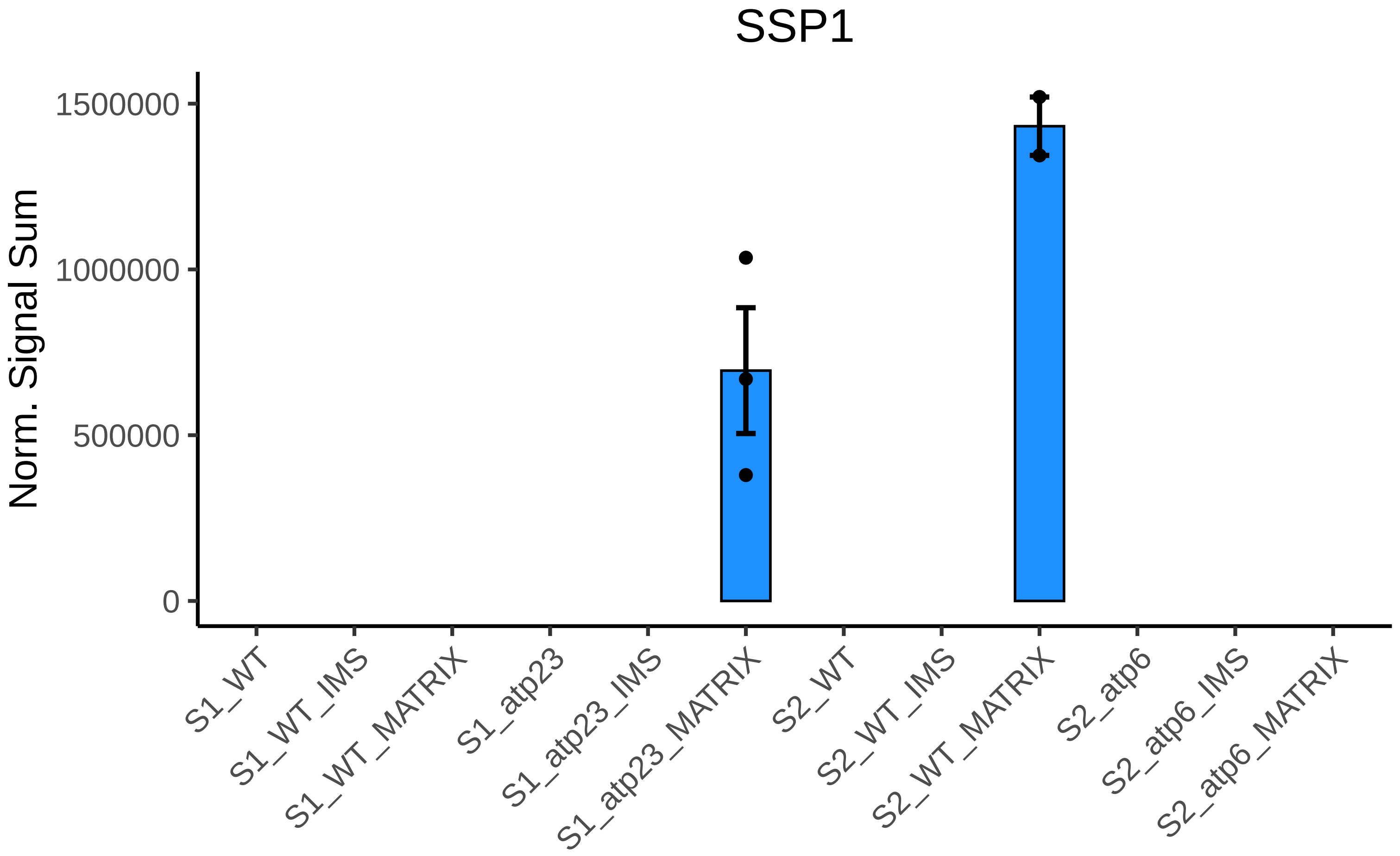

SSP1
Norm. Signal Sum
1500000
1000000
500000
0
S1_WT
S1_WT_IMS
S1_WT_MATRIX
S1_atp23
S1_atp23_IMS
S1_atp23_MATRIX
S2_WT
S2_WT_IMS
S2_WT_MATRIX
S2_atp6
S2_atp6_IMS
S2_atp6_MATRIX

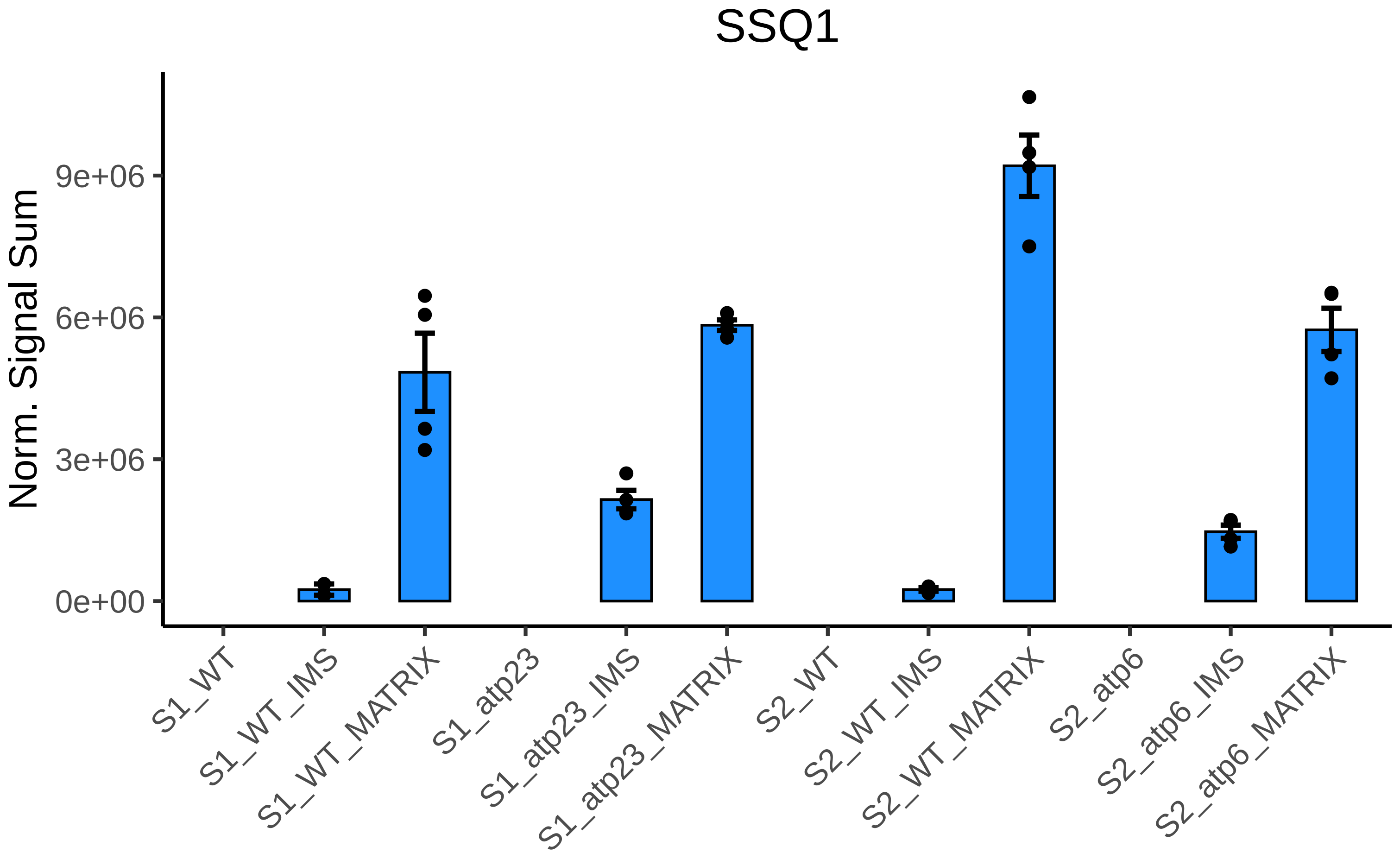
SSQ1
Norm. Signal Sum
9e+06
6e+06
3e+06
0e+00
S1_WT
S1_WT_IMS
S1_WT_MATRIX
S1_atp23
S1_atp23_IMS
S1_atp23_MATRIX
S2_WT
S2_WT_IMS
S2_WT_MATRIX
S2_atp6
S2_atp6_IMS
S2_atp6_MATRIX

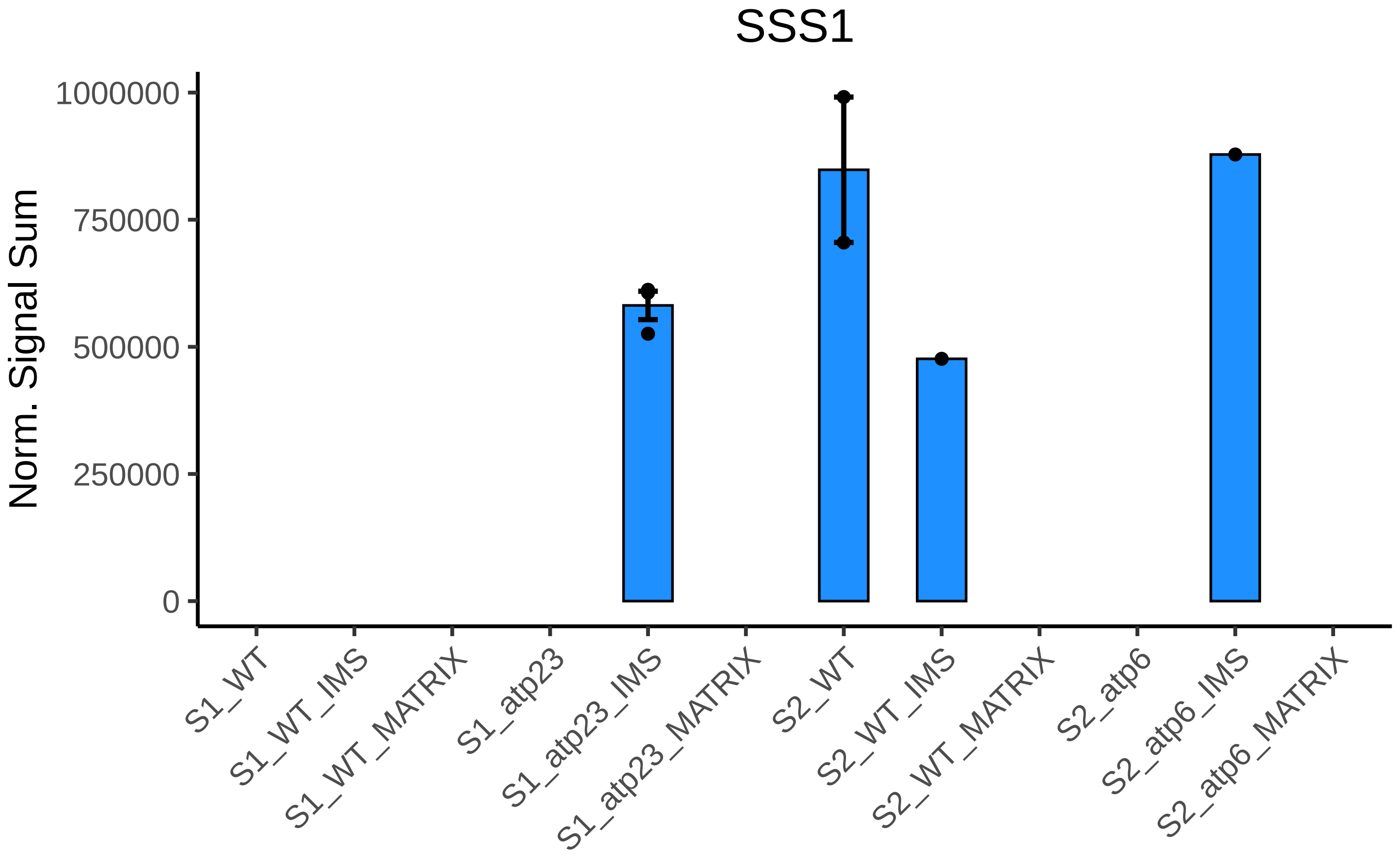
SSS1
Norm. Signal Sum
1000000
750000
500000
250000
0
S1_WT
S1_WT_IMS
S1_WT_MATRIX
S1_atp23
S1_atp23_IMS
S1_atp23_MATRIX
S2_WT
S2_WT_IMS
S2_WT_MATRIX
S2_atp6
S2_atp6_IMS
S2_atp6_MATRIX

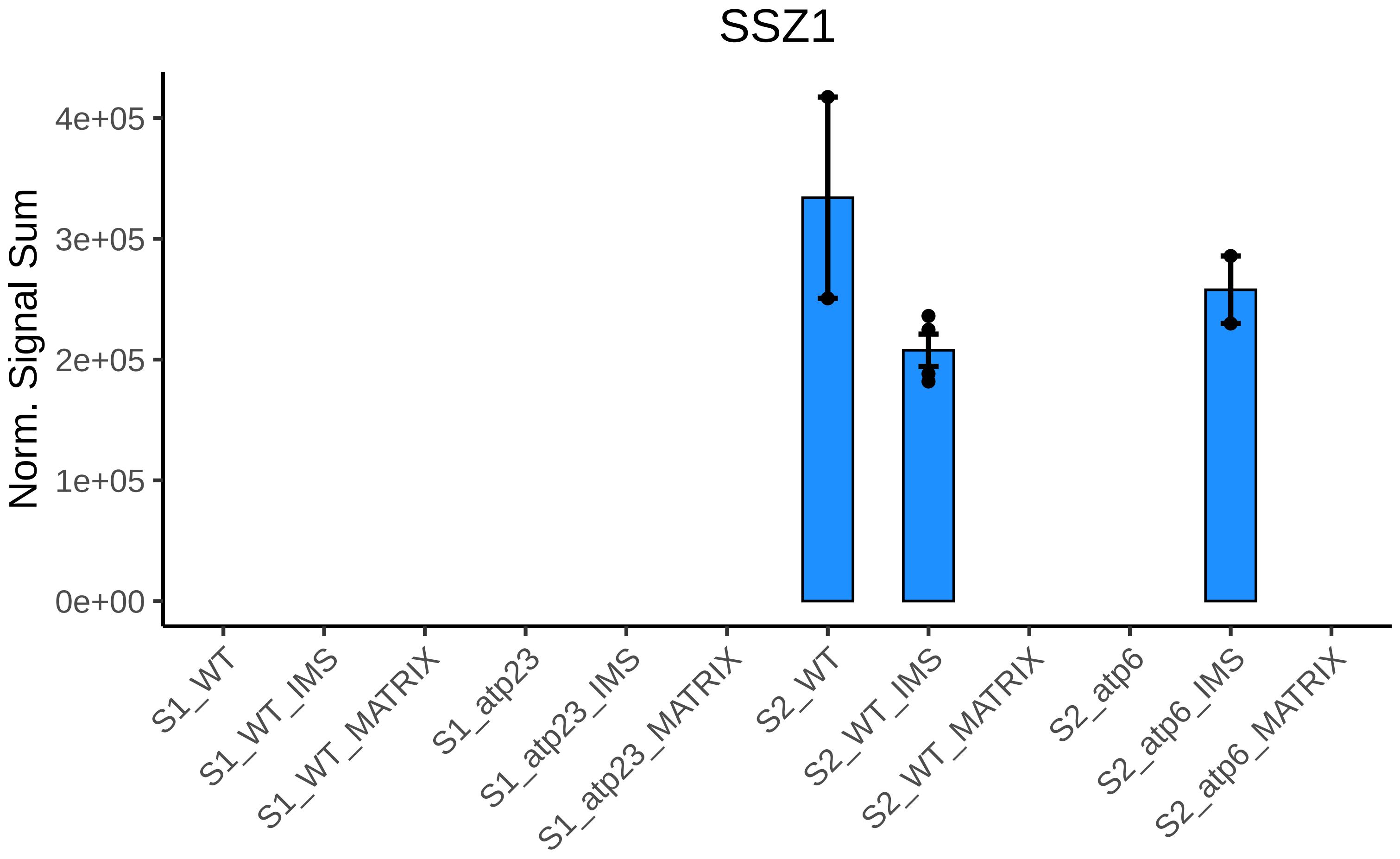

SSZ1
Norm. Signal Sum
4e+05
3e+05
2e+05
1e+05
0e+00
S1_WT
S1_WT_IMS
S1_WT_MATRIX
S1_atp23
S1_atp23_IMS
S1_atp23_MATRIX
S2_WT
S2_WT_IMS
S2_WT_MATRIX
S2_atp6
S2_atp6_IMS
S2_atp6_MATRIX

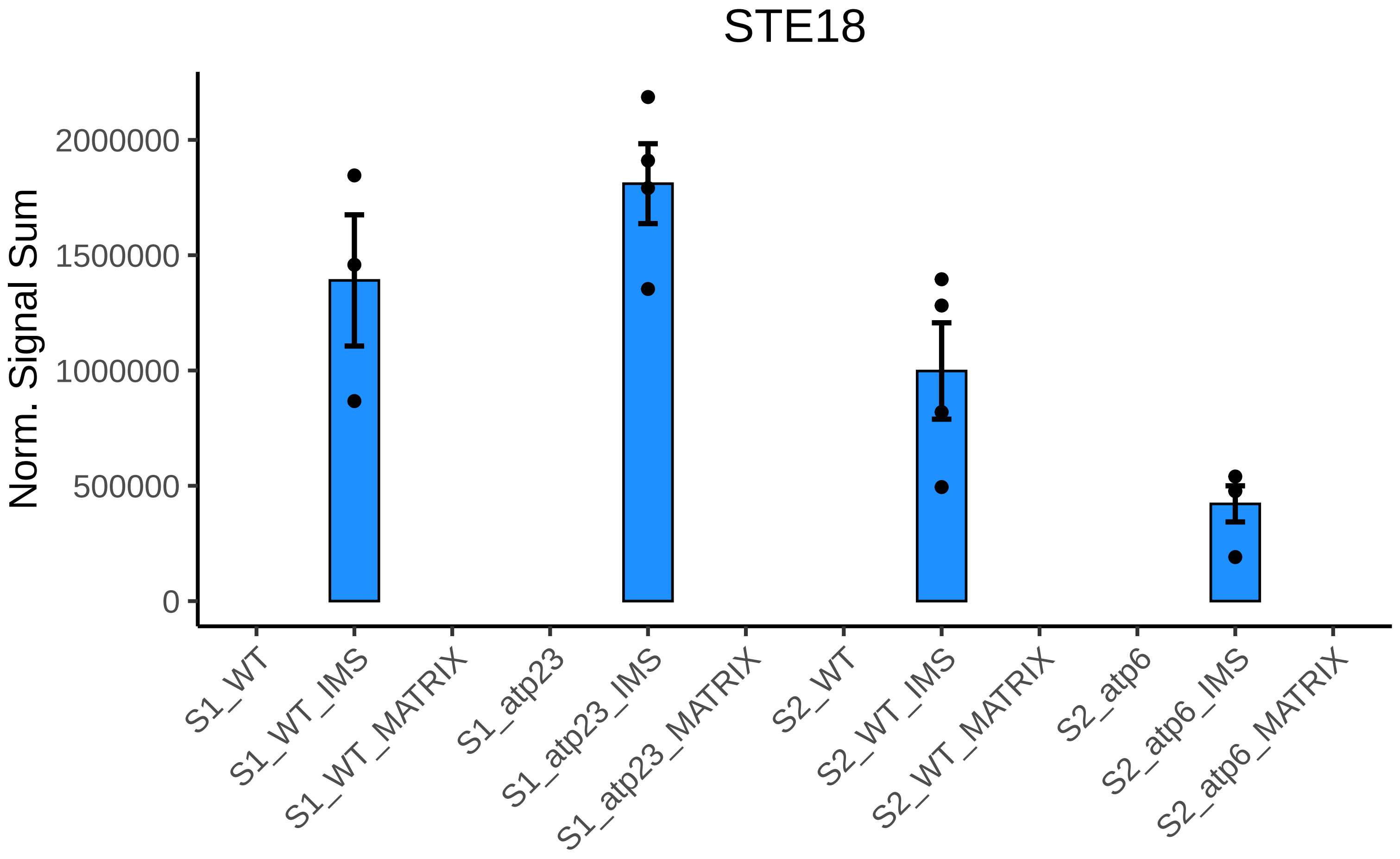
STE18
Norm. Signal Sum
2000000
1500000
1000000
500000
0
S1_WT
S1_WT_IMS
S1_WT_MATRIX
S1_atp23
S1_atp23_IMS
S1_atp23_MATRIX
S2_WT
S2_WT_IMS
S2_WT_MATRIX
S2_atp6
S2_atp6_IMS
S2_atp6_MATRIX

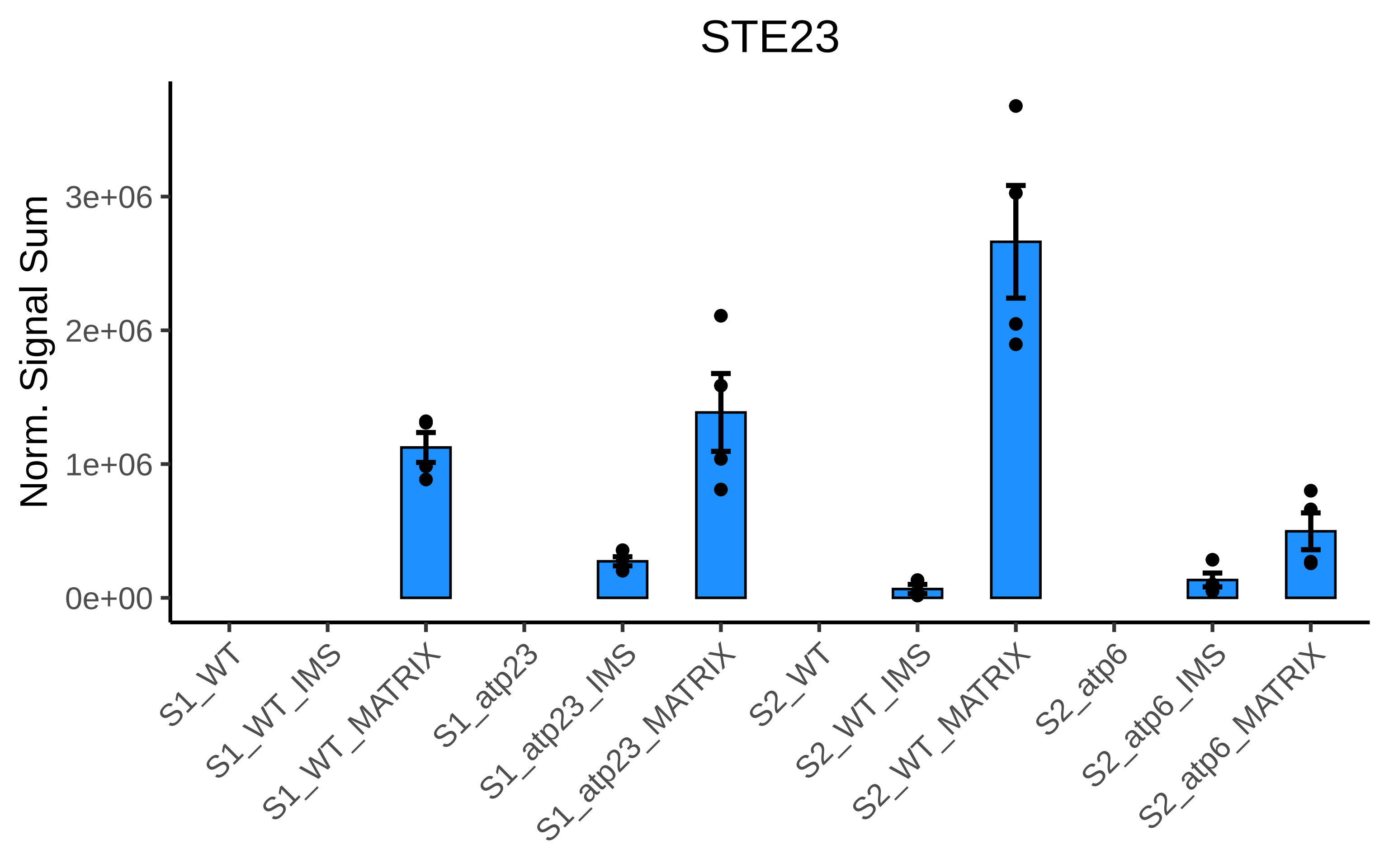
STE23
Norm. Signal Sum
3e+06
2e+06
1e+06
0e+00
S1_WT
S1_WT_IMS
S1_WT_MATRIX
S1_atp23
S1_atp23_IMS
S1_atp23_MATRIX
S2_WT
S2_WT_IMS
S2_WT_MATRIX
S2_atp6
S2_atp6_IMS
S2_atp6_MATRIX

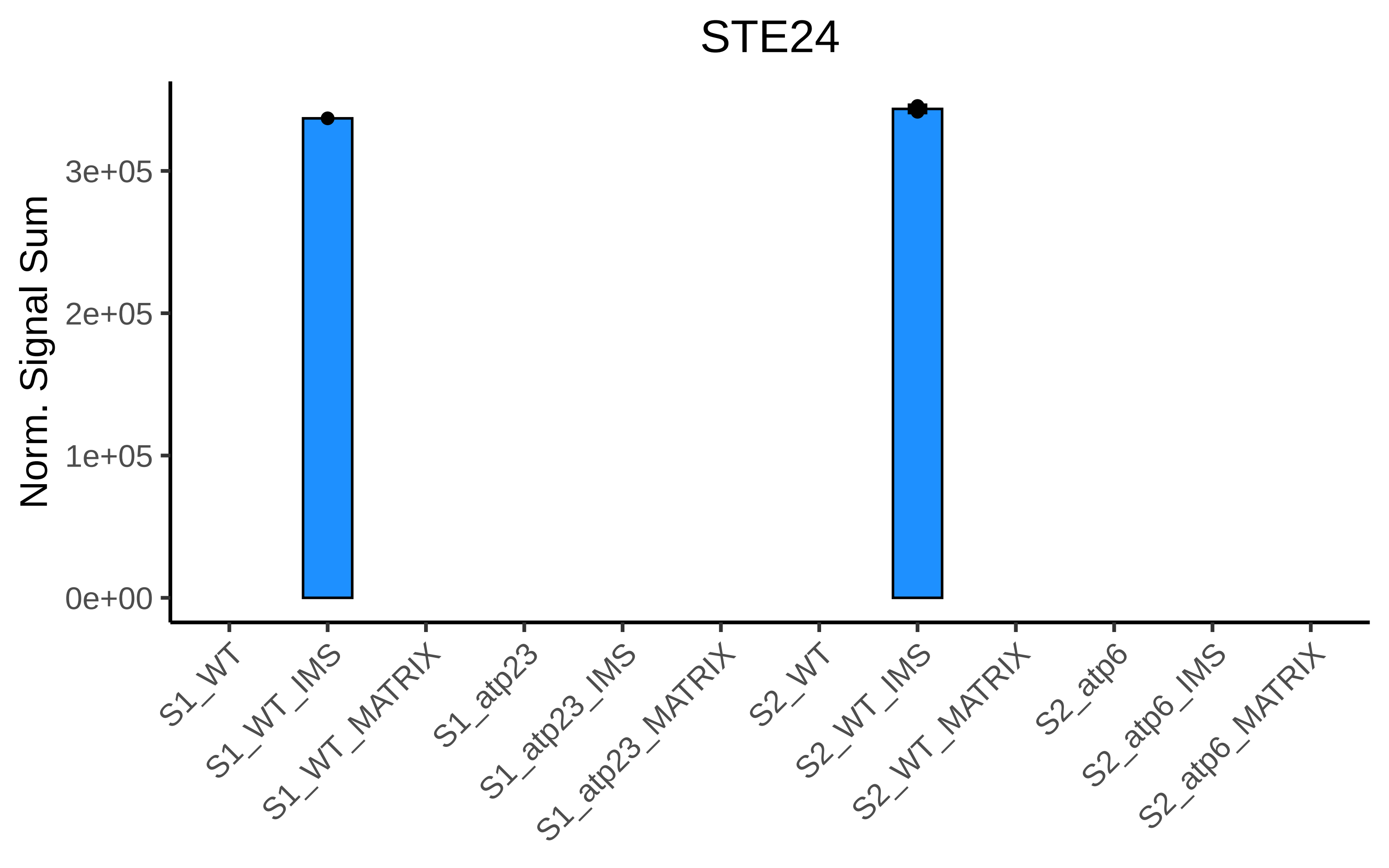

STE24
Norm. Signal Sum
3e+05
2e+05
1e+05
0e+00
S1_WT
S1_WT_IMS
S1_WT_MATRIX
S1_atp23
S1_atp23_IMS
S1_atp23_MATRIX
S2_WT
S2_WT_IMS
S2_WT_MATRIX
S2_atp6
S2_atp6_IMS
S2_atp6_MATRIX

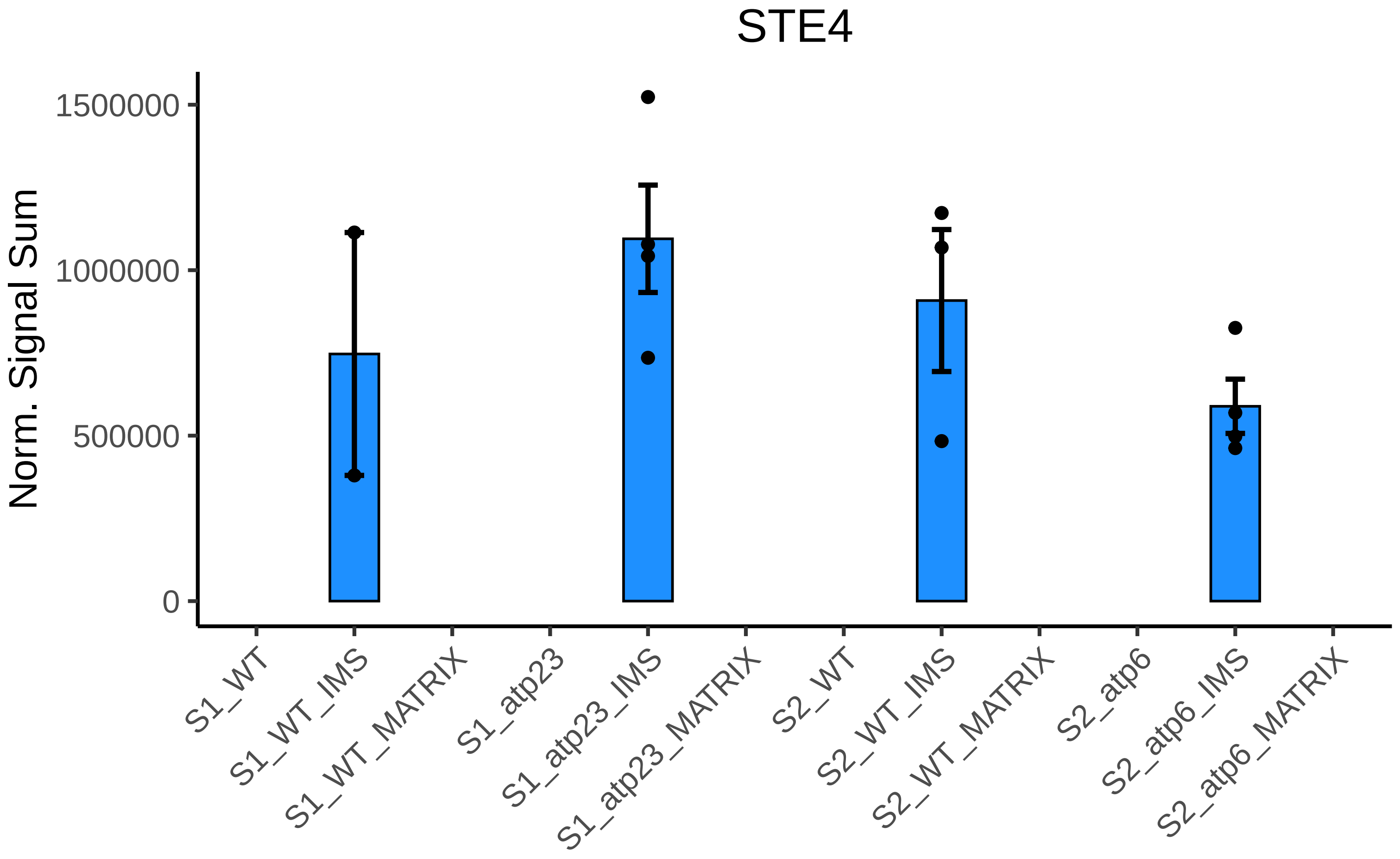
STE4
Norm. Signal Sum
1500000
1000000
500000
0
S1_WT
S1_WT_IMS
S1_WT_MATRIX
S1_atp23
S1_atp23_IMS
S1_atp23_MATRIX
S2_WT
S2_WT_IMS
S2_WT_MATRIX
S2_atp6
S2_atp6_IMS
S2_atp6_MATRIX

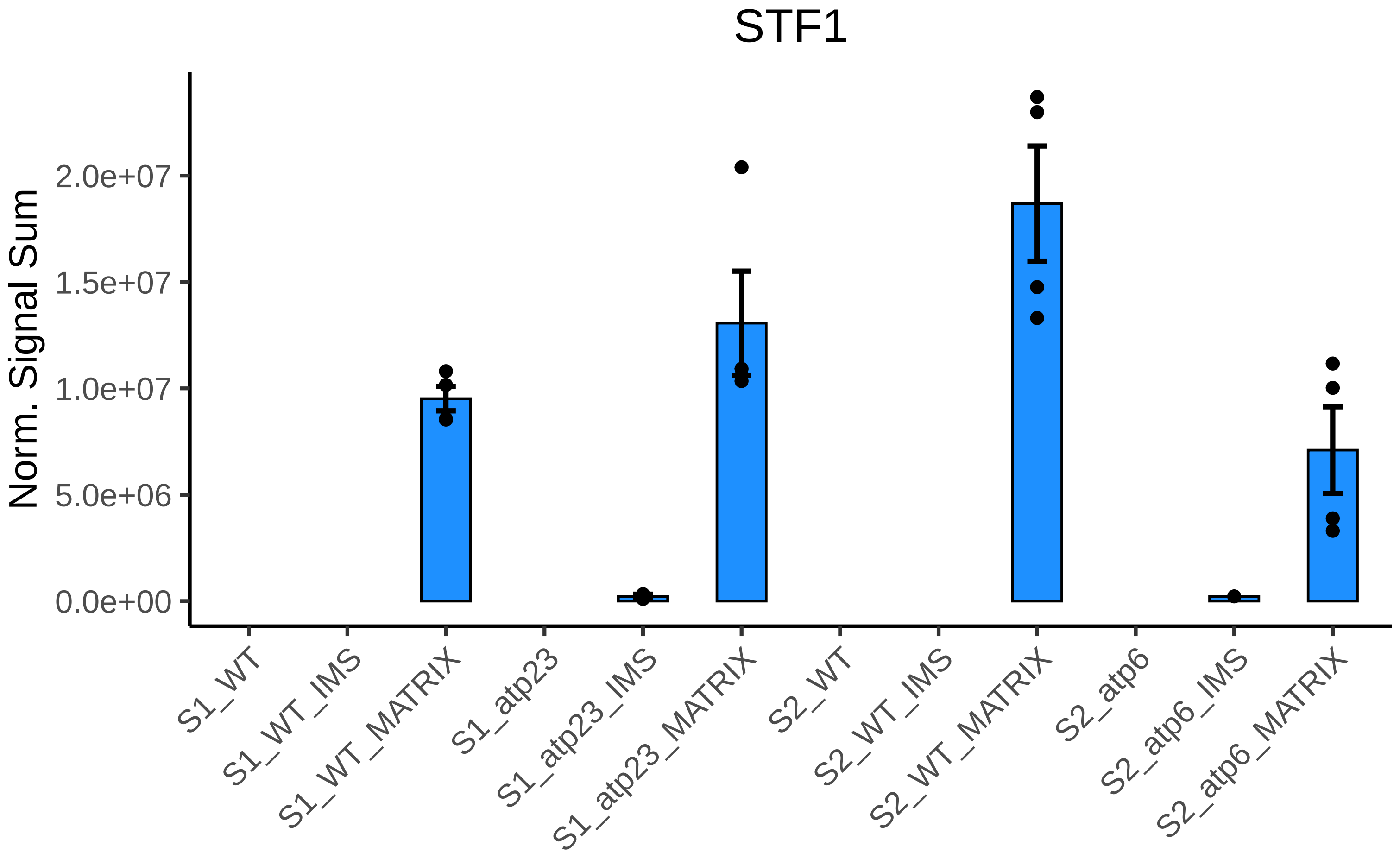
STF1
Norm. Signal Sum
2.0e+07
1.5e+07
1.0e+07
5.0e+06
0.0e+00
S1_WT
S1_WT_IMS
S1_WT_MATRIX
S1_atp23
S1_atp23_IMS
S1_atp23_MATRIX
S2_WT
S2_WT_IMS
S2_WT_MATRIX
S2_atp6
S2_atp6_IMS
S2_atp6_MATRIX

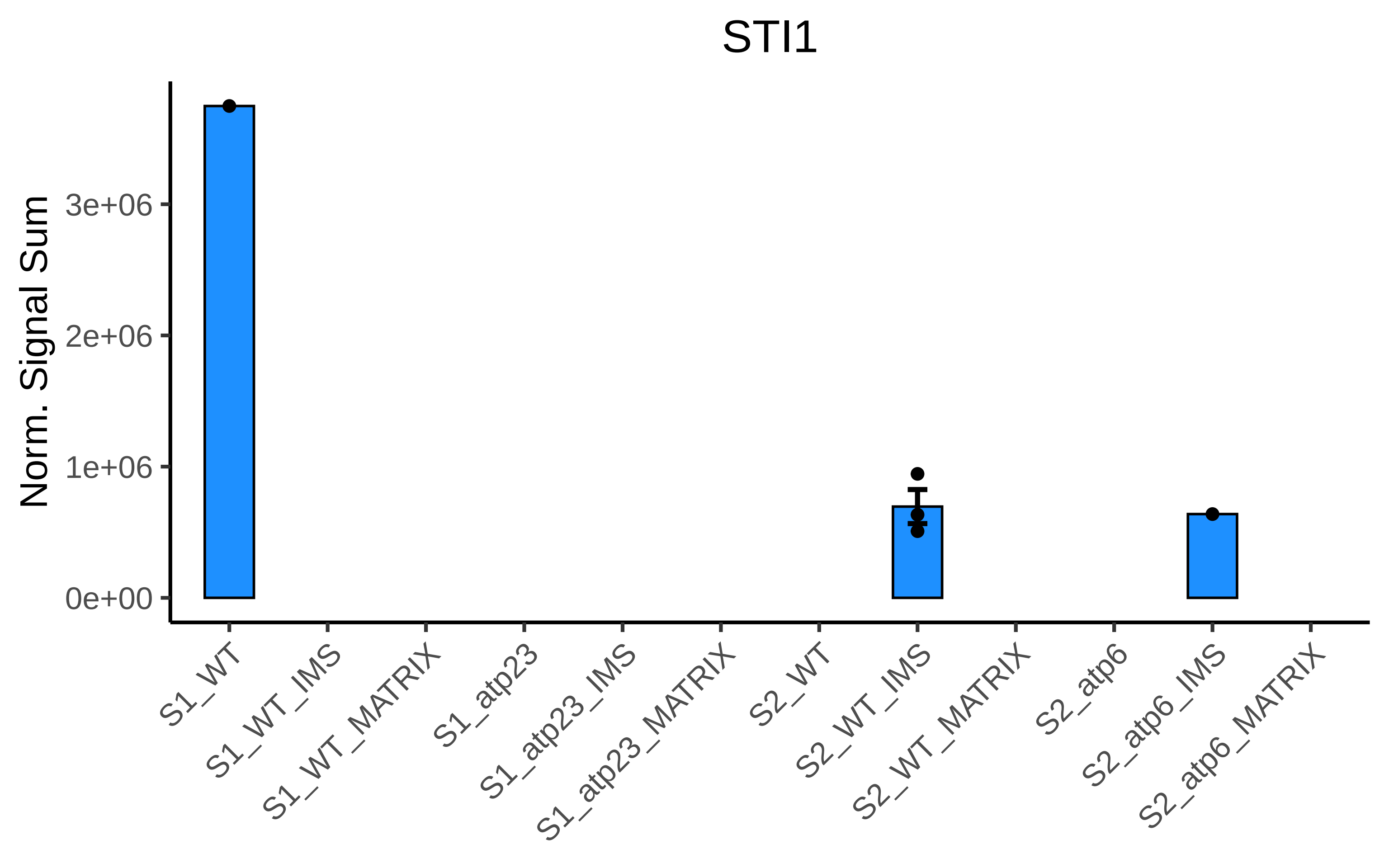
STI1
Norm. Signal Sum
3e+06
2e+06
1e+06
0e+00
S1_WT
S1_WT_IMS
S1_WT_MATRIX
S1_atp23
S1_atp23_IMS
S1_atp23_MATRIX
S2_WT
S2_WT_IMS
S2_WT_MATRIX
S2_atp6
S2_atp6_IMS
S2_atp6_MATRIX

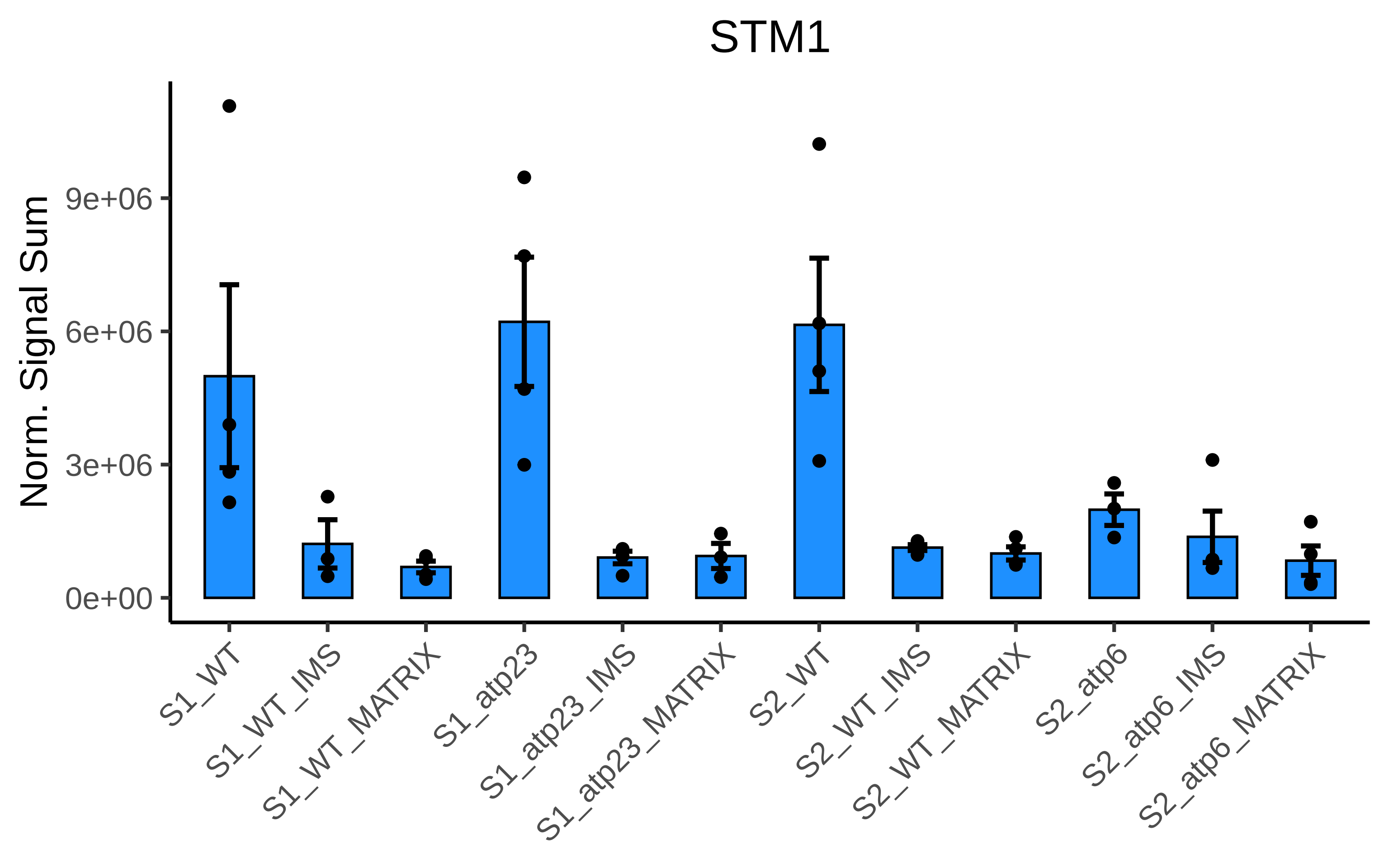
STM1
Norm. Signal Sum
9e+06
6e+06
3e+06
0e+00
S1_WT
S1_WT_IMS
S1_WT_MATRIX
S1_atp23
S1_atp23_IMS
S1_atp23_MATRIX
S2_WT
S2_WT_IMS
S2_WT_MATRIX
S2_atp6
S2_atp6_IMS
S2_atp6_MATRIX

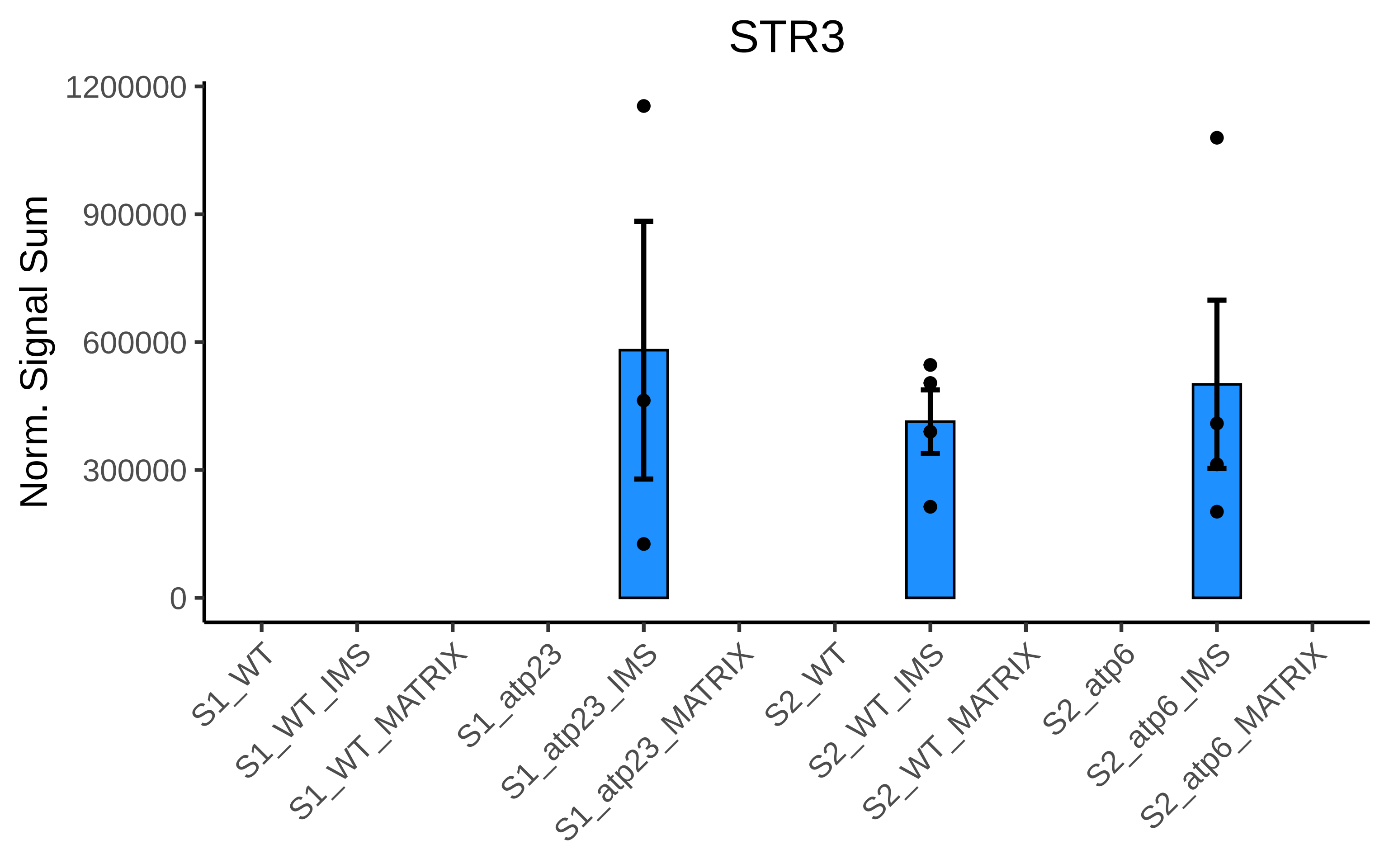

STR3
Norm. Signal Sum
1200000
900000
600000
300000
0
S1_WT
S1_WT_IMS
S1_WT_MATRIX
S1_atp23
S1_atp23_IMS
S1_atp23_MATRIX
S2_WT
S2_WT_IMS
S2_WT_MATRIX
S2_atp6
S2_atp6_IMS
S2_atp6_MATRIX

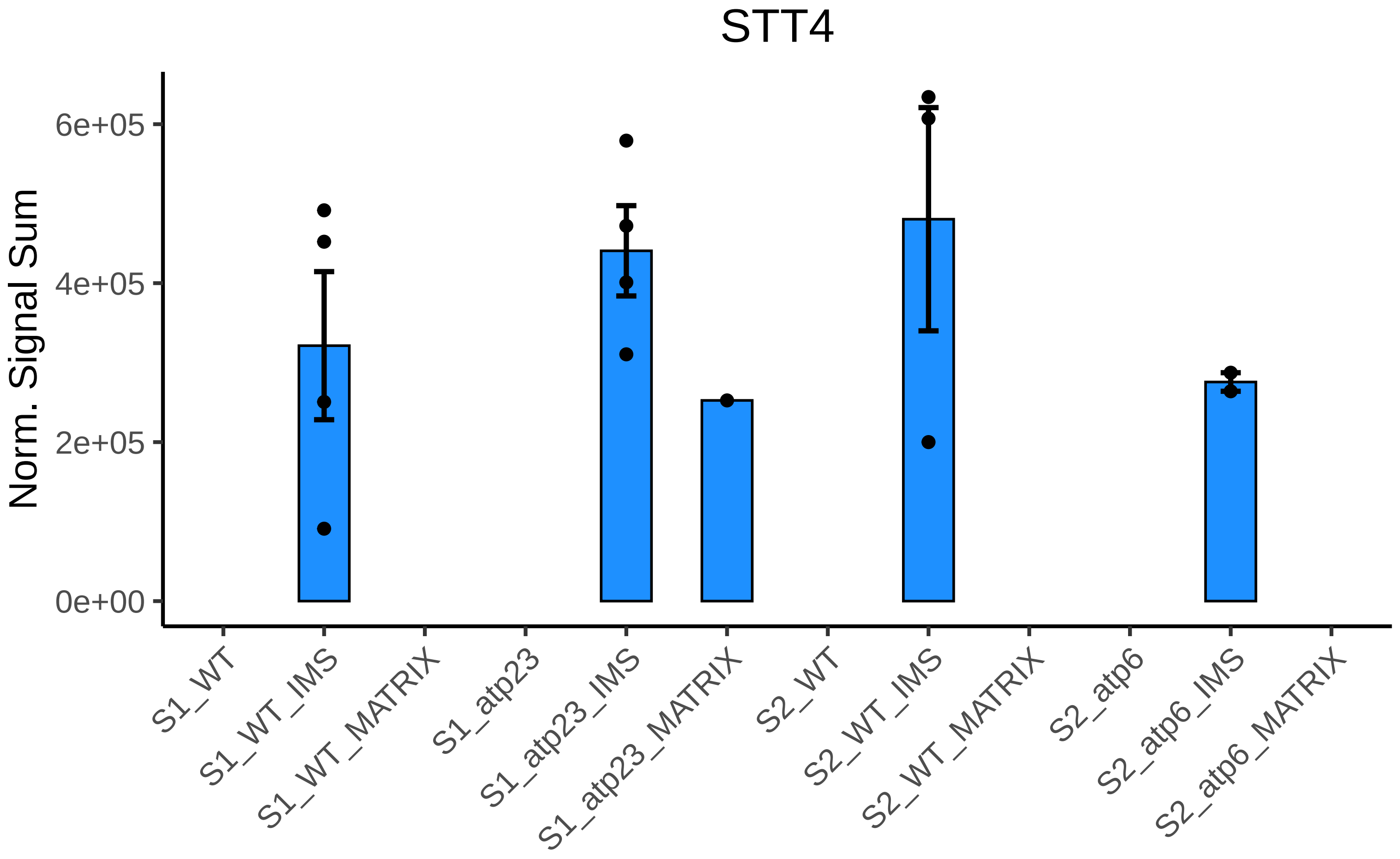

STT4
Norm. Signal Sum
6e+05
4e+05
2e+05
0e+00
S1_WT
S1_WT_IMS
S1_WT_MATRIX
S1_atp23
S1_atp23_IMS
S1_atp23_MATRIX
S2_WT
S2_WT_IMS
S2_WT_MATRIX
S2_atp6
S2_atp6_IMS
S2_atp6_MATRIX

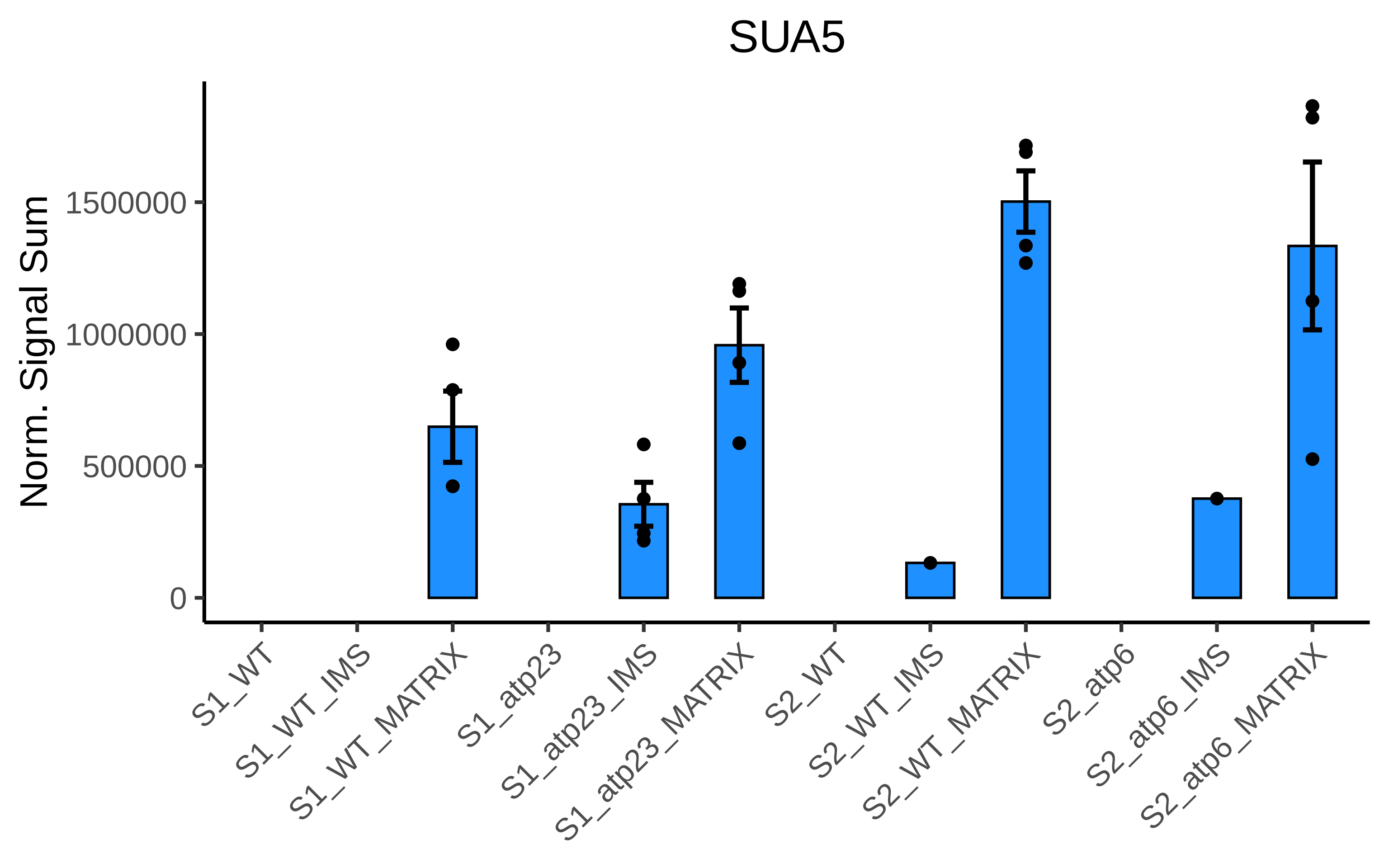
SUA5
Norm. Signal Sum
1500000
1000000
500000
0
S1_WT
S1_WT_IMS
S1_WT_MATRIX
S1_atp23
S1_atp23_IMS
S1_atp23_MATRIX
S2_WT
S2_WT_IMS
S2_WT_MATRIX
S2_atp6
S2_atp6_IMS
S2_atp6_MATRIX

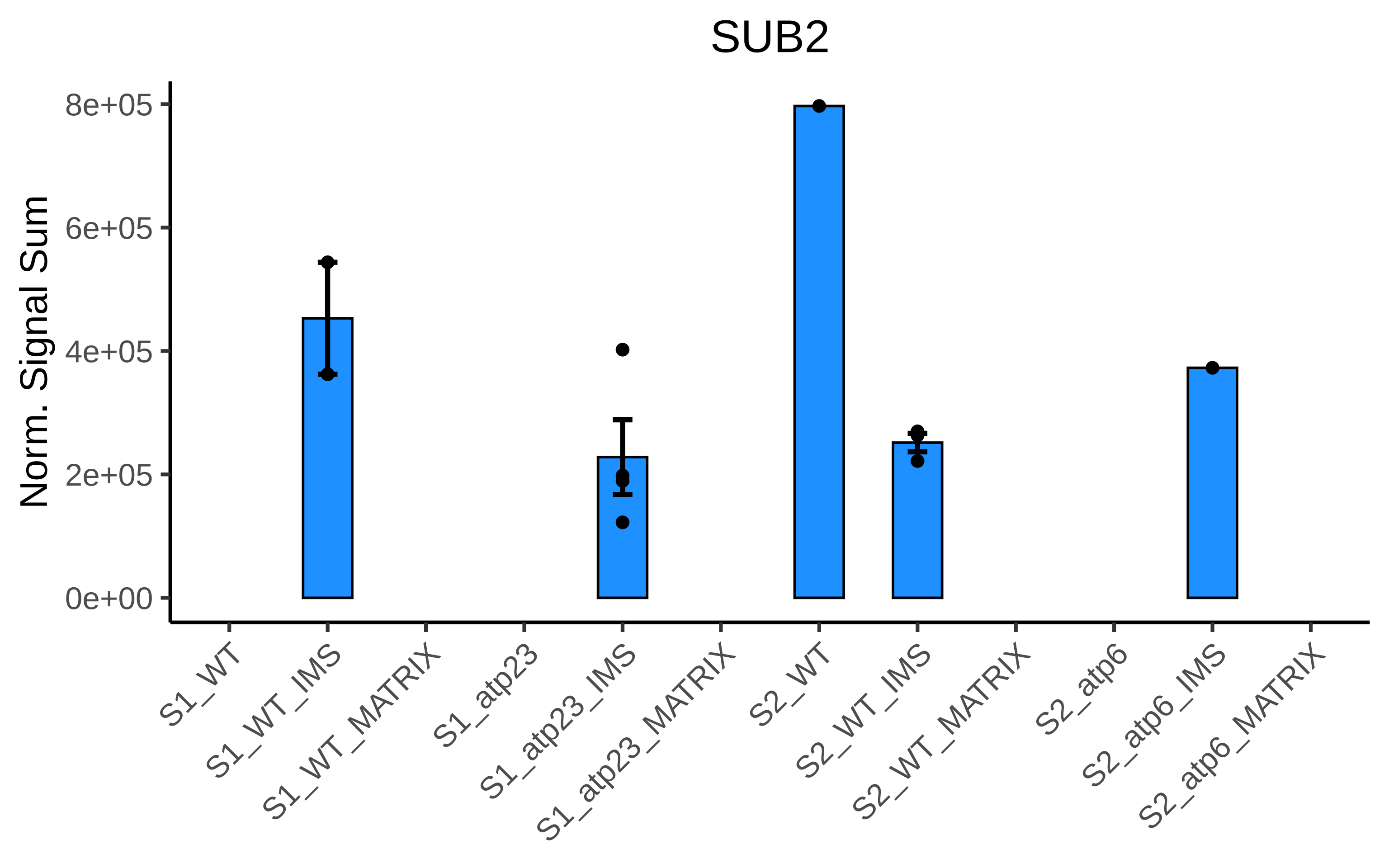

SUB2
Norm. Signal Sum
8e+05
6e+05
4e+05
2e+05
0e+00
S1_WT
S1_WT_IMS
S1_WT_MATRIX
S1_atp23
S1_atp23_IMS
S1_atp23_MATRIX
S2_WT
S2_WT_IMS
S2_WT_MATRIX
S2_atp6
S2_atp6_IMS
S2_atp6_MATRIX

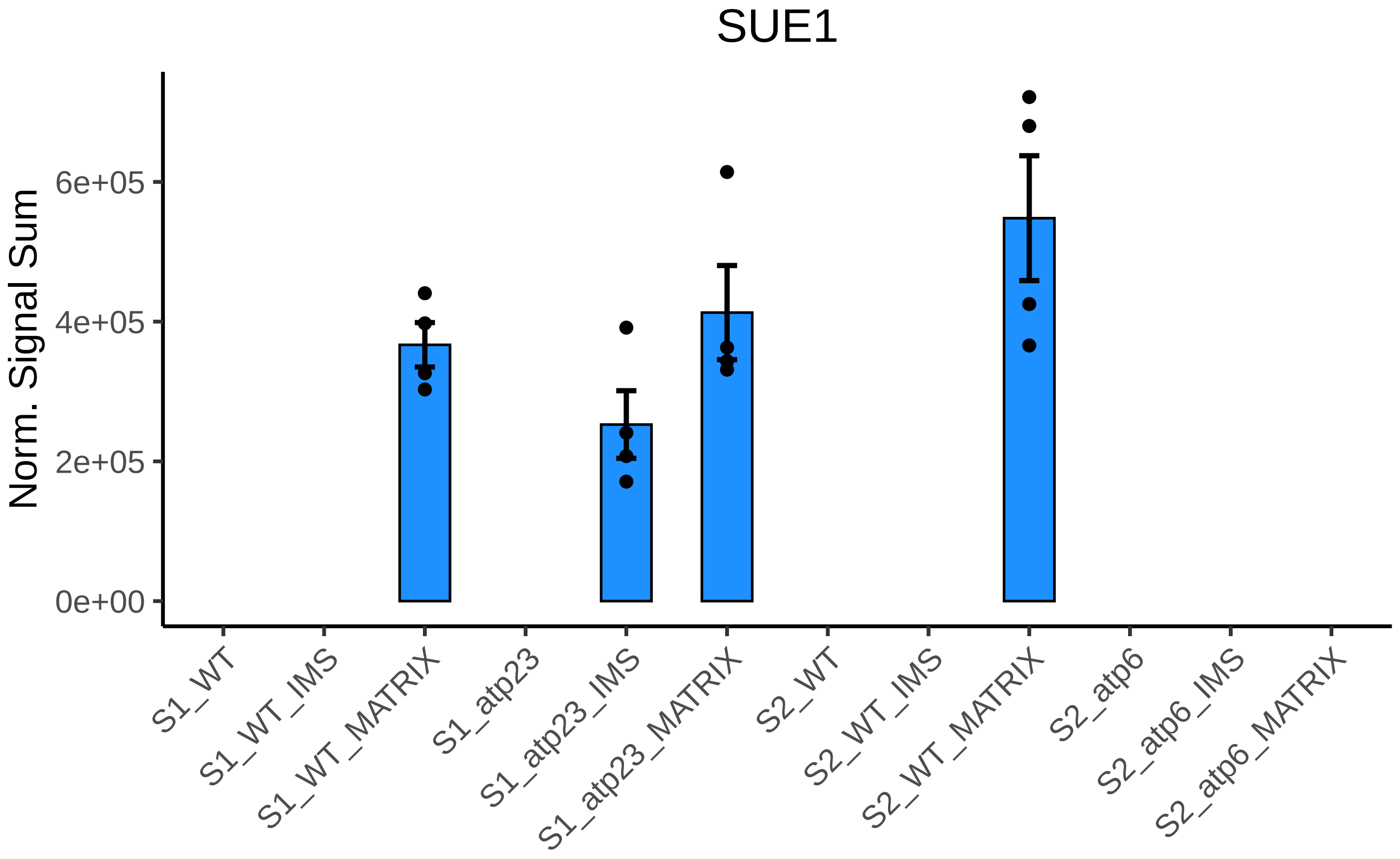
SUE1
Norm. Signal Sum
0e+00
2e+05
4e+05
6e+05
S1_WT
S1_WT_IMS
S1_WT_MATRIX
S1_atp23
S1_atp23_IMS
S1_atp23_MATRIX
S2_WT
S2_WT_IMS
S2_WT_MATRIX
S2_atp6
S2_atp6_IMS
S2_atp6_MATRIX

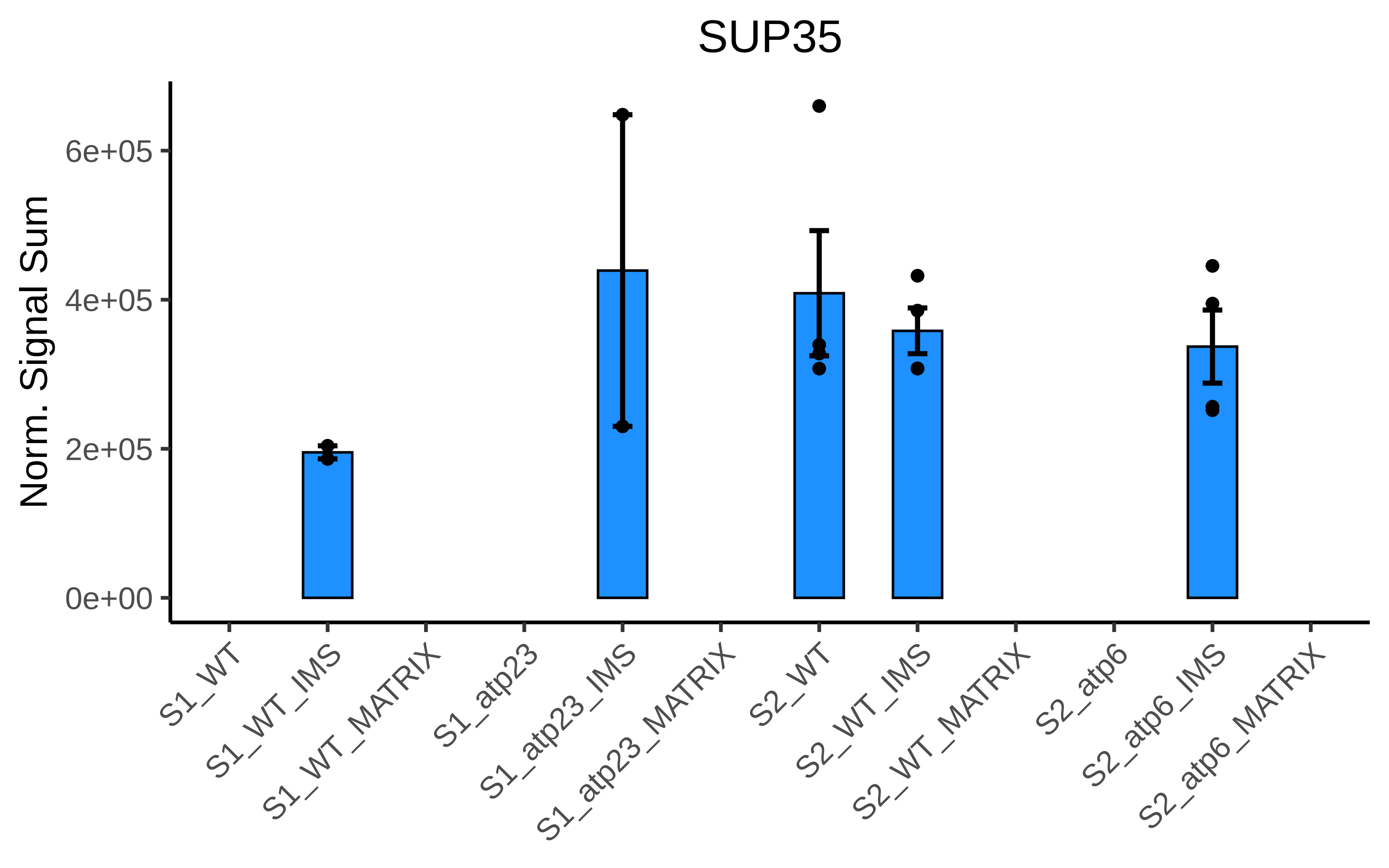
SUP35
Norm. Signal Sum
6e+05
4e+05
2e+05
0e+00
S1_WT
S1_WT_IMS
S1_WT_MATRIX
S1_atp23
S1_atp23_IMS
S1_atp23_MATRIX
S2_WT
S2_WT_IMS
S2_WT_MATRIX
S2_atp6
S2_atp6_IMS
S2_atp6_MATRIX

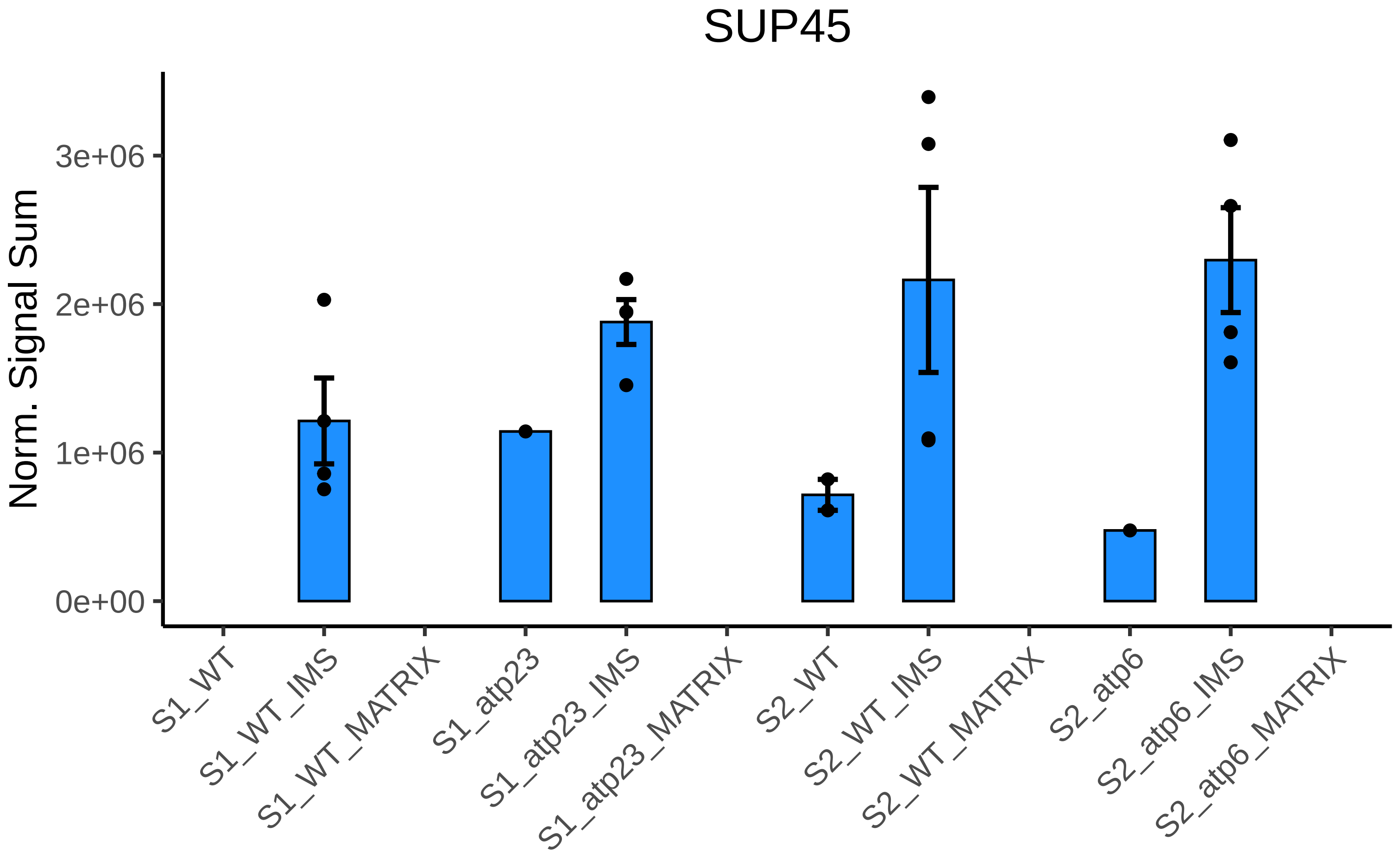

SUP45
Norm. Signal Sum
3e+06
2e+06
1e+06
0e+00
S1_WT
S1_WT_IMS
S1_WT_MATRIX
S1_atp23
S1_atp23_IMS
S1_atp23_MATRIX
S2_WT
S2_WT_IMS
S2_WT_MATRIX
S2_atp6
S2_atp6_IMS
S2_atp6_MATRIX

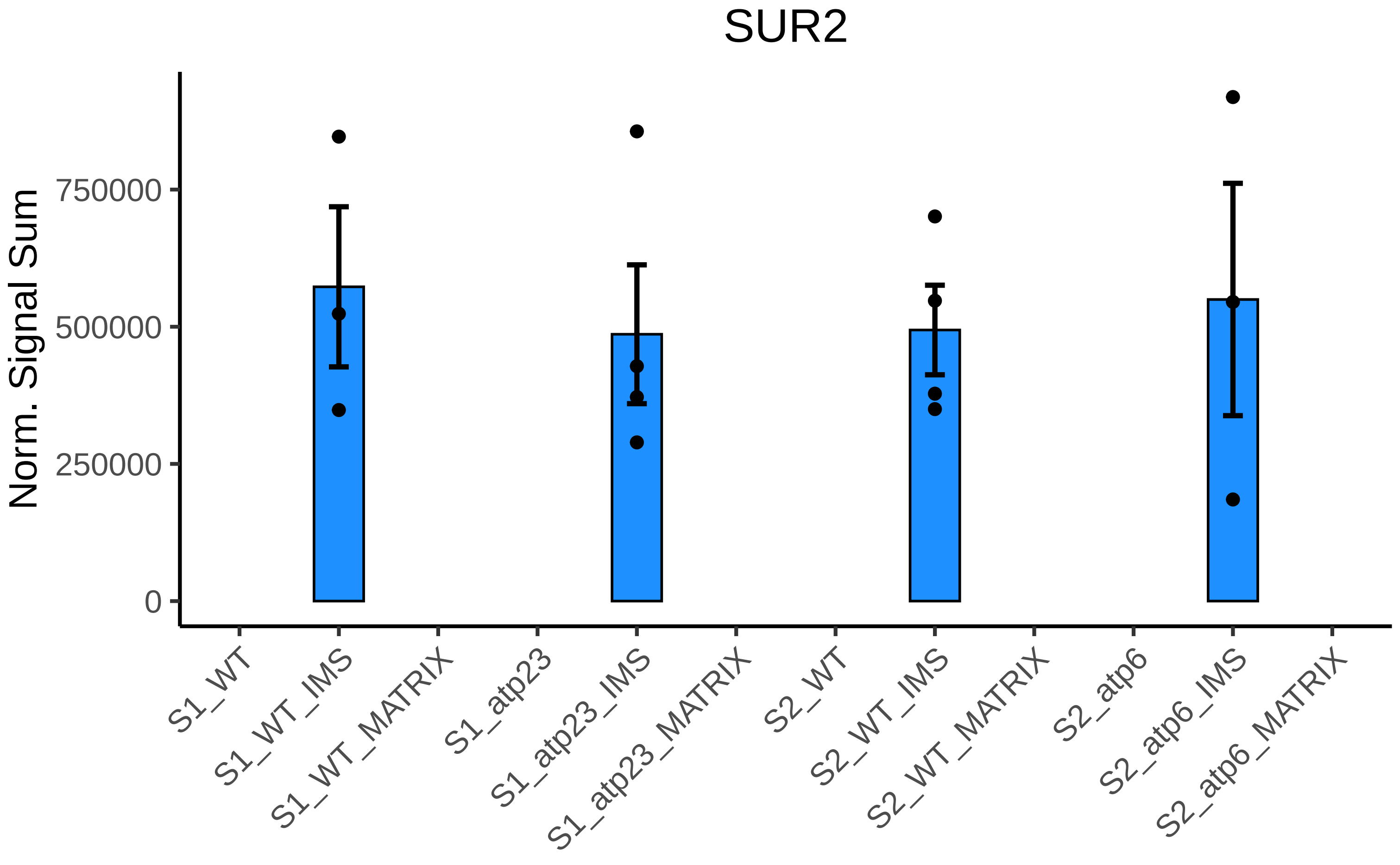

SUR2
Norm. Signal Sum
750000
500000
250000
0
S1_WT
S1_WT_IMS
S1_WT_MATRIX
S1_atp23
S1_atp23_IMS
S1_atp23_MATRIX
S2_WT
S2_WT_IMS
S2_WT_MATRIX
S2_atp6
S2_atp6_IMS
S2_atp6_MATRIX

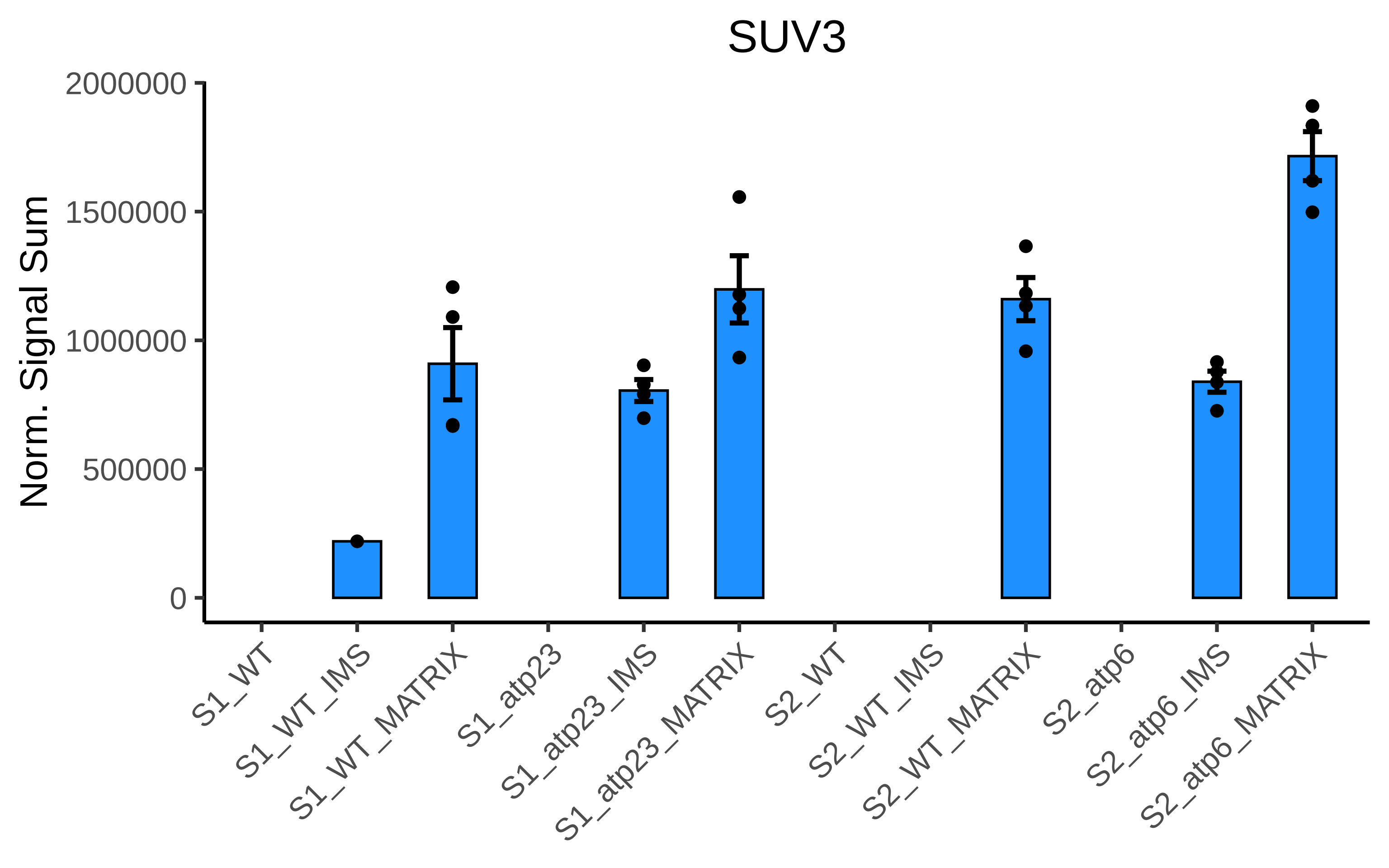
SUV3
Norm. Signal Sum
2000000
1500000
1000000
500000
0
S1_WT
S1_WT_IMS
S1_WT_MATRIX
S1_atp23
S1_atp23_IMS
S1_atp23_MATRIX
S2_WT
S2_WT_IMS
S2_WT_MATRIX
S2_atp6
S2_atp6_IMS
S2_atp6_MATRIX

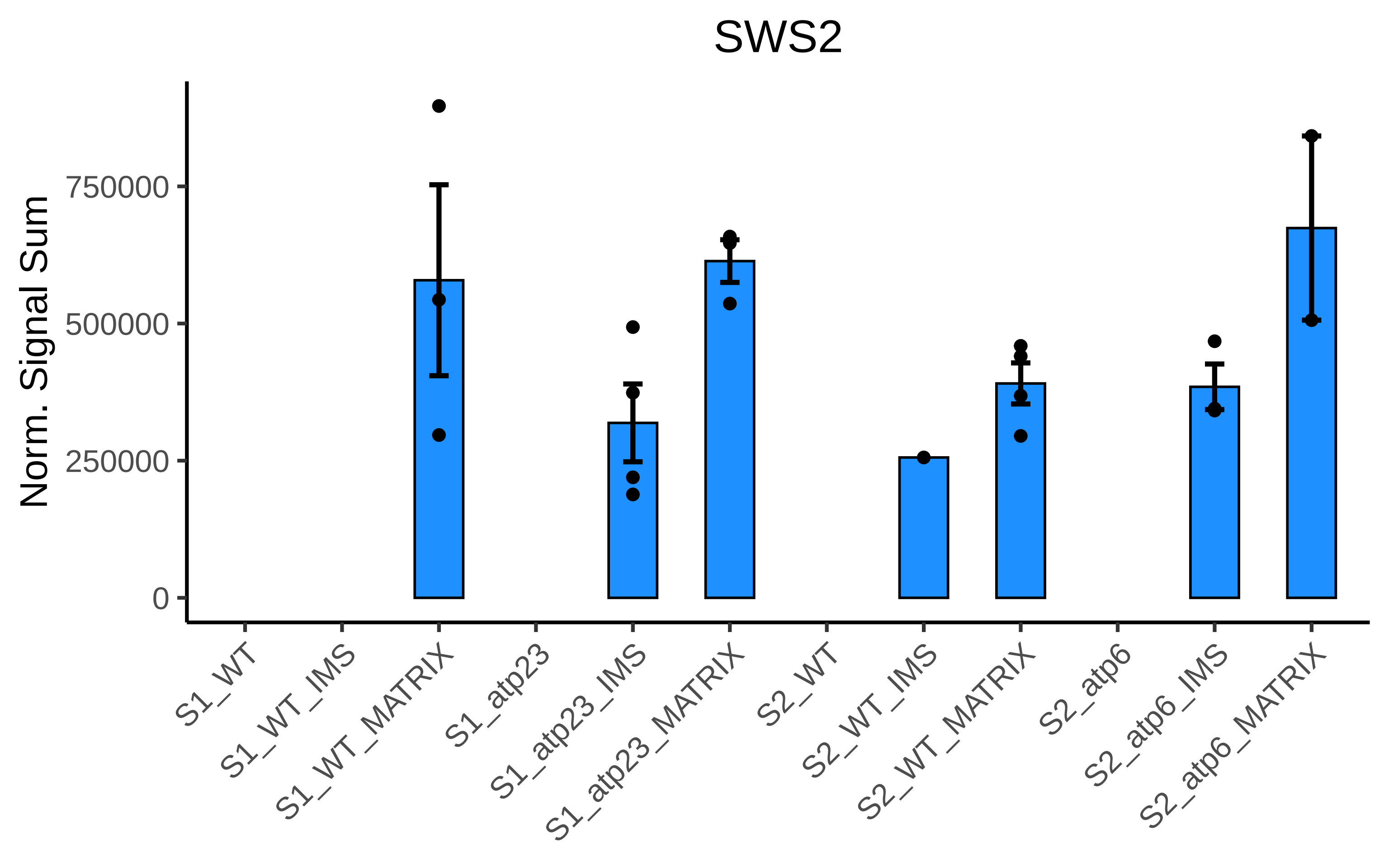
SWS2
Norm. Signal Sum
750000
500000
250000
0
S1_WT
S1_WT_IMS
S1_WT_MATRIX
S1_atp23
S1_atp23_IMS
S1_atp23_MATRIX
S2_WT
S2_WT_IMS
S2_WT_MATRIX
S2_atp6
S2_atp6_IMS
S2_atp6_MATRIX

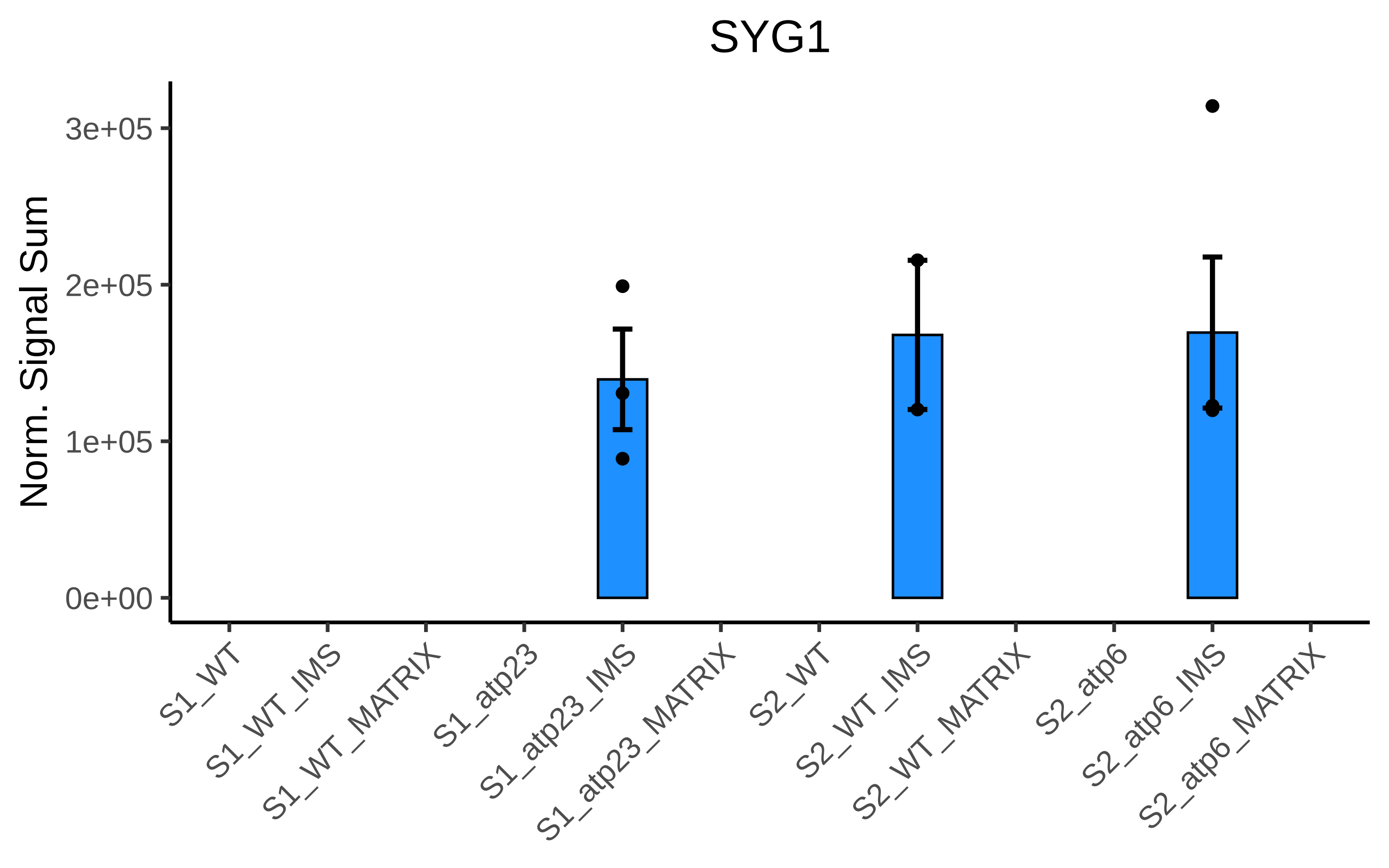
SYG1
Norm. Signal Sum
3e+05
2e+05
1e+05
0e+00
S1_WT
S1_WT_IMS
S1_WT_MATRIX
S1_atp23
S1_atp23_IMS
S1_atp23_MATRIX
S2_WT
S2_WT_IMS
S2_WT_MATRIX
S2_atp6
S2_atp6_IMS
S2_atp6_MATRIX

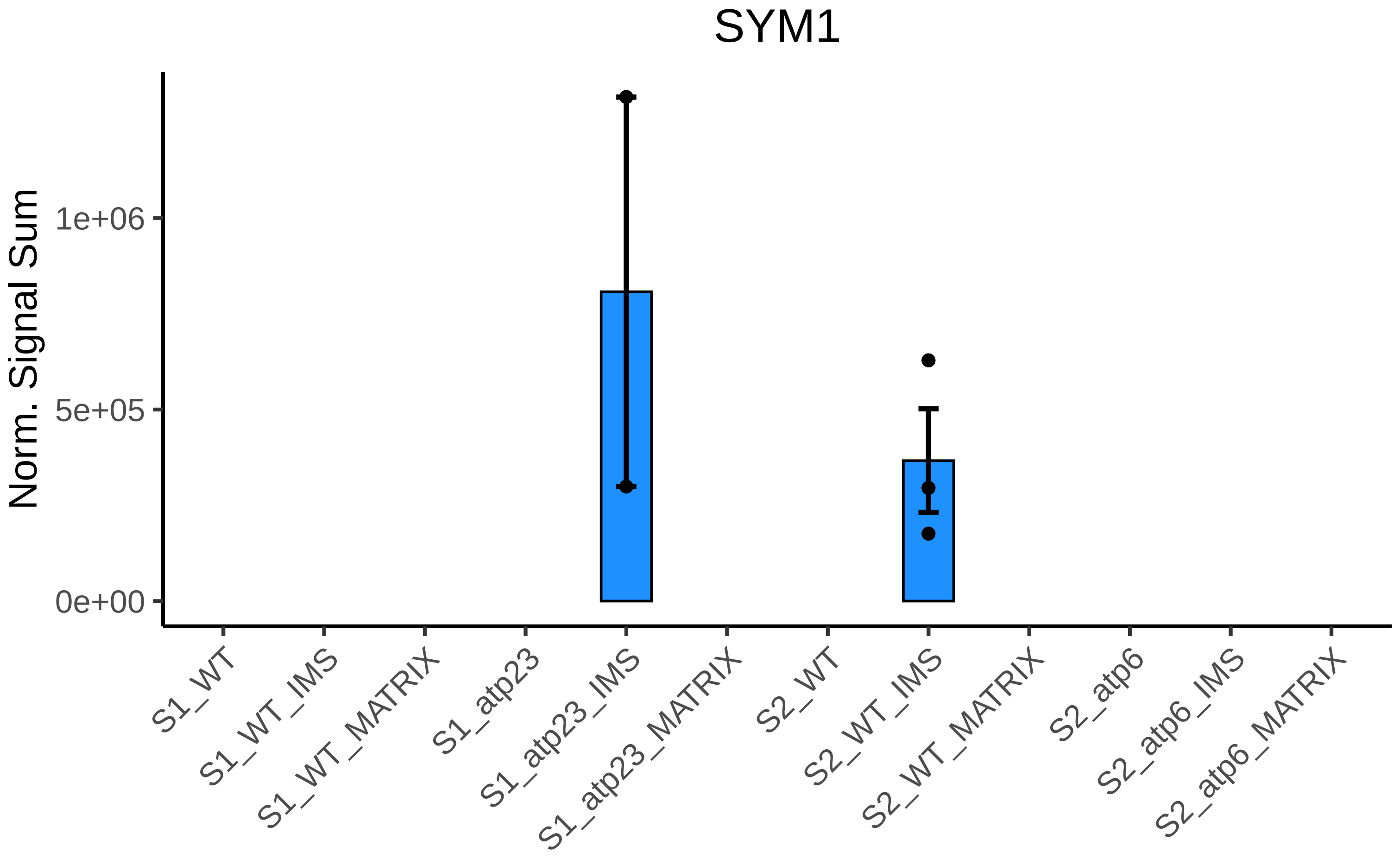

SYM1
Norm. Signal Sum
1e+06
5e+05
0e+00
S1_WT
S1_WT_IMS
S1_WT_MATRIX
S1_atp23
S1_atp23_IMS
S1_atp23_MATRIX
S2_WT
S2_WT_IMS
S2_WT_MATRIX
S2_atp6
S2_atp6_IMS
S2_atp6_MATRIX

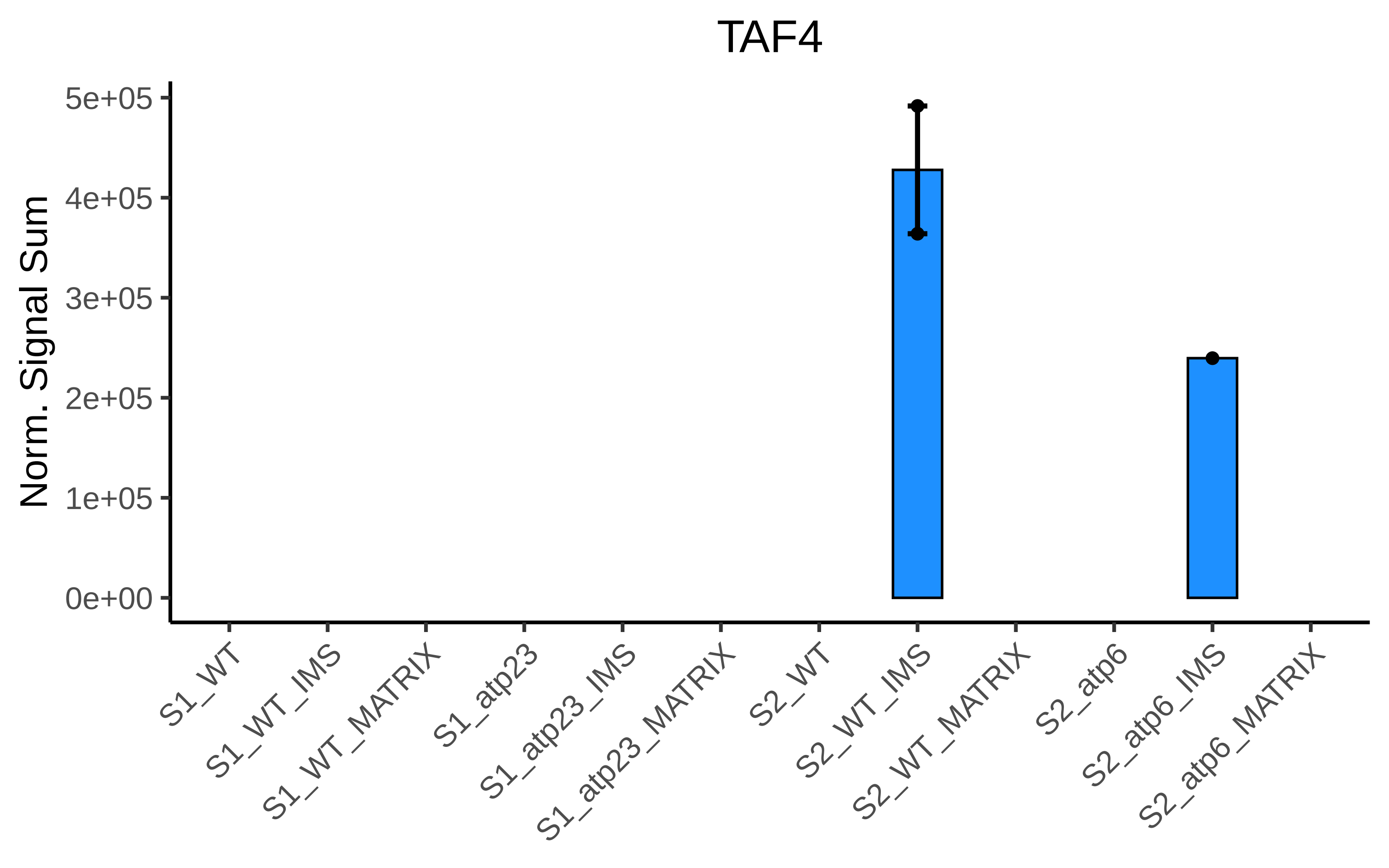
TAF4
Norm. Signal Sum
5e+05
4e+05
3e+05
2e+05
1e+05
0e+00
S1_WT
S1_WT_IMS
S1_WT_MATRIX
S1_atp23
S1_atp23_IMS
S1_atp23_MATRIX
S2_WT
S2_WT_IMS
S2_WT_MATRIX
S2_atp6
S2_atp6_IMS
S2_atp6_MATRIX

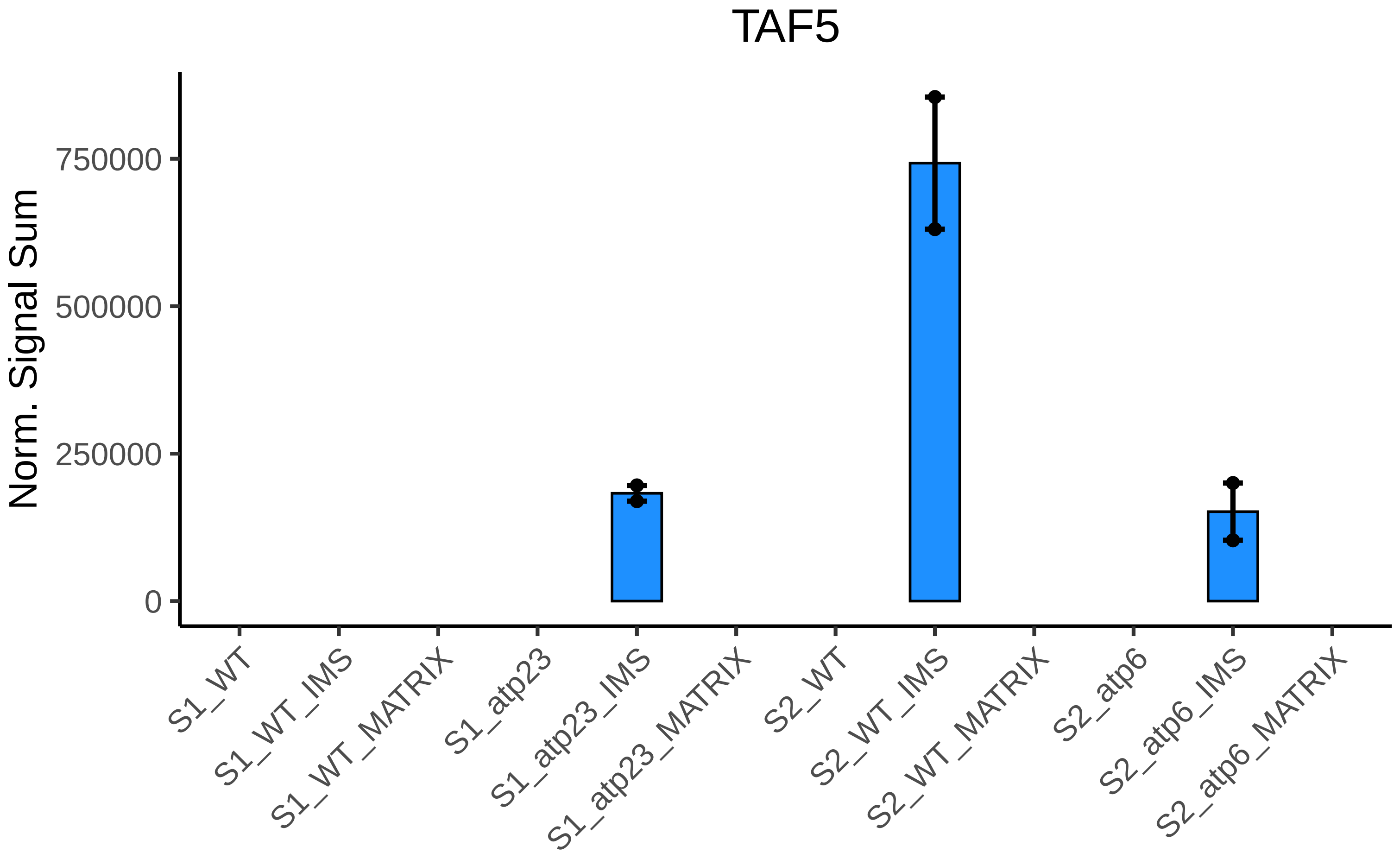

TAF5
Norm. Signal Sum
750000
500000
250000
0
S1_WT
S1_WT_IMS
S1_WT_MATRIX
S1_atp23
S1_atp23_IMS
S1_atp23_MATRIX
S2_WT
S2_WT_IMS
S2_WT_MATRIX
S2_atp6
S2_atp6_IMS
S2_atp6_MATRIX

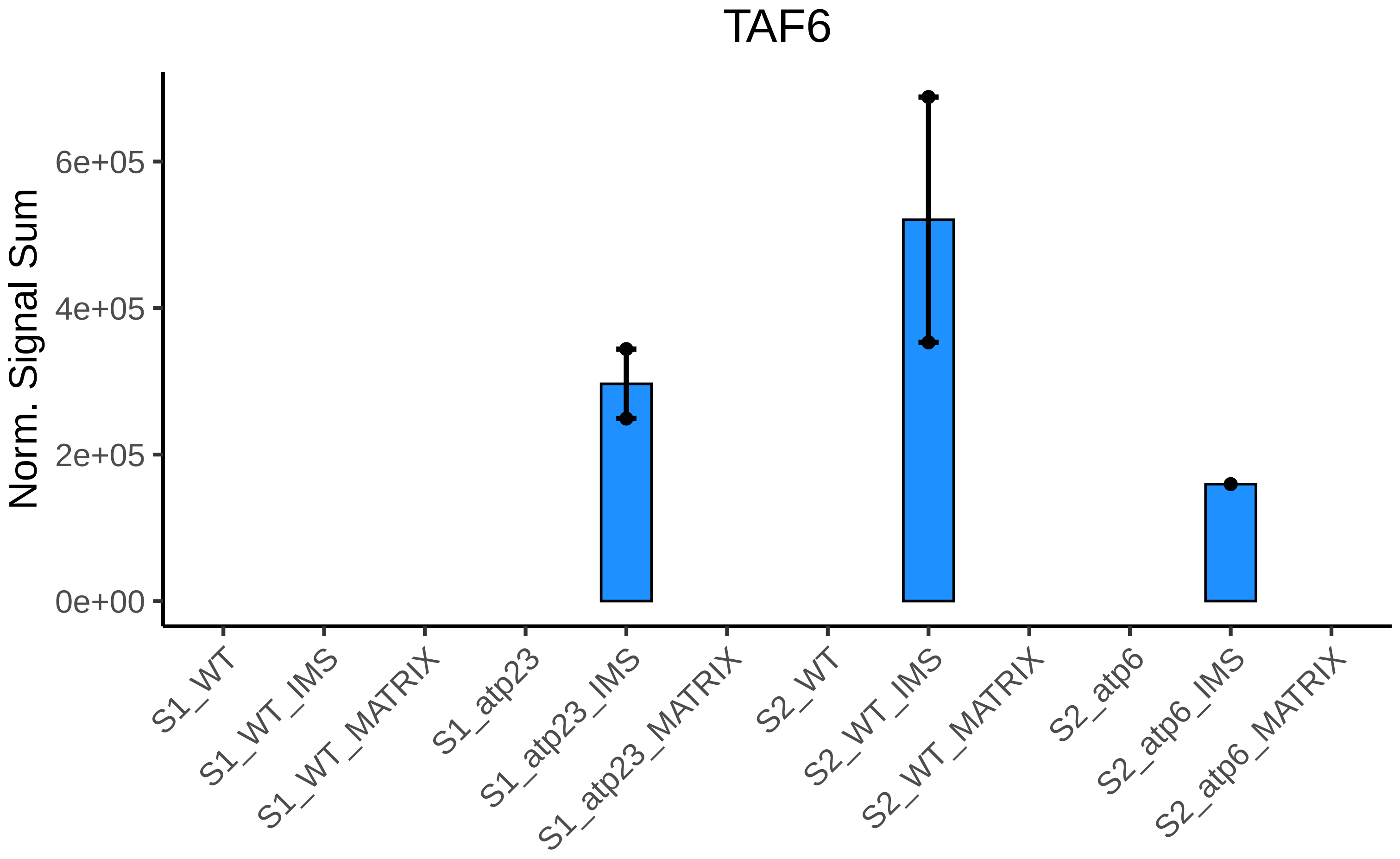

TAF6
Norm. Signal Sum
6e+05
4e+05
2e+05
0e+00
S1_WT
S1_WT_IMS
S1_WT_MATRIX
S1_atp23
S1_atp23_IMS
S1_atp23_MATRIX
S2_WT
S2_WT_IMS
S2_WT_MATRIX
S2_atp6
S2_atp6_IMS
S2_atp6_MATRIX

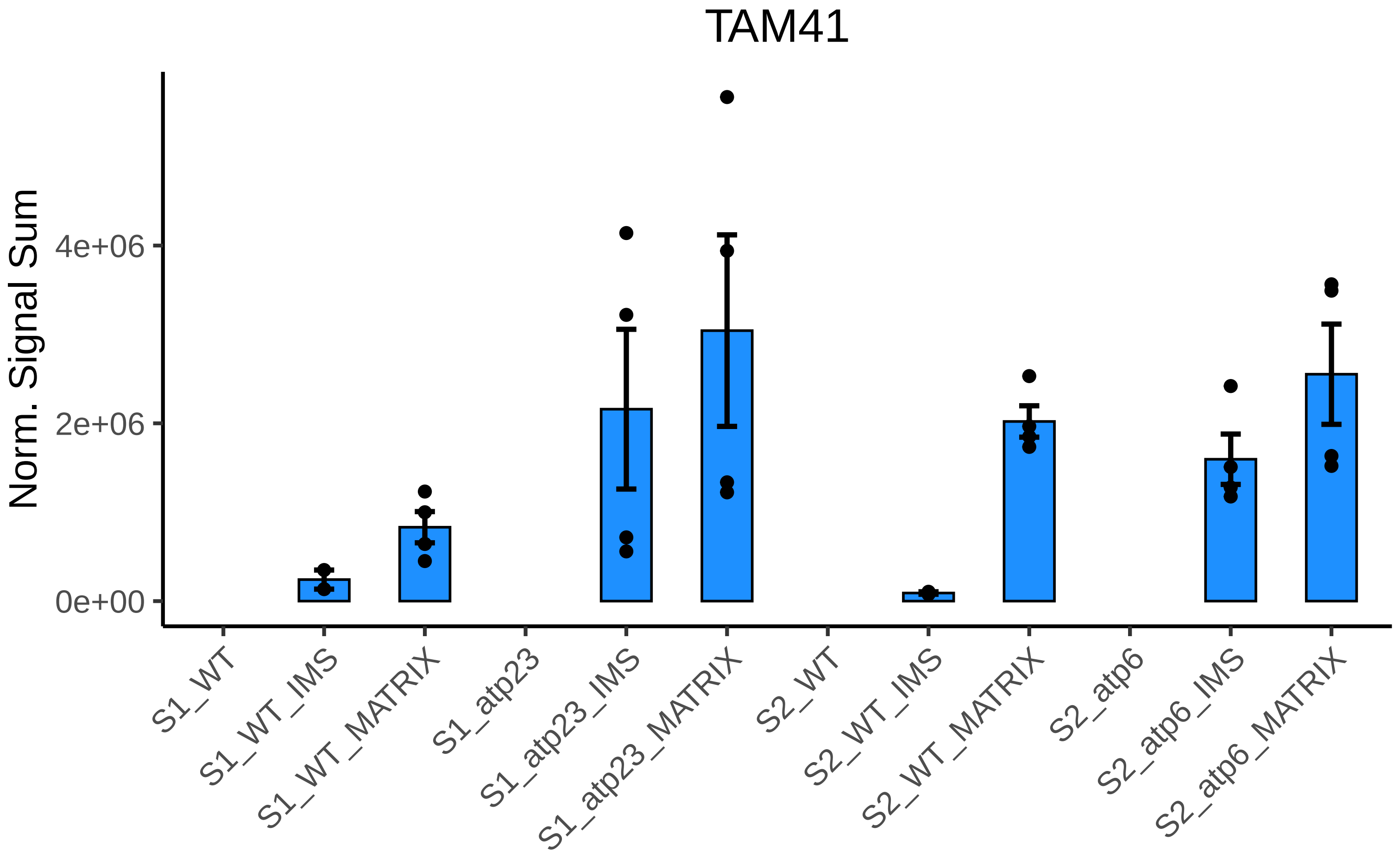

TAM41
Norm. Signal Sum
4e+06
2e+06
0e+00
S1_WT
S1_WT_IMS
S1_WT_MATRIX
S1_atp23
S1_atp23_IMS
S1_atp23_MATRIX
S2_WT
S2_WT_IMS
S2_WT_MATRIX
S2_atp6
S2_atp6_IMS
S2_atp6_MATRIX

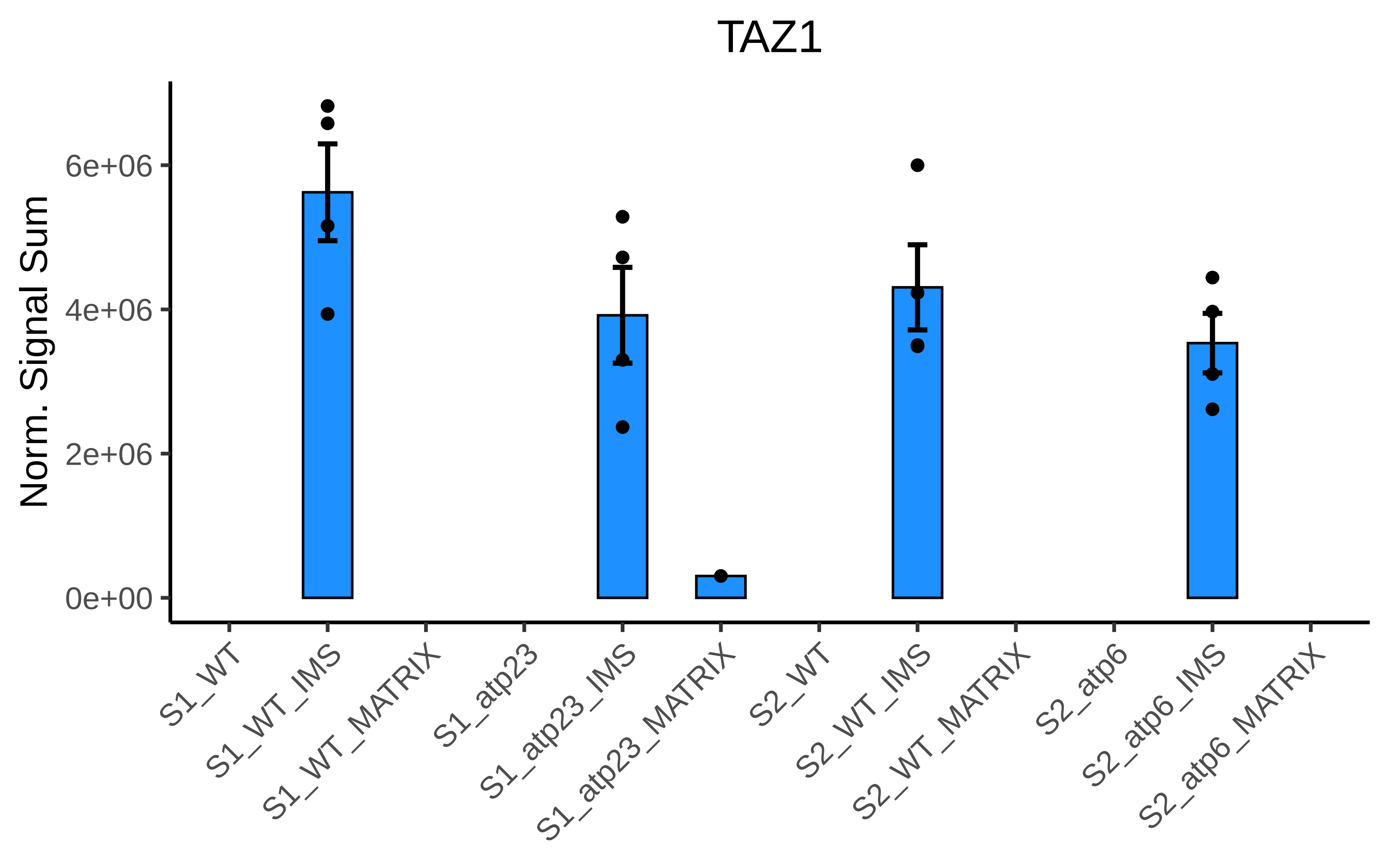

TAZ1
Norm. Signal Sum
6e+06
4e+06
2e+06
0e+00
S1_WT
S1_WT_IMS
S1_WT_MATRIX
S1_atp23
S1_atp23_IMS
S1_atp23_MATRIX
S2_WT
S2_WT_IMS
S2_WT_MATRIX
S2_atp6
S2_atp6_IMS
S2_atp6_MATRIX

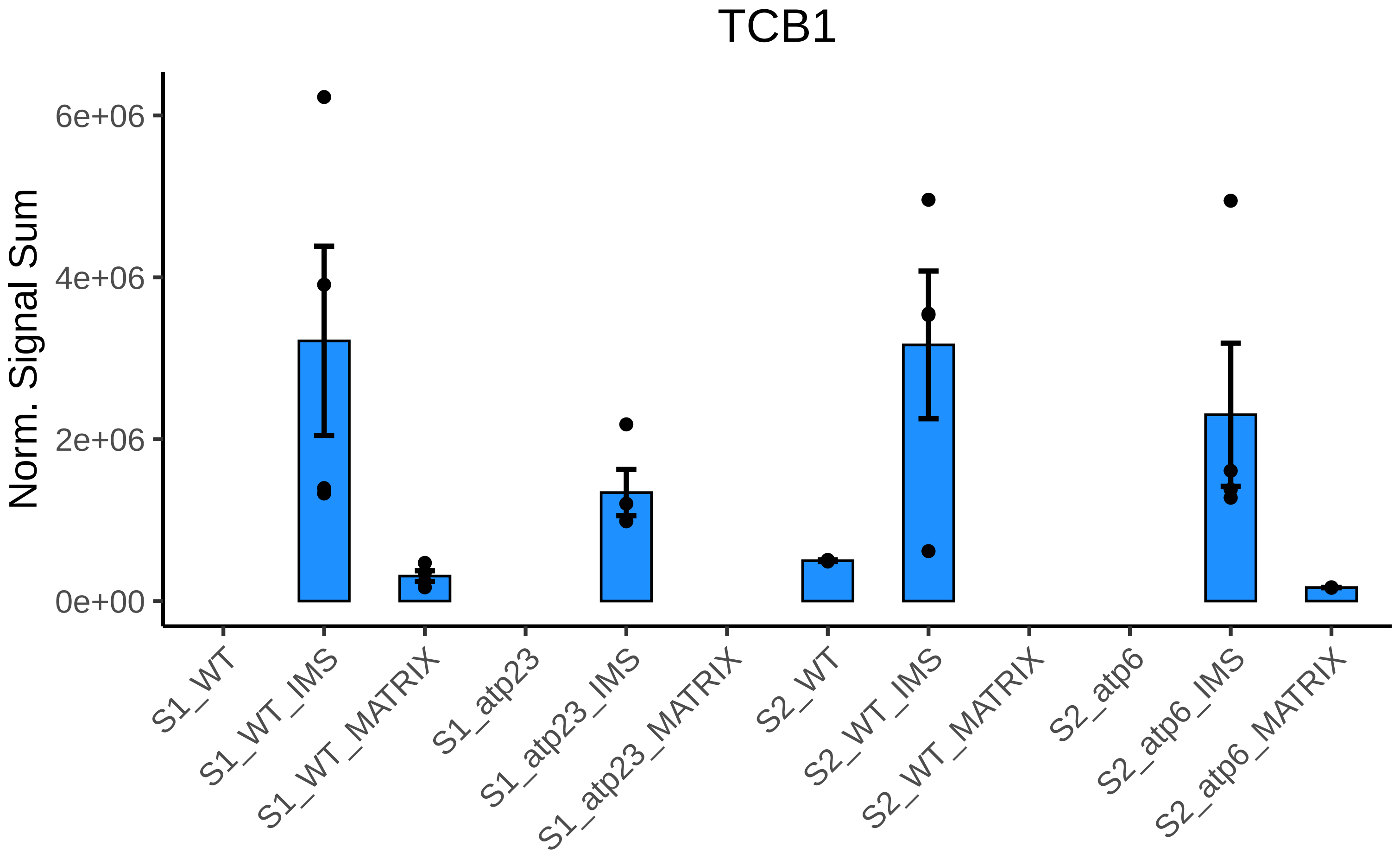
TCB1
Norm. Signal Sum
6e+06
4e+06
2e+06
0e+00
S1_WT
S1_WT_IMS
S1_WT_MATRIX
S1_atp23
S1_atp23_IMS
S1_atp23_MATRIX
S2_WT
S2_WT_IMS
S2_WT_MATRIX
S2_atp6
S2_atp6_IMS
S2_atp6_MATRIX

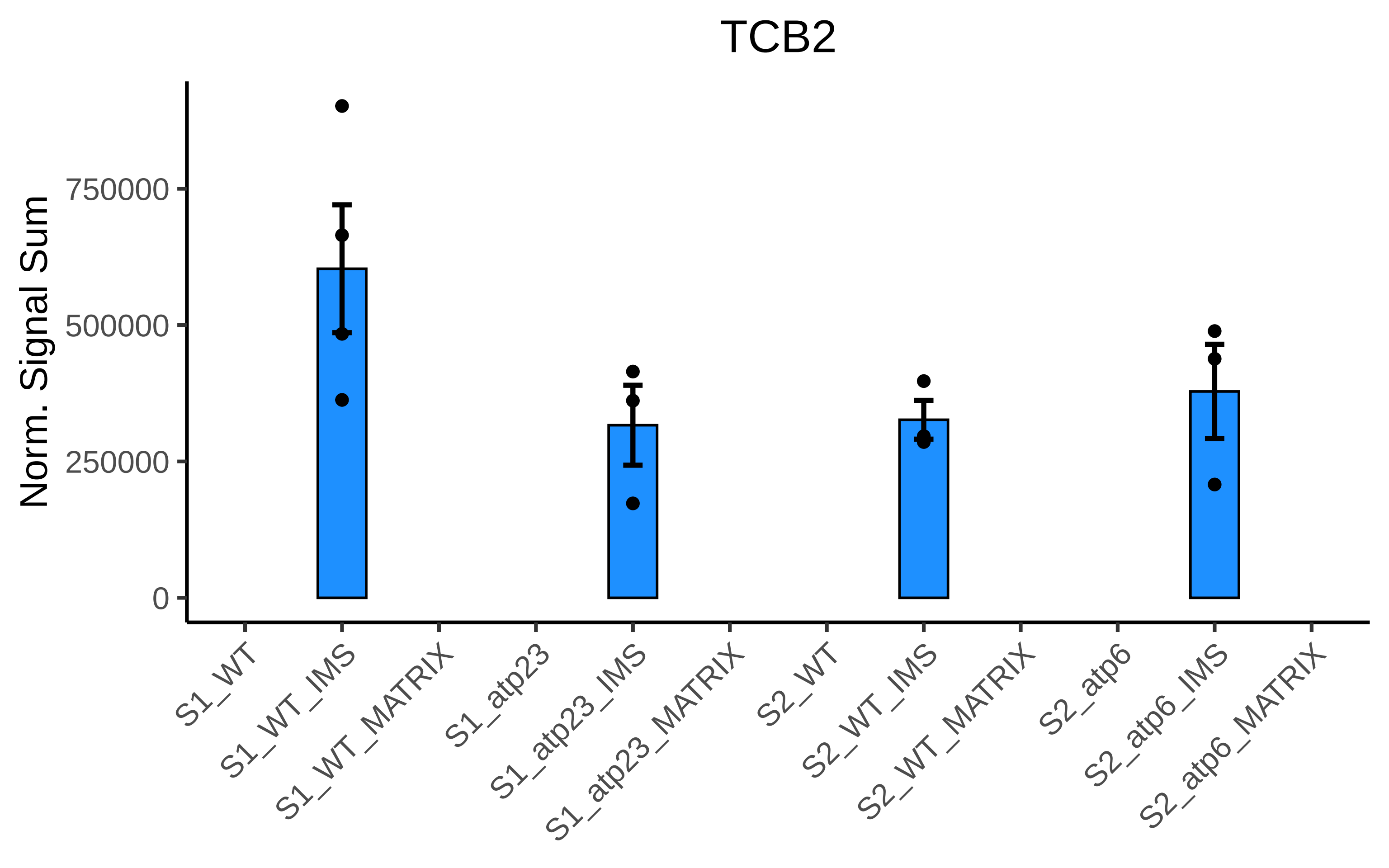

TCB2
Norm. Signal Sum
750000
500000
250000
0
S1_WT
S1_WT_IMS
S1_WT_MATRIX
S1_atp23
S1_atp23_IMS
S1_atp23_MATRIX
S2_WT
S2_WT_IMS
S2_WT_MATRIX
S2_atp6
S2_atp6_IMS
S2_atp6_MATRIX

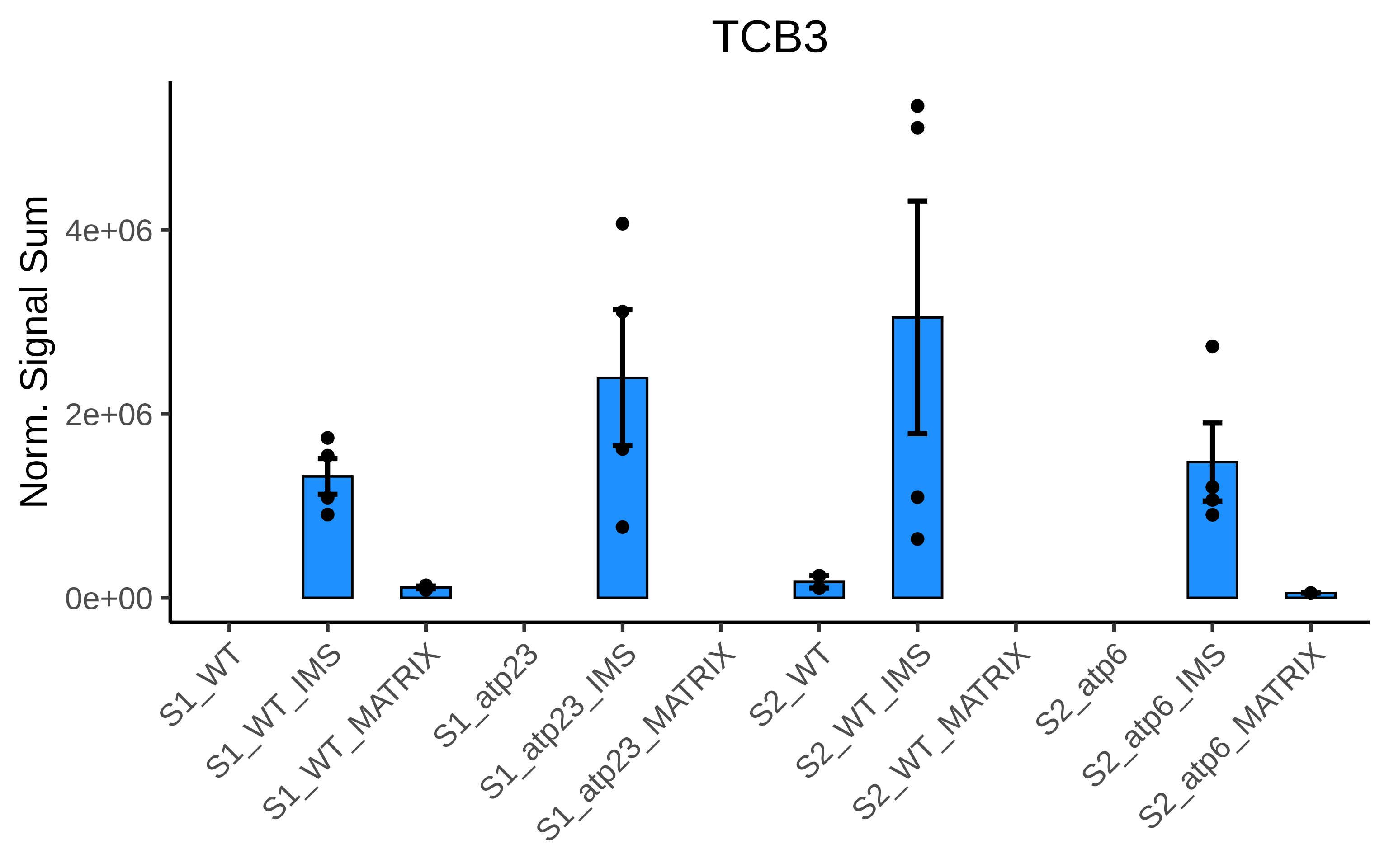
TCB3
Norm. Signal Sum
4e+06
2e+06
0e+00
S1_WT
S1_WT_IMS
S1_WT_MATRIX
S1_atp23
S1_atp23_IMS
S1_atp23_MATRIX
S2_WT
S2_WT_IMS
S2_WT_MATRIX
S2_atp6
S2_atp6_IMS
S2_atp6_MATRIX

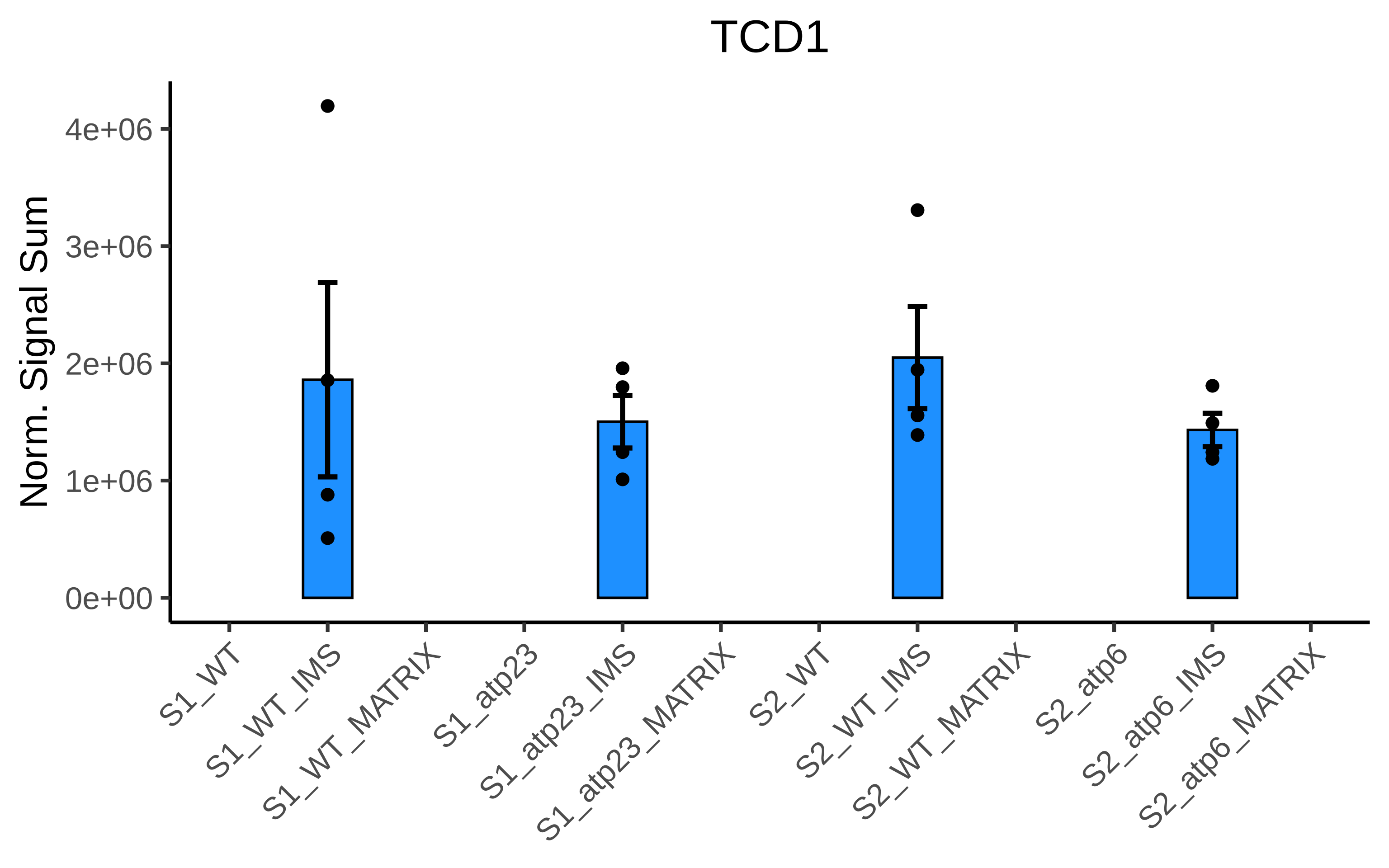

TCD1
Norm. Signal Sum
4e+06
3e+06
2e+06
1e+06
0e+00
S1_WT
S1_WT_IMS
S1_WT_MATRIX
S1_atp23
S1_atp23_IMS
S1_atp23_MATRIX
S2_WT
S2_WT_IMS
S2_WT_MATRIX
S2_atp6
S2_atp6_IMS
S2_atp6_MATRIX

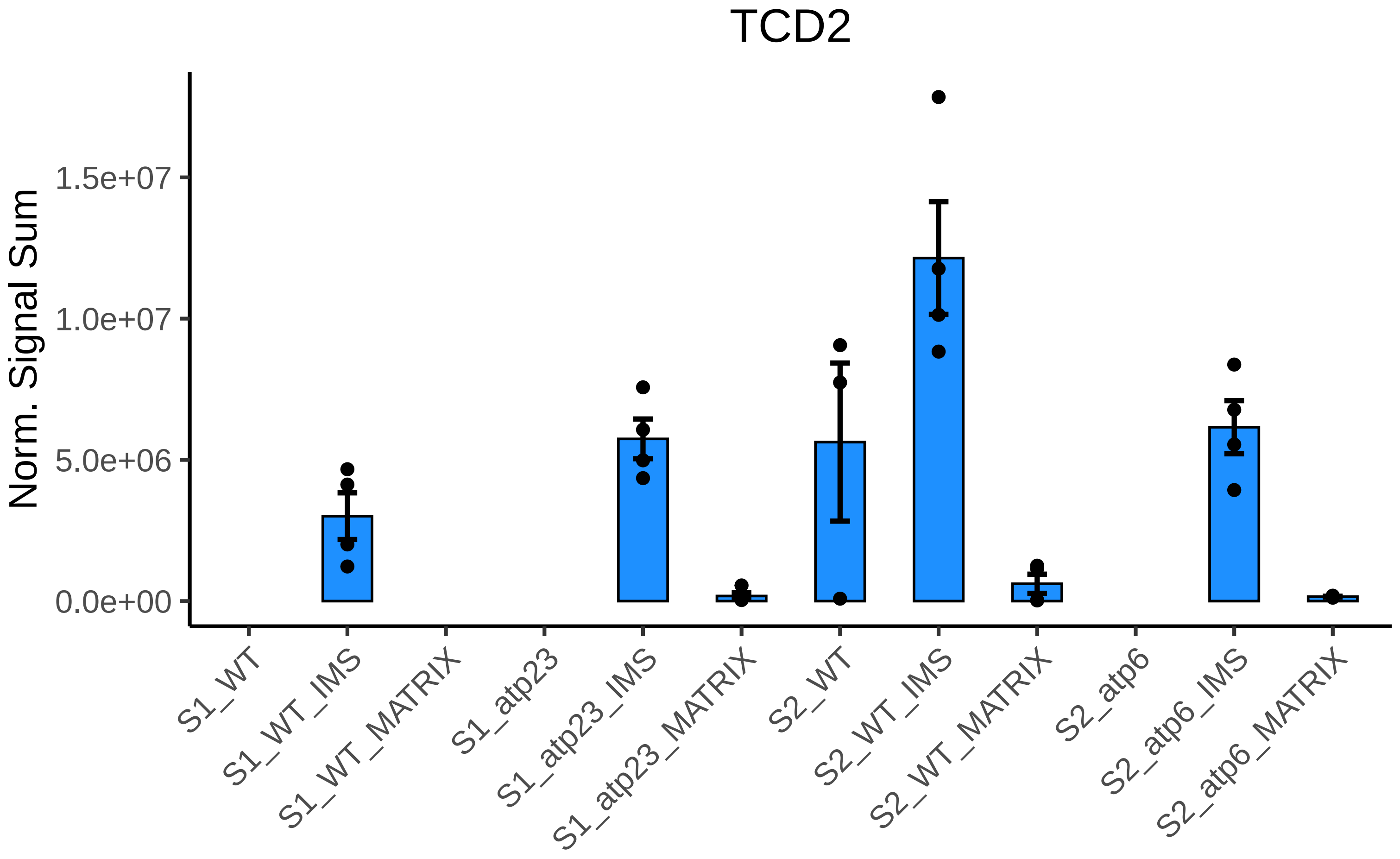

TCD2
Norm. Signal Sum
1.5e+07
1.0e+07
5.0e+06
0.0e+00
S1_WT
S1_WT_IMS
S1_WT_MATRIX
S1_atp23
S1_atp23_IMS
S1_atp23_MATRIX
S2_WT
S2_WT_IMS
S2_WT_MATRIX
S2_atp6
S2_atp6_IMS
S2_atp6_MATRIX

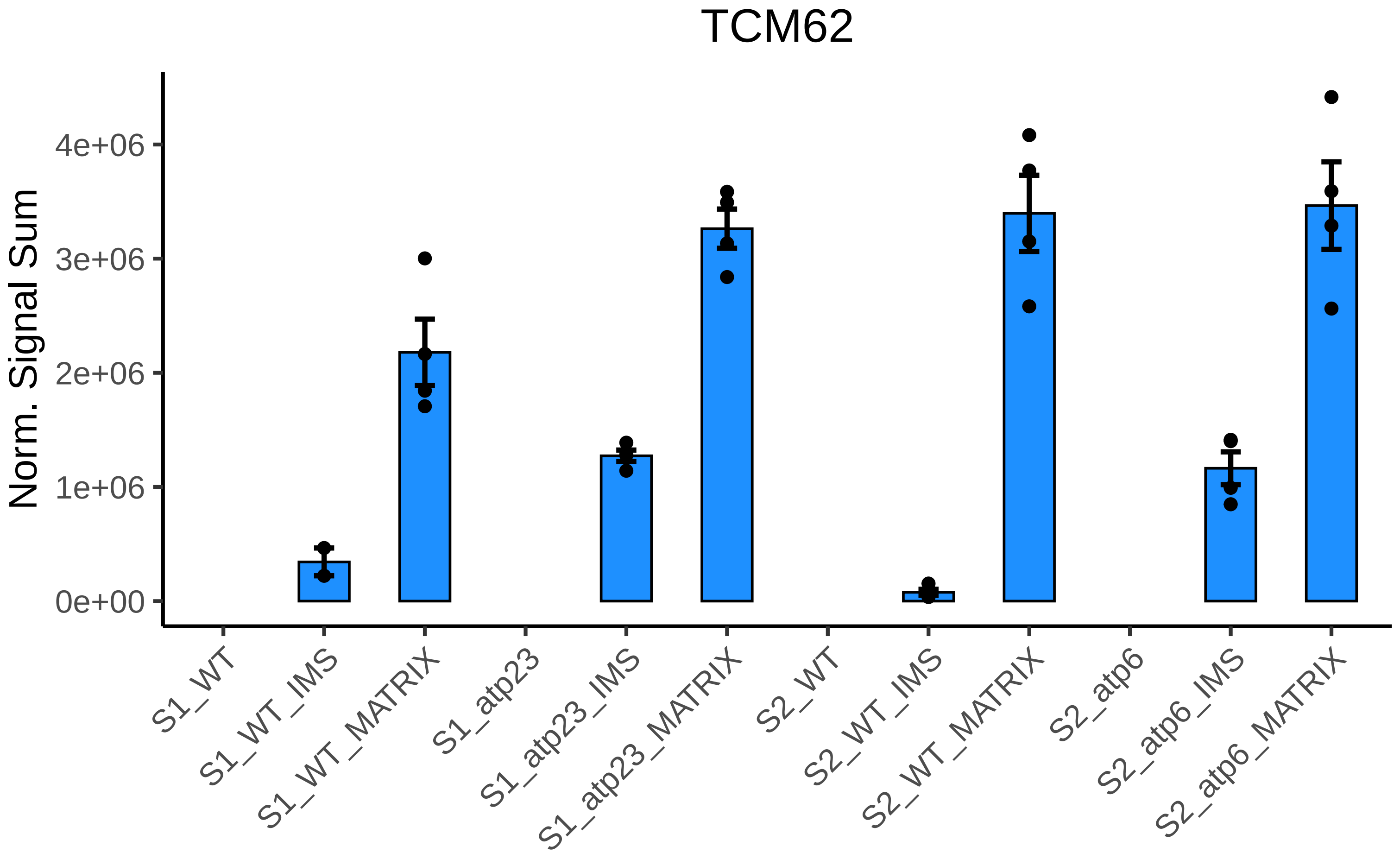
TCM62
Norm. Signal Sum
4e+06
3e+06
2e+06
1e+06
0e+00
S1_WT
S1_WT_IMS
S1_WT_MATRIX
S1_atp23
S1_atp23_IMS
S1_atp23_MATRIX
S2_WT
S2_WT_IMS
S2_WT_MATRIX
S2_atp6
S2_atp6_IMS
S2_atp6_MATRIX

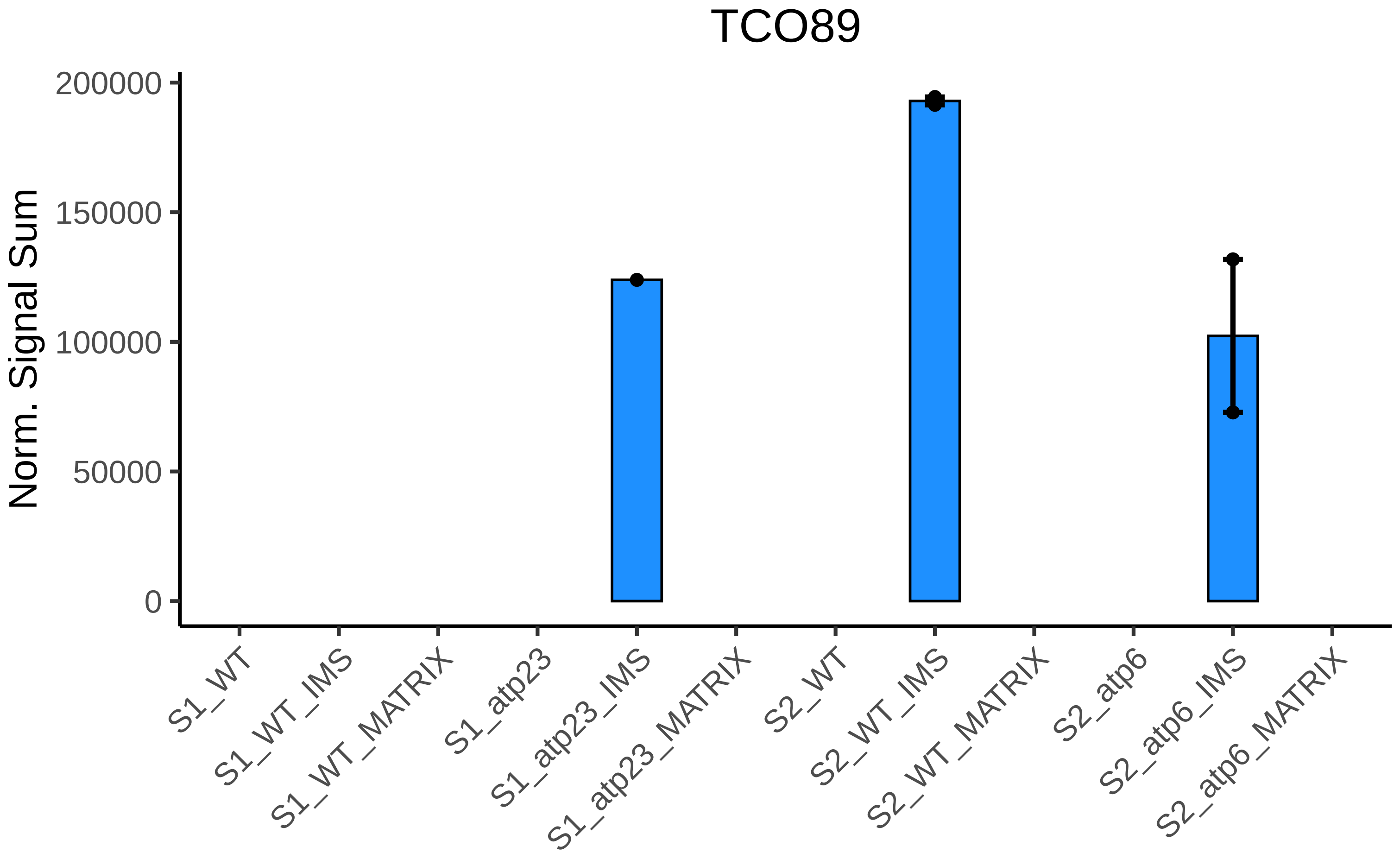

TCO89
Norm. Signal Sum
200000
150000
100000
50000
0
S1_WT
S1_WT_IMS
S1_WT_MATRIX
S1_atp23
S1_atp23_IMS
S1_atp23_MATRIX
S2_WT
S2_WT_IMS
S2_WT_MATRIX
S2_atp6
S2_atp6_IMS
S2_atp6_MATRIX

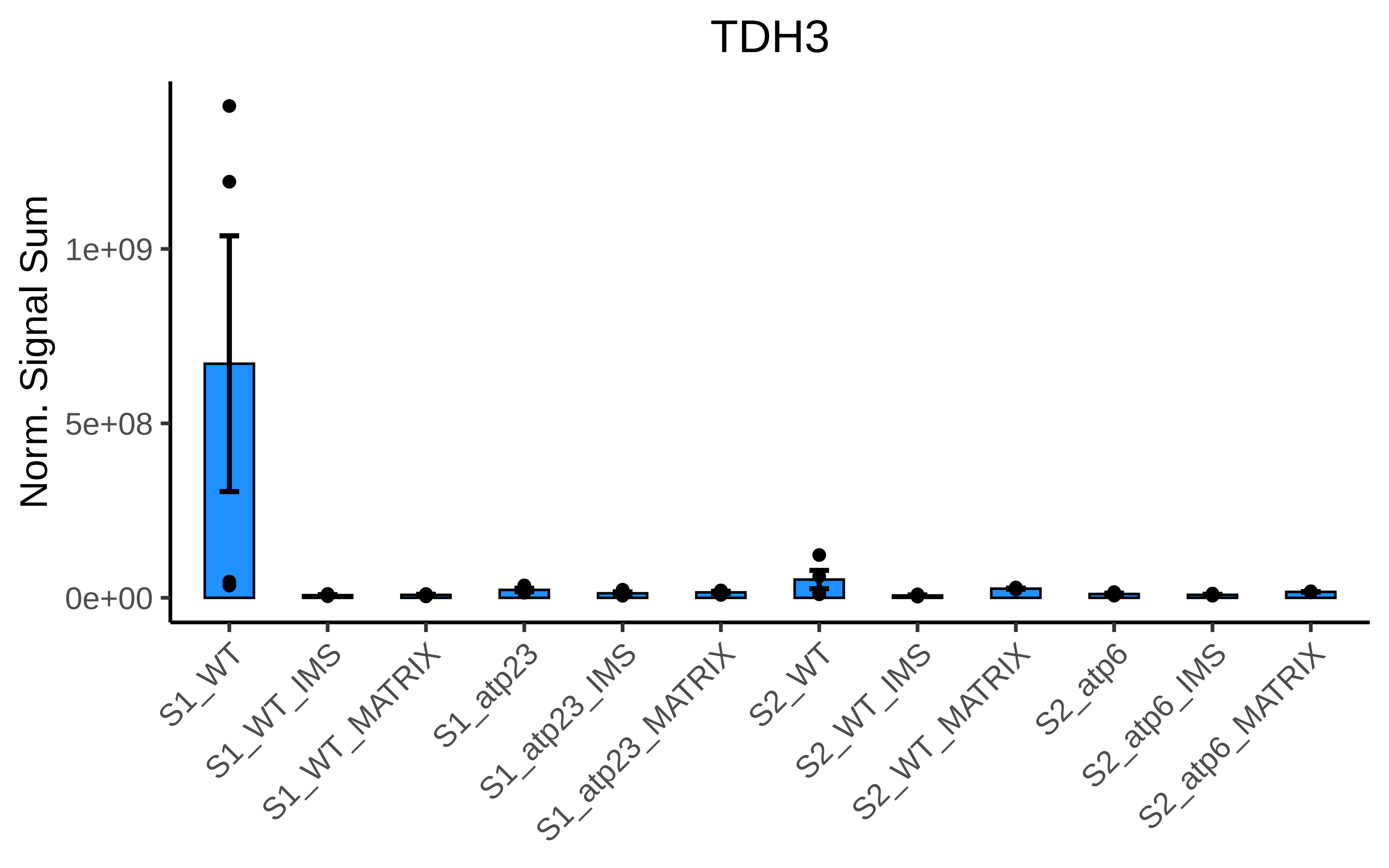

TDH3
Norm. Signal Sum
1e+09
5e+08
0e+00
S1_WT
S1_WT_IMS
S1_WT_MATRIX
S1_atp23
S1_atp23_IMS
S1_atp23_MATRIX
S2_WT
S2_WT_IMS
S2_WT_MATRIX
S2_atp6
S2_atp6_IMS
S2_atp6_MATRIX

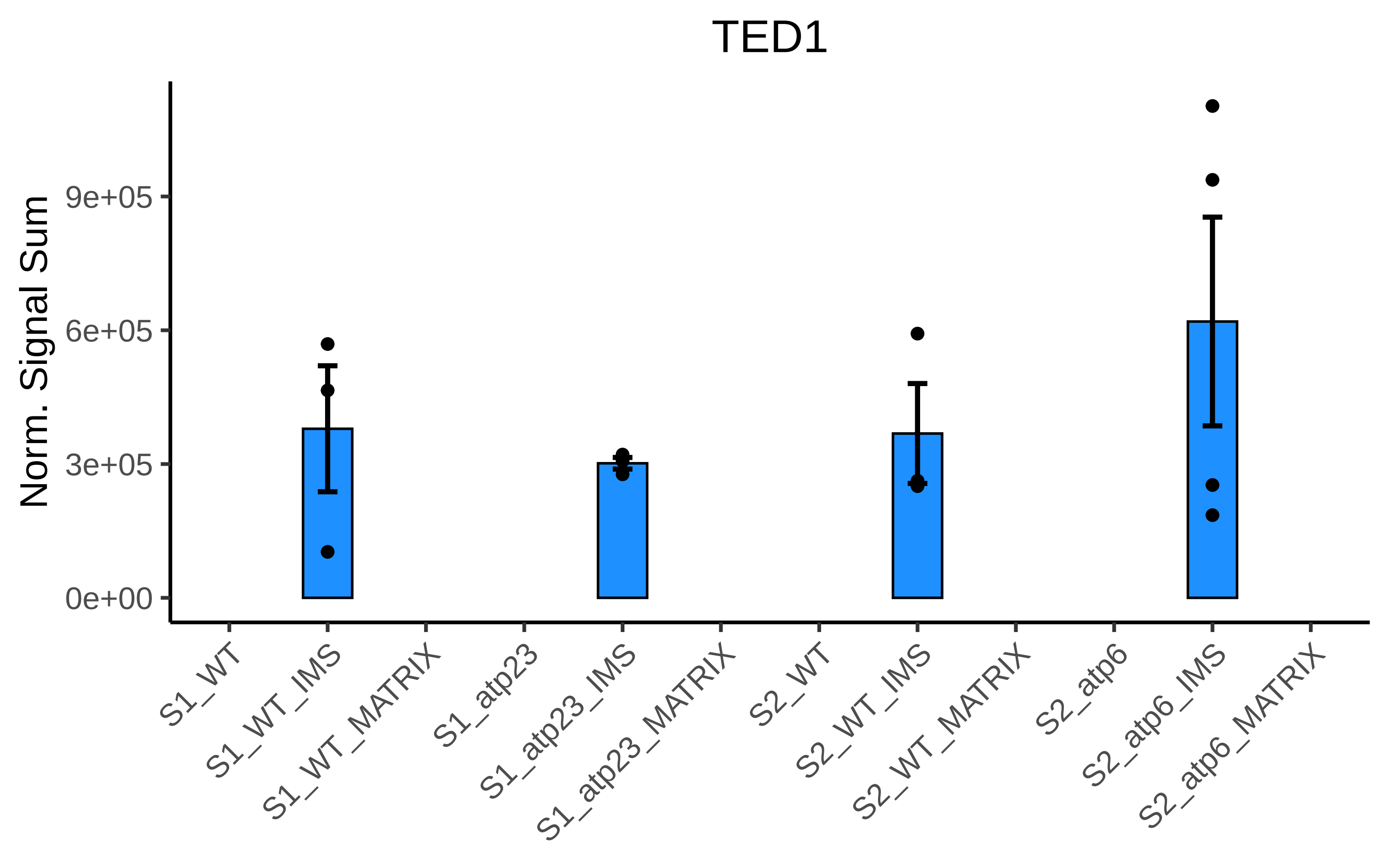
TED1
Norm. Signal Sum
0e+00
3e+05
6e+05
9e+05
S1_WT
S1_WT_IMS
S1_WT_MATRIX
S1_atp23
S1_atp23_IMS
S1_atp23_MATRIX
S2_WT
S2_WT_IMS
S2_WT_MATRIX
S2_atp6
S2_atp6_IMS
S2_atp6_MATRIX

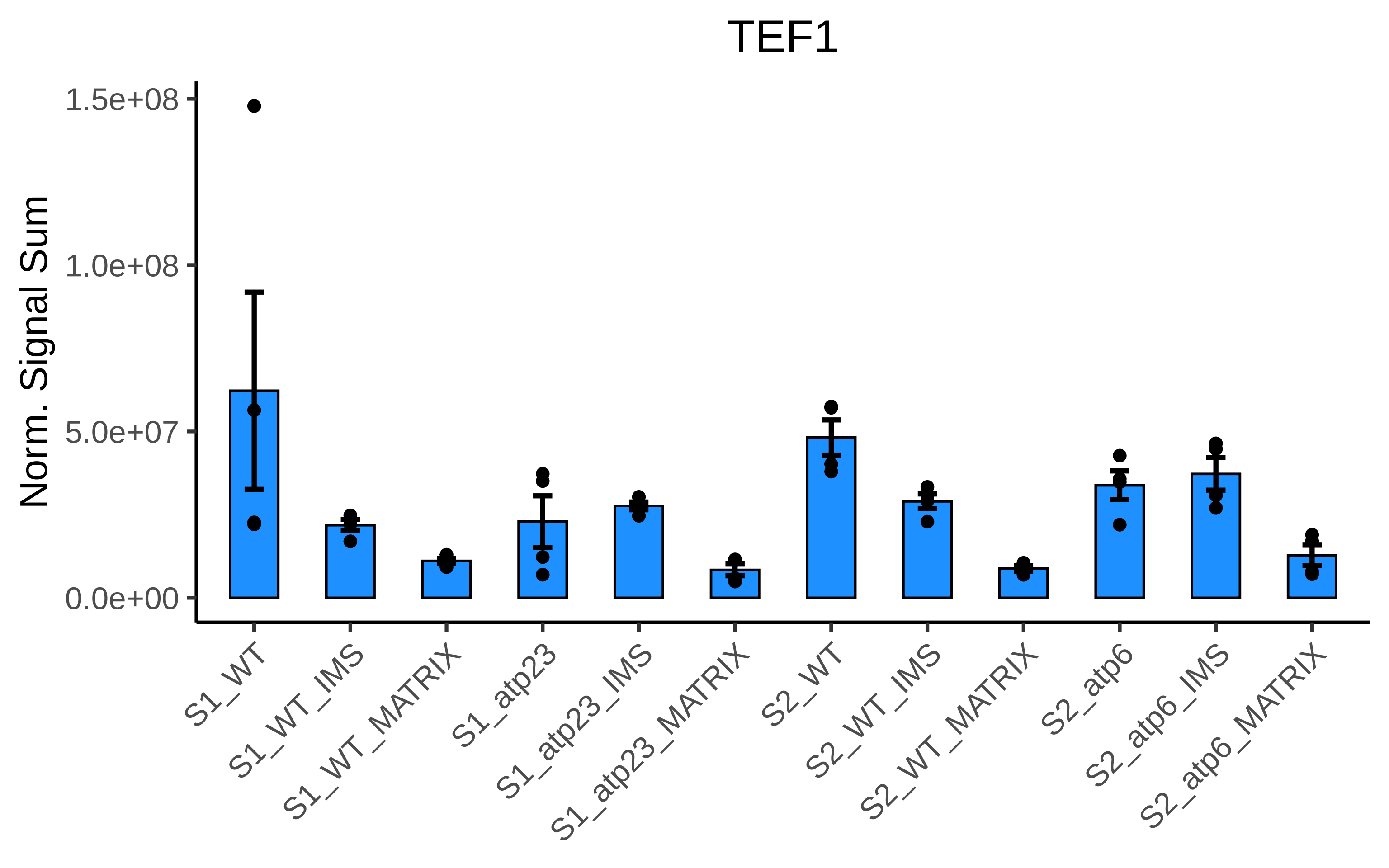

TEF1
Norm. Signal Sum
1.5e+08
1.0e+08
5.0e+07
0.0e+00
S1_WT
S1_WT_IMS
S1_WT_MATRIX
S1_atp23
S1_atp23_IMS
S1_atp23_MATRIX
S2_WT
S2_WT_IMS
S2_WT_MATRIX
S2_atp6
S2_atp6_IMS
S2_atp6_MATRIX

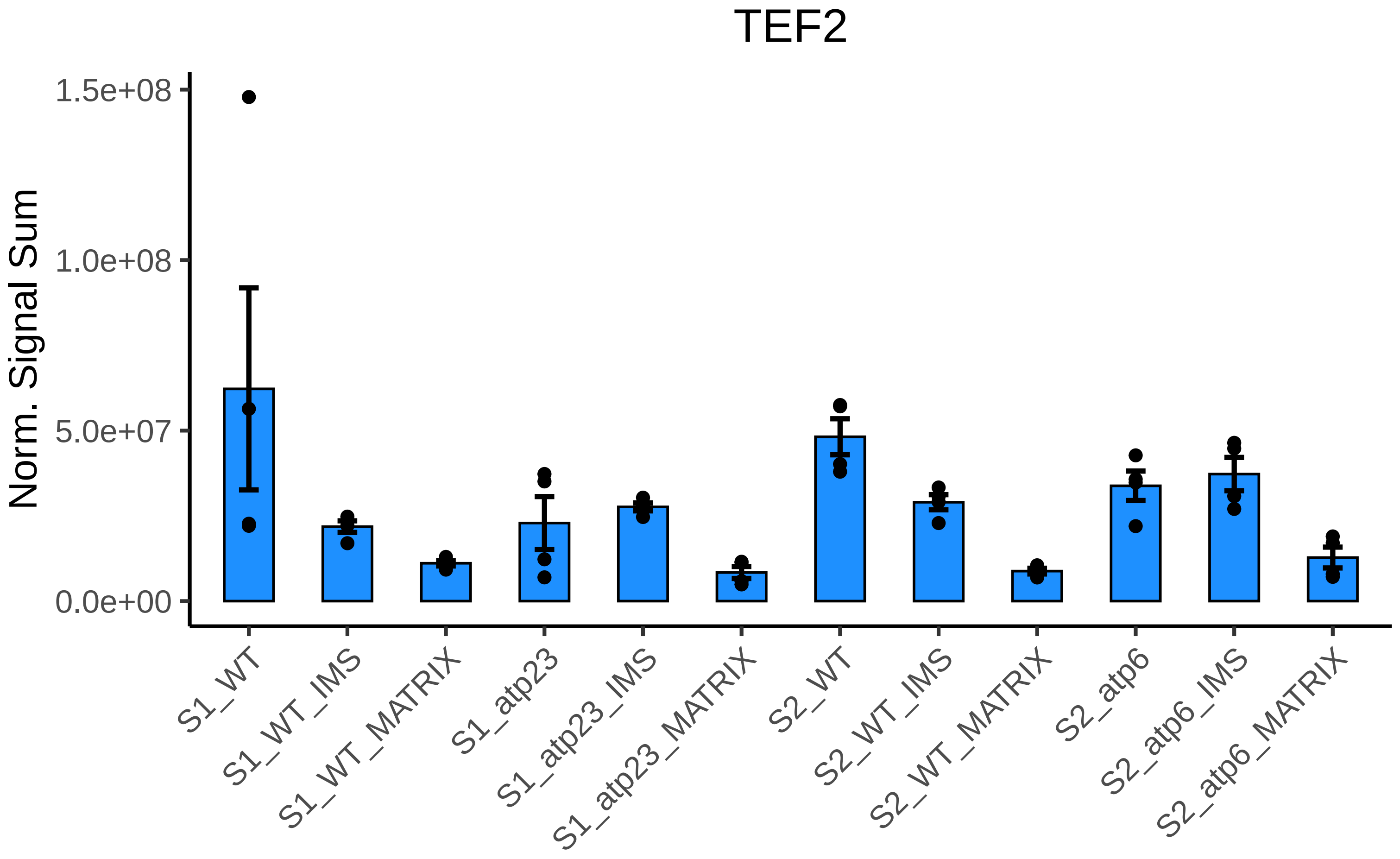

TEF2
Norm. Signal Sum
1.5e+08
1.0e+08
5.0e+07
0.0e+00
S1_WT
S1_WT_IMS
S1_WT_MATRIX
S1_atp23
S1_atp23_IMS
S1_atp23_MATRIX
S2_WT
S2_WT_IMS
S2_WT_MATRIX
S2_atp6
S2_atp6_IMS
S2_atp6_MATRIX

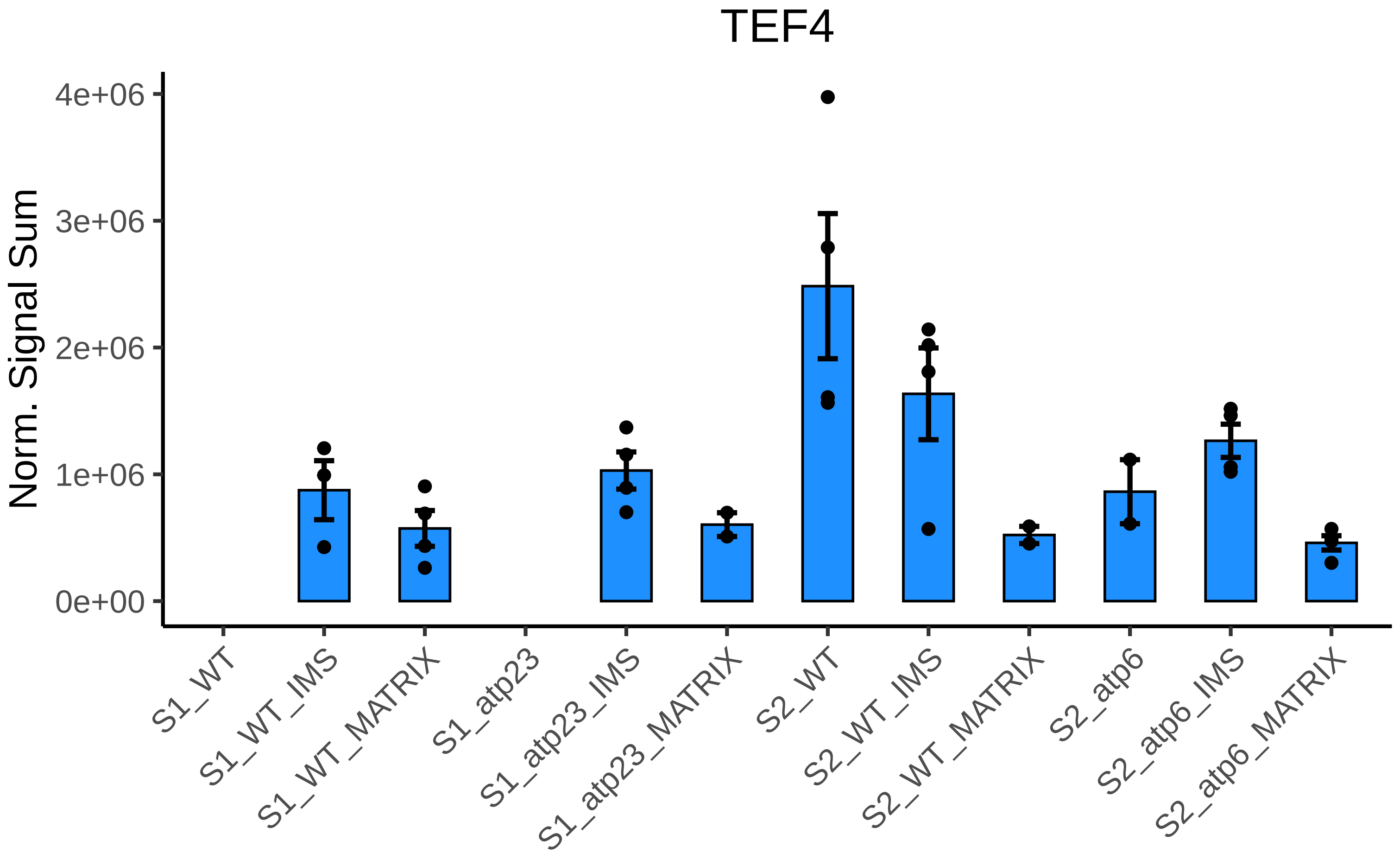

TEF4
Norm. Signal Sum
4e+06
3e+06
2e+06
1e+06
0e+00
S1_WT
S1_WT_IMS
S1_WT_MATRIX
S1_atp23
S1_atp23_IMS
S1_atp23_MATRIX
S2_WT
S2_WT_IMS
S2_WT_MATRIX
S2_atp6
S2_atp6_IMS
S2_atp6_MATRIX

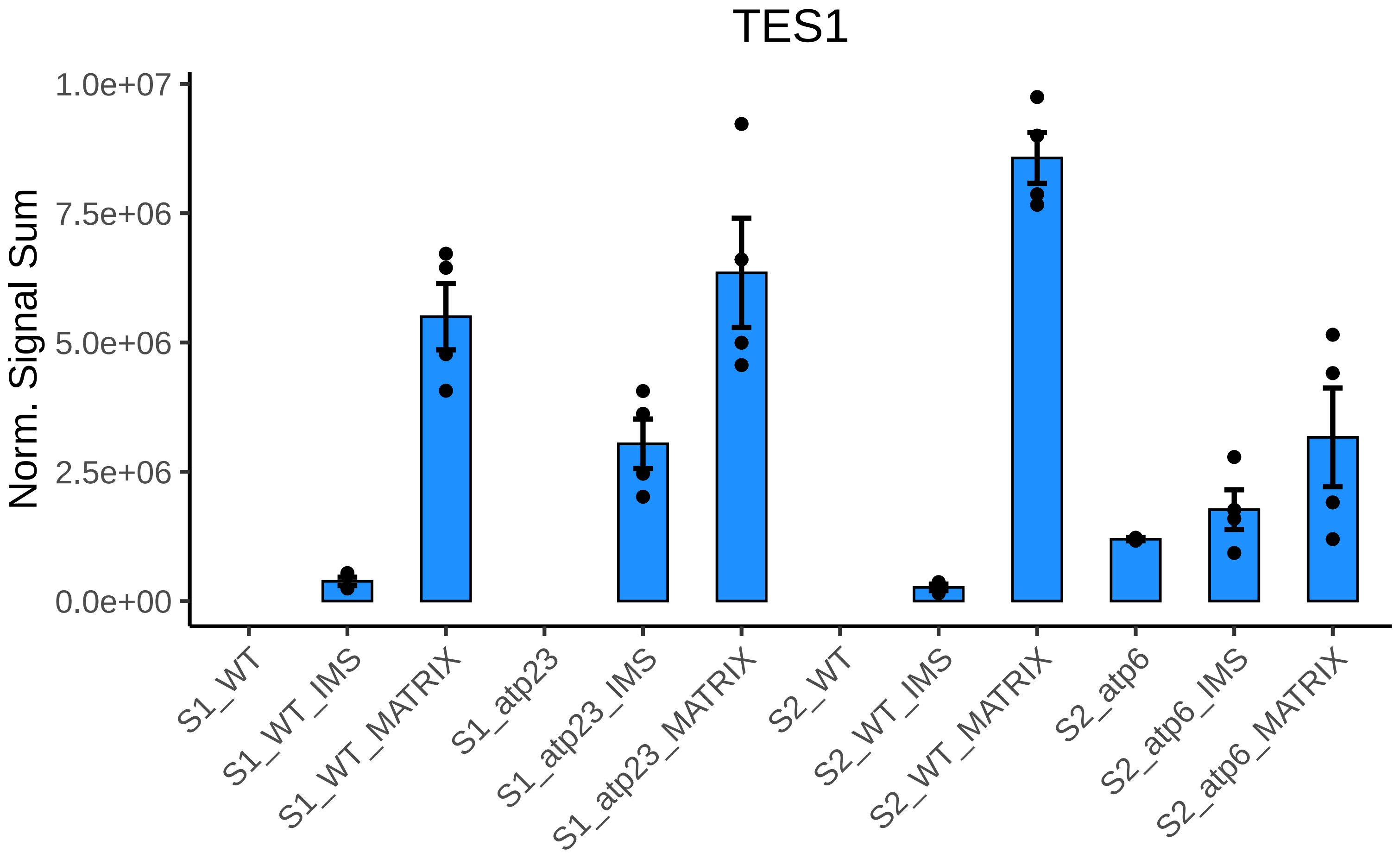
TES1
Norm. Signal Sum
1.0e+07
7.5e+06
5.0e+06
2.5e+06
0.0e+00
S1_WT
S1_WT_IMS
S1_WT_MATRIX
S1_atp23
S1_atp23_IMS
S1_atp23_MATRIX
S2_WT
S2_WT_IMS
S2_WT_MATRIX
S2_atp6
S2_atp6_IMS
S2_atp6_MATRIX

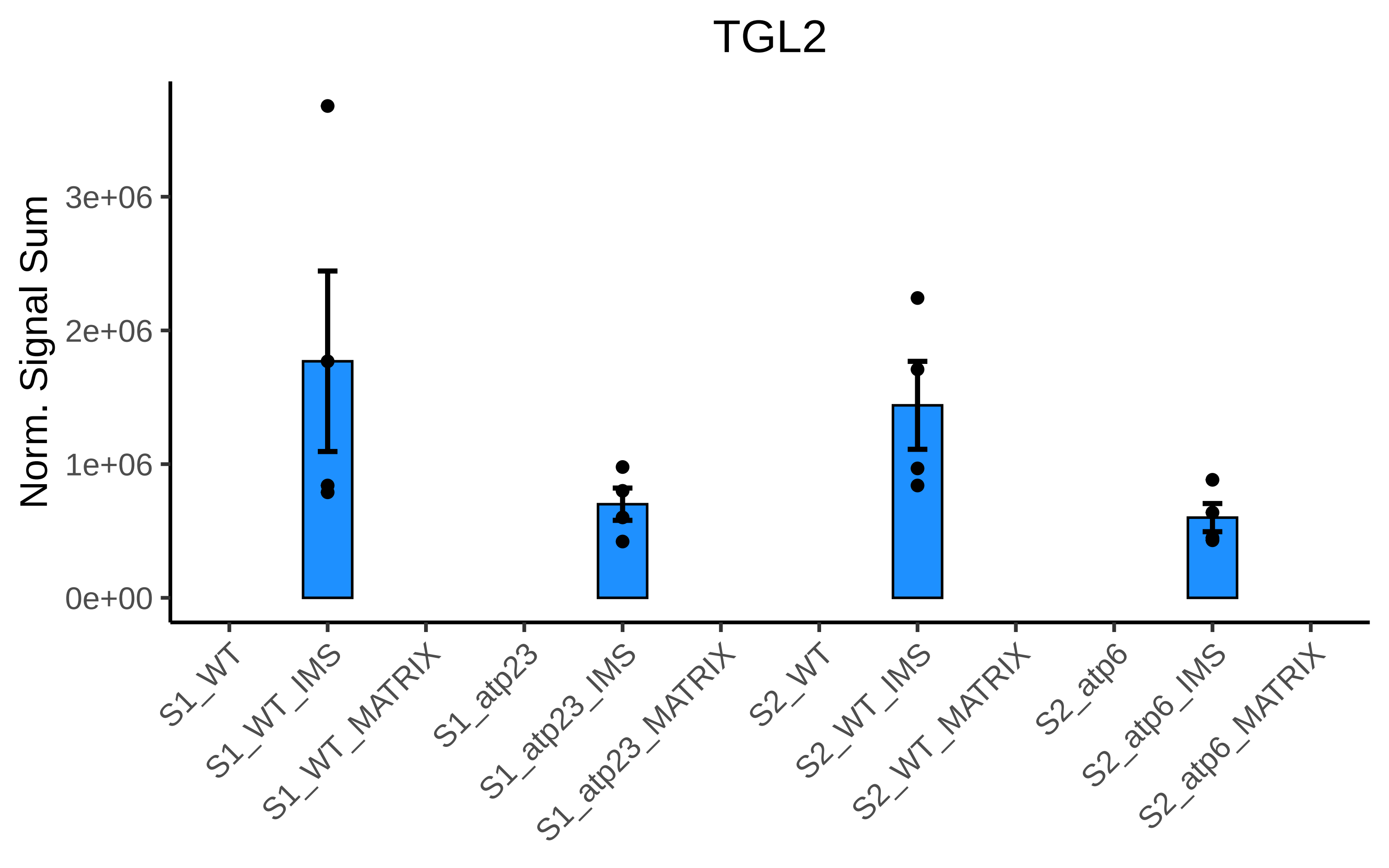

TGL2
Norm. Signal Sum
3e+06
2e+06
1e+06
0e+00
S1_WT
S1_WT_IMS
S1_WT_MATRIX
S1_atp23
S1_atp23_IMS
S1_atp23_MATRIX
S2_WT
S2_WT_IMS
S2_WT_MATRIX
S2_atp6
S2_atp6_IMS
S2_atp6_MATRIX

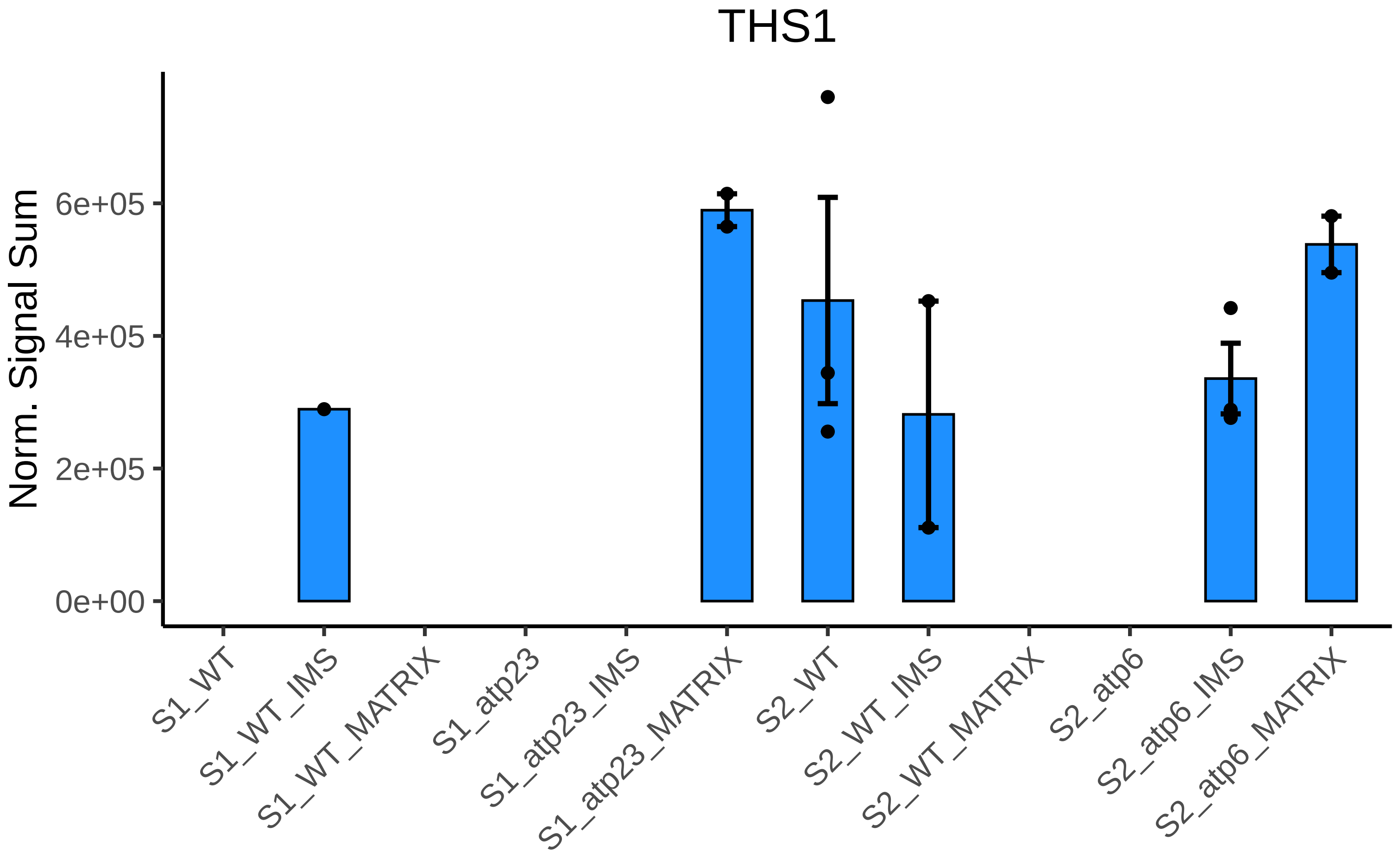
THS1
Norm. Signal Sum
6e+05
4e+05
2e+05
0e+00
S1_WT
S1_WT_IMS
S1_WT_MATRIX
S1_atp23
S1_atp23_IMS
S1_atp23_MATRIX
S2_WT
S2_WT_IMS
S2_WT_MATRIX
S2_atp6
S2_atp6_IMS
S2_atp6_MATRIX

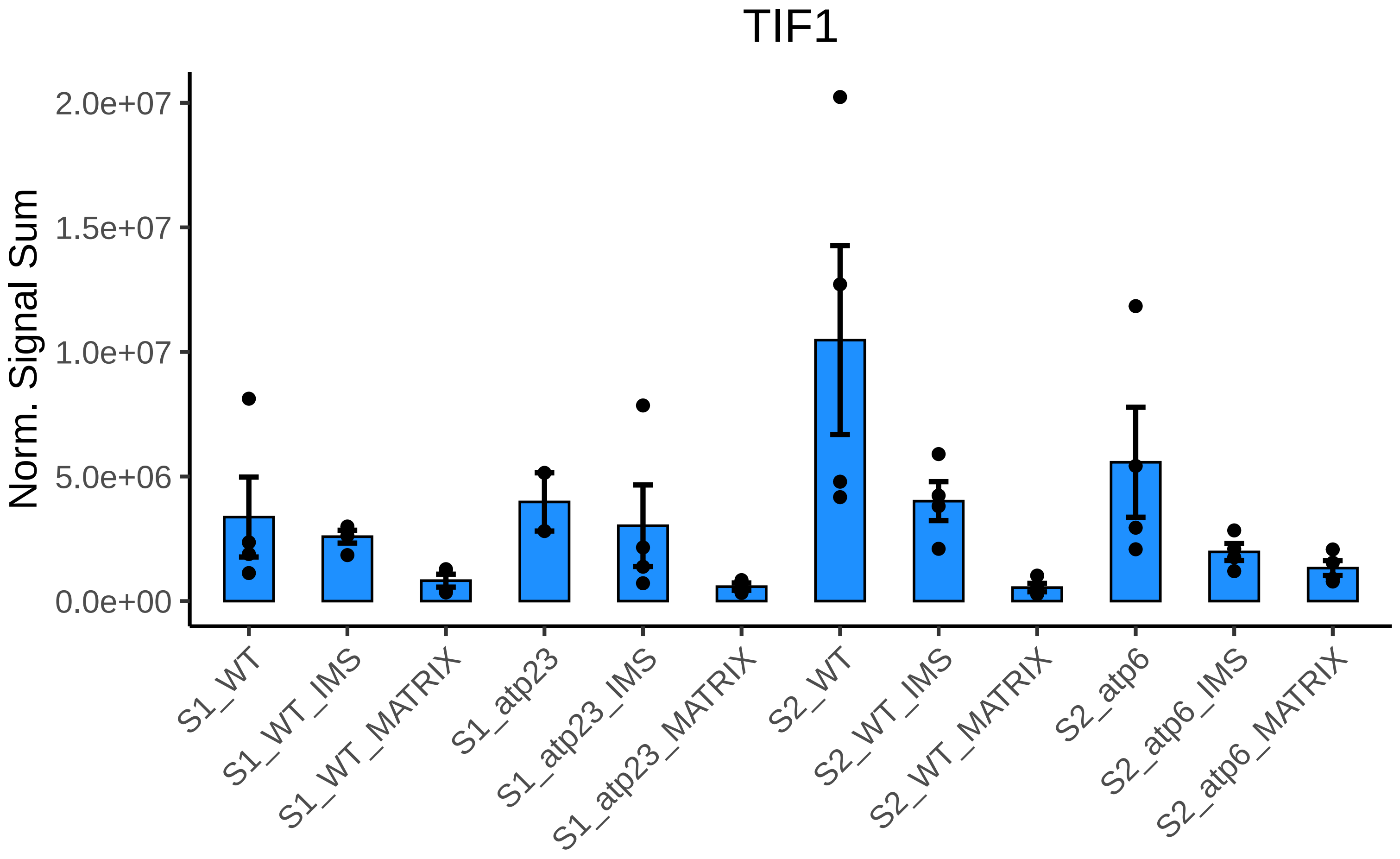

TIF1
Norm. Signal Sum
2.0e+07
1.5e+07
1.0e+07
5.0e+06
0.0e+00
S1_WT
S1_WT_IMS
S1_WT_MATRIX
S1_atp23
S1_atp23_IMS
S1_atp23_MATRIX
S2_WT
S2_WT_IMS
S2_WT_MATRIX
S2_atp6
S2_atp6_IMS
S2_atp6_MATRIX

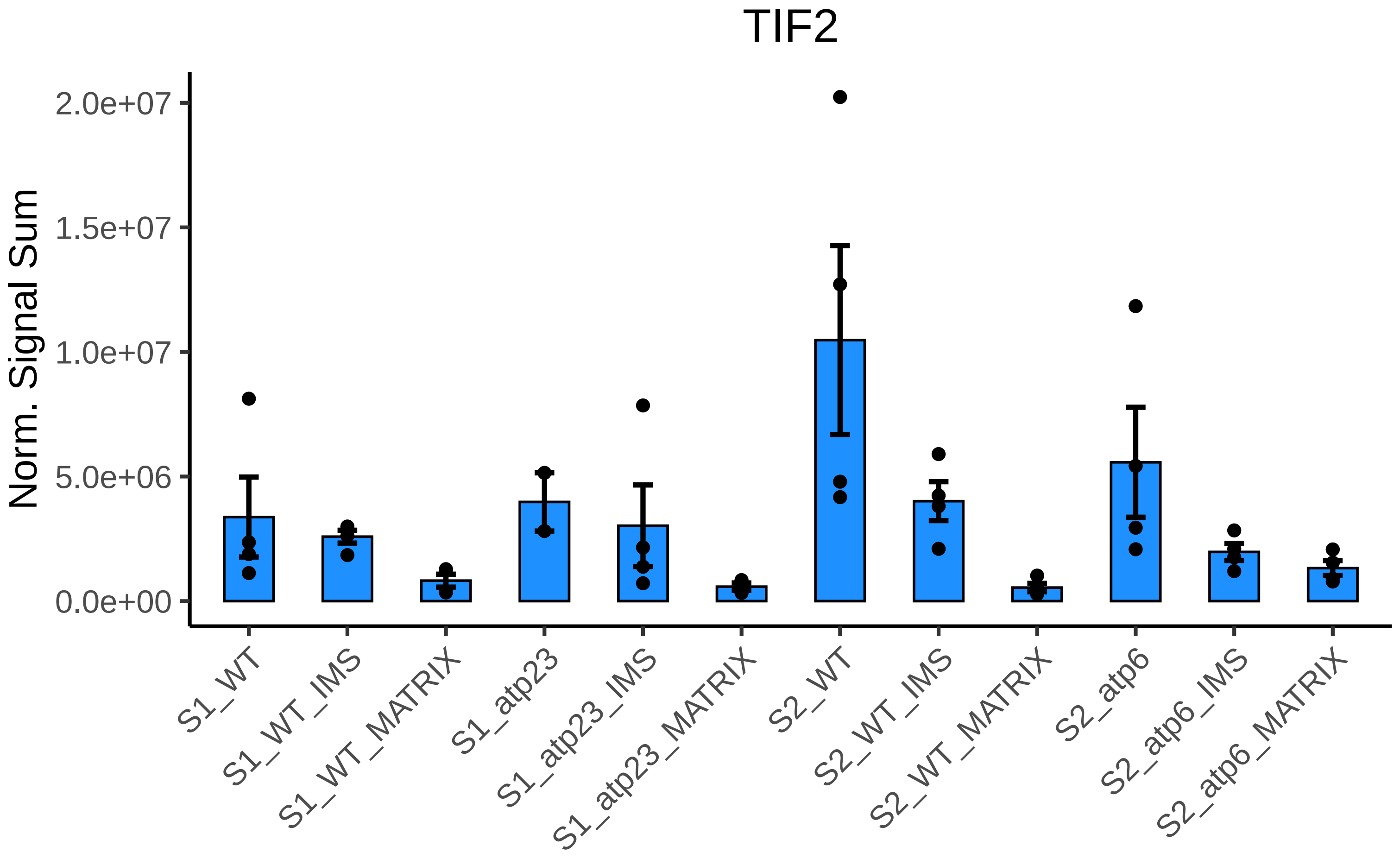
TIF2
Norm. Signal Sum
2.0e+07
1.5e+07
1.0e+07
5.0e+06
0.0e+00
S1_WT
S1_WT_IMS
S1_WT_MATRIX
S1_atp23
S1_atp23_IMS
S1_atp23_MATRIX
S2_WT
S2_WT_IMS
S2_WT_MATRIX
S2_atp6
S2_atp6_IMS
S2_atp6_MATRIX

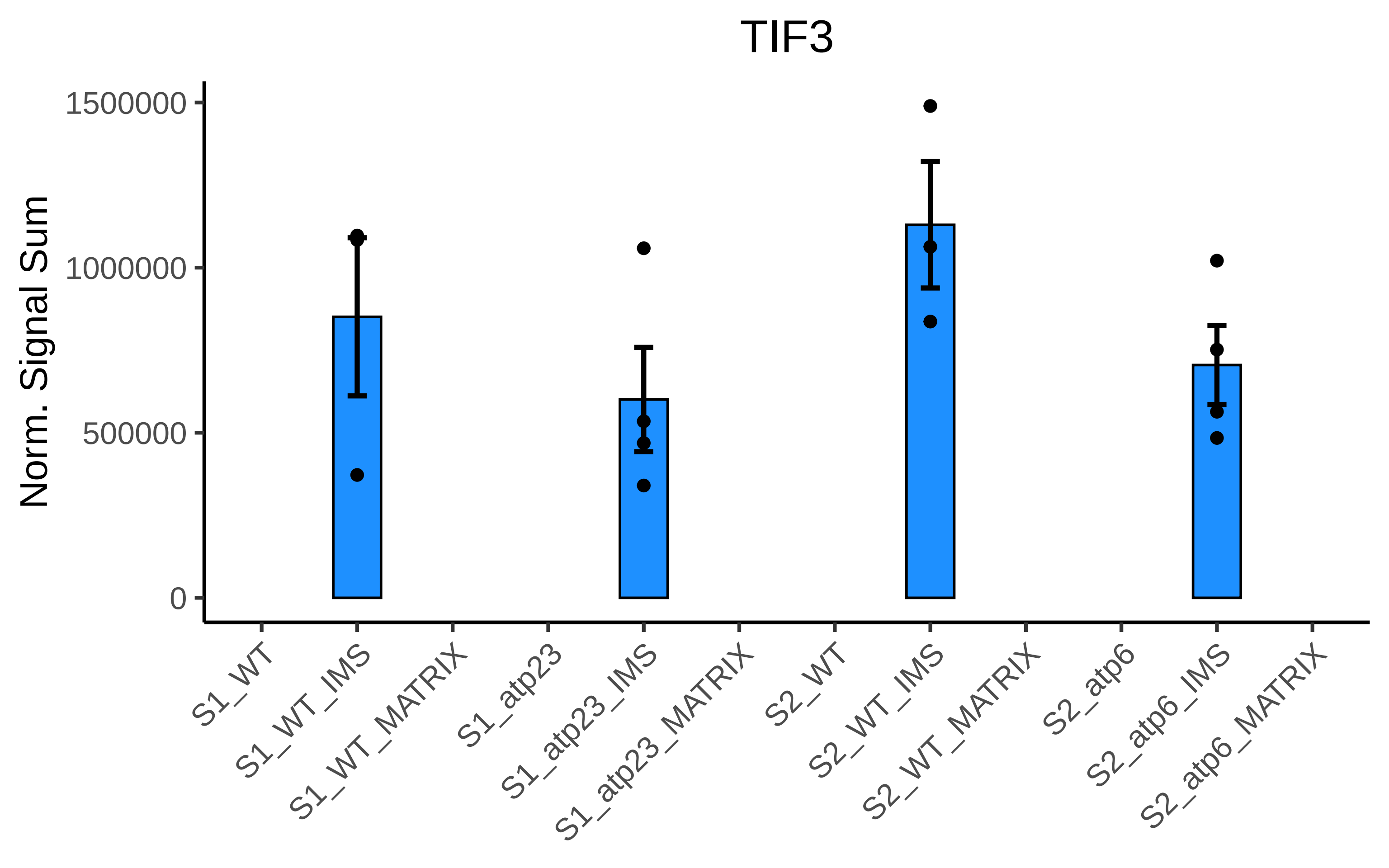
TIF3
Norm. Signal Sum
1500000
1000000
500000
0
S1_WT
S1_WT_IMS
S1_WT_MATRIX
S1_atp23
S1_atp23_IMS
S1_atp23_MATRIX
S2_WT
S2_WT_IMS
S2_WT_MATRIX
S2_atp6
S2_atp6_IMS
S2_atp6_MATRIX

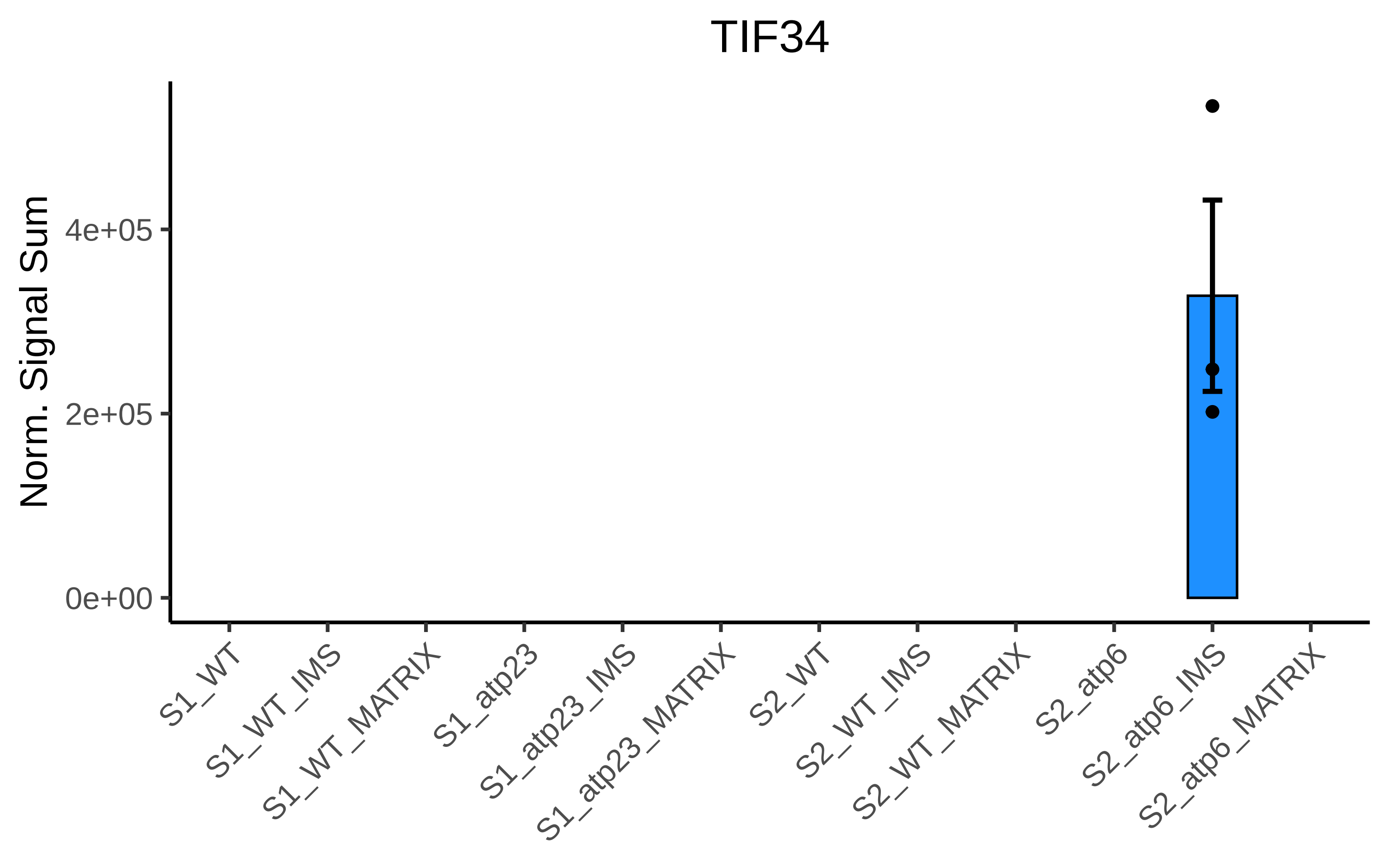

TIF34
Norm. Signal Sum
4e+05
2e+05
0e+00
S1_WT
S1_WT_IMS
S1_WT_MATRIX
S1_atp23
S1_atp23_IMS
S1_atp23_MATRIX
S2_WT
S2_WT_IMS
S2_WT_MATRIX
S2_atp6
S2_atp6_IMS
S2_atp6_MATRIX

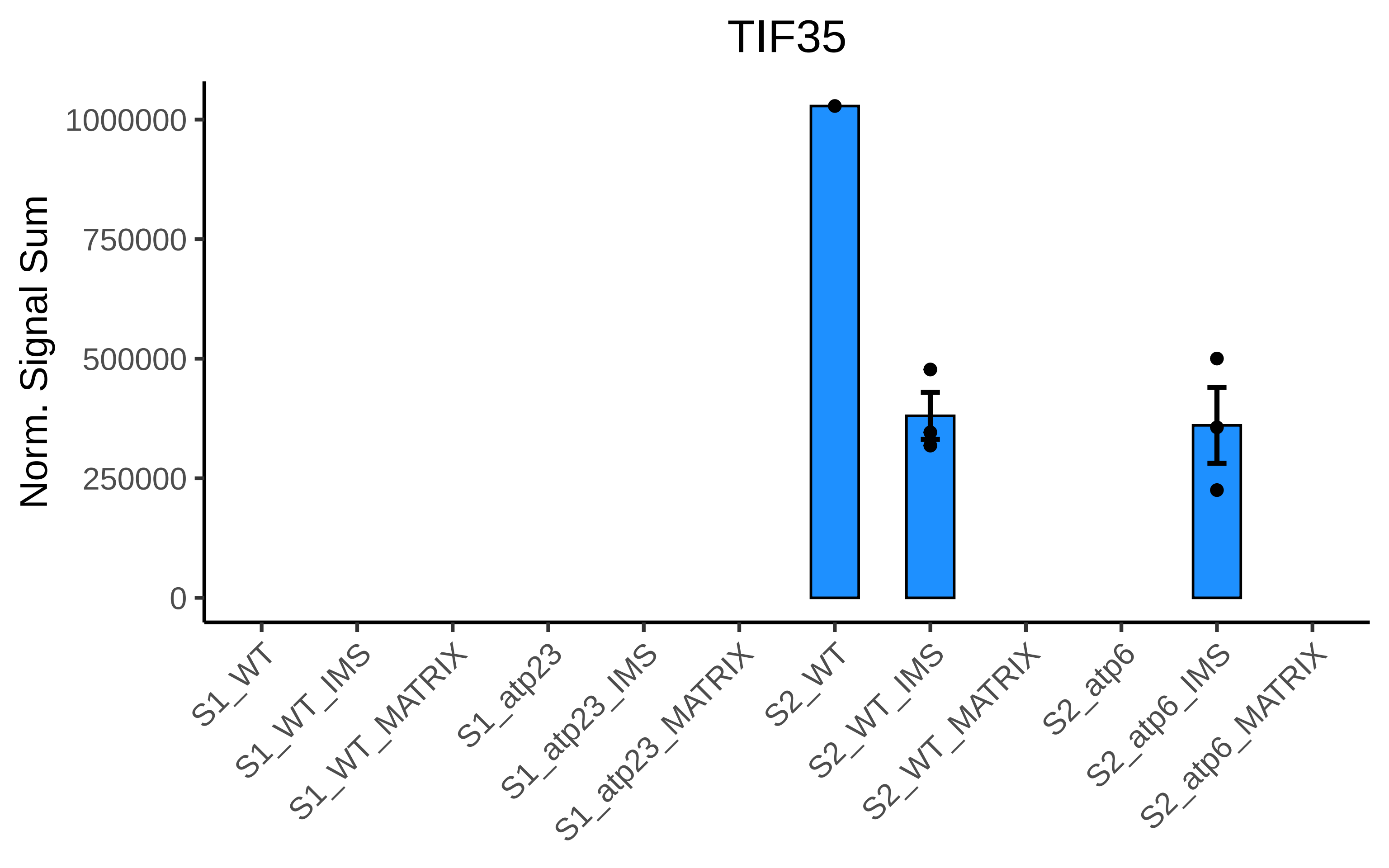
TIF35
Norm. Signal Sum
1000000
750000
500000
250000
0
S1_WT
S1_WT_IMS
S1_WT_MATRIX
S1_atp23
S1_atp23_IMS
S1_atp23_MATRIX
S2_WT
S2_WT_IMS
S2_WT_MATRIX
S2_atp6
S2_atp6_IMS
S2_atp6_MATRIX

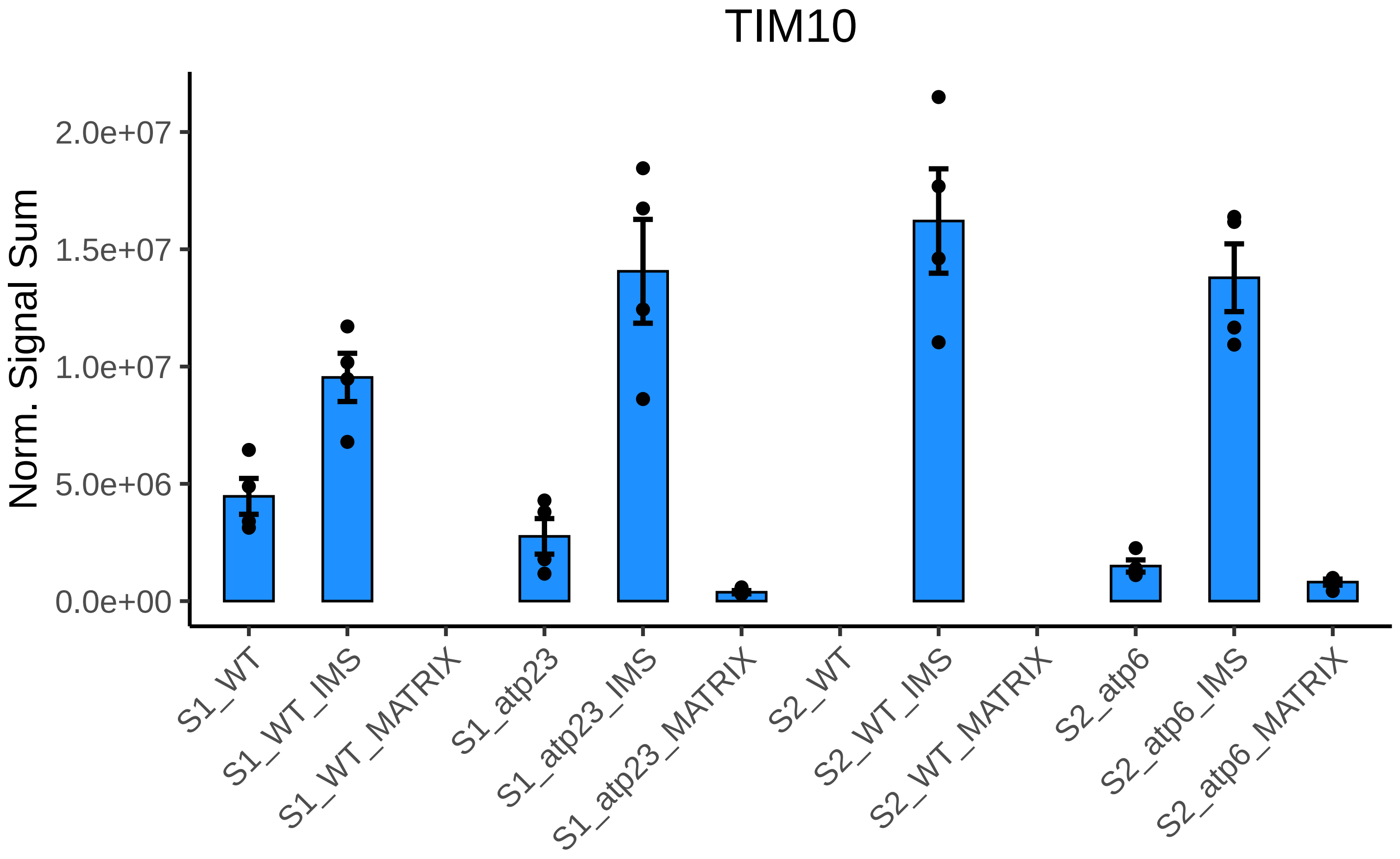
TIM10
Norm. Signal Sum
2.0e+07
1.5e+07
1.0e+07
5.0e+06
0.0e+00
S1_WT
S1_WT_IMS
S1_WT_MATRIX
S1_atp23
S1_atp23_IMS
S1_atp23_MATRIX
S2_WT
S2_WT_IMS
S2_WT_MATRIX
S2_atp6
S2_atp6_IMS
S2_atp6_MATRIX

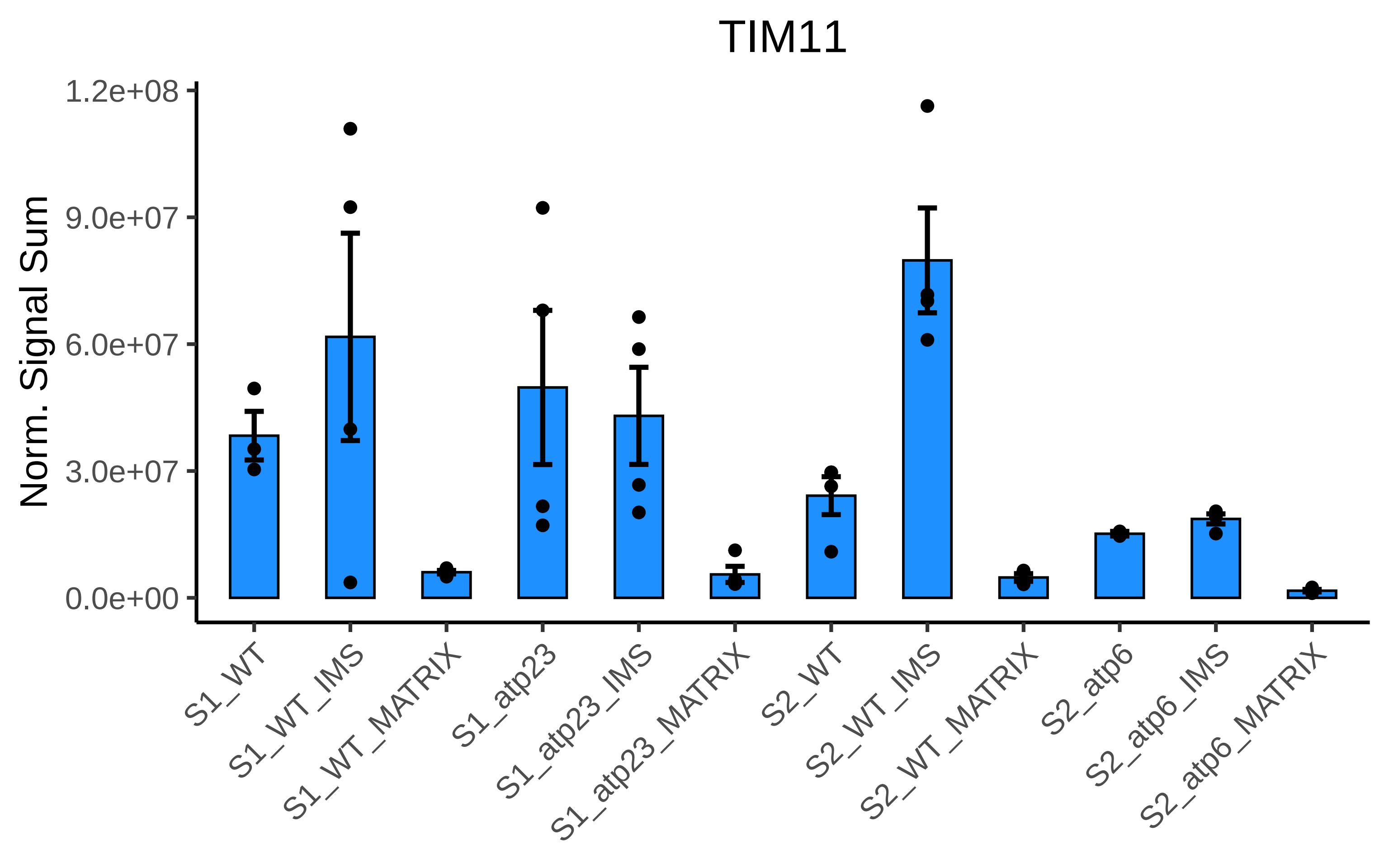

TIM11
Norm. Signal Sum
1.2e+08
9.0e+07
6.0e+07
3.0e+07
0.0e+00
S1_WT
S1_WT_IMS
S1_WT_MATRIX
S1_atp23
S1_atp23_IMS
S1_atp23_MATRIX
S2_WT
S2_WT_IMS
S2_WT_MATRIX
S2_atp6
S2_atp6_IMS
S2_atp6_MATRIX

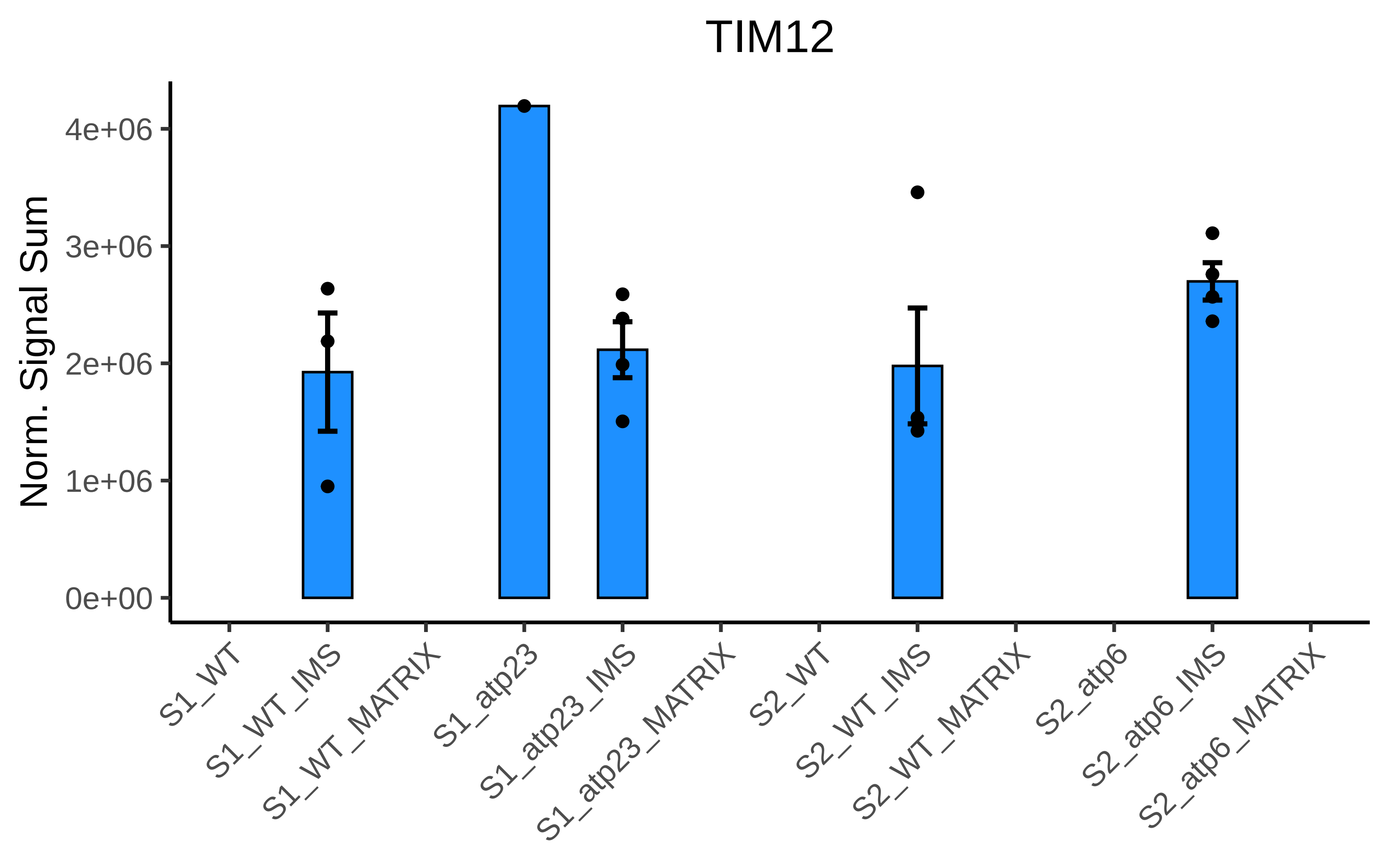

TIM12
Norm. Signal Sum
4e+06
3e+06
2e+06
1e+06
0e+00
S1_WT
S1_WT_IMS
S1_WT_MATRIX
S1_atp23
S1_atp23_IMS
S1_atp23_MATRIX
S2_WT
S2_WT_IMS
S2_WT_MATRIX
S2_atp6
S2_atp6_IMS
S2_atp6_MATRIX

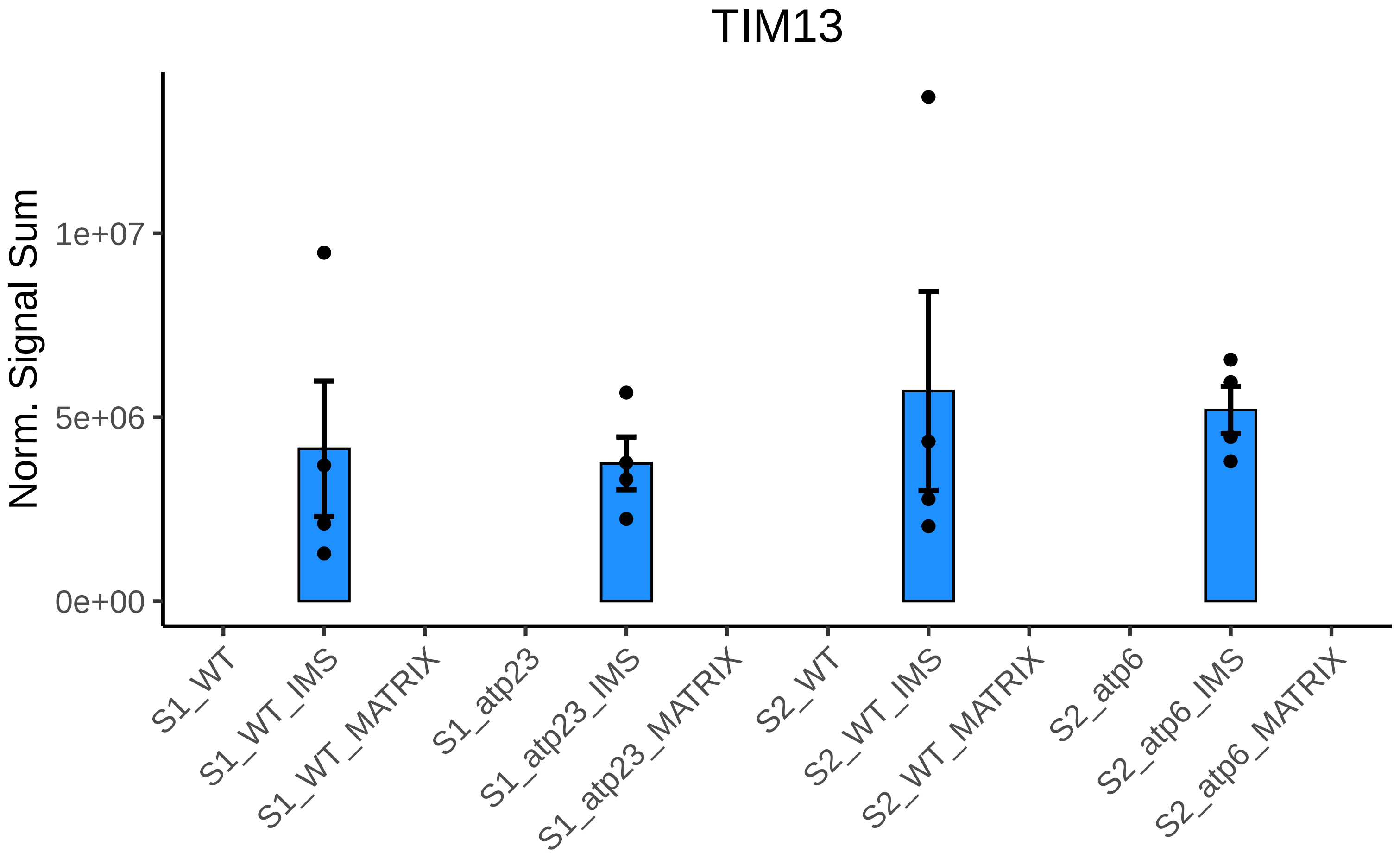
TIM13
Norm. Signal Sum
1e+07
5e+06
0e+00
S1_WT
S1_WT_IMS
S1_WT_MATRIX
S1_atp23
S1_atp23_IMS
S1_atp23_MATRIX
S2_WT
S2_WT_IMS
S2_WT_MATRIX
S2_atp6
S2_atp6_IMS
S2_atp6_MATRIX

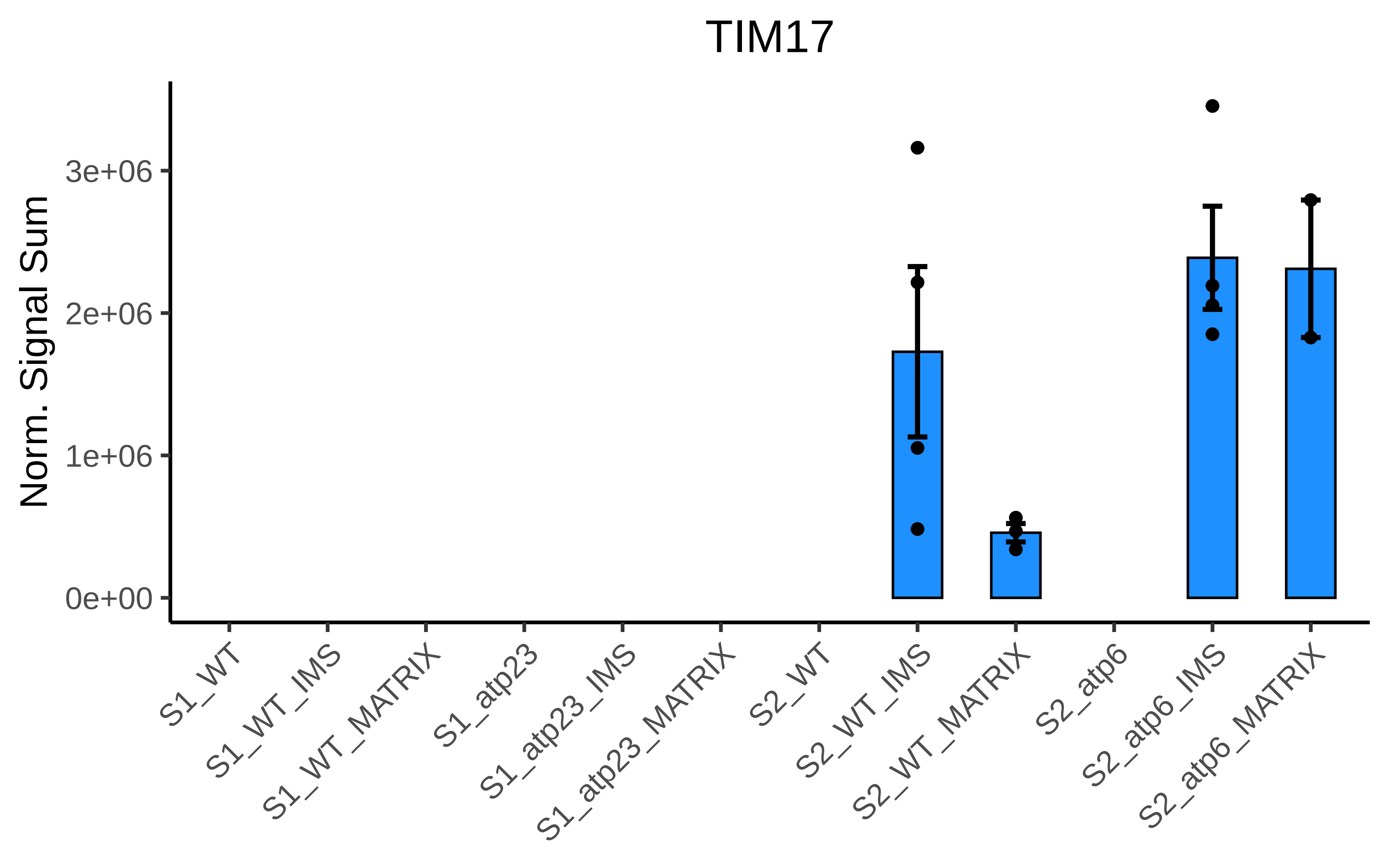
TIM17
Norm. Signal Sum
3e+06
2e+06
1e+06
0e+00
S1_WT
S1_WT_IMS
S1_WT_MATRIX
S1_atp23
S1_atp23_IMS
S1_atp23_MATRIX
S2_WT
S2_WT_IMS
S2_WT_MATRIX
S2_atp6
S2_atp6_IMS
S2_atp6_MATRIX

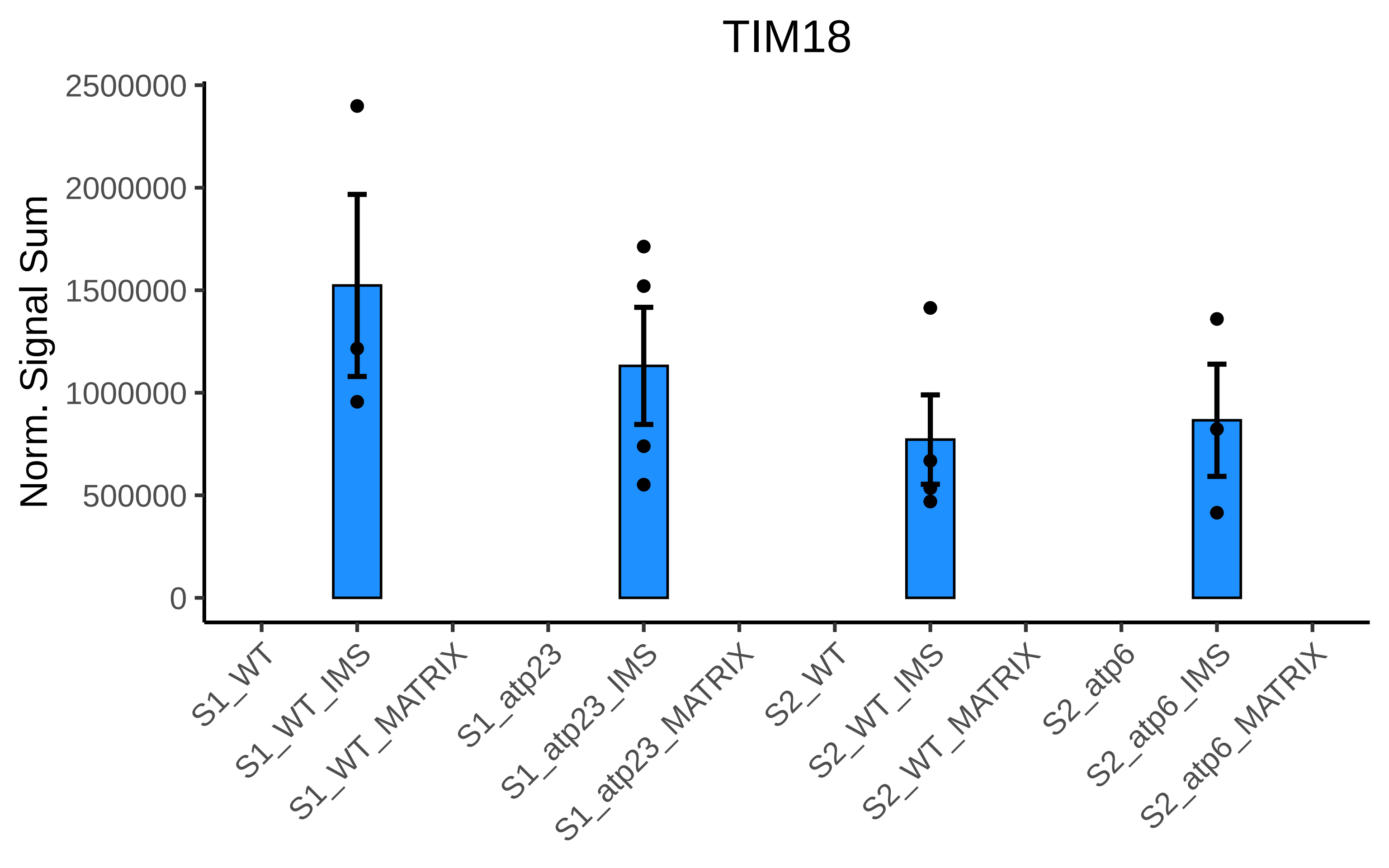

TIM18
Norm. Signal Sum
2500000
2000000
1500000
1000000
500000
0
S1_WT
S1_WT_IMS
S1_WT_MATRIX
S1_atp23
S1_atp23_IMS
S1_atp23_MATRIX
S2_WT
S2_WT_IMS
S2_WT_MATRIX
S2_atp6
S2_atp6_IMS
S2_atp6_MATRIX

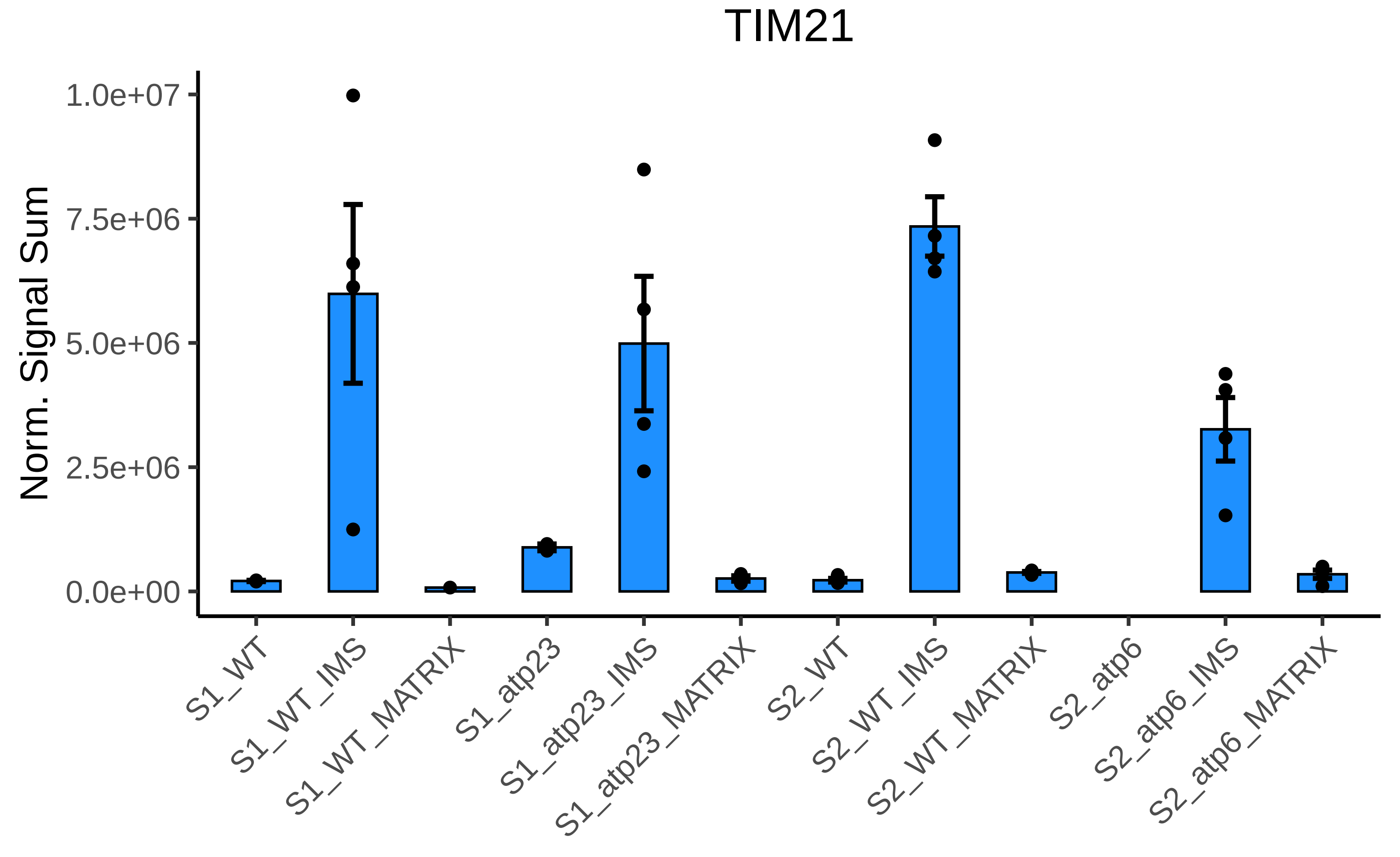
TIM21
Norm. Signal Sum
1.0e+07
7.5e+06
5.0e+06
2.5e+06
0.0e+00
S1_WT
S1_WT_IMS
S1_WT_MATRIX
S1_atp23
S1_atp23_IMS
S1_atp23_MATRIX
S2_WT
S2_WT_IMS
S2_WT_MATRIX
S2_atp6
S2_atp6_IMS
S2_atp6_MATRIX

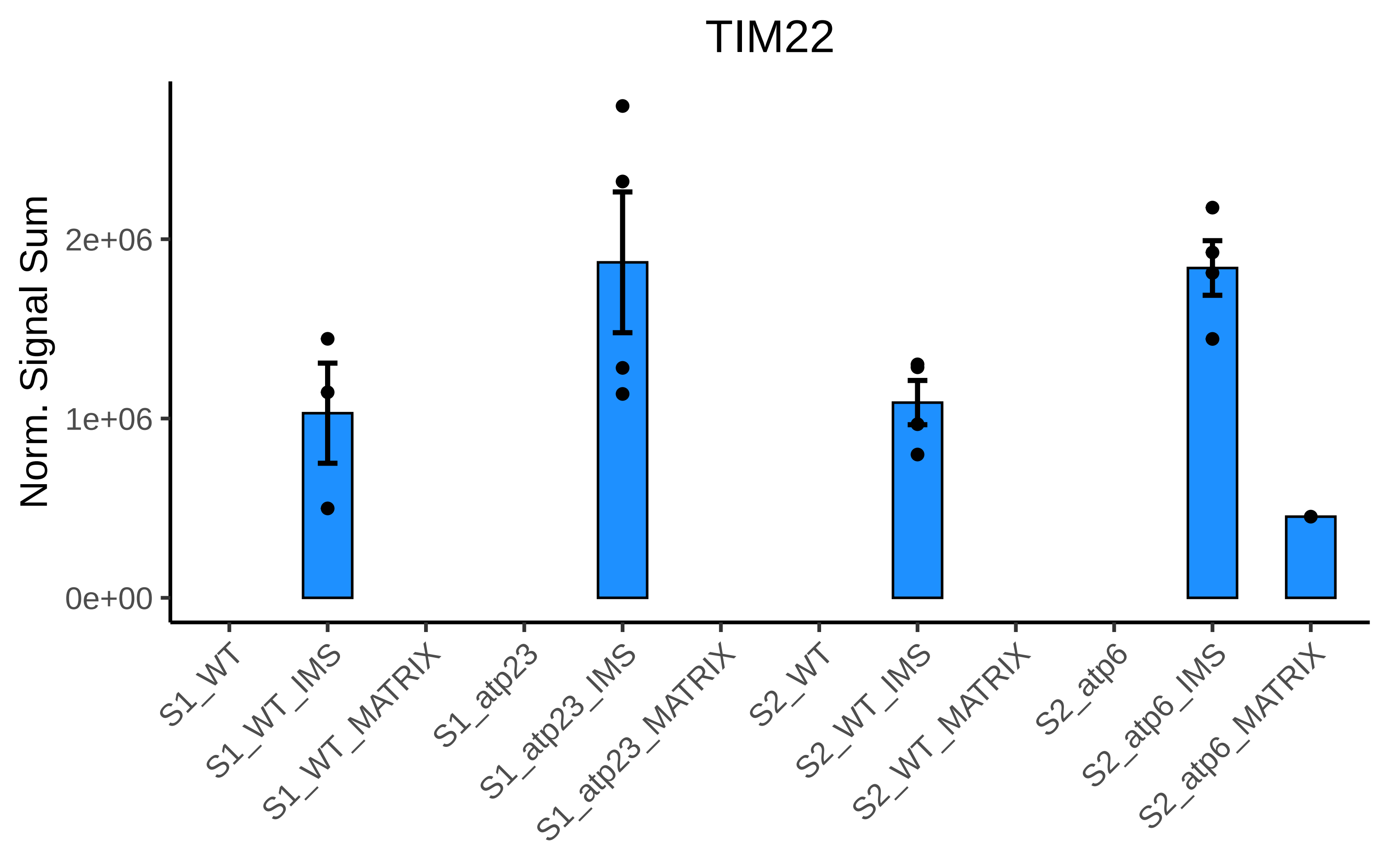

TIM22
Norm. Signal Sum
2e+06
1e+06
0e+00
S1_WT
S1_WT_IMS
S1_WT_MATRIX
S1_atp23
S1_atp23_IMS
S1_atp23_MATRIX
S2_WT
S2_WT_IMS
S2_WT_MATRIX
S2_atp6
S2_atp6_IMS
S2_atp6_MATRIX

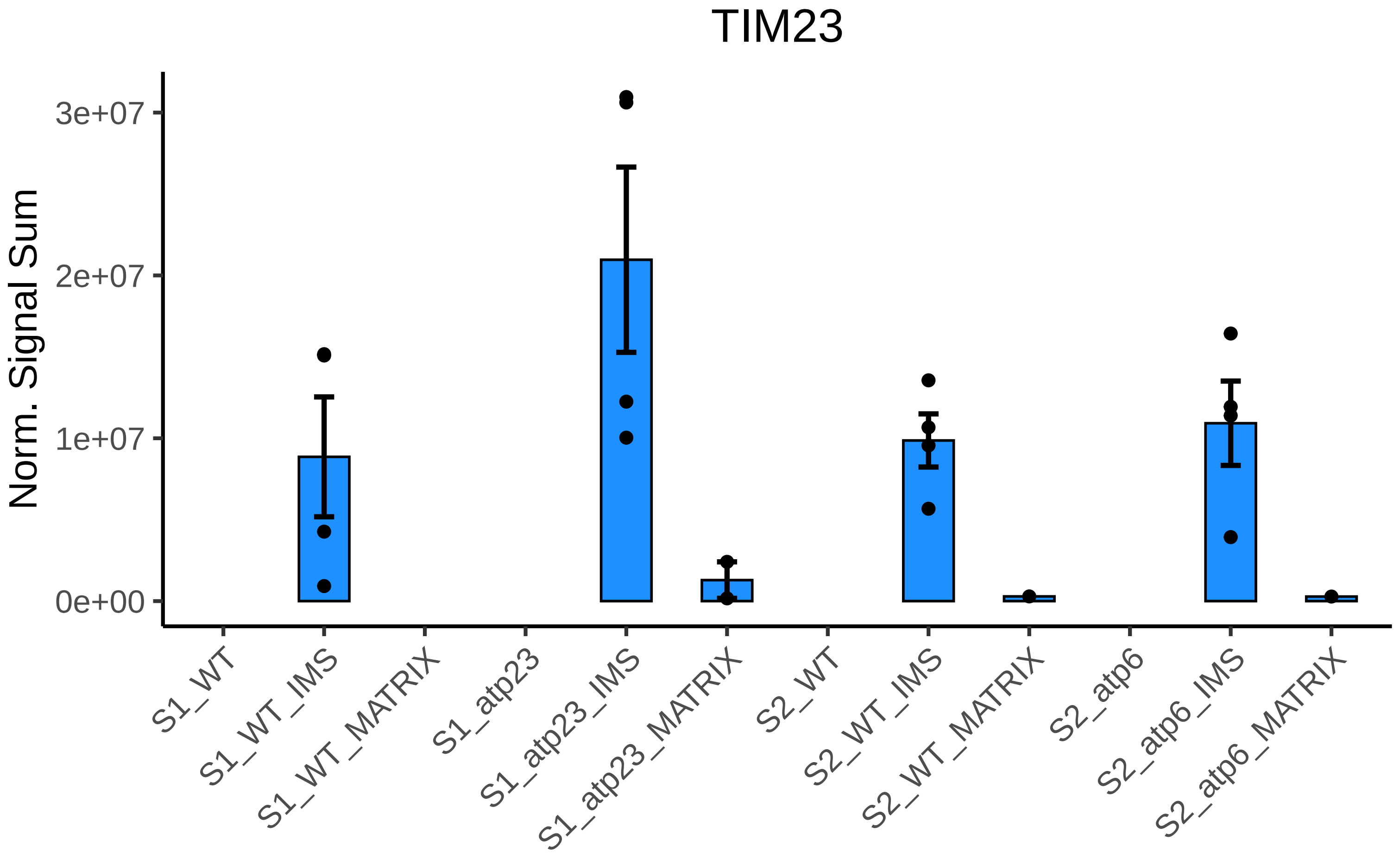

TIM23
Norm. Signal Sum
3e+07
2e+07
1e+07
0e+00
S1_WT
S1_WT_IMS
S1_WT_MATRIX
S1_atp23
S1_atp23_IMS
S1_atp23_MATRIX
S2_WT
S2_WT_IMS
S2_WT_MATRIX
S2_atp6
S2_atp6_IMS
S2_atp6_MATRIX

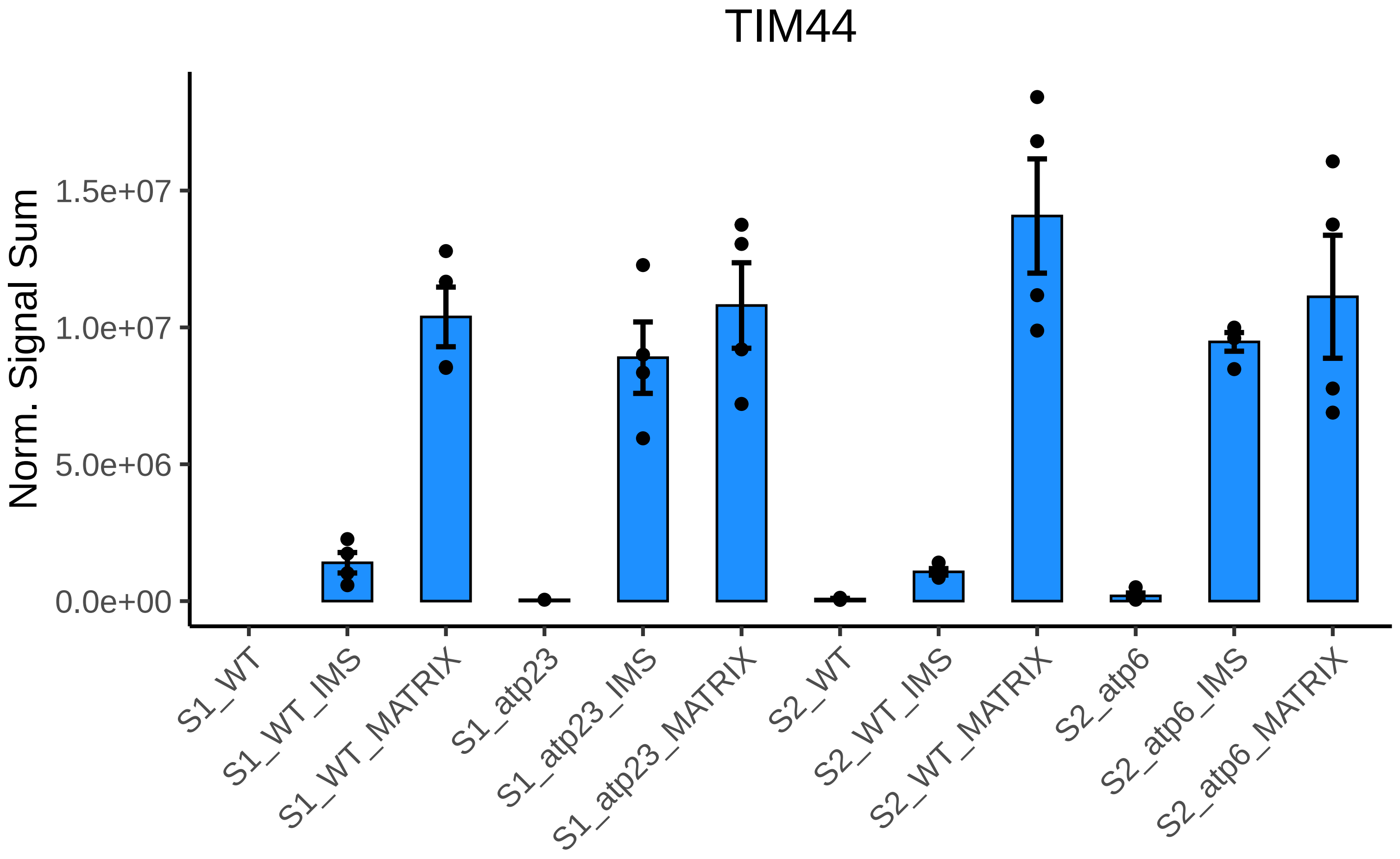
TIM44
Norm. Signal Sum
1.5e+07
1.0e+07
5.0e+06
0.0e+00
S1_WT
S1_WT_IMS
S1_WT_MATRIX
S1_atp23
S1_atp23_IMS
S1_atp23_MATRIX
S2_WT
S2_WT_IMS
S2_WT_MATRIX
S2_atp6
S2_atp6_IMS
S2_atp6_MATRIX

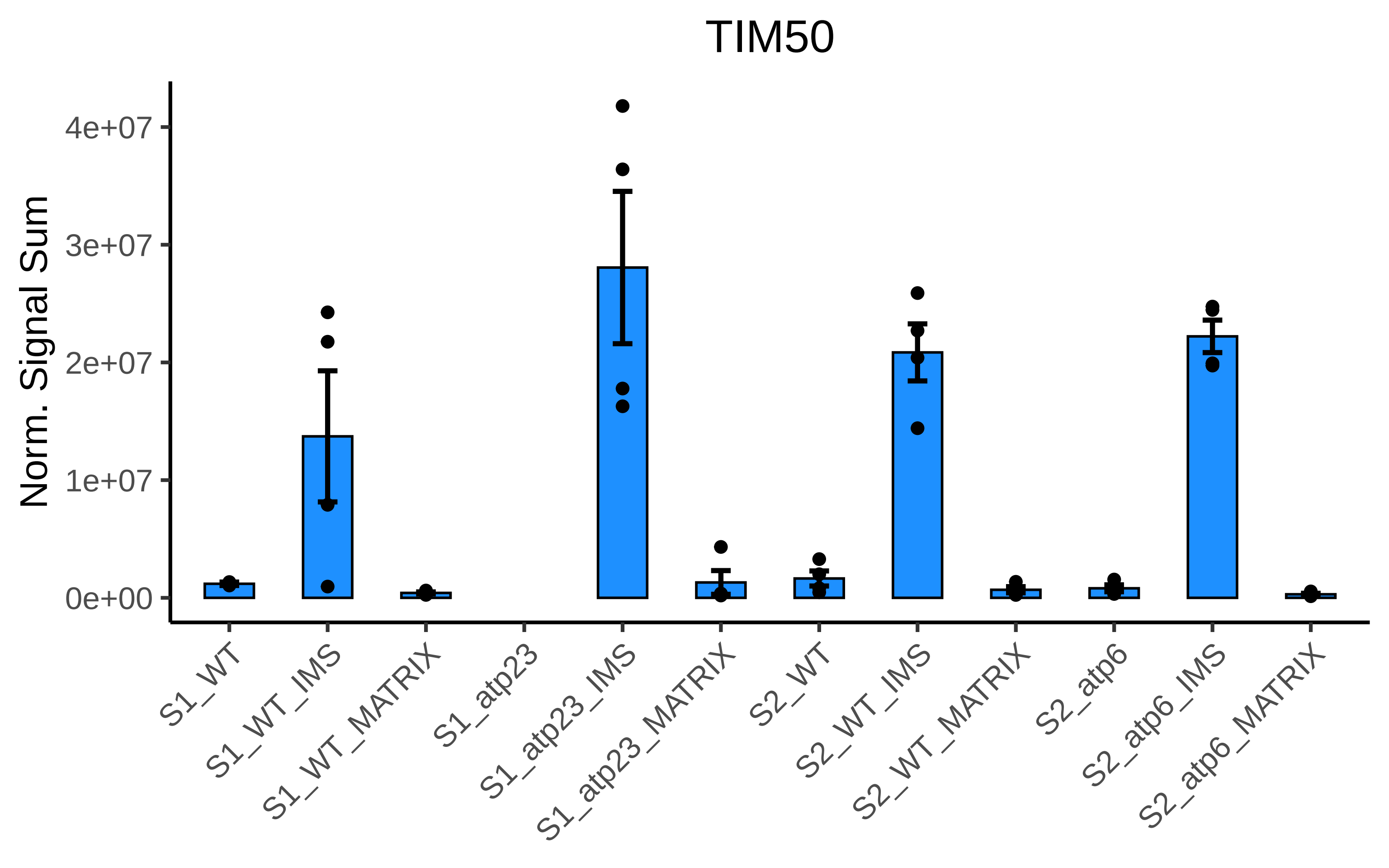

TIM50
Norm. Signal Sum
4e+07
3e+07
2e+07
1e+07
0e+00
S1_WT
S1_WT_IMS
S1_WT_MATRIX
S1_atp23
S1_atp23_IMS
S1_atp23_MATRIX
S2_WT
S2_WT_IMS
S2_WT_MATRIX
S2_atp6
S2_atp6_IMS
S2_atp6_MATRIX

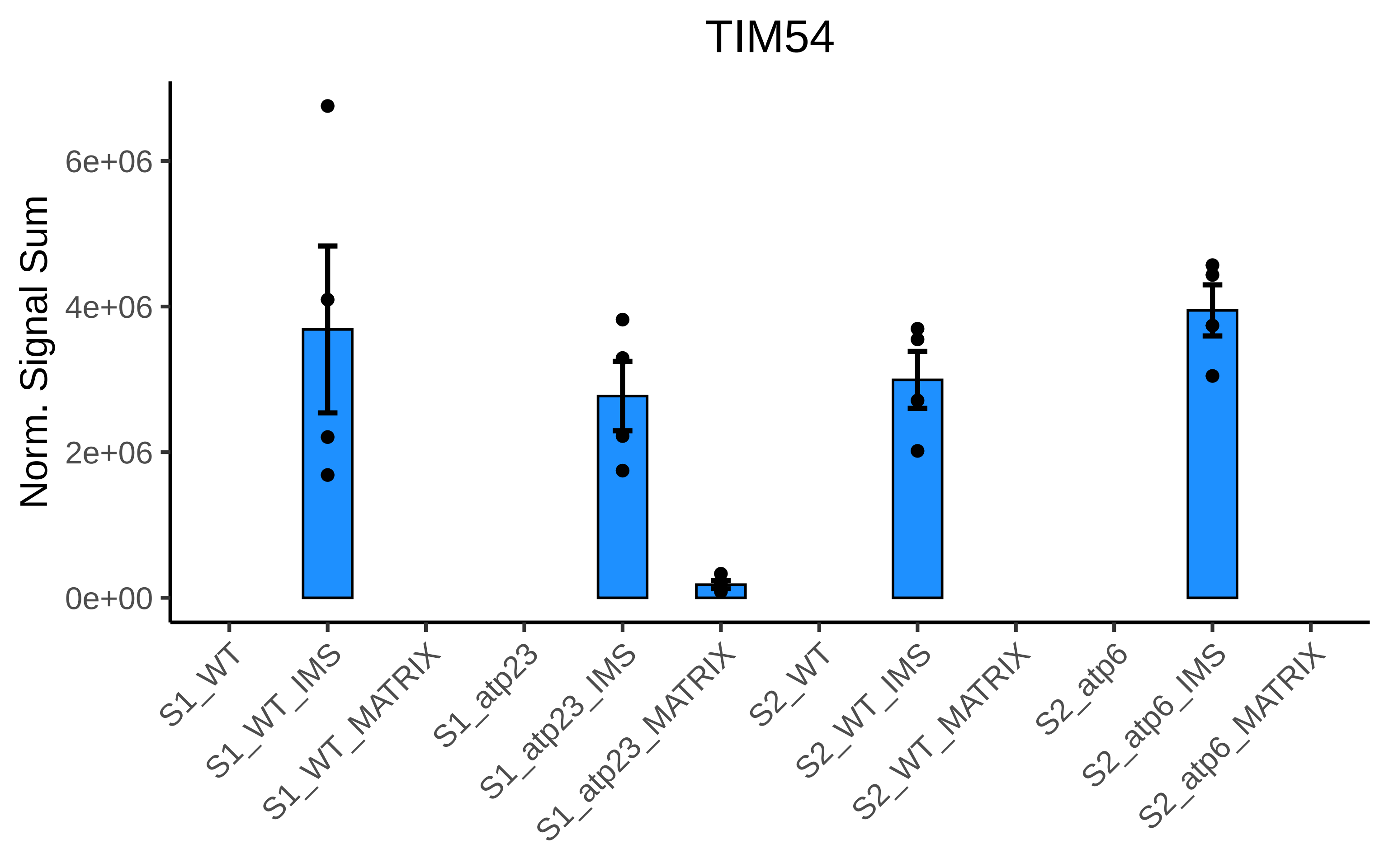

TIM54
Norm. Signal Sum
6e+06
4e+06
2e+06
0e+00
S1_WT
S1_WT_IMS
S1_WT_MATRIX
S1_atp23
S1_atp23_IMS
S1_atp23_MATRIX
S2_WT
S2_WT_IMS
S2_WT_MATRIX
S2_atp6
S2_atp6_IMS
S2_atp6_MATRIX

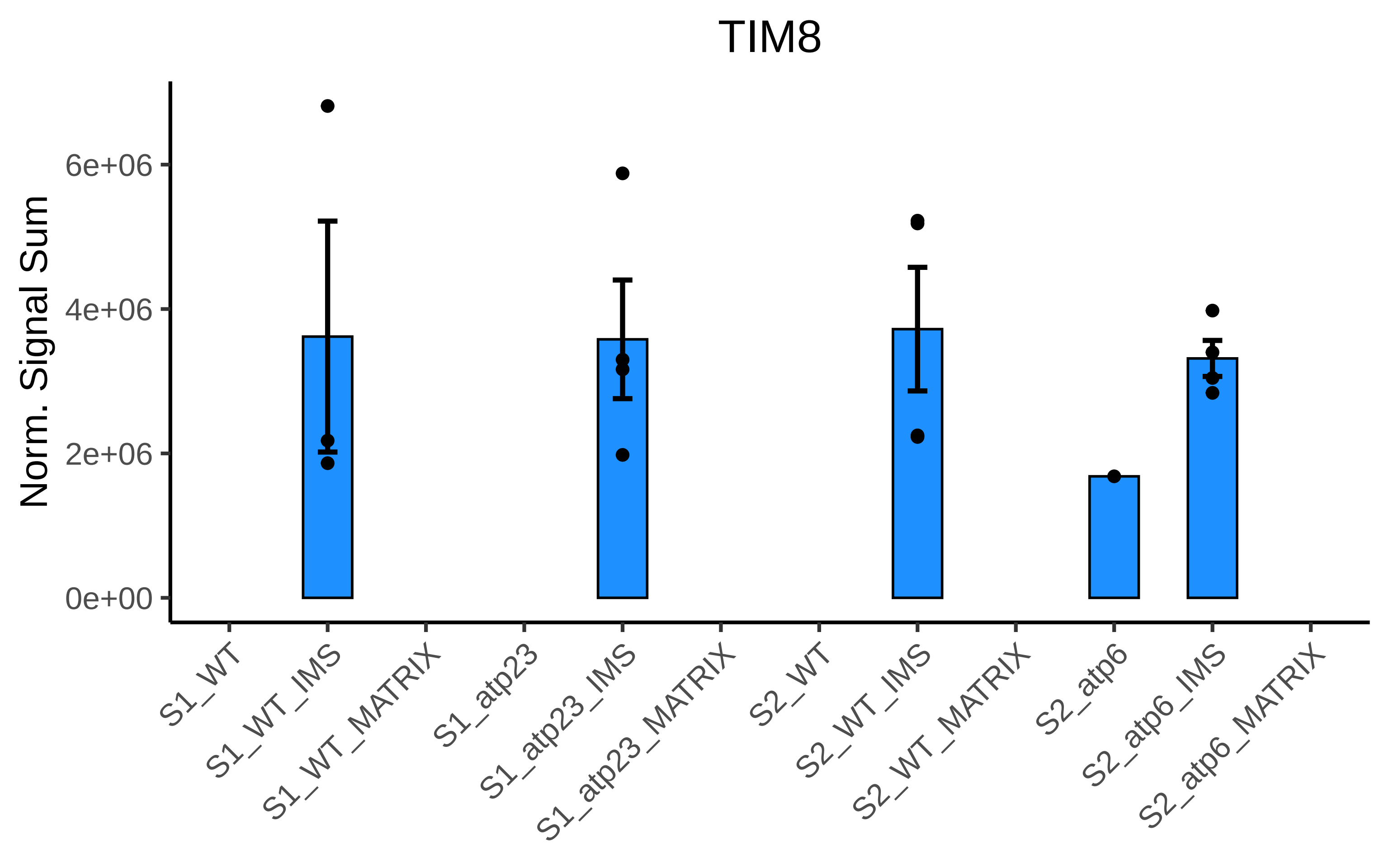
TIM8
Norm. Signal Sum
6e+06
4e+06
2e+06
0e+00
S1_WT
S1_WT_IMS
S1_WT_MATRIX
S1_atp23
S1_atp23_IMS
S1_atp23_MATRIX
S2_WT
S2_WT_IMS
S2_WT_MATRIX
S2_atp6
S2_atp6_IMS
S2_atp6_MATRIX

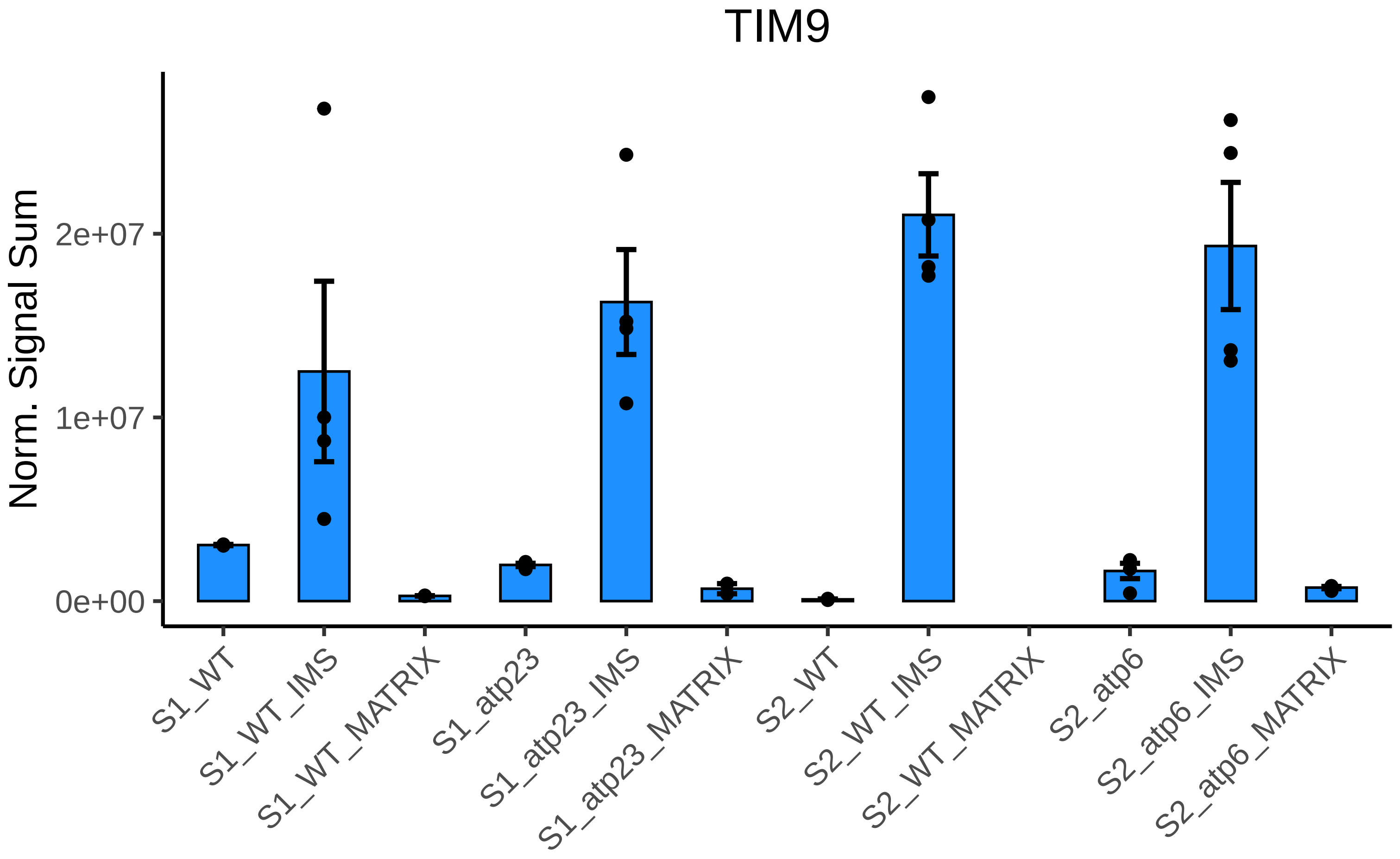

TIM9
Norm. Signal Sum
2e+07
1e+07
0e+00
S1_WT
S1_WT_IMS
S1_WT_MATRIX
S1_atp23
S1_atp23_IMS
S1_atp23_MATRIX
S2_WT
S2_WT_IMS
S2_WT_MATRIX
S2_atp6
S2_atp6_IMS
S2_atp6_MATRIX

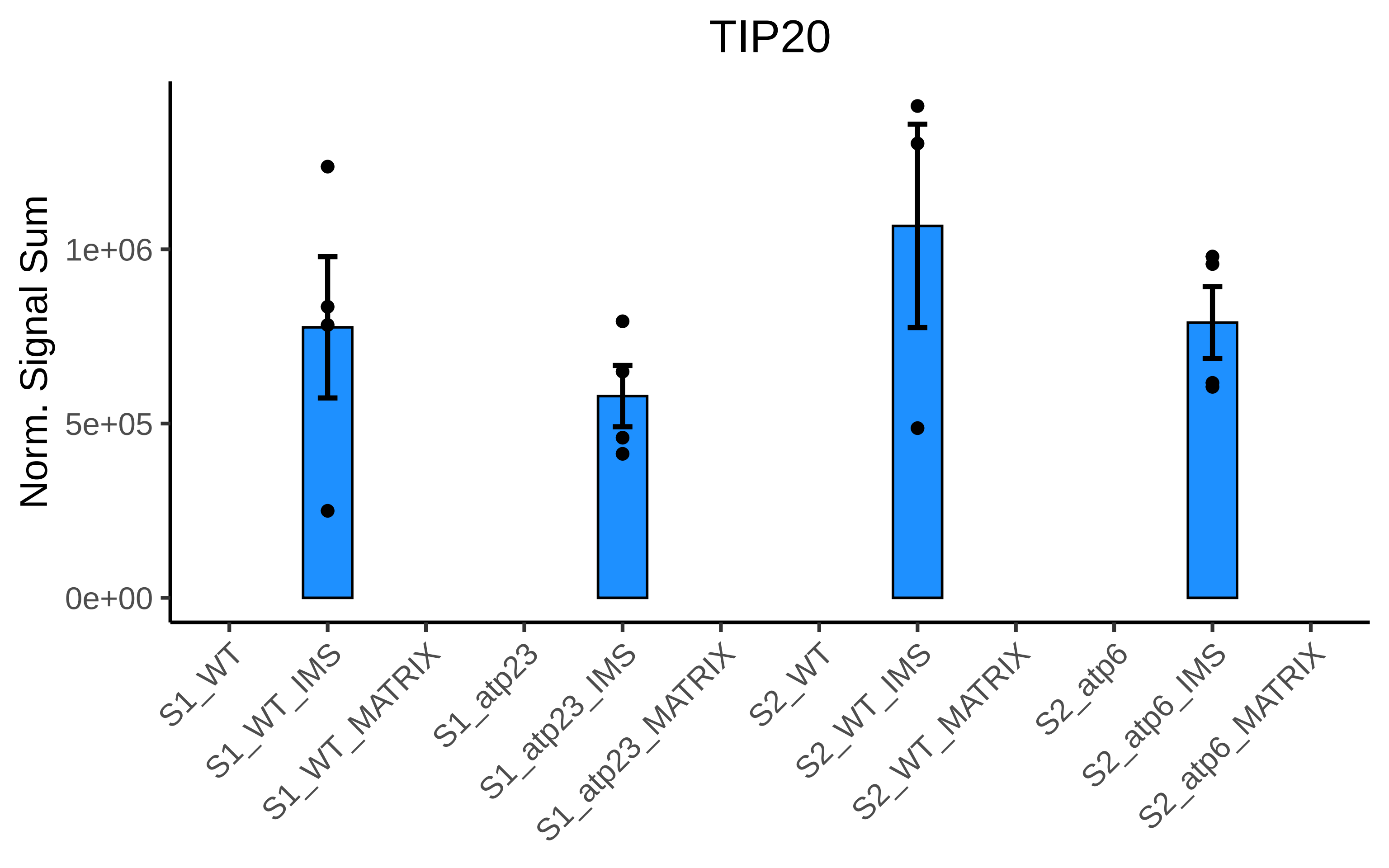
TIP20
Norm. Signal Sum
1e+06
5e+05
0e+00
S1_WT
S1_WT_IMS
S1_WT_MATRIX
S1_atp23
S1_atp23_IMS
S1_atp23_MATRIX
S2_WT
S2_WT_IMS
S2_WT_MATRIX
S2_atp6
S2_atp6_IMS
S2_atp6_MATRIX

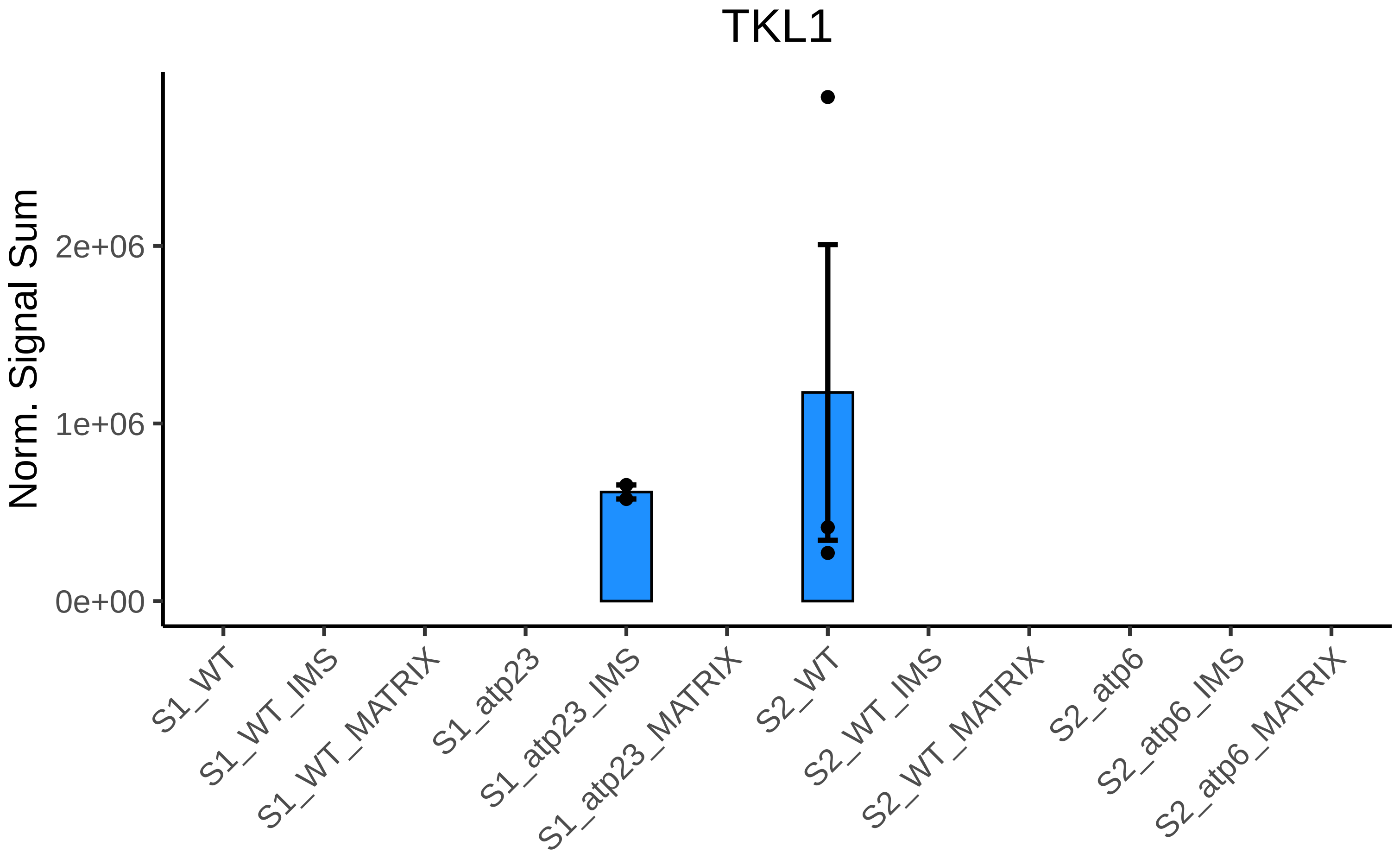

TKL1
Norm. Signal Sum
2e+06
1e+06
0e+00
S1_WT
S1_WT_IMS
S1_WT_MATRIX
S1_atp23
S1_atp23_IMS
S1_atp23_MATRIX
S2_WT
S2_WT_IMS
S2_WT_MATRIX
S2_atp6
S2_atp6_IMS
S2_atp6_MATRIX

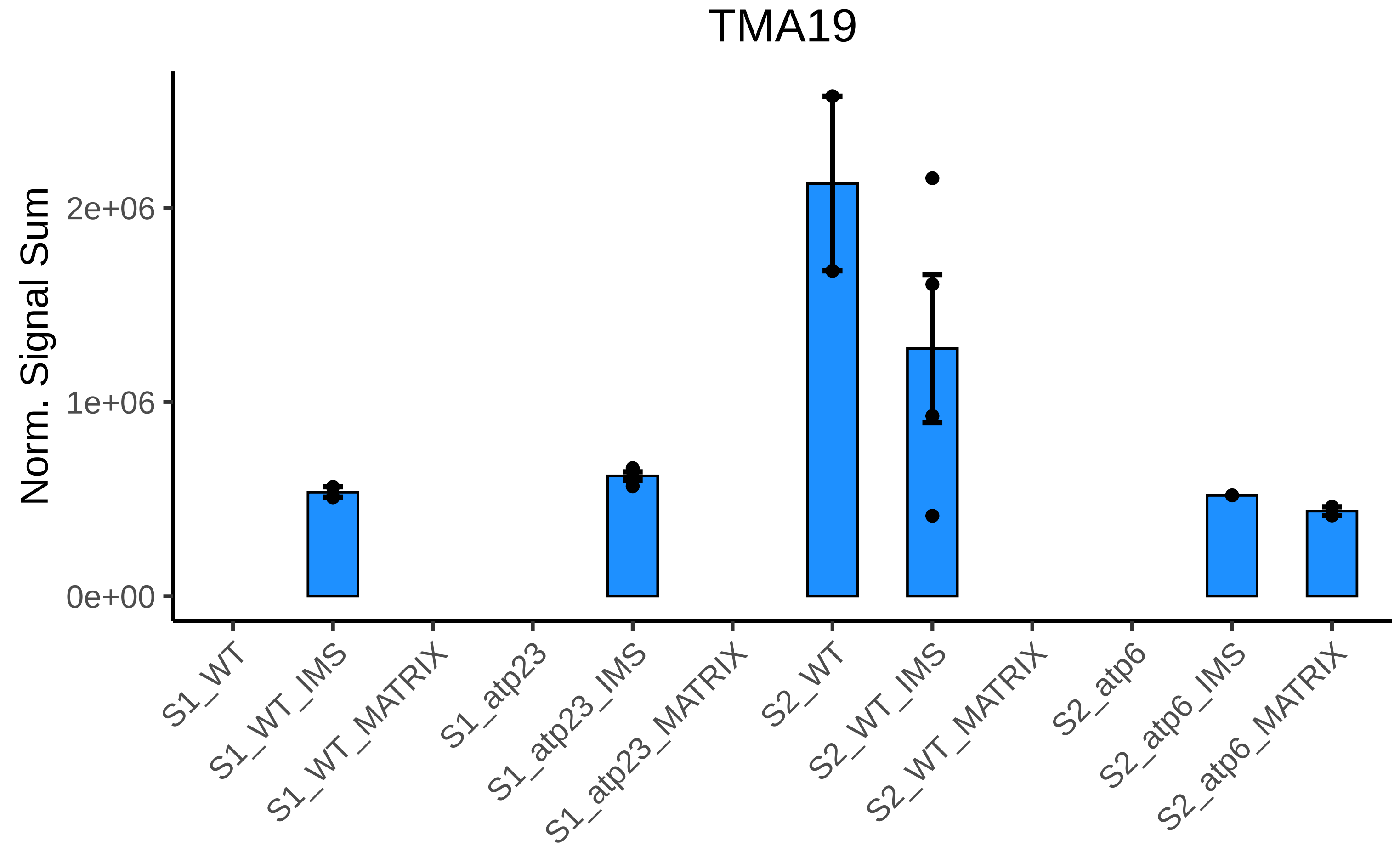

TMA19
Norm. Signal Sum
2e+06
1e+06
0e+00
S1_WT
S1_WT_IMS
S1_WT_MATRIX
S1_atp23
S1_atp23_IMS
S1_atp23_MATRIX
S2_WT
S2_WT_IMS
S2_WT_MATRIX
S2_atp6
S2_atp6_IMS
S2_atp6_MATRIX

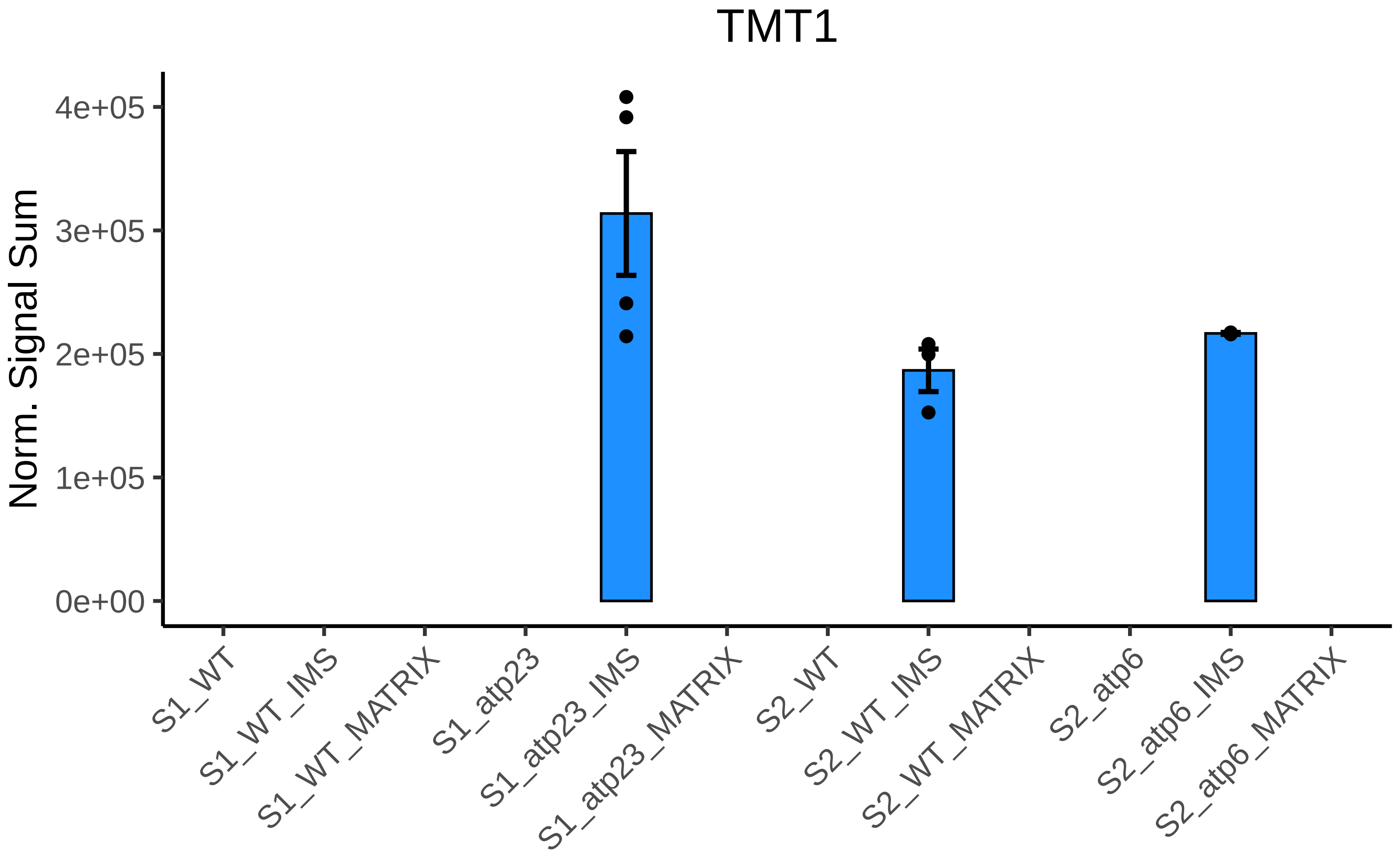
TMT1
Norm. Signal Sum
4e+05
3e+05
2e+05
1e+05
0e+00
S1_WT
S1_WT_IMS
S1_WT_MATRIX
S1_atp23
S1_atp23_IMS
S1_atp23_MATRIX
S2_WT
S2_WT_IMS
S2_WT_MATRIX
S2_atp6
S2_atp6_IMS
S2_atp6_MATRIX

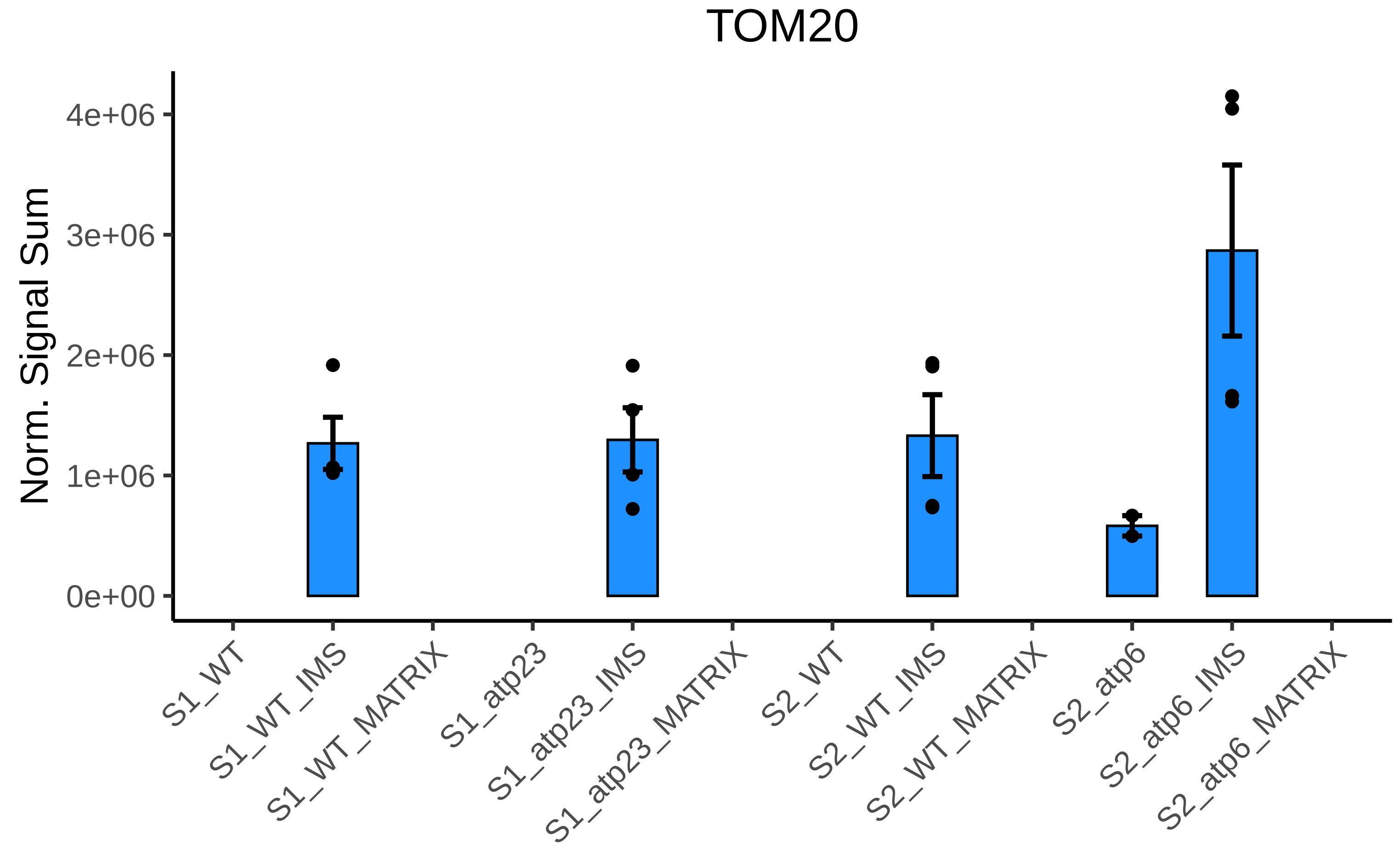
TOM20
Norm. Signal Sum
4e+06
3e+06
2e+06
1e+06
0e+00
S1_WT
S1_WT_IMS
S1_WT_MATRIX
S1_atp23
S1_atp23_IMS
S1_atp23_MATRIX
S2_WT
S2_WT_IMS
S2_WT_MATRIX
S2_atp6
S2_atp6_IMS
S2_atp6_MATRIX

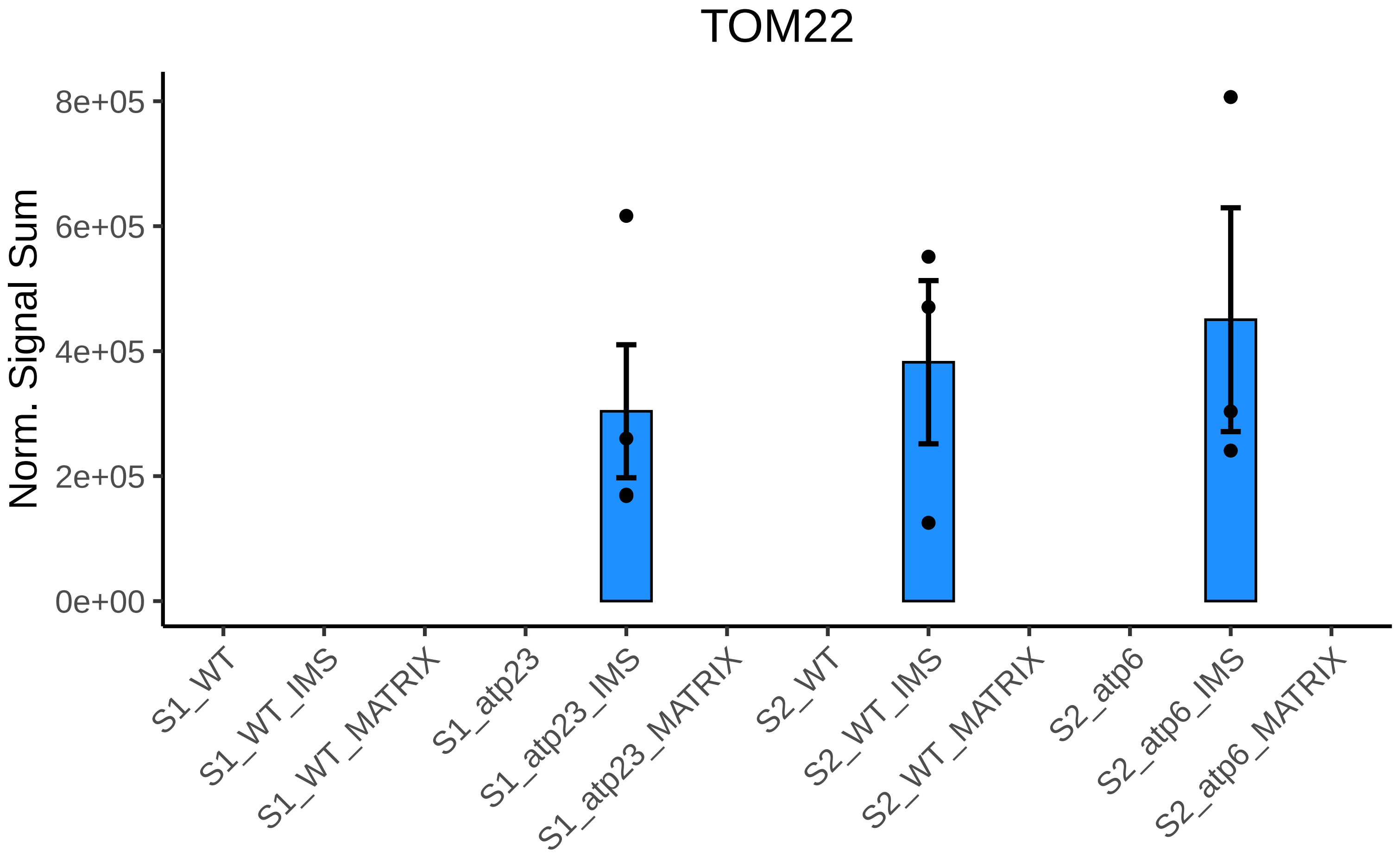
TOM22
Norm. Signal Sum
8e+05
6e+05
4e+05
2e+05
0e+00
S1_WT
S1_WT_IMS
S1_WT_MATRIX
S1_atp23
S1_atp23_IMS
S1_atp23_MATRIX
S2_WT
S2_WT_IMS
S2_WT_MATRIX
S2_atp6
S2_atp6_IMS
S2_atp6_MATRIX

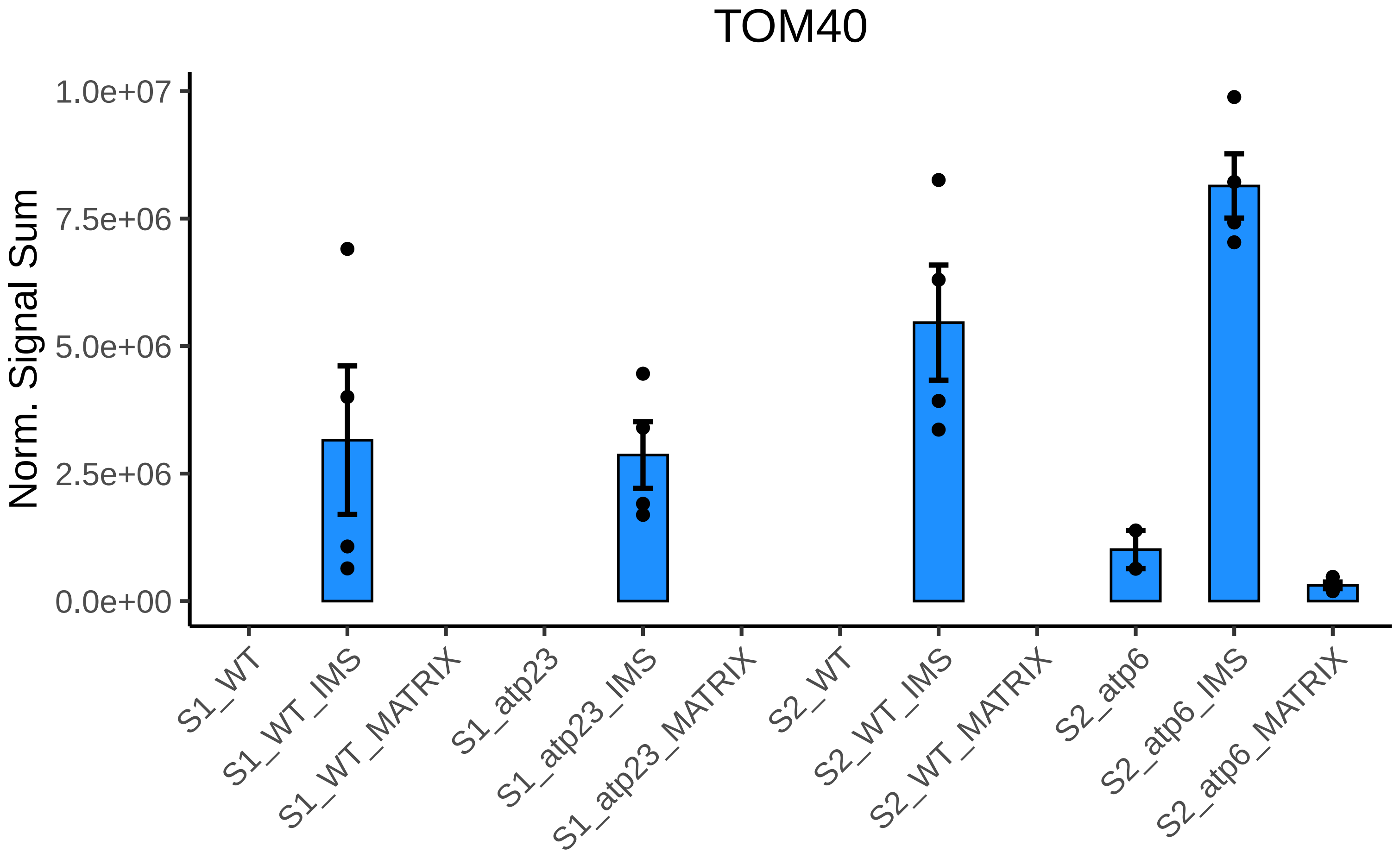

TOM40
Norm. Signal Sum
1.0e+07
7.5e+06
5.0e+06
2.5e+06
0.0e+00
S1_WT
S1_WT_IMS
S1_WT_MATRIX
S1_atp23
S1_atp23_IMS
S1_atp23_MATRIX
S2_WT
S2_WT_IMS
S2_WT_MATRIX
S2_atp6
S2_atp6_IMS
S2_atp6_MATRIX

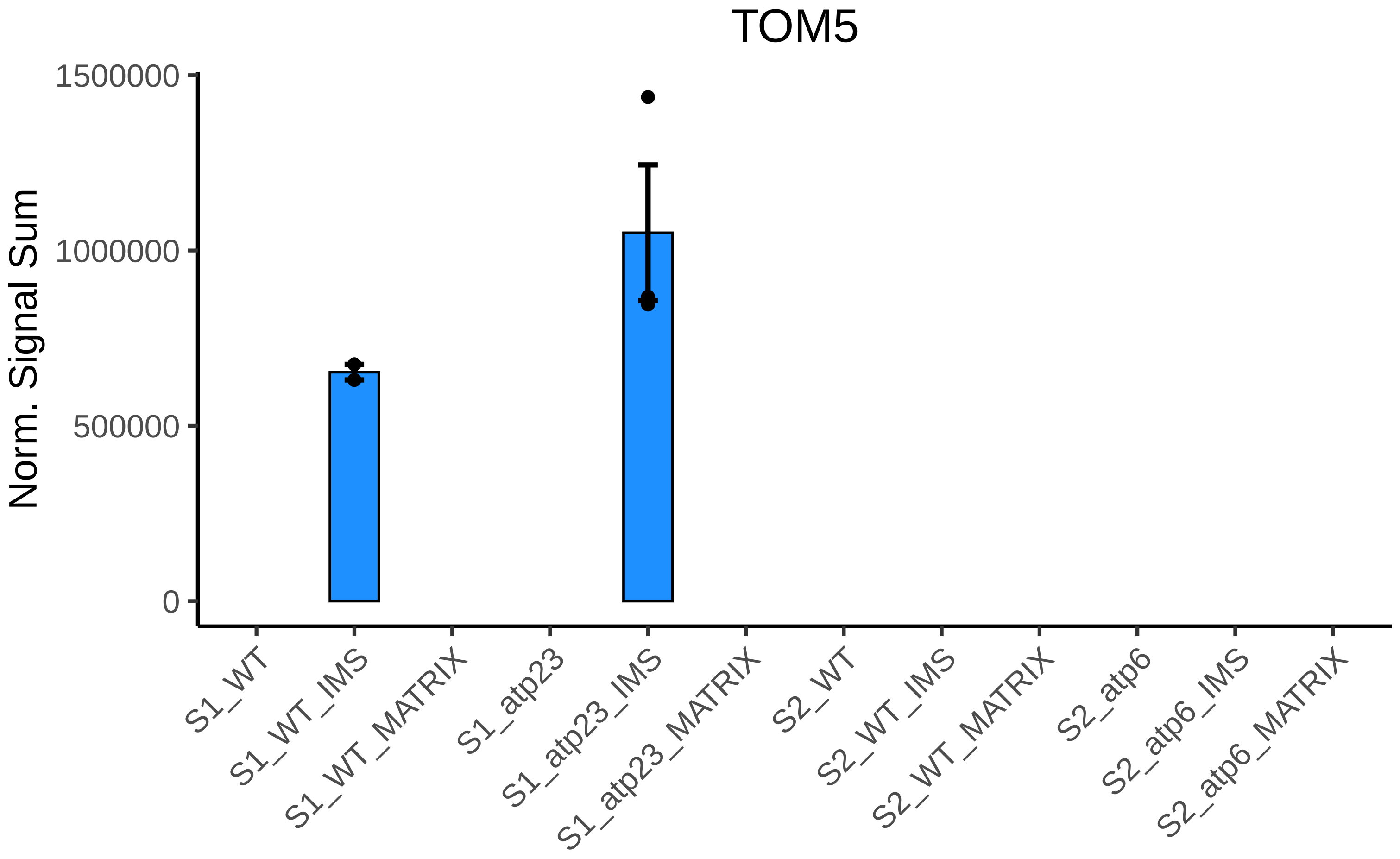

TOM5
Norm. Signal Sum
1500000
1000000
500000
0
S1_WT
S1_WT_IMS
S1_WT_MATRIX
S1_atp23
S1_atp23_IMS
S1_atp23_MATRIX
S2_WT
S2_WT_IMS
S2_WT_MATRIX
S2_atp6
S2_atp6_IMS
S2_atp6_MATRIX

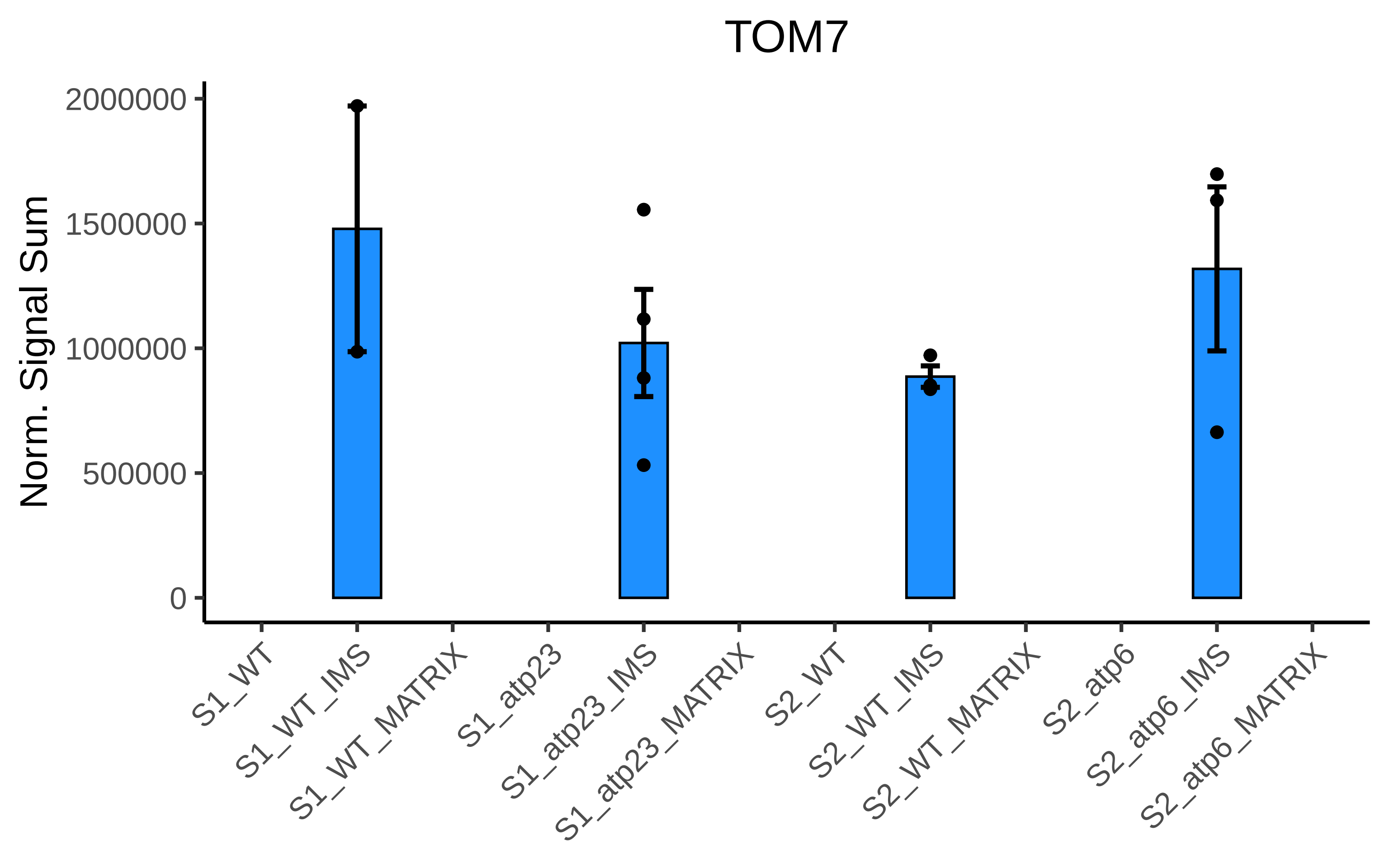

TOM7
Norm. Signal Sum
2000000
1500000
1000000
500000
0
S1_WT
S1_WT_IMS
S1_WT_MATRIX
S1_atp23
S1_atp23_IMS
S1_atp23_MATRIX
S2_WT
S2_WT_IMS
S2_WT_MATRIX
S2_atp6
S2_atp6_IMS
S2_atp6_MATRIX

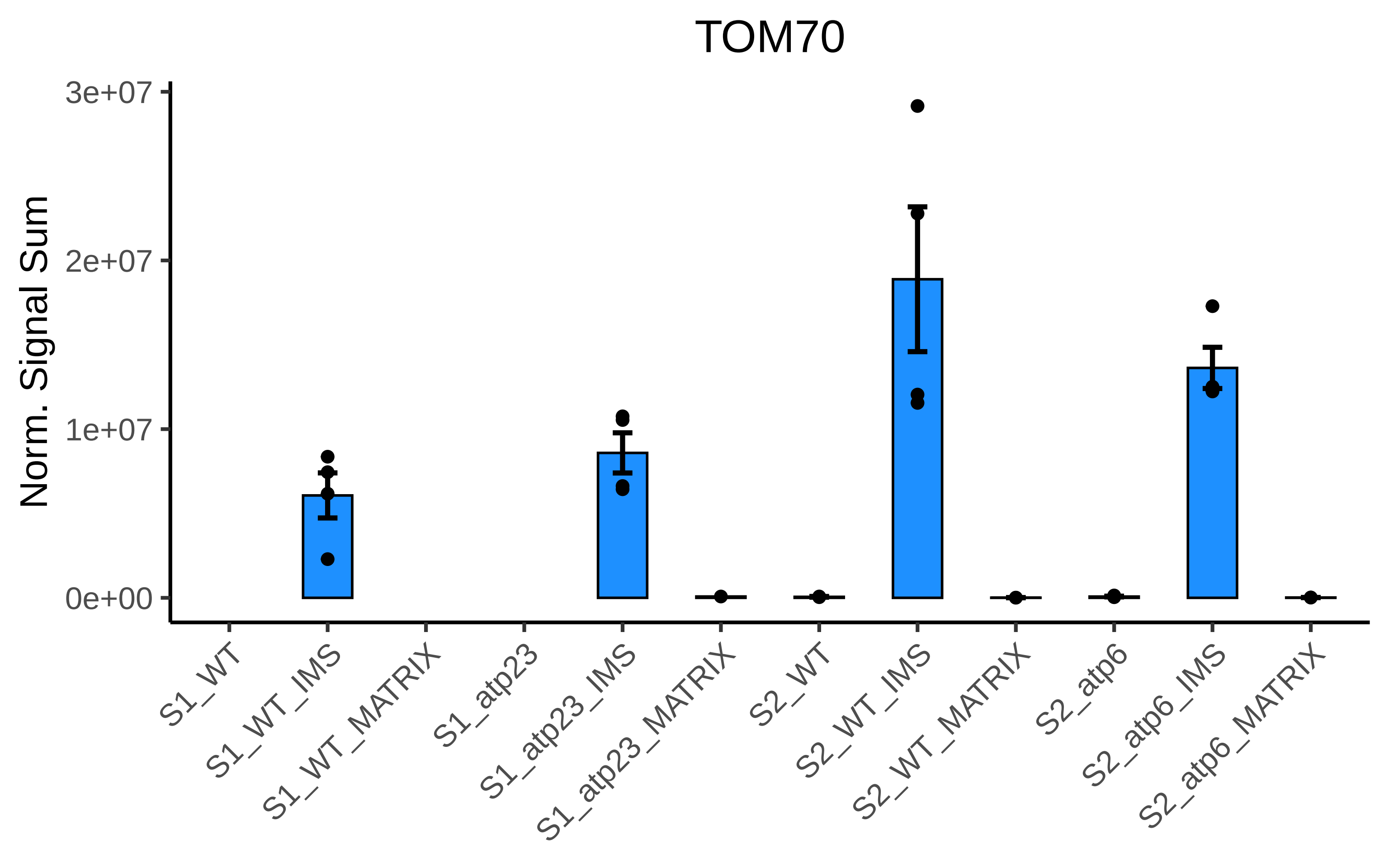

TOM70
Norm. Signal Sum
3e+07
2e+07
1e+07
0e+00
S1_WT
S1_WT_IMS
S1_WT_MATRIX
S1_atp23
S1_atp23_IMS
S1_atp23_MATRIX
S2_WT
S2_WT_IMS
S2_WT_MATRIX
S2_atp6
S2_atp6_IMS
S2_atp6_MATRIX

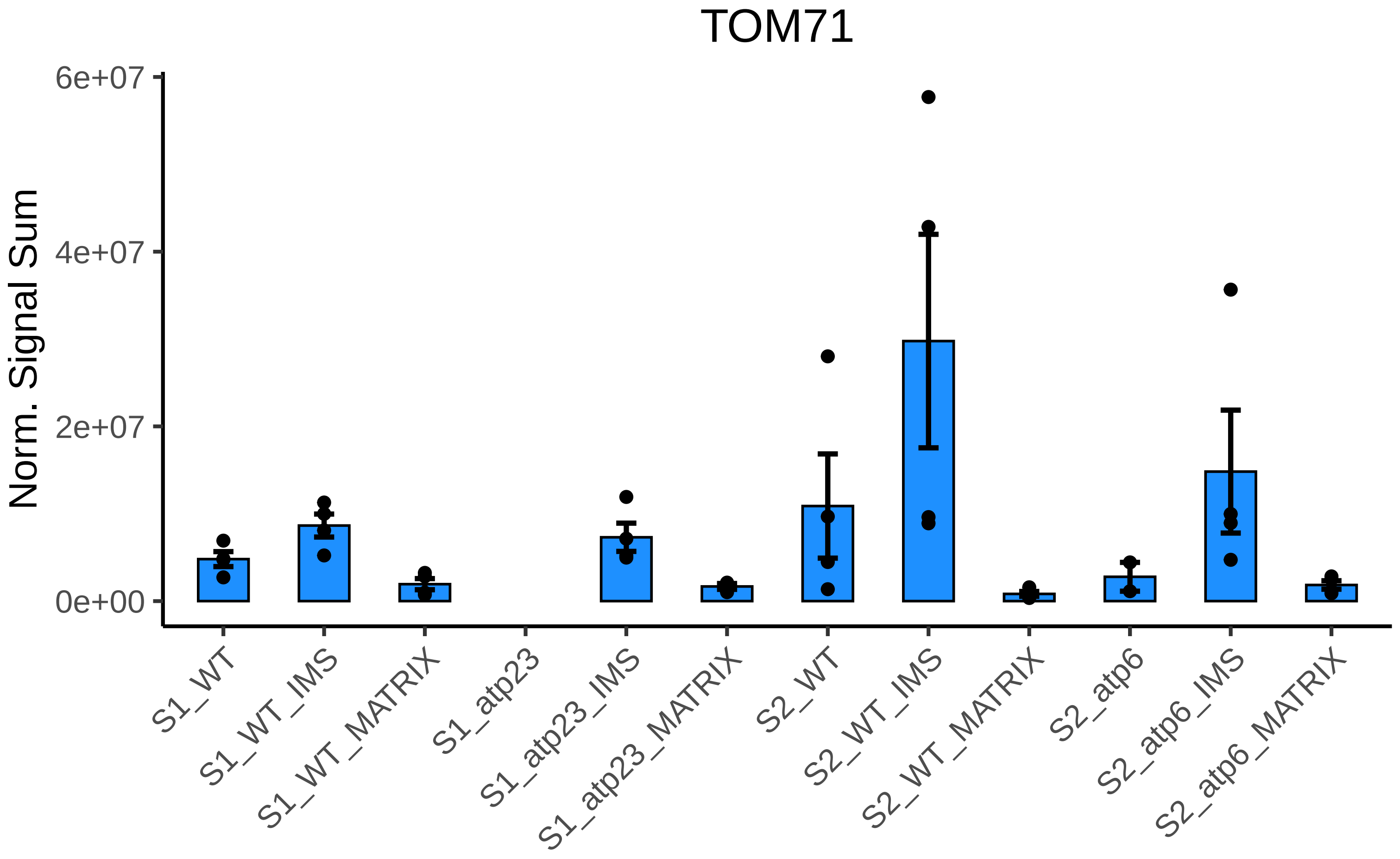

TOM71
Norm. Signal Sum
6e+07
4e+07
2e+07
0e+00
S1_WT
S1_WT_IMS
S1_WT_MATRIX
S1_atp23
S1_atp23_IMS
S1_atp23_MATRIX
S2_WT
S2_WT_IMS
S2_WT_MATRIX
S2_atp6
S2_atp6_IMS
S2_atp6_MATRIX

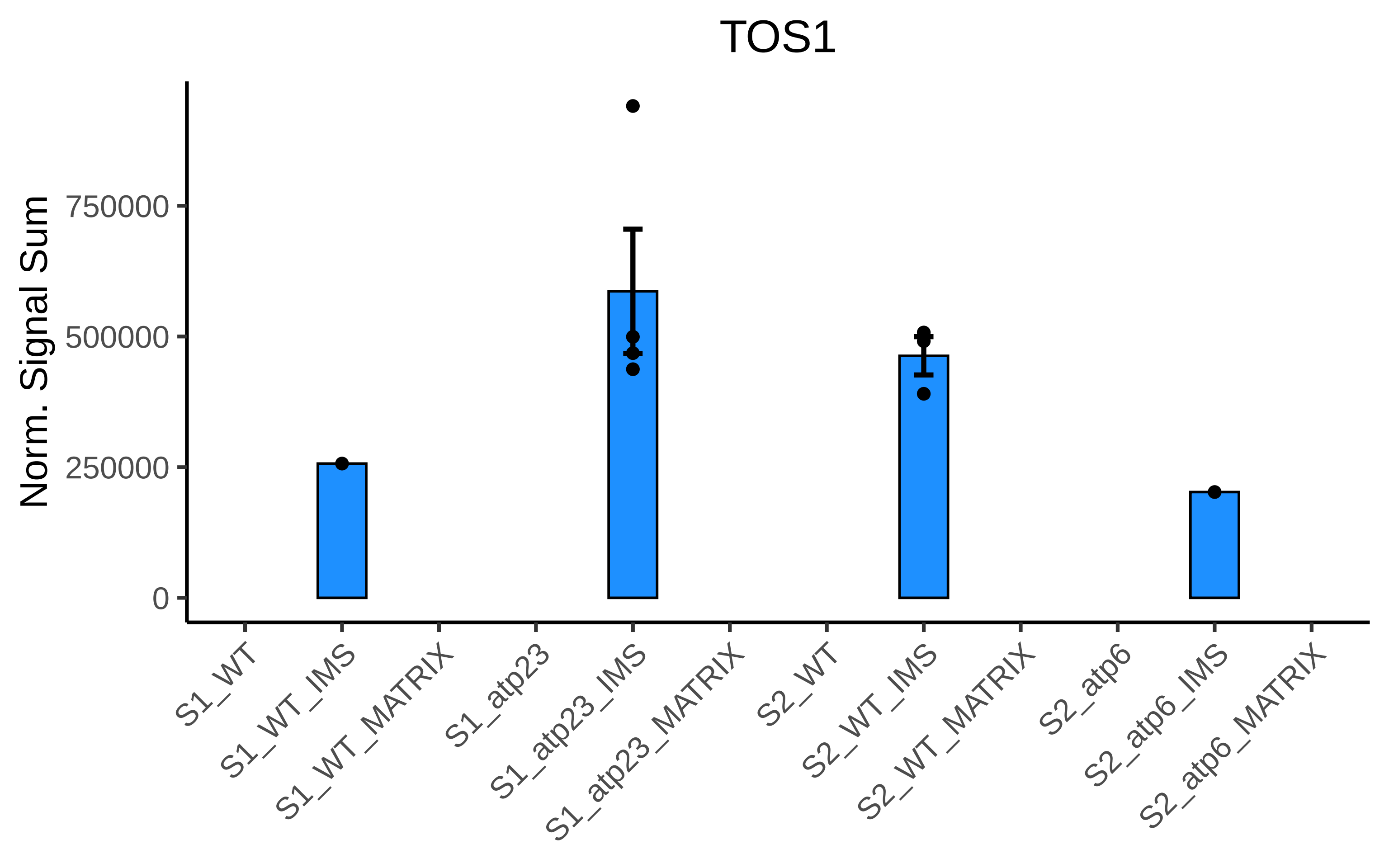
TOS1
Norm. Signal Sum
750000
500000
250000
0
S1_WT
S1_WT_IMS
S1_WT_MATRIX
S1_atp23
S1_atp23_IMS
S1_atp23_MATRIX
S2_WT
S2_WT_IMS
S2_WT_MATRIX
S2_atp6
S2_atp6_IMS
S2_atp6_MATRIX

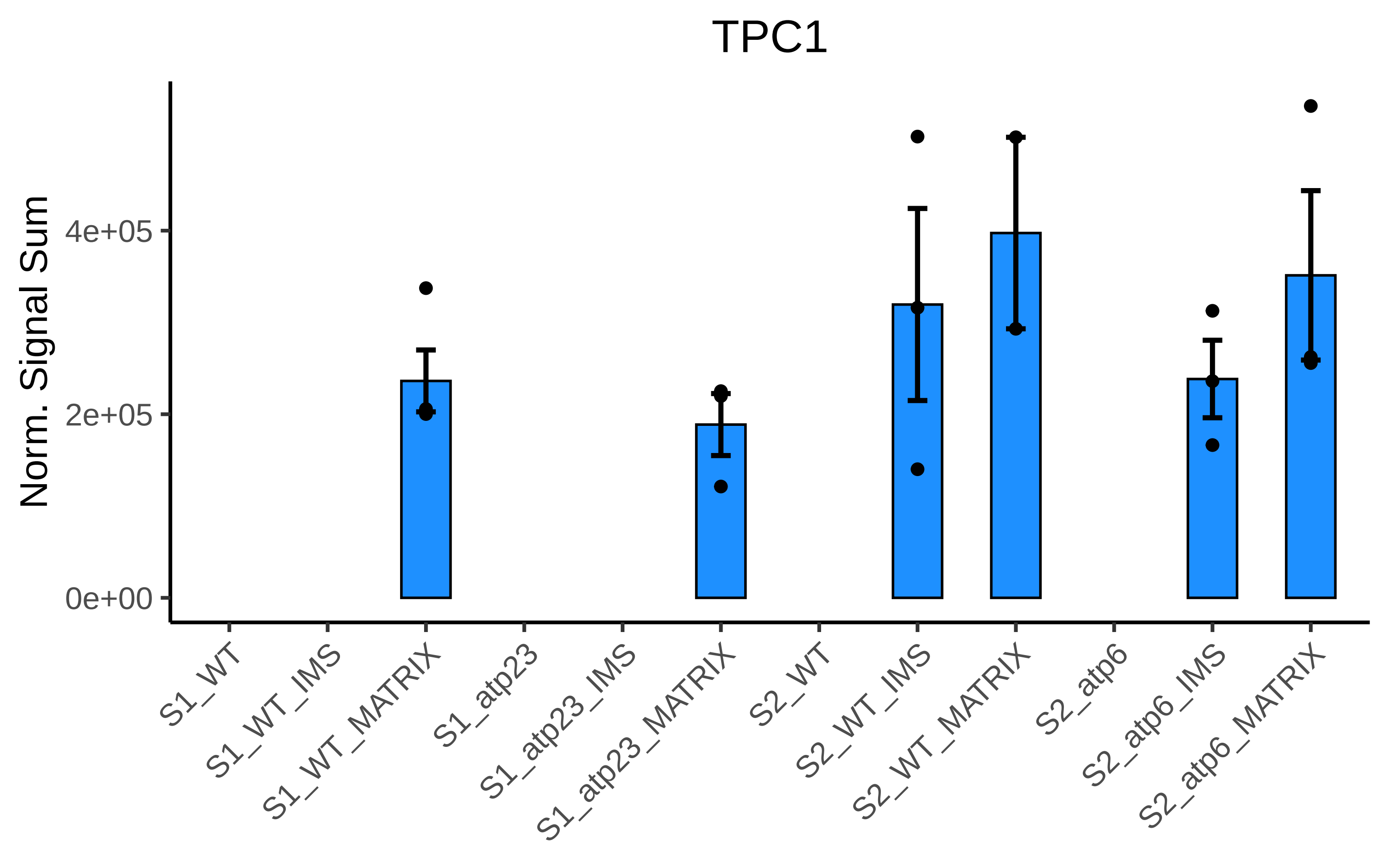
TPC1
Norm. Signal Sum
4e+05
2e+05
0e+00
S1_WT
S1_WT_IMS
S1_WT_MATRIX
S1_atp23
S1_atp23_IMS
S1_atp23_MATRIX
S2_WT
S2_WT_IMS
S2_WT_MATRIX
S2_atp6
S2_atp6_IMS
S2_atp6_MATRIX

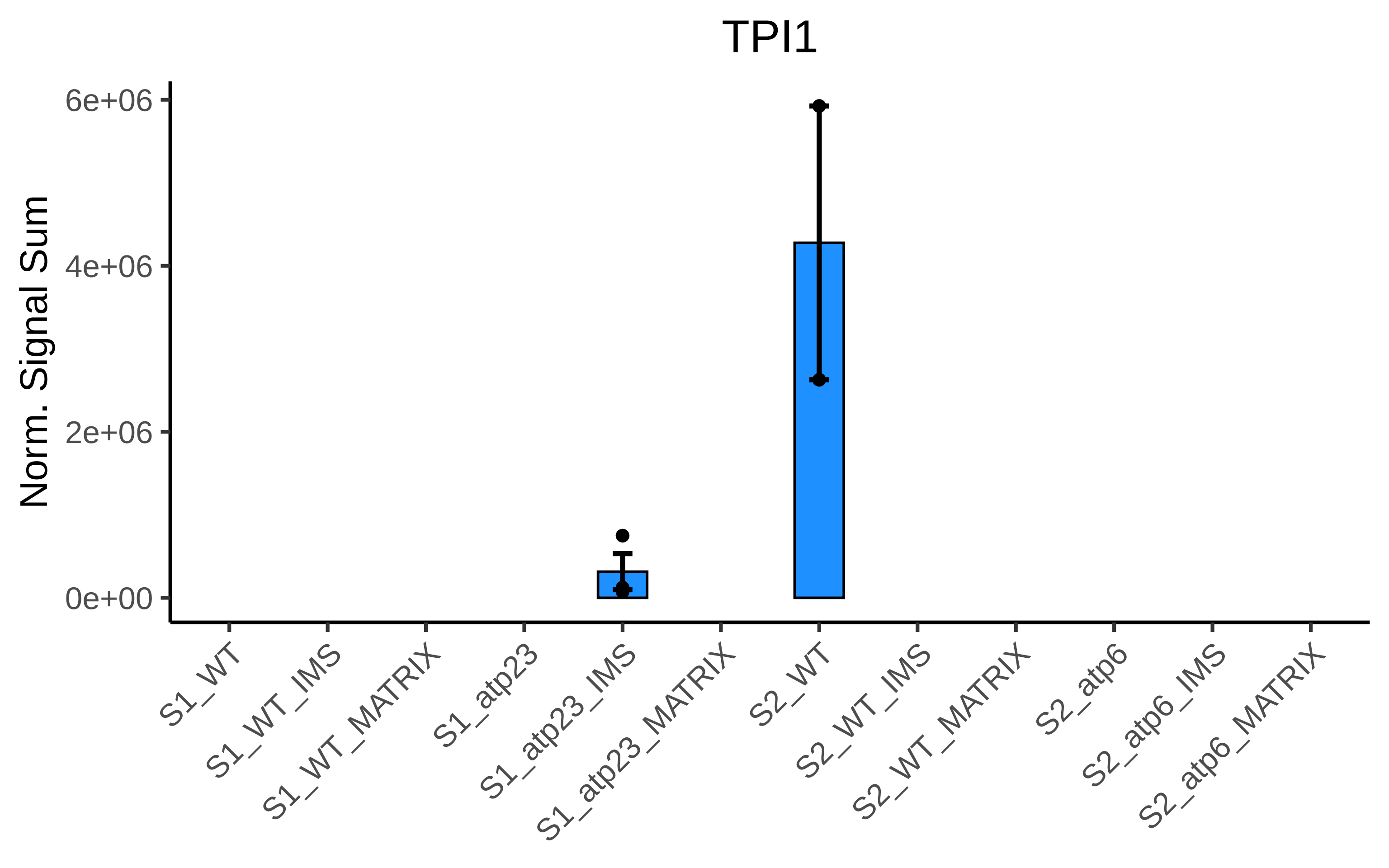

TPI1
Norm. Signal Sum
6e+06
4e+06
2e+06
0e+00
S1_WT
S1_WT_IMS
S1_WT_MATRIX
S1_atp23
S1_atp23_IMS
S1_atp23_MATRIX
S2_WT
S2_WT_IMS
S2_WT_MATRIX
S2_atp6
S2_atp6_IMS
S2_atp6_MATRIX

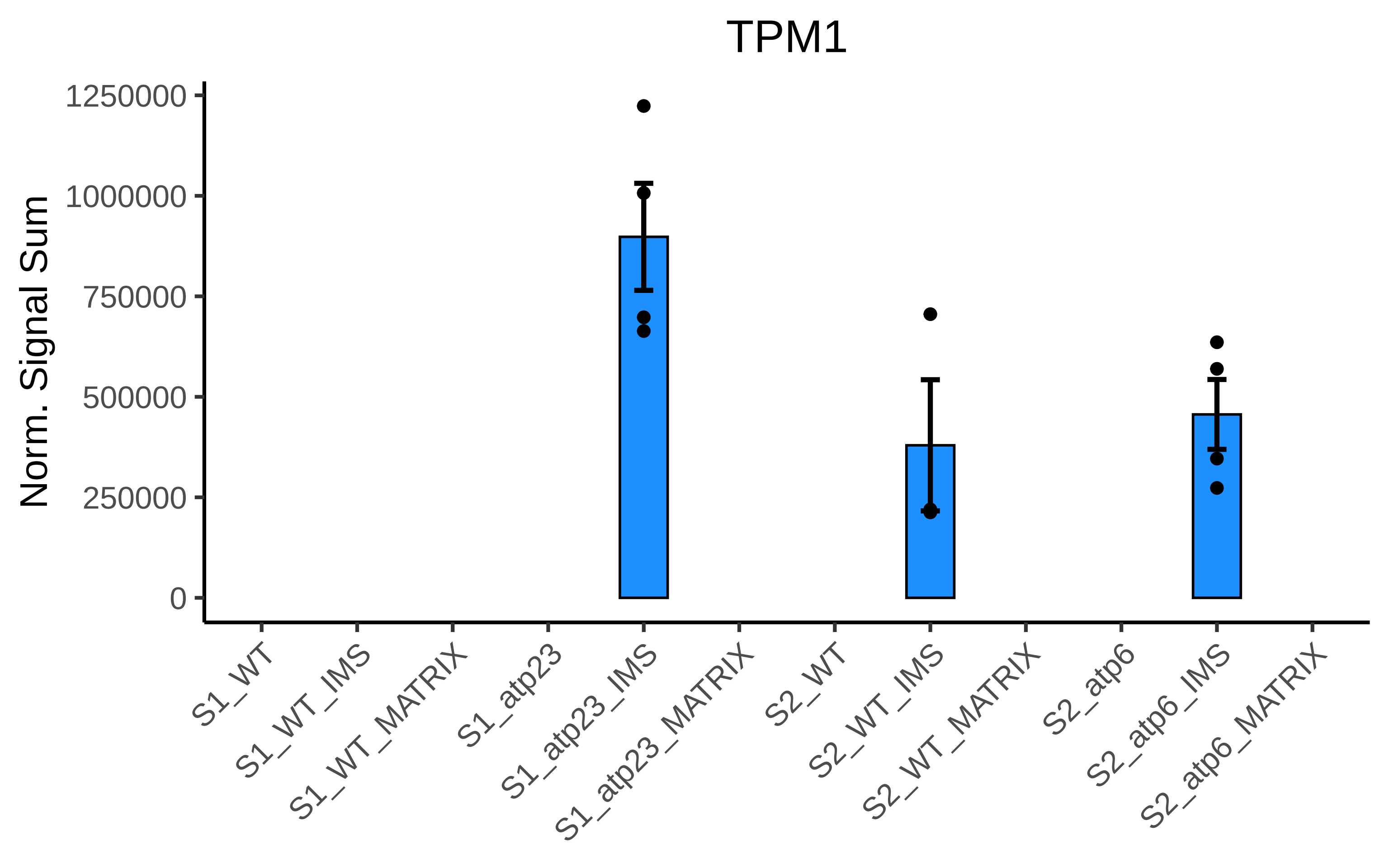
TPM1
Norm. Signal Sum
1250000
1000000
750000
500000
250000
0
S1_WT
S1_WT_IMS
S1_WT_MATRIX
S1_atp23
S1_atp23_IMS
S1_atp23_MATRIX
S2_WT
S2_WT_IMS
S2_WT_MATRIX
S2_atp6
S2_atp6_IMS
S2_atp6_MATRIX

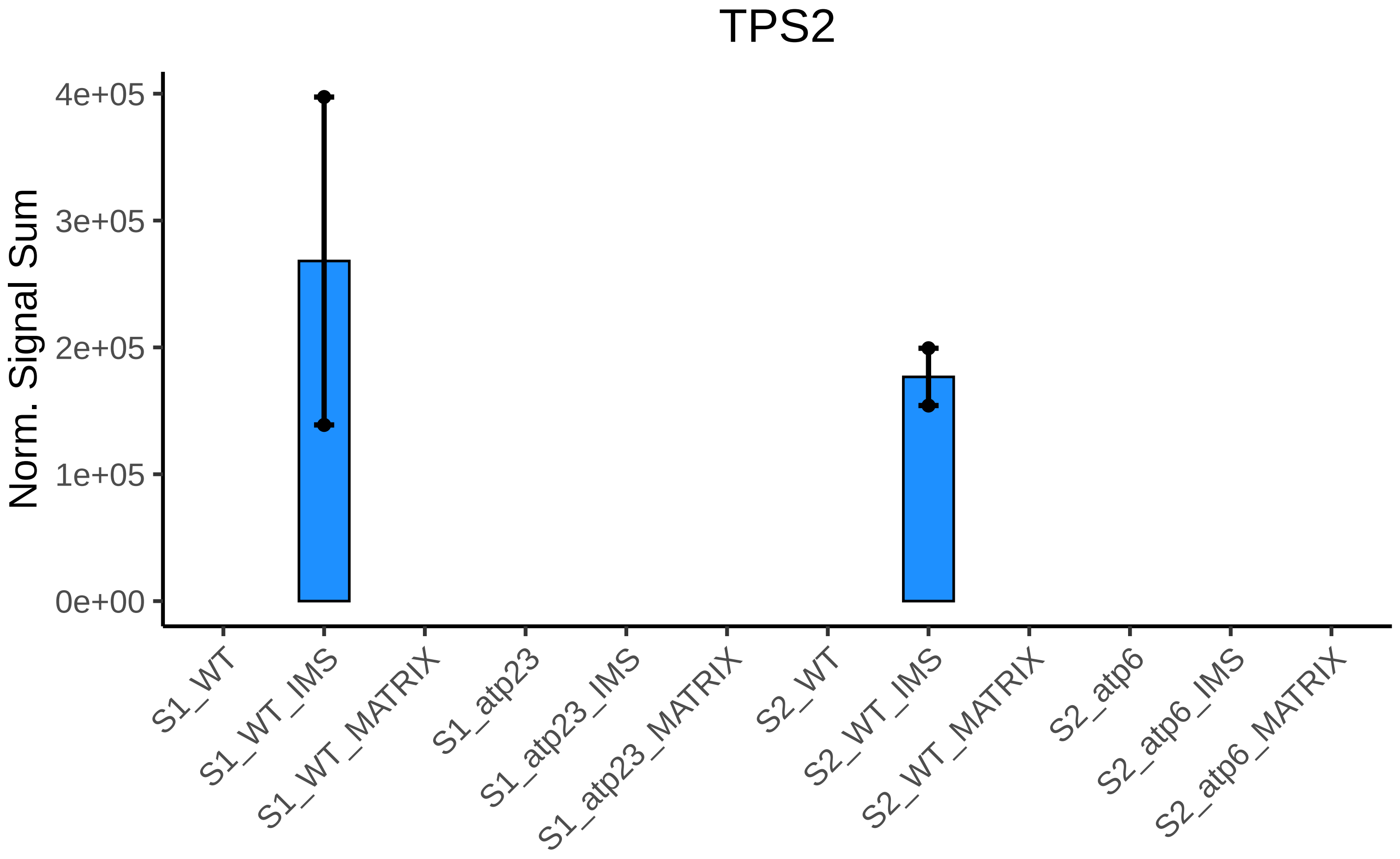

TPS2
Norm. Signal Sum
4e+05
3e+05
2e+05
1e+05
0e+00
S1_WT
S1_WT_IMS
S1_WT_MATRIX
S1_atp23
S1_atp23_IMS
S1_atp23_MATRIX
S2_WT
S2_WT_IMS
S2_WT_MATRIX
S2_atp6
S2_atp6_IMS
S2_atp6_MATRIX

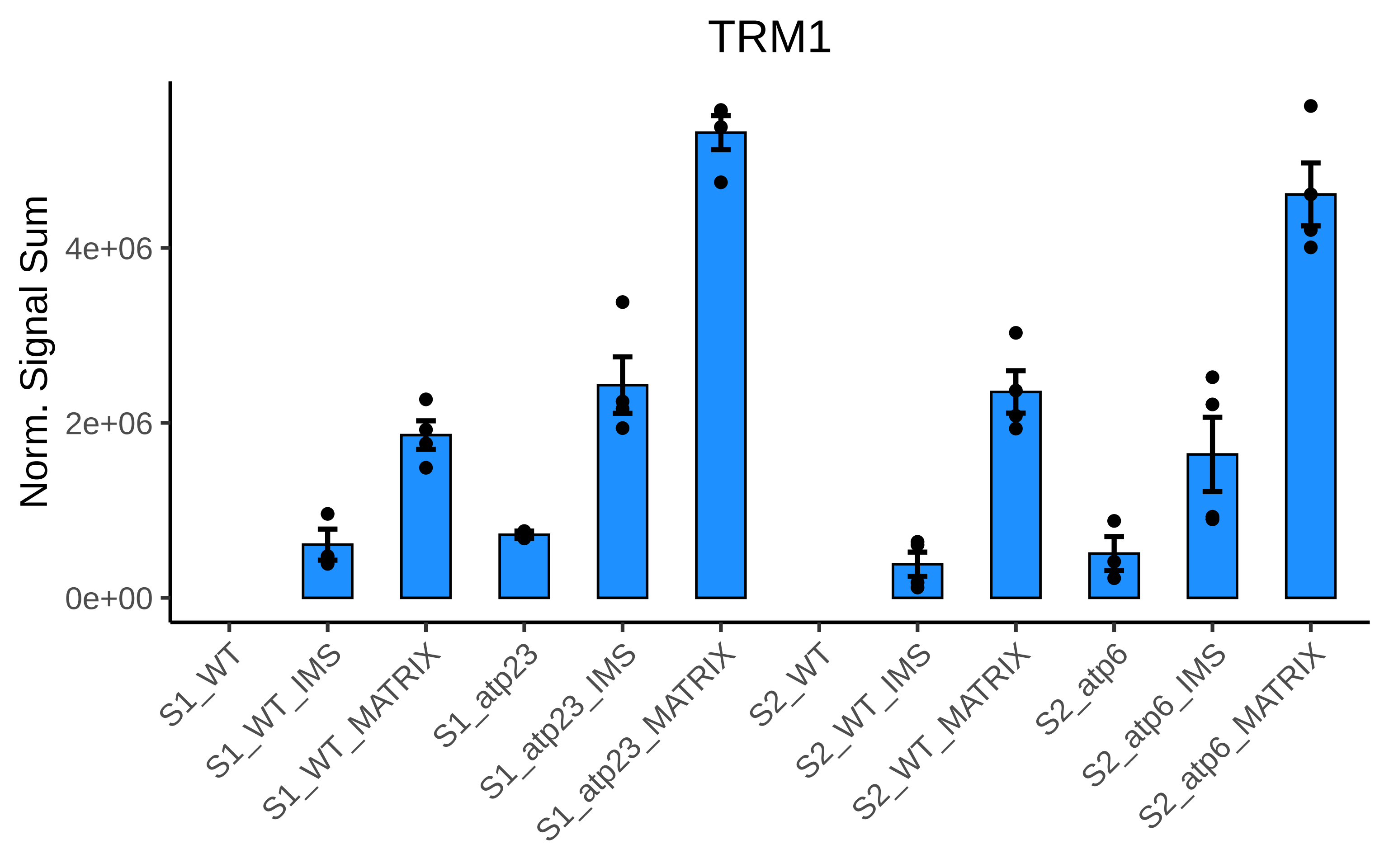

TRM1
Norm. Signal Sum
0e+00
2e+06
4e+06
S1_WT
S1_WT_IMS
S1_WT_MATRIX
S1_atp23
S1_atp23_IMS
S1_atp23_MATRIX
S2_WT
S2_WT_IMS
S2_WT_MATRIX
S2_atp6
S2_atp6_IMS
S2_atp6_MATRIX

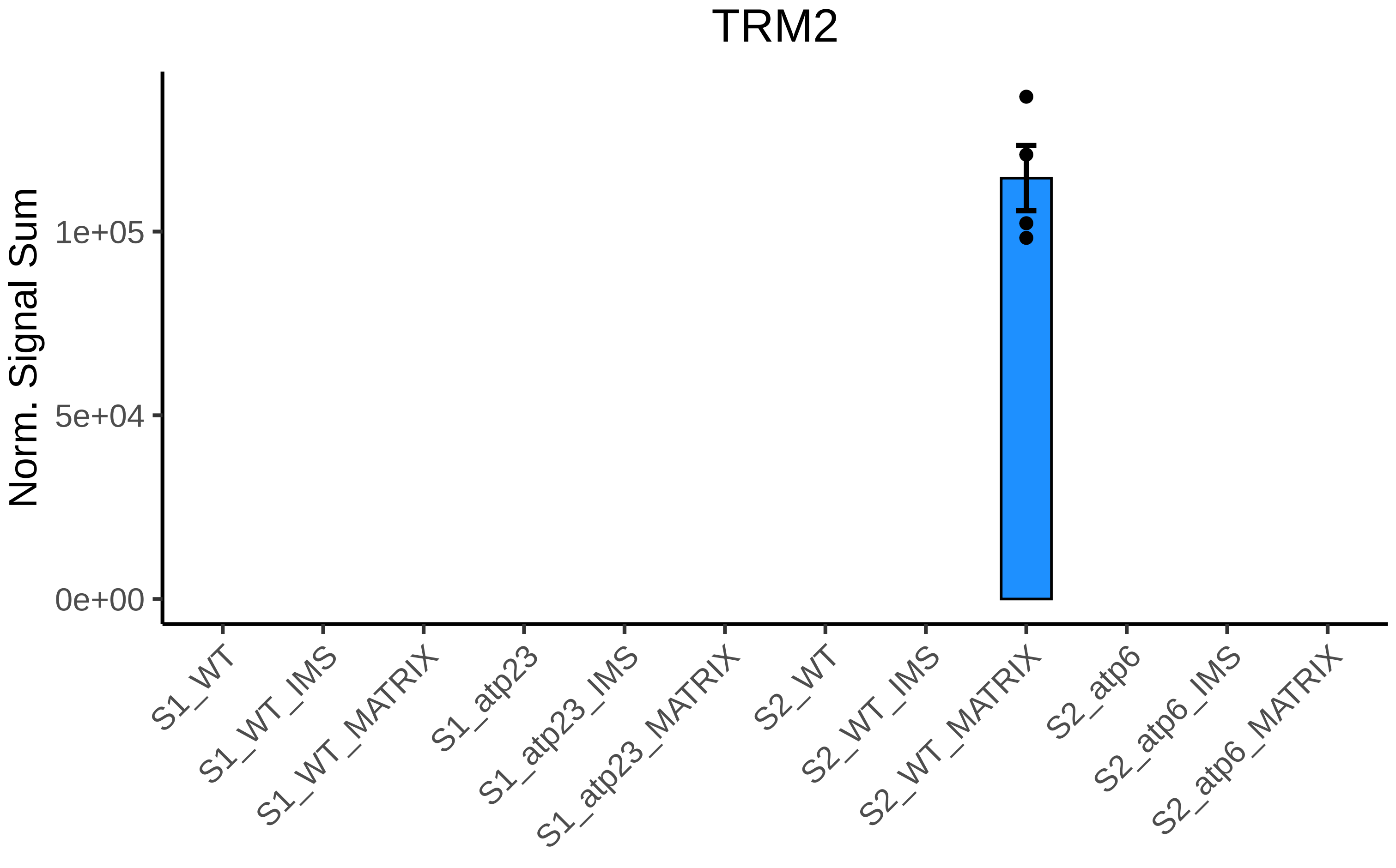

TRM2
Norm. Signal Sum
1e+05
5e+04
0e+00
S1_WT
S1_WT_IMS
S1_WT_MATRIX
S1_atp23
S1_atp23_IMS
S1_atp23_MATRIX
S2_WT
S2_WT_IMS
S2_WT_MATRIX
S2_atp6
S2_atp6_IMS
S2_atp6_MATRIX

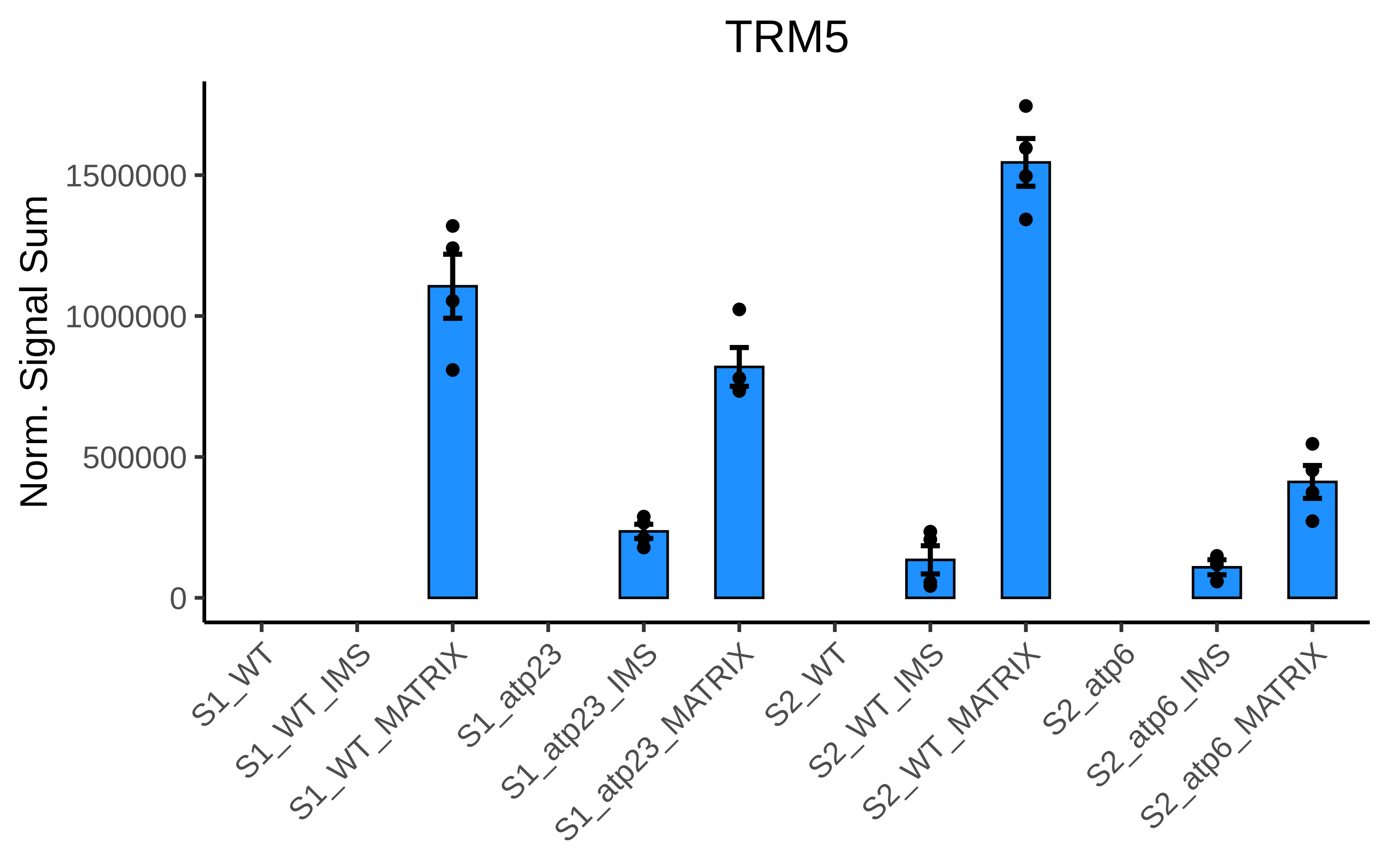

TRM5
Norm. Signal Sum
1500000
1000000
500000
0
S1_WT
S1_WT_IMS
S1_WT_MATRIX
S1_atp23
S1_atp23_IMS
S1_atp23_MATRIX
S2_WT
S2_WT_IMS
S2_WT_MATRIX
S2_atp6
S2_atp6_IMS
S2_atp6_MATRIX

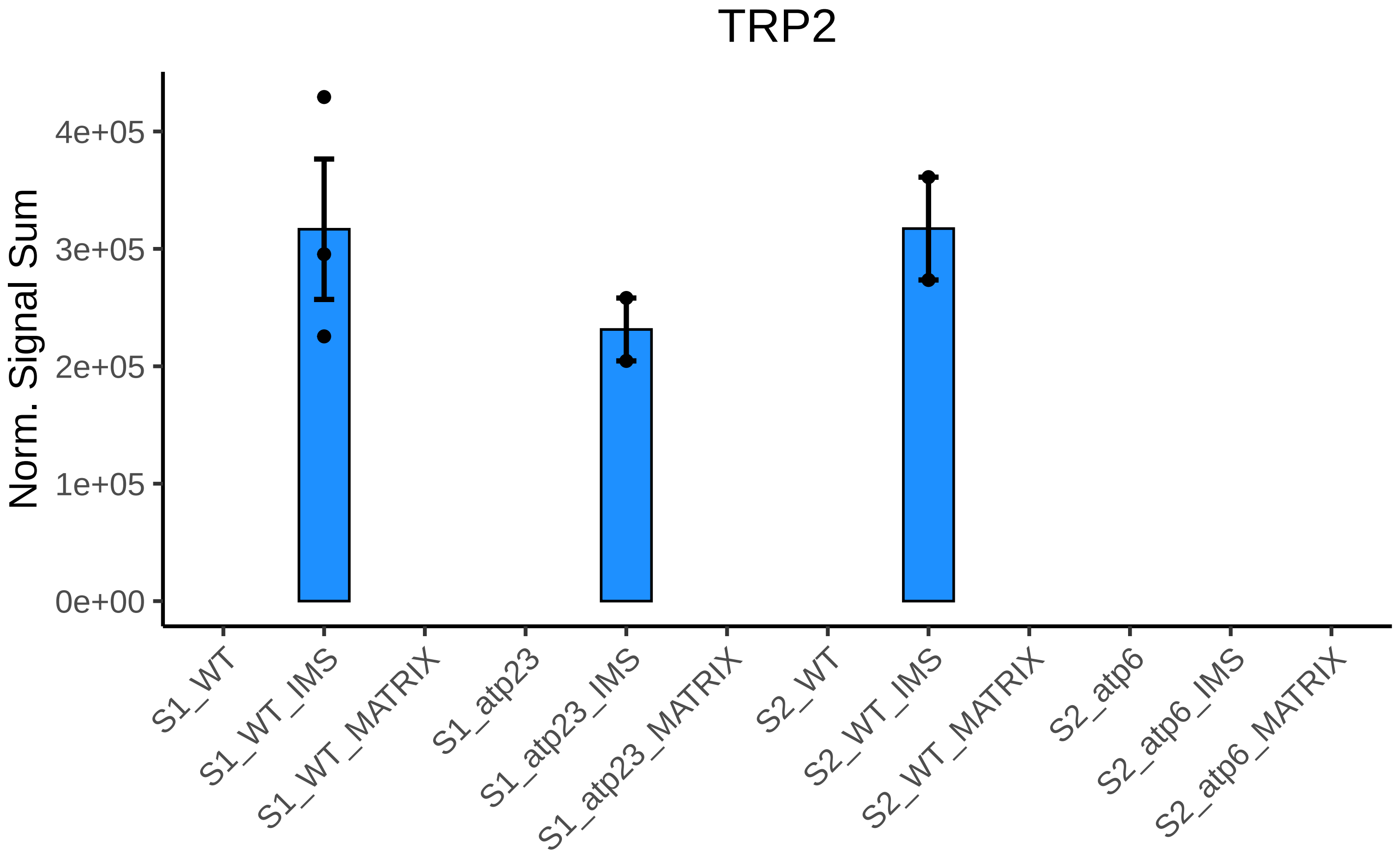
TRP2
Norm. Signal Sum
0e+00
1e+05
2e+05
3e+05
4e+05
S1_WT
S1_WT_IMS
S1_WT_MATRIX
S1_atp23
S1_atp23_IMS
S1_atp23_MATRIX
S2_WT
S2_WT_IMS
S2_WT_MATRIX
S2_atp6
S2_atp6_IMS
S2_atp6_MATRIX

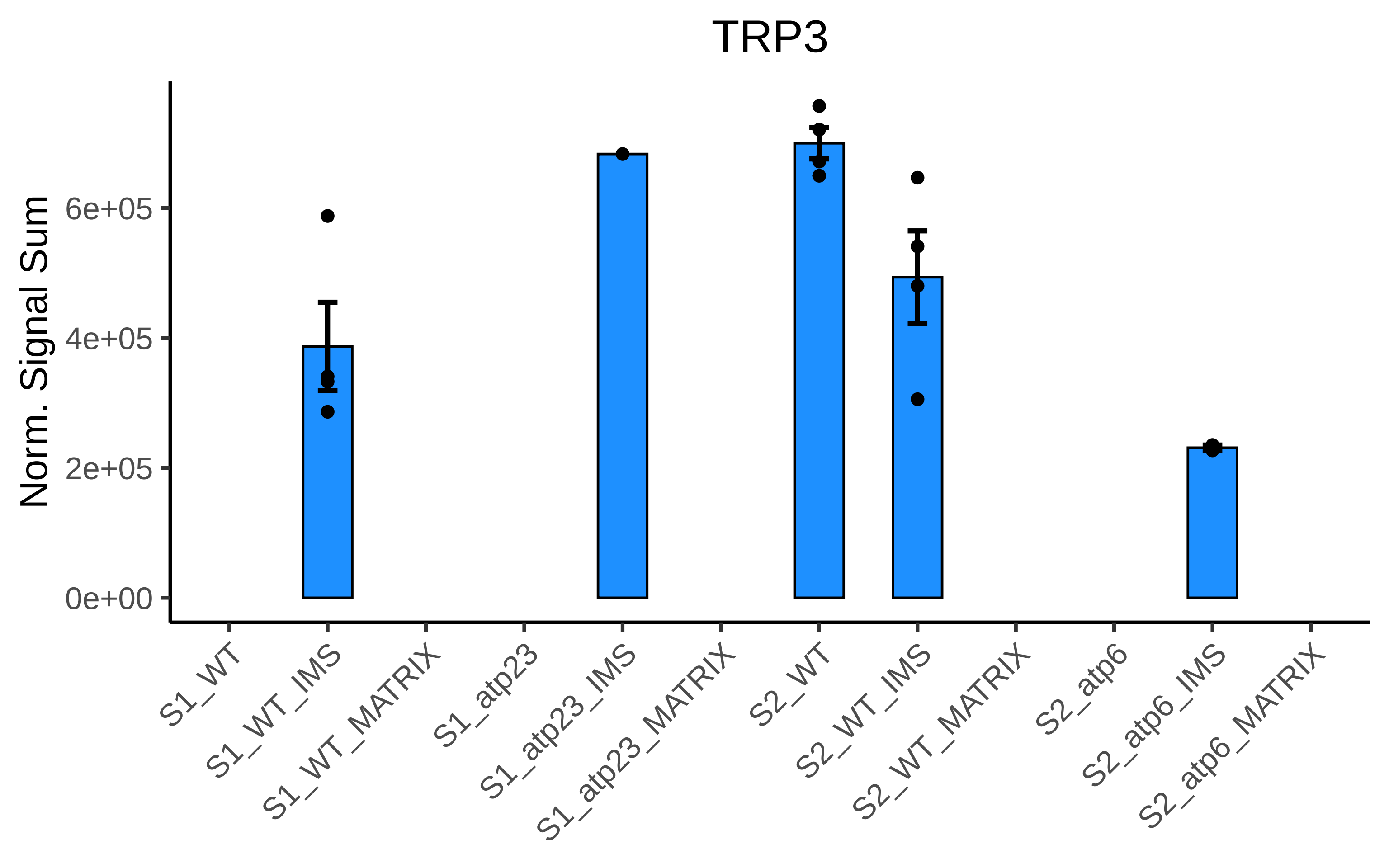

TRP3
Norm. Signal Sum
6e+05
4e+05
2e+05
0e+00
S1_WT
S1_WT_IMS
S1_WT_MATRIX
S1_atp23
S1_atp23_IMS
S1_atp23_MATRIX
S2_WT
S2_WT_IMS
S2_WT_MATRIX
S2_atp6
S2_atp6_IMS
S2_atp6_MATRIX

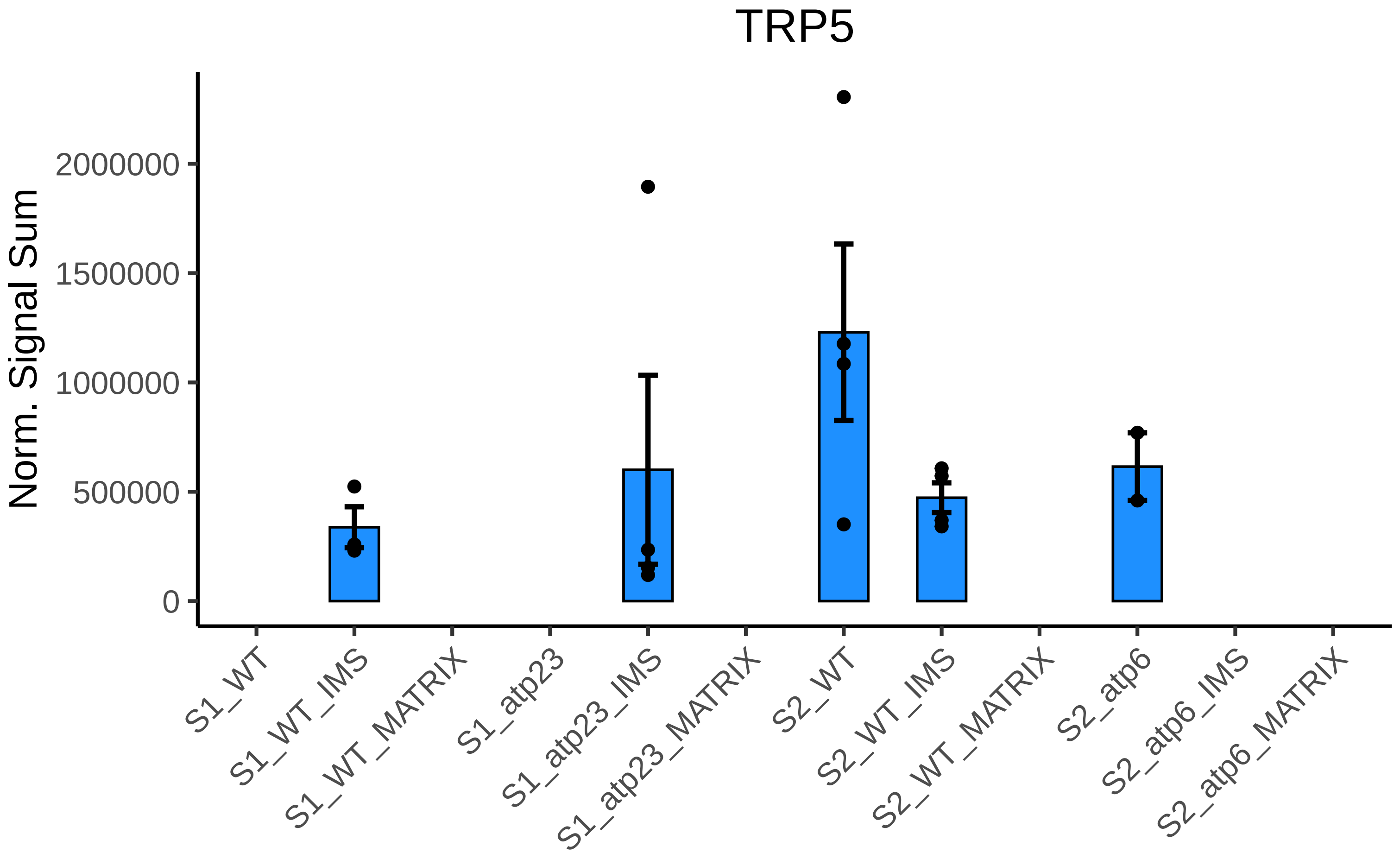
TRP5
Norm. Signal Sum
2000000
1500000
1000000
500000
0
S1_WT
S1_WT_IMS
S1_WT_MATRIX
S1_atp23
S1_atp23_IMS
S1_atp23_MATRIX
S2_WT
S2_WT_IMS
S2_WT_MATRIX
S2_atp6
S2_atp6_IMS
S2_atp6_MATRIX

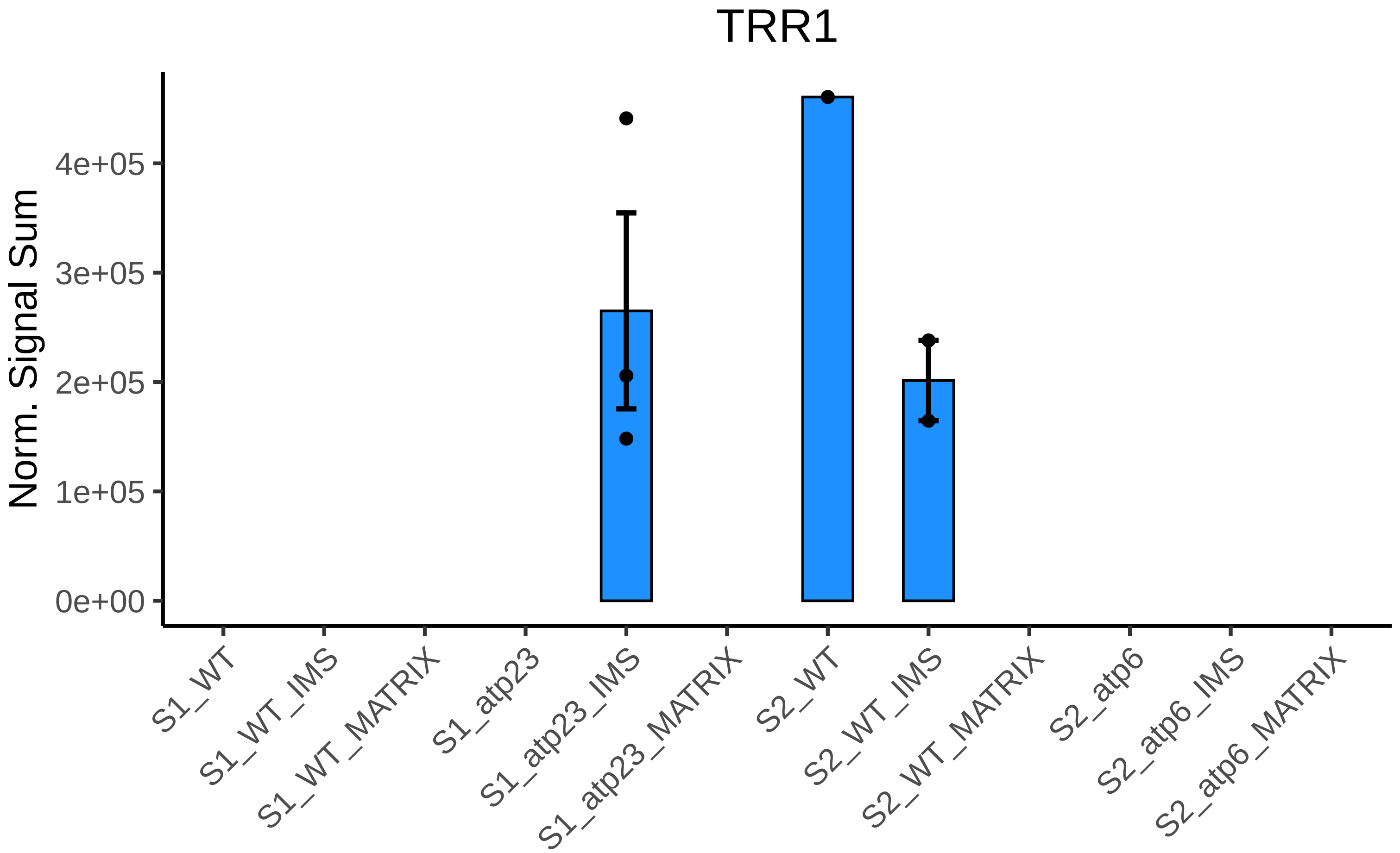
TRR1
Norm. Signal Sum
4e+05
3e+05
2e+05
1e+05
0e+00
S1_WT
S1_WT_IMS
S1_WT_MATRIX
S1_atp23
S1_atp23_IMS
S1_atp23_MATRIX
S2_WT
S2_WT_IMS
S2_WT_MATRIX
S2_atp6
S2_atp6_IMS
S2_atp6_MATRIX

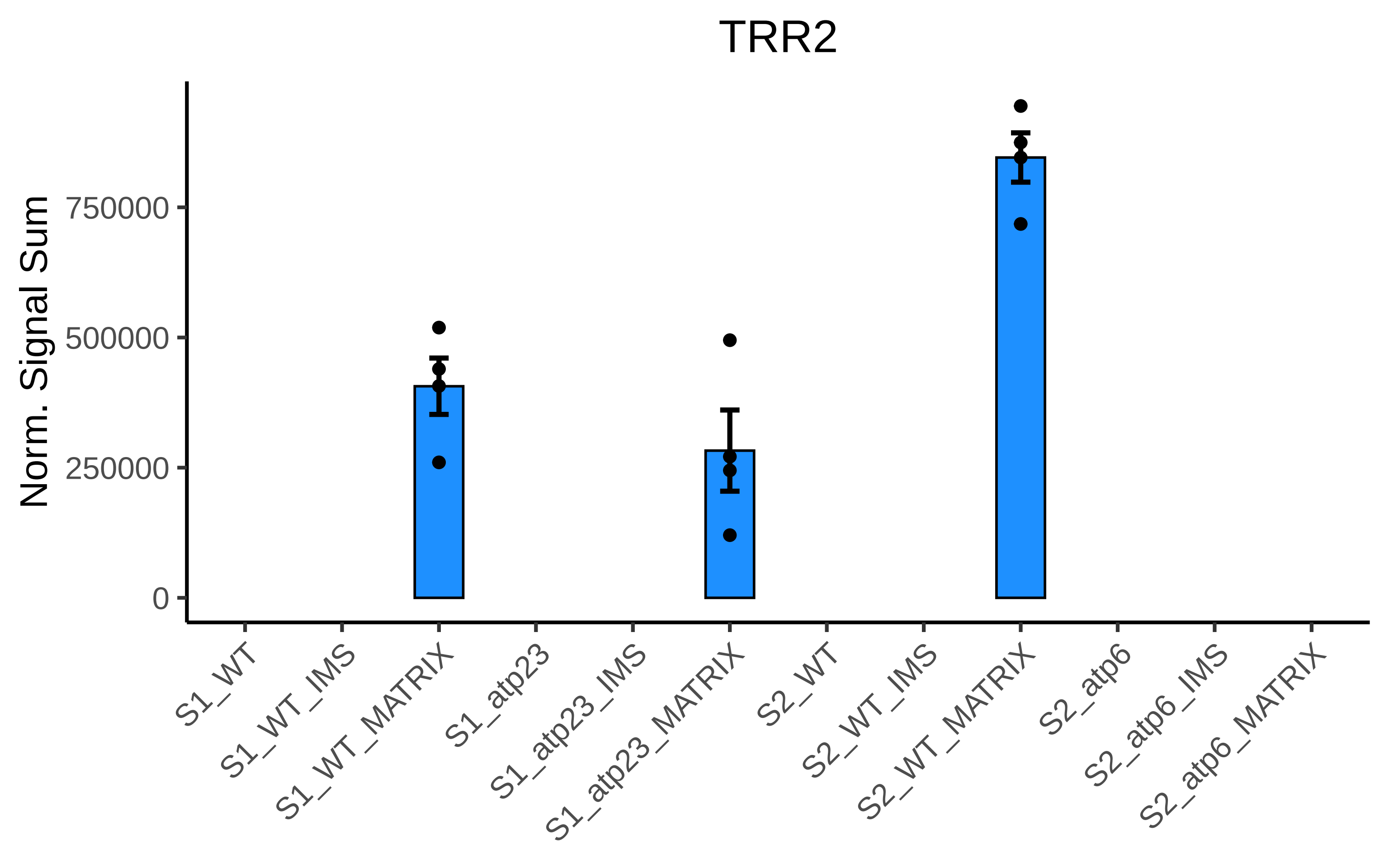
TRR2
Norm. Signal Sum
750000
500000
250000
0
S1_WT
S1_WT_IMS
S1_WT_MATRIX
S1_atp23
S1_atp23_IMS
S1_atp23_MATRIX
S2_WT
S2_WT_IMS
S2_WT_MATRIX
S2_atp6
S2_atp6_IMS
S2_atp6_MATRIX

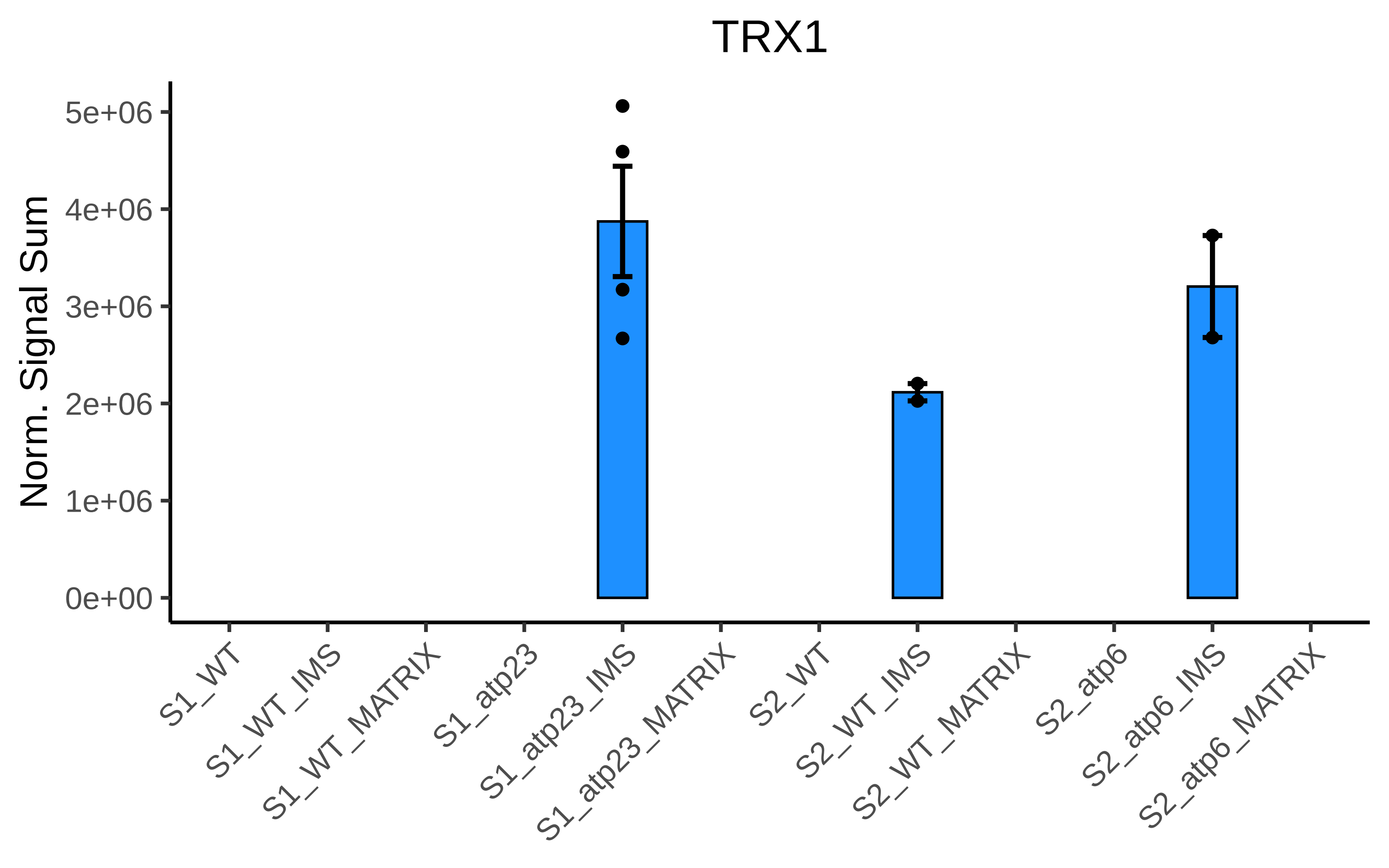
TRX1
Norm. Signal Sum
0e+00
1e+06
2e+06
3e+06
4e+06
5e+06
S1_WT
S1_WT_IMS
S1_WT_MATRIX
S1_atp23
S1_atp23_IMS
S1_atp23_MATRIX
S2_WT
S2_WT_IMS
S2_WT_MATRIX
S2_atp6
S2_atp6_IMS
S2_atp6_MATRIX

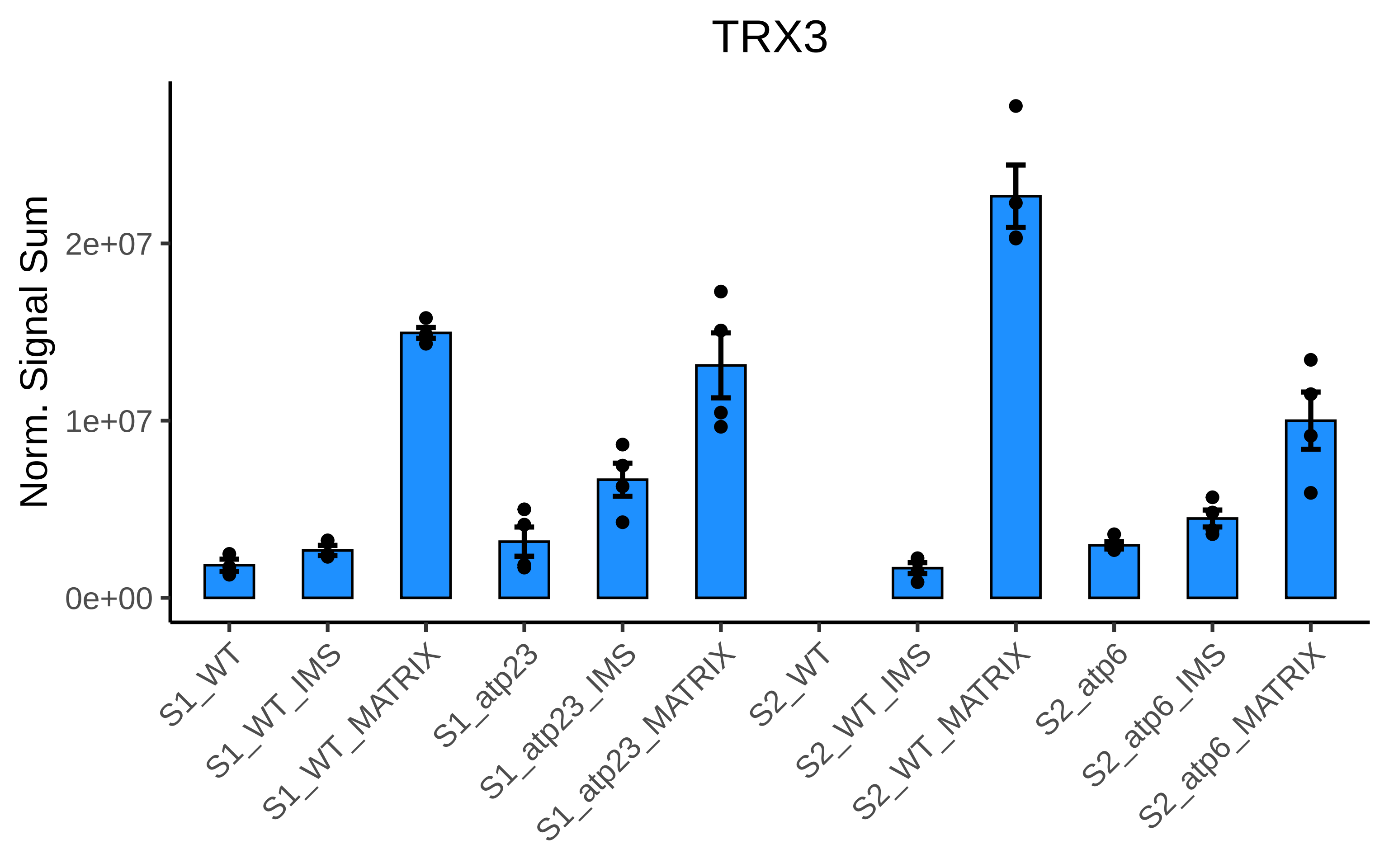

TRX3
Norm. Signal Sum
2e+07
1e+07
0e+00
S1_WT
S1_WT_IMS
S1_WT_MATRIX
S1_atp23
S1_atp23_IMS
S1_atp23_MATRIX
S2_WT
S2_WT_IMS
S2_WT_MATRIX
S2_atp6
S2_atp6_IMS
S2_atp6_MATRIX

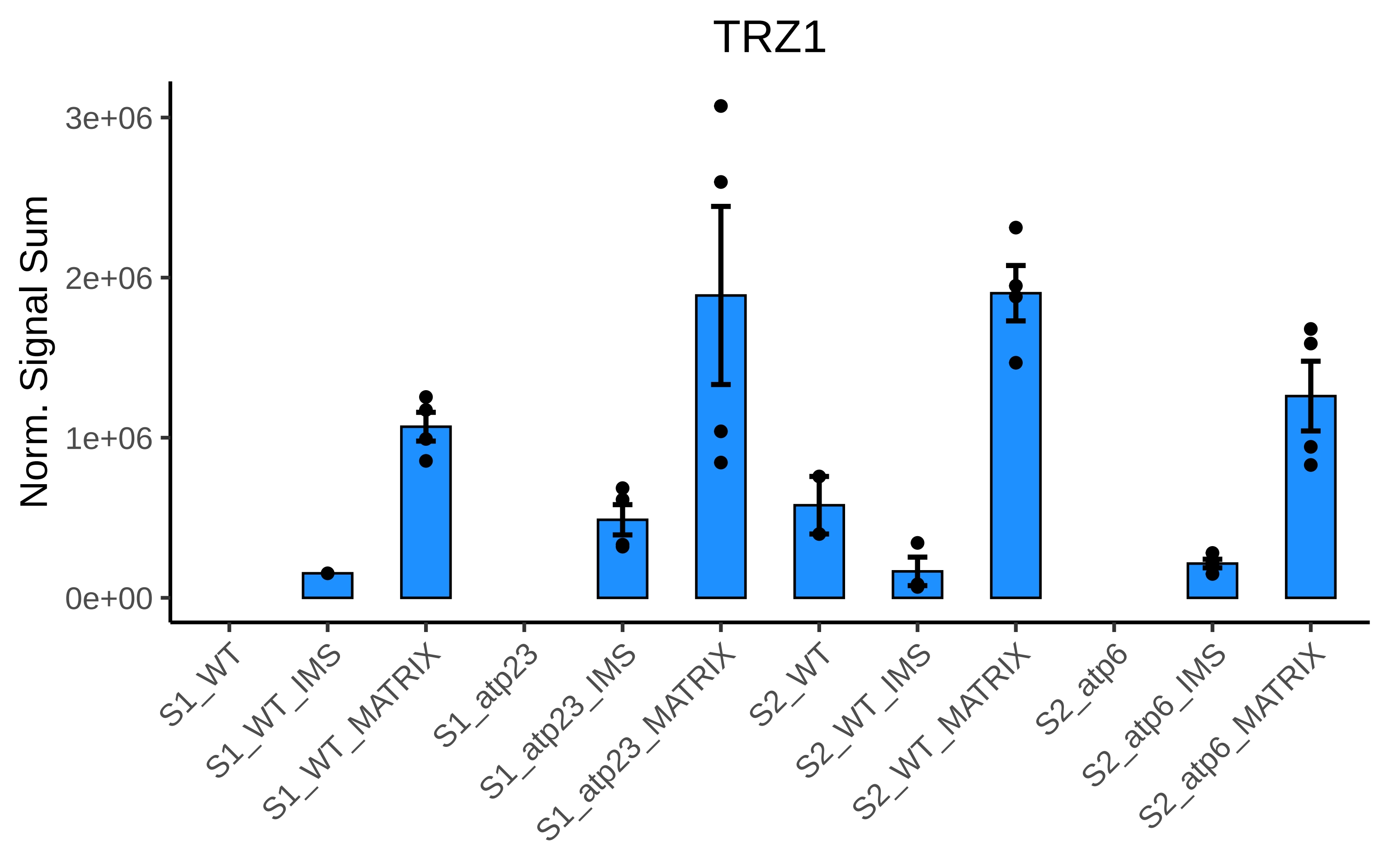
TRZ1
Norm. Signal Sum
3e+06
2e+06
1e+06
0e+00
S1_WT
S1_WT_IMS
S1_WT_MATRIX
S1_atp23
S1_atp23_IMS
S1_atp23_MATRIX
S2_WT
S2_WT_IMS
S2_WT_MATRIX
S2_atp6
S2_atp6_IMS
S2_atp6_MATRIX

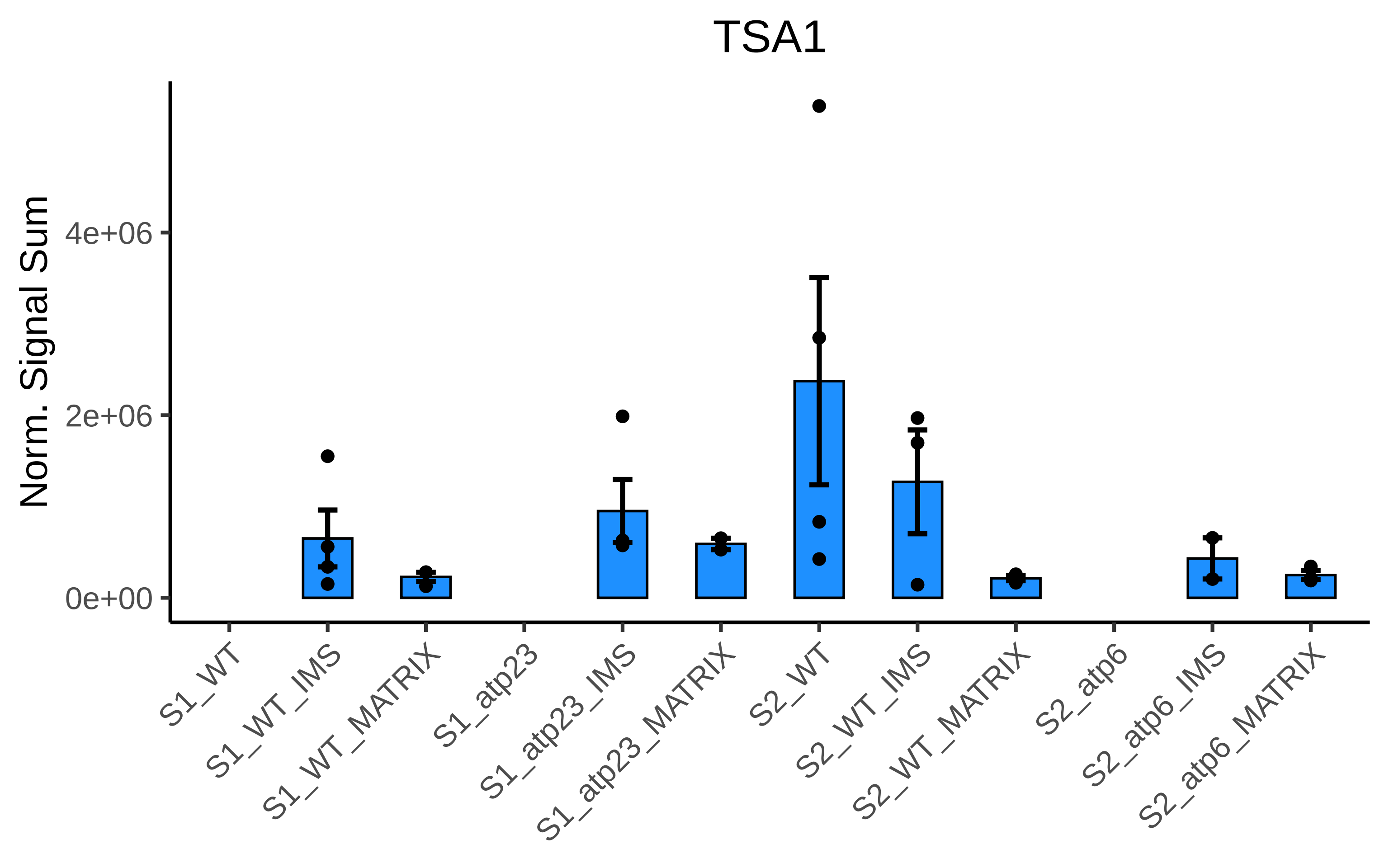
TSA1
Norm. Signal Sum
4e+06
2e+06
0e+00
S1_WT
S1_WT_IMS
S1_WT_MATRIX
S1_atp23
S1_atp23_IMS
S1_atp23_MATRIX
S2_WT
S2_WT_IMS
S2_WT_MATRIX
S2_atp6
S2_atp6_IMS
S2_atp6_MATRIX

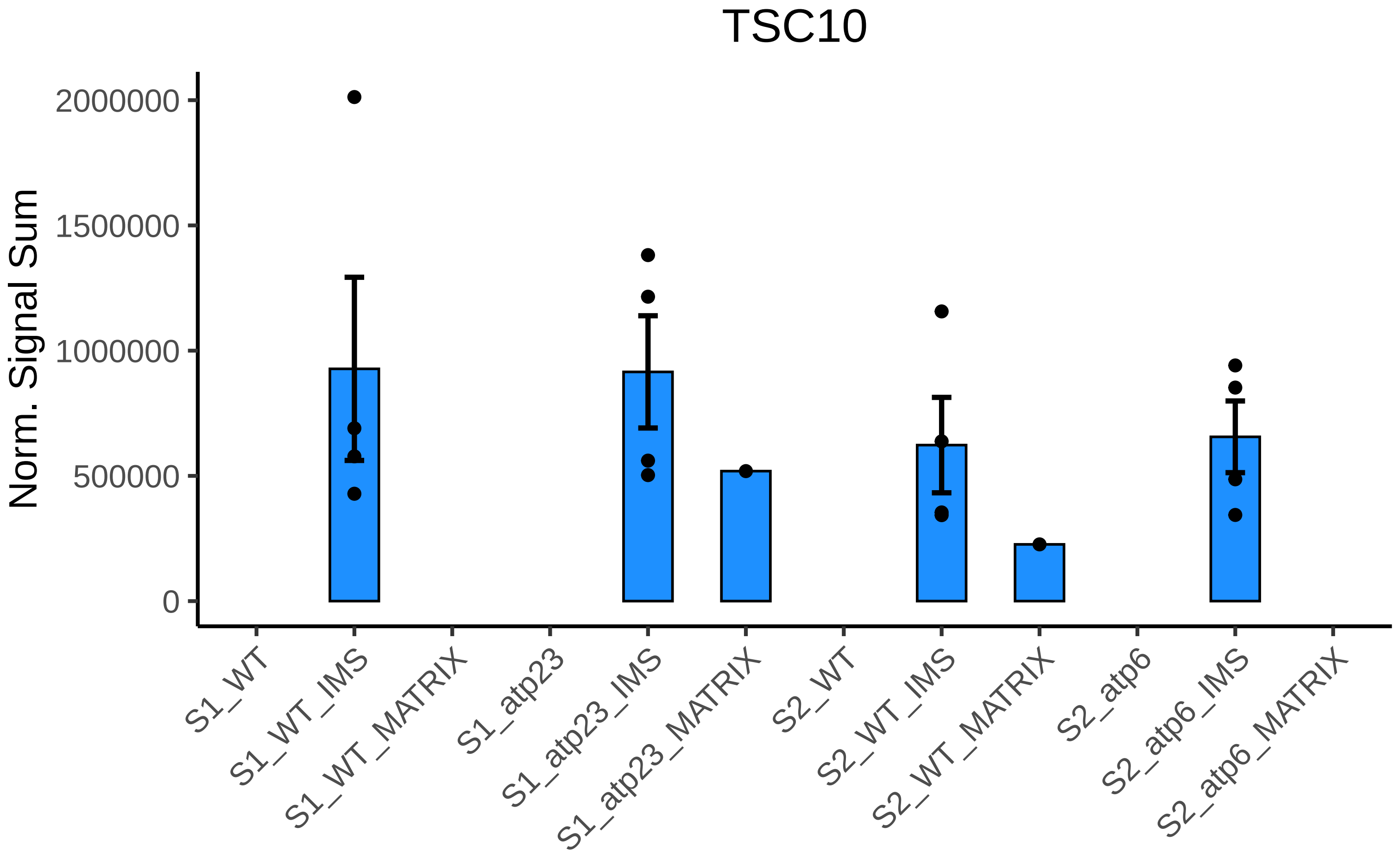

TSC10
Norm. Signal Sum
2000000
1500000
1000000
500000
0
S1_WT
S1_WT_IMS
S1_WT_MATRIX
S1_atp23
S1_atp23_IMS
S1_atp23_MATRIX
S2_WT
S2_WT_IMS
S2_WT_MATRIX
S2_atp6
S2_atp6_IMS
S2_atp6_MATRIX

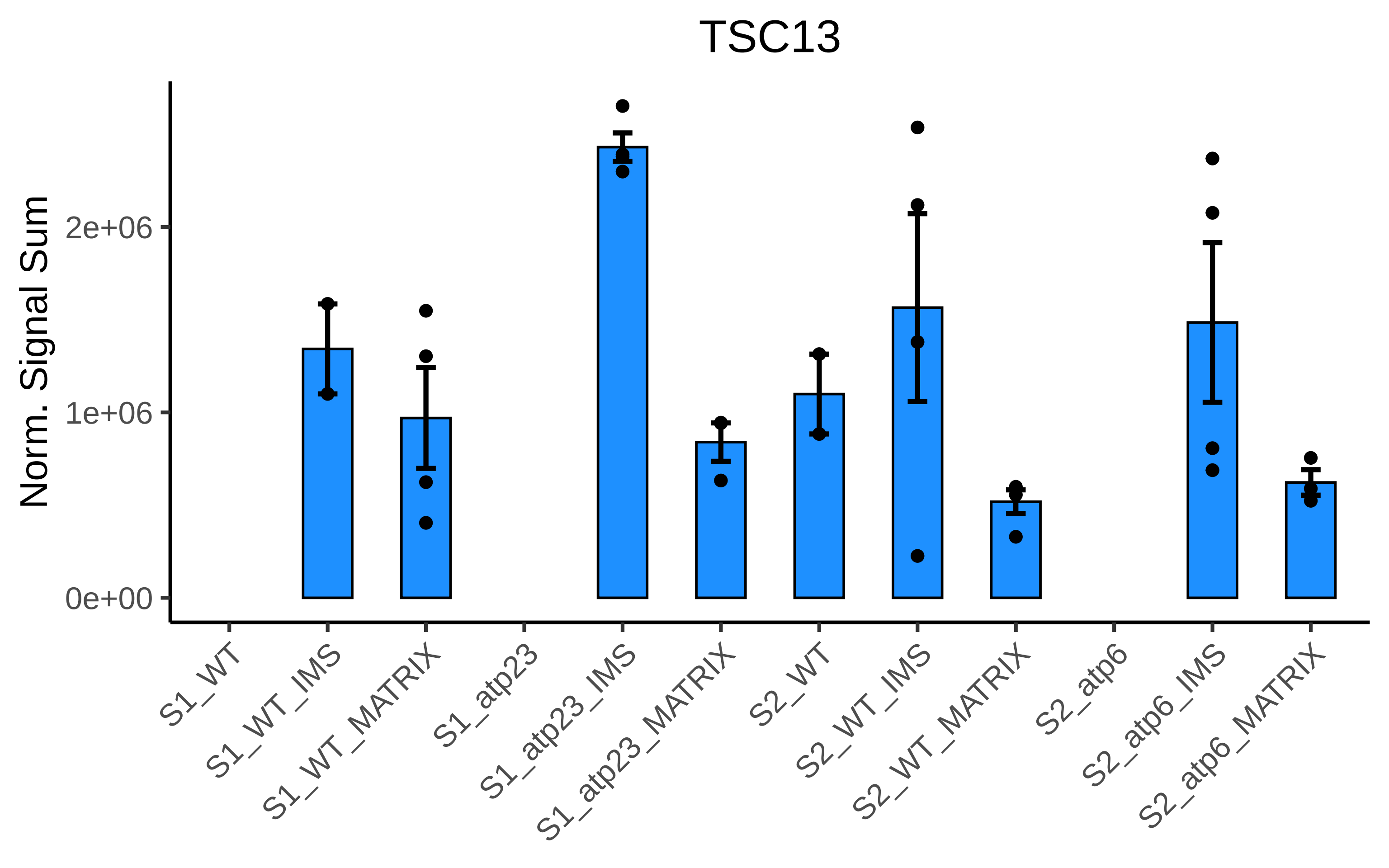

TSC13
Norm. Signal Sum
2e+06
1e+06
0e+00
S1_WT
S1_WT_IMS
S1_WT_MATRIX
S1_atp23
S1_atp23_IMS
S1_atp23_MATRIX
S2_WT
S2_WT_IMS
S2_WT_MATRIX
S2_atp6
S2_atp6_IMS
S2_atp6_MATRIX

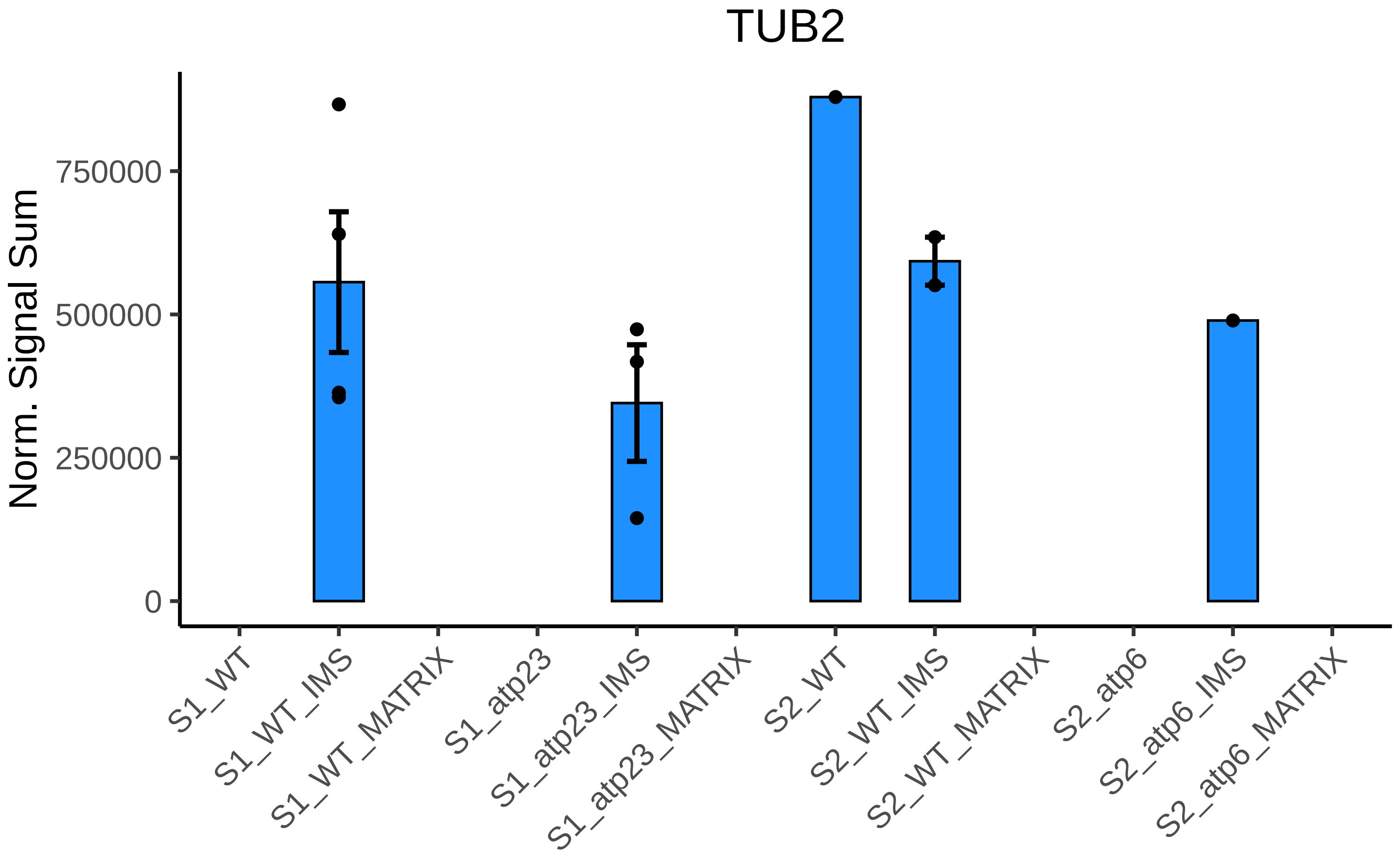
TUB2
Norm. Signal Sum
750000
500000
250000
0
S1_WT
S1_WT_IMS
S1_WT_MATRIX
S1_atp23
S1_atp23_IMS
S1_atp23_MATRIX
S2_WT
S2_WT_IMS
S2_WT_MATRIX
S2_atp6
S2_atp6_IMS
S2_atp6_MATRIX

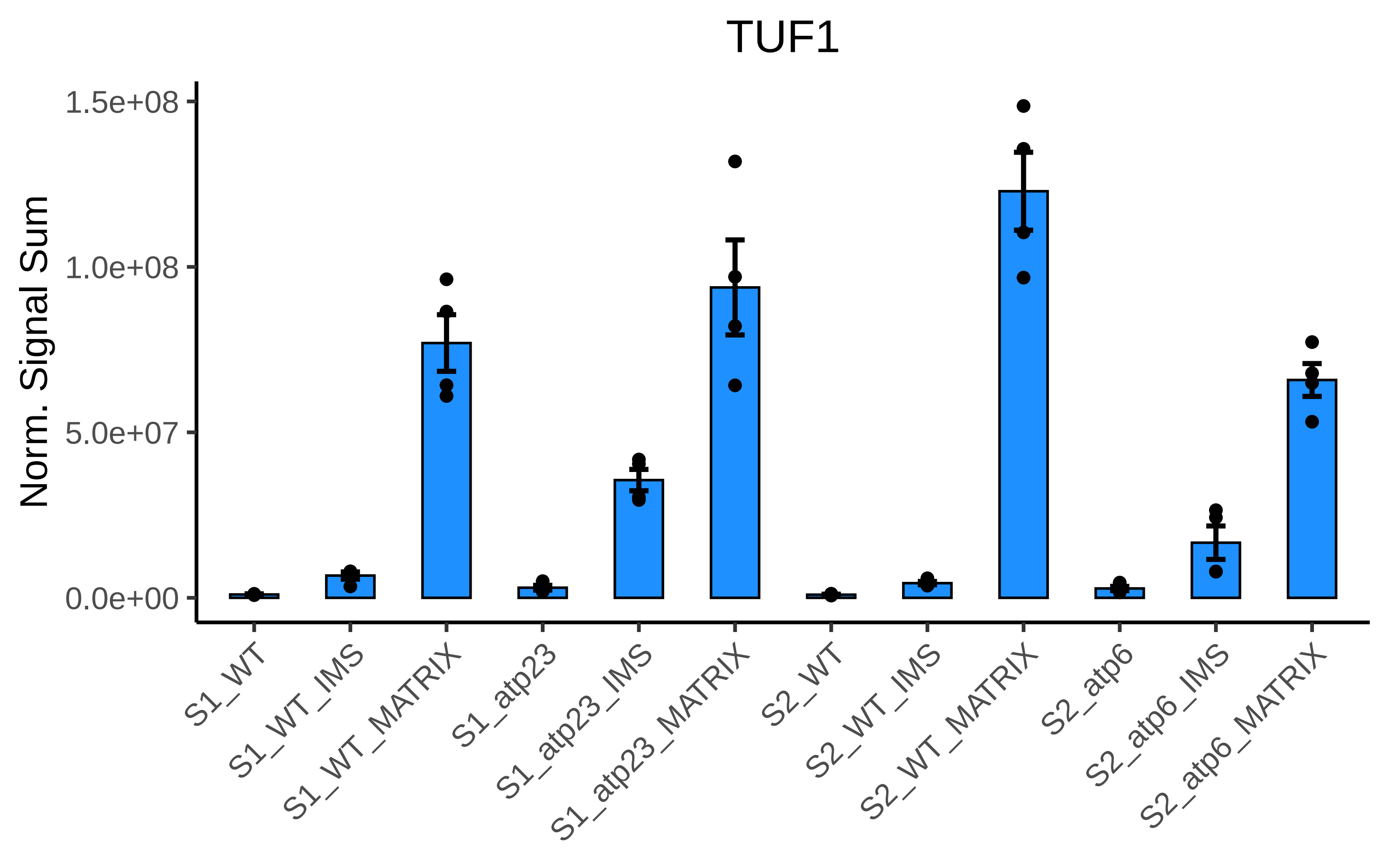
TUF1
Norm. Signal Sum
1.5e+08
1.0e+08
5.0e+07
0.0e+00
S1_WT
S1_WT_IMS
S1_WT_MATRIX
S1_atp23
S1_atp23_IMS
S1_atp23_MATRIX
S2_WT
S2_WT_IMS
S2_WT_MATRIX
S2_atp6
S2_atp6_IMS
S2_atp6_MATRIX

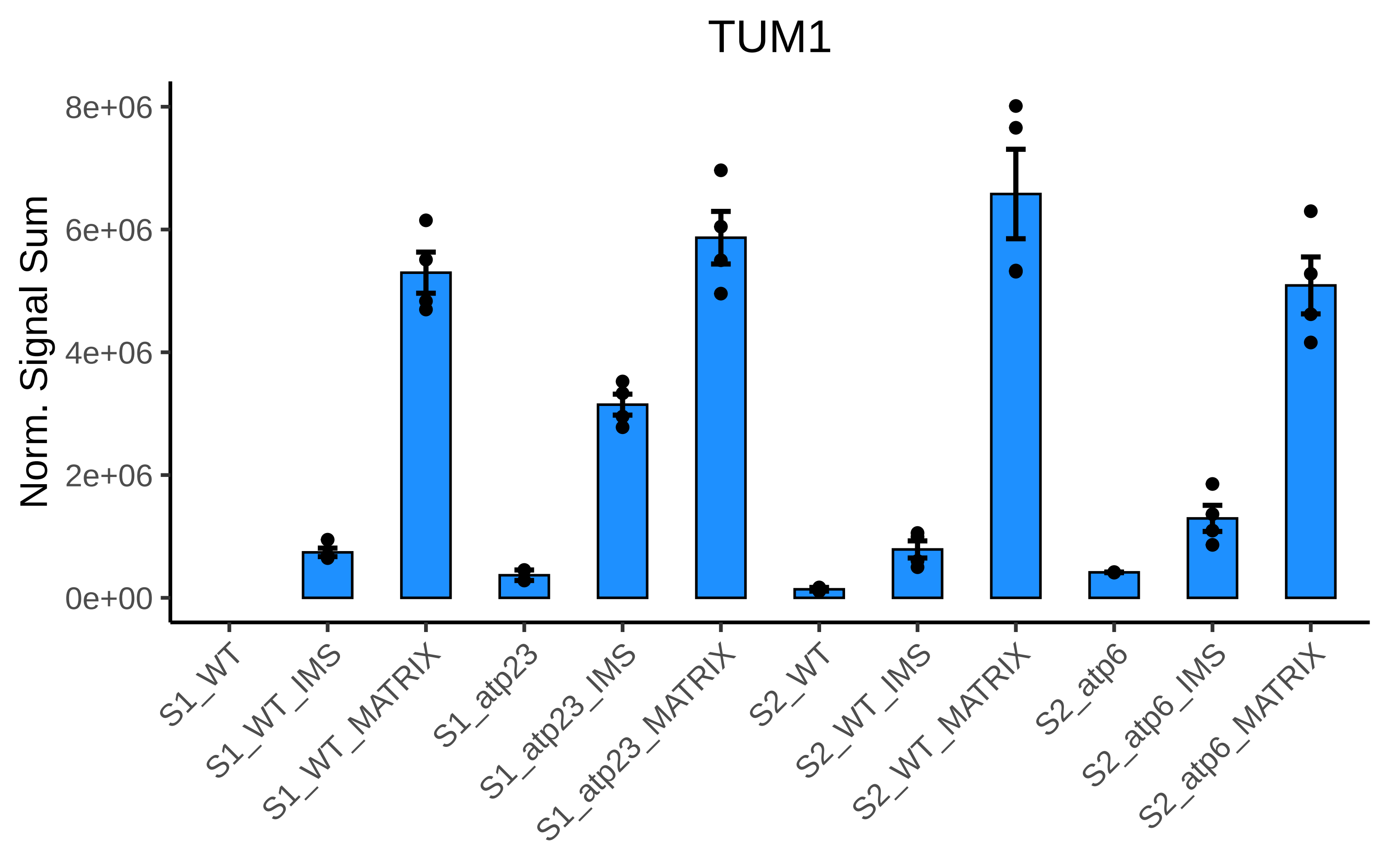
TUM1
Norm. Signal Sum
8e+06
6e+06
4e+06
2e+06
0e+00
S1_WT
S1_WT_IMS
S1_WT_MATRIX
S1_atp23
S1_atp23_IMS
S1_atp23_MATRIX
S2_WT
S2_WT_IMS
S2_WT_MATRIX
S2_atp6
S2_atp6_IMS
S2_atp6_MATRIX

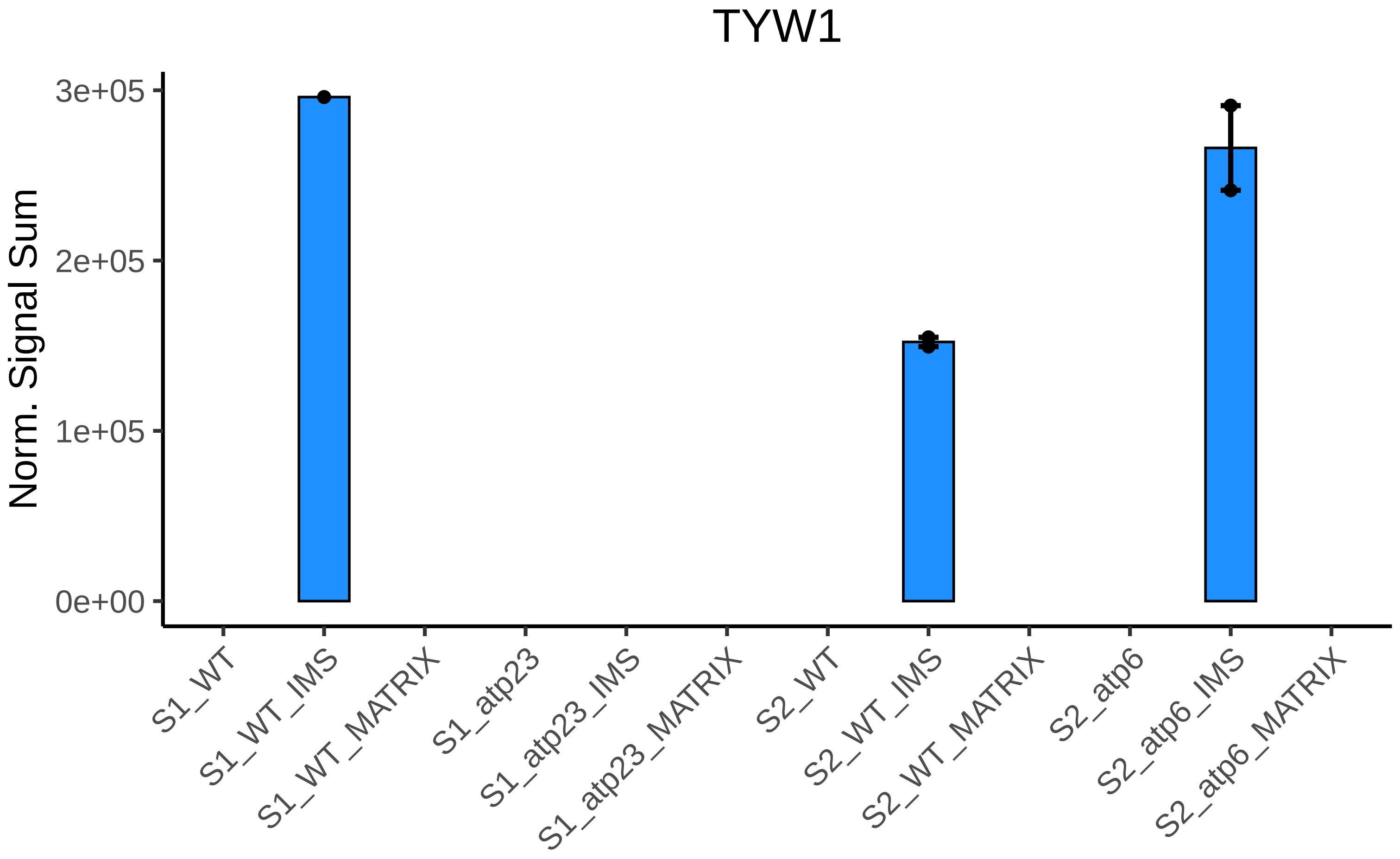

TYW1
Norm. Signal Sum
3e+05
2e+05
1e+05
0e+00
S1_WT
S1_WT_IMS
S1_WT_MATRIX
S1_atp23
S1_atp23_IMS
S1_atp23_MATRIX
S2_WT
S2_WT_IMS
S2_WT_MATRIX
S2_atp6
S2_atp6_IMS
S2_atp6_MATRIX

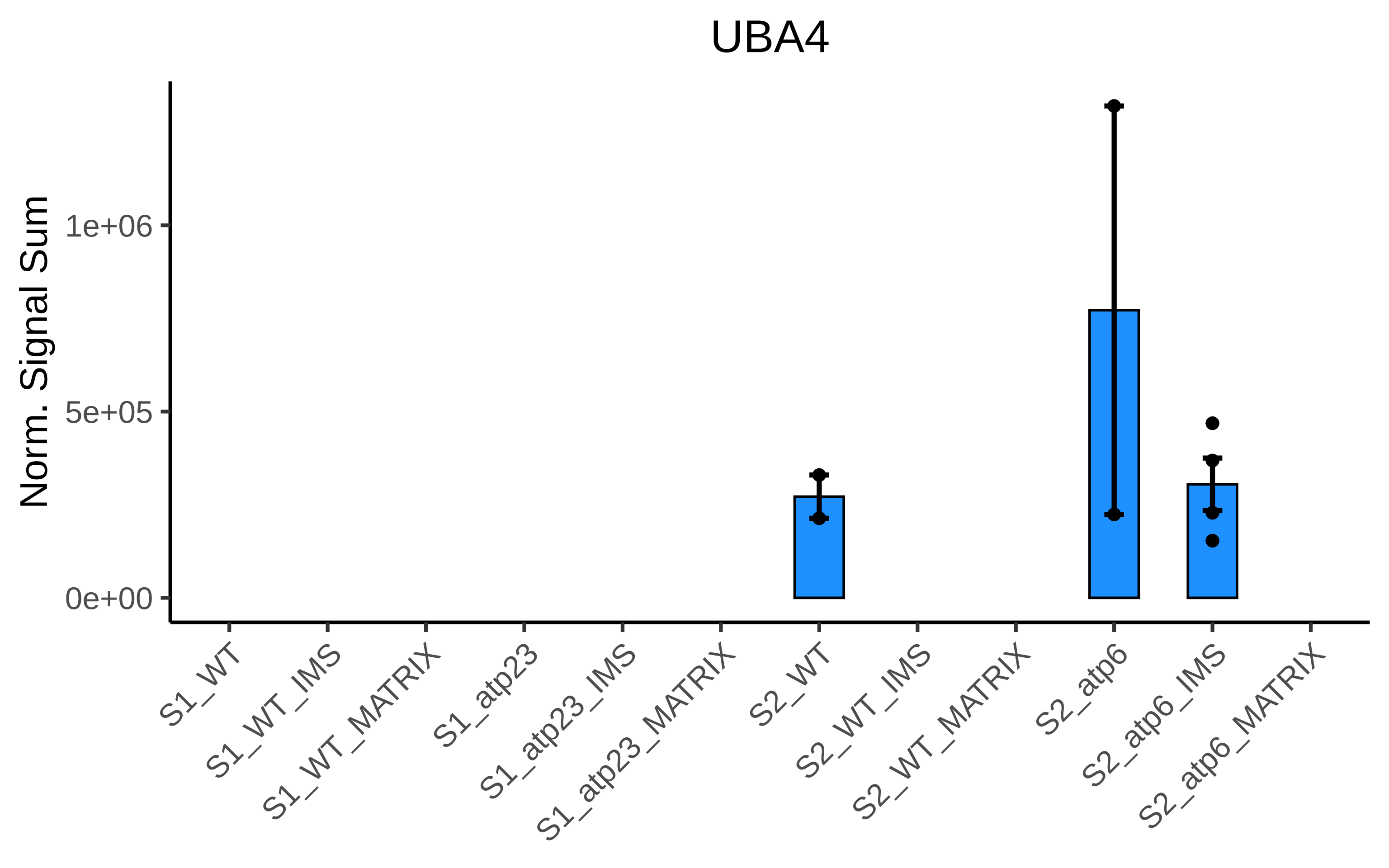
UBA4
Norm. Signal Sum
1e+06
5e+05
0e+00
S1_WT
S1_WT_IMS
S1_WT_MATRIX
S1_atp23
S1_atp23_IMS
S1_atp23_MATRIX
S2_WT
S2_WT_IMS
S2_WT_MATRIX
S2_atp6
S2_atp6_IMS
S2_atp6_MATRIX

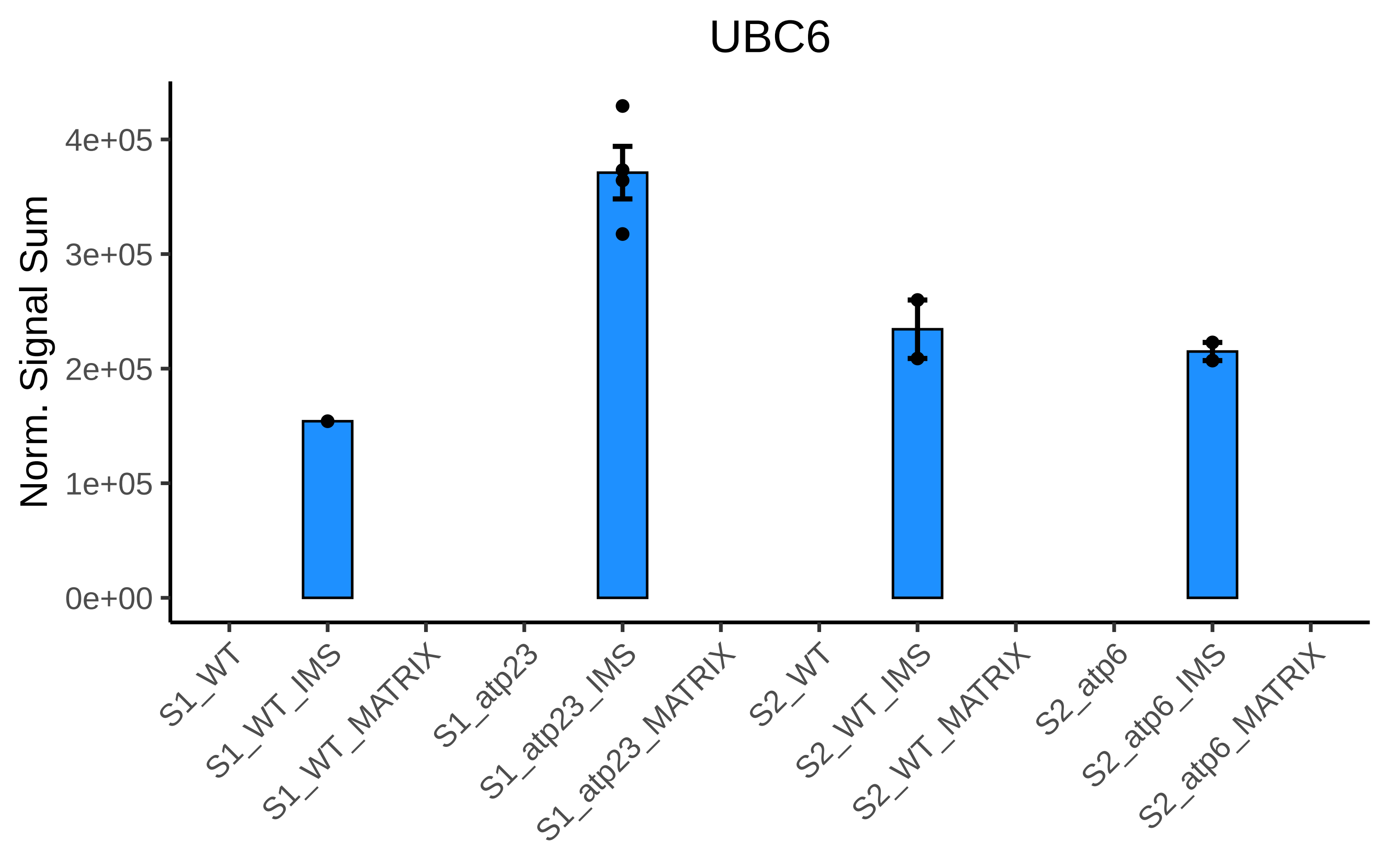
UBC6
Norm. Signal Sum
4e+05
3e+05
2e+05
1e+05
0e+00
S1_WT
S1_WT_IMS
S1_WT_MATRIX
S1_atp23
S1_atp23_IMS
S1_atp23_MATRIX
S2_WT
S2_WT_IMS
S2_WT_MATRIX
S2_atp6
S2_atp6_IMS
S2_atp6_MATRIX

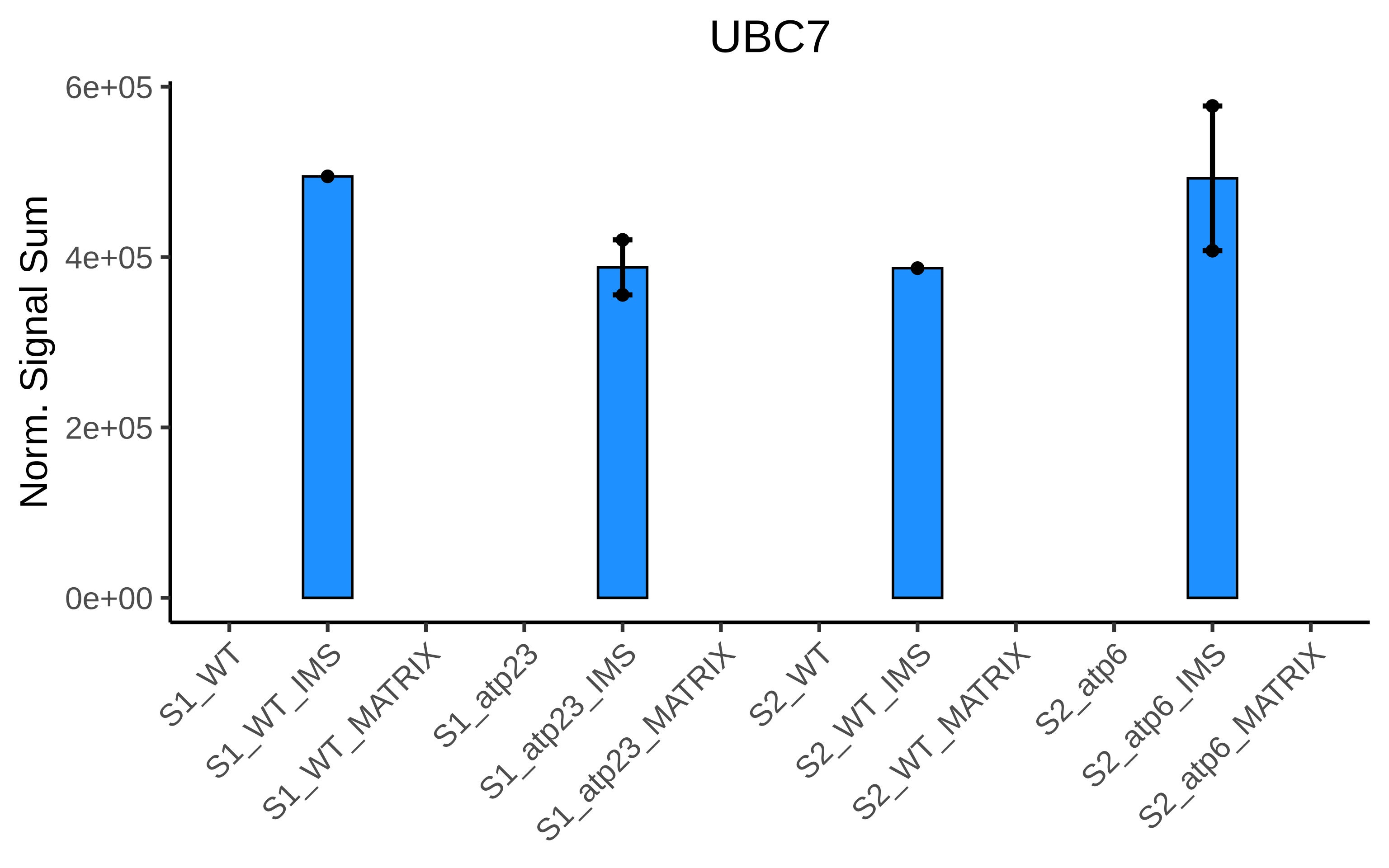

UBC7
Norm. Signal Sum
6e+05
4e+05
2e+05
0e+00
S1_WT
S1_WT_IMS
S1_WT_MATRIX
S1_atp23
S1_atp23_IMS
S1_atp23_MATRIX
S2_WT
S2_WT_IMS
S2_WT_MATRIX
S2_atp6
S2_atp6_IMS
S2_atp6_MATRIX

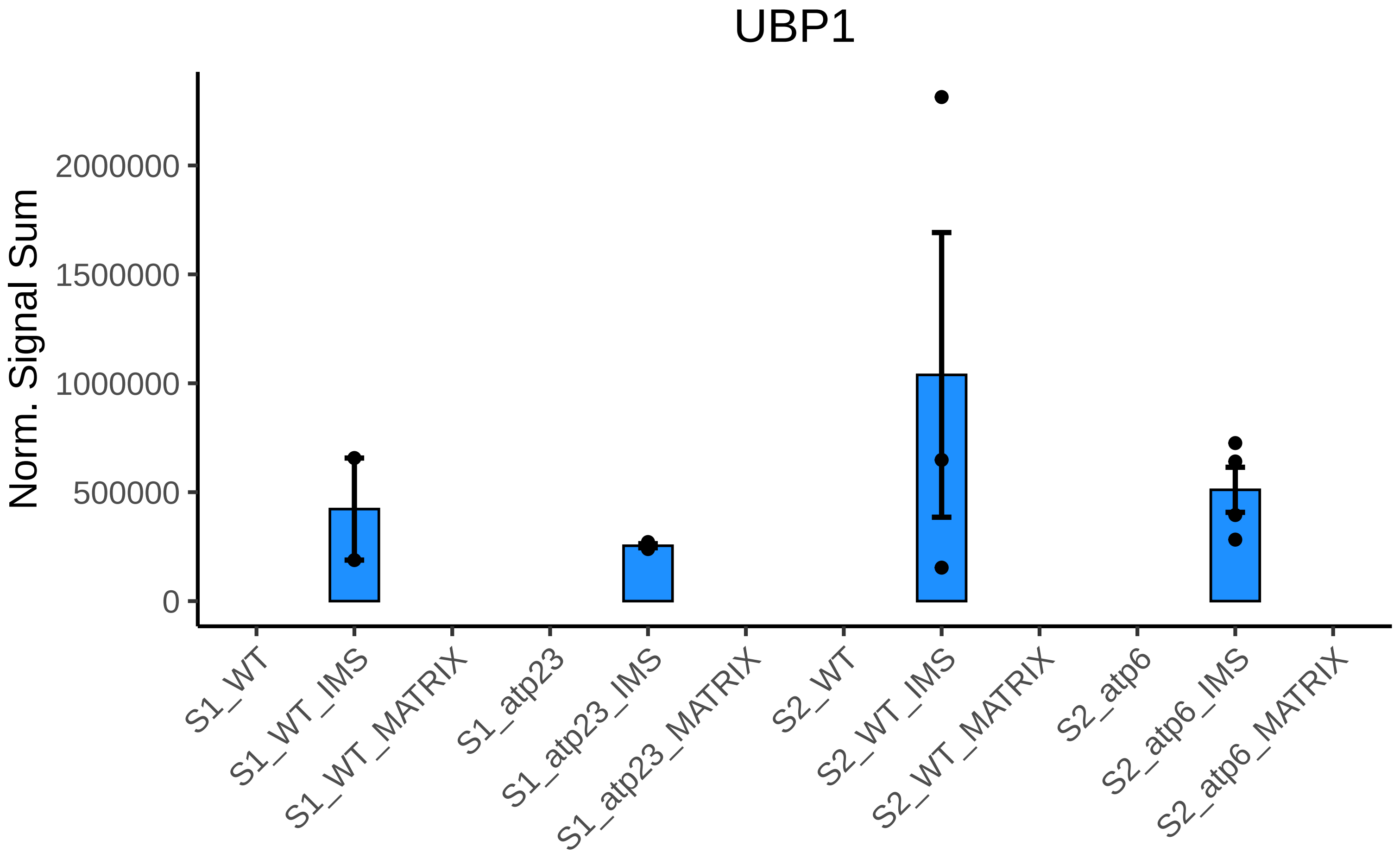
UBP1
Norm. Signal Sum
2000000
1500000
1000000
500000
0
S1_WT
S1_WT_IMS
S1_WT_MATRIX
S1_atp23
S1_atp23_IMS
S1_atp23_MATRIX
S2_WT
S2_WT_IMS
S2_WT_MATRIX
S2_atp6
S2_atp6_IMS
S2_atp6_MATRIX

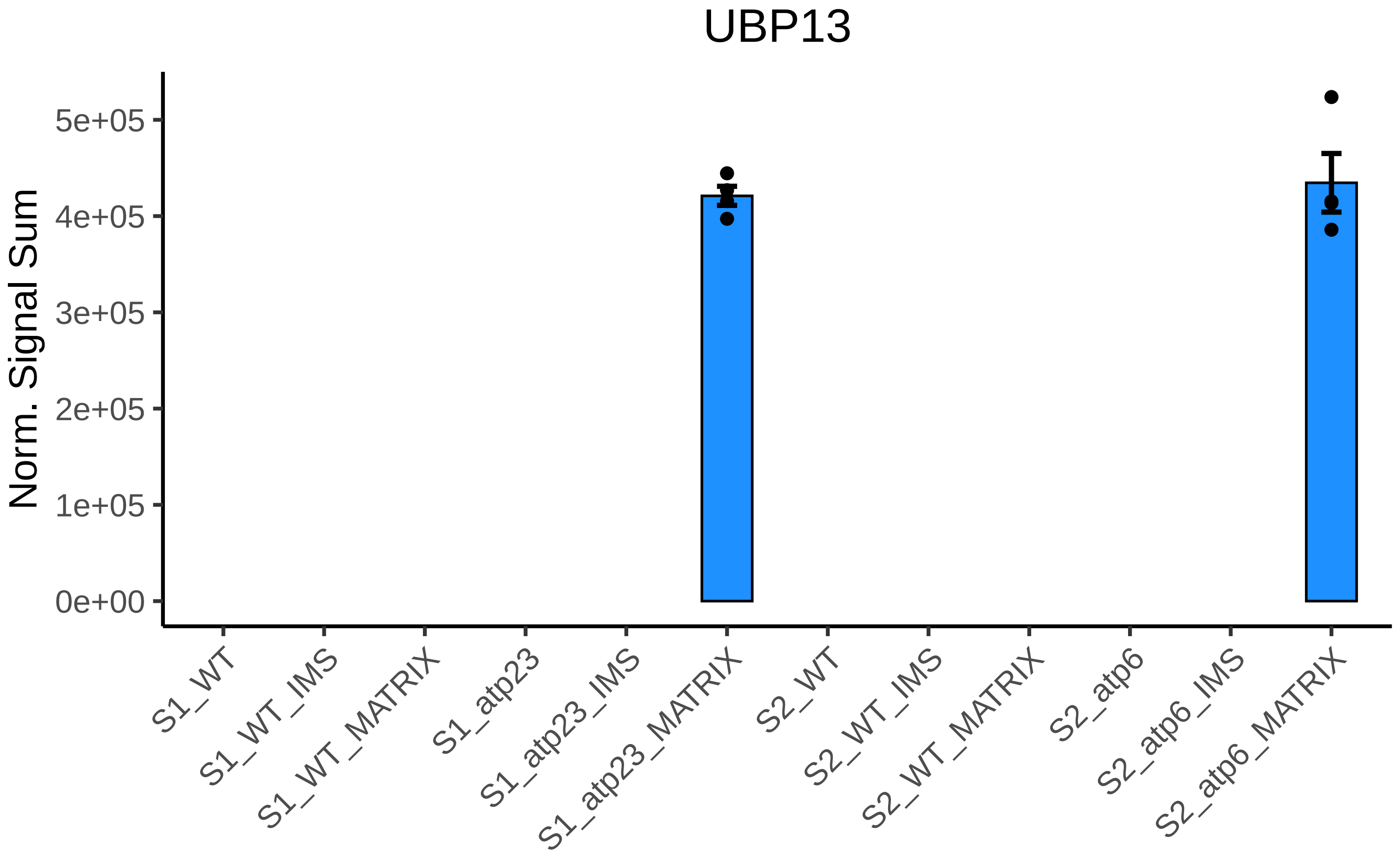
UBP13
Norm. Signal Sum
5e+05
4e+05
3e+05
2e+05
1e+05
0e+00
S1_WT
S1_WT_IMS
S1_WT_MATRIX
S1_atp23
S1_atp23_IMS
S1_atp23_MATRIX
S2_WT
S2_WT_IMS
S2_WT_MATRIX
S2_atp6
S2_atp6_IMS
S2_atp6_MATRIX

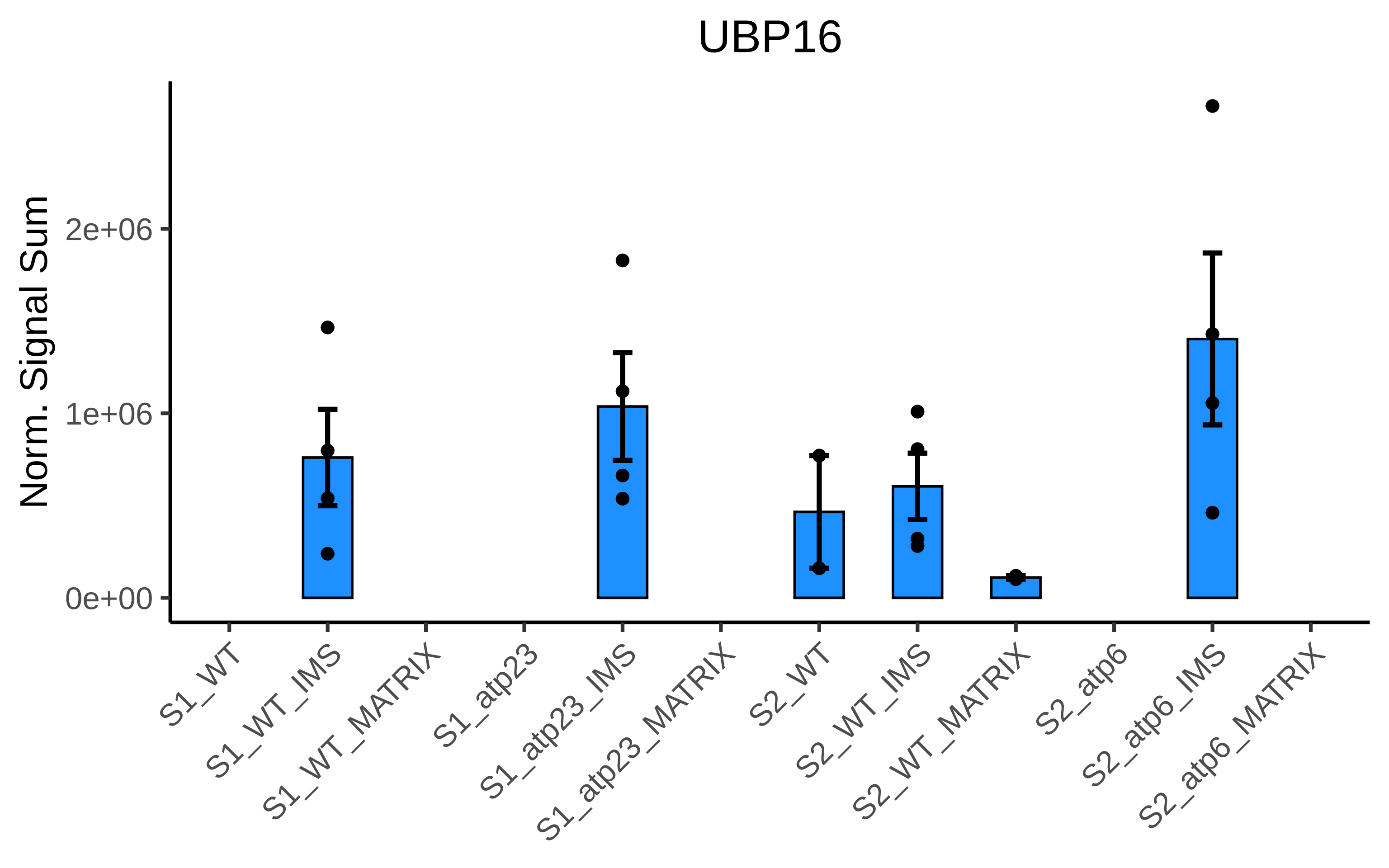

UBP16
Norm. Signal Sum
2e+06
1e+06
0e+00
S1_WT
S1_WT_IMS
S1_WT_MATRIX
S1_atp23
S1_atp23_IMS
S1_atp23_MATRIX
S2_WT
S2_WT_IMS
S2_WT_MATRIX
S2_atp6
S2_atp6_IMS
S2_atp6_MATRIX

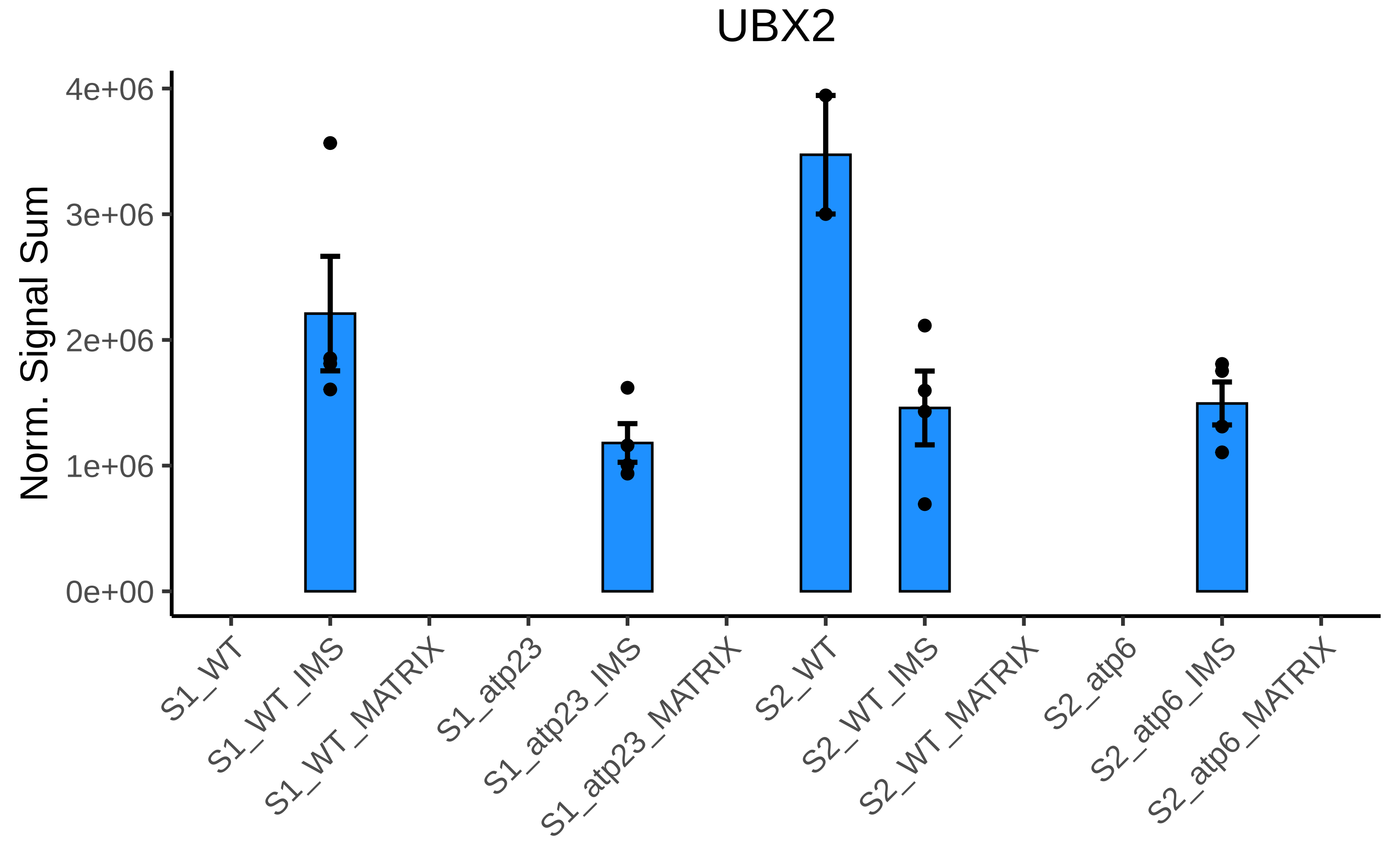

UBX2
Norm. Signal Sum
4e+06
3e+06
2e+06
1e+06
0e+00
S1_WT
S1_WT_IMS
S1_WT_MATRIX
S1_atp23
S1_atp23_IMS
S1_atp23_MATRIX
S2_WT
S2_WT_IMS
S2_WT_MATRIX
S2_atp6
S2_atp6_IMS
S2_atp6_MATRIX

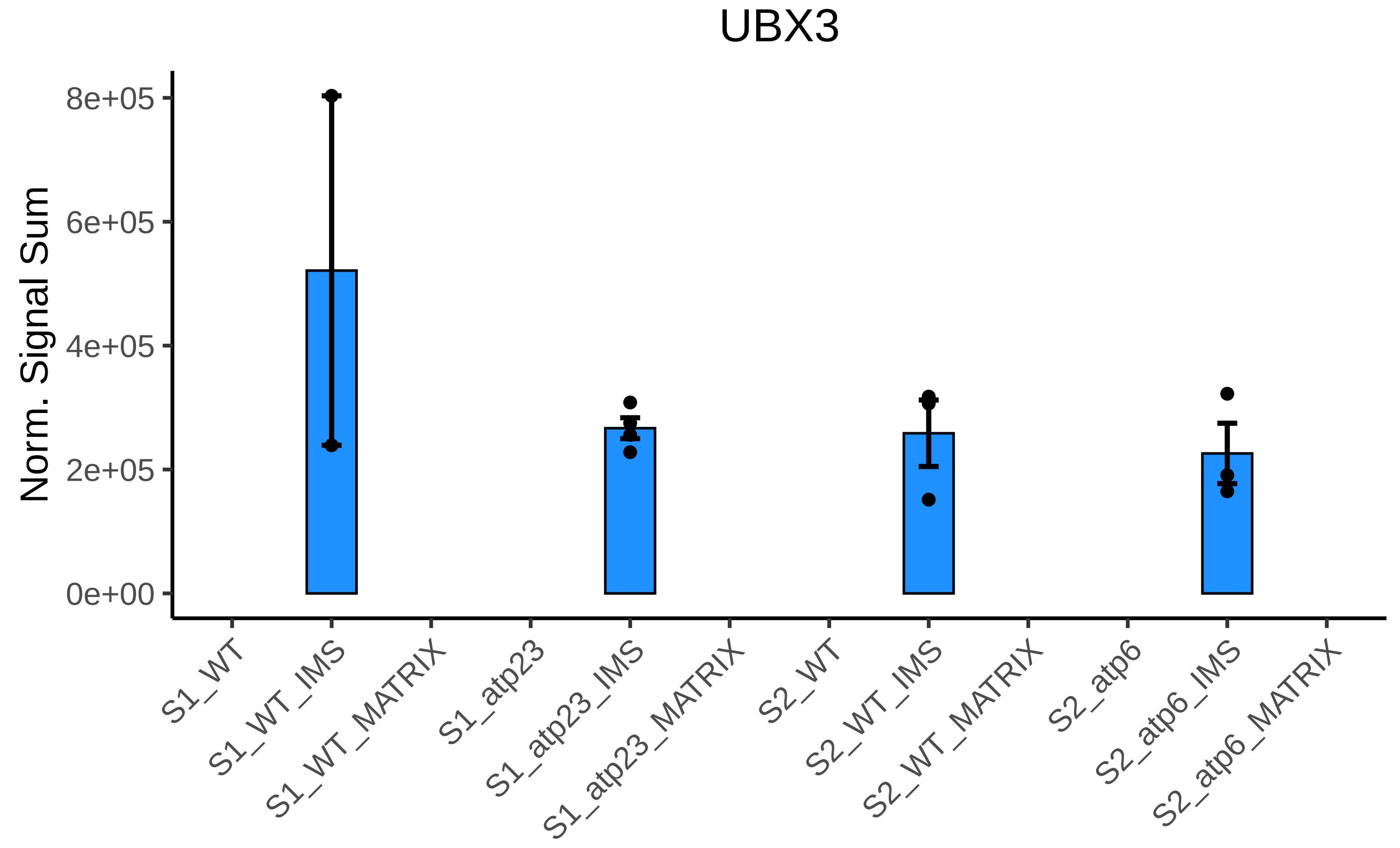

UBX3
Norm. Signal Sum
8e+05
6e+05
4e+05
2e+05
0e+00
S1_WT
S1_WT_IMS
S1_WT_MATRIX
S1_atp23
S1_atp23_IMS
S1_atp23_MATRIX
S2_WT
S2_WT_IMS
S2_WT_MATRIX
S2_atp6
S2_atp6_IMS
S2_atp6_MATRIX

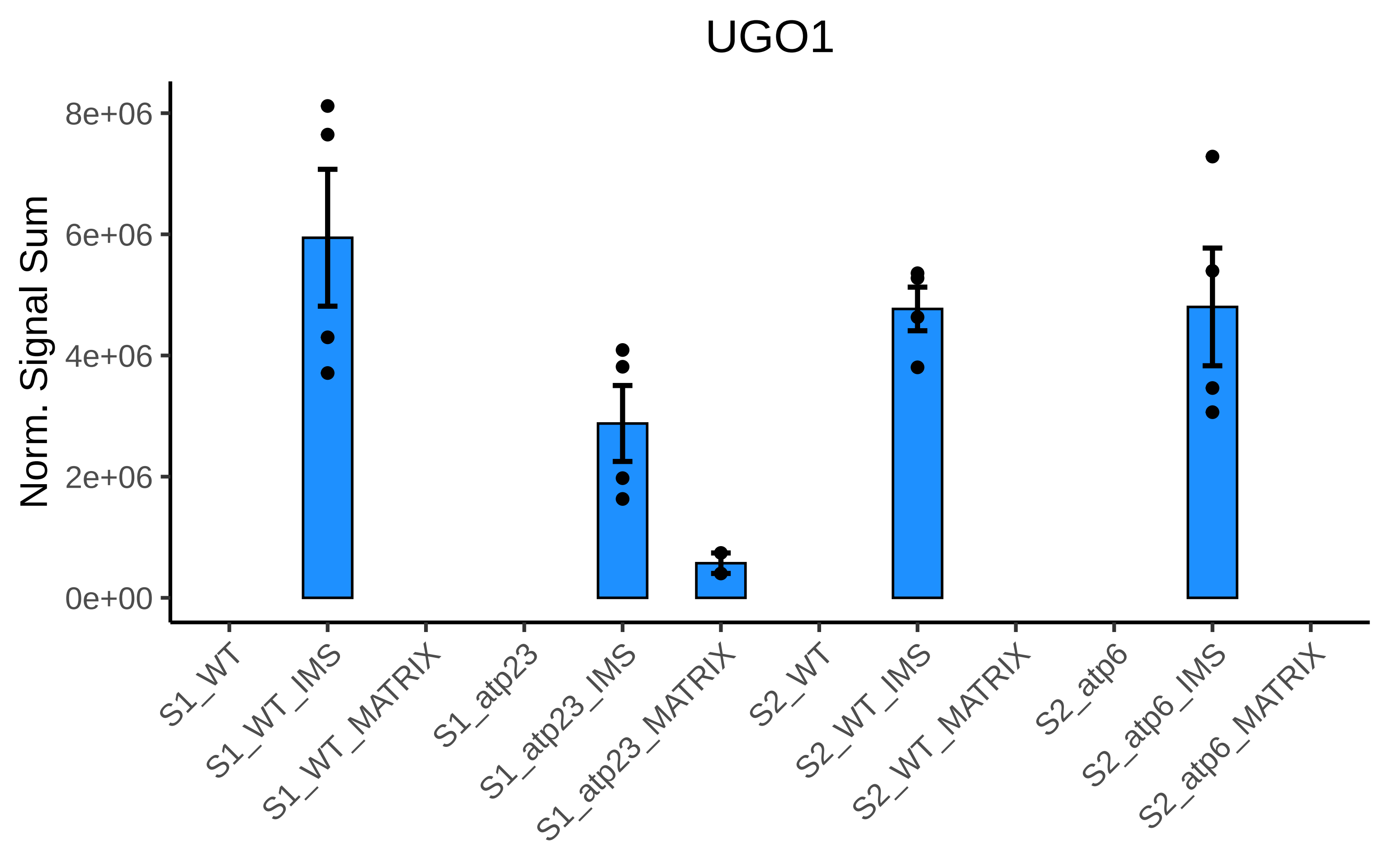

UGO1
Norm. Signal Sum
8e+06
6e+06
4e+06
2e+06
0e+00
S1_WT
S1_WT_IMS
S1_WT_MATRIX
S1_atp23
S1_atp23_IMS
S1_atp23_MATRIX
S2_WT
S2_WT_IMS
S2_WT_MATRIX
S2_atp6
S2_atp6_IMS
S2_atp6_MATRIX

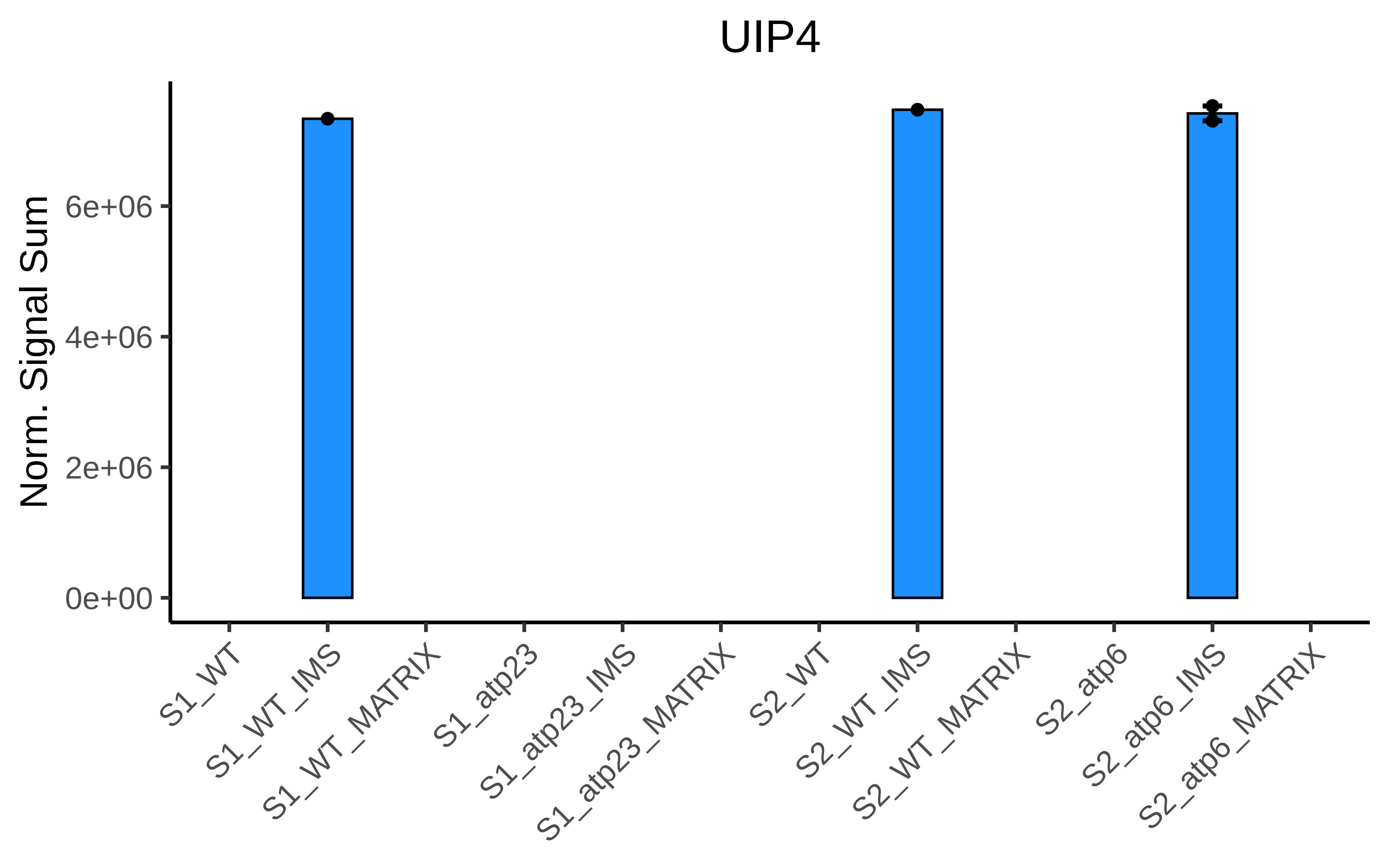
UIP4
Norm. Signal Sum
6e+06
4e+06
2e+06
0e+00
S1_WT
S1_WT_IMS
S1_WT_MATRIX
S1_atp23
S1_atp23_IMS
S1_atp23_MATRIX
S2_WT
S2_WT_IMS
S2_WT_MATRIX
S2_atp6
S2_atp6_IMS
S2_atp6_MATRIX

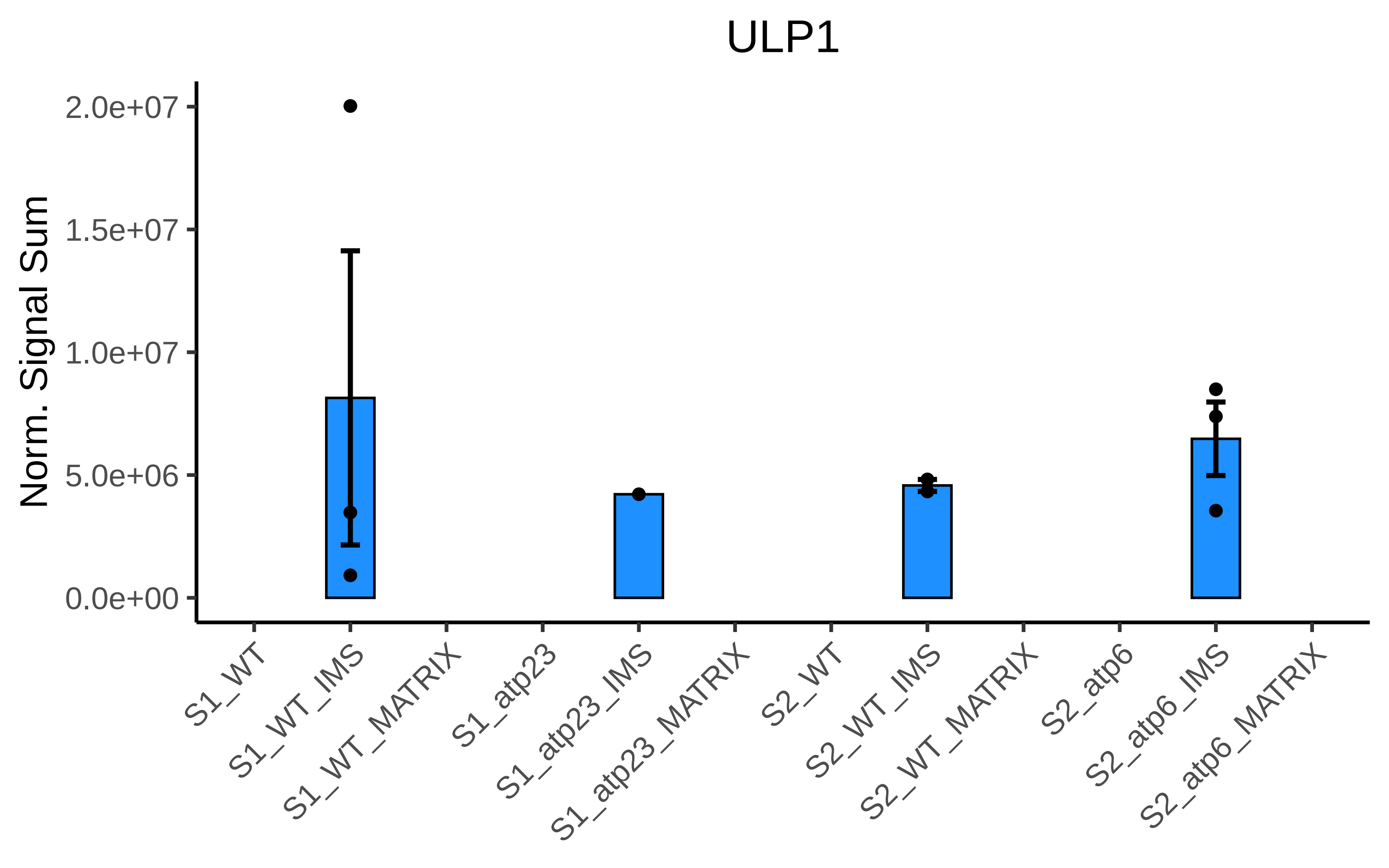
ULP1
Norm. Signal Sum
2.0e+07
1.5e+07
1.0e+07
5.0e+06
0.0e+00
S1_WT
S1_WT_IMS
S1_WT_MATRIX
S1_atp23
S1_atp23_IMS
S1_atp23_MATRIX
S2_WT
S2_WT_IMS
S2_WT_MATRIX
S2_atp6
S2_atp6_IMS
S2_atp6_MATRIX

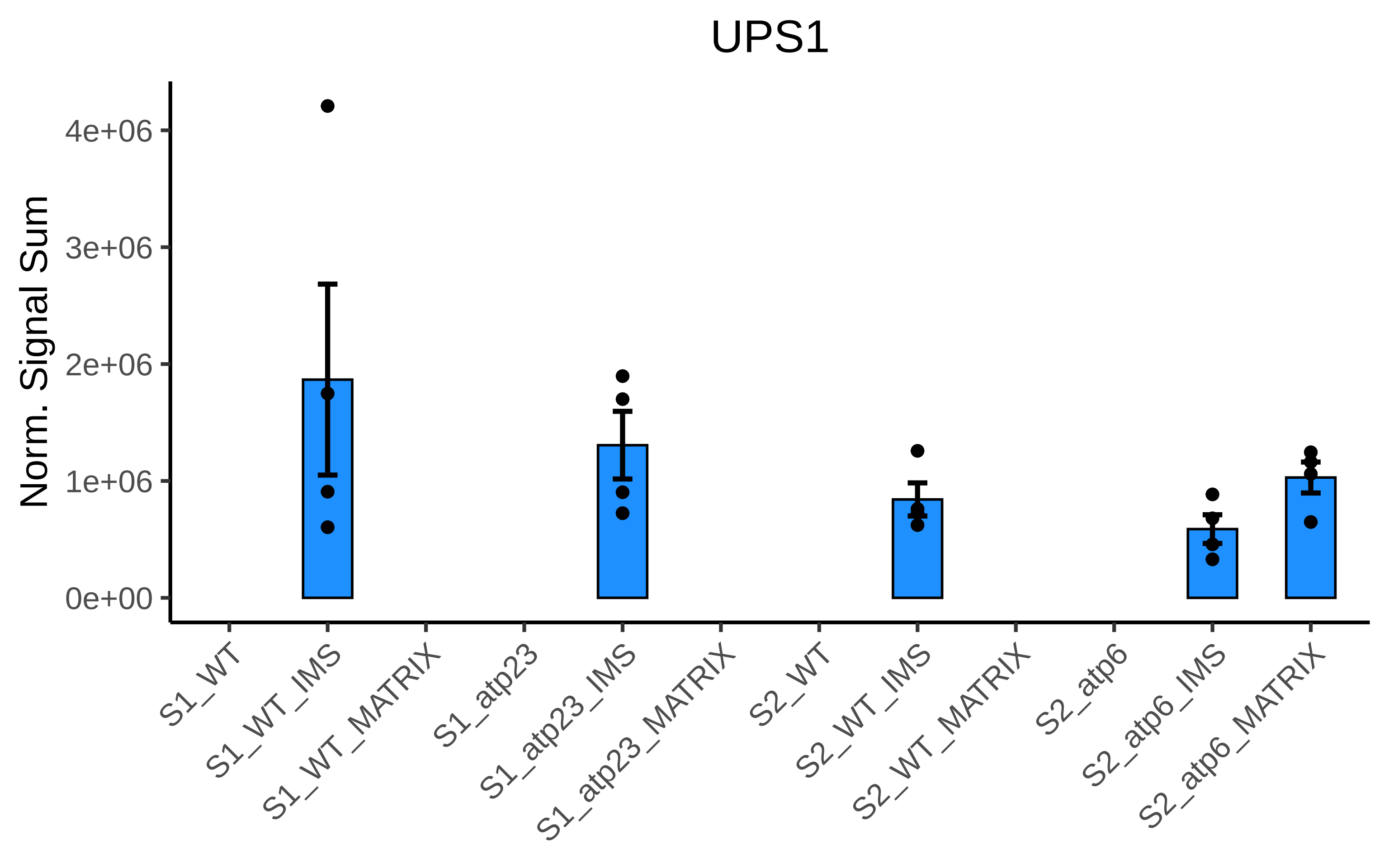
UPS1
Norm. Signal Sum
4e+06
3e+06
2e+06
1e+06
0e+00
S1_WT
S1_WT_IMS
S1_WT_MATRIX
S1_atp23
S1_atp23_IMS
S1_atp23_MATRIX
S2_WT
S2_WT_IMS
S2_WT_MATRIX
S2_atp6
S2_atp6_IMS
S2_atp6_MATRIX

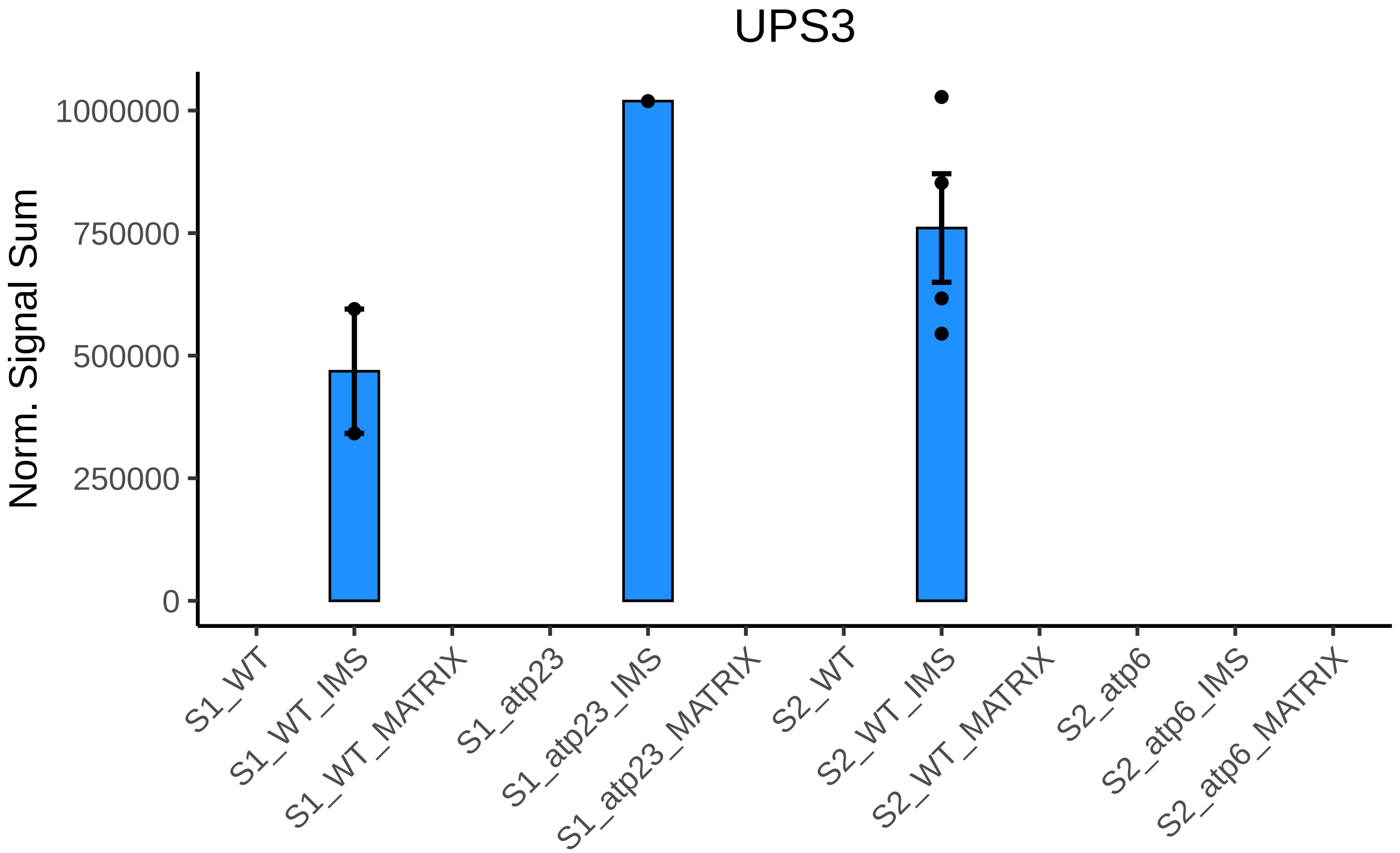
UPS3
Norm. Signal Sum
1000000
750000
500000
250000
0
S1_WT
S1_WT_IMS
S1_WT_MATRIX
S1_atp23
S1_atp23_IMS
S1_atp23_MATRIX
S2_WT
S2_WT_IMS
S2_WT_MATRIX
S2_atp6
S2_atp6_IMS
S2_atp6_MATRIX

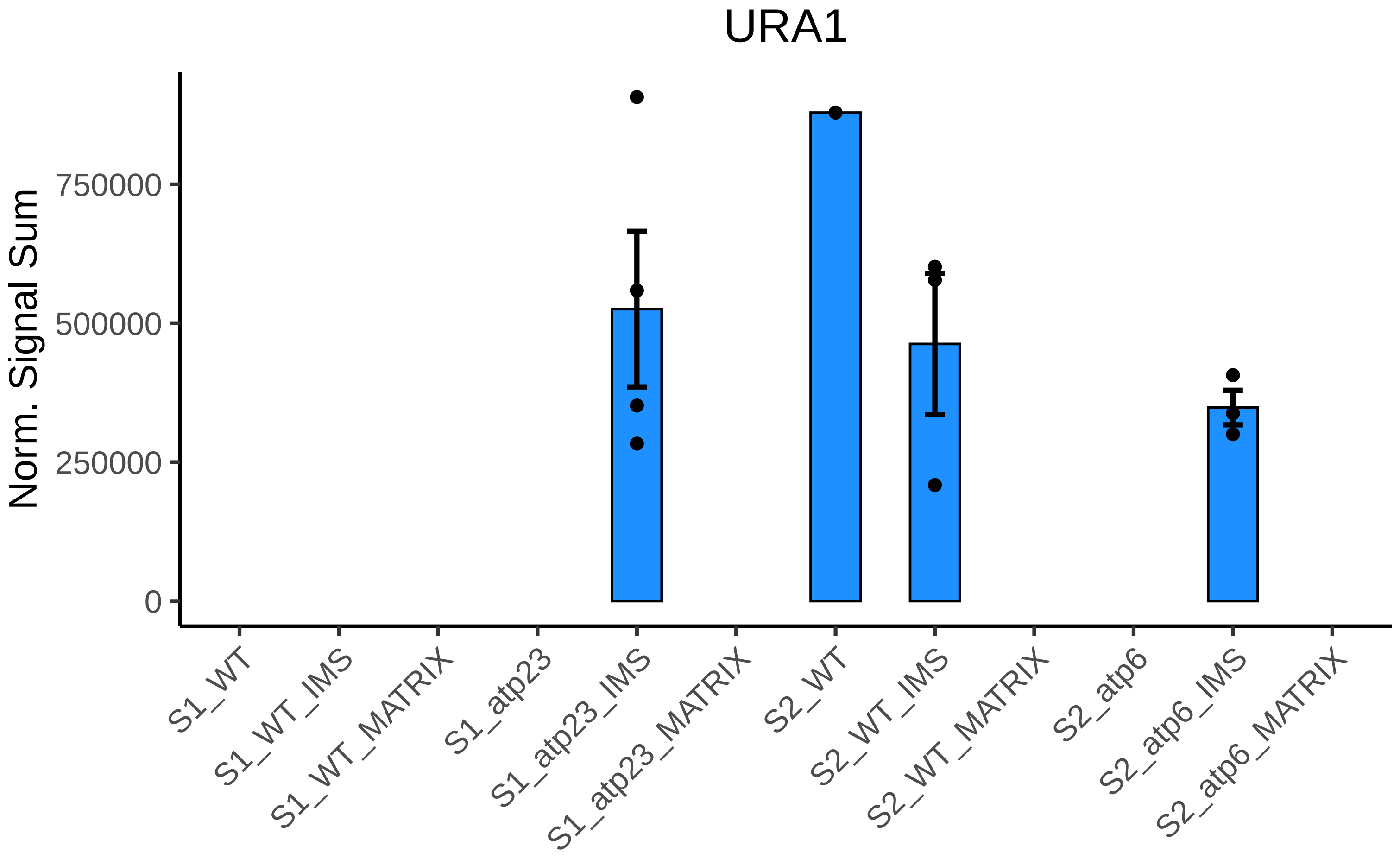
URA1
Norm. Signal Sum
750000
500000
250000
0
S1_WT
S1_WT_IMS
S1_WT_MATRIX
S1_atp23
S1_atp23_IMS
S1_atp23_MATRIX
S2_WT
S2_WT_IMS
S2_WT_MATRIX
S2_atp6
S2_atp6_IMS
S2_atp6_MATRIX

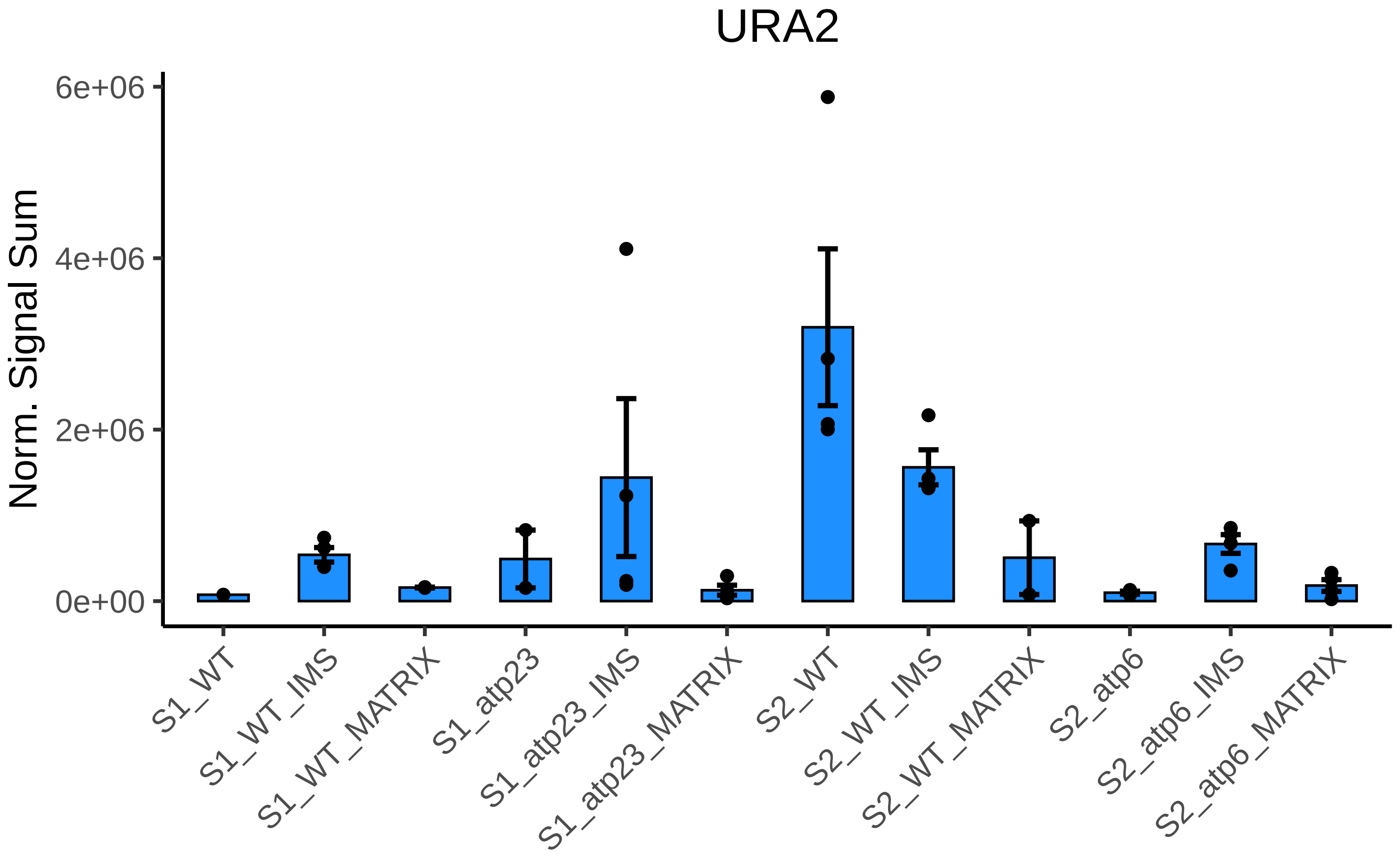

URA2
Norm. Signal Sum
6e+06
4e+06
2e+06
0e+00
S1_WT
S1_WT_IMS
S1_WT_MATRIX
S1_atp23
S1_atp23_IMS
S1_atp23_MATRIX
S2_WT
S2_WT_IMS
S2_WT_MATRIX
S2_atp6
S2_atp6_IMS
S2_atp6_MATRIX

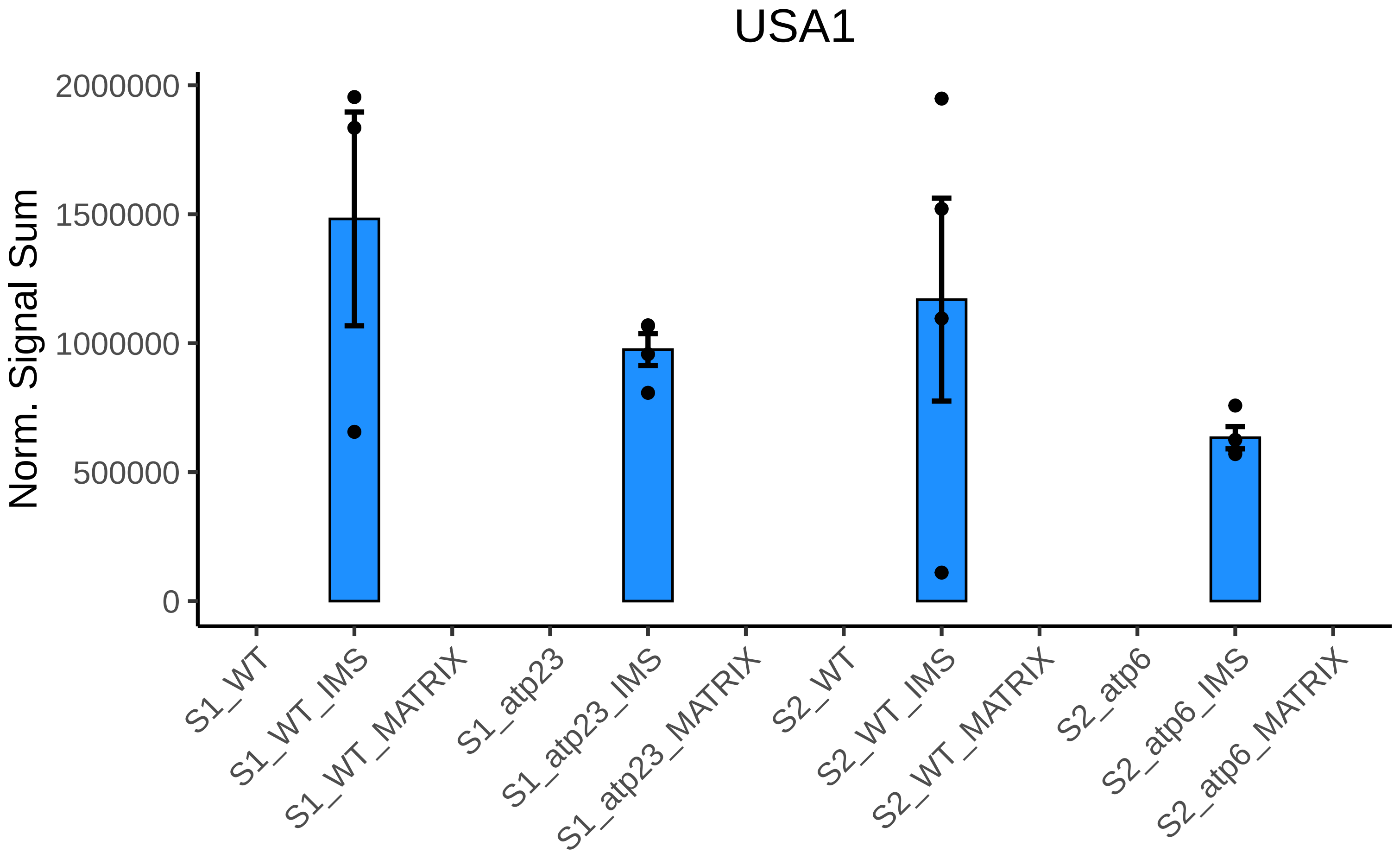
USA1
Norm. Signal Sum
2000000
1500000
1000000
500000
0
S1_WT
S1_WT_IMS
S1_WT_MATRIX
S1_atp23
S1_atp23_IMS
S1_atp23_MATRIX
S2_WT
S2_WT_IMS
S2_WT_MATRIX
S2_atp6
S2_atp6_IMS
S2_atp6_MATRIX

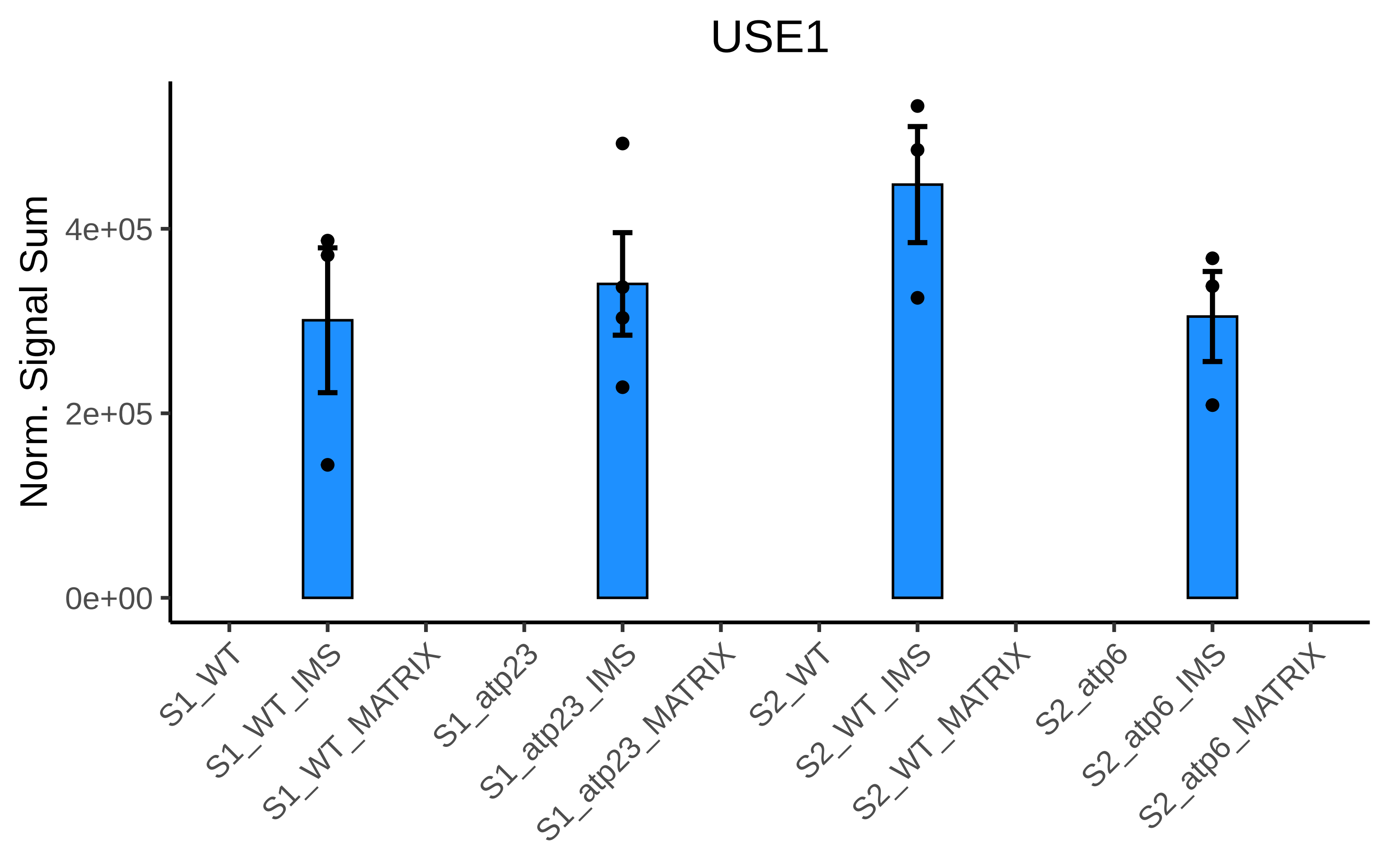
USE1
Norm. Signal Sum
4e+05
2e+05
0e+00
S1_WT
S1_WT_IMS
S1_WT_MATRIX
S1_atp23
S1_atp23_IMS
S1_atp23_MATRIX
S2_WT
S2_WT_IMS
S2_WT_MATRIX
S2_atp6
S2_atp6_IMS
S2_atp6_MATRIX

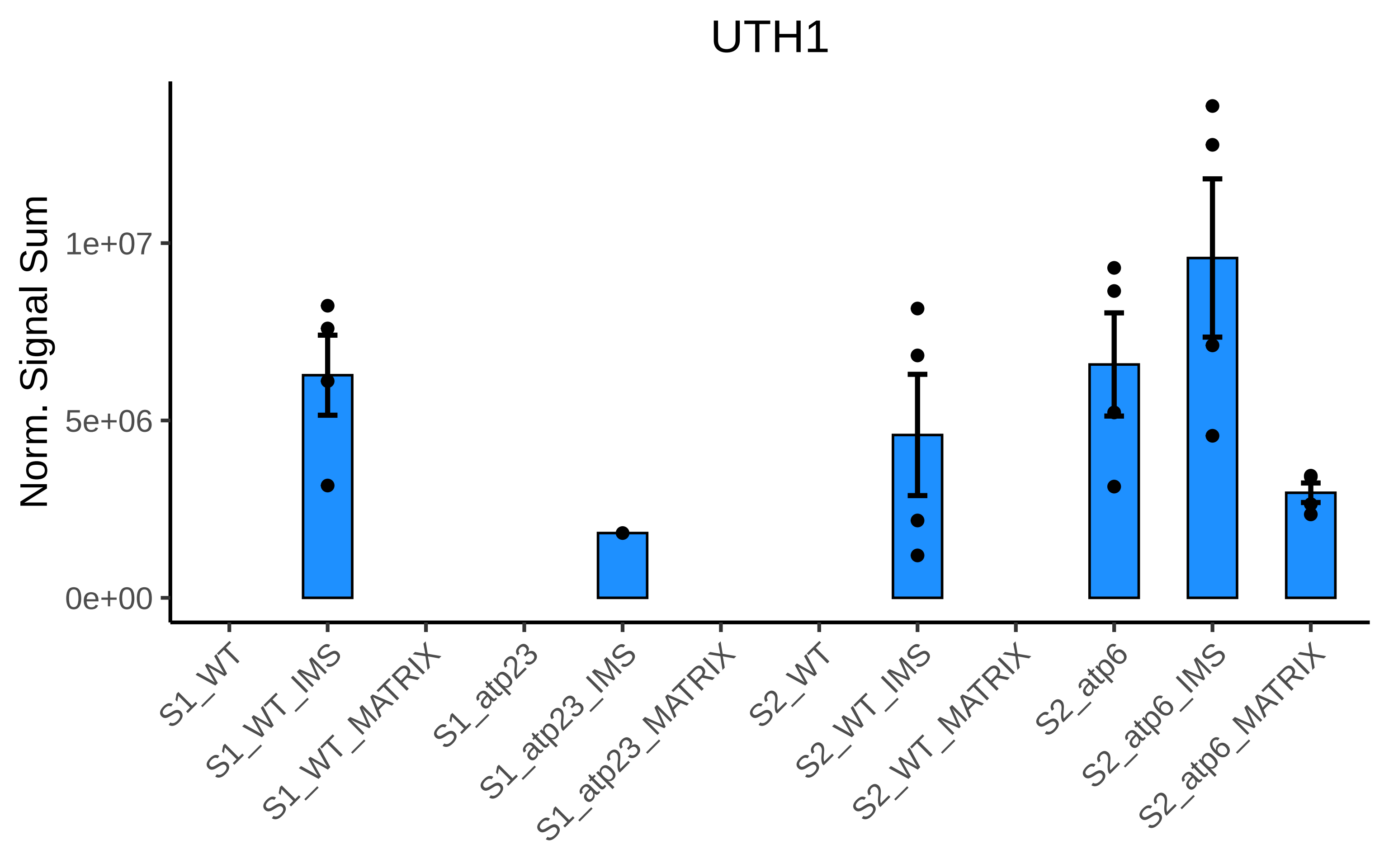

UTH1
Norm. Signal Sum
1e+07
5e+06
0e+00
S1_WT
S1_WT_IMS
S1_WT_MATRIX
S1_atp23
S1_atp23_IMS
S1_atp23_MATRIX
S2_WT
S2_WT_IMS
S2_WT_MATRIX
S2_atp6
S2_atp6_IMS
S2_atp6_MATRIX

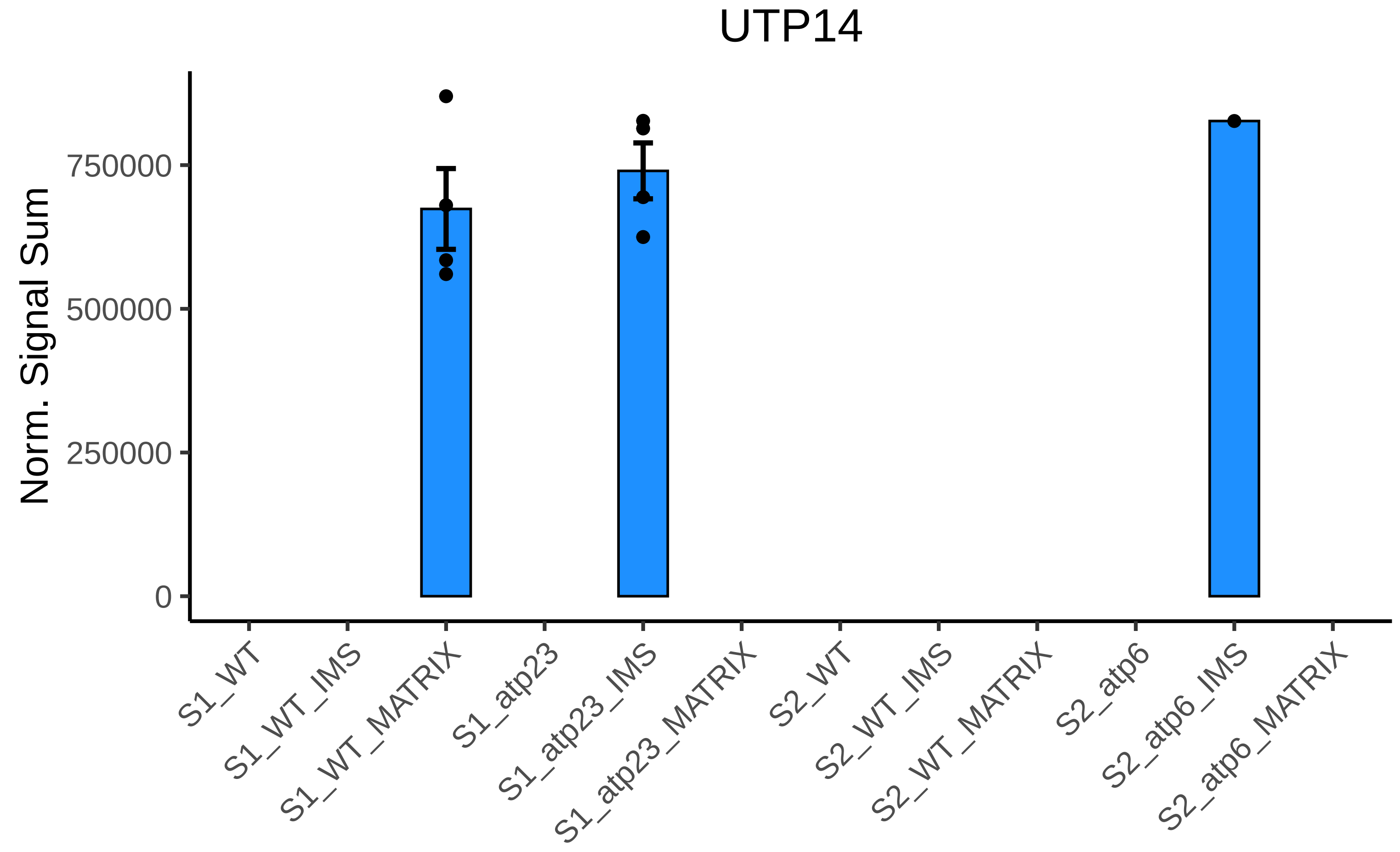

UTP14
Norm. Signal Sum
750000
500000
250000
0
S1_WT
S1_WT_IMS
S1_WT_MATRIX
S1_atp23
S1_atp23_IMS
S1_atp23_MATRIX
S2_WT
S2_WT_IMS
S2_WT_MATRIX
S2_atp6
S2_atp6_IMS
S2_atp6_MATRIX

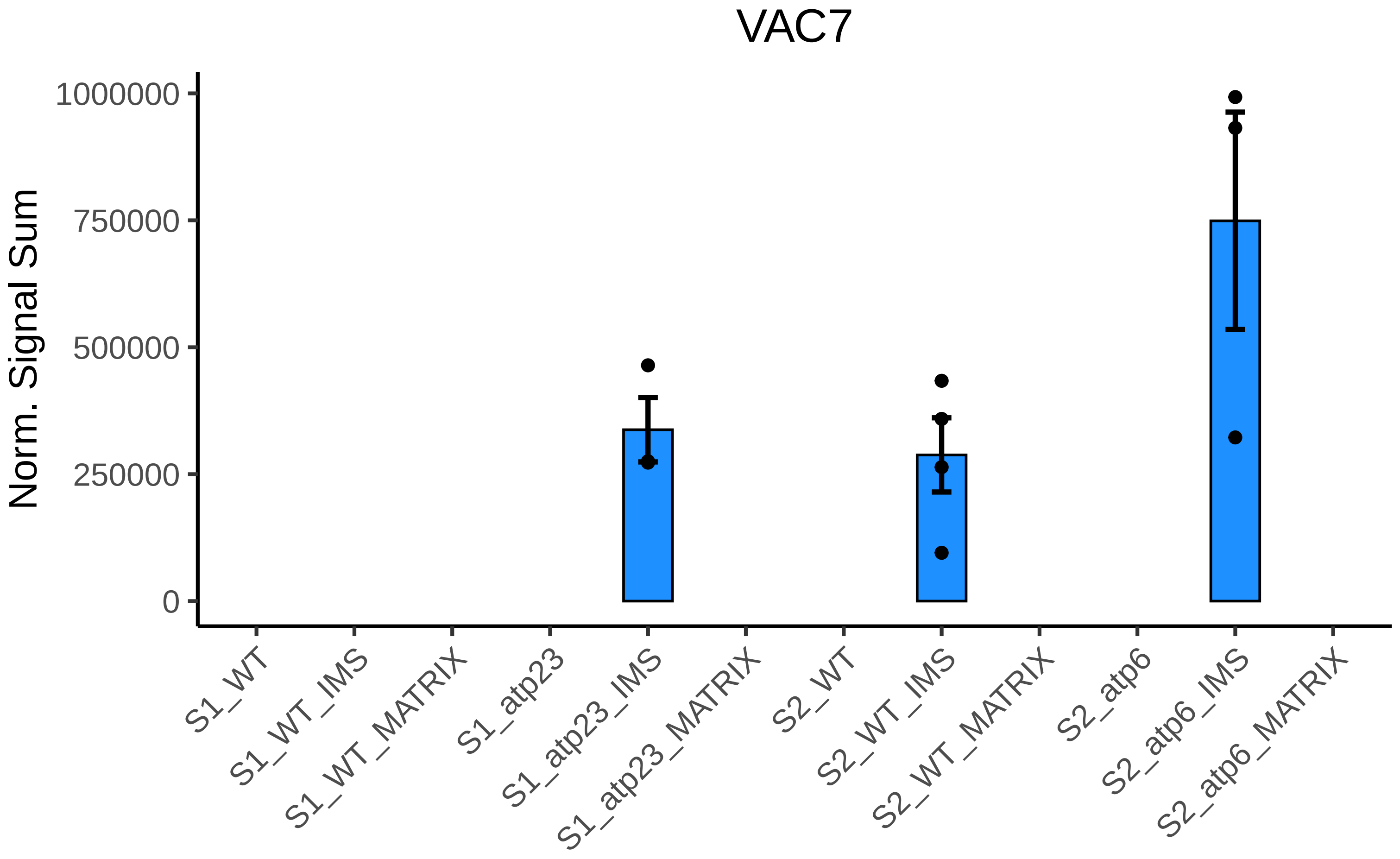

VAC7
Norm. Signal Sum
1000000
750000
500000
250000
0
S1_WT
S1_WT_IMS
S1_WT_MATRIX
S1_atp23
S1_atp23_IMS
S1_atp23_MATRIX
S2_WT
S2_WT_IMS
S2_WT_MATRIX
S2_atp6
S2_atp6_IMS
S2_atp6_MATRIX

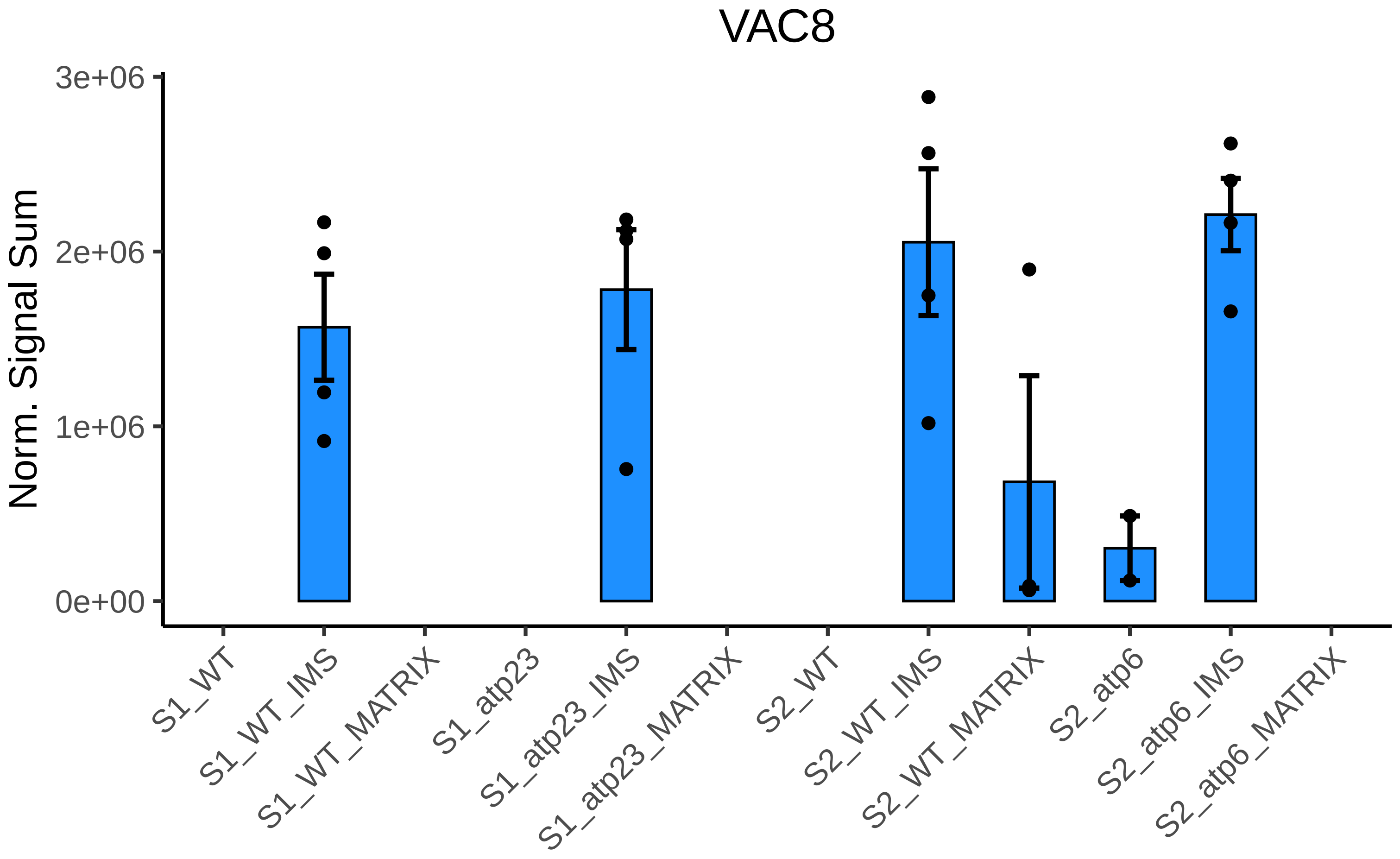

VAC8
Norm. Signal Sum
3e+06
2e+06
1e+06
0e+00
S1_WT
S1_WT_IMS
S1_WT_MATRIX
S1_atp23
S1_atp23_IMS
S1_atp23_MATRIX
S2_WT
S2_WT_IMS
S2_WT_MATRIX
S2_atp6
S2_atp6_IMS
S2_atp6_MATRIX

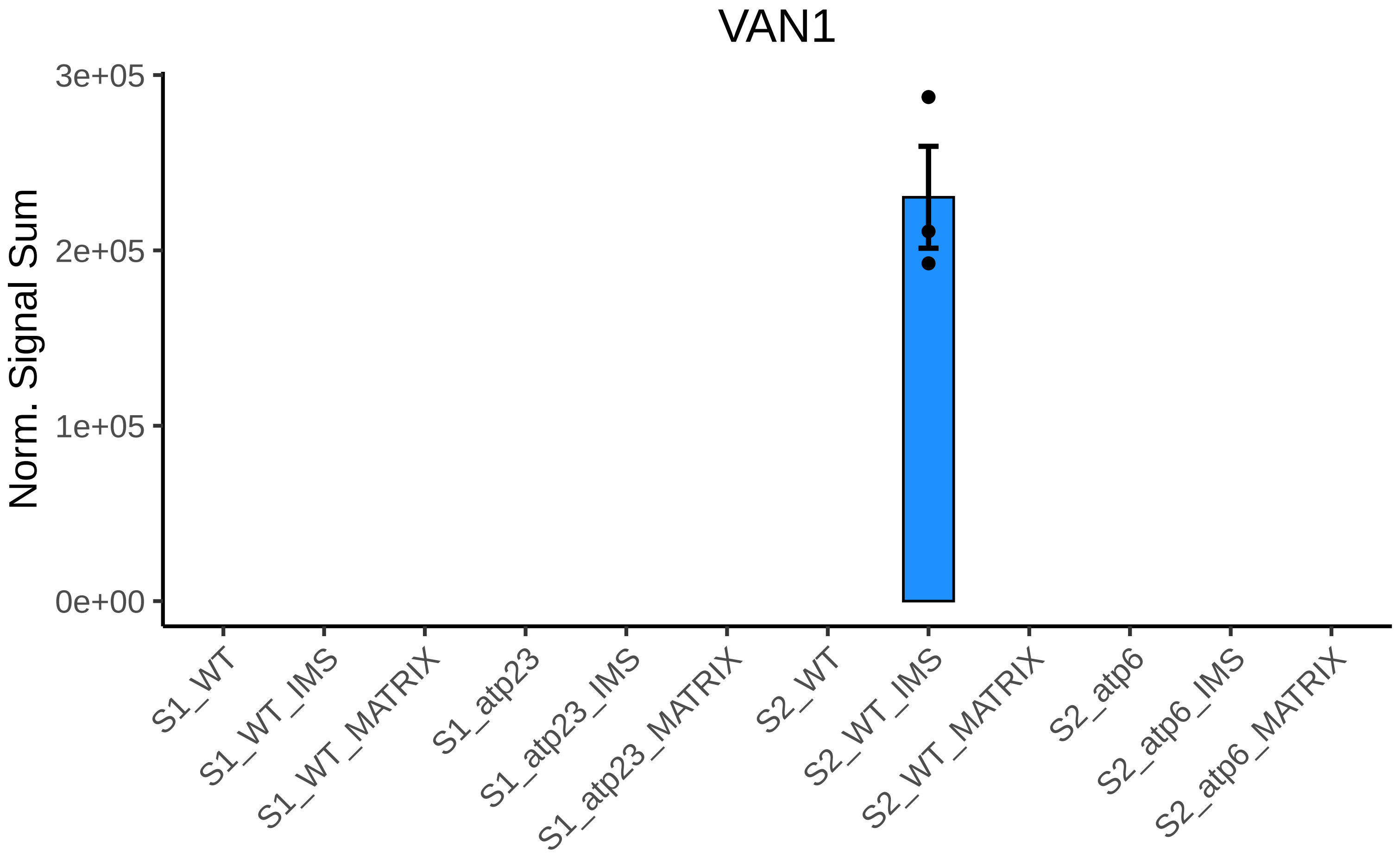
VAN1
Norm. Signal Sum
3e+05
2e+05
1e+05
0e+00
S1_WT
S1_WT_IMS
S1_WT_MATRIX
S1_atp23
S1_atp23_IMS
S1_atp23_MATRIX
S2_WT
S2_WT_IMS
S2_WT_MATRIX
S2_atp6
S2_atp6_IMS
S2_atp6_MATRIX

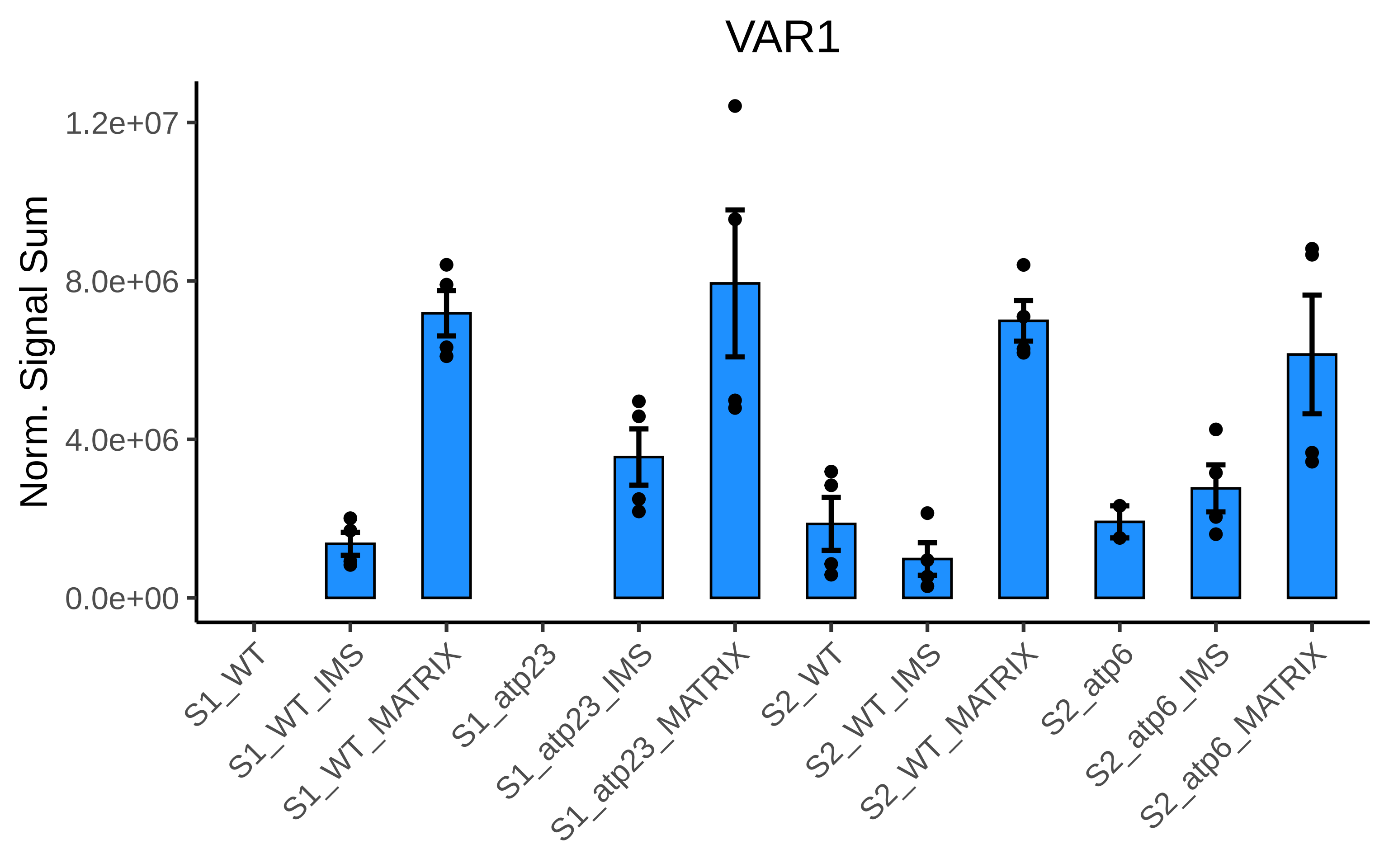

VAR1
Norm. Signal Sum
1.2e+07
8.0e+06
4.0e+06
0.0e+00
S1_WT
S1_WT_IMS
S1_WT_MATRIX
S1_atp23
S1_atp23_IMS
S1_atp23_MATRIX
S2_WT
S2_WT_IMS
S2_WT_MATRIX
S2_atp6
S2_atp6_IMS
S2_atp6_MATRIX

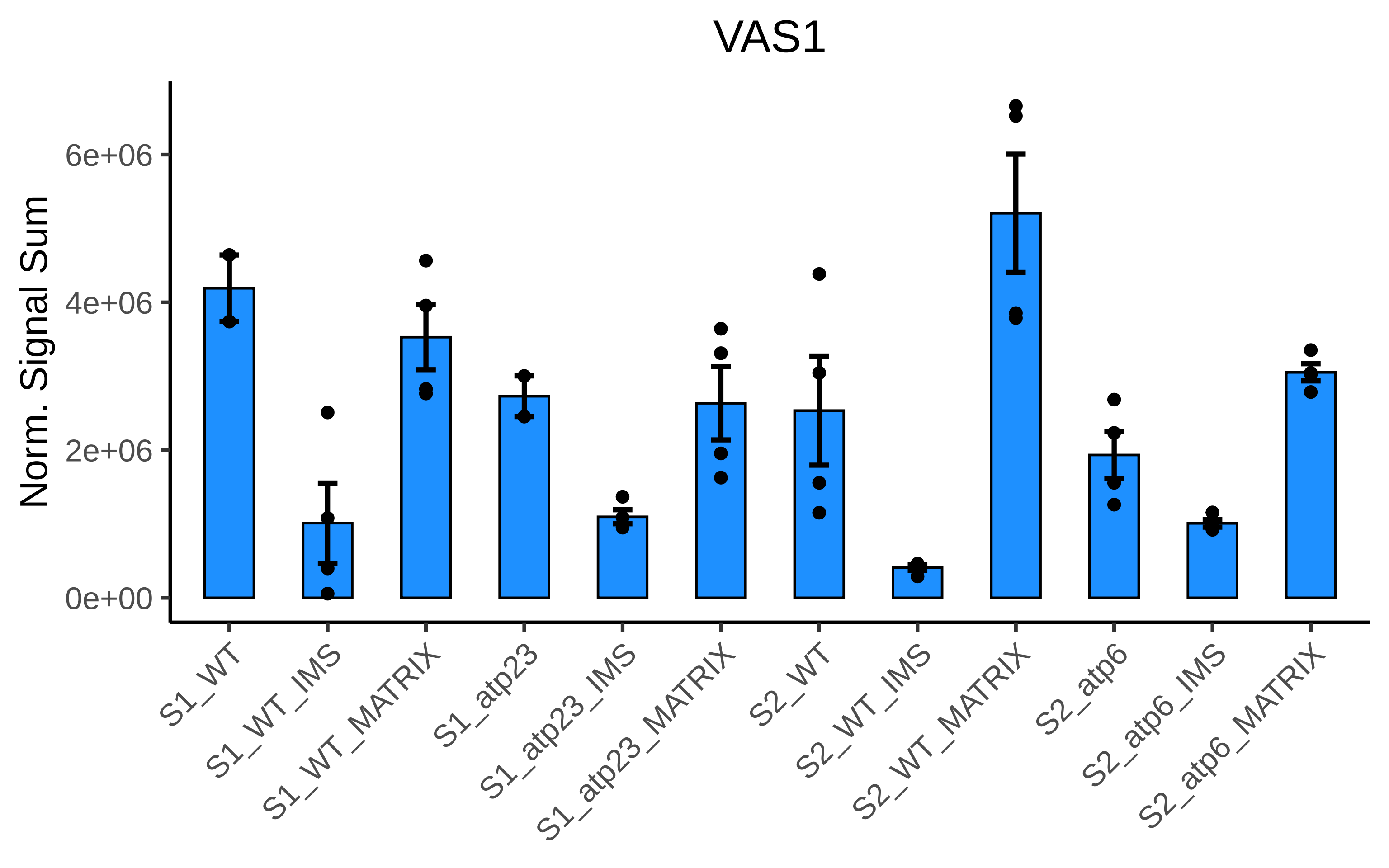

VAS1
Norm. Signal Sum
6e+06
4e+06
2e+06
0e+00
S1_WT
S1_WT_IMS
S1_WT_MATRIX
S1_atp23
S1_atp23_IMS
S1_atp23_MATRIX
S2_WT
S2_WT_IMS
S2_WT_MATRIX
S2_atp6
S2_atp6_IMS
S2_atp6_MATRIX

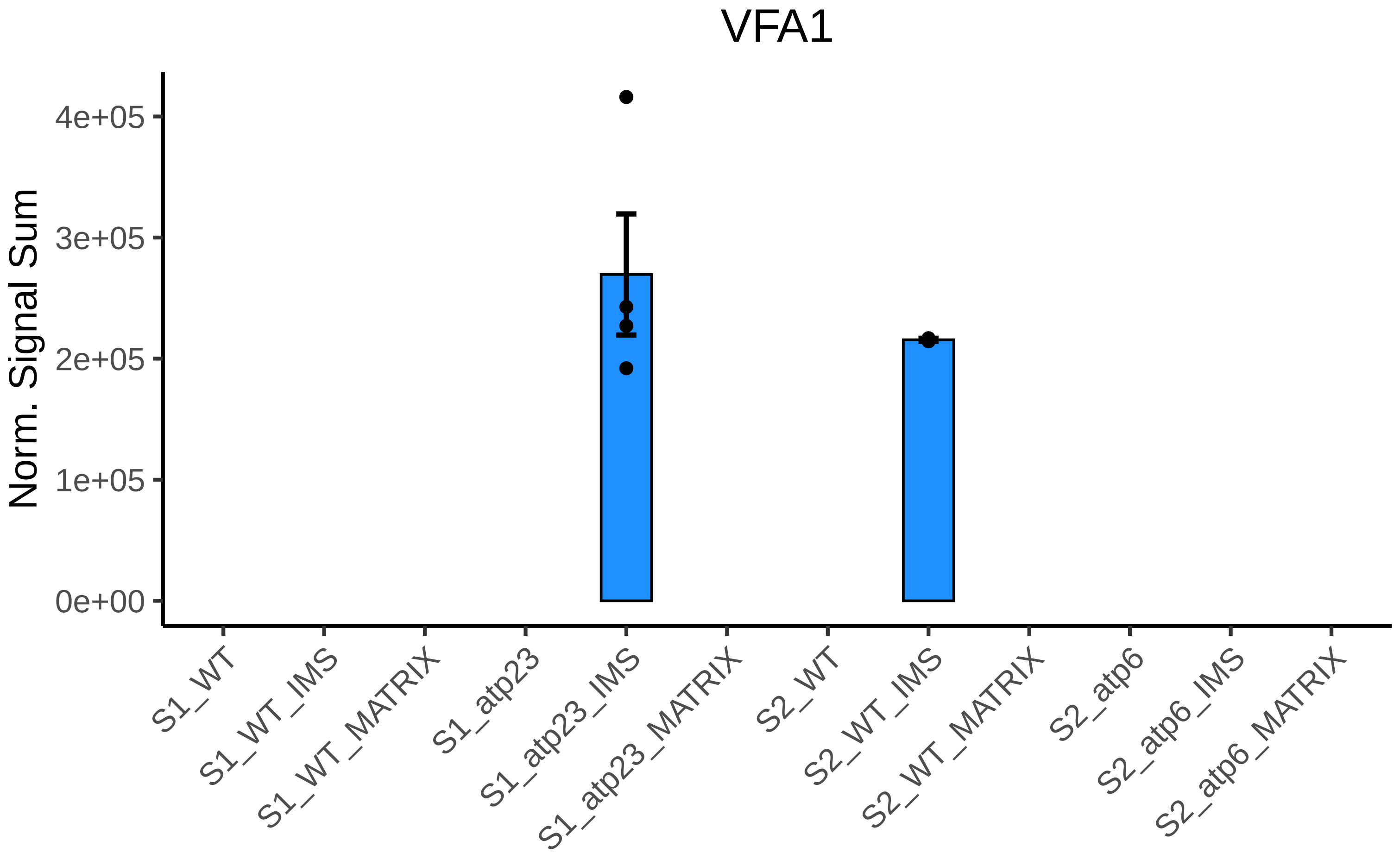

VFA1
Norm. Signal Sum
0e+00
1e+05
2e+05
3e+05
4e+05
S1_WT
S1_WT_IMS
S1_WT_MATRIX
S1_atp23
S1_atp23_IMS
S1_atp23_MATRIX
S2_WT
S2_WT_IMS
S2_WT_MATRIX
S2_atp6
S2_atp6_IMS
S2_atp6_MATRIX

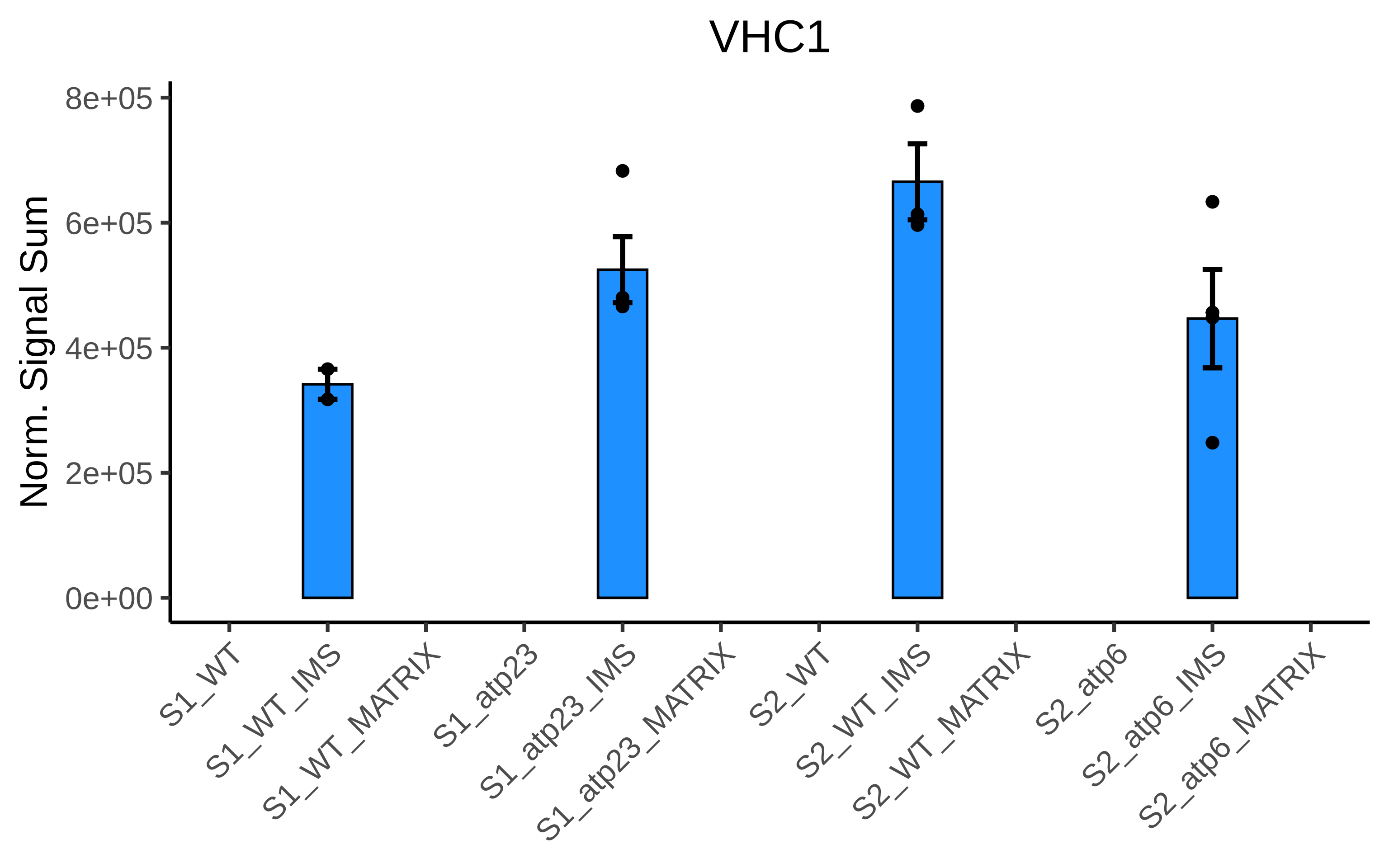

VHC1
Norm. Signal Sum
8e+05
6e+05
4e+05
2e+05
0e+00
S1_WT
S1_WT_IMS
S1_WT_MATRIX
S1_atp23
S1_atp23_IMS
S1_atp23_MATRIX
S2_WT
S2_WT_IMS
S2_WT_MATRIX
S2_atp6
S2_atp6_IMS
S2_atp6_MATRIX

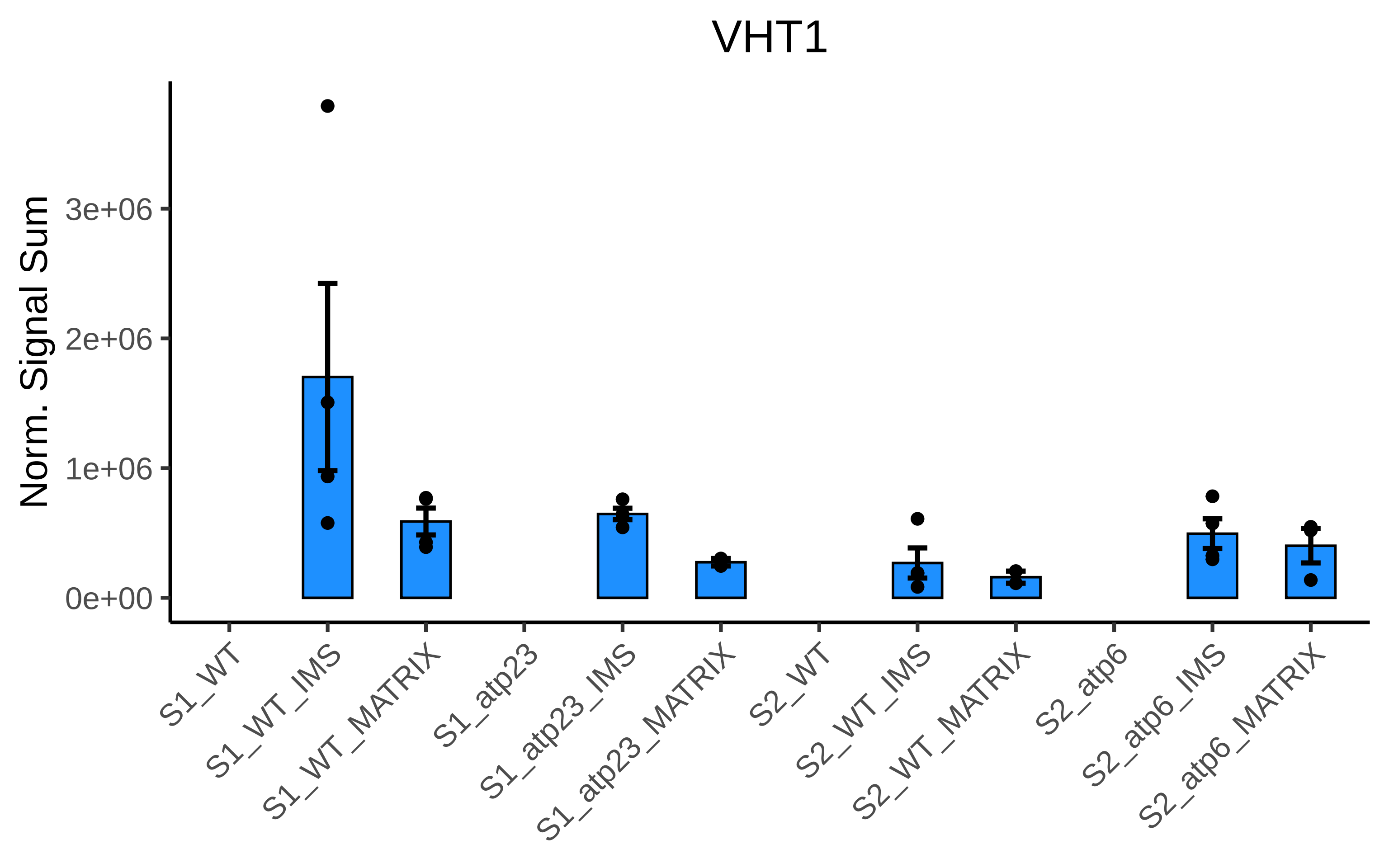
VHT1
Norm. Signal Sum
3e+06
2e+06
1e+06
0e+00
S1_WT
S1_WT_IMS
S1_WT_MATRIX
S1_atp23
S1_atp23_IMS
S1_atp23_MATRIX
S2_WT
S2_WT_IMS
S2_WT_MATRIX
S2_atp6
S2_atp6_IMS
S2_atp6_MATRIX

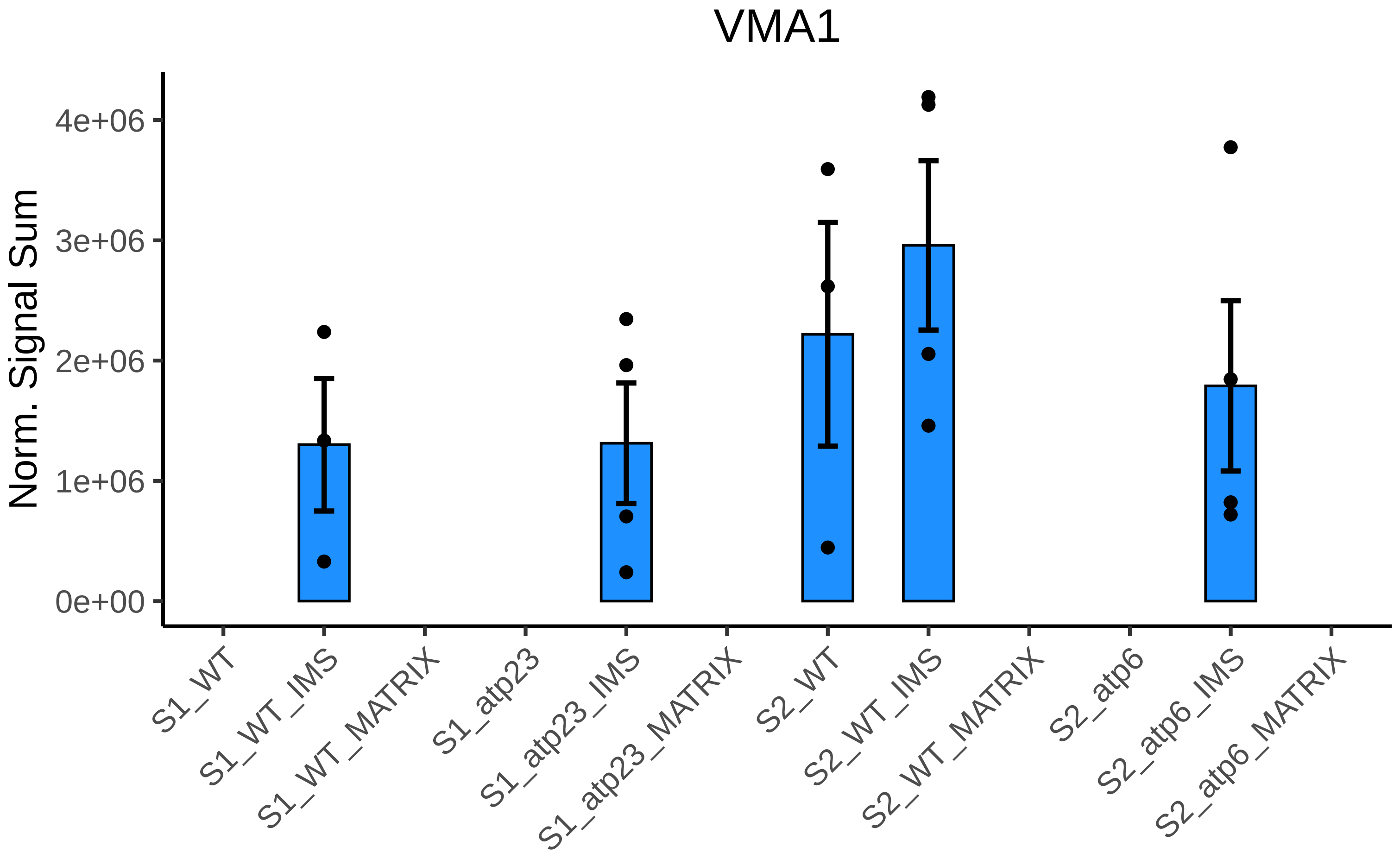
VMA1
Norm. Signal Sum
4e+06
3e+06
2e+06
1e+06
0e+00
S1_WT
S1_WT_IMS
S1_WT_MATRIX
S1_atp23
S1_atp23_IMS
S1_atp23_MATRIX
S2_WT
S2_WT_IMS
S2_WT_MATRIX
S2_atp6
S2_atp6_IMS
S2_atp6_MATRIX

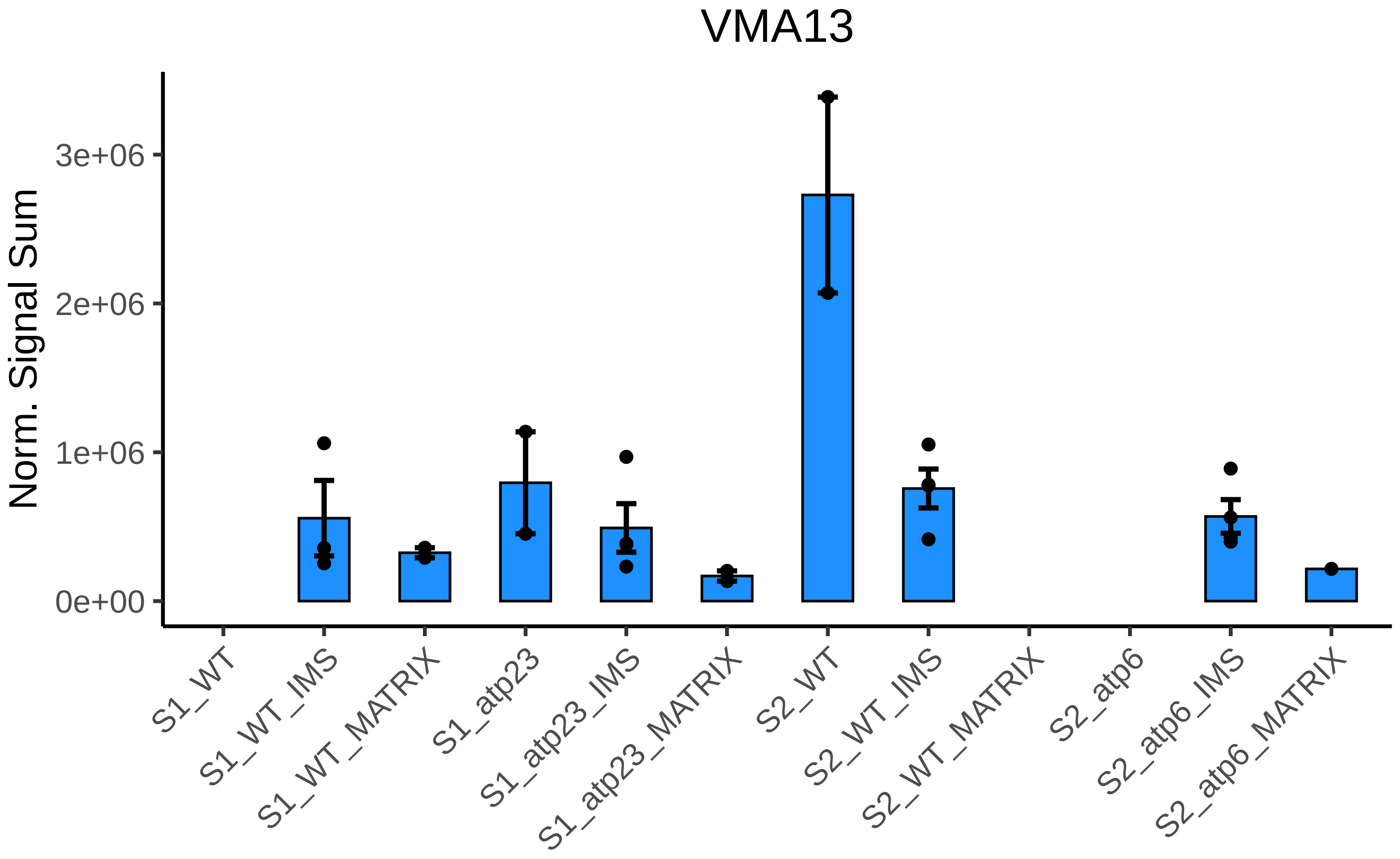
VMA13
Norm. Signal Sum
3e+06
2e+06
1e+06
0e+00
S1_WT
S1_WT_IMS
S1_WT_MATRIX
S1_atp23
S1_atp23_IMS
S1_atp23_MATRIX
S2_WT
S2_WT_IMS
S2_WT_MATRIX
S2_atp6
S2_atp6_IMS
S2_atp6_MATRIX

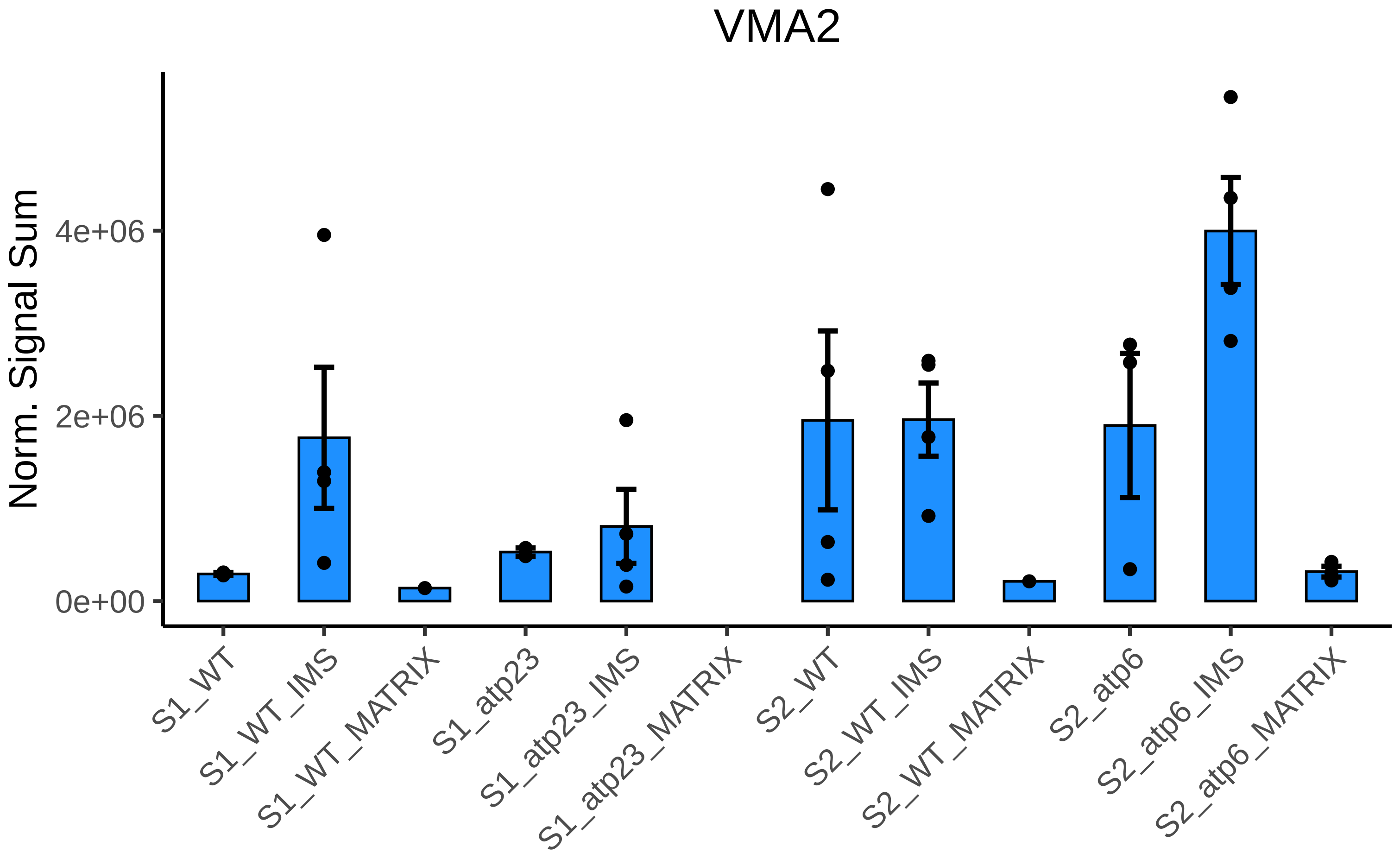

VMA2
Norm. Signal Sum
4e+06
2e+06
0e+00
S1_WT
S1_WT_IMS
S1_WT_MATRIX
S1_atp23
S1_atp23_IMS
S1_atp23_MATRIX
S2_WT
S2_WT_IMS
S2_WT_MATRIX
S2_atp6
S2_atp6_IMS
S2_atp6_MATRIX

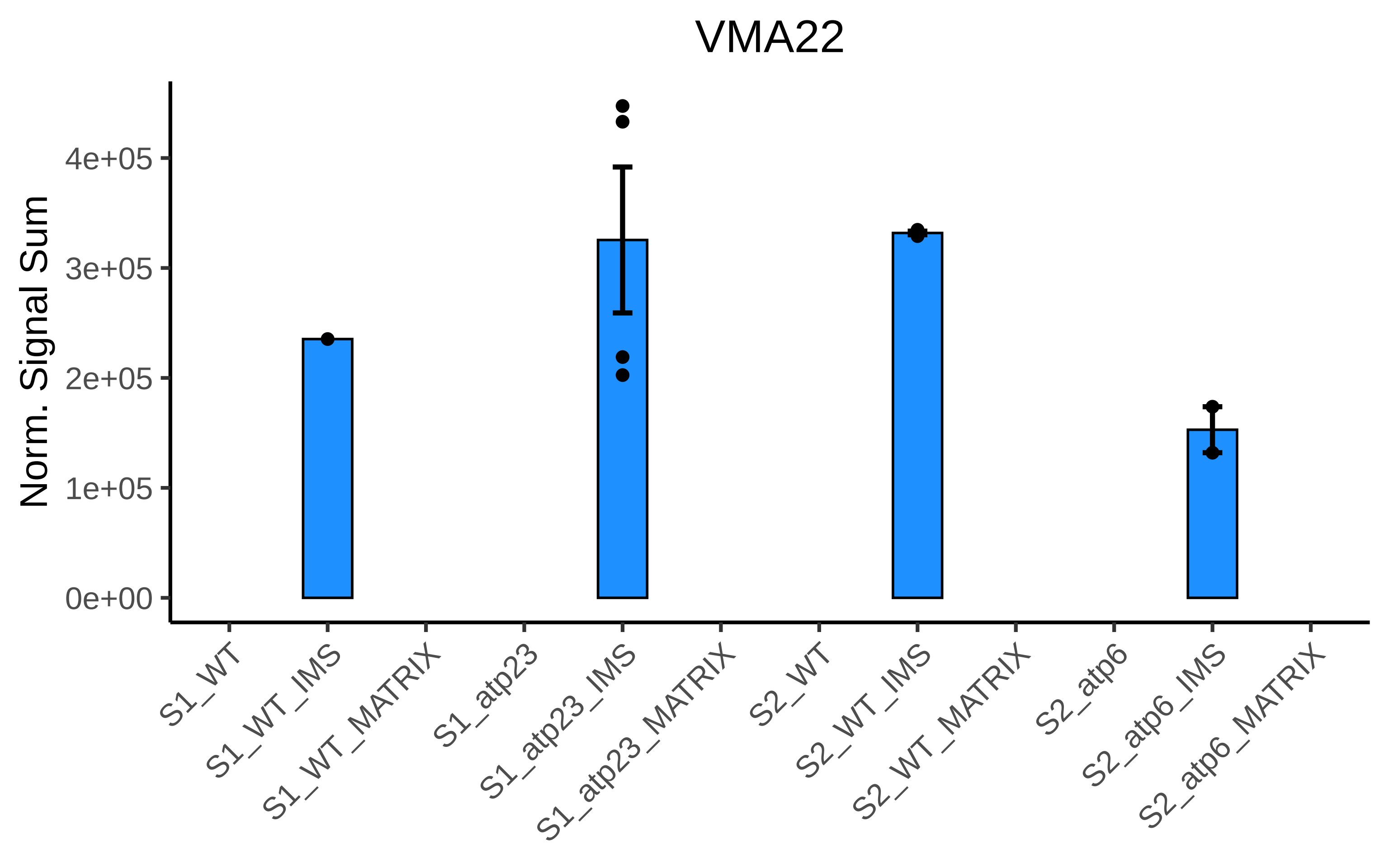
VMA22
Norm. Signal Sum
4e+05
3e+05
2e+05
1e+05
0e+00
S1_WT
S1_WT_IMS
S1_WT_MATRIX
S1_atp23
S1_atp23_IMS
S1_atp23_MATRIX
S2_WT
S2_WT_IMS
S2_WT_MATRIX
S2_atp6
S2_atp6_IMS
S2_atp6_MATRIX

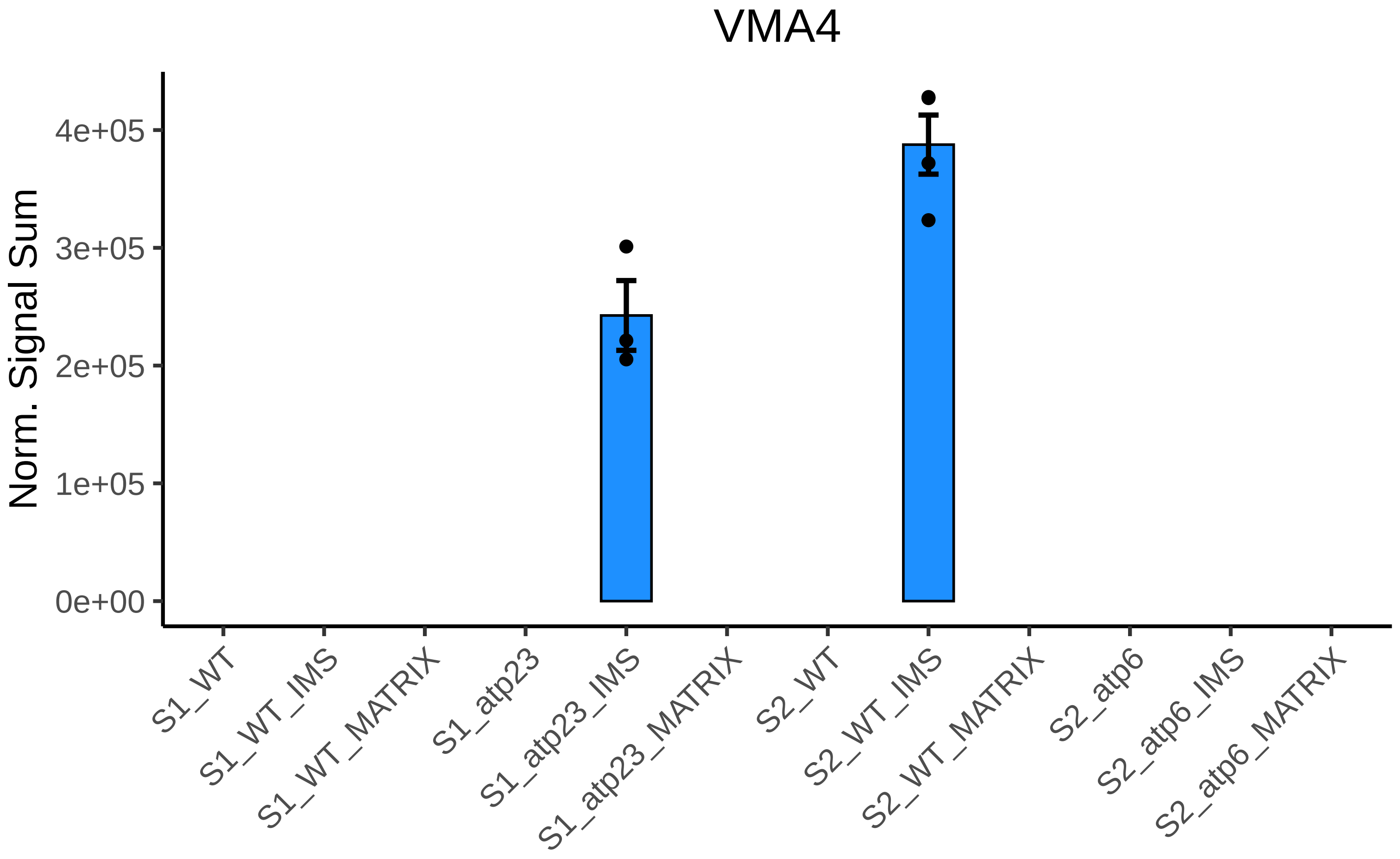

VMA4
Norm. Signal Sum
4e+05
3e+05
2e+05
1e+05
0e+00
S1_WT
S1_WT_IMS
S1_WT_MATRIX
S1_atp23
S1_atp23_IMS
S1_atp23_MATRIX
S2_WT
S2_WT_IMS
S2_WT_MATRIX
S2_atp6
S2_atp6_IMS
S2_atp6_MATRIX

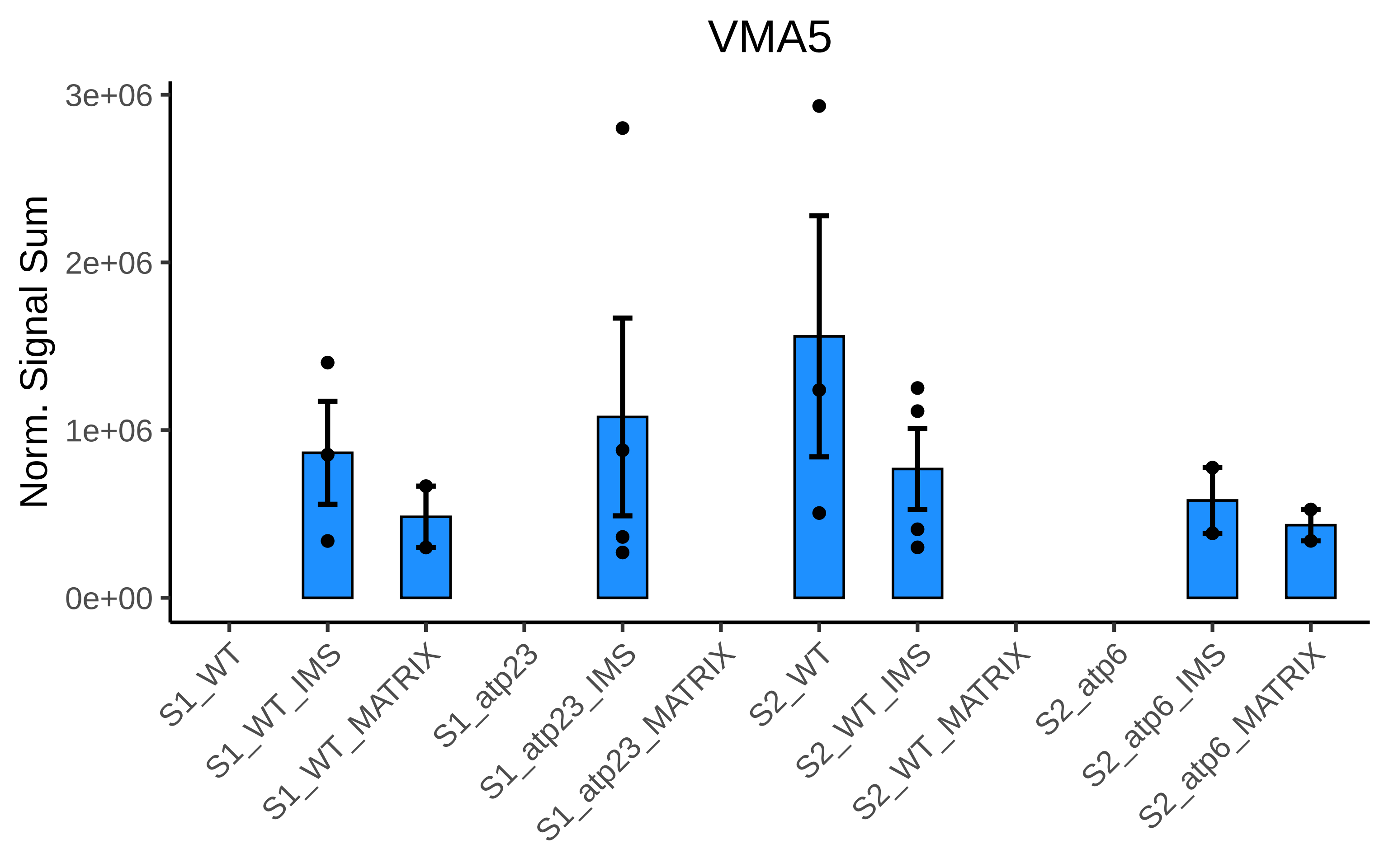
VMA5
Norm. Signal Sum
3e+06
2e+06
1e+06
0e+00
S1_WT
S1_WT_IMS
S1_WT_MATRIX
S1_atp23
S1_atp23_IMS
S1_atp23_MATRIX
S2_WT
S2_WT_IMS
S2_WT_MATRIX
S2_atp6
S2_atp6_IMS
S2_atp6_MATRIX

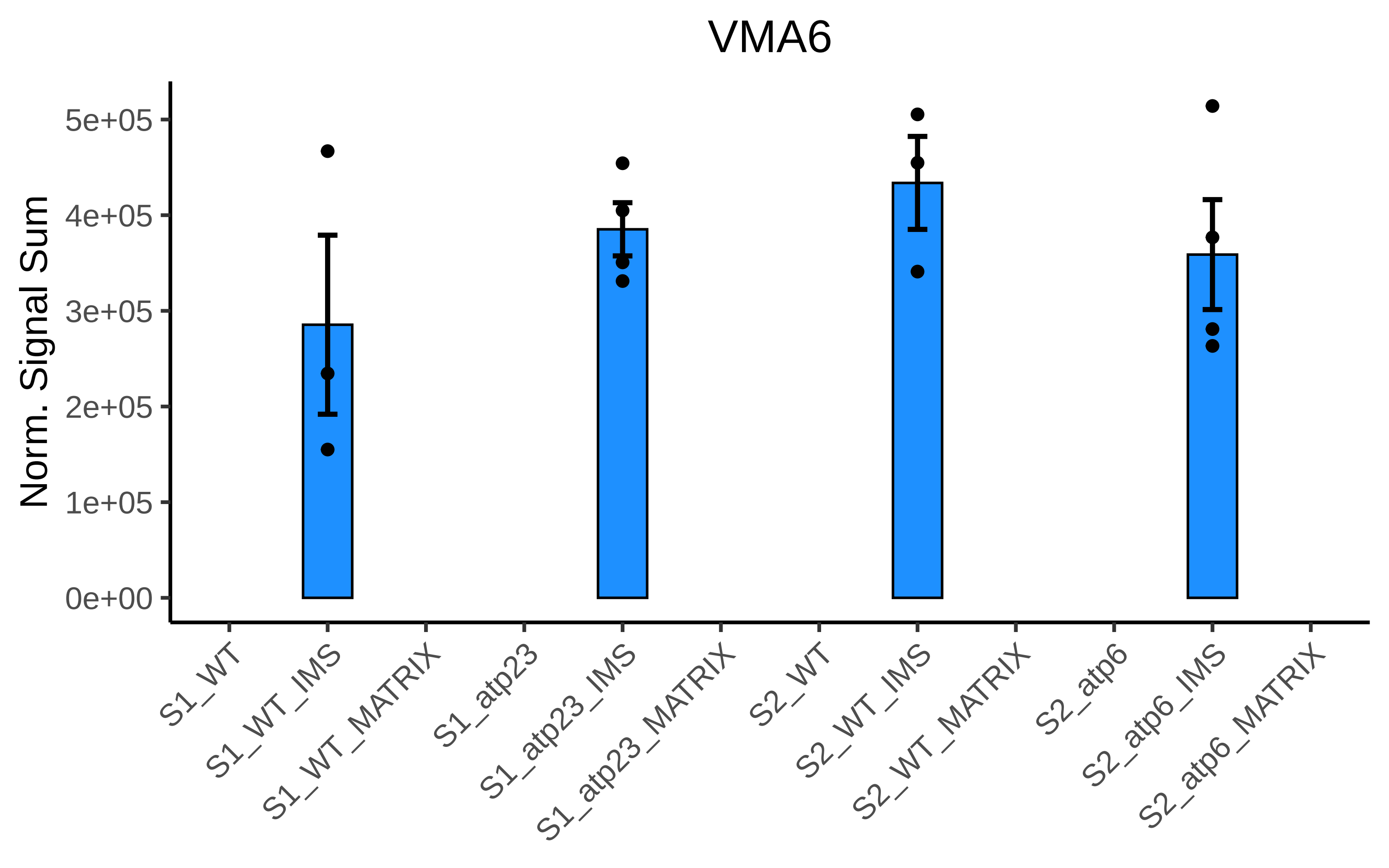

VMA6
Norm. Signal Sum
5e+05
4e+05
3e+05
2e+05
1e+05
0e+00
S1_WT
S1_WT_IMS
S1_WT_MATRIX
S1_atp23
S1_atp23_IMS
S1_atp23_MATRIX
S2_WT
S2_WT_IMS
S2_WT_MATRIX
S2_atp6
S2_atp6_IMS
S2_atp6_MATRIX

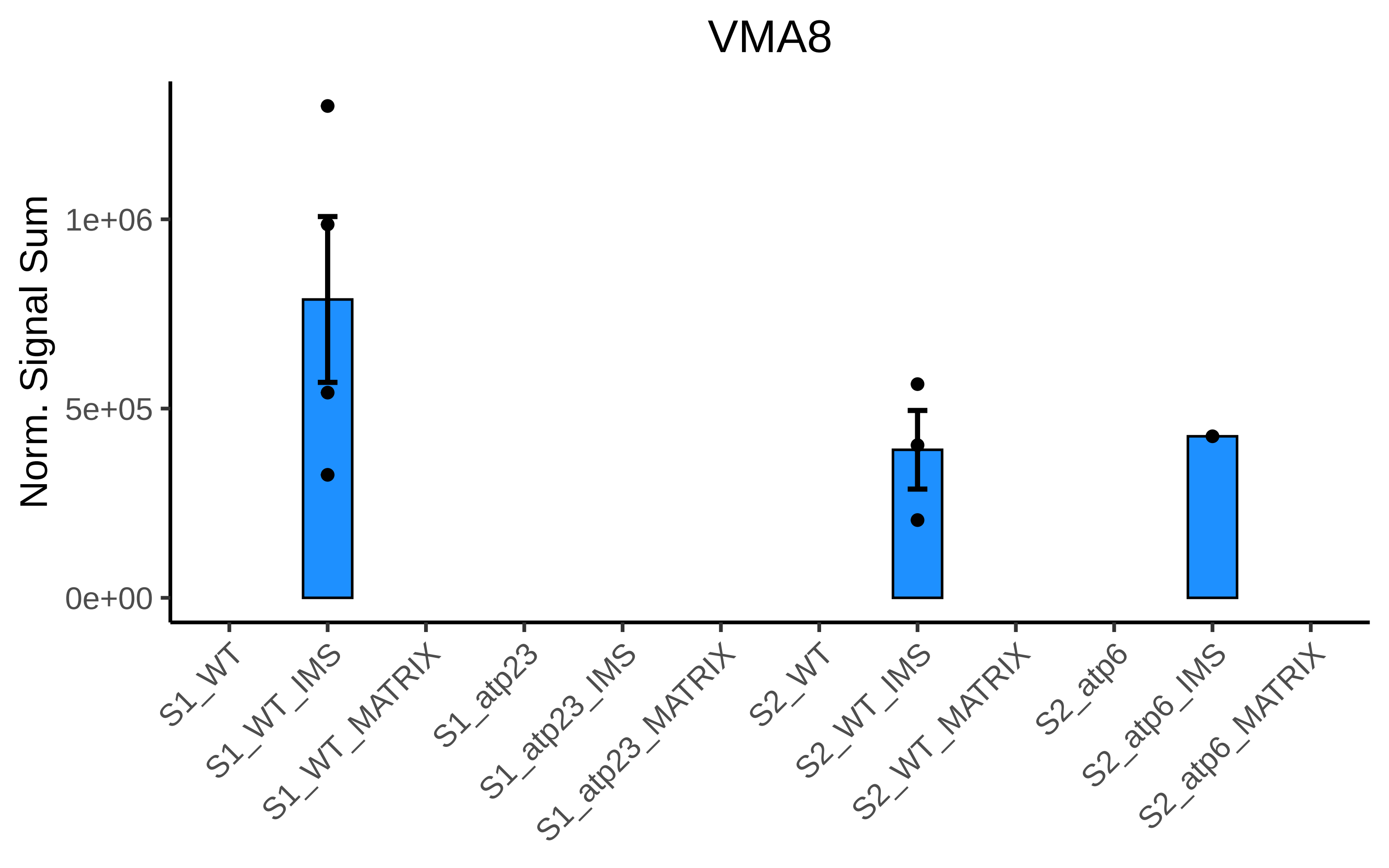

VMA8
Norm. Signal Sum
1e+06
5e+05
0e+00
S1_WT
S1_WT_IMS
S1_WT_MATRIX
S1_atp23
S1_atp23_IMS
S1_atp23_MATRIX
S2_WT
S2_WT_IMS
S2_WT_MATRIX
S2_atp6
S2_atp6_IMS
S2_atp6_MATRIX

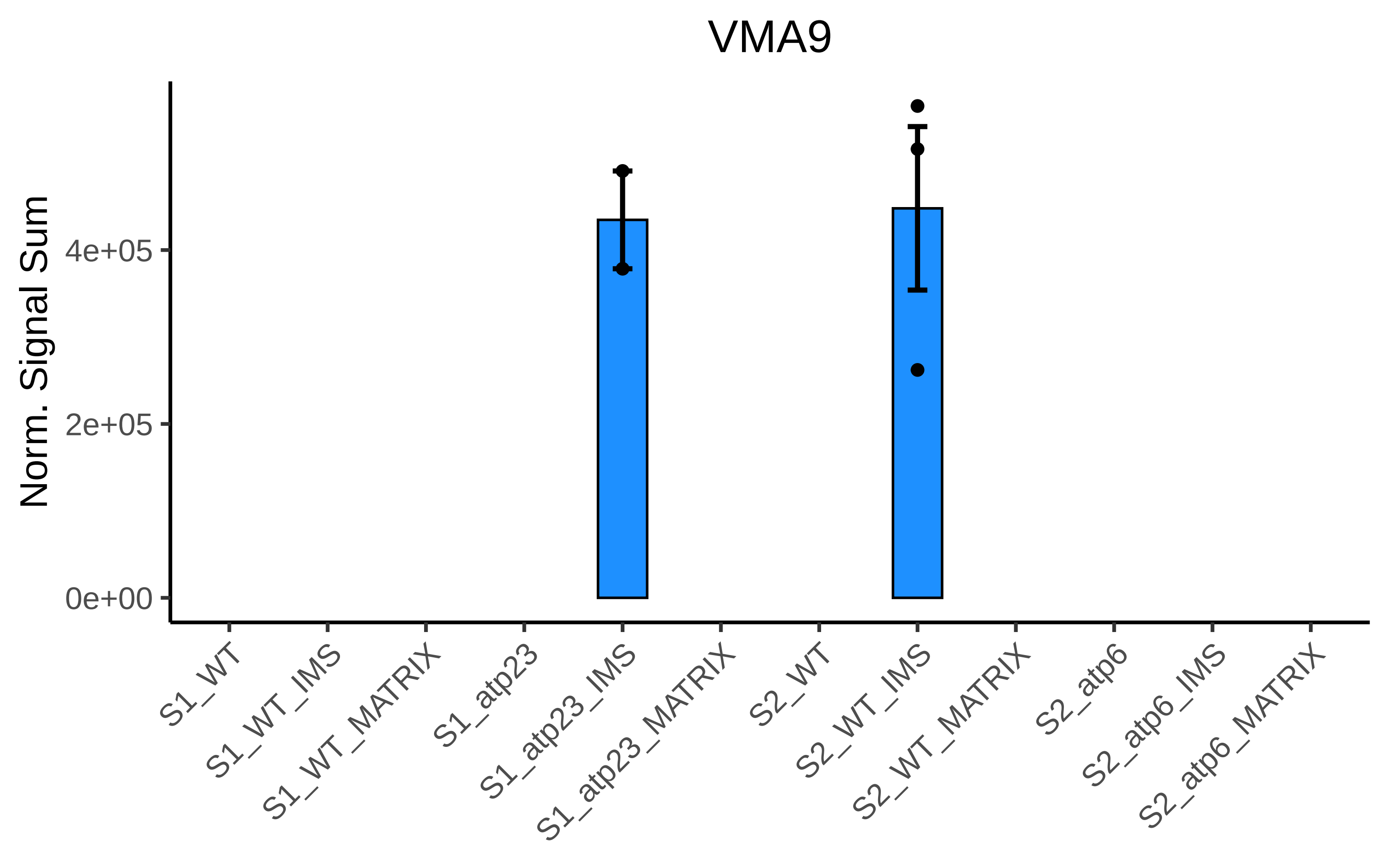

VMA9
Norm. Signal Sum
0e+00
2e+05
4e+05
S1_WT
S1_WT_IMS
S1_WT_MATRIX
S1_atp23
S1_atp23_IMS
S1_atp23_MATRIX
S2_WT
S2_WT_IMS
S2_WT_MATRIX
S2_atp6
S2_atp6_IMS
S2_atp6_MATRIX

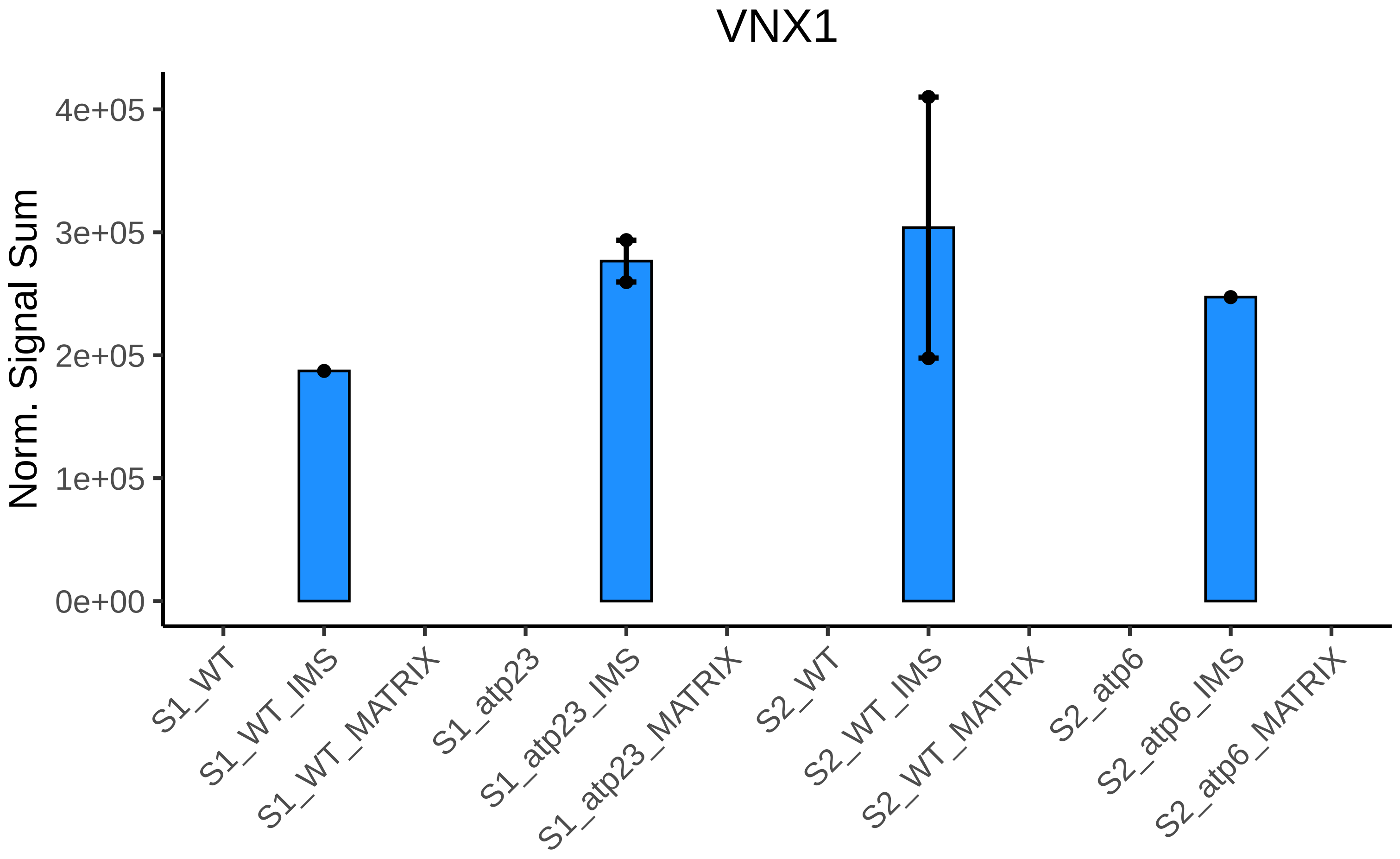
VNX1
Norm. Signal Sum
4e+05
3e+05
2e+05
1e+05
0e+00
S1_WT
S1_WT_IMS
S1_WT_MATRIX
S1_atp23
S1_atp23_IMS
S1_atp23_MATRIX
S2_WT
S2_WT_IMS
S2_WT_MATRIX
S2_atp6
S2_atp6_IMS
S2_atp6_MATRIX

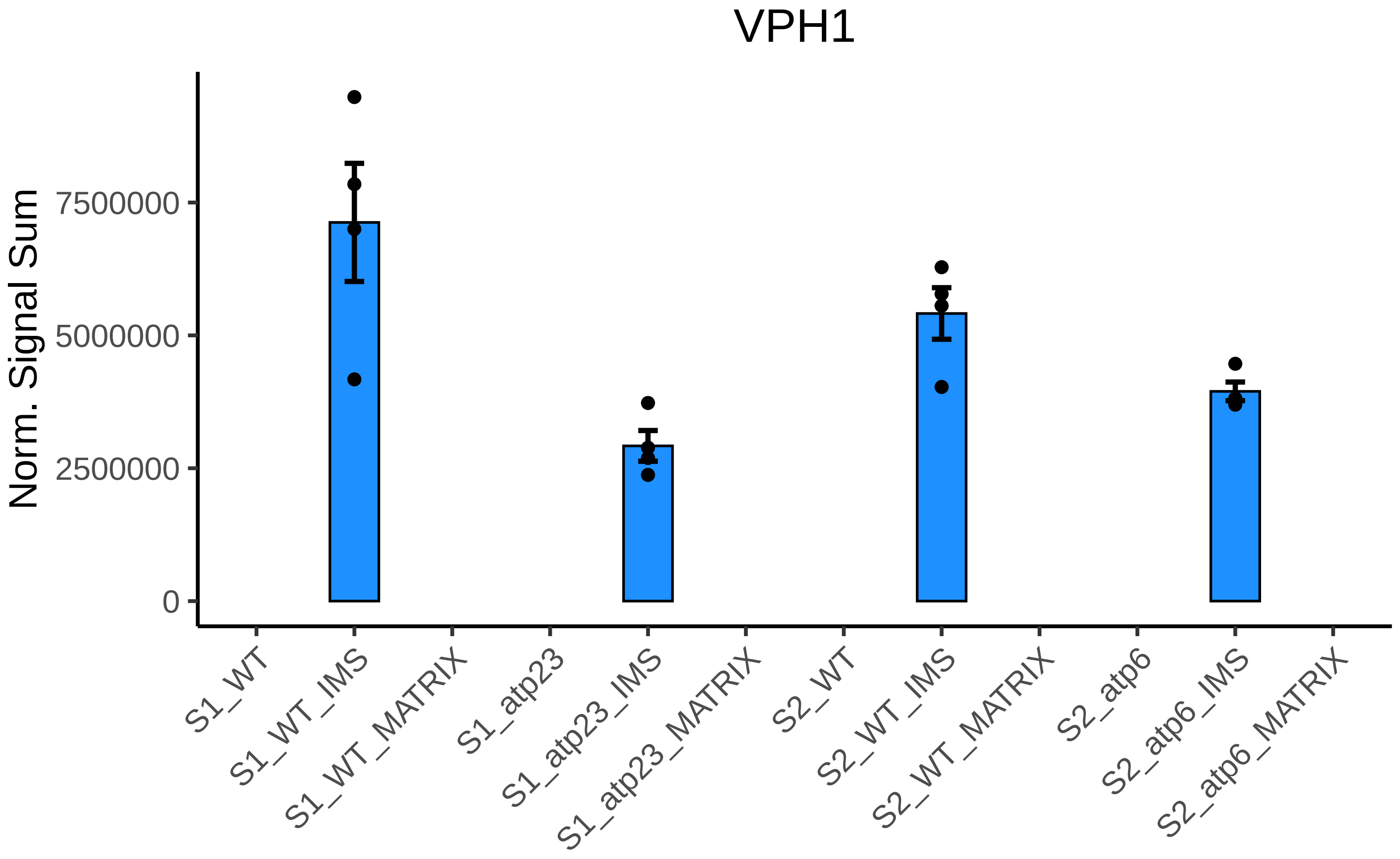
VPH1
Norm. Signal Sum
7500000
5000000
2500000
0
S1_WT
S1_WT_IMS
S1_WT_MATRIX
S1_atp23
S1_atp23_IMS
S1_atp23_MATRIX
S2_WT
S2_WT_IMS
S2_WT_MATRIX
S2_atp6
S2_atp6_IMS
S2_atp6_MATRIX

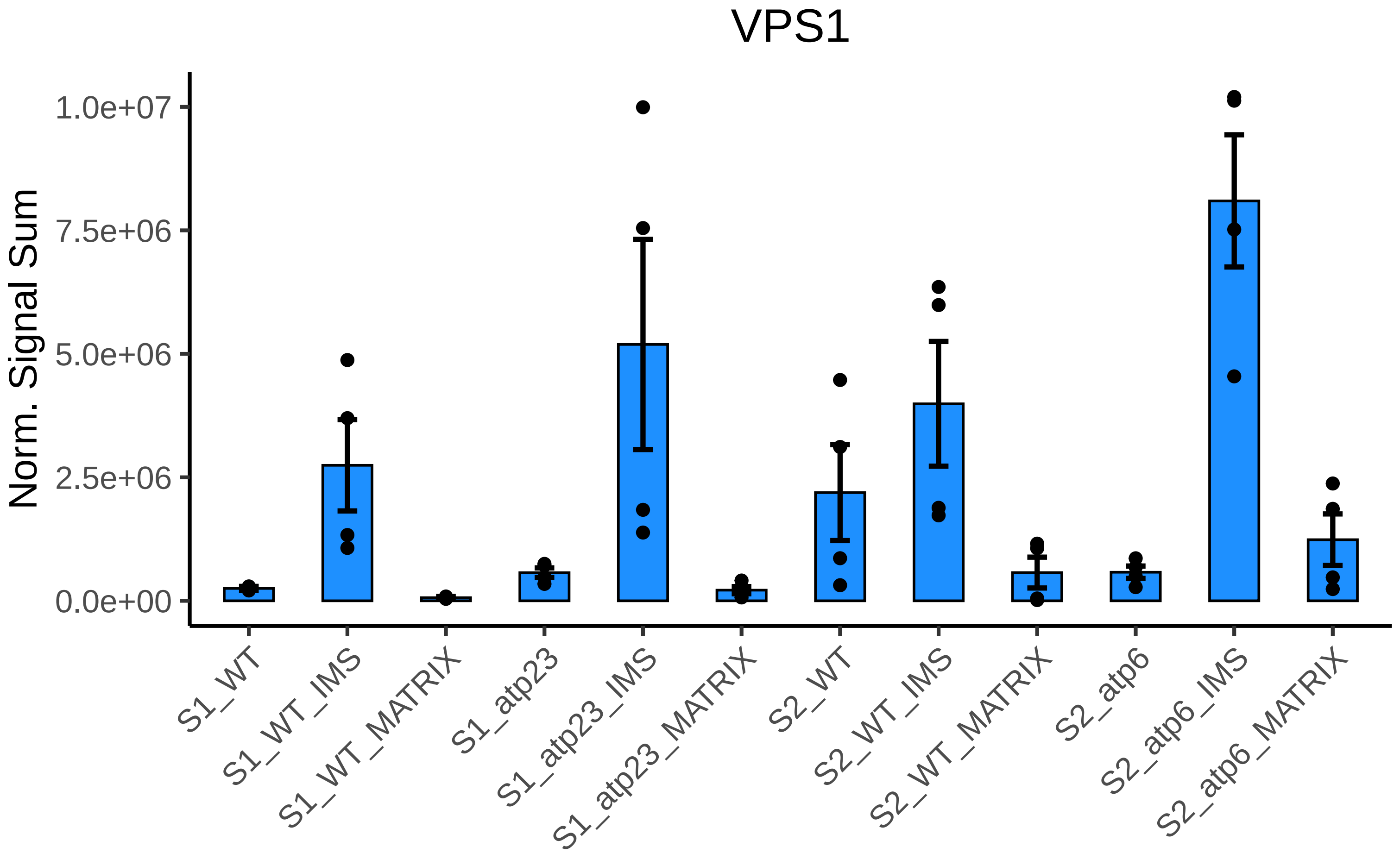
VPS1
Norm. Signal Sum
1.0e+07
7.5e+06
5.0e+06
2.5e+06
0.0e+00
S1_WT
S1_WT_IMS
S1_WT_MATRIX
S1_atp23
S1_atp23_IMS
S1_atp23_MATRIX
S2_WT
S2_WT_IMS
S2_WT_MATRIX
S2_atp6
S2_atp6_IMS
S2_atp6_MATRIX

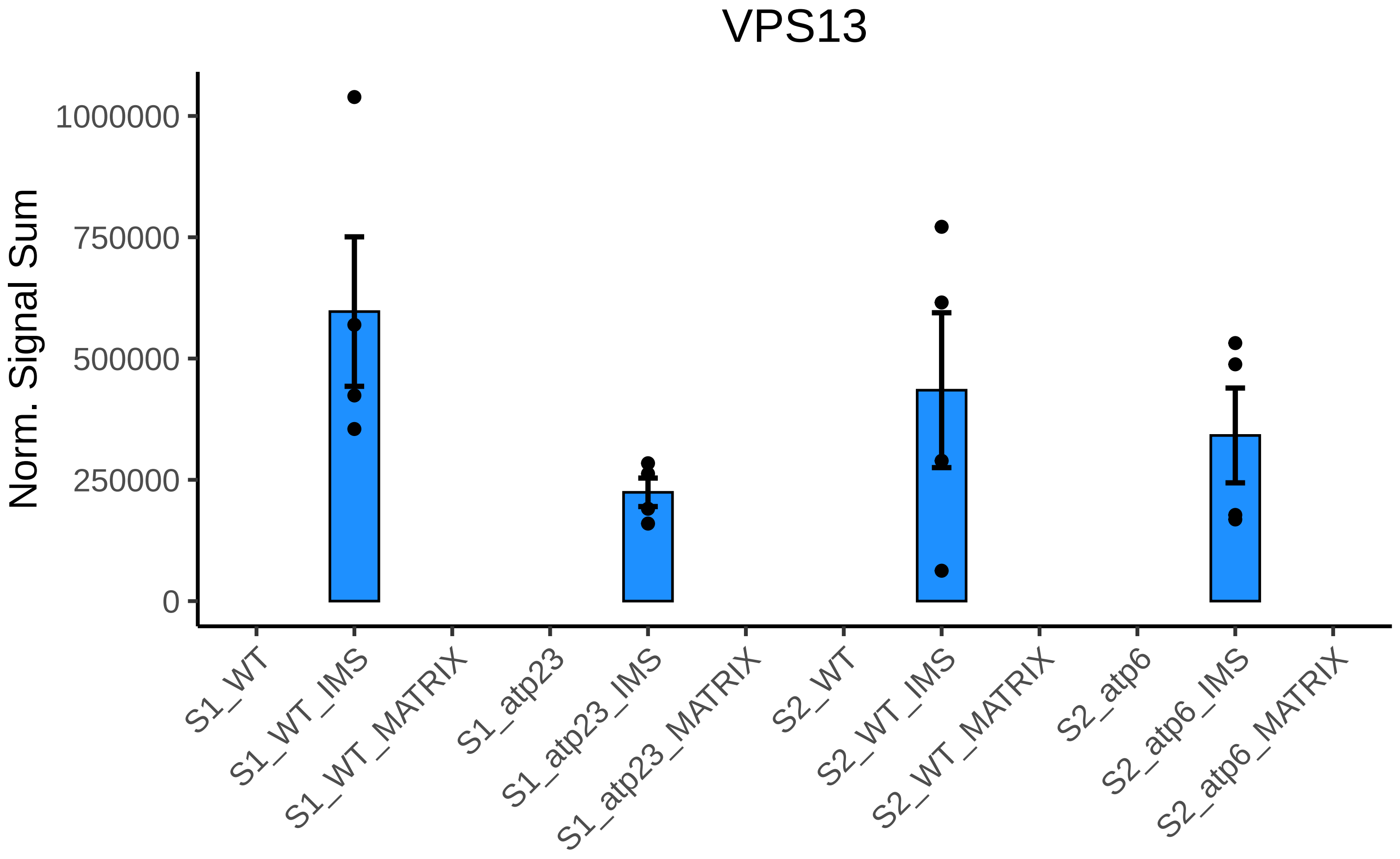

VPS13
Norm. Signal Sum
1000000
750000
500000
250000
0
S1_WT
S1_WT_IMS
S1_WT_MATRIX
S1_atp23
S1_atp23_IMS
S1_atp23_MATRIX
S2_WT
S2_WT_IMS
S2_WT_MATRIX
S2_atp6
S2_atp6_IMS
S2_atp6_MATRIX

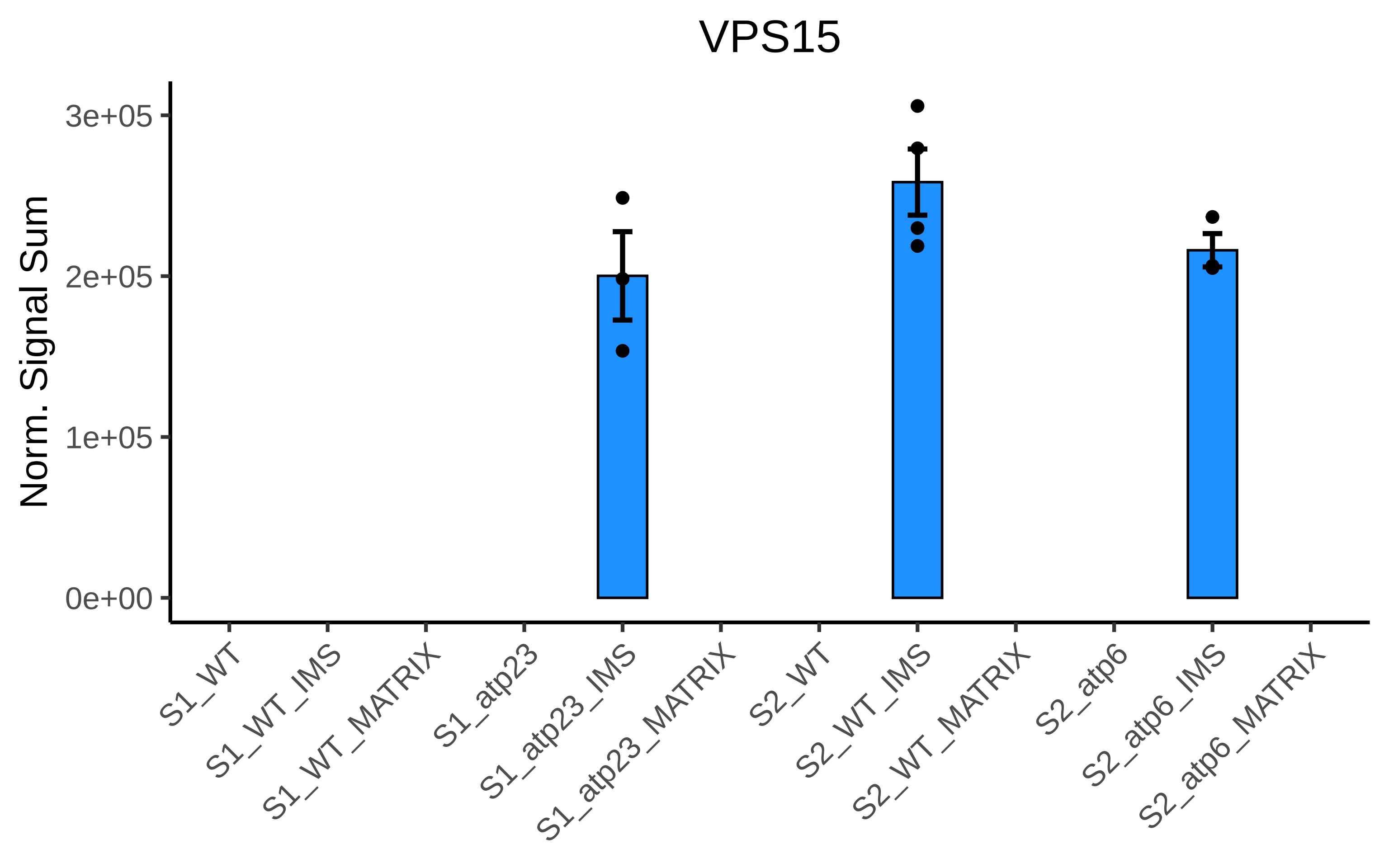

VPS15
Norm. Signal Sum
3e+05
2e+05
1e+05
0e+00
S1_WT
S1_WT_IMS
S1_WT_MATRIX
S1_atp23
S1_atp23_IMS
S1_atp23_MATRIX
S2_WT
S2_WT_IMS
S2_WT_MATRIX
S2_atp6
S2_atp6_IMS
S2_atp6_MATRIX

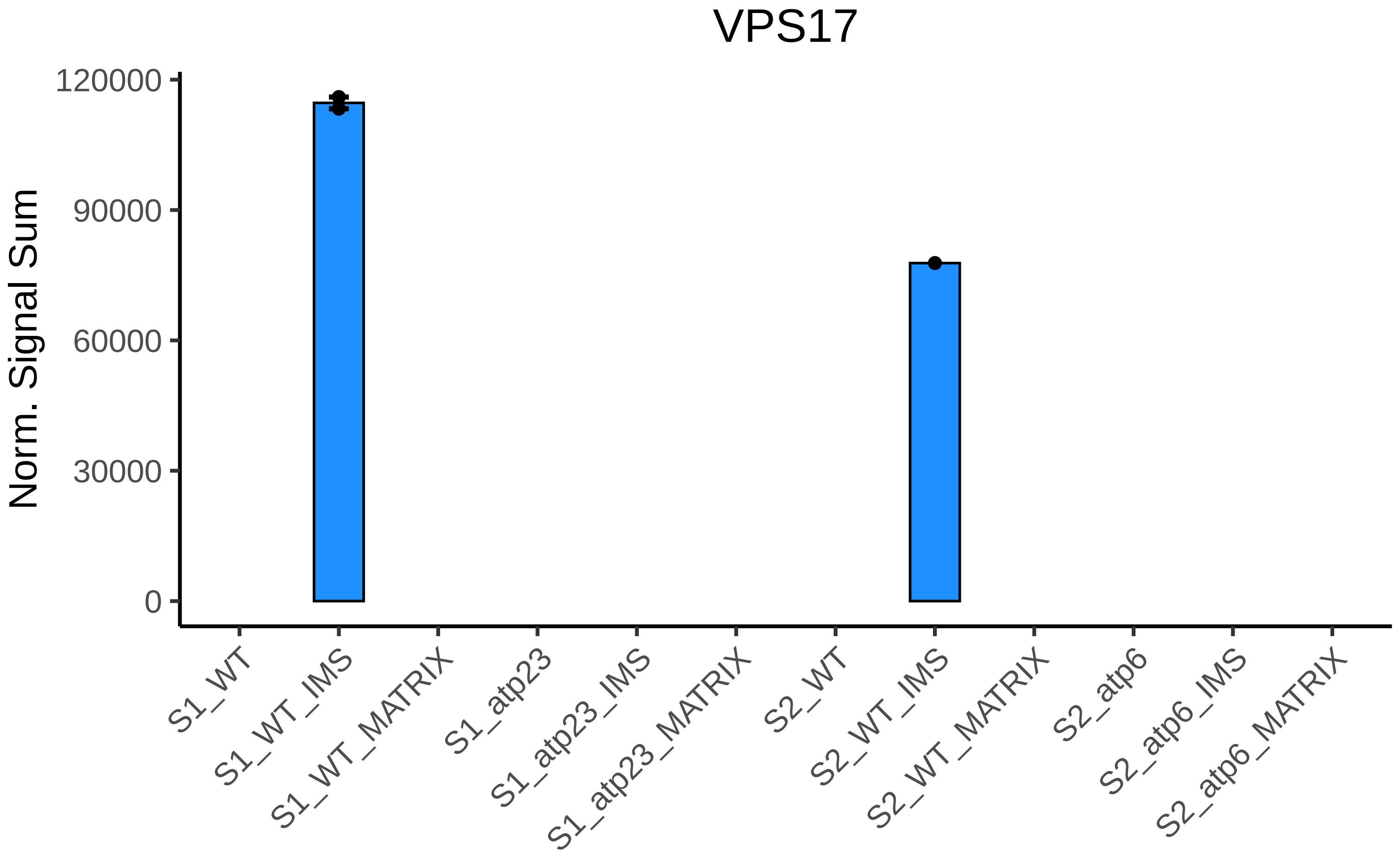

VPS17
Norm. Signal Sum
120000
90000
60000
30000
0
S1_WT
S1_WT_IMS
S1_WT_MATRIX
S1_atp23
S1_atp23_IMS
S1_atp23_MATRIX
S2_WT
S2_WT_IMS
S2_WT_MATRIX
S2_atp6
S2_atp6_IMS
S2_atp6_MATRIX

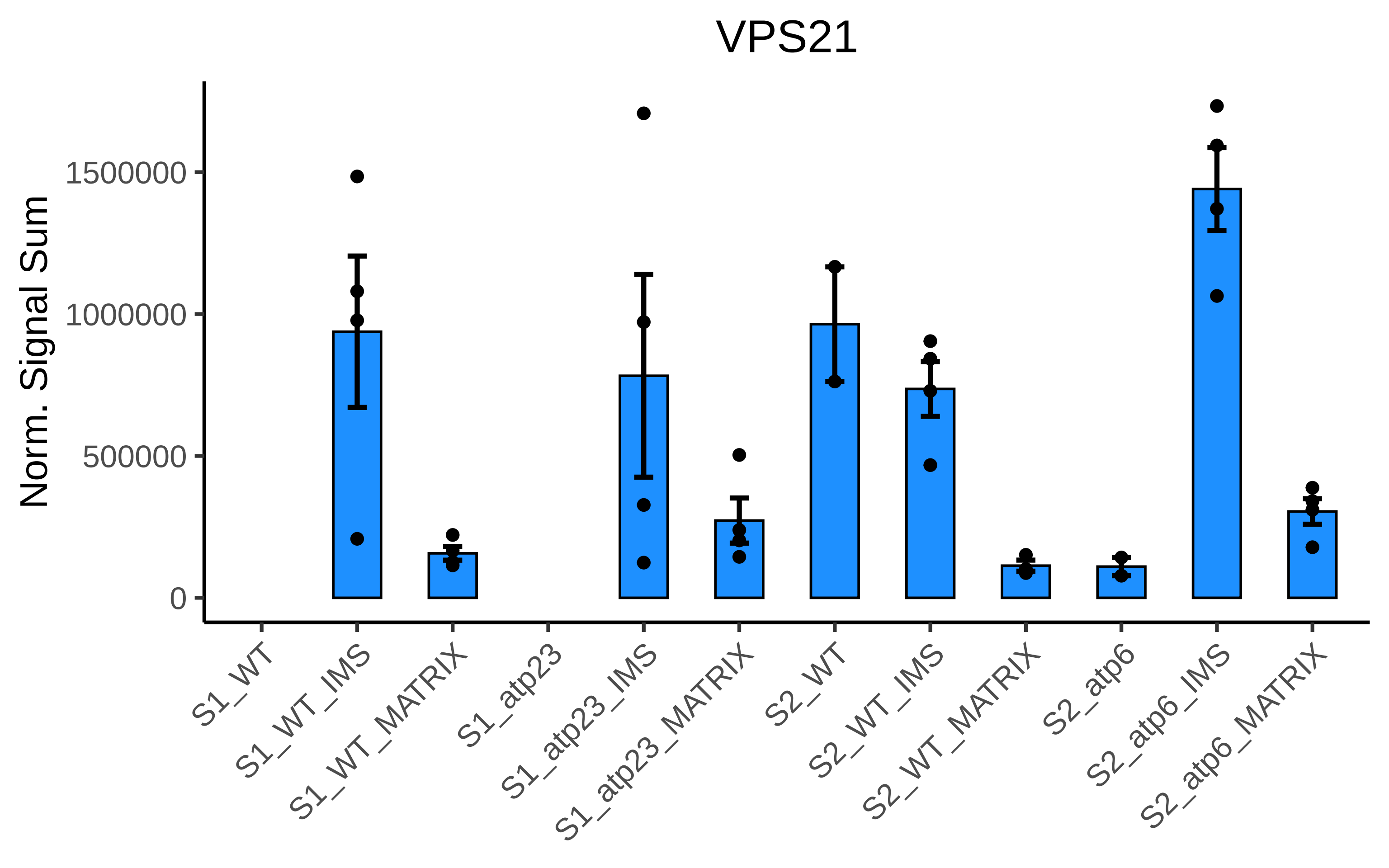

VPS21
Norm. Signal Sum
1500000
1000000
500000
0
S1_WT
S1_WT_IMS
S1_WT_MATRIX
S1_atp23
S1_atp23_IMS
S1_atp23_MATRIX
S2_WT
S2_WT_IMS
S2_WT_MATRIX
S2_atp6
S2_atp6_IMS
S2_atp6_MATRIX

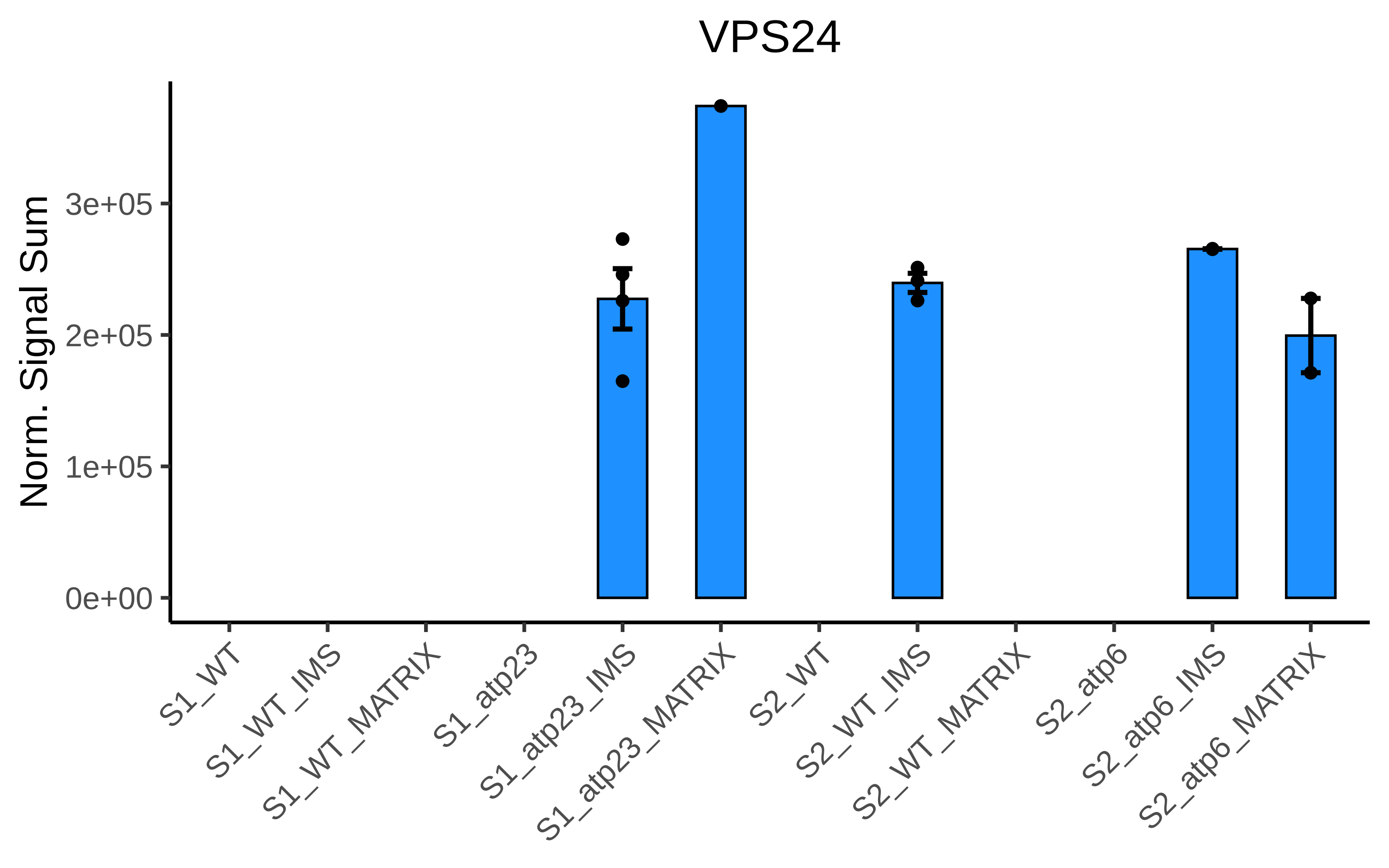

VPS24
Norm. Signal Sum
3e+05
2e+05
1e+05
0e+00
S1_WT
S1_WT_IMS
S1_WT_MATRIX
S1_atp23
S1_atp23_IMS
S1_atp23_MATRIX
S2_WT
S2_WT_IMS
S2_WT_MATRIX
S2_atp6
S2_atp6_IMS
S2_atp6_MATRIX

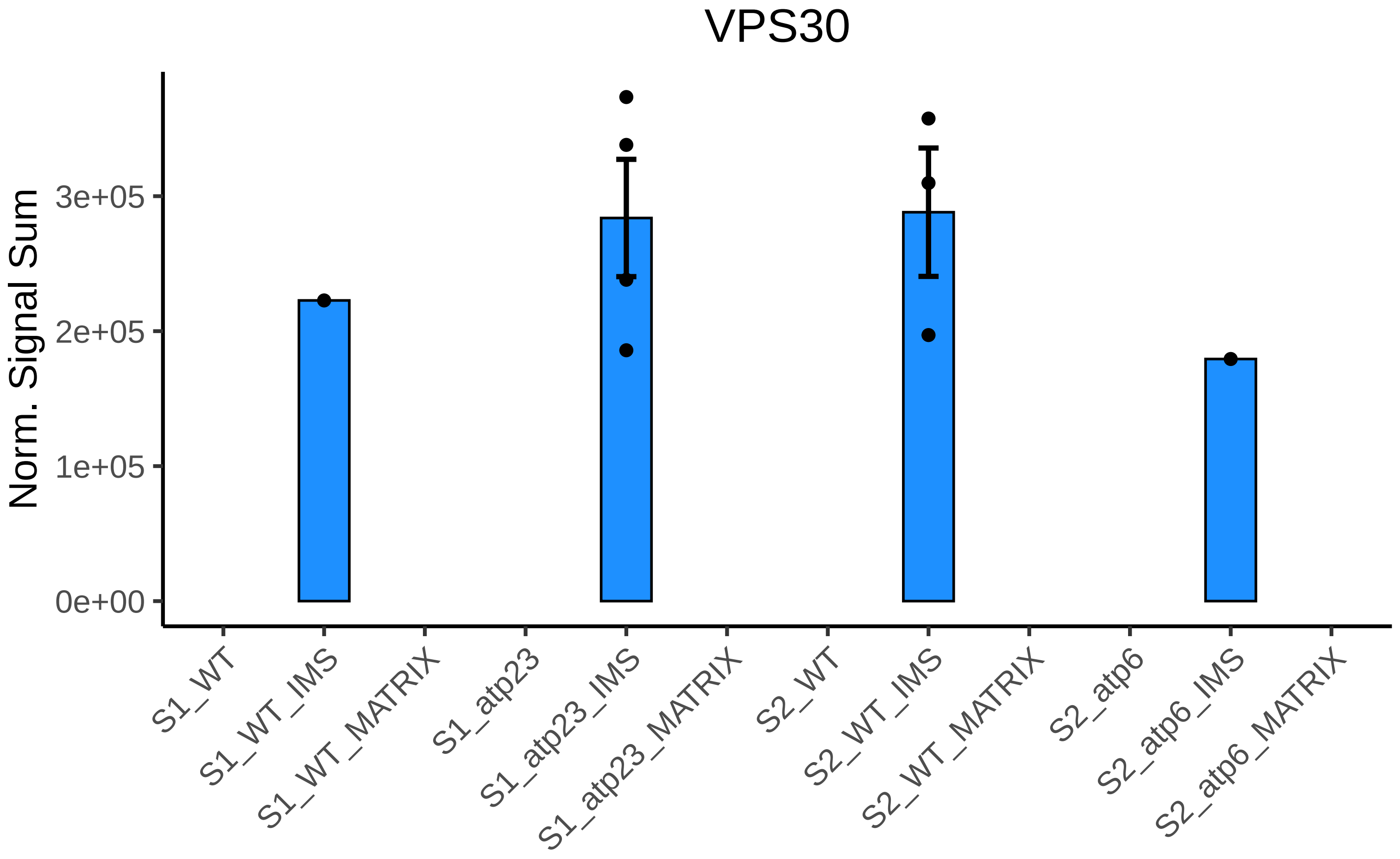
VPS30
Norm. Signal Sum
3e+05
2e+05
1e+05
0e+00
S1_WT
S1_WT_IMS
S1_WT_MATRIX
S1_atp23
S1_atp23_IMS
S1_atp23_MATRIX
S2_WT
S2_WT_IMS
S2_WT_MATRIX
S2_atp6
S2_atp6_IMS
S2_atp6_MATRIX

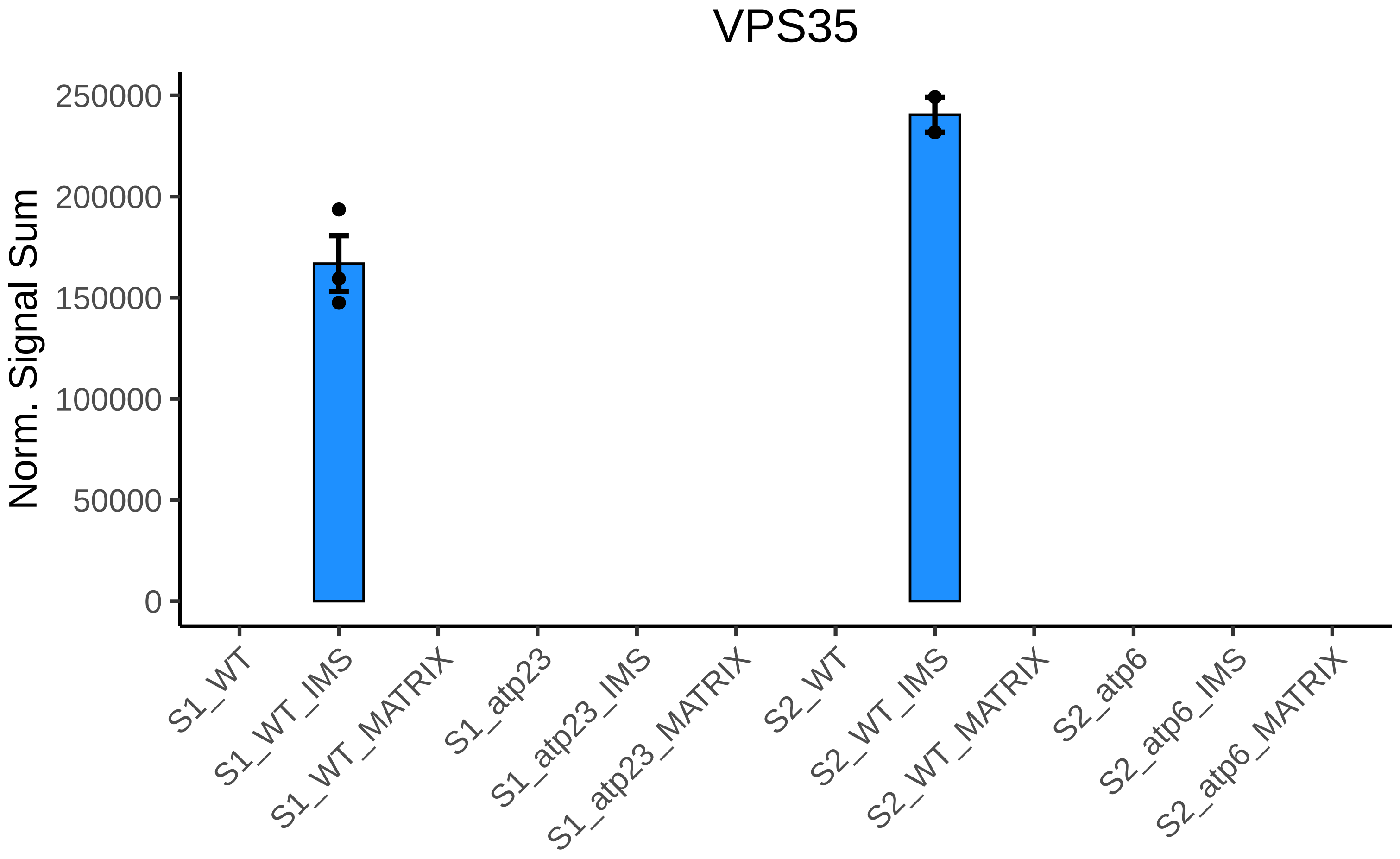
VPS35
Norm. Signal Sum
250000
200000
150000
100000
50000
0
S1_WT
S1_WT_IMS
S1_WT_MATRIX
S1_atp23
S1_atp23_IMS
S1_atp23_MATRIX
S2_WT
S2_WT_IMS
S2_WT_MATRIX
S2_atp6
S2_atp6_IMS
S2_atp6_MATRIX

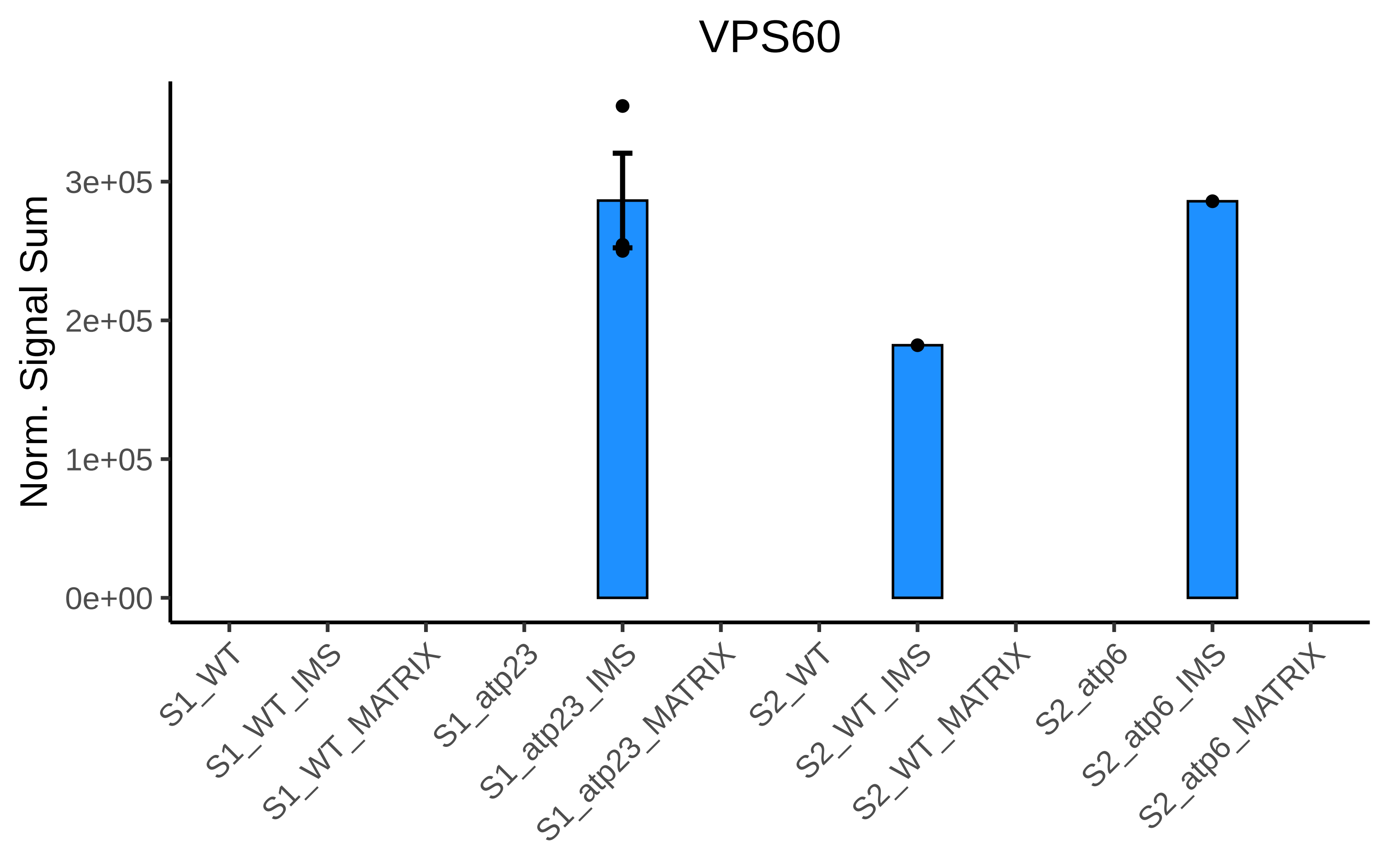

VPS60
Norm. Signal Sum
3e+05
2e+05
1e+05
0e+00
S1_WT
S1_WT_IMS
S1_WT_MATRIX
S1_atp23
S1_atp23_IMS
S1_atp23_MATRIX
S2_WT
S2_WT_IMS
S2_WT_MATRIX
S2_atp6
S2_atp6_IMS
S2_atp6_MATRIX

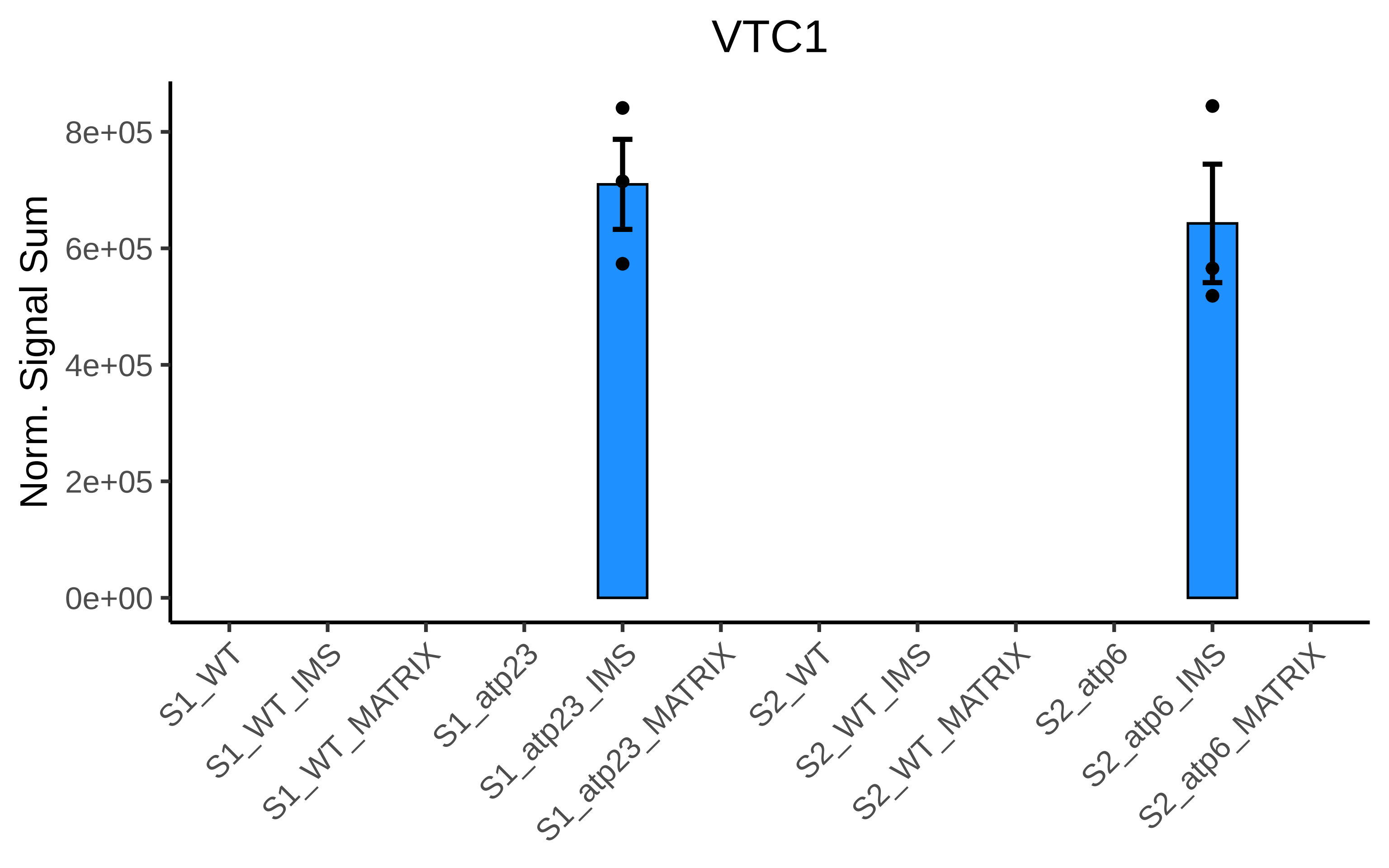

VTC1
Norm. Signal Sum
8e+05
6e+05
4e+05
2e+05
0e+00
S1_WT
S1_WT_IMS
S1_WT_MATRIX
S1_atp23
S1_atp23_IMS
S1_atp23_MATRIX
S2_WT
S2_WT_IMS
S2_WT_MATRIX
S2_atp6
S2_atp6_IMS
S2_atp6_MATRIX

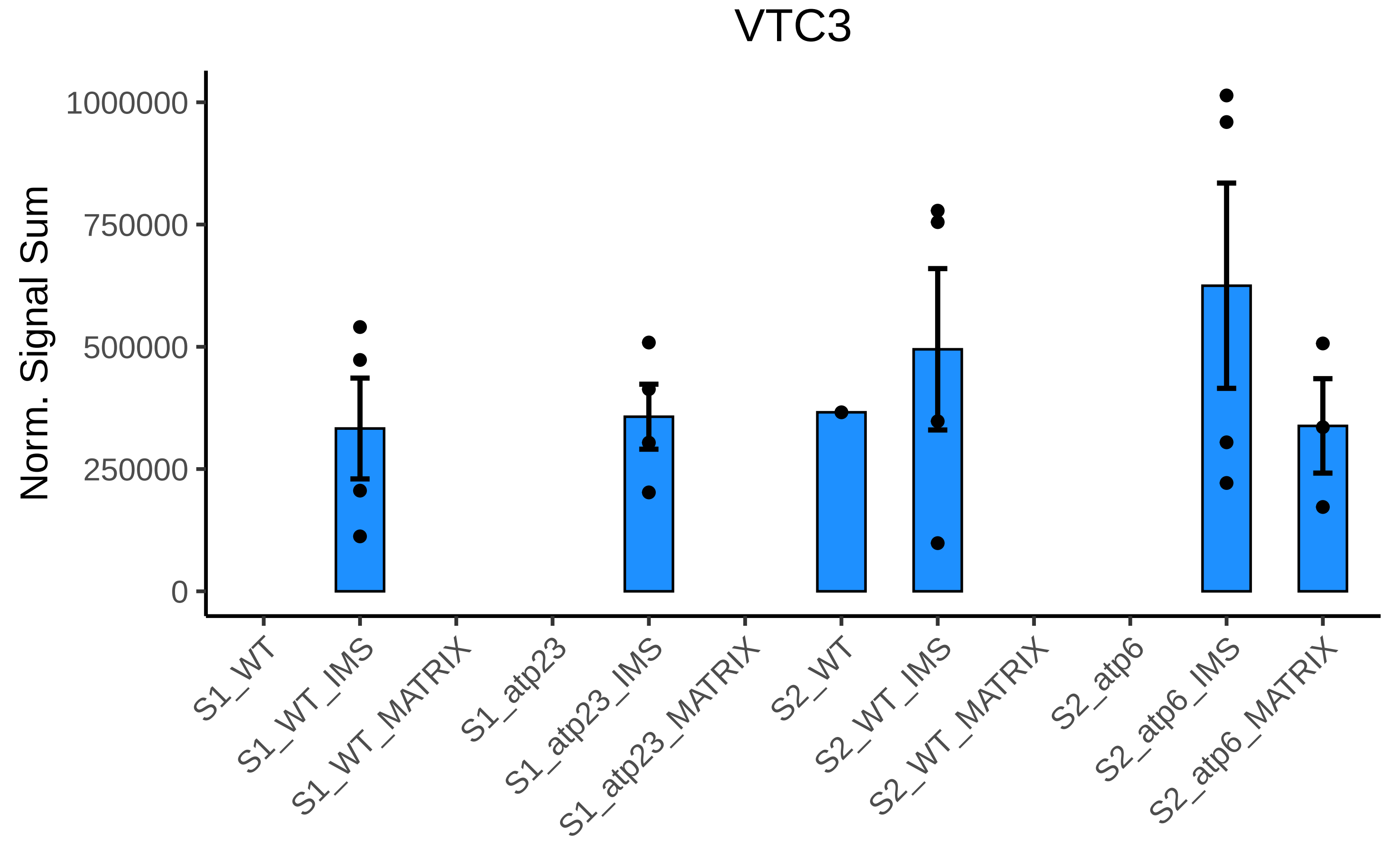

VTC3
Norm. Signal Sum
1000000
750000
500000
250000
0
S1_WT
S1_WT_IMS
S1_WT_MATRIX
S1_atp23
S1_atp23_IMS
S1_atp23_MATRIX
S2_WT
S2_WT_IMS
S2_WT_MATRIX
S2_atp6
S2_atp6_IMS
S2_atp6_MATRIX

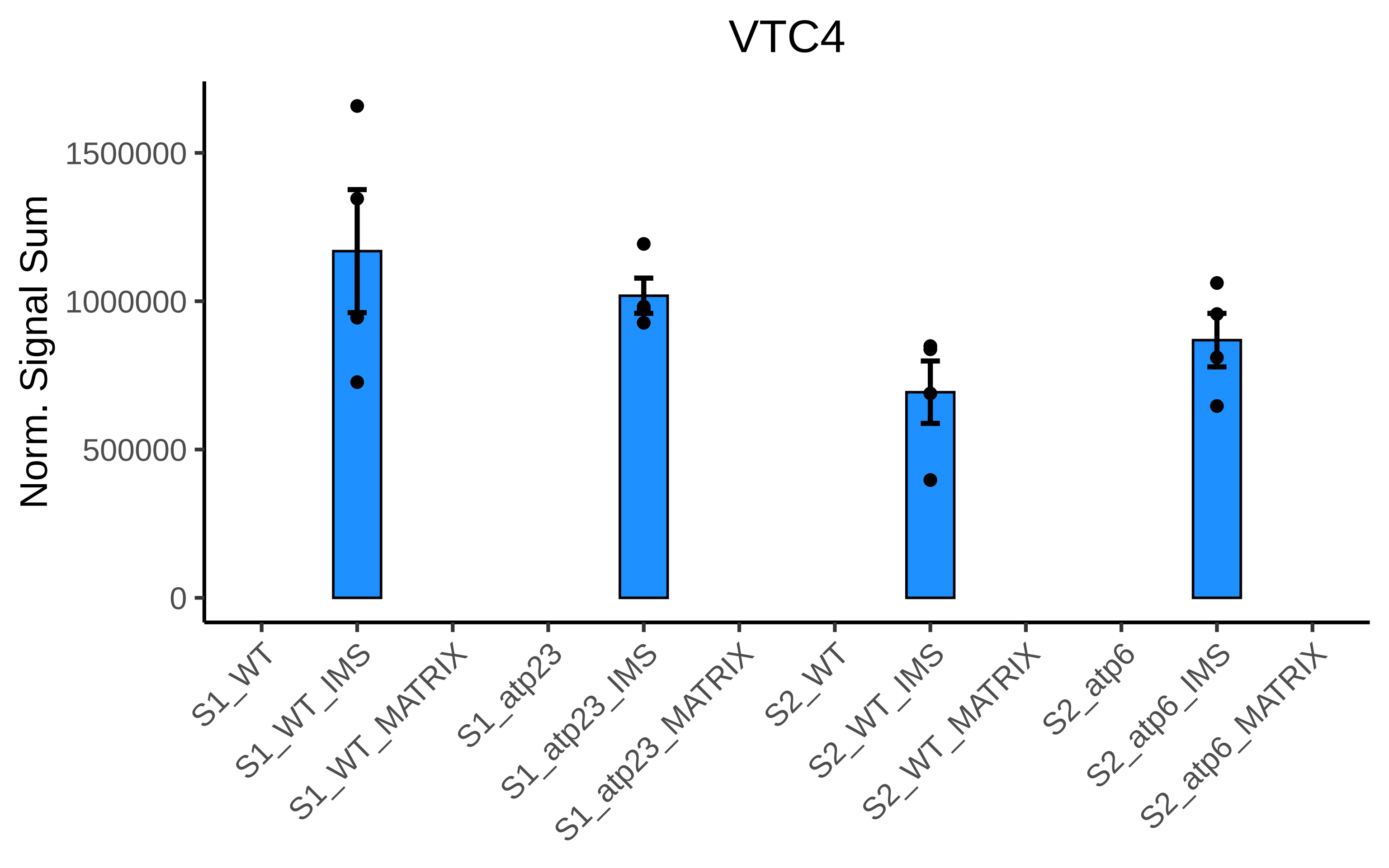
VTC4
Norm. Signal Sum
0
500000
1000000
1500000
S1_WT
S1_WT_IMS
S1_WT_MATRIX
S1_atp23
S1_atp23_IMS
S1_atp23_MATRIX
S2_WT
S2_WT_IMS
S2_WT_MATRIX
S2_atp6
S2_atp6_IMS
S2_atp6_MATRIX

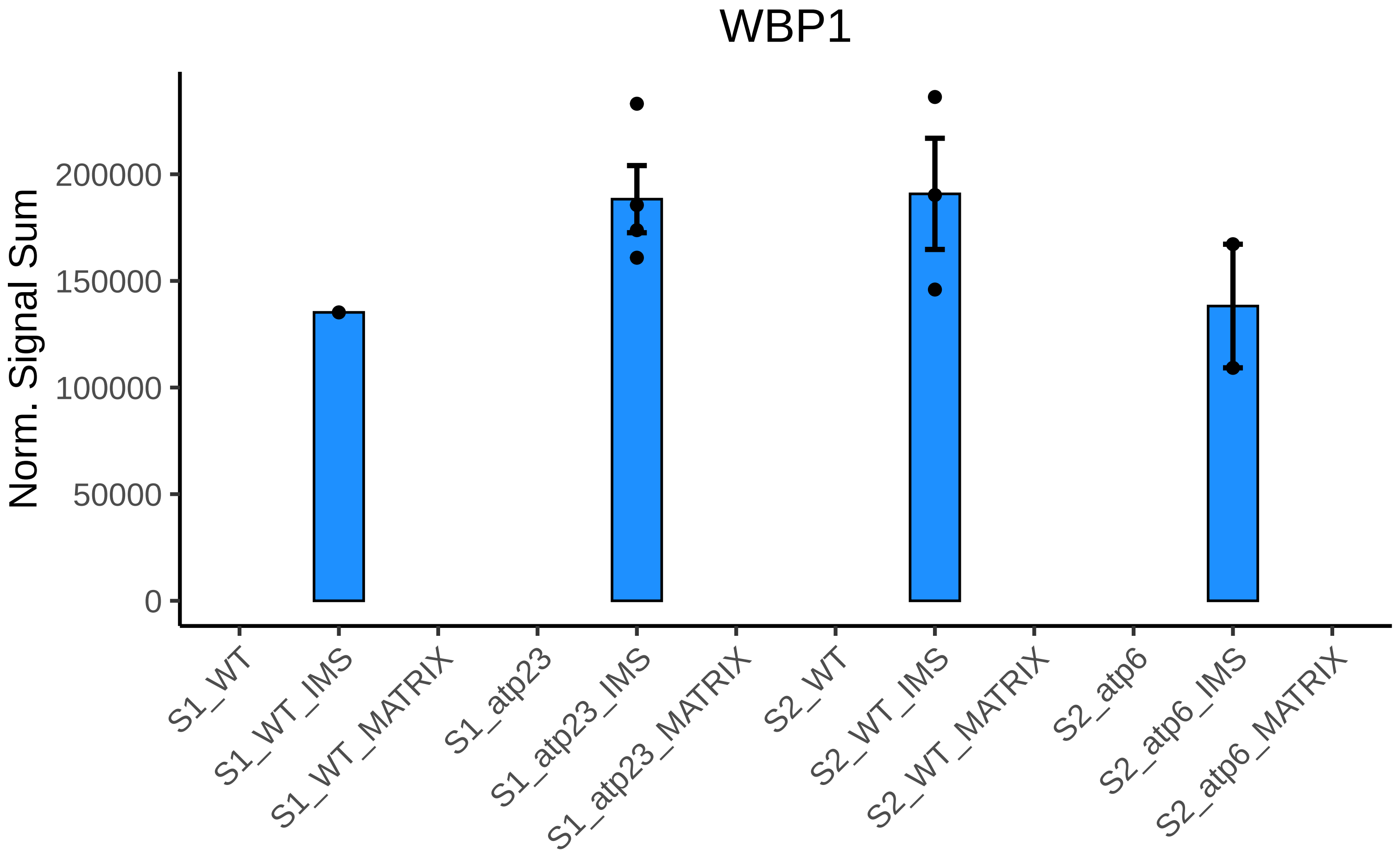
WBP1
Norm. Signal Sum
200000
150000
100000
50000
0
S1_WT
S1_WT_IMS
S1_WT_MATRIX
S1_atp23
S1_atp23_IMS
S1_atp23_MATRIX
S2_WT
S2_WT_IMS
S2_WT_MATRIX
S2_atp6
S2_atp6_IMS
S2_atp6_MATRIX

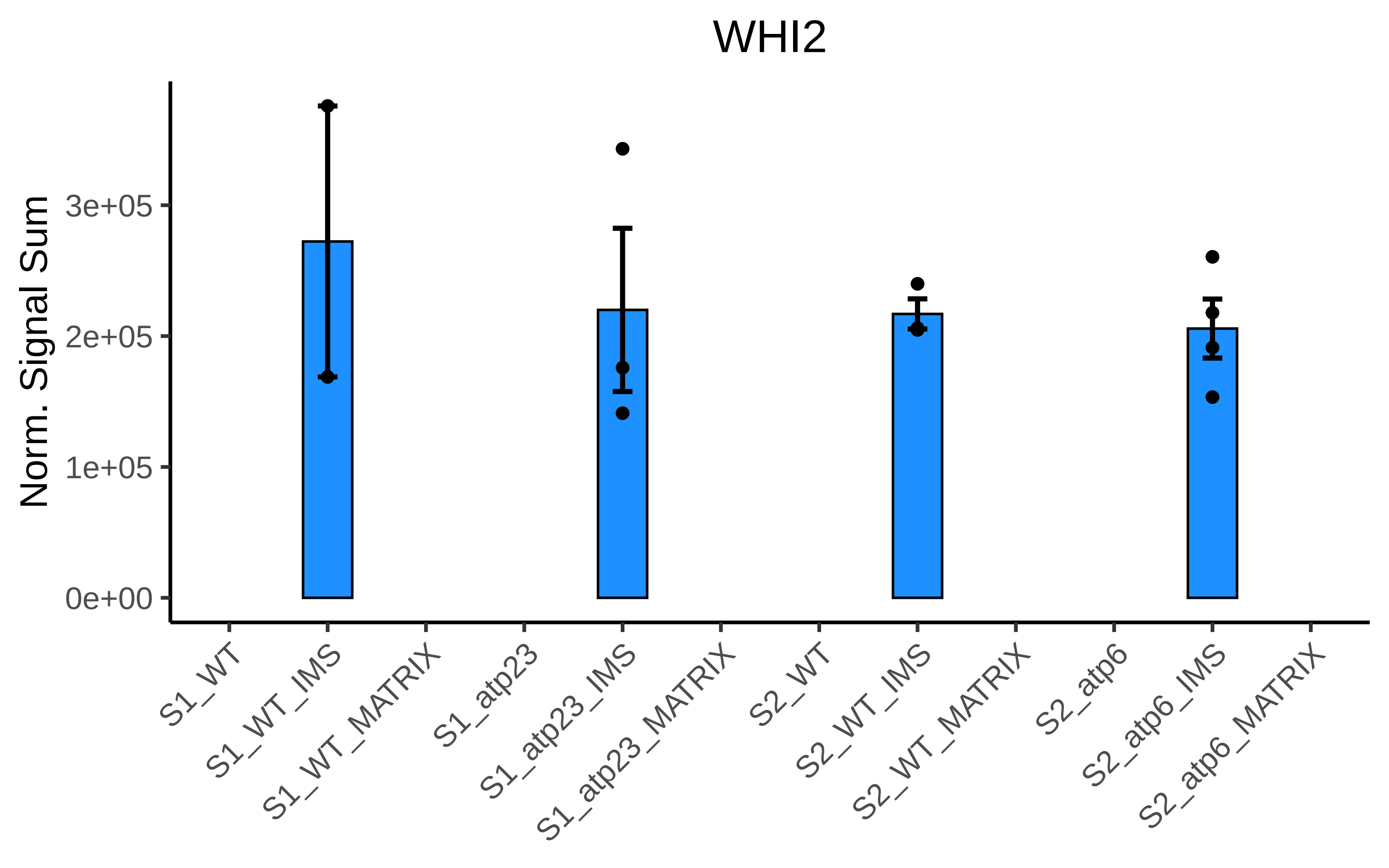

WHI2
Norm. Signal Sum
3e+05
2e+05
1e+05
0e+00
S1_WT
S1_WT_IMS
S1_WT_MATRIX
S1_atp23
S1_atp23_IMS
S1_atp23_MATRIX
S2_WT
S2_WT_IMS
S2_WT_MATRIX
S2_atp6
S2_atp6_IMS
S2_atp6_MATRIX

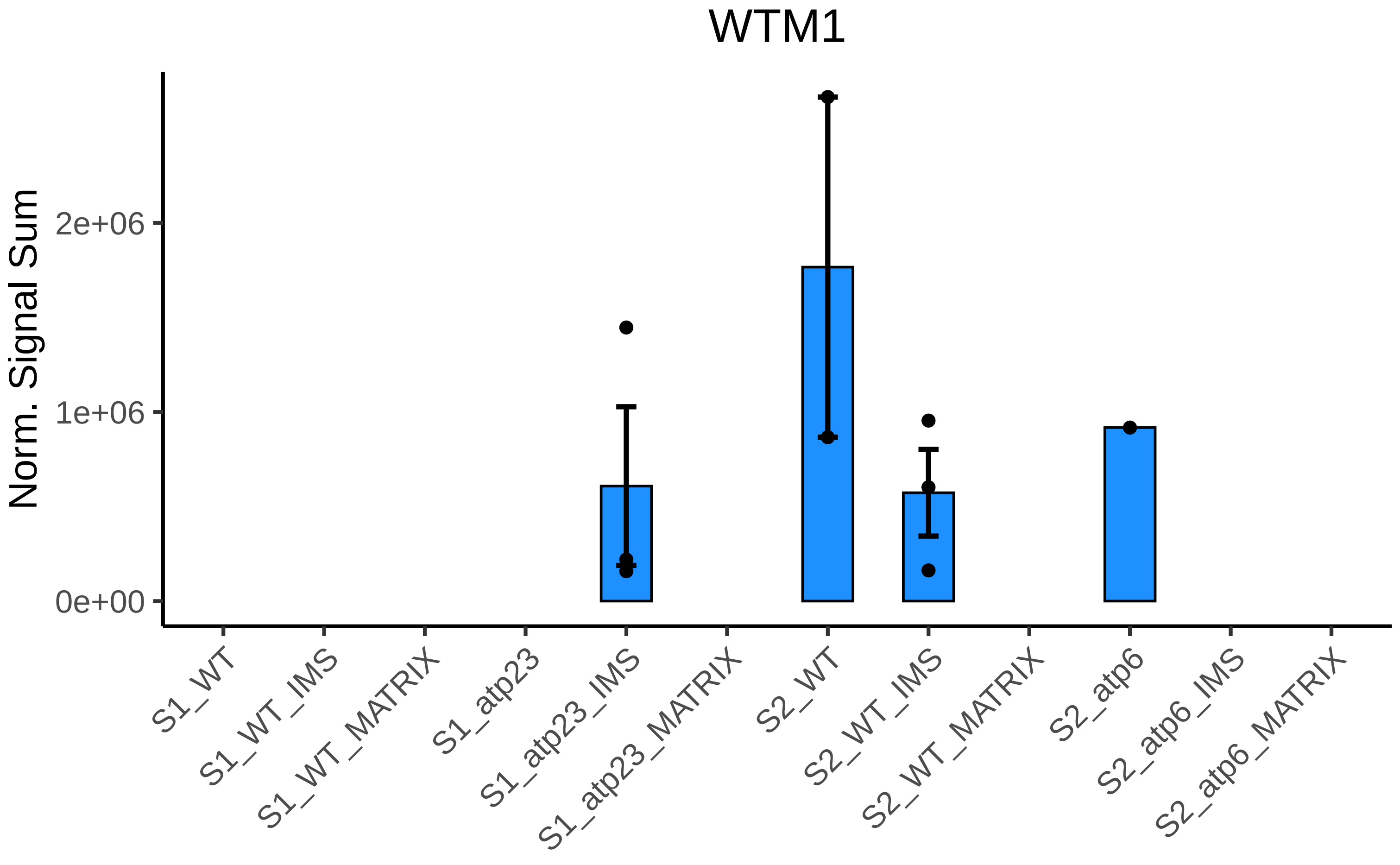

WTM1
Norm. Signal Sum
2e+06
1e+06
0e+00
S1_WT
S1_WT_IMS
S1_WT_MATRIX
S1_atp23
S1_atp23_IMS
S1_atp23_MATRIX
S2_WT
S2_WT_IMS
S2_WT_MATRIX
S2_atp6
S2_atp6_IMS
S2_atp6_MATRIX

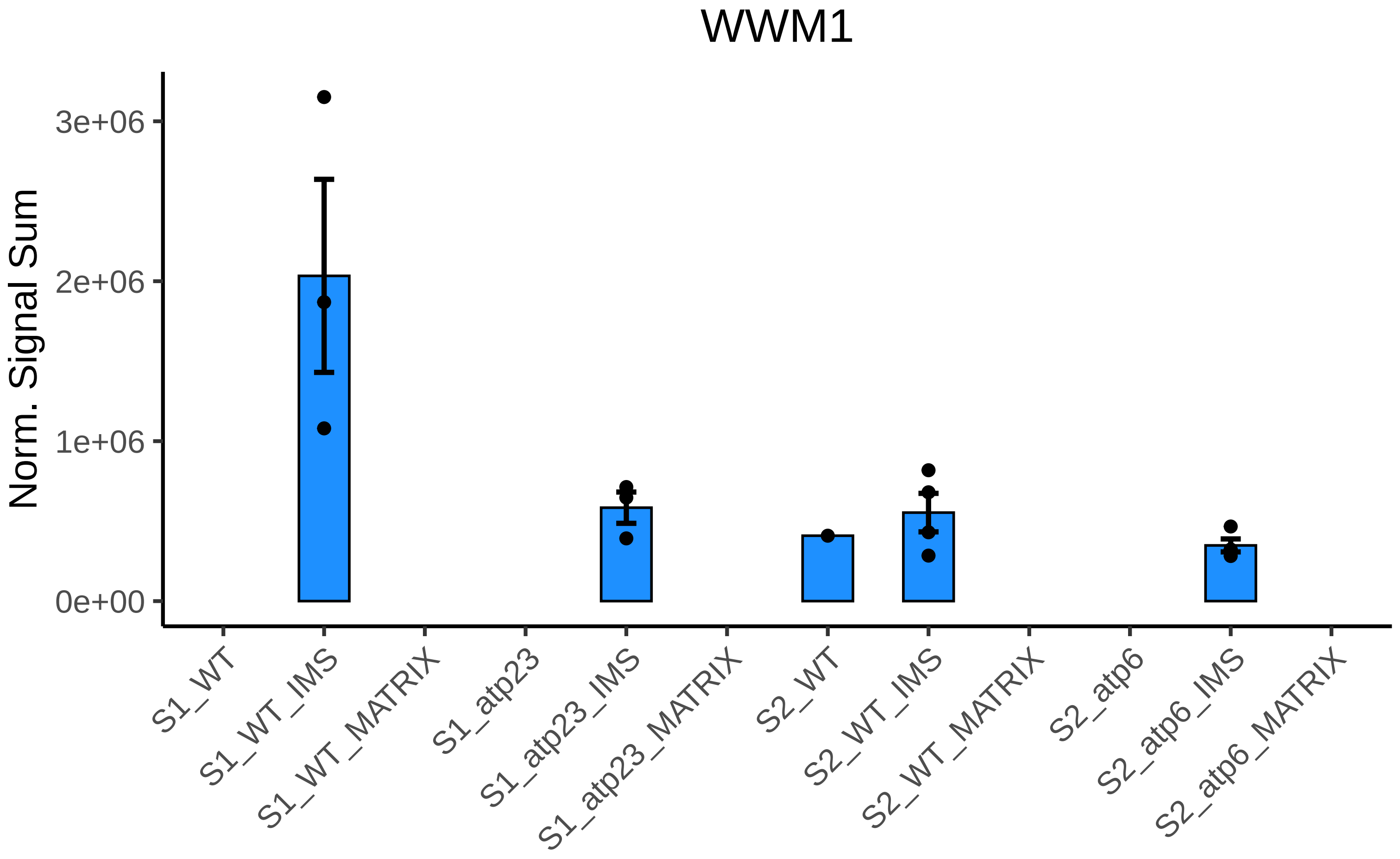

WWM1
Norm. Signal Sum
3e+06
2e+06
1e+06
0e+00
S1_WT
S1_WT_IMS
S1_WT_MATRIX
S1_atp23
S1_atp23_IMS
S1_atp23_MATRIX
S2_WT
S2_WT_IMS
S2_WT_MATRIX
S2_atp6
S2_atp6_IMS
S2_atp6_MATRIX

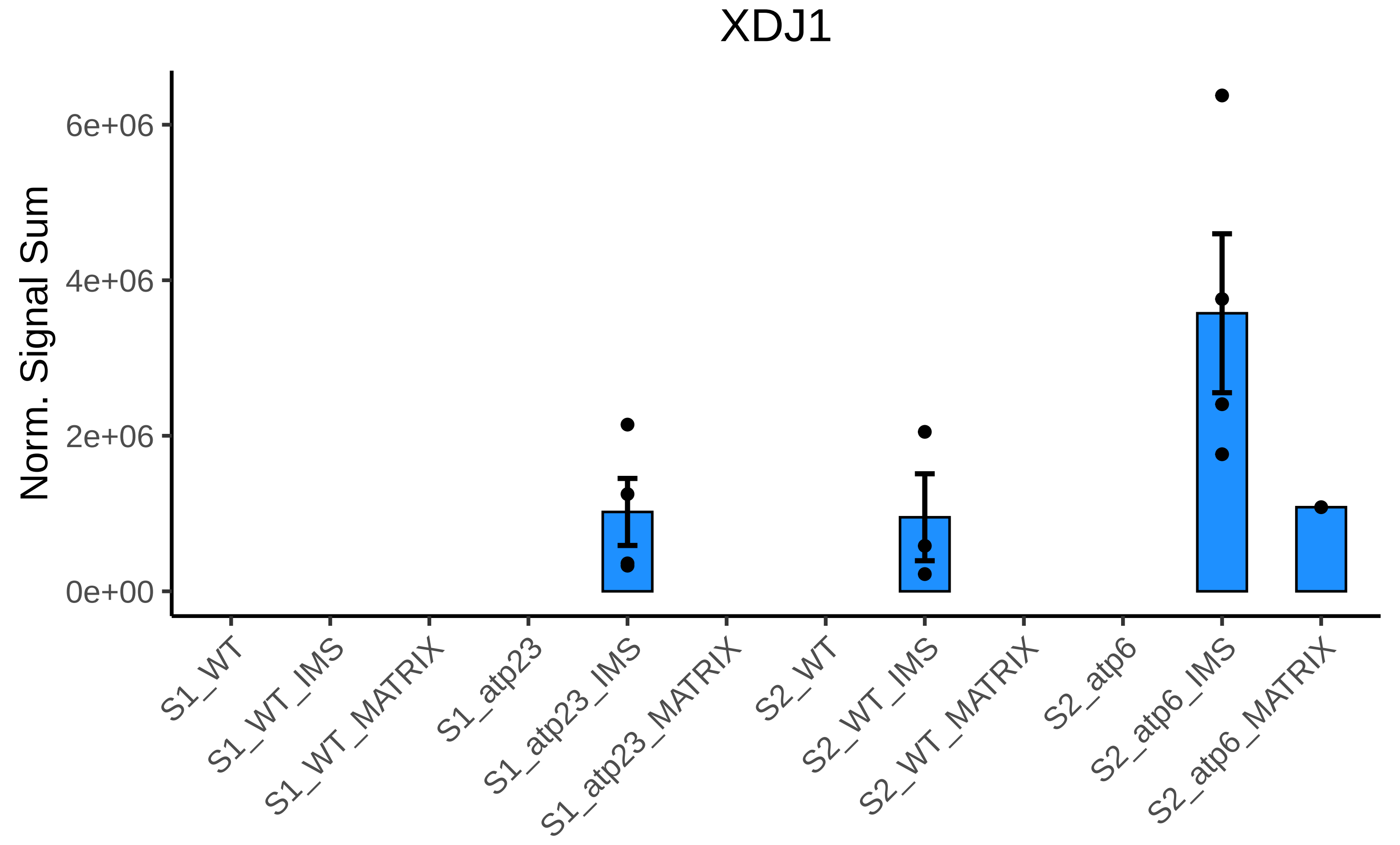

XDJ1
Norm. Signal Sum
6e+06
4e+06
2e+06
0e+00
S1_WT
S1_WT_IMS
S1_WT_MATRIX
S1_atp23
S1_atp23_IMS
S1_atp23_MATRIX
S2_WT
S2_WT_IMS
S2_WT_MATRIX
S2_atp6
S2_atp6_IMS
S2_atp6_MATRIX

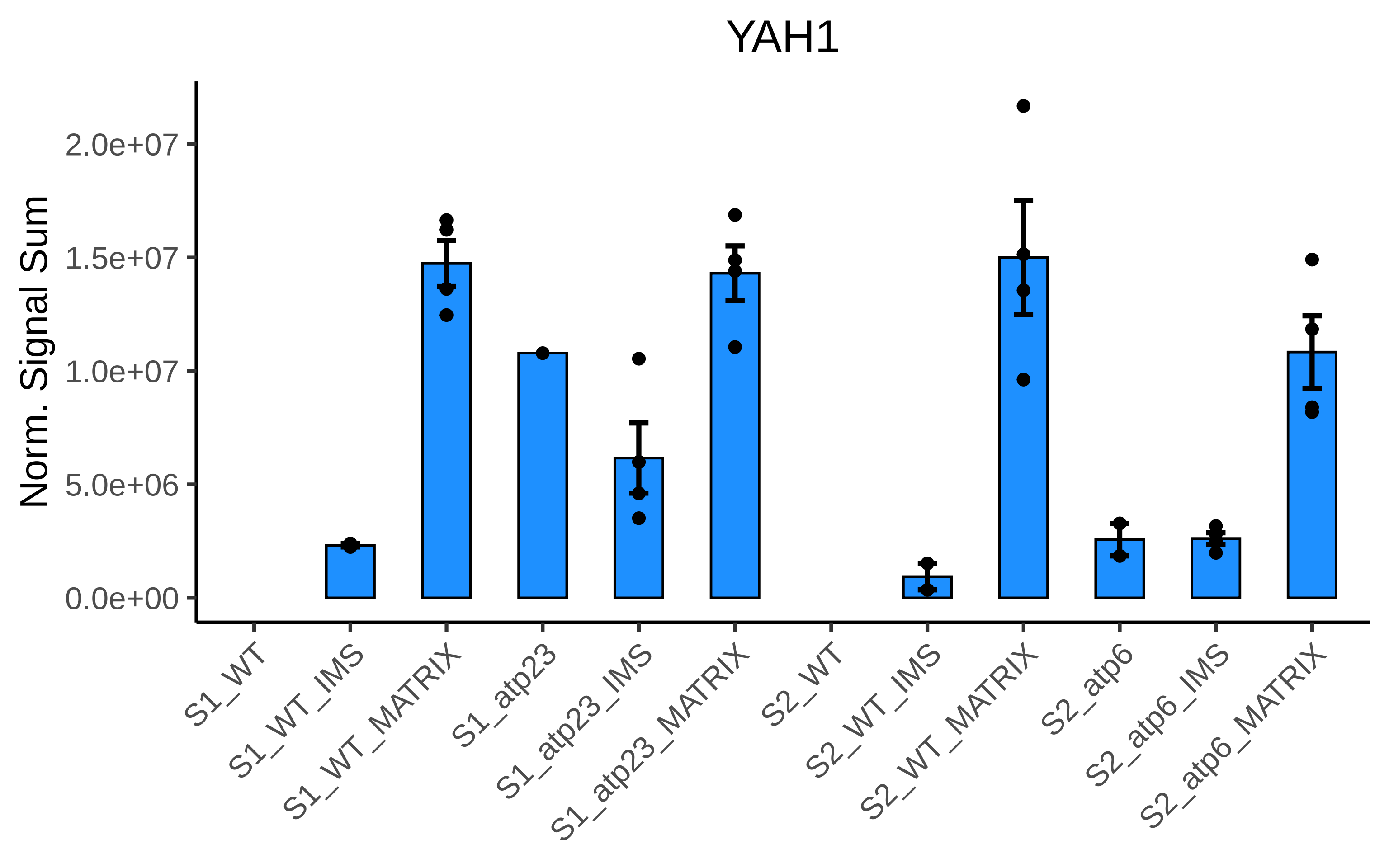

YAH1
Norm. Signal Sum
2.0e+07
1.5e+07
1.0e+07
5.0e+06
0.0e+00
S1_WT
S1_WT_IMS
S1_WT_MATRIX
S1_atp23
S1_atp23_IMS
S1_atp23_MATRIX
S2_WT
S2_WT_IMS
S2_WT_MATRIX
S2_atp6
S2_atp6_IMS
S2_atp6_MATRIX

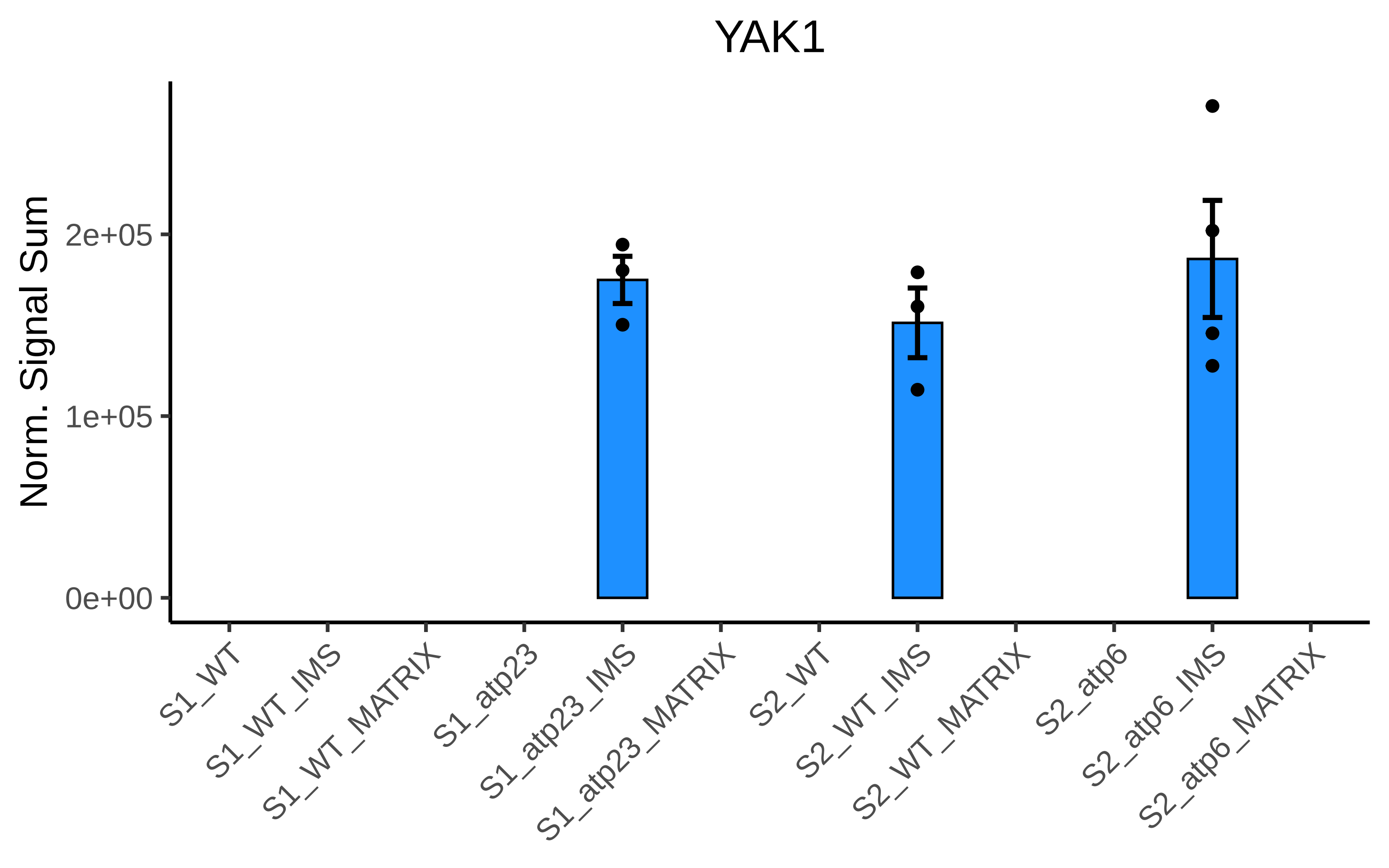

YAK1
Norm. Signal Sum
2e+05
1e+05
0e+00
S1_WT
S1_WT_IMS
S1_WT_MATRIX
S1_atp23
S1_atp23_IMS
S1_atp23_MATRIX
S2_WT
S2_WT_IMS
S2_WT_MATRIX
S2_atp6
S2_atp6_IMS
S2_atp6_MATRIX

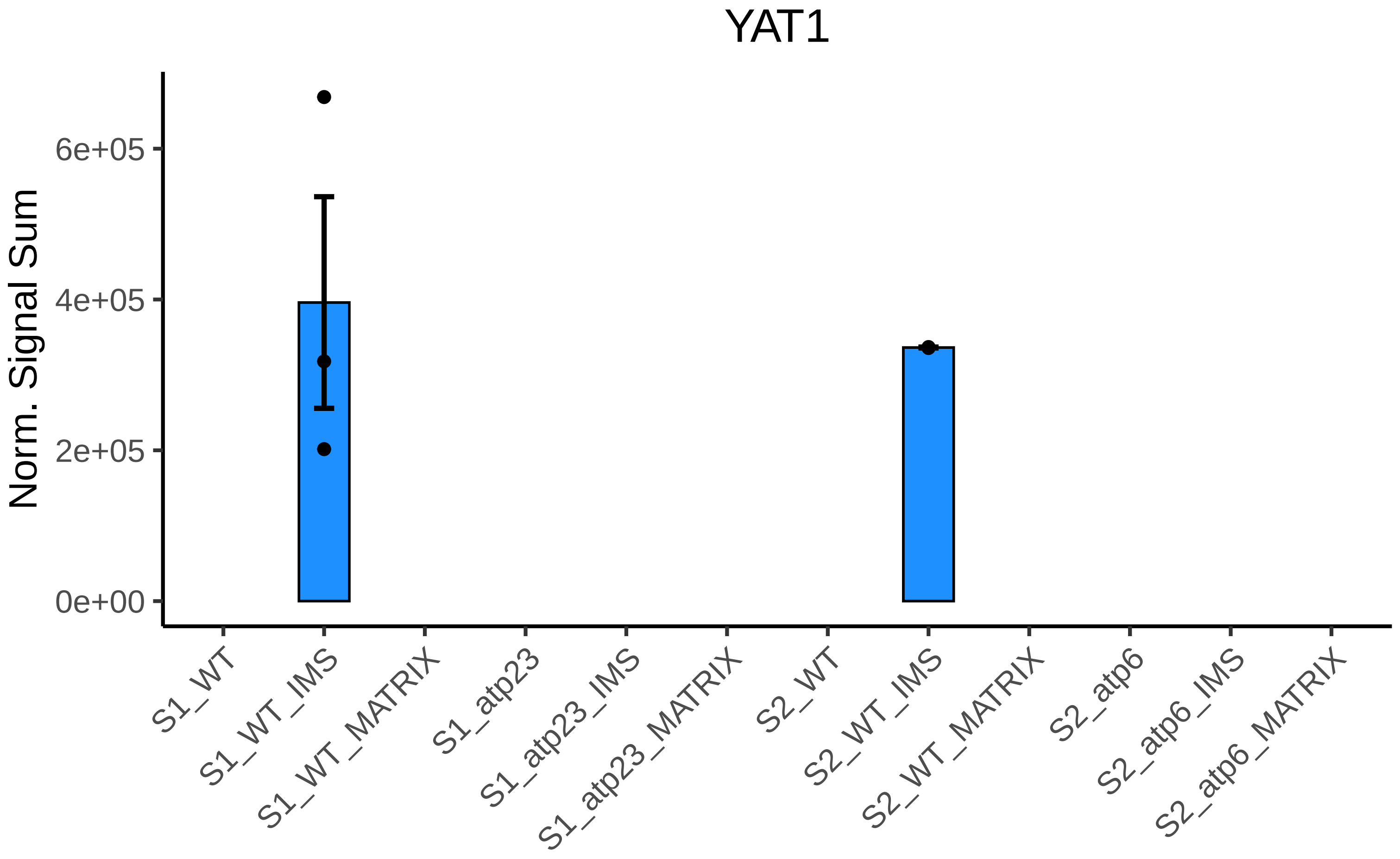

YAT1
Norm. Signal Sum
6e+05
4e+05
2e+05
0e+00
S1_WT
S1_WT_IMS
S1_WT_MATRIX
S1_atp23
S1_atp23_IMS
S1_atp23_MATRIX
S2_WT
S2_WT_IMS
S2_WT_MATRIX
S2_atp6
S2_atp6_IMS
S2_atp6_MATRIX

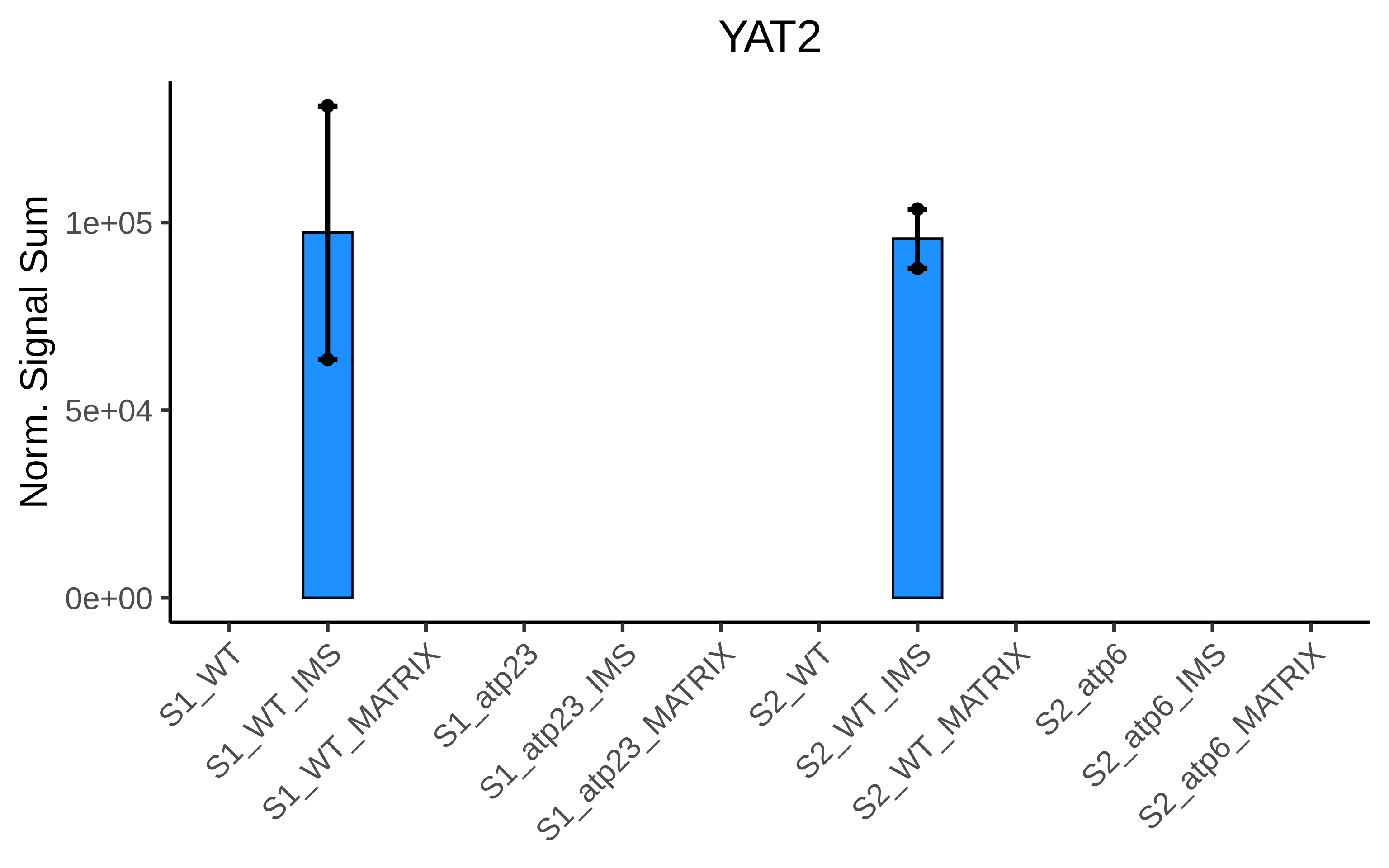
YAT2
Norm. Signal Sum
1e+05
5e+04
0e+00
S1_WT
S1_WT_IMS
S1_WT_MATRIX
S1_atp23
S1_atp23_IMS
S1_atp23_MATRIX
S2_WT
S2_WT_IMS
S2_WT_MATRIX
S2_atp6
S2_atp6_IMS
S2_atp6_MATRIX

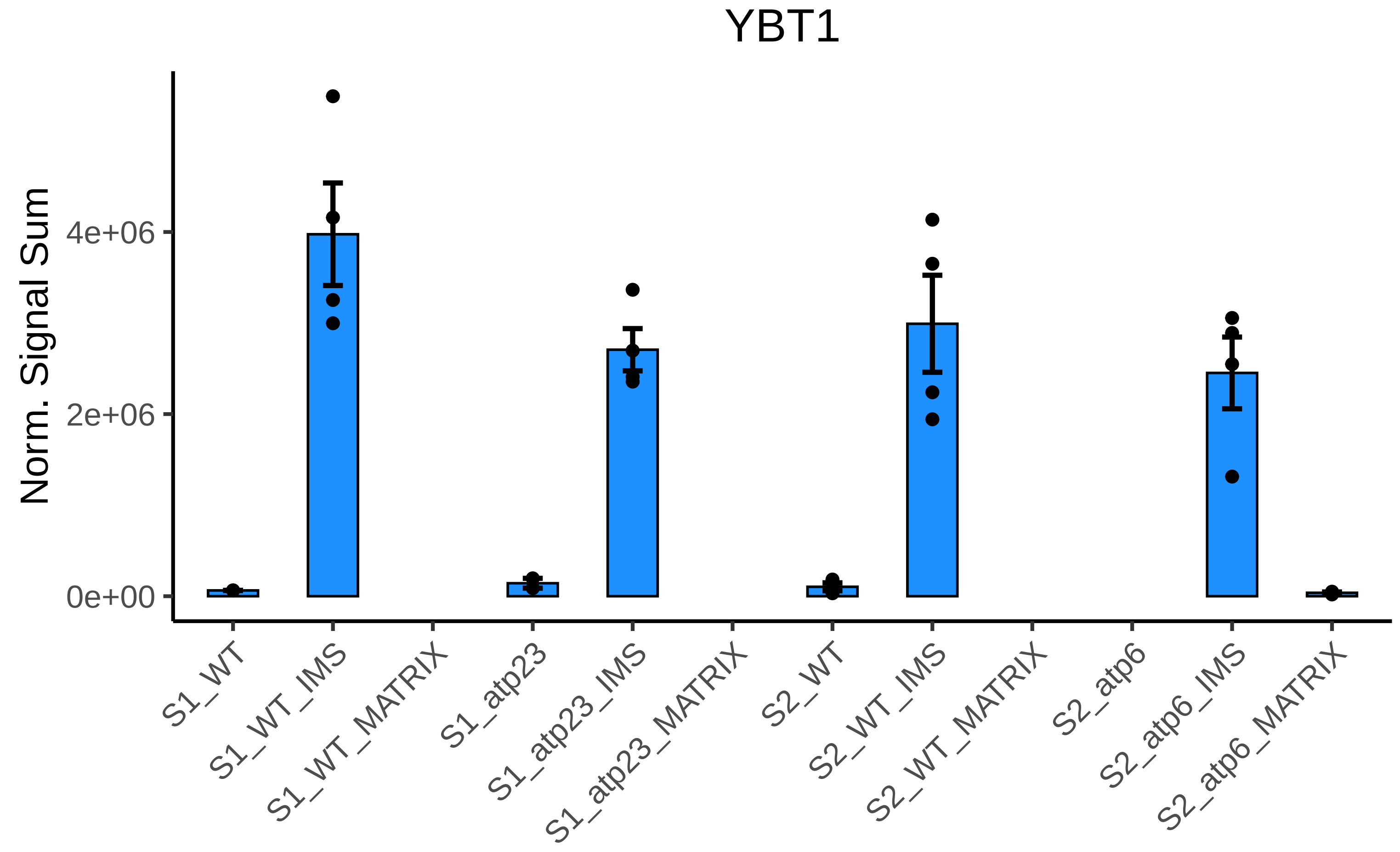

YBT1
Norm. Signal Sum
4e+06
2e+06
0e+00
S1_WT
S1_WT_IMS
S1_WT_MATRIX
S1_atp23
S1_atp23_IMS
S1_atp23_MATRIX
S2_WT
S2_WT_IMS
S2_WT_MATRIX
S2_atp6
S2_atp6_IMS
S2_atp6_MATRIX

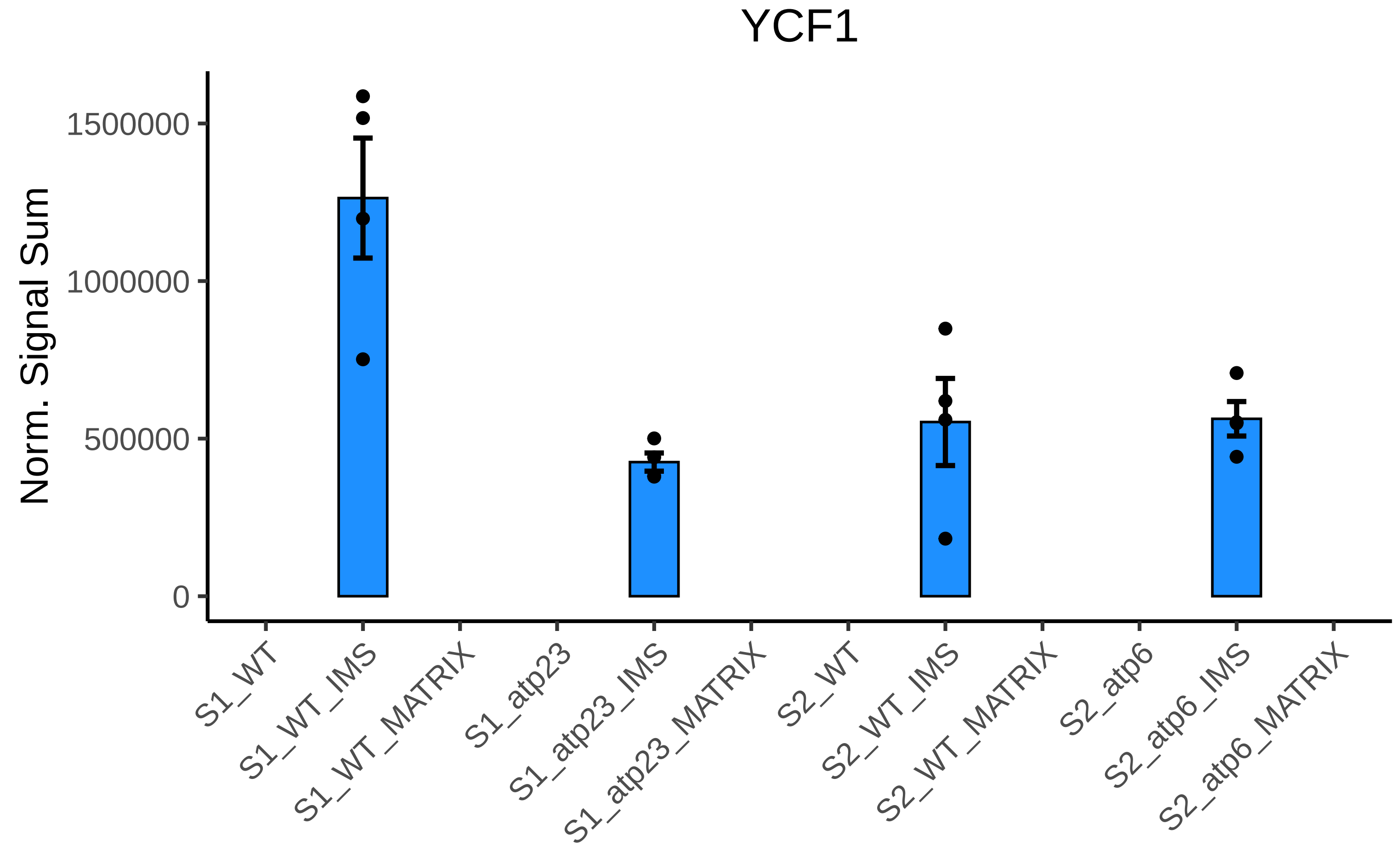

YCF1
Norm. Signal Sum
1500000
1000000
500000
0
S1_WT
S1_WT_IMS
S1_WT_MATRIX
S1_atp23
S1_atp23_IMS
S1_atp23_MATRIX
S2_WT
S2_WT_IMS
S2_WT_MATRIX
S2_atp6
S2_atp6_IMS
S2_atp6_MATRIX

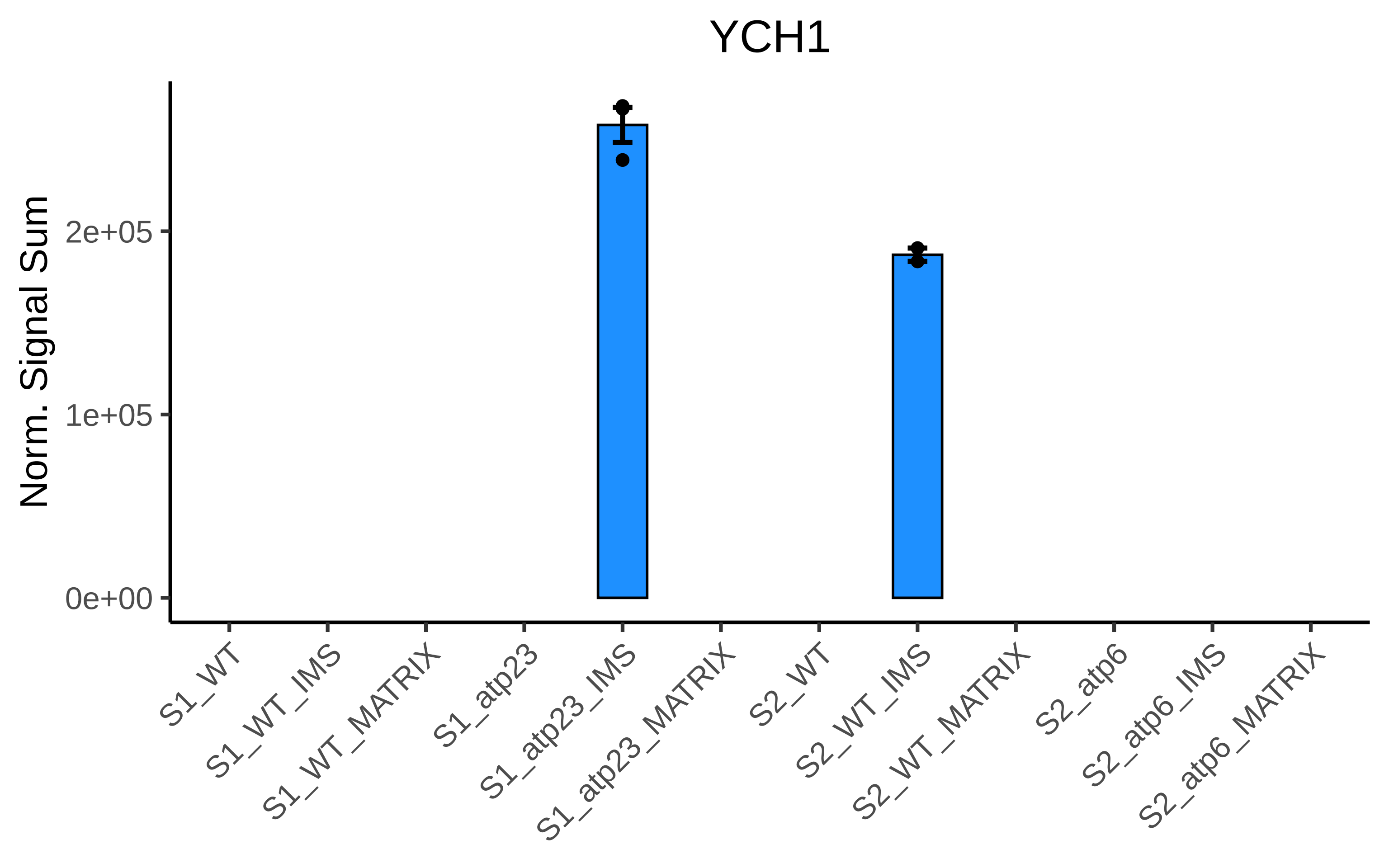

YCH1
Norm. Signal Sum
2e+05
1e+05
0e+00
S1_WT
S1_WT_IMS
S1_WT_MATRIX
S1_atp23
S1_atp23_IMS
S1_atp23_MATRIX
S2_WT
S2_WT_IMS
S2_WT_MATRIX
S2_atp6
S2_atp6_IMS
S2_atp6_MATRIX

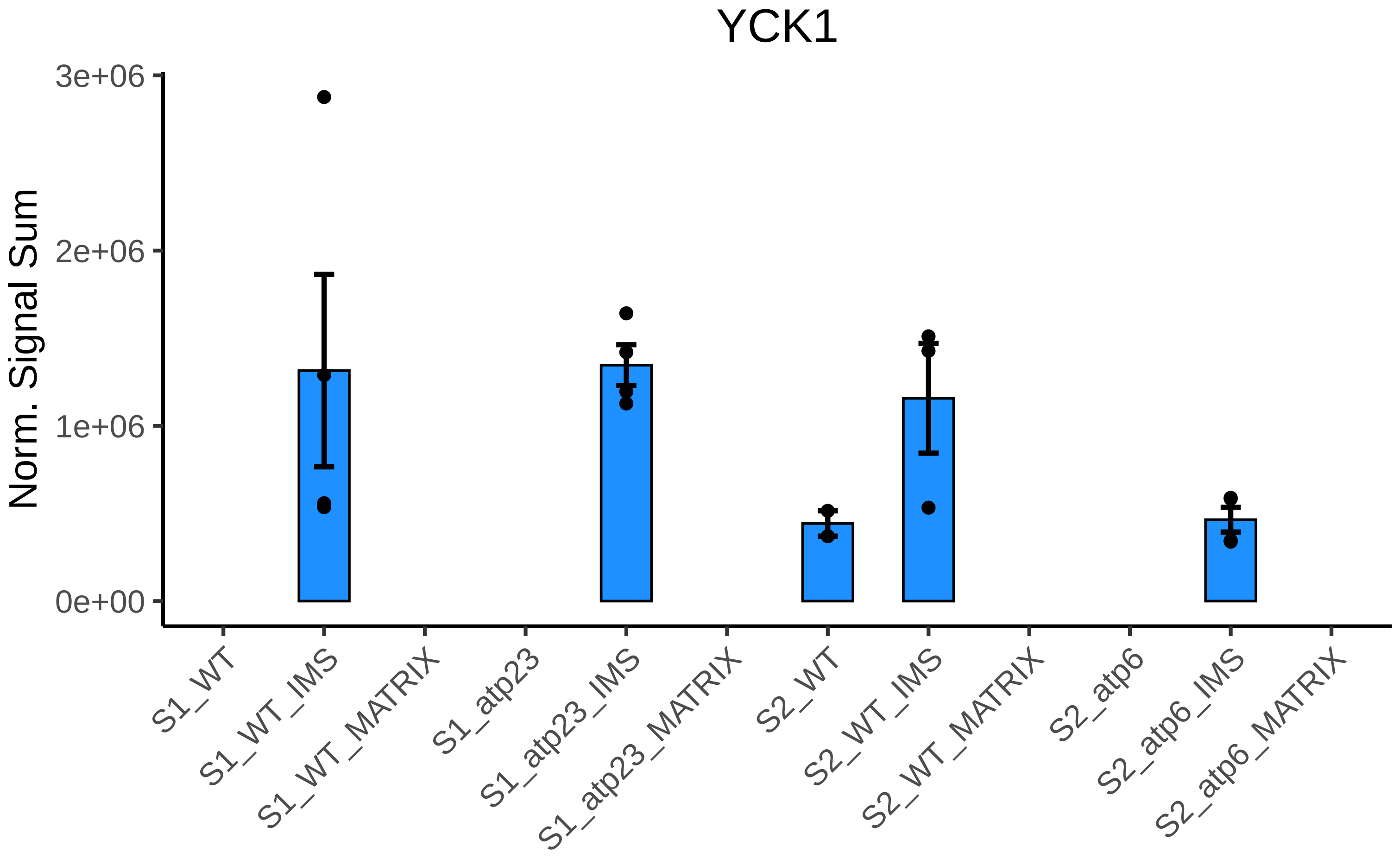
YCK1
Norm. Signal Sum
3e+06
2e+06
1e+06
0e+00
S1_WT
S1_WT_IMS
S1_WT_MATRIX
S1_atp23
S1_atp23_IMS
S1_atp23_MATRIX
S2_WT
S2_WT_IMS
S2_WT_MATRIX
S2_atp6
S2_atp6_IMS
S2_atp6_MATRIX

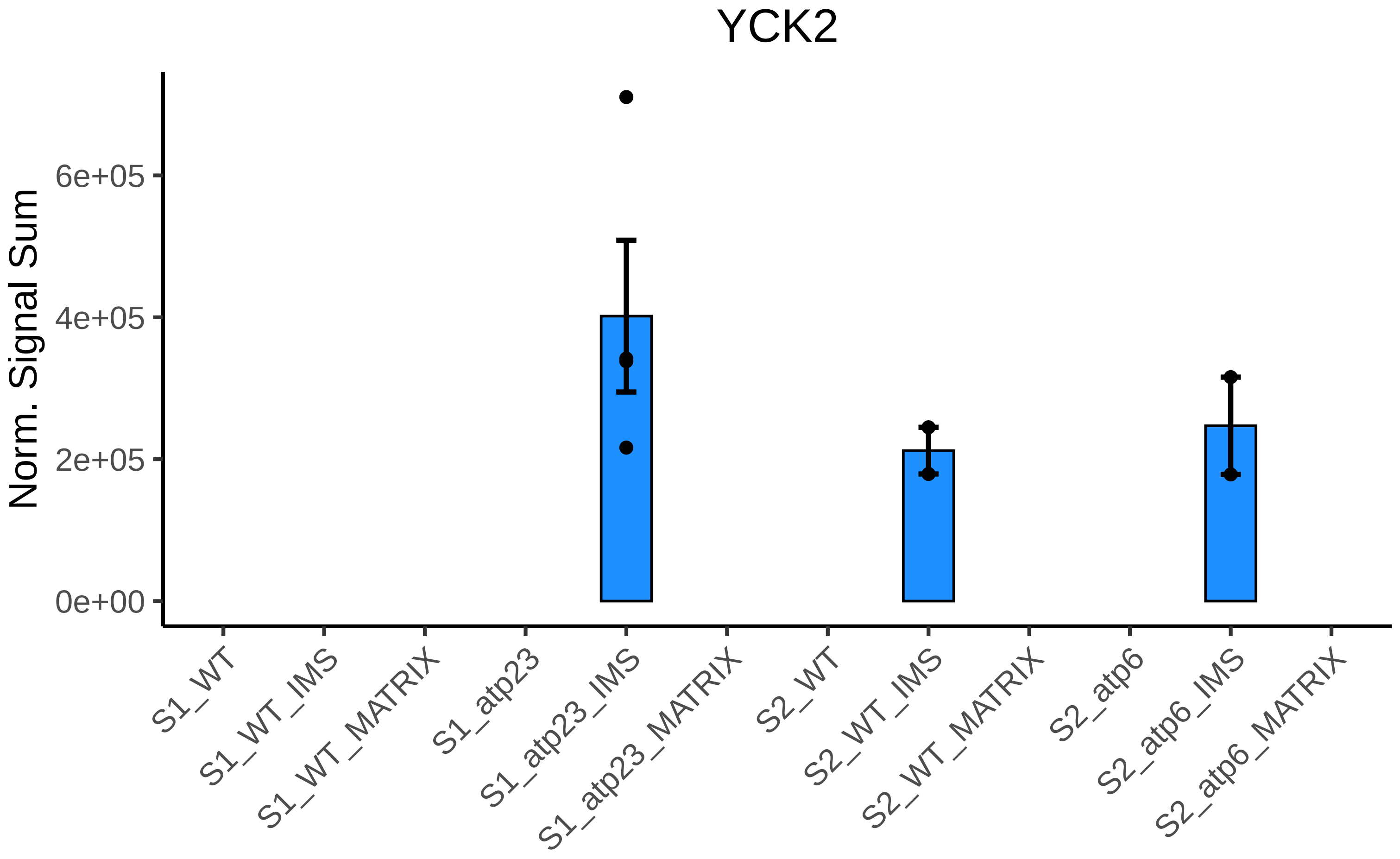

YCK2
Norm. Signal Sum
6e+05
4e+05
2e+05
0e+00
S1_WT
S1_WT_IMS
S1_WT_MATRIX
S1_atp23
S1_atp23_IMS
S1_atp23_MATRIX
S2_WT
S2_WT_IMS
S2_WT_MATRIX
S2_atp6
S2_atp6_IMS
S2_atp6_MATRIX

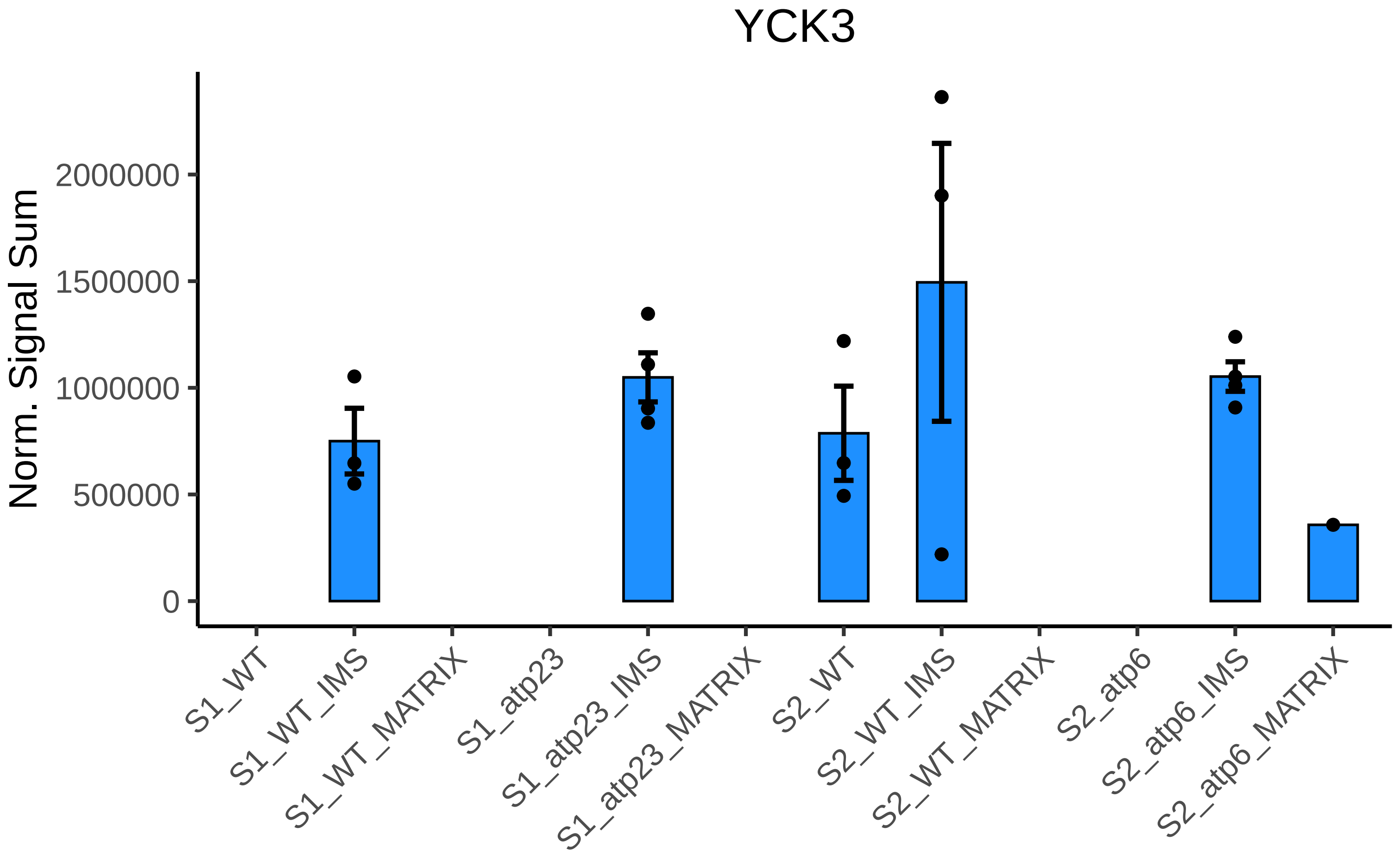
YCK3
Norm. Signal Sum
2000000
1500000
1000000
500000
0
S1_WT
S1_WT_IMS
S1_WT_MATRIX
S1_atp23
S1_atp23_IMS
S1_atp23_MATRIX
S2_WT
S2_WT_IMS
S2_WT_MATRIX
S2_atp6
S2_atp6_IMS
S2_atp6_MATRIX

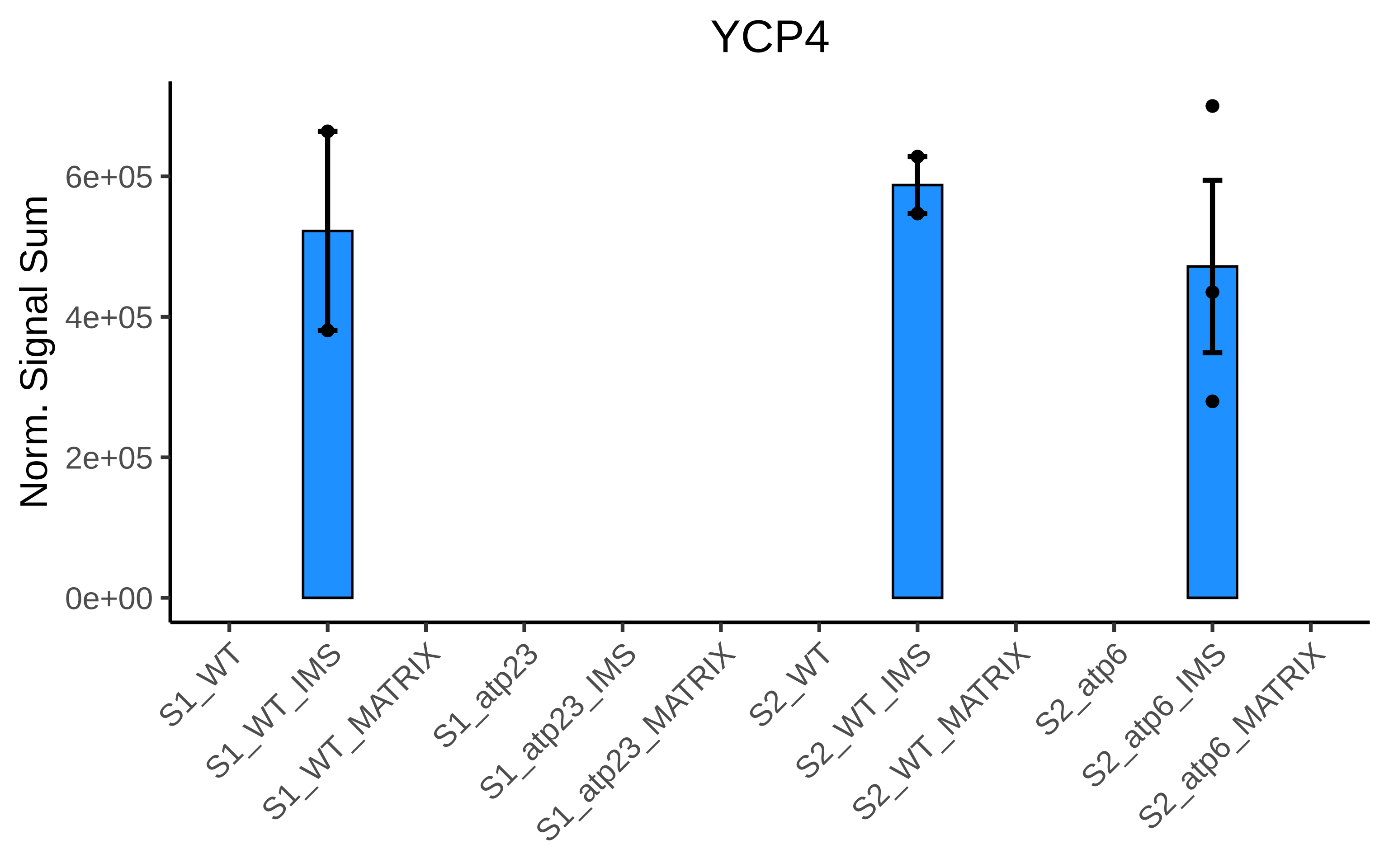

YCP4
Norm. Signal Sum
6e+05
4e+05
2e+05
0e+00
S1_WT
S1_WT_IMS
S1_WT_MATRIX
S1_atp23
S1_atp23_IMS
S1_atp23_MATRIX
S2_WT
S2_WT_IMS
S2_WT_MATRIX
S2_atp6
S2_atp6_IMS
S2_atp6_MATRIX

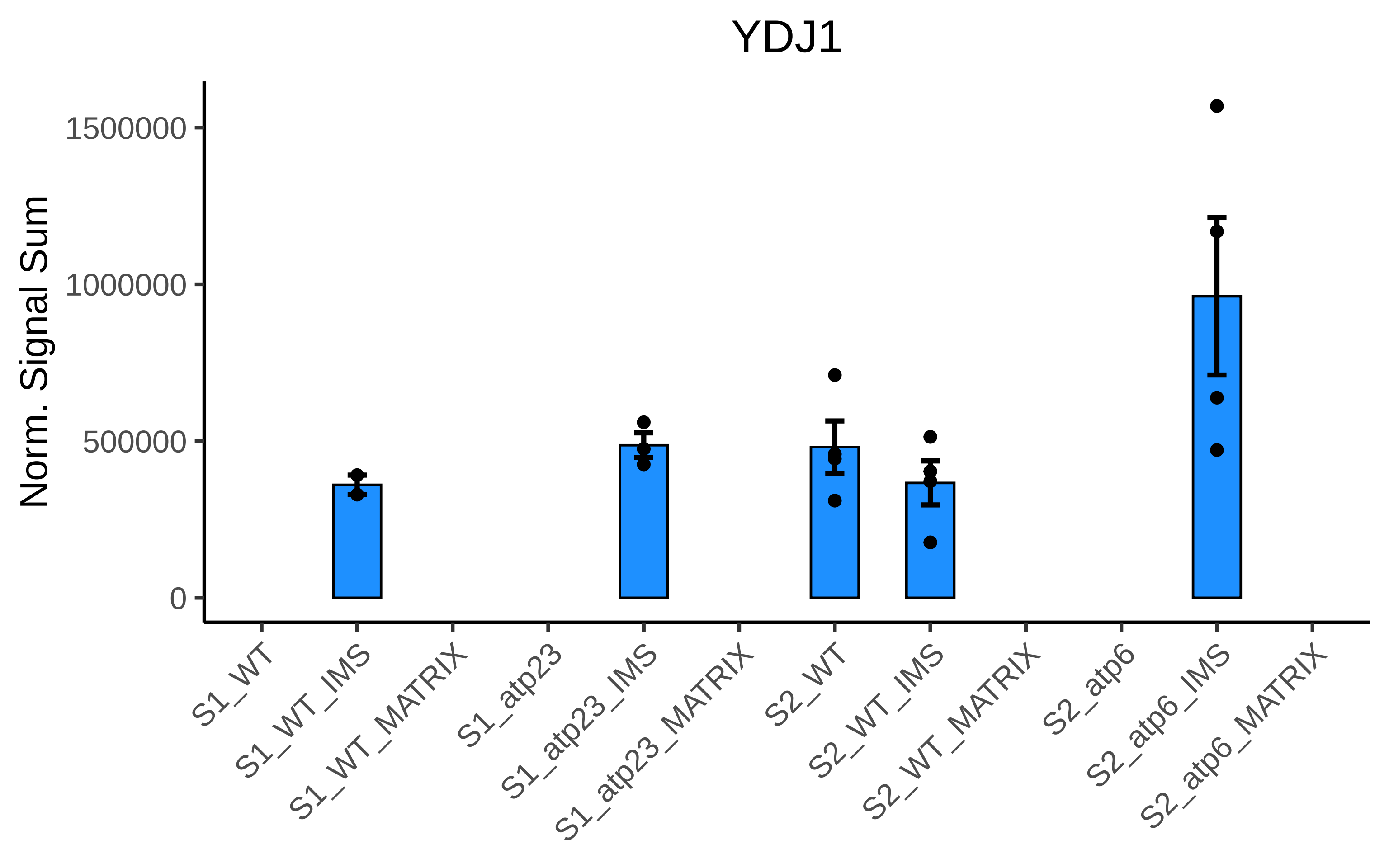

YDJ1
Norm. Signal Sum
1500000
1000000
500000
0
S1_WT
S1_WT_IMS
S1_WT_MATRIX
S1_atp23
S1_atp23_IMS
S1_atp23_MATRIX
S2_WT
S2_WT_IMS
S2_WT_MATRIX
S2_atp6
S2_atp6_IMS
S2_atp6_MATRIX

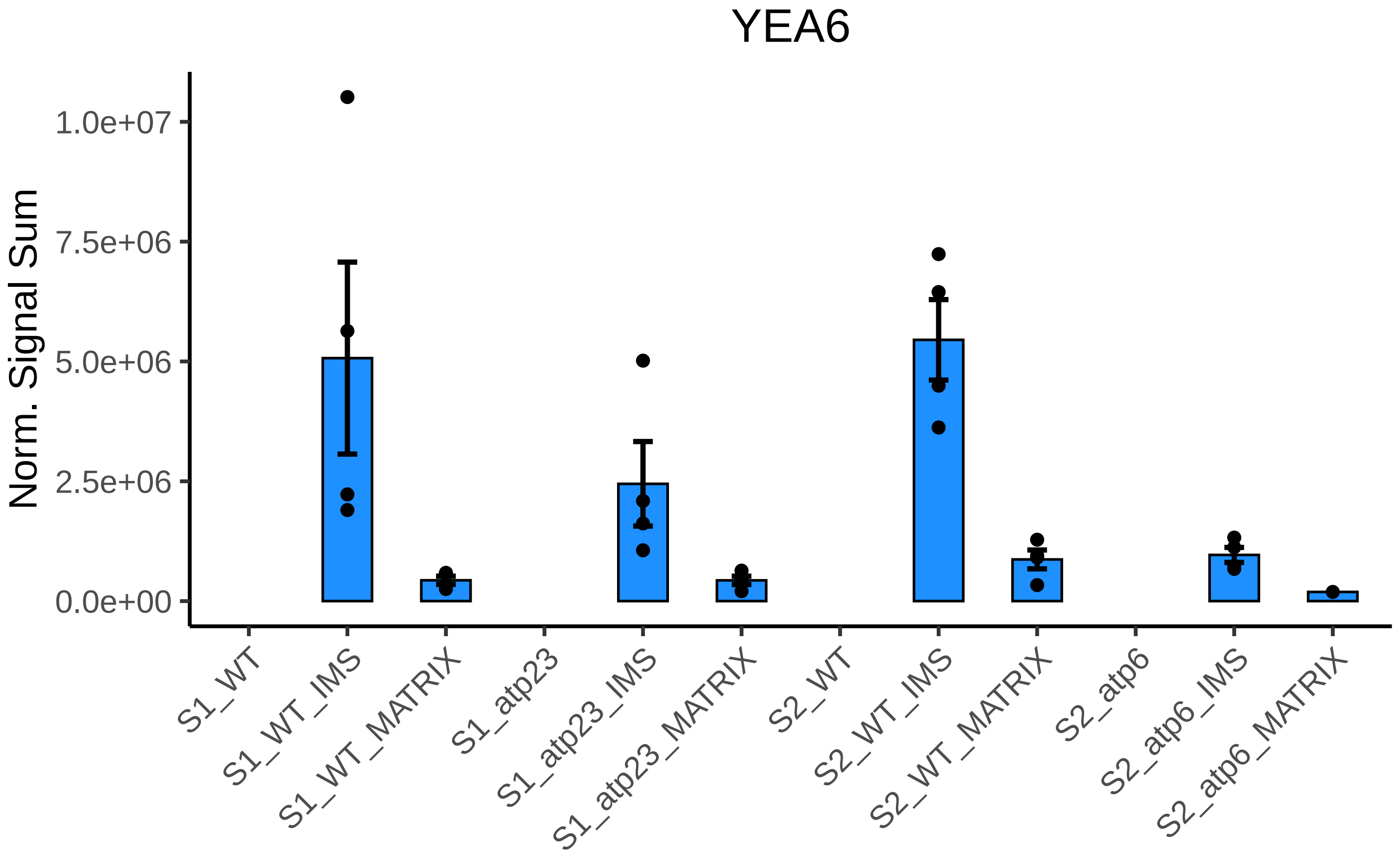
YEA6
Norm. Signal Sum
1.0e+07
7.5e+06
5.0e+06
2.5e+06
0.0e+00
S1_WT
S1_WT_IMS
S1_WT_MATRIX
S1_atp23
S1_atp23_IMS
S1_atp23_MATRIX
S2_WT
S2_WT_IMS
S2_WT_MATRIX
S2_atp6
S2_atp6_IMS
S2_atp6_MATRIX

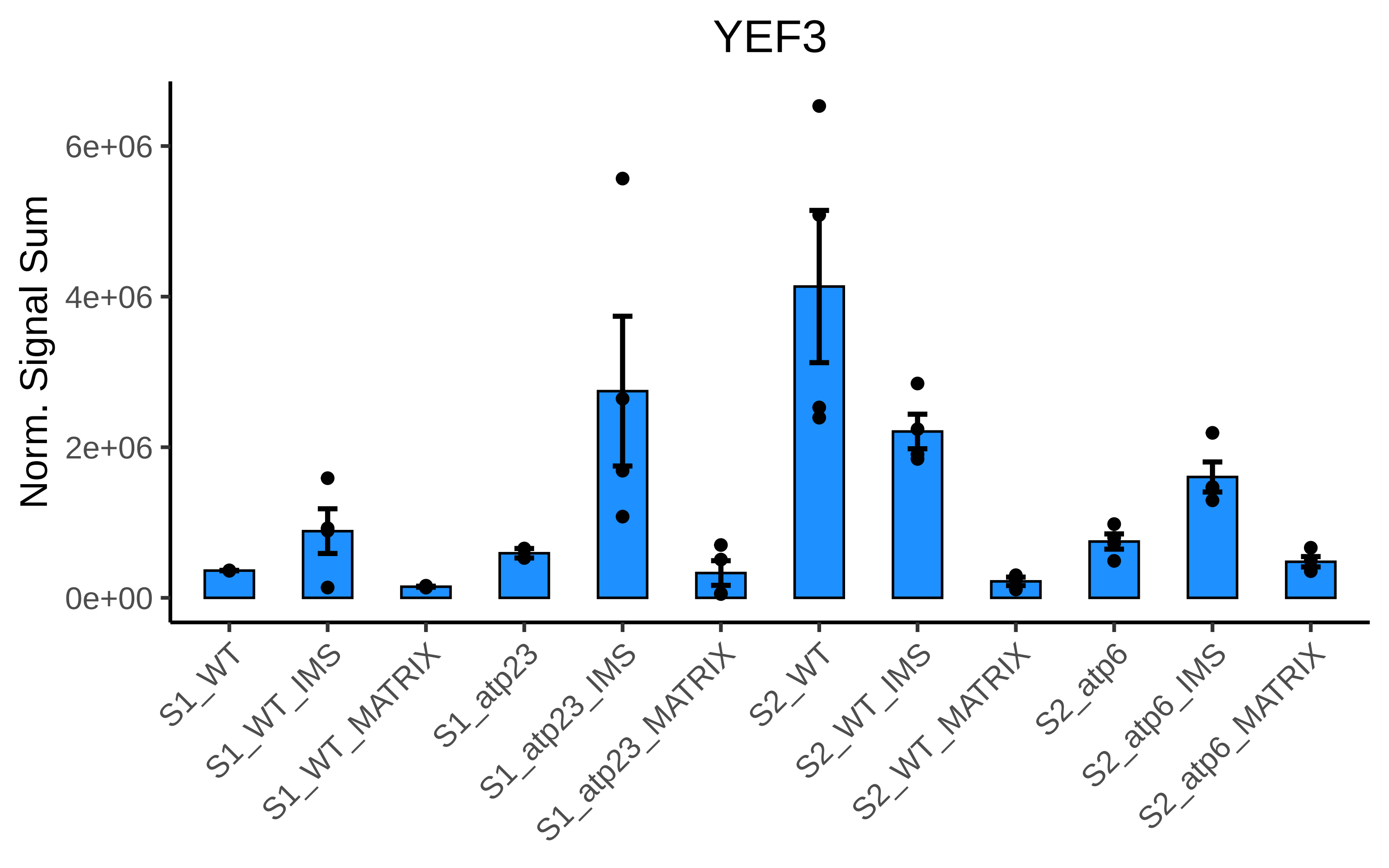
YEF3
Norm. Signal Sum
6e+06
4e+06
2e+06
0e+00
S1_WT
S1_WT_IMS
S1_WT_MATRIX
S1_atp23
S1_atp23_IMS
S1_atp23_MATRIX
S2_WT
S2_WT_IMS
S2_WT_MATRIX
S2_atp6
S2_atp6_IMS
S2_atp6_MATRIX

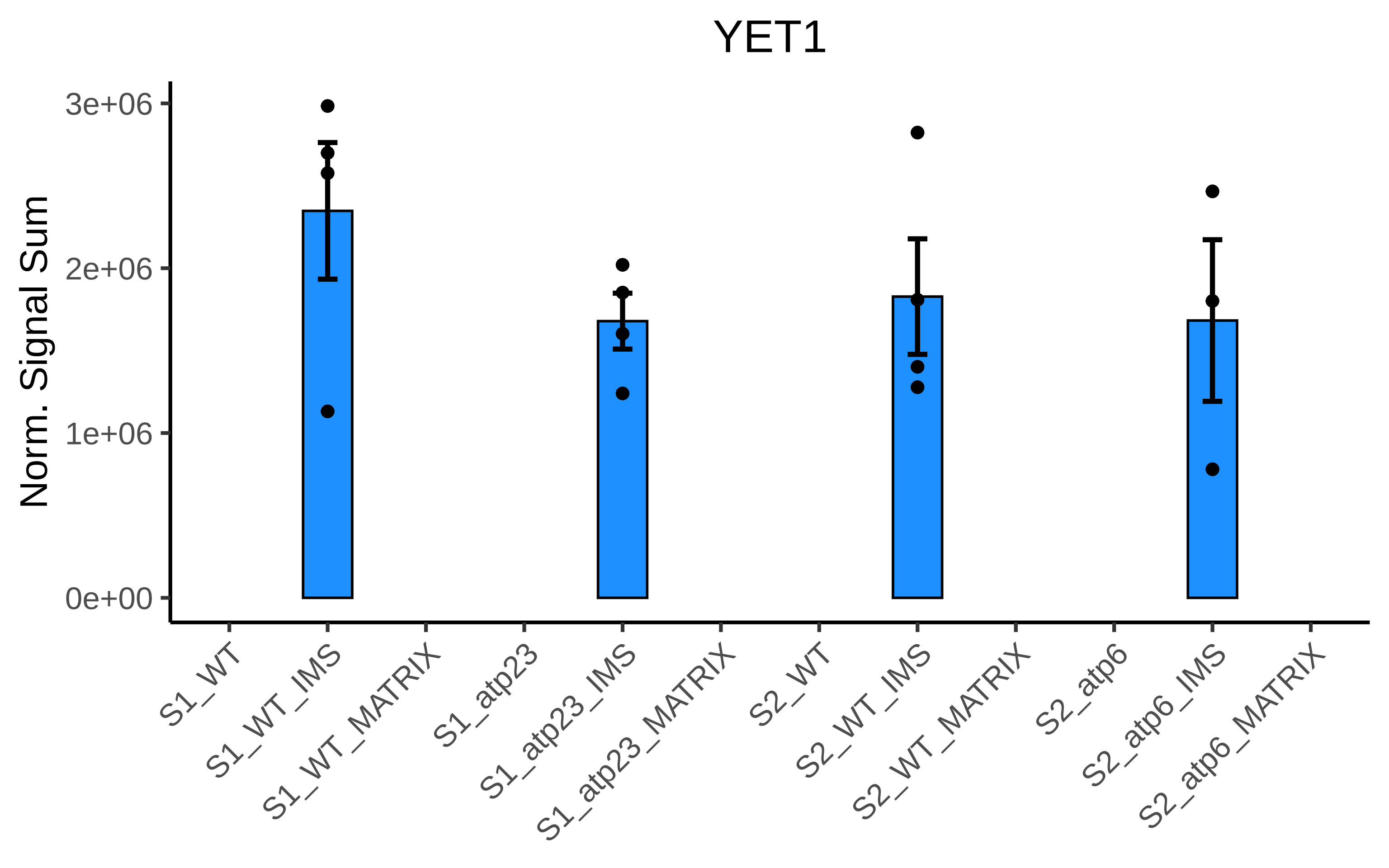

YET1
Norm. Signal Sum
3e+06
2e+06
1e+06
0e+00
S1_WT
S1_WT_IMS
S1_WT_MATRIX
S1_atp23
S1_atp23_IMS
S1_atp23_MATRIX
S2_WT
S2_WT_IMS
S2_WT_MATRIX
S2_atp6
S2_atp6_IMS
S2_atp6_MATRIX

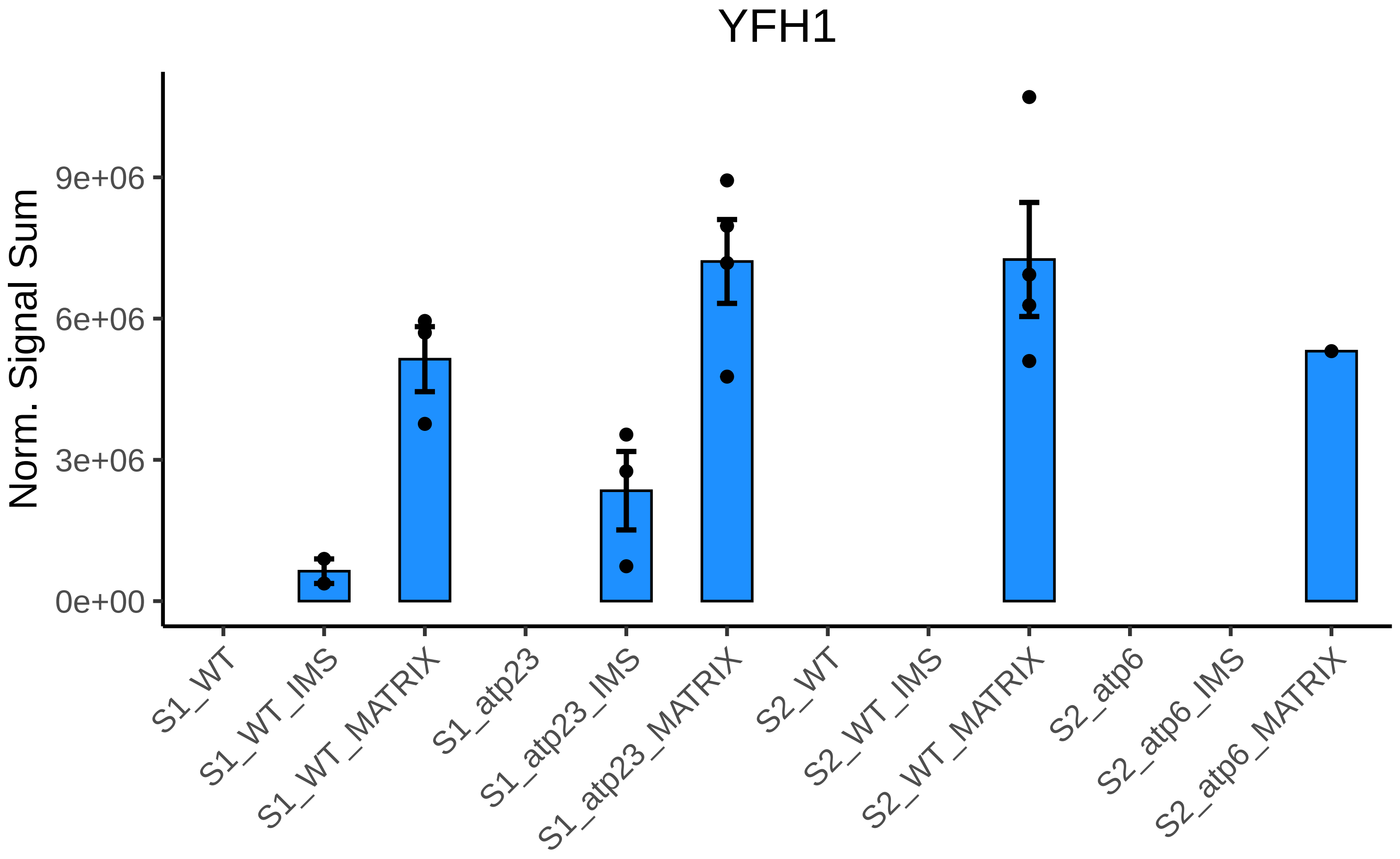

YFH1
Norm. Signal Sum
9e+06
6e+06
3e+06
0e+00
S1_WT
S1_WT_IMS
S1_WT_MATRIX
S1_atp23
S1_atp23_IMS
S1_atp23_MATRIX
S2_WT
S2_WT_IMS
S2_WT_MATRIX
S2_atp6
S2_atp6_IMS
S2_atp6_MATRIX

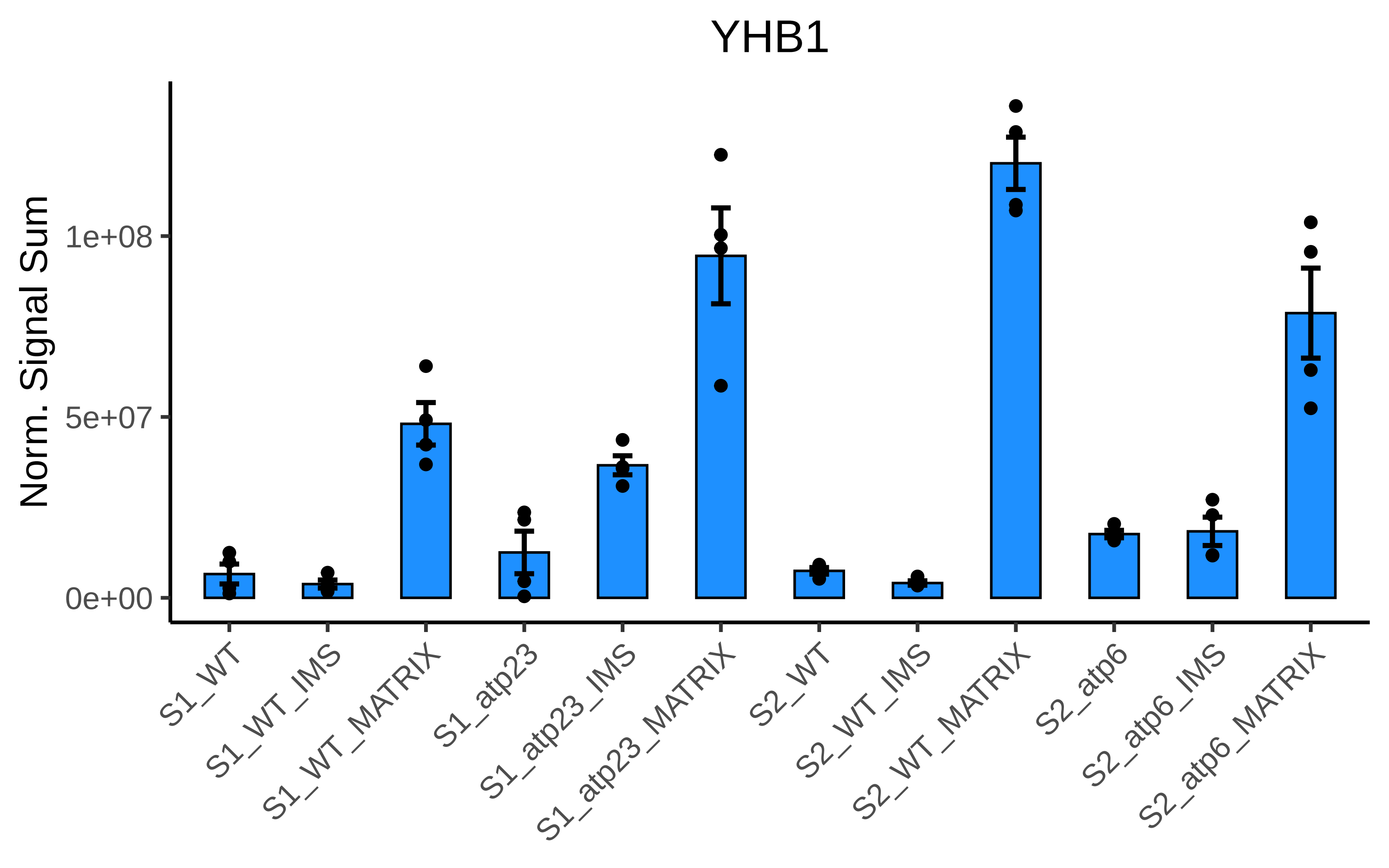

YHB1
Norm. Signal Sum
1e+08
5e+07
0e+00
S1_WT
S1_WT_IMS
S1_WT_MATRIX
S1_atp23
S1_atp23_IMS
S1_atp23_MATRIX
S2_WT
S2_WT_IMS
S2_WT_MATRIX
S2_atp6
S2_atp6_IMS
S2_atp6_MATRIX

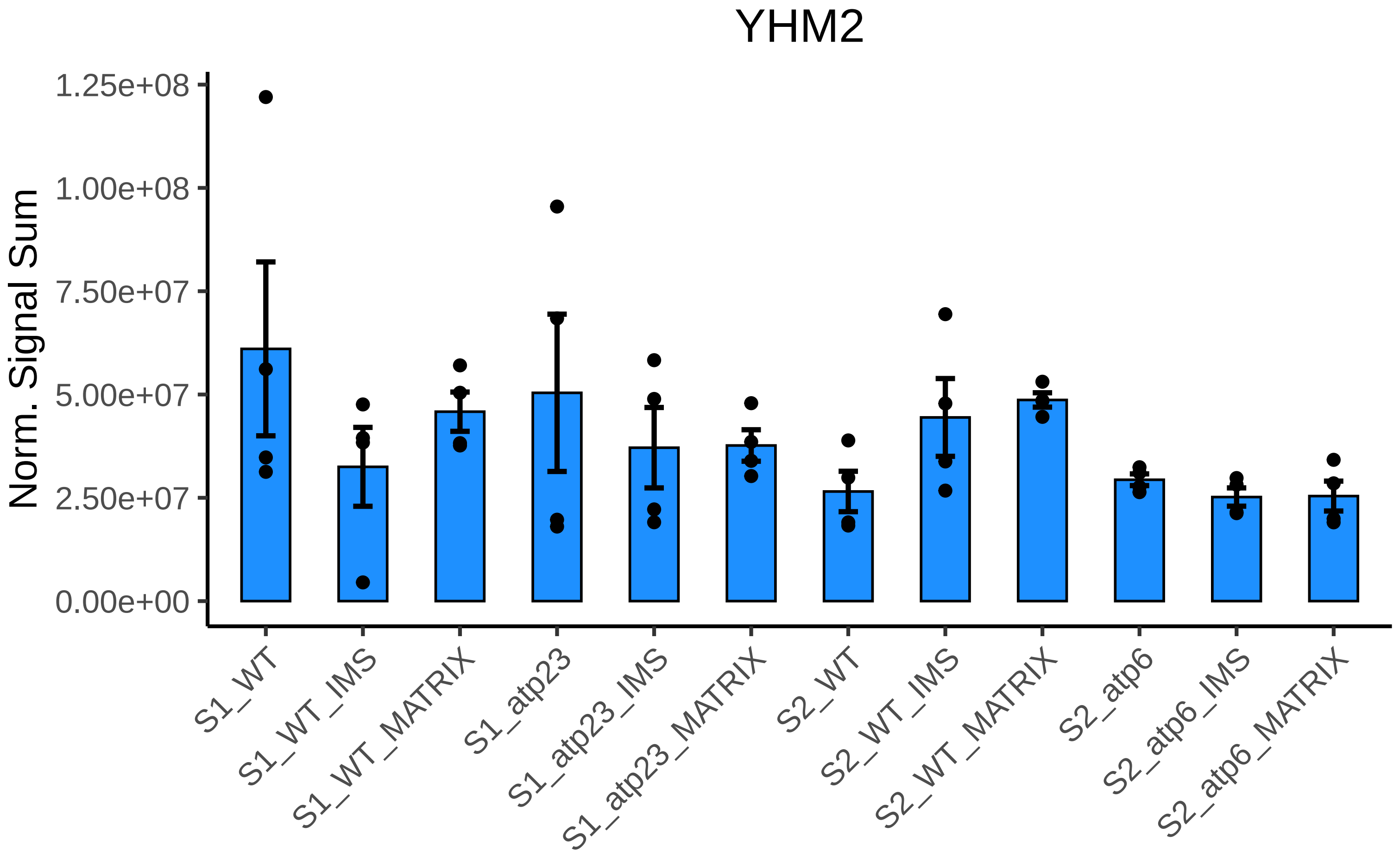

YHM2
Norm. Signal Sum
1.25e+08
1.00e+08
7.50e+07
5.00e+07
2.50e+07
0.00e+00
S1_WT
S1_WT_IMS
S1_WT_MATRIX
S1_atp23
S1_atp23_IMS
S1_atp23_MATRIX
S2_WT
S2_WT_IMS
S2_WT_MATRIX
S2_atp6
S2_atp6_IMS
S2_atp6_MATRIX

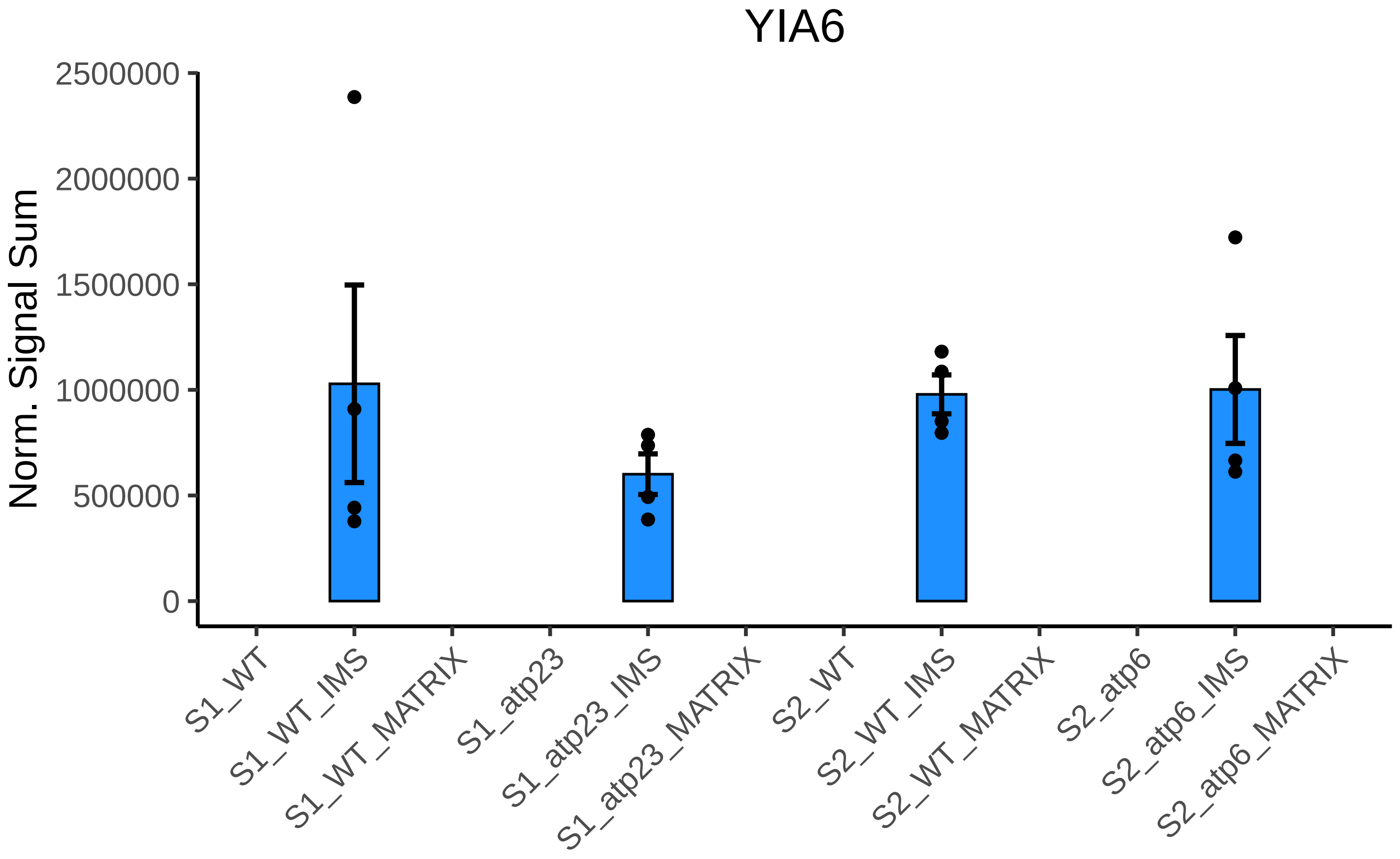
YIA6
Norm. Signal Sum
2500000
2000000
1500000
1000000
500000
0
S1_WT
S1_WT_IMS
S1_WT_MATRIX
S1_atp23
S1_atp23_IMS
S1_atp23_MATRIX
S2_WT
S2_WT_IMS
S2_WT_MATRIX
S2_atp6
S2_atp6_IMS
S2_atp6_MATRIX

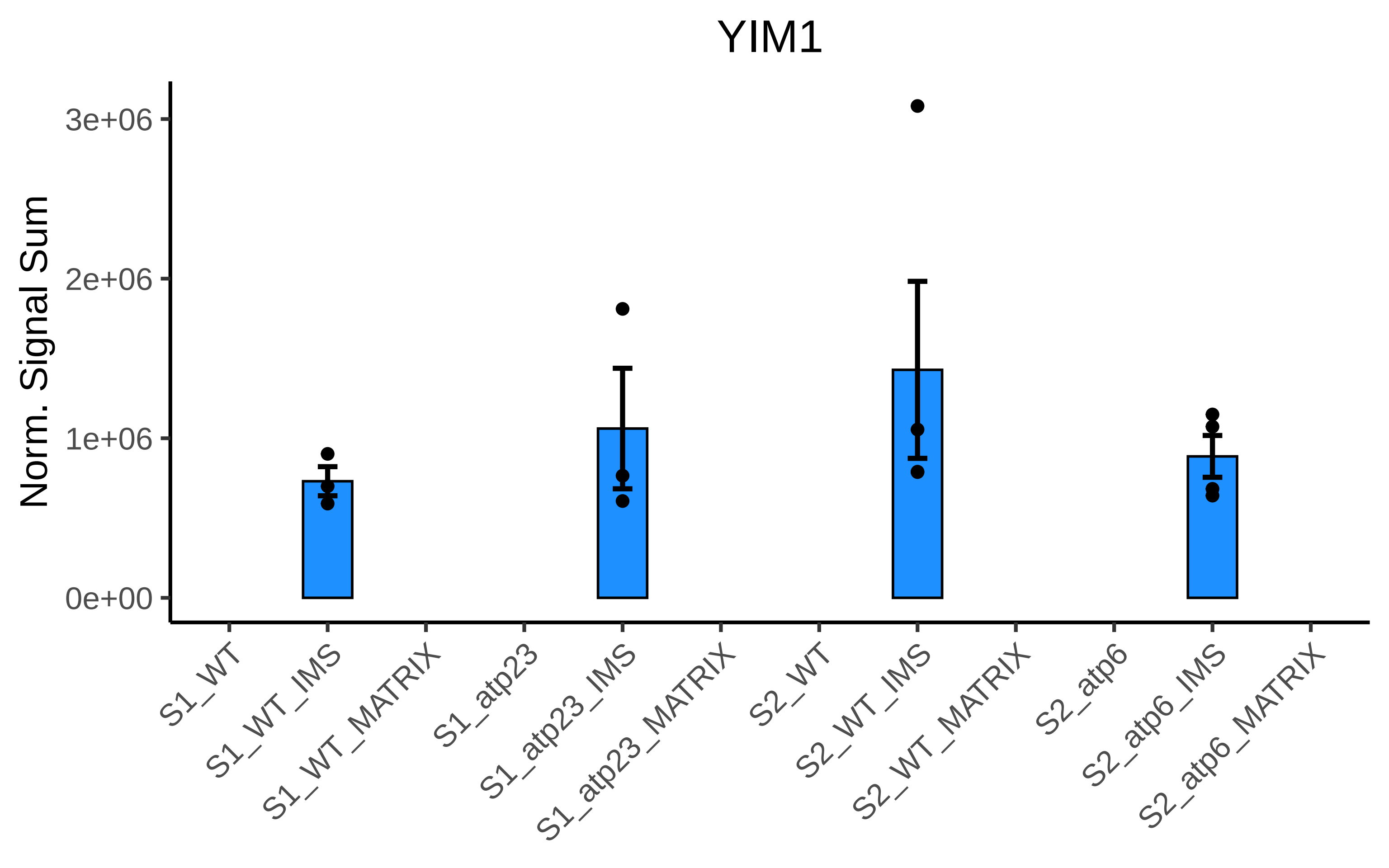
YIM1
Norm. Signal Sum
3e+06
2e+06
1e+06
0e+00
S1_WT
S1_WT_IMS
S1_WT_MATRIX
S1_atp23
S1_atp23_IMS
S1_atp23_MATRIX
S2_WT
S2_WT_IMS
S2_WT_MATRIX
S2_atp6
S2_atp6_IMS
S2_atp6_MATRIX

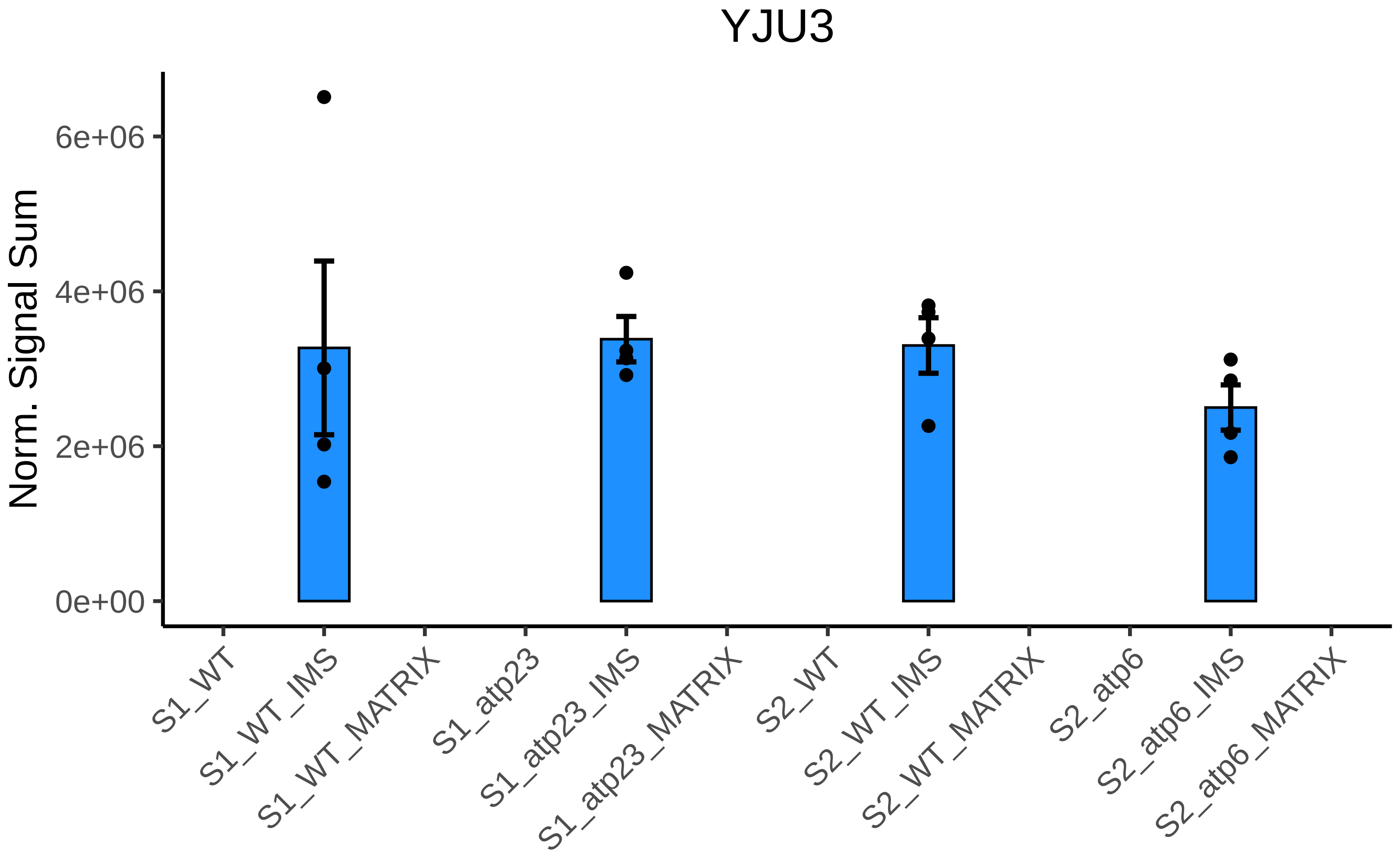
YJU3
Norm. Signal Sum
6e+06
4e+06
2e+06
0e+00
S1_WT
S1_WT_IMS
S1_WT_MATRIX
S1_atp23
S1_atp23_IMS
S1_atp23_MATRIX
S2_WT
S2_WT_IMS
S2_WT_MATRIX
S2_atp6
S2_atp6_IMS
S2_atp6_MATRIX

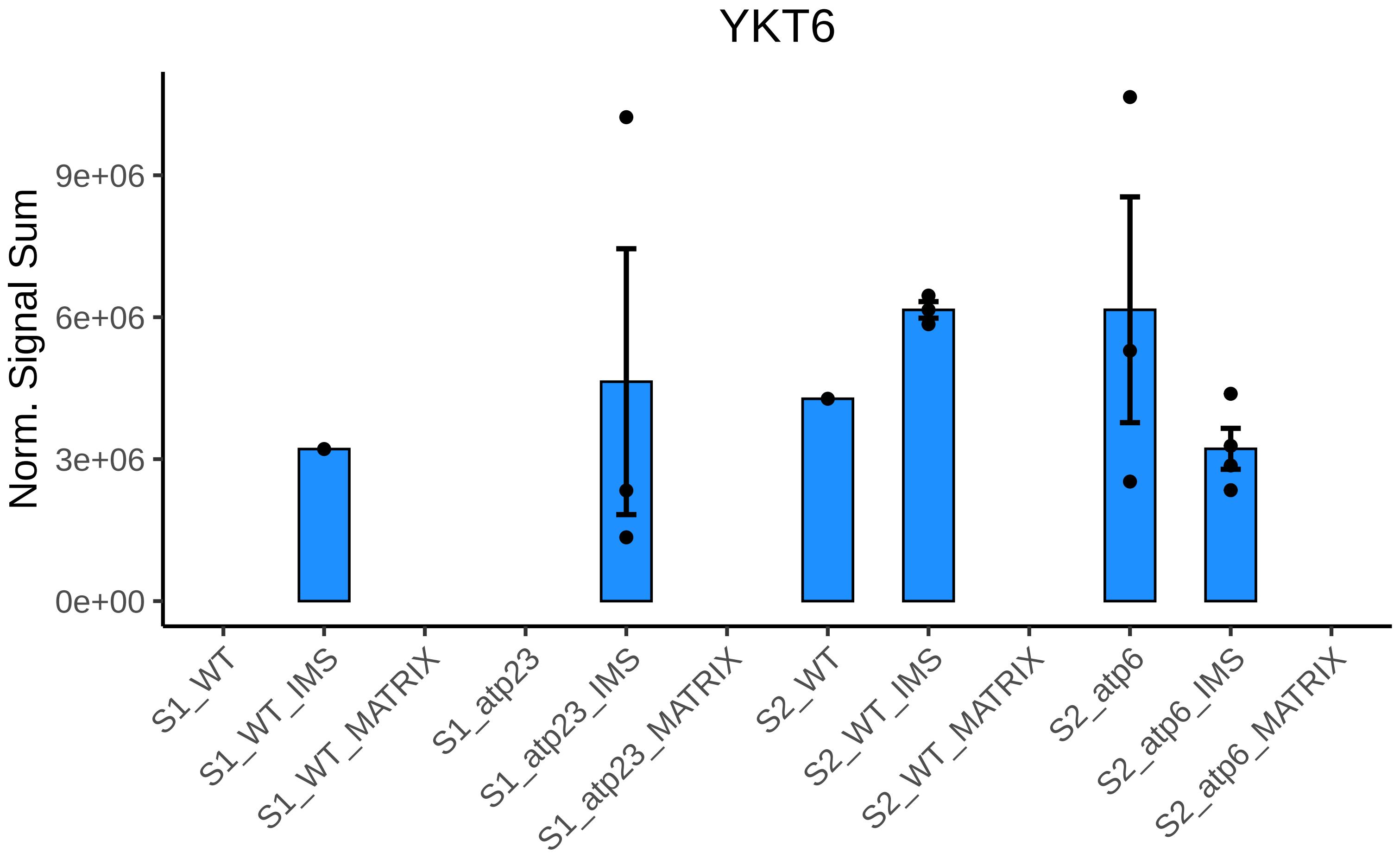

YKT6
Norm. Signal Sum
9e+06
6e+06
3e+06
0e+00
S1_WT
S1_WT_IMS
S1_WT_MATRIX
S1_atp23
S1_atp23_IMS
S1_atp23_MATRIX
S2_WT
S2_WT_IMS
S2_WT_MATRIX
S2_atp6
S2_atp6_IMS
S2_atp6_MATRIX

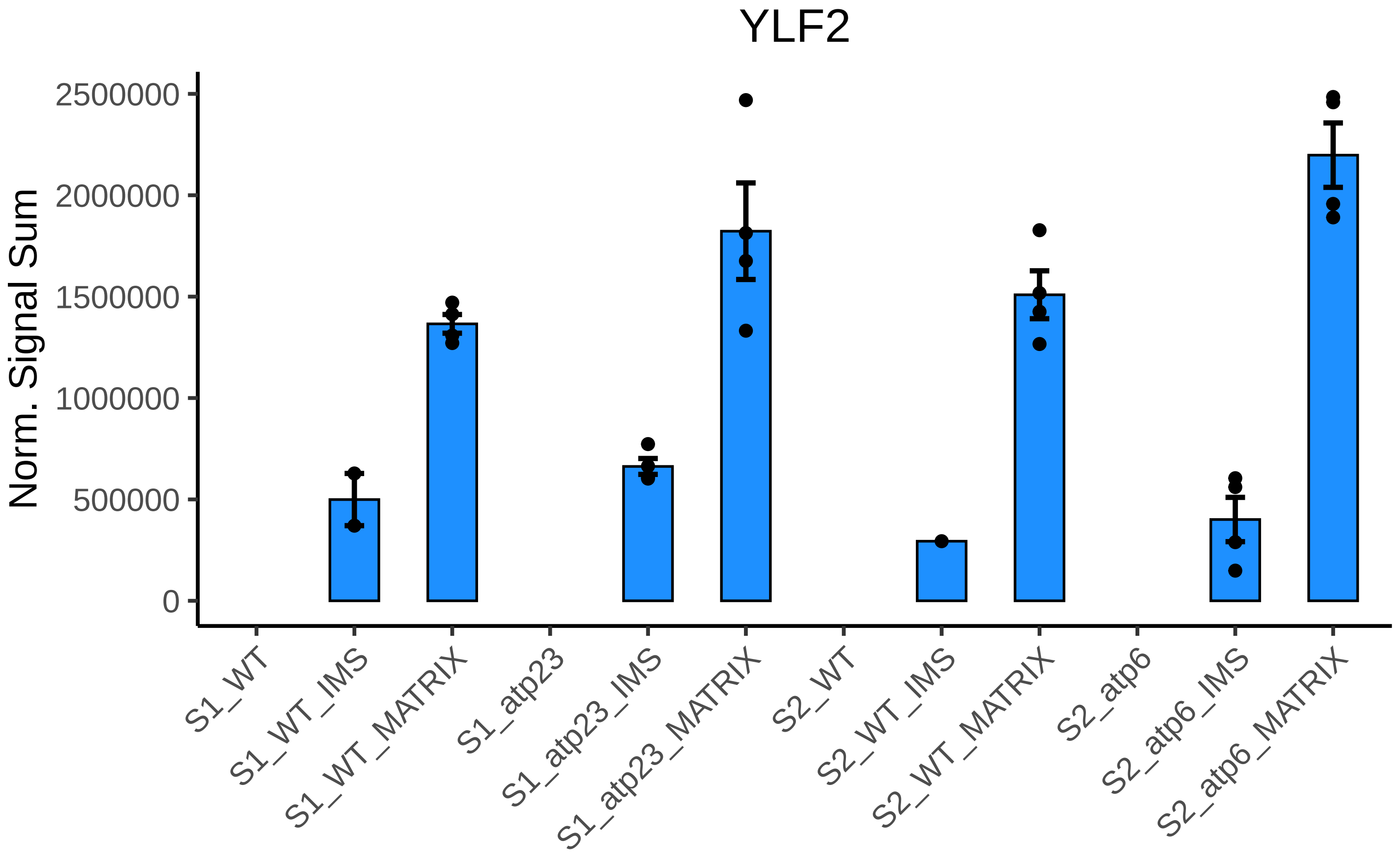
YLF2
Norm. Signal Sum
2500000
2000000
1500000
1000000
500000
0
S1_WT
S1_WT_IMS
S1_WT_MATRIX
S1_atp23
S1_atp23_IMS
S1_atp23_MATRIX
S2_WT
S2_WT_IMS
S2_WT_MATRIX
S2_atp6
S2_atp6_IMS
S2_atp6_MATRIX

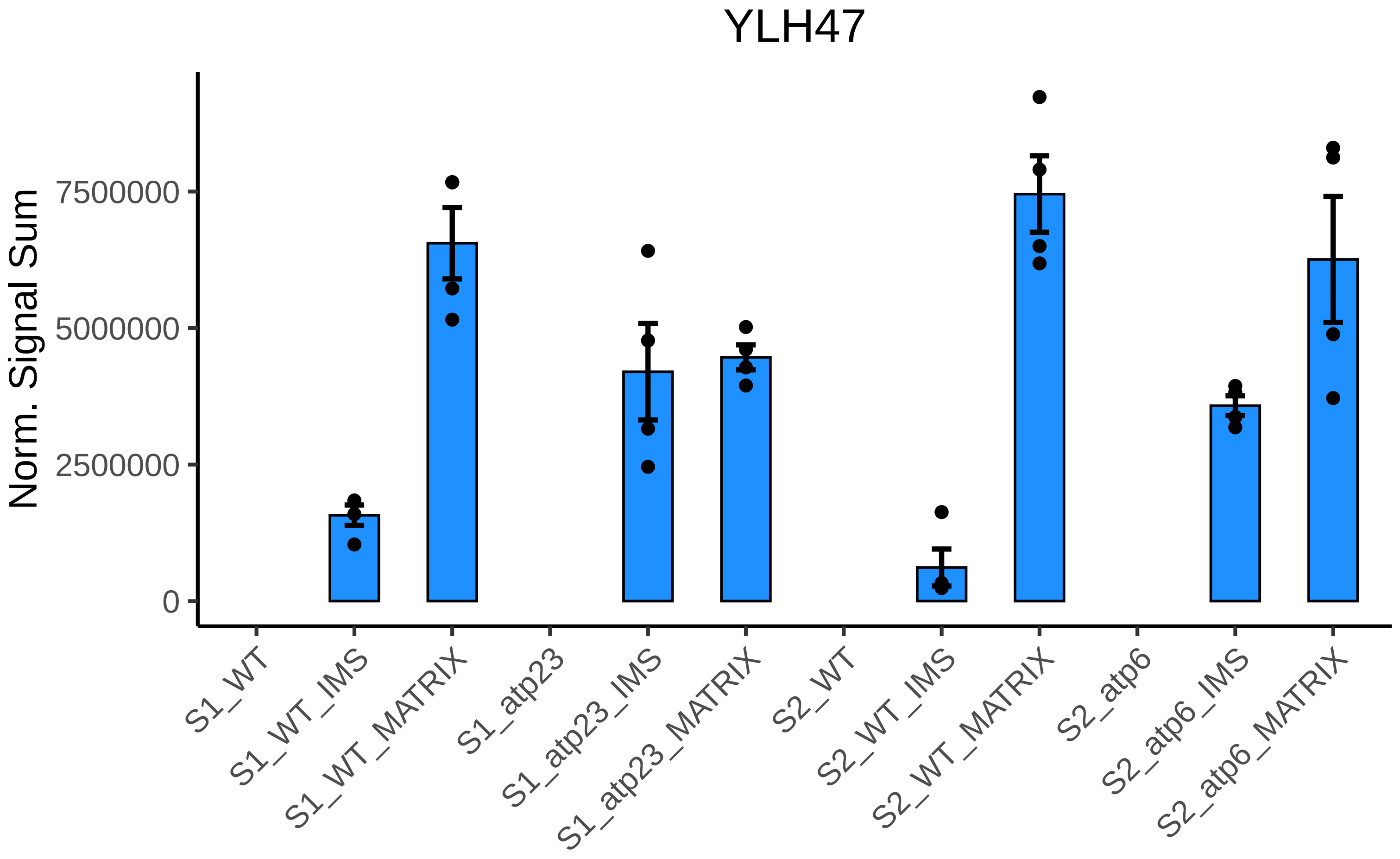
YLH47
Norm. Signal Sum
7500000
5000000
2500000
0
S1_WT
S1_WT_IMS
S1_WT_MATRIX
S1_atp23
S1_atp23_IMS
S1_atp23_MATRIX
S2_WT
S2_WT_IMS
S2_WT_MATRIX
S2_atp6
S2_atp6_IMS
S2_atp6_MATRIX

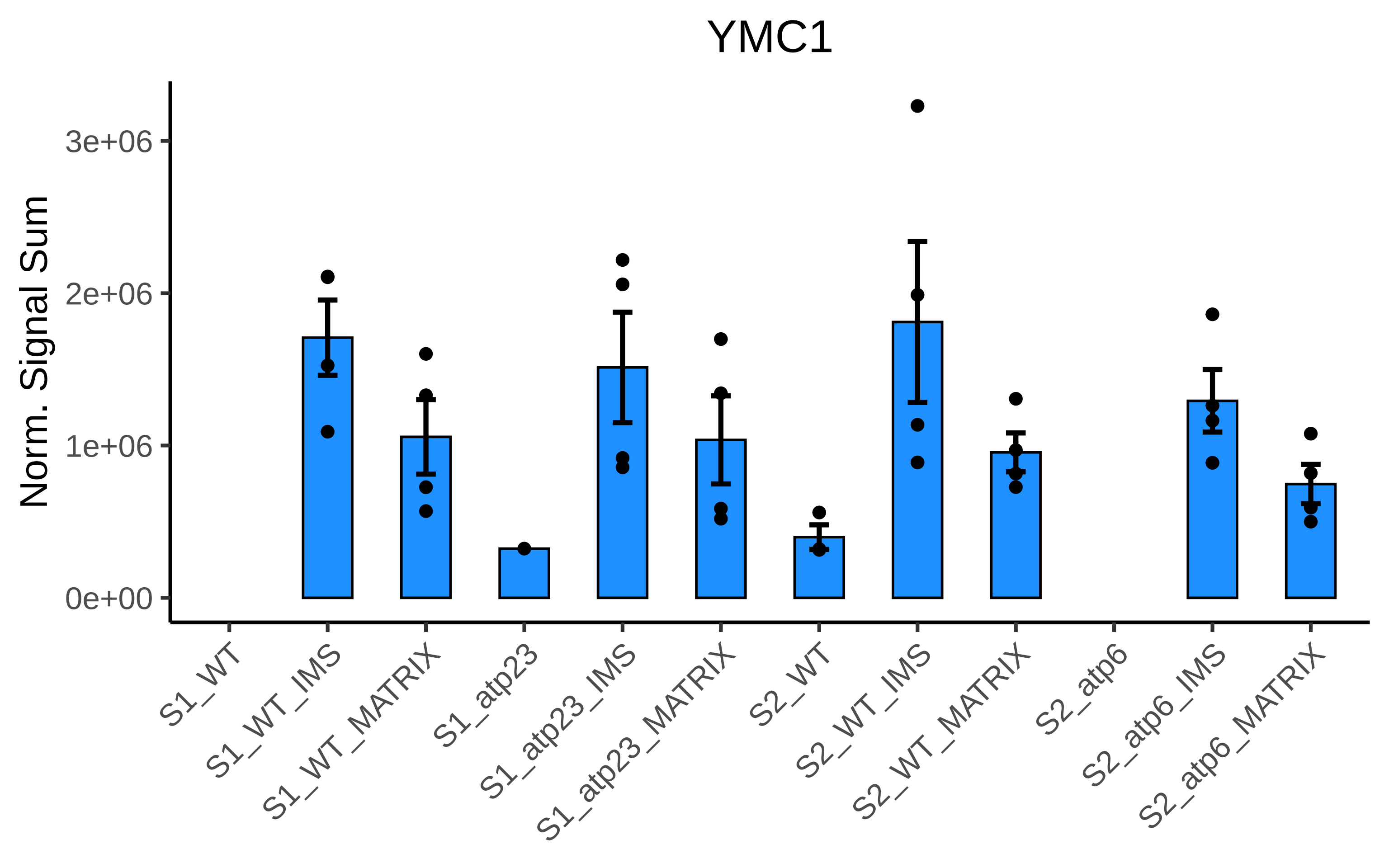

YMC1
Norm. Signal Sum
3e+06
2e+06
1e+06
0e+00
S1_WT
S1_WT_IMS
S1_WT_MATRIX
S1_atp23
S1_atp23_IMS
S1_atp23_MATRIX
S2_WT
S2_WT_IMS
S2_WT_MATRIX
S2_atp6
S2_atp6_IMS
S2_atp6_MATRIX

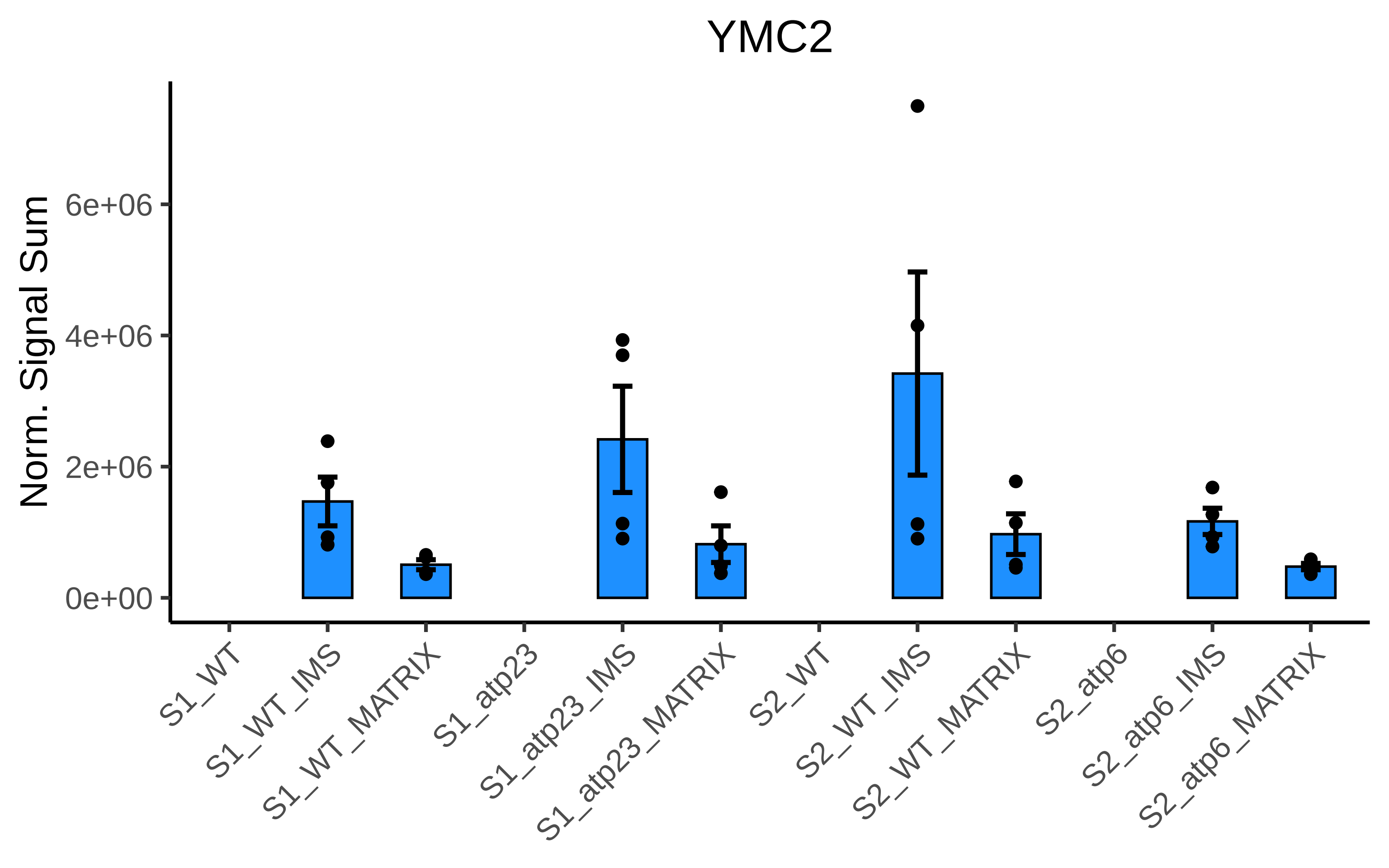
YMC2
Norm. Signal Sum
6e+06
4e+06
2e+06
0e+00
S1_WT
S1_WT_IMS
S1_WT_MATRIX
S1_atp23
S1_atp23_IMS
S1_atp23_MATRIX
S2_WT
S2_WT_IMS
S2_WT_MATRIX
S2_atp6
S2_atp6_IMS
S2_atp6_MATRIX

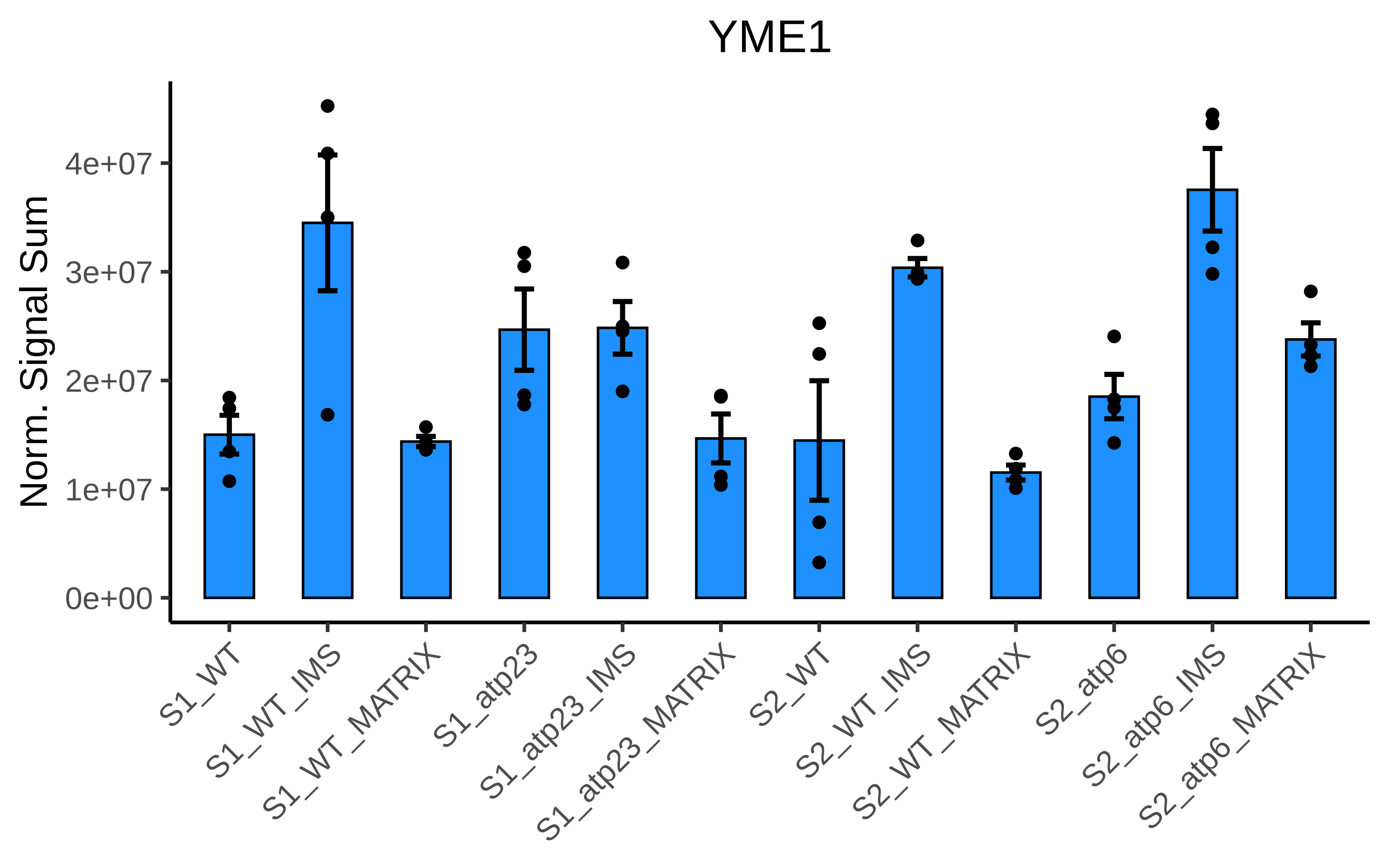

YME1
Norm. Signal Sum
0e+00
1e+07
2e+07
3e+07
4e+07
S1_WT
S1_WT_IMS
S1_WT_MATRIX
S1_atp23
S1_atp23_IMS
S1_atp23_MATRIX
S2_WT
S2_WT_IMS
S2_WT_MATRIX
S2_atp6
S2_atp6_IMS
S2_atp6_MATRIX

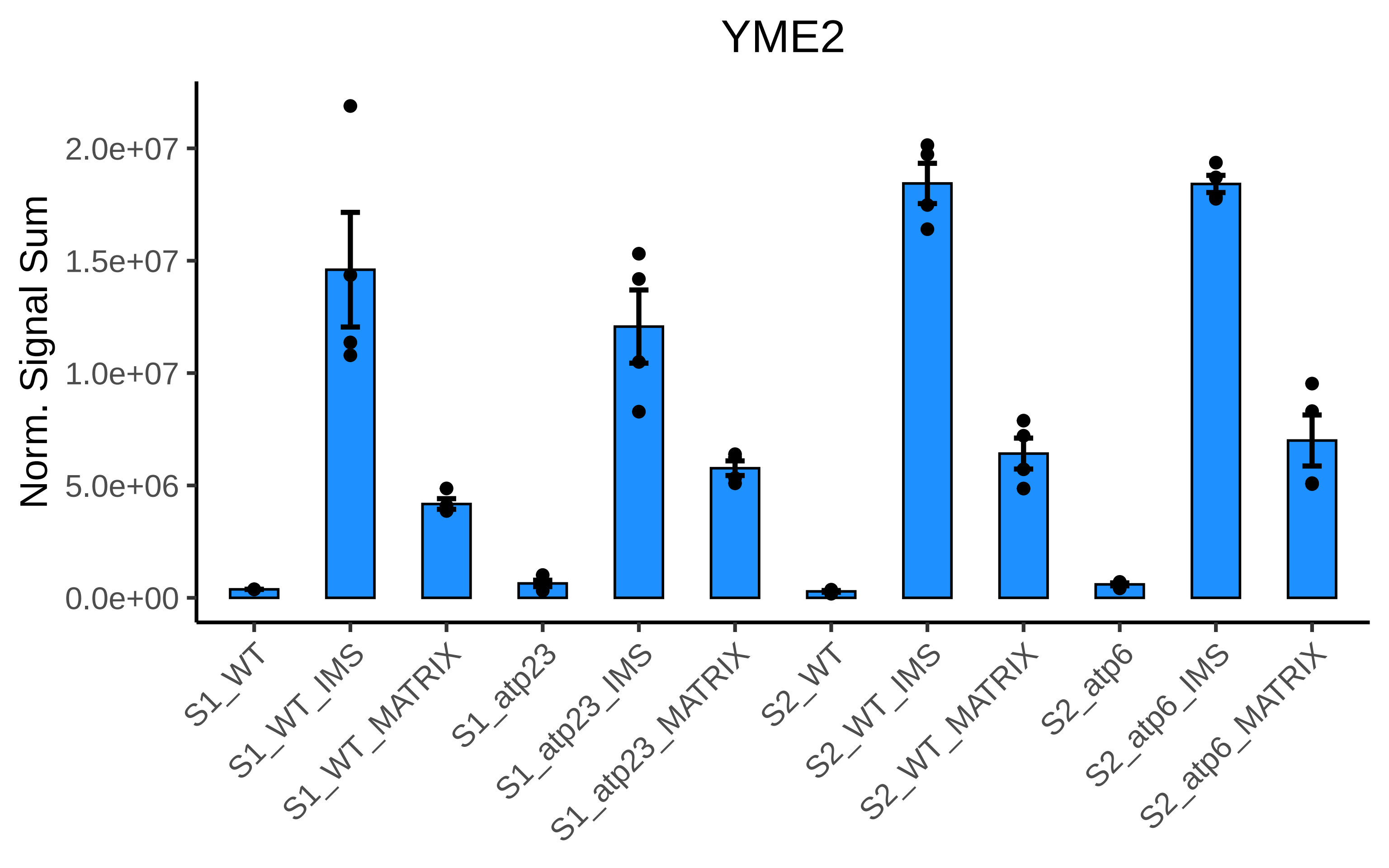
YME2
Norm. Signal Sum
2.0e+07
1.5e+07
1.0e+07
5.0e+06
0.0e+00
S1_WT
S1_WT_IMS
S1_WT_MATRIX
S1_atp23
S1_atp23_IMS
S1_atp23_MATRIX
S2_WT
S2_WT_IMS
S2_WT_MATRIX
S2_atp6
S2_atp6_IMS
S2_atp6_MATRIX

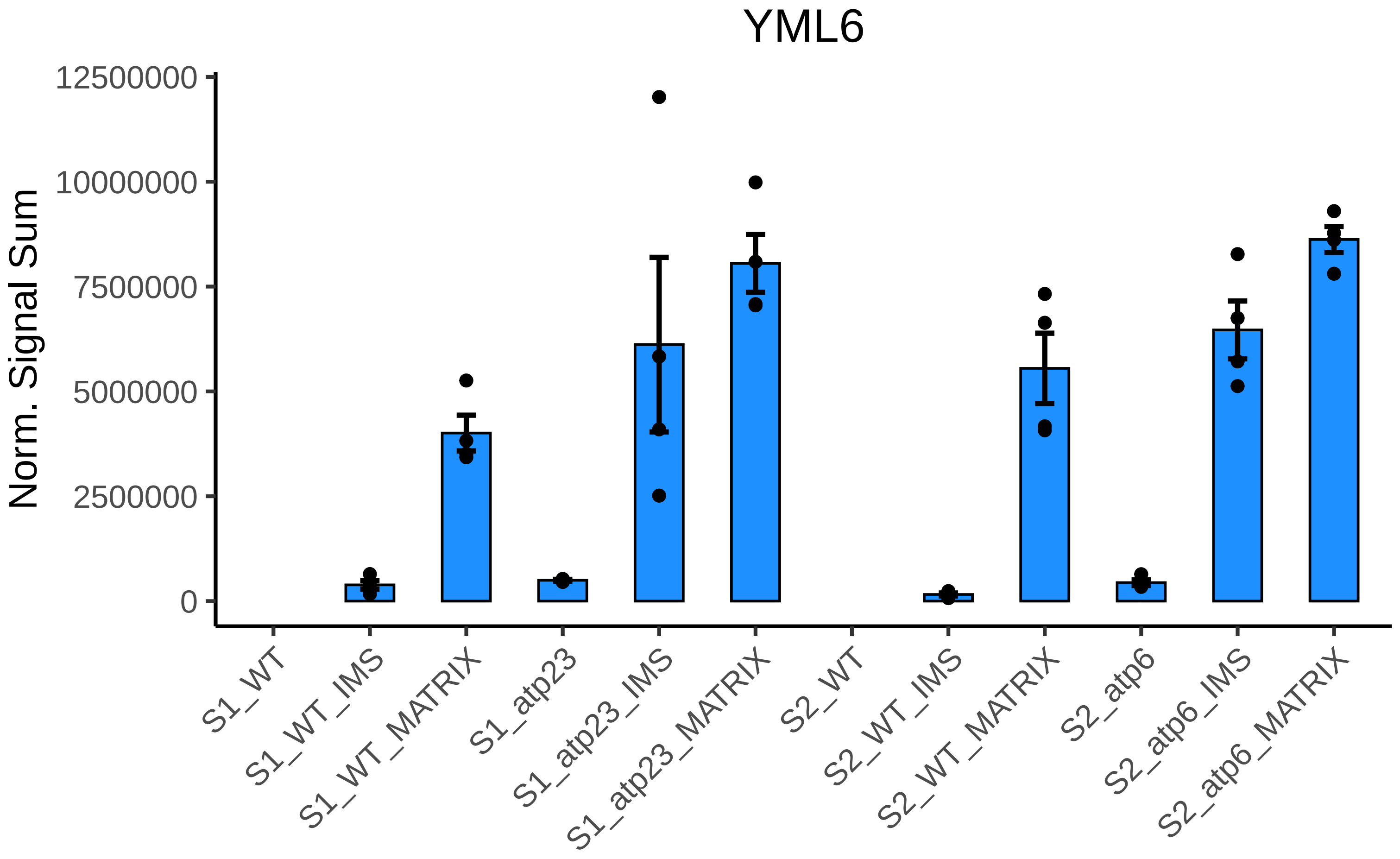

YML6
Norm. Signal Sum
12500000
10000000
7500000
5000000
2500000
0
S1_WT
S1_WT_IMS
S1_WT_MATRIX
S1_atp23
S1_atp23_IMS
S1_atp23_MATRIX
S2_WT
S2_WT_IMS
S2_WT_MATRIX
S2_atp6
S2_atp6_IMS
S2_atp6_MATRIX

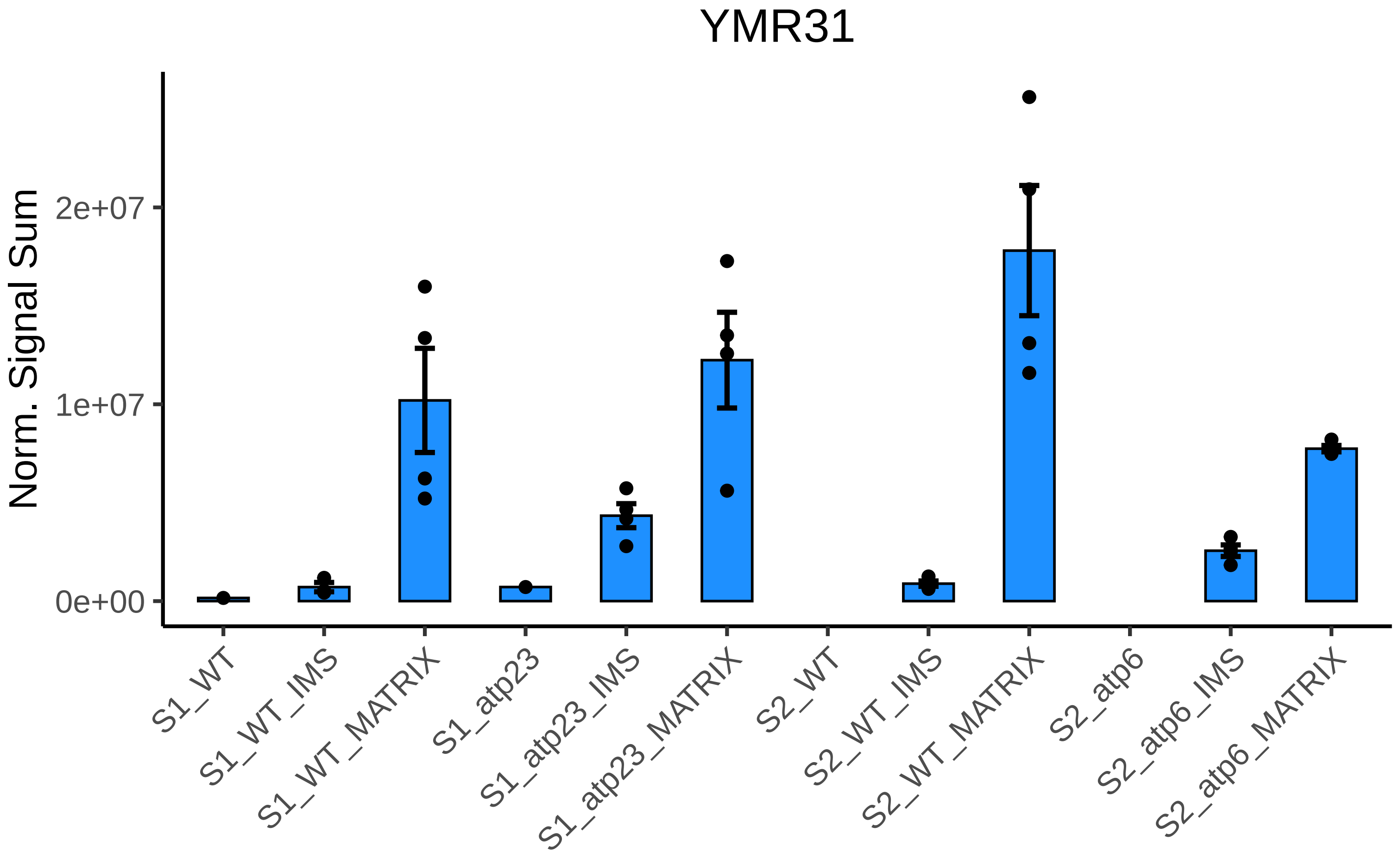
YMR31
Norm. Signal Sum
2e+07
1e+07
0e+00
S1_WT
S1_WT_IMS
S1_WT_MATRIX
S1_atp23
S1_atp23_IMS
S1_atp23_MATRIX
S2_WT
S2_WT_IMS
S2_WT_MATRIX
S2_atp6
S2_atp6_IMS
S2_atp6_MATRIX

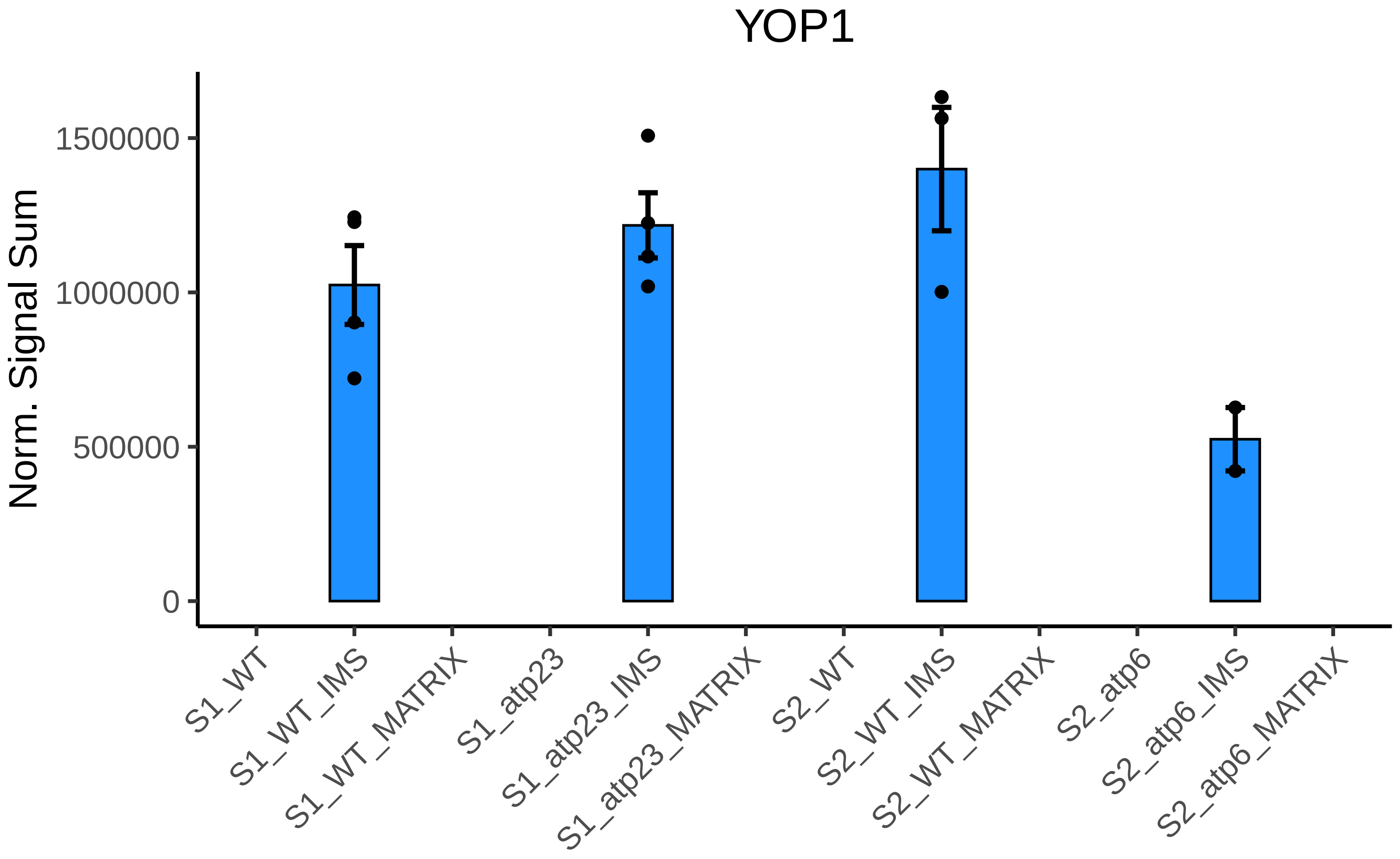
YOP1
Norm. Signal Sum
1500000
1000000
500000
0
S1_WT
S1_WT_IMS
S1_WT_MATRIX
S1_atp23
S1_atp23_IMS
S1_atp23_MATRIX
S2_WT
S2_WT_IMS
S2_WT_MATRIX
S2_atp6
S2_atp6_IMS
S2_atp6_MATRIX

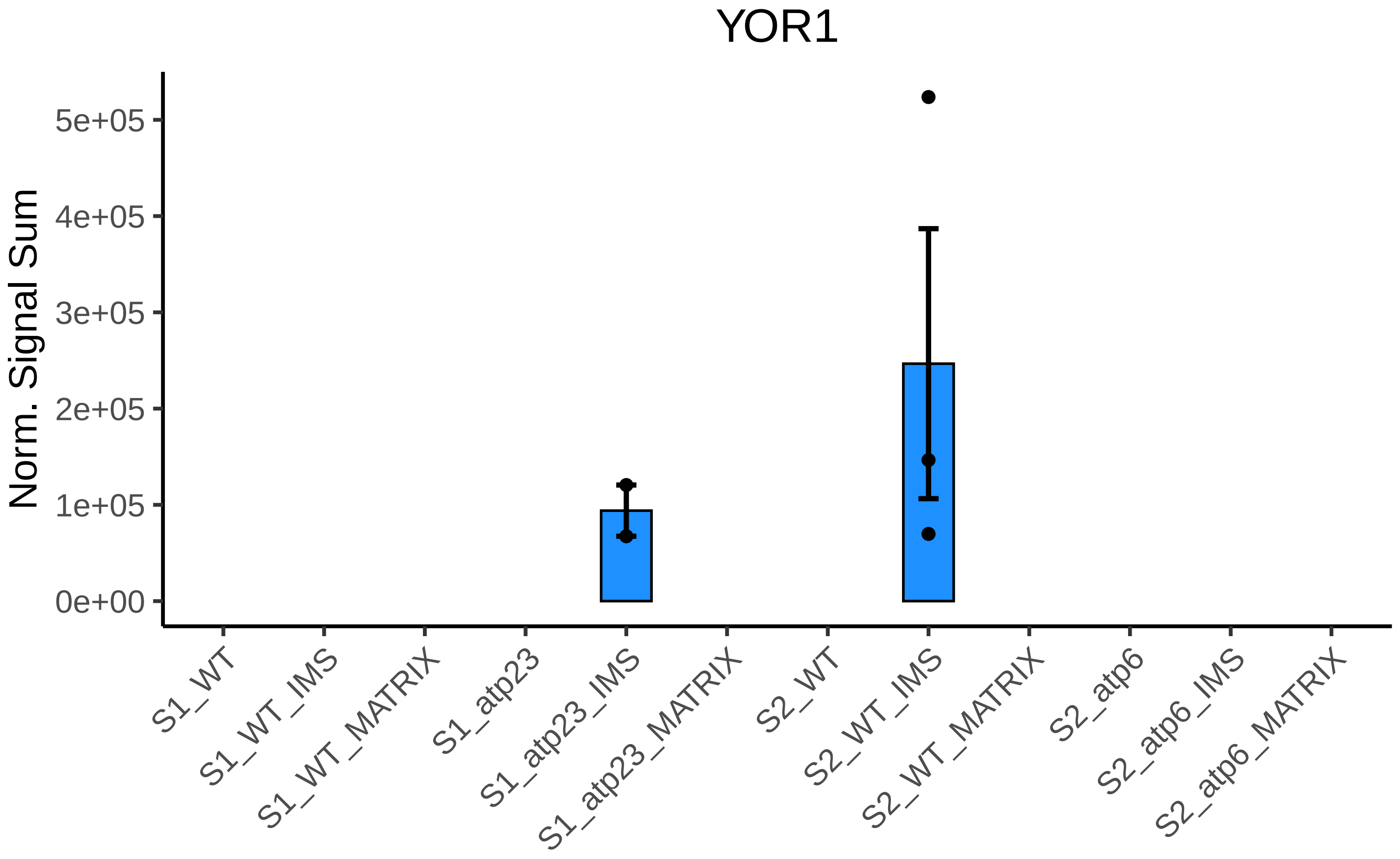
YOR1
Norm. Signal Sum
5e+05
4e+05
3e+05
2e+05
1e+05
0e+00
S1_WT
S1_WT_IMS
S1_WT_MATRIX
S1_atp23
S1_atp23_IMS
S1_atp23_MATRIX
S2_WT
S2_WT_IMS
S2_WT_MATRIX
S2_atp6
S2_atp6_IMS
S2_atp6_MATRIX

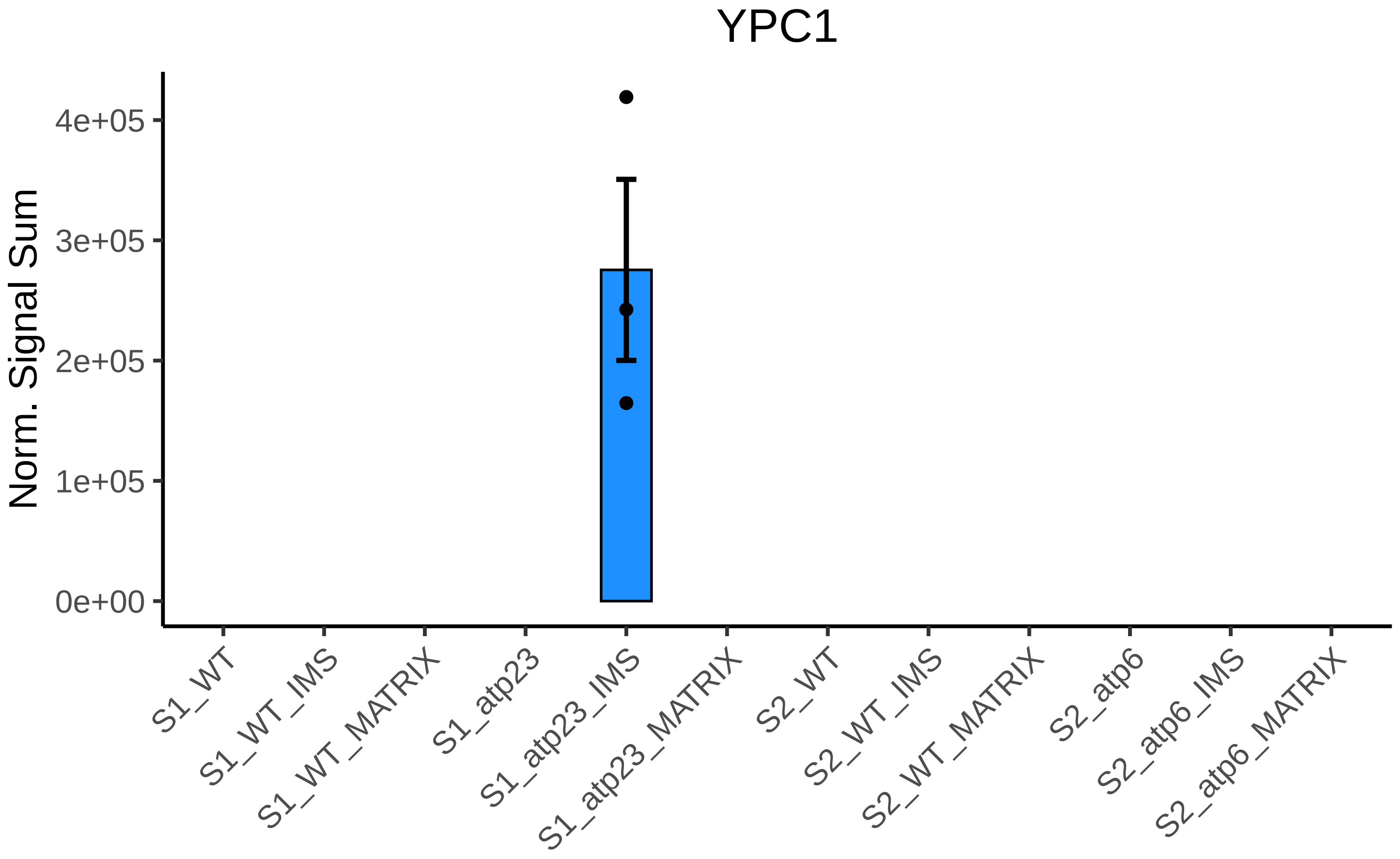
YPC1
Norm. Signal Sum
0e+00
1e+05
2e+05
3e+05
4e+05
S1_WT
S1_WT_IMS
S1_WT_MATRIX
S1_atp23
S1_atp23_IMS
S1_atp23_MATRIX
S2_WT
S2_WT_IMS
S2_WT_MATRIX
S2_atp6
S2_atp6_IMS
S2_atp6_MATRIX

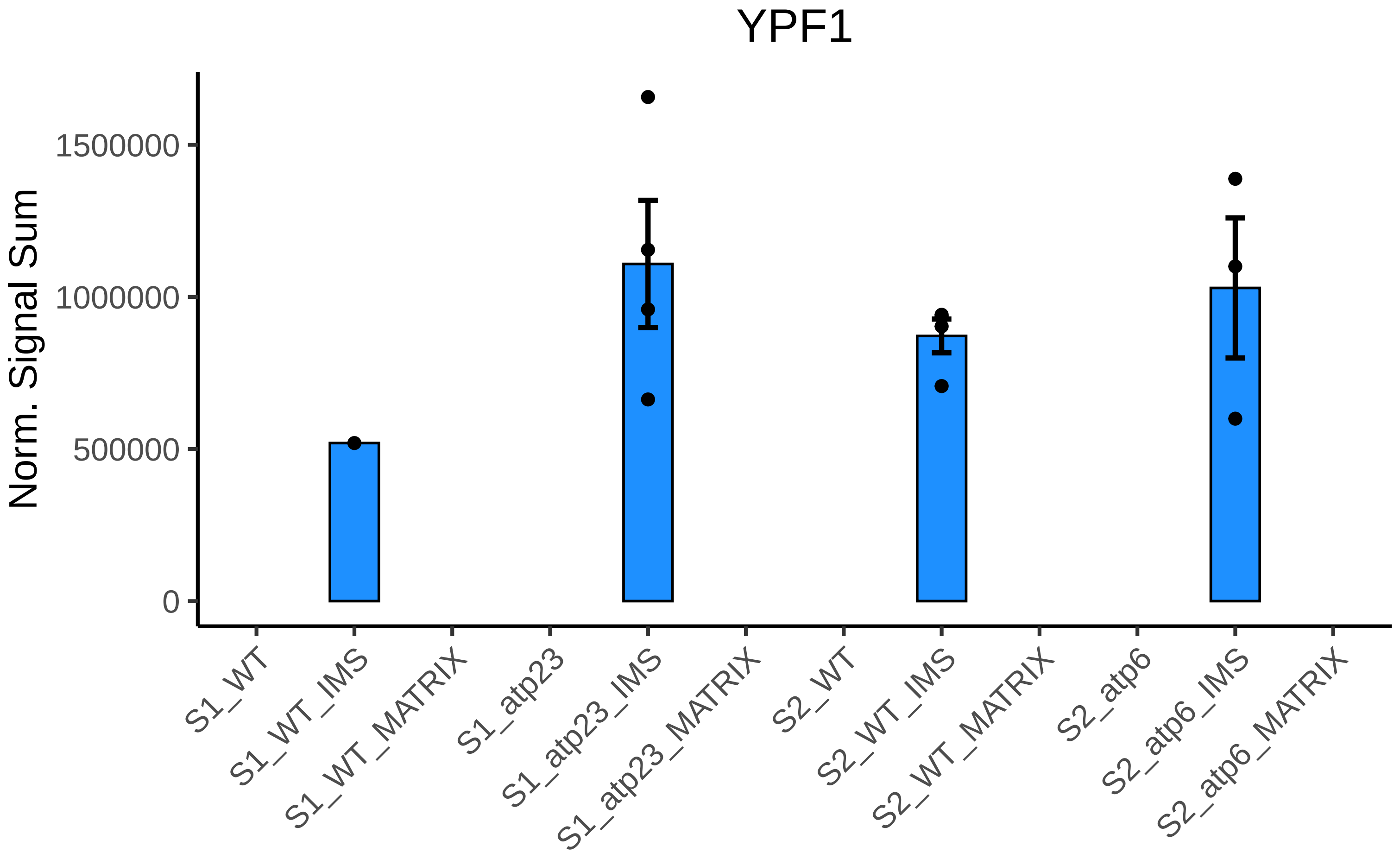
YPF1
Norm. Signal Sum
1500000
1000000
500000
0
S1_WT
S1_WT_IMS
S1_WT_MATRIX
S1_atp23
S1_atp23_IMS
S1_atp23_MATRIX
S2_WT
S2_WT_IMS
S2_WT_MATRIX
S2_atp6
S2_atp6_IMS
S2_atp6_MATRIX

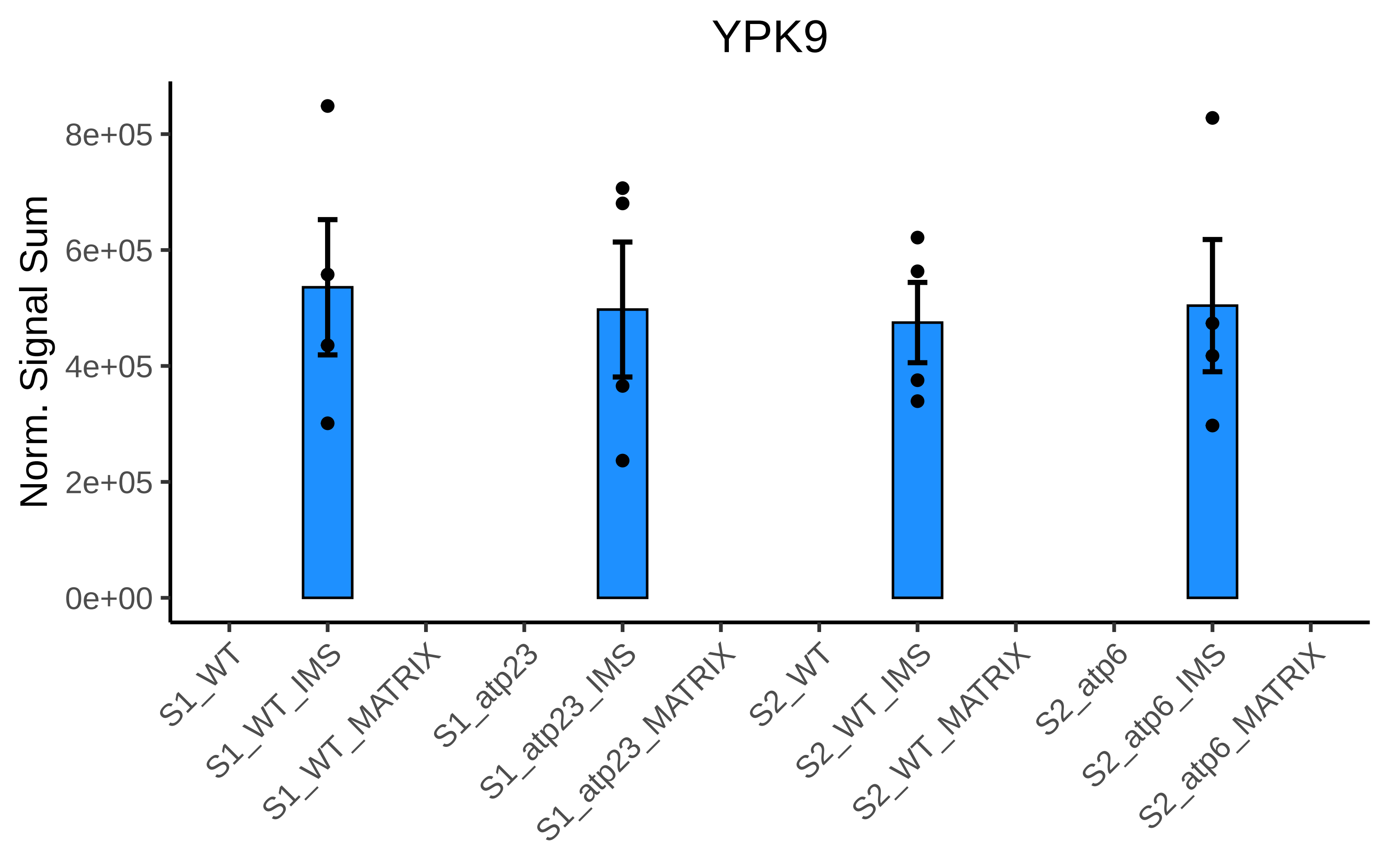

YPK9
Norm. Signal Sum
8e+05
6e+05
4e+05
2e+05
0e+00
S1_WT
S1_WT_IMS
S1_WT_MATRIX
S1_atp23
S1_atp23_IMS
S1_atp23_MATRIX
S2_WT
S2_WT_IMS
S2_WT_MATRIX
S2_atp6
S2_atp6_IMS
S2_atp6_MATRIX

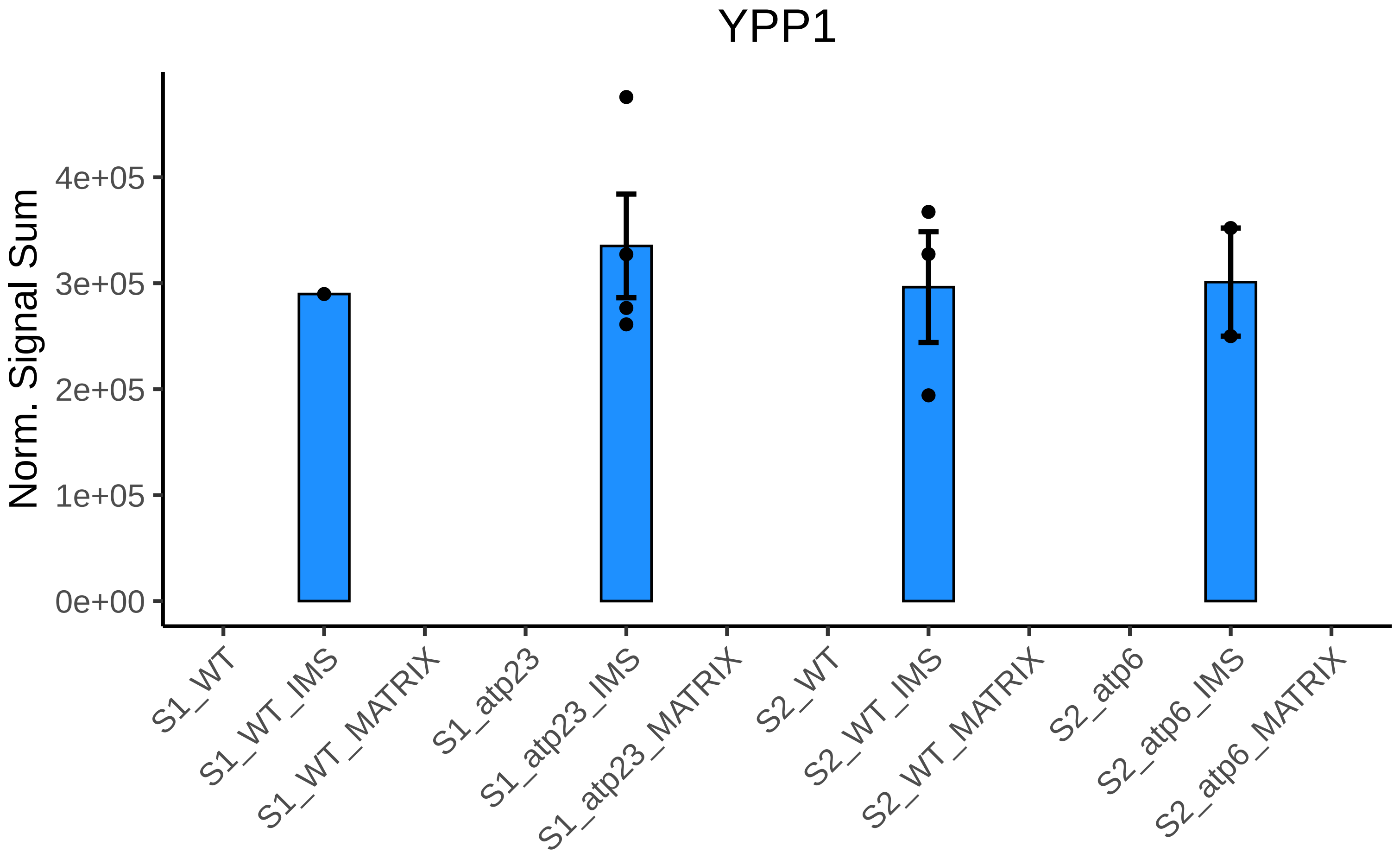
YPP1
Norm. Signal Sum
0e+00
1e+05
2e+05
3e+05
4e+05
S1_WT
S1_WT_IMS
S1_WT_MATRIX
S1_atp23
S1_atp23_IMS
S1_atp23_MATRIX
S2_WT
S2_WT_IMS
S2_WT_MATRIX
S2_atp6
S2_atp6_IMS
S2_atp6_MATRIX

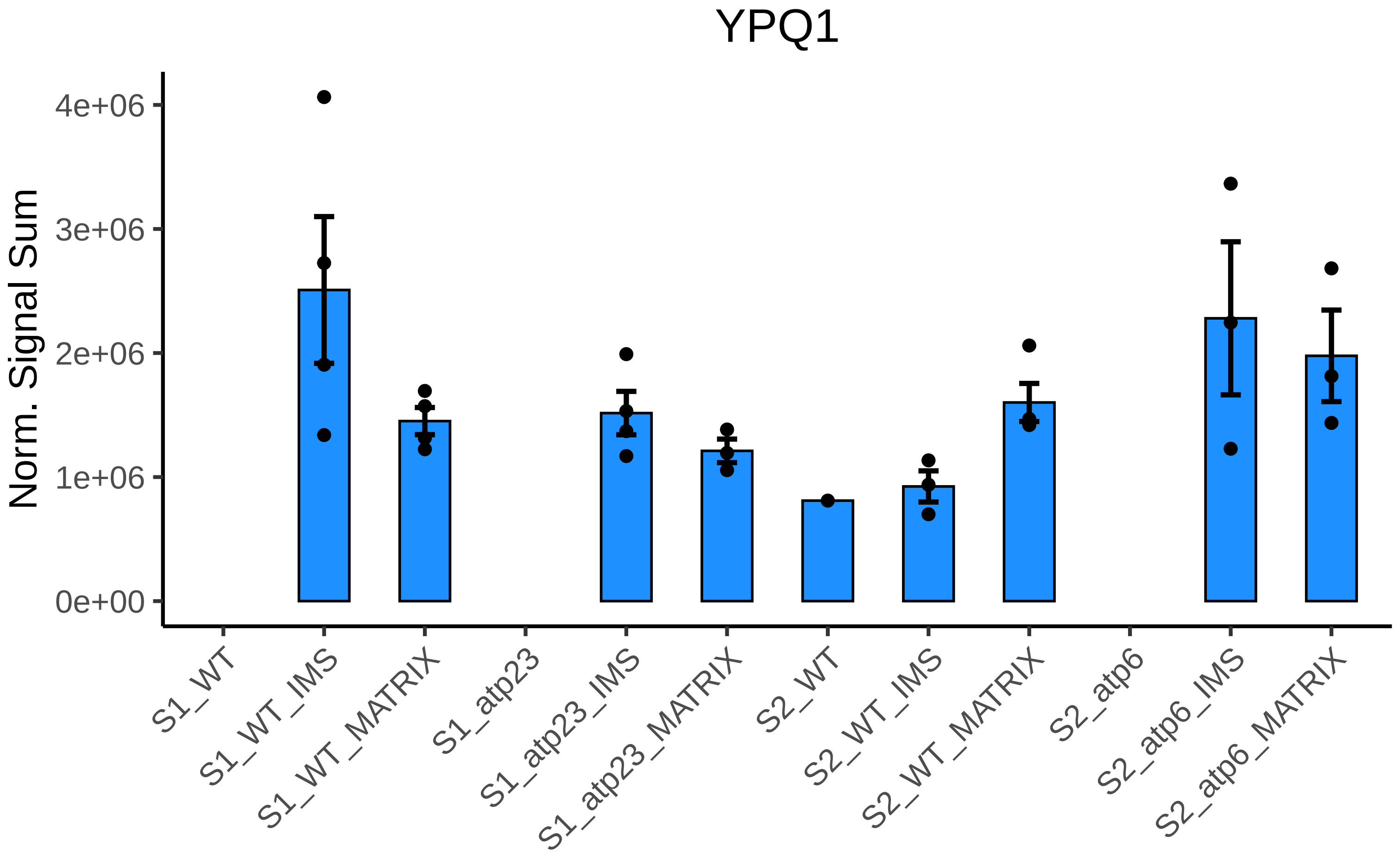

YPQ1
Norm. Signal Sum
4e+06
3e+06
2e+06
1e+06
0e+00
S1_WT
S1_WT_IMS
S1_WT_MATRIX
S1_atp23
S1_atp23_IMS
S1_atp23_MATRIX
S2_WT
S2_WT_IMS
S2_WT_MATRIX
S2_atp6
S2_atp6_IMS
S2_atp6_MATRIX

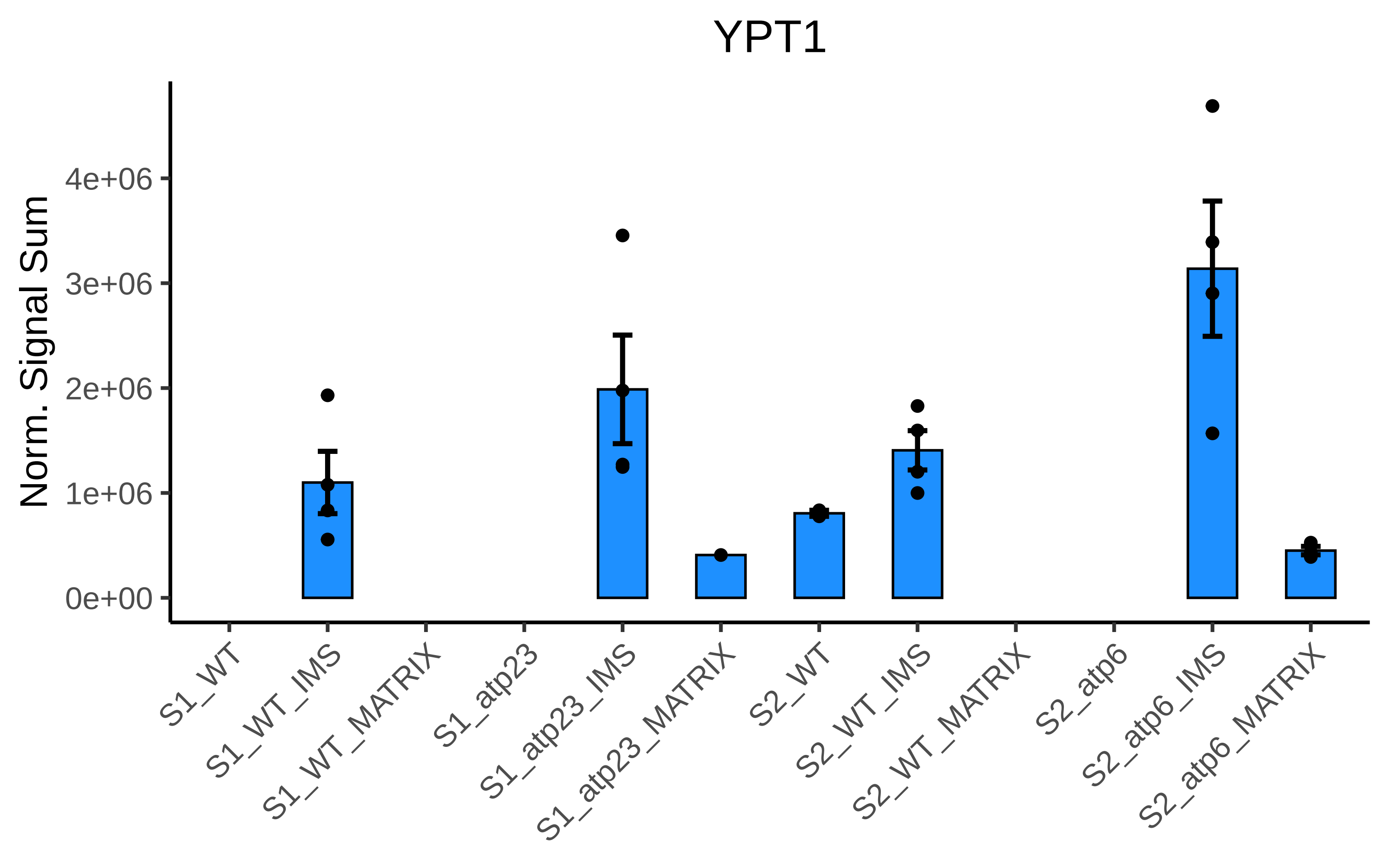

YPT1
Norm. Signal Sum
4e+06
3e+06
2e+06
1e+06
0e+00
S1_WT
S1_WT_IMS
S1_WT_MATRIX
S1_atp23
S1_atp23_IMS
S1_atp23_MATRIX
S2_WT
S2_WT_IMS
S2_WT_MATRIX
S2_atp6
S2_atp6_IMS
S2_atp6_MATRIX

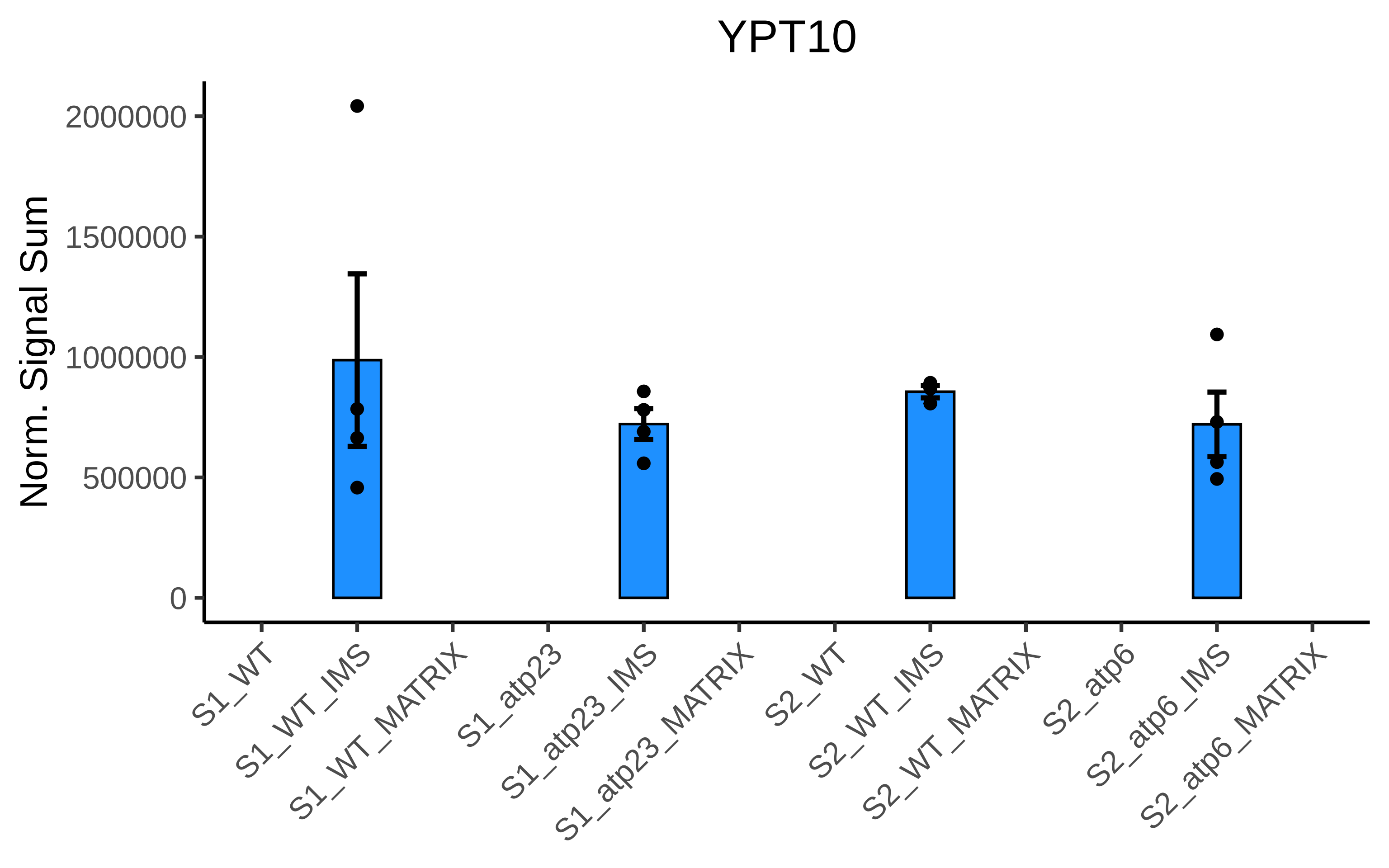

YPT10
Norm. Signal Sum
2000000
1500000
1000000
500000
0
S1_WT
S1_WT_IMS
S1_WT_MATRIX
S1_atp23
S1_atp23_IMS
S1_atp23_MATRIX
S2_WT
S2_WT_IMS
S2_WT_MATRIX
S2_atp6
S2_atp6_IMS
S2_atp6_MATRIX

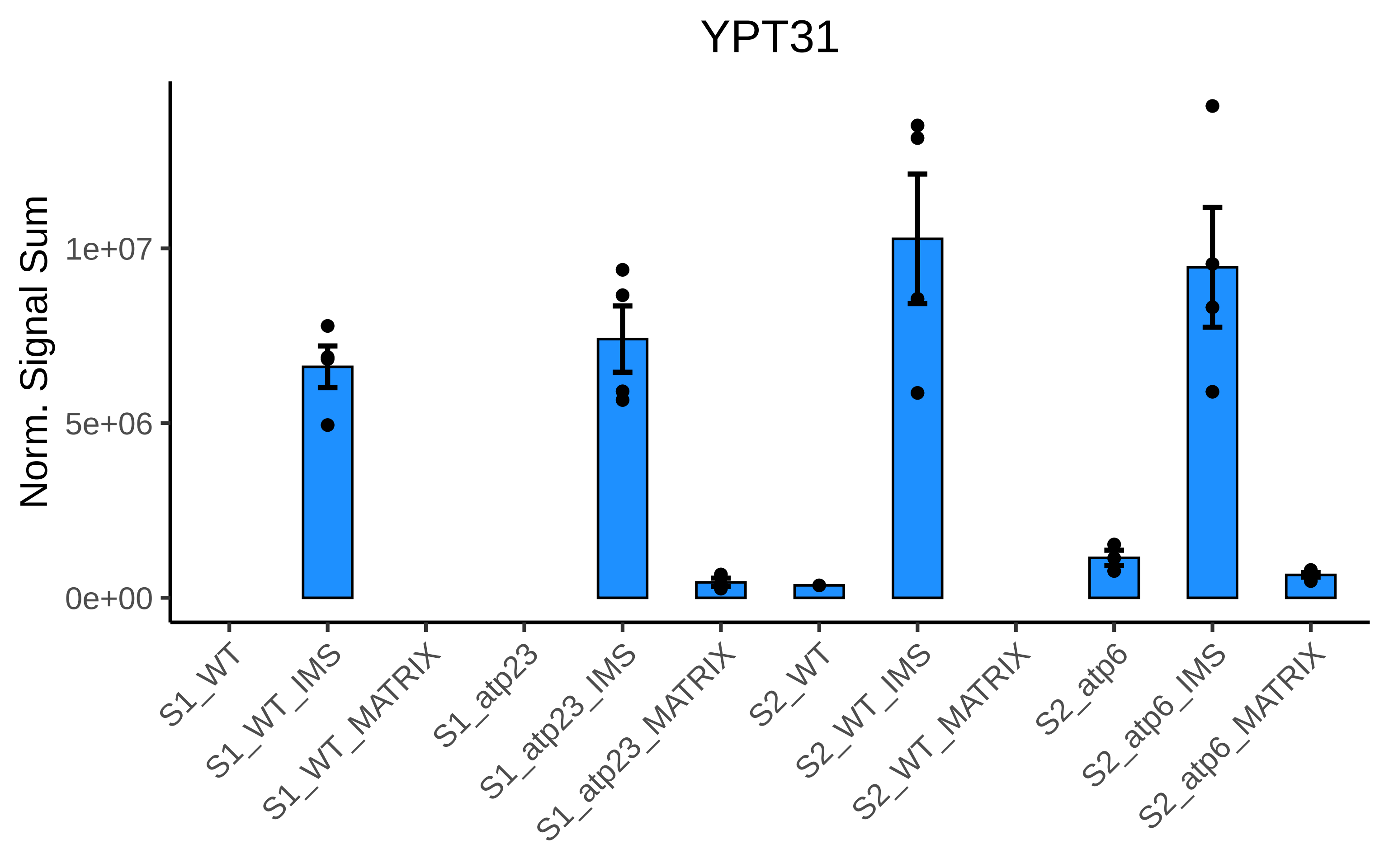

YPT31
Norm. Signal Sum
1e+07
5e+06
0e+00
S1_WT
S1_WT_IMS
S1_WT_MATRIX
S1_atp23
S1_atp23_IMS
S1_atp23_MATRIX
S2_WT
S2_WT_IMS
S2_WT_MATRIX
S2_atp6
S2_atp6_IMS
S2_atp6_MATRIX

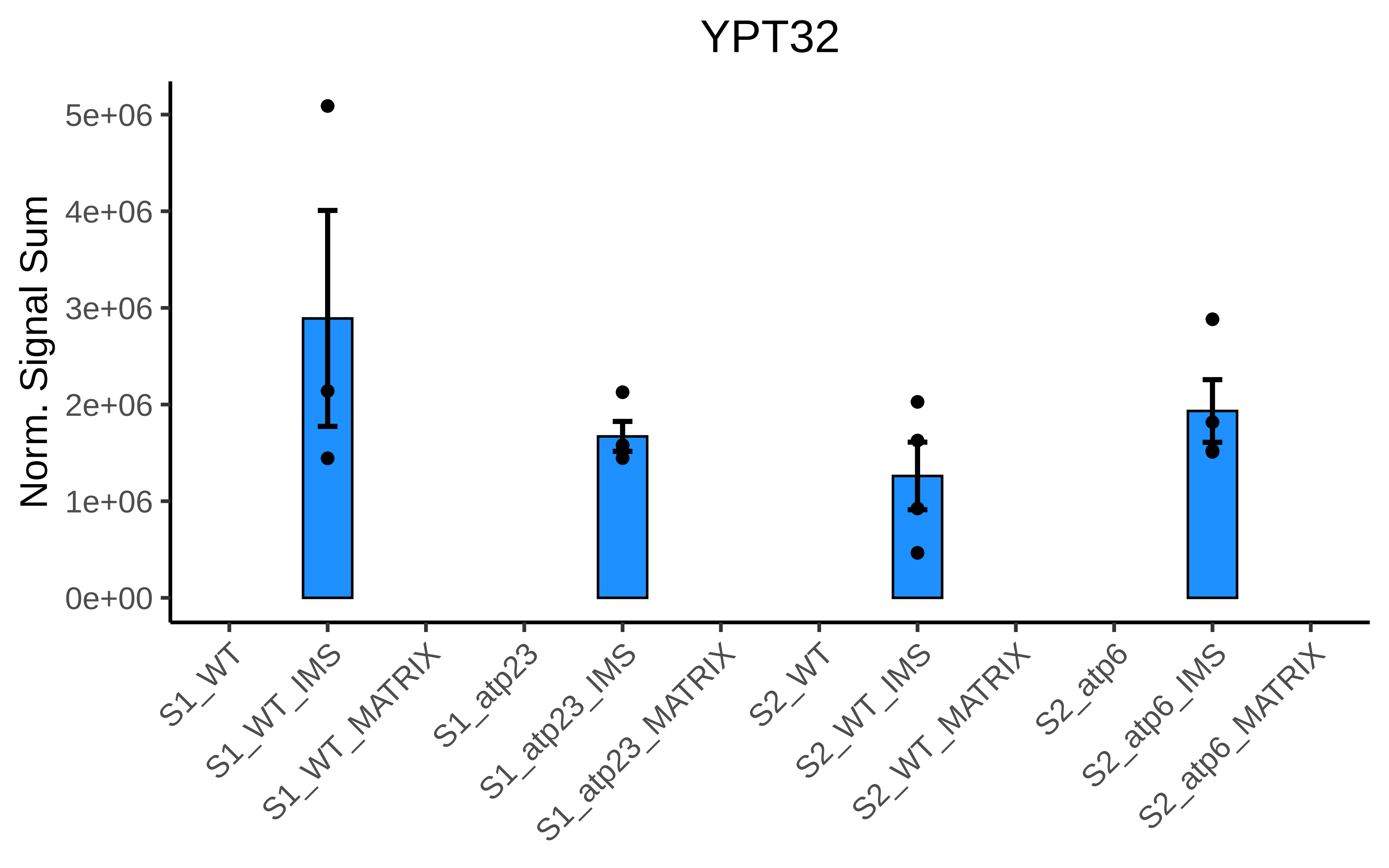

YPT32
Norm. Signal Sum
5e+06
4e+06
3e+06
2e+06
1e+06
0e+00
S1_WT
S1_WT_IMS
S1_WT_MATRIX
S1_atp23
S1_atp23_IMS
S1_atp23_MATRIX
S2_WT
S2_WT_IMS
S2_WT_MATRIX
S2_atp6
S2_atp6_IMS
S2_atp6_MATRIX

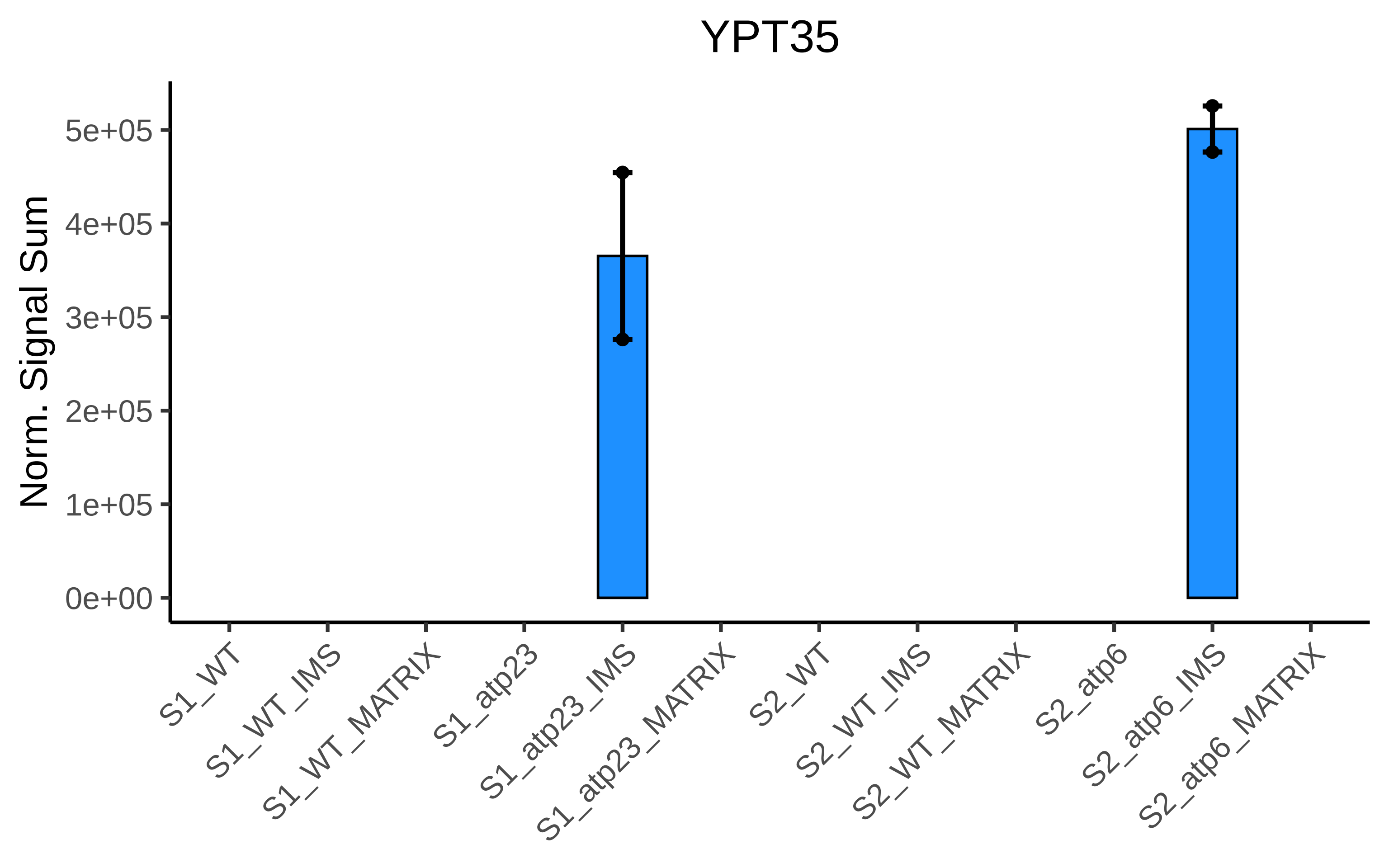
YPT35
Norm. Signal Sum
5e+05
4e+05
3e+05
2e+05
1e+05
0e+00
S1_WT
S1_WT_IMS
S1_WT_MATRIX
S1_atp23
S1_atp23_IMS
S1_atp23_MATRIX
S2_WT
S2_WT_IMS
S2_WT_MATRIX
S2_atp6
S2_atp6_IMS
S2_atp6_MATRIX

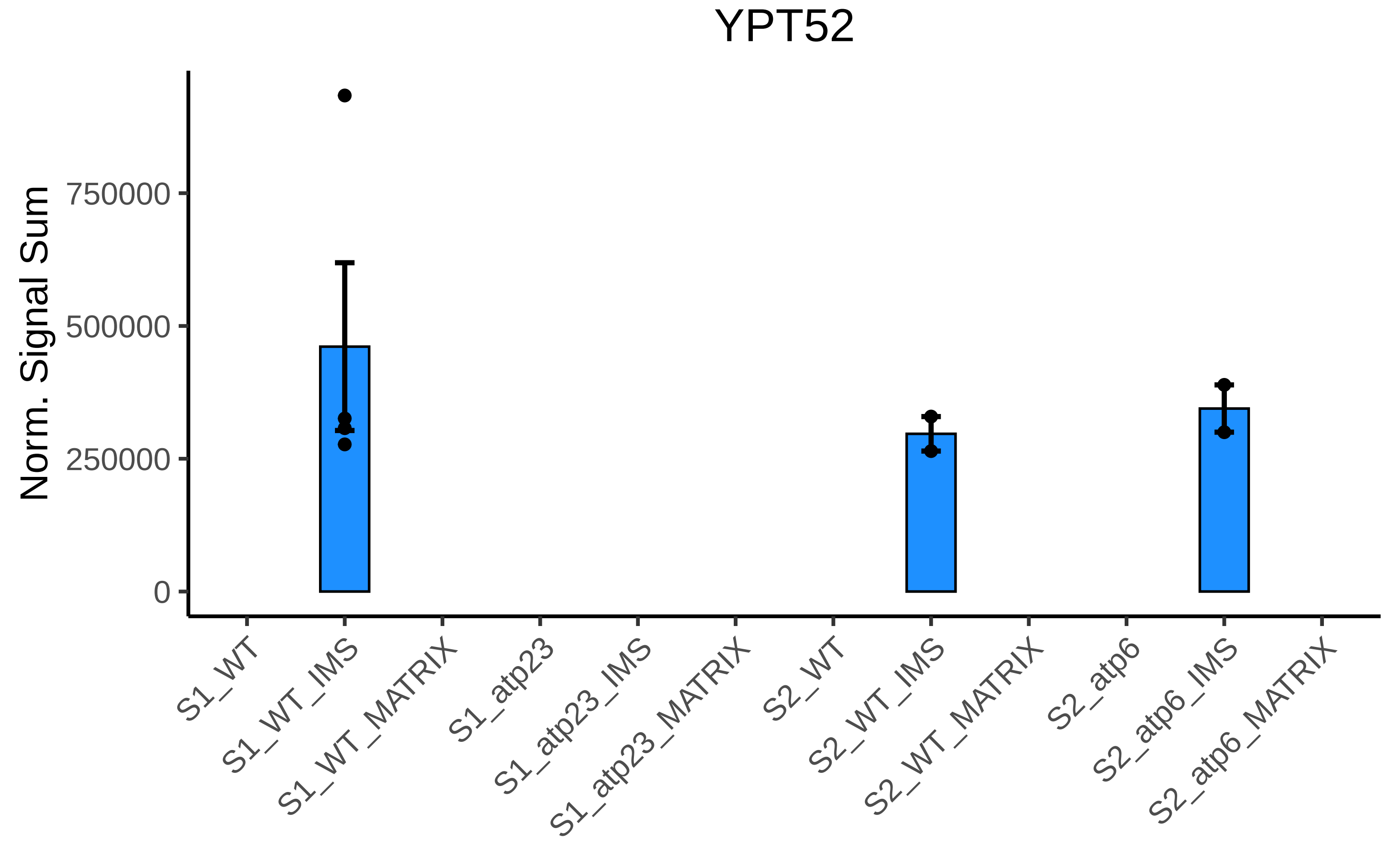
YPT52
Norm. Signal Sum
750000
500000
250000
0
S1_WT
S1_WT_IMS
S1_WT_MATRIX
S1_atp23
S1_atp23_IMS
S1_atp23_MATRIX
S2_WT
S2_WT_IMS
S2_WT_MATRIX
S2_atp6
S2_atp6_IMS
S2_atp6_MATRIX

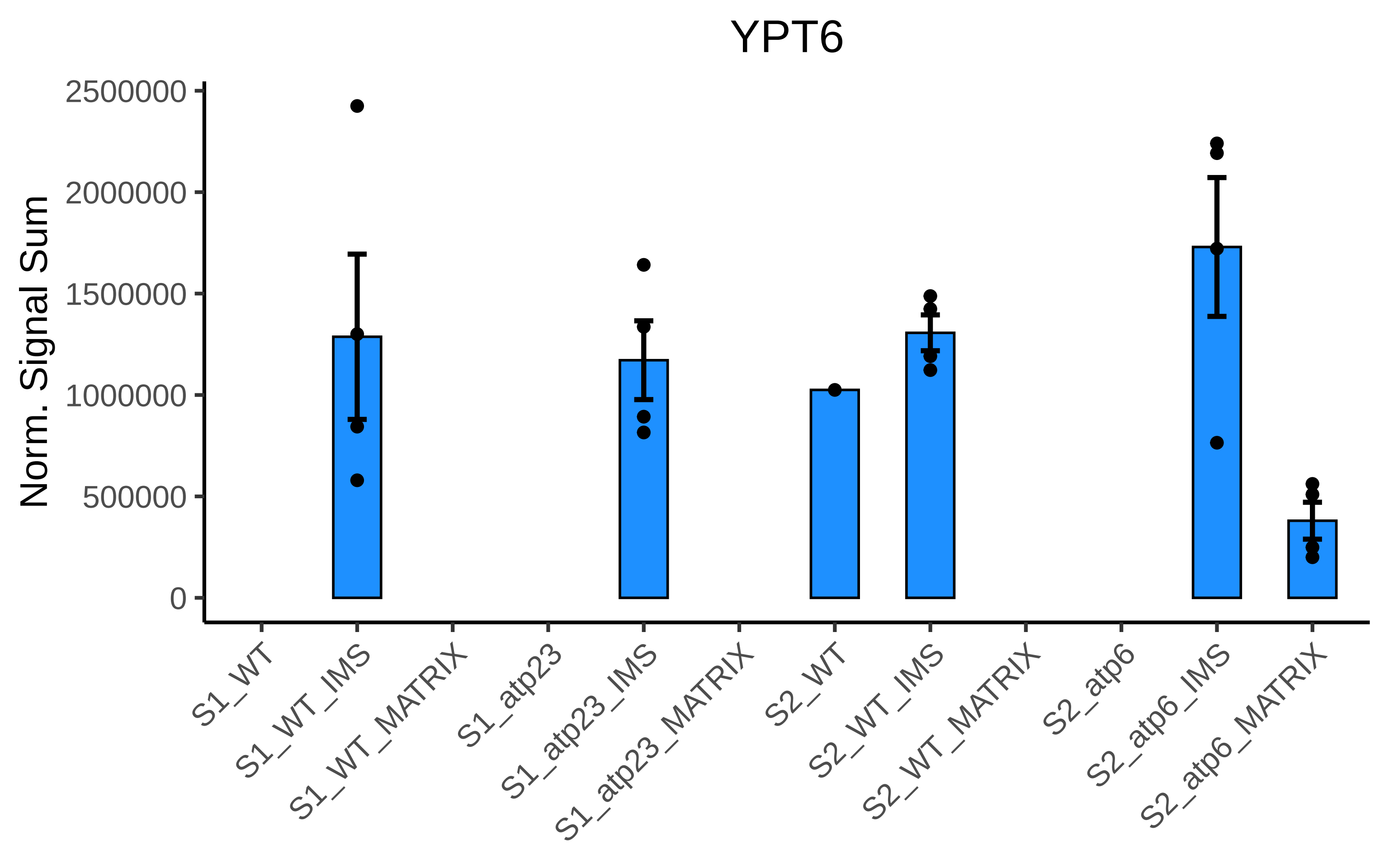

YPT6
Norm. Signal Sum
2500000
2000000
1500000
1000000
500000
0
S1_WT
S1_WT_IMS
S1_WT_MATRIX
S1_atp23
S1_atp23_IMS
S1_atp23_MATRIX
S2_WT
S2_WT_IMS
S2_WT_MATRIX
S2_atp6
S2_atp6_IMS
S2_atp6_MATRIX

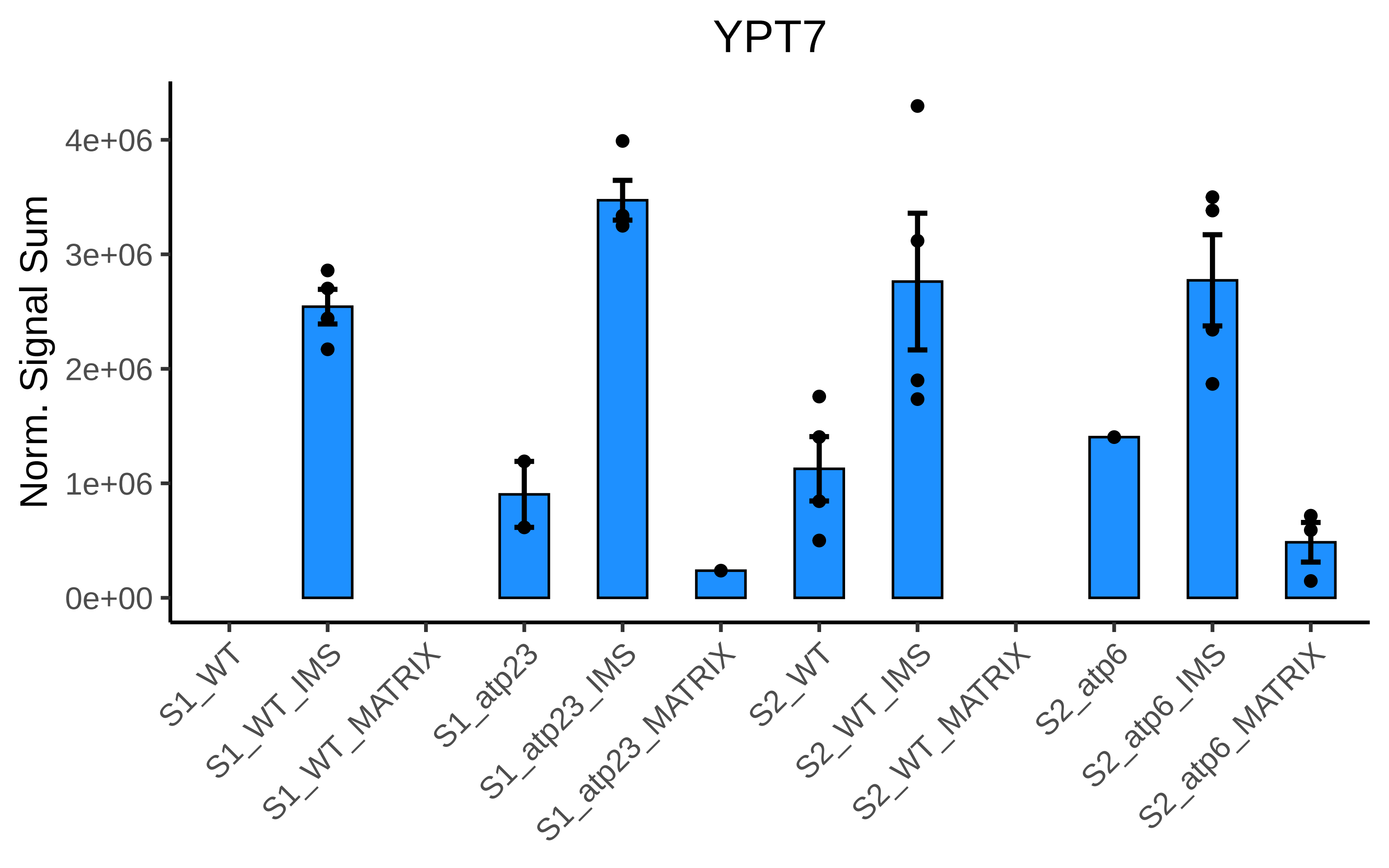
YPT7
Norm. Signal Sum
4e+06
3e+06
2e+06
1e+06
0e+00
S1_WT
S1_WT_IMS
S1_WT_MATRIX
S1_atp23
S1_atp23_IMS
S1_atp23_MATRIX
S2_WT
S2_WT_IMS
S2_WT_MATRIX
S2_atp6
S2_atp6_IMS
S2_atp6_MATRIX

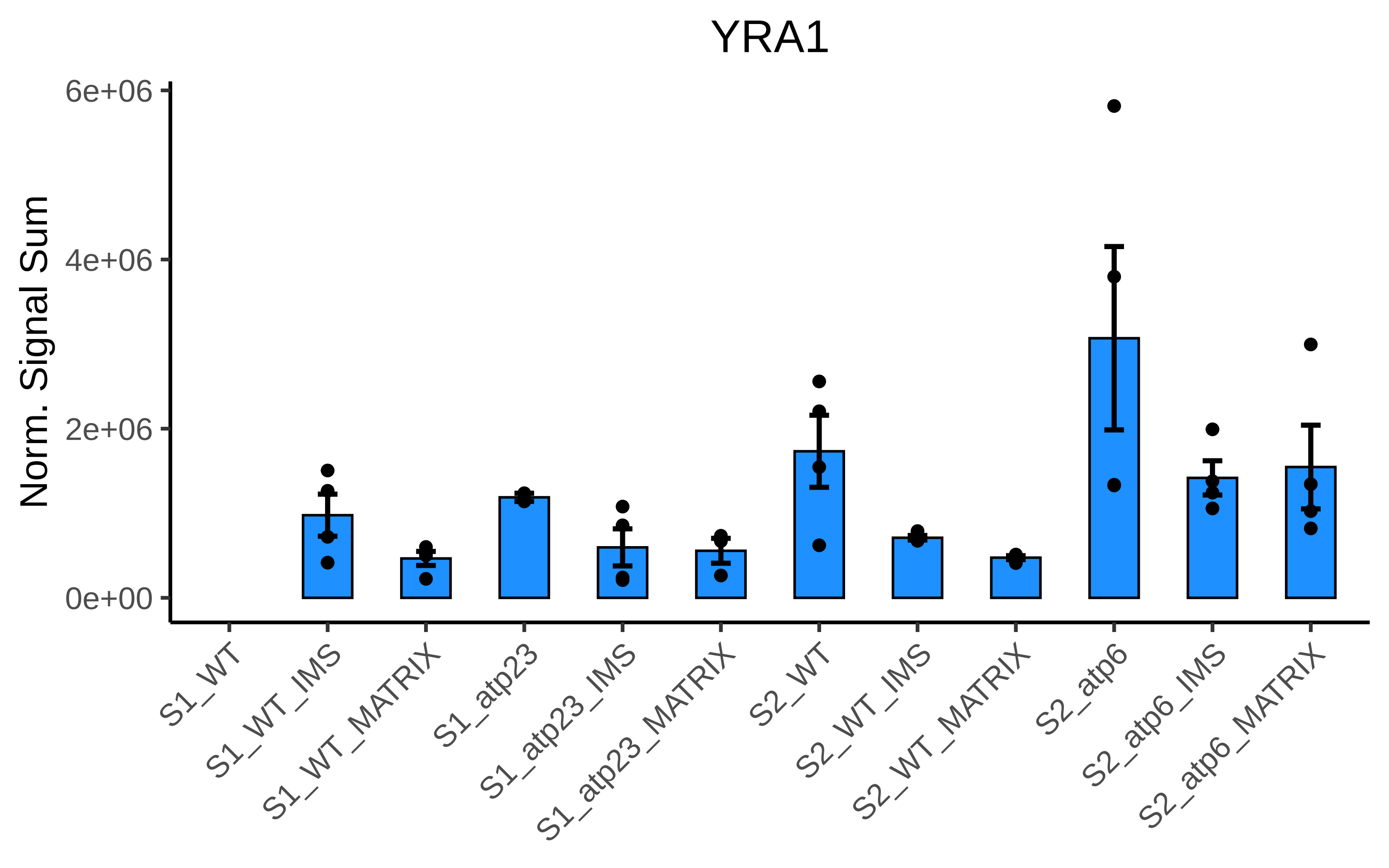
YRA1
Norm. Signal Sum
6e+06
4e+06
2e+06
0e+00
S1_WT
S1_WT_IMS
S1_WT_MATRIX
S1_atp23
S1_atp23_IMS
S1_atp23_MATRIX
S2_WT
S2_WT_IMS
S2_WT_MATRIX
S2_atp6
S2_atp6_IMS
S2_atp6_MATRIX

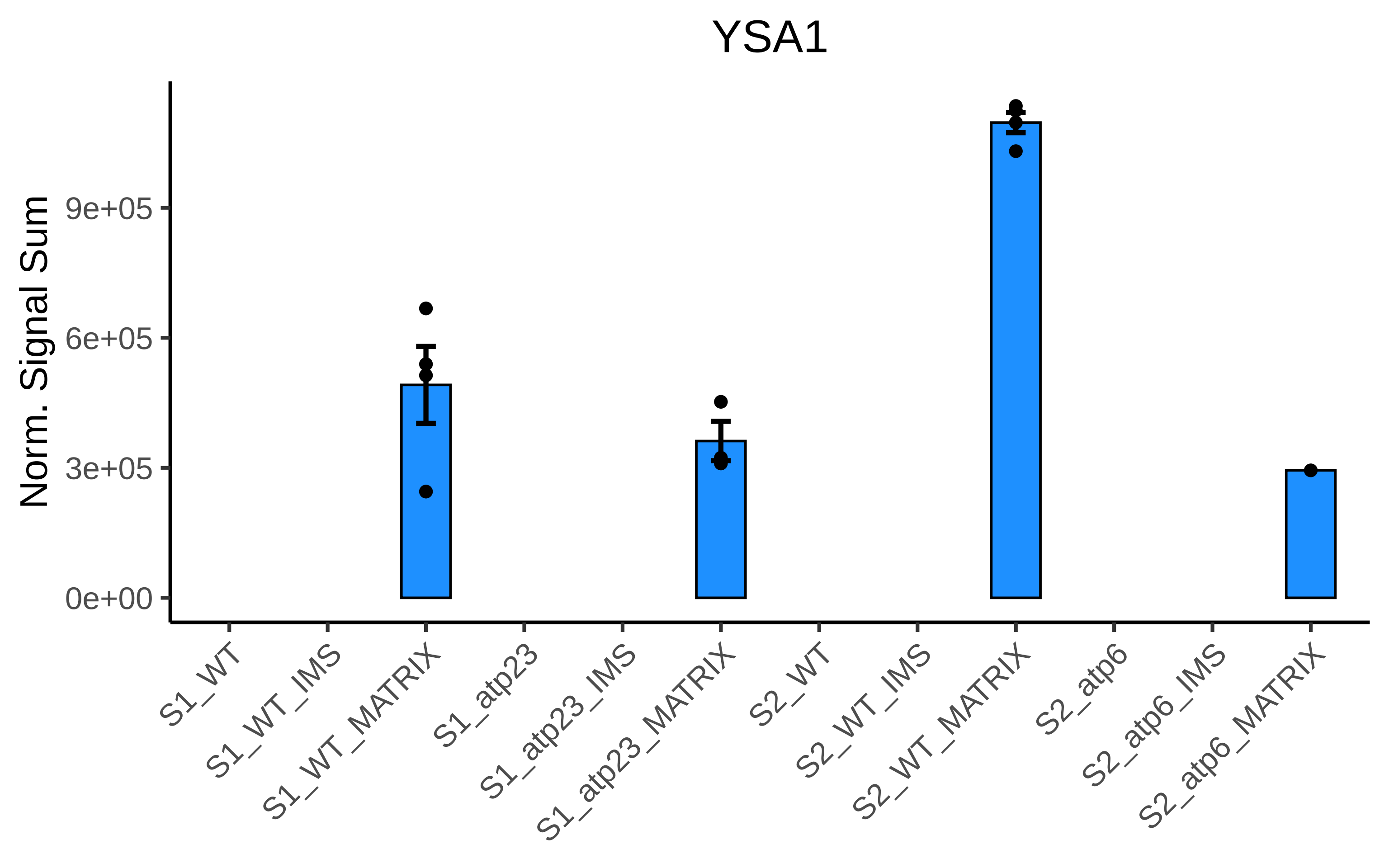

YSA1
Norm. Signal Sum
9e+05
6e+05
3e+05
0e+00
S1_WT
S1_WT_IMS
S1_WT_MATRIX
S1_atp23
S1_atp23_IMS
S1_atp23_MATRIX
S2_WT
S2_WT_IMS
S2_WT_MATRIX
S2_atp6
S2_atp6_IMS
S2_atp6_MATRIX

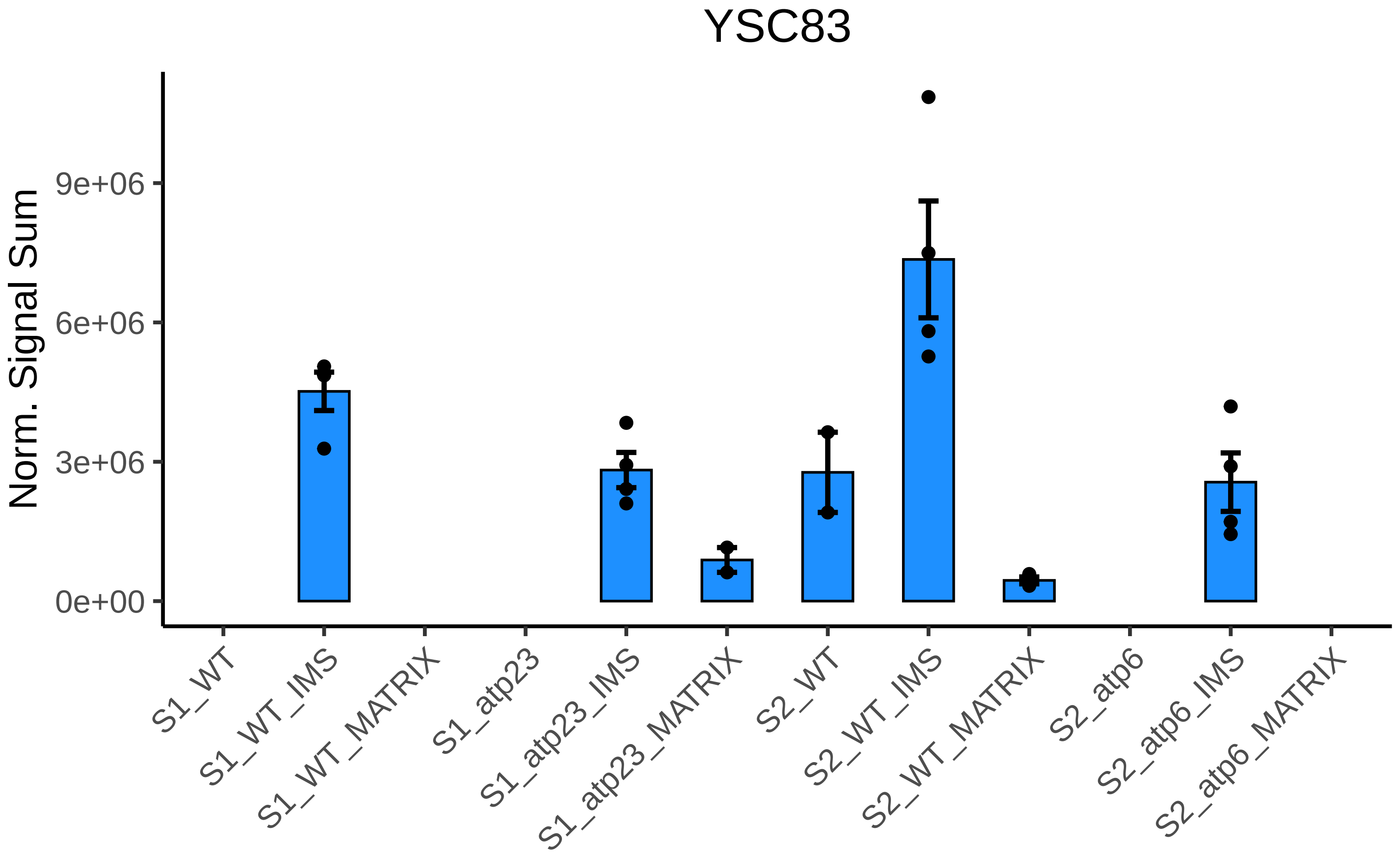
YSC83
Norm. Signal Sum
9e+06
6e+06
3e+06
0e+00
S1_WT
S1_WT_IMS
S1_WT_MATRIX
S1_atp23
S1_atp23_IMS
S1_atp23_MATRIX
S2_WT
S2_WT_IMS
S2_WT_MATRIX
S2_atp6
S2_atp6_IMS
S2_atp6_MATRIX

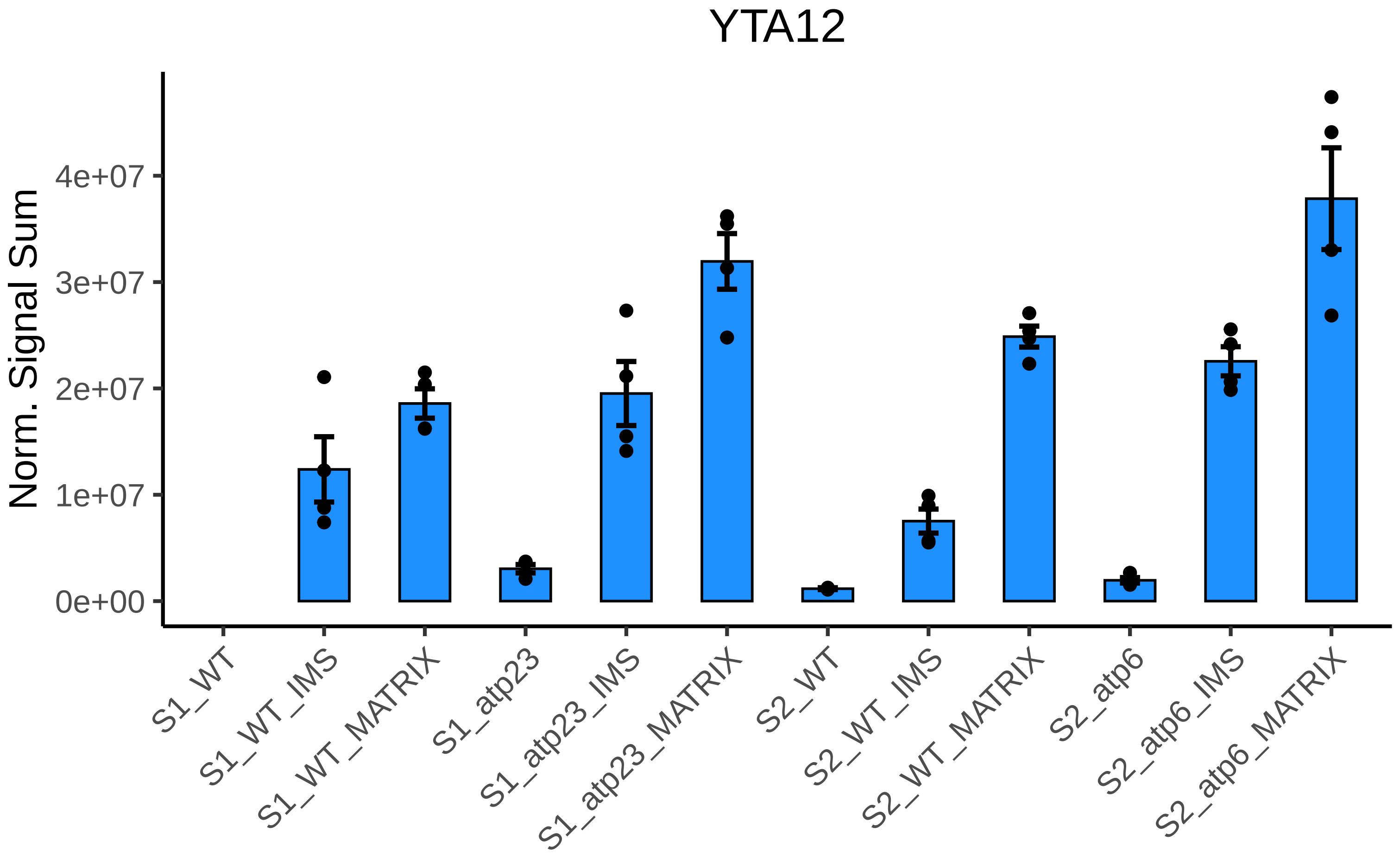

YTA12
Norm. Signal Sum
4e+07
3e+07
2e+07
1e+07
0e+00
S1_WT
S1_WT_IMS
S1_WT_MATRIX
S1_atp23
S1_atp23_IMS
S1_atp23_MATRIX
S2_WT
S2_WT_IMS
S2_WT_MATRIX
S2_atp6
S2_atp6_IMS
S2_atp6_MATRIX

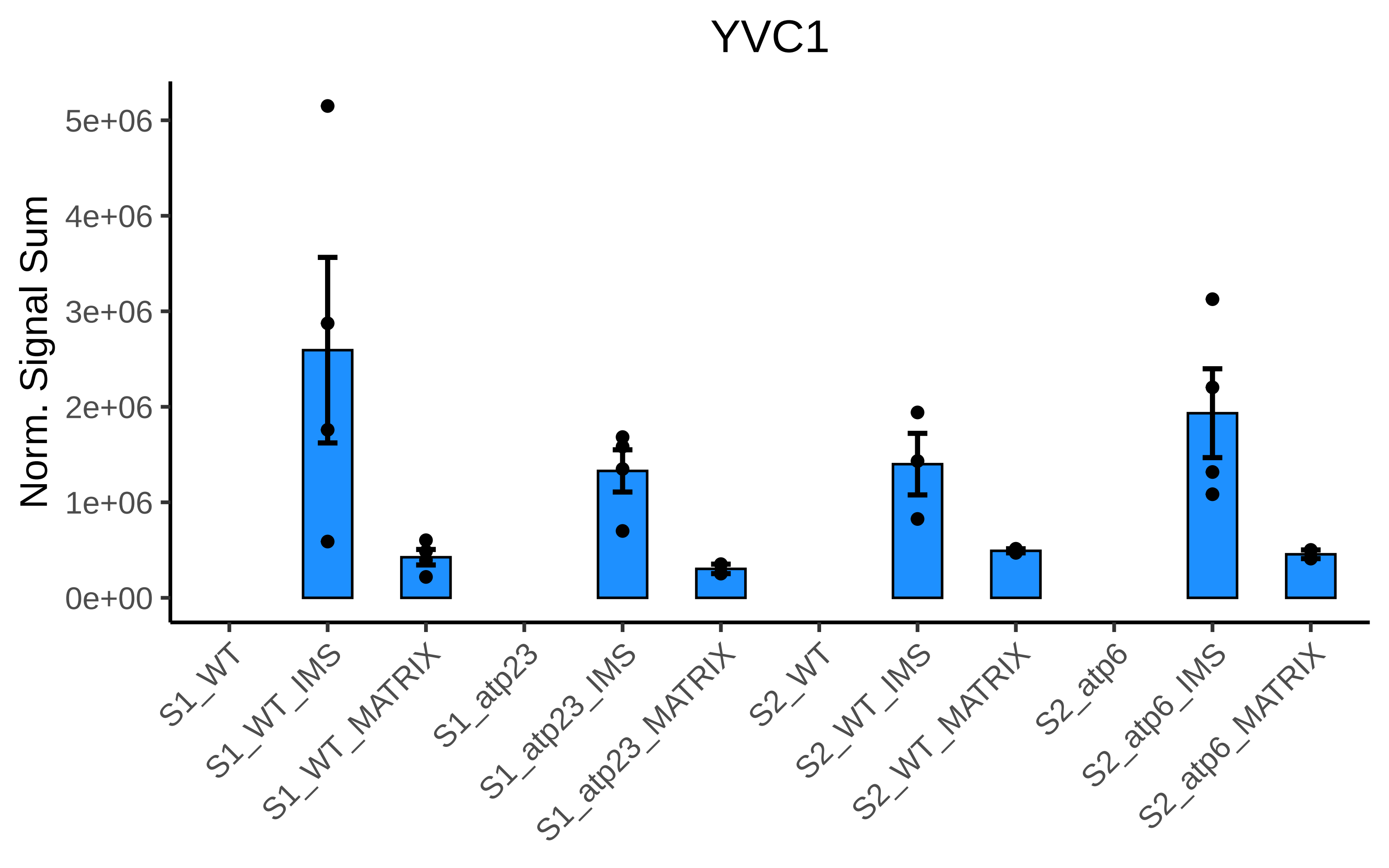

YVC1
Norm. Signal Sum
5e+06
4e+06
3e+06
2e+06
1e+06
0e+00
S1_WT
S1_WT_IMS
S1_WT_MATRIX
S1_atp23
S1_atp23_IMS
S1_atp23_MATRIX
S2_WT
S2_WT_IMS
S2_WT_MATRIX
S2_atp6
S2_atp6_IMS
S2_atp6_MATRIX

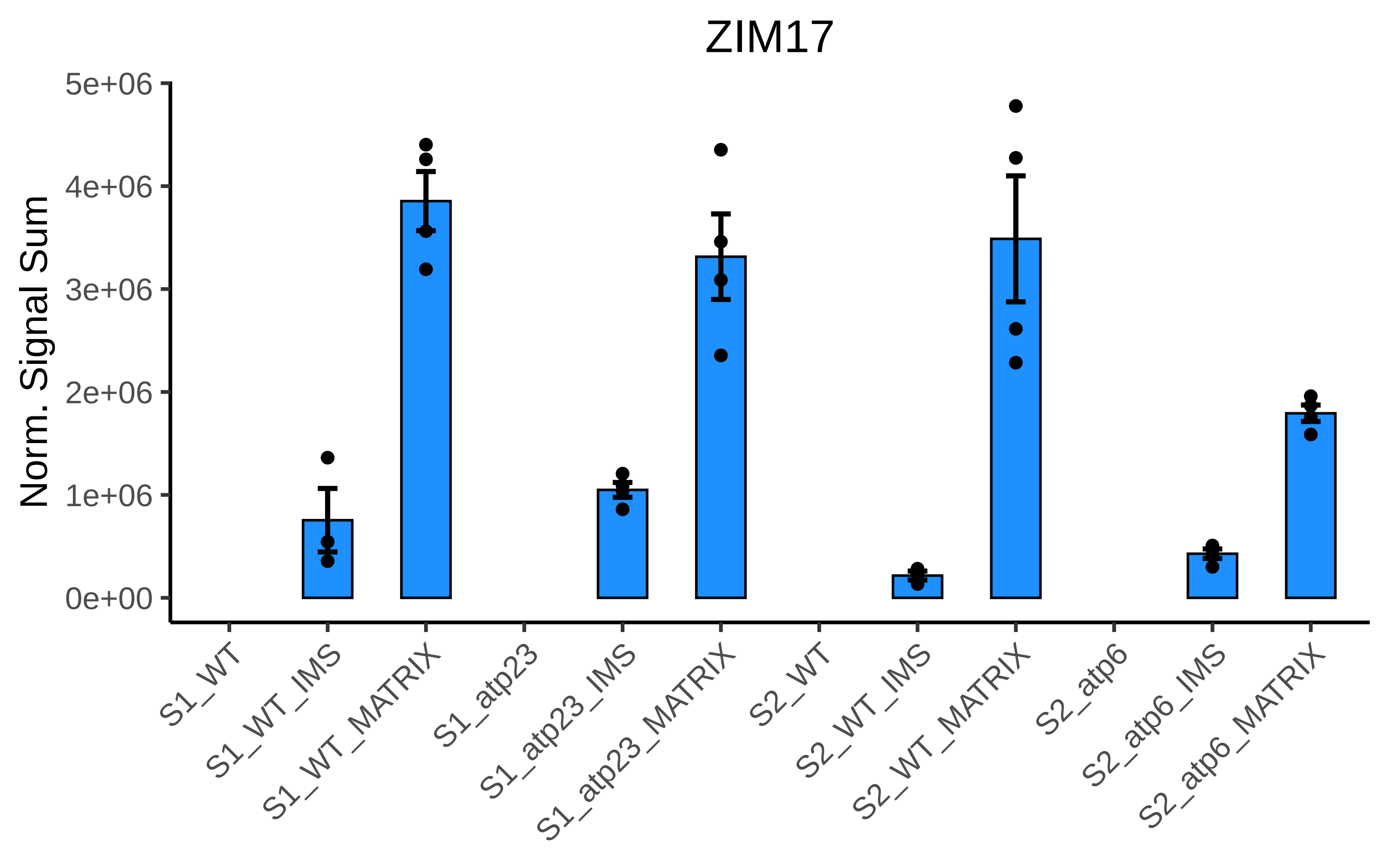
ZIM17
Norm. Signal Sum
5e+06
4e+06
3e+06
2e+06
1e+06
0e+00
S1_WT
S1_WT_IMS
S1_WT_MATRIX
S1_atp23
S1_atp23_IMS
S1_atp23_MATRIX
S2_WT
S2_WT_IMS
S2_WT_MATRIX
S2_atp6
S2_atp6_IMS
S2_atp6_MATRIX

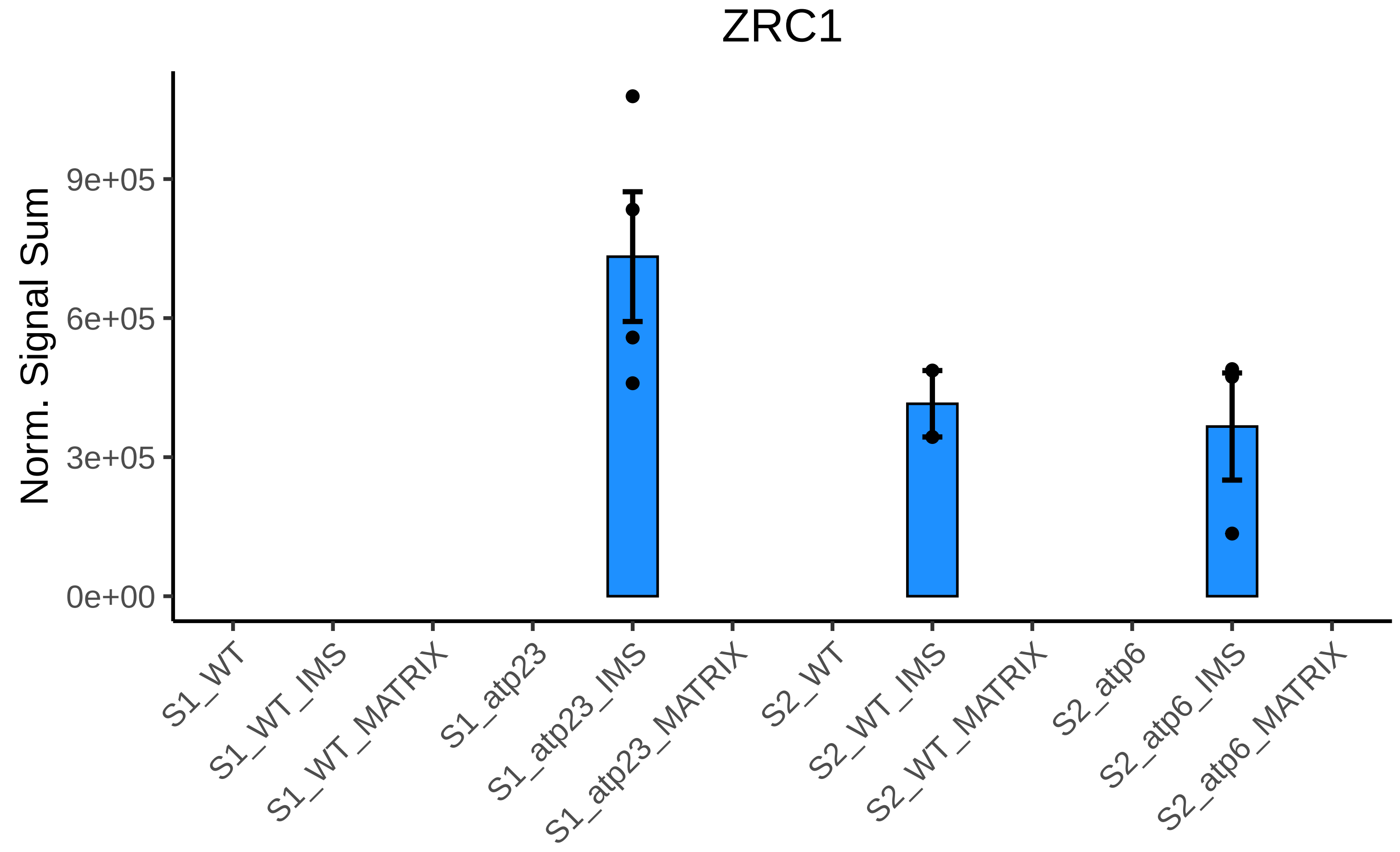

ZRC1
Norm. Signal Sum
9e+05
6e+05
3e+05
0e+00
S1_WT
S1_WT_IMS
S1_WT_MATRIX
S1_atp23
S1_atp23_IMS
S1_atp23_MATRIX
S2_WT
S2_WT_IMS
S2_WT_MATRIX
S2_atp6
S2_atp6_IMS
S2_atp6_MATRIX

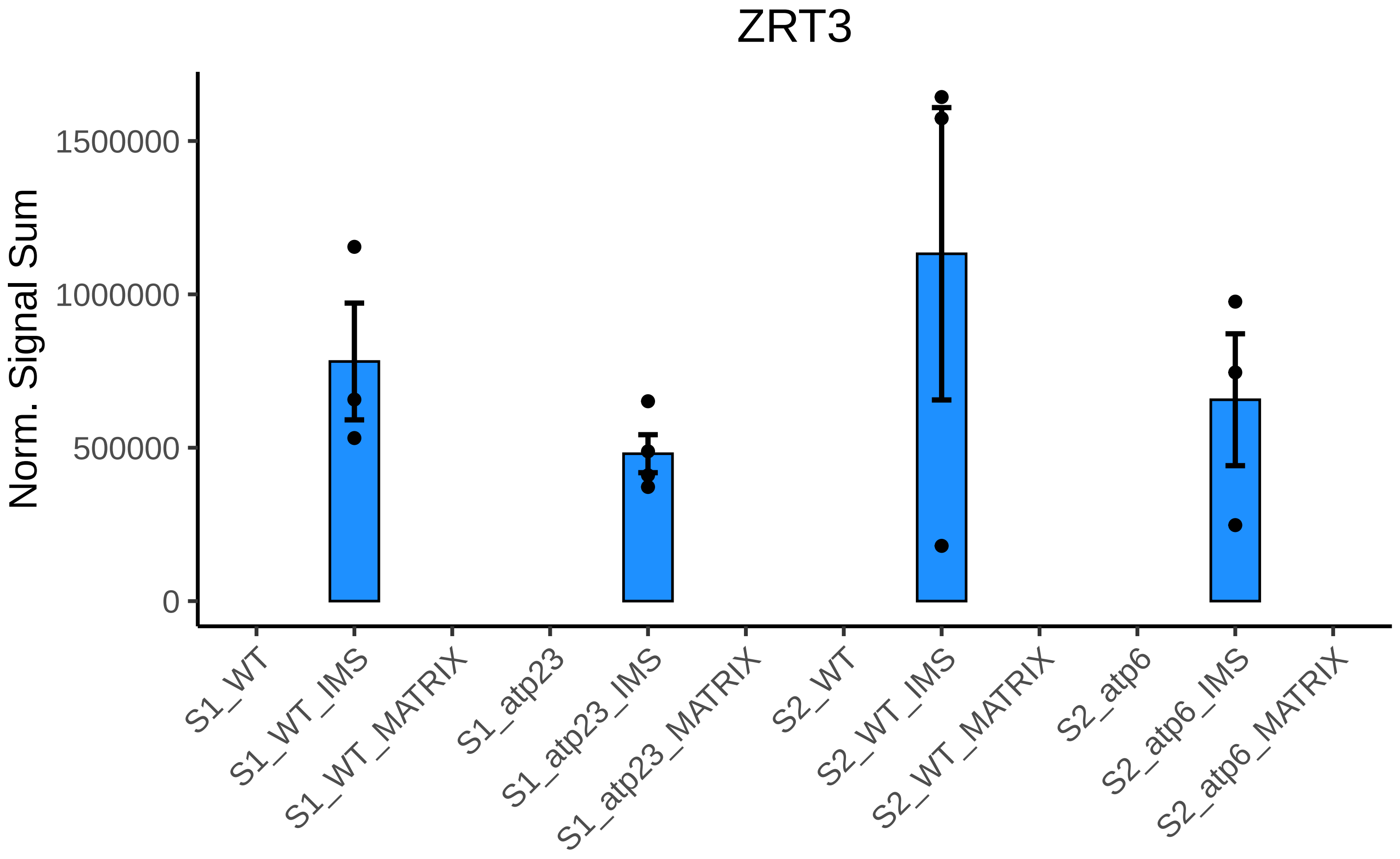
ZRT3
Norm. Signal Sum
1500000
1000000
500000
0
S1_WT
S1_WT_IMS
S1_WT_MATRIX
S1_atp23
S1_atp23_IMS
S1_atp23_MATRIX
S2_WT
S2_WT_IMS
S2_WT_MATRIX
S2_atp6
S2_atp6_IMS
S2_atp6_MATRIX

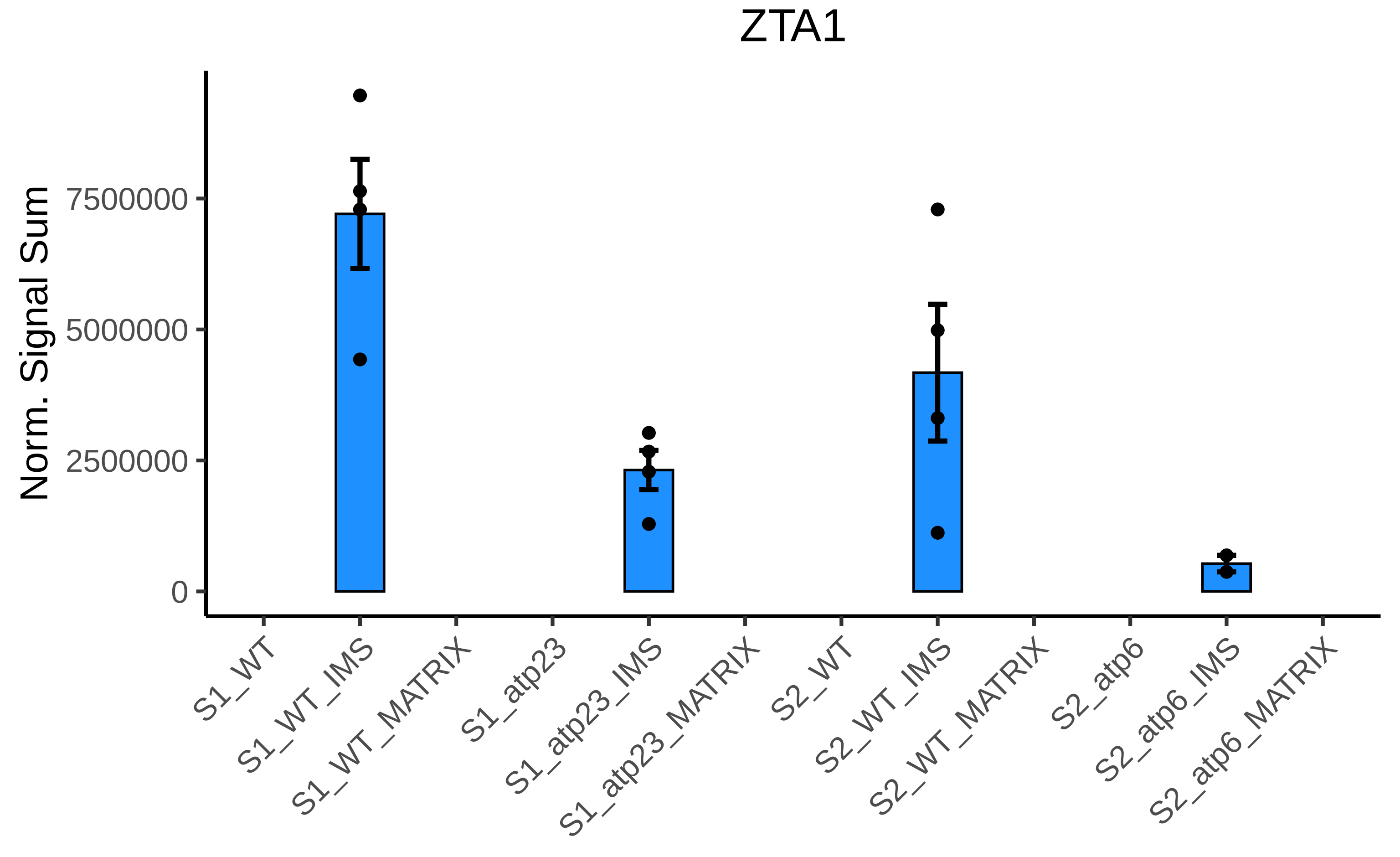
ZTA1
Norm. Signal Sum
0
2500000
5000000
7500000
S1_WT
S1_WT_IMS
S1_WT_MATRIX
S1_atp23
S1_atp23_IMS
S1_atp23_MATRIX
S2_WT
S2_WT_IMS
S2_WT_MATRIX
S2_atp6
S2_atp6_IMS
S2_atp6_MATRIX

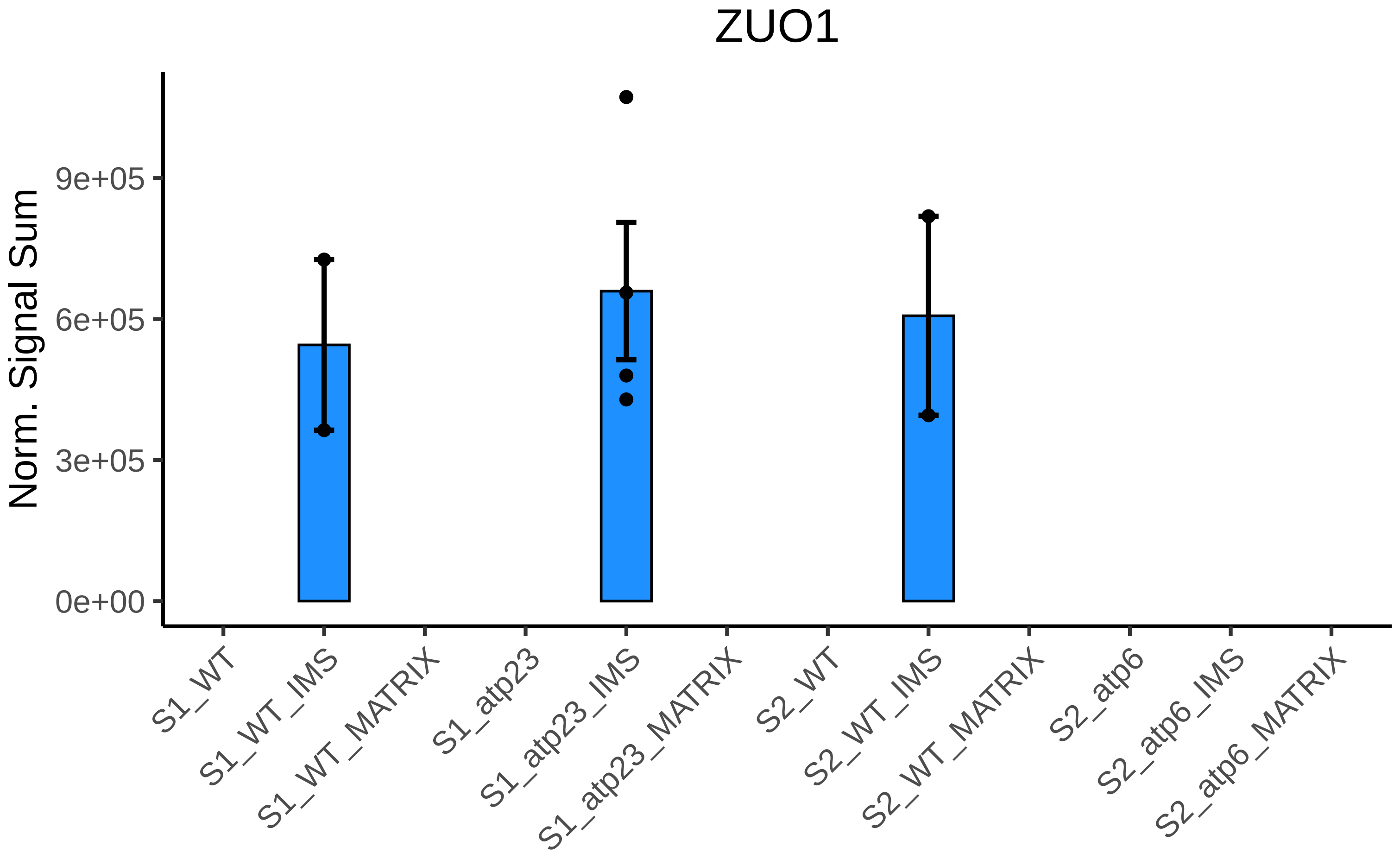
ZUO1
Norm. Signal Sum
9e+05
6e+05
3e+05
0e+00
S1_WT
S1_WT_IMS
S1_WT_MATRIX
S1_atp23
S1_atp23_IMS
S1_atp23_MATRIX
S2_WT
S2_WT_IMS
S2_WT_MATRIX
S2_atp6
S2_atp6_IMS
S2_atp6_MATRIX
